# Unsupervised Deconvolution of Dynamic Imaging Reveals Intratumor Vascular Heterogeneity and Repopulation Dynamics

**DOI:** 10.1371/journal.pone.0112143

**Published:** 2014-11-07

**Authors:** Li Chen, Peter L. Choyke, Niya Wang, Robert Clarke, Zaver M. Bhujwalla, Elizabeth M. C. Hillman, Ge Wang, Yue Wang

**Affiliations:** 1 Genetics Branch, National Cancer Institute, National Institutes of Health, Bethesda, MD 20892, United States of America; 2 Molecular Imaging Program, National Cancer Institute, National Institutes of Health, Bethesda, MD 20892, United States of America; 3 Department of Electrical and Computer Engineering, Virginia Polytechnic Institute and State University, Arlington, VA 22203, United States of America; 4 Lombardi Comprehensive Cancer Center, Georgetown University, Washington, D. C. 20057, United States of America; 5 Department of Radiology and Radiological Science, Johns Hopkins University School of Medicine, Baltimore, MD 21205, United States of America; 6 Department of Biomedical Engineering, Columbia University, New York, NY 10027, United States of America; 7 Department of Biomedical Engineering, Biomedical Imaging Center, Rensselaer Polytechnic Institute, Troy, NY 12180, United States of America; Stanford University School of Medicine, United States of America

## Abstract

With the existence of biologically distinctive malignant cells originated within the same tumor, intratumor functional heterogeneity is present in many cancers and is often manifested by the intermingled vascular compartments with distinct pharmacokinetics. However, intratumor vascular heterogeneity cannot be resolved directly by most *in vivo* dynamic imaging. We developed multi-tissue compartment modeling (MTCM), a completely unsupervised method of deconvoluting dynamic imaging series from heterogeneous tumors that can improve vascular characterization in many biological contexts. Applying MTCM to dynamic contrast-enhanced magnetic resonance imaging of breast cancers revealed characteristic intratumor vascular heterogeneity and therapeutic responses that were otherwise undetectable. MTCM is readily applicable to other dynamic imaging modalities for studying intratumor functional and phenotypic heterogeneity, together with a variety of foreseeable applications in the clinic.

## Introduction

Intratumor genetic or epigenetic heterogeneity has been found in many cancers as evidenced by deep sequencing selectively applied to different parts of the same tumor [1], [2]. Consequently, cancer cells display remarkable phenotypic variability, including ability to induce angiogenesis, seed metastases, and survive therapy [3]–[5]. Advanced solid tumors often contain vascular compartments with distinct pharmacokinetics, comprising hypoxic regions and spatially intermingled irregular vasculature that is leaky and inefficient [6]–[8]. The complexity of heterogeneity has clinical implications. A more heterogeneous tumor is more likely to fail therapy due to increased drug-resistant variants [3], [5], and characteristics of the dominant cell type will not necessarily predict the behaviors of interest rooted in specific cells [4].

Dynamic contrast-enhanced magnetic resonance imaging (DCE-MRI) provides a noninvasive *in vivo* method to evaluate tumor vasculature architectures based on contrast accumulation and washout [7], [9]. While DCE-MRI can potentially depict the intratumor heterogeneity of vascular permeability [10], the quantitative application of DCE-MRI has been hindered by its inability to accurately resolve vascular compartments with distinct pharmacokinetics due to limited imaging resolution [7], [11]. We emphasize that identification of spatially mixed multiple vascular cytotypes is principally different from imaging an inhomogeneously distributed single vascular cytotype, and it is the former scenario that presents significant technological challenges to portraying tumor cytotypes. This indistinction among the contributions of different compartments to the mixed tracer signals can confound compartment modeling and deep phenotyping for association studies [4], [12], [13]. The goal of the present work was to discern vascular heterogeneity and its changes in tumors using DCE-MRI and novel mathematical models, for personalized cancer diagnosis and treatment.

**Figure 1:**
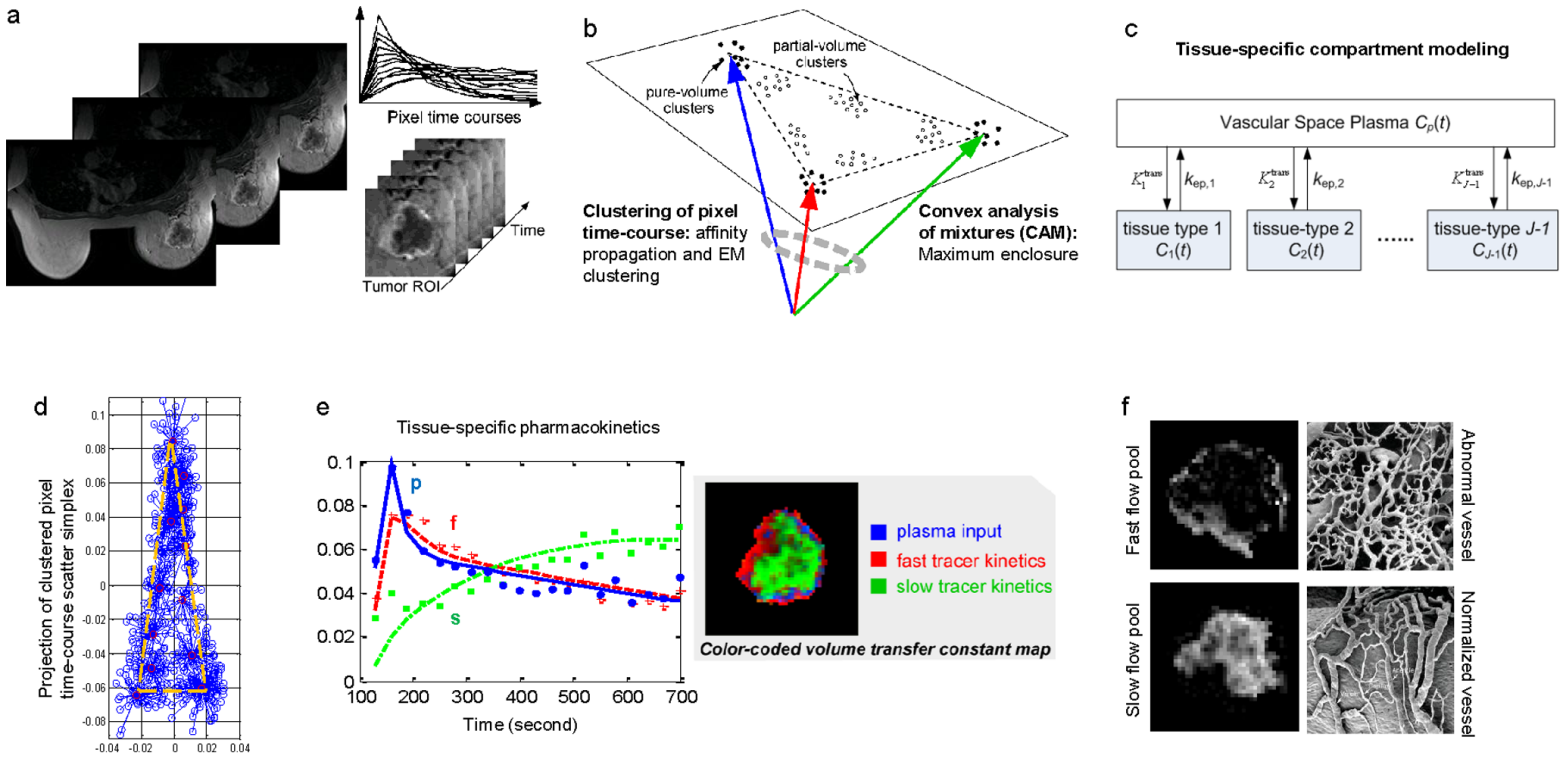
The proposed multi-tissue compartment modeling pipeline for uncovering intratumor vascular heterogeneity. (a) On the DCE-MRI sequence, tumor region is extracted using a digital mask. Then, pixel time-courses are collected and normalized over time. (b) Pixel time-courses are grouped into clusters with initialization-free multivariate clustering techniques. On the simplex of pixel time-courses, the clusters present at the vertices are identified by a convex analysis of mixtures. (c) Using pure-volume pixels, multi-compartment modeling is performed to estimate tissue-specific flux rate constants and volume transfer constants. (d) Scatter simplex of real DCE-MRI data from an advanced breast cancer. (e) Estimated tissue-specific compartmental time-activity curves: ‘blue’ – plasma input function; ‘red’ – fast flow kinetics; ‘green’ – slow flow kinetics; and example images of the associated local volume transfer constants. (f) Illustrative microscopic images of normal and abnormal vessel architecture (McDonald and Choyke, *Nat Med* **9**, 2003).

We developed a computational method (multi-tissue compartment modeling - MTCM) for deconvolving intratumor vascular heterogeneity and identifying pharmacokinetics changes in many biological contexts [5], [14], [15]. MTCM works by applying a convex analysis of mixtures that enables geometrically-principled delineation of distinct vascular structures from DCE-MRI data (**Fig. 1a–c**). A formal mathematical description of the method and its detailed implementation is available in Methods.

## Results

### Overview of MTCM

Tumors to be analyzed by MTCM contain unknown numbers of distinct vascular compartments. The pixel-wise tracer concentration in a particular vascular compartment is modeled as being proportional to the local volume transfer constant of the vascular compartment (Method). Because there are often significant numbers of partial-volume pixels, MTCM instead estimates pharmacokinetic parameters (flux rate constants) via the time-courses of pure-volume pixels (pixels whose signal is highly enriched in a particular vascular compartment). Convex analysis of mixtures identifies those pure-volume pixels present at the vertices of the clustered pixel time series scatter simplex, without any knowledge of compartment distribution (Method). When the number of underlying vascular compartments is detected using the minimum description length (MDL) criterion, MTCM provides a completely unsupervised approach to characterize intratumor heterogeneity (**Methods** and **Appendix S1 in File S1**).

**Figure 2:**
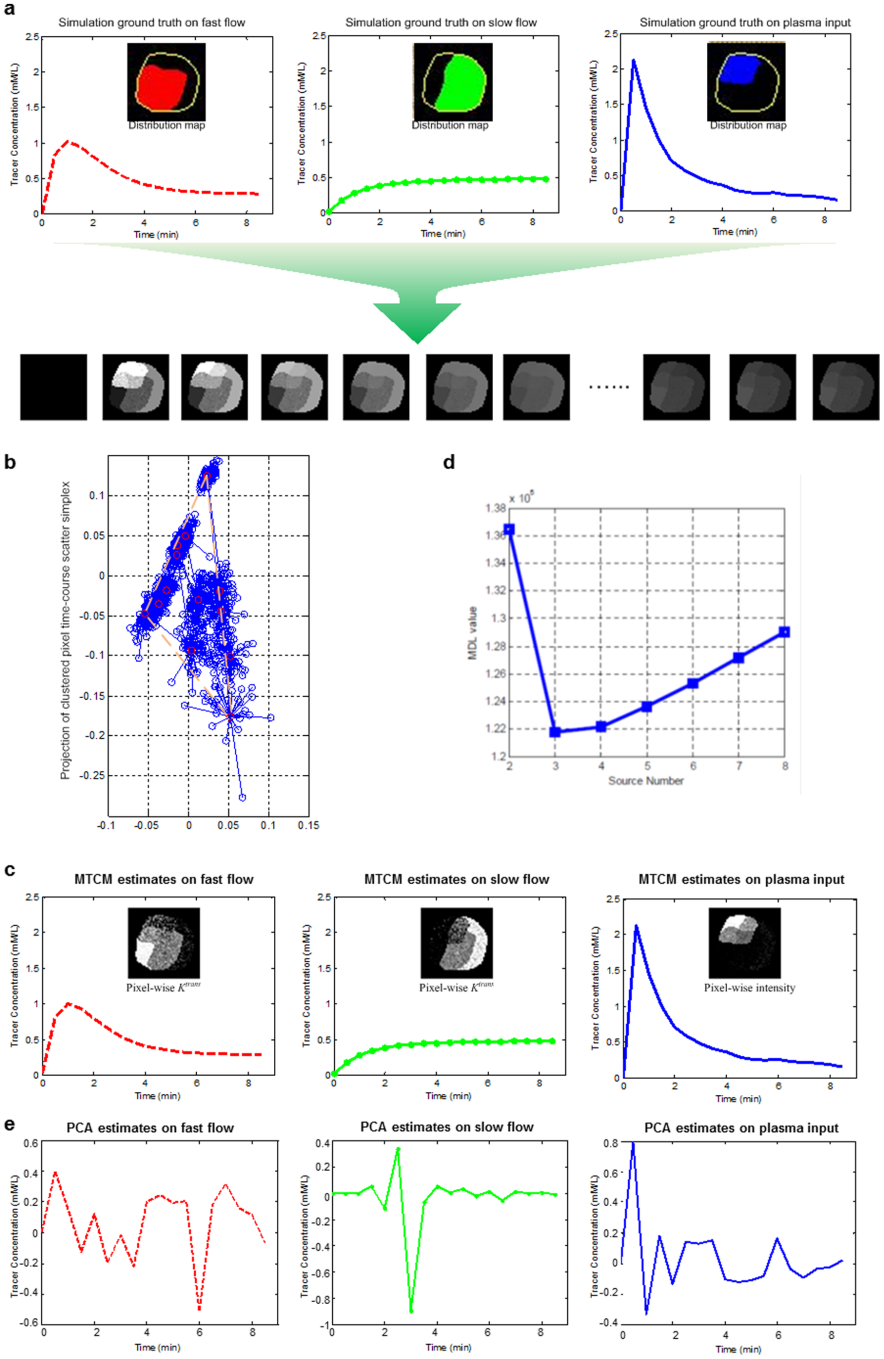
MTCM estimates time-activity curves in multiple vascular compartments simultaneously and quantitatively reconstructs tissue-specific local volume transfer constants - synthetic DCE-MRI experiments: (a) synthesis of image series; (b) scatter simplex of synthesized image series; (c) tissue-specific compartmental tracer concentration curves and local volume transfer constant maps, estimated by MTCM; (d) MDL model selection to detect the number of compartments; (e) tissue-specific compartmental tracer concentration curves estimated by principle component analysis (PCA).

Modeling the pharmacokinetics of each vascular compartment using pure-volume pixel time-courses allowed us to estimate individual compartment flux rate constants (**Fig. 1d–e**). Non-negative least-square estimation yielded pixel-wise local volume transfer constants (Methods and **Fig. 1f**). Using synthetic and mouse DCE-MRI experiments, we showed that MTCM can be used to estimate pharmacokinetic parameters in several vascular compartments simultaneously and to quantitatively reconstruct tissue-specific local volume transfer constants (**Data S1–S2,** **Figs. 2** **, S1–S2** and **Tables S1–S2**). Furthermore, MTCM enabled quantitation of differences in tissue-specific vascular permeability across time (for example, therapeutic responses in longitudinal studies; Methods). Thus, the change in values of flux rate constants in a given vascular compartment could be determined, despite an expected difference in that vascular compartment's relative abundance.

We also analyzed the same realistic synthetic dataset using a “traditional” way of principal component analysis - PCA. By a comparison of the tracer concentration extracted by PCA (**Fig. 2e**) to that estimated by MTCM (**Fig. 2d**), we can see that tracer concentration curves estimated by PCA are highly fluctuant and significant deviated from the ground truth. In fact, similar unsatisfactory results produced by PCA or classic factor analysis have been observed in the earlier studies by us and others (Cinotti, Bazin et al. 1991, Zhou, Huang et al. 1997, Hillman and Moore 2007, Hillman, Amoozegar et al. 2011). We should clarify that MTCM consists of two major analytic parts: convex analysis of mixtures (CAM) and compartment modeling (CM), where the CAM is a critical step that automatically identifies the pure tissue pixels and their time activity curves, followed by the CM that estimates the pharmacokinetics parameters without being contaminated by the partial-volume effect. In contrast, since PCA does not enforce the nonnegative constraint for tracer concentration estimation, a subsequent compartment modeling cannot be performed to estimate pharmacokinetic parameters.

### Intratumor vascular heterogeneity in breast cancer revealed by MTCM

In keeping with our goal to use MTCM to better uncover vascular heterogeneity in human tumors, we applied MTCM to DCE-MRI sequence data obtained from a case of advanced breast cancer (**Fig. 1a**). In this breast tumor [7], vascular heterogeneity is characterized by active angiogenesis in the peripheral “rim” and concurrent inner-core hypoxia. Upon preliminary analysis using MDL, we found that a two-tissue compartment model of the fast and slow tracer clearance rates was sufficient to account for the variable permeability at the majority of pixels (Methods). Thus, we used pure-volume pixels associated with these two vascular pools to estimate tissue-specific flux rate constants and to reconstruct local volume transfer constant maps (Methods). MTCM reveals two vascular compartments with distinct flux rate constants (**Fig. 1e**). Accordingly, we detected distinct spatial patterns of specific local volume transfer constant in the two vascular compartments (**Fig. 1f**) with a significant fraction of partial volume pixels.

Intratumor vascular heterogeneity identified by MTCM is consistent with the knowledge obtained from *ex vivo* microscopic and molecular studies [7], [13]. Defective endothelial barrier function is one of the better documented abnormalities of tumor vessels, resulting in functional heterogeneity in vascular permeability to macromolecules [7], [11]. As a tumor rapidly outgrows its blood supply, it requires neovessel maturation, often leaving an inner core of the tumor with regions where blood flow and oxygen concentration are significantly lower than in normal tissues [6]. MTCM reconstructed local volume transfer constant maps correlate well with the differential gene expression known to regulate angiogenesis [7], [13].

### Changes in intratumor vascular heterogeneity in longitudinal studies

**Figure 3:**
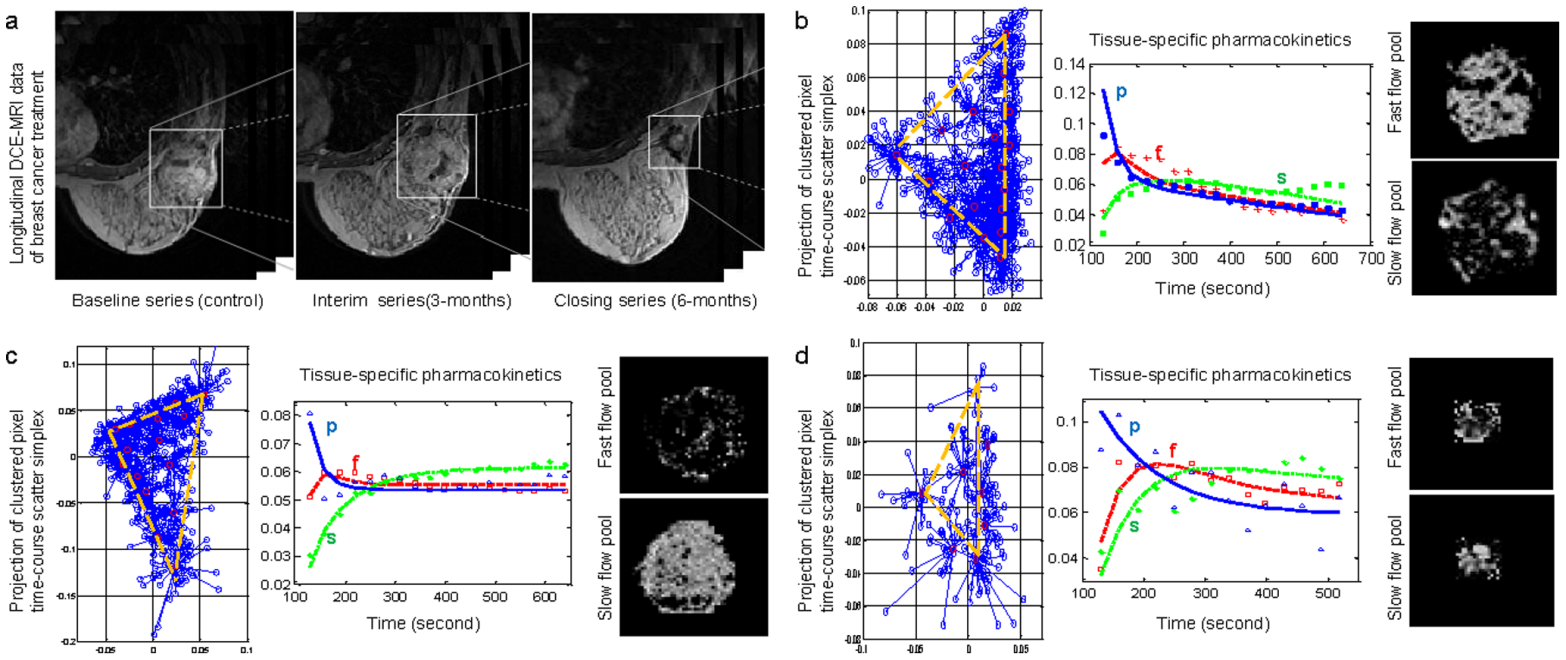
Quantitative estimates of tissue-specific pharmacokinetic parameters in a longitudinal breast cancer study reveal changes in tumor vascular behavior in response to hybrid anti-angiogenesis chemotherapy. While tumor size regression (largely determined by bulk tumor populations rather than rarer cancer stem cells) is clearly observed, together with a transient “normalization”, the detected tumor islands of persistent enhancement predict the confirmed recurrence despite the dramatic size changes. (a) Snapshots of DCE-MRI sequences taken from the same tumor before, during, and after therapy. (b) Scatter simplex of baseline DCE-MRI data taken before therapy; estimated tissue-specific compartmental time-activity curves; and example images of the associated local volume transfer constants. (c) Scatter simplex of interim DCE-MRI data taken during therapy; estimated tissue-specific compartmental time-activity curves; and example images of the associated local volume transfer constants. (d) Scatter simplex of closing DCE-MRI data taken after therapy; estimated tissue-specific compartmental time-activity curves; and example images of the associated local volume transfer constants.

We also detected changes in pharmacokinetic patterns among longitudinal DCE-MRI data from breast cancer acquired before, during, and after treatment (**Fig. 3a**), quantified as different flux rate constants over time (Methods and **Table S3**). For example, the two vascular compartment time-activity curves revealed by MTCM in the baseline data are highly distinct (**Fig. 3b**). We detected significantly higher permeability in a fast-flow pool and slightly lower permeability in a slow-flow pool when compared with the normal state. In contrast, the interim response (**Fig. 3c**) exhibits vascular compartment time-activity curves that are distinct but much closer to each other, whereas the closing response (**Fig. 3d**) shows a significant decrease in permeability of the fast-flow pool. We also detected different local volume transfer constant maps (**Fig. 3b–d**) and changes in the fractions of partial-volume pixels (**Table S4**).

### Comparative studies using standard compartment modeling

We compared tissue-specific pharmacokinetics detected with MTCM to the results of a standard compartment analysis of (total) vascular pool within the region of interest. Total time-activity curves were indistinct across time (**Fig. S2**) owing to therapeutic effects in some parts of the tumor but not in others and large fractions of partial-volume pixels. In this longitudinal study, we deconvolved total time-activity curves into two phased therapeutic effects using MTCM: a transient “normalization” of abnormal yet surviving tumor vasculature together with the significant and consistent drop in the relative volume transfer constants [6], [16]. In contrast, standard analysis may not return informative results when both the flux rate constant and volume transfer constant change heterogeneously in response to therapy. These examples illustrate the ability of MTCM to discover intratumor vascular heterogeneity and to detect changes in each vascular compartment over time. Finally, we tested the applicability of MTCM to dynamic fluorescence imaging data acquired on a mouse after bolus injection of indocyanine green dye by deconvolving biodistribution dynamics of the major organs [17] (**Fig. S3**). The dissected tissue compartments constitute anatomical structures of the mouse that agree well with a digital anatomical mouse atlas.

## Discussion

Several previous studies have discussed the problem of intratumor vascular heterogeneity in compartment modelling [7], [11], [16], [18], a major outstanding issue for the characterization of complex phenotypes and therapeutic responses. Some methods have addressed the estimation of multi-compartment pharmacokinetics in the presence of varying partial-volume effects, relying on known regions of pure-volume pixels and number of compartments [10], [13], [16], [17]. The significant advantage of our strategy is its ability to detect and quantify intratumor vascular heterogeneity without any type of external information. The benefits of such a method include its wide applicability, sensitive detection of heterogeneity dynamics, and reliance on longitudinal data from one single subject (**Appendix S1 in File S1**).

We have identified differential and heterogeneous changes in tissue-specific vascular pharmacokinetics in tumors during treatment that were undetected using standard analysis, including tumor islands of persistent enhancement that have escaped the effects of therapy [18]. These results are particularly intriguing when considered together with recent imaging studies describing foci of resistant and more aggressive clones within a tumor [5], [13]. While it is not yet possible to assign causality, these *in vivo* results allowed us to propose new hypotheses regarding the complex relationships between intratumor heterogeneity, clonal repopulation, cancer stem-cell, and therapeutic efficacy [1], [3], [5], [10], [19].

In metastatic disease, recent studies have revealed the emergence of treatment-resistant subclones that were present at a minor frequency in the primary tumour [20]. Thus, modeling cancer diagnosis and treatment in the future should involve characterization of subpopulations within the primary tumour, monitoring of clonal dynamics during treatment and eradication of treatment-emergent clones [21]. To prospectively assess intratumor heterogeneity, profiling of multiregional tumour samples would be required. However, it is impractical and potentially risky to take multiple ‘random’ biopsies in every patient, owing to both sampling bias and the inability to resolve intermingled heterogeneity [22]. MTCM would not only make longitudinal in vivo surveillance possible but also enable imaging-informed selective biopsies.

The future challenges of applying MTCM lie in the gap between research experiments and clinical practice. Unlike high-quality data in well-designed research studies, clinical data are usually with limited spatial and/or temporal resolution, accompanied by higher noise level (**Fig. S4**). Lower spatial resolution results in less pure-volume pixels and thus reduces the accuracy of MTCM; while limited temporal resolution prevents accurate differentiation and estimation of pharmacokinetic parameters associated with distinctive vascular compartments.

So far we have tested MTCM method on DCE-MRI data [7], dynamic contrast-enhanced optical imaging data [17], [23], and dynamic PET imaging data [24], acquired from both human tissue/organ and whole-body mouse model (e.g., **Fig. S4**). Theoretically, the MTCM method can produce confident estimation on any ‘dynamic contrast-enhanced’ imaging data with sufficient quality (e.g., spatial and temporal resolution) [25], [26]. However, we should emphasize that there are a few fundamental assumptions behind the MTCM methodology, as specified in the newly proved theorems (e.g., linear convex combination, existence of pure-tissue pixels). As in most medical imaging analysis, object motion constitutes a major source of error and can significantly confound the modeling results. Currently, MTCM is limited to ‘parallel’ compartment models, while the CAM part of the MTCM algorithm is applicable to resolving partial-volume contamination problem independent of the compartment models being used for subsequent parameter estimation.

## Methods

### Multi-tissue compartment modeling of DCE-MRI series

Let us consider *J*-tissue compartment model of DCE-MRI series (the *J*th tissue compartment corresponds to tracer plasma input, indexed by *p*), whose tracer concentration kinetics are governed by a set of first-order differential equations (**Fig. 1c**) [27], [28]

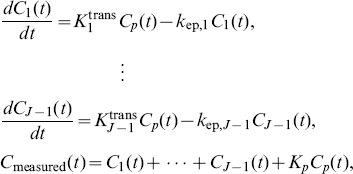
(1)where 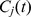 is the tracer concentrations in the interstitial space weighted by the fractional interstitial volume in tissue-type *j* at time 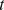 for *j* = 1,…, *J*, where *J* is the total number of vascular compartments;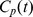 is the tracer concentration in plasma (tracer input function); 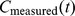 is the measured tracer concentration; 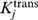 is the unidirectional volume transfer constant (/min)from plasma to tissue-type *j*; 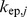 is the flux rate constants (/min) in tissue-type *j*; and 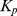 is the plasma volume[28].

Solving (1) leads to 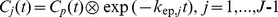, where ∶denotes the mathematical convolution, and 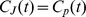. The spatial-temporal patterns of tracer concentrations (pixel time-course) can be expressed as[29]

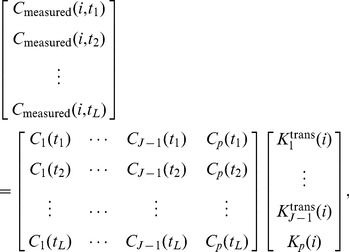
(2)where 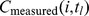 is the tracer concentration at time 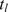 at pixel 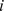, 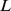 is the number of sampled time points, 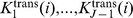 are the local volume transfer constants of the tissue-types 1 to (*J*-1), at pixel *i*, respectively; and 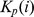 is the local plasma volume at pixel 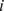.

### Parallelism between multi-tissue compartment modeling and the theory of convex sets

Apply a sum-based normalization to pixel time-course 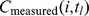 and using vector-matrix notation, we can re-express (2) as 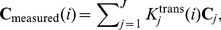
(3)where 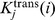 is accordingly normalized over 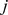, 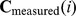 and 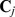 are the vector notations (over time) of pixel time course 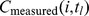 and compartment time course 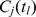, respectively. Since 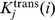 is always non-negative, as a non-negative linear combination of 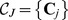, the set of pixel time-course 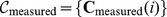 forms a subset of the *convex set* readily defined by the set of 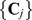




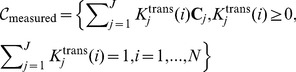
(4)MTCM exploits the strong parallelism between the multi-compartment model (3) and the theory of convex set. The fundamental principle is that *latent* compartments are pure vasculatures in a tumor whose pharmacokinetics 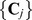 reside at the extremities of the scatter simplex occupied by the pixel time-courses, and accordingly, the interior of the simplex is occupied by the partial-volume pixels (linear non-negative mixtures of compartments) (**Fig. 1b**). Estimates of compartment pharmacokinetics may then be derived from the vertices of the multifaceted simplex that most tightly encloses the pixel time-courses and has the same number of compartments as vertices (**Fig. 1d**) [30]. MTCM algorithm is supported theoretically by a well-grounded mathematical frameworkas summarised below (see formal proofs in **Appendix S2 in File S1**).

### Theorem 1 (Convexity of pixel time-course)

*Suppose that the J compartment pharmacokinetics* 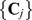 *are linearly independent, and* 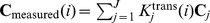 *where local volume transfer constants* 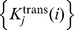 *are non-negative and have at least one pixel whose signal is highly and exclusively enriched in a particular vascular compartment, then,* 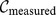 *uniquely specifies a convex set* 
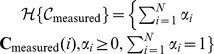
 *which is in fact identical to the convex set* 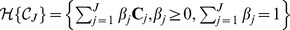 *, whose vertices are the J compartment pharmacokinetics* 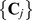.

### Theorem 2 (Optimum source dominance)

*Suppose that the local volume transfer constants* 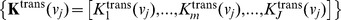 *are the vertices of the pixel time-course scatter simplex. Then the MTCM solution based on these vertices* 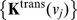 *achieves the maximum source dominance in the sense of* 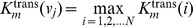.

From Theorems 1 and 2, there is a mathematical solution uniquely identifying the compartment model (3) based on the observed set of pixel time-course 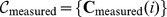 (identifiability and optimality): The *vertices* of convex set 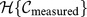 are the *J* compartment pharmacokinetics 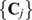 when there is at least one pixel whose signal is highly and exclusively enriched in a particular vascular compartment(**Fig. 1b**). This means that, in principle, under a noise-free scenario, we can directly estimate 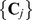 by locating the *vertices* of 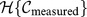 (**Fig. 1d**).

### Data preprocessing

First, the tumor area is extracted by masking out the non-tumor tissues surrounding the tumor site [31] (**Fig. 1a**). Second, the first few image frames, such as the four initial images of DCE-MRI sequences in our experiments, are removed because they correspond to the time prior to sufficient onsite tracer uptake. Third, pixels whose temporal average signal intensity is lower than 5% of the maximum value, or whose temporal dynamic variation is lower than 5% of the maximum value, are eliminated, because these non-informative pixels could have a negative impact on subsequent analyses. Fourth, the pixel time series is normalized over time using a sum-based normalization scheme, focusing the analysis on the “shape” of pharmacokinetics rather than on absolute tracer concentration.

It is true that accurate extraction of tumor region is critical to any image-based analysis that is focused on tumor characterization, where non-tumor tissue would constitute a confounding factor. Theoretically, MTCM method can handle well such situation since it is a completely unsupervised approach, relying on the MDL-based model selection. Specifically, since MTCM is specifically designed to work on multiple tissue compartment modeling, when a significant portion of the surrounding healthy tissue is included in the processed ‘tumor’ area, the healthy tissue will be considered as an additional/individual compartment in Eq. (1) and **Fig. 1c**. The MDL-based model selection procedure will statistically determine the number of underlying tissue compartments in the processed area, e.g., whether the contribution of surrounding healthy tissues is significant to be considered as an independent compartment. Though MTCM methodology can accept the processed area extracted by any image segmentation methods, the tumor region in our study can be outlined by a collaborative effort by computer scientists and clinicians (Wang, Adali et al. 1998, Xuan, Adali et al. 2000, Li, Wang et al. 2001). In the case of heavy noise and fuzzy boundary, a consensus approach may be adopted that surveys the results of multiple methods.

### Clustering of pixel time-course

To reduce the impact of noise/outlier data points, improve the efficiency of subsequent convex analysis of mixtures, and permit an automated determination of the number of underlying vascular compartments using the minimum description length (MDL) criterion, we aggregated pixel time-courses into representative clusters using a combined affinity propagation and expectation-maximization clustering [32] (**Fig. 1b**, **Appendix S2 in File S1**).As an initialization-free and near-global-optimum clustering method, affinity propagation clustering (APC) simultaneously considers all data points as potential exemplars and recursively exchange real-valued messages between data points until a high-quality set of exemplars and corresponding clusters gradually emerges. Let the “similarity” 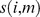 indicate how well the *m*th data point is suited to be the exemplar for *i*th data point; the “responsibility” 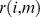 reflects the accumulated evidence for how well-suited the *m*th data point is to serve as the exemplar for the *i*th data point; the “availability” 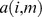 reflects the accumulated evidence for how appropriate the *i*th data point chooses *m*th data point as its exemplar. Then, supposing that there are *N* data points (e.g., pixels) in total, the responsibilities 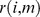 are computed based on 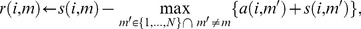
(5)where the availabilities 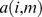 are initialized to zero and the competitive update rule (5) is purely data-driven. Whereas the responsibility update (5) allows all candidate exemplars to compete for ownership of a data point, the availability update rule
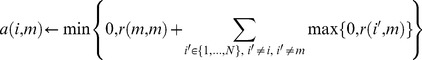
(6)collects evidence from data points to support a good exemplar, where the “self-availability” is updated differently 
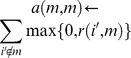
. Then, the availabilities and responsibilities are combined to identify exemplars 
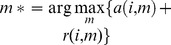
. The update rules are repeated iteratively and terminated when no change occurs for 10 iterations [32].

### Convex analysis of mixtures

To identify the *vertices* of convex set 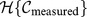, we performed convex analysis of mixtures (CAM) on the obtained *M* cluster centers 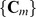 (**Fig. 1d**). We assumed *J* vascular compartments and conducted an exhaustive combinatorial search (with total 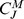 combinations), based on a convex-hull-to-data fitting criterion, to identify the most probable 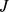 vertices (**Appendix S2 in File S1**). We used the margin-of-error
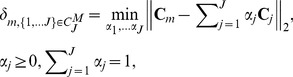
(7)to quantify the distance between 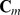 and convex set 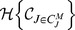, where we have 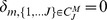 if 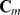 is inside 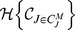. We then selected the most probable *J* vertices when the corresponding sum of the margin between the convex hull and the remaining “exterior” cluster centers reaches its minimum:



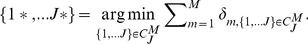
(8)


### Model selection procedure

One important discovery step concerning MTCM is to detect the number *J* of the underlying tissue compartments. We used MDL, a widely-adopted and consistent information theoretic criterion, to guide model selection (**Appendix S2 in File S1**). We performed CAM on several competing candidates, and selected the optimal model that assigns high probabilities to the observed data while at the same time whose parameters are not too complex to encode [33]. Specifically, a model is selected with *J* tissue compartments by minimizing the total description code length defined by 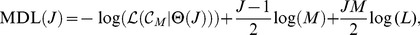
(9)where 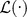 denotes the joint likelihood function of the clustered compartment model, 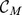 denotes the set of *M* cluster centers, and 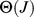 denotes the set of freely adjustable parameters in the clustered compartment model (see **Appendix S2 in File S1**).

### Estimation of pharmacokinetics parameters in MTCM

Having determined the pure-volume pixels associated with the vertices of 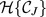 and the value of *J*, we estimated the values of tissue-specific vascular compartment pharmacokinetics parameters, i.e., flux rate constants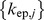 and volume transfer constants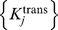, where the vertex of fastest tracer enhancement (reaching its peak most rapidly) is identified as 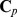 (**Appendix S2 in File S1**). We constructed the Toeplitz matrix 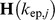 (sampled system impulse response) and solved the following optimization problem



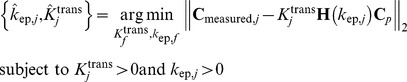
(10),for 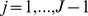. Subsequently, we estimated local volume transfer constants by solving
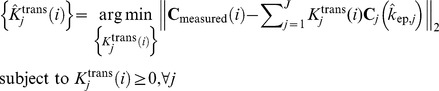
(11)which readily reveals the intratumor vascular heterogeneity.

### Synthetic DCE-MRI datasets

We first validated MTCM-generated estimates of tissue-specific vascular pharmacokinetics parameters using a set of realistic synthetic DCE-MRI data with known parameter values. We set *J* = 3, indexing two tissue compartments and one plasma input. We generated a large number of synthetic DCE-MRI time series by multiplying the customized local volume transfer constant maps 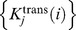 with known compartment pharmacokinetics 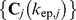. Synthetic data were comprised of 50 replicated datasets generated for each of the 12 parameter settings (**Data S1**). We performed MTCM on all the datasets and compared the estimates of tissue-specific kinetic parameters produced by MTCM with the ground truth, in terms of both biases (accuracy) and variance (reproducibility) of the estimates, measured over 50 replicated datasets. For comparison purposes, we also evaluated the three most relevant methods (**Table S1**). To determine whether the proposed MDL criterion detects the correct number of underlying tissue compartments, we calculated the MDL values for 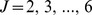 and identified the most probable value of *J* when MDL achieves its minimum value(s) (**Fig. 2**).

### Characterization of differential vascular pharmacokinetics in advanced breast cancer case

In the second application, we analyzed the real DCE-MRI data of an advanced breast tumor using both MTCM and the classic method. The T1-weighted gadolinium-enhanced (Gd-DTPA) DCE-MRI data set was acquired by three-dimensional scans performed every 30 seconds for a total of 11 minutes after the injection, on a 1.5 Tesla magnet using three-dimensional spoiled gradient-echo sequences (TR<7 msec, TE<1.5 msec, flip angle  = 30°, matrix  = 192×256, 0.5 averages). Typically, 12–15 slices are obtained and 15–18 time frames are acquired for each case. We visually examined the convexity of projected pixel time-course via the top two convexity-preserved projections where the margin between the “exterior” data points and the convex hull is minimized. We observed that two-tissue compartments (a three-vertex convex set) were sufficient to describe the observed pixel time-course scatter simplex. While additional compartments can be used to account for outlier vertices, these compartments become difficult to interpret. We analyzed the dataset by setting 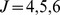 and observed noise-like and biologically implausible pharmacokinetics patterns. The minimum value of MDL confirmed *J* = 3. The number of clusters 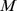 takes values between 12 and 18, determined automatically by the APC algorithm.

### Characterizing longitudinal changes of differential vascular pharmacokinetics in treating angiogenic-active breast cancer case

Vascular pharmacokinetics parameter values estimated by MTCM reveal longitudinal changes that may serve as the evidence of differential and heterogeneous responses to therapy. We analyzed the data sets arising from a longitudinal study of tumor response to anti-angiogenic therapy using similar imaging protocols (**Data S3**). Three sets of DCE-MRI data were acquired during standard treatment, each three months apart, serving as the potential endpoints in assessing the response to therapy. To detect various yet potentially hidden changes accounting for intratumor heterogeneity, we applied the same MTCM and MDL (as well as the classic method) to the three data sets separately.

### Open source multiplatform standalone MTCM Java-R software

Java GUI supported MTCM was implemented in both R and MATLAB, and runs on both Microsoft Windows and Linux platforms (http://mloss.org/software/view/437/). MTCM takes input the.mat data files that record the pixel time-course of DCE-MRI data in matrices. Each row corresponds to a time frame and each column corresponds to a pixel. Results of MTCM are provided to the users via a Java-based GUI (**Fig. S5**).

## Supporting Information

Figure S1 **MTCM estimates time-activity curves in multiple vascular compartments simultaneously and quantitatively reconstructs tissue-specific local volume transfer constants – mouse DCE-MRI experimental data.** (a) Snapshots of DCE-MRI sequence taken from the same tumor at 26 time points. Time point 1 is pre-contrast, and time points 2-26 are post-contrast. The first two time points are removed in the experiment. Each time point contains 4 sections from the same tumor. (b) The MDL curve of model selection and 3 is the optimal choice corresponding to the minimum MDL value. (c) Estimated tissue-specific compartmental time-activity curves: ‘blue’ - plasma input function; ‘red’ – fast flow kinetics; ‘green’ – slow flow kinetics. (d) Estimated maps of local volume transfer constants from four sections in the same tumor.
(TIFF)

Figure S2 **Comparison of time-activity curves of total vascular pool within the region of interests and tissue-specific time-activity curves estimated by MTCM, in a longitudinal DCE-MRI study on a breast cancer tumor: (a) – (c) time-activity curves of total vascular pool; (d) – (f) MTCM-estimated time-activity curves of fast flow pool; (g) – (i) MTCM-estimated time-activity curves of slow flow pool; (j) – (l) MTCM-estimated time-activity curves of plasma input function.**
(TIFF)

Figure S3 **MTCM dissects tissue compartments into anatomical structures of the mouse using dynamic fluorescence molecular imaging data acquired on a mouse after bolus injection of indocyanine green dye, allowing the longitudinal identification of the internal organs.** (a) Physiologically interpretable biodistribution dynamics of the major organs with ten fluorescence time courses showing distinct patterns of circulating, accumulating, or metabolizing the dye in different organs. (b) The merged and color-coded maps of the dissected tissue compartments agree well with a digital anatomical mouse atlas. (c) The gray-scale maps of the dissected individual tissue compartment (Kidney: *K* ^trans^ = 1.0004, *k*_ep_ = 0.0134; Spine: *K* ^trans^ = 1.0269, *k*_ep_ = 0.0241; Antipose: *K* ^trans^ = 0.7333, *k*_ep_ = 0.0100; Large intestine: *K* ^trans^ = 0.7808, *k*_ep_ = 0.0203; Nodes: *K* ^trans^ = 0.6719, *k*_ep_ = 0.0049; Blood vessels: *K* ^trans^ = 0.9891, *k*_ep_ = 0.0222; Liver: *K* ^trans^ = 0.7839, *k*_ep_ = 0.0128; Brain: *K* ^trans^ = 0.7553, *k*_ep_ = 0.0258; Stomach: *K* ^trans^ = 0.8955, *k*_ep_ = 0.0143; Lung: *K* ^trans^ = 0.6656, *k*_ep_ = 0.0167).
(TIFF)

Figure S4 **MTCM estimates time-activity curves in multiple DCE-MRI data produced in clinical practice.** (a) – (c) show raw image series, scatter simplex of image series and estimated tissue-specific compartmental time-activity curves and local volume transfer constant maps, respectively for a case; (d) – (f) display the same things for another case.
(TIFF)

Figure S5 **MTCM software package in R and Java is developed to implement MTCM algorithm, as well as the other algorithms widely used in blind source separation.** The user-friendly Java GUI (a) can generate the tissue-specific local volume transfer constants and pharmacokinetic parameters on the right. Two pop-up windows (b) will show the projection of clustered pixels on the simplex, and (c) will display the estimated tissue-specific compartmental time-activity curves.
(TIFF)

Table S1 **Comparison of tissue-specific kinetic parameter estimation by MTCM and three most relevant methods, based on synthetic DCE-MRI experimental data.**
(DOCX)

Table S2 **Tissue-specific kinetic parameter estimates by MTCM on mouse DCE-MRI experimental data.**
(DOCX)

Table S3 **MTCM estimates of flux rate constants and volume transfer constants of a breast cancer tumor before, during, and after treatment in the longitudinal study.**
(DOCX)

Table S4 **Fractions of partial-volume pixels before, during, and after treatment in the longitudinal study.**
(DOCX)

Table S5 **MTCM estimated tissue heterogeneity score before, during, and after treatment in the longitudinal study.**
(DOCX)

Data S1 **Synthetic datasets generated for 12 parameter settings.**
(PDF)

Data S2 **DCE-MRI data sets arising from mouse DCE-MRI experiments.**
(PDF)

Data S3 **DCE-MRI data sets arising from a longitudinal study of tumor response to anti-angiogenic therapy.**
(PDF)

File S1 **Supplementary discussion (appendix S1) and supplementary method (appendix S2).**
(DOCX)
