# Supplementary figures and images for: Unsupervised Deconvolution of Dynamic Imaging Reveals Intratumor Vascular Heterogeneity and Repopulation Dynamics
Source: PLoS One. 2014 Nov 7;9(11):e112143. doi: 10.1371/journal.pone.0112143 (PMC4224420; doi:10.1371/journal.pone.0112143)

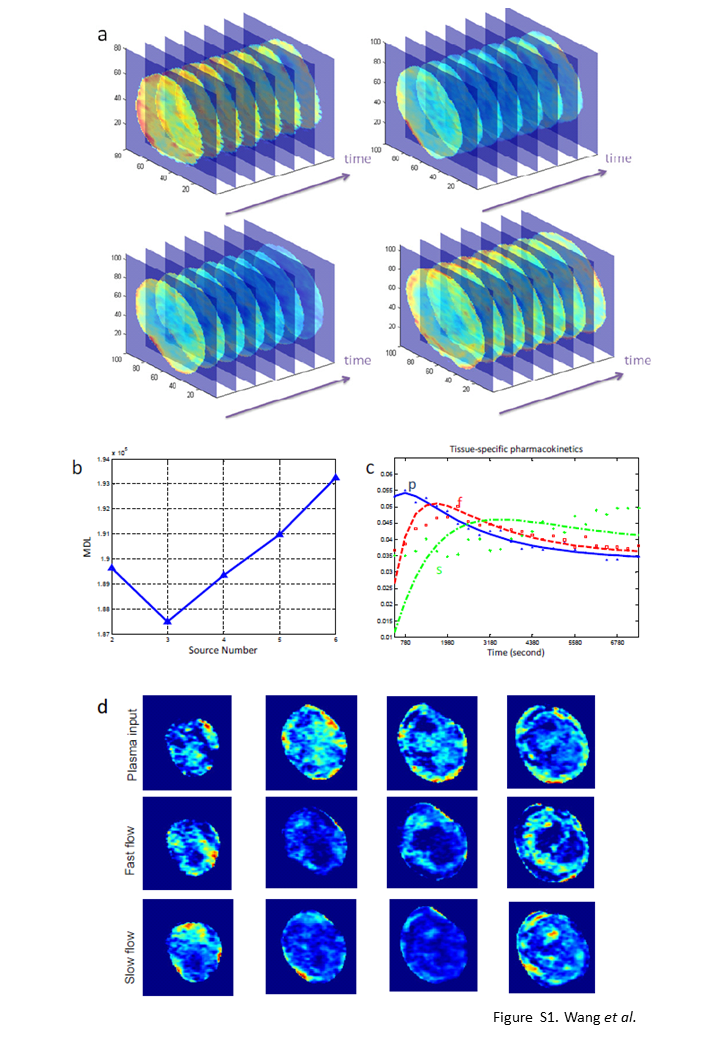

Supplement: Figure S1 — MTCM estimates time-activity curves in multiple vascular compartments simultaneously and quantitatively reconstructs tissue-specific local volume transfer constants – mouse DCE-MRI experimental data. (a) Snapshots of DCE-MRI sequence taken from the same tumor at 26 time points. Time point 1 is pre-contrast, and time points 2-26 are post-contrast. The first two time points are removed in the experiment. Each time point contains 4 sections from the same tumor. (b) The MDL curve of model selection and 3 is the optimal choice corresponding to the minimum MDL value. (c) Estimated tissue-specific compartmental time-activity curves: ‘blue’ - plasma input function; ‘red’ – fast flow kinetics; ‘green’ – slow flow kinetics. (d) Estimated maps of local volume transfer constants from four sections in the same tumor. (TIFF) [file pone.0112143.s001.tiff]

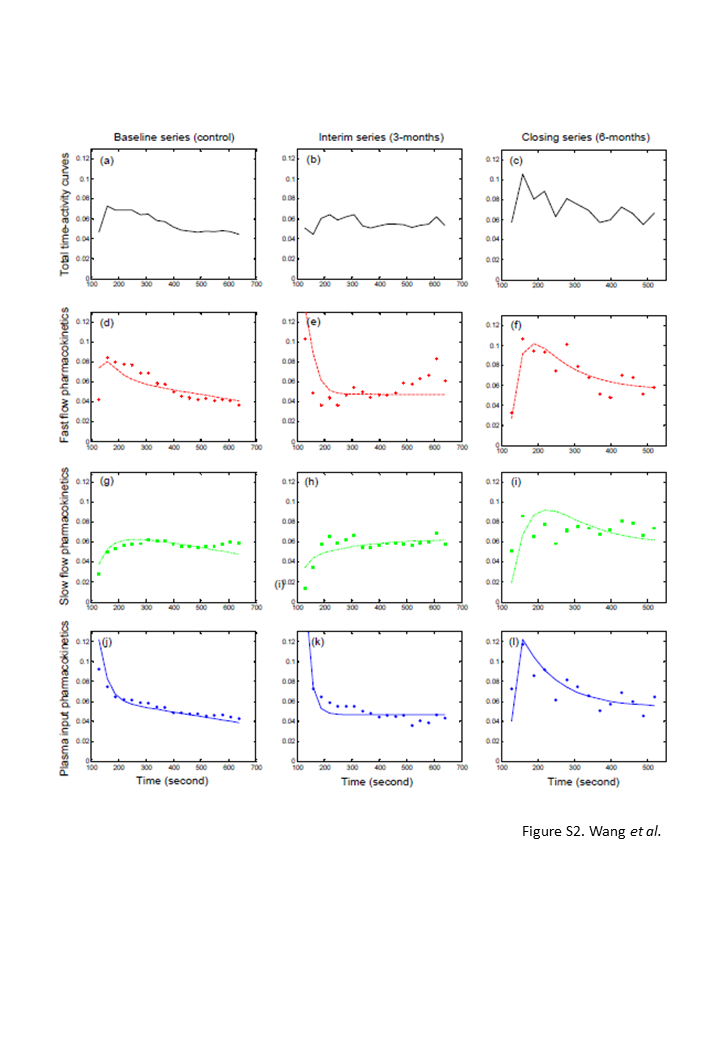

Supplement: Figure S2 — Comparison of time-activity curves of total vascular pool within the region of interests and tissue-specific time-activity curves estimated by MTCM, in a longitudinal DCE-MRI study on a breast cancer tumor: (a) – (c) time-activity curves of total vascular pool; (d) – (f) MTCM-estimated time-activity curves of fast flow pool; (g) – (i) MTCM-estimated time-activity curves of slow flow pool; (j) – (l) MTCM-estimated time-activity curves of plasma input function. (TIFF) [file pone.0112143.s002.tiff]

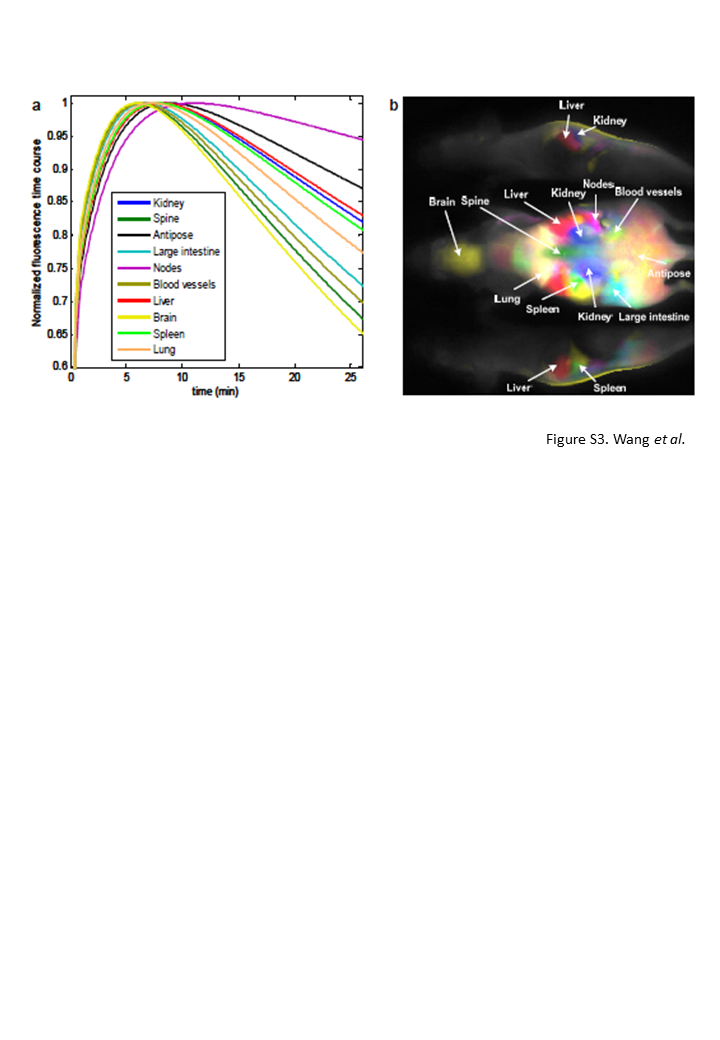

Supplement: Figure S3 — MTCM dissects tissue compartments into anatomical structures of the mouse using dynamic fluorescence molecular imaging data acquired on a mouse after bolus injection of indocyanine green dye, allowing the longitudinal identification of the internal organs. (a) Physiologically interpretable biodistribution dynamics of the major organs with ten fluorescence time courses showing distinct patterns of circulating, accumulating, or metabolizing the dye in different organs. (b) The merged and color-coded maps of the dissected tissue compartments agree well with a digital anatomical mouse atlas. (c) The gray-scale maps of the dissected individual tissue compartment (Kidney: K trans = 1.0004, k ep = 0.0134; Spine: K trans = 1.0269, k ep = 0.0241; Antipose: K trans = 0.7333, k ep = 0.0100; Large intestine: K trans = 0.7808, k ep = 0.0203; Nodes: K trans = 0.6719, k ep = 0.0049; Blood vessels: K trans = 0.9891, k ep = 0.0222; Liver: K trans = 0.7839, k ep = 0.0128; Brain: K trans = 0.7553, k ep = 0.0258; Stomach: K trans = 0.8955, k ep = 0.0143; Lung: K trans = 0.6656, k ep = 0.0167). (TIFF) [file pone.0112143.s003.tiff]

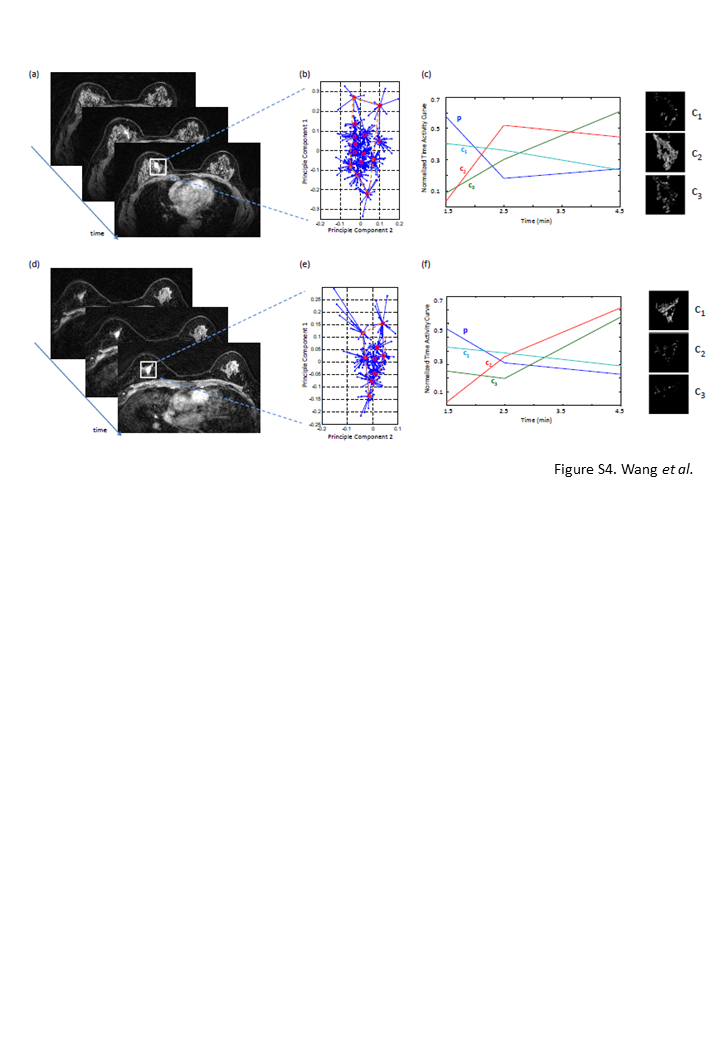

Supplement: Figure S4 — MTCM estimates time-activity curves in multiple DCE-MRI data produced in clinical practice. (a) – (c) show raw image series, scatter simplex of image series and estimated tissue-specific compartmental time-activity curves and local volume transfer constant maps, respectively for a case; (d) – (f) display the same things for another case. (TIFF) [file pone.0112143.s004.tiff]

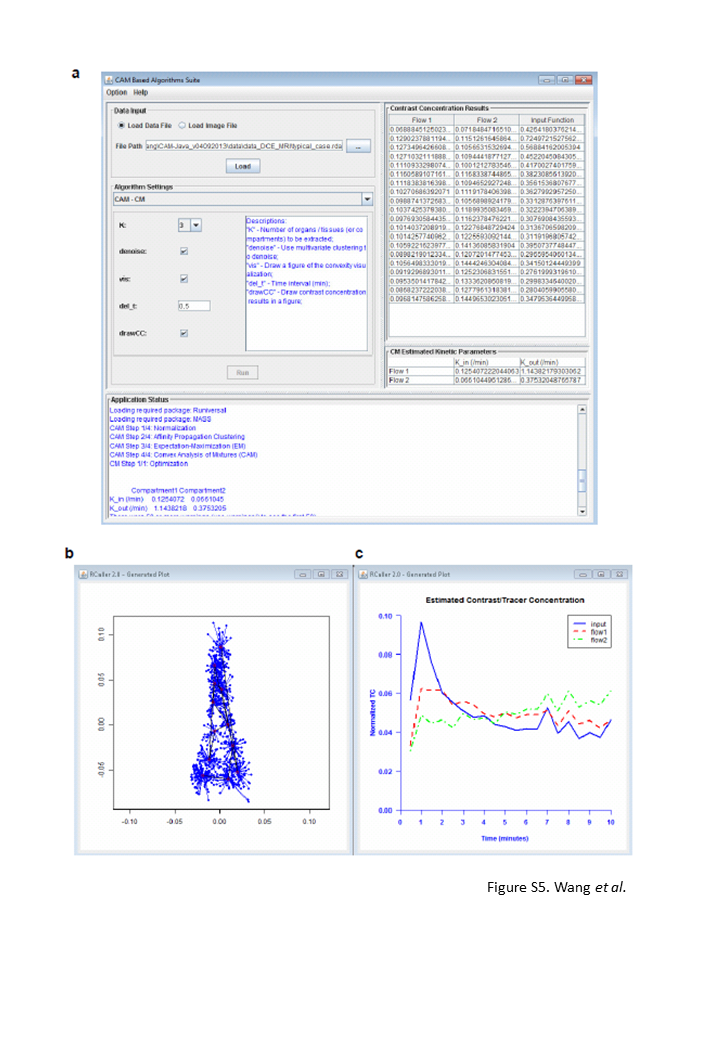

Supplement: Figure S5 — MTCM software package in R and Java is developed to implement MTCM algorithm, as well as the other algorithms widely used in blind source separation. The user-friendly Java GUI (a) can generate the tissue-specific local volume transfer constants and pharmacokinetic parameters on the right. Two pop-up windows (b) will show the projection of clustered pixels on the simplex, and (c) will display the estimated tissue-specific compartmental time-activity curves. (TIFF) [file pone.0112143.s005.tiff]
