# Supplementary material for: Unsupervised Deconvolution of Dynamic Imaging Reveals Intratumor Vascular Heterogeneity and Repopulation Dynamics
Source: PLoS One. 2014 Nov 7;9(11):e112143. doi: 10.1371/journal.pone.0112143 (PMC4224420; doi:10.1371/journal.pone.0112143)
Supplement: Table S1 — Comparison of tissue-specific kinetic parameter estimation by MTCM and three most relevant methods, based on synthetic DCE-MRI experimental data. (DOCX) [file pone.0112143.s006.docx]

Table S1. Comparison of tissue-specific kinetic parameter estimation by MTCM, classic CM [[1](#_ENREF_1),[2](#_ENREF_2)], and IQML [[3](#_ENREF_3),[4](#_ENREF_4)], based on synthetic DCE-MRI experiments.

| Ground truth parameter values | | SNR=20dB | | | SNR=15dB | | | SNR=10dB | | |
| --- | --- | --- | --- | --- | --- | --- | --- | --- | --- | --- |
|  |  | CAM | CM | IQML | CAM | CM | IQML | CAM | CM | IQML |
| **Scenario 1** | $K_{s}^{\text{trans}}=0.03$ /min | **0.035**  ±0.002 | 0.048  ±0.001 | 0.061  ±0.040 | **0.033**  ±0.006 | 0.047  ±0.001 | 0.066  ±0.037 | **0.037**  ±0.005 | 0.046  ±0.001 | 0.079  ±0.043 |
|  | $k_{\text{ep},s}=0.1$ /min | **0.104**  ±0.003 | 0.226  ±0.001 | 0.208  ±0.016 | **0.120**  ±0.022 | 0.231  ±0.001 | 0.232  ±0.081 | **0.098**  ±0.030 | 0.226  ±0.003 | 0.235  ±0.077 |
|  | $K_{f}^{\text{trans}}=0.03$ /min | **0.035**  ±0.002 | 0.094  ±0.001 | 0.096  ±0.064 | **0.034**  ±0.005 | 0.083  ±0.001 | 0.098  ±0.056 | **0.038**  ±0.004 | 0.084  ±0.001 | 0.123  ±0.067 |
|  | $k_{\text{ep},f}=0.5$ /min | **0.498**  ±0.020 | 0.913  ±0.002 | 0.606  ±0.047 | **0.489**  ±0.031 | 0.805  ±0.001 | 0.645  ±0.324 | **0.415**  ±0.057 | 0.820  ±0.008 | 0.674  ±0.272 |
| **Scenario 2** | $K_{s}^{\text{trans}}=0.03$ /min | **0.043**  ±0.009 | 0.051  ±0.001 | 0.076  ±0.043 | **0.042**  ±0.010 | 0.050  ±0.001 | 0.079  ±0.036 | **0.042**  ±0.005 | 0.0049  ±0.001 | 0.076  ±0.036 |
|  | $k_{\text{ep},s}=0.1$ /min | **0.124**  ±0.019 | 0.265  ±0.001 | 0.154  ±0.002 | **0.116**  ±0.023 | 0.258  ±0.001 | 0.192  ±0.057 | **0.088**  ±0.031 | 0.247  ±0.003 | 0.171  ±0.048 |
|  | $K_{f}^{\text{trans}}=0.05$ /min | **0.074**  ±0.017 | 0.125  ±0.001 | 0.116  ±0.067 | **0.069**  ±0.017 | 0.123  ±0.008 | 0.129  ±0.061 | **0.067**  ±0.007 | 0.133  ±0.001 | 0.132  ±0.063 |
|  | $k_{\text{ep},f}=1.2$ /min | **1.286**  ±0.058 | 1.587  ±0.005 | 0.508  ±0.007 | **1.232**  ±0.074 | 1.545  ±0.006 | 0.654  ±0.229 | **1.003**  ±0.182 | 1.708  ±0.019 | 0.648  ±0.235 |
| **Scenario 3** | $K_{s}^{\text{trans}}=0.06$ /min | **0.085**  ±0.011 | 0.095  ±0.001 | 0.118  ±0.042 | **0.084**  ±0.010 | 0.092  ±0.001 | 0.122  ±0.033 | **0.080**  ±0.006 | 0.096  ±0.003 | 0.202  ±0.181 |
|  | $k_{\text{ep},s}=0.5$ /min | **0.516**  ±0.014 | 0.758  ±0.001 | 0.728  ±0.133 | **0.510**  ±0.030 | 0.743  ±0.002 | 0.735  ±0.106 | **0.526**  ±0.012 | 0.763  ±0.035 | 0.467  ±0.185 |
|  | $K_{f}^{\text{trans}}=0.05$ /min | **0.071**  ±0.010 | 0.134  ±0.001 | 0.149  ±0.054 | **0.071**  ±0.009 | 0.132  ±0.001 | 0.158  ±0.043 | **0.072**  ±0.006 | 0.1401  ±0.008 | 0.2615  ±0.269 |
|  | $k_{\text{ep},f}=1.2$ /min | **1.256**  ±0.043 | 1.729  ±0.001 | 1.386  ±0.319 | **1.256**  ±0.076 | 1.736  ±0.004 | 1.435  ±0.278 | **0.901**  ±0.126 | 1.737  ±0.133 | 0.799  ±0.313 |
| **Scenario 4** | $K_{s}^{\text{trans}}=0.05$ /min | **0.068**  ±0.009 | 0.101  ±0.001 | 0.112  ±0.030 | **0.066**  ±0.009 | 0.096  ±0.001 | 0.112  ±0.033 | **0.076**  ±0.006 | 0.1010  ±0.003 | 0.181  ±0.289 |
|  | $k_{\text{ep},s}=0.6$ /min | **0.625**  ±0.011 | 1.047  ±0.001 | 0.795  ±0.201 | **0.627**  ±0.038 | 1.000  ±0.002 | 0.784  ±0.179 | **0.649**  ±0.018 | 1.026  ±0.045 | 0.520  ±0.198 |
|  | $K_{f}^{\text{trans}}=0.08$ /min | **0.109**  ±0.015 | 0.168  ±0.001 | 0.169  ±0.045 | **0.100**  ±0.017 | 0.154  ±0.001 | 0.165  ±0.048 | **0.099**  ±0.008 | 0.170  ±0.007 | 0.275  ±0.438 |
|  | $k_{\text{ep},f}=1.5$ /min | **1.582**  ±0.040 | 2.104  ±0.004 | 1.335  ±0.401 | **1.425**  ±0.183 | 1.957  ±0.006 | 1.309  ±0.371 | **1.237**  ±0.102 | 2.050  ±0.126 | 0.793  ±0.297 |

1. Tofts PS, Brix G, Buckley DL, Evelhoch JL, Henderson E, et al. (1999) Estimating kinetic parameters from dynamic contrast-enhanced T1-weighted MRI of a diffusable tracer: Standardized quantities and symbols. J Magn Reson Imaging 10: 223-232.

2. Chen L, Choyke PL, Chan TH, Chi CY, Wang G, et al. (2011) Tissue-specific compartmental analysis for dynamic contrast-enhanced MR imaging of complex tumors. IEEE Trans Med Imaging 30: 2044-2058.

3. Wang ZJ, Zhu H, Liu KJR, Wang Y. Simultaneous estimation of kinetic parameters and the input function from DCE-MRI data: theory and simulation; 2004. pp. 996-999.

4. Riabkov DY, Di Bella EVR (2002) Estimation of kinetic parameters without input functions: analysis of three methods for multichannel blind identification. Biomedical Engineering, IEEE Transactions on 49: 1318-1327.
