# Supplementary material for: Unsupervised Deconvolution of Dynamic Imaging Reveals Intratumor Vascular Heterogeneity and Repopulation Dynamics
Source: PLoS One. 2014 Nov 7;9(11):e112143. doi: 10.1371/journal.pone.0112143 (PMC4224420; doi:10.1371/journal.pone.0112143)
Supplement: Table S2 — Tissue-specific kinetic parameter estimates by MTCM on mouse DCE-MRI experimental data. (DOCX) [file pone.0112143.s007.docx]

Table S2. Tissue-specific kinetic parameter estimates by MTCM on mouse DCE-MRI experimental data.

|  | $K_{f}^{\text{trans}}$(/min) | $K_{s}^{\text{trans}}$(/min) | $k_{\text{ep},f}$(/min) | $k_{\text{ep},s}$(/min) |
| --- | --- | --- | --- | --- |
| Kinetic parameter | 0.190 | 0.052 | 0.652 | 0.205 |
