# Supplementary material for: Unsupervised Deconvolution of Dynamic Imaging Reveals Intratumor Vascular Heterogeneity and Repopulation Dynamics
Source: PLoS One. 2014 Nov 7;9(11):e112143. doi: 10.1371/journal.pone.0112143 (PMC4224420; doi:10.1371/journal.pone.0112143)
Supplement: Table S3 — MTCM estimates of flux rate constants and volume transfer constants of a breast cancer tumor before, during, and after treatment in the longitudinal study. (DOCX) [file pone.0112143.s008.docx]

Table S3. MTCM estimates of flux rate constants and volume transfer constants of a breast cancer tumor before, during, and after treatment in the longitudinal study.

|  | $K_{f}^{\text{trans}}$ (/min) | $K_{s}^{\text{trans}}$(/min) | $k_{\text{ep},f}$ (/min) | $k_{\text{ep},s}$ (/min) |
| --- | --- | --- | --- | --- |
| Before Treatment | 1.780 | 0.270 | 2.093 | 0.695 |
| During Treatment | 0.412 | 0.057 | 1.781 | 0.749 |
| After Treatment | 0.289 | 0.055 | 1.068 | 0.582 |
