# Supplementary material for: Unsupervised Deconvolution of Dynamic Imaging Reveals Intratumor Vascular Heterogeneity and Repopulation Dynamics
Source: PLoS One. 2014 Nov 7;9(11):e112143. doi: 10.1371/journal.pone.0112143 (PMC4224420; doi:10.1371/journal.pone.0112143)
Supplement: Table S4 — Fractions of partial-volume pixels before, during, and after treatment in the longitudinal study. (DOCX) [file pone.0112143.s009.docx]

Table S4. MTCM estimated fractions of partial-volume pixels before, during, and after treatment in the longitudinal study.

|  | Fraction of Partial-Volume Pixels |
| --- | --- |
| Before Treatment | 61.2% |
| During Treatment | 70.3% |
| After Treatment | 66.8% |
