# Supplementary material for: Unsupervised Deconvolution of Dynamic Imaging Reveals Intratumor Vascular Heterogeneity and Repopulation Dynamics
Source: PLoS One. 2014 Nov 7;9(11):e112143. doi: 10.1371/journal.pone.0112143 (PMC4224420; doi:10.1371/journal.pone.0112143)
Supplement: Table S5 — MTCM estimated tissue heterogeneity score before, during, and after treatment in the longitudinal study. (DOCX) [file pone.0112143.s010.docx]

Table S5. MTCM estimated tissue heterogeneity score before, during, and after treatment in the longitudinal study.

|  | Tissue Heterogeneity Score |
| --- | --- |
| Before Treatment | 0.1445 |
| During Treatment | 0.0253 |
| After Treatment | 0.0983 |
