# Supplementary material for: Unsupervised Deconvolution of Dynamic Imaging Reveals Intratumor Vascular Heterogeneity and Repopulation Dynamics
Source: PLoS One. 2014 Nov 7;9(11):e112143. doi: 10.1371/journal.pone.0112143 (PMC4224420; doi:10.1371/journal.pone.0112143)
Supplement: Data S2 — DCE-MRI data sets arising from mouse DCE-MRI experiments. (PDF) [file pone.0112143.s012.pdf]

Data S2

Demo_CAM_MDL.R:

```
rm(list=ls())
setwd('F:/Niya Wang/DCE-MRI')

library(compositions)
library(apcluster)
library(geometry)
library(nnls)

source('measure_conv.R')

#################################################
X <- read.table('MouseData.txt')
X <- matrix(unlist(X),1609,24)
X <- t(X)

max_pixel <- max(X)
X <- X/max_pixel
X <- X*2^12

data_size<- dim(X)[2]
L <- dim(X)[1]

##### use APC to cluster the observation #####
cat("Performing APC to cluster data\n")

denom <- as.matrix(colSums(X))
num <- dim(X)[1]
denom <- t(denom[,rep(1,num)])

X_proj <- X/denom

sim <- negDistMat(t(X_proj),r=2)
apres <- apcluster(sim,q=0.1)
cluster <- as.vector(unlist(apres@exemplars))

small_cluster <- matrix(numeric(0),0,0)
for (k in 1:length(cluster)){
  if
(length(as.vector(unlist(apres@clusters[k])))<0.1*dim(X)[2]/length(clu
ster))
    small_cluster <- c(small_cluster,k)
}

if (length(small_cluster)==0){
  cluster <- cluster
} else {
  cluster <- cluster[-small_cluster]
}
```

```
J <- length(cluster)


J_out <- J
corner <- c(1:J)

##### estimate A and S and calculate MDL#####
cat("Calculating MDL\n")
MDL1 <- matrix(numeric(0), 0,0)
likelihood_all1 <- matrix(numeric(0), 0,0)
penalty_all1 <- matrix(numeric(0), 0,0)
sigma_all1 <- matrix(numeric(0), 0,0)

likelihood_all2 <- matrix(numeric(0), 0,0)
penalty_all2 <- matrix(numeric(0), 0,0)
sigma_all2 <- matrix(numeric(0), 0,0)
MDL2 <- matrix(numeric(0), 0,0)

for (K_est in 2:6){
  if (K_est==J_out){
    A_est <- X_proj[,cluster[corner]]
    ind <- cluster[corner]
  }
  else {
    cornerResult <- measure_conv(X_proj[,cluster[corner]], K_est)

    A_est <- cornerResult[[1]]
    ind <- cornerResult[[2]]
  }


  S_est <- matrix(0,nrow=dim(A_est)[2],ncol=dim(X_proj)[2])
  for (i in 1:ncol(X_proj)){
    S_est[,i] <- coef(nnls(A_est,X[,i]))
  }

  likelihood1 <- (L*J)/2*log(var(as.vector(X[,cluster]-
A_est%*%S_est[,cluster])))
  likelihood2 <- (L*data_size)/2*log(var(as.vector(X-A_est%*%S_est)))
  sigma_all1 <- c(sigma_all1,var(as.vector(X[,cluster]-
A_est%*%S_est[,cluster])))
  sigma_all2 <- c(sigma_all2,var(as.vector(X-A_est%*%S_est)))


  likelihood_all1 <- c(likelihood_all1,likelihood1)
  likelihood_all2 <- c(likelihood_all2,likelihood2)

  penalty1 <- (K_est*L)/2*log(J)+(K_est*J)/2*log(L) #+log(J*L)/2
  penalty2 <- (K_est*L)/2*log(data_size)+(K_est*data_size)/2*log(L)
#+log(J*L)/2

  penalty_all1 <- c(penalty_all1,penalty1)
```

```
  penalty_all2 <- c(penalty_all2,penalty2)
  MDL1 <- c(MDL1,(likelihood1+penalty1))
  MDL2 <- c(MDL2,(likelihood2+penalty2))
}


rm(sim)
save(list=ls(all=TRUE),file="DCEMRIresult.RData")
```

Measure_conv.R:

```
measure_conv <- function(X,N){
  # This function needs the "nnls" package in R, you can use the
command:
  # install.packages("nnls")
  # in R interface to install that package if you don't have it.
  require("nnls")

  M <- nrow(X); L <- ncol(X)
  total_index <- t(combn(1:L,N))
  comb <- nrow(total_index)

  error <- matrix(0,comb,1)
  for (p in 1:comb) {
    A <- X[,total_index[p,]]
    Others <- X[,-total_index[p,]]
    Ae <- rbind(1e-5*A, matrix(1,1,N))
    Oe <- rbind(1e-5*Others, matrix(1,1,L-N))
    alpha <- matrix(0,N,L-N)
    for (i in 1:(L-N))
      alpha[,i] <- nnls(Ae, Oe[,i])$x
    error[p] <- norm(Others - A %*% alpha, 'f')^2
  }
  val <- min(error)
  ind <- which.min(error)

  eA <- X[,total_index[ind,]]
  cornerind <- total_index[ind,]
  return(list(eA,cornerind))
}
```

MouseData:

```
  3.7961918e-004  3.9077081e-004  4.0952338e-004  4.2059364e-004
4.6419575e-004  4.4030764e-004  5.1684025e-004  4.0533002e-004
4.6528018e-004  3.7823797e-004  3.6511117e-004  4.1231186e-004
4.8629763e-004  3.8848764e-004  3.5524868e-004  3.6753861e-004
4.1236217e-004  3.5929834e-004  4.4060208e-004  3.3659664e-004
4.1440939e-004  3.9706365e-004  3.6700569e-004  3.8439753e-004
  5.4247386e-004  5.2576381e-004  5.2448147e-004  5.1865385e-004
6.3719142e-004  5.9663456e-004  6.3368700e-004  6.1148708e-004
5.4040962e-004  6.2143234e-004  5.5260137e-004  5.2016271e-004
5.8637111e-004  4.5676707e-004  5.6944748e-004  5.1972763e-004
4.9038953e-004  5.5264028e-004  5.0003625e-004  5.5225154e-004
5.9781273e-004  4.8751148e-004  4.8767730e-004  4.6764311e-004
  5.8793480e-004  5.4991896e-004  5.8994838e-004  6.4630329e-004
6.7775080e-004  6.0528617e-004  6.9390512e-004  5.9357734e-004
6.7413905e-004  5.3801156e-004  5.4126426e-004  6.3369152e-004
6.7465697e-004  5.8717266e-004  5.5178115e-004  5.7966468e-004
5.6249323e-004  5.4286000e-004  6.0608324e-004  5.3906513e-004
5.3276480e-004  5.8497305e-004  4.9842964e-004  5.5153501e-004
  5.8211891e-004  5.1306683e-004  6.4469042e-004  6.2889323e-004
6.5500138e-004  6.0622566e-004  6.1889637e-004  7.0819512e-004
6.2554846e-004  6.0636348e-004  5.9286239e-004  6.2414812e-004
6.0639413e-004  5.3678062e-004  5.9785003e-004  5.9769377e-004
6.0082297e-004  5.5949624e-004  5.4613953e-004  5.7938856e-004
4.9182647e-004  5.3633272e-004  4.8982352e-004  5.7574142e-004
  5.8402332e-004  5.6168118e-004  5.7444663e-004  5.7610912e-004
6.5081997e-004  6.9047441e-004  7.0072908e-004  6.5398219e-004
6.5189584e-004  6.4901289e-004  5.6900934e-004  5.8581542e-004
6.2372589e-004  5.3781969e-004  6.0291114e-004  5.6874953e-004
4.9784003e-004  6.0317641e-004  5.5498017e-004  6.0505059e-004
6.8638718e-004  5.0247012e-004  5.2074082e-004  5.3267818e-004
  6.3352413e-004  5.3165959e-004  6.2979732e-004  5.9269469e-004
6.1529968e-004  6.1173021e-004  6.3035896e-004  6.4883895e-004
6.0790640e-004  6.4711456e-004  5.1448360e-004  5.0979083e-004
5.8693149e-004  5.5613100e-004  5.3373575e-004  5.5623880e-004
5.0093723e-004  5.5958788e-004  5.8091809e-004  5.5363895e-004
5.4070233e-004  5.1757731e-004  6.0046335e-004  5.0205830e-004
  4.0752511e-004  4.9901991e-004  4.1567336e-004  4.9723053e-004
3.9849776e-004  4.4936777e-004  5.0218099e-004  5.8077724e-004
4.2049048e-004  4.2404109e-004  4.3413985e-004  4.3982501e-004
4.6676995e-004  4.3399368e-004  4.3898083e-004  4.0948186e-004
4.1877726e-004  3.8369472e-004  4.5771283e-004  4.3668774e-004
3.9148710e-004  4.4373564e-004  4.8349927e-004  4.0690005e-004
  6.0226262e-004  5.8223253e-004  5.8379676e-004  6.4437451e-004
6.3379959e-004  6.3079101e-004  6.8816078e-004  5.5314080e-004
7.1860536e-004  4.9141423e-004  5.4483413e-004  5.9349985e-004
7.0800941e-004  5.5006313e-004  5.4786867e-004  5.3868555e-004
5.8977304e-004  5.3838111e-004  6.1613282e-004  4.9101692e-004
6.0946542e-004  5.5326735e-004  4.8658047e-004  5.4667708e-004
```

6.3269580e-004	5.6027048e-004	6.9870955e-004	6.3875834e-004
6.6491243e-004	6.5473870e-004	6.4362805e-004	6.9863426e-004
6.6805503e-004	5.8645855e-004	5.8277790e-004	6.7252909e-004
6.3420417e-004	5.8999289e-004	5.9550526e-004	6.3489965e-004
5.6762889e-004	5.9003921e-004	5.5302195e-004	5.8969554e-004
5.1740291e-004	5.5439850e-004	5.3571597e-004	6.0945164e-004
5.6205461e-004	5.1765690e-004	5.6699575e-004	5.8643507e-004
6.2690930e-004	6.1780884e-004	6.3994352e-004	7.4461798e-004
6.3999459e-004	6.2063560e-004	5.8318280e-004	5.8656115e-004
5.9852401e-004	5.9910658e-004	6.0360694e-004	6.1270974e-004
5.7985415e-004	5.8843425e-004	5.7289087e-004	6.3762596e-004
5.7337611e-004	5.2192507e-004	4.9940010e-004	5.6480846e-004
6.4035341e-004	5.4739940e-004	6.8251889e-004	6.5858191e-004
7.0638300e-004	6.9545409e-004	7.0277964e-004	6.6190895e-004
7.0877887e-004	6.3774543e-004	5.4810315e-004	5.9671562e-004
6.3630333e-004	6.0902660e-004	6.2870745e-004	6.4204169e-004
5.2945845e-004	5.7735280e-004	5.9178741e-004	6.4229540e-004
6.6279150e-004	5.2548309e-004	6.0498087e-004	5.6335512e-004
5.6503211e-004	6.2273006e-004	5.8675469e-004	6.3022357e-004
5.8001888e-004	6.5570591e-004	6.2651922e-004	7.5010685e-004
5.7841778e-004	6.1118715e-004	5.4793083e-004	5.3369791e-004
6.1633061e-004	5.9237893e-004	5.2025019e-004	5.6029993e-004
5.3572572e-004	5.1681396e-004	6.1061300e-004	5.5405172e-004
5.1040071e-004	5.3175044e-004	6.6233176e-004	5.1003392e-004
6.0529918e-004	5.7931904e-004	6.1025794e-004	6.5978739e-004
6.4032026e-004	6.1485793e-004	6.7148560e-004	5.7536387e-004
7.7593734e-004	5.6239752e-004	5.6965608e-004	6.4130260e-004
7.2526022e-004	6.0546446e-004	6.1445950e-004	5.6713642e-004
6.7965698e-004	5.6374299e-004	6.1835584e-004	5.4491527e-004
6.7600256e-004	5.5236946e-004	5.4810272e-004	5.8157321e-004
6.1640079e-004	5.5465363e-004	7.0568714e-004	6.1808636e-004
6.3746744e-004	6.6681903e-004	6.5314617e-004	7.0665311e-004
6.5292210e-004	5.8250366e-004	5.7691104e-004	6.3431861e-004
6.2116182e-004	5.8868785e-004	6.0476321e-004	6.2746405e-004
5.6412070e-004	5.7555152e-004	5.2019138e-004	6.1462006e-004
5.3732886e-004	5.2814138e-004	5.4620518e-004	6.0113095e-004
5.4925546e-004	5.2345685e-004	5.5850151e-004	5.8153921e-004
6.0039257e-004	6.0581091e-004	6.2827981e-004	7.2051378e-004
6.4723732e-004	5.8272289e-004	5.6331926e-004	5.7971664e-004
5.7962572e-004	6.4697149e-004	5.8800375e-004	6.0763770e-004
5.7953325e-004	5.8263025e-004	5.8078954e-004	6.4492512e-004
5.7059956e-004	5.2279027e-004	5.1290907e-004	5.8732231e-004
6.1837137e-004	5.6630479e-004	6.9078026e-004	6.5391583e-004
7.0614551e-004	6.8660297e-004	7.1741809e-004	6.8227854e-004
6.9718700e-004	6.2024787e-004	5.6650417e-004	6.1162327e-004
6.4368625e-004	6.3195055e-004	6.5716830e-004	6.7318519e-004
5.4483660e-004	5.6323440e-004	6.1316114e-004	6.4595716e-004
6.5446703e-004	5.5310984e-004	6.3919977e-004	5.6510072e-004
5.6368081e-004	6.4624706e-004	6.0545836e-004	6.7234971e-004
6.2000334e-004	6.9806978e-004	6.2366424e-004	7.2301978e-004
6.1093952e-004	5.8574215e-004	5.6009788e-004	5.4420697e-004
6.0693160e-004	6.3473693e-004	5.2921560e-004	6.1573571e-004

5.6311267e-004	5.0644351e-004	6.0648097e-004	5.6432901e-004
5.0516318e-004	4.9658272e-004	6.7663172e-004	5.3906371e-004
5.9184457e-004	6.6083073e-004	6.6385820e-004	7.2432772e-004
5.9595681e-004	5.6895266e-004	6.4383636e-004	6.7911635e-004
5.8778869e-004	5.8921520e-004	6.1376867e-004	5.8166859e-004
6.1769504e-004	5.9129350e-004	6.5306071e-004	5.2053426e-004
6.1374150e-004	5.7732478e-004	5.6704458e-004	5.5628641e-004
5.9296106e-004	5.6102022e-004	6.7497831e-004	5.4759257e-004
5.4406439e-004	4.8296448e-004	5.2788526e-004	5.1373284e-004
5.0213863e-004	4.2795776e-004	5.3442567e-004	5.6537591e-004
5.3541870e-004	5.7585991e-004	4.2186112e-004	4.5709906e-004
5.8046178e-004	4.4490731e-004	5.3283592e-004	4.5401591e-004
4.7048951e-004	4.1990018e-004	4.7925163e-004	5.1518031e-004
4.8273194e-004	4.8451642e-004	5.8478636e-004	4.5742882e-004
5.9261531e-004	5.9092995e-004	6.4464689e-004	6.5547277e-004
6.2864588e-004	6.3163464e-004	5.8987580e-004	5.8199470e-004
8.0139685e-004	5.7817703e-004	5.6331651e-004	6.3267439e-004
6.7792859e-004	6.2952442e-004	6.0124946e-004	5.6657831e-004
6.6028755e-004	5.9312023e-004	6.1731522e-004	5.6465408e-004
7.1253588e-004	5.3078303e-004	5.7830480e-004	6.1294841e-004
5.6984795e-004	6.2443703e-004	6.3266786e-004	6.0869253e-004
6.7172819e-004	6.5017026e-004	6.9993116e-004	6.7751161e-004
6.6851152e-004	5.9900351e-004	5.6940036e-004	6.0673644e-004
6.3558352e-004	6.5900927e-004	6.3742069e-004	6.2537192e-004
5.2009805e-004	5.7777342e-004	6.4509179e-004	6.3743127e-004
6.6897967e-004	5.7152381e-004	6.1355846e-004	5.6058411e-004
5.9322511e-004	6.1891669e-004	6.2155370e-004	6.8234061e-004
6.6607050e-004	7.2224016e-004	6.5667857e-004	7.0797134e-004
6.3707971e-004	5.8615096e-004	6.0283601e-004	5.5718444e-004
6.0531133e-004	6.3897842e-004	5.6695308e-004	7.0618544e-004
6.1622150e-004	5.0714309e-004	6.3204534e-004	5.6742525e-004
5.1456207e-004	5.1514134e-004	6.9396150e-004	5.8474604e-004
6.2372469e-004	7.2085350e-004	7.0114392e-004	7.7564245e-004
6.4008479e-004	6.4528419e-004	6.6132174e-004	6.9275478e-004
5.9823369e-004	6.0388206e-004	6.3990826e-004	6.0046307e-004
6.1762163e-004	6.2373099e-004	6.5082015e-004	5.3140518e-004
6.0340374e-004	5.9430511e-004	5.5839375e-004	5.7415508e-004
5.6579932e-004	5.6077719e-004	6.8163448e-004	5.6499674e-004
6.8767105e-004	6.3014518e-004	6.7751099e-004	6.7702653e-004
6.5155866e-004	6.1235289e-004	6.5994030e-004	7.0578262e-004
6.6965426e-004	7.3642742e-004	5.2505031e-004	5.5507116e-004
7.5750118e-004	5.5929452e-004	6.9640338e-004	5.7675678e-004
6.2571903e-004	5.3854749e-004	5.9004471e-004	6.3283389e-004
6.1106091e-004	6.2677609e-004	7.4143094e-004	5.7468953e-004
5.7504856e-004	5.8645059e-004	6.5291004e-004	6.7368669e-004
6.1154935e-004	6.4817433e-004	5.7802696e-004	6.1547250e-004
7.5771633e-004	5.8155238e-004	5.7014372e-004	6.4371017e-004
6.5114435e-004	6.5380994e-004	5.5932466e-004	5.7135703e-004
6.0129243e-004	6.1628791e-004	6.1533932e-004	5.8656980e-004
7.0333919e-004	5.4218799e-004	5.6795255e-004	6.4024971e-004
5.6026608e-004	5.1723963e-004	6.3502944e-004	6.1609907e-004
5.7523092e-004	6.2282108e-004	7.0547024e-004	6.2031495e-004

6.5805425e-004	5.7446503e-004	5.8657111e-004	6.2135130e-004
5.5555630e-004	5.8955346e-004	5.8296436e-004	5.5541558e-004
6.1030348e-004	5.4718571e-004	5.0530893e-004	6.3294196e-004
5.6678941e-004	5.3219375e-004	5.7520493e-004	6.0302661e-004
5.3069106e-004	6.3742223e-004	5.5588558e-004	5.8317212e-004
6.2501238e-004	5.9622632e-004	6.5282809e-004	6.7908550e-004
6.3612907e-004	5.7278130e-004	5.4583176e-004	5.9634451e-004
6.1489543e-004	6.6809462e-004	5.8822024e-004	5.6918027e-004
5.1828806e-004	5.8334577e-004	6.5866216e-004	6.3219437e-004
6.4872000e-004	5.6713120e-004	5.7240541e-004	5.7620698e-004
6.0614894e-004	6.1941607e-004	5.9148693e-004	6.8827016e-004
6.6962233e-004	7.1457280e-004	6.7756980e-004	6.9446377e-004
6.4298927e-004	6.1781888e-004	6.7755942e-004	6.0409239e-004
6.2177534e-004	5.9357683e-004	6.2962603e-004	7.2085163e-004
6.2507229e-004	5.2683806e-004	6.7177176e-004	5.4757443e-004
5.3068498e-004	5.6761860e-004	6.8416094e-004	5.8982817e-004
6.2466890e-004	7.1450911e-004	7.1910009e-004	7.3593768e-004
6.9182925e-004	7.1487266e-004	6.7771730e-004	6.6272906e-004
6.3214266e-004	5.7777861e-004	6.6371528e-004	5.9148941e-004
6.0704203e-004	6.3876016e-004	6.0657051e-004	5.5282184e-004
6.0117432e-004	5.5113695e-004	5.6092451e-004	5.9464449e-004
5.3148193e-004	5.5814389e-004	6.7399186e-004	6.0272205e-004
6.6899903e-004	6.8258752e-004	6.6539364e-004	7.2514842e-004
6.6244305e-004	6.6273508e-004	6.3457660e-004	7.1046685e-004
6.3460086e-004	7.2135453e-004	5.1924346e-004	5.4937489e-004
7.2839306e-004	5.6002273e-004	6.9225066e-004	5.9875606e-004
5.9699191e-004	5.6901808e-004	5.3562566e-004	6.2522048e-004
6.1689226e-004	6.4045252e-004	7.0442981e-004	5.8157326e-004
5.5088529e-004	5.3857554e-004	6.4903240e-004	7.1054224e-004
5.7687647e-004	6.5505823e-004	6.1017041e-004	6.9378960e-004
6.3477099e-004	6.2795071e-004	6.4240239e-004	6.2183090e-004
6.5570249e-004	6.3240754e-004	5.8337692e-004	6.4182402e-004
5.9120322e-004	6.5054846e-004	5.7983884e-004	7.1500649e-004
6.5193085e-004	6.3039309e-004	5.6388881e-004	6.4630338e-004
5.6778680e-004	6.1223103e-004	6.2949663e-004	6.5056187e-004
5.9506646e-004	6.4130541e-004	5.6473917e-004	5.9916882e-004
7.3208335e-004	6.3189618e-004	5.9370004e-004	6.1707155e-004
6.3661698e-004	6.7725642e-004	6.1879891e-004	5.7136759e-004
5.9992140e-004	6.6522567e-004	5.9099682e-004	6.2351847e-004
7.2519714e-004	5.4707032e-004	5.9306303e-004	6.6141083e-004
6.0218449e-004	6.5576140e-004	5.8873332e-004	6.8407904e-004
6.5612062e-004	7.3049299e-004	6.7774775e-004	6.6478216e-004
6.4340877e-004	5.9470230e-004	6.7168663e-004	5.8359407e-004
5.8812326e-004	5.7828071e-004	5.9315961e-004	6.9208888e-004
6.0907329e-004	4.9269734e-004	6.4005665e-004	5.2880340e-004
4.9236337e-004	5.7317156e-004	6.4993394e-004	5.9093912e-004
5.8744135e-004	6.5950156e-004	7.4596463e-004	7.1251861e-004
7.1953675e-004	7.3746433e-004	6.4264692e-004	6.5075347e-004
5.9886937e-004	5.6897661e-004	6.3684901e-004	5.7656651e-004
6.5796354e-004	5.9480588e-004	6.1647453e-004	4.8709183e-004
5.9752628e-004	5.6098869e-004	5.0523438e-004	6.1972452e-004
5.4865425e-004	5.8888811e-004	6.4171254e-004	6.0049747e-004

6.2138846e-004	7.1771808e-004	6.2795181e-004	6.9386279e-004
6.0468605e-004	5.7402253e-004	6.0391278e-004	7.1999180e-004
6.2056894e-004	6.5925448e-004	5.2774712e-004	5.6726138e-004
6.7251384e-004	5.6223433e-004	6.5994359e-004	6.3905398e-004
4.7822219e-004	5.7486350e-004	5.0143793e-004	6.4750040e-004
6.4398602e-004	6.4824024e-004	6.4556372e-004	6.1827604e-004
5.5741167e-004	5.6344348e-004	6.8196785e-004	7.0278578e-004
5.9348390e-004	6.3608903e-004	5.9845502e-004	7.1113050e-004
6.0298418e-004	5.8836480e-004	6.6865029e-004	6.1669841e-004
6.6494863e-004	6.1244327e-004	5.8508596e-004	6.2001804e-004
5.9778284e-004	6.4219051e-004	5.8215302e-004	7.3510088e-004
6.5421713e-004	6.1290267e-004	5.7192279e-004	6.5157759e-004
5.6952618e-004	5.6532523e-004	5.9090366e-004	5.7743704e-004
6.0357415e-004	6.2484035e-004	7.5288676e-004	5.7910154e-004
6.8673732e-004	5.9029042e-004	5.7209442e-004	6.1512842e-004
5.7701510e-004	6.1290707e-004	5.6297284e-004	5.4440396e-004
5.7845986e-004	5.7355361e-004	5.1601540e-004	6.1465273e-004
5.8266519e-004	5.8826465e-004	5.6124298e-004	6.0028698e-004
5.7735362e-004	6.1890212e-004	5.0909910e-004	6.8051496e-004
6.0725160e-004	7.0807210e-004	6.5143521e-004	6.4457439e-004
6.4033907e-004	6.3043273e-004	6.8417357e-004	6.0499732e-004
5.9178386e-004	5.5904801e-004	6.1910541e-004	6.5134431e-004
5.9184905e-004	5.1093641e-004	6.5446201e-004	5.1759805e-004
4.9606242e-004	5.7448226e-004	6.1689403e-004	5.5601208e-004
5.4210835e-004	6.2673748e-004	7.0867552e-004	6.4426627e-004
7.0887605e-004	7.3648705e-004	6.3467366e-004	6.1801722e-004
6.0166161e-004	5.3360887e-004	6.0594131e-004	5.5319568e-004
6.1195272e-004	5.6964607e-004	5.9696428e-004	5.0485034e-004
5.3593390e-004	5.1209894e-004	4.8505959e-004	5.9456033e-004
4.9565710e-004	5.7956515e-004	6.0307335e-004	6.1172344e-004
5.9014432e-004	6.1501600e-004	6.5819657e-004	6.8483330e-004
6.9734172e-004	6.5361572e-004	5.8911645e-004	6.3841935e-004
5.9552841e-004	5.8784176e-004	5.1100202e-004	5.4735591e-004
6.8631041e-004	5.5230065e-004	5.7979312e-004	5.9876988e-004
5.5828360e-004	5.6949741e-004	4.2547465e-004	5.9633925e-004
5.9429542e-004	6.0158854e-004	6.3758204e-004	5.5566186e-004
6.0333573e-004	6.7281647e-004	6.0645863e-004	6.9958239e-004
5.8785539e-004	5.3053928e-004	6.0611482e-004	6.5287243e-004
6.1198871e-004	6.0619276e-004	5.4065223e-004	5.7109651e-004
6.4319618e-004	5.5481291e-004	6.1203210e-004	6.0985006e-004
4.9548788e-004	5.8185865e-004	4.9317039e-004	6.7839836e-004
6.3756225e-004	6.1507088e-004	6.3180472e-004	6.2459933e-004
5.3669003e-004	5.9806411e-004	5.6030985e-004	5.8874013e-004
5.2426854e-004	5.0955173e-004	5.5006189e-004	4.9588017e-004
5.3702491e-004	5.8189502e-004	5.3871889e-004	4.7633884e-004
5.1533864e-004	5.4450039e-004	5.5198959e-004	5.7104051e-004
6.2913794e-004	5.0347100e-004	5.1985231e-004	6.1854163e-004
6.4400729e-004	6.2808668e-004	6.0040870e-004	6.0035723e-004
5.6608624e-004	5.6143224e-004	6.6326790e-004	6.6609604e-004
6.1482808e-004	6.1169756e-004	5.8574231e-004	7.2685622e-004
6.2226491e-004	5.6699064e-004	6.8549173e-004	6.2675105e-004
6.6595647e-004	6.1247543e-004	5.8648390e-004	6.3732812e-004

6.0762269e-004	6.3690967e-004	5.7425293e-004	7.2450457e-004
6.6775514e-004	5.7835515e-004	5.8570986e-004	6.3930212e-004
5.7419260e-004	5.7311478e-004	5.9652937e-004	5.8140430e-004
6.1157485e-004	6.0551176e-004	7.3796681e-004	5.6520644e-004
7.1258674e-004	6.0164813e-004	5.5886060e-004	5.9507335e-004
5.7678756e-004	6.3469776e-004	5.6852269e-004	5.3943483e-004
5.3183232e-004	5.8944906e-004	5.0874452e-004	6.0882911e-004
5.8298992e-004	5.9654418e-004	5.3940246e-004	6.1405687e-004
5.2487793e-004	5.8036768e-004	4.9504436e-004	5.9962150e-004
5.3645554e-004	5.1851164e-004	6.3111450e-004	6.6147441e-004
6.4784577e-004	5.7086436e-004	5.7028086e-004	6.3312324e-004
6.2423838e-004	5.6191409e-004	5.7182147e-004	5.3525706e-004
5.7425769e-004	5.5613348e-004	6.4506644e-004	6.0520097e-004
6.3462327e-004	5.4622807e-004	5.3769727e-004	5.7677177e-004
5.4782581e-004	5.8656101e-004	5.0025831e-004	6.6302305e-004
6.0638961e-004	6.9011989e-004	6.4723873e-004	6.2482196e-004
6.5255981e-004	6.2658347e-004	6.4692237e-004	6.0124412e-004
5.8252751e-004	5.8018509e-004	6.0539979e-004	6.1065397e-004
5.8143213e-004	5.1132884e-004	6.3106768e-004	5.3652549e-004
4.9018821e-004	5.6024303e-004	5.9953220e-004	5.3697506e-004
5.1349960e-004	5.9075348e-004	6.9402192e-004	6.2779765e-004
6.9691928e-004	7.0181207e-004	6.0998775e-004	6.1684332e-004
6.0784680e-004	5.2910576e-004	5.6640977e-004	5.3697587e-004
6.0266908e-004	5.6212521e-004	6.0607889e-004	5.0824135e-004
5.0205855e-004	5.0214048e-004	4.5408830e-004	5.9177507e-004
4.7125824e-004	5.5897799e-004	5.8250922e-004	5.9946590e-004
5.4896195e-004	5.7680144e-004	6.3054517e-004	6.0632857e-004
6.7603780e-004	6.0336934e-004	5.6919802e-004	6.0720566e-004
5.7288211e-004	5.2341741e-004	4.9545865e-004	5.6118918e-004
6.6545112e-004	5.4679820e-004	5.4808689e-004	5.7237365e-004
5.2314787e-004	5.5583351e-004	4.0131997e-004	5.7838208e-004
5.6597353e-004	5.7737480e-004	5.7584039e-004	5.2733316e-004
5.7973944e-004	6.4112060e-004	5.7253001e-004	6.9312293e-004
5.6598260e-004	5.0848776e-004	5.8961363e-004	6.2198976e-004
6.0307185e-004	5.8193140e-004	5.1948498e-004	5.8626028e-004
6.6018914e-004	5.3978020e-004	5.9394864e-004	6.0828535e-004
5.2193581e-004	5.6544475e-004	4.7065861e-004	6.7998990e-004
6.1029030e-004	5.7690981e-004	6.2886649e-004	6.0595277e-004
5.1456082e-004	5.9892549e-004	5.6157637e-004	6.1401147e-004
5.5625464e-004	4.8823940e-004	5.5680443e-004	5.0441914e-004
5.5873078e-004	5.7924313e-004	5.6953449e-004	4.9462086e-004
5.4881106e-004	5.7030359e-004	5.6786762e-004	5.9777468e-004
6.5003948e-004	4.9682967e-004	5.2846524e-004	6.2635506e-004
6.5295880e-004	6.4515024e-004	6.3333944e-004	5.9242451e-004
5.7876478e-004	5.6147749e-004	6.4595547e-004	6.3836247e-004
6.2886832e-004	6.0115801e-004	5.8561671e-004	7.3493813e-004
6.5859094e-004	5.7247483e-004	7.0714118e-004	6.3441909e-004
6.8489488e-004	6.1911404e-004	6.3087685e-004	6.7572713e-004
6.3999603e-004	6.4320522e-004	5.9814357e-004	7.3086937e-004
7.0468150e-004	5.6771970e-004	6.1215265e-004	6.3138151e-004
5.4959874e-004	6.0913744e-004	6.2735571e-004	6.4609126e-004
6.9276756e-004	5.2329039e-004	6.3804330e-004	5.9226548e-004

6.5251117e-004	6.4495781e-004	5.8860971e-004	5.8776759e-004
6.3172099e-004	5.9938774e-004	6.0135877e-004	6.1848419e-004
5.7861990e-004	5.2259475e-004	6.3259056e-004	6.0895966e-004
5.3086961e-004	5.4216341e-004	6.2892903e-004	5.9453427e-004
5.6735500e-004	5.6949838e-004	6.1655928e-004	6.0119579e-004
5.9339176e-004	5.6550566e-004	6.9856484e-004	5.6104244e-004
7.2932037e-004	6.1551451e-004	5.4533708e-004	5.6665119e-004
5.6824030e-004	6.3567728e-004	5.7515800e-004	5.2766608e-004
4.8369368e-004	5.8985037e-004	5.0291452e-004	6.0425894e-004
5.8737800e-004	5.8248561e-004	5.2231472e-004	6.1257937e-004
5.1667701e-004	5.6034156e-004	5.1063597e-004	6.0916734e-004
5.1343620e-004	4.9809758e-004	6.5388136e-004	6.4656716e-004
6.5566174e-004	5.6999708e-004	5.6541986e-004	6.4229329e-004
6.1632213e-004	5.8138125e-004	5.8455607e-004	5.3880189e-004
5.7251143e-004	5.5902323e-004	6.0041485e-004	5.8945574e-004
6.3942549e-004	5.3279598e-004	4.9982449e-004	5.4691306e-004
5.3156124e-004	5.5721603e-004	5.1136677e-004	6.5601175e-004
6.3827912e-004	6.6625449e-004	6.5772774e-004	6.2122430e-004
6.7784476e-004	6.2678954e-004	6.2064042e-004	6.0934752e-004
5.9175983e-004	6.1157350e-004	5.9234479e-004	5.8728167e-004
5.9216681e-004	5.2326950e-004	6.2846858e-004	5.7745200e-004
4.9788655e-004	5.6489282e-004	6.0431642e-004	5.4031739e-004
5.1486613e-004	5.6336833e-004	6.7584839e-004	6.3876944e-004
6.8744595e-004	6.6491993e-004	6.0051313e-004	6.2798853e-004
6.2997542e-004	5.5213529e-004	5.5058293e-004	5.3242680e-004
5.9394221e-004	5.7978547e-004	6.1576375e-004	5.2773517e-004
5.0067724e-004	5.0311311e-004	4.5362190e-004	6.0220205e-004
4.6327204e-004	5.5189515e-004	5.8502109e-004	5.8672213e-004
5.1438367e-004	5.4792852e-004	5.9857575e-004	5.5378683e-004
6.5415622e-004	5.6575180e-004	5.4998434e-004	5.8625804e-004
5.6329409e-004	4.9422017e-004	4.8695035e-004	5.7442070e-004
6.4354189e-004	5.3180430e-004	5.2931486e-004	5.4396527e-004
5.2009766e-004	5.3282893e-004	4.1640586e-004	5.6716993e-004
5.3174351e-004	5.4474843e-004	5.2890355e-004	5.0434207e-004
5.5643977e-004	5.9642260e-004	5.3961668e-004	6.7535872e-004
5.5037401e-004	4.8004102e-004	5.6146191e-004	6.0318167e-004
5.8935075e-004	5.5970500e-004	5.0106742e-004	5.9555669e-004
6.6830215e-004	5.2187766e-004	5.6710036e-004	5.9943563e-004
5.4639470e-004	5.5310036e-004	4.6253287e-004	6.6338442e-004
5.7422568e-004	5.3350700e-004	6.3499392e-004	5.7507816e-004
4.9210068e-004	5.6516635e-004	5.6480359e-004	6.2292386e-004
5.8027588e-004	4.5911986e-004	5.4292221e-004	5.0630084e-004
5.8669622e-004	5.6659809e-004	5.6963097e-004	5.0955248e-004
5.5045502e-004	5.7841747e-004	5.6942305e-004	5.9287218e-004
6.2748564e-004	4.8598954e-004	5.2787471e-004	5.9366276e-004
6.1808614e-004	6.3417199e-004	6.3654699e-004	5.7307525e-004
4.3491760e-004	5.1558347e-004	5.3652701e-004	4.9866819e-004
4.6969280e-004	4.3702606e-004	4.5522142e-004	3.9003893e-004
4.1080521e-004	4.8086169e-004	5.1903119e-004	4.6751365e-004
4.4183648e-004	4.5440735e-004	4.5766506e-004	4.4343357e-004
4.9379319e-004	5.0812969e-004	5.4654117e-004	5.7548620e-004
5.6773938e-004	5.7560395e-004	5.3353015e-004	6.1923185e-004

5.5941513e-004	5.6246623e-004	6.6185024e-004	5.9376219e-004
6.3263800e-004	6.1943570e-004	5.8237834e-004	7.1482465e-004
6.5878160e-004	5.7406540e-004	6.9287758e-004	6.4158748e-004
7.0882315e-004	6.2487248e-004	7.1153200e-004	7.0948723e-004
6.6201788e-004	6.6416587e-004	6.5082982e-004	7.2565961e-004
7.4491688e-004	6.1025628e-004	6.7508759e-004	6.3088717e-004
5.5183136e-004	5.4879172e-004	6.2580577e-004	5.9312997e-004
5.9981878e-004	5.3675113e-004	6.2293155e-004	5.9038035e-004
7.2312157e-004	6.4515647e-004	5.3599771e-004	5.3338805e-004
5.4603144e-004	5.9738601e-004	5.6545246e-004	5.0536426e-004
4.6704614e-004	5.5200702e-004	5.0636622e-004	5.9676111e-004
5.5470728e-004	5.3895309e-004	5.4194431e-004	5.8009541e-004
5.1253436e-004	5.6008523e-004	5.9318509e-004	6.4610716e-004
5.0289546e-004	4.8530405e-004	6.8467524e-004	6.1966146e-004
6.5998592e-004	5.5516273e-004	5.7007327e-004	6.2839990e-004
6.3238734e-004	6.1547190e-004	5.8428432e-004	5.7244804e-004
5.3959685e-004	5.6246892e-004	5.5912192e-004	5.6535235e-004
6.5564577e-004	5.4093245e-004	4.4611943e-004	5.5460217e-004
5.2335289e-004	5.2794832e-004	4.9589096e-004	6.3264745e-004
6.3851794e-004	6.2805475e-004	6.7908841e-004	6.2913483e-004
7.1965349e-004	6.1992854e-004	6.0094953e-004	6.4283836e-004
6.0837979e-004	6.2924802e-004	5.9512076e-004	5.7212265e-004
6.2397060e-004	5.4187914e-004	6.0942827e-004	6.1887414e-004
5.3429005e-004	5.8658633e-004	5.8982706e-004	5.4280082e-004
5.4024479e-004	5.5292541e-004	6.1873313e-004	6.8620523e-004
6.7810460e-004	6.3283245e-004	6.0622852e-004	6.3167899e-004
6.6352446e-004	6.0028424e-004	5.7195155e-004	5.4262363e-004
5.7061912e-004	6.0974850e-004	6.0428541e-004	5.4689257e-004
5.4303530e-004	5.4323742e-004	5.2514153e-004	6.2940540e-004
4.8867022e-004	5.6312754e-004	6.0491321e-004	5.7907238e-004
4.8745932e-004	5.1449182e-004	6.0334928e-004	5.2916387e-004
6.4409066e-004	5.7440334e-004	5.6340288e-004	5.8358013e-004
5.9037894e-004	5.1250457e-004	5.0317950e-004	5.7169360e-004
6.3935430e-004	5.3813605e-004	5.3886673e-004	5.2956228e-004
5.1948585e-004	5.0135219e-004	4.5818989e-004	5.5955918e-004
4.7792959e-004	5.2097614e-004	5.0598403e-004	5.1395031e-004
5.4378209e-004	5.9282045e-004	5.3845522e-004	6.3082078e-004
5.6531889e-004	5.0934280e-004	5.3967806e-004	6.0316229e-004
5.5708183e-004	5.3704796e-004	4.9420114e-004	6.0719154e-004
6.5433369e-004	5.0932425e-004	5.5136137e-004	5.6821133e-004
5.8391682e-004	5.6161714e-004	4.4531992e-004	6.3873272e-004
5.3566782e-004	5.0437457e-004	6.1964207e-004	5.2635952e-004
4.8664188e-004	5.0422519e-004	5.4515444e-004	6.4819005e-004
5.8697614e-004	4.1638007e-004	5.1355085e-004	5.4721416e-004
6.0149096e-004	5.5048750e-004	5.3284492e-004	5.0323586e-004
5.6558521e-004	5.4209201e-004	5.4149896e-004	5.8494446e-004
5.3609893e-004	4.7893459e-004	5.1281103e-004	5.4815452e-004
5.8007668e-004	5.8162691e-004	6.4965311e-004	5.5710824e-004
4.7471619e-004	5.9925609e-004	6.3304137e-004	6.0017401e-004
5.7444803e-004	4.8236504e-004	5.1160611e-004	4.4361565e-004
5.3104558e-004	5.6494214e-004	5.9189934e-004	5.7962927e-004
5.0821617e-004	5.4408533e-004	5.3502790e-004	5.0925031e-004

6.0752509e-004	5.7647655e-004	6.4303141e-004	6.4073133e-004
6.0763328e-004	6.5047505e-004	6.1475825e-004	6.6015485e-004
4.1324535e-004	4.0188794e-004	3.8844693e-004	3.3910802e-004
3.4792376e-004	3.4132204e-004	4.1338121e-004	4.0240760e-004
3.7871565e-004	3.5716199e-004	3.6872170e-004	3.3645896e-004
3.7735463e-004	4.3599854e-004	4.2984538e-004	4.1743196e-004
4.1005514e-004	4.6178205e-004	4.7022738e-004	5.1073822e-004
4.7351047e-004	5.2195194e-004	4.7476628e-004	4.7387180e-004
5.4549723e-004	5.3925852e-004	6.3619595e-004	5.1407427e-004
6.2930296e-004	5.8245340e-004	5.7985431e-004	6.0541579e-004
6.3800007e-004	5.8274723e-004	5.6488643e-004	6.3673253e-004
6.6626293e-004	6.1196192e-004	7.1828195e-004	6.3064777e-004
6.5826678e-004	6.3376698e-004	6.3579438e-004	6.5274710e-004
6.8376625e-004	6.7322813e-004	6.8547111e-004	6.4567549e-004
6.4352660e-004	5.6532637e-004	5.9136441e-004	6.5245490e-004
6.1840451e-004	6.0094056e-004	6.2337685e-004	6.9128553e-004
7.3288991e-004	6.3659112e-004	6.6475649e-004	5.8571354e-004
6.7436269e-004	6.0304959e-004	7.2434309e-004	6.2991247e-004
7.0533951e-004	6.2952680e-004	6.7413422e-004	7.4702255e-004
7.4212295e-004	5.7470096e-004	6.5697647e-004	6.0091807e-004
5.2150592e-004	5.7422544e-004	6.4676019e-004	6.2427917e-004
5.2145994e-004	5.1392340e-004	6.3517876e-004	5.9475529e-004
6.8705232e-004	5.8273204e-004	5.4673888e-004	5.6700414e-004
6.1853936e-004	6.0101257e-004	5.7916284e-004	5.7275113e-004
4.5475290e-004	5.6520576e-004	5.4067599e-004	6.0371187e-004
6.1194292e-004	5.1720265e-004	4.7935746e-004	5.5307480e-004
5.1087327e-004	5.0246828e-004	5.3175869e-004	6.0713361e-004
5.4042772e-004	5.2856194e-004	6.8879542e-004	6.2559242e-004
6.9195229e-004	5.6637249e-004	5.9256636e-004	6.4145015e-004
5.8631394e-004	6.2024670e-004	5.6645252e-004	5.5921855e-004
5.7875917e-004	5.5149254e-004	5.2497059e-004	5.8228879e-004
6.0172107e-004	5.4270937e-004	4.8776819e-004	5.1467851e-004
5.1654993e-004	5.4611760e-004	5.4145617e-004	6.7978149e-004
6.7305972e-004	6.1185050e-004	6.6787752e-004	6.2820587e-004
6.9858770e-004	6.1255273e-004	6.0959355e-004	5.9111179e-004
5.9642309e-004	6.1078854e-004	6.0083684e-004	5.8912401e-004
6.3590978e-004	5.7242486e-004	6.5051546e-004	6.4533316e-004
5.2978705e-004	6.2067476e-004	5.9392740e-004	5.9009026e-004
5.4246375e-004	5.3306576e-004	6.2464371e-004	6.1499039e-004
6.5215603e-004	6.2909088e-004	5.9406341e-004	6.2465211e-004
6.5163511e-004	6.1605331e-004	5.6278335e-004	5.7175022e-004
6.3725920e-004	5.9816052e-004	5.8913339e-004	5.5857617e-004
5.7021355e-004	5.2316458e-004	4.7648449e-004	5.9505785e-004
4.7603175e-004	5.6833512e-004	5.7172976e-004	5.3014203e-004
5.2809204e-004	4.7568962e-004	5.4398678e-004	6.4741923e-004
5.7106480e-004	4.3566484e-004	5.3008246e-004	6.0962416e-004
5.9326558e-004	5.2410734e-004	5.0573340e-004	5.5616409e-004
6.1241504e-004	5.0548984e-004	5.3708171e-004	5.6143742e-004
5.2278851e-004	5.6117186e-004	5.0127384e-004	5.8891896e-004
5.5271473e-004	5.1993791e-004	6.7511659e-004	5.6202139e-004
4.8021306e-004	5.7775948e-004	6.0505731e-004	6.1198392e-004
6.3419912e-004	4.5077204e-004	5.2564028e-004	4.8185625e-004

6.1547531e-004	5.6128188e-004	5.7185070e-004	5.6170832e-004
5.2983203e-004	5.6319372e-004	5.6361340e-004	5.2919795e-004
5.8418250e-004	5.3216884e-004	6.0160879e-004	5.6004536e-004
5.1325984e-004	6.1477495e-004	6.1198670e-004	5.7169894e-004
4.9245504e-004	5.7340553e-004	5.9927475e-004	5.4418426e-004
5.1668071e-004	4.7461329e-004	5.4472756e-004	5.2691263e-004
5.2911810e-004	5.1273633e-004	5.3406396e-004	5.3058402e-004
5.2516640e-004	5.5015048e-004	5.4267026e-004	5.5373360e-004
5.6969024e-004	6.1334470e-004	6.5674214e-004	6.7899050e-004
6.4834465e-004	6.6601284e-004	6.6561243e-004	6.6072089e-004
5.4701163e-004	5.2576412e-004	6.3839968e-004	5.1631838e-004
6.4646463e-004	5.4674961e-004	5.5299600e-004	5.9030984e-004
6.1894738e-004	5.8497507e-004	5.4416333e-004	6.2510784e-004
6.7639013e-004	5.8371092e-004	7.2483844e-004	5.8482730e-004
6.6701441e-004	6.2524405e-004	6.5837624e-004	6.7645202e-004
6.5883331e-004	6.8181568e-004	6.8286277e-004	6.4252512e-004
6.4119861e-004	5.6000774e-004	6.0011249e-004	6.3298701e-004
6.0912137e-004	5.9965163e-004	6.2168267e-004	6.5575886e-004
6.9190554e-004	6.2309680e-004	6.1536988e-004	5.5908374e-004
6.4812410e-004	5.8718964e-004	7.2040383e-004	5.8130913e-004
6.8260000e-004	6.3353038e-004	6.8330474e-004	7.3118357e-004
7.0535118e-004	5.6188539e-004	6.6877079e-004	5.9267192e-004
5.2097996e-004	5.7115864e-004	6.3594594e-004	5.8783859e-004
5.1461087e-004	5.1573184e-004	6.2968524e-004	6.2185795e-004
6.6261314e-004	5.7384837e-004	5.4248307e-004	5.6270635e-004
6.2121660e-004	5.8174107e-004	5.7717129e-004	5.9209807e-004
4.5370696e-004	5.4212034e-004	5.4458818e-004	6.1629106e-004
5.9592922e-004	4.8730723e-004	4.9644363e-004	5.3335744e-004
5.1395289e-004	5.0726303e-004	5.5664044e-004	6.2312752e-004
5.4202659e-004	5.4204208e-004	6.9669427e-004	6.3394477e-004
6.9588474e-004	5.6756596e-004	6.2353327e-004	6.2068120e-004
5.8629204e-004	6.2133725e-004	5.5373645e-004	5.8168627e-004
5.9037737e-004	5.4411789e-004	5.3013263e-004	5.8270592e-004
6.0225166e-004	5.6170709e-004	5.0412078e-004	5.2753236e-004
4.9932045e-004	5.4863549e-004	5.2589169e-004	6.4441960e-004
6.2909009e-004	5.8683244e-004	6.5843543e-004	6.1907382e-004
6.9296912e-004	5.8876189e-004	6.0547369e-004	5.8674115e-004
6.0245779e-004	6.0318550e-004	6.0819088e-004	5.9311523e-004
6.4771950e-004	5.7988231e-004	6.4152900e-004	6.2886074e-004
5.5316451e-004	6.3536143e-004	5.4670747e-004	5.7730170e-004
5.3937449e-004	4.6362210e-004	5.6959048e-004	6.2751818e-004
5.9101664e-004	4.6567831e-004	5.5457195e-004	6.1345643e-004
5.9089424e-004	5.1514345e-004	5.2933562e-004	5.4394324e-004
5.8387246e-004	4.9584215e-004	5.5298164e-004	5.4543177e-004
5.1117349e-004	5.8866317e-004	5.2914226e-004	5.8470412e-004
5.4631865e-004	5.4237535e-004	6.5258620e-004	5.6364904e-004
4.8579939e-004	5.9748594e-004	6.0202830e-004	6.2448556e-004
6.4650376e-004	4.5680424e-004	5.4773857e-004	5.1126860e-004
6.1433783e-004	5.6397061e-004	5.7391924e-004	5.6707607e-004
5.4692815e-004	5.5240828e-004	5.5507659e-004	5.4147533e-004
5.5952505e-004	5.5343618e-004	6.2089752e-004	5.6111033e-004
5.0055290e-004	6.0240335e-004	5.9957654e-004	5.7219820e-004

5.0595899e-004	5.6471612e-004	6.2217662e-004	5.7015652e-004
5.5670997e-004	4.9233629e-004	5.8129489e-004	5.4329148e-004
5.7803198e-004	5.1437069e-004	5.2246012e-004	5.5698057e-004
5.5666058e-004	5.7130368e-004	5.6249212e-004	5.8669764e-004
5.8615599e-004	6.0891584e-004	6.5075156e-004	6.8994606e-004
6.4156322e-004	6.5459787e-004	6.8867509e-004	6.5947004e-004
4.2935762e-004	4.6026389e-004	4.3567472e-004	4.0492770e-004
4.3330131e-004	3.8851370e-004	4.6962732e-004	4.1397072e-004
4.9418450e-004	4.1210684e-004	4.3046699e-004	3.9051273e-004
5.0554763e-004	4.9012955e-004	4.9080210e-004	4.5423675e-004
4.4235617e-004	4.8443102e-004	5.1745417e-004	5.6115554e-004
5.3485116e-004	5.6758513e-004	5.4785955e-004	5.2299159e-004
4.9470186e-004	4.8544695e-004	5.8406507e-004	5.1900115e-004
6.1910343e-004	4.6594734e-004	5.2991251e-004	5.6174141e-004
5.7492798e-004	5.8267701e-004	5.1547812e-004	5.8819453e-004
6.2422996e-004	5.4319046e-004	6.5180979e-004	5.2945627e-004
6.1157712e-004	5.9564692e-004	6.1335679e-004	6.1965840e-004
6.2482097e-004	6.5450284e-004	6.3265358e-004	6.1138149e-004
6.3233620e-004	5.4002917e-004	6.3215892e-004	5.8060708e-004
6.2474287e-004	5.9217970e-004	5.7923666e-004	6.0644803e-004
5.9910581e-004	5.7020751e-004	5.6932219e-004	5.5661821e-004
6.3008445e-004	5.6804183e-004	7.0882119e-004	5.4894341e-004
6.3939564e-004	6.3860220e-004	6.8052950e-004	7.2637085e-004
6.5250177e-004	5.5918236e-004	6.5280743e-004	6.1918730e-004
5.3628361e-004	5.8063577e-004	6.0536237e-004	5.8513976e-004
5.4867834e-004	5.3348158e-004	6.2063760e-004	6.4448372e-004
6.5212362e-004	5.7760373e-004	5.3214961e-004	5.4325863e-004
5.8497280e-004	5.5527987e-004	5.9153672e-004	5.5864752e-004
4.5242767e-004	5.0428572e-004	5.4777277e-004	6.0917753e-004
5.4265512e-004	4.4444183e-004	5.2050473e-004	5.3759257e-004
5.3403389e-004	5.3138004e-004	5.9568140e-004	6.0064730e-004
5.0589329e-004	5.3375783e-004	7.0308321e-004	6.4560687e-004
6.5723184e-004	5.7662070e-004	6.0832729e-004	5.9571986e-004
5.8928937e-004	6.2879534e-004	5.5283449e-004	6.3003809e-004
5.8795237e-004	5.3297740e-004	5.3794720e-004	5.8558390e-004
6.2153005e-004	5.6009268e-004	5.2746756e-004	5.4285784e-004
4.7433649e-004	5.3983639e-004	5.1394642e-004	6.3355954e-004
5.9287797e-004	5.6497665e-004	6.3449063e-004	6.2714363e-004
7.1077165e-004	5.5467234e-004	6.0908757e-004	6.0661855e-004
6.0873422e-004	5.9283363e-004	5.8643421e-004	5.8841737e-004
6.4311645e-004	5.7436328e-004	6.2199745e-004	6.1716582e-004
5.8057024e-004	6.3006686e-004	5.3382586e-004	5.6701007e-004
5.3870000e-004	5.9626283e-004	6.4933275e-004	6.5109153e-004
6.1562138e-004	5.1530445e-004	6.2486464e-004	5.3211037e-004
6.3006395e-004	5.4549708e-004	5.3622290e-004	5.9001862e-004
5.4174944e-004	5.9211197e-004	5.8195405e-004	5.8554625e-004
5.7767193e-004	6.0158770e-004	6.4254773e-004	6.7265506e-004
5.9116739e-004	6.3237667e-004	6.5527596e-004	6.4659087e-004
5.7743950e-004	5.9556172e-004	6.1918680e-004	5.5642793e-004
5.8842606e-004	5.3628277e-004	6.8029686e-004	5.8440407e-004
7.1259351e-004	6.0031492e-004	6.2354303e-004	5.9528129e-004
7.1415033e-004	6.6911979e-004	6.6031840e-004	6.4990707e-004

6.2749677e-004	6.4663161e-004	6.7942961e-004	8.0509207e-004
7.6811867e-004	7.5562234e-004	7.4388340e-004	7.2008747e-004
4.5179979e-004	3.9846359e-004	4.4358921e-004	4.6385032e-004
5.4119412e-004	3.9299033e-004	4.7238242e-004	4.8792511e-004
4.7352130e-004	5.3318263e-004	4.8312693e-004	4.9197570e-004
5.0138987e-004	4.9417463e-004	5.1766829e-004	4.9463563e-004
5.1944087e-004	5.3656577e-004	5.1437815e-004	5.4077182e-004
5.2988690e-004	5.6722743e-004	5.1597536e-004	5.4416277e-004
5.6631745e-004	5.2110881e-004	6.4601336e-004	4.8603876e-004
6.2199794e-004	5.2091067e-004	5.4213739e-004	5.6212094e-004
5.6758826e-004	5.4881760e-004	5.3220738e-004	5.7689428e-004
6.1343930e-004	5.2957392e-004	6.7804931e-004	5.4010923e-004
5.9983933e-004	5.9701837e-004	6.4936826e-004	6.8542572e-004
5.9632190e-004	6.1660234e-004	6.4453571e-004	5.9311336e-004
5.3158628e-004	5.5391106e-004	5.8238021e-004	5.6529393e-004
4.7968356e-004	5.0565117e-004	6.7271965e-004	6.1266942e-004
6.1351675e-004	5.6749459e-004	5.1980445e-004	5.5951194e-004
5.8080946e-004	5.8376655e-004	5.6322486e-004	6.3325121e-004
4.9744564e-004	5.0645642e-004	5.2331164e-004	5.9849751e-004
5.9692965e-004	4.9534070e-004	5.4508660e-004	5.3621487e-004
5.6715890e-004	5.4363477e-004	6.4143899e-004	6.0623410e-004
6.0347830e-004	5.4065627e-004	6.7233392e-004	5.9433760e-004
6.9047515e-004	5.9096042e-004	5.7542339e-004	6.0902823e-004
6.5668891e-004	6.4516866e-004	6.5766573e-004	6.6888929e-004
6.1221571e-004	6.1064384e-004	6.2373675e-004	7.1651601e-004
6.8336753e-004	6.8058878e-004	7.2697563e-004	6.9037810e-004
6.4845163e-004	5.6713425e-004	6.0585863e-004	5.8646670e-004
6.3523157e-004	6.1701336e-004	6.9050094e-004	6.3992472e-004
7.0357410e-004	6.0771954e-004	6.9195268e-004	6.6231738e-004
6.0874923e-004	6.5302843e-004	6.4251458e-004	6.2481099e-004
6.7272346e-004	6.2150467e-004	6.3126326e-004	8.0943132e-004
7.2206501e-004	7.6004952e-004	6.9073265e-004	7.3302869e-004
4.7598328e-004	3.9006134e-004	4.2988931e-004	4.0622647e-004
5.3551375e-004	3.9714457e-004	4.6034023e-004	4.6065680e-004
4.6595013e-004	5.4602821e-004	4.9469920e-004	5.1596555e-004
5.0834472e-004	4.7885788e-004	5.1630716e-004	5.1715019e-004
5.0279183e-004	5.3849071e-004	5.0681426e-004	5.1979346e-004
5.1069348e-004	5.4157463e-004	5.1947821e-004	5.5092139e-004
5.3525696e-004	5.2528910e-004	5.8605413e-004	6.0858812e-004
6.1751332e-004	5.0363371e-004	6.3320175e-004	5.6601227e-004
6.3567911e-004	5.8114021e-004	5.4988246e-004	5.0753509e-004
5.3173100e-004	4.9408302e-004	5.4961652e-004	5.8950129e-004
4.8969022e-004	5.5985208e-004	5.9400688e-004	5.9122157e-004
5.6090284e-004	4.7984414e-004	5.1745876e-004	5.7678764e-004
5.8656117e-004	5.4535338e-004	6.3687230e-004	6.3645252e-004
6.0259200e-004	5.7534131e-004	7.0053340e-004	6.1108042e-004
7.1323387e-004	6.2440554e-004	6.2172419e-004	6.1205050e-004
6.4585720e-004	6.6355524e-004	6.6731281e-004	6.7136390e-004
6.1649281e-004	6.0283288e-004	6.0581426e-004	7.0348410e-004
6.6339292e-004	6.6907961e-004	7.1351627e-004	6.8416595e-004
6.7309832e-004	5.8843628e-004	6.4955885e-004	5.9466646e-004
6.7492838e-004	6.5085328e-004	7.3342507e-004	7.0381571e-004

7.4623183e-004	6.5132762e-004	7.0503484e-004	7.1700236e-004
6.1332599e-004	6.7952527e-004	6.7654462e-004	6.2415763e-004
6.9740346e-004	6.4434670e-004	6.2195769e-004	7.9259298e-004
7.2508337e-004	7.4027554e-004	6.9459611e-004	7.6619729e-004
5.2458017e-004	4.2766756e-004	4.4620751e-004	3.9923822e-004
5.2822523e-004	4.8225520e-004	4.9836270e-004	4.6094711e-004
5.1541976e-004	5.2100156e-004	5.0420398e-004	5.5919318e-004
5.2925863e-004	4.6956742e-004	5.0681474e-004	5.4750816e-004
5.2082545e-004	5.5123030e-004	5.1894255e-004	5.1534748e-004
5.4665434e-004	5.4868537e-004	5.5151149e-004	6.0744023e-004
5.6745311e-004	5.5814834e-004	5.9469417e-004	5.1534464e-004
6.2645494e-004	4.5598954e-004	5.5622365e-004	5.4301806e-004
5.8954298e-004	6.4493735e-004	6.4287236e-004	5.8592622e-004
5.6530914e-004	5.6418749e-004	6.7592430e-004	6.1335691e-004
5.7567576e-004	6.4133393e-004	6.3873564e-004	6.4901685e-004
5.9530258e-004	6.7333430e-004	6.3209003e-004	5.7189715e-004
5.4221142e-004	5.3864034e-004	6.0102409e-004	5.9097406e-004
6.2199455e-004	5.0437280e-004	6.0881479e-004	5.3674870e-004
6.2192269e-004	5.9740356e-004	5.2217471e-004	5.1932669e-004
5.0464796e-004	4.8682745e-004	5.3530926e-004	5.9317464e-004
4.8333534e-004	5.4773316e-004	5.7577885e-004	5.7325567e-004
5.9093659e-004	4.5976122e-004	4.9578925e-004	5.3275901e-004
6.1828525e-004	5.6505954e-004	6.6032134e-004	6.0508271e-004
6.3489073e-004	6.2608704e-004	7.4823026e-004	6.6679186e-004
7.5285546e-004	6.5742177e-004	6.5848608e-004	6.7207744e-004
6.0869924e-004	6.8232954e-004	6.5759191e-004	5.8349888e-004
6.5393848e-004	6.0415856e-004	6.0391707e-004	7.3065552e-004
6.7831785e-004	6.5703159e-004	6.7724413e-004	7.1504759e-004
4.5876071e-004	4.1036754e-004	4.1686316e-004	3.5365325e-004
4.6787862e-004	4.1295466e-004	4.1443906e-004	3.8829986e-004
4.3772320e-004	4.7912961e-004	5.0532839e-004	4.7536838e-004
4.9932505e-004	4.3456523e-004	5.2833782e-004	4.9106284e-004
4.6780720e-004	4.8471301e-004	5.1390478e-004	4.8869155e-004
4.7676003e-004	5.3877341e-004	5.8040179e-004	5.1968699e-004
5.8318037e-004	5.8957914e-004	6.0970387e-004	5.5347968e-004
6.4508026e-004	4.7086608e-004	5.6579517e-004	5.6789395e-004
6.2600101e-004	6.5908510e-004	6.4331609e-004	5.9197235e-004
5.3697485e-004	5.8925847e-004	6.4509313e-004	6.3326219e-004
6.0758690e-004	6.5228515e-004	6.4454437e-004	6.2683289e-004
5.7815875e-004	6.3816784e-004	6.0235173e-004	5.2862256e-004
5.8998409e-004	5.4146013e-004	6.3882084e-004	5.8873890e-004
6.1449915e-004	5.3056072e-004	5.3674815e-004	5.5540219e-004
5.1948871e-004	5.4693292e-004	5.1643520e-004	5.6281082e-004
5.1397685e-004	5.2176884e-004	5.0722349e-004	5.2713511e-004
4.9438050e-004	5.1312123e-004	4.8392988e-004	4.2777592e-004
5.7412931e-004	5.0949439e-004	4.5771116e-004	5.0030914e-004
5.5391542e-004	5.5476648e-004	5.8301243e-004	5.7349198e-004
6.2547166e-004	5.1018348e-004	6.5336785e-004	5.3986111e-004
6.4742974e-004	6.1492831e-004	5.2849803e-004	5.5424018e-004
4.9130948e-004	4.9720379e-004	5.3758173e-004	5.8654978e-004
4.7969568e-004	5.3707055e-004	5.5120475e-004	5.5475377e-004
5.7763960e-004	4.8140095e-004	5.4354398e-004	5.0104356e-004

4.8202573e-004	4.2929738e-004	4.6431123e-004	4.1887303e-004
5.1420565e-004	4.5940738e-004	4.4399619e-004	4.1942349e-004
5.3323909e-004	5.1265789e-004	5.8131450e-004	5.1942108e-004
5.6119070e-004	4.8844540e-004	5.6303136e-004	5.6355682e-004
5.2199486e-004	5.3291407e-004	5.9374904e-004	5.5409641e-004
5.4994205e-004	6.3030614e-004	6.4072962e-004	5.6552953e-004
5.7286775e-004	5.7610399e-004	6.0859730e-004	5.7311021e-004
6.3367383e-004	4.7030183e-004	5.5523316e-004	5.6556900e-004
6.1707557e-004	6.5358254e-004	6.4203162e-004	5.7921206e-004
5.3729549e-004	6.0312208e-004	6.5465523e-004	6.0648152e-004
6.2938976e-004	6.3765141e-004	6.0809794e-004	6.1515394e-004
6.0819052e-004	6.3636623e-004	6.0578357e-004	5.2032332e-004
5.9413094e-004	5.6223581e-004	6.4179457e-004	6.0342527e-004
6.2535638e-004	5.3101283e-004	5.4807460e-004	5.6731229e-004
5.3137599e-004	5.5614785e-004	5.4904340e-004	5.5634869e-004
5.0437742e-004	5.2266540e-004	5.0812349e-004	5.2607193e-004
5.3482092e-004	5.0674665e-004	4.9153381e-004	4.3720836e-004
5.6508226e-004	5.0188284e-004	4.6666271e-004	5.0069502e-004
5.6465983e-004	5.5593014e-004	5.7121809e-004	5.6519076e-004
6.3195113e-004	5.2456296e-004	6.5985833e-004	5.6492323e-004
6.4237393e-004	6.1155585e-004	5.5104426e-004	5.8144676e-004
4.9145705e-004	5.0947208e-004	5.4744251e-004	5.8630684e-004
4.8807802e-004	5.1812742e-004	5.3335339e-004	5.3505675e-004
5.6260833e-004	4.9566091e-004	5.6187746e-004	4.7377521e-004
5.8303510e-004	5.4782737e-004	5.9198777e-004	5.8300982e-004
5.6095456e-004	6.0998257e-004	6.5084297e-004	6.2900942e-004
6.0998811e-004	6.1249392e-004	5.1437520e-004	5.7446961e-004
5.0825620e-004	5.6171292e-004	5.5860299e-004	5.3112554e-004
5.1780863e-004	5.3648352e-004	5.1355304e-004	4.9471857e-004
5.3412092e-004	5.1774233e-004	5.7534709e-004	4.9381296e-004
4.8038400e-004	4.2559659e-004	4.9095931e-004	4.6097199e-004
5.2624591e-004	4.6460400e-004	4.4575170e-004	4.5488980e-004
5.5830241e-004	4.7093777e-004	5.4729843e-004	4.8894392e-004
5.4319560e-004	4.9555729e-004	5.2175413e-004	5.9537582e-004
5.2370818e-004	5.0113768e-004	6.0781452e-004	5.5357111e-004
5.7442213e-004	6.1799771e-004	6.1097553e-004	5.8308556e-004
5.4458134e-004	5.5230550e-004	5.8176107e-004	5.6955831e-004
5.9141830e-004	4.6499085e-004	5.2226114e-004	5.3413373e-004
6.0006026e-004	6.0625417e-004	6.4354508e-004	5.8325360e-004
5.8979676e-004	5.9682484e-004	6.5911694e-004	5.6285162e-004
6.1646225e-004	6.3979556e-004	5.8373841e-004	6.3473072e-004
6.4506220e-004	6.7907342e-004	6.7200902e-004	5.5619456e-004
5.3710851e-004	6.0282070e-004	5.6339243e-004	6.3441492e-004
5.4123061e-004	5.5159647e-004	5.9939810e-004	5.6974259e-004
5.5499666e-004	5.3891014e-004	4.8925492e-004	5.5774087e-004
5.0199206e-004	5.3450464e-004	5.4466820e-004	4.5555800e-004
5.5155168e-004	4.8674150e-004	5.2493584e-004	5.1294817e-004
4.8447018e-004	5.6139979e-004	5.0877356e-004	4.5831320e-004
6.0339260e-004	5.7578750e-004	6.4565401e-004	6.2631737e-004
6.0241217e-004	5.2372900e-004	5.8128445e-004	5.6802484e-004
5.3516115e-004	5.9070483e-004	5.7692484e-004	5.4244502e-004
5.1605421e-004	5.1505044e-004	5.2855920e-004	5.1605963e-004

5.5634386e-004	4.8644119e-004	5.1026441e-004	4.9245717e-004
5.3742904e-004	5.0216416e-004	4.6602578e-004	5.1743388e-004
5.5002206e-004	5.4605507e-004	5.6352775e-004	5.8112082e-004
6.3829318e-004	5.3161892e-004	6.1555389e-004	5.8462513e-004
6.1206119e-004	5.8252453e-004	5.9244709e-004	5.7098024e-004
4.8312038e-004	5.2515458e-004	5.4709995e-004	5.9037090e-004
5.1409121e-004	5.0242702e-004	5.0528515e-004	5.2125226e-004
5.2763433e-004	4.7061389e-004	5.4418288e-004	4.8049771e-004
5.7952970e-004	5.3179715e-004	5.8637523e-004	5.5049968e-004
5.4878219e-004	5.5801029e-004	6.5244552e-004	6.5246160e-004
6.0778597e-004	5.8848591e-004	5.0895842e-004	5.8459314e-004
5.1257576e-004	5.5482828e-004	5.2500195e-004	4.9362577e-004
4.9617566e-004	5.0271502e-004	5.0221623e-004	4.8334818e-004
5.2596291e-004	5.1546720e-004	5.3480340e-004	4.5809882e-004
5.1153135e-004	4.8605984e-004	5.4298042e-004	5.5262596e-004
6.1167817e-004	5.0782786e-004	5.2361084e-004	5.4162752e-004
6.6366754e-004	5.5224508e-004	6.3113483e-004	5.7375718e-004
6.3101728e-004	5.6851466e-004	6.2926432e-004	7.0807153e-004
6.0630120e-004	6.3534277e-004	6.8006464e-004	6.9394354e-004
7.0639381e-004	7.1465639e-004	7.3079852e-004	6.8579413e-004
5.2442128e-004	5.1637636e-004	5.9735752e-004	5.7932863e-004
5.8270889e-004	4.9315360e-004	5.1664059e-004	5.4977092e-004
5.5228006e-004	5.4652120e-004	5.8228728e-004	5.6429354e-004
5.7016309e-004	6.1815888e-004	6.0471694e-004	5.7329766e-004
6.5494640e-004	6.0884682e-004	5.4808315e-004	6.2066490e-004
6.7596344e-004	6.4055782e-004	6.3279197e-004	5.6082265e-004
5.5569934e-004	5.4678097e-004	5.4781928e-004	5.6072367e-004
6.0870621e-004	5.4687730e-004	6.3510190e-004	6.3623952e-004
6.0981643e-004	5.7385706e-004	5.9227883e-004	5.7942374e-004
5.2546377e-004	5.3514339e-004	5.4277244e-004	5.4774252e-004
5.1491863e-004	4.8033254e-004	4.8154811e-004	5.2737771e-004
5.0354419e-004	4.7324158e-004	5.5352043e-004	4.8045788e-004
5.5991474e-004	5.0230764e-004	5.3791490e-004	5.7386905e-004
5.2650445e-004	5.5312032e-004	6.2057903e-004	6.5264580e-004
5.7114827e-004	5.7672954e-004	5.1368961e-004	5.3624864e-004
4.9890974e-004	5.3553922e-004	5.1066796e-004	5.0483834e-004
4.9206285e-004	4.8314723e-004	5.0284178e-004	4.6937060e-004
4.9664245e-004	4.7806461e-004	4.7897454e-004	4.5759507e-004
5.0336977e-004	4.4092543e-004	5.0519042e-004	4.5763041e-004
5.0495302e-004	4.6822639e-004	4.8986121e-004	4.5178477e-004
5.3871362e-004	4.5062490e-004	4.1694841e-004	4.8589362e-004
4.9786924e-004	4.7533854e-004	5.0321719e-004	5.6335503e-004
5.4697107e-004	4.6683813e-004	5.2067313e-004	5.0616825e-004
5.7313748e-004	5.9571422e-004	5.1917475e-004	5.4318445e-004
5.1054177e-004	5.0744511e-004	5.3951162e-004	5.5830662e-004
5.7971822e-004	4.6593303e-004	5.4672998e-004	5.5669904e-004
6.1528487e-004	5.2709036e-004	6.0320598e-004	5.7912804e-004
6.3457311e-004	5.6159342e-004	6.1909281e-004	6.9560757e-004
6.0739298e-004	6.8128685e-004	6.4004205e-004	7.1986766e-004
6.8351786e-004	7.0539476e-004	7.5497213e-004	6.5966738e-004
5.3406731e-004	4.9643229e-004	6.0089110e-004	6.0217459e-004
5.8602802e-004	5.1371442e-004	4.9751620e-004	5.3485627e-004

5.1631761e-004	5.2784957e-004	5.5145833e-004	5.7807304e-004
5.2909086e-004	6.0874123e-004	5.5418548e-004	5.9446243e-004
6.5469173e-004	5.9423233e-004	5.4048127e-004	6.0468684e-004
6.6367259e-004	5.8294917e-004	6.1995428e-004	5.6884729e-004
4.4690214e-004	5.4944665e-004	5.4384534e-004	5.6859727e-004
5.8233833e-004	4.8011142e-004	5.4931310e-004	5.5875171e-004
5.3596821e-004	4.9367929e-004	5.1624280e-004	4.8386420e-004
4.6303050e-004	4.7577299e-004	4.7598512e-004	4.6528200e-004
4.7821492e-004	4.8143891e-004	5.0162063e-004	5.2596335e-004
5.1740666e-004	5.3824855e-004	4.3220937e-004	4.1836124e-004
5.4508247e-004	5.2560828e-004	5.5180171e-004	5.3111579e-004
5.5959661e-004	5.1635649e-004	6.0699538e-004	6.3321447e-004
5.5309077e-004	5.2558872e-004	5.3549281e-004	5.4485725e-004
5.2020313e-004	5.1329225e-004	5.0706758e-004	4.9165534e-004
4.8645301e-004	4.6979151e-004	4.5304707e-004	5.0363065e-004
4.8486153e-004	4.5299152e-004	5.0537491e-004	4.6348709e-004
5.3446807e-004	4.8763415e-004	4.9875459e-004	5.7955636e-004
5.1421690e-004	5.4178959e-004	5.9377876e-004	6.1709824e-004
5.1137844e-004	5.7761648e-004	5.0054949e-004	4.8004889e-004
4.9104676e-004	4.9357869e-004	5.0393363e-004	5.1299951e-004
5.0289600e-004	4.5049399e-004	4.9234319e-004	4.5684005e-004
4.6898579e-004	4.5400569e-004	4.7741435e-004	4.4807292e-004
5.3604301e-004	5.3069775e-004	5.6155131e-004	6.7155116e-004
6.1336828e-004	5.9717658e-004	6.0961919e-004	5.8786345e-004
5.2223836e-004	6.1728620e-004	5.6896612e-004	5.2689973e-004
5.2640756e-004	5.9696812e-004	5.6382657e-004	5.2600732e-004
5.9314602e-004	5.1498341e-004	4.8400100e-004	5.4120277e-004
5.0122504e-004	5.1138625e-004	5.8080975e-004	5.3538555e-004
4.8624245e-004	4.4100736e-004	5.2356371e-004	5.1806310e-004
5.6346272e-004	5.1663741e-004	5.2587499e-004	4.9536557e-004
5.6074411e-004	5.1017847e-004	4.7685478e-004	5.3383297e-004
5.4122476e-004	5.6639919e-004	5.8362794e-004	6.3673141e-004
5.6754916e-004	5.7468763e-004	5.9217447e-004	5.8996030e-004
6.4441870e-004	6.4880653e-004	6.1586484e-004	6.8369692e-004
4.9810953e-004	5.1518108e-004	5.4325449e-004	5.4383234e-004
5.3309098e-004	4.4907787e-004	5.5666467e-004	5.4222462e-004
5.3810365e-004	4.8498360e-004	5.3576154e-004	5.4559112e-004
6.0692488e-004	5.6732376e-004	5.7357430e-004	6.6905755e-004
6.3292988e-004	6.6332730e-004	5.8998751e-004	6.8393047e-004
6.6200944e-004	6.8231180e-004	7.2850620e-004	6.3378700e-004
4.8234711e-004	5.3228712e-004	5.3294144e-004	5.9749627e-004
5.7686240e-004	4.5740550e-004	5.1759741e-004	5.2027678e-004
5.0656732e-004	5.0752973e-004	4.9846196e-004	4.9399795e-004
4.2776325e-004	4.4171230e-004	4.7786118e-004	4.6785602e-004
4.7384419e-004	4.7016671e-004	4.4968597e-004	5.0177619e-004
4.9391576e-004	5.2890994e-004	4.0111688e-004	4.0248296e-004
5.4840899e-004	5.3381483e-004	5.8332045e-004	6.9961122e-004
6.1723504e-004	6.1553632e-004	6.3547745e-004	6.0015255e-004
5.6226752e-004	6.1095812e-004	5.9147447e-004	5.4964430e-004
5.5834962e-004	6.3268245e-004	5.7129962e-004	5.4466015e-004
5.8235068e-004	5.4067712e-004	5.0387525e-004	5.5796288e-004
5.4119572e-004	5.2503402e-004	5.7621810e-004	5.2964468e-004

3.8670396e-004	3.5178013e-004	4.1738649e-004	4.1901117e-004
4.5804480e-004	4.1097614e-004	4.2107507e-004	3.9917696e-004
4.4227647e-004	4.2952673e-004	4.0743143e-004	4.3768340e-004
4.3698194e-004	4.7917698e-004	4.8796745e-004	5.1704401e-004
4.5057845e-004	5.0787946e-004	5.1439584e-004	4.8920650e-004
5.1204071e-004	5.2668741e-004	5.3623498e-004	5.9354783e-004
5.0646978e-004	5.3163251e-004	5.4575385e-004	5.5013793e-004
5.1841373e-004	4.4694429e-004	5.6880618e-004	5.2943868e-004
5.0993691e-004	4.8309044e-004	5.0243336e-004	5.1387701e-004
5.6517216e-004	5.6700505e-004	5.7477412e-004	6.6080387e-004
6.4210304e-004	6.3961391e-004	5.7120791e-004	6.4882735e-004
6.4481632e-004	6.5449826e-004	7.0172752e-004	6.4465409e-004
7.0131588e-004	6.1477433e-004	6.1337922e-004	6.5221198e-004
5.8638927e-004	5.6007545e-004	5.7843160e-004	6.6694360e-004
5.6585978e-004	5.6235251e-004	5.9131844e-004	5.5414550e-004
5.9332739e-004	5.5835074e-004	6.6212101e-004	5.6730639e-004
6.0525423e-004	5.6738980e-004	5.8510467e-004	6.5298428e-004
5.0836490e-004	5.6856150e-004	5.3973720e-004	6.3293525e-004
4.9300106e-004	5.4011355e-004	5.2913232e-004	6.1123959e-004
5.6760601e-004	4.6535750e-004	5.1065195e-004	5.0181509e-004
4.8998693e-004	4.9896103e-004	4.8129076e-004	5.0980102e-004
4.2689804e-004	4.1989504e-004	4.8046934e-004	4.7494945e-004
4.6348062e-004	4.5734229e-004	4.4053448e-004	5.0049695e-004
4.8534722e-004	5.3452970e-004	3.9448537e-004	3.9742886e-004
4.8652137e-004	5.0221718e-004	4.8609289e-004	5.6713598e-004
5.1960042e-004	4.9837487e-004	5.1010372e-004	5.1501520e-004
4.9952207e-004	4.4384874e-004	4.6964797e-004	4.0309913e-004
4.6344766e-004	4.5318266e-004	4.3842720e-004	4.5661880e-004
3.8686465e-004	4.3004497e-004	4.6834331e-004	5.1226378e-004
4.1921114e-004	4.3127823e-004	4.4409640e-004	3.8267465e-004
4.7995122e-004	4.7102801e-004	5.0499137e-004	4.7865076e-004
4.7669653e-004	4.3070834e-004	5.1385388e-004	5.2173428e-004
4.0525591e-004	4.1420096e-004	4.0586506e-004	3.9595985e-004
4.2499814e-004	4.3361852e-004	4.0640731e-004	4.3112473e-004
3.9297339e-004	3.8722313e-004	4.0916738e-004	4.3288217e-004
4.1078369e-004	3.7731755e-004	3.6659240e-004	3.9615593e-004
5.6606899e-004	5.8400410e-004	6.8056591e-004	6.0061782e-004
6.1302990e-004	6.3432880e-004	6.5001649e-004	6.7817695e-004
6.4643093e-004	6.3894122e-004	6.8409008e-004	6.8075373e-004
7.1843321e-004	6.6258735e-004	6.0605034e-004	6.2977604e-004
6.6080927e-004	5.9329916e-004	7.4143955e-004	6.3743298e-004
6.4495379e-004	6.2901355e-004	6.3469967e-004	5.6885056e-004
4.7124399e-004	4.4936137e-004	4.9825701e-004	4.8201109e-004
5.1125992e-004	4.5275398e-004	5.0164563e-004	4.8843891e-004
5.0093562e-004	4.9491807e-004	4.8880922e-004	5.0035208e-004
5.1661219e-004	5.5407762e-004	5.6098316e-004	5.7660391e-004
5.3941863e-004	6.0191809e-004	6.3008266e-004	5.8139440e-004
5.5629137e-004	6.0743265e-004	6.7004761e-004	6.3580935e-004
5.3748261e-004	5.5684919e-004	5.6953050e-004	6.0168239e-004
5.3599185e-004	4.7738115e-004	5.5805067e-004	5.1619699e-004
5.1306070e-004	5.0242755e-004	4.9923822e-004	5.0802687e-004
5.0887131e-004	5.4638533e-004	5.7373825e-004	6.2862606e-004

6.4417576e-004	5.8617283e-004	5.4231135e-004	5.9180004e-004
6.8094766e-004	6.1628036e-004	6.3841183e-004	6.4217512e-004
5.0077560e-004	5.1570823e-004	5.4102203e-004	5.9219345e-004
5.4280390e-004	4.7896225e-004	4.9444012e-004	4.7553077e-004
4.4926189e-004	4.5842879e-004	4.5598455e-004	5.0523869e-004
4.3435166e-004	3.9022080e-004	4.8811970e-004	4.6933933e-004
4.2774116e-004	4.4905583e-004	4.1915700e-004	4.7356594e-004
4.3764688e-004	5.0542466e-004	4.0527346e-004	3.9773135e-004
4.8639973e-004	4.9716862e-004	5.2683013e-004	5.5744400e-004
5.3109968e-004	4.6492948e-004	5.2789100e-004	5.0627604e-004
4.7984003e-004	4.2607481e-004	4.3764577e-004	4.3848663e-004
4.5374397e-004	4.9208428e-004	4.4185635e-004	3.9901278e-004
3.9908163e-004	4.7309250e-004	4.5521147e-004	4.7725155e-004
3.9840233e-004	4.3277520e-004	4.2766608e-004	3.7558356e-004
4.4543447e-004	4.5757751e-004	4.7532789e-004	4.5211607e-004
4.3377495e-004	3.8997688e-004	4.7566442e-004	4.7788088e-004
3.5862386e-004	3.8430796e-004	3.7290635e-004	3.5893760e-004
3.7167069e-004	3.8540576e-004	3.5685181e-004	3.9509241e-004
3.5883689e-004	3.4536084e-004	3.8822572e-004	3.9578153e-004
3.7553666e-004	3.3945586e-004	3.2241688e-004	3.5618745e-004
5.7160847e-004	5.9759886e-004	6.6138928e-004	6.2832686e-004
6.1237732e-004	6.6907694e-004	6.5988029e-004	7.0227463e-004
6.4799259e-004	6.4175173e-004	7.0998611e-004	7.1028690e-004
6.9824055e-004	6.8004568e-004	5.9438568e-004	6.3326927e-004
6.4278721e-004	6.4460734e-004	7.9156914e-004	6.8893033e-004
6.8291953e-004	6.4284509e-004	6.5415718e-004	6.2309127e-004
4.2863169e-004	4.0543229e-004	4.4968703e-004	4.3010927e-004
4.5379123e-004	3.7538131e-004	4.3458038e-004	4.1884240e-004
4.4237641e-004	4.2650625e-004	4.0264140e-004	4.3490565e-004
4.5431578e-004	4.7822898e-004	4.7928726e-004	4.9059200e-004
4.9491240e-004	5.3467108e-004	5.6455682e-004	5.0447926e-004
4.9611412e-004	5.4106436e-004	6.0824292e-004	5.5017421e-004
5.5814144e-004	5.9496687e-004	5.7546647e-004	6.2097341e-004
5.4979079e-004	5.0601955e-004	5.7032710e-004	5.5171442e-004
5.4415561e-004	5.3043177e-004	5.1082223e-004	5.1759418e-004
4.9747358e-004	5.2992301e-004	5.9692220e-004	6.2022678e-004
6.5011339e-004	5.7322712e-004	5.5381170e-004	5.9592038e-004
7.0734421e-004	6.1851500e-004	6.1524395e-004	6.3791360e-004
5.0707514e-004	5.0869089e-004	5.5361948e-004	5.7011111e-004
5.1788092e-004	4.9531004e-004	4.9534746e-004	4.6164421e-004
4.5375301e-004	4.4603482e-004	4.4317787e-004	5.0683331e-004
4.4549090e-004	3.9680429e-004	4.8123639e-004	4.8153879e-004
4.1349117e-004	4.3723763e-004	4.1543878e-004	4.7207621e-004
4.2427510e-004	4.8763422e-004	4.0292355e-004	3.9941989e-004
4.6141660e-004	4.7104259e-004	5.1108398e-004	5.2342909e-004
5.0819021e-004	4.2279910e-004	5.0281042e-004	4.7807214e-004
4.5712322e-004	4.0723823e-004	4.2520552e-004	4.3629915e-004
4.2937793e-004	4.7517866e-004	4.2560297e-004	3.8987811e-004
3.8774491e-004	4.3028872e-004	4.2832242e-004	4.4453337e-004
3.8122565e-004	3.9939005e-004	3.8863944e-004	3.5283741e-004
4.4552677e-004	4.3614904e-004	4.5555091e-004	4.5735721e-004
4.0626269e-004	4.2138407e-004	4.6623498e-004	4.1689076e-004

3.9874262e-004	3.7305780e-004	3.9068216e-004	3.6196536e-004
3.7110494e-004	3.6723927e-004	3.8397132e-004	3.9024831e-004
3.1061941e-004	3.2714788e-004	3.9245692e-004	4.1323066e-004
3.3136597e-004	3.5176059e-004	3.5663840e-004	3.2558131e-004
4.2394946e-004	4.3509069e-004	4.4807489e-004	4.2369268e-004
3.9205594e-004	3.5315048e-004	4.3297684e-004	4.3354466e-004
3.4087072e-004	3.5798245e-004	3.5545662e-004	3.4415854e-004
3.3751509e-004	3.5103523e-004	3.3136438e-004	3.6316427e-004
3.2854461e-004	3.2975135e-004	3.5748409e-004	3.6952107e-004
3.4316958e-004	3.0123381e-004	3.0288992e-004	3.1652196e-004
5.8911137e-004	5.9425132e-004	6.6443956e-004	6.5990479e-004
6.2247558e-004	6.6747784e-004	6.6106237e-004	7.1825918e-004
6.5693843e-004	6.5422598e-004	6.9764994e-004	7.1774711e-004
6.8879247e-004	6.9931953e-004	5.9747887e-004	6.4716428e-004
6.5636837e-004	6.7162233e-004	7.8663761e-004	7.0450573e-004
6.7378138e-004	6.3456343e-004	6.5714067e-004	6.5076923e-004
3.9181761e-004	3.9748844e-004	4.3335407e-004	4.0503929e-004
4.2810103e-004	3.6505196e-004	4.1761411e-004	4.0162399e-004
4.2942107e-004	3.9920641e-004	3.7582096e-004	4.1156298e-004
4.3911066e-004	4.4446327e-004	4.1177222e-004	4.2515624e-004
4.8056470e-004	5.0540168e-004	5.3690928e-004	4.5108285e-004
5.0406457e-004	5.1539378e-004	5.5763389e-004	5.4333128e-004
5.7787140e-004	5.9770868e-004	5.7381955e-004	5.9985106e-004
5.4295683e-004	5.1882550e-004	5.5719161e-004	5.7675516e-004
5.6787913e-004	5.1841925e-004	4.9410319e-004	5.2726890e-004
5.0712310e-004	5.1999424e-004	6.1083976e-004	5.9593694e-004
6.5659660e-004	5.8508670e-004	5.8140232e-004	6.2604394e-004
6.6737749e-004	6.4204205e-004	6.1236900e-004	6.0382712e-004
4.4612863e-004	4.5280787e-004	5.0197759e-004	5.0862463e-004
5.1353256e-004	4.2768809e-004	4.9243837e-004	4.3997062e-004
4.2431820e-004	3.9986015e-004	4.1994303e-004	4.5134282e-004
4.1464817e-004	4.3554798e-004	4.1409284e-004	4.1324906e-004
3.8494714e-004	4.0715377e-004	4.0462625e-004	4.2375051e-004
3.8571310e-004	3.7569382e-004	3.5427380e-004	3.5945328e-004
4.4971262e-004	4.2831727e-004	4.6874395e-004	4.3905427e-004
3.9799084e-004	4.0516186e-004	4.5642334e-004	4.2522469e-004
4.1640205e-004	3.7075301e-004	3.7292988e-004	3.6538561e-004
3.6666681e-004	3.7188710e-004	3.9175792e-004	3.5607582e-004
3.0518008e-004	3.1038313e-004	3.8265794e-004	3.7690187e-004
3.2510734e-004	3.5084964e-004	3.3046459e-004	3.3221253e-004
4.1919546e-004	4.3476595e-004	4.5988447e-004	4.7784750e-004
4.6501971e-004	4.4383945e-004	4.6109705e-004	4.8553079e-004
4.4725895e-004	4.4967410e-004	4.4934770e-004	4.5901801e-004
4.9283732e-004	4.7896514e-004	4.4353331e-004	4.5342653e-004
5.2272121e-004	5.1801767e-004	5.9105255e-004	5.1239927e-004
5.4833625e-004	5.2899784e-004	5.7776004e-004	5.8101763e-004
5.6702145e-004	5.6203677e-004	5.6822813e-004	5.8250198e-004
5.3793228e-004	5.3888706e-004	5.4848226e-004	5.8620602e-004
5.9853590e-004	4.9443874e-004	4.8733431e-004	5.4418622e-004
5.5977090e-004	5.2723811e-004	5.9839149e-004	5.6593269e-004
6.5823949e-004	6.0611077e-004	6.0799766e-004	6.3234796e-004
6.2525674e-004	6.8359716e-004	6.1278774e-004	5.7074544e-004

4.4715282e-004	4.3761809e-004	5.1122366e-004	4.9320713e-004
5.0314438e-004	4.6285900e-004	4.8410342e-004	4.2209426e-004
4.1524427e-004	4.0945931e-004	3.9924459e-004	4.5775253e-004
4.0242048e-004	3.9595961e-004	4.0947898e-004	4.2720544e-004
3.7017914e-004	3.8326987e-004	3.9820626e-004	4.1743834e-004
3.9610858e-004	3.7153543e-004	3.4577509e-004	3.6718336e-004
4.4158395e-004	4.4161934e-004	4.6684959e-004	4.4738055e-004
4.0878860e-004	3.8007653e-004	4.2804866e-004	4.2374413e-004
4.2431492e-004	3.7465453e-004	3.8015471e-004	3.6886331e-004
3.6585736e-004	3.9682440e-004	3.8466552e-004	3.5230182e-004
3.1020295e-004	3.2294699e-004	3.5958522e-004	3.4009755e-004
3.3985648e-004	3.4786272e-004	3.1908446e-004	3.3754758e-004
5.5588463e-004	5.1397595e-004	5.6525340e-004	5.4859519e-004
5.2280886e-004	5.6488892e-004	5.4701199e-004	5.8719853e-004
5.8824779e-004	4.9085762e-004	5.1275715e-004	5.6226407e-004
6.1270417e-004	5.2788551e-004	5.5586122e-004	5.5158629e-004
6.6415500e-004	6.2351869e-004	5.9443721e-004	6.1326676e-004
6.0768299e-004	7.1692430e-004	6.3689638e-004	5.7217815e-004
6.4613331e-004	6.4508460e-004	6.6802588e-004	7.2275435e-004
6.6298664e-004	6.8433896e-004	7.0733619e-004	7.1030355e-004
6.9502828e-004	6.6424842e-004	6.0965012e-004	6.3372949e-004
6.3255822e-004	6.7953453e-004	6.1200000e-004	6.7716504e-004
6.2402821e-004	6.1145805e-004	5.7866602e-004	5.4310927e-004
5.4855498e-004	5.9817212e-004	5.9863047e-004	5.9892423e-004
4.6505684e-004	3.9864928e-004	4.8542317e-004	4.3498779e-004
4.3166670e-004	5.0633216e-004	4.5380856e-004	4.9426304e-004
4.7281753e-004	4.2906064e-004	4.6579468e-004	4.8218671e-004
5.3754664e-004	4.4903012e-004	4.2892183e-004	4.8069729e-004
5.5837094e-004	5.3086693e-004	4.6509242e-004	5.2948999e-004
5.1157077e-004	6.1311950e-004	5.7196885e-004	4.6778859e-004
4.0956533e-004	3.7339083e-004	3.5867816e-004	4.2970814e-004
3.7231510e-004	3.6617064e-004	3.5299144e-004	3.3901937e-004
3.3939457e-004	3.1637103e-004	3.6860273e-004	3.0556721e-004
2.9661289e-004	3.1358923e-004	2.6653277e-004	2.9661126e-004
2.9748018e-004	2.8927136e-004	2.9289962e-004	2.8777084e-004
2.8381241e-004	2.7885305e-004	3.1558338e-004	2.8413927e-004
6.2224291e-004	6.4786653e-004	6.5141970e-004	7.2157242e-004
6.8015872e-004	6.9665044e-004	6.9110650e-004	6.8564119e-004
7.0151185e-004	6.7262042e-004	6.2722688e-004	6.3481263e-004
6.1678370e-004	6.7607703e-004	6.4104055e-004	6.7237458e-004
6.2048626e-004	5.8477854e-004	5.8809867e-004	5.7193376e-004
5.4915313e-004	5.8892240e-004	5.7711510e-004	6.0980680e-004
6.2291694e-004	5.7432969e-004	6.3503510e-004	7.1391124e-004
6.7917457e-004	7.0823517e-004	7.0158417e-004	7.1290285e-004
6.6743130e-004	7.1296882e-004	6.9755131e-004	6.5037116e-004
6.4694734e-004	6.6336525e-004	6.1893383e-004	6.8842660e-004
6.7402524e-004	6.0487835e-004	6.1424183e-004	5.8388816e-004
5.9316661e-004	5.9169283e-004	6.0114241e-004	6.1126454e-004
4.6532555e-004	4.2533929e-004	4.9183510e-004	4.8718669e-004
4.6729139e-004	5.3332560e-004	4.5387676e-004	5.0768298e-004
4.9058846e-004	4.7580736e-004	4.7770987e-004	5.3433640e-004
5.5194605e-004	4.8598625e-004	4.6519849e-004	5.4638600e-004

5.9798865e-004	5.5236482e-004	4.8726114e-004	5.8543269e-004
5.2759344e-004	5.9572668e-004	6.1156048e-004	4.7944194e-004
4.0934196e-004	3.8649150e-004	3.4889443e-004	4.1876556e-004
3.6096592e-004	3.7034394e-004	3.3079552e-004	3.2551528e-004
3.2019600e-004	3.0513993e-004	3.5078388e-004	3.0618023e-004
2.8304263e-004	3.0786897e-004	2.7557682e-004	2.7266293e-004
2.8355206e-004	2.7282955e-004	3.0090152e-004	2.6973166e-004
2.7682798e-004	2.6717811e-004	3.1182549e-004	2.7608474e-004
6.2769995e-004	6.5981425e-004	6.5565594e-004	7.1380314e-004
6.8816126e-004	6.9432995e-004	6.8603718e-004	6.7859492e-004
6.9273783e-004	6.8171252e-004	6.4283764e-004	6.2264405e-004
6.1293774e-004	6.6887432e-004	6.4777886e-004	6.6301200e-004
6.1788120e-004	5.7797501e-004	5.8117500e-004	5.9271371e-004
5.4616979e-004	5.7820645e-004	5.7565997e-004	6.0188495e-004
6.2996619e-004	6.3308174e-004	6.7695399e-004	7.5473747e-004
7.1610746e-004	7.3801224e-004	7.4210211e-004	7.2470240e-004
7.1232658e-004	7.3812618e-004	7.3289943e-004	6.7222849e-004
6.6854950e-004	6.7666297e-004	6.2420550e-004	7.0188528e-004
6.8688230e-004	6.1602844e-004	6.4512853e-004	5.9939871e-004
5.9862147e-004	6.0878615e-004	6.0121127e-004	6.2121778e-004
4.1124164e-004	3.6610961e-004	3.8365190e-004	3.8960706e-004
3.7817314e-004	3.6110855e-004	3.3963726e-004	3.6180568e-004
3.6824736e-004	2.7686455e-004	3.3290254e-004	3.0688750e-004
2.8757167e-004	3.1038556e-004	3.0031498e-004	2.9720129e-004
2.7913877e-004	2.7361828e-004	2.8259366e-004	2.8888535e-004
2.8494571e-004	3.0262717e-004	2.7021516e-004	2.6261898e-004
4.0757706e-004	3.9384660e-004	3.5245347e-004	4.0836096e-004
3.5360151e-004	3.6779237e-004	3.3132667e-004	3.1590518e-004
3.0497946e-004	3.0390247e-004	3.3395493e-004	3.1019367e-004
2.7293146e-004	3.1041158e-004	2.9427736e-004	2.6339816e-004
2.7963693e-004	2.6494155e-004	3.0954029e-004	2.6470199e-004
2.7489486e-004	2.5436063e-004	3.0522704e-004	2.6998741e-004
6.2828828e-004	6.5519723e-004	6.5606674e-004	7.0151748e-004
6.8750029e-004	6.7647362e-004	6.7486991e-004	6.7781085e-004
6.6883270e-004	6.7744641e-004	6.5402029e-004	6.0664654e-004
6.0270053e-004	6.4086552e-004	6.4176072e-004	6.3865868e-004
6.0485458e-004	5.8062734e-004	5.7693257e-004	6.0373904e-004
5.3741837e-004	5.7382815e-004	5.7421843e-004	5.7768804e-004
6.3600525e-004	6.7683734e-004	7.0858726e-004	8.0112075e-004
7.5544966e-004	7.6156827e-004	7.8193778e-004	7.4391559e-004
7.5561317e-004	7.4474774e-004	7.4917046e-004	6.8382658e-004
6.8058119e-004	6.9374326e-004	6.3506134e-004	6.9572056e-004
6.9304176e-004	6.2458288e-004	6.7917249e-004	6.2241995e-004
6.0592429e-004	6.2892273e-004	6.0366259e-004	6.3955502e-004
4.9713977e-004	6.0025544e-004	6.2207684e-004	6.5542133e-004
6.1883347e-004	6.6373138e-004	6.2738387e-004	6.6360891e-004
5.9344280e-004	6.5473028e-004	6.1960921e-004	6.1985147e-004
6.3162245e-004	6.1620468e-004	6.6982965e-004	6.1729790e-004
6.2993930e-004	6.8939236e-004	6.2349845e-004	7.1260342e-004
7.0416696e-004	6.7203645e-004	7.0193350e-004	6.8210198e-004
4.1375015e-004	3.7492786e-004	3.9978030e-004	4.0639092e-004
3.7491123e-004	3.6657351e-004	3.3166408e-004	3.4925740e-004

3.6178109e-004	2.8693011e-004	3.1561981e-004	3.0555011e-004
2.9714721e-004	3.1599443e-004	3.0072747e-004	2.9292807e-004
2.7876470e-004	2.6186638e-004	2.7885258e-004	2.9134475e-004
2.8230632e-004	2.8924863e-004	2.7008446e-004	2.5728574e-004
4.0553141e-004	3.9037258e-004	3.6601369e-004	4.0284009e-004
3.5602661e-004	3.6775357e-004	3.5258749e-004	3.1162864e-004
3.0239248e-004	3.1365060e-004	3.2253789e-004	3.1466710e-004
2.7015231e-004	3.1847272e-004	3.1324739e-004	2.7593661e-004
2.7846592e-004	2.6910882e-004	3.1479882e-004	2.7251605e-004
2.8093410e-004	2.4791198e-004	2.9936641e-004	2.6874648e-004
6.3747614e-004	6.9295823e-004	7.0956965e-004	8.2430014e-004
7.8624586e-004	7.7279980e-004	7.9696058e-004	7.6223664e-004
7.7429890e-004	7.2755011e-004	7.4278883e-004	6.7996036e-004
6.8224332e-004	7.1308830e-004	6.5312517e-004	6.7694926e-004
6.8760234e-004	6.1549133e-004	6.9724928e-004	6.3934317e-004
6.1606208e-004	6.3983630e-004	6.0648515e-004	6.5655369e-004
4.8426354e-004	5.4522758e-004	5.5039779e-004	6.1205431e-004
5.7813950e-004	6.0297034e-004	5.7019602e-004	5.9078481e-004
5.6850503e-004	5.7107907e-004	5.4977679e-004	5.8138405e-004
5.3987157e-004	5.2087801e-004	6.3236667e-004	5.6435959e-004
5.7086913e-004	6.2862803e-004	5.6533783e-004	6.5301522e-004
6.2657554e-004	6.5214452e-004	6.5495326e-004	6.5358855e-004
5.1263590e-004	6.1794088e-004	6.9455312e-004	6.4883057e-004
6.1282687e-004	6.3611419e-004	6.8818679e-004	6.6898191e-004
5.8741322e-004	6.8532467e-004	6.5600623e-004	6.4241729e-004
6.7492059e-004	6.8781634e-004	7.1776098e-004	6.7430768e-004
6.6471529e-004	6.8928346e-004	7.2409595e-004	6.7306963e-004
6.5091599e-004	6.5759751e-004	7.4081523e-004	6.6107836e-004
6.2665479e-004	6.9714284e-004	6.4979769e-004	7.0415535e-004
7.5418710e-004	6.8385110e-004	7.2116072e-004	7.5464588e-004
6.3291100e-004	6.7563911e-004	7.1392173e-004	6.4139995e-004
6.6420792e-004	7.1834253e-004	6.8483163e-004	5.7760997e-004
6.3274780e-004	6.3118344e-004	6.0806677e-004	6.6474744e-004
6.0596825e-004	6.2965327e-004	6.0686114e-004	6.2748160e-004
4.4115475e-004	4.1222701e-004	4.3587722e-004	4.4481326e-004
3.8886492e-004	3.9214567e-004	3.5609347e-004	3.5863454e-004
3.7753709e-004	3.2462805e-004	3.3051509e-004	3.2089312e-004
3.3806844e-004	3.4466766e-004	3.1986822e-004	3.0285210e-004
3.0926909e-004	2.8067449e-004	2.9736232e-004	3.2621339e-004
3.1413951e-004	3.0997339e-004	3.0598297e-004	2.7794914e-004
3.3391669e-004	3.6664295e-004	3.7895728e-004	4.1055180e-004
3.8651428e-004	3.3984793e-004	3.1794306e-004	3.1537490e-004
3.2379329e-004	2.6588381e-004	2.7159243e-004	2.9396435e-004
2.6775734e-004	3.0305857e-004	2.8791260e-004	3.1475956e-004
2.6362461e-004	2.4815785e-004	2.7709507e-004	2.5738664e-004
2.4663324e-004	2.3562687e-004	2.6830279e-004	2.6455902e-004
6.5279315e-004	6.9519972e-004	7.0910132e-004	7.9018109e-004
7.9568147e-004	7.6034392e-004	7.5018610e-004	7.8010703e-004
7.3360125e-004	6.8327916e-004	7.1855845e-004	6.5391106e-004
6.8379429e-004	7.0511838e-004	6.6918899e-004	6.3878779e-004
6.4925532e-004	5.9985623e-004	6.9131104e-004	6.4318982e-004
6.0422270e-004	6.3045883e-004	5.7714277e-004	6.1583267e-004

5.0034292e-004	5.9290024e-004	5.4427009e-004	5.9648417e-004
5.9411328e-004	4.9455089e-004	5.9228965e-004	5.0846488e-004
5.4432362e-004	5.1740071e-004	5.1459602e-004	5.1107970e-004
5.1443776e-004	5.0483918e-004	5.6343044e-004	5.5307271e-004
5.6481951e-004	5.1362043e-004	5.5660709e-004	5.1395018e-004
6.2477855e-004	5.8400576e-004	6.6710458e-004	5.9644844e-004
4.8542685e-004	5.3431245e-004	5.3937483e-004	5.9583859e-004
5.5727060e-004	5.8103171e-004	5.4981188e-004	5.6419129e-004
5.6257211e-004	5.5101777e-004	5.3464591e-004	5.6568954e-004
5.0346605e-004	5.0998184e-004	6.0910195e-004	5.5201161e-004
5.6545979e-004	6.1516720e-004	5.6283840e-004	6.4095273e-004
6.1172742e-004	6.7067498e-004	6.6050772e-004	6.4417682e-004
5.2431019e-004	6.0851137e-004	6.8241481e-004	6.3012459e-004
5.9556479e-004	6.3015927e-004	6.7257824e-004	6.7486799e-004
6.1329037e-004	6.5267904e-004	6.5953353e-004	6.4354013e-004
6.8051732e-004	6.7203138e-004	7.2931567e-004	6.7396143e-004
6.7631766e-004	6.9314346e-004	7.1787891e-004	6.6769631e-004
6.5470739e-004	6.5551880e-004	7.2653568e-004	6.5603838e-004
5.1892696e-004	5.3507541e-004	5.6243553e-004	5.8335825e-004
6.1668601e-004	5.9235581e-004	6.5661996e-004	6.4733822e-004
6.0977536e-004	6.5274554e-004	6.0501376e-004	6.2808381e-004
6.8993276e-004	6.8839293e-004	7.1584049e-004	6.3163454e-004
6.6035749e-004	6.3807545e-004	6.6178161e-004	6.7121124e-004
6.3934078e-004	6.6627986e-004	6.6387119e-004	6.4106884e-004
6.1137146e-004	7.0189137e-004	6.4112469e-004	7.2667933e-004
7.7944050e-004	6.7658460e-004	7.3653818e-004	7.5725917e-004
6.5708620e-004	6.7306470e-004	7.1707388e-004	6.4150579e-004
6.7379950e-004	7.2732798e-004	6.7514637e-004	6.0275047e-004
6.2295419e-004	6.4019975e-004	5.9860184e-004	6.7761196e-004
6.2117167e-004	6.1485385e-004	6.0619896e-004	6.3124778e-004
6.2682008e-004	6.9665798e-004	7.1552716e-004	7.1359111e-004
7.5459341e-004	6.5352296e-004	7.1932035e-004	6.4916539e-004
7.0014584e-004	6.8274337e-004	6.6408078e-004	7.0918097e-004
6.4747690e-004	6.9697991e-004	6.4589571e-004	6.4418984e-004
6.1910768e-004	5.9677694e-004	6.4623168e-004	6.8044741e-004
5.7697196e-004	6.2959471e-004	5.6428181e-004	6.7576169e-004
4.9744317e-004	5.2825961e-004	5.4672203e-004	5.8059336e-004
4.9687332e-004	4.8496026e-004	5.3299865e-004	5.0724598e-004
4.4324775e-004	4.5594863e-004	4.4083773e-004	4.6138931e-004
4.5529544e-004	4.5318536e-004	4.6289118e-004	4.3619973e-004
4.5164111e-004	3.9366828e-004	4.4062581e-004	4.4751936e-004
4.3271536e-004	4.6455588e-004	4.0994081e-004	4.3890805e-004
4.5872260e-004	4.3311080e-004	4.7282502e-004	4.7399633e-004
4.0734007e-004	4.0610016e-004	3.6469172e-004	3.7026293e-004
3.8361490e-004	3.4181675e-004	3.2817743e-004	3.2141471e-004
3.4831600e-004	3.5928672e-004	3.2414424e-004	3.0669360e-004
3.2481691e-004	2.9555400e-004	3.0937417e-004	3.3354897e-004
3.3015101e-004	3.1807069e-004	3.1700894e-004	2.9237274e-004
3.4536879e-004	3.9843335e-004	3.8822654e-004	4.2978506e-004
3.9409421e-004	3.4816991e-004	3.3059733e-004	3.1730958e-004
3.2606592e-004	2.7142816e-004	2.7903118e-004	3.0101088e-004
2.8342154e-004	3.2731827e-004	2.9406040e-004	3.2095063e-004

2.6946814e-004	2.6667977e-004	2.8899329e-004	2.6904706e-004
2.5718383e-004	2.5205380e-004	2.8888286e-004	2.8392632e-004
6.4663387e-004	6.9178584e-004	6.8063215e-004	7.6767501e-004
7.8974116e-004	7.3651480e-004	7.2543597e-004	7.6399629e-004
7.0942908e-004	6.6042622e-004	6.9991907e-004	6.5528160e-004
6.8081294e-004	7.1517796e-004	6.7172770e-004	6.2708600e-004
6.4750170e-004	5.8861174e-004	6.9566713e-004	6.4190407e-004
6.0494659e-004	6.2952620e-004	5.8291943e-004	5.9522614e-004
4.9305722e-004	5.6279259e-004	5.5631220e-004	6.0239225e-004
6.0343622e-004	4.8854860e-004	5.9418404e-004	5.0748803e-004
5.5477960e-004	5.0545123e-004	5.2822925e-004	5.1010762e-004
5.2522335e-004	5.1007080e-004	5.8434754e-004	5.3937477e-004
5.5832467e-004	5.3883861e-004	5.5970874e-004	5.2684973e-004
6.4484510e-004	5.9748215e-004	6.7288379e-004	6.2958340e-004
4.9037328e-004	5.3502816e-004	5.5012424e-004	5.8890278e-004
5.4675987e-004	5.6958682e-004	5.3211699e-004	5.5396010e-004
5.6733988e-004	5.4300969e-004	5.3508779e-004	5.6292849e-004
4.8700443e-004	5.1910982e-004	5.8297919e-004	5.4875379e-004
5.6449806e-004	6.0563099e-004	5.6540944e-004	6.4750714e-004
6.1467847e-004	6.9765307e-004	6.6953426e-004	6.3528749e-004
5.3510332e-004	5.4975752e-004	5.6045031e-004	5.8921819e-004
6.2951898e-004	5.9389608e-004	6.6056501e-004	6.2830855e-004
6.0441293e-004	6.7369379e-004	6.1787781e-004	6.4226297e-004
6.8432077e-004	6.9267341e-004	7.2301872e-004	6.3640349e-004
6.7263510e-004	6.7242289e-004	6.8034965e-004	6.9839030e-004
6.4352053e-004	7.0886382e-004	6.6905530e-004	6.6163347e-004
5.9083910e-004	6.9136145e-004	6.4526233e-004	7.4191866e-004
7.9364477e-004	6.8174041e-004	7.5572924e-004	7.5524754e-004
6.8852803e-004	6.7313747e-004	7.0903852e-004	6.4460390e-004
6.8162070e-004	7.2457551e-004	6.7147170e-004	6.3915175e-004
6.2467079e-004	6.4513198e-004	5.9051301e-004	6.9049851e-004
6.4258827e-004	6.0506382e-004	6.1531733e-004	6.3029202e-004
6.3218312e-004	7.1427402e-004	7.3312703e-004	7.4163312e-004
7.8497037e-004	6.5989245e-004	7.2377358e-004	6.7499807e-004
7.1193751e-004	6.9056860e-004	6.8704663e-004	7.3215374e-004
6.7311959e-004	7.0754735e-004	6.7483351e-004	6.6769155e-004
6.3689570e-004	6.2140606e-004	6.4356330e-004	6.9174284e-004
5.8283798e-004	6.3742626e-004	5.8106579e-004	6.7522028e-004
4.9539899e-004	5.4219285e-004	5.8371526e-004	6.0383268e-004
5.1575717e-004	5.1033718e-004	5.6070281e-004	5.5243242e-004
4.8366391e-004	4.9146634e-004	4.6309295e-004	4.9611975e-004
4.8833023e-004	4.8622667e-004	5.0052295e-004	4.6164288e-004
4.8420257e-004	4.2690188e-004	4.6668353e-004	4.7209052e-004
4.7468003e-004	4.9959579e-004	4.1961473e-004	4.6639646e-004
4.7590017e-004	4.5737863e-004	5.1099032e-004	5.0296581e-004
4.3057818e-004	4.1948548e-004	3.8128268e-004	3.9073665e-004
4.0019184e-004	3.6797755e-004	3.4385768e-004	3.2931705e-004
3.5838175e-004	3.8704156e-004	3.3004933e-004	3.2225295e-004
3.5029365e-004	3.1962061e-004	3.2591317e-004	3.4360562e-004
3.4621992e-004	3.4088474e-004	3.3330639e-004	3.2247960e-004
3.7241151e-004	4.1916114e-004	4.0687713e-004	4.4581016e-004
4.0466953e-004	3.5613833e-004	3.5816449e-004	3.2915472e-004

3.3563109e-004	2.9455154e-004	2.9787809e-004	3.1298000e-004
2.9837501e-004	3.5099776e-004	3.0413541e-004	3.3009212e-004
2.8020101e-004	2.9195865e-004	3.0825352e-004	2.9151977e-004
2.7691831e-004	2.8723666e-004	3.1605346e-004	3.1239835e-004
6.4069238e-004	6.9226725e-004	6.4911519e-004	7.3398878e-004
7.5860039e-004	7.1203466e-004	6.9667953e-004	7.2485575e-004
6.8709647e-004	6.4449471e-004	6.8139150e-004	6.5810348e-004
6.7785358e-004	7.0109851e-004	6.6131834e-004	6.1336417e-004
6.5500893e-004	5.8928071e-004	6.9515650e-004	6.2333612e-004
5.9988769e-004	6.1359772e-004	5.8298810e-004	5.7257056e-004
5.4138237e-004	5.5606668e-004	5.3846465e-004	5.1491209e-004
5.0610376e-004	4.8240756e-004	4.8295896e-004	4.8306616e-004
4.4343062e-004	4.4677559e-004	5.2795938e-004	4.6056771e-004
3.8819161e-004	4.4595932e-004	4.9554726e-004	4.2175366e-004
5.0266709e-004	4.2239790e-004	4.6768817e-004	4.6112284e-004
5.0188739e-004	4.0574049e-004	5.0185969e-004	5.1163603e-004
4.9653406e-004	5.2653893e-004	5.5366650e-004	5.9226732e-004
6.0135339e-004	4.8085784e-004	5.7905313e-004	5.0305518e-004
5.5677741e-004	4.8858800e-004	5.4943205e-004	5.1671197e-004
5.3113348e-004	5.0880843e-004	5.8349581e-004	5.1081937e-004
5.3568868e-004	5.4219525e-004	5.4559045e-004	5.2484053e-004
6.3692383e-004	6.0518977e-004	6.6759186e-004	6.3398895e-004
4.9108332e-004	5.3886487e-004	5.6550982e-004	5.8751536e-004
5.4182301e-004	5.5426704e-004	5.1361804e-004	5.5252726e-004
5.7368274e-004	5.4661695e-004	5.4091318e-004	5.6321724e-004
4.8320823e-004	5.2580878e-004	5.5092703e-004	5.5105083e-004
5.5997967e-004	5.8794174e-004	5.7119962e-004	6.5506261e-004
6.1641245e-004	7.1641790e-004	6.7358707e-004	6.2948056e-004
5.6622429e-004	5.5501040e-004	6.4091358e-004	6.1906116e-004
5.9216091e-004	6.3419608e-004	6.2301090e-004	6.7277362e-004
6.6849944e-004	5.9147153e-004	6.4203650e-004	6.2714498e-004
6.7368888e-004	6.4279385e-004	7.3859551e-004	6.7754605e-004
6.7187384e-004	6.5933794e-004	7.3296984e-004	6.8866802e-004
6.9712778e-004	6.7157669e-004	7.0566667e-004	6.9588880e-004
5.5839930e-004	5.5182372e-004	5.6393462e-004	5.9471646e-004
6.3199057e-004	5.9625761e-004	6.5509834e-004	6.1217713e-004
5.9697035e-004	6.7169098e-004	6.2578553e-004	6.4303131e-004
6.5406536e-004	6.7048655e-004	7.1114625e-004	6.4452345e-004
6.6810899e-004	6.8668228e-004	6.8606186e-004	7.2327210e-004
6.4012459e-004	7.3187603e-004	6.6238605e-004	6.6373493e-004
6.1517590e-004	5.6704723e-004	5.9134269e-004	6.6236577e-004
6.9077389e-004	6.3607300e-004	6.8362673e-004	6.8222718e-004
6.8556992e-004	7.3365218e-004	6.9682377e-004	7.0973270e-004
6.6134725e-004	7.2500003e-004	7.4319360e-004	7.0351183e-004
7.1757182e-004	7.0028630e-004	7.0497143e-004	7.3619072e-004
6.8377196e-004	6.7685889e-004	6.9179287e-004	6.8818382e-004
5.8271774e-004	6.7023898e-004	6.6328820e-004	7.5560009e-004
7.9870019e-004	7.0286675e-004	7.7478499e-004	7.5533029e-004
7.2290360e-004	6.8263766e-004	6.9666865e-004	6.4784488e-004
6.8649060e-004	7.2794565e-004	6.8168594e-004	6.8094270e-004
6.4253586e-004	6.5064442e-004	5.8831906e-004	7.0043778e-004
6.6231815e-004	6.0547267e-004	6.3236905e-004	6.3105414e-004

6.2434169e-004	7.3143910e-004	7.4412524e-004	7.7736021e-004
8.0143621e-004	6.7295904e-004	7.1522636e-004	7.0726810e-004
7.2867003e-004	6.9351637e-004	6.9873476e-004	7.3245622e-004
6.9435476e-004	7.0911286e-004	7.0363707e-004	6.9298952e-004
6.6644938e-004	6.5400638e-004	6.4490525e-004	6.9740479e-004
5.9954452e-004	6.4017797e-004	6.1290734e-004	6.6109440e-004
4.9271656e-004	4.8561060e-004	5.4506068e-004	5.3289361e-004
4.5778502e-004	4.3306928e-004	4.0926948e-004	4.1371577e-004
4.3217425e-004	4.0495655e-004	3.7875734e-004	3.5764402e-004
3.7743679e-004	4.2522683e-004	3.4927511e-004	3.5177451e-004
3.8223917e-004	3.5535335e-004	3.4943952e-004	3.6876286e-004
3.6201550e-004	3.8146503e-004	3.5882074e-004	3.6489180e-004
4.1307762e-004	4.2984723e-004	4.3576126e-004	4.5750863e-004
4.1805054e-004	3.7016952e-004	4.0023935e-004	3.5795914e-004
3.5280253e-004	3.3385745e-004	3.3194132e-004	3.3805633e-004
3.1876714e-004	3.7624396e-004	3.2468746e-004	3.5111897e-004
2.9948146e-004	3.2690248e-004	3.3507763e-004	3.2858830e-004
3.0468290e-004	3.3056636e-004	3.4917505e-004	3.4566575e-004
6.3740709e-004	6.8383076e-004	6.1966783e-004	6.9845677e-004
7.2595953e-004	7.0060439e-004	6.7555279e-004	6.7754135e-004
6.7006877e-004	6.3274792e-004	6.6310595e-004	6.6313798e-004
6.7794863e-004	6.6668108e-004	6.4088831e-004	5.9703886e-004
6.6465154e-004	5.9661345e-004	6.8259011e-004	5.9748437e-004
5.9142814e-004	5.9067195e-004	5.7331969e-004	5.6375822e-004
5.2633388e-004	5.2478582e-004	4.9882091e-004	4.6400102e-004
4.7640695e-004	4.2074437e-004	4.5033002e-004	4.2355611e-004
3.8332401e-004	3.9536773e-004	4.7222045e-004	4.2677746e-004
3.4913689e-004	3.9235483e-004	4.3875539e-004	3.5290914e-004
4.5044981e-004	3.7980334e-004	3.9874473e-004	4.1715601e-004
4.2742529e-004	3.6232832e-004	4.4344626e-004	4.2578546e-004
5.1423113e-004	5.0402129e-004	5.4080921e-004	5.7330690e-004
5.9344264e-004	4.8254439e-004	5.4891393e-004	4.8436897e-004
5.4566287e-004	4.8528450e-004	5.6369102e-004	5.1869841e-004
5.1882616e-004	5.1098044e-004	5.5809163e-004	4.7774278e-004
5.0907879e-004	5.1451773e-004	5.1979133e-004	5.0559981e-004
6.0691843e-004	6.0013388e-004	6.4675973e-004	6.0768105e-004
4.8969836e-004	5.3276417e-004	5.6006567e-004	5.8490879e-004
5.3104074e-004	5.3035884e-004	4.9203850e-004	5.4219646e-004
5.6648506e-004	5.5084705e-004	5.3679307e-004	5.5242618e-004
4.8110353e-004	5.1497412e-004	5.1561231e-004	5.5477317e-004
5.5185889e-004	5.6016148e-004	5.7853833e-004	6.4801612e-004
6.0477608e-004	7.0721772e-004	6.6342832e-004	6.2054186e-004
5.6651513e-004	5.3337644e-004	6.1644608e-004	6.1425699e-004
6.0249845e-004	6.1455341e-004	5.9662407e-004	6.4421653e-004
6.6870551e-004	5.6490318e-004	6.3797394e-004	6.1026288e-004
6.4181456e-004	6.2701984e-004	7.2703428e-004	6.8076490e-004
6.5009083e-004	6.4615157e-004	7.3530120e-004	6.9882302e-004
7.1838236e-004	6.8002728e-004	7.0191183e-004	7.1644432e-004
5.7501085e-004	5.4962504e-004	5.7216229e-004	5.9023395e-004
6.2603231e-004	6.0225288e-004	6.4261317e-004	6.0130584e-004
5.9849702e-004	6.4905635e-004	6.3027731e-004	6.3332824e-004
6.1453794e-004	6.3211891e-004	6.8772986e-004	6.5554376e-004

6.5361582e-004	6.7372278e-004	6.7873427e-004	7.3668301e-004
6.3559618e-004	7.2684289e-004	6.4938144e-004	6.5093978e-004
5.8756335e-004	6.4913231e-004	6.8518304e-004	7.6800281e-004
7.9860333e-004	7.2978428e-004	7.7903150e-004	7.5387472e-004
7.4909411e-004	6.9508654e-004	6.8327616e-004	6.4892283e-004
6.8777395e-004	7.3896439e-004	6.9348588e-004	7.1271486e-004
6.5721417e-004	6.4980550e-004	5.9170306e-004	7.0108725e-004
6.6813007e-004	6.1123834e-004	6.4726213e-004	6.3592350e-004
6.1550702e-004	7.4710064e-004	7.4878507e-004	8.1205952e-004
8.0915554e-004	6.9446707e-004	7.1172534e-004	7.3323768e-004
7.5246349e-004	6.9437257e-004	7.0664559e-004	7.2481819e-004
7.1361910e-004	7.1852143e-004	7.2585524e-004	7.1554688e-004
6.9631984e-004	6.8390275e-004	6.5875790e-004	7.0369450e-004
6.2294319e-004	6.5029877e-004	6.5487290e-004	6.5556879e-004
5.1397088e-004	5.1881861e-004	5.7337600e-004	5.6558297e-004
4.9073157e-004	4.5541477e-004	4.4751565e-004	4.4296135e-004
4.7949789e-004	4.4664876e-004	4.2470899e-004	4.1007358e-004
4.1128844e-004	4.6769404e-004	3.8980450e-004	3.9401421e-004
4.1672419e-004	4.0474954e-004	3.8532354e-004	4.1403488e-004
3.8946939e-004	4.3737760e-004	3.9454403e-004	4.1554773e-004
6.4348233e-004	6.6126852e-004	6.0167174e-004	6.7570742e-004
7.1648702e-004	7.0641711e-004	6.6693939e-004	6.3885844e-004
6.6155536e-004	6.2424672e-004	6.4125944e-004	6.7469703e-004
6.8136456e-004	6.3793297e-004	6.1862025e-004	5.8589345e-004
6.6754948e-004	6.0729575e-004	6.5642536e-004	5.7831212e-004
5.8669139e-004	5.7457114e-004	5.6097614e-004	5.7423621e-004
5.9974367e-004	5.6170265e-004	5.4071406e-004	5.2505801e-004
5.5161765e-004	4.4180181e-004	4.7710088e-004	4.0342919e-004
3.9967930e-004	4.4212664e-004	5.0862314e-004	4.8379645e-004
3.9741115e-004	4.2545982e-004	4.5678754e-004	3.4748550e-004
4.6684855e-004	4.2139832e-004	4.1576068e-004	4.3115107e-004
4.3782873e-004	4.2639441e-004	4.6379869e-004	4.3528861e-004
4.8967675e-004	4.8035477e-004	5.0386804e-004	5.2815652e-004
5.5631185e-004	4.9647109e-004	5.1219866e-004	4.7283620e-004
5.5329534e-004	5.0090470e-004	5.3289405e-004	5.0206573e-004
4.9813250e-004	5.2730764e-004	5.1953174e-004	4.7579209e-004
5.0994608e-004	4.9263991e-004	5.3203734e-004	5.2738900e-004
5.8772623e-004	6.0932618e-004	6.5115649e-004	5.9883295e-004
5.1710507e-004	5.2133294e-004	5.4578565e-004	5.8303538e-004
5.2034710e-004	5.2148948e-004	4.7590679e-004	5.2903408e-004
5.5083867e-004	5.3277904e-004	5.2548651e-004	5.1935317e-004
4.8069888e-004	5.0068188e-004	5.1405111e-004	5.7592757e-004
5.4460725e-004	5.5370375e-004	5.9204764e-004	6.2509220e-004
6.0432128e-004	6.5499829e-004	6.1208007e-004	6.0777755e-004
5.7673328e-004	5.4369295e-004	6.0181503e-004	6.0529631e-004
6.1993764e-004	5.8206709e-004	5.8375158e-004	6.2385059e-004
6.5136720e-004	5.6279983e-004	6.3925631e-004	6.0157664e-004
6.0872894e-004	6.1401821e-004	7.2994317e-004	6.8044083e-004
6.2876068e-004	6.4058857e-004	7.0672117e-004	6.9872297e-004
7.0402199e-004	6.8777347e-004	6.8257653e-004	7.0314416e-004
6.0753817e-004	6.8076437e-004	7.5175524e-004	8.2349774e-004
8.2934715e-004	7.6458367e-004	7.7178425e-004	7.7375327e-004

7.7895528e-004	7.0552518e-004	6.7377734e-004	6.7616929e-004
7.1486109e-004	7.4601121e-004	7.0827850e-004	7.1991555e-004
6.4769687e-004	6.6487226e-004	6.2518423e-004	6.9233931e-004
6.5410696e-004	6.2090546e-004	6.5499893e-004	6.4490025e-004
5.2483527e-004	5.2856571e-004	5.7710064e-004	5.6862710e-004
4.9817442e-004	4.6520005e-004	4.7202913e-004	4.4553608e-004
4.8765324e-004	4.4786560e-004	4.6139769e-004	4.4346425e-004
4.2230439e-004	4.8901563e-004	4.3464954e-004	4.3700003e-004
4.3027900e-004	4.4688522e-004	4.0484665e-004	4.3464429e-004
4.0738389e-004	4.5984189e-004	4.1502446e-004	4.3462774e-004
6.3726530e-004	7.0910840e-004	6.5099052e-004	7.4076913e-004
7.8662914e-004	7.0400091e-004	7.1278468e-004	7.0533276e-004
6.7288402e-004	6.6605480e-004	6.4713343e-004	6.5913211e-004
6.6962335e-004	6.5114375e-004	6.4764067e-004	6.3029841e-004
6.7284008e-004	6.1187145e-004	6.0390400e-004	6.4925182e-004
6.4145729e-004	5.8299908e-004	6.5424379e-004	6.2154538e-004
6.0453407e-004	5.6192948e-004	5.2780635e-004	5.8250903e-004
6.1010540e-004	6.2751141e-004	5.7189974e-004	5.1161596e-004
5.6026065e-004	5.3123278e-004	5.3200132e-004	6.1837817e-004
5.8901632e-004	5.5342264e-004	5.1859641e-004	4.9084685e-004
5.8317109e-004	5.4582838e-004	5.6745743e-004	4.6249649e-004
4.9395418e-004	4.9023750e-004	4.6882910e-004	5.0503061e-004
5.3265951e-004	4.7932662e-004	4.7567609e-004	4.7471688e-004
5.2721054e-004	3.7792002e-004	4.0834710e-004	3.2169649e-004
3.5182909e-004	4.3226481e-004	4.5794238e-004	4.0737184e-004
3.5402472e-004	3.7762612e-004	3.9135651e-004	3.0920897e-004
3.9380137e-004	3.6399914e-004	3.5166724e-004	3.7088167e-004
3.9498564e-004	4.1732237e-004	3.9447254e-004	3.9266027e-004
4.9032306e-004	4.7989311e-004	4.5527656e-004	5.0278970e-004
4.9732343e-004	5.0243040e-004	4.7185968e-004	4.4806453e-004
5.1794172e-004	4.8854545e-004	4.6300833e-004	4.7594035e-004
4.7360507e-004	5.0719892e-004	4.8134903e-004	4.9420996e-004
5.3739444e-004	4.6620531e-004	5.5530060e-004	5.5472782e-004
5.6761818e-004	5.6264129e-004	6.3068375e-004	5.8349910e-004
6.2639544e-004	7.2467684e-004	7.9035845e-004	8.4713756e-004
8.4615184e-004	7.6546420e-004	7.6935382e-004	7.8188571e-004
7.8507316e-004	6.9050834e-004	7.1102091e-004	7.1937147e-004
7.2704427e-004	7.4620180e-004	7.2422897e-004	7.2496504e-004
6.6776188e-004	6.7967786e-004	6.8572137e-004	6.9279961e-004
6.5588845e-004	6.4922604e-004	6.9077067e-004	6.8061291e-004
6.9481198e-004	5.9791045e-004	5.8667302e-004	6.2505696e-004
6.9000777e-004	5.9312153e-004	5.7720474e-004	4.5904473e-004
5.1713378e-004	5.9706985e-004	5.5376830e-004	5.9366976e-004
5.6070366e-004	5.6320088e-004	5.1387306e-004	4.9756490e-004
5.8109443e-004	5.4447465e-004	5.3451306e-004	4.7518947e-004
5.2960624e-004	5.4327885e-004	4.8354097e-004	5.4356631e-004
6.2532970e-004	7.1471803e-004	7.9754634e-004	8.1948981e-004
8.4399803e-004	7.7036369e-004	7.9929276e-004	7.8040539e-004
7.7082041e-004	6.9408427e-004	7.4075375e-004	7.4628717e-004
7.2739988e-004	7.4849433e-004	7.2438573e-004	7.3052014e-004
6.7209637e-004	6.6976856e-004	7.0932784e-004	6.9688571e-004
6.7126715e-004	6.6808418e-004	7.0107145e-004	7.0767423e-004

5.7922442e-004	4.9672721e-004	4.7177153e-004	4.9825312e-004
5.8622825e-004	4.7077125e-004	4.7269021e-004	3.7374887e-004
3.8912689e-004	4.9745083e-004	4.5664726e-004	4.7693679e-004
4.5263137e-004	4.6848637e-004	4.0130335e-004	4.4248131e-004
4.6530829e-004	4.6992755e-004	4.2435012e-004	3.9773796e-004
4.5078372e-004	4.5042457e-004	3.8451908e-004	4.4679688e-004
6.3438607e-004	5.6458669e-004	5.4968618e-004	5.7475506e-004
5.8276216e-004	5.2060348e-004	5.4744606e-004	4.5067626e-004
4.6606741e-004	5.0714800e-004	4.3177376e-004	4.4552608e-004
4.7560572e-004	4.8304052e-004	4.7123221e-004	4.7357847e-004
5.4022171e-004	4.3733185e-004	5.2497564e-004	4.8898759e-004
5.3228997e-004	4.7077321e-004	5.0490589e-004	5.4282835e-004
6.0238865e-004	6.6376136e-004	7.6328812e-004	7.6353579e-004
8.2009863e-004	7.7247369e-004	8.2903534e-004	7.7949044e-004
7.5496896e-004	7.2511380e-004	7.5623745e-004	7.5149329e-004
7.3532094e-004	7.4145023e-004	7.2353886e-004	7.4006360e-004
6.8575288e-004	6.6801799e-004	7.1844949e-004	6.9362705e-004
6.8825044e-004	6.8019511e-004	6.8936552e-004	7.1285275e-004
6.2002390e-004	6.0844602e-004	6.7789126e-004	7.4142350e-004
7.2466569e-004	6.4476582e-004	6.9200201e-004	7.3289695e-004
7.0454269e-004	6.9386059e-004	6.6425897e-004	6.1740658e-004
6.4187040e-004	5.9070336e-004	6.2117758e-004	6.0438903e-004
6.3894695e-004	5.8376600e-004	6.3276147e-004	6.6100211e-004
6.2297555e-004	6.1252930e-004	6.7028470e-004	6.1665065e-004
6.4558763e-004	5.5211761e-004	5.5738034e-004	5.7728089e-004
6.6461260e-004	5.2232283e-004	5.4489567e-004	4.8137747e-004
4.4763007e-004	5.6508274e-004	5.3902116e-004	5.2235767e-004
5.1091745e-004	5.4445085e-004	4.6781768e-004	5.4688361e-004
5.4291338e-004	5.3955762e-004	4.9060422e-004	4.8228933e-004
5.4871167e-004	5.3150085e-004	4.7675721e-004	5.2263919e-004
7.0284944e-004	6.3417149e-004	6.2578816e-004	6.7708654e-004
6.3065058e-004	5.5198319e-004	6.2067773e-004	5.0802505e-004
4.9469726e-004	5.2595102e-004	4.5993584e-004	4.8730173e-004
5.1649923e-004	4.8179208e-004	5.0450045e-004	4.8648236e-004
5.3226040e-004	4.6241401e-004	5.3019499e-004	4.9558056e-004
5.3384108e-004	4.8596146e-004	4.9877857e-004	5.3667430e-004
6.0954038e-004	6.4365577e-004	7.2989348e-004	7.3359355e-004
7.7819474e-004	7.5605010e-004	8.4748086e-004	7.5864168e-004
7.4599951e-004	7.4659527e-004	7.5589627e-004	7.5321652e-004
7.3275898e-004	7.2197564e-004	7.3284455e-004	7.3989257e-004
7.0366779e-004	6.9059306e-004	7.3513611e-004	7.0351931e-004
7.0029697e-004	7.0648626e-004	6.8211414e-004	7.1073888e-004
5.8103644e-004	6.1576602e-004	6.5038621e-004	7.3103847e-004
6.8477578e-004	6.7399179e-004	7.4511602e-004	7.6996289e-004
7.6051484e-004	7.0638910e-004	6.8664916e-004	7.2709056e-004
7.1168684e-004	7.0436197e-004	6.8081419e-004	6.9538657e-004
6.6714035e-004	6.8675516e-004	6.9531123e-004	6.7513902e-004
6.7932042e-004	6.5267472e-004	6.5828170e-004	6.3258949e-004
6.3398093e-004	6.0116671e-004	6.8279372e-004	7.5968758e-004
7.3589249e-004	6.5657614e-004	6.8427056e-004	7.1019902e-004
6.9749528e-004	6.8335731e-004	6.5863255e-004	6.0303765e-004
6.4015145e-004	5.7415600e-004	6.0180589e-004	5.9042369e-004

6.3562109e-004	5.9171673e-004	6.3266771e-004	6.6009373e-004
6.1992148e-004	6.1974680e-004	6.6563355e-004	6.2024093e-004
6.4445185e-004	5.9616692e-004	5.9800252e-004	6.4152924e-004
6.6735226e-004	5.6433606e-004	6.0893098e-004	5.2504498e-004
5.1367898e-004	5.6252048e-004	5.7791820e-004	5.3967620e-004
5.5821165e-004	5.3690038e-004	5.1407199e-004	5.6382805e-004
5.6965042e-004	5.4142447e-004	5.1752726e-004	5.3497848e-004
5.4128466e-004	5.5085609e-004	5.0064801e-004	5.3480263e-004
5.7584836e-004	5.6409863e-004	5.0838420e-004	5.4961628e-004
5.0966372e-004	5.3936383e-004	5.0006277e-004	4.3111771e-004
4.7867667e-004	4.3865813e-004	4.3513050e-004	4.3085305e-004
4.6917289e-004	4.6052555e-004	4.8501191e-004	3.8529270e-004
4.6534411e-004	4.5518306e-004	4.5779785e-004	5.0458930e-004
4.4377582e-004	4.9959996e-004	4.5364431e-004	4.2383888e-004
6.2628268e-004	6.3157575e-004	7.0289410e-004	7.3220879e-004
7.5485045e-004	7.1922269e-004	8.4851847e-004	7.3237944e-004
7.2750711e-004	7.2789597e-004	7.3197456e-004	7.3222206e-004
7.0943750e-004	6.7298529e-004	7.3544223e-004	7.0746806e-004
6.8590439e-004	6.8842118e-004	7.1615280e-004	7.1136729e-004
6.9975006e-004	7.0271127e-004	6.5369460e-004	6.9146632e-004
6.4731551e-004	5.9469939e-004	6.9005325e-004	7.6254731e-004
7.3409105e-004	6.7870080e-004	6.6176655e-004	6.6743563e-004
6.6511396e-004	6.7919453e-004	6.3284178e-004	5.8712474e-004
6.1495023e-004	5.7326033e-004	5.9221088e-004	5.6919239e-004
6.0478732e-004	5.8349398e-004	6.2178030e-004	6.2164853e-004
6.0727670e-004	6.1124824e-004	6.3318717e-004	5.8919463e-004
7.0634841e-004	7.1917544e-004	7.0511258e-004	7.5791181e-004
6.2983434e-004	6.6033071e-004	6.9250763e-004	6.1430390e-004
6.1866531e-004	5.9895225e-004	6.1492028e-004	6.1469107e-004
6.0626846e-004	5.1139484e-004	6.0681959e-004	5.6318616e-004
5.6381942e-004	5.4292884e-004	5.6929455e-004	5.9952696e-004
5.7204293e-004	5.7836169e-004	5.6921251e-004	5.7284853e-004
6.2550372e-004	6.2266302e-004	6.8159131e-004	7.7590777e-004
7.4872619e-004	6.9072876e-004	8.2950783e-004	7.2976977e-004
6.9462287e-004	6.6517136e-004	7.0740901e-004	7.1324775e-004
6.8081570e-004	6.3765913e-004	7.3849453e-004	6.8601764e-004
6.7243607e-004	6.5400921e-004	6.9386479e-004	6.9749427e-004
6.8237595e-004	6.7936984e-004	6.2122531e-004	6.9052106e-004
6.0218791e-004	6.1146103e-004	6.7171401e-004	7.4176237e-004
6.8312944e-004	7.0963633e-004	7.7519957e-004	7.2995167e-004
7.2612552e-004	6.5144424e-004	7.0470330e-004	6.7192460e-004
6.9285436e-004	7.0151855e-004	6.6195297e-004	7.2436925e-004
6.5532043e-004	6.2037910e-004	6.6157893e-004	7.1842941e-004
6.4079728e-004	6.8095306e-004	6.5896136e-004	6.1148996e-004
5.5965278e-004	6.0008229e-004	6.3370376e-004	6.9292269e-004
6.8690050e-004	6.6757094e-004	7.1100498e-004	6.7174126e-004
6.1944734e-004	6.2145629e-004	6.0514502e-004	6.0844710e-004
6.1272281e-004	6.1433791e-004	5.5916740e-004	5.9008627e-004
5.9096960e-004	6.1111180e-004	5.6453780e-004	6.0982288e-004
5.8813438e-004	5.6830252e-004	5.7345344e-004	5.6406425e-004
6.2564545e-004	5.8473658e-004	6.5359524e-004	7.2366689e-004
6.9751896e-004	6.2973473e-004	6.6170213e-004	6.2759467e-004

5.9690868e-004	5.7964669e-004	6.1676733e-004	5.5386074e-004
5.9290139e-004	5.2322293e-004	5.2858019e-004	5.5679875e-004
6.0539380e-004	5.6966835e-004	5.9870198e-004	6.1500707e-004
5.7541435e-004	5.5392277e-004	5.8815035e-004	5.9423862e-004
6.7216496e-004	6.5000693e-004	7.1738352e-004	7.4406964e-004
7.4330541e-004	7.3571409e-004	6.9660138e-004	7.0269968e-004
7.0420316e-004	7.5789682e-004	5.9741426e-004	6.2952649e-004
6.4196128e-004	6.4991208e-004	6.5085085e-004	6.3910167e-004
6.1348212e-004	6.2736479e-004	6.5648851e-004	6.3315290e-004
6.1711225e-004	6.0151919e-004	6.4300279e-004	5.7881454e-004
6.7751588e-004	7.0256343e-004	6.6992817e-004	6.8879424e-004
6.0916074e-004	6.3693360e-004	6.5482584e-004	6.4107947e-004
6.2546377e-004	6.2173391e-004	6.0901853e-004	6.2634671e-004
5.8059172e-004	5.4983294e-004	6.1715250e-004	5.5805500e-004
5.5607004e-004	5.6331810e-004	5.8690702e-004	5.7332221e-004
5.8210287e-004	5.6721423e-004	5.8373067e-004	5.8166709e-004
5.8350860e-004	5.8426047e-004	5.7936685e-004	5.8264508e-004
5.4846641e-004	4.9369928e-004	4.9763021e-004	5.0459462e-004
4.7548329e-004	4.7934895e-004	4.8324336e-004	5.1523355e-004
4.6905337e-004	4.7127584e-004	4.9718340e-004	4.1005276e-004
5.0077579e-004	4.6928467e-004	4.5061081e-004	5.2574302e-004
4.8403936e-004	5.0053251e-004	4.5100812e-004	4.5374886e-004
6.1439585e-004	5.7401316e-004	6.1418059e-004	6.1174665e-004
6.0419540e-004	5.5534615e-004	6.0144498e-004	5.5659056e-004
5.4117462e-004	5.0019907e-004	5.0424189e-004	4.6951088e-004
4.9261803e-004	5.1192482e-004	5.9559933e-004	5.0505024e-004
5.5807635e-004	5.2965870e-004	5.4733318e-004	5.5181551e-004
5.1151793e-004	5.4770747e-004	5.4321189e-004	4.7638777e-004
6.2829841e-004	6.2628170e-004	6.7388307e-004	7.9335664e-004
7.3418803e-004	7.0176118e-004	8.1373255e-004	7.3383664e-004
6.9659147e-004	6.6968540e-004	6.8447304e-004	7.2092055e-004
6.7280242e-004	6.3976337e-004	7.2507077e-004	6.9528773e-004
6.6175974e-004	6.5471786e-004	6.8487805e-004	6.9221301e-004
6.8466784e-004	6.8789744e-004	6.3696582e-004	6.8146891e-004
6.0503211e-004	6.1153273e-004	6.7030532e-004	7.6827197e-004
7.0635577e-004	7.1661759e-004	7.8524515e-004	7.4221531e-004
7.3571506e-004	6.6678002e-004	7.1486745e-004	7.0750897e-004
7.0316815e-004	7.2978792e-004	6.7367611e-004	7.2997245e-004
6.7290678e-004	6.2792114e-004	6.8385039e-004	7.5550939e-004
6.5653950e-004	6.9902085e-004	6.7047495e-004	6.2893459e-004
5.7161567e-004	5.8600864e-004	6.3531945e-004	6.8751066e-004
6.9205422e-004	6.7017792e-004	7.1931118e-004	6.7406608e-004
6.3272723e-004	6.2703397e-004	6.1411504e-004	6.1044237e-004
6.0732852e-004	6.2438645e-004	5.6221723e-004	6.0202551e-004
5.8793231e-004	6.1438742e-004	5.6229399e-004	6.0094809e-004
6.0830936e-004	5.7459346e-004	5.7715773e-004	5.6354424e-004
6.2595203e-004	5.9033705e-004	6.5969188e-004	7.2120476e-004
6.8812656e-004	6.2953896e-004	6.7069416e-004	6.0415322e-004
5.7848648e-004	5.6440827e-004	6.2200139e-004	5.4931674e-004
5.6955194e-004	5.2587703e-004	5.2698841e-004	5.6054539e-004
6.0169556e-004	5.4465962e-004	5.7997054e-004	5.9266148e-004
5.6718953e-004	5.3549657e-004	5.8434674e-004	5.7858137e-004

6.4890595e-004	6.3466858e-004	6.9393760e-004	7.4385651e-004
6.7490064e-004	7.1947408e-004	6.9695413e-004	6.7302395e-004
6.4322533e-004	6.8248544e-004	5.5936044e-004	6.0608223e-004
6.1696518e-004	5.9080245e-004	5.7194625e-004	6.2168446e-004
5.7376920e-004	5.5342376e-004	5.9470157e-004	5.8904820e-004
5.6064551e-004	5.2081010e-004	5.9469753e-004	5.2996827e-004
8.2243203e-004	8.7891074e-004	7.9312926e-004	8.7834326e-004
7.9023134e-004	8.1987810e-004	7.4723853e-004	7.6062369e-004
7.4917266e-004	7.3775640e-004	6.9118431e-004	7.6459761e-004
6.8338058e-004	7.0868988e-004	7.0510562e-004	6.6280781e-004
6.3206494e-004	6.7515708e-004	6.4570690e-004	6.8538492e-004
6.6638649e-004	6.5178625e-004	6.5604017e-004	6.9579887e-004
6.9429551e-004	6.8892495e-004	6.4183172e-004	7.0708609e-004
6.5314166e-004	6.3004365e-004	6.2335657e-004	5.5927981e-004
5.5788849e-004	5.7285027e-004	5.3912723e-004	5.3128651e-004
5.1478905e-004	5.3129724e-004	5.4232505e-004	4.4544333e-004
4.9857193e-004	5.1397526e-004	4.4825784e-004	5.6098650e-004
4.8010471e-004	5.4967831e-004	4.5794425e-004	4.6683007e-004
6.1705535e-004	5.8358131e-004	6.2871675e-004	6.1000613e-004
5.9719200e-004	4.9716855e-004	5.5371755e-004	5.4379308e-004
5.1310069e-004	5.1140167e-004	5.4985183e-004	5.5995805e-004
5.1543280e-004	5.1110792e-004	4.7861804e-004	4.7516614e-004
5.0993050e-004	5.0879667e-004	5.0545709e-004	5.5901185e-004
5.4506962e-004	5.0467477e-004	4.6956751e-004	5.1656561e-004
6.0585448e-004	5.9609118e-004	6.3749515e-004	6.9307163e-004
6.4476288e-004	5.9924855e-004	6.7026942e-004	5.8464210e-004
5.5464590e-004	5.2731433e-004	5.5327758e-004	4.9390076e-004
4.8223369e-004	5.2315913e-004	5.4902679e-004	5.1841878e-004
5.2086023e-004	5.3015551e-004	5.3154466e-004	5.3914446e-004
5.1239001e-004	5.3233410e-004	5.4174883e-004	5.0146140e-004
5.8212850e-004	5.9742495e-004	6.5022908e-004	7.5718961e-004
7.1048435e-004	7.0836977e-004	7.5170811e-004	7.3801381e-004
7.0663670e-004	6.7522026e-004	7.2614467e-004	7.0034602e-004
7.0044416e-004	7.5380394e-004	7.0903965e-004	6.9801278e-004
6.7609716e-004	6.1590428e-004	7.5837683e-004	7.4678741e-004
6.3314996e-004	6.8061291e-004	7.0448498e-004	6.5801045e-004
6.0328588e-004	6.1175640e-004	6.7916967e-004	7.3412088e-004
7.1811452e-004	7.2184965e-004	7.7156442e-004	7.5410478e-004
7.5922233e-004	6.7778377e-004	7.0530000e-004	7.4205433e-004
7.1961779e-004	7.1042974e-004	6.6670749e-004	7.3044487e-004
7.0507538e-004	6.4029987e-004	6.8338661e-004	7.6428388e-004
6.9243339e-004	6.8460760e-004	6.3311000e-004	6.5564254e-004
5.5528803e-004	5.6842885e-004	6.1295861e-004	7.0617160e-004
7.2624568e-004	7.0010840e-004	7.3658187e-004	6.8046515e-004
6.7697161e-004	6.4753593e-004	6.3221178e-004	6.5136548e-004
6.1311472e-004	6.2080655e-004	5.9052301e-004	6.2293409e-004
5.5153226e-004	6.1764057e-004	5.7690410e-004	5.8395906e-004
6.0256204e-004	6.0587721e-004	5.7964551e-004	5.6271169e-004
7.2318216e-004	7.7796698e-004	7.2521629e-004	8.0005956e-004
7.3272190e-004	8.2488796e-004	7.2101388e-004	7.2001035e-004
6.9872809e-004	6.9136241e-004	6.3829825e-004	7.1922298e-004
6.7230433e-004	6.7990496e-004	6.6733332e-004	6.5903004e-004

6.3482559e-004	6.4809343e-004	6.5434349e-004	6.6257572e-004
6.2263534e-004	5.9739288e-004	6.3084484e-004	6.8170391e-004
6.4707345e-004	6.2440515e-004	6.3398058e-004	6.6380810e-004
6.2571809e-004	5.3705875e-004	5.8700776e-004	5.6592314e-004
5.2903430e-004	5.1217664e-004	5.5616611e-004	5.5920781e-004
5.2614869e-004	5.1850341e-004	5.0018560e-004	4.8405510e-004
5.1374795e-004	4.9246012e-004	5.0264019e-004	5.6243884e-004
5.2605498e-004	5.0058823e-004	4.7052393e-004	4.9703515e-004
6.1971635e-004	6.1179171e-004	6.3342610e-004	7.1315144e-004
6.6389788e-004	6.2779484e-004	6.8418324e-004	5.9893835e-004
5.6369332e-004	5.5485022e-004	5.7768614e-004	5.2013573e-004
4.9386729e-004	5.4266976e-004	5.4603373e-004	5.4186139e-004
5.2170604e-004	5.3018644e-004	5.2894394e-004	5.4973223e-004
5.4559822e-004	5.2536154e-004	5.3837748e-004	5.2041920e-004
5.8684283e-004	6.0059180e-004	6.6178404e-004	7.6942686e-004
7.2582890e-004	7.1255940e-004	7.4943545e-004	7.3056905e-004
6.8087906e-004	6.7473658e-004	7.1539212e-004	6.5304260e-004
6.9147666e-004	7.2751341e-004	6.9628085e-004	6.6836389e-004
6.8625351e-004	6.0149643e-004	7.5453076e-004	7.3731625e-004
6.1503668e-004	6.6947084e-004	6.9542995e-004	6.5892728e-004
5.9030599e-004	6.0198190e-004	6.6885169e-004	7.4980235e-004
7.2599680e-004	6.9513099e-004	7.7702387e-004	7.5335312e-004
7.5606452e-004	6.9373996e-004	7.0910575e-004	7.5687343e-004
7.0965788e-004	7.2127985e-004	6.7664123e-004	7.1844202e-004
6.9549512e-004	6.3534371e-004	6.9166317e-004	7.6590074e-004
6.9982366e-004	7.1250217e-004	6.6023311e-004	6.5747644e-004
5.3887682e-004	5.9480999e-004	6.1286100e-004	7.2102150e-004
7.3087156e-004	7.2150362e-004	7.2129992e-004	6.8541989e-004
6.9465986e-004	6.6190427e-004	6.5578219e-004	6.5754907e-004
6.3556297e-004	6.1577372e-004	6.2856344e-004	6.3406254e-004
5.7881974e-004	6.4106229e-004	6.0858901e-004	6.1849185e-004
6.1026613e-004	6.1834996e-004	6.0001176e-004	5.9154381e-004
6.6923566e-004	6.7586502e-004	7.3250653e-004	7.9853531e-004
6.8873205e-004	8.1216122e-004	6.7464334e-004	6.5700729e-004
6.1397679e-004	6.2393778e-004	5.8844216e-004	6.3850539e-004
6.0899297e-004	6.0719503e-004	5.8233692e-004	6.0529770e-004
5.8433958e-004	6.2270819e-004	5.8825176e-004	5.9318409e-004
5.4814966e-004	5.3094443e-004	5.8858911e-004	6.2133774e-004
8.2658949e-004	7.7589105e-004	8.1418597e-004	8.8826596e-004
8.7085327e-004	8.6694969e-004	7.9790533e-004	7.1525446e-004
7.4432585e-004	7.7069768e-004	7.2432963e-004	7.0370475e-004
7.0001346e-004	7.0206505e-004	7.1302806e-004	6.3726505e-004
6.2729862e-004	7.0706554e-004	6.3542220e-004	7.0722328e-004
6.1733949e-004	7.0809224e-004	5.9668787e-004	6.6552999e-004
6.7757032e-004	6.5322798e-004	6.4902708e-004	6.9513991e-004
6.6322292e-004	5.8657801e-004	6.4052150e-004	6.0428714e-004
5.6374677e-004	5.3459067e-004	5.8329826e-004	5.6279567e-004
5.5472393e-004	5.4184004e-004	5.2953484e-004	5.3272783e-004
5.2765997e-004	5.0293867e-004	5.3072466e-004	5.7975980e-004
5.4088239e-004	5.1599969e-004	5.0644891e-004	5.1233830e-004
6.5550042e-004	6.1982602e-004	6.3236149e-004	7.4201374e-004
6.7957817e-004	6.6008161e-004	7.0287018e-004	6.1742462e-004

5.7796542e-004	5.8564049e-004	5.9211475e-004	5.2859336e-004
5.0350977e-004	5.6272953e-004	5.7117647e-004	5.6837432e-004
5.4192000e-004	5.3027000e-004	5.3745888e-004	5.5603188e-004
5.7029871e-004	5.2849101e-004	5.5108311e-004	5.3231412e-004
6.3766219e-004	6.1936940e-004	5.9721626e-004	6.8662097e-004
6.5136066e-004	6.0861553e-004	6.1040225e-004	5.8430491e-004
6.0570863e-004	5.5712269e-004	5.7429295e-004	5.2602352e-004
5.3080059e-004	5.9003645e-004	6.0921891e-004	5.3876019e-004
5.8018978e-004	5.1914726e-004	5.4425980e-004	6.0083612e-004
5.1030426e-004	5.8370466e-004	6.1613241e-004	5.6533990e-004
5.9173674e-004	6.2062113e-004	6.9007213e-004	8.0854091e-004
7.6160915e-004	7.1244598e-004	7.7372113e-004	7.4196587e-004
6.8978235e-004	6.8690570e-004	7.2018160e-004	6.5466150e-004
6.8473764e-004	7.0646037e-004	6.9129281e-004	6.5538361e-004
6.8547044e-004	6.0079840e-004	7.3957194e-004	7.4202684e-004
6.3696763e-004	6.8246596e-004	6.9825230e-004	6.5520374e-004
5.8266423e-004	6.0905005e-004	6.6617804e-004	7.4683019e-004
7.2822966e-004	6.8839025e-004	7.8200395e-004	7.4709731e-004
7.5613268e-004	7.1876010e-004	7.2136910e-004	7.6376645e-004
7.1742889e-004	7.3205460e-004	7.0844077e-004	7.1268402e-004
6.9503042e-004	6.4177137e-004	7.0364130e-004	7.5629009e-004
7.1297036e-004	7.1902740e-004	6.7444495e-004	6.7238926e-004
5.7573763e-004	6.1351455e-004	6.4108458e-004	6.7512927e-004
7.1063296e-004	7.2976925e-004	7.8072463e-004	7.1619767e-004
7.2994993e-004	7.1867723e-004	7.1703694e-004	7.4083831e-004
7.0301224e-004	6.9668930e-004	7.2152601e-004	7.3552463e-004
6.9851437e-004	6.6401919e-004	7.0979173e-004	7.0942294e-004
6.5943901e-004	6.7041091e-004	6.7187737e-004	6.9470369e-004
5.4327177e-004	6.1075034e-004	6.3153885e-004	7.2266333e-004
7.3358344e-004	7.3839119e-004	7.3463014e-004	6.9688969e-004
7.0679028e-004	6.7334407e-004	6.5925963e-004	6.6136594e-004
6.4981995e-004	6.2185980e-004	6.4455278e-004	6.3505759e-004
5.9935925e-004	6.5525339e-004	6.2505393e-004	6.5317177e-004
6.1872887e-004	6.2248178e-004	6.0453208e-004	6.0249415e-004
5.7480978e-004	5.8723813e-004	6.1666159e-004	6.8113579e-004
7.0790402e-004	7.4106040e-004	7.0847396e-004	6.7457766e-004
6.6357045e-004	6.5726839e-004	6.6099767e-004	6.3348720e-004
6.2926269e-004	6.5807134e-004	6.4018308e-004	6.3857870e-004
6.1368161e-004	6.1508629e-004	5.7307244e-004	6.1174680e-004
5.9163768e-004	5.9566068e-004	6.0577849e-004	6.2239818e-004
7.7049328e-004	7.2410051e-004	8.2506124e-004	8.7475548e-004
8.4842846e-004	9.0902878e-004	7.5687272e-004	7.1236226e-004
7.2697071e-004	7.3182540e-004	6.9635484e-004	7.1127981e-004
6.9404608e-004	7.0112348e-004	6.7365027e-004	6.7117323e-004
6.4121200e-004	7.1428942e-004	6.7030636e-004	6.6764848e-004
6.2101197e-004	6.5209184e-004	6.4589700e-004	7.0064666e-004
7.4173257e-004	7.1984890e-004	7.3124304e-004	8.1074853e-004
8.1847947e-004	7.1115632e-004	7.6023498e-004	6.9558409e-004
6.9529674e-004	6.6709089e-004	7.0019464e-004	6.3262427e-004
6.5370090e-004	6.0223826e-004	6.5302172e-004	5.9265211e-004
5.9860207e-004	6.1468279e-004	6.2248940e-004	6.4592088e-004
5.9853304e-004	6.3040781e-004	5.5220049e-004	5.7876407e-004

7.1097213e-004	6.4114200e-004	6.2775766e-004	6.8849663e-004
6.7241876e-004	6.4399751e-004	6.7823727e-004	6.3309973e-004
5.7244643e-004	5.6170789e-004	5.9836150e-004	5.5363124e-004
5.4784011e-004	5.7995148e-004	5.7201511e-004	5.8163694e-004
5.6124854e-004	5.2135717e-004	5.5841095e-004	5.8132761e-004
6.0687502e-004	5.3618387e-004	5.6836427e-004	5.5702435e-004
6.5681667e-004	6.0503616e-004	6.2786807e-004	7.5613685e-004
6.8030463e-004	6.4765195e-004	6.8561913e-004	6.1482561e-004
6.2110252e-004	6.2368806e-004	6.1965210e-004	5.3890931e-004
5.2666113e-004	5.8749037e-004	6.0053765e-004	5.8266592e-004
5.6453714e-004	5.4796258e-004	5.4714210e-004	5.9515871e-004
5.2879570e-004	5.7002185e-004	5.9970112e-004	5.4930896e-004
6.7889379e-004	6.2626072e-004	6.8823688e-004	8.1008894e-004
7.7114849e-004	7.5698688e-004	7.6885635e-004	7.4129998e-004
7.0727976e-004	6.9397622e-004	6.7343253e-004	6.5508309e-004
6.5830341e-004	6.4782804e-004	6.3614947e-004	6.8808137e-004
6.4111134e-004	6.0330180e-004	6.5327893e-004	7.0439478e-004
6.4279120e-004	6.6174816e-004	6.5562388e-004	6.2052938e-004
5.8803220e-004	6.6367656e-004	7.2334736e-004	8.1581390e-004
7.6566114e-004	7.0495850e-004	8.2122942e-004	7.5788054e-004
7.3796550e-004	7.3918123e-004	7.7736218e-004	7.2013102e-004
6.9969110e-004	7.4284959e-004	7.4428656e-004	6.8017113e-004
6.9813582e-004	6.4038983e-004	7.3941857e-004	7.6901546e-004
7.0924459e-004	7.2739925e-004	7.1648917e-004	6.7317157e-004
5.7483566e-004	6.0861374e-004	6.4353678e-004	7.1382889e-004
7.3799620e-004	7.0966377e-004	7.8824250e-004	7.2780743e-004
7.7307735e-004	7.4359150e-004	7.3360647e-004	7.8956023e-004
7.1912030e-004	7.2434417e-004	7.4083253e-004	7.5084264e-004
7.1911403e-004	6.6918576e-004	7.2902975e-004	7.3807937e-004
7.0269886e-004	7.0355370e-004	6.8055920e-004	7.0185887e-004
5.8141837e-004	6.4109909e-004	6.6226996e-004	7.2000695e-004
7.7975087e-004	7.6705744e-004	7.8243292e-004	7.4242230e-004
7.3784785e-004	7.3520871e-004	7.2258864e-004	7.1940981e-004
7.1832549e-004	6.8641522e-004	7.3867656e-004	7.0009028e-004
6.9017100e-004	7.0357029e-004	7.1297413e-004	7.3996284e-004
6.5570704e-004	6.5641823e-004	6.5331208e-004	6.8606288e-004
5.6016754e-004	5.7770860e-004	6.2509777e-004	6.6572306e-004
7.0633998e-004	7.4373090e-004	7.0762105e-004	6.7528037e-004
6.7152608e-004	6.5209706e-004	6.3282602e-004	6.5302339e-004
6.1341231e-004	6.2682599e-004	6.1462157e-004	6.1761204e-004
5.8489649e-004	6.3406523e-004	5.9386248e-004	6.1365667e-004
5.7136544e-004	5.9937839e-004	5.6529122e-004	5.8952819e-004
6.8361837e-004	6.8478398e-004	7.3709109e-004	8.5974000e-004
8.5030182e-004	8.6140929e-004	8.3803560e-004	7.3379109e-004
7.1657404e-004	7.0154456e-004	7.3661914e-004	6.8749762e-004
7.0211038e-004	6.7479903e-004	7.0663792e-004	6.9545503e-004
6.9098761e-004	6.3702924e-004	6.2232313e-004	6.9179294e-004
6.3801417e-004	6.6541340e-004	7.1039898e-004	6.7373508e-004
7.7623942e-004	7.3271951e-004	7.4207925e-004	8.3944385e-004
8.6815024e-004	7.5790640e-004	8.1378823e-004	7.5628520e-004
7.6142642e-004	7.3214866e-004	7.4691973e-004	6.7163759e-004
6.8888119e-004	6.3963783e-004	7.1446535e-004	6.6140000e-004

6.5934837e-004	6.6298599e-004	6.8868433e-004	6.7625253e-004
6.5671460e-004	6.7007255e-004	6.1122798e-004	6.2430995e-004
7.4533555e-004	6.5812841e-004	6.6084917e-004	7.1231426e-004
7.1067073e-004	6.7030350e-004	6.9907054e-004	6.5918629e-004
6.1805327e-004	6.0746545e-004	6.3156741e-004	5.7531077e-004
5.9218766e-004	6.2502670e-004	5.9979616e-004	5.9950647e-004
6.0139611e-004	5.4800996e-004	5.9154991e-004	5.9552131e-004
6.4533831e-004	5.8066606e-004	6.1816654e-004	5.9100596e-004
6.6981466e-004	6.1226291e-004	6.3970908e-004	7.3060986e-004
6.8112135e-004	6.3522848e-004	6.8643130e-004	6.2834866e-004
6.3930337e-004	6.5768589e-004	6.3570468e-004	5.5607688e-004
5.4600673e-004	5.9872914e-004	6.0904201e-004	5.8910731e-004
5.6786243e-004	5.8299153e-004	5.6491750e-004	6.1088830e-004
5.5263267e-004	5.7712833e-004	6.0766070e-004	5.6245014e-004
6.8537194e-004	6.4212689e-004	7.2260924e-004	8.1095650e-004
7.8443777e-004	7.5832703e-004	7.8399142e-004	7.3938032e-004
7.1608847e-004	7.0040161e-004	6.8798354e-004	6.5800516e-004
6.5464171e-004	6.4423124e-004	6.4570676e-004	6.8244246e-004
6.2874230e-004	6.0431797e-004	6.4478564e-004	7.0766055e-004
6.6797147e-004	6.6942908e-004	6.5499797e-004	6.1531928e-004
6.1594894e-004	6.9219068e-004	7.5538603e-004	8.3999717e-004
7.9014689e-004	7.4209625e-004	8.4850482e-004	7.7565285e-004
7.6919195e-004	7.8060162e-004	8.1255144e-004	7.4608554e-004
7.0960211e-004	7.5670431e-004	7.8038177e-004	7.0432987e-004
7.2586932e-004	6.6280730e-004	7.5587955e-004	7.7927786e-004
7.4005421e-004	7.4414334e-004	7.4042019e-004	6.9105049e-004
5.8600647e-004	6.2617446e-004	6.6124833e-004	7.5648009e-004
7.7499425e-004	7.4076296e-004	8.3104265e-004	7.5589286e-004
7.8911052e-004	7.8557078e-004	7.7304999e-004	8.1047638e-004
7.3047198e-004	7.5632305e-004	7.9510357e-004	7.7059693e-004
7.3912491e-004	6.9871442e-004	7.6575291e-004	7.5602400e-004
7.1263201e-004	7.2626462e-004	7.0164436e-004	7.1693445e-004
6.0533840e-004	6.6517336e-004	7.0096001e-004	7.4238564e-004
8.5032076e-004	7.8552797e-004	8.1720595e-004	7.6964153e-004
7.6588869e-004	7.6460364e-004	7.5777400e-004	7.5787488e-004
7.5359657e-004	7.3154968e-004	7.6289082e-004	7.2709948e-004
7.2725404e-004	7.3472271e-004	7.3776938e-004	7.7191590e-004
6.7979643e-004	6.6447137e-004	6.7604422e-004	7.0619446e-004
5.7896189e-004	5.9863518e-004	6.3650680e-004	6.8293621e-004
7.1784243e-004	7.5049470e-004	7.2300019e-004	6.9097520e-004
6.8602680e-004	6.5266844e-004	6.4179917e-004	6.7875616e-004
6.3955462e-004	6.3086202e-004	6.3490075e-004	6.2303941e-004
6.3220336e-004	6.4103279e-004	6.1593289e-004	6.4466421e-004
5.6033481e-004	6.1212064e-004	5.7297450e-004	5.9950227e-004
7.7877854e-004	7.0225137e-004	7.4637284e-004	7.7391653e-004
8.1971071e-004	7.0941723e-004	7.8541556e-004	7.3417470e-004
7.2383601e-004	7.0461516e-004	7.2417448e-004	6.6033012e-004
6.8884653e-004	7.0319117e-004	6.4491006e-004	6.9300231e-004
6.8434990e-004	6.1859410e-004	6.8191551e-004	6.6508855e-004
6.8990525e-004	6.7589006e-004	6.6856186e-004	6.6148781e-004
7.5618540e-004	6.7171606e-004	6.8529288e-004	7.4195724e-004
7.3070030e-004	6.6991975e-004	7.2180071e-004	6.8416445e-004

6.7563682e-004	7.0720143e-004	6.7401862e-004	5.9435559e-004
6.0833516e-004	6.5157686e-004	6.3686515e-004	5.9750539e-004
6.1563376e-004	6.2943532e-004	6.1089323e-004	6.1157711e-004
6.4641392e-004	6.2044529e-004	6.5397842e-004	6.1967992e-004
6.6442878e-004	6.9604994e-004	7.8106637e-004	8.3618345e-004
8.3094684e-004	7.8419007e-004	8.4720607e-004	7.6504127e-004
7.6837011e-004	7.4034212e-004	7.6537699e-004	7.1037000e-004
6.8848538e-004	6.9242719e-004	7.1059994e-004	6.8081928e-004
6.7807788e-004	6.4209393e-004	7.2749484e-004	7.6443561e-004
7.3048526e-004	7.1334906e-004	7.2511537e-004	6.7230863e-004
6.1107436e-004	6.6875435e-004	7.4506189e-004	8.5254497e-004
7.9391227e-004	7.6694314e-004	8.5966258e-004	7.9405404e-004
8.0677004e-004	8.5395372e-004	8.2783193e-004	8.3621767e-004
7.6039619e-004	8.0931913e-004	8.7788551e-004	7.7044663e-004
7.6719234e-004	7.1230475e-004	8.0551287e-004	7.8200962e-004
7.5933369e-004	7.8419926e-004	7.6758297e-004	7.4242720e-004
6.2657945e-004	6.7258463e-004	7.0962327e-004	7.9323151e-004
9.1108799e-004	8.3338041e-004	9.2803039e-004	8.2989044e-004
8.2166982e-004	8.2702082e-004	8.4591925e-004	8.2949596e-004
7.5496359e-004	8.1499465e-004	8.3374865e-004	8.3306943e-004
8.0221373e-004	7.6884268e-004	8.1855939e-004	8.1835697e-004
7.3293372e-004	7.2384646e-004	7.3461150e-004	7.4064423e-004
6.0977026e-004	6.6957883e-004	7.1410918e-004	7.5395440e-004
8.1129797e-004	7.4844678e-004	7.9554463e-004	7.6013885e-004
7.5188073e-004	7.2481626e-004	7.1264722e-004	7.4223003e-004
7.4296194e-004	7.0042870e-004	7.0094953e-004	6.7640470e-004
7.1627052e-004	7.1012846e-004	6.7600529e-004	7.3077760e-004
6.4241615e-004	6.5641680e-004	6.3379091e-004	6.6282622e-004
6.2640723e-004	6.6222355e-004	6.8762380e-004	8.0901216e-004
8.8045964e-004	8.8927583e-004	8.3099061e-004	7.6337488e-004
7.6096812e-004	7.2654575e-004	7.6511140e-004	7.4472987e-004
7.7654609e-004	7.1030344e-004	7.3258483e-004	7.1136114e-004
7.3351878e-004	6.8671750e-004	6.8840261e-004	7.1566623e-004
6.3387313e-004	6.9567135e-004	7.0720672e-004	6.5016739e-004
7.9940683e-004	7.2510293e-004	7.9886979e-004	8.1322128e-004
8.7492727e-004	7.7556052e-004	8.5771267e-004	7.7275270e-004
8.1248645e-004	7.8057825e-004	7.9902945e-004	7.2950526e-004
7.5696735e-004	7.8087795e-004	7.0725333e-004	7.5997988e-004
7.6704596e-004	6.8669644e-004	7.3687233e-004	7.3112360e-004
7.4551236e-004	7.5370328e-004	7.3866089e-004	7.4490381e-004
7.8247050e-004	7.0558638e-004	7.2487183e-004	7.6703398e-004
7.6483580e-004	6.9319564e-004	7.3255917e-004	7.0504828e-004
7.1242823e-004	7.4505313e-004	7.1017700e-004	6.3991791e-004
6.4759655e-004	6.9995498e-004	6.5821547e-004	6.0996457e-004
6.5198247e-004	6.6517507e-004	6.3730277e-004	6.6033110e-004
6.6127586e-004	6.6858355e-004	7.0016594e-004	6.7265077e-004
6.8648953e-004	7.0364150e-004	7.8264602e-004	8.2576610e-004
8.4110523e-004	7.9337099e-004	8.5877074e-004	7.5706861e-004
7.8536484e-004	7.4300043e-004	7.7061674e-004	7.2317623e-004
7.1177953e-004	7.1505918e-004	7.2861201e-004	7.0467517e-004
6.9255849e-004	6.6904181e-004	7.5109667e-004	7.8430623e-004
7.5341450e-004	7.3197516e-004	7.5580119e-004	7.0676964e-004

6.2218044e-004	6.7787676e-004	7.8791958e-004	8.7441865e-004
8.2558487e-004	8.0658433e-004	8.9154528e-004	8.2464531e-004
8.2693354e-004	8.8386705e-004	8.6533675e-004	8.8652776e-004
7.8920911e-004	8.3641353e-004	9.1203545e-004	8.1122507e-004
7.9862637e-004	7.4161356e-004	8.4836376e-004	8.0495139e-004
8.0057606e-004	8.3021077e-004	8.1701895e-004	7.7120960e-004
6.4948772e-004	6.8754252e-004	7.4783304e-004	8.4544212e-004
9.6541361e-004	8.7766307e-004	9.9532296e-004	8.8201155e-004
8.4635301e-004	8.7183507e-004	8.8743256e-004	8.7047275e-004
7.9004107e-004	8.7269528e-004	8.7888607e-004	8.7825638e-004
8.4550002e-004	7.9592575e-004	8.5569205e-004	8.6507649e-004
7.9737425e-004	7.7263529e-004	7.8837472e-004	7.6011433e-004
6.4421896e-004	6.8577551e-004	7.4840544e-004	8.0765880e-004
8.7262224e-004	7.7888665e-004	8.4649738e-004	8.2369958e-004
7.9856411e-004	7.7316965e-004	7.5588559e-004	7.7108073e-004
8.1004477e-004	7.5002437e-004	7.4893035e-004	7.2462039e-004
7.8683779e-004	7.4803273e-004	7.0959727e-004	7.7478086e-004
6.8243366e-004	6.9726490e-004	6.8358454e-004	7.0721928e-004
6.3072637e-004	6.9740061e-004	7.1662548e-004	8.3889578e-004
9.4008711e-004	9.0493065e-004	8.8716271e-004	8.1459906e-004
8.3345492e-004	7.3585278e-004	8.1864628e-004	7.7851498e-004
8.4581484e-004	7.5977833e-004	7.7721807e-004	7.3509917e-004
8.0520503e-004	7.4537535e-004	7.5066528e-004	7.6951896e-004
6.9774313e-004	7.3464900e-004	7.7527454e-004	6.7494404e-004
5.7098250e-004	5.8046042e-004	6.0882066e-004	6.7893823e-004
7.4014514e-004	7.1989198e-004	7.6112477e-004	6.3834470e-004
7.0845178e-004	6.5489242e-004	7.2295470e-004	5.9163635e-004
5.7762119e-004	5.8953615e-004	6.0792290e-004	6.4075250e-004
6.3985334e-004	6.0524005e-004	5.7366055e-004	6.0979533e-004
5.9451060e-004	6.2196999e-004	6.0260767e-004	6.0545797e-004
8.2257827e-004	7.5793124e-004	8.4580798e-004	8.6760093e-004
8.8139781e-004	8.5253558e-004	9.0004601e-004	7.9741458e-004
8.5983411e-004	8.1455510e-004	8.2822838e-004	7.8178129e-004
8.2133406e-004	8.3672800e-004	7.5525648e-004	7.6440525e-004
8.0887812e-004	7.5574136e-004	7.3609479e-004	7.6840480e-004
7.6918471e-004	7.8376550e-004	8.1584655e-004	7.6979771e-004
7.4305659e-004	6.7988978e-004	7.5594135e-004	7.9870289e-004
7.6405790e-004	7.3362094e-004	7.8714328e-004	6.8100201e-004
7.4297513e-004	7.2079845e-004	7.0696442e-004	6.3211851e-004
6.7977363e-004	6.7834189e-004	6.7952396e-004	6.7975797e-004
6.6101796e-004	6.4523502e-004	6.3578560e-004	7.2440958e-004
7.0547620e-004	6.7389567e-004	6.6186492e-004	6.4211563e-004
6.8429558e-004	6.9799449e-004	7.7371672e-004	8.1583184e-004
8.4729482e-004	7.9816612e-004	8.7749370e-004	7.9116253e-004
8.0423616e-004	7.7928050e-004	8.2153687e-004	8.0353954e-004
7.8283624e-004	7.8904597e-004	7.9885821e-004	7.7661057e-004
7.6768968e-004	7.2584971e-004	8.3072617e-004	8.4849789e-004
8.2760525e-004	8.0319439e-004	8.4196035e-004	7.7989713e-004
6.4019948e-004	6.9030069e-004	8.2200991e-004	9.0129334e-004
8.5983335e-004	8.5384879e-004	9.4643574e-004	8.6628043e-004
8.5055015e-004	9.0514767e-004	8.8744145e-004	9.3436416e-004
8.4837440e-004	8.7925600e-004	9.4706011e-004	8.8015564e-004

8.2707558e-004	7.8826258e-004	9.0501644e-004	8.4236603e-004
8.9958585e-004	9.0644785e-004	8.5915549e-004	8.1653043e-004
6.7809368e-004	6.9584120e-004	7.7325750e-004	8.6154963e-004
1.0527570e-003	9.0425744e-004	1.0154001e-003	9.3192827e-004
8.8635375e-004	9.2244803e-004	8.9386573e-004	8.9709640e-004
8.6173297e-004	9.0044765e-004	9.0784578e-004	9.1040453e-004
9.0199978e-004	8.4870087e-004	8.6291574e-004	9.0516861e-004
8.6262614e-004	8.0916942e-004	8.6902673e-004	7.9574127e-004
6.7181879e-004	6.6201667e-004	7.6447274e-004	8.4821165e-004
8.7670828e-004	8.0863260e-004	8.9949528e-004	9.0876519e-004
8.3354891e-004	7.6803871e-004	7.9460436e-004	7.7323451e-004
8.5886980e-004	7.8285279e-004	7.9367534e-004	7.4390086e-004
8.7942810e-004	7.5797332e-004	7.4124803e-004	8.3176185e-004
7.0414129e-004	7.5339258e-004	7.3998193e-004	7.4283891e-004
5.8163070e-004	5.8374556e-004	6.4637748e-004	6.7562039e-004
7.4199011e-004	7.0304585e-004	7.4320422e-004	6.6943636e-004
7.0314377e-004	6.1621760e-004	6.7292455e-004	6.5926121e-004
7.0416684e-004	6.5767405e-004	6.2913141e-004	6.1748073e-004
6.6940385e-004	6.2344327e-004	6.2492292e-004	6.3207656e-004
6.1074337e-004	6.1868103e-004	6.5850359e-004	5.8630484e-004
8.4775363e-004	7.7827483e-004	8.5599073e-004	9.1341611e-004
8.7023668e-004	8.8305655e-004	8.6584978e-004	8.2128434e-004
8.1549950e-004	8.5911297e-004	8.0491613e-004	8.1194486e-004
7.5787785e-004	8.4359986e-004	7.7504593e-004	7.1932645e-004
7.6238826e-004	7.9257833e-004	7.1627229e-004	7.9978385e-004
7.4048353e-004	7.9862974e-004	8.0890227e-004	7.8156582e-004
7.6163342e-004	7.2646998e-004	7.6002414e-004	8.2985987e-004
8.6458778e-004	7.7710858e-004	8.1493144e-004	7.5398377e-004
7.8980482e-004	8.0283913e-004	7.4530872e-004	7.4485756e-004
6.8038281e-004	7.2750109e-004	7.2982306e-004	7.1522893e-004
7.3349076e-004	6.9927375e-004	7.0783362e-004	7.7848473e-004
7.0855144e-004	7.1760676e-004	7.0496683e-004	7.5817561e-004
7.8912909e-004	7.3072183e-004	8.1393641e-004	8.6775018e-004
8.9658276e-004	8.0893970e-004	8.6628061e-004	7.4029512e-004
8.0263570e-004	7.6262726e-004	7.4556045e-004	7.2204048e-004
7.4146796e-004	7.4685832e-004	7.6014057e-004	7.4523986e-004
7.1894277e-004	7.0990087e-004	7.7340165e-004	8.4804712e-004
8.0127273e-004	7.6905217e-004	8.0584647e-004	7.5230669e-004
6.4587795e-004	6.8722357e-004	7.9975923e-004	8.1633675e-004
8.5931351e-004	8.0089590e-004	9.0133406e-004	8.8918496e-004
8.3107335e-004	8.4498995e-004	9.2030042e-004	9.4007472e-004
8.8261151e-004	8.9648990e-004	9.3853162e-004	8.8457073e-004
8.8458009e-004	7.9852532e-004	9.6151336e-004	9.0847015e-004
9.4638129e-004	9.4136092e-004	9.3020011e-004	8.7313031e-004
7.0234798e-004	6.9848886e-004	7.5006420e-004	8.7123715e-004
1.0505823e-003	9.1162123e-004	9.7809151e-004	9.4709665e-004
9.1336507e-004	9.3831097e-004	8.2643563e-004	8.6048478e-004
9.5179120e-004	8.3135870e-004	9.1960921e-004	8.8764276e-004
9.5128703e-004	8.7500461e-004	8.1805457e-004	8.8836481e-004
8.4479712e-004	8.2065185e-004	9.0552626e-004	8.4128525e-004
6.1163559e-004	5.6893442e-004	6.7644221e-004	7.2233387e-004
6.8064951e-004	7.0337675e-004	7.4514927e-004	7.0940490e-004

6.5469947e-004	6.4942481e-004	6.3845078e-004	6.3975750e-004
6.4038785e-004	6.9072496e-004	6.4883706e-004	5.8124786e-004
6.5171430e-004	6.4874267e-004	5.8974999e-004	6.6342623e-004
5.7908490e-004	6.2105199e-004	6.2249974e-004	6.1303418e-004
8.1897470e-004	7.6341959e-004	8.2572921e-004	9.2638795e-004
9.2002435e-004	9.0728587e-004	8.3199135e-004	8.4626640e-004
8.5454083e-004	8.8159519e-004	8.5674277e-004	8.4724504e-004
7.3692531e-004	8.1639133e-004	8.1205850e-004	7.7062509e-004
7.7101585e-004	7.5933448e-004	7.6873587e-004	8.4954091e-004
7.6457486e-004	8.6047944e-004	7.9662782e-004	8.0870217e-004
8.0516607e-004	7.6712095e-004	8.3832466e-004	9.2409262e-004
9.4792404e-004	8.6402186e-004	9.0310384e-004	8.1270658e-004
8.4166504e-004	8.4910973e-004	7.9691589e-004	8.1433171e-004
6.9618902e-004	7.9259319e-004	7.9323845e-004	7.6547147e-004
8.3992485e-004	7.6447657e-004	7.7739116e-004	8.2998040e-004
7.8100582e-004	7.7240594e-004	7.9601993e-004	8.6876568e-004
7.3890504e-004	7.2299907e-004	7.9225996e-004	8.3665179e-004
8.8910674e-004	7.8773638e-004	8.6805181e-004	8.8121662e-004
8.2289935e-004	7.7480146e-004	8.1403120e-004	8.6238155e-004
8.6785421e-004	8.6812327e-004	8.6069315e-004	8.5456073e-004
8.4851465e-004	8.0276642e-004	9.3672275e-004	1.0059428e-003
9.5395742e-004	9.2644354e-004	9.6581392e-004	9.0191377e-004
5.7894421e-004	5.8267821e-004	6.2309965e-004	6.8652986e-004
8.3079007e-004	7.8375332e-004	8.3081199e-004	8.1937632e-004
7.5843984e-004	8.0918517e-004	7.6049881e-004	8.2111675e-004
7.2233118e-004	8.0156240e-004	7.9358969e-004	7.6647962e-004
8.3209689e-004	8.4406942e-004	7.9448395e-004	8.6809978e-004
8.2255596e-004	7.9973804e-004	8.5181096e-004	7.6649683e-004
6.7915842e-004	6.3126426e-004	6.9750304e-004	7.8622047e-004
7.5695121e-004	7.9399969e-004	6.9235156e-004	7.1999189e-004
7.1787200e-004	7.4126218e-004	7.2209055e-004	7.2559930e-004
6.0576224e-004	6.9578124e-004	7.0562681e-004	6.4531167e-004
6.4957888e-004	6.5555298e-004	6.5149216e-004	7.0435379e-004
6.4475936e-004	7.4718248e-004	6.7654379e-004	6.6115834e-004
8.4043536e-004	8.5859896e-004	8.9913807e-004	1.0438689e-003
1.1233523e-003	9.7645812e-004	1.0439158e-003	9.2550021e-004
9.6783493e-004	9.6029036e-004	8.9958934e-004	9.0244977e-004
7.7993055e-004	8.9689092e-004	9.0176269e-004	8.7490237e-004
9.0410333e-004	8.7813550e-004	8.6079929e-004	9.2334356e-004
8.6969257e-004	8.6875400e-004	9.0004940e-004	9.9485443e-004
8.2816360e-004	8.0092555e-004	8.4965987e-004	8.8607364e-004
9.4087078e-004	8.6209101e-004	8.8442587e-004	8.8447593e-004
8.5629581e-004	7.8484598e-004	7.7982203e-004	8.9282010e-004
8.6772141e-004	8.6423042e-004	9.0378885e-004	8.4546593e-004
8.4061309e-004	8.5875700e-004	9.4414920e-004	1.0631623e-003
9.8334393e-004	9.4215997e-004	1.0268735e-003	9.2057783e-004
5.3719746e-004	5.3160114e-004	5.6247447e-004	6.1065218e-004
6.7730536e-004	6.4493476e-004	6.8299881e-004	6.5020959e-004
6.1673568e-004	6.4804507e-004	6.2682477e-004	6.8082339e-004
6.1831553e-004	6.5758268e-004	6.5029101e-004	6.5276577e-004
6.7376877e-004	6.9032519e-004	6.6068201e-004	7.1387106e-004
7.0307915e-004	6.8036698e-004	7.3555138e-004	6.5511995e-004

7.7705531e-004	8.0298689e-004	8.3113447e-004	9.6456696e-004
9.7417974e-004	9.2917020e-004	9.7400413e-004	8.8174589e-004
8.7923846e-004	8.9646925e-004	8.0886751e-004	8.3872078e-004
7.0590498e-004	8.5774083e-004	8.4602358e-004	8.4765081e-004
8.3350542e-004	8.3099662e-004	8.2908889e-004	8.5607434e-004
7.9679837e-004	7.9928551e-004	8.5920839e-004	9.3187009e-004
8.9406446e-004	8.2593034e-004	9.2835867e-004	9.7496850e-004
9.6123372e-004	1.0119160e-003	1.0111279e-003	9.8389036e-004
9.2063165e-004	9.1289270e-004	9.0210960e-004	9.8689453e-004
8.6878995e-004	8.8992642e-004	9.2771968e-004	8.7207755e-004
8.6198542e-004	9.2501432e-004	9.4438881e-004	9.8562564e-004
8.8876074e-004	9.8768272e-004	9.3323654e-004	9.6787862e-004
9.4064539e-004	9.2860596e-004	9.8183262e-004	9.2570548e-004
9.6790798e-004	9.0203459e-004	8.8968215e-004	8.9352233e-004
9.0683174e-004	8.9053839e-004	8.6033625e-004	9.8336418e-004
8.4623136e-004	9.2613608e-004	9.5149233e-004	8.6468033e-004
8.5555881e-004	9.2507800e-004	8.9192776e-004	9.4743371e-004
9.4014499e-004	9.4804948e-004	1.0321219e-003	8.9339742e-004
6.3819711e-004	6.7475051e-004	6.8628499e-004	7.0252793e-004
7.5695194e-004	6.7035560e-004	7.0439525e-004	7.7301463e-004
7.0845710e-004	6.3186137e-004	6.4870967e-004	7.6986787e-004
7.3478059e-004	7.4558984e-004	7.6101827e-004	7.0857792e-004
7.2793354e-004	7.4524620e-004	8.1039862e-004	8.9917409e-004
8.3909548e-004	8.0937013e-004	8.9354394e-004	8.0388223e-004
5.3644067e-004	5.3701896e-004	4.7233827e-004	5.6880669e-004
5.0547919e-004	5.6673399e-004	5.1654202e-004	4.8357957e-004
4.8674572e-004	5.0919186e-004	4.6534773e-004	4.7587742e-004
4.3447583e-004	4.8009944e-004	4.0975213e-004	4.4277479e-004
4.6631322e-004	3.4617011e-004	4.8593243e-004	4.4596078e-004
4.3626555e-004	4.8695421e-004	4.2228524e-004	4.5735005e-004
6.8300236e-004	6.3000007e-004	6.4212676e-004	6.6265326e-004
6.1973956e-004	5.0930007e-004	4.8568453e-004	5.0322609e-004
5.1174268e-004	4.9990693e-004	4.8336498e-004	4.9185120e-004
4.6987012e-004	4.6330997e-004	4.8466254e-004	4.5671186e-004
4.0078610e-004	4.5221373e-004	4.6308652e-004	5.2116495e-004
4.5318384e-004	4.6676482e-004	4.8002275e-004	5.0527792e-004
5.0842846e-004	5.0679252e-004	5.4388707e-004	4.8205537e-004
4.7038229e-004	4.2877471e-004	4.5004102e-004	4.5978248e-004
3.8375785e-004	3.9328041e-004	3.4390586e-004	3.6820046e-004
4.0466539e-004	3.4538263e-004	4.2307057e-004	3.9646179e-004
3.6658320e-004	4.1954384e-004	3.9842530e-004	4.5628547e-004
3.9517362e-004	4.0337092e-004	4.0057924e-004	4.7189114e-004
5.5465661e-004	4.7556048e-004	4.4116232e-004	5.3944629e-004
5.3011818e-004	5.4448246e-004	4.4115694e-004	4.5291322e-004
5.4518654e-004	5.6829614e-004	5.0466676e-004	4.2021977e-004
4.4492443e-004	4.9713346e-004	4.1769775e-004	4.2483331e-004
4.7278120e-004	4.1937963e-004	5.1659056e-004	4.7358649e-004
4.5681239e-004	4.7861120e-004	4.5856489e-004	4.8576304e-004
6.0279931e-004	6.0548942e-004	6.1730339e-004	6.8080542e-004
5.9907210e-004	5.4635975e-004	5.1980054e-004	5.3303059e-004
5.5450670e-004	4.9214303e-004	4.8283589e-004	5.1260536e-004
4.6056497e-004	5.0376132e-004	4.9569576e-004	4.8829539e-004

4.6044501e-004	3.9597972e-004	4.9787002e-004	4.7111065e-004
4.5839864e-004	5.0808593e-004	4.7839485e-004	4.7909357e-004
6.7901774e-004	6.6062949e-004	6.6580284e-004	6.9319768e-004
6.4208200e-004	5.1640067e-004	5.0003939e-004	4.9504458e-004
5.2876415e-004	5.5858462e-004	4.9250144e-004	4.9537337e-004
5.0634450e-004	4.8566853e-004	4.9158371e-004	4.4116264e-004
4.5423262e-004	4.8853325e-004	4.5846612e-004	5.0803361e-004
4.7621285e-004	4.6880194e-004	4.7420389e-004	4.9146233e-004
5.8375599e-004	6.0007451e-004	6.4026953e-004	5.8203681e-004
5.7741512e-004	5.1866856e-004	5.2116674e-004	5.4679989e-004
4.6308670e-004	4.5953372e-004	4.4415631e-004	4.4581395e-004
4.7927245e-004	4.1139009e-004	4.8908136e-004	4.7000377e-004
4.5599180e-004	4.9804992e-004	4.7419395e-004	5.1911418e-004
4.6652374e-004	4.7166670e-004	4.7671911e-004	5.2310133e-004
4.9872615e-004	5.1965655e-004	5.1365983e-004	4.6206066e-004
4.2705273e-004	4.8118960e-004	4.1482823e-004	4.2987178e-004
4.0318973e-004	4.5409720e-004	4.6107747e-004	4.4088947e-004
4.5955038e-004	4.7453187e-004	4.4439067e-004	5.4272178e-004
5.5626625e-004	5.1173776e-004	5.7000614e-004	5.8469579e-004
4.7187827e-004	5.4688510e-004	5.6220769e-004	5.7312430e-004
6.1165789e-004	5.2045249e-004	4.8110847e-004	5.8889519e-004
5.8300422e-004	5.5823357e-004	4.6217019e-004	4.7629797e-004
5.8507640e-004	5.8752092e-004	5.7056005e-004	4.8123471e-004
5.0315475e-004	5.4256950e-004	4.5961410e-004	4.8013579e-004
5.2163596e-004	4.7367167e-004	5.5035678e-004	5.0660601e-004
5.0025575e-004	5.2051661e-004	4.9581510e-004	5.3604861e-004
6.2011708e-004	6.0710379e-004	6.4958265e-004	7.1426878e-004
6.4560000e-004	5.6842563e-004	5.4950672e-004	5.8022329e-004
5.5667966e-004	5.2676040e-004	5.3414010e-004	5.3118634e-004
4.8212182e-004	5.0432812e-004	5.1214203e-004	5.1601346e-004
4.8865003e-004	4.4668550e-004	5.0564361e-004	4.9084038e-004
4.6573841e-004	4.8899030e-004	5.0193933e-004	4.8625480e-004
6.5817645e-004	6.5442968e-004	7.0154251e-004	7.1415216e-004
6.6234151e-004	5.3140351e-004	5.6449797e-004	5.4505109e-004
6.1584693e-004	5.8302759e-004	4.8293914e-004	5.1651325e-004
5.5083826e-004	5.3396079e-004	5.0054954e-004	4.7302237e-004
4.8779715e-004	4.7053102e-004	4.6849248e-004	5.0451617e-004
4.8649509e-004	5.0750573e-004	4.7909939e-004	4.7413793e-004
5.8467976e-004	6.5442373e-004	6.7851464e-004	6.1212110e-004
6.3519423e-004	5.5394373e-004	5.3174297e-004	5.2040958e-004
5.0511490e-004	4.8313724e-004	5.0077860e-004	4.7004393e-004
4.6338704e-004	4.2780211e-004	4.9488568e-004	4.6113423e-004
4.6834970e-004	5.0282285e-004	4.7354001e-004	5.0737591e-004
4.6850729e-004	4.3278414e-004	4.8588067e-004	4.9383537e-004
5.8786466e-004	5.9864725e-004	6.2246092e-004	5.7521572e-004
5.1306987e-004	5.7947103e-004	4.9734560e-004	5.4330176e-004
4.7063010e-004	5.1557002e-004	5.4015586e-004	5.1236889e-004
5.3254198e-004	5.1701958e-004	5.0528368e-004	6.2489184e-004
5.9471650e-004	5.5694794e-004	6.0309237e-004	6.0615128e-004
5.0553444e-004	5.8419528e-004	5.8725326e-004	6.0816748e-004
5.4848017e-004	5.8540457e-004	5.7457841e-004	5.1792825e-004
5.7905627e-004	5.5726912e-004	5.7177814e-004	5.0975474e-004

5.2504428e-004	5.5639204e-004	5.1726619e-004	5.4351376e-004
5.5166112e-004	5.6552433e-004	4.8483381e-004	6.1131222e-004
6.0126987e-004	5.7415097e-004	5.8899816e-004	7.1960458e-004
5.5893292e-004	5.3670540e-004	6.1683280e-004	6.2178919e-004
6.2642453e-004	5.9963943e-004	6.9495749e-004	7.2418507e-004
6.6275269e-004	6.0686748e-004	5.7659010e-004	6.1727314e-004
5.5676499e-004	5.5321609e-004	5.8032587e-004	5.5014374e-004
5.1799421e-004	5.0423278e-004	5.2827369e-004	5.3467655e-004
4.9271005e-004	5.0665214e-004	5.1804393e-004	5.1694470e-004
4.9444169e-004	4.8373896e-004	5.2335925e-004	4.9063518e-004
6.6323116e-004	6.6680972e-004	7.1541965e-004	7.1154626e-004
6.9569003e-004	5.5231430e-004	6.3194247e-004	5.8544290e-004
6.4952990e-004	5.9743172e-004	5.0714424e-004	5.5028271e-004
5.7575183e-004	5.6337312e-004	5.2049471e-004	5.1302307e-004
5.3950834e-004	4.7807395e-004	4.7821712e-004	5.3718188e-004
4.9133744e-004	5.2142730e-004	4.8341974e-004	4.7374098e-004
5.9111508e-004	6.7994275e-004	7.0504012e-004	6.3008731e-004
6.4968846e-004	5.8116342e-004	5.6671680e-004	5.4529251e-004
5.4427247e-004	5.1486564e-004	5.3489493e-004	4.9969762e-004
4.8226042e-004	4.7404016e-004	5.1877265e-004	4.9736509e-004
5.0076473e-004	5.2463495e-004	4.8058851e-004	5.0463279e-004
4.9752959e-004	4.4633824e-004	4.9799114e-004	4.9324754e-004
6.4411451e-004	7.1273268e-004	6.8894209e-004	6.4674651e-004
6.8238619e-004	6.4639741e-004	6.8638154e-004	5.9296675e-004
6.2677856e-004	6.6195994e-004	6.2793762e-004	6.1983452e-004
6.3238569e-004	6.4195554e-004	5.5324886e-004	7.2373543e-004
6.4660978e-004	6.2730705e-004	6.3778498e-004	8.2267345e-004
6.3910603e-004	5.9816454e-004	6.6802288e-004	6.7923970e-004
6.0444939e-004	6.4389384e-004	6.2862609e-004	6.8126462e-004
5.5414368e-004	6.6760770e-004	5.7450226e-004	6.4166581e-004
5.6912990e-004	5.6834407e-004	6.3600051e-004	5.6441945e-004
6.4153881e-004	6.9051109e-004	6.6471046e-004	5.8506003e-004
6.1314474e-004	7.3554581e-004	6.8594016e-004	7.4975721e-004
6.9038400e-004	6.9233711e-004	6.7600617e-004	6.4212155e-004
6.4425776e-004	5.9792906e-004	5.9147844e-004	6.4347114e-004
6.7341393e-004	6.2700939e-004	5.6471888e-004	5.5483761e-004
6.0264659e-004	5.8490217e-004	6.6592125e-004	5.8359366e-004
5.5119187e-004	6.0238430e-004	5.5539991e-004	5.7307989e-004
5.1784817e-004	5.6645570e-004	5.6813826e-004	5.1045547e-004
5.2915808e-004	5.4213361e-004	5.2235818e-004	5.5455307e-004
6.1287735e-004	6.1071382e-004	7.1956210e-004	7.1935052e-004
6.7846623e-004	6.4194684e-004	6.1316218e-004	6.4965968e-004
5.6578038e-004	5.7489613e-004	5.8202939e-004	5.6420655e-004
5.5313860e-004	5.0567903e-004	5.2885966e-004	5.5414031e-004
5.2613478e-004	5.3336490e-004	5.3676857e-004	5.6154873e-004
5.2916069e-004	4.8633895e-004	5.3553695e-004	4.9979604e-004
6.6788535e-004	6.8372968e-004	7.1002039e-004	6.8933070e-004
7.0297229e-004	5.8221527e-004	6.7398296e-004	5.9933429e-004
6.6080970e-004	5.8798007e-004	5.5137176e-004	5.8672311e-004
5.5456731e-004	5.9714529e-004	5.5235504e-004	5.5491226e-004
5.7656979e-004	5.0235660e-004	4.9506492e-004	5.7161812e-004
5.0027378e-004	5.2052292e-004	4.8082759e-004	4.8760105e-004

6.3566041e-004	6.9736016e-004	7.1962502e-004	6.5907842e-004
6.4890859e-004	6.0976183e-004	6.1087792e-004	6.0773085e-004
5.9222170e-004	5.7390674e-004	5.7062188e-004	5.2784543e-004
5.4885166e-004	5.2487360e-004	5.3479036e-004	5.3197506e-004
5.2674108e-004	5.5761501e-004	4.7223589e-004	5.0446915e-004
5.4952644e-004	4.9191312e-004	5.2472357e-004	5.1643520e-004
6.1506874e-004	6.9909435e-004	7.2010883e-004	6.9226386e-004
6.5447905e-004	6.5223184e-004	6.4353009e-004	6.3420637e-004
5.4068083e-004	5.3045133e-004	6.0957836e-004	5.6132698e-004
5.7808032e-004	6.0263145e-004	5.2475095e-004	6.3018550e-004
6.1055819e-004	5.4318951e-004	5.8404609e-004	6.3624531e-004
5.4508004e-004	5.7186047e-004	6.0719316e-004	6.2408473e-004
6.8732411e-004	7.5186731e-004	7.3571215e-004	7.0516100e-004
7.0080251e-004	6.6287766e-004	7.1216666e-004	6.3237367e-004
6.6635381e-004	6.9929987e-004	6.7789121e-004	6.1541230e-004
6.4621124e-004	6.7195676e-004	5.8854974e-004	7.0455752e-004
6.0792864e-004	6.3621004e-004	6.2609912e-004	8.0491886e-004
6.5060341e-004	6.1215408e-004	6.5291613e-004	6.6201459e-004
6.8346167e-004	6.7035056e-004	7.2735504e-004	7.6071618e-004
6.1982930e-004	6.7569864e-004	6.2581734e-004	7.2401235e-004
6.6387573e-004	5.8257200e-004	6.8880268e-004	6.4072618e-004
6.2873753e-004	7.3207283e-004	6.9526414e-004	6.4839218e-004
7.0068998e-004	7.6601328e-004	7.6936823e-004	7.6917352e-004
7.4577684e-004	7.0830078e-004	7.0080382e-004	6.7747077e-004
5.7458077e-004	6.6706627e-004	6.7075474e-004	5.7110778e-004
5.9912149e-004	5.9856850e-004	6.6478323e-004	5.8589925e-004
5.4180397e-004	5.3651500e-004	5.6471863e-004	5.4872067e-004
5.7933926e-004	5.3384488e-004	5.2549889e-004	5.6283294e-004
5.0516674e-004	5.4852768e-004	5.5166661e-004	6.0417890e-004
5.2511819e-004	5.6937065e-004	5.6786023e-004	5.9219486e-004
6.0485837e-004	5.9521853e-004	6.2528747e-004	6.1250528e-004
6.5757656e-004	6.7356866e-004	5.8000983e-004	5.9252299e-004
5.9081636e-004	5.3237633e-004	6.1438448e-004	5.3013989e-004
5.0275547e-004	5.4541818e-004	5.2791693e-004	6.2758845e-004
5.6125693e-004	5.4680479e-004	5.9241995e-004	6.1332533e-004
6.1719191e-004	5.1588092e-004	5.4886146e-004	5.4291308e-004
6.2825084e-004	6.0227262e-004	6.1803120e-004	6.3313254e-004
7.1049691e-004	6.5201412e-004	5.8934883e-004	5.7551505e-004
5.9250367e-004	5.7679496e-004	6.6561897e-004	5.9400026e-004
5.3320586e-004	6.1403752e-004	5.7132817e-004	5.8570798e-004
5.0715078e-004	5.6869973e-004	5.7920527e-004	5.0822506e-004
5.3977253e-004	5.2845081e-004	5.2377729e-004	5.4185060e-004
5.9862410e-004	6.2740383e-004	6.9036976e-004	7.0124252e-004
6.7977693e-004	6.7783511e-004	6.3343822e-004	6.3700514e-004
5.6682507e-004	5.7637510e-004	6.0587969e-004	5.7646903e-004
5.4502168e-004	5.3831111e-004	5.3389605e-004	5.8810915e-004
5.5738740e-004	5.7193229e-004	5.7843031e-004	5.8121129e-004
5.5356677e-004	5.0491063e-004	5.4400759e-004	5.2928794e-004
6.6520426e-004	6.7024656e-004	6.9607272e-004	7.0610143e-004
7.0301942e-004	6.2238173e-004	6.9696187e-004	6.3298001e-004
6.6991127e-004	5.9894500e-004	5.9027268e-004	6.1902833e-004
5.8660208e-004	6.2455247e-004	5.6883482e-004	5.7514128e-004

5.9612237e-004	5.2483655e-004	4.9925303e-004	6.0190728e-004
5.0305769e-004	5.4120646e-004	5.0217244e-004	5.1814245e-004
6.6312330e-004	7.1123151e-004	7.2613610e-004	6.7550146e-004
6.6127678e-004	6.1827464e-004	6.3253408e-004	6.2964995e-004
6.4137147e-004	6.0686165e-004	5.9552600e-004	5.3377128e-004
5.6071873e-004	5.6409364e-004	5.5706866e-004	5.3631926e-004
5.2101282e-004	5.6855082e-004	4.7318563e-004	5.1167348e-004
5.6737833e-004	5.1753038e-004	5.2296101e-004	5.3544441e-004
6.3644767e-004	7.1728816e-004	7.5198508e-004	7.3717066e-004
6.9401655e-004	6.6126198e-004	6.7519411e-004	6.7186639e-004
5.7885681e-004	5.4955309e-004	6.3719241e-004	5.4614172e-004
6.0275607e-004	6.1085577e-004	5.3054639e-004	6.1455500e-004
6.1541059e-004	5.4012661e-004	5.4261526e-004	6.2073857e-004
5.5309807e-004	5.6695567e-004	5.8326645e-004	6.0539316e-004
7.2492221e-004	7.7288895e-004	7.7392168e-004	7.3542973e-004
7.4691520e-004	6.7989597e-004	7.3149086e-004	6.4970127e-004
6.8885711e-004	6.9968279e-004	6.8555569e-004	6.1617965e-004
6.4067902e-004	6.8826554e-004	5.9895348e-004	6.7943754e-004
5.7817067e-004	6.2056728e-004	6.2094943e-004	7.5858124e-004
6.3136652e-004	5.9709529e-004	6.1770591e-004	6.6056463e-004
6.7806889e-004	6.5434011e-004	7.3272047e-004	7.7776470e-004
6.5126302e-004	6.7540706e-004	6.4161043e-004	7.3706586e-004
6.6961969e-004	6.1905157e-004	6.9232158e-004	6.2451982e-004
6.3552933e-004	7.5296100e-004	6.4256032e-004	6.7096877e-004
6.8486790e-004	7.3480468e-004	7.5608715e-004	7.3842391e-004
7.1341963e-004	6.9400913e-004	6.7429607e-004	6.7327271e-004
5.6357847e-004	5.6058887e-004	5.4412612e-004	5.7700380e-004
4.6329918e-004	4.6030302e-004	5.0808019e-004	5.2372961e-004
5.5557198e-004	5.2827703e-004	5.5638554e-004	6.3281901e-004
5.6630484e-004	5.5664271e-004	5.7394804e-004	6.1976985e-004
6.0910250e-004	5.8354731e-004	6.3748463e-004	6.1726648e-004
6.4572103e-004	5.7854949e-004	6.2238042e-004	6.2932868e-004
5.8224164e-004	7.1637466e-004	7.2290409e-004	6.2132385e-004
6.3188758e-004	6.4345835e-004	6.8062365e-004	6.2518497e-004
5.9400717e-004	5.5928068e-004	6.0598485e-004	5.7714662e-004
6.3312831e-004	5.5462150e-004	5.7049943e-004	5.9466685e-004
5.3794209e-004	5.6169060e-004	5.5922565e-004	6.4538351e-004
5.3012294e-004	5.7741870e-004	5.7338593e-004	5.9411577e-004
6.1421082e-004	6.3833475e-004	6.5747726e-004	6.6979157e-004
6.6655647e-004	7.1195849e-004	6.1027118e-004	6.0344084e-004
6.1996000e-004	5.4112695e-004	6.2569142e-004	5.4444975e-004
5.0760950e-004	5.3373334e-004	5.2694957e-004	6.3385056e-004
5.4510504e-004	5.4682679e-004	5.7499652e-004	5.9976424e-004
6.0908969e-004	5.2116170e-004	5.4065645e-004	5.2682473e-004
6.0507278e-004	5.8568575e-004	6.3155808e-004	6.3387958e-004
7.2012415e-004	6.6903942e-004	5.9943463e-004	5.7680276e-004
5.9259949e-004	5.6794973e-004	6.4035614e-004	5.9001760e-004
5.1101259e-004	6.1254724e-004	5.8333050e-004	5.8125512e-004
4.9543181e-004	5.5280808e-004	5.8319875e-004	5.0528032e-004
5.3847961e-004	5.1204628e-004	5.0981860e-004	5.2254323e-004
5.7883770e-004	6.3483795e-004	6.5250426e-004	6.7800720e-004
6.7219279e-004	6.9037480e-004	6.3550766e-004	6.0353120e-004

5.6523815e-004	5.6575185e-004	6.0683001e-004	5.7582319e-004
5.2858697e-004	5.6749501e-004	5.3463090e-004	6.0455882e-004
5.7744025e-004	5.8410119e-004	6.0454344e-004	5.8891455e-004
5.5415722e-004	5.2106645e-004	5.4417965e-004	5.4834492e-004
6.3544762e-004	6.5096297e-004	6.5976013e-004	7.0228522e-004
6.8372868e-004	6.3876703e-004	6.7799765e-004	6.4013719e-004
6.5328370e-004	6.1172937e-004	6.2314770e-004	6.2316006e-004
6.0272825e-004	6.2122291e-004	5.6433307e-004	5.7285454e-004
6.0418258e-004	5.4809642e-004	5.1468762e-004	6.1277025e-004
5.0351144e-004	5.5675184e-004	5.2219877e-004	5.4947626e-004
6.6773185e-004	7.1033383e-004	7.0853182e-004	6.7801966e-004
6.6577816e-004	6.1914767e-004	6.2971908e-004	6.3921521e-004
6.6473969e-004	6.1795568e-004	6.1321796e-004	5.4065378e-004
5.5514317e-004	5.8389120e-004	5.7573791e-004	5.4027622e-004
5.2808229e-004	5.7949105e-004	4.8496282e-004	5.2979072e-004
5.6153232e-004	5.3397832e-004	5.0965988e-004	5.5181938e-004
6.4283216e-004	7.0190374e-004	7.6304319e-004	7.4754134e-004
7.1142007e-004	6.7464910e-004	6.8365979e-004	6.8856319e-004
6.0843579e-004	5.6564598e-004	6.4669612e-004	5.3614727e-004
6.1112196e-004	5.9010473e-004	5.3685228e-004	6.0512526e-004
6.0995358e-004	5.4888790e-004	5.0758891e-004	6.0738919e-004
5.4357112e-004	5.6886017e-004	5.4983229e-004	5.8223344e-004
7.3092290e-004	7.4660942e-004	7.6027095e-004	7.1557910e-004
7.5097846e-004	6.8183967e-004	7.2337085e-004	6.5044078e-004
6.8782333e-004	6.8070779e-004	6.7092742e-004	6.0314416e-004
6.4183040e-004	7.0666631e-004	5.9641741e-004	6.4892368e-004
5.5543943e-004	6.0260978e-004	6.1709905e-004	6.9716401e-004
6.1239167e-004	5.9531731e-004	5.8173621e-004	6.6199386e-004
6.2741214e-004	6.5935971e-004	6.0863393e-004	7.0810014e-004
5.6514524e-004	5.8262185e-004	6.0882793e-004	6.2291214e-004
6.6558695e-004	6.5071831e-004	6.5860512e-004	7.7142329e-004
6.5925461e-004	6.4592526e-004	6.6430429e-004	7.2771324e-004
6.7812372e-004	6.8612907e-004	7.3210578e-004	7.5269534e-004
7.3443956e-004	6.8119516e-004	7.0609264e-004	7.3090511e-004
5.8832786e-004	7.3043084e-004	7.3843825e-004	6.8157795e-004
6.5851874e-004	7.1860456e-004	6.3248214e-004	6.1659139e-004
6.1312444e-004	5.5777634e-004	6.3786069e-004	6.0546797e-004
5.8714759e-004	5.2418326e-004	5.4859972e-004	5.9786484e-004
5.6530238e-004	5.7106539e-004	5.7000403e-004	6.4738918e-004
5.4046120e-004	5.3289761e-004	5.4547336e-004	5.4179880e-004
6.2330214e-004	6.1602383e-004	6.5300624e-004	7.3279678e-004
7.1818070e-004	7.2901933e-004	6.4446832e-004	5.6933847e-004
6.5098172e-004	5.6182515e-004	6.2078438e-004	5.5088993e-004
5.0671543e-004	5.9076051e-004	5.5811666e-004	6.1660126e-004
5.3303482e-004	5.5170545e-004	5.7826425e-004	5.3876462e-004
5.7713648e-004	5.4041279e-004	5.0222036e-004	5.1268388e-004
5.4193808e-004	6.4007004e-004	6.2053552e-004	6.5747496e-004
6.3950323e-004	6.4705010e-004	6.5580527e-004	6.0979537e-004
5.7029827e-004	5.5592092e-004	6.1904838e-004	5.8634022e-004
5.6456729e-004	5.8077140e-004	5.0112986e-004	5.8842974e-004
5.9780282e-004	5.6708348e-004	5.8864687e-004	6.3180884e-004
5.0755024e-004	5.6884218e-004	5.2694099e-004	5.4885935e-004

5.9836595e-004	6.3135241e-004	6.2317660e-004	6.5229453e-004
6.6324743e-004	6.2404765e-004	6.1138645e-004	6.2263895e-004
6.3547648e-004	6.1323086e-004	6.1310688e-004	5.6747277e-004
5.4856310e-004	5.9357416e-004	5.9357671e-004	5.2704170e-004
5.8537269e-004	5.8147655e-004	5.0500103e-004	5.4528730e-004
4.9938012e-004	5.2672416e-004	4.9402995e-004	5.8470228e-004
6.7035184e-004	7.0774555e-004	7.1638111e-004	7.2217753e-004
6.7976894e-004	6.3975333e-004	6.3796468e-004	6.8189246e-004
6.4355382e-004	6.0027647e-004	6.3578736e-004	5.7370598e-004
5.6685094e-004	5.8403546e-004	5.8819496e-004	6.1394196e-004
6.0098903e-004	5.5394802e-004	5.0486250e-004	6.1186322e-004
5.4978400e-004	5.8024121e-004	5.1398468e-004	5.3568264e-004
6.5809842e-004	6.5658271e-004	7.3383670e-004	6.7506639e-004
7.1478037e-004	6.7846039e-004	6.9678410e-004	6.5087173e-004
6.2875231e-004	5.8317563e-004	6.3612981e-004	5.5329160e-004
6.2179625e-004	5.8829474e-004	5.1281870e-004	5.8160024e-004
5.3909962e-004	5.8622587e-004	5.3273908e-004	5.9477267e-004
5.3576699e-004	5.4072347e-004	5.0013207e-004	6.0228560e-004
6.3412418e-004	6.7008124e-004	5.9363665e-004	7.4929213e-004
6.2278072e-004	6.4371097e-004	6.5283666e-004	7.0762372e-004
6.7837957e-004	5.7738404e-004	6.7531382e-004	6.5309904e-004
6.2403074e-004	6.8887943e-004	6.2594136e-004	7.0744650e-004
6.0801962e-004	6.5917859e-004	7.1267934e-004	7.0908340e-004
7.2508787e-004	6.9137887e-004	6.0934911e-004	6.7380613e-004
5.1440470e-004	5.7636460e-004	5.9202009e-004	6.0018386e-004
5.3599042e-004	5.8594864e-004	5.7786224e-004	5.3627996e-004
5.5740702e-004	6.4813643e-004	5.8439182e-004	7.0365259e-004
5.5959333e-004	4.8282036e-004	5.6283431e-004	6.4024936e-004
5.7581247e-004	6.6333609e-004	6.3484101e-004	6.7472366e-004
5.5866608e-004	6.2018301e-004	5.7194935e-004	5.8846155e-004
6.2287656e-004	7.4364367e-004	7.5747946e-004	7.0226293e-004
6.7792545e-004	7.1823683e-004	6.3767564e-004	6.4220197e-004
6.1556586e-004	5.8433549e-004	6.7074975e-004	6.2246063e-004
5.8016334e-004	5.6154688e-004	5.4449381e-004	5.9642160e-004
6.1702634e-004	5.9030723e-004	5.8485600e-004	6.5664024e-004
5.3058678e-004	5.5443990e-004	5.4755577e-004	5.3399007e-004
6.0870156e-004	6.1082582e-004	6.5053409e-004	7.3009974e-004
6.8391225e-004	7.2252775e-004	6.5651778e-004	5.5962433e-004
6.3730638e-004	5.5056642e-004	6.0132383e-004	5.5633092e-004
5.1878275e-004	5.7946239e-004	5.5666920e-004	6.0960888e-004
5.3696922e-004	5.5954351e-004	5.7259046e-004	5.4364883e-004
5.4156102e-004	5.4639633e-004	4.7630053e-004	5.1140148e-004
4.9084554e-004	6.1227998e-004	5.8385855e-004	6.1719133e-004
5.9796654e-004	6.1709915e-004	6.0171081e-004	5.9044956e-004
5.2332463e-004	5.2031082e-004	5.9432775e-004	5.6481118e-004
5.3535342e-004	5.5415530e-004	4.7681262e-004	5.6985415e-004
5.7945981e-004	5.4873066e-004	5.9169861e-004	6.1314728e-004
4.8141930e-004	5.7310097e-004	5.0219931e-004	5.2669689e-004
6.3671927e-004	6.6377994e-004	6.8458539e-004	6.9907415e-004
6.5785984e-004	6.4364626e-004	6.1094236e-004	6.8275271e-004
6.1435263e-004	5.8519786e-004	6.2307938e-004	5.8544196e-004
5.3062123e-004	5.5176922e-004	6.0054948e-004	6.3721393e-004

5.9874033e-004	5.5076079e-004	5.1099167e-004	6.1904799e-004
5.1553002e-004	5.7849460e-004	4.8825216e-004	5.2791044e-004
6.2079074e-004	6.0498899e-004	6.5557269e-004	6.0713509e-004
6.4675228e-004	6.3655665e-004	6.6747272e-004	6.3108625e-004
5.9818935e-004	5.6156330e-004	5.9777102e-004	5.4257498e-004
5.9154291e-004	5.6271812e-004	5.0209946e-004	5.4023550e-004
5.2433238e-004	5.7458445e-004	5.2627958e-004	5.5391248e-004
5.2189122e-004	5.4380960e-004	4.8610917e-004	5.8063975e-004
6.0963928e-004	6.7041609e-004	5.7575690e-004	7.5091684e-004
6.2274298e-004	6.6063492e-004	6.6007649e-004	6.9474381e-004
6.7427589e-004	5.8445669e-004	6.6005409e-004	6.3442205e-004
6.0896509e-004	6.7094390e-004	6.2392476e-004	6.6805657e-004
5.7036779e-004	6.2713597e-004	6.7952614e-004	7.1489801e-004
6.8179000e-004	6.7090927e-004	5.9413747e-004	6.3001235e-004
5.1625122e-004	6.3487842e-004	6.3924531e-004	6.1834857e-004
5.9496968e-004	6.2643296e-004	6.1672325e-004	5.9841305e-004
6.0909071e-004	7.0254916e-004	6.1518032e-004	7.1790240e-004
5.5851952e-004	5.1525651e-004	6.0814913e-004	6.7868085e-004
5.9584343e-004	7.0586483e-004	6.5180370e-004	7.1146093e-004
5.6695583e-004	6.2803480e-004	5.9242394e-004	6.1470029e-004
6.4179521e-004	7.3009517e-004	7.4203147e-004	6.9472502e-004
6.7690927e-004	6.8997823e-004	6.3659534e-004	6.5677456e-004
5.9640693e-004	5.9564059e-004	6.7265567e-004	6.2504334e-004
5.5317068e-004	5.8303330e-004	5.1855297e-004	5.8317080e-004
6.4718528e-004	5.9471473e-004	5.8652567e-004	6.3622362e-004
5.1454658e-004	5.6907651e-004	5.3954755e-004	5.1645674e-004
5.9248901e-004	6.0593509e-004	6.3559490e-004	6.9342069e-004
6.5569016e-004	6.9194815e-004	6.5639861e-004	5.7299765e-004
6.0509687e-004	5.3161284e-004	5.9195404e-004	5.5856482e-004
5.2550997e-004	5.7746905e-004	5.4292717e-004	6.0741260e-004
5.5835996e-004	5.7667658e-004	5.7842628e-004	5.4386782e-004
5.1328925e-004	5.5610083e-004	4.5659939e-004	5.1234209e-004
4.6119108e-004	5.7752841e-004	5.5551488e-004	5.8023978e-004
5.6317377e-004	5.8929973e-004	5.6104951e-004	5.8782032e-004
4.8099978e-004	4.9484405e-004	5.5460040e-004	5.5152342e-004
5.1535146e-004	5.3959343e-004	4.5655233e-004	5.5745408e-004
5.6205856e-004	5.2734918e-004	5.7518558e-004	5.8944055e-004
4.5853315e-004	5.6921791e-004	4.7872952e-004	5.0747764e-004
5.9528719e-004	6.0774408e-004	6.3240677e-004	6.5673763e-004
6.2034176e-004	6.2542650e-004	5.8252981e-004	6.6368237e-004
5.7508326e-004	5.6502905e-004	5.9850907e-004	5.7992082e-004
4.9927452e-004	5.2340425e-004	5.8832260e-004	6.4344112e-004
5.8103778e-004	5.4005533e-004	5.1945672e-004	5.9898857e-004
4.9015085e-004	5.6617205e-004	4.7196459e-004	5.1464408e-004
6.0462690e-004	6.8017069e-004	5.9404866e-004	7.3837204e-004
6.2850831e-004	6.6332996e-004	6.4978415e-004	6.8637828e-004
6.8619412e-004	5.9080140e-004	6.4286382e-004	6.1172305e-004
6.0737239e-004	6.5783093e-004	6.3304484e-004	6.4338928e-004
5.6009310e-004	5.9330430e-004	6.4606944e-004	7.2058784e-004
6.3361230e-004	6.4200343e-004	5.8722950e-004	5.9400381e-004
4.7968348e-004	6.3288397e-004	6.2861474e-004	5.8440886e-004
5.9166085e-004	6.1494588e-004	5.9655732e-004	6.0404054e-004

6.2067682e-004	6.8165973e-004	5.9367865e-004	6.7735616e-004
5.4707342e-004	5.3523014e-004	6.1240076e-004	6.6300803e-004
5.9409640e-004	6.7432002e-004	6.1503668e-004	6.8722860e-004
5.5573197e-004	5.9275767e-004	5.7344693e-004	6.0108709e-004
6.3433668e-004	7.0079282e-004	7.0629561e-004	6.6987825e-004
6.5946784e-004	6.4141759e-004	6.3533518e-004	6.5977350e-004
5.9584039e-004	5.7643343e-004	6.5133507e-004	6.2942614e-004
5.5997765e-004	5.9261669e-004	5.1088350e-004	5.6685242e-004
6.3307652e-004	5.6718016e-004	5.6383449e-004	5.7929767e-004
5.0185670e-004	5.7080147e-004	5.2129258e-004	5.0411130e-004
5.9768217e-004	6.3651755e-004	6.1798605e-004	6.7187792e-004
6.3829940e-004	6.4502731e-004	6.3027824e-004	6.0992372e-004
5.5614767e-004	5.3226318e-004	5.9932476e-004	5.4347153e-004
5.1494857e-004	5.6344463e-004	4.9238823e-004	5.8771487e-004
5.9017006e-004	5.9947859e-004	5.5915493e-004	5.3033172e-004
4.8313401e-004	5.6494656e-004	4.5172899e-004	4.9893052e-004
5.3442108e-004	5.4324110e-004	6.3139551e-004	5.2318595e-004
6.2675375e-004	6.6360633e-004	6.1812243e-004	6.4056416e-004
5.8603525e-004	5.1676616e-004	5.4716933e-004	5.5654746e-004
5.4430102e-004	5.3395538e-004	5.5722446e-004	5.8767342e-004
5.5359842e-004	5.4027277e-004	5.9895517e-004	5.5767597e-004
5.0625589e-004	5.4807114e-004	5.0330612e-004	5.0895776e-004
6.0313049e-004	6.5992623e-004	6.0955228e-004	7.0546378e-004
6.3937533e-004	6.2528654e-004	5.9517517e-004	6.9612193e-004
6.8463656e-004	5.9245549e-004	5.9759156e-004	5.3327173e-004
6.0434773e-004	6.1086127e-004	6.0937146e-004	6.1070388e-004
5.2973762e-004	5.7481416e-004	6.2773796e-004	6.8997729e-004
5.5958239e-004	6.1255667e-004	5.5863310e-004	5.8111145e-004
5.4267581e-004	6.4875817e-004	6.0999965e-004	5.4482123e-004
6.1200085e-004	5.7632168e-004	5.5465165e-004	6.6974974e-004
5.3981606e-004	6.4114410e-004	6.4208964e-004	5.7021303e-004
5.2601273e-004	5.5716926e-004	6.1438822e-004	5.7372167e-004
5.9695056e-004	6.8021753e-004	5.6563911e-004	6.4211839e-004
5.5791906e-004	6.0774093e-004	5.7518503e-004	6.7614882e-004
6.3352652e-004	6.5044813e-004	6.3426800e-004	6.5457841e-004
6.6288040e-004	6.0808116e-004	6.2738982e-004	6.3433708e-004
6.0191668e-004	5.7707631e-004	6.1245475e-004	5.8981711e-004
5.8593987e-004	5.9716284e-004	5.6175220e-004	5.3294204e-004
5.6848821e-004	5.4741715e-004	5.2031533e-004	5.3225437e-004
5.0284212e-004	5.7757089e-004	5.1161089e-004	5.1769784e-004
5.9897713e-004	6.5811707e-004	6.4823145e-004	6.1839826e-004
6.0682371e-004	5.9149698e-004	5.9475194e-004	6.6766112e-004
5.3120939e-004	5.4897222e-004	5.8211335e-004	5.3936696e-004
4.8992832e-004	5.4938668e-004	4.4333202e-004	5.5965359e-004
5.7408607e-004	5.6919940e-004	5.3322992e-004	5.1479300e-004
4.5138594e-004	5.3823859e-004	4.6603527e-004	4.7222594e-004
5.4032869e-004	5.5016905e-004	6.2249204e-004	5.6587392e-004
6.0599763e-004	6.2461552e-004	6.1422843e-004	6.0647159e-004
5.7445891e-004	5.0072003e-004	5.8641183e-004	5.4539178e-004
5.1470175e-004	5.3598722e-004	5.1411056e-004	5.9263679e-004
5.6104240e-004	5.4052140e-004	5.9045661e-004	5.1263381e-004
5.0303838e-004	5.6454254e-004	4.4068378e-004	4.7621634e-004

6.1298296e-004	6.5981329e-004	6.3834894e-004	6.9381685e-004
6.4868039e-004	6.6168334e-004	6.4323716e-004	6.3793230e-004
7.3627972e-004	5.8159584e-004	6.2912503e-004	6.0789826e-004
6.4568505e-004	6.4607189e-004	6.5055046e-004	6.1928842e-004
6.4302074e-004	5.3889885e-004	5.9320928e-004	7.0178448e-004
5.7471191e-004	5.9082056e-004	5.7206581e-004	5.9451541e-004
6.0657686e-004	5.6100912e-004	5.0696515e-004	5.5266891e-004
5.7630687e-004	4.9221653e-004	5.7276608e-004	5.8564736e-004
5.0808140e-004	5.7345246e-004	6.2226901e-004	4.8738073e-004
4.4015924e-004	5.2088077e-004	5.6591402e-004	5.3034069e-004
5.2744302e-004	6.0595605e-004	5.5876165e-004	6.2733583e-004
5.3799059e-004	5.6325446e-004	5.4867064e-004	6.7555239e-004
6.3573668e-004	6.1479610e-004	6.1026310e-004	6.8718057e-004
6.7205899e-004	6.0097969e-004	6.1700537e-004	5.8146204e-004
6.1993166e-004	5.6943898e-004	5.7578010e-004	5.7612726e-004
5.4767918e-004	5.7701473e-004	5.8745944e-004	5.3505755e-004
5.4334194e-004	5.0898155e-004	5.1135595e-004	5.6852191e-004
5.1749877e-004	5.6335033e-004	4.7599811e-004	5.2061130e-004
5.8814826e-004	6.4579654e-004	6.1925023e-004	5.9692953e-004
5.9892118e-004	5.4742643e-004	6.0268471e-004	6.4199728e-004
5.6399188e-004	5.1958091e-004	5.8219985e-004	5.4905338e-004
5.5335923e-004	5.4171735e-004	4.7133248e-004	5.3166255e-004
5.3987881e-004	5.1674310e-004	5.3907813e-004	4.7986026e-004
4.6131768e-004	5.4427622e-004	4.4650074e-004	4.8223030e-004
5.3579074e-004	5.8861994e-004	5.9545248e-004	5.9058005e-004
5.9085619e-004	5.5693937e-004	5.5054718e-004	6.2207281e-004
5.4032214e-004	5.1452711e-004	5.5833880e-004	5.0594246e-004
4.8914037e-004	5.1431532e-004	4.4868364e-004	5.5715001e-004
5.2889713e-004	5.3010324e-004	5.2241565e-004	4.6639355e-004
4.7250604e-004	5.0867042e-004	4.0776282e-004	4.3856433e-004
6.0183389e-004	6.2608907e-004	5.9506956e-004	6.5318478e-004
6.1381045e-004	6.0937148e-004	5.7091523e-004	6.4935668e-004
6.7095723e-004	5.5414393e-004	5.8674393e-004	5.3974530e-004
6.1592033e-004	6.0664339e-004	5.4994231e-004	6.0667569e-004
5.5593163e-004	5.2161426e-004	5.8763013e-004	6.4223488e-004
5.2115640e-004	5.6215251e-004	5.0552210e-004	5.8197415e-004
5.5634888e-004	5.2528371e-004	4.6738407e-004	5.2564110e-004
5.1654768e-004	4.7362821e-004	5.2395550e-004	4.7524744e-004
5.0936038e-004	4.8146395e-004	5.6294702e-004	4.9946336e-004
4.0991320e-004	5.0421080e-004	5.0990749e-004	5.0296654e-004
5.0502887e-004	5.5151352e-004	5.8564033e-004	5.9861968e-004
5.1759576e-004	5.7823244e-004	5.3020105e-004	6.0853393e-004
5.9560112e-004	6.1549053e-004	6.3769832e-004	6.6140406e-004
6.6899044e-004	5.8582274e-004	6.2854979e-004	5.6309772e-004
6.0205754e-004	5.2901922e-004	5.7940773e-004	5.6401535e-004
5.5706791e-004	5.6129892e-004	5.5184294e-004	5.2186528e-004
5.2700136e-004	5.2627511e-004	4.9800658e-004	5.4454397e-004
4.8705025e-004	5.3632006e-004	4.6462716e-004	4.9008651e-004
5.6870459e-004	6.0351279e-004	6.0766423e-004	5.6911650e-004
5.6984252e-004	5.1641878e-004	5.8606807e-004	6.4972258e-004
5.6082518e-004	5.3062399e-004	5.6196749e-004	5.0946846e-004
5.3480355e-004	5.2626285e-004	4.6176086e-004	5.3305953e-004

5.0486885e-004 4.9845646e-004 5.3957452e-004 4.6803742e-004
4.4729281e-004 5.0977601e-004 4.2237710e-004 4.8697479e-004
5.1562762e-004 5.4793089e-004 5.8874823e-004 5.7154454e-004
5.6701859e-004 5.3912192e-004 5.1912814e-004 5.8883215e-004
5.4712042e-004 5.1066237e-004 5.3986392e-004 5.0536168e-004
5.0205253e-004 4.7271066e-004 4.5245211e-004 5.1960509e-004
5.1342667e-004 4.8717493e-004 5.2423769e-004 4.5603438e-004
4.8698557e-004 5.1364717e-004 4.0834908e-004 4.2575330e-004
6.0400488e-004 6.1414670e-004 5.9498067e-004 6.6397263e-004
6.4449364e-004 6.3613369e-004 6.1765643e-004 6.3598862e-004
6.9684714e-004 5.3415832e-004 5.9086188e-004 5.9915906e-004
6.2335512e-004 6.0827729e-004 5.8339085e-004 5.9238978e-004
6.0058973e-004 5.3116135e-004 5.8833770e-004 6.4059079e-004
5.3655775e-004 5.7765118e-004 4.9855440e-004 5.9220794e-004
5.6123614e-004 5.9468270e-004 6.2790253e-004 6.3355142e-004
6.4039728e-004 5.7680256e-004 6.4164822e-004 5.6476262e-004
5.8733096e-004 5.1665577e-004 5.9939257e-004 5.3406322e-004
5.5784491e-004 5.3700173e-004 5.1712845e-004 5.3458272e-004
5.1271604e-004 5.3126611e-004 5.1816858e-004 5.2629207e-004
4.8058438e-004 5.2017259e-004 4.6074424e-004 4.8093550e-004
5.4612057e-004 5.6808988e-004 5.8670821e-004 5.6623574e-004
5.8401375e-004 5.1401785e-004 5.7624646e-004 6.3494063e-004
5.8589673e-004 5.6343012e-004 5.5119340e-004 5.1842857e-004
5.2941602e-004 5.1440323e-004 4.7914411e-004 5.2851951e-004
4.9458833e-004 4.8762174e-004 5.4500773e-004 4.8259872e-004
4.6083075e-004 4.7729936e-004 4.2675617e-004 4.7984856e-004
4.7849330e-004 5.0208320e-004 5.5207267e-004 5.4205631e-004
5.1847644e-004 5.2553843e-004 4.9212955e-004 5.6820476e-004
5.1844145e-004 4.5704123e-004 5.5526123e-004 5.4214282e-004
4.5761570e-004 4.9101485e-004 5.5409845e-004 5.0126347e-004
6.4427017e-004 5.5501652e-004 5.2921699e-004 5.7489693e-004
5.4868799e-004 5.7913880e-004 5.6229188e-004 6.1082365e-004
5.5509064e-004 5.7168322e-004 6.4724701e-004 6.1750757e-004
6.5159565e-004 5.6771479e-004 6.3041312e-004 5.3219740e-004
5.8747748e-004 5.0513758e-004 5.8115045e-004 5.4269966e-004
5.1797769e-004 5.1616035e-004 5.2238876e-004 5.4600830e-004
5.0196714e-004 5.2195163e-004 4.7493431e-004 5.1337223e-004
4.9116189e-004 4.8987768e-004 4.9193169e-004 4.7415042e-004
5.4316667e-004 5.4583671e-004 5.5666589e-004 5.9108061e-004
6.1237042e-004 5.3669908e-004 6.2692644e-004 6.2487036e-004
6.0130672e-004 5.7821663e-004 5.8636299e-004 5.2157151e-004
5.4782080e-004 5.1609444e-004 5.2262002e-004 5.4623623e-004
5.0932245e-004 5.0754847e-004 5.8498337e-004 5.2448925e-004
4.8920041e-004 5.1513351e-004 4.4083774e-004 5.2934101e-004
5.1372514e-004 5.2841227e-004 5.4862838e-004 5.8710453e-004
5.6727724e-004 4.9950932e-004 5.2016166e-004 5.6473602e-004
5.4092648e-004 5.4032871e-004 4.7302807e-004 5.1645072e-004
5.0020422e-004 4.8903190e-004 4.4601761e-004 4.5951900e-004
5.0417970e-004 4.5833994e-004 5.0060189e-004 4.6852417e-004
4.6420579e-004 4.8230816e-004 4.1320326e-004 4.2962707e-004
5.2511510e-004 5.5942420e-004 5.1009762e-004 6.0245660e-004
5.5527729e-004 5.3310698e-004 5.3837340e-004 5.2894856e-004

4.8859218e-004	4.7248307e-004	4.9211940e-004	4.6538129e-004
4.5852465e-004	4.7043428e-004	4.1919537e-004	4.8397887e-004
4.6255210e-004	4.7866428e-004	4.2831407e-004	4.8825626e-004
4.9685221e-004	4.4696603e-004	4.4372322e-004	4.8200221e-004
5.6866564e-004	5.7830350e-004	6.7869497e-004	6.5297120e-004
6.2207723e-004	5.5399827e-004	5.3154667e-004	7.1765314e-004
5.8174337e-004	5.2553575e-004	6.0737254e-004	6.6035803e-004
5.5814872e-004	5.9089686e-004	6.8063251e-004	6.3178196e-004
7.1695626e-004	6.9401356e-004	6.5009060e-004	6.5051315e-004
6.5530135e-004	7.3821082e-004	7.3775418e-004	7.6493251e-004
5.3636391e-004	5.6192063e-004	6.2417297e-004	5.9681111e-004
6.4583642e-004	5.5912625e-004	6.2328271e-004	5.3958115e-004
5.9190847e-004	5.1088291e-004	5.7490976e-004	5.3653985e-004
4.9804390e-004	5.0987651e-004	5.1877176e-004	5.5864545e-004
4.9046293e-004	5.1882607e-004	4.5741431e-004	4.8655321e-004
4.9357652e-004	4.7457821e-004	5.0109554e-004	4.8004936e-004
5.3204125e-004	5.1678129e-004	5.1230051e-004	5.9767820e-004
6.2449127e-004	5.6147162e-004	6.3675262e-004	6.1954756e-004
6.1169379e-004	5.8636395e-004	5.6761075e-004	5.4346549e-004
5.3877037e-004	5.1575463e-004	5.6104883e-004	5.6767663e-004
5.0523916e-004	5.1377977e-004	6.0452112e-004	5.4909274e-004
5.0583672e-004	5.1589603e-004	4.5603989e-004	5.4936801e-004
5.2171819e-004	5.2677668e-004	5.3413527e-004	5.8411671e-004
5.7554737e-004	5.0153854e-004	5.4260450e-004	5.6032604e-004
5.4373543e-004	5.4678399e-004	4.5562189e-004	5.3554916e-004
4.9100543e-004	5.1617817e-004	4.5588001e-004	4.6008307e-004
5.0554487e-004	4.6379068e-004	4.9821864e-004	4.8557625e-004
4.6180838e-004	4.7688653e-004	4.2316192e-004	4.3707120e-004
5.6407742e-004	5.8626992e-004	6.8185260e-004	6.6023598e-004
6.3711834e-004	5.5308627e-004	5.2884710e-004	7.0902436e-004
5.9364956e-004	5.2235933e-004	5.8681816e-004	6.6010376e-004
5.6286853e-004	6.1643358e-004	7.0025471e-004	6.5696752e-004
6.9272397e-004	7.1398054e-004	6.4187706e-004	6.5933097e-004
6.5184677e-004	7.2492170e-004	7.2536858e-004	7.4049563e-004
4.7284234e-004	5.1189781e-004	5.7606996e-004	5.5438376e-004
5.3582906e-004	4.5767729e-004	4.9590728e-004	5.2423524e-004
4.9726772e-004	5.0032622e-004	5.6042567e-004	4.9296734e-004
5.3607442e-004	5.1015088e-004	5.6647291e-004	5.4583450e-004
5.9731350e-004	5.8537371e-004	5.6154034e-004	6.3942561e-004
5.5231468e-004	6.1809927e-004	6.2771800e-004	6.5247163e-004
5.2930643e-004	5.6822618e-004	6.0001932e-004	5.9099557e-004
6.4322469e-004	5.3939056e-004	6.1755403e-004	5.5174496e-004
5.8663038e-004	5.3447818e-004	5.5636495e-004	5.4840439e-004
4.9347422e-004	5.1730832e-004	5.2571877e-004	5.6219116e-004
5.0151585e-004	5.2324029e-004	4.5889301e-004	4.8524194e-004
4.9362524e-004	4.7224793e-004	5.1482889e-004	4.9012484e-004
5.3333637e-004	5.1457404e-004	4.9386951e-004	6.2067973e-004
6.2664009e-004	6.0213327e-004	6.2277970e-004	6.1957544e-004
6.0957897e-004	5.8204819e-004	5.6364374e-004	5.7857521e-004
5.3267409e-004	5.2762825e-004	5.9533368e-004	5.8971811e-004
5.0559506e-004	5.1998845e-004	5.9230537e-004	5.4403668e-004
5.1146594e-004	5.1994753e-004	4.7782765e-004	5.6700538e-004

5.3438931e-004	5.0464794e-004	5.1599854e-004	5.6208079e-004
5.8823955e-004	5.1693857e-004	5.7880956e-004	5.7401958e-004
5.6279570e-004	5.4429476e-004	4.6245073e-004	5.6070006e-004
4.7446749e-004	5.2910780e-004	4.8843968e-004	5.1100743e-004
4.8626433e-004	4.7445024e-004	5.3042766e-004	5.1243849e-004
4.6774944e-004	4.6276809e-004	4.2604988e-004	4.6610241e-004
4.9206287e-004	6.0765297e-004	5.6969665e-004	6.2177914e-004
6.6366068e-004	5.3331174e-004	5.6705643e-004	6.4096053e-004
5.6215381e-004	5.3180056e-004	5.7333011e-004	6.2794617e-004
5.0756451e-004	5.8396127e-004	5.2621168e-004	5.4138385e-004
5.6850123e-004	5.3523453e-004	5.3181170e-004	5.5597977e-004
5.4135828e-004	5.1365369e-004	5.0678328e-004	5.2350611e-004
5.7010696e-004	6.2380660e-004	6.7354970e-004	6.7689427e-004
7.0775751e-004	6.2464285e-004	6.0279343e-004	6.4252924e-004
6.1232562e-004	6.8273616e-004	6.1187221e-004	6.0401298e-004
5.5070322e-004	5.6389788e-004	5.7557032e-004	6.0858080e-004
5.8122590e-004	5.8493418e-004	5.7711615e-004	6.3819048e-004
5.4699907e-004	5.5701390e-004	5.6732267e-004	6.1848327e-004
5.2437779e-004	5.7368436e-004	6.3097794e-004	6.1259925e-004
6.3142621e-004	6.1832098e-004	6.3998647e-004	7.1197456e-004
6.4141523e-004	6.1083029e-004	6.0042801e-004	5.8406439e-004
5.7885829e-004	5.2000484e-004	5.9440990e-004	5.9462569e-004
5.8878819e-004	5.8699115e-004	5.6191045e-004	6.7249975e-004
5.3727324e-004	5.7097938e-004	5.6071162e-004	5.5374388e-004
5.5017238e-004	5.7683878e-004	6.2726100e-004	6.1331094e-004
6.0703266e-004	5.5393066e-004	5.1848937e-004	6.7043003e-004
5.7707859e-004	5.1291600e-004	5.4987067e-004	6.2504008e-004
5.4978665e-004	5.9187201e-004	6.5707098e-004	6.1872922e-004
6.0869186e-004	6.9114532e-004	6.0352073e-004	6.1053908e-004
6.2073991e-004	6.4337444e-004	6.3307515e-004	6.2747991e-004
5.4468722e-004	5.4013811e-004	5.3616013e-004	6.1003389e-004
6.3338577e-004	5.8478866e-004	6.0315178e-004	5.9524468e-004
6.1096958e-004	5.7416478e-004	5.6201081e-004	5.8966201e-004
5.2380213e-004	5.5262732e-004	5.4669703e-004	5.8117732e-004
5.0053780e-004	5.1435693e-004	4.9398283e-004	4.8012066e-004
4.9143747e-004	4.8572894e-004	4.8186786e-004	5.3357236e-004
5.3737219e-004	4.6657123e-004	4.9221479e-004	5.8964713e-004
6.0267284e-004	5.6156948e-004	6.0119708e-004	6.2033908e-004
6.0093479e-004	5.5148006e-004	5.0693834e-004	5.8086337e-004
5.0015234e-004	5.3706201e-004	5.7301459e-004	5.8226944e-004
4.8608063e-004	5.0059219e-004	6.0555605e-004	5.4920631e-004
4.9139276e-004	4.8964595e-004	4.4693544e-004	5.2585547e-004
4.3106982e-004	5.0663787e-004	4.8270663e-004	5.2666051e-004
5.2182128e-004	5.3305220e-004	4.5972999e-004	4.5257093e-004
4.7883594e-004	4.0810092e-004	4.6713467e-004	4.6784448e-004
4.3750321e-004	4.0667532e-004	4.2104783e-004	4.1344418e-004
3.9907926e-004	4.3902453e-004	3.7820496e-004	4.4324797e-004
4.0171143e-004	4.1979774e-004	3.7932992e-004	4.7007880e-004
5.0570049e-004	4.8395406e-004	5.4176270e-004	5.1046189e-004
5.5077857e-004	4.7033570e-004	4.5920697e-004	4.3019437e-004
4.4270734e-004	4.5185641e-004	4.1206215e-004	4.3379318e-004
4.5403648e-004	4.9743923e-004	3.8799135e-004	4.2953310e-004

4.1649273e-004 3.7621496e-004 4.1046594e-004 4.4605895e-004
4.1739433e-004 3.9867496e-004 4.0184345e-004 4.2603612e-004
5.6777571e-004 5.9652957e-004 6.9916913e-004 6.8528789e-004
7.1265429e-004 6.8064533e-004 6.5103055e-004 6.9589901e-004
6.4977586e-004 6.6655593e-004 6.6782569e-004 6.0645405e-004
5.9094319e-004 5.7154203e-004 5.9243450e-004 6.0931986e-004
6.1365677e-004 6.1760300e-004 5.7393523e-004 7.1074330e-004
5.5520669e-004 5.6984626e-004 5.7345998e-004 6.2107827e-004
5.7221942e-004 6.1209319e-004 6.6783125e-004 6.5875128e-004
6.4123483e-004 5.4822535e-004 5.3348001e-004 6.5450490e-004
5.7509985e-004 5.7845621e-004 5.6868192e-004 6.0568668e-004
5.6360317e-004 6.1708335e-004 6.6352777e-004 6.7451948e-004
6.2827949e-004 6.8658698e-004 6.2553774e-004 6.8236519e-004
6.1906893e-004 6.9185486e-004 6.8270475e-004 7.0478641e-004
4.8897728e-004 5.3552580e-004 5.5836439e-004 4.7446838e-004
4.9264101e-004 4.6510568e-004 4.5012507e-004 4.9592484e-004
4.8024972e-004 5.3440083e-004 5.0272814e-004 4.3286982e-004
4.8183787e-004 5.1461354e-004 5.4029810e-004 5.5185545e-004
5.9958403e-004 5.8394228e-004 6.3643304e-004 6.4313056e-004
6.2574100e-004 6.6692885e-004 6.6846730e-004 7.3312762e-004
5.6130221e-004 5.2638515e-004 5.3273901e-004 6.2226884e-004
6.3457112e-004 5.8564125e-004 6.0514521e-004 6.0423752e-004
6.2455647e-004 6.0587573e-004 5.6912327e-004 6.1968510e-004
5.5123669e-004 5.8442731e-004 5.4455904e-004 5.8733832e-004
5.1949681e-004 5.1833893e-004 5.1139905e-004 5.0277668e-004
4.9045942e-004 4.9668492e-004 4.8019746e-004 5.3316209e-004
5.5134448e-004 4.6459343e-004 5.1051204e-004 6.1010681e-004
6.0112176e-004 5.5798830e-004 5.8952057e-004 6.1974583e-004
5.8951000e-004 5.4030134e-004 5.3689341e-004 5.8744490e-004
4.9667079e-004 5.3843217e-004 5.7043135e-004 5.8687999e-004
4.9228637e-004 5.0863366e-004 5.8934589e-004 5.4960445e-004
4.8654302e-004 4.9740353e-004 4.4758184e-004 5.1274522e-004
4.2042239e-004 5.2486737e-004 4.8562785e-004 5.2131272e-004
5.1119005e-004 5.4543719e-004 4.6212717e-004 4.4901713e-004
4.6992181e-004 4.1437430e-004 4.7621199e-004 4.6870863e-004
4.3265051e-004 3.9703347e-004 4.1192958e-004 3.8739239e-004
3.9963268e-004 4.4759759e-004 3.8436224e-004 4.3558681e-004
3.9083317e-004 4.3791313e-004 3.6306240e-004 4.3678382e-004
4.9697160e-004 4.7902772e-004 5.6857533e-004 5.2315845e-004
5.5877800e-004 4.7610899e-004 5.0231269e-004 4.3244344e-004
4.4183844e-004 4.4406162e-004 4.3749437e-004 4.3320849e-004
4.5148319e-004 4.8883848e-004 4.0167929e-004 4.6293122e-004
4.3620329e-004 3.7360038e-004 4.2568542e-004 4.5470885e-004
4.0223384e-004 3.9995684e-004 4.3043342e-004 4.4219723e-004
5.4562031e-004 5.7614765e-004 6.8117683e-004 6.9336277e-004
6.9805099e-004 6.7615521e-004 6.5966403e-004 6.7330743e-004
6.3333138e-004 6.7226606e-004 6.5240491e-004 5.9285956e-004
5.8699641e-004 5.7271350e-004 5.7305561e-004 6.1067146e-004
6.1527699e-004 6.1245984e-004 5.8012319e-004 6.8651751e-004
5.4748278e-004 5.5676208e-004 5.5603353e-004 6.1323986e-004
5.7639061e-004 4.9878197e-004 5.2088275e-004 6.4513159e-004
6.2405707e-004 6.0909934e-004 6.1194213e-004 6.2160719e-004

6.1564863e-004	6.2195047e-004	6.0955888e-004	6.4215092e-004
5.5489817e-004	6.0572720e-004	5.9372098e-004	6.0306954e-004
5.3228543e-004	5.4264444e-004	5.7034277e-004	5.5979055e-004
5.2800588e-004	5.3161860e-004	4.6536414e-004	5.3813795e-004
5.6839867e-004	4.9253002e-004	5.4640639e-004	6.0193021e-004
6.1749345e-004	5.2237249e-004	5.5594652e-004	5.6690005e-004
5.5518901e-004	5.0731889e-004	5.2862459e-004	5.6870926e-004
4.6607959e-004	4.8632945e-004	5.0840249e-004	5.6022026e-004
4.8467887e-004	4.9275671e-004	5.1214293e-004	5.1254357e-004
4.6373400e-004	4.6971838e-004	4.4747664e-004	5.0798274e-004
4.3384362e-004	5.2219855e-004	4.8173434e-004	5.2468099e-004
4.8532996e-004	5.1043935e-004	4.5912065e-004	4.2362055e-004
5.0539703e-004	4.5697850e-004	4.6238296e-004	4.5166497e-004
4.4106766e-004	4.2317830e-004	4.0787984e-004	3.6119455e-004
3.9793973e-004	4.2181332e-004	4.2512923e-004	4.3537212e-004
4.0536486e-004	4.3650198e-004	3.5273957e-004	4.0251787e-004
5.6755902e-004	5.4155677e-004	6.0551861e-004	6.7273248e-004
6.3371941e-004	6.2934363e-004	6.3947499e-004	6.9122083e-004
6.4828043e-004	6.4690181e-004	6.2481794e-004	5.8476887e-004
5.4848050e-004	5.5064335e-004	5.9292100e-004	5.8990948e-004
6.0782972e-004	5.7734762e-004	5.7723772e-004	6.4872690e-004
5.2724511e-004	5.3975508e-004	5.4434832e-004	5.8913625e-004
5.6778769e-004	5.9445380e-004	6.2360373e-004	5.6834925e-004
5.9427894e-004	5.5025004e-004	5.3189037e-004	5.5595765e-004
5.4085064e-004	6.0827826e-004	5.5875862e-004	5.8131439e-004
5.6476177e-004	5.7159136e-004	5.7768750e-004	6.3831247e-004
6.4728124e-004	7.0395456e-004	6.8342380e-004	7.0220229e-004
6.5015268e-004	7.1160687e-004	6.9776711e-004	7.7824981e-004
5.7220199e-004	5.0236392e-004	5.3787079e-004	6.4213987e-004
6.2061159e-004	5.7948148e-004	6.1311474e-004	6.0365017e-004
6.0896503e-004	6.0924397e-004	6.3432691e-004	6.1698881e-004
5.5208415e-004	5.9907335e-004	5.7815485e-004	5.9169711e-004
5.3674295e-004	5.5808527e-004	5.4808153e-004	5.6591460e-004
5.4054848e-004	5.3125994e-004	4.6324953e-004	5.2274692e-004
5.6050826e-004	5.6639218e-004	6.0357041e-004	5.7081808e-004
6.0995972e-004	5.7334712e-004	5.5467912e-004	5.4676065e-004
5.5531207e-004	6.0867282e-004	5.7442073e-004	6.0152897e-004
5.5963399e-004	5.5637296e-004	5.5754452e-004	6.2823604e-004
6.4825160e-004	7.0094288e-004	6.7804090e-004	6.9529102e-004
6.4643360e-004	7.2240864e-004	6.7745402e-004	7.5312193e-004
5.3357404e-004	5.3164306e-004	5.2058858e-004	4.6717499e-004
4.4972173e-004	4.8764000e-004	4.8448247e-004	4.5615016e-004
4.5875770e-004	5.0509343e-004	5.0284290e-004	4.9888290e-004
4.6980933e-004	4.7627486e-004	4.6797626e-004	5.2129789e-004
4.8621489e-004	5.2842527e-004	6.1520591e-004	6.0307018e-004
5.5787428e-004	6.6517114e-004	6.1775041e-004	6.8460341e-004
5.7289089e-004	5.5808962e-004	5.8738288e-004	6.1125550e-004
6.6398537e-004	6.1928621e-004	6.2312707e-004	6.2605634e-004
6.1844673e-004	6.5167902e-004	6.4964728e-004	6.5088917e-004
5.9411635e-004	5.8673238e-004	5.7313914e-004	6.2326768e-004
6.7513726e-004	7.0555360e-004	6.8948462e-004	7.1566076e-004
6.8555964e-004	7.3299900e-004	6.8847428e-004	7.0312236e-004

5.3574494e-004	4.8303549e-004	4.6355273e-004	3.8167647e-004
4.2681085e-004	4.2685003e-004	4.1415457e-004	3.7857820e-004
4.0599729e-004	3.6986839e-004	3.8619270e-004	3.9534840e-004
3.8928648e-004	3.9899629e-004	4.2868373e-004	4.2781743e-004
4.0488820e-004	4.6116950e-004	4.4951005e-004	4.5026297e-004
4.2033023e-004	5.1883687e-004	4.8576323e-004	5.6347968e-004
5.9776173e-004	6.0377335e-004	5.5278649e-004	5.9963450e-004
6.0660017e-004	5.1032603e-004	5.3120035e-004	6.0692677e-004
5.5859738e-004	5.6842141e-004	5.3972204e-004	5.0276477e-004
5.0796800e-004	5.4953869e-004	5.1680155e-004	4.7179277e-004
4.2164792e-004	5.0933522e-004	5.1882408e-004	5.0599602e-004
5.2366882e-004	5.3031760e-004	4.8288507e-004	5.0628791e-004
6.1312248e-004	6.5275657e-004	6.4380786e-004	6.8410143e-004
6.1546148e-004	5.9761816e-004	5.8668906e-004	6.5017409e-004
5.7819039e-004	6.0954443e-004	6.4369741e-004	6.8691807e-004
6.5409453e-004	7.0166965e-004	5.9244414e-004	6.6115444e-004
7.2172401e-004	7.3190812e-004	7.3591500e-004	7.9765399e-004
7.7588414e-004	8.3409414e-004	7.7613279e-004	7.9860172e-004
5.3403666e-004	4.8565514e-004	4.8056053e-004	4.0169933e-004
5.0201013e-004	4.5629352e-004	4.4304925e-004	3.8483671e-004
3.9779005e-004	3.8364050e-004	3.6609681e-004	4.0017576e-004
3.9884516e-004	4.1217916e-004	3.6550841e-004	3.3817824e-004
3.4780470e-004	3.9214144e-004	3.8156420e-004	3.0127884e-004
3.3673263e-004	3.5046232e-004	2.9389825e-004	3.5841279e-004
5.9881335e-004	5.8984086e-004	5.6028548e-004	6.4973879e-004
6.5302644e-004	5.7275392e-004	5.7032079e-004	6.0258270e-004
5.7775577e-004	5.5403622e-004	5.7264732e-004	5.3181025e-004
5.1653793e-004	5.3002828e-004	5.1173875e-004	5.0437007e-004
4.6112714e-004	5.6564089e-004	5.4313598e-004	5.1825483e-004
5.0968819e-004	5.4799239e-004	4.6234148e-004	5.2997404e-004
5.2110163e-004	4.9394914e-004	4.6219301e-004	4.4307913e-004
4.1835551e-004	4.0792243e-004	3.7252231e-004	4.0489980e-004
3.6661087e-004	3.4712143e-004	3.4065915e-004	3.4153555e-004
3.6073854e-004	4.0916824e-004	3.8830754e-004	3.6359154e-004
3.6779859e-004	4.4258443e-004	3.8220095e-004	4.5400550e-004
3.8332085e-004	4.1415854e-004	4.4799883e-004	5.0936003e-004
5.2862419e-004	5.5351292e-004	5.5754220e-004	6.6439467e-004
6.5849730e-004	5.5514369e-004	5.7642504e-004	6.0894836e-004
5.8162809e-004	6.4532881e-004	5.7410607e-004	5.3383202e-004
5.2988778e-004	5.5915158e-004	5.9769247e-004	5.9699226e-004
5.6864155e-004	5.6951874e-004	5.3133319e-004	5.9891051e-004
4.8248150e-004	5.2413180e-004	5.6297969e-004	5.3597655e-004
6.0225443e-004	5.6228224e-004	5.3751720e-004	5.5437697e-004
5.8204512e-004	5.2488658e-004	5.0478968e-004	5.7229320e-004
5.0041369e-004	4.9506638e-004	5.1419967e-004	4.7197867e-004
4.9961286e-004	4.9373626e-004	4.9345200e-004	4.6504598e-004
4.1356447e-004	5.2715458e-004	4.8620360e-004	4.4594290e-004
4.7110263e-004	4.9620120e-004	4.5164077e-004	4.6837208e-004
5.9099507e-004	5.6131228e-004	5.5852257e-004	6.6397828e-004
6.4677221e-004	5.8734739e-004	6.0962877e-004	6.0269385e-004
5.9806491e-004	5.4628239e-004	6.2222300e-004	5.5227400e-004
5.4265639e-004	5.3642552e-004	5.1420424e-004	5.3940759e-004

5.1710475e-004	5.5909075e-004	5.2462157e-004	5.3542900e-004
5.0769655e-004	5.6892964e-004	4.8397450e-004	5.5889784e-004
5.8438833e-004	6.3234002e-004	5.7523645e-004	5.6853276e-004
5.4238481e-004	5.1650062e-004	4.8816542e-004	5.7534983e-004
5.2418050e-004	5.1596112e-004	5.0370328e-004	5.5805860e-004
5.6919832e-004	5.7285218e-004	5.7751626e-004	6.1525432e-004
6.0692450e-004	6.5405470e-004	6.6653048e-004	7.4144623e-004
6.3869618e-004	6.9156826e-004	7.2657652e-004	7.4283819e-004
4.9279367e-004	4.9101282e-004	4.5065705e-004	4.5291081e-004
4.1723221e-004	3.9867422e-004	3.7301277e-004	4.1320229e-004
3.5356805e-004	3.5901073e-004	3.5488472e-004	3.4745520e-004
3.7228242e-004	4.0064997e-004	3.7354902e-004	3.5054814e-004
3.4890985e-004	4.2098158e-004	3.8866325e-004	4.4631800e-004
3.7346127e-004	3.9971438e-004	4.1872269e-004	4.6606228e-004
5.3728234e-004	5.5560560e-004	5.6833190e-004	6.6552845e-004
6.5942545e-004	5.4599720e-004	5.7051390e-004	6.0638288e-004
5.7832422e-004	6.2027390e-004	5.8013370e-004	5.4996905e-004
5.3425091e-004	5.5867810e-004	5.9501476e-004	5.8095207e-004
5.6057329e-004	5.6769140e-004	5.1181617e-004	5.9176273e-004
4.6806547e-004	5.1654081e-004	5.3671651e-004	5.2174425e-004
4.7136394e-004	4.7805817e-004	4.5537770e-004	4.3609789e-004
5.0500761e-004	4.0749565e-004	4.1546463e-004	4.0290652e-004
3.9400908e-004	3.8756990e-004	4.0010955e-004	4.0734498e-004
3.3142301e-004	4.0831080e-004	3.4853416e-004	3.6759357e-004
3.6345265e-004	3.6935866e-004	3.8896887e-004	3.6360271e-004
3.4703302e-004	3.5306302e-004	3.6449487e-004	3.4410690e-004
5.6956780e-004	5.6270885e-004	5.5102476e-004	6.5461735e-004
6.2821063e-004	5.6412171e-004	6.1561934e-004	6.0519532e-004
5.9135364e-004	5.2344154e-004	6.3791685e-004	5.5769498e-004
5.3087658e-004	5.3070069e-004	4.9833040e-004	5.2834654e-004
5.2120097e-004	5.3658776e-004	5.1271582e-004	5.3466579e-004
4.8482630e-004	5.6739047e-004	4.9775669e-004	5.5626981e-004
5.8239608e-004	6.4483576e-004	5.9579691e-004	6.0086521e-004
5.7387153e-004	5.2554416e-004	5.1300223e-004	5.8429837e-004
5.4526878e-004	5.3385350e-004	5.2296832e-004	5.6674414e-004
5.8473440e-004	5.8131687e-004	5.7457070e-004	6.5462622e-004
6.0854258e-004	6.4384847e-004	6.4854893e-004	7.3896383e-004
6.2974314e-004	6.6245292e-004	6.8318410e-004	6.8592603e-004
5.4293837e-004	5.0796499e-004	4.9273574e-004	5.1352320e-004
4.5771778e-004	4.4017746e-004	4.2996263e-004	4.2462440e-004
3.9838318e-004	4.6310238e-004	3.9766941e-004	4.0105091e-004
3.7186879e-004	4.3526059e-004	3.9572497e-004	3.7877371e-004
4.0037855e-004	4.2429352e-004	3.8312331e-004	3.8054615e-004
3.7780767e-004	3.5367422e-004	3.8920639e-004	4.0572719e-004
5.4187818e-004	5.5167358e-004	5.8522253e-004	6.7406180e-004
6.4550746e-004	5.4321943e-004	5.7671750e-004	6.0207650e-004
5.7679031e-004	5.9858579e-004	5.9942012e-004	5.6845305e-004
5.4321834e-004	5.7632386e-004	5.7609312e-004	5.6512225e-004
5.3888763e-004	5.7545956e-004	5.3017244e-004	5.7994777e-004
4.6550463e-004	5.0492594e-004	5.0154827e-004	5.3012650e-004
5.7132203e-004	5.8148355e-004	5.7539447e-004	6.2185458e-004
5.5279238e-004	4.4008277e-004	4.8583944e-004	4.9711535e-004

5.0458925e-004	4.9788037e-004	5.0992849e-004	4.9978458e-004
5.2640673e-004	5.4250658e-004	5.3045755e-004	6.0722062e-004
5.5720717e-004	6.0321671e-004	6.2137938e-004	6.6493946e-004
5.8410551e-004	6.5249082e-004	6.3256973e-004	6.5185010e-004
5.1345601e-004	5.0575905e-004	4.8411026e-004	5.0332609e-004
4.3510470e-004	4.4163194e-004	4.2083742e-004	4.4047808e-004
3.8328824e-004	4.3975165e-004	3.6902323e-004	3.5270149e-004
3.2997813e-004	4.0850363e-004	3.9706971e-004	3.5501333e-004
3.3657092e-004	3.5183975e-004	3.3762575e-004	3.5834180e-004
3.3457112e-004	3.0150874e-004	3.7783382e-004	3.6578315e-004
5.3036760e-004	6.1052052e-004	6.3267510e-004	6.7292474e-004
6.8840712e-004	6.4238529e-004	6.8072994e-004	7.2208302e-004
7.0092361e-004	6.1950296e-004	6.9930601e-004	6.6891740e-004
6.2970535e-004	6.4485881e-004	6.5855747e-004	6.5459106e-004
6.0493877e-004	5.7523411e-004	6.5248410e-004	6.2291682e-004
5.8274175e-004	6.6055880e-004	5.7411678e-004	6.5821211e-004
6.1197529e-004	6.5841546e-004	6.0299506e-004	6.7613070e-004
6.0019111e-004	5.0134462e-004	5.0599031e-004	5.6023442e-004
5.3870191e-004	5.4571210e-004	5.4276313e-004	5.5206021e-004
5.4233945e-004	6.0839530e-004	5.6305686e-004	6.7442092e-004
6.1235513e-004	6.3629962e-004	6.4457768e-004	7.0099279e-004
6.1647354e-004	6.7784671e-004	6.2304002e-004	6.3398910e-004
5.3354399e-004	5.6546251e-004	6.0800965e-004	6.1456581e-004
6.1968301e-004	5.3107728e-004	5.3022358e-004	5.3338462e-004
5.8503011e-004	5.1139930e-004	5.3842727e-004	5.5844127e-004
4.9135216e-004	5.4258111e-004	5.2101740e-004	4.8296623e-004
4.8293460e-004	5.4881794e-004	4.8812071e-004	5.0699920e-004
4.6978749e-004	4.7766945e-004	4.6640886e-004	4.8201082e-004
5.6799511e-004	6.6514980e-004	6.3435176e-004	6.5679458e-004
6.7820369e-004	6.4992576e-004	7.0000796e-004	7.2392894e-004
7.0207995e-004	6.1894553e-004	7.2372858e-004	6.7859871e-004
6.5757189e-004	6.6215209e-004	6.7333957e-004	6.2242658e-004
6.5587614e-004	5.7342213e-004	6.2505991e-004	6.1898660e-004
5.7040910e-004	6.3192738e-004	5.6295496e-004	6.3085904e-004
6.4091866e-004	7.1715290e-004	6.5006551e-004	7.4360412e-004
6.3755154e-004	6.3269188e-004	6.0430181e-004	6.6050456e-004
6.1404775e-004	6.5734726e-004	6.4208855e-004	6.4935659e-004
6.2689607e-004	7.0990421e-004	6.2847896e-004	7.5177227e-004
6.6805820e-004	6.9980269e-004	6.9318450e-004	7.5965242e-004
6.8247710e-004	7.4648527e-004	6.3582908e-004	6.6980318e-004
5.1863364e-004	5.6257806e-004	5.5296117e-004	5.8772566e-004
5.6314448e-004	4.4841064e-004	4.7225595e-004	4.6891173e-004
4.6326625e-004	4.3858602e-004	4.4660832e-004	4.9400538e-004
4.5340701e-004	4.9438911e-004	5.0374474e-004	5.1835776e-004
5.4021216e-004	5.3501560e-004	5.5237600e-004	5.3293144e-004
5.5555419e-004	5.5205107e-004	5.4624372e-004	5.4422148e-004
5.6040632e-004	5.8998704e-004	5.1398001e-004	5.9960682e-004
4.8773668e-004	5.0640830e-004	4.7620181e-004	4.4831061e-004
4.5255443e-004	4.8300299e-004	4.3431788e-004	4.6766014e-004
4.3245006e-004	4.3341478e-004	4.7078950e-004	4.5267764e-004
4.4579006e-004	4.0549038e-004	4.0088309e-004	4.0767736e-004
4.1389626e-004	4.2193717e-004	4.5242603e-004	4.3770907e-004

5.7234553e-004	6.6682925e-004	6.3545107e-004	6.5872839e-004
6.8559175e-004	6.6621098e-004	7.0943906e-004	7.2436512e-004
7.0056431e-004	6.3361428e-004	7.0555268e-004	7.0740852e-004
6.3392812e-004	6.6801799e-004	6.5759939e-004	6.1992172e-004
6.4214841e-004	5.4844981e-004	6.0975442e-004	5.9980985e-004
5.5335544e-004	6.0252732e-004	5.7305279e-004	6.5010549e-004
5.8672533e-004	6.5526752e-004	6.0244618e-004	6.5342225e-004
6.3570766e-004	5.2633404e-004	5.1539190e-004	5.5308489e-004
5.1167681e-004	5.1388075e-004	5.3415127e-004	5.6639997e-004
5.2738082e-004	6.0716327e-004	5.7632637e-004	5.9480206e-004
6.2164268e-004	6.1157401e-004	6.1365469e-004	6.2656817e-004
6.2439522e-004	6.4787333e-004	5.7557155e-004	5.8671426e-004
5.6916857e-004	5.8813284e-004	5.3216697e-004	6.1055851e-004
5.3451530e-004	5.2426704e-004	5.1855753e-004	4.5092616e-004
4.7152738e-004	4.7532613e-004	4.2808110e-004	4.9505581e-004
4.3812489e-004	4.3685940e-004	4.6450982e-004	4.6578032e-004
4.7838429e-004	4.1182592e-004	4.3907190e-004	4.2458183e-004
4.5144393e-004	4.4878172e-004	4.6092263e-004	4.5007134e-004
5.4656006e-004	6.4291757e-004	6.0872239e-004	6.4526222e-004
6.5429868e-004	6.3729897e-004	6.7797612e-004	6.6722144e-004
6.5123373e-004	6.3304570e-004	6.4936979e-004	6.7832512e-004
6.0739713e-004	6.5590286e-004	6.0679436e-004	5.8025215e-004
6.0770534e-004	5.3539411e-004	5.8141077e-004	5.5370585e-004
5.4685437e-004	5.5199776e-004	5.6583017e-004	6.5365013e-004
6.4981011e-004	7.0949440e-004	6.5288771e-004	7.2625864e-004
6.9682035e-004	6.0198391e-004	5.7036897e-004	6.3929764e-004
5.6587887e-004	6.0912513e-004	6.4025196e-004	6.5338863e-004
6.0660734e-004	7.1952616e-004	6.5004532e-004	6.7919273e-004
6.8402498e-004	7.0050587e-004	7.0322097e-004	7.2744278e-004
6.8801340e-004	7.2730114e-004	6.2083524e-004	6.4785637e-004
5.9316623e-004	5.8122986e-004	5.6375022e-004	6.5415277e-004
5.6251342e-004	5.4184454e-004	5.3420016e-004	4.9377693e-004
4.9661745e-004	5.1694446e-004	4.8735949e-004	5.2435725e-004
4.7693382e-004	4.7731639e-004	4.7232852e-004	4.9472482e-004
5.2820006e-004	4.4494744e-004	5.0008282e-004	4.4096314e-004
4.6386895e-004	5.0740378e-004	5.2187237e-004	5.3893660e-004
5.9771922e-004	6.8387098e-004	6.6166559e-004	6.6657555e-004
7.0607054e-004	6.6179520e-004	6.7413898e-004	7.0860107e-004
6.3334045e-004	6.6948975e-004	6.9057095e-004	7.2448590e-004
6.4139895e-004	6.6981409e-004	6.3612337e-004	6.1009079e-004
6.1629832e-004	5.7736077e-004	6.3182862e-004	6.1689198e-004
5.5572206e-004	5.8917333e-004	5.6873448e-004	6.3506000e-004
6.3615458e-004	6.0037107e-004	6.1400833e-004	6.7247692e-004
6.1553534e-004	5.7296512e-004	5.5481221e-004	5.3491445e-004
5.2101607e-004	5.2755637e-004	4.8837425e-004	5.2864639e-004
4.9307538e-004	4.7723161e-004	4.8783810e-004	4.8585085e-004
5.5220223e-004	4.3583738e-004	5.3646688e-004	4.5013062e-004
4.6875242e-004	5.1042776e-004	5.2433397e-004	5.3612416e-004
4.6089831e-004	5.1285668e-004	5.3432856e-004	5.1803557e-004
5.1537936e-004	5.1775575e-004	4.9998877e-004	5.3169653e-004
4.6071543e-004	4.0610795e-004	4.7852442e-004	4.6199725e-004
4.0164361e-004	4.4008337e-004	4.0438176e-004	4.4487706e-004

4.4598028e-004	4.3471554e-004	4.5661167e-004	4.5790050e-004
3.8558490e-004	4.1834849e-004	4.1484225e-004	4.1242938e-004
4.3508772e-004	4.3393982e-004	3.8201217e-004	4.2978492e-004
3.7786004e-004	3.7506602e-004	3.5325197e-004	3.8852698e-004
3.2082295e-004	3.4724392e-004	3.3101300e-004	3.3438222e-004
3.1390909e-004	3.1179085e-004	3.0413314e-004	3.1496541e-004
2.9921934e-004	3.3685998e-004	3.1032588e-004	3.3977937e-004
2.8786716e-004	3.0794053e-004	2.8303250e-004	3.2356178e-004
6.0681970e-004	5.5430355e-004	5.9489007e-004	6.1488077e-004
5.8045963e-004	5.2244031e-004	5.0140067e-004	5.1671104e-004
4.8762983e-004	4.6003030e-004	4.4332049e-004	4.6218312e-004
4.6299060e-004	4.3698270e-004	4.6125716e-004	4.4339070e-004
5.0715362e-004	4.0227179e-004	5.0436209e-004	4.4006466e-004
4.3491957e-004	4.5476903e-004	4.8321429e-004	4.8876795e-004
4.7213092e-004	5.2959113e-004	5.5700324e-004	5.4688996e-004
5.2781081e-004	5.3579728e-004	4.9260709e-004	5.2005147e-004
4.7635326e-004	4.2786406e-004	4.7390445e-004	4.6685772e-004
4.1570626e-004	4.2662683e-004	4.1388028e-004	4.4204422e-004
4.3787160e-004	4.5658711e-004	4.5000148e-004	4.6633178e-004
4.1720424e-004	3.9962125e-004	4.1773774e-004	4.0813640e-004
4.4890297e-004	5.4177070e-004	4.9596539e-004	5.3101085e-004
5.8678321e-004	4.8643010e-004	4.8349456e-004	4.7601396e-004
4.4675603e-004	4.2713719e-004	4.6774701e-004	4.3140768e-004
4.0905082e-004	4.2737908e-004	4.3485622e-004	4.2781854e-004
4.1173025e-004	4.1360370e-004	4.3558393e-004	4.6056727e-004
4.1547844e-004	4.4311965e-004	4.1850004e-004	4.2250542e-004
4.4930184e-004	4.0892094e-004	3.6550416e-004	4.1990278e-004
3.5954546e-004	3.4645084e-004	3.2410658e-004	3.5707652e-004
3.1472386e-004	3.0891965e-004	3.3821593e-004	3.2404067e-004
2.9517128e-004	2.7921385e-004	3.0783371e-004	3.0340224e-004
3.0051797e-004	3.0984790e-004	2.9950408e-004	3.2294771e-004
2.8075936e-004	2.6718003e-004	2.4626602e-004	3.0599374e-004
5.8591428e-004	6.3528799e-004	6.9444263e-004	7.0042282e-004
7.0609536e-004	6.8411842e-004	6.1346935e-004	6.7852475e-004
6.2717190e-004	6.5638087e-004	7.0578729e-004	6.8498975e-004
6.2348280e-004	6.4161443e-004	6.0408629e-004	6.1749660e-004
6.3329258e-004	6.0421274e-004	6.6524123e-004	6.4114887e-004
5.5184800e-004	5.5097122e-004	5.6249941e-004	6.0902082e-004
6.0903422e-004	5.7277584e-004	6.0278011e-004	6.2848082e-004
5.8864863e-004	5.2342373e-004	5.0818169e-004	5.4307166e-004
4.7870667e-004	4.4109930e-004	4.5365584e-004	4.4188560e-004
4.6161966e-004	4.5477684e-004	4.8646727e-004	4.8219647e-004
5.0613090e-004	4.1551863e-004	5.2135330e-004	4.8129756e-004
4.6413486e-004	4.6090827e-004	4.7992177e-004	5.1749241e-004
5.4950996e-004	5.5638465e-004	5.4465530e-004	5.8622703e-004
5.0881481e-004	4.7723536e-004	4.9530443e-004	4.9503887e-004
4.8993134e-004	4.5653700e-004	4.5693744e-004	4.7295009e-004
4.2905736e-004	4.4112885e-004	4.5904618e-004	4.5361476e-004
4.3057005e-004	4.4950498e-004	4.2933862e-004	4.5543424e-004
4.4521425e-004	4.2040258e-004	4.1398485e-004	4.5470298e-004
4.8260584e-004	5.3171556e-004	5.5303682e-004	5.6072573e-004
5.2988368e-004	5.4183380e-004	4.7296836e-004	5.0789496e-004

4.7133729e-004	4.4177728e-004	4.6753434e-004	4.6268851e-004
4.1430119e-004	4.2538495e-004	4.1512628e-004	4.2877377e-004
4.1638326e-004	4.5445699e-004	4.3918479e-004	4.6184740e-004
4.1698096e-004	3.9655464e-004	4.0954061e-004	4.0759806e-004
4.5588476e-004	5.5436087e-004	5.1232317e-004	5.5134243e-004
5.8176505e-004	4.8392232e-004	4.8333241e-004	4.7906747e-004
4.5650006e-004	4.3291354e-004	4.8353872e-004	4.3772462e-004
4.1381503e-004	4.2965153e-004	4.5396393e-004	4.2765297e-004
4.0108068e-004	4.1548863e-004	4.4666511e-004	4.7190309e-004
4.3077747e-004	4.4324341e-004	4.3253190e-004	4.2359126e-004
4.4311905e-004	3.9908686e-004	3.5788970e-004	3.9650293e-004
3.5435719e-004	3.3556284e-004	3.1755873e-004	3.3610017e-004
3.1224970e-004	2.9710771e-004	3.2708957e-004	3.1787106e-004
2.7839526e-004	2.6900732e-004	2.9996052e-004	2.9134210e-004
3.0109423e-004	3.0370864e-004	2.7483243e-004	3.2424212e-004
2.7718076e-004	2.5494441e-004	2.2222429e-004	2.8199348e-004
3.6389900e-004	3.7163707e-004	3.9699378e-004	3.8470256e-004
3.4074039e-004	3.3852523e-004	3.0511231e-004	3.1750884e-004
3.3552523e-004	3.1014333e-004	2.9397265e-004	2.7873422e-004
2.6636684e-004	2.8930400e-004	2.5029486e-004	2.7432096e-004
2.6677367e-004	3.0511012e-004	2.4525682e-004	2.6989051e-004
2.4731461e-004	2.7502862e-004	2.6198468e-004	2.6749709e-004
5.9803533e-004	6.1676496e-004	6.8822487e-004	7.1248463e-004
6.9158102e-004	6.8173648e-004	5.9443037e-004	6.5125497e-004
6.2807901e-004	6.3514797e-004	6.8043265e-004	6.6564673e-004
5.9940795e-004	6.2983752e-004	5.8600389e-004	6.0039895e-004
6.2850905e-004	5.9093157e-004	6.3501159e-004	6.2786027e-004
5.4522413e-004	5.2705767e-004	5.4030716e-004	5.9889159e-004
5.5064258e-004	5.2799046e-004	5.4241474e-004	5.8597173e-004
5.4096524e-004	4.9123404e-004	4.8201319e-004	5.1659116e-004
4.3887081e-004	4.0168270e-004	4.3083772e-004	3.9314699e-004
4.1738811e-004	4.2682939e-004	4.7374381e-004	4.6356370e-004
4.6675679e-004	3.9288511e-004	4.9878653e-004	4.4220706e-004
4.3481568e-004	4.3520428e-004	4.2707237e-004	4.9285361e-004
5.5381424e-004	5.6772544e-004	5.5884825e-004	6.0939166e-004
5.3604171e-004	4.9888232e-004	5.2418759e-004	5.0930686e-004
5.0930392e-004	4.6560558e-004	4.7028218e-004	4.7708074e-004
4.5016559e-004	4.6114748e-004	4.6800769e-004	4.5997286e-004
4.6391301e-004	4.6492443e-004	4.4573266e-004	4.6222388e-004
4.5958584e-004	4.3208081e-004	4.3609303e-004	4.7083015e-004
4.9634397e-004	5.3168029e-004	5.5031152e-004	5.6722757e-004
5.3731672e-004	5.3067206e-004	4.6418258e-004	5.1120501e-004
4.5792788e-004	4.6176765e-004	4.7078601e-004	4.7041451e-004
4.2748352e-004	4.3956850e-004	4.1431645e-004	4.2640043e-004
4.0405408e-004	4.5428664e-004	4.3640708e-004	4.5496402e-004
4.1931298e-004	4.0235034e-004	4.0038618e-004	4.1071555e-004
4.6443334e-004	5.5020512e-004	5.1568613e-004	5.5851518e-004
5.5405915e-004	4.7637593e-004	4.7681918e-004	4.8848744e-004
4.6024325e-004	4.3413781e-004	4.9469270e-004	4.4912080e-004
4.1743481e-004	4.3509164e-004	4.4923218e-004	4.2674770e-004
3.8919361e-004	4.1786093e-004	4.5415469e-004	4.8441329e-004
4.3379745e-004	4.3523315e-004	4.4186629e-004	4.1614618e-004

4.1406656e-004	3.9650976e-004	3.6232093e-004	3.4262062e-004
3.1965753e-004	2.5591141e-004	3.0984376e-004	3.2161950e-004
2.8313641e-004	2.8963945e-004	2.9765574e-004	2.5859173e-004
2.6600706e-004	2.6875536e-004	2.4575234e-004	2.6190848e-004
2.6902554e-004	2.8093688e-004	2.6146798e-004	3.0980701e-004
2.7420877e-004	2.5757273e-004	2.8508400e-004	2.8762143e-004
4.3676778e-004	3.8890661e-004	3.5322746e-004	3.8608089e-004
3.5966495e-004	3.4275249e-004	3.2595760e-004	3.2327507e-004
3.1310162e-004	2.9621023e-004	3.2008074e-004	3.1774255e-004
2.7344556e-004	2.6746551e-004	2.9022620e-004	2.8833524e-004
3.0079321e-004	3.0662050e-004	2.5326614e-004	3.2375885e-004
2.7663332e-004	2.6003748e-004	2.1301076e-004	2.6718059e-004
3.8224088e-004	3.6137000e-004	4.1483920e-004	3.8565369e-004
3.4445387e-004	3.3520112e-004	3.0117539e-004	3.1086939e-004
3.2750762e-004	3.0465613e-004	2.9141989e-004	2.7290097e-004
2.5583543e-004	2.7175381e-004	2.4615669e-004	2.8154441e-004
2.7282663e-004	3.0568754e-004	2.4157118e-004	2.5363391e-004
2.4199823e-004	2.7061328e-004	2.5622162e-004	2.5331247e-004
6.2844668e-004	6.1252892e-004	6.8361701e-004	7.2028440e-004
6.8595738e-004	6.6937097e-004	5.8904785e-004	6.1587371e-004
6.2493180e-004	6.2045967e-004	6.6153424e-004	6.5827710e-004
5.8983009e-004	6.2687809e-004	5.7553555e-004	5.9083279e-004
6.1605375e-004	5.8033808e-004	6.0053708e-004	6.1940147e-004
5.4326198e-004	5.2380400e-004	5.2208822e-004	5.8806194e-004
5.8290413e-004	5.7029154e-004	5.7171349e-004	6.3041846e-004
5.7458796e-004	5.3018476e-004	5.3889533e-004	5.5765134e-004
4.7149097e-004	4.3381598e-004	4.6678827e-004	4.1476713e-004
4.2717002e-004	4.5871078e-004	5.2611851e-004	5.0621663e-004
4.8765927e-004	4.3891298e-004	5.3323277e-004	4.5844196e-004
4.6295940e-004	4.8187428e-004	4.4993814e-004	5.3370968e-004
5.5579566e-004	5.9334915e-004	5.5969763e-004	5.7332936e-004
5.8698922e-004	5.3899534e-004	5.2733466e-004	5.4620254e-004
5.0270349e-004	5.0525085e-004	5.0829712e-004	5.1097506e-004
4.7837096e-004	4.5550901e-004	4.8431050e-004	4.6803759e-004
5.0119116e-004	4.5888795e-004	4.7566240e-004	4.9072510e-004
4.3273489e-004	4.4286635e-004	4.3272044e-004	5.0048154e-004
4.9738908e-004	5.2650510e-004	5.6058411e-004	5.5953530e-004
5.5079255e-004	5.1135124e-004	4.7650627e-004	5.2064336e-004
4.6058380e-004	4.6982746e-004	4.9454649e-004	4.7147099e-004
4.4999621e-004	4.5457230e-004	4.2037478e-004	4.2786254e-004
3.9494599e-004	4.5768954e-004	4.3790088e-004	4.6272705e-004
4.2952931e-004	4.1350618e-004	4.0576908e-004	4.1422748e-004
4.0628853e-004	3.8154137e-004	3.4856673e-004	3.6016343e-004
3.1971556e-004	2.5859884e-004	3.0756742e-004	3.1237528e-004
2.8008128e-004	2.7841863e-004	3.0573926e-004	2.6754463e-004
2.7210692e-004	2.6425544e-004	2.6153343e-004	2.6059752e-004
2.5839065e-004	2.7096427e-004	2.5365272e-004	2.9093875e-004
2.5748880e-004	2.3458115e-004	2.7600215e-004	2.7226532e-004
3.6647899e-004	3.4591999e-004	3.7328730e-004	3.6062573e-004
3.5013760e-004	3.2988339e-004	2.9962752e-004	3.1387473e-004
3.2290437e-004	2.6318443e-004	3.1751339e-004	2.9424038e-004
2.4724356e-004	2.8250410e-004	2.4918088e-004	3.2568820e-004

2.8599202e-004	2.4805866e-004	2.3433848e-004	2.8596129e-004
2.4455265e-004	2.5618073e-004	2.2126073e-004	2.6949125e-004
4.3488706e-004	3.8788474e-004	3.6535771e-004	3.9016316e-004
3.7369295e-004	3.5399245e-004	3.4512437e-004	3.1083300e-004
3.2785801e-004	3.2224829e-004	3.2159738e-004	3.1484311e-004
2.7933362e-004	2.8062183e-004	2.9333567e-004	2.7861690e-004
2.9105863e-004	3.1511654e-004	2.5022842e-004	3.0195268e-004
2.7317658e-004	2.9423157e-004	2.2275038e-004	2.5664389e-004
3.9284743e-004	3.4971070e-004	4.2586234e-004	3.7473174e-004
3.5047759e-004	3.2964977e-004	2.9824438e-004	3.1309020e-004
3.0526359e-004	2.8462129e-004	2.8443544e-004	2.7585415e-004
2.5924126e-004	2.6138593e-004	2.4414490e-004	2.9101811e-004
2.8599780e-004	3.0877367e-004	2.3586674e-004	2.4836165e-004
2.4249954e-004	2.5038189e-004	2.5500311e-004	2.7174094e-004
6.7555242e-004	6.2720670e-004	6.9300141e-004	7.3835002e-004
7.0040008e-004	6.5017577e-004	5.9331568e-004	5.7470368e-004
6.0294612e-004	6.0722056e-004	6.5342152e-004	6.5700129e-004
6.0711296e-004	6.2099719e-004	5.8321745e-004	6.1444343e-004
6.2147855e-004	5.9763495e-004	6.1301979e-004	6.3126897e-004
5.5454692e-004	5.6094113e-004	5.5396726e-004	6.0323784e-004
6.2083530e-004	6.1148278e-004	5.9910072e-004	6.5239980e-004
6.0931646e-004	5.5068688e-004	5.7266112e-004	5.7148786e-004
5.0342254e-004	4.7316662e-004	4.9875613e-004	4.7656307e-004
4.4390135e-004	5.1095884e-004	5.4070455e-004	5.3852025e-004
5.0926464e-004	4.9307883e-004	5.5638723e-004	4.7356813e-004
4.8428148e-004	5.0171884e-004	4.9121789e-004	5.5197692e-004
5.7211834e-004	5.9581829e-004	5.8340728e-004	6.2007189e-004
6.2593236e-004	5.4977108e-004	5.4660572e-004	5.4549125e-004
5.2902049e-004	5.0415835e-004	5.2810932e-004	5.3454268e-004
4.9212418e-004	4.8773432e-004	4.8101531e-004	4.8278886e-004
5.0509674e-004	4.7450658e-004	4.8846806e-004	4.9109577e-004
4.4034863e-004	4.6430447e-004	4.5929683e-004	5.0778990e-004
4.9494441e-004	5.9159486e-004	5.8113903e-004	6.2117763e-004
5.7490748e-004	5.3887769e-004	5.3815266e-004	5.3225570e-004
5.2886740e-004	4.7192367e-004	5.4143490e-004	4.6834919e-004
4.7406123e-004	4.9952479e-004	4.7744035e-004	4.7226866e-004
5.1579975e-004	4.7872509e-004	4.4716924e-004	4.8124234e-004
5.0519652e-004	4.8042629e-004	4.4499514e-004	4.7576585e-004
4.9648355e-004	5.3130732e-004	5.5676688e-004	5.6567990e-004
5.5292661e-004	4.9179840e-004	4.5474286e-004	5.0300804e-004
4.1478824e-004	4.6037265e-004	5.0383745e-004	4.9469677e-004
4.6502029e-004	4.5519668e-004	4.4460007e-004	4.4686026e-004
4.3827920e-004	4.4455607e-004	4.3573586e-004	4.1668058e-004
4.2222178e-004	4.3794369e-004	3.9630026e-004	4.4819262e-004
4.5617312e-004	4.5480944e-004	4.2839206e-004	4.3919236e-004
4.3391412e-004	4.1835825e-004	4.1882561e-004	4.2905318e-004
3.9413082e-004	3.9648540e-004	3.9267616e-004	3.8796067e-004
3.3867130e-004	3.8345889e-004	3.2255886e-004	3.6382573e-004
3.5866143e-004	3.8001285e-004	3.2982042e-004	3.4018110e-004
3.5763995e-004	3.3442885e-004	3.1813581e-004	3.3947582e-004
4.1739296e-004	3.9421851e-004	3.6960692e-004	3.7157675e-004
3.3130941e-004	3.1528066e-004	3.3122338e-004	3.3534723e-004

3.1597801e-004	2.8938281e-004	3.0543264e-004	2.7739996e-004
2.7697952e-004	2.9543170e-004	2.5156407e-004	2.6812930e-004
2.7069659e-004	2.9364107e-004	2.6181294e-004	3.1483517e-004
2.6504891e-004	2.8411456e-004	2.9258261e-004	2.6801704e-004
3.7899759e-004	3.4495434e-004	3.7735059e-004	3.6317288e-004
3.6025759e-004	3.4818108e-004	3.1374682e-004	3.2162218e-004
3.2242251e-004	2.8260827e-004	3.2073991e-004	3.0149771e-004
2.7072520e-004	2.8856454e-004	2.6840851e-004	3.3198408e-004
2.9020489e-004	2.7738718e-004	2.2497219e-004	2.8192369e-004
2.3775337e-004	2.4923901e-004	2.2336112e-004	2.7856974e-004
4.1786510e-004	3.8871272e-004	3.9528790e-004	3.9389420e-004
3.7698795e-004	3.4084523e-004	3.3344504e-004	3.0899894e-004
3.2383481e-004	3.3149041e-004	3.1486993e-004	2.9608268e-004
2.7512039e-004	2.7596106e-004	2.8838492e-004	2.7252314e-004
2.7771729e-004	2.9974533e-004	2.5269899e-004	2.7373282e-004
2.6070788e-004	3.0587534e-004	2.3787217e-004	2.6032082e-004
4.0880205e-004	3.5687903e-004	4.1355646e-004	3.8625282e-004
3.5464769e-004	3.2022022e-004	3.0389746e-004	3.1682269e-004
2.9372007e-004	2.7446919e-004	2.8938918e-004	2.9264742e-004
2.7018345e-004	2.7632213e-004	2.5553303e-004	2.8176737e-004
2.8794943e-004	2.9935879e-004	2.3731803e-004	2.6755889e-004
2.6053442e-004	2.5674628e-004	2.5765572e-004	2.9204164e-004
6.6010929e-004	6.3978262e-004	6.0452268e-004	6.3909755e-004
6.3960724e-004	5.9242616e-004	5.9343646e-004	6.0444702e-004
5.5116778e-004	5.3533920e-004	5.3198342e-004	5.5417885e-004
4.9358496e-004	5.5632560e-004	5.1067785e-004	5.5474959e-004
5.5639033e-004	5.5754556e-004	5.8636256e-004	5.4410678e-004
5.0124872e-004	5.6430581e-004	5.5898291e-004	5.9828333e-004
5.4311105e-004	6.1487024e-004	6.1610879e-004	6.5907493e-004
6.3667599e-004	5.7769491e-004	5.7861670e-004	5.5591774e-004
5.4428489e-004	5.1453129e-004	5.7084361e-004	5.2544543e-004
5.1887763e-004	5.2450862e-004	4.8791161e-004	5.0803332e-004
5.3421660e-004	5.1184552e-004	4.9202783e-004	5.0176115e-004
4.8894121e-004	5.0073410e-004	4.8299912e-004	5.2037014e-004
5.0255926e-004	5.9633479e-004	5.9272071e-004	6.2897844e-004
5.9684445e-004	5.4341671e-004	5.2408571e-004	5.3350464e-004
5.1941810e-004	4.6778288e-004	5.4082464e-004	5.1352361e-004
4.9155384e-004	5.1850238e-004	4.8209578e-004	4.6941845e-004
4.9074784e-004	4.7751568e-004	4.6164546e-004	4.6800447e-004
4.6952099e-004	4.8261507e-004	4.3400472e-004	4.8575053e-004
5.1266891e-004	5.1315823e-004	5.8081970e-004	5.5007984e-004
5.7182471e-004	4.9488666e-004	4.8773442e-004	5.1654823e-004
4.3906801e-004	4.9232381e-004	5.2852418e-004	4.7556638e-004
4.8778880e-004	4.4670932e-004	4.6365395e-004	4.6084449e-004
4.4263399e-004	4.6095761e-004	4.4801466e-004	4.5979733e-004
4.2814025e-004	4.3922996e-004	4.0343536e-004	4.4797089e-004
4.8488693e-004	4.9510816e-004	4.8200677e-004	4.8222618e-004
4.5732308e-004	4.4285945e-004	4.4539437e-004	4.6537165e-004
4.3662400e-004	4.3010978e-004	4.2804021e-004	4.2765273e-004
3.9639138e-004	4.1550706e-004	3.5606367e-004	3.6991805e-004
3.5579157e-004	3.7192178e-004	4.0011328e-004	3.8144708e-004
4.0162624e-004	3.7646145e-004	3.5439351e-004	3.7443764e-004

4.1894683e-004	3.6398019e-004	3.7358208e-004	3.9873842e-004
3.3980325e-004	3.0129269e-004	3.1482424e-004	3.2458561e-004
2.9769276e-004	2.7840497e-004	2.9296929e-004	2.7833552e-004
2.9673510e-004	2.8006656e-004	2.7598072e-004	2.6981383e-004
2.6122310e-004	2.8481090e-004	2.5173364e-004	2.9614067e-004
2.4677311e-004	2.3445827e-004	2.7914941e-004	2.6386429e-004
3.9969444e-004	3.6219926e-004	3.6212291e-004	3.7711715e-004
3.7392986e-004	3.4999462e-004	3.3438204e-004	3.2368866e-004
3.0598507e-004	3.3345795e-004	3.1719569e-004	3.1584628e-004
2.9469602e-004	2.8901165e-004	2.9949102e-004	2.9370352e-004
2.8548203e-004	3.0560120e-004	2.3880259e-004	2.8360976e-004
2.5651112e-004	2.5553340e-004	2.2964894e-004	2.8362035e-004
3.8765171e-004	3.6989399e-004	4.0905544e-004	3.9123662e-004
3.7743845e-004	3.1461980e-004	3.0228395e-004	3.1176263e-004
2.9650997e-004	2.8803013e-004	2.9227127e-004	2.6213175e-004
2.5974459e-004	2.7018176e-004	2.6546229e-004	2.7736984e-004
2.5769738e-004	2.7540767e-004	2.3635849e-004	2.6507612e-004
2.3559709e-004	2.8505488e-004	2.5013008e-004	2.7207447e-004
4.1968889e-004	3.5477317e-004	4.0355945e-004	3.8417050e-004
3.2461454e-004	3.1639484e-004	3.0747162e-004	3.1656979e-004
2.8788325e-004	2.7195193e-004	2.8683260e-004	3.1313439e-004
2.6132351e-004	2.9260244e-004	2.7569953e-004	2.6736751e-004
2.7920188e-004	2.7424366e-004	2.6424976e-004	2.7092439e-004
3.0265965e-004	2.9235358e-004	2.7414769e-004	3.1571533e-004
5.8528488e-004	6.1599807e-004	5.7109764e-004	6.4429063e-004
5.8583036e-004	5.8011994e-004	5.7024154e-004	5.7699210e-004
5.1150300e-004	4.6996916e-004	4.8836043e-004	4.7924570e-004
4.4573627e-004	5.1226765e-004	5.0668670e-004	5.2198851e-004
4.6481625e-004	4.8373866e-004	5.0183562e-004	5.1414605e-004
4.2515952e-004	5.1056754e-004	4.7906065e-004	5.2500116e-004
5.6363856e-004	5.7913756e-004	5.9917054e-004	6.2701786e-004
6.4644561e-004	5.5162184e-004	5.3593917e-004	5.5121194e-004
5.3241930e-004	5.0221184e-004	5.6696373e-004	5.2842335e-004
4.9565154e-004	5.0737083e-004	4.6957372e-004	4.8703921e-004
4.9068657e-004	4.7054711e-004	4.9022448e-004	5.1448494e-004
4.6152592e-004	4.8871013e-004	4.7004068e-004	5.0610388e-004
5.1747476e-004	6.5000961e-004	6.5537359e-004	6.9393929e-004
6.7096978e-004	6.1838201e-004	6.1729801e-004	5.8937749e-004
5.9462235e-004	5.3338769e-004	5.9710312e-004	5.4627105e-004
5.4165327e-004	5.4915585e-004	5.3205025e-004	5.2120595e-004
5.4773799e-004	5.2987972e-004	5.0134696e-004	5.2601998e-004
5.2247933e-004	5.2781051e-004	4.6518620e-004	5.1849349e-004
5.2453892e-004	5.6094260e-004	5.9415618e-004	5.9467212e-004
5.8844736e-004	5.2870417e-004	5.1838320e-004	5.3906406e-004
4.7368064e-004	4.9290606e-004	5.4074830e-004	5.0101960e-004
5.1211115e-004	4.9290427e-004	4.9458589e-004	4.5802398e-004
4.7212059e-004	4.6856479e-004	4.7660268e-004	4.7092245e-004
4.3158515e-004	4.5764136e-004	4.3618114e-004	4.8105422e-004
4.0075962e-004	3.8538321e-004	3.8343652e-004	4.1672605e-004
3.5363928e-004	3.4637338e-004	3.1248749e-004	3.3023835e-004
3.4326097e-004	2.8192470e-004	2.9669594e-004	2.9808136e-004
2.9685719e-004	2.8111132e-004	2.7552059e-004	2.9679837e-004

2.9623306e-004	3.1190464e-004	2.5754311e-004	2.9333418e-004
2.5261190e-004	2.7145370e-004	3.0231183e-004	2.8509290e-004
3.7842750e-004	3.8728647e-004	3.8013484e-004	3.7578661e-004
3.7362692e-004	3.2756981e-004	3.2628435e-004	3.2329750e-004
3.1313857e-004	3.3556524e-004	3.1107950e-004	2.9182057e-004
2.7819510e-004	2.8632935e-004	2.9702757e-004	2.7132756e-004
2.5552673e-004	2.7455056e-004	2.5761394e-004	2.7396250e-004
2.5489065e-004	2.8126327e-004	2.4597412e-004	2.8888596e-004
3.9731180e-004	3.4864188e-004	3.7392716e-004	3.8873847e-004
3.5878053e-004	3.0272745e-004	3.0287346e-004	3.0425962e-004
2.8082679e-004	2.5659325e-004	2.9883212e-004	2.7961958e-004
2.5035834e-004	2.8209949e-004	2.5453058e-004	2.6647191e-004
2.5467899e-004	2.5798914e-004	2.3539583e-004	2.7030955e-004
2.6114938e-004	2.7590989e-004	2.5234143e-004	2.8687859e-004
7.3430981e-004	7.5603269e-004	7.0986518e-004	7.2713636e-004
7.1884161e-004	7.0282498e-004	6.6945141e-004	6.7998190e-004
6.6682324e-004	6.2991799e-004	6.3955202e-004	6.2380189e-004
5.7145934e-004	5.7961035e-004	5.5778312e-004	5.9304161e-004
6.0627338e-004	5.9671137e-004	5.9551591e-004	6.2955332e-004
5.8284485e-004	5.9717526e-004	6.1664738e-004	6.6974055e-004
5.4416813e-004	6.5006677e-004	6.7272481e-004	6.8630105e-004
6.9409241e-004	6.2501951e-004	6.2363131e-004	6.1222061e-004
5.8500690e-004	5.5666204e-004	6.1192877e-004	5.6928280e-004
5.6319511e-004	5.4609982e-004	5.5128759e-004	5.3358781e-004
5.5619098e-004	5.4637082e-004	5.1405012e-004	5.3700837e-004
5.2932816e-004	5.3315346e-004	4.8821460e-004	5.3260579e-004
5.5459731e-004	6.0961093e-004	6.1963742e-004	6.5758121e-004
6.3468373e-004	5.8371839e-004	5.8758023e-004	5.9151236e-004
5.5722087e-004	5.1089426e-004	5.6661152e-004	5.3461095e-004
5.5652382e-004	5.5154391e-004	5.2425124e-004	4.7976577e-004
5.0496092e-004	5.1503954e-004	5.2741273e-004	5.2136849e-004
4.6256842e-004	4.9240020e-004	4.6159852e-004	5.0521627e-004
4.2222852e-004	3.3570831e-004	3.9802676e-004	4.2164142e-004
3.4306252e-004	3.4023502e-004	3.3129211e-004	3.1547495e-004
3.2034339e-004	2.7679552e-004	2.8650460e-004	2.9357217e-004
3.1556413e-004	2.8999944e-004	2.8604240e-004	2.9994471e-004
2.6672715e-004	2.9551733e-004	2.7723148e-004	2.8290525e-004
2.6536649e-004	2.5995947e-004	2.7709494e-004	2.9795442e-004
3.7362431e-004	3.4813000e-004	3.7693189e-004	3.9448512e-004
3.4309744e-004	3.4457870e-004	3.3056008e-004	3.0690872e-004
2.9097452e-004	3.0752391e-004	2.9671560e-004	3.0601832e-004
2.8146182e-004	3.2644775e-004	2.8359483e-004	2.9762317e-004
2.2918229e-004	2.9085062e-004	2.6913515e-004	2.3371873e-004
2.6234356e-004	2.5819335e-004	2.5573001e-004	2.7167189e-004
3.8169581e-004	3.4898303e-004	3.5892741e-004	3.7311031e-004
3.7984275e-004	3.1273730e-004	2.9887406e-004	3.0565241e-004
2.8853532e-004	2.6495387e-004	2.9723541e-004	2.5833047e-004
2.4170465e-004	2.8223915e-004	2.5663037e-004	2.6581439e-004
2.3902287e-004	2.5879972e-004	2.3864605e-004	2.7477862e-004
2.5247804e-004	2.8123377e-004	2.5481245e-004	2.7697508e-004
6.7196706e-004	7.2990338e-004	6.9144146e-004	7.2514174e-004
6.6949652e-004	6.9114377e-004	6.4991196e-004	6.8879886e-004

6.6082684e-004	5.9953114e-004	6.3025293e-004	5.8238235e-004
5.5092105e-004	5.5379233e-004	5.5807898e-004	5.5597044e-004
5.7498667e-004	5.5557308e-004	5.6847736e-004	6.0923546e-004
5.4304205e-004	5.6123245e-004	5.6084582e-004	6.2020916e-004
7.3003132e-004	6.8572744e-004	6.7093343e-004	6.9081204e-004
6.5334671e-004	6.2256405e-004	5.7573343e-004	5.2900657e-004
5.6503506e-004	5.7392122e-004	5.5832021e-004	5.4852651e-004
5.3876646e-004	5.5075131e-004	5.5441391e-004	5.6427578e-004
6.1033487e-004	5.6024374e-004	5.9132153e-004	6.0803046e-004
6.1812277e-004	5.7750350e-004	6.2080043e-004	6.2369213e-004
5.7314713e-004	6.2096838e-004	6.5642338e-004	6.4980767e-004
6.7324737e-004	6.1059624e-004	5.9706223e-004	6.1982645e-004
5.6340291e-004	5.6496532e-004	6.0409748e-004	5.7676471e-004
5.5294611e-004	5.4070457e-004	5.5159982e-004	5.3656934e-004
5.4324191e-004	5.5076466e-004	5.1847805e-004	5.4998291e-004
5.3259976e-004	5.2681699e-004	5.0605004e-004	5.4428213e-004
5.7614197e-004	6.4386206e-004	6.6182896e-004	7.0806120e-004
6.7997597e-004	6.4267572e-004	6.4735048e-004	6.3996800e-004
6.1885580e-004	5.3497624e-004	5.9315958e-004	5.6872971e-004
5.9701208e-004	5.7769405e-004	5.6365559e-004	5.0551219e-004
5.4549001e-004	5.5370056e-004	5.5955582e-004	5.5466473e-004
5.0095078e-004	5.2513697e-004	4.8060028e-004	5.2566394e-004
4.2535301e-004	3.4838299e-004	4.0478817e-004	4.3717393e-004
3.6021987e-004	3.4678366e-004	3.2315507e-004	3.3146630e-004
3.4064716e-004	2.9739426e-004	2.9270433e-004	3.1526119e-004
3.1405005e-004	2.7267275e-004	2.9358129e-004	3.1256115e-004
2.9215992e-004	3.1992893e-004	2.6841096e-004	2.9845161e-004
2.6686835e-004	2.6923166e-004	3.1730178e-004	3.0358870e-004
4.0124942e-004	3.5981022e-004	3.7344966e-004	3.9943506e-004
3.7140626e-004	3.3544376e-004	3.4721064e-004	3.0359461e-004
3.0122143e-004	2.9724914e-004	2.9472774e-004	2.9783645e-004
2.8859867e-004	3.0932081e-004	2.9486167e-004	2.9237520e-004
2.3786146e-004	2.9002746e-004	2.8234583e-004	2.4648539e-004
2.7194588e-004	2.6496725e-004	2.5669391e-004	2.9068062e-004
6.2653420e-004	6.9509770e-004	6.5676974e-004	7.1127410e-004
6.2499444e-004	6.4939437e-004	5.9504068e-004	6.3075151e-004
6.3450865e-004	5.4688050e-004	6.0180439e-004	5.3775909e-004
5.1060831e-004	5.4439709e-004	5.6883404e-004	5.2905903e-004
5.6275789e-004	5.3780607e-004	5.5487141e-004	5.8358947e-004
5.2661609e-004	5.3684875e-004	5.3311376e-004	5.6811902e-004
8.0422102e-004	7.5816407e-004	7.7347952e-004	7.9442821e-004
7.3181459e-004	7.2858411e-004	6.6273561e-004	6.0003375e-004
6.4481352e-004	6.4510180e-004	6.3998647e-004	6.2377828e-004
6.1665296e-004	6.2462769e-004	6.3503992e-004	6.3091170e-004
6.7725181e-004	6.3194669e-004	6.6604243e-004	6.8216116e-004
6.9489029e-004	6.5327309e-004	7.0150381e-004	6.9724100e-004
5.8939133e-004	6.4790447e-004	6.7367307e-004	7.2093273e-004
6.8646162e-004	6.6702253e-004	6.5914264e-004	6.6737695e-004
6.2133893e-004	5.4799157e-004	5.9553157e-004	5.8009874e-004
6.0668339e-004	5.7948753e-004	5.7266386e-004	5.1600613e-004
5.6889097e-004	5.6139304e-004	5.6149205e-004	5.6159614e-004
5.2384617e-004	5.2669267e-004	4.8792150e-004	5.3375209e-004

5.5681219e-004	5.9215032e-004	5.7918952e-004	6.3040134e-004
6.1971226e-004	5.7001070e-004	5.7187554e-004	5.4843978e-004
4.9378241e-004	5.1958446e-004	5.4989896e-004	5.0482762e-004
5.1296021e-004	5.1601024e-004	4.9831829e-004	5.0355367e-004
5.1985584e-004	4.9311878e-004	4.8674556e-004	5.1227368e-004
4.5998414e-004	4.9700548e-004	4.8215649e-004	4.8283148e-004
4.0803458e-004	3.7086951e-004	3.7519510e-004	3.9065725e-004
3.9541890e-004	3.2967250e-004	3.4516461e-004	3.1800927e-004
3.0739512e-004	2.9001560e-004	2.9879016e-004	3.0334339e-004
2.8465958e-004	2.9972466e-004	3.1184896e-004	2.8994049e-004
2.6209306e-004	2.9946788e-004	2.7907925e-004	2.5859270e-004
2.7713099e-004	2.6628034e-004	2.6655232e-004	3.0610253e-004
5.5523110e-004	6.1294466e-004	5.8007085e-004	6.2632504e-004
5.6518620e-004	5.7125570e-004	5.2481021e-004	5.6767900e-004
5.6399137e-004	4.8813900e-004	5.5748205e-004	4.8171074e-004
4.6167901e-004	5.0713681e-004	5.3502990e-004	4.7250988e-004
5.0322239e-004	5.0866852e-004	5.1142768e-004	5.2354739e-004
4.8914186e-004	4.9917242e-004	4.9049295e-004	5.0217592e-004
8.6900551e-004	8.2425719e-004	8.8610675e-004	9.0154319e-004
8.3449855e-004	8.4985465e-004	7.8180924e-004	6.9694432e-004
7.4606939e-004	7.3938661e-004	7.6451342e-004	7.2701172e-004
7.3498735e-004	7.3258691e-004	7.4041978e-004	7.3286294e-004
7.8642154e-004	7.3797930e-004	7.8464221e-004	7.9511537e-004
8.1708832e-004	7.8532394e-004	8.3346033e-004	8.2333297e-004
5.9821438e-004	6.6024275e-004	6.8999945e-004	7.2559041e-004
6.9167577e-004	6.8713503e-004	6.5771952e-004	6.8235851e-004
6.2119974e-004	5.6679128e-004	5.9420928e-004	5.9212663e-004
6.0758847e-004	5.7647422e-004	5.7479895e-004	5.3351160e-004
5.9000927e-004	5.6434535e-004	5.5324122e-004	5.6901466e-004
5.5253119e-004	5.2983688e-004	4.8955315e-004	5.3932986e-004
5.8766778e-004	6.2565639e-004	6.0751877e-004	6.6288440e-004
6.2651420e-004	6.0654739e-004	5.9456483e-004	5.7107363e-004
5.2450980e-004	5.3889754e-004	5.6547628e-004	5.1880108e-004
5.2184345e-004	5.4226673e-004	5.0663816e-004	5.1674014e-004
5.3733985e-004	5.2145051e-004	4.9373665e-004	5.2905624e-004
4.9292333e-004	5.2761348e-004	4.9615702e-004	4.9285820e-004
5.4743393e-004	5.5530584e-004	5.0498478e-004	5.5884443e-004
5.4874104e-004	5.2845321e-004	4.9830124e-004	4.9667176e-004
4.9024610e-004	4.4432665e-004	4.8137162e-004	4.9517636e-004
4.4110836e-004	4.5086554e-004	4.4947023e-004	4.7333460e-004
4.5678726e-004	4.5047001e-004	4.2282740e-004	4.7597793e-004
4.3056767e-004	4.1595774e-004	4.2743689e-004	4.5459811e-004
8.3890512e-004	7.9019302e-004	8.4745281e-004	8.3498762e-004
8.0153860e-004	8.1882323e-004	7.7843663e-004	6.9804495e-004
7.2799555e-004	7.1123056e-004	7.3070945e-004	7.1808608e-004
7.4492836e-004	7.0311598e-004	7.0558615e-004	7.3509660e-004
7.8059842e-004	7.2300297e-004	7.8597905e-004	8.0267875e-004
7.7646833e-004	7.9602971e-004	8.4431350e-004	8.0861086e-004
6.0969684e-004	5.8192911e-004	5.7338153e-004	6.0881201e-004
5.6531973e-004	5.5158888e-004	5.0875798e-004	4.9730986e-004
4.9612558e-004	4.7834282e-004	4.8354802e-004	4.6170711e-004
4.6833014e-004	4.7954247e-004	4.6727350e-004	4.8880787e-004

5.0108225e-004	4.8461533e-004	4.9461504e-004	5.2612677e-004
4.9870909e-004	5.1329567e-004	5.2016588e-004	5.2689933e-004
6.3622298e-004	6.5834129e-004	6.8108462e-004	6.6811875e-004
6.7374627e-004	6.8218711e-004	6.2020562e-004	6.3041356e-004
6.1127941e-004	6.0240051e-004	5.5489219e-004	6.4562292e-004
5.7915237e-004	5.6782148e-004	6.1202708e-004	5.7167152e-004
5.8115985e-004	6.0310154e-004	5.3018387e-004	6.0696967e-004
5.7521573e-004	5.5896028e-004	5.3318606e-004	5.9818881e-004
6.0104240e-004	6.5733968e-004	6.9193557e-004	7.1530699e-004
6.7493107e-004	6.7527620e-004	6.2580247e-004	6.6826015e-004
5.9799587e-004	5.6628187e-004	5.8790980e-004	5.5612788e-004
5.8384199e-004	5.5935587e-004	5.4954355e-004	5.3636641e-004
5.8050020e-004	5.3526131e-004	5.2983464e-004	5.6580381e-004
5.5546589e-004	5.3219436e-004	4.8299968e-004	5.1413809e-004
5.9937674e-004	6.4728230e-004	6.1679484e-004	6.7048862e-004
6.1559114e-004	6.1365546e-004	6.1838215e-004	5.9056193e-004
5.6331418e-004	5.6431380e-004	5.6793245e-004	5.4441352e-004
5.2069691e-004	5.5813436e-004	5.2445606e-004	5.2936007e-004
5.6080516e-004	5.6397294e-004	5.1517892e-004	5.3486771e-004
5.1795164e-004	5.5303847e-004	5.1602308e-004	5.0894333e-004
5.3218724e-004	5.6247189e-004	4.9330160e-004	5.3403375e-004
5.3960983e-004	5.1243584e-004	5.0253377e-004	4.9188401e-004
4.9372240e-004	4.4897390e-004	4.9683300e-004	4.9548283e-004
4.2909570e-004	4.4871651e-004	4.4094072e-004	4.4311919e-004
4.4286515e-004	4.3679130e-004	4.2311349e-004	4.6322979e-004
4.2028128e-004	4.1197897e-004	4.2362166e-004	4.4326825e-004
7.6971856e-004	6.8787363e-004	7.3155561e-004	7.2114696e-004
7.0502379e-004	6.9522897e-004	6.5761759e-004	6.1811246e-004
6.4553430e-004	5.8573672e-004	6.1665947e-004	5.9502688e-004
6.1513048e-004	6.0506328e-004	5.8658469e-004	6.0139809e-004
6.4667877e-004	6.2086192e-004	6.2487304e-004	6.9464129e-004
6.1762022e-004	6.4088075e-004	6.8081410e-004	6.7147829e-004
5.7761748e-004	5.7424844e-004	5.6735039e-004	6.0984808e-004
5.9217686e-004	5.2720572e-004	4.9609764e-004	5.0902266e-004
4.9288170e-004	4.7389401e-004	4.6945554e-004	4.7581194e-004
4.5852180e-004	4.7330526e-004	4.6712788e-004	4.9210442e-004
4.9831803e-004	4.7523934e-004	4.7920604e-004	5.1967353e-004
4.7594374e-004	4.9605544e-004	4.8242330e-004	5.1223012e-004
6.2804880e-004	6.4303512e-004	6.1397014e-004	6.3626927e-004
6.1440637e-004	6.2715745e-004	5.2525225e-004	5.6213590e-004
5.5429716e-004	5.4575207e-004	5.2327501e-004	5.6966189e-004
5.0762027e-004	5.6286668e-004	5.5877391e-004	5.1171811e-004
5.0683451e-004	5.5448565e-004	5.0306907e-004	5.6262933e-004
5.1597389e-004	5.3004484e-004	5.0973160e-004	5.6726325e-004
6.1703952e-004	6.7504837e-004	6.9814034e-004	6.6594602e-004
6.8595023e-004	6.7533671e-004	6.3860105e-004	6.3126039e-004
6.1249528e-004	6.0547593e-004	5.6143486e-004	6.1898741e-004
5.8878685e-004	5.4129066e-004	5.6654532e-004	5.7549794e-004
5.9013325e-004	5.7729241e-004	5.3309310e-004	5.8601834e-004
5.7035730e-004	5.4371753e-004	4.9000415e-004	5.4658465e-004
6.1589990e-004	6.5165483e-004	6.7139995e-004	6.8389348e-004
6.4421514e-004	6.4796674e-004	6.1117751e-004	6.3424189e-004

5.8006859e-004	5.7566914e-004	5.8486107e-004	5.4091516e-004
5.5281539e-004	5.4323020e-004	5.4022956e-004	5.3578200e-004
5.7537730e-004	5.3540590e-004	5.1192829e-004	5.6614628e-004
5.4682246e-004	5.4807896e-004	5.1510699e-004	4.9799024e-004
5.4304439e-004	5.9861942e-004	5.5066519e-004	5.7800260e-004
5.8627337e-004	5.5944629e-004	5.4992727e-004	5.3178942e-004
5.1948563e-004	4.8075545e-004	5.1794751e-004	5.3958190e-004
4.7762200e-004	4.6321727e-004	4.8920179e-004	4.5875391e-004
4.6692848e-004	4.6719930e-004	4.7433247e-004	5.0062558e-004
4.4942524e-004	4.5564234e-004	4.3666359e-004	4.7922805e-004
9.0177519e-004	8.1628199e-004	9.1254832e-004	9.1867827e-004
9.0403293e-004	8.8369389e-004	9.0639348e-004	8.2050433e-004
8.7062828e-004	8.1346136e-004	8.5711289e-004	8.2836152e-004
8.5534081e-004	8.2591725e-004	8.0280621e-004	8.2494784e-004
8.9637854e-004	8.7314246e-004	8.7984609e-004	9.4702469e-004
8.5547304e-004	8.8393384e-004	9.5933303e-004	9.3636994e-004
6.5695345e-004	6.0285407e-004	6.0853564e-004	6.6526455e-004
6.1556343e-004	5.6944253e-004	5.2971423e-004	5.3686874e-004
5.2808530e-004	4.8952230e-004	5.1007627e-004	4.9076279e-004
4.8132460e-004	4.9232806e-004	4.7908357e-004	5.0770905e-004
5.2230339e-004	5.0821842e-004	5.0480440e-004	5.4918787e-004
4.9486511e-004	5.2178511e-004	5.0127711e-004	5.2432393e-004
6.1889515e-004	6.3057907e-004	5.9287880e-004	6.1955670e-004
5.9765276e-004	5.8339103e-004	4.8964153e-004	5.2992256e-004
5.1090060e-004	5.1002402e-004	5.0636404e-004	5.1562334e-004
4.8538460e-004	5.2895062e-004	5.1413139e-004	4.7599200e-004
4.8725543e-004	5.0314057e-004	4.7959590e-004	5.1572814e-004
4.7533909e-004	5.0409212e-004	4.8192447e-004	5.2078025e-004
6.3624568e-004	6.5349006e-004	6.6322568e-004	6.2729123e-004
6.4637429e-004	6.3536390e-004	6.0761254e-004	5.6107249e-004
5.8049046e-004	5.7986254e-004	5.2794954e-004	6.0032982e-004
5.4875054e-004	5.1648525e-004	5.2710350e-004	5.5559023e-004
5.5538067e-004	5.6868400e-004	5.1046399e-004	5.6353306e-004
5.2613505e-004	5.3341831e-004	4.8711515e-004	5.2860622e-004
6.0342879e-004	6.5127593e-004	6.8876136e-004	6.6976126e-004
6.5172628e-004	6.4675065e-004	6.1305695e-004	6.4845537e-004
5.7861520e-004	5.7975747e-004	5.7755984e-004	5.5260700e-004
5.6373698e-004	5.2251169e-004	5.5119520e-004	5.4211047e-004
5.8053818e-004	5.1730406e-004	5.2124109e-004	5.7094272e-004
5.4423255e-004	5.4695517e-004	5.1488378e-004	5.0807398e-004
8.9215332e-004	7.9778639e-004	9.1188958e-004	9.4445507e-004
9.2217856e-004	8.9594105e-004	8.6568038e-004	8.0138291e-004
8.5083387e-004	8.0439368e-004	8.4878420e-004	8.0114772e-004
7.9367259e-004	8.4205082e-004	8.0386433e-004	7.8101573e-004
8.6189900e-004	8.6183535e-004	8.3116240e-004	9.4446047e-004
8.2229845e-004	8.2763382e-004	8.8673475e-004	9.2329511e-004
6.4376167e-004	6.2034591e-004	6.1279547e-004	6.6029503e-004
6.1537795e-004	5.7395361e-004	5.2238340e-004	5.6119695e-004
5.2695601e-004	5.0429704e-004	5.1455160e-004	4.9646125e-004
4.8221126e-004	4.9844736e-004	4.8512247e-004	4.9987187e-004
5.1875031e-004	4.9252883e-004	5.0193672e-004	5.4025852e-004
4.9312705e-004	5.1074982e-004	4.9206721e-004	5.2035690e-004

6.5153523e-004	6.3659890e-004	6.0551289e-004	6.2548545e-004
5.9033088e-004	5.7546014e-004	5.0824671e-004	5.1289188e-004
5.1677298e-004	5.0417858e-004	5.1299411e-004	5.0905169e-004
4.8420233e-004	5.1314820e-004	4.8193922e-004	4.6799684e-004
4.8471678e-004	4.9407889e-004	4.6893503e-004	4.8616117e-004
4.4698648e-004	4.9466897e-004	4.7521621e-004	4.9458261e-004
6.2457686e-004	6.6011027e-004	6.6151483e-004	6.2976498e-004
6.4307304e-004	6.3383597e-004	6.0645568e-004	5.7508661e-004
5.5909950e-004	5.7804112e-004	5.4638241e-004	5.6471583e-004
5.3851759e-004	5.0057160e-004	5.2082770e-004	5.6279299e-004
5.6895051e-004	5.4882905e-004	5.2286944e-004	5.5896673e-004
5.2574069e-004	5.2796917e-004	4.8361361e-004	5.1199822e-004
5.9685735e-004	6.3929544e-004	6.7377453e-004	6.5400275e-004
6.4816474e-004	6.3937713e-004	6.1676769e-004	6.4407084e-004
5.9176127e-004	5.8955549e-004	5.7508447e-004	5.7200147e-004
5.5351369e-004	5.1850958e-004	5.5483676e-004	5.3219423e-004
5.8132895e-004	5.1859852e-004	5.2274292e-004	5.6489538e-004
5.2327442e-004	5.3097805e-004	5.2134677e-004	5.1050616e-004
7.9139571e-004	7.3945311e-004	7.8015833e-004	8.4255160e-004
7.7195962e-004	7.6228927e-004	6.8573236e-004	6.9692901e-004
6.7636668e-004	6.6684528e-004	6.8227125e-004	6.3901006e-004
6.1893184e-004	6.7705307e-004	6.2961536e-004	6.2874556e-004
6.7554065e-004	6.4482445e-004	6.6530883e-004	7.2808757e-004
6.3120891e-004	6.6504821e-004	6.4441972e-004	7.0264307e-004
6.3359058e-004	6.4225041e-004	6.1379644e-004	6.3253992e-004
6.2756061e-004	5.7234771e-004	5.1807394e-004	5.5856863e-004
5.0756828e-004	5.2078119e-004	5.0834063e-004	4.8618138e-004
4.9462579e-004	5.0333016e-004	4.8183464e-004	4.5881313e-004
4.8938087e-004	4.6631282e-004	4.7790476e-004	5.0348652e-004
4.5846538e-004	4.7959795e-004	4.9068054e-004	4.9397923e-004
6.5731218e-004	6.5902204e-004	6.3600432e-004	6.3525587e-004
6.0153424e-004	5.8791472e-004	5.7030196e-004	5.1642166e-004
5.2910441e-004	5.3224087e-004	5.4729106e-004	5.3486474e-004
4.8695169e-004	4.9183251e-004	4.8758530e-004	5.2203868e-004
5.3333098e-004	5.3797362e-004	4.9297216e-004	5.1442163e-004
4.7333490e-004	4.9854523e-004	4.7286714e-004	4.8571938e-004
6.0970220e-004	6.4202986e-004	6.6086951e-004	6.5097584e-004
6.4513963e-004	6.5656519e-004	5.9732096e-004	6.2356563e-004
5.5790126e-004	5.7137263e-004	5.6690053e-004	5.5806738e-004
5.4640982e-004	5.1425674e-004	5.3507149e-004	5.3832815e-004
5.7426550e-004	5.1298294e-004	5.2931448e-004	5.5932168e-004
5.1415125e-004	5.3273434e-004	5.0769084e-004	5.3518021e-004
6.0922321e-004	6.2873650e-004	6.4739387e-004	6.5927955e-004
6.3666058e-004	5.9093077e-004	6.1004616e-004	6.0427241e-004
6.0313563e-004	5.8560750e-004	5.5499801e-004	5.6450351e-004
5.3406115e-004	5.4145069e-004	5.1595230e-004	5.3429826e-004
5.3632664e-004	5.1754474e-004	5.1535390e-004	5.3078326e-004
5.0340682e-004	4.8833810e-004	4.8432254e-004	4.9217683e-004
7.6735344e-004	7.2453546e-004	8.0026709e-004	8.2556428e-004
7.8651086e-004	7.9845191e-004	7.2941361e-004	7.0684660e-004
6.7634096e-004	7.1239266e-004	6.9501832e-004	6.4744569e-004
6.4169165e-004	7.3365054e-004	6.4428654e-004	6.6533024e-004

7.0189728e-004	6.4747638e-004	6.9778704e-004	7.5605822e-004
6.6840906e-004	7.0480185e-004	6.6322892e-004	7.4451780e-004
6.8195330e-004	7.1019662e-004	6.7215157e-004	7.1493220e-004
6.9600464e-004	6.2763874e-004	5.8277286e-004	6.3178352e-004
5.6858013e-004	5.8731869e-004	5.5250595e-004	5.5381950e-004
5.4245940e-004	5.5105111e-004	5.3569101e-004	5.1124056e-004
5.5805435e-004	5.1513143e-004	5.3847075e-004	5.7472396e-004
5.1914967e-004	5.2037861e-004	5.5402971e-004	5.5874122e-004
6.7306210e-004	6.8726153e-004	6.4500437e-004	6.5336807e-004
6.2127576e-004	5.8773192e-004	5.6418644e-004	5.3060359e-004
5.2664425e-004	5.2829662e-004	5.5735228e-004	5.1497256e-004
4.7961699e-004	4.9526556e-004	4.9000393e-004	5.0001442e-004
5.1790395e-004	5.1215610e-004	4.8658813e-004	5.1243570e-004
4.6078493e-004	4.8191116e-004	4.8103578e-004	4.8407864e-004
6.2575232e-004	6.5059132e-004	6.5778798e-004	6.6822126e-004
6.4029633e-004	6.5593836e-004	5.9512327e-004	6.0922439e-004
5.4708324e-004	5.6296932e-004	5.7340116e-004	5.5721708e-004
5.4149397e-004	5.2872952e-004	5.3967463e-004	5.5488410e-004
5.8170232e-004	5.4286499e-004	5.3223720e-004	5.4469214e-004
5.1017916e-004	5.3059465e-004	4.9930704e-004	5.3466792e-004
6.1810682e-004	6.3984646e-004	6.5171758e-004	6.6852898e-004
6.4830996e-004	6.0509382e-004	6.0689732e-004	6.1407450e-004
5.9852585e-004	5.8485501e-004	5.6592382e-004	5.7808927e-004
5.3495038e-004	5.4108504e-004	5.2221239e-004	5.2457745e-004
5.3492161e-004	5.1720935e-004	5.0605097e-004	5.4307736e-004
4.9013489e-004	4.9380834e-004	4.8412303e-004	5.0839638e-004
5.8771759e-004	5.7908699e-004	5.7819922e-004	6.0006310e-004
6.0139595e-004	5.4218408e-004	5.4084837e-004	5.0681536e-004
5.2427981e-004	5.1801332e-004	4.8023350e-004	4.9696158e-004
4.6488228e-004	5.2038429e-004	4.7295107e-004	4.9426652e-004
4.7226350e-004	4.7952671e-004	4.7498025e-004	4.9686377e-004
4.7734291e-004	4.4010181e-004	4.6536654e-004	5.0594255e-004
5.9854871e-004	5.6337894e-004	6.1340009e-004	6.3077697e-004
6.0947664e-004	5.5020560e-004	5.2130734e-004	5.0826807e-004
5.0125444e-004	5.0410756e-004	4.7449738e-004	5.2832543e-004
4.7222735e-004	5.0243138e-004	5.1636789e-004	4.7108466e-004
5.0491314e-004	4.7627452e-004	4.8553534e-004	5.5831160e-004
4.9970426e-004	5.0879740e-004	5.3840053e-004	5.3639706e-004
8.2538485e-004	8.0609211e-004	8.9171364e-004	9.4044527e-004
8.8475377e-004	9.2247759e-004	8.4330930e-004	8.1377039e-004
7.5663957e-004	8.3636634e-004	7.6163494e-004	7.4788996e-004
7.3979569e-004	8.7845723e-004	7.7180509e-004	7.5323562e-004
8.1914385e-004	7.3246510e-004	8.0755060e-004	8.6412669e-004
7.7421298e-004	8.1186446e-004	7.7077052e-004	8.7943273e-004
7.4301216e-004	8.0298969e-004	7.4839348e-004	8.2062584e-004
7.9484566e-004	7.1096352e-004	6.6181000e-004	7.0668793e-004
6.4723373e-004	6.7558106e-004	6.1990415e-004	6.4323000e-004
6.1856717e-004	6.2361723e-004	6.1431988e-004	5.8038380e-004
6.4066800e-004	5.8742483e-004	6.1296574e-004	6.4857384e-004
5.8911613e-004	5.8049134e-004	6.2491739e-004	6.3648926e-004
6.8258449e-004	7.0647836e-004	6.6621284e-004	6.7256101e-004
6.4842317e-004	6.0718753e-004	5.8280969e-004	5.5790591e-004

5.3854738e-004	5.4745840e-004	5.6760279e-004	5.3045840e-004
4.9261435e-004	5.0639651e-004	5.1807157e-004	5.1583430e-004
5.3097635e-004	5.2106936e-004	4.9683478e-004	5.3608109e-004
4.8238332e-004	4.8970836e-004	4.9300543e-004	4.9976179e-004
6.2773541e-004	6.5309655e-004	6.5229347e-004	6.8208438e-004
6.4172801e-004	6.5137129e-004	5.9122169e-004	6.1074368e-004
5.5184220e-004	5.6579357e-004	5.8107264e-004	5.6689199e-004
5.4309620e-004	5.4524880e-004	5.5531556e-004	5.5531891e-004
5.8189267e-004	5.5357977e-004	5.3488350e-004	5.3943488e-004
5.0444507e-004	5.3312704e-004	4.9339249e-004	5.3722782e-004
6.2082330e-004	6.3879031e-004	6.3556489e-004	6.7542828e-004
6.5033283e-004	6.0444421e-004	5.9556110e-004	6.0216682e-004
5.9047161e-004	5.7435289e-004	5.6478094e-004	5.7356217e-004
5.2394789e-004	5.5191474e-004	5.1478096e-004	5.2378156e-004
5.2133591e-004	5.2108915e-004	4.9972212e-004	5.4339133e-004
4.7814276e-004	4.9390100e-004	4.7752996e-004	5.1175839e-004
5.8984385e-004	5.9105959e-004	5.8879321e-004	5.9939521e-004
6.0691932e-004	5.5913303e-004	5.4262366e-004	5.1437681e-004
5.2416134e-004	5.1752455e-004	4.7620208e-004	4.9120509e-004
4.5890488e-004	5.1786182e-004	4.6989398e-004	4.7778212e-004
4.7754367e-004	4.7575955e-004	4.5943960e-004	4.8093771e-004
4.5963669e-004	4.3664701e-004	4.6968076e-004	5.1052535e-004
5.6726435e-004	5.8901598e-004	5.9748565e-004	5.9545298e-004
6.3912508e-004	5.7063060e-004	5.8201920e-004	5.6876830e-004
5.5548549e-004	5.1247003e-004	5.4468679e-004	5.5977328e-004
5.4218677e-004	4.9297676e-004	4.8615280e-004	5.0257523e-004
5.2581138e-004	5.2203108e-004	4.8967879e-004	5.4360474e-004
5.0855691e-004	4.8118504e-004	4.8271965e-004	4.7918152e-004
6.1079640e-004	5.8311773e-004	6.1323511e-004	6.3229581e-004
6.2919281e-004	5.6923080e-004	5.0996847e-004	5.0336629e-004
5.0429933e-004	4.8072053e-004	4.6152019e-004	5.1300776e-004
4.5295560e-004	4.9438958e-004	4.9849197e-004	4.6345820e-004
4.8974917e-004	4.6687386e-004	4.7650014e-004	5.4382233e-004
4.7588621e-004	5.0471273e-004	5.3462114e-004	5.3964248e-004
8.3065338e-004	9.1457357e-004	9.1416534e-004	1.0520163e-003
9.7495678e-004	9.7334318e-004	9.2560444e-004	9.1709516e-004
8.1232923e-004	9.1621104e-004	8.2316467e-004	8.3669275e-004
8.2388313e-004	9.7994639e-004	8.8535855e-004	8.1456715e-004
9.3570799e-004	7.9212691e-004	8.3864100e-004	9.2117309e-004
8.3571796e-004	8.6553144e-004	8.6650690e-004	9.3595737e-004
7.7783575e-004	8.4753988e-004	7.8698575e-004	8.7753897e-004
8.4021376e-004	7.6849099e-004	6.9217462e-004	7.2403083e-004
6.9299609e-004	7.1695920e-004	6.6323992e-004	6.8822643e-004
6.4171386e-004	6.4123273e-004	6.4621444e-004	6.1350012e-004
6.6119447e-004	6.2544603e-004	6.4028044e-004	6.6637839e-004
6.1017062e-004	5.9603341e-004	6.2872903e-004	6.5310902e-004
6.7819540e-004	6.9595543e-004	6.6448182e-004	6.8097323e-004
6.5723923e-004	6.1334304e-004	5.9281812e-004	5.8207502e-004
5.4140987e-004	5.6549186e-004	5.7287862e-004	5.5243396e-004
5.1506080e-004	5.1718039e-004	5.5563124e-004	5.4364260e-004
5.5468964e-004	5.3806370e-004	5.1179093e-004	5.4833914e-004
5.0939361e-004	5.1062231e-004	4.9071629e-004	5.1271418e-004

6.1463725e-004	6.4129646e-004	6.3459236e-004	6.7156396e-004
6.4398262e-004	6.3908345e-004	5.7814951e-004	6.0459234e-004
5.6010467e-004	5.6803761e-004	5.8326825e-004	5.7685073e-004
5.3697906e-004	5.5537217e-004	5.4815752e-004	5.3979129e-004
5.7198180e-004	5.4293871e-004	5.3631629e-004	5.3791700e-004
4.8869527e-004	5.3098956e-004	4.8638427e-004	5.3942706e-004
6.1393823e-004	6.4057090e-004	6.2953618e-004	6.7975378e-004
6.6123718e-004	6.0565825e-004	5.9055031e-004	5.8433297e-004
5.8836068e-004	5.5980816e-004	5.4748340e-004	5.5268906e-004
5.0140539e-004	5.5780359e-004	5.0060976e-004	5.2423463e-004
5.0145557e-004	5.1688102e-004	4.7990024e-004	5.3634745e-004
4.6235522e-004	4.7354519e-004	4.7986517e-004	5.0309012e-004
5.6780610e-004	5.9327517e-004	5.8233214e-004	5.8393173e-004
6.0236426e-004	5.7884752e-004	5.3976531e-004	5.3369657e-004
5.2664864e-004	5.1162933e-004	4.6756217e-004	5.0109206e-004
4.6126911e-004	5.0957411e-004	4.7627730e-004	4.7678240e-004
4.8800794e-004	4.8638807e-004	4.6058001e-004	4.5210921e-004
4.6403531e-004	4.5693706e-004	4.7478473e-004	5.0333962e-004
5.6259776e-004	5.8114988e-004	5.7381441e-004	5.9845376e-004
6.0525682e-004	5.7397720e-004	5.9215444e-004	5.7464972e-004
5.5006819e-004	5.2867047e-004	5.1453464e-004	5.4463130e-004
4.9272965e-004	4.8358794e-004	4.5741790e-004	4.6661675e-004
4.8240603e-004	5.0765307e-004	4.7050392e-004	5.0447607e-004
4.8164447e-004	4.9098473e-004	4.4923566e-004	4.8580389e-004
5.8929126e-004	6.2464891e-004	6.3299414e-004	6.4819610e-004
6.6600960e-004	6.2265803e-004	5.9847152e-004	6.2010274e-004
6.0640545e-004	5.4534707e-004	5.7096576e-004	6.0372658e-004
5.9101611e-004	5.2228222e-004	5.1369752e-004	5.4103404e-004
5.4701067e-004	5.4091451e-004	5.1593064e-004	5.7944808e-004
5.3980545e-004	5.1615460e-004	5.0287372e-004	5.1157951e-004
6.9363380e-004	6.8502080e-004	6.6643159e-004	7.2173859e-004
7.2681003e-004	6.3711959e-004	6.5057733e-004	6.6795376e-004
6.1276194e-004	6.2296788e-004	6.1937855e-004	6.0875402e-004
5.8336341e-004	5.8689316e-004	5.7794404e-004	5.9147942e-004
5.5706275e-004	5.9763236e-004	5.8252864e-004	6.3645830e-004
5.7444295e-004	6.0758737e-004	5.6055302e-004	6.1777292e-004
7.1218362e-004	7.7023878e-004	7.2544155e-004	8.3630472e-004
8.2610255e-004	7.8700735e-004	6.9039618e-004	6.9200359e-004
6.5991307e-004	6.5724771e-004	6.4116321e-004	6.4971963e-004
6.1717768e-004	7.2326673e-004	6.8708156e-004	6.4512431e-004
7.1886530e-004	6.2720486e-004	6.2170314e-004	7.0381722e-004
6.2176676e-004	6.8619525e-004	7.0608501e-004	7.1660477e-004
9.2190615e-004	1.0653029e-003	9.6577306e-004	1.1704135e-003
1.0885692e-003	1.0105868e-003	9.4256716e-004	9.8749848e-004
9.3398108e-004	9.8163345e-004	8.9874541e-004	9.5331980e-004
8.6276649e-004	9.1749152e-004	9.1568056e-004	8.7024720e-004
9.5303891e-004	8.5901564e-004	8.8353617e-004	9.1908335e-004
8.3502202e-004	8.3826046e-004	8.5561664e-004	8.9446006e-004
7.1230726e-004	7.4985481e-004	6.9924051e-004	7.3968497e-004
7.1707397e-004	6.8771483e-004	6.1978499e-004	6.2193976e-004
5.7830603e-004	6.0237339e-004	6.0511038e-004	5.9098561e-004
5.3606996e-004	5.5534592e-004	5.7298198e-004	5.5872834e-004

5.7437761e-004	5.4062132e-004	5.3006971e-004	5.7056401e-004
5.3855863e-004	5.3750127e-004	5.1906650e-004	5.4429819e-004
6.2793573e-004	6.5659851e-004	6.3548748e-004	6.7299949e-004
6.5493741e-004	6.2268489e-004	5.7188807e-004	5.9062772e-004
5.5075734e-004	5.7369116e-004	5.6662051e-004	5.7483687e-004
5.3712926e-004	5.5039508e-004	5.6627706e-004	5.4951469e-004
5.7612677e-004	5.4706819e-004	5.3355661e-004	5.3097753e-004
4.9424284e-004	5.1959482e-004	4.8368992e-004	5.3080420e-004
6.0602235e-004	6.2738686e-004	6.1987151e-004	6.7767745e-004
6.7659419e-004	6.1485104e-004	5.7655948e-004	5.9675562e-004
5.8705237e-004	5.6844152e-004	5.6412964e-004	5.6184846e-004
5.0869549e-004	5.5845060e-004	5.2422177e-004	5.3941421e-004
5.2589487e-004	5.3697567e-004	5.0933081e-004	5.4459539e-004
4.7242089e-004	5.0744686e-004	4.9232817e-004	5.0951322e-004
5.7184690e-004	6.1346139e-004	6.1452901e-004	6.1524284e-004
6.3607554e-004	5.8923055e-004	5.6125078e-004	5.4398348e-004
5.4893715e-004	5.2823512e-004	5.0305275e-004	4.9827481e-004
4.6687733e-004	5.1945249e-004	4.8095830e-004	4.8524458e-004
4.9586115e-004	5.0048166e-004	4.5977896e-004	4.7590584e-004
4.5723397e-004	4.5532529e-004	4.7994640e-004	5.0246155e-004
5.5962455e-004	5.9049006e-004	5.6788318e-004	5.9300722e-004
6.0696510e-004	5.9373258e-004	5.7467743e-004	5.7866672e-004
5.5747993e-004	5.2408177e-004	5.0095813e-004	5.4638286e-004
4.8547491e-004	4.9448830e-004	4.6858570e-004	4.7314695e-004
4.7407715e-004	5.0821035e-004	4.6366150e-004	4.7776426e-004
4.8120553e-004	4.8962313e-004	4.3896338e-004	4.8525431e-004
6.0574879e-004	6.4714000e-004	6.5944548e-004	6.9562776e-004
6.8489018e-004	6.2532793e-004	6.1483619e-004	6.3258174e-004
6.0854379e-004	5.7341403e-004	5.8318635e-004	6.0311439e-004
6.0163507e-004	5.4036130e-004	5.0875653e-004	5.3477718e-004
5.3309152e-004	5.5132982e-004	5.0972414e-004	5.8637590e-004
5.3939685e-004	5.2651574e-004	5.1474870e-004	5.2228857e-004
7.0341023e-004	7.0526690e-004	6.6273069e-004	7.2915410e-004
7.2793284e-004	6.3949745e-004	6.5651660e-004	6.6910268e-004
5.8782044e-004	6.2150513e-004	6.3735079e-004	5.9454919e-004
6.0025789e-004	5.8178118e-004	5.8123410e-004	5.8259837e-004
5.9104615e-004	6.1833909e-004	5.7797194e-004	6.2196938e-004
5.7788038e-004	6.0556289e-004	5.4189721e-004	6.3886345e-004
7.2398348e-004	8.3176773e-004	7.4434235e-004	8.9165282e-004
8.4315072e-004	8.2835511e-004	7.3498222e-004	7.7187787e-004
7.0858590e-004	6.8855496e-004	6.8932713e-004	7.0661123e-004
6.2840881e-004	6.8678584e-004	6.7484965e-004	6.8429847e-004
7.3004262e-004	6.5689426e-004	6.6076737e-004	7.2472663e-004
6.5117608e-004	7.3771355e-004	7.1537324e-004	7.2795652e-004
7.0270633e-004	7.4711817e-004	6.8312851e-004	7.4502145e-004
7.2163904e-004	6.8558057e-004	6.1773026e-004	6.2593262e-004
5.7230651e-004	5.9787284e-004	6.1109438e-004	5.9617733e-004
5.5220627e-004	5.6560742e-004	5.7339990e-004	5.6200859e-004
5.8440308e-004	5.4645170e-004	5.4086611e-004	5.6056558e-004
5.4498228e-004	5.4792719e-004	5.1465308e-004	5.3537783e-004
6.1654468e-004	6.3774089e-004	6.3164911e-004	6.5635462e-004
6.6917011e-004	6.1650580e-004	5.6069823e-004	5.8689404e-004

5.5342322e-004	5.6806261e-004	5.6136376e-004	5.6315778e-004
5.2554042e-004	5.4650875e-004	5.4868382e-004	5.4334367e-004
5.6835114e-004	5.3329028e-004	5.3366223e-004	5.3114578e-004
4.8526483e-004	5.1449292e-004	4.8829389e-004	5.2893201e-004
6.0028264e-004	6.3658255e-004	6.4426685e-004	6.9286718e-004
7.0030219e-004	6.2609965e-004	5.7970609e-004	5.9631761e-004
6.0414951e-004	5.7457190e-004	5.6269475e-004	5.5137058e-004
5.1530822e-004	5.5023695e-004	5.2202159e-004	5.4123215e-004
5.1655175e-004	5.4140923e-004	5.0666949e-004	5.3933774e-004
4.7265419e-004	4.9076738e-004	4.9462438e-004	4.9980697e-004
5.6441460e-004	5.9598754e-004	6.0882994e-004	6.0845622e-004
6.3956833e-004	5.9889158e-004	5.7299361e-004	5.8068137e-004
5.6229369e-004	5.3901297e-004	5.2288103e-004	5.2748504e-004
4.9727368e-004	5.2926366e-004	4.9787204e-004	5.1784937e-004
5.1285789e-004	5.4355561e-004	4.8525124e-004	4.8097675e-004
4.8301071e-004	4.9217966e-004	4.7666662e-004	5.0733278e-004
5.7779142e-004	6.1047247e-004	5.8288955e-004	6.3924049e-004
6.4093288e-004	5.9504541e-004	5.7776504e-004	5.7494912e-004
5.6633436e-004	5.5724164e-004	5.2959514e-004	5.5110340e-004
5.0891169e-004	5.0711437e-004	4.8335906e-004	4.7340077e-004
4.8271300e-004	5.2167941e-004	4.5344502e-004	5.1666815e-004
4.8761067e-004	4.9369004e-004	4.4451141e-004	4.8664009e-004
6.3682950e-004	6.8300820e-004	6.9031517e-004	7.5217882e-004
7.2537150e-004	6.7803756e-004	6.6499654e-004	6.8560400e-004
6.5278496e-004	6.1126862e-004	6.3706526e-004	6.5825529e-004
6.5011636e-004	5.7869461e-004	5.3874883e-004	5.9624488e-004
5.6905266e-004	5.8350422e-004	5.4881132e-004	6.1566017e-004
5.8918775e-004	5.8582499e-004	5.6093946e-004	5.6198509e-004
6.9891859e-004	7.0846266e-004	6.6696486e-004	7.3085913e-004
6.9537864e-004	6.5346531e-004	6.3692019e-004	6.6734401e-004
5.5025700e-004	5.8054054e-004	6.2231167e-004	5.6795107e-004
5.8045023e-004	5.2994092e-004	5.5224176e-004	5.3818055e-004
5.9661882e-004	5.8217339e-004	5.7310033e-004	6.3003079e-004
5.8747466e-004	6.3567396e-004	5.4432632e-004	6.4323764e-004
7.8242175e-004	9.1498831e-004	8.6420927e-004	1.0546478e-003
9.0037734e-004	9.2239562e-004	8.2891934e-004	9.4025078e-004
8.3824422e-004	8.3084642e-004	8.1370932e-004	8.9847495e-004
7.4038290e-004	8.1624377e-004	7.9429458e-004	8.0399315e-004
8.0309259e-004	7.8029912e-004	7.6233325e-004	7.9004804e-004
7.5051711e-004	7.7560166e-004	7.5971143e-004	7.9034238e-004
6.6137438e-004	7.1238524e-004	6.5900403e-004	7.1233399e-004
6.9818742e-004	6.3896929e-004	5.6970438e-004	6.0542418e-004
5.5576608e-004	5.7153100e-004	5.7260800e-004	5.7396453e-004
5.5284990e-004	5.5241487e-004	5.5378185e-004	5.4667865e-004
5.7176244e-004	5.4045225e-004	5.2854021e-004	5.3018512e-004
5.0518131e-004	5.1876481e-004	5.0045447e-004	5.2371270e-004
6.1532493e-004	6.2593752e-004	6.4291104e-004	6.6980280e-004
7.0357966e-004	6.3846457e-004	5.8015842e-004	6.0500635e-004
5.8482290e-004	5.8432171e-004	5.8169864e-004	5.5655859e-004
5.2559664e-004	5.5591235e-004	5.4195082e-004	5.4729145e-004
5.4908736e-004	5.4075924e-004	5.5065802e-004	5.4604527e-004
4.9904513e-004	5.1678986e-004	5.0479569e-004	5.2210642e-004

5.9140610e-004	6.2301730e-004	6.6980350e-004	6.9282769e-004
6.9980667e-004	6.3036665e-004	6.0408098e-004	6.1768929e-004
6.1712280e-004	5.7877472e-004	5.7126356e-004	5.5839610e-004
5.4667894e-004	5.5120107e-004	5.2322322e-004	5.4982258e-004
5.3055990e-004	5.7350077e-004	5.1602446e-004	5.3729049e-004
4.8488445e-004	4.9265824e-004	4.8507978e-004	5.1238686e-004
5.5903610e-004	5.8945434e-004	5.8277397e-004	6.2504652e-004
6.4074749e-004	6.1555284e-004	5.7112200e-004	5.9929947e-004
5.7139486e-004	5.5895658e-004	5.5593743e-004	5.6317465e-004
5.1869397e-004	5.3823198e-004	5.1215905e-004	5.3740015e-004
5.0920570e-004	5.7044400e-004	4.8922312e-004	5.1742709e-004
5.1851114e-004	5.1688450e-004	4.6693886e-004	4.9945775e-004
6.0966346e-004	6.5829570e-004	6.4618078e-004	7.1773285e-004
6.9449235e-004	6.1555642e-004	6.0685810e-004	6.0576880e-004
5.9691505e-004	6.0428810e-004	5.7252603e-004	5.8494159e-004
5.6549581e-004	5.4902650e-004	5.0727461e-004	5.1619894e-004
5.2864473e-004	5.4466770e-004	4.8235016e-004	5.5537690e-004
5.0501538e-004	5.4069982e-004	5.0659321e-004	5.0959944e-004
6.5454688e-004	6.7783165e-004	6.7833529e-004	7.5787092e-004
7.1859393e-004	7.0388481e-004	7.0311067e-004	7.0258249e-004
6.6166847e-004	6.5869126e-004	6.7908801e-004	6.8423901e-004
6.7950491e-004	6.1217285e-004	5.9078507e-004	6.4395489e-004
6.2300236e-004	6.1316798e-004	6.0313888e-004	6.6076939e-004
6.4824829e-004	6.2289741e-004	5.8086164e-004	6.1104738e-004
6.7068484e-004	7.0612502e-004	6.4773999e-004	7.3239599e-004
6.6845833e-004	6.2280105e-004	6.4684029e-004	6.4065312e-004
5.6897450e-004	5.7562154e-004	5.9845936e-004	5.6716967e-004
5.6056173e-004	5.2444829e-004	5.7373063e-004	5.5893080e-004
6.3922525e-004	6.2393122e-004	5.4887807e-004	5.9594748e-004
5.7117781e-004	5.8431461e-004	5.5025777e-004	6.3574573e-004
7.1854409e-004	8.0857424e-004	8.2731539e-004	9.1596144e-004
8.0138483e-004	8.2973974e-004	7.5873689e-004	8.1299276e-004
6.9361094e-004	7.3259271e-004	7.4854660e-004	7.7678887e-004
7.1042028e-004	7.1075340e-004	6.9515091e-004	6.8412774e-004
7.0745412e-004	7.0629187e-004	7.1450901e-004	7.1458921e-004
7.2060439e-004	7.4440743e-004	6.8699068e-004	7.2249815e-004
6.9426371e-004	7.5230982e-004	7.0351372e-004	7.7098636e-004
7.3812256e-004	6.8161957e-004	6.0582787e-004	6.5430564e-004
5.9279798e-004	5.9169650e-004	6.0597040e-004	5.9581221e-004
5.8574446e-004	5.8645986e-004	5.6822499e-004	5.6948749e-004
5.9251512e-004	5.6444846e-004	5.5155443e-004	5.5560344e-004
5.2415739e-004	5.4073769e-004	5.3386985e-004	5.4686496e-004
6.2178218e-004	6.3198581e-004	6.6200369e-004	6.8306738e-004
7.0665256e-004	6.5087040e-004	5.9669628e-004	6.1921393e-004
5.9433829e-004	5.9378531e-004	5.8713312e-004	5.7116235e-004
5.4762953e-004	5.6038250e-004	5.4187018e-004	5.4748308e-004
5.5299854e-004	5.4453137e-004	5.7028441e-004	5.4806884e-004
5.1467778e-004	5.0745607e-004	5.0619479e-004	5.2351247e-004
6.0531234e-004	6.2114143e-004	6.8682901e-004	7.3009596e-004
7.2773277e-004	6.6297218e-004	6.3756238e-004	6.6161352e-004
6.4302256e-004	6.1053516e-004	6.0143218e-004	5.9550822e-004
6.0253860e-004	5.8174226e-004	5.5873601e-004	5.7831722e-004

5.6240015e-004	6.1844406e-004	5.5707127e-004	5.7930179e-004
5.1752283e-004	5.2159990e-004	4.9940233e-004	5.4503593e-004
6.0066416e-004	6.6831880e-004	6.3994089e-004	7.0970559e-004
6.9510828e-004	6.0954961e-004	6.0289017e-004	5.9781779e-004
5.8342343e-004	6.0542524e-004	5.6562832e-004	5.7512676e-004
5.4483298e-004	5.5194189e-004	5.1559451e-004	5.1897616e-004
5.3232114e-004	5.3757078e-004	4.8756249e-004	5.4080304e-004
4.9427313e-004	5.3388895e-004	5.0036888e-004	5.0173625e-004
6.5094059e-004	6.9403522e-004	6.6248812e-004	7.3317056e-004
6.4547716e-004	6.2326490e-004	6.3589387e-004	6.2292237e-004
5.5120104e-004	5.4516020e-004	5.6675044e-004	5.4972095e-004
5.5113503e-004	5.0380251e-004	5.6732538e-004	5.4445933e-004
6.3108040e-004	5.8864788e-004	5.4286659e-004	5.9245397e-004
5.7691813e-004	5.8232137e-004	5.5250972e-004	6.0427631e-004
6.6166406e-004	7.0493798e-004	6.9557552e-004	7.2217519e-004
7.1945776e-004	6.7167945e-004	5.9870881e-004	6.5263952e-004
5.8556219e-004	5.7337651e-004	5.8571956e-004	5.7301603e-004
5.7029980e-004	5.5882141e-004	5.4092616e-004	5.4373898e-004
5.6536282e-004	5.4195171e-004	5.6632041e-004	5.3517701e-004
5.0477998e-004	4.9770305e-004	4.9814257e-004	5.2058775e-004
6.2744012e-004	6.1708932e-004	6.9916561e-004	7.4356463e-004
7.4491907e-004	6.7560059e-004	6.5404490e-004	6.6868648e-004
6.5954434e-004	6.4284969e-004	6.1676234e-004	6.3544526e-004
6.4611806e-004	6.0427006e-004	5.9485155e-004	5.9500219e-004
5.8617799e-004	6.1758912e-004	6.0148623e-004	6.0244337e-004
5.5850408e-004	5.2894246e-004	5.4003387e-004	5.8099465e-004
5.7349566e-004	6.1926761e-004	6.0422503e-004	6.7148250e-004
6.5576789e-004	6.3082487e-004	5.5644460e-004	5.8436247e-004
5.6134982e-004	5.9398191e-004	5.6907031e-004	5.6768752e-004
5.2848978e-004	5.6817450e-004	5.2016412e-004	5.3169639e-004
5.2401664e-004	5.5039255e-004	4.7986704e-004	5.4753483e-004
5.0807122e-004	5.0732840e-004	4.8640282e-004	4.9725040e-004
6.1866258e-004	6.9617524e-004	6.9306900e-004	7.4090112e-004
7.2058770e-004	6.3193990e-004	6.6674448e-004	6.2215068e-004
6.1501823e-004	6.2779046e-004	6.0006844e-004	6.3288902e-004
5.8293495e-004	5.6384889e-004	5.5393203e-004	5.5456176e-004
5.3556978e-004	5.6683231e-004	5.4742880e-004	5.6920203e-004
5.5188844e-004	5.5679854e-004	5.2615219e-004	5.2831721e-004
5.9830392e-004	6.4448416e-004	6.3234042e-004	7.2502573e-004
6.7473488e-004	6.1477281e-004	6.5379679e-004	6.2294700e-004
5.7585089e-004	5.5073420e-004	5.4699896e-004	5.4325956e-004
5.5290473e-004	5.3255515e-004	5.6420147e-004	5.3984702e-004
5.7184354e-004	5.6455204e-004	5.1663704e-004	5.7118465e-004
5.2152813e-004	5.3480084e-004	5.2101476e-004	5.4854836e-004
7.0304808e-004	6.9083690e-004	7.5342409e-004	7.8545607e-004
7.2961955e-004	7.1627037e-004	6.8878988e-004	6.7022537e-004
5.8682953e-004	6.0310899e-004	6.4834775e-004	6.4126110e-004
6.4492754e-004	5.7192303e-004	5.9272630e-004	5.8386723e-004
6.5575588e-004	6.1072830e-004	6.3729362e-004	6.4613977e-004
6.7230842e-004	6.4400141e-004	6.1550110e-004	6.4102602e-004
6.8692562e-004	7.3117691e-004	7.2888068e-004	7.6578044e-004
7.6048731e-004	7.1032092e-004	6.4967530e-004	7.0215880e-004

6.3018441e-004	5.9480322e-004	6.1508708e-004	6.1481936e-004
6.1405775e-004	5.8841959e-004	5.7191233e-004	5.7382316e-004
5.9429571e-004	5.7166682e-004	5.9836311e-004	5.5966149e-004
5.3746290e-004	5.1092940e-004	5.2539815e-004	5.4325828e-004
6.2898164e-004	6.1613545e-004	6.9597890e-004	7.4604360e-004
7.4933002e-004	6.7665429e-004	6.7625911e-004	6.8799355e-004
6.7830928e-004	6.5408653e-004	6.2801070e-004	6.6665195e-004
6.7204244e-004	6.2999774e-004	6.2035107e-004	6.1914399e-004
6.2284026e-004	6.4573660e-004	6.2857225e-004	6.2697392e-004
5.9257974e-004	5.4732847e-004	5.7329735e-004	6.1701548e-004
5.8023757e-004	6.3524627e-004	6.2997353e-004	6.8964006e-004
6.6086939e-004	6.4303369e-004	5.7034266e-004	5.9466681e-004
5.7118853e-004	6.0316852e-004	5.8093373e-004	5.8156456e-004
5.3929910e-004	5.9022850e-004	5.3188954e-004	5.5426471e-004
5.3496131e-004	5.6622530e-004	4.9497985e-004	5.6643823e-004
5.1979376e-004	5.1090065e-004	5.0341363e-004	5.2580853e-004
6.0617803e-004	6.9520215e-004	7.0794011e-004	7.4846103e-004
7.1017708e-004	6.2348061e-004	6.3924673e-004	6.1262128e-004
6.0119217e-004	6.1946387e-004	5.7437972e-004	6.2893219e-004
5.6570602e-004	5.5941984e-004	5.5977899e-004	5.4529333e-004
5.2814030e-004	5.6743617e-004	5.5333020e-004	5.5813628e-004
5.4882687e-004	5.3962755e-004	4.9753789e-004	5.1014175e-004
6.0364738e-004	6.3900542e-004	6.2591165e-004	7.3814033e-004
6.8083519e-004	6.2012143e-004	6.4052772e-004	6.3125894e-004
5.6262205e-004	5.2449320e-004	5.4021490e-004	5.2175435e-004
5.3008807e-004	5.1142037e-004	5.4390907e-004	5.2225310e-004
5.4903809e-004	5.4130481e-004	4.9873753e-004	5.5330143e-004
5.0121237e-004	5.2352672e-004	4.9884582e-004	5.0657785e-004
6.3848935e-004	6.3484658e-004	6.8751193e-004	7.2553031e-004
7.4289723e-004	6.6284416e-004	6.7965584e-004	6.8533256e-004
6.7516990e-004	6.4962245e-004	6.2343286e-004	6.7185669e-004
6.5654573e-004	6.1881584e-004	6.0729249e-004	6.1448140e-004
6.2543784e-004	6.3634990e-004	6.3041229e-004	6.1080790e-004
6.0328498e-004	5.4359801e-004	5.9123398e-004	6.1708313e-004
5.8195872e-004	6.3747208e-004	6.6313405e-004	7.0186402e-004
6.7326556e-004	6.7451459e-004	6.0326714e-004	6.1188242e-004
6.0266556e-004	6.2668862e-004	6.3211263e-004	6.1085442e-004
5.7637511e-004	6.1975935e-004	5.5115874e-004	5.9464894e-004
5.6363645e-004	5.9990361e-004	5.3667874e-004	6.2829300e-004
5.6104004e-004	5.4220321e-004	5.3690789e-004	5.7384845e-004
6.0593612e-004	7.0479269e-004	7.1669779e-004	7.7184608e-004
7.1254949e-004	6.2218092e-004	6.1664775e-004	6.3066474e-004
5.8816484e-004	6.0838423e-004	5.6011468e-004	6.1461559e-004
5.5869936e-004	5.7132440e-004	5.6580843e-004	5.6542580e-004
5.5186165e-004	5.8161677e-004	5.5751389e-004	5.4578429e-004
5.5395956e-004	5.3124649e-004	4.9364105e-004	5.2053719e-004
6.2798132e-004	6.6858574e-004	7.3970156e-004	7.3219919e-004
6.8064437e-004	6.6566405e-004	6.4024756e-004	6.4213020e-004
6.8002410e-004	6.8757476e-004	6.4018210e-004	6.6082140e-004
6.4896444e-004	6.2780935e-004	6.2503829e-004	6.0498559e-004
6.0928403e-004	6.4026740e-004	5.9754113e-004	6.1046824e-004
5.7694444e-004	5.8935855e-004	5.5562181e-004	5.8268892e-004

5.9621393e-004	5.8899268e-004	6.0703550e-004	7.2791570e-004
6.8136350e-004	6.0992126e-004	6.2600599e-004	6.3486479e-004
5.9159069e-004	5.2326734e-004	5.5297316e-004	5.2525320e-004
5.1737188e-004	5.1996090e-004	5.4449632e-004	5.3625560e-004
5.3560553e-004	5.4980998e-004	5.0096999e-004	5.3742388e-004
4.9500552e-004	5.2804033e-004	4.8766232e-004	5.0558017e-004
7.0799395e-004	7.6455253e-004	7.7661833e-004	8.5950008e-004
8.9993671e-004	8.1214498e-004	7.9728678e-004	8.3815274e-004
7.5621357e-004	6.6951098e-004	7.1928604e-004	7.3385563e-004
7.1582588e-004	6.8681697e-004	6.7843919e-004	6.7567185e-004
6.9486466e-004	6.6401393e-004	6.8783558e-004	6.7120012e-004
6.4556116e-004	5.9814280e-004	6.4266006e-004	6.5110812e-004
6.2209518e-004	6.7990395e-004	7.0839695e-004	7.6381247e-004
7.1179333e-004	6.8180084e-004	6.3361108e-004	6.3496812e-004
6.2759459e-004	6.3390797e-004	6.0628451e-004	6.1569920e-004
5.8886173e-004	6.0208702e-004	5.5635931e-004	6.0168585e-004
5.7614231e-004	6.0682317e-004	5.5150966e-004	6.2306956e-004
5.6029357e-004	5.4601187e-004	5.4054693e-004	5.9204997e-004
6.1933244e-004	7.1678827e-004	7.6199970e-004	8.1288391e-004
7.2167092e-004	6.6479179e-004	6.4714102e-004	6.4587279e-004
6.3673597e-004	6.4290709e-004	5.9017914e-004	6.5010208e-004
5.9996343e-004	6.0118901e-004	6.0684739e-004	5.9182289e-004
5.8026047e-004	6.2861794e-004	5.9156984e-004	5.8408688e-004
5.8688446e-004	5.6384721e-004	5.1560775e-004	5.4628517e-004
6.2770011e-004	6.9588309e-004	7.4434732e-004	7.4224094e-004
7.0684617e-004	6.6904965e-004	6.5709873e-004	6.6220167e-004
6.7997750e-004	6.9099596e-004	6.5035149e-004	6.5288958e-004
6.7205465e-004	6.2827220e-004	6.3962227e-004	6.2532275e-004
6.3255988e-004	6.3659916e-004	5.9939951e-004	6.1207989e-004
5.8500491e-004	5.8493482e-004	5.8583604e-004	6.0556049e-004
5.2158171e-004	6.4600096e-004	6.0260693e-004	6.7111106e-004
6.5704522e-004	5.9966836e-004	6.1074049e-004	6.6356597e-004
6.0703646e-004	6.0607342e-004	5.7923321e-004	6.0053405e-004
5.8776610e-004	5.8634978e-004	5.6156803e-004	5.3301998e-004
5.4429522e-004	5.6418726e-004	5.3434797e-004	5.4043710e-004
5.2005032e-004	5.6528323e-004	5.1760754e-004	5.3054586e-004
6.3644242e-004	5.9679158e-004	6.2978441e-004	7.7441862e-004
7.0264023e-004	6.3085377e-004	6.2277781e-004	6.5062757e-004
5.8855364e-004	5.0179717e-004	5.5785167e-004	5.1511078e-004
4.8407040e-004	5.0282544e-004	5.2990080e-004	5.5986719e-004
5.4559404e-004	5.4728251e-004	4.8997730e-004	5.3108549e-004
4.9173922e-004	5.5117939e-004	4.9747268e-004	4.8549040e-004
6.2746959e-004	6.7397727e-004	7.2208696e-004	7.7275527e-004
7.2925400e-004	7.1524315e-004	6.6676029e-004	6.5145419e-004
6.5992781e-004	6.5351484e-004	6.4318583e-004	6.4012207e-004
6.2591242e-004	6.2602606e-004	5.8304035e-004	6.2626165e-004
6.1583627e-004	6.1656395e-004	5.9783047e-004	6.6949773e-004
5.9774877e-004	5.7193385e-004	5.7695234e-004	6.2318622e-004
6.4203369e-004	7.3091534e-004	7.6970406e-004	8.6039235e-004
7.7137342e-004	6.8740465e-004	6.8563630e-004	6.8893406e-004
6.5619656e-004	6.4842690e-004	6.2629333e-004	6.4964242e-004
6.2448184e-004	6.1619512e-004	6.2021676e-004	6.3799552e-004

6.2735108e-004 6.6248135e-004 6.1682660e-004 6.1455450e-004
6.1015469e-004 5.9194319e-004 5.5630773e-004 5.9822408e-004
6.6930847e-004 7.4302968e-004 7.8782639e-004 7.8840893e-004
7.3708371e-004 6.9227070e-004 6.8169164e-004 6.7202759e-004
6.9953936e-004 6.9715979e-004 6.3838980e-004 6.6571949e-004
6.7248588e-004 6.2527983e-004 6.4626175e-004 6.1388024e-004
6.2542201e-004 6.5277277e-004 5.8426423e-004 6.1099268e-004
5.9902215e-004 5.8191744e-004 5.7846516e-004 6.1173817e-004
6.3961063e-004 5.4606996e-004 6.4751896e-004 7.5653481e-004
6.9276741e-004 6.1715080e-004 6.2844844e-004 6.3969291e-004
6.2384654e-004 5.3858773e-004 5.7138092e-004 5.4673445e-004
5.1526893e-004 5.2074475e-004 5.7269202e-004 5.8824482e-004
5.4274793e-004 5.8930002e-004 5.5652496e-004 5.5765420e-004
5.2519535e-004 5.6499214e-004 5.6878477e-004 5.1867901e-004
6.7472644e-004 7.1497080e-004 7.6328147e-004 8.4468636e-004
7.9226433e-004 7.3587587e-004 7.1590494e-004 6.7896623e-004
6.9278492e-004 6.5459043e-004 6.4006672e-004 6.5356909e-004
6.4143431e-004 6.2210827e-004 5.9836856e-004 6.2766267e-004
6.5197550e-004 6.2234075e-004 6.2655353e-004 6.6132846e-004
6.1676545e-004 5.8063418e-004 5.8334197e-004 6.4306171e-004
6.7454358e-004 7.5549804e-004 7.7694413e-004 8.9507634e-004
7.9500138e-004 7.4584171e-004 7.5155971e-004 7.2219071e-004
7.0981217e-004 6.9019747e-004 6.8396571e-004 6.7781584e-004
6.7256157e-004 6.5279200e-004 6.6719062e-004 6.5520301e-004
6.7180143e-004 7.0101061e-004 6.4467192e-004 6.5810238e-004
6.4257508e-004 6.3159029e-004 6.0831332e-004 6.3931977e-004
6.8449583e-004 7.4358868e-004 7.8422671e-004 8.2125604e-004
7.8730664e-004 7.0932932e-004 7.0878658e-004 7.1839418e-004
7.0279976e-004 6.8148426e-004 6.6046619e-004 6.7581526e-004
6.8646236e-004 6.4644650e-004 6.4403056e-004 6.4911148e-004
6.3490200e-004 6.5725966e-004 5.8882839e-004 6.3167134e-004
6.1219480e-004 5.8664283e-004 6.0230443e-004 6.3073395e-004
6.1795035e-004 5.8799194e-004 6.8419185e-004 7.8518882e-004
8.3159607e-004 6.8571368e-004 7.6006181e-004 7.3584339e-004
7.1723931e-004 6.1680622e-004 6.9247103e-004 6.8422950e-004
6.4962881e-004 6.0225888e-004 6.3120697e-004 6.6068402e-004
6.2010350e-004 6.3579544e-004 6.5784232e-004 6.4467834e-004
6.0681326e-004 6.1797578e-004 6.8593907e-004 6.2340862e-004
6.6782437e-004 7.0565318e-004 6.9003532e-004 7.8913161e-004
8.8613015e-004 7.8167388e-004 8.1898652e-004 7.9490599e-004
7.9766889e-004 7.3247611e-004 7.4878092e-004 7.1649275e-004
7.1887464e-004 6.8973665e-004 7.3550828e-004 7.0675192e-004
6.7456451e-004 6.6780219e-004 7.0038658e-004 6.9896804e-004
6.5138220e-004 6.6996111e-004 6.6505370e-004 6.8938754e-004
6.8846776e-004 6.9686539e-004 7.4610126e-004 8.2439445e-004
7.6895782e-004 7.4807603e-004 7.3670440e-004 6.8991426e-004
6.9937033e-004 6.4749310e-004 6.6436575e-004 6.6196414e-004
6.5819391e-004 6.5907495e-004 6.1662037e-004 6.3691354e-004
6.7801306e-004 6.3064176e-004 6.4288460e-004 6.7896585e-004
6.3793437e-004 5.9076589e-004 6.1814015e-004 6.7326719e-004
6.9107611e-004 7.6058944e-004 7.7497926e-004 9.3896642e-004
8.5988310e-004 7.6270066e-004 7.9244822e-004 7.5552544e-004

7.1945705e-004	6.8447291e-004	7.2175352e-004	6.9371315e-004
6.8343521e-004	6.6266871e-004	6.6498849e-004	6.7973240e-004
7.1606229e-004	6.9453294e-004	6.6790266e-004	6.8433891e-004
6.7184110e-004	6.4887026e-004	6.3657488e-004	6.7895868e-004
7.4459478e-004	7.9373417e-004	8.3391451e-004	8.9963694e-004
8.4548019e-004	7.6879744e-004	7.7603842e-004	7.6890664e-004
7.6255326e-004	7.1531643e-004	6.9974568e-004	7.0822063e-004
7.1343304e-004	6.8403779e-004	6.6929102e-004	6.7370273e-004
6.7565839e-004	6.9123742e-004	6.2144843e-004	6.6330250e-004
6.3766843e-004	6.1930191e-004	6.4331743e-004	6.6445128e-004
5.9915995e-004	5.7252194e-004	5.3053239e-004	6.5585594e-004
6.6882152e-004	5.5137697e-004	5.7186343e-004	6.2659198e-004
6.1811633e-004	6.4695241e-004	6.7889590e-004	6.8548009e-004
6.5015271e-004	6.4069717e-004	6.7316671e-004	6.1018052e-004
5.9105027e-004	6.1679585e-004	6.7484577e-004	6.9361801e-004
6.7563668e-004	5.6545805e-004	6.4669304e-004	6.4571343e-004
6.6939098e-004	6.6993394e-004	6.5932046e-004	7.3634584e-004
8.5101805e-004	7.0616293e-004	7.6248733e-004	7.2854527e-004
7.7160152e-004	7.1271276e-004	6.6822623e-004	7.3185051e-004
6.7179697e-004	6.3057957e-004	7.1173403e-004	6.4305740e-004
5.9368991e-004	6.6500076e-004	6.7846093e-004	6.6164914e-004
5.8991587e-004	6.1235460e-004	6.7601664e-004	6.5115398e-004
7.3308182e-004	7.5537969e-004	7.7979667e-004	9.0167315e-004
8.5149100e-004	7.5593304e-004	7.8860659e-004	7.4559556e-004
7.2287998e-004	6.5769834e-004	7.0479666e-004	6.9223036e-004
6.6382591e-004	6.6895252e-004	6.3141141e-004	6.4623254e-004
7.2854167e-004	6.4727663e-004	6.5426332e-004	6.7430598e-004
6.6414716e-004	6.1346828e-004	6.3287342e-004	6.8840508e-004
7.4854556e-004	7.7107367e-004	8.2199802e-004	9.7013255e-004
8.5157239e-004	8.3688102e-004	8.3603733e-004	8.0167303e-004
7.8280245e-004	7.0907267e-004	7.3881997e-004	7.3750645e-004
7.2585311e-004	7.0652802e-004	7.0445638e-004	6.9200830e-004
7.2956308e-004	6.9780211e-004	6.9211674e-004	6.9197368e-004
6.7651318e-004	6.6955222e-004	6.9493383e-004	7.0395123e-004
7.3317520e-004	7.8666856e-004	8.0903780e-004	8.8887441e-004
8.6729111e-004	7.5493784e-004	7.8519482e-004	7.7129286e-004
7.7363422e-004	7.2275028e-004	7.2348643e-004	7.4802477e-004
7.2514550e-004	7.2047194e-004	6.5897411e-004	7.4105123e-004
6.7472724e-004	7.1972552e-004	6.4940139e-004	7.1268011e-004
6.5758075e-004	6.5233573e-004	6.5932861e-004	6.9095033e-004
5.3841846e-004	5.7925105e-004	5.8214084e-004	6.1388480e-004
6.3790900e-004	5.6955246e-004	5.8952373e-004	6.4609134e-004
6.1528830e-004	6.2423566e-004	7.3592542e-004	6.3703173e-004
7.0774960e-004	6.3616794e-004	6.4458211e-004	6.4457122e-004
6.7526041e-004	6.3961952e-004	6.5864260e-004	6.7954217e-004
7.0593083e-004	6.0366138e-004	6.5524579e-004	6.9235293e-004
6.0796941e-004	5.9528381e-004	5.3690402e-004	6.3913918e-004
7.3928502e-004	5.7521259e-004	6.2548205e-004	5.9918858e-004
6.3033780e-004	6.5518700e-004	5.9021400e-004	6.6675332e-004
6.2306485e-004	5.9300823e-004	6.6433863e-004	5.6168230e-004
5.3318701e-004	5.8711303e-004	6.3254042e-004	6.3225305e-004
5.6939090e-004	5.3692453e-004	6.4105314e-004	5.9643573e-004

6.7528053e-004	6.8664615e-004	6.7606567e-004	7.7193091e-004
8.3482101e-004	7.5636125e-004	7.2704465e-004	7.7653678e-004
6.9486989e-004	6.3748271e-004	6.5377529e-004	6.6829519e-004
6.9607090e-004	7.0720205e-004	7.0865898e-004	7.1062232e-004
6.7899453e-004	6.4638171e-004	6.3143879e-004	7.0767741e-004
6.1660126e-004	6.9039458e-004	6.6959461e-004	6.9748465e-004
7.5655436e-004	7.4893830e-004	7.7142920e-004	8.9240861e-004
8.2785703e-004	7.6552817e-004	7.8394788e-004	7.7810451e-004
7.3205233e-004	6.5470842e-004	7.1648340e-004	7.0566068e-004
6.6797997e-004	6.7891160e-004	6.4278347e-004	6.4593660e-004
7.3010132e-004	6.5369326e-004	6.4912454e-004	6.7406338e-004
6.7068655e-004	6.2833790e-004	6.4571758e-004	7.0170479e-004
7.5355459e-004	7.5369283e-004	8.2227145e-004	9.7339768e-004
8.3962151e-004	8.3533078e-004	8.1542175e-004	7.9315195e-004
7.8486398e-004	6.7937061e-004	7.3442214e-004	7.3926274e-004
7.1324881e-004	7.0605533e-004	6.9990716e-004	6.9022074e-004
7.2968923e-004	6.6428995e-004	6.9718923e-004	6.7599362e-004
6.6535659e-004	6.6459083e-004	6.9900139e-004	7.1053641e-004
7.6728353e-004	8.4377399e-004	8.4400795e-004	9.3275346e-004
9.0883038e-004	7.8811857e-004	8.3246223e-004	7.8463226e-004
8.1829497e-004	7.7201385e-004	7.5534837e-004	7.9150889e-004
7.4944775e-004	7.4770200e-004	6.8909221e-004	7.7754210e-004
7.1691044e-004	7.5070666e-004	6.9139071e-004	7.5367006e-004
6.9122540e-004	6.8843346e-004	6.9935046e-004	7.3031234e-004
5.6901635e-004	6.1375812e-004	6.0823179e-004	6.2707736e-004
6.8028636e-004	5.9933250e-004	6.0900267e-004	6.5160205e-004
6.2413297e-004	6.4629252e-004	7.4947657e-004	6.3414247e-004
7.1935613e-004	6.6197438e-004	6.8089640e-004	6.5484586e-004
7.1121314e-004	6.7060502e-004	6.7272168e-004	6.9183878e-004
7.3186573e-004	6.3539168e-004	6.9969201e-004	7.2703547e-004
7.7060195e-004	7.2145803e-004	7.5064665e-004	8.5573492e-004
7.9003116e-004	7.5738911e-004	7.7300878e-004	8.0487375e-004
7.3598351e-004	6.6075950e-004	7.2578214e-004	7.1293859e-004
6.9719442e-004	6.9366353e-004	6.6673689e-004	6.5299087e-004
7.0577188e-004	6.7603959e-004	6.6613678e-004	6.9366256e-004
6.8495513e-004	6.4742856e-004	6.4682940e-004	7.1106567e-004
7.4577202e-004	7.1538219e-004	7.7539214e-004	9.4557989e-004
8.1242954e-004	7.9855121e-004	7.7811003e-004	7.7157660e-004
7.4592418e-004	6.4090168e-004	7.1750965e-004	7.0672533e-004
6.7826302e-004	6.8539583e-004	6.7803955e-004	6.6783508e-004
7.0438985e-004	6.1470544e-004	6.5816096e-004	6.2380014e-004
6.4743112e-004	6.4909976e-004	6.6524844e-004	7.1155175e-004
7.8430098e-004	8.8161767e-004	8.6468978e-004	9.8090220e-004
9.0791762e-004	8.2906659e-004	8.3768173e-004	7.7125621e-004
8.4454860e-004	7.8189433e-004	7.7041329e-004	7.9759056e-004
7.4177446e-004	7.3598788e-004	7.1273085e-004	7.6260832e-004
7.4834384e-004	7.4570278e-004	7.1088655e-004	7.5087996e-004
7.0101308e-004	6.7742924e-004	7.0513552e-004	7.3039530e-004
7.6244019e-004	6.4616637e-004	6.9414343e-004	8.6059452e-004
7.3599791e-004	7.5474443e-004	7.7685238e-004	7.7933265e-004
6.6432709e-004	7.1041315e-004	7.6443082e-004	7.6250259e-004
7.2884418e-004	7.3368428e-004	6.9637370e-004	7.3823894e-004

6.8947433e-004	6.7094571e-004	7.4270942e-004	7.0086973e-004
7.0338907e-004	6.6395898e-004	6.8343188e-004	7.1708909e-004
7.9692247e-004	7.6199332e-004	7.7861419e-004	9.2677154e-004
8.4182656e-004	8.0578207e-004	7.8109580e-004	8.6998515e-004
8.0311119e-004	6.7694840e-004	7.5841663e-004	7.4593909e-004
7.5850196e-004	7.1780619e-004	7.3411611e-004	6.9496680e-004
7.1498350e-004	6.7801011e-004	6.4835606e-004	6.9903476e-004
7.0812718e-004	6.8428233e-004	6.4492360e-004	7.3017571e-004
7.6781875e-004	7.5572415e-004	8.0309027e-004	9.5447300e-004
8.3028381e-004	8.2065459e-004	7.5499614e-004	7.5315566e-004
7.7175623e-004	6.5636150e-004	7.3540676e-004	6.8920002e-004
6.9284502e-004	7.0383050e-004	6.9532898e-004	6.9263460e-004
7.0405020e-004	6.4068493e-004	6.7877493e-004	6.3819120e-004
6.5582160e-004	6.7027246e-004	6.7395894e-004	7.3192336e-004
7.4962999e-004	8.7391441e-004	8.3626594e-004	9.3909781e-004
8.9149253e-004	8.1227589e-004	8.6693397e-004	7.5431450e-004
8.2107641e-004	7.9809623e-004	7.8267345e-004	8.0364074e-004
7.1757112e-004	7.2938293e-004	7.2196544e-004	7.8365572e-004
7.3505727e-004	7.5336570e-004	7.1073492e-004	7.5280729e-004
7.4385351e-004	6.8350318e-004	7.0002498e-004	7.5319350e-004
6.6892007e-004	5.9829016e-004	6.1457267e-004	7.4633732e-004
6.7771451e-004	6.2649939e-004	6.7208819e-004	7.4312983e-004
6.3343793e-004	5.8470937e-004	6.4596131e-004	6.3897045e-004
6.7480656e-004	6.2209053e-004	6.4716573e-004	6.2230566e-004
5.8104839e-004	5.7176749e-004	6.0339920e-004	6.3385641e-004
6.3414678e-004	5.7887362e-004	5.2304555e-004	6.0122130e-004
7.6885289e-004	8.8710731e-004	8.5353306e-004	9.8476456e-004
9.0235932e-004	8.5839872e-004	8.1543246e-004	7.3290481e-004
8.3541588e-004	7.6219485e-004	8.0910324e-004	7.2856063e-004
7.1628227e-004	7.2584429e-004	7.5441280e-004	7.7021160e-004
7.4059464e-004	7.2816803e-004	7.2488263e-004	7.6063539e-004
7.4698472e-004	6.9962682e-004	6.4916190e-004	7.6794749e-004
7.3470379e-004	8.0935053e-004	8.1216209e-004	8.9865031e-004
8.8708161e-004	7.7264404e-004	8.5641394e-004	8.4360047e-004
7.7761023e-004	8.1615793e-004	8.5211918e-004	8.2240596e-004
7.2963398e-004	7.9145005e-004	7.5475201e-004	8.4739074e-004
7.8021034e-004	7.9423175e-004	7.5313775e-004	7.3101302e-004
8.0212927e-004	7.4287732e-004	7.7669002e-004	8.7220767e-004
7.5177560e-004	7.2697963e-004	7.0835213e-004	8.4463772e-004
8.3734613e-004	7.2368512e-004	7.9146861e-004	8.0435746e-004
7.3700624e-004	7.2034940e-004	8.0879714e-004	7.1377051e-004
7.3051905e-004	8.1131139e-004	7.9817837e-004	8.3616325e-004
7.6057031e-004	7.6269025e-004	7.7900497e-004	8.2846561e-004
7.2834654e-004	7.3787376e-004	7.6402808e-004	8.6336622e-004
6.4849328e-004	7.6318337e-004	7.2982527e-004	8.1207031e-004
7.6871090e-004	7.4649401e-004	7.6064370e-004	6.4101903e-004
7.2371634e-004	6.8746202e-004	7.3050226e-004	6.6466896e-004
6.2219528e-004	6.4834808e-004	6.7272606e-004	6.9683595e-004
6.4612937e-004	6.4481571e-004	6.2558927e-004	7.0252112e-004
6.9818104e-004	6.1592426e-004	5.7593014e-004	6.9299142e-004
5.8586373e-004	6.1556229e-004	6.2071234e-004	7.1412804e-004
6.8202759e-004	5.7567774e-004	6.5013591e-004	6.8690492e-004

5.8959698e-004	6.5648281e-004	6.9618627e-004	6.3846713e-004
5.8573540e-004	6.4787210e-004	6.0908495e-004	6.9102480e-004
6.3702247e-004	6.3840035e-004	6.1808428e-004	5.5372420e-004
6.3917760e-004	5.9531287e-004	6.3175992e-004	7.1864843e-004
4.0730696e-004	4.5658623e-004	4.4790166e-004	3.8943910e-004
3.6885522e-004	3.8663128e-004	3.3824107e-004	3.7670787e-004
3.5011335e-004	4.0683827e-004	3.5677795e-004	3.1820200e-004
3.5274080e-004	4.0040194e-004	3.9058152e-004	3.7696905e-004
3.6478870e-004	3.9171002e-004	4.0652262e-004	4.0215941e-004
4.1584177e-004	4.2143181e-004	5.1259417e-004	4.6921290e-004
6.7098300e-004	6.3686512e-004	6.0179125e-004	5.5553699e-004
5.7793649e-004	5.4328094e-004	4.9630216e-004	4.8746446e-004
5.0946516e-004	5.0423113e-004	4.4676764e-004	4.9728258e-004
4.7783427e-004	4.7681566e-004	4.4972452e-004	4.4996077e-004
4.2882851e-004	3.9189395e-004	4.4420893e-004	4.1400940e-004
5.1204580e-004	4.3891261e-004	4.8288114e-004	4.7133773e-004
6.5773519e-004	7.0193721e-004	6.7244000e-004	6.0206333e-004
4.8312525e-004	5.6514241e-004	4.6356628e-004	5.6065619e-004
4.6228689e-004	4.9893818e-004	5.3560048e-004	4.5206660e-004
4.6421305e-004	5.1948598e-004	5.2505759e-004	4.8076236e-004
4.6999429e-004	4.7539583e-004	5.4014407e-004	4.7768526e-004
5.4438481e-004	5.0971512e-004	5.4278623e-004	5.8134676e-004
5.1499382e-004	5.4838510e-004	5.6282211e-004	4.9519330e-004
4.7091777e-004	5.0633417e-004	4.8787215e-004	4.7440789e-004
4.3662406e-004	5.3166665e-004	4.5348607e-004	4.4042302e-004
4.5610546e-004	5.3558200e-004	5.2101146e-004	4.8790682e-004
5.0939279e-004	5.1715282e-004	5.4518768e-004	5.6201126e-004
5.4731233e-004	5.6308538e-004	7.1126074e-004	6.4468315e-004
5.1523449e-004	5.7915572e-004	6.0828682e-004	5.8177876e-004
4.8009036e-004	4.8653145e-004	5.1142839e-004	5.1730156e-004
5.3104909e-004	5.3396295e-004	5.2445498e-004	5.2049068e-004
4.9928920e-004	5.8004318e-004	5.9328284e-004	5.0958498e-004
5.5551269e-004	5.3661711e-004	6.1045733e-004	7.0075164e-004
5.4951107e-004	6.3733869e-004	6.9859636e-004	6.3419476e-004
5.9318847e-004	5.4176718e-004	5.7405398e-004	5.5299315e-004
5.3699961e-004	5.0836404e-004	5.2309496e-004	5.2167688e-004
5.4877799e-004	4.7863377e-004	4.9766632e-004	4.0237366e-004
5.0851484e-004	5.1770281e-004	4.8640492e-004	4.4177181e-004
4.5421405e-004	4.5348738e-004	4.6976098e-004	4.7889825e-004
4.8560335e-004	4.6719328e-004	4.2450372e-004	5.4098039e-004
6.6581646e-004	5.6545153e-004	5.9929694e-004	6.2383796e-004
5.9249590e-004	5.5430233e-004	5.0558311e-004	5.2165004e-004
5.4791533e-004	5.3791825e-004	4.5658913e-004	5.1682125e-004
4.9437761e-004	5.1072577e-004	4.8409016e-004	4.4322876e-004
4.9376881e-004	4.7046581e-004	4.7814070e-004	4.4556304e-004
5.5766210e-004	5.0186379e-004	4.9341816e-004	4.7834248e-004
6.8421786e-004	6.7328004e-004	6.7313190e-004	5.6522146e-004
5.5773809e-004	5.6033816e-004	4.8281477e-004	5.2198079e-004
4.6794510e-004	4.7822706e-004	4.9734799e-004	4.7822609e-004
4.5974377e-004	4.7507224e-004	4.9420034e-004	4.6579211e-004
4.3054399e-004	4.0016036e-004	5.0041129e-004	4.5943209e-004
5.0590228e-004	4.7565834e-004	4.8169403e-004	5.3079361e-004

5.6599280e-004	5.9286789e-004	5.8046087e-004	5.5825146e-004
4.6880389e-004	5.4222282e-004	4.7937044e-004	4.8910858e-004
4.3013618e-004	5.1126161e-004	4.7399284e-004	4.4766215e-004
4.3024384e-004	5.3126109e-004	4.8538233e-004	4.7813053e-004
4.7569486e-004	4.7839811e-004	5.4311489e-004	4.9739809e-004
5.3400866e-004	5.2468509e-004	5.9256236e-004	5.9994601e-004
4.7726646e-004	5.2209912e-004	5.7174388e-004	5.1877505e-004
4.5330264e-004	4.9618363e-004	5.2257841e-004	4.9278973e-004
4.7283660e-004	5.2010748e-004	4.9138194e-004	4.7759131e-004
4.3172702e-004	5.6857623e-004	5.5351095e-004	4.7606808e-004
5.4080391e-004	5.1170524e-004	5.6123674e-004	6.3056545e-004
5.3184826e-004	5.5914828e-004	6.9872828e-004	6.3967277e-004
5.8920363e-004	5.9187520e-004	6.4059522e-004	6.0021348e-004
5.2091941e-004	5.3658994e-004	5.4008103e-004	5.3373688e-004
5.8997663e-004	5.9597059e-004	6.0088445e-004	6.2009081e-004
6.1587837e-004	6.1523551e-004	6.4884042e-004	5.9165739e-004
6.1788260e-004	5.9137643e-004	6.8500264e-004	6.5769133e-004
6.8399661e-004	6.9823167e-004	6.9536318e-004	7.0033026e-004
4.8561562e-004	5.0791921e-004	4.8886497e-004	5.1484699e-004
4.9896232e-004	5.1278518e-004	4.9547899e-004	4.4768551e-004
5.0061129e-004	5.4396145e-004	5.5893201e-004	5.3503760e-004
5.2598464e-004	5.3490508e-004	5.6563239e-004	5.0000728e-004
5.8109943e-004	5.1580684e-004	5.5739054e-004	5.6964708e-004
5.8969330e-004	5.4629375e-004	6.5449269e-004	6.3380157e-004
5.8854213e-004	5.5842857e-004	5.7632258e-004	5.3756417e-004
5.6146691e-004	5.2065342e-004	4.7244475e-004	4.9311812e-004
4.5005714e-004	4.3799476e-004	4.5796036e-004	5.0494119e-004
4.5929203e-004	5.0714023e-004	4.6461493e-004	4.4178159e-004
4.7432741e-004	4.8119467e-004	5.3231901e-004	4.5760679e-004
4.9245630e-004	4.6934274e-004	4.5368653e-004	5.9835402e-004
6.3097710e-004	5.7176009e-004	5.8970391e-004	6.1262841e-004
5.9385816e-004	5.5248580e-004	5.0434450e-004	5.0266080e-004
4.9730647e-004	5.0907288e-004	4.5074430e-004	5.1431546e-004
4.3204880e-004	4.6781356e-004	4.7205204e-004	4.6126998e-004
4.5368283e-004	4.5783974e-004	4.5198650e-004	4.3771623e-004
4.9569850e-004	4.7707147e-004	4.8024476e-004	4.9471447e-004
6.4462580e-004	6.4809941e-004	6.5270277e-004	5.9890958e-004
5.4746967e-004	5.8495979e-004	4.9619920e-004	5.1214526e-004
4.6024065e-004	4.9084516e-004	5.2354447e-004	4.6437149e-004
4.4005274e-004	5.0282444e-004	4.9579301e-004	4.7071790e-004
4.4749001e-004	4.4042127e-004	5.2968156e-004	4.7123361e-004
5.2906378e-004	4.8905783e-004	4.8540465e-004	5.1804261e-004
5.5155554e-004	5.2281777e-004	5.3600314e-004	5.2862366e-004
4.6511380e-004	5.2098610e-004	5.0765150e-004	4.5793635e-004
4.4371895e-004	5.1619878e-004	4.4784091e-004	4.6037426e-004
4.4764611e-004	5.2574884e-004	4.9202314e-004	4.8890489e-004
5.1310545e-004	5.0439627e-004	5.3987728e-004	5.2806338e-004
5.3519668e-004	5.3651724e-004	6.1298179e-004	6.2214716e-004
4.9527855e-004	5.5247660e-004	5.8113236e-004	5.3300243e-004
4.8063489e-004	5.1362232e-004	5.2013947e-004	5.0767267e-004
5.1231719e-004	5.3039551e-004	5.4228056e-004	5.0903415e-004
4.5376262e-004	5.7142631e-004	5.5655875e-004	4.9499857e-004

5.4938412e-004 5.1631435e-004 5.9733108e-004 6.2401593e-004
5.4575200e-004 5.6992008e-004 6.6971345e-004 6.4350702e-004
6.5725690e-004 6.9981307e-004 6.1915492e-004 7.0153447e-004
6.8551609e-004 6.7347384e-004 6.4382225e-004 6.5284839e-004
6.6597210e-004 7.0999382e-004 7.2459770e-004 6.5976205e-004
7.4742583e-004 6.9825278e-004 7.4994563e-004 6.4178667e-004
7.3824273e-004 7.0273125e-004 7.7168107e-004 8.1223088e-004
7.5303657e-004 6.8623001e-004 8.1728310e-004 8.3180024e-004
4.9494121e-004 4.7183588e-004 4.7412578e-004 5.6059931e-004
4.6933589e-004 5.7368483e-004 4.7520042e-004 4.7807641e-004
4.3206326e-004 5.0739724e-004 4.7916932e-004 5.4107819e-004
4.9191460e-004 6.0071648e-004 4.9338386e-004 4.6052246e-004
5.4285230e-004 5.2218069e-004 5.9524263e-004 5.4314394e-004
5.9352689e-004 5.2040669e-004 5.7636588e-004 5.6712030e-004
6.3190486e-004 5.9130143e-004 5.9763498e-004 5.5955439e-004
5.8341162e-004 5.5144253e-004 4.9022793e-004 5.1724295e-004
4.9256043e-004 4.5879522e-004 5.0234237e-004 5.3365173e-004
4.9415772e-004 5.6059973e-004 5.0762337e-004 4.7894201e-004
5.3714574e-004 5.3515893e-004 6.0110932e-004 5.0390034e-004
5.6628742e-004 5.3570465e-004 5.1863597e-004 6.3281645e-004
5.8882311e-004 5.7160000e-004 5.7771702e-004 6.0977061e-004
5.8663510e-004 5.5828771e-004 5.0966226e-004 5.0463175e-004
4.9019128e-004 5.1844461e-004 4.6728132e-004 5.0479580e-004
4.3227519e-004 4.6229715e-004 4.8610269e-004 4.7129585e-004
4.5494117e-004 4.6446120e-004 4.4117895e-004 4.4907525e-004
4.7955740e-004 4.8506961e-004 4.8016677e-004 4.9065849e-004
6.4548885e-004 6.2591718e-004 6.4608030e-004 6.3078698e-004
5.7008595e-004 5.9528287e-004 5.3636932e-004 5.0477799e-004
4.9308340e-004 5.2286706e-004 5.3855650e-004 4.8429328e-004
4.6043042e-004 5.1201853e-004 4.9347853e-004 4.8046730e-004
4.7860225e-004 4.7326973e-004 5.2375945e-004 4.7967085e-004
5.2902605e-004 4.9672853e-004 4.8821475e-004 5.0923255e-004
5.1331745e-004 5.7563835e-004 5.8767915e-004 5.3281429e-004
5.0766474e-004 5.4710978e-004 5.4457653e-004 5.0575205e-004
5.3182671e-004 5.4507340e-004 5.5932396e-004 5.3288062e-004
4.7303702e-004 5.7078780e-004 5.5559022e-004 5.2275266e-004
5.5792395e-004 5.2701488e-004 6.1231307e-004 6.0114134e-004
5.5862362e-004 5.7108297e-004 6.5496939e-004 6.6077804e-004
5.4456242e-004 5.6845596e-004 5.4925401e-004 6.5284535e-004
5.8600029e-004 6.5419749e-004 5.7802621e-004 5.8200125e-004
5.8774100e-004 5.7857647e-004 6.1027886e-004 6.0408651e-004
5.5597442e-004 6.3553458e-004 5.9645685e-004 5.7332769e-004
6.9694550e-004 6.3614204e-004 7.0878701e-004 6.5147256e-004
6.8665612e-004 6.0323503e-004 6.5197337e-004 7.1898644e-004
5.9182163e-004 5.5694619e-004 5.8292103e-004 6.3056487e-004
4.8878542e-004 5.9305438e-004 5.2747293e-004 4.7427230e-004
4.5046869e-004 5.5189665e-004 5.2959850e-004 6.2242810e-004
5.3370099e-004 7.1225507e-004 5.2469489e-004 4.7793595e-004
5.9963827e-004 5.8568810e-004 6.3277429e-004 5.6749159e-004
6.6938259e-004 6.4291619e-004 6.4901791e-004 6.0022954e-004
5.8691396e-004 5.6703015e-004 5.5272297e-004 6.1965889e-004
5.8825008e-004 5.7486276e-004 5.1870754e-004 5.3948525e-004

5.1110425e-004	5.0409822e-004	4.9379242e-004	4.7059788e-004
4.7981679e-004	4.7423103e-004	5.4043725e-004	4.3501065e-004
5.1017738e-004	4.2388568e-004	4.7193417e-004	4.3838437e-004
4.8421208e-004	4.9939926e-004	5.2175803e-004	4.5456061e-004
5.8097037e-004	5.9615390e-004	5.9672067e-004	6.1435932e-004
5.7220162e-004	5.6966979e-004	5.2802583e-004	5.1571042e-004
5.2549582e-004	5.6521979e-004	5.0768946e-004	4.9964589e-004
4.5172404e-004	4.6166516e-004	5.0594693e-004	4.9665503e-004
4.7662162e-004	4.9673200e-004	4.4214105e-004	4.6887042e-004
4.6394947e-004	4.8512186e-004	4.8359089e-004	5.0046419e-004
6.5061581e-004	6.2902512e-004	6.5261855e-004	6.8828993e-004
5.8555028e-004	6.1220360e-004	6.0646651e-004	5.2717731e-004
5.2195429e-004	5.5886674e-004	5.6656657e-004	5.3030789e-004
5.1669169e-004	5.4453687e-004	4.7707378e-004	4.8738009e-004
5.3606674e-004	5.0160980e-004	5.5281714e-004	5.0822172e-004
5.5419705e-004	5.2188996e-004	5.2896613e-004	5.2656137e-004
6.1552253e-004	6.4808750e-004	6.9632271e-004	7.4678395e-004
7.4151820e-004	8.0070124e-004	7.7500490e-004	6.9885714e-004
7.6320012e-004	6.9862188e-004	8.0402178e-004	7.2384690e-004
6.7868484e-004	7.7211372e-004	6.9768407e-004	6.6080909e-004
8.3300153e-004	7.3201030e-004	7.1741053e-004	7.4338198e-004
7.2651349e-004	7.1506886e-004	7.2990793e-004	8.1400173e-004
5.4135753e-004	5.9195120e-004	5.6681369e-004	6.2426880e-004
4.6899521e-004	5.3598350e-004	5.2556894e-004	4.6707423e-004
4.4909901e-004	5.4717755e-004	5.1523236e-004	6.0410840e-004
5.1796161e-004	6.6767052e-004	5.3739945e-004	4.9404906e-004
6.2457536e-004	6.2223502e-004	6.2098571e-004	5.8197019e-004
6.7518448e-004	6.5921906e-004	6.4149476e-004	6.3392706e-004
5.5661095e-004	5.3933407e-004	5.1833259e-004	4.8838654e-004
5.2568159e-004	5.1073400e-004	4.4349166e-004	4.9274162e-004
5.1538368e-004	4.5199042e-004	4.8902892e-004	4.9550518e-004
5.0264208e-004	5.4204601e-004	5.2390489e-004	5.3096477e-004
5.5063765e-004	5.6554964e-004	6.3314372e-004	5.1203897e-004
5.8771555e-004	5.7221844e-004	5.6305235e-004	5.9375538e-004
6.1439165e-004	5.6481829e-004	5.4948367e-004	6.1155092e-004
6.0482982e-004	5.9110703e-004	5.2934631e-004	5.9168912e-004
5.3149971e-004	5.2785844e-004	5.3442283e-004	5.1167983e-004
4.8808406e-004	5.0638246e-004	5.8445062e-004	4.8046104e-004
5.5223738e-004	4.6940509e-004	5.4412059e-004	4.6873794e-004
4.9887962e-004	5.4127969e-004	5.6278230e-004	5.4414549e-004
5.7578470e-004	6.2580469e-004	6.1014159e-004	6.5073984e-004
5.7602180e-004	6.0260236e-004	5.4423543e-004	5.1636781e-004
5.1863870e-004	5.8933249e-004	5.0892620e-004	5.0502871e-004
4.9411619e-004	4.6839857e-004	5.1100636e-004	4.7802735e-004
4.6318814e-004	4.8540195e-004	4.4064558e-004	4.8724653e-004
4.7736231e-004	4.8695944e-004	4.9564770e-004	4.3985820e-004
6.6260148e-004	6.3677407e-004	6.6976242e-004	7.2282388e-004
6.0094309e-004	6.1506129e-004	6.3629016e-004	5.6212237e-004
5.5802311e-004	5.8892323e-004	5.9729716e-004	5.4286903e-004
5.2232166e-004	5.1358285e-004	5.0322998e-004	4.9705743e-004
5.6467963e-004	5.1299922e-004	5.2456621e-004	4.9809416e-004
5.3626541e-004	5.1570864e-004	5.1733949e-004	5.3089103e-004

6.1072383e-004	7.0740387e-004	7.4942443e-004	7.2562341e-004
6.8776362e-004	7.6632005e-004	6.9453841e-004	6.7010302e-004
6.2063007e-004	7.1656259e-004	6.6452743e-004	6.5082463e-004
6.8454584e-004	6.4464769e-004	5.7465474e-004	5.7126356e-004
6.7355628e-004	6.1870148e-004	6.7603230e-004	6.3297882e-004
6.8056733e-004	6.2165665e-004	6.4163901e-004	6.3520124e-004
6.1560715e-004	6.4954687e-004	6.9849442e-004	7.3117105e-004
7.2557743e-004	7.7969667e-004	7.6810124e-004	7.0624157e-004
7.0520827e-004	6.5137139e-004	7.6151153e-004	6.7980625e-004
6.4445120e-004	7.4219897e-004	6.6690742e-004	6.1210505e-004
7.8444420e-004	7.0134866e-004	6.9576305e-004	6.6942642e-004
6.8149732e-004	6.6827488e-004	6.7400452e-004	7.1286547e-004
4.9337783e-004	6.3525530e-004	6.5678793e-004	6.9572352e-004
6.8192164e-004	6.6907411e-004	7.8139048e-004	7.1757608e-004
7.1749596e-004	7.0173180e-004	7.6664321e-004	6.9208568e-004
6.8197552e-004	6.4668043e-004	6.5312161e-004	6.8219841e-004
7.3077210e-004	7.2530143e-004	6.8542990e-004	7.1623780e-004
6.6576320e-004	5.6567857e-004	6.8939531e-004	7.9634448e-004
5.4975961e-004	6.4978719e-004	5.7052702e-004	5.7965977e-004
4.9138881e-004	5.3087070e-004	5.4857659e-004	4.4995372e-004
4.7613923e-004	5.1235114e-004	5.5913241e-004	5.3783354e-004
5.2029473e-004	5.8528212e-004	5.4140417e-004	5.4575758e-004
5.9393853e-004	5.8962793e-004	6.4821198e-004	6.0824546e-004
6.7690843e-004	6.1581553e-004	6.0208346e-004	6.4234374e-004
4.9015830e-004	5.5042440e-004	5.3813652e-004	5.5523940e-004
4.6902122e-004	5.0926339e-004	4.9008336e-004	4.7370371e-004
4.6173150e-004	5.3003144e-004	4.9567376e-004	5.4681039e-004
5.3902535e-004	6.4206628e-004	5.4221461e-004	5.8123718e-004
6.3153348e-004	6.4979416e-004	6.3375635e-004	5.9913626e-004
6.4183271e-004	6.5732913e-004	6.4677582e-004	6.3620458e-004
6.4009478e-004	6.2154334e-004	6.2056801e-004	6.9238468e-004
6.5962522e-004	6.4807186e-004	5.7924545e-004	6.0954434e-004
5.5576678e-004	5.8409918e-004	5.5892103e-004	5.3877322e-004
5.2986201e-004	5.5661002e-004	5.9909603e-004	4.9277375e-004
5.4386813e-004	4.9420710e-004	5.4153642e-004	4.9995611e-004
4.9354151e-004	5.4035200e-004	5.4742325e-004	4.9484295e-004
5.8321447e-004	6.2716611e-004	6.2751948e-004	6.8172172e-004
5.5352808e-004	6.0957469e-004	5.8778947e-004	5.3915556e-004
5.4733205e-004	6.2949938e-004	5.3848280e-004	5.4039601e-004
5.1654606e-004	4.5542743e-004	5.1965271e-004	5.0846966e-004
4.8196500e-004	5.1593798e-004	4.6332979e-004	5.0148470e-004
4.8057700e-004	4.7881385e-004	5.0467790e-004	4.8224415e-004
6.2332445e-004	6.3255799e-004	6.7170041e-004	7.3488915e-004
6.1304682e-004	6.2063576e-004	6.6673182e-004	6.2511628e-004
5.6353393e-004	5.9182687e-004	6.3524215e-004	5.5489611e-004
5.4553911e-004	5.2232575e-004	4.7972257e-004	4.8488360e-004
5.9353259e-004	5.2824286e-004	5.6262454e-004	5.3249703e-004
5.5044222e-004	5.1827807e-004	5.3906256e-004	5.3070755e-004
6.5685489e-004	7.0967837e-004	6.6423524e-004	6.4391051e-004
6.5267028e-004	7.0441804e-004	6.7350197e-004	5.6483761e-004
6.2797485e-004	6.9620699e-004	6.2649864e-004	5.9720954e-004
5.8771543e-004	6.6053662e-004	5.2774555e-004	5.8878191e-004

5.7622389e-004	5.7016562e-004	6.8638261e-004	5.7297075e-004
5.9443506e-004	5.7605130e-004	5.7366748e-004	6.5956877e-004
6.4015861e-004	7.2444660e-004	7.4211280e-004	8.0290034e-004
7.6109849e-004	7.8541081e-004	7.0725821e-004	7.1737239e-004
6.6882608e-004	7.0083952e-004	6.5704774e-004	6.5635338e-004
6.7110025e-004	6.3627174e-004	6.1092069e-004	5.8399331e-004
6.5955265e-004	6.2849505e-004	6.2263101e-004	6.2032242e-004
6.5920728e-004	6.2380626e-004	6.5378139e-004	6.2362033e-004
5.9208555e-004	6.5261454e-004	7.1552067e-004	7.1403860e-004
7.2683817e-004	7.5827235e-004	7.7297163e-004	7.0908311e-004
6.8696719e-004	6.6711348e-004	7.2995544e-004	6.5936062e-004
6.2989233e-004	7.0714013e-004	6.3933377e-004	5.9621251e-004
7.1657555e-004	6.9381711e-004	6.2683558e-004	6.4243449e-004
6.2708199e-004	6.1165924e-004	6.3016551e-004	6.8561200e-004
5.0986726e-004	6.3840067e-004	6.2523516e-004	7.0222918e-004
6.6014389e-004	7.0725629e-004	7.2623231e-004	6.8529563e-004
6.8171728e-004	6.5710570e-004	7.3012168e-004	6.4669822e-004
5.9568578e-004	6.4077356e-004	5.7182148e-004	6.5527263e-004
7.1352451e-004	6.6470970e-004	6.7971263e-004	6.3844747e-004
6.1734648e-004	5.2410325e-004	6.2417796e-004	7.5084448e-004
5.1411885e-004	6.1297576e-004	5.6739229e-004	5.3226400e-004
4.4986593e-004	4.7749942e-004	5.4153043e-004	4.4917953e-004
4.7701383e-004	4.8030725e-004	5.0865296e-004	4.7806495e-004
5.1870307e-004	5.5985711e-004	5.0761848e-004	5.4509329e-004
5.2393537e-004	5.0141379e-004	6.0817545e-004	5.6653367e-004
5.8893328e-004	5.4362959e-004	6.5192919e-004	6.3606328e-004
4.7564171e-004	5.7636424e-004	5.1349381e-004	5.5569285e-004
4.7817578e-004	5.1042334e-004	5.1829341e-004	4.8977646e-004
4.4596072e-004	5.3487519e-004	5.1925537e-004	5.3198777e-004
5.3497101e-004	6.0045873e-004	5.7122626e-004	5.8371118e-004
6.4553437e-004	6.5400676e-004	6.7563105e-004	6.3460467e-004
6.6969475e-004	6.4028527e-004	6.1472550e-004	6.6728263e-004
4.9548021e-004	5.5527948e-004	5.4338881e-004	5.1322897e-004
5.4004423e-004	5.4031007e-004	5.1658748e-004	5.4573765e-004
5.5221102e-004	5.3574420e-004	5.3246862e-004	5.5976388e-004
5.6276713e-004	6.1066831e-004	6.0157689e-004	6.5775636e-004
6.2894366e-004	6.7035900e-004	6.5439890e-004	5.8723372e-004
6.3449974e-004	6.3660144e-004	6.6493830e-004	6.4421001e-004
5.8845359e-004	6.3551619e-004	6.5700807e-004	6.8828322e-004
5.8230736e-004	6.2385871e-004	5.9324752e-004	5.5902005e-004
5.4584577e-004	6.0304932e-004	5.2551959e-004	5.4188304e-004
5.1519874e-004	5.1026613e-004	5.4370300e-004	5.1075559e-004
4.6258551e-004	5.1076063e-004	4.8355599e-004	5.3011351e-004
4.7093430e-004	4.6978566e-004	4.9996694e-004	4.2195599e-004
5.8206625e-004	6.3939980e-004	6.2777464e-004	7.4722206e-004
5.7494176e-004	6.1546626e-004	6.5673421e-004	6.0841626e-004
5.4870306e-004	6.0928777e-004	6.1723296e-004	5.8401185e-004
5.4117438e-004	4.7413528e-004	5.1399930e-004	5.0851853e-004
5.5493139e-004	5.3036938e-004	5.0895338e-004	5.2326424e-004
5.1845031e-004	5.0369069e-004	5.4019692e-004	5.5699261e-004
5.7278828e-004	5.8654146e-004	6.1769610e-004	6.7339285e-004
6.3844286e-004	6.0719262e-004	6.2498798e-004	6.6214005e-004

5.9311932e-004	5.9632894e-004	6.3424467e-004	5.3289562e-004
5.4917538e-004	5.0923092e-004	5.0080646e-004	5.0215098e-004
5.8800986e-004	5.4518585e-004	5.8769176e-004	5.6531635e-004
5.0746288e-004	5.0838089e-004	5.0280434e-004	5.2222805e-004
6.3411816e-004	6.2676662e-004	6.3176578e-004	6.8870535e-004
6.5505016e-004	6.2738720e-004	6.2723203e-004	5.5014572e-004
6.1330365e-004	6.5043784e-004	6.3654012e-004	5.5556488e-004
5.6407394e-004	6.2625247e-004	5.2193686e-004	5.6208225e-004
5.6452061e-004	5.4956392e-004	6.0555438e-004	5.3800270e-004
5.6431491e-004	5.5589093e-004	5.3643872e-004	6.0374769e-004
6.7396526e-004	7.6240829e-004	6.8969185e-004	7.2921501e-004
7.3716381e-004	7.5541019e-004	6.8856974e-004	6.6570153e-004
6.8206628e-004	7.1384378e-004	6.7822647e-004	6.1563363e-004
6.4555192e-004	6.4183772e-004	5.6808967e-004	6.0519886e-004
6.2585081e-004	6.1840517e-004	6.3597814e-004	6.0283915e-004
6.0835127e-004	5.9467790e-004	5.9628456e-004	5.9907082e-004
6.2080762e-004	6.6454914e-004	6.6060229e-004	7.3122204e-004
7.4435408e-004	7.2000247e-004	7.4507398e-004	6.8646843e-004
7.0530424e-004	6.8919214e-004	6.4685621e-004	6.2380661e-004
6.0887926e-004	6.2230804e-004	6.4160238e-004	5.8695526e-004
6.4794261e-004	6.1564246e-004	6.6448006e-004	6.0460491e-004
6.0501223e-004	5.7684015e-004	6.3018610e-004	6.5435049e-004
5.7752551e-004	6.2502714e-004	6.7550169e-004	6.9534442e-004
7.1011612e-004	7.4131189e-004	7.8331493e-004	7.0392927e-004
6.8760607e-004	6.4653212e-004	6.8052310e-004	6.4592388e-004
6.2836347e-004	6.8977494e-004	6.5505205e-004	6.0540636e-004
6.9804341e-004	6.7112262e-004	6.8408746e-004	6.2625403e-004
6.6413550e-004	5.9302518e-004	6.1567138e-004	6.1752147e-004
5.6515587e-004	6.6270013e-004	6.5928409e-004	7.1529171e-004
6.8799935e-004	7.3968628e-004	7.4121213e-004	6.7794803e-004
6.4681607e-004	6.8462352e-004	6.6697650e-004	6.2702964e-004
5.7236102e-004	6.1741717e-004	5.7891487e-004	6.0689694e-004
6.5704137e-004	6.5375498e-004	6.1433947e-004	6.4377252e-004
5.7567284e-004	5.3196667e-004	5.6486424e-004	6.8790672e-004
5.7058491e-004	5.7017365e-004	5.7816849e-004	6.1257704e-004
6.0766120e-004	6.8353802e-004	6.6945182e-004	6.3709693e-004
6.5179443e-004	5.8260273e-004	6.5682787e-004	5.4072346e-004
5.3689462e-004	6.1499999e-004	5.2784942e-004	5.7848354e-004
6.6461077e-004	5.8717684e-004	6.4431690e-004	5.7310091e-004
5.9770716e-004	5.1988346e-004	6.1559674e-004	7.0820302e-004
5.4663848e-004	5.7891499e-004	5.7702984e-004	5.3663231e-004
5.0611194e-004	4.9381884e-004	4.8911537e-004	4.7817198e-004
4.9908237e-004	4.3384059e-004	4.6590606e-004	4.8491529e-004
4.8628363e-004	5.1712418e-004	5.2324405e-004	4.9894790e-004
5.0690083e-004	5.1044134e-004	5.3203313e-004	5.1967239e-004
5.5418772e-004	5.6442468e-004	6.4311869e-004	6.1151922e-004
5.0559052e-004	5.6809896e-004	4.9712520e-004	5.2135193e-004
4.6082833e-004	4.9866582e-004	5.2268066e-004	5.0457095e-004
4.5774405e-004	5.1599520e-004	5.4643066e-004	5.0265749e-004
5.6779152e-004	5.5911120e-004	5.5798264e-004	5.9129509e-004
6.1523795e-004	6.1867513e-004	6.8884352e-004	6.4244335e-004
6.6433506e-004	6.0090139e-004	6.5885790e-004	6.7902575e-004

4.5973517e-004	5.7057320e-004	5.4518197e-004	5.7314090e-004
5.4757664e-004	5.6914835e-004	5.8181379e-004	5.6161857e-004
5.3629139e-004	5.8730767e-004	5.7264544e-004	5.7412848e-004
5.8429594e-004	6.7344979e-004	6.2152791e-004	6.6104977e-004
7.0806322e-004	7.1033643e-004	6.8467397e-004	6.4659177e-004
6.5226536e-004	6.6602988e-004	6.5762513e-004	7.0047392e-004
6.1209266e-004	6.2648008e-004	6.7294481e-004	7.1682429e-004
6.4607039e-004	6.3989393e-004	6.4188652e-004	6.5141977e-004
5.6082629e-004	5.9622801e-004	5.8516554e-004	5.8942130e-004
5.4690383e-004	6.3036320e-004	5.9759806e-004	5.5150465e-004
5.3455587e-004	5.5065745e-004	5.6983159e-004	5.6025825e-004
5.2306813e-004	5.1868767e-004	5.4729580e-004	4.9941352e-004
5.3787856e-004	6.1063645e-004	5.9620397e-004	6.8432625e-004
5.3582124e-004	6.0442166e-004	6.3222383e-004	5.5517010e-004
5.3117999e-004	5.7406767e-004	5.7428022e-004	5.6987368e-004
5.2645638e-004	4.6915350e-004	5.2848499e-004	5.1756024e-004
4.8300604e-004	5.1072346e-004	4.9336559e-004	5.1592643e-004
4.8393390e-004	4.7460206e-004	5.1157533e-004	5.2482394e-004
5.5564854e-004	6.1214126e-004	6.3987864e-004	7.1948169e-004
6.0586893e-004	6.1828180e-004	6.6099213e-004	6.8495906e-004
5.6788881e-004	5.9809934e-004	6.4800591e-004	5.6809477e-004
5.2649685e-004	5.2316768e-004	5.0641154e-004	4.8329635e-004
5.4323385e-004	5.8558618e-004	5.4125635e-004	5.4303177e-004
5.3961713e-004	4.8575057e-004	5.3334587e-004	5.1625576e-004
5.6314052e-004	6.0610846e-004	5.9527070e-004	6.9139966e-004
6.5281504e-004	5.8863369e-004	6.0865234e-004	6.2105501e-004
5.9720321e-004	6.2120390e-004	6.3997178e-004	5.3216869e-004
5.3649073e-004	5.1404203e-004	5.1241049e-004	5.3924711e-004
5.6341283e-004	5.4204553e-004	5.6558012e-004	5.5544671e-004
5.1839152e-004	5.4084975e-004	4.9270185e-004	5.3543669e-004
6.4239666e-004	7.0227608e-004	6.5334736e-004	7.0878933e-004
6.9514180e-004	7.1070352e-004	6.7667451e-004	6.1421345e-004
6.5773250e-004	7.1428508e-004	6.6953778e-004	5.7645813e-004
6.0874177e-004	6.7434428e-004	5.5565426e-004	5.8346921e-004
5.9940321e-004	5.7232181e-004	6.2707777e-004	5.8760347e-004
5.5285964e-004	5.4602724e-004	5.6836593e-004	6.1956077e-004
6.7450492e-004	7.0626453e-004	7.1186027e-004	7.7121364e-004
7.6370257e-004	7.3206623e-004	7.0474278e-004	7.0072142e-004
7.0226472e-004	6.9710192e-004	6.5936957e-004	6.1909070e-004
6.2634403e-004	6.2081638e-004	6.1683629e-004	6.1530428e-004
6.1898521e-004	6.1998796e-004	6.0506584e-004	5.8626221e-004
6.0054750e-004	5.8914298e-004	6.0399991e-004	6.1241075e-004
5.7818153e-004	6.2384455e-004	6.2720936e-004	6.5742408e-004
7.2347623e-004	7.0056533e-004	7.8633316e-004	6.4778059e-004
6.9870401e-004	6.6519927e-004	6.5718056e-004	6.2106834e-004
6.1854640e-004	6.2384765e-004	6.6566524e-004	6.2095339e-004
6.8983375e-004	6.2755228e-004	6.9010115e-004	6.2147605e-004
6.2087897e-004	5.7495866e-004	5.8795593e-004	6.2079843e-004
5.6872051e-004	6.2602748e-004	7.0874386e-004	7.2579409e-004
7.1032823e-004	7.6350184e-004	7.5619660e-004	6.9665133e-004
6.9949997e-004	7.0254717e-004	6.6890669e-004	6.5439379e-004
6.1191517e-004	6.7485342e-004	6.4019172e-004	6.3385250e-004

6.7064092e-004 6.6665738e-004 6.3879818e-004 6.3674949e-004
6.3633577e-004 5.6245014e-004 6.3906093e-004 6.3779909e-004
5.7418381e-004 6.5161931e-004 6.1761278e-004 7.1651318e-004
6.6713100e-004 7.3749328e-004 6.6876414e-004 6.3567757e-004
6.3812878e-004 6.1358453e-004 6.2242582e-004 5.7534566e-004
5.2871887e-004 6.1734841e-004 5.4673858e-004 5.6516643e-004
6.5814338e-004 5.9134442e-004 6.2157599e-004 5.9924339e-004
5.7097850e-004 5.1981417e-004 5.5386151e-004 6.6940900e-004
5.9151035e-004 5.6915905e-004 5.8887510e-004 5.8897903e-004
5.8618585e-004 6.4737590e-004 6.3345224e-004 6.2067922e-004
6.3687607e-004 6.0725003e-004 6.2530343e-004 5.3079883e-004
5.8864334e-004 5.7285854e-004 5.5504224e-004 5.6735399e-004
5.9748315e-004 6.5554328e-004 6.8022285e-004 6.6992512e-004
6.5699863e-004 6.1122380e-004 6.9223535e-004 7.1560880e-004
6.1526938e-004 5.4693330e-004 6.0331103e-004 5.4829218e-004
5.7556310e-004 5.4312624e-004 5.0095740e-004 5.1916599e-004
5.3350916e-004 4.2952624e-004 4.6842275e-004 5.1134219e-004
5.0138678e-004 5.3161563e-004 5.1356351e-004 5.0580214e-004
5.5835064e-004 5.5788627e-004 5.3214856e-004 5.4627438e-004
5.7372996e-004 6.3600691e-004 6.3134915e-004 6.3142461e-004
5.0578308e-004 5.3764138e-004 5.0095026e-004 5.1493107e-004
4.4939599e-004 4.9122533e-004 5.2247202e-004 5.2425625e-004
4.8159445e-004 5.1700808e-004 5.5237036e-004 5.1239421e-004
5.9208732e-004 5.5076975e-004 5.7525329e-004 5.7646607e-004
6.0148208e-004 6.2047482e-004 6.5912554e-004 6.2522425e-004
6.5962141e-004 5.9436241e-004 6.7869917e-004 6.6549581e-004
4.6527020e-004 5.7902290e-004 5.4843317e-004 6.0537379e-004
5.8160834e-004 6.0047135e-004 6.3053150e-004 6.0584021e-004
5.5285975e-004 6.0425262e-004 6.0528058e-004 5.7982624e-004
6.0496338e-004 6.7894885e-004 6.2194074e-004 6.5300046e-004
7.2291669e-004 7.1108920e-004 7.3331047e-004 6.5236626e-004
6.5693281e-004 6.4154336e-004 6.5633537e-004 7.1336521e-004
5.9499528e-004 6.2241871e-004 6.9726774e-004 7.2503257e-004
6.7948340e-004 6.2710370e-004 6.7876487e-004 6.6272251e-004
5.5182780e-004 5.9464198e-004 6.3263447e-004 6.0927417e-004
5.6020204e-004 6.7419214e-004 5.7946728e-004 5.6423535e-004
5.4671544e-004 5.6440209e-004 5.9463132e-004 5.7003145e-004
5.7866081e-004 5.3575182e-004 5.7645008e-004 5.5105268e-004
5.2885058e-004 6.0387106e-004 6.0404671e-004 6.5339194e-004
5.3715710e-004 5.8531904e-004 6.2959544e-004 5.5941206e-004
5.3647987e-004 5.1910225e-004 5.7391083e-004 5.5309651e-004
5.0600481e-004 5.0163248e-004 5.3238508e-004 5.2543003e-004
4.7497286e-004 4.9283807e-004 4.9489807e-004 5.0555277e-004
4.7325106e-004 4.6463296e-004 4.9939589e-004 5.0918509e-004
5.3809138e-004 6.2221529e-004 6.0609511e-004 7.0121381e-004
5.7773027e-004 6.1461840e-004 6.5047073e-004 6.6291209e-004
5.3488724e-004 5.8637718e-004 6.2012835e-004 5.7034160e-004
5.3347389e-004 5.2623298e-004 5.4412329e-004 4.9278518e-004
5.0692972e-004 5.6441197e-004 5.0007012e-004 5.1328742e-004
5.1146913e-004 4.8319630e-004 5.3370206e-004 5.2241165e-004
5.3428223e-004 5.9168974e-004 6.0392509e-004 6.9748428e-004
6.4431639e-004 6.1759481e-004 6.3589173e-004 6.5663665e-004

6.1083282e-004	6.1525751e-004	6.3157843e-004	5.3274976e-004
5.0346056e-004	4.9778927e-004	5.2121365e-004	5.2040360e-004
5.2370915e-004	5.5084568e-004	5.5476998e-004	5.6012681e-004
5.2282459e-004	5.2128120e-004	4.9432933e-004	5.2978998e-004
5.9429627e-004	6.6644402e-004	6.6149686e-004	7.4043815e-004
6.8300101e-004	6.8167195e-004	6.6954346e-004	6.3439086e-004
6.3272670e-004	7.0208553e-004	6.6669754e-004	5.8067056e-004
5.9215272e-004	6.4234114e-004	5.5939695e-004	5.7411869e-004
5.9975241e-004	5.6360997e-004	5.8582397e-004	5.8921114e-004
5.4607469e-004	5.4396068e-004	5.5218818e-004	5.9759332e-004
6.7390918e-004	7.0250985e-004	6.9498437e-004	7.3438587e-004
7.3084373e-004	6.9388282e-004	6.9599872e-004	6.7830722e-004
6.8914243e-004	6.9349495e-004	6.7522457e-004	6.1586233e-004
6.0673208e-004	6.1684067e-004	6.2033719e-004	6.3411728e-004
6.0018893e-004	6.1684597e-004	6.0435828e-004	5.9451631e-004
5.6896219e-004	5.7245715e-004	5.9604357e-004	6.0007576e-004
6.0377478e-004	6.1654017e-004	6.6483168e-004	6.9338916e-004
7.3358799e-004	6.8928151e-004	7.5108628e-004	6.3515996e-004
6.8946404e-004	6.6758812e-004	6.4247783e-004	6.0071156e-004
6.1269067e-004	6.0901036e-004	6.3708461e-004	6.2303571e-004
7.0553138e-004	6.1225105e-004	6.5989325e-004	5.9417506e-004
5.8632276e-004	5.7287101e-004	5.8344689e-004	6.1200159e-004
5.4473439e-004	6.1563287e-004	6.8422958e-004	7.1759711e-004
7.0936841e-004	7.6069582e-004	7.5754590e-004	6.9033686e-004
7.1384988e-004	7.0140520e-004	6.6433064e-004	6.5638112e-004
6.0850888e-004	6.6471839e-004	6.7903876e-004	6.5242928e-004
6.7331322e-004	6.4043829e-004	6.5991996e-004	6.3284402e-004
6.6342925e-004	5.6975508e-004	6.4616634e-004	6.0999113e-004
5.9415621e-004	6.8326145e-004	6.4874296e-004	7.2681681e-004
6.9896110e-004	7.4871590e-004	6.8309468e-004	6.3243689e-004
6.2926760e-004	6.3391089e-004	6.2580600e-004	6.0004406e-004
5.4533537e-004	6.2492627e-004	5.9063609e-004	5.8941361e-004
6.4413709e-004	5.9005800e-004	6.1553939e-004	6.3334781e-004
5.7433061e-004	5.4431008e-004	5.6766433e-004	6.4028325e-004
5.9327843e-004	5.3703797e-004	5.9489861e-004	5.5505551e-004
5.7070787e-004	5.3963810e-004	5.0479568e-004	5.2629170e-004
5.3109849e-004	4.4974846e-004	4.6287229e-004	5.1527828e-004
5.1528450e-004	5.3011766e-004	5.0674018e-004	5.0478424e-004
5.5255434e-004	5.6134006e-004	5.5332633e-004	5.6749143e-004
5.8782440e-004	6.2852791e-004	6.2160737e-004	6.2881246e-004
4.9150006e-004	5.0281671e-004	4.9420115e-004	5.2167679e-004
4.7152942e-004	5.0638364e-004	5.3385325e-004	5.5532451e-004
5.1058062e-004	5.5829248e-004	5.9395027e-004	5.3418138e-004
6.2029687e-004	5.5753786e-004	6.0717427e-004	5.8100100e-004
6.0843500e-004	6.4990515e-004	6.6414705e-004	6.2734800e-004
6.7691065e-004	6.0006345e-004	6.7272027e-004	6.5809554e-004
4.8662293e-004	6.1453348e-004	6.0921532e-004	6.7681201e-004
6.4960142e-004	6.6438990e-004	7.0785133e-004	6.8037295e-004
6.2290714e-004	6.3866659e-004	6.4681553e-004	6.1776757e-004
6.3245292e-004	6.9796672e-004	6.2810309e-004	6.5633516e-004
7.2581688e-004	7.1029579e-004	7.4660699e-004	6.4882824e-004
6.5206666e-004	6.3499827e-004	6.5692179e-004	7.0625907e-004

5.8480672e-004	6.8126628e-004	7.1874420e-004	6.9692236e-004
7.4332743e-004	6.7289076e-004	7.4333383e-004	7.1507086e-004
6.6372122e-004	6.7497014e-004	6.7508177e-004	6.6314996e-004
5.8755660e-004	6.2042133e-004	6.4140285e-004	6.2836178e-004
5.7503648e-004	5.9817451e-004	6.3845476e-004	5.6770498e-004
6.1580157e-004	5.4530941e-004	6.2523995e-004	6.1614667e-004
5.8668698e-004	6.3132786e-004	7.2055872e-004	7.3792315e-004
6.9058454e-004	6.2579583e-004	6.9914488e-004	6.4873266e-004
5.4086442e-004	5.9229854e-004	6.3623294e-004	5.9967078e-004
5.5913713e-004	6.6654466e-004	5.4503628e-004	5.5907189e-004
5.3246455e-004	5.5130981e-004	5.7467177e-004	5.4630147e-004
5.7609112e-004	5.1430778e-004	5.5909026e-004	5.3340140e-004
5.1993050e-004	5.9768056e-004	6.0351541e-004	6.2660656e-004
5.5783904e-004	5.8242296e-004	6.3259677e-004	5.6058791e-004
5.4737307e-004	5.0510646e-004	5.9419431e-004	5.4570187e-004
5.1467767e-004	5.1384594e-004	5.3634949e-004	5.2432837e-004
4.8779729e-004	4.7503476e-004	5.0000018e-004	4.9101712e-004
4.7379413e-004	4.7315660e-004	4.9807061e-004	5.1784996e-004
5.3951201e-004	6.1984108e-004	5.9854376e-004	6.7360859e-004
5.7732651e-004	6.3056748e-004	6.5687993e-004	6.6503549e-004
5.3465046e-004	5.7982100e-004	5.9901970e-004	5.5340455e-004
5.2057454e-004	5.4396447e-004	5.7811377e-004	5.0254712e-004
4.8427816e-004	5.5793134e-004	4.8859801e-004	4.9529834e-004
4.9204869e-004	4.8386693e-004	5.2468565e-004	5.2020757e-004
5.2169501e-004	5.8680789e-004	5.9803703e-004	7.0246939e-004
6.4057059e-004	6.2723736e-004	6.4255015e-004	6.3973472e-004
6.1997240e-004	5.9047225e-004	6.1646735e-004	5.2729702e-004
4.9080929e-004	4.9480824e-004	5.2890609e-004	5.2214785e-004
5.0239224e-004	5.2237687e-004	5.3380076e-004	5.5165270e-004
5.2359750e-004	5.1498706e-004	4.9712083e-004	5.2155217e-004
5.7240028e-004	6.6175987e-004	6.7797044e-004	7.4529975e-004
6.5630944e-004	6.6989916e-004	6.7279312e-004	6.6144794e-004
6.1468488e-004	6.7967974e-004	6.5935374e-004	5.8797050e-004
5.8958474e-004	6.1221594e-004	5.7432243e-004	5.8029963e-004
6.1197579e-004	5.6072191e-004	5.6151765e-004	5.9160253e-004
5.4032910e-004	5.3282212e-004	5.5064748e-004	5.9761196e-004
6.4742174e-004	6.6834668e-004	6.8278951e-004	7.2388747e-004
7.0764271e-004	6.6755974e-004	6.9666754e-004	6.7925351e-004
6.7691840e-004	6.9372104e-004	6.5701682e-004	6.2886057e-004
5.9065281e-004	6.1017410e-004	6.2245125e-004	6.3702727e-004
5.9186047e-004	6.1125842e-004	6.2017790e-004	5.9150522e-004
5.5515252e-004	5.7096756e-004	5.9580323e-004	5.9874402e-004
6.1291833e-004	6.1020102e-004	6.7594940e-004	7.1604847e-004
7.1763645e-004	6.8810843e-004	7.2700062e-004	6.3891288e-004
6.6906336e-004	6.4325775e-004	6.2928567e-004	5.9706901e-004
6.0010699e-004	6.0461414e-004	6.3281503e-004	6.3307663e-004
7.1028979e-004	6.1275113e-004	6.3337345e-004	5.8584515e-004
5.9412504e-004	5.8795283e-004	5.8666430e-004	5.8801570e-004
5.4742263e-004	6.1744747e-004	6.6532820e-004	7.0259688e-004
7.0393938e-004	7.3571937e-004	7.3471204e-004	6.8284821e-004
6.9569050e-004	6.9329195e-004	6.6819687e-004	6.5397787e-004
5.9717431e-004	6.4335078e-004	6.9476830e-004	6.5608673e-004

6.5739849e-004	6.2211491e-004	6.5110698e-004	6.2666957e-004
6.5763298e-004	5.7444473e-004	6.4681377e-004	5.9824572e-004
5.9128022e-004	6.8741043e-004	6.5807093e-004	7.1060895e-004
7.1542828e-004	7.4300285e-004	6.7785200e-004	6.4651383e-004
6.2573819e-004	6.2891215e-004	6.4180956e-004	6.0922829e-004
5.5539828e-004	6.2436861e-004	6.2228828e-004	6.0320302e-004
6.2322579e-004	5.8789800e-004	6.1565520e-004	6.4137056e-004
5.7898957e-004	5.7213685e-004	5.9547825e-004	6.2733752e-004
5.0852889e-004	5.8748558e-004	5.4101644e-004	5.8524858e-004
5.3684112e-004	6.1273473e-004	6.2500093e-004	6.0958922e-004
6.0038018e-004	6.0346362e-004	6.0990207e-004	5.8502197e-004
5.9927002e-004	5.9230942e-004	5.5200067e-004	6.0050611e-004
6.2319019e-004	6.0670305e-004	6.0769640e-004	7.1557624e-004
7.2173045e-004	6.2307575e-004	7.1344900e-004	6.5020847e-004
6.2033025e-004	5.3904565e-004	6.5006597e-004	5.5057800e-004
5.7865868e-004	6.2668037e-004	5.6072309e-004	5.3299154e-004
5.7511806e-004	4.9098714e-004	4.7243663e-004	5.1765213e-004
5.0221615e-004	4.9262188e-004	4.7031839e-004	5.3526988e-004
5.1722036e-004	5.0869800e-004	5.0156069e-004	5.1571531e-004
5.2740188e-004	4.8480689e-004	5.5893399e-004	5.5235566e-004
5.2003475e-004	5.2860442e-004	5.2900499e-004	5.4591747e-004
5.0783475e-004	4.9288712e-004	4.7351223e-004	5.0645844e-004
4.9540788e-004	4.8894378e-004	4.7055448e-004	5.2216291e-004
5.4511823e-004	5.2824186e-004	5.6112539e-004	5.1819427e-004
5.3626960e-004	5.7437568e-004	5.9608811e-004	5.9735158e-004
6.4691172e-004	6.1169174e-004	6.3333019e-004	6.4695037e-004
4.6932438e-004	4.9533838e-004	5.2121024e-004	5.6331269e-004
5.3958355e-004	5.6972509e-004	6.0770758e-004	6.2872104e-004
5.7931736e-004	6.4018727e-004	6.7926307e-004	5.8904881e-004
6.4339509e-004	6.0037103e-004	6.1539060e-004	6.2189245e-004
6.3425960e-004	6.7164542e-004	6.9629797e-004	6.4688447e-004
6.6156159e-004	5.9915768e-004	6.4647330e-004	6.5982566e-004
5.5323473e-004	6.9986226e-004	7.1734207e-004	8.0251147e-004
7.4084217e-004	7.6385487e-004	8.1012447e-004	7.6447321e-004
7.0529901e-004	6.9173741e-004	6.8977647e-004	6.6736550e-004
6.5999087e-004	7.0277428e-004	6.5469676e-004	6.4515900e-004
7.0289042e-004	6.9335381e-004	7.1119376e-004	6.3229750e-004
6.4289031e-004	6.2792757e-004	6.3218076e-004	6.6250812e-004
5.5781603e-004	6.0132970e-004	6.9102783e-004	7.0418464e-004
6.4078692e-004	5.9578248e-004	6.6222789e-004	5.9733744e-004
5.3313834e-004	5.6789322e-004	5.9954295e-004	5.5598345e-004
5.5163342e-004	6.2030644e-004	5.0154325e-004	5.3163449e-004
5.1766695e-004	5.0870387e-004	5.4202868e-004	4.9587770e-004
5.2619488e-004	4.9000583e-004	5.2251670e-004	4.8443789e-004
5.2721107e-004	5.9978341e-004	6.1325880e-004	6.0039751e-004
5.9047437e-004	6.1151382e-004	6.5274193e-004	5.8589426e-004
5.4565273e-004	5.3131123e-004	6.0540553e-004	5.5346708e-004
5.2629426e-004	5.2145438e-004	5.7979136e-004	5.2632471e-004
4.9839748e-004	4.9403599e-004	5.0263826e-004	4.9809756e-004
4.6806508e-004	4.9547041e-004	4.9972879e-004	5.4669970e-004
5.4662378e-004	6.0596246e-004	5.7969286e-004	6.5554270e-004
5.7808674e-004	6.5762106e-004	6.6842593e-004	6.4712373e-004

5.8586993e-004	5.6743105e-004	5.8663817e-004	5.3072063e-004
4.7902665e-004	5.5505543e-004	5.8154855e-004	5.1331424e-004
4.7521201e-004	5.4238891e-004	4.9451655e-004	4.8455288e-004
4.8195742e-004	4.7716549e-004	5.1906602e-004	4.9069435e-004
4.9984581e-004	5.9045346e-004	5.9609258e-004	6.8927214e-004
6.4759476e-004	6.1179303e-004	6.2830512e-004	6.2969467e-004
6.0541027e-004	5.6433775e-004	6.0599782e-004	5.1563804e-004
5.2262046e-004	4.8054499e-004	5.3899951e-004	5.2649781e-004
5.2087563e-004	4.8861808e-004	5.2710949e-004	5.5696434e-004
5.2507441e-004	5.1593581e-004	4.8033670e-004	5.2727634e-004
5.8255266e-004	6.5668653e-004	6.8923655e-004	7.3478815e-004
6.1839858e-004	6.5169051e-004	6.7832081e-004	6.7335053e-004
6.0401381e-004	6.8020700e-004	6.4163387e-004	5.9745439e-004
6.1946979e-004	6.1493876e-004	5.9619253e-004	6.0912488e-004
6.1494061e-004	5.7001610e-004	5.5748747e-004	5.8149913e-004
5.3229052e-004	5.2062665e-004	5.7588282e-004	6.0827463e-004
6.0461737e-004	6.1992930e-004	6.6400379e-004	7.2069380e-004
6.9762474e-004	6.5079275e-004	6.9073812e-004	6.7785576e-004
6.8686140e-004	6.6637415e-004	6.1717772e-004	6.3909923e-004
5.6254315e-004	5.9058727e-004	6.0102902e-004	6.2521393e-004
5.9179719e-004	6.0335903e-004	6.3451001e-004	5.6784779e-004
5.6364326e-004	5.8703504e-004	5.6863374e-004	5.7261089e-004
6.1571288e-004	6.8624462e-004	6.1367772e-004	6.6493021e-004
6.9944176e-004	6.9619657e-004	7.3450006e-004	7.0411171e-004
6.5801106e-004	6.7865287e-004	6.5692527e-004	6.7829603e-004
6.5599203e-004	7.0116244e-004	6.2285690e-004	6.9687080e-004
7.3867184e-004	6.5668994e-004	6.9992074e-004	8.1075549e-004
7.9586569e-004	7.1559477e-004	7.6948971e-004	7.7070232e-004
4.7393253e-004	5.0393405e-004	5.1575068e-004	5.8227392e-004
5.2856881e-004	5.4019979e-004	5.6468177e-004	5.9436685e-004
5.7755149e-004	6.5419640e-004	6.2973493e-004	5.8035972e-004
6.5822345e-004	5.9613820e-004	6.7578918e-004	6.0251020e-004
6.2351595e-004	6.5589097e-004	6.7670956e-004	6.5293508e-004
7.3053125e-004	6.3244112e-004	6.7371482e-004	6.7350043e-004
5.2049722e-004	6.2503529e-004	6.4571654e-004	7.5144137e-004
6.7448859e-004	7.1900299e-004	7.6586854e-004	7.6591937e-004
6.9195425e-004	6.9422823e-004	7.0537509e-004	6.6677740e-004
6.7531693e-004	6.8407071e-004	6.0707345e-004	6.7062964e-004
6.6994605e-004	6.8225974e-004	7.2610425e-004	6.7002825e-004
6.3660675e-004	6.2170293e-004	6.2935469e-004	6.6768118e-004
6.1307248e-004	6.9253454e-004	7.4116209e-004	7.5381635e-004
7.5163749e-004	6.9455903e-004	7.5016677e-004	6.9889419e-004
6.0396890e-004	6.2786808e-004	6.4623148e-004	6.0491126e-004
5.7517993e-004	6.5096786e-004	5.4302497e-004	5.5970944e-004
5.1600700e-004	5.7044248e-004	6.1643209e-004	5.2096952e-004
5.6821364e-004	5.1785579e-004	5.6438272e-004	5.5637738e-004
5.0871577e-004	5.7763164e-004	6.2703773e-004	6.0721369e-004
6.1616780e-004	5.7900375e-004	6.0223251e-004	5.6099729e-004
5.7655184e-004	5.3828718e-004	5.8660886e-004	5.3547940e-004
5.3764194e-004	5.4476630e-004	5.1891684e-004	5.2840590e-004
5.0079770e-004	4.6849214e-004	5.1253029e-004	4.8410952e-004
4.7168168e-004	4.7986284e-004	4.9463218e-004	4.8154826e-004

5.4551219e-004	6.1045386e-004	6.2591456e-004	6.1739759e-004
5.8842762e-004	6.6766761e-004	6.8941852e-004	6.2650369e-004
5.3201029e-004	5.5838337e-004	5.8025632e-004	5.3413362e-004
5.0673923e-004	5.4448137e-004	6.0705162e-004	5.2386503e-004
4.8635575e-004	5.5126146e-004	4.9003468e-004	4.8796676e-004
4.4982836e-004	5.2160138e-004	5.0202783e-004	5.3201945e-004
5.1751394e-004	5.5965713e-004	5.4502832e-004	6.4555115e-004
6.1098233e-004	6.2429132e-004	6.2909302e-004	6.1166585e-004
6.3716414e-004	5.4285728e-004	5.9325960e-004	5.1315717e-004
4.9060024e-004	5.2976239e-004	5.6160589e-004	5.2923808e-004
5.0132925e-004	4.6937260e-004	5.3171287e-004	5.1509106e-004
5.0217388e-004	4.5431720e-004	5.1768564e-004	4.7603851e-004
5.1911767e-004	6.0487747e-004	6.2978828e-004	6.6469688e-004
5.9744630e-004	6.2139108e-004	6.3915188e-004	6.6206545e-004
5.9745161e-004	5.6186733e-004	5.9650981e-004	5.4054640e-004
5.7743372e-004	5.1797603e-004	5.7094396e-004	5.4112880e-004
5.7849511e-004	5.3688042e-004	5.3542403e-004	5.6801956e-004
5.3617881e-004	5.3880572e-004	4.9762319e-004	5.8055970e-004
5.6367429e-004	6.9232580e-004	6.6092589e-004	6.6566833e-004
7.1075355e-004	7.3856766e-004	6.8884200e-004	7.0038273e-004
6.5276551e-004	6.8596241e-004	6.9751510e-004	6.5843541e-004
5.8257747e-004	6.1845129e-004	6.6750689e-004	6.5420668e-004
5.9104573e-004	6.1206313e-004	6.7342361e-004	6.3280250e-004
5.9265539e-004	5.8658005e-004	6.2705463e-004	6.2326835e-004
6.3589364e-004	6.2076477e-004	6.1863933e-004	6.7156466e-004
7.4109362e-004	6.5846923e-004	6.8911626e-004	6.3977730e-004
6.4926075e-004	6.5882878e-004	6.0111303e-004	6.1803900e-004
6.1481923e-004	6.4799632e-004	6.0634717e-004	6.2595028e-004
6.7379793e-004	6.1855480e-004	6.4961292e-004	7.1448467e-004
7.2753583e-004	7.1269769e-004	6.8108915e-004	7.7325602e-004
6.0405376e-004	5.9192180e-004	5.9370842e-004	5.1945865e-004
5.8955261e-004	6.0719832e-004	5.4599453e-004	4.8090761e-004
5.1189779e-004	4.9618773e-004	4.8212288e-004	4.8053870e-004
4.8330597e-004	4.4277114e-004	4.8946724e-004	5.3539925e-004
4.8091925e-004	4.8162525e-004	4.6401982e-004	5.0866829e-004
4.8449320e-004	4.5793996e-004	5.2806047e-004	5.5527528e-004
4.7928547e-004	4.6598577e-004	4.8971517e-004	5.0161661e-004
4.8206478e-004	4.6855002e-004	4.6362163e-004	4.9182746e-004
4.6790842e-004	4.6296495e-004	4.6932512e-004	5.3546998e-004
5.2798413e-004	5.0548837e-004	5.3220376e-004	5.3737452e-004
5.1556162e-004	5.5125162e-004	5.7107581e-004	5.6900426e-004
6.0605654e-004	5.5457454e-004	5.6780049e-004	6.1122800e-004
4.6530772e-004	5.2786634e-004	5.6519829e-004	6.3238751e-004
5.8786815e-004	6.1255257e-004	6.7078709e-004	6.7977305e-004
6.5846823e-004	7.4214344e-004	7.0361682e-004	6.4351637e-004
6.8924983e-004	6.5685245e-004	6.7967494e-004	6.4563313e-004
6.6129453e-004	6.6068256e-004	7.0068424e-004	6.6874643e-004
7.0152764e-004	6.3535366e-004	6.6388599e-004	6.6522321e-004
5.9816963e-004	7.0243025e-004	7.1822226e-004	8.4078482e-004
7.1711397e-004	7.8521372e-004	8.0834184e-004	7.9626126e-004
7.2251305e-004	7.1003260e-004	6.9569894e-004	6.9529285e-004
7.1579457e-004	6.8838883e-004	6.3187760e-004	6.5921064e-004

6.6588729e-004	6.7201476e-004	6.9410696e-004	6.6143375e-004
6.3871389e-004	6.1852463e-004	6.1162849e-004	6.4336933e-004
5.6579486e-004	6.0116407e-004	6.7032040e-004	6.8655130e-004
6.6389935e-004	6.4260952e-004	6.5935658e-004	6.4727894e-004
5.8378688e-004	5.9983938e-004	5.8524300e-004	5.5526092e-004
5.6530691e-004	6.3489437e-004	5.2732797e-004	5.2386608e-004
5.1255703e-004	5.5889483e-004	5.9458101e-004	4.8445231e-004
5.2773622e-004	5.1470295e-004	5.3031947e-004	5.1991848e-004
5.1293659e-004	5.7508628e-004	6.1378747e-004	5.8450011e-004
6.3998562e-004	6.0549047e-004	6.1953474e-004	5.7333337e-004
5.7013484e-004	5.4480057e-004	5.8609052e-004	5.4867705e-004
5.4156376e-004	5.4521572e-004	5.5835085e-004	5.3918852e-004
4.9838674e-004	4.9897625e-004	5.1386311e-004	5.0188543e-004
4.4580457e-004	4.9169724e-004	4.9421473e-004	5.1108450e-004
5.2601413e-004	5.9010356e-004	5.9625556e-004	6.0826163e-004
5.6571225e-004	6.7668875e-004	6.7224477e-004	6.0664247e-004
5.5368447e-004	5.4166267e-004	5.6920112e-004	5.1752992e-004
4.8716966e-004	5.3981121e-004	5.7334748e-004	5.3972966e-004
5.0027579e-004	5.2587425e-004	5.0362854e-004	4.5754045e-004
4.3176705e-004	5.0968801e-004	5.0498471e-004	4.6630028e-004
4.7785956e-004	5.3673860e-004	5.2633102e-004	6.0416105e-004
6.2737915e-004	5.8883052e-004	6.1226373e-004	6.0019566e-004
6.0579313e-004	5.4076868e-004	5.8080241e-004	5.0224493e-004
5.3156112e-004	5.2538422e-004	5.6301053e-004	5.2774762e-004
5.2193747e-004	4.6314327e-004	5.5128662e-004	5.3724099e-004
5.1825774e-004	4.5630293e-004	5.1912694e-004	4.9537824e-004
5.7087015e-004	6.7604176e-004	6.5347443e-004	6.6633109e-004
6.9729339e-004	7.2415668e-004	6.6567761e-004	6.7567368e-004
6.0819228e-004	6.5993021e-004	6.8460456e-004	6.2102670e-004
5.5215023e-004	5.8108140e-004	6.4961611e-004	5.9965038e-004
5.7692430e-004	6.0740273e-004	6.3378505e-004	6.0398089e-004
5.5393262e-004	5.8403186e-004	5.8569593e-004	5.9884736e-004
6.0735369e-004	6.2545779e-004	5.9406324e-004	6.4536720e-004
6.5923596e-004	5.7644518e-004	6.2749385e-004	6.0516673e-004
4.9811720e-004	5.5889373e-004	5.4566526e-004	6.0363188e-004
5.6413074e-004	5.5493551e-004	4.9951280e-004	5.7818844e-004
6.5403580e-004	5.7068287e-004	5.3751632e-004	6.5540221e-004
6.3516435e-004	6.0017708e-004	6.2547547e-004	5.8407550e-004
6.1458861e-004	5.9195023e-004	5.9732648e-004	5.4100763e-004
6.0223675e-004	5.7633957e-004	5.2922409e-004	4.6491051e-004
4.9391786e-004	5.2506226e-004	4.8644771e-004	4.7410802e-004
4.8313224e-004	4.7478310e-004	4.8858807e-004	5.2486502e-004
4.9070061e-004	4.7102978e-004	4.5334109e-004	5.0943332e-004
4.7074910e-004	4.5780744e-004	5.3059437e-004	5.4101760e-004
4.6829973e-004	5.2874465e-004	5.6335931e-004	6.3198766e-004
6.0207593e-004	5.9199505e-004	6.3104709e-004	6.6500493e-004
6.4380716e-004	7.1100048e-004	6.6521570e-004	6.5048746e-004
6.8828209e-004	6.5650362e-004	6.8725704e-004	6.4206040e-004
6.6848691e-004	6.4038325e-004	6.9144528e-004	6.4766688e-004
7.1053150e-004	6.3961916e-004	6.7219409e-004	6.8066220e-004
5.6415594e-004	6.6346550e-004	6.7202772e-004	7.7273998e-004
6.7964687e-004	7.1876901e-004	7.8272802e-004	7.6747040e-004

7.0128465e-004	7.0879269e-004	6.8798939e-004	6.9306817e-004
7.2045646e-004	6.8433648e-004	6.3793607e-004	6.3868389e-004
6.4642016e-004	6.4040613e-004	6.7507690e-004	6.6473296e-004
6.3211098e-004	6.0917399e-004	6.3542146e-004	6.2657055e-004
6.5824208e-004	6.7906706e-004	6.7988422e-004	8.0405010e-004
7.2976480e-004	7.6517487e-004	7.4580561e-004	6.9008341e-004
6.7730914e-004	7.0683297e-004	6.6381319e-004	6.5184131e-004
6.3929627e-004	6.1265554e-004	6.1515463e-004	6.0638005e-004
6.2714927e-004	6.1259027e-004	5.9228871e-004	5.5850190e-004
5.5875791e-004	5.4603234e-004	5.5072008e-004	5.6958418e-004
5.9675043e-004	5.9904032e-004	6.4081851e-004	6.7339103e-004
6.4633430e-004	6.5425951e-004	6.3241669e-004	6.3574853e-004
5.7577407e-004	6.0365169e-004	5.8321960e-004	5.5904723e-004
5.6136806e-004	6.2645454e-004	5.5563875e-004	5.0809339e-004
5.2461444e-004	5.5617990e-004	6.0839386e-004	4.9720141e-004
5.0443391e-004	5.1956181e-004	5.4035711e-004	5.5148387e-004
4.9477910e-004	5.5443325e-004	6.0221009e-004	5.9280952e-004
6.2454783e-004	5.8069876e-004	5.8337639e-004	5.8989234e-004
5.6622192e-004	5.5303134e-004	5.5840930e-004	5.4055866e-004
5.5692704e-004	5.4622402e-004	5.4626779e-004	5.4076611e-004
4.8064246e-004	5.2442022e-004	5.0933346e-004	4.8884215e-004
4.6021320e-004	4.8479799e-004	4.7730761e-004	5.0170143e-004
5.0917455e-004	5.5391069e-004	5.9127581e-004	5.7638909e-004
5.7670838e-004	6.6703303e-004	6.7156526e-004	5.7894189e-004
5.2884547e-004	5.2325394e-004	5.6656751e-004	5.1585737e-004
4.9745459e-004	5.4016584e-004	5.5440608e-004	5.5319851e-004
5.1584936e-004	5.2320076e-004	5.2143683e-004	4.6804966e-004
4.3314061e-004	5.1602945e-004	5.1907575e-004	4.7195379e-004
5.8379027e-004	7.0162953e-004	6.7225128e-004	6.8484111e-004
6.9142415e-004	7.4631906e-004	6.9703664e-004	6.9952972e-004
6.7455492e-004	7.2365470e-004	6.9921513e-004	6.5561231e-004
5.8973793e-004	6.3089193e-004	6.8495137e-004	6.3160578e-004
6.0029954e-004	6.2887426e-004	6.5723481e-004	6.2832209e-004
5.9385128e-004	5.8259077e-004	5.9075560e-004	6.3705868e-004
6.4645497e-004	6.5632077e-004	7.2180844e-004	7.0776429e-004
7.0274326e-004	6.6223177e-004	6.7059293e-004	6.7767962e-004
6.2341279e-004	6.4199534e-004	6.1526248e-004	5.8353826e-004
6.0356320e-004	5.8004836e-004	5.7875452e-004	5.7621645e-004
6.1448840e-004	5.8487325e-004	6.3600765e-004	6.6154222e-004
6.9163875e-004	6.4448132e-004	6.7113654e-004	7.1686578e-004
4.5501951e-004	4.5608550e-004	4.8664725e-004	4.8930964e-004
5.0056014e-004	4.7045958e-004	4.9332852e-004	5.2196818e-004
4.9511602e-004	5.1714898e-004	5.4096447e-004	5.7540091e-004
5.7754426e-004	5.6528099e-004	5.9362644e-004	6.0169844e-004
5.7858265e-004	5.9159152e-004	6.1520412e-004	5.9230476e-004
6.0921912e-004	5.8442731e-004	6.0746414e-004	6.3170891e-004
6.0430312e-004	6.7731060e-004	6.9982342e-004	7.7057275e-004
6.8459185e-004	7.1030160e-004	7.7101865e-004	7.3014265e-004
6.9350745e-004	6.8073245e-004	6.5258463e-004	6.8892799e-004
7.1095083e-004	6.5407269e-004	6.4496907e-004	6.2446212e-004
6.4037877e-004	6.2435657e-004	6.4726830e-004	6.3819147e-004
6.1788334e-004	5.9833231e-004	6.0726522e-004	5.9673248e-004

6.2892814e-004	6.0486999e-004	6.3698745e-004	7.1878108e-004
6.6132746e-004	6.7462775e-004	6.6444075e-004	6.2980871e-004
6.0890146e-004	6.6085014e-004	6.0816031e-004	5.9345298e-004
5.8780659e-004	5.8449800e-004	5.7671274e-004	5.8076762e-004
5.9008024e-004	5.8679339e-004	5.8961558e-004	5.1694388e-004
5.1600327e-004	5.4165175e-004	5.3236740e-004	5.6711462e-004
4.6190371e-004	5.1286913e-004	5.2974129e-004	5.5028591e-004
5.2137070e-004	6.1403916e-004	6.2132918e-004	5.5259530e-004
4.9496727e-004	4.9039190e-004	5.2325173e-004	4.7728535e-004
4.6867738e-004	5.0965333e-004	4.9568072e-004	5.5944457e-004
5.1262291e-004	4.7300207e-004	5.2729680e-004	4.2453637e-004
4.2590464e-004	4.7666021e-004	5.1047800e-004	4.1923166e-004
6.0321089e-004	6.0527062e-004	6.3908650e-004	6.8122760e-004
6.3888224e-004	5.6272242e-004	6.4784182e-004	6.0516554e-004
4.6534186e-004	5.7648710e-004	5.5396899e-004	5.6854537e-004
5.5518761e-004	5.1994249e-004	5.3559130e-004	5.7015936e-004
6.3418280e-004	5.7616598e-004	5.1955156e-004	5.8987871e-004
5.8120076e-004	5.6904275e-004	6.1326291e-004	5.7280174e-004
6.5007516e-004	7.2220706e-004	7.5315687e-004	7.4404608e-004
7.0579446e-004	6.8689835e-004	6.2874662e-004	6.8541539e-004
6.3812951e-004	6.3503052e-004	6.3334049e-004	5.7509058e-004
5.9175470e-004	5.6309646e-004	5.9804770e-004	5.5489670e-004
5.6982269e-004	5.8675646e-004	6.5668208e-004	6.2491697e-004
6.5998256e-004	6.0671440e-004	7.2421251e-004	7.2322582e-004
6.4476722e-004	7.0594130e-004	6.7164031e-004	6.7191542e-004
6.4614117e-004	5.8691076e-004	5.7315961e-004	6.1201853e-004
5.6077510e-004	6.1736591e-004	5.6094049e-004	5.8394322e-004
6.0349339e-004	5.9000644e-004	5.4034503e-004	5.7405327e-004
5.7209736e-004	5.8848579e-004	5.4212634e-004	5.5916448e-004
5.5018785e-004	5.5008457e-004	6.0931979e-004	5.9804447e-004
4.4967517e-004	4.7598822e-004	5.1034134e-004	4.9651717e-004
5.1683326e-004	4.8377780e-004	5.2436972e-004	5.3674063e-004
5.2056331e-004	5.5026556e-004	5.7401436e-004	5.8312956e-004
5.8693561e-004	5.9266185e-004	6.2408115e-004	6.2492168e-004
5.8608563e-004	6.0666841e-004	6.2949637e-004	6.1853697e-004
5.9768623e-004	5.9742278e-004	6.3290278e-004	6.2596238e-004
6.0080728e-004	6.6031031e-004	6.8812368e-004	7.2974479e-004
6.6582287e-004	6.8006950e-004	7.4487989e-004	6.9460818e-004
6.8085187e-004	6.6208871e-004	6.3101556e-004	6.6477212e-004
6.7963317e-004	6.2963628e-004	6.4227771e-004	6.0530439e-004
6.2905934e-004	6.0273366e-004	6.2891035e-004	6.2269652e-004
6.0386184e-004	5.8983956e-004	5.9359043e-004	5.7105585e-004
4.2933493e-004	4.7660487e-004	4.8722061e-004	5.1826116e-004
4.8909528e-004	5.5052390e-004	5.8260577e-004	5.1018340e-004
4.4058239e-004	4.5643566e-004	4.7975490e-004	4.3839445e-004
4.2998741e-004	4.7269546e-004	4.5272875e-004	5.3397946e-004
4.8652855e-004	4.4476124e-004	4.9963314e-004	4.0637997e-004
4.1841193e-004	4.4993764e-004	4.9214710e-004	4.0776828e-004
5.5844199e-004	5.8127707e-004	6.0664256e-004	6.7083587e-004
7.0428652e-004	6.6525851e-004	6.9221712e-004	6.4814977e-004
6.8077641e-004	7.0215423e-004	6.1443850e-004	6.2103660e-004
5.7095761e-004	6.3568639e-004	6.1571191e-004	5.8128868e-004

6.2926937e-004	6.2187666e-004	6.2321291e-004	5.9483775e-004
5.4353890e-004	6.0976147e-004	6.0065321e-004	6.0555644e-004
5.9940082e-004	6.0946762e-004	6.6639250e-004	7.0512232e-004
6.3222401e-004	5.9077078e-004	6.6242537e-004	6.1814797e-004
4.9279831e-004	6.0016999e-004	5.7972175e-004	5.5320407e-004
5.5294285e-004	5.1954019e-004	5.7549422e-004	5.7595996e-004
6.1985561e-004	5.9027031e-004	5.3281273e-004	5.7660145e-004
5.6835425e-004	5.6253609e-004	6.0634124e-004	5.9071061e-004
6.3379975e-004	7.3107259e-004	7.2503903e-004	7.4076559e-004
6.7639965e-004	6.4404748e-004	5.8812455e-004	6.3512815e-004
6.0241610e-004	5.9203962e-004	5.9573994e-004	5.4070991e-004
5.3483638e-004	5.2559401e-004	5.8037972e-004	5.2046304e-004
5.2421272e-004	5.5633626e-004	6.1098364e-004	5.7068333e-004
6.1372595e-004	5.4671637e-004	6.9068283e-004	6.6890183e-004
6.0804905e-004	7.7802641e-004	6.7266594e-004	7.2637436e-004
6.8983022e-004	6.1359884e-004	6.1850612e-004	6.2602698e-004
6.1008013e-004	6.3059350e-004	6.2375142e-004	6.1328554e-004
6.3829344e-004	6.1291239e-004	5.8327132e-004	6.1462108e-004
6.1538135e-004	6.5939716e-004	6.1448571e-004	6.3014190e-004
6.3260788e-004	6.2357689e-004	6.8113582e-004	6.7475789e-004
6.0839028e-004	5.6051207e-004	5.7929514e-004	5.2612190e-004
5.7434694e-004	5.0545283e-004	4.7509274e-004	4.6142953e-004
4.6309976e-004	5.2846982e-004	4.5348316e-004	4.4538092e-004
4.8704047e-004	4.7882149e-004	4.4942020e-004	4.8674348e-004
4.6912268e-004	4.8361080e-004	4.4376740e-004	4.4026583e-004
4.7070530e-004	4.4815389e-004	4.9138042e-004	4.9851188e-004
4.4120138e-004	4.4388771e-004	4.7194248e-004	4.4027316e-004
4.6522865e-004	4.5466543e-004	4.5044909e-004	4.5971458e-004
4.6378268e-004	4.6281007e-004	4.9201240e-004	4.8162472e-004
4.9208833e-004	5.2988475e-004	5.4284121e-004	5.5951261e-004
5.1437292e-004	5.6571836e-004	5.6564670e-004	5.5997720e-004
5.2347069e-004	5.3035900e-004	5.9403195e-004	5.5950301e-004
4.7452040e-004	5.3288288e-004	5.6795529e-004	5.6315365e-004
5.4705677e-004	5.6711533e-004	5.9073592e-004	6.0876383e-004
4.9423140e-004	5.8306109e-004	5.0886828e-004	5.3868724e-004
5.7869536e-004	5.2142316e-004	5.2864260e-004	4.9794666e-004
4.6586296e-004	5.5987782e-004	4.7311024e-004	4.5096291e-004
5.0235914e-004	4.9285479e-004	4.7023350e-004	4.8694228e-004
5.9054546e-004	6.0577932e-004	6.5325793e-004	7.1078571e-004
7.0223417e-004	7.0955516e-004	7.2177035e-004	6.9026605e-004
6.8460222e-004	7.7798709e-004	6.8761376e-004	6.7949109e-004
6.2970766e-004	6.5743925e-004	6.9608523e-004	6.0769236e-004
6.8232951e-004	6.4420151e-004	5.9687036e-004	6.2794589e-004
5.7173283e-004	6.3751235e-004	6.1829270e-004	6.7784521e-004
6.2064033e-004	7.1199413e-004	7.2438047e-004	7.5725606e-004
6.5710678e-004	6.3399723e-004	6.1881947e-004	6.0845511e-004
5.5145349e-004	5.9768591e-004	5.8016660e-004	5.2309332e-004
5.1911248e-004	4.9835793e-004	5.9205735e-004	5.4381843e-004
5.4589322e-004	5.7375978e-004	5.4760124e-004	5.4885863e-004
6.0208807e-004	4.8820895e-004	5.9041897e-004	6.0405416e-004
6.0103628e-004	7.2506968e-004	7.0040490e-004	7.5901678e-004
6.9751359e-004	6.5449357e-004	5.6687818e-004	5.8463438e-004

5.9379174e-004	5.7289589e-004	6.1095378e-004	5.7876104e-004
5.5254848e-004	5.5919314e-004	5.8182527e-004	5.4537180e-004
5.6067885e-004	5.9676741e-004	6.3360557e-004	6.0294660e-004
5.7626962e-004	6.4949493e-004	7.3035966e-004	6.8393120e-004
4.5044364e-004	4.9725773e-004	5.2379319e-004	4.9932099e-004
5.0795911e-004	5.0346761e-004	5.3261590e-004	5.3194047e-004
5.4335195e-004	5.6300037e-004	5.7972262e-004	5.6194732e-004
5.5244218e-004	5.9745956e-004	6.2104303e-004	6.2100891e-004
5.5987002e-004	6.1134362e-004	6.1454029e-004	6.3388915e-004
5.6071971e-004	5.7960518e-004	6.4463139e-004	5.8701169e-004
6.3286506e-004	7.1427437e-004	7.0583752e-004	7.5227945e-004
6.6079298e-004	6.3133275e-004	5.8695208e-004	5.9111658e-004
5.7441810e-004	5.7974369e-004	5.6817784e-004	5.2408739e-004
5.0306373e-004	5.0007761e-004	5.7740501e-004	5.2569286e-004
5.1357773e-004	5.4811635e-004	5.5609258e-004	5.0776505e-004
5.8125252e-004	4.7807361e-004	6.1003111e-004	6.0159453e-004
5.6290516e-004	6.3831324e-004	6.6162128e-004	6.7123030e-004
6.2394381e-004	6.4016091e-004	5.3891541e-004	5.1248150e-004
5.4086235e-004	5.2503598e-004	5.6473496e-004	5.6704626e-004
5.4771321e-004	5.6083941e-004	5.3015327e-004	5.3204093e-004
5.5596351e-004	5.8417731e-004	5.8795616e-004	5.9867361e-004
5.5447497e-004	6.3132698e-004	6.9150902e-004	6.4269928e-004
6.3916056e-004	6.8215979e-004	6.8686335e-004	7.4178595e-004
6.6307071e-004	6.2583981e-004	5.9223339e-004	5.9330191e-004
5.7852412e-004	5.8854405e-004	5.7081877e-004	5.4481019e-004
5.2150264e-004	5.2375301e-004	5.7383096e-004	5.3852412e-004
5.4010520e-004	5.4087589e-004	5.6511057e-004	5.0232057e-004
5.7594082e-004	5.0518555e-004	6.1196694e-004	6.0002516e-004
5.9567373e-004	6.2431538e-004	6.7983320e-004	6.4712597e-004
6.1500267e-004	6.4855121e-004	5.5441748e-004	5.2499698e-004
5.3019656e-004	5.2046365e-004	5.6768279e-004	5.7049900e-004
5.4757571e-004	5.6667301e-004	5.2126969e-004	5.3676594e-004
5.7282412e-004	5.8158023e-004	5.7995957e-004	6.0342763e-004
5.6355135e-004	6.3314936e-004	7.1317751e-004	6.3565459e-004
5.1951129e-004	5.2739882e-004	5.8598923e-004	6.2578109e-004
5.5894909e-004	6.4724284e-004	6.7765948e-004	6.7654348e-004
5.8892055e-004	6.6091333e-004	5.8024270e-004	5.7321705e-004
5.5763716e-004	6.0342204e-004	5.8103807e-004	5.8005698e-004
5.7319501e-004	5.9484696e-004	6.2994509e-004	5.7925988e-004
5.3619650e-004	5.5442601e-004	5.4011818e-004	5.8687293e-004
6.1976031e-004	5.9637273e-004	6.1907437e-004	6.3648757e-004
6.2059087e-004	5.5500385e-004	5.3954909e-004	5.4489676e-004
5.1367470e-004	4.9913417e-004	5.3063195e-004	5.1020330e-004
4.9662306e-004	4.9235533e-004	5.0842897e-004	4.8545292e-004
5.2811301e-004	4.9158702e-004	5.3704758e-004	4.9660480e-004
4.8868013e-004	5.2987659e-004	6.4677229e-004	5.8986696e-004
6.0124428e-004	6.0846638e-004	6.8725420e-004	6.4293623e-004
5.7212675e-004	6.8546455e-004	6.1796114e-004	5.7895870e-004
5.2779041e-004	5.7203365e-004	5.7535234e-004	5.6063716e-004
5.7088850e-004	5.9276196e-004	5.2361103e-004	5.7266669e-004
6.2781216e-004	5.8921221e-004	5.8520168e-004	6.4788472e-004
6.2561122e-004	6.9314096e-004	7.0496050e-004	6.5295612e-004

7.5842079e-004	7.3708102e-004	7.1688333e-004	7.0921714e-004
7.1483322e-004	7.2318621e-004	7.4636412e-004	6.6216901e-004
6.7010997e-004	7.4330829e-004	6.7905203e-004	6.5575833e-004
6.3200543e-004	6.5856767e-004	6.5487735e-004	5.7595246e-004
6.6261923e-004	6.1709054e-004	6.2467903e-004	6.5010953e-004
6.4216609e-004	6.5039034e-004	7.1019487e-004	6.5393012e-004
4.9710706e-004	5.2347619e-004	5.8858632e-004	6.0717048e-004
5.6289068e-004	6.3318423e-004	6.6877941e-004	6.3509474e-004
5.8335831e-004	6.8964166e-004	5.9986896e-004	5.8747150e-004
5.9682257e-004	5.6269474e-004	5.9114907e-004	5.6424987e-004
5.8837356e-004	6.2409448e-004	6.5463762e-004	5.9614389e-004
5.5224377e-004	5.5352334e-004	5.4900967e-004	5.9963881e-004
6.2135844e-004	5.6246301e-004	6.1595037e-004	6.1696050e-004
6.0623439e-004	5.5652430e-004	5.4288805e-004	5.3105329e-004
4.9041914e-004	4.9069808e-004	5.1016572e-004	4.9945125e-004
5.0284336e-004	4.9461797e-004	4.9624024e-004	4.7111391e-004
5.3629244e-004	5.0222568e-004	5.3144107e-004	5.1660126e-004
4.8852586e-004	5.5147178e-004	6.4081841e-004	5.7147529e-004
6.0605595e-004	5.7963147e-004	6.4593954e-004	6.4228458e-004
5.4966605e-004	6.5683213e-004	5.9999927e-004	5.7086918e-004
5.1308636e-004	5.5854982e-004	5.8021543e-004	5.2457013e-004
5.4563197e-004	5.4748752e-004	5.2912346e-004	5.5049520e-004
6.3874773e-004	5.9625020e-004	5.9566112e-004	6.1438500e-004
6.1670968e-004	7.1691853e-004	6.7491856e-004	6.9098927e-004
7.2467195e-004	6.9360988e-004	6.7281276e-004	6.7329682e-004
6.7894928e-004	6.7216626e-004	6.9657918e-004	6.0932286e-004
6.3750352e-004	6.5635474e-004	6.1595395e-004	6.0619754e-004
5.8551646e-004	6.0238360e-004	5.9216145e-004	5.3904312e-004
5.9416221e-004	5.6075360e-004	5.7405336e-004	5.8065543e-004
5.8099586e-004	5.7882719e-004	6.3279223e-004	5.8686666e-004
5.4094846e-004	5.2468065e-004	5.7285625e-004	6.3407928e-004
6.1654855e-004	6.4639597e-004	6.2574580e-004	6.0195492e-004
5.7891448e-004	5.9737150e-004	5.8388604e-004	5.7984822e-004
5.8669434e-004	5.5775596e-004	5.9246607e-004	5.2381062e-004
5.1203402e-004	5.7159834e-004	5.5720903e-004	5.1640181e-004
4.9389077e-004	4.9929106e-004	5.3242972e-004	5.1530580e-004
4.9330515e-004	5.1431735e-004	5.7685358e-004	6.1251812e-004
5.7717881e-004	6.2744753e-004	6.7197523e-004	6.4820610e-004
5.9894664e-004	6.9336013e-004	6.2042304e-004	5.9722955e-004
6.0949684e-004	5.8277014e-004	5.9569096e-004	5.6630261e-004
6.0319289e-004	6.1880240e-004	6.4676048e-004	6.1396265e-004
5.5898131e-004	5.6273837e-004	5.4915500e-004	5.8795625e-004
5.3866000e-004	6.0603453e-004	6.1836456e-004	6.7320956e-004
6.7559301e-004	6.4855472e-004	6.9087511e-004	5.9070917e-004
5.9763838e-004	6.0947755e-004	6.0022918e-004	6.1709015e-004
6.0204050e-004	5.7868414e-004	6.6274265e-004	6.0910934e-004
5.7642032e-004	6.2598629e-004	5.7142806e-004	5.8631577e-004
5.3994084e-004	6.1674516e-004	5.3745057e-004	5.2175524e-004
6.3625720e-004	5.5820246e-004	6.0950824e-004	6.2386505e-004
6.0425383e-004	5.5751322e-004	5.7179123e-004	5.3789295e-004
4.9545521e-004	5.0121982e-004	4.9266504e-004	4.9599742e-004
5.0490474e-004	5.0406909e-004	5.1262190e-004	4.7356815e-004

5.3683766e-004	5.1384747e-004	5.3666369e-004	5.2239114e-004
5.0129402e-004	5.4929945e-004	6.1626542e-004	5.7704801e-004
6.5072664e-004	6.5146177e-004	7.1385373e-004	6.8670641e-004
6.2349101e-004	6.9428957e-004	6.3705225e-004	6.0649238e-004
5.7643122e-004	5.8338654e-004	6.4037350e-004	5.8593570e-004
5.8349637e-004	5.7774423e-004	5.6388546e-004	5.9582930e-004
6.9398966e-004	6.5923833e-004	6.7374851e-004	6.2932755e-004
6.7601893e-004	7.5869294e-004	7.1046017e-004	7.5895817e-004
7.0557599e-004	6.7112048e-004	6.4495407e-004	6.5183530e-004
6.5213199e-004	6.4714358e-004	6.7198124e-004	5.8813285e-004
6.4100180e-004	6.1570577e-004	5.8973714e-004	5.8639164e-004
5.7143487e-004	5.8241420e-004	5.5904551e-004	5.3779021e-004
5.6407051e-004	5.5070312e-004	5.5891132e-004	5.5482425e-004
5.6041746e-004	5.5238858e-004	5.8529860e-004	5.7199210e-004
5.3313673e-004	5.1994149e-004	5.7448682e-004	6.2955938e-004
6.2247508e-004	6.4985885e-004	6.3284335e-004	6.1879831e-004
5.9267240e-004	6.0442094e-004	5.8275892e-004	5.9155974e-004
5.8845428e-004	5.7122315e-004	6.0630646e-004	5.2658260e-004
5.1224745e-004	5.7751109e-004	5.5160205e-004	5.2525250e-004
5.0972935e-004	4.9012015e-004	5.3774245e-004	5.2563468e-004
5.0949975e-004	5.1725579e-004	5.7374871e-004	6.2282772e-004
5.9803952e-004	6.2897912e-004	6.7166604e-004	6.5927843e-004
6.1728542e-004	6.9210159e-004	6.4305188e-004	5.9880716e-004
6.1502201e-004	6.0746376e-004	6.0347532e-004	5.7889853e-004
6.0421633e-004	5.9911676e-004	6.2585244e-004	6.1687616e-004
5.4664476e-004	5.7004720e-004	5.6180955e-004	5.6761458e-004
5.3413241e-004	5.4788543e-004	6.0609354e-004	6.3891242e-004
6.5523494e-004	6.7261443e-004	6.7212964e-004	6.1123745e-004
6.0726910e-004	5.9003333e-004	5.4563952e-004	6.0785048e-004
6.0740521e-004	5.9752744e-004	5.3434246e-004	5.6187717e-004
5.5964840e-004	6.1705129e-004	5.6354950e-004	5.1050590e-004
5.4684814e-004	5.5831240e-004	5.8923554e-004	5.4268297e-004
5.3518394e-004	6.1284957e-004	6.2563783e-004	6.8058383e-004
6.6752850e-004	6.5722874e-004	7.1548586e-004	6.0868861e-004
6.3388645e-004	6.1316116e-004	6.0870236e-004	6.3311792e-004
5.9460185e-004	6.0851872e-004	6.4872381e-004	6.0250128e-004
5.7006245e-004	6.4168277e-004	5.7702088e-004	5.9070633e-004
5.6224839e-004	6.0653427e-004	5.3740120e-004	5.3167465e-004
6.3010685e-004	6.7558316e-004	7.2036502e-004	6.7291899e-004
6.2701787e-004	6.5469868e-004	6.0883230e-004	6.0365383e-004
5.9487665e-004	5.5551052e-004	6.1564628e-004	5.8815008e-004
5.6005301e-004	5.6137535e-004	5.3380235e-004	5.8744617e-004
6.6411168e-004	6.4490046e-004	6.6166779e-004	5.9956325e-004
6.7104091e-004	7.1191129e-004	6.8109787e-004	7.1968829e-004
5.2647885e-004	5.2387158e-004	5.6646887e-004	6.2600088e-004
6.1661305e-004	6.4366678e-004	6.5004759e-004	6.4928284e-004
6.0293818e-004	6.6375468e-004	6.0572011e-004	6.0441355e-004
5.9839037e-004	6.0750279e-004	6.0961922e-004	5.4428350e-004
5.3556850e-004	5.6850251e-004	5.7569111e-004	5.9246643e-004
5.3102527e-004	5.1696922e-004	5.7433265e-004	5.4903790e-004
5.3414675e-004	5.8609298e-004	5.9384467e-004	6.7691439e-004
6.9513080e-004	6.8067784e-004	6.9877128e-004	6.5360867e-004

6.3197761e-004	6.1561064e-004	5.8062718e-004	6.6652749e-004
6.2950520e-004	6.5555894e-004	6.0339524e-004	6.1129344e-004
5.8296013e-004	6.3553009e-004	5.7453111e-004	5.7320570e-004
5.6804719e-004	5.9895667e-004	5.7836753e-004	5.5799264e-004
5.4162140e-004	5.8434079e-004	6.5385510e-004	6.8255764e-004
5.8397211e-004	6.3154908e-004	7.2879181e-004	6.0422497e-004
6.3404447e-004	6.3174011e-004	6.6197706e-004	5.7485839e-004
5.7395146e-004	6.2389360e-004	5.9572246e-004	5.7068103e-004
6.1294873e-004	6.4949671e-004	5.6846405e-004	5.8539376e-004
5.7150681e-004	5.6151293e-004	5.4113514e-004	5.3971294e-004
7.8738498e-004	8.5087995e-004	8.4428168e-004	7.8423052e-004
7.8509338e-004	8.0692746e-004	7.7937749e-004	8.1404165e-004
8.1257196e-004	7.1679002e-004	7.5544957e-004	7.4880759e-004
7.1996677e-004	7.6347006e-004	7.0122779e-004	7.4266712e-004
7.8899320e-004	7.9868259e-004	8.4776043e-004	7.5597929e-004
8.1693022e-004	8.2188344e-004	7.8581658e-004	8.9998943e-004
6.7820335e-004	6.5721167e-004	6.3005820e-004	6.4396379e-004
6.1940834e-004	6.4037391e-004	6.5989979e-004	5.7485761e-004
6.4206262e-004	5.9600451e-004	5.7117292e-004	5.8186006e-004
5.6316412e-004	5.7512217e-004	5.4452111e-004	5.4081966e-004
5.5703962e-004	5.7062575e-004	5.5014893e-004	5.4096987e-004
5.4730717e-004	5.4289813e-004	5.1871128e-004	5.7442452e-004
5.3633325e-004	5.1696431e-004	5.8491871e-004	6.3089642e-004
6.4834053e-004	6.5863144e-004	6.7925626e-004	6.8752619e-004
6.2815716e-004	6.2250516e-004	6.1906285e-004	6.2143806e-004
6.0092333e-004	5.8991706e-004	6.3350005e-004	5.8409949e-004
5.4329635e-004	5.7305797e-004	5.5701306e-004	5.5113986e-004
5.4473797e-004	5.0539275e-004	5.7331847e-004	5.6052698e-004
5.1683666e-004	5.4325751e-004	5.4487017e-004	6.5727632e-004
6.5194849e-004	6.2991308e-004	6.4498349e-004	6.3257299e-004
6.2970122e-004	5.8115067e-004	5.4450618e-004	6.4608615e-004
6.1102631e-004	6.3773079e-004	5.5002111e-004	5.5377162e-004
5.5644134e-004	6.1893823e-004	5.1979937e-004	5.2998305e-004
5.4022456e-004	5.2573610e-004	5.8680211e-004	5.4296805e-004
5.2990759e-004	5.6797017e-004	6.3627632e-004	6.5941831e-004
6.5186928e-004	6.7197310e-004	7.2971007e-004	6.4631787e-004
6.5310860e-004	6.4228965e-004	6.5691693e-004	6.4776644e-004
6.0626891e-004	6.3416915e-004	6.0646978e-004	5.7004328e-004
6.1388165e-004	6.7327765e-004	5.8516786e-004	5.8901713e-004
5.8309938e-004	5.9803092e-004	5.3616389e-004	5.5919986e-004
5.7213507e-004	6.6328692e-004	6.5624698e-004	6.3665121e-004
6.1287121e-004	6.0560515e-004	5.6078391e-004	5.8781009e-004
5.6893748e-004	4.9327061e-004	5.4184423e-004	5.3880777e-004
5.0888878e-004	5.2548547e-004	5.1857663e-004	5.6066464e-004
5.7480371e-004	5.8997757e-004	5.9688462e-004	5.8053208e-004
6.1950491e-004	6.5207163e-004	6.0890260e-004	6.4823274e-004
8.0064978e-004	8.6027895e-004	8.4858968e-004	8.1274884e-004
8.0991305e-004	8.8033197e-004	8.3039062e-004	8.2612237e-004
8.6312336e-004	7.6438302e-004	7.6013988e-004	7.6287803e-004
7.5919245e-004	7.7689801e-004	7.2402781e-004	7.1662823e-004
7.7908077e-004	7.8343475e-004	8.1083919e-004	7.2013120e-004
7.6047266e-004	7.8573293e-004	7.5137736e-004	8.4954212e-004

5.4231165e-004	5.2264376e-004	5.7357713e-004	6.3233254e-004
6.3762142e-004	6.6122047e-004	6.8941823e-004	7.0488300e-004
6.3511480e-004	6.4823694e-004	6.3340044e-004	6.2459506e-004
6.0119785e-004	6.1155438e-004	6.1774084e-004	6.0072949e-004
5.6506635e-004	5.6748337e-004	5.8137106e-004	5.7101125e-004
5.5947872e-004	5.3009477e-004	5.9568754e-004	5.7873552e-004
5.1389405e-004	5.2840018e-004	5.1708415e-004	6.4024875e-004
6.6330780e-004	6.0145072e-004	6.4670030e-004	6.4141663e-004
6.1079959e-004	6.0828993e-004	5.8379643e-004	6.3206755e-004
6.1103221e-004	6.5698588e-004	5.6139607e-004	5.6321186e-004
5.8048982e-004	6.2448140e-004	5.1454663e-004	5.6802208e-004
5.4249287e-004	5.3202933e-004	5.7518159e-004	5.5775279e-004
5.2730781e-004	5.4705270e-004	6.4277490e-004	6.5549389e-004
6.4069850e-004	6.4719169e-004	7.2045629e-004	6.4802206e-004
6.4259798e-004	6.6365709e-004	6.8370793e-004	6.2323162e-004
6.1837598e-004	5.9029294e-004	5.7294365e-004	5.2094656e-004
6.4713655e-004	6.5108789e-004	5.8923256e-004	5.7351087e-004
5.7519582e-004	5.8809026e-004	5.2972320e-004	5.5951178e-004
6.4085603e-004	7.5213739e-004	7.0324572e-004	7.2901229e-004
7.2435045e-004	6.9873042e-004	6.3111912e-004	6.9030822e-004
6.2099609e-004	5.4822121e-004	5.7691167e-004	5.8197956e-004
5.8175954e-004	5.6761102e-004	5.8505108e-004	6.1932484e-004
6.3710443e-004	6.2444273e-004	6.6829794e-004	6.1383167e-004
6.5708248e-004	7.1519079e-004	6.5602275e-004	7.2669439e-004
7.5288888e-004	8.4414137e-004	8.0743989e-004	8.3634240e-004
8.1465147e-004	8.6567088e-004	8.4839910e-004	7.7276689e-004
8.3853652e-004	7.5938902e-004	7.5520779e-004	7.4131902e-004
7.6780948e-004	7.2992541e-004	7.2694781e-004	6.8221270e-004
7.5816507e-004	7.6337577e-004	8.0132192e-004	6.8112194e-004
7.1573556e-004	7.7123834e-004	7.4498955e-004	7.8874842e-004
5.9902178e-004	6.2830682e-004	5.9301437e-004	6.2981335e-004
5.8250455e-004	5.7928298e-004	6.0314298e-004	5.3441076e-004
5.5401842e-004	5.3148642e-004	5.1764060e-004	5.2304333e-004
5.0930882e-004	5.1107410e-004	5.2044078e-004	5.0029391e-004
5.1025992e-004	5.1703366e-004	5.0120140e-004	4.9210590e-004
5.0643311e-004	5.0556950e-004	4.8994938e-004	5.2346879e-004
5.4450502e-004	5.2544747e-004	5.6365508e-004	6.3032878e-004
6.2723724e-004	6.5175297e-004	6.9089775e-004	7.0230186e-004
6.4515921e-004	6.5429698e-004	6.4689589e-004	6.2006887e-004
5.9191979e-004	6.2225960e-004	6.1340556e-004	6.1039024e-004
5.7621421e-004	5.6834849e-004	5.9635045e-004	5.7540345e-004
5.7232121e-004	5.4747677e-004	5.9902790e-004	5.9863181e-004
4.2952522e-004	4.2143003e-004	4.1004728e-004	4.2459164e-004
4.6144488e-004	5.1502850e-004	4.8796410e-004	4.8817154e-004
5.0067966e-004	5.2835807e-004	4.9325125e-004	5.5628659e-004
4.5924899e-004	5.2187002e-004	4.9325995e-004	4.9609756e-004
5.0329573e-004	5.4477711e-004	5.1825555e-004	5.4813201e-004
5.2452741e-004	5.2741363e-004	5.6687949e-004	5.4754507e-004
5.2127758e-004	5.2427777e-004	6.1493587e-004	6.3506160e-004
6.6189059e-004	6.2830810e-004	6.9455605e-004	6.4422318e-004
6.3343858e-004	6.6433918e-004	6.7176072e-004	6.1213769e-004
6.2438370e-004	5.6712473e-004	5.6161432e-004	5.0798366e-004

6.3969106e-004	5.9341275e-004	5.8721101e-004	5.6347444e-004
5.6465008e-004	5.7780232e-004	5.2871200e-004	5.5817878e-004
6.3561673e-004	7.2155482e-004	6.5324568e-004	7.0724967e-004
7.3000073e-004	6.5932242e-004	6.0814746e-004	6.4711925e-004
5.7701510e-004	5.4462341e-004	5.6013792e-004	5.7030757e-004
5.6912577e-004	5.3931895e-004	6.0305691e-004	5.9742549e-004
6.1313063e-004	6.0186694e-004	6.4861441e-004	6.0541420e-004
6.2717540e-004	6.8551242e-004	6.3311106e-004	7.1946418e-004
7.6482311e-004	8.7181885e-004	8.0332754e-004	8.7000463e-004
8.5966535e-004	8.3690687e-004	8.9251158e-004	7.6114884e-004
8.2575086e-004	7.6106712e-004	7.6180970e-004	7.4706992e-004
8.0132668e-004	7.1352528e-004	7.5623264e-004	7.0416532e-004
7.6886696e-004	7.8242699e-004	8.3687644e-004	6.9484906e-004
7.4116548e-004	7.8041355e-004	7.7692312e-004	8.0201928e-004
5.9847512e-004	6.4183537e-004	6.0582979e-004	6.4248471e-004
5.9639060e-004	5.8893699e-004	6.1200505e-004	5.4383383e-004
5.5706946e-004	5.2960479e-004	5.2858971e-004	5.2961565e-004
5.1849028e-004	5.1513391e-004	5.2245770e-004	5.0018282e-004
5.1327356e-004	5.1627798e-004	5.0426456e-004	5.0375528e-004
5.1284673e-004	5.1815419e-004	5.0921609e-004	5.3691540e-004
4.1661865e-004	4.3209924e-004	4.1776874e-004	4.1147956e-004
4.4609427e-004	4.9230451e-004	4.8681954e-004	4.8663362e-004
4.8411315e-004	5.0366320e-004	4.8522305e-004	5.1028843e-004
4.4709706e-004	5.0874544e-004	4.7699286e-004	4.9759398e-004
5.0076869e-004	5.5203114e-004	5.1603685e-004	5.6294708e-004
5.1943487e-004	5.1778273e-004	5.6793694e-004	5.6556625e-004
5.1591055e-004	5.1268767e-004	5.8633689e-004	6.1775316e-004
6.6647358e-004	6.0943396e-004	6.6694111e-004	6.3087532e-004
6.2462923e-004	6.5584856e-004	6.3293203e-004	5.9613716e-004
6.0493982e-004	5.5459511e-004	5.5531348e-004	5.1423820e-004
6.0297039e-004	5.3487603e-004	5.7451839e-004	5.5404365e-004
5.6037078e-004	5.5688263e-004	5.2996671e-004	5.5691115e-004
6.5029191e-004	6.8225026e-004	6.2887052e-004	6.7726288e-004
7.1508138e-004	6.0530707e-004	5.8086813e-004	6.0653991e-004
5.4987802e-004	5.5484277e-004	5.4507077e-004	5.6169865e-004
5.5177539e-004	5.2662208e-004	6.0851469e-004	5.6761535e-004
5.9703512e-004	5.8821162e-004	6.3634764e-004	5.9026649e-004
6.0633036e-004	6.5255034e-004	6.2004413e-004	7.1139878e-004
7.6879984e-004	8.5248519e-004	7.7881101e-004	8.4279568e-004
8.6752675e-004	7.6766423e-004	8.9383015e-004	7.2914759e-004
7.8464096e-004	7.4240277e-004	7.2603895e-004	7.2116045e-004
7.9472191e-004	6.7916693e-004	7.5301327e-004	7.0889083e-004
7.5330537e-004	7.7324633e-004	8.2472168e-004	6.9156555e-004
7.4799656e-004	7.4721755e-004	7.7451633e-004	7.9957354e-004
5.9678021e-004	6.5300359e-004	6.1885105e-004	6.5437313e-004
6.1225070e-004	6.0074535e-004	6.2262412e-004	5.5649762e-004
5.6295762e-004	5.3625713e-004	5.3931431e-004	5.3629509e-004
5.2831139e-004	5.1906006e-004	5.2321624e-004	5.0228056e-004
5.1822824e-004	5.1562442e-004	5.1126314e-004	5.1882191e-004
5.2430099e-004	5.3323258e-004	5.2815430e-004	5.5261017e-004
5.9539903e-004	5.9762223e-004	6.3936393e-004	6.5132856e-004
6.2108123e-004	6.1993567e-004	6.1528278e-004	5.8107692e-004

5.5584848e-004	5.4734379e-004	5.6491226e-004	5.6797298e-004
5.1211016e-004	5.2527842e-004	5.4686630e-004	5.2879690e-004
5.4343179e-004	5.3554580e-004	5.1520362e-004	5.2048097e-004
4.9617568e-004	5.1395463e-004	5.2296532e-004	5.2621795e-004
4.8915517e-004	5.1701655e-004	5.5004934e-004	5.9545349e-004
6.1247061e-004	6.5871284e-004	6.7366924e-004	6.5041591e-004
6.1005188e-004	6.7488533e-004	5.9803539e-004	6.5228331e-004
6.4710568e-004	5.9501905e-004	5.7268601e-004	5.7134737e-004
5.9122415e-004	5.8545677e-004	6.0530790e-004	6.2190942e-004
5.8228493e-004	5.7633287e-004	5.8616711e-004	5.9794635e-004
4.7527477e-004	4.6825948e-004	5.5154548e-004	5.6448236e-004
5.7618786e-004	5.8528527e-004	6.1575420e-004	6.1925531e-004
6.3230735e-004	6.3298401e-004	6.1071981e-004	6.1690010e-004
5.9904291e-004	5.6195732e-004	6.1026168e-004	6.2648336e-004
6.0123105e-004	6.2150041e-004	5.8440422e-004	5.8926117e-004
6.5867568e-004	6.1323500e-004	6.0984858e-004	6.1029813e-004
4.1644273e-004	4.4735712e-004	4.3882388e-004	4.2382047e-004
4.5193319e-004	4.8066413e-004	5.0177791e-004	4.9825126e-004
4.9288955e-004	4.8685309e-004	4.9435299e-004	4.8614091e-004
4.3856071e-004	5.0826353e-004	4.7137060e-004	5.0360156e-004
5.0524898e-004	5.4628933e-004	5.2127012e-004	5.8246082e-004
5.1625070e-004	5.0703048e-004	5.6013573e-004	5.7445691e-004
6.6775782e-004	6.4433822e-004	6.2690048e-004	6.5014797e-004
6.8492255e-004	5.6259579e-004	5.6315995e-004	5.7693558e-004
5.4053609e-004	5.6458121e-004	5.3129245e-004	5.4718494e-004
5.3762352e-004	5.2746850e-004	5.9126040e-004	5.3769938e-004
5.8788309e-004	5.8335345e-004	6.2929462e-004	5.7661452e-004
5.9535213e-004	6.2800647e-004	6.1101645e-004	6.9672789e-004
8.1171149e-004	8.4784787e-004	7.9519737e-004	8.3751732e-004
8.9673564e-004	7.6191353e-004	9.1007176e-004	7.4702872e-004
7.9889696e-004	7.6616609e-004	7.2470029e-004	7.3093594e-004
8.0556043e-004	6.8518266e-004	7.7356466e-004	7.4437668e-004
7.6381819e-004	8.0443370e-004	8.2583133e-004	7.1970011e-004
7.8980313e-004	7.6458692e-004	8.0117212e-004	8.3959963e-004
5.9771432e-004	6.5761986e-004	6.2844713e-004	6.6470716e-004
6.2858412e-004	6.1419273e-004	6.3062007e-004	5.7283715e-004
5.7205244e-004	5.5315332e-004	5.4985730e-004	5.4624332e-004
5.4156969e-004	5.2920154e-004	5.3534879e-004	5.1375873e-004
5.2788817e-004	5.2508471e-004	5.2691202e-004	5.3811898e-004
5.4312045e-004	5.5247142e-004	5.4705641e-004	5.7017668e-004
5.8777588e-004	5.9633878e-004	6.3811541e-004	6.4950870e-004
6.3046654e-004	6.2115656e-004	6.2295786e-004	5.7580293e-004
5.6322108e-004	5.5856217e-004	5.6734504e-004	5.7567061e-004
5.2383495e-004	5.2262914e-004	5.4941329e-004	5.3565309e-004
5.4473798e-004	5.2871695e-004	5.2066203e-004	5.1819861e-004
4.9230509e-004	5.0616160e-004	5.1501401e-004	5.2446385e-004
4.7700933e-004	4.9848860e-004	5.5347814e-004	6.0010109e-004
6.1538059e-004	6.4142329e-004	6.7800541e-004	6.4109731e-004
6.0190800e-004	6.7029899e-004	5.9908215e-004	6.3916606e-004
6.6270214e-004	5.9452748e-004	5.7432956e-004	5.9249335e-004
5.9792737e-004	5.8354747e-004	5.9991090e-004	6.1562104e-004
5.8612936e-004	5.7132391e-004	5.8674037e-004	5.8801760e-004

4.6768429e-004	4.5723998e-004	5.4157771e-004	5.6836722e-004
5.6245027e-004	5.7116141e-004	6.1259764e-004	6.0715352e-004
6.1497602e-004	6.4322213e-004	6.3013050e-004	6.2026388e-004
6.0330119e-004	5.7108037e-004	6.0076696e-004	6.2186107e-004
6.1963606e-004	6.2850237e-004	5.9691114e-004	5.8949692e-004
6.7524629e-004	6.1963531e-004	6.1018053e-004	6.0960687e-004
6.7218060e-004	6.1873879e-004	6.2958608e-004	6.3676435e-004
6.5337603e-004	5.4373109e-004	5.6590262e-004	5.5976730e-004
5.4340252e-004	5.6165052e-004	5.2594913e-004	5.3269535e-004
5.3508139e-004	5.3802827e-004	5.6423157e-004	5.2247949e-004
5.8487926e-004	5.8469770e-004	6.2934318e-004	5.7474010e-004
5.9281979e-004	6.1874947e-004	6.0852102e-004	6.7825499e-004
7.7378750e-004	7.8530220e-004	7.3799230e-004	7.6570749e-004
8.3761757e-004	7.3768866e-004	8.2364414e-004	7.1922076e-004
7.4560005e-004	7.2812523e-004	6.7700136e-004	6.8538045e-004
7.6164193e-004	6.5306074e-004	7.4655961e-004	7.0603972e-004
7.2226470e-004	8.0100940e-004	7.9017953e-004	7.0852225e-004
7.6598498e-004	7.6914599e-004	7.9210846e-004	8.2682088e-004
6.0091422e-004	6.1932269e-004	6.5491238e-004	6.6074215e-004
6.6054368e-004	6.4513729e-004	6.4354707e-004	5.9836631e-004
5.8224773e-004	5.8523190e-004	5.8338341e-004	5.9527151e-004
5.6539325e-004	5.4151750e-004	5.6443349e-004	5.6242355e-004
5.5544327e-004	5.4637183e-004	5.4572165e-004	5.3209164e-004
5.0798651e-004	5.1222352e-004	5.2811499e-004	5.2880854e-004
4.6526391e-004	4.7008933e-004	5.5269226e-004	5.9794901e-004
5.6694777e-004	5.9375874e-004	6.4206969e-004	6.1575207e-004
6.0411055e-004	6.6901381e-004	6.6558649e-004	6.4625686e-004
6.3469479e-004	6.0519537e-004	5.8400551e-004	6.3271834e-004
6.4183817e-004	6.3299552e-004	6.0498364e-004	6.0809433e-004
6.7146451e-004	6.1101653e-004	6.0374086e-004	6.0746577e-004
3.8482442e-004	3.6446955e-004	3.6360584e-004	3.8748597e-004
3.9781185e-004	3.6378688e-004	3.7102771e-004	3.7679361e-004
3.7658331e-004	4.3054906e-004	4.4331126e-004	4.4201050e-004
4.3868311e-004	4.1112513e-004	4.7741667e-004	4.5258035e-004
4.5884292e-004	4.5871139e-004	5.0587153e-004	4.7885496e-004
5.1995379e-004	4.7675234e-004	5.0523920e-004	5.2558845e-004
4.7870754e-004	5.4906926e-004	5.7029033e-004	6.5010486e-004
6.1492836e-004	6.3468698e-004	6.6041427e-004	5.9160871e-004
6.3149301e-004	6.0525678e-004	5.1638896e-004	5.8237591e-004
5.5686236e-004	5.6951902e-004	5.2734048e-004	5.0959995e-004
5.2592870e-004	5.2570499e-004	5.4988300e-004	5.2931314e-004
5.3829838e-004	5.1109506e-004	5.2755582e-004	5.4248054e-004
6.9622285e-004	6.9405601e-004	6.5743008e-004	6.8841792e-004
7.1990884e-004	6.4937705e-004	6.4986589e-004	6.0795498e-004
5.9441445e-004	6.0282620e-004	5.4798667e-004	5.6244651e-004
6.2669293e-004	5.5431681e-004	6.1490311e-004	5.6700545e-004
6.0435328e-004	6.6193864e-004	6.6457946e-004	6.1918616e-004
6.2092170e-004	7.0639579e-004	6.9453540e-004	7.3009929e-004
4.6879589e-004	4.8427845e-004	5.5204297e-004	5.6995932e-004
5.7698021e-004	5.8671442e-004	6.4866735e-004	5.9393269e-004
5.7817207e-004	6.5417732e-004	6.3502660e-004	6.3996844e-004
6.3574720e-004	6.0791225e-004	5.6250373e-004	6.1352871e-004

5.9902664e-004	6.0240172e-004	5.7585270e-004	5.8715428e-004
6.0364483e-004	5.6238235e-004	5.7604945e-004	5.6565902e-004
4.1375029e-004	4.0429652e-004	4.2919391e-004	5.0538428e-004
4.6624201e-004	4.7086090e-004	5.0251722e-004	4.9231382e-004
4.9719221e-004	5.6493017e-004	5.8093307e-004	5.7485369e-004
5.4866579e-004	5.5009426e-004	5.4520536e-004	5.6084518e-004
6.0835959e-004	5.6771761e-004	6.2632025e-004	5.6703278e-004
6.2917304e-004	5.8594340e-004	5.8041363e-004	6.1669093e-004
3.5453091e-004	3.4352419e-004	3.3268343e-004	3.6682612e-004
3.5418499e-004	3.2935890e-004	3.1226073e-004	3.1668766e-004
3.0861291e-004	3.3604227e-004	3.7684954e-004	3.5794330e-004
3.9097502e-004	3.4682550e-004	4.1065154e-004	3.7676560e-004
3.8193203e-004	3.8501008e-004	4.6355313e-004	4.4170975e-004
4.6629789e-004	4.2438919e-004	4.5221196e-004	4.6832419e-004
4.4466949e-004	4.5980780e-004	5.0584312e-004	5.1408004e-004
5.4363949e-004	5.2127898e-004	6.4077834e-004	5.3196217e-004
5.6306645e-004	5.2693332e-004	5.4733292e-004	5.4159958e-004
4.9009133e-004	5.2697073e-004	5.5000394e-004	5.3687704e-004
5.7027371e-004	5.8913232e-004	5.4309065e-004	6.2875797e-004
5.4823482e-004	5.1501929e-004	5.3863681e-004	5.7250254e-004
6.1431027e-004	5.8494853e-004	5.8990571e-004	6.2949304e-004
6.0101404e-004	5.3473686e-004	5.6875240e-004	5.3036324e-004
5.2981315e-004	5.0533508e-004	5.1159327e-004	5.0224747e-004
5.3361089e-004	5.3536208e-004	5.2427400e-004	5.2510918e-004
5.5145097e-004	5.6977324e-004	6.1041611e-004	5.5853340e-004
5.7129361e-004	6.2270103e-004	6.1261635e-004	6.5783144e-004
4.1530217e-004	4.3212291e-004	4.5124887e-004	5.3234658e-004
4.7365879e-004	4.9279199e-004	5.3056595e-004	5.0513167e-004
5.0379643e-004	5.8475841e-004	6.1074215e-004	6.0035924e-004
5.7362577e-004	5.8320047e-004	5.4811919e-004	5.8786564e-004
6.2922755e-004	5.8555794e-004	6.2163423e-004	5.8519687e-004
6.3398411e-004	5.7752118e-004	6.0199572e-004	6.0897373e-004
3.5579058e-004	3.5102291e-004	3.4157332e-004	3.7344291e-004
3.6984679e-004	3.5192534e-004	3.3032795e-004	3.3902127e-004
3.5878701e-004	3.9084915e-004	4.3050275e-004	4.0008676e-004
4.2855619e-004	4.0112918e-004	4.2748690e-004	4.2594269e-004
4.4506512e-004	4.3544514e-004	5.3322611e-004	4.8819826e-004
4.9365815e-004	4.8548458e-004	4.7810545e-004	5.2791677e-004
5.6280979e-004	6.0691306e-004	6.0846946e-004	6.2889648e-004
5.9920541e-004	5.8006016e-004	5.6121573e-004	5.2723128e-004
5.1023523e-004	5.5929884e-004	4.7664229e-004	4.8377076e-004
5.4115290e-004	4.9957080e-004	5.0954238e-004	4.9653907e-004
4.9047644e-004	5.6524143e-004	5.6340908e-004	5.6479790e-004
5.3793186e-004	6.4795624e-004	6.0074679e-004	6.5192024e-004
6.6240049e-004	6.5469141e-004	7.3950413e-004	7.1392870e-004
7.5840091e-004	7.2218466e-004	7.3613833e-004	7.4653723e-004
7.1410494e-004	6.9626555e-004	7.8666330e-004	7.4324070e-004
7.1646084e-004	7.4576180e-004	7.7366436e-004	7.6225969e-004
7.1999634e-004	7.8861577e-004	8.3111305e-004	8.0995701e-004
7.7738009e-004	7.8768400e-004	7.9785835e-004	8.6028493e-004
4.1790263e-004	4.5553980e-004	4.7320867e-004	5.2189053e-004
4.7009004e-004	5.0041186e-004	5.3446454e-004	5.1675765e-004

5.0104482e-004 5.9232152e-004 6.0343705e-004 5.8775635e-004
5.7094246e-004 5.7247600e-004 5.5585416e-004 5.8713538e-004
6.0684246e-004 5.8750183e-004 5.8736026e-004 5.7346166e-004
6.0922079e-004 5.5717156e-004 6.0139844e-004 5.7734810e-004
3.5952176e-004 3.5672341e-004 3.6768844e-004 4.0283103e-004
3.8510315e-004 3.7596832e-004 3.5982793e-004 3.7100386e-004
3.9766638e-004 4.2813906e-004 4.6411998e-004 4.3773306e-004
4.5968036e-004 4.4713965e-004 4.4335320e-004 4.8240125e-004
5.0340275e-004 4.8328558e-004 5.6660722e-004 5.2964345e-004
5.0723651e-004 5.2971427e-004 5.0606489e-004 5.4569548e-004
3.2175570e-004 3.6420665e-004 3.1182479e-004 3.9151525e-004
3.4763092e-004 3.3985596e-004 3.4215781e-004 3.1956193e-004
3.0694946e-004 3.0707711e-004 2.9692272e-004 3.5438390e-004
3.4736542e-004 3.2942180e-004 3.7402071e-004 3.5306755e-004
3.5977751e-004 3.5044283e-004 4.2693053e-004 4.1977800e-004
4.0789546e-004 4.1785660e-004 4.4652566e-004 4.4216328e-004
3.9231464e-004 3.9347432e-004 3.7816909e-004 4.0170472e-004
3.4033663e-004 3.4028942e-004 3.6745515e-004 2.7464233e-004
2.8695903e-004 3.1572310e-004 2.9136074e-004 2.6367236e-004
3.2022842e-004 2.8449445e-004 2.5910500e-004 2.5128777e-004
2.9730075e-004 2.8844667e-004 3.0575859e-004 3.1146313e-004
2.5474481e-004 2.7665973e-004 2.5873269e-004 2.8435397e-004
5.0287956e-004 5.4156563e-004 5.9459565e-004 6.6800797e-004
6.4586807e-004 6.3569634e-004 6.6801472e-004 6.3879263e-004
6.7905791e-004 6.4757276e-004 5.6315718e-004 5.9848886e-004
5.8212937e-004 6.2489002e-004 5.7818886e-004 5.6533725e-004
5.8840332e-004 6.2996976e-004 5.8409144e-004 5.8887331e-004
5.8734820e-004 5.4903670e-004 5.4824265e-004 5.7008550e-004
5.2209450e-004 5.3522273e-004 5.7698993e-004 5.9046299e-004
5.2086271e-004 5.1781260e-004 5.0738129e-004 4.6568768e-004
4.8199815e-004 4.7874375e-004 4.5069306e-004 4.5483818e-004
4.9228984e-004 4.9062155e-004 4.8667540e-004 4.9844863e-004
4.7952880e-004 5.1835426e-004 5.4702139e-004 5.3011434e-004
5.2644526e-004 6.2194013e-004 5.8743056e-004 6.2026229e-004
6.7717175e-004 6.9119534e-004 7.9366022e-004 7.6054691e-004
8.1182874e-004 7.7488898e-004 7.6520297e-004 7.7204421e-004
7.6204297e-004 7.4094391e-004 8.2051737e-004 7.6905507e-004
7.5848339e-004 7.5813293e-004 8.1642373e-004 8.3197550e-004
7.6932361e-004 8.4456491e-004 9.0918170e-004 8.7879213e-004
8.3078133e-004 8.6133051e-004 8.8138200e-004 9.5146864e-004
4.1823016e-004 4.4477647e-004 4.8136688e-004 4.9862509e-004
4.6136454e-004 4.9557293e-004 5.3507739e-004 5.3954181e-004
5.1785247e-004 6.0655805e-004 5.9427637e-004 5.8899788e-004
5.6424025e-004 5.7146267e-004 5.7182949e-004 5.8016925e-004
5.8617150e-004 6.0034024e-004 5.6524659e-004 5.7381442e-004
5.8663536e-004 5.5719225e-004 5.7790724e-004 5.6811811e-004
3.6010277e-004 3.6114335e-004 3.8446682e-004 4.3121629e-004
3.9297381e-004 4.0003442e-004 3.7527260e-004 3.9398683e-004
4.1200042e-004 4.4192276e-004 4.6491688e-004 4.5706263e-004
4.8514780e-004 4.7350092e-004 4.6331969e-004 5.1365366e-004
5.3637292e-004 5.0908986e-004 5.6548537e-004 5.4286403e-004
5.1438661e-004 5.3076017e-004 5.4963671e-004 5.2585713e-004

3.0688147e-004	3.6921344e-004	3.2145117e-004	3.8102421e-004
3.5477536e-004	3.4707718e-004	3.3703510e-004	3.0583238e-004
3.1168906e-004	3.3860711e-004	3.1518027e-004	3.4654443e-004
3.7277651e-004	3.4098952e-004	3.7580795e-004	3.8184156e-004
3.9498523e-004	3.7282617e-004	4.6052763e-004	4.4638836e-004
4.0821030e-004	4.4164182e-004	4.4543195e-004	4.6492869e-004
3.9705697e-004	3.9432840e-004	3.8040488e-004	4.0681527e-004
3.3365537e-004	3.5477235e-004	3.6679104e-004	2.9195570e-004
2.7325387e-004	3.2246106e-004	3.0337556e-004	2.7914225e-004
3.1896762e-004	2.8881808e-004	2.6616818e-004	2.4648584e-004
2.9348766e-004	2.9331611e-004	2.9237247e-004	2.8763817e-004
2.7121167e-004	2.7106093e-004	2.7097290e-004	2.7412287e-004
5.4159084e-004	5.4955477e-004	6.0122041e-004	6.1350115e-004
5.3447096e-004	5.3783227e-004	5.4081107e-004	5.0385782e-004
5.1595015e-004	4.8575555e-004	5.0272248e-004	4.7016827e-004
5.1989920e-004	5.3263282e-004	5.3891949e-004	5.6455868e-004
5.4696755e-004	5.5143238e-004	6.0646133e-004	5.6281523e-004
5.9351439e-004	6.2882349e-004	6.2524508e-004	6.5198568e-004
5.3568893e-004	5.6749267e-004	6.4262864e-004	6.1261467e-004
6.1426161e-004	5.8587675e-004	5.5550829e-004	5.3589667e-004
5.5261182e-004	5.4664612e-004	5.3835848e-004	5.3877343e-004
5.2466957e-004	5.0516643e-004	5.3641132e-004	5.8406283e-004
5.3974262e-004	5.6618887e-004	6.2064775e-004	5.9437097e-004
5.6603937e-004	6.3065178e-004	6.3322919e-004	6.8154534e-004
7.0093341e-004	7.2124851e-004	8.0051094e-004	7.9010607e-004
7.9967189e-004	8.2084788e-004	8.0321719e-004	8.3134578e-004
7.9821449e-004	7.6000461e-004	8.3581200e-004	8.3100908e-004
8.2284540e-004	7.9054374e-004	8.8664396e-004	8.7872957e-004
8.8122253e-004	8.8059559e-004	9.1542082e-004	9.4802896e-004
8.7572772e-004	9.1546182e-004	9.2574037e-004	9.8524586e-004
4.4318126e-004	4.5385141e-004	4.9662987e-004	5.1959841e-004
5.1117662e-004	5.0597842e-004	5.8006539e-004	5.7885000e-004
5.7174002e-004	6.1343516e-004	5.8368298e-004	6.0148277e-004
5.5447893e-004	5.9268260e-004	5.7948253e-004	5.6751126e-004
5.8395664e-004	6.0308883e-004	5.5740910e-004	5.7798806e-004
5.5729755e-004	5.2921676e-004	5.5212948e-004	5.3639330e-004
3.8237888e-004	3.8885343e-004	3.9112248e-004	4.4658477e-004
3.9895147e-004	4.3749953e-004	4.1825959e-004	4.3908287e-004
4.3962648e-004	4.8923995e-004	4.9417798e-004	4.9023101e-004
5.1780821e-004	5.1493622e-004	5.1265346e-004	5.2202679e-004
5.7284691e-004	5.4077092e-004	5.5754583e-004	5.3865665e-004
5.5027402e-004	5.4138537e-004	5.9179075e-004	5.4488925e-004
3.2295701e-004	3.5690417e-004	3.6204144e-004	3.8835943e-004
3.6618005e-004	3.7057908e-004	3.2005434e-004	3.1729277e-004
3.3479409e-004	3.8834303e-004	3.6690432e-004	3.5921893e-004
3.9773675e-004	3.6417139e-004	3.9971201e-004	4.3170330e-004
4.3359683e-004	4.1616027e-004	4.8940738e-004	4.8878538e-004
4.3255245e-004	4.6087083e-004	4.5641010e-004	4.5949548e-004
3.8582882e-004	3.6373633e-004	3.6320731e-004	3.9640062e-004
3.0878683e-004	3.1915507e-004	3.3587420e-004	2.7887058e-004
2.6233878e-004	2.8742157e-004	2.6866627e-004	2.4973211e-004
2.6280692e-004	2.6789099e-004	2.2156550e-004	2.2352646e-004

2.6873713e-004	2.6268486e-004	2.7420609e-004	3.0086271e-004
2.8008893e-004	2.5583955e-004	2.5100951e-004	2.5630485e-004
5.3863434e-004	5.5958123e-004	6.0160685e-004	5.9810810e-004
5.3421065e-004	5.0452359e-004	4.8967030e-004	4.6149997e-004
4.7848131e-004	4.8134198e-004	4.4433657e-004	4.5369919e-004
4.4043675e-004	4.4993978e-004	4.6147744e-004	5.0616403e-004
4.6635372e-004	4.7219816e-004	5.2409889e-004	5.0484244e-004
5.1840940e-004	5.8021219e-004	5.5453266e-004	5.7808317e-004
3.9180880e-004	3.8710803e-004	3.8939754e-004	4.2625327e-004
3.8627350e-004	4.3591054e-004	4.3718016e-004	4.4764770e-004
4.4969450e-004	4.9763399e-004	4.9863012e-004	4.9901249e-004
5.1839378e-004	5.1794009e-004	5.2511032e-004	5.1386887e-004
5.5667423e-004	5.4536203e-004	5.5342732e-004	5.3369079e-004
5.6390808e-004	5.3130979e-004	5.5748757e-004	5.5218983e-004
3.2562992e-004	3.5118892e-004	3.6987557e-004	3.9183426e-004
3.6699812e-004	3.7577662e-004	3.0460130e-004	3.3593617e-004
3.4285610e-004	3.7469269e-004	3.7226444e-004	3.6906578e-004
4.0037175e-004	3.7904514e-004	4.1513457e-004	4.2391546e-004
4.4163386e-004	4.2341188e-004	4.7474341e-004	4.7053330e-004
4.3809522e-004	4.4451982e-004	4.6664744e-004	4.4408977e-004
3.8244423e-004	3.7804005e-004	3.6629537e-004	4.0034507e-004
3.2013775e-004	3.1873359e-004	3.4376440e-004	2.9781013e-004
2.7531327e-004	2.8685921e-004	2.7991131e-004	2.6033600e-004
2.7896581e-004	2.6167124e-004	2.2739543e-004	2.2059719e-004
2.7683653e-004	2.7377608e-004	2.6534555e-004	2.8554962e-004
2.8821719e-004	2.3914902e-004	2.5471093e-004	2.4648352e-004
6.9706752e-004	7.3565259e-004	8.2140605e-004	7.7464423e-004
7.8477185e-004	8.4863984e-004	7.8042963e-004	7.7348503e-004
7.9579394e-004	7.4119872e-004	7.5211438e-004	7.8382002e-004
7.9524647e-004	7.3408041e-004	8.5353272e-004	8.4006314e-004
8.7395304e-004	8.1866408e-004	8.7433335e-004	8.7278002e-004
8.6047596e-004	9.2805717e-004	9.1271441e-004	1.0058072e-003
7.3017634e-004	7.5757962e-004	7.8689128e-004	8.3525207e-004
8.1621518e-004	8.3216259e-004	8.2326683e-004	8.0785036e-004
7.8432022e-004	8.0738516e-004	8.0862614e-004	8.3615354e-004
8.4934101e-004	8.0592236e-004	8.7642454e-004	8.3963449e-004
8.6527089e-004	8.6741844e-004	8.5225797e-004	8.7582541e-004
8.5779616e-004	8.7497804e-004	9.0022165e-004	9.6095882e-004
4.0251533e-004	3.9208390e-004	3.9759606e-004	4.2189191e-004
3.8843690e-004	4.3787446e-004	4.6751818e-004	4.4888339e-004
4.7477279e-004	5.0030074e-004	4.9504326e-004	5.2044251e-004
5.2419204e-004	5.2064428e-004	5.2476858e-004	5.0887871e-004
5.3727318e-004	5.5360254e-004	5.5110244e-004	5.3573131e-004
5.5543303e-004	5.0017568e-004	5.1433366e-004	5.4046723e-004
3.2855202e-004	3.5976183e-004	3.6805332e-004	3.9545305e-004
3.6820104e-004	3.7551478e-004	3.1492001e-004	3.5197656e-004
3.4852736e-004	3.5296016e-004	3.7995269e-004	3.7051082e-004
4.0039091e-004	3.9964907e-004	4.2907295e-004	4.0915108e-004
4.6364052e-004	4.3420536e-004	4.7202505e-004	4.3746427e-004
4.5713735e-004	4.4986571e-004	4.8036857e-004	4.5102570e-004
3.6335696e-004	3.7924150e-004	3.6019780e-004	3.9241818e-004
3.3699739e-004	3.3058209e-004	3.4978636e-004	3.2281290e-004

2.8471890e-004	2.8409628e-004	2.8981729e-004	2.6759159e-004
2.8150155e-004	2.5930301e-004	2.4707991e-004	2.4022419e-004
2.7395518e-004	2.7329379e-004	2.7200425e-004	2.7289137e-004
2.8323841e-004	2.4384133e-004	2.7093329e-004	2.5822441e-004
3.8040411e-004	3.1721846e-004	3.8067668e-004	3.7624373e-004
2.9564449e-004	2.6791401e-004	2.9938461e-004	2.8765472e-004
2.8070374e-004	2.8186856e-004	2.6303266e-004	2.5290787e-004
2.4679423e-004	2.5527494e-004	2.2979304e-004	2.3888298e-004
2.4146156e-004	2.4729219e-004	2.6556494e-004	2.6131794e-004
2.7300693e-004	2.4898515e-004	2.6725615e-004	2.4737747e-004
7.1029340e-004	7.3970812e-004	7.6828935e-004	7.4571058e-004
7.4482759e-004	7.7328530e-004	7.2938603e-004	6.7733038e-004
7.2357911e-004	7.1005744e-004	6.7373944e-004	7.0466712e-004
7.2260432e-004	6.7397004e-004	7.8546196e-004	7.4611493e-004
7.8119810e-004	7.5056681e-004	7.8580833e-004	7.6673567e-004
7.9170326e-004	8.7383966e-004	8.3502954e-004	9.6078505e-004
7.6264406e-004	8.0625585e-004	8.4652673e-004	9.2650228e-004
8.9701630e-004	9.1551570e-004	9.0702985e-004	8.9232743e-004
8.7110155e-004	8.9713266e-004	8.8845909e-004	9.4113627e-004
9.5480961e-004	9.0824980e-004	9.8072324e-004	9.4302501e-004
9.5868374e-004	9.7917566e-004	9.6262981e-004	9.7091083e-004
9.7087859e-004	9.7307186e-004	1.0192365e-003	1.1060544e-003
4.0829037e-004	4.0214189e-004	3.9510438e-004	4.2404206e-004
3.9342721e-004	4.3503085e-004	4.6746337e-004	4.2751288e-004
4.6779267e-004	4.6213038e-004	4.6947825e-004	5.0131683e-004
5.0008469e-004	4.9004189e-004	5.0178583e-004	4.9210586e-004
5.0633946e-004	5.3796628e-004	5.4106461e-004	5.1161795e-004
5.2317185e-004	4.6833618e-004	4.8774637e-004	5.1292781e-004
6.3941807e-004	6.2632318e-004	6.0129052e-004	5.6727706e-004
5.9104495e-004	5.5035138e-004	5.1112675e-004	4.6500388e-004
4.7359342e-004	4.7016453e-004	4.5188282e-004	4.1561985e-004
4.5189776e-004	4.3199226e-004	4.7655844e-004	4.6029838e-004
4.7909514e-004	4.2913237e-004	4.5278122e-004	4.5219314e-004
4.9455667e-004	5.0910292e-004	5.0446876e-004	5.2162825e-004
5.3432967e-004	5.7530154e-004	5.7990743e-004	6.1811696e-004
6.2764423e-004	5.9200569e-004	6.1358654e-004	5.7229043e-004
5.7511700e-004	5.3718348e-004	5.7257601e-004	5.3804791e-004
4.9920206e-004	5.1110136e-004	5.0019139e-004	5.0806585e-004
5.0362079e-004	5.2442335e-004	5.0382817e-004	5.0466321e-004
5.1406011e-004	5.1384557e-004	4.7108279e-004	5.0725704e-004
7.1145016e-004	6.7938518e-004	6.4605335e-004	6.6845131e-004
6.5521697e-004	6.1615219e-004	5.6747581e-004	5.1246818e-004
4.9933478e-004	5.0237113e-004	4.8899009e-004	4.4153054e-004
5.0746192e-004	4.7818598e-004	5.2389917e-004	5.1009837e-004
4.9914941e-004	4.6031253e-004	4.6316590e-004	4.9155286e-004
5.3182039e-004	5.2708016e-004	5.4098571e-004	5.4222951e-004
6.8442449e-004	6.8109634e-004	7.5005369e-004	7.5594713e-004
6.9723633e-004	6.8445144e-004	7.0768957e-004	6.1734037e-004
5.8668469e-004	6.0188120e-004	6.2909360e-004	6.2105978e-004
6.3045330e-004	6.1487660e-004	6.4508517e-004	6.4518943e-004
6.8231679e-004	6.4913508e-004	6.6955731e-004	6.8556803e-004
6.7917476e-004	6.2871788e-004	6.8891239e-004	7.1236670e-004

7.0844113e-004	6.7690854e-004	6.5037921e-004	7.2180422e-004
6.6966176e-004	6.5153364e-004	5.9468207e-004	5.2983404e-004
5.0597214e-004	5.0222866e-004	4.9182338e-004	4.4983492e-004
5.3067360e-004	4.8853426e-004	5.3030500e-004	5.3312189e-004
4.8117969e-004	4.7782643e-004	4.6882240e-004	5.0547755e-004
5.4901009e-004	5.2558977e-004	5.4873845e-004	5.4130217e-004
5.3411706e-004	5.5084855e-004	5.5340939e-004	5.9292064e-004
6.0809312e-004	5.8547486e-004	6.0166046e-004	5.5028623e-004
5.5099091e-004	5.0804884e-004	5.3648953e-004	5.0761996e-004
4.7816285e-004	5.0248652e-004	4.9080261e-004	5.0493021e-004
5.1476729e-004	4.8448735e-004	4.8739606e-004	4.8615678e-004
4.7778032e-004	4.7706586e-004	4.6640509e-004	4.6971947e-004
6.8884301e-004	6.8954479e-004	7.1391776e-004	7.3541365e-004
6.7813190e-004	6.6784189e-004	7.0329646e-004	5.7569912e-004
5.6090900e-004	5.5200031e-004	5.7167672e-004	5.8418374e-004
5.9533547e-004	5.6394036e-004	5.9698676e-004	6.1486936e-004
6.2745508e-004	5.9445344e-004	6.3195698e-004	6.4596569e-004
6.2347236e-004	5.9738058e-004	6.4605944e-004	6.6079154e-004
7.3106115e-004	7.1586420e-004	7.7514109e-004	9.1671658e-004
8.6136410e-004	8.2740061e-004	8.2171048e-004	7.5716691e-004
7.4157469e-004	7.3054668e-004	7.1560575e-004	7.2619737e-004
7.8296778e-004	7.2756402e-004	7.8207697e-004	7.9412681e-004
7.4403964e-004	7.5013318e-004	7.7547732e-004	7.8673322e-004
8.4759187e-004	7.3627359e-004	8.8056378e-004	8.4697855e-004
6.8326487e-004	7.1230618e-004	8.0851220e-004	8.5427907e-004
8.8529909e-004	8.8183378e-004	8.9604671e-004	8.8532527e-004
8.5808532e-004	8.5241241e-004	8.4377101e-004	8.7949680e-004
8.4954726e-004	8.8111269e-004	9.1856523e-004	8.7122536e-004
8.8076855e-004	9.3157979e-004	9.3901409e-004	9.4106435e-004
9.5975067e-004	8.7961313e-004	9.4071747e-004	1.0078394e-003
6.6489042e-004	6.8203718e-004	6.5429691e-004	7.0853546e-004
6.6641509e-004	6.7434076e-004	6.7191632e-004	5.4647432e-004
5.1274982e-004	4.9250469e-004	4.9038914e-004	4.9813418e-004
5.2597240e-004	4.8598503e-004	5.1833263e-004	5.5311665e-004
4.9451025e-004	5.2959750e-004	5.4515486e-004	5.4420492e-004
5.4583448e-004	5.2924239e-004	5.5065047e-004	5.4211899e-004
6.0285526e-004	6.4319011e-004	6.9494330e-004	8.3779471e-004
8.2503709e-004	8.1393767e-004	8.1504034e-004	7.6441187e-004
7.8178372e-004	7.6725670e-004	7.1493709e-004	7.2641062e-004
8.0663816e-004	7.4643429e-004	7.9211491e-004	7.4160245e-004
7.5467618e-004	8.0901387e-004	8.2201008e-004	8.2085391e-004
8.7846112e-004	7.4913359e-004	8.5818286e-004	8.7882469e-004
6.5641987e-004	6.9514901e-004	6.6485931e-004	6.8353245e-004
6.4291694e-004	6.3866432e-004	6.6189891e-004	5.5622518e-004
5.3555458e-004	5.4908029e-004	5.3179024e-004	5.6646750e-004
5.5950989e-004	5.3050877e-004	5.9191752e-004	5.8343516e-004
5.9461211e-004	5.6774094e-004	6.2306595e-004	5.9494555e-004
5.5648301e-004	5.7377543e-004	6.1875237e-004	5.9586703e-004
5.9562644e-004	6.3228414e-004	6.1483070e-004	7.3272784e-004
6.6561556e-004	6.7285744e-004	6.6676088e-004	5.4573477e-004
5.3746014e-004	5.6123771e-004	4.7516573e-004	4.6562029e-004
5.2707150e-004	4.6462053e-004	4.8173558e-004	4.8597635e-004

4.2147484e-004	5.2538780e-004	5.4513176e-004	5.2276435e-004
5.1515928e-004	4.6147859e-004	5.1130418e-004	4.8794763e-004
5.2513303e-004	5.3422566e-004	5.6154495e-004	6.0611064e-004
6.1633930e-004	5.6449263e-004	6.0457279e-004	5.5106265e-004
5.2622539e-004	5.2889090e-004	5.3505472e-004	5.0021456e-004
5.1803236e-004	5.1106155e-004	5.3155824e-004	4.9481539e-004
4.8996704e-004	4.7353571e-004	4.8688160e-004	4.9031138e-004
4.9129182e-004	4.7731082e-004	4.5938028e-004	4.6440577e-004
6.1249762e-004	6.6933512e-004	6.0037377e-004	6.4169368e-004
6.1022741e-004	6.3060121e-004	6.0853201e-004	5.2151586e-004
4.7480471e-004	4.9309734e-004	4.8725405e-004	5.0954513e-004
4.9077179e-004	5.0124816e-004	5.0736764e-004	5.1662531e-004
4.7380665e-004	5.2444030e-004	5.5686827e-004	5.2840555e-004
4.9208797e-004	4.8940469e-004	5.1152815e-004	4.9047398e-004
6.1554844e-004	6.3661362e-004	6.7843470e-004	7.3475153e-004
7.4152047e-004	8.2528390e-004	7.6838970e-004	8.0664351e-004
7.4590402e-004	7.5072594e-004	7.2476986e-004	7.0909238e-004
7.6083228e-004	7.2206743e-004	7.8007647e-004	7.5817779e-004
7.0269815e-004	8.3399487e-004	8.2817388e-004	8.1755892e-004
8.4170709e-004	7.7232561e-004	7.8420265e-004	8.8313259e-004
5.9825847e-004	6.5867133e-004	5.8659962e-004	6.1864241e-004
5.9483502e-004	5.8892414e-004	5.7669598e-004	5.1440934e-004
4.7493374e-004	4.9066294e-004	5.0031278e-004	5.1133501e-004
4.9301903e-004	5.0973644e-004	5.2166348e-004	5.0669113e-004
4.9324952e-004	5.3043119e-004	5.5799480e-004	5.1978469e-004
4.9456383e-004	5.1281658e-004	5.2367058e-004	5.0412842e-004
7.1245336e-004	7.4460560e-004	7.9689010e-004	8.3033144e-004
8.2797807e-004	9.3749270e-004	8.4866754e-004	8.8200859e-004
8.0212066e-004	8.3674696e-004	8.3264969e-004	8.2334648e-004
7.9508125e-004	7.8725890e-004	8.3100429e-004	8.3639281e-004
7.7006007e-004	8.7911449e-004	8.9699456e-004	9.0723365e-004
8.7035474e-004	8.4337231e-004	8.0895297e-004	9.1133820e-004
5.2438164e-004	5.4840666e-004	5.8347787e-004	6.4262560e-004
6.2598531e-004	6.1798001e-004	6.3898416e-004	5.9462246e-004
5.6058304e-004	5.8823396e-004	5.5776029e-004	5.4469558e-004
5.5247471e-004	5.4567856e-004	5.6607914e-004	5.3426645e-004
5.1626882e-004	5.3541180e-004	5.2386260e-004	5.0595752e-004
5.0428743e-004	5.0124488e-004	4.8515813e-004	4.8880457e-004
6.0354295e-004	6.5318750e-004	5.8587026e-004	6.0924819e-004
5.9668699e-004	6.0404291e-004	5.8880517e-004	5.3046447e-004
4.9023582e-004	4.9812427e-004	4.9943854e-004	5.1080890e-004
4.7301663e-004	5.1594996e-004	4.8012639e-004	5.0067262e-004
4.4773464e-004	4.9638873e-004	5.2602151e-004	5.0684046e-004
4.7652396e-004	4.7241018e-004	4.7962160e-004	4.9362837e-004
7.8426200e-004	8.2105475e-004	9.4069382e-004	1.0646335e-003
1.0342985e-003	1.0575750e-003	1.0270092e-003	9.9258270e-004
9.4879609e-004	1.0148853e-003	9.7430112e-004	9.9313368e-004
9.4927588e-004	9.1651281e-004	9.8853136e-004	9.9786989e-004
9.5145269e-004	9.9129020e-004	9.9649200e-004	1.0323260e-003
9.9104505e-004	9.7639967e-004	9.9215060e-004	1.0464993e-003
5.2291578e-004	5.6428362e-004	5.5097030e-004	6.0137067e-004
5.8252331e-004	5.7779375e-004	5.9652195e-004	5.4694821e-004

5.2098586e-004	5.1772808e-004	5.3793426e-004	5.1080804e-004
4.8554519e-004	4.9116583e-004	5.0701461e-004	4.8875483e-004
4.9908634e-004	4.9229096e-004	4.7258662e-004	4.8702870e-004
4.8384695e-004	4.3201993e-004	4.7549044e-004	4.7979240e-004
6.4484841e-004	7.1375596e-004	6.8394070e-004	7.6364979e-004
7.3900107e-004	7.8677430e-004	7.7083994e-004	7.1297451e-004
6.6206949e-004	6.9159963e-004	6.3118225e-004	6.7195788e-004
6.1574296e-004	6.4934972e-004	6.2643707e-004	6.5249250e-004
5.8500150e-004	6.0457465e-004	6.0950717e-004	6.5203189e-004
6.1441893e-004	5.9946491e-004	6.2017614e-004	6.6189760e-004
8.2338267e-004	8.1812093e-004	9.4298595e-004	1.0866848e-003
1.0392137e-003	1.0085515e-003	1.0682324e-003	1.0079288e-003
9.8623737e-004	1.0175297e-003	1.0047236e-003	1.0268768e-003
1.0362032e-003	9.4905742e-004	1.0193951e-003	1.0395249e-003
1.0123714e-003	1.0397611e-003	1.0072652e-003	1.0209538e-003
1.0424673e-003	9.8494107e-004	1.0335332e-003	1.0713970e-003
5.4426298e-004	5.7760945e-004	6.1712411e-004	6.6331330e-004
6.2859585e-004	6.6714530e-004	6.7404741e-004	6.3903476e-004
5.9924813e-004	6.4695030e-004	5.9649245e-004	5.7954241e-004
5.9543822e-004	5.8958711e-004	5.9772993e-004	5.5737884e-004
5.5014284e-004	5.8695737e-004	5.6991001e-004	5.2816264e-004
5.1696436e-004	5.2340800e-004	5.2167242e-004	5.1716204e-004
6.3543365e-004	6.5124006e-004	6.2080787e-004	6.5640699e-004
6.4586924e-004	6.5586629e-004	6.5347228e-004	5.7711275e-004
5.5386423e-004	5.4229787e-004	5.1933830e-004	5.3964783e-004
4.9759696e-004	5.3621648e-004	4.7647995e-004	5.0901077e-004
4.6839177e-004	4.9108575e-004	5.3464973e-004	4.9826479e-004
4.9948382e-004	4.8917929e-004	5.0306048e-004	5.0891501e-004
7.4915119e-004	7.6571766e-004	8.5644248e-004	1.0303781e-003
9.7763129e-004	9.5807729e-004	1.0169774e-003	9.8825063e-004
9.4000562e-004	9.8741213e-004	9.6052051e-004	9.6169327e-004
1.0196653e-003	8.9546460e-004	9.6607687e-004	1.0011413e-003
9.6735136e-004	9.8458319e-004	9.4787544e-004	1.0050099e-003
9.8158188e-004	9.1950463e-004	1.0057274e-003	1.0518716e-003
5.9857940e-004	6.2208434e-004	6.4712185e-004	6.8929231e-004
6.4767852e-004	7.0088357e-004	7.0221220e-004	6.7638885e-004
6.2693155e-004	6.7687065e-004	6.4769469e-004	6.2647722e-004
6.4203904e-004	6.3627149e-004	6.3834578e-004	5.8930612e-004
6.0492918e-004	6.2130194e-004	6.1152149e-004	5.5538882e-004
5.4712995e-004	5.6038256e-004	5.5692052e-004	5.5940643e-004
5.1666511e-004	5.6574710e-004	5.7548014e-004	6.2393055e-004
5.9625533e-004	6.1967634e-004	6.2679216e-004	5.7607224e-004
5.5557625e-004	5.2812654e-004	5.5125511e-004	5.3405854e-004
5.2005958e-004	5.2283023e-004	5.1527005e-004	5.1030552e-004
4.9270803e-004	5.2248080e-004	5.0185498e-004	5.1345151e-004
5.0390916e-004	4.7139590e-004	4.9753686e-004	4.9562917e-004
5.8306131e-004	5.7552801e-004	5.8345505e-004	6.4401008e-004
5.9565055e-004	6.0334057e-004	5.8763105e-004	5.1841063e-004
5.3428592e-004	5.1670111e-004	5.1508973e-004	5.1812441e-004
4.9684683e-004	5.1068537e-004	4.8202519e-004	4.7946853e-004
4.8258810e-004	5.0011007e-004	5.2331178e-004	4.9782507e-004
5.1304475e-004	4.9597871e-004	5.0687363e-004	4.9915339e-004

8.0426811e-004	8.2491737e-004	9.0408878e-004	1.0025967e-003
9.7743064e-004	9.8414200e-004	1.0603621e-003	9.8957054e-004
9.4327795e-004	9.5383760e-004	9.6263672e-004	9.3966573e-004
1.0304912e-003	9.1182234e-004	9.6193685e-004	9.4711441e-004
9.7712368e-004	9.8645374e-004	9.6895636e-004	9.4245718e-004
9.3752188e-004	9.2927671e-004	9.7738302e-004	9.9023880e-004
6.2111719e-004	6.2936761e-004	6.7195135e-004	7.0424785e-004
6.5839675e-004	7.1411747e-004	7.1585616e-004	6.9694448e-004
6.6241203e-004	6.9037346e-004	6.6107365e-004	6.3484709e-004
6.4340522e-004	6.4935886e-004	6.3726830e-004	5.9991791e-004
5.9050297e-004	6.3123067e-004	6.1959465e-004	5.6800446e-004
5.7504178e-004	5.7642869e-004	5.7852507e-004	5.7051069e-004
5.7631102e-004	5.9719564e-004	6.3629339e-004	6.6061546e-004
6.1911975e-004	6.5309319e-004	6.9296420e-004	6.3502832e-004
5.9022367e-004	5.9549695e-004	5.9215781e-004	5.6986634e-004
5.3811519e-004	5.7160044e-004	5.8623038e-004	5.7566934e-004
5.6986511e-004	5.7858649e-004	5.5163197e-004	5.5175736e-004
5.8271077e-004	5.1533704e-004	5.3637716e-004	4.9710742e-004
8.6311308e-004	8.9733265e-004	8.7148328e-004	9.3940302e-004
9.4998560e-004	9.5065069e-004	1.1110112e-003	9.3245004e-004
9.2678127e-004	9.3323017e-004	8.8672567e-004	9.2341595e-004
9.1625133e-004	8.7945878e-004	9.2796591e-004	9.1147228e-004
9.3768314e-004	9.3298408e-004	8.6310298e-004	8.5288988e-004
8.6221381e-004	8.9471777e-004	8.6207515e-004	8.8294498e-004
6.8961257e-004	6.8343894e-004	7.0798298e-004	7.5392917e-004
7.0993369e-004	7.5004977e-004	7.5751092e-004	7.3536318e-004
7.1039693e-004	7.4180136e-004	7.2669344e-004	6.9750172e-004
6.7862521e-004	7.0024372e-004	6.7359899e-004	6.5720289e-004
6.5430027e-004	6.7363742e-004	6.6668849e-004	6.1238999e-004
6.2597750e-004	6.1833920e-004	6.1259647e-004	6.1115020e-004
6.1508466e-004	6.0365615e-004	6.6755386e-004	6.7525955e-004
6.0285692e-004	6.7840554e-004	7.0885889e-004	6.5229996e-004
5.9990252e-004	6.0801934e-004	6.0505254e-004	5.8061727e-004
5.7203468e-004	5.7770597e-004	6.0426300e-004	5.9454805e-004
5.8086398e-004	5.9900404e-004	5.8249595e-004	5.6318429e-004
6.1389503e-004	5.4556370e-004	5.5984387e-004	5.2195384e-004
5.4966629e-004	5.7250666e-004	6.0406383e-004	6.5311011e-004
6.3409498e-004	6.2769026e-004	6.6760655e-004	5.9079184e-004
5.8484262e-004	5.7676902e-004	5.6172961e-004	5.7271993e-004
5.6561315e-004	5.2722538e-004	5.5683994e-004	5.3553464e-004
5.1964729e-004	5.7994394e-004	5.2380194e-004	5.5378718e-004
4.9866467e-004	5.0229735e-004	5.1836080e-004	5.1395097e-004
6.1021479e-004	6.0187389e-004	6.0623279e-004	6.8325688e-004
6.2424019e-004	5.9777212e-004	5.8796523e-004	5.2280053e-004
5.2801600e-004	4.9781116e-004	4.9986304e-004	5.2711394e-004
5.0364577e-004	4.9507873e-004	4.9960746e-004	4.4428034e-004
4.8553968e-004	4.8087051e-004	4.7412063e-004	4.8322056e-004
5.1371679e-004	4.5339199e-004	4.9070588e-004	4.4993797e-004
9.4243110e-004	9.9992128e-004	9.5058205e-004	1.0411587e-003
1.1042432e-003	1.0728382e-003	1.2924948e-003	1.0633768e-003
1.0857664e-003	1.0693028e-003	9.9381979e-004	1.0512897e-003
1.0172596e-003	9.7615947e-004	1.0448858e-003	1.0541457e-003

1.0814102e-003	1.0601761e-003	9.6127203e-004	9.5449797e-004
9.7590167e-004	9.9122434e-004	9.8209815e-004	1.0108893e-003
7.3017120e-004	7.0464361e-004	7.3651905e-004	7.8772107e-004
7.4328545e-004	7.6827176e-004	7.9099117e-004	7.4666168e-004
7.4240744e-004	7.7967966e-004	7.6669082e-004	7.3825037e-004
7.0132221e-004	7.1617049e-004	6.9392919e-004	7.0317493e-004
6.8551533e-004	7.0129207e-004	6.9362151e-004	6.4428253e-004
6.7371223e-004	6.4835684e-004	6.4142102e-004	6.4086959e-004
6.4074543e-004	6.1752103e-004	6.7448223e-004	6.8337431e-004
6.0093556e-004	6.8960333e-004	7.0901452e-004	6.4947394e-004
5.9316761e-004	6.0698861e-004	5.9788430e-004	5.8480985e-004
5.7909697e-004	5.7190118e-004	6.0572405e-004	5.9409779e-004
5.7618783e-004	6.0910362e-004	5.9435352e-004	5.6643048e-004
6.0819044e-004	5.4730642e-004	5.6252199e-004	5.3295985e-004
5.5874964e-004	5.7297153e-004	6.1778631e-004	6.5556260e-004
6.3570293e-004	6.1263221e-004	6.6750952e-004	5.8774350e-004
5.8541083e-004	5.8528427e-004	5.6058010e-004	5.6598263e-004
5.6736778e-004	5.3152752e-004	5.8542412e-004	5.4008920e-004
5.2936874e-004	5.9547335e-004	5.3107073e-004	5.6288604e-004
5.0454325e-004	5.1192433e-004	5.1405834e-004	5.3040564e-004
6.4775658e-004	6.4014194e-004	6.3825367e-004	7.0567220e-004
6.6061974e-004	6.1009193e-004	6.1672580e-004	5.4752121e-004
5.3117737e-004	4.9145462e-004	4.8995568e-004	5.2412438e-004
5.0462778e-004	4.9227916e-004	4.9280879e-004	4.4989955e-004
4.8973576e-004	4.6809640e-004	4.5091432e-004	4.8866713e-004
5.0241203e-004	4.4529376e-004	4.8392351e-004	4.4655633e-004
9.7519137e-004	1.0096448e-003	9.7195625e-004	1.0783061e-003
1.1361365e-003	1.0633626e-003	1.2577797e-003	1.0831097e-003
1.1117871e-003	1.0997712e-003	1.0280951e-003	1.0460979e-003
1.0116871e-003	1.0044031e-003	1.0295493e-003	1.0684452e-003
1.0744959e-003	1.0432896e-003	9.6250587e-004	9.6424289e-004
1.0074618e-003	9.3298437e-004	9.5763976e-004	9.9756231e-004
7.3938329e-004	7.0147245e-004	7.4134616e-004	7.7876484e-004
7.2351522e-004	7.5553883e-004	7.9469208e-004	7.0755869e-004
7.1451794e-004	7.6383630e-004	7.4763627e-004	7.3198474e-004
6.9276226e-004	6.7226299e-004	6.8386742e-004	7.0729571e-004
6.6665925e-004	6.8435179e-004	6.9821801e-004	6.2537378e-004
6.6876275e-004	6.4139364e-004	6.3454993e-004	6.2371365e-004
6.2767598e-004	6.1331538e-004	6.6523469e-004	6.7802101e-004
6.0971174e-004	6.7122481e-004	6.8967624e-004	6.2167345e-004
5.8435272e-004	5.9262937e-004	5.6738700e-004	5.6361455e-004
5.5885837e-004	5.6781068e-004	5.9471006e-004	5.6751506e-004
5.5138611e-004	6.1352734e-004	5.7208045e-004	5.5907556e-004
5.6148077e-004	5.2860443e-004	5.3578401e-004	5.4152899e-004
6.1579734e-004	6.6358941e-004	6.8598917e-004	7.1313571e-004
6.4904641e-004	6.2060961e-004	6.2237661e-004	5.5982685e-004
5.6821073e-004	5.6844763e-004	5.5695095e-004	5.4308391e-004
5.5968456e-004	5.2604008e-004	5.3682995e-004	5.3977747e-004
5.4978939e-004	5.7761349e-004	5.1819311e-004	5.4882334e-004
5.4928861e-004	5.1862068e-004	5.7142353e-004	5.3856923e-004
7.0863865e-004	7.0368964e-004	6.8712489e-004	7.5681976e-004
7.7623151e-004	7.1145306e-004	7.7521204e-004	6.6452158e-004

6.4900932e-004	5.8001542e-004	5.5947264e-004	6.1218259e-004
5.8673229e-004	5.8560429e-004	5.6748169e-004	5.6970276e-004
5.9032026e-004	5.5398483e-004	5.1659265e-004	5.6278495e-004
5.6998210e-004	5.1429540e-004	5.3738863e-004	5.3794220e-004
9.4079608e-004	9.6058664e-004	9.6640663e-004	1.0940961e-003
1.0882527e-003	1.0103287e-003	1.1579555e-003	1.0228998e-003
1.0156744e-003	1.0763595e-003	1.0308107e-003	9.9806895e-004
9.6227371e-004	9.7674473e-004	1.0068607e-003	1.0467720e-003
1.0139682e-003	1.0162058e-003	9.5215783e-004	9.7181427e-004
1.0128814e-003	8.9704797e-004	9.3890146e-004	9.6364206e-004
7.3774441e-004	7.1592753e-004	7.5515607e-004	7.7462930e-004
7.0052494e-004	7.4487797e-004	7.9025429e-004	6.8322575e-004
6.7942422e-004	7.2292441e-004	7.1658081e-004	7.0464358e-004
6.6752553e-004	6.3685156e-004	6.7028096e-004	6.8889637e-004
6.4564367e-004	6.6991795e-004	7.0626559e-004	6.0914182e-004
6.3398377e-004	6.2993403e-004	6.2325614e-004	6.0715810e-004
5.9379540e-004	5.9765202e-004	6.5888501e-004	6.8551692e-004
6.2989857e-004	6.5174665e-004	6.7210497e-004	6.0980837e-004
5.8615563e-004	5.9013737e-004	5.6077309e-004	5.4790432e-004
5.4688919e-004	5.7687504e-004	6.1306385e-004	5.6106529e-004
5.3670230e-004	6.1609667e-004	5.6406680e-004	5.5441170e-004
5.4366981e-004	5.3119818e-004	5.2601496e-004	5.6119161e-004
6.3685421e-004	6.7092448e-004	6.7246142e-004	7.0075803e-004
6.4984435e-004	6.0824388e-004	6.1914022e-004	5.4172253e-004
5.4459595e-004	5.3449491e-004	5.2699656e-004	5.2549253e-004
5.3238258e-004	5.1076228e-004	5.0261242e-004	5.0919128e-004
5.2525441e-004	5.2235724e-004	4.7958380e-004	5.1661698e-004
5.1266962e-004	4.8763641e-004	5.3704118e-004	5.0941219e-004
8.4396655e-004	8.7256129e-004	8.7862286e-004	1.0067202e-003
9.2129184e-004	8.9005676e-004	1.0129189e-003	8.6310125e-004
8.5061161e-004	9.4453416e-004	9.3382940e-004	9.0732024e-004
8.4513992e-004	8.0130421e-004	8.9143067e-004	9.0790137e-004
8.5425330e-004	8.4534411e-004	8.4308998e-004	8.1963887e-004
8.3782089e-004	8.1081665e-004	8.0687595e-004	8.1134606e-004
6.7144503e-004	6.6541876e-004	7.3454405e-004	7.5855482e-004
6.8206631e-004	7.1188180e-004	7.2588022e-004	6.6657118e-004
6.0969108e-004	6.2639723e-004	6.1905639e-004	6.1243598e-004
5.8698210e-004	6.0553191e-004	6.2498794e-004	6.0794193e-004
5.7211508e-004	6.4796041e-004	6.4753641e-004	5.8899609e-004
5.7498015e-004	5.8206693e-004	5.8609384e-004	5.9332888e-004
6.4610394e-004	6.7210726e-004	6.8322642e-004	7.1756379e-004
6.6104575e-004	6.1855972e-004	6.2160428e-004	5.6472032e-004
5.9921131e-004	5.9216480e-004	5.6115321e-004	5.8535561e-004
5.6411749e-004	5.6107370e-004	5.5231288e-004	5.5382968e-004
5.4407748e-004	5.8115445e-004	5.2428708e-004	5.5327905e-004
5.5434681e-004	5.5507584e-004	5.7138274e-004	5.5713940e-004
7.3189396e-004	7.3861450e-004	8.0311700e-004	8.4688903e-004
7.5681205e-004	7.7700884e-004	8.0131652e-004	7.4333699e-004
6.8328118e-004	7.0338791e-004	7.1082893e-004	7.1662577e-004
6.6801267e-004	6.4675082e-004	6.8149070e-004	6.9410464e-004
6.4288154e-004	6.9184735e-004	7.2053642e-004	6.5203964e-004
6.1861258e-004	6.5298223e-004	6.5307321e-004	6.4987534e-004

6.6530138e-004	6.6546346e-004	6.6270411e-004	7.2158920e-004
6.6422457e-004	6.3350597e-004	6.3695027e-004	5.9359315e-004
6.0048389e-004	6.0010493e-004	5.6176404e-004	5.9515316e-004
5.7693204e-004	5.7037936e-004	5.8444234e-004	5.7048570e-004
5.4696621e-004	5.8646142e-004	5.4574311e-004	5.6367548e-004
5.6574529e-004	5.7645221e-004	5.7208065e-004	5.6919642e-004
6.4594240e-004	6.8578946e-004	6.8708713e-004	7.3223775e-004
6.8330402e-004	6.7228338e-004	6.5402070e-004	5.7983467e-004
5.4680486e-004	5.4603940e-004	5.5561002e-004	5.3350876e-004
5.1678348e-004	5.2701497e-004	5.0788374e-004	5.1201684e-004
5.2519303e-004	4.9912356e-004	4.7612902e-004	4.9087005e-004
4.9614613e-004	5.0812878e-004	5.3269148e-004	4.9729824e-004
7.5105439e-004	7.7301842e-004	8.1568499e-004	8.9189965e-004
8.0285236e-004	8.1283481e-004	8.2103413e-004	7.9386895e-004
7.0366978e-004	7.2616845e-004	7.4276872e-004	7.6154248e-004
7.0600539e-004	6.6188160e-004	7.1525016e-004	7.4082415e-004
6.8087722e-004	7.2900254e-004	7.3515596e-004	6.9108384e-004
6.5345816e-004	6.7828271e-004	6.8835040e-004	6.8080942e-004
6.7942080e-004	6.6158200e-004	6.5467969e-004	7.1806459e-004
6.6907899e-004	6.4807794e-004	6.5169315e-004	6.1186749e-004
5.9077035e-004	6.0066576e-004	5.5997143e-004	5.8725533e-004
5.7425327e-004	5.7086363e-004	5.8938717e-004	5.8155575e-004
5.5342390e-004	5.8669833e-004	5.5801393e-004	5.6256857e-004
5.7811744e-004	5.7864733e-004	5.7089214e-004	5.7749204e-004
6.4518770e-004	7.0998880e-004	7.0272238e-004	7.5758304e-004
7.0218531e-004	6.9728904e-004	6.8449156e-004	6.1317995e-004
5.7051066e-004	5.6993730e-004	5.8016292e-004	5.4163812e-004
5.2603956e-004	5.3328500e-004	5.2365362e-004	5.2150125e-004
5.3428981e-004	5.1633317e-004	4.9714326e-004	4.8758974e-004
5.1219984e-004	5.1971723e-004	5.5173277e-004	5.0818367e-004
6.6177441e-004	6.3690201e-004	6.5510764e-004	6.8922073e-004
6.7377351e-004	6.4499567e-004	6.3405969e-004	6.0320787e-004
5.7445928e-004	5.5048673e-004	5.3891787e-004	5.5424169e-004
5.2094631e-004	5.3311627e-004	5.2952407e-004	5.4445429e-004
5.2836940e-004	5.3059323e-004	5.1565450e-004	4.8350250e-004
5.4150249e-004	5.3530248e-004	5.4319609e-004	5.4363985e-004
7.3469462e-004	8.3966462e-004	8.3404376e-004	9.1490349e-004
8.6285968e-004	9.6707413e-004	8.9568688e-004	8.1409533e-004
7.2193508e-004	7.1418063e-004	7.7248365e-004	7.3544760e-004
6.9879243e-004	7.0581560e-004	7.5954459e-004	7.1373087e-004
7.2858298e-004	7.3244334e-004	6.6733426e-004	6.8935737e-004
7.1266084e-004	6.6956169e-004	7.1478313e-004	6.9321801e-004
7.8283409e-004	8.9780804e-004	8.1386349e-004	9.7856496e-004
8.8412658e-004	9.0131606e-004	8.6408627e-004	8.7016026e-004
7.4580635e-004	7.6462969e-004	8.0754026e-004	8.2571979e-004
7.6271621e-004	7.0939935e-004	7.9088642e-004	8.4741520e-004
7.7680408e-004	8.2427061e-004	7.7865327e-004	7.7165259e-004
7.8794771e-004	7.5947406e-004	7.5407543e-004	7.5322596e-004
6.9172675e-004	6.5781399e-004	6.3955250e-004	7.1951645e-004
6.7696482e-004	6.6343223e-004	6.7507137e-004	6.2258165e-004
5.7820033e-004	6.1509691e-004	5.6423777e-004	5.4861909e-004
5.7639250e-004	5.7175018e-004	5.8452255e-004	5.9653787e-004

5.7520816e-004	5.8355271e-004	5.9228443e-004	5.6725617e-004
5.9329651e-004	5.8743177e-004	5.8434087e-004	5.9903486e-004
7.1163128e-004	7.2462282e-004	7.5490880e-004	8.0247463e-004
7.7457540e-004	8.0319388e-004	7.8150349e-004	7.0831453e-004
6.5500966e-004	6.3155201e-004	6.4268718e-004	6.4054354e-004
6.1071210e-004	6.0822882e-004	6.4469624e-004	6.0447316e-004
6.2398924e-004	6.2637161e-004	5.8810755e-004	5.5702862e-004
6.2002623e-004	6.0063237e-004	6.2350491e-004	6.1810850e-004
7.7721495e-004	9.5883659e-004	8.4022788e-004	1.0563785e-003
9.2736729e-004	9.4639379e-004	9.1556922e-004	9.1336759e-004
7.7741985e-004	7.9991693e-004	8.2987464e-004	8.5038619e-004
7.9042029e-004	7.4810542e-004	8.2206224e-004	8.9403777e-004
8.3174987e-004	8.8086064e-004	8.2543406e-004	8.3166952e-004
8.6487304e-004	8.3409621e-004	7.9050235e-004	8.0633369e-004
6.9102264e-004	6.5472107e-004	6.3220570e-004	7.1513213e-004
6.7771167e-004	6.5618528e-004	6.6851143e-004	6.0254625e-004
5.7740374e-004	5.9957012e-004	5.5746276e-004	5.2233912e-004
5.6396994e-004	5.4890623e-004	5.7330218e-004	5.8867550e-004
5.6856676e-004	5.6530794e-004	5.8809074e-004	5.6409709e-004
5.8324057e-004	5.8252932e-004	5.9100382e-004	5.9441300e-004
6.3756403e-004	6.1050720e-004	6.4992601e-004	7.1116301e-004
6.5661648e-004	6.6845547e-004	6.9283435e-004	6.3350391e-004
5.6104680e-004	5.6365861e-004	5.2151956e-004	5.3085758e-004
5.1436211e-004	5.1303524e-004	5.3978233e-004	5.0141722e-004
5.2495603e-004	5.4181326e-004	5.2848288e-004	4.5266908e-004
5.2703689e-004	5.2836933e-004	5.2587377e-004	5.3936854e-004
6.6676219e-004	8.6689215e-004	7.4213401e-004	9.7916242e-004
8.3041158e-004	8.2989667e-004	8.1025741e-004	8.0375247e-004
7.3191708e-004	7.2747444e-004	7.1741709e-004	7.2948707e-004
7.1642650e-004	6.9250762e-004	7.1896684e-004	7.7851639e-004
7.4387093e-004	7.8630364e-004	7.6200689e-004	7.6227685e-004
7.5083185e-004	7.7075922e-004	7.0000136e-004	7.3878674e-004
7.0978099e-004	6.3592949e-004	6.4540972e-004	7.0302196e-004
6.9294154e-004	6.7132224e-004	6.8138188e-004	5.9944842e-004
5.8754641e-004	5.6867275e-004	5.1935708e-004	5.0988431e-004
5.2931275e-004	5.0814230e-004	5.5459523e-004	5.6494944e-004
5.4043016e-004	5.3605554e-004	5.6337757e-004	5.2633348e-004
5.5308573e-004	5.4022964e-004	5.9148030e-004	5.6466220e-004
6.3500619e-004	7.0291299e-004	6.8921456e-004	8.3567538e-004
7.5910684e-004	7.2639757e-004	7.5598191e-004	7.0375949e-004
6.5575517e-004	6.7466754e-004	5.9670614e-004	6.1597697e-004
6.0776698e-004	6.0090945e-004	6.2583968e-004	6.2053589e-004
6.2139244e-004	6.7719015e-004	6.6206338e-004	6.2450339e-004
6.1623432e-004	6.6085163e-004	6.0141993e-004	6.4310392e-004
7.3977566e-004	6.3256094e-004	6.8072176e-004	7.1080802e-004
7.2572927e-004	6.8870740e-004	7.0512675e-004	5.9638935e-004
6.0387533e-004	5.7016096e-004	5.2118405e-004	5.0973949e-004
5.2337724e-004	4.9811932e-004	5.5736316e-004	5.8127145e-004
5.4435381e-004	5.4551198e-004	5.7337662e-004	5.4770095e-004
5.5574774e-004	5.2797796e-004	6.1378913e-004	5.6981572e-004
5.4478919e-004	5.7176863e-004	6.2082988e-004	6.8233242e-004
6.8587245e-004	6.0180723e-004	6.4334246e-004	5.9277951e-004

5.8886720e-004	6.4566535e-004	5.3411999e-004	5.6385330e-004
5.5256385e-004	5.4735858e-004	5.7287533e-004	5.6076708e-004
5.6909164e-004	6.2979182e-004	6.1370816e-004	5.6710119e-004
5.3230550e-004	5.9977802e-004	5.4435507e-004	5.6759366e-004
6.7225909e-004	6.4580595e-004	6.3198034e-004	6.6527570e-004
6.4137251e-004	6.0840952e-004	6.1131295e-004	5.4653615e-004
5.3842871e-004	5.2381869e-004	5.3684521e-004	5.0575527e-004
5.2826328e-004	5.1083778e-004	5.0867458e-004	5.4992395e-004
5.4365172e-004	5.5608562e-004	5.4689300e-004	5.6215623e-004
5.3559103e-004	5.6898429e-004	5.6876990e-004	5.9519782e-004
6.5283193e-004	5.3963524e-004	6.1491555e-004	6.1549010e-004
6.7552498e-004	6.1034287e-004	6.3382306e-004	5.4073382e-004
5.4922084e-004	5.4840447e-004	4.5005233e-004	4.6350253e-004
4.5728240e-004	4.3828023e-004	4.8616876e-004	5.2998310e-004
4.7831619e-004	5.0642725e-004	5.3069862e-004	5.0490656e-004
4.9376387e-004	4.5252640e-004	5.4304159e-004	4.9255181e-004
5.9963403e-004	5.7172946e-004	6.9723779e-004	6.8684248e-004
7.2000825e-004	6.2001345e-004	6.8836792e-004	6.1217404e-004
6.1267949e-004	6.8596835e-004	5.8441091e-004	5.9449283e-004
5.6828053e-004	5.8217137e-004	6.0206848e-004	6.0092070e-004
6.0777448e-004	6.4143004e-004	6.4413769e-004	5.6716824e-004
5.5197986e-004	5.9158630e-004	5.7125618e-004	5.9162607e-004
5.3533721e-004	6.0854807e-004	7.0204245e-004	6.8539759e-004
6.6556844e-004	6.5442675e-004	7.3960805e-004	6.6963573e-004
6.6334225e-004	6.9473704e-004	6.7655431e-004	6.2748601e-004
6.1994565e-004	6.0362485e-004	6.4390388e-004	6.0773583e-004
6.3888697e-004	6.1194232e-004	6.5920035e-004	5.9791212e-004
6.1258578e-004	5.8884552e-004	6.1476284e-004	6.1923089e-004
7.3086872e-004	6.6228845e-004	6.6776671e-004	6.8020351e-004
7.0795014e-004	6.6775549e-004	6.4783015e-004	5.8379013e-004
5.7136964e-004	5.4932972e-004	5.3555332e-004	5.3481197e-004
5.4157572e-004	5.2947033e-004	5.1005265e-004	5.8813857e-004
5.5813917e-004	5.7783288e-004	5.8843980e-004	5.6217334e-004
5.5349595e-004	5.6557372e-004	5.9802746e-004	5.8811587e-004
6.2582632e-004	5.1566847e-004	5.9946445e-004	6.2574909e-004
6.5358894e-004	5.8010993e-004	6.1942452e-004	5.7751599e-004
5.5260350e-004	5.9488260e-004	4.6982433e-004	4.8816236e-004
4.5838843e-004	4.7598390e-004	4.9518888e-004	5.3061891e-004
5.0021751e-004	5.3993995e-004	5.3627689e-004	4.9517952e-004
4.6648954e-004	4.7876606e-004	4.8035867e-004	4.9028040e-004
5.8041935e-004	5.9800180e-004	7.4926513e-004	6.7775199e-004
7.2105395e-004	6.5662025e-004	7.4780789e-004	6.3071374e-004
5.9395281e-004	6.8211646e-004	6.3150471e-004	6.0489123e-004
5.7849451e-004	5.9877351e-004	6.3891757e-004	6.1853949e-004
6.2249546e-004	6.1411734e-004	6.5934275e-004	5.6842148e-004
6.0333544e-004	5.7278760e-004	5.8346093e-004	6.2172710e-004
7.1373167e-004	7.0130768e-004	6.9884052e-004	6.7876685e-004
6.8056998e-004	6.4361587e-004	6.3365632e-004	6.0054136e-004
5.6228883e-004	5.4146914e-004	5.5354695e-004	5.4766978e-004
5.4857637e-004	5.2573958e-004	4.9996868e-004	5.4601637e-004
5.4928094e-004	5.7635762e-004	5.7250143e-004	5.4763322e-004
5.5914027e-004	5.6493517e-004	5.6590184e-004	5.8240131e-004

6.7150925e-004	5.7766058e-004	6.3055663e-004	6.5962067e-004
6.7845722e-004	6.5587064e-004	6.4854092e-004	5.9360325e-004
5.7746258e-004	5.8684747e-004	5.1497895e-004	5.3080848e-004
4.9592586e-004	5.2720198e-004	5.0851976e-004	6.0153189e-004
5.3820758e-004	5.7547520e-004	5.6935240e-004	5.3401072e-004
5.0244341e-004	5.3286786e-004	5.0912291e-004	5.4070568e-004
6.2936732e-004	6.1388640e-004	7.5691137e-004	6.9245028e-004
7.3793977e-004	6.7366860e-004	7.6333316e-004	6.3419984e-004
5.8388796e-004	6.4410835e-004	5.9330060e-004	5.7964314e-004
5.3801591e-004	6.0410131e-004	6.4590426e-004	6.2265155e-004
6.0783955e-004	6.0360218e-004	6.4824799e-004	5.5200998e-004
5.9915475e-004	5.6151047e-004	5.6286070e-004	6.0891433e-004
5.7211645e-004	6.1839704e-004	6.3960647e-004	6.0134631e-004
6.0057432e-004	5.9557889e-004	6.0401185e-004	5.6038854e-004
5.6152436e-004	5.3292267e-004	5.2613635e-004	4.9206766e-004
5.3237933e-004	5.0001579e-004	5.4060602e-004	5.2085389e-004
5.1980416e-004	5.2842351e-004	4.9387247e-004	5.3224116e-004
4.9038117e-004	5.1356593e-004	5.1334468e-004	4.9537930e-004
7.5561651e-004	7.3875421e-004	7.5663398e-004	7.4576383e-004
7.4619967e-004	6.9887021e-004	6.7750255e-004	6.5936116e-004
6.0659059e-004	5.8092223e-004	5.9245550e-004	5.7964957e-004
5.8381014e-004	5.5125507e-004	5.2131238e-004	5.7330965e-004
5.7900650e-004	6.2025015e-004	6.0327142e-004	5.6101382e-004
5.8631704e-004	5.7973982e-004	5.9299100e-004	5.9594218e-004
6.1513361e-004	5.4704726e-004	6.5843826e-004	6.6624469e-004
6.6756895e-004	6.6326152e-004	7.1316348e-004	5.8651633e-004
5.7368821e-004	5.6884206e-004	5.4319404e-004	5.2890479e-004
4.4774601e-004	5.6428895e-004	5.7207586e-004	6.2388232e-004
5.7316394e-004	5.7818993e-004	5.7217312e-004	4.9509754e-004
5.2117118e-004	5.5387399e-004	4.8243770e-004	5.3627330e-004
6.3029697e-004	6.2685943e-004	7.1849339e-004	6.9326963e-004
6.9585083e-004	6.4500001e-004	7.2277703e-004	6.6033674e-004
6.1658693e-004	6.3659053e-004	5.7343072e-004	5.7362090e-004
5.1101446e-004	5.8179330e-004	6.1863460e-004	5.8946176e-004
5.6503875e-004	5.8733519e-004	6.2181424e-004	5.9981573e-004
5.9792813e-004	5.3855072e-004	5.8194539e-004	5.9617123e-004
7.6253299e-004	7.9756760e-004	7.9759180e-004	7.8298720e-004
8.0312145e-004	7.4910188e-004	6.9042332e-004	6.4374358e-004
6.6872868e-004	6.4013990e-004	6.3370402e-004	6.3182367e-004
6.1582266e-004	5.7519034e-004	5.6985604e-004	6.2664285e-004
5.9128353e-004	6.2615055e-004	6.0145416e-004	5.9555305e-004
6.0332943e-004	5.7962209e-004	6.4143419e-004	6.3614335e-004
6.2561739e-004	5.6751318e-004	6.8273565e-004	6.7937907e-004
6.7293160e-004	6.5712744e-004	7.1001774e-004	6.0582011e-004
5.7945140e-004	5.4669785e-004	5.5348754e-004	5.2338764e-004
4.4957306e-004	5.5216152e-004	5.4589492e-004	5.8221054e-004
5.6004087e-004	5.6758245e-004	5.7668442e-004	4.8218507e-004
5.3502334e-004	5.3330197e-004	5.3064919e-004	5.2616177e-004
6.6674202e-004	6.6802790e-004	7.0857232e-004	7.4610134e-004
7.0039417e-004	6.5786124e-004	7.4412128e-004	6.7705463e-004
6.3564339e-004	6.1349860e-004	5.6631341e-004	5.5783664e-004
4.7959956e-004	5.8290672e-004	6.0336677e-004	5.7889440e-004

5.5306259e-004 5.9286025e-004 5.8894746e-004 6.0471278e-004
6.0682403e-004 5.4602997e-004 6.0475746e-004 5.7884397e-004
5.9498985e-004 6.3677608e-004 6.4280610e-004 6.6176106e-004
6.5310055e-004 6.1495554e-004 6.4554457e-004 6.1087322e-004
5.9210714e-004 5.7091180e-004 5.5653413e-004 5.3695310e-004
5.4146205e-004 5.3890044e-004 5.5523968e-004 5.3818099e-004
5.5187542e-004 5.4774679e-004 5.2209244e-004 5.6170077e-004
5.3080937e-004 5.3141272e-004 5.2978400e-004 5.3511771e-004
7.7436506e-004 8.0785614e-004 8.3529817e-004 8.1845500e-004
8.2916141e-004 7.8159086e-004 7.3038552e-004 6.6832300e-004
7.0021511e-004 6.6787680e-004 6.7105444e-004 6.5534070e-004
6.4265754e-004 5.7615511e-004 5.8140782e-004 6.4712652e-004
6.0733310e-004 6.5405837e-004 6.1604305e-004 6.1137695e-004
6.0443551e-004 5.8650000e-004 6.6298855e-004 6.6150399e-004
6.7101434e-004 6.8315113e-004 6.7298657e-004 7.5321815e-004
6.8612963e-004 6.6386689e-004 7.7186522e-004 6.6724779e-004
6.3626816e-004 5.9710542e-004 5.9508372e-004 5.5207394e-004
4.5286099e-004 5.6479020e-004 5.7888353e-004 5.6202285e-004
5.7102990e-004 5.9784199e-004 5.6680168e-004 5.6692320e-004
6.0373370e-004 5.5922991e-004 6.4757219e-004 5.2970528e-004
6.3330607e-004 6.5843519e-004 6.6623796e-004 7.0420626e-004
6.9951803e-004 6.6375542e-004 6.7269233e-004 6.3976008e-004
6.2843735e-004 5.9681732e-004 5.8871020e-004 5.7385521e-004
5.6899838e-004 5.6808422e-004 5.8012854e-004 5.6551998e-004
5.5876320e-004 5.6960905e-004 5.5579256e-004 5.7057662e-004
5.3858694e-004 5.4098995e-004 5.3724894e-004 5.5081217e-004
7.7431684e-004 7.8465817e-004 8.1661435e-004 8.1498343e-004
8.2237834e-004 7.8628471e-004 7.8373251e-004 6.9509793e-004
7.1114246e-004 6.5898424e-004 7.0644288e-004 6.5703242e-004
6.3199448e-004 6.1079959e-004 6.1664302e-004 6.3479893e-004
6.5893844e-004 6.6162381e-004 6.3496451e-004 6.4638376e-004
6.2649036e-004 6.0988420e-004 6.9444299e-004 6.4638150e-004
6.6216532e-004 7.9370353e-004 6.7867409e-004 8.6349917e-004
7.4327188e-004 7.3715972e-004 8.1657295e-004 7.2279893e-004
7.1489948e-004 6.2290559e-004 6.7999608e-004 6.4220246e-004
5.4584199e-004 5.8672363e-004 6.2314159e-004 6.3188247e-004
6.0720941e-004 6.9925295e-004 5.7812803e-004 6.6076043e-004
6.5303272e-004 6.6195213e-004 6.6045046e-004 5.9717977e-004
6.5689758e-004 6.4572436e-004 6.6603493e-004 7.2149829e-004
6.8269408e-004 6.6926231e-004 6.5795396e-004 6.3231430e-004
6.1733332e-004 5.8578623e-004 6.0847990e-004 6.0307095e-004
5.6606739e-004 5.7014367e-004 5.7310791e-004 5.8001032e-004
5.4362926e-004 5.9904621e-004 5.6601995e-004 5.3459509e-004
5.5205355e-004 5.5044128e-004 5.2149491e-004 5.5001615e-004
6.5473316e-004 6.9798743e-004 7.1470432e-004 7.3356002e-004
7.3832452e-004 6.7668813e-004 7.2372943e-004 6.7288954e-004
6.4073250e-004 6.3379871e-004 6.0485810e-004 6.0515075e-004
5.9451576e-004 5.9615534e-004 6.0376343e-004 6.1587330e-004
5.9854558e-004 5.9853523e-004 5.9169278e-004 5.9951787e-004
5.8106940e-004 5.6374290e-004 5.7915444e-004 5.7693299e-004
8.0073802e-004 7.8148198e-004 8.2654228e-004 8.4405059e-004
9.1290379e-004 8.2483624e-004 7.9646352e-004 7.2689120e-004

7.4178426e-004	7.0316334e-004	7.1950223e-004	7.1030413e-004
7.1664862e-004	7.0960981e-004	6.9250865e-004	6.7125619e-004
7.8098201e-004	6.5634091e-004	6.9855276e-004	7.0594058e-004
6.8368592e-004	6.8598025e-004	7.2267864e-004	6.9071011e-004
5.4486151e-004	6.8630828e-004	5.8626800e-004	7.4594394e-004
6.5450733e-004	6.4168870e-004	7.1347121e-004	6.2571916e-004
6.2288391e-004	5.3723071e-004	6.2033997e-004	5.7466298e-004
4.9886883e-004	5.0508019e-004	5.5463432e-004	5.6878267e-004
5.2770031e-004	6.2119620e-004	5.0578259e-004	5.8014174e-004
5.7550464e-004	5.9156322e-004	5.6070965e-004	5.1705044e-004
6.7057072e-004	6.7102823e-004	6.8571346e-004	7.3529736e-004
6.8501674e-004	6.8679896e-004	6.8078574e-004	6.4125611e-004
6.2368649e-004	5.8719967e-004	6.0617608e-004	6.1166349e-004
5.6901437e-004	5.7327194e-004	5.7267449e-004	5.8032482e-004
5.4186357e-004	5.9994034e-004	5.6865413e-004	5.3708073e-004
5.5755606e-004	5.4847923e-004	5.2168174e-004	5.3813345e-004
6.8037018e-004	7.2375001e-004	7.4253578e-004	7.5368784e-004
7.5913296e-004	7.0822280e-004	7.5403239e-004	6.7599314e-004
6.5175867e-004	6.5593576e-004	6.1741921e-004	6.1729846e-004
6.1614696e-004	6.0057058e-004	6.1757093e-004	6.3655019e-004
5.8976335e-004	6.0482384e-004	5.9580003e-004	5.9729173e-004
5.8168419e-004	5.5921801e-004	5.8694488e-004	5.8286858e-004
6.8349917e-004	7.0756525e-004	7.4960169e-004	7.8849209e-004
7.9001732e-004	7.1214452e-004	7.4569075e-004	6.8670335e-004
6.7623670e-004	6.5155556e-004	6.6651293e-004	6.6283115e-004
6.4597549e-004	6.7143363e-004	6.6663502e-004	6.8364305e-004
7.0030031e-004	6.2563449e-004	6.5904770e-004	6.7470482e-004
6.4567171e-004	6.6043957e-004	6.2895879e-004	6.6224086e-004
6.4871697e-004	7.0545459e-004	6.8268472e-004	7.0390276e-004
6.9490907e-004	7.1633958e-004	6.6115374e-004	6.3713327e-004
6.1631046e-004	6.2628697e-004	6.6937484e-004	6.1532548e-004
5.9126393e-004	5.7955789e-004	6.0808235e-004	5.9509394e-004
6.1676287e-004	6.3558984e-004	5.8800090e-004	5.8162607e-004
5.4376222e-004	5.9994069e-004	5.7887370e-004	6.4132581e-004
7.0608080e-004	7.1081474e-004	7.5232744e-004	7.7728232e-004
7.1808125e-004	7.2705082e-004	7.4957812e-004	6.6284223e-004
6.6994112e-004	6.1865036e-004	6.1246201e-004	6.2328928e-004
5.9995704e-004	5.8102110e-004	5.9978610e-004	5.9982894e-004
5.4900019e-004	5.9193930e-004	5.8567806e-004	5.6884748e-004
5.7035500e-004	5.4219988e-004	5.3512861e-004	5.3882979e-004
6.9253283e-004	7.2829278e-004	7.4912481e-004	7.6134919e-004
7.6876262e-004	7.2530712e-004	7.6554366e-004	6.8422718e-004
6.5361125e-004	6.7182614e-004	6.3981515e-004	6.3833196e-004
6.3088248e-004	6.2283536e-004	6.2395156e-004	6.4627583e-004
6.0761876e-004	6.3343282e-004	6.0695867e-004	5.9141598e-004
5.9472882e-004	5.6829194e-004	6.2939867e-004	5.9938662e-004
6.9764274e-004	7.2109053e-004	7.8097192e-004	7.3169224e-004
6.9115650e-004	6.9475948e-004	6.9491923e-004	6.6188071e-004
6.5670611e-004	6.4647735e-004	6.3083849e-004	6.2712752e-004
6.4115207e-004	6.1409995e-004	6.1353647e-004	6.0089030e-004
6.1448770e-004	6.5294087e-004	6.1990524e-004	6.0478975e-004
6.1811121e-004	6.0494550e-004	6.0279424e-004	6.5568907e-004

7.3557680e-004	8.1058101e-004	7.8265660e-004	8.1612701e-004
7.9421538e-004	8.2028761e-004	7.9283142e-004	7.5102077e-004
6.9555965e-004	6.8115286e-004	7.2766139e-004	7.0000700e-004
6.3432356e-004	6.7370242e-004	6.7148867e-004	6.6010309e-004
6.6063796e-004	6.9008001e-004	6.4293772e-004	6.3854724e-004
6.1632362e-004	6.4939786e-004	6.2910878e-004	6.7912981e-004
7.1116416e-004	7.2551551e-004	7.6911281e-004	7.7216605e-004
7.8605271e-004	7.5226276e-004	7.9430865e-004	7.1229333e-004
6.8815498e-004	6.7536684e-004	6.8047860e-004	6.6768836e-004
6.4399469e-004	6.5730723e-004	6.3297599e-004	6.4071111e-004
6.5383360e-004	6.6627486e-004	6.1745169e-004	6.0485610e-004
6.1134892e-004	5.9284261e-004	6.6949945e-004	6.2294249e-004
6.7227155e-004	7.0080323e-004	7.1844569e-004	7.5138018e-004
7.3167596e-004	7.0362022e-004	7.1219000e-004	6.7369700e-004
6.6070534e-004	6.5185709e-004	6.3925698e-004	6.2715028e-004
5.8644173e-004	5.9591384e-004	5.9691819e-004	6.3127332e-004
6.3080530e-004	6.4797966e-004	6.3493912e-004	6.0893375e-004
5.9615220e-004	6.2596078e-004	6.2253900e-004	6.2576120e-004
7.1892525e-004	8.0469294e-004	8.0617455e-004	8.5642359e-004
8.5966883e-004	8.0118772e-004	8.7020497e-004	7.3588424e-004
7.7590795e-004	7.4635462e-004	7.3579486e-004	7.2462652e-004
6.9392742e-004	7.2892808e-004	7.2758143e-004	6.8254417e-004
6.9853527e-004	6.9146088e-004	7.4733443e-004	6.9730770e-004
6.9829891e-004	6.6299377e-004	7.2620212e-004	7.0710231e-004
7.9923013e-004	8.3272177e-004	9.0547520e-004	9.9748705e-004
9.8907154e-004	1.0208034e-003	1.0620848e-003	9.5844382e-004
8.9611507e-004	8.6753015e-004	8.9847925e-004	8.7026210e-004
9.0334083e-004	8.6654031e-004	8.3492264e-004	8.8238755e-004
9.0245968e-004	8.9814128e-004	8.6011163e-004	8.6907689e-004
8.6135710e-004	8.1653245e-004	8.4608795e-004	8.6615492e-004
8.2490646e-004	8.4603381e-004	9.3183872e-004	9.8092876e-004
9.6299182e-004	8.8845743e-004	9.7811983e-004	9.4386961e-004
8.7208663e-004	8.7583266e-004	8.5932569e-004	8.7969902e-004
8.4228894e-004	8.4625067e-004	8.3277165e-004	8.2351895e-004
8.2183798e-004	8.6885323e-004	8.6311718e-004	8.3898360e-004
8.7904114e-004	8.6044584e-004	8.7277738e-004	9.2521467e-004
7.2107585e-004	8.1298084e-004	7.9915125e-004	8.2687331e-004
8.2927152e-004	8.3483756e-004	7.9922710e-004	7.4284429e-004
7.0724146e-004	6.9443323e-004	7.5761648e-004	7.0893105e-004
6.3188205e-004	6.6322257e-004	6.9090500e-004	6.4813838e-004
6.8956026e-004	7.0977722e-004	6.5045005e-004	6.4338539e-004
6.2438790e-004	6.7306303e-004	6.5304998e-004	7.3928542e-004
7.9237079e-004	7.7668611e-004	8.5439542e-004	8.4118749e-004
8.7991808e-004	8.3602547e-004	8.9610154e-004	7.8857190e-004
7.5681847e-004	7.7898813e-004	7.6997168e-004	7.3223941e-004
7.1148940e-004	7.3021956e-004	7.2108940e-004	6.8552964e-004
6.9199024e-004	7.2232248e-004	6.7394214e-004	6.4969317e-004
6.4416253e-004	6.2990276e-004	7.0141887e-004	6.7957694e-004
7.3298087e-004	7.5141568e-004	8.1869926e-004	8.2733152e-004
8.1360198e-004	7.7680104e-004	8.1112083e-004	7.7031035e-004
7.3109531e-004	7.0707638e-004	7.1745171e-004	6.8428616e-004
6.2306476e-004	6.4956611e-004	6.3140833e-004	6.5970475e-004

6.9762611e-004	7.0912713e-004	6.3618938e-004	6.5330167e-004
6.3392911e-004	6.5332869e-004	6.6330133e-004	6.6181978e-004
7.4609228e-004	8.5578927e-004	8.7992096e-004	9.3126210e-004
9.4573572e-004	8.9875766e-004	9.7136425e-004	8.3341895e-004
8.7719162e-004	8.3555812e-004	8.1754898e-004	8.2461296e-004
7.7708911e-004	7.9277896e-004	7.7666131e-004	7.2677298e-004
7.5288556e-004	7.5573722e-004	8.1584418e-004	7.3297645e-004
7.6568773e-004	7.3349394e-004	7.7335520e-004	7.5823310e-004
7.8261878e-004	8.2902837e-004	9.6425136e-004	9.6760431e-004
9.7855834e-004	9.9744358e-004	1.0853828e-003	1.0007787e-003
8.9915644e-004	8.4638839e-004	8.4738056e-004	8.7990909e-004
8.7415072e-004	8.1723735e-004	8.1416348e-004	8.6283962e-004
8.3980874e-004	8.3060004e-004	8.2074675e-004	8.1234874e-004
8.5364585e-004	8.0627963e-004	8.2090621e-004	8.7451337e-004
7.6736535e-004	7.8769155e-004	8.8525483e-004	8.6541560e-004
8.6444712e-004	8.3622434e-004	9.1474790e-004	8.3090660e-004
7.8963707e-004	7.5074990e-004	7.9009209e-004	7.4436114e-004
6.7454837e-004	7.2512565e-004	6.6853222e-004	6.7168401e-004
7.6531339e-004	7.6156763e-004	6.3656276e-004	7.0183308e-004
6.6651520e-004	6.6822561e-004	7.1654245e-004	6.8980055e-004
7.6737821e-004	8.7172707e-004	9.3272144e-004	9.7252394e-004
9.9143584e-004	9.6463198e-004	1.0250620e-003	9.1923725e-004
9.1162469e-004	8.8885525e-004	8.6139502e-004	8.6423913e-004
8.1402593e-004	7.9945238e-004	7.9473978e-004	7.6272861e-004
7.6812548e-004	8.1125583e-004	8.4522959e-004	7.3081799e-004
7.8401457e-004	7.9067494e-004	7.7864515e-004	7.8245265e-004
7.3468831e-004	7.0967665e-004	6.7875527e-004	6.8504692e-004
6.1907170e-004	6.0579543e-004	6.5766677e-004	6.2982451e-004
6.3908809e-004	6.8502309e-004	5.8048799e-004	5.8058121e-004
6.8037663e-004	5.5852579e-004	5.8483583e-004	6.4075478e-004
5.7697554e-004	6.3454609e-004	6.2897083e-004	5.9402705e-004
6.1265752e-004	6.1069394e-004	6.1200077e-004	6.5159025e-004
7.8184792e-004	6.5271049e-004	6.7575760e-004	6.8471555e-004
5.8956494e-004	5.9198102e-004	6.7057901e-004	6.8310963e-004
6.8204924e-004	6.1151301e-004	5.5893070e-004	5.9400807e-004
5.7248145e-004	6.4008484e-004	6.1854832e-004	6.4663275e-004
6.6452334e-004	6.4638753e-004	6.4971935e-004	6.5287203e-004
5.7733658e-004	6.0855069e-004	5.3892375e-004	5.4520970e-004
6.6944472e-004	6.9666476e-004	6.1509278e-004	7.0354156e-004
6.2421140e-004	6.4764085e-004	5.7162487e-004	5.6401918e-004
6.8295516e-004	6.3011108e-004	6.0406330e-004	5.2249183e-004
6.7380107e-004	5.5387504e-004	5.9261530e-004	6.2155881e-004
5.8731936e-004	5.7801139e-004	6.4395586e-004	6.3221346e-004
5.4812471e-004	6.0379806e-004	6.1441204e-004	6.0102475e-004
7.1748010e-004	7.2427276e-004	6.8225496e-004	6.8239009e-004
6.2647194e-004	6.2215584e-004	6.3271359e-004	6.1411633e-004
6.0891081e-004	6.5847921e-004	5.5728268e-004	5.3624180e-004
6.3674958e-004	5.2285468e-004	5.6644778e-004	5.9331831e-004
5.5326366e-004	5.8739916e-004	5.9225944e-004	5.7281010e-004
5.6881378e-004	5.7150693e-004	5.6901606e-004	5.7917273e-004
7.3271876e-004	6.3094354e-004	6.5142242e-004	6.6203350e-004
5.6855778e-004	5.4410807e-004	5.9093114e-004	6.1991667e-004

6.0159394e-004	5.5953127e-004	4.9879412e-004	5.4487769e-004
5.0084412e-004	5.7999427e-004	5.0989559e-004	5.5684434e-004
5.5330584e-004	5.5341612e-004	5.5265732e-004	5.7103550e-004
4.9341162e-004	5.2404150e-004	4.8027325e-004	5.0531611e-004
6.5866631e-004	6.9444584e-004	6.8786764e-004	6.8967789e-004
6.7145961e-004	6.6084122e-004	6.8857867e-004	7.0035516e-004
6.3566146e-004	5.9846099e-004	6.1719974e-004	6.1034718e-004
6.2688273e-004	5.5471745e-004	6.0319973e-004	6.4531606e-004
5.6798242e-004	6.1869455e-004	6.3594941e-004	5.2385858e-004
6.0352733e-004	6.3244936e-004	6.8687816e-004	5.9116851e-004
6.0181310e-004	7.4378893e-004	6.9915455e-004	7.5825465e-004
6.9551650e-004	6.8307553e-004	7.7294216e-004	7.9793826e-004
7.2278084e-004	6.8818097e-004	6.4116232e-004	6.9446577e-004
7.2904618e-004	6.8710047e-004	7.1904757e-004	7.3556014e-004
7.5849821e-004	6.9614399e-004	7.5921111e-004	7.4729491e-004
7.6554765e-004	6.8524553e-004	8.0357687e-004	7.2430614e-004
6.3911722e-004	6.2408623e-004	6.2777218e-004	7.0289829e-004
5.8938483e-004	6.2028259e-004	5.3003478e-004	5.2359881e-004
6.2055749e-004	5.7889895e-004	5.7580572e-004	5.0043932e-004
5.9472167e-004	5.4178505e-004	5.4477407e-004	5.2794347e-004
5.5578623e-004	5.1008497e-004	6.0001494e-004	5.9174566e-004
5.0059698e-004	5.6985256e-004	5.6350596e-004	5.5386192e-004
6.8958377e-004	7.3186525e-004	6.6818025e-004	6.8642816e-004
6.3234117e-004	6.6086256e-004	6.1194295e-004	6.2318669e-004
6.1086855e-004	6.2858233e-004	5.6016009e-004	5.0246163e-004
6.1576658e-004	4.8162359e-004	5.5554880e-004	5.6259109e-004
5.3843872e-004	5.5782842e-004	5.6763092e-004	5.5343633e-004
5.3917301e-004	5.5555048e-004	5.6773033e-004	5.6750001e-004
6.7803403e-004	6.4671088e-004	6.2982649e-004	6.3033153e-004
5.6214236e-004	5.3215310e-004	5.3066049e-004	5.4270640e-004
5.2265033e-004	5.1699137e-004	4.7709583e-004	4.9111332e-004
4.7818555e-004	5.2549611e-004	4.5249338e-004	5.0309638e-004
4.7903297e-004	4.7200054e-004	4.9008919e-004	5.3106979e-004
4.5970237e-004	4.7083128e-004	4.6551966e-004	4.8901173e-004
5.9756065e-004	6.9500139e-004	6.5768234e-004	7.4232623e-004
6.6906189e-004	6.2979016e-004	6.7410475e-004	6.8481189e-004
6.3880063e-004	5.9008620e-004	5.5224968e-004	5.7166722e-004
6.2048498e-004	5.5754029e-004	5.8894686e-004	6.2399280e-004
6.1033075e-004	5.6879548e-004	6.2282239e-004	5.6765256e-004
6.1705660e-004	5.8095958e-004	6.2414685e-004	5.7981949e-004
6.2437655e-004	6.7356789e-004	6.9110691e-004	6.5175984e-004
7.0732913e-004	6.4871908e-004	8.0392507e-004	6.5780288e-004
7.1089392e-004	7.7408565e-004	7.3813061e-004	7.1632872e-004
7.3081370e-004	7.3609343e-004	7.5033604e-004	7.3041837e-004
8.0322979e-004	7.5352959e-004	7.4593870e-004	7.5053927e-004
7.2505986e-004	7.5224742e-004	7.8383647e-004	7.3462218e-004
7.4771969e-004	7.6450757e-004	7.1468286e-004	7.3322561e-004
6.9260559e-004	6.4679966e-004	5.9396337e-004	6.5663565e-004
6.0328604e-004	6.4865011e-004	6.0033852e-004	6.1116065e-004
6.0214862e-004	6.0596537e-004	5.8186347e-004	6.0891356e-004
6.2853616e-004	5.4655023e-004	6.1601846e-004	6.2829655e-004
5.5309017e-004	6.0647834e-004	6.1636625e-004	6.2005261e-004

6.0472444e-004	5.6838806e-004	6.0223974e-004	6.6706505e-004
5.7550878e-004	5.9431639e-004	4.9830491e-004	4.9664444e-004
5.6649734e-004	5.3923368e-004	5.3583780e-004	4.7491859e-004
5.2206973e-004	4.9439917e-004	4.9105207e-004	4.7510659e-004
5.0522318e-004	4.6409161e-004	5.3803726e-004	5.4575404e-004
4.7500582e-004	5.2100496e-004	5.3468050e-004	5.2342365e-004
6.5522223e-004	6.8809201e-004	6.6244655e-004	6.6428086e-004
6.0639699e-004	6.5293016e-004	5.7197372e-004	5.7896705e-004
5.7135496e-004	5.7387754e-004	5.3598041e-004	4.6282907e-004
5.4655653e-004	4.4909369e-004	5.1182463e-004	5.1504199e-004
5.1665703e-004	5.1325528e-004	5.1758393e-004	5.2938133e-004
4.9268864e-004	5.1078207e-004	5.2967182e-004	5.3467749e-004
6.2270259e-004	6.0885363e-004	5.8654380e-004	5.9852807e-004
5.4668248e-004	5.1109273e-004	4.8578170e-004	4.9733583e-004
4.5680146e-004	4.6838408e-004	4.4600038e-004	4.4035088e-004
4.5120437e-004	4.6734545e-004	4.1851765e-004	4.5333173e-004
4.3482571e-004	4.2116163e-004	4.4994194e-004	4.9568123e-004
4.3422241e-004	4.3771868e-004	4.5613456e-004	4.6570153e-004
5.7074577e-004	6.2603784e-004	6.1176802e-004	6.6978110e-004
6.2218604e-004	5.6460989e-004	5.9245116e-004	5.7821500e-004
5.7108872e-004	5.4349079e-004	5.0011066e-004	4.7790002e-004
5.5005320e-004	4.7550422e-004	5.0724491e-004	5.3109670e-004
5.0916283e-004	4.8493494e-004	5.4117426e-004	4.8455629e-004
5.0740970e-004	5.0629770e-004	5.1994302e-004	5.0230925e-004
7.0371958e-004	7.4545562e-004	7.3864956e-004	7.9460119e-004
7.1589705e-004	8.2108671e-004	8.0743963e-004	8.1480498e-004
8.4253978e-004	8.5355606e-004	9.0638585e-004	8.5352499e-004
8.2558380e-004	9.1805497e-004	8.2391103e-004	9.1965306e-004
8.6491083e-004	9.0450284e-004	8.8696777e-004	8.7252776e-004
8.7934118e-004	8.7077789e-004	9.8534621e-004	8.8603102e-004
6.4227788e-004	6.3537445e-004	6.6626997e-004	6.6063362e-004
5.9735175e-004	6.0500499e-004	5.1616963e-004	5.6521023e-004
5.3381544e-004	5.3406553e-004	5.0229616e-004	5.0879145e-004
4.8397472e-004	5.1786098e-004	4.9556323e-004	5.0445932e-004
5.1054834e-004	4.8507361e-004	5.1405287e-004	5.1785956e-004
4.8297206e-004	5.1687877e-004	5.0987688e-004	5.2634595e-004
5.8096545e-004	5.5118886e-004	5.8374199e-004	6.2610328e-004
5.7277457e-004	5.8368550e-004	4.9393060e-004	4.8214551e-004
5.2472109e-004	5.1029267e-004	4.9466539e-004	4.6404766e-004
4.6873707e-004	4.4426898e-004	4.6874155e-004	4.5810111e-004
4.8900019e-004	4.5316966e-004	4.7969789e-004	5.2486303e-004
4.5391525e-004	4.9351977e-004	5.3594257e-004	5.1838848e-004
6.1700340e-004	6.2579433e-004	6.5282689e-004	6.3735548e-004
5.8523705e-004	6.1109044e-004	5.4697233e-004	5.2005627e-004
5.0143754e-004	5.1754005e-004	5.1495260e-004	4.0902843e-004
4.8033193e-004	4.3251402e-004	4.5699411e-004	4.7971556e-004
4.7749995e-004	4.6582389e-004	4.6434692e-004	4.9750731e-004
4.5920652e-004	4.5199249e-004	4.8025110e-004	4.8444806e-004
5.6244510e-004	5.3916335e-004	5.1191092e-004	5.3399095e-004
5.0031926e-004	4.5964930e-004	4.4533920e-004	4.4762577e-004
4.2028818e-004	4.0698270e-004	3.8963071e-004	4.1634888e-004
4.3713196e-004	4.1887329e-004	4.0050041e-004	4.1722613e-004

4.0876293e-004 3.9877078e-004 4.3649360e-004 4.5849584e-004
4.1109785e-004 4.2558292e-004 4.5869021e-004 4.5679376e-004
5.3828517e-004 5.8490716e-004 5.9028673e-004 6.0859611e-004
6.0404719e-004 5.1097713e-004 5.7517816e-004 5.2898253e-004
5.4050813e-004 5.6233321e-004 4.9712374e-004 4.5811435e-004
5.2311382e-004 4.5235803e-004 4.9392747e-004 4.8067717e-004
4.4944350e-004 4.8377458e-004 5.1546062e-004 4.8233018e-004
4.7375845e-004 4.8726373e-004 5.0747408e-004 4.8571693e-004
7.2097419e-004 7.5102708e-004 7.4437211e-004 7.9558737e-004
7.4235450e-004 8.7201828e-004 8.0028734e-004 8.8632957e-004
8.3503821e-004 7.9912189e-004 8.2806830e-004 8.3239964e-004
7.7955310e-004 8.9220128e-004 8.1400883e-004 8.6879989e-004
8.2693006e-004 8.6069130e-004 8.0322626e-004 8.5321635e-004
8.1079113e-004 8.5178037e-004 9.2056252e-004 8.8055144e-004
7.4446225e-004 7.4162980e-004 6.5586247e-004 6.7153797e-004
6.8449618e-004 7.2825974e-004 6.2240385e-004 7.1995452e-004
7.2554441e-004 6.3376226e-004 6.3277918e-004 6.7884162e-004
7.3204875e-004 6.6554590e-004 6.9511833e-004 6.9075113e-004
7.2863481e-004 6.1323673e-004 6.8517495e-004 7.4382803e-004
6.7905478e-004 7.3220760e-004 7.2333684e-004 8.0105272e-004
6.6498450e-004 6.4310741e-004 6.6242096e-004 6.6778014e-004
6.1528569e-004 6.2693571e-004 5.5810269e-004 5.6081770e-004
5.5086637e-004 5.3588868e-004 4.9252507e-004 5.6542461e-004
5.0074250e-004 5.1888340e-004 5.4866529e-004 5.7347545e-004
5.5553552e-004 5.1140187e-004 5.6539663e-004 5.4523042e-004
5.1919984e-004 5.3421319e-004 5.1991895e-004 5.5666405e-004
5.6181567e-004 5.2230098e-004 5.7927189e-004 5.8417265e-004
5.3764686e-004 5.3256539e-004 4.6246872e-004 4.4662029e-004
4.7696032e-004 4.5620071e-004 4.5487410e-004 4.3538024e-004
4.4762639e-004 4.6303377e-004 4.3667591e-004 4.2884378e-004
4.2482262e-004 4.4074969e-004 4.3571093e-004 4.7566945e-004
4.5144690e-004 4.6970098e-004 4.9572239e-004 4.6156154e-004
5.9326665e-004 5.8172636e-004 6.2717905e-004 6.1347656e-004
5.6036613e-004 6.0437697e-004 5.1199963e-004 5.1316239e-004
5.1246458e-004 5.0023389e-004 4.9299205e-004 4.4288048e-004
4.4983371e-004 4.0240661e-004 4.5510365e-004 4.6913893e-004
4.9465316e-004 4.6428979e-004 4.4459684e-004 4.8599950e-004
4.3735647e-004 4.6447587e-004 5.0023488e-004 5.1311369e-004
5.7442869e-004 5.5816938e-004 5.7112984e-004 5.8951265e-004
5.5642461e-004 5.1810896e-004 4.9293634e-004 4.7603837e-004
4.0631650e-004 4.3578049e-004 4.4802175e-004 3.7840442e-004
4.2623720e-004 4.1008602e-004 4.2612688e-004 4.3621683e-004
4.1857950e-004 3.9770346e-004 4.4500361e-004 4.5986519e-004
4.2570983e-004 4.2835156e-004 4.6598523e-004 4.7060784e-004
4.5583197e-004 4.3485846e-004 4.2398025e-004 4.4734012e-004
4.5350397e-004 4.4229575e-004 4.3357452e-004 4.1914757e-004
4.3271933e-004 4.5126310e-004 4.3977531e-004 4.4346759e-004
4.8415098e-004 4.3589842e-004 4.9012902e-004 4.8689466e-004
4.6221054e-004 5.1474932e-004 4.9548859e-004 5.5366919e-004
4.9169691e-004 5.4831646e-004 5.9186164e-004 5.6445366e-004
5.2999138e-004 5.7013126e-004 6.0616879e-004 6.2087728e-004
5.9977249e-004 5.2601301e-004 5.4583763e-004 5.3263867e-004

5.3466960e-004	5.0472057e-004	4.8019428e-004	4.3174709e-004
5.0452175e-004	4.3478383e-004	4.5775827e-004	4.7895544e-004
4.4993145e-004	4.8733808e-004	5.0261249e-004	4.8130599e-004
4.3592037e-004	4.9528582e-004	4.9221758e-004	4.6733031e-004
7.9904676e-004	7.7681428e-004	8.3312286e-004	8.3493140e-004
8.4750290e-004	9.6173627e-004	8.2034055e-004	9.2224467e-004
8.8941661e-004	7.7625700e-004	8.1348415e-004	8.1979575e-004
8.1909355e-004	8.3552039e-004	8.1773791e-004	8.2637790e-004
8.4991779e-004	8.0574238e-004	7.9681346e-004	9.1846676e-004
8.0619137e-004	9.1972956e-004	8.9446091e-004	9.7147895e-004
6.6339438e-004	6.3867847e-004	6.2438173e-004	6.2969644e-004
5.9364352e-004	6.1231657e-004	5.4361059e-004	5.4248841e-004
5.4873416e-004	5.0773301e-004	4.8454996e-004	5.3447972e-004
5.0786236e-004	4.9553007e-004	5.6351287e-004	5.5380262e-004
5.3938330e-004	4.9702411e-004	5.4028718e-004	5.2310782e-004
5.3489724e-004	5.3399634e-004	5.3100640e-004	5.5392257e-004
5.6879313e-004	5.4637004e-004	6.0036370e-004	5.9611654e-004
5.7671973e-004	5.7831552e-004	5.0987703e-004	4.8985091e-004
4.9706098e-004	4.8870587e-004	4.7765548e-004	4.5221941e-004
4.3596510e-004	4.0995373e-004	4.5411954e-004	4.6606468e-004
4.7921881e-004	4.6475398e-004	4.3467819e-004	4.7978076e-004
4.4701363e-004	4.6627023e-004	5.0613646e-004	4.8684613e-004
5.8119207e-004	5.7615672e-004	6.2298603e-004	6.3470927e-004
5.7725701e-004	5.5562238e-004	5.2419319e-004	5.0687859e-004
4.3748381e-004	4.5414535e-004	4.7492001e-004	3.9364067e-004
4.3488867e-004	4.1260685e-004	4.3153975e-004	4.6815528e-004
4.4067792e-004	4.2199923e-004	4.4192169e-004	4.4574273e-004
4.3685032e-004	4.5540069e-004	4.6340018e-004	4.8284410e-004
4.6162691e-004	4.7402624e-004	4.3141786e-004	4.5285197e-004
4.5610073e-004	4.6359856e-004	4.3930578e-004	4.3825676e-004
4.6190919e-004	4.8396846e-004	4.7624877e-004	4.6861769e-004
5.0315967e-004	4.2705172e-004	5.0486387e-004	5.2069286e-004
4.6809495e-004	5.3940903e-004	5.1303853e-004	5.6738510e-004
4.8696303e-004	5.3944391e-004	5.7991182e-004	5.6497137e-004
4.3781391e-004	4.3849926e-004	4.6038251e-004	4.8028580e-004
5.1881574e-004	4.8361510e-004	4.9216208e-004	4.4735950e-004
4.7364202e-004	4.5151228e-004	4.4056161e-004	4.8891708e-004
4.9504286e-004	5.1669827e-004	5.0381752e-004	5.5026553e-004
4.6464547e-004	5.2352995e-004	4.8084625e-004	5.6803662e-004
4.9651751e-004	5.7228997e-004	5.9770778e-004	5.8277408e-004
5.0507753e-004	5.3252681e-004	5.9338556e-004	6.2003394e-004
6.0543141e-004	5.6299180e-004	5.3508893e-004	5.4106033e-004
5.3607809e-004	4.9612022e-004	4.8383070e-004	4.4990987e-004
5.0008549e-004	4.4956442e-004	4.6996847e-004	5.0029083e-004
4.7141981e-004	5.1398521e-004	4.9806507e-004	5.2660343e-004
4.3850352e-004	5.3288783e-004	5.2865208e-004	4.8597736e-004
5.1586921e-004	5.8148868e-004	6.0505526e-004	6.4049287e-004
6.0674781e-004	5.4783393e-004	6.1127711e-004	5.5044175e-004
5.4429693e-004	5.7727256e-004	5.1263624e-004	4.7445875e-004
4.9849578e-004	4.7092898e-004	4.7690723e-004	4.8575598e-004
4.4246931e-004	5.0571846e-004	5.1461316e-004	5.0836822e-004
5.0044277e-004	5.0744096e-004	5.2599589e-004	4.9684626e-004

6.7938476e-004	6.6147777e-004	7.1412341e-004	7.5052890e-004
7.3240457e-004	7.7923982e-004	7.0211917e-004	7.5679798e-004
7.1813937e-004	6.3059240e-004	6.5401400e-004	6.3972978e-004
6.1888614e-004	6.6717507e-004	6.2988588e-004	6.7783175e-004
6.5094353e-004	6.3397274e-004	6.1883996e-004	7.2935135e-004
6.2814244e-004	7.0304335e-004	6.8111591e-004	7.4947337e-004
7.1457295e-004	6.3396050e-004	7.0284995e-004	7.2936525e-004
7.1731208e-004	7.4473819e-004	7.5014670e-004	8.0631717e-004
7.4693668e-004	6.9152442e-004	7.2236003e-004	6.9126018e-004
7.3698354e-004	7.1862178e-004	7.5150095e-004	6.9337114e-004
7.9629291e-004	8.0532306e-004	7.2997742e-004	8.1053453e-004
8.1348127e-004	8.6960083e-004	8.3866389e-004	9.1475107e-004
7.8640264e-004	7.3703640e-004	6.5311349e-004	7.2326798e-004
6.2443397e-004	6.5018079e-004	6.2967201e-004	6.3277021e-004
6.4089547e-004	6.5242087e-004	5.6946924e-004	6.3948841e-004
6.8772932e-004	6.6364036e-004	7.1754145e-004	6.4723087e-004
6.2805494e-004	6.5505464e-004	6.3161322e-004	6.4625875e-004
6.5868668e-004	6.7911156e-004	6.8646460e-004	7.0560449e-004
5.5976918e-004	5.5570395e-004	5.9512418e-004	6.0023190e-004
6.0622177e-004	5.8664974e-004	5.3803281e-004	5.0274395e-004
5.0440271e-004	4.9551284e-004	4.9158286e-004	4.6585637e-004
4.4154634e-004	4.2293278e-004	4.6852208e-004	4.9212914e-004
4.8039944e-004	4.7657097e-004	4.4395400e-004	4.7388778e-004
4.6130025e-004	4.7559813e-004	4.9880489e-004	4.7548389e-004
6.0486096e-004	6.0657605e-004	6.5370393e-004	6.7343465e-004
6.0021711e-004	5.7239018e-004	5.3536805e-004	5.3760187e-004
4.5357428e-004	4.6970273e-004	4.9183794e-004	4.3695726e-004
4.4961498e-004	4.3159743e-004	4.5033259e-004	4.9495503e-004
4.6203140e-004	4.3750431e-004	4.5508228e-004	4.4336340e-004
4.4789790e-004	4.9618196e-004	4.6333025e-004	4.8629928e-004
4.8372702e-004	4.8941129e-004	5.1450855e-004	5.4362013e-004
5.7193005e-004	5.2340254e-004	5.1751790e-004	4.7615136e-004
5.1992945e-004	5.0257437e-004	4.7526145e-004	5.3472574e-004
5.1715999e-004	5.6606016e-004	5.4576858e-004	5.9171440e-004
4.9794419e-004	5.6106866e-004	4.9483081e-004	6.0688077e-004
5.4666275e-004	5.9899141e-004	6.0629319e-004	6.1669727e-004
5.3090715e-004	5.5230821e-004	6.2091145e-004	6.6941643e-004
6.4935813e-004	6.1628599e-004	5.6870440e-004	5.7392310e-004
5.6673981e-004	5.3902804e-004	5.0698670e-004	4.7708928e-004
5.2136496e-004	4.6787392e-004	4.9944608e-004	5.2634539e-004
4.8651571e-004	5.3828888e-004	5.2053459e-004	5.6164310e-004
4.6863486e-004	5.6159496e-004	5.5509970e-004	5.0146765e-004
5.4272426e-004	6.1088178e-004	6.3132659e-004	6.9440436e-004
6.4363216e-004	6.1935160e-004	6.4152884e-004	5.9753114e-004
5.7549668e-004	5.9624398e-004	5.3463457e-004	5.1565710e-004
5.2128552e-004	5.0710704e-004	4.8591639e-004	5.2166983e-004
4.8059255e-004	5.0795558e-004	5.2570696e-004	5.3521914e-004
5.1789092e-004	5.1984924e-004	5.3929252e-004	5.1498225e-004
6.2113897e-004	6.0259875e-004	6.6794347e-004	6.9797228e-004
6.7606076e-004	6.9535278e-004	6.5045281e-004	6.8069779e-004
6.4001915e-004	5.7949350e-004	5.9522493e-004	5.7945795e-004
5.5883327e-004	6.1633255e-004	5.7232150e-004	6.3568575e-004

5.9385615e-004 5.8829150e-004 5.7699584e-004 6.6592141e-004
5.7798349e-004 6.4959157e-004 6.1593228e-004 6.8648963e-004
7.1408856e-004 6.3340686e-004 7.0560646e-004 7.4372394e-004
7.2506684e-004 7.6501543e-004 7.5180973e-004 7.7232518e-004
7.3545154e-004 6.9369785e-004 7.1308814e-004 6.7904938e-004
7.2606266e-004 7.1146687e-004 7.6371797e-004 7.1201977e-004
7.6908538e-004 7.9548206e-004 7.3835693e-004 8.1070358e-004
8.1282924e-004 8.5557655e-004 8.1966541e-004 8.9464909e-004
7.2235063e-004 6.7122726e-004 5.9917051e-004 6.4739837e-004
5.6767552e-004 6.0403277e-004 5.7246481e-004 5.5810154e-004
5.8322681e-004 5.6429642e-004 5.0442279e-004 5.4192873e-004
5.5673924e-004 5.4794436e-004 6.1554775e-004 5.4193720e-004
5.4741553e-004 5.5830092e-004 5.7327215e-004 5.7031630e-004
5.9448931e-004 6.2749448e-004 5.9859832e-004 6.1070881e-004
5.9614900e-004 6.2256034e-004 6.4529186e-004 6.7531374e-004
6.5984482e-004 6.4556244e-004 5.9843351e-004 5.5056282e-004
5.2796339e-004 5.1665328e-004 5.4711875e-004 5.2114612e-004
4.6062597e-004 4.5960109e-004 4.9438798e-004 5.4076064e-004
4.9411561e-004 4.8919897e-004 4.6634172e-004 4.7060854e-004
4.8461306e-004 4.9723926e-004 4.8870555e-004 4.8444280e-004
6.5481394e-004 6.2521208e-004 6.5864046e-004 6.6900272e-004
6.3475221e-004 5.7952857e-004 5.4034579e-004 5.6561451e-004
4.7132374e-004 5.1021928e-004 5.2627374e-004 4.8863603e-004
4.9363569e-004 4.8015877e-004 4.9833567e-004 5.2228803e-004
5.0432138e-004 4.6910007e-004 5.0481218e-004 4.7307784e-004
4.7241163e-004 5.4123024e-004 4.9436453e-004 4.9279187e-004
6.0358275e-004 6.5623365e-004 6.9216889e-004 7.6941871e-004
7.2013726e-004 7.0057153e-004 6.6480861e-004 6.4808934e-004
6.2366841e-004 6.1853099e-004 5.6750438e-004 5.3236643e-004
5.6477335e-004 4.9705770e-004 5.5175582e-004 5.4678629e-004
5.0379695e-004 5.6243158e-004 5.6109151e-004 5.9125345e-004
5.1682277e-004 5.7610530e-004 5.7355234e-004 5.0658274e-004
5.8732108e-004 6.5354750e-004 6.3110223e-004 7.2326387e-004
6.7248971e-004 6.8134094e-004 6.5133530e-004 6.4189489e-004
5.9845170e-004 6.0597663e-004 5.5091224e-004 5.5684103e-004
5.5029709e-004 5.7339131e-004 4.9611905e-004 5.6702038e-004
5.4759985e-004 4.9541700e-004 5.3638211e-004 5.6203335e-004
5.3263051e-004 5.2611314e-004 5.3934527e-004 5.4147509e-004
6.7526296e-004 6.3491777e-004 6.5195031e-004 7.1604170e-004
6.7305217e-004 7.2620850e-004 6.8312233e-004 6.6804183e-004
7.2430726e-004 6.6730347e-004 6.9215614e-004 6.2820401e-004
6.8942796e-004 6.6949009e-004 7.3864000e-004 6.8845515e-004
7.3050478e-004 7.3233263e-004 7.0021648e-004 7.7115408e-004
7.9880228e-004 8.0782101e-004 7.9076328e-004 8.4402732e-004
7.3908384e-004 8.0504826e-004 7.0798089e-004 7.7429171e-004
7.2177043e-004 6.7643385e-004 6.1054624e-004 6.5677332e-004
7.0905172e-004 6.9629103e-004 6.0485674e-004 7.0350728e-004
6.6996099e-004 6.2199336e-004 6.1405184e-004 6.3428917e-004
6.0265162e-004 6.4955059e-004 6.5671167e-004 6.1654633e-004
6.4422970e-004 6.5862472e-004 6.9323540e-004 6.6509952e-004
6.4906651e-004 6.6062206e-004 7.0146192e-004 7.6157133e-004
7.1865128e-004 6.8204773e-004 6.1764765e-004 5.9495303e-004

5.4410743e-004	5.3245397e-004	5.7850893e-004	5.8402585e-004
5.0696943e-004	5.0768192e-004	5.3062705e-004	5.8938592e-004
5.2132315e-004	5.0029796e-004	4.9445526e-004	4.8338307e-004
5.0737867e-004	5.3915026e-004	4.9730223e-004	4.9750971e-004
6.7809883e-004	6.2875787e-004	6.6499681e-004	6.2448274e-004
6.6449739e-004	6.0501939e-004	5.6052984e-004	5.9843319e-004
5.1440515e-004	5.7560770e-004	5.7530528e-004	5.4812519e-004
5.4102630e-004	5.4834205e-004	5.4493893e-004	5.7309044e-004
5.5308199e-004	5.1868577e-004	5.6035443e-004	5.3182797e-004
5.1771651e-004	5.5594908e-004	5.3354561e-004	5.2081996e-004
6.5751249e-004	7.2976759e-004	7.3602131e-004	7.9315301e-004
7.6793478e-004	7.5897254e-004	7.2098646e-004	7.0064706e-004
6.6288443e-004	6.7887887e-004	6.0598763e-004	6.0689624e-004
5.8074895e-004	5.4408958e-004	5.8997910e-004	5.6163578e-004
5.5792196e-004	5.6227879e-004	5.7249465e-004	6.0806597e-004
5.6669298e-004	5.6861031e-004	5.7917810e-004	5.3688383e-004
6.1360987e-004	6.9979661e-004	6.2557973e-004	7.6271596e-004
6.9700526e-004	7.1315390e-004	6.5684452e-004	6.8273705e-004
6.3362197e-004	6.1424026e-004	5.7482017e-004	5.8519567e-004
5.7762716e-004	6.3403370e-004	5.4474930e-004	6.0031394e-004
5.9331705e-004	5.1787133e-004	5.4621238e-004	5.9387772e-004
5.3304245e-004	5.5880862e-004	5.5367584e-004	5.6778846e-004
6.7252408e-004	6.4921718e-004	6.2712730e-004	7.1302327e-004
6.8310036e-004	7.1007731e-004	6.6428867e-004	6.1469591e-004
7.3243554e-004	6.2471228e-004	6.7214615e-004	5.8871481e-004
6.4710258e-004	6.4607353e-004	7.0767199e-004	6.7842726e-004
6.9901437e-004	6.9881754e-004	6.8081068e-004	7.5666339e-004
7.8511203e-004	7.7383762e-004	7.7871022e-004	8.1203091e-004
6.4393018e-004	6.4677744e-004	7.0526057e-004	6.5292071e-004
7.0346535e-004	7.2228898e-004	7.5665541e-004	6.7857126e-004
7.4671983e-004	6.6630995e-004	6.9134013e-004	6.3568527e-004
6.7235169e-004	7.4320991e-004	6.8774927e-004	7.7297741e-004
7.3751617e-004	6.8949434e-004	6.7849921e-004	7.8063292e-004
7.4750950e-004	7.3365415e-004	7.7069021e-004	8.2160893e-004
7.9287305e-004	8.8763810e-004	7.3871901e-004	8.3461436e-004
8.0096156e-004	7.6944884e-004	6.8337805e-004	7.4408774e-004
7.6173975e-004	7.2217608e-004	6.5944379e-004	7.1863092e-004
7.4165172e-004	7.0025091e-004	6.7311370e-004	7.1665872e-004
6.9902583e-004	6.7200867e-004	6.5874965e-004	6.9130219e-004
6.9387852e-004	6.6525480e-004	7.5580174e-004	6.6579657e-004
6.0349966e-004	6.2770238e-004	6.0993215e-004	6.8293125e-004
6.6880081e-004	6.7941681e-004	6.3439144e-004	6.0438101e-004
5.6357154e-004	5.5663158e-004	5.6780690e-004	5.7544210e-004
5.5836684e-004	5.6495356e-004	6.0328435e-004	6.3238462e-004
5.5070825e-004	5.5868241e-004	5.3276100e-004	5.2823254e-004
5.3367225e-004	5.3531447e-004	5.5985217e-004	5.3591882e-004
7.3451213e-004	6.5639855e-004	7.5345584e-004	7.6104735e-004
7.6975870e-004	6.6674632e-004	5.9202213e-004	6.0977040e-004
5.5642793e-004	5.8108666e-004	5.8689679e-004	6.1120436e-004
5.7025366e-004	5.5776072e-004	5.5267251e-004	6.3735525e-004
5.6216901e-004	5.0806279e-004	5.4705710e-004	5.2016080e-004
5.3385773e-004	5.6603941e-004	5.0026324e-004	5.2350489e-004

6.9199088e-004	7.2353301e-004	7.0381460e-004	7.0207664e-004
7.2618976e-004	7.4403576e-004	6.8230506e-004	6.9367077e-004
6.2599001e-004	6.5301808e-004	6.1958989e-004	5.9034914e-004
5.9986065e-004	5.4594134e-004	5.9465779e-004	6.0876703e-004
5.8564410e-004	5.4219781e-004	5.6722903e-004	6.0957298e-004
5.4479489e-004	5.5004626e-004	5.5292493e-004	5.2939113e-004
6.6156912e-004	7.3465188e-004	7.2421618e-004	7.9513680e-004
7.6357397e-004	7.3621556e-004	6.9165199e-004	6.8713339e-004
6.7870686e-004	6.8882198e-004	5.9546603e-004	6.6500189e-004
5.7658348e-004	6.3105830e-004	5.9033318e-004	5.7100842e-004
6.1868000e-004	5.5395238e-004	5.5329844e-004	6.0740249e-004
6.1576358e-004	5.5053740e-004	5.8751351e-004	5.7895912e-004
5.9827339e-004	7.0223026e-004	6.1408606e-004	7.5855709e-004
6.6318631e-004	7.0017921e-004	6.4991377e-004	7.0599197e-004
6.7809461e-004	6.3407542e-004	6.3988600e-004	6.1425612e-004
6.0768839e-004	6.3979071e-004	6.3501994e-004	6.3508131e-004
5.9224661e-004	5.9801755e-004	5.8088067e-004	6.2280039e-004
5.5568728e-004	6.3739027e-004	6.0770247e-004	6.5103536e-004
4.7917438e-004	5.8650298e-004	6.1189196e-004	5.8901633e-004
6.2587831e-004	6.3457221e-004	5.9000663e-004	6.0393591e-004
5.8278273e-004	5.8973144e-004	5.9183255e-004	6.0022080e-004
5.9479805e-004	6.6566134e-004	6.4105177e-004	6.6996375e-004
5.9244569e-004	6.1811187e-004	6.6008839e-004	6.4120759e-004
5.9703451e-004	6.5303397e-004	6.2773026e-004	7.2924381e-004
5.0602563e-004	5.4353388e-004	5.4996517e-004	5.9715507e-004
5.6912944e-004	6.1837933e-004	5.8103669e-004	5.5575033e-004
5.9174522e-004	5.1959624e-004	5.7596341e-004	5.6101309e-004
5.4315574e-004	5.7849949e-004	6.0746248e-004	6.0347599e-004
5.9024026e-004	6.0092648e-004	6.0436067e-004	6.5845823e-004
6.6358100e-004	6.6262265e-004	6.1132332e-004	7.0042907e-004
7.1403953e-004	7.2904534e-004	7.4772119e-004	7.2986646e-004
7.4842166e-004	7.3322481e-004	7.3120519e-004	6.5124528e-004
7.6174166e-004	6.5706625e-004	6.8193859e-004	6.1436406e-004
6.3394001e-004	7.1962000e-004	7.0593516e-004	7.3927423e-004
7.1816065e-004	7.4270304e-004	7.0948746e-004	8.0686658e-004
7.8720080e-004	7.6314829e-004	8.2402656e-004	8.1825849e-004
6.6847686e-004	7.1845410e-004	6.3232388e-004	6.8917159e-004
6.6914152e-004	6.2948656e-004	5.5140811e-004	5.6683135e-004
5.7746169e-004	5.7222415e-004	5.0632832e-004	5.4867299e-004
5.4203524e-004	5.0329273e-004	5.0001920e-004	5.2725932e-004
5.3104119e-004	5.3337059e-004	5.2457635e-004	5.3114582e-004
5.5168213e-004	5.1917326e-004	5.9123477e-004	5.3193109e-004
6.7675805e-004	6.6631002e-004	6.8807507e-004	7.8730402e-004
7.1434170e-004	7.4424114e-004	6.5486887e-004	6.7202219e-004
5.9397190e-004	5.9837841e-004	6.0457866e-004	6.4472313e-004
6.2562941e-004	6.0042601e-004	6.3824597e-004	7.0203633e-004
5.9493222e-004	5.7808222e-004	5.5542045e-004	5.6403759e-004
5.7023978e-004	5.9202935e-004	5.9341996e-004	5.8032127e-004
6.9763011e-004	6.2929872e-004	7.1352307e-004	6.7462295e-004
7.1714493e-004	6.3173773e-004	5.9401465e-004	5.9925068e-004
5.6273198e-004	6.0222385e-004	5.9431439e-004	6.0564936e-004
5.9446342e-004	5.7113156e-004	5.5340728e-004	6.5265756e-004

5.7462192e-004	5.2576655e-004	5.6512262e-004	5.6865145e-004
5.5509200e-004	5.5744953e-004	5.2984437e-004	5.4894495e-004
6.8829380e-004	7.1078872e-004	6.9828505e-004	6.6237446e-004
6.9229781e-004	7.2336545e-004	6.6671384e-004	6.9319470e-004
6.2844281e-004	6.6399787e-004	6.0881407e-004	6.0855637e-004
5.7248402e-004	5.6445315e-004	6.2217718e-004	5.8265844e-004
6.1137819e-004	5.3940772e-004	5.4807108e-004	5.9503126e-004
5.4084236e-004	5.3292022e-004	5.2339982e-004	5.3568934e-004
6.6781760e-004	7.3595691e-004	6.9124198e-004	8.3496408e-004
7.4433476e-004	7.0683470e-004	6.7161999e-004	6.4656520e-004
6.7045266e-004	6.6962439e-004	6.0750246e-004	6.6180878e-004
5.8377125e-004	6.8055264e-004	6.1106886e-004	5.7140726e-004
5.9846655e-004	5.6617986e-004	5.5518572e-004	6.0267933e-004
6.0761781e-004	5.6851158e-004	6.0050706e-004	5.8028665e-004
4.4441265e-004	5.9461591e-004	5.9245156e-004	5.9471095e-004
6.4727997e-004	6.5271581e-004	5.9627247e-004	6.3065664e-004
5.9930277e-004	5.9553437e-004	5.9444945e-004	6.3533561e-004
6.0006727e-004	6.7439792e-004	6.5038921e-004	6.6660142e-004
6.3235937e-004	6.4191263e-004	6.6941664e-004	6.6486630e-004
6.1436162e-004	6.5866427e-004	6.2942918e-004	7.4355377e-004
5.4950303e-004	5.7122023e-004	5.3945700e-004	6.1100165e-004
5.6722804e-004	5.9539439e-004	5.7146576e-004	5.5405665e-004
6.1191509e-004	5.2946825e-004	5.5855347e-004	5.5839767e-004
5.6073620e-004	5.5026665e-004	6.1922648e-004	6.0653890e-004
6.0178628e-004	6.1402046e-004	6.1034122e-004	6.6723663e-004
6.9701954e-004	6.8986783e-004	6.1808233e-004	7.1635577e-004
7.4660309e-004	7.8126561e-004	7.8837316e-004	7.0959846e-004
7.1762827e-004	6.7622900e-004	7.2283244e-004	6.5554955e-004
7.1063392e-004	6.3572981e-004	6.4823081e-004	6.3484142e-004
6.1933475e-004	7.2895964e-004	7.0433415e-004	7.1458888e-004
7.1286180e-004	7.3840974e-004	6.9382906e-004	7.8433983e-004
7.4486394e-004	7.6864098e-004	7.5810133e-004	7.6666582e-004
6.6894788e-004	6.9504320e-004	6.2011089e-004	6.5026717e-004
6.7215673e-004	6.1858044e-004	5.6512524e-004	5.6452365e-004
5.7297774e-004	5.4147590e-004	5.1294164e-004	5.2020584e-004
5.3531471e-004	5.0008111e-004	4.9564036e-004	5.2335122e-004
5.2355867e-004	4.9802887e-004	5.0268874e-004	5.1559937e-004
5.1464125e-004	5.1304647e-004	5.5859785e-004	5.1571575e-004
6.6679336e-004	6.7029448e-004	7.0238365e-004	7.9605855e-004
7.2920653e-004	7.6970572e-004	6.8139202e-004	6.8609408e-004
6.1457728e-004	6.4714333e-004	6.1864359e-004	6.3135310e-004
6.4663729e-004	6.4532692e-004	6.6867650e-004	7.3593413e-004
6.3140923e-004	6.1761617e-004	5.7145176e-004	6.0500081e-004
5.9844503e-004	5.9886073e-004	6.2116854e-004	6.5704338e-004
6.9945431e-004	6.3150440e-004	7.2651450e-004	7.2521609e-004
6.7855389e-004	6.5877177e-004	6.0095193e-004	6.2409849e-004
5.6737626e-004	5.9272314e-004	5.6573872e-004	6.0339096e-004
6.0950234e-004	5.5895683e-004	5.8922803e-004	6.6875736e-004
5.7938544e-004	5.2649739e-004	5.5599275e-004	5.8665565e-004
5.3352118e-004	5.8054841e-004	5.5224140e-004	5.5653069e-004
4.7948172e-004	5.5351538e-004	6.3507201e-004	6.3860770e-004
6.1736368e-004	6.5359382e-004	6.5124211e-004	6.3573830e-004

7.0725518e-004	5.9674493e-004	5.9214448e-004	6.3680481e-004
5.3525479e-004	6.1808339e-004	6.0905717e-004	5.9865738e-004
5.7821648e-004	5.5912104e-004	5.6463179e-004	5.4193349e-004
5.6071980e-004	5.5215879e-004	5.7682574e-004	5.6007327e-004
6.5290704e-004	6.5745211e-004	6.5105227e-004	6.1519396e-004
6.3410825e-004	7.0954331e-004	6.5462296e-004	6.5744971e-004
5.9271300e-004	6.5101088e-004	6.2506393e-004	6.0451229e-004
5.8835500e-004	5.7781120e-004	6.3351321e-004	6.1834951e-004
6.2229131e-004	5.6158727e-004	5.4209959e-004	5.9562179e-004
5.2855932e-004	5.2549492e-004	5.3315877e-004	5.6655449e-004
6.6356797e-004	7.1322975e-004	6.8174282e-004	7.6268462e-004
6.7982325e-004	6.8641143e-004	6.1411464e-004	6.1969688e-004
6.6117027e-004	6.6046539e-004	5.9086327e-004	6.8894983e-004
5.7660424e-004	6.5295950e-004	6.1878994e-004	5.4116271e-004
5.8082027e-004	5.6711909e-004	5.5016579e-004	5.7480700e-004
6.0105979e-004	5.6510663e-004	5.7987039e-004	5.8112245e-004
6.0296597e-004	6.9446825e-004	6.3476016e-004	7.9391002e-004
6.9500106e-004	6.9831575e-004	6.6292731e-004	6.7687186e-004
7.2655585e-004	6.7676479e-004	6.6342879e-004	6.3397644e-004
6.0604214e-004	6.6319697e-004	6.9305186e-004	5.9926535e-004
5.8383252e-004	5.8957479e-004	5.9284050e-004	6.1417119e-004
5.6330253e-004	6.0852086e-004	5.8290927e-004	6.5811112e-004
4.9969677e-004	6.6572480e-004	6.2510645e-004	6.8013369e-004
7.0393900e-004	7.2099535e-004	6.3792706e-004	6.8556108e-004
6.8107732e-004	6.6234454e-004	6.3310645e-004	6.7120908e-004
6.6490113e-004	6.9830846e-004	6.7305822e-004	6.8169383e-004
6.5002617e-004	6.7749142e-004	6.9753545e-004	6.7863279e-004
6.2740335e-004	6.8237515e-004	6.5051828e-004	7.5584344e-004
5.0672730e-004	5.7092367e-004	5.3596051e-004	5.9517390e-004
5.8145952e-004	5.9627176e-004	5.8667193e-004	6.0223395e-004
6.1748103e-004	5.6052138e-004	5.7120534e-004	6.2801826e-004
5.8920123e-004	5.6315030e-004	6.3450894e-004	6.2498222e-004
6.4239168e-004	6.2316959e-004	6.3975365e-004	6.7050678e-004
7.0221935e-004	6.9249117e-004	6.0151200e-004	7.1360708e-004
6.5085037e-004	6.7603128e-004	6.6221911e-004	6.3533441e-004
6.1883059e-004	5.8029348e-004	5.8602837e-004	5.8837929e-004
6.0330120e-004	5.3551195e-004	5.4998025e-004	5.6972553e-004
5.6230009e-004	6.3375778e-004	6.3015839e-004	6.3920196e-004
6.2071400e-004	6.5710611e-004	6.4624081e-004	7.0680318e-004
6.9395741e-004	7.2100895e-004	6.9515109e-004	6.8973590e-004
8.9587484e-004	8.7440519e-004	7.9866125e-004	8.1762619e-004
7.9166243e-004	7.7628202e-004	8.2428841e-004	7.4721935e-004
7.6116428e-004	7.4908651e-004	7.7731325e-004	7.5667776e-004
7.3247041e-004	8.0005203e-004	7.8068911e-004	7.9707887e-004
8.2817693e-004	8.3474192e-004	7.8617618e-004	8.2212609e-004
7.6735814e-004	8.8433317e-004	6.8915637e-004	8.7268891e-004
6.8050587e-004	6.7958183e-004	6.3904653e-004	6.1297926e-004
6.7266698e-004	6.1178702e-004	5.7426165e-004	5.7771971e-004
5.7974607e-004	5.2520785e-004	5.3702389e-004	5.2253556e-004
5.3841773e-004	5.0369276e-004	5.0418611e-004	5.3941651e-004
5.0958616e-004	4.8176218e-004	4.9592440e-004	5.1722225e-004
4.9687000e-004	5.2112432e-004	5.3114512e-004	5.2039223e-004

5.3789136e-004	5.0381722e-004	5.1486189e-004	5.2680842e-004
5.1825056e-004	5.5494285e-004	5.8347393e-004	5.4842960e-004
5.6229232e-004	5.4677739e-004	5.5351543e-004	5.7411064e-004
5.2480922e-004	5.1191309e-004	5.8188908e-004	6.0857879e-004
5.8715417e-004	6.0058807e-004	6.0344777e-004	6.5585681e-004
5.5397670e-004	6.4142438e-004	6.6018314e-004	5.9315302e-004
6.6842315e-004	6.7050735e-004	7.3503281e-004	8.0732815e-004
8.1027730e-004	8.1015105e-004	7.3391128e-004	7.0913816e-004
6.6246391e-004	7.2329841e-004	6.5137629e-004	6.3827330e-004
6.5126341e-004	6.6552042e-004	6.8501706e-004	7.2953867e-004
6.4010121e-004	6.3764465e-004	5.7627236e-004	6.2931167e-004
5.9869203e-004	6.0668046e-004	6.5050533e-004	6.9323574e-004
6.7791381e-004	6.1848295e-004	7.1690152e-004	7.5867336e-004
6.2305732e-004	6.8168413e-004	5.8952282e-004	6.5213026e-004
5.7236163e-004	5.8344132e-004	5.5897668e-004	6.0015077e-004
5.9376023e-004	5.6820254e-004	6.2957421e-004	6.5600294e-004
5.9605853e-004	5.6086047e-004	5.4050040e-004	6.2197408e-004
5.3842870e-004	5.8778185e-004	5.8557048e-004	5.9363942e-004
4.8324645e-004	5.6471703e-004	6.0451667e-004	6.0449324e-004
5.9708561e-004	6.4193060e-004	6.5060567e-004	6.1210622e-004
7.0715434e-004	5.7581837e-004	5.8179650e-004	6.0724768e-004
5.2972374e-004	6.2744747e-004	5.7921540e-004	5.7830982e-004
5.9131373e-004	5.1017213e-004	5.4845035e-004	5.1507026e-004
5.5661279e-004	5.3142983e-004	5.4580817e-004	5.0889885e-004
6.4560429e-004	6.9129570e-004	6.4034770e-004	6.7941686e-004
6.2807507e-004	6.8741695e-004	5.9061854e-004	6.5304668e-004
6.5500769e-004	6.5387848e-004	5.9445230e-004	6.8185275e-004
5.8803208e-004	6.1386294e-004	6.1443012e-004	5.5482099e-004
5.9145321e-004	5.5789712e-004	5.6489102e-004	5.6928508e-004
5.5635482e-004	5.6582778e-004	5.6671766e-004	5.7661759e-004
6.1163900e-004	6.7152787e-004	6.3833938e-004	7.9768889e-004
7.0201753e-004	6.6392319e-004	6.3675949e-004	6.2454266e-004
6.7967182e-004	6.4157044e-004	6.1321692e-004	6.1695807e-004
5.7312692e-004	6.5674918e-004	6.8033235e-004	5.6610767e-004
5.5953774e-004	5.6567950e-004	5.8908360e-004	5.9531875e-004
5.5666371e-004	5.8696492e-004	5.5595656e-004	5.9130260e-004
5.7721658e-004	7.2029427e-004	6.6484638e-004	7.4908658e-004
7.3029578e-004	7.7007999e-004	6.8150890e-004	7.2510268e-004
7.3634747e-004	7.1165913e-004	6.6532369e-004	6.8258049e-004
6.8172040e-004	6.9077549e-004	6.7985019e-004	6.6785087e-004
6.4479894e-004	6.5521908e-004	6.5274388e-004	6.6939116e-004
6.0723245e-004	6.5780297e-004	6.2111689e-004	7.0340502e-004
5.6244149e-004	5.6745109e-004	5.6671601e-004	5.9125180e-004
5.6476197e-004	5.3681766e-004	5.3608797e-004	5.7609133e-004
5.7701848e-004	5.2731856e-004	5.2417243e-004	5.6372806e-004
5.7886352e-004	5.7742868e-004	6.0347399e-004	6.2972418e-004
6.1377062e-004	6.2507627e-004	6.4887089e-004	6.7985155e-004
6.7895500e-004	7.1329779e-004	6.5525605e-004	7.0439526e-004
7.4376019e-004	7.6012495e-004	6.9516210e-004	7.1103206e-004
6.7488635e-004	6.4208261e-004	6.6031764e-004	6.4498848e-004
6.1557506e-004	5.9848854e-004	5.6473454e-004	6.1219056e-004
5.7657724e-004	6.8058503e-004	6.6108264e-004	6.6819201e-004

7.0651766e-004 7.1103664e-004 6.8926527e-004 7.3340109e-004
6.7304246e-004 7.6658074e-004 6.1884374e-004 7.0925544e-004
7.8665590e-004 8.1189751e-004 7.7667463e-004 8.1413012e-004
7.7069888e-004 7.7355524e-004 8.2038113e-004 8.4432172e-004
6.7409765e-004 7.3678011e-004 8.8109296e-004 8.4839153e-004
7.7073126e-004 8.2444571e-004 7.1118036e-004 8.5884280e-004
6.9897817e-004 7.6662219e-004 7.9368566e-004 8.3528718e-004
7.9943131e-004 8.6359863e-004 6.8800895e-004 8.4284932e-004
8.6655616e-004 8.8829323e-004 9.2833089e-004 7.1243249e-004
7.5349571e-004 8.4376280e-004 7.6505638e-004 8.6394098e-004
8.4185254e-004 8.4367761e-004 8.0591653e-004 7.7844699e-004
8.4685937e-004 7.8268530e-004 8.4327564e-004 8.5666326e-004
7.9207304e-004 8.1244070e-004 7.8059331e-004 8.4325900e-004
7.7718437e-004 7.8313411e-004 7.9032122e-004 7.7209994e-004
5.8207990e-004 5.7426537e-004 5.1795656e-004 5.3423802e-004
5.5598855e-004 4.9968899e-004 4.7253157e-004 4.5364547e-004
4.6834341e-004 4.3665462e-004 4.2893473e-004 4.2882413e-004
4.4075011e-004 4.1864463e-004 4.1947720e-004 4.4384304e-004
4.3301134e-004 4.2234618e-004 4.1133886e-004 4.4851917e-004
4.1437404e-004 4.4595654e-004 4.6848611e-004 4.5048546e-004
6.1066171e-004 6.3591739e-004 6.8570764e-004 6.8033581e-004
7.4311538e-004 7.3684125e-004 6.3655319e-004 6.9867409e-004
6.5936503e-004 6.4913878e-004 5.9667123e-004 6.1184210e-004
6.1520890e-004 6.4262685e-004 6.3141403e-004 6.0258312e-004
5.3540758e-004 5.8762207e-004 5.5451842e-004 5.7917240e-004
5.3755032e-004 5.6097085e-004 6.0333402e-004 5.9435057e-004
6.8175978e-004 6.8245873e-004 7.8498619e-004 8.6840177e-004
7.9614320e-004 7.9914147e-004 7.3867888e-004 7.1288117e-004
6.7128528e-004 7.2753051e-004 6.6144120e-004 6.9809395e-004
6.4493729e-004 6.6063010e-004 7.1143266e-004 7.2176926e-004
6.7126603e-004 6.5677817e-004 5.8011561e-004 6.8127098e-004
6.1415802e-004 6.4636636e-004 6.8142804e-004 7.3452879e-004
5.0542898e-004 5.5955336e-004 6.4109781e-004 6.0981086e-004
6.1555755e-004 6.5476613e-004 6.3755336e-004 6.2359082e-004
6.7239990e-004 5.6302006e-004 5.5096693e-004 6.2578135e-004
5.0645015e-004 5.9539445e-004 5.6889764e-004 5.4953688e-004
5.7058921e-004 4.9394212e-004 5.7480401e-004 5.1218028e-004
5.0744793e-004 5.1004053e-004 5.2840005e-004 5.0981390e-004
6.2018925e-004 6.7607850e-004 6.2111810e-004 6.9898933e-004
6.2364919e-004 6.9956527e-004 6.0177094e-004 6.4601320e-004
6.6996239e-004 6.3142000e-004 5.8825838e-004 6.5623580e-004
5.8297419e-004 6.2449700e-004 5.9951967e-004 5.5280107e-004
5.6745534e-004 5.5738873e-004 5.9563978e-004 5.7331818e-004
5.5098084e-004 5.8620467e-004 5.7015597e-004 5.7596881e-004
5.9763461e-004 6.7665555e-004 6.3166796e-004 7.7043171e-004
6.7859917e-004 6.6299879e-004 6.3885175e-004 6.5928609e-004
6.9355856e-004 6.2950086e-004 6.0959336e-004 6.1165486e-004
6.2066989e-004 6.2214815e-004 6.9885288e-004 6.0910665e-004
5.9665107e-004 5.8378630e-004 5.8297451e-004 5.9973430e-004
5.4033146e-004 5.8065922e-004 5.4004531e-004 5.8668750e-004
6.3540642e-004 7.5973173e-004 7.5606765e-004 8.2293771e-004
8.2827160e-004 7.8656361e-004 7.2718595e-004 7.6525641e-004

7.5928288e-004	7.2960788e-004	7.0498456e-004	7.1426250e-004
6.7949381e-004	6.8367734e-004	6.6526781e-004	6.4881309e-004
6.6629189e-004	6.4626959e-004	6.6087740e-004	7.0410031e-004
6.1175157e-004	6.5980136e-004	6.3076125e-004	6.5755121e-004
5.6183421e-004	6.2686739e-004	5.8909999e-004	6.7032718e-004
6.1839253e-004	6.6054010e-004	6.1823451e-004	6.4933402e-004
6.7759033e-004	6.5249184e-004	6.0120359e-004	6.8529767e-004
6.5237847e-004	5.7497562e-004	6.8344100e-004	6.4663748e-004
7.0461852e-004	6.6796513e-004	7.1446080e-004	7.0441418e-004
7.1360118e-004	7.2495383e-004	6.5090237e-004	7.1723122e-004
5.3684256e-004	5.5943755e-004	5.5746762e-004	5.9151127e-004
5.6734779e-004	5.2485314e-004	5.3008726e-004	5.8968784e-004
5.3693110e-004	4.9560857e-004	4.9872970e-004	5.5932438e-004
5.5995207e-004	5.9098419e-004	5.8988547e-004	6.1958008e-004
6.2311602e-004	6.2883359e-004	6.5113445e-004	6.8379336e-004
6.6520796e-004	7.1530223e-004	6.3527274e-004	7.0451949e-004
7.2470860e-004	7.3707735e-004	6.7710520e-004	6.9650539e-004
6.6816677e-004	6.3688887e-004	6.2809692e-004	6.2955829e-004
5.6569977e-004	6.0368342e-004	5.6384579e-004	5.9168406e-004
5.5015048e-004	6.4061329e-004	6.1373413e-004	6.4610488e-004
6.3345185e-004	6.6632373e-004	6.6576191e-004	7.1266149e-004
6.6023393e-004	7.3719044e-004	5.9018601e-004	6.6675556e-004
7.6677313e-004	8.7701739e-004	7.6829975e-004	7.9130175e-004
7.3883907e-004	7.7875386e-004	7.9203653e-004	8.7593896e-004
6.9249276e-004	7.5067190e-004	8.0016172e-004	8.0555183e-004
7.5103562e-004	7.8577273e-004	7.2816329e-004	8.7094514e-004
7.2541815e-004	7.7072848e-004	7.7220476e-004	8.3823377e-004
7.9427414e-004	8.3240369e-004	7.6585970e-004	8.4810574e-004
8.3123615e-004	8.5127323e-004	8.7315869e-004	7.0818642e-004
7.1480920e-004	7.6628863e-004	7.3431901e-004	8.0034374e-004
7.5476141e-004	7.4283516e-004	7.0469242e-004	7.0671703e-004
7.3578610e-004	7.1996140e-004	7.5312024e-004	7.4045570e-004
7.2209478e-004	7.1354597e-004	6.9053024e-004	7.4284294e-004
6.7365047e-004	7.2498392e-004	7.1818136e-004	7.0479121e-004
5.5310664e-004	5.4625051e-004	5.3267060e-004	5.7777035e-004
5.2711311e-004	5.5098765e-004	5.4022657e-004	5.7185587e-004
5.8923132e-004	5.8090889e-004	5.4776960e-004	5.7168943e-004
5.9194781e-004	5.5998190e-004	6.0952281e-004	6.1497446e-004
5.8475961e-004	6.3943335e-004	5.9414269e-004	6.5273068e-004
5.9711911e-004	6.6648528e-004	6.8744423e-004	6.5754450e-004
5.2775744e-004	5.7165162e-004	5.8400976e-004	6.4492390e-004
6.0586774e-004	6.6908824e-004	6.7475472e-004	6.8361439e-004
6.4984347e-004	6.2982080e-004	6.3360925e-004	6.7235602e-004
6.3521537e-004	6.2939934e-004	6.7665579e-004	6.7633492e-004
6.5518552e-004	7.2677412e-004	6.4124957e-004	7.3571572e-004
6.5813758e-004	6.7376879e-004	7.1975680e-004	6.3856103e-004
6.3130695e-004	6.7249143e-004	7.4198535e-004	7.1503563e-004
7.8353770e-004	7.6291915e-004	6.6357147e-004	7.2329782e-004
6.7848679e-004	6.5242499e-004	6.2211005e-004	6.4266961e-004
6.1698114e-004	6.7690715e-004	6.4649574e-004	5.9180465e-004
5.3573915e-004	6.0053503e-004	5.6951970e-004	5.8027534e-004
5.3689095e-004	5.6942627e-004	6.0461620e-004	5.6755729e-004

6.6554455e-004 6.6852376e-004 8.0249623e-004 8.3730290e-004
7.8457153e-004 7.6845738e-004 7.3945623e-004 7.0717810e-004
6.7412658e-004 7.3487638e-004 6.5180738e-004 7.1627069e-004
6.2823940e-004 6.4073602e-004 7.0129523e-004 6.7176145e-004
6.5849817e-004 6.4262139e-004 5.8402382e-004 6.5896781e-004
5.9328343e-004 6.3156019e-004 6.6501455e-004 7.1777278e-004
5.1274835e-004 5.5477810e-004 6.2155680e-004 6.0143411e-004
6.1190764e-004 6.4231755e-004 6.3087976e-004 6.2139842e-004
6.3588652e-004 5.7003947e-004 5.3879610e-004 6.3406368e-004
5.1535812e-004 5.8347934e-004 5.5074652e-004 5.4878175e-004
5.6957811e-004 4.8392525e-004 5.7912740e-004 5.1591663e-004
4.9702285e-004 5.3007460e-004 5.1386189e-004 5.0048601e-004
5.9702768e-004 5.9621736e-004 6.4877885e-004 6.2996030e-004
6.5086994e-004 7.5113733e-004 6.3221322e-004 6.9419137e-004
6.6450595e-004 7.1190052e-004 6.7944593e-004 6.6860479e-004
6.5611479e-004 6.6629439e-004 6.4599889e-004 6.8528041e-004
6.4852010e-004 6.2332982e-004 5.7849532e-004 6.2672174e-004
5.7215084e-004 5.6967231e-004 5.8511242e-004 6.1728971e-004
6.0426501e-004 6.4803597e-004 5.9566797e-004 6.8022500e-004
6.2226788e-004 7.1932294e-004 6.2191230e-004 6.7752268e-004
6.8946918e-004 6.3110136e-004 5.9219229e-004 6.2177630e-004
5.9617787e-004 6.0958420e-004 5.9826428e-004 5.5647526e-004
5.8352400e-004 5.6126899e-004 5.9483049e-004 5.8296164e-004
5.4307435e-004 5.9006055e-004 5.7597374e-004 5.8260409e-004
5.9688801e-004 6.7271279e-004 6.4941160e-004 7.5394400e-004
6.7931366e-004 6.5754967e-004 6.2917211e-004 6.7797772e-004
6.8768991e-004 6.0564543e-004 6.0543370e-004 6.0520449e-004
6.3651848e-004 5.9747881e-004 6.8731404e-004 6.3681141e-004
6.1245677e-004 5.9459361e-004 5.8227631e-004 5.9216436e-004
5.3134569e-004 5.9159113e-004 5.3605794e-004 5.5608328e-004
5.0856764e-004 5.3421569e-004 5.3346729e-004 5.8124606e-004
5.6373687e-004 5.3012763e-004 5.2170111e-004 5.9661282e-004
5.3110278e-004 5.0795917e-004 4.9772549e-004 5.4904203e-004
5.5613826e-004 5.8313333e-004 6.0153504e-004 6.2916634e-004
6.3934903e-004 6.2824434e-004 6.5940467e-004 6.9328240e-004
6.5080386e-004 7.1003790e-004 6.3210525e-004 7.1892944e-004
6.5507085e-004 6.8274574e-004 6.3223729e-004 6.4071859e-004
6.1203618e-004 5.7644890e-004 5.5755187e-004 5.7621802e-004
5.0809710e-004 5.5596334e-004 5.0771367e-004 5.3714648e-004
5.1331820e-004 5.8448312e-004 5.7626360e-004 6.0430547e-004
5.7602393e-004 6.2233572e-004 6.3339475e-004 6.7413670e-004
6.2671899e-004 6.6653320e-004 5.8826411e-004 6.2581513e-004
7.4520208e-004 8.8085765e-004 7.8135361e-004 7.8506480e-004
6.7725113e-004 7.8496750e-004 7.2322044e-004 8.3574498e-004
6.5916048e-004 6.7520810e-004 7.0201258e-004 7.4907894e-004
6.7277277e-004 6.4822586e-004 6.1415292e-004 7.6957753e-004
6.2755749e-004 6.5794153e-004 6.7685565e-004 7.5895035e-004
6.9800977e-004 7.1408704e-004 7.2933229e-004 7.6467732e-004
5.7457734e-004 6.0510007e-004 5.6594081e-004 5.4063422e-004
5.2392353e-004 4.9743678e-004 4.8852308e-004 4.8581596e-004
4.5254990e-004 4.2611886e-004 4.2232577e-004 4.3287639e-004
4.2857382e-004 4.0985543e-004 4.2986045e-004 4.5085034e-004

4.2004275e-004	3.9972403e-004	4.2473148e-004	4.4292113e-004
4.0918601e-004	4.5148528e-004	4.5381459e-004	4.6403278e-004
5.1686210e-004	5.9064593e-004	5.9796507e-004	6.8326142e-004
6.0920090e-004	6.8056918e-004	6.6272076e-004	6.8494219e-004
7.1734423e-004	6.7867058e-004	6.5383135e-004	7.0178257e-004
6.9285453e-004	6.6221238e-004	7.4382762e-004	7.3664750e-004
7.0691202e-004	8.1008561e-004	7.2151130e-004	7.8648776e-004
7.0052480e-004	7.3415012e-004	8.0700786e-004	7.4330858e-004
5.6979120e-004	6.6638124e-004	7.0057251e-004	7.0967551e-004
6.8057340e-004	6.9085919e-004	6.4871495e-004	7.1633708e-004
6.3987223e-004	6.1764418e-004	6.2361531e-004	6.3527937e-004
6.4886974e-004	6.6204183e-004	6.6255860e-004	6.0012302e-004
5.8820310e-004	6.5548798e-004	5.7180846e-004	6.3156190e-004
5.9019173e-004	6.1890804e-004	6.1231750e-004	5.5729509e-004
4.9863413e-004	5.7637224e-004	6.0812368e-004	6.2636074e-004
5.8851478e-004	6.2368924e-004	6.7316192e-004	6.4406547e-004
6.8005237e-004	6.3224410e-004	5.6234688e-004	6.1655833e-004
5.9847371e-004	5.4635336e-004	5.7200139e-004	5.7211579e-004
5.7953727e-004	5.1634610e-004	5.8116266e-004	5.2214474e-004
5.5626413e-004	5.8098264e-004	5.0863652e-004	4.9251244e-004
5.4745888e-004	5.5938451e-004	6.0513013e-004	6.3965666e-004
6.5064998e-004	6.8445009e-004	5.9306753e-004	6.4765253e-004
6.3260831e-004	6.5258797e-004	6.9175044e-004	6.7049179e-004
6.4811073e-004	6.0305382e-004	6.0956675e-004	6.6389643e-004
5.8883458e-004	5.6307924e-004	5.6494889e-004	5.8617548e-004
5.2133299e-004	5.4562894e-004	5.4927477e-004	5.6917118e-004
5.8401763e-004	6.3759580e-004	6.1085544e-004	6.6642945e-004
6.4263464e-004	7.4512775e-004	6.5000501e-004	7.2386564e-004
7.0174398e-004	6.8972394e-004	6.2194366e-004	6.2478828e-004
6.4174295e-004	6.3354496e-004	6.2894092e-004	6.2154941e-004
6.2287405e-004	5.8864547e-004	5.7558564e-004	6.2416651e-004
5.5163479e-004	5.8556889e-004	5.9034534e-004	6.0716456e-004
6.6178117e-004	7.7269533e-004	7.7355866e-004	7.9166732e-004
7.5095697e-004	7.3814718e-004	6.9614481e-004	7.6872464e-004
7.3279311e-004	6.9280058e-004	6.8546082e-004	6.4330862e-004
6.7791117e-004	5.9741229e-004	6.6784992e-004	6.9312265e-004
6.4206640e-004	6.3450867e-004	5.9910669e-004	6.4622321e-004
5.3979504e-004	5.9741166e-004	5.6728091e-004	5.8799726e-004
5.5598207e-004	5.8954724e-004	5.7225527e-004	6.6043640e-004
6.1580341e-004	6.6076372e-004	5.9328576e-004	6.8172996e-004
6.3068657e-004	6.1416246e-004	5.7345060e-004	6.0650134e-004
5.9148004e-004	5.8688159e-004	6.9780511e-004	6.7836768e-004
6.9097137e-004	6.7967155e-004	7.4449051e-004	7.0914425e-004
6.8399819e-004	7.5574448e-004	6.8478419e-004	7.4027760e-004
5.1934083e-004	5.7345095e-004	5.2853097e-004	5.6712075e-004
5.4211062e-004	5.1097511e-004	4.7954958e-004	5.3227266e-004
4.8438427e-004	5.0565139e-004	4.5783819e-004	4.9827584e-004
5.3055889e-004	5.7555482e-004	5.9395301e-004	6.0749028e-004
6.0672220e-004	6.0994584e-004	6.2131291e-004	6.8669899e-004
5.9484702e-004	6.3762061e-004	6.2303116e-004	6.6328282e-004
6.9135295e-004	7.2695393e-004	6.9267183e-004	6.6447460e-004
6.0121725e-004	6.0509620e-004	6.1441436e-004	6.3437583e-004

5.2156384e-004	5.4512435e-004	5.5903089e-004	5.7273652e-004
5.4702183e-004	5.4038542e-004	5.4310741e-004	6.3080648e-004
5.0377216e-004	5.6709124e-004	5.8565428e-004	6.2392889e-004
6.1715132e-004	6.2335095e-004	5.9428670e-004	6.2387031e-004
6.8218580e-004	7.8505352e-004	7.5080334e-004	7.2786737e-004
6.6963893e-004	7.9029953e-004	7.0240907e-004	8.2865180e-004
7.3055555e-004	6.8459456e-004	6.9773682e-004	7.4614586e-004
6.8324724e-004	6.7682214e-004	6.3814512e-004	6.8267222e-004
6.4612716e-004	7.1453617e-004	6.6850630e-004	7.6633254e-004
6.7318477e-004	7.1919832e-004	7.7343865e-004	7.3288764e-004
5.2351841e-004	5.6355729e-004	5.1362385e-004	5.0983058e-004
4.8453098e-004	4.5595872e-004	4.6124130e-004	4.4679868e-004
4.1648894e-004	3.9747454e-004	3.9204331e-004	4.1067370e-004
4.0215354e-004	3.8443903e-004	4.1485736e-004	4.3754655e-004
3.9515959e-004	3.8714227e-004	4.1978067e-004	4.3443425e-004
4.0084458e-004	4.4195852e-004	4.5250257e-004	4.6262236e-004
5.0047673e-004	4.8869790e-004	4.9948396e-004	5.2792561e-004
4.7856347e-004	4.9911945e-004	4.6254537e-004	4.7383208e-004
4.9022454e-004	4.9848494e-004	4.8249202e-004	5.2610836e-004
5.4532920e-004	5.3214062e-004	5.4754718e-004	5.9963287e-004
5.2118660e-004	5.5435222e-004	5.6839649e-004	5.9215998e-004
5.6289943e-004	5.8524734e-004	6.1996198e-004	5.8285151e-004
5.0389371e-004	6.1287718e-004	6.3555531e-004	7.1564842e-004
6.3286330e-004	7.0676768e-004	6.6006945e-004	6.9081500e-004
7.2538502e-004	6.8440147e-004	6.6553860e-004	7.1823271e-004
7.1417457e-004	6.8967638e-004	7.5906774e-004	7.4287970e-004
7.2511034e-004	8.2254554e-004	7.3636763e-004	7.8750293e-004
6.9171598e-004	7.3258825e-004	8.0817791e-004	7.4599328e-004
5.7709237e-004	6.7213576e-004	7.4666250e-004	7.1921624e-004
6.9582858e-004	6.9576340e-004	6.5246418e-004	7.2219370e-004
6.5595632e-004	6.3670077e-004	6.3025732e-004	6.3319158e-004
6.6876739e-004	6.4900672e-004	6.7795735e-004	6.1171535e-004
5.9717684e-004	6.2984666e-004	5.8258821e-004	6.1514348e-004
5.7962653e-004	6.0567709e-004	6.0439033e-004	5.6811333e-004
6.5881237e-004	6.6993771e-004	8.1645390e-004	7.2286928e-004
7.9073908e-004	8.1638405e-004	7.0713149e-004	7.4785903e-004
6.6617424e-004	7.0463590e-004	6.3693285e-004	7.0432694e-004
6.5811793e-004	6.4104378e-004	6.4237897e-004	5.9598059e-004
6.0167952e-004	6.0287211e-004	6.0452356e-004	5.8143034e-004
5.4505643e-004	5.6135024e-004	6.1139188e-004	6.0131324e-004
5.0711799e-004	5.7317063e-004	6.0278316e-004	6.2998662e-004
5.8063613e-004	6.3628652e-004	6.6797793e-004	6.2405314e-004
6.7432047e-004	6.5719360e-004	5.6225911e-004	6.3636097e-004
6.1174914e-004	5.3367757e-004	5.8272509e-004	5.8534463e-004
5.6703409e-004	5.3029042e-004	5.9082746e-004	5.3213707e-004
5.5656581e-004	5.7914278e-004	5.2423615e-004	5.1472869e-004
5.3765140e-004	5.6769013e-004	6.1680742e-004	6.2596332e-004
6.5029167e-004	6.5034625e-004	5.8342906e-004	6.5050367e-004
6.1933216e-004	6.2539482e-004	6.4785716e-004	6.4215092e-004
6.1899198e-004	5.6717338e-004	5.9479906e-004	6.3034538e-004
5.6492531e-004	5.2458938e-004	5.5164653e-004	5.5719604e-004
5.1136521e-004	5.3357704e-004	5.0757476e-004	5.5674829e-004

6.1271097e-004	5.9297439e-004	6.1363340e-004	6.5271069e-004
6.8567222e-004	7.1511208e-004	6.2912873e-004	6.8080630e-004
6.8989300e-004	5.9085204e-004	6.0958641e-004	5.9150146e-004
6.3218767e-004	5.7994114e-004	6.2021284e-004	5.6148588e-004
6.0665146e-004	5.9630512e-004	5.7508789e-004	5.8329474e-004
5.6638182e-004	5.9298333e-004	5.6985192e-004	5.4473472e-004
6.6521282e-004	7.6155715e-004	7.8114116e-004	7.8447150e-004
7.5542035e-004	7.5341733e-004	6.9721072e-004	7.8158348e-004
7.3303199e-004	6.9876184e-004	6.8115132e-004	6.3648375e-004
6.8264098e-004	5.9716111e-004	6.5151407e-004	6.8851301e-004
6.2834320e-004	6.4982427e-004	5.9320734e-004	6.5162724e-004
5.4234600e-004	5.9554688e-004	5.7970654e-004	5.8994789e-004
5.8667469e-004	6.1405347e-004	6.1648780e-004	6.9797784e-004
6.4324797e-004	6.8663897e-004	6.1506015e-004	7.1635656e-004
6.5343252e-004	6.1788718e-004	5.8697676e-004	6.0023450e-004
5.9066663e-004	6.1740512e-004	7.1979327e-004	6.7847414e-004
6.8498135e-004	6.8655025e-004	7.3345324e-004	7.0060755e-004
6.7004160e-004	7.5116698e-004	6.6380091e-004	7.0605653e-004
5.0859067e-004	5.5730646e-004	5.1759518e-004	5.5533454e-004
5.3564650e-004	5.1315819e-004	4.9214753e-004	5.2972774e-004
4.9193817e-004	5.1667800e-004	4.8819251e-004	5.1165489e-004
5.6553796e-004	5.8557918e-004	6.2822031e-004	6.3551543e-004
6.2164241e-004	6.1139531e-004	6.4085743e-004	6.9637832e-004
5.9216791e-004	6.3539520e-004	6.7064323e-004	6.8541634e-004
6.5696977e-004	6.7497366e-004	6.6804752e-004	6.4709432e-004
5.7612338e-004	5.6639570e-004	5.7388672e-004	5.7339338e-004
5.0556376e-004	5.0999687e-004	5.3039772e-004	5.3943072e-004
5.4237562e-004	5.2331391e-004	5.1948604e-004	6.0823656e-004
4.8660667e-004	5.5198753e-004	5.6571281e-004	5.9330446e-004
5.8196354e-004	5.6511637e-004	6.0051971e-004	6.0072768e-004
7.0097393e-004	7.4982100e-004	7.7087742e-004	7.5820262e-004
6.8030210e-004	7.5002498e-004	7.1709105e-004	7.9926985e-004
7.2927203e-004	6.5518740e-004	6.6124601e-004	6.9318367e-004
6.4432657e-004	6.4284868e-004	6.2205532e-004	6.2471184e-004
5.8974618e-004	6.5749839e-004	6.2878475e-004	7.1185290e-004
6.4705651e-004	6.8055674e-004	7.2143273e-004	7.0309683e-004
5.5307313e-004	5.7813918e-004	5.4851282e-004	5.3454423e-004
5.1316817e-004	4.8431142e-004	4.8766705e-004	4.6655500e-004
4.4802119e-004	4.1633884e-004	4.0160767e-004	4.3093486e-004
4.1011344e-004	4.0083574e-004	4.4513859e-004	4.5190361e-004
4.1299848e-004	4.0604504e-004	4.2967311e-004	4.5020694e-004
4.0922127e-004	4.5481416e-004	4.6615802e-004	4.7560388e-004
4.6308793e-004	4.7997037e-004	4.7455179e-004	4.9987612e-004
4.4562796e-004	4.6936529e-004	4.5739480e-004	4.5206089e-004
4.2936404e-004	4.6003825e-004	4.4216929e-004	5.0221660e-004
5.1404707e-004	4.9077029e-004	5.1846583e-004	5.9033059e-004
4.9730275e-004	5.1709123e-004	5.6641790e-004	5.9007029e-004
5.3953810e-004	5.6085740e-004	5.9845028e-004	5.7081785e-004
5.1577727e-004	6.2029000e-004	6.5304033e-004	7.0473707e-004
6.4601447e-004	7.1051389e-004	6.3908494e-004	6.5985988e-004
6.9952905e-004	6.7815300e-004	6.5765416e-004	7.1595382e-004
6.9475950e-004	7.0896387e-004	7.6091189e-004	7.4343063e-004

7.2366159e-004 7.8518543e-004 7.2874613e-004 7.4978327e-004
6.6788867e-004 7.1821587e-004 7.7707290e-004 7.4587145e-004
6.4291219e-004 6.9783381e-004 8.0258037e-004 7.0080180e-004
7.5549549e-004 7.8107553e-004 6.7784132e-004 7.4267709e-004
6.5757605e-004 6.6928196e-004 6.2101391e-004 6.7498264e-004
6.4685401e-004 6.4106293e-004 6.2944765e-004 6.1155089e-004
5.8525053e-004 5.9558479e-004 5.9191961e-004 5.9529868e-004
5.5590297e-004 5.7764380e-004 5.9379103e-004 5.8021412e-004
6.0291063e-004 5.7755780e-004 5.6895967e-004 6.1944463e-004
6.5792157e-004 7.1610267e-004 6.4250252e-004 6.6628034e-004
6.8685674e-004 5.8923030e-004 5.9517793e-004 5.7956153e-004
5.9966289e-004 5.6434100e-004 5.8787623e-004 5.4938390e-004
5.7020977e-004 5.8212268e-004 5.5294581e-004 5.8717125e-004
5.5471866e-004 5.5249750e-004 5.6455590e-004 5.5560849e-004
6.3561609e-004 6.7347969e-004 7.0589913e-004 7.1235697e-004
7.1211120e-004 7.3524522e-004 6.5260258e-004 7.3301811e-004
6.9487696e-004 6.6926373e-004 6.6247204e-004 6.0405940e-004
6.7747357e-004 5.9588783e-004 6.3038734e-004 6.7008626e-004
6.1174302e-004 6.2168050e-004 5.7592175e-004 6.0945250e-004
5.3136020e-004 5.8484722e-004 5.6981918e-004 5.9904052e-004
6.5715246e-004 7.0911084e-004 6.9433518e-004 8.2024561e-004
6.9602451e-004 7.4600583e-004 6.7786739e-004 7.6903202e-004
7.0493095e-004 6.4727185e-004 6.1148805e-004 6.1433713e-004
6.1797097e-004 6.3435946e-004 7.2919439e-004 6.5761946e-004
6.6285545e-004 6.9328338e-004 6.9336819e-004 6.5058962e-004
6.3833444e-004 7.1175247e-004 6.1273715e-004 6.0785082e-004
5.2105071e-004 5.3834749e-004 5.2530960e-004 5.7000187e-004
5.7258473e-004 5.4776274e-004 5.4520425e-004 5.7792560e-004
5.4911902e-004 5.5906227e-004 5.4047171e-004 5.6319790e-004
6.2420664e-004 6.3605158e-004 7.0138348e-004 6.9490816e-004
6.7672344e-004 6.4184610e-004 6.8546084e-004 7.3915222e-004
6.0892792e-004 6.9728366e-004 7.2069568e-004 7.3756505e-004
6.0511670e-004 6.1479704e-004 6.2812655e-004 6.3947134e-004
5.6226645e-004 5.3805446e-004 5.4457270e-004 5.1774931e-004
4.9460577e-004 4.8985791e-004 5.2783154e-004 5.2980523e-004
5.6306209e-004 5.6763024e-004 5.4782434e-004 6.1442342e-004
5.3989838e-004 6.0088687e-004 6.1394686e-004 6.1951682e-004
5.9308148e-004 5.5351431e-004 6.4744322e-004 6.1570964e-004
6.5537033e-004 6.7717768e-004 7.0356252e-004 6.8400278e-004
5.9481802e-004 6.2658584e-004 6.1335806e-004 6.3398173e-004
5.8904852e-004 5.4616999e-004 5.5121013e-004 5.5194461e-004
5.1649829e-004 5.1136515e-004 5.2249967e-004 5.4188046e-004
4.4500710e-004 5.2059355e-004 5.1695448e-004 5.9152808e-004
5.4426405e-004 5.4535993e-004 6.1513133e-004 5.8598458e-004
4.8049569e-004 5.2711569e-004 5.3841616e-004 5.4556660e-004
5.0777555e-004 5.6504450e-004 5.4572465e-004 5.2809347e-004
5.1634879e-004 5.4699271e-004 5.2822047e-004 5.9558827e-004
5.9191365e-004 6.0645228e-004 6.2628486e-004 6.9136982e-004
5.9353754e-004 6.2056821e-004 6.6241492e-004 6.8565451e-004
6.1592448e-004 6.2133350e-004 6.6258771e-004 6.5388661e-004
5.6107885e-004 6.7389297e-004 7.4046255e-004 7.6670098e-004
7.0420816e-004 7.6975061e-004 6.8446674e-004 6.8507406e-004

7.0304565e-004	6.8969552e-004	6.7259801e-004	7.1619112e-004
7.2049099e-004	7.1507870e-004	7.6310018e-004	7.5680074e-004
7.5209037e-004	7.5393388e-004	7.2541789e-004	7.7449637e-004
6.2497071e-004	7.0811815e-004	7.6405707e-004	7.2705916e-004
6.0698578e-004	6.6814128e-004	7.6373050e-004	7.1562725e-004
7.0988901e-004	7.0813610e-004	6.6958767e-004	7.1282873e-004
6.6470800e-004	6.4749106e-004	6.2857391e-004	6.4673739e-004
6.7953578e-004	6.1200540e-004	6.4773366e-004	6.4700437e-004
6.1832792e-004	5.9319664e-004	5.8258389e-004	5.8894360e-004
5.5070574e-004	5.9233179e-004	6.0984616e-004	6.0018645e-004
6.0788539e-004	6.6325653e-004	6.9590368e-004	6.6691764e-004
6.7329197e-004	7.2987004e-004	6.6585797e-004	7.1325722e-004
6.1053952e-004	6.3400764e-004	5.7273330e-004	6.3593115e-004
6.3188553e-004	6.0426614e-004	5.8288725e-004	5.9500847e-004
5.6609826e-004	5.8862897e-004	5.6525566e-004	5.8982208e-004
5.4247689e-004	5.6449341e-004	5.8931761e-004	5.5533521e-004
5.1087484e-004	5.5247052e-004	5.9637328e-004	5.3298952e-004
6.1021560e-004	5.9699797e-004	5.8237028e-004	6.0620384e-004
5.6594654e-004	5.5343119e-004	5.5135923e-004	5.4936134e-004
5.3487468e-004	5.2396445e-004	5.4688851e-004	5.6352063e-004
5.1733801e-004	4.7718784e-004	5.1622542e-004	5.1546024e-004
4.8214269e-004	5.0488666e-004	4.2668195e-004	5.5182654e-004
6.6049167e-004	6.9540412e-004	6.9183591e-004	7.4030302e-004
7.0038317e-004	7.7230137e-004	7.0515335e-004	7.4046254e-004
7.0784845e-004	6.7491176e-004	6.4532504e-004	6.2982130e-004
6.7860899e-004	6.2678887e-004	5.9809486e-004	6.7144605e-004
5.9192789e-004	6.4653358e-004	5.6461869e-004	6.3399551e-004
5.6307328e-004	5.8696943e-004	5.9573207e-004	5.9887506e-004
5.4048025e-004	5.6670100e-004	5.4133307e-004	5.9839543e-004
5.9012142e-004	5.5509120e-004	5.6690031e-004	5.7749402e-004
5.5920563e-004	5.5636784e-004	5.8715903e-004	5.9733150e-004
6.7010325e-004	6.6123402e-004	7.2923049e-004	7.2674608e-004
7.0733092e-004	6.8641924e-004	7.0189017e-004	7.4365361e-004
6.2545986e-004	6.7694734e-004	7.7702392e-004	7.4225773e-004
6.4255376e-004	6.4761414e-004	6.3862051e-004	6.5761840e-004
5.6092598e-004	5.5737457e-004	5.6273954e-004	5.2049800e-004
5.0881342e-004	4.9642860e-004	5.5581340e-004	5.2748436e-004
5.4302962e-004	5.6754515e-004	5.5303365e-004	6.1829107e-004
5.2141536e-004	5.8097203e-004	5.8211135e-004	6.2908216e-004
5.7464975e-004	5.5528035e-004	6.3465696e-004	5.9075840e-004
6.3708448e-004	6.3670074e-004	6.9543815e-004	6.7418093e-004
6.1209498e-004	5.9240310e-004	6.1522015e-004	6.1201310e-004
5.8583080e-004	5.4377412e-004	5.1151068e-004	5.2258807e-004
4.7776771e-004	5.0067720e-004	5.2274943e-004	4.8575731e-004
4.4357590e-004	4.8008038e-004	4.9933937e-004	5.3854942e-004
5.2399225e-004	5.1233250e-004	5.5795361e-004	5.6960018e-004
4.5352121e-004	4.8765862e-004	4.4626690e-004	4.7385255e-004
4.4563071e-004	4.2650013e-004	4.3091239e-004	4.2911211e-004
3.9879171e-004	3.9571003e-004	3.9521194e-004	4.3920738e-004
4.0955553e-004	3.9816835e-004	4.7205065e-004	4.7420197e-004
4.5845934e-004	4.5942538e-004	4.8708021e-004	5.1620973e-004
4.7148650e-004	5.2666550e-004	5.6184083e-004	5.5449727e-004

4.8505470e-004	5.6905472e-004	5.7009287e-004	5.6277001e-004
5.4320529e-004	5.9581477e-004	5.8852808e-004	5.7035990e-004
5.4322070e-004	5.7331357e-004	5.5694316e-004	6.2472923e-004
6.1603614e-004	6.3733220e-004	6.5965642e-004	7.0481066e-004
6.2239743e-004	6.4896949e-004	6.8771787e-004	7.2213489e-004
6.3569786e-004	6.4247061e-004	6.7299031e-004	6.7104354e-004
6.2052839e-004	7.1855770e-004	8.0216661e-004	8.0136418e-004
7.4719038e-004	8.0869308e-004	7.1221500e-004	6.9136104e-004
7.0095067e-004	6.9880992e-004	6.8606176e-004	7.1255301e-004
7.1329363e-004	7.1724725e-004	7.4409929e-004	7.6481489e-004
7.4528591e-004	7.3114841e-004	7.0768945e-004	7.7577768e-004
6.0631604e-004	6.9469490e-004	7.4005239e-004	7.0677837e-004
6.0295225e-004	6.5965741e-004	7.4199535e-004	7.1420173e-004
7.1061448e-004	7.1966954e-004	6.7950526e-004	6.9668151e-004
6.6862328e-004	6.4299848e-004	6.4633702e-004	6.6651807e-004
6.6962650e-004	6.0570228e-004	6.1862607e-004	6.5052958e-004
6.1498526e-004	6.0889751e-004	5.7867667e-004	5.9606144e-004
5.3900213e-004	6.0166985e-004	6.3272774e-004	5.9832410e-004
5.7045644e-004	6.3866439e-004	6.3916169e-004	6.2950386e-004
6.2507427e-004	6.7660587e-004	6.3844566e-004	6.7523419e-004
6.1059611e-004	6.0066588e-004	5.5316556e-004	5.9536473e-004
6.0605873e-004	5.7697837e-004	5.4967376e-004	5.8763211e-004
5.3097974e-004	5.7660212e-004	5.5018383e-004	5.7174647e-004
5.1769480e-004	5.5604406e-004	5.6384226e-004	5.4042469e-004
6.5208181e-004	6.5826211e-004	6.3339679e-004	7.0276682e-004
6.6248736e-004	7.5059771e-004	6.8224405e-004	6.9971148e-004
6.9018436e-004	6.5049405e-004	6.2523769e-004	6.3413837e-004
6.6767748e-004	6.3921403e-004	5.7565006e-004	6.7256010e-004
5.8408472e-004	6.2758826e-004	5.4736568e-004	6.1619619e-004
5.7101634e-004	5.8617028e-004	5.8537646e-004	5.8558426e-004
5.7275315e-004	5.9020618e-004	5.6436031e-004	6.2354658e-004
6.2041551e-004	5.7579111e-004	5.9266562e-004	6.2275537e-004
6.0050323e-004	5.8804422e-004	6.4629257e-004	6.3774615e-004
7.1437474e-004	7.0666661e-004	7.6870863e-004	7.6785541e-004
7.4820349e-004	7.3587357e-004	7.0692674e-004	7.5471547e-004
6.4616992e-004	6.9535722e-004	8.0628689e-004	7.6178240e-004
6.7017996e-004	6.7235074e-004	6.5123677e-004	6.7479048e-004
5.7437252e-004	5.6959889e-004	5.8148942e-004	5.4140922e-004
5.3355977e-004	5.0808731e-004	5.8831909e-004	5.4341613e-004
5.5604632e-004	6.0859926e-004	6.0908044e-004	6.4373578e-004
5.6072879e-004	6.0479745e-004	6.1305607e-004	6.7447146e-004
5.8758624e-004	5.9487768e-004	6.6261206e-004	6.1250116e-004
6.1340589e-004	6.0872124e-004	6.5559315e-004	6.3087558e-004
5.8918937e-004	5.5526740e-004	5.7468245e-004	5.5711559e-004
5.4581807e-004	5.0709891e-004	4.8192570e-004	4.8013469e-004
4.3813689e-004	4.6996112e-004	4.9661905e-004	4.6501693e-004
4.2631734e-004	4.4509226e-004	4.7352422e-004	5.0176588e-004
4.9220845e-004	4.7792196e-004	5.1018704e-004	5.3196054e-004
8.7395360e-004	8.6380460e-004	8.0188345e-004	8.3670432e-004
8.0517627e-004	8.7553884e-004	8.1737252e-004	8.4136231e-004
8.2359093e-004	8.0991564e-004	7.7070751e-004	7.4163351e-004
7.6123589e-004	7.6427151e-004	7.3082435e-004	7.4803292e-004

7.2510654e-004	7.0543190e-004	7.2400940e-004	7.4220037e-004
7.3988216e-004	7.4875532e-004	8.0875098e-004	7.2624205e-004
7.4221789e-004	7.3117205e-004	6.7194666e-004	7.0595551e-004
7.0796011e-004	7.1081748e-004	6.7715957e-004	6.7303186e-004
6.4254305e-004	6.4665719e-004	6.2555654e-004	5.9350502e-004
6.0323197e-004	5.8475824e-004	6.0042716e-004	6.3222661e-004
6.2272975e-004	5.9422691e-004	6.1238561e-004	6.3634831e-004
6.0901681e-004	6.3142375e-004	6.5319055e-004	6.1614522e-004
4.4445583e-004	4.8735522e-004	4.4362294e-004	4.7721642e-004
4.6435985e-004	4.4327052e-004	4.3238934e-004	4.4926500e-004
4.2222898e-004	4.1853577e-004	4.3464587e-004	4.7230154e-004
4.3466403e-004	4.4459789e-004	5.1454409e-004	5.0090671e-004
5.1213660e-004	5.0634190e-004	5.2398695e-004	5.7279050e-004
5.2251338e-004	5.8046660e-004	6.1590150e-004	5.9667893e-004
5.0170146e-004	6.1475924e-004	6.1346899e-004	5.9582994e-004
5.8888116e-004	6.1997565e-004	6.2683837e-004	6.2174159e-004
5.8625329e-004	6.0275872e-004	5.9550547e-004	6.4747427e-004
6.4522534e-004	6.6162631e-004	6.8569279e-004	7.0644650e-004
6.4873506e-004	6.7025666e-004	7.0195382e-004	7.4482040e-004
6.4900397e-004	6.6877768e-004	6.7953877e-004	6.8065614e-004
6.7018818e-004	7.5847337e-004	8.3480777e-004	8.2783583e-004
7.6679215e-004	8.1917098e-004	7.2713010e-004	6.9807546e-004
6.9995827e-004	6.9896667e-004	6.9896280e-004	6.9958412e-004
6.9593050e-004	7.1233093e-004	7.0226667e-004	7.4534022e-004
7.0740574e-004	7.1988384e-004	6.8570934e-004	7.7042292e-004
5.9873889e-004	6.7134104e-004	7.0375847e-004	6.7474575e-004
5.7578259e-004	6.4184671e-004	7.0925959e-004	7.0322333e-004
6.9889918e-004	7.1923260e-004	6.7504603e-004	6.7167509e-004
6.7497256e-004	6.3675985e-004	6.6062115e-004	6.6706592e-004
6.4781363e-004	5.9774648e-004	6.0485916e-004	6.4401457e-004
5.9396827e-004	6.2492726e-004	5.8389545e-004	6.0657035e-004
5.2791328e-004	6.0581455e-004	6.4647387e-004	5.7718040e-004
6.3025614e-004	6.2211181e-004	5.9481581e-004	6.6858380e-004
6.2743960e-004	7.1756398e-004	6.4542738e-004	6.6424215e-004
6.6567092e-004	6.3206158e-004	6.1546938e-004	6.4192076e-004
6.5429455e-004	6.4466432e-004	5.6120050e-004	6.7203958e-004
5.8271213e-004	6.0392919e-004	5.3110985e-004	6.0560288e-004
5.7276232e-004	5.8369635e-004	5.7351332e-004	5.6614772e-004
7.3742670e-004	7.3477035e-004	7.1716997e-004	7.9043224e-004
7.2724672e-004	7.9388644e-004	8.0855310e-004	8.3308114e-004
7.3272781e-004	6.5404330e-004	7.1179995e-004	6.9317775e-004
6.6834588e-004	6.4694247e-004	6.4164374e-004	6.6883836e-004
6.4356413e-004	6.0742919e-004	6.4182525e-004	6.4226682e-004
5.6439161e-004	5.8978995e-004	6.2195746e-004	5.6569604e-004
5.9810352e-004	6.1054072e-004	5.8971872e-004	6.3251831e-004
6.3872014e-004	5.9517955e-004	6.0690382e-004	6.6862932e-004
6.3236768e-004	6.1940739e-004	7.0622929e-004	6.6620171e-004
7.4283048e-004	7.4076455e-004	7.8702225e-004	7.8832122e-004
7.6296348e-004	7.6604790e-004	6.9785576e-004	7.4714923e-004
6.6274501e-004	7.0182605e-004	8.1855939e-004	7.6658381e-004
7.0874077e-004	7.0713127e-004	6.7404486e-004	6.9207253e-004
6.0108415e-004	5.8658613e-004	6.0375185e-004	5.7918479e-004

5.7366770e-004	5.2742010e-004	6.2031356e-004	5.6933701e-004
5.8653345e-004	6.4754474e-004	6.6874010e-004	6.7279194e-004
6.0063785e-004	6.2683994e-004	6.4754359e-004	7.1212275e-004
5.9563707e-004	6.3685716e-004	6.9532591e-004	6.4755943e-004
6.0275133e-004	5.9190925e-004	6.2012282e-004	6.0494238e-004
5.7478332e-004	5.3720668e-004	5.4285559e-004	5.3429583e-004
5.3022047e-004	4.8265106e-004	4.7944296e-004	4.6423318e-004
4.2786515e-004	4.6009701e-004	4.8072405e-004	4.6851217e-004
4.2965277e-004	4.4236729e-004	4.7035604e-004	4.8409656e-004
4.7726822e-004	4.7354386e-004	4.8457770e-004	5.1176443e-004
4.5363744e-004	5.0782525e-004	5.1298334e-004	5.0252063e-004
5.4055432e-004	5.0745023e-004	5.1858903e-004	5.3762517e-004
5.1235046e-004	5.1484245e-004	5.4586955e-004	5.6597822e-004
5.5803136e-004	5.7794302e-004	6.4719700e-004	6.1816380e-004
6.2894309e-004	6.0030544e-004	6.2261774e-004	7.0718335e-004
6.0859868e-004	6.7301538e-004	6.8261127e-004	6.7857788e-004
6.4515005e-004	8.0396427e-004	8.1274590e-004	8.3383278e-004
7.3918924e-004	7.8716458e-004	7.3385581e-004	7.6382900e-004
7.3275778e-004	7.1539648e-004	7.0668801e-004	7.3357453e-004
7.3025200e-004	7.5740041e-004	7.0457008e-004	7.4232614e-004
6.8662188e-004	7.4750280e-004	7.3125691e-004	7.8532373e-004
6.6463427e-004	7.0659378e-004	6.9933107e-004	6.8993462e-004
6.3203427e-004	6.7274681e-004	7.3432548e-004	7.3113505e-004
7.3552382e-004	7.3879602e-004	7.0711413e-004	6.4868687e-004
6.6434390e-004	6.5030155e-004	6.8476300e-004	6.3348914e-004
6.4185746e-004	6.4207274e-004	6.4249776e-004	6.5510893e-004
5.9588217e-004	6.6031201e-004	6.3599963e-004	6.8326796e-004
5.2037610e-004	5.9807725e-004	6.3557488e-004	5.7362075e-004
6.3138882e-004	6.2995931e-004	6.1198327e-004	6.7349037e-004
6.4897088e-004	7.2572460e-004	7.0107542e-004	7.0930349e-004
6.7656070e-004	6.2384653e-004	6.8152034e-004	6.9805671e-004
6.4850530e-004	6.5536784e-004	5.8300710e-004	6.6123057e-004
6.0884614e-004	5.9201065e-004	6.1192850e-004	6.2028920e-004
5.3889942e-004	5.4378233e-004	5.9206600e-004	5.7454314e-004
6.5645338e-004	7.0797696e-004	7.1414684e-004	7.9010165e-004
7.4265797e-004	7.5985733e-004	7.7025639e-004	7.8105420e-004
6.9915925e-004	6.8365052e-004	7.0308305e-004	6.6499165e-004
6.9326389e-004	6.2836257e-004	6.6940319e-004	6.6582546e-004
5.9884672e-004	6.2802425e-004	6.3410547e-004	6.3626555e-004
5.7902353e-004	6.1905073e-004	5.9951202e-004	5.9366161e-004
6.0797660e-004	5.9860540e-004	6.0157495e-004	6.6389790e-004
6.5805872e-004	5.8622079e-004	6.0063860e-004	6.9692326e-004
6.7664751e-004	6.7115824e-004	7.4668361e-004	6.8876286e-004
7.1277028e-004	7.4822355e-004	7.6949634e-004	7.4784922e-004
7.4391018e-004	7.5257252e-004	6.7809607e-004	6.8634565e-004
6.4146126e-004	6.8981168e-004	7.3360633e-004	7.3455759e-004
6.7607865e-004	7.1923299e-004	6.9136059e-004	7.0403998e-004
6.6937772e-004	6.5999063e-004	6.6487066e-004	6.8635539e-004
6.5117577e-004	6.0786243e-004	7.2408478e-004	6.6991271e-004
7.1780686e-004	7.5994880e-004	7.5736009e-004	7.4951950e-004
6.9078589e-004	7.2988019e-004	7.2302324e-004	7.8456091e-004
6.3276746e-004	6.9241569e-004	8.1639933e-004	7.2912610e-004

6.6077971e-004	6.1385237e-004	6.6089765e-004	6.3926530e-004
5.9025297e-004	6.0620491e-004	5.7766068e-004	5.7040936e-004
5.7465215e-004	5.4162685e-004	5.0591520e-004	5.1515194e-004
4.8497102e-004	4.8798244e-004	4.9797475e-004	4.8188346e-004
4.5876925e-004	4.3933812e-004	4.7841676e-004	4.9223678e-004
4.6342127e-004	4.7567598e-004	4.8117161e-004	5.1543719e-004
8.2255181e-004	7.9517321e-004	8.3581112e-004	8.8619824e-004
7.8995708e-004	7.7355448e-004	7.7640462e-004	8.5077953e-004
7.8320806e-004	7.3627464e-004	7.5816354e-004	7.2796451e-004
7.3362315e-004	7.6988029e-004	6.8516875e-004	6.6988888e-004
6.5297965e-004	6.5487203e-004	6.6223045e-004	6.9547725e-004
6.4348557e-004	6.5925981e-004	7.0800964e-004	6.6827745e-004
6.5714812e-004	6.7608505e-004	6.4458788e-004	6.6510614e-004
6.6133228e-004	6.4202549e-004	6.1246323e-004	6.1693025e-004
5.6280416e-004	5.9169562e-004	5.8716350e-004	5.3331867e-004
5.4693569e-004	5.2675690e-004	5.4563330e-004	5.7746716e-004
5.5396779e-004	5.5487541e-004	5.7877625e-004	6.0353082e-004
5.6364956e-004	5.6494494e-004	6.0100909e-004	5.7446750e-004
4.3415032e-004	4.8433179e-004	4.7236160e-004	5.0032348e-004
5.0828043e-004	4.9844424e-004	4.6214467e-004	4.9165582e-004
4.8644042e-004	4.7649552e-004	5.2374393e-004	5.4459382e-004
5.0186188e-004	5.5449893e-004	5.9544941e-004	5.6261710e-004
5.9343738e-004	5.8222370e-004	5.8902752e-004	6.8247483e-004
6.0805607e-004	6.6474975e-004	6.8975160e-004	6.7776813e-004
6.8552746e-004	7.6171591e-004	7.8064416e-004	7.9801260e-004
7.1635994e-004	7.5318506e-004	6.8832740e-004	6.7597345e-004
6.7458094e-004	6.5919511e-004	6.7205929e-004	6.4724217e-004
6.6164523e-004	6.9471898e-004	6.1348043e-004	6.5060538e-004
6.0102879e-004	6.7656192e-004	6.4897211e-004	7.2484986e-004
5.9310648e-004	6.1091853e-004	6.2074712e-004	6.1216702e-004
6.1212612e-004	6.5079205e-004	6.4935868e-004	7.0677456e-004
7.1157955e-004	7.4360624e-004	7.1144673e-004	7.2621337e-004
6.7963339e-004	5.9896283e-004	6.5565330e-004	6.5643009e-004
6.3097090e-004	6.2673149e-004	6.0836764e-004	6.3716582e-004
5.9862153e-004	5.9472850e-004	6.4853139e-004	6.2250251e-004
5.5683036e-004	5.4399093e-004	5.9301096e-004	5.9969982e-004
6.0230037e-004	6.1093189e-004	6.1882491e-004	6.1264775e-004
6.3719663e-004	6.3735690e-004	6.2327237e-004	6.9599116e-004
6.6050827e-004	6.4937561e-004	7.8439200e-004	6.9553919e-004
7.4408858e-004	7.4860343e-004	7.5466090e-004	7.5399831e-004
7.2613849e-004	7.4966699e-004	7.0566758e-004	6.9923987e-004
6.6518656e-004	6.7866738e-004	7.7739383e-004	7.1129546e-004
6.0927411e-004	5.7950652e-004	5.9866921e-004	6.0143042e-004
5.7156017e-004	5.3365812e-004	5.1989370e-004	5.3589061e-004
5.3322079e-004	4.7769698e-004	5.0733348e-004	4.7969622e-004
4.4706267e-004	4.7591721e-004	4.8686173e-004	5.0100802e-004
4.6289994e-004	4.8967148e-004	4.9047738e-004	4.8113781e-004
4.6481244e-004	5.1161052e-004	4.9090433e-004	5.1723228e-004
6.8875651e-004	6.7884757e-004	7.1543148e-004	7.0271907e-004
6.3234105e-004	6.5808500e-004	6.2318399e-004	6.5428992e-004
6.5411015e-004	5.7600469e-004	5.8782843e-004	5.7686931e-004
5.4365657e-004	5.7052891e-004	5.2405726e-004	5.2854746e-004

4.9340689e-004	4.9057349e-004	5.0035435e-004	5.2692165e-004
4.8972604e-004	4.9416095e-004	5.2450108e-004	5.4484392e-004
7.4925990e-004	7.7451002e-004	8.1448456e-004	8.2664430e-004
8.1155633e-004	7.4815183e-004	7.7367977e-004	8.2062210e-004
7.8751514e-004	7.6815096e-004	7.7314276e-004	7.3969130e-004
7.6676722e-004	7.2897600e-004	7.3638171e-004	6.8492467e-004
6.9403794e-004	6.9644589e-004	6.5142362e-004	7.7424073e-004
6.9912107e-004	7.0342634e-004	7.3368894e-004	6.8646861e-004
4.4443014e-004	4.8812972e-004	5.0812627e-004	5.1775948e-004
5.2249894e-004	5.3015609e-004	4.8560914e-004	5.1406424e-004
5.1649893e-004	5.0534007e-004	5.4955509e-004	5.7675282e-004
5.3372980e-004	5.8622139e-004	6.2154296e-004	5.8944740e-004
6.0739064e-004	6.1275373e-004	6.1730544e-004	7.1892199e-004
6.3912594e-004	6.8528432e-004	7.1874858e-004	7.1178064e-004
6.6011163e-004	7.2470674e-004	7.3471971e-004	7.5311471e-004
6.7531280e-004	7.1685897e-004	6.5500700e-004	6.4947033e-004
6.5922068e-004	6.3139504e-004	6.4171397e-004	6.3034525e-004
6.5470238e-004	6.7835556e-004	5.9392610e-004	6.2313094e-004
5.7667919e-004	6.4602453e-004	6.2617140e-004	6.9834615e-004
5.8234013e-004	6.0234714e-004	6.0488579e-004	6.0069421e-004
6.2039874e-004	6.7950996e-004	6.8292397e-004	7.4395486e-004
7.5159094e-004	7.2407609e-004	7.0697827e-004	7.4756799e-004
6.6327277e-004	6.9888523e-004	7.6348077e-004	6.6943941e-004
6.6817961e-004	6.6402432e-004	6.8811762e-004	6.6100303e-004
6.3523735e-004	6.7289945e-004	6.4284931e-004	6.3325127e-004
5.9379905e-004	6.5245808e-004	6.3628072e-004	6.4082946e-004
5.9293519e-004	6.0118423e-004	6.1588756e-004	6.0958287e-004
6.2523759e-004	6.5065348e-004	6.3050292e-004	6.8121344e-004
6.7295870e-004	6.5170339e-004	7.8036249e-004	6.9496443e-004
7.2297773e-004	7.3025201e-004	7.2503644e-004	7.2429684e-004
6.9960627e-004	7.1545747e-004	7.1429812e-004	6.7750615e-004
6.4411275e-004	6.6611503e-004	7.1644610e-004	6.6626754e-004
6.4254036e-004	6.6143600e-004	6.9596409e-004	6.6228508e-004
6.1826136e-004	6.4220556e-004	5.9197983e-004	6.3327031e-004
6.2585245e-004	5.3546972e-004	5.6870771e-004	5.3442136e-004
5.1485664e-004	5.2353485e-004	4.9580276e-004	4.9981127e-004
4.6618645e-004	4.9064806e-004	4.7741218e-004	5.0562479e-004
4.6557243e-004	4.8402322e-004	4.9845561e-004	5.2374406e-004
7.0214011e-004	7.4726791e-004	8.0640960e-004	7.7719218e-004
7.6299014e-004	7.2638207e-004	7.4689515e-004	7.7400284e-004
7.5806706e-004	7.3347757e-004	7.3224356e-004	7.2115347e-004
7.1917729e-004	6.7003811e-004	7.2139954e-004	6.4628763e-004
6.5554171e-004	6.4647033e-004	5.8977794e-004	7.3983872e-004
6.7216853e-004	6.7387060e-004	6.5780318e-004	6.3736644e-004
4.5877240e-004	4.9492201e-004	5.1313235e-004	5.1647153e-004
5.0190232e-004	5.2102660e-004	4.7372287e-004	5.1043021e-004
4.8910952e-004	5.0103979e-004	5.2843768e-004	5.5480799e-004
5.1850869e-004	5.5560123e-004	5.8237958e-004	5.7012361e-004
5.7732707e-004	6.1280490e-004	6.0535128e-004	6.8787282e-004
6.3889976e-004	6.4968977e-004	7.1379950e-004	7.0068749e-004
6.5921195e-004	7.2592324e-004	7.3788731e-004	7.4779242e-004
6.7357748e-004	7.2031005e-004	6.5423133e-004	6.8618101e-004

6.5693098e-004	6.3247309e-004	6.4125445e-004	6.5949082e-004
6.7368629e-004	6.8807862e-004	5.9306658e-004	6.1117623e-004
6.0309244e-004	6.4821790e-004	6.2166947e-004	6.8164936e-004
5.8343154e-004	6.4151861e-004	6.2684847e-004	6.2447754e-004
6.1304895e-004	7.0082767e-004	7.0556620e-004	7.4979644e-004
7.4091839e-004	7.4402530e-004	7.0740732e-004	7.1294037e-004
6.5095631e-004	6.8240993e-004	7.0464186e-004	6.4624332e-004
6.5469970e-004	6.3430048e-004	6.5582008e-004	6.5115454e-004
5.9793574e-004	6.4668636e-004	6.4726987e-004	6.2297095e-004
5.9423005e-004	6.2654854e-004	6.0383805e-004	6.2964068e-004
5.9908620e-004	6.0628794e-004	6.5970972e-004	6.0706207e-004
5.7297618e-004	6.1316902e-004	5.4857966e-004	5.9055458e-004
5.5453499e-004	4.8763454e-004	5.3542679e-004	4.6241761e-004
4.6375933e-004	4.6846915e-004	4.5138514e-004	4.6995916e-004
4.3367584e-004	4.7161341e-004	4.5754954e-004	4.8062807e-004
4.2999295e-004	4.7881675e-004	4.8780098e-004	5.1081469e-004
7.1293428e-004	7.1867328e-004	7.5211561e-004	7.6013047e-004
7.0923338e-004	6.8969731e-004	6.7188012e-004	6.8904155e-004
6.7110562e-004	6.1667932e-004	6.4694679e-004	6.4300475e-004
5.8902957e-004	5.7433354e-004	6.1575200e-004	5.3598019e-004
5.5112605e-004	5.6102990e-004	5.2596501e-004	5.8786526e-004
5.8925034e-004	6.0191652e-004	5.5072966e-004	5.6573677e-004
6.6983611e-004	7.5254607e-004	7.9224445e-004	7.4039167e-004
7.4107491e-004	7.9584113e-004	7.9880390e-004	7.8859201e-004
7.5526521e-004	7.4874636e-004	7.1163294e-004	6.6877644e-004
7.6682879e-004	6.4658910e-004	7.5376434e-004	7.3888843e-004
6.6233674e-004	6.9002539e-004	6.2371595e-004	8.1436688e-004
6.3500322e-004	6.8423852e-004	7.2567520e-004	6.8637088e-004
4.7408680e-004	5.0112540e-004	5.1459916e-004	5.3949430e-004
4.9502332e-004	5.1958137e-004	4.7296165e-004	5.0910275e-004
4.7595900e-004	4.9994415e-004	5.0931900e-004	5.2961981e-004
5.1329873e-004	5.3096352e-004	5.4541742e-004	5.4894453e-004
5.4864406e-004	5.9763939e-004	5.7932177e-004	6.4989082e-004
6.0748776e-004	6.0597439e-004	6.8327364e-004	6.6196323e-004
6.6976702e-004	7.3249731e-004	7.1967457e-004	7.5915591e-004
6.9161276e-004	7.2554206e-004	6.6419896e-004	7.0471170e-004
6.6268154e-004	6.3407197e-004	6.5250996e-004	6.7613527e-004
6.7448214e-004	6.7836247e-004	6.0526199e-004	6.0284737e-004
6.2437648e-004	6.4178453e-004	6.0957945e-004	6.6129061e-004
5.9009939e-004	6.6957413e-004	6.3194970e-004	6.3981824e-004
5.8046090e-004	6.6947285e-004	7.2087742e-004	7.3228957e-004
7.4813567e-004	7.5636491e-004	7.2955848e-004	7.6037615e-004
7.3346318e-004	7.3162914e-004	7.3200779e-004	6.7371664e-004
6.9085801e-004	7.1731152e-004	6.7999476e-004	7.1297736e-004
6.8057705e-004	6.2838041e-004	6.7318002e-004	6.6608573e-004
6.0717693e-004	6.6152198e-004	6.3881191e-004	6.1770978e-004
5.7370486e-004	5.8006737e-004	6.1024210e-004	5.7309394e-004
5.1919115e-004	5.7058985e-004	5.1119342e-004	5.3958753e-004
4.9420453e-004	4.7024028e-004	5.3016869e-004	4.3429797e-004
4.4182279e-004	4.6612770e-004	4.5081443e-004	4.7064322e-004
4.4759043e-004	4.6174925e-004	4.7681843e-004	4.8779562e-004
4.2933851e-004	4.8919932e-004	5.0755332e-004	5.1261937e-004

6.7451459e-004	6.8554305e-004	7.0453319e-004	7.0131863e-004
6.3349983e-004	6.3295495e-004	6.2617607e-004	6.3675780e-004
6.0806834e-004	5.3409948e-004	5.7636086e-004	5.6342808e-004
5.2358481e-004	4.9747422e-004	5.1898778e-004	4.7647927e-004
4.9013776e-004	5.2489196e-004	4.9091107e-004	5.1201216e-004
5.0792922e-004	5.4107064e-004	5.1325231e-004	4.9622877e-004
7.5101995e-004	7.8861542e-004	8.3751953e-004	7.9734458e-004
7.9352065e-004	8.2650853e-004	8.4597073e-004	8.0826278e-004
7.7984511e-004	7.5652865e-004	7.4618305e-004	7.0707961e-004
7.9664552e-004	6.5666101e-004	8.1961906e-004	7.3832892e-004
6.7347896e-004	6.8604921e-004	6.6881954e-004	7.8546068e-004
6.7234518e-004	7.2784013e-004	7.1539757e-004	7.0657348e-004
5.4055516e-004	5.7241764e-004	6.0022491e-004	5.9487343e-004
5.9565650e-004	5.9321204e-004	5.7345040e-004	6.1558647e-004
6.0634845e-004	5.7854589e-004	5.7760607e-004	6.3400959e-004
5.9957417e-004	6.3473548e-004	6.5120996e-004	6.7030107e-004
6.2208898e-004	6.5843522e-004	6.7000664e-004	7.1090586e-004
6.8172325e-004	6.9503495e-004	7.4813701e-004	7.0079439e-004
6.6853312e-004	7.4204800e-004	7.2446665e-004	7.7323771e-004
7.5158464e-004	7.2255600e-004	7.0881554e-004	6.8491307e-004
6.9093151e-004	6.5109394e-004	6.7744569e-004	6.6775212e-004
6.6295193e-004	6.4260449e-004	6.1690586e-004	6.0127473e-004
6.2045573e-004	6.2685124e-004	6.0711724e-004	6.5172258e-004
6.0841232e-004	6.5072524e-004	6.5690473e-004	6.1580281e-004
5.7854642e-004	7.0359232e-004	7.2518538e-004	7.5705176e-004
7.4123272e-004	7.8608250e-004	7.1837599e-004	7.6122931e-004
7.2057144e-004	7.2198670e-004	7.0352147e-004	6.5110243e-004
6.8884474e-004	6.7473546e-004	6.7615375e-004	7.1555200e-004
6.6007144e-004	6.2601898e-004	6.8080259e-004	6.7502985e-004
6.1287392e-004	6.1364642e-004	6.1752093e-004	6.3846407e-004
6.2845301e-004	6.1331973e-004	6.2077938e-004	6.3436282e-004
5.4839481e-004	5.6475347e-004	5.4081035e-004	5.6974226e-004
5.2911594e-004	4.6266722e-004	5.2100905e-004	4.8669621e-004
4.6812439e-004	4.4338284e-004	4.4121684e-004	4.4654998e-004
4.5546488e-004	5.0567179e-004	4.8521788e-004	4.8962711e-004
4.5263502e-004	5.2499623e-004	4.9577861e-004	4.9732112e-004
7.6204534e-004	7.1798750e-004	7.1495101e-004	7.3700803e-004
7.0748857e-004	6.8504739e-004	6.7760321e-004	6.6699438e-004
6.0838959e-004	6.0391209e-004	6.2462868e-004	6.0985515e-004
5.5513532e-004	5.3311746e-004	5.9590330e-004	5.4395490e-004
5.4571933e-004	5.5089605e-004	5.7124537e-004	5.4700284e-004
5.8238959e-004	5.8879600e-004	5.6684963e-004	5.4779745e-004
5.8172468e-004	6.0782440e-004	6.9597911e-004	6.2472983e-004
6.3628724e-004	6.8432347e-004	6.8623792e-004	6.4466767e-004
6.2941700e-004	5.9922387e-004	5.8036785e-004	5.5215541e-004
6.3103718e-004	5.3477121e-004	6.4962651e-004	6.1071703e-004
5.4180346e-004	5.6654324e-004	5.6640473e-004	6.5792409e-004
5.3761226e-004	6.0982646e-004	5.9560726e-004	5.8087819e-004
5.0686950e-004	5.0567409e-004	5.4499693e-004	5.9881121e-004
5.2891178e-004	5.4388556e-004	5.1240537e-004	5.2924098e-004
5.1304516e-004	5.0980487e-004	5.1658101e-004	5.2793007e-004
5.4899791e-004	5.3350269e-004	5.4071560e-004	5.6098221e-004

5.4333603e-004	5.7489543e-004	5.4557731e-004	6.3190317e-004
5.6436216e-004	6.1399977e-004	6.4358313e-004	6.5154188e-004
5.6856974e-004	7.4687162e-004	7.3154496e-004	7.7005033e-004
7.3808948e-004	8.0782888e-004	7.4647137e-004	7.7136891e-004
7.6208820e-004	7.4346554e-004	7.1970506e-004	6.5803539e-004
7.4257381e-004	7.0486593e-004	6.9040305e-004	7.2809591e-004
6.8496205e-004	6.0766083e-004	6.9734718e-004	6.8489814e-004
6.1608652e-004	6.2208217e-004	6.2711269e-004	6.4733301e-004
6.5016258e-004	6.3074983e-004	6.3771897e-004	6.6500045e-004
5.7275240e-004	5.6558840e-004	5.7298951e-004	5.7396338e-004
5.3582825e-004	4.7759835e-004	5.0297191e-004	5.2337124e-004
4.6629497e-004	4.3344233e-004	4.4886966e-004	4.4212861e-004
4.7060737e-004	5.1578721e-004	5.0458215e-004	4.9053922e-004
4.7611780e-004	5.2925771e-004	4.9569727e-004	4.7229329e-004
7.7252700e-004	7.2662765e-004	7.3006559e-004	7.4309696e-004
7.2226257e-004	6.9854523e-004	6.7592844e-004	6.9209664e-004
5.9193964e-004	6.1269030e-004	6.5129634e-004	6.0007788e-004
5.3421584e-004	5.5117480e-004	6.1863213e-004	5.6938191e-004
5.7238794e-004	5.5554803e-004	5.9594045e-004	5.6177717e-004
5.9656402e-004	5.8750447e-004	5.7454604e-004	5.5303761e-004
5.5454085e-004	5.4830599e-004	6.2342474e-004	6.5482972e-004
5.9201593e-004	5.8274741e-004	6.0931705e-004	6.4210886e-004
6.3309284e-004	5.9809188e-004	6.0330062e-004	6.1546023e-004
6.5795420e-004	6.5742483e-004	6.6161355e-004	6.8007848e-004
6.5087082e-004	6.4563016e-004	6.6828199e-004	7.1566102e-004
6.4790889e-004	6.9565301e-004	7.2122717e-004	7.0108092e-004
6.3013642e-004	6.6525192e-004	7.1019666e-004	7.1313434e-004
7.2965762e-004	6.7623642e-004	6.7245726e-004	6.6021337e-004
6.9667025e-004	6.2973007e-004	6.3719843e-004	6.4714796e-004
6.2967413e-004	5.9661588e-004	6.1566415e-004	6.0961966e-004
5.4395730e-004	5.7951095e-004	5.7517775e-004	5.9701886e-004
5.7585051e-004	6.0605882e-004	6.1201725e-004	5.6623261e-004
5.8097293e-004	7.4661750e-004	7.3696823e-004	7.6948635e-004
7.2701107e-004	7.9271733e-004	7.6807127e-004	7.4066121e-004
7.5807725e-004	7.3117345e-004	7.7473823e-004	7.0768772e-004
7.6525562e-004	7.1715737e-004	6.8013852e-004	7.0700888e-004
6.7125520e-004	6.2669873e-004	6.8013594e-004	6.8988783e-004
6.3642587e-004	6.6122090e-004	6.5199842e-004	6.6013388e-004
6.9918922e-004	6.8301537e-004	6.7681957e-004	6.8638168e-004
6.4609879e-004	6.0470252e-004	6.3704465e-004	5.9073533e-004
5.4269165e-004	5.3459223e-004	5.2135906e-004	5.4078254e-004
4.6178768e-004	4.5742693e-004	4.9132659e-004	4.8946829e-004
4.9418774e-004	5.0591212e-004	5.1582934e-004	4.9751496e-004
4.9221834e-004	5.3004108e-004	5.1326012e-004	4.6427855e-004
6.5565831e-004	6.3090416e-004	6.8704929e-004	6.8570882e-004
6.6748128e-004	6.5179206e-004	6.1615329e-004	6.2892490e-004
5.8003537e-004	5.5181654e-004	5.8772654e-004	5.4973743e-004
5.2751738e-004	5.3572934e-004	5.9706408e-004	5.5105152e-004
5.2787917e-004	5.5601865e-004	5.7152785e-004	6.0747747e-004
5.5558013e-004	6.1238823e-004	5.8286710e-004	5.7014573e-004
5.7757330e-004	5.6308057e-004	6.5571645e-004	7.1134273e-004
6.0864742e-004	5.9114506e-004	5.8573252e-004	6.1833426e-004

6.0674534e-004 5.9313401e-004 5.9585321e-004 5.8335898e-004
6.1343849e-004 6.0882121e-004 6.4368254e-004 6.6714102e-004
6.2998709e-004 6.4461911e-004 6.0917005e-004 7.0779533e-004
6.1639727e-004 6.9811524e-004 6.9200569e-004 7.2330248e-004
3.7546526e-004 4.1502019e-004 3.2120941e-004 4.1543492e-004
4.0798397e-004 3.0082486e-004 3.1306168e-004 3.2639230e-004
3.5468828e-004 2.9322226e-004 3.1364949e-004 3.1588406e-004
3.0791233e-004 2.7237427e-004 2.7303476e-004 2.8247375e-004
2.6694409e-004 2.7287453e-004 2.4703688e-004 2.7920405e-004
2.8634649e-004 2.8363634e-004 2.8361093e-004 2.8505854e-004
5.8740047e-004 7.3999175e-004 6.8926105e-004 7.4825723e-004
7.0264997e-004 7.7236637e-004 7.0317557e-004 7.3140195e-004
7.5087135e-004 7.0923741e-004 7.3481203e-004 6.5133754e-004
7.3486270e-004 6.9261543e-004 6.9551726e-004 7.2171370e-004
6.5565406e-004 6.1294643e-004 6.7642219e-004 6.8763655e-004
6.2325921e-004 6.2127206e-004 6.0731971e-004 6.5806547e-004
6.3031042e-004 6.2916584e-004 5.8314796e-004 6.2198780e-004
5.8464396e-004 5.5340739e-004 5.6122446e-004 5.3394884e-004
5.1490352e-004 4.8202489e-004 4.7245259e-004 5.0399789e-004
4.5054445e-004 4.2165136e-004 4.6906533e-004 4.4766417e-004
4.7209054e-004 5.0823305e-004 5.0849545e-004 4.9765151e-004
4.7919925e-004 5.0934912e-004 4.8559953e-004 4.8262238e-004
7.2267094e-004 6.9982051e-004 7.1916830e-004 7.1218889e-004
7.0053498e-004 6.6646560e-004 6.5221153e-004 6.5741857e-004
5.6387871e-004 5.6746362e-004 5.8907834e-004 5.5620978e-004
4.9410961e-004 5.1681644e-004 5.5699970e-004 5.2706382e-004
5.3999361e-004 5.2678521e-004 5.2962502e-004 5.4501095e-004
5.0960199e-004 5.6905566e-004 5.4512265e-004 5.0047118e-004
6.1993586e-004 6.3372227e-004 7.2255210e-004 7.4075691e-004
6.8842320e-004 6.7183864e-004 6.0524326e-004 6.4293362e-004
6.0405052e-004 5.9927082e-004 6.0879865e-004 5.7830823e-004
5.6214871e-004 5.8726192e-004 6.3972909e-004 6.2314636e-004
5.8577092e-004 6.4849874e-004 5.9426894e-004 6.6465692e-004
5.9379215e-004 7.0259317e-004 6.5511130e-004 6.7283680e-004
6.1811888e-004 6.5658672e-004 7.0815352e-004 7.2590425e-004
7.1167084e-004 6.7935348e-004 6.4951269e-004 6.5986789e-004
6.6892753e-004 6.3537952e-004 6.4450802e-004 6.4304339e-004
6.2101647e-004 6.0867792e-004 5.9428658e-004 6.0492136e-004
5.3264661e-004 5.8353836e-004 5.5482297e-004 5.8539228e-004
5.5904550e-004 6.0902274e-004 5.8169317e-004 5.4951214e-004
3.8225253e-004 4.1600506e-004 3.5633469e-004 4.0730108e-004
3.9240819e-004 3.0895110e-004 3.3563464e-004 3.1347276e-004
3.4466652e-004 2.8178161e-004 3.1536025e-004 3.1659102e-004
3.0767175e-004 3.0560904e-004 2.6482505e-004 2.9599253e-004
2.6205189e-004 2.4387617e-004 2.4821797e-004 2.8239495e-004
2.9044836e-004 2.8473895e-004 2.7450518e-004 2.6519172e-004
6.2534474e-004 6.9440836e-004 7.2105580e-004 7.7559699e-004
7.3725502e-004 7.8452179e-004 7.1232245e-004 7.7005479e-004
7.5702289e-004 7.1867713e-004 7.9795947e-004 6.9473106e-004
7.2635885e-004 6.9094048e-004 6.8951710e-004 7.0931752e-004
6.8052778e-004 6.4893540e-004 6.6120959e-004 6.9828800e-004
6.1466965e-004 6.7101838e-004 6.3284663e-004 6.5518678e-004

7.2266544e-004	6.8804080e-004	6.5905528e-004	6.8917382e-004
6.8196710e-004	6.1237205e-004	6.4845043e-004	6.1054391e-004
5.4640228e-004	5.5060953e-004	5.2985402e-004	5.3940082e-004
4.9007023e-004	4.8476145e-004	5.0914491e-004	5.0618769e-004
5.1393846e-004	5.1701552e-004	5.2014703e-004	5.3568649e-004
4.9098998e-004	5.3084630e-004	5.1936340e-004	4.9039367e-004
6.4458478e-004	6.8114138e-004	7.1716766e-004	6.9048285e-004
6.8433893e-004	7.0784440e-004	6.1483348e-004	6.5617767e-004
5.8282418e-004	5.7751799e-004	6.1009834e-004	5.6148740e-004
5.2146005e-004	5.4542662e-004	5.7303291e-004	5.2580622e-004
5.3025613e-004	5.6143699e-004	5.4142404e-004	5.7603145e-004
5.1201835e-004	6.1406456e-004	5.7167654e-004	5.4543785e-004
6.5278827e-004	6.8040934e-004	7.8292700e-004	8.5152588e-004
7.4802099e-004	7.2198801e-004	6.6985629e-004	7.3461204e-004
7.0159942e-004	7.1407165e-004	6.8462719e-004	6.8916107e-004
6.6594467e-004	7.3947367e-004	7.8679486e-004	7.9111365e-004
7.3765998e-004	7.8513297e-004	7.0347710e-004	7.9544634e-004
6.8225847e-004	7.8005271e-004	7.6918602e-004	8.1809203e-004
6.6463528e-004	7.2584249e-004	7.5028113e-004	7.8390491e-004
7.3439737e-004	7.2585151e-004	6.8872220e-004	7.4227573e-004
7.1534359e-004	6.8843574e-004	6.8857617e-004	7.2753016e-004
6.9365084e-004	6.8963796e-004	6.9905807e-004	6.8151062e-004
6.4315138e-004	6.4414191e-004	6.2863373e-004	7.0371362e-004
5.9630329e-004	6.6875879e-004	6.5477306e-004	6.1967548e-004
3.8244791e-004	4.3586216e-004	3.5639906e-004	4.4261290e-004
4.0061647e-004	3.1714536e-004	3.4873299e-004	3.2059786e-004
3.9634990e-004	3.0328413e-004	3.2968985e-004	3.1503556e-004
3.0484392e-004	2.9726349e-004	2.7167009e-004	3.0070552e-004
2.4220164e-004	2.6074889e-004	2.6007739e-004	2.9060242e-004
3.1223753e-004	2.9839647e-004	2.6549535e-004	2.7725038e-004
6.1321734e-004	6.1981654e-004	7.0042392e-004	7.5581370e-004
7.0478223e-004	7.4298389e-004	6.5049593e-004	7.0103527e-004
6.8455491e-004	6.6913787e-004	7.4160426e-004	6.6713092e-004
6.6503974e-004	6.2661260e-004	6.6671301e-004	6.2061817e-004
6.5526106e-004	6.4218169e-004	6.3232477e-004	6.2350623e-004
5.8892877e-004	6.2901180e-004	6.5001461e-004	6.0235096e-004
6.6099888e-004	6.5271975e-004	6.4292987e-004	6.3624195e-004
6.1955686e-004	6.2702264e-004	6.0347412e-004	6.2353321e-004
6.2245550e-004	5.8609858e-004	5.6381289e-004	5.6081761e-004
5.3478256e-004	5.8280171e-004	6.1203259e-004	5.2450935e-004
5.2537073e-004	5.4748630e-004	5.2679169e-004	5.8455949e-004
5.3351590e-004	5.5311824e-004	5.7536267e-004	5.5019365e-004
6.3326722e-004	6.4315753e-004	5.4340959e-004	6.0413986e-004
5.8831538e-004	5.5748330e-004	5.5307295e-004	5.3285804e-004
5.1434104e-004	4.8123514e-004	4.7899965e-004	5.0240491e-004
4.7133188e-004	4.4363446e-004	4.8060853e-004	4.6345452e-004
4.6743795e-004	4.9671773e-004	5.0831368e-004	5.1447543e-004
4.6584959e-004	4.8818381e-004	4.8491140e-004	4.7820186e-004
7.2364362e-004	6.7783732e-004	7.0014823e-004	6.8028999e-004
6.9619151e-004	6.6462673e-004	6.6263379e-004	6.5352287e-004
5.7763355e-004	5.7894280e-004	5.6896884e-004	5.4686271e-004
5.0961463e-004	5.3736696e-004	5.3717784e-004	4.9823680e-004

5.2589102e-004	5.2851965e-004	5.0375183e-004	5.4931591e-004
4.6846664e-004	5.4725054e-004	5.0874176e-004	4.9382641e-004
5.0563766e-004	5.9387914e-004	6.0659100e-004	5.9140454e-004
5.7131472e-004	6.0512382e-004	5.2188009e-004	5.2584376e-004
5.1687063e-004	5.1204699e-004	5.0431625e-004	4.9135669e-004
4.4204586e-004	5.0499713e-004	5.0688135e-004	5.2746968e-004
4.4428747e-004	4.7295805e-004	4.8640478e-004	4.6851148e-004
4.5421782e-004	5.3532892e-004	4.9360420e-004	5.0410906e-004
6.6366444e-004	7.0576214e-004	7.6388913e-004	8.4693306e-004
7.8243527e-004	7.4344969e-004	7.1160453e-004	7.8906529e-004
7.4119956e-004	7.6143560e-004	7.1692676e-004	7.6372277e-004
7.2574374e-004	8.2450997e-004	8.3268117e-004	8.2105015e-004
7.7646712e-004	8.1603602e-004	7.3450783e-004	8.2941409e-004
7.1639630e-004	7.8998586e-004	7.8614763e-004	8.1447994e-004
6.7791814e-004	7.3625303e-004	7.4718389e-004	7.8776549e-004
7.3140195e-004	7.3507232e-004	6.7174756e-004	7.2583748e-004
6.8842428e-004	6.7378224e-004	6.7347116e-004	6.9658998e-004
6.5441483e-004	6.5133347e-004	6.5742017e-004	6.4502461e-004
6.0375442e-004	6.2508463e-004	5.9816326e-004	6.3935085e-004
5.7349761e-004	6.4179311e-004	6.0903400e-004	5.8023981e-004
4.8744818e-004	5.4408931e-004	5.0806226e-004	6.0814936e-004
5.9959607e-004	6.1210080e-004	6.4093005e-004	6.2269828e-004
6.7464518e-004	6.1779357e-004	6.6505876e-004	6.1393595e-004
6.6281759e-004	6.4843945e-004	6.5767932e-004	6.5234164e-004
6.1975869e-004	6.5501260e-004	6.1786016e-004	6.8273026e-004
5.5555237e-004	6.4293219e-004	6.1789597e-004	6.1483841e-004
3.9911716e-004	4.2967436e-004	3.8025555e-004	4.3781620e-004
3.9884156e-004	3.2363425e-004	3.6341039e-004	3.1329518e-004
3.9456113e-004	3.1929144e-004	3.4131533e-004	3.1198992e-004
3.0694951e-004	3.0717753e-004	2.8193837e-004	3.2110717e-004
2.5455124e-004	2.4277993e-004	2.7586312e-004	3.0074517e-004
3.2647412e-004	3.0239103e-004	2.5960133e-004	2.6470666e-004
6.2172093e-004	6.5441418e-004	6.6560729e-004	7.0729289e-004
7.1248641e-004	7.2826688e-004	6.8030183e-004	7.4179660e-004
6.8416169e-004	7.0246910e-004	7.2418332e-004	6.6465735e-004
6.6944391e-004	6.6699125e-004	6.5776361e-004	6.9460987e-004
6.5651628e-004	6.5885429e-004	6.0043090e-004	6.7463043e-004
5.7894117e-004	6.3244025e-004	5.7728056e-004	6.2303561e-004
6.6305566e-004	6.6685508e-004	6.1233692e-004	6.1855822e-004
6.1537178e-004	6.1858368e-004	5.7495068e-004	5.7109068e-004
5.7929780e-004	5.3258560e-004	5.2041364e-004	5.2216054e-004
5.0910214e-004	5.3683764e-004	5.6577512e-004	4.8479556e-004
5.0467394e-004	5.1984775e-004	5.0990185e-004	5.4568741e-004
5.1230724e-004	5.0841302e-004	5.3185607e-004	5.1549458e-004
6.7927617e-004	6.4943984e-004	5.8942626e-004	6.4057138e-004
6.3057280e-004	5.8291593e-004	5.9446001e-004	5.8174576e-004
5.3288436e-004	5.0677036e-004	5.0285333e-004	5.2919430e-004
5.0733089e-004	4.9256179e-004	4.9736931e-004	5.1210004e-004
4.8006749e-004	5.2473121e-004	5.2963608e-004	5.4187873e-004
4.7357075e-004	4.9812560e-004	5.0952737e-004	4.8392188e-004
6.9722017e-004	6.9631754e-004	7.1673999e-004	6.6164431e-004
6.6489739e-004	6.8805888e-004	6.5302926e-004	6.3463050e-004

6.1029878e-004	5.9619917e-004	5.9327616e-004	5.5002699e-004
5.0178136e-004	5.5468791e-004	5.6614567e-004	5.1731489e-004
5.2591543e-004	5.2372714e-004	5.1586308e-004	5.3346636e-004
4.7736842e-004	5.4362697e-004	5.1315310e-004	5.0744602e-004
5.0388090e-004	6.1222565e-004	6.1593052e-004	6.4630565e-004
6.0597873e-004	6.3045326e-004	5.4902191e-004	5.6485851e-004
5.4747914e-004	5.6946011e-004	5.3295696e-004	5.3792653e-004
4.8494524e-004	5.9135992e-004	5.8778067e-004	6.0771132e-004
4.9037301e-004	5.3899261e-004	5.3978210e-004	5.1357555e-004
5.0817399e-004	5.7555423e-004	5.4680345e-004	5.8721792e-004
6.7542160e-004	6.9455337e-004	7.2069035e-004	7.4856466e-004
6.9119925e-004	7.1150376e-004	6.5936486e-004	6.9480494e-004
6.6168433e-004	6.4275498e-004	6.5013350e-004	6.4264353e-004
6.4069188e-004	6.3064216e-004	6.3282806e-004	6.2493174e-004
5.9004390e-004	6.1400680e-004	5.9156067e-004	6.0230181e-004
5.7939900e-004	6.2035807e-004	5.9546360e-004	5.7930425e-004
4.9802724e-004	5.2572839e-004	5.4587603e-004	5.8801135e-004
5.9452467e-004	6.6221683e-004	6.6790759e-004	6.3252772e-004
6.6789627e-004	6.0881888e-004	6.3682999e-004	6.0867694e-004
6.3770231e-004	6.4075588e-004	6.5088600e-004	6.3183655e-004
5.9530795e-004	6.2408962e-004	5.7630154e-004	6.7540335e-004
5.4531502e-004	6.2593598e-004	6.2436795e-004	6.1772430e-004
4.7514156e-004	5.1992794e-004	4.8852887e-004	5.8847696e-004
5.5765605e-004	5.5010885e-004	5.8684716e-004	5.9651464e-004
6.4714059e-004	5.8577261e-004	6.5891756e-004	5.9701393e-004
6.2768464e-004	6.5574865e-004	6.5261821e-004	6.4961352e-004
6.1992813e-004	6.6511604e-004	6.0648863e-004	6.6844176e-004
5.7211844e-004	6.5195166e-004	6.2156381e-004	6.0512075e-004
4.1393839e-004	4.4406227e-004	3.8274540e-004	4.2538704e-004
4.0477390e-004	3.3235444e-004	3.6772712e-004	3.1365608e-004
3.8579561e-004	3.3820088e-004	3.5580402e-004	3.2376438e-004
3.1388077e-004	3.2038417e-004	3.1126069e-004	3.3563196e-004
2.9119853e-004	2.6081828e-004	3.0715048e-004	3.0729442e-004
3.3641917e-004	2.9985813e-004	2.8213413e-004	2.7229155e-004
6.9262298e-004	7.2251655e-004	7.5523302e-004	7.5570005e-004
6.8259897e-004	7.6360648e-004	7.0474768e-004	7.0819493e-004
6.8075872e-004	6.7028290e-004	6.8176168e-004	5.9796475e-004
6.3187428e-004	6.6801165e-004	6.4949153e-004	6.2785800e-004
6.1084409e-004	5.8276588e-004	6.1740303e-004	6.0156779e-004
6.2136211e-004	6.2854704e-004	6.5073359e-004	5.9965806e-004
6.4334513e-004	6.6978630e-004	5.9477576e-004	6.0606876e-004
6.0000997e-004	5.8943298e-004	5.5363493e-004	5.3141451e-004
5.3337265e-004	4.8810334e-004	4.9043810e-004	5.0087447e-004
4.7643561e-004	5.0211306e-004	5.0652642e-004	4.7396787e-004
4.9898662e-004	5.0869576e-004	5.1245128e-004	5.2782807e-004
4.9567914e-004	4.9688142e-004	5.2191616e-004	4.9732102e-004
6.9980085e-004	6.4943371e-004	6.3814443e-004	6.5974894e-004
6.5678263e-004	6.1970744e-004	6.2317913e-004	6.1647339e-004
5.6292238e-004	5.2908563e-004	5.2052275e-004	5.5459702e-004
5.3392329e-004	5.3143712e-004	5.3407414e-004	5.4165354e-004
4.9584200e-004	5.5712757e-004	5.4621395e-004	5.5538953e-004
4.8619568e-004	5.1239035e-004	5.0858767e-004	5.0318241e-004

7.2376065e-004	7.6083143e-004	7.6081978e-004	7.1630355e-004
6.8002561e-004	7.2479427e-004	6.7347621e-004	6.2118868e-004
6.5989225e-004	6.1863622e-004	6.3315767e-004	5.7802760e-004
5.0153565e-004	5.8521771e-004	6.2285559e-004	5.9624451e-004
5.4980222e-004	5.3027729e-004	5.4382524e-004	5.3662795e-004
5.2625715e-004	5.4829264e-004	5.6194844e-004	5.3882349e-004
6.1918666e-004	7.0421117e-004	7.6960211e-004	8.1590208e-004
8.0074109e-004	7.6713657e-004	7.4907904e-004	8.2674732e-004
7.5701645e-004	8.3063899e-004	7.7683815e-004	7.9822222e-004
7.9825032e-004	9.1230257e-004	8.6482996e-004	8.2645621e-004
7.7388677e-004	8.2544515e-004	7.8622055e-004	8.0174390e-004
7.4611336e-004	8.1419318e-004	7.9993662e-004	7.8557472e-004
4.4769031e-004	5.2882658e-004	5.0254586e-004	5.9536549e-004
5.9450844e-004	6.0119127e-004	6.2405749e-004	6.3235853e-004
6.8022124e-004	6.1683309e-004	6.9553019e-004	6.4999335e-004
6.5612444e-004	7.0183573e-004	6.8486189e-004	6.8152396e-004
6.4043807e-004	7.0515506e-004	6.2447636e-004	7.0189814e-004
6.1094032e-004	6.8620738e-004	6.7412916e-004	6.5299534e-004
4.1402860e-004	4.2070277e-004	3.8276499e-004	4.2914220e-004
4.0236233e-004	3.3967877e-004	3.8238883e-004	3.2520819e-004
4.0588423e-004	3.3642042e-004	3.5650281e-004	3.3527777e-004
3.0018634e-004	3.1165632e-004	3.2316468e-004	3.3244870e-004
3.0845156e-004	2.8671305e-004	3.1925729e-004	3.1432923e-004
3.4386828e-004	3.1204642e-004	2.9301399e-004	2.7981688e-004
6.6343049e-004	7.1028716e-004	6.9036434e-004	6.8740247e-004
6.6273961e-004	6.7425068e-004	6.3163227e-004	5.8385616e-004
5.8460890e-004	5.6194350e-004	5.4386470e-004	5.3321384e-004
5.0646975e-004	5.8002477e-004	5.5376105e-004	5.2782359e-004
5.5881521e-004	5.3938627e-004	5.7435654e-004	5.6604133e-004
5.3891410e-004	5.3272409e-004	6.0298895e-004	5.2595157e-004
7.3342418e-004	7.1200231e-004	7.4207304e-004	6.8823369e-004
6.8907675e-004	7.0479691e-004	6.5012513e-004	6.4323003e-004
6.1692485e-004	5.9914790e-004	5.9169283e-004	5.5756541e-004
5.1577210e-004	5.5236492e-004	6.1538465e-004	5.6766368e-004
5.3902862e-004	5.4332016e-004	5.4538902e-004	5.5038807e-004
5.1961627e-004	5.1772392e-004	5.2133236e-004	5.2289102e-004
4.6500254e-004	5.4226053e-004	5.3297324e-004	5.9880117e-004
6.2846630e-004	6.5939360e-004	6.8726811e-004	6.7504287e-004
7.1815480e-004	6.4380087e-004	6.9024641e-004	6.6164609e-004
6.8276470e-004	7.0663347e-004	6.8713914e-004	6.6685858e-004
6.3754564e-004	6.6532294e-004	6.2724641e-004	6.9766976e-004
5.8198944e-004	6.8364065e-004	6.7508455e-004	6.3478625e-004
4.1637948e-004	4.7479109e-004	4.2837379e-004	4.8466989e-004
4.4668274e-004	4.6836838e-004	4.7861107e-004	4.7592603e-004
5.2252592e-004	5.0259212e-004	5.5316715e-004	5.5471901e-004
5.5815427e-004	5.6583292e-004	5.7975009e-004	5.8434907e-004
5.7473093e-004	6.3748589e-004	5.5887946e-004	6.0536843e-004
5.8826222e-004	6.1268477e-004	6.2323720e-004	6.2394708e-004
4.6378372e-004	4.1686553e-004	4.2950927e-004	4.1630143e-004
4.8880659e-004	4.5181123e-004	4.5376914e-004	4.8337856e-004
5.0305069e-004	5.2584706e-004	5.0786732e-004	4.7568276e-004
5.5313965e-004	5.1943937e-004	5.0178079e-004	5.4014728e-004

5.5793460e-004 5.0110187e-004 5.2157845e-004 5.3222991e-004
5.0105795e-004 5.4414013e-004 5.6682175e-004 5.4239332e-004
5.4018931e-004 5.6685746e-004 5.6057438e-004 6.4533054e-004
6.4044364e-004 6.5878147e-004 6.2480352e-004 6.4632930e-004
6.1016258e-004 6.5168116e-004 6.2732089e-004 5.2293611e-004
6.2395533e-004 6.0378446e-004 6.7788053e-004 6.0433998e-004
5.9685610e-004 5.4948287e-004 5.4907124e-004 6.3065210e-004
5.6236716e-004 5.2949268e-004 5.7186055e-004 6.0407570e-004
6.6253667e-004 7.2480238e-004 7.5133992e-004 7.4104880e-004
7.0089898e-004 7.4532317e-004 7.0484319e-004 6.3835339e-004
6.4759751e-004 6.5357068e-004 6.1701798e-004 5.8510592e-004
6.0220050e-004 6.3990944e-004 5.9949860e-004 6.0835056e-004
6.3183419e-004 5.5693304e-004 6.2982144e-004 5.9057787e-004
5.9531492e-004 5.8521277e-004 6.4265115e-004 5.9450387e-004
6.1088071e-004 6.5746537e-004 6.1028350e-004 6.2093644e-004
5.9633814e-004 5.5666496e-004 5.4669296e-004 5.3341473e-004
5.1601966e-004 4.8991603e-004 4.9633841e-004 5.2258297e-004
4.8747775e-004 5.1601901e-004 4.9963995e-004 5.1849961e-004
5.2422794e-004 5.4282809e-004 5.5262429e-004 5.7266979e-004
5.2186692e-004 5.4718905e-004 5.8625068e-004 5.1872180e-004
6.7936214e-004 6.4942741e-004 6.6397959e-004 6.3296616e-004
6.5846152e-004 6.4203131e-004 5.9633098e-004 6.2071475e-004
5.6527124e-004 5.3021335e-004 5.3070474e-004 5.5555256e-004
5.2738034e-004 5.2875275e-004 5.7582135e-004 5.2933660e-004
5.1676561e-004 5.5182053e-004 5.5704857e-004 5.6565633e-004
5.3153653e-004 5.3759351e-004 5.2199309e-004 5.3769517e-004
4.4893881e-004 5.4097035e-004 5.8986440e-004 6.5401451e-004
6.0311349e-004 5.8756853e-004 5.6795146e-004 6.0059929e-004
6.0516159e-004 6.4880218e-004 6.3566642e-004 6.3727346e-004
6.4566914e-004 6.6490074e-004 6.9233663e-004 6.5603058e-004
5.9943036e-004 6.3723408e-004 6.2336523e-004 6.2337404e-004
5.8033808e-004 6.2409943e-004 6.6984915e-004 5.5492822e-004
4.5105924e-004 5.2312884e-004 5.2115261e-004 5.7190763e-004
6.0795344e-004 6.1712689e-004 6.5071584e-004 6.6801022e-004
7.0821072e-004 6.4312325e-004 7.0436588e-004 6.8220102e-004
6.7363925e-004 6.9001583e-004 6.8674468e-004 7.0123917e-004
6.3983147e-004 6.8331271e-004 6.4427708e-004 7.1259176e-004
6.1157734e-004 7.0352339e-004 7.0377067e-004 6.7021856e-004
3.8332196e-004 4.4233081e-004 4.0265678e-004 4.2588859e-004
3.7641530e-004 4.0527926e-004 4.1101177e-004 3.9741382e-004
4.3774056e-004 4.3053899e-004 4.3732804e-004 4.8011960e-004
5.0080862e-004 4.7021179e-004 5.1275649e-004 4.9590243e-004
5.0600886e-004 5.6492806e-004 5.2765028e-004 5.2982165e-004
5.5023297e-004 5.4067721e-004 5.6776206e-004 5.8341264e-004
4.2258087e-004 4.1693245e-004 3.8767363e-004 4.2811406e-004
4.0488720e-004 3.7108640e-004 3.7649935e-004 3.2624078e-004
3.6030145e-004 3.5825610e-004 3.1143461e-004 3.2284892e-004
3.2129235e-004 3.1519998e-004 3.0639118e-004 3.0140198e-004
3.1780817e-004 3.0503231e-004 3.0574023e-004 3.1822557e-004
2.8097048e-004 2.7715621e-004 2.8020116e-004 3.1797920e-004
4.5947544e-004 4.4775800e-004 4.4465740e-004 4.3267739e-004
5.1356958e-004 4.8945652e-004 4.9129123e-004 5.2856892e-004

5.1805176e-004	5.6936451e-004	5.6561246e-004	4.9684608e-004
5.9456223e-004	5.6634603e-004	5.5918140e-004	5.7150821e-004
5.9242500e-004	5.2791872e-004	5.4873202e-004	5.6410598e-004
5.3314562e-004	5.5087559e-004	5.7538364e-004	5.4713011e-004
6.4438589e-004	7.1057663e-004	7.3489992e-004	7.4416590e-004
6.8240872e-004	6.8681843e-004	7.0962381e-004	6.1267585e-004
6.3459318e-004	6.1199060e-004	5.6961155e-004	5.9642752e-004
5.6920365e-004	6.3963137e-004	6.0846812e-004	6.0584588e-004
6.2606440e-004	5.6936493e-004	6.2398872e-004	6.1441104e-004
5.8139737e-004	5.9099024e-004	6.4002645e-004	5.7638959e-004
7.1166139e-004	6.7738982e-004	6.8809948e-004	6.4379776e-004
6.8742345e-004	6.6214689e-004	6.1272373e-004	6.3987150e-004
5.6583781e-004	5.5184025e-004	5.3862543e-004	5.5568731e-004
5.2337768e-004	5.4123350e-004	5.8985694e-004	5.4016600e-004
5.3017579e-004	5.4223786e-004	5.4339021e-004	5.9463774e-004
5.5236419e-004	5.3313300e-004	5.4505387e-004	5.4449459e-004
5.5391336e-004	6.1662096e-004	6.9197480e-004	7.5591684e-004
6.8768422e-004	6.8617145e-004	6.8136401e-004	6.9333955e-004
7.3653125e-004	7.3089839e-004	7.2419491e-004	7.5838459e-004
7.1928735e-004	7.0553143e-004	7.7149511e-004	7.3193669e-004
6.7024029e-004	7.2775406e-004	7.0807274e-004	7.1534270e-004
6.3292500e-004	6.6237483e-004	7.5511869e-004	6.2587824e-004
7.0558347e-004	7.3133016e-004	7.2939717e-004	7.3175241e-004
7.0110091e-004	7.3218372e-004	7.5022265e-004	7.1910647e-004
6.9467150e-004	6.5180060e-004	7.0200828e-004	6.8697565e-004
7.3764112e-004	6.6656491e-004	6.6788758e-004	6.5113808e-004
6.5161171e-004	6.1652526e-004	6.4125203e-004	6.7582182e-004
5.7123632e-004	6.2788288e-004	6.2902532e-004	6.3848248e-004
4.2315496e-004	4.7303348e-004	4.8562014e-004	5.1549324e-004
5.4147414e-004	5.2929806e-004	5.6703116e-004	6.0464854e-004
6.4997947e-004	5.9245506e-004	6.4752632e-004	6.5253288e-004
6.4464600e-004	6.3364395e-004	6.6672689e-004	6.7761893e-004
6.2085179e-004	6.8247586e-004	6.3261368e-004	6.8845393e-004
6.2856438e-004	6.9985552e-004	7.1060215e-004	6.9858071e-004
3.7106220e-004	4.2228769e-004	3.8409111e-004	4.0536608e-004
3.4980359e-004	3.6828635e-004	3.8005355e-004	3.5665374e-004
3.9977444e-004	3.7984895e-004	3.7107947e-004	4.1962749e-004
4.4287082e-004	4.0862008e-004	4.5200768e-004	4.4106789e-004
4.4138695e-004	4.9997883e-004	5.0381117e-004	4.7773519e-004
5.1639623e-004	4.8959465e-004	5.1047937e-004	5.2873803e-004
4.2057160e-004	4.4094431e-004	4.0732051e-004	4.3844349e-004
3.9700182e-004	3.5775093e-004	3.7856133e-004	3.5091444e-004
3.7225250e-004	3.5363068e-004	3.0513803e-004	3.0529658e-004
3.3467145e-004	3.0288035e-004	3.2677274e-004	3.0566552e-004
3.1629345e-004	3.0436186e-004	2.8942717e-004	3.1134571e-004
2.7651243e-004	2.5004510e-004	3.0224534e-004	3.0637592e-004
4.2649029e-004	4.5497727e-004	4.0288680e-004	4.4031066e-004
4.2772049e-004	3.5700078e-004	4.0343353e-004	3.4965930e-004
4.0735968e-004	3.2824245e-004	3.2769639e-004	3.5076085e-004
2.9180222e-004	3.3745450e-004	3.1345520e-004	3.3463979e-004
3.5046464e-004	3.6370134e-004	3.3089569e-004	3.1701733e-004
3.2847177e-004	3.3459703e-004	3.2948610e-004	3.3511350e-004

3.9637749e-004	4.2086884e-004	3.7166625e-004	3.7049169e-004
4.2936012e-004	3.7724929e-004	3.8534772e-004	4.3919750e-004
4.4420768e-004	4.2648033e-004	4.3764069e-004	4.5499037e-004
4.6436577e-004	4.6384553e-004	4.7146560e-004	4.9675185e-004
5.3473549e-004	5.0244600e-004	4.9168683e-004	5.4571561e-004
4.8998228e-004	5.3397904e-004	5.7912576e-004	5.1808655e-004
4.5058598e-004	4.6444312e-004	4.4983118e-004	4.4687109e-004
4.8276672e-004	4.9518761e-004	4.9973979e-004	5.3431980e-004
4.9960770e-004	5.6464455e-004	5.6511381e-004	4.9176007e-004
5.7995236e-004	5.5952770e-004	5.7063923e-004	5.5805536e-004
5.8106882e-004	5.2940744e-004	5.5561776e-004	5.6747749e-004
5.4523042e-004	5.2428094e-004	5.5419009e-004	5.2952750e-004
5.9190455e-004	6.8434444e-004	6.5707955e-004	6.7579554e-004
7.1664467e-004	7.1830006e-004	6.2748468e-004	6.4765026e-004
6.7395915e-004	6.6029247e-004	6.2621501e-004	5.7692324e-004
6.5800038e-004	5.7659522e-004	6.0484624e-004	6.4264506e-004
6.2329897e-004	5.7025104e-004	6.0017500e-004	5.9803695e-004
6.3389470e-004	5.8699379e-004	5.6135745e-004	6.0148259e-004
6.2370667e-004	6.8546853e-004	7.0426003e-004	7.3712340e-004
6.5975141e-004	6.4521468e-004	6.8977766e-004	6.0483626e-004
6.1711253e-004	5.8251678e-004	5.4942128e-004	6.1990558e-004
5.7237380e-004	6.3260621e-004	6.2495880e-004	6.2197399e-004
6.1585101e-004	5.8663360e-004	6.0838879e-004	6.4282111e-004
5.9115708e-004	6.0944034e-004	6.4303991e-004	5.8257791e-004
7.5585852e-004	7.3702617e-004	7.2132320e-004	7.0798077e-004
7.3302191e-004	6.9277224e-004	6.5924758e-004	6.6507427e-004
5.8731134e-004	5.8504021e-004	5.6078062e-004	5.7572314e-004
5.4187534e-004	5.5639687e-004	6.0333562e-004	5.7385763e-004
5.5177959e-004	5.4241615e-004	5.3925706e-004	6.2688065e-004
5.7188347e-004	5.3946969e-004	5.7727007e-004	5.6918172e-004
7.1289544e-004	8.0937532e-004	7.8314839e-004	8.9704584e-004
8.0447934e-004	7.8836812e-004	7.6289465e-004	7.5995208e-004
7.8006547e-004	7.2398331e-004	7.0034267e-004	7.6418124e-004
6.5996982e-004	6.4700465e-004	6.8344382e-004	6.7158723e-004
6.6724375e-004	7.1545965e-004	6.1090299e-004	6.6526666e-004
6.5912324e-004	6.6268778e-004	7.0918203e-004	6.5473388e-004
6.9438759e-004	7.4659304e-004	7.3743174e-004	7.5055790e-004
7.4575929e-004	7.8397884e-004	7.6893459e-004	7.5235973e-004
7.7483476e-004	7.0683262e-004	7.5110067e-004	7.4934916e-004
7.5360193e-004	6.9259149e-004	7.1859984e-004	7.4629710e-004
6.5677754e-004	6.7660616e-004	6.9431309e-004	7.3151561e-004
5.9181586e-004	6.4648876e-004	6.9339157e-004	6.5433477e-004
6.3007606e-004	6.8418824e-004	6.7118231e-004	6.9494442e-004
6.6371198e-004	6.9960636e-004	6.8879216e-004	6.6085916e-004
6.2815983e-004	5.9704199e-004	6.2912377e-004	6.1796325e-004
6.3381181e-004	5.9375718e-004	5.9909216e-004	5.9118989e-004
6.0378971e-004	5.7467620e-004	5.9165187e-004	6.1262804e-004
5.2422000e-004	5.7953822e-004	5.7985021e-004	5.6920886e-004
4.7419300e-004	4.9458627e-004	5.1503390e-004	5.4204544e-004
5.4905198e-004	5.7344782e-004	6.5342904e-004	6.4263622e-004
6.7676828e-004	6.4143512e-004	6.6640229e-004	6.5303294e-004
6.8626505e-004	6.4770275e-004	6.8060703e-004	6.9160671e-004

6.2702330e-004	6.6150547e-004	6.6340568e-004	6.7222471e-004
6.2659777e-004	6.6845132e-004	6.7078458e-004	6.5273281e-004
3.7270070e-004	3.9778779e-004	4.0674695e-004	4.3184966e-004
3.9431678e-004	3.8176257e-004	4.0885412e-004	4.4961759e-004
4.6658032e-004	4.2950063e-004	4.5306508e-004	4.9396911e-004
5.2801506e-004	4.8379525e-004	5.6505161e-004	5.3392808e-004
5.1920740e-004	6.0253772e-004	5.4250151e-004	5.6115221e-004
5.9611861e-004	6.1002893e-004	6.0153972e-004	6.3877732e-004
4.2425498e-004	4.1757770e-004	3.8340707e-004	4.2513744e-004
3.6136776e-004	3.4797534e-004	3.6433824e-004	3.1979027e-004
3.4857885e-004	3.7672905e-004	3.1221486e-004	3.0023995e-004
3.4675666e-004	2.9350131e-004	3.2121823e-004	3.1776376e-004
2.9393270e-004	2.8433370e-004	2.7251355e-004	3.2070631e-004
2.9086696e-004	2.7253673e-004	3.1855873e-004	3.1906220e-004
3.9502519e-004	4.5969291e-004	3.9619066e-004	4.2621750e-004
3.9679718e-004	3.3213531e-004	3.5718248e-004	3.5896763e-004
3.9745055e-004	3.1730148e-004	3.0733563e-004	3.1508124e-004
3.0997313e-004	3.0299061e-004	3.2839044e-004	3.2920874e-004
3.1138580e-004	3.3745196e-004	2.9151177e-004	3.0870353e-004
2.8769058e-004	2.9445688e-004	3.1401918e-004	3.1081183e-004
4.1979279e-004	4.4929950e-004	3.9310287e-004	3.6542436e-004
4.2289424e-004	4.2617513e-004	4.1503964e-004	4.8579710e-004
4.4831164e-004	4.6292065e-004	4.9712882e-004	4.8014171e-004
5.3707416e-004	5.3077823e-004	5.1907368e-004	5.3197730e-004
5.9181835e-004	5.4251858e-004	5.5303521e-004	5.8928748e-004
5.2807810e-004	5.5960144e-004	5.9481098e-004	5.4406477e-004
6.4372945e-004	6.3508187e-004	6.3422503e-004	6.1134406e-004
6.4830011e-004	6.0622339e-004	5.4524830e-004	5.8756485e-004
5.1990547e-004	4.9803965e-004	4.9234860e-004	5.2496282e-004
5.2119388e-004	5.1239209e-004	5.5635598e-004	5.1796892e-004
4.9967134e-004	5.3153669e-004	5.3011744e-004	5.8219934e-004
5.3900828e-004	5.4776002e-004	5.7284501e-004	5.6118902e-004
5.1479509e-004	5.7409651e-004	5.8526782e-004	6.4199381e-004
5.9180047e-004	6.3796678e-004	6.5017957e-004	6.5412200e-004
6.6737215e-004	6.2483547e-004	6.0541634e-004	6.7957188e-004
5.9418699e-004	5.9527478e-004	6.3170876e-004	6.1821309e-004
5.5702380e-004	6.3645514e-004	6.0598199e-004	6.3389078e-004
5.3697594e-004	5.5488681e-004	6.5785850e-004	5.1684892e-004
6.9364165e-004	7.5764168e-004	7.3279007e-004	7.2504237e-004
7.1095515e-004	7.8120288e-004	7.5210790e-004	7.1647015e-004
7.0210922e-004	6.4208765e-004	6.9746938e-004	6.5670765e-004
7.1860588e-004	6.5846437e-004	6.4614842e-004	6.6745821e-004
6.4150146e-004	6.2148519e-004	6.3303218e-004	6.8279541e-004
5.4720223e-004	6.2985001e-004	6.3634332e-004	6.2047363e-004
5.0500692e-004	5.2402561e-004	4.8107025e-004	5.5650795e-004
5.2905036e-004	5.5823934e-004	6.0373571e-004	6.0634663e-004
5.9333666e-004	5.8674114e-004	6.0033766e-004	5.7323532e-004
6.4943569e-004	5.7321330e-004	6.1145447e-004	6.5721202e-004
5.9712121e-004	6.3185551e-004	6.2211195e-004	6.3227188e-004
5.9015265e-004	6.0928601e-004	6.1003813e-004	6.2324927e-004
3.6988447e-004	3.8454936e-004	3.7606317e-004	3.9584244e-004
3.4686008e-004	3.3533323e-004	3.7034686e-004	3.4923228e-004

3.8550883e-004	3.4748181e-004	3.5782759e-004	3.8110065e-004
4.0444771e-004	3.9010174e-004	4.4588959e-004	4.3163016e-004
4.2664792e-004	4.7556889e-004	4.6332082e-004	4.7531175e-004
5.3029461e-004	5.2036221e-004	5.0015254e-004	5.2579919e-004
4.1138677e-004	4.5469051e-004	3.9144971e-004	4.3929942e-004
3.7892067e-004	3.2710891e-004	3.4173627e-004	3.4133267e-004
3.5315256e-004	3.4432954e-004	2.9022367e-004	2.8674473e-004
3.3781938e-004	2.7995052e-004	3.3806967e-004	3.2991011e-004
2.7929249e-004	2.9040828e-004	2.5000191e-004	3.1086645e-004
2.7853138e-004	2.5019401e-004	3.0651111e-004	2.9305949e-004
4.1301888e-004	4.6233406e-004	3.8937126e-004	3.9349270e-004
4.2014159e-004	4.1961532e-004	4.3219061e-004	4.5768113e-004
4.7440604e-004	4.4554562e-004	4.7496518e-004	5.1240249e-004
5.0695686e-004	5.1418608e-004	5.3869076e-004	5.3465708e-004
5.4611305e-004	5.2097836e-004	5.4349820e-004	5.9205962e-004
5.0090686e-004	5.6120304e-004	5.7309934e-004	5.2673066e-004
6.8654303e-004	6.9529835e-004	6.7978601e-004	6.8749356e-004
6.9009690e-004	6.2146637e-004	6.1378205e-004	6.2589695e-004
5.4833024e-004	5.5066602e-004	5.1940938e-004	5.3340988e-004
5.3054337e-004	5.2568055e-004	5.5352156e-004	5.2922526e-004
5.0798030e-004	5.1734140e-004	5.0560595e-004	5.7332510e-004
5.2799957e-004	5.1960198e-004	5.6499784e-004	5.7202317e-004
6.6014623e-004	7.5804698e-004	7.5225856e-004	7.9345155e-004
7.5672533e-004	7.2795387e-004	7.4645120e-004	7.1683399e-004
6.7068706e-004	6.6002307e-004	6.2469056e-004	6.3247851e-004
5.9248294e-004	5.8695855e-004	6.1377127e-004	5.7395644e-004
6.1459528e-004	6.1805797e-004	5.6342960e-004	6.1411579e-004
5.7298755e-004	5.8829453e-004	6.0479406e-004	6.0080407e-004
6.9051853e-004	7.8332413e-004	7.5089371e-004	7.6147183e-004
7.3588709e-004	8.2696974e-004	7.9050550e-004	7.8051483e-004
7.8291948e-004	6.9834733e-004	7.0155092e-004	7.6076931e-004
7.2761367e-004	6.9360119e-004	7.0271909e-004	7.5222703e-004
6.6656528e-004	6.7564230e-004	6.9845414e-004	7.6150887e-004
5.8977053e-004	6.6462372e-004	6.8892955e-004	6.4119547e-004
6.0097773e-004	6.7331144e-004	6.8560202e-004	6.8607248e-004
6.6424158e-004	7.2623123e-004	6.7604413e-004	6.5728280e-004
6.3267929e-004	5.9768285e-004	6.4129735e-004	5.8198963e-004
6.3106255e-004	5.9308768e-004	6.1714090e-004	5.9785803e-004
5.9038437e-004	5.7248850e-004	5.5887147e-004	6.0999230e-004
5.2343872e-004	6.1439625e-004	5.7834467e-004	5.8253959e-004
4.4164782e-004	4.5803810e-004	4.5714913e-004	4.6725081e-004
4.5897791e-004	4.6544683e-004	5.2686113e-004	5.1849987e-004
5.7268841e-004	5.5892786e-004	5.5993127e-004	5.7718701e-004
6.3138602e-004	5.9346463e-004	6.2860959e-004	6.2520876e-004
5.8745930e-004	6.1029250e-004	6.4616102e-004	6.3792324e-004
6.2352975e-004	6.4447604e-004	6.5551327e-004	6.4909876e-004
3.6545657e-004	3.6305658e-004	3.8363840e-004	4.1523247e-004
3.8122534e-004	3.4963844e-004	3.7430862e-004	4.1655422e-004
4.2287126e-004	3.8095428e-004	3.9781695e-004	4.3966473e-004
4.4283009e-004	4.6301189e-004	5.2104996e-004	4.8714115e-004
4.6419310e-004	5.4178787e-004	5.0161329e-004	5.6514890e-004
5.5361439e-004	5.8231009e-004	5.6757350e-004	5.7850676e-004

4.0178700e-004	4.2539143e-004	3.9275087e-004	3.8234859e-004
3.8365673e-004	3.1055351e-004	3.5034729e-004	3.2349757e-004
3.6496984e-004	3.3740664e-004	3.5173642e-004	3.4969880e-004
3.6589602e-004	3.6611989e-004	3.8110513e-004	3.8582013e-004
3.7670293e-004	4.2410543e-004	4.0159847e-004	4.3474336e-004
4.5400846e-004	4.7607979e-004	4.8255757e-004	4.7397467e-004
3.9594100e-004	4.2318290e-004	3.8397119e-004	4.1993165e-004
3.5263942e-004	3.3936708e-004	3.4600725e-004	3.1252726e-004
3.4919876e-004	3.4493193e-004	2.8763222e-004	3.0024699e-004
3.3423024e-004	2.7671564e-004	3.1585008e-004	3.1359361e-004
2.8218683e-004	2.6265236e-004	2.6426914e-004	3.1334259e-004
2.8568609e-004	2.7906167e-004	3.2817257e-004	3.1048615e-004
3.8622995e-004	4.7019843e-004	3.9879465e-004	4.4975440e-004
4.1838805e-004	3.5235433e-004	3.2192192e-004	3.6979903e-004
3.8166131e-004	3.4859578e-004	3.2749699e-004	3.2143276e-004
3.5489006e-004	3.0909891e-004	3.4646069e-004	3.4797446e-004
3.0359082e-004	3.5447367e-004	3.0535236e-004	3.1158692e-004
3.1390802e-004	3.2221593e-004	3.1073162e-004	3.0702689e-004
4.3666954e-004	4.7617502e-004	3.9999329e-004	3.8635878e-004
4.0470746e-004	4.2941522e-004	4.3387204e-004	4.6866169e-004
4.5156168e-004	4.5265521e-004	4.5379288e-004	4.7263626e-004
5.2193864e-004	5.1210926e-004	5.2204394e-004	5.2154861e-004
5.3118123e-004	5.4299836e-004	5.3973409e-004	5.7497420e-004
5.0356658e-004	5.0703118e-004	5.4981270e-004	5.2266423e-004
7.1474233e-004	7.6937252e-004	7.9123586e-004	7.6426065e-004
7.1322975e-004	8.1992228e-004	7.3337501e-004	7.1458142e-004
7.1338113e-004	6.5048778e-004	6.6585785e-004	6.7271201e-004
6.8149091e-004	6.5183635e-004	6.7100530e-004	6.9053806e-004
6.4491942e-004	6.1822966e-004	6.3784712e-004	6.8673685e-004
5.6631817e-004	6.6205183e-004	6.3448258e-004	6.4411701e-004
3.6437462e-004	3.7972624e-004	3.6437248e-004	3.8332994e-004
3.4574255e-004	3.0993347e-004	3.5039908e-004	3.5856974e-004
3.5869897e-004	3.3923934e-004	3.4581232e-004	3.7023114e-004
3.6470452e-004	3.7195246e-004	4.1724358e-004	4.1867675e-004
3.9667777e-004	4.3364782e-004	4.2561252e-004	4.9199444e-004
4.8093178e-004	5.1713752e-004	5.0132004e-004	4.9970733e-004
4.0311093e-004	4.3348904e-004	3.6238306e-004	4.4311306e-004
3.8738180e-004	3.3332929e-004	3.0223091e-004	3.3672756e-004
3.3375822e-004	3.3192783e-004	2.7279191e-004	2.9689090e-004
3.3760729e-004	2.8025653e-004	3.1559917e-004	3.3308019e-004
2.7378368e-004	2.8429218e-004	2.8202814e-004	3.0878519e-004
2.9186934e-004	2.8457581e-004	2.9907830e-004	2.7861225e-004
4.0877416e-004	4.6691600e-004	4.2017944e-004	4.2424398e-004
4.2408460e-004	4.4370329e-004	4.8174581e-004	4.8572297e-004
5.1095221e-004	4.7296366e-004	5.2125142e-004	5.6001481e-004
5.7946039e-004	5.3526642e-004	5.8374611e-004	5.6207615e-004
5.1462904e-004	5.3727888e-004	5.6875410e-004	5.8402753e-004
4.8582815e-004	5.3752256e-004	5.3770025e-004	4.9053964e-004
6.3220816e-004	6.4832320e-004	6.2565230e-004	6.4250230e-004
6.1946463e-004	5.6893224e-004	5.7968186e-004	5.6492149e-004
5.1235137e-004	5.2647157e-004	5.0953424e-004	4.9033615e-004
5.2377023e-004	5.0904565e-004	5.0806721e-004	4.9602542e-004

4.7622407e-004	5.1249479e-004	4.6813737e-004	5.1209116e-004
4.9593568e-004	4.9014515e-004	5.5387951e-004	5.3280512e-004
6.6863665e-004	7.5499770e-004	7.5951294e-004	7.2572977e-004
6.6354138e-004	7.3956158e-004	6.4771520e-004	7.3495855e-004
6.6952525e-004	5.9110665e-004	6.0060685e-004	6.6598121e-004
6.3172178e-004	6.0386624e-004	6.3749542e-004	6.6137633e-004
6.1347230e-004	6.0391724e-004	6.2277681e-004	6.7894744e-004
5.4628024e-004	6.1380076e-004	5.9266405e-004	6.1811667e-004
7.0128926e-004	7.2015471e-004	7.6648942e-004	7.5745920e-004
6.6715652e-004	7.5771307e-004	6.7032798e-004	6.4733208e-004
6.6613810e-004	6.1239825e-004	6.4123652e-004	6.2712670e-004
6.4111278e-004	6.1911493e-004	6.5628153e-004	6.4680587e-004
5.8471620e-004	5.7867423e-004	6.0496441e-004	6.2852906e-004
5.3953475e-004	6.5068387e-004	6.0848004e-004	6.2058671e-004
4.4764694e-004	4.4371527e-004	4.3981235e-004	4.3975375e-004
4.2675979e-004	4.3678699e-004	4.3211167e-004	4.2551843e-004
4.6342744e-004	4.7093452e-004	4.6710305e-004	4.8455581e-004
5.0351031e-004	4.9129667e-004	5.1961979e-004	5.2083345e-004
5.1820073e-004	5.0791695e-004	5.5451562e-004	5.4952622e-004
5.3213166e-004	5.5856086e-004	5.7377896e-004	5.6719134e-004
3.6490426e-004	3.6925881e-004	3.8432526e-004	4.1394430e-004
3.9126940e-004	3.7591163e-004	3.8163353e-004	4.0397717e-004
4.0828407e-004	4.0221017e-004	4.0969602e-004	4.5882082e-004
4.1293033e-004	4.6687503e-004	5.3200081e-004	4.8795791e-004
4.6403622e-004	5.2763415e-004	5.3242153e-004	6.0988004e-004
5.4450247e-004	5.9687767e-004	5.8780576e-004	5.7568554e-004
3.7387840e-004	4.0653606e-004	3.6494319e-004	3.6652145e-004
3.4449231e-004	2.9846983e-004	3.2775128e-004	3.5560298e-004
3.4265422e-004	3.3721943e-004	3.2803490e-004	3.4812521e-004
3.5403961e-004	3.3436621e-004	3.7800058e-004	3.8863811e-004
3.6494873e-004	3.9956490e-004	3.8885938e-004	4.5693264e-004
4.3175556e-004	4.8459549e-004	4.8183837e-004	4.7112236e-004
3.8655060e-004	4.0876308e-004	4.2399469e-004	3.9693734e-004
3.3554158e-004	3.4790597e-004	3.2705131e-004	3.3033438e-004
3.4310631e-004	3.2957662e-004	2.9567119e-004	3.0596400e-004
3.0652367e-004	2.7503500e-004	2.8368640e-004	2.8323667e-004
2.9788948e-004	2.8006954e-004	2.9227237e-004	2.8473152e-004
2.7198327e-004	2.8442984e-004	2.9657446e-004	2.9028807e-004
4.0445640e-004	4.3544664e-004	3.6549385e-004	4.5685234e-004
4.0814478e-004	3.5101279e-004	2.9566330e-004	3.6217067e-004
3.3695366e-004	3.4517598e-004	3.0411479e-004	3.2339423e-004
3.5884568e-004	3.0600886e-004	3.2487145e-004	3.4320040e-004
3.0917065e-004	3.1190700e-004	3.2737213e-004	3.1738925e-004
3.1291998e-004	3.2407466e-004	3.1777634e-004	2.9354435e-004
4.1035633e-004	4.6475223e-004	4.1703098e-004	4.0709905e-004
4.1736434e-004	4.1959729e-004	4.5515468e-004	4.8041461e-004
4.8616071e-004	4.6238350e-004	4.8199602e-004	5.2516986e-004
5.5712540e-004	5.1980353e-004	5.6812602e-004	5.3597651e-004
4.9253264e-004	5.3525822e-004	5.3937039e-004	5.5167967e-004
4.7587380e-004	4.9327140e-004	5.0454210e-004	4.8091864e-004
6.7844428e-004	7.2539102e-004	6.9518242e-004	7.2264964e-004
7.0618192e-004	6.5233103e-004	6.4570844e-004	5.9973184e-004

5.9123246e-004	5.6916238e-004	5.5771755e-004	5.2548880e-004
5.6856147e-004	5.5123536e-004	5.2877460e-004	5.3021847e-004
4.9525999e-004	5.1595669e-004	4.9249279e-004	5.5002521e-004
5.0340462e-004	4.8515869e-004	5.7315476e-004	5.4935277e-004
7.6444354e-004	7.9242142e-004	8.3423888e-004	8.2199226e-004
7.2764997e-004	7.7122840e-004	6.5486513e-004	7.5228850e-004
7.0593474e-004	6.2663823e-004	6.5424226e-004	6.7271093e-004
6.4613424e-004	6.2778185e-004	6.3183498e-004	6.8009300e-004
6.1911564e-004	6.0767766e-004	6.4806729e-004	6.8032023e-004
5.7316748e-004	6.5466830e-004	5.9842183e-004	6.7898980e-004
6.6887682e-004	6.8217899e-004	7.1797750e-004	7.2256873e-004
6.3313534e-004	6.9449167e-004	6.3134808e-004	5.9448575e-004
6.4193587e-004	5.8089512e-004	6.3059539e-004	6.0808504e-004
6.2318076e-004	6.0164268e-004	6.4682440e-004	6.0783897e-004
5.3994518e-004	5.5916856e-004	6.0395905e-004	6.1465568e-004
5.2414543e-004	6.2925041e-004	5.9432173e-004	6.0256715e-004
4.2909171e-004	4.1353323e-004	4.1830022e-004	4.2460730e-004
4.1896348e-004	4.2614564e-004	3.9245043e-004	4.0059097e-004
4.3456010e-004	4.3177084e-004	4.2937746e-004	4.5822190e-004
4.5767683e-004	4.7367574e-004	5.0684382e-004	4.8094204e-004
5.1460295e-004	4.7746237e-004	5.3475572e-004	5.5152023e-004
5.3041561e-004	5.5593817e-004	5.7505404e-004	5.7096481e-004
3.5150324e-004	3.6786485e-004	3.6673259e-004	4.0122762e-004
3.5882081e-004	3.5945442e-004	3.7388722e-004	3.8253448e-004
3.6705659e-004	3.6972629e-004	3.8354597e-004	4.2155684e-004
3.7596375e-004	4.1285968e-004	4.7214598e-004	4.5290801e-004
4.2232960e-004	4.8036081e-004	4.7513560e-004	5.5890305e-004
4.9220213e-004	5.5130399e-004	5.2826547e-004	5.3568286e-004
3.8444105e-004	4.0329703e-004	4.1278603e-004	4.0256813e-004
3.4085054e-004	3.3836718e-004	3.0390181e-004	3.4107142e-004
3.3201920e-004	3.1817436e-004	2.7976344e-004	3.0786634e-004
3.0020191e-004	2.7669733e-004	2.8350719e-004	2.9109058e-004
2.8119236e-004	2.7511280e-004	3.0466470e-004	2.8184223e-004
2.5149143e-004	2.8303736e-004	2.7747274e-004	2.6774508e-004
3.8697448e-004	4.3571089e-004	4.4812657e-004	4.2085996e-004
4.3145879e-004	4.3390341e-004	5.0746804e-004	5.1197247e-004
5.0297295e-004	4.7588629e-004	5.1251713e-004	5.4252365e-004
5.4381552e-004	5.1293997e-004	5.4609041e-004	5.2028407e-004
4.7981067e-004	4.6921658e-004	5.0176374e-004	5.0270013e-004
4.1833504e-004	4.3402440e-004	4.9464226e-004	4.4439958e-004
4.2169785e-004	4.0776779e-004	4.0346824e-004	4.2082011e-004
4.1110350e-004	4.2080323e-004	3.9360746e-004	3.9822121e-004
4.1298811e-004	4.0430452e-004	4.0384166e-004	4.3931697e-004
4.2956477e-004	4.5031042e-004	4.9541845e-004	4.7500808e-004
5.0633406e-004	4.7898377e-004	5.0935960e-004	5.5611355e-004
5.1921718e-004	5.5351935e-004	5.5436075e-004	5.7011066e-004
3.5170659e-004	3.7400686e-004	3.6577135e-004	3.8313684e-004
3.4055088e-004	3.4836759e-004	3.5342876e-004	3.6046681e-004
3.4370236e-004	3.5030734e-004	3.4801070e-004	3.6519352e-004
3.5595014e-004	3.6868272e-004	4.1591965e-004	4.0563508e-004
3.9588749e-004	4.2910251e-004	4.2327039e-004	4.9596321e-004
4.4124123e-004	5.0015721e-004	4.7215969e-004	4.8582892e-004

7.3414367e-004	6.9478666e-004	7.0440819e-004	6.5665875e-004
6.6739482e-004	6.1026309e-004	5.6227666e-004	5.4801800e-004
5.4133587e-004	4.7591797e-004	4.9450334e-004	5.0861768e-004
4.6800271e-004	5.3975846e-004	4.9479266e-004	5.6060249e-004
5.1127585e-004	5.2893041e-004	4.8051833e-004	4.9678169e-004
4.6734234e-004	5.2924278e-004	5.0253823e-004	5.4305425e-004
8.4243850e-004	7.9629257e-004	8.4015820e-004	8.6553050e-004
7.3745284e-004	7.2739618e-004	6.8605012e-004	6.9689739e-004
7.0947269e-004	6.4508461e-004	6.6264694e-004	6.7290798e-004
6.5030367e-004	6.2162572e-004	6.0696488e-004	6.2043837e-004
5.8537665e-004	5.8687734e-004	6.6360817e-004	6.4133547e-004
5.8134671e-004	6.6849528e-004	6.0547922e-004	6.4830334e-004
4.1546384e-004	4.0933656e-004	3.9151578e-004	4.1325593e-004
3.8929733e-004	4.0351431e-004	4.0116220e-004	3.9766887e-004
3.8890762e-004	3.9284914e-004	3.9086402e-004	4.0442147e-004
4.0341250e-004	4.1418015e-004	4.7257014e-004	4.7145013e-004
4.8876368e-004	4.7200815e-004	4.7518312e-004	5.4758064e-004
4.7678529e-004	5.3223748e-004	5.1935067e-004	5.4518873e-004
4.0312221e-004	4.0476047e-004	4.0232629e-004	3.8106432e-004
3.6772838e-004	3.3320941e-004	3.0680286e-004	3.4438033e-004
3.1201916e-004	3.0912577e-004	3.1091776e-004	2.9052926e-004
3.0634245e-004	2.8450369e-004	2.8796502e-004	3.0593624e-004
2.6892154e-004	2.9700098e-004	2.9631748e-004	2.9726622e-004
2.5808910e-004	2.8695299e-004	2.7734898e-004	2.6834651e-004
6.1908868e-004	6.5285777e-004	6.1974351e-004	6.1760208e-004
5.8282448e-004	5.9032494e-004	5.3538237e-004	5.1458182e-004
5.1827658e-004	4.9074881e-004	4.8637838e-004	4.7299827e-004
4.9707251e-004	4.8735889e-004	4.7117466e-004	4.8350602e-004
4.5964758e-004	4.8114079e-004	4.6255734e-004	4.8940215e-004
5.0218594e-004	4.7538755e-004	5.3240900e-004	5.0225131e-004
7.1334167e-004	6.8927479e-004	6.6858619e-004	6.8971602e-004
6.5967365e-004	5.9442405e-004	5.9154014e-004	5.9090550e-004
5.5855473e-004	5.5416308e-004	5.3302648e-004	5.7915496e-004
5.2238570e-004	5.8791827e-004	5.3105444e-004	5.6984632e-004
5.3539287e-004	5.4598933e-004	5.3298721e-004	5.2255724e-004
5.1858992e-004	5.7314022e-004	5.4800558e-004	6.0777593e-004
8.2215643e-004	7.8460587e-004	8.2199181e-004	8.1862469e-004
7.2499452e-004	7.2711852e-004	6.7560203e-004	6.5099813e-004
6.9739509e-004	6.1473752e-004	6.3153751e-004	6.5286153e-004
6.4217305e-004	6.0179549e-004	6.2427999e-004	5.8706763e-004
5.7278841e-004	5.8080741e-004	6.3677307e-004	6.3728640e-004
5.9605137e-004	6.1772269e-004	5.9299411e-004	6.0365465e-004
4.0940390e-004	4.0793537e-004	3.9473049e-004	4.0706957e-004
3.7569161e-004	3.8436192e-004	3.9858287e-004	3.8659765e-004
3.7165703e-004	3.8799520e-004	3.8200193e-004	3.6501674e-004
3.7693385e-004	3.7683135e-004	4.3459993e-004	4.3725209e-004
4.5572546e-004	4.4333954e-004	4.3234071e-004	5.1805128e-004
4.2086787e-004	4.8919658e-004	4.8609525e-004	4.9245106e-004
7.1971009e-004	7.1867323e-004	7.2806214e-004	7.8331034e-004
7.2918185e-004	7.0570646e-004	6.4041061e-004	6.1374394e-004
5.9419280e-004	5.7145594e-004	5.3296337e-004	5.9208497e-004
5.3497778e-004	6.0400205e-004	5.4716586e-004	5.9085889e-004

5.1119443e-004	5.8420478e-004	5.0154801e-004	5.6200845e-004
5.4091620e-004	5.7723653e-004	5.2639802e-004	5.7928800e-004
7.2869165e-004	6.8745516e-004	7.0765670e-004	7.3444257e-004
6.7235989e-004	6.4272359e-004	6.3976474e-004	6.3509232e-004
6.3151987e-004	6.3244232e-004	6.0732827e-004	6.4065577e-004
6.1197585e-004	5.8855140e-004	5.9946481e-004	5.8514121e-004
5.7943344e-004	5.6721817e-004	6.2392247e-004	6.1739045e-004
5.8607236e-004	6.1151320e-004	6.0813149e-004	6.2675314e-004
7.3712764e-004	7.6056109e-004	7.4148018e-004	7.3718980e-004
6.6521887e-004	7.3122776e-004	6.2322507e-004	6.0903981e-004
6.3290769e-004	5.7172276e-004	5.7430482e-004	5.9400024e-004
6.1531104e-004	5.7403448e-004	6.1843137e-004	5.3345882e-004
5.4645445e-004	5.6140701e-004	5.7040330e-004	5.9728818e-004
5.6437010e-004	5.5117333e-004	5.8288632e-004	5.5662563e-004
8.0291730e-004	8.1784520e-004	8.2163951e-004	8.2856696e-004
7.1366783e-004	7.5522505e-004	6.8362232e-004	6.6599868e-004
6.8720069e-004	6.2071593e-004	6.3192708e-004	6.5042326e-004
6.4674469e-004	6.1301624e-004	6.5440941e-004	5.7671266e-004
5.7657239e-004	6.0853278e-004	5.9702978e-004	6.3834635e-004
6.1800847e-004	5.8103463e-004	6.3485510e-004	5.7458341e-004
7.5179334e-004	7.5903750e-004	7.9237182e-004	7.9949052e-004
6.9981486e-004	7.1290134e-004	6.9162138e-004	6.6336105e-004
6.8599118e-004	6.4229629e-004	6.4511497e-004	6.4547214e-004
6.4093026e-004	6.2814931e-004	6.7140702e-004	5.9961875e-004
5.7646114e-004	6.3681575e-004	6.0813520e-004	6.4930992e-004
6.2396792e-004	6.2152770e-004	6.6448368e-004	5.7565801e-004
6.7363333e-004	7.6650994e-004	7.3083786e-004	7.4102810e-004
6.4791193e-004	7.0219142e-004	6.2510827e-004	6.4336514e-004
6.1197863e-004	6.1718431e-004	5.8832899e-004	5.8248843e-004
5.8635716e-004	5.7803275e-004	6.1408209e-004	5.4434354e-004
5.5039497e-004	5.3627066e-004	5.3750277e-004	5.8676211e-004
5.5283576e-004	5.7166962e-004	6.0269258e-004	5.5556909e-004
6.7653247e-004	6.2097159e-004	6.7872757e-004	7.0550590e-004
7.1166692e-004	6.1349698e-004	6.1735170e-004	5.8269766e-004
5.8533628e-004	5.4727341e-004	5.0311358e-004	5.6473148e-004
5.2576292e-004	5.5599253e-004	5.4943237e-004	5.3865208e-004
4.4663946e-004	5.3712328e-004	5.0884279e-004	5.3671784e-004
5.2732698e-004	5.0335455e-004	5.4306877e-004	5.6387512e-004
6.7677003e-004	7.3959949e-004	7.2564764e-004	7.5601686e-004
7.2954111e-004	7.3140285e-004	6.7507049e-004	6.4616895e-004
6.4611145e-004	5.9314570e-004	5.7153907e-004	5.9723995e-004
5.7142985e-004	5.7181971e-004	5.6406773e-004	5.4688468e-004
5.2090919e-004	5.8953944e-004	5.1934224e-004	5.5875433e-004
5.2920239e-004	5.2742845e-004	5.6411901e-004	5.3475096e-004
5.8992759e-004	6.3905834e-004	6.3683736e-004	7.0984717e-004
6.9683224e-004	7.1733640e-004	7.2153760e-004	6.9399666e-004
6.7514276e-004	6.6904270e-004	6.4383766e-004	6.7598646e-004
6.3738591e-004	6.1226251e-004	6.4295190e-004	6.3317968e-004
6.0232228e-004	6.1947451e-004	6.2933051e-004	6.4415242e-004
5.5722475e-004	5.9435474e-004	5.8035964e-004	6.0576394e-004
6.5207131e-004	7.4364519e-004	7.1805270e-004	7.2628885e-004
7.0337704e-004	7.1481038e-004	6.5722379e-004	6.4906159e-004

6.4376938e-004	5.9093628e-004	5.8243427e-004	5.9055054e-004
5.7980821e-004	5.7598176e-004	5.7572278e-004	5.7511621e-004
5.4042504e-004	5.8729402e-004	5.3931706e-004	5.9002535e-004
5.4281934e-004	5.5868718e-004	5.6709594e-004	5.5403204e-004
7.1722751e-004	8.0663354e-004	7.9301574e-004	8.3087455e-004
7.9985031e-004	7.8512818e-004	7.6403329e-004	7.2500916e-004
7.1241250e-004	6.6278302e-004	6.7429409e-004	6.4519784e-004
6.3809108e-004	6.7003105e-004	6.7477717e-004	6.8679079e-004
6.3369640e-004	6.6391063e-004	6.4459706e-004	6.8162387e-004
6.0383919e-004	6.8286326e-004	6.8321434e-004	6.0313386e-004
7.3773766e-004	8.0472436e-004	7.4960024e-004	7.6536360e-004
6.9484740e-004	7.2149654e-004	6.5005822e-004	6.7830029e-004
6.5971713e-004	6.5653818e-004	6.5029165e-004	6.2428693e-004
6.0669730e-004	6.1911353e-004	6.5881365e-004	6.2720677e-004
6.0695133e-004	6.0186579e-004	5.8315943e-004	6.2853940e-004
5.8172800e-004	5.9277668e-004	6.3010119e-004	5.8847376e-004
5.1392326e-004	5.5777568e-004	5.4488012e-004	6.1793122e-004
5.6415651e-004	5.5301099e-004	5.0451954e-004	5.3547120e-004
5.2658358e-004	5.5623770e-004	5.4330145e-004	5.0401605e-004
5.2019327e-004	4.9846772e-004	4.7232436e-004	4.8043553e-004
5.1847236e-004	4.6290241e-004	4.4644122e-004	4.7994132e-004
4.7004597e-004	4.3984441e-004	4.6255079e-004	4.5248104e-004
6.6394445e-004	7.5336067e-004	7.2156209e-004	7.3023340e-004
7.0941160e-004	7.2344928e-004	6.6486202e-004	6.7042577e-004
6.5354613e-004	6.1834283e-004	6.1204960e-004	5.9291963e-004
5.8416024e-004	5.9619678e-004	6.0280789e-004	5.9795671e-004
5.7203813e-004	5.9743749e-004	5.6651705e-004	6.2593006e-004
5.6422310e-004	5.9321702e-004	6.0235469e-004	5.7464109e-004
6.5382196e-004	7.2068734e-004	7.1627802e-004	7.5580724e-004
7.4002029e-004	7.4357567e-004	7.3121891e-004	6.8924359e-004
6.8583504e-004	6.5892877e-004	6.6130150e-004	6.2057501e-004
6.1337250e-004	6.4479843e-004	6.5503946e-004	6.9260527e-004
6.4236323e-004	6.4702229e-004	6.3066982e-004	6.9482176e-004
6.0344518e-004	6.8784835e-004	6.6882398e-004	5.9678643e-004
7.7264734e-004	8.2366550e-004	7.7805739e-004	8.1042362e-004
7.5405766e-004	7.5077383e-004	6.9855191e-004	7.1268374e-004
6.9089032e-004	6.3933035e-004	6.8273766e-004	6.5198472e-004
6.3266429e-004	6.2604358e-004	6.7253941e-004	6.6747230e-004
6.2505422e-004	6.3750964e-004	6.3190454e-004	6.4638462e-004
5.8952627e-004	5.9701996e-004	6.5750408e-004	5.7947678e-004
7.1157768e-004	7.7998746e-004	7.3036704e-004	7.4765894e-004
7.2722060e-004	7.4847866e-004	6.6846314e-004	6.8529471e-004
6.6765933e-004	6.4991289e-004	6.3703931e-004	6.0239098e-004
5.9323751e-004	6.1730979e-004	6.3454885e-004	6.1081351e-004
6.0006998e-004	6.1052341e-004	5.9818763e-004	6.4778203e-004
5.8290246e-004	6.2400500e-004	6.4312488e-004	6.0048109e-004
6.6053054e-004	7.4572983e-004	7.3512371e-004	8.1102161e-004
7.6999092e-004	7.6152472e-004	7.5201591e-004	7.3724015e-004
7.2265588e-004	6.7812292e-004	6.9537554e-004	6.4724256e-004
6.7090474e-004	6.6094834e-004	6.7610091e-004	7.4086715e-004
6.7351031e-004	6.6433556e-004	6.8204754e-004	7.2597458e-004
6.2436420e-004	6.9904847e-004	7.0530402e-004	6.3270725e-004

7.5403608e-004	7.6606519e-004	7.2221443e-004	7.7790122e-004
7.1962590e-004	7.1393425e-004	6.6711742e-004	6.9196583e-004
6.5725404e-004	6.1942342e-004	6.7857983e-004	6.5331554e-004
6.2771727e-004	6.2762326e-004	6.7097839e-004	6.6306055e-004
6.1008761e-004	6.3383472e-004	6.1559609e-004	6.4504621e-004
5.8652845e-004	5.9337572e-004	6.3570983e-004	5.7308111e-004
6.7859318e-004	7.2357274e-004	7.1564270e-004	7.5823991e-004
7.3739038e-004	7.6184295e-004	6.7073833e-004	6.7775963e-004
6.5348936e-004	6.3977082e-004	6.1782806e-004	5.9606315e-004
5.8088175e-004	5.8900450e-004	5.8764989e-004	5.9774921e-004
5.7623984e-004	5.7223109e-004	5.6896263e-004	6.2398186e-004
5.3917208e-004	5.8933088e-004	6.0971413e-004	5.6411495e-004
6.7135305e-004	7.3790825e-004	7.1633996e-004	8.1964562e-004
7.4919667e-004	7.3383514e-004	7.2757427e-004	7.4583506e-004
7.2730753e-004	6.7002545e-004	7.0481478e-004	6.4846838e-004
6.8377607e-004	6.6576646e-004	6.8097765e-004	7.3739923e-004
6.4891543e-004	6.6683622e-004	6.8384086e-004	7.1215857e-004
6.2947463e-004	6.5783264e-004	7.0166396e-004	6.3779288e-004
7.5154520e-004	7.7719495e-004	7.0981575e-004	7.2720592e-004
7.0072072e-004	7.3092119e-004	6.3394714e-004	6.7498458e-004
6.6173062e-004	6.6365622e-004	6.2383473e-004	6.2486842e-004
6.1163014e-004	6.1096385e-004	6.3587917e-004	5.9178197e-004
6.1263528e-004	5.9385887e-004	6.0937565e-004	6.2703730e-004
5.7117507e-004	6.3233981e-004	6.4557594e-004	6.2150072e-004
6.7602312e-004	7.0101693e-004	7.4073496e-004	8.0792001e-004
7.7879563e-004	8.2513035e-004	7.5888948e-004	7.6784435e-004
6.5319746e-004	6.9501053e-004	6.3689756e-004	6.2581199e-004
6.3672564e-004	6.5872222e-004	5.9101431e-004	6.7258903e-004
6.2585108e-004	6.2816307e-004	5.7349064e-004	5.6572979e-004
5.9213281e-004	6.0318185e-004	5.7602230e-004	6.5750953e-004
7.1862663e-004	7.3650721e-004	6.8575186e-004	7.6948684e-004
7.0757524e-004	6.7864053e-004	6.7542115e-004	6.9510765e-004
6.6093226e-004	6.1967036e-004	6.7636234e-004	6.2941345e-004
6.4961385e-004	6.3643864e-004	6.6128920e-004	6.8549332e-004
5.7904418e-004	6.4645040e-004	6.4118706e-004	6.4714905e-004
6.0803753e-004	5.7890754e-004	6.5407630e-004	6.0298840e-004
7.3404163e-004	7.6153831e-004	7.1737470e-004	7.7091090e-004
7.4997454e-004	7.6376226e-004	6.7663391e-004	6.6982731e-004
6.6378660e-004	6.4644875e-004	6.2217629e-004	6.1562363e-004
5.8364071e-004	5.9503537e-004	6.0337910e-004	5.8548352e-004
5.8078828e-004	5.5590650e-004	5.7513532e-004	6.1397617e-004
5.3620810e-004	5.8482176e-004	5.9143300e-004	5.7323099e-004
7.1560020e-004	7.5849882e-004	7.1580583e-004	7.4112268e-004
7.0769052e-004	7.4356272e-004	6.6246116e-004	6.6817731e-004
6.7384125e-004	6.6114943e-004	6.1281965e-004	6.2812417e-004
6.1410034e-004	6.1024724e-004	6.1148865e-004	6.0172597e-004
6.0334025e-004	5.9312404e-004	5.9609648e-004	6.2181611e-004
5.6869579e-004	6.1863924e-004	6.2305971e-004	6.0366148e-004
7.3936474e-004	6.5534485e-004	7.5297627e-004	8.5795313e-004
7.6227606e-004	8.0225666e-004	7.5696541e-004	7.7729001e-004
7.5036935e-004	7.5529121e-004	7.0652161e-004	6.6328983e-004
6.5543242e-004	6.4916250e-004	6.6777757e-004	6.5052447e-004

6.4614312e-004	6.1263994e-004	5.7895756e-004	5.8638427e-004
6.5553159e-004	6.3670975e-004	6.2843905e-004	6.8578341e-004
7.0629972e-004	6.6616226e-004	6.6630743e-004	7.1097967e-004
6.7162806e-004	6.7321517e-004	6.5119099e-004	7.2581207e-004
6.7846330e-004	6.3560261e-004	6.6106138e-004	5.7293018e-004
5.9220662e-004	6.2123153e-004	6.3071364e-004	6.1693632e-004
5.6024290e-004	5.8537093e-004	6.0303345e-004	6.4464301e-004
5.8666349e-004	6.0354058e-004	6.4524201e-004	5.9326694e-004
6.9798472e-004	7.4954455e-004	7.2904155e-004	7.5980842e-004
7.2619269e-004	7.5633245e-004	6.8462160e-004	6.8014578e-004
6.8581854e-004	6.6882122e-004	6.2952825e-004	6.3946488e-004
6.2186034e-004	6.1509055e-004	6.0814134e-004	6.1617196e-004
6.0225412e-004	6.0622323e-004	5.9532770e-004	6.3221203e-004
5.7691330e-004	6.1713948e-004	6.1600406e-004	6.0046226e-004
5.6661989e-004	4.9295937e-004	5.7079734e-004	6.2987069e-004
5.4986445e-004	6.0488661e-004	5.7713158e-004	5.6687248e-004
5.9490554e-004	5.7412090e-004	5.3439324e-004	5.0529457e-004
4.9859892e-004	4.6759214e-004	5.0509119e-004	4.8827176e-004
4.8865904e-004	4.7436159e-004	4.3360948e-004	4.4540167e-004
5.0278533e-004	4.6984353e-004	4.6638665e-004	5.0994430e-004
7.4610779e-004	6.9246888e-004	7.1375656e-004	7.1048499e-004
7.0270587e-004	6.9858732e-004	6.5461828e-004	7.1482542e-004
6.7327132e-004	6.3651404e-004	6.6306429e-004	5.5917747e-004
5.6910700e-004	6.0315162e-004	6.2173810e-004	5.9404311e-004
5.5078515e-004	5.6357807e-004	5.8014270e-004	6.3393234e-004
5.6774073e-004	5.9915746e-004	6.3225793e-004	5.8074198e-004
6.8892042e-004	7.0327841e-004	7.3125845e-004	7.5238947e-004
7.3249573e-004	6.8700805e-004	6.8153132e-004	6.7826525e-004
6.5396543e-004	5.8178014e-004	6.3540178e-004	5.9021790e-004
6.0718617e-004	6.2415075e-004	6.5209020e-004	6.1768424e-004
5.9585377e-004	6.0123252e-004	6.2387486e-004	6.1017463e-004
5.6946358e-004	6.1339374e-004	6.3713781e-004	5.9668903e-004
6.9282520e-004	7.4800770e-004	7.4424672e-004	7.8767775e-004
7.5720689e-004	7.7250454e-004	7.0796536e-004	7.0211307e-004
7.0446613e-004	6.8315554e-004	6.5767248e-004	6.5954213e-004
6.3622165e-004	6.2640123e-004	6.1548977e-004	6.3326651e-004
6.0854755e-004	6.2628601e-004	6.0133956e-004	6.4688553e-004
5.8681959e-004	6.2300969e-004	6.1380440e-004	6.0472208e-004
7.8294966e-004	7.3795773e-004	8.1210317e-004	7.5560923e-004
7.9210327e-004	7.6062408e-004	6.8843455e-004	7.3172101e-004
7.0857444e-004	6.8040263e-004	6.9382800e-004	5.7933069e-004
5.8313442e-004	6.1033272e-004	6.3128599e-004	6.1002641e-004
5.7923654e-004	5.8408015e-004	5.8181201e-004	6.4832442e-004
5.8055831e-004	6.1200866e-004	6.4908856e-004	6.0718157e-004
7.4198488e-004	7.4910816e-004	8.1755080e-004	8.2793235e-004
8.1445894e-004	7.6986018e-004	7.2940200e-004	7.3571259e-004
6.9901826e-004	6.2446648e-004	6.7163997e-004	6.2640218e-004
6.2822842e-004	6.4383178e-004	6.8457665e-004	6.4273382e-004
6.3058545e-004	6.0930527e-004	6.4356958e-004	6.4094324e-004
5.7755357e-004	6.2411051e-004	6.5748206e-004	6.1783967e-004
6.9908748e-004	7.5674095e-004	7.5946777e-004	8.2404642e-004
7.9611399e-004	7.9252970e-004	7.3110384e-004	7.3017557e-004

7.2890789e-004	7.0226415e-004	6.8704976e-004	6.8033961e-004
6.5465829e-004	6.4129010e-004	6.3313718e-004	6.5034744e-004
6.2010712e-004	6.4649081e-004	6.1249570e-004	6.5748101e-004
5.9498400e-004	6.3255423e-004	6.1476954e-004	6.1353314e-004
7.4008007e-004	7.5521374e-004	8.6426147e-004	8.5651275e-004
8.6890005e-004	8.1999165e-004	7.6766778e-004	7.8871279e-004
7.4096194e-004	6.8876176e-004	7.0578491e-004	6.5148791e-004
6.6464856e-004	6.6300685e-004	7.0885124e-004	6.7822996e-004
6.7035595e-004	6.4090747e-004	6.5162739e-004	6.8113524e-004
5.9821393e-004	6.4285257e-004	6.6452308e-004	6.4265373e-004
6.8243960e-004	7.0044493e-004	7.8224717e-004	8.0584595e-004
8.0607779e-004	7.7351002e-004	7.5406235e-004	7.5171044e-004
7.3671312e-004	6.9704710e-004	7.0087062e-004	6.5246277e-004
6.7066884e-004	6.7181668e-004	6.8080374e-004	6.8977698e-004
6.8239174e-004	6.4878786e-004	6.4389178e-004	6.6411345e-004
6.1819649e-004	6.5661984e-004	6.6268607e-004	6.4029101e-004
6.9357792e-004	7.5044483e-004	7.5393092e-004	8.2211092e-004
7.9999794e-004	7.8578874e-004	7.1702633e-004	7.3762907e-004
7.2550666e-004	7.0090852e-004	7.0097015e-004	6.7436379e-004
6.6975370e-004	6.3979386e-004	6.7213408e-004	6.6710604e-004
6.4499567e-004	6.6352272e-004	6.4166638e-004	6.6651450e-004
6.0348550e-004	6.3758576e-004	6.3972593e-004	6.3099190e-004
7.3286763e-004	7.4894339e-004	8.1660380e-004	8.6463246e-004
8.6789290e-004	8.1131575e-004	7.7848422e-004	7.7987880e-004
7.4650494e-004	6.9423683e-004	6.9803586e-004	6.5064647e-004
6.5479227e-004	6.2614858e-004	6.7842151e-004	6.6652120e-004
6.8236231e-004	6.4900698e-004	6.1548236e-004	6.6445163e-004
5.8022894e-004	6.2872840e-004	6.3029039e-004	6.1268343e-004
6.1286619e-004	6.4636829e-004	6.3326210e-004	6.7341991e-004
6.4878227e-004	6.2452933e-004	6.0012755e-004	5.8255211e-004
5.8313804e-004	5.6779459e-004	5.6561144e-004	5.4608178e-004
5.4762569e-004	5.2270449e-004	5.7173060e-004	5.3396939e-004
5.1241441e-004	5.1850671e-004	5.5606366e-004	5.8797847e-004
4.7508401e-004	5.5125513e-004	5.1660191e-004	5.2363843e-004
7.3807713e-004	7.4908763e-004	7.7327722e-004	8.2012861e-004
7.6996537e-004	7.5732222e-004	7.4844867e-004	7.1231263e-004
7.0427642e-004	6.4935096e-004	6.4971368e-004	6.3736892e-004
6.1638416e-004	5.8287810e-004	6.1315131e-004	6.0028896e-004
6.2522395e-004	6.3364758e-004	5.6192667e-004	6.0864068e-004
5.5001111e-004	5.6872347e-004	5.9777196e-004	5.4155281e-004
6.4166130e-004	6.9034084e-004	6.6157131e-004	6.7747144e-004
6.5277638e-004	6.4653541e-004	6.0221217e-004	6.0424089e-004
6.1729248e-004	5.6342057e-004	5.7259231e-004	5.6149728e-004
5.5322694e-004	5.3937925e-004	5.7560093e-004	5.4085994e-004
5.3354733e-004	5.4137238e-004	5.5495473e-004	5.9465035e-004
4.8333521e-004	5.5744952e-004	5.2200066e-004	5.4097381e-004
6.6674690e-004	7.4946786e-004	7.5731715e-004	7.6933955e-004
7.6627193e-004	7.2847464e-004	6.9953763e-004	6.9106198e-004
6.9242578e-004	6.5377129e-004	6.7149664e-004	6.4658502e-004
6.3277151e-004	5.9851611e-004	6.5051310e-004	6.2289969e-004
6.1799264e-004	6.2206310e-004	6.4031809e-004	6.3366562e-004
5.8621040e-004	6.0274504e-004	6.2275902e-004	6.0110219e-004

7.6628935e-004	7.8948900e-004	8.0412306e-004	8.9460418e-004
8.2318961e-004	8.4382819e-004	8.0721603e-004	7.9265340e-004
7.8922255e-004	7.4737499e-004	7.6244876e-004	6.9615305e-004
7.0574645e-004	6.6338171e-004	6.6505335e-004	6.5555063e-004
6.5951621e-004	7.0753740e-004	6.6555117e-004	7.0258028e-004
6.3060349e-004	6.3305281e-004	6.5973707e-004	6.0441370e-004
7.4155448e-004	7.5115293e-004	7.5295984e-004	7.9877910e-004
7.5683932e-004	7.4240720e-004	7.3595413e-004	6.8892342e-004
6.8295044e-004	6.5096286e-004	6.2061324e-004	6.1472227e-004
6.0299282e-004	5.9839542e-004	5.7169569e-004	6.1723958e-004
6.0132990e-004	6.2442731e-004	5.8797801e-004	5.8914095e-004
5.6555525e-004	5.5247845e-004	5.8447952e-004	5.4757508e-004
6.2008666e-004	6.5812391e-004	6.1594683e-004	6.2912267e-004
6.0902585e-004	5.9242868e-004	5.5690047e-004	5.8386449e-004
5.8126428e-004	5.2526061e-004	5.3204894e-004	5.4379120e-004
5.1070391e-004	5.1255448e-004	5.3274594e-004	5.2192587e-004
5.3042398e-004	5.1761458e-004	5.2268256e-004	5.4597505e-004
4.8705115e-004	5.2167710e-004	5.0486466e-004	5.0689481e-004
6.7541931e-004	6.6999775e-004	7.3617636e-004	8.2739875e-004
8.0261241e-004	7.6861777e-004	7.6076985e-004	7.5037317e-004
7.1921691e-004	6.7839655e-004	7.0749300e-004	6.3858929e-004
6.1982650e-004	6.1629833e-004	5.8587088e-004	6.0090824e-004
6.1471563e-004	6.4154651e-004	5.9169847e-004	5.9276234e-004
5.9477131e-004	5.7166945e-004	5.8866027e-004	5.9910472e-004
7.0913453e-004	7.4185317e-004	6.9718476e-004	7.4513357e-004
7.8120790e-004	7.2534769e-004	7.0090880e-004	7.2072280e-004
6.9194443e-004	6.7081556e-004	6.3446960e-004	6.3365309e-004
6.4146338e-004	6.3558738e-004	6.1919847e-004	6.3463865e-004
6.1393381e-004	6.1825178e-004	6.4659440e-004	6.3196143e-004
5.7969155e-004	5.7092907e-004	5.9936508e-004	5.8254604e-004
6.5730476e-004	6.7784602e-004	6.7331035e-004	6.9666958e-004
6.7016049e-004	6.7543841e-004	6.1649564e-004	6.4109267e-004
6.3975765e-004	5.7305650e-004	6.0608535e-004	5.8253304e-004
5.5895420e-004	5.7551122e-004	5.9812127e-004	5.7973938e-004
5.8535233e-004	5.7332627e-004	5.6620772e-004	6.0047474e-004
5.3481381e-004	5.6473176e-004	5.5797085e-004	5.5195502e-004
7.4924239e-004	7.3575439e-004	7.0328856e-004	7.1943749e-004
8.0743016e-004	7.6998625e-004	7.3316581e-004	7.6025768e-004
7.2625586e-004	6.8594517e-004	6.7047152e-004	6.5384292e-004
6.6055506e-004	6.7329337e-004	6.4858595e-004	6.6330176e-004
6.3022785e-004	6.4345174e-004	6.5646810e-004	6.7108530e-004
5.9615630e-004	6.1217218e-004	6.3451914e-004	6.1282123e-004
6.5175963e-004	6.5948711e-004	6.5128782e-004	6.8392607e-004
6.5647189e-004	6.6176156e-004	6.2257738e-004	6.3270175e-004
6.1200680e-004	5.6747404e-004	6.0512009e-004	5.7636591e-004
5.5124427e-004	5.7468102e-004	5.8208253e-004	5.7281778e-004
5.8466292e-004	5.5910459e-004	5.5676109e-004	5.8296979e-004
5.4588310e-004	5.4960807e-004	5.6794759e-004	5.4513292e-004
7.0582984e-004	7.5284639e-004	7.2518387e-004	7.7030870e-004
7.7829900e-004	7.3234455e-004	7.2592885e-004	6.9832760e-004
6.9666577e-004	6.5709786e-004	6.5130916e-004	6.7264610e-004
6.3059975e-004	6.3269665e-004	6.3988764e-004	6.5756316e-004

6.2502979e-004	6.3357984e-004	6.1334617e-004	6.5848665e-004
6.1232274e-004	6.0520570e-004	6.1808106e-004	6.0865530e-004
8.0078424e-004	8.0204617e-004	7.6635314e-004	7.9285305e-004
8.6805134e-004	7.9955968e-004	7.7887910e-004	7.8971121e-004
7.4621374e-004	6.8331645e-004	6.5192419e-004	6.3613425e-004
6.6529526e-004	6.6547538e-004	6.3842080e-004	6.6534735e-004
6.2438761e-004	6.2157563e-004	6.6459688e-004	6.9073237e-004
5.9893999e-004	6.0712468e-004	6.4193354e-004	5.7471664e-004
7.1167710e-004	6.9639950e-004	7.2983727e-004	7.2467628e-004
7.9544443e-004	8.4453498e-004	7.8585117e-004	7.8493341e-004
7.5687398e-004	7.3325093e-004	7.3037353e-004	6.7218977e-004
7.1996292e-004	7.0091884e-004	6.7903521e-004	6.9177014e-004
6.9548517e-004	7.1184989e-004	6.7246769e-004	7.0063630e-004
6.5495845e-004	7.0389446e-004	7.0748712e-004	6.9194840e-004
6.9069536e-004	6.7968717e-004	7.0573649e-004	7.4890253e-004
7.2487413e-004	7.6261054e-004	6.9696402e-004	6.9772796e-004
6.7445970e-004	6.3501344e-004	6.8277318e-004	6.2081059e-004
6.0944608e-004	6.3931840e-004	6.4727791e-004	6.3752112e-004
6.3604080e-004	6.2190781e-004	6.1141521e-004	6.4263534e-004
6.0159882e-004	6.2117180e-004	6.1244388e-004	6.0403439e-004
7.2212257e-004	7.5113958e-004	7.2982306e-004	7.8572556e-004
7.9896838e-004	7.5760274e-004	7.3619928e-004	7.3281745e-004
7.2900110e-004	6.8402344e-004	6.9845047e-004	6.8501162e-004
6.5537317e-004	6.5582905e-004	6.7717838e-004	6.8605485e-004
6.4023434e-004	6.5780962e-004	6.3976517e-004	6.6751325e-004
6.4702070e-004	6.4277714e-004	6.3630426e-004	6.3752659e-004
8.5550371e-004	8.0623577e-004	8.2438353e-004	8.0406290e-004
9.0000468e-004	8.4532888e-004	8.2482091e-004	8.4162562e-004
7.7963003e-004	7.3317655e-004	7.1021938e-004	6.6460595e-004
7.0473389e-004	6.9432991e-004	6.8111942e-004	7.0267083e-004
6.6746082e-004	6.6316965e-004	7.0059714e-004	7.2584252e-004
6.3470079e-004	6.7648396e-004	6.7910113e-004	6.2785056e-004
7.3136832e-004	7.3690660e-004	7.7072693e-004	7.8578024e-004
8.2358545e-004	8.7150528e-004	8.2561908e-004	8.0716000e-004
7.7327620e-004	7.5968668e-004	7.3091460e-004	6.7472151e-004
7.2073673e-004	6.9679164e-004	6.8171293e-004	6.7346614e-004
6.9604680e-004	7.1304533e-004	6.8784841e-004	6.8896651e-004
6.5376781e-004	6.9991840e-004	6.9721694e-004	6.8764792e-004
6.9945141e-004	6.9352130e-004	6.9468039e-004	7.4141106e-004
7.1391460e-004	7.5907694e-004	7.1099556e-004	6.9357286e-004
6.6224010e-004	6.4648477e-004	6.7755692e-004	6.3056886e-004
6.1595430e-004	6.1934760e-004	6.3579741e-004	6.2720450e-004
6.1676561e-004	6.1145343e-004	6.0025573e-004	6.3868791e-004
5.8312991e-004	6.1605123e-004	6.0583650e-004	6.1367870e-004
7.5015078e-004	7.6505603e-004	7.6174182e-004	8.2937532e-004
8.5567067e-004	7.9272524e-004	7.8221141e-004	7.3703878e-004
7.6913663e-004	7.1995474e-004	7.0670627e-004	7.1671867e-004
6.6261517e-004	6.5983225e-004	6.6504761e-004	6.8216422e-004
6.5524181e-004	6.4210071e-004	6.4593035e-004	6.8928677e-004
6.3665250e-004	6.1990270e-004	6.1091296e-004	6.1350137e-004
8.6045459e-004	8.0429633e-004	8.5910606e-004	8.2904869e-004
8.8884997e-004	9.0611821e-004	8.5699193e-004	8.9335012e-004

7.8809253e-004	8.0134813e-004	7.3733070e-004	7.0978925e-004
7.5010421e-004	6.9844832e-004	7.0170971e-004	7.3288854e-004
7.1808364e-004	7.1047363e-004	7.1710871e-004	7.0588657e-004
6.6922166e-004	7.2251553e-004	7.2419067e-004	7.0792121e-004
7.7879360e-004	7.8373437e-004	7.9524865e-004	8.5448918e-004
8.2998371e-004	8.9380502e-004	8.4545252e-004	8.1103410e-004
7.8980334e-004	7.6690428e-004	7.5377844e-004	6.9663470e-004
6.9128312e-004	7.0538759e-004	7.0526327e-004	6.8965461e-004
6.8061457e-004	7.1148531e-004	6.8858825e-004	7.0164674e-004
6.3853232e-004	6.9285111e-004	6.6479662e-004	6.9408698e-004
7.4866803e-004	7.4363066e-004	7.7214463e-004	8.0475232e-004
7.9493696e-004	7.9889213e-004	7.6534681e-004	7.2805355e-004
7.4103667e-004	6.9191694e-004	6.9330946e-004	7.1531317e-004
6.7626422e-004	6.7756101e-004	7.1275792e-004	7.0874127e-004
6.9272514e-004	6.9929690e-004	6.8046850e-004	7.1097334e-004
6.6199614e-004	6.8643299e-004	7.0945919e-004	6.4409856e-004
7.3350828e-004	7.4069209e-004	7.7442954e-004	7.9585235e-004
8.0945928e-004	7.8717250e-004	7.5838044e-004	7.2547061e-004
7.5561945e-004	7.1690445e-004	7.1053598e-004	6.9759600e-004
6.5246911e-004	6.4892664e-004	6.8443359e-004	6.7234164e-004
6.6192866e-004	6.5965652e-004	6.6784062e-004	6.6132470e-004
6.6850921e-004	6.5142989e-004	6.3728300e-004	6.5270887e-004
6.5710787e-004	6.6043611e-004	6.2616832e-004	7.3829475e-004
7.2591126e-004	6.5412192e-004	6.9224116e-004	6.0281490e-004
6.3739674e-004	6.1576375e-004	5.5129464e-004	6.2344741e-004
5.6988243e-004	5.5930158e-004	5.5070705e-004	5.5936702e-004
5.6979566e-004	5.3057759e-004	5.5340370e-004	6.1490638e-004
5.0829442e-004	4.8550578e-004	4.9744934e-004	4.4135368e-004
9.1295010e-004	8.7161666e-004	9.4802414e-004	9.3798689e-004
9.9571580e-004	9.8979217e-004	9.2509817e-004	1.0026152e-003
8.5889020e-004	8.9339707e-004	8.0589129e-004	7.8929802e-004
8.1620204e-004	7.6554569e-004	7.7473164e-004	8.1250048e-004
7.9792805e-004	7.6692310e-004	7.7321388e-004	7.6872310e-004
7.4517047e-004	7.8555309e-004	7.8555303e-004	7.9470556e-004
7.9164319e-004	8.2313486e-004	7.9874779e-004	8.8341878e-004
8.3446024e-004	8.8318060e-004	8.4522531e-004	8.0788007e-004
7.9125620e-004	7.7703352e-004	7.4681186e-004	7.0221083e-004
6.8838173e-004	6.9250524e-004	6.9723360e-004	6.9556735e-004
6.6502477e-004	7.0683678e-004	6.7426030e-004	6.8766763e-004
6.2758723e-004	6.6107934e-004	6.5968855e-004	6.8245795e-004
7.5300051e-004	7.8187078e-004	7.9722232e-004	7.9840074e-004
7.9711463e-004	7.8937270e-004	7.4195262e-004	7.7474509e-004
7.4478249e-004	7.0383251e-004	7.3411631e-004	7.0449829e-004
6.9129711e-004	6.7653622e-004	6.9112668e-004	7.0117120e-004
6.7388716e-004	6.9319317e-004	6.7213055e-004	6.8981411e-004
6.3487282e-004	7.0609785e-004	7.0664917e-004	6.6291001e-004
7.7989807e-004	7.8230574e-004	8.1035735e-004	8.6073954e-004
8.2965693e-004	8.4256809e-004	8.0369948e-004	7.4959941e-004
7.6313395e-004	7.1825250e-004	7.0682455e-004	7.2292284e-004
6.9763817e-004	6.8507691e-004	7.3208332e-004	7.1890172e-004
7.1230919e-004	7.1028279e-004	6.7917023e-004	7.3079026e-004
6.7527850e-004	7.0462293e-004	7.2246928e-004	6.5644133e-004

7.2460409e-004 7.3941055e-004 7.7086041e-004 7.8259462e-004
7.8981015e-004 7.8147536e-004 7.6208101e-004 7.0140290e-004
7.6202548e-004 7.1269815e-004 6.7743825e-004 6.8603984e-004
6.1278691e-004 6.2866689e-004 6.5178366e-004 6.4790939e-004
6.5616160e-004 6.5248087e-004 6.5973533e-004 6.6215014e-004
6.4015064e-004 6.2945099e-004 6.2094512e-004 6.3531980e-004
7.2275240e-004 7.4769316e-004 7.6407319e-004 8.5642956e-004
8.8560680e-004 7.8364060e-004 8.3247559e-004 7.9759861e-004
7.6238034e-004 7.5512918e-004 6.9774335e-004 6.8616959e-004
6.1913759e-004 6.9744104e-004 6.6605845e-004 6.4395902e-004
6.9116197e-004 6.2159965e-004 6.5352394e-004 7.2490778e-004
6.0060653e-004 6.6788349e-004 6.0148421e-004 6.2108150e-004
7.8185541e-004 8.3650822e-004 7.9234202e-004 9.0471252e-004
8.5166770e-004 8.6757888e-004 8.3353239e-004 8.0176907e-004
7.9185990e-004 7.7437545e-004 7.5003483e-004 7.1285246e-004
6.9479394e-004 6.9156974e-004 7.0135236e-004 7.2008240e-004
6.5956372e-004 6.9965230e-004 6.6984326e-004 6.7928166e-004
6.4086288e-004 6.3251889e-004 6.6741263e-004 6.5614062e-004
6.6641434e-004 6.8137964e-004 6.3670475e-004 6.5660823e-004
6.4588273e-004 6.7619962e-004 6.2677545e-004 6.1959567e-004
6.0963485e-004 6.1129128e-004 6.1824489e-004 5.8364296e-004
5.6298071e-004 5.6332837e-004 5.4790522e-004 5.6808199e-004
5.2346141e-004 5.5420357e-004 5.7261762e-004 5.7046307e-004
5.2530214e-004 5.4893813e-004 5.4745398e-004 5.4461384e-004
6.3162381e-004 6.7808744e-004 6.6691417e-004 6.3499034e-004
6.4136981e-004 6.2472495e-004 6.0689697e-004 5.7885190e-004
5.5856170e-004 5.5255905e-004 5.5039594e-004 5.3084290e-004
5.4167508e-004 5.5477316e-004 5.5386550e-004 5.2904349e-004
5.2264423e-004 5.1417459e-004 5.1781620e-004 5.5132246e-004
5.0247632e-004 5.4200825e-004 5.4243421e-004 5.1259503e-004
8.0859593e-004 8.4713633e-004 8.7906170e-004 8.8133977e-004
8.9892709e-004 8.6027902e-004 8.2158853e-004 8.3686711e-004
8.0991791e-004 7.6248623e-004 7.9013318e-004 7.5024427e-004
7.4270148e-004 7.1718573e-004 7.3877872e-004 7.4478051e-004
7.1265937e-004 7.3935221e-004 7.0807488e-004 7.3770506e-004
6.7789409e-004 7.4805575e-004 7.4623596e-004 7.0800276e-004
7.9951908e-004 8.0974663e-004 8.3621398e-004 9.1398381e-004
8.5081384e-004 8.7428415e-004 8.3269457e-004 7.6810872e-004
7.8037799e-004 7.4933977e-004 7.2585235e-004 7.2514043e-004
7.1955932e-004 6.9042236e-004 7.4739120e-004 7.2557387e-004
7.3038190e-004 7.2083360e-004 6.6944219e-004 7.4901896e-004
6.8265019e-004 7.1205333e-004 7.2503176e-004 6.6682014e-004
6.8774026e-004 7.3783465e-004 7.3781738e-004 7.6616196e-004
7.6407858e-004 7.6679549e-004 7.5021217e-004 6.7383125e-004
7.6564615e-004 6.8736472e-004 6.4095021e-004 6.5089260e-004
5.5703902e-004 5.9805602e-004 6.0585522e-004 6.1529773e-004
6.3092992e-004 6.3709506e-004 6.3973911e-004 6.5435217e-004
5.9367504e-004 5.9740967e-004 6.1108083e-004 6.0454656e-004
7.2072774e-004 7.7026042e-004 7.6899240e-004 9.0994846e-004
8.9641770e-004 8.2672689e-004 8.3157330e-004 8.0885935e-004
7.8940556e-004 7.8932964e-004 7.3232176e-004 6.9418635e-004
6.7159015e-004 7.2177371e-004 7.0104355e-004 7.2195879e-004

6.8405057e-004	6.7040058e-004	6.4326038e-004	6.8721508e-004
6.7218034e-004	6.0970508e-004	6.5332171e-004	6.2299623e-004
7.3780899e-004	7.7868348e-004	7.1193179e-004	7.4959184e-004
7.1989369e-004	7.6577664e-004	7.0799900e-004	6.7399358e-004
7.0270742e-004	6.6038739e-004	6.7472296e-004	6.4923751e-004
6.0180882e-004	5.9721942e-004	6.0624628e-004	6.3465236e-004
5.7042821e-004	6.1203190e-004	6.1512853e-004	6.1041678e-004
5.6124989e-004	5.8295362e-004	5.9965806e-004	5.6562223e-004
6.4644724e-004	6.8330784e-004	6.8598830e-004	6.9505008e-004
6.9695299e-004	6.9343526e-004	6.7390484e-004	6.6027232e-004
6.3501501e-004	6.1382244e-004	6.1876229e-004	6.0898345e-004
5.9516879e-004	6.0732683e-004	6.0166934e-004	5.8484265e-004
5.8474179e-004	5.7386019e-004	6.0845558e-004	5.8590293e-004
5.6948552e-004	5.8167345e-004	5.9340002e-004	5.7456087e-004
6.8130207e-004	7.3971754e-004	7.1448981e-004	6.9310586e-004
6.8030578e-004	6.5629937e-004	6.3866386e-004	6.2320179e-004
6.0725650e-004	5.9512840e-004	5.8098054e-004	5.6818618e-004
5.8637775e-004	5.9628952e-004	5.8827932e-004	5.8674124e-004
5.6825881e-004	5.6682837e-004	5.4497135e-004	5.9615407e-004
5.3965826e-004	5.8314868e-004	5.8756951e-004	5.5143566e-004
7.6847382e-004	8.1660185e-004	8.3854201e-004	8.4119326e-004
8.7870017e-004	8.3456580e-004	8.1939496e-004	8.5212545e-004
7.9853709e-004	7.4715287e-004	7.6688784e-004	7.5137145e-004
7.5103118e-004	7.3559073e-004	7.5645426e-004	7.6937770e-004
6.9951978e-004	7.4846727e-004	6.9587927e-004	7.4135687e-004
6.9177753e-004	7.4117238e-004	7.6494762e-004	6.9953804e-004
6.8563163e-004	7.5882877e-004	7.4152570e-004	8.0705089e-004
7.5801258e-004	7.9585191e-004	7.7042822e-004	6.6075778e-004
7.4268422e-004	6.8383985e-004	6.5777832e-004	6.5436531e-004
6.0159455e-004	6.0949891e-004	6.4607007e-004	6.4987749e-004
6.5311549e-004	6.5470539e-004	6.1603985e-004	6.8940490e-004
6.3497292e-004	6.1619050e-004	6.6064406e-004	6.1403531e-004
6.2046250e-004	6.7074815e-004	6.3815709e-004	7.5996418e-004
7.4872512e-004	7.1376554e-004	6.9372357e-004	6.7306846e-004
7.3761799e-004	6.3428604e-004	6.0645540e-004	5.7509141e-004
5.0232369e-004	5.8932908e-004	5.7828899e-004	5.7542494e-004
5.5598768e-004	5.8741902e-004	5.8365049e-004	5.9162699e-004
5.3445240e-004	5.3369586e-004	5.4736395e-004	5.1959320e-004
7.4663664e-004	8.0191801e-004	7.5722809e-004	7.9726195e-004
7.6862914e-004	7.9219938e-004	7.5262343e-004	7.1801797e-004
7.4720410e-004	6.9676018e-004	7.1584059e-004	6.9400285e-004
6.2374656e-004	6.3569083e-004	6.5696980e-004	6.6976939e-004
6.1211911e-004	6.3938342e-004	6.6886701e-004	6.5244859e-004
6.1117983e-004	6.2212435e-004	6.4438219e-004	5.9062227e-004
6.8017599e-004	6.7671994e-004	6.7802348e-004	6.9590339e-004
6.8710548e-004	6.8666502e-004	6.3130590e-004	6.6338571e-004
6.0963204e-004	6.2776409e-004	5.9183354e-004	6.0459715e-004
5.8017280e-004	6.0215998e-004	5.8622559e-004	5.9657372e-004
5.5533081e-004	5.8518616e-004	6.0889206e-004	5.8956703e-004
5.7081933e-004	5.8779900e-004	5.8934177e-004	5.8240512e-004
6.8729506e-004	7.1892876e-004	7.1278670e-004	7.4520515e-004
7.3794145e-004	7.5044359e-004	7.1636715e-004	7.0967902e-004

6.8080115e-004	6.6105944e-004	6.5651299e-004	6.5379824e-004
6.3999345e-004	6.4331408e-004	6.5235618e-004	6.3465312e-004
6.1786131e-004	6.1745057e-004	6.4373818e-004	6.2672267e-004
6.0937804e-004	6.2199809e-004	6.3393165e-004	6.1701283e-004
7.2423511e-004	7.9123952e-004	7.7658230e-004	7.7213478e-004
7.4577679e-004	7.2504357e-004	6.9424961e-004	6.8078253e-004
6.6251273e-004	6.5075645e-004	6.2805701e-004	6.2206302e-004
6.3836786e-004	6.4316379e-004	6.3574786e-004	6.3465157e-004
6.0800805e-004	6.0738703e-004	5.8583575e-004	6.3745260e-004
5.6969625e-004	6.3005442e-004	6.2605552e-004	5.9070645e-004
8.1210672e-004	8.5895037e-004	9.0329860e-004	9.0431278e-004
9.6414314e-004	8.9987033e-004	8.8460781e-004	9.2413025e-004
8.5089800e-004	8.0559302e-004	8.0842292e-004	7.9938845e-004
8.1355701e-004	7.8660871e-004	7.9629764e-004	8.0422373e-004
7.4166100e-004	7.8858674e-004	7.3185543e-004	7.8605204e-004
7.2355977e-004	7.8002131e-004	8.0318218e-004	7.3477508e-004
6.5119921e-004	7.5360629e-004	7.0893666e-004	7.9126403e-004
7.2403824e-004	7.6416734e-004	7.2449954e-004	6.2019718e-004
7.0810631e-004	6.4760525e-004	6.3169617e-004	6.0562928e-004
5.6761760e-004	5.7336130e-004	6.1451213e-004	5.9770784e-004
6.2382538e-004	6.4441514e-004	5.8387525e-004	6.4923985e-004
6.2400405e-004	5.8535644e-004	6.3119680e-004	5.7777745e-004
5.8634159e-004	6.7601100e-004	6.3460030e-004	7.2116716e-004
6.8873444e-004	6.9364141e-004	6.7185586e-004	6.5324615e-004
7.1062746e-004	6.0997676e-004	6.2118119e-004	5.9950689e-004
5.0933941e-004	5.5170917e-004	5.8570912e-004	5.7043806e-004
5.4954887e-004	5.8031509e-004	6.0941290e-004	5.9142154e-004
5.3742237e-004	5.3792418e-004	5.8324030e-004	5.0139961e-004
7.4105905e-004	7.5718744e-004	7.4055213e-004	7.5562524e-004
7.6359110e-004	7.3956243e-004	6.8972465e-004	6.9179398e-004
6.6413518e-004	6.6811629e-004	6.3525797e-004	6.5846321e-004
6.1236384e-004	6.3856132e-004	6.2845525e-004	6.1490180e-004
5.8206865e-004	6.1809228e-004	6.4803072e-004	6.4735481e-004
5.8959002e-004	6.2748016e-004	6.1241243e-004	6.1220073e-004
7.2924099e-004	7.9101196e-004	7.9336260e-004	8.2534994e-004
7.8795957e-004	8.0772121e-004	7.4845912e-004	7.4047588e-004
7.1683960e-004	7.0726713e-004	6.7598636e-004	6.7738898e-004
6.9962081e-004	6.9313074e-004	7.0557771e-004	6.7963869e-004
6.5156593e-004	6.6088223e-004	6.4365381e-004	6.8415079e-004
6.2005545e-004	6.8237499e-004	6.7263543e-004	6.4831784e-004
7.5586938e-004	7.9646110e-004	7.9237801e-004	8.0484919e-004
7.9918631e-004	7.6885168e-004	7.2046585e-004	7.1662136e-004
6.9044787e-004	6.7193374e-004	6.6522002e-004	6.5623692e-004
6.4804680e-004	6.5557795e-004	6.5760154e-004	6.7539803e-004
6.3633683e-004	6.4625288e-004	6.2408086e-004	6.7618693e-004
5.9139213e-004	6.4084074e-004	6.5272300e-004	6.1993636e-004
7.1331109e-004	7.4439566e-004	7.4131165e-004	7.4128156e-004
7.1066656e-004	7.1763998e-004	6.9048535e-004	6.6499849e-004
6.5844660e-004	6.2126525e-004	6.2801539e-004	6.3216862e-004
6.2147420e-004	6.0349863e-004	6.2733790e-004	6.1111964e-004
6.0347575e-004	6.3029921e-004	6.1049903e-004	6.3675953e-004
6.0212714e-004	6.2892698e-004	6.4518429e-004	6.1387538e-004

7.6017580e-004	7.8259704e-004	7.5586697e-004	8.0562934e-004
8.1393513e-004	7.9452814e-004	7.4683192e-004	7.6272975e-004
7.0642956e-004	6.9159544e-004	6.8128793e-004	6.6305933e-004
6.9376934e-004	6.6272925e-004	6.8568392e-004	6.5365581e-004
6.4063013e-004	6.7188617e-004	6.6263747e-004	6.5491459e-004
6.2734130e-004	6.7459403e-004	6.8339262e-004	6.7572723e-004
8.3022783e-004	8.5829472e-004	9.2595637e-004	9.1664474e-004
9.5164032e-004	9.3536232e-004	8.6963948e-004	9.0798522e-004
8.6297303e-004	8.1620592e-004	8.1816166e-004	7.7825104e-004
8.2522992e-004	7.9298642e-004	8.0242261e-004	7.8731358e-004
7.5627492e-004	7.8004152e-004	7.2998035e-004	7.9307544e-004
7.3336385e-004	7.9123737e-004	8.2233083e-004	7.4719732e-004
7.3473259e-004	7.5736085e-004	7.2945573e-004	8.6322705e-004
8.0869999e-004	7.8809238e-004	7.6059812e-004	6.8000585e-004
6.7158935e-004	7.1024596e-004	6.9344153e-004	6.1905036e-004
6.8605256e-004	6.0005172e-004	6.7064213e-004	6.1994806e-004
6.3330842e-004	6.7817303e-004	6.1488865e-004	6.3087800e-004
6.3684506e-004	6.1918322e-004	6.5207493e-004	5.6625462e-004
6.2884306e-004	7.1020730e-004	6.6807659e-004	7.0338739e-004
7.0409750e-004	6.8715406e-004	6.6925682e-004	6.2030162e-004
6.4442153e-004	5.9494537e-004	5.9103683e-004	6.1373191e-004
5.2275011e-004	5.6986608e-004	5.7823872e-004	5.4301778e-004
5.6237038e-004	5.8360888e-004	5.9464652e-004	6.0653988e-004
5.6040757e-004	5.7622383e-004	5.8531568e-004	5.7366495e-004
7.1355174e-004	7.5636456e-004	7.2720792e-004	7.9489929e-004
7.7282616e-004	7.7165855e-004	6.9791522e-004	7.4641604e-004
6.5819031e-004	7.0102543e-004	6.6132388e-004	6.6669038e-004
6.3576807e-004	6.5951019e-004	6.5659785e-004	6.5934943e-004
6.0681876e-004	6.8681684e-004	6.7086428e-004	7.0852099e-004
6.2084534e-004	6.7433543e-004	6.7534250e-004	6.6590837e-004
7.4818046e-004	7.6887402e-004	7.4195581e-004	8.1048475e-004
8.1022814e-004	8.5013479e-004	7.6829939e-004	7.7043334e-004
7.3042708e-004	7.3688713e-004	6.9979372e-004	6.9943897e-004
7.0870909e-004	6.8354936e-004	7.1666202e-004	6.9258553e-004
6.6088891e-004	7.0946522e-004	6.8461458e-004	7.0662353e-004
6.5938216e-004	6.7388904e-004	6.8916900e-004	6.8126002e-004
7.6184064e-004	8.3949196e-004	8.3622809e-004	8.8395564e-004
8.5241480e-004	8.4207096e-004	7.6237296e-004	7.9172559e-004
7.4470275e-004	7.5252278e-004	7.1510515e-004	7.0751972e-004
7.2245557e-004	7.2078695e-004	7.1857688e-004	7.1363765e-004
6.7241263e-004	6.9158082e-004	6.5028650e-004	7.1917950e-004
6.2623312e-004	7.0186490e-004	6.8416528e-004	6.6308459e-004
7.6484260e-004	7.7682312e-004	8.0956769e-004	8.1006553e-004
8.3330231e-004	7.9328519e-004	7.4066136e-004	7.2210176e-004
7.2555983e-004	6.9096193e-004	6.8201356e-004	6.5951071e-004
6.4069661e-004	6.6062316e-004	6.6740268e-004	6.7313875e-004
6.4488981e-004	6.5423149e-004	6.4941855e-004	6.9236811e-004
6.0909710e-004	6.6009052e-004	6.6713235e-004	6.2669658e-004
7.4013589e-004	7.6658219e-004	7.6254831e-004	7.6519615e-004
7.3038434e-004	7.7175011e-004	7.1932135e-004	6.9781716e-004
7.1898851e-004	6.5170665e-004	6.6231462e-004	6.5147581e-004
6.3980311e-004	6.3725447e-004	6.5823650e-004	6.3984968e-004

6.2829307e-004 6.3581554e-004 6.1276725e-004 6.6807553e-004
6.0142048e-004 6.4616579e-004 6.5948174e-004 6.2406201e-004
8.0706204e-004 8.4163586e-004 8.4427264e-004 8.6722705e-004
8.9424735e-004 8.4884983e-004 8.0147442e-004 8.0676719e-004
7.4067102e-004 7.2841748e-004 7.2405359e-004 7.3196791e-004
7.1650454e-004 6.9521574e-004 6.9108358e-004 6.7760501e-004
6.6158864e-004 7.0823453e-004 6.8604050e-004 6.8638349e-004
6.5521253e-004 6.9711317e-004 7.1695495e-004 7.1346908e-004
8.6265296e-004 9.0454519e-004 9.1871870e-004 9.8235473e-004
9.7719337e-004 9.8703172e-004 8.8702892e-004 9.0587843e-004
8.5247243e-004 8.3185024e-004 8.2207736e-004 7.8235142e-004
8.2218469e-004 7.8777378e-004 8.1025901e-004 7.5539749e-004
7.4217501e-004 7.6640291e-004 7.5612262e-004 7.7634850e-004
7.2118765e-004 7.8266510e-004 7.8775863e-004 7.5851842e-004
8.2410414e-004 8.4659972e-004 9.2496193e-004 9.0989142e-004
1.0008963e-003 9.1857371e-004 8.5511577e-004 9.3658800e-004
8.7976316e-004 8.0283423e-004 7.9615392e-004 7.8190188e-004
8.0809480e-004 7.8345134e-004 7.4846158e-004 7.5362674e-004
7.4232367e-004 7.3350745e-004 7.0279439e-004 7.6422421e-004
7.2189342e-004 7.6892352e-004 7.7058819e-004 7.1691102e-004
8.0288234e-004 7.5736029e-004 7.9503169e-004 8.6421199e-004
8.6331737e-004 8.3289857e-004 7.6520407e-004 7.5832006e-004
7.2375678e-004 6.9288431e-004 7.0573627e-004 6.7755977e-004
6.7334446e-004 6.3492125e-004 6.6287136e-004 6.1852977e-004
6.3934701e-004 6.3035362e-004 6.3126974e-004 6.5110149e-004
5.7826903e-004 6.6735050e-004 6.7008018e-004 6.3801759e-004
6.4273182e-004 6.8890268e-004 6.3874574e-004 7.0926002e-004
7.1738627e-004 7.0134624e-004 6.7039576e-004 6.2887445e-004
5.7261456e-004 6.5150445e-004 6.0579194e-004 6.0830033e-004
6.0004444e-004 5.8255649e-004 6.1847195e-004 5.6264250e-004
5.5496465e-004 6.2385372e-004 5.9530618e-004 5.9024207e-004
5.7641977e-004 5.9528166e-004 5.9289804e-004 5.5726367e-004
6.9292798e-004 7.7712955e-004 7.1086449e-004 8.0899592e-004
7.8798260e-004 7.9961686e-004 7.2432375e-004 7.8158357e-004
6.8865519e-004 7.1833926e-004 7.0960587e-004 6.6513545e-004
6.6956902e-004 6.6871563e-004 6.6329655e-004 6.8874059e-004
6.3123045e-004 7.2907441e-004 6.9695292e-004 7.4814790e-004
6.4193619e-004 6.8914206e-004 7.1497195e-004 7.0081862e-004
7.5253291e-004 7.8126923e-004 7.6944908e-004 8.2711599e-004
8.5608225e-004 8.9503344e-004 7.7031834e-004 7.7433037e-004
7.5255200e-004 7.6770932e-004 7.0215944e-004 7.0924504e-004
7.3641012e-004 7.0456469e-004 7.4195565e-004 7.0494419e-004
6.7272982e-004 7.4879128e-004 6.8598025e-004 7.3220345e-004
6.7368192e-004 6.9256833e-004 6.9329856e-004 6.9353850e-004
7.9950075e-004 8.7567278e-004 8.4853836e-004 9.1939876e-004
9.1912647e-004 8.8693103e-004 7.9837813e-004 8.3750029e-004
7.7172267e-004 7.8432269e-004 7.6060912e-004 7.4545432e-004
7.3546289e-004 7.4120416e-004 7.4237795e-004 7.6010333e-004
7.0532085e-004 7.3119052e-004 6.8435463e-004 7.5788214e-004
6.3025941e-004 6.9408666e-004 7.1540581e-004 6.8982520e-004
7.7559563e-004 7.8963640e-004 8.4923768e-004 8.3628482e-004
8.6122360e-004 8.3205773e-004 7.6990572e-004 7.4971731e-004

7.6161162e-004	7.0707673e-004	7.0893212e-004	6.8760179e-004
6.5438106e-004	6.9383185e-004	6.8603577e-004	6.7777192e-004
6.6057449e-004	6.6666301e-004	6.5495311e-004	7.0103254e-004
6.3036120e-004	6.8080770e-004	6.7270848e-004	6.4882319e-004
7.8299378e-004	8.2311235e-004	8.0628255e-004	8.3928903e-004
8.0600983e-004	8.5156874e-004	8.0464841e-004	7.8846367e-004
7.9513455e-004	7.1513683e-004	7.0921631e-004	7.1225260e-004
6.9649015e-004	6.7111028e-004	7.0656773e-004	6.8118668e-004
6.5142113e-004	6.7018051e-004	6.5571028e-004	7.3068849e-004
6.4620728e-004	6.6483101e-004	7.0792512e-004	6.5824392e-004
6.8919484e-004	6.7993114e-004	6.6860215e-004	7.3935078e-004
7.1930821e-004	7.2360582e-004	6.3846762e-004	6.5659541e-004
6.1728866e-004	5.9078040e-004	6.0563395e-004	5.9767137e-004
5.5011401e-004	5.5471736e-004	5.9350632e-004	5.4420430e-004
5.6469630e-004	5.5156645e-004	5.6745110e-004	5.5477859e-004
5.2231588e-004	5.8314682e-004	5.8267270e-004	5.8030509e-004
7.0596214e-004	7.8855923e-004	6.9634466e-004	7.9839242e-004
8.1526660e-004	8.4182819e-004	7.5999231e-004	7.7917171e-004
7.0001936e-004	7.3025725e-004	7.2649390e-004	6.7298704e-004
6.9255171e-004	6.7115772e-004	6.7831187e-004	6.8700935e-004
6.4266309e-004	7.5176891e-004	7.1551961e-004	7.4347130e-004
6.6700953e-004	6.7443191e-004	7.1040247e-004	7.1295722e-004
7.4049180e-004	7.9898446e-004	7.6138102e-004	8.3202329e-004
8.8146534e-004	8.9828903e-004	7.5747177e-004	7.7328605e-004
7.6188793e-004	7.7347006e-004	7.0358385e-004	7.0189034e-004
7.1956945e-004	7.2186805e-004	7.4362173e-004	7.0272348e-004
6.5996828e-004	7.6058207e-004	6.6616834e-004	7.2914909e-004
6.6727139e-004	6.9031802e-004	6.8046075e-004	6.8639440e-004
8.1146297e-004	8.7318145e-004	8.4547204e-004	9.0716736e-004
9.5324278e-004	9.0544463e-004	8.1694849e-004	8.4416417e-004
7.8025728e-004	7.8829128e-004	7.7565000e-004	7.6283517e-004
7.2454101e-004	7.5796482e-004	7.5023923e-004	7.7177421e-004
7.1803488e-004	7.5700080e-004	7.0641848e-004	7.6059031e-004
6.3037644e-004	6.7886122e-004	7.4961116e-004	7.0129918e-004
7.7343047e-004	7.9673385e-004	8.4386252e-004	8.5435811e-004
8.6638155e-004	8.5768764e-004	7.9989046e-004	7.7435659e-004
7.7408314e-004	6.9340084e-004	7.2592493e-004	7.0848997e-004
6.6630039e-004	6.9316338e-004	6.9017230e-004	6.8239296e-004
6.6682625e-004	6.8840217e-004	6.5292495e-004	6.9813681e-004
6.3993200e-004	6.9441684e-004	6.7698595e-004	6.6488996e-004
7.8957080e-004	8.2623244e-004	8.0733515e-004	8.6460065e-004
8.5194474e-004	8.7560910e-004	8.2237608e-004	8.2588661e-004
8.2448736e-004	7.4808186e-004	7.1188244e-004	7.3347792e-004
7.1625032e-004	6.7242655e-004	7.0363098e-004	6.7038127e-004
6.3867432e-004	6.6557352e-004	6.6293226e-004	7.4979830e-004
6.5942194e-004	6.5197535e-004	7.2018882e-004	6.6913657e-004
5.8435528e-004	5.8751750e-004	5.3845804e-004	6.0580928e-004
6.2690146e-004	6.5669674e-004	5.6089048e-004	5.5711048e-004
5.2423307e-004	5.3263871e-004	5.2184980e-004	5.2920116e-004
4.6753671e-004	4.7330060e-004	5.3265876e-004	4.6311188e-004
4.9058876e-004	5.0978082e-004	5.1074843e-004	4.9037765e-004
4.9034501e-004	4.8196443e-004	4.7867691e-004	5.1486913e-004

7.1974100e-004	8.0900475e-004	7.1284740e-004	8.0943039e-004
8.6675979e-004	8.8087832e-004	7.3481018e-004	7.5545345e-004
7.3426377e-004	7.6166821e-004	6.9285276e-004	6.7911780e-004
6.7702777e-004	7.0440985e-004	7.2257024e-004	6.7740132e-004
6.2872043e-004	7.5608127e-004	6.5090091e-004	6.9766268e-004
6.4027807e-004	6.5885033e-004	6.5486912e-004	6.7047129e-004
7.9499748e-004	8.5633599e-004	7.9642319e-004	8.7170857e-004
9.3524779e-004	8.9048300e-004	8.1073279e-004	8.2626706e-004
7.5251448e-004	7.6751540e-004	7.6363128e-004	7.4892941e-004
7.0016907e-004	7.5949826e-004	7.3111815e-004	7.5050422e-004
7.2295892e-004	7.7582455e-004	6.8739797e-004	7.2021944e-004
6.1943757e-004	6.8370142e-004	7.5865308e-004	7.0266926e-004
7.6200660e-004	7.8138105e-004	8.2732568e-004	8.7515479e-004
8.7344061e-004	8.6525873e-004	8.2045259e-004	7.8419276e-004
7.6856290e-004	6.9135622e-004	7.3402531e-004	7.1079370e-004
6.7011295e-004	6.8227858e-004	6.7918480e-004	6.7726651e-004
6.6478553e-004	7.1320878e-004	6.5650890e-004	6.8833574e-004
6.3828517e-004	7.0322309e-004	6.9498269e-004	6.6353865e-004
7.7520336e-004	7.9270604e-004	7.5297024e-004	8.4668219e-004
8.2467748e-004	8.4879098e-004	8.0026498e-004	7.9629447e-004
8.0198607e-004	7.1534542e-004	6.7264847e-004	7.1479668e-004
6.9643218e-004	6.3692465e-004	6.6663491e-004	6.2499763e-004
6.0600949e-004	6.4386980e-004	6.4324170e-004	7.2844871e-004
6.4293464e-004	6.2703049e-004	6.8158207e-004	6.4857246e-004
7.5765261e-004	7.6534765e-004	8.0711918e-004	9.6809273e-004
8.2007521e-004	9.1738637e-004	7.2498732e-004	7.6736030e-004
7.7527607e-004	7.4541436e-004	7.5944928e-004	7.2389015e-004
6.9792960e-004	7.2971337e-004	7.0913778e-004	6.9061785e-004
6.9894223e-004	6.5044229e-004	7.1008541e-004	6.5181874e-004
7.0150809e-004	6.4271371e-004	7.0009085e-004	7.2043105e-004
5.6221546e-004	5.9316696e-004	6.7934403e-004	6.3235249e-004
6.6231307e-004	6.5458092e-004	5.6744260e-004	5.9000188e-004
6.4504810e-004	4.9517197e-004	5.3687644e-004	5.0202127e-004
5.3683885e-004	5.7308168e-004	4.9365767e-004	4.9640030e-004
4.8648749e-004	4.7681503e-004	4.6187188e-004	4.5102170e-004
4.6093421e-004	5.4802775e-004	5.1500635e-004	4.9950535e-004
5.7581178e-004	6.6804745e-004	5.5117966e-004	6.5434098e-004
6.8348803e-004	6.8961916e-004	5.8636115e-004	6.0884730e-004
5.7584438e-004	6.1075424e-004	5.6537364e-004	5.4323297e-004
5.3049575e-004	5.7068639e-004	5.7903448e-004	5.4278797e-004
4.9979773e-004	6.1118347e-004	5.2757579e-004	5.5147895e-004
5.0793932e-004	5.2578122e-004	5.2430523e-004	5.4337470e-004
7.5074338e-004	8.0333046e-004	7.3542025e-004	8.0820626e-004
8.6418191e-004	8.3891318e-004	7.8968459e-004	7.7108634e-004
6.9351781e-004	7.2854663e-004	7.1392146e-004	6.9771132e-004
6.5436923e-004	7.2558205e-004	6.8020182e-004	6.9207216e-004
7.0614167e-004	7.5561111e-004	6.4034820e-004	6.4837807e-004
5.8371753e-004	6.7199532e-004	7.3745010e-004	6.6555139e-004
7.4103063e-004	7.6322719e-004	7.8843793e-004	8.9220708e-004
8.6379673e-004	8.5158434e-004	8.2511530e-004	7.7055291e-004
7.4111449e-004	6.8409261e-004	7.4304087e-004	6.8793021e-004
6.6605129e-004	6.5416354e-004	6.4755924e-004	6.5562561e-004

6.5938088e-004	7.2981452e-004	6.4210242e-004	6.6283489e-004
6.0945898e-004	7.0854279e-004	7.0792506e-004	6.4680821e-004
7.8095197e-004	7.7619990e-004	7.3764945e-004	8.4443321e-004
8.0640509e-004	8.5108775e-004	7.9641516e-004	7.7240070e-004
7.6757694e-004	6.8057595e-004	6.4300906e-004	7.1994263e-004
6.9163737e-004	6.1575040e-004	6.4032928e-004	5.9001445e-004
5.9455965e-004	6.4488496e-004	6.3131715e-004	7.1849360e-004
6.4221100e-004	6.2174341e-004	6.4869462e-004	6.5137005e-004
6.4645533e-004	6.4768426e-004	6.7031567e-004	8.2238899e-004
6.7605136e-004	7.5135654e-004	6.1487958e-004	6.4788036e-004
6.3976507e-004	6.4029088e-004	6.5083275e-004	6.2453905e-004
6.0619794e-004	6.2129227e-004	6.0099576e-004	5.8508191e-004
5.9526102e-004	5.5992565e-004	6.2422201e-004	5.4374960e-004
5.8831600e-004	5.4730872e-004	5.9305677e-004	6.4057787e-004
5.2584218e-004	5.1736600e-004	5.6541592e-004	6.0852713e-004
5.9891256e-004	5.9357900e-004	6.2284927e-004	5.3403686e-004
4.9473695e-004	5.0515826e-004	5.1161821e-004	4.9626434e-004
4.8400556e-004	5.1083238e-004	4.7153408e-004	4.5501329e-004
5.1632027e-004	5.2324383e-004	4.5735409e-004	4.4711794e-004
4.2240064e-004	5.0824799e-004	5.4521459e-004	4.4839599e-004
7.3737181e-004	7.8217270e-004	7.6668066e-004	8.7681330e-004
7.9519687e-004	8.4214403e-004	7.1643663e-004	7.3249299e-004
7.5359469e-004	6.3657423e-004	6.8456470e-004	6.6547943e-004
6.7017580e-004	5.8187767e-004	5.8170223e-004	6.1405639e-004
5.8352143e-004	7.1054359e-004	6.1725553e-004	6.7431344e-004
5.8052431e-004	6.7844098e-004	6.3716233e-004	6.6653886e-004
6.8463950e-004	6.7405430e-004	6.8596973e-004	7.2559845e-004
7.4894323e-004	7.7022292e-004	7.0691045e-004	6.7196526e-004
6.4562485e-004	5.7799336e-004	5.8726237e-004	6.5513462e-004
6.0905542e-004	5.5034638e-004	5.7275176e-004	5.1413112e-004
5.4590654e-004	5.6749437e-004	5.6580808e-004	6.1506779e-004
5.9550395e-004	5.4130885e-004	5.4631885e-004	5.7210501e-004
4.9447491e-004	5.4001009e-004	4.9356285e-004	5.4167819e-004
5.0090547e-004	5.1283548e-004	4.5399707e-004	5.0218592e-004
4.4903347e-004	4.8101247e-004	4.5833224e-004	4.6884544e-004
4.3414834e-004	4.1615205e-004	4.2322060e-004	3.8803644e-004
4.0360192e-004	4.3456825e-004	4.3404195e-004	4.3012617e-004
3.9772473e-004	4.1962319e-004	3.7701386e-004	4.1308729e-004

MouseDataResult

A=[0.0366 0.0532 0.0352

0.0387 0.0551 0.035

0.0434 0.0513 0.0365

0.0443 0.0527 0.0402

0.0465 0.0503 0.0365

0.0468 0.0487 0.0347

0.0502 0.0447 0.0355

0.0454 0.0433 0.04

0.0442 0.0414 0.0402

0.0447 0.0425 0.0366

0.0439 0.0427 0.037

0.043 0.0393 0.0402

0.0426 0.0371 0.0412

0.0413 0.0376 0.0424

0.04 0.0367 0.0456

0.0412 0.0372 0.0434

0.0399 0.039 0.0422

0.0376 0.0368 0.0473

0.0409 0.0358 0.0447

0.038 0.0376 0.049

0.0376 0.0336 0.0477

0.0376 0.0338 0.0496

0.0372 0.0349 0.0496

0.0381 0.0347 0.0497];

```
Aorg=[ 0.035	0.051	0.0333
0.037	0.0528	0.0331
0.0419	0.0498	0.035
0.0421	0.0502	0.0379
0.0447	0.0484	0.0348
0.0434	0.0453	0.0319
0.0487	0.0435	0.0342
0.0436	0.0417	0.038
0.0428	0.0402	0.0386
0.0429	0.0409	0.0348
0.0434	0.0424	0.0363
0.0432	0.0396	0.0401
0.0421	0.0368	0.0404
0.0414	0.0378	0.0422
0.0421	0.0387	0.0475
0.0425	0.0385	0.0445
0.0404	0.0397	0.0424
0.0396	0.0389	0.0494
0.0422	0.0371	0.0458
0.0404	0.04	0.0516
0.0402	0.036	0.0505
0.0406	0.0366	0.0531
0.0392	0.0368	0.0518
0.0408	0.0373	0.0528];
```

```
eCf=[ 0.0263    0.0405  0.0473  0.0501  0.0505  0.0498  0.0486  0.0472  0.0457  0.0444  0.0432  0.0421
      0.0411  0.0402  0.0395  0.0389  0.0383  0.0378  0.0374  0.0371  0.0368  0.0365  0.0363  0.0361];

eCs=[ 0.0114    0.0208  0.0283  0.034   0.0383  0.0413  0.0434  0.0448  0.0456  0.046   0.0461  0.046
      0.0458  0.0454  0.045   0.0445  0.0441  0.0436  0.0432  0.0427  0.0424  0.042   0.0416  0.0413];

eCp=[ 0.0532    0.0541  0.0532  0.0514  0.0495  0.0476  0.0458  0.0442  0.0428  0.0416  0.0405  0.0396
      0.0388  0.0381  0.0375  0.037   0.0366  0.0362  0.0358  0.0356  0.0353  0.0351  0.0349  0.0347];

eKtrans=[ 0.1897        0.0524];

eKep=[ 0.6521   0.2053];

del_t=5;
```
