# Supplementary material for: Unsupervised Deconvolution of Dynamic Imaging Reveals Intratumor Vascular Heterogeneity and Repopulation Dynamics
Source: PLoS One. 2014 Nov 7;9(11):e112143. doi: 10.1371/journal.pone.0112143 (PMC4224420; doi:10.1371/journal.pone.0112143)
Supplement: File S1 — Supplementary discussion (appendix S1) and supplementary method (appendix S2). (DOCX) [file pone.0112143.s014.docx]

**Appendix S1. Supplementary Discussion**

**Dynamic intratumor heterogeneity: clonal repopulation & multi-compartment model.**

For the characterization of complex phenotypes and therapeutic responses ^[1-3](#_ENREF_1)^, a major outstanding issue is how to accurately quantify intratumor vascular heterogeneity that may be severely confounded by the varying partial-volume effect [^3^](#_ENREF_3),[^4^](#_ENREF_4). Specifically, to capture the changes (that may reflect the underlying ‘clonal’ repopulation dynamics ^34^) in (1) local volume transfer maps, (2) compartment pharmacokinetics, and (3) number of distinctive compartments, a completely unsupervised learning method is required to solve the multi-compartment model based on only observed DCE-MRI data.

MTCM using DCE-MRI has the potential to reveal functional intratumor vascular heterogeneity, without any type of external information, thus is an unbiased and data-driven approach. This advantage has significant implications. The recent results of Kreso *et al*. strongly suggest that biological differences between tumor cells can be due to additional mechanisms, other than genetic heterogeneities ^34^. It has been reported, despite validated genetic homogeneity, the different cancer ‘clones’ observed in a tumor, displayed notable differences in behavior during the experiments ^34^. More interestingly, some of these unusual clones remained inactive initially but reemerged at later stage; therapeutic drugs preferentially eliminated persistent clones while increased the proportion of clones that were initially dormant ^34^. A more likely explanation about this clonal repopulation is the involvement of one or more distinct semistable epigenetic states, on which MTCM can help to reveal quantitatively at phenotypic and functional level. To our best knowledge, MTCM is the first tool of its type to address the suggestion that “Improved mathematical models built on actual clinical and experimental observations, will likely allow us to construct these pictures in the not-so-distant future ^34^.” For example, the results of MTCM may provide mechanistic models of tumor progression and responses to refine and optimize biopsy sampling, in terms of timing, number, and location of biopsies. Furthermore, some of form of intratumor heterogeneity (ITH) index may be subsequently defined as a predictor of tumor history and behaviour ^38^.

In complex tissues, functional heterogeneity is of great interest since it represents the integration of various upstream factors. Since cell-cell signalling plays a critical role in tumor development and evolution, cellular heterogeneity may constitutes only a partial picture, and dissection based on cell types may provide limited information since functional aspect is largely missing ^39^. MTCM (a completely unsupervised method empowered by MDL based model selection) and DCE-MRI provide a powerful and in vivo method to reveal and quantify functional heterogeneity. In contrast, many conventional analytic methods (relying on prior knowledge) may miss the low-frequency yet critical subclone(s).

**Detecting the number of tissue compartment (model selection).**

To discover and characterize intratumor vascular heterogeneity, the true number of the underlying tissue compartments is an unknown ‘structural’ model parameter and must be estimated from the data [^5^](#_ENREF_5),[^6^](#_ENREF_6). To assure that MTCM provides a completely unsupervised machine learning method, we exploit the information theoretical criterion called minimum description length (MDL) [^7^](#_ENREF_7),[^8^](#_ENREF_8), to detect the most appropriate number of tissue compartments in our analysis. From the MDL principle, we derived the specific MDL for MTCM model as Eq. (9) (see Supplementary Method).

MDL calculates the total number of ‘bits’ that are required to encode/explain both the ‘data’ and ‘model’. When the model is given (or estimated), only information about ‘mismatch’ between model and data needs to be explained (or encoded). The first term (negative joint likelihood) in the MDL determines exactly the ‘bits’ for explaining the ‘data’ conditioned on the given model. The second and third terms represents the ‘penalty’ on the model complexity, that is, the total number of bits for explaining the model. Each of these terms involves two multiplicative factors: the number of free-adjustable parameters and the original data points used to estimate the parameter (or the original data points the parameter is used to or can ‘explain’). Specifically, when estimating the compartment TCs (the column vectors of mixing matrix) parameterized by $J-1$ independent $k_{\text{ep}}$, we use some form of vector-average operation (i.e., $\min_{\alpha_{m_{1}},...\alpha_{m_{J}}} \left\|\mathbf{\mu}_{\mathbf{C}_{\text{measured}},m}-\sum_{j=1}^{J} \alpha_{m_{j}}\mathbf{\mu}_{\mathbf{C}_{\text{measured}},m_{j}}\right\|_{2}$; where nonnegative $\alpha_{m_{1}}, ... , \alpha_{m_{J}}$, with sum equal to 1, are the coefficients for defining a convex hull), the scalar entry $C_{j}\left(t_{l}\right)$ in $\mathbf{C}_{j}$is estimated involving only $M$ scalar entries $\mu_{\mathbf{C}_{\text{measured}}}\left(m,t_{l}\right)$ for a given $t_{l}$, contributing total $\left(J-1\right)\log\left(M\right)/2$ bits. Similarly, when estimating the local volume transfer constants (the row vectors of sources) $\mathbf{K}^{\text{trans}}(m)$ with total $JM$ entries, we use some form of vector-average operation (i.e., solving linear equations), where the scalar entry $K_{j}^{\text{trans}}(m)$ in $\mathbf{K}^{\text{trans}}(m)$ is estimated involving only $L$ scalar entries $\mu_{\mathbf{C}_{\text{measured}}}\left(m,t_{l}\right)$ for a given $m$, contributing total $JM\log(L)/2$ bits.

**Data quality control (QC).**

Quality control should be applied to reduce error in all image parameters. The impact of motion should be assessed and tumours for which parameter estimates are unreliable should be rejected. The level of bulk motion can be assessed for each tumor by first extracting an averaged time series plot for each tumor region of interest (ROI) on each slice in the imaging volume and then by visual assessment of the dynamic time series images. In- and through-plane motion can be investigated and a categorical score can be assigned for each tumor based on the evaluations of bulk motion. Tumors with a motion assessment score higher than a pre-specified threshold should be excluded ^38^.

**Appendix S2. Supplementary Method**

**Parallelism between multi-compartment modeling and the theory of convex sets.**

We now discuss the identifiability of the compartment model (2) and the required conditions via the following definitions and theorems.

***Definition* 1.** Given a set of compartment TCs , we denote the convex set it specifies by

. (S1)

***Definition* 2.** A compartment TC vector $\mathbf{C}_{j}$ is a vertex point of the convex set if it can only be expressed as a trivial convex combination of $\mathbf{C}_{1},...,\mathbf{C}_{J}$.

**Lemma 1 (Convex envelope of pixel time series).** *Suppose that the J compartment pharmacokinetics* $\left\{\mathbf{C}_{j}\right\}$ *are linearly independent, and* $\mathbf{C}_{\text{measured}}(i)=\sum_{j=1}^{J} K_{j}^{\text{trans}}(i)\mathbf{C}_{j}$*where spatially-distributed local volume transfer constants* $\left\{K_{j}^{\text{trans}}(i)\right\}$ *are non-negative and normalized. Then, the elements of (the pixel time series) are confined within a convex set*  *whose vertices are the J compartment TCs* $\mathbf{C}_{1},...,\mathbf{C}_{J}$.

***Definition* 3.** Any pixel whose associated normalized spatially-distributed volume transfer constants are in the form of $\mathbf{K}^{\text{trans}}(i_{\text{WGP(}j\text{)}})\approx\mathbf{e}_{j}$ is called a well-grounded point (WGP) and corresponds to a pure-volume pixel, where $\left\{\mathbf{e}_{j}\right\}$ is the standard basis of *J*-dimensional real space (the axes of the first quadrant). In other words, we define pure volume pixels (or well-grounded pixels) as the pixels that are occupied by only a single compartment tissue type.

*Proof of Lemma 1*. By the definition of convex set ^43^, the fact that, $\forall\left\{i,j\right\}\text{ }K_{j}^{\text{trans}}(i)\geq0$, $\sum_{j=1}^{J} K_{j}^{\text{trans}}(i)=1$ and $\mathbf{C}_{\text{measured}}(i)=\sum_{j=1}^{J} K_{j}^{\text{trans}}(i)\mathbf{C}_{j}$ readily yield where

. (S2)

Since $\mathbf{C}_{1},...,\mathbf{C}_{J}$ are linearly independent, it follows that

$\sum_{j=1}^{J} \alpha_{j}\mathbf{C}_{j}=\mathbf{0}\text{ iff }\alpha_{j}=0\text{ }\forall j$ (S3)

that also implies that ∀*j*

$\mathbf{C}_{j}\text{=}\sum_{j=1}^{J} \alpha_{j}^{'}\mathbf{C}_{j}\text{ iff [}\alpha_{1}^{'},...,\alpha_{J}^{'}]^{T}=\mathbf{e}_{j}\text{ }\forall j$ (S4)

*i.e.*, $\mathbf{C}_{j}$ can only be a trivial convex combination of $\mathbf{C}_{1},...,\mathbf{C}_{J}$. Hence, by Definition 2, $\mathbf{C}_{1},...,\mathbf{C}_{J}$ are therefore the vertices of convex set .

*Proof of Theorem 1*. Since ∃, ∀*j*, and $\mathbf{C}_{\text{measured}}(i)=\sum_{j=1}^{J} K_{j}^{\text{trans}}(i)\mathbf{C}_{j}$, we have

$\mathbf{C}_{\text{measured}}(i_{\text{WGP(}j\text{)}})=\mathbf{C}_{j}$. (S5)

Then, for any , we have

$\begin{matrix} \mathbf{z}=\sum_{j=1}^{J} \alpha_{j}\mathbf{C}_{j} \\ \text{ }=\sum_{j=1}^{J} \alpha_{j}\mathbf{C}_{\text{measured}}(i_{\text{WGP(}j\text{)}}) \\ \text{ }=\sum_{i=1}^{N} \alpha_{i}^{'}\mathbf{C}_{\text{measured}}(i),\text{ where }\alpha_{i}^{'}=\left\{\begin{matrix} \alpha_{j}, & i\in\left\{i_{\text{WGP(}j\text{)}}\right\}, \\ 0, & i\notin\left\{i_{\text{WGP(}j\text{)}}\right\}, \end{matrix}\right. \end{matrix}$ (S6)

that implies , *i.e.*, . On the other hand, for any , we have

$\begin{matrix} \mathbf{z}=\sum_{i=1}^{N} \alpha_{i}\mathbf{C}_{\text{measured}}(i) \\ \text{ }=\sum_{i=1}^{N} \alpha_{i}\sum_{j=1}^{J} K_{j}^{\text{trans}}(i)\mathbf{C}_{j} \\ \text{ }=\sum_{j=1}^{J} \left[\sum_{i=1}^{N} \alpha_{i}K_{j}^{\text{trans}}(i)\right]\mathbf{C}_{j} \\ \text{ = }\sum_{j=1}^{J} \beta_{j}\mathbf{C}_{j},\text{ where }\beta_{j}=\sum_{i=1}^{N} \alpha_{i}K_{j}^{\text{trans}}(i)\text{ and }\sum_{j=1}^{J} \beta_{j}=1, \end{matrix}$ (S7)

that implies , *i.e.*, . Combining and gives , and together with Lemma 1 readily completes the proof of Theorem 1.

*Proof of theorem 2.* Consider the pixel $\mathbf{K}^{\text{trans}}(i*)=\sum_{j=1}^{J} \alpha_{j}\left(i*\right)\mathbf{K}^{\text{trans}}(v_{j})$ of the convex hull defined by the vertices $\left\{\mathbf{K}^{\text{trans}}(v_{j})\right\}$ whose $m\text{th}$entry is the largest among all pixels, i.e., $K_{m}^{\text{trans}}(i*)=\max_{i=1,2,...N} K_{m}^{\text{trans}}(i)$. Since $\sum_{j=1}^{J} \alpha_{j}\left(i*\right)=1$, we may therefore write

$K_{m}^{\text{trans}}(i*)=\left(\sum_{j=1}^{J} \alpha_{j}\left(i*\right)\right)K_{m}^{\text{trans}}(i*)=\sum_{j=1}^{J} \alpha_{j}\left(i*\right)K_{m}^{\text{trans}}(i*)$. (S8)

Alternatively, the $m\text{th}$entry of $\mathbf{K}^{\text{trans}}(i*)$ can be expressed as

$K_{m}^{\text{trans}}(i*)=\sum_{j=1}^{J} \alpha_{j}\left(i*\right)K_{m}^{\text{trans}}(v_{j})$. (S9)

By the unique convex expression of $K_{m}^{\text{trans}}(i*)$, we have

$\sum_{j=1}^{J} \alpha_{j}\left(i*\right)\left(K_{m}^{\text{trans}}(i*)-K_{m}^{\text{trans}}(v_{j})\right)=0$, (S10)

which, together with the fact $\alpha_{j}\left(i*\right)\geq0\text{ and }K_{m}^{\text{trans}}(i*)-K_{m}^{\text{trans}}(v_{j})\geq0$, implies $i*\in\left\{v_{j}\right\}$.

**Clustering of Pixel Time Series**

The purpose of multivariate clustering of normalized pixel time series is three-fold: 1) data clustering has proven to be an effective tool for reducing the impact of noise/outlier data points on model learning ^44-46^; 2) aggregation of pixel time series into a few clusters improves the efficiency of subsequent convex analysis of mixtures [^9^](#_ENREF_9),[^10^](#_ENREF_10); 3) the resultant clustered compartment model permits an automated determination of the number of underlying tissue compartments using the minimum description length (MDL) criterion [^5^](#_ENREF_5),[^11^](#_ENREF_11),[^12^](#_ENREF_12).

There has been considerable success in using SFNMs to model clustered data sets such as DCE-MRI data, taking a sum of the following general form ^[13-15](#_ENREF_13)^:

$p\left(\mathbf{K}^{\text{trans}}(i)\right)=\sum_{m=1}^{J} \pi_{m} g\left(\mathbf{K}^{\text{trans}}(i)|\mathbf{e}_{m},\mathbf{\Sigma}_{\mathbf{K}^{\text{trans}},m}\right)+\sum_{m=J+1}^{M} \pi_{m} g\left(\mathbf{K}^{\text{trans}}(i)|\mathbf{\mu}_{\mathbf{K}^{\text{trans}},m},\mathbf{\Sigma}_{\mathbf{K}^{\text{trans}},m}\right)$, (S11)

where the first term corresponds to the clusters of pure volume pixels , the second term corresponds to the clusters of partial volume pixels , $M$ is the total number of pixel clusters, is the mixing factor, is the Gaussian kernel, $\mathbf{e}_{m}$ denotes the *m*th natural basis vector corresponding to the mean vector of the *m*th pure tissue compartment, and and $\mathbf{\Sigma}_{\mathbf{K}^{\text{trans}},m}$ are the mean vector and covariance matrix of cluster , respectively. It is worth noting that SFNMs are a flexible and powerful statistical modeling tool and can adequately model clustered structure with essentially arbitrary complexity by introducing a sufficient number of mixture components. Thus, strict adherence of the (in general, unknown) ground-truth data distribution to the form in (9) is not required in most real-world applications [^13^](#_ENREF_13),[^14^](#_ENREF_14). By incorporating (7) into (9), the SFNM model for pixel time series becomes:

(S12)

where $\mathbf{\Sigma}_{\mathbf{C}_{\text{measured}},m}=\mathbf{A\Sigma}_{\mathbf{K}^{\text{trans}},m}\mathbf{A}^{T}$ and $\mathbf{\mu}_{\mathbf{C}_{\text{measured}},m}=\mathbf{A\mu}_{\mathbf{K}^{\text{trans}},m}$, with $\mathbf{C}=\left[\mathbf{C}_{1},...,\mathbf{C}_{J}\right]$. Accordingly, the first term of (S12) represents the corner clusters and the second term of (S12) represents the interior clusters (as shown in Fig. 1). From Theorem 1 and SFNM model (S12), the clustered pixel time series set is (approximately) confined within a convex set whose corner centers are the *J* compartment TCs $\mathbf{C}_{1},...,\mathbf{C}_{J}$.

It has been shown that significant computational savings can be achieved by using the EM algorithm to allow a mixture of the form (S12) to be fitted to the data [^13^](#_ENREF_13),[^14^](#_ENREF_14). Determination of the parameters of the model (S12) can be viewed as a “missing data” problem in which the missing information corresponds to pixel labels $l_{im}=\mathbf{I}\left(i,m\right)$ specifying which cluster generated each data point with $\mathbf{I}\left(i,m\right)$ denoting the indicator function. When no information about $l_{im}$ is available, the log-likelihood for the model (10) takes the form

, (S13)

where denotes the joint likelihood function of SFNM and $\mathbf{\Theta}=\{\pi_{m},\mathbf{\mu}_{\mathbf{C}_{\text{measured}},m},\mathbf{\Sigma}_{\mathbf{C}_{\text{measured}},m},\forall m\}$. If, however, we were given a set of already clustered data with specified pixel labels, then the log likelihood (known as the “complete” data log-likelihood) becomes

, (S14)

where $\mathbf{L}=\{l_{im}|i=1,...,N;m=1,...,M\}$. Actually, we only have indirect, probabilistic, information in the form of the posterior responsibilities $z_{im}$ for each model *m* having generated the pixel time series $\mathbf{C}_{\text{measured}}(i)$. Taking the expectation of (S14), we then obtain the complete data log likelihood in the form

, (S15)

in which the $z_{im}=\Pr\left(l_{im}=1|\mathbf{C}_{\text{measured}}(i)\right)$ are constants, and $\mathbf{Z}=\{z_{im}|i=1,...,N;m=1,...,M\}$.

Maximization of (S15) can be performed using the two-stage form of the EM algorithm, where the pixel labels $l_{im}$ are treated as missing data as aforementioned. At each complete cycle of the algorithm we commence with an “old” set of parameter values $\mathbf{\Theta}$. We first use these parameters in the E-step to evaluate the posterior probabilities $z_{im}$ using Bayes theorem [^16^](#_ENREF_16)

$z_{im}=\Pr\left(l_{im}=1|\mathbf{C}_{\text{measured}}(i)\right)=\frac{\pi_{m}g\left[\mathbf{C}_{\text{measured}}(i)|\mathbf{\mu}_{\mathbf{C}_{\text{measured}},m},\mathbf{\Sigma}_{\mathbf{C}_{\text{measured}},m}\right]}{\sum_{m\text{*}=1}^{M} \pi_{m\text{*}}g\left[\mathbf{C}_{\text{measured}}(i)|\mathbf{\mu}_{\mathbf{C}_{\text{measured}},m\text{*}},\mathbf{\Sigma}_{\mathbf{C}_{\text{measured}},m\text{*}}\right]},\text{ }m\in\left\{1,...,M\right\}$. (S16)

These posterior probabilities are then used in the M-step to obtain “new” values $\mathbf{\Theta}$ using the following re-estimation formulas

$\pi_{m}=\frac{1}{N}\sum_{i=1}^{N} z_{im}$, (S17)

$\mathbf{\mu}_{\mathbf{C}_{\text{measured}},m}=\frac{\sum_{i=1}^{N} z_{im}\mathbf{C}_{\text{measured}}(i)}{\sum_{i=1}^{N} z_{im}}$, (S18)

$\mathbf{\Sigma}_{\mathbf{C}_{\text{measured}},m}=\frac{\sum_{i=1}^{N} z_{im}\left(\mathbf{C}_{\text{measured}}(i)-\mathbf{\mu}_{\mathbf{C}_{\text{measured}},m}\right)\left(\mathbf{C}_{\text{measured}}(i)-\mathbf{\mu}_{\mathbf{C}_{\text{measured}},m}\right)^{T}}{\sum_{i=1}^{N} z_{im}}$. (S19)

**Convex Analysis of Mixtures**

At this point in our analysis procedure, each pixel time-course is represented by a group of cluster centers, where both the dimensionality and noise/outlier effect are significantly reduced. Given the obtained *M* cluster centers $\mathbf{\mu}_{\mathbf{C}_{\text{measured}},1},...,\mathbf{\mu}_{\mathbf{C}_{\text{measured}},M}$, CAM is applied to separate pure-volume clusters from partial-volume clusters by detecting the “corners” of the convex hull containing all clusters of pixel TCs (theoretically supported by Lemma 1 and Theorem 1). Assuming the number of compartments $J$ is known a priori, an exhaustive combinatorial search (with total $C_{J}^{M}$ combinations), based on a convex-hull-to-data fitting criterion, is performed to identify the most probable $J$ corners. This explicitly maps pure-volume pixels to the corners and partial-volume pixels to the interior clusters of the convex hull.

Let $\left\{\mathbf{\mu}_{\mathbf{C}_{\text{measured}},m_{1}},...,\mathbf{\mu}_{\mathbf{C}_{\text{measured}},m_{J}}\right\}$ be any size-*J* subset of $\left\{\mathbf{\mu}_{\mathbf{C}_{\text{measured}},1},...,\mathbf{\mu}_{\mathbf{C}_{\text{measured}},M}\right\}$. Then, the margin (*i.e.*, distance) between $\mathbf{\mu}_{\mathbf{C}_{\text{measured}},m}$ and the convex hull is computed by

$\delta_{m,\left(m_{1},...,m_{J}\right)}=\min_{\alpha_{m_{1}},...\alpha_{m_{J}}} \left\|\mathbf{\mu}_{\mathbf{C}_{\text{measured}},m}-\sum_{j=1}^{J} \alpha_{m_{j}}\mathbf{\mu}_{\mathbf{C}_{\text{measured}},m_{j}}\right\|_{2},$ (S20)

where $\alpha_{m_{j}}\geq0,\text{ }\sum_{j=1}^{J} \alpha_{m_{j}}=1$. It shall be noted that if $\mathbf{\mu}_{\mathbf{C}_{\text{measured}},m}$ is inside then $\delta_{m,\left(m_{1},...,m_{J}\right)}=0$. Next, we define the convex-hull-to-data fitting error as the sum of the margin between the convex hull and the “exterior” cluster centers and detect the most probably *J* corners with cluster indices $\left(m_{1}^{*},...m_{J}^{*}\right)$ when the criterion function reaches its minimum:

$\left(m_{1}^{*},...m_{J}^{*}\right)\text{=}\mathop{\mathrm{argmin}}\limits_{\left(m_{1},...,m_{J}\right)} \sum_{m=1}^{M} \delta_{m,\left(m_{1},...,m_{J}\right)}$. (S21)

The optimization problems of (22) and (23) can be solved by advanced convex optimization procedure described in ^43^ and an exhaustive combinatorial search (for realistic values of *J* and *M*, in practice), respectively.

**Model selection procedure**.

One important issue concerning MTCM method is the detection of the structural parameter *J* in the model (the number of underlying tissue compartments or types), often called model selection [^8^](#_ENREF_8),[^17^](#_ENREF_17),[^18^](#_ENREF_18). This is indeed particularly critical in real-world applications where the true structure of the compartment models may be unknown a priori. We propose to use a widely-adopted and consistent information theoretic criterion, namely the minimum description length (MDL) [^8^](#_ENREF_8),[^12^](#_ENREF_12),[^17^](#_ENREF_17), to guide model selection. The major thrust of this approach is the formulation of a model fitting procedure in which an optimal model is selected from several competing candidates, such that the selected model best fits the observed data. MDL formulates the problem explicitly as an information coding problem in which the best model fit is measured such that it assigns high probabilities to the observed data while at the same time the model itself is not too complex to describe.

However, when the number of pixels is large as in DCE-MRI application, direct use of MDL may underestimate the value of *J*, due to the lack of “structure” in classical compartment models (over-parameterization) [^5^](#_ENREF_5),[^11^](#_ENREF_11),[^12^](#_ENREF_12). We therefore propose to naturally adopt and extend the clustered compartment models into the MDL formulation ^52^. The proposed clustered compartment model allows all pixels belonging to the same cluster to share a common $\mathbf{K}_{m}^{\text{trans}}$, thus greatly reducing model complexity for a given value of *J* (the number of convex hull corners). Specifically, a model is selected with *J* tissue compartments by minimizing the total description length defined by [^5^](#_ENREF_5),[^17^](#_ENREF_17)

(S22)

where denotes the joint likelihood function of the clustered compartment model, denotes the set of *M* cluster centers, and $\Theta\left(J\right)$ denotes the set of freely adjustable parameters in the clustered compartment model.

Our aim herein is to use MDL criterion [^5^](#_ENREF_5),[^17^](#_ENREF_17) and the MTCM estimates to select the best value of *J* automatically (the number of convex hull corners or tissue compartments). In the clustered *J*-tissue compartment model, we allow all pixels belonging to the same cluster to share a common local volume transfer constant, namely $\mathbf{K}_{m}^{\text{trans}}$ with length *J*, $m=1,...,M$. Letting $\mathbf{C}_{\text{measured},m}$ be the *m*th cluster center associated with $\mathbf{K}_{m}^{\text{trans}}$, from (4), we can express the clustered compartment model as follows:

$\mathbf{C}_{\text{measured},m}=\left[\mathbf{C}_{1},...,\mathbf{C}_{J}\right]\mathbf{K}_{m}^{\text{trans}}+\mathbf{n}$, (S23)

where $\mathbf{n}$ is the modeling residual noise assumed to follow zero-mean white Gaussian distribution $\mathbf{n}\sim N(\mathbf{0},\sigma^{2}\cdot\mathbf{I})$ with variance $\sigma^{2}$.

We specify $\Theta\left(J\right)$ as follows. From equations (3)-(4), $\mathbf{C}_{1},...,\mathbf{C}_{J-1}$ are parameterized by $k_{\text{ep},j},j=1,2,...,J-1$. Furthermore, $\mathbf{C}_{p}$ is parameterized by $\{\lambda_{1},\lambda_{2},\lambda_{3},\alpha_{2},\alpha_{3}\}$ based on the well-known exponential model [^11^](#_ENREF_11),[^19^](#_ENREF_19). Then, together with $\mathbf{K}_{1}^{\text{trans}},...,\mathbf{K}_{M}^{\text{trans}}$ and $\sigma$, we have $\Theta\left(J\right)=\{\lambda_{1},\lambda_{2},\lambda_{3},\alpha_{2},\alpha_{3},k_{\text{ep},1},k_{\text{ep},2},...k_{\text{ep},J-1},\mathbf{K}_{1}^{\text{trans}},...,\mathbf{K}_{M}^{\text{trans}},\sigma\}$.

Based on MTCM estimated $\mathbf{C}_{1},...,\mathbf{C}_{J}$ determined by $\hat{\mathbf{C}}_{p}$ and $\left\{\hat{k}_{\text{ep},j}, j=1,...,J-1\right\}$, and $\mathbf{K}_{m}^{\text{trans}}$ and $\sigma$ obtained by the maximum-likelihood estimation,

,

we can express the joint likelihood function in the MDL given by (S22) as

. (S24)

**Estimation of pharmacokinetics parameters in MTCM.**

Having determined the probabilistic pixel memberships associated with pure-volume compartments, $z_{ij}$ for $j=1,...,J$, $i=1,...,N$, we can then estimate the tissue-specific compartmental parameters, namely $K_{j}^{\text{trans}}$and $k_{\text{ep},j}$, $j=1,...,J$, directly from DCE-MRI pixel time series $\mathbf{C}_{\text{measured}}(i)$, in which various compartment modeling techniques can be readily applied.

To specify which “exterior” cluster is associated with which compartment, we investigate the temporal enhancement patterns of the “exterior” cluster centers. As aforementioned, $C_{p}$ is associated with the cluster of the fastest enhancement (reaching its peak most rapidly); $C_{j}$ is associated with the cluster of *j*th tissue type. We then compute $\mathbf{C}_{p}$ and $\mathbf{C}_{j}$ via

$\mathbf{C}_{p}=\frac{\sum_{i=1}^{N} z_{iJ}\mathbf{C}_{\text{measured}}(i)}{\sum_{i=1}^{N} z_{iJ}},\text{ }\mathbf{C}_{j}=\frac{\sum_{i=1}^{N} z_{ij}\mathbf{C}_{\text{measured}}(i)}{\sum_{i=1}^{N} z_{ij}}\text{, }j=1,...,J-1$ . (S25)

We then recall the relationship $C_{j}(t)=K_{j}^{\text{trans}}C_{p}(t)\otimes\exp(-k_{\text{ep},j}t)$, and discretize the convolution (with discretization interval $\Delta t$ (/min)) to the following vector-matrix notation

$\mathbf{C}_{\text{measured},j}=K_{j}^{\text{trans}}\mathbf{H}\left(k_{\text{ep},j}\right)\mathbf{C}_{p},\text{ }j=1,...,J-1$ (S26)

by constructing a Toeplitz matrix

$\mathbf{H}\left(k_{\text{ep},j}\right)=\left[\begin{matrix} e^{-k_{\text{ep},j}t_{1}} & 0 & 0 & ... & 0 \\ e^{-k_{\text{ep},j}t_{2}} & e^{-k_{\text{ep},j}t_{1}} & 0 & ... & 0 \\ ... & ... & ... & ... & ... \\ e^{-k_{\text{ep},j}t_{L}} & e^{-k_{\text{ep},j}t_{L-1}} & e^{-k_{\text{ep},j}t_{L-2}} & ... & e^{-k_{\text{ep},j}t_{1}} \end{matrix}\right]\Delta t$ (S27)

that is the sampled system impulse response. Then, the estimate of $k_{\text{ep},j}$ and $K_{j}^{\text{trans}}$ can be obtained by solving the following optimization problem

$\begin{matrix} \left\{\hat{k}_{\text{ep},j},\hat{K}_{j}^{\text{trans}}\right\}=\mathop{\mathrm{argmin}}\limits_{K_{f}^{\text{trans}},k_{\text{ep},f}} \left\|\mathbf{C}_{\text{measured},j}-K_{j}^{\text{trans}}\mathbf{H}\left(k_{\text{ep},j}\right)\mathbf{C}_{p}\right\|_{2} \\ \text{ }s.t. K_{j}^{\text{trans}}>0, k_{\text{ep},j}>0 \end{matrix}$ (S28)

for $j=1,...,J-1$. Finally, we can calculate the compartment TCs $\mathbf{C}\left(\mathbf{C}_{p},\hat{k}_{\text{ep},1},...,\hat{k}_{\text{ep},J-1}\right)$ based on $\mathbf{C}_{p}$and $\hat{k}_{\text{ep},1},...,\hat{k}_{\text{ep},J-1}$, and then estimate the local volume transfer constants $\mathbf{K}^{\text{trans}}(i)=\left[K_{1}^{\text{trans}}(i),...,K_{J-1}^{\text{trans}}(i),K_{p}(i)\right]^{T}$ based on equation (4) via

$\begin{matrix} \hat{\mathbf{K}}^{\text{trans}}(i)=\mathop{\mathrm{argmin}}\limits_{\mathbf{K}^{\text{trans}}\left(i\right)} \left\|\mathbf{C}_{\text{measured}}\left(i\right)-\mathbf{C}\left(\mathbf{C}_{p},\hat{k}_{\text{ep},1},...,\hat{k}_{\text{ep},J-1}\right)\mathbf{K}^{\text{trans}}\left(i\right)\right\|_{2} \\ \text{ }s.t. K_{1}^{\text{trans}}\left(i\right)\geq0,...,K_{J-1}^{\text{trans}}\left(i\right)\geq0,K_{p}\left(i\right)\geq0, \end{matrix}$ (S29)

that reflects the spatial heterogeneity of vascular permeability ^53^.

**References**

1. Kreso, A.*, et al.* Variable clonal repopulation dynamics influence chemotherapy response in colorectal cancer. *Science* **339**, 543-548 (2013).

2. Kim, E.*, et al.* Multiscale imaging and computational modeling of blood flow in the tumor vasculature. *Ann Biomed Eng* **40**(2012).

3. McDonald, D.M. & Choyke, P.L. Imaging of angiogenesis: from microscope to clinic. *Nat Med* **9**, 713-725 (2003).

4. Li, K.-L.*, et al.* Heterogeneity in the angiogenic response of a BT474 human breast cancer to a novel vascular endothelial growth factor-receptor tyrosine kinase inhibitor: Assessment by voxel analysis of dynamic contrast-enhanced MRI. *J Magn Reson Imaging* **22**, 511-519 (2005).

5. Hawkins, R.A., Phelps, M.E. & Huang, S.-C. Effects of temporal sampling, glucose metabolic rates, and disruptions of the blood-brain barrier on the FDG model with and without a vascular compartment: studies in human brain tumors with PET. *Journal of Cerehral Blood Flow and Metaholism* **6**, 170-183 (1986).

6. Chen, L.*, et al.* Tissue-specific compartmental analysis for dynamic contrast-enhanced MR imaging of complex tumors. *IEEE Trans Med Imaging* **30**, 2044-2058 (2011).

7. Cover, T.M. & Thomas, J.A. *Elements of Information Theory*, (Wiley-Interscience, New York, 2006).

8. Wax, M. & Kailath, T. Detection of signals by information theoretic criteria. *IEEE Trans Acoustics, Speech, and Signal Processing* **33**, 387-392 (1985).

9. Zhu, Y., Chan, T.-H., Hoffman, E.P. & Wang, Y. Gene expression dissection by non-negative well-grounded source separation. in *IEEE Intl Workshop on Machine Learning for Signal Processing* 255-260 (Cancún, Mexico, 2008).

10. Chen, L.*, et al.* Convex analysis and separation of composite signals in DCE-MRI. in *Biomedical Imaging: From Nano to Macro, 2008. ISBI 2008. 5th IEEE International Symposium on* 1557-1560 (2008).

11. Wang, Z.J., Zhu, H., Liu, K.J.R. & Wang, Y. Simultaneous estimation of kinetic parameters and the input function from DCE-MRI data: theory and simulation. in *Biomedical Imaging: Nano to Macro, 2004. IEEE International Symposium on*, Vol. 1 996-999 (2004).

12. Graham, M.W. & Miller, D.J. Unsupervised learning of parsimonious mixtures on large spaces with integrated feature and component selection. *IEEE Trans Signal Processing* **54**, 1289-1303 (2006).

13. Titterington, D.M., Smith, A.F.M. & Markov, U.E. *Statistical Analysis of Finite Mixture Distributions*, (John Wiley, New York, 1985).

14. Chen, S., A., B.C. & J., L.M. Clustered components analysis for functional MRI. *Medical Imaging, IEEE Transactions on* **23**, 85-98 (2004).

15. Wang, Y., Xuan, J., Srikanchana, R. & Choyke, P.L. Modeling and reconstruction of mixed functional and molecular patterns. *Intl. J. Biomed. Imaging*, ID29707 (2006).

16. Bayes, T. & Price, R. An Essay towards solving a Problem in the Doctrine of Chance. By the late Rev. Mr. Bayes, communicated by Mr. Price, in a letter to John Canton, A. M. F. R. S. *Philosophical Transactions of the Royal Society of London* **53**, 370-418 (1763).

17. Rissanen, J. Modeling by shortest data description. *Automatica* **14**, 465-471 (1978).

18. Wang, Y., Lin, S.H., Li, H. & Kung, S.Y. Data mapping by probabilistic modular networks and information theoretic criteria *IEEE Trans. Signal Processing* **46**, 3378-3397 (1998).

19. Riabkov, D.Y. & Di Bella, E.V.R. Estimation of kinetic parameters without input functions: analysis of three methods for multichannel blind identification. *Biomedical Engineering, IEEE Transactions on* **49**, 1318-1327 (2002).
