# Supplementary material for: Unsupervised Deconvolution of Dynamic Imaging Reveals Intratumor Vascular Heterogeneity and Repopulation Dynamics
Source: PLoS One. 2014 Nov 7;9(11):e112143. doi: 10.1371/journal.pone.0112143 (PMC4224420; doi:10.1371/journal.pone.0112143)
Supplement: Data S1 — Synthetic datasets generated for 12 parameter settings. (PDF) [file pone.0112143.s011.pdf]

Data S1

Demo_for_simulation.m:

```
clear;close all;clc;
load 'simdataS1SNR10'


%=================================================================
%----------------------CAM-------------------
[A_est S_est]=CAM(X_mask,3,1,0);
% ------------------end CAM-----------------
%=================================================================



%=================================================================
%-----Decide which curve correspond to which tissue type-----
K=3;
for k=1:K,
    [peak,peakIX(k)]=max(A_est(:,k));
end
[peakind,inds]=max(peakIX); %% the slow flow has latest come of peak
[peakind,indp]=min(peakIX); %% the input function has earliest come of
peak
indf=setdiff([1,2,3],[inds,indp]);
TC_f=A_est(:,indf);
TC_s=A_est(:,inds);
TC_p=A_est(:,indp);
TCs=[TC_f,TC_s,TC_p];
% ------show TCs-------------
h=figure;
x=[1:size(TC_p,1)]*0.5;   %0.5 minutes per frame
hl1 = line(x,TC_p,'LineStyle','--','Color','b');legend('input')
ax1 = gca;
set(ax1,'XColor','b','YColor','b')
xlabel('Time (minutes)')
ylabel('Tracer Concentration (mM/L)')
text(size(TC_p,1)/2,max(TC_p)/2,'Estimated TCs');%%to show title of
the figure
ax2 =
axes('Position',get(ax1,'Position'),'XAxisLocation','top','YAxisLocati
on','right','Color','none','XColor','r','YColor','r');
hl2 = line(x,TC_f,'Color','r','LineStyle','-','Parent',ax2);
hold on;
hl3 =
line(x,TC_s,'Color','r','LineStyle',':','Parent',ax2);legend('fast','s
low')
ylabel('Tracer Concentration (mM/L)')
saveas(h,'Estimated TCs.png')

h=figure;
hl1 = line(x,gtTCp,'LineStyle','--','Color','b');legend('input')
```

```
ax1 = gca;
set(ax1,'XColor','b','YColor','b')
xlabel('Time (minutes)')
ylabel('Tracer Concentration (mM/L)')
text(size(TC_p,1)/2,max(gtTCp)/2,'Ground Truth TCs');%%to show title
of the figure
ax2 =
axes('Position',get(ax1,'Position'),'XAxisLocation','top','YAxisLocati
on','right','Color','none','XColor','r','YColor','r');
hl2 = line(x,gtTCf,'Color','r','LineStyle','-','Parent',ax2);
hold on;
hl3 =
line(x,gtTCs,'Color','r','LineStyle',':','Parent',ax2);legend('fast','
slow')
ylabel('Tracer Concentration (mM/L)')
saveas(h,'Ground Truth TCs.png')
% --------------------end--------------------
%===============================================================


%===============================================================
% ----------------perform CM--------------------
% random initialization
kis=0.1*rand(1);
kos=kis+rand(1);
kif=0.1*rand(1);
kof=kif+rand(1);
initk=[kis,kos,kif,kof];
[eKtrans_s,eKep_s,eKtrans_f,eKep_f,pixelwise_Ktran]=CM(X_mask,TCs,init
k,del_t);
save simresult eKtrans_s eKep_s eKtrans_f eKep_f Ktrans_s Kep_s
Ktrans_f Kep_f
% ----------------end perform CM--------------------
% =============================================================
```

Ground truth TCs:

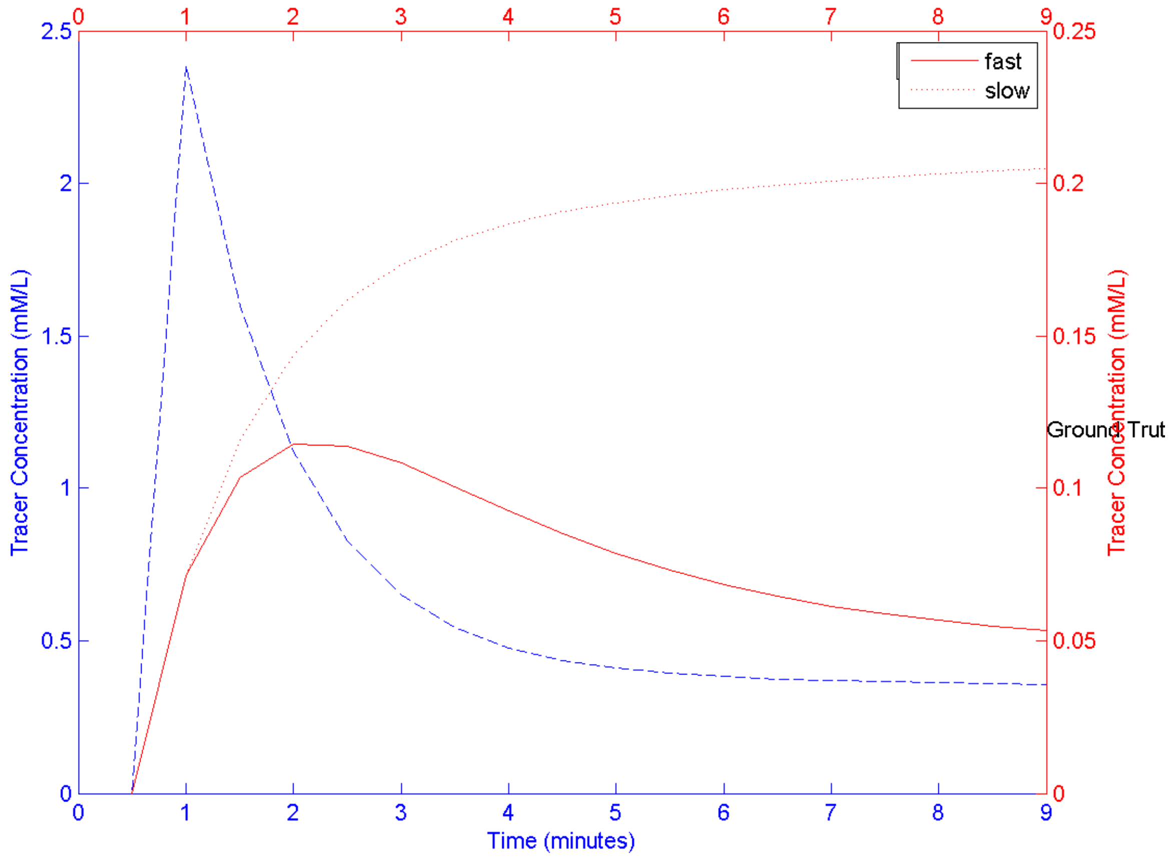

Estimated TCs

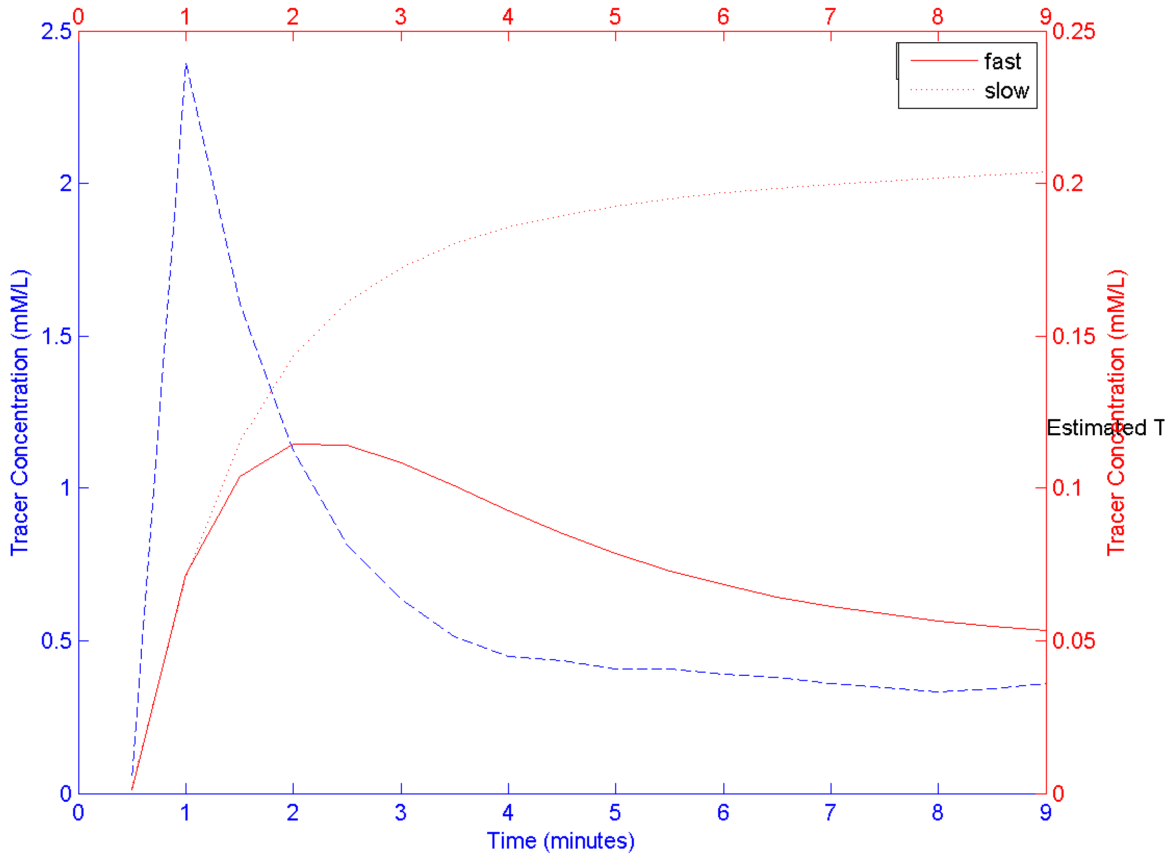

ROI_fast:

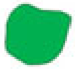

ROI_slow:

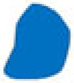

ROI_input

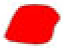

Simulation data S1SNR10 result:

Kep_f=0.5

Kep_s=0.1

Ktrans_f=0.03

Ktrans_s=0.03

eKep_f=0.4875

eKep_s=0.0962

eKtrans_f=0.0296

eKtrans_s=0.0296

simulator.m:

```
clear;clc
close all


% DCE-MRI data simulation, generate the data "simdata.mat"
% make sure you set the current directory to the one containing "Simulator.m"

% % % % % % % % ==============parameter setting
============================================
NumTframe=18;       % number of time frames for Dynamic imaging data
del_t=0.5;          % the sampling interval between time frames (/min)
SNR=10;             % the signal to noise ratio for the simulated data
Ktrans_s = 0.05;    % volume trasfer rate for slow flow
Kep_s =0.6;         % flux rate for slow flow
Ktrans_f = 0.08;    % volume trasfer rate for fast flow
Kep_f = 1.5;        % flux rate for fast flow
% Note: The spatial disbtribution of differnt tissue types are in the figures:
ROI_fast.jpg, ROI_slow.jpg, ROI_input.jpg
% % % % % % % % ==========================================================



%============distribution maps for different tissue types=============
%----------slow volume transfer
R1 = double(imread('ROI_fast.jpg'));  R1=R1(:,:,1)/255; [m n]=size(R1); R1 =
ones(m,n)-R1;
R1(find(R1>0.5))=1; R1(find(R1<=0.5))=0;
R1(R1==1) = imnoise(R1(R1==1),'gaussian',0,0.05);   %add gaussian noise on
the spatial distribution
%----------plasma volume
R2 = double(imread('ROI_input.jpg'));  R2=R2(:,:,2)/255; R2 = ones(m,n)-R2;
R2(find(R2>0.5))=1; R2(find(R2<=0.5))=0;
R2(R2==1) = imnoise(R2(R2==1),'gaussian',0,0.05);
%---------fast volume transfer
R3 = double(imread('ROI_slow.jpg')); R3=R3(:,:,1)/255; R3 = ones(m,n)-R3;
R3(find(R3>0.5))=1; R3(find(R3<=0.5))=0;
R3(R3==1) = imnoise(R3(R3==1),'gaussian',0,0.05);

%===========Time activity curves=============
%-------Plasma input function---------
time=0:0.5:15; % mintues
Cp=0.5*(3.99*exp(-1*time)+0.78*exp(-0.0111*time));
%-------Associated fast volume transfer compartment---------
Cf = Ktrans_f*conv(Cp,exp(-Kep_f*time));
Cf = Cf(1:length(time));
%-------Associated slow volume transfer compartment---------
Cs = Ktrans_s*conv(Cp,exp(-Kep_s*time));
Cs = Cs(1:length(time));

Cp=[0,Cp(1:NumTframe-1)];
Cs=[0,Cs(1:NumTframe-1)];
Cf=[0,Cf(1:NumTframe-1)];
```

```
% %-------show Cp, Cs, Cf-----------------
% timeslot=[0:NumTframe-1]*del_t;
% figure; plot(timeslot, Cp, '-b','LineWidth', 4);
% hold on; plot(timeslot, Cs,':g', 'LineWidth', 4);
% hold on; plot(timeslot, Cf,'--r','LineWidth', 4);
% legend ('plasma input','slow flow','fast flow', 'fontsize', 18, 1);
% xlabel('time (min)','fontsize', 18);
% ylabel('Tracer Concentration (mM/L)','fontsize', 18);
% set (gcf,'Position',[300,300,400,300], 'color','w');
% %-------show normalized Cp, Cs, Cf-----------------
% figure; plot(timeslot, Cp/sum(Cp), '-b','LineWidth', 4);
% hold on; plot(timeslot, Cs/sum(Cs),':g', 'LineWidth', 4);
% hold on; plot(timeslot, Cf/sum(Cf),'--r','LineWidth', 4);
% legend ('plasma input','slow flow','fast flow', 'fontsize', 18, 1);
% xlabel('time (min)','fontsize', 18);
% ylabel('Normalized TC','fontsize', 18);
% set(gca,'fontsize',18)


%========Data generation=========
sumR=R1(:)+R2(:)+R3(:)+eps;
S = [R1(:)'./sumR'; R2(:)'./sumR'; R3(:)'./sumR'];
A = [Cs' Cp' Cf'];
X = A*S;
%------------loading the mask------------------
index=find(sum(S,1)>0);
index=index';
N=length(index); % N is the number of the pixels in masked ROI
M=NumTframe;

X_mask=X(:,index);
Rn=eye(M);
alpha=0.9;
for i=1:M
    for j=1:M
        Rn(i,j)=alpha^(abs(i-j));
    end
end
R=chol(Rn);

for i=1:N,
    sigma=std(X_mask(:,i))*10^(-SNR/10);
    X_mask(:,i)=X_mask(:,i)+sigma*R'*randn(M,1);
end
X_mask(find(X_mask<0))=eps;
origX=X(:,index);
gtS=S(:,index);  gtSs=gtS(1,:); gtSp=gtS(2,:); gtSf=gtS(3,:);
gtTCp=Cp';gtTCf=Cf';gtTCs=Cs';
save simdata gtTCf gtTCs gtTCp gtSs gtSp gtSf del_t Ktrans_f Kep_f Ktrans_s
Kep_s X_mask
```

simulation data

```
#simdataS1SNR10:

Kep_f=0.5
Kep_f=0.5
Kep_s=0.1
Ktrans_f=0.03
Ktrans_s=0.03
del_t=0.5
gtSf=0          0          0          0          0          0
0          0          0          0          0          0
0          0          0          0          0          0
0          0          0          0          0          0
0          0          0          0          0          0
0          0          0          0          0          0
0          0          0          0          0          0
0          0          0          0          0          0
0          0          0          0          0          0
0          0          0          0          0          0
0          0          0          0          0          0
0          0          0          0          0          0
0          0          0          0          0          0
0          0          0          0          0          0
0          0          0          0          0          0
0          0          0          0          0          0
0          0          0          0          0          0
0          0          0          0          0          0
0          0          0          0          0          0
0          0          0          0          0          0
0          0          0          0          0          0
0          0          0          0          0          0
0          0          0          0          0          0
0          0          0          0          0          0
0          0          0          0          0          0
0          0          0          0          0          0
0          0          0          0          0          0
0          0          0          0          0          0
0          0          0          0          0          0
0          0          0          0          0          0
0          0          0          0          0          0
0          0          0          0          0          0
0          0          0          0          0          0
0          0          0          0          0          0
0          0          0     0.5593    0.35312    0.51442
0          0          0          0          0          0
0          0          0          0          0          0
0          0          0          0          0          0
0          0          0          0          0          0
```

0	0	0	0	0	0
0.5	0.46736	0.4634	0.5	0.52245	0.4886
0	0	0	0	0	0
0	0	0	0	0	0
0	0	0	0	0	0
0	0	0	0	0	0
0	0	0	0	0.49022	0.57984
0.41857	0.37374	0.5	0.54093	0.5	0.57661
0.5	0	0	0	0	0
0	0	0	0	0	0
0	0	0	0	0	0
0	0	0	0	0	0
0	0	0	0.49138	0.47198	0.5
0.58079	0.5	0.50888	0.56456	0.60103	0.53759
0.52786	0.54348	0.41996	0	0	0
0	0	0	0	0	0
0	0	0	0	0	0
0	0	0	0	0	0
0	0.47515	0.46177	0.48304	0.46769	0.50609
0.57877	0.5	0.4066	0.47317	0.45989	0.53221
0.5	0.49707	0.41003	0.57031	0	0
0	0	0	0	0	0
0	0	0	0	0	0
0	0	0	0	0	0
0.5	0.5085	0.5	0.46075	0.5342	0.49722
0.5	0.52192	0.45687	0.5	0.51491	0.40948
0.65548	0.58223	0.5	0.5	0.59748	0.5
0	0	0	0	0	0
0	0	0	0	0	0.34131
0.29458	0.30372	0.37115	0.37492	0.26713	0.38387
0.33803	0.44792	0.496	0.5	0.48695	0.46982
0.5	0.4778	0.50319	0.48983	0.56002	0.50014
0.35275	0.5	0.49097	0.5	0.5	0.44565
0.4977	0.48922	0	0	0	0
0	0	0	0	0	0.50705
0.30936	0.29768	0.28929	0.36855	0.33333	0.32306
0.25054	0.26664	0.33655	0.33728	0.5265	0.43687
0.39823	0.49444	0.48603	0.48632	0.5	0.5
0.50093	0.55883	0.23132	0.56067	0.50146	0.65757
0.48994	0.44525	0.5	0.51636	0	0
0	0	0	0	0	0.53001
0.509	0.49879	0.33333	0.36575	0.33633	0.27239
0.33453	0.33874	0.33333	0.33926	0.34324	0.28438
0.68184	0.45931	0.56814	0.5	0.52791	0.4179
0.5	0.46543	0.52031	0.5	0.49534	0.48094
0.42707	0.5	0.48776	0.45348	0.5	0.55252
0	0	0	0	0	0
0.55814	0.5	0.5	0.5	0.51276	0.27307
0.3564	0.33333	0.32483	0.33726	0.36491	0.3364
0.42017	0.34039	0.44679	0.51322	0.59962	0.38591
0.43854	0.5	0.46699	0.5426	0.5	0.51752
0.44191	0.58394	0.46671	0.417	0.5	0.56451

0.5	0.5	0	0	0	0
0	0.45399	0.55224	0.5	0.40293	0.46445
0.44886	0.31825	0.40739	0.26596	0.33707	0.38413
0.35651	0.28775	0.38273	0.3526	0.45907	0.5
0.46707	0.38639	0.42286	0.5	0.41797	0.5
0.5	0.51749	0.5	0.39871	0.56759	0.50127
0.50829	0.55528	0.5	0.49056	0	0
0	0.46695	0.5	0.52821	0.44401	0.41725
0.5	0.44412	0.51833	0.33333	0.34242	0.27339
0.31995	0.36287	0.35858	0.36496	0.30718	0.34928
0.54226	0.52175	0.52146	0.46746	0.5632	0.5
0.5	0.37305	0.5	0.58453	0.52485	0.5326
0.53875	0.5331	0.55172	0.44858	0.5	0.45056
0	0.35561	0.46489	0.5	0.48327	0.41698
0.54751	0.5	0.48743	0.46501	0.34119	0.38883
0.20008	0.35015	0.28411	0.2614	0.26973	0.45708
0.32724	0.47467	0.5	0.49999	0.53253	0.50152
0.46812	0.53366	0.48796	0.44676	0.46675	0.33116
0.53908	0.38963	0.46469	0.50585	0.47543	0.48188
1	0.50574	0.47828	0.51905	0.42334	0.42155
0.5	0.52639	0.62511	0.5	0.53928	0.30627
0.3109	0.33811	0.3571	0.35143	0.32371	0.25506
0.32528	0.34024	0.48026	0.5	0.5	0.41324
0.50672	0.46018	0.49728	0.50519	0.52716	0.59241
0.48647	0.46144	0.55328	0.48448	0.43001	0.53994
0.49547	1	0.5	0.5	0.51416	0.50594
0.4961	0.47434	0.48871	0.5	0.5	0.52609
0.30335	0.25654	0.39804	0.38267	0.40913	0.21581
0.37017	0.34479	0.52286	0.5	0.5249	0.54172
0.64004	0.49975	0.55522	0.52135	0.49118	0.45786
0.38338	0.41524	0.52251	0.49164	0.5	0.48189
0.51867	0.54541	1	0.46733	0.5	0.47521
0.52	0.48142	0.45067	0.47865	0.6147	0.53689
0.43313	0.27266	0.40651	0.34571	0.34031	0.32909
0.26615	0.30899	0.31487	0.48655	0.57853	0.5
0.57737	0.52092	0.5	0.5	0.48164	0.2879
0.4749	0.4967	0.55365	0.54106	0.4772	0.56969
0.43741	0.41026	1	1	1	0.55253
0.44011	0.45628	0.50539	0.49549	0.81754	0.61035
0.52339	0.5	0.33333	0.33333	0.36645	0.22683
0.30328	0.33333	0.63393	0.5	0.5	0.5
0.49157	0.5	0.44523	0.62166	0.5	0.54115
0.57537	0.34631	0.47505	0.5	0.56466	0.49448
0.53735	0.5	1	1	1	1
0.42914	0.5	0.5	0.44567	0.49111	0.5
0.5	0.5	0.5	0.30146	0.29276	0.33787
0.50187	0.50146	0.12723	0.53647	0.43661	0.5
0.54198	0.5	0.51439	0.5576	0.37509	0.45721
0.5	0.55761	0.44074	0.5	0.53646	0.45554
0.5	0.58788	1	1	1	1
1	1	0.50281	0.5	0.52082	0.4906
0.5	0.5	0.46119	0.47794	0.43427	0.5

0.4783	0.50882	0.51741	0.40747	0.5	0.5
0.5	0.53779	0.44735	0.65213	0.36086	0.60575
0.51519	0.47514	0.34258	0.49384	0.5038	0.52425
0.5	0.45202	0.6917	1	1	1
1	1	1	0.40918	0.5	0.5
0.5	0.5	1	1	0.48122	0.598
0.54879	0.494	0.54911	0.5	0.60735	0.57373
0.43443	0.5	0.5	0.4273	0.5	0.51327
0.49877	0.51953	0.5	0.47661	0.46599	0.51107
0.62196	1	1	1	1	1
1	1	1	1	1	0.60315
0.50762	1	1	1	1	0.43168
0.5583	0.48139	0.4727	0.36067	0.39925	0.39244
0.49449	0.5	0.549	0.57531	0.5	0.48595
0.49116	0.57761	0.63776	0.46638	0.5	0.48241
1	1	1	1	1	1
1	1	1	1	1	1
1	1	1	1	1	0.34648
0.47105	0.55691	0.5	0.57461	0.5	0.61161
0.5	0.6051	0.55362	0.5	0.51629	0.5
0.5	0.5	0.51336	1	1	1
1	1	1	1	1	1
1	1	1	1	1	1
1	1	1	1	0.43028	0.48971
0.52856	0.48393	0.50813	0.57845	0.37024	0.56798
0.26832	0.49586	0.5	0.55188	0.48305	1
1	1	1	1	1	1
1	1	1	1	1	1
1	1	1	1	1	1
1	1	0.5	0.5	0.5	0.45052
0.50225	0.49206	0.49959	0.5	0.44081	1
1	1	1	1	1	1
1	1	1	1	1	1
1	1	1	1	1	1
1	1	1	1	1	1
0.54256	0.46966	0.50318	0.49809	1	1
1	1	1	1	1	1
1	1	1	1	1	1
1	1	1	1	1	1
1	1	1	1	1	1
1	1	1	1	1	1
1	1	1	1	1	1
1	1	1	1	1	1
1	1	1	1	1	1
1	1	1	1	1	1
1	1	1	1	1	1
1	1	1	1	1	1
1	1	1	1	1	1
1	1	1	1	1	1
1	1	1	1	1	1
1	1	1	1	1	1
1	1	1	1	1	1

1	1	1	1	1	1
1	1	1	1	1	1
1	1	1	1	1	1
1	1	1	1	1	1
1	1	1	1	1	1
1	1	1	1	1	1
1	1	1	1	1	1
1	1	1	1	1	1
1	1	1	1	1	1
1	1	1	1	1	1
1	1	1	1	1	1
1	1	1	1	1	1
1	1	1	1	1	1
1	1	1	1	1	1
1	1	1	1	1	1
1	1	1	1	1	1
1					
gtSs=1	1	1	1	1	1
1	1	1	1	1	1
1	1	1	1	1	1
1	1	0.42836	0.4788	0.47053	0.50637
1	1	1	1	1	1
1	1	1	1	1	1
1	1	1	0	0.49645	0.49671
0.50492	0.52901	0.5	0.49199	1	1
1	1	1	1	1	1
1	1	1	1	1	1
1	0	0	0.54259	0.4154	0.5
0.46973	0.48501	0.43905	0.66366	0.4839	1
1	1	1	1	1	1
1	1	1	1	1	1
1	1	0	0	0	0.51849
0.48634	0.58601	0.50608	0.5	0.56976	0.67163
0.49326	0.5	1	1	1	1
1	1	1	1	1	1
1	1	1	1	1	1
0	0	0	0	0.41688	0.60648
0.59026	0.5032	0.5	0.5252	0.47334	0.5
0.49158	0.49133	1	1	1	1
1	1	1	1	1	1
1	1	1	1	1	1
0	0	0	0	0	0
0.48372	0.43016	0.55212	0.49644	0.5	0.5
0.5	0.5	0.52921	0.41594	1	1
1	1	1	1	1	1
1	1	1	1	1	1
1	1	1	0	0	0
0	0	0	0.66941	0.5	0.56393
0.5	0.49266	0.54063	0.53974	0.44355	0.52109
0.47686	0.4557	1	1	1	1
1	1	1	1	1	1
1	1	1	0.4407	0.64688	0.48558

1	0	0	0	0	0
0	0	0.46085	0.50722	0.38179	0.4921
0.4461	0.40522	0.44416	0.58802	0.41052	0.48945
0.53199	1	1	1	1	1
1	1	1	1	1	1
0.5	0.53264	0.5366	0.5	0.47755	0.5114
1	0	0	0	0	0
0	0	0.5	0.40446	0.41206	0.4912
0.5	0.5	0.5	0.50185	0.5	0.50788
0.54295	1	1	1	1	1
1	1	1	1	0.50978	0.42016
0.58143	0.62626	0.5	0.45907	0.5	0.42339
0.5	1	0	0	0	0
0	0	0	0.54929	0.49298	0.5
0.55717	0.5	0.50252	0.5	0.3353	0.5
0.51478	0.5055	0.38605	1	1	1
1	1	1	0.50862	0.52802	0.5
0.41921	0.5	0.49112	0.43544	0.39897	0.46241
0.47214	0.45652	0.58004	0	0	0
0	0	0	0	0.70065	0.43767
0.52246	0.5	0.39289	0.52409	0.45618	0.57275
0.51302	0.5	0.5	0.50502	1	1
1	0.52485	0.53823	0.51696	0.53231	0.49391
0.42123	0.5	0.5934	0.52683	0.54011	0.46779
0.5	0.50293	0.58997	0.42969	0	0
0	0	0	0	0	0
0	0.4412	0.45804	0.56263	0.49764	0.5
0.50105	0.49794	0.49742	0.49911	0.47735	0.52504
0.5	0.4915	0.5	0.53925	0.4658	0.50278
0.5	0.47808	0.54313	0.5	0.48509	0.59052
0.34452	0.41777	0.5	0.5	0.40252	0.5
0	0	0	0	0	0
0	0	0	0.58635	0.45237	0.26797
0.37019	0.34814	0.29133	0.29178	0.36643	0.33279
0.28566	0.23479	0.504	0.5	0.51305	0.53018
0.5	0.5222	0.49681	0.51017	0.43998	0.49986
0.64725	0.5	0.50903	0.5	0.5	0.55435
0.5023	0.51078	0	0	0	0
0	0	0	0	0	0
0.34532	0.35116	0.3535	0.25942	0.33333	0.33847
0.35406	0.42044	0.30239	0.32544	0.4735	0.56313
0.60177	0.50556	0.51397	0.51368	0.5	0.5
0.49907	0.44117	0.76868	0.43933	0.49854	0.34243
0.51006	0.55475	0.5	0.48364	0	0
0	0	0	0	0	0
0	0	0.33333	0.2685	0.33633	0.35482
0.3434	0.34484	0.33333	0.31669	0.31351	0.35781
0.31816	0.54069	0.43186	0.5	0.47209	0.5821
0.5	0.53457	0.47969	0.5	0.50466	0.51906
0.57293	0.5	0.51224	0.54652	0.5	0.44748
0	0	0	0	0	0
0	0	0	0	0	0.36346

0.35794	0.33333	0.3204	0.32549	0.27019	0.3364
0.33417	0.34039	0.55321	0.48678	0.40038	0.61409
0.56146	0.5	0.53301	0.4574	0.5	0.48248
0.55809	0.41606	0.53329	0.583	0.5	0.43549
0.5	0.5	0	0	0	0
0	0	0	0	0	0
0	0.37604	0.37413	0.36702	0.32586	0.33673
0.35651	0.41616	0.24485	0.21336	0.54093	0.5
0.53293	0.61361	0.57714	0.5	0.58203	0.5
0.5	0.48251	0.5	0.60129	0.43241	0.49873
0.49171	0.44472	0.5	0.50944	0	0
0	0	0	0	0	0
0	0	0	0.33333	0.41636	0.3633
0.36755	0.34828	0.22236	0.23084	0.35626	0.30144
0.45774	0.47825	0.47854	0.53254	0.4368	0.5
0.5	0.62695	0.5	0.41547	0.47515	0.4674
0.46125	0.4669	0.44828	0.55142	0.5	0.54944
0	0	0	0	0	0
0	0	0	0	0.30128	0.28575
0.34876	0.35015	0.3774	0.36469	0.41159	0.18269
0.33638	0.52533	0.5	0.50001	0.46747	0.49848
0.53188	0.46634	0.51204	0.55324	0.53325	0.66884
0.46092	0.61037	0.53531	0.49415	0.52457	0.51812
0	0	0	0	0	0
0	0	0	0	0	0.34384
0.40322	0.33516	0.33796	0.29715	0.38546	0.37247
0.33736	0.31952	0.51974	0.5	0.5	0.58676
0.49328	0.53982	0.50272	0.49481	0.47284	0.40759
0.51353	0.53856	0.44672	0.51552	0.56999	0.46006
0.50453	0	0	0	0	0
0	0	0	0	0	0
0.34637	0.37173	0.29195	0.38267	0.38562	0.34992
0.33838	0.31042	0.47714	0.5	0.4751	0.45828
0.35996	0.50025	0.44478	0.47865	0.50882	0.54214
0.61662	0.58476	0.47749	0.50836	0.5	0.51811
0.48133	0.45459	0	0	0	0
0	0	0	0	0	0
0	0.32829	0.36191	0.34416	0.34031	0.35558
0.36772	0.3455	0.36911	0.51345	0.42147	0.5
0.42263	0.47908	0.5	0.5	0.51836	0.7121
0.5251	0.5033	0.44635	0.45894	0.5228	0.43031
0.56259	0.58974	0	0	0	0
0	0	0	0	0	0
0	0	0.33333	0.33333	0.36645	0.38785
0.34836	0.33333	0.36607	0.5	0.5	0.5
0.50843	0.5	0.55477	0.37834	0.5	0.45885
0.42463	0.65369	0.52495	0.5	0.43534	0.50552
0.46265	0.5	0	0	0	0
0	0	0	0	0	0
0	0	0	0.34933	0.36768	0.32385
0.49813	0.49854	0.87277	0.46353	0.56339	0.5
0.45802	0.5	0.48561	0.4424	0.62491	0.54279

0.5	0.44239	0.55926	0.5	0.46354	0.54446
0.5	0.41212	0	0	0	0
0	0	0	0	0	0
0	0	0	0	0.56573	0.5
0.5217	0.49118	0.48259	0.59253	0.5	0.5
0.5	0.46221	0.55265	0.34787	0.63914	0.39425
0.48481	0.52486	0.65742	0.50616	0.4962	0.47575
0.5	0.54798	0.3083	0	0	0
0	0	0	0	0	0
0	0	0	0	0.51878	0.402
0.45121	0.506	0.45089	0.5	0.39265	0.42627
0.56557	0.5	0.5	0.5727	0.5	0.48673
0.50123	0.48047	0.5	0.52339	0.53401	0.48893
0.37804	0	0	0	0	0
0	0	0	0	0	0
0	0	0	0	0	0.56832
0.4417	0.51861	0.5273	0.63933	0.60075	0.60756
0.50551	0.5	0.451	0.42469	0.5	0.51405
0.50884	0.42239	0.36224	0.53362	0.5	0.51759
0	0	0	0	0	0
0	0	0	0	0	0
0	0	0	0	0	0.65352
0.52895	0.44309	0.5	0.42539	0.5	0.38839
0.5	0.3949	0.44638	0.5	0.48371	0.5
0.5	0.5	0.48664	0	0	0
0	0	0	0	0	0
0	0	0	0	0	0
0	0	0	0	0.56972	0.51029
0.47144	0.51607	0.49187	0.42155	0.62976	0.43202
0.73168	0.50414	0.5	0.44812	0.51695	0
0	0	0	0	0	0
0	0	0	0	0	0
0	0	0	0	0	0
0	0	0.5	0.5	0.5	0.54948
0.49775	0.50794	0.50041	0.5	0.55919	0
0	0	0	0	0	0
0	0	0	0	0	0
0	0	0	0	0	0
0	0	0	0	0	0
0.45744	0.53034	0.49682	0.50191	0	0
0	0	0	0	0	0
0	0	0	0	0	0
0	0	0	0	0	0
0	0	0	0	0	0
0	0	0	0	0	0
0	0	0	0	0	0
0	0	0	0	0	0
0	0	0	0	0	0
0	0	0	0	0	0
0	0	0	0	0	0
0	0	0	0	0	0
0	0	0	0	0	0

```
0 0 0 0 0 0
0 0 0 0 0 0
0 0 0 0 0 0
0 0 0 0 0 0
0 0 0 0 0 0
0 0 0 0 0 0
0 0 0 0 0 0
0 0 0 0 0 0
0 0 0 0 0 0
0 0 0 0 0 0
0 0 0 0 0 0
0 0 0 0 0 0
0 0 0 0 0 0
0 0 0 0 0 0
0 0 0 0 0 0
0 0 0 0 0 0
0 0 0 0 0 0
0 0 0 0 0 0
0 0 0 0 0 0
0 0 0 0 0 0
0
gtSp=0 0 0 0 0 0
0 0 0 0 0 0
0 0 0 0 0 0
0 0 0.57164 0.5212 0.52947 0.49363
0 0 0 0 0 0
0 0 0 0 0 0
0 0 0 1 0.50355 0.50329
0.49508 0.47099 0.5 0.50801 0 0
0 0 0 0 0 0
0 0 0 0 0 0
0 1 1 0.45741 0.5846 0.5
0.53027 0.51499 0.56095 0.33634 0.5161 0
0 0 0 0 0 0
0 0 0 0 0 0
0 0 1 1 1 0.48151
0.51366 0.41399 0.49392 0.5 0.43024 0.32837
0.50674 0.5 0 0 0 0
0 0 0 0 0 0
0 0 0 0 0 0
1 1 1 1 0.58312 0.39352
0.40974 0.4968 0.5 0.4748 0.52666 0.5
0.50842 0.50867 0 0 0 0
0 0 0 0 0 0
0 0 0 0 0 0
1 1 1 1 1 1
0.51628 0.56984 0.44788 0.50356 0.5 0.5
0.5 0.5 0.47079 0.58406 0 0
0 0 0 0 0 0
0 0 0 0 0 0
0 0 0 1 1 1
1 1 1 0.33059 0.5 0.43607
```

0.5	0.50734	0.45937	0.46026	0.55645	0.47891
0.52314	0.5443	0	0	0	0
0	0	0	0	0	0
0	0	0	0	0	0
0	1	1	1	1	1
1	1	0.53915	0.49278	0.61821	0.5079
0.5539	0.59478	0.55584	0.41198	0.58948	0.51055
0.46801	0	0	0	0	0
0	0	0	0	0	0
0	0	0	0	0	0
0	1	1	1	1	1
1	1	0.5	0.59554	0.58794	0.5088
0.5	0.5	0.5	0.49815	0.5	0.49212
0.45705	0	0	0	0	0
0	0	0	0	0	0
0	0	0	0	0	0
0	0	1	1	1	1
1	1	1	0.45071	0.50702	0.5
0.44283	0.5	0.49748	0.5	0.6647	0.5
0.48522	0.4945	0.61395	0	0	0
0	0	0	0	0	0
0	0	0	0	0	0
0	0	0	1	1	1
1	1	1	1	0.29935	0.56233
0.47754	0.5	0.60711	0.47591	0.54382	0.42725
0.48698	0.5	0.5	0.49498	0	0
0	0	0	0	0	0
0	0	0	0	0	0
0	0	0	0	1	1
1	1	1	1	1	1
1	0.5588	0.54196	0.43737	0.50236	0.5
0.49895	0.50206	0.50258	0.50089	0.52265	0.47496
0	0	0	0	0	0
0	0	0	0	0	0
0	0	0	0	0	0
1	1	1	1	1	1
1	1	1	0.41365	0.54763	0.39072
0.33523	0.34814	0.33752	0.3333	0.36643	0.28335
0.37631	0.31729	0	0	0	0
0	0	0	0	0	0
0	0	0	0	0	0
0	0	1	1	1	1
1	1	1	1	1	0.49295
0.34532	0.35116	0.35722	0.37203	0.33333	0.33847
0.3954	0.31293	0.36106	0.33728	0	0
0	0	0	0	0	0
0	0	0	0	0	0
0	0	0	0	1	1
1	1	1	1	1	0.46999
0.491	0.50121	0.33333	0.36575	0.32734	0.37279
0.32207	0.31642	0.33333	0.34405	0.34324	0.35781
0	0	0	0	0	0

0	0	0	0	0	0
0	0	0	0	0	0
1	1	1	1	1	1
0.44186	0.5	0.5	0.5	0.48724	0.36346
0.28566	0.33333	0.35477	0.33726	0.36491	0.3272
0.24566	0.31921	0	0	0	0
0	0	0	0	0	0
0	0	0	0	0	0
0	0	1	1	1	1
1	0.54601	0.44776	0.5	0.59707	0.53555
0.55114	0.30571	0.21848	0.36702	0.33707	0.27914
0.28697	0.29609	0.37242	0.43404	0	0
0	0	0	0	0	0
0	0	0	0	0	0
0	0	0	0	1	1
1	0.53305	0.5	0.47179	0.55599	0.58275
0.5	0.55588	0.48167	0.33333	0.24121	0.3633
0.3125	0.28885	0.41906	0.4042	0.33656	0.34928
0	0	0	0	0	0
0	0	0	0	0	0
0	0	0	0	0	0
1	0.64439	0.53511	0.5	0.51673	0.58302
0.45249	0.5	0.51257	0.53499	0.35753	0.32541
0.45116	0.2997	0.3385	0.37391	0.31868	0.36023
0.33638	0	0	0	0	0
0	0	0	0	0	0
0	0	0	0	0	0
0	0.49426	0.52172	0.48095	0.57666	0.57845
0.5	0.47361	0.37489	0.5	0.46072	0.34989
0.28588	0.32673	0.30494	0.35143	0.29082	0.37247
0.33736	0.34024	0	0	0	0
0	0	0	0	0	0
0	0	0	0	0	0
0	0	0.5	0.5	0.48584	0.49406
0.5039	0.52566	0.51129	0.5	0.5	0.47391
0.35029	0.37173	0.31002	0.23465	0.20525	0.43427
0.29145	0.34479	0	0	0	0
0	0	0	0	0	0
0	0	0	0	0	0
0	0	0	0.53267	0.5	0.52479
0.48	0.51858	0.54933	0.52135	0.3853	0.46311
0.56687	0.39905	0.23158	0.31013	0.31939	0.31532
0.36613	0.3455	0.31602	0	0	0
0	0	0	0	0	0
0	0	0	0	0	0
0	0	0	0	0	0.44747
0.55989	0.54372	0.49461	0.50451	0.18246	0.38965
0.47661	0.5	0.33333	0.33333	0.2671	0.38531
0.34836	0.33333	0	0	0	0
0	0	0	0	0	0
0	0	0	0	0	0
0	0	0	0	0	0

0.57086	0.5	0.5	0.55433	0.50889	0.5
0.5	0.5	0.5	0.34921	0.33956	0.33828
0	0	0	0	0	0
0	0	0	0	0	0
0	0	0	0	0	0
0	0	0	0	0	0
0	0	0.49719	0.5	0.47918	0.5094
0.5	0.5	0.53881	0.52206	0	0
0	0	0	0	0	0
0	0	0	0	0	0
0	0	0	0	0	0
0	0	0	0	0	0
0	0	0	0.59082	0.5	0.5
0.5	0.5	0	0	0	0
0	0	0	0	0	0
0	0	0	0	0	0
0	0	0	0	0	0
0	0	0	0	0	0
0	0	0	0	0	0.39685
0.49238	0	0	0	0	0
0	0	0	0	0	0
0	0	0	0	0	0
0	0	0	0	0	0
0	0	0	0	0	0
0	0	0	0	0	0
0	0	0	0	0	0
0	0	0	0	0	0
0	0	0	0	0	0
0	0	0	0	0	0
0	0	0	0	0	0
0	0	0	0	0	0
0	0	0	0	0	0
0	0	0	0	0	0
0	0	0	0	0	0
0	0	0	0	0	0
0	0	0	0	0	0
0	0	0	0	0	0
0	0	0	0	0	0
0	0	0	0	0	0
0	0	0	0	0	0
0	0	0	0	0	0
0	0	0	0	0	0
0	0	0	0	0	0
0	0	0	0	0	0
0	0	0	0	0	0
0	0	0	0	0	0
0	0	0	0	0	0
0	0	0	0	0	0
0	0	0	0	0	0
0	0	0	0	0	0
0	0	0	0	0	0
0	0	0	0	0	0

0	0	0	0	0	0
0	0	0	0	0	0
0	0	0	0	0	0
0	0	0	0	0	0
0	0	0	0	0	0
0	0	0	0	0	0
0	0	0	0	0	0
0	0	0	0	0	0
0	0	0	0	0	0
0	0	0	0	0	0
0	0	0	0	0	0
0	0	0	0	0	0
0	0	0	0	0	0
0	0	0	0	0	0
0	0	0	0	0	0
0	0	0	0	0	0
0	0	0	0	0	0
0	0	0	0	0	0
0	0	0	0	0	0
0	0	0	0	0	0
0	0	0	0	0	0
0	0	0	0	0	0
0	0	0	0	0	0
0	0	0	0	0	0
0					

gtTCf=
0
0.07155
0.10366
0.11432
0.11389
0.10824
0.10059
0.092638
0.085208
0.078648
0.073046
0.06836
0.06449
0.06132
0.058731
0.05662
0.054894
0.053478

gtTCs=
0
0.07155
0.116
0.14393
0.16177

0.17342
0.18126
0.18671
0.19067
0.19366
0.19601
0.19792
0.19952
0.20088
0.20206
0.20309
0.20398
0.20476

gtTCp=
0
2.385
1.5979
1.1196
0.8287
0.65143
0.54309
0.47655
0.43538
0.4096
0.39316
0.38239
0.37506
0.36982
0.36585
0.36266
0.35995
0.35753

X_mask=
0.0022484,2.2204e-016,0.0033748,2.2204e-016,2.2204e-016,2.2204e-016,2.2204e-016,2.2204e-016,0.0033904,0.0072767,2.2204e-016,0.0025844,2.2204e-016,2.2204e-016,2.2204e-016,2.2204e-016,0.0081626,2.2204e-016,0.0040083,2.2204e-016,2.2204e-016,0.0689,2.2204e-016,0.025188,0.00090851,0.0040193,0.0032049,2.2204e-016,2.2204e-016,2.2204e-016,2.2204e-016,0.0020517,0.0091332,0.0051866,0.0072337,2.2204e-016,2.2204e-016,2.2204e-016,0.0028636,0.064381,2.2204e-016,2.2204e-016,2.2204e-016,2.2204e-016,0.027218,0.023869,0.001601,0.0013081,2.2204e-016,2.2204e-016,0.0038673,0.0015719,0.0039682,2.2204e-016,2.2204e-016,2.2204e-016,0.0032644,2.2204e-016,0.0030937,0.0026613,2.2204e-016,0.091546,2.2204e-016,0.0070206,0.018826,2.2204e-016,0.036677,0.012133,2.2204e-016,2.2204e-016,0.038699,0.0077043,2.2204e-016,0.0044587,0.00065803,2.2204e-016,0.0070225,2.2204e-016,0.0019301,2.2204e-016,0.0073831,0.0026638,0.00035379,2.2204e-016,0.0048386,2.2204e-

016,2.2204e-016,2.2204e-016,2.2204e-016,2.2204e-
016,0.029546,0.01737,2.2204e-016,2.2204e-016,2.2204e-016,2.2204e-
016,0.011752,0.078041,0.010882,2.2204e-016,0.0022451,2.2204e-
016,2.2204e-016,2.2204e-016,2.2204e-016,0.0059298,2.2204e-
016,0.00074562,0.00010981,0.0042638,0.0012151,0.0031365,0.0054541,0.00
50368,0.025076,2.2204e-016,0.080776,2.2204e-016,2.2204e-
016,0.0032023,2.2204e-016,2.2204e-016,0.0020948,2.2204e-016,2.2204e-
016,2.2204e-016,0.0067711,2.2204e-016,2.2204e-016,2.2204e-
016,0.0060687,0.0014114,2.2204e-016,2.2204e-016,2.2204e-016,2.2204e-
016,0.0055236,0.0027509,2.2204e-016,0.0023322,0.0030161,2.2204e-
016,2.2204e-016,2.2204e-016,0.020995,2.2204e-016,0.086036,2.2204e-
016,2.2204e-016,2.2204e-
016,0.025459,0.0058511,0.032885,0.011673,2.2204e-
016,0.020375,0.028986,2.2204e-016,0.011804,0.031125,2.2204e-
016,2.2204e-016,0.0056297,2.2204e-016,0.0047471,2.2204e-016,2.2204e-
016,0.0040121,0.0035533,0.0018302,2.2204e-
016,0.00071181,0.0026275,2.2204e-016,2.2204e-016,2.2204e-016,2.2204e-
016,2.2204e-016,2.2204e-016,2.2204e-016,2.2204e-
016,0.044698,0.074097,2.2204e-016,0.024473,2.2204e-
016,0.024806,0.0056565,2.2204e-016,0.033305,2.2204e-
016,0.024244,2.2204e-016,2.2204e-016,2.2204e-
016,0.001566,0.0044695,0.0035469,2.2204e-016,2.2204e-016,4.6725e-
005,2.2204e-016,2.2204e-016,2.2204e-016,2.2204e-
016,0.0020137,0.0056467,0.0021767,2.2204e-016,2.2204e-016,2.2204e-
016,2.2204e-016,2.2204e-016,2.2204e-016,0.050388,2.2204e-
016,0.011187,2.2204e-016,0.0091767,0.02253,2.2204e-
016,0.017905,2.2204e-
016,0.0052956,0.0074485,0.024828,0.015581,0.0077825,0.041253,2.2204e-
016,2.2204e-016,2.2204e-016,2.2204e-016,0.010104,2.2204e-
016,0.0095448,0.004941,2.2204e-016,0.0011046,2.2204e-016,2.2204e-
016,2.2204e-016,0.0040279,2.2204e-016,2.2204e-016,0.0032189,2.2204e-
016,2.2204e-016,0.10436,0.025494,2.2204e-016,2.2204e-
016,0.039705,2.2204e-016,0.023373,0.012988,0.025176,2.2204e-
016,0.015912,2.2204e-016,2.2204e-
016,0.012266,0.014304,0.0067384,0.0047374,2.2204e-016,2.2204e-
016,2.2204e-016,0.0014952,2.2204e-
016,0.0025421,0.01094,0.0003749,0.0063387,2.2204e-016,2.2204e-
016,2.2204e-016,2.2204e-016,0.0085379,0.0011607,2.2204e-
016,0.0031604,2.2204e-016,0.0090529,0.0055232,2.2204e-
016,0.035073,0.0060492,2.2204e-016,2.2204e-
016,0.0011137,0.0082315,2.2204e-016,0.01764,2.2204e-
016,0.024236,0.0051028,2.2204e-016,0.011648,0.031404,2.2204e-
016,2.2204e-016,2.2204e-016,2.2204e-016,0.0059022,2.2204e-016,2.2204e-
016,0.0038284,0.0073895,2.2204e-
016,0.0063664,0.0047578,0.0016242,0.0021264,2.2204e-
016,0.0014513,2.2204e-016,2.2204e-016,2.2204e-016,2.2204e-
016,0.0029392,2.2204e-016,2.2204e-016,0.019961,0.050624,2.2204e-
016,2.2204e-016,0.079524,2.2204e-016,2.2204e-016,0.021995,2.2204e-
016,2.2204e-016,2.2204e-016,0.0044307,2.2204e-016,2.2204e-
016,0.025154,2.2204e-016,2.2204e-016,2.2204e-016,2.2204e-016,2.2204e-
016,0.002227,0.0080358,0.001333,2.2204e-016,2.2204e-
016,0.00075391,0.0037536,0.0036369,0.0016803,9.7979e-

005,0.0037614,0.0087774,2.2204e-016,2.2204e-
016,0.0016919,0.026193,0.012996,0.063104,2.2204e-016,2.2204e-
016,2.2204e-016,0.025646,0.0079718,2.2204e-016,0.037519,2.2204e-
016,0.0051063,2.2204e-016,0.027174,2.2204e-016,2.2204e-016,2.2204e-
016,2.2204e-016,0.0079457,2.2204e-016,2.2204e-016,0.0011452,2.2204e-
016,0.00019208,0.0036164,0.0055147,2.2204e-
016,0.0088094,0.0036161,0.0066894,2.2204e-016,0.011157,9.9742e-
005,2.2204e-016,2.2204e-016,0.00086911,0.00013777,2.2204e-
016,0.057526,2.2204e-
016,0.067276,0.099942,0.055629,0.075538,0.034363,2.2204e-016,2.2204e-
016,0.02285,2.2204e-016,0.011586,2.2204e-
016,0.0017367,0.020727,0.026907,0.0055377,2.2204e-016,0.04182,2.2204e-
016,0.00012818,0.0054423,0.00018004,0.00014433,0.00085781,0.0087797,2.
2204e-016,2.2204e-016,2.2204e-016,2.2204e-016,2.2204e-016,2.2204e-
016,2.2204e-016,2.2204e-016,0.0012118,0.0050122,2.2204e-016,2.2204e-
016,2.2204e-016,0.018331,0.075393,2.2204e-016,0.044119,2.2204e-
016,2.2204e-016,2.2204e-016,0.040234,2.2204e-016,2.2204e-016,2.2204e-
016,0.029478,0.023814,2.2204e-016,0.022558,2.2204e-
016,0.013961,2.2204e-016,0.02945,0.0018836,0.0052651,2.2204e-
016,0.001488,0.001298,2.2204e-016,0.0041071,2.2204e-
016,0.0027494,0.0022966,2.2204e-016,2.2204e-016,2.2204e-
016,0.0035016,0.0017142,0.0061537,0.00054875,2.2204e-016,2.2204e-
016,0.085403,2.2204e-016,2.2204e-016,2.2204e-016,2.2204e-
016,0.038386,0.015547,2.2204e-016,2.2204e-016,2.2204e-
016,0.010912,2.2204e-016,0.017938,2.2204e-016,2.2204e-
016,0.00085114,2.2204e-016,2.2204e-016,0.032499,0.0019669,2.2204e-
016,2.2204e-016,0.00048469,0.0024615,0.0009354,2.2204e-
016,0.0033516,0.00038173,0.0012051,0.0017326,2.2204e-016,2.2204e-
016,2.2204e-016,2.2204e-016,0.001541,0.00097911,2.2204e-016,2.2204e-
016,0.0072597,2.2204e-016,2.2204e-016,2.2204e-016,2.2204e-
016,0.0063423,2.2204e-016,2.2204e-016,2.2204e-016,2.2204e-
016,0.0076384,2.2204e-016,0.0056087,0.016963,0.0072978,2.2204e-
016,2.2204e-016,2.2204e-016,2.2204e-016,0.0011046,2.2204e-
016,0.0018708,0.0044797,2.2204e-016,2.2204e-016,2.2204e-016,2.2204e-
016,0.00031218,2.2204e-016,0.0043732,0.00062187,0.008985,2.2204e-
016,2.2204e-016,2.2204e-
016,0.0018141,0.0075072,0.077169,0.030165,2.2204e-
016,0.033854,0.12118,0.013176,0.048258,0.043096,0.047995,0.021702,2.22
04e-016,0.025461,2.2204e-016,0.0042441,2.2204e-
016,0.00082458,0.0085843,0.0023502,0.022731,2.2204e-016,2.2204e-
016,0.0052551,0.0027915,0.0037117,0.0030038,0.0025526,0.0015746,2.2204
e-016,2.2204e-016,2.2204e-016,0.0040803,2.2204e-016,2.2204e-
016,2.2204e-016,0.0021453,0.0057076,2.2204e-016,2.2204e-016,2.2204e-
016,2.2204e-016,0.068425,0.0034446,2.2204e-
016,0.00069565,0.0091966,2.2204e-016,2.2204e-016,2.2204e-
016,0.029086,0.0060246,2.2204e-016,0.024798,2.2204e-016,2.2204e-
016,2.2204e-016,2.2204e-016,0.030976,0.030521,2.2204e-
016,0.00082957,2.2204e-016,2.2204e-016,0.0020535,2.2204e-016,8.1685e-
006,2.2204e-016,0.0012901,0.001778,2.2204e-
016,0.0027,0.0037729,2.2204e-016,2.2204e-
016,0.00044386,0.0033072,2.2204e-016,0.070265,0.022598,2.2204e-
016,0.012432,0.027089,0.044594,2.2204e-

016,0.013846,0.0066245,0.020703,0.0046888,2.2204e-
016,0.020588,0.017776,2.2204e-016,0.0042056,2.2204e-016,2.2204e-
016,2.2204e-016,0.0010216,0.00066516,2.2204e-
016,0.0060225,0.0052445,0.00035761,2.2204e-
016,0.0011986,0.0070753,2.2204e-016,0.00030627,0.0013472,2.2204e-
016,2.2204e-016,0.0038038,0.004175,2.2204e-016,2.2204e-016,2.2204e-
016,0.0058949,2.2204e-016,2.2204e-016,0.0055208,2.2204e-016,2.2204e-
016,2.2204e-016,2.2204e-016,0.026083,2.2204e-
016,0.01734,0.0072841,2.2204e-016,0.025813,2.2204e-016,2.2204e-
016,2.2204e-016,0.019564,0.0028039,0.0025758,2.2204e-
016,0.0018672,0.00032161,0.0070365,2.2204e-016,2.2204e-
016,0.0023288,0.010226,0.0014876,2.2204e-016,2.2204e-
016,0.0021558,2.2204e-016,2.2204e-016,2.2204e-016,2.2204e-
016,0.0088222,2.2204e-016,2.2204e-016,2.2204e-016,2.2204e-
016,0.024726,2.2204e-016,0.0019336,0.022367,0.028818,2.2204e-
016,2.2204e-016,0.0042197,2.2204e-016,2.2204e-016,0.00034618,2.2204e-
016,2.2204e-016,2.2204e-016,2.2204e-016,2.2204e-016,2.2204e-
016,0.0025489,2.2204e-016,0.0029642,0.0043573,0.005879,2.2204e-
016,0.0038554,2.2204e-016,2.2204e-016,2.2204e-016,0.0046509,2.2204e-
016,0.0022798,0.0017062,0.0011034,0.053409,0.022947,0.012959,2.2204e-
016,0.057479,2.2204e-016,0.017126,2.2204e-016,0.0063845,2.2204e-
016,0.022967,0.0068025,2.2204e-016,2.2204e-016,0.041428,2.2204e-
016,0.0096642,2.2204e-016,0.0012235,0.0020811,2.2204e-016,2.2204e-
016,0.00268,0.0038212,0.0052801,0.0041703,2.2204e-016,2.2204e-
016,0.0019534,0.0033982,2.2204e-016,0.0028245,2.2204e-
016,0.0029213,1.8672e-005,0.0053597,2.2204e-016,0.0011605,2.2204e-
016,2.2204e-016,2.2204e-016,2.2204e-016,2.2204e-
016,0.0050859,0.029891,0.026379,0.024945,2.2204e-016,2.2204e-
016,2.2204e-016,2.2204e-016,0.04228,0.007759,2.2204e-016,2.2204e-
016,2.2204e-016,2.2204e-016,0.0032997,2.2204e-
016,0.00047996,0.00043765,2.2204e-016,2.2204e-016,2.2204e-
016,0.0038471,0.0014047,0.0038909,2.2204e-016,0.0016372,2.2204e-
016,0.00064941,2.2204e-
016,0.0023029,0.00053307,0.00068496,0.0082998,0.0029294,0.0018727,2.22
04e-016,2.2204e-016,0.0069818,0.024111,2.2204e-016,2.2204e-
016,0.014421,0.011536,0.023711,0.0049519,0.0022132,2.2204e-
016,0.0021411,0.0010477,0.0067525,2.2204e-016,2.2204e-016,2.2204e-
016,0.0033353,0.0034154,0.0018069,0.00062285,2.2204e-
016,0.0064197,2.2204e-016,2.2204e-
016,0.0024349,0.0014785,0.0027713,2.2204e-016,2.2204e-016,2.2204e-
016,2.2204e-016,2.2204e-016,0.0016041,2.2204e-016,2.2204e-016,2.2204e-
016,0.010012,0.03158,2.2204e-016,0.017719,0.022135,2.2204e-
016,0.0032445,0.0027723,2.2204e-016,0.0047169,2.2204e-016,2.2204e-
016,0.0055745,0.0059223,2.2204e-016,0.0042797,2.2204e-016,2.2204e-
016,0.00061649,2.2204e-016,2.2204e-016,0.00093654,0.00063886,2.2204e-
016,2.2204e-016,0.0006934,2.2204e-016,2.2204e-016,0.001658,2.2204e-
016,0.0026536,0.002507,0.00054585,2.2204e-016,2.2204e-
016,0.030186,0.013337,0.011048,2.2204e-
016,0.0078947,0.00035224,0.00093651,2.2204e-016,2.2204e-
016,0.001714,2.2204e-016,2.2204e-
016,0.0047764,0.00086618,0.00026522,0.0081887,2.2204e-016,2.2204e-
016,0.0015789,2.2204e-016,2.2204e-016,2.2204e-

016,0.0054997,0.0085495,0.00084381,0.0052648,0.0010947,0.0028558,2.2204e-016,0.0031097,2.2204e-016,0.00021527,2.2204e-016,2.2204e-016,0.0034967,2.2204e-016,2.2204e-016,2.2204e-016,0.028495,2.2204e-016,0.0050194,2.2204e-016,0.00063375,2.2204e-016,0.0010082,2.2204e-016,2.2204e-016,0.0037492,2.2204e-016,2.2204e-016,0.0058464,0.0019312,0.0065867,2.2204e-016,2.2204e-016,2.2204e-016,2.2204e-016,2.2204e-016,0.0034365,2.2204e-016,0.0013249,2.2204e-016,0.00040566,0.00073284,2.2204e-016,0.00052855,0.0013165,8.6148e-005,0.0026822,2.2204e-016,2.2204e-016,0.0061937,0.0015945,2.2204e-016,0.0027804,0.0024011,0.0037182,2.2204e-016,0.0078392,2.2204e-016,0.0010863,0.0037613,2.2204e-016,0.00085597,2.2204e-016,2.2204e-016,2.2204e-016,0.0026929,2.2204e-016,8.5756e-005,2.2204e-016,0.0012636,0.0016328,0.0017029,0.0035474,0.00015203,0.0042224,0.0050575,0.0012606,2.2204e-016,0.0014989,0.001972,2.2204e-016,0.0015981,2.2204e-016,2.2204e-016,2.2204e-016,2.2204e-016,2.2204e-016,0.0043844,2.2204e-016,0.0022398,2.2204e-016,0.0026255,0.0047824,2.2204e-016,2.2204e-016,0.00038936,2.2204e-016,2.2204e-016,0.0018797,2.2204e-016,0.0028727,2.9722e-005,2.2204e-016,0.0020799,2.2204e-016,0.0051043,2.2204e-016,2.2204e-016,0.0019284,2.2204e-016,0.00073328,2.2204e-016,0.0032117,2.2204e-016,0.0045385,2.2204e-016,0.0015304,0.005461,0.0023979,0.00084965,2.2204e-016,0.0010242,0.0045125,2.2204e-016,2.2204e-016,2.2204e-016,0.00016435,0.0017287,0.0025064,2.2204e-016,0.0071128,2.2204e-016,0.0045412,2.2204e-016,0.0033492,0.0013911,0.0043254,0.00067861,0.00040749,0.0047798,0.00015893,0.0027497,2.2204e-016,0.0030322,2.2204e-016,2.2204e-016,2.2204e-016,2.2204e-016,2.2204e-016,0.0026964,2.2204e-016,0.0044205,2.2204e-016,0.0034589,0.0042935,0.00094795,2.2204e-016,0.0024449,2.2204e-016,0.0025398,2.2204e-016,2.2204e-016,0.00026284,9.343e-006,0.0053549,2.2204e-016,2.2204e-016,2.7803e-005,2.2204e-016,2.2204e-016,0.0007859,0.0012445,2.2204e-016,2.2204e-016,0.0037996,0.0025058,0.0018712,0.0045724,0.00080901,2.2204e-016,2.2204e-016,0.0045604,2.2204e-016,2.2204e-016,6.7996e-005,0.0011647,2.2204e-016,2.2204e-016,2.2204e-016,0.0010378,0.0035374,0.0038504,0.0044828,2.2204e-016,0.002731,0.0013783,0.00030325,2.2204e-016,2.2204e-016,0.0015729,2.2204e-016,0.0022186,0.00042734,0.0017681,2.2204e-016,2.2204e-016,0.0067858,2.2204e-016,2.2204e-016,2.2204e-016,2.2204e-016,2.2204e-016,0.00067385,0.00038905,0.0037154,2.2204e-016,2.2204e-016,2.2204e-016,0.0022979,0.00089273,2.2204e-016,9.2551e-005,2.2204e-016,0.0037352,2.2204e-016,0.0020225,2.2204e-016,0.0018792,0.0016318,0.00091268,2.2204e-016,2.2204e-016,2.2204e-016,2.2204e-016,0.0039901,2.2204e-016,0.0059167,0.0023221,0.0053391,0.0012609,2.2204e-016,0.0010101,0.00093719,2.2204e-016,0.00055523,0.003535,2.2204e-016,2.2204e-016,0.0028869,0.00060503,0.0043412,0.0026378,1.0387e-005,2.2204e-016,2.2204e-016,2.2204e-016,0.0020271,2.2204e-016,0.0024479,0.0058865,2.2204e-016,2.2204e-016,2.2204e-016,2.2204e-016,0.0018554,2.2204e-016,0.0036754,2.2204e-016,2.2204e-016,2.2204e-016,0.0049514,0.0033721,2.2204e-016,2.2204e-016,0.0020871,2.2204e-016,2.2204e-016,2.2204e-016,0.0011743,0.00075418,2.2204e-016,2.2204e-

016,2.2204e-016,2.2204e-016,0.004432,8.944e-
005,0.0010191,0.0025466,2.2204e-016,0.0018382,2.2204e-
016,0.0005954,2.2204e-016,2.2204e-
016,0.0025657,0.0024755,0.0033843,0.0010546,2.2204e-
016,0.0028503,0.0034081,2.2204e-
016,0.0032044,0.0081752,0.004199,2.2204e-016,0.00067334,9.8559e-
005,2.2204e-016,0.0028342,2.2204e-016,2.2204e-016,2.2204e-
016,0.00016964,0.0015863,2.2204e-016,2.2204e-
016,0.0029823,0.0038041,2.2204e-016,0.00060526,2.2204e-
016,0.0022203,0.0034299,0.00073952,2.2204e-016,0.0034266,2.2204e-
016,0.00076175,2.2204e-016,2.2204e-016,2.2204e-016,0.0010023,2.2204e-
016,0.0023834,2.2204e-016,0.0028828,2.2204e-016,2.2204e-
016,0.0022286,0.0025531,0.0014244,2.2204e-016,2.2204e-
016,0.00064109,0.0045715,2.2204e-016,0.0052799,0.0027845,2.2204e-
016,2.2204e-016,2.2204e-016,0.0046829,2.2204e-
016,0.005141,0.000963,2.2204e-016,0.00098093,0.0013942
0.074981,0.061099,0.075901,0.065157,0.072091,0.071078,0.063982,0.06203
9,0.075527,0.081841,0.06594,0.069856,0.072328,0.069498,0.070531,0.0649
01,0.073873,0.066362,0.073165,0.06838,1.3595,1.3187,1.2837,1.2583,0.07
5439,0.075249,0.075576,0.073407,0.064216,0.066589,0.069157,0.073325,0.
077144,0.077488,0.076087,0.068475,0.06263,0.068525,0.074865,2.4162,1.2
419,1.1992,1.1963,1.1384,1.2615,1.2667,0.070549,0.070384,0.067733,0.06
1984,0.076415,0.070357,0.07545,0.067101,0.066123,0.069978,0.073467,0.0
71357,0.075854,0.07251,0.070095,2.4347,2.321,1.1315,1.4621,1.202,1.342
,1.2671,1.3646,0.86126,1.291,0.079867,0.065728,0.074154,0.079385,0.069
335,0.07629,0.067862,0.075372,0.072864,0.072734,0.070406,0.075428,0.07
1578,0.07488,0.065945,2.2795,2.3525,2.3863,1.1423,1.284,1.0392,1.2136,
1.204,1.0388,0.81076,1.2625,1.2861,0.077729,0.070899,0.075449,0.072021
,0.072508,0.069163,0.069629,0.077254,0.062226,0.068695,0.072506,0.0752
69,0.073534,0.074836,0.079708,0.080602,2.4266,2.3314,2.4766,2.4276,1.4
078,0.98977,0.99716,1.1984,1.2236,1.1274,1.2609,1.1813,1.2622,1.247,0.
069101,0.069703,0.078222,0.075683,0.066636,0.067997,0.05827,0.062787,0
.073635,0.072768,0.067396,0.078587,0.072318,0.06656,0.069372,0.071882,
2.4189,2.3447,2.4602,2.3383,2.3737,2.347,1.289,1.3777,1.135,1.2543,1.1
856,1.2392,1.2741,1.2149,1.1647,1.4418,0.073206,0.067631,0.075066,0.06
7255,0.073821,0.059008,0.071445,0.072952,0.07427,0.072165,0.067134,0.0
71424,0.074568,0.072991,0.068423,0.066126,0.070616,2.4099,2.332,2.3459
,2.3449,2.4366,2.4591,0.83095,1.243,1.0353,1.252,1.271,1.1093,1.1705,1
.3437,1.2022,1.2618,1.3061,0.067676,0.068657,0.068298,0.073526,0.06467
9,0.070681,0.070952,0.062051,0.064399,0.070615,0.069195,0.074547,0.075
736,0.073966,0.071412,0.070298,0.061356,2.3346,2.3521,2.4217,2.4023,2.
2664,2.3706,2.3093,1.3335,1.2529,1.4821,1.2706,1.3808,1.4697,1.3694,1.
0474,1.4489,1.2657,1.1833,0.069146,0.066116,0.073539,0.068646,0.078204
,0.06279,0.080235,0.075594,0.070312,0.071047,0.071636,0.068128,0.06263
6,0.074046,0.073408,0.069154,0.07562,0.064747,2.2861,2.484,2.4361,2.33
49,2.3257,2.4176,2.3462,1.2355,1.4595,1.4513,1.2238,1.2477,1.2078,1.19
55,1.2544,1.2371,1.2134,1.1448,0.065524,0.068477,0.062823,0.075732,0.0
57707,0.075691,0.083811,0.073136,0.075936,0.07124,0.069688,0.071117,0.
069991,0.079385,0.072373,0.0695,0.075241,0.068619,0.081661,2.3683,2.34
11,2.392,2.3556,2.3284,2.3477,2.3825,1.1112,1.2195,1.2757,1.097,1.258,
1.2122,1.2186,1.627,1.2676,1.1945,1.196,1.459,0.070577,0.073555,0.0702
47,0.068947,0.073813,0.07593,0.065284,0.07645,0.079089,0.075251,0.0725

23,0.069953,0.070735,0.064041,0.067977,0.07134,0.071558,0.076103,2.4017,2.3251,2.3968,2.4137,2.366,2.2704,2.4756,0.75578,1.3549,1.2017,1.1815,1.4765,1.1679,1.3337,1.0539,1.1445,1.2396,1.2157,1.2022,0.067375,0.069793,0.064856,0.076384,0.077842,0.074455,0.072164,0.071265,0.071018,0.074832,0.076433,0.072623,0.069577,0.075042,0.082004,0.072542,0.066563,0.072417,2.4327,2.3458,2.475,2.3032,2.3779,2.3968,2.3966,2.4077,2.4154,1.3853,1.2684,1.0765,1.2,1.264,1.2281,1.2321,1.2124,1.2231,1.2854,1.1492,0.070458,0.071833,0.068296,0.071758,0.073267,0.076688,0.071049,0.080956,0.072962,0.0763,0.07283,0.086378,0.071595,0.067274,0.068826,0.072792,0.070078,0.07087,2.4556,2.3833,2.4566,2.4424,2.4341,2.4215,2.3944,2.3706,2.3801,1.0582,1.2742,1.0037,0.84248,0.87586,0.86478,0.86969,0.9189,0.71329,0.97389,0.78983,0.071792,0.075245,0.072032,0.073642,0.073138,0.078893,0.069548,0.068543,0.069173,0.069497,0.071606,0.070056,0.071162,0.072337,0.071838,0.078357,0.070109,0.070248,2.3646,2.3696,2.4211,2.3031,2.4935,2.2971,2.3175,2.3902,2.4262,1.2016,0.85818,0.86965,0.92542,0.9639,0.83553,0.88853,0.96545,0.81415,0.8925,0.89173,0.072593,0.076743,0.063565,0.070201,0.072459,0.068075,0.07713,0.070528,0.073859,0.072944,0.065604,0.069255,0.072113,0.074314,0.072676,0.079007,0.072714,0.064158,2.4,2.4308,2.2788,2.34,2.3885,2.3894,2.4101,1.1649,1.1861,1.1971,0.83975,0.9261,0.81506,0.94939,0.81758,0.78725,0.84932,0.85507,0.85203,0.91923,0.074482,0.067658,0.068837,0.071122,0.075616,0.075404,0.067648,0.073813,0.071021,0.073436,0.074687,0.066433,0.067733,0.071309,0.069655,0.073361,0.070578,0.066528,2.287,2.3666,2.4029,2.4052,2.3516,2.2841,1.1192,1.2063,1.2155,1.2314,1.1715,0.91305,0.71415,0.83364,0.91381,0.86518,0.89752,0.82826,0.6312,0.80924,0.072799,0.072144,0.072727,0.071193,0.070473,0.068289,0.072184,0.072084,0.07367,0.065866,0.077257,0.073055,0.080348,0.066794,0.066124,0.068483,0.071032,0.078689,2.4214,2.4354,2.3846,2.4111,2.4962,1.3486,1.1441,1.2692,1.4967,1.3256,1.3063,0.78736,0.56489,0.91665,0.82949,0.72444,0.74718,0.75528,0.95876,1.0434,0.070317,0.077129,0.069769,0.073298,0.074383,0.074587,0.072856,0.06952,0.067566,0.069636,0.075181,0.071559,0.067418,0.071492,0.074009,0.07641,0.068493,0.074222,2.2923,2.4266,2.4413,1.3351,1.2039,1.1589,1.3727,1.3568,1.2073,1.3773,1.2143,0.84883,0.61268,0.92568,0.79119,0.72783,1.0391,0.9942,0.87784,0.9017,0.067699,0.072045,0.067627,0.070306,0.073297,0.07115,0.071534,0.069056,0.07221,0.075317,0.070412,0.07428,0.074976,0.065865,0.069592,0.073803,0.072228,0.072582,2.4582,1.595,1.2601,1.226,1.2847,1.4576,1.1264,1.2373,1.2576,1.3314,0.89962,0.81054,1.1295,0.78483,0.82304,0.94028,0.79999,0.90379,0.85171,0.072888,0.072687,0.067854,0.078374,0.073666,0.072713,0.074094,0.071973,0.077418,0.072799,0.067853,0.071748,0.068427,0.072319,0.074557,0.07464,0.070097,0.067678,1.1913,1.2596,1.1636,1.3841,1.3983,1.2008,1.1338,0.93535,1.2112,1.158,0.87013,0.75073,0.83272,0.78631,0.92001,0.73103,0.91603,0.85009,0.86693,0.075544,0.077378,0.06842,0.072903,0.072083,0.079131,0.069678,0.067911,0.074657,0.084054,0.07447,0.072244,0.07173,0.075583,0.06657,0.07035,0.064743,0.07114,1.2353,1.1927,1.1929,1.1549,1.1989,1.3018,1.2488,1.221,1.2506,1.1701,0.86458,0.90007,0.79277,0.59803,0.54356,1.0855,0.71616,0.83883,0.06296,0.072804,0.070053,0.072336,0.074374,0.071254,0.074762,0.075127,0.076062,0.063193,0.075488,0.067586,0.068045,0.063826,0.074715,0.069902,0.074652,0.074051,0.07187,1.3612,1.2529,1.2965,1.1913,1.3371,1.3476,1.3139,0.90983,1.1641,1.3446,1.0131,0.61282,0.78971,0.78477,0.83515,0.91809,0.88957,0.79778,0.073233,0.072386,0.070216,0.067995,0.075244,0.072876,0.076596,0.07595,0.071857,0.064487,0.073242,0.075401,0.

069925,0.072889,0.069144,0.072887,0.072236,0.075779,0.070834,0.073908,
1.0782,1.3507,1.2659,1.2009,1.2313,0.49885,0.99808,1.1967,1.2433,0.821
12,0.84191,0.67603,0.95378,0.90837,0.85381,0.06904,0.065463,0.066099,0
.069655,0.074234,0.070791,0.070434,0.072041,0.070498,0.069278,0.069694
,0.07304,0.07229,0.075834,0.072177,0.075963,0.068113,0.072231,0.072251
,0.072742,0.069534,0.072477,1.4121,1.2397,1.2043,1.3326,1.2345,1.2481,
1.2528,1.2263,1.2289,0.89519,0.85644,0.87941,0.080109,0.075077,0.06737
9,0.073373,0.072521,0.076908,0.071162,0.071165,0.067201,0.078785,0.072
233,0.075803,0.072245,0.069296,0.077528,0.06711,0.070363,0.071888,0.07
1557,0.071354,0.072244,0.071571,0.069879,0.066612,0.072701,0.072401,1.
201,1.2179,1.1285,1.2525,1.2448,1.2121,1.3155,1.2954,0.069981,0.072985
,0.076697,0.072718,0.077764,0.069422,0.06656,0.075768,0.075864,0.06876
9,0.074691,0.069955,0.067555,0.072113,0.070356,0.065425,0.073366,0.074
051,0.069128,0.065783,0.07121,0.070334,0.070658,0.070465,0.074089,0.07
3128,0.076124,0.070809,0.069345,1.3127,1.2642,1.2411,1.2585,1.2147,0.0
76478,0.07195,0.073666,0.073554,0.070522,0.074239,0.06819,0.071842,0.0
74664,0.073149,0.073783,0.078118,0.068435,0.069465,0.070605,0.066419,0
.069477,0.068048,0.075541,0.080583,0.072093,0.074144,0.07158,0.074787,
0.070307,0.074181,0.070602,0.071236,0.070441,0.069512,0.074714,0.06965
6,0.071033,0.95688,1.2247,0.072653,0.074466,0.065856,0.072235,0.068262
,0.07361,0.068558,0.064765,0.074487,0.067115,0.070547,0.075222,0.07259
8,0.07579,0.071717,0.072095,0.069497,0.069521,0.068828,0.076957,0.0696
96,0.072205,0.074641,0.071496,0.071982,0.067551,0.074738,0.072818,0.07
0155,0.07272,0.070349,0.069616,0.077181,0.073456,0.06768,0.073951,0.07
5367,0.075278,0.069779,0.079325,0.067546,0.073383,0.074353,0.068903,0.
072335,0.071373,0.069481,0.071779,0.074642,0.067577,0.073201,0.068066,
0.072851,0.075405,0.071265,0.073919,0.072991,0.076336,0.075946,0.07180
1,0.06809,0.073575,0.073498,0.071959,0.073266,0.073742,0.069169,0.0708
05,0.068714,0.064757,0.075859,0.069644,0.074162,0.066393,0.072342,0.07
5916,0.068539,0.068961,0.070351,0.065994,0.070646,0.072379,0.069018,0.
071014,0.073189,0.064913,0.073803,0.070757,0.075362,0.069839,0.071939,
0.074673,0.069092,0.071625,0.072461,0.073616,0.071716,0.075301,0.07076
1,0.073967,0.075976,0.072887,0.073826,0.067718,0.071611,0.075838,0.071
492,0.068914,0.073738,0.071684,0.074299,0.074122,0.068205,0.079908,0.0
71281,0.074617,0.071687,0.073117,0.072388,0.075372,0.072321,0.075629,0
.075208,0.070916,0.075605,0.069548,0.073898,0.070882,0.070523,0.070911
,0.071038,0.069805,0.074174,0.069127,0.076795,0.070545,0.075689,0.0763
05,0.070568,0.069767,0.073935,0.071184,0.072951,0.068805,0.069218,0.07
2092,0.069463,0.076468,0.067947,0.066117,0.071911,0.070224,0.069597,0.
073398,0.072304,0.068253,0.069967,0.07587,0.076905,0.069907,0.076926,0
.072097,0.068737,0.068171,0.073853,0.069887,0.071133,0.07144,0.072812,
0.068003,0.069413,0.067322,0.074012,0.075982,0.074009,0.076186,0.07087
8,0.073189,0.072822,0.07086,0.069351,0.070876,0.071115,0.07032,0.07349
9,0.071387,0.073579,0.071496,0.070326,0.078258,0.065645,0.071413,0.064
996,0.06925,0.069897,0.071248,0.071879,0.073496,0.072411,0.070747,0.06
7899,0.071776,0.072221,0.06655,0.071605,0.068476,0.073403,0.069671,0.0
74866,0.066303,0.071356,0.072978,0.072472,0.069505,0.070557,0.07126,0.
065406,0.074421,0.072113,0.077101,0.075793,0.075101,0.075172,0.06805,0
.070309,0.071871,0.067976,0.072735,0.075515,0.066088,0.070698,0.074628
,0.071467,0.072919,0.076812,0.072222,0.070336,0.072038,0.070883,0.0727
26,0.069325,0.072415,0.076434,0.067457,0.069757,0.067643,0.071822,0.07
1808,0.070779,0.077838,0.071323,0.068846,0.069412,0.073663,0.074352,0.

071832,0.06691,0.075005,0.069766,0.072587,0.069338,0.07178,0.070486,0.
070513,0.067134,0.068011,0.071218,0.074628,0.071294,0.071144,0.074053,
0.072546,0.07339,0.071438,0.07306,0.070849,0.071884,0.075859,0.075621,
0.074078,0.071397,0.07075,0.074589,0.076603,0.06481,0.075278,0.079548,
0.074477,0.066868,0.070591,0.072189,0.068373,0.076875,0.069085,0.06881
3,0.065149,0.072629,0.073515,0.071719,0.071613,0.076184,0.07406,0.0671
73,0.071665,0.069853,0.073287,0.074489,0.073591,0.068666,0.073741,0.07
1342,0.073578,0.066175,0.07144,0.066157,0.071982,0.067686,0.074558,0.0
69767,0.073433,0.065804,0.072467,0.075155,0.072138,0.074874,0.066854,0
.070053,0.071,0.07761,0.069927,0.076291,0.07616,0.064897,0.072604,0.07
1377,0.075301,0.06735,0.077233,0.072953,0.070044,0.070688,0.071137
0.11793,0.10244,0.1208,0.11082,0.11609,0.11423,0.10912,0.10788,0.11977
,0.12651,0.11091,0.12014,0.11768,0.11096,0.11882,0.11097,0.11895,0.117
07,0.11598,0.1094,0.92385,0.92332,0.8741,0.88599,0.12173,0.11921,0.120
91,0.11612,0.10585,0.108,0.11425,0.11593,0.12181,0.12305,0.12009,0.114
38,0.10844,0.11372,0.11828,1.6648,0.86552,0.82736,0.84134,0.81164,0.88
615,0.86686,0.11381,0.11577,0.11071,0.10672,0.12273,0.11265,0.12156,0.
11085,0.11155,0.11747,0.11903,0.11904,0.12288,0.1183,0.11352,1.6598,1.
5576,0.79517,1.0031,0.82546,0.94354,0.88877,0.96079,0.62309,0.91373,0.
12276,0.10776,0.11586,0.12319,0.1145,0.12229,0.11241,0.11755,0.11844,0
.11015,0.11471,0.12058,0.11325,0.11851,0.11734,1.526,1.582,1.5802,0.77
432,0.88147,0.76019,0.84478,0.81223,0.71529,0.58802,0.89323,0.91438,0.
12299,0.11872,0.12022,0.11363,0.11311,0.11394,0.11147,0.12252,0.1066,0
.10994,0.11805,0.12094,0.11795,0.11822,0.12608,0.1197,1.6021,1.5106,1.
6271,1.5813,0.96024,0.70052,0.7173,0.85336,0.85046,0.77363,0.86395,0.8
1497,0.88071,0.85151,0.11829,0.11337,0.11893,0.1173,0.10886,0.11034,0.
10418,0.10428,0.1203,0.11538,0.11501,0.1254,0.11359,0.10961,0.11924,0.
1153,1.6372,1.5898,1.6597,1.5587,1.582,1.5956,0.91716,0.94398,0.80863,
0.88025,0.82004,0.86898,0.89338,0.81958,0.81703,1.0082,0.11875,0.11364
,0.12049,0.11342,0.12297,0.10205,0.11655,0.11545,0.12014,0.11809,0.112
81,0.11484,0.1168,0.1179,0.1147,0.11103,0.11877,1.6296,1.5345,1.5314,1
.6015,1.6449,1.634,0.58753,0.8597,0.71698,0.88715,0.88874,0.779,0.8443
1,0.91906,0.83743,0.90162,0.88822,0.11105,0.11801,0.11351,0.11944,0.11
364,0.11969,0.11493,0.10675,0.1073,0.11393,0.11675,0.11596,0.11537,0.1
1133,0.11204,0.10895,0.10321,1.5525,1.5641,1.6087,1.6075,1.4854,1.6238
,1.5323,0.92078,0.89379,1.0312,0.88741,0.95875,1.0017,0.96704,0.76328,
0.99641,0.87614,0.8368,0.11315,0.11398,0.11694,0.11574,0.11993,0.10755
,0.1197,0.11882,0.11718,0.11443,0.12018,0.10611,0.098865,0.1133,0.1126
5,0.10906,0.11329,0.1129,1.5216,1.7055,1.6178,1.5438,1.5345,1.6692,1.5
204,0.85867,1.0126,1.0009,0.85508,0.88106,0.84972,0.83757,0.88277,0.87
21,0.83391,0.81783,0.10675,0.1187,0.11129,0.11675,0.10073,0.11824,0.12
993,0.11734,0.12079,0.11062,0.10788,0.10901,0.10966,0.12043,0.11017,0.
10941,0.11251,0.10722,0.12377,1.5592,1.5592,1.5787,1.5638,1.5459,1.584
2,1.5638,0.77783,0.84216,0.90216,0.77496,0.91142,0.84825,0.85267,1.136
9,0.89328,0.83453,0.83596,1.0022,0.11718,0.11846,0.1133,0.10932,0.1152
4,0.12267,0.10292,0.11615,0.11699,0.11159,0.11123,0.10539,0.10913,0.10
201,0.10491,0.10861,0.10989,0.11358,1.6207,1.5005,1.6366,1.6398,1.5494
,1.5059,1.6573,0.53908,0.91967,0.85614,0.81224,1.0274,0.82569,0.93139,
0.74419,0.79772,0.86797,0.84459,0.84086,0.11275,0.11494,0.1085,0.11375
,0.11407,0.10988,0.11244,0.10987,0.11003,0.11467,0.11465,0.10964,0.108
33,0.11101,0.11779,0.10889,0.10635,0.11132,1.6285,1.5556,1.6647,1.5327
,1.5783,1.5669,1.591,1.6313,1.5582,0.96145,0.86661,0.74211,0.80697,0.8

7692,0.86981,0.85238,0.83168,0.84847,0.91258,0.80335,0.10755,0.11023,0
.10662,0.11011,0.11238,0.11316,0.10933,0.11905,0.11367,0.11414,0.11046
,0.12324,0.10856,0.10665,0.10697,0.10949,0.10921,0.10767,1.6653,1.6122
,1.6225,1.6285,1.6362,1.6166,1.614,1.5609,1.6209,0.74672,0.84976,0.714
54,0.6113,0.61832,0.61907,0.62814,0.64844,0.51895,0.70661,0.55948,0.10
914,0.11279,0.10929,0.11207,0.10994,0.11302,0.10933,0.10767,0.10414,0.
10765,0.10973,0.10663,0.10857,0.11202,0.10883,0.11682,0.10824,0.10883,
1.5645,1.5788,1.6301,1.5135,1.7094,1.5279,1.545,1.5673,1.637,0.80818,0
.61434,0.62502,0.67704,0.70436,0.60165,0.63814,0.67577,0.59431,0.62727
,0.6475,0.10881,0.11557,0.10678,0.10762,0.11177,0.10494,0.11584,0.1110
9,0.11369,0.10943,0.10836,0.10689,0.10977,0.11102,0.11016,0.11552,0.11
168,0.10342,1.594,1.6627,1.5225,1.5949,1.5861,1.6059,1.6039,0.80978,0.
82583,0.82163,0.6095,0.6645,0.58796,0.68657,0.58087,0.56824,0.60456,0.
62213,0.61259,0.65064,0.11071,0.10645,0.10755,0.10852,0.11562,0.11298,
0.10628,0.1149,0.11194,0.10989,0.11005,0.10386,0.107,0.11036,0.10871,0
.11742,0.11027,0.10402,1.4891,1.5805,1.6173,1.6188,1.5128,1.5107,0.776
6,0.85388,0.84627,0.84722,0.80538,0.65509,0.52646,0.59483,0.6587,0.608
26,0.63725,0.59562,0.47466,0.59276,0.11222,0.11019,0.10771,0.1125,0.10
894,0.10616,0.10861,0.10903,0.11057,0.10472,0.11743,0.10971,0.11667,0.
10646,0.10559,0.10626,0.10971,0.11598,1.6247,1.6304,1.5531,1.5948,1.71
93,0.93442,0.8122,0.88699,1.0464,0.91287,0.91255,0.57558,0.42323,0.656
88,0.59202,0.53491,0.53648,0.55345,0.69791,0.73208,0.10756,0.11399,0.1
0966,0.11325,0.11391,0.11345,0.11384,0.10794,0.10756,0.1077,0.11247,0.
11069,0.10499,0.1103,0.11086,0.11219,0.10687,0.11395,1.5065,1.652,1.63
62,0.93703,0.83206,0.80142,0.96712,0.92077,0.82272,0.94272,0.86621,0.6
1874,0.45837,0.66354,0.56755,0.53199,0.73263,0.69811,0.63522,0.65183,0
.10231,0.11148,0.10668,0.11206,0.1127,0.11209,0.10958,0.11303,0.10987,
0.11519,0.10892,0.11344,0.11384,0.10567,0.10692,0.11153,0.10993,0.1109
6,1.6672,1.0913,0.85476,0.82887,0.87363,0.99877,0.79207,0.84936,0.8759
9,0.92584,0.62205,0.57577,0.80428,0.56832,0.59083,0.6564,0.5907,0.6563
2,0.61128,0.11142,0.10954,0.10558,0.11528,0.11273,0.11115,0.11238,0.11
002,0.11471,0.111,0.10883,0.10773,0.10901,0.10991,0.11277,0.11024,0.10
582,0.098133,0.82077,0.85818,0.81629,0.93536,0.94264,0.84468,0.76625,0
.65785,0.84619,0.79691,0.63281,0.55723,0.60359,0.58408,0.66237,0.52545
,0.64549,0.59871,0.61782,0.11382,0.11389,0.10689,0.11395,0.11065,0.116
39,0.10746,0.10451,0.11158,0.11843,0.11515,0.11142,0.10971,0.11446,0.1
0509,0.11088,0.10462,0.10626,0.8581,0.8406,0.84807,0.77282,0.82392,0.8
7771,0.86587,0.86205,0.87268,0.8228,0.61796,0.61583,0.57246,0.45042,0.
41395,0.78685,0.52472,0.59458,0.10024,0.11184,0.11193,0.11069,0.11089,
0.10923,0.11289,0.11445,0.11342,0.10316,0.11448,0.1078,0.10694,0.10532
,0.11597,0.11022,0.11143,0.10819,0.10431,0.94643,0.87862,0.90077,0.825
02,0.94177,0.93289,0.91136,0.62264,0.80474,0.90894,0.7126,0.45529,0.57
457,0.55811,0.60308,0.66596,0.63927,0.57007,0.11372,0.11003,0.11115,0.
10522,0.11101,0.11099,0.11553,0.11179,0.11001,0.099955,0.11046,0.11305
,0.10934,0.1087,0.10773,0.11169,0.1135,0.10868,0.10261,0.10741,0.74521
,0.91062,0.86369,0.83465,0.85252,0.37541,0.71043,0.85751,0.8738,0.5896
6,0.57702,0.50433,0.68279,0.65352,0.60106,0.10678,0.10561,0.1032,0.106
9,0.1138,0.1087,0.11324,0.10792,0.10902,0.10725,0.10663,0.11525,0.1131
8,0.11296,0.11159,0.11573,0.1068,0.10901,0.10306,0.10423,0.10036,0.102
77,0.97109,0.87628,0.84574,0.90713,0.85294,0.88859,0.88471,0.83901,0.8
7732,0.62577,0.6168,0.63604,0.11784,0.11251,0.10896,0.11081,0.11175,0.
11722,0.10632,0.1088,0.1075,0.11707,0.11354,0.11455,0.10842,0.10694,0.

11457,0.10528,0.1079,0.11152,0.10926,0.10829,0.1036,0.10358,0.10192,0.
099648,0.10458,0.10708,0.82437,0.82837,0.78482,0.87637,0.87095,0.83489
,0.90206,0.89628,0.10977,0.10808,0.11575,0.11016,0.11507,0.11127,0.104
14,0.11515,0.11326,0.10684,0.11192,0.10835,0.10774,0.11021,0.10915,0.1
0548,0.11304,0.11017,0.10784,0.10478,0.1123,0.10954,0.10612,0.10351,0.
10669,0.10426,0.10691,0.10351,0.10267,0.85543,0.87529,0.86766,0.86459,
0.86004,0.10815,0.10143,0.11076,0.11036,0.10563,0.11391,0.10719,0.1111
6,0.10854,0.11065,0.11207,0.11534,0.10854,0.11017,0.111,0.10401,0.1076
3,0.10742,0.11575,0.12108,0.11201,0.10981,0.10464,0.10684,0.10374,0.10
417,0.10201,0.1027,0.10163,0.10156,0.10721,0.10275,0.10348,0.66785,0.8
5303,0.10378,0.10465,0.099539,0.10454,0.10804,0.1124,0.10522,0.10423,0
.11423,0.10609,0.11136,0.11289,0.11148,0.11261,0.10971,0.11014,0.10703
,0.10803,0.10548,0.11404,0.11011,0.11208,0.11484,0.10212,0.10438,0.102
76,0.10631,0.1067,0.10291,0.10596,0.10058,0.099035,0.10691,0.10505,0.1
0243,0.10557,0.10638,0.10605,0.10188,0.11142,0.10475,0.11335,0.10964,0
.10708,0.11044,0.11033,0.10724,0.11008,0.11121,0.10534,0.1135,0.10407,
0.11182,0.11068,0.10969,0.10953,0.10313,0.11149,0.10782,0.10239,0.0988
62,0.10494,0.1055,0.10489,0.10497,0.10596,0.10138,0.10479,0.10285,0.09
7786,0.10625,0.10175,0.10416,0.099928,0.10583,0.11597,0.10664,0.10757,
0.10779,0.1043,0.10986,0.11269,0.1064,0.11445,0.11061,0.10452,0.11374,
0.11012,0.10411,0.10053,0.1035,0.10692,0.10258,0.10327,0.10297,0.10404
,0.10383,0.10916,0.10452,0.10323,0.10597,0.10752,0.10461,0.10096,0.101
55,0.10759,0.10487,0.10275,0.10474,0.11082,0.11296,0.11097,0.10806,0.1
1526,0.11089,0.113,0.10954,0.11264,0.10339,0.10685,0.10454,0.1072,0.10
561,0.10141,0.10754,0.10352,0.10682,0.10493,0.10171,0.10362,0.10303,0.
10309,0.10611,0.099947,0.10673,0.10387,0.10827,0.10883,0.10387,0.10233
,0.10582,0.10154,0.10618,0.10819,0.10563,0.10746,0.10972,0.10691,0.098
979,0.097904,0.10393,0.10342,0.098697,0.1037,0.10318,0.10105,0.10307,0
.10588,0.10724,0.10263,0.11123,0.103,0.1019,0.099469,0.10445,0.1024,0.
1028,0.10568,0.10618,0.10074,0.10275,0.097769,0.1034,0.10577,0.10508,0
.10784,0.10341,0.10632,0.10631,0.10429,0.10169,0.10261,0.10181,0.10218
,0.1047,0.10413,0.10622,0.10329,0.103,0.1106,0.099206,0.10273,0.098277
,0.10227,0.1019,0.10348,0.10353,0.10322,0.10522,0.10361,0.10063,0.1040
4,0.10335,0.10088,0.10504,0.10043,0.1061,0.10202,0.10832,0.10184,0.102
91,0.10548,0.10586,0.10259,0.1022,0.10385,0.097611,0.10641,0.10432,0.1
1005,0.10789,0.10798,0.10823,0.10152,0.1032,0.10409,0.099955,0.10527,0
.10747,0.098296,0.10083,0.10624,0.10341,0.10422,0.10852,0.1051,0.1007,
0.10074,0.10328,0.10378,0.10218,0.10436,0.1087,0.10134,0.10113,0.09960
4,0.10306,0.10234,0.10411,0.10876,0.10051,0.10043,0.10169,0.10575,0.10
407,0.10369,0.099247,0.10598,0.10283,0.10321,0.10102,0.1071,0.10258,0.
1028,0.10016,0.099972,0.10365,0.10874,0.10371,0.1029,0.10668,0.10327,0
.10509,0.10237,0.10598,0.10324,0.10423,0.10713,0.10681,0.1062,0.10317,
0.10283,0.10496,0.10732,0.09914,0.10745,0.11104,0.10522,0.096571,0.103
61,0.10399,0.10275,0.10732,0.10211,0.10111,0.098027,0.10467,0.10548,0.
10241,0.10395,0.10673,0.10628,0.098783,0.10336,0.10183,0.10479,0.10772
,0.10366,0.10106,0.10788,0.10448,0.10481,0.099578,0.10494,0.097416,0.1
0382,0.10077,0.10313,0.099789,0.10394,0.099327,0.1046,0.10602,0.10439,
0.10728,0.10047,0.10279,0.10237,0.11053,0.10195,0.10841,0.10633,0.0966
62,0.10203,0.10255,0.10707,0.10051,0.10993,0.10354,0.10352,0.10304,0.1
0546
0.1465,0.13137,0.14536,0.14086,0.1391,0.1396,0.13724,0.13635,0.14772,0
.15535,0.14164,0.15067,0.14387,0.14143,0.14447,0.13997,0.14562,0.14205

,0.14099,0.13881,0.63549,0.69246,0.67235,0.65468,0.14822,0.14693,0.147
01,0.14369,0.13212,0.13815,0.14228,0.14215,0.15191,0.15346,0.1502,0.14
075,0.1393,0.14094,0.14572,1.1935,0.61504,0.59191,0.60227,0.61461,0.65
708,0.61879,0.14633,0.14181,0.13979,0.1359,0.14469,0.14673,0.14735,0.1
3624,0.13661,0.14866,0.14126,0.14747,0.14664,0.14795,0.14127,1.1852,1.
1123,0.57562,0.73912,0.62441,0.70251,0.64156,0.67244,0.47998,0.69677,0
.14941,0.13792,0.14578,0.15175,0.14527,0.14806,0.14085,0.14971,0.14383
,0.13794,0.14497,0.14937,0.14192,0.14793,0.14548,1.0794,1.1263,1.1071,
0.57235,0.65917,0.56806,0.61503,0.58222,0.51771,0.46992,0.67618,0.6897
7,0.14965,0.14567,0.14966,0.14044,0.13761,0.13832,0.14112,0.15003,0.13
758,0.13838,0.14722,0.14809,0.14187,0.15165,0.15637,0.14772,1.1487,1.0
549,1.1808,1.0693,0.68268,0.53104,0.54332,0.64526,0.63452,0.56232,0.63
882,0.59707,0.65622,0.62596,0.14316,0.14541,0.14866,0.14826,0.14281,0.
13831,0.13357,0.13239,0.146,0.14646,0.14947,0.15108,0.14187,0.13967,0.
14768,0.13702,1.1765,1.1153,1.1636,1.091,1.0897,1.1045,0.69854,0.6757,
0.61339,0.67048,0.60715,0.64336,0.67528,0.58866,0.60861,0.71878,0.1474
8,0.14238,0.14609,0.14154,0.14789,0.12753,0.14423,0.1457,0.14631,0.145
85,0.14048,0.14596,0.14709,0.14872,0.1453,0.13664,0.14302,1.1372,1.073
9,1.0593,1.1061,1.1589,1.1612,0.45688,0.62365,0.53328,0.66275,0.66596,
0.58102,0.64459,0.64983,0.62005,0.65891,0.65026,0.14186,0.1478,0.14127
,0.14425,0.14083,0.14728,0.1405,0.13361,0.13992,0.14334,0.14661,0.1400
6,0.14179,0.13182,0.13524,0.12707,0.13395,1.0636,1.1045,1.1016,1.1422,
1.0083,1.1561,1.0812,0.67402,0.66979,0.7277,0.65406,0.6802,0.73297,0.7
082,0.58142,0.74187,0.66331,0.62953,0.14196,0.14473,0.14677,0.14155,0.
15019,0.1359,0.15233,0.14289,0.14451,0.14391,0.14408,0.12552,0.11962,0
.13226,0.1305,0.12693,0.1308,0.14339,1.041,1.21,1.1358,0.99693,1.0489,
1.2226,1.0316,0.62795,0.75188,0.74128,0.62506,0.66496,0.61597,0.62681,
0.65659,0.64066,0.60406,0.62399,0.13914,0.14781,0.14,0.14288,0.13386,0
.14269,0.15529,0.14305,0.1462,0.13238,0.12431,0.1309,0.13152,0.13355,0
.12841,0.12911,0.13055,0.12549,0.14758,1.0839,1.0698,1.0607,1.127,1.02
6,1.1318,1.0783,0.59714,0.6138,0.69053,0.592,0.68116,0.65499,0.63073,0
.8058,0.67667,0.62585,0.61864,0.69617,0.14919,0.14987,0.1402,0.1378,0.
14226,0.1476,0.1214,0.13558,0.13971,0.13125,0.1303,0.12606,0.12845,0.1
1689,0.12283,0.12624,0.12876,0.13502,1.1682,1.0198,1.14,1.169,1.087,1.
0443,1.1652,0.42167,0.64031,0.64373,0.61309,0.75614,0.60514,0.67309,0.
56395,0.58429,0.64341,0.63793,0.59685,0.14597,0.14572,0.13519,0.13411,
0.132,0.1288,0.1326,0.12956,0.12746,0.13414,0.13549,0.13085,0.13082,0.
12946,0.13597,0.13197,0.12721,0.12966,1.1643,1.0518,1.148,1.0622,1.069
7,1.1189,1.1266,1.1118,1.1025,0.67041,0.61584,0.54333,0.5798,0.65206,0
.64814,0.6261,0.60589,0.61902,0.6618,0.60592,0.12759,0.13037,0.12684,0
.1307,0.13021,0.13409,0.12895,0.13687,0.1338,0.13277,0.12652,0.14317,0
.12495,0.12571,0.12293,0.12832,0.12785,0.12633,1.1253,1.1448,1.1769,1.
1653,1.1226,1.1103,1.1377,1.0966,1.1324,0.5662,0.58888,0.55224,0.46604
,0.46882,0.47217,0.48153,0.50265,0.39855,0.52784,0.42734,0.12957,0.132
61,0.1273,0.1294,0.13233,0.1309,0.12684,0.12826,0.12305,0.12687,0.1353
,0.12574,0.12957,0.13013,0.13053,0.13823,0.12763,0.12727,1.1144,1.1254
,1.1647,1.0146,1.2444,1.0478,1.0532,1.0829,1.1406,0.58982,0.46909,0.46
432,0.49844,0.51412,0.44877,0.48248,0.48952,0.4534,0.47008,0.48952,0.1
2746,0.13767,0.12879,0.12689,0.13043,0.12396,0.13335,0.13108,0.13134,0
.12625,0.13166,0.12291,0.12949,0.12751,0.12892,0.13569,0.13166,0.12086
,1.1569,1.134,1.0582,1.094,1.16,1.1024,1.1192,0.58108,0.61658,0.59269,
0.46922,0.50445,0.4397,0.52225,0.43167,0.42768,0.46155,0.4782,0.47239,

0.49843,0.12708,0.12499,0.12761,0.1275,0.13202,0.13229,0.12643,0.13421
,0.13106,0.12933,0.13063,0.12685,0.1276,0.12928,0.12852,0.13653,0.1301
2,0.12158,1.0482,1.1234,1.113,1.1249,1.0612,1.0806,0.56282,0.60518,0.6
036,0.64036,0.58978,0.50379,0.39694,0.4582,0.48794,0.46414,0.48198,0.4
4876,0.38063,0.45604,0.13143,0.12873,0.12557,0.13261,0.13099,0.12696,0
.13043,0.12636,0.12745,0.12178,0.1371,0.12799,0.13584,0.12774,0.12464,
0.12534,0.12976,0.135,1.1444,1.1343,1.0908,1.136,1.1966,0.67061,0.5937
7,0.65624,0.76114,0.6541,0.66506,0.44498,0.32431,0.48835,0.45549,0.407
47,0.4064,0.43635,0.51683,0.5322,0.12717,0.13203,0.12752,0.13696,0.132
68,0.13198,0.13245,0.12793,0.12651,0.12716,0.1308,0.13217,0.12208,0.12
742,0.12965,0.13017,0.12635,0.13169,1.0098,1.1832,1.1805,0.6742,0.5860
4,0.56549,0.72275,0.64293,0.57422,0.67264,0.64175,0.45973,0.36436,0.50
799,0.43756,0.40629,0.53351,0.51027,0.49217,0.5157,0.12114,0.13182,0.1
2606,0.13019,0.12976,0.13084,0.12902,0.13373,0.12998,0.13193,0.1261,0.
13443,0.13486,0.12575,0.12385,0.12992,0.128,0.1305,1.1692,0.79383,0.61
649,0.62091,0.64287,0.71997,0.57356,0.61275,0.64211,0.67086,0.46296,0.
44355,0.60065,0.43668,0.44463,0.48815,0.43773,0.49322,0.46447,0.13121,
0.13099,0.12663,0.1346,0.13135,0.13325,0.12989,0.1294,0.13148,0.13166,
0.12911,0.12642,0.12752,0.13189,0.13204,0.1276,0.12715,0.10779,0.60712
,0.61749,0.61721,0.66751,0.66642,0.60853,0.55146,0.4839,0.61793,0.5852
4,0.48134,0.43714,0.46568,0.44605,0.50784,0.39114,0.48122,0.4632,0.476
53,0.13427,0.13211,0.12535,0.1335,0.13026,0.13448,0.12605,0.12375,0.13
023,0.1336,0.13484,0.12955,0.12836,0.13413,0.1262,0.13013,0.12752,0.11
808,0.63469,0.61152,0.63366,0.53134,0.59722,0.61207,0.60766,0.63522,0.
64037,0.5833,0.48612,0.45924,0.44995,0.3596,0.33457,0.59501,0.40449,0.
42801,0.12064,0.12934,0.13099,0.13061,0.12758,0.12576,0.13078,0.1324,0
.13208,0.12637,0.13396,0.1296,0.12594,0.12626,0.13511,0.12964,0.12736,
0.1249,0.11433,0.68318,0.63784,0.65952,0.60962,0.69366,0.67816,0.64843
,0.45961,0.5693,0.65593,0.53352,0.35726,0.43202,0.41321,0.47255,0.5302
8,0.49055,0.42652,0.13331,0.12583,0.13301,0.12118,0.12633,0.12962,0.13
429,0.12996,0.1323,0.12019,0.13209,0.13005,0.12778,0.12723,0.12393,0.1
3173,0.13584,0.12002,0.11372,0.11874,0.52841,0.68635,0.6231,0.58908,0.
6241,0.29875,0.5278,0.64129,0.62344,0.43714,0.41385,0.39192,0.51641,0.
49745,0.44077,0.12273,0.12593,0.12296,0.12503,0.13255,0.1301,0.1288,0.
12875,0.12847,0.12411,0.12466,0.13614,0.13302,0.1307,0.12915,0.13308,0
.12583,0.12502,0.11353,0.11549,0.11206,0.11376,0.69973,0.65074,0.63431
,0.6764,0.62797,0.65251,0.6404,0.6262,0.64584,0.46928,0.44728,0.48115,
0.13787,0.13294,0.13458,0.12994,0.12998,0.13738,0.12451,0.12824,0.1271
9,0.1355,0.1363,0.13238,0.12875,0.1237,0.13472,0.12531,0.12557,0.13229
,0.12829,0.12444,0.11373,0.11563,0.11318,0.11022,0.11423,0.1189,0.5737
8,0.59129,0.54352,0.65708,0.64024,0.60467,0.66243,0.66224,0.12991,0.12
811,0.1338,0.12976,0.13306,0.13361,0.12442,0.13607,0.13491,0.12837,0.1
3341,0.12513,0.1299,0.12569,0.12898,0.12689,0.13287,0.12986,0.12974,0.
12439,0.1309,0.13013,0.12394,0.11451,0.11898,0.11502,0.11507,0.11515,0
.11404,0.61383,0.63169,0.66859,0.6074,0.63038,0.11848,0.11391,0.13093,
0.13007,0.12459,0.13362,0.12246,0.13008,0.12479,0.12841,0.13094,0.1327
3,0.12809,0.13037,0.13048,0.12681,0.12507,0.12845,0.13308,0.14026,0.13
294,0.13096,0.12211,0.11674,0.11511,0.1152,0.11057,0.11407,0.11111,0.1
1004,0.11558,0.11261,0.11436,0.4915,0.6175,0.11335,0.11555,0.11244,0.1
1382,0.12954,0.13145,0.12566,0.12546,0.13646,0.12977,0.1318,0.13178,0.
13176,0.13163,0.12964,0.12907,0.12731,0.12924,0.12123,0.12815,0.13084,
0.13096,0.1326,0.11279,0.11586,0.11182,0.11584,0.11837,0.11316,0.11777

,0.11023,0.11173,0.11721,0.11599,0.1141,0.11693,0.11484,0.11442,0.1123
2,0.12153,0.13028,0.13488,0.12724,0.12953,0.12911,0.12759,0.12582,0.13
252,0.12779,0.1246,0.13356,0.12351,0.13108,0.12914,0.128,0.12969,0.114
98,0.12143,0.11876,0.11184,0.1096,0.11702,0.11451,0.11318,0.11547,0.11
597,0.10995,0.11595,0.11718,0.10953,0.11692,0.11307,0.11458,0.11131,0.
11685,0.13467,0.12798,0.1253,0.12796,0.12503,0.12703,0.13409,0.12392,0
.13889,0.1294,0.12415,0.12984,0.12989,0.11509,0.11177,0.11332,0.11788,
0.11333,0.11307,0.11262,0.11484,0.11507,0.11795,0.11466,0.11434,0.1176
6,0.11894,0.11473,0.11389,0.1114,0.11634,0.11529,0.11208,0.11436,0.130
38,0.13295,0.12841,0.12852,0.13466,0.12863,0.1328,0.1288,0.13396,0.112
38,0.11513,0.11632,0.11686,0.11267,0.11299,0.11774,0.11722,0.11832,0.1
1411,0.11253,0.11343,0.11346,0.11416,0.11573,0.11273,0.11722,0.11583,0
.1179,0.11897,0.11414,0.11443,0.11706,0.1134,0.11796,0.1272,0.12514,0.
12692,0.12964,0.11645,0.11119,0.10811,0.11477,0.11463,0.10999,0.1123,0
.11279,0.11175,0.113,0.11496,0.11606,0.11223,0.12077,0.11309,0.11358,0
.11144,0.11582,0.11413,0.11328,0.11491,0.11509,0.11118,0.11398,0.11092
,0.116,0.11758,0.11533,0.11646,0.1165,0.11604,0.11488,0.11341,0.11331,
0.11237,0.11238,0.11377,0.11524,0.11738,0.11489,0.11435,0.11549,0.1193
9,0.1101,0.11381,0.11119,0.11343,0.11415,0.11367,0.11364,0.11364,0.114
78,0.11514,0.1122,0.1155,0.11242,0.11143,0.11623,0.10987,0.11581,0.112
12,0.11672,0.11468,0.11362,0.11716,0.11806,0.11457,0.11303,0.11242,0.1
0946,0.11817,0.11445,0.1198,0.11876,0.11862,0.11852,0.11357,0.11375,0.
11553,0.11243,0.116,0.11834,0.11072,0.11304,0.11676,0.11496,0.11359,0.
1193,0.11624,0.11311,0.11095,0.11284,0.11602,0.11435,0.11498,0.12067,0
.11431,0.11059,0.11031,0.11392,0.115,0.11628,0.11984,0.11403,0.11212,0
.11233,0.11703,0.11508,0.11404,0.11002,0.11532,0.11337,0.11457,0.11314
,0.11759,0.11148,0.11466,0.11021,0.11217,0.1148,0.11949,0.11389,0.1129
4,0.11787,0.11301,0.11268,0.11412,0.11906,0.11194,0.11472,0.11716,0.11
885,0.11626,0.11326,0.11348,0.11694,0.11816,0.10915,0.11692,0.12021,0.
11569,0.10589,0.11562,0.11412,0.11215,0.11642,0.11574,0.11206,0.11003,
0.11452,0.11429,0.11292,0.11412,0.11567,0.11579,0.10699,0.11157,0.1139
8,0.11429,0.11884,0.11581,0.11082,0.11903,0.11504,0.11678,0.11318,0.11
494,0.10811,0.11382,0.11292,0.11393,0.11079,0.11249,0.10806,0.11779,0.
11789,0.11488,0.11754,0.11207,0.1148,0.11127,0.11938,0.11269,0.12059,0
.11718,0.10718,0.11306,0.11154,0.11635,0.11155,0.1193,0.1147,0.1141,0.
11208,0.11435
0.16315,0.147,0.1608,0.16181,0.15643,0.15881,0.15477,0.15495,0.16494,0
.17076,0.16038,0.16981,0.16078,0.15767,0.15675,0.1582,0.16104,0.15752,
0.1567,0.15846,0.45373,0.54178,0.52328,0.4937,0.16683,0.16858,0.16755,
0.16191,0.15262,0.15424,0.15661,0.16201,0.16962,0.16965,0.16451,0.1584
8,0.15699,0.16143,0.1656,0.88799,0.47338,0.4659,0.48099,0.47633,0.5126
1,0.46578,0.16442,0.15977,0.15895,0.15505,0.16328,0.16867,0.16486,0.15
542,0.15225,0.16639,0.1556,0.1668,0.16813,0.1652,0.15736,0.89686,0.762
57,0.459,0.57783,0.50789,0.55814,0.51823,0.52955,0.39142,0.56956,0.166
78,0.14819,0.16002,0.17042,0.16656,0.1673,0.15515,0.16717,0.16071,0.16
134,0.16182,0.16709,0.15997,0.15819,0.16337,0.8116,0.84729,0.84278,0.4
3826,0.5341,0.42844,0.47555,0.45882,0.42313,0.3923,0.52533,0.5472,0.16
933,0.16212,0.16453,0.15737,0.15619,0.1595,0.15535,0.1655,0.15548,0.15
394,0.16375,0.16755,0.15949,0.16809,0.17216,0.16762,0.85232,0.7518,0.8
4363,0.78417,0.53845,0.41538,0.45472,0.51804,0.49554,0.43061,0.49206,0
.46163,0.50584,0.47862,0.15994,0.16461,0.16488,0.16422,0.16067,0.16047
,0.15083,0.15288,0.16478,0.16226,0.16937,0.16717,0.15845,0.15869,0.161

63,0.15753,0.87799,0.82855,0.88684,0.77927,0.81582,0.82632,0.56593,0.4
9931,0.48308,0.53614,0.45708,0.51038,0.52983,0.43895,0.48764,0.53879,0
.16224,0.16428,0.16581,0.15752,0.16736,0.14498,0.16454,0.15913,0.16542
,0.16631,0.15842,0.16056,0.1671,0.16445,0.15981,0.15277,0.15889,0.8756
4,0.82128,0.76764,0.83776,0.83405,0.82886,0.37309,0.47892,0.41827,0.52
224,0.512,0.4723,0.51297,0.49209,0.47857,0.52213,0.48606,0.15618,0.166
24,0.15784,0.16575,0.16214,0.16539,0.15419,0.15171,0.16172,0.15612,0.1
6339,0.15827,0.161,0.13966,0.14404,0.13823,0.15018,0.7909,0.80958,0.82
798,0.83961,0.71713,0.86705,0.78021,0.52277,0.53148,0.54306,0.53222,0.
5217,0.5695,0.55018,0.4791,0.56989,0.51235,0.49958,0.15902,0.16445,0.1
6198,0.15842,0.16713,0.15378,0.17311,0.1608,0.16393,0.162,0.16348,0.13
763,0.13129,0.14194,0.14105,0.13326,0.13872,0.16582,0.77134,0.95761,0.
82094,0.71438,0.79008,0.91414,0.75434,0.48083,0.59384,0.57293,0.49767,
0.53849,0.47233,0.47239,0.49723,0.50033,0.47325,0.51878,0.15878,0.1665
2,0.15604,0.15809,0.15079,0.15976,0.17133,0.16099,0.16534,0.14263,0.13
169,0.14004,0.14227,0.13919,0.13459,0.13916,0.13696,0.13612,0.16552,0.
76697,0.81498,0.77408,0.88455,0.76496,0.83561,0.79967,0.45807,0.45751,
0.54159,0.45859,0.53369,0.50957,0.50362,0.62571,0.5421,0.49103,0.48662
,0.52719,0.17034,0.16877,0.15739,0.15295,0.15805,0.16365,0.13027,0.144
46,0.14714,0.13684,0.13714,0.13562,0.13532,0.1237,0.1323,0.13345,0.138
57,0.14464,0.88552,0.71763,0.86252,0.91216,0.79004,0.76135,0.83885,0.3
5139,0.49513,0.49138,0.49241,0.59235,0.45532,0.51241,0.44528,0.44614,0
.51043,0.49489,0.4645,0.16237,0.16686,0.15449,0.14177,0.14127,0.13939,
0.14023,0.13741,0.13397,0.14401,0.14606,0.13817,0.13703,0.13995,0.1420
7,0.14101,0.13887,0.13581,0.8694,0.73251,0.8647,0.7682,0.75773,0.8596,
0.79714,0.84021,0.81825,0.49845,0.48555,0.43976,0.44258,0.51839,0.5177
5,0.49998,0.45992,0.50427,0.54256,0.4839,0.13753,0.13909,0.13707,0.138
23,0.13854,0.14125,0.13919,0.14404,0.14396,0.14168,0.1335,0.15058,0.13
245,0.13122,0.13288,0.13931,0.13515,0.13624,0.86406,0.88677,0.89244,0.
90372,0.88286,0.83424,0.83346,0.8135,0.83717,0.46737,0.42833,0.42903,0
.37549,0.37306,0.39276,0.38646,0.39848,0.32189,0.43614,0.33946,0.13745
,0.13977,0.13715,0.13838,0.14284,0.13948,0.1359,0.13638,0.13279,0.1347
6,0.14342,0.13344,0.13859,0.13723,0.13747,0.14648,0.13919,0.13729,0.82
609,0.74511,0.87288,0.71921,0.94506,0.72824,0.79833,0.83213,0.82613,0.
45292,0.37467,0.37847,0.39877,0.41504,0.34417,0.38964,0.38007,0.38205,
0.37057,0.41241,0.13379,0.14544,0.14029,0.13483,0.13859,0.13522,0.1421
1,0.14023,0.14068,0.1335,0.14421,0.13168,0.14031,0.13358,0.13538,0.146
41,0.13707,0.13216,0.83171,0.86089,0.78665,0.80051,0.89809,0.80821,0.8
5496,0.44425,0.45824,0.46203,0.37653,0.40475,0.35739,0.42971,0.33358,0
.34934,0.37697,0.37368,0.37155,0.39456,0.13373,0.13693,0.13453,0.13886
,0.14026,0.14425,0.13415,0.13909,0.14133,0.13758,0.13599,0.13453,0.138
49,0.13969,0.14126,0.1462,0.13899,0.12823,0.78174,0.82755,0.88055,0.83
111,0.75332,0.7295,0.45833,0.46749,0.46087,0.47442,0.45199,0.40371,0.3
2134,0.37536,0.37951,0.36925,0.38195,0.35282,0.32145,0.38369,0.13936,0
.1362,0.13124,0.14122,0.14071,0.13397,0.13735,0.13556,0.13734,0.13119,
0.14321,0.13791,0.14494,0.13753,0.13293,0.13525,0.1418,0.14242,0.90347
,0.82421,0.80319,0.82729,0.91722,0.49427,0.45333,0.52592,0.58606,0.490
03,0.49552,0.37152,0.27908,0.38848,0.35542,0.33084,0.3358,0.35641,0.39
717,0.4254,0.13698,0.14103,0.1359,0.14482,0.14161,0.14257,0.14134,0.13
683,0.13379,0.13612,0.13702,0.14496,0.13088,0.13569,0.13734,0.13663,0.
13322,0.1369,0.75375,0.85216,0.85908,0.5044,0.44281,0.42144,0.55561,0.
4646,0.44182,0.53005,0.50293,0.36887,0.2918,0.42059,0.34638,0.32618,0.

40209,0.40652,0.41431,0.41653,0.12937,0.13972,0.13539,0.14056,0.1366,0
.14005,0.13855,0.14382,0.13654,0.13806,0.13434,0.14162,0.14451,0.13179
,0.13026,0.13993,0.13729,0.13998,0.853,0.59849,0.47552,0.46058,0.48431
,0.5579,0.44165,0.46954,0.46549,0.50392,0.36677,0.35722,0.48471,0.3491
9,0.33207,0.38349,0.35261,0.39585,0.36883,0.14124,0.13949,0.13334,0.14
347,0.14072,0.14408,0.13672,0.13897,0.14182,0.14209,0.14346,0.13562,0.
13772,0.14336,0.14033,0.13988,0.13481,0.11012,0.47067,0.46279,0.46891,
0.54002,0.52632,0.48705,0.41648,0.3917,0.46633,0.45195,0.39217,0.3671,
0.37826,0.37878,0.40247,0.31101,0.38737,0.3649,0.36905,0.14459,0.13929
,0.13595,0.14177,0.13926,0.14261,0.1344,0.13182,0.1382,0.13785,0.14153
,0.1386,0.13532,0.14313,0.13355,0.13761,0.13548,0.11623,0.4928,0.45196
,0.48782,0.37731,0.47338,0.44737,0.44203,0.49847,0.49989,0.46343,0.393
51,0.35524,0.36889,0.29192,0.28322,0.47866,0.32224,0.336,0.1272,0.1382
9,0.13791,0.13697,0.13604,0.13522,0.13853,0.13764,0.14078,0.136,0.1421
,0.13952,0.13382,0.13385,0.14537,0.1374,0.13355,0.13325,0.11331,0.5670
3,0.49859,0.50757,0.47841,0.55254,0.50713,0.47607,0.36866,0.44205,0.48
494,0.42453,0.30668,0.34444,0.33322,0.37569,0.4423,0.39842,0.34183,0.1
432,0.13291,0.1434,0.12867,0.13447,0.13944,0.14231,0.1403,0.14621,0.13
216,0.14049,0.13727,0.13713,0.13563,0.13158,0.1394,0.14789,0.11768,0.1
137,0.11722,0.39593,0.52572,0.46081,0.46819,0.47867,0.24164,0.4121,0.4
9807,0.47819,0.34944,0.32935,0.31113,0.39704,0.38859,0.34053,0.1301,0.
13587,0.13396,0.13339,0.13943,0.13807,0.13819,0.13499,0.13507,0.13288,
0.13115,0.14747,0.14084,0.13889,0.13666,0.14114,0.13362,0.13457,0.1126
1,0.11421,0.1128,0.1142,0.54641,0.49182,0.49357,0.53336,0.4924,0.50516
,0.51108,0.49873,0.51918,0.3741,0.36264,0.4029,0.14488,0.14118,0.14882
,0.13532,0.13924,0.14608,0.13194,0.13389,0.13381,0.14319,0.14552,0.142
02,0.13626,0.13121,0.14254,0.13521,0.13395,0.13972,0.13689,0.13254,0.1
1333,0.11553,0.11318,0.10879,0.11333,0.11791,0.42332,0.45119,0.38893,0
.51146,0.46926,0.46047,0.5202,0.52547,0.14022,0.13943,0.14299,0.13961,
0.14377,0.14124,0.13446,0.14299,0.14357,0.13796,0.14144,0.1314,0.14066
,0.1328,0.13646,0.1349,0.14402,0.14027,0.13738,0.13486,0.1387,0.13827,
0.12891,0.11479,0.12081,0.11416,0.1158,0.11268,0.1121,0.44443,0.47416,
0.52351,0.45099,0.48368,0.11726,0.11349,0.1416,0.13619,0.13291,0.14446
,0.12804,0.13945,0.13272,0.13513,0.13995,0.14281,0.13597,0.13972,0.139
04,0.13291,0.13375,0.13699,0.14293,0.14791,0.14244,0.13743,0.12984,0.1
1487,0.11411,0.11271,0.11215,0.1111,0.11141,0.10855,0.1143,0.1124,0.11
376,0.37461,0.47384,0.11332,0.1158,0.11323,0.11317,0.13964,0.13557,0.1
3672,0.13333,0.14842,0.14181,0.14167,0.13884,0.13956,0.13769,0.13744,0
.13995,0.13712,0.13721,0.12854,0.13326,0.13876,0.13684,0.1421,0.11278,
0.11362,0.1132,0.11746,0.11975,0.11029,0.11694,0.11059,0.11152,0.11748
,0.11378,0.11241,0.11762,0.11349,0.11351,0.11418,0.11847,0.14179,0.143
62,0.13214,0.1415,0.13564,0.13729,0.13329,0.14107,0.13412,0.13283,0.14
415,0.13233,0.13864,0.13897,0.13703,0.14162,0.11545,0.12205,0.11541,0.
11042,0.10869,0.11323,0.11389,0.11326,0.11626,0.11615,0.11104,0.11387,
0.11561,0.10844,0.1168,0.11147,0.1135,0.11096,0.11763,0.14244,0.13437,
0.13568,0.13983,0.13339,0.13297,0.14348,0.13116,0.1538,0.13792,0.13192
,0.13975,0.14031,0.11564,0.11158,0.11284,0.11476,0.11236,0.11434,0.113
01,0.11497,0.11639,0.11877,0.11434,0.11296,0.11878,0.1173,0.11488,0.11
664,0.1123,0.11883,0.11584,0.11198,0.11485,0.13895,0.139,0.13762,0.138
94,0.14358,0.14066,0.14299,0.13873,0.14306,0.11152,0.11432,0.11471,0.1
1735,0.1116,0.11114,0.11658,0.11693,0.11743,0.1142,0.11296,0.11092,0.1
1235,0.11384,0.11551,0.11354,0.1186,0.11532,0.11639,0.11584,0.11497,0.

11352,0.11513,0.11234,0.11795,0.13623,0.1366,0.13603,0.13704,0.11698,0
.11021,0.10802,0.11366,0.11387,0.11041,0.11427,0.11431,0.11186,0.11157
,0.11253,0.11534,0.11302,0.1196,0.11438,0.11366,0.11106,0.11633,0.1159
5,0.11289,0.11487,0.11648,0.111,0.11396,0.11131,0.1129,0.11675,0.11443
,0.11512,0.11369,0.11497,0.11437,0.11169,0.11609,0.11251,0.11281,0.113
79,0.11329,0.11486,0.11462,0.11598,0.11467,0.118,0.11019,0.11366,0.111
79,0.11249,0.11361,0.11342,0.11331,0.11063,0.1165,0.11386,0.1115,0.113
47,0.11499,0.1105,0.11676,0.10896,0.11569,0.11357,0.11439,0.11474,0.11
431,0.11686,0.11776,0.11539,0.11289,0.11362,0.10975,0.11679,0.11527,0.
11923,0.11918,0.11641,0.116,0.11134,0.11273,0.11533,0.11252,0.11331,0.
11774,0.10954,0.11127,0.1158,0.1167,0.114,0.11821,0.11374,0.11388,0.11
133,0.11146,0.11328,0.11352,0.11419,0.11827,0.11419,0.11136,0.10923,0.
11451,0.11429,0.11716,0.11934,0.11316,0.1121,0.11236,0.11709,0.11486,0
.11445,0.10972,0.11445,0.11253,0.11246,0.11365,0.11574,0.1107,0.11545,
0.10953,0.11201,0.11512,0.11963,0.11353,0.11206,0.11648,0.11279,0.1117
8,0.11411,0.11794,0.11031,0.11203,0.1163,0.1177,0.11623,0.11314,0.1144
,0.11522,0.11812,0.1098,0.11745,0.12075,0.11421,0.10569,0.11505,0.1135
8,0.11145,0.11579,0.11489,0.11203,0.10837,0.11308,0.11383,0.112,0.1119
3,0.11524,0.11516,0.10782,0.11248,0.11083,0.11148,0.11646,0.1153,0.112
55,0.11789,0.1139,0.11702,0.113,0.11441,0.10694,0.11609,0.11132,0.1130
1,0.11008,0.11294,0.10999,0.11594,0.11651,0.11597,0.11873,0.11235,0.11
487,0.11066,0.12056,0.11391,0.11829,0.11627,0.10786,0.11223,0.1101,0.1
1317,0.1126,0.11939,0.11453,0.11299,0.11048,0.11179
0.17264,0.15626,0.16955,0.17463,0.16751,0.16976,0.16685,0.16613,0.1777
5,0.18187,0.1713,0.18169,0.17026,0.16757,0.16996,0.16907,0.17418,0.168
34,0.1703,0.16847,0.38812,0.45684,0.42161,0.394,0.17421,0.18002,0.1789
8,0.17611,0.16595,0.1632,0.16602,0.17082,0.18043,0.17784,0.17754,0.167
85,0.16977,0.1742,0.17555,0.7258,0.39305,0.36934,0.40405,0.41613,0.424
31,0.41016,0.17448,0.17195,0.16913,0.16695,0.17303,0.18032,0.1747,0.17
115,0.16295,0.17697,0.16672,0.17574,0.17892,0.17894,0.16998,0.73979,0.
55544,0.40342,0.47811,0.42642,0.44473,0.44795,0.45843,0.33479,0.48945,
0.17841,0.16474,0.17319,0.18102,0.17598,0.17823,0.16761,0.17513,0.1700
9,0.17174,0.1732,0.17633,0.17182,0.1721,0.17723,0.65489,0.67573,0.6401
4,0.3662,0.44917,0.36622,0.38646,0.37873,0.36581,0.34316,0.4465,0.4861
3,0.17596,0.17227,0.17543,0.16557,0.16448,0.16948,0.17106,0.17848,0.16
61,0.16964,0.17026,0.17913,0.1692,0.17676,0.18392,0.17557,0.68813,0.63
151,0.66364,0.62112,0.43762,0.36204,0.40199,0.42744,0.41822,0.35904,0.
38676,0.38669,0.41193,0.39166,0.17,0.17497,0.17472,0.17589,0.17424,0.1
6905,0.16317,0.16545,0.17369,0.17496,0.18123,0.18049,0.17131,0.17324,0
.17226,0.16597,0.69848,0.60938,0.68423,0.63161,0.6183,0.65031,0.47481,
0.39477,0.40216,0.45094,0.36635,0.43034,0.42993,0.35358,0.403,0.43,0.1
7031,0.17769,0.17605,0.16675,0.17941,0.15528,0.17064,0.16891,0.17848,0
.18323,0.1677,0.17072,0.18126,0.17602,0.17042,0.16409,0.17099,0.68944,
0.6483,0.59481,0.65094,0.65694,0.66047,0.3246,0.39616,0.3431,0.4286,0.
42814,0.40315,0.4227,0.40758,0.40918,0.43012,0.39329,0.16645,0.17888,0
.1699,0.17726,0.17417,0.17746,0.16008,0.16234,0.17243,0.16865,0.17759,
0.17411,0.17241,0.1425,0.15158,0.14,0.16219,0.61824,0.66798,0.64544,0.
64769,0.55705,0.67741,0.63702,0.45351,0.44371,0.4166,0.44555,0.4324,0.
45828,0.45554,0.4136,0.475,0.43046,0.42474,0.17366,0.17603,0.17234,0.1
72,0.17755,0.16807,0.18402,0.17123,0.17493,0.17503,0.17455,0.13932,0.1
3367,0.14422,0.14388,0.13829,0.14001,0.1756,0.57092,0.75424,0.68855,0.
51887,0.61562,0.75258,0.63524,0.40229,0.49852,0.48566,0.43777,0.43894,

0.40101,0.39134,0.41408,0.41717,0.39218,0.42782,0.16602,0.17996,0.1662
6,0.16892,0.16587,0.17153,0.183,0.17031,0.18116,0.14553,0.13286,0.1445
4,0.14576,0.1414,0.1377,0.14182,0.13935,0.1384,0.17611,0.57708,0.65567
,0.63075,0.6725,0.56943,0.61873,0.63968,0.38049,0.36814,0.46242,0.3810
1,0.43943,0.42586,0.41986,0.52141,0.46104,0.42152,0.40229,0.41826,0.17
807,0.18123,0.16953,0.16381,0.17205,0.17473,0.1339,0.14747,0.15038,0.1
3801,0.14056,0.13895,0.13657,0.12654,0.13196,0.13658,0.1419,0.15095,0.
70076,0.59669,0.68936,0.71198,0.57821,0.60771,0.64431,0.30521,0.38136,
0.4025,0.4186,0.48211,0.35759,0.41226,0.38003,0.37378,0.42343,0.41635,
0.3905,0.17631,0.17753,0.17115,0.14671,0.14484,0.14005,0.14015,0.14109
,0.13722,0.1452,0.14964,0.14069,0.13991,0.14019,0.14553,0.14351,0.1444
1,0.13864,0.71763,0.56818,0.67441,0.56862,0.58389,0.68954,0.63667,0.67
875,0.61703,0.39621,0.42442,0.35631,0.36703,0.42055,0.42315,0.41743,0.
37679,0.45587,0.43517,0.40885,0.14011,0.13884,0.13968,0.1419,0.14178,0
.14281,0.14271,0.14477,0.1476,0.14453,0.13682,0.15691,0.13252,0.13281,
0.13619,0.14281,0.138,0.13661,0.70632,0.712,0.6839,0.73616,0.68749,0.6
6903,0.66778,0.64952,0.62703,0.40204,0.31817,0.37455,0.29688,0.3165,0.
33822,0.30746,0.3321,0.28095,0.36451,0.2873,0.14118,0.14355,0.14108,0.
14311,0.14433,0.14287,0.14036,0.142,0.13605,0.13718,0.14708,0.13678,0.
14219,0.14055,0.14077,0.14792,0.14199,0.14267,0.68532,0.57149,0.7091,0
.54986,0.78904,0.57022,0.62888,0.62681,0.59283,0.36139,0.31737,0.31609
,0.33033,0.33001,0.28613,0.33441,0.30562,0.33336,0.31237,0.35322,0.138
19,0.1479,0.14878,0.1383,0.14321,0.13768,0.14462,0.14398,0.1429,0.1357
5,0.15156,0.13486,0.14253,0.13329,0.13837,0.14578,0.13908,0.13553,0.68
296,0.65474,0.6381,0.62888,0.71189,0.61798,0.6339,0.34417,0.36736,0.38
307,0.32866,0.33232,0.30481,0.33687,0.28672,0.30481,0.33353,0.30066,0.
31343,0.3123,0.13393,0.14297,0.13628,0.14519,0.14381,0.14975,0.13636,0
.14509,0.14434,0.1404,0.13945,0.13819,0.14438,0.1398,0.1438,0.15098,0.
14181,0.13177,0.61321,0.6364,0.66573,0.63197,0.58675,0.5216,0.36055,0.
38909,0.37188,0.38744,0.37738,0.34548,0.2889,0.31235,0.30726,0.31655,0
.32062,0.2952,0.26894,0.3203,0.14358,0.14012,0.13133,0.14536,0.14632,0
.13798,0.14054,0.13693,0.13959,0.13272,0.15041,0.13894,0.14943,0.14341
,0.13809,0.13528,0.14538,0.14451,0.71661,0.64708,0.59941,0.6937,0.7503
2,0.41809,0.37534,0.41489,0.50587,0.35962,0.38004,0.31037,0.24491,0.33
533,0.31087,0.28165,0.27499,0.3066,0.33778,0.33229,0.14096,0.14126,0.1
3894,0.14819,0.14472,0.14412,0.14706,0.13989,0.13634,0.13782,0.14311,0
.15024,0.13238,0.13857,0.13968,0.13824,0.13769,0.14094,0.54842,0.67024
,0.70034,0.39025,0.3704,0.34427,0.46519,0.34776,0.34571,0.41495,0.4147
7,0.31217,0.25922,0.33677,0.29643,0.27642,0.32139,0.33142,0.3559,0.362
64,0.13189,0.14193,0.13709,0.14501,0.14031,0.14148,0.13917,0.14644,0.1
4016,0.13946,0.13497,0.14286,0.14715,0.13423,0.13367,0.14571,0.14241,0
.14235,0.76416,0.49808,0.38268,0.37319,0.41678,0.45811,0.36037,0.37332
,0.36623,0.42946,0.29288,0.30199,0.41285,0.29688,0.2769,0.31209,0.3042
5,0.33577,0.31782,0.14598,0.14684,0.14044,0.14384,0.14378,0.14887,0.13
753,0.14228,0.14456,0.1433,0.15116,0.13899,0.14348,0.1456,0.13974,0.14
308,0.14064,0.10534,0.35793,0.35304,0.3737,0.4311,0.43643,0.40105,0.33
844,0.32722,0.3623,0.3617,0.30567,0.32148,0.32018,0.31775,0.33949,0.27
693,0.32469,0.31088,0.30072,0.14735,0.1425,0.13887,0.14635,0.14014,0.1
4675,0.13819,0.13698,0.14134,0.14025,0.1449,0.14321,0.13771,0.1471,0.1
3847,0.13969,0.13695,0.11077,0.41687,0.36252,0.4024,0.29691,0.37646,0.
35921,0.35629,0.41963,0.40203,0.36573,0.32976,0.30533,0.30868,0.26594,
0.24774,0.3909,0.27009,0.29282,0.13277,0.14004,0.13997,0.13972,0.13381

,0.13847,0.14078,0.1386,0.1409,0.14364,0.14993,0.1415,0.13562,0.13694,
0.14629,0.14164,0.13656,0.13706,0.10582,0.47185,0.41653,0.4125,0.37831
,0.47656,0.39946,0.39318,0.29714,0.35693,0.38648,0.35354,0.25544,0.285
32,0.29493,0.33148,0.36328,0.33603,0.28822,0.14694,0.13482,0.14697,0.1
2931,0.13829,0.13986,0.14353,0.14472,0.15321,0.13362,0.14212,0.13923,0
.13961,0.13715,0.13446,0.14505,0.15046,0.111,0.10746,0.11044,0.3273,0.
42691,0.37049,0.37487,0.3953,0.20879,0.34716,0.4003,0.38442,0.29175,0.
2755,0.26585,0.33619,0.32372,0.29095,0.12971,0.13634,0.13787,0.13925,0
.14235,0.14089,0.14173,0.1338,0.14125,0.13426,0.13447,0.15248,0.14512,
0.14359,0.13726,0.14435,0.13592,0.13843,0.10553,0.10785,0.1076,0.10829
,0.43576,0.38934,0.42674,0.41691,0.38064,0.40034,0.41698,0.40639,0.424
74,0.32758,0.29346,0.3411,0.14582,0.14308,0.16057,0.14338,0.14143,0.14
923,0.13243,0.13686,0.13723,0.14403,0.14988,0.14684,0.14058,0.13415,0.
14637,0.14044,0.13324,0.1446,0.13913,0.13307,0.10964,0.10867,0.10483,0
.1041,0.10988,0.11082,0.34981,0.36318,0.31076,0.40556,0.3723,0.3683,0.
42062,0.42237,0.14421,0.14493,0.14763,0.14366,0.14757,0.14413,0.13798,
0.14541,0.14741,0.13931,0.14416,0.13304,0.14695,0.13222,0.13893,0.1372
5,0.15055,0.14495,0.141,0.14011,0.14276,0.14383,0.12957,0.11008,0.1134
2,0.11,0.10952,0.10836,0.10531,0.35074,0.39567,0.44138,0.36583,0.37659
,0.11027,0.10954,0.14528,0.13791,0.13488,0.14987,0.13299,0.14265,0.134
02,0.13571,0.14406,0.14624,0.13863,0.14436,0.14169,0.13643,0.13706,0.1
402,0.14548,0.15219,0.1446,0.14237,0.13048,0.10975,0.10798,0.10592,0.1
0785,0.10653,0.10538,0.1034,0.10675,0.10803,0.10834,0.31443,0.38111,0.
10881,0.11022,0.1071,0.10547,0.14601,0.13693,0.13896,0.13921,0.15124,0
.14746,0.14561,0.14378,0.14371,0.13855,0.13799,0.14456,0.13931,0.14263
,0.13156,0.13404,0.14647,0.13961,0.14555,0.10859,0.10826,0.10774,0.111
06,0.11351,0.10531,0.11212,0.10342,0.10529,0.11335,0.1084,0.10856,0.11
254,0.10871,0.10973,0.11033,0.11302,0.1436,0.14807,0.13397,0.14237,0.1
375,0.14046,0.13755,0.14303,0.13484,0.13414,0.14871,0.13469,0.14259,0.
13986,0.14102,0.1443,0.10945,0.11421,0.10933,0.10439,0.10361,0.10728,0
.10963,0.10889,0.11188,0.11096,0.10664,0.10929,0.10695,0.10465,0.10995
,0.1055,0.10797,0.10681,0.11279,0.14625,0.13658,0.13789,0.14373,0.1347
2,0.13446,0.15238,0.13193,0.15716,0.14088,0.13297,0.14219,0.14339,0.10
939,0.10578,0.107,0.10876,0.10678,0.1099,0.10819,0.10923,0.11135,0.111
81,0.10856,0.10783,0.11366,0.11274,0.10977,0.1115,0.1067,0.1125,0.1099
3,0.10626,0.10675,0.14163,0.1426,0.1407,0.14324,0.14838,0.14329,0.1447
5,0.14433,0.14836,0.10453,0.11138,0.10783,0.11146,0.10734,0.10621,0.11
024,0.11332,0.10975,0.10769,0.10643,0.10673,0.10777,0.10831,0.10785,0.
10911,0.11236,0.11148,0.10833,0.10863,0.11003,0.10646,0.11083,0.10558,
0.11324,0.13819,0.14056,0.13872,0.14093,0.11256,0.10561,0.10085,0.1092
3,0.10797,0.10256,0.11158,0.10845,0.10811,0.10572,0.10623,0.10914,0.10
621,0.11155,0.10797,0.10689,0.10551,0.11145,0.1116,0.10734,0.1103,0.11
161,0.10705,0.10665,0.10423,0.10783,0.111,0.11013,0.10793,0.10334,0.10
926,0.10911,0.10562,0.10962,0.10591,0.1062,0.10863,0.10719,0.10765,0.1
0776,0.11068,0.10994,0.11179,0.1037,0.11033,0.10682,0.10657,0.10951,0.
10856,0.1072,0.10324,0.1074,0.10838,0.10725,0.10735,0.10803,0.10627,0.
10985,0.10224,0.11063,0.10708,0.10672,0.10743,0.10838,0.11148,0.11214,
0.10963,0.10662,0.10858,0.10431,0.10949,0.11151,0.11094,0.11417,0.1094
1,0.11009,0.10768,0.10735,0.10811,0.10804,0.1072,0.10961,0.10212,0.104
7,0.11048,0.10847,0.10966,0.11302,0.10862,0.10784,0.10343,0.1061,0.107
5,0.11094,0.1085,0.11267,0.10884,0.10648,0.10353,0.10963,0.10741,0.112
85,0.11052,0.10919,0.108,0.10754,0.11211,0.10982,0.10968,0.10677,0.108

41,0.10741,0.10659,0.10739,0.10883,0.10537,0.10826,0.10496,0.10743,0.1
0931,0.11251,0.10661,0.10714,0.10979,0.10773,0.10693,0.10729,0.11172,0
.10419,0.10744,0.11213,0.11188,0.11224,0.10858,0.10578,0.10859,0.11136
,0.10585,0.1112,0.11334,0.10802,0.10132,0.10819,0.1068,0.10633,0.1094,
0.1092,0.10521,0.10372,0.10788,0.10662,0.10476,0.107,0.11,0.10921,0.10
274,0.10572,0.1051,0.10754,0.11033,0.10958,0.10804,0.11464,0.10875,0.1
1049,0.1059,0.10905,0.10328,0.11144,0.1049,0.10693,0.10521,0.10973,0.1
0501,0.10811,0.10886,0.10965,0.11244,0.10627,0.10898,0.10458,0.11482,0
.10794,0.10998,0.11177,0.10126,0.10708,0.10329,0.10858,0.10733,0.11316
,0.11075,0.10785,0.10419,0.1043
0.18049,0.16146,0.1784,0.17853,0.17145,0.17532,0.1757,0.17501,0.18559,
0.19366,0.17909,0.18703,0.1794,0.17501,0.17843,0.18182,0.18362,0.17485
,0.1762,0.17388,0.3196,0.41424,0.37508,0.35054,0.18253,0.1854,0.18733,
0.1901,0.17313,0.17048,0.175,0.17902,0.1896,0.18792,0.18413,0.17966,0.
17536,0.18261,0.18356,0.66648,0.35809,0.3335,0.35462,0.36339,0.38536,0
.34776,0.18119,0.17877,0.18126,0.17045,0.17813,0.18687,0.1818,0.18112,
0.17163,0.18166,0.1753,0.18687,0.18279,0.18644,0.17794,0.6085,0.45808,
0.37069,0.42807,0.38951,0.38117,0.37181,0.40478,0.30898,0.41833,0.1899
2,0.17104,0.18119,0.18552,0.1838,0.18657,0.17572,0.18193,0.17677,0.178
4,0.17984,0.18442,0.17863,0.18489,0.18174,0.54987,0.54287,0.53837,0.30
834,0.40024,0.32429,0.36293,0.32459,0.3147,0.31504,0.42425,0.42557,0.1
8293,0.17729,0.18075,0.17531,0.1735,0.17339,0.17703,0.18734,0.17669,0.
17827,0.17682,0.18966,0.1775,0.18218,0.18691,0.1797,0.61081,0.51486,0.
54856,0.48377,0.37881,0.31466,0.36848,0.36194,0.38311,0.31401,0.32157,
0.35115,0.37504,0.32717,0.17682,0.18297,0.18109,0.18462,0.1797,0.17889
,0.17635,0.16969,0.18288,0.18053,0.18821,0.18768,0.18088,0.17993,0.179
79,0.17516,0.60161,0.44729,0.61909,0.50444,0.55333,0.52174,0.43405,0.3
5744,0.3457,0.39625,0.32022,0.38793,0.38518,0.30703,0.35694,0.40723,0.
17728,0.18517,0.18509,0.17269,0.18665,0.16642,0.17975,0.17852,0.18859,
0.19106,0.17543,0.17804,0.1909,0.18362,0.17685,0.17611,0.17999,0.5912,
0.51808,0.52424,0.48289,0.49134,0.52242,0.30839,0.35725,0.30321,0.3761
9,0.37522,0.37066,0.38061,0.33969,0.34284,0.39407,0.3223,0.1762,0.1879
8,0.18205,0.18278,0.18358,0.18167,0.16911,0.17474,0.18003,0.17775,0.18
396,0.18419,0.17944,0.14032,0.15731,0.13813,0.17243,0.47766,0.54609,0.
56074,0.50455,0.50683,0.54463,0.49419,0.37053,0.36886,0.35101,0.38849,
0.35741,0.40401,0.4154,0.36655,0.43317,0.38397,0.37219,0.18375,0.18171
,0.18221,0.18005,0.18546,0.17856,0.1936,0.18194,0.18306,0.18121,0.1797
1,0.1404,0.13186,0.14558,0.13952,0.13822,0.14058,0.18117,0.44635,0.616
31,0.60915,0.43191,0.48796,0.66665,0.51517,0.37863,0.4289,0.39243,0.37
838,0.39034,0.34627,0.32875,0.35948,0.34931,0.34247,0.376,0.17577,0.18
78,0.17146,0.17816,0.17342,0.18116,0.18921,0.17969,0.18621,0.14499,0.1
3191,0.14762,0.14764,0.14142,0.13549,0.14248,0.13912,0.13708,0.18259,0
.49934,0.562,0.50091,0.56486,0.42766,0.53375,0.54303,0.35213,0.31664,0
.40354,0.33847,0.38254,0.37598,0.35024,0.44793,0.39522,0.37884,0.36348
,0.3739,0.18604,0.18545,0.1771,0.17429,0.18023,0.18256,0.13549,0.14733
,0.14949,0.13745,0.1402,0.1393,0.13466,0.12644,0.13183,0.13569,0.14202
,0.15346,0.60531,0.46956,0.56422,0.57594,0.50647,0.51665,0.50554,0.274
79,0.35625,0.35912,0.38123,0.39254,0.30561,0.35471,0.34875,0.32296,0.3
5642,0.35073,0.34622,0.18187,0.18435,0.1819,0.14692,0.14889,0.13942,0.
14315,0.14032,0.13664,0.14396,0.15136,0.14118,0.14246,0.14063,0.14638,
0.14173,0.14735,0.13678,0.58264,0.43787,0.55816,0.46079,0.50272,0.5542
4,0.50225,0.56672,0.49387,0.35756,0.37656,0.31855,0.31587,0.38016,0.36

541,0.38298,0.32651,0.39475,0.387,0.38045,0.14141,0.13827,0.14294,0.14
004,0.14299,0.14289,0.14297,0.14316,0.14711,0.14399,0.13652,0.15559,0.
12925,0.1326,0.13744,0.14231,0.13752,0.13818,0.61118,0.59792,0.5468,0.
61294,0.58527,0.58793,0.54987,0.56481,0.50821,0.34044,0.26099,0.31983,
0.26261,0.27308,0.30268,0.25964,0.30378,0.253,0.32305,0.24999,0.14048,
0.14415,0.14013,0.14451,0.1472,0.14324,0.14031,0.13912,0.13721,0.13776
,0.15112,0.13684,0.13802,0.14066,0.14218,0.15075,0.13873,0.14585,0.572
65,0.44254,0.58489,0.4482,0.66276,0.41376,0.4793,0.51354,0.46547,0.309
05,0.26561,0.2803,0.29122,0.29831,0.25906,0.30532,0.26248,0.30546,0.27
415,0.32001,0.13981,0.14766,0.14947,0.13952,0.14221,0.13945,0.1462,0.1
4307,0.14056,0.13541,0.15918,0.13412,0.14375,0.12985,0.14164,0.14707,0
.13715,0.13421,0.5611,0.51831,0.54349,0.50626,0.68312,0.51604,0.5532,0
.28313,0.32148,0.30763,0.30246,0.28759,0.28169,0.28681,0.24741,0.28357
,0.27431,0.27106,0.27629,0.27852,0.12927,0.14292,0.13654,0.14299,0.142
82,0.15208,0.13859,0.14521,0.14149,0.14084,0.14204,0.13815,0.14483,0.1
4221,0.14254,0.14992,0.14186,0.12981,0.51801,0.49411,0.54904,0.56034,0
.47006,0.43586,0.31303,0.31155,0.33178,0.3369,0.32727,0.30124,0.25506,
0.27913,0.26673,0.2776,0.28297,0.25818,0.22794,0.29184,0.14463,0.14085
,0.12884,0.14485,0.14631,0.14042,0.14191,0.13565,0.14001,0.1343,0.1480
7,0.13792,0.15321,0.14531,0.13751,0.13553,0.14493,0.14717,0.58388,0.53
256,0.50184,0.60209,0.63166,0.34813,0.31896,0.36327,0.4374,0.31371,0.3
0879,0.28179,0.2209,0.31042,0.26941,0.2475,0.24395,0.26501,0.29256,0.2
8257,0.14219,0.13987,0.14103,0.15061,0.14602,0.14373,0.1473,0.14048,0.
13678,0.13695,0.14536,0.15189,0.13383,0.13926,0.1402,0.13898,0.14156,0
.14141,0.47062,0.54591,0.5455,0.34302,0.3334,0.28338,0.40197,0.30411,0
.28718,0.342,0.33513,0.26943,0.2289,0.30987,0.26864,0.23895,0.27705,0.
28911,0.30931,0.31779,0.13186,0.14532,0.13686,0.14496,0.13732,0.14361,
0.13892,0.14551,0.14095,0.14006,0.13401,0.14378,0.14638,0.13371,0.1340
2,0.1458,0.14162,0.14159,0.63961,0.4135,0.31568,0.29196,0.35786,0.3764
,0.3181,0.31426,0.30758,0.34978,0.26311,0.26057,0.35781,0.26658,0.2491
2,0.27784,0.27091,0.2909,0.27516,0.14905,0.14303,0.14038,0.14436,0.144
22,0.15085,0.13312,0.14352,0.14761,0.1453,0.15575,0.13754,0.1445,0.145
4,0.13816,0.14318,0.14376,0.099011,0.29874,0.28799,0.31957,0.35685,0.3
5898,0.32388,0.28238,0.31029,0.28872,0.32633,0.27848,0.2878,0.28747,0.
28219,0.28829,0.2434,0.29318,0.28561,0.2544,0.14872,0.14287,0.1384,0.1
4446,0.14294,0.14867,0.1403,0.13841,0.14017,0.13926,0.14338,0.14273,0.
13696,0.14828,0.14049,0.14036,0.14017,0.10093,0.33906,0.32155,0.34071,
0.2469,0.33539,0.29939,0.31242,0.35913,0.36492,0.32052,0.29906,0.2648,
0.28556,0.23965,0.2284,0.3447,0.22767,0.25315,0.13142,0.14019,0.13982,
0.14028,0.13158,0.14009,0.13999,0.13676,0.14099,0.14403,0.1538,0.14544
,0.13889,0.13799,0.14754,0.14149,0.13559,0.1386,0.097072,0.4063,0.3710
2,0.35298,0.31654,0.3985,0.31687,0.33627,0.25351,0.29847,0.32793,0.293
76,0.22245,0.24899,0.27272,0.30535,0.32934,0.30552,0.24597,0.14676,0.1
3076,0.14653,0.1313,0.13605,0.13804,0.13948,0.14488,0.15553,0.13458,0.
1413,0.13962,0.13878,0.13874,0.13643,0.14678,0.15125,0.10483,0.10032,0
.10285,0.27289,0.36611,0.28525,0.32434,0.34035,0.17658,0.29657,0.32344
,0.32157,0.24396,0.24655,0.24569,0.29048,0.28417,0.25805,0.12721,0.136
05,0.13733,0.13681,0.14594,0.13911,0.14474,0.13168,0.14209,0.13284,0.1
3305,0.15345,0.14518,0.14104,0.13573,0.14292,0.13552,0.14229,0.09783,0
.097831,0.10176,0.09963,0.37117,0.33528,0.3885,0.32591,0.31763,0.32524
,0.34692,0.34514,0.37112,0.28004,0.24709,0.30088,0.14241,0.13963,0.166
7,0.14316,0.14285,0.14872,0.1318,0.13877,0.13686,0.14197,0.15164,0.149

19,0.14081,0.1337,0.15142,0.13945,0.135,0.1472,0.14008,0.13354,0.10127
,0.0997,0.097672,0.094333,0.10416,0.10314,0.30575,0.30732,0.24494,0.35
472,0.3225,0.31693,0.37399,0.34969,0.14629,0.14442,0.14493,0.14429,0.1
4938,0.14633,0.14107,0.14471,0.14693,0.13697,0.14555,0.13037,0.14934,0
.13243,0.14061,0.13685,0.15354,0.14462,0.13884,0.13936,0.14269,0.14512
,0.12579,0.1013,0.10561,0.10307,0.10107,0.1015,0.09709,0.29952,0.34359
,0.41331,0.28547,0.33218,0.10265,0.10171,0.14553,0.13482,0.13474,0.150
08,0.13405,0.14392,0.13059,0.13505,0.14546,0.14503,0.13917,0.14423,0.1
4262,0.13523,0.13628,0.13843,0.14776,0.15157,0.14489,0.14234,0.12954,0
.10052,0.098837,0.098188,0.097915,0.098281,0.097335,0.09613,0.1007,0.1
003,0.099556,0.27181,0.30331,0.10195,0.10045,0.10139,0.095717,0.1476,0
.13469,0.13881,0.14055,0.15476,0.1484,0.15216,0.14529,0.14086,0.13896,
0.13637,0.14493,0.14027,0.14161,0.13167,0.12937,0.14736,0.1405,0.14202
,0.099642,0.10011,0.099825,0.10299,0.10238,0.098374,0.10352,0.096577,0
.096416,0.10655,0.1004,0.10329,0.10481,0.10334,0.10293,0.10116,0.10422
,0.1485,0.14721,0.13285,0.14608,0.13687,0.14235,0.13816,0.14146,0.1340
8,0.13181,0.14664,0.1353,0.14175,0.14097,0.14273,0.14113,0.10047,0.105
74,0.10262,0.09591,0.099223,0.10009,0.10196,0.10051,0.10414,0.10365,0.
09725,0.10276,0.099324,0.097186,0.1013,0.097865,0.10025,0.10056,0.1056
2,0.1496,0.13502,0.13756,0.14143,0.13545,0.13173,0.15378,0.13271,0.161
66,0.14059,0.13307,0.14108,0.14353,0.10008,0.098006,0.097334,0.10171,0
.098725,0.10443,0.099138,0.10254,0.1037,0.10266,0.10112,0.10017,0.1052
1,0.10425,0.10189,0.10176,0.098551,0.10522,0.10226,0.099654,0.099957,0
.14005,0.14224,0.13875,0.14311,0.14839,0.14282,0.14453,0.14626,0.14928
,0.096205,0.10463,0.10109,0.10212,0.10043,0.097629,0.10318,0.10558,0.1
0261,0.10096,0.098918,0.099113,0.098779,0.10058,0.10106,0.10067,0.1052
5,0.10309,0.10025,0.10252,0.10265,0.097797,0.10075,0.098123,0.10447,0.
13857,0.14007,0.14029,0.13921,0.10381,0.095422,0.091486,0.099753,0.100
81,0.095622,0.10221,0.10174,0.10136,0.098884,0.099026,0.10142,0.098817
,0.10156,0.10221,0.097678,0.098916,0.10374,0.10248,0.097943,0.10284,0.
10353,0.10068,0.099995,0.097719,0.10255,0.10248,0.10193,0.10175,0.0971
97,0.1017,0.10205,0.098274,0.10355,0.099216,0.098307,0.1014,0.099241,0
.098835,0.10307,0.10251,0.10107,0.10459,0.094864,0.10099,0.098055,0.09
8851,0.10187,0.10166,0.098501,0.096261,0.10234,0.10064,0.099804,0.1012
2,0.10053,0.10043,0.10311,0.096157,0.10151,0.10128,0.097247,0.1007,0.0
99775,0.1019,0.10614,0.10447,0.10073,0.098629,0.099192,0.10339,0.10071
,0.1025,0.10556,0.10375,0.10386,0.10056,0.10176,0.10197,0.10252,0.1010
8,0.1012,0.095009,0.096702,0.10263,0.10098,0.10266,0.10574,0.10139,0.0
99785,0.096595,0.098608,0.10041,0.10248,0.1006,0.10465,0.10153,0.09956
6,0.094259,0.10224,0.10096,0.10615,0.10132,0.10161,0.099292,0.09853,0.
10317,0.10304,0.1048,0.096696,0.10294,0.10145,0.098595,0.10086,0.10163
,0.097492,0.10133,0.097362,0.10072,0.10279,0.10271,0.099628,0.10044,0.
10164,0.099715,0.098711,0.099578,0.10439,0.098581,0.10038,0.10431,0.10
52,0.10599,0.10078,0.098419,0.099743,0.10358,0.097159,0.10444,0.10547,
0.097612,0.093722,0.10214,0.10017,0.095761,0.1003,0.10086,0.097609,0.0
94106,0.098806,0.098801,0.097059,0.10095,0.10092,0.10153,0.096006,0.09
9034,0.097866,0.10237,0.10158,0.10113,0.099839,0.10743,0.10076,0.10111
,0.10079,0.10152,0.096738,0.10333,0.096406,0.09948,0.096757,0.10162,0.
096369,0.10046,0.10069,0.10244,0.1049,0.10165,0.10095,0.095725,0.10839
,0.10219,0.1029,0.10231,0.095566,0.10035,0.097561,0.098936,0.10063,0.1
0476,0.10379,0.10037,0.097051,0.094263

0.18585,0.16896,0.18252,0.18447,0.18156,0.17997,0.18137,0.17706,0.1938
5,0.19404,0.18243,0.19081,0.18349,0.17837,0.18236,0.18297,0.18831,0.18
392,0.18014,0.17871,0.28462,0.37748,0.35206,0.32342,0.18688,0.1875,0.1
9412,0.19686,0.17778,0.17631,0.17645,0.18694,0.19619,0.19698,0.1913,0.
18514,0.17949,0.188,0.19377,0.5588,0.32423,0.29361,0.31692,0.35851,0.3
5923,0.31481,0.18636,0.17963,0.18353,0.17712,0.1859,0.19247,0.18685,0.
18466,0.17657,0.18885,0.17843,0.19194,0.1893,0.18741,0.18212,0.54355,0
.37254,0.3572,0.38296,0.36257,0.35736,0.35186,0.35736,0.30796,0.3746,0
.19859,0.17747,0.18601,0.18827,0.18957,0.19366,0.18216,0.19052,0.18161
,0.18158,0.18483,0.18948,0.18278,0.18711,0.18632,0.47354,0.45524,0.452
54,0.2818,0.3665,0.30668,0.3206,0.28501,0.28363,0.30394,0.38202,0.3784
1,0.18546,0.1836,0.18573,0.18152,0.18159,0.18218,0.18267,0.19122,0.177
96,0.18629,0.18286,0.18906,0.18502,0.18962,0.19361,0.18484,0.55402,0.4
3607,0.46542,0.46989,0.33772,0.28637,0.34351,0.33867,0.33881,0.29502,0
.29641,0.32755,0.34126,0.29646,0.18532,0.18822,0.18465,0.19679,0.1858,
0.1815,0.18133,0.17453,0.18881,0.18207,0.19069,0.19238,0.19125,0.18233
,0.18635,0.17909,0.54959,0.39911,0.58996,0.4367,0.49058,0.45282,0.3928
5,0.29891,0.32453,0.37252,0.30109,0.37445,0.35096,0.2857,0.3136,0.3936
,0.18242,0.18918,0.18703,0.18082,0.19048,0.17274,0.18682,0.18282,0.191
94,0.19218,0.18586,0.18604,0.19142,0.18778,0.17743,0.18449,0.18526,0.5
0278,0.46429,0.51435,0.41255,0.38802,0.45714,0.29179,0.31712,0.28095,0
.33793,0.32654,0.34369,0.36604,0.30903,0.30636,0.36596,0.30097,0.18072
,0.1995,0.18885,0.18667,0.185,0.18194,0.18073,0.1832,0.18792,0.17971,0
.18914,0.18818,0.18715,0.13946,0.16058,0.13656,0.17734,0.39163,0.51156
,0.47529,0.4455,0.41492,0.50613,0.39901,0.34684,0.3633,0.31603,0.37176
,0.32827,0.36825,0.35886,0.34233,0.39111,0.35224,0.36163,0.19134,0.185
87,0.18144,0.18721,0.19416,0.19119,0.19994,0.18809,0.18848,0.18671,0.1
8191,0.1381,0.13032,0.14322,0.13809,0.13532,0.14115,0.18705,0.38677,0.
49325,0.50111,0.42006,0.48392,0.58247,0.42828,0.34435,0.40284,0.35561,
0.3508,0.36778,0.31521,0.29998,0.31738,0.30828,0.32746,0.32741,0.17986
,0.19635,0.17737,0.18421,0.17884,0.18738,0.19547,0.18676,0.19544,0.142
73,0.12933,0.14824,0.14697,0.13887,0.13497,0.1403,0.13587,0.1373,0.185
76,0.43227,0.46573,0.41794,0.49224,0.3615,0.47101,0.42273,0.32036,0.29
192,0.36173,0.30554,0.33874,0.33586,0.32412,0.41027,0.36448,0.3252,0.3
1272,0.33776,0.19124,0.19448,0.18889,0.17641,0.18909,0.19468,0.13428,0
.14439,0.14845,0.13671,0.13891,0.14159,0.13437,0.12502,0.13042,0.13456
,0.14063,0.15577,0.54132,0.38973,0.51186,0.48182,0.40738,0.48797,0.457
32,0.25719,0.33982,0.32594,0.35075,0.3677,0.29391,0.3201,0.32894,0.289
93,0.3471,0.31032,0.34145,0.19035,0.19038,0.18389,0.14532,0.14863,0.14
103,0.1422,0.13468,0.13252,0.14295,0.15224,0.13997,0.14275,0.13791,0.1
4329,0.14021,0.14923,0.13532,0.46795,0.40777,0.5115,0.4562,0.4294,0.50
062,0.46579,0.52043,0.41798,0.32445,0.35098,0.31297,0.29467,0.35438,0.
33128,0.34477,0.28307,0.35347,0.33403,0.34429,0.14197,0.13654,0.14302,
0.13814,0.14078,0.14022,0.1441,0.1434,0.15006,0.14483,0.1374,0.15495,0
.12564,0.13058,0.13996,0.14256,0.13253,0.13823,0.54273,0.53581,0.49173
,0.53495,0.48614,0.51598,0.4934,0.5108,0.40761,0.30029,0.23237,0.28289
,0.22975,0.23849,0.28355,0.23414,0.27971,0.23862,0.28064,0.22461,0.137
59,0.14505,0.13874,0.14346,0.14463,0.14287,0.13777,0.13653,0.13314,0.1
365,0.15151,0.13677,0.13684,0.13859,0.14179,0.15167,0.13899,0.14524,0.
54796,0.38148,0.5036,0.38472,0.58305,0.35349,0.40879,0.43656,0.38627,0
.29061,0.22291,0.25007,0.27645,0.2619,0.23229,0.28518,0.24618,0.27755,
0.25202,0.28571,0.13672,0.14911,0.14966,0.13605,0.14252,0.14161,0.1427

9,0.13956,0.13783,0.13256,0.15887,0.13177,0.14059,0.12749,0.13986,0.14
739,0.13727,0.13426,0.48481,0.44611,0.48627,0.3958,0.60444,0.47226,0.4
4487,0.25202,0.27829,0.25696,0.26512,0.27295,0.25468,0.27017,0.22295,0
.25628,0.25198,0.25312,0.26129,0.25275,0.12543,0.1412,0.1347,0.1414,0.
14088,0.1504,0.13947,0.14588,0.13942,0.14161,0.13842,0.13964,0.14376,0
.14305,0.14104,0.14766,0.14047,0.12893,0.41119,0.44836,0.51952,0.48644
,0.36661,0.41808,0.28859,0.28391,0.29223,0.31096,0.26748,0.27185,0.236
79,0.25385,0.25316,0.26254,0.26753,0.23584,0.20658,0.27404,0.14539,0.1
4063,0.12762,0.14533,0.1461,0.13849,0.14305,0.13348,0.13913,0.13416,0.
14749,0.13483,0.15629,0.14715,0.13623,0.13251,0.14377,0.14613,0.57544,
0.47615,0.42693,0.51129,0.58261,0.32156,0.27334,0.3082,0.39425,0.28517
,0.27091,0.256,0.20663,0.27745,0.23495,0.23001,0.21372,0.25027,0.25921
,0.2538,0.13954,0.14038,0.14195,0.1534,0.14524,0.13895,0.1489,0.13987,
0.13686,0.13709,0.14554,0.15233,0.13172,0.13925,0.13826,0.1363,0.14096
,0.13979,0.43349,0.4712,0.45301,0.30389,0.28443,0.2618,0.35842,0.24725
,0.26415,0.31105,0.293,0.22846,0.20367,0.286,0.24483,0.22437,0.24945,0
.2545,0.29738,0.29158,0.12905,0.14506,0.13749,0.14439,0.13417,0.14321,
0.13894,0.14615,0.14037,0.13757,0.13288,0.14213,0.14264,0.1312,0.13187
,0.14463,0.14139,0.13943,0.58364,0.36332,0.29464,0.25469,0.30112,0.356
27,0.29553,0.29737,0.27481,0.3291,0.24089,0.2508,0.31529,0.25208,0.219
29,0.26699,0.25255,0.27971,0.25456,0.14698,0.14082,0.14045,0.14274,0.1
4139,0.15012,0.13342,0.14353,0.14704,0.14463,0.16144,0.13585,0.14204,0
.14457,0.13756,0.1428,0.14468,0.091055,0.25818,0.25136,0.27803,0.31869
,0.31156,0.27515,0.25343,0.27424,0.25338,0.30277,0.25005,0.26084,0.274
14,0.26143,0.25542,0.2118,0.2767,0.24906,0.23385,0.14761,0.14042,0.136
69,0.14155,0.14298,0.14792,0.14046,0.13754,0.13667,0.13789,0.14547,0.1
4263,0.13436,0.14718,0.1424,0.13702,0.14035,0.093741,0.29755,0.26165,0
.29068,0.21321,0.2899,0.2475,0.30384,0.3054,0.3181,0.27196,0.26339,0.2
246,0.25976,0.22521,0.21221,0.31737,0.21608,0.21552,0.12986,0.13983,0.
13832,0.13723,0.12718,0.13951,0.13908,0.1335,0.13926,0.14228,0.15258,0
.14842,0.13934,0.13673,0.14484,0.13632,0.13478,0.13703,0.090767,0.3468
5,0.33517,0.30998,0.27982,0.3499,0.2672,0.32353,0.21298,0.26045,0.2833
3,0.26639,0.21572,0.21601,0.25178,0.28776,0.31237,0.29137,0.22129,0.14
597,0.12889,0.14288,0.13095,0.13477,0.13845,0.13919,0.14408,0.15654,0.
13419,0.13988,0.13583,0.13821,0.14052,0.13246,0.14476,0.152,0.095987,0
.091815,0.092648,0.24402,0.33237,0.26899,0.27834,0.2968,0.15249,0.2590
5,0.28353,0.26911,0.22235,0.23557,0.23175,0.27688,0.25753,0.23102,0.12
579,0.13776,0.13398,0.13466,0.14408,0.13706,0.1465,0.13003,0.14257,0.1
3269,0.13049,0.15314,0.14606,0.14161,0.13427,0.14197,0.13581,0.14308,0
.090893,0.09161,0.09029,0.092644,0.29568,0.30002,0.35421,0.30313,0.281
26,0.30421,0.29961,0.30468,0.33525,0.24767,0.22255,0.27475,0.13976,0.1
3802,0.17208,0.1415,0.14473,0.14836,0.13103,0.13698,0.13321,0.13988,0.
15208,0.1467,0.13953,0.13166,0.1503,0.13743,0.13447,0.1459,0.13731,0.1
3121,0.093475,0.090233,0.089121,0.087086,0.094938,0.093878,0.26516,0.2
5161,0.22995,0.30817,0.27152,0.27626,0.3472,0.30189,0.14758,0.14539,0.
14494,0.14376,0.14545,0.14634,0.14008,0.14282,0.14585,0.13486,0.14294,
0.12665,0.15358,0.1275,0.13901,0.1377,0.15243,0.14359,0.13872,0.13803,
0.13927,0.14467,0.12304,0.095488,0.096194,0.095443,0.092361,0.092788,0
.0913,0.25648,0.30825,0.36465,0.26632,0.31335,0.094692,0.092077,0.1455
1,0.13477,0.13386,0.15027,0.13099,0.1425,0.12718,0.13299,0.14333,0.142
71,0.13648,0.14494,0.14184,0.13549,0.13466,0.13728,0.14586,0.1483,0.14
514,0.13995,0.12483,0.092039,0.090757,0.090514,0.089846,0.090915,0.091

217,0.089974,0.092991,0.09242,0.090539,0.24122,0.28793,0.095279,0.0901
56,0.096302,0.088286,0.14287,0.13251,0.14076,0.14175,0.14955,0.14781,0
.14872,0.14258,0.14212,0.13764,0.13302,0.14419,0.13828,0.14086,0.12915
,0.12603,0.14386,0.14137,0.13907,0.088824,0.091917,0.092791,0.095448,0
.096053,0.090385,0.094482,0.088001,0.087574,0.097002,0.092208,0.095552
,0.095838,0.094099,0.094745,0.094636,0.094428,0.15129,0.14814,0.13171,
0.14485,0.1338,0.14006,0.13735,0.13736,0.13091,0.1309,0.14478,0.13475,
0.1384,0.14099,0.13959,0.14141,0.093445,0.097632,0.097007,0.089639,0.0
9218,0.091778,0.095746,0.092084,0.09489,0.094693,0.09035,0.094253,0.09
2934,0.089646,0.0933,0.090252,0.092932,0.093984,0.098733,0.14807,0.131
86,0.13583,0.1419,0.13553,0.13075,0.15114,0.13228,0.1621,0.13909,0.131
34,0.13806,0.13959,0.092527,0.089426,0.089257,0.094446,0.090581,0.0963
02,0.09144,0.095446,0.094513,0.094392,0.09341,0.092317,0.095931,0.0971
27,0.093418,0.094878,0.090729,0.098207,0.094669,0.092259,0.092324,0.14
327,0.14021,0.13846,0.14157,0.14436,0.14167,0.14305,0.14385,0.14617,0.
08835,0.096083,0.096479,0.094027,0.093808,0.089239,0.095952,0.098519,0
.094872,0.091645,0.090069,0.092286,0.09151,0.093911,0.093148,0.090799,
0.096319,0.094701,0.092532,0.093028,0.094596,0.091467,0.092897,0.09074
6,0.095485,0.13639,0.13823,0.13988,0.13889,0.094337,0.086858,0.086405,
0.092513,0.092534,0.086909,0.094203,0.092926,0.092413,0.091911,0.09120
9,0.093377,0.089984,0.092448,0.094502,0.090358,0.093194,0.094265,0.093
428,0.092023,0.093916,0.094121,0.093246,0.091137,0.091084,0.093373,0.0
94266,0.092666,0.092883,0.089166,0.094718,0.093161,0.092201,0.095648,0
.09018,0.091633,0.093544,0.092494,0.089923,0.096156,0.092335,0.093405,
0.096245,0.087861,0.092562,0.091208,0.089807,0.094643,0.095431,0.09029
,0.087214,0.094502,0.092672,0.090375,0.093759,0.09482,0.091806,0.09521
8,0.089695,0.090969,0.092477,0.087943,0.091685,0.092373,0.096696,0.099
087,0.097235,0.093152,0.091215,0.090136,0.094624,0.092203,0.094784,0.0
96143,0.096799,0.095638,0.091313,0.094452,0.093242,0.091555,0.093578,0
.092629,0.089267,0.089496,0.093674,0.092019,0.093921,0.097123,0.093162
,0.092312,0.089614,0.090319,0.093974,0.093592,0.092419,0.096213,0.0928
32,0.090845,0.085393,0.094347,0.091908,0.097528,0.094486,0.092591,0.09
0532,0.088742,0.094651,0.095885,0.096145,0.088643,0.093524,0.092737,0.
09025,0.095843,0.094849,0.08917,0.093852,0.088687,0.09237,0.093236,0.0
93388,0.091165,0.0964,0.093692,0.093819,0.091846,0.090242,0.099342,0.0
90415,0.092746,0.094399,0.095954,0.098309,0.093131,0.091529,0.092166,0
.095501,0.090118,0.096206,0.09421,0.091362,0.085639,0.095147,0.091407,
0.088679,0.093208,0.094686,0.088807,0.085503,0.092998,0.091439,0.09090
4,0.093286,0.094008,0.093179,0.0904,0.091242,0.090705,0.092814,0.09214
6,0.094259,0.092213,0.097927,0.09298,0.095979,0.092509,0.091368,0.0879
23,0.094729,0.088685,0.092355,0.088685,0.094126,0.087336,0.090514,0.09
2404,0.094851,0.096301,0.09426,0.091513,0.086972,0.097201,0.093149,0.0
93988,0.094585,0.087684,0.090739,0.091445,0.090134,0.090532,0.096781,0
.093533,0.093265,0.089618,0.088169
0.19287,0.17507,0.18731,0.19053,0.18426,0.18291,0.18718,0.1835,0.19822
,0.19665,0.18423,0.19164,0.18607,0.18223,0.18517,0.18826,0.19197,0.186
33,0.18315,0.18071,0.2822,0.34508,0.33154,0.30396,0.18957,0.19396,0.19
533,0.20012,0.18661,0.18518,0.18137,0.19265,0.20048,0.19724,0.19248,0.
18878,0.18286,0.19157,0.19834,0.47002,0.28625,0.28161,0.31721,0.34197,
0.31773,0.31355,0.19283,0.18707,0.18593,0.1825,0.19042,0.19961,0.1941,
0.18702,0.1787,0.19228,0.17678,0.19567,0.19459,0.18994,0.18674,0.50459
,0.38895,0.32073,0.34283,0.34338,0.35272,0.329,0.33176,0.29527,0.35297

,0.2014,0.18573,0.19115,0.18913,0.19344,0.19651,0.18928,0.19306,0.1872
8,0.18609,0.18644,0.18991,0.18682,0.19178,0.19129,0.42555,0.37921,0.40
236,0.26948,0.33009,0.28936,0.30576,0.27361,0.27157,0.29888,0.3456,0.3
5465,0.1884,0.19053,0.18843,0.18624,0.18564,0.18281,0.18586,0.19511,0.
18649,0.19136,0.1873,0.1906,0.18614,0.19063,0.1958,0.1885,0.52987,0.38
72,0.44351,0.40037,0.31116,0.27236,0.32132,0.31356,0.31562,0.26569,0.2
8929,0.31993,0.30676,0.26283,0.18698,0.19341,0.19037,0.20226,0.19241,0
.18481,0.18747,0.18214,0.19787,0.18019,0.19477,0.19683,0.19426,0.18485
,0.1892,0.1842,0.52122,0.3935,0.53216,0.42725,0.41042,0.43553,0.35088,
0.29243,0.30187,0.35712,0.27381,0.33714,0.35217,0.2736,0.29948,0.37003
,0.18265,0.19036,0.18881,0.18781,0.19174,0.17501,0.1914,0.18464,0.1958
2,0.19717,0.18918,0.1915,0.19722,0.19309,0.18246,0.19278,0.18822,0.497
27,0.41398,0.48287,0.38645,0.30862,0.42335,0.29424,0.29586,0.24198,0.3
3248,0.31822,0.30376,0.36052,0.31446,0.30353,0.34626,0.29314,0.18407,0
.1981,0.19012,0.18912,0.1905,0.18486,0.18237,0.18551,0.1896,0.18258,0.
19855,0.18969,0.19157,0.13911,0.15676,0.13488,0.18266,0.38038,0.47966,
0.43212,0.42038,0.34062,0.46966,0.38164,0.32401,0.36038,0.31539,0.3424
3,0.30945,0.32115,0.31923,0.33547,0.3862,0.33857,0.33738,0.19188,0.191
46,0.18227,0.18888,0.19692,0.19496,0.20014,0.19201,0.19345,0.18962,0.1
8593,0.13384,0.12994,0.14315,0.13517,0.13428,0.14063,0.18675,0.41429,0
.47728,0.4485,0.38213,0.45369,0.50193,0.39835,0.33558,0.40411,0.32587,
0.31823,0.35916,0.26734,0.28281,0.29155,0.29845,0.31431,0.3129,0.18293
,0.20042,0.18679,0.18706,0.17996,0.19315,0.2016,0.19294,0.19965,0.1399
5,0.1279,0.14953,0.1475,0.13918,0.1352,0.13883,0.13281,0.13643,0.19032
,0.39818,0.44695,0.3614,0.46092,0.35557,0.42305,0.41012,0.2948,0.27986
,0.34537,0.28596,0.34099,0.32564,0.31053,0.361,0.34632,0.31482,0.27759
,0.29703,0.19376,0.19964,0.18777,0.1826,0.19062,0.20145,0.13242,0.1433
7,0.14502,0.13176,0.13955,0.13938,0.13423,0.12443,0.12973,0.13221,0.13
673,0.15469,0.51423,0.35028,0.49153,0.44252,0.38585,0.45331,0.39005,0.
25093,0.3151,0.31273,0.32927,0.32513,0.27884,0.32042,0.29667,0.29279,0
.32059,0.28733,0.33232,0.19158,0.1976,0.18501,0.14185,0.14748,0.13839,
0.14135,0.13345,0.13113,0.14221,0.14985,0.13789,0.13972,0.13659,0.1389
5,0.13825,0.1493,0.13142,0.45094,0.39145,0.48059,0.44841,0.40092,0.460
99,0.44098,0.46759,0.36421,0.31765,0.34115,0.30825,0.27724,0.32023,0.3
2669,0.31278,0.26508,0.34525,0.32526,0.31306,0.14106,0.13471,0.1416,0.
13731,0.13773,0.13777,0.14224,0.14346,0.14907,0.14033,0.13398,0.14934,
0.12337,0.12776,0.13982,0.14156,0.12938,0.13558,0.51583,0.46478,0.4263
5,0.4596,0.44552,0.43744,0.45806,0.46665,0.35009,0.28237,0.23355,0.261
94,0.21456,0.21467,0.26975,0.22393,0.26878,0.22902,0.2746,0.19981,0.13
474,0.14219,0.13659,0.14286,0.14151,0.1405,0.13577,0.13592,0.13303,0.1
3579,0.15213,0.13363,0.13499,0.13583,0.14006,0.15076,0.13883,0.14267,0
.51978,0.32879,0.47902,0.33024,0.50649,0.32236,0.41378,0.35614,0.3455,
0.26216,0.19795,0.24094,0.25032,0.22922,0.22269,0.26244,0.2447,0.26684
,0.25471,0.26584,0.13334,0.15138,0.15186,0.13285,0.13862,0.14054,0.141
21,0.13791,0.13555,0.13187,0.16449,0.12942,0.13825,0.12488,0.13895,0.1
4383,0.13578,0.13088,0.45532,0.50851,0.4278,0.32817,0.56876,0.42091,0.
3835,0.23446,0.25785,0.23576,0.24397,0.24751,0.24306,0.26339,0.21517,0
.23869,0.25214,0.24299,0.22148,0.24241,0.12127,0.13955,0.13265,0.13948
,0.1378,0.14812,0.13628,0.14276,0.13795,0.14057,0.13524,0.13848,0.1413
7,0.1424,0.14076,0.14417,0.1382,0.12576,0.38908,0.42537,0.43262,0.4425
3,0.34061,0.3462,0.25303,0.25714,0.25765,0.28066,0.23403,0.2676,0.2235
3,0.23501,0.24769,0.23748,0.24814,0.23468,0.20098,0.24716,0.14481,0.13

85,0.12506,0.14517,0.14118,0.13548,0.14164,0.13288,0.13489,0.1327,0.14
632,0.13127,0.1525,0.14774,0.13703,0.13084,0.14198,0.14688,0.5288,0.43
153,0.4172,0.45148,0.54479,0.3065,0.23281,0.28342,0.34163,0.26206,0.24
891,0.23462,0.19546,0.25108,0.22308,0.22444,0.19295,0.23187,0.24818,0.
22591,0.13713,0.14111,0.1416,0.15269,0.14638,0.13749,0.14691,0.13809,0
.13525,0.13607,0.14628,0.15482,0.12744,0.13526,0.13528,0.13425,0.13767
,0.13826,0.41072,0.40332,0.41642,0.28164,0.26712,0.23613,0.35077,0.244
4,0.23418,0.28938,0.25879,0.22622,0.19443,0.26463,0.23296,0.20669,0.23
275,0.23293,0.2879,0.29828,0.1273,0.14008,0.13591,0.14285,0.12755,0.13
955,0.13751,0.14833,0.13915,0.13508,0.13391,0.13841,0.13906,0.13119,0.
12715,0.14454,0.14065,0.13919,0.50476,0.32984,0.26133,0.2279,0.27912,0
.32167,0.27591,0.26182,0.23915,0.31167,0.21366,0.23666,0.30268,0.24158
,0.22682,0.2471,0.23654,0.23484,0.23703,0.1449,0.13518,0.13808,0.13763
,0.13785,0.14662,0.13321,0.1427,0.1466,0.14098,0.16398,0.13447,0.1413,
0.1447,0.13647,0.14226,0.14217,0.083795,0.24168,0.22775,0.2557,0.2959,
0.28591,0.25921,0.23224,0.27443,0.21614,0.26369,0.23229,0.25138,0.2493
4,0.25858,0.25629,0.19929,0.25795,0.22326,0.21889,0.14863,0.13761,0.13
636,0.1386,0.13956,0.14633,0.13953,0.13625,0.13507,0.13516,0.14622,0.1
4159,0.13516,0.146,0.14007,0.13523,0.1403,0.085182,0.26855,0.23005,0.2
5928,0.20495,0.28382,0.21796,0.29802,0.27259,0.29684,0.23511,0.25282,0
.21787,0.24169,0.21166,0.2031,0.307,0.20337,0.20299,0.12851,0.13864,0.
13601,0.13543,0.12498,0.14275,0.13698,0.13105,0.13594,0.1413,0.15211,0
.14468,0.13796,0.13347,0.1398,0.1334,0.13457,0.13627,0.081949,0.32624,
0.29883,0.26341,0.23792,0.3025,0.2421,0.2967,0.18138,0.22612,0.24464,0
.25352,0.20436,0.20506,0.23334,0.27271,0.31103,0.27536,0.20729,0.1437,
0.12723,0.14271,0.12816,0.13248,0.13403,0.13606,0.14169,0.15827,0.1336
8,0.13751,0.13059,0.13704,0.13951,0.12989,0.14213,0.14916,0.087523,0.0
83636,0.084038,0.22125,0.29516,0.24975,0.28009,0.25799,0.1278,0.23677,
0.26343,0.25477,0.19273,0.21837,0.22704,0.2882,0.25252,0.22602,0.1233,
0.13669,0.13486,0.13349,0.14173,0.13675,0.14467,0.12562,0.13822,0.1328
3,0.12807,0.15171,0.1438,0.14047,0.13243,0.14045,0.13335,0.14288,0.082
749,0.084692,0.082203,0.087433,0.2764,0.28523,0.32271,0.28163,0.27675,
0.26523,0.26549,0.29068,0.28971,0.24494,0.20967,0.25383,0.13634,0.1350
6,0.17347,0.13875,0.1444,0.14635,0.1321,0.13518,0.13104,0.13577,0.1518
1,0.14294,0.13752,0.12792,0.15093,0.13816,0.13115,0.14074,0.13378,0.12
916,0.084284,0.081103,0.080835,0.081563,0.086084,0.08418,0.25454,0.240
7,0.22263,0.30085,0.24973,0.24836,0.30114,0.2821,0.1481,0.13901,0.1423
7,0.14135,0.14611,0.14486,0.14062,0.13937,0.14299,0.1337,0.14261,0.120
6,0.15365,0.12335,0.13851,0.13694,0.15275,0.14148,0.14002,0.13537,0.13
856,0.14376,0.12021,0.086973,0.087892,0.087765,0.086511,0.084279,0.081
371,0.24376,0.26462,0.33274,0.21992,0.28806,0.086601,0.086644,0.1442,0
.13265,0.13129,0.14619,0.13102,0.14158,0.12395,0.1297,0.14365,0.13814,
0.13689,0.14331,0.1397,0.13274,0.13237,0.13503,0.14486,0.14756,0.14276
,0.13951,0.12184,0.082878,0.082647,0.085026,0.084631,0.083603,0.083361
,0.080728,0.085713,0.083372,0.08382,0.21836,0.25151,0.088767,0.083405,
0.087278,0.081012,0.14176,0.13043,0.14099,0.1384,0.15032,0.14793,0.148
72,0.14123,0.14202,0.13423,0.1312,0.14045,0.13571,0.13759,0.12612,0.11
932,0.1405,0.13833,0.14255,0.081044,0.084722,0.085907,0.087492,0.08849
7,0.083201,0.084272,0.081021,0.07938,0.086477,0.08527,0.088549,0.09097
6,0.085947,0.087115,0.086601,0.088993,0.15033,0.14568,0.12978,0.14093,
0.13046,0.1362,0.13545,0.13576,0.12652,0.12959,0.14231,0.13324,0.1365,
0.14173,0.13977,0.14095,0.086445,0.092482,0.091287,0.081905,0.082975,0

.084166,0.087523,0.086702,0.087274,0.088227,0.082935,0.087872,0.086576
,0.082764,0.086234,0.082943,0.084672,0.087041,0.090621,0.14498,0.13194
,0.13177,0.14115,0.13272,0.12608,0.15229,0.13141,0.16187,0.13954,0.130
57,0.1353,0.13892,0.08622,0.081362,0.082436,0.085786,0.082934,0.087713
,0.083553,0.087108,0.088056,0.088358,0.087743,0.085794,0.089337,0.0882
5,0.087214,0.086732,0.083704,0.089878,0.087946,0.084179,0.087026,0.140
78,0.13689,0.13603,0.14084,0.14056,0.14204,0.14046,0.1414,0.14536,0.07
9118,0.087838,0.088602,0.086697,0.085249,0.082137,0.087271,0.090298,0.
089005,0.086449,0.084254,0.087161,0.085048,0.083722,0.083677,0.085856,
0.089097,0.087534,0.081698,0.086346,0.088199,0.083932,0.084667,0.08374
7,0.088431,0.13397,0.13837,0.14027,0.13665,0.087415,0.078677,0.076752,
0.084957,0.08508,0.079787,0.08806,0.085486,0.08778,0.084127,0.083196,0
.08394,0.08104,0.086431,0.088051,0.0844,0.083907,0.085979,0.085605,0.0
85897,0.085563,0.084324,0.083977,0.084677,0.084621,0.086713,0.088072,0
.08482,0.085146,0.081075,0.086761,0.083891,0.085737,0.088669,0.082165,
0.084464,0.086644,0.086071,0.082756,0.087233,0.084229,0.087651,0.08682
2,0.082002,0.085407,0.083005,0.084032,0.08699,0.086445,0.083927,0.0770
64,0.088898,0.086564,0.082801,0.085404,0.088591,0.083257,0.087852,0.08
2393,0.084497,0.086754,0.079881,0.086372,0.086436,0.091305,0.089379,0.
090721,0.086889,0.083705,0.083488,0.087766,0.083361,0.086557,0.087387,
0.090571,0.088501,0.083681,0.086917,0.08449,0.083532,0.086444,0.084153
,0.083124,0.083146,0.088726,0.085227,0.086492,0.091495,0.086418,0.0842
76,0.083589,0.083076,0.083605,0.085992,0.084979,0.086508,0.084691,0.08
221,0.077943,0.085512,0.082842,0.090489,0.090054,0.085115,0.083117,0.0
80294,0.086906,0.089918,0.088622,0.081236,0.086405,0.085763,0.08215,0.
088063,0.087884,0.081773,0.084339,0.082686,0.085073,0.084748,0.084591,
0.084466,0.089191,0.086925,0.086598,0.082962,0.082454,0.091054,0.08192
7,0.083854,0.084223,0.086574,0.090977,0.089478,0.085337,0.082396,0.088
72,0.083473,0.089175,0.087249,0.083326,0.07881,0.088418,0.084811,0.079
463,0.087211,0.087325,0.080822,0.079632,0.085948,0.084607,0.085353,0.0
86787,0.086461,0.085572,0.084005,0.084006,0.081888,0.086472,0.085031,0
.086045,0.082762,0.089266,0.085216,0.086189,0.084535,0.086221,0.082031
,0.08856,0.081785,0.08332,0.082943,0.087602,0.077505,0.082394,0.082668
,0.086576,0.088228,0.086282,0.083655,0.081281,0.090781,0.084488,0.0859
12,0.08846,0.080638,0.081481,0.083165,0.080046,0.08141,0.088013,0.0856
11,0.084558,0.083818,0.079438
0.198,0.182,0.19212,0.19671,0.18879,0.18853,0.18879,0.19099,0.19775,0.
19878,0.18428,0.19683,0.18878,0.18798,0.18815,0.19369,0.19733,0.19336,
0.1868,0.18643,0.26331,0.33943,0.32629,0.30343,0.19128,0.19899,0.19605
,0.20074,0.19019,0.18524,0.18622,0.19512,0.19976,0.20141,0.1915,0.1935
3,0.18909,0.19326,0.19541,0.44756,0.27587,0.27446,0.29432,0.33525,0.30
041,0.29331,0.19866,0.19482,0.1893,0.18503,0.19176,0.20244,0.1988,0.19
518,0.18402,0.1998,0.18133,0.19606,0.19835,0.19047,0.18522,0.4679,0.36
755,0.28604,0.32686,0.33556,0.33629,0.33433,0.3244,0.28466,0.3338,0.20
448,0.19312,0.19742,0.1914,0.19613,0.19261,0.193,0.19405,0.18889,0.188
79,0.19125,0.19362,0.19048,0.19402,0.1946,0.40479,0.40537,0.42017,0.26
855,0.33126,0.27952,0.29489,0.25403,0.24401,0.28879,0.34469,0.32793,0.
19408,0.1939,0.19129,0.18849,0.18874,0.18956,0.1922,0.19766,0.19234,0.
19465,0.18984,0.18912,0.18749,0.19674,0.19622,0.18843,0.51616,0.35777,
0.43147,0.40858,0.31448,0.27643,0.30955,0.32238,0.33282,0.25862,0.2684
2,0.30516,0.30399,0.23341,0.19039,0.19274,0.19298,0.20289,0.19788,0.19
045,0.1903,0.18295,0.20263,0.18766,0.19761,0.19871,0.19708,0.18936,0.1

8898,0.19171,0.49133,0.34553,0.48007,0.38724,0.37657,0.40315,0.33743,0
.30393,0.29092,0.33349,0.29052,0.32564,0.33431,0.27921,0.27356,0.35585
,0.18685,0.19572,0.19062,0.19413,0.19209,0.18221,0.19085,0.18644,0.200
51,0.19706,0.18897,0.1939,0.20193,0.19726,0.18586,0.19079,0.19128,0.45
867,0.37358,0.48043,0.35313,0.22953,0.40442,0.29437,0.27758,0.24069,0.
30882,0.29035,0.30147,0.34037,0.31793,0.29386,0.32384,0.28236,0.18529,
0.2006,0.19315,0.19143,0.19531,0.18778,0.18555,0.19267,0.18914,0.18487
,0.19889,0.19101,0.19336,0.13855,0.15717,0.13206,0.18875,0.34593,0.460
07,0.37506,0.42278,0.37159,0.38858,0.33221,0.30639,0.3305,0.3162,0.316
55,0.31161,0.30455,0.31142,0.31415,0.35948,0.31713,0.32611,0.19335,0.1
9509,0.1876,0.19261,0.19918,0.19733,0.20425,0.19676,0.19844,0.19228,0.
18861,0.1307,0.13073,0.14079,0.13695,0.13109,0.13625,0.19149,0.36465,0
.43665,0.40456,0.38462,0.45755,0.478,0.37714,0.31577,0.3856,0.33238,0.
28469,0.34856,0.25939,0.2695,0.29188,0.27167,0.29354,0.30489,0.18356,0
.20522,0.19149,0.18983,0.18551,0.19281,0.20504,0.19647,0.19788,0.1386,
0.12528,0.14717,0.14841,0.13992,0.13376,0.134,0.13183,0.13341,0.19449,
0.39786,0.43966,0.32731,0.42729,0.34926,0.42052,0.36935,0.28716,0.2716
3,0.3273,0.29748,0.2984,0.31813,0.29048,0.34055,0.33111,0.29307,0.2669
4,0.27194,0.19415,0.20297,0.19221,0.18359,0.19314,0.20408,0.13189,0.14
303,0.14054,0.12737,0.13571,0.13631,0.13198,0.12352,0.12756,0.13304,0.
13409,0.15448,0.52764,0.34276,0.43519,0.4017,0.38835,0.45673,0.37927,0
.2479,0.28769,0.28753,0.3154,0.29259,0.24721,0.29449,0.2953,0.28246,0.
33382,0.2806,0.31486,0.19439,0.20133,0.18692,0.14197,0.14416,0.1381,0.
14013,0.13121,0.12775,0.13801,0.14793,0.13584,0.13717,0.13581,0.13675,
0.13393,0.15003,0.12792,0.42175,0.39493,0.41528,0.41282,0.42103,0.4716
7,0.41088,0.41752,0.37254,0.29631,0.33248,0.27594,0.28156,0.30169,0.31
38,0.33494,0.23061,0.34204,0.31282,0.29406,0.13749,0.13377,0.1398,0.13
74,0.1357,0.13865,0.14189,0.13992,0.14566,0.13948,0.13141,0.14867,0.12
024,0.12587,0.1384,0.13793,0.12789,0.13252,0.44659,0.45182,0.39551,0.4
407,0.4426,0.40591,0.41937,0.39089,0.33434,0.30298,0.23391,0.25578,0.2
0255,0.21085,0.27278,0.23087,0.2541,0.22593,0.25471,0.19258,0.13218,0.
14106,0.1346,0.1416,0.14012,0.13912,0.13548,0.1324,0.1307,0.13501,0.15
226,0.13398,0.13169,0.13344,0.13898,0.15104,0.13742,0.1414,0.53093,0.2
7237,0.4581,0.32153,0.43955,0.28904,0.37257,0.3473,0.34897,0.24519,0.1
9078,0.2362,0.23065,0.20298,0.21036,0.24772,0.23553,0.26496,0.23898,0.
26377,0.12921,0.15243,0.14887,0.13186,0.13758,0.14102,0.13892,0.13845,
0.13371,0.13057,0.16574,0.12608,0.13727,0.12041,0.13754,0.14634,0.1315
6,0.12742,0.42956,0.46388,0.41554,0.30517,0.52937,0.37969,0.34491,0.21
991,0.23663,0.2233,0.22757,0.23826,0.23998,0.24268,0.22005,0.22543,0.2
2859,0.22422,0.20931,0.24294,0.11585,0.13802,0.12633,0.13845,0.13727,0
.14818,0.13331,0.14139,0.13675,0.13553,0.13684,0.13761,0.13991,0.13967
,0.13876,0.14152,0.13743,0.12485,0.32883,0.41704,0.37721,0.38492,0.315
59,0.33738,0.22905,0.26845,0.2345,0.25027,0.20915,0.26364,0.21134,0.22
79,0.24932,0.2327,0.22674,0.23481,0.19273,0.23505,0.14498,0.13598,0.12
133,0.14333,0.14303,0.13559,0.14066,0.13233,0.13018,0.13081,0.14287,0.
12785,0.15049,0.1448,0.13383,0.13049,0.13883,0.14435,0.51259,0.38684,0
.42409,0.41772,0.50131,0.29496,0.22169,0.27932,0.33677,0.26661,0.21941
,0.22089,0.1851,0.25238,0.19527,0.21127,0.18992,0.22473,0.23632,0.2360
5,0.13592,0.13852,0.13725,0.15342,0.1488,0.13538,0.14626,0.13608,0.134
4,0.13304,0.14468,0.15415,0.12491,0.13477,0.13656,0.13278,0.133,0.1348
4,0.36117,0.37575,0.3783,0.26711,0.24995,0.21384,0.32256,0.24258,0.204
75,0.2553,0.25083,0.21541,0.18632,0.25416,0.22482,0.20122,0.24501,0.22

397,0.2764,0.29142,0.12663,0.13819,0.13209,0.14011,0.12554,0.13774,0.1
3724,0.14573,0.13789,0.13054,0.12987,0.13851,0.13848,0.12951,0.12675,0
.14363,0.13919,0.13882,0.47155,0.30957,0.22679,0.23218,0.25375,0.29383
,0.25371,0.27303,0.2084,0.28712,0.20273,0.21856,0.29054,0.22436,0.2144
9,0.22607,0.22612,0.21895,0.22069,0.14425,0.13454,0.13526,0.13349,0.13
397,0.1449,0.13242,0.14244,0.14523,0.13854,0.16172,0.1324,0.14123,0.14
188,0.13385,0.14221,0.14087,0.078055,0.22381,0.21767,0.24375,0.29671,0
.28427,0.2383,0.19934,0.26146,0.20639,0.26238,0.22685,0.25119,0.23328,
0.2499,0.22713,0.19065,0.25021,0.21964,0.2145,0.14636,0.13558,0.13354,
0.14143,0.13696,0.14258,0.13816,0.13317,0.13212,0.13236,0.14346,0.1394
2,0.13335,0.14272,0.1388,0.13128,0.13922,0.077747,0.23388,0.22312,0.24
264,0.19009,0.28315,0.19042,0.28525,0.23278,0.26932,0.20007,0.2379,0.2
1999,0.23733,0.19862,0.20317,0.28108,0.19341,0.2066,0.12643,0.13724,0.
13654,0.13357,0.12014,0.14157,0.13479,0.13132,0.13213,0.13823,0.14899,
0.14374,0.13753,0.13391,0.13684,0.13098,0.1338,0.13268,0.076157,0.3118
3,0.30098,0.24014,0.23133,0.27445,0.2429,0.28938,0.16825,0.19303,0.237
48,0.24711,0.1942,0.20751,0.2273,0.26584,0.27779,0.26265,0.20437,0.140
95,0.12588,0.14288,0.12648,0.13176,0.13232,0.1348,0.13996,0.15884,0.13
266,0.13664,0.12925,0.13663,0.13789,0.12834,0.14115,0.14656,0.079807,0
.077451,0.078129,0.20707,0.28203,0.24498,0.24785,0.24698,0.1273,0.2261
8,0.24165,0.20492,0.17989,0.21463,0.2177,0.27618,0.22899,0.21761,0.120
95,0.13606,0.13296,0.13261,0.13778,0.13614,0.1429,0.1243,0.1353,0.1311
7,0.12654,0.15297,0.14172,0.14093,0.13065,0.13874,0.1325,0.13898,0.077
506,0.080608,0.074883,0.079305,0.25142,0.27011,0.29461,0.2529,0.25402,
0.25434,0.23718,0.2703,0.2702,0.22828,0.20419,0.2203,0.13558,0.13329,0
.17768,0.13379,0.14313,0.14475,0.13059,0.13361,0.1297,0.13144,0.15065,
0.1408,0.13498,0.12642,0.14712,0.1359,0.13027,0.14028,0.13095,0.12756,
0.077583,0.074538,0.074602,0.073986,0.078329,0.07924,0.22918,0.20483,0
.20161,0.27279,0.21918,0.23406,0.26137,0.27257,0.14661,0.13502,0.14115
,0.14,0.14309,0.1422,0.13928,0.13686,0.14128,0.12915,0.14127,0.11702,0
.15152,0.11962,0.13798,0.13629,0.15076,0.13809,0.1364,0.13569,0.13609,
0.14186,0.11759,0.078819,0.081499,0.081174,0.078466,0.077311,0.073375,
0.21169,0.24149,0.29523,0.20306,0.25975,0.079709,0.077916,0.14163,0.12
867,0.13132,0.1414,0.12825,0.13865,0.12181,0.12806,0.14296,0.13521,0.1
3445,0.14055,0.13778,0.13011,0.13053,0.13094,0.14095,0.14383,0.14046,0
.13709,0.11796,0.075703,0.076072,0.078953,0.079327,0.078057,0.075386,0
.075818,0.078061,0.076611,0.077515,0.19699,0.23032,0.082063,0.077938,0
.080666,0.076327,0.14093,0.13034,0.14139,0.13786,0.15376,0.14695,0.151
56,0.13946,0.14023,0.13072,0.12788,0.13839,0.1318,0.13613,0.12279,0.11
548,0.14007,0.13541,0.14183,0.075277,0.078221,0.079358,0.081669,0.0808
08,0.076373,0.077812,0.076556,0.072337,0.078946,0.080081,0.081352,0.08
5209,0.081555,0.081667,0.079126,0.084334,0.15173,0.14145,0.12621,0.140
76,0.12703,0.13483,0.13254,0.13353,0.12541,0.12894,0.13977,0.13227,0.1
3698,0.13998,0.13708,0.14066,0.08181,0.084482,0.085448,0.074424,0.0760
99,0.078798,0.080165,0.078277,0.078905,0.080447,0.076752,0.081108,0.08
0203,0.07825,0.080215,0.074603,0.079374,0.081268,0.083876,0.14148,0.13
117,0.12807,0.13845,0.13436,0.12429,0.15084,0.12929,0.16061,0.13924,0.
12852,0.13079,0.13891,0.07814,0.075927,0.075785,0.077615,0.075518,0.08
179,0.077614,0.080702,0.081116,0.082274,0.081694,0.079103,0.080796,0.0
81773,0.078954,0.078605,0.079505,0.081589,0.080473,0.080584,0.079513,0
.13635,0.13594,0.13377,0.14067,0.13847,0.14147,0.13782,0.14208,0.14595
,0.072348,0.081654,0.083274,0.080313,0.078493,0.075787,0.080831,0.0837

15,0.081414,0.081217,0.076128,0.081476,0.078839,0.077189,0.077769,0.08
0241,0.081856,0.083445,0.073105,0.080071,0.079891,0.077259,0.077847,0.
078265,0.081009,0.13216,0.13464,0.13573,0.13405,0.081738,0.073303,0.06
9922,0.079723,0.077481,0.073222,0.082685,0.077707,0.080262,0.076117,0.
075953,0.078449,0.07617,0.079577,0.079974,0.077995,0.076776,0.079791,0
.077377,0.079302,0.079046,0.07688,0.078021,0.076014,0.07694,0.079715,0
.081854,0.079127,0.078016,0.074301,0.079704,0.075556,0.078246,0.078259
,0.075457,0.079566,0.077017,0.079581,0.077807,0.079149,0.077457,0.0823
57,0.081138,0.073405,0.078965,0.074976,0.077917,0.079403,0.078282,0.07
7112,0.071416,0.081922,0.080886,0.075393,0.077418,0.081041,0.076367,0.
081039,0.075573,0.077712,0.080006,0.074746,0.078319,0.078564,0.084577,
0.08321,0.084623,0.081405,0.078394,0.076379,0.078125,0.077483,0.081916
,0.080248,0.085635,0.080146,0.077629,0.07947,0.079499,0.076346,0.07895
1,0.077628,0.077928,0.07812,0.08494,0.079098,0.080854,0.083175,0.07837
1,0.078038,0.078305,0.075891,0.078358,0.079898,0.07756,0.07998,0.07726
2,0.075866,0.071176,0.078658,0.07698,0.082752,0.085353,0.079439,0.0773
1,0.074102,0.0786,0.084167,0.082569,0.074341,0.078879,0.079592,0.07612
9,0.079121,0.07988,0.07527,0.078397,0.076386,0.079044,0.078476,0.07734
9,0.077819,0.082633,0.080833,0.081937,0.077537,0.076302,0.082187,0.075
972,0.078024,0.07973,0.080787,0.086652,0.08121,0.077086,0.074491,0.083
001,0.078343,0.082551,0.079939,0.08034,0.072606,0.081646,0.076309,0.07
3508,0.079798,0.079391,0.072993,0.07433,0.080094,0.077659,0.078271,0.0
78599,0.078957,0.076033,0.077477,0.078046,0.075893,0.078554,0.078268,0
.079564,0.075849,0.080487,0.079226,0.079597,0.078838,0.078598,0.076241
,0.082315,0.073281,0.075665,0.076069,0.08085,0.072488,0.076836,0.07587
3,0.079207,0.078827,0.079817,0.075282,0.075106,0.081946,0.079732,0.079
265,0.083132,0.072605,0.075691,0.076216,0.073883,0.073133,0.079074,0.0
80295,0.078032,0.076276,0.073454
0.20048,0.18431,0.19466,0.19957,0.19252,0.19408,0.19348,0.19278,0.2011
5,0.19867,0.18877,0.19481,0.19502,0.19018,0.18719,0.19644,0.20223,0.19
748,0.18974,0.18941,0.22963,0.34682,0.34027,0.30713,0.19367,0.20248,0.
19739,0.20243,0.18949,0.18797,0.18819,0.19704,0.2028,0.20382,0.19613,0
.19799,0.1905,0.19491,0.19225,0.42076,0.27812,0.26558,0.28744,0.31531,
0.2751,0.30737,0.19825,0.19534,0.19014,0.19181,0.19053,0.20783,0.20183
,0.19939,0.18819,0.20229,0.18665,0.19989,0.20081,0.1888,0.19003,0.5032
9,0.36649,0.26633,0.31601,0.33461,0.3153,0.32796,0.30662,0.28028,0.347
09,0.20612,0.19112,0.20058,0.19196,0.20144,0.19326,0.19591,0.20152,0.1
9579,0.18764,0.19263,0.19315,0.18962,0.19112,0.1951,0.4049,0.38217,0.4
1828,0.26671,0.32746,0.28313,0.28035,0.26677,0.23523,0.27457,0.34655,0
.31567,0.20034,0.20181,0.1987,0.18967,0.19401,0.19785,0.19887,0.20295,
0.19489,0.19435,0.19125,0.19385,0.18934,0.1977,0.1999,0.18971,0.48592,
0.36044,0.44943,0.39983,0.31055,0.27839,0.31022,0.32212,0.32077,0.2627
9,0.27774,0.30523,0.30221,0.25373,0.19116,0.19793,0.1956,0.19888,0.202
74,0.19497,0.193,0.18475,0.20417,0.18723,0.19862,0.19574,0.19942,0.193
94,0.19228,0.19426,0.48825,0.32214,0.46185,0.39987,0.37089,0.359,0.325
81,0.30056,0.28451,0.32746,0.28875,0.31513,0.31114,0.25336,0.27195,0.3
4937,0.18968,0.20061,0.19178,0.19376,0.19379,0.18534,0.19082,0.19129,0
.20293,0.19717,0.19038,0.19406,0.20331,0.19701,0.18688,0.19181,0.19229
,0.43321,0.36962,0.4494,0.3674,0.22018,0.4079,0.28773,0.28671,0.2446,0
.31509,0.27661,0.28123,0.31448,0.30608,0.27861,0.32956,0.27664,0.18503
,0.20085,0.19806,0.19337,0.20058,0.19382,0.18856,0.19554,0.18974,0.190
28,0.19956,0.19332,0.19145,0.13609,0.15743,0.13088,0.19448,0.36733,0.4

5741,0.36177,0.41915,0.37543,0.38106,0.30846,0.30112,0.33966,0.32798,0
.30064,0.27892,0.32209,0.29859,0.30699,0.35099,0.33667,0.3125,0.19124,
0.19845,0.18401,0.18989,0.20013,0.19479,0.20536,0.19594,0.20581,0.1953
,0.19122,0.12924,0.13225,0.13905,0.13461,0.12887,0.13412,0.19397,0.360
77,0.45442,0.36176,0.37606,0.44651,0.44283,0.34502,0.30897,0.36044,0.3
3276,0.28923,0.33379,0.25783,0.27686,0.27366,0.25512,0.28205,0.29719,0
.18623,0.20827,0.19255,0.18705,0.18738,0.19352,0.20321,0.19594,0.20181
,0.13641,0.12232,0.1459,0.14863,0.13945,0.13276,0.13044,0.12932,0.1312
,0.19329,0.3765,0.42059,0.29729,0.43229,0.32327,0.38374,0.37648,0.2628
6,0.26421,0.30917,0.29172,0.28522,0.32355,0.26722,0.31165,0.34854,0.29
249,0.27835,0.23464,0.20154,0.20624,0.19785,0.18682,0.19851,0.20593,0.
12784,0.14041,0.13686,0.12475,0.1347,0.13406,0.13065,0.123,0.12355,0.1
2968,0.13123,0.15376,0.54208,0.37347,0.42684,0.38257,0.39736,0.42033,0
.33804,0.23759,0.3069,0.26849,0.32013,0.26743,0.26898,0.29311,0.29126,
0.28436,0.32987,0.25498,0.30603,0.19547,0.20342,0.19139,0.14275,0.1413
7,0.13896,0.13944,0.12807,0.12487,0.13395,0.14504,0.13369,0.13683,0.13
262,0.1355,0.12998,0.1519,0.12692,0.43644,0.38238,0.38254,0.37719,0.40
006,0.45841,0.43034,0.41161,0.3532,0.2974,0.30874,0.27438,0.2753,0.302
17,0.32261,0.33104,0.22949,0.33453,0.29011,0.27482,0.13539,0.13411,0.1
3683,0.1344,0.13393,0.1406,0.13848,0.13692,0.14197,0.137,0.1291,0.147,
0.11639,0.12323,0.13567,0.13766,0.12483,0.12943,0.40005,0.43976,0.3559
7,0.42039,0.43685,0.40435,0.38191,0.374,0.32999,0.29411,0.23909,0.2411
3,0.207,0.21921,0.27423,0.23079,0.24431,0.2195,0.24685,0.19899,0.13096
,0.13836,0.13358,0.14176,0.14045,0.13735,0.13545,0.13181,0.12801,0.135
54,0.15087,0.13383,0.12771,0.13475,0.13955,0.15086,0.13574,0.14029,0.5
4622,0.2865,0.41558,0.35493,0.44243,0.29516,0.3931,0.35102,0.35434,0.2
4993,0.18634,0.22907,0.21563,0.19143,0.22328,0.22728,0.22147,0.25658,0
.22126,0.25483,0.12906,0.14838,0.14685,0.12934,0.13504,0.1379,0.13596,
0.13534,0.1311,0.12774,0.16393,0.12493,0.13645,0.11853,0.13644,0.14293
,0.13009,0.12412,0.43154,0.42307,0.37347,0.32291,0.55497,0.37463,0.344
1,0.19201,0.22693,0.17885,0.21517,0.23203,0.22875,0.23468,0.21807,0.21
451,0.2195,0.207,0.19804,0.24746,0.11388,0.1378,0.1244,0.1395,0.13725,
0.14975,0.13295,0.13797,0.13321,0.13203,0.13115,0.13197,0.13569,0.1385
5,0.13617,0.13829,0.13624,0.12033,0.30668,0.44511,0.38686,0.3574,0.323
87,0.32128,0.20784,0.25739,0.23566,0.24236,0.21654,0.26696,0.20681,0.2
2498,0.24798,0.23124,0.22149,0.23057,0.18258,0.21954,0.14231,0.13161,0
.12229,0.14407,0.14082,0.13485,0.13811,0.1295,0.12766,0.13306,0.13851,
0.12356,0.14665,0.14496,0.13358,0.12926,0.13732,0.14293,0.46431,0.3581
5,0.38695,0.38991,0.44203,0.27991,0.21643,0.24763,0.32259,0.23352,0.23
483,0.21033,0.19119,0.23051,0.18683,0.20583,0.18555,0.20971,0.23666,0.
23228,0.13472,0.1357,0.13549,0.15095,0.14791,0.13487,0.1481,0.13437,0.
13484,0.13137,0.14115,0.15044,0.12393,0.13347,0.136,0.12723,0.13253,0.
1347,0.3493,0.33565,0.38421,0.266,0.23897,0.19861,0.28526,0.24057,0.21
314,0.20903,0.24898,0.21896,0.17517,0.25781,0.21789,0.20154,0.23618,0.
20905,0.26755,0.28068,0.12418,0.13405,0.13152,0.13848,0.12266,0.13601,
0.1378,0.14442,0.13776,0.12453,0.12879,0.134,0.13566,0.12932,0.12628,0
.14455,0.13931,0.13649,0.46231,0.29076,0.21721,0.21388,0.24504,0.25858
,0.23488,0.26235,0.19742,0.27826,0.19283,0.22134,0.27247,0.21655,0.220
53,0.22594,0.21816,0.21989,0.2074,0.14173,0.13292,0.135,0.13124,0.1305
5,0.14289,0.12876,0.14312,0.14716,0.13556,0.16253,0.13141,0.14299,0.13
89,0.13116,0.14271,0.14166,0.072596,0.24403,0.21486,0.23065,0.2701,0.2
7069,0.24207,0.17848,0.2316,0.18976,0.22583,0.22562,0.24158,0.23046,0.

25056,0.23127,0.18999,0.24293,0.21315,0.21231,0.14431,0.13734,0.13152,
0.14053,0.13371,0.14063,0.13827,0.13258,0.13186,0.12917,0.14064,0.1384
3,0.12862,0.13764,0.13711,0.12946,0.13518,0.074114,0.21698,0.24106,0.2
4301,0.19589,0.27189,0.19059,0.25435,0.23049,0.26712,0.20581,0.2314,0.
22052,0.23452,0.19223,0.19729,0.26955,0.1877,0.20725,0.12538,0.13513,0
.13142,0.13137,0.11747,0.13972,0.13118,0.12641,0.12725,0.138,0.14695,0
.13974,0.13538,0.13489,0.1352,0.1335,0.13339,0.13093,0.070839,0.27721,
0.2896,0.22175,0.21681,0.27152,0.20945,0.27092,0.15623,0.19195,0.21709
,0.22355,0.19214,0.2005,0.22024,0.24969,0.26489,0.25475,0.19992,0.1380
1,0.12365,0.14133,0.12539,0.13041,0.13288,0.13099,0.13808,0.15866,0.12
943,0.13707,0.12572,0.13599,0.1333,0.12543,0.13998,0.1458,0.073847,0.0
76043,0.073262,0.19998,0.25245,0.25722,0.22526,0.2396,0.12368,0.21467,
0.23745,0.19702,0.17994,0.20178,0.20823,0.26959,0.23642,0.21302,0.1192
3,0.1333,0.13356,0.12917,0.1346,0.13518,0.13625,0.12106,0.13408,0.1287
,0.12261,0.15188,0.1427,0.13774,0.12788,0.13453,0.13189,0.13762,0.0739
94,0.075242,0.070922,0.072606,0.24977,0.27619,0.26023,0.24345,0.22799,
0.24259,0.24411,0.24764,0.24423,0.23554,0.2036,0.21757,0.1344,0.13311,
0.17736,0.12999,0.14365,0.13948,0.12926,0.13204,0.13088,0.12854,0.1487
7,0.13952,0.1307,0.1245,0.14696,0.13415,0.12942,0.14092,0.12903,0.1252
7,0.071285,0.070917,0.06969,0.07031,0.072483,0.072733,0.19796,0.17556,
0.19073,0.24024,0.22653,0.21726,0.23877,0.24366,0.14338,0.13289,0.1384
6,0.13708,0.14006,0.14427,0.13815,0.13369,0.14102,0.12549,0.14018,0.11
506,0.15053,0.11843,0.13713,0.12877,0.15079,0.13551,0.13465,0.13447,0.
13458,0.14107,0.11501,0.073833,0.07537,0.076743,0.073983,0.07315,0.067
815,0.22817,0.22592,0.29192,0.19221,0.23166,0.07455,0.071403,0.1414,0.
12434,0.13136,0.13838,0.12496,0.13981,0.12046,0.1278,0.14006,0.13431,0
.13399,0.13715,0.13485,0.12938,0.13157,0.1282,0.13951,0.14546,0.13863,
0.13686,0.11509,0.0711,0.070431,0.072854,0.075356,0.071568,0.06861,0.0
71588,0.071875,0.072155,0.071298,0.19639,0.24623,0.076902,0.071366,0.0
76901,0.071945,0.14066,0.1278,0.13982,0.13446,0.15534,0.14637,0.15184,
0.13736,0.13816,0.12864,0.12498,0.13628,0.13338,0.13269,0.12421,0.1129
1,0.13737,0.13482,0.14033,0.069905,0.072784,0.075571,0.073232,0.073382
,0.069603,0.070872,0.070689,0.066795,0.072739,0.073148,0.075077,0.0781
01,0.075204,0.075209,0.072075,0.078696,0.15158,0.1393,0.12456,0.13765,
0.12384,0.13637,0.13077,0.13281,0.12187,0.12456,0.13878,0.13337,0.1351
8,0.13977,0.13363,0.13592,0.075136,0.078722,0.078373,0.067327,0.070703
,0.072299,0.073247,0.074016,0.071339,0.07568,0.069782,0.074114,0.07379
7,0.070493,0.072589,0.070201,0.074694,0.074833,0.07892,0.14337,0.13012
,0.12755,0.13737,0.13208,0.12093,0.14896,0.12667,0.16061,0.13522,0.126
79,0.1272,0.13875,0.072483,0.069958,0.071421,0.073081,0.071738,0.07752
1,0.07081,0.076311,0.074481,0.075814,0.075488,0.073226,0.07601,0.07617
,0.074907,0.073588,0.073615,0.075885,0.074769,0.074163,0.072594,0.1352
3,0.13469,0.13158,0.13838,0.13526,0.13696,0.137,0.13963,0.14372,0.0679
2,0.07557,0.078784,0.074681,0.073496,0.068275,0.075606,0.079175,0.0755
16,0.076535,0.071431,0.075359,0.074136,0.071797,0.072261,0.074756,0.07
6499,0.076125,0.069356,0.070893,0.073402,0.071507,0.070646,0.07276,0.0
75482,0.13215,0.13313,0.13324,0.13241,0.076243,0.066497,0.063701,0.075
109,0.071265,0.066451,0.077032,0.0721,0.076305,0.069402,0.072285,0.073
777,0.071555,0.075455,0.075568,0.069872,0.070932,0.074343,0.072076,0.0
72413,0.072675,0.071634,0.071339,0.071681,0.071047,0.074367,0.07607,0.
073002,0.073319,0.067392,0.07199,0.069207,0.075166,0.073017,0.06788,0.
07255,0.071336,0.073255,0.07339,0.073377,0.073399,0.076253,0.073961,0.

067516,0.074322,0.070144,0.07159,0.072589,0.073457,0.071745,0.065646,0
.075484,0.077346,0.069769,0.07235,0.07571,0.071125,0.075192,0.067983,0
.072531,0.07394,0.071094,0.072442,0.072041,0.079599,0.076877,0.078798,
0.073617,0.073739,0.069038,0.072401,0.074109,0.076672,0.07233,0.078592
,0.073055,0.072207,0.073431,0.073525,0.070964,0.072587,0.071783,0.0743
68,0.072991,0.077371,0.071031,0.074701,0.080469,0.072976,0.072766,0.07
1559,0.0702,0.072324,0.076215,0.072619,0.075038,0.069864,0.071942,0.06
614,0.074397,0.068624,0.07544,0.078791,0.073961,0.073989,0.06741,0.072
788,0.07908,0.079414,0.069021,0.073222,0.072828,0.071333,0.073001,0.07
4191,0.070379,0.073167,0.068909,0.073375,0.071363,0.071192,0.07086,0.0
76843,0.07644,0.074377,0.072557,0.070304,0.07792,0.069081,0.074009,0.0
7323,0.075601,0.07824,0.075898,0.073514,0.06877,0.07604,0.070964,0.076
348,0.073402,0.075347,0.066338,0.080574,0.071563,0.066809,0.071627,0.0
73648,0.068517,0.06787,0.075508,0.072154,0.072478,0.075197,0.073756,0.
070991,0.072372,0.073362,0.070796,0.075159,0.070955,0.073831,0.068882,
0.07414,0.074304,0.073451,0.073248,0.07204,0.07197,0.077528,0.068046,0
.069316,0.070079,0.074059,0.067935,0.068818,0.071985,0.075287,0.072279
,0.073211,0.06823,0.069756,0.074937,0.075614,0.073665,0.076718,0.06895
3,0.070572,0.069208,0.069253,0.068202,0.074353,0.075514,0.072095,0.071
489,0.069211
0.19962,0.18642,0.19851,0.2064,0.19252,0.19603,0.19249,0.1905,0.20332,
0.19879,0.18673,0.20106,0.19756,0.19129,0.19273,0.19949,0.2048,0.20191
,0.1926,0.1919,0.24703,0.34615,0.32614,0.31134,0.19496,0.20442,0.19605
,0.20517,0.1967,0.18638,0.19449,0.20097,0.20595,0.19933,0.19877,0.1988
5,0.19293,0.1958,0.19203,0.37693,0.28757,0.25054,0.29663,0.3082,0.2699
2,0.27083,0.19655,0.19832,0.19338,0.19355,0.19521,0.20836,0.2026,0.203
21,0.1911,0.20183,0.1901,0.20441,0.20299,0.18763,0.19044,0.50337,0.347
38,0.27455,0.30826,0.32058,0.30125,0.32784,0.29089,0.263,0.34281,0.207
39,0.19163,0.19804,0.19524,0.20014,0.19284,0.19388,0.20535,0.1963,0.18
425,0.19537,0.19078,0.19562,0.19778,0.20052,0.425,0.3652,0.36996,0.256
61,0.30709,0.26829,0.27861,0.26323,0.24472,0.26967,0.34892,0.31997,0.2
0255,0.20424,0.19907,0.19347,0.19447,0.20051,0.20663,0.20586,0.19773,0
.19849,0.19123,0.19541,0.19483,0.20274,0.2035,0.19473,0.44454,0.31008,
0.43086,0.39708,0.30578,0.27799,0.29372,0.31171,0.3061,0.26881,0.26425
,0.30745,0.3104,0.26225,0.19659,0.20243,0.19413,0.20155,0.20196,0.1976
4,0.19742,0.18849,0.20603,0.19338,0.20075,0.20102,0.19729,0.19577,0.19
623,0.194,0.44764,0.3242,0.44099,0.38274,0.36506,0.38087,0.33146,0.324
48,0.29513,0.30805,0.28264,0.30946,0.29305,0.24078,0.27295,0.31861,0.1
9214,0.20411,0.1955,0.19647,0.19492,0.18287,0.1966,0.1937,0.2052,0.199
23,0.19129,0.19547,0.20517,0.19831,0.19143,0.19784,0.19848,0.4549,0.36
895,0.43402,0.35881,0.21748,0.40934,0.29036,0.25912,0.23633,0.2986,0.2
6711,0.27405,0.32037,0.29691,0.25839,0.3259,0.2821,0.18594,0.2045,0.20
166,0.19685,0.19903,0.19444,0.1902,0.19378,0.18998,0.19024,0.20018,0.1
9782,0.19744,0.13317,0.15739,0.12968,0.1965,0.35359,0.46313,0.40308,0.
39522,0.34506,0.33528,0.27837,0.27834,0.32092,0.3454,0.32033,0.28125,0
.33513,0.28932,0.31112,0.33315,0.30521,0.2818,0.19323,0.20242,0.18751,
0.1901,0.20023,0.19542,0.20479,0.19295,0.20748,0.19592,0.1923,0.12852,
0.13055,0.13695,0.13457,0.12841,0.13396,0.19881,0.35538,0.4343,0.37375
,0.37126,0.40335,0.38787,0.32459,0.33038,0.36176,0.31335,0.29105,0.329
35,0.26524,0.26597,0.23639,0.26392,0.27899,0.31062,0.1899,0.208,0.1974
5,0.18903,0.18811,0.19735,0.20282,0.19831,0.20645,0.1332,0.11878,0.143
37,0.14645,0.13942,0.13036,0.12773,0.12388,0.12992,0.19803,0.38748,0.4

4272,0.31756,0.40845,0.31872,0.40754,0.3679,0.25136,0.28587,0.29424,0.
30472,0.28992,0.32681,0.25696,0.29288,0.33323,0.27339,0.28197,0.25959,
0.20205,0.20634,0.19843,0.19063,0.202,0.20606,0.1261,0.13813,0.13285,0
.12107,0.13522,0.13418,0.12698,0.12102,0.1221,0.13101,0.13225,0.15067,
0.51817,0.35621,0.41729,0.38785,0.39821,0.42685,0.35661,0.233,0.29912,
0.2636,0.31424,0.24217,0.2701,0.28222,0.26934,0.27956,0.31718,0.2714,0
.28927,0.19694,0.2059,0.19611,0.14183,0.13771,0.1396,0.13483,0.12592,0
.12051,0.13241,0.14694,0.13344,0.13801,0.12792,0.13608,0.131,0.15,0.12
724,0.41234,0.36172,0.44195,0.36996,0.37937,0.45777,0.40203,0.38319,0.
31172,0.30838,0.30012,0.29193,0.2931,0.30381,0.31972,0.32712,0.24399,0
.3196,0.3022,0.26701,0.1337,0.13308,0.13545,0.13301,0.1333,0.14236,0.1
3498,0.13418,0.14353,0.13483,0.12731,0.14598,0.11665,0.12215,0.13439,0
.14027,0.12316,0.12854,0.35945,0.42289,0.36052,0.3688,0.37874,0.38428,
0.38439,0.37702,0.34632,0.29537,0.24275,0.23931,0.2041,0.22317,0.27079
,0.23506,0.23018,0.21937,0.24026,0.19791,0.12947,0.13524,0.13185,0.140
94,0.13689,0.13536,0.13284,0.1282,0.12368,0.13326,0.14935,0.1328,0.125
53,0.13351,0.13561,0.14969,0.13218,0.13788,0.55615,0.30869,0.42905,0.2
934,0.39197,0.27641,0.36768,0.33836,0.32861,0.24408,0.18845,0.21752,0.
22533,0.19194,0.20507,0.22769,0.21238,0.25391,0.21771,0.24674,0.12481,
0.14625,0.14689,0.12764,0.13228,0.13636,0.13501,0.13369,0.12922,0.1261
9,0.16431,0.1235,0.1356,0.11407,0.13791,0.14402,0.12887,0.12559,0.4504
2,0.40343,0.33582,0.33103,0.58299,0.36877,0.32684,0.1594,0.23263,0.189
37,0.23236,0.21078,0.22621,0.24167,0.20877,0.21137,0.21079,0.19764,0.1
8184,0.23168,0.10943,0.13723,0.12344,0.13876,0.13543,0.14702,0.13226,0
.13902,0.13252,0.12648,0.12861,0.13071,0.13547,0.13848,0.13397,0.13555
,0.13518,0.11868,0.36054,0.40461,0.38649,0.33156,0.33868,0.28943,0.187
2,0.25048,0.23299,0.22503,0.21149,0.25664,0.21622,0.22369,0.25711,0.22
167,0.21505,0.22875,0.18126,0.22027,0.14019,0.13131,0.11901,0.14452,0.
13849,0.13489,0.13642,0.12541,0.12669,0.13372,0.14,0.12187,0.14639,0.1
4456,0.1286,0.12868,0.137,0.14064,0.4162,0.37694,0.37216,0.35912,0.436
78,0.27776,0.21935,0.2321,0.31801,0.22536,0.21634,0.20081,0.19989,0.22
158,0.19816,0.19579,0.18335,0.20064,0.25299,0.24775,0.13157,0.13472,0.
13459,0.14742,0.14232,0.13231,0.14764,0.13331,0.13327,0.13066,0.13839,
0.15041,0.12168,0.13106,0.13428,0.12436,0.13018,0.13391,0.3557,0.3112,
0.40486,0.27749,0.22131,0.19227,0.25079,0.22178,0.21114,0.22624,0.2266
3,0.20621,0.17488,0.26009,0.21519,0.19239,0.21921,0.21514,0.2752,0.262
56,0.12261,0.13041,0.12898,0.133,0.12385,0.1334,0.1369,0.14472,0.14062
,0.12346,0.12784,0.13581,0.12978,0.12821,0.12459,0.14376,0.13816,0.135
99,0.45896,0.26902,0.23097,0.21126,0.23604,0.24027,0.21753,0.26375,0.1
8214,0.2777,0.19212,0.22001,0.26587,0.22405,0.2109,0.21139,0.20312,0.2
0957,0.21375,0.13912,0.13197,0.13334,0.12929,0.12862,0.14671,0.13087,0
.13924,0.1448,0.13577,0.16032,0.12882,0.1412,0.13464,0.13279,0.14241,0
.13791,0.067953,0.22895,0.20148,0.20823,0.25108,0.29022,0.24314,0.1890
5,0.19704,0.20985,0.21424,0.23284,0.23809,0.23153,0.25215,0.22577,0.18
598,0.23874,0.21065,0.20851,0.14172,0.13707,0.13087,0.14119,0.13205,0.
13903,0.13366,0.13302,0.12907,0.1274,0.14126,0.13751,0.12783,0.13581,0
.13541,0.12658,0.13232,0.067568,0.21668,0.24622,0.22509,0.18821,0.2527
9,0.19004,0.25492,0.22732,0.25753,0.20859,0.23177,0.19765,0.22276,0.19
277,0.18805,0.27783,0.18185,0.20855,0.12752,0.13014,0.13165,0.12818,0.
11566,0.13691,0.1302,0.12549,0.12589,0.13693,0.14586,0.13804,0.13221,0
.13297,0.13356,0.1326,0.13157,0.12998,0.064967,0.27208,0.27919,0.2231,
0.21648,0.26181,0.22336,0.28789,0.14587,0.1885,0.2152,0.21718,0.191,0.

20297,0.21544,0.23423,0.25733,0.24282,0.20622,0.13716,0.12276,0.14021,
0.12396,0.1292,0.13164,0.13122,0.13713,0.15882,0.1307,0.13447,0.12381,
0.13415,0.13225,0.12506,0.13862,0.14538,0.068768,0.071944,0.070526,0.1
9534,0.26871,0.26393,0.218,0.22894,0.11355,0.20519,0.24997,0.19381,0.1
8274,0.20233,0.21125,0.24297,0.23641,0.21162,0.11668,0.13293,0.13155,0
.12769,0.13567,0.13422,0.13589,0.11894,0.13122,0.12619,0.11709,0.14854
,0.14227,0.13644,0.12676,0.1334,0.12852,0.13212,0.069145,0.070462,0.06
6918,0.067601,0.22383,0.26699,0.25917,0.24359,0.23989,0.24562,0.22714,
0.25516,0.24026,0.23075,0.19879,0.22465,0.13395,0.1315,0.17471,0.12972
,0.14203,0.13835,0.1263,0.13092,0.1277,0.12642,0.14744,0.13754,0.12943
,0.12428,0.14753,0.13443,0.12673,0.13909,0.12925,0.12217,0.067928,0.06
6781,0.065611,0.065146,0.068962,0.070732,0.19006,0.16568,0.18276,0.210
7,0.22672,0.20768,0.22494,0.21534,0.14039,0.13255,0.13664,0.13588,0.13
703,0.14473,0.13867,0.13482,0.13966,0.12509,0.13895,0.11323,0.14958,0.
11631,0.13559,0.13016,0.15151,0.13547,0.13218,0.13285,0.13205,0.13987,
0.11197,0.070773,0.070097,0.071607,0.069147,0.067189,0.063654,0.20099,
0.20698,0.2775,0.17563,0.22938,0.070399,0.068424,0.13976,0.12138,0.129
36,0.13725,0.12168,0.139,0.11683,0.12622,0.14016,0.13367,0.13345,0.135
06,0.13531,0.1275,0.13176,0.13137,0.13677,0.14434,0.13777,0.13311,0.11
184,0.064973,0.067118,0.067625,0.070623,0.066561,0.064725,0.067184,0.0
66513,0.065947,0.067981,0.18935,0.21648,0.071567,0.065588,0.071502,0.0
67271,0.14072,0.12805,0.13692,0.13257,0.15417,0.14532,0.14942,0.13679,
0.13639,0.12618,0.12235,0.13668,0.1314,0.1303,0.1237,0.11294,0.13866,0
.13462,0.13885,0.066417,0.068762,0.070679,0.069577,0.069473,0.064271,0
.066785,0.067181,0.062634,0.067448,0.069264,0.069418,0.073775,0.068223
,0.069745,0.06579,0.07,0.15144,0.13972,0.12363,0.13644,0.12507,0.13446
,0.12912,0.13031,0.12002,0.12364,0.13621,0.13142,0.13451,0.13761,0.132
78,0.13229,0.072173,0.074303,0.07215,0.065557,0.065403,0.06623,0.07002
7,0.06747,0.065392,0.069594,0.06543,0.068081,0.067845,0.064464,0.06898
2,0.067478,0.068966,0.069415,0.072456,0.14134,0.13349,0.12819,0.1391,0
.13093,0.11899,0.14861,0.12702,0.16202,0.13465,0.1251,0.12687,0.13857,
0.065809,0.065236,0.065721,0.067195,0.065603,0.072374,0.066107,0.07238
6,0.069062,0.071847,0.070533,0.067055,0.07044,0.069231,0.06924,0.07049
4,0.071696,0.072069,0.06795,0.070925,0.068348,0.13436,0.13029,0.13107,
0.13943,0.13211,0.13697,0.13559,0.1348,0.14236,0.063819,0.07061,0.0736
19,0.071479,0.070485,0.061681,0.070518,0.073717,0.070878,0.071234,0.06
7217,0.070278,0.069152,0.068595,0.067394,0.070368,0.071482,0.070382,0.
06474,0.065141,0.068449,0.066242,0.067132,0.070799,0.070266,0.13032,0.
13374,0.13533,0.12917,0.071451,0.063288,0.060132,0.07026,0.064561,0.06
2387,0.072391,0.067883,0.072892,0.063095,0.066966,0.070229,0.067446,0.
070941,0.073853,0.064275,0.064656,0.069961,0.068582,0.069893,0.068447,
0.064839,0.066419,0.067805,0.065271,0.069066,0.07237,0.065987,0.071011
,0.063378,0.069473,0.063746,0.069423,0.068856,0.063044,0.068479,0.0674
64,0.067655,0.06944,0.070119,0.068886,0.07109,0.067553,0.062479,0.0690
09,0.067409,0.067163,0.067145,0.067901,0.068637,0.06192,0.070887,0.072
89,0.065521,0.068527,0.072121,0.06642,0.069208,0.064414,0.066039,0.070
853,0.066041,0.067666,0.065974,0.074514,0.071234,0.073336,0.069958,0.0
69152,0.064582,0.068248,0.068945,0.071189,0.068095,0.071906,0.066259,0
.066739,0.067461,0.066483,0.065723,0.067963,0.067625,0.071453,0.065843
,0.071221,0.067984,0.071177,0.074834,0.068462,0.068584,0.067322,0.0648
07,0.068487,0.072377,0.067845,0.071588,0.067126,0.066704,0.059339,0.06
9722,0.063694,0.070691,0.073919,0.067065,0.068359,0.064787,0.067514,0.

073206,0.074517,0.062432,0.068217,0.069335,0.067981,0.070837,0.069736,
0.065454,0.068189,0.066487,0.069254,0.066401,0.067982,0.066689,0.07228
3,0.071091,0.072292,0.066964,0.065517,0.07224,0.064186,0.071694,0.0688
88,0.070065,0.073391,0.071609,0.067298,0.06448,0.068059,0.065893,0.070
328,0.068918,0.070494,0.063289,0.075213,0.066988,0.063301,0.066349,0.0
68676,0.064723,0.064859,0.068798,0.067899,0.068143,0.071271,0.070953,0
.069064,0.068816,0.067288,0.066469,0.070848,0.064021,0.067947,0.068173
,0.069017,0.07,0.068258,0.066507,0.067635,0.069153,0.071432,0.063751,0
.067537,0.066049,0.068473,0.063421,0.062763,0.067206,0.07407,0.068051,
0.069158,0.06566,0.066383,0.069568,0.071762,0.06959,0.072714,0.067222,
0.065599,0.066013,0.064544,0.064657,0.069073,0.071877,0.068142,0.06593
7,0.06271
0.19996,0.18738,0.2002,0.20565,0.1962,0.1995,0.1988,0.19359,0.20381,0.
19945,0.19345,0.20462,0.19729,0.19135,0.18923,0.19911,0.2053,0.20218,0
.19406,0.19557,0.2647,0.33855,0.33906,0.28908,0.19755,0.20343,0.20035,
0.20538,0.20247,0.18919,0.19476,0.20207,0.20656,0.20087,0.19949,0.2029
,0.19622,0.19409,0.1923,0.36753,0.28911,0.26263,0.30342,0.28423,0.2716
7,0.26927,0.19743,0.19805,0.19753,0.19316,0.19453,0.21038,0.2031,0.205
83,0.19189,0.20108,0.19212,0.20857,0.20536,0.1903,0.19154,0.5025,0.325
84,0.24854,0.2971,0.3224,0.31135,0.29753,0.26618,0.25387,0.31289,0.206
68,0.19374,0.19857,0.19516,0.1994,0.19608,0.198,0.20384,0.19499,0.1858
2,0.19564,0.19675,0.19815,0.19914,0.20715,0.33198,0.36216,0.34204,0.24
754,0.3018,0.27312,0.27928,0.24603,0.24637,0.26736,0.32593,0.30788,0.2
0345,0.20695,0.1959,0.19543,0.19587,0.20521,0.20997,0.2023,0.1981,0.20
207,0.19483,0.1983,0.19585,0.20591,0.20337,0.19245,0.44348,0.28471,0.4
5195,0.37334,0.31153,0.25998,0.309,0.32253,0.27628,0.22774,0.2846,0.29
778,0.30921,0.26339,0.20182,0.20251,0.19692,0.19827,0.20109,0.20022,0.
20331,0.18862,0.2091,0.19847,0.20114,0.2044,0.19474,0.1983,0.19802,0.1
9456,0.47084,0.34452,0.42138,0.37343,0.3627,0.39519,0.34598,0.31851,0.
29219,0.33082,0.27623,0.31402,0.28272,0.25367,0.2924,0.29624,0.1922,0.
20667,0.20009,0.20039,0.19942,0.18417,0.19627,0.19537,0.20686,0.20231,
0.19118,0.19583,0.20885,0.1984,0.19777,0.20067,0.20321,0.44886,0.42284
,0.4243,0.29279,0.18684,0.35621,0.28554,0.25421,0.22746,0.30031,0.2716
7,0.26588,0.30545,0.29234,0.24851,0.32328,0.3001,0.18859,0.20264,0.201
91,0.19826,0.19586,0.1968,0.1909,0.19677,0.19025,0.18875,0.20053,0.199
46,0.2035,0.133,0.15543,0.12871,0.19775,0.35445,0.42463,0.38962,0.4115
8,0.34088,0.33102,0.28612,0.27468,0.31443,0.33039,0.30635,0.28972,0.29
237,0.29483,0.31182,0.3164,0.29556,0.28309,0.19145,0.19939,0.19143,0.1
9371,0.20322,0.19666,0.2039,0.19536,0.20556,0.19698,0.19811,0.12657,0.
12909,0.13846,0.13187,0.12642,0.13494,0.1998,0.35655,0.45617,0.38836,0
.35617,0.42023,0.37649,0.37681,0.32073,0.36863,0.32098,0.27275,0.29473
,0.26117,0.26182,0.22551,0.26612,0.27271,0.31826,0.19722,0.21527,0.199
97,0.19333,0.18551,0.19887,0.20493,0.20094,0.20629,0.13369,0.11869,0.1
4526,0.14733,0.13602,0.12821,0.12775,0.12173,0.12693,0.19669,0.3658,0.
41085,0.34825,0.43463,0.28863,0.36884,0.31538,0.25825,0.25886,0.29439,
0.31288,0.29706,0.32316,0.26353,0.29787,0.34878,0.27006,0.29733,0.2540
7,0.2002,0.20655,0.20293,0.19332,0.20016,0.21023,0.12722,0.13573,0.131
35,0.11992,0.13242,0.13344,0.12674,0.12116,0.121,0.12914,0.13143,0.153
21,0.51824,0.37413,0.38788,0.40077,0.37545,0.3919,0.31943,0.23829,0.30
446,0.26532,0.32241,0.2712,0.25243,0.30224,0.26399,0.25314,0.30043,0.2
6423,0.29903,0.19686,0.20528,0.20087,0.13971,0.13725,0.14076,0.13402,0
.12651,0.12111,0.12966,0.1425,0.13244,0.13699,0.1274,0.1332,0.12956,0.

15064,0.12635,0.4267,0.35843,0.42925,0.39278,0.37158,0.41901,0.37944,0
.3784,0.29938,0.30193,0.30805,0.2712,0.29462,0.31256,0.30483,0.32987,0
.24686,0.30472,0.31927,0.27121,0.13138,0.1328,0.135,0.13625,0.13086,0.
14194,0.13323,0.13328,0.14243,0.134,0.12659,0.14737,0.11464,0.12234,0.
13262,0.1376,0.11895,0.12926,0.34934,0.40659,0.35425,0.34197,0.35809,0
.37924,0.37678,0.38838,0.33065,0.27435,0.21645,0.23442,0.21057,0.21271
,0.25978,0.22587,0.22046,0.21413,0.24616,0.19419,0.1288,0.13394,0.1316
2,0.14056,0.13433,0.13026,0.13212,0.12824,0.12546,0.13421,0.15305,0.13
306,0.1217,0.13195,0.13232,0.14742,0.13133,0.1366,0.5686,0.31642,0.394
42,0.31996,0.38942,0.24799,0.36234,0.33954,0.29764,0.23415,0.19273,0.2
2014,0.21422,0.20274,0.20085,0.21704,0.21788,0.25213,0.19947,0.23078,0
.1256,0.14326,0.14686,0.12643,0.12998,0.13619,0.13401,0.13307,0.12703,
0.12615,0.16308,0.12192,0.13394,0.11199,0.13712,0.1448,0.12677,0.12622
,0.40776,0.42613,0.28334,0.31007,0.58642,0.36345,0.29786,0.16849,0.225
93,0.19501,0.2306,0.21721,0.2067,0.23665,0.20961,0.20556,0.20698,0.194
31,0.1815,0.22696,0.10756,0.13756,0.12556,0.13979,0.13146,0.14391,0.13
103,0.1374,0.13339,0.12401,0.12577,0.12969,0.13558,0.13644,0.13131,0.1
3384,0.13513,0.11939,0.34611,0.42016,0.34618,0.3044,0.32914,0.3262,0.1
9164,0.23509,0.21009,0.20696,0.19748,0.25413,0.218,0.2195,0.23405,0.20
01,0.22153,0.22077,0.1788,0.20534,0.14065,0.13059,0.11824,0.14728,0.13
241,0.13035,0.13365,0.12472,0.12268,0.13147,0.13916,0.11937,0.14356,0.
14503,0.12509,0.12645,0.13787,0.13946,0.36204,0.40294,0.36074,0.36212,
0.43974,0.25805,0.19649,0.20885,0.33843,0.20554,0.22489,0.20226,0.1943
3,0.22151,0.19385,0.18665,0.19948,0.20369,0.23323,0.24377,0.13056,0.13
275,0.1359,0.1457,0.14427,0.13191,0.14518,0.13042,0.13244,0.12843,0.13
639,0.14955,0.12099,0.12851,0.13123,0.12142,0.13107,0.13287,0.35057,0.
26956,0.393,0.28813,0.21628,0.20004,0.23131,0.21398,0.19299,0.22674,0.
20921,0.19216,0.1707,0.24289,0.21375,0.18769,0.22326,0.20997,0.26706,0
.24854,0.12106,0.12876,0.12834,0.13089,0.12417,0.13322,0.13489,0.14659
,0.13938,0.12218,0.1287,0.13691,0.12946,0.12912,0.12318,0.14498,0.1380
5,0.13566,0.39766,0.26121,0.25783,0.21394,0.26392,0.22114,0.19261,0.25
368,0.17777,0.2482,0.19269,0.2085,0.26878,0.21945,0.19679,0.20379,0.21
209,0.21153,0.20524,0.1364,0.13128,0.13376,0.12905,0.12816,0.14323,0.1
3005,0.1362,0.14065,0.13633,0.16082,0.12583,0.14421,0.13363,0.13002,0.
1431,0.13671,0.06303,0.22812,0.1888,0.21519,0.24909,0.29341,0.25076,0.
16325,0.19208,0.17462,0.19704,0.21669,0.2258,0.22241,0.25025,0.21739,0
.18238,0.22243,0.20099,0.21319,0.13983,0.13545,0.12945,0.14103,0.13073
,0.13464,0.13477,0.1324,0.12578,0.12442,0.14121,0.13623,0.12763,0.1363
3,0.13387,0.12701,0.13249,0.062044,0.20374,0.22971,0.2175,0.19332,0.25
411,0.19073,0.24998,0.21662,0.2511,0.18668,0.23064,0.20226,0.2298,0.18
626,0.18331,0.25605,0.18553,0.21593,0.12808,0.12984,0.12778,0.12646,0.
11486,0.13488,0.12791,0.1249,0.12727,0.13762,0.14603,0.14169,0.13119,0
.13243,0.13085,0.12939,0.12951,0.12675,0.06138,0.25229,0.27547,0.22112
,0.2027,0.24912,0.21261,0.27712,0.14911,0.17078,0.24697,0.19864,0.1908
5,0.21436,0.21384,0.2322,0.24107,0.24964,0.20909,0.13206,0.12062,0.137
73,0.12156,0.12944,0.13039,0.13069,0.13384,0.16178,0.12994,0.13075,0.1
2389,0.13143,0.12985,0.12235,0.14089,0.14379,0.064304,0.066458,0.06569
,0.18754,0.25489,0.23936,0.21674,0.22639,0.10947,0.21168,0.22689,0.177
82,0.18178,0.20398,0.21392,0.23079,0.21914,0.20859,0.11548,0.13029,0.1
2901,0.1284,0.13116,0.13364,0.13622,0.11756,0.12957,0.1243,0.11609,0.1
4874,0.1399,0.13559,0.12739,0.13291,0.12659,0.13131,0.064054,0.066958,
0.065379,0.065344,0.19367,0.26073,0.26681,0.26846,0.2308,0.23879,0.243

65,0.24498,0.23786,0.21141,0.18638,0.23153,0.13297,0.13007,0.1756,0.13
045,0.13934,0.13616,0.12478,0.12907,0.12667,0.1253,0.14362,0.13621,0.1
2973,0.12189,0.14767,0.13025,0.12677,0.13843,0.13165,0.11992,0.064589,
0.062576,0.063369,0.061245,0.064363,0.067524,0.18254,0.16732,0.19507,0
.21352,0.1979,0.21035,0.2066,0.22302,0.13921,0.13359,0.13585,0.13232,0
.13589,0.14699,0.13836,0.13352,0.13651,0.12413,0.1386,0.11298,0.14612,
0.11551,0.13634,0.12805,0.15155,0.13726,0.13017,0.13093,0.13042,0.1382
8,0.10857,0.066126,0.065717,0.070083,0.065882,0.064379,0.059692,0.2025
8,0.19082,0.28843,0.15949,0.22319,0.065091,0.064437,0.13605,0.1199,0.1
2631,0.13783,0.1203,0.13834,0.11448,0.12486,0.13775,0.13117,0.12906,0.
13435,0.13208,0.12868,0.1312,0.13145,0.13684,0.14294,0.13516,0.12796,0
.11004,0.061544,0.061948,0.064804,0.06858,0.063023,0.060641,0.065047,0
.06256,0.061623,0.064892,0.21067,0.21598,0.067583,0.060266,0.06559,0.0
62508,0.13786,0.12427,0.13717,0.13018,0.15322,0.14544,0.14873,0.13571,
0.13399,0.12474,0.1209,0.13686,0.13079,0.13182,0.12238,0.1127,0.13809,
0.13375,0.13439,0.062026,0.063126,0.066049,0.066128,0.067749,0.063169,
0.062634,0.062902,0.061491,0.06436,0.065034,0.066157,0.070103,0.062875
,0.064916,0.063122,0.067053,0.15179,0.14031,0.12635,0.13549,0.12329,0.
1341,0.12882,0.12853,0.11931,0.1243,0.1355,0.12771,0.13155,0.13569,0.1
315,0.13078,0.068604,0.067978,0.069294,0.063863,0.064325,0.061684,0.06
6537,0.062813,0.061237,0.065449,0.061447,0.063882,0.063174,0.059484,0.
064978,0.061834,0.065266,0.065416,0.069053,0.14057,0.1353,0.12534,0.13
907,0.13074,0.11839,0.1445,0.12421,0.16067,0.1347,0.12399,0.12537,0.14
059,0.0627,0.061742,0.063055,0.063487,0.064341,0.067543,0.062009,0.066
799,0.065011,0.068034,0.066456,0.062177,0.065143,0.06408,0.066198,0.06
6551,0.065114,0.066699,0.064523,0.064551,0.062461,0.13663,0.13223,0.13
072,0.1339,0.13144,0.13328,0.13421,0.1325,0.1451,0.061553,0.068573,0.0
70461,0.066388,0.068166,0.056259,0.065537,0.068981,0.067028,0.06579,0.
063681,0.068719,0.063374,0.06312,0.064904,0.063767,0.067023,0.064607,0
.059508,0.06248,0.066292,0.063001,0.062792,0.066048,0.06681,0.12866,0.
13205,0.13414,0.12726,0.065769,0.060219,0.05617,0.067128,0.062007,0.05
9819,0.068627,0.061477,0.069825,0.058556,0.064543,0.065864,0.06469,0.0
65503,0.069529,0.061793,0.061448,0.065897,0.06437,0.067548,0.064775,0.
060476,0.060956,0.066777,0.061278,0.066529,0.067675,0.060216,0.065825,
0.061719,0.067983,0.060577,0.067035,0.065217,0.059599,0.066817,0.06461
2,0.062885,0.064764,0.064745,0.0635,0.066964,0.063531,0.05807,0.066162
,0.064168,0.065359,0.063452,0.062419,0.065687,0.058619,0.066355,0.0684
18,0.059315,0.065647,0.068576,0.065028,0.06573,0.062963,0.062688,0.066
497,0.063875,0.060753,0.063168,0.071214,0.067713,0.067904,0.065654,0.0
65813,0.061254,0.063358,0.066182,0.067209,0.062369,0.068204,0.062445,0
.062715,0.063742,0.062774,0.060121,0.064955,0.063363,0.067957,0.062215
,0.065914,0.063013,0.066748,0.069289,0.064593,0.06398,0.064353,0.06329
,0.06607,0.067121,0.062961,0.065327,0.063521,0.061254,0.056048,0.06430
4,0.059119,0.067936,0.069602,0.063793,0.066491,0.059291,0.064428,0.069
016,0.071644,0.060253,0.063324,0.065637,0.061749,0.066396,0.068747,0.0
63325,0.064871,0.062959,0.065657,0.061973,0.066005,0.064769,0.064988,0
.065668,0.070065,0.063534,0.061204,0.067864,0.059104,0.0663,0.065294,0
.064631,0.06885,0.067914,0.064795,0.062181,0.066459,0.06324,0.065149,0
.063549,0.068357,0.061127,0.070017,0.063462,0.060127,0.061356,0.064238
,0.060407,0.06048,0.064577,0.063234,0.066369,0.068052,0.064912,0.06511
2,0.065124,0.063941,0.064227,0.065876,0.060151,0.062895,0.063268,0.062
081,0.067178,0.065295,0.062867,0.06389,0.066606,0.066596,0.060691,0.06

4562,0.064557,0.06312,0.06108,0.058686,0.064798,0.069119,0.06409,0.065
935,0.063678,0.062768,0.064813,0.066509,0.066214,0.070659,0.061966,0.0
62129,0.062723,0.06035,0.06178,0.065245,0.069404,0.064061,0.062919,0.0
56714
0.20432,0.18883,0.20256,0.20034,0.19564,0.20015,0.1989,0.1968,0.20439,
0.2012,0.19779,0.20809,0.19944,0.19177,0.18793,0.20305,0.20589,0.20521
,0.1978,0.19868,0.28289,0.34534,0.34859,0.29157,0.19791,0.20591,0.2005
8,0.20538,0.20194,0.19278,0.19551,0.20254,0.20657,0.2041,0.20339,0.202
85,0.19955,0.19317,0.19447,0.37275,0.30646,0.25423,0.30492,0.29683,0.2
5856,0.27303,0.19849,0.19845,0.20241,0.19626,0.19471,0.21477,0.203,0.2
0723,0.19769,0.2025,0.19361,0.21109,0.2067,0.18803,0.19252,0.52204,0.2
8995,0.26016,0.31854,0.30841,0.30066,0.30667,0.28816,0.24478,0.30034,0
.20495,0.19752,0.20353,0.19367,0.20267,0.19648,0.19534,0.19979,0.19808
,0.1876,0.19774,0.19473,0.20085,0.20355,0.20694,0.34965,0.35367,0.3151
8,0.26011,0.28513,0.25958,0.27772,0.24006,0.23344,0.27216,0.29177,0.29
584,0.2011,0.20996,0.19384,0.19745,0.19292,0.20838,0.20836,0.20433,0.2
0049,0.20605,0.19685,0.19835,0.19617,0.20135,0.20366,0.19593,0.44647,0
.2966,0.47795,0.38467,0.29222,0.26315,0.30322,0.3232,0.28227,0.23355,0
.28057,0.29343,0.31414,0.25693,0.20507,0.20317,0.1994,0.19815,0.20032,
0.20192,0.20525,0.18987,0.20658,0.19612,0.20397,0.20355,0.19855,0.2022
8,0.19934,0.19398,0.46345,0.33009,0.40007,0.33735,0.39865,0.4295,0.344
18,0.30505,0.28695,0.31456,0.27395,0.32713,0.2879,0.24331,0.27452,0.31
613,0.19724,0.20331,0.20161,0.19891,0.20044,0.183,0.19482,0.19841,0.20
449,0.20493,0.19285,0.20083,0.21109,0.20077,0.19883,0.19998,0.20485,0.
4141,0.42478,0.38963,0.27343,0.21858,0.31235,0.27978,0.26542,0.2352,0.
31289,0.27593,0.27063,0.30697,0.31221,0.23846,0.31795,0.29012,0.19123,
0.20597,0.20519,0.19771,0.19933,0.199,0.18947,0.1922,0.19408,0.19121,0
.20121,0.20234,0.20107,0.13269,0.15363,0.12856,0.20529,0.30671,0.43458
,0.38194,0.40151,0.34403,0.33891,0.29315,0.26743,0.31618,0.33669,0.306
98,0.29006,0.28886,0.2883,0.30584,0.31325,0.27828,0.28307,0.18995,0.20
203,0.19165,0.19625,0.20664,0.19499,0.20566,0.19719,0.20293,0.19782,0.
19484,0.12267,0.13073,0.13696,0.13133,0.1249,0.13624,0.19924,0.38491,0
.45119,0.36939,0.37298,0.41354,0.38767,0.37907,0.33748,0.3549,0.3236,0
.27861,0.30649,0.27416,0.27847,0.22852,0.25973,0.26324,0.27625,0.20099
,0.21006,0.19821,0.19724,0.18356,0.20539,0.2091,0.20467,0.20508,0.1322
,0.11607,0.14324,0.14684,0.13773,0.12387,0.12652,0.12006,0.12741,0.199
54,0.32531,0.38807,0.37014,0.42851,0.25798,0.3332,0.30515,0.24838,0.23
036,0.29818,0.33051,0.28304,0.32858,0.25908,0.30591,0.33951,0.26971,0.
29801,0.26372,0.19961,0.20388,0.20149,0.19132,0.19894,0.21043,0.12567,
0.13402,0.13168,0.11862,0.13036,0.13125,0.12581,0.12193,0.11844,0.1270
4,0.13023,0.15303,0.47398,0.33929,0.37139,0.39772,0.3621,0.36568,0.301
83,0.24442,0.34823,0.25984,0.31685,0.26816,0.25243,0.29352,0.25826,0.2
6045,0.30244,0.27151,0.30078,0.19929,0.2022,0.20028,0.13802,0.13474,0.
14093,0.13393,0.1268,0.12047,0.13103,0.14383,0.13318,0.13488,0.1279,0.
1323,0.12884,0.14922,0.12326,0.42042,0.35042,0.42238,0.36429,0.36709,0
.42246,0.39846,0.39733,0.32375,0.29609,0.29879,0.26799,0.31538,0.31558
,0.30784,0.33047,0.28078,0.32577,0.3217,0.24397,0.13015,0.13015,0.1342
6,0.13489,0.13014,0.13888,0.13211,0.13224,0.14206,0.1339,0.12684,0.147
47,0.11137,0.12165,0.13299,0.13483,0.1224,0.1273,0.35854,0.43899,0.361
82,0.33673,0.36317,0.35129,0.34367,0.35199,0.32451,0.25976,0.24582,0.2
2,0.20588,0.21241,0.25509,0.21267,0.21728,0.21398,0.24563,0.20168,0.12
679,0.13403,0.1302,0.13619,0.13292,0.13227,0.13397,0.13025,0.12264,0.1

3295,0.1506,0.1308,0.12474,0.13031,0.13286,0.14764,0.13232,0.13349,0.5
7343,0.31729,0.41641,0.3214,0.36536,0.22809,0.34396,0.32945,0.31247,0.
21019,0.19547,0.2118,0.20743,0.19318,0.20173,0.2177,0.21135,0.25552,0.
20022,0.22205,0.12588,0.14097,0.14953,0.12619,0.13009,0.13429,0.13431,
0.13293,0.12644,0.12561,0.16852,0.11935,0.13353,0.10946,0.13604,0.143,
0.12627,0.12757,0.37355,0.42065,0.23939,0.31171,0.51388,0.37861,0.2772
,0.15613,0.21726,0.21287,0.21543,0.20875,0.20879,0.24101,0.20369,0.205
74,0.20384,0.20953,0.18709,0.21997,0.1043,0.13762,0.12448,0.13965,0.13
085,0.14281,0.13258,0.13483,0.13178,0.12458,0.12555,0.13004,0.13473,0.
13492,0.12967,0.13255,0.13624,0.11995,0.31412,0.42506,0.33777,0.30604,
0.32754,0.33341,0.18672,0.21762,0.18679,0.19501,0.21799,0.24045,0.2135
5,0.22438,0.21965,0.20172,0.20933,0.20931,0.18451,0.19153,0.13837,0.12
684,0.11953,0.14541,0.13195,0.13005,0.13456,0.12621,0.12345,0.13032,0.
13745,0.12108,0.14148,0.14562,0.12513,0.12394,0.1358,0.1391,0.36366,0.
39419,0.33778,0.35033,0.41841,0.2627,0.18796,0.21766,0.35505,0.2034,0.
22661,0.19527,0.19013,0.21983,0.20117,0.18336,0.19907,0.20589,0.23403,
0.2396,0.13026,0.13554,0.13492,0.14609,0.14614,0.13192,0.14574,0.12811
,0.13173,0.12793,0.13084,0.14688,0.11835,0.12477,0.12902,0.12109,0.127
7,0.13199,0.36129,0.30381,0.38108,0.29052,0.19844,0.19691,0.22544,0.19
859,0.20833,0.24437,0.20187,0.18523,0.17233,0.234,0.2144,0.1772,0.2271
4,0.19983,0.26532,0.23835,0.11996,0.12849,0.12671,0.13131,0.12167,0.13
1,0.13158,0.14847,0.13782,0.11899,0.12845,0.13408,0.1295,0.12742,0.121
48,0.14591,0.13323,0.1346,0.42113,0.24879,0.24253,0.2138,0.27364,0.217
62,0.18363,0.27987,0.19414,0.24776,0.17345,0.20607,0.26939,0.21173,0.1
9398,0.20362,0.20769,0.21365,0.19988,0.13239,0.13017,0.13345,0.12774,0
.12983,0.14229,0.12769,0.13627,0.1395,0.13724,0.16387,0.1236,0.14398,0
.13279,0.13131,0.14321,0.13455,0.061594,0.22378,0.1697,0.22567,0.25511
,0.30336,0.22532,0.16024,0.19482,0.18251,0.18235,0.20422,0.23757,0.219
88,0.24685,0.21192,0.185,0.21119,0.19538,0.22454,0.13598,0.13557,0.129
,0.1413,0.12872,0.13258,0.13336,0.13115,0.12675,0.12099,0.13796,0.1358
9,0.12652,0.13662,0.1324,0.12726,0.13183,0.060025,0.20682,0.20934,0.21
747,0.20095,0.28832,0.20226,0.22363,0.20115,0.22987,0.17639,0.21628,0.
20424,0.23108,0.18798,0.1824,0.24863,0.18472,0.21951,0.1256,0.13275,0.
12753,0.12466,0.11385,0.13158,0.12448,0.12329,0.12672,0.13583,0.14601,
0.14007,0.12848,0.13115,0.13069,0.12821,0.12809,0.12453,0.057666,0.248
31,0.28166,0.22673,0.18919,0.24087,0.20535,0.26441,0.14925,0.17379,0.2
3704,0.18432,0.19189,0.22162,0.22664,0.23573,0.25407,0.24625,0.20701,0
.12788,0.12081,0.1358,0.12105,0.12948,0.13095,0.12924,0.13127,0.16479,
0.13059,0.12996,0.12192,0.12823,0.12754,0.12224,0.14186,0.14391,0.0610
43,0.061013,0.062291,0.1773,0.23714,0.23513,0.2026,0.22439,0.10694,0.2
1277,0.23174,0.16457,0.18158,0.20141,0.20858,0.2213,0.21353,0.21149,0.
11566,0.1326,0.12931,0.12838,0.13069,0.13412,0.13442,0.11416,0.12689,0
.12266,0.11727,0.15144,0.13944,0.13232,0.1266,0.13062,0.125,0.12847,0.
059374,0.063764,0.063932,0.061877,0.19073,0.24951,0.26279,0.26102,0.21
059,0.24196,0.23995,0.23289,0.2394,0.22368,0.1924,0.2256,0.13307,0.129
5,0.17591,0.12949,0.14107,0.13285,0.12401,0.13109,0.12708,0.12295,0.14
357,0.13831,0.12784,0.12292,0.14538,0.13213,0.12465,0.13895,0.13108,0.
12002,0.061039,0.059365,0.059749,0.057593,0.062117,0.062996,0.18476,0.
15616,0.18839,0.19628,0.21448,0.20624,0.19668,0.22329,0.13857,0.13089,
0.13544,0.13124,0.133,0.14419,0.13725,0.13065,0.13248,0.12424,0.13958,
0.10959,0.14385,0.11377,0.13465,0.12733,0.15271,0.13751,0.13095,0.1314
,0.1291,0.13877,0.10791,0.065073,0.062326,0.066005,0.062397,0.062482,0

.056065,0.18035,0.20755,0.2785,0.15499,0.22122,0.061829,0.061833,0.135
39,0.11865,0.12268,0.1356,0.11974,0.13497,0.11398,0.12204,0.1377,0.130
88,0.12845,0.13426,0.1302,0.12579,0.13149,0.12956,0.13509,0.14042,0.13
629,0.12638,0.1105,0.058133,0.059268,0.06171,0.067104,0.059066,0.05710
8,0.062862,0.05932,0.058739,0.063478,0.16905,0.21421,0.06267,0.058343,
0.063226,0.059626,0.13767,0.12204,0.13569,0.13171,0.15551,0.14627,0.14
922,0.13309,0.13198,0.12453,0.11996,0.13468,0.13243,0.13114,0.12047,0.
11015,0.13609,0.13463,0.13297,0.05932,0.058333,0.060865,0.065227,0.064
449,0.060458,0.060497,0.060647,0.05604,0.060025,0.06034,0.061763,0.066
926,0.059537,0.062671,0.059452,0.063389,0.1519,0.14048,0.12532,0.13511
,0.12254,0.13327,0.12797,0.12817,0.11862,0.12228,0.13263,0.1287,0.1316
7,0.13503,0.12804,0.13018,0.065898,0.063323,0.067817,0.062541,0.062013
,0.057846,0.06286,0.06172,0.059562,0.062543,0.057632,0.059486,0.060254
,0.05624,0.063894,0.059636,0.062463,0.064416,0.064442,0.1417,0.13412,0
.12629,0.13834,0.1273,0.11741,0.14142,0.12316,0.16477,0.13223,0.12398,
0.12244,0.14038,0.058877,0.058132,0.060537,0.060366,0.0609,0.062214,0.
057761,0.065471,0.061395,0.065426,0.059561,0.060276,0.063214,0.060712,
0.059818,0.061567,0.060578,0.062728,0.062339,0.059796,0.058914,0.13423
,0.1291,0.12966,0.13562,0.13075,0.13125,0.13391,0.13088,0.14806,0.0595
12,0.065195,0.067933,0.064462,0.065584,0.052497,0.062598,0.067463,0.06
5153,0.061456,0.06203,0.065773,0.060835,0.061253,0.062512,0.060105,0.0
6425,0.062048,0.055314,0.060033,0.062421,0.061563,0.058208,0.064263,0.
066989,0.1263,0.13409,0.13246,0.12627,0.062135,0.05735,0.05341,0.06407
2,0.058479,0.058611,0.065571,0.055962,0.064884,0.056044,0.060603,0.062
368,0.061318,0.06208,0.066229,0.059066,0.058246,0.063854,0.061101,0.06
5367,0.06036,0.055173,0.057488,0.065315,0.058396,0.063237,0.06387,0.05
7362,0.064308,0.05815,0.0642,0.05696,0.063048,0.062398,0.055976,0.0631
95,0.061382,0.059053,0.063103,0.06344,0.060285,0.063463,0.0607,0.05679
2,0.062564,0.063076,0.059393,0.061051,0.058304,0.06181,0.055048,0.0629
11,0.064442,0.05676,0.06247,0.065676,0.062528,0.063164,0.059097,0.0604
95,0.065474,0.062296,0.058101,0.058822,0.067049,0.064749,0.063593,0.06
3695,0.060575,0.059605,0.060378,0.064388,0.064512,0.059035,0.063804,0.
059626,0.058936,0.06057,0.058329,0.057692,0.061833,0.058743,0.065054,0
.060571,0.059754,0.059158,0.062739,0.064746,0.063171,0.06399,0.06135,0
.059646,0.063549,0.064467,0.061142,0.060267,0.060876,0.058658,0.055549
,0.058843,0.054076,0.065188,0.065825,0.05935,0.06298,0.057897,0.062677
,0.064828,0.068267,0.057741,0.059323,0.061654,0.059581,0.0645,0.065199
,0.058452,0.060575,0.058248,0.06219,0.058507,0.063477,0.059531,0.06171
3,0.062515,0.067676,0.060751,0.057927,0.0672,0.05547,0.062822,0.064622
,0.06096,0.065875,0.062948,0.062217,0.059908,0.064508,0.059756,0.06162
3,0.059645,0.063702,0.057667,0.067796,0.061198,0.057991,0.060817,0.059
39,0.057376,0.057441,0.061591,0.059881,0.065312,0.063932,0.062623,0.06
309,0.060673,0.060373,0.060653,0.062867,0.054818,0.058121,0.060138,0.0
5814,0.064222,0.06373,0.06227,0.062011,0.063459,0.063733,0.059453,0.06
1637,0.060963,0.060182,0.059024,0.055871,0.06297,0.065006,0.060721,0.0
60899,0.061322,0.060986,0.061289,0.060244,0.061701,0.065138,0.058739,0
.060481,0.058892,0.05574,0.05955,0.062105,0.065874,0.062196,0.061215,0
.054365
0.20901,0.18688,0.20691,0.20045,0.19798,0.20036,0.20122,0.1934,0.20459
,0.19801,0.19827,0.20568,0.19829,0.19704,0.18874,0.20075,0.21241,0.201
43,0.19643,0.20086,0.3039,0.33778,0.35123,0.30593,0.19687,0.20722,0.19
942,0.20574,0.20154,0.19521,0.19881,0.20363,0.20636,0.20605,0.2044,0.2

0286,0.2002,0.19428,0.19512,0.37901,0.31111,0.24945,0.29264,0.27437,0.
25303,0.27841,0.20225,0.19608,0.20484,0.19658,0.19409,0.2174,0.20355,0
.20509,0.19903,0.20568,0.19926,0.21182,0.20292,0.19273,0.194,0.4836,0.
31069,0.24868,0.31346,0.30234,0.31126,0.2856,0.28142,0.2419,0.29744,0.
20135,0.1964,0.20562,0.19096,0.20327,0.20256,0.19649,0.20016,0.20027,0
.18962,0.20062,0.19488,0.20073,0.20364,0.20801,0.35741,0.35101,0.32467
,0.26547,0.26834,0.26022,0.26863,0.2494,0.25125,0.28167,0.27283,0.2814
4,0.20781,0.21113,0.19289,0.2012,0.19531,0.21175,0.21373,0.20803,0.200
81,0.20873,0.19397,0.19972,0.2004,0.19736,0.20292,0.19718,0.42047,0.32
259,0.46123,0.38104,0.29864,0.26956,0.30455,0.32768,0.29143,0.23772,0.
27886,0.28418,0.30264,0.26521,0.21113,0.20439,0.20074,0.20067,0.19911,
0.19834,0.20921,0.1864,0.20847,0.19885,0.20496,0.20579,0.19708,0.20216
,0.20074,0.19656,0.38484,0.32894,0.41479,0.3235,0.40734,0.40272,0.3230
4,0.30259,0.29492,0.31408,0.27642,0.34229,0.29614,0.25704,0.26136,0.32
943,0.20207,0.20334,0.20397,0.19675,0.20283,0.18185,0.19769,0.20054,0.
20424,0.20905,0.19928,0.20391,0.21016,0.20097,0.19717,0.202,0.20868,0.
36898,0.41079,0.37194,0.23775,0.26359,0.3403,0.27984,0.27055,0.24072,0
.29935,0.26806,0.26225,0.30985,0.31074,0.21345,0.30179,0.29351,0.19693
,0.20136,0.20577,0.19718,0.19884,0.19902,0.18818,0.19351,0.19183,0.190
29,0.20076,0.20101,0.2002,0.13227,0.15114,0.12792,0.20664,0.33167,0.42
471,0.35659,0.35392,0.30873,0.3543,0.27802,0.26309,0.31033,0.34238,0.3
0671,0.29467,0.28754,0.29451,0.29811,0.32276,0.28135,0.28959,0.19625,0
.20683,0.19724,0.19896,0.20545,0.1926,0.20686,0.19654,0.207,0.20159,0.
19348,0.12032,0.1312,0.13499,0.12898,0.12347,0.13192,0.19954,0.40028,0
.44731,0.37656,0.36351,0.43667,0.38673,0.37575,0.32388,0.34916,0.29674
,0.26185,0.29508,0.274,0.29077,0.2311,0.27012,0.25757,0.28206,0.20397,
0.21202,0.19794,0.19911,0.1903,0.20334,0.20851,0.20466,0.20721,0.13379
,0.11613,0.14177,0.14947,0.13485,0.12512,0.12331,0.12065,0.12887,0.197
26,0.30108,0.3479,0.42003,0.44228,0.2619,0.38814,0.35879,0.25791,0.238
42,0.29968,0.30655,0.26341,0.33276,0.27646,0.33946,0.34055,0.27645,0.3
0648,0.24892,0.19997,0.20517,0.19944,0.19212,0.2031,0.21192,0.12566,0.
13447,0.13025,0.11875,0.12849,0.12956,0.12592,0.11951,0.1172,0.12762,0
.1299,0.15412,0.39881,0.31957,0.37704,0.38296,0.33125,0.34506,0.29793,
0.24723,0.33622,0.25203,0.29834,0.27513,0.25557,0.29605,0.25903,0.2465
8,0.29958,0.27313,0.30617,0.20032,0.20538,0.20119,0.13879,0.13708,0.14
112,0.13686,0.1263,0.12064,0.13076,0.14407,0.13121,0.13171,0.12745,0.1
3131,0.12708,0.14942,0.1215,0.42255,0.32388,0.41057,0.35336,0.3642,0.4
2192,0.32764,0.38501,0.32048,0.29703,0.29283,0.25738,0.30339,0.29771,0
.29871,0.34258,0.27045,0.30693,0.31935,0.25253,0.12707,0.12934,0.13279
,0.13415,0.12977,0.13859,0.13246,0.13048,0.14159,0.13274,0.12396,0.146
18,0.10954,0.12049,0.13282,0.136,0.1215,0.12467,0.38331,0.40465,0.3706
5,0.3783,0.3614,0.37206,0.31847,0.34818,0.34022,0.25464,0.25059,0.2264
9,0.21902,0.2071,0.23899,0.22194,0.21699,0.20368,0.25066,0.18952,0.124
1,0.13545,0.12709,0.13371,0.13166,0.1306,0.13363,0.13097,0.12067,0.132
32,0.15468,0.12974,0.12415,0.13241,0.13196,0.14465,0.12985,0.13128,0.5
6325,0.2984,0.40498,0.33766,0.38072,0.24447,0.31348,0.31842,0.30879,0.
22417,0.19015,0.21555,0.20919,0.19215,0.19709,0.21017,0.20598,0.24678,
0.18714,0.21783,0.12796,0.1419,0.15019,0.12626,0.13043,0.13611,0.13062
,0.13172,0.12709,0.12474,0.16701,0.11825,0.13215,0.10711,0.13304,0.139
19,0.12588,0.12784,0.40865,0.40165,0.26374,0.29885,0.50274,0.38,0.2606
1,0.16382,0.22004,0.18951,0.2344,0.20083,0.20683,0.23375,0.20401,0.216
94,0.19632,0.21692,0.18862,0.20522,0.10171,0.13748,0.12284,0.13917,0.1

2867,0.14081,0.13163,0.13324,0.12914,0.12621,0.12608,0.13028,0.13487,0
.13418,0.1279,0.13227,0.13267,0.12173,0.33578,0.38953,0.38723,0.29314,
0.37687,0.29983,0.19687,0.21004,0.18583,0.19196,0.21798,0.22957,0.2056
2,0.21699,0.21271,0.19561,0.21183,0.21022,0.17491,0.18452,0.13631,0.12
846,0.12027,0.14676,0.13296,0.12976,0.13475,0.12442,0.12254,0.13056,0.
13794,0.12315,0.13768,0.14206,0.12662,0.12453,0.13804,0.13905,0.38091,
0.35753,0.34348,0.34861,0.36894,0.24569,0.19038,0.21261,0.3522,0.19036
,0.22838,0.183,0.19223,0.22058,0.20252,0.19003,0.1793,0.20344,0.22614,
0.24263,0.13133,0.13464,0.13335,0.14298,0.14711,0.12961,0.14385,0.1281
2,0.12936,0.12846,0.13246,0.14665,0.11734,0.12506,0.1291,0.11909,0.123
63,0.13177,0.37751,0.25444,0.35475,0.28984,0.20781,0.21828,0.21992,0.1
9734,0.21078,0.22427,0.20181,0.17122,0.16335,0.22572,0.2154,0.17792,0.
23189,0.19245,0.25786,0.2173,0.11943,0.12846,0.12636,0.13115,0.12309,0
.13346,0.13084,0.1481,0.13563,0.11877,0.12633,0.13137,0.12843,0.12635,
0.11933,0.14822,0.13033,0.13661,0.4235,0.22694,0.23859,0.22459,0.26565
,0.19953,0.1964,0.25709,0.1948,0.22305,0.18959,0.20227,0.28228,0.22744
,0.19359,0.21731,0.20811,0.20977,0.19153,0.1313,0.12766,0.13342,0.1258
3,0.13006,0.1406,0.12669,0.13733,0.13878,0.13612,0.16368,0.12321,0.143
47,0.13316,0.13082,0.13848,0.13487,0.06086,0.2155,0.17652,0.22681,0.25
2,0.3155,0.22006,0.14437,0.17719,0.17685,0.1803,0.20815,0.23061,0.2255
2,0.23351,0.20355,0.18444,0.19781,0.21103,0.21067,0.13383,0.1355,0.127
21,0.14321,0.12616,0.1317,0.13375,0.12995,0.12566,0.11984,0.13754,0.13
557,0.12462,0.13565,0.1338,0.12741,0.13298,0.055945,0.21472,0.19131,0.
2173,0.1999,0.2638,0.19227,0.22419,0.18216,0.18928,0.17794,0.20776,0.2
1137,0.22368,0.1905,0.18842,0.25025,0.19026,0.20836,0.12447,0.13272,0.
1274,0.12522,0.10988,0.12865,0.1241,0.12291,0.12573,0.13401,0.14555,0.
13959,0.12617,0.12856,0.13107,0.12676,0.12732,0.12256,0.056228,0.2372,
0.28406,0.20896,0.17494,0.23137,0.2,0.25768,0.14717,0.17224,0.25874,0.
19352,0.17881,0.21238,0.22679,0.23926,0.2417,0.2373,0.19545,0.12676,0.
11736,0.1344,0.12197,0.12762,0.12945,0.13149,0.13179,0.16569,0.13045,0
.12836,0.12217,0.12684,0.12551,0.12277,0.14001,0.14383,0.058954,0.0600
2,0.059945,0.18352,0.22993,0.22248,0.19838,0.2221,0.11284,0.18783,0.21
754,0.15098,0.18464,0.20533,0.20469,0.21642,0.18966,0.21623,0.11427,0.
13293,0.12949,0.1256,0.13055,0.13409,0.13587,0.11136,0.12525,0.12487,0
.11857,0.15078,0.14174,0.12744,0.12682,0.13183,0.12819,0.12578,0.05694
,0.061908,0.062475,0.061415,0.17207,0.24074,0.24297,0.26188,0.19295,0.
24585,0.23515,0.24275,0.23398,0.21124,0.1886,0.21331,0.13157,0.12816,0
.17895,0.12901,0.14098,0.12984,0.1252,0.12888,0.1241,0.12127,0.1459,0.
13947,0.12766,0.12044,0.14364,0.13262,0.12385,0.13934,0.13182,0.11645,
0.060144,0.057219,0.057044,0.054381,0.059174,0.059,0.19418,0.1617,0.18
175,0.19762,0.2109,0.20146,0.20594,0.20503,0.14003,0.13065,0.13768,0.1
3107,0.13044,0.14641,0.13276,0.13043,0.13238,0.1263,0.13569,0.10666,0.
14382,0.1109,0.1335,0.1298,0.15379,0.13425,0.13007,0.12871,0.12937,0.1
3917,0.10601,0.062027,0.06106,0.060995,0.05953,0.058411,0.053726,0.191
95,0.20038,0.27914,0.17051,0.21907,0.061012,0.060156,0.13445,0.11607,0
.1198,0.1354,0.12054,0.13155,0.11387,0.12333,0.1391,0.12944,0.12997,0.
13465,0.12764,0.12594,0.13013,0.13023,0.13544,0.13853,0.13764,0.12597,
0.10994,0.0555,0.05688,0.057272,0.063009,0.055756,0.054106,0.062816,0.
056879,0.058643,0.061885,0.16198,0.21747,0.060836,0.053929,0.059339,0.
057698,0.13782,0.12208,0.135,0.12913,0.15163,0.14484,0.14658,0.13217,0
.13194,0.12291,0.11831,0.1317,0.13503,0.13024,0.11917,0.11059,0.13572,
0.13404,0.13064,0.057312,0.057835,0.058182,0.062249,0.060971,0.0575,0.

056647,0.059195,0.055649,0.0573,0.059149,0.060084,0.065327,0.055608,0.
06084,0.054906,0.0586,0.1511,0.13989,0.12559,0.13398,0.12158,0.1327,0.
12725,0.1293,0.1176,0.12204,0.13172,0.12864,0.12806,0.13785,0.12826,0.
13049,0.06358,0.060374,0.066386,0.058281,0.060232,0.054963,0.059733,0.
057784,0.0597,0.061828,0.057045,0.056388,0.05877,0.054348,0.06303,0.05
6916,0.057647,0.061024,0.061015,0.14103,0.13192,0.12556,0.13814,0.1275
,0.11526,0.14218,0.11917,0.16598,0.13275,0.12542,0.1231,0.1357,0.05809
3,0.05624,0.057492,0.057569,0.061173,0.059886,0.054225,0.062612,0.0595
96,0.063826,0.056158,0.058282,0.061738,0.059515,0.05915,0.059557,0.057
887,0.059571,0.060987,0.057179,0.05697,0.1316,0.12645,0.13008,0.13407,
0.12889,0.12848,0.1323,0.12962,0.14488,0.058254,0.061925,0.064986,0.06
2406,0.061594,0.049695,0.061706,0.064709,0.061739,0.057616,0.060099,0.
063685,0.062153,0.060159,0.057502,0.059909,0.061059,0.058646,0.054975,
0.058365,0.059897,0.060444,0.056628,0.0618,0.062234,0.12826,0.13295,0.
12935,0.126,0.059005,0.056153,0.051479,0.059812,0.054789,0.056222,0.06
2917,0.054109,0.065315,0.052785,0.058054,0.056894,0.058023,0.060981,0.
063892,0.057071,0.056267,0.06057,0.056844,0.062273,0.057552,0.053915,0
.055994,0.061411,0.05595,0.060628,0.060214,0.055498,0.061862,0.057215,
0.062491,0.055777,0.061032,0.058464,0.052654,0.060933,0.058062,0.05658
1,0.060518,0.060456,0.057426,0.060019,0.056431,0.052068,0.062081,0.060
856,0.058602,0.058895,0.058176,0.060997,0.052922,0.057194,0.062174,0.0
51587,0.059918,0.060241,0.058678,0.05972,0.055142,0.057392,0.063717,0.
060231,0.05488,0.053828,0.063256,0.064856,0.061072,0.059612,0.056557,0
.05568,0.054587,0.063313,0.063467,0.056297,0.059748,0.055333,0.056451,
0.059876,0.056279,0.053755,0.058118,0.057371,0.06058,0.05884,0.058575,
0.056476,0.063028,0.061798,0.060995,0.059947,0.057605,0.056589,0.06031
,0.060645,0.057286,0.059225,0.059108,0.055534,0.054793,0.056209,0.0517
34,0.060443,0.061647,0.05797,0.061939,0.058418,0.060221,0.061913,0.065
248,0.054148,0.057694,0.057969,0.054249,0.061157,0.060865,0.053956,0.0
58833,0.056413,0.058608,0.056903,0.060898,0.058573,0.060712,0.060941,0
.065135,0.056931,0.055678,0.06344,0.05197,0.059044,0.062197,0.058942,0
.063635,0.060856,0.06054,0.057037,0.061961,0.059675,0.058778,0.055645,
0.059138,0.054431,0.06476,0.058045,0.053778,0.058168,0.058027,0.054599
,0.056331,0.058683,0.057233,0.061239,0.061188,0.061253,0.060507,0.0568
23,0.060667,0.057936,0.06167,0.052409,0.058024,0.056155,0.058859,0.060
404,0.060095,0.059577,0.059712,0.063263,0.062284,0.05743,0.060305,0.05
8125,0.058961,0.056821,0.055674,0.059112,0.059837,0.05949,0.057665,0.0
58323,0.057552,0.056677,0.058351,0.060345,0.062895,0.054273,0.056521,0
.055538,0.054603,0.057274,0.059661,0.062177,0.058472,0.058652,0.052948
0.2114,0.18877,0.21067,0.20171,0.19715,0.20202,0.20617,0.19727,0.20565
,0.20083,0.19757,0.20451,0.20028,0.19457,0.19091,0.1999,0.21252,0.2002
1,0.20089,0.20414,0.31059,0.34698,0.33328,0.3071,0.19989,0.20813,0.203
88,0.20108,0.19912,0.19278,0.2027,0.20341,0.20435,0.20665,0.20207,0.20
368,0.20017,0.19318,0.19877,0.35558,0.29399,0.24379,0.29368,0.281,0.24
867,0.29979,0.20532,0.19967,0.20665,0.20263,0.19657,0.21956,0.20345,0.
20734,0.20331,0.20529,0.2064,0.21232,0.20539,0.19442,0.19149,0.45141,0
.32399,0.23317,0.2982,0.30896,0.30601,0.29373,0.26965,0.24516,0.29209,
0.19978,0.19517,0.20682,0.19493,0.20146,0.20442,0.20056,0.20173,0.2003
8,0.18912,0.19861,0.19738,0.19867,0.20691,0.20767,0.28824,0.35789,0.34
13,0.26044,0.27945,0.26585,0.27927,0.25726,0.26399,0.27834,0.26114,0.2
8259,0.20669,0.20965,0.19081,0.2041,0.19811,0.20906,0.21393,0.21038,0.
20494,0.21124,0.19483,0.20012,0.20259,0.20104,0.20569,0.199,0.38657,0.

29194,0.49415,0.37879,0.29826,0.26478,0.315,0.29922,0.25916,0.23662,0.
26825,0.29268,0.30086,0.25305,0.21277,0.20249,0.20546,0.19995,0.19975,
0.2004,0.20907,0.18712,0.20787,0.1995,0.20443,0.20454,0.19462,0.20433,
0.20255,0.19512,0.38668,0.31066,0.45641,0.32162,0.4043,0.41486,0.33635
,0.29286,0.29494,0.30204,0.25962,0.34161,0.30382,0.24977,0.25479,0.339
36,0.20659,0.20335,0.2049,0.19961,0.20597,0.18564,0.20104,0.20359,0.20
541,0.20699,0.20172,0.20403,0.20682,0.20161,0.19609,0.20464,0.20616,0.
36545,0.39568,0.3951,0.2285,0.24244,0.33746,0.26693,0.25916,0.2453,0.3
0777,0.28348,0.2641,0.30907,0.31376,0.21193,0.31163,0.28443,0.20212,0.
20001,0.20678,0.1976,0.20087,0.19657,0.19074,0.1964,0.19405,0.19917,0.
20303,0.20126,0.20214,0.13197,0.15121,0.12544,0.20566,0.35086,0.42954,
0.34362,0.32105,0.32552,0.36884,0.26136,0.25295,0.31318,0.32218,0.3004
8,0.28583,0.28275,0.2582,0.28261,0.30718,0.27073,0.29059,0.19782,0.208
85,0.19889,0.20196,0.20665,0.19484,0.20382,0.19798,0.20931,0.19947,0.1
9304,0.11854,0.13304,0.13622,0.12675,0.1235,0.13049,0.20265,0.35425,0.
40827,0.35378,0.33014,0.47189,0.33662,0.36741,0.32617,0.31818,0.28295,
0.26504,0.28205,0.27042,0.28436,0.23973,0.2797,0.24336,0.28089,0.20737
,0.20829,0.20218,0.19928,0.20117,0.20529,0.21071,0.20689,0.20521,0.131
77,0.11476,0.14454,0.15017,0.13618,0.12249,0.12365,0.11799,0.12737,0.1
9907,0.25165,0.34626,0.39264,0.41409,0.28864,0.36681,0.3286,0.25756,0.
22911,0.30429,0.30889,0.25073,0.32827,0.26442,0.35079,0.34485,0.28153,
0.31508,0.24061,0.19816,0.20474,0.20039,0.19434,0.20123,0.21324,0.1251
2,0.13565,0.1305,0.11728,0.12908,0.13032,0.12357,0.11682,0.11584,0.128
95,0.12568,0.15173,0.40354,0.3046,0.39905,0.39148,0.34016,0.34378,0.28
915,0.23895,0.32947,0.26702,0.30365,0.28189,0.23339,0.27622,0.26527,0.
25268,0.29146,0.25161,0.31571,0.19908,0.20387,0.20323,0.13739,0.13749,
0.14204,0.13996,0.12598,0.11861,0.13292,0.14328,0.13154,0.12989,0.1266
5,0.13301,0.12667,0.14729,0.12087,0.37741,0.33339,0.41397,0.35536,0.32
294,0.38469,0.30125,0.39861,0.29085,0.27208,0.28186,0.26371,0.30554,0.
30307,0.28986,0.34961,0.28249,0.2911,0.30665,0.24855,0.12864,0.12862,0
.1354,0.13223,0.12765,0.13472,0.13096,0.12977,0.14376,0.12983,0.12542,
0.14489,0.10808,0.12004,0.13072,0.13649,0.12089,0.1265,0.40171,0.39608
,0.34664,0.3608,0.3182,0.35626,0.34866,0.31563,0.30306,0.2609,0.25949,
0.23812,0.21435,0.19167,0.25768,0.21864,0.21182,0.19745,0.22669,0.1972
2,0.12447,0.13424,0.12516,0.13612,0.13237,0.13111,0.13179,0.13296,0.12
154,0.13519,0.15149,0.13193,0.12154,0.13074,0.1306,0.14166,0.12807,0.1
2989,0.51625,0.28582,0.40553,0.3541,0.39747,0.27607,0.31355,0.32421,0.
27773,0.2118,0.1944,0.22851,0.17662,0.17943,0.19903,0.22341,0.22832,0.
25473,0.18358,0.20338,0.12748,0.1424,0.14544,0.12621,0.13025,0.13107,0
.13317,0.12934,0.12891,0.12404,0.16623,0.11936,0.1327,0.10668,0.13216,
0.13759,0.12743,0.12663,0.39842,0.37219,0.27874,0.28374,0.51009,0.3918
5,0.2415,0.16228,0.21976,0.21161,0.21397,0.21073,0.21592,0.22182,0.191
38,0.21907,0.19405,0.20452,0.19979,0.20075,0.10031,0.13823,0.12255,0.1
3721,0.1263,0.13749,0.13094,0.13227,0.13038,0.12671,0.12613,0.12959,0.
13663,0.13411,0.12819,0.13301,0.1345,0.12323,0.31575,0.41131,0.39904,0
.26975,0.35537,0.32654,0.20576,0.22354,0.18142,0.20581,0.20076,0.23254
,0.21058,0.2051,0.21727,0.20448,0.21649,0.21057,0.16693,0.17597,0.1335
3,0.12804,0.1173,0.15076,0.13416,0.13177,0.13262,0.12501,0.12198,0.128
95,0.13924,0.11989,0.13651,0.14233,0.1262,0.1265,0.13484,0.13676,0.418
61,0.33726,0.33239,0.34426,0.37339,0.2528,0.19187,0.19624,0.33813,0.15
683,0.20433,0.17976,0.19632,0.21744,0.1912,0.17511,0.18199,0.20158,0.2
1896,0.24013,0.12933,0.13434,0.13309,0.14527,0.1464,0.12674,0.14571,0.

1273,0.12814,0.12758,0.13352,0.14312,0.11475,0.12844,0.12955,0.1177,0.
1207,0.13326,0.3469,0.2472,0.35709,0.27876,0.18819,0.2025,0.20354,0.18
66,0.19798,0.25142,0.18813,0.16934,0.16244,0.2129,0.2274,0.18553,0.229
3,0.18354,0.25874,0.19771,0.11829,0.12591,0.12762,0.13231,0.12133,0.13
256,0.12975,0.1499,0.13265,0.11935,0.12596,0.12937,0.1284,0.12491,0.11
909,0.14522,0.13062,0.13597,0.37363,0.22814,0.22113,0.22775,0.26542,0.
20081,0.20352,0.27628,0.19793,0.20861,0.17845,0.19368,0.28519,0.22156,
0.19677,0.21429,0.21763,0.20334,0.1814,0.13006,0.12596,0.13391,0.12467
,0.13278,0.14229,0.12529,0.13567,0.13797,0.13484,0.16376,0.12559,0.146
95,0.13387,0.1289,0.13666,0.13318,0.058383,0.21646,0.20031,0.22379,0.2
3485,0.30367,0.21335,0.13928,0.16647,0.15561,0.15915,0.21398,0.23455,0
.235,0.2243,0.2093,0.18093,0.19793,0.21169,0.22096,0.1316,0.13598,0.12
382,0.14329,0.12588,0.13149,0.13172,0.12834,0.12633,0.11899,0.1368,0.1
3396,0.12366,0.13484,0.13283,0.12595,0.13311,0.05558,0.2084,0.2106,0.2
3219,0.21123,0.26232,0.18227,0.22485,0.18684,0.18374,0.1615,0.20533,0.
20941,0.2188,0.18799,0.18018,0.24531,0.1879,0.20455,0.12512,0.13242,0.
12729,0.12582,0.11117,0.12751,0.1225,0.12625,0.12305,0.13543,0.14832,0
.1374,0.12593,0.12659,0.1317,0.12763,0.12697,0.12298,0.053227,0.23694,
0.2792,0.22244,0.16589,0.25618,0.19467,0.2698,0.13754,0.15615,0.24362,
0.21825,0.1647,0.21529,0.21878,0.23349,0.23874,0.25578,0.20234,0.12741
,0.11345,0.13027,0.12089,0.12576,0.13061,0.12955,0.13317,0.16251,0.130
69,0.1271,0.12082,0.12567,0.12674,0.12219,0.1395,0.14268,0.05723,0.059
512,0.059759,0.1786,0.23627,0.2034,0.19576,0.20518,0.10218,0.184,0.207
14,0.14378,0.18848,0.20611,0.19462,0.23186,0.20735,0.20996,0.11217,0.1
3509,0.12921,0.12624,0.12939,0.13386,0.13375,0.11498,0.12433,0.12484,0
.11807,0.15266,0.14083,0.12631,0.12577,0.13161,0.12834,0.12611,0.05528
5,0.056567,0.061244,0.059067,0.17446,0.23805,0.24196,0.25577,0.18106,0
.25693,0.21377,0.22023,0.23246,0.20841,0.17259,0.2161,0.13117,0.1298,0
.18343,0.1289,0.14163,0.12919,0.12711,0.12945,0.12552,0.12279,0.14516,
0.13845,0.12818,0.12032,0.14514,0.13209,0.12145,0.13839,0.12824,0.1151
1,0.059829,0.056138,0.053423,0.052616,0.056267,0.057628,0.19454,0.1495
7,0.18966,0.19824,0.23138,0.20053,0.19607,0.22003,0.1399,0.12885,0.133
02,0.1316,0.13089,0.14746,0.13328,0.12861,0.13394,0.12457,0.1345,0.106
04,0.14447,0.1087,0.13373,0.13037,0.153,0.13404,0.13207,0.13092,0.1296
2,0.1387,0.10529,0.058558,0.058713,0.05951,0.057617,0.055709,0.05181,0
.19751,0.22954,0.25488,0.16438,0.22016,0.059724,0.059087,0.13237,0.113
05,0.11924,0.13591,0.11785,0.12861,0.11404,0.12061,0.14007,0.12851,0.1
2745,0.136,0.12879,0.1262,0.12886,0.13037,0.13277,0.13838,0.1365,0.124
97,0.10955,0.054758,0.054803,0.058926,0.060198,0.054334,0.051645,0.061
502,0.054284,0.056015,0.058711,0.17174,0.22364,0.059294,0.054227,0.057
98,0.054235,0.13789,0.11912,0.13323,0.12747,0.15197,0.1436,0.14524,0.1
3194,0.13149,0.12351,0.11896,0.12958,0.13497,0.132,0.11732,0.11149,0.1
3343,0.13136,0.13252,0.055463,0.056914,0.055903,0.059951,0.056034,0.05
4244,0.054711,0.058634,0.054534,0.053663,0.055611,0.05619,0.064517,0.0
50947,0.057739,0.053217,0.05707,0.15181,0.13708,0.12385,0.13434,0.1191
,0.13134,0.12499,0.13044,0.12061,0.12272,0.13039,0.1274,0.12803,0.1380
1,0.13009,0.13048,0.060808,0.058977,0.064577,0.053851,0.057615,0.05309
8,0.056633,0.057162,0.05752,0.059331,0.056962,0.055478,0.057125,0.0533
48,0.059457,0.054608,0.053319,0.057496,0.059447,0.14256,0.13259,0.1269
3,0.13642,0.12708,0.1151,0.14263,0.11852,0.16585,0.13497,0.12439,0.122
95,0.13525,0.056212,0.055766,0.055433,0.055779,0.058985,0.054853,0.052
783,0.059918,0.058633,0.061235,0.053371,0.056403,0.060787,0.057638,0.0

55218,0.057517,0.056066,0.056571,0.059011,0.055411,0.05586,0.13386,0.1
2703,0.12985,0.1325,0.12746,0.12891,0.13286,0.13019,0.1432,0.055341,0.
057698,0.063924,0.060429,0.058982,0.049096,0.058422,0.062262,0.05902,0
.056744,0.056198,0.058834,0.057884,0.057589,0.054914,0.056771,0.059353
,0.05507,0.054317,0.054218,0.057398,0.057687,0.055452,0.058806,0.06009
1,0.12766,0.13212,0.12756,0.1236,0.057262,0.056264,0.048443,0.056431,0
.053309,0.05305,0.05815,0.050227,0.061603,0.051095,0.055232,0.054906,0
.056301,0.057788,0.059657,0.05617,0.053933,0.058572,0.056615,0.06046,0
.055989,0.052281,0.052285,0.059585,0.05586,0.056576,0.05717,0.052141,0
.058221,0.055147,0.058566,0.055534,0.059174,0.057309,0.049749,0.05766,
0.056898,0.056253,0.060241,0.056288,0.055235,0.057636,0.053014,0.04904
8,0.058734,0.059052,0.056091,0.057581,0.055353,0.057321,0.052896,0.055
98,0.05991,0.051752,0.056505,0.057108,0.054177,0.056854,0.054164,0.055
525,0.058953,0.056737,0.0536,0.052595,0.060842,0.059549,0.05864,0.0588
94,0.054877,0.054379,0.049694,0.060268,0.059379,0.055156,0.057351,0.05
3855,0.05557,0.059326,0.05588,0.052642,0.056727,0.058183,0.058081,0.05
7355,0.055582,0.055458,0.06148,0.059246,0.058483,0.057224,0.0537,0.054
267,0.057897,0.058262,0.055938,0.058021,0.058658,0.05327,0.054192,0.05
3864,0.051529,0.055808,0.059631,0.056327,0.061841,0.054843,0.05732,0.0
6037,0.065,0.051327,0.05701,0.053903,0.052596,0.057328,0.058028,0.0529
67,0.057787,0.054312,0.055767,0.055973,0.057817,0.055306,0.056598,0.06
0544,0.065119,0.058032,0.053024,0.06075,0.048601,0.057842,0.060064,0.0
56619,0.061169,0.0582,0.056428,0.055863,0.058083,0.056505,0.057249,0.0
52984,0.056296,0.053571,0.060816,0.055455,0.051219,0.055262,0.055082,0
.051046,0.053901,0.057939,0.052887,0.058378,0.058824,0.05791,0.059333,
0.054437,0.061302,0.056024,0.05953,0.050885,0.05594,0.054585,0.055447,
0.059017,0.058414,0.057145,0.057398,0.06081,0.060462,0.054787,0.058624
,0.05447,0.057582,0.054403,0.05287,0.055162,0.057236,0.05748,0.055865,
0.057191,0.055371,0.053999,0.056214,0.057506,0.059489,0.053108,0.05478
5,0.05364,0.054753,0.052624,0.056224,0.05856,0.055922,0.055917,0.05088
4
0.21173,0.18811,0.2134,0.20015,0.19825,0.19917,0.21154,0.19725,0.20598
,0.20165,0.19869,0.20048,0.20051,0.19741,0.19277,0.19692,0.21236,0.203
37,0.19852,0.20297,0.29208,0.3574,0.32862,0.31214,0.20367,0.20491,0.20
21,0.20145,0.20261,0.19466,0.20745,0.20552,0.20449,0.20953,0.20658,0.2
0555,0.20129,0.19554,0.19819,0.32484,0.2941,0.25132,0.2975,0.29184,0.2
3186,0.32812,0.20585,0.20392,0.20686,0.20342,0.19857,0.21855,0.20803,0
.20483,0.20237,0.2061,0.20392,0.21001,0.20651,0.19675,0.19342,0.45474,
0.33228,0.24984,0.29671,0.28179,0.30752,0.30591,0.28692,0.24365,0.3052
2,0.20018,0.20058,0.20624,0.19753,0.2043,0.20953,0.2033,0.20786,0.2019
6,0.19183,0.19979,0.19987,0.20323,0.2085,0.21099,0.29455,0.3496,0.3516
5,0.26968,0.28471,0.26479,0.27661,0.27759,0.26822,0.27825,0.26663,0.30
229,0.20355,0.21379,0.19178,0.20278,0.20049,0.21209,0.21221,0.21172,0.
20556,0.20871,0.19505,0.20629,0.20176,0.20236,0.2063,0.20537,0.34268,0
.29531,0.46795,0.35156,0.29095,0.27847,0.30865,0.2904,0.27574,0.25936,
0.25942,0.3015,0.29636,0.25556,0.21303,0.20589,0.20658,0.20081,0.20151
,0.20117,0.20269,0.19227,0.20911,0.20226,0.20492,0.20572,0.1946,0.2058
4,0.20032,0.19787,0.3546,0.33603,0.46853,0.32867,0.37453,0.38502,0.346
49,0.30356,0.29211,0.30353,0.26279,0.32838,0.3106,0.2581,0.26679,0.308
62,0.20917,0.20677,0.20108,0.20247,0.20743,0.18566,0.20359,0.20395,0.2
069,0.21007,0.2042,0.19868,0.20519,0.20014,0.19687,0.20402,0.20699,0.3
5026,0.37452,0.40624,0.22794,0.25852,0.33311,0.26987,0.2388,0.24555,0.

30818,0.28917,0.2664,0.31078,0.31487,0.22072,0.31092,0.27637,0.2032,0.
20021,0.2076,0.20426,0.20278,0.19675,0.19508,0.1968,0.19515,0.19853,0.
20431,0.2023,0.20363,0.13182,0.15099,0.12492,0.20722,0.33324,0.41424,0
.32365,0.34533,0.32653,0.39133,0.31325,0.2771,0.30911,0.31035,0.29641,
0.28399,0.25182,0.25431,0.28793,0.2902,0.27351,0.28099,0.20015,0.20702
,0.20183,0.20322,0.21015,0.19614,0.20927,0.20047,0.2112,0.20258,0.1976
1,0.11707,0.13042,0.13423,0.12811,0.12188,0.12773,0.20091,0.34298,0.41
344,0.32463,0.31225,0.45965,0.33206,0.35434,0.33222,0.33939,0.28932,0.
2568,0.29715,0.26043,0.28553,0.23102,0.25972,0.23623,0.2868,0.20505,0.
20817,0.19974,0.20082,0.1995,0.2034,0.2162,0.20711,0.20668,0.13141,0.1
1465,0.14372,0.14929,0.13357,0.12022,0.12563,0.11795,0.12897,0.20318,0
.26327,0.34243,0.3906,0.41735,0.32099,0.34922,0.36265,0.27116,0.22674,
0.30536,0.32491,0.25213,0.33962,0.25555,0.34718,0.31699,0.28601,0.3015
7,0.22583,0.19814,0.20735,0.20035,0.1927,0.2026,0.2123,0.12602,0.13314
,0.13112,0.11512,0.12889,0.1286,0.11889,0.11578,0.1161,0.12741,0.12553
,0.15216,0.39647,0.34921,0.40822,0.34815,0.3436,0.30457,0.36121,0.2408
3,0.31695,0.26899,0.2992,0.27494,0.23832,0.27962,0.25247,0.24992,0.298
16,0.24967,0.31182,0.1953,0.20455,0.20316,0.13682,0.13781,0.14103,0.14
022,0.12474,0.1188,0.13126,0.14578,0.12972,0.12932,0.12526,0.13123,0.1
2541,0.14429,0.11868,0.37306,0.3404,0.4041,0.38028,0.30296,0.38523,0.3
1668,0.3789,0.27193,0.27495,0.28893,0.27074,0.32258,0.29962,0.29295,0.
36247,0.27225,0.28901,0.32189,0.25142,0.13075,0.12909,0.1348,0.13016,0
.12873,0.13251,0.12907,0.12773,0.14153,0.12932,0.12304,0.14341,0.10543
,0.11913,0.13037,0.13395,0.11971,0.12749,0.3701,0.41437,0.33655,0.3611
,0.31566,0.34519,0.34826,0.32844,0.3134,0.26877,0.25088,0.23502,0.2166
2,0.18947,0.24781,0.22057,0.21549,0.19257,0.21653,0.19073,0.12399,0.13
441,0.12568,0.13495,0.1328,0.13188,0.12974,0.13364,0.1227,0.13391,0.15
072,0.12988,0.12216,0.13039,0.13018,0.14001,0.12721,0.1311,0.52617,0.2
2604,0.37458,0.35469,0.4137,0.29861,0.34138,0.28923,0.31326,0.22899,0.
1822,0.21377,0.18243,0.19201,0.19268,0.22388,0.22794,0.24269,0.19381,0
.19381,0.12726,0.14186,0.14517,0.1241,0.13174,0.13164,0.13066,0.12953,
0.12912,0.12171,0.16773,0.1212,0.13254,0.10582,0.13451,0.13576,0.12735
,0.12685,0.33049,0.39459,0.30247,0.29438,0.48441,0.3892,0.22377,0.1544
7,0.22945,0.22321,0.21419,0.20774,0.20685,0.21451,0.19141,0.21785,0.18
635,0.20334,0.19904,0.20644,0.10016,0.13771,0.12159,0.13507,0.12623,0.
13886,0.13132,0.13113,0.12569,0.12891,0.12973,0.12947,0.13762,0.13616,
0.12819,0.1307,0.13303,0.12185,0.30605,0.41207,0.40857,0.29316,0.33365
,0.29456,0.20783,0.23874,0.17558,0.18373,0.17904,0.22978,0.21681,0.211
22,0.20599,0.18723,0.22325,0.20971,0.16725,0.17166,0.13148,0.1268,0.11
587,0.14926,0.13501,0.13062,0.13406,0.12418,0.12299,0.13085,0.13813,0.
12028,0.13564,0.14155,0.12851,0.1263,0.13407,0.13519,0.44713,0.32623,0
.32207,0.32003,0.40346,0.24862,0.19757,0.21042,0.32596,0.17374,0.22065
,0.18194,0.19713,0.214,0.18275,0.18205,0.18882,0.19272,0.21904,0.23935
,0.12964,0.13595,0.13531,0.14529,0.14573,0.1285,0.1466,0.12831,0.12816
,0.1282,0.13141,0.14068,0.11533,0.1308,0.12951,0.11781,0.1193,0.1329,0
.36004,0.28347,0.35423,0.28926,0.18497,0.21024,0.20077,0.2088,0.21366,
0.245,0.20161,0.17726,0.16021,0.21903,0.2379,0.19531,0.20477,0.1932,0.
24241,0.2003,0.11675,0.12478,0.13071,0.13123,0.11904,0.13365,0.13099,0
.1492,0.13457,0.11983,0.12588,0.12955,0.12853,0.12419,0.11848,0.14176,
0.13042,0.13344,0.30134,0.20635,0.19655,0.22929,0.2506,0.17,0.19404,0.
28439,0.19785,0.21318,0.18775,0.18328,0.28332,0.20609,0.19885,0.21733,
0.22825,0.20264,0.17624,0.12853,0.12801,0.13388,0.12491,0.13353,0.1406

8,0.12372,0.13769,0.13782,0.13308,0.16323,0.12522,0.14582,0.13714,0.13
008,0.13507,0.13143,0.054618,0.21849,0.20383,0.22221,0.24356,0.27827,0
.20314,0.12216,0.16271,0.16478,0.15299,0.22524,0.22038,0.24551,0.21522
,0.19986,0.17699,0.20367,0.2015,0.23158,0.13089,0.13249,0.12376,0.1433
,0.12442,0.13092,0.13136,0.12835,0.12398,0.11782,0.13619,0.13238,0.121
2,0.13303,0.13318,0.12442,0.13451,0.055236,0.21061,0.21043,0.22431,0.2
0462,0.25175,0.17639,0.23313,0.20513,0.17447,0.16471,0.20591,0.21297,0
.21358,0.1893,0.18342,0.23379,0.20004,0.19121,0.12572,0.13037,0.12815,
0.12792,0.11195,0.12866,0.12054,0.12537,0.12701,0.13492,0.14803,0.1380
1,0.12684,0.12664,0.13042,0.12737,0.12362,0.12048,0.052371,0.22422,0.2
6309,0.19009,0.17125,0.27687,0.19611,0.2787,0.14131,0.14631,0.22822,0.
2199,0.16661,0.20795,0.21229,0.22375,0.23967,0.24603,0.21006,0.13085,0
.11503,0.12894,0.11915,0.12212,0.13258,0.12816,0.1298,0.15991,0.13248,
0.12896,0.11984,0.12619,0.12945,0.12105,0.13878,0.14332,0.057496,0.058
679,0.055326,0.17229,0.24714,0.18772,0.17221,0.19948,0.095261,0.17911,
0.21435,0.15933,0.18687,0.22251,0.19301,0.23059,0.22285,0.21714,0.1143
8,0.13327,0.12644,0.12713,0.12937,0.13242,0.1334,0.11426,0.12282,0.125
8,0.11778,0.15402,0.1379,0.12549,0.12248,0.1315,0.12985,0.12453,0.0564
16,0.054874,0.059384,0.056268,0.17213,0.22997,0.25381,0.26866,0.18729,
0.25364,0.21605,0.2076,0.24104,0.19022,0.17965,0.20168,0.12867,0.12636
,0.18302,0.12728,0.14066,0.13205,0.12614,0.13186,0.12637,0.12141,0.146
03,0.13679,0.12954,0.12162,0.1459,0.12931,0.12175,0.13636,0.12968,0.11
577,0.057902,0.052909,0.050981,0.050678,0.054787,0.054405,0.17274,0.17
344,0.17079,0.18779,0.23242,0.20174,0.22448,0.22463,0.13955,0.12877,0.
13381,0.13188,0.13082,0.14636,0.13118,0.12838,0.13287,0.12464,0.13419,
0.10601,0.14336,0.10884,0.13133,0.12941,0.15278,0.13352,0.13255,0.1284
,0.13031,0.14,0.10577,0.057413,0.05603,0.056767,0.054298,0.053881,0.04
9775,0.22051,0.22173,0.2571,0.1862,0.21793,0.05623,0.057891,0.13279,0.
11187,0.116,0.13441,0.11806,0.12662,0.11316,0.11906,0.13888,0.12872,0.
12823,0.13627,0.12849,0.12549,0.12763,0.12681,0.13347,0.13919,0.13595,
0.12289,0.10736,0.052254,0.053128,0.054732,0.059167,0.052164,0.05103,0
.059041,0.052927,0.055412,0.0571,0.16939,0.23373,0.057867,0.057058,0.0
54687,0.051287,0.13817,0.11697,0.13481,0.128,0.14682,0.14565,0.14385,0
.13049,0.12957,0.12234,0.11831,0.12894,0.1324,0.13346,0.11531,0.11142,
0.13326,0.13072,0.12737,0.052313,0.05464,0.055804,0.058477,0.054758,0.
05228,0.052098,0.058494,0.054721,0.052253,0.052675,0.055014,0.063064,0
.051555,0.056053,0.051735,0.057341,0.15455,0.13645,0.12216,0.13377,0.1
1904,0.13222,0.12478,0.12929,0.12002,0.12279,0.12987,0.12654,0.12617,0
.13635,0.12916,0.12797,0.059141,0.05552,0.063225,0.051881,0.054723,0.0
51934,0.053465,0.056789,0.054179,0.057246,0.054455,0.052057,0.054954,0
.052476,0.056335,0.054109,0.050779,0.055861,0.056787,0.14369,0.13026,0
.12564,0.13639,0.12422,0.11573,0.1424,0.11912,0.16677,0.13277,0.12842,
0.12262,0.13797,0.057088,0.053375,0.055539,0.052245,0.060632,0.052982,
0.050794,0.056946,0.056864,0.059171,0.05249,0.053366,0.058597,0.056848
,0.054099,0.057386,0.054202,0.053379,0.055744,0.052934,0.052493,0.1336
1,0.12748,0.13039,0.13395,0.12712,0.12934,0.13258,0.12979,0.14244,0.05
3713,0.054907,0.060342,0.057872,0.058128,0.046094,0.05801,0.060293,0.0
55931,0.055629,0.057532,0.056468,0.053748,0.055494,0.054352,0.053766,0
.057506,0.054584,0.054257,0.05344,0.057747,0.056037,0.055024,0.055851,
0.059698,0.12675,0.13366,0.12483,0.12234,0.054432,0.055471,0.046704,0.
05636,0.053376,0.049427,0.055176,0.04952,0.060578,0.049569,0.053378,0.
05336,0.053597,0.05581,0.05665,0.054358,0.051782,0.055901,0.053725,0.0

5759,0.052578,0.053396,0.05129,0.056558,0.054092,0.054579,0.054829,0.0
5246,0.057373,0.052076,0.054219,0.05605,0.057561,0.055531,0.048316,0.0
56268,0.054956,0.052147,0.05664,0.05764,0.054468,0.054587,0.052486,0.0
47923,0.056486,0.05622,0.053588,0.056079,0.052042,0.054303,0.052616,0.
05294,0.060079,0.051586,0.057623,0.056867,0.054525,0.055702,0.053895,0
.054331,0.056997,0.056094,0.052645,0.050346,0.059872,0.057059,0.056899
,0.05771,0.051053,0.055232,0.050808,0.058267,0.057883,0.053088,0.05531
1,0.050884,0.055229,0.058894,0.054198,0.049533,0.056777,0.055428,0.055
799,0.056966,0.053457,0.054705,0.058213,0.058374,0.05665,0.055275,0.05
2443,0.05156,0.05579,0.05632,0.054263,0.056817,0.055795,0.052469,0.052
819,0.053762,0.05146,0.056208,0.058522,0.056234,0.059976,0.052239,0.05
4651,0.057133,0.063472,0.050006,0.055085,0.050386,0.051772,0.055623,0.
057443,0.05191,0.057633,0.052502,0.055069,0.054952,0.055024,0.050844,0
.055071,0.058506,0.061697,0.054271,0.050718,0.05688,0.046251,0.057629,
0.056512,0.054101,0.05885,0.057502,0.054911,0.052973,0.054162,0.05499,
0.054248,0.053539,0.054245,0.050641,0.058211,0.052908,0.04967,0.053228
,0.05475,0.052366,0.052789,0.055525,0.051938,0.054793,0.054231,0.05617
6,0.056045,0.052946,0.058624,0.053268,0.055905,0.051018,0.053746,0.052
519,0.052026,0.056896,0.055557,0.05287,0.055993,0.057442,0.056858,0.05
1958,0.056654,0.052621,0.058105,0.054823,0.050517,0.053301,0.057533,0.
055707,0.055297,0.053986,0.054305,0.051079,0.053638,0.055663,0.058433,
0.05155,0.053456,0.049653,0.050908,0.05224,0.053416,0.056388,0.054144,
0.05494,0.050564
0.21383,0.19246,0.21448,0.2001,0.20041,0.19758,0.21181,0.19842,0.20515
,0.20303,0.2017,0.20259,0.20214,0.19582,0.194,0.20187,0.21579,0.20329,
0.19958,0.20359,0.30717,0.34185,0.32485,0.30293,0.20296,0.2074,0.20504
,0.20306,0.2063,0.19899,0.20795,0.20196,0.20278,0.21088,0.20901,0.2055
8,0.2002,0.19481,0.19906,0.29277,0.29997,0.25244,0.30512,0.26729,0.235
92,0.32466,0.20471,0.20457,0.20946,0.20416,0.19557,0.219,0.20875,0.205
1,0.20699,0.20432,0.20609,0.2108,0.20934,0.2001,0.19602,0.44597,0.3776
2,0.2511,0.298,0.27382,0.31127,0.282,0.2671,0.24027,0.29028,0.19901,0.
20113,0.20747,0.19883,0.20921,0.20975,0.20783,0.21095,0.20269,0.19314,
0.19583,0.19921,0.20166,0.20578,0.21393,0.29261,0.3366,0.35613,0.26672
,0.29512,0.25279,0.26629,0.27795,0.26298,0.27241,0.27934,0.28443,0.207
63,0.21232,0.19257,0.2036,0.20647,0.21277,0.21379,0.21173,0.20595,0.21
106,0.19672,0.21198,0.20298,0.19903,0.2103,0.20746,0.3715,0.30377,0.44
473,0.35558,0.29431,0.26688,0.30954,0.30998,0.27505,0.27213,0.25943,0.
29863,0.27888,0.26945,0.21218,0.2043,0.20652,0.20052,0.20599,0.20314,0
.20309,0.19333,0.21203,0.203,0.20701,0.20686,0.20075,0.20389,0.19899,0
.19739,0.37115,0.35441,0.46875,0.3413,0.39054,0.38632,0.3239,0.2883,0.
29,0.30069,0.2579,0.34242,0.31502,0.26654,0.2714,0.30938,0.21231,0.208
91,0.20211,0.20402,0.20622,0.18631,0.20352,0.20368,0.21144,0.21412,0.1
9791,0.20223,0.20649,0.20434,0.19194,0.20462,0.20593,0.30957,0.39591,0
.44102,0.24621,0.27442,0.33839,0.26696,0.22738,0.22714,0.2962,0.27846,
0.25172,0.29267,0.30467,0.21935,0.32186,0.29861,0.20784,0.20258,0.2079
,0.20395,0.20308,0.20303,0.19903,0.19845,0.20119,0.19904,0.20304,0.201
29,0.20821,0.1302,0.15038,0.12482,0.20947,0.30782,0.42148,0.29276,0.36
334,0.34712,0.42452,0.25883,0.27991,0.31095,0.32311,0.31231,0.285,0.25
492,0.24705,0.28242,0.2803,0.27822,0.26638,0.20158,0.20784,0.20306,0.2
0549,0.21289,0.19557,0.21256,0.20377,0.20926,0.20177,0.20057,0.11721,0
.12982,0.13423,0.12759,0.12296,0.12775,0.19871,0.36264,0.45334,0.36235
,0.33698,0.47656,0.36339,0.33261,0.3243,0.31965,0.31635,0.28467,0.3077

7,0.24461,0.27043,0.2393,0.26179,0.24218,0.28198,0.20459,0.20466,0.201
66,0.20424,0.20091,0.20251,0.21711,0.20811,0.20513,0.13497,0.11311,0.1
4295,0.15027,0.13055,0.12189,0.12631,0.11801,0.12997,0.20306,0.25704,0
.38416,0.35445,0.40148,0.30964,0.3351,0.3984,0.26757,0.24701,0.28955,0
.32164,0.25728,0.31606,0.25749,0.3312,0.30576,0.28023,0.30678,0.23864,
0.19705,0.20995,0.20397,0.19534,0.20262,0.21145,0.12651,0.13282,0.1302
5,0.11505,0.12874,0.12939,0.12055,0.11633,0.11549,0.12577,0.12611,0.15
098,0.37575,0.35451,0.44176,0.29399,0.32563,0.30201,0.31212,0.23052,0.
30071,0.26795,0.30473,0.27699,0.24008,0.28025,0.25418,0.23556,0.31119,
0.24165,0.30772,0.19755,0.20132,0.19698,0.13598,0.13764,0.14013,0.1408
,0.12574,0.12013,0.1305,0.14599,0.12643,0.12797,0.12351,0.13201,0.1283
1,0.14444,0.12008,0.36137,0.33935,0.38336,0.42982,0.33875,0.35971,0.31
468,0.34463,0.27878,0.25349,0.33383,0.2683,0.32514,0.295,0.29672,0.350
07,0.25303,0.28985,0.31839,0.25092,0.12813,0.13285,0.13613,0.13169,0.1
2794,0.13223,0.12797,0.12594,0.144,0.13137,0.12141,0.14353,0.10524,0.1
1844,0.12601,0.13134,0.11632,0.12832,0.41298,0.3999,0.39952,0.35985,0.
33368,0.36234,0.33031,0.35201,0.34203,0.26991,0.25455,0.25397,0.22345,
0.18793,0.23274,0.20715,0.23529,0.20914,0.21733,0.19847,0.12472,0.1361
,0.12372,0.13265,0.1323,0.12989,0.13146,0.13309,0.12193,0.13297,0.1518
7,0.13003,0.12347,0.13101,0.13145,0.1411,0.12698,0.13001,0.49081,0.210
79,0.37559,0.40167,0.42817,0.32995,0.33413,0.29598,0.33083,0.22479,0.1
9897,0.20406,0.19614,0.19718,0.1969,0.2191,0.2141,0.22919,0.19025,0.19
695,0.1269,0.13899,0.14409,0.12518,0.13103,0.13398,0.12746,0.12806,0.1
3034,0.1196,0.16928,0.12046,0.13282,0.10455,0.13516,0.13655,0.12772,0.
1294,0.34063,0.42702,0.36381,0.28324,0.44454,0.39851,0.27609,0.16437,0
.20452,0.21172,0.21684,0.20308,0.20345,0.19311,0.1928,0.21232,0.18146,
0.20805,0.21036,0.20244,0.099011,0.14016,0.12263,0.13515,0.12768,0.140
69,0.1279,0.13332,0.12668,0.12765,0.13138,0.12673,0.13818,0.1322,0.129
79,0.13034,0.13106,0.12007,0.29654,0.41191,0.3296,0.31143,0.33197,0.29
316,0.19752,0.26433,0.16058,0.15031,0.19243,0.20868,0.21659,0.21006,0.
20459,0.19056,0.21821,0.20781,0.16905,0.1716,0.13285,0.127,0.11514,0.1
4951,0.13698,0.12787,0.13381,0.12257,0.12181,0.12778,0.13655,0.11922,0
.13322,0.14202,0.12653,0.12706,0.13502,0.13218,0.43976,0.32191,0.3057,
0.34112,0.38731,0.23734,0.20174,0.21247,0.3075,0.18659,0.22969,0.20012
,0.18568,0.22187,0.17745,0.18255,0.189,0.19147,0.22488,0.22765,0.13105
,0.13442,0.1368,0.14364,0.1432,0.12751,0.14537,0.12741,0.12482,0.12651
,0.13069,0.1432,0.11491,0.13133,0.12752,0.11745,0.12199,0.13189,0.3435
,0.33315,0.35971,0.28149,0.19023,0.1971,0.22321,0.20792,0.19082,0.2482
7,0.19411,0.17091,0.15856,0.20776,0.24174,0.19498,0.19429,0.19493,0.24
034,0.18989,0.11726,0.12381,0.12885,0.13048,0.12197,0.13251,0.1288,0.1
4614,0.13612,0.11917,0.12513,0.129,0.12708,0.12297,0.11779,0.13937,0.1
308,0.13606,0.34492,0.22718,0.18433,0.20677,0.22779,0.14912,0.19538,0.
2723,0.19149,0.22241,0.19639,0.18242,0.28718,0.20461,0.20089,0.21939,0
.2325,0.19849,0.18046,0.12652,0.13142,0.13193,0.12446,0.13175,0.13809,
0.12352,0.13698,0.13801,0.13226,0.16321,0.12685,0.14663,0.13796,0.1282
6,0.13689,0.13168,0.05536,0.19162,0.21072,0.22533,0.24065,0.28399,0.22
37,0.11804,0.16013,0.17663,0.17315,0.21373,0.22659,0.25269,0.2166,0.18
449,0.17796,0.20733,0.21431,0.2225,0.13166,0.13028,0.12198,0.14226,0.1
2487,0.13117,0.13111,0.12961,0.12382,0.11761,0.13684,0.13476,0.12063,0
.13495,0.13395,0.12227,0.13645,0.052695,0.2158,0.20405,0.19503,0.20152
,0.22907,0.14138,0.22178,0.20845,0.15739,0.17091,0.21109,0.21237,0.201
58,0.192,0.17989,0.219,0.19511,0.18992,0.12469,0.12976,0.12908,0.12575

,0.1116,0.12931,0.11928,0.12441,0.13274,0.13421,0.14877,0.13892,0.1253
9,0.129,0.1291,0.12942,0.12329,0.12095,0.051825,0.20947,0.25163,0.1980
7,0.16636,0.27079,0.19981,0.24393,0.138,0.17562,0.25226,0.22157,0.1667
5,0.20358,0.21305,0.21536,0.23743,0.22836,0.20606,0.13215,0.11291,0.12
707,0.11924,0.12223,0.13203,0.12702,0.13268,0.15931,0.13246,0.1296,0.1
2007,0.12799,0.1283,0.12016,0.14001,0.14314,0.054746,0.055207,0.052814
,0.16047,0.24347,0.19029,0.16924,0.20611,0.0985,0.18076,0.21209,0.1731
5,0.17006,0.21925,0.20173,0.22705,0.2178,0.21079,0.11419,0.13071,0.127
54,0.12804,0.12803,0.1313,0.13737,0.11177,0.1249,0.12652,0.11939,0.156
59,0.13578,0.12453,0.1211,0.13059,0.1279,0.12313,0.053004,0.054021,0.0
58112,0.056336,0.18936,0.21536,0.25275,0.26763,0.18114,0.24788,0.20312
,0.20383,0.2507,0.19239,0.18641,0.20656,0.12767,0.12712,0.18477,0.1285
1,0.14217,0.1319,0.12741,0.13042,0.12502,0.12104,0.14885,0.13649,0.130
6,0.12065,0.14598,0.128,0.12248,0.13888,0.12869,0.11497,0.055367,0.050
729,0.049484,0.050558,0.05297,0.053435,0.18754,0.17124,0.17034,0.18745
,0.21857,0.1941,0.23386,0.21868,0.14076,0.13191,0.13284,0.1299,0.13189
,0.1445,0.12802,0.12903,0.13462,0.12172,0.13177,0.10606,0.14379,0.1069
9,0.133,0.13082,0.15086,0.13191,0.13042,0.12706,0.12817,0.13914,0.1046
4,0.057782,0.054809,0.05611,0.05304,0.051937,0.047255,0.20846,0.20281,
0.24147,0.20855,0.23894,0.054486,0.059017,0.13321,0.11192,0.11616,0.13
329,0.12146,0.12516,0.11256,0.12067,0.13743,0.12663,0.12651,0.13389,0.
12835,0.12463,0.12932,0.12627,0.13064,0.13886,0.13737,0.12344,0.10478,
0.053049,0.050977,0.050181,0.057668,0.05005,0.047417,0.057782,0.051696
,0.055194,0.054679,0.16504,0.20996,0.055603,0.053301,0.053061,0.051768
,0.13765,0.1144,0.13418,0.12911,0.14431,0.1481,0.14105,0.12687,0.12911
,0.12333,0.11776,0.12944,0.13301,0.13165,0.11524,0.10949,0.13431,0.131
1,0.12726,0.05038,0.054801,0.055969,0.057665,0.054293,0.051608,0.05367
2,0.0571,0.053801,0.051491,0.051714,0.054609,0.059125,0.052152,0.05483
3,0.051832,0.05538,0.15554,0.13719,0.12302,0.13293,0.11555,0.13494,0.1
2068,0.12985,0.11842,0.12252,0.12938,0.1258,0.12535,0.13536,0.13257,0.
12501,0.056703,0.056435,0.060953,0.051398,0.052889,0.049595,0.052987,0
.052806,0.054462,0.055957,0.052206,0.050995,0.055484,0.050312,0.054609
,0.054098,0.050359,0.053636,0.055574,0.14268,0.12843,0.12589,0.13593,0
.12348,0.11699,0.14058,0.11908,0.16825,0.13394,0.12857,0.12346,0.13768
,0.053658,0.052942,0.054017,0.049745,0.058464,0.054122,0.048581,0.0579
21,0.056385,0.058358,0.051805,0.051922,0.056164,0.056368,0.05348,0.055
644,0.051579,0.053724,0.055617,0.052084,0.050977,0.13139,0.12934,0.130
08,0.13547,0.1287,0.1283,0.13197,0.12825,0.14179,0.053351,0.051891,0.0
56787,0.056609,0.054621,0.045441,0.05477,0.061064,0.056404,0.056471,0.
052568,0.057089,0.052873,0.05418,0.052803,0.052645,0.055091,0.052061,0
.05387,0.050878,0.057303,0.053294,0.051929,0.0534,0.056924,0.12415,0.1
3299,0.125,0.12357,0.053241,0.053428,0.046083,0.055974,0.053218,0.0485
72,0.053569,0.046652,0.058224,0.047542,0.050181,0.053024,0.053579,0.05
3431,0.055444,0.053528,0.049474,0.054406,0.051071,0.055177,0.051297,0.
05328,0.049648,0.057102,0.052153,0.052153,0.053716,0.049847,0.055693,0
.048423,0.052692,0.056856,0.054724,0.054052,0.046889,0.055676,0.054493
,0.050069,0.057338,0.055805,0.05528,0.05491,0.053459,0.045792,0.056763
,0.054232,0.051332,0.054159,0.048091,0.052455,0.053738,0.054315,0.0568
16,0.050822,0.055818,0.055994,0.053801,0.054421,0.051196,0.055797,0.05
5728,0.054644,0.050327,0.050486,0.057644,0.056344,0.055013,0.052336,0.
049633,0.05323,0.051047,0.057329,0.054463,0.050342,0.052002,0.050896,0
.056056,0.056808,0.055786,0.051051,0.052283,0.053946,0.055022,0.055364

,0.050569,0.055658,0.054811,0.05682,0.053012,0.055701,0.048103,0.05037
3,0.056992,0.052864,0.052498,0.055891,0.053371,0.048957,0.052939,0.052
989,0.050528,0.054467,0.058748,0.05181,0.057413,0.051332,0.051684,0.05
5394,0.059814,0.05039,0.055412,0.048907,0.048671,0.052679,0.056178,0.0
53106,0.056088,0.050609,0.052059,0.053157,0.053285,0.050568,0.052836,0
.055232,0.060998,0.052271,0.051145,0.056188,0.044973,0.054377,0.054548
,0.053263,0.056424,0.056397,0.052614,0.052522,0.054674,0.055606,0.0523
16,0.051597,0.052111,0.0492,0.057564,0.052261,0.047829,0.051944,0.0545
55,0.052391,0.050466,0.05338,0.049607,0.05293,0.054268,0.054061,0.0543
16,0.051136,0.057673,0.053009,0.054607,0.05101,0.054578,0.053318,0.051
155,0.055719,0.053912,0.051825,0.054485,0.056988,0.05524,0.049172,0.05
5174,0.051152,0.055542,0.052937,0.04955,0.052238,0.05672,0.054739,0.05
3611,0.052052,0.050498,0.049031,0.051977,0.055008,0.052283,0.051759,0.
051491,0.049214,0.051506,0.051096,0.052894,0.05651,0.053316,0.05213,0.
050182

#simdataS1SNR15:

Kep_f=0.5
Kep_f=0.5
Kep_s=0.1
Ktrans_f=0.03
Ktrans_s=0.03
del_t=0.5

gtSf=0	0	0	0	0	0
0	0	0	0	0	0
0	0	0	0	0	0
0	0	0	0	0	0
0	0	0	0	0	0
0	0	0	0	0	0
0	0	0	0	0	0
0	0	0	0	0	0
0	0	0	0	0	0
0	0	0	0	0	0
0	0	0	0	0	0
0	0	0	0	0	0
0	0	0	0	0	0
0	0	0	0	0	0
0	0	0	0	0	0
0	0	0	0	0	0
0	0	0	0	0	0
0	0	0	0	0	0
0	0	0	0	0	0
0	0	0	0	0	0
0	0	0	0	0	0
0	0	0	0	0	0
0	0	0	0	0	0
0	0	0	0	0	0
0	0	0	0	0	0
0	0	0	0	0	0

0	0	0	0	0	0
0	0	0	0	0	0
0	0	0	0	0	0
0	0	0	0	0	0
0	0	0	0	0	0
0	0	0	0	0	0
0	0	0	0	0	0
0	0	0	0.5	0.46419	0.44109
0	0	0	0	0	0
0	0	0	0	0	0
0	0	0	0	0	0
0	0	0	0	0	0
0	0	0	0	0	0
0.57237	0.45312	0.5	0.6107	0.56282	0.49425
0	0	0	0	0	0
0	0	0	0	0	0
0	0	0	0	0	0
0	0	0	0	0	0
0	0	0	0	0.55987	0.53749
0.5275	0.53043	0.53198	0.61494	0.5	0.47748
0.51258	0	0	0	0	0
0	0	0	0	0	0
0	0	0	0	0	0
0	0	0	0	0	0
0	0	0	0.54522	0.50819	0.48728
0.44735	0.43204	0.5	0.5	0.4573	0.48777
0.35764	0.52237	0.5	0	0	0
0	0	0	0	0	0
0	0	0	0	0	0
0	0	0	0	0	0
0	0.48282	0.48911	0.50532	0.50726	0.46558
0.5	0.3431	0.4977	0.39281	0.52896	0.53205
0.47302	0.47664	0.51891	0.41606	0	0
0	0	0	0	0	0
0	0	0	0	0	0
0	0	0	0	0	0
0.45679	0.44552	0.51086	0.4445	0.56508	0.47447
0.5	0.55228	0.48247	0.46033	0.50438	0.57074
0.33442	0.51616	0.46163	0.5436	0.5	0.52304
0	0	0	0	0	0
0	0	0	0	0	0.39567
0.35317	0.26352	0.3652	0.29742	0.33135	0.33622
0.29626	0.3532	0.5569	0.42652	0.61357	0.52928
0.5	0.49854	0.65245	0.51819	0.49771	0.5
0.47696	0.48808	0.54283	0.5126	0.5	0.54183
0.37323	0.43958	0	0	0	0
0	0	0	0	0	0.46595
0.37563	0.30888	0.27294	0.33333	0.33973	0.35098
0.26266	0.31161	0.35651	0.3624	0.44297	0.54435
0.47426	0.53168	0.47887	0.45128	0.40908	0.49321
0.53576	0.5907	0.50837	0.45413	0.54529	0.48219
0.47635	0.51303	0.52763	0.48439	0	0

0	0	0	0	0	0.5
0.54795	0.48903	0.42349	0.33333	0.29041	0.39718
0.36046	0.33333	0.26573	0.33333	0.37923	0.36231
0.56881	0.5	0.5046	0.47784	0.44472	0.5
0.77894	0.49857	0.43177	0.5	0.5	0.55746
0.51082	0.54066	0.44008	0.49498	0.5	0.52599
0	0	0	0	0	0
0.5	0.46109	0.45548	0.51307	0.50249	0.3821
0.33333	0.34668	0.34225	0.24452	0.38733	0.30251
0.30335	0.30639	0.38114	0.5	0.5	0.5671
0.5614	0.46513	0.46085	0.54258	0.50066	0.5
0.5	0.5	0.51582	0.49734	0.49008	0.45883
0.36315	0.43041	0	0	0	0
0	0.586	0.52933	0.53191	0.46629	0.5
0.53966	0.33333	0.36608	0.37874	0.33333	0.36011
0.38862	0.31877	0.37906	0.3337	0.5	0.5
0.53753	0.54789	0.50224	0.5	0.41734	0.57341
0.41473	0.48123	0.5	0.5	0.53538	0.5
0.5	0.5	0.46942	0.45638	0	0
0	0.3408	0.52564	0.34637	0.48836	0.50422
0.5	0.54671	0.47777	0.29169	0.28981	0.38355
0.29806	0.34593	0.24734	0.29512	0.33333	0.15593
0.46251	0.49961	0.45718	0.49541	0.5	0.39931
0.47744	0.51198	0.43345	0.58256	0.42296	0.4838
0.45135	0.5719	0.38409	0.46578	0.49407	0.5
0	0.5	0.54308	0.526	0.47113	0.45047
0.5	0.54049	0.47277	0.44198	0.33333	0.33432
0.35727	0.33333	0.31557	0.35886	0.37778	0.3827
0.31894	0.4859	0.50018	0.58121	0.45933	0.5
0.5	0.46612	0.5	0.56707	0.4726	0.52817
0.5051	0.47764	0.46196	0.5012	0.55713	0.45355
1	0.53664	0.5	0.61545	0.5	0.5
0.45007	0.55576	0.45612	0.5	0.55842	0.33333
0.34034	0.34984	0.33333	0.37896	0.31821	0.20974
0.33333	0.37706	0.55997	0.5	0.5	0.54886
0.5	0.48411	0.52083	0.5	0.49763	0.5
0.5	0.61181	0.50862	0.5	0.53636	0.58292
0.5	1	0.51719	0.49308	0.54026	0.74727
0.48526	0.49306	0.47833	0.45067	0.43982	0.5
0.26715	0.29551	0.3514	0.28263	0.40636	0.28872
0.34667	0.36623	0.5	0.5	0.52414	0.5
0.47129	0.48515	0.51212	0.42948	0.55411	0.60195
0.67231	0.5	0.53693	0.4876	0.57651	0.58677
0.53523	0.44857	1	0.45766	0.5	0.5
0.5121	0.49815	0.5	0.45705	0.5	0.6064
0.46966	0.22332	0.33333	0.31261	0.43138	0.24969
0.33716	0.37152	0.27781	0.47331	0.48962	0.49155
0.51861	0.46612	0.45529	0.44889	0.52749	0.5
0.43212	0.65371	0.47712	0.63036	0.46791	0.51271
0.57609	0.49191	1	1	1	0.53693
0.39841	0.51861	0.5	0.5595	0.51833	0.62264
0.54384	0.47819	0.20831	0.36313	0.33708	0.38041

0.33387	0.33333	0.41659	0.50173	0.5	0.51262
0.61037	0.44634	0.5	0.45632	0.53512	0.46048
0.51422	0.51906	0.5	0.40747	0.42797	0.5
0.43891	0.5	1	1	1	1
0.47567	0.5	0.5	0.84311	0.49588	0.49144
0.64584	0.42946	0.5	0.33366	0.3725	0.35091
0.44014	0.59622	0.54759	0.5	0.5	0.38955
0.52752	0.54885	0.48965	0.4922	0.38561	0.46161
0.49797	0.49002	0.59364	0.46544	0.5	0.49065
0.42516	0.47575	1	1	1	1
1	1	0.5	0.46797	0.55551	0.65424
0.46431	0.45101	0.5	0.59185	0.46186	0.62586
0.44945	0.48025	0.5	0.36218	0.5	0.55334
0.47386	0.5	0.51404	0.5	0.48939	0.55445
0.50691	0.477	0.54685	0.5	0.31342	0.5
0.48346	0.5	0.46605	1	1	1
1	1	1	0.5	0.44741	0.6825
0.55499	0.46519	1	1	0.47109	0.5532
0.37752	0.5	0.49338	0.5	0.5	0.46737
0.48254	0.5	0.51301	0.49024	0.63159	0.577
0.5	0.36781	0.4133	0.43581	0.5	0.45245
0.57755	1	1	1	1	1
1	1	1	1	1	0.5
0.47606	1	1	1	1	0.52174
0.47929	0.46001	0.34787	0.53051	0.53335	0.44323
0.46528	0.57027	0.5	0.64367	0.43558	0.47677
0.50262	0.37202	0.5	0.5	0.5	0.5
1	1	1	1	1	1
1	1	1	1	1	1
1	1	1	1	1	0.5
0.5	0.48868	0.42127	0.56426	0.5	0.30995
0.5	0.49586	0.54744	0.47493	0.5	0.57004
0.48379	0.5189	0.51258	1	1	1
1	1	1	1	1	1
1	1	1	1	1	1
1	1	1	1	0.62345	0.5
0.50565	0.47326	0.47634	0.5	0.45789	0.52789
0.5	0.54316	0.5	0.52434	0.49675	1
1	1	1	1	1	1
1	1	1	1	1	1
1	1	1	1	1	1
1	1	0.44342	0.54371	0.50459	0.52179
0.57367	0.43134	0.38057	0.42482	0.5	1
1	1	1	1	1	1
1	1	1	1	1	1
1	1	1	1	1	1
1	1	1	1	1	1
0.54583	0.5	0.63539	0.55631	1	1
1	1	1	1	1	1
1	1	1	1	1	1
1	1	1	1	1	1
1	1	1	1	1	1

1 1 1 1 1 1
1 1 1 1 1 1
1 1 1 1 1 1
1 1 1 1 1 1
1 1 1 1 1 1
1 1 1 1 1 1
1 1 1 1 1 1
1 1 1 1 1 1
1 1 1 1 1 1
1 1 1 1 1 1
1 1 1 1 1 1
1 1 1 1 1 1
1 1 1 1 1 1
1 1 1 1 1 1
1 1 1 1 1 1
1 1 1 1 1 1
1 1 1 1 1 1
1 1 1 1 1 1
1 1 1 1 1 1
1 1 1 1 1 1
1 1 1 1 1 1
1 1 1 1 1 1
1 1 1 1 1 1
1 1 1 1 1 1
1 1 1 1 1 1
1 1 1 1 1 1
1 1 1 1 1 1
1 1 1 1 1 1
1
gtSs=1 1 1 1 1 1
1 1 1 1 1 1
1 1 1 1 1 1
1 1 0.50117 0.5082 0.5 0.45348
1 1 1 1 1 1
1 1 1 1 1 1
1 1 1 0 0.50395 0.5196
0.5 0.52659 0.5 0.38173 1 1
1 1 1 1 1 1
1 1 1 1 1 1
1 0 0 0.43225 0.4668 0.4739
0.45221 0.516 0.5 0.51445 0.44741 1
1 1 1 1 1 1
1 1 1 1 1 1
1 1 0 0 0 0.49127
0.50353 0.60358 0.5 0.46505 0.45123 0.49771
0.48908 0.44685 1 1 1 1
1 1 1 1 1 1
1 1 1 1 1 1
0 0 0 0 0.56022 0.56148
0.49508 0.52741 0.47723 0.5 0.5 0.5
0.47392 0.55533 1 1 1 1
1 1 1 1 1 1

1	1	1	1	1	1
0	0	0	0	0	0
0.46265	0.4102	0.5	0.53127	0.5	0.49064
0.47921	0.4974	0.5	0.5	1	1
1	1	1	1	1	1
1	1	1	1	1	1
1	1	1	0	0	0
0	0	0	0.44415	0.23125	0.52448
0.60108	0.53948	0.47094	0.5231	0.5	0.5
0.45245	0.42415	1	1	1	1
1	1	1	1	1	1
1	1	1	0.5	0.53581	0.55891
1	0	0	0	0	0
0	0	0.5	0.49836	0.50877	0.44977
0.5	0.5	0.5323	0.52512	0.4852	0.55776
0.59705	1	1	1	1	1
1	1	1	1	1	1
0.42763	0.54688	0.5	0.3893	0.43718	0.50575
1	0	0	0	0	0
0	0	0.48412	0.56829	0.46183	0.67387
0.53788	0.5	0.51805	0.39711	0.58892	0.5
0.53768	1	1	1	1	1
1	1	1	1	0.44013	0.46251
0.4725	0.46957	0.46802	0.38506	0.5	0.52252
0.48742	1	0	0	0	0
0	0	0	0.5	0.48134	0.50312
0.54863	0.54029	0.51931	0.55294	0.39475	0.5
0.5	0.5	0.5	1	1	1
1	1	1	0.45478	0.49181	0.51272
0.55265	0.56796	0.5	0.5	0.5427	0.51223
0.64236	0.47763	0.5	0	0	0
0	0	0	0	0.5	0.49901
0.48742	0.53057	0.48743	0.43946	0.55948	0.5
0.62499	0.48424	0.57133	0.44045	1	1
1	0.51718	0.51089	0.49468	0.49274	0.53442
0.5	0.6569	0.5023	0.60719	0.47104	0.46795
0.52698	0.52336	0.48109	0.58394	0	0
0	0	0	0	0	0
0	0.43408	0.48348	0.5	0.5	0.49483
0.54473	0.5	0.46552	0.5	0.50699	0.60622
0.54321	0.55448	0.48914	0.5555	0.43492	0.52553
0.5	0.44772	0.51753	0.53967	0.49562	0.42926
0.66558	0.48384	0.53837	0.4564	0.5	0.47696
0	0	0	0	0	0
0	0	0	0.5	0.51979	0.39567
0.33442	0.36824	0.36618	0.35129	0.29588	0.33622
0.347	0.29361	0.4431	0.57348	0.38643	0.47072
0.5	0.50146	0.34755	0.48181	0.50229	0.5
0.52304	0.51192	0.45717	0.4874	0.5	0.45817
0.62677	0.56042	0	0	0	0
0	0	0	0	0	0
0.24874	0.38325	0.32818	0.33333	0.33973	0.36478

0.36867	0.37508	0.28697	0.2895	0.55703	0.45565
0.52574	0.46832	0.52113	0.54872	0.59092	0.50679
0.46424	0.4093	0.49163	0.54587	0.45471	0.51781
0.52365	0.48697	0.47237	0.51561	0	0
0	0	0	0	0	0
0	0	0.15302	0.33333	0.387	0.27261
0.27909	0.33333	0.39387	0.33333	0.39137	0.27537
0.43119	0.5	0.4954	0.52216	0.55528	0.5
0.22106	0.50143	0.56823	0.5	0.5	0.44254
0.48918	0.45934	0.55992	0.50502	0.5	0.47401
0	0	0	0	0	0
0	0	0	0	0	0.27486
0.33333	0.30664	0.34225	0.43766	0.38733	0.36531
0.34832	0.36041	0.61886	0.5	0.5	0.4329
0.4386	0.53487	0.53915	0.45742	0.49934	0.5
0.5	0.5	0.48418	0.50266	0.50992	0.54117
0.63685	0.56959	0	0	0	0
0	0	0	0	0	0
0	0.33333	0.36608	0.23173	0.33333	0.36594
0.29735	0.34061	0.28463	0.3326	0.5	0.5
0.46247	0.45211	0.49776	0.5	0.58266	0.42659
0.58527	0.51877	0.5	0.5	0.46462	0.5
0.5	0.5	0.53058	0.54362	0	0
0	0	0	0	0	0
0	0	0	0.32815	0.3551	0.26271
0.35527	0.30814	0.35348	0.34597	0.33333	0.42203
0.53749	0.50039	0.54282	0.50459	0.5	0.60069
0.52256	0.48802	0.56655	0.41744	0.57704	0.5162
0.54865	0.4281	0.61591	0.53422	0.50593	0.5
0	0	0	0	0	0
0	0	0	0	0.33333	0.31185
0.28546	0.33333	0.27813	0.35886	0.37207	0.26847
0.34511	0.5141	0.49982	0.41879	0.54067	0.5
0.5	0.53388	0.5	0.43293	0.5274	0.47183
0.4949	0.52236	0.53804	0.4988	0.44287	0.54645
0	0	0	0	0	0
0	0	0	0	0	0.33333
0.31961	0.33934	0.33333	0.37896	0.3409	0.39513
0.33333	0.29322	0.44003	0.5	0.5	0.45114
0.5	0.51589	0.47917	0.5	0.50237	0.5
0.5	0.38819	0.49138	0.5	0.46364	0.41708
0.5	0	0	0	0	0
0	0	0	0	0	0
0.38705	0.35225	0.3514	0.35868	0.31854	0.35625
0.34667	0.26755	0.5	0.5	0.47586	0.5
0.52871	0.51485	0.48788	0.57052	0.44589	0.39805
0.32769	0.5	0.46307	0.5124	0.42349	0.41323
0.46477	0.55143	0	0	0	0
0	0	0	0	0	0
0	0.38834	0.33333	0.38318	0.26459	0.35173
0.32569	0.37323	0.33053	0.52669	0.51038	0.50845
0.48139	0.53388	0.54471	0.55111	0.47251	0.5

0.56788 0.34629 0.52288 0.36964 0.53209 0.48729
0.42391 0.50809 0 0 0 0
0 0 0 0 0 0
0 0 0.39315 0.36313 0.21436 0.20968
0.3232 0.33333 0.58341 0.49827 0.5 0.48738
0.38963 0.55366 0.5 0.54368 0.46488 0.53952
0.48578 0.48094 0.5 0.59253 0.57203 0.5
0.56109 0.5 0 0 0 0
0 0 0 0 0 0
0 0 0 0.33268 0.28038 0.32367
0.55986 0.40378 0.45241 0.5 0.5 0.61045
0.47248 0.45115 0.51035 0.5078 0.61439 0.53839
0.50203 0.50998 0.40636 0.53456 0.5 0.50935
0.57484 0.52425 0 0 0 0
0 0 0 0 0 0
0 0 0 0 0.53814 0.37414
0.55055 0.51975 0.5 0.63782 0.5 0.44666
0.52614 0.5 0.48596 0.5 0.51061 0.44555
0.49309 0.523 0.45315 0.5 0.68658 0.5
0.51654 0.5 0.53395 0 0 0
0 0 0 0 0 0
0 0 0 0 0.52891 0.4468
0.62248 0.5 0.50662 0.5 0.5 0.53263
0.51746 0.5 0.48699 0.50976 0.36841 0.423
0.5 0.63219 0.5867 0.56419 0.5 0.54755
0.42245 0 0 0 0 0
0 0 0 0 0 0
0 0 0 0 0 0.47826
0.52071 0.53999 0.65213 0.46949 0.46665 0.55677
0.53472 0.42973 0.5 0.35633 0.56442 0.52323
0.49738 0.62798 0.5 0.5 0.5 0.5
0 0 0 0 0 0
0 0 0 0 0 0
0 0 0 0 0 0.5
0.5 0.51132 0.57873 0.43574 0.5 0.69005
0.5 0.50414 0.45256 0.52507 0.5 0.42996
0.51621 0.4811 0.48742 0 0 0
0 0 0 0 0 0
0 0 0 0 0 0
0 0 0 0 0.37655 0.5
0.49435 0.52674 0.52366 0.5 0.54211 0.47211
0.5 0.45684 0.5 0.47566 0.50325 0
0 0 0 0 0 0
0 0 0 0 0 0
0 0 0 0 0 0
0 0 0.55658 0.45629 0.49541 0.47821
0.42633 0.56866 0.61943 0.57518 0.5 0
0 0 0 0 0 0
0 0 0 0 0 0
0 0 0 0 0 0
0 0 0 0 0 0
0.45417 0.5 0.36461 0.44369 0 0

0 0 0 0 0 0
0 0 0 0 0 0
0 0 0 0 0 0
0 0 0 0 0 0
0 0 0 0 0 0
0 0 0 0 0 0
0 0 0 0 0 0
0 0 0 0 0 0
0 0 0 0 0 0
0 0 0 0 0 0
0 0 0 0 0 0
0 0 0 0 0 0
0 0 0 0 0 0
0 0 0 0 0 0
0 0 0 0 0 0
0 0 0 0 0 0
0 0 0 0 0 0
0 0 0 0 0 0
0 0 0 0 0 0
0 0 0 0 0 0
0 0 0 0 0 0
0 0 0 0 0 0
0 0 0 0 0 0
0 0 0 0 0 0
0 0 0 0 0 0
0 0 0 0 0 0
0 0 0 0 0 0
0 0 0 0 0 0
0 0 0 0 0 0
0 0 0 0 0 0
0 0 0 0 0 0
0 0 0 0 0 0
0
gtSp=0 0 0 0 0 0
0 0 0 0 0 0
0 0 0 0 0 0
0 0 0.49883 0.4918 0.5 0.54652
0 0 0 0 0 0
0 0 0 0 0 0
0 0 0 1 0.49605 0.4804
0.5 0.47341 0.5 0.61827 0 0
0 0 0 0 0 0
0 0 0 0 0 0
0 1 1 0.56775 0.5332 0.5261
0.54779 0.484 0.5 0.48555 0.55259 0
0 0 0 0 0 0
0 0 0 0 0 0
0 0 1 1 1 0.50873
0.49647 0.39642 0.5 0.53495 0.54877 0.50229
0.51092 0.55315 0 0 0 0
0 0 0 0 0 0
0 0 0 0 0 0

1	1	1	1	0.43978	0.43852
0.50492	0.47259	0.52277	0.5	0.5	0.5
0.52608	0.44467	0	0	0	0
0	0	0	0	0	0
0	0	0	0	0	0
1	1	1	1	1	1
0.53735	0.5898	0.5	0.46873	0.5	0.50936
0.52079	0.5026	0.5	0.5	0	0
0	0	0	0	0	0
0	0	0	0	0	0
0	0	0	1	1	1
1	1	1	0.55585	0.76875	0.47552
0.39892	0.46052	0.52906	0.4769	0.5	0.5
0.54755	0.57585	0	0	0	0
0	0	0	0	0	0
0	0	0	0	0	0
0	1	1	1	1	1
1	1	0.5	0.50164	0.49123	0.55023
0.5	0.5	0.4677	0.47488	0.5148	0.44224
0.40295	0	0	0	0	0
0	0	0	0	0	0
0	0	0	0	0	0
0	1	1	1	1	1
1	1	0.51588	0.43171	0.53817	0.32613
0.46212	0.5	0.48195	0.60289	0.41108	0.5
0.46232	0	0	0	0	0
0	0	0	0	0	0
0	0	0	0	0	0
0	0	1	1	1	1
1	1	1	0.5	0.51866	0.49688
0.45137	0.45971	0.48069	0.44706	0.60525	0.5
0.5	0.5	0.5	0	0	0
0	0	0	0	0	0
0	0	0	0	0	0
0	0	0	1	1	1
1	1	1	1	0.5	0.50099
0.51258	0.46943	0.51257	0.56054	0.44052	0.5
0.37501	0.51576	0.42867	0.55955	0	0
0	0	0	0	0	0
0	0	0	0	0	0
0	0	0	0	1	1
1	1	1	1	1	1
1	0.56592	0.51652	0.5	0.5	0.50517
0.45527	0.5	0.53448	0.5	0.49301	0.39378
0	0	0	0	0	0
0	0	0	0	0	0
0	0	0	0	0	0
1	1	1	1	1	1
1	1	1	0.5	0.48021	0.20867
0.31241	0.36824	0.26862	0.35129	0.37277	0.32756
0.35674	0.3532	0	0	0	0
0	0	0	0	0	0

0	0	0	0	0	0
0	0	1	1	1	1
1	1	1	1	1	0.53405
0.37563	0.30787	0.39888	0.33333	0.32053	0.28425
0.36867	0.31331	0.35651	0.3481	0	0
0	0	0	0	0	0
0	0	0	0	0	0
0	0	0	0	1	1
1	1	1	1	1	0.5
0.45205	0.51097	0.42349	0.33333	0.32259	0.33021
0.36046	0.33333	0.3404	0.33333	0.2294	0.36231
0	0	0	0	0	0
0	0	0	0	0	0
0	0	0	0	0	0
1	1	1	1	1	1
0.5	0.53891	0.54452	0.48693	0.49751	0.34303
0.33333	0.34668	0.31551	0.31782	0.22534	0.33219
0.34832	0.3332	0	0	0	0
0	0	0	0	0	0
0	0	0	0	0	0
0	0	1	1	1	1
1	0.414	0.47067	0.46809	0.53371	0.5
0.46034	0.33333	0.26784	0.38953	0.33333	0.27395
0.31402	0.34061	0.33631	0.3337	0	0
0	0	0	0	0	0
0	0	0	0	0	0
0	0	0	0	1	1
1	0.6592	0.47436	0.65363	0.51164	0.49578
0.5	0.45329	0.52223	0.38016	0.3551	0.35375
0.34667	0.34593	0.39918	0.35892	0.33333	0.42203
0	0	0	0	0	0
0	0	0	0	0	0
0	0	0	0	0	0
1	0.5	0.45692	0.474	0.52887	0.54953
0.5	0.45951	0.52723	0.55802	0.33333	0.35383
0.35727	0.33333	0.40631	0.28229	0.25016	0.34883
0.33595	0	0	0	0	0
0	0	0	0	0	0
0	0	0	0	0	0
0	0.46336	0.5	0.38455	0.5	0.5
0.54993	0.44424	0.54388	0.5	0.44158	0.33333
0.34005	0.31082	0.33333	0.24207	0.3409	0.39513
0.33333	0.32972	0	0	0	0
0	0	0	0	0	0
0	0	0	0	0	0
0	0	0.48281	0.50692	0.45974	0.25273
0.51474	0.50694	0.52167	0.54933	0.56018	0.5
0.34581	0.35225	0.29721	0.35868	0.2751	0.35503
0.30666	0.36623	0	0	0	0
0	0	0	0	0	0
0	0	0	0	0	0
0	0	0	0.54234	0.5	0.5

0.4879	0.50185	0.5	0.54295	0.5	0.3936
0.53034	0.38834	0.33333	0.30421	0.30402	0.39858
0.33716	0.25524	0.39167	0	0	0
0	0	0	0	0	0
0	0	0	0	0	0
0	0	0	0	0	0.46307
0.60159	0.48139	0.5	0.4405	0.48167	0.37736
0.45616	0.52181	0.39854	0.27373	0.44856	0.40991
0.34293	0.33333	0	0	0	0
0	0	0	0	0	0
0	0	0	0	0	0
0	0	0	0	0	0
0.52433	0.5	0.5	0.15689	0.50412	0.50856
0.35416	0.57054	0.5	0.33366	0.34712	0.32542
0	0	0	0	0	0
0	0	0	0	0	0
0	0	0	0	0	0
0	0	0	0	0	0
0	0	0.5	0.53203	0.44449	0.34576
0.53569	0.54899	0.5	0.40815	0	0
0	0	0	0	0	0
0	0	0	0	0	0
0	0	0	0	0	0
0	0	0	0	0	0
0	0	0	0.5	0.55259	0.3175
0.44501	0.53481	0	0	0	0
0	0	0	0	0	0
0	0	0	0	0	0
0	0	0	0	0	0
0	0	0	0	0	0
0	0	0	0	0	0.5
0.52394	0	0	0	0	0
0	0	0	0	0	0
0	0	0	0	0	0
0	0	0	0	0	0
0	0	0	0	0	0
0	0	0	0	0	0
0	0	0	0	0	0
0	0	0	0	0	0
0	0	0	0	0	0
0	0	0	0	0	0
0	0	0	0	0	0
0	0	0	0	0	0
0	0	0	0	0	0
0	0	0	0	0	0
0	0	0	0	0	0
0	0	0	0	0	0
0	0	0	0	0	0
0	0	0	0	0	0
0	0	0	0	0	0
0	0	0	0	0	0
0	0	0	0	0	0
0	0	0	0	0	0

0	0	0	0	0	0
0	0	0	0	0	0
0	0	0	0	0	0
0	0	0	0	0	0
0	0	0	0	0	0
0	0	0	0	0	0
0	0	0	0	0	0
0	0	0	0	0	0
0	0	0	0	0	0
0	0	0	0	0	0
0	0	0	0	0	0
0	0	0	0	0	0
0	0	0	0	0	0
0	0	0	0	0	0
0	0	0	0	0	0
0	0	0	0	0	0
0	0	0	0	0	0
0	0	0	0	0	0
0	0	0	0	0	0
0	0	0	0	0	0
0	0	0	0	0	0
0	0	0	0	0	0
0	0	0	0	0	0
0	0	0	0	0	0
0	0	0	0	0	0
0	0	0	0	0	0
0	0	0	0	0	0
0	0	0	0	0	0
0	0	0	0	0	0
0	0	0	0	0	0
0	0	0	0	0	0
0	0	0	0	0	0
0	0	0	0	0	0
0	0	0	0	0	0
0	0	0	0	0	0
0	0	0	0	0	0
0					

gtTCf=
0
0.07155
0.10366
0.11432
0.11389
0.10824
0.10059
0.092638
0.085208
0.078648
0.073046
0.06836
0.06449

0.06132
0.058731
0.05662
0.054894
0.053478

gtTCs=
0
0.07155
0.116
0.14393
0.16177
0.17342
0.18126
0.18671
0.19067
0.19366
0.19601
0.19792
0.19952
0.20088
0.20206
0.20309
0.20398
0.20476

gtTCp=
0
2.385
1.5979
1.1196
0.8287
0.65143
0.54309
0.47655
0.43538
0.4096
0.39316
0.38239
0.37506
0.36982
0.36585
0.36266
0.35995
0.35753

X_mask=
2.2204e-016,0.00015702,0.0026107,2.2204e-016,1.041e-005,0.00055343,2.2204e-016,2.2204e-016,0.0017086,2.2204e-016,9.2897e-005,2.2204e-016,2.2204e-016,0.00075279,2.2204e-016,2.2204e-016,2.2204e-016,0.0030329,0.001438,2.2204e-016,0.017955,2.2204e-016,2.2204e-016,2.2204e-016,2.2204e-016,0.00041569,2.2204e-

016,2.2204e-016,2.2204e-016,0.00013707,0.00015742,0.0014064,2.2204e-016,2.2204e-016,0.0017082,0.0018947,0.0014796,2.2204e-016,0.00060087,2.2204e-016,2.2204e-016,0.01241,0.0015053,2.2204e-016,0.0024267,0.0069177,2.2204e-016,0.00036427,2.2204e-016,2.2204e-016,0.0012304,0.00060621,2.2204e-016,2.2204e-016,2.2204e-016,0.0010119,0.0011917,0.0014705,2.2204e-016,0.0024831,0.00036703,8.2919e-005,0.017603,0.0039722,2.2204e-016,2.2204e-016,0.0071468,2.2204e-016,0.0081691,0.0054022,2.2204e-016,2.2204e-016,2.2204e-016,2.2204e-016,2.2204e-016,2.2204e-016,2.2204e-016,0.0017635,0.0015307,2.2204e-016,2.2204e-016,2.2204e-016,2.2204e-016,2.2204e-016,2.2204e-016,2.2204e-016,0.017143,0.01626,2.2204e-016,0.0028764,0.0027075,2.2204e-016,0.001553,2.2204e-016,0.003553,0.0038841,2.2204e-016,0.0028385,0.0019088,0.0011269,0.00093725,0.0021512,2.2204e-016,0.0013659,0.0004513,0.0019933,2.2204e-016,0.00012626,2.2204e-016,2.2204e-016,0.0011422,0.00022948,2.2204e-016,2.2204e-016,0.013853,2.2204e-016,2.2204e-016,0.021168,2.2204e-016,2.2204e-016,0.016518,0.013773,0.0012378,0.0097199,2.2204e-016,0.011788,0.010204,2.2204e-016,2.2204e-016,0.00031706,2.2204e-016,0.00085738,0.00092517,2.2204e-016,0.0021448,2.2204e-016,2.2204e-016,0.0019308,0.0025147,0.00014086,0.0008253,2.2204e-016,0.0017765,2.2204e-016,2.2204e-016,0.005148,0.0017275,0.012357,0.00095341,2.2204e-016,2.2204e-016,2.2204e-016,2.2204e-016,2.2204e-016,2.2204e-016,2.2204e-016,0.008764,0.021862,2.2204e-016,0.00065806,2.2204e-016,0.0015886,2.2204e-016,2.2204e-016,0.0019515,2.2204e-016,2.2204e-016,0.00079658,2.2204e-016,0.001504,0.0038716,2.2204e-016,0.0051929,2.2204e-016,0.0012565,0.0014676,2.2204e-016,2.2204e-016,0.0066957,0.027714,0.009024,0.0050441,2.2204e-016,0.0044853,0.001361,0.0074919,0.0031899,2.2204e-016,2.2204e-016,2.2204e-016,2.2204e-016,2.2204e-016,0.0035414,0.011366,0.0010373,0.00031317,0.0019048,2.2204e-016,0.001453,0.00039701,2.2204e-016,0.0013322,0.0014486,2.2204e-016,2.2204e-016,2.2204e-016,2.2204e-016,0.000849,0.0013045,0.00074011,0.0016548,2.2204e-016,0.0099232,0.0030652,0.0065567,2.2204e-016,0.012622,0.0041976,2.2204e-016,0.0020932,0.013314,0.013058,0.012155,2.2204e-016,0.0023897,0.0013796,0.0026749,0.0059669,2.2204e-016,2.2204e-016,2.2204e-016,0.00054102,2.2204e-016,2.2204e-016,0.0028198,0.0030973,0.0018653,2.2204e-016,0.0011892,0.0012081,9.3005e-005,0.00097575,2.2204e-016,0.00011685,0.00063366,2.2204e-016,0.0040263,2.2204e-016,0.0066783,2.2204e-016,2.2204e-016,0.012233,2.2204e-016,0.013414,0.0017098,0.0037985,0.011114,2.2204e-016,0.0075292,3.7053e-005,2.2204e-016,4.2213e-005,0.01305,0.007347,2.2204e-016,2.2204e-016,2.2204e-016,2.2204e-016,0.0026662,0.00018063,0.0045053,0.0016291,0.0011656,2.2204e-016,0.00077062,2.2204e-016,0.001277,0.003693,0.001415,0.0014289,2.2204e-016,2.2204e-016,2.2204e-016,2.2204e-016,2.2204e-016,0.00015249,2.2204e-016,0.012245,2.2204e-016,2.2204e-016,2.2204e-016,2.2204e-

016,0.0057911,0.00051073,2.2204e-016,0.008065,0.0082763,2.2204e-
016,2.2204e-016,2.2204e-016,0.0063637,2.2204e-016,0.017176,2.2204e-
016,0.00095267,2.2204e-016,2.2204e-016,2.2204e-016,2.2204e-
016,2.2204e-016,2.2204e-016,2.2204e-016,2.2204e-
016,0.0005003,0.0016367,2.2204e-
016,0.0013331,0.00073176,0.0010097,0.00071939,2.2204e-016,2.2204e-
016,0.021976,2.2204e-
016,0.034087,0.004687,0.017068,0.0048666,0.016059,2.2204e-016,2.2204e-
016,0.0067051,0.015109,2.2204e-
016,0.007047,0.014633,0.0027695,0.0018643,0.0087587,0.010114,0.0014381
,0.0032229,0.0011263,2.2204e-
016,0.0016741,0.00010017,0.00091126,2.2204e-016,2.2204e-
016,0.0005315,2.2204e-016,2.2204e-016,2.2204e-016,0.0015208,2.2204e-
016,2.2204e-016,0.00039843,0.0034132,2.2204e-016,2.2204e-
016,0.0099817,2.2204e-016,2.2204e-016,0.012171,2.2204e-016,2.2204e-
016,2.2204e-016,0.0029329,2.2204e-016,0.0043,0.0010388,2.2204e-
016,2.2204e-016,2.2204e-016,2.2204e-016,0.015594,2.2204e-016,2.2204e-
016,0.0011305,2.2204e-016,0.00082451,2.2204e-016,4.3159e-
005,0.00027069,2.2204e-016,0.00034963,2.2204e-016,2.481e-
005,0.00045334,0.0012346,0.0012647,0.00057358,1.9912e-
005,0.00021206,0.00063505,2.2204e-
016,0.0017315,0.012601,0.017162,2.2204e-016,0.020549,2.2204e-
016,0.01925,2.2204e-016,0.015164,2.2204e-016,2.2204e-016,2.2204e-
016,2.2204e-016,0.011499,0.0022386,2.2204e-016,2.2204e-
016,0.0034538,2.2204e-016,2.2204e-
016,0.001031,0.00077525,0.00013868,2.2204e-016,0.00028592,2.2204e-
016,2.2204e-016,2.2204e-016,0.000421,0.00080442,2.2204e-
016,0.00029576,0.00076326,2.2204e-016,0.00013908,0.00060107,2.2204e-
016,2.2204e-016,0.021968,2.2204e-016,2.2204e-016,2.2204e-
016,0.012667,0.0036261,2.2204e-016,0.0081843,0.0050092,2.2204e-
016,2.2204e-016,2.2204e-016,0.0046098,0.0036792,2.2204e-016,2.2204e-
016,2.2204e-016,2.2204e-016,0.0022268,0.00026097,2.2204e-
016,0.00081265,2.2204e-016,0.00084967,2.2204e-016,2.2204e-
016,0.0018949,0.0008113,0.0013556,0.0010581,2.2204e-016,2.2204e-
016,0.00036417,2.2204e-016,2.2204e-016,0.00022185,2.2204e-016,2.2204e-
016,0.010053,8.1666e-005,0.021813,0.013323,0.010826,0.010104,2.2204e-
016,0.011033,2.2204e-016,2.2204e-016,0.00083283,2.2204e-016,2.2204e-
016,2.2204e-016,0.007674,0.0032605,0.00024149,2.2204e-016,2.2204e-
016,2.2204e-016,0.00083643,0.0006881,2.2204e-016,2.2204e-016,2.2204e-
016,0.0011444,0.00097898,0.00037399,0.0012183,2.2204e-016,2.2204e-
016,2.2204e-016,0.00020659,2.2204e-016,2.2204e-016,0.0012397,2.2204e-
016,2.2204e-016,2.2204e-
016,0.0024711,0.019461,0.0012612,0.0034358,2.2204e-016,2.2204e-
016,0.0094687,0.0077184,2.2204e-016,2.2204e-016,2.2204e-
016,0.010079,2.2204e-016,0.0088828,2.2204e-016,0.0020168,2.3261e-
006,2.2204e-016,2.2204e-016,0.00073683,0.00016474,2.2204e-
016,0.0014247,2.2204e-016,0.0021357,2.2204e-
016,0.0012159,0.0003605,0.00070338,2.2204e-016,2.2204e-
016,0.0014329,0.0011282,2.2204e-016,2.2204e-016,2.2204e-
016,0.00089714,2.2204e-016,2.2204e-016,2.2204e-016,0.017399,2.2204e-
016,0.0036632,2.2204e-016,2.2204e-016,0.0050437,2.2204e-
016,0.0038116,0.0031156,0.0023766,2.2204e-

016,0.0018534,0.0022748,0.0013906,2.2204e-
016,0.0010871,0.0024989,2.2204e-016,0.00091273,2.2204e-
016,0.0012889,0.00079729,0.0010058,2.2204e-016,2.2204e-016,2.2204e-
016,0.0016561,2.2204e-016,0.0013877,0.00051207,2.2204e-
016,0.0003797,2.2204e-016,0.0011926,2.2204e-
016,0.010611,0.01064,2.2204e-016,2.2204e-
016,0.020249,0.0057349,0.0013462,2.2204e-016,0.0060815,2.2204e-
016,2.2204e-016,2.2204e-016,0.0014419,0.010499,2.2204e-
016,0.0027794,2.2204e-016,0.0020449,2.2204e-016,2.2204e-016,2.2204e-
016,2.2204e-016,2.2204e-016,0.0006905,2.2204e-016,2.2204e-016,2.2204e-
016,2.2204e-016,2.2204e-016,2.2204e-
016,0.00021144,0.00043662,0.00036417,0.00043635,2.2204e-016,2.2204e-
016,2.2204e-016,2.2204e-016,2.2204e-016,0.001339,2.2204e-016,2.2204e-
016,0.016007,0.0089574,2.2204e-016,2.2204e-016,2.2204e-
016,0.018916,0.0050183,0.0057731,0.012216,2.2204e-016,2.2204e-
016,2.2204e-016,0.0049046,0.0085048,2.2204e-016,2.2204e-016,2.2204e-
016,0.00019606,2.296e-005,0.0010711,2.2204e-
016,0.00057678,0.00024452,0.00059458,2.2204e-016,2.2204e-
016,0.0020224,0.00046837,2.2204e-016,2.2204e-
016,0.0010129,0.00033217,2.2204e-
016,0.011787,0.0080629,0.012143,0.0080728,0.018521,0.001777,2.2204e-
016,3.8839e-005,2.2204e-016,2.2204e-
016,0.0029865,0.00054335,0.0032649,2.2204e-016,0.0011957,2.2204e-
016,2.2204e-016,0.0025356,0.001756,2.2204e-016,2.2204e-016,8.6578e-
005,2.2204e-016,0.00047996,2.2204e-016,0.0021448,0.00019156,2.2204e-
016,2.2204e-016,2.2204e-016,0.00098806,2.2204e-016,2.2204e-
016,2.2204e-016,2.2204e-016,2.2204e-016,2.2204e-
016,0.0088283,0.00071351,0.0074633,0.0042341,0.0062558,2.2204e-
016,2.2204e-016,0.0048833,0.0059054,2.2204e-016,2.2204e-
016,0.00050226,0.0046637,2.2204e-016,0.0021776,2.2204e-016,2.2204e-
016,2.2204e-016,0.00011844,2.2204e-016,2.2204e-016,2.2204e-
016,2.2204e-016,0.00063695,0.00031657,2.2204e-016,2.2204e-
016,0.001323,2.2204e-016,0.00047935,0.00095355,2.2204e-016,2.2204e-
016,2.2204e-016,0.00090903,2.2204e-016,0.0008255,0.0027238,2.2204e-
016,2.2204e-016,2.2204e-016,2.2204e-016,0.0029076,2.2204e-
016,0.013414,2.2204e-016,2.2204e-016,2.2204e-016,2.2204e-016,2.2204e-
016,0.0073461,0.0033803,0.0068301,2.2204e-016,0.010574,2.2204e-
016,2.2204e-016,0.001152,0.0002641,0.00078162,0.00045113,2.2204e-
016,0.00078622,2.2204e-016,0.00051419,0.0001995,2.2204e-016,2.2204e-
016,0.00029005,0.0011483,0.00045344,2.2204e-016,0.00048304,2.2204e-
016,2.2204e-016,0.011752,2.2204e-016,2.2204e-016,0.0091565,2.2204e-
016,2.2204e-016,2.2204e-016,2.2204e-016,0.0055758,2.2204e-
016,0.010239,0.00075518,0.0013626,6.9293e-005,2.2204e-016,2.2204e-
016,0.00033659,0.00093789,0.00010939,0.00054397,0.0016812,0.00070168,2
.2204e-016,0.00038856,0.00040625,2.2204e-016,2.2204e-
016,0.0014363,0.00082597,2.2204e-016,2.2204e-016,2.2204e-016,2.2204e-
016,2.2204e-016,0.0004083,2.2204e-016,0.00090716,2.2204e-
016,0.017215,2.2204e-016,0.0053896,0.012576,0.0024245,2.2204e-
016,2.2204e-016,0.0024408,2.2204e-016,0.0093069,2.2204e-
016,0.0013265,0.00035277,0.0034972,2.085e-005,2.2204e-016,2.2204e-
016,2.2204e-016,2.2204e-016,2.2204e-016,2.2204e-016,0.001306,2.2204e-
016,0.00059117,0.00043791,2.2204e-016,2.2204e-

016,0.0011391,0.00083803,0.0002815,2.2204e-016,0.00014129,9.0886e-005,2.2204e-016,2.2204e-016,0.00068891,0.00052922,2.2204e-016,0.010208,2.2204e-016,0.0050374,2.2204e-016,0.021099,2.2204e-016,0.0068055,0.00075321,2.2204e-016,2.2204e-016,2.2204e-016,2.2204e-016,2.2204e-016,2.2204e-016,2.2204e-016,2.2204e-016,0.0015495,2.2204e-016,0.0017238,2.2204e-016,0.00013791,0.0016821,0.00074587,2.2204e-016,0.00051203,0.0017525,0.00069625,0.0022776,0.0003675,0.0014553,2.2204e-016,2.2204e-016,0.00016139,2.2204e-016,2.2204e-016,2.2204e-016,0.0091351,0.0063555,3.2535e-005,0.0030562,2.2204e-016,0.00082778,0.0005205,0.00057811,0.00071812,2.2204e-016,2.2204e-016,0.00045078,0.0023081,2.2204e-016,0.0030945,0.00077352,2.2204e-016,2.2204e-016,0.0011407,0.0029587,2.2204e-016,4.4489e-005,0.001176,2.2204e-016,0.00019623,0.00032453,2.2204e-016,0.0018564,0.00086072,2.2204e-016,0.00060998,0.00073873,2.2204e-016,2.2204e-016,2.2204e-016,0.0013917,0.0013087,0.00016781,0.0074086,0.0016653,2.2204e-016,5.0212e-005,2.2204e-016,0.0014961,2.2204e-016,2.2204e-016,0.0018032,0.00030372,0.0012383,2.2204e-016,2.2204e-016,2.2204e-016,2.2204e-016,0.00025482,2.2204e-016,2.2204e-016,2.2204e-016,2.2204e-016,0.001623,2.2204e-016,2.2204e-016,2.2204e-016,0.0023606,2.2204e-016,0.001545,0.0017621,0.00086032,0.0013375,0.0010728,0.00052604,2.2204e-016,0.0008491,0.00097673,2.2204e-016,0.00035228,0.0007292,0.0024685,2.2204e-016,2.2204e-016,7.718e-005,0.0019711,2.2204e-016,2.2204e-016,0.0003809,2.2204e-016,0.0010419,0.00083946,2.2204e-016,2.2204e-016,2.2204e-016,0.00039615,2.2204e-016,0.00034921,0.0015624,2.2204e-016,2.2204e-016,0.0001457,2.2204e-016,0.0010481,2.2204e-016,2.2204e-016,2.2204e-016,2.2204e-016,0.00091877,7.0312e-005,0.0003132,0.0010091,2.2204e-016,0.0017411,0.0013843,0.0011085,2.2204e-016,2.2204e-016,0.0016346,2.2204e-016,0.00035785,0.001311,0.00095053,2.2204e-016,2.3312e-005,2.2204e-016,0.00074276,0.0016552,0.0004785,2.2204e-016,2.2204e-016,2.2204e-016,2.2204e-016,2.2204e-016,0.00085909,2.2204e-016,2.2204e-016,2.2204e-016,2.2204e-016,2.2204e-016,2.2204e-016,0.00077311,2.2204e-016,5.0414e-005,2.2204e-016,2.2204e-016,0.0010691,2.2204e-016,0.00078844,0.00072594,2.2204e-016,2.2204e-016,2.2204e-016,2.2204e-016,2.2204e-016,0.001516,2.2204e-016,0.00089308,0.00031222,0.001045,0.0009393,2.2204e-016,0.00074411,0.00067531,2.2204e-016,2.2204e-016,0.001142,2.2204e-016,0.00012053,0.0019126,0.00014749,2.2204e-016,2.2204e-016,2.2204e-016,0.0004688,0.0010228,0.0011735,0.0014532,2.2204e-016,0.00056491,0.00068583,2.2204e-016,2.2204e-016,0.0016804,0.00066254,2.2204e-016,0.0001349,0.00083991,2.2204e-016,2.2204e-016,0.00076545,2.2204e-016,0.00017112,0.00039035,0.0020808,0.0017677,2.2204e-016,2.2204e-016,0.00089008,0.00065578,2.2204e-016,2.2204e-016,0.00039029,2.2204e-016,0.00044121,2.2204e-016,2.2204e-016,2.2204e-016,0.00028217,0.0016697,2.2204e-016,5.9243e-005,0.00086979,0.00047967,2.2204e-016,2.2204e-016,2.2204e-016,2.2204e-016,2.2204e-016,2.2204e-016,0.00050062,0.00072728,4.213e-005,4.6473e-005,2.2204e-016,0.00046489,0.00020925,2.2204e-016,2.2204e-

016,0.00043722,0.00025817,0.0010825,2.2204e-016,2.2204e-
016,0.001109,0.00035657,2.2204e-016,0.00049333,2.2204e-
016,0.00042834,2.2204e-016,2.2204e-016,2.2204e-016,0.0012855,2.2204e-
016,0.00014939,2.2204e-016,2.2204e-016,0.00076616,0.001438,2.2204e-
016,2.2204e-016,0.00052661,2.2204e-016,2.2204e-016,2.2204e-
016,2.2204e-016,2.2204e-016,0.0012436,2.2204e-016,2.2204e-016,2.2204e-
016,2.2204e-016,2.2204e-016,5.3602e-005,0.0012067,0.0002857,2.2204e-
016,0.00031481,0.001032,0.0003871,0.0012371,0.0017247,0.00058803,0.000
9119,0.00065241,0.00021408,2.2204e-016,2.2204e-016,2.2204e-
016,2.2204e-016,0.00068495,2.2204e-016,2.2204e-016,2.2204e-
016,2.2204e-016,2.2204e-016,0.0013381,2.2204e-016,2.2204e-016,2.2204e-
016,2.2204e-016,2.2204e-016,2.2204e-
016,0.0015425,0.00037835,0.00042331,0.0021613,2.2204e-016,2.2204e-
016,0.00025682,0.00030652,2.2204e-016,2.2204e-016,2.2204e-016,2.2204e-
016,0.0003164,0.00048478,2.2204e-016,2.2204e-016,4.2469e-
005,0.0012935,2.2204e-016,2.2204e-016,2.2204e-
016,0.0011061,0.00093271,0.0017918,0.00011096,2.2204e-016,7.0424e-
005,2.2204e-016,0.0005622,0.00018798,0.00079344,6.2002e-
005,0.0005419,0.00066581,2.2204e-
016,0.00068885,0.00072481,0.00074508,2.2204e-016,0.00065054,2.2204e-
016,2.2204e-016,0.00012369,0.00044184,0.00010578,2.2204e-016,2.2204e-
016,0.001142,2.2204e-016,0.00017023,2.2204e-016,2.2204e-016,7.9262e-
005,2.2204e-016,2.2204e-016,0.00098643,0.00037086,2.2204e-016,2.2204e-
016,2.2204e-016,2.2204e-016,2.2204e-
016,0.00031044,0.00015878,0.0003702,2.2204e-016,2.2204e-016,2.2204e-
016,0.0005058,0.0017032,2.2204e-016,0.00044416,2.2204e-016,2.2204e-
016,2.2204e-016,2.2204e-016,2.2204e-016,9.692e-
005,0.00068352,0.00042014,2.2204e-016,2.2204e-016,0.00049216,2.2204e-
016,2.2204e-016,2.2204e-016,2.2204e-016,2.1097e-
005,0.00066843,0.00029371,0.00058035,0.00034721,0.00018088
0.070343,0.073717,0.074786,0.06979,0.07314,0.071476,0.070493,0.071291,
0.073718,0.06902,0.071762,0.06823,0.071056,0.071272,0.071209,0.069123,
0.070454,0.075141,0.072774,0.06985,1.2406,1.2081,1.2218,1.3257,0.07166
,0.072359,0.066686,0.0703,0.071564,0.07011,0.070999,0.072866,0.068475,
0.07008,0.072651,0.073521,0.073511,0.068746,0.072552,2.3787,1.2077,1.1
925,1.2286,1.1623,1.2245,1.5113,0.071707,0.071742,0.06847,0.070103,0.0
72298,0.07414,0.071077,0.070039,0.071078,0.071876,0.072374,0.072769,0.
069867,0.072837,0.071669,2.3641,2.4077,1.3893,1.2951,1.2753,1.346,1.19
17,1.2316,1.1959,1.347,0.069879,0.071324,0.070043,0.066747,0.071047,0.
070906,0.073702,0.073281,0.069111,0.071915,0.069589,0.070171,0.071211,
0.071172,0.066884,2.4046,2.4102,2.3742,1.2491,1.2202,0.97507,1.2285,1.
3073,1.349,1.2389,1.2565,1.3507,0.072144,0.072515,0.071826,0.073425,0.
07035,0.07305,0.07189,0.074046,0.071096,0.071603,0.069288,0.071958,0.0
73124,0.072322,0.069214,0.07242,2.4042,2.3677,2.376,2.4034,1.0849,1.07
49,1.2558,1.1684,1.2824,1.2405,1.2166,1.2435,1.3004,1.0901,0.070654,0.
071552,0.068451,0.07176,0.073424,0.071513,0.07321,0.069785,0.070907,0.
072028,0.074112,0.073134,0.07237,0.069329,0.071926,0.069909,2.3661,2.3
809,2.3772,2.4018,2.3948,2.3833,1.3086,1.4219,1.2315,1.1497,1.2167,1.2
417,1.2813,1.2525,1.2208,1.2248,0.06871,0.073386,0.072163,0.069998,0.0
72333,0.071496,0.068436,0.071071,0.068712,0.073661,0.075552,0.07227,0.
076761,0.072369,0.07377,0.07229,0.069016,2.3839,2.3955,2.3989,2.3779,2
.3691,2.3768,1.3579,1.8436,1.1675,0.99451,1.1312,1.2966,1.1609,1.2239,

1.2238,1.3422,1.4116,0.07173,0.071974,0.073246,0.071079,0.072586,0.073
702,0.0684,0.07402,0.073319,0.070184,0.067848,0.069343,0.072445,0.0726
4,0.072812,0.072021,0.07311,2.353,2.3962,2.3834,2.3854,2.3866,2.3995,2
.3865,1.2255,1.2288,1.2171,1.3605,1.2439,1.2132,1.1573,1.1737,1.2693,1
.0961,0.99739,0.072187,0.070411,0.071705,0.06967,0.067535,0.073264,0.0
73639,0.073354,0.070515,0.07085,0.071831,0.070649,0.072131,0.070624,0.
071651,0.071956,0.069736,0.075332,2.3795,2.4088,2.3916,2.3706,2.3907,2
.3779,2.3989,1.2642,1.0761,1.3235,0.81249,1.1462,1.2239,1.1825,1.4603,
1.0409,1.2329,1.1379,0.068267,0.070202,0.06826,0.074018,0.070962,0.075
883,0.071971,0.074214,0.070557,0.072627,0.070949,0.071763,0.074184,0.0
73444,0.072782,0.070224,0.071606,0.071846,0.071959,2.3594,2.3966,2.364
9,2.4037,2.3688,2.3807,2.374,1.2231,1.2763,1.2214,1.1113,1.1424,1.188,
1.0876,1.4715,1.2179,1.2319,1.2317,1.245,0.071888,0.073336,0.069986,0.
070444,0.070026,0.069241,0.071867,0.068956,0.071626,0.070866,0.072224,
0.073943,0.068664,0.072772,0.072242,0.072023,0.071724,0.070118,2.3866,
2.4108,2.3751,2.4216,2.3994,2.4153,2.3938,1.2486,1.2223,1.2463,1.1576,
1.2717,1.3623,1.0912,1.2352,0.94089,1.2655,1.0709,1.3745,0.073555,0.07
4406,0.073241,0.071122,0.072975,0.071655,0.07174,0.07056,0.070069,0.07
1678,0.071482,0.070546,0.071445,0.07375,0.072436,0.071027,0.072191,0.0
74341,2.3553,2.3767,2.4041,2.359,2.377,2.4078,2.3806,2.3727,2.3649,1.3
816,1.261,1.2349,1.2305,1.2341,1.1202,1.2196,1.2949,1.244,1.187,0.9776
4,0.073408,0.072059,0.073115,0.070135,0.071526,0.071955,0.070011,0.071
211,0.070066,0.070965,0.071488,0.073279,0.073105,0.071782,0.071315,0.0
72178,0.072053,0.071561,2.3803,2.3935,2.4017,2.357,2.4112,2.3644,2.393
1,2.3762,2.3954,1.2253,1.1748,0.54983,0.7917,0.93803,0.69568,0.88043,0
.92979,0.83058,0.89244,0.88572,0.073664,0.071968,0.070749,0.071049,0.0
70933,0.071227,0.070885,0.071376,0.072821,0.071829,0.071022,0.07178,0.
071841,0.071617,0.071874,0.071356,0.071525,0.071679,2.4041,2.3802,2.4,
2.3805,2.4031,2.3857,2.3546,2.4043,2.3843,1.2984,0.93997,0.78292,0.998
67,0.84365,0.81228,0.7262,0.92233,0.79223,0.89437,0.87681,0.070182,0.0
72487,0.070605,0.072599,0.071813,0.071842,0.073145,0.071442,0.073402,0
.072153,0.070772,0.069137,0.072018,0.07031,0.071044,0.07145,0.070771,0
.070332,2.3842,2.3906,2.3905,2.4082,2.3933,2.4042,2.3807,1.2363,1.1159
,1.2467,1.0514,0.83692,0.8084,0.83041,0.91766,0.8507,0.85809,0.83539,0
.59991,0.90786,0.072251,0.072292,0.071151,0.071384,0.071083,0.072618,0
.072384,0.071833,0.073296,0.071175,0.069434,0.071721,0.072244,0.071036
,0.07048,0.072691,0.06845,0.070572,2.3838,2.379,2.416,2.3821,2.3898,2.
3585,1.2095,1.3306,1.3398,1.1797,1.2215,0.86356,0.85195,0.87124,0.811,
0.80613,0.59429,0.83997,0.88439,0.84032,0.07235,0.071384,0.071686,0.07
2276,0.070886,0.073954,0.070533,0.072381,0.071602,0.072388,0.071617,0.
070585,0.073301,0.073228,0.070453,0.06905,0.07163,0.071758,2.3705,2.38
38,2.358,2.3958,2.3705,1.0365,1.154,1.1524,1.3077,1.2297,1.1407,0.8418
6,0.69491,0.96148,0.84415,0.70817,0.79732,0.85297,0.85602,0.84732,0.07
1186,0.072802,0.072249,0.073268,0.071904,0.072024,0.070898,0.070953,0.
07049,0.073002,0.070864,0.073359,0.072026,0.072592,0.071936,0.071496,0
.072378,0.070414,2.3876,2.3934,2.37,1.5694,1.186,1.5828,1.2571,1.2212,
1.2354,1.1145,1.2608,0.95076,0.89186,0.90035,0.87686,0.87451,0.99215,0
.90524,0.84832,1.0474,0.070239,0.071246,0.069797,0.072043,0.070431,0.0
70905,0.070916,0.070879,0.071073,0.071305,0.072583,0.071892,0.072379,0
.072108,0.071186,0.071295,0.071104,0.070473,2.3861,1.2264,1.1133,1.168
6,1.3169,1.357,1.2151,1.1332,1.2831,1.3849,0.84341,0.89177,0.90913,0.8
4202,1.0135,0.72457,0.65413,0.88991,0.84109,0.070762,0.070189,0.070865

,0.071305,0.072374,0.069541,0.072285,0.071451,0.071953,0.071922,0.0699
07,0.073435,0.071752,0.070999,0.070163,0.071744,0.070742,0.070982,1.15
14,1.2385,0.97551,1.2374,1.2448,1.3475,1.095,1.331,1.2326,1.0824,0.843
82,0.86111,0.79528,0.8448,0.6325,0.8498,0.98825,0.84643,0.83728,0.0705
73,0.071732,0.07133,0.071859,0.071504,0.069679,0.073949,0.07146,0.0713
19,0.071538,0.071451,0.072519,0.0694,0.070465,0.070516,0.071575,0.0697
45,0.070668,1.196,1.2478,1.1433,0.65988,1.2633,1.2225,1.2701,1.3445,1.
3652,1.2222,0.86677,0.88782,0.7614,0.895,0.71345,0.89275,0.77635,0.916
63,0.071777,0.071006,0.071071,0.070733,0.069235,0.072693,0.071378,0.07
0965,0.071153,0.071845,0.07098,0.07199,0.072576,0.071723,0.071207,0.07
0705,0.071636,0.070726,0.071723,1.3316,1.2276,1.2257,1.1829,1.2206,1.2
393,1.3018,1.2391,0.97912,1.2915,0.96448,0.84021,0.77197,0.78046,0.992
4,0.85497,0.65638,0.98634,0.070124,0.070869,0.072611,0.071277,0.072746
,0.071516,0.070926,0.071484,0.070717,0.072226,0.071319,0.06968,0.07014
9,0.071489,0.072254,0.07327,0.071736,0.072627,0.072056,0.070707,1.1564
,1.4587,1.1757,1.2397,1.0881,1.1861,0.94214,1.1129,1.2877,0.98649,0.71
316,1.1075,1.0178,0.86879,0.83068,0.070177,0.07181,0.073161,0.071128,0
.072212,0.073053,0.07229,0.070804,0.07197,0.072435,0.070267,0.070893,0
.072709,0.071248,0.070171,0.070966,0.07129,0.071195,0.070246,0.070887,
0.070529,0.072401,1.2726,1.2438,1.2248,0.43857,1.2551,1.2528,0.88321,1
.3699,1.2337,0.83964,0.87966,0.81348,0.073031,0.071648,0.075594,0.0714
18,0.070972,0.071468,0.071357,0.071308,0.071171,0.069411,0.072705,0.07
0954,0.072269,0.071282,0.071637,0.070126,0.072823,0.071571,0.072374,0.
070223,0.071662,0.070957,0.070534,0.070619,0.072118,0.071981,1.2237,1.
3076,1.099,0.87731,1.3088,1.3583,1.2269,1.022,0.072763,0.070993,0.0703
59,0.072035,0.070486,0.071345,0.071415,0.07043,0.071357,0.072775,0.070
606,0.07339,0.069841,0.071356,0.073061,0.072132,0.071187,0.07119,0.072
437,0.071953,0.073075,0.071043,0.073469,0.07145,0.071035,0.071447,0.07
0883,0.070119,0.07029,1.2386,1.366,0.80578,1.0991,1.3051,0.071815,0.07
1909,0.071358,0.071706,0.070466,0.070652,0.072626,0.073433,0.071763,0.
074411,0.072887,0.071858,0.070186,0.072267,0.073861,0.070436,0.071334,
0.072937,0.07225,0.071579,0.071884,0.070196,0.07355,0.072406,0.07032,0
.071989,0.072154,0.071267,0.072323,0.070226,0.072499,0.072955,0.071671
,1.2296,1.277,0.071091,0.071783,0.070502,0.072393,0.070018,0.071343,0.
07395,0.071882,0.072264,0.070408,0.071006,0.069463,0.06978,0.072446,0.
070255,0.071648,0.071254,0.070878,0.072864,0.070702,0.072369,0.070929,
0.073582,0.070719,0.072264,0.073732,0.071777,0.072259,0.072503,0.07224
6,0.071215,0.072567,0.073348,0.070419,0.072043,0.071961,0.074067,0.070
84,0.070751,0.071253,0.073603,0.06999,0.071795,0.071894,0.071463,0.072
356,0.071945,0.071563,0.071559,0.070554,0.072428,0.070355,0.072166,0.0
72774,0.070407,0.070875,0.071316,0.070259,0.071911,0.071093,0.071389,0
.070423,0.071346,0.073202,0.07175,0.071953,0.07264,0.070399,0.073429,0
.072394,0.072252,0.070581,0.070553,0.073519,0.071135,0.07185,0.072566,
0.072449,0.070404,0.07011,0.071151,0.072373,0.073012,0.071532,0.07055,
0.071177,0.070379,0.071029,0.070592,0.07229,0.070838,0.070561,0.070773
,0.071059,0.070714,0.071583,0.07245,0.070866,0.07124,0.071561,0.070986
,0.072606,0.071028,0.072699,0.072438,0.07085,0.071839,0.070118,0.07085
7,0.071129,0.0741,0.070295,0.072391,0.071915,0.07266,0.071885,0.070551
,0.072036,0.072358,0.071014,0.070969,0.072474,0.070651,0.071612,0.0734
34,0.071275,0.070958,0.070519,0.070777,0.071824,0.072494,0.072427,0.07
2915,0.071381,0.072014,0.071905,0.071176,0.070381,0.073018,0.072381,0.
070441,0.07167,0.072033,0.071139,0.069854,0.072811,0.071156,0.071596,0

.071485,0.07353,0.072491,0.071515,0.06942,0.072341,0.072788,0.071315,0
.071351,0.071426,0.070881,0.071085,0.071382,0.069903,0.071014,0.071877
,0.073385,0.069532,0.07179,0.072179,0.071508,0.071247,0.070926,0.07094
9,0.071384,0.069852,0.070226,0.071935,0.071714,0.071859,0.071626,0.071
608,0.07167,0.071273,0.0707,0.071736,0.072371,0.071413,0.072311,0.0709
39,0.071385,0.07246,0.0719,0.070968,0.072082,0.071839,0.072008,0.07153
,0.071651,0.071667,0.072147,0.071435,0.071651,0.071656,0.070726,0.0727
78,0.072857,0.070832,0.07052,0.072079,0.070514,0.07032,0.071428,0.0714
79,0.070215,0.072729,0.069938,0.070895,0.071839,0.07189,0.070428,0.071
093,0.072852,0.071974,0.07151,0.072391,0.071946,0.072669,0.072616,0.07
2856,0.07202,0.072474,0.072224,0.072166,0.071578,0.070955,0.071129,0.0
71305,0.071835,0.070605,0.071381,0.071285,0.071259,0.071782,0.073227,0
.071386,0.071067,0.070415,0.070983,0.070426,0.07104,0.072921,0.072134,
0.071911,0.072819,0.070108,0.070315,0.071902,0.072279,0.071213,0.07028
2,0.071196,0.071282,0.071827,0.071993,0.070606,0.070205,0.071225,0.072
732,0.070594,0.070984,0.07166,0.072674,0.072436,0.073866,0.072253,0.07
2061,0.07184,0.07113,0.072467,0.071585,0.072731,0.071433,0.07194,0.072
302,0.069981,0.072035,0.072448,0.072628,0.071755,0.071982,0.070429,0.0
70905,0.071541,0.071646,0.071432,0.070262,0.070938,0.072238,0.070288,0
.071811,0.069943,0.071054,0.071405,0.071019,0.071307,0.073318,0.072381
,0.071138,0.071124,0.070597,0.071555,0.070335,0.072317,0.072335,0.0713
65,0.070251,0.071719,0.071089,0.072035,0.07279,0.070523,0.072485,0.070
308,0.071374,0.070812,0.07005,0.07189,0.070896,0.072481,0.071939,0.070
832,0.070656,0.072426,0.071334,0.071016,0.071256,0.071221,0.071279,0.0
72355,0.071973,0.072143,0.072288,0.072108
0.11549,0.11801,0.11909,0.11431,0.11725,0.11778,0.11421,0.11692,0.1180
7,0.11337,0.11615,0.11264,0.11586,0.11515,0.11499,0.11384,0.11458,0.12
1,0.11753,0.11491,0.864,0.84643,0.84687,0.91808,0.11619,0.118,0.11133,
0.11524,0.11652,0.11464,0.11642,0.11739,0.11394,0.11407,0.11772,0.1167
2,0.11728,0.11362,0.11617,1.5895,0.83349,0.83211,0.85556,0.81351,0.855
13,1.0315,0.11691,0.11615,0.11324,0.11574,0.11671,0.11648,0.11562,0.11
475,0.11657,0.11697,0.1175,0.1177,0.11557,0.11808,0.11491,1.5874,1.613
1,0.96613,0.89579,0.88342,0.93947,0.82911,0.84831,0.83568,0.93243,0.11
472,0.11779,0.1143,0.11223,0.11548,0.11549,0.11661,0.1179,0.11322,0.11
653,0.11313,0.11564,0.11622,0.11647,0.11199,1.6216,1.621,1.5869,0.8716
4,0.85332,0.69221,0.8661,0.90746,0.93343,0.86965,0.87382,0.93203,0.117
1,0.11611,0.11515,0.11757,0.11489,0.1178,0.11521,0.11859,0.11495,0.115
35,0.11331,0.11498,0.11921,0.11788,0.11405,0.11733,1.613,1.5811,1.5906
,1.6129,0.76135,0.75842,0.88404,0.81977,0.89193,0.87166,0.84011,0.8702
6,0.9067,0.76778,0.11546,0.11683,0.11318,0.11696,0.11868,0.11605,0.117
77,0.11478,0.1151,0.11586,0.11905,0.11826,0.11606,0.11342,0.11728,0.11
521,1.5841,1.5997,1.5765,1.6086,1.6027,1.5942,0.90776,0.98007,0.8615,0
.80721,0.85053,0.8582,0.88834,0.87364,0.84682,0.85331,0.11435,0.11731,
0.11638,0.1151,0.11576,0.11596,0.11195,0.11553,0.11448,0.11746,0.12016
,0.11777,0.12191,0.11604,0.11738,0.11613,0.11379,1.603,1.5942,1.6126,1
.591,1.5884,1.5771,0.93991,1.2503,0.81939,0.70752,0.78426,0.89958,0.81
225,0.85789,0.85449,0.92856,0.97942,0.11662,0.11549,0.11676,0.11662,0.
11769,0.11698,0.11272,0.11774,0.11831,0.11517,0.11317,0.11446,0.11737,
0.11074,0.112,0.11043,0.1175,1.5715,1.6005,1.5903,1.5813,1.5983,1.6192
,1.6002,0.8516,0.86176,0.85075,0.94395,0.87768,0.8386,0.81445,0.81844,
0.88954,0.76803,0.70439,0.11644,0.11387,0.11487,0.11371,0.11172,0.1167
6,0.11714,0.11778,0.11631,0.11466,0.11605,0.10921,0.11051,0.10831,0.10

869,0.10884,0.10847,0.11833,1.6009,1.6219,1.5956,1.5956,1.5968,1.6011,
1.6113,0.87689,0.76087,0.91569,0.58608,0.81013,0.85497,0.83056,1.0035,
0.73942,0.85906,0.79994,0.11323,0.115,0.11207,0.11915,0.11532,0.11902,
0.11666,0.11873,0.1151,0.10982,0.10884,0.11005,0.11226,0.11047,0.10978
,0.10925,0.10949,0.11115,0.11607,1.5814,1.5994,1.5909,1.5989,1.5722,1.
5883,1.5887,0.85046,0.88633,0.84726,0.78365,0.79931,0.83511,0.75867,1.
015,0.84138,0.86043,0.8577,0.87248,0.1177,0.11785,0.11287,0.11482,0.11
536,0.11442,0.10892,0.10773,0.10983,0.11078,0.11233,0.11169,0.10748,0.
11115,0.11019,0.11177,0.11025,0.10822,1.5975,1.6331,1.5921,1.641,1.616
7,1.6205,1.5977,0.87678,0.84768,0.86467,0.81282,0.88571,0.94184,0.7688
1,0.86231,0.67469,0.88299,0.75926,0.95487,0.11822,0.1177,0.11767,0.11,
0.11079,0.10955,0.10972,0.10909,0.10768,0.1122,0.11006,0.1097,0.10903,
0.1117,0.11069,0.10982,0.11083,0.11331,1.5555,1.5802,1.6122,1.5691,1.5
725,1.629,1.5956,1.5982,1.5742,0.95647,0.87602,0.86265,0.86492,0.85966
,0.79455,0.85094,0.89708,0.86616,0.82918,0.69265,0.11163,0.11049,0.111
25,0.10851,0.10912,0.11061,0.10825,0.10891,0.1081,0.1096,0.10982,0.11,
0.11374,0.11028,0.10967,0.10899,0.11118,0.10979,1.5977,1.5939,1.6154,1
.57,1.6255,1.575,1.6092,1.5921,1.6134,0.85286,0.81494,0.41519,0.5706,0
.6695,0.51273,0.63084,0.65839,0.60074,0.63878,0.63412,0.11163,0.11164,
0.10793,0.10921,0.11003,0.10922,0.10694,0.10974,0.11083,0.11014,0.1097
5,0.1102,0.10876,0.10985,0.10942,0.10949,0.11143,0.11105,1.6102,1.6032
,1.6083,1.582,1.6066,1.5936,1.5713,1.6083,1.6085,0.89267,0.66361,0.565
32,0.70687,0.61093,0.58913,0.53109,0.65781,0.57504,0.64279,0.62754,0.1
0934,0.10967,0.10852,0.11146,0.11028,0.11071,0.11208,0.10933,0.11179,0
.1094,0.1086,0.10823,0.11033,0.10862,0.10916,0.10985,0.10916,0.10864,1
.5895,1.6095,1.5867,1.6217,1.6149,1.6038,1.5958,0.85773,0.77566,0.8597
6,0.73593,0.60254,0.58275,0.59493,0.65686,0.61103,0.61927,0.59698,0.44
63,0.64592,0.10985,0.11045,0.10936,0.11011,0.11063,0.11094,0.10719,0.1
1,0.1121,0.10907,0.10816,0.1091,0.11045,0.10848,0.10971,0.11028,0.1071
9,0.10826,1.5892,1.5836,1.6364,1.5878,1.5852,1.5726,0.8332,0.91928,0.9
2756,0.81605,0.84696,0.61217,0.6152,0.61895,0.58901,0.58684,0.44878,0.
60366,0.63309,0.60247,0.11195,0.10929,0.10967,0.10926,0.10745,0.11298,
0.10892,0.11133,0.1101,0.11024,0.10942,0.10989,0.11139,0.11108,0.10882
,0.10748,0.11127,0.11071,1.5899,1.6049,1.5551,1.611,1.5974,0.72601,0.8
0458,0.79823,0.9023,0.85564,0.79732,0.60519,0.51097,0.67755,0.60481,0.
52091,0.5773,0.60827,0.6173,0.60868,0.11024,0.11073,0.10938,0.11028,0.
10983,0.11089,0.11022,0.1085,0.10971,0.11037,0.10858,0.11126,0.10963,0
.11104,0.10971,0.1093,0.11045,0.10874,1.595,1.6099,1.5874,1.0655,0.831
22,1.0843,0.87309,0.84381,0.85918,0.77723,0.86651,0.67877,0.63951,0.64
085,0.6301,0.62412,0.70986,0.65077,0.61082,0.74003,0.10971,0.109,0.108
63,0.11038,0.10879,0.11011,0.10979,0.10849,0.10989,0.10817,0.11232,0.1
101,0.11078,0.10921,0.11082,0.10963,0.10993,0.1085,1.6024,0.85097,0.77
813,0.80884,0.91221,0.94741,0.83932,0.79014,0.8822,0.95323,0.60385,0.6
36,0.64689,0.60319,0.72221,0.53235,0.48982,0.63689,0.59969,0.10851,0.1
0906,0.1085,0.11025,0.11118,0.10805,0.11121,0.11029,0.1092,0.11014,0.1
0852,0.11146,0.11073,0.10891,0.10834,0.10928,0.11,0.10261,0.79648,0.86
332,0.69466,0.84504,0.86923,0.9325,0.76286,0.91895,0.8576,0.75744,0.60
467,0.6143,0.57356,0.60762,0.47026,0.60759,0.70257,0.60443,0.60425,0.1
083,0.1099,0.10986,0.10955,0.10986,0.10889,0.11195,0.11017,0.11012,0.1
0999,0.1095,0.10973,0.10753,0.10887,0.10812,0.10956,0.10846,0.10327,0.
82932,0.86426,0.79666,0.48789,0.87721,0.84368,0.87279,0.92112,0.92818,
0.84493,0.62081,0.63704,0.55294,0.63926,0.52133,0.6371,0.56712,0.65169

,0.11067,0.10805,0.10794,0.10976,0.10867,0.11104,0.10893,0.10957,0.109
39,0.10914,0.10732,0.10914,0.10959,0.11018,0.10833,0.10772,0.10885,0.1
0916,0.10424,0.92024,0.84239,0.84859,0.81446,0.84457,0.86271,0.88664,0
.85947,0.69906,0.89033,0.67716,0.60117,0.55617,0.56471,0.7025,0.61168,
0.48136,0.70539,0.10928,0.10879,0.11043,0.10974,0.11138,0.1103,0.11056
,0.10877,0.10885,0.11182,0.10851,0.10793,0.10714,0.11035,0.11177,0.110
64,0.11015,0.10406,0.10387,0.1028,0.80784,0.99953,0.8151,0.86094,0.755
21,0.82373,0.66582,0.76328,0.89842,0.69887,0.52657,0.77239,0.71923,0.6
2223,0.59608,0.10978,0.11036,0.11131,0.10928,0.10959,0.11301,0.10988,0
.10939,0.10916,0.1118,0.10801,0.10806,0.11098,0.11144,0.11035,0.10935,
0.11003,0.1094,0.10229,0.10252,0.10316,0.10461,0.86816,0.86934,0.84405
,0.33997,0.86972,0.86682,0.62774,0.93989,0.85071,0.6031,0.62924,0.5845
9,0.11135,0.1082,0.11353,0.10952,0.10882,0.11035,0.10876,0.1092,0.1101
8,0.10803,0.11189,0.11,0.10976,0.11048,0.10834,0.10896,0.11097,0.10983
,0.11132,0.10899,0.10308,0.10291,0.10228,0.10343,0.1046,0.10343,0.8490
2,0.90836,0.76621,0.62901,0.90213,0.94818,0.85089,0.71582,0.11193,0.10
722,0.10975,0.11094,0.10888,0.11147,0.10842,0.10758,0.10977,0.11097,0.
10867,0.11144,0.10878,0.10902,0.1114,0.11062,0.1095,0.1101,0.1125,0.11
001,0.11163,0.1098,0.11228,0.10355,0.10313,0.10337,0.10387,0.10212,0.1
0162,0.85324,0.94121,0.57675,0.77029,0.89889,0.10432,0.10406,0.10989,0
.1085,0.11136,0.10924,0.11115,0.11158,0.11056,0.11296,0.11131,0.10929,
0.10844,0.10993,0.10978,0.10744,0.11041,0.11267,0.11095,0.11022,0.1099
5,0.10933,0.1116,0.10507,0.10252,0.10464,0.10424,0.10297,0.1047,0.1026
7,0.10443,0.10499,0.10391,0.85536,0.8805,0.1035,0.10402,0.10272,0.1043
2,0.10801,0.1099,0.11256,0.11173,0.1105,0.1091,0.11,0.10775,0.10766,0.
11094,0.1073,0.11088,0.1102,0.10879,0.1125,0.10859,0.11129,0.10906,0.1
1173,0.10327,0.10391,0.1056,0.10341,0.10398,0.10432,0.10442,0.1036,0.1
0427,0.10522,0.10283,0.10421,0.10408,0.10636,0.10326,0.1028,0.10205,0.
11213,0.10821,0.11012,0.1113,0.10884,0.11121,0.11253,0.10933,0.10993,0
.10776,0.11051,0.10888,0.10973,0.11089,0.10887,0.10877,0.10377,0.10348
,0.1032,0.10287,0.10377,0.10247,0.10394,0.10555,0.10481,0.10435,0.1044
6,0.10249,0.10548,0.10393,0.10409,0.10263,0.10294,0.10595,0.10316,0.10
851,0.11021,0.11082,0.10908,0.10881,0.1095,0.1107,0.11068,0.10998,0.10
8,0.10954,0.10808,0.10996,0.10287,0.10377,0.10325,0.10323,0.1033,0.102
98,0.10247,0.10377,0.10437,0.10281,0.10269,0.10327,0.10348,0.10468,0.1
0292,0.10422,0.10448,0.10204,0.10408,0.10218,0.10307,0.1094,0.1114,0.1
0859,0.1106,0.10922,0.11161,0.11055,0.10928,0.11117,0.10434,0.10363,0.
10315,0.10501,0.10296,0.10424,0.10552,0.10322,0.10293,0.10327,0.10292,
0.10405,0.10443,0.10453,0.10469,0.10389,0.10374,0.10385,0.10345,0.1028
7,0.10524,0.10447,0.10275,0.10389,0.10474,0.10887,0.10775,0.10922,0.10
856,0.10377,0.1032,0.10592,0.10418,0.10347,0.10193,0.10454,0.10465,0.1
0374,0.10243,0.10388,0.1032,0.10342,0.10369,0.10215,0.10359,0.10482,0.
10581,0.10168,0.1035,0.10414,0.10426,0.10366,0.10337,0.10324,0.1037,0.
10191,0.10244,0.10357,0.10348,0.10412,0.10367,0.1035,0.10393,0.10317,0
.10237,0.10399,0.10483,0.10367,0.10448,0.10348,0.10287,0.10448,0.10355
,0.10341,0.10346,0.10401,0.10357,0.10372,0.10298,0.10412,0.10402,0.103
81,0.10395,0.1037,0.10304,0.10502,0.10437,0.10265,0.10249,0.1044,0.101
91,0.10277,0.1034,0.10391,0.10222,0.10469,0.10224,0.10323,0.10361,0.10
38,0.10287,0.10312,0.10502,0.10442,0.10364,0.10438,0.10431,0.10435,0.1
0414,0.10551,0.10412,0.10409,0.10466,0.10424,0.10417,0.103,0.10353,0.1
0324,0.10338,0.10329,0.10339,0.10303,0.10358,0.10343,0.10559,0.10383,0
.1036,0.10258,0.10325,0.10297,0.1027,0.10473,0.10423,0.10461,0.10558,0

.10307,0.10223,0.10342,0.10387,0.10296,0.10303,0.1032,0.10426,0.10444,
0.10396,0.10226,0.10248,0.10331,0.10527,0.10234,0.10339,0.10383,0.1056
2,0.1047,0.10599,0.10403,0.1044,0.10339,0.10316,0.10477,0.10362,0.1043
7,0.10418,0.10385,0.10426,0.10174,0.10447,0.10496,0.10542,0.10393,0.10
404,0.10238,0.10298,0.10396,0.1044,0.10348,0.10262,0.10337,0.1036,0.10
305,0.10434,0.10217,0.10341,0.10362,0.10291,0.10341,0.10545,0.10503,0.
10338,0.10272,0.10342,0.10384,0.10312,0.10422,0.10556,0.10411,0.1029,0
.10391,0.10273,0.10473,0.10377,0.1025,0.10486,0.10239,0.10338,0.10312,
0.10269,0.1036,0.10323,0.10413,0.10365,0.10349,0.10285,0.10476,0.10315
,0.1027,0.1036,0.10334,0.10327,0.10426,0.10469,0.10461,0.10374,0.10377
0.14408,0.14578,0.14715,0.14165,0.14423,0.14477,0.14284,0.14388,0.1458
8,0.14049,0.14524,0.14154,0.14427,0.14137,0.14399,0.14252,0.14343,0.14
827,0.14605,0.14194,0.63567,0.62582,0.62305,0.66814,0.14318,0.14466,0.
13877,0.14305,0.14569,0.14246,0.14496,0.14682,0.14226,0.142,0.14595,0.
1445,0.14604,0.14058,0.14431,1.115,0.6102,0.61611,0.62955,0.59608,0.62
71,0.74806,0.14396,0.14408,0.14117,0.14409,0.14618,0.14482,0.14266,0.1
4329,0.14437,0.14436,0.14461,0.14548,0.14285,0.14662,0.14148,1.1119,1.
1331,0.70529,0.65909,0.64432,0.69348,0.6065,0.62191,0.61555,0.68377,0.
14307,0.14471,0.1429,0.13952,0.14395,0.14411,0.14408,0.14572,0.14191,0
.14456,0.14306,0.14334,0.14391,0.14379,0.14021,1.1401,1.137,1.1124,0.6
443,0.63038,0.51998,0.64054,0.6656,0.67955,0.63705,0.6386,0.68203,0.14
502,0.14454,0.14392,0.14629,0.14291,0.14646,0.14355,0.14464,0.14319,0.
14344,0.14171,0.1435,0.14709,0.14509,0.14206,0.14562,1.1437,1.087,1.10
56,1.1455,0.56818,0.56562,0.64997,0.61553,0.65079,0.6461,0.61416,0.651
65,0.66966,0.57181,0.14456,0.14449,0.14097,0.14596,0.14665,0.14389,0.1
4444,0.14211,0.14297,0.14349,0.14601,0.14532,0.14213,0.14149,0.14552,0
.14238,1.1142,1.1302,1.0976,1.1145,1.1363,1.1234,0.66027,0.72281,0.633
7,0.59898,0.62375,0.62759,0.64836,0.65187,0.61998,0.63177,0.14207,0.14
527,0.14405,0.14338,0.14381,0.1425,0.14067,0.14443,0.14077,0.14484,0.1
4709,0.14589,0.1498,0.1452,0.14594,0.14421,0.14207,1.1301,1.1153,1.122
5,1.1154,1.1115,1.1005,0.69339,0.88711,0.60698,0.53114,0.58844,0.66472
,0.59901,0.63578,0.63211,0.68162,0.72431,0.14534,0.14351,0.14346,0.143
68,0.14725,0.14539,0.14058,0.14597,0.14692,0.14272,0.14116,0.14294,0.1
4463,0.12948,0.13194,0.13058,0.14571,1.0909,1.113,1.1069,1.0959,1.1147
,1.1379,1.118,0.63459,0.63833,0.63272,0.69442,0.65134,0.61667,0.6066,0
.60345,0.65762,0.57399,0.53056,0.14617,0.14216,0.14382,0.14227,0.13979
,0.14572,0.14471,0.14564,0.14361,0.14327,0.14336,0.12736,0.13046,0.127
42,0.12598,0.1279,0.12806,0.14554,1.1131,1.1542,1.1178,1.1066,1.1215,1
.1081,1.1311,0.64676,0.57254,0.67367,0.45251,0.60732,0.62839,0.61217,0
.72569,0.55917,0.63419,0.5999,0.14353,0.14374,0.14026,0.14658,0.14341,
0.14694,0.14442,0.14654,0.14336,0.12804,0.12743,0.1285,0.13079,0.12918
,0.12698,0.12863,0.1292,0.12995,0.14321,1.1035,1.1259,1.1123,1.1267,1.
0814,1.1064,1.0981,0.62737,0.65219,0.63131,0.57927,0.59481,0.62111,0.5
6766,0.74067,0.61656,0.63263,0.63444,0.64238,0.14495,0.1465,0.1403,0.1
4283,0.14326,0.14213,0.12769,0.12699,0.12952,0.13059,0.13266,0.13049,0
.12735,0.1312,0.12972,0.13311,0.12935,0.12709,1.1276,1.1443,1.1201,1.1
592,1.1519,1.1369,1.114,0.65006,0.62814,0.63707,0.61114,0.65037,0.6848
,0.5765,0.63568,0.51677,0.6605,0.57228,0.69984,0.14603,0.14487,0.146,0
.12973,0.1303,0.12813,0.12844,0.1297,0.12648,0.13385,0.1296,0.13037,0.
12758,0.13025,0.13097,0.13009,0.12961,0.13373,1.0969,1.1108,1.1425,1.0
921,1.1057,1.1365,1.1102,1.1257,1.1004,0.69315,0.64604,0.63622,0.63483
,0.62985,0.58934,0.62354,0.66005,0.63709,0.61138,0.52223,0.1317,0.1300

8,0.13069,0.12895,0.12739,0.13073,0.12791,0.12745,0.1276,0.1297,0.1296
9,0.12815,0.13606,0.12927,0.12969,0.1272,0.12998,0.12856,1.1172,1.1192
,1.1282,1.114,1.1409,1.1062,1.1305,1.1143,1.1341,0.6225,0.60144,0.3333
,0.43244,0.50366,0.39913,0.47872,0.49108,0.45515,0.48159,0.48178,0.129
49,0.13218,0.125,0.12857,0.12947,0.12807,0.12327,0.12913,0.12964,0.130
39,0.12867,0.12979,0.12719,0.12864,0.12903,0.12716,0.13275,0.13042,1.1
266,1.1159,1.1305,1.1049,1.1231,1.1189,1.0892,1.1315,1.134,0.64595,0.4
9518,0.43359,0.52099,0.46397,0.45269,0.41101,0.49332,0.44094,0.48606,0
.47533,0.12984,0.12842,0.1289,0.13017,0.12925,0.13022,0.13225,0.1289,0
.13043,0.12753,0.12808,0.12842,0.12828,0.12853,0.1291,0.12931,0.12795,
0.12843,1.1033,1.1275,1.109,1.1568,1.1393,1.1401,1.1144,0.62199,0.5697
5,0.62233,0.54314,0.45691,0.44379,0.44935,0.49331,0.46543,0.47307,0.45
432,0.35439,0.48403,0.12803,0.13077,0.13016,0.12918,0.13018,0.12954,0.
12118,0.1297,0.13262,0.12841,0.12807,0.12638,0.1306,0.12681,0.12977,0.
12956,0.1271,0.12673,1.119,1.1119,1.1577,1.0983,1.1047,1.0939,0.60373,
0.66728,0.66586,0.59147,0.61388,0.4607,0.4699,0.46249,0.45096,0.45233,
0.35719,0.46071,0.48034,0.45688,0.13333,0.12938,0.12895,0.127,0.12613,
0.13251,0.12901,0.12942,0.12966,0.12876,0.12899,0.12921,0.13065,0.1295
2,0.12809,0.12779,0.13328,0.13176,1.1154,1.1307,1.0887,1.1279,1.1229,0
.52767,0.5859,0.57731,0.6498,0.62546,0.58291,0.46229,0.39589,0.50676,0
.45764,0.40146,0.44043,0.45848,0.47062,0.46255,0.13003,0.13002,0.12759
,0.1287,0.12928,0.12971,0.13097,0.1268,0.13156,0.13009,0.12834,0.12934
,0.1289,0.13059,0.12945,0.12826,0.13069,0.12884,1.1228,1.1374,1.1188,0
.76697,0.60647,0.77037,0.63921,0.61283,0.61852,0.56545,0.62774,0.50446
,0.48087,0.48047,0.47484,0.46919,0.53187,0.48869,0.46653,0.54831,0.129
39,0.1283,0.12869,0.13053,0.12821,0.13188,0.12901,0.12765,0.13025,0.12
594,0.13309,0.12987,0.13113,0.12754,0.13223,0.12991,0.12957,0.12836,1.
1271,0.61077,0.56741,0.58439,0.65878,0.68885,0.60717,0.57207,0.63548,0
.68589,0.45811,0.48004,0.48626,0.45617,0.54344,0.40947,0.38381,0.48469
,0.44997,0.12825,0.12813,0.12698,0.13033,0.13059,0.12767,0.12988,0.129
57,0.12765,0.12981,0.12758,0.1312,0.13104,0.12911,0.12794,0.12741,0.13
088,0.11352,0.58548,0.63016,0.51542,0.60838,0.63655,0.67732,0.55229,0.
66261,0.62771,0.55667,0.46151,0.46022,0.43386,0.46191,0.36937,0.45424,
0.52765,0.45732,0.45738,0.12643,0.1297,0.12871,0.12793,0.12881,0.12934
,0.12929,0.1305,0.12904,0.12922,0.12957,0.12631,0.12602,0.12774,0.1270
6,0.1282,0.12883,0.11423,0.59775,0.62589,0.58065,0.37603,0.62872,0.615
89,0.62846,0.66382,0.67225,0.61683,0.4752,0.48309,0.42513,0.48074,0.40
559,0.47745,0.43234,0.48765,0.12964,0.12753,0.12637,0.12924,0.12869,0.
13059,0.1275,0.13054,0.12803,0.12647,0.12335,0.12866,0.1282,0.12888,0.
12604,0.12615,0.12722,0.12865,0.1147,0.66096,0.61058,0.61393,0.5858,0.
60804,0.62911,0.63418,0.62814,0.51408,0.63935,0.50463,0.4554,0.4224,0.
43232,0.52392,0.46342,0.37538,0.53134,0.12872,0.12815,0.12915,0.12873,
0.13063,0.13033,0.13011,0.12766,0.12837,0.13167,0.12537,0.12784,0.1245
5,0.13036,0.13068,0.12855,0.13019,0.11439,0.11472,0.11359,0.58909,0.71
143,0.58734,0.62865,0.55003,0.60462,0.4959,0.54631,0.64828,0.52174,0.4
0461,0.56495,0.53445,0.47495,0.44659,0.13015,0.12951,0.13144,0.12812,0
.12705,0.13283,0.12856,0.1292,0.12805,0.1314,0.12652,0.12719,0.13037,0
.13369,0.13047,0.12916,0.12953,0.12819,0.11298,0.11289,0.11415,0.1148,
0.62961,0.63996,0.60644,0.27282,0.64008,0.63134,0.46559,0.67928,0.6155
6,0.45239,0.47109,0.44316,0.13115,0.12606,0.13142,0.12918,0.12789,0.13
128,0.12778,0.12807,0.12898,0.12714,0.13289,0.13015,0.12877,0.13019,0.
12536,0.12891,0.13048,0.13029,0.13199,0.12914,0.11392,0.11327,0.11318,

0.11412,0.11501,0.11377,0.61657,0.66149,0.55417,0.47076,0.65652,0.6968
4,0.62335,0.52128,0.13144,0.12486,0.13003,0.13024,0.1294,0.13276,0.128
25,0.127,0.12902,0.13026,0.12743,0.13048,0.12791,0.12778,0.1312,0.1310
7,0.12726,0.12967,0.13578,0.1292,0.13107,0.12823,0.13126,0.11456,0.114
15,0.11341,0.11518,0.11361,0.11251,0.6258,0.67516,0.42874,0.56068,0.65
421,0.11507,0.11441,0.12959,0.12733,0.13276,0.12892,0.13055,0.13061,0.
12943,0.13291,0.13132,0.12886,0.12733,0.12949,0.12672,0.12518,0.12972,
0.13461,0.13109,0.13078,0.12852,0.12989,0.12953,0.11541,0.11353,0.1162
5,0.11536,0.11325,0.11565,0.11388,0.11503,0.11515,0.11443,0.61629,0.63
885,0.11452,0.11442,0.11385,0.11527,0.12691,0.13014,0.13234,0.1335,0.1
2916,0.12829,0.13047,0.12772,0.12576,0.12902,0.12441,0.13088,0.12952,0
.12808,0.13471,0.12772,0.13063,0.12831,0.13008,0.1138,0.11537,0.11517,
0.11413,0.11494,0.11419,0.1146,0.11383,0.11475,0.11672,0.11358,0.11475
,0.11441,0.1165,0.11382,0.11426,0.11252,0.13171,0.12738,0.12851,0.1322
3,0.12682,0.13018,0.13484,0.12776,0.12988,0.12598,0.13012,0.12817,0.12
798,0.13036,0.12772,0.12858,0.11398,0.11471,0.11382,0.1141,0.11433,0.1
1386,0.11594,0.1162,0.11512,0.11502,0.11512,0.11313,0.11629,0.11415,0.
11468,0.11282,0.11313,0.11651,0.11421,0.12522,0.12904,0.13053,0.1286,0
.12885,0.12895,0.13043,0.12995,0.12931,0.12799,0.12884,0.12829,0.12928
,0.11373,0.11436,0.11424,0.11377,0.11365,0.1139,0.11397,0.11454,0.1150
9,0.11359,0.11307,0.11404,0.11433,0.11592,0.11298,0.11461,0.11529,0.11
331,0.11427,0.11332,0.11368,0.13039,0.12971,0.12803,0.12995,0.12754,0.
13123,0.13215,0.12973,0.13036,0.11495,0.11416,0.11354,0.11576,0.11406,
0.11417,0.11542,0.11396,0.11358,0.1149,0.11419,0.11471,0.1153,0.11514,
0.11565,0.11427,0.11448,0.11488,0.11449,0.11347,0.11595,0.11463,0.1129
9,0.11584,0.11594,0.12702,0.12751,0.12642,0.1263,0.11447,0.11394,0.116
73,0.1148,0.11488,0.11273,0.11544,0.11485,0.1138,0.11356,0.11467,0.113
14,0.11346,0.11437,0.11358,0.11407,0.11539,0.11581,0.11182,0.11369,0.1
1446,0.11477,0.11428,0.11377,0.11371,0.11432,0.11307,0.11329,0.11468,0
.11409,0.11509,0.11456,0.11424,0.11424,0.11416,0.11305,0.11499,0.11555
,0.11473,0.11538,0.11375,0.11464,0.11494,0.11447,0.11404,0.11408,0.114
88,0.11439,0.11459,0.11395,0.11452,0.11489,0.1142,0.11454,0.11428,0.11
418,0.11531,0.11481,0.11272,0.11313,0.11469,0.11244,0.11383,0.11399,0.
1145,0.1128,0.11526,0.11307,0.11356,0.11499,0.115,0.1132,0.11359,0.115
72,0.11468,0.11411,0.1149,0.11477,0.11488,0.1148,0.11643,0.11439,0.114
16,0.1155,0.11498,0.11485,0.11419,0.11502,0.11306,0.11317,0.11438,0.11
385,0.11396,0.11409,0.11419,0.1164,0.11411,0.11401,0.11329,0.11381,0.1
14,0.11416,0.11504,0.11538,0.11521,0.11532,0.11404,0.11322,0.11416,0.1
1462,0.1132,0.11362,0.11337,0.11514,0.11504,0.11487,0.11351,0.11391,0.
11411,0.11579,0.11305,0.11389,0.11454,0.11641,0.11521,0.11636,0.11499,
0.11495,0.11403,0.11368,0.11491,0.114,0.11429,0.11439,0.11423,0.11496,
0.1128,0.11524,0.11574,0.11618,0.11467,0.11479,0.1135,0.11425,0.11503,
0.11533,0.11399,0.11281,0.11399,0.11424,0.11323,0.11521,0.11344,0.1144
6,0.11398,0.11414,0.11385,0.11581,0.11532,0.11399,0.11386,0.11437,0.11
461,0.11331,0.11428,0.1157,0.11372,0.11378,0.11482,0.11364,0.11506,0.1
1465,0.11351,0.11539,0.11345,0.11372,0.11372,0.11244,0.11405,0.11397,0
.11492,0.11486,0.11444,0.11321,0.11589,0.11378,0.11335,0.11382,0.11375
,0.11376,0.11512,0.11509,0.11581,0.11409,0.11447
0.16202,0.1635,0.16535,0.16027,0.16214,0.16123,0.16178,0.16188,0.16331
,0.1592,0.16262,0.15901,0.16206,0.15909,0.16189,0.16051,0.16168,0.1660
7,0.16471,0.16056,0.49307,0.49497,0.49209,0.51432,0.16109,0.16225,0.15
62,0.16067,0.16425,0.15934,0.16351,0.16454,0.1604,0.16044,0.16325,0.16

312,0.16333,0.15897,0.16232,0.827,0.47895,0.48475,0.49467,0.46913,0.49
179,0.5792,0.16177,0.16149,0.15835,0.16116,0.16422,0.16323,0.15966,0.1
6098,0.16143,0.16263,0.16412,0.16276,0.16088,0.1662,0.15909,0.81281,0.
83757,0.55178,0.51841,0.49864,0.53717,0.47711,0.48578,0.48338,0.53145,
0.16123,0.1624,0.16131,0.15779,0.1628,0.16063,0.16184,0.16327,0.1607,0
.16221,0.16033,0.16078,0.16171,0.16083,0.15772,0.85188,0.82822,0.81743
,0.50486,0.49348,0.41782,0.51412,0.52596,0.5235,0.49521,0.49788,0.5304
,0.1627,0.16218,0.16199,0.16437,0.16133,0.16418,0.16129,0.16314,0.1609
2,0.16132,0.15891,0.15993,0.16469,0.16224,0.15998,0.16337,0.84528,0.79
497,0.82189,0.85225,0.44589,0.44812,0.50809,0.48935,0.50979,0.50977,0.
48434,0.50918,0.52903,0.45407,0.16183,0.16401,0.1591,0.16347,0.16529,0
.16177,0.16373,0.16015,0.1618,0.16151,0.16533,0.16394,0.16,0.15998,0.1
6308,0.16019,0.81419,0.83799,0.8111,0.8189,0.84504,0.84148,0.51604,0.5
5466,0.49974,0.47248,0.47674,0.48773,0.50208,0.50954,0.48266,0.49354,0
.15825,0.16311,0.16122,0.16153,0.16135,0.1625,0.15824,0.16198,0.15804,
0.16283,0.16491,0.16297,0.16756,0.16342,0.16307,0.16252,0.16016,0.8329
2,0.81545,0.82284,0.81681,0.82297,0.81677,0.5418,0.6678,0.47954,0.4267
5,0.4629,0.51251,0.46587,0.49889,0.4958,0.53014,0.56198,0.16349,0.1615
3,0.16142,0.1619,0.16413,0.16312,0.15881,0.16298,0.16483,0.15997,0.157
61,0.15888,0.163,0.13796,0.14121,0.13977,0.1632,0.79772,0.81583,0.8197
,0.80085,0.83061,0.83889,0.83371,0.49501,0.49614,0.49431,0.53805,0.508
37,0.48425,0.48692,0.48155,0.51842,0.45827,0.42932,0.16447,0.16032,0.1
6224,0.1601,0.1577,0.16454,0.16124,0.16308,0.16134,0.16112,0.16211,0.1
3467,0.14,0.13683,0.13321,0.135,0.13734,0.16297,0.81674,0.8599,0.82775
,0.80299,0.82797,0.83102,0.83066,0.49983,0.45429,0.52448,0.37506,0.485
85,0.49432,0.47981,0.55625,0.44893,0.49691,0.47801,0.16163,0.16139,0.1
585,0.16416,0.16102,0.16468,0.16218,0.16366,0.16277,0.13534,0.13581,0.
13732,0.13782,0.1383,0.13393,0.1378,0.13844,0.1379,0.16065,0.82085,0.8
1442,0.82895,0.83759,0.78945,0.82386,0.80996,0.4913,0.51491,0.50093,0.
45826,0.47381,0.4907,0.44837,0.57519,0.48283,0.49845,0.48969,0.50336,0
.1635,0.16391,0.15932,0.15982,0.16261,0.15993,0.13519,0.13493,0.13935,
0.14052,0.14231,0.13964,0.13617,0.14143,0.13836,0.14362,0.13675,0.1358
8,0.83539,0.84458,0.83012,0.86567,0.86616,0.85507,0.81993,0.50438,0.48
828,0.49408,0.48063,0.50717,0.53016,0.46146,0.49915,0.41949,0.51792,0.
45291,0.5416,0.16217,0.16227,0.1638,0.13875,0.13913,0.13786,0.13687,0.
1397,0.1351,0.14478,0.13872,0.14151,0.13611,0.13794,0.14072,0.13922,0.
13773,0.14452,0.81908,0.82017,0.84998,0.79964,0.82409,0.85273,0.81089,
0.82185,0.82097,0.53945,0.50411,0.49598,0.49763,0.49288,0.46948,0.4859
2,0.51159,0.50044,0.48075,0.4192,0.14181,0.13972,0.13843,0.13813,0.135
12,0.13982,0.13648,0.13552,0.13698,0.13941,0.13749,0.13564,0.14829,0.1
379,0.13928,0.13494,0.13877,0.13657,0.82115,0.8264,0.83731,0.83373,0.8
4206,0.81231,0.83952,0.82669,0.83059,0.48648,0.47327,0.28285,0.35135,0
.40093,0.32382,0.38144,0.38755,0.36538,0.38383,0.38219,0.1365,0.14188,
0.13131,0.13662,0.13761,0.13679,0.12946,0.13746,0.13875,0.13956,0.1375
9,0.13838,0.13551,0.13785,0.13763,0.13535,0.14473,0.13985,0.8363,0.820
14,0.83934,0.82394,0.82651,0.82942,0.79932,0.84858,0.81327,0.48557,0.3
9247,0.34665,0.41478,0.37419,0.36599,0.33348,0.38876,0.35363,0.38926,0
.38094,0.14013,0.13638,0.13783,0.13832,0.13876,0.1394,0.14304,0.13862,
0.13804,0.13435,0.13666,0.13783,0.13648,0.13812,0.13842,0.13795,0.1358
5,0.13693,0.82322,0.83249,0.82007,0.86884,0.84781,0.85728,0.83251,0.47
622,0.434,0.47213,0.42519,0.366,0.35428,0.36006,0.39002,0.37272,0.3775
4,0.3625,0.29354,0.38392,0.13597,0.13958,0.13958,0.13829,0.14022,0.138

35,0.12469,0.13883,0.1431,0.13706,0.13733,0.1335,0.13898,0.1346,0.1393
5,0.1383,0.13564,0.13518,0.82751,0.8198,0.85979,0.80789,0.81737,0.7961
1,0.45623,0.50852,0.5005,0.44591,0.46564,0.36958,0.38099,0.37307,0.364
47,0.36491,0.29918,0.37281,0.38242,0.36573,0.14433,0.13824,0.13764,0.1
3484,0.13387,0.14131,0.13807,0.13664,0.13878,0.13732,0.13794,0.13763,0
.13884,0.13885,0.13741,0.13792,0.1441,0.14095,0.81378,0.84416,0.80339,
0.83154,0.83026,0.39727,0.44267,0.44134,0.49733,0.47522,0.44491,0.3735
1,0.32265,0.39552,0.37052,0.32896,0.34907,0.37132,0.37775,0.36908,0.13
862,0.13828,0.13563,0.13645,0.13893,0.1391,0.14049,0.13376,0.1411,0.13
921,0.13657,0.13761,0.13719,0.14006,0.13784,0.13676,0.13977,0.13836,0.
82976,0.82045,0.82067,0.57881,0.46439,0.5886,0.49112,0.47391,0.48082,0
.43368,0.48095,0.39801,0.38205,0.37945,0.38137,0.37308,0.41737,0.38495
,0.37507,0.43028,0.13875,0.13715,0.13852,0.13958,0.13672,0.14292,0.138
43,0.1357,0.13955,0.13303,0.14225,0.13893,0.14017,0.13504,0.14256,0.13
98,0.1378,0.13744,0.83585,0.46399,0.43784,0.44267,0.49337,0.53112,0.46
852,0.42909,0.48941,0.52399,0.37052,0.3843,0.38486,0.36577,0.4265,0.32
868,0.31606,0.38876,0.36067,0.13731,0.13777,0.13338,0.14012,0.13956,0.
13683,0.13969,0.13809,0.1348,0.13868,0.1357,0.13974,0.13968,0.13843,0.
13721,0.1349,0.14088,0.11304,0.45647,0.48199,0.40016,0.46819,0.48694,0
.51154,0.42653,0.50188,0.48114,0.42833,0.37123,0.37178,0.34773,0.36959
,0.30753,0.36353,0.41743,0.36029,0.36992,0.13447,0.13821,0.13837,0.135
44,0.1371,0.13881,0.13708,0.13962,0.13721,0.13729,0.13888,0.13313,0.13
537,0.13635,0.13518,0.13607,0.13871,0.11358,0.45988,0.48339,0.44546,0.
30478,0.47605,0.47066,0.47979,0.50312,0.50718,0.47156,0.38086,0.38479,
0.34133,0.38346,0.33222,0.38235,0.34763,0.38712,0.13798,0.1357,0.13451
,0.13764,0.13734,0.13918,0.13651,0.14061,0.13563,0.13349,0.12893,0.136
92,0.13576,0.13819,0.13352,0.13365,0.13457,0.1387,0.1141,0.50893,0.467
67,0.46037,0.4505,0.45877,0.48198,0.46976,0.48624,0.39787,0.48271,0.39
996,0.36421,0.33925,0.34958,0.41484,0.37317,0.30528,0.42399,0.13832,0.
13674,0.13816,0.13683,0.14009,0.13976,0.1405,0.13595,0.13726,0.14068,0
.13147,0.13774,0.13135,0.13979,0.13976,0.13455,0.13951,0.11374,0.11457
,0.11313,0.45328,0.5337,0.44808,0.48686,0.41494,0.46124,0.38877,0.4087
1,0.49385,0.41487,0.32969,0.43718,0.41915,0.37598,0.35573,0.13976,0.13
827,0.1404,0.1361,0.13379,0.14232,0.13699,0.1396,0.13667,0.14123,0.135
09,0.1356,0.13822,0.14384,0.14132,0.13832,0.13841,0.13726,0.11304,0.11
198,0.11366,0.11469,0.47947,0.49918,0.46442,0.22714,0.49229,0.4859,0.3
6306,0.52548,0.46106,0.36034,0.37719,0.35781,0.14136,0.1329,0.13833,0.
13787,0.13726,0.14181,0.136,0.13621,0.13747,0.1358,0.14266,0.14118,0.1
3748,0.1389,0.13251,0.13808,0.13846,0.13897,0.1413,0.13829,0.11389,0.1
1291,0.11258,0.11293,0.1146,0.1135,0.47204,0.50215,0.42854,0.36885,0.4
9923,0.53259,0.47424,0.40386,0.14048,0.13182,0.13996,0.1385,0.1382,0.1
4463,0.13702,0.13472,0.13875,0.13932,0.13575,0.13867,0.13739,0.13537,0
.13987,0.13977,0.13457,0.13823,0.14766,0.13777,0.1392,0.13677,0.14079,
0.11399,0.11353,0.11273,0.11483,0.11295,0.11229,0.48312,0.5149,0.33738
,0.42982,0.49341,0.11444,0.11347,0.13841,0.13518,0.14385,0.13794,0.139
38,0.13941,0.13809,0.14142,0.14073,0.13716,0.13563,0.13882,0.13334,0.1
3341,0.13914,0.14408,0.14142,0.14173,0.13687,0.14016,0.13596,0.11539,0
.1127,0.11541,0.11434,0.11316,0.11521,0.11384,0.11442,0.11471,0.11424,
0.46877,0.48924,0.11414,0.11481,0.11387,0.11438,0.13558,0.13864,0.1416
1,0.14517,0.13733,0.13601,0.13993,0.13754,0.13328,0.1371,0.13068,0.141
,0.13913,0.13728,0.14541,0.13651,0.13943,0.13728,0.1381,0.11375,0.1142
8,0.11474,0.11434,0.11416,0.11351,0.11476,0.11344,0.11421,0.11676,0.11

319,0.11464,0.11402,0.1161,0.11332,0.11329,0.11252,0.14039,0.1366,0.13
789,0.14148,0.13426,0.13784,0.14768,0.1368,0.13842,0.13406,0.13956,0.1
3743,0.13605,0.1391,0.13659,0.13737,0.11352,0.11407,0.11338,0.11358,0.
11397,0.11369,0.11471,0.1154,0.11463,0.11407,0.11416,0.11274,0.116,0.1
1373,0.11411,0.11295,0.11325,0.11586,0.11424,0.13243,0.13751,0.13992,0
.13888,0.13786,0.13774,0.13999,0.13833,0.13861,0.13699,0.138,0.13588,0
.13943,0.11356,0.11363,0.11439,0.11303,0.11303,0.11395,0.11352,0.11382
,0.11462,0.11334,0.11322,0.11298,0.1135,0.11535,0.11275,0.11443,0.1151
2,0.11285,0.11418,0.1132,0.11351,0.14091,0.13753,0.13659,0.13798,0.135
43,0.14075,0.14296,0.14031,0.13888,0.11449,0.11381,0.11347,0.11522,0.1
1336,0.11371,0.11499,0.11344,0.11314,0.11384,0.11391,0.11457,0.11499,0
.11478,0.11447,0.1138,0.11387,0.11385,0.11454,0.1134,0.11573,0.11404,0
.11245,0.11509,0.11552,0.13458,0.1367,0.13209,0.1351,0.1138,0.1135,0.1
1573,0.11418,0.11439,0.11259,0.11451,0.11457,0.11307,0.1132,0.11382,0.
11283,0.11288,0.11391,0.11309,0.11264,0.11507,0.11518,0.11113,0.11294,
0.11359,0.11354,0.11416,0.1139,0.11259,0.11342,0.11331,0.11283,0.11455
,0.11378,0.11522,0.11418,0.11356,0.1133,0.11297,0.11259,0.11494,0.1149
7,0.11447,0.1148,0.11336,0.11448,0.11434,0.1141,0.11412,0.1135,0.11415
,0.11306,0.11425,0.11379,0.11421,0.11458,0.11389,0.11411,0.11386,0.114
19,0.11463,0.1137,0.11309,0.11272,0.11426,0.11212,0.11345,0.11347,0.11
409,0.11233,0.11432,0.11307,0.11267,0.11404,0.11457,0.11256,0.1135,0.1
1501,0.11449,0.11313,0.11485,0.11462,0.1146,0.11427,0.11588,0.11362,0.
11302,0.1152,0.11474,0.11422,0.11373,0.11479,0.11237,0.11297,0.11427,0
.11436,0.11309,0.11318,0.11394,0.1157,0.11382,0.11365,0.11301,0.11328,
0.11366,0.114,0.11466,0.11518,0.1146,0.11448,0.1137,0.11359,0.11409,0.
11383,0.11288,0.11275,0.11259,0.11518,0.11443,0.11404,0.11241,0.11348,
0.114,0.11533,0.11344,0.11388,0.11433,0.1158,0.11456,0.11569,0.11395,0
.11463,0.11352,0.1129,0.11435,0.11365,0.11349,0.11385,0.11417,0.1149,0
.11266,0.1143,0.11521,0.1154,0.11338,0.11495,0.11289,0.11356,0.11469,0
.11468,0.11426,0.11313,0.11391,0.11415,0.11234,0.11458,0.11317,0.11434
,0.11367,0.11378,0.11333,0.11562,0.11512,0.11363,0.11368,0.11417,0.114
01,0.11346,0.11368,0.11582,0.11385,0.1134,0.11407,0.11289,0.1152,0.114
67,0.11351,0.11417,0.11344,0.1129,0.11336,0.11186,0.11374,0.11353,0.11
42,0.11496,0.11447,0.11287,0.1152,0.11302,0.11322,0.11331,0.11297,0.11
381,0.11403,0.11476,0.11497,0.11326,0.11362
0.17478,0.17464,0.17651,0.17051,0.17395,0.1717,0.1735,0.17386,0.17534,
0.1711,0.17394,0.17002,0.17407,0.17152,0.17422,0.17321,0.17341,0.17694
,0.17513,0.17297,0.41698,0.41748,0.40869,0.42806,0.1733,0.17395,0.1690
2,0.17282,0.17628,0.17161,0.17566,0.17521,0.17274,0.17246,0.17478,0.17
451,0.17651,0.17065,0.17381,0.65722,0.40244,0.40187,0.41133,0.38848,0.
40244,0.48266,0.17304,0.17369,0.16934,0.17372,0.17563,0.17439,0.17113,
0.17303,0.17302,0.17425,0.17515,0.17383,0.17266,0.17724,0.17067,0.6422
1,0.66937,0.45296,0.42529,0.40529,0.43799,0.39833,0.40067,0.40542,0.44
535,0.17286,0.17426,0.17375,0.171,0.17423,0.17294,0.17407,0.17434,0.17
186,0.17293,0.17059,0.17252,0.17305,0.17231,0.17024,0.67501,0.647,0.62
885,0.41796,0.41462,0.35181,0.42748,0.43269,0.442,0.40945,0.41107,0.43
281,0.17317,0.1737,0.17442,0.17533,0.17313,0.17525,0.17241,0.1743,0.17
455,0.17181,0.16966,0.17158,0.17558,0.17327,0.17119,0.17364,0.65814,0.
624,0.65684,0.67446,0.37361,0.38167,0.42145,0.40656,0.42716,0.42427,0.
40249,0.42975,0.43949,0.39117,0.17308,0.17567,0.17144,0.17471,0.17733,
0.17283,0.17593,0.17203,0.1731,0.17375,0.17557,0.17639,0.17037,0.17266
,0.17504,0.1735,0.64607,0.65679,0.62679,0.63807,0.67704,0.66522,0.4276

7,0.45278,0.4187,0.39473,0.39287,0.40915,0.41304,0.42191,0.39498,0.412
19,0.17171,0.17479,0.17346,0.17244,0.17193,0.1743,0.16792,0.173,0.1710
7,0.1753,0.17624,0.17519,0.17927,0.17435,0.17423,0.17367,0.17274,0.662
87,0.63377,0.63489,0.64515,0.65287,0.63507,0.44684,0.53728,0.40201,0.3
6134,0.38534,0.42943,0.39068,0.42029,0.41803,0.4348,0.46434,0.17439,0.
1722,0.17558,0.17431,0.17563,0.17624,0.17171,0.17388,0.17644,0.17102,0
.16964,0.17159,0.1729,0.14079,0.14361,0.14447,0.1754,0.626,0.62216,0.6
3323,0.62682,0.64393,0.65171,0.6589,0.41335,0.41613,0.41059,0.44253,0.
42557,0.40118,0.40873,0.39914,0.43331,0.38635,0.36833,0.1755,0.17155,0
.1743,0.17235,0.16901,0.17396,0.17355,0.17459,0.17312,0.17474,0.17402,
0.13627,0.14361,0.13977,0.13366,0.13633,0.13987,0.17362,0.63061,0.6923
7,0.64872,0.61671,0.65192,0.65929,0.66595,0.41441,0.38355,0.43239,0.32
314,0.41028,0.4169,0.39944,0.44684,0.38788,0.4132,0.40145,0.17262,0.17
2,0.1695,0.17618,0.17212,0.17552,0.17513,0.17549,0.17336,0.13737,0.138
03,0.13957,0.14044,0.14047,0.13502,0.14094,0.1415,0.14012,0.17403,0.65
489,0.64112,0.6483,0.66482,0.61797,0.6601,0.64963,0.40617,0.42703,0.41
516,0.38839,0.39736,0.4088,0.37448,0.46994,0.39898,0.4157,0.40948,0.41
438,0.17615,0.17592,0.17053,0.1707,0.17379,0.16975,0.137,0.13863,0.142
46,0.14452,0.14655,0.14337,0.13906,0.14492,0.14129,0.14887,0.13959,0.1
3885,0.67401,0.65443,0.64907,0.68703,0.68637,0.66121,0.63971,0.42103,0
.40529,0.41215,0.4027,0.42201,0.44411,0.39162,0.41022,0.35853,0.43207,
0.38478,0.44414,0.17323,0.17399,0.17574,0.14175,0.14238,0.14065,0.1402
7,0.14302,0.13957,0.15072,0.14109,0.1467,0.13879,0.14009,0.1443,0.1413
,0.14004,0.14848,0.63414,0.64119,0.6759,0.63535,0.64719,0.67554,0.6594
7,0.66069,0.64566,0.44466,0.41575,0.41624,0.41889,0.41187,0.39412,0.40
363,0.42131,0.41949,0.39979,0.35858,0.1449,0.14432,0.14134,0.14186,0.1
3704,0.14334,0.13952,0.13822,0.14109,0.14311,0.14054,0.13749,0.15474,0
.14041,0.14277,0.13723,0.14191,0.1395,0.64788,0.63495,0.6643,0.67033,0
.6631,0.63388,0.66414,0.63577,0.65798,0.40477,0.39882,0.24839,0.29998,
0.34011,0.28227,0.32315,0.32441,0.30845,0.32657,0.31687,0.13871,0.1464
6,0.13317,0.13891,0.14001,0.14008,0.12946,0.13994,0.14125,0.14246,0.14
106,0.14163,0.13809,0.1409,0.14072,0.13725,0.15,0.14276,0.65876,0.6352
,0.65437,0.64277,0.64893,0.64434,0.62946,0.6586,0.63174,0.39585,0.3308
2,0.29298,0.35222,0.31273,0.31457,0.28629,0.32885,0.29973,0.32942,0.31
714,0.14511,0.139,0.14023,0.14072,0.14247,0.14384,0.14804,0.14145,0.14
045,0.13554,0.13893,0.14205,0.1385,0.14162,0.14108,0.14058,0.13857,0.1
411,0.65065,0.67291,0.65296,0.68266,0.67292,0.67598,0.65059,0.3823,0.3
4202,0.38316,0.34552,0.31027,0.30257,0.30358,0.32312,0.31281,0.3212,0.
31213,0.25383,0.32034,0.13784,0.14278,0.1424,0.1419,0.1442,0.14165,0.1
2341,0.14181,0.14765,0.13957,0.14094,0.13619,0.14149,0.13801,0.1434,0.
14072,0.1388,0.13646,0.65268,0.65031,0.67151,0.62854,0.65122,0.63121,0
.36271,0.41663,0.39416,0.36125,0.37068,0.30932,0.32137,0.31272,0.31147
,0.31281,0.25857,0.31786,0.32409,0.31386,0.14883,0.14126,0.14011,0.136
92,0.13607,0.14511,0.14166,0.1388,0.14181,0.14038,0.14132,0.14038,0.14
151,0.14249,0.14058,0.14213,0.14977,0.1453,0.62817,0.66131,0.63323,0.6
4877,0.66088,0.32369,0.35599,0.35435,0.40217,0.38011,0.3613,0.31323,0.
27977,0.33078,0.31142,0.28296,0.2907,0.31351,0.3171,0.31241,0.14166,0.
14073,0.1389,0.13827,0.14205,0.14208,0.14507,0.13523,0.14593,0.1421,0.
14005,0.14166,0.14015,0.14375,0.14094,0.13963,0.14465,0.14224,0.65449,
0.66072,0.64114,0.45541,0.37396,0.47126,0.40506,0.37625,0.39152,0.3482
6,0.37987,0.33203,0.32185,0.31634,0.32092,0.31077,0.3458,0.32887,0.312
6,0.36405,0.14237,0.13986,0.14221,0.14191,0.13953,0.14674,0.14203,0.13

921,0.14485,0.13464,0.14771,0.14156,0.14499,0.13713,0.14814,0.14376,0.
14106,0.14048,0.6568,0.37128,0.35261,0.35802,0.3998,0.4232,0.37631,0.3
4751,0.39455,0.42321,0.3164,0.32041,0.32216,0.30875,0.35772,0.2815,0.2
7436,0.32599,0.30284,0.14076,0.13952,0.13535,0.14445,0.14111,0.14028,0
.14285,0.14182,0.137,0.14214,0.13805,0.14261,0.14368,0.14159,0.14028,0
.13726,0.14498,0.10764,0.37293,0.39211,0.32638,0.37657,0.39476,0.41714
,0.35327,0.40446,0.38549,0.34422,0.31166,0.31414,0.29599,0.30655,0.266
21,0.30347,0.35627,0.30519,0.31602,0.13594,0.14073,0.14129,0.13769,0.1
4042,0.14242,0.13938,0.14304,0.14018,0.14091,0.14132,0.13373,0.13799,0
.13972,0.13726,0.1376,0.14129,0.10854,0.37248,0.3801,0.3585,0.25534,0.
38289,0.37947,0.38316,0.40753,0.41434,0.37656,0.32386,0.32264,0.28763,
0.32266,0.28302,0.32107,0.29727,0.3199,0.14126,0.13983,0.13754,0.14065
,0.14098,0.14161,0.13905,0.14502,0.13762,0.13466,0.12922,0.1389,0.1378
4,0.14178,0.13542,0.13564,0.13783,0.14208,0.10867,0.4068,0.37952,0.364
45,0.36375,0.37324,0.39142,0.36881,0.38829,0.32961,0.38672,0.34056,0.3
0829,0.28939,0.29226,0.34647,0.31639,0.26604,0.3471,0.14189,0.14041,0.
14151,0.13957,0.14391,0.143,0.14365,0.13866,0.14082,0.14571,0.13187,0.
14128,0.13163,0.14456,0.14232,0.13641,0.14187,0.10835,0.10835,0.10791,
0.36629,0.43328,0.36242,0.39661,0.33133,0.37075,0.31773,0.33292,0.3967
2,0.3437,0.28447,0.36119,0.34653,0.31633,0.30041,0.1446,0.14089,0.1434
7,0.13978,0.13409,0.1464,0.13937,0.14351,0.13978,0.14546,0.13812,0.138
83,0.14175,0.14827,0.14598,0.14122,0.14253,0.14,0.10796,0.10664,0.1077
4,0.10931,0.38708,0.40347,0.37915,0.19472,0.3917,0.39259,0.29167,0.428
35,0.37536,0.30391,0.31482,0.30544,0.14593,0.13485,0.14026,0.14052,0.1
407,0.14662,0.13886,0.13814,0.14035,0.13896,0.14748,0.14491,0.14045,0.
14136,0.13318,0.1421,0.1414,0.14216,0.1453,0.14211,0.10741,0.10649,0.1
0731,0.10708,0.10859,0.10852,0.37878,0.39596,0.35102,0.30409,0.39588,0
.42552,0.38011,0.32785,0.1437,0.13261,0.14357,0.14143,0.14045,0.15018,
0.14003,0.13743,0.1431,0.14211,0.13883,0.14148,0.14085,0.13717,0.14288
,0.14306,0.1368,0.14143,0.15354,0.14075,0.14232,0.13986,0.14535,0.1075
6,0.10817,0.1077,0.10894,0.10727,0.10683,0.39357,0.41141,0.27633,0.350
62,0.39169,0.10961,0.10785,0.1424,0.13676,0.14921,0.14066,0.14266,0.14
208,0.14091,0.14502,0.1434,0.14014,0.13891,0.14295,0.13433,0.13431,0.1
4255,0.14952,0.14585,0.14628,0.13998,0.14491,0.13754,0.10958,0.10718,0
.1092,0.10939,0.10792,0.10931,0.10761,0.1094,0.10895,0.10834,0.37968,0
.393,0.10806,0.1091,0.10824,0.10882,0.13822,0.14103,0.1447,0.15044,0.1
398,0.13847,0.1439,0.14228,0.13504,0.14068,0.13104,0.14505,0.14246,0.1
4018,0.15016,0.13949,0.14256,0.14003,0.14142,0.1078,0.10885,0.10881,0.
10799,0.10909,0.1083,0.10869,0.10723,0.10863,0.11071,0.10768,0.10882,0
.10769,0.11069,0.10778,0.10767,0.10655,0.14323,0.14009,0.14119,0.14554
,0.13612,0.14103,0.15333,0.13951,0.14146,0.13667,0.14259,0.14083,0.138
35,0.14299,0.1389,0.14058,0.10808,0.10815,0.10758,0.10774,0.10821,0.10
813,0.10882,0.10927,0.10916,0.10815,0.10845,0.10749,0.11008,0.10819,0.
10924,0.10784,0.10739,0.10925,0.10825,0.13292,0.14003,0.14237,0.1432,0
.14072,0.14072,0.14399,0.14085,0.14097,0.13887,0.14043,0.13823,0.14254
,0.108,0.10793,0.1089,0.10736,0.1078,0.10825,0.10755,0.10776,0.10944,0
.10836,0.10725,0.10773,0.10767,0.10938,0.10712,0.10881,0.10937,0.1081,
0.10853,0.10756,0.10785,0.14516,0.13921,0.1403,0.13998,0.13622,0.14526
,0.14761,0.14407,0.1411,0.10847,0.10821,0.10812,0.10927,0.10763,0.1084
2,0.10926,0.10719,0.1074,0.10713,0.10878,0.10902,0.10898,0.10891,0.109
04,0.10862,0.10825,0.10791,0.109,0.1072,0.10993,0.10826,0.10704,0.1095
,0.10978,0.13687,0.14059,0.13274,0.13662,0.1082,0.10768,0.11005,0.1085

9,0.10927,0.1072,0.10898,0.10935,0.1073,0.10806,0.1082,0.10733,0.1083,
0.10822,0.10755,0.10753,0.10962,0.1099,0.10589,0.10786,0.10769,0.10745
,0.1085,0.10819,0.10685,0.10751,0.10747,0.10771,0.10823,0.10788,0.1093
7,0.10885,0.10841,0.10761,0.10767,0.10747,0.10958,0.10941,0.10847,0.10
952,0.10754,0.10847,0.10854,0.10805,0.10849,0.10835,0.10845,0.10769,0.
10819,0.10941,0.10916,0.10828,0.10782,0.10874,0.10763,0.10818,0.10891,
0.10796,0.10737,0.10652,0.1094,0.10594,0.10745,0.10706,0.10804,0.10751
,0.10838,0.10748,0.10691,0.10813,0.10839,0.1073,0.10862,0.10883,0.1080
6,0.10813,0.10933,0.10962,0.10971,0.10853,0.11034,0.10852,0.10737,0.10
969,0.10882,0.10909,0.10783,0.10881,0.10701,0.10761,0.10855,0.10828,0.
10761,0.10756,0.10866,0.11002,0.10863,0.10777,0.10764,0.10764,0.10754,
0.109,0.10887,0.10881,0.10902,0.10769,0.1085,0.10813,0.10847,0.10807,0
.10752,0.10705,0.10738,0.10874,0.10881,0.10808,0.10626,0.10745,0.10804
,0.10919,0.10842,0.10777,0.10941,0.11079,0.10894,0.11011,0.10873,0.108
48,0.10771,0.10765,0.10858,0.10822,0.10779,0.10872,0.10783,0.10926,0.1
075,0.1084,0.10943,0.10943,0.10766,0.10926,0.10732,0.10707,0.10882,0.1
0851,0.10849,0.10773,0.10822,0.108,0.10751,0.10847,0.10822,0.10829,0.1
0805,0.10801,0.10815,0.10979,0.10917,0.10828,0.10792,0.10789,0.10859,0
.1074,0.10838,0.10957,0.10856,0.10825,0.10858,0.10749,0.10875,0.108,0.
10774,0.10865,0.10802,0.10717,0.10802,0.10618,0.10805,0.10767,0.10856,
0.10945,0.10886,0.10723,0.10892,0.10754,0.10755,0.10744,0.1076,0.10862
,0.1083,0.10886,0.10896,0.10706,0.10824
0.18157,0.18285,0.18534,0.17937,0.18115,0.17988,0.18297,0.17983,0.1816
2,0.18014,0.18086,0.17982,0.18148,0.17902,0.18227,0.18158,0.18222,0.18
384,0.18504,0.18042,0.36604,0.37109,0.35658,0.37823,0.18164,0.18185,0.
17799,0.18126,0.18428,0.17976,0.18305,0.18289,0.18052,0.1807,0.18099,0
.18189,0.1849,0.17945,0.18184,0.55343,0.35046,0.3536,0.36322,0.34408,0
.3546,0.42096,0.18129,0.18145,0.17791,0.18177,0.18213,0.18213,0.17935,
0.18128,0.17895,0.18161,0.18132,0.18159,0.18078,0.18547,0.17891,0.5376
,0.56021,0.39397,0.37719,0.35344,0.37898,0.34828,0.34941,0.35009,0.389
36,0.18073,0.18405,0.18072,0.18173,0.18212,0.18144,0.18208,0.18315,0.1
7998,0.17989,0.17918,0.17948,0.18079,0.17993,0.17799,0.56301,0.52333,0
.51715,0.37108,0.36081,0.3115,0.37252,0.37395,0.3864,0.35719,0.35951,0
.37562,0.18075,0.18252,0.18124,0.18209,0.18051,0.18342,0.17996,0.18302
,0.18165,0.1797,0.17773,0.17783,0.18363,0.18003,0.17944,0.18109,0.5485
8,0.51896,0.55223,0.57383,0.33036,0.34559,0.37276,0.3579,0.37302,0.369
27,0.35217,0.37648,0.39158,0.34775,0.18159,0.18292,0.17844,0.18121,0.1
8574,0.18058,0.18374,0.17956,0.18174,0.18287,0.18515,0.18417,0.17896,0
.1807,0.18197,0.18061,0.53371,0.53623,0.53025,0.52957,0.56326,0.57206,
0.37047,0.39201,0.36956,0.34908,0.33818,0.35834,0.36313,0.37032,0.3492
7,0.36033,0.17941,0.18299,0.18096,0.18121,0.1813,0.18261,0.17601,0.183
04,0.17973,0.18325,0.18359,0.18263,0.18656,0.18235,0.18125,0.18212,0.1
801,0.53208,0.52658,0.54408,0.52672,0.53946,0.52048,0.38837,0.45901,0.
35685,0.32718,0.33611,0.37693,0.34583,0.3686,0.36864,0.38122,0.40953,0
.18098,0.18063,0.18265,0.18275,0.1835,0.18343,0.17934,0.18234,0.18261,
0.17849,0.17839,0.17936,0.18029,0.14074,0.14443,0.14638,0.18211,0.5157
2,0.51221,0.52893,0.52676,0.54378,0.55489,0.5447,0.36402,0.37136,0.364
39,0.38645,0.37136,0.34901,0.36105,0.34859,0.37863,0.34588,0.32811,0.1
8373,0.18018,0.1813,0.1803,0.17642,0.18158,0.18204,0.181,0.17993,0.183
02,0.18068,0.13543,0.14425,0.13983,0.13149,0.13509,0.13953,0.18166,0.5
2585,0.58223,0.54531,0.52026,0.54047,0.54686,0.55518,0.36749,0.34572,0
.3759,0.29439,0.36152,0.3683,0.35001,0.38501,0.34124,0.36022,0.35124,0

.17958,0.17898,0.17775,0.1831,0.18017,0.1828,0.18297,0.18312,0.18113,0
.13622,0.13774,0.13829,0.13931,0.13929,0.13237,0.14147,0.14147,0.14043
,0.18228,0.54753,0.53278,0.52101,0.5423,0.50932,0.54855,0.53005,0.3566
6,0.36951,0.36746,0.34327,0.35497,0.36041,0.33137,0.41041,0.34929,0.36
125,0.35817,0.36692,0.18432,0.18316,0.17857,0.17868,0.18172,0.1785,0.1
3724,0.13859,0.14198,0.14549,0.14754,0.14333,0.14014,0.14563,0.14203,0
.15166,0.13893,0.14016,0.56187,0.54841,0.53101,0.55575,0.57098,0.54949
,0.5274,0.36692,0.35466,0.35981,0.35092,0.36628,0.38328,0.33919,0.3563
3,0.32264,0.38099,0.33967,0.38738,0.18106,0.18201,0.18226,0.1416,0.142
22,0.14166,0.13994,0.14379,0.14051,0.15295,0.14168,0.14852,0.13753,0.1
3972,0.14517,0.1419,0.14051,0.14981,0.5297,0.52343,0.5713,0.52224,0.54
628,0.55529,0.56063,0.54892,0.53825,0.38231,0.36296,0.36517,0.37119,0.
36167,0.3483,0.35003,0.36575,0.36667,0.35007,0.32392,0.1445,0.1452,0.1
4118,0.14287,0.1358,0.14375,0.14026,0.13745,0.14131,0.14364,0.14033,0.
13725,0.15664,0.14046,0.14383,0.13683,0.14216,0.13935,0.54246,0.53943,
0.55973,0.56224,0.55982,0.53643,0.55613,0.53683,0.55163,0.35737,0.3580
4,0.22482,0.26493,0.30323,0.25623,0.28164,0.2821,0.27276,0.28735,0.274
37,0.13796,0.14782,0.13164,0.13948,0.14012,0.13994,0.12728,0.13985,0.1
4113,0.14318,0.14169,0.142,0.13799,0.14062,0.141,0.13741,0.15122,0.143
61,0.55031,0.5348,0.53999,0.54939,0.55968,0.52547,0.5269,0.5459,0.5189
9,0.33195,0.2895,0.2611,0.31071,0.27189,0.2769,0.25265,0.28857,0.26477
,0.28876,0.27748,0.14567,0.13859,0.14086,0.13998,0.14288,0.1446,0.1498
9,0.14137,0.14061,0.13407,0.13893,0.14249,0.13779,0.14205,0.14151,0.14
099,0.13803,0.14196,0.53458,0.56339,0.54262,0.58959,0.56758,0.56841,0.
54369,0.32465,0.29086,0.324,0.30131,0.27497,0.27184,0.26894,0.28223,0.
27485,0.28279,0.27533,0.23016,0.27584,0.13676,0.14257,0.14279,0.14283,
0.14513,0.14136,0.119,0.14153,0.14917,0.14041,0.14139,0.1351,0.14125,0
.13827,0.14425,0.14092,0.13907,0.13664,0.54986,0.55103,0.56436,0.51933
,0.53209,0.53036,0.30066,0.35581,0.33342,0.30494,0.31131,0.27687,0.285
07,0.27675,0.27385,0.27971,0.23411,0.28361,0.28248,0.27766,0.15111,0.1
408,0.13974,0.13601,0.13533,0.14539,0.14353,0.13715,0.14096,0.14032,0.
14107,0.14007,0.14105,0.14267,0.14072,0.143,0.15234,0.14678,0.52203,0.
55509,0.52831,0.54864,0.56412,0.27774,0.29922,0.29933,0.33619,0.32812,
0.30648,0.28025,0.25489,0.28955,0.2753,0.253,0.25798,0.27538,0.27875,0
.27506,0.1425,0.14128,0.13833,0.13724,0.14167,0.1419,0.14674,0.13398,0
.14763,0.14282,0.13959,0.141,0.13923,0.14414,0.14156,0.14019,0.14395,0
.14258,0.54298,0.54382,0.51678,0.37954,0.3227,0.39041,0.35099,0.31812,
0.32903,0.29738,0.31979,0.28929,0.28525,0.27421,0.28565,0.27784,0.3041
7,0.29434,0.27837,0.31776,0.14299,0.14012,0.14291,0.14163,0.14015,0.14
851,0.14227,0.13872,0.14468,0.13349,0.14909,0.14182,0.14489,0.13531,0.
14924,0.14491,0.14078,0.13977,0.55487,0.31007,0.29844,0.30956,0.34478,
0.35817,0.31136,0.28591,0.33687,0.35926,0.27776,0.28118,0.28837,0.2737
6,0.30987,0.25229,0.24761,0.28176,0.27101,0.14026,0.13989,0.13491,0.14
437,0.14108,0.14001,0.14303,0.14121,0.13601,0.1415,0.13779,0.14197,0.1
4416,0.14249,0.14083,0.13628,0.14608,0.10069,0.32156,0.32794,0.27903,0
.32576,0.33592,0.36166,0.30249,0.34267,0.3291,0.29095,0.27331,0.27817,
0.26215,0.27046,0.24047,0.26775,0.3104,0.27161,0.28121,0.13515,0.14077
,0.14138,0.13669,0.14116,0.14231,0.13874,0.14345,0.14078,0.14117,0.141
77,0.13194,0.13853,0.14032,0.13633,0.13619,0.14112,0.1014,0.32713,0.31
827,0.30147,0.22201,0.32743,0.3208,0.32258,0.34714,0.34383,0.32778,0.2
8766,0.28647,0.25379,0.28162,0.25474,0.28208,0.26958,0.27849,0.14084,0
.14083,0.13716,0.13996,0.14088,0.14195,0.13904,0.14667,0.13714,0.13329

,0.12654,0.13922,0.1369,0.14123,0.13457,0.13356,0.13829,0.14291,0.1009
3,0.34248,0.32213,0.30362,0.30893,0.32321,0.33548,0.30882,0.32713,0.27
594,0.33075,0.2997,0.2713,0.25873,0.25737,0.30466,0.28223,0.2389,0.306
35,0.1428,0.1411,0.14211,0.13981,0.14479,0.14415,0.14548,0.138,0.14137
,0.14704,0.12889,0.14208,0.1303,0.14509,0.14293,0.13501,0.14148,0.1008
1,0.10096,0.10024,0.30551,0.36377,0.30316,0.33266,0.2796,0.30958,0.268
7,0.28471,0.33365,0.30585,0.253,0.31497,0.30422,0.28241,0.26443,0.1461
6,0.14137,0.14264,0.13951,0.13266,0.14646,0.13999,0.14407,0.13924,0.14
538,0.13875,0.13838,0.14165,0.14902,0.14704,0.1414,0.14444,0.14011,0.0
99859,0.09976,0.10006,0.10175,0.33466,0.34499,0.31859,0.17056,0.33465,
0.33009,0.25157,0.36504,0.30652,0.26684,0.27484,0.27301,0.14744,0.1337
8,0.13911,0.14066,0.14049,0.14804,0.13896,0.13787,0.14104,0.13956,0.15
005,0.14602,0.1404,0.14168,0.13246,0.14242,0.14149,0.14294,0.14638,0.1
4233,0.098977,0.098783,0.099696,0.10028,0.1008,0.10062,0.32718,0.33625
,0.29304,0.26045,0.32947,0.37247,0.31874,0.27987,0.14488,0.12997,0.144
62,0.14255,0.14008,0.15209,0.14056,0.1359,0.14314,0.1425,0.13867,0.141
5,0.14044,0.13622,0.14155,0.14326,0.13653,0.14211,0.1574,0.14148,0.142
89,0.13996,0.14533,0.10016,0.10043,0.1001,0.10102,0.099236,0.099203,0.
33646,0.3463,0.23415,0.30057,0.33743,0.10183,0.10076,0.14235,0.13642,0
.15126,0.14037,0.14318,0.14238,0.14115,0.14534,0.14322,0.1404,0.13872,
0.14346,0.13241,0.13322,0.14203,0.15172,0.1478,0.14725,0.14035,0.14526
,0.13757,0.10144,0.099427,0.10155,0.10154,0.10058,0.10188,0.099518,0.1
0099,0.10193,0.10095,0.31887,0.33394,0.10011,0.10104,0.10047,0.10079,0
.1381,0.14123,0.14548,0.15308,0.13911,0.13835,0.14519,0.1432,0.13394,0
.14065,0.12898,0.14621,0.14285,0.1407,0.15264,0.13878,0.14273,0.13949,
0.14204,0.10062,0.10189,0.10094,0.10014,0.10117,0.10056,0.10103,0.0995
57,0.10104,0.10317,0.10002,0.10091,0.10006,0.10258,0.099791,0.10025,0.
098355,0.14463,0.13995,0.14153,0.14713,0.13527,0.14046,0.15586,0.13974
,0.14201,0.13668,0.14314,0.14151,0.13771,0.14344,0.13923,0.14077,0.100
23,0.10106,0.1004,0.099396,0.10029,0.10072,0.1019,0.10226,0.10232,0.10
065,0.1009,0.099684,0.10226,0.10071,0.10103,0.099965,0.099992,0.10178,
0.10037,0.13086,0.14079,0.14233,0.14324,0.14067,0.14075,0.14493,0.1407
6,0.141,0.13827,0.13974,0.13764,0.14263,0.099307,0.10001,0.10136,0.100
07,0.10126,0.10045,0.10026,0.099602,0.10185,0.10052,0.098928,0.099672,
0.099998,0.10186,0.099255,0.1013,0.10157,0.10036,0.10063,0.099867,0.10
013,0.14534,0.13798,0.14006,0.14009,0.13544,0.14671,0.14982,0.14578,0.
14144,0.10068,0.10095,0.10001,0.10127,0.099622,0.10065,0.10145,0.10034
,0.10009,0.099773,0.10086,0.10103,0.10159,0.10147,0.10181,0.10143,0.10
068,0.10072,0.10145,0.099258,0.10228,0.10137,0.098879,0.10123,0.10174,
0.13644,0.14046,0.13033,0.13605,0.10052,0.10022,0.103,0.10084,0.10185,
0.10047,0.10073,0.10094,0.10013,0.10044,0.10037,0.099345,0.099999,0.10
019,0.099927,0.099776,0.1022,0.10255,0.098167,0.099843,0.099682,0.0998
26,0.1005,0.10008,0.09943,0.10027,0.099947,0.1004,0.10051,0.10037,0.10
143,0.10108,0.101,0.10045,0.099778,0.099934,0.10151,0.10119,0.10138,0.
10143,0.10014,0.10056,0.10074,0.10044,0.10156,0.10109,0.10036,0.099634
,0.10103,0.10152,0.10121,0.10019,0.10008,0.1005,0.099734,0.10077,0.100
85,0.10041,0.099967,0.099236,0.10087,0.098684,0.10043,0.099269,0.10017
,0.10031,0.10058,0.099761,0.098848,0.10092,0.10072,0.098826,0.10039,0.
10127,0.099693,0.1007,0.10175,0.10221,0.10133,0.10038,0.10237,0.1004,0
.10032,0.10187,0.10095,0.10107,0.10069,0.10135,0.09943,0.09956,0.10097
,0.10034,0.099446,0.10012,0.10144,0.10212,0.10051,0.10007,0.10008,0.09
9782,0.10023,0.10125,0.10076,0.10068,0.10118,0.10033,0.10102,0.10057,0

.10046,0.10112,0.10014,0.099339,0.10036,0.10097,0.10137,0.09982,0.0984
62,0.10047,0.10042,0.10141,0.10074,0.10015,0.1013,0.10325,0.10134,0.10
21,0.10129,0.10067,0.1005,0.1001,0.099985,0.10061,0.099486,0.10118,0.1
0075,0.10089,0.099295,0.099994,0.10213,0.1012,0.10023,0.10172,0.099594
,0.099419,0.10117,0.10083,0.1006,0.099894,0.10105,0.10022,0.10079,0.10
079,0.10091,0.10086,0.10028,0.10069,0.10013,0.10127,0.10132,0.10073,0.
099992,0.10025,0.10096,0.099793,0.10115,0.10124,0.10096,0.10107,0.1004
4,0.10057,0.10195,0.10014,0.10014,0.10168,0.10014,0.099545,0.10042,0.0
98753,0.10097,0.099817,0.10064,0.10152,0.10112,0.099819,0.10138,0.1001
9,0.099849,0.10055,0.10061,0.10113,0.10116,0.10149,0.10139,0.10018,0.1
0058
0.18648,0.18634,0.19143,0.18405,0.1872,0.18575,0.18855,0.18561,0.18743
,0.18598,0.18702,0.18682,0.18844,0.18505,0.18785,0.18672,0.1879,0.1876
5,0.19033,0.18599,0.3341,0.33684,0.32056,0.34101,0.18642,0.18817,0.184
44,0.18598,0.18873,0.1856,0.18778,0.18854,0.18601,0.18578,0.18588,0.18
831,0.19061,0.18382,0.18683,0.48153,0.31769,0.32435,0.32516,0.31879,0.
3234,0.38009,0.18748,0.18687,0.18352,0.18829,0.18856,0.18848,0.18606,0
.18577,0.18502,0.18667,0.18704,0.18696,0.186,0.19057,0.18441,0.46586,0
.49271,0.35567,0.34586,0.32588,0.34079,0.31247,0.32034,0.32307,0.34935
,0.18513,0.18981,0.18722,0.18734,0.18839,0.18762,0.18691,0.18931,0.185
83,0.18387,0.18517,0.18718,0.18466,0.18516,0.18288,0.50104,0.45414,0.4
7015,0.33815,0.32656,0.288,0.33733,0.34294,0.34705,0.32915,0.32461,0.3
4107,0.18608,0.18818,0.18803,0.18734,0.18551,0.18834,0.18565,0.18808,0
.18742,0.18534,0.1842,0.18258,0.18782,0.18558,0.18374,0.18701,0.48129,
0.45177,0.46817,0.50261,0.30114,0.31836,0.34605,0.323,0.3481,0.33695,0
.31721,0.33662,0.36167,0.31683,0.187,0.18745,0.18501,0.18672,0.19084,0
.18572,0.18927,0.18682,0.18733,0.18875,0.18964,0.19049,0.18494,0.18552
,0.18783,0.18838,0.46728,0.46382,0.46019,0.44477,0.498,0.52513,0.33632
,0.34982,0.34586,0.31336,0.31223,0.33319,0.33023,0.34002,0.31233,0.335
29,0.18794,0.18835,0.18725,0.18765,0.18694,0.18811,0.18202,0.18807,0.1
852,0.18861,0.18855,0.18902,0.1926,0.1882,0.18575,0.18813,0.1855,0.467
97,0.45147,0.47879,0.45653,0.47243,0.45018,0.35323,0.41185,0.3319,0.31
021,0.30656,0.33988,0.3138,0.33761,0.33723,0.34926,0.37727,0.18563,0.1
8583,0.18825,0.18816,0.18964,0.18799,0.18307,0.18822,0.18919,0.18568,0
.18416,0.18401,0.18648,0.14009,0.143,0.14511,0.18695,0.46064,0.44689,0
.46431,0.47276,0.47806,0.49589,0.46295,0.33605,0.3379,0.33797,0.35744,
0.33791,0.31976,0.33528,0.32328,0.34521,0.3175,0.30144,0.19055,0.18527
,0.18689,0.18484,0.18262,0.18636,0.18824,0.18553,0.18688,0.18824,0.184
86,0.13415,0.14335,0.13799,0.12873,0.13314,0.13801,0.18714,0.45838,0.5
1271,0.48301,0.46431,0.46016,0.47468,0.49498,0.33425,0.32399,0.34377,0
.2796,0.33493,0.33755,0.32553,0.35464,0.31448,0.32909,0.32049,0.18509,
0.18351,0.18352,0.1891,0.18647,0.18662,0.18922,0.1889,0.18615,0.13469,
0.13605,0.13646,0.13825,0.1376,0.12935,0.14048,0.14102,0.13864,0.18811
,0.48359,0.47167,0.47522,0.48915,0.45348,0.48859,0.45408,0.32978,0.334
86,0.33191,0.31651,0.3281,0.32962,0.30662,0.36936,0.32141,0.32853,0.32
605,0.33889,0.18892,0.18768,0.18425,0.18455,0.18784,0.1857,0.13535,0.1
3752,0.14128,0.14514,0.14748,0.14222,0.13911,0.14473,0.14107,0.15311,0
.13852,0.13915,0.49793,0.4923,0.46863,0.4863,0.49952,0.49744,0.45638,0
.33784,0.32365,0.33509,0.32478,0.3383,0.34628,0.31483,0.32792,0.30207,
0.3434,0.31263,0.35759,0.18548,0.18749,0.18762,0.14045,0.14142,0.14056
,0.13872,0.14285,0.13956,0.1535,0.14109,0.14942,0.13577,0.13805,0.1437
8,0.14068,0.13873,0.1506,0.45664,0.45432,0.50188,0.45948,0.47712,0.496

17,0.48751,0.49525,0.47788,0.34737,0.32879,0.33409,0.32997,0.33173,0.32051,0.3198,0.33003,0.33396,0.31907,0.2967,0.14294,0.1449,0.13962,0.14236,0.13313,0.14267,0.1394,0.13579,0.14114,0.14373,0.13892,0.13476,0.15648,0.13853,0.14415,0.13488,0.14015,0.13833,0.48281,0.49421,0.48709,0.50044,0.49,0.48009,0.4836,0.46385,0.49171,0.32428,0.32552,0.21148,0.24761,0.27628,0.23799,0.25995,0.25965,0.24933,0.26304,0.24826,0.13503,0.14697,0.12915,0.1377,0.13838,0.13912,0.12403,0.13828,0.14022,0.14207,0.14089,0.14135,0.13588,0.13972,0.13967,0.13508,0.1521,0.14302,0.48641,0.47383,0.47194,0.49144,0.48122,0.46354,0.45621,0.48604,0.43424,0.29693,0.26206,0.2398,0.28142,0.2502,0.25119,0.23103,0.26113,0.24251,0.26169,0.25297,0.14607,0.13668,0.13985,0.13787,0.14161,0.14459,0.1496,0.14113,0.13823,0.13157,0.13775,0.14236,0.13581,0.14021,0.14056,0.13894,0.13643,0.141,0.47447,0.49758,0.47714,0.52142,0.49516,0.48677,0.47183,0.28566,0.25656,0.2917,0.26499,0.24944,0.24989,0.24626,0.25293,0.25308,0.25969,0.24788,0.21333,0.24622,0.13415,0.14181,0.14007,0.14159,0.14462,0.14008,0.11412,0.14004,0.14895,0.13949,0.14005,0.13354,0.13995,0.13669,0.14367,0.13949,0.13731,0.13567,0.48939,0.48772,0.49128,0.46722,0.45633,0.46288,0.26542,0.30924,0.29618,0.27096,0.27472,0.25306,0.25761,0.25127,0.25096,0.2611,0.21892,0.26392,0.25605,0.25769,0.15099,0.13919,0.13877,0.13426,0.13359,0.14434,0.14298,0.13544,0.13949,0.13826,0.14038,0.13935,0.13965,0.14105,0.13842,0.1416,0.15339,0.14728,0.4473,0.48739,0.47371,0.48225,0.48163,0.24759,0.26819,0.27042,0.30102,0.28807,0.27645,0.25575,0.23721,0.26469,0.25019,0.23511,0.24035,0.25184,0.25645,0.25252,0.14117,0.14022,0.13614,0.1351,0.14036,0.14018,0.1466,0.13111,0.14759,0.14163,0.13841,0.14033,0.13721,0.1417,0.14027,0.13852,0.14245,0.1424,0.46629,0.4806,0.46072,0.33132,0.28447,0.34382,0.3096,0.28536,0.2951,0.262,0.28088,0.26338,0.26078,0.25438,0.25794,0.24934,0.27691,0.27143,0.25622,0.29447,0.14338,0.13884,0.14314,0.1401,0.13892,0.14687,0.14156,0.13811,0.14491,0.1306,0.14909,0.14088,0.14327,0.13275,0.14966,0.14423,0.13952,0.13901,0.48878,0.2762,0.26704,0.27181,0.30143,0.31691,0.27595,0.25015,0.30094,0.31876,0.25304,0.25811,0.25868,0.24752,0.28215,0.23241,0.22444,0.25668,0.24643,0.13942,0.13819,0.13356,0.14302,0.13969,0.13852,0.1421,0.13973,0.13358,0.1413,0.1362,0.14001,0.14239,0.14193,0.13945,0.13425,0.14539,0.092504,0.28817,0.28628,0.24911,0.28807,0.29814,0.3136,0.26328,0.30469,0.28373,0.25958,0.24966,0.25423,0.24488,0.24781,0.22401,0.24632,0.2797,0.24658,0.25701,0.13346,0.13959,0.1403,0.13445,0.13946,0.14071,0.13721,0.14229,0.13874,0.13947,0.14038,0.12941,0.13721,0.14009,0.13477,0.13461,0.14013,0.093223,0.2906,0.28971,0.26697,0.19699,0.28961,0.2799,0.27515,0.30811,0.30156,0.29126,0.26605,0.25901,0.23451,0.25526,0.23707,0.26451,0.24645,0.25518,0.13934,0.13953,0.13587,0.13822,0.14037,0.14111,0.13675,0.14649,0.13521,0.13035,0.12232,0.13842,0.13478,0.14066,0.13264,0.13112,0.1364,0.1425,0.092529,0.30212,0.28565,0.26512,0.26756,0.28889,0.2985,0.27475,0.29051,0.24454,0.28677,0.27262,0.24695,0.23352,0.23525,0.27741,0.25676,0.22098,0.28235,0.14221,0.14078,0.14117,0.13816,0.14356,0.14298,0.14484,0.13689,0.13992,0.14639,0.12543,0.14135,0.12712,0.14415,0.1415,0.13249,0.14096,0.091838,0.09397,0.092447,0.26558,0.31979,0.26875,0.29415,0.25051,0.27476,0.24001,0.25074,0.28548,0.27988,0.23455,0.2797,0.2762,0.25738,0.2427,0.14603,0.13975,0.14161,0.1392,0.12997,0.14532,0.13858,0.14341,0.13727,0.14451,0.13754,0.13768,0.13974,0.14896,0.14657,0.13938,0.14379,0.13918,0.091844,0.091407,0.091889,0.093286,0.2929,0.29939,0.28448,0.15417,0.29572,0.29582,0.22159,0.31736,0.26513,0.24597,0.24965,0.24839,0.14638,0.13

181,0.13684,0.13928,0.13887,0.14837,0.13769,0.13611,0.14022,0.13805,0.
15101,0.14438,0.13876,0.14074,0.13015,0.14141,0.14048,0.14247,0.1462,0
.14147,0.091185,0.090915,0.092041,0.091666,0.092796,0.091996,0.29262,0
.28971,0.25727,0.23271,0.29194,0.32647,0.28053,0.24455,0.14469,0.12704
,0.14449,0.14237,0.13891,0.15308,0.13885,0.13413,0.14159,0.14077,0.137
42,0.1397,0.13944,0.13391,0.14003,0.1423,0.13531,0.14004,0.15898,0.140
53,0.14206,0.13951,0.14438,0.092561,0.092751,0.0915,0.092595,0.091224,
0.091401,0.29958,0.30312,0.20616,0.27033,0.29673,0.09388,0.092958,0.14
049,0.13381,0.15197,0.13883,0.14183,0.14121,0.13996,0.14447,0.14178,0.
13993,0.13752,0.14217,0.1295,0.1316,0.14045,0.1528,0.14708,0.14693,0.1
393,0.14504,0.13519,0.093457,0.091125,0.093342,0.093613,0.092255,0.093
443,0.092089,0.092812,0.09392,0.093211,0.27696,0.30119,0.092449,0.0924
14,0.092603,0.092579,0.13689,0.14031,0.14461,0.15308,0.13755,0.13566,0
.14482,0.14136,0.13176,0.13922,0.12581,0.14561,0.14251,0.14051,0.1527,
0.13802,0.14089,0.13826,0.14087,0.09226,0.093854,0.093202,0.092983,0.0
93482,0.092823,0.093516,0.092048,0.092676,0.094873,0.092783,0.092752,0
.092491,0.094095,0.092469,0.092431,0.090768,0.14306,0.13895,0.14013,0.
14635,0.1334,0.13984,0.15647,0.13884,0.141,0.13421,0.14224,0.14025,0.1
3592,0.14307,0.13783,0.13923,0.092891,0.093188,0.092197,0.091011,0.091
602,0.092507,0.093923,0.09462,0.094094,0.092248,0.09319,0.091624,0.094
425,0.092994,0.092942,0.091463,0.091688,0.093781,0.093219,0.12768,0.13
933,0.14068,0.14217,0.13943,0.13907,0.14315,0.13922,0.13962,0.13573,0.
13941,0.13628,0.14148,0.091007,0.091944,0.093851,0.091345,0.093012,0.0
92658,0.093399,0.092241,0.093897,0.092308,0.091707,0.092083,0.092384,0
.093269,0.090968,0.092878,0.093584,0.092625,0.092822,0.092273,0.091934
,0.14501,0.13596,0.1389,0.13846,0.13252,0.14635,0.15051,0.14518,0.1396
1,0.09285,0.092662,0.092408,0.092927,0.092057,0.093525,0.093435,0.0922
73,0.092248,0.091753,0.092648,0.092688,0.093346,0.093008,0.093421,0.09
3251,0.092708,0.092417,0.093426,0.091961,0.094546,0.093878,0.091576,0.
093026,0.093482,0.13481,0.1395,0.12752,0.13376,0.092918,0.092201,0.095
27,0.092699,0.094132,0.092854,0.092464,0.093601,0.092397,0.092741,0.09
2466,0.091499,0.091939,0.092244,0.092198,0.091403,0.093223,0.093971,0.
090135,0.091891,0.092315,0.092016,0.09284,0.092675,0.091526,0.092388,0
.093072,0.092833,0.092356,0.092699,0.09347,0.092891,0.092635,0.092998,
0.0924,0.091723,0.093985,0.093203,0.092813,0.094037,0.092263,0.092545,
0.092591,0.092251,0.093805,0.09333,0.092626,0.091124,0.092775,0.093458
,0.093313,0.092419,0.092228,0.092772,0.092575,0.092787,0.093484,0.0921
75,0.091817,0.092217,0.092703,0.09121,0.092166,0.091304,0.09309,0.0924
5,0.093025,0.092703,0.090319,0.093174,0.093517,0.091046,0.092068,0.093
053,0.092479,0.093073,0.094355,0.094552,0.093432,0.093097,0.093657,0.0
92483,0.091687,0.093872,0.09362,0.09361,0.092097,0.092804,0.091624,0.0
91314,0.09332,0.092559,0.091847,0.092693,0.093225,0.094159,0.092542,0.
092535,0.091603,0.09191,0.092973,0.093647,0.093325,0.0929,0.093194,0.0
92792,0.093429,0.092968,0.092879,0.094032,0.091716,0.091311,0.093719,0
.092482,0.093656,0.091953,0.09129,0.092676,0.092389,0.093182,0.092761,
0.091653,0.093308,0.094602,0.093095,0.093971,0.093265,0.092739,0.09204
7,0.092419,0.092768,0.092418,0.092032,0.093162,0.092518,0.092417,0.090
848,0.091775,0.094357,0.093116,0.091932,0.093965,0.091807,0.091318,0.0
93832,0.092746,0.092135,0.09197,0.093014,0.09262,0.092804,0.092813,0.0
92343,0.093343,0.092561,0.092241,0.092339,0.093656,0.093208,0.091907,0
.092563,0.09252,0.093116,0.091965,0.092355,0.09271,0.092756,0.092659,0
.092789,0.093024,0.093349,0.091916,0.092351,0.093344,0.092705,0.091347

,0.093072,0.090711,0.092376,0.092487,0.092541,0.093464,0.093037,0.0923
77,0.09315,0.091288,0.092182,0.093057,0.092087,0.092382,0.093384,0.092
946,0.093314,0.092033,0.092663
0.19042,0.19175,0.19455,0.18782,0.19179,0.18983,0.19231,0.18847,0.1916
4,0.19049,0.19073,0.19043,0.19235,0.18958,0.19105,0.19119,0.19113,0.19
081,0.19336,0.19013,0.32104,0.32416,0.30171,0.31958,0.18977,0.19215,0.
18802,0.18883,0.19205,0.18963,0.19151,0.1925,0.19,0.1893,0.1909,0.1925
8,0.19434,0.18837,0.19056,0.44949,0.30446,0.30632,0.3112,0.30387,0.305
89,0.35082,0.19118,0.19055,0.18771,0.19225,0.19144,0.19196,0.18865,0.1
8958,0.18894,0.19053,0.1915,0.1911,0.19051,0.19462,0.18903,0.42989,0.4
5845,0.33124,0.33221,0.30894,0.32077,0.29622,0.30432,0.30774,0.32268,0
.19045,0.19361,0.19137,0.19095,0.19314,0.19131,0.19188,0.19294,0.19071
,0.18753,0.18885,0.19233,0.18911,0.19135,0.18848,0.45574,0.41337,0.426
81,0.33096,0.3076,0.274,0.31614,0.31981,0.32126,0.31367,0.30871,0.3215
1,0.18991,0.19373,0.19237,0.1914,0.19121,0.19158,0.18862,0.19192,0.190
85,0.18782,0.18921,0.18777,0.19195,0.19017,0.18848,0.19121,0.44493,0.4
1002,0.43043,0.45776,0.28807,0.30323,0.3258,0.30756,0.32356,0.31822,0.
3015,0.3181,0.33818,0.29924,0.19141,0.19065,0.1881,0.1913,0.19248,0.18
939,0.19362,0.18986,0.19064,0.19378,0.19445,0.19349,0.18897,0.18926,0.
19221,0.19232,0.42975,0.42091,0.428,0.41149,0.46587,0.47003,0.32104,0.
32658,0.32991,0.29292,0.29505,0.31382,0.31006,0.31947,0.29538,0.3198,0
.19094,0.19266,0.19111,0.19217,0.19153,0.19108,0.18718,0.19155,0.19,0.
19235,0.19189,0.19263,0.19566,0.19119,0.1902,0.19185,0.1905,0.43445,0.
42575,0.44332,0.42986,0.43118,0.40919,0.33786,0.37894,0.31144,0.30133,
0.29188,0.32695,0.29391,0.32207,0.31898,0.33355,0.35973,0.18985,0.1916
3,0.19148,0.19279,0.19279,0.19198,0.18767,0.19258,0.19303,0.19038,0.18
803,0.18752,0.19062,0.1381,0.14177,0.14376,0.19075,0.42884,0.40753,0.4
2557,0.44137,0.44548,0.46268,0.41891,0.32338,0.31391,0.32119,0.3338,0.
32061,0.30708,0.31893,0.30275,0.32172,0.29884,0.28682,0.19408,0.18962,
0.19145,0.18943,0.1877,0.18963,0.19099,0.18867,0.19062,0.1927,0.18984,
0.1307,0.14256,0.13556,0.12584,0.13127,0.13664,0.18962,0.41623,0.46512
,0.44343,0.43447,0.41352,0.42992,0.45668,0.30899,0.30569,0.31956,0.271
49,0.31919,0.3179,0.30751,0.33385,0.29433,0.31637,0.30477,0.18949,0.18
763,0.18934,0.19243,0.18927,0.18902,0.19322,0.19309,0.191,0.1323,0.134
03,0.1343,0.13569,0.13431,0.12674,0.13922,0.13966,0.13612,0.19271,0.44
588,0.42295,0.44215,0.45992,0.41272,0.42782,0.40683,0.30432,0.31576,0.
30842,0.30042,0.31613,0.30862,0.28882,0.34716,0.30062,0.3102,0.31483,0
.32476,0.19115,0.19119,0.18794,0.18868,0.1918,0.19018,0.13326,0.13621,
0.13892,0.1433,0.1466,0.14053,0.13849,0.14374,0.13991,0.15317,0.13653,
0.13697,0.44514,0.44792,0.41729,0.4384,0.46271,0.46255,0.42038,0.32089
,0.30854,0.31312,0.31242,0.3273,0.32144,0.29588,0.31113,0.29073,0.3243
1,0.29838,0.33877,0.18909,0.19245,0.19041,0.13901,0.13952,0.13857,0.13
637,0.14124,0.13767,0.15305,0.13946,0.14875,0.1337,0.13644,0.14344,0.1
3901,0.13642,0.14951,0.41424,0.426,0.45067,0.42315,0.45201,0.46621,0.4
4991,0.44957,0.43542,0.32744,0.31163,0.31731,0.30724,0.30865,0.30463,0
.29929,0.30958,0.31432,0.30148,0.2852,0.14186,0.14379,0.13738,0.14218,
0.1308,0.14154,0.13795,0.13265,0.13952,0.14321,0.13638,0.13178,0.15707
,0.13688,0.1421,0.13347,0.13831,0.1367,0.44903,0.45173,0.44445,0.45286
,0.44946,0.43771,0.44107,0.41426,0.45065,0.3072,0.30355,0.19966,0.2320
1,0.26224,0.22581,0.24837,0.24415,0.23406,0.25117,0.23115,0.13245,0.14
581,0.12631,0.13516,0.13687,0.13876,0.12103,0.13611,0.13846,0.13958,0.
14001,0.14105,0.13402,0.13779,0.13778,0.133,0.15224,0.14264,0.4357,0.4

2434,0.42347,0.4511,0.43635,0.41994,0.40944,0.44209,0.38657,0.26909,0.
24493,0.23044,0.26198,0.23573,0.23851,0.21794,0.24715,0.23014,0.24631,
0.23555,0.14501,0.13524,0.13777,0.13514,0.14079,0.14286,0.14957,0.1402
9,0.13628,0.12856,0.13638,0.14117,0.13418,0.13953,0.13945,0.13739,0.13
399,0.13959,0.42949,0.45292,0.43015,0.47663,0.46548,0.46356,0.42458,0.
26044,0.22904,0.27161,0.24669,0.234,0.23439,0.23524,0.234,0.23395,0.24
191,0.23238,0.20228,0.23134,0.13197,0.13997,0.13805,0.1393,0.14306,0.1
3782,0.10916,0.13834,0.14841,0.1369,0.13759,0.13094,0.13671,0.13362,0.
1424,0.13809,0.13651,0.1335,0.44819,0.45071,0.44174,0.43837,0.4193,0.4
085,0.23567,0.27731,0.26871,0.2498,0.25032,0.23583,0.24466,0.236,0.235
3,0.24612,0.20711,0.24827,0.24832,0.23835,0.1508,0.13706,0.13754,0.132
6,0.13101,0.14277,0.14209,0.13331,0.13741,0.13694,0.13815,0.13832,0.13
835,0.139,0.13692,0.14033,0.15356,0.14556,0.41879,0.44039,0.42385,0.44
387,0.44652,0.22949,0.24549,0.25494,0.27117,0.26273,0.25579,0.23914,0.
22568,0.24661,0.23692,0.22118,0.22782,0.23845,0.2442,0.24173,0.13922,0
.13863,0.13493,0.13233,0.13858,0.13783,0.14539,0.1286,0.14655,0.14037,
0.13739,0.13946,0.1348,0.13949,0.13916,0.1371,0.14078,0.14185,0.43318,
0.44929,0.4085,0.30483,0.26201,0.30429,0.27916,0.25727,0.27121,0.23562
,0.2586,0.24806,0.24582,0.23922,0.24334,0.23302,0.25671,0.2538,0.23811
,0.27752,0.14217,0.13765,0.14208,0.13852,0.13678,0.14638,0.14026,0.136
3,0.14455,0.128,0.14743,0.13898,0.14233,0.13004,0.14856,0.14308,0.1379
6,0.13708,0.44859,0.25579,0.24206,0.25151,0.27908,0.29236,0.24799,0.22
378,0.27593,0.29479,0.23838,0.24542,0.24281,0.23227,0.26835,0.21828,0.
21099,0.23826,0.23566,0.13853,0.13739,0.13076,0.14155,0.13913,0.1365,0
.1411,0.13715,0.13107,0.14015,0.13427,0.13824,0.14078,0.141,0.13768,0.
13171,0.14354,0.085138,0.2611,0.26338,0.23111,0.26437,0.26823,0.28195,
0.24899,0.28084,0.26031,0.24019,0.23555,0.23816,0.22721,0.23582,0.2156
6,0.23842,0.26944,0.2326,0.23675,0.13002,0.13806,0.13771,0.13221,0.138
04,0.13953,0.1355,0.13997,0.13715,0.13766,0.13856,0.12598,0.13588,0.13
894,0.13216,0.13117,0.13821,0.086311,0.26753,0.25452,0.23995,0.17788,0
.26332,0.26094,0.24659,0.27651,0.27404,0.26412,0.25121,0.24005,0.22185
,0.24374,0.22343,0.24673,0.23017,0.24127,0.13753,0.13662,0.13424,0.136
4,0.13842,0.14039,0.13549,0.14594,0.13314,0.12725,0.11905,0.13676,0.13
305,0.13816,0.12899,0.12773,0.13505,0.14108,0.085534,0.28301,0.26148,0
.23526,0.23772,0.2654,0.27811,0.2466,0.26338,0.21978,0.26356,0.25919,0
.23224,0.22357,0.21936,0.26318,0.24357,0.20638,0.26318,0.14016,0.13949
,0.14,0.13678,0.1421,0.14225,0.14347,0.13463,0.13789,0.14534,0.12273,0
.13966,0.12383,0.14268,0.13916,0.1308,0.13959,0.084147,0.086725,0.0849
05,0.24179,0.29717,0.2509,0.26207,0.22625,0.25276,0.2168,0.22719,0.258
98,0.26352,0.22017,0.2581,0.26076,0.24034,0.22704,0.14553,0.13745,0.14
037,0.13749,0.12639,0.14435,0.13602,0.14264,0.13486,0.14261,0.13447,0.
13591,0.13808,0.14775,0.14499,0.13845,0.14296,0.13696,0.084537,0.08408
7,0.084366,0.086399,0.26496,0.28014,0.25823,0.14122,0.27501,0.27203,0.
20352,0.29088,0.23712,0.22742,0.23659,0.23292,0.14496,0.12918,0.1337,0
.13718,0.1367,0.14911,0.13573,0.13381,0.13912,0.13667,0.1502,0.14382,0
.13815,0.14001,0.12702,0.14029,0.1386,0.14034,0.14499,0.13968,0.08404,
0.084233,0.08544,0.084538,0.085759,0.085128,0.26792,0.26797,0.23558,0.
20865,0.28003,0.2949,0.26382,0.22644,0.14324,0.12467,0.14312,0.14107,0
.13672,0.15384,0.13729,0.13201,0.14053,0.13918,0.13573,0.13739,0.13684
,0.13201,0.13726,0.14017,0.13366,0.13856,0.15916,0.13865,0.14006,0.137
66,0.14286,0.085091,0.085565,0.083988,0.085409,0.083874,0.084168,0.275
3,0.28941,0.19281,0.25464,0.27318,0.086115,0.085637,0.13915,0.13202,0.

15163,0.13728,0.13929,0.13968,0.13942,0.14364,0.13955,0.13792,0.135,0.
14091,0.12627,0.12907,0.13905,0.15198,0.14607,0.14562,0.13694,0.14372,
0.13282,0.085612,0.084383,0.085967,0.086416,0.08467,0.08505,0.08482,0.
084839,0.086961,0.086007,0.25353,0.27324,0.085031,0.084837,0.085405,0.
085513,0.13507,0.13925,0.14308,0.15388,0.13529,0.13394,0.14437,0.13969
,0.12877,0.13707,0.12296,0.14503,0.14079,0.13877,0.15249,0.13638,0.137
96,0.13562,0.13889,0.085,0.085957,0.086096,0.085387,0.086857,0.085632,
0.086694,0.084601,0.085453,0.087319,0.084874,0.084606,0.08538,0.086693
,0.085239,0.084382,0.083648,0.14081,0.13731,0.13787,0.14661,0.13124,0.
13841,0.15675,0.1367,0.1395,0.13108,0.1408,0.1382,0.13302,0.14134,0.13
468,0.13712,0.085334,0.085296,0.084304,0.083514,0.084489,0.08422,0.086
482,0.087145,0.086897,0.085072,0.085874,0.084257,0.086636,0.085816,0.0
85642,0.083693,0.084553,0.086411,0.086105,0.12482,0.13859,0.1384,0.140
9,0.13843,0.1378,0.14141,0.13687,0.13707,0.13252,0.13846,0.13397,0.139
85,0.084285,0.083853,0.086491,0.083792,0.085337,0.084378,0.085943,0.08
5124,0.086145,0.084306,0.085072,0.084382,0.084952,0.085049,0.083609,0.
085218,0.08626,0.085382,0.085823,0.084961,0.084379,0.14343,0.13304,0.1
3749,0.13616,0.13021,0.14496,0.15103,0.1447,0.13794,0.08512,0.085529,0
.085133,0.085593,0.084489,0.086515,0.085844,0.085159,0.084522,0.084447
,0.085319,0.085562,0.08554,0.08551,0.086102,0.085514,0.085715,0.084692
,0.085976,0.084596,0.086802,0.086288,0.083568,0.08542,0.08593,0.13209,
0.13738,0.1243,0.13119,0.085593,0.084863,0.086944,0.085904,0.086225,0.
08567,0.085467,0.085858,0.084657,0.084559,0.085176,0.084156,0.084167,0
.084868,0.085188,0.084362,0.085739,0.086053,0.083142,0.084108,0.084463
,0.084979,0.085073,0.084978,0.084021,0.085973,0.084696,0.08567,0.08532
1,0.085487,0.085753,0.085844,0.08505,0.085125,0.084944,0.084232,0.0862
87,0.085643,0.085972,0.086362,0.084964,0.085145,0.084691,0.085149,0.08
6555,0.085368,0.084779,0.08407,0.08552,0.086414,0.085617,0.084631,0.08
4476,0.085688,0.084818,0.08531,0.085666,0.084762,0.084362,0.084592,0.0
85893,0.084391,0.084342,0.084495,0.086174,0.085196,0.08555,0.085179,0.
083139,0.086286,0.085941,0.083812,0.085464,0.085483,0.085368,0.085242,
0.08632,0.087397,0.085966,0.085988,0.086159,0.084697,0.084448,0.08625,
0.085273,0.086673,0.085477,0.085041,0.084264,0.083751,0.086113,0.08461
4,0.084191,0.085383,0.085875,0.0854,0.084849,0.085323,0.083961,0.08438
7,0.085677,0.085555,0.085825,0.085509,0.08561,0.084858,0.086579,0.0853
46,0.085753,0.086287,0.084376,0.083457,0.086115,0.084868,0.086279,0.08
4437,0.083895,0.085025,0.085033,0.086114,0.085504,0.084131,0.085843,0.
086013,0.085176,0.087174,0.086214,0.085963,0.084956,0.084483,0.084951,
0.084874,0.084629,0.085388,0.085185,0.084687,0.08371,0.083935,0.086866
,0.086171,0.084908,0.086091,0.084238,0.084143,0.086871,0.084906,0.0851
51,0.085124,0.085214,0.085571,0.085079,0.084851,0.085182,0.08602,0.084
846,0.085221,0.084684,0.086748,0.085403,0.084952,0.085448,0.08465,0.08
5255,0.084662,0.085148,0.085264,0.085395,0.085196,0.085444,0.08587,0.0
85747,0.084134,0.084698,0.085627,0.085302,0.084445,0.084855,0.083784,0
.084712,0.085369,0.084532,0.085921,0.085691,0.084726,0.085282,0.083426
,0.0845,0.08571,0.084231,0.084913,0.08611,0.086117,0.086181,0.08484,0.
084162
0.19318,0.19424,0.19786,0.1912,0.19559,0.1937,0.19567,0.19165,0.19337,
0.19296,0.19333,0.19342,0.19629,0.1919,0.19426,0.19325,0.19342,0.19515
,0.19694,0.19301,0.30694,0.30915,0.29327,0.3092,0.19157,0.19629,0.1912
5,0.1917,0.19522,0.1926,0.19367,0.19669,0.19351,0.19264,0.19364,0.1963
5,0.19732,0.19281,0.19139,0.41913,0.29136,0.29518,0.29856,0.29408,0.29

46,0.33053,0.19399,0.19341,0.19011,0.19428,0.19444,0.19427,0.19141,0.1
9178,0.19279,0.19382,0.19362,0.19487,0.1932,0.19693,0.19283,0.39564,0.
41973,0.3236,0.32093,0.2986,0.30555,0.27983,0.28358,0.30341,0.31311,0.
19296,0.19588,0.19381,0.19394,0.19611,0.19273,0.19459,0.19603,0.19358,
0.19216,0.19149,0.1954,0.19134,0.19398,0.19211,0.41034,0.39405,0.41088
,0.31784,0.29903,0.2657,0.30816,0.30402,0.31118,0.29971,0.29826,0.3069
5,0.19244,0.19633,0.19545,0.19456,0.19242,0.19352,0.19113,0.19512,0.19
302,0.1916,0.19186,0.19134,0.19642,0.19362,0.1931,0.19442,0.41328,0.37
972,0.41814,0.4312,0.27722,0.29331,0.30992,0.3007,0.31122,0.31688,0.29
008,0.30513,0.32744,0.28644,0.19373,0.19292,0.19061,0.19512,0.19458,0.
19269,0.19759,0.19278,0.19335,0.19702,0.1985,0.19707,0.19185,0.19258,0
.19568,0.19663,0.39479,0.40046,0.39587,0.38516,0.45669,0.44147,0.31008
,0.31484,0.32425,0.28684,0.2898,0.30502,0.29602,0.30266,0.28047,0.3051
4,0.19485,0.19523,0.19276,0.19351,0.19535,0.19407,0.18829,0.19504,0.19
264,0.19563,0.19547,0.19416,0.19802,0.19433,0.19199,0.19514,0.19284,0.
40893,0.39883,0.42603,0.40239,0.39928,0.37862,0.32029,0.35239,0.29962,
0.29499,0.28386,0.31927,0.28966,0.30702,0.30935,0.33042,0.34278,0.1931
9,0.19487,0.19433,0.19642,0.19576,0.19547,0.19042,0.19557,0.19484,0.19
206,0.19176,0.19026,0.19282,0.13688,0.14085,0.14183,0.19392,0.40519,0.
3768,0.40677,0.4138,0.41233,0.42929,0.3956,0.31604,0.30748,0.30658,0.3
226,0.30818,0.2949,0.30044,0.28441,0.31515,0.28621,0.27715,0.19715,0.1
9417,0.19411,0.19245,0.19089,0.19088,0.19391,0.19175,0.19235,0.19527,0
.19379,0.12797,0.14104,0.13442,0.123,0.12934,0.13559,0.19196,0.40249,0
.4299,0.42966,0.4005,0.38521,0.40639,0.42788,0.30023,0.29372,0.30366,0
.26068,0.305,0.30341,0.29068,0.32265,0.28551,0.31286,0.29092,0.19264,0
.19127,0.19294,0.19587,0.19245,0.19234,0.19689,0.19609,0.19423,0.13019
,0.13191,0.13271,0.13355,0.13241,0.12349,0.13693,0.13823,0.13349,0.196
36,0.41867,0.38998,0.41448,0.44147,0.38142,0.3937,0.36576,0.29199,0.30
536,0.29764,0.28902,0.30757,0.293,0.28102,0.33149,0.28215,0.29971,0.30
339,0.31853,0.19276,0.19537,0.19268,0.19312,0.19463,0.193,0.13123,0.13
358,0.13649,0.14169,0.14573,0.13742,0.13629,0.14345,0.13936,0.15258,0.
1343,0.135,0.424,0.41488,0.38995,0.39193,0.42765,0.44526,0.39565,0.308
28,0.29426,0.29764,0.29999,0.31144,0.3094,0.28966,0.29826,0.28204,0.30
882,0.28622,0.32746,0.19319,0.19649,0.19389,0.13776,0.13616,0.13647,0.
13515,0.13961,0.13549,0.15266,0.13811,0.14886,0.13224,0.13454,0.14227,
0.13803,0.13403,0.14822,0.39346,0.39904,0.43129,0.38885,0.41806,0.4404
5,0.4238,0.43067,0.40693,0.31717,0.30569,0.3057,0.2963,0.29738,0.29447
,0.2894,0.30175,0.30567,0.30583,0.28124,0.13998,0.14198,0.13588,0.1411
,0.12901,0.13985,0.13724,0.13009,0.13841,0.14228,0.13462,0.12942,0.156
77,0.13453,0.14095,0.13125,0.13696,0.13471,0.41533,0.42645,0.42068,0.4
1525,0.41981,0.41012,0.42522,0.39191,0.40268,0.30208,0.29282,0.19185,0
.22443,0.25378,0.21565,0.24008,0.23402,0.22882,0.23335,0.2248,0.12997,
0.14453,0.12382,0.13263,0.13613,0.13627,0.11834,0.13354,0.13674,0.1382
2,0.13866,0.13921,0.13201,0.13591,0.13563,0.12997,0.15108,0.14168,0.40
177,0.38723,0.40266,0.41708,0.41607,0.38735,0.37774,0.40334,0.37152,0.
25343,0.23476,0.22352,0.24801,0.23083,0.22694,0.21165,0.2337,0.22149,0
.23244,0.22848,0.14304,0.13312,0.13612,0.13323,0.1386,0.14209,0.14848,
0.13844,0.13414,0.12574,0.13471,0.13895,0.13163,0.13829,0.13754,0.1353
,0.13149,0.13741,0.40124,0.42363,0.40484,0.4433,0.44173,0.43899,0.398,
0.24599,0.21388,0.25947,0.23155,0.22906,0.22683,0.22577,0.22597,0.2257
7,0.23855,0.21725,0.19604,0.21728,0.12846,0.13833,0.13589,0.13796,0.14
143,0.13573,0.10437,0.13708,0.14632,0.13601,0.13587,0.12863,0.1343,0.1

3206,0.14102,0.13633,0.13501,0.13092,0.41557,0.41948,0.41876,0.41769,0
.3973,0.38252,0.21874,0.2554,0.25314,0.23399,0.23612,0.22477,0.23712,0
.22124,0.22197,0.24015,0.19992,0.23542,0.23745,0.22914,0.15092,0.13513
,0.13535,0.1303,0.12883,0.14109,0.14189,0.13192,0.1361,0.13459,0.13644
,0.13677,0.13641,0.13686,0.13504,0.13819,0.15295,0.14513,0.40719,0.413
19,0.39807,0.42057,0.42065,0.21316,0.23565,0.23571,0.24996,0.24649,0.2
3336,0.22695,0.2161,0.24098,0.22996,0.21099,0.21558,0.22806,0.23106,0.
23007,0.13839,0.13693,0.13298,0.12975,0.13633,0.13592,0.14467,0.12752,
0.14614,0.13818,0.13596,0.13726,0.13331,0.13754,0.13732,0.13561,0.1387
8,0.14014,0.41368,0.41267,0.39701,0.28433,0.24518,0.2875,0.26158,0.242
29,0.2568,0.2213,0.23911,0.23511,0.23661,0.22939,0.23163,0.22437,0.247
,0.24973,0.23225,0.26811,0.1414,0.13642,0.14106,0.13669,0.13408,0.1452
8,0.13819,0.134,0.14358,0.12549,0.14607,0.13754,0.14091,0.128,0.14735,
0.14133,0.13615,0.1347,0.40837,0.23588,0.21807,0.23793,0.25508,0.27846
,0.23712,0.21247,0.25546,0.2775,0.23126,0.23488,0.22898,0.22175,0.2566
5,0.20946,0.20329,0.23009,0.23174,0.13752,0.13605,0.12774,0.13989,0.13
743,0.13493,0.13911,0.13512,0.12873,0.13922,0.13284,0.13601,0.13859,0.
13931,0.13523,0.12897,0.14156,0.0779,0.24058,0.24667,0.22084,0.24603,0
.25206,0.26844,0.23085,0.26352,0.24586,0.22863,0.22725,0.22709,0.22277
,0.22728,0.20975,0.22605,0.25956,0.22399,0.22421,0.12776,0.13577,0.136
13,0.13009,0.1363,0.13805,0.13335,0.1379,0.13536,0.13597,0.13587,0.124
03,0.13421,0.13682,0.13056,0.12833,0.13624,0.079623,0.24036,0.23658,0.
22881,0.16356,0.25032,0.23989,0.23694,0.26232,0.26141,0.24593,0.2411,0
.22875,0.21477,0.23386,0.21547,0.23288,0.22003,0.23165,0.13518,0.13482
,0.13215,0.13471,0.13693,0.1387,0.13328,0.14346,0.13202,0.12477,0.1153
6,0.13599,0.13044,0.13571,0.12619,0.12498,0.13294,0.13913,0.079015,0.2
6913,0.2463,0.22235,0.21871,0.2568,0.2606,0.22505,0.24437,0.20441,0.24
484,0.25086,0.22391,0.21529,0.21128,0.25409,0.23327,0.19737,0.24845,0.
13879,0.13754,0.13801,0.1346,0.1405,0.14097,0.14307,0.13276,0.13637,0.
1435,0.11905,0.13799,0.12078,0.14147,0.13693,0.12834,0.13755,0.077914,
0.080078,0.078684,0.22327,0.29013,0.23851,0.24486,0.21101,0.23519,0.20
492,0.20519,0.24976,0.25749,0.21061,0.24048,0.24882,0.22937,0.21954,0.
1446,0.13583,0.13781,0.13477,0.12315,0.14239,0.13413,0.14051,0.13243,0
.14083,0.13306,0.13378,0.13608,0.1469,0.14275,0.13615,0.14138,0.13472,
0.077773,0.078208,0.077356,0.079976,0.25062,0.26504,0.24492,0.13246,0.
25721,0.25931,0.19523,0.27232,0.2205,0.21436,0.22455,0.22143,0.14391,0
.12552,0.13116,0.13511,0.13525,0.14777,0.13339,0.13073,0.13667,0.13567
,0.14916,0.14116,0.13644,0.13831,0.12357,0.1384,0.13702,0.13881,0.1442
3,0.13867,0.077587,0.077701,0.079293,0.078627,0.079315,0.07905,0.25364
,0.24415,0.22427,0.196,0.26432,0.27197,0.24716,0.21206,0.14182,0.12258
,0.14227,0.13949,0.13539,0.1537,0.13555,0.1298,0.13958,0.13759,0.1347,
0.13572,0.13503,0.12969,0.13554,0.13908,0.13186,0.1373,0.15933,0.13651
,0.13847,0.13612,0.14166,0.078325,0.078539,0.077807,0.078639,0.077488,
0.078039,0.26126,0.27867,0.17782,0.23778,0.25518,0.079529,0.078509,0.1
3771,0.12961,0.1514,0.13499,0.13799,0.13757,0.13749,0.14161,0.13846,0.
1358,0.13434,0.13874,0.12288,0.12589,0.13746,0.15202,0.14531,0.14444,0
.1344,0.14188,0.12974,0.078556,0.077665,0.078865,0.080228,0.078563,0.0
77783,0.078691,0.078788,0.080479,0.078858,0.23417,0.26079,0.07904,0.07
8744,0.07841,0.078809,0.13313,0.13853,0.14145,0.15404,0.133,0.13215,0.
1421,0.1389,0.12513,0.13585,0.11924,0.14367,0.1391,0.13668,0.152,0.134
86,0.13582,0.13373,0.13655,0.078099,0.078874,0.07937,0.079618,0.079775
,0.079323,0.080141,0.077858,0.07863,0.081708,0.078525,0.078037,0.07909

5,0.080114,0.077895,0.077606,0.07722,0.1392,0.13589,0.13651,0.14543,0.
12854,0.13659,0.15641,0.13463,0.13838,0.12955,0.13993,0.13604,0.13049,
0.14016,0.13297,0.13538,0.07885,0.078318,0.078038,0.077138,0.077641,0.
077462,0.080253,0.080126,0.080123,0.078466,0.07968,0.077144,0.08029,0.
079384,0.079008,0.077637,0.077756,0.079665,0.078758,0.12175,0.1367,0.1
3672,0.13986,0.13766,0.13633,0.1406,0.13475,0.13484,0.13031,0.13702,0.
13254,0.13755,0.078138,0.076458,0.080188,0.077778,0.078456,0.078127,0.
079519,0.078514,0.080715,0.078455,0.079098,0.077865,0.078272,0.078044,
0.07753,0.078607,0.079806,0.078526,0.078802,0.078737,0.078033,0.14211,
0.13065,0.13536,0.13398,0.12772,0.14322,0.15086,0.14454,0.13597,0.0796
35,0.078903,0.078514,0.079721,0.077968,0.080071,0.080177,0.078904,0.07
7503,0.077849,0.078673,0.078868,0.079026,0.078779,0.07908,0.079131,0.0
78681,0.077606,0.079145,0.078052,0.080483,0.080259,0.077613,0.078962,0
.079338,0.1305,0.1358,0.12103,0.12898,0.078984,0.078112,0.080152,0.079
318,0.079805,0.079685,0.078855,0.079154,0.078508,0.078284,0.079544,0.0
7731,0.077541,0.078626,0.078738,0.07734,0.079119,0.079147,0.076386,0.0
77735,0.077959,0.078178,0.077646,0.078313,0.078061,0.079189,0.078261,0
.078853,0.079243,0.079302,0.078994,0.080174,0.078602,0.077838,0.078908
,0.077988,0.079181,0.078423,0.078455,0.079843,0.078481,0.07838,0.07861
1,0.078465,0.07945,0.078879,0.078248,0.077789,0.078913,0.079275,0.0792
65,0.077863,0.077954,0.07939,0.078297,0.078559,0.07928,0.078062,0.0781
93,0.078036,0.07928,0.077516,0.077602,0.077645,0.079337,0.07909,0.0794
69,0.078651,0.077252,0.079618,0.07991,0.077193,0.078937,0.078763,0.077
585,0.079398,0.080046,0.08058,0.079853,0.078505,0.080324,0.078618,0.07
8034,0.079239,0.078238,0.079427,0.07946,0.078428,0.078338,0.077217,0.0
79748,0.077851,0.077154,0.078732,0.079463,0.078651,0.077665,0.078978,0
.077259,0.077878,0.079842,0.078764,0.07969,0.079764,0.078712,0.079498,
0.079933,0.079004,0.080212,0.079494,0.078195,0.077831,0.07936,0.078233
,0.079502,0.077796,0.077285,0.078187,0.078651,0.079907,0.078926,0.0781
44,0.078171,0.079272,0.078368,0.080525,0.079321,0.079147,0.077919,0.07
7942,0.078106,0.07836,0.077286,0.078599,0.078412,0.078294,0.077438,0.0
77662,0.080502,0.080157,0.079122,0.078941,0.077314,0.077259,0.080489,0
.07853,0.078698,0.078718,0.078257,0.078895,0.078291,0.078159,0.079189,
0.079125,0.078124,0.078849,0.078647,0.079956,0.078505,0.078077,0.07836
5,0.077922,0.07822,0.078098,0.079071,0.078101,0.078927,0.079121,0.0789
25,0.079248,0.079542,0.077444,0.07798,0.079399,0.078443,0.07771,0.0785
78,0.077256,0.07814,0.079621,0.078213,0.079212,0.078989,0.07804,0.0790
64,0.076672,0.07819,0.079091,0.077856,0.077677,0.078894,0.078933,0.079
762,0.078048,0.077921
0.1947,0.1965,0.20131,0.19445,0.19733,0.19589,0.198,0.19522,0.19578,0.
19595,0.19568,0.19718,0.19787,0.19525,0.19844,0.19486,0.19547,0.19664,
0.19773,0.19624,0.29562,0.30668,0.28278,0.30213,0.19552,0.19753,0.1935
9,0.19387,0.19714,0.19488,0.19734,0.19924,0.19561,0.19487,0.19677,0.19
89,0.19753,0.19386,0.19578,0.4038,0.28308,0.28306,0.28904,0.29185,0.28
796,0.32323,0.19692,0.19634,0.19251,0.19572,0.19615,0.19754,0.19325,0.
19276,0.19605,0.19537,0.19329,0.19769,0.19662,0.19968,0.19457,0.38648,
0.41152,0.30189,0.30211,0.29544,0.29558,0.27443,0.28014,0.30046,0.3105
1,0.19541,0.19856,0.19619,0.19548,0.19836,0.1937,0.19746,0.1979,0.1959
1,0.19511,0.19383,0.19762,0.1942,0.19691,0.19522,0.39807,0.37738,0.390
39,0.31205,0.2911,0.26025,0.30618,0.29476,0.30891,0.28833,0.29324,0.30
575,0.1943,0.19788,0.19559,0.19614,0.19533,0.19541,0.19395,0.19684,0.1
9621,0.19433,0.19372,0.193,0.19868,0.19698,0.19544,0.19754,0.40512,0.3

6291,0.4018,0.41408,0.27449,0.28925,0.29674,0.2921,0.30369,0.30956,0.2
8563,0.29918,0.31668,0.27548,0.19618,0.1937,0.19363,0.19786,0.19653,0.
19418,0.19912,0.19507,0.19507,0.1998,0.19982,0.19957,0.19452,0.19535,0
.19735,0.19877,0.37855,0.39112,0.3774,0.37099,0.43728,0.42072,0.29331,
0.30237,0.31116,0.28759,0.28682,0.29882,0.29029,0.29984,0.2709,0.30122
,0.19755,0.19639,0.19593,0.19626,0.19902,0.19594,0.19121,0.19595,0.195
5,0.19763,0.1972,0.19732,0.20185,0.19685,0.1945,0.19658,0.19533,0.3862
9,0.39536,0.41057,0.38248,0.3927,0.35323,0.30807,0.34314,0.29154,0.288
14,0.27772,0.30997,0.28615,0.29765,0.30427,0.33107,0.3325,0.19566,0.19
599,0.1964,0.19865,0.19795,0.19879,0.19313,0.19827,0.19613,0.19389,0.1
9499,0.19268,0.19567,0.13582,0.13921,0.1409,0.19769,0.3779,0.37797,0.3
8967,0.39528,0.39281,0.39948,0.3861,0.31281,0.30269,0.30106,0.31722,0.
29804,0.28726,0.29355,0.276,0.30549,0.27703,0.26925,0.19934,0.19692,0.
19664,0.19465,0.19319,0.19261,0.19713,0.19317,0.19399,0.19881,0.19519,
0.12651,0.13994,0.13275,0.12115,0.12787,0.13394,0.19435,0.38787,0.4124
7,0.40462,0.39497,0.38009,0.37656,0.41102,0.29272,0.28499,0.29149,0.25
687,0.29787,0.29578,0.28159,0.31956,0.27645,0.30069,0.27726,0.19563,0.
19413,0.19437,0.19847,0.19646,0.19451,0.19889,0.19768,0.19537,0.128,0.
13011,0.1311,0.13054,0.13087,0.12117,0.13508,0.13677,0.13171,0.19776,0
.39456,0.38092,0.3953,0.43221,0.37006,0.37229,0.35359,0.29126,0.2985,0
.28606,0.27698,0.29869,0.28708,0.2771,0.33136,0.27516,0.2937,0.28769,0
.30746,0.19557,0.19749,0.19523,0.19554,0.19646,0.19598,0.1294,0.1314,0
.13578,0.13996,0.14492,0.13539,0.13493,0.14195,0.13828,0.15277,0.13192
,0.13313,0.4063,0.40768,0.37634,0.38057,0.40971,0.41707,0.39025,0.3059
1,0.28608,0.29329,0.29055,0.30856,0.30396,0.28141,0.2944,0.2783,0.3001
8,0.27817,0.31864,0.19512,0.19895,0.19614,0.13552,0.13448,0.13482,0.13
429,0.13808,0.1336,0.15295,0.13652,0.14875,0.13084,0.13208,0.14123,0.1
3583,0.13149,0.14695,0.36924,0.37209,0.42287,0.3739,0.40862,0.41531,0.
41463,0.41854,0.37553,0.3054,0.29832,0.30617,0.29333,0.29477,0.28707,0
.28465,0.29394,0.29704,0.2919,0.27801,0.1389,0.14081,0.13517,0.13984,0
.12646,0.13768,0.13546,0.1276,0.13704,0.14022,0.13291,0.12716,0.15636,
0.13277,0.13941,0.12896,0.13589,0.13249,0.40744,0.40437,0.40535,0.3873
7,0.39866,0.40046,0.41197,0.38359,0.38649,0.29038,0.28507,0.1855,0.216
56,0.2469,0.20825,0.23572,0.23416,0.22123,0.23075,0.21543,0.12838,0.14
346,0.12185,0.12948,0.13465,0.13509,0.11516,0.13173,0.13496,0.13661,0.
13713,0.13842,0.1294,0.13401,0.13498,0.1277,0.15042,0.14006,0.38398,0.
38282,0.39357,0.3966,0.3898,0.37571,0.36256,0.38006,0.35599,0.23414,0.
22468,0.22431,0.24163,0.2272,0.21693,0.20731,0.22587,0.21857,0.22594,0
.21841,0.14199,0.12983,0.13514,0.13183,0.13818,0.14092,0.1475,0.13643,
0.13167,0.12313,0.13345,0.13861,0.12935,0.1369,0.13623,0.13326,0.1293,
0.13594,0.39348,0.40044,0.38823,0.42398,0.43282,0.40745,0.39704,0.2336
9,0.20466,0.24858,0.22134,0.22044,0.22432,0.21802,0.2172,0.21901,0.229
12,0.21587,0.19078,0.21026,0.12596,0.13637,0.1333,0.136,0.13985,0.1337
,0.1001,0.13563,0.14495,0.13394,0.13414,0.12685,0.13244,0.12982,0.1397
5,0.13396,0.13243,0.12922,0.40197,0.40456,0.40809,0.40988,0.38277,0.37
869,0.21032,0.24284,0.24293,0.22864,0.22494,0.21717,0.23022,0.21779,0.
2154,0.2329,0.1936,0.22653,0.23557,0.22592,0.15068,0.1339,0.13343,0.12
824,0.12673,0.14056,0.14013,0.12892,0.13435,0.13283,0.13464,0.13466,0.
13424,0.1352,0.13424,0.13755,0.15279,0.14475,0.39589,0.39976,0.38393,0
.41085,0.40116,0.19908,0.22681,0.22673,0.23885,0.23524,0.22386,0.21622
,0.20994,0.23342,0.22158,0.20901,0.20924,0.21971,0.22029,0.22527,0.137
51,0.13505,0.13042,0.12735,0.1342,0.13345,0.14446,0.12558,0.14499,0.13

677,0.13466,0.13607,0.13081,0.13573,0.13559,0.13413,0.1364,0.13993,0.3
9394,0.3932,0.38329,0.27361,0.23954,0.27403,0.25138,0.23355,0.24724,0.
21389,0.23019,0.22784,0.23131,0.22417,0.22243,0.2192,0.23886,0.23784,0
.22751,0.2634,0.13923,0.13475,0.14037,0.13432,0.133,0.1439,0.13638,0.1
3259,0.1434,0.1235,0.14483,0.13475,0.13973,0.1249,0.14581,0.139,0.1342
6,0.13339,0.40334,0.21737,0.20784,0.22208,0.23666,0.25519,0.23274,0.20
19,0.2385,0.26691,0.22601,0.2298,0.22237,0.21629,0.24657,0.20498,0.196
35,0.22317,0.22576,0.13657,0.13356,0.12497,0.1388,0.13608,0.13347,0.13
7,0.13433,0.12608,0.13767,0.13084,0.13419,0.13596,0.13864,0.13327,0.12
66,0.13982,0.071754,0.23203,0.23929,0.21221,0.23491,0.23645,0.2528,0.2
1525,0.25291,0.2362,0.21341,0.22376,0.22397,0.21599,0.21956,0.2014,0.2
1635,0.25229,0.21724,0.22014,0.12534,0.13387,0.13434,0.12898,0.13498,0
.13651,0.13202,0.1355,0.13369,0.13474,0.13425,0.12203,0.13234,0.13524,
0.12932,0.12629,0.13369,0.07401,0.22904,0.22496,0.22215,0.15362,0.2362
5,0.23202,0.22892,0.24769,0.24819,0.23222,0.23542,0.2264,0.21116,0.225
09,0.20701,0.22783,0.21383,0.22561,0.13304,0.13281,0.13016,0.13326,0.1
3501,0.13659,0.13199,0.14248,0.12943,0.12208,0.11296,0.13485,0.12807,0
.13439,0.12386,0.12279,0.13054,0.1386,0.073554,0.25509,0.2327,0.21692,
0.20023,0.24447,0.25202,0.2154,0.23724,0.19794,0.23188,0.24082,0.21754
,0.20998,0.20693,0.25012,0.22789,0.19293,0.24492,0.13776,0.13716,0.136
86,0.13288,0.1384,0.13974,0.14143,0.1305,0.13552,0.14223,0.11601,0.136
77,0.11833,0.13906,0.13527,0.12544,0.13573,0.072403,0.074423,0.073491,
0.21899,0.28197,0.226,0.23185,0.20634,0.21907,0.19411,0.19798,0.23422,
0.2546,0.20413,0.23437,0.23795,0.22349,0.21397,0.1426,0.13398,0.13603,
0.13259,0.12056,0.14139,0.13202,0.1397,0.13078,0.13916,0.13178,0.13334
,0.13415,0.1462,0.14257,0.13498,0.14049,0.13302,0.072508,0.072493,0.07
2199,0.074511,0.24695,0.2561,0.23573,0.12453,0.23572,0.25397,0.18936,0
.25359,0.20892,0.20931,0.2196,0.21761,0.1424,0.1236,0.12901,0.13413,0.
13393,0.14675,0.13287,0.12956,0.13515,0.13341,0.14861,0.13955,0.13511,
0.13747,0.12104,0.13676,0.13589,0.13683,0.14341,0.13676,0.071663,0.071
817,0.074198,0.072936,0.073404,0.073066,0.23759,0.23561,0.21591,0.1848
8,0.24697,0.2612,0.23448,0.20603,0.14038,0.11962,0.14112,0.13746,0.134
19,0.15331,0.13408,0.1273,0.13846,0.13578,0.13225,0.13398,0.13429,0.12
781,0.13429,0.13745,0.12925,0.13563,0.1583,0.13503,0.13704,0.1343,0.13
983,0.073181,0.07286,0.072519,0.072508,0.072131,0.072081,0.24805,0.272
32,0.16571,0.22472,0.24452,0.073503,0.073108,0.13654,0.12766,0.15031,0
.13411,0.137,0.13541,0.13518,0.14064,0.13725,0.13466,0.13282,0.13692,0
.11989,0.12345,0.13558,0.15149,0.14485,0.14257,0.13285,0.1405,0.12692,
0.073237,0.072107,0.073823,0.07431,0.073313,0.071814,0.073287,0.072779
,0.075037,0.073048,0.22416,0.25526,0.073547,0.073834,0.073492,0.073384
,0.1321,0.13682,0.14001,0.15324,0.13106,0.13083,0.14071,0.13741,0.1233
4,0.13433,0.11639,0.14231,0.13743,0.1345,0.15136,0.13294,0.13354,0.131
87,0.13478,0.073089,0.073135,0.073237,0.074155,0.074302,0.073844,0.073
901,0.072776,0.073188,0.075636,0.073064,0.072467,0.073327,0.074206,0.0
72029,0.072448,0.070665,0.13713,0.13505,0.13559,0.14385,0.12698,0.1351
5,0.15601,0.13281,0.13637,0.12766,0.13802,0.13489,0.12744,0.13866,0.13
152,0.13292,0.074034,0.07333,0.071753,0.07141,0.071714,0.071932,0.0746
21,0.074607,0.073631,0.073316,0.073825,0.071639,0.074396,0.074347,0.07
4056,0.072298,0.072419,0.07358,0.073173,0.11938,0.13495,0.13491,0.1385
4,0.13641,0.13384,0.13883,0.13231,0.13318,0.12845,0.13431,0.13062,0.13
598,0.072114,0.071291,0.075158,0.072145,0.073404,0.072495,0.073945,0.0
72989,0.075575,0.073329,0.073681,0.071436,0.07224,0.072235,0.072178,0.

073081,0.073622,0.072376,0.073111,0.072611,0.073168,0.142,0.12885,0.13
37,0.13138,0.1255,0.1411,0.14993,0.14323,0.13411,0.074411,0.072479,0.0
73476,0.074048,0.07276,0.074007,0.074532,0.073595,0.071862,0.07206,0.0
73047,0.073335,0.073024,0.073021,0.07305,0.073492,0.073845,0.071964,0.
073534,0.072222,0.074226,0.074696,0.071938,0.073113,0.073286,0.1288,0.
134,0.11886,0.12697,0.073536,0.073172,0.074892,0.073742,0.074499,0.074
211,0.073629,0.073696,0.073116,0.073066,0.073935,0.072277,0.072356,0.0
72773,0.073548,0.071879,0.07421,0.073304,0.070591,0.072622,0.072471,0.
072567,0.072199,0.072685,0.072475,0.073421,0.072492,0.073321,0.073903,
0.073655,0.072964,0.073811,0.072952,0.071326,0.073323,0.072159,0.07358
2,0.072508,0.07309,0.074326,0.073318,0.072267,0.073384,0.072912,0.0740
13,0.073121,0.072667,0.071865,0.07293,0.073967,0.073834,0.071793,0.072
168,0.073463,0.072041,0.072425,0.073214,0.072015,0.072937,0.073046,0.0
73299,0.072326,0.072002,0.072295,0.073369,0.073442,0.073899,0.072481,0
.071412,0.073811,0.074777,0.071776,0.073568,0.073308,0.072322,0.073401
,0.074565,0.074903,0.074528,0.072586,0.074466,0.073642,0.072831,0.0734
13,0.07203,0.073357,0.074163,0.073228,0.072526,0.071804,0.074521,0.072
426,0.072166,0.073098,0.07404,0.072292,0.072505,0.07371,0.071563,0.072
531,0.073782,0.073038,0.073853,0.07468,0.072886,0.07416,0.074048,0.073
305,0.07458,0.074142,0.072642,0.072697,0.073598,0.072398,0.074193,0.07
2558,0.071346,0.072077,0.073224,0.074281,0.072618,0.072489,0.072656,0.
072374,0.07304,0.07487,0.074106,0.073543,0.072476,0.071987,0.072659,0.
073033,0.071095,0.073443,0.072405,0.072785,0.071544,0.072227,0.074543,
0.074187,0.073457,0.073326,0.07183,0.071446,0.074551,0.073318,0.072569
,0.073403,0.072414,0.073536,0.072396,0.072782,0.074049,0.07417,0.07244
1,0.073548,0.072931,0.074068,0.072948,0.07262,0.072717,0.072591,0.0728
58,0.072496,0.073711,0.072844,0.072977,0.073844,0.073332,0.073564,0.07
4655,0.072207,0.071958,0.073453,0.072821,0.072506,0.072399,0.071673,0.
072354,0.074143,0.072605,0.072982,0.073958,0.072142,0.0736,0.071821,0.
072517,0.073056,0.072767,0.072116,0.073711,0.073321,0.074377,0.072471,
0.071686
0.19693,0.19745,0.20256,0.19567,0.19879,0.19816,0.20022,0.1981,0.19672
,0.19824,0.19816,0.19802,0.19924,0.19649,0.2,0.19591,0.19716,0.1981,0.
19979,0.19852,0.28641,0.30242,0.2804,0.30368,0.19622,0.19864,0.19586,0
.19514,0.19982,0.19761,0.19811,0.20127,0.1988,0.19741,0.19634,0.19976,
0.19877,0.19794,0.19728,0.37315,0.27767,0.27962,0.28775,0.28772,0.2828
,0.32211,0.19899,0.19861,0.19452,0.19863,0.19908,0.19902,0.19503,0.196
6,0.19957,0.19692,0.19603,0.19939,0.19815,0.20167,0.19622,0.38393,0.39
501,0.30028,0.29615,0.2917,0.28879,0.27197,0.276,0.29374,0.3024,0.1981
3,0.19872,0.19736,0.19593,0.19891,0.1961,0.19923,0.19859,0.19766,0.197
83,0.19625,0.19938,0.19768,0.19962,0.19688,0.39213,0.36356,0.38648,0.3
1318,0.28612,0.25769,0.30625,0.2951,0.30111,0.28229,0.2869,0.30131,0.1
9558,0.19859,0.1966,0.19824,0.19801,0.19671,0.19625,0.19831,0.19785,0.
19497,0.19547,0.19554,0.20026,0.19965,0.19677,0.19923,0.3804,0.34728,0
.3982,0.40021,0.27832,0.28885,0.29292,0.28541,0.30253,0.30528,0.28337,
0.29123,0.31343,0.27253,0.19839,0.1972,0.1965,0.20002,0.19796,0.19603,
0.20081,0.19662,0.19511,0.20098,0.20221,0.20032,0.19547,0.19702,0.1994
,0.2004,0.37068,0.38349,0.36734,0.35992,0.42209,0.41882,0.29018,0.3035
4,0.31038,0.279,0.28389,0.29026,0.28903,0.29543,0.27649,0.29522,0.1991
7,0.19741,0.19912,0.1983,0.20003,0.1973,0.19352,0.19865,0.19906,0.1998
3,0.19944,0.1995,0.203,0.19829,0.19658,0.19876,0.1965,0.38336,0.3843,0
.41395,0.37118,0.38231,0.34751,0.30481,0.34167,0.28325,0.28354,0.26534

,0.30406,0.28239,0.29283,0.29592,0.31389,0.33063,0.19853,0.19922,0.198
76,0.20037,0.19844,0.20127,0.19646,0.19871,0.19828,0.19525,0.19693,0.1
9537,0.19757,0.13359,0.13766,0.14013,0.1988,0.37549,0.36777,0.37227,0.
38519,0.38463,0.38582,0.38069,0.29828,0.29627,0.28797,0.30575,0.29644,
0.28342,0.28743,0.27358,0.30477,0.27518,0.2643,0.20036,0.19936,0.19741
,0.19798,0.19476,0.19551,0.19917,0.19572,0.19666,0.20081,0.19704,0.125
06,0.13934,0.13151,0.11895,0.12581,0.13317,0.19565,0.37869,0.40005,0.3
9925,0.38284,0.36839,0.36294,0.40007,0.29259,0.28097,0.28335,0.2523,0.
30072,0.29445,0.2822,0.3097,0.27371,0.29756,0.27383,0.19732,0.197,0.19
611,0.20097,0.19817,0.1954,0.20073,0.19947,0.19764,0.12561,0.12818,0.1
2934,0.1294,0.12902,0.11884,0.13389,0.13555,0.13056,0.19901,0.37643,0.
37861,0.37592,0.40634,0.36352,0.35466,0.34352,0.28636,0.29327,0.28075,
0.27437,0.29936,0.2866,0.27184,0.33448,0.27464,0.2886,0.28259,0.29763,
0.19792,0.1994,0.19638,0.19816,0.19807,0.19637,0.12762,0.12991,0.13467
,0.13861,0.14334,0.13501,0.13362,0.13925,0.13571,0.1525,0.12989,0.1320
7,0.39887,0.39057,0.36893,0.36809,0.41339,0.41778,0.38072,0.30413,0.28
154,0.2871,0.28088,0.30622,0.29894,0.27843,0.29304,0.26943,0.29066,0.2
772,0.31604,0.19749,0.19891,0.19817,0.13468,0.13378,0.13329,0.13268,0.
13676,0.13249,0.15171,0.13509,0.14826,0.12964,0.13098,0.1405,0.13447,0
.13008,0.14578,0.37246,0.3646,0.39806,0.36132,0.39872,0.40139,0.39724,
0.39988,0.37206,0.29814,0.29743,0.30411,0.28301,0.29718,0.28266,0.2795
7,0.29602,0.30008,0.28478,0.27792,0.13701,0.14031,0.13389,0.13894,0.12
457,0.13689,0.13414,0.12606,0.13609,0.1395,0.13032,0.12535,0.15612,0.1
3212,0.13886,0.12765,0.13486,0.1306,0.38573,0.39436,0.40671,0.37461,0.
37763,0.39516,0.40115,0.38116,0.36953,0.28633,0.28391,0.18251,0.20877,
0.24167,0.2048,0.22926,0.22914,0.21562,0.22287,0.20766,0.12623,0.14248
,0.11951,0.12784,0.13304,0.13328,0.1132,0.13034,0.13415,0.13512,0.1357
3,0.13658,0.12706,0.13311,0.13369,0.12589,0.14984,0.13907,0.38105,0.37
295,0.38448,0.38505,0.37266,0.36643,0.34462,0.3782,0.34962,0.22555,0.2
1708,0.22113,0.23732,0.22154,0.21276,0.20418,0.22104,0.21313,0.22221,0
.2127,0.14055,0.12843,0.13368,0.12956,0.13664,0.14016,0.14719,0.13591,
0.12949,0.12109,0.13256,0.13783,0.12847,0.13552,0.13547,0.13234,0.1282
2,0.13471,0.3686,0.38997,0.37644,0.42117,0.40666,0.39954,0.39082,0.225
67,0.19865,0.24184,0.21902,0.21714,0.21948,0.21243,0.21553,0.20998,0.2
2382,0.20831,0.18792,0.20969,0.12401,0.13481,0.13252,0.13522,0.13936,0
.13238,0.097659,0.13391,0.14378,0.13272,0.13335,0.12511,0.13168,0.1279
3,0.13892,0.13299,0.13093,0.12821,0.38531,0.4027,0.40499,0.39414,0.368
15,0.37377,0.2038,0.23205,0.23585,0.21618,0.22034,0.21475,0.22905,0.21
098,0.21228,0.23152,0.18994,0.21989,0.22978,0.22043,0.14923,0.13311,0.
13222,0.12665,0.12436,0.13882,0.13846,0.12671,0.13374,0.13199,0.13272,
0.13284,0.13246,0.1336,0.13338,0.13728,0.15164,0.14399,0.37938,0.38654
,0.37351,0.39615,0.38764,0.19469,0.22429,0.22108,0.23453,0.22802,0.218
05,0.21246,0.20445,0.22833,0.21464,0.20492,0.20657,0.21697,0.21069,0.2
1949,0.13573,0.13428,0.1285,0.12542,0.13324,0.13195,0.14426,0.12346,0.
14426,0.13471,0.13223,0.13528,0.12917,0.13419,0.13424,0.13351,0.13614,
0.13923,0.39136,0.38566,0.37443,0.26893,0.23257,0.27215,0.23949,0.2232
5,0.23715,0.2061,0.23132,0.22057,0.22837,0.22088,0.22166,0.21481,0.236
38,0.2294,0.2251,0.25761,0.13835,0.13444,0.13886,0.13297,0.13196,0.143
56,0.13521,0.13171,0.14301,0.12188,0.14426,0.13427,0.13903,0.12395,0.1
4603,0.13841,0.13316,0.13177,0.41255,0.20431,0.19934,0.21285,0.2199,0.
24252,0.22656,0.19669,0.22717,0.25731,0.22476,0.22493,0.21811,0.21613,
0.23999,0.20082,0.19601,0.2149,0.21941,0.13424,0.13224,0.124,0.1383,0.

13436,0.13243,0.13587,0.13341,0.1241,0.13667,0.13017,0.13226,0.13437,0
.1373,0.13202,0.12407,0.13912,0.066643,0.224,0.23259,0.20522,0.23138,0
.22612,0.2416,0.20858,0.24342,0.22561,0.20684,0.22118,0.21645,0.21056,
0.21463,0.19664,0.21318,0.24816,0.21868,0.21622,0.12409,0.13322,0.1325
5,0.12778,0.13379,0.13534,0.13055,0.13336,0.13194,0.13286,0.13358,0.12
101,0.13138,0.13352,0.12744,0.12369,0.13282,0.069315,0.22331,0.21293,0
.21287,0.14847,0.22474,0.22544,0.22676,0.2362,0.24329,0.22367,0.23203,
0.22364,0.20997,0.21955,0.204,0.21784,0.2074,0.22175,0.13022,0.1315,0.
12811,0.13178,0.1343,0.13563,0.1311,0.1413,0.12771,0.11964,0.11032,0.1
336,0.12656,0.13306,0.12187,0.12085,0.12943,0.13811,0.068494,0.24214,0
.23441,0.20946,0.19605,0.22985,0.23689,0.21221,0.22689,0.19321,0.22106
,0.2386,0.21235,0.20469,0.20214,0.24551,0.22211,0.18953,0.24071,0.1359
1,0.13595,0.13559,0.13165,0.13739,0.13872,0.1404,0.12888,0.13357,0.142
1,0.11328,0.13636,0.11612,0.13758,0.13324,0.12323,0.13425,0.06788,0.06
9667,0.06957,0.21307,0.27962,0.22361,0.21877,0.20229,0.2084,0.18915,0.
19123,0.22705,0.2506,0.20018,0.22844,0.22881,0.2187,0.21057,0.14258,0.
13326,0.13561,0.13221,0.11878,0.1404,0.1309,0.13947,0.1286,0.13732,0.1
3025,0.13238,0.13302,0.14446,0.14145,0.13386,0.13998,0.13182,0.067207,
0.067695,0.066876,0.069427,0.23789,0.24044,0.22956,0.11998,0.2276,0.24
87,0.18686,0.2478,0.20487,0.20441,0.21381,0.21126,0.1415,0.12158,0.127
48,0.13265,0.1324,0.14644,0.13096,0.12805,0.13414,0.13177,0.14812,0.13
847,0.13375,0.13541,0.11933,0.13604,0.13392,0.13547,0.14259,0.13541,0.
067412,0.067699,0.068622,0.067828,0.068403,0.068173,0.23059,0.22421,0.
20448,0.17574,0.23977,0.24308,0.22306,0.19574,0.13896,0.11788,0.14004,
0.13528,0.13266,0.15379,0.1327,0.12579,0.13762,0.13442,0.1305,0.13306,
0.13293,0.12606,0.13244,0.13677,0.1277,0.13404,0.15745,0.13313,0.13592
,0.13381,0.13813,0.069094,0.067656,0.067483,0.068795,0.067242,0.067622
,0.23722,0.25888,0.16109,0.21823,0.23052,0.068926,0.068334,0.13471,0.1
2582,0.14951,0.13258,0.1361,0.13428,0.13323,0.13939,0.13595,0.13356,0.
1311,0.13582,0.11683,0.12121,0.13475,0.15144,0.14363,0.14125,0.13185,0
.13883,0.12439,0.068389,0.066962,0.069261,0.069303,0.068017,0.067452,0
.068772,0.068257,0.07021,0.06882,0.21746,0.24649,0.068514,0.069118,0.0
68652,0.06789,0.12983,0.13599,0.13888,0.15246,0.12921,0.12909,0.1393,0
.13656,0.12208,0.13366,0.11396,0.14081,0.13558,0.13231,0.15041,0.13188
,0.13248,0.13085,0.13239,0.067952,0.068437,0.068445,0.069599,0.069381,
0.068114,0.069032,0.068056,0.068918,0.070404,0.069116,0.067869,0.06883
4,0.069412,0.067226,0.067252,0.066589,0.13545,0.13331,0.1342,0.14312,0
.12501,0.1336,0.15603,0.13155,0.13545,0.12581,0.13697,0.13291,0.12617,
0.13772,0.13033,0.1316,0.069141,0.068556,0.067205,0.066652,0.067692,0.
067217,0.070044,0.070179,0.068664,0.068339,0.069388,0.067251,0.069001,
0.069791,0.068842,0.067791,0.068237,0.068674,0.068437,0.11784,0.13341,
0.13295,0.13676,0.13548,0.1324,0.13831,0.13048,0.13215,0.12669,0.13352
,0.12876,0.13392,0.067534,0.066299,0.069612,0.068259,0.068845,0.067764
,0.068954,0.068361,0.070742,0.068409,0.068469,0.066836,0.067546,0.0671
51,0.068231,0.067345,0.068813,0.067419,0.068136,0.068616,0.068316,0.14
173,0.12704,0.1319,0.12967,0.12393,0.14125,0.14942,0.14254,0.1327,0.06
9828,0.06704,0.068602,0.069915,0.068141,0.070351,0.069708,0.068766,0.0
67413,0.067612,0.068368,0.068094,0.068153,0.068288,0.068403,0.068175,0
.068828,0.067517,0.06901,0.067215,0.06988,0.068864,0.067382,0.067858,0
.068596,0.12686,0.13269,0.11672,0.12552,0.069108,0.068609,0.069895,0.0
68811,0.069872,0.068922,0.069098,0.068642,0.067554,0.06854,0.06898,0.0
67496,0.067881,0.067654,0.068893,0.067019,0.069773,0.068666,0.066555,0

.068048,0.068481,0.068312,0.068201,0.068398,0.067861,0.068558,0.067751
,0.06818,0.069577,0.068775,0.06843,0.069062,0.068254,0.066698,0.069145
,0.06757,0.068652,0.06821,0.068415,0.070124,0.069118,0.068124,0.068246
,0.06825,0.069177,0.068202,0.067766,0.067618,0.067951,0.069521,0.06906
,0.06745,0.067293,0.068575,0.067297,0.067998,0.068174,0.06744,0.068536
,0.068335,0.068155,0.06716,0.066673,0.067698,0.068927,0.068415,0.06898
2,0.067476,0.066847,0.068987,0.070027,0.066861,0.069007,0.068833,0.068
138,0.068597,0.070285,0.070506,0.069614,0.067506,0.0694,0.069066,0.068
374,0.06857,0.066867,0.067802,0.069101,0.069118,0.067874,0.067285,0.06
9917,0.068009,0.067448,0.0682,0.069067,0.067979,0.067273,0.068448,0.06
7327,0.068276,0.068655,0.068055,0.069024,0.070384,0.068482,0.069101,0.
069826,0.068871,0.06992,0.069185,0.06929,0.068713,0.069473,0.067775,0.
069589,0.068187,0.067041,0.06734,0.068108,0.068474,0.068399,0.067646,0
.068581,0.068312,0.067941,0.070466,0.069713,0.068813,0.067819,0.067594
,0.067797,0.068127,0.066752,0.06896,0.067682,0.068526,0.067325,0.06758
1,0.069842,0.068901,0.069064,0.069093,0.066827,0.066831,0.069982,0.068
412,0.067696,0.069192,0.067962,0.069048,0.067632,0.068044,0.069012,0.0
69139,0.068082,0.068524,0.068594,0.070159,0.068328,0.067337,0.068098,0
.067881,0.068225,0.068384,0.068881,0.067825,0.068358,0.069554,0.068985
,0.068602,0.070461,0.067699,0.067571,0.068823,0.06882,0.067811,0.06834
2,0.067219,0.067505,0.069726,0.068339,0.068008,0.068972,0.067492,0.068
995,0.067669,0.067856,0.068689,0.067606,0.06758,0.069138,0.069288,0.06
9475,0.067918,0.066835
0.19809,0.19769,0.20391,0.19865,0.20106,0.19986,0.20203,0.20031,0.1989
,0.19988,0.20081,0.20064,0.20066,0.19862,0.20285,0.19657,0.19778,0.199
26,0.20183,0.20028,0.27844,0.29988,0.27561,0.29964,0.19763,0.20037,0.1
9813,0.19926,0.20141,0.19934,0.20014,0.20122,0.20115,0.19776,0.19788,0
.20061,0.2008,0.19817,0.19923,0.3698,0.27824,0.27535,0.29077,0.28463,0
.28196,0.32191,0.20078,0.2004,0.19637,0.20022,0.20142,0.19918,0.19665,
0.1986,0.19966,0.1997,0.19787,0.20066,0.19949,0.20279,0.19896,0.36874,
0.36999,0.30159,0.29111,0.2861,0.28606,0.27308,0.2718,0.29029,0.29495,
0.19831,0.20111,0.19979,0.19734,0.1995,0.1982,0.20137,0.20032,0.1992,0
.20004,0.19861,0.20117,0.19881,0.20016,0.1999,0.38272,0.36194,0.37857,
0.306,0.28916,0.2622,0.30279,0.29167,0.29397,0.28691,0.28317,0.29137,0
.19797,0.19975,0.19937,0.19936,0.20102,0.19989,0.19743,0.20115,0.19922
,0.19758,0.19799,0.19698,0.2008,0.20116,0.19835,0.20114,0.37828,0.3354
9,0.37943,0.3757,0.27838,0.28303,0.28836,0.28223,0.2957,0.30259,0.2860
8,0.28485,0.30696,0.26698,0.19985,0.19794,0.19727,0.2005,0.19884,0.198
15,0.20245,0.19906,0.19747,0.20162,0.20238,0.20225,0.19692,0.19899,0.2
0066,0.20192,0.36096,0.38369,0.36071,0.34424,0.40604,0.40446,0.28847,0
.30589,0.30018,0.27647,0.28538,0.29181,0.28426,0.29204,0.27635,0.2853,
0.20104,0.19878,0.20027,0.19807,0.20176,0.19829,0.19546,0.20054,0.2009
8,0.20175,0.20151,0.20194,0.20516,0.20027,0.19959,0.19906,0.19844,0.36
344,0.38169,0.39592,0.35157,0.36762,0.33965,0.2983,0.34665,0.27934,0.2
7723,0.25927,0.301,0.276,0.29501,0.29038,0.31289,0.32505,0.19979,0.200
72,0.20143,0.20135,0.19918,0.20368,0.19936,0.20085,0.20038,0.19684,0.1
9832,0.19751,0.19919,0.13249,0.1367,0.13927,0.2002,0.37776,0.37491,0.3
6005,0.38289,0.37526,0.38003,0.36539,0.2928,0.29072,0.28378,0.30489,0.
29108,0.28638,0.28377,0.2692,0.30237,0.27406,0.26266,0.20245,0.20222,0
.19873,0.19867,0.19592,0.19662,0.19995,0.1977,0.19894,0.20332,0.1977,0
.12273,0.13859,0.13058,0.11717,0.12379,0.13225,0.19823,0.37212,0.38588
,0.3879,0.3756,0.3671,0.35851,0.38341,0.28821,0.27948,0.28558,0.25359,

0.29682,0.29514,0.28352,0.30535,0.272,0.29117,0.26558,0.19902,0.19867,
0.19687,0.20142,0.20077,0.19708,0.20136,0.20104,0.20035,0.12465,0.1271
7,0.12765,0.12833,0.12717,0.11711,0.13271,0.1346,0.12939,0.19998,0.376
25,0.36416,0.36957,0.40355,0.37242,0.34476,0.3438,0.28917,0.29047,0.27
6,0.27251,0.29816,0.28265,0.26801,0.3261,0.27429,0.29078,0.28258,0.291
04,0.19983,0.19958,0.19621,0.19931,0.2006,0.19913,0.12603,0.12818,0.13
368,0.13755,0.14133,0.13376,0.13235,0.13882,0.13352,0.15259,0.12884,0.
13047,0.39012,0.38164,0.34431,0.36242,0.39822,0.42323,0.37272,0.30005,
0.28118,0.28616,0.28361,0.29973,0.29876,0.27605,0.2931,0.26681,0.29614
,0.2755,0.31585,0.19956,0.20077,0.19952,0.13314,0.13282,0.13211,0.1317
,0.13534,0.13107,0.15145,0.13375,0.14699,0.12804,0.12871,0.13967,0.133
89,0.12919,0.14381,0.37523,0.34607,0.38817,0.34113,0.38585,0.40253,0.4
0294,0.39005,0.36457,0.29051,0.30271,0.29314,0.27755,0.29377,0.28453,0
.27696,0.29586,0.29445,0.28414,0.27985,0.13664,0.13878,0.13332,0.13834
,0.12325,0.13557,0.13329,0.12477,0.13547,0.13879,0.12946,0.12363,0.155
69,0.13059,0.13901,0.12731,0.13347,0.12865,0.37664,0.36846,0.40593,0.3
7249,0.35949,0.37419,0.38149,0.36785,0.3524,0.28183,0.28125,0.18083,0.
20443,0.23961,0.20033,0.22623,0.22275,0.21384,0.21946,0.20437,0.12487,
0.14207,0.11732,0.12585,0.13105,0.13235,0.11107,0.12921,0.13365,0.1337
1,0.135,0.1366,0.12599,0.13172,0.13234,0.12449,0.14903,0.13966,0.36241
,0.36379,0.37763,0.38222,0.36928,0.36076,0.34025,0.36289,0.33516,0.216
53,0.2119,0.21834,0.23363,0.22247,0.21121,0.20637,0.21963,0.20787,0.21
516,0.21073,0.14002,0.12672,0.13278,0.12845,0.1358,0.13877,0.14647,0.1
3428,0.12828,0.11948,0.13149,0.13701,0.12713,0.13514,0.13494,0.13143,0
.12727,0.13329,0.36974,0.38225,0.3686,0.39844,0.4136,0.39387,0.38684,0
.21109,0.1906,0.23408,0.21592,0.2146,0.21606,0.20785,0.20894,0.20914,0
.22019,0.2073,0.18738,0.20583,0.12227,0.13352,0.13197,0.13443,0.13849,
0.13058,0.094483,0.13325,0.14388,0.13209,0.13217,0.12325,0.1307,0.1264
5,0.13831,0.13204,0.13014,0.12732,0.38257,0.37964,0.40483,0.37665,0.36
177,0.36819,0.20001,0.22779,0.2307,0.20865,0.2121,0.20909,0.22627,0.20
997,0.21167,0.23142,0.18627,0.21762,0.22434,0.2147,0.14824,0.13136,0.1
3066,0.12476,0.123,0.13791,0.13746,0.12565,0.13237,0.1313,0.13209,0.13
135,0.13054,0.13313,0.13264,0.13661,0.15087,0.14271,0.36329,0.36806,0.
35711,0.38435,0.38954,0.18715,0.21628,0.21457,0.22888,0.22231,0.21134,
0.21105,0.20318,0.22896,0.20774,0.19883,0.20118,0.213,0.2099,0.21671,0
.1355,0.13351,0.12711,0.12421,0.13207,0.1313,0.14356,0.121,0.14318,0.1
3288,0.13051,0.13335,0.12865,0.13355,0.13254,0.13278,0.13524,0.13755,0
.37874,0.39661,0.36717,0.26038,0.2297,0.27362,0.23383,0.21504,0.23061,
0.20073,0.23042,0.22207,0.22369,0.21883,0.22575,0.21449,0.23347,0.2304
4,0.22472,0.25687,0.13691,0.13325,0.13758,0.13177,0.1309,0.14326,0.134
51,0.1307,0.14299,0.12078,0.14387,0.13322,0.13792,0.12307,0.1457,0.136
65,0.13299,0.13042,0.4073,0.20061,0.18972,0.20757,0.21449,0.24516,0.22
246,0.19583,0.22555,0.2508,0.2221,0.2158,0.21593,0.21301,0.24152,0.199
98,0.19348,0.21025,0.21394,0.1333,0.13058,0.12205,0.13812,0.13325,0.13
144,0.13496,0.13136,0.12244,0.1361,0.12866,0.13086,0.13375,0.13788,0.1
3118,0.12241,0.13898,0.063093,0.21992,0.22693,0.19643,0.22888,0.22139,
0.23562,0.19832,0.23034,0.22152,0.20562,0.2191,0.20834,0.20915,0.21735
,0.1956,0.21271,0.24615,0.215,0.21069,0.12283,0.13192,0.13149,0.12585,
0.13266,0.13503,0.12852,0.13266,0.13057,0.13136,0.13247,0.11895,0.1296
4,0.13361,0.12649,0.12164,0.13194,0.065647,0.21591,0.21012,0.20957,0.1
4454,0.21692,0.22115,0.222,0.23446,0.23842,0.22403,0.22833,0.22038,0.2
0665,0.21205,0.1981,0.21482,0.20307,0.22095,0.12904,0.12991,0.12689,0.

13068,0.13411,0.13397,0.13007,0.14002,0.12638,0.11766,0.10775,0.13226,
0.12579,0.13148,0.12049,0.11988,0.12794,0.13766,0.064844,0.23661,0.227
87,0.20889,0.18681,0.22485,0.23022,0.21144,0.2206,0.18737,0.22172,0.23
785,0.20911,0.20487,0.1947,0.23639,0.21775,0.19035,0.23611,0.13515,0.1
3465,0.13369,0.12965,0.13681,0.13742,0.13901,0.12668,0.13194,0.14077,0
.11119,0.1352,0.11444,0.13636,0.1323,0.12135,0.13313,0.065187,0.066147
,0.065595,0.21368,0.27055,0.20749,0.20685,0.19902,0.19806,0.18627,0.18
699,0.21787,0.248,0.19995,0.22765,0.2238,0.21293,0.20643,0.14205,0.131
66,0.13458,0.13102,0.11635,0.1406,0.13037,0.13895,0.12743,0.13604,0.12
893,0.13053,0.13131,0.1436,0.1413,0.13285,0.13951,0.13024,0.063121,0.0
63473,0.063702,0.065063,0.23725,0.23485,0.22246,0.11391,0.21628,0.2451
4,0.18841,0.24162,0.19949,0.19711,0.21522,0.20671,0.14099,0.11917,0.12
622,0.13204,0.13146,0.14635,0.12949,0.12613,0.13292,0.1304,0.14771,0.1
376,0.13228,0.13315,0.11853,0.13433,0.13274,0.1345,0.142,0.1348,0.0630
54,0.064146,0.064572,0.064168,0.063849,0.065185,0.21803,0.21538,0.1950
3,0.17046,0.22346,0.23878,0.21713,0.19123,0.13802,0.11591,0.13929,0.13
458,0.13122,0.15319,0.13167,0.12506,0.13583,0.13339,0.12937,0.13152,0.
13232,0.1238,0.13227,0.13567,0.12574,0.13313,0.15714,0.1321,0.13434,0.
13218,0.13758,0.065033,0.064143,0.063773,0.06483,0.063532,0.063725,0.2
3197,0.25375,0.15541,0.21463,0.22334,0.06486,0.064321,0.13339,0.12366,
0.14871,0.13055,0.13528,0.13315,0.13161,0.1373,0.13457,0.13223,0.13,0.
13485,0.11459,0.12018,0.13387,0.15102,0.14323,0.14118,0.13085,0.1376,0
.12372,0.063761,0.06261,0.066095,0.065554,0.064462,0.062832,0.064636,0
.06488,0.064568,0.065114,0.20946,0.23906,0.063874,0.064983,0.065189,0.
064035,0.12797,0.13495,0.13707,0.15202,0.1284,0.12731,0.13837,0.13632,
0.1216,0.13273,0.11235,0.13996,0.13397,0.13084,0.14967,0.13081,0.131,0
.13026,0.13088,0.064232,0.064351,0.064343,0.065775,0.065575,0.063795,0
.064649,0.063934,0.065043,0.06565,0.065399,0.064218,0.065174,0.065599,
0.06307,0.063795,0.063506,0.13476,0.13244,0.13329,0.14186,0.12311,0.13
219,0.15625,0.13111,0.13408,0.12393,0.13536,0.13221,0.12412,0.13557,0.
12952,0.13025,0.064936,0.064637,0.063056,0.063678,0.063682,0.063429,0.
065714,0.066088,0.064291,0.064467,0.065496,0.06336,0.064182,0.066286,0
.064475,0.063877,0.063331,0.06402,0.064608,0.11564,0.13199,0.1306,0.13
65,0.13556,0.13102,0.13787,0.12924,0.1314,0.12431,0.13223,0.12656,0.13
325,0.06408,0.062925,0.065478,0.064508,0.06497,0.06336,0.065115,0.0652
71,0.066717,0.064576,0.064425,0.06389,0.064412,0.062601,0.063941,0.062
961,0.065218,0.06366,0.064108,0.064871,0.064283,0.14017,0.12576,0.1308
2,0.1287,0.12189,0.1402,0.14876,0.14173,0.13172,0.065879,0.062946,0.06
436,0.065337,0.064584,0.065575,0.064994,0.064976,0.06335,0.063402,0.06
4269,0.063837,0.064161,0.064732,0.064261,0.064647,0.064966,0.063866,0.
064731,0.06311,0.06613,0.065082,0.063649,0.06369,0.065265,0.12599,0.13
246,0.11454,0.12389,0.064975,0.064436,0.065525,0.064704,0.065693,0.064
593,0.064646,0.064687,0.063396,0.065215,0.065268,0.064379,0.064037,0.0
6356,0.064458,0.062584,0.066021,0.064475,0.062445,0.063544,0.064215,0.
063953,0.063851,0.064952,0.063655,0.064358,0.063682,0.064002,0.065034,
0.064747,0.064406,0.065362,0.065183,0.062795,0.06534,0.063382,0.064194
,0.064457,0.064086,0.065764,0.064328,0.064482,0.064574,0.064607,0.0659
69,0.065478,0.063581,0.063537,0.06365,0.065342,0.065691,0.063792,0.063
426,0.064327,0.063346,0.06405,0.064332,0.063359,0.064645,0.064436,0.06
3582,0.062959,0.06336,0.064199,0.064955,0.06476,0.064558,0.063574,0.06
289,0.064885,0.066417,0.062969,0.065459,0.064699,0.064485,0.064704,0.0
65729,0.066355,0.065846,0.063855,0.065332,0.065321,0.064865,0.064794,0

.062912,0.063733,0.064988,0.06446,0.063686,0.063234,0.066336,0.064178,
0.063677,0.064144,0.065252,0.064435,0.063257,0.064962,0.063758,0.06403
9,0.064349,0.064201,0.06527,0.066269,0.064682,0.06549,0.066179,0.06532
,0.065863,0.064572,0.065364,0.064801,0.066479,0.06402,0.065948,0.06413
7,0.062949,0.063226,0.064949,0.064759,0.064146,0.064139,0.064792,0.064
156,0.064901,0.06622,0.065826,0.064962,0.064484,0.06377,0.064473,0.064
393,0.062516,0.065216,0.063399,0.064868,0.063446,0.064047,0.065989,0.0
64892,0.065267,0.064588,0.062619,0.062958,0.065762,0.063998,0.063293,0
.064937,0.064188,0.065271,0.063502,0.064444,0.065087,0.064849,0.063723
,0.06527,0.064403,0.065721,0.064884,0.062864,0.064522,0.064088,0.06436
8,0.064248,0.064722,0.064283,0.065043,0.06601,0.065853,0.065176,0.0664
57,0.06412,0.064389,0.064498,0.064537,0.064475,0.064508,0.063251,0.063
524,0.06623,0.064715,0.063555,0.065581,0.063764,0.065313,0.06399,0.064
321,0.064419,0.064368,0.063785,0.065214,0.065828,0.065462,0.064256,0.0
63404
0.19902,0.19872,0.20491,0.20109,0.20257,0.19986,0.20359,0.20045,0.2003
8,0.2013,0.20266,0.20113,0.2028,0.2003,0.20395,0.19783,0.19805,0.20112
,0.2027,0.20148,0.28501,0.2972,0.27325,0.30061,0.19875,0.20099,0.20021
,0.2012,0.20282,0.20135,0.20169,0.20244,0.20128,0.19955,0.19894,0.2026
1,0.20348,0.19933,0.20049,0.37939,0.27986,0.27193,0.28715,0.28475,0.28
265,0.31369,0.2022,0.20256,0.19681,0.20307,0.20303,0.20059,0.19863,0.1
9978,0.20183,0.20168,0.19904,0.20153,0.19925,0.20437,0.20072,0.36511,0
.37183,0.30666,0.27969,0.28198,0.28854,0.26916,0.272,0.28365,0.29845,0
.20093,0.20322,0.20041,0.19778,0.20051,0.1998,0.20268,0.20166,0.19995,
0.2011,0.19966,0.20293,0.19989,0.20058,0.20197,0.37606,0.34999,0.38222
,0.3085,0.28856,0.2618,0.30057,0.28956,0.29526,0.28128,0.27972,0.2879,
0.19915,0.20219,0.20056,0.20084,0.20089,0.20035,0.19886,0.20241,0.2010
1,0.19945,0.19927,0.19814,0.20226,0.20244,0.19988,0.20266,0.36803,0.32
624,0.37643,0.3738,0.27831,0.29076,0.28398,0.27913,0.29064,0.29739,0.2
7967,0.2816,0.30369,0.26401,0.20033,0.19966,0.19956,0.20197,0.20022,0.
19998,0.20403,0.20076,0.19848,0.20335,0.20419,0.20464,0.19955,0.20034,
0.20113,0.20187,0.35682,0.38801,0.34863,0.33914,0.39786,0.39225,0.2792
9,0.29838,0.29777,0.27447,0.28173,0.29266,0.27974,0.28704,0.27696,0.28
499,0.20131,0.19931,0.20211,0.19958,0.20212,0.20049,0.1976,0.20217,0.2
0118,0.20214,0.20183,0.20295,0.20579,0.20119,0.20049,0.20165,0.19998,0
.35256,0.38349,0.37966,0.34764,0.3717,0.35106,0.29268,0.34908,0.27757,
0.27287,0.25713,0.3008,0.27199,0.30071,0.28509,0.30429,0.32611,0.20087
,0.20191,0.2029,0.20225,0.20103,0.20351,0.20121,0.20338,0.20156,0.1994
7,0.19956,0.19798,0.2003,0.13114,0.1358,0.1382,0.20209,0.36764,0.36914
,0.36053,0.3909,0.36761,0.36014,0.3665,0.28369,0.28327,0.28441,0.30619
,0.29295,0.28038,0.27998,0.27373,0.30695,0.27194,0.25957,0.20334,0.202
92,0.20133,0.19912,0.19798,0.19933,0.20032,0.19893,0.20033,0.20401,0.1
9985,0.12153,0.13856,0.12934,0.11599,0.12192,0.13063,0.20035,0.37963,0
.37665,0.37416,0.37468,0.37134,0.355,0.37528,0.28897,0.27373,0.28458,0
.25146,0.29434,0.29213,0.27249,0.30069,0.26842,0.28365,0.26667,0.2019,
0.20163,0.19785,0.20354,0.20276,0.19844,0.20204,0.20299,0.20122,0.1240
3,0.12616,0.12604,0.12803,0.12604,0.11566,0.13129,0.13367,0.129,0.2017
3,0.36259,0.36846,0.34977,0.38662,0.35671,0.33616,0.33564,0.28489,0.29
321,0.27935,0.26627,0.29223,0.28125,0.26736,0.32597,0.26886,0.28934,0.
28477,0.284,0.20246,0.20179,0.1987,0.20084,0.20193,0.19995,0.12482,0.1
2702,0.13236,0.13705,0.14051,0.132,0.13148,0.13801,0.13198,0.15285,0.1
2803,0.12861,0.38892,0.3754,0.33763,0.36695,0.39528,0.41246,0.37411,0.

29796,0.27958,0.28503,0.27854,0.2986,0.29187,0.27602,0.28548,0.26198,0
.29002,0.27772,0.31243,0.20091,0.20134,0.19969,0.13134,0.13235,0.13145
,0.13034,0.13467,0.13013,0.15108,0.13249,0.14658,0.12633,0.12715,0.138
5,0.13371,0.1277,0.14244,0.36923,0.35254,0.38032,0.33651,0.37155,0.397
77,0.37904,0.393,0.37772,0.29034,0.29165,0.28678,0.27321,0.28592,0.279
7,0.26798,0.29546,0.29454,0.28265,0.28074,0.13678,0.13882,0.13201,0.13
846,0.12239,0.13465,0.13283,0.12319,0.13346,0.1385,0.12944,0.1223,0.15
534,0.12947,0.13765,0.12538,0.13231,0.12721,0.38236,0.35878,0.39558,0.
35122,0.36838,0.36856,0.38412,0.37031,0.34462,0.27848,0.28265,0.17765,
0.20049,0.23546,0.19548,0.22507,0.22103,0.21358,0.21767,0.20297,0.1241
4,0.1416,0.11563,0.12609,0.13091,0.13137,0.10986,0.12789,0.13228,0.133
66,0.13415,0.13495,0.12505,0.13057,0.13126,0.12258,0.14927,0.13994,0.3
5304,0.36213,0.37033,0.37005,0.36682,0.36519,0.33861,0.36721,0.33962,0
.21215,0.21143,0.2122,0.23371,0.21598,0.20853,0.20705,0.21916,0.20519,
0.21552,0.21484,0.13936,0.12486,0.13223,0.127,0.13484,0.13756,0.14635,
0.13259,0.12748,0.11801,0.13049,0.13768,0.12622,0.13437,0.13489,0.1295
,0.12649,0.13209,0.35585,0.38076,0.35553,0.38392,0.40149,0.38761,0.379
02,0.20292,0.18585,0.22639,0.21602,0.21431,0.21366,0.20079,0.2103,0.20
674,0.21958,0.20588,0.18608,0.20222,0.12096,0.13245,0.13044,0.13343,0.
13831,0.12907,0.092621,0.13197,0.14269,0.13235,0.13154,0.12258,0.12993
,0.12433,0.13886,0.13083,0.13,0.12614,0.37218,0.36777,0.39031,0.38051,
0.34928,0.3661,0.19458,0.2263,0.22489,0.20454,0.21173,0.20793,0.2214,0
.20862,0.20906,0.22658,0.18441,0.21969,0.2221,0.211,0.14805,0.13082,0.
13018,0.1237,0.12173,0.13692,0.13651,0.12501,0.13148,0.13114,0.13001,0
.13086,0.12895,0.1324,0.1309,0.13591,0.15042,0.14158,0.35175,0.3667,0.
36351,0.36866,0.38791,0.18248,0.2073,0.2166,0.22688,0.22148,0.20728,0.
20837,0.20087,0.22378,0.20744,0.19547,0.1979,0.21022,0.20841,0.21599,0
.13474,0.1323,0.12665,0.12411,0.13124,0.13057,0.14321,0.11983,0.1429,0
.13242,0.13017,0.13204,0.127,0.13262,0.13153,0.13159,0.13483,0.13691,0
.3599,0.38359,0.37156,0.25752,0.2279,0.26838,0.22834,0.22029,0.21804,0
.19969,0.22516,0.21907,0.22404,0.21474,0.2167,0.21545,0.22951,0.2287,0
.21854,0.25418,0.1356,0.1319,0.1363,0.13095,0.12961,0.14331,0.13391,0.
12944,0.14198,0.11969,0.14366,0.13258,0.13735,0.12194,0.14507,0.13617,
0.13194,0.12977,0.39831,0.20005,0.19127,0.21272,0.21831,0.23455,0.2196
9,0.19688,0.22661,0.23972,0.21997,0.21242,0.21312,0.21232,0.23635,0.19
5,0.19059,0.20171,0.21249,0.13148,0.12973,0.12019,0.13799,0.13324,0.13
07,0.13426,0.13101,0.12086,0.13551,0.12731,0.12975,0.13215,0.13711,0.1
2997,0.12159,0.13813,0.060452,0.21361,0.22137,0.19747,0.2187,0.21696,0
.22753,0.19309,0.22629,0.21529,0.20772,0.21623,0.20796,0.21001,0.21457
,0.19557,0.20809,0.24304,0.21109,0.20848,0.12188,0.131,0.1308,0.12425,
0.13151,0.13379,0.12762,0.13091,0.13003,0.12921,0.13122,0.11731,0.1284
4,0.13254,0.12557,0.12071,0.13096,0.062067,0.21647,0.2087,0.20729,0.13
965,0.21863,0.22086,0.214,0.23221,0.23293,0.22133,0.22689,0.21851,0.20
12,0.20965,0.19646,0.21257,0.20204,0.21842,0.12855,0.12954,0.12676,0.1
3016,0.13416,0.1332,0.13009,0.13951,0.12481,0.11636,0.10669,0.13118,0.
12443,0.13116,0.11827,0.11841,0.12695,0.13772,0.062084,0.22947,0.21669
,0.20638,0.18184,0.22511,0.22202,0.21228,0.21919,0.18497,0.21229,0.237
3,0.20793,0.19988,0.19332,0.22728,0.2164,0.18912,0.2412,0.13515,0.1336
1,0.13243,0.12929,0.13599,0.13665,0.1377,0.12582,0.13018,0.13989,0.109
38,0.13351,0.11374,0.13517,0.13182,0.12084,0.13213,0.061781,0.062251,0
.062665,0.21272,0.2656,0.20031,0.20465,0.18932,0.19709,0.18708,0.18733
,0.20905,0.24405,0.19963,0.22541,0.2195,0.21029,0.205,0.1412,0.13167,0

.13321,0.12935,0.1147,0.13976,0.13063,0.13753,0.1271,0.1351,0.1278,0.1
2957,0.13116,0.14319,0.14131,0.1315,0.13823,0.1298,0.061181,0.060922,0
.060973,0.061468,0.22438,0.22921,0.2238,0.10999,0.20817,0.23558,0.1833
8,0.24324,0.19536,0.1946,0.21193,0.20519,0.14094,0.11715,0.12413,0.131
6,0.13063,0.14508,0.12799,0.12439,0.13282,0.1304,0.14712,0.1373,0.1314
3,0.13209,0.11749,0.13321,0.13127,0.13313,0.14125,0.13438,0.059718,0.0
61356,0.061297,0.061198,0.061262,0.062585,0.20929,0.21294,0.18557,0.16
615,0.2139,0.22988,0.21514,0.18293,0.1372,0.11451,0.13861,0.13447,0.12
99,0.15289,0.13103,0.12379,0.13446,0.13208,0.12836,0.13101,0.13131,0.1
2323,0.13123,0.13367,0.12485,0.13164,0.15788,0.13029,0.13337,0.13122,0
.1369,0.061155,0.059898,0.060768,0.06138,0.061444,0.060989,0.22056,0.2
4288,0.15488,0.20966,0.21771,0.062165,0.061097,0.13283,0.12155,0.14878
,0.13062,0.13441,0.13223,0.12969,0.13649,0.13402,0.13088,0.12983,0.133
72,0.11266,0.11877,0.13312,0.14982,0.143,0.14019,0.12962,0.13673,0.122
13,0.060458,0.059487,0.062638,0.061899,0.060867,0.060171,0.062019,0.06
1613,0.060959,0.062098,0.20735,0.2332,0.060445,0.061762,0.062702,0.060
651,0.12722,0.13416,0.13563,0.15269,0.12694,0.12565,0.13781,0.136,0.11
976,0.13205,0.11147,0.13975,0.13405,0.13042,0.14829,0.12937,0.12984,0.
12974,0.1304,0.061619,0.06209,0.061088,0.061784,0.062668,0.060345,0.06
1236,0.06097,0.061671,0.062407,0.062108,0.061537,0.06151,0.062423,0.05
9973,0.060695,0.060296,0.13407,0.13065,0.13327,0.14098,0.12189,0.13156
,0.15668,0.13023,0.13364,0.12235,0.13462,0.13099,0.12301,0.13452,0.127
37,0.12942,0.062269,0.06141,0.060775,0.06024,0.060796,0.060414,0.06192
7,0.063019,0.060801,0.060798,0.062001,0.060062,0.061093,0.063292,0.062
216,0.060527,0.060646,0.061488,0.062081,0.11363,0.1312,0.12987,0.13525
,0.1354,0.13041,0.13829,0.12784,0.13045,0.12386,0.13157,0.12538,0.1322
9,0.060867,0.060719,0.062255,0.060801,0.061679,0.060037,0.062355,0.062
181,0.063462,0.061331,0.061282,0.060712,0.060381,0.059761,0.060556,0.0
60349,0.061632,0.06022,0.061193,0.061345,0.06132,0.1393,0.12529,0.1298
,0.12957,0.12101,0.13984,0.14818,0.14094,0.1311,0.061938,0.06009,0.061
259,0.062056,0.061651,0.062358,0.061571,0.061847,0.060345,0.060605,0.0
60994,0.060498,0.060899,0.061717,0.060616,0.06176,0.061172,0.060742,0.
06141,0.060373,0.063573,0.061915,0.060604,0.061149,0.061991,0.12431,0.
13187,0.11294,0.12305,0.061455,0.060809,0.062154,0.061369,0.062331,0.0
61666,0.061257,0.06158,0.05999,0.062202,0.062241,0.061067,0.06049,0.06
0882,0.061071,0.059355,0.062902,0.061538,0.058994,0.060123,0.061531,0.
060791,0.061211,0.061871,0.060729,0.060985,0.059812,0.061231,0.062364,
0.060917,0.061328,0.061557,0.061993,0.059874,0.061954,0.060669,0.06109
1,0.061735,0.060575,0.062182,0.061573,0.061818,0.062012,0.062174,0.063
265,0.062846,0.060305,0.060714,0.060633,0.062472,0.062645,0.060825,0.0
60452,0.061217,0.059935,0.060665,0.061795,0.06007,0.061975,0.061655,0.
060625,0.059861,0.06043,0.060897,0.062065,0.061754,0.060903,0.060072,0
.060067,0.062163,0.06281,0.059563,0.061475,0.061132,0.061336,0.061886,
0.062023,0.063241,0.062931,0.061341,0.061462,0.061944,0.060955,0.06115
6,0.059784,0.060666,0.06258,0.060813,0.059776,0.060804,0.062602,0.0612
6,0.060266,0.060982,0.062238,0.061382,0.060209,0.061137,0.060078,0.061
152,0.061568,0.060685,0.062217,0.062885,0.061912,0.062229,0.062294,0.0
62208,0.062942,0.061885,0.061931,0.061749,0.063312,0.060278,0.063031,0
.060631,0.059676,0.060875,0.061522,0.061824,0.060715,0.061126,0.061319
,0.061098,0.062449,0.062786,0.062626,0.061729,0.061112,0.060123,0.0615
82,0.062056,0.059957,0.061803,0.061033,0.061474,0.060184,0.061411,0.06
2734,0.061241,0.06241,0.061384,0.060023,0.060117,0.062907,0.061264,0.0

60204,0.062126,0.06078,0.061576,0.060308,0.062049,0.061817,0.061623,0.
060198,0.061903,0.060656,0.062,0.06185,0.059877,0.06169,0.060886,0.061
039,0.061079,0.061944,0.061445,0.062083,0.06258,0.062632,0.061852,0.06
3837,0.060786,0.061391,0.061733,0.061658,0.061899,0.061233,0.059965,0.
060591,0.062494,0.062305,0.060614,0.062506,0.061094,0.061094,0.060398,
0.060966,0.061309,0.061067,0.060556,0.06228,0.061954,0.062548,0.061051
,0.061386
0.20079,0.19898,0.20623,0.20341,0.20254,0.20158,0.20539,0.20254,0.2017
6,0.20266,0.2042,0.20098,0.20441,0.20133,0.2044,0.19941,0.20024,0.2022
9,0.20299,0.20187,0.27539,0.2958,0.27059,0.29877,0.19901,0.20094,0.201
02,0.20264,0.20323,0.20229,0.20412,0.2031,0.20187,0.20017,0.20055,0.20
322,0.20439,0.20066,0.20122,0.36987,0.27538,0.2741,0.28009,0.28857,0.2
8015,0.30686,0.2031,0.20509,0.1986,0.20409,0.20289,0.20219,0.1992,0.20
121,0.2028,0.20171,0.2002,0.2019,0.20077,0.20411,0.20236,0.35348,0.369
4,0.30098,0.28753,0.27885,0.2756,0.26866,0.26905,0.28486,0.30114,0.202
53,0.20397,0.20246,0.19829,0.20255,0.20149,0.204,0.20206,0.20167,0.202
45,0.20149,0.20355,0.20116,0.20365,0.20338,0.39848,0.33973,0.36781,0.3
0365,0.28002,0.26204,0.29421,0.28461,0.29519,0.27415,0.28499,0.28588,0
.20112,0.20316,0.20225,0.20278,0.202,0.2016,0.20103,0.20343,0.20202,0.
20169,0.20089,0.20001,0.20468,0.2042,0.20091,0.20257,0.34927,0.32151,0
.37225,0.37402,0.27302,0.28616,0.27905,0.28183,0.29289,0.29404,0.27818
,0.27361,0.29543,0.26828,0.20171,0.20054,0.20067,0.20341,0.20254,0.201
21,0.20502,0.20157,0.20029,0.20369,0.20667,0.20552,0.20216,0.20271,0.2
0277,0.20241,0.34906,0.37093,0.33821,0.33165,0.39203,0.39231,0.27939,0
.29952,0.29701,0.27792,0.28257,0.28979,0.28051,0.29177,0.27505,0.28534
,0.20188,0.20024,0.20318,0.20122,0.20268,0.20213,0.1984,0.20288,0.2026
5,0.20366,0.20342,0.20504,0.20723,0.20222,0.20162,0.20151,0.20109,0.34
014,0.38223,0.3732,0.34194,0.36263,0.35886,0.29621,0.33528,0.27627,0.2
7172,0.25474,0.3043,0.26983,0.30323,0.28716,0.29933,0.3139,0.2013,0.20
225,0.20599,0.20369,0.20249,0.20429,0.20233,0.20414,0.20165,0.20109,0.
20119,0.19953,0.20096,0.13031,0.13507,0.13724,0.20428,0.37491,0.36914,
0.33885,0.38659,0.36404,0.36474,0.38162,0.28562,0.27698,0.28547,0.2975
8,0.28839,0.27517,0.27526,0.26068,0.30491,0.27476,0.25832,0.20458,0.20
301,0.20139,0.1996,0.19908,0.19941,0.20185,0.20072,0.20164,0.20388,0.2
0045,0.12038,0.13828,0.12949,0.11517,0.1216,0.12993,0.20148,0.37848,0.
37693,0.35985,0.37243,0.35924,0.35263,0.38102,0.29098,0.27262,0.28569,
0.25437,0.29116,0.29762,0.2697,0.30045,0.27037,0.28214,0.27075,0.20461
,0.20312,0.19907,0.20363,0.20427,0.19996,0.20325,0.20515,0.20149,0.121
77,0.12489,0.12503,0.12783,0.12577,0.11469,0.13116,0.13337,0.12868,0.2
0244,0.35871,0.36353,0.34838,0.38008,0.35334,0.34365,0.3335,0.28287,0.
28939,0.28008,0.26305,0.28697,0.27785,0.26077,0.32081,0.27061,0.28617,
0.28316,0.28088,0.2029,0.20276,0.20045,0.20259,0.20313,0.20041,0.124,0
.12757,0.13133,0.13701,0.13978,0.13123,0.13029,0.13781,0.1315,0.15331,
0.12636,0.12867,0.37684,0.3817,0.35128,0.37493,0.37467,0.4111,0.36814,
0.29389,0.28518,0.28062,0.26593,0.29931,0.29101,0.27702,0.27942,0.2638
4,0.29079,0.2751,0.31495,0.20167,0.20229,0.20159,0.13098,0.13151,0.130
63,0.12993,0.13518,0.12938,0.15131,0.13141,0.14625,0.1252,0.1259,0.137
47,0.13306,0.12705,0.14188,0.35614,0.34545,0.3729,0.33995,0.35886,0.39
11,0.37913,0.38452,0.37839,0.27887,0.28079,0.28562,0.27217,0.28521,0.2
835,0.26762,0.29775,0.29121,0.2762,0.2828,0.13626,0.13843,0.13037,0.13
843,0.12069,0.1339,0.13221,0.12226,0.13283,0.13785,0.12939,0.12112,0.1
5465,0.12811,0.1371,0.12452,0.13141,0.1258,0.37436,0.34587,0.38552,0.3

4914,0.3778,0.37236,0.38253,0.3706,0.33732,0.27825,0.27845,0.17591,0.2
0257,0.23346,0.19393,0.22199,0.21679,0.20576,0.21953,0.20137,0.12282,0
.14103,0.11437,0.12655,0.13038,0.13107,0.10855,0.1278,0.13137,0.13188,
0.1337,0.13493,0.12513,0.12966,0.1305,0.12195,0.14913,0.13906,0.35289,
0.35509,0.3675,0.36822,0.36312,0.35995,0.34548,0.3579,0.35139,0.20982,
0.21482,0.20932,0.22646,0.2116,0.20746,0.20772,0.22095,0.20658,0.2142,
0.21187,0.13918,0.12451,0.13223,0.12696,0.1344,0.13731,0.14514,0.13121
,0.12605,0.11694,0.12996,0.13796,0.1247,0.13281,0.13506,0.12866,0.1248
7,0.13237,0.36683,0.38186,0.36194,0.38512,0.39588,0.37681,0.38423,0.19
971,0.18635,0.22167,0.21471,0.21729,0.21141,0.20197,0.20652,0.20177,0.
21677,0.20122,0.18512,0.20042,0.11933,0.13239,0.12957,0.13311,0.13741,
0.12815,0.09123,0.13194,0.14134,0.13183,0.13006,0.12192,0.12906,0.1242
3,0.13859,0.13077,0.1296,0.1252,0.36675,0.36023,0.38692,0.36965,0.3437
,0.37268,0.19563,0.22384,0.22666,0.19865,0.21286,0.20787,0.21596,0.206
32,0.20898,0.21821,0.1859,0.21166,0.22008,0.21281,0.14832,0.13044,0.13
,0.12223,0.12136,0.13609,0.13712,0.12386,0.13073,0.13015,0.1295,0.1306
3,0.12824,0.13253,0.12999,0.13618,0.14964,0.1417,0.33552,0.37268,0.366
24,0.37734,0.37374,0.18122,0.20529,0.21254,0.22725,0.21537,0.19871,0.2
0608,0.19486,0.218,0.20275,0.19484,0.19576,0.20917,0.20695,0.21894,0.1
3376,0.1313,0.12549,0.12274,0.13012,0.12963,0.1425,0.11933,0.14199,0.1
3173,0.12951,0.13156,0.12611,0.13149,0.13106,0.13049,0.13347,0.1359,0.
35318,0.37601,0.36949,0.2476,0.22073,0.26789,0.22041,0.21904,0.21753,0
.19306,0.22665,0.22145,0.22092,0.20927,0.21631,0.21134,0.22892,0.22884
,0.21469,0.25087,0.13501,0.13131,0.13562,0.13005,0.12837,0.14353,0.133
53,0.1287,0.14075,0.11794,0.14328,0.13197,0.13716,0.1204,0.14481,0.134
86,0.13145,0.12951,0.39331,0.19746,0.1872,0.20521,0.22013,0.22376,0.21
872,0.19544,0.21416,0.24641,0.21809,0.20719,0.21234,0.20822,0.23434,0.
19606,0.1913,0.20278,0.2106,0.13113,0.12928,0.11836,0.13751,0.13215,0.
13031,0.13306,0.13011,0.12037,0.13473,0.12589,0.12875,0.13148,0.13617,
0.13002,0.12076,0.13809,0.057351,0.20749,0.22296,0.1928,0.21807,0.2172
4,0.22229,0.1907,0.21754,0.20888,0.20658,0.21058,0.20449,0.20566,0.211
87,0.19304,0.21042,0.24199,0.20815,0.20693,0.12131,0.13158,0.13055,0.1
229,0.13078,0.13222,0.1268,0.13009,0.12901,0.12849,0.12997,0.11646,0.1
2739,0.1325,0.12446,0.12023,0.13024,0.059694,0.20948,0.20781,0.19925,0
.13882,0.2153,0.21744,0.21025,0.22421,0.23327,0.22014,0.22691,0.21433,
0.20254,0.21344,0.19877,0.20709,0.2021,0.21926,0.12825,0.12946,0.12671
,0.13042,0.13326,0.13231,0.12924,0.13987,0.12416,0.11541,0.10559,0.130
75,0.12368,0.13116,0.1171,0.11818,0.12556,0.1374,0.059742,0.23067,0.21
928,0.20001,0.1821,0.21986,0.21897,0.21312,0.22049,0.17507,0.20606,0.2
3999,0.21058,0.20214,0.18864,0.22554,0.21128,0.18455,0.24201,0.13499,0
.13282,0.13141,0.12812,0.13577,0.13668,0.13706,0.12629,0.12861,0.13997
,0.1089,0.13354,0.1122,0.13525,0.13028,0.11933,0.13123,0.059316,0.0598
77,0.060041,0.20733,0.26245,0.19802,0.20594,0.18654,0.1943,0.17943,0.1
874,0.20921,0.24068,0.19524,0.22521,0.21673,0.20817,0.20132,0.13978,0.
13002,0.13224,0.12894,0.11342,0.13906,0.13147,0.1372,0.12642,0.1345,0.
1273,0.12792,0.13106,0.14265,0.14009,0.1305,0.13729,0.12845,0.05874,0.
058376,0.058618,0.058504,0.22045,0.21822,0.22143,0.10844,0.20083,0.236
03,0.17314,0.23484,0.19326,0.19693,0.20657,0.20189,0.14039,0.11631,0.1
2326,0.13034,0.12897,0.14548,0.12781,0.12362,0.13136,0.12889,0.14687,0
.1367,0.13021,0.13173,0.11643,0.13249,0.13042,0.13319,0.13971,0.13351,
0.057146,0.059064,0.057961,0.05896,0.058564,0.059342,0.20212,0.20797,0
.18804,0.16305,0.20996,0.22352,0.20938,0.18089,0.13631,0.11314,0.1385,

0.13397,0.12935,0.15225,0.1303,0.12283,0.13405,0.13164,0.12789,0.13086
,0.13068,0.12223,0.12984,0.13333,0.12448,0.13126,0.15875,0.12976,0.133
49,0.12992,0.13581,0.058877,0.057659,0.057958,0.059062,0.059143,0.0584
59,0.21684,0.24965,0.14691,0.20503,0.21715,0.059899,0.058863,0.13404,0
.12038,0.14826,0.13059,0.13392,0.13155,0.12894,0.13624,0.13311,0.13025
,0.12875,0.13301,0.11152,0.1183,0.13247,0.14956,0.1423,0.13967,0.12866
,0.13603,0.12105,0.058282,0.056755,0.060343,0.058564,0.058857,0.057212
,0.059737,0.059459,0.058461,0.059743,0.19858,0.22401,0.057895,0.05961,
0.060226,0.058405,0.12783,0.13324,0.13557,0.15235,0.12574,0.12444,0.13
714,0.13588,0.11848,0.13123,0.10983,0.13974,0.13285,0.1298,0.14737,0.1
2975,0.12927,0.12854,0.12999,0.058702,0.059659,0.058556,0.059239,0.060
338,0.057722,0.058582,0.058065,0.059514,0.059483,0.058974,0.059268,0.0
58405,0.060371,0.057691,0.058326,0.058027,0.1331,0.13004,0.13295,0.140
58,0.12154,0.13126,0.15657,0.12918,0.13209,0.12323,0.13486,0.13052,0.1
2191,0.13426,0.1266,0.12842,0.059671,0.057977,0.057659,0.058081,0.0582
34,0.057797,0.059729,0.060748,0.058703,0.058075,0.059376,0.057664,0.05
8297,0.059701,0.05979,0.057863,0.058156,0.058751,0.059076,0.11275,0.13
041,0.12933,0.13443,0.13507,0.12994,0.13749,0.12678,0.12971,0.12282,0.
13151,0.12447,0.13098,0.058484,0.058972,0.059634,0.058056,0.059465,0.0
57328,0.060767,0.059646,0.060774,0.058688,0.058133,0.058972,0.057606,0
.056948,0.057799,0.057681,0.059237,0.057707,0.058871,0.059311,0.058858
,0.13835,0.12536,0.12937,0.12844,0.12041,0.14077,0.14722,0.1403,0.1309
3,0.059484,0.057302,0.059079,0.059427,0.058819,0.059489,0.05873,0.0591
18,0.057282,0.057543,0.058847,0.05822,0.058744,0.059039,0.05809,0.0590
76,0.058094,0.058022,0.059474,0.0581,0.061211,0.059433,0.058013,0.0579
06,0.059401,0.12415,0.13157,0.1108,0.12237,0.058297,0.057833,0.059153,
0.058161,0.05949,0.059023,0.058267,0.058457,0.058039,0.059966,0.059561
,0.059359,0.058446,0.057733,0.057973,0.05615,0.060523,0.058561,0.05655
,0.058742,0.059459,0.058169,0.059072,0.059113,0.057627,0.059128,0.0572
82,0.058168,0.059997,0.058431,0.058999,0.059514,0.05928,0.057821,0.059
508,0.058566,0.057896,0.059479,0.057055,0.059218,0.059055,0.058964,0.0
59547,0.059328,0.06104,0.0605,0.05792,0.057702,0.058127,0.059416,0.059
976,0.058019,0.057325,0.059077,0.058178,0.058376,0.05934,0.057736,0.05
942,0.058921,0.057969,0.057567,0.057839,0.058292,0.05981,0.059439,0.05
7944,0.057359,0.057281,0.059798,0.060296,0.057105,0.058414,0.058471,0.
057866,0.059525,0.059063,0.059759,0.060694,0.058974,0.059086,0.0591,0.
058642,0.058407,0.05806,0.058268,0.059944,0.058017,0.05778,0.058474,0.
05984,0.058985,0.05822,0.057947,0.060039,0.058515,0.057966,0.059238,0.
057434,0.057969,0.059343,0.058066,0.059335,0.059715,0.05931,0.05966,0.
059511,0.059786,0.060416,0.058723,0.059032,0.058956,0.06068,0.057821,0
.06095,0.058546,0.057515,0.058004,0.058343,0.059202,0.058622,0.059033,
0.0585,0.059047,0.059617,0.059998,0.059708,0.059229,0.058816,0.057223,
0.059162,0.058855,0.057595,0.059948,0.058338,0.059082,0.057515,0.05901
,0.059472,0.058885,0.060246,0.058634,0.057489,0.058691,0.060393,0.0586
09,0.058053,0.059425,0.057755,0.05866,0.057715,0.059441,0.059006,0.059
637,0.057795,0.058899,0.058191,0.058708,0.059157,0.05781,0.058837,0.05
8603,0.058961,0.058498,0.059776,0.058964,0.05947,0.0595,0.05914,0.0592
32,0.060574,0.058176,0.059841,0.058731,0.05931,0.059019,0.058334,0.056
459,0.057291,0.060036,0.059068,0.058212,0.059639,0.05815,0.058679,0.05
7656,0.058085,0.05895,0.057675,0.058196,0.059508,0.05926,0.06026,0.058
696,0.058619

0.20306,0.20155,0.20676,0.20487,0.20344,0.2026,0.20562,0.20384,0.2017,
0.20269,0.20482,0.20237,0.20527,0.20289,0.20452,0.20117,0.20119,0.2034
,0.20465,0.20185,0.27819,0.29575,0.27099,0.29999,0.19907,0.20188,0.202
03,0.20377,0.20464,0.20319,0.20519,0.20367,0.203,0.20155,0.20089,0.204
75,0.20596,0.20227,0.20282,0.36464,0.27267,0.27486,0.26876,0.28384,0.2
8227,0.3056,0.20412,0.20612,0.19883,0.20447,0.20345,0.2026,0.20019,0.2
011,0.20431,0.2034,0.20218,0.20204,0.2019,0.20342,0.20308,0.35491,0.35
862,0.2973,0.27998,0.28261,0.27886,0.26905,0.26902,0.27286,0.30939,0.2
0278,0.20524,0.20449,0.19917,0.20297,0.20131,0.20604,0.20306,0.20314,0
.20354,0.20167,0.20513,0.20099,0.20426,0.20363,0.40389,0.33789,0.37844
,0.30699,0.27536,0.26669,0.29129,0.27898,0.29162,0.27804,0.28724,0.283
33,0.20208,0.20339,0.20222,0.20383,0.20345,0.20284,0.20156,0.20426,0.2
0162,0.20168,0.20232,0.20121,0.20497,0.20601,0.20205,0.20379,0.33727,0
.31952,0.36405,0.37494,0.26559,0.28362,0.2796,0.28351,0.29381,0.2993,0
.27413,0.27051,0.29261,0.27117,0.20273,0.20325,0.20136,0.20466,0.2026,
0.20188,0.20571,0.20258,0.20165,0.20521,0.20663,0.20629,0.20263,0.2033
9,0.20413,0.20346,0.35179,0.37216,0.33249,0.32555,0.37476,0.39334,0.28
031,0.29147,0.29812,0.27645,0.27871,0.287,0.28585,0.29306,0.27627,0.28
739,0.20299,0.20158,0.20331,0.20291,0.20432,0.20384,0.20132,0.2037,0.2
0364,0.20505,0.20485,0.20504,0.20681,0.20198,0.20198,0.20296,0.20143,0
.3322,0.38425,0.3638,0.3343,0.34814,0.36063,0.29587,0.33602,0.28153,0.
26816,0.25598,0.30143,0.27186,0.29822,0.28691,0.2916,0.30788,0.20273,0
.20298,0.20597,0.20396,0.20284,0.2044,0.20282,0.20501,0.20146,0.20187,
0.20078,0.19974,0.20137,0.12959,0.13449,0.13742,0.2055,0.37164,0.35438
,0.34143,0.36362,0.35883,0.365,0.37284,0.28529,0.27617,0.2845,0.29833,
0.28413,0.27835,0.27705,0.25715,0.29796,0.27083,0.25957,0.20501,0.2046
1,0.20392,0.20088,0.20118,0.20074,0.20367,0.20206,0.20227,0.20552,0.20
259,0.11948,0.13667,0.12911,0.11444,0.12052,0.12997,0.20315,0.38111,0.
36988,0.35675,0.36005,0.35882,0.35048,0.39585,0.2907,0.27381,0.28898,0
.25195,0.29414,0.2944,0.26897,0.30096,0.27417,0.28383,0.26954,0.2048,0
.20295,0.20014,0.20405,0.20468,0.20056,0.20432,0.20699,0.20212,0.12153
,0.12447,0.12509,0.12698,0.12526,0.11349,0.13125,0.13305,0.12841,0.202
37,0.36986,0.37004,0.34331,0.38345,0.35853,0.32793,0.34199,0.2845,0.29
173,0.28212,0.25499,0.28155,0.27804,0.25948,0.31355,0.27395,0.28532,0.
27829,0.27247,0.20429,0.20418,0.20217,0.20297,0.20529,0.20133,0.12332,
0.12761,0.13106,0.13604,0.1391,0.13066,0.1306,0.13846,0.13069,0.15275,
0.12572,0.12887,0.37429,0.38178,0.34779,0.36049,0.38353,0.40295,0.3655
9,0.29191,0.28337,0.28549,0.2688,0.29908,0.28785,0.2819,0.28006,0.2628
,0.29645,0.27581,0.30177,0.20161,0.20318,0.20259,0.13092,0.13189,0.129
56,0.1288,0.13432,0.12973,0.15098,0.1306,0.14652,0.12391,0.12584,0.136
43,0.13289,0.12626,0.14171,0.35379,0.33207,0.37503,0.32886,0.34618,0.3
8035,0.37952,0.37663,0.37443,0.27711,0.27301,0.28499,0.27701,0.28402,0
.28279,0.26414,0.29695,0.28655,0.27532,0.28348,0.13644,0.13795,0.12869
,0.13767,0.12014,0.13385,0.13176,0.12067,0.13221,0.13703,0.12939,0.119
81,0.15411,0.12794,0.13633,0.12393,0.13079,0.12511,0.37566,0.34301,0.3
8815,0.34319,0.36762,0.3748,0.37168,0.37167,0.33605,0.27633,0.27325,0.
17388,0.20223,0.2307,0.19565,0.21733,0.21757,0.20017,0.21921,0.20531,0
.12175,0.1396,0.11349,0.12628,0.1291,0.13032,0.10806,0.12666,0.13071,0
.13187,0.13258,0.13509,0.12416,0.1284,0.12967,0.12194,0.14906,0.13892,
0.34155,0.34989,0.36884,0.37071,0.34787,0.36178,0.33712,0.35387,0.3593
9,0.20734,0.21567,0.20871,0.2239,0.20658,0.20407,0.20729,0.22087,0.207
55,0.21435,0.20947,0.13816,0.12411,0.13188,0.12661,0.13347,0.13776,0.1

4434,0.13075,0.12626,0.11527,0.12929,0.13736,0.12384,0.13244,0.13382,0
.12785,0.1247,0.13183,0.36554,0.37891,0.35273,0.38973,0.39326,0.38045,
0.38323,0.19601,0.18641,0.21728,0.21617,0.21442,0.20814,0.20344,0.2009
5,0.20024,0.21833,0.20236,0.18338,0.19971,0.11895,0.13072,0.12929,0.13
267,0.13804,0.12787,0.089289,0.13117,0.14031,0.13084,0.12991,0.12097,0
.12911,0.12286,0.13838,0.13043,0.1291,0.12424,0.37321,0.35664,0.37063,
0.35271,0.35461,0.37379,0.19526,0.21968,0.22381,0.19648,0.21256,0.2038
6,0.21598,0.20567,0.21018,0.21664,0.18814,0.21197,0.21852,0.21482,0.14
869,0.12932,0.13015,0.12195,0.12046,0.13522,0.13654,0.12342,0.13003,0.
12918,0.12855,0.13029,0.12752,0.13168,0.12931,0.1367,0.14925,0.14079,0
.32641,0.37428,0.36272,0.3609,0.36341,0.176,0.20865,0.20438,0.22258,0.
21602,0.19551,0.20874,0.19603,0.21743,0.20389,0.19376,0.19306,0.21046,
0.2059,0.21756,0.13328,0.13086,0.12479,0.12125,0.12903,0.12986,0.14155
,0.11846,0.14165,0.13186,0.12922,0.13133,0.12501,0.13083,0.13028,0.129
61,0.13357,0.13605,0.34436,0.3701,0.35934,0.24796,0.22065,0.26294,0.21
81,0.21764,0.21441,0.18656,0.21998,0.21664,0.21801,0.20973,0.21263,0.2
0662,0.23003,0.22714,0.21295,0.24612,0.13467,0.12985,0.13462,0.12911,0
.12743,0.14406,0.13213,0.12791,0.14005,0.11763,0.14287,0.13092,0.13614
,0.11919,0.14518,0.13384,0.13057,0.12939,0.37899,0.1961,0.18582,0.2073
5,0.22614,0.22262,0.21277,0.18834,0.20809,0.24032,0.21627,0.20635,0.21
22,0.20534,0.2296,0.19308,0.1906,0.20456,0.2074,0.13071,0.1283,0.1174,
0.13709,0.13091,0.13059,0.13425,0.12885,0.12054,0.13416,0.12503,0.1276
9,0.1307,0.13704,0.13024,0.12013,0.1373,0.055264,0.21027,0.21441,0.188
95,0.21212,0.21289,0.21999,0.18904,0.21561,0.20303,0.20743,0.20862,0.2
0493,0.20573,0.20666,0.19055,0.21076,0.24567,0.20821,0.2079,0.12162,0.
13111,0.1305,0.12177,0.12982,0.13179,0.12648,0.12978,0.12846,0.12815,0
.12962,0.11516,0.12766,0.13137,0.12334,0.11887,0.13025,0.057033,0.2051
3,0.20863,0.19325,0.13892,0.21706,0.21451,0.21316,0.22747,0.23229,0.21
582,0.22543,0.21363,0.20074,0.21133,0.19821,0.20705,0.20124,0.21477,0.
12774,0.12845,0.12621,0.12983,0.13259,0.132,0.12781,0.13992,0.12335,0.
11408,0.10442,0.12997,0.12318,0.13063,0.11669,0.1174,0.12511,0.13716,0
.056527,0.22624,0.2181,0.20537,0.18414,0.21842,0.21835,0.20919,0.21739
,0.17301,0.20806,0.23816,0.20706,0.20194,0.18959,0.22297,0.20817,0.186
77,0.24063,0.13467,0.13194,0.131,0.12633,0.1353,0.13619,0.13694,0.1253
7,0.12784,0.13996,0.10783,0.13284,0.11132,0.13507,0.12917,0.11791,0.13
052,0.057406,0.057796,0.058354,0.20548,0.25571,0.20115,0.20414,0.18568
,0.19086,0.17174,0.18226,0.21021,0.24311,0.19092,0.23028,0.2132,0.2030
5,0.20421,0.13999,0.12995,0.13178,0.1281,0.11202,0.13844,0.13089,0.136
85,0.12584,0.13435,0.1279,0.12759,0.12977,0.14195,0.13951,0.12998,0.13
686,0.12721,0.056297,0.05635,0.056476,0.056564,0.22011,0.21385,0.21805
,0.10609,0.19353,0.23639,0.1719,0.23154,0.19208,0.20148,0.20828,0.2034
3,0.14008,0.1152,0.12307,0.12944,0.12894,0.14438,0.12699,0.12368,0.130
86,0.12809,0.14617,0.13675,0.12902,0.13158,0.1163,0.13228,0.12966,0.13
256,0.13914,0.1325,0.05534,0.057002,0.05646,0.056751,0.055927,0.05659,
0.20169,0.20279,0.18771,0.15606,0.21571,0.22332,0.20325,0.17711,0.1354
7,0.11245,0.138,0.1337,0.12796,0.15264,0.13033,0.12149,0.13441,0.13111
,0.12743,0.12981,0.13086,0.1214,0.12844,0.1321,0.12388,0.13011,0.15829
,0.12891,0.13221,0.13011,0.13509,0.056998,0.056249,0.055675,0.056904,0
.057787,0.05611,0.20962,0.23987,0.14579,0.20076,0.21437,0.056768,0.057
032,0.13383,0.11978,0.14891,0.12997,0.13317,0.13186,0.12866,0.13622,0.
13268,0.12974,0.12865,0.13154,0.11066,0.11769,0.13178,0.14931,0.14224,
0.13888,0.12749,0.13538,0.11948,0.055795,0.054311,0.058248,0.056402,0.

05609,0.055301,0.057635,0.057328,0.056561,0.057288,0.19761,0.22157,0.0
55774,0.057241,0.058812,0.055605,0.12711,0.13278,0.13608,0.15284,0.125
41,0.12404,0.13759,0.13534,0.1173,0.13142,0.10887,0.13916,0.13209,0.12
931,0.14686,0.12902,0.12855,0.12888,0.12949,0.056498,0.057773,0.05675,
0.057327,0.058202,0.055745,0.056975,0.057055,0.057725,0.057691,0.05716
3,0.057102,0.057162,0.058416,0.055478,0.056679,0.05578,0.13292,0.1296,
0.13276,0.13962,0.12121,0.13057,0.15573,0.12949,0.13116,0.12305,0.1349
3,0.12945,0.12157,0.13314,0.12615,0.12722,0.057523,0.055498,0.056861,0
.055436,0.056559,0.055585,0.057735,0.058439,0.056157,0.055098,0.056898
,0.055869,0.056442,0.057529,0.057001,0.055981,0.056012,0.056759,0.0571
61,0.11113,0.12956,0.129,0.13385,0.13471,0.12926,0.13716,0.12608,0.129
34,0.12158,0.13154,0.12443,0.13017,0.056321,0.055954,0.057181,0.056609
,0.056796,0.056468,0.058848,0.057159,0.058335,0.056329,0.055919,0.0567
03,0.055607,0.055376,0.055211,0.05583,0.056993,0.056214,0.056786,0.056
987,0.057088,0.1379,0.12436,0.1281,0.12745,0.11986,0.14078,0.14735,0.1
3948,0.12986,0.057424,0.055068,0.056381,0.057288,0.057077,0.057728,0.0
5657,0.056632,0.055812,0.056322,0.05649,0.056315,0.056277,0.057483,0.0
56208,0.056283,0.055605,0.056118,0.057798,0.056262,0.058739,0.057073,0
.056326,0.055693,0.057179,0.12332,0.13122,0.11013,0.12184,0.056256,0.0
55686,0.056729,0.055975,0.057436,0.056281,0.056157,0.056199,0.056244,0
.057527,0.057487,0.057263,0.056284,0.055217,0.056143,0.055167,0.058455
,0.056619,0.054361,0.057186,0.056522,0.056407,0.056767,0.05658,0.05518
5,0.056678,0.055848,0.056694,0.057538,0.055574,0.056783,0.057012,0.057
315,0.056273,0.058086,0.056922,0.05583,0.057378,0.054393,0.057103,0.05
7202,0.058102,0.057849,0.056852,0.058206,0.058281,0.056117,0.055799,0.
056515,0.057511,0.057417,0.056135,0.056152,0.057166,0.055279,0.056099,
0.057238,0.056105,0.056758,0.057362,0.056409,0.055607,0.055342,0.05660
1,0.056729,0.056978,0.055535,0.055545,0.055467,0.057708,0.057895,0.054
969,0.056204,0.056163,0.055947,0.056915,0.056386,0.05802,0.058783,0.05
6672,0.056104,0.057059,0.056375,0.055691,0.055975,0.056781,0.058676,0.
05657,0.056938,0.056099,0.056912,0.057378,0.056719,0.055734,0.05729,0.
056319,0.055417,0.057157,0.055525,0.056186,0.057297,0.056255,0.057838,
0.057376,0.057199,0.057181,0.056664,0.057326,0.05749,0.056955,0.057005
,0.057045,0.059019,0.055757,0.058248,0.056143,0.055928,0.055865,0.0564
53,0.056773,0.057096,0.057091,0.057548,0.057308,0.057248,0.057892,0.05
733,0.057407,0.056612,0.055716,0.057049,0.056422,0.05594,0.058426,0.05
6217,0.056428,0.054378,0.057062,0.057152,0.056728,0.058054,0.056541,0.
055833,0.056206,0.058297,0.05646,0.056252,0.057661,0.05519,0.057147,0.
056266,0.057186,0.056272,0.057219,0.056045,0.0569,0.056966,0.056455,0.
056699,0.055817,0.056557,0.056582,0.056336,0.056269,0.057245,0.057262,
0.057076,0.05733,0.057947,0.056452,0.057881,0.056461,0.057438,0.05681,
0.057246,0.056713,0.056333,0.054429,0.055059,0.057972,0.056158,0.05604
5,0.057846,0.055829,0.056343,0.055666,0.055804,0.057137,0.055263,0.055
822,0.057444,0.057134,0.058336,0.056285,0.056617
0.20401,0.20273,0.2065,0.20517,0.20485,0.20396,0.20577,0.20581,0.20253
,0.20263,0.20584,0.20248,0.20541,0.203,0.20434,0.20246,0.20297,0.2035,
0.20656,0.20369,0.27722,0.29295,0.27406,0.29476,0.20136,0.20364,0.2022
2,0.20504,0.20599,0.20445,0.20588,0.20455,0.20485,0.20078,0.20231,0.20
453,0.20531,0.20382,0.20372,0.36735,0.27453,0.27379,0.26863,0.28049,0.
27564,0.30636,0.20434,0.20713,0.19826,0.20496,0.20378,0.20451,0.20225,
0.20262,0.20573,0.2042,0.20248,0.20333,0.20295,0.20436,0.20423,0.36145
,0.34168,0.28999,0.27458,0.2808,0.28946,0.2708,0.27448,0.27142,0.29632

,0.20449,0.20549,0.20536,0.20009,0.2043,0.20239,0.20787,0.20401,0.2049
,0.20523,0.20268,0.20576,0.20297,0.20562,0.20367,0.40311,0.32925,0.376
99,0.30336,0.2746,0.26485,0.28592,0.27478,0.28993,0.27132,0.28908,0.27
734,0.20411,0.20417,0.20343,0.20471,0.20576,0.20262,0.20313,0.20394,0.
20212,0.2024,0.20366,0.20099,0.20596,0.20708,0.20366,0.20548,0.34007,0
.31533,0.36238,0.36479,0.26843,0.28095,0.28182,0.28138,0.28759,0.29537
,0.27777,0.26701,0.29036,0.27365,0.20275,0.20357,0.20257,0.20366,0.203
73,0.20352,0.20591,0.20269,0.20183,0.20614,0.20683,0.20669,0.20458,0.2
0483,0.20421,0.20299,0.35163,0.36744,0.34055,0.32786,0.37414,0.37973,0
.28127,0.28736,0.29908,0.26791,0.27529,0.2894,0.27719,0.29059,0.27825,
0.28211,0.2035,0.20221,0.20324,0.20372,0.20497,0.20379,0.20142,0.20514
,0.20456,0.20506,0.20553,0.20506,0.20795,0.20269,0.20307,0.2043,0.2023
,0.34474,0.36757,0.36013,0.33867,0.34991,0.35998,0.29002,0.33213,0.276
62,0.2719,0.25467,0.29497,0.26985,0.29177,0.28015,0.2943,0.30658,0.203
13,0.20311,0.20654,0.20611,0.20363,0.20495,0.20347,0.20492,0.20269,0.2
0317,0.20118,0.19997,0.20266,0.12914,0.13555,0.1384,0.206,0.36996,0.34
95,0.34531,0.35362,0.3441,0.35317,0.38458,0.28543,0.2676,0.2816,0.2973
1,0.28158,0.2804,0.28016,0.25808,0.28861,0.26795,0.25809,0.20651,0.206
89,0.20491,0.20278,0.20256,0.20332,0.20326,0.20212,0.20302,0.20519,0.2
0211,0.11893,0.13744,0.12862,0.11354,0.11969,0.12936,0.20446,0.37051,0
.36476,0.3527,0.36086,0.35564,0.3496,0.39129,0.28877,0.26637,0.29155,0
.25307,0.2903,0.28897,0.26636,0.30162,0.26975,0.27949,0.26956,0.20679,
0.20393,0.20319,0.20496,0.20618,0.20106,0.2052,0.2065,0.20324,0.1215,0
.12489,0.12503,0.12681,0.12425,0.11238,0.13122,0.13245,0.12776,0.20213
,0.35628,0.35633,0.33511,0.36658,0.36007,0.32123,0.36211,0.28816,0.286
29,0.27004,0.25722,0.28611,0.27882,0.25725,0.31195,0.27537,0.2887,0.27
901,0.26763,0.20605,0.20555,0.20281,0.20448,0.2071,0.20201,0.12262,0.1
2718,0.13115,0.13524,0.13907,0.1304,0.12964,0.13738,0.13024,0.15269,0.
12514,0.12795,0.36266,0.36837,0.34069,0.3588,0.36095,0.39755,0.34079,0
.28429,0.2822,0.28917,0.26756,0.29258,0.28606,0.27764,0.27922,0.26305,
0.28614,0.27338,0.30631,0.20289,0.20481,0.20242,0.13006,0.13175,0.1290
1,0.12888,0.13379,0.12919,0.15052,0.13035,0.14584,0.12328,0.12558,0.13
619,0.13305,0.1258,0.14129,0.34872,0.33366,0.35656,0.32805,0.35724,0.3
8208,0.37182,0.36998,0.37113,0.27824,0.26953,0.28459,0.27669,0.28006,0
.28358,0.2673,0.29219,0.29186,0.27923,0.27729,0.13619,0.13776,0.12805,
0.13831,0.11976,0.13343,0.13091,0.12053,0.13141,0.13542,0.12968,0.1191
2,0.15507,0.12775,0.13618,0.1241,0.13033,0.12557,0.37772,0.34635,0.396
16,0.3552,0.36318,0.37393,0.36871,0.36555,0.3431,0.27614,0.27666,0.175
71,0.20138,0.23082,0.1959,0.21597,0.21707,0.2006,0.21432,0.20025,0.120
49,0.13952,0.11268,0.12678,0.12821,0.12906,0.1071,0.12605,0.1301,0.131
38,0.13212,0.13468,0.12282,0.12844,0.12798,0.12249,0.14923,0.13827,0.3
5143,0.36254,0.37961,0.36607,0.35722,0.36065,0.33609,0.34927,0.34073,0
.20307,0.20756,0.20883,0.22116,0.20424,0.20206,0.20798,0.22388,0.20828
,0.21195,0.20654,0.13855,0.12316,0.13151,0.1262,0.13332,0.1381,0.14424
,0.13031,0.12551,0.11442,0.1288,0.13661,0.12299,0.13218,0.13317,0.1273
2,0.12419,0.13168,0.35713,0.37465,0.34671,0.37787,0.3821,0.37697,0.373
63,0.19715,0.1849,0.21447,0.21426,0.21619,0.20817,0.20413,0.20047,0.20
443,0.21753,0.2012,0.18431,0.19856,0.11876,0.13031,0.1284,0.13203,0.13
831,0.12793,0.08868,0.13087,0.13963,0.12988,0.12901,0.1205,0.12842,0.1
2215,0.13881,0.12936,0.12902,0.12391,0.37528,0.35686,0.36687,0.35403,0
.35454,0.36498,0.19705,0.2195,0.22009,0.19653,0.20485,0.19842,0.2121,0
.20508,0.2064,0.2148,0.18633,0.20925,0.216,0.21528,0.14832,0.12938,0.1

2999,0.12048,0.12052,0.13543,0.13652,0.12309,0.13007,0.12795,0.12866,0
.13015,0.12674,0.13113,0.12923,0.13659,0.14953,0.14088,0.31951,0.37415
,0.3688,0.36696,0.36794,0.17349,0.2004,0.20288,0.22018,0.20977,0.19702
,0.20991,0.19324,0.21913,0.19945,0.18827,0.19016,0.20947,0.20482,0.212
94,0.13245,0.12964,0.12386,0.1217,0.12875,0.12979,0.142,0.11805,0.1404
9,0.1318,0.1283,0.13132,0.12485,0.13068,0.13028,0.12907,0.13216,0.1360
2,0.34945,0.36216,0.3556,0.23918,0.21259,0.25958,0.21767,0.21448,0.208
96,0.18132,0.21901,0.21631,0.21878,0.20847,0.21066,0.20478,0.22495,0.2
2448,0.20353,0.24889,0.13371,0.12993,0.13408,0.12944,0.12729,0.14413,0
.13242,0.1274,0.13977,0.11584,0.14337,0.13176,0.13624,0.11824,0.1457,0
.13371,0.13052,0.12875,0.37742,0.19686,0.19039,0.20149,0.22528,0.22035
,0.21034,0.18463,0.21129,0.23145,0.21299,0.20331,0.21533,0.2006,0.2291
,0.19263,0.1878,0.20281,0.20658,0.13024,0.12777,0.11669,0.13698,0.1299
3,0.12932,0.13433,0.1287,0.11966,0.13449,0.12471,0.12769,0.13054,0.137
02,0.12956,0.11909,0.13698,0.053194,0.20285,0.21358,0.18433,0.20501,0.
21175,0.21696,0.18082,0.21466,0.20354,0.20889,0.21156,0.20588,0.20427,
0.20236,0.1883,0.2088,0.2443,0.2078,0.20304,0.12036,0.12999,0.12974,0.
12114,0.13,0.13092,0.12578,0.12916,0.12844,0.12778,0.12922,0.11475,0.1
2635,0.13029,0.12367,0.11794,0.12891,0.055022,0.20147,0.20594,0.19483,
0.13414,0.20371,0.2153,0.21231,0.22308,0.22769,0.2059,0.22205,0.20532,
0.19909,0.20878,0.19257,0.20948,0.20147,0.20834,0.12826,0.12816,0.1248
6,0.1291,0.13285,0.1317,0.12771,0.13942,0.12325,0.1136,0.10346,0.12879
,0.12316,0.13035,0.116,0.11736,0.12428,0.13688,0.054501,0.22511,0.2107
8,0.20386,0.18625,0.21529,0.21642,0.2067,0.21823,0.16988,0.20217,0.238
73,0.2087,0.19656,0.18883,0.21765,0.20262,0.18519,0.24087,0.13423,0.13
167,0.13096,0.12662,0.13452,0.13593,0.13696,0.12613,0.12721,0.13941,0.
107,0.13224,0.11053,0.13401,0.12911,0.11677,0.13013,0.055945,0.057017,
0.057081,0.20217,0.24384,0.19637,0.19822,0.19191,0.18943,0.16885,0.186
59,0.2079,0.23971,0.19121,0.22952,0.21172,0.2012,0.19983,0.14,0.12868,
0.13139,0.12839,0.11144,0.13701,0.12965,0.13623,0.12554,0.13443,0.1270
4,0.12669,0.12981,0.14231,0.13928,0.12966,0.13711,0.12739,0.054317,0.0
55114,0.054685,0.055652,0.21917,0.21529,0.2099,0.10473,0.18379,0.22406
,0.17012,0.23294,0.19182,0.20209,0.20408,0.20237,0.13965,0.11462,0.123
01,0.12886,0.12876,0.14401,0.12678,0.12335,0.13136,0.12814,0.14691,0.1
3685,0.12943,0.13162,0.11517,0.13195,0.12912,0.13142,0.13857,0.13175,0
.053909,0.055248,0.054805,0.055235,0.054936,0.054611,0.19769,0.19614,0
.18617,0.15244,0.21481,0.22407,0.20499,0.17915,0.13496,0.11202,0.13794
,0.13378,0.12794,0.15345,0.12943,0.12041,0.13434,0.12931,0.12726,0.129
77,0.13021,0.12138,0.12935,0.13125,0.12316,0.13023,0.15737,0.12868,0.1
3196,0.12924,0.13512,0.055394,0.054371,0.053959,0.055195,0.056,0.05446
7,0.21172,0.23334,0.14768,0.19811,0.21787,0.055162,0.055055,0.13376,0.
11842,0.14872,0.12916,0.13223,0.13157,0.12837,0.13709,0.13228,0.12885,
0.12839,0.1307,0.1096,0.11694,0.13085,0.14892,0.14202,0.13801,0.12726,
0.13505,0.11881,0.053486,0.052942,0.056291,0.055044,0.054906,0.053729,
0.055925,0.05598,0.054539,0.05529,0.19274,0.21828,0.054599,0.056019,0.
056953,0.053848,0.12651,0.13238,0.13533,0.15292,0.12503,0.12335,0.1376
7,0.13489,0.11727,0.13076,0.10845,0.13839,0.13217,0.12799,0.14646,0.12
833,0.12755,0.12837,0.12822,0.054521,0.0562,0.054911,0.055267,0.056802
,0.055041,0.054877,0.055209,0.055797,0.056331,0.055329,0.055239,0.0555
75,0.055693,0.053854,0.054853,0.054266,0.1319,0.12929,0.13166,0.14002,
0.12075,0.12996,0.15502,0.12904,0.13061,0.12224,0.13404,0.12868,0.1214
1,0.13306,0.12516,0.12682,0.055822,0.054002,0.055358,0.053607,0.054337

,0.053413,0.05629,0.056681,0.053999,0.053927,0.055318,0.054439,0.0546,
0.055243,0.054914,0.053993,0.054628,0.055043,0.05555,0.11041,0.12986,0
.12844,0.13389,0.13326,0.12945,0.13763,0.12552,0.1297,0.12147,0.13122,
0.12402,0.1304,0.053481,0.053797,0.055341,0.054912,0.054777,0.055044,0
.057442,0.055702,0.055836,0.054826,0.054407,0.05537,0.053599,0.053873,
0.053919,0.053676,0.055221,0.054793,0.055061,0.055631,0.055226,0.13767
,0.12407,0.12832,0.12554,0.11904,0.14058,0.14672,0.14055,0.12942,0.055
314,0.053233,0.054345,0.055548,0.055497,0.0562,0.054355,0.055093,0.053
861,0.054338,0.054436,0.054743,0.054257,0.056143,0.054381,0.054701,0.0
53914,0.054817,0.055945,0.053919,0.057245,0.055143,0.054944,0.053907,0
.055785,0.12348,0.13022,0.1098,0.12163,0.054713,0.054126,0.055109,0.05
4015,0.056315,0.054587,0.05426,0.054283,0.054307,0.056236,0.055601,0.0
56213,0.055001,0.053402,0.054032,0.05417,0.056755,0.055164,0.052956,0.
055982,0.054751,0.05479,0.054473,0.054952,0.053648,0.054542,0.054466,0
.055229,0.055719,0.053709,0.055092,0.056222,0.056013,0.054184,0.056879
,0.055295,0.054452,0.055194,0.053048,0.055416,0.055491,0.055977,0.0565
07,0.054972,0.056452,0.056108,0.054639,0.053875,0.054076,0.056145,0.05
4951,0.054367,0.053607,0.055597,0.053347,0.054501,0.056481,0.054787,0.
055131,0.05536,0.055115,0.054439,0.054159,0.05486,0.054338,0.055455,0.
053628,0.054012,0.053929,0.056585,0.056487,0.053986,0.054606,0.054071,
0.053514,0.055417,0.054848,0.055762,0.057107,0.054801,0.054438,0.05593
1,0.054363,0.054023,0.054475,0.054655,0.055936,0.054296,0.054445,0.054
551,0.055669,0.055216,0.055414,0.054183,0.055035,0.054601,0.054548,0.0
55855,0.053755,0.05412,0.055763,0.054514,0.056091,0.055024,0.055903,0.
055752,0.054646,0.056086,0.055706,0.054873,0.055295,0.055943,0.056943,
0.054138,0.057219,0.053999,0.05385,0.053863,0.055051,0.054828,0.055513
,0.055527,0.055358,0.055494,0.055785,0.055711,0.056025,0.054831,0.0545
33,0.054571,0.055887,0.054652,0.054306,0.057298,0.054657,0.05536,0.053
165,0.054581,0.054439,0.054974,0.055752,0.054986,0.054091,0.0543,0.055
864,0.054896,0.053495,0.056429,0.053498,0.055647,0.054336,0.054924,0.0
54618,0.055299,0.054374,0.054548,0.055618,0.055106,0.054877,0.054514,0
.054911,0.055126,0.054242,0.054905,0.055817,0.054911,0.055904,0.055316
,0.056248,0.05414,0.055118,0.054499,0.055394,0.055012,0.055714,0.05583
6,0.054316,0.052761,0.053941,0.056694,0.054231,0.054141,0.055581,0.054
713,0.054402,0.053875,0.053796,0.055422,0.052884,0.054893,0.056109,0.0
55458,0.056745,0.053954,0.055188
0.20493,0.20386,0.20852,0.2064,0.20604,0.20544,0.20735,0.20586,0.20336
,0.20323,0.20644,0.2036,0.20572,0.20353,0.20553,0.20279,0.20287,0.2037
6,0.20795,0.2055,0.2771,0.29123,0.27178,0.28762,0.20152,0.20514,0.2025
7,0.20637,0.20683,0.2057,0.20474,0.20619,0.2061,0.2017,0.20315,0.20527
,0.20654,0.20367,0.20568,0.37612,0.27728,0.27717,0.26783,0.27409,0.281
19,0.30906,0.20524,0.20766,0.19964,0.20442,0.2057,0.2056,0.20334,0.202
4,0.20668,0.20557,0.20469,0.20446,0.2047,0.20493,0.20541,0.35435,0.339
57,0.28072,0.28199,0.27751,0.28842,0.27148,0.27162,0.26864,0.29171,0.2
0402,0.20741,0.20617,0.20291,0.2053,0.2021,0.20728,0.20384,0.20585,0.2
0695,0.20415,0.20628,0.20328,0.20543,0.20491,0.38564,0.332,0.35493,0.3
0375,0.27355,0.26376,0.28615,0.27979,0.28883,0.26918,0.28649,0.27319,0
.20527,0.20497,0.20319,0.2049,0.20584,0.20248,0.20372,0.20573,0.20221,
0.20407,0.20393,0.20116,0.20525,0.20824,0.20448,0.20506,0.34706,0.2906
3,0.36261,0.35691,0.26881,0.28201,0.28178,0.28315,0.28722,0.2847,0.276
85,0.26355,0.29757,0.26696,0.20408,0.20458,0.20425,0.20451,0.20395,0.2
0504,0.20558,0.20262,0.20413,0.20482,0.20686,0.20811,0.20455,0.20457,0

.20576,0.20355,0.34666,0.37646,0.34707,0.32088,0.3612,0.36822,0.2842,0
.28529,0.30635,0.27065,0.27808,0.29598,0.27185,0.28679,0.27795,0.28443
,0.20547,0.20279,0.20509,0.20411,0.20615,0.20298,0.20331,0.20628,0.204
58,0.20625,0.2049,0.20486,0.20794,0.20409,0.20317,0.20522,0.20372,0.35
359,0.36389,0.36165,0.33316,0.34314,0.35929,0.29164,0.33182,0.27752,0.
26694,0.25295,0.30008,0.26645,0.28667,0.27589,0.29576,0.30073,0.20293,
0.20407,0.20737,0.20805,0.20598,0.20619,0.20359,0.20689,0.20357,0.2045
7,0.20303,0.20186,0.20336,0.1286,0.13474,0.13852,0.20623,0.3757,0.3526
6,0.33907,0.34629,0.34879,0.33351,0.37673,0.27979,0.2768,0.27405,0.295
46,0.27802,0.27917,0.27776,0.25935,0.28839,0.27255,0.25637,0.20755,0.2
0738,0.20593,0.20275,0.20343,0.20319,0.20462,0.20176,0.20444,0.20677,0
.20305,0.11855,0.13695,0.12855,0.11251,0.11964,0.12913,0.20616,0.37441
,0.34115,0.35176,0.36085,0.35598,0.34413,0.37313,0.28536,0.27017,0.290
41,0.25459,0.29043,0.28485,0.26712,0.30605,0.26894,0.27897,0.26577,0.2
076,0.20401,0.20325,0.20523,0.20497,0.20082,0.20443,0.2071,0.20314,0.1
2141,0.12593,0.12505,0.12699,0.1248,0.11146,0.13119,0.13231,0.12749,0.
20347,0.35269,0.35651,0.32117,0.3606,0.3594,0.32049,0.3677,0.28935,0.2
8651,0.28062,0.25862,0.29098,0.28103,0.25476,0.31329,0.27246,0.29017,0
.27608,0.27343,0.20565,0.20635,0.20316,0.2055,0.20679,0.20341,0.12229,
0.12619,0.13118,0.13542,0.13878,0.12994,0.12928,0.13687,0.13021,0.1530
3,0.12512,0.12755,0.35853,0.35685,0.35319,0.36924,0.35117,0.38781,0.33
708,0.27316,0.27694,0.29554,0.27749,0.29583,0.28455,0.27324,0.27973,0.
25746,0.28651,0.274,0.31242,0.20351,0.20703,0.20358,0.12981,0.13108,0.
12836,0.12945,0.13303,0.12858,0.15114,0.12985,0.14508,0.1234,0.12505,0
.13671,0.13321,0.12632,0.14039,0.3363,0.32727,0.34703,0.34488,0.35364,
0.38824,0.35733,0.36776,0.37468,0.27843,0.27085,0.27884,0.28005,0.2808
1,0.27533,0.26602,0.29352,0.29235,0.27699,0.28342,0.13592,0.13659,0.12
777,0.13815,0.11893,0.13238,0.13087,0.12114,0.13159,0.13525,0.12919,0.
1187,0.15615,0.12742,0.13551,0.12442,0.13029,0.12534,0.3731,0.35076,0.
39161,0.36366,0.35648,0.35913,0.36238,0.34872,0.3356,0.2741,0.28283,0.
17634,0.19956,0.23,0.19297,0.21352,0.21409,0.19487,0.21163,0.20077,0.1
2008,0.13902,0.11295,0.12599,0.12714,0.12858,0.10662,0.12524,0.1302,0.
13033,0.13169,0.13333,0.12208,0.12798,0.12758,0.12334,0.1492,0.13762,0
.34271,0.3428,0.38079,0.36655,0.337,0.3563,0.33357,0.34435,0.34691,0.1
9768,0.20645,0.20701,0.22117,0.20256,0.20117,0.20611,0.22617,0.21081,0
.21238,0.20516,0.13843,0.12269,0.13192,0.12553,0.13322,0.13858,0.14351
,0.13022,0.12508,0.11411,0.12826,0.13548,0.12279,0.1317,0.13273,0.1276
7,0.1239,0.13148,0.36088,0.38229,0.34787,0.37102,0.37534,0.38511,0.375
21,0.19484,0.18569,0.21108,0.21376,0.20714,0.20654,0.20391,0.20564,0.2
0816,0.2173,0.1949,0.18243,0.19752,0.11866,0.1291,0.12803,0.13163,0.13
892,0.12764,0.087264,0.13044,0.13906,0.1289,0.12857,0.12063,0.12722,0.
12204,0.13791,0.12922,0.1284,0.1237,0.37518,0.33759,0.36039,0.34228,0.
35284,0.36771,0.19156,0.21559,0.22279,0.19322,0.19541,0.19841,0.21041,
0.20228,0.20401,0.21691,0.18819,0.20718,0.2095,0.2136,0.14843,0.12947,
0.12946,0.12071,0.12047,0.13496,0.13548,0.12162,0.1288,0.12741,0.12794
,0.12948,0.12659,0.13064,0.12932,0.13701,0.14939,0.14012,0.33113,0.380
21,0.35991,0.3635,0.35524,0.17746,0.19826,0.1974,0.2187,0.21262,0.1949
8,0.20858,0.19207,0.21782,0.19733,0.18837,0.18644,0.20952,0.20546,0.21
043,0.13165,0.12893,0.1239,0.12196,0.12766,0.12872,0.14228,0.11859,0.1
4101,0.13165,0.12829,0.13113,0.12499,0.1301,0.12992,0.1285,0.1317,0.13
544,0.35849,0.35998,0.35633,0.23616,0.2102,0.25952,0.22099,0.21295,0.2
0163,0.1838,0.21158,0.21461,0.21787,0.2075,0.20904,0.20215,0.21998,0.2

2321,0.20115,0.24319,0.1343,0.12967,0.13356,0.12887,0.12733,0.14453,0.
13136,0.1277,0.13968,0.11519,0.14452,0.13098,0.13652,0.11764,0.14528,0
.13365,0.13003,0.12841,0.37395,0.19399,0.19525,0.20065,0.22027,0.21426
,0.20926,0.18432,0.20773,0.2322,0.21217,0.20603,0.20946,0.20375,0.2273
6,0.19254,0.18363,0.1978,0.20234,0.12986,0.12619,0.11608,0.13636,0.130
12,0.12865,0.13476,0.12824,0.11849,0.13421,0.12426,0.12745,0.13105,0.1
3716,0.1295,0.11875,0.13612,0.051924,0.20439,0.21223,0.18196,0.20345,0
.20745,0.21122,0.18286,0.20786,0.19998,0.2055,0.2083,0.20143,0.2016,0.
19898,0.18848,0.21384,0.23909,0.20852,0.20038,0.11962,0.1296,0.12902,0
.12032,0.12921,0.13071,0.12533,0.12896,0.12772,0.1279,0.12845,0.11403,
0.12635,0.12988,0.12342,0.11704,0.12877,0.053512,0.19961,0.20579,0.202
06,0.13028,0.20621,0.21474,0.20964,0.22434,0.22741,0.20671,0.22733,0.2
0758,0.19624,0.20468,0.19322,0.21238,0.19696,0.20066,0.12752,0.12841,0
.12551,0.12871,0.13306,0.13056,0.12672,0.13919,0.12279,0.1126,0.102,0.
12852,0.12266,0.13027,0.11534,0.11654,0.12393,0.13722,0.053194,0.22294
,0.20269,0.19828,0.18425,0.2197,0.20597,0.20759,0.2176,0.16322,0.20176
,0.23102,0.20914,0.19371,0.18597,0.22078,0.2019,0.18706,0.23766,0.1331
4,0.13104,0.13022,0.12629,0.13487,0.13547,0.13631,0.12555,0.12677,0.13
904,0.10613,0.13186,0.11008,0.13414,0.12884,0.11682,0.13056,0.054647,0
.055463,0.055203,0.20252,0.24775,0.19129,0.19375,0.19255,0.18438,0.163
04,0.18211,0.20885,0.23347,0.19075,0.22661,0.21491,0.19634,0.19872,0.1
4099,0.13001,0.13158,0.12835,0.1107,0.13769,0.12862,0.13534,0.12511,0.
13364,0.12702,0.12649,0.13023,0.14204,0.13909,0.12985,0.13624,0.12692,
0.052907,0.053327,0.052838,0.054134,0.22193,0.20923,0.20264,0.10292,0.
19001,0.22195,0.17275,0.23075,0.19557,0.20014,0.20272,0.19703,0.1389,0
.11359,0.12253,0.12787,0.1283,0.1443,0.12632,0.12289,0.13106,0.12768,0
.14694,0.13772,0.12875,0.13121,0.11367,0.13228,0.12904,0.1312,0.13849,
0.13205,0.052636,0.05382,0.052793,0.052695,0.052546,0.053345,0.20084,0
.19949,0.18795,0.14706,0.21945,0.22978,0.20366,0.18267,0.1345,0.11062,
0.13748,0.13285,0.12669,0.15314,0.12928,0.12056,0.13496,0.12887,0.1268
1,0.12926,0.12993,0.12127,0.12887,0.13135,0.12294,0.12974,0.15796,0.12
908,0.13236,0.12866,0.1354,0.053449,0.053088,0.052108,0.053902,0.05420
4,0.05388,0.2064,0.23473,0.14657,0.19449,0.21598,0.053026,0.053571,0.1
3304,0.11779,0.14849,0.12961,0.13186,0.13116,0.12794,0.13647,0.13121,0
.1284,0.12874,0.13054,0.10919,0.11684,0.13068,0.14846,0.14192,0.13793,
0.12808,0.1351,0.11881,0.05266,0.051841,0.055033,0.054324,0.052753,0.0
52798,0.053827,0.05406,0.052724,0.053987,0.19304,0.22084,0.053133,0.05
3863,0.055762,0.053134,0.12624,0.13226,0.1353,0.15386,0.1252,0.12365,0
.13742,0.13485,0.11652,0.13058,0.10783,0.13881,0.13156,0.12727,0.14686
,0.12831,0.12707,0.1286,0.1273,0.0537,0.054619,0.053433,0.053664,0.055
179,0.053771,0.053545,0.053648,0.054801,0.054782,0.053695,0.054217,0.0
54666,0.054298,0.052346,0.05308,0.05295,0.13136,0.12881,0.13062,0.1388
4,0.12035,0.12982,0.15537,0.12878,0.13052,0.12253,0.13391,0.12839,0.12
034,0.13287,0.12511,0.12663,0.05497,0.052698,0.053427,0.052574,0.05360
5,0.052251,0.054351,0.055015,0.052988,0.052984,0.054068,0.053468,0.052
597,0.054221,0.053452,0.052898,0.053181,0.053621,0.053629,0.11013,0.12
954,0.12748,0.13364,0.13266,0.12924,0.13777,0.12481,0.12951,0.12184,0.
13055,0.124,0.13038,0.051927,0.053048,0.053927,0.053469,0.053755,0.054
145,0.055782,0.054357,0.05464,0.05394,0.05324,0.054436,0.051905,0.0527
41,0.052681,0.052899,0.054002,0.053642,0.053737,0.054393,0.05399,0.137
37,0.12376,0.12705,0.12464,0.11846,0.14052,0.14647,0.14011,0.12933,0.0
53373,0.051878,0.052867,0.053574,0.05389,0.054873,0.053565,0.053578,0.

052518,0.053299,0.052661,0.053307,0.053804,0.054514,0.052627,0.053298,
0.053035,0.053783,0.053302,0.052783,0.055803,0.053768,0.053079,0.05205
,0.053941,0.12317,0.13022,0.10948,0.12059,0.054525,0.052741,0.053901,0
.052728,0.055385,0.052875,0.053269,0.052527,0.052657,0.054772,0.054075
,0.053902,0.053679,0.052519,0.052183,0.052512,0.054897,0.052901,0.0522
84,0.053943,0.053098,0.05294,0.053225,0.053776,0.052964,0.053661,0.053
368,0.053603,0.053734,0.053034,0.053915,0.05443,0.054065,0.052901,0.05
5069,0.053759,0.053086,0.053152,0.050995,0.054029,0.053791,0.054343,0.
055157,0.054196,0.055194,0.054043,0.053046,0.052096,0.053102,0.054238,
0.052759,0.053045,0.052409,0.054095,0.051988,0.053414,0.055204,0.05321
9,0.053698,0.055179,0.053842,0.053282,0.053245,0.054023,0.053333,0.053
59,0.052294,0.052543,0.051962,0.055078,0.054988,0.052782,0.053954,0.05
3049,0.052397,0.05338,0.053361,0.053747,0.055393,0.0537,0.05258,0.0541
47,0.052779,0.052298,0.053227,0.053321,0.054639,0.052434,0.052188,0.05
3562,0.053721,0.053435,0.05345,0.053063,0.054212,0.05362,0.053318,0.05
4601,0.052375,0.053309,0.05389,0.0533,0.054117,0.053259,0.054214,0.054
174,0.053223,0.054853,0.054231,0.053394,0.053696,0.05474,0.055529,0.05
2724,0.056015,0.052312,0.052096,0.052166,0.053965,0.052767,0.054188,0.
054212,0.053516,0.05337,0.054584,0.053606,0.055004,0.053187,0.052833,0
.053259,0.054244,0.053168,0.052286,0.055608,0.053362,0.053121,0.051497
,0.053177,0.052622,0.053254,0.054723,0.053355,0.051795,0.05334,0.05480
3,0.053528,0.051702,0.055434,0.051583,0.054499,0.052631,0.053449,0.053
33,0.054611,0.053239,0.053053,0.05383,0.054342,0.053623,0.052225,0.053
629,0.053674,0.053047,0.05288,0.053815,0.052913,0.054788,0.053583,0.05
4543,0.053,0.053887,0.05314,0.053846,0.053121,0.054304,0.054402,0.0534
28,0.052263,0.053427,0.055482,0.05292,0.052294,0.053989,0.05289,0.0530
2,0.05292,0.052374,0.053402,0.052153,0.053591,0.054431,0.053872,0.0549
31,0.052903,0.053443

#simdataS1SNR20:

Kep_f=0.5
Kep_f=0.5
Kep_s=0.1
Ktrans_f=0.03
Ktrans_s=0.03
del_t=0.5
gtSf=0 0 0 0 0 0
0 0 0 0 0 0
0 0 0 0 0 0
0 0 0 0 0 0
0 0 0 0 0 0
0 0 0 0 0 0
0 0 0 0 0 0
0 0 0 0 0 0
0 0 0 0 0 0
0 0 0 0 0 0
0 0 0 0 0 0
0 0 0 0 0 0
0 0 0 0 0 0
0 0 0 0 0 0

0	0	0	0	0	0
0	0	0	0	0	0
0	0	0	0	0	0
0	0	0	0	0	0
0	0	0	0	0	0
0	0	0	0	0	0
0	0	0	0	0	0
0	0	0	0	0	0
0	0	0	0	0	0
0	0	0	0	0	0
0	0	0	0	0	0
0	0	0	0	0	0
0	0	0	0	0	0
0	0	0	0	0	0
0	0	0	0	0	0
0	0	0	0	0	0
0	0	0	0	0	0
0	0	0	0	0	0
0	0	0	0	0	0
0	0	0	0	0	0
0	0	0	0.51431	0.44964	0.48192
0	0	0	0	0	0
0	0	0	0	0	0
0	0	0	0	0	0
0	0	0	0	0	0
0	0	0	0	0	0
0.49252	0.66023	0.5231	0.5	0.5	0.58239
0	0	0	0	0	0
0	0	0	0	0	0
0	0	0	0	0	0
0	0	0	0	0	0
0	0	0	0	0.5	0.5
0.47505	0.5	0.5018	0.50284	0.48852	0.51513
0.4547	0	0	0	0	0
0	0	0	0	0	0
0	0	0	0	0	0
0	0	0	0	0	0
0	0	0	0.5	0.51446	0.51027
0.5	0.44609	0.42259	0.5	0.61287	0.50114
0.48993	0.4888	0.64102	0	0	0
0	0	0	0	0	0
0	0	0	0	0	0
0	0	0	0	0	0
0	0.41538	0.54464	0.44022	0.5	0.5
0.53822	0.55706	0.5	0.44161	0.48912	0.58601
0.56863	0.4529	0.5	0.5	0	0
0	0	0	0	0	0
0	0	0	0	0	0
0	0	0	0	0	0
0.50641	0.53028	0.47817	0.56045	0.5	0.5
0.47913	0.48211	0.55669	0.70309	0.45989	0.59863
0.48513	0.49307	0.5	0.45738	0.46694	0.51383

0	0	0	0	0	0
0	0	0	0	0	0.28658
0.44138	0.3436	0.35899	0.33333	0.34611	0.33333
0.34821	0.27493	0.41093	0.48316	0.48792	0.5
0.60203	0.5	0.54127	0.52239	0.40316	0.5
0.56696	0.5	0.60869	0.46443	0.45027	0.5
0.5	0.6033	0	0	0	0
0	0	0	0	0	0.48422
0.30253	0.33333	0.2888	0.33388	0.36277	0.34444
0.36573	0.33811	0.25374	0.31956	0.48663	0.41821
0.46702	0.5	0.55204	0.50385	0.45603	0.48763
0.48956	0.51572	0.44375	0.65303	0.5	0.44749
0.41553	0.43459	0.39291	0.53859	0	0
0	0	0	0	0	0.5
0.54515	0.62254	0.32443	0.35416	0.33333	0.27287
0.39777	0.27581	0.28109	0.3638	0.35352	0.3547
0.44696	0.354	0.59324	0.50561	0.46651	0.50183
0.56493	0.4758	0.48207	0.62766	0.47702	0.5857
0.5659	0.62498	0.50788	0.48943	0.33587	0.49726
0	0	0	0	0	0
0.5	0.6392	0.4644	0.30586	0.5	0.31675
0.35232	0.22561	0.28123	0.33333	0.20696	0.38577
0.36308	0.32545	0.48059	0.42245	0.5895	0.5
0.56797	0.53058	0.54212	0.46192	0.5	0.49024
0.51745	0.5	0.55693	0.51017	0.49118	0.47355
0.5	0.5	0	0	0	0
0	0.5	0.53356	0.5	0.46503	0.5
0.5	0.30229	0.36021	0.33333	0.33333	0.35527
0.29398	0.26434	0.32317	0.34238	0.5901	0.62484
0.53613	0.5	0.47747	0.413	0.46349	0.49292
0.46494	0.4827	0.43487	0.4393	0.5	0.4978
0.50944	0.36275	0.50596	0.5	0	0
0	0.58902	0.46039	0.49745	0.56227	0.46674
0.5	0.5074	0.51386	0.37506	0.35796	0.35283
0.33333	0.38168	0.31769	0.36014	0.32918	0.33549
0.54503	0.5	0.5	0.43151	0.50605	0.4437
0.58377	0.5578	0.5	0.5	0.41767	0.5
0.5804	0.60257	0.46961	0.59132	0.47204	0.5
0	0.5	0.49856	0.39093	0.37252	0.52553
0.52491	0.46541	0.49739	0.49552	0.27693	0.27459
0.29531	0.30009	0.3141	0.37078	0.35653	0.36552
0.33692	0.47412	0.43426	0.35065	0.48953	0.4792
0.46657	0.69423	0.48721	0.50367	0.5	0.5
0.5	0.35852	0.43207	0.36813	0.5	0.46115
1	0.47891	0.5	0.45152	0.5	0.56515
0.55212	0.49747	0.56074	0.52534	0.41343	0.33333
0.35144	0.28428	0.25745	0.33333	0.34334	0.3664
0.39004	0.34625	0.43989	0.54286	0.47785	0.52353
0.49914	0.5	0.44297	0.45622	0.78929	0.49356
0.49321	0.4578	0.49317	0.47678	0.65295	0.61443
0.49228	1	0.41873	0.40851	0.51115	0.57288
0.59289	0.52531	0.49715	0.5	0.5	0.5

0.33672	0.33333	0.34426	0.34169	0.34899	0.33333
0.36894	0.37074	0.50292	0.64655	0.5	0.47089
0.5	0.58211	0.5	0.52419	0.53854	0.51288
0.50066	0.5	0.49415	0.46491	0.57635	0.49101
0.45496	0.54819	1	0.58608	0.4562	0.40469
0.5	0.5	0.5	0.46409	0.49242	0.56105
0.5	0.36543	0.37906	0.33935	0.33336	0.31417
0.34301	0.35062	0.38578	0.54024	0.48451	0.5
0.57831	0.29076	0.51372	0.5	0.5	0.50893
0.43629	0.51802	0.46134	0.42968	0.58786	0.48483
0.5	0.5067	1	1	1	0.68316
0.5	0.55897	0.5	0.58676	0.5	0.5152
0.45803	0.44952	0.346	0.35074	0.35776	0.38727
0.34476	0.33741	0.53026	0.41775	0.47614	0.5
0.5	0.39109	0.5836	0.5427	0.5	0.5
0.33764	0.46858	0.56999	0.56609	0.5	0.39852
0.51471	0.5	1	1	1	1
0.5	0.57512	0.48209	0.5	0.5	0.50054
0.46252	0.50678	0.5	0.28811	0.33698	0.35909
0.50025	0.47962	0.49393	0.58641	0.46185	0.5
0.5	0.32572	0.5	0.5	0.53435	0.14103
0.60283	0.47233	0.5	0.49063	0.52395	0.5
0.67115	0.5	1	1	1	1
1	1	0.57002	0.64005	0.45265	0.50489
0.51696	0.48565	0.42715	0.5	0.57636	0.5726
0.48513	0.41066	0.54011	0.48656	0.49349	0.54122
0.41919	0.50986	0.57237	0.40119	0.5	0.45974
0.46943	0.5	0.52057	0.5	0.53659	0.4395
0.49296	0.5142	0.43975	1	1	1
1	1	1	0.37411	0.51086	0.5
0.41781	0.44664	1	1	0.48802	0.50328
0.43385	0.5286	0.5	0.60044	0.577	0.54476
0.47992	0.53083	0.58746	0.53996	0.45074	0.48324
0.5	0.45544	0.46409	0.42621	0.62948	0.53509
0.5	1	1	1	1	1
1	1	1	1	1	0.48079
0.50183	1	1	1	1	0.5
0.48059	0.51614	0.5311	0.64524	0.40575	0.46099
0.5	0.5	0.5	0.5	0.50294	0.62186
0.54602	0.45327	0.5	0.5	0.40481	0.53061
1	1	1	1	1	1
1	1	1	1	1	1
1	1	1	1	1	0.5
0.52075	0.34375	0.41301	0.5138	0.49534	0.55203
0.37638	0.38655	0.54385	0.45544	0.49391	0.50064
0.5	0.49805	0.5	1	1	1
1	1	1	1	1	1
1	1	1	1	1	1
1	1	1	1	0.62302	0.5
0.68084	0.49395	0.5	0.5	0.5063	0.58951
0.49973	0.5	0.45565	0.5	0.56105	1
1	1	1	1	1	1

```
1 1 1 1 1 1
1 1 1 1 1 1
1 1 0.5 0.5 0.5 0.46668
0.54738 0.4183 0.5 0.43522 0.43481 1
1 1 1 1 1 1
1 1 1 1 1 1
1 1 1 1 1 1
1 1 1 1 1 1
0.42938 0.45918 0.55079 0.55349 1 1
1 1 1 1 1 1
1 1 1 1 1 1
1 1 1 1 1 1
1 1 1 1 1 1
1 1 1 1 1 1
1 1 1 1 1 1
1 1 1 1 1 1
1 1 1 1 1 1
1 1 1 1 1 1
1 1 1 1 1 1
1 1 1 1 1 1
1 1 1 1 1 1
1 1 1 1 1 1
1 1 1 1 1 1
1 1 1 1 1 1
1 1 1 1 1 1
1 1 1 1 1 1
1 1 1 1 1 1
1 1 1 1 1 1
1 1 1 1 1 1
1 1 1 1 1 1
1 1 1 1 1 1
1 1 1 1 1 1
1 1 1 1 1 1
1 1 1 1 1 1
1 1 1 1 1 1
1 1 1 1 1 1
1 1 1 1 1 1
1 1 1 1 1 1
1 1 1 1 1 1
1 1 1 1 1 1
1 1 1 1 1 1
1
gtSs=1 1 1 1 1 1
1 1 1 1 1 1
1 1 1 1 1 1
1 1 0.52999 0.41984 0.47847 0.52128
1 1 1 1 1 1
1 1 1 1 1 1
1 1 1 0 0.5 0.40531
0.50605 0.5339 0.47385 0.48377 1 1
1 1 1 1 1 1
1 1 1 1 1 1
```

1	0	0	0.53963	0.67874	0.5
0.63632	0.50603	0.48267	0.5116	0.50532	1
1	1	1	1	1	1
1	1	1	1	1	1
1	1	0	0	0	0.50778
0.40924	0.55703	0.48921	0.5	0.5	0.46027
0.48625	0.46159	1	1	1	1
1	1	1	1	1	1
1	1	1	1	1	1
0	0	0	0	0.42179	0.4481
0.52744	0.43047	0.5306	0.60702	0.4675	0.48713
0.63393	0.55944	1	1	1	1
1	1	1	1	1	1
1	1	1	1	1	1
0	0	0	0	0	0
0.5	0.47586	0.54709	0.45337	0.5108	0.43799
0.38637	0.48563	0.28975	0.5	1	1
1	1	1	1	1	1
1	1	1	1	1	1
1	1	1	0	0	0
0	0	0	0.48009	0.43531	0.51247
0.5	0.5008	0.5	0.50203	0.43367	0.40231
0.41314	0.44735	1	1	1	1
1	1	1	1	1	1
1	1	1	0.48569	0.55036	0.51808
1	0	0	0	0	0
0	0	0.5	0.5	0.43709	0.5
0.58091	0.58682	0.48016	0.55973	0.58448	0.59864
0.42413	1	1	1	1	1
1	1	1	1	1	1
0.50748	0.33977	0.4769	0.5	0.5	0.41761
1	0	0	0	0	0
0	0	0.39549	0.48896	0.48682	0.46032
0.58909	0.34691	0.44371	0.5	0.5	0.52701
0.42367	1	1	1	1	1
1	1	1	1	0.5	0.5
0.52495	0.5	0.4982	0.49716	0.51148	0.48487
0.5453	1	0	0	0	0
0	0	0	0.45233	0.49726	0.47203
0.5	0.40902	0.372	0.53364	0.44155	0.4987
0.41564	0.5	0.63888	1	1	1
1	1	1	0.5	0.48554	0.48973
0.5	0.55391	0.57741	0.5	0.38713	0.49886
0.51007	0.5112	0.35898	0	0	0
0	0	0	0	0.55598	0.51302
0.38431	0.5	0.59306	0.45463	0.45552	0.41901
0.55711	0.33674	0.45841	0.46478	1	1
1	0.58462	0.45536	0.55978	0.5	0.5
0.46178	0.44294	0.5	0.55839	0.51088	0.41399
0.43137	0.5471	0.5	0.5	0	0
0	0	0	0	0	0
0	0.5	0.46475	0.50063	0.42508	0.5

0.50407	0.5	0.5	0.44774	0.35373	0.47515
0.49359	0.46972	0.52183	0.43955	0.5	0.5
0.52087	0.51789	0.44331	0.29691	0.54011	0.40137
0.51487	0.50693	0.5	0.54262	0.53306	0.48617
0	0	0	0	0	0
0	0	0	0.5	0.68067	0.37566
0.25567	0.32409	0.33915	0.33333	0.30777	0.33333
0.27221	0.40656	0.58907	0.51684	0.51208	0.5
0.39797	0.5	0.45873	0.47761	0.59684	0.5
0.43304	0.5	0.39131	0.53557	0.54973	0.5
0.5	0.3967	0	0	0	0
0	0	0	0	0	0
0.35687	0.33333	0.37201	0.33388	0.29529	0.34444
0.36573	0.33811	0.41299	0.31694	0.51337	0.58179
0.53298	0.5	0.44796	0.49615	0.54397	0.51237
0.51044	0.48428	0.55625	0.34697	0.5	0.55251
0.58447	0.56541	0.60709	0.46141	0	0
0	0	0	0	0	0
0	0	0.34654	0.35592	0.33333	0.35969
0.20446	0.35444	0.31653	0.2724	0.28879	0.2906
0.55304	0.646	0.40676	0.49439	0.53349	0.49817
0.43507	0.5242	0.51793	0.37234	0.52298	0.4143
0.4341	0.37502	0.49212	0.51057	0.66413	0.50274
0	0	0	0	0	0
0	0	0	0	0	0.41966
0.35232	0.3872	0.44948	0.33333	0.39652	0.22846
0.27384	0.31551	0.51941	0.57755	0.4105	0.5
0.43203	0.46942	0.45788	0.53808	0.5	0.50976
0.48255	0.5	0.44307	0.48983	0.50882	0.52645
0.5	0.5	0	0	0	0
0	0	0	0	0	0
0	0.3793	0.28061	0.33333	0.33333	0.32397
0.33944	0.36809	0.40219	0.28784	0.4099	0.37516
0.46387	0.5	0.52253	0.587	0.53651	0.50708
0.53506	0.5173	0.56513	0.5607	0.5	0.5022
0.49056	0.63725	0.49404	0.5	0	0
0	0	0	0	0	0
0	0	0	0.24989	0.35796	0.29435
0.33333	0.37901	0.37334	0.36014	0.39943	0.32903
0.45497	0.5	0.5	0.56849	0.49395	0.5563
0.41623	0.4422	0.5	0.5	0.58233	0.5
0.4196	0.39743	0.53039	0.40868	0.52796	0.5
0	0	0	0	0	0
0	0	0	0	0.34355	0.31472
0.35234	0.37816	0.34295	0.31111	0.28695	0.26896
0.33692	0.52588	0.56574	0.64935	0.51047	0.5208
0.53343	0.30577	0.51279	0.49633	0.5	0.5
0.5	0.64148	0.56793	0.63187	0.5	0.53885
0	0	0	0	0	0
0	0	0	0	0	0.33333
0.29687	0.3546	0.35482	0.33333	0.34334	0.24783
0.23007	0.34343	0.56011	0.45714	0.52215	0.47647

0.50086 0.5 0.55703 0.54378 0.21071 0.50644
0.50679 0.5422 0.50683 0.52322 0.34705 0.38557
0.50772 0 0 0 0 0
0 0 0 0 0 0
0.33672 0.33333 0.34426 0.34169 0.34899 0.33333
0.36181 0.32016 0.49708 0.35345 0.5 0.52911
0.5 0.41789 0.5 0.47581 0.46146 0.48712
0.49934 0.5 0.50585 0.53509 0.42365 0.50899
0.54504 0.45181 0 0 0 0
0 0 0 0 0 0
0 0.32236 0.24188 0.30757 0.32023 0.36599
0.31399 0.29875 0.23884 0.45976 0.51549 0.5
0.42169 0.70924 0.48628 0.5 0.5 0.49107
0.56371 0.48198 0.53866 0.57032 0.41214 0.51517
0.5 0.4933 0 0 0 0
0 0 0 0 0 0
0 0 0.25888 0.38828 0.35776 0.30376
0.33683 0.32517 0.46974 0.58225 0.52386 0.5
0.5 0.60891 0.4164 0.4573 0.5 0.5
0.66236 0.53142 0.43001 0.43391 0.5 0.60148
0.48529 0.5 0 0 0 0
0 0 0 0 0 0
0 0 0 0.40582 0.32604 0.35909
0.49975 0.52038 0.50607 0.41359 0.53815 0.5
0.5 0.67428 0.5 0.5 0.46565 0.85897
0.39717 0.52767 0.5 0.50937 0.47605 0.5
0.32885 0.5 0 0 0 0
0 0 0 0 0 0
0 0 0 0 0.42364 0.4274
0.51487 0.58934 0.45989 0.51344 0.50651 0.45878
0.58081 0.49014 0.42763 0.59881 0.5 0.54026
0.53057 0.5 0.47943 0.5 0.46341 0.5605
0.50704 0.4858 0.56025 0 0 0
0 0 0 0 0 0
0 0 0 0 0.51198 0.49672
0.56615 0.4714 0.5 0.39956 0.423 0.45524
0.52008 0.46917 0.41254 0.46004 0.54926 0.51676
0.5 0.54456 0.53591 0.57379 0.37052 0.46491
0.5 0 0 0 0 0
0 0 0 0 0 0
0 0 0 0 0 0.5
0.51941 0.48386 0.4689 0.35476 0.59425 0.53901
0.5 0.5 0.5 0.5 0.49706 0.37814
0.45398 0.54673 0.5 0.5 0.59519 0.46939
0 0 0 0 0 0
0 0 0 0 0 0
0 0 0 0 0 0.5
0.47925 0.65625 0.58699 0.4862 0.50466 0.44797
0.62362 0.61345 0.45615 0.54456 0.50609 0.49936
0.5 0.50195 0.5 0 0 0
0 0 0 0 0 0
0 0 0 0 0 0

0	0	0	0	0.37698	0.5
0.31916	0.50605	0.5	0.5	0.4937	0.41049
0.50027	0.5	0.54435	0.5	0.43895	0
0	0	0	0	0	0
0	0	0	0	0	0
0	0	0	0	0	0
0	0	0.5	0.5	0.5	0.53332
0.45262	0.5817	0.5	0.56478	0.56519	0
0	0	0	0	0	0
0	0	0	0	0	0
0	0	0	0	0	0
0	0	0	0	0	0
0.57062	0.54082	0.44921	0.44651	0	0
0	0	0	0	0	0
0	0	0	0	0	0
0	0	0	0	0	0
0	0	0	0	0	0
0	0	0	0	0	0
0	0	0	0	0	0
0	0	0	0	0	0
0	0	0	0	0	0
0	0	0	0	0	0
0	0	0	0	0	0
0	0	0	0	0	0
0	0	0	0	0	0
0	0	0	0	0	0
0	0	0	0	0	0
0	0	0	0	0	0
0	0	0	0	0	0
0	0	0	0	0	0
0	0	0	0	0	0
0	0	0	0	0	0
0	0	0	0	0	0
0	0	0	0	0	0
0	0	0	0	0	0
0	0	0	0	0	0
0	0	0	0	0	0
0	0	0	0	0	0
0	0	0	0	0	0
0	0	0	0	0	0
0	0	0	0	0	0
0	0	0	0	0	0
0	0	0	0	0	0
0	0	0	0	0	0
0	0	0	0	0	0
0					
gtSp=0	0	0	0	0	0
0	0	0	0	0	0
0	0	0	0	0	0
0	0	0.47001	0.58016	0.52153	0.47872
0	0	0	0	0	0
0	0	0	0	0	0

0	0	0	1	0.5	0.59469
0.49395	0.4661	0.52615	0.51623	0	0
0	0	0	0	0	0
0	0	0	0	0	0
0	1	1	0.46037	0.32126	0.5
0.36368	0.49397	0.51733	0.4884	0.49468	0
0	0	0	0	0	0
0	0	0	0	0	0
0	0	1	1	1	0.49222
0.59076	0.44297	0.51079	0.5	0.5	0.53973
0.51375	0.53841	0	0	0	0
0	0	0	0	0	0
0	0	0	0	0	0
1	1	1	1	0.57821	0.5519
0.47256	0.56953	0.4694	0.39298	0.5325	0.51287
0.36607	0.44056	0	0	0	0
0	0	0	0	0	0
0	0	0	0	0	0
1	1	1	1	1	1
0.5	0.52414	0.45291	0.54663	0.4892	0.56201
0.61363	0.51437	0.71025	0.5	0	0
0	0	0	0	0	0
0	0	0	0	0	0
0	0	0	1	1	1
1	1	1	0.51991	0.56469	0.48753
0.5	0.4992	0.5	0.49797	0.56633	0.59769
0.58686	0.55265	0	0	0	0
0	0	0	0	0	0
0	0	0	0	0	0
0	1	1	1	1	1
1	1	0.5	0.5	0.56291	0.5
0.41909	0.41318	0.51984	0.44027	0.41552	0.40136
0.57587	0	0	0	0	0
0	0	0	0	0	0
0	0	0	0	0	0
0	1	1	1	1	1
1	1	0.60451	0.51104	0.51318	0.53968
0.41091	0.65309	0.55629	0.5	0.5	0.47299
0.57633	0	0	0	0	0
0	0	0	0	0	0
0	0	0	0	0	0
0	0	1	1	1	1
1	1	1	0.54767	0.50274	0.52797
0.5	0.59098	0.628	0.46636	0.55845	0.5013
0.58436	0.5	0.36112	0	0	0
0	0	0	0	0	0
0	0	0	0	0	0
0	0	0	1	1	1
1	1	1	1	0.44402	0.48698
0.61569	0.5	0.40694	0.54537	0.54448	0.58099
0.44289	0.66326	0.54159	0.53522	0	0
0	0	0	0	0	0

0	0	0	0	0	0
0	0	0	0	1	1
1	1	1	1	1	1
1	0.5	0.53525	0.49937	0.57492	0.5
0.49593	0.5	0.5	0.55226	0.64627	0.52485
0	0	0	0	0	0
0	0	0	0	0	0
0	0	0	0	0	0
1	1	1	1	1	1
1	1	1	0.5	0.31933	0.33775
0.30295	0.33231	0.30186	0.33333	0.34611	0.33333
0.37959	0.31851	0	0	0	0
0	0	0	0	0	0
0	0	0	0	0	0
0	0	1	1	1	1
1	1	1	1	1	0.51578
0.3406	0.33333	0.33919	0.33224	0.34193	0.31113
0.26853	0.32378	0.33327	0.36349	0	0
0	0	0	0	0	0
0	0	0	0	0	0
0	0	0	0	1	1
1	1	1	1	1	0.5
0.45485	0.37746	0.32904	0.28992	0.33333	0.36744
0.39777	0.36975	0.40238	0.3638	0.35769	0.3547
0	0	0	0	0	0
0	0	0	0	0	0
0	0	0	0	0	0
1	1	1	1	1	1
0.5	0.3608	0.5356	0.69414	0.5	0.26359
0.29535	0.3872	0.2693	0.33333	0.39652	0.38577
0.36308	0.35904	0	0	0	0
0	0	0	0	0	0
0	0	0	0	0	0
0	0	1	1	1	1
1	0.5	0.46644	0.5	0.53497	0.5
0.5	0.31841	0.35918	0.33333	0.33333	0.32075
0.36659	0.36757	0.27463	0.36978	0	0
0	0	0	0	0	0
0	0	0	0	0	0
0	0	0	0	1	1
1	0.41098	0.53961	0.50255	0.43773	0.53326
0.5	0.4926	0.48614	0.37506	0.28409	0.35283
0.33333	0.23931	0.30897	0.27971	0.27139	0.33549
0	0	0	0	0	0
0	0	0	0	0	0
0	0	0	0	0	0
1	0.5	0.50144	0.60907	0.62748	0.47447
0.47509	0.53459	0.50261	0.50448	0.37952	0.41068
0.35234	0.32175	0.34295	0.31811	0.35653	0.36552
0.32615	0	0	0	0	0
0	0	0	0	0	0
0	0	0	0	0	0

0	0.52109	0.5	0.54848	0.5	0.43485
0.44788	0.50253	0.43926	0.47466	0.58657	0.33333
0.35169	0.36111	0.38773	0.33333	0.31332	0.38577
0.37988	0.31032	0	0	0	0
0	0	0	0	0	0
0	0	0	0	0	0
0	0	0.58127	0.59149	0.48885	0.42712
0.40711	0.47469	0.50285	0.5	0.5	0.5
0.32656	0.33333	0.31148	0.31663	0.30202	0.33333
0.26926	0.3091	0	0	0	0
0	0	0	0	0	0
0	0	0	0	0	0
0	0	0	0.41392	0.5438	0.59531
0.5	0.5	0.5	0.53591	0.50758	0.43895
0.5	0.31221	0.37906	0.35307	0.34641	0.31983
0.34301	0.35062	0.37538	0	0	0
0	0	0	0	0	0
0	0	0	0	0	0
0	0	0	0	0	0.31684
0.5	0.44103	0.5	0.41324	0.5	0.4848
0.54197	0.55048	0.39513	0.26098	0.28448	0.30897
0.31841	0.33741	0	0	0	0
0	0	0	0	0	0
0	0	0	0	0	0
0	0	0	0	0	0
0.5	0.42488	0.51791	0.5	0.5	0.49946
0.53748	0.49322	0.5	0.30606	0.33698	0.28182
0	0	0	0	0	0
0	0	0	0	0	0
0	0	0	0	0	0
0	0	0	0	0	0
0	0	0.42998	0.35995	0.54735	0.49511
0.48304	0.51435	0.57285	0.5	0	0
0	0	0	0	0	0
0	0	0	0	0	0
0	0	0	0	0	0
0	0	0	0	0	0
0	0	0	0.62589	0.48914	0.5
0.58219	0.55336	0	0	0	0
0	0	0	0	0	0
0	0	0	0	0	0
0	0	0	0	0	0
0	0	0	0	0	0
0	0	0	0	0	0.51921
0.49817	0	0	0	0	0
0	0	0	0	0	0
0	0	0	0	0	0
0	0	0	0	0	0
0	0	0	0	0	0
0	0	0	0	0	0
0	0	0	0	0	0
0	0	0	0	0	0

0	0	0	0	0	0
0	0	0	0	0	0
0	0	0	0	0	0
0	0	0	0	0	0
0	0	0	0	0	0
0	0	0	0	0	0
0	0	0	0	0	0
0	0	0	0	0	0
0	0	0	0	0	0
0	0	0	0	0	0
0	0	0	0	0	0
0	0	0	0	0	0
0	0	0	0	0	0
0	0	0	0	0	0
0	0	0	0	0	0
0	0	0	0	0	0
0	0	0	0	0	0
0	0	0	0	0	0
0	0	0	0	0	0
0	0	0	0	0	0
0	0	0	0	0	0
0	0	0	0	0	0
0	0	0	0	0	0
0	0	0	0	0	0
0	0	0	0	0	0
0	0	0	0	0	0
0	0	0	0	0	0
0	0	0	0	0	0
0	0	0	0	0	0
0	0	0	0	0	0
0	0	0	0	0	0
0	0	0	0	0	0
0	0	0	0	0	0
0	0	0	0	0	0
0	0	0	0	0	0
0	0	0	0	0	0
0	0	0	0	0	0
0	0	0	0	0	0
0	0	0	0	0	0
0	0	0	0	0	0
0	0	0	0	0	0
0	0	0	0	0	0
0	0	0	0	0	0
0	0	0	0	0	0
0	0	0	0	0	0
0	0	0	0	0	0
0	0	0	0	0	0
0	0	0	0	0	0
0	0	0	0	0	0
0					

gtTCf=

0
0.07155
0.10366
0.11432
0.11389
0.10824
0.10059
0.092638
0.085208
0.078648
0.073046
0.06836
0.06449
0.06132
0.058731
0.05662
0.054894
0.053478

gtTCs=
0
0.07155
0.116
0.14393
0.16177
0.17342
0.18126
0.18671
0.19067
0.19366
0.19601
0.19792
0.19952
0.20088
0.20206
0.20309
0.20398
0.20476

gtTCp=
0
2.385
1.5979
1.1196
0.8287
0.65143
0.54309
0.47655
0.43538
0.4096
0.39316
0.38239

0.37506
0.36982
0.36585
0.36266
0.35995
0.35753

X_mask=
3.4884e-006,0.00044924,0.00044134,2.2204e-016,2.2204e-
016,0.00073602,0.0001353,2.2204e-
016,0.00034075,0.00038145,0.00032156,2.2204e-
016,0.00025396,0.00068856,2.2204e-016,0.00038344,2.2204e-016,2.2204e-
016,2.2204e-016,0.0006038,2.2204e-016,0.0041082,2.2204e-
016,0.0025854,0.0012658,0.0002949,2.2204e-016,2.2204e-
016,0.00028977,2.2204e-016,0.00068612,0.00097969,2.2204e-016,2.2204e-
016,2.2204e-016,2.2204e-016,0.00076182,0.00030883,2.2204e-
016,0.0043199,2.2204e-016,2.2204e-016,2.2204e-016,2.2204e-016,2.2204e-
016,2.2204e-016,0.00016136,0.00059284,2.2204e-016,2.2204e-
016,0.00092861,2.2204e-016,6.7177e-005,2.2204e-016,2.2204e-
016,2.2204e-016,2.2204e-016,0.00053311,2.2204e-016,2.2204e-
016,0.0012008,2.2204e-016,2.2204e-016,0.0012827,2.2204e-
016,0.0078569,2.2204e-016,2.2204e-016,2.2204e-
016,0.0014638,0.0015729,0.00034619,0.00093581,0.0010491,2.2204e-
016,0.00037037,2.2204e-016,0.00062269,2.2204e-016,2.2204e-016,2.2204e-
016,0.00055703,6.8809e-005,0.00019239,0.00032171,0.00040926,2.2204e-
016,2.2204e-016,2.2204e-016,0.0018926,2.2204e-016,0.0025917,2.2204e-
016,2.2204e-016,0.00081974,0.001805,0.0019616,2.2204e-016,2.2204e-
016,0.00050855,0.00025252,2.0531e-005,2.2204e-016,2.2204e-016,2.2204e-
016,0.00057023,2.2204e-016,2.2204e-016,0.00040869,8.4154e-005,3.5505e-
005,0.0012805,2.2204e-016,0.00030399,2.2204e-
016,0.00093024,0.0051633,0.0032936,2.2204e-016,0.00020104,2.2204e-
016,0.0016132,0.0012762,0.0039043,2.2204e-016,0.0010202,2.2204e-
016,2.2204e-016,0.00024071,2.2204e-016,2.2204e-016,5.698e-005,2.2204e-
016,0.00030534,0.00038723,5.0031e-005,6.3027e-005,0.00011797,2.2204e-
016,0.00024116,2.2204e-016,2.2204e-016,1.2968e-005,2.2204e-
016,2.2204e-016,0.0016916,0.0037389,2.2204e-016,2.2204e-
016,0.0033421,0.0019111,2.2204e-016,0.0034709,2.2204e-
016,0.0013825,0.0058213,2.2204e-016,0.00035463,2.2204e-016,2.2204e-
016,2.2204e-016,0.0004251,2.2204e-016,0.00054945,0.00050982,2.2204e-
016,0.00012031,0.00038128,0.00081199,2.2204e-
016,0.00019073,0.00022708,0.00011342,2.2204e-
016,0.00023326,0.00014827,0.00031178,2.2204e-016,2.2204e-016,2.2204e-
016,2.2204e-016,0.00096967,0.0012225,2.2204e-016,2.2204e-
016,0.00017222,2.2204e-016,0.0011207,0.0069471,2.2204e-016,2.2204e-
016,2.2204e-016,0.00040279,2.2204e-016,0.0011423,2.2204e-
016,0.00060027,2.2204e-016,0.00046469,2.2204e-
016,0.00031252,0.0003221,2.2204e-016,2.2204e-016,2.2204e-
016,0.00076329,9.6879e-005,0.00054146,2.2204e-016,7.8116e-
005,0.00045828,0.0030453,2.2204e-
016,0.0024178,0.0080559,0.0044865,0.0074759,2.2204e-016,2.2204e-
016,2.2204e-016,0.00090346,0.0030378,0.00079143,5.9872e-006,2.2204e-
016,0.0047141,0.00101,0.0010209,0.00071705,2.2204e-016,2.2204e-

016,0.00044299,2.2204e-016,2.2204e-016,2.2204e-016,2.2204e-
016,2.2204e-016,0.00040456,2.2204e-016,0.00016698,2.2204e-
016,0.00010769,2.2204e-016,0.0004884,0.00050066,0.0004126,2.2204e-
016,2.2204e-016,0.012491,0.0048756,0.0025456,2.2204e-016,2.2204e-
016,2.2204e-016,0.00021839,0.0016954,2.2204e-016,2.2204e-016,9.8419e-
005,2.2204e-016,2.2204e-016,2.2204e-
016,0.0044178,0.00075908,0.0039708,2.2204e-016,2.2204e-
016,0.00033552,2.2204e-016,0.00058655,0.00035013,2.2204e-016,2.2204e-
016,0.00011458,2.2204e-016,2.2204e-016,2.2204e-016,2.2204e-
016,0.00019906,0.0004941,2.2204e-016,6.553e-005,2.2204e-
016,0.00065597,0.0090775,2.2204e-016,2.2204e-016,2.2204e-
016,0.00092544,2.2204e-016,0.0016536,0.0032096,2.2204e-016,2.2204e-
016,0.0031921,2.2204e-016,4.6789e-005,0.0057189,2.2204e-016,2.2204e-
016,2.2204e-016,0.0032404,0.0011337,2.2204e-016,0.00069774,2.2204e-
016,8.9534e-005,0.00037533,2.2204e-016,2.844e-005,2.2204e-
016,0.00018812,7.3697e-005,2.2204e-016,0.00010956,2.2204e-
016,0.00035753,9.7038e-
005,0.0001339,0.00071954,0.00052966,0.0016888,2.2204e-016,2.2204e-
016,2.2204e-016,0.0048909,0.00073524,0.011361,0.0041031,2.2204e-
016,0.00019347,0.00037065,2.2204e-016,2.2204e-016,0.0015542,2.2204e-
016,0.00097422,0.0081449,0.0010549,2.2204e-
016,0.00023136,0.00026268,2.2204e-016,2.2204e-016,2.2204e-
016,0.00040084,2.2204e-016,0.00012501,0.00018556,2.1476e-
005,0.00063059,2.2204e-016,2.2204e-016,2.2204e-016,2.2204e-
016,0.00012511,2.2204e-016,2.2204e-016,2.2204e-016,0.0040828,2.2204e-
016,2.2204e-016,2.2204e-016,2.2204e-016,2.2204e-
016,0.0062907,0.0079013,2.2204e-016,0.0025232,2.2204e-
016,0.0034067,0.00010173,2.2204e-016,2.2204e-016,2.2204e-016,2.2204e-
016,0.00030113,2.2204e-016,2.2204e-016,2.2204e-016,0.00042382,2.2204e-
016,2.2204e-016,2.2204e-016,2.2204e-016,2.2204e-016,9.844e-
005,2.2204e-016,0.00054613,2.2204e-016,2.2204e-016,2.2204e-
016,0.00013891,2.2204e-016,0.00079112,0.00042315,2.2204e-016,2.2204e-
016,0.0029342,3.9666e-
005,0.0051733,0.002712,0.0021208,0.0038294,2.2204e-016,2.2204e-
016,2.2204e-016,0.00010165,2.2204e-016,2.2204e-016,2.2204e-
016,0.00011655,0.000856,2.9608e-005,2.2204e-
016,0.0016889,0.00042199,4.1366e-006,0.00021243,2.2204e-016,2.2204e-
016,2.2204e-016,2.2204e-016,2.2204e-016,0.00035083,2.2204e-
016,0.0005589,0.00010623,0.00020582,2.2204e-
016,0.0003709,0.00065188,0.00014275,2.2204e-
016,0.0063931,0.0010504,2.2204e-016,2.2204e-
016,0.0011122,0.0073953,2.2204e-016,2.2204e-016,2.2204e-016,2.2204e-
016,0.0013517,0.0035961,0.0010329,2.2204e-016,0.0042806,2.2204e-
016,0.0036328,0.00084126,2.2204e-016,3.2817e-005,0.00041599,2.2204e-
016,2.2204e-016,2.2204e-016,2.2204e-016,2.2204e-016,2.2204e-
016,0.00012844,0.00027312,5.3882e-005,0.00037682,2.2204e-016,2.2204e-
016,7.836e-005,2.2463e-005,2.2204e-016,2.2204e-016,2.2204e-
016,2.2204e-016,0.0020721,0.0026567,0.0037837,2.2204e-
016,0.0031271,2.2204e-016,2.2204e-016,2.2204e-016,0.0012977,2.2204e-
016,2.2204e-016,0.0014227,0.0010146,2.2204e-016,0.00093252,2.2204e-
016,2.2204e-016,2.2204e-016,2.2204e-016,0.00018916,2.2204e-
016,0.00031276,2.2204e-016,0.00025698,0.00054959,2.2204e-016,4.9657e-

005,0.00058785,0.00026558,0.00051152,7.7086e-005,2.2204e-016,7.5962e-
005,0.00019871,2.2204e-016,0.00022308,2.2204e-
016,0.0043399,0.0019582,0.00010752,2.2204e-016,0.0024011,2.2204e-
016,0.0026434,0.0028116,0.0057922,2.2204e-
016,0.0018496,0.0024176,2.2204e-016,0.0026106,2.2204e-
016,0.0015536,2.2204e-016,2.2204e-016,0.0044748,2.2204e-016,5.1167e-
005,2.2204e-016,2.2204e-016,2.2204e-016,4.0745e-006,2.2204e-
016,2.2204e-016,0.000299,2.2204e-016,2.2204e-016,0.00032173,2.2204e-
016,0.00057472,2.0725e-005,2.2204e-016,0.00018754,7.6909e-005,2.2204e-
016,0.00064995,0.0016555,2.2204e-016,0.002644,0.0084117,2.2204e-
016,2.2204e-016,0.0024867,0.0017054,2.2204e-016,0.0035758,2.2204e-
016,0.0034884,2.2204e-016,2.2204e-016,2.2204e-016,0.0039159,2.2204e-
016,0.0025583,2.2204e-016,3.2172e-005,2.2204e-016,1.706e-005,2.2204e-
016,0.00057737,0.00026153,7.3555e-006,2.2204e-016,2.2204e-
016,0.00027497,2.2204e-016,0.00043225,2.2204e-016,2.2204e-016,2.2204e-
016,0.00026873,2.2204e-016,2.2204e-016,0.00072757,2.2204e-
016,0.006564,0.00043551,2.2204e-016,0.0020297,2.2204e-016,2.2204e-
016,2.2204e-016,2.2204e-016,2.2204e-016,2.2204e-016,2.2204e-
016,2.2204e-016,0.00025029,2.2204e-
016,0.0037498,0.00039374,0.0020462,2.2204e-016,2.2204e-016,1.285e-
005,2.2204e-016,0.00022794,6.0168e-005,2.2204e-016,2.2204e-
016,0.00012758,2.2204e-016,2.2204e-016,3.908e-005,2.2204e-016,2.2204e-
016,2.2204e-016,0.00026756,1.266e-005,0.00048599,2.2204e-016,2.2204e-
016,2.2204e-016,2.2204e-016,2.2204e-016,2.2204e-
016,0.0018082,0.0010585,0.0079103,0.0060932,2.2204e-
016,0.00080563,0.0014462,2.2204e-016,2.2204e-016,2.2204e-
016,0.0031232,2.2204e-016,2.2204e-016,2.2204e-016,2.2204e-016,2.2204e-
016,2.2204e-016,2.2204e-016,2.2204e-016,0.00053982,2.2204e-
016,2.2204e-016,5.9323e-006,2.2204e-016,2.2204e-016,2.2204e-
016,0.00042044,2.2204e-016,2.2204e-016,2.2204e-016,2.2204e-
016,1.7357e-005,2.2204e-016,0.0022304,2.2204e-016,0.0025421,2.2204e-
016,0.002929,2.2204e-016,0.0014554,2.2204e-016,2.2204e-016,2.2204e-
016,2.2204e-016,0.0019269,0.0033245,0.00034547,0.001281,2.2204e-
016,0.00076158,0.0019006,0.00011799,0.00014372,0.00037853,0.00025493,8
.6467e-005,0.00027979,2.2204e-016,2.2204e-016,2.2204e-016,9.4901e-
005,2.2204e-016,8.3699e-005,0.00013355,2.2204e-016,2.2204e-
016,0.00030027,2.2204e-
016,0.00064997,0.0046878,0.0041981,0.0017096,5.5943e-
005,0.0012087,0.002161,0.0045952,0.0019002,0.0045897,0.00049328,0.0011
999,9.9903e-005,0.00041873,2.2204e-016,0.0018173,0.0017138,2.2204e-
016,2.2204e-016,2.2204e-016,0.00060664,1.4128e-006,2.2204e-
016,0.00033586,2.2204e-016,5.7284e-005,0.00030148,2.9442e-
005,0.00047942,2.2204e-016,2.2204e-016,2.2204e-016,3.9274e-
005,2.2204e-016,0.00017391,2.2204e-016,2.2204e-016,2.2204e-
016,0.0018371,0.0023636,0.0018362,2.2204e-016,0.0010025,2.2204e-
016,2.2204e-016,2.2204e-016,0.00083533,2.2204e-016,0.0012174,3.0674e-
005,0.00025478,2.2204e-016,0.0012437,2.2204e-
016,0.00071035,0.00092584,2.2204e-016,0.0001655,6.1184e-
007,0.00044017,2.2204e-016,0.00010236,2.2204e-016,2.2204e-
016,0.00018854,0.00010818,2.9647e-005,2.2204e-016,0.00039242,2.2204e-
016,2.6818e-005,2.6011e-006,0.00023243,0.00036888,0.00020657,5.2534e-
005,0.0042533,2.2204e-016,2.2204e-016,2.2204e-016,2.2204e-

016,0.0020529,0.0024036,0.0054706,0.0035546,0.00033442,0.0021729,0.000
97989,2.2204e-016,0.0014483,0.00063856,0.00036566,1.9003e-005,6.039e-
005,3.7312e-005,0.00098735,0.00027994,0.00039902,7.0557e-
007,0.00033577,2.2204e-016,0.00033417,2.2204e-016,2.2204e-016,2.2204e-
016,0.00044713,0.00013302,2.2204e-016,0.00027869,2.2204e-016,2.2204e-
016,0.00014799,2.2204e-016,2.2204e-016,0.0023332,0.00089826,2.2204e-
016,2.2204e-016,2.2204e-016,0.0065426,2.2204e-
016,0.0031464,0.0011815,2.2204e-
016,0.00016475,0.00052443,0.00022158,7.7565e-005,2.2204e-
016,0.00036811,0.00011868,0.00037276,2.2204e-
016,0.00024659,0.00018235,0.00054825,2.2204e-016,2.2204e-
016,0.00023268,0.00022199,2.2204e-016,2.2204e-016,0.0002142,2.2204e-
016,2.2204e-016,2.2204e-016,0.00020039,0.00043804,2.2204e-
016,0.00023962,2.2204e-016,0.00037647,0.003744,2.2204e-
016,0.005222,2.2204e-016,2.2204e-016,0.0037716,2.2204e-016,2.2204e-
016,0.00017555,0.00026612,0.00017926,7.2636e-005,0.00038657,2.2204e-
016,2.2204e-016,3.5527e-005,0.00018852,0.00042228,1.2682e-005,2.2204e-
016,2.2204e-016,0.00011399,2.2204e-016,0.00013475,2.2204e-016,2.2204e-
016,0.00027049,2.2204e-016,0.00027736,2.2204e-016,8.8831e-005,2.2204e-
016,2.2601e-006,2.839e-
005,0.00025916,0.0002647,0.0018646,0.00252,2.2204e-016,2.2204e-
016,0.00062345,2.2204e-016,0.00028259,2.2204e-016,2.2204e-016,2.2204e-
016,2.2204e-016,2.2204e-016,0.00045678,2.2204e-016,0.00013351,2.2204e-
016,2.2204e-016,2.2204e-016,2.2204e-016,0.00012196,2.2204e-
016,0.00011318,7.8592e-005,2.2204e-016,2.2204e-
016,0.00050411,0.00047255,2.2204e-016,0.00025569,0.00014721,9.5237e-
005,2.2204e-016,2.2204e-016,0.0001681,2.2204e-016,0.00020557,2.2204e-
016,0.00010111,2.2204e-016,0.0032769,0.00015499,2.2204e-016,2.2204e-
016,4.2818e-005,2.2204e-016,0.00031188,2.2204e-016,2.2204e-
016,8.7196e-006,1.8592e-006,8.5274e-007,5.4694e-
005,0.00018753,2.2204e-016,2.2204e-016,6.9618e-005,0.00019076,2.2204e-
016,2.2204e-016,0.0001414,0.00010967,0.00016899,2.2204e-
016,0.00015127,5.4787e-005,2.2204e-016,0.00053651,2.2204e-016,2.2204e-
016,0.00047967,2.2204e-016,0.00014945,8.0643e-005,2.2204e-016,2.2204e-
016,2.2204e-016,0.00022176,0.00016886,0.00040988,0.00031495,2.2204e-
016,2.2204e-016,0.00023959,0.00010182,0.00048003,2.2204e-016,2.2204e-
016,0.00042574,1.6572e-005,2.2204e-
016,0.00014155,0.00022288,0.00036205,6.5488e-005,2.2204e-016,2.2204e-
016,2.2204e-016,2.2204e-016,2.2204e-016,2.2204e-016,2.2204e-
016,5.0397e-005,2.2204e-016,2.2204e-016,0.00022078,2.2204e-
016,0.0002377,2.2204e-016,0.00026706,0.00016651,0.00042351,3.6029e-
005,2.2204e-016,0.00058186,2.2204e-016,0.00016963,2.2204e-
016,0.00045719,2.2204e-016,0.00034431,0.00044978,2.2204e-
016,0.00037976,9.8043e-005,2.2204e-016,0.00034044,2.2204e-016,2.2204e-
016,2.2204e-016,0.00017679,2.2204e-016,2.2204e-016,1.1194e-
005,4.8099e-005,2.2204e-016,2.2204e-016,2.2204e-016,2.2204e-
016,2.2204e-016,2.2204e-016,9.5547e-005,2.2204e-
016,0.00037976,0.00034854,4.0472e-005,0.0001993,2.2204e-
016,0.00010518,0.00014204,0.00078449,0.00033482,2.2204e-
016,0.00046834,0.00038878,0.0007424,0.00063808,0.00031004,0.00021955,0
.00012522,9.3383e-005,2.2204e-016,9.583e-006,0.00011669,8.5306e-
005,2.2204e-016,0.00022178,2.2204e-016,0.00037747,2.2204e-016,2.2204e-

016,0.00038307,0.00040122,2.517e-005,0.00028228,2.2204e-016,2.2204e-
016,0.00020049,0.0005651,0.00010746,2.2204e-016,2.2204e-016,2.2204e-
016,0.00019848,2.2204e-016,2.2204e-016,0.00015682,2.2204e-016,2.2204e-
016,0.00021011,0.00017328,0.00037796,2.2204e-016,5.8238e-006,2.2204e-
016,2.2204e-016,2.2204e-016,0.00034196,2.2204e-016,0.00013319,2.2204e-
016,0.0004026,2.2204e-016,0.00036335,2.2204e-016,2.2204e-016,9.6427e-
005,2.2204e-016,0.00036026,9.5882e-005,2.2204e-016,2.2204e-
016,2.2204e-016,2.2204e-016,2.0136e-005,0.00022524,2.2204e-
016,0.00023229,8.367e-005,2.2204e-016,2.2204e-016,7.7199e-
005,0.00034,0.00023592,9.6665e-005,2.2204e-016,0.00034126,2.2204e-
016,0.0002494,2.2204e-016,0.00032416,2.2204e-016,0.00026864,2.2204e-
016,2.2204e-016,0.00032287,0.00012997,0.00042494,2.2204e-016,2.2204e-
016,2.2204e-016,2.2204e-016,2.2204e-016,0.00038049,2.2204e-
016,0.00023408,2.2204e-016,0.00013889,2.2204e-016,0.0005769,2.2204e-
016,6.1111e-005,2.2204e-016,0.00011496,2.2204e-016,2.2204e-
016,2.2204e-016,2.2204e-016,0.00028329,2.6368e-005,2.2204e-
016,0.00027295,0.00036435,9.8515e-005,5.2988e-005,2.2204e-016,2.2204e-
016,2.2204e-016,7.4946e-005,2.2204e-016,0.00069446,2.2204e-
016,2.2204e-016,2.2204e-016,0.00056021,2.2204e-
016,0.00019923,0.00013041,0.00027304,0.00021054,0.00016635,2.2204e-
016,0.00012672,2.2204e-016,2.5113e-005,2.2204e-016,1.6912e-
005,2.2204e-016,4.3475e-006,7.7531e-006,0.00047996,3.0965e-
005,6.9488e-005,2.2204e-016,0.00014794,2.2204e-016,1.2493e-
005,0.00015261,2.2204e-016,2.2204e-016,0.00025783,2.2204e-016,9.0315e-
005,0.0006352,0.00015965,0.00020836,2.2204e-016,2.2204e-016,2.2204e-
016,2.2204e-016,2.2204e-016,2.2204e-016,0.00021162,0.00023886,2.2204e-
016,0.0001034,0.00029744,0.0001191,2.2204e-016,0.00054636,2.2204e-
016,0.00049811,2.2204e-016,0.00021365,2.2204e-016,2.2204e-016,2.2204e-
016,1.7702e-005,2.2204e-016,0.00015654,0.00019894,2.2204e-
016,0.00015924,8.6877e-005,2.2204e-016,2.2204e-016,2.2204e-
016,2.2204e-016,0.00026918,0.00073106,2.2204e-
016,0.00021572,0.00045548,0.00025748,5.3711e-005,2.2204e-016,2.2204e-
016,2.2204e-016,2.2204e-016,2.2204e-016,2.2204e-
016,0.00030089,0.00054389,8.5479e-005,0.00014312,2.2204e-
016,0.00042042,6.6172e-005,2.2204e-016,0.00050461,4.5826e-005,7.9263e-
005,6.3393e-005,8.2188e-005,0.00027254,2.2204e-016,2.2204e-
016,2.2204e-016,2.2204e-016,0.00030437,2.2204e-016,2.2204e-
016,2.2204e-016,8.9235e-005,0.00023036,7.4465e-006,2.2204e-
016,2.2204e-016,0.00038516,2.2204e-016,2.2204e-016,2.2204e-
016,2.2204e-016
0.071567,0.071964,0.072056,0.071198,0.070608,0.072482,0.071249,0.07037
1,0.071819,0.072134,0.071571,0.07142,0.071873,0.072132,0.071052,0.0721
03,0.070775,0.071607,0.071131,0.071998,1.1552,1.4194,1.2762,1.1802,0.0
72566,0.07211,0.071462,0.071418,0.07144,0.071117,0.07189,0.072764,0.07
1145,0.071121,0.07151,0.071332,0.072276,0.071593,0.070773,2.3896,1.229
,1.4476,1.2104,1.1501,1.2853,1.2653,0.071961,0.072101,0.070983,0.07134
8,0.072622,0.071095,0.072078,0.071399,0.07079,0.070853,0.071515,0.0724
38,0.071448,0.071555,0.072258,2.3833,2.3786,1.1381,0.81248,1.236,0.912
38,1.2103,1.267,1.2025,1.2184,0.071578,0.072372,0.071973,0.071237,0.07
1712,0.071178,0.072125,0.071072,0.070769,0.071358,0.071707,0.071277,0.
071607,0.07134,0.071852,2.3785,2.3789,2.3879,1.2135,1.4356,1.0994,1.24
73,1.2282,1.2295,1.3195,1.262,1.3157,0.071672,0.071882,0.071902,0.0713

03,0.071094,0.070794,0.071006,0.07219,0.069735,0.070788,0.072499,0.071
857,0.071522,0.072773,0.071563,0.072106,2.3861,2.3896,2.3908,2.3889,1.
4045,1.3501,1.1637,1.3896,1.1599,0.98213,1.3004,1.2581,0.92001,1.0914,
0.071953,0.071172,0.07105,0.071733,0.071524,0.07192,0.071751,0.071466,
0.071829,0.071727,0.0702,0.071995,0.070388,0.07074,0.071669,0.071538,2
.3815,2.3855,2.3884,2.3787,2.3849,2.3859,1.2285,1.2798,1.1225,1.3328,1
.2053,1.3766,1.4898,1.2637,1.7111,1.2259,0.071189,0.071932,0.071271,0.
072225,0.07173,0.071105,0.071922,0.071729,0.072115,0.071175,0.0719,0.0
72174,0.071453,0.071485,0.071171,0.071609,0.072178,2.3756,2.3753,2.374
6,2.3797,2.3862,2.3883,1.2675,1.3773,1.1992,1.2279,1.2266,1.2351,1.220
6,1.3795,1.4562,1.4288,1.3465,0.072697,0.070985,0.072525,0.070927,0.07
1807,0.071423,0.072055,0.071793,0.071086,0.071329,0.071185,0.07251,0.0
71463,0.07198,0.071321,0.071623,0.071577,2.3883,2.3737,2.3851,2.389,2.
389,2.3908,2.3843,1.2277,1.2238,1.3748,1.2326,1.0431,1.0265,1.2712,1.0
929,1.0329,1.0021,1.403,0.070485,0.07065,0.072196,0.070243,0.070008,0.
071458,0.071656,0.070923,0.071785,0.070767,0.071815,0.07156,0.071707,0
.071228,0.071892,0.071572,0.071737,0.070521,2.3838,2.3999,2.3909,2.385
7,2.3782,2.3718,2.3836,1.4693,1.2538,1.2584,1.3207,1.0229,1.5795,1.354
3,1.2268,1.2317,1.1684,1.4089,0.071201,0.070647,0.072096,0.071291,0.07
1926,0.0715,0.071481,0.070417,0.071698,0.070882,0.071279,0.071316,0.07
1004,0.071609,0.071911,0.071626,0.071759,0.071234,0.072036,2.3937,2.38
46,2.3782,2.3839,2.3837,2.3798,2.3887,1.3413,1.2344,1.2893,1.2305,1.43
53,1.5247,1.156,1.3629,1.2294,1.4208,1.234,0.90808,0.072181,0.072187,0
.071604,0.0716,0.072082,0.070997,0.071828,0.0711,0.071828,0.071375,0.0
71336,0.071805,0.071319,0.071957,0.071735,0.072016,0.07226,0.071971,2.
388,2.3834,2.3826,2.3808,2.3912,2.3848,2.3928,1.1026,1.195,1.4948,1.22
84,1.0096,1.3305,1.3326,1.4127,1.097,1.6114,1.3268,1.3074,0.071552,0.0
71869,0.071564,0.070812,0.071254,0.072023,0.071062,0.071562,0.071537,0
.071467,0.072083,0.07151,0.071453,0.07142,0.071397,0.071486,0.071591,0
.071174,2.3864,2.3829,2.3835,2.3838,2.3844,2.3904,2.3863,2.3927,2.3926
,1.2275,1.3119,1.226,1.4051,1.2302,1.2186,1.2277,1.2262,1.3495,1.5671,
1.2832,0.071477,0.071463,0.072384,0.071464,0.071319,0.070901,0.071137,
0.071144,0.071793,0.071283,0.071824,0.071597,0.071787,0.071278,0.07192
3,0.071782,0.072332,0.071803,2.3831,2.3804,2.3907,2.3856,2.3894,2.389,
2.3861,2.3859,2.3755,1.2293,0.81108,0.85215,0.77088,0.84077,0.7702,0.8
426,0.87291,0.84287,0.94853,0.81106,0.072006,0.071422,0.071869,0.07122
6,0.071143,0.071488,0.070841,0.071465,0.072072,0.071069,0.071767,0.071
888,0.071674,0.071296,0.072116,0.072293,0.071836,0.071795,2.3935,2.386
,2.3849,2.3776,2.3854,2.3882,2.3861,2.3751,2.3816,1.2633,0.86123,0.844
47,0.85835,0.84026,0.86694,0.79072,0.69604,0.82101,0.8411,0.91311,0.07
2142,0.071503,0.070906,0.071536,0.071293,0.071745,0.071314,0.071822,0.
071886,0.071512,0.071761,0.071381,0.071575,0.071688,0.071626,0.071425,
0.070925,0.070655,2.3899,2.3878,2.3842,2.3871,2.3817,2.386,2.375,1.226
,1.1233,0.94633,0.83359,0.7397,0.84378,0.92275,0.99254,0.928,1.0033,0.
91399,0.89861,0.89064,0.071928,0.071257,0.071777,0.071456,0.071902,0.0
72171,0.071441,0.072047,0.072053,0.071476,0.071749,0.071725,0.071337,0
.071616,0.072002,0.071013,0.071652,0.071502,2.3932,2.3853,2.3815,2.387
7,2.3867,2.3825,1.2304,0.90719,1.3161,1.6752,1.2291,0.68329,0.75251,0.
97,0.69386,0.84465,0.98879,0.9614,0.91602,0.90222,0.071529,0.071412,0.
071614,0.07114,0.071405,0.071315,0.070984,0.071765,0.071508,0.071275,0
.071594,0.071519,0.072176,0.071417,0.07117,0.071747,0.071664,0.071636,
2.3838,2.3827,2.3852,2.3919,2.3972,1.228,1.1481,1.2291,1.3088,1.2265,1

.2294,0.80587,0.90579,0.84016,0.84145,0.81165,0.92243,0.91872,0.7093,0
.92508,0.071609,0.071473,0.071524,0.070981,0.071879,0.071609,0.071697,
0.071321,0.071205,0.071896,0.071672,0.072032,0.071473,0.07168,0.070836
,0.071827,0.071707,0.071323,2.3836,2.3825,2.3876,1.0245,1.3178,1.2361,
1.0827,1.304,1.2255,1.2073,1.1958,0.93764,0.72962,0.88525,0.84271,0.62
538,0.78926,0.71801,0.70072,0.84551,0.071332,0.07164,0.071287,0.071863
,0.071713,0.071524,0.071279,0.071781,0.071363,0.071122,0.071406,0.0714
06,0.071352,0.071451,0.071892,0.071933,0.071946,0.07167,2.3845,1.2275,
1.2276,1.4808,1.5162,1.1705,1.1698,1.3156,1.241,1.2387,0.9508,1.0223,0
.88513,0.81639,0.86221,0.81056,0.89198,0.91617,0.82526,0.071183,0.0715
79,0.070388,0.071666,0.071376,0.072054,0.071283,0.071385,0.07161,0.071
075,0.071382,0.071464,0.071907,0.071279,0.07166,0.070912,0.071201,0.07
1666,1.2795,1.2298,1.34,1.2302,1.0746,1.1099,1.2321,1.0904,1.1624,1.42
42,0.84237,0.8846,0.90924,0.97113,0.84356,0.79655,0.96343,0.95178,0.79
026,0.071762,0.071717,0.071998,0.071642,0.071699,0.071741,0.071649,0.0
71303,0.071516,0.071461,0.071289,0.071187,0.071853,0.071611,0.071397,0
.071912,0.071102,0.072146,1.4212,1.4436,1.205,1.0578,1.0142,1.1714,1.2
39,1.2302,1.2325,1.2297,0.8286,0.84228,0.79205,0.80459,0.77239,0.8448,
0.6928,0.78675,0.071319,0.071994,0.07146,0.071829,0.071962,0.071158,0.
071587,0.071894,0.071535,0.072092,0.071471,0.07148,0.071329,0.071686,0
.071259,0.071624,0.071439,0.071427,0.071421,1.0305,1.331,1.4515,1.2239
,1.2285,1.2285,1.3101,1.2455,1.086,1.2253,0.7941,0.94906,0.88881,0.872
15,0.81333,0.86454,0.88233,0.94144,0.0715,0.071677,0.071719,0.07183,0.
071035,0.0717,0.071224,0.07136,0.071795,0.07137,0.071675,0.071206,0.07
1935,0.071466,0.071532,0.071786,0.071657,0.071815,0.07175,0.071608,0.8
0756,1.2289,1.0919,1.2253,1.0259,1.2318,1.1979,1.3297,1.3489,0.98523,0
.67719,0.73161,0.78478,0.81013,0.85201,0.071872,0.071282,0.071708,0.07
1498,0.07244,0.071741,0.071936,0.071385,0.072004,0.071393,0.071778,0.0
71201,0.07128,0.071491,0.072333,0.071545,0.070891,0.07173,0.071686,0.0
71384,0.071764,0.071474,1.2252,1.0581,1.2727,1.2277,1.227,1.2274,1.319
4,1.2119,1.2318,0.78044,0.85058,0.72308,0.071953,0.071852,0.071416,0.0
71438,0.071842,0.071249,0.071949,0.071348,0.071761,0.071641,0.071969,0
.07118,0.071411,0.071562,0.071694,0.071131,0.071185,0.071866,0.071257,
0.071561,0.070847,0.071715,0.07187,0.071455,0.071713,0.071045,1.0657,0
.90828,1.3368,1.22,1.1856,1.2566,1.4019,1.2279,0.071374,0.071842,0.071
654,0.071722,0.071812,0.071716,0.070921,0.07147,0.071769,0.071511,0.07
18,0.071453,0.071345,0.071192,0.071693,0.071418,0.071544,0.071513,0.07
1746,0.071764,0.071557,0.072027,0.071408,0.071577,0.071112,0.071449,0.
071514,0.071685,0.071915,1.5208,1.2077,1.2301,1.4178,1.3515,0.071379,0
.072066,0.071008,0.070878,0.071673,0.071319,0.070672,0.071835,0.071542
,0.071844,0.071548,0.07115,0.0715,0.071455,0.071513,0.071484,0.071743,
0.071652,0.071315,0.071666,0.072033,0.07202,0.071461,0.071709,0.071624
,0.071583,0.071529,0.071139,0.071713,0.071415,0.071907,0.071527,0.0716
06,1.2698,1.2254,0.071746,0.071447,0.071238,0.071586,0.070592,0.072041
,0.071172,0.071241,0.071729,0.071719,0.071611,0.071616,0.071499,0.0711
85,0.071107,0.07162,0.071503,0.071556,0.071433,0.071577,0.071556,0.071
85,0.07154,0.071812,0.071674,0.07142,0.072091,0.071171,0.071208,0.0719
75,0.071595,0.071584,0.071564,0.071391,0.071467,0.071243,0.071866,0.07
1496,0.07185,0.071892,0.071565,0.071817,0.071695,0.071773,0.071918,0.0
71355,0.071244,0.072163,0.071578,0.071443,0.071725,0.072066,0.072113,0
.071692,0.071476,0.071649,0.071209,0.071565,0.071358,0.071438,0.071281
,0.071713,0.071594,0.071635,0.071949,0.071233,0.071742,0.071238,0.0717

27,0.071793,0.071966,0.07168,0.071682,0.072032,0.071514,0.071447,0.071
521,0.072096,0.071264,0.071998,0.072186,0.070606,0.071913,0.071752,0.0
71318,0.071976,0.071357,0.071191,0.071401,0.071841,0.071287,0.071552,0
.071598,0.071589,0.071524,0.07158,0.071467,0.071478,0.071296,0.071153,
0.071388,0.071425,0.072081,0.071989,0.07163,0.071693,0.071672,0.071547
,0.071915,0.072319,0.071889,0.071388,0.072061,0.071728,0.072144,0.0719
65,0.071863,0.071884,0.071757,0.071564,0.071477,0.071362,0.071594,0.07
1562,0.070912,0.071863,0.071492,0.071865,0.071398,0.071332,0.07174,0.0
71946,0.071534,0.071781,0.071573,0.071511,0.071594,0.071722,0.071433,0
.071148,0.071306,0.071129,0.071566,0.071799,0.071394,0.071799,0.071826
,0.07145,0.071841,0.071856,0.072094,0.071288,0.0714,0.071468,0.071559,
0.07146,0.071892,0.071409,0.071586,0.071109,0.071826,0.070975,0.072052
,0.070807,0.071603,0.071558,0.07125,0.071838,0.071615,0.071366,0.07142
3,0.070823,0.071534,0.071639,0.071563,0.071354,0.071765,0.071519,0.071
602,0.071589,0.071552,0.071901,0.071728,0.071484,0.07137,0.072096,0.07
1495,0.071534,0.071199,0.071816,0.071125,0.071729,0.07122,0.071375,0.0
71679,0.071776,0.072121,0.071608,0.071491,0.071471,0.071282,0.071441,0
.071873,0.071324,0.071646,0.071246,0.07146,0.071182,0.07205,0.071417,0
.071496,0.071278,0.071435,0.071229,0.071558,0.071089,0.071601,0.071778
,0.071546,0.071326,0.071984,0.071775,0.071591,0.071609,0.071439,0.0712
59,0.07153,0.071459,0.071548,0.072315,0.071216,0.07134,0.071291,0.0721
12,0.07115,0.071932,0.071546,0.071732,0.071639,0.071791,0.071725,0.071
856,0.071258,0.071821,0.071219,0.071579,0.071208,0.071411,0.071511,0.0
72062,0.071426,0.071476,0.071626,0.071584,0.071307,0.071737,0.071535,0
.070968,0.071069,0.071908,0.07178,0.071464,0.072092,0.071667,0.071908,
0.071105,0.071219,0.071108,0.071611,0.071421,0.0713,0.071749,0.071746,
0.07133,0.071697,0.071957,0.07168,0.07111,0.072044,0.071416,0.072072,0
.071438,0.071648,0.071161,0.071197,0.071146,0.071486,0.071603,0.07161,
0.071714,0.071567,0.071597,0.071578,0.071369,0.070978,0.071404,0.07143
7,0.071651,0.072324,0.07148,0.071679,0.07192,0.071971,0.071554,0.07120
2,0.071402,0.071033,0.071198,0.071529,0.07153,0.071772,0.071983,0.0716
75,0.071702,0.071249,0.071774,0.071559,0.071184,0.071955,0.071642,0.07
1634,0.071639,0.071613,0.071787,0.071603,0.07141,0.071252,0.071336,0.0
71753,0.070865,0.071395,0.071216,0.071418,0.071806,0.071632,0.071539,0
.071646,0.071963,0.071354,0.071521,0.071271,0.07103
0.11557,0.11604,0.11629,0.11566,0.1148,0.11708,0.11552,0.11524,0.11625
,0.11729,0.11636,0.11585,0.11646,0.11672,0.11554,0.11666,0.11565,0.116
2,0.11583,0.11671,0.80848,0.9783,0.88482,0.82476,0.117,0.11603,0.11611
,0.11585,0.11588,0.11557,0.11629,0.117,0.11585,0.11556,0.11568,0.11574
,0.11663,0.11547,0.1154,1.5994,0.85935,0.99414,0.84477,0.8048,0.89283,
0.87991,0.11689,0.11649,0.11546,0.11603,0.11701,0.11545,0.11637,0.1159
9,0.11532,0.11555,0.11602,0.11683,0.11598,0.11592,0.11677,1.596,1.5932
,0.79779,0.59073,0.86541,0.65432,0.8454,0.88337,0.83884,0.8509,0.11616
,0.11705,0.11678,0.11573,0.11612,0.11534,0.11677,0.1153,0.11539,0.1155
5,0.11594,0.11557,0.1162,0.11534,0.11619,1.5912,1.593,1.6016,0.84653,0
.98702,0.77527,0.86736,0.85671,0.85703,0.91313,0.87727,0.91136,0.11609
,0.11648,0.11642,0.11571,0.11595,0.11527,0.11561,0.11645,0.11414,0.115
22,0.11712,0.1163,0.11616,0.11723,0.11601,0.11615,1.6017,1.603,1.5976,
1.6013,0.96776,0.93213,0.81487,0.96109,0.81374,0.70151,0.90384,0.87756
,0.65947,0.77052,0.11637,0.11571,0.11561,0.11638,0.11634,0.11612,0.115
61,0.11594,0.11596,0.11629,0.11455,0.11635,0.11478,0.11503,0.1164,0.11
582,1.5909,1.5988,1.6035,1.5909,1.6034,1.6033,0.8561,0.88765,0.79031,0

.92173,0.84399,0.95246,1.0253,0.88227,1.165,0.85467,0.11577,0.11653,0.
11563,0.11669,0.11615,0.11546,0.11599,0.11631,0.11621,0.11573,0.11651,
0.11678,0.11568,0.11611,0.11568,0.11607,0.11643,1.583,1.5868,1.5888,1.
5972,1.5998,1.6035,0.87909,0.95363,0.84037,0.85527,0.85594,0.86339,0.8
5204,0.95329,1.0047,0.98695,0.93089,0.11712,0.11531,0.11661,0.11516,0.
11651,0.11602,0.11618,0.11613,0.11587,0.11586,0.11584,0.11685,0.1157,0
.11006,0.11026,0.1103,0.11555,1.6005,1.586,1.5979,1.6008,1.5996,1.6099
,1.602,0.85645,0.85305,0.95082,0.86117,0.73828,0.7269,0.88431,0.77095,
0.73255,0.71345,0.96801,0.1149,0.11517,0.11632,0.11435,0.1149,0.11596,
0.11649,0.11536,0.11661,0.11532,0.11627,0.10999,0.10806,0.10909,0.1104
5,0.10974,0.10871,0.11504,1.5973,1.6131,1.602,1.5984,1.5924,1.5827,1.5
949,1.012,0.87322,0.87741,0.91759,0.72499,1.082,0.93526,0.85641,0.8587
2,0.82204,0.97515,0.11569,0.11502,0.11657,0.11568,0.11626,0.11604,0.11
594,0.11485,0.11622,0.10934,0.10946,0.10995,0.10957,0.10987,0.11028,0.
11007,0.1099,0.11047,0.11583,1.6074,1.5947,1.5877,1.5965,1.5986,1.5905
,1.6056,0.9331,0.85926,0.89581,0.85817,0.99246,1.0464,0.81119,0.94203,
0.85932,0.97978,0.86248,0.65253,0.1166,0.11672,0.11604,0.11596,0.11666
,0.11572,0.11002,0.10946,0.1099,0.10976,0.11022,0.11097,0.1096,0.10873
,0.10993,0.11043,0.11059,0.10829,1.5976,1.5987,1.5958,1.5944,1.6045,1.
5984,1.6063,0.77908,0.83448,1.0279,0.85649,0.71564,0.9231,0.92432,0.97
406,0.7738,1.1028,0.91829,0.9056,0.11622,0.11641,0.116,0.11011,0.109,0
.11115,0.10925,0.10981,0.10923,0.10903,0.11019,0.11054,0.10999,0.10868
,0.10881,0.11046,0.10958,0.10922,1.5998,1.5984,1.5979,1.5973,1.5969,1.
6007,1.6044,1.6045,1.603,0.85733,0.90983,0.85423,0.97022,0.85824,0.852
05,0.85568,0.85537,0.93621,1.0768,0.88939,0.10983,0.1096,0.11091,0.108
74,0.10966,0.10924,0.10974,0.10951,0.1093,0.10713,0.11045,0.10881,0.11
04,0.10994,0.11013,0.11051,0.1111,0.1099,1.5978,1.5946,1.6015,1.5956,1
.6004,1.5994,1.5997,1.5999,1.5875,0.85823,0.58995,0.61216,0.559,0.6053
2,0.55862,0.60628,0.62562,0.6058,0.67253,0.5861,0.11133,0.10995,0.1101
9,0.1094,0.10822,0.11024,0.10845,0.1097,0.11149,0.10969,0.1091,0.10989
,0.10834,0.10996,0.11097,0.11042,0.11018,0.10879,1.6061,1.5984,1.5987,
1.5953,1.5972,1.6012,1.5953,1.5888,1.5976,0.87318,0.61908,0.60729,0.61
692,0.60425,0.62221,0.57224,0.51212,0.59151,0.60494,0.65124,0.11072,0.
11091,0.1097,0.1098,0.10923,0.10984,0.11011,0.11019,0.11004,0.10968,0.
11035,0.10795,0.10964,0.11055,0.11098,0.11065,0.11066,0.10851,1.5992,1
.6016,1.5925,1.6041,1.5939,1.5982,1.5885,0.8497,0.78155,0.66856,0.6002
3,0.53934,0.6063,0.6579,0.70215,0.66192,0.71036,0.6516,0.64099,0.63754
,0.11082,0.1114,0.10887,0.10957,0.11061,0.11046,0.10925,0.1105,0.1107,
0.10863,0.1102,0.10878,0.10875,0.10844,0.11012,0.10936,0.11212,0.10968
,1.6045,1.6021,1.5922,1.6004,1.5999,1.5956,0.85308,0.64319,0.91038,1.1
367,0.84874,0.50501,0.54642,0.69066,0.51133,0.60853,0.70026,0.68127,0.
65225,0.6445,0.11011,0.11057,0.10906,0.10952,0.10882,0.10927,0.10901,0
.11053,0.11009,0.10957,0.10966,0.10947,0.10952,0.10952,0.10962,0.11029
,0.10979,0.10991,1.5935,1.5997,1.5941,1.6051,1.6085,0.84989,0.8,0.8526
5,0.90189,0.84954,0.8516,0.58257,0.64708,0.60353,0.60587,0.58526,0.658
83,0.65393,0.52208,0.65845,0.10867,0.10831,0.10922,0.10921,0.11024,0.1
105,0.11044,0.10954,0.11002,0.11051,0.11072,0.11082,0.10953,0.10993,0.
10897,0.11207,0.1099,0.10994,1.595,1.6,1.6022,0.71923,0.90884,0.85692,
0.75786,0.89728,0.8484,0.83754,0.83019,0.66373,0.53397,0.6317,0.60516,
0.46524,0.5733,0.52485,0.51639,0.60693,0.10913,0.10978,0.10957,0.11078
,0.10979,0.11047,0.10842,0.10954,0.10963,0.10935,0.11051,0.10982,0.108
46,0.10847,0.11065,0.10894,0.11039,0.11,1.5971,0.85022,0.85052,1.0138,

1.0339,0.81658,0.81252,0.90848,0.8593,0.85762,0.67562,0.72097,0.63241,
0.58896,0.61772,0.58516,0.63716,0.6526,0.59445,0.10987,0.11046,0.11094
,0.11012,0.10988,0.11082,0.10731,0.11002,0.10988,0.10945,0.10988,0.109
7,0.1118,0.11031,0.11168,0.10916,0.10991,0.10352,0.88484,0.85252,0.922
25,0.85117,0.75071,0.77525,0.85334,0.76279,0.80731,0.97565,0.60538,0.6
3349,0.65001,0.69105,0.60623,0.57576,0.68035,0.67624,0.57214,0.11069,0
.10936,0.11051,0.10956,0.1101,0.10988,0.1107,0.11003,0.10625,0.10988,0
.1097,0.10998,0.11018,0.11022,0.10775,0.1087,0.10948,0.10417,0.97645,0
.99024,0.83781,0.74113,0.71141,0.81619,0.85851,0.85308,0.85465,0.85223
,0.59633,0.60638,0.57271,0.58134,0.56064,0.60771,0.50825,0.57018,0.109
6,0.10817,0.11,0.11065,0.11011,0.1085,0.10987,0.10983,0.10916,0.11008,
0.10976,0.10988,0.10979,0.11039,0.10867,0.11007,0.11031,0.1091,0.10347
,0.72363,0.9171,0.99597,0.84549,0.85131,0.85077,0.9019,0.86412,0.75866
,0.84906,0.57464,0.6731,0.63647,0.62429,0.58855,0.61969,0.62972,0.6684
4,0.10925,0.11015,0.1101,0.10912,0.1119,0.10969,0.10938,0.10989,0.1099
6,0.11061,0.10972,0.1101,0.11115,0.1085,0.11015,0.11,0.10971,0.10373,0
.10391,0.10376,0.57996,0.85183,0.76153,0.84992,0.71983,0.85061,0.83261
,0.91897,0.92718,0.69687,0.50006,0.53514,0.56856,0.58517,0.61073,0.109
65,0.11061,0.11028,0.10987,0.11051,0.11129,0.1092,0.1093,0.11018,0.109
43,0.11212,0.11003,0.10863,0.109,0.11055,0.1112,0.10919,0.10996,0.1035
5,0.10336,0.10372,0.10344,0.84885,0.7398,0.87801,0.8508,0.84898,0.8502
4,0.90928,0.83844,0.85655,0.56687,0.61,0.52894,0.11035,0.11042,0.10997
,0.10857,0.1107,0.10938,0.11025,0.11204,0.11011,0.11013,0.10984,0.1137
1,0.10838,0.11034,0.10993,0.10974,0.10922,0.11007,0.10772,0.10983,0.10
324,0.10361,0.10398,0.10378,0.10405,0.1031,0.746,0.64602,0.92121,0.846
82,0.82225,0.86768,0.9652,0.84851,0.10868,0.10927,0.10988,0.11111,0.10
937,0.11004,0.10929,0.10906,0.11109,0.10992,0.109,0.1109,0.10981,0.110
01,0.11023,0.10992,0.10978,0.10983,0.10964,0.11087,0.10991,0.10985,0.1
1053,0.10379,0.10337,0.10351,0.1036,0.10375,0.10398,1.0399,0.83913,0.8
5091,0.97169,0.93137,0.10342,0.1039,0.10937,0.10905,0.11107,0.10919,0.
109,0.10903,0.10893,0.10962,0.10991,0.10916,0.10853,0.10919,0.11041,0.
10992,0.1101,0.11041,0.10992,0.11093,0.1085,0.10979,0.10965,0.10377,0.
10367,0.10359,0.10366,0.1034,0.1038,0.10369,0.10381,0.10365,0.10352,0.
87521,0.84969,0.1039,0.10348,0.10356,0.10379,0.10883,0.11056,0.10932,0
.10922,0.10821,0.11103,0.11042,0.10984,0.10969,0.10931,0.10951,0.10965
,0.10826,0.1093,0.11035,0.10982,0.10979,0.11125,0.10935,0.10386,0.1035
2,0.10348,0.10416,0.10329,0.10332,0.10396,0.10391,0.10367,0.10361,0.10
338,0.1037,0.10334,0.10394,0.10362,0.10397,0.10392,0.10984,0.10983,0.1
1171,0.11087,0.1101,0.10969,0.10897,0.11174,0.11128,0.10911,0.11059,0.
11021,0.11014,0.10974,0.10963,0.10994,0.10345,0.10357,0.10327,0.1035,0
.10328,0.10385,0.10374,0.10368,0.10402,0.10346,0.10365,0.1033,0.10395,
0.10382,0.10398,0.10399,0.10378,0.10402,0.10388,0.10845,0.10993,0.1081
7,0.10978,0.10996,0.11018,0.10897,0.10891,0.11039,0.10969,0.11094,0.10
965,0.10885,0.10354,0.10376,0.10353,0.10362,0.1038,0.10384,0.10357,0.1
0371,0.10341,0.10356,0.10354,0.10335,0.1035,0.10359,0.10425,0.10421,0.
10366,0.10391,0.1037,0.10349,0.10395,0.11056,0.1098,0.10958,0.11066,0.
10943,0.1113,0.11013,0.1111,0.11116,0.10405,0.10379,0.10353,0.10358,0.
10387,0.10369,0.10286,0.10389,0.10354,0.1042,0.1035,0.10323,0.10379,0.
10388,0.10361,0.10377,0.10349,0.10368,0.10379,0.10411,0.10374,0.10329,
0.10346,0.10323,0.10375,0.11082,0.11023,0.10946,0.10949,0.10342,0.1039
2,0.10403,0.10393,0.10362,0.10357,0.10356,0.10354,0.1035,0.10392,0.103
77,0.10387,0.10329,0.10371,0.10307,0.10423,0.10303,0.10384,0.10352,0.1

0345,0.10421,0.10389,0.1035,0.10364,0.10313,0.10369,0.10364,0.10365,0.
10321,0.10389,0.10359,0.10359,0.1036,0.10361,0.10394,0.10388,0.10358,0
.10357,0.10418,0.10351,0.10352,0.10323,0.10386,0.10329,0.10376,0.10338
,0.10347,0.10358,0.10393,0.10406,0.10384,0.1035,0.10356,0.10342,0.1034
5,0.10365,0.10337,0.10349,0.10334,0.10365,0.10346,0.10411,0.10335,0.10
343,0.10317,0.10361,0.10322,0.10372,0.10333,0.10409,0.10374,0.10371,0.
10352,0.10418,0.10385,0.10376,0.10361,0.10367,0.1035,0.10363,0.10369,0
.10371,0.10441,0.10324,0.10336,0.10337,0.10407,0.10344,0.10402,0.10355
,0.10386,0.10365,0.10377,0.10384,0.10418,0.10314,0.10394,0.10342,0.103
74,0.10327,0.10362,0.10366,0.104,0.10363,0.10361,0.10384,0.10365,0.103
23,0.10376,0.10387,0.10333,0.10321,0.10385,0.10394,0.10333,0.10437,0.1
0395,0.1041,0.10313,0.10323,0.10334,0.10369,0.10323,0.1034,0.10414,0.1
04,0.10354,0.10385,0.10391,0.10407,0.10317,0.10422,0.10351,0.10418,0.1
0355,0.10372,0.10337,0.10327,0.10329,0.10338,0.10379,0.10352,0.10361,0
.10344,0.10351,0.10356,0.10366,0.10316,0.10348,0.10369,0.10345,0.10422
,0.10344,0.1037,0.1039,0.10405,0.10355,0.10343,0.10352,0.10309,0.10328
,0.10363,0.10377,0.10381,0.10407,0.10392,0.1039,0.10334,0.10382,0.1037
5,0.10315,0.10402,0.10372,0.10387,0.10369,0.10359,0.10415,0.10371,0.10
328,0.10352,0.10337,0.10407,0.10291,0.10351,0.10329,0.10336,0.10404,0.
10389,0.10385,0.10383,0.10429,0.10371,0.10355,0.10348,0.10304
0.1434,0.14401,0.1443,0.14386,0.14246,0.14527,0.14356,0.14332,0.14434,
0.14525,0.14432,0.14337,0.1439,0.14452,0.14326,0.14459,0.1441,0.14431,
0.14367,0.14442,0.5995,0.7122,0.64869,0.61149,0.14474,0.14383,0.14374,
0.14405,0.14376,0.14394,0.14412,0.14425,0.14405,0.1439,0.14375,0.14429
,0.14451,0.14318,0.14339,1.1221,0.63392,0.71928,0.62273,0.59798,0.6550
3,0.64744,0.14516,0.14456,0.14338,0.14412,0.14519,0.1428,0.14404,0.143
78,0.14353,0.14358,0.144,0.14466,0.14401,0.14348,0.14487,1.1183,1.1173
,0.59155,0.45825,0.63958,0.49784,0.62375,0.64845,0.62,0.62711,0.14427,
0.14474,0.14484,0.14378,0.14391,0.1431,0.14491,0.14335,0.14362,0.14339
,0.14379,0.14349,0.14439,0.14364,0.1441,1.113,1.1174,1.1234,0.62341,0.
71623,0.58001,0.63803,0.62979,0.63193,0.66762,0.64467,0.66654,0.14381,
0.14421,0.14428,0.14322,0.14449,0.14317,0.14341,0.14403,0.14254,0.1429
7,0.14538,0.14439,0.14421,0.14489,0.14372,0.14367,1.1242,1.1273,1.1202
,1.1278,0.70282,0.67969,0.60439,0.69871,0.60334,0.52933,0.66233,0.6474
1,0.50216,0.57453,0.14431,0.14391,0.14383,0.14459,0.14407,0.14398,0.14
334,0.14366,0.14355,0.14396,0.14264,0.14443,0.14274,0.1431,0.14435,0.1
4366,1.1135,1.1185,1.125,1.1131,1.1252,1.1281,0.62839,0.65188,0.58955,
0.67442,0.62187,0.69705,0.74108,0.64916,0.83496,0.62841,0.14396,0.1440
9,0.14348,0.14485,0.14435,0.14353,0.14376,0.14433,0.14398,0.1437,0.144
13,0.14461,0.1431,0.14407,0.14366,0.14429,0.14413,1.1033,1.1114,1.1071
,1.1191,1.1195,1.1236,0.64411,0.69231,0.62073,0.63043,0.63056,0.63774,
0.62957,0.69552,0.72974,0.71929,0.68043,0.14531,0.14341,0.14471,0.1429
9,0.1445,0.14394,0.14435,0.1441,0.1441,0.14399,0.14378,0.14445,0.14377
,0.12869,0.13009,0.13017,0.14372,1.1198,1.1085,1.1171,1.1255,1.1201,1.
1319,1.127,0.63067,0.62715,0.69506,0.63785,0.55552,0.54518,0.64805,0.5
7379,0.55015,0.53673,0.70485,0.14285,0.1431,0.14411,0.14263,0.14298,0.
14371,0.14489,0.14363,0.14444,0.14365,0.14357,0.12939,0.12457,0.12787,
0.13006,0.12893,0.12664,0.14321,1.1189,1.1358,1.1206,1.1194,1.1155,1.1
056,1.121,0.73345,0.64114,0.64525,0.6706,0.54564,0.77693,0.68177,0.632
04,0.63365,0.61037,0.70976,0.14383,0.14325,0.14403,0.1436,0.14398,0.14
4,0.1435,0.14249,0.14387,0.12852,0.12871,0.12968,0.12889,0.12919,0.129
49,0.12951,0.12913,0.13034,0.14396,1.1235,1.1188,1.1058,1.12,1.1205,1.

1123,1.1278,0.68334,0.63108,0.65497,0.63308,0.72177,0.75504,0.60372,0.
68872,0.63063,0.71426,0.63698,0.49712,0.14478,0.14462,0.14437,0.14411,
0.14474,0.14397,0.12922,0.12843,0.1291,0.12924,0.13057,0.13186,0.1286,
0.12587,0.1294,0.12997,0.13003,0.12541,1.1188,1.1242,1.1143,1.1177,1.1
286,1.1168,1.1296,0.5835,0.61689,0.74324,0.63029,0.53763,0.67522,0.675
9,0.70619,0.57798,0.7945,0.66981,0.66075,0.14436,0.1443,0.14402,0.1311
2,0.12771,0.13151,0.12857,0.1291,0.12794,0.12729,0.12931,0.13105,0.129
33,0.1264,0.12686,0.13069,0.1286,0.1289,1.1181,1.1203,1.1203,1.1202,1.
1169,1.1164,1.1246,1.1241,1.1232,0.63193,0.66558,0.63263,0.70586,0.633
55,0.63012,0.63055,0.63056,0.683,0.77729,0.64992,0.12906,0.12854,0.130
67,0.12693,0.12878,0.12879,0.1296,0.12937,0.12759,0.12284,0.13051,0.12
605,0.13002,0.12944,0.12943,0.13051,0.13093,0.12894,1.1164,1.1186,1.12
31,1.1153,1.1212,1.1197,1.1193,1.1231,1.106,0.63306,0.45608,0.46455,0.
42655,0.45903,0.42708,0.45819,0.47236,0.45814,0.5022,0.44748,0.1322,0.
12955,0.12958,0.12862,0.12606,0.12983,0.12701,0.12883,0.13238,0.12871,
0.12722,0.12894,0.12599,0.12992,0.13128,0.1297,0.12966,0.12611,1.1286,
1.118,1.1247,1.1148,1.1199,1.1221,1.1163,1.1101,1.1219,0.63192,0.46993
,0.45994,0.46879,0.45934,0.47102,0.43626,0.3987,0.44952,0.46064,0.4876
1,0.13009,0.13172,0.12964,0.12925,0.12761,0.1292,0.13055,0.1296,0.1295
9,0.1286,0.13056,0.12461,0.12883,0.1305,0.13146,0.13118,0.1321,0.12719
,1.1223,1.1245,1.116,1.1306,1.117,1.1175,1.1095,0.61422,0.56802,0.4953
2,0.45468,0.41456,0.46044,0.49529,0.52117,0.49856,0.53061,0.48805,0.48
09,0.47934,0.13126,0.13351,0.1267,0.12905,0.13046,0.12973,0.12742,0.13
006,0.13034,0.12562,0.12998,0.12641,0.12665,0.12551,0.1293,0.1286,0.13
419,0.1289,1.1295,1.1258,1.1119,1.1221,1.1218,1.1171,0.62012,0.47721,0
.65758,0.8083,0.61546,0.39416,0.41977,0.5189,0.39797,0.46367,0.5238,0.
50714,0.48929,0.4855,0.12997,0.1315,0.12694,0.12878,0.12701,0.12794,0.
12771,0.13025,0.12926,0.12902,0.12887,0.12861,0.12794,0.12851,0.12906,
0.13005,0.12919,0.12909,1.1178,1.1222,1.114,1.1338,1.13,0.61584,0.5810
1,0.61893,0.65182,0.61646,0.61755,0.44444,0.48644,0.45907,0.45971,0.44
524,0.49581,0.49187,0.4056,0.49412,0.12658,0.12535,0.1279,0.12846,0.12
975,0.13143,0.13044,0.12885,0.12982,0.13011,0.13114,0.13085,0.12866,0.
12918,0.12834,0.13387,0.12919,0.1293,1.1166,1.1178,1.1164,0.52887,0.65
563,0.62142,0.55338,0.64774,0.61408,0.60827,0.60238,0.49513,0.41243,0.
4757,0.45955,0.36545,0.44035,0.40559,0.40091,0.46014,0.1276,0.12915,0.
12887,0.13104,0.12908,0.13085,0.12633,0.12774,0.12916,0.12887,0.13129,
0.12878,0.12635,0.12613,0.13031,0.12638,0.13013,0.12924,1.1178,0.61572
,0.616,0.72496,0.73611,0.59736,0.59108,0.65606,0.62379,0.62061,0.50661
,0.53746,0.47672,0.44789,0.46577,0.44692,0.47903,0.49126,0.45225,0.129
48,0.13076,0.13278,0.1295,0.12973,0.13072,0.12314,0.12959,0.12916,0.12
87,0.1289,0.12891,0.13371,0.13073,0.13323,0.12847,0.12989,0.11417,0.63
914,0.6193,0.66668,0.61822,0.54844,0.56795,0.61879,0.55699,0.58815,0.6
9991,0.45981,0.47858,0.48791,0.51833,0.45843,0.43922,0.50694,0.50386,0
.43741,0.13124,0.12804,0.13013,0.12833,0.12956,0.12927,0.13074,0.13013
,0.12056,0.12947,0.12896,0.12984,0.12933,0.12995,0.12458,0.12591,0.129
2,0.11485,0.70163,0.71132,0.60978,0.54177,0.52529,0.59617,0.62126,0.62
035,0.62002,0.6159,0.4524,0.4596,0.43727,0.4437,0.42972,0.46067,0.3935
6,0.43448,0.12917,0.12482,0.12922,0.1304,0.12926,0.12596,0.12889,0.128
45,0.12792,0.12927,0.12891,0.12926,0.12958,0.13007,0.12668,0.12945,0.1
2999,0.12774,0.11413,0.5329,0.65982,0.7137,0.61393,0.61522,0.61868,0.6
5106,0.62665,0.55595,0.61575,0.43644,0.50225,0.4804,0.47249,0.45023,0.
4684,0.47378,0.49854,0.12758,0.12991,0.12913,0.12717,0.1351,0.12874,0.

12851,0.1293,0.12908,0.13128,0.12869,0.13007,0.1318,0.12638,0.12966,0.
12931,0.12884,0.1144,0.11449,0.11426,0.43678,0.61788,0.55686,0.61605,0
.52647,0.6166,0.60536,0.66597,0.66724,0.51804,0.39024,0.41211,0.43329,
0.44602,0.46146,0.12814,0.13142,0.13009,0.12911,0.12968,0.13244,0.1269
4,0.12776,0.12943,0.12889,0.13426,0.12975,0.12691,0.12691,0.12972,0.13
224,0.12812,0.12942,0.11403,0.11394,0.11458,0.11411,0.61617,0.54037,0.
63373,0.6159,0.61526,0.61631,0.65583,0.61014,0.62054,0.43622,0.46208,0
.40746,0.12937,0.12986,0.12945,0.12627,0.13061,0.12872,0.1292,0.13445,
0.12943,0.12927,0.12859,0.13915,0.12596,0.13008,0.12922,0.12937,0.1280
9,0.12923,0.12391,0.12909,0.11409,0.11421,0.11473,0.1146,0.11461,0.113
78,0.54723,0.48165,0.66478,0.61626,0.59609,0.6274,0.69512,0.6188,0.126
6,0.1276,0.12976,0.13179,0.12802,0.12945,0.12864,0.12773,0.13184,0.129
13,0.12708,0.13164,0.12914,0.13005,0.12992,0.12922,0.12874,0.12916,0.1
2857,0.13122,0.12932,0.12891,0.13103,0.11444,0.11409,0.11442,0.11429,0
.11452,0.1144,0.74859,0.60975,0.61794,0.69977,0.67016,0.11426,0.11443,
0.1288,0.12832,0.13138,0.1282,0.12852,0.12645,0.12716,0.12802,0.12963,
0.12792,0.12653,0.12792,0.13063,0.1295,0.1293,0.13042,0.13006,0.13169,
0.12582,0.12852,0.12919,0.11433,0.11411,0.11422,0.11425,0.11407,0.1143
7,0.11449,0.11444,0.11442,0.11444,0.63385,0.61862,0.11461,0.11404,0.11
457,0.11442,0.1282,0.12991,0.1284,0.12805,0.12473,0.13208,0.13045,0.12
905,0.12927,0.12863,0.12857,0.12901,0.12543,0.12802,0.13059,0.12919,0.
12882,0.13206,0.12831,0.1147,0.11438,0.114,0.1149,0.11413,0.11407,0.11
462,0.11445,0.11432,0.11438,0.11388,0.11448,0.11408,0.11468,0.11422,0.
11466,0.11484,0.12925,0.12859,0.1335,0.13197,0.12958,0.12928,0.12717,0
.1333,0.13282,0.12756,0.13058,0.12959,0.12948,0.12913,0.12887,0.12944,
0.11404,0.11422,0.11366,0.11415,0.11382,0.11452,0.11434,0.11431,0.1147
6,0.11423,0.11401,0.1142,0.11454,0.11447,0.11472,0.11477,0.11459,0.114
62,0.11451,0.12557,0.12926,0.1244,0.12926,0.12934,0.12956,0.12818,0.12
671,0.12966,0.12923,0.13112,0.12875,0.12707,0.11415,0.11436,0.11438,0.
11438,0.11425,0.11455,0.11427,0.11426,0.11418,0.11423,0.11413,0.11421,
0.11417,0.11443,0.11501,0.11455,0.11451,0.11436,0.11431,0.11422,0.1146
3,0.12996,0.12898,0.12907,0.13062,0.12802,0.1317,0.12946,0.13139,0.131
61,0.11466,0.11457,0.11426,0.11404,0.11433,0.1144,0.11369,0.11451,0.11
402,0.11477,0.11423,0.1139,0.11439,0.11455,0.11416,0.11445,0.11419,0.1
1428,0.11441,0.11463,0.11461,0.11397,0.11427,0.11373,0.1146,0.13134,0.
1306,0.12809,0.12792,0.11417,0.11452,0.11474,0.11437,0.11435,0.11425,0
.11414,0.11445,0.11413,0.11451,0.11429,0.11445,0.11383,0.11457,0.11384
,0.11486,0.11376,0.11458,0.1141,0.11407,0.11495,0.11444,0.11401,0.1142
6,0.11381,0.11434,0.11441,0.11421,0.11407,0.1145,0.11448,0.11445,0.114
25,0.11435,0.11462,0.11461,0.11435,0.11452,0.11475,0.11407,0.11419,0.1
1395,0.11447,0.11391,0.11421,0.11391,0.11397,0.11416,0.11454,0.11477,0
.11437,0.11417,0.1143,0.11399,0.11429,0.11441,0.11397,0.11432,0.11393,
0.11419,0.11403,0.11479,0.11392,0.1142,0.11386,0.11432,0.11401,0.11408
,0.11392,0.1148,0.11449,0.11433,0.11421,0.11487,0.11428,0.1145,0.11421
,0.11446,0.11419,0.11428,0.11445,0.11453,0.11502,0.11416,0.1138,0.1143
1,0.11494,0.11387,0.11467,0.1142,0.11456,0.11426,0.11433,0.11453,0.114
6,0.11383,0.11458,0.11432,0.11436,0.11429,0.11418,0.1142,0.11481,0.114
3,0.11427,0.11445,0.1143,0.11372,0.11447,0.11466,0.11401,0.11395,0.114
66,0.11473,0.11415,0.11491,0.11454,0.11474,0.11398,0.11385,0.1141,0.11
433,0.1138,0.1142,0.11472,0.11434,0.11432,0.11433,0.11475,0.11451,0.11
401,0.11489,0.11424,0.11465,0.11422,0.11447,0.11411,0.11393,0.11404,0.
11412,0.11435,0.11428,0.1144,0.11419,0.1142,0.11431,0.114,0.11397,0.11

406,0.11432,0.11413,0.11493,0.11407,0.1143,0.11452,0.1147,0.11444,0.11
393,0.11403,0.11412,0.11404,0.11426,0.11462,0.11435,0.11467,0.11478,0.
1148,0.11383,0.11438,0.11433,0.11395,0.11461,0.11449,0.11467,0.11419,0
.11425,0.11486,0.11444,0.11425,0.11414,0.11399,0.1146,0.11367,0.11404,
0.11412,0.1142,0.1147,0.11463,0.11454,0.1144,0.11499,0.11438,0.11423,0
.11385,0.11358
0.1614,0.16199,0.16145,0.16162,0.1605,0.16309,0.16138,0.16074,0.16213,
0.16286,0.16227,0.16098,0.16188,0.16217,0.16115,0.16275,0.16143,0.1621
8,0.16161,0.16207,0.47266,0.55051,0.50644,0.48008,0.16249,0.16158,0.16
141,0.16166,0.16149,0.16139,0.1618,0.16264,0.1616,0.16169,0.16161,0.16
176,0.16211,0.16093,0.16161,0.82908,0.49765,0.55448,0.48851,0.47281,0.
51087,0.5069,0.16286,0.16212,0.16087,0.16212,0.16312,0.16033,0.16195,0
.16165,0.1615,0.16159,0.16184,0.16249,0.16215,0.16143,0.16252,0.8308,0
.82594,0.46876,0.37732,0.5016,0.40435,0.49,0.50751,0.4881,0.49264,0.16
186,0.16223,0.16254,0.16199,0.16196,0.16109,0.1626,0.16107,0.16186,0.1
6122,0.16186,0.16127,0.1621,0.1612,0.16165,0.82362,0.82689,0.83491,0.4
9073,0.55324,0.45932,0.50047,0.49466,0.49443,0.51782,0.50459,0.5172,0.
16175,0.16226,0.16213,0.16141,0.1623,0.16161,0.16169,0.16185,0.16076,0
.16075,0.16344,0.16237,0.16161,0.16285,0.16162,0.16195,0.83507,0.83514
,0.83058,0.83403,0.54048,0.52862,0.47666,0.53936,0.4764,0.42646,0.5184
2,0.50505,0.40742,0.45635,0.16206,0.16184,0.16181,0.1621,0.16203,0.161
73,0.16124,0.16157,0.16136,0.16189,0.16064,0.162,0.16073,0.16103,0.162
38,0.16127,0.8242,0.83127,0.83149,0.82218,0.83212,0.83582,0.49265,0.50
933,0.46556,0.52569,0.48851,0.5415,0.56943,0.50866,0.63428,0.49151,0.1
6172,0.16213,0.16174,0.16258,0.16186,0.16152,0.16161,0.16202,0.16163,0
.16154,0.16163,0.16252,0.16122,0.16204,0.16138,0.16202,0.1615,0.81499,
0.82405,0.81563,0.82673,0.82389,0.83534,0.50345,0.53475,0.48894,0.4958
,0.49559,0.50012,0.49276,0.53932,0.56472,0.55582,0.52757,0.16317,0.161
27,0.16205,0.16107,0.16229,0.16144,0.16235,0.16165,0.16183,0.16212,0.1
6181,0.1629,0.16168,0.13698,0.1399,0.13935,0.16166,0.82553,0.81764,0.8
3082,0.83259,0.83181,0.84212,0.83541,0.49273,0.49302,0.53935,0.50307,0
.443,0.43652,0.50405,0.45508,0.43916,0.43159,0.54603,0.16089,0.16089,0
.16218,0.16068,0.16056,0.16172,0.16246,0.16139,0.16221,0.16161,0.16146
,0.13828,0.13029,0.13637,0.13893,0.13761,0.1339,0.16085,0.83118,0.8447
3,0.83301,0.83002,0.82138,0.818,0.82999,0.56255,0.50143,0.50449,0.5221
8,0.43832,0.59349,0.52987,0.49371,0.49691,0.48228,0.54856,0.16204,0.16
111,0.16201,0.16199,0.16177,0.16206,0.1612,0.1606,0.16165,0.1373,0.137
5,0.13874,0.1376,0.13781,0.13822,0.1386,0.13723,0.13989,0.16173,0.8333
4,0.83249,0.81897,0.82618,0.82737,0.8209,0.83719,0.53266,0.49497,0.510
31,0.49449,0.5577,0.57874,0.4786,0.53476,0.49376,0.55098,0.49958,0.402
72,0.16285,0.16243,0.16215,0.16216,0.16255,0.16175,0.13807,0.13675,0.1
3764,0.13774,0.14017,0.14216,0.13718,0.13252,0.13792,0.1387,0.13887,0.
1315,0.82922,0.83503,0.82286,0.82893,0.8342,0.82501,0.83727,0.46232,0.
48431,0.57024,0.49573,0.42998,0.52463,0.52573,0.54513,0.46014,0.60541,
0.51886,0.51308,0.16228,0.16222,0.16211,0.14136,0.13591,0.14123,0.1374
8,0.13782,0.13597,0.13502,0.1376,0.1408,0.13831,0.13337,0.1342,0.14014
,0.13736,0.1374,0.82548,0.82785,0.82865,0.827,0.82387,0.82447,0.83396,
0.83661,0.8323,0.49501,0.51867,0.49654,0.54599,0.49802,0.49398,0.49343
,0.49326,0.53103,0.5935,0.50605,0.13771,0.13661,0.13962,0.13468,0.1375
9,0.13745,0.13852,0.13845,0.13529,0.12798,0.13992,0.13304,0.13881,0.13
83,0.13785,0.14007,0.13994,0.13727,0.83029,0.82893,0.83166,0.82193,0.8
3016,0.82599,0.8283,0.83004,0.81363,0.4954,0.37572,0.37401,0.34127,0.3

6726,0.34518,0.36557,0.37628,0.36593,0.39667,0.36285,0.14251,0.13856,0
.13841,0.1375,0.13282,0.13852,0.13509,0.13678,0.14277,0.1376,0.13444,0
.1377,0.13269,0.13926,0.14072,0.13855,0.13843,0.13309,0.83169,0.82322,
0.83393,0.82516,0.82956,0.83312,0.8245,0.81766,0.83223,0.48144,0.3749,
0.36886,0.37722,0.36896,0.37526,0.35117,0.32669,0.36223,0.37104,0.3878
9,0.13904,0.14201,0.13901,0.13793,0.13533,0.13791,0.14001,0.13812,0.13
829,0.13719,0.14029,0.13056,0.13756,0.14017,0.14154,0.14099,0.14284,0.
13504,0.82782,0.83647,0.82212,0.84272,0.82699,0.82464,0.81741,0.46729,
0.43758,0.38582,0.36531,0.33695,0.36932,0.39309,0.40877,0.39605,0.4203
4,0.38641,0.38109,0.3812,0.14098,0.14492,0.13367,0.13765,0.13963,0.138
27,0.1347,0.1391,0.13932,0.13207,0.13908,0.13362,0.13446,0.13192,0.137
65,0.13737,0.14611,0.13796,0.83826,0.83421,0.82194,0.83444,0.82977,0.8
2776,0.47602,0.36955,0.50126,0.60676,0.47139,0.32349,0.33999,0.41252,0
.32756,0.37121,0.41583,0.39847,0.38913,0.38571,0.13898,0.14155,0.13377
,0.13752,0.13438,0.13606,0.13592,0.13942,0.13797,0.13807,0.13729,0.137
55,0.13532,0.13707,0.13802,0.13912,0.13787,0.13776,0.82976,0.83255,0.8
2279,0.84264,0.83901,0.47203,0.44608,0.47051,0.49504,0.46975,0.47264,0
.35914,0.38622,0.36782,0.36881,0.35871,0.3956,0.39313,0.33205,0.3913,0
.13362,0.13175,0.13619,0.13715,0.13916,0.14181,0.13971,0.13767,0.13904
,0.13896,0.14096,0.14069,0.13727,0.13808,0.1371,0.14475,0.13805,0.1381
3,0.82846,0.82911,0.82583,0.40852,0.49985,0.47629,0.42445,0.4919,0.470
04,0.46411,0.46013,0.39057,0.33618,0.37799,0.36741,0.30347,0.35518,0.3
3195,0.3279,0.36989,0.13572,0.13782,0.13766,0.14081,0.13806,0.14037,0.
13384,0.13526,0.13798,0.13787,0.14147,0.13725,0.13361,0.13287,0.13949,
0.13344,0.13936,0.13791,0.82594,0.47176,0.46992,0.54845,0.55338,0.4596
6,0.45399,0.50019,0.4757,0.47437,0.40216,0.42278,0.3812,0.35935,0.3721
7,0.35915,0.38171,0.39051,0.36382,0.13868,0.14086,0.14457,0.13832,0.13
873,0.13988,0.12828,0.13832,0.13765,0.13767,0.13759,0.13778,0.14492,0.
14083,0.14423,0.13735,0.13931,0.11383,0.48822,0.47498,0.50752,0.47227,
0.42306,0.43565,0.47186,0.43022,0.45217,0.52695,0.36793,0.38081,0.3898
6,0.41194,0.36716,0.35418,0.40105,0.39618,0.35239,0.14103,0.13599,0.13
915,0.13682,0.13818,0.13766,0.14063,0.13981,0.12397,0.1384,0.1379,0.13
931,0.13818,0.13925,0.13023,0.13249,0.13818,0.11432,0.53205,0.53757,0.
46722,0.41789,0.40567,0.45541,0.47463,0.47451,0.47566,0.46859,0.36269,
0.3683,0.35334,0.3583,0.34839,0.36932,0.32229,0.3504,0.1376,0.13081,0.
13808,0.13961,0.13768,0.13334,0.13765,0.13651,0.13615,0.1372,0.13752,0
.13766,0.13846,0.13926,0.13404,0.13819,0.13927,0.13549,0.11364,0.41002
,0.50192,0.53765,0.46949,0.47125,0.4754,0.49376,0.47762,0.42626,0.4715
3,0.35073,0.394,0.38285,0.37717,0.36373,0.37578,0.37816,0.39313,0.1356
8,0.13875,0.1377,0.1345,0.14732,0.13753,0.13759,0.13786,0.1375,0.14136
,0.13705,0.13934,0.1416,0.13349,0.13847,0.1379,0.13754,0.11397,0.11397
,0.11374,0.34428,0.47106,0.4297,0.47042,0.40677,0.47313,0.46512,0.5067
1,0.5057,0.40786,0.3207,0.33626,0.34808,0.35958,0.36922,0.1364,0.14171
,0.13942,0.13746,0.13857,0.14301,0.13395,0.13561,0.13826,0.13765,0.145
88,0.13926,0.13457,0.13448,0.13826,0.14234,0.13662,0.13841,0.11365,0.1
1363,0.11405,0.11393,0.4684,0.41552,0.48428,0.46979,0.47033,0.47181,0.
50113,0.46583,0.47627,0.35482,0.37061,0.33123,0.13807,0.13913,0.13802,
0.13317,0.14011,0.13735,0.13816,0.14639,0.1378,0.13792,0.13667,0.15447
,0.13271,0.13937,0.13795,0.13822,0.13629,0.13784,0.12917,0.1377,0.1135
9,0.11363,0.11432,0.11424,0.11424,0.11364,0.42199,0.37731,0.50591,0.47
297,0.45553,0.47858,0.52547,0.47236,0.13378,0.13506,0.13885,0.14231,0.
13583,0.13824,0.1375,0.13564,0.1421,0.13782,0.13459,0.14188,0.13775,0.

13946,0.13943,0.138,0.13718,0.13784,0.13648,0.14088,0.13807,0.13728,0.
1409,0.11392,0.11376,0.11414,0.11379,0.11405,0.11373,0.56513,0.46776,0
.47004,0.52697,0.50773,0.11363,0.11393,0.13775,0.13704,0.14119,0.13642
,0.13731,0.13336,0.13444,0.13609,0.13849,0.13622,0.13381,0.13601,0.140
26,0.13863,0.13804,0.13975,0.13936,0.14165,0.13191,0.13642,0.13805,0.1
138,0.11381,0.11376,0.11362,0.11336,0.11388,0.11391,0.11394,0.11398,0.
11408,0.48145,0.47271,0.1142,0.1136,0.11408,0.11418,0.13691,0.13896,0.
13695,0.1361,0.1307,0.14262,0.13975,0.13771,0.13797,0.1374,0.1372,0.13
767,0.13198,0.13573,0.14005,0.13795,0.13772,0.14257,0.13652,0.11429,0.
11383,0.11367,0.11432,0.11362,0.11355,0.11402,0.11397,0.11385,0.11393,
0.11359,0.11419,0.11357,0.11421,0.1139,0.11402,0.1145,0.13815,0.13675,
0.1451,0.14215,0.13775,0.13804,0.13529,0.14424,0.14333,0.13552,0.14007
,0.13836,0.13817,0.13781,0.13764,0.13818,0.11383,0.11383,0.11319,0.113
53,0.11344,0.11393,0.11376,0.11384,0.11425,0.11371,0.11371,0.1137,0.11
408,0.11401,0.11416,0.1143,0.11398,0.1142,0.11405,0.13183,0.13796,0.12
966,0.13845,0.13785,0.13829,0.13694,0.13387,0.13844,0.13792,0.14032,0.
13746,0.13457,0.11382,0.11367,0.11399,0.11414,0.11375,0.11425,0.11374,
0.11387,0.1137,0.1139,0.11366,0.11364,0.11372,0.11401,0.11441,0.11414,
0.11426,0.11383,0.11377,0.11388,0.11435,0.13827,0.13767,0.138,0.13999,
0.13588,0.14223,0.13834,0.14116,0.14157,0.11415,0.1142,0.11388,0.11356
,0.11406,0.11397,0.11329,0.1139,0.11374,0.11437,0.11368,0.11347,0.1139
2,0.11427,0.11383,0.1139,0.11387,0.1138,0.11396,0.1141,0.1144,0.1135,0
.11376,0.11339,0.11403,0.14149,0.13997,0.13581,0.13552,0.11381,0.11421
,0.1144,0.11375,0.11409,0.11377,0.11387,0.11404,0.11363,0.11398,0.1138
3,0.11398,0.11345,0.11417,0.1134,0.11446,0.11351,0.11416,0.11357,0.113
74,0.11442,0.11398,0.1135,0.11386,0.11336,0.11384,0.11404,0.1136,0.113
77,0.11414,0.11391,0.11393,0.11382,0.1138,0.11411,0.11413,0.11403,0.11
413,0.11436,0.11346,0.11384,0.11348,0.11413,0.11346,0.1138,0.11344,0.1
1357,0.11384,0.11414,0.11417,0.11394,0.11379,0.11387,0.1136,0.11389,0.
11401,0.11371,0.11378,0.11344,0.11364,0.11369,0.1143,0.1133,0.11367,0.
11351,0.11389,0.11365,0.1136,0.11356,0.11448,0.114,0.11385,0.11393,0.1
1447,0.11404,0.11417,0.11396,0.11417,0.11358,0.11382,0.11403,0.11416,0
.11478,0.11365,0.11349,0.11383,0.11435,0.11331,0.11425,0.11377,0.11387
,0.11363,0.11385,0.11414,0.11414,0.1136,0.1141,0.11398,0.11398,0.1138,
0.11367,0.1138,0.11436,0.11402,0.11402,0.11389,0.11384,0.11339,0.11389
,0.11417,0.11375,0.11354,0.11416,0.11437,0.11368,0.1144,0.11391,0.1140
4,0.11343,0.11341,0.11376,0.11382,0.11341,0.11359,0.11419,0.1141,0.113
83,0.11398,0.11444,0.11399,0.11363,0.11445,0.1138,0.11422,0.1138,0.114
25,0.11363,0.11356,0.11356,0.11372,0.114,0.11385,0.11402,0.11362,0.113
82,0.11382,0.11371,0.11377,0.11373,0.11418,0.11388,0.11436,0.11379,0.1
1395,0.11396,0.11417,0.11412,0.1136,0.11358,0.1137,0.11347,0.11402,0.1
142,0.11412,0.11434,0.11434,0.11432,0.11351,0.11386,0.11381,0.11356,0.
11422,0.11399,0.11418,0.11394,0.1138,0.11441,0.11407,0.11394,0.11374,0
.11341,0.11436,0.11366,0.11372,0.1135,0.11377,0.11417,0.1141,0.11395,0
.11403,0.11462,0.1139,0.1139,0.11356,0.11324
0.1731,0.17376,0.17361,0.17314,0.17277,0.17483,0.17293,0.17267,0.17324
,0.17477,0.17336,0.17252,0.1739,0.17374,0.17274,0.17439,0.17336,0.1737
,0.17308,0.17357,0.3952,0.45298,0.41869,0.40095,0.17369,0.17352,0.1731
3,0.17365,0.17338,0.17307,0.17321,0.17438,0.17312,0.17331,0.17356,0.17
355,0.17358,0.17286,0.17313,0.65017,0.41519,0.45607,0.40732,0.39803,0.
42366,0.42278,0.1743,0.17411,0.17222,0.17325,0.17462,0.17246,0.17356,0
.17317,0.17345,0.17322,0.17339,0.17436,0.17338,0.17326,0.17365,0.65034

,0.6528,0.39504,0.32944,0.41845,0.34775,0.40827,0.4187,0.41045,0.41096
,0.17351,0.174,0.1741,0.17382,0.17336,0.17265,0.17429,0.17294,0.17357,
0.17269,0.17338,0.17291,0.1735,0.17289,0.17303,0.64148,0.65499,0.65717
,0.40753,0.44991,0.38855,0.41507,0.41231,0.41172,0.4279,0.41932,0.4254
9,0.17349,0.17382,0.17388,0.17251,0.1735,0.17336,0.17333,0.17355,0.172
6,0.17249,0.17509,0.17386,0.17293,0.17475,0.17281,0.17374,0.65718,0.65
956,0.65212,0.65574,0.44364,0.43511,0.39823,0.44371,0.39987,0.36369,0.
42849,0.41929,0.35009,0.38586,0.17372,0.17376,0.17316,0.174,0.17326,0.
17341,0.17312,0.1732,0.17312,0.17355,0.17242,0.17387,0.17272,0.17278,0
.17372,0.17289,0.64897,0.65486,0.6541,0.64864,0.65608,0.65522,0.41099,
0.42281,0.39202,0.43461,0.40713,0.44541,0.46489,0.42385,0.51198,0.4069
8,0.17336,0.17402,0.17339,0.17406,0.17377,0.17297,0.17302,0.1743,0.173
49,0.17315,0.17329,0.17424,0.17271,0.1737,0.173,0.17384,0.17288,0.6379
1,0.64949,0.63828,0.65118,0.64861,0.65434,0.41681,0.44147,0.41101,0.41
04,0.41353,0.41615,0.41064,0.44418,0.46265,0.45476,0.43404,0.17477,0.1
729,0.17364,0.17254,0.17375,0.17322,0.17441,0.17335,0.17381,0.17396,0.
17352,0.17436,0.1736,0.13972,0.14394,0.14258,0.17316,0.64669,0.64345,0
.65446,0.6532,0.65879,0.66345,0.65513,0.4096,0.40752,0.4451,0.41726,0.
37337,0.36935,0.41781,0.38519,0.3719,0.36774,0.44951,0.17282,0.17282,0
.17383,0.17226,0.17197,0.17319,0.17418,0.17301,0.17379,0.17345,0.17345
,0.14149,0.13074,0.13887,0.14176,0.14054,0.13551,0.17255,0.65406,0.670
04,0.65299,0.65175,0.64854,0.64286,0.65183,0.45823,0.41711,0.41935,0.4
319,0.37068,0.48069,0.43673,0.41119,0.41379,0.40412,0.45177,0.17424,0.
17287,0.17357,0.17372,0.17348,0.17351,0.17263,0.17242,0.17336,0.14032,
0.14052,0.14235,0.14079,0.14073,0.14114,0.14192,0.14001,0.14387,0.1737
5,0.65372,0.65546,0.63959,0.64834,0.65164,0.64493,0.6635,0.43796,0.412
09,0.42053,0.4104,0.4573,0.47436,0.4015,0.44033,0.41029,0.45376,0.4155
1,0.34651,0.17475,0.17448,0.17383,0.17374,0.17435,0.17335,0.14094,0.13
958,0.14032,0.14039,0.14441,0.14652,0.14031,0.13339,0.14109,0.14189,0.
14186,0.13206,0.65098,0.65265,0.64713,0.65146,0.65692,0.64966,0.66302,
0.39059,0.40531,0.46557,0.41398,0.36479,0.43292,0.43237,0.4472,0.38797
,0.49129,0.43046,0.42433,0.17433,0.17372,0.17362,0.14576,0.13801,0.145
29,0.14076,0.14082,0.13805,0.13721,0.14053,0.14479,0.14147,0.13503,0.1
3602,0.14405,0.14065,0.14074,0.6498,0.65147,0.65354,0.64745,0.64628,0.
64214,0.65639,0.65974,0.65789,0.41296,0.43018,0.41308,0.45128,0.41525,
0.41091,0.40929,0.41018,0.43808,0.48013,0.41679,0.14043,0.1393,0.14283
,0.13671,0.14061,0.14071,0.14193,0.14182,0.13756,0.12756,0.14348,0.134
17,0.14218,0.14128,0.14106,0.14391,0.1434,0.14026,0.64984,0.65327,0.65
723,0.64866,0.65236,0.6482,0.65443,0.65487,0.63893,0.41286,0.32816,0.3
1659,0.28935,0.3105,0.29413,0.30867,0.31907,0.30847,0.33143,0.30842,0.
14696,0.14166,0.14138,0.14075,0.13414,0.14155,0.13741,0.13949,0.14706,
0.14074,0.13628,0.14071,0.13357,0.143,0.14426,0.14147,0.14126,0.13435,
0.65265,0.64497,0.65317,0.64854,0.65105,0.65375,0.64697,0.6426,0.65887
,0.38707,0.31501,0.31347,0.32048,0.3116,0.31626,0.29941,0.28023,0.3066
6,0.31572,0.32496,0.14212,0.14645,0.14256,0.14091,0.13741,0.14105,0.14
379,0.14136,0.14165,0.13977,0.14437,0.13083,0.14082,0.14407,0.14584,0.
14527,0.1479,0.13762,0.65033,0.65907,0.64532,0.66417,0.65155,0.64857,0
.64178,0.37802,0.35573,0.31423,0.31032,0.28852,0.31072,0.33131,0.33839
,0.33396,0.34915,0.32181,0.32099,0.31956,0.14486,0.15032,0.13523,0.140
36,0.14299,0.14124,0.13637,0.14271,0.14246,0.13276,0.14234,0.13531,0.1
3658,0.13238,0.14052,0.14064,0.15207,0.14091,0.66479,0.65923,0.64786,0
.65603,0.65151,0.64813,0.38362,0.30046,0.40463,0.48256,0.37794,0.2805,

0.28996,0.34645,0.28319,0.31421,0.35026,0.33107,0.3258,0.32377,0.14231
,0.14604,0.13515,0.14053,0.13641,0.13863,0.13835,0.14296,0.14096,0.141
05,0.14007,0.14031,0.13734,0.13978,0.14122,0.14253,0.14091,0.14121,0.6
4742,0.65357,0.64657,0.66122,0.65916,0.38022,0.36049,0.37925,0.39771,0
.37909,0.3815,0.307,0.32362,0.3103,0.3115,0.30231,0.33329,0.3299,0.285
53,0.32699,0.135,0.1327,0.13866,0.14016,0.1425,0.14627,0.14344,0.14093
,0.14268,0.14254,0.14551,0.14459,0.14046,0.14114,0.13986,0.14984,0.140
94,0.14089,0.65576,0.65005,0.64523,0.33285,0.40173,0.38531,0.34488,0.3
9453,0.37897,0.37574,0.36945,0.32403,0.28852,0.31589,0.31101,0.26194,0
.30285,0.28487,0.28144,0.31203,0.1379,0.14077,0.14056,0.14495,0.14083,
0.14441,0.13569,0.13725,0.14144,0.14077,0.14607,0.14049,0.13519,0.1342
9,0.143,0.13447,0.14252,0.14064,0.64999,0.37977,0.37717,0.43902,0.4423
6,0.37194,0.36623,0.4035,0.38211,0.38168,0.33876,0.35158,0.3224,0.3055
,0.31734,0.30339,0.32025,0.32781,0.30728,0.14229,0.14516,0.15019,0.141
41,0.14208,0.14345,0.12792,0.1418,0.14059,0.14063,0.1406,0.141,0.15019
,0.14495,0.14952,0.14028,0.14317,0.10813,0.39329,0.3809,0.40915,0.3825
9,0.3405,0.35229,0.37978,0.35018,0.36762,0.42135,0.30984,0.32057,0.327
12,0.34556,0.31103,0.30016,0.33414,0.33124,0.30021,0.14514,0.1384,0.14
26,0.13957,0.14125,0.14058,0.14462,0.14339,0.12212,0.14171,0.14102,0.1
431,0.14136,0.14292,0.13049,0.13356,0.14137,0.10875,0.42696,0.43095,0.
37769,0.33815,0.33038,0.36891,0.38155,0.38188,0.38583,0.3773,0.3062,0.
31195,0.30077,0.30231,0.29668,0.31281,0.27715,0.29635,0.14065,0.13159,
0.14108,0.14321,0.1408,0.13507,0.14041,0.13901,0.13866,0.14004,0.14056
,0.14073,0.1415,0.14265,0.1355,0.14118,0.14305,0.1377,0.10784,0.33351,
0.40272,0.42812,0.37888,0.38057,0.38332,0.39725,0.38524,0.34634,0.3794
,0.2974,0.32724,0.32247,0.3164,0.30944,0.31775,0.31762,0.32749,0.1382,
0.14199,0.14067,0.13601,0.15391,0.14067,0.14084,0.14076,0.14018,0.1453
6,0.13981,0.14303,0.14568,0.13516,0.1416,0.14115,0.14068,0.10834,0.108
25,0.10795,0.28368,0.38092,0.34648,0.37962,0.332,0.38176,0.37522,0.405
96,0.40539,0.33881,0.2764,0.28863,0.29463,0.30493,0.31245,0.13893,0.14
61,0.14261,0.14053,0.14142,0.14781,0.13558,0.13782,0.14124,0.14071,0.1
514,0.14282,0.13637,0.13644,0.14151,0.14741,0.13955,0.14138,0.1079,0.1
0816,0.1084,0.10817,0.37898,0.33791,0.39032,0.38091,0.38004,0.38147,0.
40282,0.37466,0.38491,0.30447,0.31186,0.28413,0.14115,0.14227,0.14119,
0.13463,0.14362,0.1404,0.14122,0.15211,0.14085,0.14092,0.13894,0.16337
,0.13394,0.14317,0.14096,0.14116,0.13897,0.14087,0.12916,0.14059,0.108
06,0.10787,0.10866,0.10839,0.10853,0.1082,0.342,0.30936,0.4069,0.38288
,0.36661,0.38397,0.42134,0.38249,0.13547,0.1369,0.14242,0.14702,0.1383
7,0.14145,0.14048,0.13773,0.14615,0.1404,0.13652,0.14672,0.14095,0.142
87,0.14318,0.14125,0.13992,0.14079,0.13868,0.14498,0.14148,0.14003,0.1
4486,0.10811,0.1079,0.10848,0.10799,0.10838,0.10801,0.45149,0.37905,0.
37953,0.4231,0.40552,0.10813,0.10839,0.14095,0.13987,0.14519,0.13915,0
.14032,0.13452,0.13625,0.13806,0.14193,0.13875,0.1354,0.13806,0.14388,
0.14213,0.14105,0.14369,0.14301,0.14573,0.13259,0.13907,0.14099,0.1079
9,0.10814,0.10793,0.10807,0.10778,0.10848,0.10819,0.10834,0.10845,0.10
813,0.38575,0.37989,0.10834,0.10798,0.10843,0.10838,0.13996,0.14228,0.
13976,0.13848,0.13106,0.14723,0.14325,0.14085,0.14108,0.14038,0.14015,
0.14043,0.13289,0.13776,0.14394,0.14109,0.1405,0.14729,0.13885,0.10846
,0.10825,0.10799,0.10867,0.10782,0.10785,0.10828,0.10834,0.10828,0.108
33,0.10797,0.10853,0.10823,0.10856,0.10832,0.10841,0.10892,0.14114,0.1
3963,0.15098,0.14685,0.14053,0.14109,0.13767,0.14949,0.14815,0.13779,0
.14406,0.14119,0.14102,0.14069,0.14073,0.14091,0.1082,0.10814,0.10748,

0.10804,0.10798,0.10793,0.1082,0.1083,0.10872,0.10812,0.10821,0.1082,0
.1085,0.10838,0.1086,0.10853,0.10852,0.10849,0.10843,0.13274,0.14079,0
.12937,0.14172,0.14081,0.14113,0.13979,0.13541,0.1414,0.14093,0.14421,
0.14057,0.13651,0.10814,0.108,0.10825,0.10836,0.10821,0.10857,0.10827,
0.1082,0.10818,0.10809,0.10801,0.10799,0.10828,0.10843,0.10891,0.10844
,0.10856,0.10831,0.10804,0.10829,0.10861,0.14125,0.1407,0.14105,0.1436
5,0.13796,0.14678,0.14126,0.14512,0.14572,0.10855,0.10874,0.10815,0.10
812,0.10825,0.10837,0.10776,0.10831,0.10815,0.10871,0.10792,0.10782,0.
10817,0.10843,0.10824,0.1083,0.10821,0.10802,0.10829,0.10841,0.10872,0
.10791,0.10819,0.10793,0.10832,0.14573,0.14351,0.13783,0.13751,0.10832
,0.10845,0.10897,0.10824,0.10851,0.1081,0.10822,0.10838,0.10814,0.1084
,0.10805,0.1084,0.10791,0.10844,0.10764,0.10872,0.10798,0.10849,0.1076
3,0.10813,0.10888,0.1081,0.10783,0.10832,0.10786,0.1083,0.10821,0.1078
1,0.1081,0.10847,0.10814,0.10802,0.1081,0.10821,0.10839,0.10845,0.1083
6,0.10829,0.10855,0.10775,0.10802,0.10785,0.10823,0.10783,0.10809,0.10
778,0.10809,0.1081,0.10841,0.10824,0.10822,0.10828,0.10836,0.10778,0.1
0806,0.10828,0.10802,0.10818,0.10774,0.10802,0.10806,0.10872,0.10763,0
.10815,0.10789,0.10831,0.1079,0.10804,0.10821,0.10868,0.10819,0.10822,
0.10825,0.10877,0.10843,0.10864,0.10814,0.1084,0.10788,0.1079,0.10843,
0.10858,0.1088,0.10796,0.10788,0.10805,0.10869,0.10784,0.10855,0.10824
,0.1083,0.10796,0.10828,0.10866,0.10857,0.10784,0.10846,0.10838,0.1081
6,0.10806,0.10824,0.10822,0.1086,0.10834,0.10824,0.10814,0.10823,0.107
71,0.10836,0.10863,0.10816,0.10787,0.10832,0.10888,0.10805,0.10896,0.1
0821,0.1084,0.108,0.1078,0.10834,0.10824,0.10791,0.10798,0.10859,0.108
62,0.1081,0.1082,0.10872,0.10809,0.10804,0.1089,0.10838,0.10855,0.1080
8,0.10858,0.1081,0.10766,0.10803,0.10826,0.1083,0.10825,0.10843,0.1080
7,0.10813,0.10814,0.10823,0.10807,0.10794,0.10861,0.10823,0.10873,0.10
818,0.10839,0.1083,0.10835,0.10849,0.10797,0.10804,0.10807,0.10789,0.1
0831,0.10878,0.10839,0.1085,0.1084,0.10864,0.10792,0.10827,0.1082,0.10
797,0.10868,0.10816,0.10869,0.10808,0.10825,0.10863,0.10848,0.10828,0.
10814,0.10789,0.10851,0.10801,0.10812,0.10786,0.10835,0.10828,0.10838,
0.10832,0.10828,0.10898,0.10836,0.10832,0.10796,0.10749
0.18136,0.18173,0.18109,0.18102,0.18071,0.18246,0.1809,0.18093,0.18084
,0.18206,0.18071,0.18063,0.18172,0.18162,0.18039,0.18217,0.18148,0.181
75,0.18106,0.1815,0.34921,0.39176,0.36435,0.35341,0.18167,0.18121,0.18
118,0.18104,0.18122,0.18118,0.18093,0.1824,0.18101,0.18119,0.1813,0.18
138,0.18138,0.18105,0.18056,0.53691,0.36286,0.39838,0.35982,0.35342,0.
37004,0.36815,0.1822,0.18202,0.18013,0.18062,0.18274,0.18041,0.18147,0
.18085,0.18134,0.18068,0.1807,0.18221,0.18141,0.18071,0.18147,0.54353,
0.54342,0.34852,0.29917,0.36708,0.31218,0.35803,0.36624,0.36046,0.3612
3,0.18146,0.18191,0.18172,0.18165,0.18129,0.18049,0.18159,0.18106,0.18
113,0.18073,0.18147,0.18107,0.18147,0.18088,0.181,0.53561,0.54366,0.54
442,0.35795,0.3902,0.34567,0.36218,0.36102,0.36059,0.37195,0.36835,0.3
7096,0.18121,0.18154,0.1816,0.18062,0.18125,0.1812,0.18134,0.18133,0.1
8061,0.18056,0.18284,0.18174,0.18083,0.18253,0.18066,0.1814,0.54858,0.
54694,0.53918,0.54153,0.38555,0.37995,0.35229,0.38562,0.35462,0.32537,
0.37737,0.36792,0.31692,0.34148,0.18182,0.18118,0.1809,0.18173,0.18139
,0.18095,0.18138,0.18162,0.18123,0.18203,0.18069,0.18147,0.18068,0.180
56,0.1812,0.18026,0.54343,0.54502,0.54866,0.5373,0.54717,0.54437,0.360
39,0.3713,0.34873,0.38106,0.3604,0.38797,0.40179,0.37304,0.43919,0.358
82,0.18077,0.18168,0.18127,0.18192,0.18168,0.18084,0.18104,0.18236,0.1
812,0.18108,0.18096,0.18241,0.18036,0.18157,0.18088,0.18145,0.18092,0.

52882,0.54128,0.53088,0.54215,0.53837,0.54287,0.36563,0.38555,0.36226,
0.36056,0.36299,0.36692,0.35912,0.3859,0.40098,0.3959,0.37757,0.18254,
0.18096,0.18126,0.18066,0.18142,0.18144,0.18203,0.18113,0.18191,0.1816
3,0.18185,0.1822,0.1817,0.13946,0.14491,0.14295,0.18128,0.53462,0.5371
3,0.54175,0.54624,0.54903,0.55746,0.54877,0.36262,0.3562,0.38901,0.365
92,0.3326,0.3291,0.36602,0.34065,0.33201,0.32928,0.39121,0.18075,0.180
66,0.18173,0.18027,0.17977,0.1811,0.18188,0.18072,0.18168,0.18162,0.18
124,0.14167,0.12835,0.13859,0.14171,0.1409,0.13455,0.18055,0.54673,0.5
614,0.54502,0.54337,0.54058,0.5367,0.54176,0.39679,0.36702,0.36808,0.3
7514,0.33229,0.41329,0.38002,0.36325,0.36385,0.35624,0.3942,0.18237,0.
18096,0.18168,0.18141,0.18111,0.1811,0.18059,0.18026,0.18101,0.14042,0
.14074,0.14281,0.14083,0.14074,0.14098,0.1421,0.14003,0.1448,0.18158,0
.54228,0.54617,0.53389,0.53879,0.53936,0.53345,0.55143,0.38157,0.36456
,0.36643,0.36074,0.39635,0.40819,0.35446,0.38416,0.36177,0.39209,0.365
91,0.31182,0.18232,0.18217,0.18156,0.18161,0.18228,0.18098,0.14118,0.1
3923,0.14018,0.14032,0.14538,0.14783,0.14048,0.13172,0.14119,0.14207,0
.14192,0.1297,0.54677,0.54528,0.541,0.54313,0.54989,0.54505,0.54843,0.
34598,0.3586,0.39979,0.36324,0.32654,0.37989,0.3772,0.38638,0.34274,0.
4198,0.37546,0.36907,0.18179,0.18159,0.18136,0.14716,0.13747,0.14629,0
.14077,0.14094,0.13734,0.1364,0.1408,0.14573,0.1417,0.13373,0.13528,0.
14478,0.14081,0.1407,0.5382,0.54207,0.54673,0.53596,0.54023,0.53539,0.
54954,0.55527,0.55353,0.36211,0.37447,0.36262,0.39176,0.36473,0.36127,
0.35776,0.35938,0.37992,0.41301,0.36235,0.14059,0.13905,0.14328,0.1358
4,0.14079,0.14095,0.14205,0.14232,0.13673,0.12459,0.14438,0.13264,0.14
245,0.14146,0.14127,0.14473,0.14402,0.14001,0.54183,0.54768,0.54887,0.
54167,0.54633,0.54145,0.54742,0.54226,0.53057,0.3604,0.29748,0.27997,0
.25597,0.27542,0.26108,0.27336,0.28045,0.27246,0.2907,0.27497,0.14835,
0.14184,0.14179,0.14048,0.13275,0.14178,0.13683,0.13921,0.14862,0.1410
4,0.13539,0.14067,0.13198,0.14352,0.14544,0.14131,0.14123,0.13279,0.54
508,0.53323,0.5412,0.53836,0.54065,0.54362,0.5407,0.53422,0.55007,0.32
851,0.27909,0.27675,0.28378,0.2774,0.27913,0.26482,0.2508,0.27061,0.28
031,0.28653,0.14246,0.14763,0.14331,0.14097,0.13672,0.14083,0.14475,0.
142,0.14195,0.13973,0.14531,0.12853,0.14091,0.14501,0.14714,0.14645,0.
15006,0.13731,0.54303,0.55029,0.53705,0.5585,0.54579,0.54296,0.53369,0
.32101,0.30009,0.26699,0.27473,0.25773,0.27359,0.29422,0.29285,0.29486
,0.30529,0.28104,0.28061,0.28014,0.14553,0.15253,0.1338,0.1402,0.14372
,0.14121,0.13557,0.14295,0.14274,0.13097,0.14287,0.13398,0.13563,0.130
57,0.1406,0.14121,0.15491,0.14082,0.55544,0.54979,0.5371,0.54819,0.543
89,0.53583,0.32818,0.25801,0.3418,0.40537,0.32163,0.25198,0.25698,0.30
466,0.25674,0.27712,0.30872,0.28882,0.28507,0.28491,0.14251,0.14738,0.
13353,0.14052,0.13548,0.13849,0.1378,0.14351,0.14116,0.14148,0.13979,0
.14046,0.13665,0.13957,0.14147,0.14302,0.14099,0.14123,0.54036,0.54647
,0.53886,0.5516,0.55052,0.32156,0.30403,0.32311,0.33668,0.3216,0.32187
,0.27258,0.28364,0.27522,0.27595,0.26578,0.29242,0.29045,0.25588,0.286
06,0.13375,0.13101,0.13815,0.14047,0.14303,0.14743,0.14414,0.14122,0.1
4339,0.14301,0.14657,0.14589,0.14076,0.14145,0.13992,0.15225,0.14101,0
.14099,0.54472,0.53852,0.53409,0.28374,0.33886,0.32691,0.28936,0.33668
,0.3207,0.3197,0.3144,0.28218,0.25796,0.27574,0.27454,0.23593,0.2698,0
.25341,0.2525,0.27482,0.13723,0.14083,0.14064,0.14621,0.14072,0.14552,
0.13434,0.13655,0.14158,0.14102,0.14748,0.14076,0.13421,0.13261,0.1434
5,0.13342,0.14323,0.14059,0.54049,0.32206,0.31986,0.37082,0.37063,0.31
516,0.31163,0.34129,0.32364,0.32249,0.29634,0.30839,0.28536,0.27236,0.

28141,0.26722,0.2826,0.28769,0.27195,0.14275,0.14662,0.15299,0.14151,0
.14252,0.144,0.12507,0.14204,0.14051,0.14064,0.14058,0.14084,0.15245,0
.1462,0.15174,0.1404,0.14379,0.10045,0.33087,0.32111,0.34312,0.32389,0
.29092,0.29861,0.31857,0.2981,0.31051,0.35499,0.27377,0.28041,0.2884,0
.3036,0.27409,0.26645,0.28919,0.28981,0.26773,0.14611,0.13777,0.14313,
0.13924,0.14124,0.14091,0.14571,0.14426,0.11773,0.14166,0.14145,0.1437
2,0.14132,0.14324,0.12829,0.13165,0.14172,0.10103,0.35974,0.36524,0.32
07,0.28858,0.28014,0.31206,0.32182,0.32104,0.3263,0.31969,0.27007,0.27
504,0.26712,0.26819,0.26444,0.27844,0.24843,0.26292,0.14073,0.12928,0.
14112,0.14356,0.14075,0.13395,0.14072,0.13859,0.13814,0.13985,0.1406,0
.14072,0.14131,0.14336,0.13454,0.14147,0.14392,0.13712,0.10009,0.2828,
0.34114,0.36225,0.31921,0.32275,0.32448,0.33407,0.32688,0.29457,0.3204
7,0.26335,0.28442,0.28257,0.27775,0.27535,0.2799,0.27934,0.28517,0.137
59,0.14183,0.1406,0.13476,0.15718,0.14047,0.14076,0.14057,0.14023,0.14
619,0.13953,0.14372,0.14696,0.13384,0.14209,0.14105,0.14077,0.10077,0.
10062,0.10041,0.24327,0.32256,0.29567,0.32272,0.28226,0.32483,0.31954,
0.34194,0.3414,0.29559,0.24897,0.25839,0.2603,0.26973,0.27601,0.13841,
0.14755,0.14284,0.14072,0.14151,0.14958,0.13456,0.1374,0.14116,0.14082
,0.15421,0.14334,0.13533,0.13559,0.14167,0.14896,0.13941,0.14161,0.100
1,0.10045,0.10085,0.10071,0.32132,0.28832,0.33075,0.32202,0.32315,0.32
053,0.33917,0.31747,0.32688,0.27198,0.27414,0.25416,0.14112,0.14277,0.
14142,0.13337,0.14443,0.14061,0.14144,0.15486,0.14104,0.14094,0.13829,
0.16938,0.13233,0.14375,0.14103,0.14139,0.13863,0.14095,0.12661,0.1406
6,0.10017,0.10022,0.10099,0.10083,0.10081,0.10043,0.29169,0.26578,0.34
51,0.3243,0.31108,0.32472,0.35473,0.32277,0.13419,0.13565,0.14292,0.14
89,0.1379,0.14171,0.14099,0.13723,0.14769,0.14039,0.13553,0.14818,0.14
107,0.14365,0.14367,0.1412,0.13966,0.14098,0.13792,0.14602,0.14151,0.1
4027,0.1457,0.10045,0.10026,0.10086,0.10019,0.10056,0.10031,0.37942,0.
32112,0.32035,0.35481,0.34276,0.1005,0.1006,0.14128,0.14013,0.14623,0.
13868,0.14063,0.13314,0.13504,0.13749,0.14217,0.13852,0.13405,0.13788,
0.14483,0.14269,0.14142,0.14426,0.14383,0.14701,0.13053,0.13883,0.1412
6,0.10016,0.10042,0.10022,0.10037,0.1003,0.10087,0.10052,0.10083,0.100
68,0.10064,0.32742,0.32281,0.10076,0.10041,0.1008,0.10066,0.14018,0.14
273,0.13958,0.13788,0.12899,0.14909,0.14386,0.1411,0.14103,0.1407,0.14
028,0.14038,0.13101,0.13712,0.14462,0.14091,0.14061,0.14898,0.13831,0.
10081,0.10079,0.10028,0.10095,0.09995,0.10026,0.10073,0.1005,0.10066,0
.10066,0.10029,0.10092,0.10074,0.10104,0.10061,0.10072,0.10125,0.1411,
0.13943,0.1536,0.14819,0.14022,0.14097,0.13703,0.1514,0.15016,0.13745,
0.14493,0.14138,0.14107,0.14091,0.14073,0.14098,0.10059,0.10036,0.1000
6,0.10061,0.10035,0.10019,0.1004,0.1007,0.10095,0.1006,0.10059,0.10041
,0.10067,0.10076,0.10078,0.10078,0.10091,0.10056,0.10066,0.13123,0.140
84,0.12652,0.14203,0.14098,0.14107,0.13973,0.13405,0.14119,0.14071,0.1
4495,0.14072,0.13552,0.1003,0.10065,0.10043,0.10073,0.10047,0.10095,0.
10068,0.10053,0.10057,0.1003,0.10046,0.10021,0.10074,0.10083,0.10111,0
.10079,0.1009,0.10069,0.10059,0.10061,0.101,0.14136,0.14092,0.14115,0.
14438,0.13744,0.14833,0.1413,0.14625,0.14671,0.10069,0.10123,0.10071,0
.10043,0.1006,0.10061,0.10014,0.10076,0.10029,0.10109,0.10062,0.1001,0
.10067,0.10095,0.1005,0.10068,0.10047,0.10015,0.10074,0.10067,0.101,0.
10026,0.10058,0.10066,0.10086,0.14682,0.14458,0.13705,0.13666,0.10054,
0.10063,0.10121,0.10052,0.10085,0.10041,0.10043,0.10071,0.10049,0.1006
9,0.1003,0.10077,0.1006,0.1008,0.10023,0.10102,0.10051,0.10066,0.09990
7,0.10049,0.10124,0.10035,0.10044,0.10069,0.10027,0.10086,0.10056,0.10

043,0.10066,0.10074,0.10054,0.10028,0.10046,0.10042,0.10063,0.10088,0.
10071,0.10055,0.10076,0.10022,0.10033,0.10011,0.10029,0.10043,0.10042,
0.10025,0.10048,0.1005,0.10055,0.10056,0.10048,0.10053,0.10071,0.10012
,0.10055,0.10062,0.10034,0.10056,0.10002,0.1003,0.10038,0.1011,0.09995
7,0.10028,0.10033,0.10068,0.10035,0.10045,0.10038,0.10128,0.10057,0.10
053,0.10063,0.10098,0.10075,0.10072,0.10061,0.10053,0.10021,0.10028,0.
1007,0.10107,0.10115,0.10016,0.10019,0.10031,0.10104,0.10006,0.10062,0
.10077,0.10062,0.10033,0.10055,0.10109,0.10092,0.10035,0.10082,0.10075
,0.10071,0.1003,0.10072,0.1007,0.10077,0.10086,0.10081,0.10047,0.10049
,0.099837,0.10055,0.10071,0.10068,0.10026,0.10049,0.10106,0.10042,0.10
141,0.10066,0.10075,0.10015,0.10019,0.10064,0.10079,0.10014,0.10022,0.
10056,0.10086,0.1004,0.10036,0.1011,0.10043,0.10032,0.10111,0.10072,0.
10084,0.10034,0.10099,0.10043,0.10009,0.10036,0.10066,0.10082,0.10062,
0.10076,0.10041,0.10057,0.10067,0.10065,0.10047,0.10025,0.10085,0.1006
7,0.10118,0.10061,0.10055,0.10052,0.10081,0.10078,0.10023,0.10034,0.10
052,0.10014,0.10081,0.10133,0.10053,0.10084,0.10072,0.10101,0.10044,0.
10067,0.10058,0.10044,0.1008,0.10066,0.1011,0.1004,0.1004,0.10102,0.10
084,0.10078,0.10044,0.10019,0.10081,0.10045,0.10041,0.10029,0.10074,0.
10074,0.10066,0.10065,0.10039,0.10133,0.10066,0.10074,0.10048,0.099853
0.18664,0.18683,0.18637,0.1865,0.18636,0.18749,0.18641,0.18654,0.18643
,0.18725,0.18654,0.18661,0.18693,0.18686,0.18637,0.18762,0.18677,0.187
31,0.18685,0.18662,0.31982,0.35712,0.33374,0.32496,0.18704,0.18666,0.1
8671,0.18661,0.18695,0.1868,0.18666,0.18766,0.18649,0.18676,0.18691,0.
18696,0.18702,0.18656,0.18619,0.47115,0.33254,0.35876,0.33014,0.32449,
0.33943,0.33607,0.18774,0.18731,0.18576,0.18623,0.1881,0.1862,0.18693,
0.18615,0.18656,0.1857,0.18602,0.1875,0.18665,0.18634,0.18632,0.47707,
0.47515,0.31905,0.28259,0.33696,0.2903,0.32635,0.33417,0.33018,0.33049
,0.18676,0.18719,0.18724,0.18714,0.18703,0.186,0.18696,0.18682,0.18674
,0.18625,0.18775,0.18641,0.18719,0.18661,0.1867,0.46694,0.47426,0.4798
3,0.32936,0.35501,0.31669,0.33061,0.33094,0.33052,0.33968,0.33596,0.33
795,0.18655,0.18704,0.18697,0.18597,0.18681,0.18661,0.18687,0.18729,0.
18597,0.18611,0.18815,0.18732,0.18672,0.18769,0.18643,0.18661,0.48108,
0.48203,0.46927,0.47676,0.34988,0.34512,0.32462,0.34981,0.32601,0.3032
8,0.34522,0.33634,0.29669,0.31329,0.18735,0.18622,0.18648,0.18716,0.18
69,0.18663,0.18678,0.18649,0.187,0.18752,0.18648,0.18696,0.18655,0.185
93,0.1864,0.18542,0.47562,0.47988,0.48265,0.46859,0.47982,0.47317,0.33
066,0.34097,0.32117,0.34749,0.32828,0.35285,0.36456,0.34079,0.39291,0.
32885,0.18606,0.18709,0.18674,0.18726,0.18723,0.18636,0.18652,0.18758,
0.18659,0.18657,0.18616,0.18764,0.18565,0.187,0.18646,0.18649,0.18642,
0.46195,0.47383,0.46597,0.4751,0.47057,0.47119,0.33379,0.34909,0.33201
,0.32949,0.33247,0.33662,0.33023,0.35025,0.3621,0.35974,0.34344,0.1878
2,0.18636,0.18678,0.18594,0.18726,0.18701,0.18739,0.18654,0.18679,0.18
695,0.18714,0.18707,0.18682,0.13806,0.14425,0.14222,0.18671,0.47087,0.
47029,0.47242,0.48129,0.48332,0.48982,0.47798,0.33276,0.32553,0.35416,
0.3355,0.3084,0.30483,0.33603,0.31401,0.30665,0.30469,0.35495,0.18678,
0.1857,0.18698,0.18573,0.18534,0.18696,0.18724,0.18585,0.18678,0.18707
,0.18693,0.14047,0.12491,0.1371,0.14035,0.13957,0.13202,0.18591,0.4809
3,0.49575,0.47887,0.47881,0.47317,0.47273,0.47732,0.35803,0.33544,0.33
696,0.34146,0.30883,0.37206,0.3454,0.3324,0.33338,0.32697,0.35931,0.18
825,0.18638,0.18696,0.1867,0.18675,0.18608,0.18589,0.18588,0.18618,0.1
3919,0.13958,0.14216,0.13974,0.13955,0.13965,0.14074,0.13835,0.14419,0
.18722,0.47357,0.4807,0.46847,0.47318,0.47415,0.47085,0.48441,0.34734,

0.33295,0.33487,0.33029,0.35923,0.37363,0.32524,0.35249,0.33108,0.3549
1,0.33275,0.291,0.18746,0.18775,0.18673,0.18716,0.18778,0.18676,0.1397
4,0.13759,0.13887,0.139,0.14466,0.14757,0.1392,0.12911,0.13984,0.14121
,0.14101,0.12652,0.47699,0.47668,0.47327,0.47454,0.48288,0.47387,0.480
74,0.31882,0.32811,0.3599,0.33194,0.30391,0.34764,0.34091,0.3494,0.313
15,0.37888,0.34343,0.33674,0.18765,0.18688,0.18639,0.14701,0.13567,0.1
4597,0.13959,0.13955,0.13555,0.13432,0.13958,0.14521,0.14066,0.13167,0
.13327,0.14419,0.13936,0.13956,0.4732,0.47792,0.4762,0.46799,0.47247,0
.46706,0.48347,0.48542,0.48418,0.33422,0.34127,0.33197,0.35673,0.33314
,0.3318,0.32675,0.32918,0.34664,0.37228,0.33112,0.13923,0.13734,0.1424
5,0.1339,0.13967,0.13961,0.14146,0.14122,0.13476,0.12061,0.14399,0.130
22,0.14114,0.14024,0.14032,0.14383,0.14311,0.13867,0.4769,0.48402,0.47
524,0.47344,0.47823,0.47594,0.48078,0.46978,0.46842,0.33059,0.28031,0.
25701,0.23362,0.25181,0.23897,0.25122,0.25589,0.25065,0.26337,0.25269,
0.14861,0.14085,0.14062,0.13937,0.13005,0.14063,0.13495,0.13787,0.1489
2,0.13995,0.13313,0.1397,0.1292,0.14292,0.14487,0.13991,0.13997,0.1300
4,0.4753,0.46819,0.47576,0.47187,0.47369,0.47471,0.4711,0.46692,0.4817
9,0.29145,0.25674,0.25298,0.26124,0.25431,0.25452,0.24418,0.23171,0.24
73,0.25756,0.26206,0.1412,0.14763,0.14244,0.13954,0.1346,0.13962,0.144
28,0.14081,0.14103,0.13865,0.14459,0.12519,0.13926,0.14456,0.14732,0.1
4588,0.15035,0.13551,0.47538,0.48315,0.47132,0.49332,0.47596,0.47838,0
.4688,0.28349,0.26676,0.23724,0.25149,0.23725,0.24991,0.2701,0.26588,0
.27018,0.27776,0.25722,0.25553,0.25516,0.14494,0.1532,0.13107,0.13889,
0.14298,0.13988,0.13354,0.14215,0.14175,0.12808,0.14202,0.13148,0.1334
9,0.12768,0.13923,0.14025,0.15582,0.13937,0.48925,0.47939,0.47057,0.48
101,0.47812,0.46955,0.29109,0.2288,0.303,0.35691,0.28379,0.23386,0.237
4,0.27921,0.23901,0.25398,0.28261,0.26098,0.25808,0.25865,0.14138,0.14
688,0.13119,0.13926,0.13309,0.13674,0.13595,0.1429,0.13989,0.14008,0.1
3862,0.13943,0.1347,0.13836,0.14019,0.14237,0.13964,0.14001,0.47432,0.
47931,0.47522,0.47983,0.47615,0.28385,0.26963,0.28458,0.29591,0.28306,
0.28303,0.25048,0.25836,0.25152,0.25147,0.24463,0.26708,0.26563,0.2365
6,0.25932,0.1314,0.12798,0.13654,0.1392,0.14215,0.14762,0.14338,0.1402
1,0.14217,0.14184,0.14623,0.14556,0.13963,0.14026,0.13852,0.15302,0.13
978,0.13982,0.4776,0.47739,0.46749,0.25166,0.30127,0.29081,0.25798,0.2
9724,0.28456,0.28295,0.27799,0.25597,0.23746,0.2519,0.25101,0.21963,0.
24884,0.23231,0.2344,0.25107,0.13519,0.13924,0.13959,0.14578,0.1394,0.
14508,0.13185,0.13431,0.14017,0.13998,0.14711,0.13969,0.13215,0.12991,
0.1428,0.13094,0.14237,0.13956,0.47304,0.28605,0.28291,0.32688,0.32601
,0.27787,0.27519,0.3032,0.28795,0.28694,0.26946,0.28001,0.26115,0.2514
4,0.25665,0.24538,0.25715,0.26058,0.24933,0.14153,0.14613,0.15357,0.14
037,0.14143,0.14336,0.12113,0.1408,0.13946,0.1396,0.13927,0.13971,0.15
317,0.146,0.15229,0.13923,0.14311,0.092579,0.29258,0.28272,0.30343,0.2
8774,0.25884,0.26552,0.28297,0.2641,0.27573,0.31421,0.24895,0.25482,0.
2631,0.27679,0.24998,0.24439,0.26137,0.26138,0.24668,0.1457,0.13612,0.
14216,0.13788,0.13989,0.13962,0.14508,0.1437,0.11275,0.14024,0.1403,0.
14312,0.14049,0.14234,0.12474,0.12905,0.14088,0.093197,0.31632,0.32437
,0.28218,0.25541,0.24928,0.27748,0.28311,0.28249,0.28999,0.28232,0.247
5,0.25155,0.24478,0.24578,0.24365,0.25466,0.2294,0.24244,0.13956,0.125
95,0.14003,0.14267,0.13953,0.1316,0.13942,0.13714,0.13625,0.13832,0.13
919,0.13945,0.14003,0.14256,0.13209,0.14022,0.14325,0.13511,0.09212,0.
25018,0.30127,0.31817,0.28191,0.28564,0.28806,0.29278,0.2875,0.26129,0
.28298,0.24201,0.25774,0.25726,0.25156,0.25222,0.25537,0.25447,0.25852

,0.1359,0.14064,0.13932,0.13244,0.15925,0.13921,0.13946,0.13943,0.1389
9,0.14565,0.13822,0.14302,0.1468,0.13132,0.14119,0.13999,0.13915,0.092
67,0.092683,0.09228,0.21662,0.28563,0.2617,0.28596,0.25021,0.28709,0.2
8248,0.30176,0.3032,0.26757,0.23037,0.23882,0.23834,0.24704,0.25294,0.
1367,0.14747,0.14196,0.13933,0.14017,0.14986,0.13215,0.13561,0.13966,0
.13948,0.15514,0.14229,0.13307,0.13363,0.14021,0.14905,0.138,0.14028,0
.091933,0.092515,0.093065,0.092642,0.28461,0.257,0.29166,0.2876,0.2860
6,0.28555,0.29937,0.28173,0.28721,0.25196,0.25172,0.23624,0.13982,0.14
178,0.14017,0.13091,0.14365,0.13945,0.14015,0.15582,0.13989,0.13943,0.
13666,0.17285,0.12973,0.14275,0.13981,0.14033,0.13718,0.13951,0.12301,
0.13972,0.092254,0.092145,0.0931,0.092761,0.09269,0.092589,0.26001,0.2
3619,0.30283,0.28381,0.27509,0.28913,0.31502,0.28619,0.13193,0.13321,0
.14175,0.14883,0.13634,0.14069,0.14002,0.13555,0.14732,0.139,0.1332,0.
14807,0.1397,0.14303,0.14293,0.13976,0.13834,0.13975,0.13626,0.14546,0
.14028,0.13859,0.14557,0.092549,0.092238,0.092833,0.092375,0.092539,0.
092323,0.33488,0.28321,0.28211,0.31251,0.30233,0.092379,0.09246,0.1403
7,0.13873,0.1459,0.13714,0.13937,0.13066,0.13285,0.13544,0.14107,0.136
99,0.1315,0.13613,0.14427,0.14179,0.13983,0.14366,0.14295,0.14675,0.12
748,0.13706,0.13986,0.092279,0.092386,0.092514,0.092429,0.092274,0.093
106,0.092785,0.092623,0.092524,0.09265,0.28956,0.28563,0.092684,0.0925
03,0.092944,0.09251,0.13868,0.14186,0.13834,0.13624,0.12578,0.14886,0.
1432,0.13959,0.1399,0.13963,0.13927,0.13891,0.12806,0.13559,0.14385,0.
13967,0.13933,0.1491,0.13669,0.092775,0.092913,0.09233,0.092974,0.0918
61,0.092468,0.09271,0.092431,0.092553,0.092692,0.092534,0.092773,0.092
794,0.092862,0.092615,0.09248,0.093378,0.13994,0.13793,0.15422,0.14839
,0.13863,0.13976,0.13515,0.15171,0.15047,0.13566,0.14458,0.1403,0.1397
9,0.13963,0.13956,0.13978,0.092605,0.092447,0.092179,0.092704,0.092442
,0.092184,0.092262,0.092842,0.093171,0.092549,0.092483,0.092491,0.0927
06,0.092916,0.092744,0.092553,0.092887,0.092567,0.092735,0.12839,0.139
74,0.12258,0.14099,0.13969,0.14001,0.13861,0.13159,0.14015,0.13949,0.1
443,0.13964,0.13394,0.09232,0.092926,0.092475,0.092637,0.092221,0.0930
78,0.092799,0.092487,0.092529,0.092435,0.092595,0.09236,0.092589,0.092
67,0.093015,0.092583,0.092818,0.092741,0.092723,0.09272,0.0931,0.14002
,0.13972,0.14,0.14334,0.13554,0.14827,0.1402,0.14586,0.1463,0.092799,0
.093053,0.092752,0.092378,0.092655,0.092883,0.092212,0.092739,0.092315
,0.093319,0.092639,0.092029,0.092717,0.092899,0.092428,0.092608,0.0924
72,0.092216,0.092682,0.092882,0.092736,0.092426,0.092916,0.092817,0.09
3096,0.14636,0.14372,0.13518,0.13485,0.092425,0.092517,0.092992,0.0924
92,0.09287,0.092507,0.092182,0.092979,0.092693,0.092826,0.092264,0.092
663,0.092733,0.092685,0.092362,0.093048,0.09267,0.092845,0.091878,0.09
2646,0.093253,0.092311,0.092555,0.092885,0.092277,0.092948,0.092698,0.
092385,0.092913,0.092797,0.092603,0.092387,0.092326,0.092442,0.092668,
0.092846,0.092832,0.092551,0.092734,0.092262,0.092188,0.092137,0.09238
7,0.092614,0.092444,0.092567,0.092239,0.092604,0.09276,0.092578,0.0924
84,0.092547,0.092781,0.092158,0.092608,0.09276,0.092535,0.092489,0.092
033,0.092525,0.092342,0.093041,0.091815,0.092304,0.092221,0.092741,0.0
92596,0.092406,0.092388,0.09328,0.09256,0.092451,0.092545,0.09287,0.09
2862,0.092577,0.09275,0.092452,0.092306,0.0924,0.092699,0.093078,0.093
224,0.092406,0.092342,0.092579,0.093012,0.092054,0.09255,0.092704,0.09
2488,0.092669,0.092571,0.09337,0.092669,0.092238,0.092871,0.092693,0.0
92772,0.092219,0.092927,0.092657,0.092808,0.092756,0.09293,0.092389,0.
092418,0.091983,0.09256,0.092833,0.09275,0.092469,0.092531,0.093102,0.

092439,0.093488,0.092774,0.092787,0.091962,0.092347,0.09257,0.09284,0.
092175,0.092325,0.092432,0.092668,0.092286,0.092476,0.093166,0.092663,
0.092406,0.09322,0.092723,0.092932,0.092456,0.092921,0.092503,0.092059
,0.092304,0.092625,0.092931,0.092737,0.092907,0.092433,0.092463,0.0925
92,0.092671,0.092619,0.09238,0.092665,0.092879,0.093107,0.092777,0.092
64,0.092492,0.092715,0.092884,0.092392,0.092561,0.092649,0.091971,0.09
2903,0.093218,0.092316,0.092947,0.0928,0.093093,0.092379,0.092677,0.09
2619,0.092421,0.092873,0.092746,0.092944,0.092482,0.092199,0.092929,0.
092767,0.092927,0.092491,0.092272,0.092747,0.092526,0.09256,0.092478,0
.092935,0.092601,0.092535,0.092411,0.092672,0.093093,0.092514,0.092854
,0.092521,0.092052
0.19044,0.19108,0.19017,0.19021,0.19079,0.19126,0.19017,0.19051,0.1904
9,0.19098,0.19086,0.19092,0.19089,0.19075,0.18993,0.19151,0.19095,0.19
132,0.1909,0.19018,0.30024,0.33611,0.31521,0.3057,0.19115,0.19028,0.19
011,0.19046,0.19079,0.19063,0.19024,0.19107,0.19019,0.19079,0.19057,0.
19072,0.19079,0.19059,0.19048,0.42753,0.31293,0.33537,0.31121,0.30773,
0.31947,0.31481,0.19135,0.19115,0.1897,0.19063,0.1923,0.18972,0.19094,
0.18983,0.19079,0.18976,0.18995,0.19129,0.19071,0.19024,0.18993,0.4379
9,0.43407,0.30231,0.27238,0.31746,0.27935,0.30851,0.31317,0.31147,0.31
009,0.19083,0.19131,0.19116,0.19105,0.19135,0.19008,0.19061,0.19061,0.
19055,0.19063,0.19174,0.1905,0.19125,0.19079,0.1905,0.42507,0.43597,0.
438,0.30962,0.33465,0.30032,0.311,0.31246,0.31403,0.31841,0.31667,0.31
519,0.19059,0.19104,0.19148,0.1902,0.19076,0.19103,0.19064,0.19109,0.1
8969,0.19035,0.19203,0.19128,0.19066,0.19179,0.19089,0.19051,0.44026,0
.44176,0.43214,0.43521,0.32693,0.32337,0.30712,0.32671,0.30656,0.28835
,0.32384,0.3173,0.28496,0.29636,0.19134,0.18956,0.19088,0.19118,0.1911
4,0.19077,0.1908,0.19018,0.19089,0.19143,0.19009,0.19062,0.19073,0.189
74,0.19008,0.1895,0.43575,0.43651,0.44005,0.42814,0.43725,0.43353,0.31
26,0.3225,0.30566,0.32642,0.30846,0.33188,0.33965,0.32304,0.36478,0.30
939,0.19011,0.19098,0.19062,0.19107,0.19074,0.19082,0.19024,0.19203,0.
19063,0.19065,0.19023,0.19154,0.18978,0.19088,0.19073,0.19064,0.19076,
0.41988,0.43435,0.42507,0.43195,0.43406,0.43125,0.31563,0.32651,0.3142
3,0.31161,0.31353,0.31569,0.31014,0.32931,0.33979,0.33713,0.32509,0.19
171,0.19024,0.19071,0.19007,0.19129,0.19076,0.19148,0.19066,0.19044,0.
19106,0.1913,0.19113,0.1906,0.13625,0.14319,0.1403,0.19078,0.43131,0.4
2537,0.43519,0.4402,0.43857,0.44815,0.43493,0.31294,0.30667,0.33047,0.
31807,0.29127,0.29154,0.31646,0.29798,0.29086,0.29049,0.33354,0.19062,
0.18994,0.19101,0.19008,0.18935,0.19072,0.19118,0.19002,0.19097,0.1910
8,0.19059,0.13881,0.1212,0.13513,0.13867,0.13783,0.12913,0.19019,0.438
21,0.45059,0.43827,0.43904,0.43486,0.4293,0.43379,0.33547,0.31436,0.31
745,0.32129,0.29263,0.34591,0.32547,0.31514,0.31719,0.30835,0.33751,0.
19179,0.19063,0.19107,0.19069,0.19058,0.1895,0.18962,0.1902,0.19053,0.
13757,0.1378,0.14073,0.13799,0.13795,0.1378,0.13907,0.13634,0.14301,0.
19083,0.43382,0.43609,0.42712,0.43404,0.43565,0.43214,0.43985,0.32522,
0.31186,0.31452,0.31218,0.33745,0.34782,0.31029,0.32987,0.3128,0.33142
,0.31319,0.27974,0.19151,0.19132,0.19085,0.19115,0.19166,0.19054,0.137
95,0.13605,0.13683,0.13746,0.14368,0.14653,0.13744,0.12601,0.13815,0.1
3945,0.13919,0.1234,0.43668,0.43369,0.42852,0.43187,0.44009,0.43487,0.
43867,0.30207,0.30831,0.33889,0.31267,0.28886,0.32728,0.31868,0.32598,
0.29899,0.35501,0.32426,0.31727,0.1917,0.19054,0.1898,0.14632,0.13319,
0.14478,0.13779,0.13787,0.13334,0.132,0.13782,0.14421,0.13901,0.129,0.
13081,0.14274,0.13759,0.13807,0.43091,0.44041,0.43791,0.43173,0.43204,

0.42471,0.4424,0.44371,0.44356,0.31634,0.32298,0.31451,0.33356,0.31405
,0.31354,0.3104,0.31052,0.32647,0.34764,0.31099,0.13745,0.13515,0.1409
4,0.13149,0.13787,0.1378,0.13991,0.13969,0.13229,0.1166,0.14267,0.1273
3,0.13993,0.13838,0.1383,0.14241,0.14192,0.13671,0.43742,0.4407,0.4312
9,0.43437,0.44437,0.43502,0.44169,0.42495,0.42586,0.31145,0.27104,0.24
221,0.21842,0.23731,0.22519,0.2366,0.24017,0.23575,0.24672,0.23963,0.1
4762,0.13927,0.13899,0.13754,0.12732,0.13875,0.13315,0.13593,0.14823,0
.13785,0.13048,0.13824,0.12595,0.14168,0.14353,0.1381,0.13826,0.12716,
0.43283,0.42919,0.43286,0.43294,0.4332,0.43389,0.42843,0.42601,0.44285
,0.2662,0.24152,0.23806,0.24702,0.23937,0.23794,0.22959,0.21885,0.2335
8,0.24381,0.24582,0.13957,0.14704,0.14093,0.13809,0.13228,0.13796,0.14
299,0.13942,0.13938,0.13623,0.14354,0.12184,0.13753,0.14354,0.14658,0.
14486,0.14962,0.13324,0.43257,0.43961,0.43134,0.45166,0.4389,0.43447,0
.42862,0.25965,0.2438,0.21815,0.23682,0.22434,0.23553,0.25426,0.24759,
0.25401,0.26094,0.24008,0.2384,0.23864,0.14384,0.15298,0.12824,0.13702
,0.14157,0.13795,0.13132,0.14056,0.14007,0.12483,0.1405,0.12875,0.1307
6,0.12452,0.13756,0.13851,0.15577,0.13756,0.44701,0.4372,0.4274,0.4384
5,0.4373,0.42753,0.2658,0.20954,0.27841,0.32958,0.25779,0.22183,0.2245
7,0.26224,0.22749,0.23834,0.2656,0.24296,0.24151,0.24399,0.13985,0.146
18,0.12836,0.13749,0.13056,0.13464,0.13374,0.14174,0.13816,0.13894,0.1
3674,0.13763,0.13228,0.13643,0.13842,0.14112,0.13814,0.13825,0.43355,0
.43831,0.43656,0.435,0.43518,0.25752,0.24648,0.25973,0.26883,0.25943,0
.25853,0.23616,0.24129,0.23562,0.23673,0.23006,0.25197,0.24998,0.22496
,0.24191,0.12864,0.12494,0.13457,0.13747,0.14053,0.14691,0.14208,0.138
81,0.14084,0.14029,0.14512,0.14434,0.13831,0.13869,0.13647,0.1527,0.13
779,0.13816,0.43508,0.43615,0.42796,0.22973,0.27663,0.26664,0.23786,0.
27241,0.26168,0.2588,0.25554,0.24006,0.22287,0.23565,0.23514,0.20741,0
.23526,0.21959,0.22203,0.23617,0.13282,0.13757,0.13775,0.14502,0.13751
,0.14399,0.12919,0.13199,0.13864,0.13814,0.14621,0.13793,0.12955,0.126
98,0.14151,0.12798,0.14113,0.13779,0.43419,0.26002,0.25688,0.29846,0.3
0029,0.25494,0.25148,0.27694,0.26277,0.26279,0.25353,0.26188,0.24745,0
.2372,0.2421,0.23025,0.24038,0.24306,0.23524,0.14032,0.14516,0.15386,0
.13885,0.13988,0.14195,0.11736,0.13912,0.13771,0.13797,0.13769,0.13773
,0.15286,0.14528,0.15208,0.13762,0.14184,0.085079,0.26605,0.2578,0.279
6,0.26412,0.23666,0.24304,0.25964,0.24176,0.25127,0.28974,0.2347,0.238
99,0.24814,0.25942,0.2352,0.23036,0.24528,0.24356,0.23134,0.14474,0.13
413,0.1406,0.13595,0.13821,0.13795,0.14401,0.14245,0.10765,0.13832,0.1
3849,0.14187,0.13882,0.14098,0.12124,0.12614,0.13905,0.085792,0.29114,
0.29974,0.25795,0.23239,0.22888,0.25545,0.26002,0.2588,0.26609,0.25628
,0.23205,0.23794,0.23004,0.2305,0.22994,0.23977,0.21683,0.22742,0.1377
7,0.12256,0.13842,0.14114,0.13793,0.12889,0.1376,0.13514,0.13412,0.136
3,0.13747,0.13778,0.13843,0.14149,0.1296,0.1389,0.14216,0.13305,0.0847
31,0.23074,0.27405,0.29062,0.25796,0.26163,0.26576,0.26792,0.26204,0.2
4007,0.25746,0.22872,0.24067,0.24152,0.23707,0.23783,0.24143,0.24022,0
.24184,0.13369,0.13936,0.13753,0.12999,0.1598,0.13724,0.13764,0.13755,
0.13724,0.1449,0.13642,0.14167,0.14582,0.1286,0.13924,0.13833,0.13725,
0.085502,0.085358,0.08475,0.19763,0.26059,0.23884,0.26398,0.22984,0.26
195,0.25599,0.27577,0.27741,0.24771,0.21822,0.22621,0.22487,0.23217,0.
23863,0.13488,0.14678,0.14044,0.13742,0.13836,0.14955,0.12948,0.13344,
0.13781,0.13766,0.15497,0.14128,0.13034,0.13126,0.13828,0.14849,0.1362
1,0.13847,0.084628,0.085327,0.085579,0.085144,0.26015,0.23644,0.2657,0
.26221,0.26078,0.2596,0.27364,0.25762,0.26265,0.23801,0.23643,0.22332,

0.13806,0.14055,0.13854,0.12847,0.14252,0.13766,0.13843,0.15612,0.1382
6,0.13791,0.13463,0.17529,0.12693,0.14132,0.13793,0.13878,0.13534,0.13
8,0.1193,0.13795,0.084801,0.084643,0.085464,0.085567,0.085079,0.08512,
0.23654,0.21504,0.27594,0.26023,0.24991,0.26344,0.28857,0.26245,0.1298
,0.13052,0.14006,0.14797,0.13425,0.13909,0.13845,0.13331,0.1465,0.1371
1,0.1305,0.14771,0.13793,0.14166,0.14133,0.13816,0.13636,0.13782,0.133
99,0.14424,0.13846,0.13663,0.14446,0.085,0.085004,0.085519,0.084933,0.
085089,0.085125,0.30808,0.26038,0.25773,0.28837,0.27637,0.084955,0.085
045,0.13889,0.13705,0.14505,0.13502,0.13765,0.12765,0.13006,0.13333,0.
13951,0.13473,0.12901,0.13392,0.14319,0.14031,0.13811,0.14244,0.14148,
0.14556,0.12436,0.13484,0.13791,0.084728,0.084964,0.085121,0.08487,0.0
84861,0.085665,0.085433,0.085183,0.085051,0.08516,0.26327,0.26381,0.08
5408,0.085179,0.085373,0.085043,0.13699,0.14008,0.13625,0.13426,0.1224
4,0.14836,0.14202,0.13764,0.13824,0.1378,0.13765,0.13717,0.1248,0.1332
8,0.14258,0.13801,0.13797,0.14842,0.13453,0.085333,0.085568,0.084673,0
.085505,0.084537,0.085253,0.085324,0.085072,0.085188,0.085122,0.084996
,0.08532,0.085212,0.085195,0.085232,0.085307,0.08583,0.13831,0.13582,0
.15412,0.14763,0.13681,0.13818,0.13271,0.15141,0.14996,0.13337,0.14344
,0.13879,0.13784,0.13769,0.13795,0.13789,0.085284,0.084802,0.084934,0.
085419,0.085215,0.084672,0.084887,0.085429,0.085638,0.084957,0.085104,
0.084916,0.085228,0.085553,0.085312,0.085023,0.085431,0.085434,0.08523
7,0.1253,0.13784,0.11878,0.13945,0.138,0.13853,0.13696,0.12887,0.13836
,0.13776,0.14282,0.13769,0.13154,0.085007,0.085509,0.084742,0.085323,0
.084814,0.085636,0.085525,0.085361,0.085034,0.08499,0.085151,0.085145,
0.084981,0.085334,0.085541,0.085214,0.08532,0.085473,0.085075,0.085317
,0.085363,0.13834,0.13791,0.13833,0.14171,0.13321,0.14727,0.13842,0.14
458,0.14517,0.085341,0.085821,0.085395,0.084799,0.085075,0.085572,0.08
4666,0.085237,0.085026,0.085908,0.085234,0.08452,0.085396,0.085403,0.0
84804,0.084963,0.084886,0.084698,0.085378,0.085578,0.085347,0.084998,0
.08555,0.085452,0.085774,0.14537,0.14242,0.13275,0.13244,0.084714,0.08
5018,0.085443,0.085075,0.085553,0.085162,0.084877,0.085661,0.085013,0.
085507,0.085199,0.085172,0.085184,0.085161,0.08485,0.085605,0.085132,0
.085379,0.084458,0.085286,0.085755,0.084886,0.085064,0.085274,0.084828
,0.085531,0.085251,0.085028,0.085539,0.085435,0.08507,0.085018,0.08503
3,0.08496,0.085192,0.085377,0.085223,0.085048,0.08519,0.084791,0.08476
,0.084679,0.085127,0.085227,0.084982,0.085353,0.085003,0.08514,0.08530
8,0.085,0.085198,0.085138,0.085241,0.084727,0.085151,0.085318,0.085116
,0.084961,0.084857,0.084903,0.084826,0.08549,0.084648,0.084909,0.08480
7,0.085188,0.08504,0.08484,0.084857,0.085632,0.085201,0.08493,0.085094
,0.085356,0.085436,0.085176,0.085043,0.085155,0.084967,0.084832,0.0851
96,0.085442,0.085643,0.08494,0.085281,0.085094,0.085511,0.084836,0.085
221,0.08517,0.085148,0.085323,0.085106,0.085774,0.085164,0.084693,0.08
555,0.085178,0.085297,0.084773,0.085447,0.085157,0.085495,0.085482,0.0
85606,0.084749,0.084911,0.08461,0.085026,0.085395,0.085143,0.084975,0.
085333,0.085366,0.084917,0.08591,0.085369,0.085393,0.08475,0.084786,0.
085148,0.085746,0.084826,0.085029,0.084827,0.085078,0.085004,0.084915,
0.085852,0.08537,0.08507,0.085774,0.085486,0.085532,0.085292,0.085501,
0.084984,0.084659,0.084627,0.08499,0.085578,0.085435,0.085572,0.08498,
0.084923,0.085147,0.085131,0.085309,0.085084,0.085152,0.085479,0.08583
7,0.085423,0.085323,0.085139,0.085417,0.085443,0.085135,0.084903,0.085
212,0.084673,0.085632,0.085707,0.084913,0.08551,0.085137,0.085627,0.08
5065,0.085144,0.085153,0.084992,0.085389,0.085236,0.085668,0.085068,0.

084638,0.085534,0.08556,0.085564,0.085037,0.085135,0.085247,0.085069,0
.084913,0.084991,0.085416,0.085316,0.085136,0.085059,0.085407,0.085707
,0.08509,0.0853,0.085085,0.084752
0.19345,0.1944,0.19318,0.19313,0.19349,0.19448,0.19311,0.19382,0.19324
,0.19396,0.19349,0.194,0.19359,0.19357,0.19305,0.19428,0.19353,0.19458
,0.19393,0.19281,0.28964,0.32278,0.303,0.29569,0.19402,0.19323,0.19315
,0.19325,0.19382,0.19373,0.19346,0.19426,0.19305,0.1939,0.19363,0.1937
6,0.1934,0.19375,0.1936,0.40582,0.30283,0.32428,0.30114,0.29646,0.3074
,0.30197,0.19423,0.19396,0.19265,0.194,0.19578,0.19268,0.19388,0.1929,
0.19365,0.19285,0.19308,0.19402,0.19362,0.19331,0.19308,0.41099,0.4107
6,0.29336,0.26483,0.3065,0.27177,0.29714,0.30112,0.30179,0.30135,0.194
13,0.1946,0.19402,0.19418,0.19441,0.19297,0.19408,0.19368,0.19348,0.19
298,0.19503,0.19332,0.1944,0.19378,0.19319,0.40144,0.41305,0.41665,0.2
9834,0.32247,0.29105,0.29866,0.30155,0.3045,0.30775,0.30656,0.30395,0.
19381,0.19408,0.19454,0.19312,0.19389,0.1943,0.19354,0.19397,0.19291,0
.19355,0.19429,0.1937,0.19356,0.19475,0.19389,0.19323,0.41634,0.4132,0
.40743,0.41163,0.31446,0.30895,0.29611,0.31253,0.29653,0.2793,0.31263,
0.30526,0.27824,0.28612,0.19435,0.1927,0.1937,0.1937,0.19403,0.19346,0
.1934,0.19327,0.19372,0.19461,0.19298,0.19346,0.19359,0.19331,0.19292,
0.19298,0.40857,0.4135,0.4143,0.40137,0.40917,0.40313,0.30209,0.30987,
0.29391,0.31118,0.29786,0.31812,0.32721,0.31327,0.34828,0.29884,0.1929
2,0.19379,0.19374,0.19372,0.19359,0.19382,0.19347,0.19502,0.19349,0.19
381,0.19373,0.19383,0.1932,0.1937,0.19355,0.19354,0.1935,0.39639,0.409
33,0.39608,0.40873,0.4065,0.40515,0.3032,0.31262,0.30366,0.29858,0.301
9,0.30589,0.29956,0.31577,0.32539,0.32128,0.3109,0.19519,0.1928,0.1935
2,0.19274,0.19402,0.19399,0.19463,0.19304,0.19361,0.19453,0.1941,0.194
12,0.19394,0.13415,0.14181,0.13867,0.19397,0.40618,0.40263,0.41296,0.4
1232,0.41474,0.42134,0.41172,0.30064,0.29558,0.31661,0.3061,0.28286,0.
28218,0.30544,0.28744,0.28242,0.28221,0.31919,0.19319,0.19283,0.19415,
0.19307,0.19211,0.19348,0.19456,0.19299,0.19413,0.19422,0.19333,0.1371
7,0.11762,0.13306,0.13659,0.136,0.12658,0.19309,0.41249,0.42469,0.4114
1,0.41376,0.41022,0.40644,0.41024,0.32221,0.30365,0.30419,0.30931,0.28
309,0.33195,0.3118,0.30283,0.30545,0.29953,0.32582,0.19443,0.19374,0.1
9411,0.19413,0.19367,0.19283,0.19202,0.19328,0.19315,0.13584,0.13613,0
.13901,0.13624,0.13607,0.1359,0.1374,0.1346,0.1416,0.19398,0.40539,0.4
0944,0.3984,0.4046,0.41171,0.4068,0.41062,0.31402,0.30012,0.30323,0.30
018,0.32267,0.33393,0.2994,0.318,0.30138,0.31977,0.30243,0.27226,0.194
72,0.19427,0.19346,0.19452,0.19444,0.19349,0.13598,0.13438,0.13488,0.1
3554,0.14247,0.14544,0.13571,0.12308,0.13655,0.13774,0.13756,0.1202,0.
4163,0.40758,0.40695,0.40499,0.41346,0.40866,0.40909,0.29213,0.29817,0
.32337,0.30157,0.27877,0.31278,0.30424,0.31509,0.28973,0.33878,0.30978
,0.30486,0.19458,0.19333,0.19305,0.14541,0.13075,0.14349,0.1359,0.1360
5,0.1313,0.12995,0.13594,0.14288,0.13739,0.12636,0.12839,0.14149,0.135
69,0.1363,0.40442,0.41464,0.40937,0.40104,0.40706,0.39554,0.41612,0.41
507,0.41577,0.304,0.31055,0.30311,0.32111,0.30236,0.30274,0.29945,0.29
93,0.31497,0.33202,0.29847,0.13538,0.13305,0.13934,0.12923,0.13609,0.1
3604,0.13851,0.13808,0.13012,0.1128,0.14149,0.12466,0.13831,0.13656,0.
13649,0.14106,0.14053,0.13451,0.41282,0.41721,0.40841,0.40274,0.41899,
0.40768,0.41615,0.39671,0.40289,0.29865,0.26455,0.2328,0.20808,0.22636
,0.2169,0.22673,0.22986,0.22555,0.2341,0.23167,0.14684,0.13774,0.13722
,0.13565,0.12471,0.13703,0.13113,0.13387,0.14734,0.13604,0.1282,0.1364
2,0.12331,0.14034,0.14232,0.13608,0.13657,0.12424,0.40859,0.40374,0.40

405,0.40354,0.40726,0.40874,0.40071,0.39916,0.41559,0.24821,0.2317,0.2
2706,0.2382,0.23046,0.22713,0.22003,0.21006,0.22332,0.23575,0.23537,0.
13804,0.14587,0.1394,0.13624,0.12991,0.13607,0.14146,0.13772,0.13777,0
.13441,0.14258,0.11889,0.13578,0.14225,0.14565,0.14351,0.1487,0.131,0.
41245,0.41333,0.40773,0.42642,0.41071,0.41156,0.40044,0.2426,0.22806,0
.20543,0.22664,0.21563,0.2254,0.24453,0.23495,0.24486,0.24996,0.23006,
0.2288,0.22893,0.14253,0.15269,0.12575,0.13524,0.14012,0.13601,0.12899
,0.1389,0.13837,0.12177,0.13924,0.12626,0.12826,0.12153,0.13541,0.1369
1,0.15526,0.13556,0.42014,0.41206,0.40279,0.41627,0.41201,0.39785,0.24
987,0.19594,0.26047,0.31323,0.24387,0.21355,0.21655,0.25136,0.21981,0.
22818,0.25629,0.2312,0.23222,0.23152,0.13814,0.14494,0.12586,0.13556,0
.12818,0.13267,0.13163,0.14037,0.1362,0.13734,0.13474,0.13569,0.12979,
0.13457,0.13673,0.13942,0.13637,0.13654,0.40674,0.41112,0.41131,0.4068
,0.40674,0.24063,0.23139,0.24314,0.25456,0.24333,0.2428,0.22867,0.2319
7,0.22645,0.22672,0.22059,0.24189,0.2391,0.21724,0.23227,0.12612,0.122
19,0.13259,0.13567,0.13895,0.14606,0.14056,0.13702,0.1393,0.13875,0.14
424,0.14279,0.13659,0.13677,0.13504,0.15209,0.13619,0.13631,0.41105,0.
41199,0.40277,0.21494,0.25828,0.24907,0.22287,0.25525,0.24549,0.24142,
0.24011,0.23068,0.21373,0.22551,0.22564,0.19953,0.22592,0.21102,0.2132
8,0.22502,0.13058,0.13552,0.13581,0.14362,0.13543,0.14292,0.12682,0.12
989,0.13662,0.13639,0.14529,0.13615,0.12686,0.12396,0.13997,0.1255,0.1
3939,0.13597,0.41099,0.244,0.23962,0.28068,0.28263,0.24012,0.23578,0.2
6121,0.24546,0.24727,0.2433,0.24963,0.23781,0.22785,0.23178,0.22061,0.
22951,0.23097,0.22427,0.13906,0.14409,0.15397,0.13705,0.13829,0.1405,0
.11379,0.13735,0.13582,0.13606,0.13601,0.13601,0.15225,0.14405,0.15157
,0.13579,0.14081,0.078524,0.25121,0.24363,0.26196,0.24676,0.22287,0.22
813,0.24312,0.22522,0.23318,0.27422,0.22507,0.22935,0.23739,0.25007,0.
22723,0.22122,0.23295,0.23147,0.22139,0.14357,0.13169,0.13897,0.13358,
0.13654,0.13619,0.14263,0.14104,0.10292,0.13661,0.13697,0.14053,0.1371
2,0.13925,0.11807,0.12329,0.13736,0.079182,0.27274,0.27936,0.24369,0.2
1917,0.21664,0.23907,0.24549,0.24238,0.24915,0.24038,0.22306,0.22885,0
.22124,0.22108,0.21994,0.23074,0.20794,0.21832,0.13597,0.11928,0.13678
,0.13986,0.13618,0.12646,0.13564,0.13327,0.13211,0.13442,0.13592,0.136
09,0.13661,0.14015,0.12712,0.13709,0.14088,0.13078,0.078213,0.21726,0.
25878,0.27301,0.24316,0.24476,0.24704,0.2514,0.24631,0.22609,0.2419,0.
21763,0.22899,0.23136,0.22878,0.22958,0.23064,0.22877,0.23198,0.13144,
0.13772,0.13573,0.12727,0.15999,0.13535,0.13599,0.13579,0.13537,0.1436
5,0.13442,0.14027,0.14473,0.12581,0.13765,0.13652,0.1354,0.079009,0.07
8588,0.078114,0.18441,0.24439,0.22192,0.24947,0.21574,0.24632,0.2387,0
.26096,0.26105,0.23627,0.21061,0.2168,0.21549,0.22299,0.22815,0.13292,
0.14543,0.13892,0.13549,0.13646,0.14871,0.12715,0.1312,0.13629,0.13613
,0.15478,0.13989,0.12788,0.12867,0.13656,0.14757,0.13432,0.1365,0.0780
47,0.078501,0.078946,0.078601,0.24428,0.2204,0.24729,0.24617,0.24599,0
.24431,0.25836,0.24321,0.24804,0.22806,0.22757,0.21511,0.13645,0.13907
,0.13688,0.12561,0.14108,0.13566,0.13672,0.15637,0.13668,0.13614,0.132
63,0.17694,0.12405,0.13988,0.1363,0.13725,0.13327,0.13624,0.11607,0.13
611,0.078461,0.078288,0.078872,0.079117,0.078468,0.07857,0.22142,0.200
51,0.25792,0.24355,0.23671,0.24741,0.27288,0.24639,0.12699,0.12796,0.1
3845,0.14732,0.13201,0.13756,0.13695,0.13129,0.1456,0.1353,0.12818,0.1
47,0.1361,0.1402,0.14003,0.13603,0.13445,0.13589,0.13192,0.14301,0.136
72,0.13479,0.1431,0.078404,0.078489,0.078802,0.07841,0.078677,0.078752
,0.28943,0.24466,0.24235,0.27054,0.26089,0.078415,0.078555,0.13715,0.1

3492,0.14393,0.13282,0.13603,0.12487,0.12745,0.13107,0.13802,0.13254,0
.12647,0.13185,0.1418,0.13861,0.1364,0.14109,0.14,0.1445,0.1215,0.1327
2,0.13624,0.078162,0.078362,0.078647,0.078288,0.07849,0.079123,0.07877
4,0.078666,0.07855,0.078567,0.24808,0.2479,0.078626,0.07851,0.078793,0
.078409,0.13519,0.13842,0.13411,0.13225,0.1191,0.14733,0.14058,0.13598
,0.13632,0.13612,0.13583,0.13534,0.12206,0.13093,0.14133,0.13601,0.136
03,0.1473,0.13249,0.078647,0.07877,0.078007,0.078871,0.078044,0.078703
,0.078765,0.078706,0.078546,0.078648,0.078197,0.078682,0.078587,0.0785
11,0.07862,0.078691,0.079031,0.13628,0.13375,0.15392,0.14659,0.13479,0
.13628,0.13037,0.15045,0.14933,0.13125,0.14194,0.13685,0.13616,0.13591
,0.13622,0.13607,0.078618,0.078345,0.07836,0.078717,0.07864,0.077914,0
.078292,0.078614,0.078974,0.078405,0.078468,0.078283,0.078584,0.079083
,0.078803,0.078521,0.07852,0.078916,0.078658,0.12208,0.13586,0.11552,0
.13762,0.13605,0.13656,0.13493,0.12627,0.13645,0.13589,0.14122,0.13607
,0.12907,0.078329,0.078816,0.078293,0.078836,0.078587,0.078808,0.07879
8,0.0787,0.078425,0.078755,0.078672,0.078638,0.078246,0.078695,0.07907
3,0.078668,0.078587,0.078843,0.07854,0.078593,0.078777,0.13673,0.13603
,0.13655,0.1403,0.13082,0.14609,0.13699,0.14333,0.14404,0.078698,0.079
133,0.078899,0.078477,0.07835,0.079076,0.078132,0.078673,0.078703,0.07
9175,0.078692,0.077721,0.079054,0.078972,0.078486,0.078552,0.078364,0.
078106,0.078763,0.078847,0.078659,0.078435,0.079104,0.078543,0.079174,
0.14439,0.14071,0.13032,0.13018,0.078001,0.078448,0.079046,0.078585,0.
078642,0.078559,0.07839,0.079031,0.078326,0.078911,0.078558,0.078654,0
.078683,0.078756,0.078308,0.078991,0.078669,0.078825,0.077862,0.078584
,0.079038,0.0783,0.078481,0.078714,0.078454,0.078921,0.078726,0.078569
,0.078936,0.078784,0.078562,0.078485,0.078439,0.078573,0.078768,0.0788
84,0.07859,0.078514,0.078622,0.078056,0.078133,0.078321,0.078656,0.078
685,0.078426,0.078894,0.078383,0.078607,0.078723,0.078314,0.078583,0.0
78726,0.07882,0.078366,0.078638,0.078678,0.078433,0.07827,0.078383,0.0
78715,0.078397,0.078904,0.07814,0.078356,0.07824,0.078565,0.078639,0.0
78318,0.078476,0.078865,0.07874,0.078521,0.0784,0.078705,0.078825,0.07
8636,0.078499,0.078444,0.078502,0.078371,0.078573,0.078797,0.079231,0.
078502,0.078824,0.07852,0.0791,0.078453,0.078635,0.078513,0.078749,0.0
78636,0.078677,0.079159,0.078481,0.078176,0.07899,0.078611,0.078732,0.
078437,0.078813,0.078607,0.078704,0.078892,0.079173,0.078034,0.078343,
0.077792,0.07851,0.078878,0.07862,0.078409,0.078903,0.078831,0.078437,
0.079496,0.079019,0.078863,0.078249,0.078251,0.078607,0.079192,0.07829
4,0.078465,0.078324,0.078262,0.078525,0.078517,0.07924,0.078533,0.0784
,0.079226,0.079302,0.078925,0.078735,0.078971,0.078733,0.078008,0.0781
,0.078602,0.079091,0.078703,0.078985,0.078727,0.078302,0.078622,0.0785
13,0.078721,0.078621,0.078788,0.079023,0.079044,0.078791,0.078745,0.07
886,0.078739,0.078734,0.078743,0.078194,0.078602,0.078143,0.079108,0.0
79157,0.078265,0.078867,0.078689,0.079208,0.078532,0.07855,0.078724,0.
078417,0.078623,0.078536,0.078977,0.078414,0.078299,0.079096,0.078987,
0.078942,0.078393,0.078689,0.078795,0.078308,0.078744,0.078428,0.07884
3,0.078717,0.078588,0.078266,0.078868,0.079018,0.078677,0.078699,0.078
445,0.078007
0.19572,0.19699,0.19563,0.19535,0.19641,0.19642,0.19543,0.19589,0.1956
4,0.19603,0.19585,0.19659,0.19593,0.19581,0.19606,0.1963,0.19636,0.197
01,0.19665,0.19539,0.28441,0.31425,0.296,0.28915,0.19671,0.19561,0.195
71,0.19654,0.19572,0.19599,0.19565,0.19696,0.1952,0.19598,0.19618,0.19
628,0.19553,0.19569,0.19603,0.39207,0.29613,0.31495,0.29376,0.28907,0.

30238,0.29547,0.1965,0.19638,0.19515,0.19644,0.19775,0.19494,0.19621,0
.19566,0.19672,0.19535,0.19577,0.19667,0.19598,0.19555,0.19562,0.3972,
0.39762,0.28752,0.26178,0.29918,0.26744,0.29149,0.2944,0.29334,0.29403
,0.19661,0.19673,0.19626,0.19653,0.19666,0.1954,0.19662,0.19573,0.1955
7,0.19504,0.19738,0.19579,0.19698,0.19574,0.19605,0.38626,0.39778,0.40
005,0.29061,0.31298,0.28325,0.29092,0.29333,0.29642,0.30027,0.30164,0.
29859,0.19601,0.19663,0.19674,0.19529,0.19615,0.1969,0.19577,0.19617,0
.19518,0.19552,0.19641,0.19586,0.19619,0.1968,0.19651,0.1955,0.39957,0
.39701,0.38817,0.39458,0.30673,0.30159,0.28967,0.30547,0.28843,0.27403
,0.30447,0.29771,0.27393,0.28084,0.19694,0.19539,0.1962,0.19624,0.1962
4,0.1952,0.1958,0.19583,0.19588,0.19729,0.19514,0.19586,0.19596,0.1961
6,0.19548,0.19574,0.39175,0.39537,0.3988,0.38678,0.38607,0.38907,0.295
15,0.30154,0.28693,0.30279,0.29051,0.31077,0.31729,0.30527,0.3358,0.29
337,0.1952,0.19629,0.19635,0.19595,0.19597,0.19605,0.19569,0.19736,0.1
9578,0.19612,0.19636,0.196,0.19569,0.19613,0.19598,0.19601,0.1961,0.37
645,0.39064,0.37663,0.38891,0.3889,0.38993,0.29947,0.30377,0.29613,0.2
9198,0.29384,0.29864,0.29214,0.30604,0.31739,0.31097,0.30078,0.19709,0
.19541,0.19601,0.19557,0.19675,0.19621,0.1968,0.19554,0.19584,0.19653,
0.19626,0.19668,0.19634,0.13256,0.14067,0.13714,0.19644,0.39024,0.3836
8,0.39649,0.39455,0.40028,0.40628,0.39671,0.2925,0.29113,0.30701,0.299
75,0.27778,0.27692,0.29615,0.2818,0.27683,0.27607,0.31259,0.19533,0.19
488,0.19662,0.19561,0.19429,0.19607,0.19715,0.19548,0.19633,0.19624,0.
19552,0.13573,0.11474,0.13143,0.13478,0.13433,0.12398,0.19525,0.39355,
0.40968,0.39441,0.40229,0.39163,0.38844,0.39571,0.31498,0.2978,0.29594
,0.30348,0.27837,0.32105,0.30562,0.29388,0.29785,0.29391,0.31533,0.196
65,0.19625,0.19635,0.19693,0.19615,0.19564,0.19447,0.196,0.19577,0.134
15,0.13488,0.1375,0.1347,0.13444,0.13404,0.13596,0.13265,0.14008,0.196
21,0.38785,0.39125,0.38511,0.38929,0.38971,0.39102,0.39273,0.30511,0.2
9426,0.29764,0.29346,0.31234,0.32227,0.29357,0.30937,0.29419,0.30965,0
.29552,0.26746,0.19704,0.19627,0.19563,0.1965,0.19729,0.19561,0.13447,
0.13269,0.1332,0.13405,0.14134,0.14442,0.13382,0.12066,0.13501,0.13605
,0.13595,0.1174,0.39843,0.39369,0.39049,0.39023,0.40026,0.39422,0.3970
3,0.28644,0.29149,0.31515,0.29364,0.27409,0.30516,0.29584,0.30568,0.28
391,0.33108,0.30362,0.29809,0.19726,0.19583,0.1956,0.14447,0.12871,0.1
4257,0.13406,0.13442,0.12963,0.12805,0.13433,0.14178,0.13566,0.12408,0
.12634,0.14033,0.1339,0.13455,0.38651,0.39474,0.39645,0.38729,0.3906,0
.3798,0.39794,0.39825,0.39727,0.29693,0.30169,0.29748,0.31442,0.29526,
0.29653,0.29179,0.29365,0.30702,0.32366,0.29308,0.13361,0.13135,0.1378
9,0.12701,0.13445,0.13458,0.13723,0.13668,0.12758,0.10951,0.13986,0.12
219,0.13663,0.13511,0.1347,0.13972,0.13917,0.13265,0.39776,0.39606,0.3
8958,0.38297,0.40166,0.39084,0.40068,0.38086,0.38807,0.29147,0.26124,0
.22641,0.20022,0.22026,0.21114,0.22192,0.22244,0.21943,0.22651,0.22643
,0.14569,0.13624,0.1354,0.13421,0.12216,0.13528,0.12906,0.13226,0.1465
8,0.13449,0.12637,0.13465,0.12093,0.13885,0.14121,0.13442,0.13468,0.12
18,0.39081,0.38644,0.39053,0.38267,0.39194,0.39421,0.3836,0.38679,0.39
975,0.23751,0.22499,0.21964,0.23165,0.22358,0.22,0.21511,0.20385,0.216
74,0.22959,0.22811,0.13637,0.14504,0.1379,0.13448,0.12783,0.13435,0.14
024,0.13631,0.13634,0.13273,0.14117,0.11612,0.13421,0.14085,0.14476,0.
1425,0.14787,0.12924,0.39243,0.39629,0.3891,0.40883,0.39403,0.39534,0.
38657,0.22934,0.2172,0.19613,0.22001,0.20923,0.21938,0.23739,0.22697,0
.23691,0.24207,0.22207,0.22351,0.22149,0.14142,0.15202,0.12324,0.13347
,0.1386,0.1344,0.12678,0.13724,0.13678,0.11916,0.13771,0.12413,0.12615

,0.11909,0.13364,0.13523,0.15523,0.1341,0.4066,0.39818,0.38622,0.40072
,0.39345,0.38228,0.23645,0.18574,0.24659,0.30028,0.23299,0.20825,0.210
55,0.2443,0.21518,0.2207,0.24912,0.22404,0.22572,0.22473,0.13677,0.143
99,0.1234,0.13394,0.12625,0.1309,0.12947,0.13884,0.13491,0.13555,0.133
06,0.13435,0.12764,0.13296,0.13521,0.13795,0.13474,0.13493,0.38816,0.3
8974,0.39218,0.39023,0.39084,0.23016,0.22162,0.23478,0.24431,0.23207,0
.23237,0.22305,0.22407,0.21945,0.2205,0.21546,0.23402,0.23252,0.21252,
0.22609,0.12368,0.11968,0.13084,0.13423,0.13742,0.14524,0.13935,0.1354
7,0.13804,0.13733,0.14301,0.14188,0.13502,0.13523,0.13339,0.15149,0.13
465,0.1347,0.39193,0.39567,0.38578,0.20529,0.24637,0.2378,0.21153,0.24
379,0.23426,0.23268,0.23174,0.22283,0.20806,0.21968,0.21928,0.19504,0.
22023,0.20498,0.20823,0.21927,0.12865,0.13403,0.13425,0.14258,0.13363,
0.14189,0.1246,0.12787,0.13505,0.13469,0.1445,0.13471,0.12439,0.12169,
0.1387,0.12308,0.13803,0.13386,0.39632,0.23318,0.22764,0.26806,0.27056
,0.22846,0.22735,0.25101,0.23253,0.23525,0.23605,0.24118,0.2307,0.2217
3,0.2259,0.21349,0.22376,0.22431,0.21691,0.13774,0.14306,0.15343,0.135
87,0.13715,0.1389,0.11048,0.13574,0.13438,0.13458,0.13465,0.13473,0.15
193,0.14291,0.15124,0.13437,0.13964,0.072849,0.24098,0.23214,0.25062,0
.2366,0.21125,0.21803,0.23399,0.21424,0.22199,0.26381,0.21792,0.22193,
0.23097,0.24112,0.22028,0.21609,0.22614,0.22326,0.21505,0.14205,0.1295
2,0.13793,0.13156,0.13482,0.1345,0.14111,0.13969,0.099058,0.13528,0.13
549,0.1392,0.13547,0.13797,0.11504,0.12066,0.13574,0.073272,0.26094,0.
26576,0.23288,0.2075,0.20768,0.22738,0.23454,0.23074,0.23768,0.22835,0
.21683,0.22325,0.21432,0.21504,0.2147,0.22435,0.20197,0.21207,0.13443,
0.11635,0.13504,0.13849,0.1346,0.12441,0.13409,0.13154,0.13024,0.13276
,0.13436,0.13428,0.13524,0.1388,0.12461,0.13556,0.13962,0.12886,0.0725
66,0.20677,0.24793,0.26156,0.23199,0.23238,0.23512,0.23763,0.23411,0.2
1598,0.22983,0.21141,0.22212,0.22525,0.22149,0.22251,0.22313,0.22207,0
.22452,0.12948,0.13621,0.13403,0.12486,0.16009,0.13371,0.13446,0.13399
,0.13363,0.14259,0.13246,0.13904,0.14384,0.1235,0.13631,0.13476,0.1339
6,0.073231,0.073112,0.072637,0.17588,0.23372,0.21272,0.23746,0.20763,0
.23452,0.22712,0.24923,0.25032,0.23003,0.2054,0.21027,0.20909,0.21682,
0.22157,0.13077,0.14429,0.13751,0.13392,0.1346,0.14755,0.12487,0.12927
,0.13443,0.13462,0.15449,0.13845,0.12576,0.12665,0.13466,0.14691,0.132
81,0.13477,0.072559,0.072691,0.073385,0.072934,0.23402,0.21278,0.23702
,0.23154,0.23557,0.23489,0.24609,0.23197,0.23431,0.22284,0.2219,0.2094
5,0.13477,0.13759,0.1354,0.12343,0.13961,0.13417,0.13529,0.15645,0.134
98,0.13454,0.13051,0.17844,0.12182,0.13849,0.1349,0.13551,0.13155,0.13
466,0.11317,0.13457,0.072908,0.072669,0.073001,0.073271,0.072961,0.073
022,0.21199,0.18981,0.24662,0.23105,0.22698,0.23492,0.26163,0.23644,0.
1248,0.12601,0.13667,0.14621,0.13002,0.13596,0.13523,0.1294,0.1448,0.1
3359,0.1259,0.14625,0.13447,0.13927,0.13864,0.13457,0.1324,0.13458,0.1
2998,0.14189,0.13535,0.13295,0.14195,0.072852,0.073097,0.073229,0.0728
31,0.072995,0.073135,0.27457,0.23411,0.23145,0.26211,0.24628,0.072949,
0.072932,0.13555,0.13305,0.14272,0.13121,0.13433,0.12267,0.12542,0.129
16,0.13663,0.13086,0.12427,0.12973,0.14033,0.13692,0.13486,0.13969,0.1
3859,0.14356,0.11873,0.13086,0.13447,0.072549,0.072646,0.07304,0.07266
6,0.073137,0.073515,0.073227,0.073251,0.072877,0.072929,0.23782,0.2360
9,0.073203,0.072757,0.07331,0.072722,0.13372,0.13668,0.13211,0.13036,0
.11626,0.14643,0.1394,0.13459,0.13468,0.13448,0.13428,0.13395,0.11927,
0.1291,0.13998,0.13439,0.13439,0.14657,0.13054,0.073174,0.072988,0.072
789,0.073273,0.07221,0.073158,0.073463,0.073201,0.073089,0.072935,0.07

2699,0.07308,0.073065,0.07271,0.073097,0.07295,0.073453,0.13489,0.1318
1,0.15341,0.14556,0.13281,0.13442,0.12837,0.14997,0.14885,0.12939,0.14
086,0.13551,0.1343,0.13437,0.13485,0.13429,0.073123,0.072619,0.072804,
0.073284,0.073101,0.072464,0.072756,0.073106,0.073525,0.072898,0.07270
8,0.072698,0.072919,0.073557,0.073411,0.072901,0.0732,0.073338,0.07309
7,0.11949,0.13424,0.11244,0.13602,0.13463,0.13478,0.13325,0.12399,0.13
462,0.13426,0.14006,0.13427,0.12694,0.072525,0.073179,0.072806,0.07321
9,0.072776,0.073212,0.073256,0.0732,0.072969,0.073192,0.073174,0.07310
9,0.072706,0.073112,0.073534,0.073062,0.072972,0.073162,0.07293,0.0728
09,0.07311,0.1351,0.13459,0.13523,0.13909,0.12901,0.14533,0.13508,0.14
227,0.14304,0.073043,0.073449,0.073313,0.072969,0.072772,0.073441,0.07
2419,0.073033,0.072943,0.07359,0.073047,0.072206,0.073297,0.073485,0.0
72969,0.072843,0.072759,0.072402,0.073157,0.073099,0.072997,0.072778,0
.073574,0.073106,0.073436,0.14338,0.13958,0.12854,0.12802,0.07261,0.07
2912,0.07321,0.072953,0.07314,0.073238,0.073053,0.07327,0.072808,0.073
053,0.073093,0.072829,0.073261,0.07321,0.072949,0.073445,0.07312,0.073
322,0.072172,0.073052,0.073532,0.07266,0.072789,0.073081,0.072878,0.07
319,0.073155,0.072842,0.073039,0.073005,0.072851,0.072888,0.072987,0.0
73016,0.073294,0.073216,0.073006,0.073033,0.073015,0.072622,0.072806,0
.072637,0.07295,0.073094,0.072786,0.073214,0.072644,0.072788,0.073174,
0.072737,0.072799,0.073276,0.073015,0.072657,0.073138,0.073143,0.07285
2,0.072726,0.072662,0.073033,0.072896,0.073241,0.072438,0.072676,0.072
768,0.072913,0.073203,0.072803,0.072862,0.073218,0.073348,0.072812,0.0
72848,0.072998,0.07313,0.072965,0.072955,0.072776,0.073096,0.072953,0.
072971,0.073356,0.073588,0.072766,0.073098,0.072938,0.073406,0.072823,
0.073042,0.073224,0.073187,0.072803,0.072996,0.073533,0.072917,0.07253
2,0.073336,0.073044,0.073218,0.072738,0.073037,0.072956,0.07306,0.0733
78,0.073444,0.072437,0.073035,0.072166,0.073068,0.073267,0.072842,0.07
2765,0.073286,0.073214,0.072409,0.07389,0.073456,0.073108,0.072657,0.0
72498,0.072665,0.073644,0.072826,0.073087,0.072985,0.072874,0.072968,0
.072833,0.073526,0.072986,0.07288,0.073578,0.073746,0.07317,0.073055,0
.07325,0.073213,0.072453,0.072601,0.073054,0.073458,0.073144,0.073196,
0.073109,0.072658,0.073117,0.072954,0.073037,0.073062,0.07323,0.073332
,0.073431,0.073243,0.073112,0.073056,0.073087,0.072984,0.073149,0.0724
42,0.073052,0.07253,0.073548,0.073578,0.072799,0.073276,0.072976,0.073
496,0.073005,0.072502,0.07302,0.072795,0.072897,0.072951,0.073499,0.07
2919,0.072515,0.073555,0.073301,0.073307,0.072853,0.072867,0.073203,0.
072655,0.073026,0.072859,0.073342,0.073274,0.072924,0.072388,0.073308,
0.073261,0.072832,0.073162,0.072771,0.072414
0.19763,0.19864,0.19745,0.19714,0.19813,0.198,0.19723,0.19758,0.19748,
0.19812,0.19787,0.19817,0.19776,0.19812,0.19775,0.19811,0.19815,0.1986
6,0.19842,0.19755,0.28118,0.31052,0.29192,0.28715,0.19867,0.19803,0.19
814,0.19836,0.19735,0.19799,0.19768,0.19808,0.19732,0.19779,0.1982,0.1
9821,0.19752,0.19764,0.1981,0.37634,0.29118,0.30873,0.28924,0.28433,0.
29636,0.29057,0.19806,0.19839,0.1973,0.19838,0.19911,0.19653,0.1982,0.
19734,0.19881,0.19668,0.19796,0.19879,0.19774,0.19709,0.19754,0.38449,
0.38309,0.28398,0.25984,0.29382,0.26478,0.28616,0.2907,0.28964,0.2899,
0.19822,0.19868,0.19833,0.198,0.19913,0.19762,0.1987,0.19758,0.19758,0
.19714,0.1993,0.19797,0.19917,0.19758,0.19777,0.37817,0.3818,0.3909,0.
28865,0.30753,0.2795,0.28615,0.29004,0.29134,0.29597,0.29749,0.29428,0
.19787,0.19866,0.19854,0.19756,0.19787,0.19878,0.19745,0.19858,0.19711
,0.19791,0.19807,0.19788,0.19794,0.19874,0.19825,0.19739,0.39038,0.386

53,0.38149,0.38489,0.30433,0.29863,0.28655,0.30066,0.28515,0.27106,0.2
9917,0.29339,0.27035,0.27745,0.19866,0.19729,0.19804,0.19811,0.19833,0
.19733,0.19772,0.1973,0.1974,0.19938,0.19735,0.19781,0.19828,0.198,0.1
976,0.19836,0.38379,0.38802,0.39371,0.37611,0.3765,0.37899,0.28963,0.2
9722,0.28143,0.29991,0.28791,0.30491,0.312,0.29796,0.32541,0.28783,0.1
9719,0.19823,0.19865,0.19761,0.19736,0.19811,0.19776,0.19934,0.198,0.1
9791,0.19812,0.19772,0.19785,0.19804,0.19804,0.19801,0.19784,0.36492,0
.37643,0.36578,0.37893,0.37513,0.37998,0.29337,0.29784,0.29192,0.2869,
0.29046,0.29687,0.28636,0.30285,0.31156,0.30544,0.2975,0.19863,0.19752
,0.19818,0.19753,0.19865,0.19803,0.19868,0.19754,0.19814,0.1985,0.1984
7,0.19849,0.19828,0.13098,0.13988,0.13589,0.19823,0.37927,0.37192,0.38
857,0.38355,0.3914,0.39184,0.38771,0.28518,0.28757,0.30115,0.29468,0.2
7528,0.2731,0.29212,0.27743,0.27495,0.27369,0.30411,0.19699,0.19702,0.
19825,0.19785,0.19646,0.1978,0.19851,0.19776,0.19848,0.19778,0.19755,0
.13436,0.11217,0.13013,0.13354,0.13301,0.12192,0.19736,0.38192,0.39859
,0.38061,0.38967,0.38112,0.37779,0.38236,0.30854,0.29313,0.29166,0.299
32,0.27453,0.31549,0.30017,0.29011,0.29162,0.28919,0.30709,0.19857,0.1
9846,0.19826,0.19857,0.19813,0.19748,0.19691,0.19789,0.19727,0.13287,0
.13315,0.1362,0.13325,0.13313,0.1326,0.13474,0.13111,0.139,0.1981,0.37
935,0.38294,0.37367,0.37703,0.38045,0.37909,0.38178,0.29882,0.28819,0.
2951,0.2901,0.30609,0.315,0.29024,0.30415,0.28974,0.30628,0.29006,0.26
543,0.19906,0.19791,0.1977,0.19822,0.19903,0.19733,0.13319,0.13105,0.1
3164,0.1327,0.14052,0.14368,0.13251,0.11882,0.13356,0.13477,0.13467,0.
11501,0.38721,0.38382,0.38236,0.37141,0.39083,0.38593,0.38715,0.28273,
0.28718,0.30802,0.28604,0.27001,0.29924,0.2927,0.30126,0.27926,0.32379
,0.29829,0.29399,0.1992,0.19743,0.19766,0.1438,0.12725,0.14128,0.13269
,0.13331,0.12806,0.12618,0.13288,0.14016,0.13451,0.12241,0.12449,0.139
37,0.13233,0.13329,0.3722,0.38586,0.39131,0.3744,0.38187,0.37028,0.384
6,0.38505,0.3872,0.29252,0.29527,0.29074,0.30825,0.29186,0.29184,0.287
1,0.28926,0.30324,0.3177,0.28802,0.13193,0.12999,0.13635,0.12517,0.133
14,0.13305,0.13631,0.13539,0.12571,0.1069,0.13864,0.12033,0.13531,0.13
373,0.13331,0.13849,0.13796,0.13136,0.38275,0.38751,0.37908,0.37344,0.
39093,0.37508,0.3894,0.37548,0.37764,0.28477,0.25852,0.22199,0.1963,0.
21471,0.20744,0.21723,0.2181,0.21444,0.22136,0.22306,0.14515,0.13496,0
.13412,0.13312,0.12019,0.13393,0.12726,0.13045,0.14619,0.133,0.12444,0
.13321,0.11884,0.13787,0.14013,0.13304,0.13329,0.11969,0.37803,0.37471
,0.37461,0.36885,0.38288,0.38663,0.37874,0.37744,0.38999,0.23079,0.221
3,0.21573,0.22828,0.21828,0.21595,0.21197,0.19996,0.21444,0.22691,0.22
281,0.13521,0.1443,0.13681,0.13297,0.12624,0.13292,0.13897,0.13509,0.1
3514,0.1313,0.14012,0.11369,0.13288,0.13992,0.14403,0.14156,0.14709,0.
12725,0.38279,0.38709,0.37987,0.39738,0.38134,0.3796,0.37344,0.22093,0
.20988,0.18816,0.21502,0.2059,0.21452,0.23165,0.22018,0.233,0.23554,0.
21676,0.21996,0.21626,0.14054,0.15177,0.12119,0.13229,0.13739,0.13321,
0.12496,0.13593,0.13549,0.11679,0.13644,0.12207,0.12423,0.11661,0.1322
3,0.13387,0.15497,0.13268,0.38881,0.38347,0.37637,0.38673,0.38532,0.36
536,0.22836,0.17863,0.2379,0.2944,0.22575,0.20516,0.20643,0.2397,0.212
44,0.21645,0.24538,0.21697,0.22129,0.22026,0.13565,0.1435,0.12141,0.13
24,0.1244,0.12925,0.12748,0.13761,0.13338,0.13426,0.13141,0.13288,0.12
566,0.13144,0.13386,0.13681,0.13348,0.13365,0.37904,0.37703,0.38243,0.
37612,0.38144,0.22176,0.21308,0.22852,0.2341,0.22462,0.22332,0.21908,0
.21955,0.21588,0.21604,0.21054,0.23036,0.22701,0.209,0.2203,0.12165,0.
11736,0.12922,0.133,0.13633,0.14475,0.13814,0.13395,0.13696,0.13607,0.

14176,0.14055,0.1335,0.13366,0.13217,0.15119,0.13334,0.13337,0.38117,0
.38512,0.37304,0.19795,0.23824,0.23099,0.20585,0.23412,0.22583,0.22406
,0.22432,0.21759,0.20487,0.2139,0.21444,0.19103,0.21422,0.20186,0.2041
1,0.21467,0.1269,0.13276,0.13287,0.14164,0.13205,0.14114,0.12265,0.126
27,0.13368,0.13333,0.14357,0.13327,0.12252,0.11948,0.13742,0.12124,0.1
3685,0.13268,0.38166,0.22419,0.21766,0.2604,0.26355,0.22139,0.22075,0.
2425,0.22786,0.2245,0.23112,0.23554,0.2257,0.21704,0.22181,0.20917,0.2
1785,0.22,0.21252,0.13673,0.14199,0.15283,0.1345,0.13597,0.1378,0.1078
4,0.13435,0.13298,0.1335,0.13336,0.13325,0.15158,0.14191,0.15052,0.132
97,0.13838,0.068089,0.23282,0.22337,0.24367,0.22868,0.20308,0.20963,0.
2274,0.20649,0.21323,0.25506,0.21263,0.21735,0.22623,0.23547,0.21551,0
.21239,0.22142,0.21827,0.21103,0.14106,0.12792,0.13641,0.12988,0.1335,
0.13329,0.14018,0.13851,0.095816,0.13385,0.13409,0.13814,0.13424,0.136
67,0.11271,0.11843,0.13432,0.068474,0.25345,0.25757,0.22439,0.20062,0.
20047,0.22027,0.22339,0.22457,0.22954,0.22157,0.21314,0.21808,0.21012,
0.21058,0.20919,0.21817,0.19767,0.20831,0.13302,0.11402,0.13365,0.1372
6,0.13326,0.12247,0.13256,0.12988,0.12855,0.13139,0.13279,0.13301,0.13
38,0.13735,0.12275,0.13424,0.13857,0.12724,0.06785,0.19896,0.24067,0.2
5366,0.22207,0.22574,0.22598,0.23311,0.22683,0.2082,0.22277,0.20671,0.
21641,0.22047,0.21705,0.21836,0.21832,0.21759,0.21951,0.12793,0.13456,
0.13291,0.12306,0.16007,0.13212,0.13307,0.13264,0.13223,0.1418,0.13099
,0.13801,0.14308,0.12156,0.13515,0.1334,0.13249,0.068805,0.0684,0.0676
16,0.17078,0.22561,0.20611,0.22857,0.20167,0.22618,0.21838,0.23967,0.2
4272,0.22313,0.20239,0.20711,0.20573,0.21296,0.21668,0.12926,0.14345,0
.13614,0.13261,0.13332,0.1467,0.12301,0.12774,0.13281,0.1334,0.15444,0
.13713,0.12406,0.12485,0.13345,0.14644,0.13128,0.13329,0.068103,0.0679
67,0.068629,0.068056,0.22622,0.20321,0.22976,0.22502,0.22683,0.22749,0
.23677,0.22567,0.22695,0.21897,0.21758,0.20399,0.13322,0.13629,0.13417
,0.12172,0.13827,0.13291,0.13368,0.15655,0.13376,0.13315,0.129,0.17919
,0.11987,0.1372,0.13341,0.13412,0.13,0.1334,0.11056,0.13318,0.068073,0
.068243,0.068184,0.068488,0.068217,0.068221,0.2046,0.18232,0.24053,0.2
2262,0.21948,0.22945,0.25343,0.22819,0.12262,0.12411,0.13536,0.14541,0
.12841,0.13449,0.13387,0.12768,0.14384,0.1321,0.12387,0.14538,0.13327,
0.13826,0.13756,0.13315,0.13088,0.13329,0.12843,0.14104,0.13402,0.1314
4,0.1411,0.068293,0.068493,0.06874,0.068247,0.06832,0.068516,0.26401,0
.2259,0.22572,0.25465,0.23701,0.068322,0.068313,0.13421,0.13181,0.1419
9,0.12957,0.13282,0.12067,0.12362,0.12761,0.13548,0.12928,0.12221,0.12
795,0.13955,0.13573,0.13335,0.13887,0.13747,0.14237,0.11671,0.1294,0.1
3281,0.068008,0.067892,0.068449,0.068034,0.06828,0.068726,0.068553,0.0
68601,0.068274,0.068294,0.22834,0.22918,0.068732,0.068044,0.068583,0.0
68258,0.13242,0.1356,0.13083,0.12885,0.11384,0.1454,0.13829,0.13337,0.
13315,0.13317,0.13311,0.13254,0.11697,0.12749,0.13889,0.13301,0.13301,
0.14579,0.12884,0.068329,0.068322,0.068153,0.068406,0.067458,0.068336,
0.068591,0.068584,0.068467,0.068204,0.068119,0.068351,0.068376,0.06806
4,0.068358,0.068369,0.068545,0.13364,0.13012,0.15309,0.14485,0.13144,0
.13315,0.12678,0.14951,0.14807,0.12769,0.13958,0.13418,0.13301,0.13311
,0.13349,0.13313,0.068657,0.067972,0.068328,0.068855,0.068338,0.067656
,0.067837,0.068409,0.068958,0.068517,0.06798,0.068054,0.068353,0.06860
7,0.069002,0.06819,0.068488,0.06874,0.068414,0.11723,0.13291,0.10986,0
.13469,0.13302,0.13343,0.13186,0.122,0.13325,0.13269,0.13897,0.13284,0
.12505,0.068225,0.068686,0.068091,0.068442,0.068318,0.068301,0.068482,
0.068451,0.068298,0.068827,0.068831,0.068403,0.068067,0.06855,0.068823

,0.068277,0.068614,0.068313,0.068211,0.068237,0.068475,0.13351,0.1334,
0.13392,0.13793,0.12747,0.14445,0.13372,0.14108,0.14227,0.068465,0.068
729,0.068699,0.068305,0.06844,0.068827,0.067611,0.068358,0.068095,0.06
8703,0.068401,0.067619,0.068873,0.068726,0.068281,0.068115,0.068236,0.
067842,0.068398,0.06847,0.068213,0.068047,0.068552,0.068527,0.068695,0
.14263,0.1384,0.12671,0.12636,0.067701,0.068224,0.068476,0.0684,0.0686
23,0.068647,0.068403,0.068432,0.068227,0.0685,0.068424,0.068153,0.0685
16,0.068572,0.068137,0.068858,0.068244,0.068456,0.067502,0.068421,0.06
8829,0.068109,0.068076,0.068359,0.068136,0.068527,0.068499,0.068043,0.
068453,0.068543,0.068102,0.068048,0.068256,0.068496,0.068544,0.068545,
0.068469,0.068353,0.068362,0.068117,0.068094,0.068074,0.068202,0.06841
3,0.067925,0.068597,0.068052,0.067893,0.068464,0.068137,0.068097,0.068
46,0.068633,0.068077,0.0686,0.068571,0.068119,0.067985,0.068107,0.0681
53,0.067963,0.068488,0.067816,0.067928,0.068042,0.068377,0.068188,0.06
827,0.068269,0.068388,0.068841,0.068268,0.068098,0.068472,0.068335,0.0
68317,0.068052,0.068278,0.068475,0.068494,0.068212,0.068741,0.069024,0
.068264,0.068481,0.06852,0.068616,0.06811,0.068384,0.068427,0.068577,0
.068208,0.068189,0.068862,0.068474,0.067984,0.068725,0.068247,0.068444
,0.068025,0.068248,0.068332,0.068466,0.068635,0.068708,0.067653,0.0685
5,0.067403,0.068408,0.068712,0.068184,0.06814,0.068494,0.06859,0.06774
9,0.069026,0.068632,0.068466,0.067926,0.067644,0.068008,0.068962,0.068
14,0.068205,0.068384,0.068304,0.068425,0.068294,0.068797,0.068149,0.06
8215,0.068754,0.069073,0.068546,0.068311,0.068437,0.068429,0.067742,0.
068067,0.068426,0.06892,0.068439,0.068468,0.068466,0.068174,0.068315,0
.067964,0.068352,0.068499,0.068473,0.068518,0.068809,0.068768,0.068256
,0.068289,0.068278,0.068166,0.068527,0.067632,0.068337,0.068166,0.0688
02,0.068907,0.068159,0.06866,0.068377,0.06888,0.068115,0.067946,0.0685
98,0.068011,0.068189,0.068323,0.068598,0.068481,0.067846,0.068802,0.06
8569,0.068386,0.068389,0.068236,0.068427,0.068051,0.068404,0.06833,0.0
68736,0.068794,0.068369,0.067742,0.068611,0.068616,0.068153,0.068426,0
.068188,0.067903
0.19926,0.20005,0.1994,0.19854,0.20006,0.19954,0.19892,0.19981,0.19897
,0.2002,0.1994,0.19962,0.19948,0.19943,0.19998,0.20008,0.19959,0.20001
,0.19956,0.19921,0.27738,0.30708,0.28793,0.28521,0.20001,0.2003,0.1996
5,0.19968,0.19908,0.19991,0.19933,0.19957,0.19899,0.19903,0.2002,0.199
58,0.19897,0.19919,0.19922,0.37091,0.28986,0.30373,0.28782,0.28036,0.2
946,0.28831,0.19958,0.19961,0.19859,0.19969,0.2005,0.19791,0.20015,0.1
9936,0.20046,0.19864,0.19929,0.20012,0.19957,0.19892,0.19922,0.37371,0
.36966,0.28031,0.26058,0.29272,0.26336,0.28174,0.28711,0.28705,0.28652
,0.19979,0.20031,0.19981,0.19969,0.2005,0.19885,0.20027,0.19974,0.1992
3,0.19886,0.20091,0.1998,0.20035,0.1993,0.19894,0.36877,0.37441,0.3817
5,0.28721,0.30199,0.27687,0.28527,0.2886,0.28716,0.29103,0.29415,0.290
13,0.19961,0.2003,0.19974,0.19921,0.19941,0.20034,0.19892,0.20013,0.19
915,0.19976,0.19945,0.19914,0.19992,0.20017,0.19986,0.1989,0.38355,0.3
8047,0.37569,0.37634,0.30025,0.29406,0.28515,0.29367,0.28099,0.26926,0
.29541,0.2901,0.26792,0.27457,0.2,0.19868,0.1997,0.20008,0.19993,0.199
24,0.1992,0.19869,0.19902,0.20138,0.19892,0.19957,0.19996,0.19977,0.19
924,0.19951,0.37692,0.37995,0.38162,0.36899,0.36769,0.37022,0.28678,0.
29283,0.28017,0.29753,0.28429,0.30114,0.30903,0.29369,0.32018,0.28461,
0.19853,0.19989,0.20019,0.1991,0.19855,0.19972,0.1998,0.20048,0.19959,
0.19941,0.19955,0.19927,0.19937,0.19999,0.19944,0.19928,0.19923,0.3587
,0.36918,0.35792,0.37908,0.36917,0.37172,0.2884,0.29541,0.2891,0.28395

,0.28812,0.29308,0.28291,0.3003,0.30527,0.30452,0.29442,0.20048,0.1989
,0.19997,0.19929,0.20014,0.19971,0.20058,0.19916,0.19912,0.20004,0.199
95,0.20017,0.20003,0.1298,0.13901,0.13506,0.20025,0.37269,0.36723,0.38
17,0.37828,0.38651,0.38499,0.37816,0.28261,0.28368,0.2979,0.29259,0.27
355,0.27052,0.28664,0.27414,0.27347,0.27146,0.29898,0.19894,0.1983,0.1
9954,0.1989,0.19828,0.19931,0.20026,0.19919,0.20008,0.19961,0.19958,0.
13321,0.11041,0.12894,0.13272,0.1319,0.12045,0.19918,0.37278,0.39375,0
.37289,0.3763,0.37709,0.37078,0.37497,0.30258,0.28892,0.28901,0.2973,0
.2708,0.31194,0.29708,0.28899,0.28842,0.28509,0.30309,0.20032,0.20025,
0.20021,0.20022,0.1996,0.19922,0.19873,0.19943,0.19867,0.13173,0.13204
,0.13529,0.13223,0.13193,0.1314,0.13337,0.12991,0.1379,0.19988,0.37335
,0.37384,0.36556,0.36789,0.37458,0.37104,0.37417,0.29544,0.28504,0.291
6,0.28678,0.30146,0.30938,0.28559,0.301,0.28586,0.30272,0.28619,0.2645
2,0.20047,0.19969,0.19913,0.19947,0.20056,0.19918,0.13193,0.1301,0.130
67,0.13169,0.13943,0.14275,0.13153,0.11706,0.13218,0.1336,0.13378,0.11
353,0.37696,0.37404,0.37641,0.36785,0.3791,0.38207,0.38021,0.27986,0.2
8364,0.30547,0.2838,0.2671,0.29769,0.28767,0.29836,0.27907,0.32043,0.2
9448,0.29119,0.20071,0.1993,0.19947,0.14323,0.12604,0.1403,0.13159,0.1
3238,0.12651,0.12475,0.13151,0.1393,0.13335,0.12082,0.12295,0.13872,0.
13139,0.13204,0.3695,0.37863,0.38426,0.36715,0.374,0.36409,0.37677,0.3
8091,0.37957,0.29043,0.29384,0.28863,0.30662,0.28997,0.28951,0.2836,0.
28721,0.30054,0.31733,0.2873,0.13076,0.12868,0.13513,0.12378,0.13199,0
.13184,0.13515,0.13441,0.1241,0.10465,0.13724,0.11871,0.1343,0.13268,0
.13184,0.13763,0.13709,0.13025,0.37516,0.38657,0.3738,0.36647,0.38301,
0.37021,0.38037,0.36421,0.36912,0.28208,0.25669,0.21762,0.19283,0.2107
9,0.20518,0.21414,0.21513,0.21052,0.2177,0.22016,0.14433,0.13364,0.133
12,0.13204,0.11825,0.1328,0.12604,0.12944,0.14553,0.13227,0.12288,0.13
214,0.11716,0.13709,0.13937,0.13205,0.13212,0.11815,0.36896,0.36528,0.
37187,0.36728,0.37595,0.38056,0.37135,0.37305,0.38573,0.22496,0.21801,
0.21277,0.22449,0.21423,0.21153,0.20793,0.19767,0.21072,0.22304,0.2192
4,0.1342,0.14375,0.1364,0.13151,0.12496,0.13191,0.13813,0.13394,0.1340
7,0.13021,0.13956,0.11158,0.13181,0.1391,0.14342,0.14049,0.1467,0.1259
,0.37717,0.37927,0.37192,0.38868,0.37853,0.37654,0.36969,0.21646,0.204
87,0.18402,0.21096,0.20216,0.21218,0.22652,0.21652,0.22903,0.23338,0.2
1248,0.21617,0.21258,0.13961,0.1517,0.11959,0.13112,0.13628,0.13198,0.
12315,0.13488,0.13456,0.11501,0.13516,0.12045,0.12258,0.11469,0.13091,
0.13278,0.15471,0.13145,0.38227,0.37824,0.36637,0.37882,0.37631,0.3590
8,0.22248,0.17383,0.23204,0.2881,0.22,0.20242,0.20305,0.23715,0.21048,
0.21297,0.24167,0.21405,0.21759,0.21623,0.13453,0.14288,0.1198,0.13162
,0.12271,0.12782,0.12634,0.13679,0.13217,0.13301,0.13017,0.13168,0.124
05,0.13006,0.13288,0.13614,0.13219,0.13278,0.376,0.37089,0.37737,0.368
46,0.37289,0.21543,0.20517,0.22372,0.22794,0.21671,0.21803,0.21709,0.2
1632,0.21203,0.21238,0.20766,0.22639,0.22457,0.20702,0.21654,0.11998,0
.1154,0.12802,0.13197,0.13535,0.14413,0.13745,0.13294,0.13601,0.13487,
0.14103,0.14013,0.13233,0.13235,0.13102,0.15121,0.13209,0.13231,0.3723
8,0.37552,0.36725,0.19303,0.23147,0.22403,0.19916,0.23182,0.21945,0.21
692,0.2171,0.21381,0.20176,0.21085,0.2124,0.18904,0.21195,0.19898,0.20
181,0.21197,0.12564,0.13153,0.13183,0.141,0.13089,0.1403,0.121,0.12466
,0.13256,0.13242,0.14281,0.13216,0.12084,0.118,0.13657,0.11939,0.13605
,0.1313,0.37165,0.22007,0.21439,0.25355,0.25557,0.21465,0.21563,0.2363
7,0.22255,0.22047,0.22815,0.23274,0.22441,0.21463,0.21968,0.2051,0.213
94,0.21673,0.20929,0.13566,0.14082,0.15253,0.13314,0.13479,0.13696,0.1

0564,0.13332,0.13188,0.13237,0.13221,0.13233,0.15124,0.14119,0.14995,0
.13194,0.13762,0.064297,0.22736,0.2177,0.23899,0.22507,0.19893,0.20598
,0.22369,0.20081,0.20782,0.25092,0.21089,0.21313,0.22313,0.23377,0.212
79,0.21008,0.21657,0.21503,0.20778,0.13989,0.1265,0.13542,0.12886,0.13
227,0.13206,0.13929,0.13794,0.093299,0.13279,0.13291,0.13705,0.13326,0
.1355,0.11082,0.11686,0.13315,0.064388,0.24556,0.25087,0.21868,0.19572
,0.19497,0.21508,0.21803,0.2182,0.22511,0.2176,0.20837,0.21552,0.20821
,0.20638,0.20602,0.21518,0.19473,0.20574,0.13176,0.1123,0.13255,0.1362
2,0.13201,0.12088,0.13135,0.1289,0.12704,0.13035,0.13164,0.13173,0.132
86,0.13648,0.12112,0.13292,0.13776,0.12577,0.063939,0.19432,0.23454,0.
24864,0.21701,0.21929,0.22054,0.22626,0.22133,0.20142,0.216,0.20477,0.
21237,0.21615,0.21416,0.21333,0.21595,0.21419,0.21551,0.1265,0.1336,0.
1315,0.1217,0.15987,0.13083,0.13174,0.13143,0.13108,0.14076,0.12976,0.
13705,0.14259,0.12013,0.13423,0.13221,0.13126,0.064886,0.064528,0.0638
62,0.16658,0.21926,0.19995,0.22212,0.19714,0.22206,0.21365,0.23348,0.2
3837,0.22025,0.19958,0.20399,0.2017,0.2085,0.21263,0.12782,0.14275,0.1
3493,0.13149,0.13227,0.14633,0.12125,0.12656,0.13144,0.13225,0.15412,0
.1363,0.12266,0.12336,0.13248,0.14562,0.12994,0.13216,0.064222,0.06419
,0.064755,0.064233,0.22132,0.19801,0.22289,0.22071,0.21969,0.22153,0.2
3002,0.21886,0.22146,0.21663,0.2152,0.20116,0.13203,0.13527,0.13297,0.
11996,0.13744,0.13194,0.13272,0.15632,0.13255,0.13186,0.12763,0.17975,
0.11829,0.13609,0.13222,0.13294,0.12864,0.13227,0.10853,0.1321,0.06437
9,0.064551,0.064399,0.064753,0.064255,0.064416,0.19946,0.17681,0.23119
,0.21618,0.21792,0.22441,0.24342,0.22287,0.12087,0.12266,0.13435,0.144
85,0.12702,0.13355,0.13269,0.12617,0.14337,0.13073,0.12239,0.14488,0.1
3251,0.13766,0.13651,0.13173,0.12964,0.13205,0.12716,0.14031,0.13271,0
.13011,0.14043,0.064454,0.064678,0.064694,0.064219,0.064464,0.064616,0
.25843,0.22043,0.2203,0.24806,0.23405,0.064431,0.064381,0.13309,0.1307
6,0.141,0.12822,0.13184,0.11904,0.12217,0.12627,0.13442,0.12791,0.1205
7,0.12677,0.13872,0.13474,0.13205,0.13814,0.13677,0.14168,0.11488,0.12
804,0.13181,0.064229,0.064309,0.064522,0.063958,0.064359,0.065106,0.06
47,0.064871,0.064348,0.064369,0.22278,0.22136,0.064905,0.064256,0.0646
82,0.064388,0.13145,0.1345,0.12963,0.12767,0.11194,0.14477,0.13754,0.1
3206,0.13193,0.13183,0.13198,0.13122,0.11542,0.12607,0.13815,0.13192,0
.13187,0.1454,0.12732,0.064325,0.064513,0.064207,0.064644,0.063951,0.0
64521,0.064705,0.064418,0.064627,0.064371,0.064342,0.064529,0.064446,0
.064067,0.064577,0.064407,0.064837,0.13241,0.12884,0.15297,0.1444,0.12
999,0.13238,0.12546,0.14924,0.14759,0.12623,0.1386,0.13287,0.13192,0.1
3188,0.13235,0.13215,0.064794,0.064214,0.064538,0.064911,0.064442,0.06
3744,0.063781,0.06485,0.065236,0.064651,0.06422,0.06424,0.064588,0.064
451,0.065262,0.064337,0.064831,0.064837,0.064668,0.1154,0.13185,0.1076
6,0.13372,0.13205,0.13217,0.13091,0.12051,0.13198,0.13147,0.1383,0.131
68,0.12358,0.064369,0.064852,0.064138,0.064651,0.064685,0.064628,0.064
684,0.064475,0.064429,0.0651,0.065236,0.064563,0.064263,0.06459,0.0649
83,0.064441,0.064628,0.064569,0.064447,0.064467,0.064775,0.13212,0.132
13,0.13267,0.13682,0.12583,0.14394,0.13261,0.1403,0.14127,0.064532,0.0
64726,0.064918,0.064729,0.064633,0.064719,0.063907,0.06448,0.064118,0.
064724,0.064615,0.063853,0.06504,0.064911,0.064517,0.064271,0.064332,0
.063843,0.064382,0.064594,0.064164,0.064323,0.064616,0.064555,0.064708
,0.14167,0.13762,0.12531,0.12485,0.064007,0.064336,0.064764,0.064619,0
.064794,0.064608,0.06462,0.064515,0.064413,0.064663,0.064422,0.064407,
0.064572,0.064867,0.064229,0.065163,0.064309,0.0648,0.063643,0.064639,

0.064906,0.064469,0.06408,0.064671,0.064212,0.064751,0.064528,0.064482
,0.06447,0.064726,0.064237,0.06421,0.064704,0.064609,0.064712,0.064401
,0.064667,0.064592,0.06454,0.064301,0.064109,0.064475,0.064534,0.06472
,0.064091,0.064733,0.06414,0.063951,0.064814,0.064331,0.064206,0.06452
3,0.06459,0.064139,0.064777,0.064577,0.064242,0.064189,0.064003,0.0641
79,0.064146,0.064669,0.063993,0.063982,0.064344,0.064582,0.064332,0.06
4505,0.064406,0.064463,0.064953,0.064113,0.064342,0.064827,0.064268,0.
064528,0.064422,0.064584,0.064523,0.064642,0.064566,0.064753,0.065186,
0.064436,0.064558,0.064693,0.064566,0.064445,0.064437,0.06452,0.064653
,0.06412,0.06412,0.065043,0.064667,0.064092,0.064732,0.064528,0.064382
,0.064232,0.064434,0.064757,0.064644,0.064735,0.064832,0.064,0.064644,
0.063581,0.064877,0.06492,0.064239,0.064256,0.064494,0.064731,0.063946
,0.065036,0.064765,0.064577,0.063944,0.063517,0.064251,0.065193,0.0642
77,0.064296,0.064441,0.064268,0.064482,0.064344,0.064838,0.064331,0.06
4597,0.064716,0.065179,0.064698,0.064407,0.064327,0.064517,0.063996,0.
064261,0.064355,0.065125,0.064589,0.064721,0.064577,0.064197,0.064394,
0.064134,0.064383,0.064563,0.064612,0.0646,0.06483,0.065035,0.064543,0
.064392,0.06437,0.064105,0.064452,0.063761,0.064531,0.064161,0.064922,
0.065057,0.064337,0.064913,0.064585,0.064942,0.064104,0.064056,0.06479
8,0.064239,0.064312,0.06431,0.064746,0.064607,0.063851,0.064844,0.0648
1,0.064444,0.064543,0.064394,0.064476,0.064037,0.064539,0.064413,0.064
793,0.064822,0.064517,0.063765,0.064818,0.064881,0.064453,0.064334,0.0
64387,0.063868
0.20055,0.20115,0.20097,0.20047,0.20091,0.20082,0.20079,0.20098,0.2002
9,0.20169,0.20094,0.20079,0.20107,0.20061,0.20104,0.20179,0.20094,0.20
112,0.20111,0.20053,0.27659,0.30265,0.28621,0.28365,0.20143,0.20182,0.
20102,0.20142,0.20088,0.20132,0.20055,0.20039,0.20055,0.20055,0.20151,
0.20106,0.20062,0.20087,0.2009,0.36544,0.28589,0.29737,0.28551,0.28004
,0.29176,0.28785,0.2011,0.20122,0.19966,0.20147,0.20158,0.19973,0.2013
6,0.20095,0.20157,0.20023,0.2007,0.20149,0.20058,0.20033,0.20047,0.367
01,0.36555,0.27826,0.25907,0.28972,0.26223,0.27806,0.28633,0.28445,0.2
8607,0.20099,0.20148,0.20138,0.20092,0.2022,0.20005,0.201,0.2011,0.201
01,0.19999,0.20218,0.20107,0.20144,0.2009,0.20039,0.36717,0.36966,0.37
71,0.28789,0.30158,0.2747,0.28172,0.28621,0.28634,0.28844,0.29045,0.28
746,0.20102,0.20169,0.20129,0.20093,0.20067,0.20179,0.20057,0.20189,0.
20067,0.20083,0.20061,0.20097,0.20159,0.2013,0.20095,0.20067,0.37636,0
.37521,0.3693,0.37265,0.2966,0.29272,0.28464,0.2911,0.27916,0.26659,0.
29479,0.28924,0.26726,0.27322,0.2012,0.20007,0.20123,0.20143,0.20126,0
.20047,0.20013,0.20012,0.20048,0.20271,0.2006,0.20088,0.20142,0.20075,
0.20074,0.20072,0.37176,0.37893,0.3794,0.36553,0.36166,0.36064,0.28753
,0.29087,0.27904,0.29368,0.28176,0.29775,0.3045,0.2903,0.31737,0.28271
,0.20012,0.2012,0.20139,0.20061,0.2002,0.20087,0.20111,0.20182,0.20045
,0.20069,0.20067,0.20067,0.20107,0.20108,0.20095,0.20048,0.2002,0.3533
8,0.3629,0.36302,0.36952,0.36257,0.36554,0.28848,0.29348,0.28676,0.280
42,0.286,0.2882,0.27865,0.29705,0.30126,0.29786,0.29149,0.20128,0.2001
1,0.20109,0.20069,0.20178,0.20133,0.20199,0.20039,0.2003,0.20165,0.201
2,0.20159,0.20125,0.12858,0.13835,0.13418,0.20196,0.3661,0.36224,0.371
56,0.37092,0.37783,0.37702,0.37341,0.28061,0.2812,0.29707,0.28953,0.27
136,0.26952,0.28518,0.27399,0.27187,0.26954,0.29741,0.20047,0.19977,0.
20078,0.20027,0.20015,0.20081,0.20109,0.20062,0.20186,0.20103,0.20084,
0.13218,0.1087,0.12804,0.13144,0.1307,0.11923,0.20036,0.36921,0.38948,
0.37253,0.37239,0.36956,0.3659,0.37055,0.29951,0.2861,0.2851,0.29558,0

.26766,0.30874,0.29499,0.28747,0.288,0.28331,0.29886,0.20171,0.20174,0
.20118,0.20221,0.20052,0.20063,0.20022,0.20041,0.20017,0.13083,0.13106
,0.13429,0.13137,0.13084,0.13114,0.13247,0.12915,0.13723,0.20149,0.372
52,0.36929,0.36074,0.36167,0.37085,0.3669,0.37507,0.29378,0.28304,0.28
927,0.28125,0.29976,0.30621,0.28523,0.29759,0.28495,0.29934,0.28404,0.
26231,0.20186,0.20099,0.20056,0.20084,0.20205,0.20098,0.13102,0.12932,
0.12964,0.13072,0.13887,0.14228,0.13054,0.11573,0.13121,0.13259,0.1330
1,0.11215,0.37182,0.36714,0.36982,0.36307,0.37401,0.37515,0.3722,0.279
36,0.28055,0.30055,0.28084,0.26632,0.29505,0.28475,0.29597,0.27646,0.3
1427,0.29067,0.28905,0.20171,0.20116,0.20068,0.14276,0.12513,0.13974,0
.13055,0.13149,0.12541,0.12362,0.13091,0.13904,0.13252,0.11945,0.12167
,0.13785,0.13034,0.13114,0.36638,0.37462,0.37903,0.36386,0.368,0.35484
,0.37463,0.37707,0.37155,0.28746,0.29234,0.28406,0.30265,0.28934,0.289
84,0.28062,0.28587,0.29816,0.3122,0.28556,0.12995,0.12756,0.13413,0.12
273,0.13121,0.13084,0.13424,0.13358,0.12279,0.10278,0.13657,0.1175,0.1
3343,0.13189,0.13099,0.13678,0.13603,0.12941,0.3677,0.3815,0.36692,0.3
6252,0.38095,0.35719,0.37001,0.36081,0.36757,0.28086,0.2557,0.21598,0.
19041,0.20917,0.20257,0.21315,0.21227,0.2078,0.21439,0.21839,0.14404,0
.1331,0.13221,0.13097,0.11681,0.13193,0.12496,0.12832,0.14531,0.13135,
0.1219,0.1312,0.11563,0.13602,0.13872,0.13116,0.1313,0.11672,0.36164,0
.36039,0.3624,0.36661,0.36784,0.37486,0.36758,0.36617,0.38156,0.22177,
0.21549,0.20975,0.2223,0.21081,0.20884,0.20429,0.19604,0.20818,0.22015
,0.21701,0.1333,0.1431,0.13577,0.13072,0.12371,0.13089,0.1376,0.13275,
0.1333,0.12932,0.1387,0.11013,0.13071,0.13851,0.14305,0.13983,0.14627,
0.12506,0.36733,0.37407,0.36279,0.3841,0.37314,0.37276,0.36273,0.21336
,0.19963,0.18111,0.20923,0.19994,0.20786,0.22438,0.21342,0.2257,0.2311
9,0.20972,0.21232,0.20702,0.13878,0.15183,0.11809,0.13028,0.13549,0.13
117,0.12211,0.13444,0.13358,0.11368,0.13431,0.11917,0.1217,0.11343,0.1
2988,0.13195,0.15432,0.1307,0.38035,0.37331,0.35695,0.37379,0.37138,0.
35139,0.21741,0.16988,0.22537,0.28373,0.21337,0.20051,0.20118,0.23344,
0.20815,0.20918,0.23899,0.2088,0.21539,0.2142,0.13404,0.14229,0.11847,
0.13074,0.12163,0.127,0.12524,0.136,0.13142,0.13189,0.12904,0.13085,0.
12329,0.12893,0.13206,0.13529,0.1312,0.13158,0.36999,0.36384,0.37477,0
.36421,0.36833,0.20972,0.20159,0.21914,0.22439,0.2136,0.21289,0.21517,
0.21367,0.21007,0.20988,0.20384,0.22424,0.2215,0.2048,0.21386,0.11864,
0.11381,0.12665,0.13109,0.13435,0.14356,0.1367,0.1318,0.13553,0.13417,
0.14031,0.13941,0.13147,0.13146,0.12981,0.15086,0.13089,0.13114,0.3666
,0.3673,0.36396,0.19092,0.22956,0.21887,0.19652,0.22484,0.21629,0.2133
3,0.21259,0.21118,0.19859,0.20943,0.21035,0.18695,0.20923,0.19712,0.20
029,0.20919,0.12469,0.13045,0.13098,0.14051,0.13002,0.13947,0.11983,0.
1234,0.13164,0.13147,0.14229,0.13127,0.11971,0.11659,0.13589,0.11801,0
.13548,0.13017,0.37118,0.21595,0.21124,0.24996,0.24965,0.21053,0.21081
,0.23078,0.21685,0.21716,0.2261,0.23101,0.22061,0.21292,0.21747,0.2015
2,0.20932,0.21245,0.2074,0.13476,0.1401,0.15225,0.13226,0.13401,0.1362
6,0.1043,0.13257,0.13086,0.13145,0.13128,0.1313,0.15084,0.14046,0.1494
8,0.13103,0.13677,0.061081,0.22379,0.21471,0.23552,0.22097,0.19191,0.2
0237,0.21981,0.19648,0.20396,0.24844,0.20886,0.20783,0.22106,0.23309,0
.2096,0.20733,0.21299,0.21138,0.2055,0.13941,0.12542,0.13473,0.12795,0
.13136,0.13101,0.13871,0.13747,0.090769,0.13191,0.13182,0.13614,0.1325
5,0.1347,0.10929,0.11548,0.13207,0.061306,0.2402,0.24622,0.21394,0.193
21,0.19113,0.21052,0.21428,0.21313,0.22064,0.21303,0.20837,0.21279,0.2
0548,0.20407,0.20307,0.21207,0.19299,0.20353,0.13075,0.11067,0.13161,0

.13562,0.13085,0.11963,0.13051,0.12791,0.12595,0.12936,0.13077,0.13067
,0.13196,0.13567,0.12024,0.13201,0.13712,0.12452,0.060865,0.18964,0.22
886,0.24463,0.21192,0.21233,0.21714,0.2201,0.21649,0.1972,0.2123,0.203
16,0.21095,0.21375,0.21428,0.21338,0.21272,0.21246,0.21327,0.12553,0.1
3305,0.13058,0.12055,0.15966,0.12989,0.13076,0.13061,0.12965,0.14004,0
.12848,0.13658,0.14186,0.11857,0.13349,0.13111,0.13041,0.061684,0.0615
92,0.06073,0.16142,0.21636,0.19722,0.21876,0.19336,0.21684,0.20829,0.2
2557,0.23524,0.21916,0.19688,0.20084,0.19963,0.20541,0.20991,0.12693,0
.14203,0.13422,0.13042,0.13115,0.14595,0.12008,0.1254,0.13046,0.1312,0
.15437,0.13564,0.1217,0.12232,0.13146,0.1453,0.12893,0.13149,0.06086,0
.060968,0.061707,0.06095,0.21757,0.19425,0.21759,0.21348,0.21531,0.217
12,0.22442,0.21565,0.21822,0.21433,0.21255,0.20052,0.13103,0.13435,0.1
3203,0.11874,0.13668,0.13096,0.13199,0.15608,0.1317,0.13089,0.12655,0.
18047,0.11691,0.13551,0.13132,0.13218,0.1276,0.13155,0.10684,0.13124,0
.061278,0.061547,0.061336,0.061526,0.061295,0.061256,0.19546,0.17314,0
.22872,0.21492,0.21344,0.21882,0.23773,0.21748,0.1198,0.12139,0.13347,
0.14446,0.1259,0.13257,0.13178,0.12471,0.14263,0.12976,0.12132,0.1446,
0.1315,0.13679,0.1355,0.13086,0.12864,0.13119,0.12614,0.13953,0.13194,
0.129,0.13988,0.061527,0.061476,0.061357,0.061111,0.061158,0.061224,0.
25487,0.2167,0.21764,0.24145,0.23026,0.061316,0.061115,0.13226,0.12993
,0.1406,0.12717,0.13083,0.11761,0.1206,0.12527,0.13367,0.12703,0.1193,
0.1256,0.13816,0.13413,0.13104,0.13723,0.13612,0.14106,0.11362,0.12683
,0.1307,0.06085,0.061332,0.061198,0.060634,0.061257,0.061927,0.061692,
0.06164,0.061355,0.061164,0.21985,0.21606,0.061845,0.061152,0.061667,0
.061257,0.13058,0.1336,0.1286,0.12694,0.1104,0.14431,0.1369,0.13135,0.
13103,0.13102,0.1311,0.13025,0.11363,0.12502,0.13748,0.13092,0.13092,0
.14468,0.12629,0.061087,0.061279,0.061174,0.061435,0.061033,0.061375,0
.061306,0.061259,0.061408,0.061185,0.061232,0.061412,0.061284,0.060844
,0.061194,0.06106,0.061634,0.13134,0.1278,0.15294,0.1438,0.12901,0.131
56,0.12422,0.14906,0.14721,0.12519,0.1378,0.13194,0.13108,0.13067,0.13
152,0.13106,0.061671,0.061164,0.061317,0.061841,0.061281,0.060582,0.06
0704,0.061611,0.061874,0.061485,0.061307,0.061203,0.061511,0.061284,0.
061811,0.061022,0.061678,0.061658,0.061659,0.11422,0.13069,0.10612,0.1
3279,0.13103,0.13112,0.12979,0.11906,0.13119,0.13047,0.13741,0.13069,0
.12232,0.061315,0.061878,0.061011,0.06145,0.061601,0.061242,0.061413,0
.061236,0.061325,0.061914,0.061863,0.061441,0.061161,0.061344,0.061769
,0.061403,0.061432,0.061354,0.06123,0.061344,0.061588,0.13141,0.13109,
0.13184,0.1359,0.12441,0.14327,0.13165,0.13972,0.14047,0.061311,0.0616
25,0.061759,0.061469,0.061419,0.061572,0.060996,0.061144,0.060947,0.06
1393,0.061226,0.060667,0.061812,0.061647,0.061326,0.061035,0.061061,0.
060864,0.061289,0.061351,0.060991,0.061121,0.061413,0.061449,0.061292,
0.14067,0.13683,0.12431,0.12359,0.061041,0.061088,0.061532,0.061462,0.
061571,0.061444,0.061436,0.061017,0.061177,0.061375,0.061089,0.061407,
0.061531,0.061654,0.061058,0.062117,0.061072,0.061747,0.060443,0.06152
4,0.061792,0.061241,0.060868,0.061447,0.060822,0.061503,0.061334,0.061
32,0.061284,0.061643,0.060956,0.060918,0.061576,0.061298,0.061643,0.06
116,0.061364,0.061332,0.061216,0.06112,0.06074,0.061222,0.061265,0.061
485,0.060984,0.061329,0.060775,0.060874,0.061818,0.061359,0.061146,0.0
61045,0.061432,0.061093,0.061563,0.061448,0.061238,0.061113,0.060854,0
.060983,0.061239,0.061409,0.060689,0.060936,0.061243,0.061388,0.061174
,0.061442,0.061239,0.061335,0.061658,0.061147,0.061258,0.061423,0.0611
44,0.061429,0.061327,0.06146,0.061398,0.061573,0.061504,0.061642,0.061

748,0.061148,0.061567,0.061616,0.061394,0.061209,0.06123,0.061338,0.06
1242,0.060941,0.060886,0.061851,0.061521,0.060991,0.061592,0.061604,0.
061291,0.061234,0.061293,0.061782,0.061451,0.061523,0.061301,0.060984,
0.061488,0.060569,0.061603,0.061715,0.06101,0.061069,0.06119,0.061615,
0.061046,0.061882,0.061704,0.061365,0.060794,0.060354,0.061106,0.06206
2,0.061255,0.061221,0.061283,0.061042,0.06155,0.061335,0.061606,0.0610
44,0.061539,0.061626,0.06195,0.061579,0.061253,0.061053,0.061124,0.060
871,0.061185,0.061153,0.061985,0.061367,0.061575,0.06152,0.061031,0.06
1352,0.06106,0.061026,0.0613,0.061243,0.061586,0.061399,0.061967,0.061
674,0.061092,0.061383,0.060988,0.061195,0.060696,0.061251,0.061043,0.0
61621,0.061841,0.061115,0.061981,0.061349,0.061681,0.061281,0.060894,0
.061533,0.061147,0.061233,0.061128,0.061529,0.061465,0.060726,0.061545
,0.06174,0.061298,0.061402,0.061403,0.061356,0.060913,0.061441,0.06128
5,0.061792,0.061601,0.061336,0.060573,0.061679,0.061775,0.061187,0.061
181,0.061163,0.061047
0.20196,0.20217,0.20237,0.20126,0.20215,0.20179,0.20184,0.20186,0.2013
3,0.20297,0.202,0.20234,0.20235,0.20187,0.20189,0.20286,0.20254,0.2022
5,0.20165,0.20175,0.27637,0.30198,0.28507,0.2831,0.20254,0.20274,0.202
43,0.20264,0.20188,0.20271,0.20142,0.2014,0.20166,0.20176,0.20295,0.20
187,0.20146,0.20202,0.20215,0.35784,0.28519,0.29435,0.2849,0.27729,0.2
9172,0.28628,0.20242,0.20251,0.20126,0.20264,0.20286,0.20094,0.20257,0
.20194,0.20261,0.20107,0.20182,0.20234,0.20198,0.20119,0.20137,0.3615,
0.35907,0.27588,0.25842,0.28803,0.26241,0.27724,0.28533,0.28181,0.2848
5,0.20223,0.20234,0.20283,0.20251,0.20337,0.20152,0.20239,0.20202,0.20
204,0.20135,0.20319,0.20244,0.20264,0.20191,0.20129,0.36906,0.3661,0.3
8009,0.28426,0.29942,0.27215,0.28022,0.28308,0.28227,0.28738,0.28991,0
.28683,0.20222,0.20256,0.20249,0.20226,0.20192,0.20271,0.20199,0.20331
,0.20203,0.2017,0.20186,0.20219,0.20257,0.2028,0.20189,0.20194,0.37035
,0.37154,0.36881,0.36758,0.29616,0.28905,0.2822,0.28804,0.27616,0.2660
5,0.29425,0.28803,0.26457,0.27134,0.20256,0.20098,0.20268,0.20271,0.20
221,0.2016,0.20125,0.20161,0.20174,0.20401,0.20173,0.2021,0.2026,0.202
05,0.20208,0.2022,0.36954,0.37888,0.37293,0.36522,0.36191,0.3605,0.284
34,0.28797,0.2788,0.29259,0.28016,0.29428,0.30337,0.28922,0.31711,0.28
238,0.20121,0.20264,0.20226,0.20155,0.20142,0.20225,0.2022,0.20335,0.2
0183,0.20179,0.20207,0.20163,0.20241,0.20212,0.20184,0.20118,0.2018,0.
34955,0.3598,0.35784,0.36133,0.36295,0.36169,0.28748,0.29146,0.2851,0.
2793,0.28517,0.28961,0.2776,0.29359,0.30123,0.295,0.29152,0.20276,0.20
109,0.20231,0.20196,0.20259,0.20229,0.2032,0.20182,0.20136,0.20296,0.2
028,0.20255,0.20276,0.12756,0.13753,0.13341,0.20323,0.36364,0.35623,0.
36952,0.36763,0.36752,0.38109,0.36654,0.28114,0.2787,0.29435,0.28827,0
.2682,0.26753,0.28404,0.27311,0.27162,0.26736,0.29577,0.20186,0.20122,
0.20147,0.20176,0.20099,0.20215,0.2022,0.20216,0.20308,0.20228,0.20193
,0.13159,0.10767,0.1273,0.13067,0.1298,0.11839,0.20162,0.3646,0.38216,
0.36723,0.36653,0.36718,0.36149,0.3703,0.29557,0.28536,0.28628,0.29434
,0.26721,0.30435,0.29445,0.28709,0.28679,0.28205,0.30011,0.20293,0.203
24,0.20242,0.20326,0.20155,0.20202,0.20143,0.20172,0.20152,0.13017,0.1
3021,0.1336,0.1307,0.13,0.13026,0.13166,0.12853,0.13653,0.20283,0.3688
3,0.36695,0.35121,0.35699,0.36795,0.36562,0.36808,0.2943,0.28028,0.288
13,0.28137,0.30084,0.30407,0.2849,0.29407,0.28201,0.29553,0.28285,0.26
031,0.2027,0.20201,0.20204,0.20191,0.20319,0.20198,0.13039,0.12864,0.1
2902,0.1305,0.13839,0.14184,0.12992,0.11447,0.13055,0.13171,0.13237,0.
11073,0.36753,0.36483,0.36938,0.36039,0.37078,0.37203,0.37055,0.2795,0

.27997,0.29741,0.2791,0.26688,0.29305,0.2839,0.29529,0.27521,0.30853,0
.28912,0.28782,0.20319,0.20235,0.20235,0.14249,0.12423,0.13884,0.12989
,0.13067,0.12457,0.12248,0.13035,0.13859,0.13206,0.11845,0.12086,0.137
18,0.12983,0.13043,0.36409,0.3745,0.37334,0.35937,0.36792,0.34921,0.36
759,0.3714,0.36569,0.28487,0.2932,0.28176,0.29943,0.28821,0.28685,0.27
994,0.28434,0.29587,0.31108,0.28271,0.12937,0.12691,0.13352,0.12175,0.
13052,0.13005,0.13377,0.13285,0.12177,0.10127,0.13593,0.11663,0.13284,
0.13142,0.13031,0.13627,0.13558,0.12867,0.36666,0.37679,0.36837,0.3597
9,0.36969,0.35791,0.36563,0.35801,0.3622,0.27868,0.25477,0.21432,0.188
03,0.20797,0.2003,0.21015,0.21049,0.20568,0.21276,0.21673,0.14353,0.13
244,0.13163,0.12996,0.11593,0.1312,0.12403,0.12742,0.14454,0.13059,0.1
209,0.13039,0.11468,0.13522,0.13827,0.13022,0.13048,0.11543,0.35835,0.
35502,0.35967,0.36563,0.36674,0.37096,0.36716,0.36459,0.37394,0.22039,
0.21342,0.20786,0.22046,0.20854,0.20777,0.20447,0.194,0.20616,0.21719,
0.21392,0.13276,0.14226,0.13495,0.13029,0.12263,0.12996,0.13715,0.1321
9,0.1327,0.12852,0.13817,0.10863,0.12984,0.13836,0.14245,0.13926,0.145
57,0.1241,0.36404,0.36812,0.35943,0.37889,0.3698,0.3722,0.36535,0.2093
2,0.19729,0.17742,0.2063,0.1981,0.20585,0.22407,0.21122,0.2227,0.22754
,0.2088,0.21033,0.20614,0.13829,0.15155,0.11711,0.12973,0.13484,0.1305
,0.12116,0.13389,0.13273,0.11237,0.13362,0.11827,0.12061,0.11209,0.129
04,0.13144,0.15413,0.13014,0.37167,0.37254,0.35383,0.37044,0.36677,0.3
5041,0.21728,0.1677,0.22125,0.27955,0.20999,0.19866,0.19983,0.23147,0.
20754,0.20666,0.23653,0.20788,0.21231,0.2115,0.13333,0.14169,0.11754,0
.13007,0.12037,0.12613,0.12437,0.13543,0.13063,0.13117,0.12831,0.13025
,0.12234,0.12827,0.1312,0.13469,0.13063,0.13086,0.3659,0.35202,0.37482
,0.35634,0.36635,0.20696,0.20061,0.21433,0.22073,0.21251,0.20914,0.213
54,0.21146,0.20726,0.20696,0.20062,0.22238,0.2197,0.20424,0.21157,0.11
788,0.11262,0.12563,0.13031,0.13372,0.14278,0.13611,0.13116,0.13505,0.
13352,0.13989,0.13895,0.13073,0.13075,0.1289,0.15042,0.13017,0.13069,0
.36548,0.36184,0.35904,0.18705,0.22519,0.21508,0.19516,0.22294,0.21068
,0.20932,0.20794,0.20851,0.19687,0.20626,0.20967,0.18609,0.20815,0.194
72,0.19877,0.20697,0.12377,0.12966,0.1302,0.13983,0.12952,0.13897,0.11
864,0.12246,0.13087,0.13047,0.14169,0.13042,0.11852,0.11553,0.13525,0.
11709,0.13479,0.12953,0.36404,0.21203,0.20865,0.24972,0.24909,0.20539,
0.20662,0.22813,0.21341,0.21318,0.2233,0.23024,0.21753,0.21106,0.21345
,0.20043,0.20625,0.20969,0.2067,0.13429,0.1398,0.15216,0.13173,0.13341
,0.13546,0.10292,0.13205,0.13017,0.13063,0.13055,0.13072,0.15061,0.140
03,0.14916,0.1303,0.13626,0.058471,0.21907,0.2113,0.23163,0.2149,0.188
89,0.19898,0.21751,0.19185,0.20145,0.24651,0.20631,0.20519,0.21979,0.2
3146,0.20829,0.20551,0.21007,0.20761,0.20346,0.13874,0.12454,0.13421,0
.12698,0.13061,0.13051,0.13833,0.13724,0.089088,0.13147,0.131,0.13572,
0.13192,0.13414,0.10809,0.1143,0.13132,0.058631,0.2366,0.24099,0.21049
,0.19047,0.18679,0.20904,0.21143,0.20896,0.218,0.20806,0.2061,0.21035,
0.20236,0.20315,0.20077,0.2103,0.19222,0.20183,0.1302,0.10932,0.13094,
0.13486,0.13006,0.1187,0.12991,0.12702,0.12514,0.1285,0.1299,0.13001,0
.13135,0.1351,0.11924,0.13127,0.13653,0.12361,0.058225,0.18535,0.22561
,0.24169,0.21026,0.21043,0.21637,0.21784,0.21327,0.19365,0.2093,0.2009
9,0.20871,0.21074,0.21164,0.21103,0.21007,0.21036,0.21064,0.12472,0.13
236,0.13013,0.1195,0.15985,0.12912,0.13026,0.13014,0.12898,0.13956,0.1
2791,0.13613,0.14138,0.11749,0.13285,0.13035,0.1299,0.058995,0.058721,
0.058232,0.15891,0.21135,0.1959,0.21696,0.19104,0.21463,0.20568,0.2225
1,0.23287,0.21729,0.19497,0.19927,0.19855,0.20453,0.20889,0.12593,0.14

168,0.1336,0.12991,0.13048,0.14555,0.11903,0.12467,0.12987,0.13057,0.1
5417,0.135,0.12068,0.12157,0.13094,0.14512,0.12825,0.13082,0.058221,0.
058412,0.058957,0.058448,0.21552,0.19072,0.21455,0.21056,0.21237,0.211
46,0.22103,0.21257,0.21559,0.21227,0.20972,0.19842,0.13032,0.13392,0.1
314,0.11782,0.13575,0.13014,0.13081,0.15611,0.13101,0.13017,0.12564,0.
18095,0.11577,0.13473,0.1306,0.13156,0.12698,0.13079,0.10548,0.13037,0
.058663,0.058831,0.058675,0.059102,0.058709,0.058627,0.19204,0.1714,0.
22525,0.21232,0.21046,0.21566,0.23309,0.2148,0.11898,0.12048,0.13286,0
.14411,0.12489,0.13196,0.13128,0.12371,0.14205,0.12923,0.12034,0.14433
,0.13085,0.13644,0.13493,0.13049,0.12793,0.13028,0.1252,0.13883,0.1313
2,0.12804,0.1391,0.058996,0.058906,0.058767,0.058667,0.058379,0.058691
,0.25147,0.2116,0.21615,0.23798,0.22717,0.058762,0.058496,0.13172,0.12
927,0.13996,0.12611,0.13039,0.11653,0.11987,0.12457,0.13279,0.12613,0.
11829,0.12476,0.1375,0.13337,0.13019,0.13651,0.13535,0.14055,0.11251,0
.12568,0.12991,0.058364,0.058833,0.058775,0.058172,0.058648,0.059228,0
.058926,0.059234,0.058632,0.058611,0.21419,0.21364,0.05923,0.058524,0.
059066,0.05885,0.12993,0.13315,0.12784,0.12594,0.10912,0.14376,0.1361,
0.13016,0.13038,0.13012,0.13067,0.12966,0.11262,0.12395,0.13672,0.1301
4,0.13028,0.14427,0.12558,0.058666,0.058637,0.058382,0.05881,0.05837,0
.058688,0.058758,0.058627,0.059035,0.058621,0.058689,0.058647,0.058609
,0.058478,0.058638,0.058483,0.058887,0.13057,0.12715,0.15248,0.14333,0
.12837,0.13074,0.12319,0.14873,0.14719,0.12417,0.13687,0.1312,0.13039,
0.1302,0.13088,0.13024,0.05897,0.058519,0.058702,0.059546,0.058874,0.0
57977,0.058172,0.059016,0.059195,0.059032,0.058663,0.058582,0.059082,0
.058584,0.059318,0.058434,0.059016,0.058934,0.058913,0.11294,0.12998,0
.10473,0.13204,0.13011,0.13029,0.12896,0.118,0.13035,0.12989,0.1369,0.
13012,0.12148,0.058352,0.059154,0.058413,0.058568,0.058928,0.058713,0.
058822,0.058707,0.058937,0.059226,0.059049,0.058737,0.058615,0.058788,
0.059152,0.058733,0.058814,0.058818,0.058711,0.058928,0.059221,0.13062
,0.13092,0.13098,0.13532,0.12369,0.14298,0.13118,0.13931,0.13998,0.058
674,0.059002,0.059065,0.058866,0.058952,0.05902,0.058371,0.058699,0.05
8328,0.059011,0.058656,0.058013,0.059206,0.05897,0.058912,0.058766,0.0
58699,0.05833,0.058777,0.05878,0.058167,0.058711,0.058523,0.05905,0.05
8634,0.14022,0.13625,0.12326,0.12263,0.05828,0.05854,0.059204,0.058992
,0.058784,0.058829,0.058677,0.058239,0.058544,0.058932,0.058204,0.0586
78,0.058825,0.058907,0.058451,0.059459,0.058386,0.059102,0.057987,0.05
8828,0.059113,0.058706,0.058352,0.059119,0.058152,0.058926,0.058946,0.
05878,0.058777,0.058917,0.058249,0.058241,0.05901,0.058528,0.05889,0.0
58678,0.058737,0.058751,0.05886,0.058516,0.058056,0.058736,0.058641,0.
058847,0.058526,0.058807,0.058248,0.058298,0.059167,0.058932,0.05878,0
.058531,0.058838,0.058708,0.058811,0.058722,0.058578,0.058464,0.058421
,0.058444,0.058686,0.058685,0.058332,0.058243,0.058471,0.058676,0.0587
07,0.058836,0.058579,0.058772,0.059126,0.058655,0.058468,0.058976,0.05
8712,0.058882,0.058646,0.058937,0.058752,0.058995,0.058813,0.059174,0.
059052,0.058644,0.05891,0.058859,0.058721,0.058651,0.05857,0.058777,0.
058639,0.058687,0.058448,0.059276,0.058726,0.058544,0.059155,0.059001,
0.058812,0.058683,0.058682,0.059082,0.058903,0.058903,0.058562,0.05851
2,0.058823,0.058029,0.05901,0.05914,0.058504,0.058569,0.058666,0.05905
6,0.058394,0.059092,0.059161,0.058876,0.058134,0.057827,0.058523,0.059
442,0.058589,0.058559,0.058649,0.058367,0.059029,0.058844,0.059071,0.0
58528,0.058909,0.058897,0.05925,0.058853,0.058551,0.058525,0.058558,0.
058195,0.058847,0.058402,0.059316,0.059044,0.058741,0.058896,0.058174,

0.05861,0.058391,0.058478,0.058818,0.058772,0.059105,0.058781,0.05941,
0.058793,0.058354,0.058746,0.05824,0.058556,0.058084,0.0587,0.058534,0
.059099,0.058999,0.05851,0.059314,0.058961,0.058989,0.058917,0.05832,0
.059088,0.058403,0.058563,0.058693,0.059065,0.058805,0.058181,0.058929
,0.059009,0.058573,0.058721,0.058571,0.058802,0.058328,0.058891,0.0585
63,0.059174,0.059015,0.0589,0.058019,0.059093,0.059305,0.058362,0.0584
18,0.058407,0.058417
0.20314,0.20312,0.20327,0.20252,0.20321,0.20273,0.20283,0.20279,0.2026
4,0.20381,0.20319,0.20339,0.20323,0.20295,0.20291,0.20382,0.20352,0.20
378,0.20239,0.20257,0.27621,0.30013,0.28308,0.2826,0.20356,0.20428,0.2
04,0.20312,0.20321,0.20371,0.20278,0.20253,0.20283,0.20307,0.20415,0.2
0305,0.20244,0.20299,0.20324,0.35366,0.28543,0.29609,0.28424,0.27768,0
.28931,0.28571,0.20347,0.20328,0.20211,0.20312,0.20368,0.20213,0.20341
,0.2028,0.20351,0.20247,0.20273,0.20354,0.20312,0.20222,0.20248,0.3583
5,0.35841,0.27537,0.25721,0.28544,0.26134,0.27689,0.28399,0.28137,0.28
355,0.203,0.20299,0.20381,0.2034,0.20402,0.20275,0.20355,0.20284,0.202
93,0.20234,0.20419,0.20355,0.20364,0.2032,0.20253,0.36284,0.36154,0.37
186,0.28253,0.29669,0.26898,0.27938,0.28129,0.28103,0.28686,0.28673,0.
28418,0.20323,0.20351,0.20355,0.20324,0.20272,0.20352,0.20297,0.20426,
0.20276,0.20257,0.20266,0.2033,0.2033,0.20348,0.2028,0.20295,0.36524,0
.36583,0.3629,0.36311,0.29515,0.28657,0.28008,0.28952,0.27684,0.26584,
0.29352,0.28878,0.26414,0.27165,0.20331,0.20244,0.20367,0.20357,0.2033
9,0.20294,0.20261,0.20255,0.20304,0.20486,0.20308,0.20318,0.2035,0.202
97,0.20272,0.20307,0.36562,0.37415,0.36875,0.36199,0.36013,0.35852,0.2
8544,0.28575,0.27776,0.29173,0.27946,0.29516,0.30059,0.28735,0.31346,0
.28154,0.20212,0.20386,0.20327,0.20243,0.20259,0.20339,0.20313,0.20425
,0.20294,0.20302,0.20274,0.20294,0.20358,0.20318,0.20312,0.20215,0.203
16,0.34963,0.35346,0.35671,0.36006,0.35785,0.36238,0.28647,0.28986,0.2
8504,0.27955,0.28505,0.28762,0.27564,0.29409,0.29932,0.29508,0.29253,0
.20391,0.20195,0.20302,0.20284,0.20367,0.20308,0.20447,0.20323,0.20271
,0.20384,0.20384,0.20377,0.20368,0.12735,0.13695,0.13284,0.20413,0.360
77,0.35345,0.36472,0.36524,0.3615,0.37249,0.36169,0.28125,0.27759,0.29
161,0.28655,0.26784,0.26687,0.28395,0.27316,0.27242,0.26653,0.29802,0.
20282,0.20283,0.20291,0.20294,0.20226,0.20347,0.20307,0.2026,0.20434,0
.20319,0.20297,0.13113,0.10671,0.12659,0.12999,0.12905,0.11753,0.20244
,0.35976,0.37782,0.36847,0.36332,0.36345,0.36011,0.36613,0.29439,0.283
99,0.28437,0.29171,0.26637,0.3019,0.29327,0.28574,0.28518,0.28195,0.29
636,0.20387,0.20377,0.20397,0.20456,0.20289,0.20294,0.20278,0.20304,0.
20281,0.12968,0.1299,0.13292,0.13002,0.12962,0.12978,0.13108,0.12796,0
.13629,0.20344,0.37138,0.36446,0.34507,0.35723,0.36133,0.36381,0.36354
,0.29296,0.27848,0.28634,0.28173,0.29987,0.30555,0.28311,0.29241,0.282
47,0.29774,0.28294,0.26104,0.20371,0.20306,0.20279,0.20273,0.20443,0.2
0306,0.12946,0.12808,0.1287,0.12985,0.13779,0.14166,0.12937,0.11341,0.
1296,0.13089,0.13194,0.10983,0.36352,0.3588,0.36667,0.36048,0.36819,0.
36614,0.36498,0.27603,0.28054,0.29684,0.2812,0.26733,0.2914,0.28615,0.
29467,0.27325,0.30738,0.28721,0.28517,0.20373,0.2035,0.20306,0.14236,0
.12314,0.13839,0.12936,0.13017,0.12398,0.1214,0.12958,0.13823,0.13126,
0.11777,0.12027,0.13696,0.12935,0.12994,0.36103,0.37093,0.37295,0.3619
,0.36453,0.34706,0.3608,0.36384,0.36304,0.28295,0.29358,0.28131,0.2979
5,0.2879,0.28626,0.27991,0.28364,0.29422,0.30978,0.28035,0.12891,0.126
14,0.13319,0.12089,0.13014,0.12954,0.13331,0.13228,0.12116,0.10022,0.1
355,0.11559,0.13201,0.13053,0.12948,0.13583,0.13512,0.12803,0.36472,0.

37009,0.36444,0.3543,0.36791,0.35105,0.36463,0.35291,0.36287,0.27831,0
.25401,0.21352,0.18634,0.20744,0.19957,0.20896,0.20988,0.20447,0.21126
,0.2147,0.14314,0.13234,0.13089,0.12952,0.115,0.13067,0.12324,0.12679,
0.1443,0.13001,0.12017,0.12982,0.11403,0.13464,0.13767,0.12973,0.13017
,0.11459,0.35779,0.35545,0.35476,0.36429,0.3628,0.3651,0.36019,0.35989
,0.36813,0.21839,0.21232,0.20732,0.21789,0.20751,0.20661,0.20339,0.191
99,0.20478,0.21701,0.21188,0.13224,0.14168,0.13445,0.12984,0.12205,0.1
2955,0.13693,0.13164,0.13209,0.12773,0.13791,0.10754,0.12967,0.13789,0
.14214,0.13901,0.14547,0.12365,0.36042,0.36574,0.35382,0.37365,0.37034
,0.37137,0.36376,0.20776,0.19633,0.1751,0.20469,0.19825,0.20649,0.2227
1,0.21059,0.22077,0.22444,0.20792,0.20824,0.20366,0.13801,0.15137,0.11
646,0.12902,0.13443,0.12978,0.12048,0.13344,0.13215,0.11144,0.13321,0.
11754,0.11991,0.11114,0.1286,0.1312,0.15418,0.12974,0.36978,0.36891,0.
34777,0.3642,0.36638,0.34835,0.21469,0.1644,0.22017,0.27735,0.20641,0.
19754,0.19816,0.23214,0.20696,0.20524,0.23623,0.2062,0.21138,0.20948,0
.13312,0.14168,0.11688,0.1293,0.1197,0.12538,0.12373,0.13512,0.12992,0
.13074,0.12747,0.12967,0.1215,0.12767,0.13055,0.13408,0.12996,0.13023,
0.36049,0.34901,0.37185,0.35164,0.36113,0.20529,0.19841,0.2126,0.21737
,0.20963,0.20709,0.21175,0.20946,0.20697,0.20519,0.1998,0.21999,0.2178
,0.20227,0.21061,0.11699,0.11156,0.12498,0.12968,0.13316,0.14243,0.135
51,0.13055,0.13512,0.13302,0.1398,0.13844,0.13027,0.13019,0.12825,0.15
039,0.12935,0.13029,0.36527,0.36015,0.35431,0.18596,0.22399,0.21241,0.
19373,0.2195,0.2093,0.20719,0.20372,0.20712,0.19616,0.20501,0.20689,0.
18515,0.20641,0.19334,0.1977,0.20623,0.12297,0.12935,0.12973,0.13942,0
.12878,0.13866,0.11768,0.12169,0.13018,0.1297,0.14146,0.12977,0.11767,
0.11493,0.13472,0.11634,0.13405,0.12918,0.3639,0.2091,0.20691,0.24687,
0.24474,0.20149,0.20298,0.22485,0.20898,0.21234,0.22185,0.22759,0.2162
,0.21065,0.21292,0.20107,0.20443,0.20755,0.20522,0.13355,0.13905,0.152
16,0.13113,0.13295,0.13503,0.10178,0.13147,0.12942,0.13013,0.12983,0.1
3001,0.15058,0.13988,0.14918,0.12967,0.13568,0.056534,0.21606,0.21001,
0.23009,0.21108,0.18594,0.19406,0.21423,0.18882,0.20149,0.2439,0.20402
,0.20523,0.21793,0.23034,0.20602,0.20509,0.2097,0.20603,0.20322,0.1382
5,0.12399,0.13354,0.12649,0.13008,0.12988,0.13792,0.13683,0.087657,0.1
3099,0.13059,0.13542,0.13138,0.13386,0.10699,0.11335,0.13085,0.05667,0
.23076,0.23806,0.20958,0.18909,0.18392,0.20499,0.20933,0.2066,0.21387,
0.20423,0.20336,0.20928,0.20128,0.20187,0.19929,0.20945,0.19054,0.2013
,0.12938,0.10821,0.13017,0.13449,0.12981,0.11792,0.12931,0.12641,0.124
53,0.12793,0.12938,0.12965,0.13117,0.13472,0.11847,0.13091,0.13616,0.1
2292,0.0561,0.18395,0.22256,0.23784,0.20695,0.20818,0.21277,0.21611,0.
21123,0.19149,0.20653,0.1996,0.20777,0.21109,0.21066,0.21097,0.20932,0
.20885,0.2093,0.12412,0.13181,0.12939,0.11872,0.15992,0.12843,0.12961,
0.12962,0.12843,0.13934,0.12725,0.13541,0.14117,0.11672,0.13236,0.1296
9,0.12919,0.056904,0.056423,0.056444,0.15575,0.20768,0.19331,0.21423,0
.1901,0.2119,0.20208,0.21872,0.2299,0.21471,0.19444,0.19872,0.19797,0.
20351,0.20805,0.12547,0.14141,0.13342,0.12956,0.12982,0.14512,0.11802,
0.12385,0.12965,0.1301,0.15406,0.13485,0.12005,0.12057,0.13041,0.14458
,0.1278,0.1303,0.056342,0.056283,0.056843,0.056421,0.2108,0.18711,0.21
353,0.20584,0.2086,0.21031,0.22075,0.20891,0.21036,0.21074,0.20823,0.1
9745,0.12979,0.13323,0.13095,0.1171,0.13518,0.12959,0.12992,0.15626,0.
13048,0.12955,0.12512,0.18144,0.11505,0.13432,0.12999,0.13097,0.12617,
0.12994,0.10417,0.12944,0.056333,0.056684,0.05658,0.056967,0.056643,0.
056496,0.1901,0.16891,0.22219,0.20852,0.20793,0.21138,0.22831,0.21216,

0.11844,0.11985,0.13234,0.14369,0.12424,0.13141,0.13066,0.12307,0.1414
7,0.12872,0.11938,0.14412,0.13019,0.13606,0.13443,0.13003,0.12727,0.12
97,0.12449,0.13851,0.13076,0.12764,0.13899,0.056862,0.056706,0.056852,
0.056375,0.056164,0.056737,0.25113,0.20724,0.21374,0.23768,0.225,0.056
748,0.05672,0.13104,0.12874,0.13964,0.12547,0.12963,0.11563,0.11888,0.
12386,0.13245,0.1257,0.11747,0.12403,0.13723,0.133,0.12975,0.13647,0.1
3491,0.14033,0.11142,0.12476,0.12954,0.056303,0.056681,0.056735,0.0561
96,0.05656,0.057013,0.056854,0.057092,0.056577,0.05652,0.21163,0.2104,
0.057108,0.056443,0.056907,0.056679,0.12956,0.13262,0.12721,0.12533,0.
1081,0.14348,0.13566,0.12974,0.12965,0.12981,0.13014,0.12892,0.11188,0
.12355,0.13633,0.12951,0.12989,0.14396,0.125,0.056352,0.05655,0.0564,0
.056696,0.056335,0.056601,0.056653,0.056553,0.056801,0.056502,0.056533
,0.056604,0.056354,0.056263,0.056473,0.056339,0.05683,0.12996,0.12637,
0.15231,0.14272,0.12759,0.13043,0.12271,0.1484,0.14688,0.1234,0.13659,
0.13055,0.12981,0.12961,0.13023,0.12965,0.056882,0.056548,0.05667,0.05
7359,0.056743,0.05591,0.05598,0.057034,0.05691,0.057157,0.056482,0.056
506,0.057227,0.056691,0.057114,0.056592,0.056902,0.057035,0.057004,0.1
1196,0.12937,0.10364,0.13135,0.12941,0.12957,0.12858,0.11723,0.12992,0
.12926,0.13655,0.12987,0.12069,0.056293,0.057045,0.05644,0.056475,0.05
691,0.056662,0.056758,0.056618,0.056851,0.057288,0.056796,0.056526,0.0
56408,0.056395,0.057035,0.056666,0.056591,0.056635,0.05684,0.057065,0.
057115,0.13011,0.13049,0.13041,0.13456,0.1231,0.1427,0.13062,0.13885,0
.13984,0.056656,0.056756,0.056854,0.056601,0.056809,0.057044,0.056179,
0.056642,0.056111,0.05695,0.056393,0.056171,0.056932,0.05696,0.056877,
0.056662,0.056499,0.056258,0.056534,0.056511,0.055921,0.056412,0.05676
5,0.056819,0.056413,0.13962,0.13574,0.12245,0.12184,0.05645,0.056199,0
.056888,0.057067,0.056698,0.056703,0.056697,0.056308,0.056359,0.056906
,0.056264,0.056618,0.056734,0.056809,0.056388,0.057367,0.056095,0.0570
75,0.056013,0.056614,0.057031,0.05679,0.05597,0.056997,0.056075,0.0567
58,0.056725,0.056758,0.056555,0.05642,0.056092,0.056111,0.056627,0.056
304,0.056748,0.056571,0.056425,0.056637,0.056602,0.05637,0.055946,0.05
6604,0.056608,0.056829,0.056355,0.056759,0.056072,0.056248,0.057089,0.
056867,0.056558,0.056342,0.056665,0.056564,0.056928,0.056605,0.056621,
0.056396,0.056365,0.056336,0.056427,0.056603,0.05627,0.056251,0.056217
,0.056441,0.056703,0.056833,0.05637,0.056762,0.057013,0.056507,0.05660
7,0.056923,0.056885,0.056792,0.056574,0.056782,0.056613,0.056917,0.056
402,0.057037,0.056942,0.056366,0.05696,0.056622,0.056524,0.056612,0.05
6252,0.056687,0.056523,0.056744,0.056291,0.057278,0.05652,0.056314,0.0
57066,0.057076,0.05675,0.056363,0.056748,0.057006,0.056989,0.056616,0.
056463,0.056351,0.056908,0.055963,0.056739,0.056973,0.056456,0.056515,
0.056488,0.05692,0.056327,0.056794,0.057099,0.056822,0.056229,0.055857
,0.056461,0.057196,0.056558,0.056465,0.056677,0.056347,0.056942,0.0566
04,0.057035,0.056221,0.056668,0.056622,0.057189,0.056688,0.056598,0.05
6748,0.056573,0.056259,0.056739,0.05625,0.05713,0.056765,0.056342,0.05
6695,0.056237,0.056858,0.056237,0.056306,0.056571,0.056673,0.056979,0.
056697,0.057189,0.056793,0.056318,0.056674,0.055997,0.056379,0.05596,0
.056393,0.0564,0.057119,0.056751,0.056404,0.057122,0.056931,0.056694,0
.056803,0.056354,0.057068,0.056259,0.056499,0.056669,0.056993,0.056627
,0.05595,0.056838,0.056817,0.056239,0.056631,0.056596,0.056771,0.05633
8,0.056955,0.056441,0.057056,0.056962,0.056871,0.055863,0.056944,0.057
058,0.056363,0.056488,0.056425,0.056104

0.20336,0.20435,0.2041,0.20381,0.20384,0.20357,0.20381,0.20392,0.20338
,0.20475,0.20413,0.20409,0.20455,0.20352,0.20384,0.20423,0.2041,0.2048
,0.20362,0.20338,0.27322,0.30177,0.28317,0.28008,0.20398,0.20506,0.204
71,0.20427,0.20457,0.20424,0.20355,0.20357,0.20402,0.20416,0.20479,0.2
0399,0.20318,0.20395,0.20399,0.35295,0.28494,0.29275,0.2825,0.27817,0.
28839,0.28556,0.20447,0.20418,0.2031,0.20349,0.20442,0.20304,0.20454,0
.20371,0.20447,0.20315,0.20345,0.20454,0.20433,0.20346,0.20331,0.35819
,0.35697,0.2773,0.25604,0.28337,0.26189,0.2773,0.28229,0.28064,0.28069
,0.20433,0.20378,0.20435,0.20423,0.20493,0.20362,0.20422,0.20371,0.204
31,0.2036,0.20438,0.20428,0.20456,0.20355,0.20368,0.36225,0.35696,0.36
694,0.28148,0.29697,0.26795,0.28196,0.28053,0.27902,0.28425,0.28636,0.
28438,0.204,0.20473,0.20453,0.20393,0.20402,0.20428,0.20397,0.20519,0.
20386,0.20384,0.20381,0.20411,0.20341,0.20441,0.20397,0.20371,0.36326,
0.35998,0.35996,0.36116,0.29435,0.28467,0.27966,0.28771,0.27527,0.2642
,0.29186,0.28611,0.26477,0.27081,0.20413,0.20301,0.2045,0.20453,0.2047
,0.20335,0.20309,0.20356,0.20425,0.20542,0.20409,0.2043,0.20466,0.2039
5,0.20363,0.20381,0.36761,0.36967,0.36477,0.36049,0.36438,0.35489,0.28
313,0.28454,0.27826,0.2889,0.27775,0.29311,0.29895,0.28647,0.31154,0.2
8057,0.20356,0.20453,0.20435,0.20363,0.2034,0.2043,0.20436,0.20507,0.2
0374,0.20381,0.20362,0.20411,0.20413,0.20395,0.20413,0.2032,0.2042,0.3
496,0.35419,0.35455,0.36125,0.35585,0.3601,0.28471,0.29007,0.28576,0.2
8,0.28489,0.28603,0.27445,0.29262,0.29567,0.29451,0.29279,0.20483,0.20
29,0.20337,0.20353,0.20419,0.20445,0.20542,0.2043,0.20403,0.20417,0.20
456,0.2047,0.20415,0.12684,0.1368,0.13246,0.20536,0.35874,0.35324,0.36
898,0.36509,0.35775,0.37141,0.362,0.27918,0.27813,0.29116,0.2848,0.267
36,0.26642,0.28193,0.27191,0.27064,0.26602,0.29499,0.20374,0.20386,0.2
0379,0.20431,0.20302,0.20419,0.20417,0.20343,0.20578,0.20383,0.20363,0
.13074,0.10583,0.12608,0.12946,0.12859,0.11695,0.20317,0.35579,0.37545
,0.3683,0.36103,0.35851,0.35719,0.36438,0.29133,0.28259,0.2832,0.28891
,0.26485,0.30357,0.29183,0.28498,0.28391,0.28,0.29685,0.20437,0.20448,
0.20483,0.20538,0.20393,0.20385,0.20395,0.20368,0.20363,0.12948,0.1295
1,0.13261,0.12945,0.12924,0.1293,0.13071,0.12748,0.13605,0.20374,0.365
72,0.36443,0.34582,0.35243,0.35909,0.35919,0.36122,0.29285,0.2782,0.28
435,0.28093,0.29868,0.30226,0.28286,0.29031,0.28329,0.29735,0.28149,0.
2613,0.20469,0.20395,0.20399,0.20333,0.20507,0.20417,0.12924,0.12753,0
.12827,0.12938,0.13734,0.14101,0.12907,0.11248,0.12904,0.13055,0.13155
,0.10897,0.36101,0.35484,0.36226,0.35229,0.36074,0.36621,0.36162,0.274
87,0.27795,0.29487,0.27912,0.26783,0.28893,0.28314,0.29405,0.27221,0.3
0716,0.28677,0.28422,0.20464,0.2041,0.2034,0.14205,0.12273,0.13793,0.1
2919,0.12989,0.12335,0.12121,0.12923,0.13809,0.13091,0.11686,0.11986,0
.13639,0.12911,0.12938,0.35553,0.36459,0.36769,0.35988,0.36466,0.3493,
0.36207,0.36081,0.35756,0.28193,0.29474,0.28166,0.29646,0.28675,0.2842
1,0.27872,0.28192,0.29045,0.30761,0.27986,0.12875,0.12559,0.13289,0.12
04,0.12964,0.12887,0.13281,0.13207,0.12061,0.099233,0.13539,0.11498,0.
13167,0.12993,0.12912,0.1357,0.13475,0.12756,0.36172,0.36609,0.35814,0
.35367,0.36541,0.35105,0.3657,0.35227,0.35566,0.2782,0.25355,0.21161,0
.18579,0.20555,0.19842,0.20742,0.20757,0.20363,0.2082,0.21289,0.14302,
0.13201,0.13048,0.1293,0.11436,0.13,0.12296,0.12617,0.14404,0.12947,0.
1194,0.12949,0.11327,0.13415,0.13734,0.1293,0.12956,0.11403,0.35408,0.
35406,0.35226,0.36165,0.3646,0.36049,0.35815,0.3566,0.36409,0.21497,0.
21208,0.20641,0.21684,0.20661,0.20606,0.20195,0.19254,0.20559,0.21572,
0.21098,0.1318,0.14143,0.13408,0.12941,0.12131,0.12905,0.13667,0.13136

,0.1316,0.12715,0.13741,0.1068,0.1293,0.13752,0.14217,0.13878,0.14519,
0.12319,0.35863,0.36471,0.34863,0.37113,0.36479,0.36765,0.35837,0.2048
,0.19324,0.1729,0.20381,0.19687,0.20352,0.21976,0.20867,0.21954,0.224,
0.20489,0.20714,0.20314,0.1375,0.15111,0.11574,0.12859,0.13426,0.1293,
0.11981,0.13293,0.13185,0.11065,0.13293,0.11688,0.11928,0.11055,0.1280
1,0.13083,0.15433,0.12968,0.36779,0.3654,0.34866,0.36086,0.3632,0.3499
6,0.21109,0.16301,0.21913,0.27354,0.20437,0.19666,0.19767,0.23071,0.20
609,0.20395,0.23569,0.20437,0.20914,0.20877,0.13284,0.14125,0.11637,0.
12861,0.11941,0.12503,0.12283,0.13482,0.12965,0.13018,0.12699,0.12892,
0.12096,0.12701,0.13017,0.13373,0.12966,0.12979,0.35833,0.35431,0.3684
6,0.34971,0.35906,0.20331,0.1969,0.21089,0.21488,0.20903,0.20541,0.210
13,0.20781,0.20563,0.20524,0.19932,0.21791,0.21717,0.20073,0.20683,0.1
1639,0.11045,0.12442,0.12958,0.13264,0.1425,0.13539,0.13004,0.13484,0.
13269,0.13933,0.13837,0.12981,0.12971,0.12761,0.15031,0.12884,0.12965,
0.36353,0.35381,0.35301,0.18385,0.22033,0.20811,0.19167,0.21698,0.2066
1,0.20457,0.20034,0.20479,0.19543,0.20332,0.20658,0.18448,0.2042,0.191
82,0.19613,0.20635,0.12244,0.12897,0.12945,0.13913,0.1286,0.13825,0.11
7,0.12113,0.12967,0.12934,0.14145,0.12956,0.11718,0.11418,0.13444,0.11
563,0.13388,0.12845,0.35747,0.20895,0.20663,0.24444,0.24273,0.19954,0.
19921,0.2232,0.20435,0.20983,0.22119,0.22612,0.21446,0.21031,0.211,0.2
0041,0.20167,0.20615,0.20457,0.13308,0.13876,0.15186,0.13079,0.13256,0
.13457,0.10059,0.13119,0.12884,0.1296,0.12934,0.12963,0.15062,0.13952,
0.14916,0.12921,0.13561,0.054755,0.21476,0.20957,0.22735,0.20838,0.183
65,0.19039,0.21237,0.18809,0.19998,0.23935,0.20385,0.20244,0.21786,0.2
2823,0.20512,0.20425,0.20875,0.2047,0.20156,0.13825,0.12342,0.13321,0.
1258,0.12946,0.1293,0.13774,0.13651,0.08642,0.13051,0.13024,0.13526,0.
13093,0.13333,0.10634,0.1126,0.13057,0.054859,0.22949,0.23613,0.20693,
0.18698,0.18126,0.20242,0.20759,0.20759,0.21178,0.20122,0.20337,0.2069
3,0.19975,0.19852,0.19887,0.20822,0.18892,0.20008,0.12895,0.10725,0.12
966,0.13399,0.12952,0.11695,0.12881,0.12598,0.12427,0.12747,0.12932,0.
12925,0.13058,0.13432,0.11766,0.13036,0.1357,0.12237,0.054369,0.18099,
0.22099,0.23699,0.20461,0.20656,0.2105,0.21461,0.21143,0.18894,0.20416
,0.19859,0.20825,0.21131,0.20897,0.21005,0.20861,0.20682,0.20831,0.123
5,0.13161,0.12888,0.11779,0.16009,0.12788,0.12945,0.1292,0.1279,0.1393
,0.12678,0.13523,0.14094,0.11612,0.13185,0.12931,0.12875,0.055157,0.05
461,0.054697,0.15276,0.20584,0.19184,0.21116,0.18641,0.21159,0.19936,0
.21676,0.22508,0.2149,0.19323,0.19757,0.19715,0.20119,0.20768,0.12495,
0.14121,0.13307,0.12916,0.12916,0.14493,0.11734,0.1234,0.12931,0.12976
,0.154,0.13464,0.11951,0.11971,0.12996,0.14449,0.12743,0.12974,0.05453
9,0.054467,0.055276,0.054481,0.20636,0.1834,0.21179,0.20366,0.20718,0.
21002,0.21794,0.20657,0.20648,0.20945,0.2064,0.19595,0.12927,0.13273,0
.13058,0.11653,0.13473,0.12922,0.12936,0.15639,0.12986,0.12893,0.1245,
0.18193,0.11435,0.13384,0.12958,0.13088,0.1256,0.1295,0.10335,0.12898,
0.054559,0.055023,0.055,0.055261,0.05509,0.054867,0.18635,0.16628,0.22
047,0.20707,0.2041,0.20958,0.22491,0.20824,0.11796,0.11922,0.13209,0.1
4322,0.12358,0.1311,0.13038,0.12269,0.14135,0.1282,0.11868,0.14404,0.1
2966,0.13567,0.13391,0.12951,0.12651,0.12927,0.12417,0.13821,0.13029,0
.12713,0.13865,0.05519,0.054822,0.055068,0.054742,0.054383,0.054855,0.
2492,0.20428,0.21052,0.23411,0.22297,0.055044,0.054793,0.13052,0.12842
,0.13914,0.12521,0.12938,0.11513,0.11825,0.12338,0.13208,0.12525,0.116
82,0.12338,0.13672,0.13228,0.12912,0.13611,0.13422,0.1402,0.1106,0.124
13,0.12919,0.05465,0.054966,0.054958,0.054325,0.054914,0.055403,0.0551

25,0.055198,0.054754,0.054883,0.20925,0.20585,0.055253,0.054744,0.0551
63,0.055069,0.12944,0.13246,0.12668,0.12518,0.10735,0.14329,0.13518,0.
12933,0.1292,0.12952,0.12977,0.12841,0.11128,0.1231,0.13618,0.12902,0.
12959,0.14379,0.12446,0.054502,0.054879,0.054627,0.054979,0.054538,0.0
54662,0.054815,0.054878,0.054835,0.054534,0.054601,0.054846,0.054667,0
.054483,0.054871,0.054692,0.055098,0.12937,0.12595,0.15227,0.14256,0.1
2736,0.13026,0.12219,0.14808,0.1466,0.12298,0.13612,0.1302,0.12963,0.1
2883,0.12979,0.12895,0.055218,0.054876,0.055109,0.055504,0.055175,0.05
4183,0.054281,0.055359,0.055192,0.055191,0.054615,0.054874,0.05537,0.0
54902,0.05528,0.054974,0.055054,0.055377,0.055174,0.11101,0.12877,0.10
26,0.13124,0.12921,0.12906,0.12798,0.11661,0.12966,0.129,0.13633,0.129
14,0.11996,0.05461,0.055242,0.054521,0.054796,0.055188,0.05498,0.05513
8,0.055066,0.055292,0.055518,0.054824,0.054826,0.054827,0.054707,0.055
335,0.05476,0.05491,0.05482,0.055259,0.055389,0.055461,0.12957,0.12995
,0.13004,0.13426,0.12245,0.14246,0.13014,0.13857,0.1395,0.055065,0.054
962,0.055107,0.055004,0.055106,0.055237,0.054627,0.054823,0.054293,0.0
55236,0.05465,0.054636,0.055371,0.054997,0.055227,0.054962,0.054713,0.
054744,0.05491,0.054915,0.053985,0.054745,0.054976,0.054967,0.054783,0
.1394,0.13562,0.12189,0.12118,0.054926,0.054428,0.055158,0.055511,0.05
4829,0.055106,0.055041,0.054624,0.054788,0.055064,0.054723,0.055031,0.
05491,0.05511,0.054843,0.055746,0.054621,0.055409,0.054357,0.054797,0.
055372,0.055246,0.054276,0.055398,0.054454,0.055135,0.055113,0.055024,
0.054993,0.05461,0.054288,0.054513,0.054936,0.0544,0.054966,0.054931,0
.054786,0.05503,0.05466,0.054423,0.054277,0.0549,0.05491,0.054861,0.05
4481,0.054907,0.05432,0.054613,0.055495,0.055318,0.055047,0.054532,0.0
54795,0.054874,0.054986,0.054787,0.055035,0.054745,0.054672,0.054789,0
.054758,0.054599,0.054503,0.054619,0.054275,0.05468,0.054756,0.055086,
0.054601,0.055057,0.055282,0.054752,0.054617,0.055276,0.055179,0.05481
6,0.054801,0.054955,0.054926,0.055234,0.054724,0.055068,0.055263,0.054
546,0.05539,0.054797,0.054805,0.055023,0.054514,0.055025,0.054875,0.05
5026,0.054477,0.055387,0.054629,0.05446,0.05542,0.055329,0.054906,0.05
4656,0.054932,0.055007,0.055201,0.054933,0.05475,0.054632,0.055321,0.0
54208,0.054882,0.055089,0.054631,0.054684,0.054631,0.055109,0.054353,0
.055214,0.055333,0.055022,0.054412,0.05404,0.054609,0.055384,0.05489,0
.054864,0.054966,0.054712,0.055284,0.054949,0.055143,0.054339,0.055019
,0.05512,0.055378,0.054682,0.055156,0.055107,0.054919,0.054405,0.05508
5,0.054326,0.055516,0.055023,0.054869,0.055055,0.054593,0.055139,0.054
536,0.054547,0.054883,0.055071,0.05525,0.054901,0.055354,0.055039,0.05
4687,0.055068,0.054198,0.054757,0.054285,0.054652,0.054676,0.05536,0.0
54935,0.054531,0.055308,0.055016,0.054871,0.055163,0.054634,0.055254,0
.054508,0.05505,0.05475,0.055076,0.054848,0.054151,0.055229,0.055183,0
.054439,0.054739,0.054978,0.054875,0.054736,0.0553,0.054652,0.055227,0
.055147,0.055152,0.054066,0.055069,0.055198,0.054686,0.054773,0.054533
,0.054419
0.20381,0.20529,0.20485,0.20454,0.2044,0.20443,0.20502,0.20478,0.20416
,0.20533,0.20523,0.20498,0.20456,0.20392,0.20434,0.20476,0.20448,0.205
57,0.20438,0.20437,0.27108,0.29856,0.28243,0.27973,0.2051,0.20578,0.20
535,0.20518,0.20551,0.205,0.20431,0.20442,0.2047,0.20523,0.20575,0.205
01,0.20425,0.20457,0.20458,0.35132,0.28323,0.29219,0.2815,0.27533,0.28
97,0.28621,0.20495,0.20488,0.20421,0.20425,0.20519,0.2043,0.20528,0.20
487,0.205,0.20399,0.20447,0.2052,0.20512,0.20467,0.20399,0.36058,0.355
65,0.27789,0.25574,0.28087,0.26289,0.27653,0.28009,0.28015,0.28118,0.2

0508,0.20473,0.20493,0.20447,0.20546,0.20413,0.2052,0.2047,0.20483,0.2
0477,0.2057,0.20525,0.20502,0.2048,0.20437,0.36094,0.35479,0.36424,0.2
8089,0.29768,0.26834,0.27997,0.27984,0.27747,0.28312,0.28697,0.28267,0
.20512,0.20531,0.20523,0.20482,0.20473,0.20507,0.20483,0.20597,0.20498
,0.20492,0.20483,0.20495,0.20407,0.20548,0.20469,0.20442,0.35973,0.354
85,0.35624,0.3578,0.29204,0.28317,0.27805,0.28692,0.27411,0.26313,0.29
086,0.28607,0.26432,0.27057,0.20465,0.20388,0.20546,0.20514,0.20544,0.
20441,0.20448,0.2041,0.20472,0.20608,0.20505,0.20509,0.20547,0.20439,0
.20428,0.20424,0.36263,0.36259,0.35389,0.35736,0.36604,0.35212,0.28062
,0.28319,0.27563,0.28742,0.27966,0.2911,0.29727,0.28428,0.30804,0.2805
6,0.20451,0.2056,0.20513,0.20403,0.20443,0.20507,0.20473,0.20558,0.205
09,0.20448,0.20455,0.20505,0.20547,0.20451,0.20488,0.20373,0.20518,0.3
4579,0.35005,0.35386,0.35873,0.35621,0.35934,0.28279,0.29056,0.28344,0
.27818,0.2841,0.28523,0.27347,0.28982,0.2947,0.29149,0.29326,0.20563,0
.20379,0.20447,0.20453,0.20498,0.20519,0.2068,0.20532,0.20489,0.20435,
0.20517,0.20555,0.20491,0.12651,0.1367,0.13217,0.20621,0.35208,0.35106
,0.3652,0.36415,0.35442,0.36986,0.36371,0.27702,0.27885,0.28796,0.2839
6,0.26826,0.26565,0.28109,0.27015,0.26891,0.26692,0.29439,0.20481,0.20
46,0.20486,0.20484,0.2042,0.20416,0.20517,0.20389,0.20637,0.20456,0.20
436,0.13025,0.10503,0.12567,0.12932,0.12838,0.11652,0.20399,0.35329,0.
37406,0.36657,0.3567,0.35473,0.35581,0.36146,0.29048,0.28081,0.28177,0
.286,0.26306,0.30313,0.28906,0.28657,0.2837,0.27706,0.29761,0.20515,0.
20495,0.20535,0.20647,0.20459,0.20445,0.20487,0.20443,0.20442,0.12924,
0.12925,0.13243,0.12916,0.12885,0.12926,0.13034,0.12721,0.13566,0.2042
4,0.36302,0.35912,0.3458,0.35201,0.35598,0.35434,0.36237,0.2898,0.2775
2,0.28299,0.27934,0.29637,0.29937,0.28176,0.28935,0.28339,0.29449,0.28
135,0.26153,0.20551,0.2048,0.20501,0.20443,0.2057,0.20474,0.129,0.127,
0.12786,0.12879,0.13716,0.14093,0.12892,0.11184,0.12889,0.13013,0.1313
,0.10812,0.35459,0.35152,0.36101,0.34768,0.35908,0.36155,0.35912,0.272
86,0.27541,0.29349,0.27603,0.26654,0.28658,0.28306,0.29286,0.27221,0.3
0707,0.28423,0.2862,0.20507,0.20493,0.20407,0.142,0.12224,0.13769,0.12
865,0.12963,0.1231,0.12056,0.12909,0.13779,0.13075,0.11624,0.11904,0.1
3596,0.1287,0.12923,0.356,0.35994,0.36278,0.35853,0.36113,0.34674,0.36
425,0.3584,0.35361,0.28116,0.29447,0.28105,0.29757,0.28381,0.28459,0.2
7667,0.28079,0.28811,0.30918,0.27941,0.12858,0.1253,0.13217,0.1202,0.1
2928,0.12861,0.13244,0.13177,0.12027,0.098768,0.13524,0.11446,0.13142,
0.1298,0.12859,0.13546,0.13429,0.12728,0.35787,0.36605,0.35454,0.35117
,0.36597,0.35074,0.36027,0.34882,0.35566,0.27826,0.25294,0.21078,0.185
26,0.20435,0.19703,0.20757,0.20572,0.20166,0.20587,0.21218,0.14276,0.1
3156,0.12998,0.12929,0.11374,0.12947,0.1227,0.12563,0.14413,0.12915,0.
11897,0.12923,0.11296,0.13375,0.13708,0.12886,0.1293,0.11363,0.3546,0.
35258,0.34986,0.3581,0.3646,0.35948,0.3533,0.35186,0.35947,0.21355,0.2
1241,0.20532,0.21509,0.20512,0.20483,0.20164,0.19186,0.20541,0.21497,0
.21033,0.13135,0.14111,0.13367,0.12904,0.12086,0.12862,0.13622,0.13114
,0.13138,0.12687,0.13709,0.1061,0.12888,0.13735,0.14189,0.13867,0.1451
8,0.12263,0.35766,0.36439,0.34775,0.36691,0.36248,0.36379,0.35319,0.20
465,0.19113,0.17252,0.20252,0.19625,0.20206,0.2184,0.20889,0.21965,0.2
2201,0.20329,0.20646,0.20324,0.13723,0.15128,0.11521,0.12854,0.13386,0
.12892,0.11941,0.13283,0.13133,0.11015,0.13265,0.11616,0.11883,0.10993
,0.1274,0.13063,0.15404,0.1292,0.36337,0.35408,0.34228,0.35696,0.36252
,0.34656,0.20942,0.16207,0.21587,0.27046,0.20176,0.19614,0.19662,0.228
77,0.20464,0.20356,0.23421,0.20261,0.20728,0.20906,0.1324,0.14091,0.11

613,0.12829,0.11877,0.12491,0.12248,0.13483,0.12934,0.12999,0.12653,0.
12873,0.1206,0.12682,0.12984,0.13348,0.12935,0.12946,0.35156,0.34951,0
.36488,0.3454,0.35744,0.20222,0.19416,0.20909,0.21314,0.20631,0.20128,
0.20906,0.20568,0.20316,0.20476,0.1978,0.21749,0.21728,0.19961,0.20626
,0.11577,0.11004,0.12404,0.12954,0.13215,0.14214,0.13522,0.12996,0.134
82,0.13249,0.13908,0.13821,0.12926,0.12941,0.12706,0.15015,0.12863,0.1
2925,0.35805,0.35536,0.35378,0.18179,0.22096,0.20619,0.18662,0.2162,0.
20686,0.20387,0.19851,0.20307,0.19375,0.20161,0.20618,0.18369,0.20358,
0.19138,0.19695,0.20423,0.1221,0.12887,0.12929,0.13894,0.12827,0.13764
,0.11654,0.12051,0.12946,0.12901,0.14134,0.12919,0.11654,0.11349,0.134
04,0.11518,0.13345,0.12804,0.35509,0.20704,0.20362,0.24298,0.24076,0.1
9662,0.19653,0.2196,0.2014,0.20611,0.22028,0.22411,0.21273,0.20886,0.2
1016,0.19965,0.20129,0.20318,0.20303,0.1329,0.13851,0.15207,0.13043,0.
13244,0.13422,0.099683,0.13094,0.12852,0.12924,0.12902,0.12927,0.1505,
0.13924,0.14922,0.1291,0.13523,0.053391,0.21166,0.20694,0.22302,0.2054
8,0.18187,0.18928,0.20942,0.18559,0.19909,0.23565,0.20242,0.20131,0.21
699,0.22538,0.20311,0.20386,0.20857,0.20293,0.20124,0.13813,0.12295,0.
13299,0.12535,0.12921,0.12912,0.13769,0.1363,0.085342,0.13,0.12983,0.1
3516,0.13046,0.13319,0.10567,0.11208,0.13051,0.053294,0.22775,0.23423,
0.20579,0.18389,0.18031,0.19925,0.20743,0.20486,0.20721,0.19925,0.2018
3,0.20608,0.19856,0.19718,0.19798,0.20713,0.18871,0.19787,0.12901,0.10
661,0.12919,0.13361,0.12915,0.11645,0.12873,0.1257,0.12361,0.12724,0.1
2903,0.12871,0.13018,0.13411,0.11725,0.13022,0.13556,0.12167,0.052761,
0.1786,0.21811,0.23376,0.20323,0.20567,0.20668,0.21259,0.2089,0.18833,
0.2028,0.19752,0.20687,0.21021,0.20828,0.20962,0.20777,0.20621,0.20749
,0.12294,0.13126,0.12861,0.11705,0.16006,0.12743,0.12907,0.12878,0.127
44,0.1392,0.12665,0.13509,0.1408,0.11576,0.13166,0.12908,0.12849,0.053
745,0.053271,0.053478,0.15188,0.20364,0.19003,0.20926,0.18354,0.21061,
0.19787,0.21463,0.22295,0.21481,0.19136,0.19554,0.19591,0.19969,0.2054
6,0.12453,0.14135,0.1327,0.12904,0.12876,0.14484,0.11688,0.12286,0.129
08,0.12908,0.15401,0.13436,0.11883,0.11906,0.12974,0.14461,0.127,0.129
34,0.053146,0.053038,0.053695,0.053389,0.2042,0.1828,0.21014,0.20277,0
.20627,0.21003,0.21512,0.20595,0.20355,0.20838,0.20591,0.19476,0.12898
,0.13244,0.13032,0.11602,0.13466,0.12874,0.12925,0.15615,0.12968,0.128
45,0.12425,0.1826,0.11372,0.13334,0.12918,0.13054,0.12536,0.1289,0.102
57,0.12864,0.053266,0.053559,0.053469,0.053921,0.053636,0.053403,0.185
42,0.16357,0.21926,0.2056,0.20319,0.20953,0.22278,0.20789,0.1171,0.118
61,0.13183,0.14323,0.12304,0.13087,0.13023,0.12239,0.14112,0.12823,0.1
1812,0.14398,0.1293,0.13548,0.13369,0.12886,0.12616,0.12899,0.12362,0.
13815,0.1299,0.12675,0.13858,0.053742,0.053493,0.053715,0.053271,0.052
859,0.053334,0.24786,0.20152,0.20893,0.23058,0.22315,0.053676,0.053567
,0.13028,0.12803,0.13901,0.12474,0.12914,0.11466,0.11763,0.12284,0.131
73,0.12469,0.11624,0.12304,0.13652,0.13183,0.12911,0.13587,0.13413,0.1
3999,0.10987,0.12371,0.12908,0.05335,0.053502,0.053402,0.052981,0.0534
71,0.054102,0.05364,0.053756,0.053455,0.053423,0.20714,0.20552,0.05365
8,0.053379,0.053553,0.053505,0.12926,0.13208,0.1265,0.12473,0.10692,0.
14309,0.13485,0.12907,0.12891,0.12918,0.12931,0.12791,0.11072,0.12271,
0.13591,0.12865,0.12918,0.14373,0.12424,0.053299,0.053502,0.053149,0.0
53617,0.053211,0.053402,0.05332,0.053315,0.053431,0.053268,0.053364,0.
053424,0.053175,0.053214,0.053756,0.053189,0.053602,0.12877,0.12571,0.
15214,0.14252,0.12693,0.13027,0.12157,0.14801,0.14628,0.12265,0.13601,
0.13012,0.12917,0.12832,0.12951,0.12888,0.053762,0.05334,0.053828,0.05

4029,0.053708,0.052721,0.053032,0.05372,0.053886,0.053658,0.052867,0.0
53425,0.054031,0.053235,0.053913,0.053589,0.053726,0.053784,0.053902,0
.11012,0.12875,0.10189,0.13073,0.12886,0.12851,0.12755,0.11589,0.1292,
0.12908,0.13587,0.12869,0.11946,0.053405,0.053825,0.052926,0.053244,0.
053479,0.053332,0.053582,0.053715,0.053752,0.053909,0.053526,0.053526,
0.05355,0.053476,0.053866,0.053198,0.053416,0.053311,0.053706,0.053779
,0.053732,0.12903,0.12965,0.12979,0.13396,0.12188,0.14228,0.12958,0.13
845,0.13923,0.053646,0.05358,0.053693,0.053511,0.053509,0.054108,0.053
303,0.053386,0.052936,0.05387,0.053415,0.053542,0.053877,0.053334,0.05
3698,0.053359,0.053297,0.053356,0.053507,0.053693,0.052637,0.053507,0.
053577,0.053594,0.05338,0.13928,0.13539,0.12154,0.1209,0.053674,0.0529
17,0.05388,0.054014,0.053418,0.053923,0.053603,0.053246,0.053431,0.053
749,0.053343,0.053537,0.05374,0.053621,0.053581,0.054108,0.05319,0.054
106,0.052879,0.053178,0.054202,0.053559,0.053023,0.054061,0.053054,0.0
53799,0.05368,0.053603,0.053493,0.053236,0.052925,0.053153,0.053429,0.
052845,0.053476,0.053509,0.053464,0.053559,0.053129,0.053054,0.052912,
0.053341,0.053327,0.053548,0.05312,0.053587,0.052957,0.053004,0.054061
,0.053983,0.053607,0.052846,0.053474,0.053458,0.05366,0.053402,0.0536,
0.053285,0.053313,0.053347,0.053304,0.053247,0.053031,0.053462,0.05281
2,0.053055,0.053665,0.053696,0.053205,0.053803,0.053772,0.053405,0.053
327,0.054051,0.05368,0.053236,0.053274,0.053767,0.053402,0.054063,0.05
3296,0.053401,0.053437,0.053288,0.053726,0.053498,0.053492,0.053729,0.
053178,0.053452,0.053477,0.053239,0.053428,0.053787,0.053347,0.053111,
0.053774,0.05386,0.053332,0.053391,0.053549,0.053518,0.053754,0.053492
,0.053161,0.053255,0.054144,0.052763,0.053527,0.053658,0.053181,0.0533
1,0.05312,0.053864,0.052937,0.053888,0.053756,0.05362,0.052992,0.05257
9,0.053214,0.053918,0.053264,0.053525,0.053451,0.053155,0.053745,0.053
518,0.053754,0.052869,0.053453,0.053514,0.053883,0.053122,0.053773,0.0
53689,0.053512,0.052951,0.053715,0.052764,0.053818,0.053522,0.053541,0
.053626,0.053098,0.053563,0.053099,0.053359,0.053301,0.053787,0.053656
,0.053331,0.054058,0.053641,0.053223,0.053436,0.052796,0.053345,0.0530
02,0.053332,0.053437,0.053978,0.053448,0.053358,0.053596,0.053776,0.05
3563,0.053598,0.053278,0.053734,0.053099,0.053828,0.053392,0.053677,0.
053591,0.052873,0.053791,0.053761,0.053018,0.053346,0.053483,0.053431,
0.05346,0.053739,0.053244,0.053624,0.053845,0.05391,0.052799,0.05377,0
.05373,0.053413,0.053249,0.053164,0.053016

#simdataS2SNR10:

Kep_f=1.2
Kep_f=1.2
Kep_s=0.1
Ktrans_f=0.05
Ktrans_s=0.03
del_t=0.5
gtSf=0 0 0 0 0 0
0 0 0 0 0 0
0 0 0 0 0 0
0 0 0 0 0 0
0 0 0 0 0 0
0 0 0 0 0 0

0	0	0	0	0	0
0	0	0	0	0	0
0	0	0	0	0	0
0	0	0	0	0	0
0	0	0	0	0	0
0	0	0	0	0	0
0	0	0	0	0	0
0	0	0	0	0	0
0	0	0	0	0	0
0	0	0	0	0	0
0	0	0	0	0	0
0	0	0	0	0	0
0	0	0	0	0	0
0	0	0	0	0	0
0	0	0	0	0	0
0	0	0	0	0	0
0	0	0	0	0	0
0	0	0	0	0	0
0	0	0	0	0	0
0	0	0	0	0	0
0	0	0	0	0	0
0	0	0	0	0	0
0	0	0	0	0	0
0	0	0	0	0	0
0	0	0	0	0	0
0	0	0	0	0	0
0	0	0	0	0	0
0	0	0	0	0	0
0	0	0	0.48788	0.4016	0.5
0	0	0	0	0	0
0	0	0	0	0	0
0	0	0	0	0	0
0	0	0	0	0	0
0	0	0	0	0	0
0.5	0.48958	0.51688	0.49748	0.5	0.5
0	0	0	0	0	0
0	0	0	0	0	0
0	0	0	0	0	0
0	0	0	0	0	0
0	0	0	0	0.49712	0.52542
0.54881	0.5	0.45786	0.58441	0.44144	0.5
0.5	0	0	0	0	0
0	0	0	0	0	0
0	0	0	0	0	0
0	0	0	0	0	0
0	0	0	0.5	0.5	0.51937
0.46427	0.46033	0.58304	0.38975	0.5	0.42868
0.49787	0.53538	0.46906	0	0	0
0	0	0	0	0	0
0	0	0	0	0	0
0	0	0	0	0	0
0	0.4956	0.5	0.49878	0.45529	0.5

0.51735	0.56124	0.36458	0.34977	0.60809	0.5
0.45641	0.52548	0.50356	0.32237	0	0
0	0	0	0	0	0
0	0	0	0	0	0
0	0	0	0	0	0
0.55319	0.57323	0.39421	0.60372	0.44338	0.40867
0.46995	0.5	0.5	0.4953	0.50638	0.47214
0.53703	0.49064	0.5	0.4385	0.57015	0.65463
0	0	0	0	0	0
0	0	0	0	0	0.29885
0.33333	0.33333	0.37346	0.33333	0.33333	0.4113
0.37962	0.32845	0.50295	0.65898	0.5	0.57887
0.48211	0.45957	0.50711	0.5	0.42856	0.52176
0.47387	0.61593	0.5521	0.5	0.51382	0.50613
0.5	0.4732	0	0	0	0
0	0	0	0	0	0.5
0.33333	0.34641	0.31335	0.33679	0.33078	0.36239
0.33333	0.26111	0.29701	0.33117	0.4902	0.56579
0.5	0.5	0.5778	0.50579	0.5	0.48379
0.55932	0.56748	0.55153	0.5	0.57006	0.6572
0.49108	0.5	0.62159	0.55882	0	0
0	0	0	0	0	0.5
0.47983	0.5	0.37143	0.27875	0.33892	0.34393
0.33333	0.33718	0.32399	0.3714	0.3565	0.33333
0.50072	0.50559	0.48237	0.57375	0.49524	0.47695
0.5	0.51865	0.52027	0.56117	0.50242	0.5
0.5	0.46403	0.49451	0.4484	0.46985	0.46359
0	0	0	0	0	0
0.48359	0.5	0.5	0.48814	0.48388	0.37028
0.29854	0.30911	0.31688	0.29846	0.23201	0.35088
0.3773	0.37421	0.43934	0.59955	0.56057	0.4964
0.47256	0.53816	0.5	0.5	0.54382	0.47829
0.5103	0.40092	0.5	0.37852	0.49696	0.53406
0.52738	0.44727	0	0	0	0
0	0.46951	0.46252	0.51922	0.5387	0.5
0.5	0.33879	0.3193	0.33333	0.38245	0.37769
0.3056	0.38699	0.33306	0.30576	0.46914	0.3817
0.5	0.5	0.45621	0.50192	0.5	0.58985
0.5	0.52221	0.5	0.41588	0.56489	0.45051
0.39597	0.50116	0.5	0.60506	0	0
0	0.46055	0.5083	0.57875	0.5	0.51104
0.46564	0.59449	0.45016	0.35741	0.36102	0.32181
0.35346	0.29176	0.32509	0.26418	0.38926	0.34484
0.56216	0.56623	0.42121	0.61252	0.45594	0.61907
0.5	0.5	0.6578	0.5	0.4081	0.5
0.5	0.5	0.5	0.51944	0.5	0.5
0	0.5	0.5246	0.58769	0.5	0.5
0.50753	0.6022	0.52153	0.5	0.33496	0.30401
0.32451	0.39694	0.3258	0.29246	0.36064	0.3642
0.33472	0.44062	0.50142	0.50702	0.5	0.49457
0.63548	0.50575	0.5	0.54678	0.55056	0.52148
0.48933	0.59137	0.5	0.51251	0.5	0.52368

1	0.51071	0.42041	0.41941	0.5484	0.42841
0.47943	0.5	0.49659	0.5	0.49792	0.32573
0.27728	0.31384	0.33333	0.40421	0.33333	0.33333
0.33333	0.32435	0.48626	0.49151	0.46002	0.5
0.60185	0.46735	0.51721	0.5556	0.49856	0.5
0.48016	0.5	0.4952	0.49675	0.48556	0.51357
0.5	1	0.5	0.43546	0.44426	0.5
0.45113	0.46201	0.53916	0.5	0.58374	0.52789
0.2516	0.34597	0.3455	0.31005	0.34964	0.34256
0.33333	0.29902	0.31537	0.5	0.50629	0.42994
0.53647	0.46446	0.4708	0.50417	0.54984	0.41404
0.53651	0.52977	0.44448	0.5	0.5	0.54104
0.5	0.45927	1	0.54427	0.53255	0.49234
0.4058	0.54147	0.5124	0.57415	0.5	0.55778
0.47409	0.26881	0.31394	0.26788	0.35136	0.39486
0.38893	0.28575	0.33974	0.5	0.57905	0.46628
0.5	0.44865	0.5	0.53992	0.5	0.53329
0.52523	0.5	0.5	0.5621	0.5	0.55267
0.45649	0.5	1	1	1	0.5
0.50036	0.5	0.62469	0.51272	0.65151	0.50324
0.4391	0.5	0.32991	0.33866	0.33333	0.33029
0.34798	0.30604	0.44142	0.5	0.46458	0.43204
0.5	0.60219	0.49922	0.47904	0.51772	0.51093
0.49432	0.60802	0.43536	0.52283	0.64106	0.50593
0.5	0.53837	1	1	1	1
0.5	0.47126	0.53011	0.52147	0.62139	0.56676
0.64262	0.45923	0.5183	0.36691	0.29924	0.38289
0.55223	0.35103	0.44313	0.5	0.4557	0.59393
0.49766	0.4748	0.43572	0.39305	0.5	0.5
0.63181	0.47635	0.51873	0.6092	0.5311	0.61869
0.53113	0.48711	1	1	1	1
1	1	0.56794	0.5	0.60584	0.53255
0.48872	0.57467	0.5	0.51988	0.51513	0.55747
0.65644	0.46825	0.49153	0.50829	0.51547	0.55986
0.50243	0.3509	0.5	0.5	0.43441	0.45751
0.3588	0.53542	0.51881	0.50296	0.51161	0.51743
0.5	0.38025	0.49649	1	1	1
1	1	1	0.40482	0.47027	0.44036
0.42049	0.49288	1	1	0.51766	0.5081
0.579	0.5	0.52693	0.50605	0.55014	0.5
0.54847	0.54504	0.5	0.59358	0.5147	0.5
0.44926	0.45984	0.47679	0.53662	0.5	0.67886
0.5061	1	1	1	1	1
1	1	1	1	1	0.57246
0.47126	1	1	1	1	0.5641
0.5	0.57012	0.5	0.62407	0.49001	0.51524
0.55315	0.50665	0.57962	0.50851	0.51792	0.5749
0.57432	0.54367	0.4548	0.5	0.45995	0.45688
1	1	1	1	1	1
1	1	1	1	1	1
1	1	1	1	1	0.44788
0.50866	0.47601	0.54675	0.53696	0.51934	0.51793

0.5 0.54436 0.5 0.47289 0.48232 0.5
0.5 0.57555 0.5 1 1 1
1 1 1 1 1 1
1 1 1 1 1 1
1 1 1 1 0.45574 0.58724
0.5 0.5803 0.57404 0.44077 0.50775 0.49038
0.45757 0.5 0.47068 0.48294 0.49407 1
1 1 1 1 1 1
1 1 1 1 1 1
1 1 1 1 1 1
1 1 0.5 0.48613 0.5 0.57569
0.388 0.5 0.48894 0.5 0.51356 1
1 1 1 1 1 1
1 1 1 1 1 1
1 1 1 1 1 1
1 1 1 1 1 1
0.52793 0.57432 0.49476 0.47425 1 1
1 1 1 1 1 1
1 1 1 1 1 1
1 1 1 1 1 1
1 1 1 1 1 1
1 1 1 1 1 1
1 1 1 1 1 1
1 1 1 1 1 1
1 1 1 1 1 1
1 1 1 1 1 1
1 1 1 1 1 1
1 1 1 1 1 1
1 1 1 1 1 1
1 1 1 1 1 1
1 1 1 1 1 1
1 1 1 1 1 1
1 1 1 1 1 1
1 1 1 1 1 1
1 1 1 1 1 1
1 1 1 1 1 1
1 1 1 1 1 1
1 1 1 1 1 1
1 1 1 1 1 1
1 1 1 1 1 1
1 1 1 1 1 1
1 1 1 1 1 1
1 1 1 1 1 1
1 1 1 1 1 1
1 1 1 1 1 1
1 1 1 1 1 1
1 1 1 1 1 1
1 1 1 1 1 1
1 1 1 1 1 1
1
gtSs=1 1 1 1 1 1
1 1 1 1 1 1

1	1	1	1	1	1
1	1	0.46302	0.49237	0.41438	0.50909
1	1	1	1	1	1
1	1	1	1	1	1
1	1	1	0	0.42851	0.50406
0.4391	0.53129	0.5	0.42881	1	1
1	1	1	1	1	1
1	1	1	1	1	1
1	0	0	0.50065	0.33799	0.5
0.5	0.5	0.46771	0.56737	0.62347	1
1	1	1	1	1	1
1	1	1	1	1	1
1	1	0	0	0	0.5
0.55049	0.58763	0.60102	0.43952	0.5	0.51178
0.57323	0.41332	1	1	1	1
1	1	1	1	1	1
1	1	1	1	1	1
0	0	0	0	0.45238	0.44329
0.463	0.5	0.52498	0.72464	0.52909	0.48391
0.37621	0.52838	1	1	1	1
1	1	1	1	1	1
1	1	1	1	1	1
0	0	0	0	0	0
0.46644	0.5	0.5	0.50283	0.40598	0.49726
0.5801	0.48939	0.5	0.5	1	1
1	1	1	1	1	1
1	1	1	1	1	1
1	1	1	0	0	0
0	0	0	0.43313	0.44056	0.48851
0.40375	0.39797	0.60925	0.53406	0.49596	0.5
0.44509	0.6674	1	1	1	1
1	1	1	1	1	1
1	1	1	0.51212	0.5984	0.5
1	0	0	0	0	0
0	0	0.50462	0.51061	0.43778	0.5
0.47913	0.5	0.49339	0.5	0.48657	0.50287
0.5	1	1	1	1	1
1	1	1	1	1	1
0.5	0.51042	0.48312	0.50252	0.5	0.5
1	0	0	0	0	0
0	0	0.5	0.51728	0.487	0.44636
0.5	0.5	0.44474	0.5	0.49363	0.5
0.60713	1	1	1	1	1
1	1	1	1	0.50288	0.47458
0.45119	0.5	0.54214	0.41559	0.55856	0.5
0.5	1	0	0	0	0
0	0	0	0.54654	0.5	0.5
0.66526	0.38678	0.53093	0.42684	0.44745	0.47417
0.46406	0.5	0.5415	1	1	1
1	1	1	0.5	0.5	0.48063
0.53573	0.53967	0.41696	0.61025	0.5	0.57132
0.50213	0.46462	0.53094	0	0	0

0	0	0	0	0.5	0.5
0.5646	0.50596	0.54039	0.51797	0.5	0.38842
0.5	0.45949	0.45868	0.63348	1	1
1	0.5044	0.5	0.50122	0.54471	0.5
0.48265	0.43876	0.63542	0.65023	0.39191	0.5
0.54359	0.47452	0.49644	0.67763	0	0
0	0	0	0	0	0
0	0.59287	0.50451	0.58063	0.45952	0.46142
0.5	0.44086	0.56001	0.42732	0.5	0.47241
0.44681	0.42677	0.60579	0.39628	0.55662	0.59133
0.53005	0.5	0.5	0.5047	0.49362	0.52786
0.46297	0.50936	0.5	0.5615	0.42985	0.34537
0	0	0	0	0	0
0	0	0	0.5	0.5	0.33359
0.33333	0.33333	0.25309	0.33333	0.33333	0.18298
0.39621	0.25281	0.49705	0.34102	0.5	0.42113
0.51789	0.54043	0.49289	0.5	0.57144	0.47824
0.52613	0.38407	0.4479	0.5	0.48618	0.49387
0.5	0.5268	0	0	0	0
0	0	0	0	0	0
0.33333	0.29752	0.34332	0.33679	0.25921	0.27522
0.33333	0.36944	0.36503	0.33442	0.5098	0.43421
0.5	0.5	0.4222	0.49421	0.5	0.51621
0.44068	0.43252	0.44847	0.5	0.42994	0.3428
0.50892	0.5	0.37841	0.44118	0	0
0	0	0	0	0	0
0	0	0.25714	0.32568	0.32216	0.31214
0.33333	0.32564	0.27816	0.4074	0.3565	0.33333
0.49928	0.49441	0.51763	0.42625	0.50476	0.52305
0.5	0.48135	0.47973	0.43883	0.49758	0.5
0.5	0.53597	0.50549	0.5516	0.53015	0.53641
0	0	0	0	0	0
0	0	0	0	0	0.32889
0.34532	0.36636	0.34156	0.26793	0.38399	0.29824
0.24638	0.33104	0.56066	0.40045	0.43943	0.5036
0.52744	0.46184	0.5	0.5	0.45618	0.52171
0.4897	0.59908	0.5	0.62148	0.50304	0.46594
0.47262	0.55273	0	0	0	0
0	0	0	0	0	0
0	0.33879	0.38293	0.33333	0.26654	0.33201
0.35798	0.25267	0.33347	0.34712	0.53086	0.6183
0.5	0.5	0.54379	0.49808	0.5	0.41015
0.5	0.47779	0.5	0.58412	0.43511	0.54949
0.60403	0.49884	0.5	0.39494	0	0
0	0	0	0	0	0
0	0	0	0.35341	0.33046	0.3391
0.35346	0.26697	0.30427	0.36791	0.32477	0.31848
0.43784	0.43377	0.57879	0.38748	0.54406	0.38093
0.5	0.5	0.3422	0.5	0.5919	0.5
0.5	0.5	0.5	0.48056	0.5	0.5
0	0	0	0	0	0
0	0	0	0	0.33007	0.32215

0.40325	0.33664	0.34643	0.41752	0.36064	0.24129
0.32089	0.55938	0.49858	0.49298	0.5	0.50543
0.36452	0.49425	0.5	0.45322	0.44944	0.47852
0.51067	0.40863	0.5	0.48749	0.5	0.47632
0	0	0	0	0	0
0	0	0	0	0	0.35377
0.36371	0.34572	0.33333	0.34416	0.33333	0.33333
0.33333	0.32695	0.51374	0.50849	0.53998	0.5
0.39815	0.53265	0.48279	0.4444	0.50144	0.5
0.51984	0.5	0.5048	0.50325	0.51444	0.48643
0.5	0	0	0	0	0
0	0	0	0	0	0
0.38516	0.36029	0.27638	0.34498	0.28878	0.31487
0.33333	0.3259	0.68463	0.5	0.49371	0.57006
0.46353	0.53554	0.5292	0.49583	0.45016	0.58596
0.46349	0.47023	0.55552	0.5	0.5	0.45896
0.5	0.54073	0	0	0	0
0	0	0	0	0	0
0	0.3656	0.34629	0.36606	0.3367	0.3428
0.3229	0.3664	0.32053	0.5	0.42095	0.53372
0.5	0.55135	0.5	0.46008	0.5	0.46671
0.47477	0.5	0.5	0.4379	0.5	0.44733
0.54351	0.5	0	0	0	0
0	0	0	0	0	0
0	0	0.39979	0.32218	0.33333	0.33151
0.32335	0.3707	0.55858	0.5	0.53542	0.56796
0.5	0.39781	0.50078	0.52096	0.48228	0.48907
0.50568	0.39198	0.56464	0.47717	0.35894	0.49407
0.5	0.46163	0	0	0	0
0	0	0	0	0	0
0	0	0	0.26618	0.31209	0.23422
0.44777	0.64897	0.55687	0.5	0.5443	0.40607
0.50234	0.5252	0.56428	0.60695	0.5	0.5
0.36819	0.52365	0.48127	0.3908	0.4689	0.38131
0.46887	0.51289	0	0	0	0
0	0	0	0	0	0
0	0	0	0	0.48487	0.44253
0.34356	0.53175	0.50847	0.49171	0.48453	0.44014
0.49757	0.6491	0.5	0.5	0.56559	0.54249
0.6412	0.46458	0.48119	0.49704	0.48839	0.48257
0.5	0.61975	0.50351	0	0	0
0	0	0	0	0	0
0	0	0	0	0.48234	0.4919
0.421	0.5	0.47307	0.49395	0.44986	0.5
0.45153	0.45496	0.5	0.40642	0.4853	0.5
0.55074	0.54016	0.52321	0.46338	0.5	0.32114
0.4939	0	0	0	0	0
0	0	0	0	0	0
0	0	0	0	0	0.4359
0.5	0.42988	0.5	0.37593	0.50999	0.48476
0.44685	0.49335	0.42038	0.49149	0.48208	0.4251
0.42568	0.45633	0.5452	0.5	0.54005	0.54312

0	0	0	0	0	0
0	0	0	0	0	0
0	0	0	0	0	0.55212
0.49134	0.52399	0.45325	0.46304	0.48066	0.48207
0.5	0.45564	0.5	0.52711	0.51768	0.5
0.5	0.42445	0.5	0	0	0
0	0	0	0	0	0
0	0	0	0	0	0
0	0	0	0	0.54426	0.41276
0.5	0.4197	0.42596	0.55923	0.49225	0.50962
0.54243	0.5	0.52932	0.51706	0.50593	0
0	0	0	0	0	0
0	0	0	0	0	0
0	0	0	0	0	0
0	0	0.5	0.51387	0.5	0.42431
0.612	0.5	0.51106	0.5	0.48644	0
0	0	0	0	0	0
0	0	0	0	0	0
0	0	0	0	0	0
0	0	0	0	0	0
0.47207	0.42568	0.50524	0.52575	0	0
0	0	0	0	0	0
0	0	0	0	0	0
0	0	0	0	0	0
0	0	0	0	0	0
0	0	0	0	0	0
0	0	0	0	0	0
0	0	0	0	0	0
0	0	0	0	0	0
0	0	0	0	0	0
0	0	0	0	0	0
0	0	0	0	0	0
0	0	0	0	0	0
0	0	0	0	0	0
0	0	0	0	0	0
0	0	0	0	0	0
0	0	0	0	0	0
0	0	0	0	0	0
0	0	0	0	0	0
0	0	0	0	0	0
0	0	0	0	0	0
0	0	0	0	0	0
0	0	0	0	0	0
0	0	0	0	0	0
0	0	0	0	0	0
0	0	0	0	0	0
0	0	0	0	0	0
0	0	0	0	0	0
0	0	0	0	0	0
0	0	0	0	0	0
0	0	0	0	0	0
0	0	0	0	0	0
0	0	0	0	0	0

0 0 0 0 0 0
0
gtSp=0 0 0 0 0 0
0 0 0 0 0 0
0 0 0 0 0 0
0 0 0.53698 0.50763 0.58562 0.49091
0 0 0 0 0 0
0 0 0 0 0 0
0 0 0 1 0.57149 0.49594
0.5609 0.46871 0.5 0.57119 0 0
0 0 0 0 0 0
0 0 0 0 0 0
0 1 1 0.49935 0.66201 0.5
0.5 0.5 0.53229 0.43263 0.37653 0
0 0 0 0 0 0
0 0 0 0 0 0
0 0 1 1 1 0.5
0.44951 0.41237 0.39898 0.56048 0.5 0.48822
0.42677 0.58668 0 0 0 0
0 0 0 0 0 0
0 0 0 0 0 0
1 1 1 1 0.54762 0.55671
0.537 0.5 0.47502 0.27536 0.47091 0.51609
0.62379 0.47162 0 0 0 0
0 0 0 0 0 0
0 0 0 0 0 0
1 1 1 1 1 1
0.53356 0.5 0.5 0.49717 0.59402 0.50274
0.4199 0.51061 0.5 0.5 0 0
0 0 0 0 0 0
0 0 0 0 0 0
0 0 0 1 1 1
1 1 1 0.56687 0.55944 0.51149
0.59625 0.60203 0.39075 0.46594 0.50404 0.5
0.55491 0.3326 0 0 0 0
0 0 0 0 0 0
0 0 0 0 0 0
0 1 1 1 1 1
1 1 0.49538 0.48939 0.56222 0.5
0.52087 0.5 0.50661 0.5 0.51343 0.49713
0.5 0 0 0 0 0
0 0 0 0 0 0
0 0 0 0 0 0
0 1 1 1 1 1
1 1 0.5 0.48272 0.513 0.55364
0.5 0.5 0.55526 0.5 0.50637 0.5
0.39287 0 0 0 0 0
0 0 0 0 0 0
0 0 0 0 0 0
0 0 1 1 1 1
1 1 1 0.45346 0.5 0.5
0.33474 0.61322 0.46907 0.57316 0.55255 0.52583

0.53594	0.5	0.4585	0	0	0
0	0	0	0	0	0
0	0	0	0	0	0
0	0	0	1	1	1
1	1	1	1	0.5	0.5
0.4354	0.49404	0.45961	0.48203	0.5	0.61158
0.5	0.54051	0.54132	0.36652	0	0
0	0	0	0	0	0
0	0	0	0	0	0
0	0	0	0	1	1
1	1	1	1	1	1
1	0.40713	0.49549	0.41937	0.54048	0.53858
0.5	0.55914	0.43999	0.57268	0.5	0.52759
0	0	0	0	0	0
0	0	0	0	0	0
0	0	0	0	0	0
1	1	1	1	1	1
1	1	1	0.5	0.5	0.36756
0.33333	0.33333	0.37346	0.33333	0.33333	0.40572
0.22417	0.41874	0	0	0	0
0	0	0	0	0	0
0	0	0	0	0	0
0	0	1	1	1	1
1	1	1	1	1	0.5
0.33333	0.35607	0.34332	0.32642	0.41001	0.36239
0.33333	0.36944	0.33797	0.33442	0	0
0	0	0	0	0	0
0	0	0	0	0	0
0	0	0	0	1	1
1	1	1	1	1	0.5
0.52017	0.5	0.37143	0.39557	0.33892	0.34393
0.33333	0.33718	0.39785	0.22121	0.287	0.33333
0	0	0	0	0	0
0	0	0	0	0	0
0	0	0	0	0	0
1	1	1	1	1	1
0.51641	0.5	0.5	0.51186	0.51612	0.30083
0.35614	0.32453	0.34156	0.43362	0.38399	0.35088
0.37632	0.29475	0	0	0	0
0	0	0	0	0	0
0	0	0	0	0	0
0	0	1	1	1	1
1	0.53049	0.53748	0.48078	0.4613	0.5
0.5	0.32242	0.29777	0.33333	0.35101	0.2903
0.33642	0.36034	0.33347	0.34712	0	0
0	0	0	0	0	0
0	0	0	0	0	0
0	0	0	0	1	1
1	0.53945	0.4917	0.42125	0.5	0.48896
0.53436	0.40551	0.54984	0.28918	0.30852	0.3391
0.29309	0.44128	0.37064	0.36791	0.28597	0.33669
0	0	0	0	0	0

0	0	0	0	0	0
0	0	0	0	0	0
1	0.5	0.4754	0.41231	0.5	0.5
0.49247	0.3978	0.47847	0.5	0.33496	0.37384
0.27224	0.26642	0.32777	0.29002	0.27873	0.39451
0.34439	0	0	0	0	0
0	0	0	0	0	0
0	0	0	0	0	0
0	0.48929	0.57959	0.58059	0.4516	0.57159
0.52057	0.5	0.50341	0.5	0.50208	0.3205
0.35901	0.34044	0.33333	0.25163	0.33333	0.33333
0.33333	0.3487	0	0	0	0
0	0	0	0	0	0
0	0	0	0	0	0
0	0	0.5	0.56454	0.55574	0.5
0.54887	0.53799	0.46084	0.5	0.41626	0.47211
0.36324	0.29374	0.37812	0.34498	0.36158	0.34256
0.33333	0.37509	0	0	0	0
0	0	0	0	0	0
0	0	0	0	0	0
0	0	0	0.45573	0.46745	0.50766
0.5942	0.45853	0.4876	0.42585	0.5	0.44222
0.52591	0.3656	0.33977	0.36606	0.31194	0.26234
0.28817	0.34785	0.33974	0	0	0
0	0	0	0	0	0
0	0	0	0	0	0
0	0	0	0	0	0.5
0.49964	0.5	0.37531	0.48728	0.34849	0.49676
0.5609	0.5	0.2703	0.33917	0.33333	0.33819
0.32867	0.32325	0	0	0	0
0	0	0	0	0	0
0	0	0	0	0	0
0	0	0	0	0	0
0.5	0.52874	0.46989	0.47853	0.37861	0.43324
0.35738	0.54077	0.4817	0.36691	0.38866	0.38289
0	0	0	0	0	0
0	0	0	0	0	0
0	0	0	0	0	0
0	0	0	0	0	0
0	0	0.43206	0.5	0.39416	0.46745
0.51128	0.42533	0.5	0.48012	0	0
0	0	0	0	0	0
0	0	0	0	0	0
0	0	0	0	0	0
0	0	0	0	0	0
0	0	0	0.59518	0.52973	0.55964
0.57951	0.50712	0	0	0	0
0	0	0	0	0	0
0	0	0	0	0	0
0	0	0	0	0	0
0	0	0	0	0	0
0	0	0	0	0	0.42754

0.52874	0	0	0	0	0
0	0	0	0	0	0
0	0	0	0	0	0
0	0	0	0	0	0
0	0	0	0	0	0
0	0	0	0	0	0
0	0	0	0	0	0
0	0	0	0	0	0
0	0	0	0	0	0
0	0	0	0	0	0
0	0	0	0	0	0
0	0	0	0	0	0
0	0	0	0	0	0
0	0	0	0	0	0
0	0	0	0	0	0
0	0	0	0	0	0
0	0	0	0	0	0
0	0	0	0	0	0
0	0	0	0	0	0
0	0	0	0	0	0
0	0	0	0	0	0
0	0	0	0	0	0
0	0	0	0	0	0
0	0	0	0	0	0
0	0	0	0	0	0
0	0	0	0	0	0
0	0	0	0	0	0
0	0	0	0	0	0
0	0	0	0	0	0
0	0	0	0	0	0
0	0	0	0	0	0
0	0	0	0	0	0
0	0	0	0	0	0
0	0	0	0	0	0
0	0	0	0	0	0
0	0	0	0	0	0
0	0	0	0	0	0
0	0	0	0	0	0
0	0	0	0	0	0
0	0	0	0	0	0
0	0	0	0	0	0
0	0	0	0	0	0
0	0	0	0	0	0
0	0	0	0	0	0
0	0	0	0	0	0
0	0	0	0	0	0
0	0	0	0	0	0
0	0	0	0	0	0
0	0	0	0	0	0
0	0	0	0	0	0
0	0	0	0	0	0
0	0	0	0	0	0

0	0	0	0	0	0
0	0	0	0	0	0
0	0	0	0	0	0
0	0	0	0	0	0
0	0	0	0	0	0
0					

gtTCf=
0
0.11925
0.14534
0.13574
0.11593
0.096197
0.079948
0.067704
0.058926
0.052819
0.048646
0.045817
0.043898
0.042582
0.041662
0.040998
0.040498
0.040102

gtTCs=
0
0.07155
0.116
0.14393
0.16177
0.17342
0.18126
0.18671
0.19067
0.19366
0.19601
0.19792
0.19952
0.20088
0.20206
0.20309
0.20398
0.20476

gtTCp=
0
2.385
1.5979
1.1196

0.8287
0.65143
0.54309
0.47655
0.43538
0.4096
0.39316
0.38239
0.37506
0.36982
0.36585
0.36266
0.35995
0.35753

X_mask=
0.0030685,2.2204e-016,2.2204e-016,2.2204e-016,2.2204e-016,2.2204e-016,0.004374,2.2204e-016,0.0011435,2.2204e-016,0.0097365,2.2204e-016,2.2204e-016,0.012221,0.00020301,0.0020754,0.0010208,0.0035671,0.012452,0.0018718,2.2204e-016,0.036644,2.2204e-016,2.2204e-016,2.2204e-016,2.2204e-016,0.010207,0.00056438,0.006216,2.2204e-016,0.0012772,0.0035536,2.2204e-016,0.0091167,0.0083212,0.0059096,2.2204e-016,0.0011077,2.2204e-016,2.2204e-016,2.2204e-016,2.2204e-016,0.013554,2.2204e-016,0.017722,2.2204e-016,2.2204e-016,0.00094705,0.0037262,0.00080915,2.2204e-016,2.2204e-016,0.0037398,2.2204e-016,2.2204e-016,2.2204e-016,0.0014895,0.0026633,0.0016327,2.2204e-016,2.2204e-016,0.0045177,2.2204e-016,0.040232,2.2204e-016,0.022117,0.019791,0.0074744,0.042956,2.2204e-016,0.0088503,2.2204e-016,0.0015828,2.2204e-016,2.2204e-016,2.2204e-016,0.0027895,0.0027944,0.0052966,2.2204e-016,2.2204e-016,2.2204e-016,0.0018904,2.2204e-016,2.2204e-016,0.0046743,0.0096896,2.2204e-016,2.2204e-016,2.2204e-016,0.033871,2.2204e-016,2.2204e-016,2.2204e-016,0.047579,2.2204e-016,2.2204e-016,2.2204e-016,2.2204e-016,0.010278,0.0012764,0.0064898,0.0095092,0.0022309,0.00050207,2.2204e-016,2.2204e-016,2.2204e-016,2.2204e-016,0.0084324,0.0057722,2.2204e-016,0.012171,2.2204e-016,0.040593,2.2204e-016,0.013132,2.2204e-016,0.060174,0.0055757,0.031659,0.014337,2.2204e-016,0.0036402,0.014674,2.2204e-016,2.2204e-016,2.2204e-016,2.2204e-016,2.2204e-016,2.2204e-016,2.2204e-016,2.2204e-016,0.003538,0.0043874,0.00018597,0.0023329,0.00012237,0.0079741,2.2204e-016,0.0049791,0.0076445,0.0038364,0.0040629,0.0081828,2.2204e-016,0.020728,0.077192,2.2204e-016,0.043128,0.036202,0.033268,0.012408,2.2204e-016,2.2204e-016,0.0048157,2.2204e-016,0.027145,2.2204e-016,0.012795,2.2204e-016,0.00045552,0.0049595,2.2204e-016,0.0085097,2.2204e-016,0.0027878,0.0024895,2.2204e-016,2.2204e-016,0.0014493,2.2204e-016,0.00060938,0.0051862,2.2204e-016,0.0010655,2.2204e-016,0.033569,0.048231,2.2204e-016,0.06976,2.2204e-016,2.2204e-016,0.0026037,2.2204e-016,2.2204e-016,2.2204e-016,2.2204e-016,2.2204e-

016,2.2204e-016,2.2204e-016,2.2204e-016,2.2204e-016,0.015118,2.2204e-
016,0.0044628,2.2204e-016,2.2204e-016,2.2204e-016,2.2204e-016,2.2204e-
016,0.0034763,0.0019999,0.00063095,2.2204e-016,0.0028808,9.4152e-
005,2.2204e-016,0.0034196,0.0022847,0.0067042,2.2204e-016,2.2204e-
016,0.10805,2.2204e-016,2.2204e-016,2.2204e-016,2.2204e-
016,0.034077,2.2204e-016,2.2204e-016,0.0046019,2.2204e-016,2.2204e-
016,0.014376,0.037084,0.01903,0.0071546,0.033264,0.0030493,2.2204e-
016,0.0036129,2.2204e-016,0.0059466,0.0065724,2.2204e-016,2.2204e-
016,2.2204e-016,0.0038595,0.0044341,0.0023314,2.2204e-016,2.2204e-
016,2.2204e-016,2.2204e-016,0.00036804,2.2204e-016,2.2204e-
016,2.2204e-016,2.2204e-016,2.2204e-016,2.2204e-016,0.090582,2.2204e-
016,2.2204e-016,2.2204e-016,2.2204e-016,2.2204e-016,2.2204e-
016,0.016866,2.2204e-016,2.2204e-016,2.2204e-016,0.037634,2.2204e-
016,0.00049778,0.010135,2.2204e-
016,0.0028273,0.0047116,0.0054795,2.2204e-016,2.2204e-
016,0.0027231,0.0029324,2.2204e-016,0.0023259,2.2204e-016,2.2204e-
016,0.0015064,0.0019775,2.2204e-016,0.0024695,2.2204e-
016,0.02171,0.020982,0.025307,0.025621,0.038599,2.2204e-
016,0.01313,0.024061,2.2204e-016,0.026365,2.2204e-
016,0.023438,2.2204e-016,2.2204e-016,0.0020372,2.2204e-016,2.2204e-
016,2.2204e-016,2.2204e-016,2.2204e-016,0.004206,2.2204e-
016,0.0044833,2.2204e-016,0.0070236,0.00023302,2.2204e-
016,0.0020582,0.00408,0.0042748,2.2204e-016,2.2204e-
016,0.00059489,0.0019834,2.2204e-016,2.2204e-
016,0.00075252,0.15154,2.2204e-016,0.080111,0.067728,0.012242,2.2204e-
016,2.2204e-016,0.02278,0.012638,0.010098,2.2204e-
016,0.0064652,2.2204e-016,2.2204e-016,0.039942,2.2204e-
016,0.047248,0.021324,2.2204e-016,0.0048948,2.2204e-016,2.2204e-
016,2.2204e-016,0.0028422,0.0058552,2.2204e-016,2.2204e-
016,0.0036445,2.2204e-016,2.2204e-016,2.2204e-016,0.0047932,2.2204e-
016,0.00045214,0.00036146,0.00037389,2.2204e-016,2.2204e-
016,0.0078897,2.2204e-016,0.017532,2.2204e-016,0.086263,2.2204e-
016,0.055307,2.2204e-016,2.2204e-016,0.035734,2.2204e-
016,0.023535,2.2204e-016,2.2204e-016,2.2204e-016,2.2204e-
016,0.036866,0.011497,0.017464,2.2204e-016,0.0007003,2.2204e-
016,2.2204e-016,2.2204e-016,2.2204e-016,2.2204e-
016,0.00040084,2.2204e-
016,0.00076639,0.0020436,0.0019496,0.0032334,0.0024711,2.2204e-
016,0.0027691,0.00089989,0.0041416,2.2204e-
016,0.052959,0.045109,0.023868,2.2204e-
016,0.042509,0.068229,0.016969,2.2204e-016,2.2204e-
016,0.031698,0.0046815,2.2204e-016,0.009106,2.2204e-
016,0.03674,2.2204e-016,0.018904,2.2204e-
016,0.044467,0.0010136,0.00073302,2.2204e-
016,0.0014016,0.00083345,2.2204e-016,0.003152,0.0032401,2.2204e-
016,2.2204e-016,0.00063865,0.0045838,0.0045664,2.2204e-016,2.2204e-
016,0.0013851,2.2204e-016,0.0041051,2.2204e-
016,0.023124,0.072425,0.051257,0.17464,2.2204e-016,0.038022,2.2204e-
016,2.2204e-016,0.041912,2.2204e-016,0.035603,0.0034473,2.2204e-
016,2.2204e-016,0.005178,2.2204e-
016,0.018765,0.019414,0.010894,0.010321,2.2204e-016,2.2204e-
016,2.2204e-016,2.2204e-016,2.2204e-016,2.2204e-

016,0.0045066,0.0021409,0.0029124,2.2204e-
016,0.0025844,0.0014978,2.2204e-016,0.0020949,2.2204e-016,2.2204e-
016,0.0025743,0.060235,2.2204e-016,2.2204e-
016,0.044056,0.042284,2.2204e-016,0.02872,0.013256,2.2204e-
016,0.0021936,2.2204e-016,2.2204e-
016,0.054713,0.0044473,0.020621,0.03333,0.0040573,0.0051484,2.2204e-
016,0.0074991,2.2204e-016,0.003131,2.2204e-016,0.0031216,2.2204e-
016,2.2204e-
016,0.00022334,0.00044499,0.0028364,0.0017623,0.0045956,2.2204e-
016,0.00072722,2.2204e-016,0.00075972,2.2204e-016,2.2204e-
016,0.0033052,0.014312,0.0014771,0.0064453,0.057773,2.2204e-
016,0.014078,0.0008548,2.2204e-016,0.020581,0.014324,2.2204e-
016,2.2204e-016,0.010451,0.016303,2.2204e-016,0.021226,2.2204e-
016,2.2204e-016,2.2204e-016,0.011545,2.2204e-016,2.2204e-
016,0.0052844,2.2204e-016,0.0036378,0.00078492,0.0025908,2.2204e-
016,0.0013165,2.2204e-016,0.0019123,2.2204e-016,2.2204e-016,2.2204e-
016,0.00018534,0.00093307,2.2204e-016,2.2204e-
016,0.056812,0.045425,0.0051524,2.2204e-
016,0.015878,0.0060945,2.2204e-016,0.0433,2.2204e-016,2.2204e-
016,2.2204e-016,2.2204e-016,2.2204e-
016,0.0061174,0.0060213,0.0015804,0.024196,0.037895,2.2204e-
016,0.0022068,2.2204e-
016,0.0014682,0.0028184,0.0002515,0.0039516,0.0059682,0.0058949,0.0015
417,0.00030008,0.0032758,0.0014431,2.2204e-016,0.0027716,2.2204e-
016,0.0012645,2.2204e-016,2.2204e-016,2.2204e-016,2.2204e-
016,0.038673,0.043208,0.038908,0.099306,0.0065362,0.013425,2.2204e-
016,0.01098,2.2204e-016,2.2204e-016,0.01695,0.023771,2.2204e-
016,0.0090705,2.2204e-016,0.020984,0.015145,2.2204e-
016,0.053983,0.0021179,2.2204e-016,2.2204e-016,2.2204e-
016,0.0013782,0.00052915,0.0010479,2.2204e-016,2.2204e-016,2.2204e-
016,0.0068392,0.0031131,2.2204e-016,2.2204e-016,0.003046,2.2204e-
016,2.2204e-016,2.2204e-016,0.034424,0.0018515,2.2204e-
016,0.027276,2.2204e-016,2.2204e-016,2.2204e-016,0.02958,2.2204e-
016,2.2204e-016,0.024828,2.2204e-016,2.2204e-016,2.2204e-016,2.2204e-
016,2.2204e-016,2.2204e-016,2.2204e-016,0.06219,0.0016565,2.2204e-
016,0.0038331,2.2204e-016,2.2204e-
016,0.0013687,0.0024013,0.0021543,0.0027958,2.2204e-016,2.2204e-
016,0.0059349,2.2204e-016,2.2204e-016,0.0024944,2.2204e-016,2.2204e-
016,0.0014041,0.035175,0.010141,0.024787,2.2204e-
016,0.053174,0.040674,2.2204e-016,0.026337,2.2204e-
016,0.012235,0.0054407,0.032988,2.2204e-016,2.2204e-016,2.2204e-
016,0.014293,2.2204e-016,0.004951,2.2204e-016,2.2204e-
016,0.0045219,0.00091885,2.2204e-016,0.0029617,2.2204e-016,2.2204e-
016,0.0026886,0.0053286,0.0043011,0.0033572,0.0031217,0.00084863,2.220
4e-016,0.0022841,0.0024899,2.2204e-016,2.2204e-016,0.015228,2.2204e-
016,2.2204e-016,2.2204e-016,0.009834,2.2204e-016,2.2204e-
016,0.042316,2.2204e-016,2.2204e-016,2.2204e-
016,0.011016,0.023328,2.2204e-016,0.0064258,0.022151,2.2204e-
016,0.0027134,2.2204e-016,2.2204e-016,0.0054936,0.0029938,2.2204e-
016,0.0028763,2.2204e-016,0.0046997,2.2204e-016,2.2204e-
016,0.0010462,0.0039261,2.2204e-016,0.0019031,2.2204e-016,2.2204e-
016,0.0038686,0.0032784,0.00042593,0.035451,0.015731,0.042779,0.031457

,0.028075,2.2204e-016,0.029428,0.025666,0.00030811,2.2204e-016,2.2204e-016,2.2204e-016,0.030472,2.2204e-016,0.029692,0.039308,0.022642,0.0073926,2.2204e-016,0.0054804,0.0039839,2.2204e-016,0.0043288,0.0016175,2.2204e-016,2.2204e-016,2.2204e-016,0.0038627,0.0038675,2.2204e-016,2.2204e-016,2.2204e-016,0.00068903,2.2204e-016,2.2204e-016,0.0029987,0.0078475,0.0093329,2.2204e-016,2.2204e-016,2.2204e-016,0.013827,0.023411,0.0076299,2.2204e-016,0.016724,2.2204e-016,2.2204e-016,2.2204e-016,2.2204e-016,2.2204e-016,0.0061785,2.2204e-016,0.0017294,0.0030031,2.2204e-016,0.0065302,2.2204e-016,2.2204e-016,2.2204e-016,0.0025491,2.2204e-016,0.0047739,2.2204e-016,0.0024763,0.00098973,2.2204e-016,0.0044746,2.2204e-016,2.2204e-016,2.2204e-016,2.2204e-016,2.2204e-016,0.0065619,0.00095811,2.2204e-016,2.2204e-016,2.2204e-016,0.024146,0.011741,0.016488,2.2204e-016,2.2204e-016,0.062593,2.2204e-016,0.0011202,0.010939,0.0035477,0.0014366,3.5091e-005,0.00079899,0.00062427,2.2204e-016,2.2204e-016,0.0019098,2.2204e-016,0.003172,0.00096879,5.9199e-006,0.0053819,2.2204e-016,2.2204e-016,0.0037379,0.0014046,2.2204e-016,0.0036114,0.0018821,0.001436,2.2204e-016,0.0027867,0.001266,2.2204e-016,0.0033505,2.2204e-016,2.2204e-016,0.0076414,2.2204e-016,0.012664,2.2204e-016,2.2204e-016,0.017153,0.0010112,2.2204e-016,0.0048121,0.002546,9.0675e-005,2.2204e-016,0.0010343,2.2204e-016,0.002154,2.2204e-016,2.2204e-016,2.2204e-016,2.2204e-016,2.2204e-016,2.2204e-016,0.00013637,2.2204e-016,2.2204e-016,2.2204e-016,0.0052861,2.2204e-016,2.2204e-016,2.2204e-016,0.0067639,2.2204e-016,0.0032181,2.2204e-016,2.2204e-016,2.2204e-016,2.2204e-016,0.011176,0.00099206,0.022779,2.2204e-016,0.0039804,0.0031404,2.2204e-016,0.0011352,2.2204e-016,2.2204e-016,0.0015354,0.0079071,2.2204e-016,0.0022237,0.0031313,0.0026836,2.2204e-016,2.2204e-016,0.0014953,2.2204e-016,2.2204e-016,0.00085627,0.0010683,2.2204e-016,2.2204e-016,0.0041698,0.00226,2.2204e-016,2.2204e-016,2.2204e-016,0.0021325,2.2204e-016,2.2204e-016,0.0057549,0.0083525,2.2204e-016,0.0016956,2.2204e-016,0.025189,2.2204e-016,2.2204e-016,0.0030239,0.003107,2.2204e-016,2.2204e-016,2.2204e-016,2.2204e-016,2.2204e-016,2.2204e-016,0.0020127,0.0030189,2.2204e-016,2.2204e-016,2.2204e-016,2.2204e-016,0.0026682,0.0010956,0.00028125,2.2204e-016,2.2204e-016,2.2204e-016,2.2204e-016,2.2204e-016,0.0032289,2.2204e-016,0.0028973,0.00081433,0.0020203,2.2204e-016,0.0064357,0.00036508,0.0089603,2.2204e-016,0.010925,2.2204e-016,2.2204e-016,2.2204e-016,0.0057535,2.2204e-016,0.0001404,0.010643,2.2204e-016,0.0015344,2.2204e-016,0.00281,2.2204e-016,2.2204e-016,2.2204e-016,2.2204e-016,2.2204e-016,6.0233e-005,0.0013796,2.2204e-016,2.2204e-016,2.2204e-016,2.2204e-016,0.0068342,0.0032938,0.0044504,2.2204e-016,2.2204e-016,2.2204e-016,2.2204e-016,0.0021831,2.2204e-016,0.00077259,2.2204e-016,2.2204e-016,2.2204e-016,2.2204e-016,2.2204e-016,2.2204e-016,2.2204e-016,0.007759,0.0037224,0.0069987,2.2204e-016,2.2204e-016,2.2204e-016,0.0033682,2.2204e-016,2.2204e-016,0.00024815,2.2204e-016,0.0013624,2.2204e-016,2.2204e-016,0.0039433,2.2204e-

016,0.0039596,2.2204e-016,0.0019664,2.2204e-016,0.00078357,2.2204e-
016,0.0020883,2.2204e-016,0.007504,2.2204e-016,2.2204e-016,2.2204e-
016,2.2204e-016,2.2204e-016,2.2204e-016,2.2204e-
016,0.001713,0.0094698,2.2204e-016,2.2204e-016,2.2204e-016,2.2204e-
016,0.0035868,0.0049329,2.2204e-016,2.2204e-016,2.2204e-016,2.2204e-
016,0.0013358,0.0034604,0.0050097,2.2204e-016,2.2204e-
016,0.0037518,2.2204e-016,0.00054526,0.0019621,2.2204e-
016,0.0042239,2.2204e-016,2.2204e-016,0.0078893,2.2204e-
016,0.00060347,2.2204e-016,2.2204e-
016,0.0030566,0.002739,0.0013653,0.00071766,0.0022973,2.2204e-
016,0.0050798,0.0042592,0.0013121,0.0012193,0.00063746,2.2204e-
016,2.2204e-016,2.2204e-016,2.2204e-016,0.0022006,2.2204e-016,2.2204e-
016,2.2204e-016,2.2204e-016,2.2204e-
016,0.010985,0.0013555,0.00026528,2.2204e-016,2.2204e-
016,0.0019861,2.2204e-016,2.2204e-016,2.2204e-
016,0.0045719,0.0006326,2.2204e-016,2.2204e-016,2.2204e-016,2.2204e-
016,0.00093542,2.2204e-016,0.00060524,0.0041798,2.2204e-
016,0.00093996,0.0034914,0.0015237,2.2204e-
016,0.0024591,0.0029091,0.0003937,2.2204e-016,0.0033718,2.2204e-
016,2.2204e-016,0.0017944,0.0020824,2.2204e-
016,0.0014675,0.0052423,2.2204e-016,2.2204e-016,2.2204e-
016,0.00047406,0.00091473,0.0004403,0.0023627,2.2204e-016,2.2204e-
016,2.2204e-016,0.0013215,3.3251e-006,0.0082803,2.2204e-
016,0.0018308,2.2204e-016,2.2204e-016,2.2204e-
016,0.0024163,0.0018022,2.2204e-016,2.2204e-016,0.0014841,2.2204e-
016,2.2204e-016,0.0020027,0.0036617,2.2204e-016,5.5292e-005,2.2204e-
016,0.0078926,2.2204e-016,2.2204e-016,0.001693,2.2204e-
016,0.0016643,0.0072206,0.010105,0.0025361,0.0054088,2.2204e-
016,2.2204e-016,0.0065656,0.0035042,0.0025087,2.2204e-
016,0.0015145,2.2204e-016,0.00096892,0.0026204,2.2204e-
016,0.0037793,2.2204e-016,2.2204e-016,0.0029207,2.2204e-
016,0.0023301,2.2204e-016,2.2204e-016,0.0053225,2.2204e-
016,0.0092346,2.2204e-016,2.2204e-
016,0.00079802,0.00079448,0.00016257,2.2204e-016,2.2204e-016,2.2204e-
016,0.0080023,2.2204e-016,2.2204e-016,2.2204e-016,2.2204e-016,2.2204e-
016,0.0017854,0.0031992,0.00094338,0.0022198,0.0039705,0.0099131,2.220
4e-016,0.0055613,0.0051987,2.2204e-016,2.2204e-016,0.0061364,2.2204e-
016,0.0055153,0.001588,0.0012244,2.2204e-016,2.2204e-
016,0.0040303,0.00066867,2.2204e-016,0.0051824,2.2204e-
016,0.00091381,2.2204e-016,0.00067634,2.2204e-016,2.2204e-
016,0.0046336,0.004478,0.00080161,2.2204e-016,2.2204e-
016,0.0047589,2.2204e-
016,0.00049626,0.0037475,0.00028147,0.001703,2.2204e-
016,0.0031452,2.2204e-016,2.2204e-016,0.004668,2.2204e-
016,0.0068466,0.00050979,2.2204e-016,0.0040545,0.0046587,2.2204e-
016,0.00043927,0.00067623,0.0016763,2.2204e-016,0.00033709,2.2204e-
016,2.2204e-016,0.0051377,0.0041248,2.2204e-
016,0.002927,0.00066479,0.0037765,2.2204e-016,0.0016742,2.2204e-
016,0.0069575,2.2204e-016,0.0031158,0.0045473,2.2204e-016,0.00018057
0.072317,0.073784,0.076856,0.075182,0.065173,0.059397,0.078953,0.06563
,0.073475,0.065839,0.079499,0.070998,0.066095,0.082367,0.070568,0.0753
37,0.070068,0.071107,0.07748,0.072463,1.3079,1.2816,1.4243,1.1776,0.06

436,0.065965,0.083209,0.07374,0.077142,0.067641,0.070407,0.073547,0.06
9978,0.077126,0.07704,0.078426,0.07226,0.075234,0.061807,2.2914,1.3637
,1.1781,1.4135,1.1206,1.2429,1.388,0.064465,0.068299,0.078468,0.07413,
0.065463,0.072781,0.074119,0.070624,0.066587,0.062633,0.073893,0.07541
9,0.06944,0.059055,0.067352,2.3433,2.3562,1.2669,1.5639,1.2274,1.2548,
1.2407,1.3335,1.0384,0.96781,0.070284,0.071748,0.064074,0.07067,0.0664
46,0.069368,0.073459,0.072384,0.07149,0.071839,0.059624,0.072191,0.070
325,0.069142,0.076958,2.4365,2.3442,2.3225,1.2353,1.1279,0.97038,0.977
79,1.3451,1.2799,1.1844,1.0454,1.3969,0.062919,0.084542,0.078263,0.080
026,0.081237,0.074702,0.074466,0.063766,0.068384,0.0737,0.063898,0.078
668,0.075967,0.060789,0.077055,0.064024,2.4194,2.309,2.4384,2.3136,1.3
816,1.3581,1.3524,1.2419,1.1668,0.70589,1.1618,1.2509,1.5017,1.1747,0.
068443,0.068223,0.063656,0.067484,0.070171,0.073063,0.074656,0.070472,
0.073678,0.074668,0.080237,0.069858,0.078484,0.080505,0.078717,0.07583
3,2.3781,2.3792,2.4138,2.4802,2.3981,2.4465,1.3434,1.2426,1.24,1.2146,
1.4222,1.2484,1.0358,1.2665,1.2229,1.2417,0.063889,0.07141,0.077347,0.
070817,0.076329,0.070357,0.072407,0.071445,0.062121,0.068375,0.074964,
0.065081,0.072611,0.081621,0.069903,0.07435,0.069648,2.4401,2.3853,2.3
496,2.4407,2.3153,2.3452,1.3914,1.2989,1.2373,1.4186,1.4365,0.98403,1.
1169,1.2123,1.2188,1.3066,0.84927,0.060761,0.076634,0.065559,0.073525,
0.074843,0.061947,0.068828,0.076782,0.076463,0.07148,0.064701,0.075213
,0.071236,0.093954,0.095589,0.098245,0.077329,2.3506,2.3399,2.4944,2.3
45,2.2639,2.3927,2.3889,1.22,1.1617,1.3524,1.2205,1.2435,1.1833,1.2363
,1.238,1.2902,1.2366,1.2821,0.076698,0.066721,0.074437,0.069904,0.0754
35,0.076867,0.068662,0.070128,0.068473,0.07503,0.076605,0.097321,0.092
842,0.097267,0.092614,0.094473,0.09925,0.064883,2.3636,2.3678,2.3686,2
.3813,2.3587,2.4957,2.3619,1.2149,1.1637,1.2399,1.3462,1.1927,1.2358,1
.303,1.2051,1.2113,1.2654,0.94727,0.068806,0.07792,0.061351,0.074774,0
.073901,0.075132,0.069147,0.066508,0.074478,0.098621,0.09426,0.098882,
0.087857,0.091377,0.098361,0.096132,0.09269,0.10076,0.070144,2.4052,2.
4208,2.4545,2.4202,2.4324,2.3538,2.3977,1.1483,1.2177,1.2613,0.81083,1
.5062,1.1515,1.3746,1.3454,1.2826,1.2619,1.2218,1.1101,0.071114,0.0755
66,0.06622,0.076043,0.067459,0.07758,0.095555,0.095299,0.097528,0.0970
96,0.096187,0.095967,0.083181,0.097309,0.098154,0.08594,0.0971,0.09595
9,2.5441,2.3619,2.4175,2.4423,2.3647,2.3759,2.317,1.2483,1.2251,1.0871
,1.2389,1.1312,1.1425,1.2118,1.4939,1.2095,1.3646,1.3518,0.90716,0.079
142,0.066412,0.066427,0.093289,0.10202,0.10134,0.086261,0.08889,0.0994
27,0.095523,0.088517,0.080687,0.10416,0.092406,0.095051,0.096404,0.094
424,0.083249,2.2536,2.3715,2.3487,2.422,2.3973,2.462,2.3359,2.4543,2.2
839,1.0209,1.2304,0.9963,1.334,1.3197,1.177,1.349,1.0353,1.4456,1.2522
,1.3037,0.095087,0.099959,0.0899,0.09991,0.087283,0.084562,0.090849,0.
098425,0.094403,0.097535,0.098205,0.097927,0.098947,0.098239,0.097483,
0.096254,0.10142,0.10722,2.3216,2.4191,2.4418,2.4098,2.3834,2.4223,2.4
552,2.4022,2.3674,1.1751,1.2701,0.93837,0.83691,0.86522,0.95736,0.9050
3,0.82834,1.0491,0.59534,1.0978,0.097246,0.1033,0.093689,0.10056,0.095
383,0.09086,0.096943,0.098664,0.090212,0.092911,0.094782,0.10373,0.103
29,0.094745,0.096555,0.098284,0.096225,0.097936,2.3907,2.4265,2.4485,2
.4373,2.5669,2.3424,2.4595,2.3607,2.3541,1.2911,0.84845,0.9545,0.88934
,0.81796,1.0011,0.92544,0.84468,0.95726,0.88432,0.86716,0.10421,0.0978
33,0.089156,0.094733,0.099318,0.09532,0.091096,0.098671,0.099491,0.099
279,0.09325,0.098594,0.10148,0.10196,0.097967,0.093596,0.098963,0.1001
9,2.3987,2.3841,2.2967,2.3763,2.4138,2.3845,2.4651,1.2609,1.3118,1.250

7,0.93177,0.97093,0.91741,0.87088,0.88556,0.88938,1.0088,0.60449,0.741
13,0.84694,0.09186,0.099414,0.09234,0.10356,0.094558,0.090767,0.094681
,0.097245,0.097043,0.097796,0.099944,0.094684,0.096207,0.093222,0.0962
7,0.092117,0.091963,0.098493,2.409,2.3944,2.3883,2.4435,2.3873,2.409,1
.2907,1.2188,1.2552,1.2885,1.2419,0.77641,0.91073,0.85712,0.86755,1.10
27,0.95243,0.86831,0.95334,0.77835,0.090346,0.10029,0.1032,0.091852,0.
097937,0.097476,0.099332,0.094406,0.0998,0.096824,0.097935,0.081614,0.
091926,0.082719,0.094492,0.098867,0.09558,0.089076,2.418,2.4136,2.4456
,2.3082,2.4033,1.3442,1.3097,1.229,1.1696,1.2005,1.2276,0.82091,0.7821
7,0.84631,0.91193,0.76036,0.89127,0.94208,0.82853,0.89889,0.094135,0.0
91492,0.097444,0.09583,0.099882,0.099991,0.10112,0.10035,0.096265,0.10
107,0.096928,0.087215,0.10084,0.089383,0.090027,0.091603,0.093201,0.09
4584,2.3019,2.4602,2.3787,1.3698,1.3086,1.0755,1.2875,1.2166,1.3529,1.
0206,1.3553,0.77775,0.82631,0.83995,0.76725,1.066,0.96233,0.94939,0.74
748,0.91794,0.099756,0.09654,0.088748,0.10068,0.096427,0.10251,0.09597
5,0.09363,0.099932,0.090631,0.097887,0.098284,0.094929,0.09125,0.09782
4,0.095784,0.090543,0.089826,2.39,1.2331,1.1899,1.0707,1.1849,1.212,1.
2157,1.0283,1.1752,1.1835,0.8796,0.94473,0.6891,0.70076,0.84368,0.7518
,0.72501,0.9833,0.93475,0.094196,0.095498,0.10104,0.0953,0.091525,0.10
267,0.097252,0.098222,0.099248,0.096688,0.095323,0.101,0.098379,0.0921
65,0.099725,0.092178,0.094992,0.12009,1.2549,1.4289,1.4989,1.1029,1.43
92,1.329,1.2163,1.2671,1.2273,1.2603,0.83123,0.94517,0.84002,0.83907,0
.67077,0.8727,0.84196,0.86954,0.87131,0.09048,0.09911,0.095324,0.08973
5,0.10271,0.094627,0.094527,0.1007,0.099835,0.099607,0.096716,0.097194
,0.094436,0.095248,0.098588,0.097899,0.091176,0.11931,1.2372,1.3761,1.
3675,1.2358,1.3903,1.324,1.1086,1.2984,1.0529,1.1657,0.91177,0.77364,0
.97286,0.86143,0.92887,0.89409,0.85853,0.96223,0.080948,0.096699,0.099
336,0.095083,0.095764,0.097951,0.088963,0.097408,0.094077,0.089793,0.0
97414,0.10229,0.086583,0.096675,0.097188,0.095789,0.095554,0.096665,0.
1199,1.1834,1.197,1.3187,1.4989,1.1891,1.2074,1.0935,1.2851,1.1141,1.3
107,0.92248,0.86287,0.94656,0.7971,0.72644,0.80425,0.9046,0.86638,0.09
2779,0.10677,0.099489,0.095119,0.095592,0.095926,0.094527,0.091747,0.0
92485,0.10008,0.099022,0.093188,0.10019,0.095239,0.098499,0.08879,0.09
316,0.11817,0.12685,0.12896,1.2017,1.2465,1.2597,0.99416,1.2427,0.9186
9,1.2402,1.4122,1.2275,0.69723,0.87057,0.86222,0.87427,0.85519,0.79993
,0.09306,0.098312,0.091214,0.096128,0.09759,0.099382,0.093343,0.095658
,0.093947,0.10132,0.094842,0.10349,0.095639,0.09398,0.10703,0.093638,0
.093727,0.096748,0.11938,0.11588,0.12595,0.11667,1.2383,1.275,1.1802,1
.2133,0.98859,1.0981,0.9022,1.3099,1.2545,0.91011,0.9813,0.98266,0.100
69,0.089528,0.090921,0.095793,0.09451,0.097234,0.093906,0.095922,0.090
475,0.0944,0.094173,0.096034,0.10748,0.087744,0.096565,0.10374,0.09884
9,0.097968,0.099978,0.094921,0.12333,0.11949,0.11842,0.11839,0.11549,0
.12189,1.0472,1.2142,1.012,1.1194,1.3121,1.0722,1.2548,1.2438,0.096198
,0.095484,0.10597,0.09948,0.094099,0.094185,0.097415,0.098164,0.098145
,0.086181,0.08937,0.094172,0.090773,0.090038,0.086166,0.09562,0.094758
,0.096615,0.096705,0.097853,0.090121,0.090724,0.094565,0.12635,0.11354
,0.12313,0.11444,0.11686,0.11744,1.4075,1.321,1.394,1.4406,1.2512,0.12
258,0.12173,0.097782,0.092455,0.097728,0.093921,0.098614,0.10323,0.093
979,0.096758,0.099789,0.10341,0.09174,0.095248,0.095852,0.092926,0.093
193,0.09528,0.095079,0.096114,0.093177,0.10728,0.098681,0.12184,0.1156
9,0.11939,0.11844,0.11969,0.117,0.12674,0.12779,0.11886,0.11922,1.0649
,1.3428,0.11556,0.11534,0.12296,0.12162,0.096787,0.093368,0.095534,0.0

93002,0.095635,0.093023,0.098028,0.10114,0.091899,0.098605,0.092629,0.
09732,0.10336,0.096476,0.097251,0.090153,0.095721,0.092864,0.0923,0.11
571,0.124,0.11792,0.12473,0.12329,0.11992,0.11266,0.12583,0.11637,0.12
702,0.11426,0.13009,0.11858,0.11488,0.11908,0.12582,0.11761,0.090521,0
.1029,0.092743,0.10025,0.094322,0.098002,0.089186,0.096372,0.094578,0.
095156,0.094326,0.093492,0.098166,0.092869,0.094736,0.092985,0.11692,0
.12529,0.1218,0.1228,0.11633,0.11109,0.11726,0.11429,0.12199,0.11981,0
.11926,0.11444,0.11971,0.11778,0.12017,0.11649,0.12042,0.11579,0.12475
,0.09667,0.10512,0.095925,0.098355,0.097746,0.094337,0.09487,0.094219,
0.092972,0.095813,0.095404,0.091611,0.093114,0.12193,0.11338,0.12237,0
.11702,0.11919,0.11378,0.11734,0.12113,0.12132,0.11533,0.12688,0.11061
,0.11442,0.11422,0.11692,0.11356,0.12132,0.11218,0.12152,0.12894,0.117
48,0.094951,0.088865,0.093237,0.099783,0.093009,0.092101,0.093513,0.09
4248,0.092836,0.12071,0.12294,0.12241,0.11378,0.11118,0.12571,0.12006,
0.12074,0.12542,0.11754,0.12419,0.11765,0.1183,0.12878,0.11865,0.11943
,0.11821,0.11775,0.12299,0.12057,0.11552,0.12062,0.12402,0.12185,0.124
63,0.1008,0.10094,0.09796,0.096366,0.11592,0.11875,0.12036,0.11503,0.1
2235,0.11779,0.1071,0.10991,0.12016,0.11471,0.12909,0.11958,0.11741,0.
11836,0.11429,0.11685,0.11536,0.1154,0.11374,0.12359,0.12005,0.11502,0
.11648,0.11701,0.11434,0.11947,0.11308,0.11557,0.12424,0.11673,0.119,0
.12107,0.12236,0.11641,0.12086,0.12,0.11759,0.11754,0.12155,0.11579,0.
11678,0.12175,0.12256,0.11574,0.11955,0.12535,0.1204,0.11553,0.11918,0
.11833,0.11995,0.11846,0.12294,0.11407,0.11783,0.11755,0.12118,0.12016
,0.12657,0.11318,0.11851,0.1149,0.11317,0.12037,0.11984,0.1203,0.11479
,0.11702,0.11959,0.1191,0.11833,0.12276,0.12391,0.11807,0.11718,0.1126
,0.12728,0.11502,0.11467,0.12057,0.11944,0.12183,0.12467,0.12953,0.124
31,0.1231,0.11631,0.1173,0.12388,0.12137,0.1212,0.11213,0.12008,0.1172
3,0.11892,0.12436,0.12194,0.12127,0.12213,0.11776,0.12208,0.10899,0.12
241,0.1181,0.11872,0.12309,0.11832,0.12655,0.11962,0.11699,0.12115,0.1
181,0.11757,0.11517,0.11862,0.11474,0.12791,0.11204,0.11478,0.11726,0.
11753,0.11436,0.12005,0.12265,0.11982,0.12354,0.1256,0.12998,0.1202,0.
1229,0.12485,0.12229,0.11694,0.12379,0.1165,0.12124,0.12038,0.119,0.11
366,0.12024,0.12213,0.12031,0.11703,0.1239,0.11655,0.1222,0.11785,0.11
956,0.11438,0.11809,0.12197,0.12073,0.11969,0.11293,0.11723,0.1241,0.1
2096,0.12184,0.12101,0.12148,0.12057,0.11299,0.12267,0.11833,0.11884,0
.12521,0.11348,0.12149,0.11746,0.11887,0.12587,0.11975,0.12054,0.11905
,0.12127,0.12263,0.11781,0.11966,0.11859,0.1163,0.12467,0.12309,0.1131
5,0.11986,0.11883,0.12209,0.11929,0.11924,0.1162,0.13107,0.11923,0.123
26,0.12231,0.11318,0.11408
0.11849,0.11846,0.11394,0.12044,0.11137,0.10535,0.12278,0.11007,0.1183
4,0.11268,0.12327,0.1146,0.10986,0.12553,0.11543,0.12143,0.11084,0.115
5,0.12238,0.11955,0.89443,0.89815,0.96844,0.8256,0.10962,0.10745,0.128
22,0.11622,0.11982,0.11287,0.11868,0.12275,0.11826,0.12084,0.1199,0.11
574,0.11934,0.12048,0.10736,1.5206,0.94563,0.7879,0.96656,0.76624,0.85
437,0.96519,0.11062,0.11473,0.12671,0.11961,0.10916,0.11642,0.11695,0.
11641,0.11258,0.10901,0.11955,0.12056,0.11415,0.10037,0.11481,1.5498,1
.5526,0.90657,1.0653,0.8721,0.87184,0.86893,0.91592,0.73807,0.68195,0.
11569,0.11377,0.10762,0.11721,0.11119,0.11902,0.11367,0.11806,0.11766,
0.11893,0.099617,0.1145,0.11463,0.11171,0.123,1.5946,1.5424,1.5279,0.8
6242,0.78232,0.67028,0.66924,0.93726,0.91313,0.82368,0.74236,0.94444,0
.10984,0.1297,0.12457,0.12739,0.12588,0.11483,0.11478,0.10849,0.11077,
0.11829,0.11061,0.12578,0.1193,0.10229,0.12073,0.11042,1.5969,1.4719,1

.6443,1.4834,0.96056,0.9571,0.95891,0.84583,0.82633,0.50896,0.81472,0.
85885,1.054,0.82905,0.11785,0.1115,0.10682,0.11495,0.1199,0.11342,0.11
64,0.11298,0.1186,0.12141,0.12627,0.11784,0.1194,0.12361,0.1196,0.1203
8,1.6099,1.606,1.6191,1.6482,1.5976,1.6519,0.92862,0.88888,0.84943,0.8
524,0.99293,0.87186,0.73463,0.88639,0.82189,0.83592,0.10807,0.11348,0.
1249,0.11565,0.12032,0.11526,0.1138,0.11688,0.10626,0.11401,0.11637,0.
11205,0.11942,0.12476,0.10774,0.1191,0.11537,1.7005,1.5837,1.5845,1.65
53,1.5534,1.618,0.94673,0.90513,0.85597,0.97077,0.97043,0.69516,0.767,
0.86001,0.85364,0.90985,0.61314,0.10426,0.12135,0.10698,0.1171,0.11318
,0.10985,0.10846,0.12071,0.11809,0.11777,0.10873,0.11651,0.11247,0.129
03,0.13338,0.13044,0.1182,1.5592,1.5514,1.7345,1.514,1.4351,1.607,1.64
1,0.84872,0.7763,0.92899,0.85715,0.86607,0.83015,0.86864,0.85894,0.885
13,0.86401,0.89749,0.1176,0.11479,0.11917,0.11714,0.11869,0.12319,0.11
467,0.11562,0.11303,0.11902,0.1211,0.1298,0.12997,0.13293,0.12994,0.12
693,0.13353,0.11365,1.5632,1.5857,1.5644,1.5882,1.5885,1.6923,1.6137,0
.84355,0.81714,0.8559,0.91823,0.78189,0.85678,0.9108,0.82492,0.81655,0
.89105,0.67017,0.11598,0.12071,0.10554,0.1191,0.11757,0.11737,0.11563,
0.11088,0.12259,0.13316,0.13035,0.1359,0.12389,0.12965,0.13053,0.13465
,0.12742,0.13529,0.11496,1.6451,1.645,1.6997,1.6351,1.5933,1.5517,1.60
57,0.82844,0.84842,0.87839,0.59383,1.0571,0.7749,0.94366,0.93939,0.880
59,0.88801,0.84031,0.80024,0.11422,0.11867,0.11125,0.11769,0.11323,0.1
2142,0.12859,0.13215,0.13454,0.13278,0.13181,0.13054,0.12022,0.12986,0
.1325,0.12094,0.13163,0.13087,1.7482,1.5631,1.6073,1.6572,1.6078,1.564
5,1.4913,0.87989,0.84938,0.756,0.83726,0.79587,0.79918,0.83115,1.0024,
0.83953,0.95954,0.96223,0.6479,0.11952,0.10864,0.11011,0.12837,0.13863
,0.13513,0.1245,0.12592,0.13493,0.12944,0.12648,0.12131,0.13354,0.1288
1,0.13174,0.13107,0.12796,0.11999,1.4562,1.5624,1.5963,1.6448,1.6269,1
.6421,1.5234,1.6782,1.5292,0.72472,0.83829,0.69432,0.95307,0.91389,0.8
0715,0.91757,0.72117,1.0244,0.88216,0.93582,0.12885,0.13361,0.12831,0.
13236,0.12363,0.12262,0.12846,0.13181,0.12865,0.13309,0.13245,0.13227,
0.13469,0.13151,0.13189,0.13308,0.13467,0.13849,1.5746,1.6174,1.6586,1
.621,1.5516,1.6325,1.6701,1.625,1.5873,0.80692,0.90768,0.67078,0.58819
,0.6193,0.69013,0.67573,0.58881,0.73308,0.44676,0.78093,0.13215,0.1370
3,0.12983,0.13426,0.12935,0.12678,0.12904,0.13374,0.13019,0.1282,0.131
86,0.13975,0.13685,0.13152,0.13155,0.13164,0.13264,0.13171,1.6085,1.64
75,1.657,1.6785,1.7484,1.5929,1.6704,1.5941,1.5919,0.91365,0.61395,0.6
8644,0.63185,0.58938,0.69154,0.65976,0.60186,0.68839,0.64708,0.63515,0
.13814,0.13294,0.12343,0.12837,0.13231,0.12896,0.12833,0.13477,0.13497
,0.13284,0.12575,0.13245,0.13585,0.13679,0.13146,0.12711,0.13161,0.131
53,1.5937,1.5575,1.4854,1.5633,1.6215,1.5859,1.6953,0.88401,0.91812,0.
86035,0.66481,0.6909,0.67226,0.6142,0.64078,0.63706,0.7105,0.46335,0.5
3752,0.60061,0.12633,0.13281,0.12835,0.13576,0.12942,0.12887,0.12762,0
.13234,0.13043,0.13139,0.13233,0.13057,0.12954,0.12974,0.13116,0.12688
,0.12865,0.13503,1.611,1.6325,1.6041,1.6431,1.5661,1.6575,0.89854,0.83
929,0.85464,0.88326,0.86332,0.57315,0.65455,0.61843,0.60463,0.77748,0.
69063,0.62125,0.68137,0.56895,0.12727,0.1312,0.13825,0.12954,0.13327,0
.13282,0.1349,0.1292,0.13257,0.1322,0.1322,0.11978,0.12812,0.12174,0.1
2929,0.13512,0.13137,0.12623,1.6622,1.6428,1.612,1.5285,1.6193,0.9314,
0.90644,0.85123,0.8136,0.82008,0.85381,0.59134,0.55854,0.61134,0.64713
,0.55871,0.64435,0.68524,0.59931,0.64529,0.12766,0.1299,0.13249,0.1315
6,0.13525,0.13484,0.13459,0.13413,0.13071,0.13725,0.13306,0.12721,0.13
483,0.12677,0.12757,0.12661,0.12867,0.12856,1.5014,1.6929,1.6058,0.952

9,0.92129,0.76047,0.91173,0.85077,0.94927,0.69315,0.9688,0.5564,0.5995
6,0.60222,0.5727,0.73075,0.69572,0.67248,0.54691,0.68595,0.13279,0.132
41,0.12627,0.13364,0.13349,0.13711,0.13029,0.12929,0.13451,0.12671,0.1
361,0.13269,0.13127,0.1263,0.13283,0.13047,0.1258,0.12554,1.5271,0.856
81,0.83779,0.77446,0.80921,0.81755,0.84926,0.72423,0.81441,0.80777,0.6
4017,0.66274,0.49878,0.51027,0.61404,0.55528,0.5273,0.71595,0.69573,0.
12899,0.13228,0.13612,0.12963,0.12668,0.13347,0.13213,0.13466,0.13154,
0.12949,0.12994,0.13252,0.13235,0.12834,0.13169,0.12607,0.12989,0.1457
,0.8875,0.98167,1.0514,0.78452,1.0067,0.93291,0.82367,0.88814,0.87796,
0.87488,0.58936,0.6716,0.59858,0.58704,0.50239,0.62323,0.59801,0.63194
,0.6285,0.126,0.13389,0.1281,0.12495,0.13708,0.131,0.13085,0.13612,0.1
3412,0.13646,0.13089,0.13264,0.13117,0.13219,0.13543,0.13333,0.1267,0.
14582,0.85389,0.92852,0.94432,0.84554,0.97012,0.91498,0.751,0.9011,0.7
3375,0.81315,0.66094,0.5556,0.71339,0.61662,0.6702,0.63922,0.61439,0.6
7494,0.11942,0.13178,0.1333,0.12947,0.13019,0.13393,0.12306,0.13434,0.
12937,0.12802,0.13285,0.13697,0.12319,0.13198,0.13169,0.13173,0.12956,
0.13378,0.14631,0.83481,0.85014,0.93149,1.0201,0.84576,0.82242,0.77455
,0.89286,0.79846,0.92357,0.64605,0.62315,0.68813,0.56651,0.5486,0.5995
3,0.66503,0.62682,0.12771,0.13816,0.13474,0.12961,0.1306,0.1322,0.1275
4,0.12716,0.12813,0.13322,0.136,0.12679,0.13548,0.13284,0.13439,0.1251
9,0.12793,0.14491,0.15281,0.1535,0.82831,0.84649,0.86367,0.71699,0.863
54,0.64539,0.8606,0.96647,0.86415,0.50499,0.64111,0.62355,0.64793,0.61
505,0.56452,0.1292,0.13386,0.12731,0.13151,0.13382,0.1331,0.13032,0.12
972,0.13236,0.1355,0.12958,0.13715,0.13078,0.12921,0.13732,0.12857,0.1
2957,0.13064,0.1442,0.13873,0.15117,0.1436,0.86976,0.85285,0.82236,0.8
3741,0.70252,0.78501,0.64247,0.8686,0.90114,0.63571,0.70195,0.7037,0.1
3514,0.12741,0.12593,0.12853,0.13216,0.13044,0.13104,0.12995,0.13003,0
.13222,0.12889,0.13171,0.14053,0.12469,0.13283,0.13758,0.13284,0.13345
,0.13355,0.12829,0.14612,0.14504,0.14318,0.14314,0.14148,0.14509,0.711
12,0.83334,0.68642,0.77062,0.91447,0.7558,0.87659,0.88204,0.13041,0.12
913,0.13728,0.13746,0.13089,0.13022,0.13294,0.13134,0.13254,0.12401,0.
12637,0.13017,0.12652,0.1287,0.1251,0.12929,0.12966,0.13206,0.13079,0.
13443,0.12672,0.12909,0.13374,0.14985,0.13911,0.15228,0.13858,0.14377,
0.1436,0.9348,0.91421,0.96677,1.008,0.86871,0.14861,0.14723,0.13107,0.
1291,0.12984,0.12973,0.13359,0.13794,0.12639,0.13191,0.13335,0.13809,0
.1287,0.13086,0.12788,0.13072,0.12792,0.13174,0.1307,0.1321,0.12809,0.
13914,0.13383,0.14774,0.14153,0.14722,0.14485,0.1472,0.1462,0.15443,0.
15325,0.14535,0.14996,0.73102,0.93724,0.14348,0.14119,0.14565,0.14578,
0.1329,0.12854,0.12997,0.12868,0.1298,0.12971,0.13009,0.13627,0.12879,
0.13056,0.1281,0.13247,0.13905,0.13013,0.1344,0.12811,0.13296,0.13054,
0.12818,0.14328,0.14835,0.14114,0.15031,0.14687,0.14596,0.13898,0.1531
2,0.14294,0.15008,0.14181,0.15454,0.14332,0.13956,0.14454,0.1499,0.141
38,0.12592,0.13908,0.12935,0.13452,0.1308,0.13095,0.12652,0.13059,0.12
75,0.13166,0.13012,0.12972,0.13418,0.12985,0.12929,0.12999,0.14242,0.1
5126,0.15019,0.14871,0.1431,0.14058,0.14256,0.14268,0.14889,0.14777,0.
14623,0.13862,0.14489,0.14389,0.14787,0.14165,0.14442,0.14221,0.14947,
0.13189,0.14048,0.1319,0.13533,0.13309,0.13174,0.12907,0.13039,0.12885
,0.13193,0.12994,0.12766,0.12623,0.14548,0.1404,0.1462,0.14265,0.14449
,0.14042,0.1406,0.146,0.15185,0.13889,0.15152,0.13525,0.14182,0.13987,
0.14357,0.14126,0.14821,0.13743,0.14405,0.15438,0.14446,0.13065,0.1260
4,0.12864,0.13506,0.12993,0.12767,0.12895,0.13034,0.12886,0.14572,0.14
616,0.14737,0.13931,0.13803,0.15153,0.14746,0.14761,0.14895,0.14376,0.

15056,0.14625,0.1422,0.1557,0.1448,0.14444,0.14848,0.1475,0.14799,0.14
295,0.14168,0.14827,0.15147,0.14825,0.15189,0.13755,0.13652,0.13286,0.
13146,0.13887,0.14777,0.14767,0.14256,0.14558,0.14721,0.13255,0.13435,
0.14532,0.14105,0.15316,0.1448,0.14245,0.14255,0.14133,0.14553,0.1433,
0.14183,0.13976,0.15138,0.1446,0.1401,0.14278,0.1428,0.14169,0.14666,0
.13789,0.14179,0.15226,0.14332,0.14374,0.14524,0.14868,0.14285,0.14636
,0.14489,0.14515,0.14397,0.14745,0.13929,0.14253,0.14628,0.14648,0.139
82,0.14461,0.15034,0.14826,0.14299,0.14808,0.14635,0.14404,0.14323,0.1
4834,0.13914,0.14522,0.14487,0.14643,0.1474,0.15051,0.14087,0.14487,0.
14066,0.13887,0.14553,0.14465,0.14383,0.14368,0.14412,0.14464,0.14789,
0.14225,0.14993,0.14966,0.14418,0.1428,0.1421,0.15613,0.14052,0.14134,
0.14493,0.14587,0.14604,0.14754,0.15215,0.14731,0.14718,0.13912,0.1438
4,0.14987,0.14699,0.14875,0.14015,0.14695,0.1441,0.14655,0.15002,0.151
84,0.14664,0.14642,0.14454,0.14771,0.13256,0.14604,0.14725,0.14668,0.1
5005,0.14584,0.14929,0.14353,0.1447,0.14564,0.14418,0.14565,0.14036,0.
14538,0.14444,0.15274,0.13704,0.14412,0.14131,0.14554,0.14232,0.14611,
0.14402,0.1466,0.14967,0.1513,0.15685,0.14694,0.1472,0.14895,0.14727,0
.1455,0.14763,0.14237,0.14678,0.14747,0.14466,0.13909,0.14467,0.14958,
0.14725,0.14226,0.15185,0.14475,0.14971,0.14464,0.14312,0.1405,0.14617
,0.14758,0.14688,0.14322,0.13673,0.14286,0.14718,0.14659,0.14666,0.145
76,0.15155,0.14663,0.13986,0.14831,0.14414,0.14538,0.15218,0.13912,0.1
4751,0.14192,0.14522,0.14854,0.14254,0.14712,0.1447,0.14713,0.14743,0.
14145,0.14547,0.14571,0.14632,0.14845,0.14712,0.13907,0.14692,0.14501,
0.14763,0.1433,0.14348,0.139,0.15639,0.14438,0.14801,0.15109,0.14036,0
.1422
0.1414,0.14512,0.14455,0.144,0.13913,0.13013,0.1526,0.13605,0.14332,0.
13845,0.15428,0.14349,0.13614,0.15097,0.14477,0.14587,0.13563,0.14127,
0.15325,0.15118,0.65103,0.69043,0.72701,0.59662,0.13816,0.13721,0.1589
8,0.14038,0.1452,0.14181,0.14567,0.15022,0.1467,0.14682,0.15062,0.1471
2,0.14236,0.1502,0.13647,1.0334,0.68,0.58108,0.72974,0.55168,0.64679,0
.70012,0.13854,0.14163,0.15261,0.15094,0.13567,0.14312,0.14414,0.14057
,0.14072,0.13575,0.1477,0.14856,0.1425,0.13161,0.14354,1.0963,1.0757,0
.66757,0.77604,0.65333,0.64029,0.65312,0.6733,0.54653,0.52043,0.13991,
0.14041,0.13372,0.14471,0.1389,0.14427,0.14077,0.14024,0.14478,0.14547
,0.13007,0.145,0.14227,0.14047,0.15123,1.1041,1.0644,1.0631,0.61338,0.
58192,0.48334,0.50585,0.69218,0.6932,0.597,0.54553,0.63856,0.13781,0.1
5633,0.14898,0.15502,0.15389,0.1451,0.14334,0.13509,0.13819,0.14813,0.
13853,0.15126,0.14506,0.12798,0.14811,0.13906,1.1427,0.99738,1.152,1.0
311,0.688,0.67741,0.71152,0.62514,0.63192,0.40107,0.61507,0.6436,0.759
83,0.61244,0.14483,0.14122,0.13191,0.14159,0.1464,0.14577,0.14425,0.13
794,0.14571,0.1479,0.15387,0.14434,0.1463,0.1538,0.14297,0.14791,1.178
5,1.1748,1.13,1.155,1.1149,1.2089,0.7109,0.6423,0.636,0.60003,0.72782,
0.6527,0.54766,0.6464,0.58914,0.59394,0.13731,0.14211,0.1519,0.148,0.1
4664,0.14649,0.14135,0.14436,0.1352,0.14411,0.14309,0.14005,0.14526,0.
153,0.13232,0.14799,0.1421,1.1758,1.1198,1.1423,1.1579,1.1243,1.1693,0
.68837,0.6807,0.61634,0.72146,0.69023,0.51843,0.55115,0.64644,0.62334,
0.66713,0.46945,0.13469,0.1484,0.13867,0.14334,0.14255,0.13981,0.13609
,0.14476,0.14537,0.1443,0.14187,0.14543,0.14405,0.13793,0.14407,0.1417
6,0.14779,1.0999,1.0628,1.2154,1.078,0.99241,1.1302,1.1862,0.60834,0.5
5263,0.68833,0.63451,0.61559,0.62288,0.65137,0.64885,0.65078,0.63503,0
.67179,0.14532,0.14162,0.14909,0.14292,0.14608,0.14638,0.13975,0.14781
,0.14078,0.14769,0.1471,0.14104,0.13985,0.14193,0.14293,0.13812,0.1425

2,0.13711,1.0646,1.1077,1.1016,1.1197,1.0684,1.2403,1.1563,0.63472,0.5
9836,0.63411,0.67534,0.57095,0.6174,0.65448,0.62909,0.57773,0.66267,0.
50963,0.14394,0.1487,0.13787,0.15025,0.14732,0.14418,0.14145,0.14025,0
.15074,0.14067,0.14061,0.13994,0.13366,0.14155,0.13586,0.1448,0.13617,
0.14582,0.1425,1.1423,1.1726,1.2371,1.154,1.0872,1.048,1.0965,0.62108,
0.62845,0.65279,0.44943,0.78561,0.54934,0.68199,0.65294,0.62549,0.6379
2,0.63193,0.58615,0.1445,0.14501,0.14159,0.14066,0.1393,0.14715,0.1391
6,0.14164,0.14218,0.14119,0.14001,0.13629,0.13448,0.13776,0.14445,0.13
003,0.13832,0.14158,1.2444,1.0826,1.1426,1.2107,1.1561,1.1218,1.0585,0
.66416,0.63859,0.55462,0.59247,0.58117,0.58673,0.61597,0.72112,0.61693
,0.695,0.71827,0.48434,0.14871,0.1338,0.13946,0.13676,0.14716,0.1456,0
.13535,0.13446,0.14039,0.13462,0.13912,0.13664,0.13735,0.13782,0.14515
,0.13815,0.13602,0.13457,1.016,1.0738,1.1223,1.1559,1.1636,1.1815,1.06
51,1.1894,1.0369,0.53643,0.61222,0.50786,0.70297,0.69624,0.55925,0.644
02,0.52096,0.76645,0.64343,0.69578,0.13838,0.13906,0.14175,0.13619,0.1
3496,0.13525,0.13903,0.14211,0.13759,0.14168,0.14032,0.14031,0.14068,0
.14009,0.14202,0.14562,0.14109,0.14204,1.0636,1.141,1.1741,1.1212,1.06
67,1.195,1.1752,1.1593,1.0928,0.5823,0.66513,0.50968,0.44614,0.46973,0
.50803,0.52211,0.45189,0.54618,0.35685,0.57439,0.14015,0.13861,0.14127
,0.14031,0.13851,0.13488,0.14031,0.14131,0.14068,0.13692,0.14327,0.145
24,0.14566,0.13969,0.14314,0.14173,0.14261,0.1418,1.1483,1.151,1.1866,
1.2244,1.2445,1.1093,1.1848,1.16,1.1138,0.66593,0.44271,0.50668,0.4723
4,0.44098,0.50443,0.4913,0.43678,0.51815,0.49973,0.48404,0.14653,0.139
13,0.13345,0.13778,0.13887,0.13849,0.13721,0.14286,0.14219,0.13795,0.1
341,0.14068,0.13896,0.13954,0.14067,0.13687,0.13507,0.13977,1.1285,1.0
892,0.98477,1.0979,1.1378,1.1312,1.2587,0.62622,0.6659,0.64175,0.48305
,0.5087,0.50589,0.4696,0.48064,0.46583,0.52887,0.36681,0.40879,0.45112
,0.13521,0.14407,0.13915,0.14071,0.13752,0.13941,0.13776,0.13871,0.137
82,0.13877,0.1425,0.14063,0.13913,0.13856,0.13899,0.14002,0.13883,0.14
544,1.1163,1.1735,1.0892,1.1878,1.0809,1.1613,0.64843,0.597,0.60935,0.
63232,0.60711,0.44007,0.4985,0.47706,0.45927,0.56506,0.50714,0.46056,0
.51613,0.41988,0.14028,0.13859,0.14479,0.14061,0.13999,0.14007,0.14201
,0.13942,0.1405,0.14285,0.14255,0.13261,0.13888,0.13663,0.13747,0.1416
9,0.13899,0.13817,1.171,1.1428,1.1654,1.0907,1.1094,0.686,0.66373,0.62
906,0.58171,0.57994,0.61283,0.43645,0.43481,0.45556,0.47907,0.42536,0.
4829,0.51482,0.46182,0.46822,0.13886,0.14589,0.14247,0.14043,0.14511,0
.14225,0.14676,0.14067,0.13986,0.14607,0.14172,0.13991,0.14195,0.13582
,0.14227,0.13681,0.13595,0.13548,1.0384,1.2147,1.1641,0.68324,0.67308,
0.55172,0.66975,0.6162,0.67097,0.52734,0.71152,0.42846,0.44998,0.45308
,0.43873,0.49917,0.51087,0.50862,0.41932,0.52802,0.14085,0.13827,0.138
45,0.13991,0.14413,0.14087,0.13816,0.13727,0.13761,0.13623,0.1472,0.14
33,0.14178,0.13842,0.14169,0.14097,0.13738,0.13512,1.0689,0.62756,0.64
092,0.5592,0.54719,0.58468,0.61519,0.50258,0.5563,0.573,0.48964,0.4896
3,0.37609,0.39324,0.46207,0.41531,0.39778,0.53087,0.52827,0.13943,0.14
188,0.14375,0.13836,0.13575,0.13743,0.1397,0.14347,0.14026,0.13757,0.1
3811,0.13989,0.13896,0.13694,0.142,0.13652,0.14078,0.13765,0.65327,0.7
0604,0.74562,0.56123,0.73307,0.66687,0.56481,0.65929,0.6304,0.59897,0.
43946,0.49977,0.45851,0.44609,0.39125,0.4666,0.46285,0.50182,0.46526,0
.13525,0.14213,0.14072,0.13622,0.14125,0.14319,0.13974,0.14125,0.14134
,0.14426,0.13998,0.14375,0.13924,0.14094,0.14428,0.14169,0.13396,0.134
65,0.60426,0.67537,0.66538,0.59761,0.72847,0.65508,0.54487,0.64651,0.5
4712,0.60667,0.50039,0.43179,0.53117,0.48244,0.51835,0.48464,0.44967,0

.5087,0.13589,0.14129,0.14202,0.14252,0.13556,0.14492,0.13394,0.14312,
0.13795,0.14201,0.14093,0.14538,0.13253,0.14208,0.13964,0.14082,0.1399
7,0.14133,0.13665,0.62262,0.62496,0.67507,0.74187,0.63161,0.5948,0.564
6,0.63509,0.59179,0.65483,0.49036,0.47434,0.5258,0.4274,0.43235,0.4673
5,0.50134,0.47648,0.13701,0.14642,0.14467,0.13806,0.13964,0.14139,0.13
595,0.13553,0.13807,0.14167,0.14545,0.13713,0.14295,0.14396,0.14074,0.
13627,0.13627,0.13791,0.14287,0.14499,0.57645,0.60729,0.61015,0.52483,
0.61131,0.48291,0.59684,0.65531,0.62747,0.38546,0.48161,0.49477,0.4915
8,0.46033,0.42801,0.14029,0.14177,0.13621,0.14378,0.14358,0.13882,0.14
262,0.14124,0.13928,0.14095,0.13755,0.14228,0.14122,0.13666,0.13896,0.
1382,0.13877,0.13748,0.1358,0.13167,0.13919,0.13194,0.59655,0.62241,0.
58328,0.60768,0.52016,0.57418,0.47896,0.59823,0.65649,0.49208,0.52202,
0.51598,0.14164,0.14286,0.13909,0.13629,0.14208,0.13635,0.13956,0.1399
1,0.14082,0.14249,0.13869,0.14286,0.14354,0.13561,0.14021,0.1424,0.142
82,0.13614,0.14153,0.13576,0.13771,0.13154,0.13309,0.13199,0.13188,0.1
3538,0.53326,0.59653,0.49435,0.54578,0.66447,0.55093,0.6389,0.63402,0.
14044,0.13678,0.14044,0.14793,0.14122,0.13909,0.13963,0.14027,0.14279,
0.13837,0.13602,0.14102,0.13978,0.13948,0.13706,0.13708,0.13915,0.1416
7,0.1411,0.14235,0.13649,0.14292,0.14291,0.13907,0.13023,0.14652,0.128
51,0.13615,0.13471,0.65738,0.66381,0.69953,0.70835,0.63702,0.13967,0.1
3427,0.13986,0.1366,0.13565,0.1406,0.14106,0.14556,0.1331,0.14102,0.14
132,0.14471,0.13953,0.13661,0.13546,0.14177,0.13979,0.14342,0.14114,0.
14009,0.13736,0.14215,0.14343,0.14052,0.13143,0.13809,0.1333,0.13882,0
.13828,0.14438,0.14166,0.13551,0.14254,0.54111,0.67726,0.13384,0.12974
,0.13883,0.1348,0.13904,0.13723,0.13876,0.1368,0.13471,0.13757,0.13854
,0.1429,0.1385,0.13704,0.13836,0.14023,0.14388,0.13547,0.1405,0.14025,
0.14228,0.14167,0.1391,0.13636,0.13866,0.13161,0.14294,0.136,0.13254,0
.12997,0.14776,0.13352,0.13786,0.13409,0.14333,0.13329,0.12892,0.13434
,0.14,0.13207,0.13689,0.14602,0.13899,0.1449,0.13762,0.13834,0.13664,0
.14049,0.13644,0.13916,0.14146,0.1375,0.14408,0.13846,0.13473,0.13961,
0.13051,0.14203,0.14101,0.13869,0.13471,0.13117,0.13552,0.13189,0.1382
5,0.13707,0.13627,0.12989,0.1373,0.13186,0.13754,0.13051,0.13611,0.135
74,0.13792,0.14534,0.14593,0.1417,0.14265,0.13816,0.1441,0.13838,0.139
06,0.14138,0.14001,0.14002,0.13579,0.13663,0.13813,0.13089,0.13677,0.1
3542,0.13627,0.13391,0.13119,0.13145,0.14303,0.12823,0.13818,0.12783,0
.12968,0.13322,0.13362,0.13038,0.1384,0.12854,0.13574,0.14312,0.13493,
0.13787,0.13789,0.13817,0.14266,0.14592,0.1371,0.14069,0.14042,0.13967
,0.13624,0.13648,0.13964,0.13044,0.13156,0.14188,0.1388,0.13757,0.1381
4,0.1366,0.1392,0.13768,0.13475,0.14649,0.13462,0.13566,0.13772,0.1383
2,0.14028,0.13071,0.13168,0.1417,0.14109,0.13618,0.14057,0.14607,0.142
12,0.14132,0.14265,0.12816,0.13771,0.13799,0.13352,0.13469,0.13715,0.1
2486,0.12466,0.13999,0.13325,0.14443,0.13649,0.13177,0.1312,0.13153,0.
13319,0.13749,0.13313,0.13026,0.14412,0.13335,0.13233,0.13459,0.13007,
0.13232,0.1356,0.13084,0.12936,0.14271,0.13363,0.1351,0.13418,0.14047,
0.13228,0.13687,0.13454,0.13572,0.13374,0.13785,0.12786,0.13612,0.1347
4,0.13664,0.1309,0.13407,0.13986,0.13636,0.13351,0.13768,0.13672,0.138
45,0.1344,0.13943,0.13053,0.13523,0.13622,0.13701,0.14121,0.14041,0.13
437,0.13965,0.13532,0.12821,0.13501,0.13447,0.13625,0.13356,0.13316,0.
13605,0.13853,0.13089,0.14165,0.13781,0.13825,0.13457,0.13271,0.14625,
0.13258,0.13474,0.13352,0.13928,0.13913,0.14012,0.14119,0.13729,0.1368
6,0.12957,0.13248,0.13843,0.13616,0.13615,0.1297,0.13477,0.13288,0.137
05,0.14135,0.14059,0.13642,0.13496,0.1357,0.13992,0.12303,0.13818,0.13

546,0.13178,0.14055,0.13591,0.14008,0.13591,0.13703,0.13603,0.13586,0.
13807,0.13134,0.13414,0.13594,0.14124,0.12605,0.13571,0.12963,0.13787,
0.13152,0.13751,0.13321,0.13675,0.13944,0.1408,0.14874,0.13658,0.13803
,0.14187,0.13383,0.13257,0.13766,0.13057,0.13675,0.13653,0.13506,0.131
12,0.13271,0.1375,0.13247,0.1316,0.14226,0.13659,0.13999,0.13553,0.133
81,0.13125,0.13719,0.13797,0.13777,0.13205,0.1269,0.13346,0.13787,0.13
753,0.13721,0.13643,0.14105,0.13716,0.13291,0.1376,0.13436,0.13477,0.1
4262,0.13169,0.13695,0.13085,0.13371,0.13794,0.13445,0.13753,0.13601,0
.14046,0.13889,0.13167,0.1319,0.13318,0.13702,0.13658,0.13829,0.13171,
0.13747,0.13301,0.13685,0.13421,0.13377,0.13287,0.14518,0.13425,0.1391
2,0.14312,0.13138,0.13341
0.16089,0.16422,0.17015,0.16762,0.15685,0.14805,0.16637,0.15153,0.1650
1,0.1585,0.17152,0.16399,0.15378,0.16831,0.16402,0.1654,0.14965,0.1541
5,0.17156,0.16738,0.51672,0.5648,0.55585,0.47834,0.15429,0.15346,0.176
6,0.16048,0.16191,0.16361,0.16363,0.17164,0.16521,0.16474,0.1685,0.169
06,0.16297,0.16817,0.15765,0.78572,0.49012,0.47155,0.59982,0.4145,0.52
201,0.5284,0.15652,0.16036,0.17312,0.16812,0.15047,0.1622,0.16193,0.15
673,0.15935,0.15285,0.16658,0.16592,0.16081,0.15005,0.16499,0.79564,0.
80997,0.50509,0.6,0.51975,0.51093,0.50522,0.53106,0.42752,0.44258,0.15
691,0.15907,0.155,0.16202,0.16011,0.16075,0.15596,0.16015,0.16666,0.16
347,0.14834,0.16098,0.16126,0.15844,0.16873,0.80591,0.77077,0.78811,0.
47864,0.465,0.40118,0.39531,0.55872,0.55034,0.46538,0.45089,0.47962,0.
15782,0.1741,0.17012,0.17047,0.17227,0.16451,0.16278,0.16097,0.15524,0
.16436,0.15852,0.17066,0.163,0.14944,0.16772,0.15975,0.8485,0.68497,0.
86205,0.77496,0.53252,0.51571,0.57195,0.47128,0.49236,0.33817,0.49746,
0.50686,0.59056,0.49601,0.16384,0.16144,0.15331,0.15772,0.1626,0.16126
,0.15915,0.15592,0.16597,0.1629,0.17142,0.16505,0.16053,0.1689,0.15913
,0.16559,0.85443,0.84352,0.88588,0.90757,0.78998,0.93024,0.58621,0.493
73,0.47807,0.4475,0.53839,0.50823,0.44186,0.50543,0.43414,0.4525,0.159
66,0.16208,0.17047,0.16019,0.17029,0.16474,0.15758,0.16143,0.15735,0.1
6636,0.16412,0.15608,0.16607,0.17074,0.1539,0.16526,0.15977,0.86551,0.
80341,0.84445,0.86922,0.81557,0.85591,0.52462,0.52618,0.47374,0.53339,
0.50388,0.41772,0.43156,0.5049,0.50269,0.5318,0.38266,0.15485,0.16279,
0.16104,0.15737,0.1628,0.1529,0.15954,0.16346,0.16139,0.1611,0.16246,0
.16549,0.16209,0.13824,0.14564,0.14075,0.16813,0.8217,0.80416,0.86715,
0.82035,0.71792,0.78935,0.90055,0.47672,0.43081,0.54109,0.52343,0.4689
,0.48303,0.50702,0.51687,0.51893,0.47667,0.52528,0.16177,0.16242,0.167
86,0.16345,0.16289,0.16742,0.15634,0.1642,0.16195,0.16849,0.16568,0.14
24,0.14062,0.1405,0.14325,0.13484,0.14441,0.15512,0.76076,0.87802,0.78
431,0.81727,0.80075,0.93722,0.82816,0.50973,0.47461,0.50505,0.5183,0.4
4191,0.49943,0.51837,0.48552,0.4194,0.52761,0.39205,0.16281,0.16635,0.
15796,0.1697,0.1661,0.16204,0.15964,0.15663,0.16017,0.14152,0.13944,0.
13761,0.13426,0.14054,0.13055,0.14536,0.13475,0.1443,0.15894,0.86271,0
.87168,0.87618,0.85822,0.82678,0.78817,0.82264,0.50781,0.49613,0.53376
,0.36929,0.60271,0.4129,0.54128,0.49107,0.47664,0.49523,0.49611,0.4759
3,0.15903,0.16354,0.16177,0.15873,0.15691,0.15931,0.13695,0.14038,0.14
021,0.14137,0.14014,0.13173,0.13671,0.13743,0.14716,0.12932,0.13773,0.
14056,0.92971,0.75505,0.79737,0.91663,0.8931,0.85345,0.74432,0.52701,0
.51934,0.43618,0.46708,0.47164,0.43206,0.48102,0.55805,0.49075,0.54278
,0.53966,0.3978,0.16635,0.15136,0.16077,0.13546,0.14445,0.14509,0.1366
,0.13462,0.13924,0.13434,0.14185,0.14152,0.13055,0.13442,0.14632,0.135
63,0.13313,0.14259,0.75406,0.76523,0.82756,0.91268,0.8996,0.8477,0.798

64,0.87125,0.76104,0.43695,0.48658,0.40063,0.55136,0.53441,0.44442,0.5
215,0.42015,0.59516,0.51599,0.56027,0.13574,0.13384,0.14449,0.13494,0.
13517,0.13771,0.13966,0.13883,0.13485,0.14024,0.13843,0.14187,0.13858,
0.13951,0.14043,0.14979,0.13578,0.13487,0.73372,0.85651,0.87902,0.8314
8,0.80213,0.90585,0.89188,0.87262,0.80962,0.46312,0.51885,0.41196,0.34
929,0.3883,0.41549,0.41627,0.36543,0.40542,0.28692,0.45047,0.13758,0.1
3065,0.14176,0.13631,0.13804,0.13661,0.13666,0.14071,0.14276,0.13443,0
.14352,0.13917,0.14066,0.13868,0.14247,0.14087,0.1399,0.13977,0.84852,
0.81116,0.91278,0.91393,0.93595,0.80202,0.87882,0.90333,0.83969,0.5208
3,0.35099,0.39528,0.37148,0.34436,0.38564,0.37687,0.3447,0.39974,0.396
08,0.38882,0.14468,0.13704,0.13065,0.13783,0.13467,0.13785,0.13305,0.1
4255,0.13938,0.1347,0.13191,0.13898,0.13564,0.1311,0.13808,0.13848,0.1
3163,0.13692,0.80678,0.84961,0.75746,0.82336,0.86864,0.84178,0.98673,0
.46105,0.51372,0.48625,0.38991,0.40779,0.40661,0.38301,0.37129,0.38586
,0.41287,0.29419,0.3211,0.36779,0.13446,0.14149,0.13826,0.13823,0.1371
6,0.13751,0.13732,0.13836,0.13592,0.13432,0.14132,0.14013,0.13871,0.13
944,0.13497,0.14293,0.14104,0.14726,0.82443,0.91528,0.79471,0.90266,0.
82441,0.86233,0.49399,0.44132,0.47033,0.47884,0.45758,0.3552,0.39284,0
.3828,0.36248,0.42635,0.38784,0.34544,0.42854,0.33294,0.14354,0.13471,
0.14324,0.14036,0.13877,0.1364,0.14154,0.13616,0.13752,0.14272,0.14202
,0.13706,0.13906,0.14014,0.13828,0.13927,0.13667,0.13987,0.90538,0.884
2,0.86556,0.81162,0.81445,0.51717,0.47434,0.46446,0.44375,0.43132,0.46
618,0.34166,0.36281,0.35472,0.3684,0.3468,0.38315,0.4052,0.36651,0.368
22,0.13729,0.14633,0.14059,0.13967,0.14429,0.14071,0.14603,0.13538,0.1
3943,0.14317,0.13974,0.14149,0.13876,0.13539,0.1451,0.13553,0.13433,0.
13171,0.70789,0.90423,0.8357,0.51251,0.49984,0.42063,0.50926,0.47776,0
.52262,0.38614,0.53134,0.33563,0.35964,0.35714,0.35365,0.37974,0.40885
,0.40842,0.32715,0.40641,0.13789,0.13234,0.14059,0.1351,0.14532,0.1347
5,0.13736,0.13752,0.13152,0.13564,0.14826,0.14399,0.14071,0.13693,0.13
941,0.13938,0.13655,0.13547,0.82049,0.46307,0.48526,0.43087,0.39468,0.
44115,0.46513,0.3803,0.41518,0.40345,0.38657,0.37681,0.30004,0.31056,0
.36484,0.32642,0.30141,0.4163,0.40726,0.14015,0.141,0.14361,0.13869,0.
13586,0.13224,0.13718,0.14244,0.13664,0.13334,0.13622,0.13993,0.1342,0
.13733,0.1426,0.13716,0.13775,0.11801,0.51297,0.52999,0.5799,0.39966,0
.55857,0.48896,0.44228,0.50034,0.47796,0.44643,0.33893,0.40274,0.37052
,0.35334,0.31932,0.35939,0.3552,0.39991,0.38505,0.13456,0.14279,0.1414
6,0.13609,0.13625,0.14405,0.14074,0.1392,0.14281,0.14343,0.13908,0.143
27,0.13779,0.13899,0.14045,0.14159,0.13346,0.11363,0.43884,0.4835,0.49
508,0.45745,0.55523,0.4969,0.40728,0.4643,0.40231,0.44527,0.3934,0.339
29,0.43674,0.38269,0.41018,0.38279,0.36791,0.39163,0.14151,0.13929,0.1
3966,0.14738,0.134,0.14575,0.13414,0.14132,0.13661,0.14669,0.1392,0.14
39,0.1343,0.14034,0.13897,0.13788,0.13885,0.14418,0.11805,0.46848,0.46
536,0.51707,0.52471,0.46679,0.43592,0.4331,0.48164,0.44699,0.48188,0.3
8803,0.37467,0.4158,0.34655,0.33628,0.38034,0.40405,0.38377,0.13804,0.
14018,0.14461,0.14084,0.1429,0.13984,0.13511,0.1352,0.13808,0.13986,0.
14275,0.13834,0.13833,0.14403,0.1379,0.13603,0.1373,0.11932,0.12075,0.
12421,0.43766,0.44985,0.4543,0.40853,0.46059,0.35599,0.44128,0.50916,0
.46493,0.30897,0.38884,0.39043,0.38828,0.36119,0.33,0.14145,0.14053,0.
13535,0.14102,0.14175,0.13511,0.1415,0.1427,0.13947,0.13915,0.13667,0.
13485,0.14218,0.13451,0.13229,0.1376,0.13653,0.13365,0.1159,0.11391,0.
12031,0.11377,0.43941,0.47607,0.43369,0.4647,0.38715,0.44037,0.36969,0
.44206,0.49909,0.38324,0.40491,0.40382,0.14003,0.14597,0.14036,0.1359,

0.14332,0.13138,0.13968,0.13912,0.14417,0.14601,0.13741,0.14174,0.1369
2,0.13832,0.14104,0.13784,0.13995,0.13095,0.13828,0.13445,0.11738,0.11
313,0.1118,0.11091,0.11446,0.11681,0.40048,0.46655,0.36657,0.40077,0.5
1922,0.41111,0.46956,0.48514,0.1427,0.13223,0.13242,0.14973,0.13991,0.
13907,0.13863,0.13705,0.14311,0.14636,0.13545,0.1379,0.14018,0.13832,0
.14207,0.13608,0.13904,0.14197,0.14098,0.13995,0.13622,0.14509,0.13568
,0.11695,0.11098,0.12386,0.10621,0.11751,0.1147,0.49299,0.5077,0.53296
,0.53845,0.4658,0.11878,0.11261,0.13911,0.13531,0.13111,0.13815,0.1374
,0.144,0.13227,0.13983,0.13914,0.1423,0.14071,0.13307,0.13823,0.13944,
0.13899,0.14374,0.13939,0.13866,0.13785,0.1331,0.14443,0.12062,0.11498
,0.11804,0.1188,0.12057,0.12063,0.12224,0.12333,0.11185,0.12099,0.4150
7,0.4953,0.11626,0.11018,0.11728,0.11631,0.13879,0.13503,0.13641,0.136
15,0.12873,0.13571,0.1384,0.13932,0.13777,0.13397,0.13736,0.13843,0.14
079,0.13219,0.14046,0.14025,0.14279,0.14186,0.14067,0.11781,0.11545,0.
11379,0.12091,0.11566,0.11204,0.11037,0.12639,0.1177,0.11743,0.1144,0.
12021,0.11151,0.10746,0.11324,0.12152,0.1151,0.14208,0.14349,0.14,0.14
348,0.13589,0.13465,0.13485,0.13965,0.13306,0.13693,0.14303,0.13828,0.
14156,0.13735,0.13194,0.13985,0.10853,0.12065,0.12284,0.12085,0.11265,
0.10955,0.11462,0.1141,0.11969,0.11764,0.11688,0.10983,0.12043,0.11388
,0.11936,0.11107,0.1176,0.11601,0.11579,0.14497,0.1394,0.13952,0.13975
,0.13392,0.14193,0.13901,0.14167,0.14017,0.13559,0.14186,0.13684,0.135
32,0.11852,0.11142,0.11846,0.11683,0.11516,0.11384,0.11081,0.11368,0.1
2007,0.11058,0.11905,0.10648,0.11103,0.11379,0.11471,0.1078,0.11856,0.
11017,0.11699,0.12433,0.11631,0.13607,0.13796,0.13545,0.13819,0.15045,
0.13601,0.13932,0.14093,0.13713,0.1154,0.11507,0.12031,0.1079,0.11289,
0.12053,0.11643,0.11971,0.11654,0.11779,0.12046,0.1175,0.11567,0.12674
,0.1145,0.11512,0.11863,0.11741,0.1211,0.1088,0.11351,0.12242,0.12517,
0.11571,0.12058,0.14196,0.13929,0.1392,0.142,0.10933,0.1162,0.1177,0.1
1521,0.11509,0.1181,0.10716,0.10724,0.1163,0.11375,0.12112,0.11622,0.1
1011,0.11472,0.11194,0.11362,0.11712,0.11355,0.11156,0.12158,0.11415,0
.11699,0.11292,0.1088,0.11063,0.11781,0.1112,0.11244,0.12008,0.11501,0
.11591,0.1154,0.1207,0.11072,0.11758,0.11326,0.1174,0.11491,0.11663,0.
11019,0.11482,0.11311,0.12001,0.1115,0.11764,0.11804,0.1173,0.11619,0.
1189,0.11778,0.11929,0.11708,0.11889,0.11007,0.11555,0.12058,0.11688,0
.12239,0.12079,0.11733,0.1176,0.11451,0.11034,0.11651,0.11378,0.11626,
0.11632,0.11361,0.11821,0.12066,0.11457,0.12004,0.12058,0.11833,0.1161
3,0.11521,0.12843,0.1159,0.11672,0.11417,0.12102,0.11861,0.12132,0.120
61,0.11572,0.11888,0.11226,0.11208,0.11889,0.12055,0.11755,0.11063,0.1
1515,0.11295,0.11392,0.12139,0.11996,0.11646,0.11225,0.11507,0.12058,0
.10435,0.1156,0.11917,0.11279,0.12013,0.11494,0.11635,0.11645,0.11775,
0.11531,0.11787,0.11894,0.11199,0.11571,0.11585,0.12342,0.10736,0.1167
7,0.11086,0.11648,0.11516,0.11663,0.11183,0.11631,0.11698,0.12141,0.12
766,0.11468,0.11769,0.12191,0.1137,0.11552,0.11389,0.10913,0.11767,0.1
1711,0.11841,0.1108,0.11351,0.11727,0.11338,0.11039,0.12174,0.11706,0.
11916,0.117,0.11526,0.11181,0.12139,0.11689,0.11857,0.1136,0.10762,0.1
1652,0.11701,0.11813,0.11477,0.11735,0.12442,0.11761,0.11345,0.11467,0
.11447,0.114,0.12285,0.10983,0.11926,0.11114,0.11387,0.11683,0.11526,0
.11693,0.11582,0.12308,0.11837,0.11098,0.11024,0.11633,0.11668,0.11747
,0.11989,0.11282,0.11871,0.11725,0.11652,0.11577,0.11539,0.11291,0.123
11,0.11172,0.11655,0.12495,0.11295,0.11444
0.17153,0.1733,0.1789,0.17475,0.17092,0.16284,0.17622,0.1643,0.17854,0
.17148,0.18336,0.17792,0.16504,0.17788,0.17915,0.17424,0.16007,0.16817

,0.18474,0.17635,0.39699,0.46936,0.41748,0.42838,0.17051,0.16537,0.191
22,0.17112,0.17717,0.17514,0.17966,0.17699,0.17551,0.17457,0.18466,0.1
765,0.16969,0.17984,0.16879,0.60613,0.3966,0.38403,0.48764,0.34667,0.4
2629,0.42409,0.17267,0.17515,0.18386,0.17871,0.16188,0.17182,0.17229,0
.16791,0.16684,0.1663,0.18185,0.17406,0.17489,0.16448,0.17493,0.66377,
0.63234,0.40907,0.46878,0.44512,0.43242,0.40617,0.43867,0.3537,0.37585
,0.1749,0.17108,0.16604,0.16972,0.1698,0.17247,0.1639,0.17491,0.178,0.
17584,0.1625,0.17386,0.17456,0.16907,0.18143,0.67478,0.59864,0.59641,0
.41266,0.3853,0.33819,0.35227,0.44277,0.45791,0.38988,0.3966,0.38066,0
.16972,0.18254,0.179,0.18117,0.17839,0.17974,0.17834,0.17478,0.17037,0
.17778,0.17028,0.18379,0.17516,0.1598,0.17643,0.17255,0.67137,0.50756,
0.65687,0.64399,0.41898,0.43438,0.45445,0.36285,0.4068,0.30758,0.42626
,0.4197,0.48274,0.4095,0.17651,0.17272,0.16691,0.17088,0.17183,0.17035
,0.1705,0.1664,0.17697,0.17603,0.18181,0.17242,0.17398,0.17898,0.17099
,0.17674,0.68375,0.64012,0.71245,0.75609,0.60993,0.75403,0.49698,0.421
02,0.39258,0.38731,0.45491,0.44141,0.37155,0.43548,0.3538,0.38882,0.16
788,0.17203,0.18103,0.17223,0.18687,0.17719,0.17059,0.17397,0.16745,0.
17693,0.17412,0.17318,0.17592,0.18086,0.16444,0.17573,0.16906,0.64903,
0.6544,0.65455,0.71731,0.61947,0.68249,0.42927,0.42825,0.39575,0.41188
,0.43214,0.35873,0.35635,0.43254,0.41409,0.44483,0.329,0.16777,0.17761
,0.17004,0.17177,0.17846,0.1638,0.1752,0.17515,0.17301,0.17116,0.17443
,0.17839,0.1724,0.13497,0.14225,0.13704,0.18249,0.62475,0.60636,0.7112
7,0.65488,0.53138,0.55641,0.74095,0.40229,0.33561,0.44128,0.42679,0.39
062,0.39469,0.41623,0.4481,0.44394,0.40073,0.46423,0.17041,0.17315,0.1
7892,0.17089,0.18019,0.17808,0.17053,0.16767,0.17679,0.17653,0.17807,0
.13976,0.13556,0.13282,0.14155,0.12938,0.14037,0.16825,0.59731,0.65498
,0.6575,0.67887,0.65698,0.71169,0.65217,0.43346,0.39846,0.42472,0.4372
9,0.36804,0.40708,0.42776,0.3922,0.35366,0.45778,0.33733,0.17337,0.174
99,0.16942,0.1837,0.1809,0.178,0.17499,0.17052,0.17488,0.13781,0.13517
,0.13236,0.12914,0.13789,0.12471,0.14383,0.13344,0.1409,0.1713,0.64946
,0.73678,0.7138,0.72829,0.63821,0.59065,0.59796,0.43148,0.38811,0.4412
4,0.31997,0.48795,0.34944,0.45023,0.3916,0.40848,0.42411,0.39916,0.402
67,0.17293,0.17232,0.17547,0.16742,0.16773,0.17042,0.13097,0.13873,0.1
3502,0.14003,0.13946,0.12665,0.13648,0.13369,0.14511,0.12452,0.1335,0.
13745,0.69366,0.59919,0.64452,0.74064,0.69482,0.61296,0.60574,0.45587,
0.445,0.34866,0.37941,0.3834,0.36376,0.40928,0.42705,0.41025,0.46681,0
.42589,0.34784,0.17702,0.16437,0.17227,0.1323,0.14149,0.1429,0.13532,0
.13113,0.13184,0.12677,0.14326,0.14416,0.12299,0.12785,0.14153,0.12937
,0.13113,0.14381,0.55984,0.60587,0.65362,0.75399,0.73037,0.66856,0.603
8,0.68197,0.59387,0.36312,0.42391,0.33415,0.43666,0.4494,0.36366,0.435
68,0.34426,0.50822,0.43566,0.46183,0.12923,0.12518,0.14409,0.12697,0.1
3268,0.13751,0.13586,0.13468,0.13099,0.1343,0.1359,0.13748,0.13528,0.1
3562,0.13379,0.14544,0.13147,0.1261,0.55852,0.66617,0.78444,0.63506,0.
60232,0.71484,0.70234,0.7313,0.61627,0.39171,0.42085,0.33873,0.2963,0.
33923,0.33977,0.33754,0.31523,0.31541,0.24701,0.36254,0.13508,0.12143,
0.137,0.12995,0.13626,0.13241,0.13527,0.13661,0.14121,0.12979,0.14069,
0.13272,0.13532,0.13547,0.13929,0.14057,0.1377,0.13712,0.67225,0.66832
,0.72483,0.751,0.72958,0.59469,0.68452,0.72238,0.68389,0.41859,0.28272
,0.33235,0.30481,0.28124,0.31084,0.31729,0.28869,0.33454,0.32528,0.336
73,0.14038,0.1292,0.12815,0.13502,0.12656,0.13351,0.13065,0.13569,0.13
222,0.12864,0.12709,0.13479,0.12823,0.11975,0.13547,0.13507,0.12414,0.
13,0.61381,0.67604,0.53435,0.67269,0.71171,0.69879,0.77154,0.37097,0.3

9924,0.38517,0.31625,0.34845,0.33192,0.32483,0.30189,0.31679,0.33427,0
.25853,0.2662,0.30464,0.13071,0.13699,0.13436,0.13046,0.13471,0.13454,
0.13357,0.13512,0.13047,0.12729,0.13625,0.13516,0.13432,0.13654,0.1299
9,0.14008,0.13879,0.14293,0.64979,0.72151,0.60504,0.72486,0.62898,0.67
62,0.37937,0.35642,0.36165,0.40255,0.35936,0.29463,0.31531,0.31617,0.2
9428,0.34556,0.30997,0.30227,0.35776,0.30357,0.14134,0.12704,0.13634,0
.13673,0.13464,0.13037,0.13671,0.13089,0.13178,0.13999,0.13807,0.13641
,0.13511,0.13858,0.13484,0.13392,0.12974,0.1372,0.71627,0.67176,0.6906
9,0.62275,0.62642,0.40782,0.38487,0.38274,0.34881,0.33344,0.38506,0.28
553,0.29394,0.29515,0.29579,0.29636,0.31707,0.32351,0.30945,0.29937,0.
13299,0.14594,0.13614,0.1364,0.14104,0.13819,0.14162,0.12793,0.13213,0
.13477,0.13859,0.13745,0.13169,0.13641,0.14644,0.13205,0.13024,0.12071
,0.5288,0.76418,0.60576,0.39129,0.38593,0.33734,0.41498,0.41409,0.4160
7,0.2894,0.41476,0.28138,0.30213,0.30899,0.2916,0.30484,0.33936,0.3381
3,0.27107,0.34707,0.13294,0.12588,0.13949,0.12833,0.14337,0.12832,0.13
244,0.1338,0.12391,0.13238,0.14994,0.13879,0.13762,0.13303,0.13475,0.1
3466,0.13361,0.13094,0.64512,0.36087,0.39589,0.34862,0.3394,0.35258,0.
36276,0.28018,0.31859,0.30522,0.32632,0.31212,0.24767,0.26941,0.31016,
0.27479,0.25725,0.34264,0.34793,0.1369,0.136,0.1414,0.13477,0.13285,0.
12481,0.13249,0.13689,0.13223,0.12911,0.1289,0.13466,0.12566,0.13303,0
.13908,0.1297,0.1322,0.096286,0.42544,0.41197,0.45841,0.31477,0.44142,
0.37141,0.32562,0.40422,0.36819,0.35316,0.2792,0.33923,0.31055,0.31034
,0.26817,0.30749,0.30303,0.33386,0.31459,0.13202,0.14197,0.13651,0.132
55,0.12874,0.14011,0.13698,0.13284,0.13843,0.13956,0.13587,0.13832,0.1
335,0.13471,0.13713,0.13827,0.12739,0.095642,0.33997,0.36515,0.3786,0.
35063,0.42997,0.41432,0.32585,0.36523,0.32511,0.36973,0.32195,0.2835,0
.34586,0.31343,0.33989,0.30321,0.30839,0.31978,0.14543,0.13462,0.1365,
0.14633,0.13282,0.14266,0.13208,0.13602,0.12922,0.14478,0.13524,0.1380
3,0.13132,0.13766,0.13565,0.13234,0.13515,0.14114,0.098755,0.37791,0.3
4565,0.40936,0.42238,0.34213,0.33366,0.35946,0.4,0.35715,0.37871,0.314
8,0.3157,0.34627,0.28687,0.29072,0.31617,0.32405,0.33454,0.13322,0.133
93,0.14312,0.13794,0.14074,0.13443,0.13144,0.13203,0.13413,0.13529,0.1
3939,0.13353,0.13298,0.13786,0.13154,0.13257,0.13195,0.099293,0.10045,
0.10023,0.31476,0.33169,0.36731,0.33545,0.37589,0.26876,0.33631,0.4114
9,0.3635,0.26395,0.32752,0.34157,0.31645,0.29267,0.26627,0.13706,0.136
37,0.13268,0.13884,0.13617,0.12763,0.13791,0.13998,0.13642,0.13735,0.1
3249,0.12717,0.13951,0.12819,0.12413,0.13379,0.13281,0.12853,0.093279,
0.093585,0.098212,0.092503,0.33306,0.37843,0.3461,0.37329,0.32328,0.35
903,0.29869,0.34172,0.38521,0.29941,0.34745,0.33377,0.13236,0.14814,0.
13907,0.13295,0.14122,0.12394,0.13548,0.13549,0.1399,0.14453,0.13081,0
.137,0.12624,0.13773,0.13605,0.1279,0.13474,0.12309,0.13369,0.13018,0.
099988,0.091492,0.092983,0.092155,0.097647,0.094903,0.3119,0.38028,0.3
0505,0.29669,0.41917,0.31103,0.36872,0.38974,0.13682,0.12516,0.12125,0
.14647,0.13614,0.13198,0.13508,0.12905,0.13954,0.14604,0.13134,0.13641
,0.1375,0.13655,0.14211,0.13085,0.13462,0.13844,0.13565,0.13446,0.1339
6,0.14522,0.1314,0.095539,0.091921,0.10372,0.088928,0.09959,0.094247,0
.36619,0.40089,0.42268,0.43151,0.39844,0.10154,0.096541,0.13361,0.1334
5,0.12532,0.13392,0.13312,0.13874,0.12676,0.13538,0.1317,0.13635,0.137
44,0.12529,0.13352,0.13481,0.13868,0.13967,0.1344,0.1322,0.13464,0.125
31,0.14059,0.10127,0.093935,0.0988,0.09888,0.099233,0.10073,0.10519,0.
10038,0.093658,0.10008,0.34717,0.39929,0.094343,0.091525,0.096946,0.09
4179,0.13224,0.13249,0.13107,0.13215,0.12083,0.1342,0.13424,0.13293,0.

13619,0.12776,0.13128,0.13431,0.13376,0.12717,0.13515,0.13751,0.13883,
0.14064,0.13824,0.10077,0.094796,0.094498,0.10053,0.097183,0.090398,0.
091592,0.10465,0.095957,0.099334,0.098527,0.10217,0.090465,0.086134,0.
093996,0.099174,0.095308,0.13734,0.1389,0.14051,0.14084,0.12853,0.1308
7,0.12949,0.13711,0.12548,0.13052,0.14061,0.13837,0.13859,0.13401,0.12
418,0.13608,0.089181,0.10165,0.10365,0.099823,0.089466,0.090187,0.0960
11,0.094263,0.10258,0.096775,0.094547,0.089441,0.10044,0.092005,0.1001
,0.089126,0.097303,0.09785,0.096541,0.14154,0.13234,0.13656,0.13352,0.
12797,0.14218,0.13364,0.13769,0.13689,0.13185,0.13687,0.13393,0.13485,
0.096281,0.091219,0.098356,0.095947,0.092628,0.095796,0.093005,0.09308
8,0.099243,0.094159,0.098575,0.087881,0.093519,0.093674,0.097698,0.089
205,0.097035,0.09051,0.096765,0.10346,0.097059,0.13075,0.13556,0.13128
,0.13102,0.14923,0.1315,0.1345,0.13699,0.13372,0.093924,0.098059,0.102
71,0.085218,0.094412,0.099194,0.098861,0.10218,0.098924,0.096817,0.101
35,0.099632,0.098182,0.10581,0.09565,0.098104,0.098688,0.097821,0.1010
5,0.087562,0.092943,0.103,0.10175,0.09689,0.10174,0.13525,0.13378,0.13
702,0.139,0.092902,0.096555,0.096583,0.094044,0.094787,0.098857,0.0901
43,0.090178,0.093422,0.093492,0.10471,0.094264,0.092315,0.09485,0.0921
88,0.093896,0.096757,0.093941,0.092745,0.09902,0.093579,0.09664,0.0926
2,0.089361,0.090235,0.096136,0.090611,0.09401,0.099966,0.09636,0.09572
7,0.096949,0.1001,0.092221,0.095655,0.091316,0.097619,0.096532,0.09534
4,0.090322,0.094878,0.093682,0.102,0.093402,0.094986,0.098269,0.099303
,0.098459,0.094509,0.094357,0.097666,0.096654,0.099035,0.091659,0.0952
69,0.10238,0.098898,0.10178,0.10341,0.097869,0.095843,0.093892,0.09001
4,0.096996,0.094834,0.098162,0.098202,0.093109,0.098513,0.099803,0.095
425,0.10271,0.099829,0.097597,0.09528,0.097637,0.10624,0.09521,0.09665
2,0.094045,0.10149,0.096753,0.10012,0.10249,0.097191,0.099009,0.092727
,0.093361,0.099107,0.10161,0.0977,0.091481,0.097967,0.093639,0.094213,
0.10246,0.101,0.098297,0.091853,0.096926,0.10098,0.08452,0.09419,0.100
54,0.09343,0.098785,0.09557,0.098703,0.097418,0.097746,0.09429,0.09769
1,0.09772,0.091219,0.098157,0.0973,0.10483,0.090185,0.092936,0.093389,
0.097032,0.095725,0.098583,0.096668,0.099295,0.098265,0.10156,0.10817,
0.095566,0.096269,0.10223,0.094006,0.098445,0.095273,0.089151,0.099486
,0.0976,0.099394,0.090053,0.093929,0.095977,0.091653,0.091187,0.1059,0
.09805,0.099083,0.095551,0.095927,0.093179,0.10133,0.096344,0.098519,0
.092697,0.091606,0.097775,0.094975,0.097075,0.094905,0.09348,0.10529,0
.097019,0.093902,0.096779,0.096055,0.092396,0.10444,0.092026,0.099744,
0.091015,0.094397,0.095552,0.09529,0.098597,0.094982,0.10264,0.10074,0
.091622,0.091879,0.098463,0.094714,0.09623,0.097999,0.093395,0.099113,
0.09727,0.095854,0.096366,0.097593,0.092228,0.10256,0.095676,0.096054,
0.10036,0.094008,0.094553
0.18292,0.18188,0.18527,0.18199,0.17868,0.17572,0.18543,0.1744,0.1843,
0.17552,0.19286,0.18538,0.16906,0.18707,0.18778,0.17992,0.16907,0.1768
7,0.19187,0.18672,0.35196,0.43395,0.36853,0.38652,0.17528,0.17368,0.19
558,0.18151,0.18877,0.18644,0.18794,0.18594,0.18408,0.18417,0.19448,0.
18681,0.17931,0.18605,0.18037,0.50958,0.36759,0.35735,0.44774,0.29967,
0.3975,0.39517,0.18057,0.18627,0.18669,0.18471,0.17463,0.17885,0.1786,
0.1743,0.17414,0.17684,0.18687,0.17834,0.18532,0.17351,0.18208,0.53326
,0.5492,0.38453,0.39358,0.39402,0.37862,0.33317,0.37344,0.3337,0.34511
,0.18729,0.17908,0.17343,0.17516,0.17388,0.18006,0.17061,0.18143,0.187
73,0.18176,0.16722,0.18302,0.18278,0.1787,0.18411,0.57603,0.52828,0.49
363,0.34205,0.32334,0.31673,0.31154,0.39645,0.42941,0.34762,0.36075,0.

31352,0.17934,0.18806,0.18491,0.18709,0.18848,0.19012,0.18804,0.18654,
0.17843,0.18597,0.17852,0.18988,0.18015,0.16644,0.18494,0.181,0.56362,
0.4026,0.53598,0.54231,0.35752,0.36114,0.39102,0.30276,0.36076,0.28968
,0.40463,0.36926,0.42507,0.3582,0.18655,0.18147,0.17831,0.179,0.18203,
0.17719,0.18179,0.17279,0.1813,0.18737,0.19052,0.1837,0.18332,0.184,0.
17557,0.18518,0.59287,0.55748,0.5894,0.65516,0.50421,0.68071,0.45505,0
.36736,0.34787,0.33585,0.40017,0.39154,0.3308,0.37211,0.31761,0.32742,
0.17259,0.17749,0.18845,0.17483,0.19426,0.18719,0.18101,0.18162,0.1781
6,0.18467,0.1878,0.18117,0.18509,0.19047,0.17384,0.18177,0.1767,0.5158
,0.54982,0.5551,0.60886,0.52072,0.55804,0.36934,0.35884,0.33089,0.3744
6,0.36433,0.32316,0.31515,0.35631,0.35102,0.38881,0.29329,0.1747,0.186
2,0.17653,0.17689,0.18708,0.17051,0.17771,0.18473,0.18214,0.17937,0.18
142,0.18957,0.18048,0.12988,0.14253,0.13335,0.18696,0.54231,0.50672,0.
5675,0.59564,0.42103,0.48687,0.61815,0.36003,0.29124,0.36018,0.3906,0.
34291,0.33536,0.38038,0.39819,0.38275,0.33991,0.42834,0.17676,0.1843,0
.1873,0.17777,0.18443,0.18448,0.17546,0.17752,0.18582,0.18265,0.18705,
0.13367,0.13034,0.12817,0.13742,0.12648,0.13585,0.17834,0.46454,0.5430
7,0.56008,0.54457,0.57197,0.54602,0.55919,0.38028,0.33985,0.37092,0.38
013,0.30941,0.34184,0.34769,0.3449,0.29648,0.39551,0.2817,0.18002,0.18
448,0.1774,0.19461,0.18782,0.18515,0.18186,0.18047,0.18243,0.13238,0.1
3161,0.12834,0.12495,0.13365,0.12073,0.13933,0.13036,0.13333,0.17706,0
.57799,0.59217,0.58841,0.63281,0.56529,0.48202,0.45396,0.37191,0.34078
,0.37217,0.28842,0.44,0.30477,0.38316,0.32079,0.36664,0.3782,0.3609,0.
35258,0.1818,0.1828,0.1844,0.1784,0.18002,0.17916,0.12593,0.13487,0.12
817,0.1366,0.13543,0.11918,0.13511,0.13034,0.14295,0.11996,0.12719,0.1
3436,0.57214,0.47809,0.49741,0.60478,0.53581,0.46938,0.5005,0.39209,0.
40569,0.30808,0.32659,0.3424,0.32457,0.3623,0.36558,0.37734,0.42123,0.
37704,0.31703,0.18196,0.17327,0.18317,0.12792,0.13712,0.13888,0.13415,
0.12581,0.12566,0.12207,0.14317,0.14032,0.11524,0.12374,0.1388,0.12354
,0.12951,0.14599,0.49195,0.46784,0.52729,0.63292,0.61801,0.57119,0.488
44,0.56217,0.49415,0.32856,0.36487,0.28704,0.38396,0.39226,0.33495,0.3
6402,0.30937,0.46765,0.3902,0.38584,0.12362,0.11933,0.14043,0.12014,0.
13127,0.13643,0.13262,0.13052,0.12757,0.13296,0.13353,0.13313,0.1288,0
.13099,0.12881,0.14117,0.12501,0.11775,0.45923,0.55896,0.67654,0.51653
,0.46711,0.61119,0.54641,0.59763,0.51704,0.33403,0.38655,0.31133,0.268
55,0.28756,0.28167,0.27442,0.27293,0.2689,0.22598,0.31782,0.13116,0.11
278,0.13244,0.12388,0.13571,0.13046,0.13152,0.13156,0.13701,0.12513,0.
13799,0.12603,0.13272,0.1302,0.13431,0.13604,0.13367,0.13318,0.54283,0
.56736,0.6417,0.66077,0.61356,0.50218,0.57752,0.63552,0.58204,0.36335,
0.25247,0.29446,0.25305,0.25577,0.26989,0.27458,0.24768,0.29918,0.2914
3,0.29309,0.13449,0.12376,0.12496,0.13142,0.12156,0.12867,0.12734,0.12
854,0.12512,0.12388,0.12481,0.13284,0.12188,0.11249,0.13179,0.1305,0.1
1755,0.12328,0.495,0.49676,0.4396,0.55009,0.58165,0.57606,0.67073,0.31
961,0.33892,0.32464,0.26568,0.3134,0.28203,0.27337,0.25327,0.27545,0.2
8661,0.23401,0.23587,0.26452,0.12976,0.13245,0.13363,0.12338,0.12962,0
.12834,0.12716,0.13187,0.12799,0.12081,0.13153,0.13108,0.12715,0.13236
,0.1251,0.13601,0.137,0.1389,0.5569,0.61076,0.54198,0.61366,0.53177,0.
58058,0.3215,0.3032,0.32252,0.32763,0.28919,0.24137,0.2686,0.28842,0.2
5537,0.3009,0.25981,0.25448,0.32265,0.27258,0.13544,0.12074,0.12929,0.
13185,0.13081,0.12571,0.1316,0.12655,0.12526,0.13924,0.1344,0.13721,0.
13199,0.13727,0.13236,0.12824,0.12705,0.137,0.58473,0.53514,0.55176,0.
55234,0.51369,0.34774,0.33404,0.30684,0.28345,0.25405,0.32468,0.25584,

0.2588,0.25688,0.27292,0.24802,0.27743,0.27212,0.26216,0.24969,0.12799
,0.14551,0.13333,0.13008,0.13785,0.13573,0.13877,0.1215,0.1307,0.13038
,0.1352,0.13485,0.12673,0.13453,0.14739,0.12849,0.1276,0.11377,0.50256
,0.6747,0.52128,0.33113,0.32934,0.27363,0.32829,0.34787,0.3423,0.21459
,0.36539,0.24055,0.25796,0.26957,0.26582,0.26729,0.30417,0.2913,0.2436
8,0.30024,0.12692,0.12074,0.13846,0.1208,0.13954,0.12131,0.12981,0.129
69,0.11668,0.1279,0.14941,0.13581,0.13397,0.12702,0.12986,0.12936,0.12
873,0.1264,0.49018,0.2991,0.3365,0.3144,0.24607,0.30583,0.27556,0.2372
,0.26002,0.24195,0.29567,0.27963,0.214,0.24098,0.27194,0.26024,0.23243
,0.29129,0.29232,0.13541,0.13258,0.13674,0.13032,0.12763,0.11779,0.131
25,0.13546,0.12929,0.12254,0.12568,0.12893,0.12073,0.13054,0.1334,0.12
797,0.12788,0.080589,0.36266,0.31437,0.37243,0.27439,0.36906,0.29565,0
.26364,0.34075,0.30848,0.28562,0.2487,0.3032,0.27536,0.26376,0.25154,0
.26394,0.2716,0.29561,0.27087,0.13044,0.13849,0.13289,0.12814,0.12311,
0.1359,0.13321,0.12874,0.13319,0.13654,0.13495,0.13532,0.13164,0.13104
,0.13443,0.13358,0.12472,0.081682,0.26523,0.29445,0.30396,0.3138,0.368
23,0.32813,0.24864,0.30496,0.28464,0.31551,0.2936,0.24724,0.29795,0.25
967,0.30046,0.25261,0.25751,0.25467,0.14905,0.13094,0.13228,0.14265,0.
12854,0.13923,0.13222,0.13203,0.1218,0.14152,0.12761,0.13531,0.13059,0
.13315,0.13044,0.12698,0.13328,0.1376,0.083331,0.31852,0.29108,0.31646
,0.34973,0.3119,0.26876,0.28915,0.33165,0.28823,0.31055,0.27232,0.2635
5,0.30539,0.2489,0.26789,0.27245,0.26743,0.30109,0.12874,0.12651,0.139
27,0.13428,0.1354,0.12992,0.12558,0.1299,0.12881,0.13267,0.13531,0.127
39,0.12609,0.13423,0.12915,0.13224,0.12777,0.082521,0.084386,0.081922,
0.24411,0.26965,0.27712,0.28367,0.30954,0.23199,0.26228,0.34046,0.2884
6,0.23221,0.29141,0.29863,0.28469,0.25621,0.22796,0.13122,0.12911,0.12
911,0.13449,0.13318,0.12139,0.13111,0.13626,0.13308,0.13279,0.12906,0.
1188,0.13821,0.12368,0.1162,0.13084,0.13016,0.12552,0.07737,0.078116,0
.081723,0.077309,0.28005,0.32118,0.27079,0.29616,0.24975,0.2942,0.2558
,0.27485,0.32455,0.27303,0.29663,0.27847,0.12657,0.1494,0.13674,0.1308
3,0.13681,0.11938,0.13175,0.1352,0.13946,0.14486,0.12563,0.13216,0.120
68,0.13321,0.13036,0.12184,0.13005,0.11677,0.12713,0.12487,0.08583,0.0
7541,0.077036,0.074836,0.082507,0.079344,0.28106,0.32733,0.25775,0.243
01,0.32929,0.23539,0.31739,0.32738,0.13124,0.12256,0.11314,0.14433,0.1
3101,0.12849,0.13094,0.12363,0.13392,0.14652,0.12798,0.13296,0.13426,0
.13187,0.1414,0.12765,0.13023,0.1348,0.12861,0.12977,0.13123,0.14425,0
.12691,0.079674,0.07687,0.087421,0.06963,0.08104,0.076541,0.29623,0.34
526,0.37082,0.37391,0.34647,0.082266,0.077669,0.12905,0.13043,0.11733,
0.12941,0.12917,0.13481,0.1208,0.13087,0.12615,0.1306,0.1313,0.11994,0
.13132,0.1295,0.13691,0.1358,0.12843,0.12733,0.12999,0.11619,0.134,0.0
85642,0.076085,0.084199,0.080243,0.082298,0.084033,0.090462,0.084784,0
.078735,0.085788,0.28492,0.35137,0.076861,0.07253,0.079737,0.076745,0.
12481,0.12936,0.12429,0.13061,0.11481,0.13272,0.13136,0.12679,0.13125,
0.12133,0.12516,0.13071,0.12688,0.12281,0.13131,0.1325,0.13407,0.13625
,0.13326,0.083361,0.077482,0.076713,0.083851,0.083077,0.074077,0.07715
7,0.087911,0.08041,0.082512,0.082453,0.084268,0.075853,0.072241,0.0784
02,0.083967,0.08039,0.1347,0.13368,0.13497,0.13488,0.12029,0.12842,0.1
2394,0.13248,0.12154,0.12632,0.13564,0.13518,0.13256,0.13027,0.12063,0
.13343,0.071794,0.084887,0.087631,0.086719,0.075105,0.072687,0.081374,
0.081146,0.087397,0.082116,0.077888,0.076563,0.081238,0.076128,0.08324
9,0.072697,0.080766,0.082561,0.081553,0.13701,0.12355,0.13333,0.12948,
0.12337,0.13943,0.12782,0.13432,0.13457,0.12728,0.13079,0.13067,0.1337

5,0.080937,0.072618,0.083481,0.079198,0.07509,0.07753,0.078786,0.07807
,0.081403,0.076186,0.080793,0.071131,0.077257,0.077185,0.07968,0.07534
8,0.080224,0.07321,0.079209,0.087743,0.080238,0.12649,0.13173,0.12599,
0.12395,0.14838,0.12825,0.13161,0.13399,0.12793,0.077665,0.081532,0.08
4089,0.069219,0.079105,0.081847,0.081014,0.086091,0.080805,0.078382,0.
083904,0.083403,0.081852,0.091099,0.080003,0.080961,0.080973,0.079629,
0.082544,0.068012,0.077836,0.084214,0.083311,0.080576,0.084085,0.13082
,0.12631,0.13452,0.13447,0.076231,0.080256,0.080085,0.080851,0.077069,
0.083754,0.07346,0.076376,0.078017,0.078572,0.087332,0.078179,0.077233
,0.079626,0.075252,0.079094,0.081066,0.078882,0.076122,0.08111,0.07836
,0.080128,0.078762,0.073909,0.077109,0.079121,0.071266,0.076846,0.0811
93,0.081262,0.077916,0.079238,0.084925,0.073678,0.081367,0.076029,0.08
4026,0.079008,0.077502,0.074248,0.077474,0.077741,0.085963,0.075887,0.
07717,0.081328,0.082861,0.08174,0.079468,0.073957,0.08401,0.076372,0.0
85004,0.077965,0.074955,0.085619,0.079865,0.085182,0.084513,0.080508,0
.082678,0.076799,0.074958,0.08181,0.081581,0.081168,0.081447,0.08005,0
.081614,0.082203,0.078542,0.085153,0.082563,0.082521,0.080234,0.080731
,0.092353,0.081918,0.082035,0.077442,0.087709,0.080867,0.08247,0.08680
3,0.080467,0.082048,0.078926,0.078384,0.080203,0.082604,0.080645,0.077
818,0.079973,0.077784,0.079903,0.085611,0.084418,0.082288,0.076568,0.0
80685,0.082223,0.07028,0.078684,0.083441,0.076398,0.083618,0.080564,0.
081105,0.08302,0.079496,0.07644,0.079806,0.084211,0.074647,0.080949,0.
076215,0.085408,0.075656,0.074461,0.079231,0.079707,0.080316,0.079983,
0.078475,0.084186,0.082636,0.079143,0.092698,0.078207,0.077501,0.08434
7,0.076193,0.080872,0.078594,0.07518,0.08339,0.081343,0.081713,0.07129
9,0.079231,0.076877,0.076643,0.0747,0.089805,0.081474,0.082329,0.07905
5,0.07893,0.077246,0.083624,0.081394,0.080912,0.073877,0.07509,0.08395
3,0.079929,0.083593,0.0787,0.080282,0.086812,0.079817,0.081176,0.08031
,0.079289,0.078066,0.086698,0.076351,0.082338,0.07291,0.078508,0.07637
3,0.081624,0.082523,0.081865,0.083981,0.081839,0.072857,0.079066,0.080
152,0.077122,0.079417,0.081489,0.078103,0.082813,0.081838,0.083844,0.0
78117,0.077621,0.076081,0.083739,0.076291,0.082638,0.084824,0.078183,0
.076342
0.18744,0.18521,0.18997,0.18652,0.18252,0.17965,0.18836,0.18192,0.1844
3,0.18074,0.1966,0.19063,0.17952,0.18935,0.19141,0.18648,0.17744,0.179
88,0.19935,0.18806,0.32002,0.38436,0.33825,0.35961,0.18279,0.17951,0.2
018,0.18736,0.18962,0.19621,0.19443,0.18959,0.18865,0.18961,0.19773,0.
19427,0.18587,0.19316,0.18311,0.4602,0.33731,0.33664,0.40057,0.26609,0
.35974,0.36546,0.18388,0.19006,0.19299,0.19227,0.17649,0.1854,0.182,0.
17816,0.17989,0.18006,0.18655,0.18419,0.19303,0.17958,0.18725,0.44647,
0.45825,0.3662,0.36255,0.35958,0.34943,0.29862,0.35623,0.31751,0.32965
,0.19747,0.18731,0.18475,0.18113,0.17865,0.1858,0.17232,0.18266,0.1883
7,0.18563,0.1712,0.18594,0.18979,0.18782,0.19153,0.49819,0.49741,0.454
76,0.31387,0.28094,0.29437,0.28952,0.36635,0.36952,0.29186,0.33434,0.2
9419,0.18615,0.19625,0.19187,0.19094,0.19383,0.1935,0.19179,0.19368,0.
18637,0.19071,0.18498,0.19162,0.18826,0.17355,0.18846,0.18206,0.48698,
0.36749,0.48215,0.49894,0.33641,0.31798,0.35399,0.27236,0.3135,0.27867
,0.36835,0.32917,0.39369,0.33555,0.19105,0.18745,0.18512,0.18932,0.185
28,0.18115,0.18695,0.18258,0.19014,0.19547,0.19558,0.19087,0.18875,0.1
9171,0.1828,0.18992,0.50086,0.47631,0.50761,0.53467,0.41991,0.56435,0.
43697,0.31768,0.34112,0.3099,0.38056,0.3403,0.29252,0.33273,0.29838,0.
28791,0.18048,0.18428,0.19326,0.18198,0.19571,0.19319,0.18633,0.18843,

0.18582,0.18449,0.18885,0.19201,0.19015,0.1939,0.17859,0.18878,0.18019
,0.49928,0.47313,0.49032,0.54451,0.42328,0.51296,0.36217,0.35141,0.302
74,0.32677,0.32383,0.29402,0.28512,0.32434,0.30208,0.36142,0.28543,0.1
8237,0.18638,0.17962,0.18477,0.19051,0.18057,0.18657,0.19332,0.18854,0
.1865,0.18976,0.19586,0.18475,0.12712,0.13804,0.13133,0.19087,0.48706,
0.42629,0.49626,0.52653,0.35097,0.45583,0.48841,0.30124,0.26853,0.2984
6,0.34909,0.31214,0.33025,0.35683,0.37513,0.35766,0.31297,0.40821,0.18
363,0.19134,0.19459,0.18396,0.18738,0.1867,0.17755,0.18197,0.19084,0.1
9139,0.19765,0.12895,0.12684,0.12573,0.13137,0.12113,0.13114,0.18358,0
.40204,0.47567,0.49067,0.47291,0.50888,0.46707,0.49782,0.32274,0.3087,
0.36312,0.35933,0.2785,0.32743,0.32342,0.31651,0.28356,0.37603,0.26574
,0.18706,0.18813,0.18135,0.19848,0.19406,0.19021,0.1874,0.1824,0.18396
,0.12946,0.12884,0.12382,0.12622,0.13194,0.11868,0.13786,0.12623,0.129
59,0.18269,0.46979,0.51496,0.50422,0.55171,0.51439,0.4074,0.38843,0.33
853,0.30364,0.32652,0.27534,0.40043,0.26557,0.35155,0.29847,0.35018,0.
34365,0.32866,0.32866,0.18822,0.18909,0.18874,0.18317,0.1887,0.18437,0
.12169,0.13263,0.12411,0.13436,0.13427,0.11411,0.13486,0.12866,0.1399,
0.11722,0.12353,0.13164,0.52752,0.42945,0.43281,0.53085,0.49998,0.4164
9,0.44777,0.35644,0.35101,0.28795,0.3028,0.34311,0.30279,0.33096,0.345
04,0.34322,0.38459,0.34143,0.29608,0.18553,0.18062,0.18879,0.12486,0.1
3414,0.13353,0.13056,0.12269,0.12192,0.11651,0.1431,0.13519,0.10932,0.
12193,0.13567,0.12174,0.12531,0.14782,0.49991,0.37916,0.50101,0.60029,
0.56675,0.49228,0.4089,0.46167,0.42687,0.30295,0.30882,0.27493,0.36732
,0.3719,0.30596,0.3803,0.28513,0.41898,0.36404,0.35013,0.12012,0.11372
,0.13836,0.11528,0.12908,0.13625,0.12984,0.12364,0.1247,0.13026,0.1295
3,0.12847,0.12766,0.12986,0.12294,0.13812,0.1188,0.11057,0.41099,0.488
55,0.59532,0.43307,0.41449,0.55095,0.49428,0.49794,0.41956,0.29887,0.3
6008,0.29418,0.2512,0.26504,0.25437,0.2401,0.23892,0.22654,0.20528,0.3
0959,0.13009,0.10518,0.13039,0.11758,0.13482,0.1291,0.12651,0.12936,0.
13399,0.12487,0.1337,0.12002,0.12881,0.12771,0.12951,0.13257,0.13066,0
.13099,0.47607,0.50555,0.54593,0.60906,0.58463,0.44753,0.51637,0.60008
,0.47453,0.33096,0.24017,0.25825,0.23394,0.22634,0.24747,0.23452,0.236
27,0.26916,0.25447,0.25048,0.13172,0.11836,0.12351,0.12456,0.11802,0.1
2399,0.12483,0.12451,0.12225,0.1182,0.11936,0.12896,0.11651,0.10619,0.
12577,0.12847,0.11237,0.11896,0.42712,0.43107,0.37877,0.45476,0.54253,
0.45242,0.61,0.26749,0.28419,0.2963,0.23435,0.27576,0.27164,0.2569,0.2
3253,0.2565,0.25785,0.2118,0.21268,0.24874,0.12699,0.12893,0.12976,0.1
1868,0.12616,0.1225,0.12098,0.12796,0.12218,0.11715,0.1294,0.1267,0.12
37,0.13,0.12166,0.13491,0.13489,0.13436,0.51403,0.52807,0.48722,0.4925
8,0.46263,0.52496,0.29418,0.26752,0.2823,0.28246,0.23628,0.21794,0.252
35,0.25714,0.24029,0.26388,0.23347,0.21518,0.2721,0.2454,0.13334,0.114
64,0.12489,0.12665,0.12648,0.12362,0.12743,0.12299,0.11937,0.1356,0.12
88,0.13393,0.12768,0.13662,0.12896,0.12387,0.12503,0.13277,0.51257,0.4
4431,0.48711,0.48132,0.45961,0.29069,0.28781,0.2853,0.24497,0.21112,0.
28256,0.24535,0.25166,0.22349,0.25465,0.22519,0.25228,0.24556,0.23577,
0.24384,0.12721,0.14191,0.12891,0.12634,0.13551,0.1326,0.13495,0.1166,
0.12605,0.12693,0.13219,0.13083,0.11938,0.13355,0.14604,0.12572,0.1228
8,0.11003,0.43996,0.57932,0.46686,0.28216,0.29557,0.23365,0.28069,0.30
759,0.29077,0.17107,0.29372,0.21684,0.2346,0.24296,0.24462,0.23823,0.2
6999,0.27111,0.22142,0.27277,0.12252,0.11586,0.1357,0.11583,0.13357,0.
11768,0.12822,0.12876,0.11263,0.12493,0.14827,0.13136,0.13029,0.12472,
0.12709,0.12352,0.12509,0.12244,0.44325,0.27386,0.27881,0.2814,0.20921

,0.25758,0.24792,0.20713,0.21533,0.20728,0.2696,0.2505,0.20555,0.21991
,0.24703,0.24598,0.2111,0.26571,0.27879,0.13255,0.13048,0.13261,0.1284
1,0.12428,0.11097,0.1267,0.1329,0.12443,0.11981,0.12074,0.12536,0.1159
7,0.12409,0.13041,0.12583,0.12402,0.067136,0.31741,0.26049,0.32363,0.2
4357,0.30613,0.27305,0.23836,0.30339,0.26214,0.23042,0.2237,0.28492,0.
23627,0.24297,0.23059,0.23632,0.24784,0.26751,0.25051,0.12871,0.13509,
0.12938,0.12448,0.11821,0.13379,0.12803,0.12341,0.12812,0.13111,0.1320
2,0.12757,0.13103,0.12812,0.13138,0.13155,0.12105,0.071865,0.22768,0.2
8279,0.27034,0.23933,0.31963,0.28244,0.18665,0.28493,0.25748,0.28896,0
.25696,0.24132,0.27184,0.24452,0.27384,0.22402,0.24113,0.22878,0.14919
,0.12768,0.12898,0.14027,0.12401,0.13556,0.13063,0.12846,0.11858,0.139
42,0.12251,0.13036,0.1269,0.13009,0.12594,0.12281,0.12876,0.13638,0.07
2266,0.2694,0.24446,0.25698,0.30744,0.25121,0.22907,0.24418,0.28725,0.
23876,0.251,0.24608,0.25077,0.28492,0.23829,0.2386,0.24675,0.22994,0.2
9036,0.12362,0.12096,0.13689,0.132,0.13664,0.12586,0.11911,0.12801,0.1
2567,0.1298,0.12998,0.12528,0.12045,0.12943,0.12559,0.12843,0.12302,0.
071232,0.074549,0.070481,0.21973,0.22671,0.23825,0.26713,0.26594,0.203
19,0.21577,0.29536,0.24203,0.20275,0.2605,0.27106,0.24296,0.23708,0.19
485,0.13071,0.12616,0.12821,0.13191,0.12877,0.11444,0.12725,0.12985,0.
13173,0.12941,0.12478,0.11196,0.13711,0.12176,0.1103,0.1289,0.12698,0.
11961,0.065116,0.068352,0.069514,0.067161,0.19784,0.27938,0.23439,0.27
115,0.24154,0.25866,0.22935,0.23088,0.27465,0.24979,0.26114,0.26423,0.
12013,0.14913,0.1368,0.13056,0.13597,0.11308,0.13011,0.13048,0.13868,0
.14464,0.12216,0.12795,0.11365,0.12927,0.12871,0.11661,0.12296,0.10963
,0.12294,0.12251,0.073416,0.065155,0.06798,0.062568,0.067045,0.067753,
0.23101,0.29806,0.21847,0.21451,0.27919,0.17827,0.27329,0.29428,0.1304
2,0.11723,0.10433,0.1416,0.12728,0.12484,0.1266,0.12027,0.12985,0.1472
,0.12282,0.128,0.13462,0.13105,0.14069,0.12244,0.12501,0.13186,0.12202
,0.12577,0.12768,0.14222,0.12457,0.065031,0.065853,0.073066,0.053162,0
.066582,0.063238,0.27181,0.31277,0.31377,0.32353,0.28942,0.070159,0.06
6619,0.12203,0.12454,0.11423,0.12599,0.12711,0.12928,0.11477,0.12795,0
.12151,0.12586,0.12741,0.11417,0.12494,0.1265,0.13567,0.13343,0.12666,
0.12293,0.12791,0.10997,0.13057,0.073739,0.064608,0.07502,0.06424,0.06
8369,0.072229,0.078806,0.06958,0.068373,0.072634,0.25106,0.30765,0.066
027,0.063752,0.066964,0.062627,0.11932,0.12642,0.11928,0.12895,0.11009
,0.13048,0.12657,0.12412,0.1278,0.11714,0.12186,0.12598,0.12231,0.1208
6,0.12449,0.1311,0.13168,0.13247,0.1312,0.069909,0.065586,0.063984,0.0
69624,0.073596,0.06176,0.062498,0.072321,0.069575,0.069514,0.070026,0.
071427,0.064975,0.062441,0.067195,0.068441,0.068213,0.13198,0.12913,0.
13071,0.12879,0.11716,0.1264,0.11893,0.12839,0.11709,0.12454,0.13286,0
.13153,0.12712,0.12771,0.11583,0.12972,0.061822,0.072012,0.077406,0.07
3549,0.065912,0.061899,0.067679,0.068564,0.072494,0.068869,0.065674,0.
064828,0.067559,0.065548,0.070572,0.062646,0.069885,0.070082,0.070876,
0.13444,0.1194,0.12925,0.12323,0.11868,0.13849,0.12339,0.1303,0.12948,
0.12436,0.12638,0.12904,0.13034,0.069883,0.061261,0.067495,0.06948,0.0
63631,0.065328,0.065077,0.063713,0.073817,0.062326,0.071324,0.060429,0
.064771,0.063078,0.066965,0.065514,0.06919,0.064554,0.06884,0.07501,0.
070624,0.1241,0.12782,0.12268,0.11707,0.14729,0.12444,0.12761,0.12739,
0.12681,0.063108,0.066566,0.071966,0.055785,0.068842,0.069323,0.068952
,0.074751,0.06626,0.06496,0.071214,0.071685,0.067333,0.07867,0.068031,
0.068958,0.069203,0.065761,0.06928,0.060525,0.063928,0.073471,0.070405
,0.068154,0.071772,0.12524,0.12058,0.13019,0.1312,0.067404,0.070359,0.

070154,0.069481,0.066273,0.070616,0.062702,0.064871,0.066992,0.065954,
0.075524,0.06498,0.064629,0.069142,0.064027,0.072653,0.071255,0.065414
,0.064391,0.068292,0.065541,0.068564,0.069918,0.062264,0.064456,0.0677
77,0.057914,0.063367,0.066954,0.06942,0.066009,0.064037,0.072853,0.062
881,0.068529,0.066539,0.06855,0.067134,0.066996,0.064096,0.067252,0.06
3828,0.072201,0.066874,0.06684,0.065642,0.071903,0.068631,0.068324,0.0
61487,0.071693,0.063631,0.073851,0.06777,0.06326,0.072909,0.068342,0.0
73087,0.071212,0.065459,0.070801,0.065308,0.064539,0.070521,0.069538,0
.070048,0.069339,0.069383,0.068061,0.070749,0.063018,0.071382,0.071055
,0.0674,0.066298,0.066087,0.07833,0.071426,0.070325,0.061532,0.076553,
0.067718,0.068767,0.074488,0.066857,0.068023,0.065511,0.065195,0.06792
9,0.071693,0.067285,0.068138,0.067876,0.062346,0.068569,0.072796,0.070
924,0.070827,0.066236,0.067194,0.066659,0.060524,0.065497,0.072317,0.0
64507,0.069494,0.06737,0.071073,0.072799,0.068624,0.063259,0.067652,0.
070616,0.064242,0.069354,0.067994,0.069894,0.065886,0.06153,0.067667,0
.065123,0.068485,0.066969,0.066614,0.071167,0.073018,0.065759,0.077936
,0.064001,0.064415,0.074085,0.062652,0.067745,0.063333,0.063241,0.0707
34,0.06735,0.068844,0.059218,0.06608,0.062397,0.063766,0.061456,0.0787
16,0.067584,0.070034,0.06558,0.068984,0.067609,0.070936,0.069563,0.069
051,0.061165,0.062921,0.069162,0.068819,0.068684,0.067364,0.067352,0.0
73571,0.070345,0.070082,0.066242,0.065511,0.065159,0.071687,0.059801,0
.070198,0.063871,0.066186,0.063103,0.068189,0.068363,0.067873,0.072456
,0.070054,0.064162,0.066612,0.067585,0.061702,0.066565,0.069171,0.0635
14,0.071296,0.069782,0.070201,0.063706,0.06772,0.063525,0.070219,0.062
557,0.072258,0.07406,0.066884,0.064187
0.1897,0.19127,0.19833,0.1887,0.18382,0.18341,0.19518,0.18762,0.18821,
0.18741,0.20127,0.19385,0.18594,0.19163,0.1979,0.19199,0.18245,0.18978
,0.20144,0.19209,0.30564,0.37963,0.32729,0.33994,0.19321,0.18208,0.209
05,0.19003,0.19416,0.19661,0.19841,0.1929,0.19138,0.19095,0.19418,0.19
742,0.19062,0.19842,0.19087,0.41148,0.32592,0.30214,0.38638,0.25156,0.
34787,0.35624,0.18674,0.19454,0.19356,0.19738,0.18049,0.19047,0.18313,
0.18153,0.18567,0.18403,0.18631,0.18982,0.1936,0.18323,0.1939,0.36974,
0.40608,0.32526,0.33338,0.35359,0.33915,0.3015,0.31668,0.30272,0.32177
,0.20213,0.18724,0.18976,0.18836,0.18375,0.18742,0.17953,0.18977,0.194
28,0.18716,0.17632,0.18602,0.19307,0.18845,0.19431,0.46808,0.45256,0.3
9939,0.30576,0.2465,0.27965,0.27533,0.33199,0.36122,0.28374,0.29676,0.
28056,0.18875,0.19937,0.1961,0.1896,0.19675,0.19691,0.19492,0.19686,0.
19134,0.19703,0.193,0.19894,0.19458,0.17892,0.19531,0.18781,0.46517,0.
33862,0.4326,0.42447,0.32326,0.30356,0.30057,0.26544,0.2983,0.27371,0.
35845,0.30787,0.3759,0.29963,0.19527,0.19262,0.18905,0.19139,0.18922,0
.18358,0.18977,0.1858,0.19418,0.19687,0.20173,0.19668,0.19571,0.19353,
0.18546,0.1948,0.45485,0.43442,0.50935,0.49571,0.43083,0.50109,0.40739
,0.28699,0.31993,0.27913,0.3711,0.31556,0.28421,0.31856,0.29085,0.2856
9,0.18435,0.185,0.19569,0.19017,0.19682,0.2,0.18582,0.19081,0.1914,0.1
8942,0.19197,0.19621,0.19491,0.19632,0.18455,0.19605,0.18627,0.41646,0
.42116,0.45426,0.47664,0.39057,0.49688,0.33461,0.32519,0.27962,0.35234
,0.27499,0.28545,0.26609,0.31808,0.29116,0.33958,0.27274,0.1917,0.1878
7,0.18038,0.18906,0.19172,0.185,0.19111,0.19326,0.19335,0.1931,0.19549
,0.19907,0.19013,0.12339,0.14027,0.13143,0.19585,0.4359,0.36937,0.4051
4,0.53615,0.34211,0.4513,0.42089,0.29095,0.25801,0.28798,0.32619,0.284
13,0.33341,0.33531,0.35099,0.32308,0.28051,0.38574,0.1927,0.19568,0.19
513,0.19026,0.18813,0.19303,0.185,0.18326,0.1986,0.19553,0.20551,0.126

86,0.1239,0.12289,0.13066,0.11742,0.12858,0.19217,0.34773,0.45805,0.44
442,0.42196,0.48874,0.40188,0.4267,0.32396,0.27011,0.34285,0.3381,0.26
716,0.32529,0.3079,0.28031,0.23776,0.35015,0.26044,0.19192,0.19802,0.1
8582,0.20089,0.19561,0.1939,0.19388,0.19063,0.18604,0.1273,0.12518,0.1
1705,0.12443,0.13064,0.11378,0.13352,0.12241,0.12705,0.18444,0.41607,0
.41116,0.44924,0.55124,0.4565,0.33465,0.37323,0.31519,0.27491,0.30519,
0.27151,0.37599,0.27126,0.33061,0.26191,0.3225,0.32191,0.29442,0.29756
,0.19006,0.19203,0.19366,0.1851,0.19685,0.19004,0.12,0.13107,0.12269,0
.13116,0.13303,0.10958,0.13587,0.12706,0.13715,0.11385,0.12078,0.13114
,0.49032,0.3593,0.44755,0.51621,0.38913,0.35963,0.43647,0.34979,0.3489
7,0.27349,0.30568,0.32787,0.29041,0.28814,0.35521,0.31974,0.34781,0.33
034,0.28039,0.18404,0.18593,0.19462,0.12269,0.13143,0.12957,0.12702,0.
12133,0.12038,0.11275,0.1405,0.13506,0.10781,0.12064,0.13391,0.11992,0
.12161,0.14886,0.46663,0.31962,0.43395,0.5685,0.52021,0.44315,0.37913,
0.41142,0.4016,0.28036,0.27951,0.25467,0.35247,0.33247,0.285,0.37463,0
.27348,0.38601,0.34384,0.31322,0.11703,0.11155,0.13631,0.11112,0.12781
,0.13415,0.12527,0.123,0.12512,0.1287,0.12704,0.12736,0.12479,0.12838,
0.12034,0.13732,0.11628,0.10594,0.40492,0.41889,0.55582,0.35644,0.3838
1,0.47104,0.46172,0.40859,0.35595,0.28802,0.345,0.28173,0.23058,0.2404
8,0.25325,0.23678,0.2255,0.19727,0.19632,0.28404,0.12819,0.1017,0.1281
7,0.11464,0.1335,0.12721,0.12422,0.12885,0.1318,0.12321,0.1296,0.11505
,0.12759,0.12423,0.12681,0.12977,0.12456,0.12924,0.44627,0.46422,0.492
56,0.55067,0.52344,0.44615,0.47958,0.53476,0.46487,0.30973,0.23003,0.2
5589,0.22084,0.21862,0.22681,0.20886,0.21642,0.25321,0.22571,0.25233,0
.12974,0.11376,0.11976,0.12248,0.11529,0.1237,0.12568,0.12457,0.1185,0
.1169,0.11507,0.1261,0.11263,0.10111,0.12453,0.12702,0.10978,0.1154,0.
41598,0.37477,0.37793,0.47155,0.52206,0.4161,0.51634,0.24381,0.25921,0
.26038,0.22235,0.26573,0.24587,0.23292,0.21331,0.23809,0.23057,0.19578
,0.20035,0.23734,0.12454,0.12604,0.12766,0.11465,0.12524,0.12069,0.118
67,0.1246,0.119,0.11548,0.12566,0.12535,0.12063,0.12638,0.11984,0.1319
6,0.13282,0.13252,0.48029,0.51121,0.41821,0.43583,0.44835,0.457,0.2721
4,0.23317,0.25605,0.27045,0.21524,0.20364,0.23013,0.24356,0.23469,0.22
063,0.23162,0.19387,0.25265,0.22582,0.13086,0.11081,0.12064,0.12374,0.
12264,0.12231,0.12576,0.12159,0.11897,0.13196,0.12546,0.13216,0.12884,
0.13614,0.12728,0.11979,0.12428,0.13074,0.43421,0.40238,0.45497,0.4440
1,0.42581,0.26055,0.253,0.26658,0.23064,0.19136,0.2464,0.24267,0.24648
,0.22093,0.22634,0.2106,0.22744,0.22691,0.22596,0.2283,0.12674,0.14522
,0.1268,0.12337,0.1312,0.1282,0.13324,0.11097,0.12283,0.12552,0.12696,
0.12945,0.1163,0.13238,0.14509,0.12367,0.12049,0.10656,0.40641,0.54156
,0.40153,0.26284,0.28812,0.22588,0.26225,0.28101,0.26085,0.13573,0.249
56,0.20177,0.21369,0.23281,0.23215,0.22344,0.24657,0.24785,0.21366,0.2
4662,0.1204,0.11293,0.13405,0.11518,0.1313,0.1127,0.1253,0.12653,0.108
96,0.12213,0.14677,0.12866,0.12653,0.12291,0.12311,0.12187,0.12277,0.1
1813,0.38813,0.25908,0.25329,0.25372,0.19285,0.24781,0.22542,0.19955,0
.20761,0.18873,0.25846,0.23266,0.19151,0.20877,0.23535,0.24169,0.19372
,0.24934,0.24549,0.13216,0.13041,0.13045,0.12761,0.12129,0.10413,0.123
01,0.13071,0.12119,0.11747,0.11772,0.12398,0.11168,0.12186,0.1281,0.12
435,0.12152,0.060184,0.29436,0.24578,0.31532,0.23516,0.27506,0.23881,0
.2195,0.2719,0.25307,0.2205,0.2109,0.26536,0.22369,0.22886,0.22601,0.2
4312,0.23504,0.2531,0.21252,0.12534,0.13282,0.127,0.12218,0.11432,0.13
15,0.12686,0.11983,0.12242,0.12655,0.13023,0.12739,0.12776,0.12267,0.1
2963,0.12707,0.12179,0.062189,0.20942,0.27754,0.23659,0.22266,0.28479,

0.23975,0.17677,0.27309,0.24164,0.25402,0.23879,0.23001,0.23774,0.2354
7,0.243,0.20062,0.22672,0.20189,0.15031,0.12528,0.12688,0.13971,0.1239
6,0.13109,0.12975,0.12514,0.11773,0.1387,0.12109,0.12671,0.12572,0.129
47,0.12366,0.12034,0.12541,0.13417,0.064185,0.24272,0.21033,0.23979,0.
27816,0.21083,0.214,0.23621,0.25823,0.2251,0.22809,0.22549,0.22883,0.2
6349,0.22626,0.2291,0.22527,0.20363,0.2766,0.11961,0.11936,0.13651,0.1
2661,0.13451,0.12533,0.11772,0.1253,0.12253,0.12639,0.12864,0.12446,0.
11822,0.12536,0.12133,0.12829,0.11897,0.063904,0.064883,0.060275,0.200
26,0.23882,0.21304,0.24358,0.26914,0.20035,0.19581,0.25855,0.1915,0.20
064,0.24928,0.25091,0.23884,0.22512,0.16893,0.13251,0.12195,0.12799,0.
13255,0.12616,0.11018,0.12339,0.12635,0.1266,0.12327,0.12379,0.10831,0
.13702,0.11891,0.10525,0.12848,0.12631,0.11773,0.058746,0.060205,0.058
099,0.057455,0.16663,0.24629,0.21221,0.25351,0.22331,0.24547,0.21257,0
.20907,0.24979,0.24095,0.23674,0.24941,0.11591,0.14968,0.1349,0.12651,
0.13487,0.1083,0.12771,0.12887,0.13722,0.14399,0.1208,0.12522,0.10897,
0.12745,0.12586,0.11264,0.12057,0.10587,0.11838,0.12077,0.064255,0.053
403,0.056097,0.054372,0.060116,0.059226,0.2227,0.27575,0.19144,0.20223
,0.26857,0.169,0.241,0.26489,0.12866,0.1155,0.099704,0.1395,0.12546,0.
1215,0.12501,0.11741,0.12806,0.14634,0.11822,0.12637,0.13043,0.12958,0
.13916,0.11983,0.1223,0.13053,0.11833,0.12417,0.12132,0.13971,0.1236,0
.055921,0.054589,0.063113,0.045466,0.060154,0.055372,0.24406,0.28886,0
.27107,0.28616,0.27005,0.059824,0.05952,0.12084,0.12407,0.11134,0.1235
6,0.12239,0.12835,0.11286,0.1244,0.12073,0.12198,0.12687,0.11122,0.123
7,0.12279,0.13424,0.12955,0.12464,0.12135,0.12559,0.10475,0.13017,0.06
3778,0.053167,0.068337,0.057022,0.057698,0.062964,0.067344,0.062685,0.
059931,0.065287,0.22086,0.27738,0.058165,0.051958,0.054571,0.053662,0.
11976,0.12415,0.11491,0.12548,0.10736,0.12839,0.12353,0.11998,0.12406,
0.1142,0.12078,0.1219,0.11825,0.11764,0.12095,0.12696,0.12778,0.13039,
0.12927,0.062216,0.05555,0.055325,0.058457,0.066899,0.055282,0.050913,
0.062727,0.05799,0.060288,0.058962,0.06547,0.057325,0.056551,0.057908,
0.0594,0.060658,0.12994,0.12641,0.12555,0.12561,0.11506,0.12276,0.1139
6,0.12399,0.1161,0.12228,0.13179,0.12878,0.12265,0.12468,0.11109,0.128
27,0.052622,0.062836,0.068089,0.063295,0.061227,0.056804,0.061914,0.06
2254,0.063826,0.058562,0.059106,0.055306,0.061097,0.05584,0.062721,0.0
55809,0.060927,0.060976,0.063019,0.1334,0.11717,0.12712,0.1216,0.11443
,0.13788,0.11759,0.1299,0.12655,0.12078,0.12691,0.12284,0.12477,0.0611
12,0.052496,0.057801,0.060005,0.054364,0.056301,0.054925,0.056692,0.06
8575,0.054014,0.061368,0.049254,0.057233,0.053943,0.057012,0.056403,0.
06232,0.056478,0.060854,0.065113,0.062762,0.1223,0.12734,0.12124,0.112
72,0.14619,0.12333,0.12645,0.12323,0.12607,0.054681,0.058855,0.061765,
0.048806,0.058093,0.061185,0.058817,0.065724,0.05523,0.054605,0.059078
,0.063565,0.05794,0.069322,0.059315,0.061836,0.059483,0.059801,0.06255
5,0.050625,0.053709,0.063838,0.060675,0.059865,0.063733,0.12245,0.1177
3,0.12729,0.12877,0.057396,0.063163,0.06056,0.058958,0.056859,0.061935
,0.055051,0.056349,0.059245,0.057321,0.066903,0.057822,0.054746,0.0626
02,0.056652,0.063553,0.062062,0.057502,0.056373,0.060598,0.05811,0.062
01,0.059825,0.054812,0.060313,0.060444,0.054291,0.053364,0.062273,0.06
3233,0.05485,0.054847,0.065551,0.056094,0.061262,0.057575,0.062238,0.0
56452,0.056042,0.058461,0.059896,0.054767,0.062193,0.058211,0.060599,0
.057243,0.060773,0.060998,0.062182,0.052405,0.06259,0.055942,0.067088,
0.060237,0.056472,0.065663,0.059783,0.063081,0.062473,0.056949,0.06280
1,0.056348,0.054136,0.062486,0.057418,0.063072,0.05925,0.061932,0.0597

02,0.062509,0.055491,0.064252,0.061485,0.057702,0.056136,0.057034,0.06
8561,0.063691,0.062405,0.052584,0.067269,0.058595,0.059309,0.061819,0.
059638,0.060465,0.058529,0.055078,0.060202,0.061941,0.057596,0.061798,
0.060204,0.0526,0.062132,0.061374,0.061844,0.061983,0.055716,0.061956,
0.060418,0.056556,0.057624,0.063489,0.056414,0.062805,0.058131,0.05808
8,0.065588,0.060851,0.059411,0.063475,0.059288,0.057206,0.061006,0.057
225,0.061858,0.054191,0.05473,0.058044,0.055144,0.058737,0.056195,0.05
9362,0.06203,0.063094,0.059001,0.068706,0.055351,0.056493,0.064246,0.0
56207,0.057452,0.055336,0.054727,0.059832,0.059618,0.059522,0.053174,0
.057489,0.052885,0.055842,0.05432,0.06746,0.059966,0.060137,0.056129,0
.060202,0.058055,0.064456,0.0635,0.060104,0.054573,0.05413,0.058308,0.
060711,0.058381,0.061182,0.060406,0.063751,0.05989,0.061271,0.058715,0
.0576,0.055013,0.064103,0.050359,0.059953,0.057446,0.056502,0.055702,0
.058742,0.05665,0.060113,0.064322,0.062118,0.056039,0.05877,0.056639,0
.051439,0.059098,0.059966,0.056267,0.062208,0.064101,0.062471,0.055213
,0.057999,0.055545,0.062618,0.053942,0.065974,0.063689,0.058347,0.0605
14
0.19178,0.1957,0.19872,0.19307,0.18594,0.18685,0.20199,0.19035,0.19033
,0.19355,0.20206,0.19259,0.19072,0.19642,0.19591,0.19368,0.18808,0.187
69,0.20254,0.19689,0.28169,0.3444,0.33615,0.32674,0.19424,0.18339,0.20
928,0.19418,0.1912,0.19495,0.20316,0.19363,0.19105,0.19002,0.20052,0.1
98,0.19357,0.20058,0.19303,0.44372,0.30613,0.28223,0.38565,0.23826,0.3
307,0.34419,0.1928,0.19588,0.19382,0.20174,0.18413,0.19385,0.18907,0.1
8362,0.18861,0.1877,0.19337,0.19276,0.19485,0.19193,0.19801,0.33382,0.
41113,0.32835,0.32759,0.31923,0.30744,0.31086,0.31865,0.30299,0.31508,
0.20235,0.19078,0.19582,0.19213,0.18428,0.19031,0.18294,0.19609,0.1928
3,0.19295,0.17901,0.18635,0.19287,0.19059,0.19531,0.46748,0.38664,0.37
862,0.30167,0.23697,0.29334,0.26164,0.3198,0.32915,0.27199,0.27537,0.2
8503,0.19199,0.20002,0.20052,0.1931,0.1977,0.20181,0.19803,0.19774,0.1
9458,0.19558,0.19562,0.19878,0.19355,0.18524,0.19817,0.19568,0.42808,0
.33014,0.44049,0.38429,0.30793,0.29603,0.29804,0.26879,0.29595,0.2677,
0.33864,0.29489,0.37361,0.27921,0.20022,0.1939,0.19177,0.19467,0.1939,
0.18658,0.19451,0.18864,0.2001,0.19797,0.2035,0.19732,0.19813,0.19209,
0.18646,0.19953,0.41636,0.40567,0.43555,0.44519,0.40974,0.5014,0.39234
,0.26893,0.30346,0.27192,0.36118,0.31235,0.29023,0.28613,0.28483,0.267
3,0.18855,0.18598,0.20028,0.19336,0.20183,0.20535,0.19034,0.19452,0.19
526,0.19513,0.19487,0.20048,0.20138,0.19574,0.18859,0.19735,0.19087,0.
39492,0.42229,0.42112,0.41773,0.40937,0.48059,0.30845,0.30976,0.26999,
0.327,0.24222,0.28484,0.25743,0.33565,0.27783,0.33321,0.27197,0.19729,
0.19125,0.18285,0.19018,0.19358,0.18674,0.18851,0.19727,0.19331,0.1940
2,0.19973,0.20337,0.19875,0.12086,0.14038,0.12821,0.203,0.40912,0.3257
5,0.37695,0.49381,0.36459,0.47812,0.39248,0.27786,0.24805,0.28494,0.30
873,0.27048,0.33997,0.33649,0.32496,0.3127,0.26652,0.36726,0.19387,0.2
004,0.19969,0.1884,0.19014,0.19841,0.18738,0.18325,0.2011,0.20186,0.20
81,0.12536,0.12419,0.11928,0.12763,0.11658,0.1271,0.19535,0.31798,0.44
798,0.39923,0.34639,0.43522,0.38451,0.3982,0.30441,0.27064,0.33411,0.3
4506,0.24325,0.31689,0.29168,0.27524,0.22304,0.33534,0.26895,0.1947,0.
19792,0.19517,0.20358,0.20237,0.19746,0.19678,0.19495,0.18402,0.12611,
0.12211,0.11726,0.12165,0.12863,0.11045,0.13209,0.1217,0.12792,0.18552
,0.38726,0.40459,0.42144,0.51206,0.41286,0.23335,0.33688,0.29543,0.266
41,0.30485,0.28577,0.36536,0.25397,0.32996,0.26335,0.3222,0.29794,0.28
139,0.29037,0.19307,0.19281,0.19817,0.18447,0.1994,0.19239,0.11939,0.1

2691,0.12165,0.12721,0.13254,0.10682,0.13353,0.1268,0.13682,0.11358,0.
11824,0.13031,0.47153,0.35209,0.41141,0.47588,0.39586,0.35026,0.47256,
0.31784,0.32452,0.27887,0.30161,0.33339,0.28463,0.25964,0.32099,0.3152
8,0.3345,0.31795,0.27153,0.19265,0.18769,0.19585,0.12015,0.12682,0.128
88,0.12614,0.12221,0.12064,0.1112,0.14162,0.13613,0.10507,0.11889,0.13
188,0.12058,0.11948,0.15011,0.43419,0.3151,0.40169,0.53214,0.4683,0.40
083,0.35445,0.38065,0.35842,0.25935,0.27917,0.25406,0.32219,0.33632,0.
27704,0.37263,0.26768,0.37204,0.33105,0.2833,0.11729,0.10806,0.13456,0
.10995,0.1277,0.13379,0.1272,0.12004,0.12397,0.12547,0.12658,0.12522,0
.12309,0.12687,0.12033,0.13645,0.11301,0.10536,0.38813,0.36905,0.50788
,0.34598,0.35501,0.45136,0.44689,0.36422,0.35377,0.28221,0.32249,0.289
15,0.21898,0.23832,0.23441,0.2471,0.23471,0.19361,0.19091,0.26071,0.12
463,0.099283,0.12588,0.1105,0.13065,0.1245,0.12417,0.12554,0.12993,0.1
2094,0.12668,0.11364,0.12627,0.12356,0.12414,0.12713,0.1251,0.12588,0.
4304,0.42082,0.44144,0.48749,0.48916,0.42085,0.45532,0.52455,0.41997,0
.27754,0.22172,0.24115,0.21459,0.2077,0.22489,0.1941,0.2097,0.22831,0.
22783,0.24443,0.12756,0.10984,0.11827,0.12208,0.11325,0.12091,0.12584,
0.12701,0.11622,0.1134,0.11453,0.12648,0.11177,0.098049,0.12407,0.1238
9,0.10975,0.11337,0.46134,0.36263,0.36796,0.44353,0.49841,0.43411,0.48
233,0.2329,0.2386,0.24773,0.20966,0.24534,0.23277,0.23365,0.22001,0.23
127,0.23172,0.1879,0.20474,0.23772,0.12257,0.1249,0.12747,0.11339,0.12
473,0.11878,0.11708,0.1217,0.11721,0.1116,0.12526,0.12511,0.11847,0.12
48,0.11896,0.13231,0.13341,0.13169,0.43814,0.50279,0.4019,0.42681,0.41
19,0.39514,0.25766,0.20693,0.23146,0.26335,0.21679,0.20663,0.20627,0.2
3738,0.21742,0.20735,0.22385,0.19775,0.2352,0.21054,0.12994,0.10895,0.
11746,0.12035,0.12092,0.11931,0.12834,0.12056,0.11883,0.13009,0.1248,0
.13581,0.12743,0.13784,0.12624,0.11779,0.12308,0.12842,0.44164,0.39145
,0.39676,0.42438,0.39104,0.22488,0.23802,0.25433,0.22921,0.17698,0.231
84,0.22537,0.23079,0.22094,0.22739,0.19346,0.22984,0.22037,0.23676,0.2
2225,0.12511,0.14682,0.1268,0.12262,0.12902,0.12648,0.12969,0.11005,0.
12365,0.12043,0.12523,0.13102,0.11307,0.13118,0.1432,0.12554,0.11802,0
.105,0.38035,0.52643,0.39201,0.24662,0.27223,0.19815,0.24395,0.27532,0
.24208,0.1252,0.24092,0.20743,0.18932,0.23253,0.22215,0.20393,0.25034,
0.25474,0.20442,0.24062,0.11783,0.11003,0.13439,0.11148,0.13195,0.1119
7,0.12571,0.12236,0.10426,0.12165,0.14351,0.12807,0.12735,0.1233,0.121
31,0.11863,0.12059,0.11785,0.3674,0.25224,0.25309,0.22087,0.19844,0.24
697,0.24704,0.20202,0.16631,0.1647,0.24453,0.22244,0.1927,0.1973,0.230
35,0.22987,0.1739,0.26282,0.23072,0.1331,0.12957,0.12846,0.1237,0.1196
6,0.10179,0.12149,0.12748,0.11959,0.11476,0.11483,0.12338,0.10758,0.11
786,0.12575,0.12443,0.12118,0.054687,0.28906,0.23125,0.30089,0.22813,0
.24049,0.20097,0.22565,0.24879,0.20302,0.21388,0.20743,0.24817,0.21487
,0.21195,0.2184,0.22844,0.2335,0.23435,0.20127,0.12412,0.12963,0.12742
,0.12048,0.1113,0.13044,0.12388,0.11658,0.12158,0.12544,0.12917,0.1257
3,0.12364,0.12303,0.12929,0.12279,0.12125,0.053812,0.20641,0.23928,0.2
3325,0.19981,0.27315,0.2282,0.15599,0.2519,0.21628,0.25184,0.22517,0.2
1759,0.23039,0.2297,0.22463,0.20912,0.21516,0.18948,0.15005,0.12167,0.
12512,0.13737,0.12407,0.12945,0.12828,0.12371,0.11575,0.13897,0.11808,
0.12337,0.12306,0.12645,0.12086,0.11656,0.12429,0.13578,0.055832,0.205
19,0.19743,0.23171,0.3091,0.20163,0.20076,0.22397,0.23329,0.2148,0.211
63,0.22193,0.21725,0.2596,0.21497,0.22677,0.20472,0.18963,0.26106,0.11
787,0.11763,0.13566,0.12376,0.132,0.12368,0.11615,0.1211,0.12021,0.124
56,0.12642,0.12274,0.1177,0.125,0.11667,0.12869,0.1195,0.056612,0.0569

5,0.055824,0.1918,0.228,0.19989,0.23576,0.25684,0.20143,0.18791,0.2154
5,0.19774,0.19565,0.2271,0.25396,0.21889,0.20845,0.17182,0.13154,0.119
48,0.12588,0.13035,0.12499,0.10712,0.12179,0.12776,0.12276,0.12243,0.1
232,0.10627,0.13718,0.11757,0.10134,0.12583,0.12649,0.11631,0.052964,0
.053988,0.054048,0.050626,0.17052,0.22095,0.19962,0.21906,0.21803,0.24
52,0.18877,0.18165,0.23794,0.22054,0.2189,0.2344,0.11424,0.15142,0.134
53,0.12495,0.13342,0.10599,0.12678,0.12843,0.13701,0.14466,0.12014,0.1
235,0.10494,0.12786,0.12179,0.10945,0.11932,0.10329,0.11679,0.12018,0.
056604,0.044356,0.046032,0.052095,0.051672,0.050605,0.22361,0.27698,0.
17367,0.17936,0.26245,0.15222,0.24222,0.24498,0.12762,0.11659,0.097383
,0.13793,0.1226,0.12106,0.12238,0.11476,0.12657,0.14793,0.11883,0.1245
8,0.13074,0.12757,0.14141,0.11718,0.12108,0.12649,0.11595,0.12436,0.11
807,0.13979,0.12159,0.052018,0.050996,0.057607,0.037084,0.054187,0.050
358,0.26385,0.26217,0.29979,0.29822,0.26387,0.053307,0.051988,0.12048,
0.12332,0.10935,0.11998,0.12266,0.12554,0.11003,0.12152,0.11732,0.1202
9,0.12395,0.10902,0.12079,0.12156,0.13233,0.12944,0.12411,0.11869,0.12
139,0.099607,0.12826,0.056317,0.044504,0.060928,0.051021,0.048743,0.05
8516,0.055245,0.057773,0.054867,0.055974,0.19413,0.27039,0.053182,0.04
6376,0.049756,0.048353,0.11751,0.12433,0.11221,0.12438,0.1045,0.1253,0
.11937,0.11618,0.12478,0.11473,0.11929,0.1219,0.11447,0.11727,0.1195,0
.12679,0.12376,0.13135,0.12986,0.053753,0.052085,0.049876,0.052019,0.0
60071,0.050442,0.046697,0.050724,0.053342,0.051399,0.053547,0.057033,0
.053406,0.051011,0.052221,0.050527,0.054208,0.12929,0.12256,0.12345,0.
12242,0.11261,0.12073,0.11292,0.12083,0.11422,0.12257,0.13058,0.1287,0
.12462,0.12377,0.1078,0.12628,0.048743,0.05562,0.060803,0.059638,0.053
403,0.047823,0.053845,0.052392,0.05677,0.052696,0.049899,0.050666,0.05
3316,0.050326,0.058922,0.04873,0.054512,0.053634,0.057526,0.13394,0.11
337,0.12699,0.1183,0.10972,0.13477,0.11948,0.12675,0.12449,0.12024,0.1
2675,0.12158,0.12482,0.052472,0.050429,0.051414,0.055717,0.049391,0.05
1062,0.051578,0.051299,0.061392,0.050834,0.054756,0.045019,0.051473,0.
047388,0.051248,0.05221,0.054141,0.04874,0.053428,0.059761,0.055805,0.
12033,0.12698,0.11942,0.11067,0.14434,0.12043,0.12438,0.12059,0.12352,
0.049153,0.053583,0.053947,0.041396,0.048928,0.055975,0.053606,0.06159
3,0.045879,0.047671,0.05289,0.057673,0.052293,0.062754,0.053219,0.0551
12,0.054774,0.053491,0.057613,0.049097,0.048876,0.059484,0.053874,0.05
2931,0.057584,0.12114,0.11238,0.12554,0.12556,0.049597,0.058122,0.0576
71,0.050448,0.052364,0.054089,0.049806,0.0481,0.051252,0.05239,0.05895
6,0.053144,0.051346,0.053618,0.05078,0.058087,0.051892,0.050897,0.0488
53,0.05568,0.050023,0.052608,0.054785,0.050478,0.05215,0.054148,0.0484
44,0.047927,0.056572,0.05637,0.051117,0.047129,0.060204,0.048556,0.055
428,0.05303,0.05368,0.048516,0.050312,0.050754,0.051893,0.04976,0.0558
49,0.051935,0.054217,0.048763,0.053178,0.056985,0.055041,0.04599,0.055
803,0.04949,0.060829,0.053108,0.051336,0.059817,0.052573,0.05882,0.056
911,0.051421,0.053865,0.050795,0.048271,0.05677,0.048712,0.057393,0.05
3782,0.053623,0.049914,0.056666,0.051644,0.056251,0.055019,0.052287,0.
048658,0.051505,0.061263,0.057748,0.055927,0.048674,0.060556,0.054725,
0.05102,0.055204,0.053722,0.054824,0.051373,0.051485,0.056334,0.052227
,0.051399,0.055035,0.05263,0.04154,0.057308,0.053935,0.055349,0.056934
,0.048315,0.054156,0.057036,0.047818,0.048678,0.05681,0.048864,0.05635
1,0.049625,0.053767,0.057962,0.056367,0.053465,0.058212,0.052016,0.052
508,0.053068,0.049077,0.055523,0.049916,0.048401,0.051533,0.048838,0.0
52991,0.047572,0.052594,0.05715,0.054576,0.050271,0.06444,0.048197,0.0

48703,0.057461,0.049718,0.054347,0.051261,0.051433,0.053592,0.053703,0
.050413,0.046318,0.054558,0.044617,0.050018,0.051611,0.059886,0.055229
,0.054103,0.051761,0.052818,0.054582,0.057085,0.05506,0.053567,0.04765
2,0.048839,0.053561,0.053763,0.051383,0.055011,0.053387,0.060814,0.052
746,0.054324,0.050955,0.050389,0.048153,0.053808,0.044334,0.051621,0.0
50182,0.05348,0.05302,0.05527,0.05005,0.051068,0.057541,0.05327,0.0480
65,0.054061,0.051335,0.043098,0.055116,0.056571,0.052254,0.056323,0.05
6327,0.053836,0.048322,0.052052,0.051,0.054594,0.046086,0.056203,0.055
326,0.052741,0.053964
0.19298,0.19557,0.20231,0.19583,0.18886,0.18826,0.2026,0.19453,0.19024
,0.20075,0.2057,0.1965,0.19304,0.19884,0.20053,0.1979,0.19267,0.19266,
0.20359,0.19644,0.27204,0.32355,0.34109,0.31743,0.1977,0.18672,0.21176
,0.20133,0.20006,0.19464,0.20371,0.19622,0.19456,0.19194,0.20155,0.202
3,0.19926,0.20554,0.19517,0.48136,0.28317,0.26066,0.39389,0.23995,0.31
685,0.33844,0.19393,0.19812,0.19616,0.20222,0.18757,0.19407,0.19173,0.
18635,0.18941,0.18723,0.19616,0.19601,0.19449,0.19266,0.20509,0.31421,
0.37599,0.3289,0.32069,0.3244,0.31187,0.31589,0.32055,0.29728,0.3155,0
.20241,0.19633,0.1998,0.19542,0.19184,0.1967,0.18364,0.19604,0.19372,0
.19523,0.18427,0.19188,0.19351,0.19233,0.1979,0.40077,0.42644,0.34368,
0.29302,0.24279,0.28703,0.26511,0.30509,0.31028,0.25292,0.28369,0.2778
2,0.19441,0.19965,0.20168,0.19406,0.20149,0.20267,0.19454,0.20125,0.19
742,0.20099,0.19544,0.19648,0.19714,0.18768,0.20021,0.19321,0.41395,0.
30662,0.43048,0.38415,0.29981,0.31277,0.30967,0.28273,0.27295,0.25673,
0.33289,0.29765,0.33483,0.27822,0.20419,0.19105,0.19631,0.19695,0.1965
1,0.18621,0.20025,0.19291,0.20177,0.2011,0.20019,0.2004,0.20028,0.1970
5,0.19333,0.20323,0.40467,0.37717,0.42898,0.44129,0.38276,0.50937,0.37
279,0.26831,0.28926,0.27304,0.35867,0.29853,0.29778,0.29243,0.2827,0.2
5981,0.19388,0.18951,0.20216,0.19788,0.19854,0.21184,0.19793,0.19473,0
.19511,0.2002,0.19677,0.19652,0.20319,0.2014,0.19395,0.19886,0.19328,0
.42591,0.39009,0.41725,0.39663,0.39047,0.47591,0.28208,0.29518,0.25097
,0.31302,0.22771,0.2779,0.26011,0.3181,0.28496,0.33392,0.27573,0.20223
,0.19242,0.18503,0.18875,0.19443,0.18771,0.19297,0.19806,0.19507,0.193
8,0.20323,0.20438,0.19994,0.11721,0.14192,0.12576,0.20546,0.39798,0.29
674,0.36961,0.4371,0.34656,0.49193,0.41615,0.27733,0.26307,0.28118,0.2
6958,0.27871,0.31739,0.32497,0.31691,0.30237,0.24588,0.36252,0.19776,0
.20674,0.2019,0.18741,0.19127,0.19876,0.19459,0.18581,0.20336,0.20754,
0.20922,0.12281,0.12339,0.11817,0.12719,0.11805,0.12428,0.19914,0.3157
5,0.41223,0.4067,0.32112,0.40654,0.38166,0.39527,0.29672,0.26946,0.320
2,0.35261,0.24021,0.31602,0.29239,0.28115,0.21633,0.32251,0.26504,0.19
561,0.20128,0.19409,0.20774,0.20782,0.19717,0.19956,0.19766,0.18347,0.
12765,0.12181,0.11696,0.12115,0.12667,0.11129,0.1328,0.12364,0.12723,0
.18676,0.36934,0.39245,0.42518,0.45389,0.41753,0.22679,0.33009,0.28253
,0.25097,0.30953,0.27131,0.35577,0.24552,0.30953,0.25945,0.31256,0.294
56,0.29371,0.28294,0.19758,0.19338,0.20017,0.18588,0.1978,0.19278,0.11
665,0.12601,0.12118,0.12746,0.13382,0.10774,0.13476,0.12643,0.1355,0.1
1219,0.11735,0.13118,0.50653,0.33249,0.3931,0.43272,0.37376,0.32782,0.
43988,0.30166,0.28351,0.26608,0.28826,0.31462,0.28166,0.26627,0.32126,
0.29797,0.33517,0.31798,0.27673,0.19627,0.18754,0.19848,0.11989,0.1267
5,0.12699,0.12587,0.12157,0.11931,0.11121,0.14414,0.1369,0.10351,0.119
2,0.13172,0.11929,0.11777,0.14824,0.4236,0.29246,0.37954,0.47395,0.428
25,0.36568,0.33395,0.40287,0.36747,0.25202,0.29284,0.25346,0.30258,0.3
4524,0.29213,0.36951,0.26126,0.33687,0.30367,0.28178,0.11763,0.11038,0

.13383,0.10694,0.12806,0.13426,0.12627,0.11749,0.12531,0.12424,0.12661
,0.12308,0.11949,0.12744,0.11944,0.13702,0.11022,0.10522,0.39398,0.371
38,0.48997,0.36219,0.37518,0.42523,0.45756,0.30682,0.3292,0.30199,0.33
014,0.2808,0.20829,0.22537,0.21737,0.24653,0.23493,0.18216,0.18613,0.2
5002,0.12493,0.097583,0.12177,0.11012,0.12884,0.12504,0.12187,0.12294,
0.12853,0.11888,0.12797,0.11098,0.12335,0.12293,0.12211,0.1252,0.12664
,0.12449,0.44168,0.40383,0.43021,0.47014,0.46625,0.40298,0.4486,0.4596
3,0.38349,0.27826,0.21518,0.24664,0.19672,0.20299,0.22878,0.20011,0.17
842,0.21188,0.21847,0.23001,0.1279,0.11054,0.1145,0.12097,0.11165,0.12
042,0.12372,0.12527,0.11438,0.11314,0.11607,0.12553,0.11061,0.095908,0
.12083,0.123,0.1081,0.11291,0.42733,0.36707,0.38734,0.42322,0.4501,0.4
0087,0.42567,0.21934,0.24774,0.23035,0.21262,0.22344,0.22675,0.22448,0
.23052,0.22357,0.23278,0.18654,0.20008,0.24017,0.12292,0.12349,0.12722
,0.11088,0.12668,0.11846,0.11678,0.1201,0.11765,0.11096,0.12803,0.1251
6,0.11632,0.12422,0.11996,0.13166,0.13333,0.12934,0.42041,0.49552,0.35
417,0.43096,0.3757,0.34377,0.24264,0.21509,0.2337,0.24118,0.21033,0.19
074,0.2065,0.23259,0.1988,0.18037,0.21325,0.19599,0.22969,0.20032,0.12
811,0.10598,0.11637,0.12027,0.12047,0.11683,0.12947,0.1205,0.11724,0.1
2875,0.12619,0.14,0.1259,0.14111,0.1248,0.11608,0.12285,0.12905,0.4397
3,0.41612,0.32228,0.39901,0.36176,0.20464,0.21334,0.2407,0.21342,0.192
63,0.20889,0.21758,0.22905,0.22655,0.2164,0.17618,0.22541,0.21796,0.24
209,0.23498,0.12548,0.14601,0.12517,0.12281,0.1295,0.12504,0.12922,0.1
089,0.12479,0.11908,0.12342,0.13107,0.11249,0.13009,0.14195,0.12378,0.
11867,0.10239,0.34516,0.4937,0.3598,0.20555,0.26886,0.20167,0.23743,0.
28332,0.24752,0.12402,0.25356,0.20946,0.18471,0.22512,0.21446,0.18302,
0.2415,0.24352,0.19619,0.23423,0.1174,0.10887,0.13435,0.10962,0.12803,
0.11007,0.12525,0.12353,0.1029,0.11957,0.14156,0.12997,0.12668,0.12391
,0.12069,0.11761,0.12189,0.11645,0.34619,0.24195,0.2429,0.21436,0.1768
,0.22418,0.22652,0.19068,0.16321,0.14315,0.24123,0.22308,0.19243,0.196
98,0.22977,0.2135,0.17364,0.24648,0.21439,0.13297,0.12759,0.12762,0.12
118,0.11946,0.10043,0.12156,0.1256,0.11633,0.11407,0.11586,0.12282,0.1
0691,0.11841,0.12223,0.12602,0.12138,0.050477,0.27292,0.21017,0.31429,
0.21931,0.20758,0.17964,0.21734,0.22987,0.20714,0.21446,0.19258,0.2222
6,0.20751,0.20482,0.21232,0.21274,0.23428,0.22502,0.2009,0.12511,0.126
84,0.12838,0.12015,0.1103,0.12958,0.12274,0.11635,0.12234,0.12477,0.12
693,0.12387,0.124,0.12307,0.12935,0.1229,0.12187,0.050526,0.20977,0.24
658,0.21268,0.17656,0.25812,0.21722,0.14188,0.24193,0.20934,0.22164,0.
2258,0.20991,0.22434,0.22449,0.22,0.20594,0.19979,0.19643,0.14944,0.12
296,0.12356,0.13576,0.12154,0.12937,0.12771,0.12717,0.11579,0.13842,0.
118,0.12103,0.12395,0.12558,0.11697,0.11791,0.12443,0.1341,0.0486,0.19
947,0.19476,0.225,0.29818,0.18814,0.19135,0.20166,0.20157,0.20186,0.19
853,0.22045,0.21727,0.26258,0.20452,0.22537,0.20158,0.19693,0.26184,0.
11768,0.11357,0.13523,0.12327,0.13082,0.12099,0.11581,0.12068,0.11823,
0.12387,0.1254,0.11961,0.1142,0.12323,0.11694,0.12968,0.11754,0.052974
,0.052889,0.049372,0.184,0.24212,0.19724,0.22803,0.24501,0.19336,0.208
53,0.21003,0.20591,0.19843,0.23203,0.2394,0.20766,0.19981,0.15961,0.13
202,0.11834,0.12486,0.13045,0.12562,0.107,0.11964,0.12462,0.12246,0.11
871,0.12345,0.10344,0.1391,0.11554,0.099723,0.12389,0.12586,0.11416,0.
047255,0.049452,0.049169,0.046702,0.16117,0.22285,0.18979,0.2262,0.201
55,0.24353,0.16833,0.17951,0.20541,0.21011,0.18521,0.23156,0.11291,0.1
4965,0.13625,0.12282,0.13281,0.10475,0.12326,0.12666,0.13501,0.14204,0
.1189,0.12194,0.10213,0.12638,0.11975,0.10786,0.11702,0.10422,0.11591,

0.1202,0.051924,0.042859,0.044389,0.050692,0.049514,0.049703,0.22848,0
.2479,0.16413,0.18366,0.25588,0.15017,0.23746,0.22157,0.12456,0.11585,
0.096589,0.13653,0.12115,0.11873,0.12108,0.11319,0.12475,0.14654,0.117
03,0.12326,0.13236,0.12916,0.14224,0.11554,0.11778,0.12402,0.11482,0.1
2336,0.11616,0.13869,0.12279,0.050168,0.048399,0.05228,0.034735,0.0510
37,0.046203,0.26141,0.24081,0.29982,0.28721,0.2557,0.048174,0.048426,0
.12011,0.12198,0.1074,0.11831,0.12023,0.12415,0.11078,0.11995,0.11444,
0.12102,0.12313,0.1078,0.11904,0.12017,0.13226,0.12652,0.12412,0.11786
,0.12138,0.098065,0.12762,0.049439,0.040677,0.058772,0.047922,0.046457
,0.053621,0.049633,0.0525,0.049962,0.049812,0.20127,0.23502,0.047227,0
.042678,0.045243,0.040059,0.11359,0.12434,0.10861,0.12219,0.10333,0.12
402,0.11741,0.11318,0.12517,0.11284,0.11719,0.12142,0.11429,0.11481,0.
11778,0.12765,0.1199,0.13358,0.12777,0.050111,0.047505,0.047985,0.0487
84,0.056808,0.046886,0.046719,0.047179,0.048064,0.048467,0.049781,0.05
1801,0.048055,0.046761,0.048209,0.048235,0.048482,0.13242,0.12015,0.12
333,0.12349,0.11219,0.11782,0.11443,0.11696,0.11363,0.12141,0.12894,0.
12616,0.12552,0.12169,0.10629,0.12608,0.046835,0.051884,0.054461,0.052
153,0.048699,0.041276,0.049924,0.049664,0.0544,0.047792,0.046328,0.044
297,0.052158,0.046344,0.054655,0.042713,0.04843,0.048177,0.052744,0.13
331,0.11402,0.12611,0.11639,0.10809,0.13593,0.12156,0.12482,0.12494,0.
11941,0.12811,0.12169,0.12445,0.046971,0.047272,0.049012,0.049931,0.04
0628,0.046402,0.048291,0.048548,0.054856,0.044545,0.050379,0.042511,0.
047994,0.043936,0.047768,0.048875,0.050395,0.042871,0.050045,0.054746,
0.050346,0.12045,0.12684,0.12027,0.10964,0.14431,0.11974,0.1249,0.1183
3,0.12146,0.045479,0.050095,0.050029,0.037352,0.047586,0.049271,0.0481
78,0.055972,0.040844,0.047463,0.048255,0.053005,0.046459,0.060667,0.04
963,0.052109,0.050538,0.04896,0.054961,0.045096,0.047626,0.058047,0.04
7848,0.047134,0.054381,0.12066,0.11099,0.12326,0.12463,0.046478,0.0527
1,0.056738,0.04601,0.049393,0.050144,0.044035,0.04099,0.044272,0.05066
2,0.053767,0.0445,0.046121,0.048767,0.049227,0.053503,0.046002,0.04428
7,0.042584,0.050484,0.044517,0.047174,0.052253,0.044846,0.049567,0.048
173,0.044686,0.042257,0.051639,0.051656,0.045644,0.04448,0.055849,0.04
4571,0.050895,0.048674,0.048581,0.046623,0.045814,0.047792,0.046795,0.
042301,0.055781,0.048034,0.050918,0.044758,0.047733,0.051505,0.049305,
0.043045,0.04855,0.047849,0.056405,0.049937,0.049359,0.053904,0.047261
,0.055588,0.050335,0.046093,0.048389,0.051076,0.044538,0.05195,0.04819
2,0.052518,0.048743,0.047105,0.045344,0.050331,0.043539,0.049942,0.049
016,0.049945,0.04517,0.048982,0.054206,0.051378,0.054437,0.045427,0.05
6258,0.050654,0.046456,0.0537,0.049006,0.05224,0.047608,0.046229,0.053
853,0.046935,0.048354,0.050435,0.045008,0.04136,0.051251,0.051232,0.05
1001,0.052697,0.045764,0.046592,0.05183,0.040954,0.043178,0.052529,0.0
467,0.050664,0.04553,0.050255,0.053022,0.050454,0.047954,0.052732,0.04
7793,0.048179,0.047981,0.046976,0.04885,0.044751,0.047495,0.047545,0.0
43759,0.055368,0.04546,0.046844,0.052523,0.047926,0.04393,0.060348,0.0
43528,0.048551,0.049802,0.048968,0.048468,0.048103,0.047102,0.048157,0
.050687,0.044971,0.042604,0.051652,0.041568,0.048266,0.04483,0.054424,
0.050336,0.048769,0.049055,0.051307,0.051634,0.05178,0.052617,0.048262
,0.043257,0.045246,0.048728,0.050976,0.048977,0.051239,0.050943,0.0553
27,0.047423,0.048185,0.047664,0.048462,0.046027,0.050756,0.039512,0.04
7469,0.04997,0.051268,0.049509,0.04789,0.046461,0.045071,0.052262,0.04
6974,0.045013,0.049772,0.043761,0.037419,0.050937,0.050906,0.046616,0.

052701,0.05072,0.049538,0.044077,0.045197,0.045694,0.050015,0.044789,0
.050593,0.048858,0.048832,0.049548
0.19715,0.19949,0.2031,0.19572,0.19138,0.1958,0.20807,0.19571,0.19036,
0.19948,0.20771,0.19787,0.19263,0.20424,0.2023,0.2006,0.19779,0.19365,
0.20418,0.19659,0.27239,0.28939,0.34229,0.29619,0.20251,0.1888,0.21128
,0.20216,0.20369,0.19809,0.20254,0.20342,0.19753,0.19851,0.20094,0.202
52,0.19786,0.20234,0.19579,0.41399,0.28763,0.26321,0.37437,0.2184,0.29
351,0.32824,0.1943,0.1996,0.19464,0.20656,0.1922,0.19121,0.19497,0.191
36,0.19262,0.18988,0.19977,0.19709,0.19387,0.19336,0.20334,0.33544,0.3
6291,0.31005,0.31786,0.32687,0.31513,0.31939,0.32165,0.29012,0.3089,0.
20111,0.19895,0.1984,0.197,0.19336,0.20052,0.18523,0.20059,0.19244,0.1
9993,0.18672,0.1934,0.1934,0.1949,0.20046,0.41428,0.42398,0.35311,0.27
978,0.23092,0.29782,0.25556,0.30123,0.30384,0.2366,0.28645,0.25909,0.1
9647,0.20478,0.20169,0.19321,0.20486,0.20261,0.19298,0.20098,0.19786,0
.20208,0.19562,0.20344,0.19906,0.18846,0.20033,0.19612,0.3894,0.34552,
0.4275,0.36768,0.29805,0.30636,0.31196,0.28155,0.28122,0.25781,0.32061
,0.28841,0.31856,0.26922,0.20377,0.19316,0.20083,0.20073,0.19733,0.189
35,0.20364,0.19743,0.20199,0.20231,0.20272,0.19622,0.20217,0.2007,0.19
037,0.20321,0.38349,0.42389,0.45149,0.45602,0.36187,0.52113,0.37323,0.
26333,0.29826,0.28117,0.36274,0.30659,0.29043,0.29987,0.28084,0.27052,
0.1957,0.19225,0.20519,0.19845,0.19755,0.21147,0.19856,0.19488,0.20135
,0.19807,0.20484,0.1951,0.2032,0.20203,0.19673,0.19881,0.19555,0.42197
,0.37885,0.4433,0.37475,0.37827,0.52974,0.29437,0.27863,0.23035,0.3059
1,0.2055,0.26681,0.25451,0.31577,0.27848,0.32955,0.27819,0.20576,0.196
47,0.18773,0.19503,0.19825,0.19073,0.19224,0.19609,0.19801,0.19882,0.2
0354,0.20455,0.20162,0.11797,0.14284,0.12802,0.20637,0.36859,0.30289,0
.33474,0.41315,0.40285,0.48238,0.40121,0.26472,0.26257,0.28157,0.28893
,0.27303,0.28526,0.30532,0.33403,0.28816,0.25968,0.35789,0.20262,0.207
38,0.20221,0.19147,0.19621,0.20331,0.19774,0.18678,0.20439,0.20878,0.2
0816,0.12169,0.11865,0.11904,0.12673,0.11645,0.1255,0.20244,0.34008,0.
40946,0.4068,0.32697,0.39776,0.37318,0.36208,0.29257,0.28285,0.31836,0
.35211,0.24346,0.298,0.28391,0.3173,0.22912,0.3105,0.27412,0.19724,0.2
0632,0.19338,0.21073,0.20505,0.19986,0.20115,0.19899,0.18439,0.12475,0
.1194,0.11486,0.11948,0.12778,0.10734,0.13518,0.12304,0.12567,0.1913,0
.34649,0.37707,0.43664,0.41921,0.3867,0.1706,0.36935,0.27818,0.23897,0
.302,0.26741,0.33637,0.26588,0.33356,0.25253,0.3307,0.296,0.27672,0.29
478,0.20107,0.19441,0.19794,0.18808,0.20237,0.19417,0.1147,0.12424,0.1
1816,0.12782,0.13368,0.10705,0.13609,0.12416,0.13638,0.11156,0.11687,0
.12965,0.4459,0.33367,0.37359,0.40829,0.40701,0.34298,0.42864,0.29062,
0.26669,0.2738,0.28873,0.30188,0.27526,0.27407,0.31146,0.30881,0.33663
,0.28916,0.26757,0.19843,0.18589,0.19905,0.1196,0.12623,0.12672,0.1287
3,0.11942,0.11835,0.11178,0.144,0.13983,0.10305,0.1166,0.13201,0.12154
,0.11656,0.14813,0.3853,0.29209,0.39333,0.42496,0.45865,0.41583,0.3444
9,0.35169,0.35689,0.26159,0.28292,0.23733,0.29849,0.33852,0.28886,0.31
669,0.25102,0.30735,0.31283,0.29139,0.11731,0.1093,0.13414,0.10482,0.1
283,0.1319,0.12707,0.11572,0.12442,0.12378,0.12429,0.12383,0.1179,0.12
602,0.11879,0.1382,0.10968,0.10286,0.38268,0.37424,0.46567,0.34489,0.3
6021,0.37157,0.47633,0.319,0.33034,0.28997,0.30535,0.26513,0.2062,0.22
996,0.18879,0.23206,0.22281,0.18514,0.19434,0.24982,0.12484,0.095054,0
.12353,0.10943,0.12647,0.12273,0.12347,0.12201,0.12961,0.11856,0.12732
,0.10859,0.12467,0.12166,0.12173,0.12607,0.1255,0.12462,0.41873,0.3724
4,0.40093,0.47279,0.42408,0.44262,0.40677,0.52677,0.38749,0.28024,0.21

93,0.23406,0.20409,0.19935,0.21544,0.20202,0.16442,0.2025,0.22866,0.23
806,0.12294,0.11224,0.11412,0.12227,0.11056,0.1206,0.12451,0.12621,0.1
1462,0.11529,0.11311,0.12367,0.10982,0.096012,0.11886,0.1224,0.10809,0
.11213,0.38716,0.3644,0.36699,0.46269,0.37127,0.34172,0.41683,0.21991,
0.21882,0.21244,0.21638,0.22053,0.23209,0.20803,0.23563,0.20193,0.2235
,0.18187,0.19038,0.22843,0.12449,0.12118,0.12653,0.11318,0.12646,0.119
31,0.11651,0.12108,0.11759,0.11116,0.12611,0.12449,0.11709,0.12672,0.1
1892,0.13085,0.1296,0.12979,0.40399,0.48588,0.34153,0.41233,0.36782,0.
35757,0.24156,0.19873,0.22222,0.2482,0.22026,0.1803,0.2129,0.21756,0.1
8968,0.18368,0.2036,0.20447,0.22483,0.19876,0.12893,0.10553,0.11517,0.
11942,0.12087,0.11468,0.12857,0.11901,0.11498,0.12892,0.12513,0.13951,
0.12451,0.14033,0.12474,0.11463,0.12037,0.1269,0.43885,0.42069,0.32294
,0.39103,0.38237,0.18356,0.204,0.23275,0.21412,0.1772,0.21681,0.21458,
0.22936,0.20933,0.23684,0.17521,0.23395,0.20299,0.22987,0.2232,0.12482
,0.14443,0.12397,0.12272,0.13028,0.12223,0.12782,0.10784,0.12611,0.117
85,0.1214,0.13035,0.1132,0.13184,0.13971,0.12369,0.11805,0.10153,0.347
91,0.49809,0.32854,0.20602,0.24841,0.18926,0.23234,0.26087,0.2401,0.12
289,0.24593,0.21115,0.19337,0.21817,0.21125,0.19702,0.22825,0.23481,0.
19416,0.22063,0.11684,0.10897,0.13269,0.10867,0.1268,0.10642,0.12384,0
.12225,0.10144,0.12101,0.13964,0.12628,0.12725,0.12356,0.12136,0.11666
,0.12273,0.11881,0.32129,0.24432,0.20473,0.20482,0.1828,0.20927,0.2282
7,0.18324,0.1505,0.15439,0.24884,0.22001,0.18711,0.19695,0.22343,0.222
5,0.16424,0.25178,0.21557,0.13202,0.12751,0.12886,0.11915,0.1195,0.100
73,0.12312,0.12504,0.11577,0.11278,0.11703,0.12058,0.10549,0.11908,0.1
2135,0.12637,0.12264,0.044927,0.28178,0.21686,0.30906,0.19461,0.21792,
0.18431,0.21729,0.23101,0.19483,0.20822,0.19241,0.22773,0.2103,0.21097
,0.21676,0.20412,0.23987,0.21584,0.20629,0.12442,0.12821,0.12733,0.121
53,0.10791,0.13093,0.12424,0.11352,0.11957,0.12504,0.12531,0.1243,0.12
313,0.12181,0.12914,0.12038,0.1221,0.044952,0.18309,0.25098,0.20497,0.
1708,0.24269,0.20552,0.14665,0.21791,0.19169,0.23081,0.23286,0.19936,0
.20741,0.2325,0.22519,0.19958,0.18691,0.19747,0.14862,0.12129,0.12333,
0.13409,0.12121,0.12959,0.1255,0.12643,0.11658,0.13793,0.11779,0.11788
,0.12566,0.12346,0.11665,0.11699,0.12217,0.13186,0.045751,0.1956,0.202
38,0.21195,0.27626,0.20819,0.1824,0.19368,0.20028,0.23173,0.20304,0.20
696,0.21862,0.25303,0.2025,0.21603,0.18453,0.19757,0.24746,0.11585,0.1
1191,0.13413,0.11967,0.12875,0.11873,0.11268,0.12182,0.11666,0.12392,0
.12377,0.1202,0.11192,0.12291,0.11487,0.13062,0.11915,0.052282,0.04861
7,0.046119,0.17373,0.23924,0.20897,0.20012,0.24754,0.19537,0.19328,0.2
1639,0.20332,0.18841,0.22167,0.22884,0.21258,0.19016,0.17882,0.13247,0
.11901,0.12693,0.13075,0.12432,0.10723,0.11988,0.12375,0.12085,0.11628
,0.12316,0.10211,0.13771,0.11445,0.09655,0.12389,0.12472,0.11384,0.043
034,0.047673,0.044174,0.041387,0.15307,0.22844,0.16698,0.23971,0.19584
,0.23573,0.15971,0.16523,0.19455,0.21922,0.20287,0.2297,0.11153,0.1513
4,0.13549,0.1197,0.13261,0.1045,0.1245,0.125,0.13274,0.14134,0.12182,0
.12226,0.10152,0.12676,0.12039,0.10673,0.11761,0.1049,0.11518,0.12058,
0.051085,0.040864,0.043448,0.049267,0.046215,0.047512,0.23945,0.23406,
0.14295,0.16914,0.22322,0.151,0.2346,0.20756,0.12423,0.11575,0.095999,
0.1343,0.12187,0.11859,0.12086,0.11072,0.12418,0.14586,0.11667,0.12508
,0.13285,0.12893,0.14391,0.11532,0.11982,0.1244,0.11314,0.12108,0.1157
9,0.14007,0.12398,0.048422,0.040997,0.048171,0.032579,0.048973,0.04097
,0.27354,0.22661,0.29266,0.28996,0.23826,0.046271,0.047746,0.12087,0.1
1975,0.10676,0.1173,0.11893,0.12146,0.11019,0.12019,0.1126,0.11981,0.1

216,0.1054,0.11845,0.12094,0.13086,0.12741,0.12492,0.11577,0.1215,0.09
7409,0.12739,0.044208,0.036548,0.057726,0.045615,0.043883,0.05039,0.04
4869,0.050476,0.046971,0.045326,0.2182,0.23551,0.044292,0.040798,0.041
827,0.037104,0.11078,0.12194,0.10825,0.12311,0.1017,0.1266,0.11702,0.1
1107,0.12138,0.1129,0.11623,0.12022,0.11348,0.11425,0.11758,0.12927,0.
12065,0.13159,0.12903,0.047796,0.043726,0.044861,0.045555,0.050585,0.0
42395,0.044805,0.042164,0.046998,0.045644,0.046358,0.04892,0.047578,0.
044415,0.042641,0.044925,0.046655,0.1315,0.11954,0.12479,0.12287,0.109
48,0.11664,0.11605,0.11708,0.11192,0.12253,0.12903,0.12587,0.12419,0.1
2245,0.10529,0.12289,0.042508,0.051084,0.052685,0.050585,0.045609,0.03
8199,0.050473,0.043399,0.055363,0.044214,0.043533,0.042133,0.047445,0.
043874,0.050978,0.039455,0.046962,0.046295,0.051358,0.13243,0.11304,0.
12491,0.11772,0.10871,0.138,0.12105,0.12594,0.12367,0.11918,0.12795,0.
123,0.12521,0.043942,0.047817,0.050092,0.045991,0.036404,0.041883,0.04
46,0.047243,0.050829,0.043733,0.048307,0.040603,0.042042,0.040703,0.04
1318,0.051382,0.048736,0.03985,0.048019,0.051649,0.051193,0.12167,0.12
566,0.1188,0.10773,0.14435,0.1203,0.12518,0.11727,0.11994,0.040682,0.0
48134,0.046573,0.03403,0.047447,0.042732,0.048162,0.051351,0.03869,0.0
44272,0.044373,0.049456,0.042406,0.056578,0.044439,0.050323,0.047895,0
.046613,0.048513,0.04274,0.046345,0.049947,0.043334,0.045498,0.049556,
0.11965,0.11232,0.12338,0.12319,0.040736,0.047709,0.051539,0.045399,0.
047125,0.047658,0.04037,0.0401,0.039194,0.04896,0.051762,0.043566,0.04
429,0.046849,0.044364,0.04993,0.044438,0.039215,0.042048,0.045958,0.03
9773,0.043683,0.048772,0.044882,0.045722,0.044629,0.041216,0.036218,0.
049388,0.048847,0.041429,0.041395,0.049454,0.040787,0.047848,0.045197,
0.048834,0.042409,0.045112,0.046216,0.043587,0.038564,0.053761,0.04372
5,0.048279,0.042552,0.042865,0.051191,0.045473,0.037913,0.045536,0.045
281,0.05285,0.045397,0.044879,0.04896,0.044868,0.050755,0.047725,0.040
6,0.046977,0.049003,0.043879,0.049149,0.044911,0.048825,0.043656,0.045
05,0.043125,0.048683,0.039427,0.045277,0.046095,0.047975,0.044712,0.04
5599,0.052172,0.049301,0.051947,0.041989,0.05201,0.049381,0.040653,0.0
51326,0.047292,0.050666,0.046706,0.041426,0.048682,0.042262,0.042063,0
.044126,0.039424,0.040403,0.046991,0.048511,0.049903,0.049355,0.044093
,0.043924,0.047441,0.036983,0.042969,0.050185,0.044501,0.047942,0.0434
78,0.04601,0.049821,0.050954,0.044202,0.049397,0.044035,0.04685,0.0458
51,0.041862,0.047261,0.044165,0.046147,0.043073,0.04296,0.055434,0.044
969,0.047603,0.048641,0.04416,0.039353,0.056991,0.043524,0.047109,0.04
5511,0.046381,0.045977,0.044466,0.045706,0.043949,0.046336,0.041876,0.
042473,0.046476,0.039438,0.044488,0.044265,0.049831,0.048722,0.04497,0
.045,0.049146,0.049476,0.050343,0.04922,0.043174,0.039261,0.040908,0.0
42313,0.047176,0.04368,0.048407,0.04919,0.051567,0.043084,0.044862,0.0
45637,0.042389,0.043778,0.050713,0.03872,0.042439,0.047229,0.049149,0.
046404,0.045865,0.042439,0.04138,0.049387,0.045988,0.040738,0.043566,0
.038259,0.036568,0.047966,0.04851,0.043569,0.049552,0.047127,0.045503,
0.041356,0.042384,0.044597,0.044817,0.043691,0.04911,0.048144,0.043963
,0.044942
0.20111,0.19762,0.20049,0.19409,0.19435,0.19576,0.2088,0.19997,0.19283
,0.20191,0.21052,0.20071,0.19122,0.20708,0.19961,0.20493,0.20086,0.195
72,0.20404,0.19746,0.2728,0.27591,0.31524,0.29474,0.20682,0.191,0.2106
3,0.20489,0.2088,0.19749,0.19731,0.20543,0.19869,0.19991,0.20666,0.202
66,0.20003,0.2053,0.19688,0.43393,0.29682,0.27893,0.35693,0.22182,0.29
487,0.31133,0.19616,0.20209,0.194,0.20814,0.19315,0.19364,0.19811,0.19

674,0.19507,0.19755,0.20322,0.19727,0.19791,0.19361,0.20732,0.35727,0.
39,0.32597,0.31724,0.33072,0.31117,0.32322,0.32554,0.30008,0.29192,0.2
0399,0.20063,0.19935,0.19561,0.19349,0.20232,0.18926,0.20266,0.1954,0.
2011,0.1907,0.19497,0.19351,0.20404,0.20398,0.39765,0.36533,0.36674,0.
29119,0.23915,0.2943,0.26886,0.29447,0.29305,0.24596,0.28743,0.25428,0
.20218,0.20045,0.20551,0.19259,0.20463,0.2053,0.19298,0.20121,0.19897,
0.1996,0.2003,0.20532,0.19929,0.19461,0.19595,0.19669,0.38281,0.35465,
0.39845,0.37174,0.31967,0.31032,0.3015,0.28578,0.29046,0.25171,0.30294
,0.28274,0.30059,0.27159,0.20717,0.19743,0.20257,0.2064,0.19584,0.1876
3,0.20749,0.20051,0.20157,0.2084,0.20608,0.19796,0.20268,0.19834,0.195
9,0.20317,0.36478,0.42802,0.43283,0.46797,0.36235,0.49254,0.34363,0.26
683,0.3018,0.27311,0.36098,0.29515,0.29301,0.27639,0.2722,0.27137,0.19
222,0.19941,0.20809,0.19901,0.20248,0.20877,0.2014,0.1968,0.20251,0.20
266,0.20745,0.19638,0.20564,0.20104,0.19842,0.2006,0.19923,0.38345,0.3
7481,0.39351,0.35315,0.3764,0.51643,0.29731,0.27588,0.22581,0.31402,0.
21137,0.26667,0.25474,0.31451,0.26907,0.33796,0.25464,0.20533,0.19894,
0.19353,0.19795,0.19554,0.19752,0.19615,0.19522,0.20244,0.19806,0.2015
4,0.20663,0.20023,0.1191,0.14447,0.12855,0.20515,0.37224,0.30083,0.358
67,0.40989,0.4099,0.44878,0.41171,0.27417,0.25306,0.27416,0.28707,0.27
715,0.30955,0.29341,0.33032,0.27031,0.23692,0.33994,0.20299,0.20979,0.
20696,0.19518,0.19971,0.20249,0.19889,0.18657,0.20504,0.21285,0.21082,
0.12201,0.11985,0.1159,0.1262,0.11903,0.12734,0.2063,0.29039,0.42058,0
.36816,0.36057,0.38927,0.44521,0.34121,0.27906,0.26647,0.32301,0.36597
,0.23968,0.30355,0.26982,0.29005,0.2423,0.29128,0.27305,0.19742,0.2097
9,0.19529,0.21074,0.20877,0.20632,0.20092,0.19984,0.18605,0.12246,0.11
993,0.11293,0.11799,0.1259,0.1062,0.13644,0.12316,0.12374,0.1916,0.360
1,0.3798,0.45548,0.39334,0.36312,0.19302,0.34083,0.26483,0.23033,0.313
95,0.26629,0.30283,0.23912,0.31195,0.24716,0.30445,0.30889,0.28633,0.2
9266,0.20645,0.19397,0.20007,0.18975,0.20541,0.19766,0.11717,0.12247,0
.1181,0.12666,0.13332,0.10718,0.13723,0.12193,0.13599,0.11375,0.11727,
0.12903,0.45494,0.33733,0.3572,0.39238,0.38467,0.34667,0.42924,0.28298
,0.24547,0.27315,0.26525,0.28698,0.27559,0.26151,0.30091,0.30425,0.345
06,0.29853,0.24838,0.20179,0.19198,0.20027,0.1159,0.12332,0.12669,0.12
886,0.11807,0.11654,0.1115,0.14366,0.1411,0.10318,0.11675,0.13128,0.11
957,0.11753,0.14889,0.39966,0.29304,0.36808,0.43272,0.4355,0.42758,0.3
347,0.34618,0.29962,0.25406,0.29025,0.23872,0.30227,0.33384,0.3059,0.2
9994,0.2312,0.32395,0.30711,0.27103,0.11372,0.10973,0.13442,0.10427,0.
12785,0.13017,0.12582,0.1161,0.12337,0.12458,0.12248,0.1262,0.12249,0.
12492,0.1198,0.13878,0.1104,0.10028,0.40242,0.39107,0.43894,0.33503,0.
32213,0.34286,0.48036,0.27442,0.36627,0.29445,0.2988,0.25131,0.20635,0
.21692,0.19333,0.22103,0.23153,0.18291,0.18771,0.23937,0.12346,0.09574
4,0.12287,0.1084,0.12556,0.12295,0.12291,0.1218,0.12953,0.11784,0.1267
3,0.10838,0.12291,0.12286,0.12221,0.12392,0.12553,0.12633,0.4206,0.369
24,0.3993,0.42253,0.44944,0.44347,0.41003,0.48909,0.37769,0.26249,0.22
866,0.21931,0.20461,0.18921,0.20674,0.20838,0.17456,0.22463,0.22236,0.
23104,0.12263,0.11258,0.11419,0.12,0.11342,0.11943,0.12334,0.12359,0.1
1597,0.11622,0.11336,0.12268,0.10881,0.095455,0.1175,0.12223,0.10619,0
.11254,0.38088,0.33089,0.3848,0.4898,0.3871,0.34663,0.38954,0.20594,0.
21698,0.18909,0.21156,0.22073,0.21571,0.19936,0.23583,0.19812,0.22145,
0.17409,0.19602,0.22862,0.1268,0.12445,0.12639,0.11158,0.12573,0.12248
,0.11304,0.12135,0.1182,0.10954,0.12609,0.12439,0.11811,0.12561,0.1184
3,0.12861,0.1278,0.13148,0.35416,0.47865,0.36672,0.39661,0.37243,0.333

2,0.21953,0.19252,0.22693,0.23865,0.24097,0.17626,0.20614,0.22191,0.18
621,0.17509,0.20022,0.21214,0.22116,0.19425,0.13042,0.104,0.11401,0.11
961,0.12222,0.11552,0.13234,0.11859,0.11375,0.13055,0.12543,0.13984,0.
1232,0.14121,0.1236,0.11437,0.11962,0.12564,0.45662,0.40238,0.31732,0.
39496,0.34633,0.17826,0.2112,0.24054,0.21567,0.175,0.20066,0.20469,0.2
2664,0.19501,0.24155,0.17526,0.23442,0.19886,0.21017,0.21718,0.12334,0
.14418,0.1215,0.12282,0.12991,0.12075,0.12776,0.1099,0.12575,0.11653,0
.12273,0.13153,0.11328,0.12978,0.13757,0.12205,0.11894,0.10169,0.34124
,0.51181,0.32449,0.20191,0.25765,0.17945,0.24734,0.26225,0.22496,0.117
,0.24631,0.21064,0.18511,0.2281,0.20542,0.1992,0.22335,0.25204,0.19175
,0.20521,0.11661,0.10774,0.13475,0.10736,0.12812,0.10569,0.12173,0.120
42,0.10166,0.12262,0.13947,0.12516,0.12737,0.12171,0.1203,0.11849,0.12
208,0.11923,0.28205,0.22816,0.1984,0.18611,0.19038,0.21908,0.22882,0.1
5577,0.1568,0.16741,0.24226,0.22126,0.18916,0.17952,0.215,0.23957,0.16
113,0.23423,0.22799,0.13177,0.12714,0.12675,0.11887,0.12129,0.10268,0.
12291,0.1262,0.11476,0.11339,0.11665,0.12162,0.10673,0.11826,0.12298,0
.12701,0.12209,0.041892,0.25414,0.20507,0.30065,0.17304,0.21751,0.1887
3,0.20678,0.21249,0.20084,0.22898,0.19655,0.22202,0.20336,0.20495,0.21
452,0.21514,0.23503,0.20422,0.18306,0.12456,0.12718,0.12633,0.12013,0.
10986,0.13022,0.12398,0.1129,0.11973,0.12366,0.12395,0.12531,0.12383,0
.12258,0.1282,0.12063,0.12447,0.045185,0.18498,0.24095,0.21156,0.17293
,0.19133,0.18908,0.15718,0.21175,0.16156,0.21259,0.23618,0.1921,0.2055
2,0.22881,0.21953,0.19782,0.18243,0.18925,0.1514,0.12293,0.12481,0.136
06,0.12004,0.12856,0.12678,0.12768,0.11903,0.13875,0.11691,0.11849,0.1
266,0.12153,0.11607,0.11784,0.11929,0.13257,0.046754,0.20586,0.18457,0
.20903,0.28768,0.19583,0.16881,0.18068,0.20006,0.22314,0.20371,0.20911
,0.21188,0.23607,0.20041,0.21793,0.17051,0.20448,0.2377,0.11471,0.1118
,0.13464,0.11858,0.13145,0.11894,0.11231,0.12191,0.11708,0.12319,0.122
91,0.12077,0.11243,0.12049,0.11671,0.13362,0.1168,0.049727,0.045997,0.
043936,0.16504,0.22945,0.17748,0.18989,0.24718,0.18157,0.19462,0.24527
,0.20867,0.18818,0.20336,0.22622,0.1958,0.18764,0.17155,0.13383,0.1176
3,0.12839,0.13037,0.12635,0.10694,0.11979,0.12436,0.11949,0.11536,0.12
249,0.10338,0.137,0.11492,0.095995,0.12434,0.12344,0.11391,0.0439,0.04
6159,0.041508,0.039485,0.15141,0.20644,0.18024,0.25088,0.1853,0.21997,
0.15038,0.18426,0.18391,0.21629,0.18616,0.23505,0.11403,0.14993,0.1348
9,0.12049,0.13151,0.10537,0.12401,0.12532,0.13219,0.14123,0.11932,0.12
295,0.10077,0.12667,0.12118,0.10899,0.11616,0.10428,0.1174,0.12093,0.0
48518,0.037794,0.043395,0.046289,0.043265,0.047965,0.23116,0.21768,0.1
6108,0.15572,0.2226,0.17386,0.23875,0.20875,0.12451,0.11516,0.096924,0
.13472,0.12362,0.11636,0.12012,0.11243,0.12455,0.14548,0.11803,0.12416
,0.13372,0.13022,0.14431,0.11711,0.11848,0.12459,0.11359,0.12231,0.116
88,0.13872,0.12193,0.045536,0.040226,0.049233,0.0331,0.046123,0.039356
,0.24839,0.20534,0.30417,0.28094,0.24094,0.043381,0.043764,0.11927,0.1
1937,0.10505,0.11723,0.11884,0.12023,0.1109,0.12206,0.11101,0.12098,0.
12474,0.10692,0.11797,0.11988,0.13218,0.12879,0.12382,0.11664,0.12266,
0.095643,0.1263,0.04301,0.036526,0.05482,0.043424,0.042703,0.046885,0.
040939,0.048249,0.044519,0.044374,0.21366,0.21081,0.045149,0.040449,0.
042951,0.035966,0.10958,0.12263,0.10825,0.12207,0.099255,0.12792,0.116
48,0.11294,0.12145,0.11044,0.11667,0.11776,0.11145,0.1122,0.11621,0.12
949,0.12054,0.13213,0.12891,0.046193,0.042628,0.042644,0.043485,0.0456
56,0.038395,0.038427,0.038182,0.048385,0.045519,0.041758,0.045263,0.04
4337,0.040956,0.043339,0.04372,0.047932,0.13246,0.11836,0.12277,0.1233

6,0.11003,0.11745,0.11739,0.11932,0.11171,0.12218,0.12789,0.12799,0.12
289,0.12497,0.10377,0.1227,0.040549,0.049447,0.049282,0.045535,0.04363
7,0.03879,0.048397,0.042198,0.052448,0.040335,0.043461,0.041628,0.0487
22,0.041959,0.048712,0.038196,0.044777,0.041703,0.047688,0.13279,0.112
74,0.12666,0.11766,0.10774,0.13707,0.12001,0.12627,0.1223,0.11957,0.12
706,0.12297,0.12479,0.042442,0.045785,0.045665,0.041574,0.036328,0.040
529,0.044598,0.046641,0.047994,0.040638,0.045412,0.039031,0.041129,0.0
41775,0.038484,0.047547,0.047632,0.035671,0.043524,0.047292,0.046587,0
.12506,0.12682,0.11944,0.10742,0.14404,0.11999,0.12796,0.11944,0.12077
,0.039867,0.044348,0.044603,0.030993,0.046728,0.041002,0.048047,0.0481
9,0.036306,0.041566,0.039846,0.047658,0.038771,0.053384,0.044988,0.046
377,0.046573,0.044563,0.045011,0.04156,0.045717,0.050339,0.043117,0.04
4186,0.047049,0.12086,0.11246,0.12607,0.12302,0.040339,0.047475,0.0506
41,0.042294,0.041386,0.04198,0.038561,0.038981,0.039307,0.048809,0.048
576,0.04514,0.042441,0.043312,0.042769,0.045362,0.043098,0.037742,0.04
,0.043591,0.037107,0.0428,0.048313,0.046661,0.042556,0.04251,0.04099,0
.035177,0.0483,0.045309,0.039148,0.039133,0.047626,0.038128,0.044086,0
.046192,0.045209,0.040539,0.044491,0.042041,0.041333,0.036459,0.052653
,0.040514,0.045372,0.039079,0.042093,0.049903,0.04253,0.035775,0.04686
7,0.047174,0.051435,0.043783,0.042064,0.047786,0.04547,0.046546,0.0483
02,0.041441,0.04638,0.044868,0.040402,0.047731,0.041943,0.04808,0.0417
2,0.037507,0.042925,0.048378,0.037875,0.043369,0.041238,0.046408,0.043
792,0.043724,0.049031,0.04502,0.05104,0.03954,0.048299,0.046412,0.0383
56,0.048489,0.045526,0.050069,0.046125,0.04236,0.048848,0.041811,0.042
664,0.040705,0.040191,0.040206,0.040599,0.049297,0.047282,0.046701,0.0
43559,0.042836,0.047516,0.034067,0.041933,0.045576,0.043759,0.046123,0
.04166,0.046929,0.045466,0.049915,0.04125,0.048685,0.04213,0.042875,0.
043748,0.043679,0.045663,0.040666,0.04291,0.038667,0.041206,0.053387,0
.04179,0.044414,0.044452,0.040909,0.037076,0.054389,0.042365,0.047434,
0.042029,0.043365,0.043058,0.041939,0.044781,0.041288,0.045928,0.03921
6,0.040372,0.042086,0.037164,0.041619,0.044541,0.044392,0.045248,0.042
593,0.044849,0.047844,0.047139,0.04748,0.046507,0.041026,0.038586,0.03
8896,0.039585,0.045078,0.04137,0.045884,0.04434,0.047114,0.038543,0.04
3518,0.042825,0.043898,0.04331,0.047972,0.0387,0.041735,0.045479,0.043
487,0.044487,0.04202,0.043455,0.040632,0.047064,0.042716,0.040872,0.04
2561,0.038453,0.035514,0.043144,0.047012,0.041163,0.045085,0.044062,0.
044601,0.039577,0.041505,0.040399,0.043656,0.038936,0.048255,0.045988,
0.042587,0.044038
0.20351,0.20022,0.20102,0.19659,0.19733,0.19725,0.21221,0.20238,0.1924
9,0.20231,0.21101,0.20907,0.19421,0.20948,0.20178,0.2065,0.20161,0.196
27,0.20374,0.1997,0.27401,0.28192,0.29532,0.27479,0.20539,0.19091,0.21
365,0.20607,0.21327,0.19881,0.19642,0.20451,0.19927,0.20262,0.21114,0.
20828,0.19896,0.20528,0.19774,0.40762,0.2941,0.30096,0.34601,0.25407,0
.29394,0.28936,0.19881,0.20334,0.19286,0.20832,0.19566,0.19376,0.20119
,0.19632,0.19763,0.19638,0.20634,0.20365,0.19716,0.192,0.20964,0.35301
,0.36724,0.30597,0.31584,0.3148,0.30689,0.30228,0.29531,0.30141,0.3082
8,0.21015,0.20118,0.1953,0.20385,0.1937,0.20304,0.18937,0.20395,0.1962
6,0.19963,0.19283,0.1983,0.19367,0.20253,0.20499,0.43151,0.40106,0.344
55,0.29681,0.24229,0.27689,0.27619,0.32373,0.30103,0.25166,0.27722,0.2
7054,0.20335,0.20244,0.20708,0.19296,0.20585,0.20678,0.19557,0.20335,0
.20126,0.20336,0.2046,0.20584,0.20028,0.19806,0.19299,0.20208,0.3852,0
.35641,0.44688,0.35553,0.31122,0.33233,0.28566,0.30154,0.27523,0.26038

,0.29659,0.28893,0.29401,0.25974,0.20768,0.19418,0.20301,0.20895,0.198
35,0.18911,0.2066,0.20293,0.20819,0.2034,0.20418,0.20092,0.20883,0.201
89,0.19453,0.20248,0.37518,0.47207,0.46428,0.45534,0.3061,0.48319,0.34
052,0.27169,0.29016,0.29712,0.32849,0.28223,0.29444,0.26828,0.24312,0.
27081,0.20015,0.19703,0.20595,0.20134,0.20534,0.20754,0.19869,0.19861,
0.20825,0.20526,0.20888,0.19693,0.20834,0.2005,0.19946,0.20232,0.19771
,0.35771,0.36731,0.39492,0.35255,0.41655,0.49523,0.29047,0.27372,0.217
37,0.31771,0.21705,0.26849,0.26114,0.31224,0.28511,0.3272,0.25929,0.20
832,0.20453,0.19591,0.19626,0.1967,0.19859,0.19905,0.19249,0.20487,0.2
0005,0.20108,0.20719,0.20201,0.11991,0.14519,0.12915,0.20668,0.36232,0
.28433,0.34814,0.39446,0.45557,0.43365,0.3803,0.27909,0.27181,0.26369,
0.27719,0.27838,0.32841,0.29751,0.31306,0.26982,0.24393,0.33554,0.2059
3,0.2093,0.20785,0.19924,0.20338,0.20364,0.20058,0.19013,0.21097,0.211
57,0.20982,0.12155,0.1206,0.11626,0.12624,0.11951,0.12654,0.20776,0.31
981,0.37577,0.35345,0.33966,0.40088,0.44303,0.38934,0.27486,0.24644,0.
29681,0.36924,0.24049,0.28578,0.27661,0.29237,0.23507,0.29652,0.26356,
0.19849,0.20852,0.19279,0.2077,0.21029,0.20251,0.19708,0.20273,0.18864
,0.12167,0.12014,0.11082,0.11759,0.12692,0.10636,0.13482,0.12279,0.122
7,0.19753,0.33471,0.42323,0.42005,0.35361,0.34154,0.18799,0.35536,0.26
721,0.2481,0.31502,0.26727,0.31916,0.25083,0.31887,0.25228,0.29133,0.2
9682,0.27625,0.29886,0.20261,0.19556,0.20317,0.1896,0.207,0.19952,0.11
677,0.12399,0.11823,0.12617,0.13287,0.10874,0.13806,0.12245,0.13627,0.
11618,0.11843,0.12811,0.405,0.36835,0.36254,0.43403,0.40029,0.36214,0.
41107,0.29348,0.23489,0.25658,0.25958,0.26763,0.28336,0.26531,0.30288,
0.27801,0.35857,0.30873,0.25341,0.20117,0.19125,0.20125,0.11681,0.1214
5,0.12521,0.12634,0.11786,0.11695,0.11184,0.14299,0.14141,0.10192,0.11
904,0.1326,0.11844,0.12072,0.14859,0.42765,0.29648,0.312,0.41225,0.404
14,0.45919,0.31411,0.36087,0.31104,0.25129,0.28303,0.243,0.30886,0.333
03,0.31084,0.29473,0.24078,0.33079,0.29993,0.27643,0.11387,0.10749,0.1
3473,0.10606,0.12709,0.13152,0.12591,0.11942,0.12418,0.12341,0.12141,0
.12453,0.1224,0.1233,0.12058,0.13696,0.10994,0.09981,0.37298,0.37346,0
.38645,0.27976,0.2995,0.37441,0.45439,0.25546,0.32692,0.3054,0.28521,0
.22366,0.20135,0.22872,0.19715,0.22522,0.2343,0.17409,0.18218,0.22995,
0.12344,0.09702,0.12292,0.10978,0.12554,0.12287,0.12081,0.12267,0.1311
5,0.11759,0.12767,0.10735,0.12361,0.12226,0.12344,0.12527,0.12546,0.12
62,0.43272,0.37095,0.39942,0.38157,0.44561,0.44211,0.40714,0.47379,0.3
5584,0.25985,0.22155,0.20226,0.20598,0.19096,0.21552,0.19937,0.17151,0
.22211,0.22318,0.24973,0.12292,0.11343,0.11471,0.11843,0.11285,0.11812
,0.12588,0.12482,0.11641,0.11558,0.11377,0.12115,0.10829,0.096131,0.11
762,0.12087,0.10693,0.11098,0.35661,0.30189,0.37502,0.51345,0.36515,0.
3294,0.38783,0.19354,0.23161,0.19749,0.21723,0.22772,0.22287,0.21574,0
.22739,0.2027,0.21907,0.17201,0.18869,0.22293,0.12572,0.1223,0.1276,0.
1112,0.12786,0.12144,0.11384,0.12112,0.11742,0.1107,0.12815,0.12492,0.
1205,0.12342,0.11911,0.12915,0.12705,0.12863,0.33732,0.42028,0.35774,0
.39789,0.34337,0.30682,0.21046,0.17994,0.21652,0.24628,0.22474,0.16781
,0.20397,0.22371,0.17857,0.17849,0.2032,0.19892,0.20695,0.19146,0.1325
8,0.10403,0.11291,0.11928,0.12072,0.11762,0.13059,0.11767,0.11446,0.13
043,0.12459,0.1408,0.1225,0.14168,0.12421,0.11356,0.11875,0.12641,0.43
662,0.38384,0.32169,0.43548,0.36979,0.16578,0.18683,0.25609,0.22665,0.
19335,0.2148,0.20187,0.23099,0.1943,0.23731,0.18181,0.22599,0.19507,0.
21963,0.22962,0.12521,0.14273,0.11992,0.12291,0.1275,0.12045,0.12904,0
.10919,0.12511,0.11883,0.12222,0.13276,0.11518,0.1319,0.13793,0.12144,

0.11975,0.10274,0.33289,0.46789,0.31106,0.18979,0.268,0.17611,0.24423,
0.25427,0.22899,0.1188,0.24427,0.19934,0.19161,0.21849,0.19628,0.19356
,0.23387,0.24465,0.2061,0.2129,0.11742,0.10642,0.13611,0.10765,0.12826
,0.10546,0.12342,0.12042,0.10046,0.12351,0.13835,0.12425,0.12785,0.121
72,0.12161,0.11704,0.1217,0.11967,0.29782,0.2184,0.1935,0.18885,0.1889
9,0.19692,0.22821,0.15226,0.15318,0.18506,0.24242,0.22898,0.19077,0.18
57,0.20249,0.23349,0.17149,0.22526,0.24585,0.12953,0.12671,0.12661,0.1
1971,0.12139,0.10112,0.12598,0.12729,0.11631,0.11233,0.11716,0.12083,0
.10803,0.1185,0.12184,0.12586,0.12283,0.037586,0.25908,0.19347,0.28635
,0.18583,0.17639,0.18718,0.20029,0.20869,0.19991,0.22177,0.18865,0.218
08,0.20619,0.19857,0.20071,0.20865,0.21958,0.19848,0.18714,0.12585,0.1
2598,0.1271,0.12108,0.10927,0.12808,0.1243,0.11105,0.12048,0.12186,0.1
2481,0.1253,0.12327,0.12253,0.12991,0.11989,0.12383,0.048171,0.19342,0
.21672,0.2219,0.17639,0.20652,0.21096,0.19017,0.21935,0.17369,0.1901,0
.23566,0.20276,0.22885,0.22127,0.21862,0.20804,0.19601,0.18749,0.15075
,0.12371,0.12271,0.13393,0.11998,0.12641,0.12567,0.12695,0.11796,0.138
18,0.11458,0.11777,0.12642,0.12158,0.11824,0.11615,0.11986,0.13143,0.0
43056,0.20345,0.1684,0.19722,0.2693,0.2065,0.16926,0.18457,0.16905,0.2
142,0.17563,0.21491,0.22228,0.22146,0.18913,0.22344,0.1773,0.20842,0.2
4199,0.11521,0.11139,0.13404,0.11792,0.13028,0.12048,0.11316,0.1216,0.
11764,0.12346,0.12226,0.12132,0.11155,0.12099,0.11595,0.13355,0.11815,
0.043585,0.044682,0.042137,0.17392,0.223,0.17646,0.18899,0.23906,0.173
8,0.1879,0.24264,0.19323,0.19606,0.19581,0.2128,0.2021,0.17826,0.18481
,0.13798,0.11907,0.12728,0.12849,0.12848,0.10654,0.12088,0.12306,0.117
38,0.11776,0.12349,0.10453,0.13721,0.11389,0.095909,0.12292,0.12213,0.
11429,0.041783,0.045147,0.039736,0.036549,0.14262,0.21782,0.15998,0.24
502,0.173,0.21371,0.14125,0.18132,0.18637,0.21871,0.18999,0.23803,0.11
626,0.14761,0.13355,0.11882,0.13035,0.10663,0.12346,0.12649,0.13074,0.
14148,0.12044,0.12209,0.102,0.12172,0.12352,0.11131,0.11532,0.1051,0.1
1768,0.12162,0.047937,0.037594,0.042712,0.044885,0.0436,0.048166,0.213
78,0.20815,0.17789,0.15654,0.19306,0.18034,0.2273,0.21705,0.12598,0.11
52,0.098749,0.13403,0.12235,0.11789,0.12011,0.11093,0.12474,0.14607,0.
1175,0.12329,0.13379,0.12933,0.14317,0.11689,0.11613,0.1217,0.11308,0.
12399,0.11808,0.1403,0.1232,0.045498,0.044239,0.048715,0.034298,0.0438
07,0.03592,0.23754,0.18301,0.2973,0.27804,0.22248,0.040542,0.046426,0.
11989,0.11755,0.10377,0.11826,0.12079,0.12076,0.11163,0.12248,0.10981,
0.11949,0.12541,0.10458,0.11926,0.11724,0.13327,0.13056,0.12488,0.1153
7,0.12013,0.095542,0.12657,0.042533,0.033189,0.051361,0.038853,0.04018
1,0.043832,0.040375,0.048991,0.044701,0.045191,0.21196,0.22498,0.04384
7,0.038492,0.042114,0.034456,0.10943,0.12075,0.10744,0.11974,0.10015,0
.12522,0.11543,0.11187,0.12175,0.11182,0.11696,0.11798,0.11228,0.11281
,0.11668,0.12791,0.12109,0.13365,0.127,0.042781,0.041874,0.03906,0.043
793,0.044112,0.038839,0.037597,0.036263,0.048556,0.043035,0.040505,0.0
44301,0.042734,0.038832,0.040905,0.043158,0.043163,0.1315,0.11718,0.12
255,0.12421,0.11175,0.11525,0.11532,0.12198,0.11119,0.12323,0.12824,0.
12588,0.1224,0.12293,0.10677,0.12027,0.037481,0.046914,0.048605,0.0439
36,0.039519,0.037577,0.045297,0.043295,0.052285,0.040744,0.042434,0.04
0203,0.04774,0.042187,0.044654,0.038261,0.046352,0.04038,0.042859,0.13
341,0.11257,0.12439,0.11663,0.10689,0.13549,0.12018,0.12521,0.12435,0.
12023,0.12621,0.12364,0.1252,0.043111,0.046216,0.043814,0.040272,0.035
294,0.040012,0.044763,0.045904,0.047065,0.038398,0.047643,0.039509,0.0
38736,0.036719,0.03688,0.046258,0.043423,0.031002,0.038515,0.046699,0.

045267,0.12438,0.1276,0.12051,0.10576,0.1451,0.12106,0.1285,0.11891,0.
1231,0.039984,0.043788,0.050315,0.031428,0.046337,0.040661,0.049329,0.
048347,0.039765,0.043094,0.039207,0.046537,0.034433,0.049638,0.045574,
0.04316,0.046414,0.041705,0.042858,0.038815,0.044298,0.04889,0.040519,
0.044088,0.04476,0.12139,0.11454,0.12399,0.1241,0.041257,0.04826,0.047
709,0.040525,0.041708,0.040625,0.037223,0.038717,0.036412,0.04662,0.04
5632,0.040744,0.040983,0.043337,0.043659,0.046164,0.042629,0.0367,0.03
8502,0.039609,0.038188,0.04065,0.043027,0.048565,0.041159,0.038751,0.0
39751,0.03704,0.04544,0.045741,0.039304,0.037229,0.045192,0.0345,0.041
816,0.046958,0.041821,0.041131,0.045014,0.039644,0.039442,0.035144,0.0
51672,0.038197,0.043835,0.036667,0.042206,0.048599,0.042145,0.034629,0
.042705,0.046876,0.049621,0.040971,0.044669,0.044252,0.042029,0.043768
,0.047757,0.040988,0.046251,0.04127,0.041237,0.044638,0.043704,0.04309
3,0.040614,0.037653,0.043634,0.044558,0.039552,0.042926,0.039385,0.044
149,0.038936,0.042049,0.045985,0.043754,0.050248,0.036747,0.045954,0.0
44027,0.039003,0.04899,0.044805,0.044474,0.045533,0.042171,0.043631,0.
041146,0.042217,0.039658,0.036638,0.038908,0.035906,0.047975,0.045361,
0.044111,0.042286,0.042846,0.046933,0.03226,0.043426,0.044582,0.043711
,0.043654,0.038135,0.045037,0.0463,0.047764,0.039724,0.047785,0.039833
,0.038089,0.041506,0.042665,0.043035,0.040884,0.042624,0.036904,0.0379
5,0.051369,0.04006,0.042734,0.043286,0.038615,0.039228,0.049891,0.0407
52,0.042808,0.044286,0.044327,0.043198,0.038423,0.044082,0.044892,0.04
6338,0.03816,0.039675,0.042627,0.034561,0.040201,0.043164,0.043335,0.0
44509,0.045918,0.04222,0.046013,0.044239,0.047896,0.045632,0.039782,0.
03575,0.038993,0.039272,0.043338,0.03887,0.042402,0.041598,0.044622,0.
038748,0.039858,0.040607,0.040745,0.039623,0.047625,0.036273,0.041182,
0.042939,0.044853,0.043057,0.039072,0.04387,0.041817,0.04994,0.040592,
0.042029,0.042504,0.03677,0.034823,0.039434,0.044457,0.036691,0.043105
,0.04224,0.045199,0.038582,0.038787,0.038198,0.045286,0.036747,0.04522
5,0.039845,0.042599,0.043182
0.20439,0.20187,0.1999,0.19977,0.20321,0.19478,0.21358,0.20313,0.19964
,0.20216,0.2102,0.21163,0.19622,0.21037,0.204,0.20617,0.20761,0.19696,
0.2033,0.20259,0.28906,0.28391,0.31048,0.27435,0.20663,0.19235,0.21194
,0.20539,0.2134,0.20017,0.19873,0.20718,0.20299,0.20597,0.20981,0.2079
4,0.19664,0.20645,0.19774,0.39242,0.26228,0.31485,0.32777,0.26166,0.29
261,0.28655,0.20384,0.2064,0.19571,0.20336,0.19968,0.19317,0.20777,0.1
9321,0.19925,0.20103,0.2073,0.20654,0.19943,0.19505,0.20931,0.35898,0.
37621,0.30142,0.29741,0.30078,0.30831,0.30373,0.29589,0.28855,0.30819,
0.20979,0.20191,0.19844,0.21267,0.19426,0.20532,0.18953,0.20164,0.2026
6,0.19938,0.19629,0.19972,0.18869,0.20195,0.20334,0.4864,0.41028,0.355
77,0.29473,0.24751,0.26079,0.28293,0.3019,0.30213,0.24072,0.27374,0.28
568,0.20837,0.20202,0.20786,0.19347,0.20668,0.20782,0.19912,0.20553,0.
19991,0.20803,0.20291,0.20584,0.20339,0.20024,0.19603,0.20742,0.36723,
0.41226,0.44693,0.34208,0.30395,0.33094,0.27765,0.30085,0.28359,0.2619
5,0.28031,0.30154,0.28682,0.25419,0.20527,0.19855,0.20458,0.2098,0.199
05,0.19444,0.20387,0.20257,0.21305,0.198,0.20595,0.20164,0.2094,0.2016
3,0.19721,0.20512,0.3596,0.4769,0.44031,0.42361,0.35613,0.46173,0.3504
3,0.28041,0.3165,0.2896,0.33772,0.28624,0.27662,0.25721,0.24578,0.2738
,0.20091,0.19528,0.2078,0.20446,0.20229,0.20584,0.19986,0.20195,0.2081
6,0.20585,0.21213,0.19415,0.2072,0.20301,0.19862,0.20579,0.19729,0.358
32,0.36748,0.38109,0.36597,0.43465,0.4919,0.30814,0.27261,0.20803,0.31
295,0.24129,0.2758,0.2689,0.31754,0.27602,0.31624,0.25286,0.20458,0.20

546,0.19782,0.20053,0.19976,0.19917,0.20172,0.1932,0.20493,0.20717,0.1
9969,0.20403,0.20418,0.12046,0.14705,0.12645,0.204,0.39424,0.28622,0.3
5524,0.38274,0.40972,0.43287,0.37956,0.28625,0.26666,0.27325,0.26699,0
.27918,0.32807,0.30436,0.30412,0.27066,0.24844,0.31536,0.20511,0.20963
,0.21193,0.2009,0.20499,0.20664,0.20066,0.18934,0.20952,0.21421,0.2079
9,0.11953,0.12049,0.11695,0.12567,0.11927,0.1263,0.20894,0.28762,0.377
04,0.37867,0.33461,0.38298,0.44024,0.41993,0.27663,0.24186,0.27567,0.3
7928,0.23381,0.27756,0.2581,0.29176,0.2455,0.2839,0.26823,0.19582,0.21
492,0.19528,0.20977,0.21419,0.20188,0.19976,0.20376,0.1912,0.12306,0.1
2033,0.1117,0.11843,0.12664,0.10564,0.13464,0.12011,0.12272,0.19808,0.
33024,0.42668,0.39893,0.35588,0.3432,0.16431,0.3379,0.2676,0.24965,0.3
187,0.27389,0.32876,0.24954,0.31482,0.24446,0.28752,0.30152,0.27396,0.
30802,0.2041,0.19969,0.20027,0.19236,0.20568,0.20461,0.11548,0.12536,0
.11841,0.12489,0.13252,0.10847,0.13982,0.12364,0.13459,0.11646,0.11678
,0.12751,0.38236,0.31783,0.37372,0.47315,0.41336,0.42598,0.42059,0.278
51,0.22449,0.26251,0.24916,0.25976,0.30133,0.25591,0.31545,0.29757,0.3
5104,0.3003,0.26186,0.20259,0.19841,0.19693,0.11899,0.12068,0.12623,0.
12501,0.11971,0.11612,0.11118,0.1434,0.14282,0.10201,0.11823,0.13161,0
.11538,0.12105,0.1464,0.41184,0.26301,0.31177,0.40832,0.43096,0.46669,
0.33604,0.3449,0.30853,0.25195,0.291,0.24471,0.30839,0.32457,0.31314,0
.28071,0.24998,0.33668,0.28677,0.25751,0.11215,0.10812,0.13413,0.10526
,0.13006,0.13153,0.12649,0.12285,0.12543,0.12282,0.12096,0.12673,0.121
04,0.12438,0.12343,0.13714,0.10504,0.099984,0.36773,0.38016,0.3732,0.2
7329,0.33314,0.38036,0.4956,0.23949,0.37272,0.30033,0.29776,0.2178,0.2
0004,0.21293,0.21147,0.20909,0.24287,0.19335,0.17605,0.21747,0.12295,0
.095976,0.12059,0.10903,0.12596,0.12389,0.12302,0.12303,0.13243,0.1184
8,0.12658,0.10693,0.12283,0.12309,0.12344,0.1234,0.1241,0.12687,0.4363
3,0.38909,0.4433,0.36763,0.40408,0.43295,0.39283,0.43476,0.30005,0.255
06,0.2177,0.19703,0.21275,0.17817,0.2121,0.18953,0.16977,0.22515,0.227
76,0.26565,0.12338,0.11335,0.11483,0.1182,0.11197,0.11663,0.12749,0.12
547,0.11412,0.1149,0.11537,0.12526,0.10658,0.097598,0.1161,0.12348,0.1
0811,0.11004,0.3616,0.29852,0.38041,0.52159,0.40412,0.3355,0.39731,0.1
9628,0.25035,0.19109,0.22354,0.22366,0.22908,0.22862,0.2315,0.21035,0.
22769,0.1685,0.18945,0.2218,0.12718,0.12073,0.12935,0.109,0.12693,0.12
069,0.11398,0.12094,0.11883,0.11097,0.12455,0.12402,0.11883,0.12397,0.
12114,0.13084,0.12682,0.128,0.34083,0.38274,0.41878,0.39572,0.33648,0.
34075,0.23888,0.20937,0.20563,0.26247,0.2273,0.17528,0.18558,0.22997,0
.19121,0.19421,0.20564,0.19288,0.21974,0.19151,0.13227,0.104,0.11589,0
.12023,0.12055,0.11617,0.1333,0.11832,0.11603,0.12871,0.12399,0.14227,
0.12366,0.14418,0.12525,0.11511,0.11794,0.12715,0.40133,0.37622,0.3768
8,0.45367,0.37395,0.17234,0.17907,0.25398,0.22489,0.19118,0.2143,0.203
87,0.23315,0.1874,0.24691,0.17441,0.21305,0.20303,0.21507,0.22752,0.12
559,0.14277,0.12114,0.1216,0.12707,0.12106,0.12772,0.10927,0.1242,0.11
824,0.12238,0.13322,0.11433,0.13203,0.13664,0.12131,0.11913,0.10365,0.
33808,0.4696,0.30791,0.17399,0.24707,0.1693,0.23974,0.23929,0.24196,0.
11954,0.22727,0.18646,0.191,0.22433,0.1957,0.20879,0.23937,0.23797,0.1
9814,0.2155,0.11376,0.10842,0.13754,0.10504,0.13038,0.1074,0.12154,0.1
2058,0.099161,0.12207,0.13726,0.12439,0.12808,0.12308,0.12131,0.11607,
0.12114,0.11934,0.28981,0.2219,0.20271,0.17185,0.19253,0.21013,0.20589
,0.14974,0.15042,0.17186,0.23594,0.22509,0.18369,0.18617,0.20009,0.214
83,0.18173,0.21911,0.23189,0.13211,0.12675,0.12729,0.12025,0.12224,0.0
99264,0.12732,0.12883,0.11569,0.11405,0.11423,0.12241,0.1099,0.12114,0

.12,0.12762,0.12484,0.038433,0.26202,0.22227,0.25291,0.18023,0.16759,0
.19876,0.18111,0.21308,0.1904,0.22008,0.20482,0.21718,0.1958,0.20953,0
.20532,0.20401,0.21026,0.19853,0.18224,0.12746,0.12447,0.12694,0.12073
,0.11123,0.12801,0.12277,0.1105,0.12041,0.12071,0.12347,0.12224,0.1198
8,0.12247,0.1317,0.11937,0.12434,0.045969,0.21535,0.20139,0.22498,0.17
469,0.18927,0.20424,0.19177,0.20189,0.16024,0.19654,0.24694,0.20329,0.
24346,0.21,0.21481,0.20597,0.20279,0.20939,0.15133,0.12418,0.12397,0.1
3365,0.11859,0.12663,0.12682,0.12708,0.11912,0.13742,0.11384,0.1161,0.
12601,0.1234,0.11895,0.11708,0.12194,0.13044,0.043093,0.2111,0.14751,0
.20069,0.28589,0.19219,0.16027,0.17961,0.17725,0.2255,0.18058,0.20114,
0.22631,0.21149,0.19716,0.22682,0.18516,0.21501,0.23293,0.11707,0.112,
0.13425,0.11994,0.13087,0.12047,0.11232,0.12007,0.11587,0.12041,0.1230
4,0.12434,0.11167,0.12218,0.11583,0.13281,0.11795,0.04244,0.04275,0.04
1803,0.17184,0.21043,0.1678,0.18059,0.21491,0.15644,0.17317,0.24212,0.
19642,0.18831,0.193,0.22117,0.20176,0.17257,0.18361,0.13851,0.11965,0.
12754,0.12876,0.13008,0.10489,0.11895,0.12222,0.11809,0.11717,0.12374,
0.10564,0.13891,0.11447,0.096086,0.12356,0.12136,0.11721,0.041549,0.04
2266,0.038627,0.036457,0.15355,0.22549,0.17879,0.20773,0.15861,0.2111,
0.12688,0.16674,0.19589,0.22877,0.17607,0.2229,0.11683,0.1474,0.13472,
0.11813,0.13061,0.10741,0.12371,0.12845,0.12927,0.14301,0.11645,0.1214
1,0.10092,0.12352,0.121,0.1105,0.11518,0.10525,0.11669,0.12331,0.04395
4,0.036532,0.039952,0.040944,0.043454,0.044487,0.20794,0.20735,0.16194
,0.17092,0.17778,0.14782,0.23433,0.21254,0.12501,0.11239,0.10111,0.134
25,0.11969,0.12058,0.12187,0.11175,0.12508,0.14865,0.11712,0.12192,0.1
3517,0.12774,0.14376,0.11783,0.1176,0.12415,0.11355,0.12196,0.11615,0.
14164,0.12308,0.048644,0.040774,0.047079,0.035549,0.040048,0.037285,0.
24699,0.17945,0.25833,0.24373,0.20649,0.037832,0.047647,0.12068,0.1176
5,0.104,0.1185,0.12219,0.11923,0.11006,0.12313,0.11083,0.11734,0.12421
,0.10594,0.11806,0.11718,0.13564,0.13124,0.12311,0.11474,0.11911,0.094
312,0.12875,0.042162,0.031083,0.051119,0.036737,0.039666,0.041441,0.03
944,0.046842,0.04453,0.044911,0.1935,0.21179,0.041646,0.035268,0.04036
8,0.035458,0.1094,0.12028,0.10689,0.11891,0.0995,0.12626,0.11717,0.112
94,0.12124,0.11279,0.11783,0.11725,0.11117,0.1127,0.11417,0.12608,0.11
983,0.13106,0.12561,0.041312,0.040656,0.040141,0.042733,0.042875,0.036
077,0.036993,0.032353,0.045864,0.04402,0.040537,0.045849,0.043007,0.03
9028,0.040237,0.044812,0.04164,0.13187,0.11802,0.12222,0.12179,0.1107,
0.11659,0.11512,0.12214,0.11094,0.12175,0.1306,0.12705,0.12128,0.12287
,0.1063,0.1201,0.036858,0.042979,0.04472,0.042027,0.039624,0.040121,0.
042312,0.043178,0.051225,0.040991,0.039949,0.039697,0.044487,0.040647,
0.04013,0.037986,0.043524,0.040324,0.040775,0.13434,0.11048,0.12415,0.
11334,0.10547,0.13298,0.11913,0.12514,0.12178,0.12111,0.12799,0.12354,
0.12533,0.041856,0.045975,0.042587,0.03989,0.035911,0.040273,0.041959,
0.045392,0.045696,0.038952,0.043472,0.039646,0.03869,0.039274,0.033866
,0.044833,0.044653,0.032245,0.038644,0.046995,0.042689,0.12517,0.12783
,0.12042,0.10468,0.14529,0.12054,0.12967,0.11881,0.12113,0.040571,0.04
3158,0.046327,0.03227,0.043703,0.037139,0.049665,0.048243,0.038933,0.0
40231,0.03663,0.044198,0.034014,0.051103,0.044837,0.041477,0.046037,0.
040633,0.043452,0.040045,0.041092,0.047924,0.043747,0.041667,0.041833,
0.12086,0.11528,0.12225,0.12541,0.03853,0.044559,0.043912,0.037597,0.0
44199,0.040276,0.036749,0.040536,0.036109,0.044275,0.04288,0.040335,0.
040226,0.040159,0.041888,0.044523,0.041761,0.034918,0.038525,0.03805,0
.035843,0.038479,0.045118,0.048051,0.041423,0.038615,0.040962,0.03796,

0.044684,0.043488,0.035914,0.035942,0.046028,0.037981,0.040006,0.04402
7,0.040372,0.039987,0.047147,0.037953,0.036144,0.035161,0.048425,0.039
102,0.044418,0.039297,0.042596,0.044009,0.042493,0.03364,0.039366,0.04
4976,0.049293,0.038474,0.046404,0.046719,0.041365,0.040706,0.045054,0.
042195,0.045777,0.042566,0.03573,0.040868,0.046496,0.042359,0.039427,0
.0381,0.043638,0.042413,0.038492,0.045228,0.039343,0.044417,0.03678,0.
042014,0.043715,0.044361,0.049823,0.034924,0.042527,0.045567,0.035824,
0.046574,0.046275,0.044069,0.041157,0.044954,0.0448,0.040355,0.043203,
0.041085,0.033574,0.037913,0.03593,0.046114,0.045002,0.042796,0.041378
,0.040195,0.045849,0.034381,0.04407,0.040752,0.042168,0.041439,0.03667
2,0.041974,0.044433,0.048811,0.037366,0.04673,0.039455,0.039447,0.0388
48,0.044454,0.041777,0.041189,0.039284,0.035847,0.037532,0.045807,0.04
013,0.042051,0.043664,0.038595,0.038408,0.054036,0.041126,0.041635,0.0
43193,0.046103,0.043334,0.035352,0.043532,0.047815,0.041971,0.039166,0
.03969,0.041585,0.033842,0.041661,0.042718,0.041345,0.042305,0.04392,0
.041639,0.045875,0.042512,0.045869,0.046167,0.03939,0.033107,0.039122,
0.04092,0.040825,0.037187,0.041362,0.037333,0.043756,0.039517,0.040087
,0.04227,0.03614,0.0387,0.049369,0.03769,0.041412,0.043913,0.04568,0.0
40181,0.039151,0.043682,0.04219,0.047055,0.040336,0.041217,0.041925,0.
035498,0.035963,0.036737,0.041367,0.034729,0.039592,0.037726,0.043388,
0.038,0.036809,0.03524,0.042932,0.037533,0.045547,0.041236,0.041886,0.
04109
0.20614,0.20366,0.2031,0.20196,0.20493,0.2031,0.21439,0.20212,0.20088,
0.20905,0.20895,0.20971,0.2005,0.20824,0.20412,0.20238,0.20708,0.19857
,0.20921,0.20346,0.29469,0.30204,0.3326,0.26911,0.20227,0.19937,0.2121
,0.2079,0.20956,0.20585,0.20042,0.20288,0.20861,0.21,0.20992,0.21437,0
.19908,0.20511,0.19583,0.38042,0.26505,0.3113,0.33908,0.26438,0.29198,
0.30393,0.20586,0.20838,0.1953,0.20702,0.20164,0.19286,0.20382,0.19334
,0.20356,0.19737,0.21215,0.20822,0.20251,0.19654,0.20682,0.36017,0.384
68,0.30071,0.2959,0.29802,0.32262,0.28883,0.30623,0.28211,0.2955,0.211
2,0.20544,0.19624,0.21392,0.19619,0.20867,0.18783,0.20029,0.20418,0.20
085,0.19582,0.20187,0.19655,0.20199,0.20392,0.51716,0.43597,0.37035,0.
28123,0.26282,0.26161,0.27281,0.30601,0.32223,0.26163,0.28003,0.26086,
0.21293,0.20604,0.20682,0.19194,0.20539,0.20846,0.19921,0.20522,0.1995
2,0.20986,0.20287,0.20672,0.20351,0.20079,0.1952,0.21021,0.38023,0.395
58,0.44298,0.32281,0.30027,0.32865,0.28061,0.29862,0.28175,0.26994,0.2
9016,0.31657,0.2936,0.26965,0.20712,0.19933,0.20402,0.20902,0.20217,0.
20158,0.2042,0.20641,0.21335,0.20024,0.20734,0.20223,0.20537,0.20089,0
.19711,0.20564,0.39204,0.40664,0.44591,0.41602,0.3913,0.51406,0.35127,
0.30087,0.31281,0.29697,0.32012,0.29347,0.27303,0.27753,0.24733,0.2753
8,0.20531,0.19567,0.20532,0.21062,0.20553,0.20007,0.19924,0.20263,0.20
698,0.20563,0.21345,0.19543,0.209,0.20248,0.19703,0.20768,0.19679,0.38
053,0.32863,0.39486,0.35426,0.43805,0.49574,0.30313,0.28535,0.21804,0.
29624,0.23617,0.27038,0.23944,0.31242,0.27359,0.30683,0.25459,0.2046,0
.20875,0.20351,0.20138,0.19806,0.19759,0.20179,0.19675,0.20606,0.20834
,0.20131,0.20389,0.20604,0.12055,0.14321,0.12563,0.20445,0.39507,0.268
6,0.37304,0.36759,0.43635,0.45197,0.39736,0.28647,0.28494,0.28971,0.25
659,0.27278,0.31336,0.31406,0.2824,0.25145,0.25308,0.30358,0.20606,0.2
1004,0.21126,0.20147,0.20761,0.20507,0.20089,0.19047,0.20926,0.21218,0
.20891,0.12118,0.12192,0.11883,0.12628,0.12136,0.12704,0.20795,0.31701
,0.34593,0.36895,0.29751,0.36267,0.44619,0.41781,0.27202,0.25135,0.291
63,0.38569,0.23461,0.24512,0.23907,0.30637,0.25073,0.29718,0.2595,0.19

315,0.21435,0.19804,0.21024,0.21798,0.20327,0.19956,0.20634,0.19213,0.
12325,0.12037,0.11308,0.11855,0.12516,0.10814,0.13289,0.12088,0.12241,
0.20128,0.31218,0.42472,0.3951,0.36794,0.37628,0.18047,0.32216,0.27176
,0.28467,0.31787,0.27352,0.31875,0.22875,0.28048,0.23821,0.29044,0.307
36,0.29039,0.30976,0.20653,0.20093,0.20222,0.1931,0.20476,0.20051,0.11
563,0.1243,0.11883,0.12587,0.13198,0.11043,0.14024,0.12278,0.13409,0.1
1481,0.1195,0.12991,0.38292,0.3057,0.40157,0.43781,0.38533,0.42707,0.4
2292,0.30005,0.22001,0.25737,0.24897,0.24461,0.30244,0.26984,0.29815,0
.30006,0.33809,0.28678,0.25141,0.2009,0.19979,0.19804,0.12096,0.12173,
0.12465,0.12778,0.12042,0.11586,0.11163,0.14416,0.14432,0.10256,0.1190
2,0.13313,0.11593,0.1227,0.14617,0.38006,0.2346,0.32402,0.41907,0.4063
7,0.46623,0.33875,0.36934,0.29912,0.2551,0.26821,0.24442,0.29021,0.308
02,0.29044,0.28128,0.25949,0.33385,0.27821,0.271,0.11252,0.10777,0.137
45,0.10604,0.13254,0.13287,0.12668,0.12403,0.12513,0.12192,0.1199,0.12
84,0.1178,0.12358,0.1236,0.13514,0.10453,0.099069,0.38274,0.37641,0.39
349,0.28071,0.31864,0.34971,0.49594,0.25773,0.4055,0.30938,0.29116,0.2
1545,0.18818,0.20492,0.20304,0.20328,0.2291,0.19172,0.17473,0.21592,0.
1215,0.0942,0.12034,0.10952,0.12664,0.12227,0.12251,0.12442,0.13043,0.
11837,0.12786,0.10554,0.12304,0.12317,0.12452,0.1209,0.12137,0.12587,0
.41982,0.34889,0.43554,0.40646,0.35464,0.36919,0.3854,0.45629,0.27632,
0.21619,0.21462,0.19472,0.24072,0.18589,0.21303,0.19496,0.16721,0.2187
3,0.22052,0.24752,0.1235,0.11416,0.11655,0.11962,0.10955,0.11849,0.126
37,0.12625,0.11528,0.11479,0.11651,0.12613,0.10851,0.095316,0.11728,0.
12197,0.10911,0.11166,0.41749,0.26458,0.40931,0.51431,0.37769,0.35379,
0.41979,0.20628,0.24052,0.18351,0.20796,0.2093,0.20658,0.22178,0.24147
,0.21059,0.22854,0.17476,0.18097,0.22095,0.12643,0.12165,0.12715,0.110
28,0.12673,0.12602,0.11492,0.11936,0.11946,0.11023,0.12345,0.12245,0.1
1741,0.12534,0.12191,0.13135,0.12807,0.12735,0.35547,0.36811,0.40177,0
.3883,0.34421,0.3588,0.21874,0.22586,0.20729,0.25792,0.22848,0.16821,0
.20012,0.22433,0.17771,0.1948,0.22952,0.18629,0.22061,0.19674,0.13467,
0.10605,0.11616,0.11938,0.12092,0.115,0.13167,0.11998,0.11685,0.12825,
0.12176,0.14197,0.12305,0.14543,0.12667,0.11542,0.11843,0.12628,0.3955
,0.37335,0.37812,0.46675,0.34722,0.17399,0.16814,0.24266,0.23527,0.200
65,0.24585,0.20383,0.23509,0.18753,0.25718,0.18446,0.20959,0.1998,0.21
788,0.23744,0.1273,0.1416,0.12194,0.1236,0.12796,0.1203,0.12589,0.1090
7,0.12346,0.12089,0.12152,0.13262,0.11284,0.12947,0.13578,0.12028,0.11
759,0.10242,0.32973,0.46127,0.34009,0.16754,0.21665,0.15508,0.23163,0.
24258,0.26682,0.12033,0.21906,0.17183,0.19477,0.24088,0.19046,0.23293,
0.22109,0.23744,0.20496,0.20001,0.10918,0.11054,0.13744,0.10469,0.1321
5,0.10669,0.12185,0.11952,0.098272,0.12365,0.13882,0.12647,0.12905,0.1
2517,0.11984,0.11621,0.12091,0.12135,0.32871,0.20894,0.19841,0.14941,0
.19161,0.2259,0.21379,0.14515,0.14964,0.19593,0.23462,0.2124,0.19375,0
.18717,0.20559,0.22067,0.17251,0.20055,0.22796,0.13358,0.12655,0.12588
,0.11834,0.12015,0.10081,0.12593,0.13061,0.11535,0.11539,0.11523,0.125
12,0.10831,0.12316,0.11785,0.12777,0.12522,0.040861,0.2583,0.21844,0.2
4737,0.18956,0.17271,0.16481,0.1733,0.22897,0.19342,0.22379,0.2137,0.2
2064,0.19025,0.21495,0.19893,0.20464,0.19011,0.21165,0.17043,0.1274,0.
12316,0.12671,0.12289,0.11103,0.12996,0.12215,0.11219,0.12208,0.12029,
0.12386,0.12233,0.11965,0.12294,0.13304,0.12049,0.12447,0.044236,0.211
24,0.18948,0.20353,0.18967,0.21581,0.20374,0.19996,0.20433,0.15958,0.1
9432,0.24418,0.20442,0.2301,0.21272,0.2247,0.21878,0.2082,0.2119,0.149
39,0.12389,0.12279,0.13112,0.11775,0.12762,0.12888,0.12678,0.11862,0.1

3537,0.11425,0.1172,0.12731,0.12093,0.12018,0.11873,0.12391,0.13058,0.
042966,0.21426,0.16041,0.20434,0.27154,0.19384,0.15772,0.18968,0.17952
,0.23799,0.1865,0.18476,0.24136,0.21084,0.18558,0.22441,0.17681,0.1988
,0.2412,0.11669,0.10975,0.13164,0.12031,0.13102,0.12218,0.11201,0.1212
5,0.11517,0.11952,0.1225,0.12407,0.11123,0.12164,0.11686,0.13118,0.119
99,0.040886,0.042993,0.040961,0.16978,0.21946,0.18131,0.17595,0.2135,0
.16875,0.17062,0.23344,0.18116,0.18398,0.1963,0.21191,0.20089,0.16846,
0.18014,0.1398,0.12152,0.12531,0.1276,0.12825,0.10707,0.11942,0.11957,
0.11924,0.11807,0.12317,0.10363,0.13985,0.11248,0.09713,0.12244,0.1243
,0.11647,0.040336,0.039042,0.036866,0.038264,0.17198,0.21104,0.17655,0
.20247,0.17107,0.21217,0.10684,0.16837,0.19346,0.22231,0.19084,0.22181
,0.11592,0.15034,0.13489,0.11946,0.1288,0.10578,0.12153,0.12871,0.1310
8,0.14622,0.1168,0.11998,0.099844,0.12698,0.12095,0.10835,0.11388,0.10
507,0.11813,0.12414,0.044134,0.034792,0.039888,0.039649,0.043017,0.042
457,0.1951,0.19524,0.16538,0.16202,0.19113,0.14529,0.24202,0.21933,0.1
2192,0.11285,0.099767,0.13321,0.12225,0.11978,0.12046,0.11183,0.12432,
0.15038,0.11816,0.12261,0.13486,0.12787,0.14494,0.11671,0.11656,0.1234
7,0.11673,0.12067,0.12047,0.14263,0.12329,0.047865,0.038753,0.046812,0
.033405,0.04152,0.035674,0.22836,0.18056,0.25605,0.25333,0.2108,0.0389
62,0.046925,0.11891,0.11843,0.10254,0.11913,0.12527,0.1203,0.11036,0.1
2255,0.11189,0.11724,0.12485,0.10648,0.11771,0.11821,0.13512,0.1324,0.
1242,0.11623,0.12051,0.09675,0.12863,0.040694,0.029463,0.050054,0.0343
96,0.040374,0.039986,0.038531,0.046791,0.043471,0.044086,0.18672,0.213
41,0.040868,0.035439,0.038232,0.03394,0.10982,0.12194,0.10934,0.12075,
0.099531,0.12788,0.11888,0.11431,0.12256,0.1115,0.11892,0.11515,0.1116
3,0.11274,0.11391,0.13051,0.12129,0.13098,0.12652,0.038075,0.038251,0.
040582,0.041076,0.039773,0.036205,0.033724,0.034407,0.042981,0.043297,
0.040332,0.047136,0.041962,0.037106,0.039948,0.045805,0.042785,0.13233
,0.11759,0.12018,0.12153,0.11065,0.11531,0.11438,0.1209,0.11056,0.1234
,0.12846,0.1271,0.121,0.12254,0.1073,0.11955,0.036448,0.044467,0.04173
8,0.041662,0.040592,0.039849,0.042913,0.04481,0.049728,0.041397,0.0412
97,0.04057,0.044119,0.038793,0.040567,0.034608,0.037755,0.040515,0.042
91,0.13298,0.10673,0.12283,0.11393,0.10448,0.13451,0.12094,0.12501,0.1
2185,0.12146,0.12588,0.12291,0.12469,0.036673,0.04543,0.041806,0.04315
9,0.034814,0.03873,0.040544,0.0455,0.046898,0.04139,0.043142,0.036524,
0.038662,0.038338,0.033703,0.046791,0.044377,0.032234,0.037888,0.04567
,0.04586,0.12548,0.12635,0.12097,0.10438,0.1464,0.11853,0.12746,0.1193
3,0.12132,0.040815,0.041509,0.04605,0.033488,0.044181,0.038203,0.04937
6,0.046675,0.037131,0.03951,0.035035,0.045571,0.035333,0.050176,0.0445
78,0.039064,0.046198,0.040574,0.042609,0.041475,0.037745,0.04589,0.042
803,0.039326,0.041241,0.1188,0.11339,0.12197,0.12838,0.03677,0.042861,
0.043061,0.0354,0.043946,0.040711,0.036498,0.038027,0.033855,0.042042,
0.04142,0.04235,0.04226,0.040843,0.039576,0.044366,0.039967,0.033546,0
.038089,0.037333,0.036915,0.0381,0.045131,0.048692,0.042469,0.037488,0
.042166,0.037042,0.047256,0.041652,0.034941,0.032939,0.048,0.037353,0.
041381,0.041103,0.040067,0.03925,0.047686,0.039914,0.036886,0.0376,0.0
44088,0.038805,0.045431,0.039179,0.042969,0.038869,0.043459,0.032598,0
.038162,0.04505,0.049815,0.037902,0.045956,0.045971,0.040146,0.040153,
0.04458,0.042867,0.046067,0.039732,0.037849,0.039921,0.046091,0.043159
,0.037174,0.035559,0.039668,0.041108,0.038217,0.042724,0.035156,0.0430
35,0.039024,0.039942,0.04187,0.043929,0.051649,0.032793,0.04256,0.0460
52,0.035412,0.043158,0.044219,0.041844,0.040639,0.043401,0.046168,0.04

0415,0.04198,0.038603,0.032565,0.040304,0.035692,0.046215,0.045102,0.0
44215,0.040699,0.041712,0.044509,0.034379,0.042813,0.042708,0.043231,0
.040941,0.036597,0.041085,0.042543,0.04654,0.038853,0.04553,0.036481,0
.039835,0.039102,0.040425,0.041436,0.044609,0.038851,0.036856,0.038571
,0.043986,0.039697,0.041397,0.042937,0.036244,0.040601,0.052083,0.0412
49,0.042042,0.042369,0.043714,0.043552,0.037492,0.040349,0.04441,0.041
739,0.040723,0.041719,0.042041,0.032678,0.042335,0.043069,0.041118,0.0
40696,0.043205,0.040726,0.043903,0.042266,0.045837,0.046932,0.039012,0
.034377,0.03739,0.040456,0.037964,0.036797,0.041579,0.03717,0.042285,0
.040394,0.038239,0.041704,0.033189,0.039891,0.046308,0.036943,0.040132
,0.043757,0.047969,0.039435,0.038981,0.043248,0.040278,0.045396,0.0412
97,0.040829,0.039508,0.031964,0.035047,0.037679,0.04224,0.036918,0.037
472,0.035775,0.045716,0.0371,0.035829,0.033972,0.042247,0.037402,0.046
652,0.040906,0.040012,0.041431
0.21001,0.20213,0.20167,0.20389,0.20179,0.20354,0.21279,0.20414,0.2018
8,0.20962,0.21167,0.20991,0.20245,0.20549,0.20242,0.20549,0.20574,0.19
812,0.20744,0.20314,0.29615,0.29264,0.31966,0.27252,0.20584,0.20097,0.
20976,0.20396,0.20851,0.20947,0.20222,0.20562,0.20587,0.21208,0.21123,
0.21062,0.19983,0.20808,0.19658,0.3504,0.26229,0.30753,0.31885,0.26699
,0.29675,0.297,0.2039,0.21043,0.19396,0.20752,0.20383,0.19473,0.20757,
0.2004,0.20262,0.19962,0.21733,0.20818,0.20507,0.19817,0.20826,0.35364
,0.38797,0.2983,0.30202,0.29935,0.31455,0.28762,0.3219,0.25788,0.28983
,0.21399,0.20581,0.19453,0.21463,0.19752,0.20962,0.18871,0.20149,0.204
4,0.20198,0.19761,0.20614,0.20034,0.20253,0.20643,0.46577,0.37728,0.37
285,0.26624,0.26217,0.26013,0.26158,0.29082,0.31888,0.25111,0.27132,0.
27954,0.21055,0.20706,0.20712,0.18884,0.20203,0.20424,0.20532,0.20882,
0.2034,0.21086,0.2029,0.21008,0.2037,0.20006,0.20093,0.2105,0.38108,0.
39205,0.45278,0.36608,0.29806,0.32185,0.25,0.31144,0.28305,0.27274,0.3
0552,0.30031,0.2937,0.27653,0.20869,0.20081,0.2034,0.20911,0.20596,0.2
0011,0.20858,0.20458,0.21086,0.20628,0.20699,0.20059,0.20507,0.20474,0
.19608,0.20416,0.39241,0.40313,0.41586,0.45898,0.39388,0.46023,0.35592
,0.29309,0.31577,0.29099,0.29009,0.29894,0.28435,0.28301,0.26126,0.278
03,0.20244,0.19679,0.20547,0.21284,0.20453,0.20175,0.19955,0.20043,0.2
072,0.20713,0.21676,0.19504,0.20723,0.20349,0.19663,0.20888,0.19707,0.
4162,0.32943,0.40226,0.34167,0.43166,0.48037,0.31062,0.314,0.22827,0.2
9114,0.27343,0.27126,0.23245,0.31929,0.27314,0.32752,0.26138,0.20581,0
.20984,0.2048,0.19968,0.20258,0.20194,0.20333,0.19754,0.20622,0.20822,
0.19757,0.20418,0.20306,0.12037,0.14053,0.12657,0.20551,0.39434,0.2628
4,0.35213,0.39786,0.44146,0.38631,0.41587,0.28778,0.27894,0.28004,0.25
58,0.27097,0.28688,0.29693,0.27159,0.27656,0.28003,0.30005,0.2083,0.21
296,0.21864,0.20214,0.20618,0.20616,0.20245,0.19206,0.20942,0.21194,0.
208,0.12082,0.1249,0.11744,0.12736,0.1207,0.1263,0.20697,0.33248,0.372
47,0.39229,0.31123,0.34672,0.43255,0.44225,0.2696,0.25621,0.29156,0.36
239,0.25685,0.24086,0.22145,0.28874,0.24376,0.29811,0.26749,0.19306,0.
21241,0.19798,0.20991,0.21997,0.20796,0.19919,0.2078,0.19155,0.12098,0
.12275,0.11395,0.11581,0.12593,0.10973,0.13494,0.12082,0.12329,0.20357
,0.32059,0.40639,0.4113,0.35726,0.3965,0.22512,0.31644,0.27335,0.26853
,0.32651,0.26876,0.32619,0.22293,0.27023,0.26163,0.30021,0.30651,0.295
51,0.30282,0.21014,0.19801,0.2022,0.19419,0.20374,0.19802,0.11506,0.12
262,0.11887,0.12763,0.13459,0.11139,0.14155,0.12283,0.13093,0.11624,0.
11861,0.13109,0.34118,0.31227,0.38265,0.38922,0.35975,0.41742,0.43596,
0.29807,0.22698,0.24425,0.24461,0.24944,0.3018,0.28364,0.29583,0.31182

,0.3278,0.27969,0.2535,0.19952,0.20695,0.19978,0.12341,0.12117,0.12643
,0.12757,0.11991,0.11648,0.11091,0.14265,0.14421,0.10185,0.11874,0.132
38,0.11297,0.12296,0.14485,0.33658,0.2428,0.33655,0.35541,0.36149,0.42
421,0.33209,0.37729,0.30586,0.2613,0.25965,0.23761,0.27077,0.31782,0.2
9407,0.27978,0.26304,0.3358,0.25903,0.2761,0.11455,0.10921,0.13846,0.1
0754,0.12959,0.13358,0.12881,0.12591,0.12521,0.11997,0.11956,0.1296,0.
11947,0.12379,0.12462,0.13724,0.10334,0.09951,0.38652,0.35082,0.37593,
0.28782,0.30027,0.39757,0.49837,0.26131,0.38331,0.298,0.29595,0.23095,
0.17826,0.20832,0.20955,0.19588,0.22424,0.18848,0.16727,0.21709,0.123,
0.092127,0.12148,0.10915,0.12749,0.12387,0.12124,0.12537,0.13014,0.118
96,0.12813,0.10683,0.12065,0.12284,0.12572,0.11891,0.1236,0.12515,0.44
904,0.3573,0.4265,0.40902,0.32265,0.34962,0.40426,0.4373,0.28507,0.190
54,0.22124,0.17728,0.24724,0.18498,0.2217,0.18838,0.16722,0.21271,0.21
703,0.24781,0.12342,0.11327,0.11778,0.11942,0.11029,0.11869,0.12565,0.
12624,0.11512,0.11544,0.11556,0.12401,0.11027,0.095092,0.11935,0.1217,
0.10841,0.11148,0.36646,0.26888,0.37924,0.49434,0.34271,0.31992,0.4151
6,0.2098,0.2288,0.20621,0.22195,0.2191,0.21074,0.22943,0.23549,0.19848
,0.22983,0.17272,0.17812,0.21606,0.12596,0.12174,0.12632,0.11166,0.128
7,0.12794,0.11483,0.11976,0.11752,0.10969,0.12549,0.1235,0.11835,0.129
04,0.12238,0.13319,0.12754,0.1275,0.40485,0.37334,0.42642,0.41358,0.29
872,0.37987,0.21601,0.21208,0.20656,0.23592,0.23309,0.19132,0.21671,0.
22254,0.18345,0.20268,0.22197,0.20335,0.22293,0.19787,0.13402,0.10618,
0.11548,0.11694,0.12209,0.11575,0.12936,0.12189,0.11693,0.12945,0.1179
8,0.13982,0.12176,0.14377,0.12637,0.1157,0.12108,0.12703,0.39321,0.364
3,0.37586,0.47822,0.30581,0.1828,0.17356,0.23766,0.21874,0.20123,0.246
35,0.19771,0.22954,0.18437,0.25327,0.18272,0.21738,0.18576,0.22477,0.2
3469,0.12607,0.14044,0.12093,0.12322,0.12657,0.11919,0.12844,0.10983,0
.12344,0.12111,0.12113,0.13386,0.11481,0.1296,0.13619,0.11927,0.11903,
0.10259,0.28767,0.47048,0.33284,0.15374,0.24465,0.16901,0.22687,0.2453
5,0.29488,0.13414,0.22744,0.16717,0.20109,0.24488,0.19271,0.22,0.20763
,0.2148,0.19123,0.20171,0.11059,0.10961,0.13685,0.1071,0.13284,0.10532
,0.12347,0.12028,0.098218,0.12517,0.14052,0.12469,0.13083,0.12386,0.12
019,0.11727,0.12197,0.12385,0.33134,0.20372,0.20711,0.15245,0.16911,0.
20666,0.21457,0.14109,0.16663,0.20272,0.21557,0.22191,0.19041,0.18449,
0.20704,0.22628,0.17193,0.21254,0.21845,0.13397,0.12617,0.12414,0.1178
3,0.11747,0.099769,0.12587,0.1304,0.11565,0.11539,0.11426,0.12655,0.11
011,0.12216,0.11932,0.13077,0.12443,0.03784,0.24036,0.23021,0.22835,0.
17305,0.18287,0.16153,0.16682,0.2243,0.20033,0.22998,0.21325,0.21892,0
.19925,0.20774,0.19536,0.20804,0.1791,0.20055,0.16857,0.12695,0.12575,
0.12694,0.12392,0.11094,0.13012,0.12103,0.1126,0.1231,0.11946,0.12369,
0.12483,0.12012,0.12309,0.13257,0.11927,0.12425,0.046887,0.20093,0.202
16,0.17824,0.18883,0.20859,0.21137,0.21251,0.19614,0.16205,0.19003,0.2
4016,0.20044,0.21561,0.20248,0.20231,0.21873,0.19918,0.22881,0.14905,0
.1222,0.1246,0.12987,0.11804,0.12949,0.13054,0.12621,0.1162,0.13371,0.
11587,0.11926,0.12812,0.12106,0.12259,0.11951,0.1252,0.13162,0.044144,
0.19793,0.16697,0.20477,0.26157,0.19342,0.14061,0.1844,0.16946,0.23576
,0.20396,0.21232,0.2383,0.2134,0.1855,0.2217,0.18356,0.19019,0.23919,0
.11772,0.1089,0.132,0.12274,0.13272,0.12093,0.11003,0.1212,0.11729,0.1
2226,0.12148,0.12088,0.10995,0.12001,0.11656,0.1318,0.12033,0.03908,0.
044355,0.041611,0.1582,0.22388,0.18209,0.17084,0.18877,0.16853,0.19105
,0.22203,0.17881,0.18161,0.18154,0.20965,0.21242,0.1748,0.17528,0.1389
4,0.12185,0.1268,0.12886,0.12919,0.10891,0.11853,0.11975,0.12049,0.118

33,0.12285,0.10346,0.13986,0.11275,0.097059,0.12247,0.1263,0.11897,0.0
38528,0.04071,0.034539,0.039374,0.15431,0.17387,0.17237,0.19701,0.1511
9,0.19629,0.095355,0.17909,0.20733,0.21211,0.19252,0.2231,0.11542,0.15
292,0.13334,0.11978,0.12898,0.10459,0.12052,0.12882,0.12936,0.1465,0.1
1738,0.12115,0.10017,0.12565,0.12059,0.10662,0.11269,0.1037,0.11668,0.
12437,0.041721,0.035329,0.037478,0.040522,0.042576,0.040321,0.21037,0.
22172,0.17277,0.1774,0.21965,0.13774,0.24042,0.21821,0.12303,0.11222,0
.099099,0.1334,0.12268,0.11976,0.12485,0.11128,0.12466,0.14902,0.11597
,0.1212,0.13738,0.12824,0.14706,0.11728,0.11691,0.12555,0.12025,0.1208
6,0.12077,0.14335,0.12563,0.046124,0.038133,0.044743,0.031876,0.039114
,0.033628,0.22199,0.17699,0.24095,0.24533,0.1925,0.038302,0.045797,0.1
1816,0.11876,0.10199,0.1209,0.1224,0.11853,0.11077,0.12374,0.11188,0.1
1861,0.12534,0.10515,0.12,0.11885,0.13356,0.13229,0.1256,0.11537,0.119
93,0.095404,0.12631,0.041163,0.029333,0.04819,0.03659,0.039691,0.03464
7,0.037934,0.041164,0.041532,0.044367,0.19294,0.19417,0.039579,0.03387
2,0.036474,0.031725,0.11066,0.12109,0.10996,0.1231,0.10117,0.12768,0.1
1806,0.11453,0.12109,0.10976,0.11797,0.11698,0.11355,0.11285,0.11167,0
.1301,0.12078,0.13056,0.12659,0.035158,0.036755,0.040406,0.038163,0.03
746,0.034843,0.035311,0.036011,0.043437,0.042758,0.036988,0.045305,0.0
39243,0.034397,0.036614,0.045134,0.043174,0.13309,0.11972,0.12378,0.11
947,0.11266,0.11473,0.11265,0.12258,0.11053,0.12344,0.13013,0.12654,0.
1204,0.12318,0.10835,0.12043,0.034247,0.044935,0.038244,0.042292,0.041
539,0.038298,0.044929,0.042816,0.050535,0.043061,0.039836,0.04217,0.04
2369,0.038375,0.039166,0.034867,0.039248,0.039448,0.043953,0.13193,0.1
0535,0.12295,0.11375,0.10612,0.13513,0.12085,0.12476,0.12068,0.12091,0
.12621,0.12284,0.12428,0.033767,0.047322,0.037826,0.040781,0.036664,0.
038544,0.038563,0.046927,0.044252,0.040738,0.042072,0.034666,0.03855,0
.037467,0.033373,0.045127,0.043561,0.032583,0.036654,0.043561,0.044511
,0.12677,0.12666,0.12322,0.10363,0.14808,0.11975,0.12532,0.11855,0.121
09,0.038791,0.040619,0.046004,0.03309,0.041548,0.036807,0.048328,0.049
151,0.036744,0.040527,0.036166,0.044508,0.03981,0.047679,0.04372,0.038
272,0.047668,0.042527,0.037599,0.043286,0.036213,0.046821,0.044497,0.0
38714,0.037802,0.11757,0.11443,0.12007,0.12837,0.034841,0.043203,0.041
118,0.034853,0.04226,0.039416,0.035234,0.038631,0.036297,0.043303,0.04
3608,0.041603,0.042301,0.036781,0.038435,0.043839,0.040335,0.035438,0.
038383,0.036559,0.037113,0.040055,0.042699,0.047069,0.039597,0.037003,
0.043056,0.039199,0.047213,0.040457,0.034852,0.033576,0.045755,0.03783
9,0.041875,0.040164,0.03896,0.03882,0.046501,0.037455,0.038313,0.03452
3,0.045471,0.041996,0.043457,0.040436,0.041315,0.039084,0.044864,0.031
069,0.03699,0.041372,0.047061,0.037995,0.045209,0.047468,0.040834,0.04
1175,0.040312,0.041609,0.044904,0.039095,0.037969,0.040913,0.046256,0.
04158,0.038474,0.030774,0.039191,0.040514,0.037777,0.045155,0.033566,0
.039883,0.042838,0.038043,0.042841,0.042853,0.050816,0.033547,0.042461
,0.045071,0.035128,0.040711,0.045476,0.039506,0.044433,0.041359,0.0471
52,0.041086,0.041893,0.034989,0.030772,0.040705,0.036834,0.04498,0.045
709,0.045531,0.042913,0.042682,0.044955,0.035003,0.0425,0.042317,0.041
648,0.037706,0.035105,0.039999,0.038908,0.045624,0.036721,0.045067,0.0
34221,0.03904,0.03797,0.038936,0.040838,0.041887,0.035382,0.03639,0.04
1932,0.044843,0.037268,0.040989,0.040492,0.036617,0.042311,0.049938,0.
040305,0.03752,0.040328,0.042632,0.043913,0.038212,0.036626,0.042146,0
.03995,0.040825,0.038844,0.041992,0.034642,0.04289,0.045313,0.041738,0
.040459,0.042996,0.042351,0.043122,0.039752,0.047757,0.043271,0.040963

,0.03623,0.040726,0.03837,0.036046,0.035885,0.041037,0.037661,0.04113,
0.041126,0.036391,0.041805,0.032671,0.041813,0.046517,0.036583,0.04073
6,0.045854,0.045607,0.040707,0.03907,0.040399,0.037807,0.046389,0.0394
43,0.041666,0.038047,0.033943,0.0355,0.037501,0.043791,0.039593,0.0386
9,0.036856,0.043591,0.035996,0.032288,0.034148,0.040291,0.036358,0.042
519,0.040587,0.039338,0.040231
0.20454,0.20162,0.20085,0.20514,0.203,0.20388,0.21563,0.20654,0.20505,
0.21061,0.20656,0.21077,0.20509,0.20214,0.20507,0.20789,0.20741,0.1995
1,0.20974,0.20425,0.26992,0.291,0.30202,0.28874,0.21026,0.204,0.21322,
0.20825,0.20733,0.20655,0.19857,0.20503,0.20469,0.21126,0.20664,0.2076
3,0.20126,0.21216,0.19904,0.32966,0.25386,0.32956,0.33863,0.24404,0.28
813,0.28344,0.20292,0.20933,0.19762,0.21317,0.20391,0.19788,0.20459,0.
2,0.20111,0.20424,0.22012,0.2066,0.20614,0.20305,0.20835,0.31613,0.350
85,0.29122,0.30183,0.31967,0.3161,0.30662,0.31463,0.2722,0.27947,0.214
16,0.20542,0.19686,0.21533,0.19876,0.20628,0.19109,0.2101,0.20453,0.19
777,0.1989,0.2094,0.19967,0.20409,0.20723,0.4864,0.36107,0.40058,0.267
57,0.27109,0.24892,0.25614,0.2931,0.31959,0.26938,0.25375,0.30438,0.21
129,0.20751,0.20637,0.19138,0.19816,0.20354,0.20868,0.20767,0.2044,0.2
1416,0.19987,0.21025,0.20351,0.19948,0.20002,0.20522,0.41895,0.43055,0
.44678,0.35694,0.30212,0.34011,0.22804,0.29218,0.28869,0.27035,0.31697
,0.28749,0.29296,0.28069,0.20918,0.20099,0.20483,0.20669,0.20245,0.201
74,0.20818,0.20558,0.20726,0.20519,0.20972,0.20454,0.20359,0.20542,0.1
9707,0.20572,0.38698,0.37028,0.37619,0.41344,0.43224,0.45855,0.35113,0
.28476,0.30277,0.28246,0.27904,0.30903,0.28343,0.30267,0.25175,0.28062
,0.20545,0.19888,0.21041,0.21215,0.2041,0.20286,0.19686,0.20204,0.2080
5,0.20386,0.21662,0.19338,0.21051,0.20446,0.19473,0.20505,0.19854,0.44
03,0.30921,0.3582,0.31677,0.41536,0.39176,0.292,0.31371,0.22996,0.2973
3,0.27347,0.27194,0.22222,0.31575,0.28182,0.32559,0.24695,0.20786,0.20
754,0.20606,0.20323,0.20327,0.20494,0.20607,0.197,0.20703,0.21087,0.20
003,0.20739,0.20432,0.12128,0.13903,0.12777,0.20227,0.38774,0.29484,0.
37052,0.36306,0.40928,0.34282,0.38318,0.29011,0.27706,0.30643,0.24218,
0.24552,0.29867,0.30238,0.28298,0.29198,0.29212,0.29948,0.20458,0.2154
4,0.21768,0.20069,0.2056,0.20735,0.20492,0.18719,0.21364,0.21065,0.211
27,0.12097,0.12494,0.11785,0.12706,0.1216,0.12614,0.20853,0.29991,0.40
733,0.4063,0.36512,0.32811,0.46256,0.43329,0.25845,0.26427,0.281,0.357
24,0.262,0.24986,0.21829,0.30825,0.2499,0.29614,0.26261,0.19745,0.2084
4,0.19901,0.20504,0.22344,0.20809,0.19955,0.2065,0.19029,0.12184,0.121
65,0.11345,0.11687,0.12768,0.10784,0.13165,0.12211,0.12238,0.20562,0.2
6471,0.39601,0.37585,0.35463,0.38398,0.21399,0.31467,0.26027,0.25573,0
.32596,0.27977,0.34331,0.22842,0.28493,0.27898,0.29212,0.30195,0.28828
,0.29973,0.20767,0.19936,0.20358,0.19606,0.20409,0.20177,0.11767,0.122
11,0.1174,0.12993,0.13463,0.1125,0.14025,0.12271,0.13107,0.11651,0.118
42,0.13096,0.35344,0.34555,0.35647,0.43449,0.32097,0.42025,0.39054,0.2
9605,0.23328,0.23935,0.24944,0.24862,0.29135,0.29336,0.27667,0.29505,0
.34962,0.27645,0.26091,0.20155,0.21256,0.19919,0.12449,0.11875,0.12623
,0.12833,0.12058,0.11758,0.11133,0.14251,0.14312,0.10183,0.1201,0.1344
2,0.11739,0.1198,0.14385,0.2885,0.31124,0.30918,0.33283,0.36149,0.4307
2,0.32912,0.41262,0.35872,0.24074,0.26336,0.23194,0.29718,0.32191,0.27
674,0.28156,0.24758,0.35459,0.25639,0.26854,0.11443,0.10727,0.13781,0.
10922,0.1309,0.13406,0.12702,0.12473,0.12424,0.1204,0.12042,0.13151,0.
11792,0.12386,0.125,0.13807,0.10397,0.099605,0.35965,0.32548,0.4148,0.
33004,0.32768,0.41161,0.45187,0.26637,0.40582,0.30566,0.29702,0.22686,

0.17409,0.20096,0.20729,0.19487,0.22918,0.17423,0.1642,0.21038,0.12385
,0.092834,0.1235,0.11033,0.12769,0.12336,0.12101,0.12583,0.1297,0.1189
8,0.12774,0.10936,0.12001,0.12281,0.12632,0.1183,0.12364,0.12443,0.436
22,0.39316,0.41459,0.41695,0.33251,0.34858,0.33977,0.46193,0.29877,0.2
0626,0.22296,0.17276,0.26661,0.17979,0.22576,0.19824,0.16903,0.19202,0
.21579,0.23951,0.12367,0.11342,0.11858,0.12124,0.11106,0.12101,0.12675
,0.12651,0.11558,0.11493,0.11487,0.12473,0.11026,0.094576,0.11855,0.12
421,0.10561,0.11213,0.39213,0.28057,0.40368,0.49386,0.32717,0.33911,0.
38452,0.20661,0.25783,0.21972,0.22204,0.23611,0.21635,0.21128,0.25316,
0.19527,0.22191,0.17757,0.18509,0.22356,0.12598,0.12128,0.1266,0.11074
,0.12759,0.12692,0.11499,0.12137,0.12,0.10943,0.12374,0.1228,0.11862,0
.13149,0.12148,0.13524,0.12671,0.12638,0.35303,0.36866,0.46495,0.39312
,0.30302,0.40288,0.22262,0.22975,0.21119,0.2269,0.236,0.1895,0.21907,0
.22632,0.17236,0.20998,0.21161,0.19145,0.22126,0.20225,0.13554,0.10697
,0.11685,0.11491,0.12142,0.1161,0.12699,0.12173,0.11803,0.1304,0.12042
,0.13949,0.12058,0.14324,0.1283,0.11645,0.11975,0.1271,0.36569,0.36687
,0.35643,0.42367,0.30323,0.20139,0.18599,0.23883,0.23033,0.19936,0.229
5,0.1991,0.23242,0.18817,0.23614,0.17857,0.20201,0.18964,0.22856,0.228
78,0.12676,0.13808,0.1226,0.12342,0.12667,0.11996,0.12842,0.10826,0.12
329,0.11981,0.11819,0.1336,0.11357,0.12896,0.13622,0.1197,0.12137,0.10
15,0.29897,0.49119,0.34072,0.14494,0.2359,0.18121,0.23244,0.22909,0.28
047,0.14195,0.24009,0.1639,0.2007,0.24016,0.19915,0.20567,0.20632,0.22
495,0.19632,0.19245,0.11043,0.11152,0.13443,0.1041,0.13345,0.10623,0.1
2188,0.12567,0.099218,0.12528,0.14141,0.12594,0.1295,0.12529,0.11788,0
.11811,0.12208,0.12344,0.33138,0.18854,0.19928,0.13246,0.18735,0.2064,
0.21632,0.14579,0.18464,0.19358,0.2317,0.21453,0.19246,0.18687,0.21579
,0.22655,0.16925,0.21784,0.21448,0.13484,0.12471,0.12225,0.11878,0.120
23,0.097324,0.1248,0.1304,0.11595,0.1169,0.11486,0.12771,0.10961,0.122
54,0.11849,0.12815,0.12451,0.037377,0.24237,0.21236,0.21985,0.15618,0.
19468,0.17389,0.1733,0.22003,0.19918,0.24076,0.21842,0.2163,0.20632,0.
21078,0.18456,0.21225,0.17004,0.19466,0.17602,0.12697,0.12414,0.12654,
0.12481,0.10866,0.13068,0.12251,0.11265,0.12378,0.11961,0.12409,0.1248
9,0.11827,0.12349,0.13041,0.12154,0.12512,0.04669,0.19681,0.19185,0.17
988,0.192,0.19657,0.21846,0.23836,0.19719,0.14871,0.20405,0.23464,0.19
897,0.23494,0.18529,0.206,0.2093,0.18881,0.22071,0.14908,0.12025,0.126
36,0.13402,0.11503,0.131,0.1328,0.12705,0.11801,0.13566,0.11588,0.1205
7,0.12844,0.11956,0.12365,0.11978,0.12521,0.12991,0.040764,0.22126,0.1
5908,0.21313,0.26531,0.18303,0.14088,0.19347,0.17565,0.24116,0.1817,0.
19918,0.23625,0.21022,0.18145,0.22139,0.19006,0.19586,0.2388,0.11861,0
.10931,0.13217,0.12313,0.13111,0.12168,0.11063,0.12194,0.11964,0.12256
,0.12277,0.12009,0.11188,0.12088,0.11769,0.13095,0.11902,0.037895,0.04
585,0.040504,0.15058,0.23415,0.17499,0.16838,0.16957,0.18005,0.20501,0
.2178,0.17263,0.18497,0.17393,0.21923,0.21904,0.17241,0.16751,0.13886,
0.12186,0.12719,0.12971,0.12934,0.10653,0.11972,0.12235,0.12113,0.1188
3,0.12227,0.10476,0.14078,0.11477,0.095152,0.12175,0.12756,0.12057,0.0
37871,0.041705,0.035632,0.040335,0.16513,0.18372,0.17015,0.18035,0.131
4,0.18781,0.095342,0.18771,0.20949,0.21181,0.18646,0.22017,0.11373,0.1
5413,0.13607,0.12106,0.1275,0.10412,0.12137,0.1272,0.12932,0.14655,0.1
1826,0.12258,0.1007,0.125,0.11996,0.10633,0.11195,0.104,0.12,0.12587,0
.041998,0.037432,0.03711,0.040778,0.041534,0.041506,0.20753,0.23294,0.
17646,0.16321,0.21495,0.13085,0.24924,0.21421,0.12127,0.11236,0.098366
,0.13319,0.12221,0.11863,0.12533,0.11113,0.12404,0.14945,0.11612,0.121

1,0.1376,0.12706,0.1461,0.1193,0.11889,0.12299,0.12144,0.12054,0.12073
,0.14136,0.12661,0.0463,0.039755,0.042834,0.030531,0.03593,0.030625,0.
20587,0.19042,0.21353,0.22593,0.19278,0.040248,0.044853,0.11887,0.1201
7,0.10337,0.12027,0.12018,0.11904,0.10946,0.12514,0.11372,0.11867,0.12
585,0.10645,0.1198,0.11859,0.13179,0.13385,0.12582,0.11592,0.12217,0.0
94849,0.12665,0.040557,0.03052,0.047983,0.032156,0.041821,0.037078,0.0
36049,0.040551,0.040058,0.044929,0.21229,0.19648,0.044271,0.034083,0.0
38251,0.030954,0.11193,0.12099,0.10958,0.12258,0.099407,0.12907,0.1173
2,0.11677,0.12366,0.1094,0.11979,0.11654,0.11573,0.11404,0.11102,0.130
13,0.12123,0.12775,0.12689,0.039918,0.035282,0.042048,0.039732,0.03496
8,0.037392,0.034966,0.035867,0.041147,0.040435,0.036222,0.043458,0.039
62,0.0354,0.035087,0.042239,0.045139,0.13472,0.11736,0.1255,0.1212,0.1
1549,0.11434,0.11446,0.12327,0.1112,0.12479,0.13182,0.12166,0.12306,0.
12278,0.10788,0.12149,0.032984,0.044812,0.038783,0.041719,0.042664,0.0
35653,0.044902,0.041947,0.04954,0.041915,0.041252,0.040688,0.041552,0.
03801,0.03803,0.035983,0.039308,0.040626,0.044772,0.1345,0.10762,0.125
17,0.11209,0.10665,0.13425,0.12324,0.12633,0.12213,0.12163,0.12595,0.1
2545,0.12474,0.036474,0.045379,0.035482,0.039547,0.038959,0.03918,0.03
7734,0.047619,0.044267,0.041061,0.040854,0.03592,0.039525,0.037796,0.0
34572,0.044505,0.042245,0.032329,0.035854,0.047186,0.047907,0.12547,0.
12574,0.12286,0.10482,0.14882,0.12246,0.12423,0.11902,0.12166,0.035605
,0.040402,0.04569,0.032454,0.042746,0.037827,0.046055,0.048937,0.03730
7,0.041908,0.033785,0.043467,0.040195,0.047429,0.042695,0.033766,0.048
146,0.043529,0.038042,0.043498,0.033232,0.044848,0.041172,0.039039,0.0
3769,0.11785,0.11362,0.12093,0.12528,0.034103,0.042706,0.04235,0.03490
3,0.039904,0.038575,0.036434,0.03939,0.035813,0.042141,0.04081,0.04162
2,0.041013,0.037814,0.040943,0.043527,0.040846,0.031717,0.038376,0.037
3,0.039672,0.041169,0.039965,0.04683,0.039496,0.035019,0.037196,0.0386
24,0.046963,0.042214,0.036413,0.03769,0.04518,0.040614,0.041884,0.0401
43,0.037456,0.04047,0.044465,0.034434,0.038979,0.036229,0.044304,0.042
659,0.042361,0.038484,0.040768,0.038986,0.043892,0.031003,0.036142,0.0
43569,0.046467,0.038919,0.044285,0.047155,0.04267,0.039068,0.040213,0.
040667,0.041443,0.036732,0.036246,0.038874,0.047043,0.04193,0.04012,0.
031428,0.040231,0.040329,0.038914,0.045384,0.035479,0.041196,0.043896,
0.036971,0.040361,0.040443,0.048247,0.032885,0.039535,0.042803,0.0348,
0.039712,0.045629,0.038847,0.045212,0.042697,0.045384,0.03989,0.040195
,0.031303,0.031216,0.039573,0.037314,0.044862,0.041597,0.04407,0.04324
9,0.041554,0.047008,0.033197,0.042605,0.041834,0.038629,0.03691,0.0347
08,0.04079,0.03613,0.04558,0.037546,0.043425,0.034294,0.038513,0.03728
8,0.039591,0.041622,0.041159,0.036815,0.035223,0.0413,0.042688,0.03502
1,0.039202,0.037836,0.034093,0.041754,0.046342,0.043492,0.037281,0.041
908,0.041048,0.041406,0.034119,0.035434,0.042253,0.040515,0.042912,0.0
39053,0.041144,0.033011,0.046561,0.045603,0.042471,0.042495,0.041547,0
.039962,0.042838,0.040519,0.047683,0.043792,0.042302,0.035206,0.037652
,0.039016,0.03596,0.036274,0.039489,0.035877,0.041559,0.043127,0.03749
3,0.044396,0.028996,0.042112,0.04192,0.036597,0.039651,0.046634,0.0429
67,0.040003,0.037122,0.041204,0.033741,0.046199,0.039162,0.041455,0.03
8709,0.033625,0.034404,0.038725,0.042335,0.037416,0.038766,0.036859,0.
043338,0.038598,0.030851,0.032737,0.040304,0.036068,0.0425,0.039654,0.
041742,0.041367

#simdataS2SNR15:

Kep_f=1.2
Kep_f=1.2
Kep_s=0.1
Ktrans_f=0.05
Ktrans_s=0.03
del_t=0.5
gtSf=0 0 0 0 0 0
0 0 0 0 0 0
0 0 0 0 0 0
0 0 0 0 0 0
0 0 0 0 0 0
0 0 0 0 0 0
0 0 0 0 0 0
0 0 0 0 0 0
0 0 0 0 0 0
0 0 0 0 0 0
0 0 0 0 0 0
0 0 0 0 0 0
0 0 0 0 0 0
0 0 0 0 0 0
0 0 0 0 0 0
0 0 0 0 0 0
0 0 0 0 0 0
0 0 0 0 0 0
0 0 0 0 0 0
0 0 0 0 0 0
0 0 0 0 0 0
0 0 0 0 0 0
0 0 0 0 0 0
0 0 0 0 0 0
0 0 0 0 0 0
0 0 0 0 0 0
0 0 0 0 0 0
0 0 0 0 0 0
0 0 0 0 0 0
0 0 0 0 0 0
0 0 0 0 0 0
0 0 0 0 0 0
0 0 0 0 0 0
0 0 0 0 0 0
0 0 0 0.4644 0.5871 0.4622
0 0 0 0 0 0
0 0 0 0 0 0
0 0 0 0 0 0
0 0 0 0 0 0
0 0 0 0 0 0
0.6249 0.5 0.5 0.50711 0.49981 0.46367
0 0 0 0 0 0
0 0 0 0 0 0
0 0 0 0 0 0

0	0	0	0	0	0
0	0	0	0	0.5	0.48525
0.41067	0.50943	0.5	0.5	0.5	0.46467
0.50993	0	0	0	0	0
0	0	0	0	0	0
0	0	0	0	0	0
0	0	0	0	0	0
0	0	0	0.54852	0.48907	0.46755
0.50748	0.38761	0.48129	0.54817	0.49418	0.49461
0.38803	0.49229	0.5	0	0	0
0	0	0	0	0	0
0	0	0	0	0	0
0	0	0	0	0	0
0	0.53529	0.3797	0.5732	0.57134	0.48624
0.51084	0.5	0.5	0.49068	0.48677	0.5
0.42862	0.46112	0.33857	0.48491	0	0
0	0	0	0	0	0
0	0	0	0	0	0
0	0	0	0	0	0
0.5	0.5	0.50657	0.51368	0.5	0.5
0.54053	0.5	0.46168	0.51399	0.58305	0.5
0.5	0.49936	0.5	0.60896	0.54338	0.47228
0	0	0	0	0	0
0	0	0	0	0	0.27461
0.38035	0.3181	0.33818	0.34804	0.27702	0.27259
0.33486	0.31473	0.46932	0.41234	0.55228	0.60093
0.5	0.38453	0.46755	0.5	0.45593	0.61859
0.51618	0.5	0.51233	0.5	0.47069	0.51655
0.5	0.52352	0	0	0	0
0	0	0	0	0	0.54958
0.33333	0.35738	0.25453	0.35236	0.24293	0.34865
0.27406	0.3641	0.33333	0.30245	0.5	0.4744
0.4223	0.5	0.51841	0.38712	0.35922	0.5
0.40917	0.49138	0.5	0.47219	0.4119	0.55084
0.57523	0.5	0.55696	0.49749	0	0
0	0	0	0	0	0.5
0.44292	0.36511	0.42216	0.3427	0.33972	0.31627
0.355	0.38578	0.31716	0.3075	0.28026	0.38472
0.5	0.5	0.43893	0.54046	0.50862	0.50705
0.49631	0.38195	0.5	0.49508	0.54683	0.48559
0.51449	0.5	0.54487	0.55139	0.51143	0.42555
0	0	0	0	0	0
0.47298	0.48523	0.5	0.52025	0.50127	0.25937
0.33382	0.33367	0.3541	0.279	0.34019	0.33882
0.34197	0.25316	0.50672	0.49514	0.50857	0.5
0.55332	0.5	0.53918	0.51123	0.4776	0.46389
0.37577	0.5	0.5	0.54611	0.42303	0.45521
0.58121	0.5	0	0	0	0
0	0.43956	0.5	0.5069	0.46393	0.56023
0.65532	0.3409	0.27168	0.35855	0.35146	0.38613
0.25389	0.37757	0.33333	0.28254	0.40842	0.58355
0.41145	0.52048	0.61592	0.49859	0.53117	0.41939

0.57825	0.5	0.55656	0.50157	0.5	0.51293
0.47783	0.43314	0.54892	0.5	0	0
0	0.5	0.5	0.5	0.46311	0.5
0.41389	0.5477	0.47856	0.30979	0.36122	0.36432
0.27088	0.37101	0.32128	0.40846	0.36306	0.35101
0.52506	0.5	0.57531	0.5	0.56182	0.45921
0.43116	0.48807	0.5	0.57	0.49321	0.49807
0.46925	0.41146	0.55282	0.5	0.5	0.5
0	0.47533	0.39133	0.5	0.55245	0.5
0.52519	0.5	0.5	0.50806	0.33333	0.33426
0.35814	0.33552	0.25467	0.3597	0.34003	0.30363
0.33333	0.5	0.5	0.49595	0.49926	0.52947
0.55048	0.5286	0.5	0.46581	0.47165	0.5
0.5	0.52555	0.46129	0.34056	0.509	0.58827
1	0.5	0.5	0.5	0.5265	0.5
0.60865	0.54003	0.41905	0.5	0.35879	0.23469
0.41424	0.38557	0.33333	0.33333	0.35099	0.31717
0.36672	0.36827	0.5	0.5	0.47917	0.50336
0.50922	0.66504	0.5	0.5613	0.46629	0.52681
0.44621	0.51817	0.50193	0.48721	0.5	0.50068
0.5	1	0.41677	0.5	0.5	0.42642
0.48726	0.56742	0.53614	0.61739	0.60865	0.48016
0.35776	0.2356	0.41043	0.41629	0.32807	0.27377
0.27727	0.21824	0.51201	0.5	0.5	0.49199
0.49888	0.59914	0.38072	0.47391	0.47479	0.48719
0.46028	0.5	0.5	0.5	0.55518	0.5
0.52357	0.5	1	0.50986	0.36088	0.5136
0.5	0.48767	0.52922	0.54751	0.45811	0.5459
0.5	0.3159	0.39207	0.35428	0.33964	0.27526
0.38799	0.41871	0.33572	0.42025	0.5	0.5
0.50848	0.50379	0.3519	0.53438	0.49778	0.51111
0.47872	0.49542	0.51634	0.5773	0.5458	0.55986
0.49907	0.41907	1	1	1	0.51116
0.52348	0.5	0.50147	0.48128	0.5	0.52999
0.50127	0.5	0.37153	0.35368	0.33333	0.43612
0.40384	0.29743	0.63267	0.47662	0.45554	0.50499
0.50223	0.5	0.54453	0.4675	0.57588	0.45317
0.5	0.5613	0.52598	0.51222	0.5499	0.55391
0.49439	0.51016	1	1	1	1
0.68241	0.50876	0.57721	0.65354	0.5	0.5
0.44378	0.41495	0.44787	0.28067	0.29832	0.28283
0.39051	0.53783	0.6052	0.56072	0.41715	0.42651
0.37723	0.55916	0.4709	0.5	0.42263	0.43923
0.49363	0.40284	0.49914	0.4911	0.47666	0.45835
0.58854	0.5	1	1	1	1
1	1	0.46149	0.5	0.38172	0.50335
0.54654	0.49123	0.53332	0.48222	0.55468	0.61871
0.68045	0.49725	0.5	0.5187	0.5	0.4799
0.67309	0.5	0.44492	0.26922	0.50784	0.51238
0.33317	0.47898	0.50466	0.66885	0.50175	0.53279
0.51926	0.53989	0.47787	1	1	1
1	1	1	0.47662	0.5	0.43275

0.5	0.46807	1	1	0.5	0.5
0.5	0.50206	0.49574	0.49737	0.51484	0.45148
0.52474	0.55215	0.51811	0.52807	0.46516	0.5
0.55603	0.5	0.48479	0.53405	0.50103	0.46525
0.48013	1	1	1	1	1
1	1	1	1	1	0.5
0.56364	1	1	1	1	0.44196
0.65894	0.5	0.48334	0.48441	0.41955	0.5
0.51838	0.48557	0.54349	0.56309	0.40418	0.4578
0.51363	0.46613	0.5	0.55698	0.43342	0.4475
1	1	1	1	1	1
1	1	1	1	1	1
1	1	1	1	1	0.51945
0.45671	0.57119	0.62919	0.5	0.53544	0.47749
0.58204	0.52978	0.50539	0.52302	0.47751	0.56278
0.5	0.45335	0.5	1	1	1
1	1	1	1	1	1
1	1	1	1	1	1
1	1	1	1	0.55494	0.54768
0.37944	0.49654	0.57098	0.49409	0.5	0.46868
0.54867	0.46811	0.46781	0.44821	0.58691	1
1	1	1	1	1	1
1	1	1	1	1	1
1	1	1	1	1	1
1	1	0.53904	0.49403	0.45106	0.5
0.43493	0.59106	0.55118	0.5	0.5	1
1	1	1	1	1	1
1	1	1	1	1	1
1	1	1	1	1	1
1	1	1	1	1	1
0.5396	0.42686	0.5	0.60244	1	1
1	1	1	1	1	1
1	1	1	1	1	1
1	1	1	1	1	1
1	1	1	1	1	1
1	1	1	1	1	1
1	1	1	1	1	1
1	1	1	1	1	1
1	1	1	1	1	1
1	1	1	1	1	1
1	1	1	1	1	1
1	1	1	1	1	1
1	1	1	1	1	1
1	1	1	1	1	1
1	1	1	1	1	1
1	1	1	1	1	1
1	1	1	1	1	1
1	1	1	1	1	1
1	1	1	1	1	1
1	1	1	1	1	1
1	1	1	1	1	1
1	1	1	1	1	1

1	1	1	1	1	1
1	1	1	1	1	1
1	1	1	1	1	1
1	1	1	1	1	1
1	1	1	1	1	1
1	1	1	1	1	1
1	1	1	1	1	1
1	1	1	1	1	1
1	1	1	1	1	1
1	1	1	1	1	1
1	1	1	1	1	1
1					
gtSs=1	1	1	1	1	1
1	1	1	1	1	1
1	1	1	1	1	1
1	1	0.41639	0.5212	0.32567	0.48958
1	1	1	1	1	1
1	1	1	1	1	1
1	1	1	0	0.49747	0.5
0.5	0.5	0.51805	0.5	1	1
1	1	1	1	1	1
1	1	1	1	1	1
1	0	0	0.5	0.43478	0.61042
0.50058	0.50173	0.50515	0.51762	0.5	1
1	1	1	1	1	1
1	1	1	1	1	1
1	1	0	0	0	0.73708
0.32107	0.50026	0.5	0.5	0.54691	0.39456
0.46722	0.5	1	1	1	1
1	1	1	1	1	1
1	1	1	1	1	1
0	0	0	0	0.49053	0.5
0.66871	0.49547	0.59411	0.5	0.55966	0.45613
0.5	0.40933	1	1	1	1
1	1	1	1	1	1
1	1	1	1	1	1
0	0	0	0	0	0
0.54584	0.41418	0.54562	0.54346	0.51741	0.47281
0.51514	0.5	0.51323	0.539	1	1
1	1	1	1	1	1
1	1	1	1	1	1
1	1	1	0	0	0
0	0	0	0.52137	0.5799	0.51226
0.43667	0.44593	0.45694	0.5	0.5	0.51816
0.5	0.48074	1	1	1	1
1	1	1	1	1	1
1	1	1	0.5356	0.4129	0.5378
1	0	0	0	0	0
0	0	0.43764	0.49022	0.48381	0.5
0.44925	0.46075	0.51437	0.5	0.48946	0.45403
0.53609	1	1	1	1	1
1	1	1	1	1	1

0.3751	0.5	0.5	0.49289	0.50019	0.53633
1	0	0	0	0	0
0	0	0.47104	0.45387	0.53684	0.47671
0.5	0.5	0.5	0.5	0.47131	0.5
0.60105	1	1	1	1	1
1	1	1	1	0.5	0.51475
0.58933	0.49057	0.5	0.5	0.5	0.53533
0.49007	1	0	0	0	0
0	0	0	0.58695	0.51435	0.52313
0.5	0.52544	0.5	0.5	0.61249	0.5
0.52734	0.50892	0.63984	1	1	1
1	1	1	0.45148	0.51093	0.53245
0.49252	0.61239	0.51871	0.45183	0.50582	0.50539
0.61197	0.50771	0.5	0	0	0
0	0	0	0	0.59367	0.5
0.5	0.5	0.5	0.47904	0.43391	0.5
0.47356	0.5	0.49871	0.5	1	1
1	0.46471	0.6203	0.4268	0.42866	0.51376
0.48916	0.5	0.5	0.50932	0.51323	0.5
0.57138	0.53888	0.66143	0.51509	0	0
0	0	0	0	0	0
0	0.49769	0.5	0.60241	0.48916	0.62986
0.54474	0.54727	0.5	0.5	0.40029	0.47668
0.5	0.5	0.49343	0.48632	0.5	0.5
0.45947	0.5	0.53832	0.48601	0.41695	0.5
0.5	0.50064	0.5	0.39104	0.45662	0.52772
0	0	0	0	0	0
0	0	0	0.45723	0.53457	0.32749
0.36822	0.34095	0.32365	0.30392	0.37539	0.31384
0.33486	0.34264	0.53068	0.58766	0.44772	0.39907
0.5	0.61547	0.53245	0.5	0.54407	0.38141
0.48382	0.5	0.48767	0.5	0.52931	0.48345
0.5	0.47648	0	0	0	0
0	0	0	0	0	0
0.33333	0.27992	0.37718	0.35236	0.37853	0.34865
0.30737	0.27181	0.33333	0.47416	0.5	0.5256
0.5777	0.5	0.48159	0.61288	0.64078	0.5
0.59083	0.50862	0.5	0.52781	0.5881	0.44916
0.42477	0.5	0.44304	0.50251	0	0
0	0	0	0	0	0
0	0	0.24716	0.3146	0.33972	0.34187
0.355	0.22844	0.3549	0.3493	0.39218	0.26614
0.5	0.5	0.56107	0.45954	0.49138	0.49295
0.50369	0.61805	0.5	0.50492	0.45317	0.51441
0.48551	0.5	0.45513	0.44861	0.48857	0.57445
0	0	0	0	0	0
0	0	0	0	0	0.31304
0.32854	0.33367	0.29179	0.3605	0.31962	0.30965
0.31606	0.37395	0.49328	0.50486	0.49143	0.5
0.44668	0.5	0.46082	0.48877	0.5224	0.53611
0.62423	0.5	0.5	0.45389	0.57697	0.54479
0.41879	0.5	0	0	0	0

0	0	0	0	0	0
0	0.3409	0.36416	0.28289	0.29709	0.22774
0.40754	0.25088	0.33333	0.35873	0.59158	0.41645
0.58855	0.47952	0.38408	0.50141	0.46883	0.58061
0.42175	0.5	0.44344	0.49843	0.5	0.48707
0.52217	0.56686	0.45108	0.5	0	0
0	0	0	0	0	0
0	0	0	0.30259	0.29549	0.27897
0.35139	0.37101	0.33936	0.19201	0.36306	0.35101
0.47494	0.5	0.42469	0.5	0.43818	0.54079
0.56884	0.51193	0.5	0.43	0.50679	0.50193
0.53075	0.58854	0.44718	0.5	0.5	0.5
0	0	0	0	0	0
0	0	0	0	0.33333	0.33148
0.26164	0.33552	0.38697	0.2806	0.31994	0.35914
0.33333	0.5	0.5	0.50405	0.50074	0.47053
0.44952	0.4714	0.5	0.53419	0.52835	0.5
0.5	0.47445	0.53871	0.65944	0.491	0.41173
0	0	0	0	0	0
0	0	0	0	0	0.38265
0.37518	0.38557	0.33333	0.33333	0.35099	0.3338
0.36672	0.26345	0.5	0.5	0.52083	0.49664
0.49078	0.33496	0.5	0.4387	0.53371	0.47319
0.55379	0.48183	0.49807	0.51279	0.5	0.49932
0.5	0	0	0	0	0
0	0	0	0	0	0
0.35776	0.34816	0.41019	0.20069	0.33596	0.36312
0.36136	0.39088	0.48799	0.5	0.5	0.50801
0.50112	0.40086	0.61928	0.52609	0.52521	0.51281
0.53972	0.5	0.5	0.5	0.44482	0.5
0.47643	0.5	0	0	0	0
0	0	0	0	0	0
0	0.34205	0.29832	0.35428	0.34278	0.36521
0.22403	0.18226	0.37794	0.57975	0.5	0.5
0.49152	0.49621	0.6481	0.46562	0.50222	0.48889
0.52128	0.50458	0.48366	0.4227	0.4542	0.44014
0.50093	0.58093	0	0	0	0
0	0	0	0	0	0
0	0	0.37153	0.35368	0.33333	0.2588
0.24147	0.35128	0.36733	0.52338	0.54446	0.49501
0.49777	0.5	0.45547	0.5325	0.42412	0.54683
0.5	0.4387	0.47402	0.48778	0.4501	0.44609
0.50561	0.48984	0	0	0	0
0	0	0	0	0	0
0	0	0	0.32234	0.42908	0.39802
0.60949	0.46217	0.3948	0.43928	0.58285	0.57349
0.62277	0.44084	0.5291	0.5	0.57737	0.56077
0.50637	0.59716	0.50086	0.5089	0.52334	0.54165
0.41146	0.5	0	0	0	0
0	0	0	0	0	0
0	0	0	0	0.44532	0.38129
0.31955	0.50275	0.5	0.4813	0.5	0.5201

0.32691	0.5	0.55508	0.73078	0.49216	0.48762
0.66683	0.52102	0.49534	0.33115	0.49825	0.46721
0.48074	0.46011	0.52213	0	0	0
0	0	0	0	0	0
0	0	0	0	0.5	0.5
0.5	0.49794	0.50426	0.50263	0.48516	0.54852
0.47526	0.44785	0.48189	0.47193	0.53484	0.5
0.44397	0.5	0.51521	0.46595	0.49897	0.53475
0.51987	0	0	0	0	0
0	0	0	0	0	0
0	0	0	0	0	0.55804
0.34106	0.5	0.51666	0.51559	0.58045	0.5
0.48162	0.51443	0.45651	0.43691	0.59582	0.5422
0.48637	0.53387	0.5	0.44302	0.56658	0.5525
0	0	0	0	0	0
0	0	0	0	0	0
0	0	0	0	0	0.48055
0.54329	0.42881	0.37081	0.5	0.46456	0.52251
0.41796	0.47022	0.49461	0.47698	0.52249	0.43722
0.5	0.54665	0.5	0	0	0
0	0	0	0	0	0
0	0	0	0	0	0
0	0	0	0	0.44506	0.45232
0.62056	0.50346	0.42902	0.50591	0.5	0.53132
0.45133	0.53189	0.53219	0.55179	0.41309	0
0	0	0	0	0	0
0	0	0	0	0	0
0	0	0	0	0	0
0	0	0.46096	0.50597	0.54894	0.5
0.56507	0.40894	0.44882	0.5	0.5	0
0	0	0	0	0	0
0	0	0	0	0	0
0	0	0	0	0	0
0	0	0	0	0	0
0.4604	0.57314	0.5	0.39756	0	0
0	0	0	0	0	0
0	0	0	0	0	0
0	0	0	0	0	0
0	0	0	0	0	0
0	0	0	0	0	0
0	0	0	0	0	0
0	0	0	0	0	0
0	0	0	0	0	0
0	0	0	0	0	0
0	0	0	0	0	0
0	0	0	0	0	0
0	0	0	0	0	0
0	0	0	0	0	0
0	0	0	0	0	0
0	0	0	0	0	0
0	0	0	0	0	0
0	0	0	0	0	0

0 0 0 0 0 0
0 0 0 0 0 0
0 0 0 0 0 0
0 0 0 0 0 0
0 0 0 0 0 0
0 0 0 0 0 0
0 0 0 0 0 0
0 0 0 0 0 0
0 0 0 0 0 0
0 0 0 0 0 0
0 0 0 0 0 0
0 0 0 0 0 0
0 0 0 0 0 0
0 0 0 0 0 0
0 0 0 0 0 0
0
gtSp=0 0 0 0 0 0
0 0 0 0 0 0
0 0 0 0 0 0
0 0 0.58361 0.4788 0.67433 0.51042
0 0 0 0 0 0
0 0 0 0 0 0
0 0 0 1 0.50253 0.5
0.5 0.5 0.48195 0.5 0 0
0 0 0 0 0 0
0 0 0 0 0 0
0 1 1 0.5 0.56522 0.38958
0.49942 0.49827 0.49485 0.48238 0.5 0
0 0 0 0 0 0
0 0 0 0 0 0
0 0 1 1 1 0.26292
0.67893 0.49974 0.5 0.5 0.45309 0.60544
0.53278 0.5 0 0 0 0
0 0 0 0 0 0
0 0 0 0 0 0
1 1 1 1 0.50947 0.5
0.33129 0.50453 0.40589 0.5 0.44034 0.54387
0.5 0.59067 0 0 0 0
0 0 0 0 0 0
0 0 0 0 0 0
1 1 1 1 1 1
0.45416 0.58582 0.45438 0.45654 0.48259 0.52719
0.48486 0.5 0.48677 0.461 0 0
0 0 0 0 0 0
0 0 0 0 0 0
0 0 0 1 1 1
1 1 1 0.47863 0.4201 0.48774
0.56333 0.55407 0.54306 0.5 0.5 0.48184
0.5 0.51926 0 0 0 0
0 0 0 0 0 0
0 0 0 0 0 0
0 1 1 1 1 1

1	1	0.56236	0.50978	0.51619	0.5
0.55075	0.53925	0.48563	0.5	0.51054	0.54597
0.46391	0	0	0	0	0
0	0	0	0	0	0
0	0	0	0	0	0
0	1	1	1	1	1
1	1	0.52896	0.54613	0.46316	0.52329
0.5	0.5	0.5	0.5	0.52869	0.5
0.39895	0	0	0	0	0
0	0	0	0	0	0
0	0	0	0	0	0
0	0	1	1	1	1
1	1	1	0.41305	0.48565	0.47687
0.5	0.47456	0.5	0.5	0.38751	0.5
0.47266	0.49108	0.36016	0	0	0
0	0	0	0	0	0
0	0	0	0	0	0
0	0	0	1	1	1
1	1	1	1	0.40633	0.5
0.5	0.5	0.5	0.52096	0.56609	0.5
0.52644	0.5	0.50129	0.5	0	0
0	0	0	0	0	0
0	0	0	0	0	0
0	0	0	0	1	1
1	1	1	1	1	1
1	0.50231	0.5	0.39759	0.51084	0.37014
0.45526	0.45273	0.5	0.5	0.59971	0.52332
0	0	0	0	0	0
0	0	0	0	0	0
0	0	0	0	0	0
1	1	1	1	1	1
1	1	1	0.54277	0.46543	0.3979
0.25143	0.34095	0.33818	0.34804	0.34759	0.41356
0.33028	0.34264	0	0	0	0
0	0	0	0	0	0
0	0	0	0	0	0
0	0	1	1	1	1
1	1	1	1	1	0.45042
0.33333	0.36269	0.36828	0.29528	0.37853	0.3027
0.41857	0.3641	0.33333	0.22338	0	0
0	0	0	0	0	0
0	0	0	0	0	0
0	0	0	0	1	1
1	1	1	1	1	0.5
0.55708	0.63489	0.33068	0.3427	0.32056	0.34187
0.29	0.38578	0.32795	0.34321	0.32756	0.34915
0	0	0	0	0	0
0	0	0	0	0	0
0	0	0	0	0	0
1	1	1	1	1	1
0.52702	0.51477	0.5	0.47975	0.49873	0.42759
0.33763	0.33266	0.3541	0.3605	0.34019	0.35153

0.34197	0.37289	0	0	0	0
0	0	0	0	0	0
0	0	0	0	0	0
0	0	1	1	1	1
1	0.56044	0.5	0.4931	0.53607	0.43977
0.34468	0.3182	0.36416	0.35855	0.35146	0.38613
0.33857	0.37155	0.33333	0.35873	0	0
0	0	0	0	0	0
0	0	0	0	0	0
0	0	0	0	1	1
1	0.5	0.5	0.5	0.53689	0.5
0.58611	0.4523	0.52144	0.38762	0.34329	0.35671
0.37773	0.25798	0.33936	0.39953	0.27388	0.29798
0	0	0	0	0	0
0	0	0	0	0	0
0	0	0	0	0	0
1	0.52467	0.60867	0.5	0.44755	0.5
0.47481	0.5	0.5	0.49194	0.33333	0.33426
0.38022	0.32897	0.35836	0.3597	0.34003	0.33723
0.33333	0	0	0	0	0
0	0	0	0	0	0
0	0	0	0	0	0
0	0.5	0.5	0.5	0.4735	0.5
0.39135	0.45997	0.58095	0.5	0.64121	0.38265
0.21058	0.22887	0.33333	0.33333	0.29802	0.34903
0.26656	0.36827	0	0	0	0
0	0	0	0	0	0
0	0	0	0	0	0
0	0	0.58323	0.5	0.5	0.57358
0.51274	0.43258	0.46386	0.38261	0.39135	0.51984
0.28447	0.41623	0.17938	0.38302	0.33596	0.36312
0.36136	0.39088	0	0	0	0
0	0	0	0	0	0
0	0	0	0	0	0
0	0	0	0.49014	0.63912	0.4864
0.5	0.51233	0.47078	0.45249	0.54189	0.4541
0.5	0.34205	0.30961	0.29145	0.31758	0.35952
0.38799	0.39903	0.28634	0	0	0
0	0	0	0	0	0
0	0	0	0	0	0
0	0	0	0	0	0.48884
0.47652	0.5	0.49853	0.51872	0.5	0.47001
0.49873	0.5	0.25693	0.29263	0.33333	0.30508
0.35469	0.35128	0	0	0	0
0	0	0	0	0	0
0	0	0	0	0	0
0	0	0	0	0	0
0.31759	0.49124	0.42279	0.34646	0.5	0.5
0.55622	0.58505	0.55213	0.39699	0.2726	0.31915
0	0	0	0	0	0
0	0	0	0	0	0
0	0	0	0	0	0

0 0 0 0 0 0
0 0 0.53851 0.5 0.61828 0.49665
0.45346 0.50877 0.46668 0.51778 0 0
0 0 0 0 0 0
0 0 0 0 0 0
0 0 0 0 0 0
0 0 0 0 0 0
0 0 0 0.52338 0.5 0.56725
0.5 0.53193 0 0 0 0
0 0 0 0 0 0
0 0 0 0 0 0
0 0 0 0 0 0
0 0 0 0 0 0
0 0 0 0 0 0.5
0.43636 0 0 0 0 0
0 0 0 0 0 0
0 0 0 0 0 0
0 0 0 0 0 0
0 0 0 0 0 0
0 0 0 0 0 0
0 0 0 0 0 0
0 0 0 0 0 0
0 0 0 0 0 0
0 0 0 0 0 0
0 0 0 0 0 0
0 0 0 0 0 0
0 0 0 0 0 0
0 0 0 0 0 0
0 0 0 0 0 0
0 0 0 0 0 0
0 0 0 0 0 0
0 0 0 0 0 0
0 0 0 0 0 0
0 0 0 0 0 0
0 0 0 0 0 0
0 0 0 0 0 0
0 0 0 0 0 0
0 0 0 0 0 0
0 0 0 0 0 0
0 0 0 0 0 0
0 0 0 0 0 0
0 0 0 0 0 0
0 0 0 0 0 0
0 0 0 0 0 0
0 0 0 0 0 0
0 0 0 0 0 0
0 0 0 0 0 0
0 0 0 0 0 0
0 0 0 0 0 0
0 0 0 0 0 0
0 0 0 0 0 0
0 0 0 0 0 0

0	0	0	0	0	0
0	0	0	0	0	0
0	0	0	0	0	0
0	0	0	0	0	0
0	0	0	0	0	0
0	0	0	0	0	0
0	0	0	0	0	0
0	0	0	0	0	0
0	0	0	0	0	0
0	0	0	0	0	0
0	0	0	0	0	0
0	0	0	0	0	0
0	0	0	0	0	0
0	0	0	0	0	0
0	0	0	0	0	0
0	0	0	0	0	0
0	0	0	0	0	0
0	0	0	0	0	0
0	0	0	0	0	0
0					

gtTCf=
0
0.11925
0.14534
0.13574
0.11593
0.096197
0.079948
0.067704
0.058926
0.052819
0.048646
0.045817
0.043898
0.042582
0.041662
0.040998
0.040498
0.040102

gtTCs=
0
0.07155
0.116
0.14393
0.16177
0.17342
0.18126
0.18671
0.19067
0.19366

0.19601
0.19792
0.19952
0.20088
0.20206
0.20309
0.20398
0.20476

gtTCp=
0
2.385
1.5979
1.1196
0.8287
0.65143
0.54309
0.47655
0.43538
0.4096
0.39316
0.38239
0.37506
0.36982
0.36585
0.36266
0.35995
0.35753

X_mask=
0.0016599,0.00053591,2.2204e-016,0.0013802,0.00064615,2.2204e-
016,0.0012221,2.2204e-016,0.0033074,8.0669e-
005,0.0013836,0.0022243,2.2204e-016,2.2204e-016,2.2204e-016,2.2204e-
016,2.2204e-016,2.2204e-016,0.00083093,0.00034843,2.2204e-
016,0.0041995,0.00026723,2.2204e-
016,0.00076709,0.00045776,0.0021593,2.2204e-
016,0.00083284,0.00092762,2.2204e-016,0.0015764,2.2204e-016,2.2204e-
016,0.0015715,2.2204e-016,2.2204e-016,2.2204e-016,2.2204e-016,2.2204e-
016,0.013316,2.2204e-016,0.0058248,2.2204e-
016,0.012708,0.011764,2.2204e-016,2.2204e-
016,0.001792,0.0038863,0.0038809,0.0011248,2.2204e-016,2.2204e-
016,0.0014345,0.00098871,0.00066243,2.6545e-006,2.2204e-
016,0.0017367,0.0022284,0.0056222,2.2204e-
016,0.001165,0.012957,0.013303,0.00019873,0.0055139,2.2204e-
016,2.2204e-016,2.2204e-016,2.2204e-016,0.0020189,0.0016679,2.2204e-
016,0.0016775,0.0013392,0.00015096,2.2204e-016,2.2204e-
016,0.0023372,2.2204e-016,2.2204e-016,2.2204e-016,2.2204e-
016,0.0018207,2.2204e-016,2.2204e-016,2.2204e-016,2.2204e-016,2.2204e-
016,0.014742,0.01628,0.020502,0.0071364,0.0044811,2.2204e-016,2.2204e-
016,0.0033338,2.2204e-016,0.00033777,2.2204e-016,0.0029297,2.2204e-
016,0.00090863,2.2204e-
016,0.0013928,0.00084417,0.0013951,0.00090933,2.2204e-016,2.2204e-

016,2.2204e-016,2.2204e-016,0.006822,2.2204e-016,2.2204e-016,2.2204e-
016,2.2204e-016,0.0088954,0.00012181,2.2204e-016,0.00078376,2.2204e-
016,0.0080258,2.2204e-
016,0.0082285,0.0012485,0.0014024,0.0020031,2.2204e-
016,0.0034176,2.2204e-016,0.0016557,2.2204e-016,0.00090817,2.2204e-
016,2.2204e-016,2.2204e-016,0.00024975,0.0014194,0.0027616,2.2204e-
016,2.2204e-016,2.2204e-016,2.2204e-016,0.015439,0.022325,2.2204e-
016,2.2204e-
016,0.00035517,0.0081728,0.0035599,0.015382,0.0043615,2.2204e-
016,2.2204e-
016,0.00247,0.0095688,0.0049214,0.0027007,0.0018162,0.0011167,0.000180
05,2.2204e-016,2.2204e-016,0.0020488,2.2204e-016,0.00066582,2.2204e-
016,0.00088779,2.2204e-016,0.00029,2.2204e-016,0.0011601,2.2204e-
016,8.3242e-005,2.2204e-016,2.2204e-016,2.2204e-
016,0.0089931,0.002421,0.016835,2.2204e-016,2.2204e-
016,0.0058525,2.2204e-016,0.0063223,0.0063613,2.2204e-016,2.2204e-
016,0.0030047,2.2204e-016,2.2204e-016,2.2204e-016,2.2204e-
016,0.00071886,2.2204e-016,2.2204e-016,2.2204e-016,2.2204e-
016,0.0016576,0.00027313,0.0013882,2.2204e-016,2.2204e-016,2.2204e-
016,2.2204e-016,2.2204e-016,2.2204e-016,0.0011663,2.2204e-016,2.2204e-
016,0.0025352,0.0053811,2.2204e-016,0.0005556,2.2204e-
016,0.0056408,2.2204e-016,0.00037688,2.2204e-016,0.020583,2.2204e-
016,2.2204e-016,2.2204e-
016,0.0049005,0.0001702,0.0075996,0.0005126,0.0028762,2.2204e-
016,2.2204e-016,2.2204e-016,0.00057733,0.00019306,0.0010293,2.2204e-
016,2.2204e-016,2.2204e-016,2.2204e-016,2.1119e-005,8.3838e-
005,2.2204e-016,2.2204e-016,5.7909e-005,2.2204e-016,2.2204e-
016,0.018916,0.013626,0.0026598,0.013897,0.0042217,0.012641,2.2204e-
016,0.0031335,2.2204e-016,0.005003,0.0082438,2.2204e-016,2.2204e-
016,2.2204e-016,2.2204e-016,2.2204e-016,2.2204e-016,2.2204e-
016,0.0010134,2.2204e-016,0.00029789,2.2204e-
016,0.0037899,0.00085128,2.2204e-016,0.0032725,2.2204e-016,2.2204e-
016,2.2204e-016,0.0013784,0.00080954,2.2204e-
016,0.0020163,0.00057737,2.2204e-
016,0.0017622,0.0041007,0.016603,0.0012112,0.016253,2.2204e-
016,2.2204e-016,2.2204e-016,2.2204e-016,2.2204e-016,2.2204e-
016,0.00031072,0.0073912,0.00074428,2.2204e-016,2.2204e-016,2.2204e-
016,0.013268,2.2204e-016,2.2204e-016,2.2204e-016,2.2204e-
016,0.00087355,0.004297,2.2204e-016,0.00099377,6.7495e-005,2.2204e-
016,0.00063241,2.2204e-016,2.2204e-016,2.2204e-
016,0.0010735,0.00087063,0.00035193,2.2204e-
016,0.0014963,0.00014409,2.2204e-016,0.0049422,2.2204e-
016,0.0053661,2.2204e-016,0.0040549,0.011134,2.2204e-
016,0.00018146,0.0086139,2.2204e-
016,0.017418,0.0022577,0.016951,0.012074,0.0038405,2.2204e-
016,2.2204e-016,2.2204e-
016,0.001917,0.00081305,0.00094645,0.00049152,0.0010719,0.00020104,0.0
008248,0.0018404,2.2204e-016,2.2204e-016,2.2204e-
016,0.00051576,2.2204e-016,2.2204e-016,2.2204e-016,2.2204e-
016,0.0027283,0.00049053,0.0082018,0.0052535,0.013868,0.0062471,2.2204
e-016,0.017813,0.02903,0.014247,0.0057897,2.2204e-016,2.2204e-
016,2.2204e-

016,0.020273,0.0053371,0.0095003,0.0060488,0.0062481,2.2204e-
016,2.2204e-016,2.2204e-016,0.0020817,0.0001894,0.00023385,2.2204e-
016,0.00031484,2.2204e-016,0.0015658,0.002027,2.2204e-016,2.2204e-
016,2.2204e-016,0.00096975,2.2204e-016,2.2204e-016,2.2204e-
016,2.2204e-016,0.00055426,0.00021082,2.2204e-016,0.014138,2.2204e-
016,2.2204e-016,0.00074934,0.019286,0.0088076,2.2204e-016,2.2204e-
016,2.2204e-016,0.00078755,0.0041624,0.00051792,2.2204e-
016,0.0051734,0.014314,2.2204e-016,2.2204e-016,2.2204e-
016,0.001714,2.2204e-016,1.314e-005,2.2204e-016,0.0010173,2.2204e-
016,2.2204e-016,0.00022161,2.2204e-016,0.0019053,2.2204e-
016,0.00047568,0.00079835,2.2204e-016,2.2204e-016,0.0011016,2.2204e-
016,2.2204e-016,0.00016422,2.2204e-016,2.2204e-016,2.2204e-
016,0.0051423,2.2204e-016,2.2204e-016,2.2204e-016,2.2204e-
016,0.019785,0.0031723,0.013103,0.0039969,2.2204e-
016,0.0030066,0.0034904,0.003156,0.0094208,0.0048364,0.0046772,2.2204e
-016,2.2204e-016,2.2204e-016,2.2204e-016,2.2204e-016,2.2204e-
016,0.00033699,0.00099899,5.827e-006,0.00065479,0.00097336,2.2204e-
016,0.00011365,2.2204e-016,2.2204e-
016,0.0018324,0.0011726,0.00061102,2.2204e-016,2.2204e-016,2.2204e-
016,0.018539,2.2204e-016,0.011272,2.2204e-016,2.2204e-
016,0.0042614,2.2204e-016,2.2204e-
016,0.006765,0.0013726,0.0010393,2.2204e-016,0.00020463,2.2204e-
016,2.2204e-016,2.2204e-016,7.186e-005,0.0074521,0.0010699,2.2204e-
016,2.2204e-016,0.00079194,0.00076143,8.1745e-005,2.2204e-016,2.2204e-
016,0.0015284,0.00068906,2.2204e-016,0.00076583,2.2204e-016,2.2204e-
016,2.2204e-016,0.0013407,2.2204e-016,0.00066178,0.010145,2.2204e-
016,0.022845,2.2204e-016,0.023352,2.2204e-016,0.0043699,2.2204e-
016,0.007862,2.2204e-016,2.2204e-016,2.2204e-016,0.0047313,2.2204e-
016,0.0040842,0.0037404,2.2204e-016,2.2204e-
016,0.0007655,0.0045137,2.2204e-
016,0.0006149,0.0002978,0.00049434,2.2204e-016,2.2204e-
016,0.00063791,2.2204e-016,0.00048428,0.00044404,0.00030653,2.2204e-
016,2.2204e-
016,0.00069562,0.001218,0.0010288,0.00030135,0.00013185,2.2204e-
016,2.2204e-016,0.025946,2.2204e-016,2.2204e-016,2.2204e-
016,0.01562,2.2204e-016,2.2204e-016,0.00023208,2.2204e-016,2.2204e-
016,2.2204e-016,2.2204e-016,2.2204e-016,0.012826,2.2204e-
016,0.0032692,0.0051265,2.2204e-016,0.00087371,2.2204e-
016,0.00058195,2.2204e-016,2.2204e-
016,0.00021538,0.0019059,0.0016671,0.0004064,2.2204e-
016,0.00075413,0.00080621,2.2204e-016,0.00043726,2.2204e-
016,0.0023786,0.00077205,0.00039523,2.2204e-
016,0.015727,0.0066993,2.2204e-016,2.2204e-
016,0.0071834,0.00018354,0.011031,2.2204e-
016,0.0043561,0.019818,0.014226,0.0084291,2.2204e-016,2.2204e-
016,0.00045975,2.2204e-016,0.0012395,2.2204e-
016,0.0010177,0.0012061,0.00098715,2.2204e-016,2.2204e-016,2.2204e-
016,0.00013269,2.2204e-016,2.2204e-016,0.0012018,0.0012647,2.2204e-
016,0.0021351,2.2204e-016,2.2204e-016,0.0005463,2.2204e-
016,0.00046531,2.2204e-016,2.2204e-016,2.2204e-016,2.2204e-
016,2.2204e-016,2.2204e-016,0.0085129,2.2204e-
016,0.0054039,0.007479,2.2204e-016,2.2204e-016,2.2204e-

016,0.0016671,2.2204e-016,0.0057051,2.2204e-
016,0.001776,0.00030869,2.2204e-016,2.2204e-
016,0.0015114,0.000672,2.2204e-016,0.00072695,2.2204e-
016,0.00080655,2.2204e-016,2.2204e-016,2.2204e-016,0.00088732,2.2204e-
016,2.2204e-016,0.00038093,0.00074384,0.0011924,2.2204e-
016,0.0011019,2.2204e-
016,0.020567,0.01375,0.0037154,0.01513,0.014715,2.2204e-016,2.2204e-
016,0.0015972,0.0037812,0.0093137,2.2204e-
016,0.00049369,0.0010827,0.0019242,2.2204e-016,2.2204e-
016,0.0052334,2.2204e-016,0.00061283,0.0006103,2.2204e-
016,0.001224,2.2204e-016,0.0023932,0.0014165,0.001575,2.2204e-
016,2.2204e-016,0.00097647,0.00099387,2.2204e-
016,0.00119,0.0005864,2.2204e-016,2.2204e-016,2.5178e-005,2.2204e-
016,2.2204e-016,0.00080466,2.2204e-016,2.2204e-016,2.2204e-
016,2.2204e-016,0.0031851,0.0037,2.2204e-016,2.2204e-
016,0.0010937,0.0033564,0.00032306,2.2204e-016,2.2204e-016,2.2204e-
016,0.0018675,2.2204e-016,0.00096355,0.001352,2.2204e-
016,0.0010375,2.2204e-016,0.00025194,2.2204e-
016,0.00093249,0.00042613,2.2204e-016,0.00029571,0.0014835,2.2204e-
016,2.2204e-
016,0.00018538,0.0002274,0.00072973,0.0017642,0.010254,2.2204e-
016,2.2204e-016,2.2204e-016,0.0058303,0.00021736,0.0031303,2.2204e-
016,2.2204e-016,0.0027574,2.2204e-016,0.0070111,2.2204e-
016,0.0010726,0.0083666,2.2204e-016,0.0024433,0.0015851,2.2204e-
016,2.2204e-016,2.2204e-016,2.2204e-016,2.2204e-
016,0.00097328,2.2204e-016,2.2204e-016,2.2204e-016,2.2204e-
016,2.2204e-016,0.00081483,0.00043616,2.2204e-016,0.00024867,2.2204e-
016,0.00047562,2.2204e-016,0.00046015,2.2204e-016,2.2204e-
016,0.0012986,2.2204e-016,0.0051979,0.0025401,2.2204e-016,2.2204e-
016,2.2204e-016,2.2204e-016,2.2204e-016,0.005269,0.0011287,2.2204e-
016,2.2204e-016,0.008103,2.2204e-016,0.0017496,0.00034043,2.2204e-
016,0.00015038,0.00051563,2.2204e-016,2.2204e-016,4.4429e-
005,0.0013412,2.2204e-016,3.0457e-005,2.2204e-016,2.2204e-016,2.2204e-
016,2.2204e-016,0.00056842,2.2204e-016,0.0016315,2.2204e-016,2.2204e-
016,2.2204e-016,2.2204e-016,0.001926,0.0023305,0.011672,2.2204e-
016,2.2204e-016,2.2204e-016,2.2204e-016,0.0074714,2.2204e-
016,0.00089792,0.0036766,0.00041054,2.2204e-016,0.0011485,4.4997e-
005,2.2204e-
016,0.00015046,0.00020291,0.00075996,0.0011392,0.0006963,2.2204e-
016,2.2204e-016,2.2204e-016,2.2204e-016,2.2204e-016,2.2204e-
016,2.2204e-016,2.2204e-016,0.00010001,9.6309e-005,2.2204e-
016,0.00104,0.00056155,2.2204e-016,2.2204e-016,0.0002682,2.2204e-
016,2.2204e-016,2.2204e-016,0.013738,2.2204e-
016,0.0020102,0.0026568,2.2204e-016,2.2204e-016,2.2204e-
016,0.0017943,0.00047021,2.2204e-016,0.0013236,2.2204e-016,2.2204e-
016,2.2204e-016,2.2204e-016,2.2204e-016,2.2204e-016,2.2204e-
016,2.2204e-016,0.0011465,0.00068743,2.2204e-016,0.0008553,2.2204e-
016,0.0012169,2.2204e-016,2.2204e-016,2.2204e-016,2.2204e-016,2.2204e-
016,2.2204e-016,2.2204e-016,2.2204e-016,0.0018678,0.01777,2.2204e-
016,2.2204e-016,0.012507,2.2204e-016,2.2204e-016,2.2204e-
016,0.0011452,0.00022593,0.00064105,2.2204e-
016,0.00016078,0.0004506,0.001466,0.00074469,2.2204e-016,2.2204e-

016,2.2204e-016,0.0011723,2.2204e-016,0.0012845,0.00045349,2.2204e-
016,0.00066962,0.00082052,0.00029555,0.0010336,0.0004947,0.0008115,0.0
0035743,2.2204e-016,0.0022946,2.2204e-016,2.2204e-
016,0.0017985,0.0018889,2.2204e-016,2.2204e-016,2.2204e-016,2.2204e-
016,0.0015852,0.0013611,2.2204e-016,0.00060614,2.2204e-016,2.2204e-
016,0.00058748,2.2204e-016,0.00010659,0.0002259,2.2204e-016,2.2204e-
016,2.2204e-016,0.00020032,0.00049596,0.00018104,2.2204e-016,2.2204e-
016,2.2204e-016,2.2204e-016,0.0015547,0.0012853,0.001179,2.2204e-
016,0.00058059,0.00081942,0.0010344,0.00053769,2.2204e-016,2.2204e-
016,2.2204e-016,2.2204e-016,2.2204e-016,2.2204e-
016,0.0013225,0.0020407,2.2204e-016,0.00056968,2.2204e-
016,0.00093384,0.00076685,0.00091591,2.2204e-016,0.0011272,2.2204e-
016,2.2204e-016,2.2204e-016,0.0028121,0.0018599,2.2204e-
016,0.00083829,0.00048274,2.2204e-016,0.0015249,2.2204e-016,2.2204e-
016,0.001643,2.2204e-016,0.001116,0.0011451,0.00088964,2.2204e-
016,2.2204e-016,0.0026573,0.0025298,0.00076189,2.2204e-
016,0.00087439,0.0014026,2.2204e-016,0.0035899,2.2204e-016,2.2204e-
016,2.2204e-016,6.9884e-005,2.2204e-016,0.00053929,2.2204e-
016,2.2204e-016,1.4188e-005,2.2204e-016,2.2204e-016,2.2204e-
016,0.0016938,2.2204e-016,0.0010462,2.2204e-016,0.00015791,2.2204e-
016,0.00026239,2.2204e-016,0.00063015,2.2204e-016,2.2204e-
016,0.00036208,0.0012371,2.2204e-
016,0.00045699,0.00069057,0.00043776,2.2204e-016,2.2204e-
016,0.0021313,2.2204e-016,2.2204e-016,2.2204e-
016,0.00066413,0.0011478,0.0013488,2.2204e-016,2.2204e-
016,0.00031331,0.0010662,9.48e-005,2.2204e-016,2.2204e-016,2.2204e-
016,2.2204e-016,2.2204e-016,0.0027278,2.2204e-
016,0.00022928,0.0017742,2.2204e-016,0.0013307,2.2204e-
016,0.0013021,0.0013911,0.0027009,2.2204e-016,2.2204e-016,2.2204e-
016,2.2204e-016,0.0013553,0.0002906,2.2204e-016,0.00058154,8.9369e-
005,0.0001541,2.2204e-016,0.001228,0.0014242,0.00081578,2.2204e-
016,0.0010068,0.00018768,2.2204e-016,2.2204e-016,2.2204e-
016,0.0011953,0.00082488,2.2204e-016,2.2204e-016,2.2204e-016,2.2204e-
016,2.2204e-016,0.00077831,0.00069343,2.2204e-016,0.0017637,2.2204e-
016,0.00074971,0.002757,2.2204e-016,0.001759,2.2204e-016,2.2204e-
016,0.0012767,2.2204e-
016,0.00062808,0.00090048,0.00047944,0.0024312,0.0018098,0.00086736,2.
2204e-016,2.2204e-016,2.2204e-016,2.2204e-
016,0.0017041,0.0035742,0.00079468,2.2204e-
016,0.0016155,0.00014175,2.2204e-016,2.2204e-016,2.2204e-
016,0.0022554,0.00021823,0.0010306,2.2204e-016,0.001131,2.2204e-
016,0.0005843,2.2204e-016,0.0012471,2.2204e-016,2.2204e-016,2.2204e-
016,2.2204e-016,2.2204e-016,0.00054787,2.2204e-016,0.0002591,2.2204e-
016,6.5411e-005,2.2204e-016,2.2204e-016,0.00070506,2.2204e-
016,0.0015124,0.0015397,2.2204e-016,2.2204e-016,2.2204e-
016,0.0019393,0.0035012,0.0022766,2.2204e-016,2.2204e-016,2.2204e-
016,2.2204e-016,0.00099208,0.00015983,0.00019842,0.00037721,2.2204e-
016,0.00055021,0.00091585,2.2204e-016,2.2204e-016,2.2204e-016,2.2204e-
016,2.2204e-016,0.0011222,2.2204e-016,2.2204e-016,2.2204e-016,2.2204e-
016,0.00031097,0.0012355,2.2204e-016,0.0001535,5.2112e-005,2.2204e-
016,2.2204e-016,2.2204e-016,0.0029634,0.00038156,2.2204e-016,2.2204e-
016,0.0016916,0.00094225,2.2204e-016,2.2204e-016,2.2204e-

016,0.0013308,0.00091046,2.2204e-016,0.0017085,2.2204e-
016,0.00068849,2.2204e-016,0.00055412,0.0017563,2.2204e-016,2.2204e-
016,0.00040869,0.0013134,0.001159,2.2204e-
016,0.0023062,0.00012278,0.0017712,0.00059091,0.00063997,2.2204e-
016,0.00045339,2.2204e-016,0.00047943,2.2204e-016,2.2204e-016,2.2204e-
016,0.00017749,0.0015514,2.2204e-016,2.2204e-
016,0.00022606,0.00077262,0.0018478,0.00017783,0.0030552,0.0039242,0.0
0017381,0.00046101,0.0021765,2.2204e-016,2.2204e-
016,0.0015992,2.2204e-016,2.2204e-016,0.0022136,2.2204e-
016,0.00089893,0.00019742,2.2204e-016,2.2204e-
016,0.0010495,0.0015481,2.2204e-016,0.00030384,0.0027268,2.2204e-
016,0.00015345,0.0019068,0.00059329,0.00067801,2.2204e-016,2.2204e-
016,0.00073036,2.2204e-016,0.00064672,2.2204e-016,2.2204e-
016,0.00048347,2.2204e-016,2.2204e-016,2.2204e-016,0.00070487,2.2204e-
016,2.2204e-016,2.2204e-016,2.2204e-
016,0.0019054,0.00094676,0.0007647,0.0025078,0.00097461,0.00033605,0.0
014338,0.0021169
0.073304,0.071256,0.070083,0.075148,0.072426,0.070514,0.071997,0.07068
3,0.074673,0.070685,0.071731,0.07336,0.070665,0.069506,0.071001,0.0707
71,0.068972,0.071791,0.074091,0.071969,1.4308,1.1799,1.6357,1.2361,0.0
70793,0.07264,0.074354,0.071171,0.072167,0.072001,0.070617,0.071791,0.
069702,0.070125,0.073608,0.068729,0.068937,0.068877,0.071923,2.3657,1.
2474,1.2274,1.2303,1.2253,1.2012,1.2428,0.068885,0.071612,0.072258,0.0
74936,0.075015,0.072649,0.070041,0.071794,0.074243,0.070785,0.071737,0
.071014,0.070754,0.073852,0.072405,2.3911,2.3714,1.2325,1.3936,0.98199
,1.2308,1.2295,1.2101,1.1796,1.2022,0.070461,0.072689,0.07248,0.070577
,0.073426,0.073317,0.070598,0.069994,0.072029,0.07359,0.070157,0.07033
9,0.070419,0.070368,0.072169,2.3648,2.3683,2.3745,0.67283,1.6324,1.238
6,1.2419,1.2438,1.1287,1.48,1.2964,1.2231,0.075623,0.070639,0.072134,0
.069276,0.074922,0.072002,0.071349,0.071657,0.072779,0.073389,0.072652
,0.072419,0.072138,0.069914,0.068843,0.069633,2.3923,2.3701,2.3827,2.3
855,1.2391,1.2319,0.8379,1.2367,1.0103,1.2128,1.1031,1.3219,1.2391,1.4
35,0.07203,0.072976,0.071132,0.074421,0.06995,0.073495,0.070738,0.0726
72,0.07065,0.070804,0.072662,0.072104,0.072094,0.073527,0.06938,0.0685
45,2.3678,2.3695,2.3994,2.4156,2.3658,2.3628,1.1188,1.4434,1.118,1.139
9,1.192,1.294,1.1767,1.2323,1.2052,1.1424,0.075204,0.072301,0.072184,0
.072018,0.06808,0.069208,0.072787,0.071157,0.072767,0.071867,0.073014,
0.069756,0.073077,0.070733,0.073593,0.068414,0.071173,2.3816,2.3788,2.
3471,2.4022,2.3838,2.4004,1.174,1.0368,1.2056,1.3673,1.3648,1.3343,1.2
19,1.2242,1.1914,1.2186,1.2655,0.070497,0.07044,0.071217,0.070228,0.07
02,0.068864,0.069439,0.07301,0.070956,0.073836,0.070971,0.069606,0.068
981,0.092943,0.098858,0.09225,0.073338,2.3607,2.3822,2.3851,2.3821,2.3
72,2.3877,2.3933,1.3696,1.2283,1.2618,1.223,1.3602,1.3113,1.1775,1.224
7,1.2549,1.345,1.1535,0.073182,0.074141,0.070127,0.069152,0.072463,0.0
71945,0.072312,0.072852,0.069075,0.070995,0.069238,0.099596,0.09478,0.
094954,0.094482,0.094449,0.09397,0.071445,2.3589,2.4108,2.4147,2.3916,
2.3889,2.3878,2.3914,1.294,1.34,1.1405,1.2863,1.2378,1.21,1.2141,1.212
2,1.2988,1.2268,0.99151,0.069753,0.073321,0.070946,0.071659,0.069582,0
.074933,0.073768,0.070771,0.074292,0.094376,0.094369,0.09059,0.097288,
0.095722,0.094535,0.097437,0.094173,0.095878,0.073523,2.3889,2.4005,2.
3954,2.4009,2.3715,2.3636,2.3722,1.0152,1.1878,1.1715,1.226,1.1771,1.2
245,1.228,0.96268,1.232,1.1803,1.2007,0.90064,0.069813,0.070618,0.0731

69,0.075872,0.070646,0.073033,0.097329,0.094321,0.094031,0.09532,0.088
553,0.093708,0.099469,0.096581,0.095736,0.0892,0.09612,0.095886,2.3817
,2.3832,2.3734,2.3944,2.3608,2.3991,2.3949,1.0132,1.2276,1.2412,1.2226
,1.2459,1.278,1.394,1.2419,1.2942,1.2252,1.222,1.2204,0.074407,0.07346
6,0.07265,0.098017,0.091732,0.098243,0.099464,0.096791,0.094289,0.0954
83,0.094844,0.095837,0.094389,0.095065,0.088966,0.092622,0.090537,0.09
414,2.3995,2.3875,2.4055,2.3963,2.3682,2.413,2.4141,2.389,2.3893,1.234
9,1.2247,0.98823,1.2744,0.9381,1.1359,1.1174,1.2307,1.2194,1.4501,1.27
18,0.097481,0.095774,0.096425,0.095729,0.095992,0.094179,0.099156,0.09
7189,0.094205,0.094981,0.098911,0.096301,0.093966,0.09412,0.095504,0.1
0049,0.098276,0.094703,2.3817,2.4193,2.3745,2.3632,2.3979,2.3921,2.397
3,2.3738,2.3583,1.3286,1.1469,1.0129,0.67106,0.87751,0.87309,0.90241,0
.88265,1.0314,0.84751,0.87898,0.092601,0.091858,0.097249,0.10108,0.094
029,0.088307,0.094023,0.094933,0.095,0.1001,0.096572,0.096796,0.094688
,0.094207,0.095054,0.09527,0.094803,0.096179,2.3638,2.3786,2.3828,2.39
86,2.3953,2.3645,2.3762,2.3649,2.3986,1.1416,0.86744,0.92796,0.93765,0
.77477,0.95735,0.78815,1.0624,0.93993,0.8653,0.60273,0.09474,0.092899,
0.091931,0.09367,0.096933,0.090311,0.09032,0.095903,0.091974,0.096318,
0.095252,0.094152,0.09115,0.097793,0.10111,0.096036,0.099098,0.094641,
2.3816,2.3544,2.4005,2.3839,2.3995,2.3794,2.367,1.2583,1.3734,1.542,0.
85864,0.88409,0.83036,0.87218,0.76312,0.97921,0.84176,0.87275,0.84273,
0.90544,0.0961,0.094992,0.091831,0.098058,0.096085,0.095394,0.092997,0
.089512,0.097156,0.095835,0.097597,0.095499,0.095307,0.094664,0.096123
,0.099242,0.094639,0.092878,2.4004,2.3564,2.4106,2.387,2.3949,2.3754,1
.3149,1.2784,1.2673,1.2066,1.2333,1.0602,0.87388,0.85176,0.91372,0.918
66,0.87297,0.90108,0.88153,0.95518,0.095018,0.096212,0.095921,0.095442
,0.097069,0.094266,0.097388,0.09542,0.094594,0.093179,0.090859,0.09526
9,0.095634,0.098423,0.093198,0.094089,0.09969,0.09464,2.3554,2.374,2.4
193,2.373,2.3735,1.3733,1.263,1.2351,1.3245,1.1202,0.89817,0.81241,0.9
211,0.92083,0.90126,0.99683,0.86104,0.94893,0.8623,0.91425,0.091925,0.
097976,0.09176,0.094736,0.10049,0.095454,0.098428,0.092625,0.1,0.09573
6,0.099269,0.096414,0.095745,0.095994,0.093061,0.094921,0.097799,0.095
14,2.3937,2.3813,2.3953,1.2442,1.2539,1.2573,1.3344,1.2609,1.4393,1.14
61,1.3201,0.99653,0.8869,0.91076,0.95404,0.68556,0.86792,1.0185,0.7176
,0.77844,0.097478,0.096519,0.097983,0.094414,0.097856,0.0939,0.090324,
0.092385,0.096441,0.099455,0.093551,0.097331,0.092736,0.090702,0.09779
3,0.094573,0.095349,0.094534,2.3769,1.2986,1.4876,1.2432,1.1292,1.2616
,1.1943,1.254,1.2579,1.2345,0.85634,0.85683,0.96579,0.84754,0.91564,0.
90995,0.87668,0.86815,0.85603,0.094396,0.097096,0.095618,0.094604,0.09
7895,0.096876,0.097756,0.095141,0.093804,0.093745,0.096016,0.094837,0.
096803,0.093103,0.088224,0.097551,0.097806,0.12,1.2429,1.2729,1.2607,1
.1896,1.2668,1.0167,1.1557,1.4254,1.2512,1.579,0.97267,0.57316,0.62018
,0.86266,0.85959,0.77392,0.88648,0.71142,0.93736,0.096457,0.096352,0.0
92771,0.096864,0.095373,0.10578,0.096331,0.099757,0.092454,0.096312,0.
093667,0.096437,0.094226,0.095248,0.09606,0.09502,0.094778,0.11828,1.4
379,1.2357,1.2471,1.4095,1.2744,1.0997,1.1571,0.98846,1.0117,1.2955,0.
73733,1.0478,0.50655,0.98382,0.86295,0.92251,0.91164,0.99148,0.095341,
0.096202,0.096857,0.095401,0.096868,0.098336,0.090041,0.093836,0.09537
9,0.095809,0.092861,0.095794,0.09622,0.094694,0.097181,0.095314,0.0968
24,0.095941,0.12013,1.2455,1.5568,1.2084,1.2462,1.2871,1.1839,1.1527,1
.3258,1.1359,1.2589,0.88136,0.81547,0.76173,0.82864,0.91974,0.98637,1.
0189,0.74937,0.091115,0.094869,0.094637,0.094948,0.093699,0.087981,0.0

96588,0.093285,0.094504,0.09344,0.093828,0.097537,0.09968,0.097158,0.0
99014,0.096364,0.092326,0.11802,0.11964,0.11828,1.2287,1.2023,1.2384,1
.2533,1.3029,1.2556,1.1855,1.237,1.2443,0.68235,0.77432,0.86046,0.7985
5,0.91001,0.90387,0.10081,0.09642,0.093992,0.095004,0.095652,0.096128,
0.097337,0.09246,0.099219,0.093648,0.09568,0.098935,0.094607,0.096202,
0.098433,0.097368,0.096521,0.094103,0.12054,0.11838,0.11691,0.11866,0.
83354,1.2345,1.0788,0.91635,1.2409,1.2437,1.3837,1.4351,1.3874,0.99527
,0.71286,0.82688,0.091159,0.097696,0.10179,0.097997,0.090676,0.091572,
0.089619,0.09895,0.095337,0.095951,0.090618,0.091311,0.095085,0.089255
,0.094387,0.094104,0.094024,0.091987,0.10001,0.096341,0.11835,0.12056,
0.11964,0.11907,0.11815,0.12007,1.3189,1.2474,1.4931,1.2525,1.1424,1.2
747,1.1719,1.2938,0.097992,0.10067,0.10567,0.095203,0.095114,0.097143,
0.092912,0.093281,0.10312,0.09517,0.091356,0.084562,0.0947,0.095404,0.
08818,0.094738,0.095894,0.10437,0.094548,0.097962,0.096987,0.096882,0.
093529,0.11792,0.1192,0.11827,0.11818,0.11795,0.12124,1.3223,1.2484,1.
4026,1.2649,1.3163,0.11886,0.11835,0.096122,0.095667,0.096681,0.095653
,0.095213,0.095811,0.097225,0.093973,0.094893,0.097451,0.095256,0.0973
85,0.093016,0.096274,0.098596,0.094283,0.094472,0.098222,0.095459,0.09
3689,0.094654,0.12013,0.11917,0.11797,0.12205,0.11956,0.11851,0.12077,
0.1219,0.11651,0.1182,1.2296,1.1109,0.12047,0.12028,0.11815,0.12015,0.
092359,0.10192,0.095817,0.094164,0.094694,0.091724,0.09428,0.095652,0.
09417,0.097536,0.098509,0.091216,0.092443,0.095345,0.093011,0.094764,0
.099376,0.093618,0.093446,0.11863,0.12036,0.12114,0.11984,0.11879,0.11
833,0.11639,0.11878,0.11782,0.11826,0.11785,0.12036,0.12136,0.11903,0.
11974,0.11695,0.11963,0.096472,0.094109,0.098295,0.10261,0.094948,0.09
6657,0.09468,0.10212,0.098141,0.094679,0.098085,0.095468,0.097694,0.09
5962,0.091231,0.094667,0.12113,0.11861,0.12004,0.12017,0.11967,0.11946
,0.11822,0.12212,0.12175,0.11967,0.11847,0.11917,0.12043,0.1184,0.1222
5,0.11789,0.11837,0.11896,0.1201,0.096729,0.097468,0.089455,0.093666,0
.099713,0.0945,0.095207,0.092658,0.098135,0.093884,0.095406,0.092729,0
.10001,0.11948,0.11956,0.11837,0.11884,0.11886,0.11863,0.11977,0.1208,
0.11898,0.11975,0.11987,0.12087,0.1185,0.11836,0.12103,0.1186,0.11978,
0.11834,0.11976,0.12117,0.12028,0.096285,0.095247,0.094113,0.096712,0.
092224,0.098927,0.096356,0.095267,0.093262,0.11831,0.12167,0.11888,0.1
1915,0.12166,0.11877,0.12016,0.11795,0.11991,0.12097,0.12142,0.11909,0
.11856,0.11923,0.11755,0.12117,0.11987,0.11826,0.11953,0.11937,0.11977
,0.11891,0.12051,0.11957,0.11941,0.097094,0.092824,0.094804,0.099526,0
.11795,0.11817,0.12017,0.12042,0.11678,0.11636,0.11892,0.11846,0.11957
,0.11953,0.12018,0.11877,0.12133,0.11997,0.12023,0.12185,0.11818,0.120
85,0.11651,0.11825,0.12013,0.11721,0.12042,0.11987,0.11979,0.12111,0.1
2112,0.1214,0.1174,0.11908,0.11806,0.11918,0.1205,0.12283,0.12003,0.11
787,0.12114,0.11827,0.11764,0.11811,0.11945,0.12107,0.11866,0.11994,0.
11787,0.12167,0.11781,0.11922,0.12008,0.12033,0.1178,0.12015,0.11818,0
.11869,0.11944,0.12005,0.11969,0.11939,0.11937,0.1188,0.11885,0.11777,
0.12011,0.11794,0.12112,0.12085,0.11932,0.11878,0.11999,0.12069,0.1215
2,0.12106,0.11855,0.11984,0.11816,0.11866,0.12049,0.11923,0.11971,0.11
947,0.1179,0.11959,0.12071,0.11616,0.11881,0.11883,0.11779,0.11832,0.1
198,0.11849,0.11775,0.1181,0.11912,0.12008,0.12102,0.11875,0.11885,0.1
1952,0.11792,0.11913,0.11904,0.12269,0.11881,0.11909,0.11831,0.11988,0
.12061,0.11748,0.11785,0.11873,0.12032,0.12022,0.11819,0.12103,0.11974
,0.11903,0.11942,0.11914,0.121,0.11754,0.11859,0.11938,0.12061,0.11995
,0.11952,0.12095,0.12014,0.1214,0.12,0.11929,0.11717,0.11913,0.11896,0

.11933,0.11803,0.11737,0.11912,0.12008,0.1211,0.11866,0.11724,0.12023,
0.11981,0.12052,0.11955,0.122,0.12312,0.11981,0.12021,0.12143,0.11855,
0.11834,0.12094,0.11834,0.11875,0.12212,0.11825,0.12044,0.11891,0.1190
5,0.11905,0.11955,0.12025,0.11806,0.12045,0.12146,0.11866,0.12023,0.12
071,0.11979,0.1207,0.1178,0.11881,0.11998,0.11887,0.12004,0.11788,0.11
772,0.11846,0.11852,0.11801,0.11726,0.1195,0.11891,0.11825,0.11871,0.1
1638,0.12112,0.12064,0.11964,0.12135,0.12065,0.11943,0.12073,0.12229
0.11749,0.11624,0.11487,0.11878,0.11632,0.11348,0.11449,0.11521,0.1181
1,0.11478,0.11751,0.11887,0.11459,0.11391,0.11654,0.11574,0.11458,0.11
638,0.11781,0.11629,0.98829,0.82679,1.1226,0.8597,0.11504,0.11706,0.11
952,0.1148,0.11684,0.11707,0.11387,0.11599,0.11404,0.1156,0.11749,0.11
283,0.11317,0.11344,0.11431,1.5762,0.87378,0.85968,0.85816,0.8572,0.84
166,0.8742,0.1134,0.11401,0.11743,0.11956,0.11863,0.11768,0.114,0.1161
6,0.11897,0.11496,0.11644,0.11643,0.11478,0.11791,0.11567,1.5968,1.583
2,0.85697,0.96567,0.69747,0.85658,0.85535,0.84273,0.8257,0.83565,0.115
75,0.11731,0.11671,0.1162,0.11782,0.11726,0.11547,0.11327,0.11584,0.11
86,0.1142,0.11622,0.11491,0.11447,0.11749,1.5911,1.5809,1.5777,0.49866
,1.1077,0.86836,0.86909,0.86808,0.7948,1.0223,0.89708,0.85601,0.11892,
0.11536,0.11619,0.11428,0.11909,0.11557,0.11566,0.11541,0.11772,0.1180
8,0.11708,0.11597,0.11741,0.11385,0.11237,0.11417,1.595,1.5937,1.5973,
1.5873,0.87009,0.86093,0.60966,0.86619,0.71979,0.83766,0.78332,0.91057
,0.86957,0.99035,0.1163,0.11781,0.11574,0.11863,0.11387,0.11867,0.1144
4,0.11678,0.11447,0.11492,0.11579,0.11568,0.116,0.11832,0.11424,0.1128
9,1.5801,1.5909,1.612,1.6305,1.5836,1.5683,0.79141,0.98674,0.78552,0.8
0475,0.83981,0.90043,0.81971,0.86327,0.83977,0.80713,0.11831,0.11647,0
.11633,0.11774,0.11132,0.11418,0.11678,0.11621,0.11775,0.11786,0.11617
,0.11522,0.11883,0.11516,0.11782,0.11266,0.11644,1.5902,1.5939,1.5676,
1.612,1.5921,1.6194,0.82722,0.73539,0.84328,0.9427,0.95431,0.92813,0.8
4642,0.85143,0.83244,0.84221,0.87568,0.11456,0.11398,0.11529,0.11505,0
.11458,0.11438,0.11245,0.11741,0.11454,0.11912,0.11454,0.11323,0.11406
,0.12922,0.13208,0.12875,0.11813,1.5801,1.6026,1.5902,1.5857,1.5907,1.
5903,1.5873,0.94181,0.84901,0.88177,0.85261,0.94782,0.90239,0.81762,0.
86042,0.87499,0.93492,0.81225,0.11736,0.11881,0.11548,0.11265,0.11557,
0.11698,0.11668,0.11767,0.11315,0.11545,0.11473,0.13345,0.12957,0.1301
8,0.1308,0.12992,0.1292,0.11763,1.5625,1.6079,1.6163,1.5965,1.6014,1.6
161,1.6013,0.89566,0.92706,0.79269,0.89487,0.86515,0.84224,0.84875,0.8
4415,0.90692,0.8599,0.69915,0.11414,0.11655,0.11525,0.11599,0.11428,0.
12001,0.11906,0.11546,0.11772,0.12946,0.12922,0.12718,0.13164,0.13059,
0.12991,0.13234,0.13047,0.13117,0.11791,1.6005,1.6098,1.623,1.6047,1.5
728,1.579,1.5816,0.71747,0.83077,0.82372,0.85614,0.82807,0.85752,0.856
12,0.68432,0.86235,0.82815,0.83474,0.64636,0.11372,0.11639,0.11886,0.1
1935,0.11493,0.11904,0.13179,0.13012,0.12936,0.13109,0.12614,0.12956,0
.13432,0.13127,0.13108,0.12742,0.13111,0.13113,1.5973,1.5892,1.5915,1.
6003,1.5898,1.6104,1.609,0.71615,0.85576,0.8656,0.84874,0.87492,0.8842
8,0.96866,0.87072,0.89748,0.85319,0.85083,0.84511,0.11977,0.11642,0.11
719,0.13293,0.12936,0.13275,0.13288,0.1329,0.13008,0.13087,0.13018,0.1
3154,0.13055,0.12987,0.12615,0.12796,0.12843,0.12928,1.6051,1.6118,1.6
187,1.6138,1.5859,1.6281,1.6215,1.5909,1.5845,0.86526,0.85764,0.70273,
0.88831,0.67697,0.80202,0.78607,0.85724,0.8543,0.99769,0.88718,0.13264
,0.13114,0.13133,0.13098,0.1315,0.12911,0.13432,0.1334,0.13011,0.12979
,0.13288,0.13249,0.12875,0.12991,0.13089,0.13395,0.13276,0.13102,1.582
1,1.6195,1.5909,1.5701,1.621,1.6016,1.6096,1.5825,1.5666,0.92092,0.805

98,0.72231,0.49812,0.63584,0.63258,0.6494,0.62922,0.7323,0.60821,0.636
11,0.12791,0.12858,0.13193,0.13424,0.12986,0.1266,0.13037,0.12999,0.13
081,0.13284,0.1314,0.1319,0.13004,0.12915,0.13123,0.12992,0.13072,0.13
121,1.5827,1.5875,1.5911,1.6078,1.6134,1.5674,1.5945,1.5786,1.6215,0.7
9684,0.62424,0.65962,0.66752,0.56664,0.68154,0.57783,0.75346,0.67353,0
.62748,0.45671,0.12941,0.12867,0.1285,0.12937,0.13229,0.12727,0.12764,
0.13219,0.12863,0.13168,0.13014,0.13004,0.12806,0.13258,0.1345,0.13072
,0.13387,0.13039,1.5853,1.5593,1.6076,1.6079,1.6086,1.5944,1.5694,0.87
868,0.95325,1.053,0.62166,0.63826,0.60014,0.63343,0.56001,0.69626,0.60
982,0.62657,0.60716,0.64856,0.13141,0.13037,0.12814,0.13203,0.13127,0.
13062,0.12961,0.12676,0.13177,0.13143,0.13256,0.13151,0.13025,0.12952,
0.13052,0.13372,0.12901,0.12991,1.6,1.5727,1.6082,1.6047,1.6166,1.584,
0.91984,0.88757,0.88511,0.83669,0.85236,0.74323,0.63221,0.61061,0.6548
6,0.65935,0.63109,0.64352,0.63569,0.68304,0.13146,0.13115,0.13162,0.13
105,0.13213,0.12938,0.13252,0.13089,0.13051,0.12893,0.12835,0.13044,0.
1305,0.13235,0.12957,0.12987,0.13323,0.12934,1.5735,1.5868,1.6181,1.58
39,1.5753,0.94474,0.88524,0.86241,0.91238,0.78878,0.64125,0.58368,0.65
773,0.65446,0.64897,0.70938,0.61793,0.67056,0.6225,0.65139,0.12903,0.1
3184,0.12799,0.13069,0.13254,0.13103,0.13309,0.12909,0.13347,0.13111,0
.1333,0.1319,0.13151,0.13131,0.12853,0.13116,0.13156,0.13051,1.599,1.5
896,1.6041,0.86607,0.86889,0.88209,0.92435,0.87844,0.99184,0.80432,0.9
2332,0.70645,0.63199,0.65277,0.67622,0.50746,0.62566,0.72761,0.52936,0
.57138,0.13217,0.13123,0.13256,0.13014,0.13183,0.13017,0.12711,0.12838
,0.13159,0.13297,0.1293,0.13251,0.12864,0.12757,0.13139,0.12925,0.1308
9,0.1301,1.6079,0.90113,1.0134,0.86504,0.79413,0.88106,0.83692,0.87523
,0.87568,0.86548,0.61242,0.6163,0.6876,0.61146,0.65908,0.6514,0.63065,
0.62428,0.61929,0.13033,0.1317,0.13085,0.1292,0.13284,0.13088,0.13286,
0.13028,0.12934,0.12914,0.13107,0.12981,0.13139,0.12937,0.12631,0.1325
5,0.13124,0.14623,0.86489,0.89657,0.88345,0.83736,0.88271,0.72833,0.80
442,0.98552,0.87726,1.0842,0.69166,0.43596,0.46827,0.62547,0.61979,0.5
6367,0.63085,0.5262,0.66905,0.13099,0.13086,0.12854,0.13131,0.13057,0.
13779,0.13106,0.13283,0.12899,0.13137,0.13012,0.13093,0.12931,0.13046,
0.13185,0.13076,0.12959,0.14431,0.99069,0.85151,0.87503,0.97119,0.8928
5,0.77823,0.81635,0.70308,0.72332,0.90055,0.53702,0.74218,0.39453,0.70
174,0.62016,0.6586,0.65732,0.70299,0.12999,0.13152,0.13174,0.1311,0.13
192,0.13265,0.12826,0.12869,0.1308,0.13079,0.12829,0.13086,0.13078,0.1
2974,0.13105,0.12995,0.13127,0.13181,0.14616,0.87525,1.0704,0.83658,0.
86984,0.89668,0.82953,0.80788,0.91186,0.7879,0.87963,0.63911,0.59528,0
.55823,0.60522,0.65897,0.69426,0.72222,0.54482,0.12832,0.13011,0.1303,
0.13111,0.12892,0.12619,0.13122,0.12864,0.13026,0.12976,0.12941,0.1325
2,0.13315,0.13192,0.13326,0.13225,0.12852,0.14409,0.14562,0.14376,0.86
127,0.84104,0.8556,0.87267,0.9022,0.8708,0.83472,0.85649,0.86434,0.507
82,0.56737,0.62359,0.57642,0.65254,0.65016,0.13401,0.13145,0.1286,0.13
066,0.13123,0.13115,0.13244,0.12887,0.13237,0.12977,0.13078,0.13305,0.
13004,0.13201,0.13337,0.13151,0.1321,0.12948,0.14598,0.14495,0.14226,0
.14536,0.60385,0.85866,0.76198,0.66147,0.86444,0.86536,0.96284,0.99314
,0.9634,0.70476,0.52238,0.59937,0.1289,0.13183,0.13469,0.13259,0.12773
,0.12873,0.12612,0.13288,0.13175,0.13204,0.12773,0.12787,0.13094,0.125
91,0.12955,0.12963,0.12933,0.12825,0.13339,0.13219,0.1447,0.14577,0.14
646,0.14504,0.14542,0.14588,0.90443,0.86894,1.0263,0.87079,0.79818,0.8
8512,0.81965,0.89353,0.13252,0.13344,0.13718,0.13054,0.1309,0.1315,0.1
2862,0.12973,0.13557,0.13039,0.1281,0.12396,0.13031,0.13041,0.12705,0.

13062,0.13078,0.13589,0.13008,0.13314,0.13145,0.13119,0.1291,0.14307,0
.14535,0.14435,0.14505,0.14351,0.14678,0.90951,0.87649,0.97105,0.87823
,0.90761,0.14472,0.14391,0.13197,0.13092,0.1317,0.13047,0.13022,0.1310
1,0.13217,0.12918,0.12998,0.13192,0.13007,0.13228,0.12936,0.1315,0.132
75,0.12957,0.12984,0.1331,0.13074,0.12921,0.13037,0.1457,0.14567,0.144
08,0.14773,0.14585,0.14521,0.14631,0.14796,0.14347,0.14409,0.85137,0.7
8108,0.14563,0.14648,0.14398,0.14568,0.12879,0.13516,0.13103,0.12942,0
.13046,0.12799,0.13027,0.13063,0.12938,0.13346,0.13243,0.12814,0.12897
,0.13053,0.12872,0.12982,0.13335,0.12971,0.12929,0.14466,0.14581,0.146
62,0.14572,0.14521,0.14417,0.14299,0.14526,0.1445,0.1441,0.14428,0.146
64,0.14777,0.14582,0.14638,0.14311,0.14584,0.13114,0.13009,0.13249,0.1
3604,0.13018,0.13128,0.13059,0.13578,0.13298,0.13007,0.13284,0.13086,0
.13192,0.13057,0.12817,0.12981,0.14709,0.14444,0.14572,0.1464,0.14534,
0.14589,0.14426,0.14832,0.14734,0.14646,0.14444,0.14482,0.14697,0.1451
,0.14868,0.14383,0.14535,0.14448,0.14695,0.13046,0.13155,0.12743,0.129
26,0.13409,0.12996,0.13,0.12885,0.13233,0.12976,0.13034,0.12891,0.1337
6,0.14593,0.145,0.14438,0.14449,0.14518,0.14552,0.14625,0.14682,0.1446
1,0.14606,0.14711,0.14711,0.14462,0.14456,0.1467,0.14435,0.14683,0.144
9,0.14513,0.14629,0.14664,0.13111,0.12958,0.12981,0.13285,0.12836,0.13
255,0.13022,0.13121,0.12834,0.14414,0.14734,0.14499,0.14599,0.1468,0.1
4547,0.14583,0.14413,0.14592,0.14636,0.14675,0.14461,0.14425,0.1447,0.
14278,0.14665,0.14611,0.14464,0.14623,0.14587,0.14605,0.14571,0.14564,
0.14531,0.14542,0.13204,0.12915,0.12947,0.13321,0.14465,0.14413,0.1452
8,0.14675,0.1425,0.14233,0.14532,0.1438,0.14609,0.14675,0.14577,0.1447
4,0.14757,0.14488,0.14621,0.14654,0.14319,0.146,0.14221,0.14394,0.1459
6,0.14363,0.14609,0.14579,0.14693,0.14753,0.14807,0.14757,0.14402,0.14
59,0.1446,0.14499,0.1471,0.14829,0.14544,0.14386,0.14749,0.1439,0.1442
4,0.14475,0.1467,0.14723,0.14565,0.14644,0.14411,0.14753,0.14422,0.145
85,0.14651,0.14699,0.14416,0.14585,0.14429,0.14454,0.14502,0.14512,0.1
4549,0.14474,0.14553,0.1447,0.14486,0.14361,0.14728,0.14385,0.14641,0.
14638,0.14534,0.1452,0.14603,0.14668,0.14711,0.14687,0.14385,0.14569,0
.14396,0.14422,0.147,0.14636,0.14599,0.14492,0.14353,0.14595,0.14638,0
.14254,0.14575,0.14491,0.14442,0.14368,0.14645,0.14416,0.1434,0.14377,
0.14554,0.14578,0.14716,0.14559,0.14507,0.14519,0.14398,0.14581,0.1451
9,0.14869,0.14535,0.14559,0.14474,0.14557,0.14654,0.14338,0.14452,0.14
393,0.14655,0.14606,0.14419,0.14583,0.14596,0.1456,0.14565,0.14576,0.1
4601,0.14328,0.14479,0.14561,0.14688,0.14568,0.14571,0.14653,0.14588,0
.14767,0.1463,0.14524,0.14333,0.1458,0.14447,0.14531,0.14502,0.14449,0
.1453,0.14569,0.14717,0.14454,0.14336,0.14586,0.14602,0.14636,0.1455,0
.1475,0.14851,0.14714,0.14586,0.14715,0.14642,0.14463,0.14672,0.14523,
0.14465,0.14855,0.14486,0.14579,0.14361,0.14556,0.14441,0.1458,0.14794
,0.1444,0.14649,0.14697,0.14522,0.14532,0.14756,0.14558,0.14594,0.1436
9,0.14486,0.14631,0.14527,0.14687,0.14294,0.14489,0.14541,0.14573,0.14
41,0.14333,0.14502,0.14557,0.14547,0.14439,0.14244,0.14752,0.14722,0.1
4511,0.1472,0.14595,0.14497,0.14653,0.14832
0.14475,0.14495,0.14198,0.147,0.14553,0.14306,0.14242,0.14308,0.14559,
0.14522,0.14538,0.14648,0.14237,0.14179,0.14513,0.14382,0.14269,0.1439
3,0.14551,0.14389,0.72687,0.6088,0.81082,0.62373,0.14321,0.14652,0.145
67,0.14216,0.14481,0.14482,0.14128,0.14343,0.14156,0.14265,0.14565,0.1
4018,0.14135,0.14173,0.14232,1.0904,0.64177,0.63224,0.62983,0.62681,0.
62783,0.64286,0.14086,0.14142,0.14496,0.14755,0.14635,0.14439,0.14245,
0.14345,0.1447,0.14401,0.14376,0.14512,0.14299,0.14522,0.14433,1.1357,

1.1055,0.6274,0.71018,0.52834,0.63918,0.6345,0.62415,0.60785,0.60428,0
.14356,0.14562,0.14515,0.1437,0.14543,0.14477,0.14426,0.14234,0.14489,
0.14747,0.14177,0.14373,0.14466,0.14359,0.14444,1.1038,1.0885,1.1043,0
.39303,0.80197,0.64205,0.64903,0.6425,0.59118,0.74337,0.65256,0.62748,
0.14705,0.14263,0.14498,0.14266,0.14671,0.1429,0.1436,0.14264,0.14564,
0.146,0.14575,0.14454,0.1453,0.14271,0.14122,0.14116,1.1219,1.1235,1.1
117,1.1044,0.63943,0.6393,0.4709,0.6375,0.54288,0.61544,0.58493,0.6611
2,0.64579,0.71716,0.1431,0.14606,0.14433,0.14616,0.14214,0.14622,0.143
36,0.14546,0.14349,0.14244,0.14368,0.14279,0.1439,0.14584,0.14139,0.14
143,1.0978,1.1102,1.1319,1.1445,1.1008,1.0958,0.59331,0.70992,0.58651,
0.60005,0.62426,0.66088,0.59891,0.63956,0.61949,0.59802,0.14646,0.1439
9,0.14465,0.14467,0.14001,0.14217,0.1457,0.14338,0.14581,0.14598,0.143
76,0.14317,0.14781,0.14244,0.14506,0.14235,0.14514,1.1025,1.1257,1.092
8,1.148,1.1135,1.141,0.61428,0.55523,0.62309,0.6887,0.70447,0.68324,0.
62194,0.61941,0.61497,0.61671,0.63692,0.14333,0.14205,0.14344,0.14189,
0.1418,0.14199,0.13963,0.14584,0.14211,0.14615,0.14392,0.13986,0.14234
,0.14006,0.13848,0.13875,0.1469,1.1032,1.1274,1.117,1.1104,1.1205,1.12
03,1.1136,0.6946,0.62113,0.65247,0.62487,0.69049,0.6592,0.60364,0.6337
,0.64611,0.67878,0.60109,0.14516,0.14626,0.14309,0.14065,0.14413,0.146
47,0.14463,0.14543,0.14135,0.14262,0.14255,0.13792,0.13867,0.13983,0.1
3985,0.13905,0.13942,0.14563,1.0844,1.1261,1.1407,1.128,1.124,1.1305,1
.1165,0.65579,0.68613,0.5896,0.6582,0.63354,0.61411,0.62409,0.62151,0.
66405,0.63149,0.52771,0.14129,0.14323,0.14347,0.14407,0.14271,0.14737,
0.14539,0.14252,0.14684,0.13898,0.13914,0.14009,0.14032,0.13972,0.1394
,0.14134,0.14078,0.14005,0.14585,1.1265,1.1183,1.135,1.1404,1.0997,1.0
925,1.109,0.53922,0.61894,0.6095,0.63284,0.61871,0.63307,0.6341,0.5187
2,0.63189,0.61498,0.61511,0.48972,0.14158,0.14517,0.14687,0.14772,0.14
245,0.14676,0.13886,0.13997,0.14037,0.13973,0.14001,0.13952,0.14138,0.
14055,0.14065,0.14091,0.1411,0.14017,1.1145,1.1246,1.1085,1.1255,1.111
7,1.1287,1.1294,0.5385,0.63262,0.6413,0.63358,0.65424,0.64587,0.70997,
0.63696,0.66017,0.63509,0.63035,0.61918,0.14931,0.14368,0.14476,0.1407
8,0.14241,0.13958,0.13914,0.14237,0.13896,0.13971,0.13954,0.14162,0.14
053,0.13982,0.13877,0.13849,0.14333,0.13901,1.1214,1.1228,1.1445,1.141
9,1.1109,1.1299,1.1339,1.1195,1.1101,0.63708,0.62766,0.53273,0.64909,0
.5174,0.5958,0.58517,0.63141,0.62363,0.72009,0.65279,0.14222,0.14022,0
.13999,0.13946,0.14033,0.13894,0.14154,0.14226,0.14158,0.1385,0.13908,
0.14068,0.13834,0.13944,0.13982,0.13901,0.1402,0.14159,1.116,1.1385,1.
113,1.0941,1.1432,1.1297,1.1202,1.1009,1.1048,0.67144,0.59709,0.53974,
0.3839,0.47874,0.4765,0.48416,0.47287,0.54309,0.45536,0.47153,0.13908,
0.14125,0.13917,0.13919,0.13865,0.14033,0.14045,0.13932,0.14131,0.1374
5,0.1401,0.14106,0.13915,0.13924,0.14122,0.13894,0.13985,0.13992,1.110
8,1.1199,1.1037,1.1281,1.1254,1.0824,1.1299,1.0965,1.145,0.5778,0.4644
7,0.49089,0.50369,0.43062,0.51035,0.43746,0.55639,0.50615,0.4727,0.357
04,0.1385,0.1388,0.14071,0.13811,0.14088,0.13979,0.14157,0.14082,0.142
16,0.14119,0.13931,0.14131,0.14038,0.13948,0.14073,0.13986,0.14152,0.1
3995,1.0981,1.0822,1.1249,1.1251,1.1389,1.1073,1.0969,0.64016,0.68295,
0.74231,0.46508,0.47985,0.45539,0.47523,0.42793,0.51558,0.46398,0.4761
7,0.46057,0.48568,0.1409,0.13929,0.1394,0.13948,0.14026,0.13837,0.1393
3,0.1407,0.14093,0.14112,0.13964,0.14128,0.13877,0.13852,0.13743,0.140
83,0.13828,0.14209,1.1158,1.0959,1.1253,1.1193,1.1466,1.1173,0.66205,0
.64372,0.64298,0.60168,0.60802,0.54148,0.47517,0.45466,0.48845,0.49554
,0.47852,0.48809,0.47529,0.51527,0.14017,0.14032,0.14048,0.14005,0.139

13,0.13838,0.1407,0.1402,0.14066,0.13952,0.14206,0.13986,0.13957,0.139
8,0.14227,0.14002,0.13933,0.13901,1.0826,1.1088,1.1258,1.1007,1.0947,0
.6778,0.64504,0.61444,0.6562,0.57566,0.47011,0.43878,0.49107,0.48716,0
.48719,0.52664,0.46356,0.49514,0.47253,0.48459,0.14155,0.13814,0.13999
,0.13921,0.13789,0.14031,0.14079,0.1415,0.13989,0.13997,0.14046,0.1412
2,0.14129,0.14116,0.13898,0.14256,0.13914,0.14041,1.121,1.117,1.1421,0
.62316,0.62369,0.63747,0.66676,0.631,0.70553,0.58076,0.66967,0.5301,0.
47318,0.48614,0.50173,0.39225,0.47125,0.53641,0.4055,0.43525,0.14037,0
.14034,0.13913,0.13946,0.13862,0.14051,0.13946,0.13752,0.14043,0.13951
,0.13922,0.14204,0.13821,0.13996,0.13913,0.13871,0.13909,0.13968,1.138
3,0.64756,0.71661,0.61145,0.57548,0.63773,0.60264,0.63193,0.62524,0.62
139,0.4635,0.46537,0.51344,0.45679,0.50057,0.48208,0.48144,0.46618,0.4
6493,0.13976,0.14094,0.13927,0.13822,0.14134,0.13825,0.14098,0.13945,0
.1395,0.13922,0.1406,0.13862,0.13924,0.1395,0.14287,0.1405,0.13723,0.1
3631,0.6295,0.65105,0.63881,0.60452,0.63602,0.53759,0.5846,0.70221,0.6
3341,0.78019,0.51624,0.34613,0.36511,0.47007,0.46758,0.4283,0.47021,0.
40544,0.49783,0.14025,0.14037,0.13864,0.14043,0.13914,0.14138,0.14078,
0.13932,0.1396,0.13917,0.14135,0.13907,0.13801,0.14033,0.14193,0.13939
,0.1393,0.13518,0.71819,0.61071,0.63427,0.69101,0.6415,0.57483,0.5941,
0.51052,0.52573,0.64266,0.41005,0.55401,0.317,0.52078,0.4646,0.48971,0
.48963,0.51863,0.13806,0.1404,0.14092,0.14123,0.14056,0.13799,0.14173,
0.13893,0.14151,0.1403,0.13873,0.14058,0.1397,0.14002,0.13795,0.13985,
0.13943,0.14088,0.13606,0.63262,0.76021,0.59705,0.62401,0.64345,0.5984
7,0.58837,0.64987,0.56637,0.64013,0.48361,0.45103,0.42691,0.45346,0.49
592,0.51368,0.5259,0.41617,0.14046,0.13979,0.13949,0.13981,0.13777,0.1
4037,0.13799,0.13872,0.13906,0.14005,0.13843,0.1413,0.13939,0.13925,0.
13954,0.14122,0.14028,0.13443,0.13548,0.13429,0.63179,0.60987,0.61282,
0.6303,0.64615,0.63746,0.60008,0.61616,0.62424,0.3919,0.43006,0.47251,
0.42882,0.4879,0.48858,0.13774,0.14116,0.13907,0.13942,0.14005,0.14076
,0.14037,0.13958,0.13872,0.1405,0.13984,0.13963,0.13839,0.14098,0.1404
6,0.13874,0.1415,0.13854,0.13703,0.13594,0.13238,0.13765,0.44293,0.620
46,0.54584,0.48507,0.61622,0.62364,0.6972,0.70579,0.69155,0.52393,0.39
995,0.45387,0.14182,0.14049,0.13976,0.13944,0.13991,0.14038,0.13928,0.
13954,0.14265,0.14082,0.13985,0.13928,0.14,0.13891,0.13868,0.13916,0.1
3897,0.13888,0.13953,0.14082,0.13426,0.13592,0.1369,0.13511,0.13525,0.
13549,0.647,0.62638,0.72286,0.62857,0.57687,0.63646,0.59315,0.64312,0.
13963,0.13777,0.1396,0.13979,0.13898,0.14024,0.13786,0.14038,0.13851,0
.13962,0.14002,0.14259,0.13929,0.13905,0.14246,0.14038,0.14004,0.13958
,0.13914,0.14121,0.13971,0.13912,0.13889,0.13334,0.13613,0.13476,0.135
4,0.13347,0.13706,0.64958,0.63249,0.69642,0.63082,0.64875,0.1351,0.134
31,0.14131,0.14003,0.14064,0.13972,0.13956,0.14035,0.13996,0.14001,0.1
388,0.14012,0.13802,0.14082,0.13968,0.14071,0.14009,0.13841,0.13978,0.
14024,0.14005,0.1396,0.14026,0.13511,0.13496,0.13451,0.13772,0.13552,0
.1353,0.13691,0.13763,0.13393,0.13428,0.61306,0.56985,0.13497,0.1373,0
.1345,0.1358,0.14089,0.13897,0.14037,0.13835,0.14001,0.14026,0.13942,0
.13918,0.1388,0.1402,0.13883,0.14059,0.14095,0.13924,0.13908,0.13913,0
.13979,0.14148,0.14004,0.1351,0.13666,0.13658,0.13637,0.13613,0.1351,0
.13352,0.13571,0.13489,0.13418,0.13526,0.137,0.13787,0.13574,0.13715,0
.13398,0.13653,0.13931,0.14128,0.13899,0.14014,0.13931,0.13992,0.13979
,0.14128,0.14083,0.13853,0.14046,0.14063,0.13905,0.13977,0.13887,0.139
26,0.13757,0.13512,0.13501,0.13721,0.13582,0.13651,0.13387,0.13795,0.1
3801,0.13655,0.1352,0.13552,0.13691,0.13543,0.13923,0.13417,0.1353,0.1

3489,0.13761,0.13857,0.13954,0.14122,0.13869,0.14091,0.13846,0.13925,0
.13897,0.13992,0.14033,0.14049,0.13958,0.13969,0.13649,0.13528,0.13459
,0.13396,0.13517,0.13623,0.13755,0.13702,0.13445,0.1361,0.13798,0.1385
5,0.13414,0.13488,0.13659,0.13521,0.1366,0.13584,0.13609,0.13734,0.136
41,0.1392,0.13905,0.1408,0.14155,0.14057,0.13927,0.13786,0.14023,0.137
38,0.13536,0.1378,0.1358,0.13648,0.13721,0.13597,0.13536,0.13436,0.136
21,0.13655,0.13675,0.13481,0.13515,0.13536,0.13371,0.13594,0.13728,0.1
3438,0.13571,0.13648,0.13625,0.13664,0.13602,0.13484,0.13648,0.13952,0
.14106,0.13857,0.13899,0.13457,0.13383,0.13539,0.13707,0.13295,0.13179
,0.13635,0.13291,0.13672,0.13768,0.13623,0.13579,0.13814,0.13565,0.137
87,0.13608,0.13379,0.13601,0.13245,0.13497,0.13648,0.134,0.13641,0.134
97,0.13733,0.13774,0.13902,0.13715,0.13456,0.13683,0.1352,0.13526,0.13
782,0.1384,0.13646,0.13474,0.13744,0.13318,0.13506,0.13499,0.13763,0.1
372,0.136,0.13722,0.13456,0.13839,0.13481,0.13673,0.13669,0.13685,0.13
492,0.13614,0.13404,0.13393,0.13487,0.13605,0.13636,0.13511,0.13541,0.
13459,0.13597,0.13386,0.1374,0.13413,0.13612,0.13626,0.13542,0.13597,0
.13672,0.13672,0.13717,0.13796,0.13401,0.13616,0.13337,0.13507,0.13645
,0.13696,0.13575,0.13458,0.13301,0.13696,0.13598,0.13274,0.13683,0.135
78,0.13455,0.13429,0.13652,0.13484,0.1339,0.13319,0.13624,0.13589,0.13
788,0.13621,0.13598,0.13587,0.13493,0.13618,0.13548,0.13865,0.1357,0.1
3587,0.1341,0.13669,0.13701,0.13449,0.1365,0.13454,0.13611,0.13661,0.1
3429,0.13665,0.136,0.13695,0.13531,0.13657,0.13631,0.1348,0.13452,0.13
56,0.13713,0.13636,0.13646,0.13719,0.13559,0.13709,0.13691,0.1352,0.13
37,0.13509,0.1343,0.13543,0.13567,0.13475,0.13502,0.13605,0.13759,0.13
478,0.13375,0.13626,0.13569,0.13684,0.13615,0.13772,0.13884,0.13692,0.
13665,0.13803,0.13595,0.1362,0.13601,0.13481,0.13538,0.13887,0.13438,0
.13621,0.13386,0.13495,0.13501,0.13602,0.13646,0.13515,0.13682,0.13687
,0.13519,0.13653,0.13678,0.13596,0.13722,0.13396,0.13587,0.1361,0.1360
1,0.13861,0.13356,0.13588,0.13597,0.13724,0.13451,0.13516,0.13407,0.13
643,0.13567,0.13569,0.13303,0.13719,0.13683,0.13466,0.13811,0.13614,0.
13591,0.13688,0.1384
0.16214,0.16281,0.16165,0.16549,0.16293,0.16255,0.15992,0.16276,0.1634
9,0.16352,0.16239,0.16573,0.15877,0.15984,0.16286,0.16365,0.16023,0.16
268,0.16383,0.16167,0.56219,0.47948,0.61816,0.48842,0.16158,0.16413,0.
16434,0.15949,0.16327,0.16349,0.1601,0.15923,0.15901,0.16135,0.1645,0.
15902,0.1577,0.15894,0.16012,0.80275,0.50622,0.49198,0.49649,0.48765,0
.49141,0.50145,0.15802,0.16066,0.16344,0.16315,0.16459,0.16254,0.15987
,0.16223,0.16045,0.1619,0.16207,0.16316,0.16184,0.16373,0.16291,0.849,
0.815,0.48982,0.55312,0.42822,0.50487,0.48995,0.48492,0.4829,0.47422,0
.16187,0.1634,0.16334,0.16171,0.16313,0.16202,0.16112,0.1604,0.16348,0
.16493,0.15945,0.16034,0.16246,0.1612,0.1634,0.81907,0.79704,0.81768,0
.33118,0.615,0.50702,0.51015,0.5049,0.46993,0.57395,0.50465,0.48441,0.
16423,0.15994,0.16215,0.16214,0.16422,0.16188,0.15945,0.16233,0.16404,
0.16319,0.16209,0.16352,0.16358,0.16104,0.15927,0.15869,0.82592,0.8364
3,0.83155,0.8131,0.50938,0.50862,0.38695,0.50077,0.43704,0.47707,0.467
36,0.51707,0.51421,0.55039,0.15976,0.16414,0.16294,0.16396,0.16043,0.1
6282,0.16165,0.16247,0.16249,0.16085,0.16083,0.1626,0.16141,0.16364,0.
15873,0.1609,0.80529,0.82706,0.84202,0.83373,0.80718,0.80197,0.46919,0
.54266,0.46445,0.47192,0.49382,0.52397,0.45963,0.49982,0.48353,0.47468
,0.1628,0.16446,0.16287,0.16236,0.15984,0.1583,0.1628,0.16158,0.16536,
0.16451,0.16071,0.16202,0.16541,0.15906,0.16294,0.16016,0.16317,0.809,
0.84431,0.80774,0.85724,0.8338,0.83658,0.48211,0.43826,0.49558,0.53033

,0.54291,0.53648,0.4866,0.48136,0.48594,0.4822,0.49787,0.16188,0.15996
,0.16086,0.16061,0.15943,0.15887,0.15855,0.16265,0.15955,0.16406,0.161
88,0.1574,0.16043,0.13994,0.13419,0.13922,0.16642,0.81476,0.83635,0.81
643,0.82502,0.82754,0.83197,0.82339,0.53246,0.48171,0.51289,0.48653,0.
53237,0.51102,0.46527,0.50169,0.50469,0.52607,0.47716,0.16112,0.164,0.
16133,0.15793,0.16053,0.1653,0.16273,0.16308,0.15901,0.15933,0.16053,0
.1319,0.13797,0.13905,0.13875,0.13739,0.14023,0.16369,0.79549,0.82454,
0.85194,0.82619,0.82972,0.8301,0.8268,0.50528,0.53933,0.46855,0.51719,
0.49489,0.48195,0.49167,0.4845,0.5159,0.4921,0.42294,0.1587,0.16125,0.
16097,0.16329,0.15894,0.16449,0.16257,0.1605,0.16401,0.13808,0.13872,0
.14322,0.13864,0.13874,0.13844,0.1399,0.14141,0.13857,0.163,0.84171,0.
83085,0.85168,0.85277,0.81801,0.80002,0.81154,0.43777,0.48144,0.4835,0
.49388,0.48607,0.49318,0.4972,0.42136,0.50003,0.47842,0.48645,0.40271,
0.16046,0.16423,0.16476,0.16492,0.15982,0.16496,0.13584,0.13825,0.1405
3,0.13839,0.14297,0.13872,0.13832,0.13964,0.1399,0.14381,0.14053,0.139
07,0.82278,0.82621,0.81978,0.83068,0.82882,0.83592,0.83809,0.43303,0.4
9184,0.50166,0.49748,0.51448,0.51488,0.55999,0.49862,0.52022,0.50274,0
.49287,0.48795,0.16766,0.16119,0.16409,0.13818,0.14448,0.13563,0.13547
,0.14213,0.1383,0.13859,0.13861,0.14106,0.13999,0.13928,0.13996,0.1391
,0.14747,0.13876,0.83744,0.82128,0.84162,0.84266,0.80673,0.83818,0.853
53,0.82566,0.8306,0.49812,0.48352,0.43087,0.5112,0.41566,0.47107,0.468
62,0.49454,0.48525,0.56026,0.50962,0.14121,0.13864,0.13905,0.1375,0.13
998,0.13815,0.13895,0.14087,0.14188,0.1372,0.13521,0.13956,0.13805,0.1
3832,0.13842,0.13368,0.13758,0.1415,0.82888,0.84425,0.83757,0.81579,0.
85783,0.83586,0.83979,0.81883,0.8183,0.52018,0.4693,0.42027,0.31131,0.
38026,0.3731,0.38162,0.37221,0.42516,0.3572,0.37233,0.13906,0.14361,0.
13552,0.13393,0.13672,0.14397,0.14094,0.1384,0.14185,0.13315,0.13864,0
.14042,0.1382,0.1381,0.14103,0.13713,0.13864,0.13757,0.81763,0.8178,0.
81434,0.84939,0.82983,0.80148,0.83925,0.81574,0.84509,0.44222,0.36957,
0.38731,0.3951,0.3434,0.40712,0.35,0.43055,0.40109,0.37878,0.29327,0.1
3784,0.1395,0.14285,0.1374,0.13899,0.14345,0.14589,0.14005,0.14464,0.1
4012,0.13901,0.14117,0.14262,0.13639,0.1375,0.13873,0.13812,0.13977,0.
80794,0.79459,0.82905,0.83331,0.85099,0.81468,0.81461,0.47902,0.50742,
0.55431,0.36241,0.37848,0.36612,0.37064,0.34439,0.39925,0.36557,0.3750
2,0.36574,0.3854,0.14017,0.13832,0.14099,0.13635,0.13893,0.13792,0.138
15,0.14481,0.13961,0.14003,0.13646,0.14074,0.13778,0.13732,0.1352,0.13
808,0.13656,0.1435,0.83475,0.8087,0.84117,0.83221,0.85082,0.82775,0.49
96,0.49148,0.48487,0.45368,0.45883,0.41829,0.37492,0.35869,0.38726,0.3
9403,0.3784,0.39178,0.37668,0.40198,0.13826,0.13929,0.13943,0.13815,0.
13601,0.13794,0.13803,0.13803,0.14089,0.1403,0.14568,0.13905,0.13925,0
.13737,0.14463,0.14105,0.13546,0.13853,0.79154,0.81589,0.83383,0.80855
,0.8041,0.50839,0.48429,0.46101,0.48296,0.44262,0.35315,0.34807,0.3838
8,0.38394,0.3808,0.40915,0.37047,0.38538,0.37761,0.38481,0.14308,0.134
31,0.14293,0.13748,0.13293,0.13935,0.13855,0.14283,0.13655,0.13886,0.1
3773,0.14002,0.13984,0.1403,0.13869,0.14288,0.137,0.13939,0.83379,0.82
343,0.85679,0.47211,0.46569,0.48367,0.5041,0.47365,0.52892,0.43754,0.5
0387,0.41922,0.37873,0.37955,0.39503,0.31722,0.36999,0.41723,0.32551,0
.3434,0.13847,0.13945,0.13499,0.13822,0.13557,0.14082,0.14162,0.13674,
0.13962,0.13592,0.13792,0.14057,0.13867,0.14246,0.13509,0.13752,0.1388
1,0.13922,0.85391,0.48366,0.5301,0.45376,0.43275,0.48269,0.45701,0.478
8,0.47387,0.47451,0.36524,0.37079,0.39665,0.36199,0.39547,0.37609,0.37
454,0.36806,0.37041,0.13811,0.14,0.13778,0.13733,0.13924,0.13553,0.139

11,0.13852,0.13989,0.13907,0.13807,0.13738,0.13756,0.1399,0.14771,0.13
906,0.13338,0.11575,0.47112,0.4984,0.48254,0.45513,0.48022,0.41037,0.4
4066,0.51733,0.47455,0.57803,0.40895,0.28458,0.29333,0.37209,0.37148,0
.33977,0.36904,0.32869,0.3853,0.13918,0.13967,0.13851,0.13862,0.13833,
0.1339,0.14054,0.13557,0.14019,0.13713,0.14265,0.13691,0.13702,0.1402,
0.14064,0.13868,0.13842,0.11577,0.53643,0.45492,0.48195,0.52424,0.4877
2,0.434,0.4457,0.38466,0.4041,0.48058,0.32758,0.42864,0.26799,0.40495,
0.3732,0.38511,0.38689,0.41258,0.13647,0.13849,0.13992,0.13983,0.13923
,0.13319,0.14442,0.13966,0.14159,0.13899,0.13946,0.13972,0.13842,0.138
58,0.13465,0.13887,0.13823,0.13983,0.11633,0.47906,0.5694,0.44652,0.47
676,0.48805,0.44575,0.44323,0.48371,0.42264,0.47957,0.38815,0.35832,0.
33577,0.3602,0.39476,0.39545,0.40315,0.33297,0.14277,0.1395,0.13813,0.
13913,0.13642,0.14488,0.13639,0.13865,0.13814,0.13994,0.13788,0.13936,
0.13519,0.13757,0.13563,0.14006,0.14153,0.11469,0.11594,0.11511,0.4763
3,0.46512,0.45813,0.47661,0.48177,0.48299,0.44659,0.46131,0.46856,0.31
424,0.34279,0.37365,0.33689,0.38464,0.38879,0.13171,0.14044,0.13948,0.
13897,0.13938,0.13959,0.13731,0.14044,0.1353,0.14143,0.1386,0.13596,0.
13634,0.13996,0.13783,0.13512,0.14061,0.13758,0.11689,0.11679,0.1126,0
.11746,0.3358,0.46645,0.40935,0.37548,0.45297,0.47014,0.52205,0.52582,
0.52017,0.40531,0.32132,0.36096,0.14452,0.13847,0.13451,0.13599,0.1425
5,0.14232,0.14214,0.13604,0.14345,0.13964,0.14166,0.14085,0.13907,0.14
171,0.13828,0.13812,0.13877,0.13933,0.1348,0.14034,0.11446,0.11588,0.1
1673,0.11511,0.11584,0.11587,0.47612,0.47567,0.5331,0.48262,0.43115,0.
47962,0.45612,0.48518,0.13639,0.13264,0.13236,0.13957,0.13715,0.13849,
0.13692,0.13948,0.13036,0.13948,0.14137,0.15047,0.13751,0.13764,0.1476
3,0.1401,0.13872,0.1327,0.13822,0.13906,0.13824,0.13707,0.13923,0.1140
9,0.116,0.1143,0.11641,0.1141,0.11725,0.47933,0.4748,0.52232,0.47027,0
.48858,0.11552,0.11448,0.14033,0.13938,0.1396,0.13907,0.1385,0.13896,0
.13831,0.141,0.13742,0.13641,0.13647,0.13847,0.14031,0.13918,0.13767,0
.13643,0.13919,0.13799,0.13947,0.14034,0.13981,0.11522,0.1157,0.11351,
0.11849,0.11451,0.1156,0.11737,0.11787,0.11413,0.1145,0.45613,0.42929,
0.11491,0.11719,0.11451,0.11577,0.14226,0.13147,0.13942,0.13807,0.1396
2,0.14248,0.13906,0.13867,0.13896,0.13649,0.13499,0.14274,0.14189,0.13
836,0.13906,0.13844,0.13651,0.14277,0.14011,0.1155,0.1175,0.11695,0.11
657,0.11583,0.11524,0.11474,0.11561,0.11519,0.1144,0.11559,0.11766,0.1
1704,0.11623,0.11795,0.11511,0.11663,0.13756,0.14156,0.13511,0.13385,0
.13841,0.13802,0.14026,0.13766,0.13847,0.13776,0.13849,0.1408,0.13608,
0.13962,0.13954,0.13874,0.11726,0.11544,0.11534,0.11725,0.11642,0.1169
1,0.11436,0.11742,0.11777,0.11686,0.11576,0.1148,0.11617,0.1153,0.1188
1,0.11562,0.11605,0.11548,0.11793,0.136,0.1372,0.14489,0.1384,0.13683,
0.13715,0.13868,0.13966,0.13707,0.13999,0.14018,0.14212,0.13551,0.1172
2,0.11614,0.11509,0.11454,0.11486,0.11533,0.11684,0.11761,0.11485,0.11
597,0.11743,0.11924,0.11442,0.11567,0.11782,0.11496,0.11684,0.11661,0.
11608,0.11717,0.11566,0.13741,0.13817,0.14148,0.14001,0.14209,0.13438,
0.13523,0.1392,0.13721,0.11548,0.11776,0.11567,0.11756,0.11713,0.11585
,0.1157,0.11484,0.11694,0.11594,0.11728,0.1162,0.11599,0.11596,0.11371
,0.11669,0.11788,0.11445,0.11659,0.11653,0.11691,0.11689,0.1162,0.1152
9,0.11714,0.13707,0.14322,0.13685,0.1343,0.11423,0.11409,0.11574,0.117
2,0.11335,0.11228,0.11586,0.11328,0.11616,0.11892,0.11627,0.1162,0.117
84,0.11631,0.11859,0.11671,0.11391,0.11514,0.11288,0.11571,0.11669,0.1
1545,0.11616,0.11511,0.11825,0.11812,0.11906,0.11701,0.1142,0.11644,0.
11545,0.1151,0.11821,0.11874,0.11678,0.11463,0.11729,0.11259,0.11563,0

.11574,0.11656,0.11726,0.11586,0.11718,0.11442,0.11803,0.1153,0.11665,
0.11636,0.11745,0.11488,0.11679,0.11469,0.11447,0.11552,0.11643,0.1165
1,0.1153,0.11567,0.1146,0.11619,0.11461,0.11796,0.11621,0.11693,0.1162
1,0.11547,0.11596,0.11704,0.11625,0.11822,0.11854,0.11358,0.11755,0.11
403,0.11486,0.11649,0.11793,0.11455,0.11537,0.11265,0.11601,0.11703,0.
11309,0.11659,0.11571,0.11466,0.11492,0.11605,0.11483,0.11443,0.11421,
0.11627,0.11537,0.11817,0.11713,0.1165,0.11594,0.11526,0.11635,0.11573
,0.11852,0.11497,0.11589,0.11437,0.11625,0.11746,0.11538,0.11689,0.115
4,0.11582,0.11691,0.1162,0.11713,0.11653,0.11637,0.11542,0.11734,0.116
15,0.11479,0.11461,0.11556,0.11746,0.11785,0.11613,0.11728,0.11511,0.1
1713,0.11682,0.11519,0.11502,0.11509,0.11335,0.11658,0.11595,0.11533,0
.11503,0.11633,0.11729,0.11445,0.11486,0.11557,0.11543,0.11736,0.11575
,0.11817,0.11859,0.11755,0.11659,0.11902,0.1167,0.11597,0.11624,0.1151
5,0.11671,0.11898,0.11468,0.11619,0.11363,0.11455,0.11454,0.11638,0.11
675,0.11514,0.11635,0.11716,0.11555,0.11645,0.11708,0.11618,0.1174,0.1
1469,0.11646,0.11634,0.11532,0.11943,0.11414,0.11648,0.11577,0.11782,0
.11522,0.11644,0.11522,0.11645,0.11543,0.11566,0.1141,0.11732,0.11685,
0.11472,0.11726,0.11603,0.11633,0.11652,0.11947
0.17391,0.17391,0.17234,0.17707,0.17287,0.17348,0.17142,0.17454,0.1752
1,0.17649,0.17418,0.1772,0.17168,0.17262,0.17417,0.17321,0.17162,0.174
16,0.17558,0.17362,0.45829,0.39776,0.50307,0.40229,0.17301,0.176,0.175
12,0.17258,0.1737,0.17494,0.17116,0.17126,0.17131,0.17203,0.17496,0.17
189,0.16958,0.17019,0.17128,0.62769,0.41544,0.41541,0.41422,0.40587,0.
41008,0.42008,0.16895,0.17083,0.17513,0.1755,0.1756,0.17367,0.17164,0.
17362,0.17213,0.17375,0.17305,0.17432,0.17442,0.17675,0.1741,0.6631,0.
62832,0.40759,0.45286,0.35979,0.42394,0.41623,0.4053,0.40518,0.39253,0
.17401,0.17463,0.17509,0.17385,0.1749,0.17355,0.17071,0.17237,0.17542,
0.17553,0.17126,0.17058,0.17372,0.17257,0.17394,0.64785,0.62994,0.6358
,0.29501,0.50807,0.42272,0.42503,0.42551,0.38934,0.4726,0.4186,0.39961
,0.17563,0.17221,0.17363,0.17246,0.17547,0.17269,0.1712,0.17428,0.1750
7,0.17576,0.17294,0.17487,0.17472,0.17273,0.1711,0.17024,0.63462,0.662
22,0.66028,0.64231,0.42467,0.42109,0.33618,0.41483,0.37265,0.39579,0.3
9636,0.42384,0.43153,0.44039,0.1723,0.1756,0.1754,0.1749,0.172,0.17408
,0.17354,0.17366,0.17457,0.1731,0.17204,0.17386,0.17397,0.17489,0.1706
2,0.17217,0.63731,0.66277,0.65845,0.65223,0.63535,0.61717,0.39647,0.43
516,0.39112,0.39627,0.41664,0.44149,0.38281,0.41166,0.39914,0.39485,0.
17549,0.17562,0.17343,0.17358,0.17131,0.17199,0.1743,0.17218,0.17561,0
.17682,0.17311,0.1745,0.1756,0.17177,0.17579,0.17157,0.17372,0.63448,0
.65768,0.64927,0.68476,0.63337,0.65288,0.4098,0.37198,0.41545,0.43579,
0.45227,0.45014,0.40609,0.39933,0.40788,0.39854,0.4105,0.17368,0.17206
,0.17219,0.17246,0.17186,0.17025,0.16941,0.17405,0.1702,0.17511,0.1737
,0.16933,0.17147,0.137,0.12733,0.13736,0.17581,0.62081,0.65458,0.6478,
0.64309,0.65783,0.65973,0.63982,0.43462,0.40201,0.42693,0.40746,0.4389
,0.4202,0.38622,0.41706,0.41311,0.42808,0.4023,0.17271,0.17668,0.17409
,0.16996,0.17186,0.17727,0.17517,0.17423,0.17148,0.17158,0.17135,0.123
96,0.13397,0.1341,0.13466,0.13324,0.13696,0.17501,0.62344,0.6345,0.658
48,0.64898,0.64895,0.64924,0.66232,0.41942,0.44882,0.38484,0.42456,0.4
1158,0.40053,0.41308,0.40809,0.42846,0.40558,0.35813,0.17165,0.17271,0
.17274,0.17477,0.16943,0.17678,0.17481,0.17308,0.17542,0.13453,0.1344,
0.14127,0.13431,0.1348,0.13326,0.13551,0.13876,0.13451,0.17508,0.66935
,0.66468,0.66911,0.66571,0.64064,0.62673,0.6381,0.37452,0.39934,0.4117
7,0.41201,0.41159,0.40936,0.41214,0.35927,0.41262,0.40365,0.40819,0.34

562,0.17229,0.17458,0.17668,0.17556,0.17316,0.17753,0.13024,0.1346,0.1
3752,0.13437,0.14277,0.13508,0.13321,0.13599,0.13565,0.14331,0.13613,0
.13556,0.66455,0.65409,0.63631,0.6644,0.65967,0.66037,0.6539,0.36896,0
.40996,0.41445,0.42053,0.42698,0.42684,0.46067,0.41944,0.44019,0.41586
,0.40538,0.40281,0.17921,0.17288,0.17463,0.13322,0.14364,0.12925,0.129
76,0.13818,0.13375,0.13473,0.1347,0.1373,0.13579,0.13517,0.1385,0.1363
8,0.14811,0.13494,0.65139,0.6401,0.65828,0.65974,0.63661,0.66892,0.675
86,0.64592,0.65727,0.41264,0.39753,0.36765,0.42374,0.35786,0.39652,0.3
9369,0.41095,0.40502,0.4541,0.42483,0.13677,0.13483,0.13447,0.1334,0.1
3565,0.13352,0.13285,0.1361,0.13902,0.13324,0.12909,0.13487,0.13467,0.
13398,0.1347,0.12704,0.1318,0.13918,0.63777,0.67482,0.66089,0.63612,0.
66847,0.66291,0.65743,0.64775,0.63576,0.43137,0.39542,0.3452,0.26688,0
.31492,0.31264,0.31318,0.31199,0.3514,0.2988,0.31154,0.13586,0.14276,0
.13013,0.12657,0.13278,0.14383,0.13794,0.134,0.1391,0.12588,0.13431,0.
13608,0.13334,0.13448,0.13799,0.13309,0.13456,0.13324,0.63308,0.62393,
0.63042,0.67668,0.65358,0.6127,0.66503,0.63689,0.66582,0.35282,0.30875
,0.31858,0.32847,0.28722,0.34242,0.29027,0.35624,0.33361,0.31901,0.249
74,0.13396,0.13559,0.14096,0.1337,0.13419,0.14437,0.14623,0.13611,0.14
29,0.13593,0.13449,0.13761,0.14099,0.13077,0.13003,0.13487,0.13193,0.1
3575,0.64197,0.61286,0.64762,0.6566,0.68287,0.65287,0.63796,0.37519,0.
39958,0.43682,0.29705,0.31678,0.30711,0.30714,0.28582,0.32348,0.30684,
0.31797,0.30634,0.31702,0.13553,0.13372,0.13868,0.13085,0.13601,0.1337
9,0.13446,0.14488,0.13564,0.13683,0.13026,0.13723,0.13318,0.13416,0.12
937,0.13217,0.13171,0.1415,0.6609,0.63039,0.67042,0.67478,0.67235,0.64
853,0.38636,0.38514,0.38772,0.35763,0.36167,0.34458,0.31334,0.29612,0.
32096,0.33206,0.31341,0.32672,0.30764,0.33537,0.13435,0.13497,0.13437,
0.1338,0.13,0.13346,0.1326,0.13249,0.1373,0.13753,0.14502,0.13569,0.13
522,0.13182,0.14322,0.13844,0.12837,0.13445,0.62222,0.64107,0.64269,0.
64379,0.64452,0.40156,0.37951,0.35559,0.37541,0.3558,0.28124,0.29285,0
.32009,0.31371,0.31205,0.33406,0.31289,0.31377,0.31342,0.32004,0.14106
,0.12795,0.14188,0.13275,0.12544,0.13566,0.13303,0.14203,0.12976,0.134
33,0.13151,0.13534,0.13612,0.13553,0.13534,0.13982,0.13042,0.13576,0.6
7506,0.64163,0.66791,0.37482,0.36971,0.39057,0.39385,0.37526,0.41761,0
.34949,0.40233,0.34668,0.30966,0.31475,0.33284,0.27077,0.31156,0.34072
,0.27505,0.28579,0.13312,0.13547,0.12889,0.13432,0.1306,0.13799,0.1397
,0.13376,0.13538,0.12878,0.13388,0.13653,0.13549,0.14232,0.12945,0.134
,0.13458,0.13511,0.66157,0.38635,0.4106,0.35913,0.34086,0.38775,0.3604
5,0.38366,0.38328,0.37408,0.30216,0.30963,0.32582,0.30155,0.3307,0.310
73,0.31252,0.30785,0.3081,0.13368,0.13587,0.13397,0.13354,0.13465,0.13
015,0.1345,0.13409,0.13716,0.13626,0.13356,0.13395,0.13296,0.13725,0.1
4835,0.13464,0.12722,0.096001,0.37479,0.39475,0.37386,0.36278,0.3734,0
.32753,0.35394,0.4093,0.37685,0.46576,0.33992,0.24227,0.25214,0.30951,
0.30929,0.28261,0.30752,0.27943,0.31153,0.13499,0.13559,0.13582,0.1344
9,0.13459,0.125,0.13628,0.12944,0.13731,0.13263,0.1401,0.13301,0.13237
,0.13698,0.1363,0.13418,0.13478,0.096141,0.42221,0.35683,0.38218,0.417
48,0.38956,0.34236,0.34596,0.30789,0.32321,0.38148,0.27506,0.3564,0.23
459,0.33357,0.30807,0.31871,0.32312,0.34273,0.13217,0.13461,0.13494,0.
13664,0.13547,0.12539,0.14422,0.13684,0.13867,0.1353,0.13757,0.13581,0
.13492,0.13503,0.12886,0.13474,0.13348,0.13601,0.095568,0.38733,0.4470
8,0.35337,0.38289,0.38341,0.34963,0.34993,0.37671,0.33339,0.38166,0.31
769,0.29853,0.28032,0.30318,0.32622,0.31852,0.3238,0.28001,0.14156,0.1
3535,0.1339,0.13505,0.13254,0.14542,0.13171,0.13417,0.13355,0.13649,0.

13315,0.13524,0.12884,0.13236,0.12992,0.13671,0.14023,0.095577,0.09683
,0.095663,0.38006,0.37495,0.3601,0.37461,0.37455,0.38179,0.35664,0.365
39,0.36695,0.26441,0.28276,0.3116,0.27402,0.31329,0.32357,0.12367,0.13
697,0.13768,0.13456,0.13466,0.13605,0.13283,0.13664,0.12812,0.13898,0.
13408,0.12962,0.13211,0.13484,0.13137,0.1295,0.13616,0.13335,0.09634,0
.096719,0.093442,0.097972,0.26308,0.36248,0.3276,0.29986,0.34924,0.370
76,0.42216,0.42272,0.41974,0.33308,0.27397,0.3018,0.14333,0.13232,0.12
736,0.13018,0.14166,0.14034,0.14207,0.12992,0.14006,0.13546,0.14012,0.
13981,0.13416,0.14159,0.13461,0.13442,0.13599,0.13699,0.1274,0.13702,0
.095045,0.096474,0.097196,0.09591,0.095083,0.096392,0.37076,0.36747,0.
42268,0.38588,0.34012,0.37327,0.35395,0.37819,0.1296,0.12539,0.12208,0
.1359,0.13358,0.13375,0.13346,0.13588,0.12057,0.13446,0.13845,0.15429,
0.13367,0.13377,0.14852,0.1365,0.135,0.12379,0.13452,0.13339,0.13323,0
.13161,0.13598,0.094492,0.096141,0.094602,0.09729,0.094554,0.098111,0.
3809,0.37321,0.4199,0.36948,0.38297,0.095874,0.095007,0.1363,0.13543,0
.13523,0.1352,0.13505,0.1351,0.13353,0.13821,0.13252,0.13045,0.13211,0
.13346,0.13652,0.13509,0.13159,0.13186,0.13585,0.13339,0.13511,0.1374,
0.13611,0.096793,0.095544,0.094585,0.098455,0.095146,0.096076,0.097741
,0.098883,0.094623,0.09447,0.35646,0.33977,0.095101,0.09607,0.09535,0.
097516,0.1405,0.12221,0.13494,0.13524,0.13661,0.1411,0.13446,0.13518,0
.13537,0.13184,0.12921,0.14188,0.13894,0.13365,0.13578,0.13522,0.13095
,0.1408,0.13782,0.09604,0.09828,0.09758,0.097569,0.096432,0.094715,0.0
95998,0.095551,0.09541,0.09445,0.095873,0.097464,0.097409,0.097298,0.0
99056,0.095508,0.096194,0.13331,0.13829,0.12855,0.12626,0.13367,0.1329
6,0.13619,0.13051,0.13448,0.13337,0.13385,0.13724,0.13037,0.13573,0.13
729,0.13554,0.097008,0.095604,0.096234,0.097021,0.097522,0.096459,0.09
6082,0.097062,0.098044,0.097641,0.095779,0.095842,0.096544,0.095866,0.
098551,0.095772,0.097,0.096,0.098692,0.13016,0.13183,0.14482,0.13453,0
.13041,0.13343,0.13491,0.1365,0.13167,0.13713,0.13724,0.13934,0.12875,
0.096995,0.096435,0.096583,0.093752,0.09542,0.094904,0.097171,0.098813
,0.095615,0.095753,0.097923,0.10021,0.095326,0.095689,0.098155,0.09504
,0.09704,0.097311,0.096018,0.096771,0.095839,0.13279,0.13547,0.1392,0.
13645,0.14051,0.12741,0.12923,0.13484,0.13375,0.095211,0.097683,0.0962
94,0.097893,0.097421,0.094986,0.095924,0.095101,0.096961,0.096783,0.09
7984,0.09554,0.09754,0.096181,0.094261,0.096529,0.09732,0.093992,0.096
955,0.096759,0.096451,0.096997,0.096544,0.096455,0.097684,0.13162,0.14
113,0.13223,0.12657,0.09439,0.095005,0.095143,0.097415,0.094051,0.0926
99,0.095595,0.093942,0.096102,0.098394,0.095848,0.09457,0.097744,0.096
788,0.098681,0.09728,0.094767,0.096022,0.092848,0.096408,0.097352,0.09
5091,0.096662,0.095397,0.098874,0.09854,0.099391,0.097404,0.094067,0.0
96873,0.096688,0.09616,0.098026,0.097853,0.09669,0.095674,0.097347,0.0
93587,0.095415,0.096301,0.097604,0.097035,0.096676,0.097521,0.09472,0.
097799,0.095882,0.097055,0.096699,0.098282,0.094863,0.097254,0.09549,0
.095469,0.094984,0.096411,0.096843,0.095666,0.096177,0.094448,0.096072
,0.095221,0.098723,0.096694,0.096741,0.096706,0.095675,0.095783,0.0969
4,0.09722,0.098383,0.099075,0.094236,0.098007,0.095098,0.096324,0.0971
93,0.09782,0.094467,0.096005,0.093514,0.097289,0.096871,0.093616,0.096
164,0.096013,0.09463,0.095879,0.095816,0.095973,0.094506,0.09504,0.096
892,0.095494,0.098894,0.096204,0.096771,0.096326,0.097194,0.096603,0.0
9557,0.097807,0.095262,0.096037,0.094227,0.096985,0.098131,0.095638,0.
097066,0.096141,0.0965,0.096731,0.096141,0.097379,0.096364,0.096292,0.
094901,0.097487,0.096172,0.095821,0.093858,0.096076,0.09754,0.097978,0

.095534,0.097612,0.095348,0.09806,0.098351,0.095211,0.095515,0.096084,
0.093348,0.096562,0.095688,0.097004,0.095166,0.097303,0.097946,0.09456
7,0.095175,0.09596,0.095299,0.096623,0.095253,0.097085,0.098986,0.0970
8,0.096845,0.098172,0.096606,0.096637,0.096151,0.095986,0.097656,0.098
236,0.094568,0.09633,0.093156,0.095054,0.095049,0.097571,0.096871,0.09
5705,0.096244,0.096723,0.09616,0.09706,0.096714,0.097357,0.096833,0.09
4734,0.097452,0.096406,0.095308,0.099322,0.093636,0.096909,0.095594,0.
097437,0.09649,0.09699,0.096059,0.097183,0.095896,0.095391,0.094303,0.
098304,0.096482,0.096224,0.097624,0.095928,0.09634,0.096707,0.099549
0.18038,0.18176,0.17935,0.18514,0.18051,0.18197,0.1805,0.18203,0.18309
,0.1834,0.18187,0.18441,0.18021,0.1821,0.18172,0.17978,0.18072,0.1822,
0.18372,0.18069,0.39473,0.34759,0.43666,0.35055,0.17902,0.18357,0.1828
6,0.18165,0.18081,0.18362,0.17935,0.17979,0.1799,0.17981,0.18202,0.180
34,0.17758,0.17823,0.17926,0.52176,0.36654,0.36129,0.36646,0.35429,0.3
614,0.36515,0.1773,0.17897,0.18405,0.18358,0.1812,0.18223,0.179,0.1816
7,0.18072,0.18197,0.18032,0.18205,0.18209,0.18349,0.18217,0.55982,0.52
203,0.35209,0.39574,0.31994,0.36722,0.36977,0.35778,0.35274,0.34581,0.
18041,0.18335,0.18353,0.18303,0.1818,0.18041,0.17929,0.17999,0.18307,0
.18213,0.17924,0.17906,0.18187,0.18125,0.18155,0.5412,0.51846,0.5284,0
.27617,0.43317,0.37022,0.36537,0.37563,0.33781,0.41503,0.3719,0.3497,0
.18329,0.18039,0.18076,0.18029,0.18308,0.18129,0.1812,0.18147,0.18291,
0.18352,0.18213,0.18245,0.1819,0.18166,0.17924,0.17796,0.51552,0.56223
,0.5504,0.54598,0.3676,0.36607,0.30577,0.36543,0.33576,0.34996,0.35479
,0.36843,0.37822,0.37535,0.18026,0.18279,0.18373,0.18213,0.17871,0.182
15,0.18146,0.18123,0.18318,0.18222,0.18043,0.18204,0.18079,0.18275,0.1
7837,0.17904,0.53624,0.55544,0.5445,0.54853,0.52104,0.51057,0.35536,0.
37393,0.3464,0.35355,0.36674,0.39069,0.33594,0.36279,0.35166,0.34819,0
.18296,0.18276,0.18161,0.18131,0.17924,0.18044,0.18076,0.17998,0.18133
,0.18462,0.18181,0.18303,0.18283,0.1804,0.1839,0.17969,0.18188,0.5288,
0.54053,0.55734,0.56546,0.5221,0.56596,0.3574,0.3291,0.36833,0.38216,0
.39067,0.395,0.35492,0.35332,0.36028,0.35369,0.36205,0.18134,0.18102,0
.18033,0.18025,0.17845,0.17741,0.17757,0.18159,0.17827,0.18136,0.18068
,0.17801,0.17914,0.13364,0.12122,0.13417,0.18311,0.51852,0.55106,0.534
64,0.54217,0.54494,0.5586,0.51995,0.37723,0.35001,0.37711,0.35352,0.38
486,0.36703,0.33828,0.37086,0.35996,0.37155,0.3564,0.18018,0.18392,0.1
8119,0.18009,0.18078,0.18339,0.18221,0.18037,0.17927,0.17985,0.17953,0
.11667,0.12979,0.12992,0.13044,0.12947,0.13303,0.18399,0.51832,0.53319
,0.54359,0.53626,0.54106,0.53723,0.55965,0.3625,0.39482,0.33698,0.3756
,0.35863,0.35568,0.36185,0.35338,0.37847,0.35738,0.31781,0.17959,0.179
97,0.18054,0.18127,0.17801,0.18557,0.18215,0.18062,0.18259,0.12981,0.1
3051,0.13924,0.13032,0.13032,0.12948,0.13133,0.13512,0.13043,0.18259,0
.57031,0.53956,0.56432,0.56377,0.53419,0.51903,0.54565,0.33712,0.35348
,0.35922,0.36793,0.3683,0.36291,0.36199,0.32535,0.3627,0.35607,0.36213
,0.30868,0.18097,0.18273,0.18336,0.18296,0.1813,0.18494,0.1244,0.13025
,0.13426,0.13065,0.142,0.13126,0.12745,0.13199,0.13175,0.14301,0.1314,
0.13158,0.55231,0.54825,0.52693,0.57421,0.54493,0.54079,0.54303,0.3336
3,0.35581,0.36223,0.36765,0.3722,0.37747,0.40217,0.37284,0.38155,0.363
1,0.35456,0.35789,0.18684,0.18096,0.18277,0.12785,0.14136,0.12289,0.12
429,0.13398,0.12864,0.13001,0.12959,0.13332,0.13137,0.13072,0.13633,0.
13345,0.14796,0.1318,0.5373,0.51918,0.53753,0.53958,0.53482,0.55434,0.
55979,0.53522,0.53968,0.36398,0.35361,0.32477,0.37183,0.31379,0.34338,
0.35444,0.35748,0.35557,0.39186,0.37269,0.13183,0.1309,0.13007,0.12891

,0.13166,0.1297,0.12805,0.13164,0.13599,0.12922,0.12245,0.13142,0.1309
6,0.12924,0.13142,0.11972,0.12631,0.13417,0.52619,0.56057,0.55905,0.53
617,0.54898,0.54463,0.54974,0.52881,0.53473,0.38492,0.34371,0.30508,0.
23317,0.27138,0.27339,0.27157,0.2764,0.30245,0.25891,0.27183,0.13229,0
.14063,0.12487,0.12027,0.12911,0.14125,0.13502,0.12979,0.13516,0.11894
,0.1294,0.13188,0.1286,0.12956,0.13451,0.12803,0.13039,0.12802,0.52701
,0.52702,0.52691,0.57578,0.54671,0.5022,0.54531,0.51602,0.55838,0.2907
4,0.26731,0.27207,0.28875,0.25193,0.29931,0.25564,0.30888,0.28429,0.28
028,0.22351,0.12943,0.13232,0.13875,0.13037,0.12983,0.14333,0.14517,0.
13114,0.14086,0.13222,0.12978,0.13412,0.1389,0.12548,0.124,0.13019,0.1
2631,0.13071,0.53604,0.51333,0.55353,0.55035,0.57464,0.54838,0.5251,0.
30994,0.32453,0.36127,0.25687,0.27453,0.27454,0.26935,0.25233,0.27643,
0.26897,0.27758,0.26762,0.27959,0.13142,0.12935,0.1362,0.12543,0.13122
,0.12995,0.13083,0.14313,0.13126,0.13136,0.12453,0.13297,0.12854,0.130
52,0.12531,0.12656,0.12651,0.13898,0.55721,0.52411,0.55454,0.57005,0.5
569,0.54261,0.31614,0.31832,0.32367,0.29455,0.30155,0.29204,0.27301,0.
26151,0.28013,0.2926,0.27637,0.28434,0.26799,0.29742,0.12961,0.13167,0
.13052,0.12935,0.12534,0.12956,0.12769,0.12837,0.13361,0.13425,0.14317
,0.13105,0.13108,0.12688,0.14072,0.13462,0.12223,0.13017,0.50954,0.526
38,0.5402,0.52735,0.54718,0.33171,0.31966,0.29709,0.32117,0.30215,0.23
601,0.25619,0.28042,0.27413,0.27551,0.28494,0.27053,0.26698,0.27478,0.
27776,0.13931,0.12164,0.14025,0.12821,0.11877,0.13107,0.1278,0.1405,0.
12406,0.12984,0.1257,0.13177,0.13149,0.13074,0.13176,0.13789,0.12523,0
.13134,0.57293,0.52921,0.54971,0.31236,0.30547,0.32394,0.33262,0.31725
,0.34765,0.28212,0.33735,0.29835,0.27252,0.26947,0.29383,0.2385,0.2715
7,0.28672,0.23985,0.25368,0.12856,0.13143,0.12255,0.13044,0.12453,0.13
541,0.13655,0.12973,0.13104,0.1224,0.13024,0.13158,0.13168,0.13919,0.1
2364,0.12949,0.13045,0.13122,0.54694,0.32234,0.32852,0.29669,0.28038,0
.32893,0.3028,0.31317,0.3148,0.31078,0.26235,0.2697,0.28091,0.26159,0.
2907,0.26879,0.27204,0.26897,0.26939,0.12949,0.13125,0.12996,0.12965,0
.12855,0.12445,0.12947,0.1299,0.13428,0.13332,0.12979,0.12964,0.12789,
0.13441,0.14682,0.1297,0.12039,0.07974,0.31398,0.33179,0.30761,0.29871
,0.30543,0.27,0.2972,0.33119,0.31499,0.38306,0.29721,0.21337,0.22545,0
.27585,0.26661,0.24796,0.26598,0.2455,0.2651,0.13074,0.13117,0.13206,0
.13095,0.1292,0.11669,0.13193,0.12377,0.13393,0.12774,0.13745,0.12792,
0.12783,0.1333,0.13143,0.12996,0.13023,0.080272,0.35341,0.29443,0.3161
2,0.34647,0.33107,0.27852,0.28779,0.26128,0.27086,0.31401,0.244,0.3078
4,0.21186,0.28363,0.26879,0.27531,0.27639,0.29677,0.12711,0.12997,0.13
052,0.13213,0.1304,0.11902,0.14256,0.13282,0.13514,0.13167,0.13414,0.1
3142,0.13024,0.13136,0.12351,0.13102,0.12878,0.13082,0.079145,0.32965,
0.37452,0.29791,0.31798,0.3232,0.29182,0.2928,0.31561,0.27423,0.31602,
0.27621,0.26372,0.24571,0.26344,0.27928,0.27864,0.27449,0.24558,0.1389
7,0.13031,0.12915,0.13013,0.1285,0.14438,0.12655,0.12886,0.12941,0.132
44,0.12888,0.13066,0.12291,0.12698,0.12421,0.13238,0.13833,0.079605,0.
081099,0.080111,0.31561,0.31034,0.30666,0.30926,0.30914,0.31507,0.2968
6,0.30226,0.30997,0.23457,0.24869,0.26994,0.24275,0.27153,0.28281,0.11
641,0.13338,0.13464,0.13098,0.13096,0.1313,0.12766,0.13331,0.12165,0.1
3638,0.13019,0.12364,0.12788,0.13074,0.12577,0.12453,0.13203,0.12827,0
.080073,0.081393,0.078353,0.08159,0.22115,0.30339,0.27577,0.25386,0.28
977,0.30988,0.35029,0.35229,0.35916,0.2948,0.23944,0.26284,0.14171,0.1
2686,0.12048,0.12448,0.13965,0.1381,0.13998,0.12419,0.1358,0.1313,0.13
719,0.13681,0.12932,0.13949,0.13065,0.13137,0.13274,0.13396,0.1215,0.1

323,0.078495,0.080332,0.081585,0.07889,0.079009,0.079619,0.31136,0.309
22,0.34939,0.31664,0.28087,0.31345,0.29381,0.31691,0.12384,0.11782,0.1
1343,0.13162,0.12899,0.12917,0.12953,0.13251,0.11153,0.13108,0.13603,0
.15487,0.12934,0.12963,0.14821,0.13302,0.13047,0.11586,0.1303,0.12803,
0.12859,0.12607,0.1325,0.079058,0.080242,0.077409,0.080831,0.078538,0.
081988,0.32069,0.31347,0.34754,0.30162,0.32267,0.079876,0.078418,0.131
82,0.13112,0.13102,0.13071,0.13131,0.13134,0.12872,0.13507,0.12749,0.1
2416,0.12695,0.12806,0.13306,0.13045,0.12638,0.12801,0.13202,0.128,0.1
3085,0.13444,0.13291,0.081303,0.07998,0.07778,0.080656,0.079101,0.0792
84,0.081564,0.082951,0.078226,0.078279,0.30275,0.28314,0.078056,0.0788
32,0.079687,0.081369,0.13684,0.11456,0.13112,0.13126,0.13296,0.13876,0
.12984,0.1301,0.13132,0.12661,0.12439,0.14007,0.13558,0.12848,0.13165,
0.13112,0.12493,0.13814,0.13415,0.080361,0.081895,0.081278,0.081697,0.
080511,0.07882,0.078754,0.079208,0.079536,0.078102,0.079762,0.080885,0
.081308,0.08143,0.083187,0.078769,0.080245,0.12934,0.13474,0.12287,0.1
1964,0.12992,0.12807,0.13292,0.12417,0.12879,0.12971,0.12922,0.13336,0
.12417,0.1316,0.13461,0.13128,0.080855,0.079695,0.078995,0.080257,0.08
1286,0.080322,0.079178,0.081026,0.081233,0.081419,0.079648,0.079996,0.
080123,0.079325,0.082379,0.080277,0.080877,0.079907,0.082977,0.1245,0.
12644,0.14406,0.13125,0.12433,0.12954,0.13014,0.13376,0.12607,0.13294,
0.13372,0.13605,0.12331,0.08158,0.081102,0.079746,0.078675,0.079506,0.
079053,0.080448,0.082236,0.080393,0.079013,0.081272,0.084179,0.079371,
0.079384,0.081748,0.078628,0.081697,0.081554,0.08093,0.080028,0.079196
,0.1274,0.13173,0.13679,0.13132,0.1375,0.12149,0.12492,0.13044,0.12926
,0.078948,0.080966,0.080877,0.081279,0.081456,0.078702,0.079857,0.0785
6,0.080441,0.081067,0.082068,0.079253,0.081799,0.079837,0.077479,0.080
08,0.081564,0.077639,0.081021,0.08033,0.079678,0.080142,0.079882,0.080
137,0.081095,0.12616,0.1382,0.12785,0.12003,0.078295,0.079118,0.079865
,0.081382,0.077562,0.076321,0.079181,0.078169,0.080416,0.081815,0.0799
42,0.078847,0.081361,0.080515,0.082714,0.080874,0.079491,0.07915,0.077
132,0.079628,0.0815,0.0783,0.080624,0.079346,0.08207,0.082831,0.082426
,0.08133,0.077613,0.081684,0.079449,0.078862,0.082632,0.081,0.080788,0
.079579,0.079929,0.077892,0.080417,0.080467,0.080656,0.081238,0.080207
,0.081212,0.078777,0.08136,0.080221,0.080167,0.080225,0.081264,0.07795
8,0.081059,0.078658,0.079036,0.079421,0.079917,0.081035,0.079982,0.080
999,0.078066,0.079882,0.078664,0.082774,0.080741,0.08045,0.080285,0.07
9774,0.079233,0.080426,0.080342,0.081683,0.084009,0.07812,0.081806,0.0
7926,0.080097,0.080698,0.080933,0.077779,0.079421,0.077061,0.080644,0.
080703,0.077462,0.079947,0.080219,0.078649,0.080354,0.079308,0.079312,
0.077944,0.07813,0.081154,0.079593,0.082674,0.079203,0.080883,0.079913
,0.080796,0.080914,0.079677,0.082041,0.079679,0.079654,0.078445,0.0800
91,0.080946,0.079942,0.081271,0.07853,0.080032,0.080443,0.080748,0.080
416,0.079951,0.079809,0.078718,0.080632,0.080151,0.079037,0.077075,0.0
79908,0.080767,0.08098,0.078893,0.081752,0.079978,0.081514,0.081905,0.
078205,0.077875,0.080695,0.076943,0.080195,0.079551,0.081419,0.079133,
0.080699,0.08168,0.077423,0.0784,0.079242,0.07882,0.078212,0.078931,0.
081357,0.082283,0.080398,0.079525,0.081492,0.080905,0.080755,0.080072,
0.079589,0.081614,0.082314,0.077239,0.080876,0.077909,0.078623,0.07866
3,0.081659,0.080524,0.079374,0.080038,0.081226,0.079821,0.08065,0.0805
44,0.08033,0.079069,0.07909,0.08134,0.080008,0.079307,0.082448,0.07734
5,0.080114,0.078911,0.081852,0.080189,0.080675,0.078616,0.081386,0.078

538,0.078928,0.078275,0.082051,0.079047,0.079858,0.081191,0.080057,0.0
79774,0.080255,0.083064
0.18301,0.18698,0.18424,0.18955,0.18574,0.18735,0.18454,0.18768,0.1873
3,0.18837,0.18802,0.18943,0.18557,0.18707,0.18712,0.18481,0.18577,0.18
907,0.18876,0.18527,0.35962,0.32191,0.3877,0.31693,0.18627,0.18915,0.1
8763,0.18712,0.18486,0.18993,0.18516,0.18583,0.18529,0.18528,0.19,0.18
564,0.18243,0.18439,0.18559,0.45945,0.33069,0.33434,0.33731,0.32685,0.
33299,0.33734,0.1826,0.18359,0.18877,0.18833,0.18629,0.18694,0.18426,0
.18645,0.18606,0.18778,0.18523,0.18687,0.18795,0.18889,0.18749,0.50778
,0.46692,0.31927,0.35883,0.29578,0.33457,0.34043,0.32415,0.32432,0.312
68,0.18515,0.18769,0.18884,0.18728,0.1867,0.18625,0.18659,0.18579,0.18
789,0.18786,0.18568,0.18439,0.18731,0.18607,0.18745,0.48246,0.44433,0.
46028,0.26157,0.38264,0.34255,0.32959,0.3441,0.31614,0.37179,0.34362,0
.32117,0.18821,0.18596,0.18644,0.18519,0.18797,0.18595,0.18597,0.1863,
0.18738,0.18927,0.18754,0.18801,0.18783,0.18749,0.18349,0.18549,0.4384
2,0.48895,0.46752,0.47077,0.33463,0.33781,0.28931,0.3379,0.30832,0.325
36,0.33056,0.33841,0.34395,0.34499,0.18692,0.18902,0.1892,0.18776,0.18
342,0.18722,0.18679,0.18708,0.18849,0.18818,0.18486,0.18778,0.1877,0.1
8884,0.18372,0.18368,0.46517,0.48346,0.46954,0.48112,0.45253,0.45188,0
.3226,0.3311,0.31691,0.32594,0.32621,0.35667,0.30915,0.32889,0.3203,0.
32265,0.18815,0.18773,0.18523,0.18578,0.18383,0.18677,0.18582,0.18649,
0.18743,0.18937,0.18907,0.18891,0.18764,0.18434,0.18844,0.18713,0.1868
,0.47266,0.4783,0.47328,0.49601,0.45388,0.49927,0.33258,0.29888,0.3366
9,0.35527,0.36303,0.3576,0.32378,0.32462,0.32519,0.32726,0.33364,0.186
07,0.18604,0.18343,0.18601,0.1845,0.18355,0.1841,0.18601,0.1839,0.1869
2,0.18619,0.18473,0.18508,0.13073,0.11696,0.13137,0.18892,0.46394,0.48
582,0.46317,0.48358,0.47557,0.49085,0.45854,0.34485,0.3191,0.34701,0.3
2758,0.35445,0.33408,0.31027,0.33804,0.33526,0.34054,0.32313,0.18567,0
.18886,0.18765,0.18667,0.18688,0.18797,0.1872,0.18504,0.18497,0.18448,
0.18635,0.11121,0.12659,0.12649,0.12676,0.12675,0.13021,0.18879,0.4410
5,0.46668,0.48472,0.46997,0.45877,0.46176,0.4884,0.32756,0.35113,0.309
82,0.34105,0.32335,0.32791,0.32736,0.32514,0.34667,0.3239,0.29652,0.18
487,0.18653,0.18703,0.18621,0.18429,0.19113,0.18679,0.18645,0.18838,0.
12639,0.1275,0.13794,0.12665,0.12752,0.12623,0.12786,0.13203,0.1267,0.
18716,0.48738,0.4663,0.50042,0.50561,0.45569,0.446,0.47367,0.31088,0.3
2642,0.32136,0.34475,0.33767,0.33562,0.33644,0.30165,0.33024,0.32947,0
.32573,0.28757,0.18574,0.18841,0.18911,0.18929,0.18686,0.19103,0.11992
,0.12713,0.13111,0.12741,0.14058,0.12729,0.12217,0.12895,0.12883,0.141
03,0.1288,0.12857,0.47044,0.48358,0.44577,0.50836,0.47537,0.47471,0.45
77,0.31259,0.32393,0.3232,0.34033,0.33795,0.34406,0.35951,0.34402,0.35
343,0.33125,0.32467,0.32462,0.19205,0.18609,0.18752,0.12408,0.14002,0.
11794,0.11909,0.13085,0.12543,0.1272,0.12597,0.13022,0.12798,0.12719,0
.13378,0.13021,0.1469,0.12785,0.46726,0.45674,0.47659,0.46545,0.47737,
0.49745,0.51074,0.4626,0.47319,0.33693,0.32375,0.30563,0.3338,0.29341,
0.32355,0.32777,0.3306,0.32499,0.34477,0.34904,0.12773,0.12825,0.12665
,0.12564,0.12821,0.12648,0.12362,0.12805,0.13318,0.12589,0.11738,0.128
12,0.12737,0.12644,0.12758,0.11446,0.12255,0.13015,0.4636,0.50268,0.49
618,0.46295,0.47982,0.47557,0.49181,0.45033,0.47109,0.34741,0.31203,0.
27806,0.21885,0.24613,0.24706,0.24242,0.2519,0.26989,0.231,0.25112,0.1
2966,0.13862,0.12064,0.11594,0.12593,0.14054,0.13156,0.12674,0.13302,0
.11312,0.12561,0.12887,0.12531,0.12597,0.13143,0.1247,0.12707,0.12436,
0.46586,0.46342,0.46023,0.51838,0.46941,0.43587,0.46837,0.43285,0.4771

4,0.24705,0.24218,0.24221,0.26751,0.22598,0.27121,0.23064,0.27391,0.25
6,0.25365,0.20574,0.12603,0.12899,0.1372,0.12727,0.12612,0.14245,0.143
88,0.12715,0.13846,0.12924,0.12587,0.13124,0.13707,0.12055,0.11952,0.1
2655,0.12213,0.12733,0.4888,0.45136,0.48708,0.49376,0.50407,0.48354,0.
45859,0.2743,0.2778,0.32201,0.23652,0.24739,0.25235,0.24771,0.22901,0.
24131,0.24583,0.25074,0.24407,0.25362,0.12769,0.12574,0.13384,0.12123,
0.12779,0.12516,0.12767,0.14229,0.12757,0.12783,0.12007,0.13062,0.1246
9,0.12681,0.12122,0.12173,0.1233,0.1367,0.48943,0.46736,0.48762,0.4990
6,0.48612,0.4684,0.27562,0.28921,0.28035,0.25462,0.26979,0.25874,0.246
6,0.23896,0.25374,0.26658,0.2507,0.25306,0.24657,0.27708,0.12639,0.128
94,0.12656,0.12613,0.12081,0.12621,0.12335,0.12474,0.13078,0.1311,0.14
118,0.12687,0.12767,0.12249,0.13813,0.13162,0.11738,0.12667,0.44135,0.
45983,0.47884,0.45846,0.47913,0.29438,0.28008,0.25523,0.28983,0.25973,
0.20397,0.23089,0.25451,0.24679,0.24716,0.25149,0.25223,0.23761,0.2478
8,0.25203,0.13818,0.11645,0.13808,0.12432,0.11408,0.12737,0.12322,0.13
824,0.11907,0.12642,0.12005,0.12863,0.12828,0.1264,0.12884,0.13535,0.1
2084,0.12798,0.50391,0.46689,0.48579,0.27878,0.26704,0.27308,0.28895,0
.27206,0.30029,0.24206,0.3,0.26959,0.2454,0.24236,0.26643,0.22035,0.24
737,0.25343,0.21853,0.22821,0.12485,0.12824,0.11755,0.12687,0.1201,0.1
3282,0.13423,0.12684,0.12798,0.11811,0.12664,0.12846,0.12854,0.13751,0
.11975,0.12594,0.12731,0.12784,0.47017,0.28132,0.29323,0.25919,0.2443,
0.28726,0.25935,0.27535,0.26984,0.27173,0.24193,0.24504,0.25246,0.2389
2,0.26194,0.24123,0.24416,0.24863,0.24648,0.12655,0.12826,0.12667,0.12
593,0.12477,0.11998,0.12475,0.12674,0.13074,0.12997,0.12584,0.12581,0.
12403,0.13219,0.14689,0.12641,0.11538,0.06704,0.26984,0.29083,0.26417,
0.25742,0.26381,0.2346,0.25812,0.29407,0.27157,0.33092,0.27529,0.19499
,0.20671,0.24974,0.24456,0.22996,0.23602,0.22322,0.23954,0.12678,0.127
7,0.12933,0.12794,0.12489,0.11072,0.1277,0.11962,0.13156,0.12464,0.134
84,0.12439,0.12432,0.13054,0.12712,0.12708,0.12659,0.067934,0.30245,0.
25126,0.27928,0.29978,0.29238,0.24452,0.25558,0.23105,0.23783,0.27721,
0.22539,0.281,0.19386,0.25251,0.23911,0.24895,0.25186,0.26931,0.12318,
0.12633,0.12694,0.12938,0.12707,0.11387,0.14064,0.12972,0.13258,0.1288
,0.13199,0.12733,0.12667,0.1282,0.12009,0.12792,0.12465,0.1277,0.06646
2,0.28414,0.33152,0.25381,0.27899,0.28699,0.24855,0.25696,0.26591,0.24
516,0.27881,0.24824,0.24079,0.22196,0.24407,0.25648,0.24645,0.24246,0.
22323,0.13592,0.12736,0.12535,0.12649,0.12567,0.14427,0.12208,0.12576,
0.12532,0.12863,0.12616,0.12673,0.11885,0.12237,0.12015,0.12891,0.1367
4,0.068058,0.069057,0.067735,0.27857,0.2749,0.27738,0.2731,0.273,0.270
5,0.26039,0.2583,0.2708,0.21461,0.22722,0.24917,0.21972,0.25015,0.2609
2,0.11041,0.131,0.1318,0.12763,0.12751,0.12768,0.12244,0.13066,0.11813
,0.13397,0.12719,0.11908,0.12411,0.12645,0.12216,0.11997,0.12799,0.124
84,0.06725,0.068652,0.066201,0.070092,0.1934,0.2638,0.24389,0.22167,0.
25655,0.26628,0.32011,0.31442,0.31056,0.26958,0.22068,0.24109,0.14028,
0.1228,0.11434,0.12041,0.13784,0.13711,0.13947,0.11986,0.13298,0.12862
,0.13492,0.13436,0.12552,0.13844,0.12729,0.12792,0.12999,0.13186,0.116
45,0.12859,0.066334,0.067484,0.068909,0.066576,0.066967,0.067198,0.266
99,0.26406,0.3038,0.28172,0.2495,0.27787,0.25459,0.27674,0.1191,0.1128
7,0.10642,0.12761,0.12551,0.12562,0.12591,0.13004,0.10529,0.12738,0.13
378,0.15564,0.12574,0.12633,0.14725,0.13023,0.12714,0.10926,0.12712,0.
12419,0.12503,0.1223,0.12954,0.066092,0.068124,0.065086,0.068595,0.065
822,0.067494,0.28814,0.27487,0.30025,0.26963,0.28771,0.068675,0.066263
,0.12864,0.12753,0.12721,0.12683,0.12813,0.1278,0.12547,0.13316,0.1233

9,0.12075,0.12435,0.12372,0.1301,0.12721,0.1214,0.12513,0.12851,0.1248
4,0.12745,0.13147,0.13014,0.068249,0.066525,0.065333,0.06856,0.066353,
0.067555,0.069207,0.069988,0.067065,0.066332,0.27078,0.24542,0.066034,
0.066606,0.067035,0.068707,0.13479,0.10824,0.12762,0.12822,0.13009,0.1
3719,0.12642,0.12621,0.12804,0.12203,0.11974,0.1389,0.13218,0.1248,0.1
2857,0.12728,0.12034,0.13609,0.13282,0.067986,0.069346,0.068968,0.0695
21,0.067646,0.066014,0.065492,0.067688,0.066969,0.066044,0.06681,0.068
978,0.068385,0.069382,0.070123,0.066127,0.067386,0.12497,0.13181,0.118
37,0.11333,0.12708,0.12334,0.12973,0.11914,0.12595,0.12602,0.1253,0.13
064,0.11983,0.12821,0.13113,0.12792,0.067724,0.068071,0.067393,0.06771
3,0.069441,0.06812,0.066156,0.06947,0.069517,0.069301,0.06772,0.0681,0
.068414,0.066393,0.069487,0.067367,0.06881,0.068191,0.071198,0.11957,0
.1225,0.14302,0.12801,0.1195,0.12683,0.12693,0.13111,0.12215,0.13062,0
.13087,0.13226,0.11835,0.069261,0.068969,0.067343,0.0653,0.068428,0.06
6586,0.067996,0.068999,0.0674,0.066805,0.068718,0.072311,0.066626,0.06
6328,0.069099,0.066683,0.070201,0.069042,0.06718,0.068405,0.066924,0.1
2373,0.12835,0.13378,0.12832,0.13468,0.11668,0.12134,0.12663,0.12573,0
.067076,0.067757,0.068802,0.069328,0.069257,0.06684,0.067921,0.066743,
0.06844,0.069311,0.069494,0.068176,0.069509,0.067171,0.065107,0.067604
,0.069856,0.065019,0.068425,0.067371,0.068128,0.067421,0.067665,0.0680
25,0.069992,0.12126,0.13623,0.12462,0.11445,0.066747,0.066518,0.067564
,0.06835,0.065366,0.064357,0.067618,0.066161,0.068313,0.068373,0.06758
,0.065991,0.068379,0.067678,0.070251,0.068621,0.066403,0.066752,0.0650
17,0.066951,0.069115,0.066231,0.068742,0.067152,0.069735,0.071317,0.07
0872,0.069963,0.064983,0.069933,0.067722,0.065239,0.070824,0.069415,0.
06863,0.06759,0.068038,0.066312,0.068023,0.069482,0.068627,0.068857,0.
068383,0.068736,0.067211,0.06935,0.067949,0.06736,0.067247,0.069001,0.
065236,0.069102,0.065779,0.067677,0.066844,0.068019,0.067862,0.067056,
0.068189,0.065727,0.06783,0.067122,0.070525,0.068445,0.068664,0.068284
,0.0679,0.067473,0.067484,0.067726,0.069859,0.072441,0.066006,0.070089
,0.065865,0.067536,0.068772,0.068878,0.065519,0.067465,0.065282,0.0682
06,0.068527,0.065321,0.067531,0.067236,0.066288,0.068615,0.067265,0.06
7789,0.066156,0.066229,0.068098,0.067133,0.068913,0.065725,0.067937,0.
068513,0.069089,0.068816,0.068596,0.068904,0.068357,0.066936,0.066455,
0.067751,0.068212,0.067041,0.068331,0.065338,0.066681,0.068081,0.06847
,0.068252,0.068287,0.066778,0.066544,0.068393,0.06752,0.066539,0.06511
2,0.06727,0.067969,0.068366,0.066642,0.069382,0.067964,0.06896,0.06996
,0.066471,0.065918,0.068529,0.065606,0.068887,0.066734,0.068169,0.0664
79,0.067553,0.069678,0.065313,0.066186,0.066712,0.066583,0.065773,0.06
6428,0.069142,0.069802,0.068465,0.067879,0.069362,0.067719,0.068544,0.
068162,0.067665,0.069832,0.070768,0.06503,0.068522,0.066651,0.066709,0
.067033,0.068991,0.068762,0.067089,0.067426,0.068899,0.06804,0.06787,0
.067843,0.068276,0.067331,0.06767,0.068284,0.067902,0.066465,0.069451,
0.065283,0.06748,0.066596,0.069334,0.068073,0.066934,0.067269,0.069184
,0.067069,0.066833,0.066359,0.070748,0.066824,0.067414,0.069201,0.0677
64,0.067578,0.06741,0.071673
0.1856,0.19104,0.18884,0.19311,0.18821,0.19192,0.18863,0.1917,0.1913,0
.19254,0.19159,0.19224,0.18935,0.19166,0.19163,0.19003,0.19003,0.19359
,0.19235,0.1888,0.3384,0.3042,0.35904,0.29631,0.19044,0.19382,0.19216,
0.19119,0.187,0.1941,0.18989,0.18988,0.19049,0.18892,0.19445,0.18919,0
.18654,0.18897,0.18897,0.41802,0.30669,0.31409,0.31392,0.30969,0.31418
,0.31916,0.18808,0.1864,0.19272,0.19187,0.18963,0.19065,0.18776,0.1910

2,0.18926,0.19067,0.18923,0.18984,0.19086,0.1919,0.19223,0.4728,0.4297
5,0.29526,0.33263,0.28258,0.31509,0.3181,0.2956,0.30434,0.29465,0.1885
4,0.19157,0.1923,0.1912,0.19011,0.18902,0.18968,0.19022,0.1939,0.19131
,0.1889,0.18933,0.1914,0.19113,0.19207,0.43037,0.40899,0.42434,0.25572
,0.35935,0.32025,0.31296,0.32937,0.30406,0.35105,0.3274,0.30748,0.1915
6,0.18937,0.1909,0.18894,0.19362,0.19055,0.19018,0.18956,0.19137,0.193
85,0.19018,0.19215,0.19145,0.19151,0.18791,0.19008,0.40706,0.45386,0.4
2622,0.43859,0.31397,0.31523,0.28202,0.31892,0.29519,0.30662,0.30945,0
.31387,0.3296,0.31985,0.19126,0.19292,0.19245,0.19133,0.18807,0.19037,
0.19082,0.19028,0.19212,0.19307,0.18969,0.18995,0.19106,0.19377,0.1873
9,0.18795,0.41621,0.42204,0.43736,0.4348,0.40767,0.41178,0.30537,0.308
33,0.29664,0.3069,0.30871,0.34032,0.29213,0.31153,0.29945,0.31016,0.19
132,0.19176,0.18876,0.18908,0.18882,0.19153,0.18946,0.19198,0.19124,0.
19289,0.19232,0.1929,0.19105,0.18831,0.1927,0.19158,0.18989,0.45384,0.
45544,0.44487,0.44485,0.41701,0.46131,0.32019,0.2862,0.31709,0.33014,0
.3388,0.34051,0.3032,0.30836,0.31059,0.3012,0.31305,0.1902,0.19141,0.1
8743,0.1889,0.18978,0.18841,0.18849,0.18947,0.1873,0.19157,0.18994,0.1
8889,0.18857,0.1289,0.11355,0.12926,0.1925,0.41642,0.44912,0.42851,0.4
4569,0.45121,0.44121,0.42496,0.32165,0.30358,0.3326,0.30955,0.34376,0.
31139,0.29547,0.31774,0.31218,0.3197,0.30168,0.18908,0.19329,0.19117,0
.19131,0.19083,0.19126,0.19265,0.18848,0.18841,0.1884,0.1907,0.10729,0
.12462,0.1245,0.12412,0.12398,0.12848,0.1928,0.40783,0.42301,0.43447,0
.43166,0.42481,0.41838,0.44629,0.30646,0.33407,0.29746,0.32111,0.30288
,0.31446,0.31106,0.31397,0.32824,0.30161,0.27925,0.18946,0.19185,0.191
4,0.18952,0.18744,0.19439,0.1921,0.19199,0.19352,0.1238,0.1252,0.13626
,0.12364,0.12474,0.1232,0.12559,0.13003,0.12401,0.19079,0.44112,0.4411
8,0.45178,0.4686,0.42179,0.40978,0.43393,0.2998,0.3052,0.30702,0.32814
,0.32021,0.31561,0.31359,0.28769,0.31493,0.31105,0.30461,0.27533,0.191
16,0.19213,0.19272,0.1937,0.19039,0.19519,0.11709,0.12467,0.1284,0.125
01,0.13936,0.12508,0.11916,0.12599,0.12604,0.14069,0.12712,0.1266,0.42
986,0.43403,0.4076,0.46897,0.43457,0.43913,0.41366,0.29299,0.30682,0.3
0793,0.31933,0.31901,0.32595,0.33651,0.32124,0.32673,0.31426,0.31282,0
.29869,0.19396,0.19123,0.19229,0.12178,0.13852,0.11429,0.11575,0.12799
,0.12356,0.12548,0.12431,0.12825,0.12519,0.1247,0.13216,0.12851,0.1468
1,0.12602,0.4275,0.42097,0.42337,0.42125,0.41735,0.44841,0.47474,0.423
65,0.4308,0.31689,0.30617,0.29165,0.31094,0.28165,0.30563,0.30709,0.31
554,0.30864,0.32364,0.33283,0.12525,0.12564,0.12321,0.12323,0.12621,0.
1246,0.12019,0.12608,0.13114,0.1234,0.11303,0.12564,0.12531,0.12352,0.
12494,0.11029,0.1192,0.12885,0.43245,0.45681,0.46187,0.42065,0.4285,0.
43716,0.46439,0.40373,0.43238,0.32478,0.29799,0.25543,0.20879,0.2366,0
.23117,0.22608,0.23173,0.25392,0.21477,0.23649,0.12664,0.13762,0.11751
,0.1121,0.12358,0.1393,0.12853,0.12432,0.13132,0.1085,0.12237,0.1258,0
.12291,0.12417,0.12875,0.1226,0.12466,0.12142,0.4273,0.41918,0.42446,0
.48371,0.41671,0.40216,0.42056,0.40346,0.42511,0.22371,0.22785,0.22703
,0.25224,0.21313,0.25252,0.21462,0.25739,0.24212,0.24207,0.19863,0.123
31,0.12661,0.13565,0.12532,0.12313,0.14181,0.14286,0.12534,0.13752,0.1
273,0.12357,0.12915,0.13602,0.11767,0.11633,0.12399,0.11868,0.12466,0.
44635,0.41368,0.44512,0.44766,0.46572,0.45064,0.39839,0.24406,0.25665,
0.29893,0.22099,0.23222,0.23814,0.22977,0.21596,0.22188,0.23141,0.2334
5,0.22759,0.23837,0.12513,0.12408,0.13277,0.11857,0.12484,0.12304,0.12
509,0.14124,0.12517,0.12548,0.11724,0.12847,0.12159,0.12414,0.11816,0.
11812,0.12131,0.13561,0.44189,0.42185,0.44846,0.45967,0.45605,0.41997,

0.24304,0.26503,0.25676,0.22415,0.24121,0.24205,0.23027,0.22097,0.2338
,0.24859,0.23642,0.24435,0.22604,0.2618,0.12384,0.12624,0.124,0.12373,
0.11742,0.12386,0.12087,0.12124,0.12863,0.12917,0.14125,0.12449,0.1253
8,0.11915,0.13701,0.12947,0.11431,0.1234,0.38946,0.4326,0.44204,0.4340
4,0.44283,0.26167,0.24739,0.23008,0.2565,0.23571,0.18596,0.21781,0.241
89,0.23279,0.23171,0.23422,0.23704,0.22272,0.2342,0.23423,0.13766,0.11
273,0.13689,0.12142,0.11004,0.12435,0.12053,0.13738,0.11552,0.12369,0.
11768,0.12593,0.12557,0.12384,0.1271,0.13373,0.1174,0.12581,0.46399,0.
42382,0.44506,0.25337,0.24865,0.2493,0.26501,0.24663,0.27656,0.21335,0
.27242,0.24677,0.22467,0.22638,0.25233,0.20816,0.231,0.2372,0.20589,0.
21628,0.12249,0.12596,0.11433,0.12483,0.11768,0.13113,0.133,0.12528,0.
12484,0.11452,0.12435,0.12521,0.12637,0.13661,0.11587,0.12415,0.12459,
0.12507,0.42643,0.25332,0.27011,0.23813,0.21513,0.26381,0.23615,0.2457
2,0.24241,0.247,0.22792,0.23622,0.23294,0.22939,0.24513,0.21834,0.2323
6,0.23137,0.23115,0.12422,0.12514,0.12479,0.12322,0.1219,0.11748,0.121
92,0.12517,0.12843,0.1277,0.12396,0.12288,0.12151,0.13009,0.14592,0.12
373,0.1114,0.058271,0.24152,0.25171,0.23863,0.23516,0.24531,0.21304,0.
2398,0.26961,0.24371,0.30951,0.25898,0.18277,0.19667,0.23334,0.22679,0
.21382,0.21385,0.21419,0.21889,0.12433,0.12501,0.12718,0.12486,0.12254
,0.10582,0.12571,0.117,0.12916,0.12172,0.13296,0.12158,0.12202,0.12874
,0.12485,0.12461,0.12417,0.059136,0.27644,0.23212,0.24566,0.28079,0.26
012,0.23147,0.23554,0.20706,0.20986,0.25435,0.21217,0.26285,0.1823,0.2
3324,0.22458,0.23374,0.23363,0.24868,0.12063,0.12393,0.12454,0.12693,0
.12502,0.11019,0.13945,0.12745,0.12977,0.12608,0.12958,0.12405,0.12392
,0.1257,0.11742,0.12575,0.12222,0.12485,0.057878,0.25328,0.29789,0.228
84,0.25511,0.25571,0.22656,0.23195,0.23902,0.22725,0.25436,0.23048,0.2
2638,0.21057,0.23195,0.24152,0.23953,0.22119,0.21238,0.13559,0.125,0.1
2246,0.12389,0.1235,0.14432,0.11903,0.12349,0.12253,0.126,0.12425,0.12
343,0.11493,0.11946,0.11779,0.12598,0.13579,0.058207,0.059871,0.058451
,0.25136,0.24894,0.25664,0.24547,0.24814,0.24897,0.2302,0.23248,0.2456
6,0.20033,0.21271,0.22851,0.20676,0.23885,0.24344,0.10616,0.12827,0.13
021,0.12459,0.12517,0.12449,0.11913,0.12828,0.11495,0.13237,0.12471,0.
11651,0.12145,0.12318,0.11929,0.11741,0.1251,0.12197,0.058868,0.059576
,0.057383,0.061015,0.17178,0.24485,0.21958,0.20554,0.23536,0.23258,0.2
9512,0.28847,0.2807,0.2434,0.21042,0.2248,0.13883,0.11962,0.11109,0.11
757,0.13693,0.13621,0.1389,0.11667,0.13015,0.12658,0.13415,0.13344,0.1
2347,0.13656,0.12491,0.12603,0.12773,0.1294,0.11316,0.12613,0.058883,0
.058755,0.059395,0.058609,0.05799,0.058189,0.24475,0.23976,0.28671,0.2
4907,0.22367,0.25014,0.23093,0.25226,0.11653,0.10923,0.10144,0.12537,0
.12338,0.12344,0.12298,0.12771,0.10101,0.1252,0.13187,0.15775,0.12343,
0.12422,0.14657,0.1273,0.12555,0.10418,0.12427,0.12117,0.12188,0.11951
,0.12778,0.057942,0.059593,0.056597,0.059536,0.057407,0.057601,0.26536
,0.25001,0.27096,0.24923,0.26164,0.058484,0.057688,0.12585,0.1242,0.12
5,0.12407,0.12523,0.12528,0.1226,0.1306,0.12121,0.11778,0.1222,0.12091
,0.12825,0.12527,0.11863,0.12288,0.12628,0.12199,0.12562,0.12944,0.127
54,0.059153,0.058226,0.055806,0.059388,0.057105,0.060071,0.060245,0.06
0833,0.058423,0.057098,0.24644,0.22338,0.057102,0.058442,0.058646,0.06
0197,0.13325,0.10399,0.12565,0.12547,0.12757,0.13492,0.12425,0.12298,0
.12553,0.1193,0.11715,0.13828,0.13001,0.122,0.12624,0.12384,0.11737,0.
13383,0.13077,0.0584,0.060545,0.060284,0.061015,0.059585,0.057925,0.05
691,0.058394,0.05878,0.057084,0.057887,0.059772,0.059883,0.060518,0.06
1303,0.057514,0.057006,0.12234,0.1296,0.11528,0.10904,0.12487,0.12069,

0.12792,0.11525,0.1233,0.1238,0.12283,0.1282,0.11619,0.12502,0.12963,0
.1253,0.059615,0.058776,0.058675,0.059434,0.061246,0.059573,0.057033,0
.06083,0.061369,0.060581,0.059271,0.059583,0.059316,0.057632,0.061989,
0.059324,0.058887,0.058609,0.062432,0.1165,0.11961,0.1421,0.12573,0.11
621,0.12452,0.12491,0.12899,0.11851,0.12808,0.12896,0.13025,0.11477,0.
059194,0.059582,0.058972,0.057221,0.059785,0.058476,0.059217,0.06047,0
.05908,0.058356,0.059853,0.062346,0.057755,0.058683,0.060493,0.058468,
0.060921,0.060144,0.058236,0.059963,0.057587,0.12029,0.12621,0.13193,0
.12638,0.13365,0.11307,0.11831,0.12393,0.12353,0.059175,0.059174,0.059
49,0.060857,0.060051,0.058227,0.058165,0.057985,0.059502,0.06073,0.060
17,0.058629,0.060939,0.059146,0.056515,0.058872,0.060674,0.05597,0.059
46,0.058532,0.059703,0.059279,0.05989,0.058135,0.061319,0.11825,0.1353
,0.12242,0.11073,0.057255,0.05817,0.058714,0.059581,0.057233,0.057094,
0.058193,0.058022,0.059221,0.059543,0.059143,0.056894,0.059513,0.05848
,0.060785,0.058549,0.058317,0.058566,0.056414,0.058279,0.060381,0.0569
39,0.059729,0.058073,0.060705,0.061936,0.062717,0.06069,0.056334,0.061
239,0.059296,0.056345,0.06196,0.06052,0.059951,0.058336,0.058343,0.058
152,0.059944,0.060201,0.059724,0.059701,0.060111,0.059805,0.05845,0.06
0981,0.059917,0.058672,0.05826,0.059698,0.056436,0.060661,0.057497,0.0
57904,0.058542,0.059218,0.059413,0.058773,0.060963,0.05802,0.059171,0.
058802,0.060885,0.059585,0.060891,0.059619,0.058192,0.058849,0.058781,
0.058638,0.060415,0.063639,0.058208,0.061392,0.056631,0.058853,0.06010
6,0.060015,0.057284,0.058406,0.056101,0.058886,0.060204,0.05777,0.0587
64,0.058691,0.057692,0.058916,0.058557,0.059159,0.057645,0.057878,0.05
9043,0.058888,0.059826,0.057465,0.058832,0.059771,0.060053,0.06026,0.0
59332,0.059948,0.059934,0.058376,0.05829,0.059955,0.059163,0.058469,0.
060099,0.05762,0.057478,0.059239,0.05907,0.059065,0.059401,0.058322,0.
057194,0.060407,0.059125,0.05761,0.056139,0.058668,0.058483,0.059554,0
.057856,0.060288,0.058103,0.060356,0.061175,0.057604,0.057139,0.059824
,0.057618,0.059683,0.05857,0.059193,0.058274,0.057805,0.061069,0.05672
8,0.058224,0.057792,0.057849,0.05704,0.057819,0.059765,0.060828,0.0597
66,0.059463,0.060335,0.058574,0.059022,0.059897,0.058673,0.062036,0.06
1306,0.056998,0.060341,0.058325,0.057439,0.058661,0.060707,0.059712,0.
059719,0.058792,0.059159,0.059212,0.059,0.058495,0.060361,0.058728,0.0
58846,0.059074,0.059601,0.057318,0.060218,0.056834,0.058454,0.058127,0
.060205,0.059421,0.058647,0.05861,0.06091,0.058534,0.05948,0.058364,0.
060974,0.057867,0.059657,0.05998,0.058955,0.059393,0.05817,0.063246
0.18848,0.19252,0.19131,0.19497,0.19216,0.19475,0.19349,0.19434,0.1944
5,0.19498,0.19439,0.19462,0.19108,0.19514,0.194,0.19314,0.19402,0.197,
0.19464,0.19158,0.33459,0.28846,0.34281,0.28196,0.19348,0.19615,0.1945
9,0.19399,0.19012,0.1961,0.1919,0.19254,0.19205,0.19108,0.19717,0.1917
9,0.1891,0.1914,0.19282,0.40092,0.28814,0.30438,0.29496,0.29698,0.3007
7,0.30332,0.19163,0.18965,0.19562,0.19578,0.19191,0.19427,0.19081,0.19
387,0.1939,0.19316,0.192,0.19359,0.19414,0.1961,0.19597,0.43651,0.3836
,0.28206,0.32151,0.27123,0.30456,0.30716,0.28706,0.29219,0.28527,0.192
97,0.1944,0.19537,0.19403,0.19305,0.19268,0.1933,0.19325,0.19527,0.194
77,0.19248,0.19145,0.1929,0.1954,0.19513,0.40094,0.37548,0.40922,0.247
38,0.33891,0.30934,0.30252,0.31316,0.29276,0.33005,0.32019,0.30219,0.1
9497,0.19333,0.19273,0.19173,0.19699,0.19429,0.19436,0.19318,0.19507,0
.19624,0.19301,0.19448,0.19469,0.19456,0.19124,0.19396,0.38763,0.42722
,0.40706,0.4265,0.30088,0.30089,0.2717,0.30693,0.28741,0.29118,0.29472
,0.30301,0.31278,0.30665,0.19488,0.19528,0.1953,0.194,0.18976,0.1927,0

.1946,0.19225,0.19669,0.19605,0.19251,0.19381,0.1939,0.19796,0.19156,0
.19087,0.3797,0.3861,0.4162,0.40518,0.38746,0.39188,0.29583,0.29609,0.
28821,0.3023,0.29627,0.3273,0.2839,0.30296,0.28681,0.30019,0.19292,0.1
939,0.19167,0.19245,0.19231,0.19508,0.1925,0.19573,0.19338,0.19723,0.1
9599,0.19742,0.19459,0.19052,0.19494,0.19469,0.19333,0.42753,0.42958,0
.41676,0.41724,0.40614,0.4355,0.306,0.27869,0.30672,0.32092,0.31661,0.
32786,0.29319,0.29384,0.29964,0.28957,0.29891,0.19285,0.19481,0.18964,
0.19196,0.19322,0.19056,0.19074,0.19275,0.18988,0.19463,0.19258,0.1908
4,0.19248,0.12757,0.1111,0.12815,0.19507,0.38982,0.41927,0.38817,0.420
53,0.4261,0.41769,0.41014,0.30964,0.28663,0.31666,0.29838,0.33515,0.29
163,0.28559,0.30154,0.3,0.30499,0.28881,0.19143,0.19497,0.19539,0.1952
9,0.19273,0.19362,0.19522,0.19125,0.19094,0.19284,0.19288,0.10368,0.12
343,0.12319,0.12281,0.12261,0.12739,0.19538,0.38204,0.4018,0.41973,0.4
0465,0.40773,0.39639,0.41182,0.29732,0.31822,0.28791,0.3079,0.28754,0.
30307,0.30851,0.30073,0.31501,0.28651,0.27252,0.1927,0.19532,0.19519,0
.19249,0.18923,0.19635,0.19503,0.19489,0.19732,0.12223,0.12427,0.13471
,0.12238,0.12296,0.12199,0.1234,0.12929,0.12272,0.19268,0.40867,0.4042
7,0.43876,0.44289,0.38772,0.39086,0.41646,0.29304,0.2952,0.3017,0.3183
6,0.29943,0.31198,0.3026,0.27817,0.30191,0.2954,0.29356,0.26865,0.1935
2,0.19375,0.19575,0.19601,0.19367,0.19689,0.11458,0.12312,0.12685,0.12
355,0.13862,0.12457,0.11704,0.12451,0.12519,0.13992,0.12586,0.12448,0.
40237,0.41478,0.40022,0.442,0.40414,0.41553,0.38992,0.28809,0.29293,0.
2999,0.30452,0.30551,0.32162,0.32043,0.31384,0.30737,0.30816,0.30746,0
.29183,0.19655,0.19372,0.19587,0.11927,0.13909,0.11239,0.11359,0.12646
,0.12143,0.12352,0.12291,0.12713,0.12418,0.12342,0.13193,0.12729,0.146
18,0.12519,0.39991,0.38568,0.40966,0.38341,0.39722,0.43107,0.44016,0.4
0644,0.40191,0.30059,0.29354,0.28407,0.29835,0.27125,0.29006,0.29983,0
.30456,0.30071,0.3088,0.31783,0.12328,0.12422,0.12126,0.1214,0.12461,0
.12251,0.11844,0.12522,0.12931,0.12125,0.11074,0.12371,0.12381,0.12226
,0.12395,0.1074,0.1173,0.12731,0.41116,0.42159,0.43891,0.40862,0.39168
,0.42055,0.43447,0.38266,0.39652,0.31568,0.2842,0.2447,0.20124,0.22576
,0.22509,0.21379,0.2175,0.23973,0.20353,0.22248,0.12452,0.13684,0.1158
3,0.10971,0.12238,0.13826,0.12742,0.12194,0.13047,0.1057,0.12004,0.124
45,0.12074,0.12261,0.12768,0.12074,0.12265,0.12113,0.40166,0.39648,0.3
9576,0.45008,0.39993,0.3919,0.41523,0.38248,0.38827,0.20916,0.22602,0.
21849,0.24192,0.2064,0.24182,0.20646,0.24288,0.23549,0.23003,0.19498,0
.12249,0.12525,0.13426,0.12371,0.12095,0.14095,0.14196,0.12273,0.13612
,0.12546,0.12197,0.12789,0.13505,0.1158,0.11414,0.12316,0.11669,0.1233
6,0.41344,0.40059,0.42645,0.4168,0.42682,0.41412,0.39423,0.22645,0.236
05,0.28951,0.20579,0.22326,0.22503,0.21907,0.20701,0.20762,0.21768,0.2
2469,0.21712,0.22508,0.12279,0.12245,0.13233,0.1164,0.12318,0.12155,0.
12358,0.14135,0.12353,0.12351,0.11583,0.12688,0.12061,0.12299,0.116,0.
11659,0.11946,0.13508,0.40961,0.39934,0.40738,0.44167,0.42439,0.39044,
0.22941,0.24868,0.23782,0.20811,0.22033,0.22998,0.21265,0.21151,0.2229
6,0.23757,0.22652,0.2354,0.21685,0.25139,0.12172,0.12516,0.12177,0.122
44,0.11493,0.12237,0.11875,0.11974,0.1279,0.12799,0.14121,0.12313,0.12
381,0.1175,0.13537,0.12858,0.11287,0.12161,0.36651,0.41411,0.42687,0.4
0467,0.42477,0.2488,0.2347,0.21534,0.24911,0.22036,0.17258,0.20735,0.2
309,0.22116,0.22088,0.22681,0.22953,0.20826,0.22303,0.21905,0.13748,0.
11088,0.13675,0.11935,0.10758,0.12262,0.11885,0.13627,0.11318,0.12167,
0.11524,0.12384,0.12324,0.12205,0.12588,0.13305,0.116,0.12384,0.4452,0
.39409,0.41433,0.23375,0.23777,0.22896,0.24821,0.22938,0.25558,0.20016

,0.2566,0.23635,0.21896,0.21674,0.24334,0.19922,0.22423,0.22434,0.1970
6,0.20684,0.12031,0.12403,0.11211,0.12312,0.1156,0.12985,0.13232,0.123
83,0.12259,0.11211,0.12296,0.12355,0.12532,0.13547,0.1136,0.12318,0.12
336,0.12339,0.39486,0.23792,0.24677,0.22465,0.20144,0.24605,0.21909,0.
23407,0.21813,0.23724,0.21891,0.22505,0.21967,0.21503,0.23718,0.21017,
0.2238,0.21985,0.22039,0.12276,0.12399,0.12352,0.1223,0.12083,0.11561,
0.11954,0.1241,0.12766,0.12724,0.12171,0.12222,0.11968,0.1295,0.14536,
0.12226,0.10853,0.052105,0.21775,0.24191,0.2215,0.22443,0.22707,0.1993
6,0.22701,0.25546,0.22261,0.29129,0.24825,0.17638,0.18888,0.22258,0.21
816,0.20588,0.20428,0.20207,0.21159,0.12277,0.12361,0.12564,0.12275,0.
12117,0.10238,0.12352,0.11495,0.12723,0.11943,0.13216,0.12033,0.12084,
0.12695,0.12306,0.12295,0.12275,0.053512,0.24834,0.2216,0.22563,0.2614
6,0.23159,0.2161,0.214,0.1976,0.194,0.23725,0.20561,0.25112,0.17446,0.
21852,0.21207,0.22376,0.22803,0.23865,0.11877,0.12117,0.12293,0.12588,
0.12414,0.10756,0.1397,0.12588,0.12728,0.1237,0.12874,0.12326,0.1228,0
.12396,0.11568,0.12476,0.12025,0.123,0.051888,0.23567,0.27746,0.21707,
0.24197,0.24754,0.21562,0.21941,0.2322,0.21677,0.23113,0.22158,0.21325
,0.20218,0.22527,0.23048,0.2286,0.20923,0.20574,0.13484,0.12252,0.1208
8,0.12237,0.12233,0.14466,0.1168,0.12233,0.12028,0.12482,0.12296,0.122
44,0.11269,0.11797,0.11594,0.12375,0.13423,0.052061,0.053682,0.051942,
0.23173,0.23056,0.24417,0.22681,0.23538,0.23453,0.21515,0.22041,0.2337
9,0.19333,0.20047,0.21731,0.19306,0.22288,0.2359,0.10428,0.12657,0.129
13,0.1231,0.12378,0.12333,0.11787,0.12665,0.11235,0.13067,0.12331,0.11
437,0.1198,0.12126,0.11733,0.11569,0.12339,0.12021,0.052254,0.053377,0
.051435,0.05555,0.16014,0.235,0.20258,0.19429,0.22952,0.2208,0.27911,0
.27688,0.26143,0.23649,0.20722,0.21869,0.13874,0.118,0.10833,0.1156,0.
13622,0.13524,0.13848,0.11514,0.12842,0.12493,0.13449,0.13244,0.12292,
0.13552,0.12377,0.12425,0.12606,0.12858,0.1108,0.12449,0.052269,0.0535
49,0.05382,0.052717,0.052181,0.052422,0.23004,0.22744,0.26516,0.23247,
0.20589,0.22402,0.21302,0.22993,0.11337,0.10606,0.09758,0.12328,0.1226
2,0.12164,0.12136,0.12698,0.098659,0.12351,0.13083,0.15849,0.12131,0.1
2211,0.14649,0.12644,0.12371,0.10108,0.1232,0.12006,0.12071,0.1171,0.1
2636,0.051755,0.052976,0.050534,0.053744,0.051197,0.051194,0.24372,0.2
3936,0.24986,0.23344,0.24333,0.052408,0.052298,0.12414,0.12262,0.12354
,0.1222,0.12301,0.12347,0.12091,0.13021,0.12006,0.11549,0.12042,0.1187
2,0.12689,0.12327,0.1162,0.12214,0.12471,0.11947,0.12438,0.12859,0.125
4,0.054051,0.052541,0.050035,0.05286,0.050498,0.05427,0.054232,0.05379
8,0.052324,0.050503,0.2346,0.20822,0.051312,0.052525,0.052735,0.053907
,0.13268,0.10101,0.12387,0.12505,0.12581,0.13402,0.12271,0.12091,0.123
92,0.1178,0.11471,0.13744,0.12863,0.12054,0.12551,0.12234,0.11512,0.13
232,0.12942,0.052219,0.054958,0.053169,0.053929,0.052249,0.052122,0.05
1291,0.051843,0.052802,0.050793,0.051501,0.054316,0.052535,0.053846,0.
054848,0.051299,0.051537,0.12029,0.12828,0.1129,0.10643,0.12375,0.1181
1,0.12672,0.11337,0.12126,0.12239,0.12048,0.12716,0.11424,0.12286,0.12
864,0.12412,0.052407,0.052499,0.052038,0.05338,0.055292,0.054298,0.051
642,0.054163,0.054946,0.054789,0.053021,0.052638,0.053079,0.051381,0.0
56049,0.052181,0.053063,0.052697,0.055788,0.11439,0.11686,0.14193,0.12
41,0.11464,0.12303,0.12386,0.12768,0.11712,0.12626,0.12805,0.13026,0.1
1217,0.052955,0.052918,0.052026,0.052194,0.053852,0.051713,0.053558,0.
05404,0.05197,0.053495,0.053804,0.056027,0.050746,0.053454,0.053375,0.
05291,0.054659,0.053525,0.052192,0.053957,0.051125,0.11812,0.12475,0.1
3154,0.12467,0.13236,0.1106,0.11633,0.12188,0.12188,0.053434,0.053718,

0.053366,0.052968,0.053038,0.052097,0.051477,0.052091,0.053578,0.05479
9,0.05345,0.052248,0.05509,0.052432,0.050168,0.052943,0.054411,0.05035
1,0.052373,0.052902,0.053655,0.052598,0.053664,0.052835,0.054857,0.115
8,0.1341,0.12143,0.10811,0.051048,0.052491,0.051822,0.052984,0.050709,
0.051946,0.05178,0.052432,0.053086,0.053892,0.053579,0.051613,0.053701
,0.051328,0.055584,0.052154,0.052756,0.051506,0.050589,0.05306,0.05419
5,0.050141,0.053104,0.051681,0.054212,0.055282,0.056787,0.055037,0.050
636,0.055658,0.054389,0.050117,0.055555,0.054794,0.053143,0.051678,0.0
51394,0.052539,0.053003,0.052997,0.053563,0.053654,0.053801,0.053391,0
.05271,0.054954,0.054743,0.052372,0.052911,0.054089,0.05099,0.054556,0
.050989,0.051562,0.05294,0.053473,0.053538,0.053205,0.054632,0.051936,
0.053195,0.052862,0.055085,0.053558,0.054808,0.054124,0.052091,0.05365
9,0.053579,0.052566,0.054466,0.057309,0.052341,0.05436,0.050179,0.0530
72,0.053619,0.054593,0.051843,0.052325,0.050755,0.053812,0.054024,0.05
1473,0.053049,0.05183,0.052407,0.052967,0.052238,0.052449,0.050536,0.0
52059,0.052948,0.050969,0.053531,0.051676,0.05291,0.053544,0.054001,0.
054554,0.054493,0.054133,0.053425,0.051934,0.052245,0.054473,0.052774,
0.053463,0.054866,0.051611,0.051686,0.053071,0.054382,0.053436,0.05347
5,0.051825,0.051284,0.053862,0.052652,0.05241,0.050294,0.05223,0.05269
7,0.053185,0.051816,0.053827,0.052834,0.053979,0.05451,0.050961,0.0507
44,0.053667,0.051208,0.053595,0.052743,0.053101,0.053276,0.051328,0.05
4135,0.050884,0.052446,0.051784,0.051975,0.051228,0.052397,0.053792,0.
053952,0.05214,0.053029,0.054129,0.050931,0.052009,0.05331,0.052021,0.
055508,0.055,0.052015,0.054011,0.052373,0.051565,0.052976,0.054642,0.0
53926,0.053135,0.053629,0.053229,0.053452,0.053228,0.053161,0.053987,0
.053242,0.052747,0.052598,0.052496,0.051652,0.053372,0.051188,0.052106
,0.052202,0.053548,0.053733,0.053141,0.05309,0.054866,0.052829,0.05379
4,0.051909,0.053887,0.051511,0.053581,0.052616,0.052565,0.053646,0.052
203,0.056882
0.1917,0.19468,0.19389,0.19657,0.19505,0.197,0.19593,0.1957,0.19608,0.
19705,0.1964,0.19628,0.19431,0.19562,0.19693,0.19473,0.19648,0.19959,0
.19713,0.1939,0.31754,0.28111,0.33465,0.26814,0.19692,0.19745,0.19679,
0.19498,0.19304,0.19891,0.19419,0.19649,0.19352,0.19406,0.19961,0.1944
5,0.1926,0.193,0.19589,0.37829,0.27396,0.29941,0.28446,0.28878,0.29136
,0.29266,0.19361,0.19261,0.1988,0.19829,0.194,0.19734,0.19426,0.19649,
0.19519,0.19631,0.19541,0.19635,0.19524,0.1979,0.19793,0.41431,0.37837
,0.26891,0.31626,0.26857,0.28954,0.30318,0.28075,0.28405,0.28366,0.196
2,0.19673,0.1978,0.19579,0.19601,0.19605,0.19647,0.19538,0.19794,0.196
94,0.19376,0.19513,0.19439,0.19777,0.19838,0.37854,0.37028,0.388,0.247
63,0.32797,0.2997,0.29134,0.30529,0.29165,0.31453,0.31152,0.29755,0.19
896,0.19614,0.19602,0.19437,0.19763,0.19647,0.19595,0.19529,0.19829,0.
19899,0.19574,0.19697,0.19767,0.19789,0.19372,0.19681,0.37265,0.41525,
0.40717,0.40776,0.29686,0.30138,0.26811,0.30396,0.28002,0.2875,0.28609
,0.29444,0.30608,0.29801,0.19702,0.19773,0.19759,0.19641,0.19276,0.194
16,0.19725,0.19446,0.19781,0.19692,0.1957,0.19521,0.19482,0.19792,0.19
35,0.19381,0.36163,0.37792,0.40587,0.39614,0.38514,0.38496,0.28631,0.2
8464,0.27938,0.29638,0.28675,0.3083,0.27666,0.29895,0.27483,0.29872,0.
19583,0.19636,0.19504,0.19452,0.19479,0.19719,0.19514,0.19757,0.19467,
0.19749,0.19823,0.19848,0.19685,0.19407,0.19721,0.19754,0.19599,0.4128
8,0.4077,0.39537,0.40571,0.38502,0.42742,0.2948,0.27181,0.30177,0.3143
2,0.31253,0.31932,0.28532,0.28604,0.29294,0.27812,0.29762,0.19522,0.19
653,0.19256,0.19346,0.19536,0.19293,0.19216,0.19635,0.19253,0.19721,0.

19529,0.19262,0.196,0.1269,0.10958,0.12735,0.19687,0.37042,0.39305,0.3
7027,0.39806,0.41753,0.40105,0.38661,0.30181,0.27619,0.3099,0.2887,0.3
28,0.28669,0.28175,0.2936,0.29202,0.29109,0.28145,0.19295,0.19821,0.19
856,0.19715,0.19408,0.19553,0.19627,0.19345,0.19424,0.19683,0.19631,0.
10233,0.122,0.12195,0.12242,0.12122,0.12672,0.19777,0.37461,0.39192,0.
40933,0.3792,0.40005,0.37046,0.39574,0.29367,0.30435,0.28931,0.30182,0
.28537,0.29498,0.29739,0.29286,0.30755,0.28673,0.26577,0.19449,0.19769
,0.19715,0.19364,0.19232,0.19858,0.19617,0.19736,0.1992,0.12135,0.1234
2,0.13525,0.12167,0.12252,0.12109,0.12243,0.12891,0.12152,0.19533,0.39
19,0.39667,0.42237,0.42147,0.38766,0.37845,0.39549,0.28467,0.28222,0.2
9537,0.31033,0.29486,0.30161,0.29917,0.27594,0.29356,0.28868,0.29095,0
.26394,0.19526,0.19571,0.19755,0.19747,0.19527,0.19942,0.11352,0.12292
,0.12622,0.12266,0.13747,0.12368,0.1158,0.12332,0.12358,0.13891,0.1249
7,0.12423,0.38861,0.40118,0.39293,0.42768,0.38832,0.38952,0.37748,0.28
247,0.28602,0.29359,0.29959,0.29669,0.31272,0.30701,0.30225,0.30228,0.
30025,0.30255,0.28254,0.19756,0.19665,0.19811,0.11786,0.13994,0.11095,
0.11183,0.12602,0.1202,0.12287,0.12261,0.12621,0.12301,0.12307,0.1313,
0.1267,0.14661,0.12373,0.37547,0.36889,0.40633,0.36546,0.38896,0.40161
,0.4394,0.38322,0.38802,0.28887,0.29435,0.27964,0.29513,0.27122,0.2781
,0.29193,0.29606,0.29652,0.30439,0.30686,0.12108,0.12347,0.12103,0.119
97,0.12318,0.12186,0.11842,0.12418,0.12887,0.1205,0.10831,0.12277,0.12
281,0.12122,0.12318,0.10602,0.11641,0.12736,0.40135,0.41243,0.4294,0.3
9489,0.37157,0.38929,0.41042,0.36126,0.37986,0.3065,0.27559,0.23438,0.
20178,0.2197,0.21889,0.20748,0.21221,0.23299,0.19668,0.21562,0.12487,0
.13602,0.11456,0.10817,0.12121,0.13827,0.12593,0.1206,0.12967,0.10446,
0.11928,0.1237,0.11989,0.12185,0.12683,0.11877,0.12131,0.11946,0.37645
,0.36734,0.39004,0.42845,0.39062,0.37999,0.39426,0.35177,0.38808,0.204
93,0.22042,0.20887,0.23568,0.19924,0.24243,0.19853,0.23608,0.22841,0.2
2166,0.19325,0.12132,0.1249,0.13411,0.12272,0.11998,0.14145,0.14135,0.
12133,0.13625,0.1243,0.12133,0.1271,0.13437,0.11444,0.11217,0.12297,0.
11477,0.12193,0.40013,0.38216,0.41034,0.39787,0.4079,0.40082,0.38604,0
.21096,0.22203,0.27303,0.20068,0.21591,0.22102,0.2091,0.19894,0.20408,
0.21162,0.21732,0.21568,0.21377,0.12129,0.12223,0.13153,0.11495,0.1224
9,0.12066,0.12274,0.14032,0.12289,0.12281,0.11456,0.12579,0.11924,0.12
205,0.11444,0.11532,0.11809,0.1346,0.37861,0.39909,0.39758,0.42052,0.4
0068,0.37497,0.21607,0.23593,0.23376,0.1953,0.21644,0.22076,0.20383,0.
2077,0.21312,0.23367,0.22132,0.22208,0.21197,0.24232,0.12113,0.12424,0
.12091,0.12161,0.11333,0.121,0.11667,0.11878,0.12731,0.12683,0.14127,0
.12211,0.12296,0.11531,0.13507,0.12827,0.11156,0.12134,0.35406,0.39887
,0.4165,0.39312,0.41078,0.24283,0.22808,0.21026,0.2321,0.20774,0.16693
,0.20408,0.22325,0.21275,0.21489,0.2203,0.2219,0.20261,0.22029,0.2159,
0.13645,0.10985,0.13632,0.11802,0.10629,0.12194,0.11828,0.13656,0.1112
9,0.12103,0.11457,0.12285,0.12175,0.12048,0.12554,0.13283,0.11451,0.12
312,0.42268,0.368,0.39942,0.21614,0.23387,0.21627,0.23528,0.21563,0.23
504,0.19684,0.23964,0.23156,0.21109,0.20612,0.23263,0.19874,0.21754,0.
21882,0.18885,0.19653,0.11846,0.12311,0.11101,0.12211,0.11449,0.12913,
0.13141,0.1233,0.12179,0.11024,0.12206,0.12314,0.12568,0.13521,0.11301
,0.12258,0.12234,0.12224,0.38566,0.23127,0.23261,0.21735,0.19044,0.233
33,0.20718,0.21399,0.19993,0.22404,0.21417,0.21894,0.21444,0.20526,0.2
3616,0.20332,0.21663,0.21594,0.21334,0.12208,0.12273,0.12211,0.12145,0
.12,0.11456,0.11764,0.12249,0.12692,0.1267,0.121,0.12123,0.11905,0.129
49,0.14642,0.12086,0.10694,0.048003,0.21092,0.22991,0.21676,0.21243,0.

21327,0.19187,0.21992,0.25076,0.205,0.27917,0.24234,0.17568,0.17981,0.
22068,0.21245,0.20369,0.19795,0.19956,0.20246,0.12201,0.12281,0.12455,
0.12193,0.11996,0.099737,0.12234,0.1138,0.12686,0.11914,0.13217,0.1189
,0.12023,0.12546,0.12229,0.1223,0.12104,0.04945,0.2339,0.21376,0.22135
,0.25529,0.22101,0.20153,0.19976,0.19135,0.17726,0.22009,0.19966,0.246
36,0.16922,0.20827,0.20572,0.2205,0.2254,0.23223,0.11832,0.12,0.12188,
0.125,0.12302,0.10632,0.13977,0.12587,0.12677,0.12234,0.12776,0.12224,
0.1216,0.12368,0.11444,0.12376,0.11836,0.12134,0.048507,0.2195,0.26056
,0.20533,0.23331,0.24316,0.20718,0.20272,0.21944,0.20682,0.2203,0.2146
2,0.20801,0.19774,0.22026,0.22487,0.22313,0.20387,0.20273,0.13405,0.12
18,0.1211,0.12158,0.12162,0.14445,0.11544,0.12161,0.11875,0.12437,0.12
211,0.12104,0.11099,0.11714,0.11491,0.12276,0.13366,0.048062,0.049137,
0.048596,0.21958,0.22025,0.2326,0.21305,0.22823,0.22379,0.21002,0.2073
6,0.22487,0.18646,0.19222,0.21316,0.19042,0.21813,0.22985,0.10351,0.12
618,0.12872,0.12311,0.12266,0.12204,0.11595,0.12623,0.11032,0.12987,0.
12259,0.11277,0.11882,0.11958,0.11639,0.11422,0.12204,0.11916,0.048231
,0.048927,0.047316,0.051302,0.15552,0.23302,0.1981,0.18659,0.22359,0.2
1219,0.26209,0.273,0.25704,0.22837,0.19953,0.21411,0.13835,0.11668,0.1
0663,0.11385,0.13538,0.13465,0.13777,0.11303,0.12716,0.12404,0.13488,0
.13173,0.12208,0.13572,0.12235,0.12403,0.12587,0.12777,0.10899,0.12367
,0.047137,0.048463,0.049333,0.049317,0.048637,0.048158,0.2192,0.22073,
0.26057,0.22585,0.19803,0.21298,0.20333,0.21989,0.11264,0.10496,0.0957
16,0.12243,0.12182,0.12035,0.12039,0.12636,0.09604,0.12291,0.1305,0.15
861,0.12074,0.12044,0.14631,0.12594,0.12233,0.098285,0.12217,0.11921,0
.11887,0.11609,0.12554,0.04897,0.04977,0.046201,0.050454,0.047408,0.04
8015,0.23859,0.22558,0.24485,0.21617,0.22585,0.048228,0.048363,0.12263
,0.12157,0.12253,0.12094,0.12194,0.12237,0.12002,0.129,0.11966,0.11422
,0.11938,0.11741,0.12617,0.12209,0.11548,0.1209,0.1237,0.11777,0.12352
,0.12695,0.12485,0.048563,0.048587,0.045389,0.048734,0.04689,0.051023,
0.049735,0.049135,0.047797,0.04595,0.22563,0.20002,0.046925,0.047145,0
.048551,0.048523,0.13303,0.098877,0.12275,0.12431,0.12472,0.13384,0.12
252,0.11946,0.12355,0.1167,0.11329,0.13791,0.12844,0.12031,0.12447,0.1
2151,0.11293,0.13141,0.12907,0.048115,0.050362,0.048369,0.050363,0.048
935,0.048588,0.048201,0.047877,0.049128,0.046321,0.047562,0.050641,0.0
48604,0.049468,0.050455,0.046816,0.047221,0.11922,0.12685,0.11167,0.10
438,0.12222,0.11665,0.12562,0.11166,0.1196,0.12196,0.11932,0.12709,0.1
1208,0.12155,0.1282,0.1222,0.048847,0.049382,0.048132,0.049051,0.05050
6,0.049486,0.047102,0.050051,0.04979,0.049624,0.050094,0.04864,0.04923
2,0.046948,0.051946,0.048155,0.049024,0.047847,0.05115,0.11348,0.11505
,0.14245,0.12258,0.11262,0.12162,0.12215,0.12748,0.11597,0.12597,0.127
84,0.12965,0.11067,0.048268,0.049096,0.047265,0.047596,0.049501,0.0483
21,0.049202,0.049841,0.047673,0.048772,0.048682,0.050973,0.047556,0.04
841,0.048485,0.048392,0.04997,0.04833,0.047771,0.049369,0.047505,0.116
69,0.12388,0.13057,0.12352,0.13154,0.10902,0.11505,0.1213,0.12088,0.04
8426,0.049181,0.047875,0.048863,0.04889,0.048088,0.046947,0.048127,0.0
4928,0.049777,0.049607,0.047845,0.051342,0.048329,0.046658,0.048228,0.
050488,0.046016,0.0482,0.048541,0.049642,0.049369,0.049925,0.047904,0.
050861,0.11483,0.13372,0.12155,0.1065,0.047037,0.048249,0.048016,0.048
887,0.046448,0.048347,0.048961,0.048265,0.048633,0.050329,0.049457,0.0
48186,0.049408,0.046838,0.051011,0.04906,0.048927,0.047106,0.046368,0.
04767,0.049661,0.046501,0.048333,0.047236,0.048873,0.050344,0.052568,0
.050411,0.046072,0.051364,0.049966,0.046074,0.05136,0.051001,0.049028,

0.047745,0.047134,0.0484,0.048753,0.048502,0.048961,0.048916,0.049561,
0.049356,0.047709,0.049512,0.051228,0.047488,0.04948,0.049592,0.04645,
0.049857,0.046065,0.048041,0.049989,0.049739,0.049897,0.048212,0.05084
7,0.048,0.048251,0.048863,0.049982,0.048836,0.050039,0.048798,0.048604
,0.049051,0.048793,0.047882,0.050531,0.052657,0.048016,0.050386,0.0452
71,0.04895,0.050312,0.049669,0.046828,0.049182,0.047421,0.05061,0.0494
84,0.046918,0.048444,0.047372,0.048623,0.048933,0.049405,0.047939,0.04
684,0.048095,0.049062,0.047009,0.049828,0.047432,0.049114,0.049866,0.0
49099,0.050061,0.04976,0.049606,0.049597,0.046349,0.047762,0.049948,0.
048478,0.049293,0.049905,0.047212,0.048801,0.048618,0.04931,0.05026,0.
049487,0.047353,0.047412,0.04965,0.048379,0.048922,0.046398,0.047955,0
.048683,0.049109,0.047802,0.049658,0.048643,0.050113,0.049659,0.047087
,0.046689,0.049721,0.047412,0.049402,0.049292,0.049127,0.048696,0.0472
73,0.050245,0.046534,0.047914,0.04697,0.048474,0.046023,0.049323,0.049
753,0.049866,0.047466,0.049404,0.049705,0.046729,0.048615,0.049685,0.0
47823,0.051132,0.050843,0.047752,0.05091,0.048937,0.047285,0.048518,0.
050317,0.050529,0.049263,0.048957,0.049191,0.048406,0.048987,0.050288,
0.049402,0.048713,0.049003,0.047992,0.048465,0.047303,0.049905,0.04664
2,0.047948,0.04767,0.049436,0.049469,0.048651,0.049006,0.049361,0.0488
37,0.050139,0.047326,0.049803,0.046861,0.050327,0.048319,0.048142,0.05
0171,0.047681,0.052436
0.19414,0.1964,0.19664,0.19837,0.19747,0.19973,0.19772,0.19656,0.19747
,0.19878,0.19819,0.19797,0.19622,0.19686,0.19953,0.19672,0.19806,0.202
13,0.19848,0.19506,0.31465,0.28197,0.3314,0.26381,0.19877,0.19913,0.19
976,0.19709,0.19391,0.20105,0.197,0.19793,0.19641,0.19559,0.20172,0.19
663,0.19417,0.19613,0.19873,0.36899,0.27106,0.29473,0.27428,0.29225,0.
28487,0.28585,0.19669,0.19457,0.19961,0.19945,0.19615,0.19859,0.196,0.
19825,0.19569,0.19949,0.19801,0.19891,0.19702,0.19965,0.19873,0.40184,
0.36333,0.26474,0.31463,0.26926,0.29095,0.2973,0.27353,0.27665,0.27927
,0.19728,0.19873,0.19995,0.19751,0.19709,0.19749,0.19873,0.19688,0.199
29,0.19822,0.19722,0.19712,0.19661,0.19857,0.19988,0.37684,0.35825,0.3
8278,0.24703,0.32346,0.297,0.28619,0.29526,0.2925,0.31268,0.29701,0.29
81,0.20159,0.19894,0.19878,0.19696,0.20018,0.19828,0.19719,0.19632,0.1
9937,0.20058,0.1986,0.19988,0.20017,0.2006,0.19497,0.20035,0.35405,0.3
9145,0.39882,0.39521,0.29585,0.3043,0.2627,0.29902,0.27864,0.27831,0.2
8482,0.29378,0.3017,0.30114,0.19936,0.19908,0.19877,0.19855,0.19591,0.
19661,0.19761,0.19603,0.19976,0.19917,0.19797,0.19854,0.19709,0.20061,
0.19502,0.19506,0.33968,0.37426,0.40031,0.37561,0.36978,0.374,0.28636,
0.27875,0.273,0.29464,0.28025,0.30398,0.26859,0.28643,0.27759,0.29246,
0.19753,0.19751,0.19762,0.19574,0.19797,0.19906,0.19611,0.19945,0.1973
,0.19918,0.19985,0.20078,0.20027,0.19674,0.19934,0.19915,0.19903,0.393
91,0.39651,0.36638,0.39991,0.36438,0.41374,0.29568,0.26861,0.30025,0.3
1278,0.30354,0.30384,0.28185,0.28381,0.28969,0.27213,0.29431,0.19795,0
.19852,0.19547,0.19549,0.19748,0.19635,0.19336,0.19852,0.19589,0.19984
,0.19847,0.19403,0.19797,0.12666,0.108,0.12656,0.1989,0.35717,0.38973,
0.365,0.40021,0.3876,0.38348,0.36631,0.29116,0.27344,0.31143,0.28617,0
.31829,0.27695,0.28421,0.29346,0.27996,0.28437,0.27844,0.19425,0.19861
,0.20015,0.19912,0.19622,0.19763,0.19862,0.19506,0.19613,0.19874,0.198
5,0.10129,0.122,0.12175,0.12179,0.12125,0.12647,0.19858,0.36278,0.3906
1,0.39884,0.37936,0.37973,0.36747,0.38609,0.28777,0.29726,0.27288,0.30
273,0.28834,0.29087,0.29251,0.28971,0.29959,0.28102,0.26029,0.19566,0.
20127,0.19803,0.19737,0.19473,0.20011,0.19913,0.19909,0.20034,0.12089,

0.12344,0.13481,0.12103,0.12155,0.12068,0.12192,0.12882,0.12079,0.1970
5,0.38026,0.3824,0.40021,0.4057,0.36949,0.37905,0.38603,0.28213,0.2781
3,0.29339,0.30226,0.29547,0.29221,0.29723,0.2751,0.2857,0.28414,0.2955
2,0.26404,0.19624,0.19781,0.19914,0.19927,0.19752,0.19987,0.11283,0.12
214,0.12582,0.12273,0.13849,0.12346,0.115,0.12263,0.12362,0.13878,0.12
336,0.12356,0.38428,0.39467,0.37574,0.41342,0.37274,0.38849,0.37992,0.
28122,0.27888,0.28938,0.30045,0.29989,0.30579,0.30072,0.29479,0.3047,0
.29416,0.30017,0.28052,0.19954,0.1974,0.20004,0.11754,0.1402,0.11059,0
.11106,0.12616,0.11953,0.12283,0.12221,0.12591,0.12327,0.1226,0.13203,
0.12596,0.14661,0.12334,0.36982,0.35091,0.39833,0.3459,0.37378,0.38706
,0.4324,0.37531,0.38299,0.28543,0.294,0.27792,0.2931,0.26965,0.28049,0
.29005,0.29391,0.288,0.29421,0.30027,0.12165,0.12263,0.12031,0.11997,0
.12264,0.12163,0.11734,0.12364,0.12842,0.11982,0.10786,0.12123,0.12177
,0.12116,0.12203,0.10448,0.11615,0.1279,0.37795,0.39517,0.43461,0.3928
3,0.36042,0.37631,0.39982,0.34993,0.37187,0.31313,0.27043,0.22652,0.19
7,0.21223,0.21291,0.20527,0.21332,0.22646,0.19648,0.2123,0.12418,0.135
65,0.1136,0.10767,0.12089,0.13925,0.12497,0.12008,0.12877,0.10328,0.11
947,0.12321,0.11964,0.12135,0.12659,0.11867,0.12132,0.11835,0.36319,0.
37318,0.39087,0.41139,0.39767,0.37041,0.39195,0.36118,0.36702,0.19709,
0.21891,0.20535,0.22692,0.1999,0.23537,0.19399,0.23156,0.22662,0.21403
,0.1912,0.12107,0.1239,0.13436,0.12216,0.1192,0.14133,0.14164,0.12103,
0.13593,0.12467,0.12081,0.1271,0.13427,0.1136,0.11201,0.12221,0.11353,
0.12214,0.3881,0.39365,0.37523,0.37901,0.38976,0.39503,0.38231,0.20455
,0.21348,0.26499,0.19762,0.2059,0.21396,0.20974,0.19839,0.20041,0.2084
2,0.21386,0.21316,0.21015,0.12112,0.12187,0.13111,0.1145,0.12242,0.120
36,0.12347,0.14088,0.12268,0.12214,0.11335,0.12501,0.11858,0.12143,0.1
1406,0.11475,0.11759,0.13497,0.3712,0.38587,0.37593,0.41363,0.38477,0.
36902,0.217,0.23277,0.23472,0.18976,0.21053,0.21491,0.19666,0.20722,0.
20603,0.23088,0.21535,0.21519,0.20852,0.23717,0.12044,0.12329,0.11984,
0.12065,0.11338,0.12118,0.11671,0.11838,0.12696,0.1264,0.1417,0.12202,
0.12185,0.11505,0.13481,0.12806,0.11048,0.12207,0.35265,0.38949,0.3914
5,0.37369,0.38534,0.23968,0.21701,0.20636,0.22293,0.20279,0.16978,0.20
209,0.22007,0.21209,0.2103,0.20935,0.21601,0.20389,0.21381,0.21008,0.1
3686,0.10911,0.13667,0.11827,0.10532,0.12167,0.11727,0.13688,0.11095,0
.12133,0.11448,0.12217,0.12122,0.11963,0.12529,0.13276,0.113,0.1228,0.
40479,0.36187,0.37899,0.21305,0.22366,0.21063,0.23186,0.20463,0.22962,
0.18602,0.23139,0.22754,0.20831,0.19829,0.22951,0.19785,0.21397,0.2166
,0.18711,0.18957,0.11748,0.12175,0.11067,0.12196,0.11342,0.12848,0.131
11,0.12226,0.12114,0.10914,0.12169,0.12241,0.12483,0.13547,0.11287,0.1
227,0.1216,0.12082,0.37378,0.22675,0.2206,0.21008,0.18521,0.22838,0.20
339,0.20727,0.18413,0.21,0.2098,0.2138,0.20951,0.20468,0.23395,0.20098
,0.21463,0.21365,0.21065,0.12195,0.12184,0.12223,0.12083,0.11874,0.113
96,0.11749,0.12258,0.12674,0.12554,0.12066,0.12073,0.11843,0.12955,0.1
4611,0.12083,0.1068,0.044622,0.21242,0.21681,0.20978,0.20485,0.20901,0
.18128,0.20953,0.24841,0.20485,0.27303,0.23811,0.17452,0.18112,0.21618
,0.21265,0.20237,0.19779,0.19473,0.19789,0.12138,0.12231,0.12392,0.121
67,0.11875,0.098058,0.12259,0.11318,0.12599,0.11892,0.13187,0.1185,0.1
2013,0.12515,0.12159,0.12077,0.12084,0.046801,0.23317,0.20506,0.21548,
0.24484,0.21291,0.18975,0.19247,0.18344,0.17158,0.2133,0.19784,0.2381,
0.1672,0.2029,0.20356,0.21771,0.22267,0.23154,0.11835,0.12046,0.12202,
0.12479,0.12259,0.10594,0.1402,0.12511,0.12661,0.12174,0.12794,0.12165
,0.12087,0.12358,0.11327,0.12367,0.11753,0.12004,0.045923,0.21356,0.25

608,0.20651,0.22557,0.23343,0.20718,0.19258,0.21297,0.19929,0.21404,0.
20863,0.20225,0.19473,0.21742,0.21559,0.21416,0.20139,0.20002,0.13414,
0.12107,0.12024,0.12098,0.12118,0.14471,0.11501,0.12079,0.11812,0.1236
1,0.12196,0.12036,0.11049,0.11665,0.11405,0.12209,0.13392,0.045053,0.0
46027,0.047027,0.21263,0.21946,0.22512,0.20843,0.2248,0.20855,0.20456,
0.20067,0.22116,0.18303,0.18896,0.21195,0.18816,0.21368,0.22637,0.1027
1,0.12527,0.12871,0.1224,0.12224,0.12113,0.11584,0.12619,0.10941,0.128
69,0.12191,0.11225,0.11932,0.11898,0.11499,0.11429,0.12141,0.1197,0.04
4931,0.046214,0.045153,0.048149,0.15083,0.22477,0.19382,0.18162,0.2211
9,0.20392,0.25312,0.26571,0.24143,0.22107,0.20007,0.2084,0.13787,0.116
2,0.10556,0.11397,0.13546,0.1347,0.13888,0.11222,0.12668,0.12344,0.134
63,0.13227,0.12149,0.13558,0.12216,0.12334,0.12564,0.12779,0.10799,0.1
2318,0.043605,0.045932,0.047004,0.046898,0.045435,0.045886,0.21973,0.2
1104,0.25907,0.22486,0.19302,0.20238,0.202,0.21419,0.11208,0.10367,0.0
94296,0.12221,0.12147,0.11902,0.1193,0.12684,0.094818,0.12257,0.13038,
0.15813,0.12018,0.11955,0.14609,0.1254,0.12137,0.096264,0.12253,0.1182
8,0.11839,0.11509,0.12499,0.04627,0.046843,0.043988,0.047689,0.044542,
0.046128,0.23581,0.21836,0.23868,0.20998,0.22386,0.04563,0.045293,0.12
229,0.12093,0.12226,0.12113,0.12135,0.12169,0.11925,0.12886,0.11875,0.
11396,0.11896,0.11656,0.12603,0.12162,0.11519,0.12054,0.12348,0.11712,
0.12361,0.12626,0.12463,0.045347,0.04549,0.042779,0.046631,0.044647,0.
047603,0.047557,0.045723,0.044912,0.044121,0.21509,0.18462,0.044107,0.
045352,0.045623,0.0461,0.13266,0.097614,0.1215,0.12365,0.12401,0.13433
,0.12277,0.1189,0.12301,0.11516,0.11216,0.13788,0.12781,0.11988,0.124,
0.12099,0.11258,0.13115,0.12782,0.045753,0.046593,0.045623,0.047577,0.
045983,0.045788,0.046706,0.045617,0.045417,0.045073,0.045607,0.048013,
0.045364,0.046532,0.047586,0.043677,0.044162,0.11846,0.12785,0.11047,0
.1035,0.12154,0.11674,0.12494,0.11049,0.11815,0.12176,0.11863,0.12679,
0.11231,0.12134,0.12858,0.12134,0.045528,0.046985,0.046091,0.046379,0.
04664,0.047057,0.044296,0.048193,0.047034,0.046727,0.04779,0.044981,0.
044996,0.044696,0.049226,0.045347,0.046219,0.045523,0.047535,0.11305,0
.1145,0.14156,0.12147,0.11193,0.12178,0.1222,0.12714,0.11577,0.12551,0
.12723,0.1301,0.10975,0.045776,0.046443,0.045201,0.045456,0.046597,0.0
46526,0.045503,0.047245,0.044596,0.044987,0.045488,0.048518,0.044797,0
.045718,0.045236,0.044839,0.04724,0.045902,0.045017,0.047054,0.043974,
0.11584,0.12314,0.1312,0.1222,0.13131,0.1086,0.11438,0.12122,0.12031,0
.046235,0.04593,0.045123,0.045603,0.045136,0.045504,0.045078,0.045736,
0.046573,0.046781,0.046485,0.044818,0.049242,0.0455,0.044426,0.044981,
0.047104,0.043889,0.044658,0.046012,0.046139,0.046059,0.047412,0.04498
7,0.047616,0.11407,0.13293,0.12113,0.10553,0.044256,0.045679,0.045119,
0.046202,0.043728,0.045469,0.045037,0.045979,0.046171,0.04734,0.046741
,0.04512,0.046401,0.043991,0.048458,0.045101,0.046216,0.044827,0.04479
,0.044907,0.046996,0.043917,0.04467,0.04438,0.046227,0.046659,0.048613
,0.047208,0.043705,0.048988,0.047168,0.043646,0.048209,0.04714,0.04540
3,0.04509,0.044981,0.045452,0.04511,0.046522,0.047102,0.04704,0.047057
,0.045808,0.044627,0.046662,0.047108,0.04403,0.046604,0.046505,0.04258
4,0.046658,0.04368,0.04578,0.047571,0.046924,0.0463,0.046411,0.046574,
0.045038,0.04577,0.046442,0.046402,0.045856,0.046212,0.046252,0.046052
,0.045659,0.046168,0.045817,0.046756,0.050118,0.045727,0.047109,0.0438
6,0.046816,0.047759,0.04681,0.044449,0.046161,0.044543,0.047547,0.0461
8,0.044744,0.045997,0.044993,0.046638,0.04614,0.04539,0.045304,0.04473
7,0.045492,0.045833,0.044941,0.047413,0.044707,0.046732,0.047303,0.046

475,0.048222,0.046716,0.047181,0.047104,0.043111,0.044961,0.048011,0.0
45495,0.045699,0.047657,0.044803,0.045121,0.04586,0.045709,0.047997,0.
046813,0.0448,0.043908,0.046989,0.046042,0.046842,0.04407,0.045281,0.0
45523,0.04698,0.044968,0.047961,0.046438,0.046594,0.046211,0.044282,0.
042816,0.047623,0.044917,0.046626,0.046204,0.046323,0.046209,0.044416,
0.047546,0.044129,0.045743,0.044283,0.045632,0.043865,0.046692,0.04649
2,0.04597,0.045201,0.047571,0.046527,0.04445,0.046062,0.045896,0.04482
3,0.047472,0.048204,0.044947,0.04744,0.045516,0.044297,0.046048,0.0467
03,0.048248,0.045993,0.045682,0.046488,0.044733,0.046101,0.047619,0.04
7044,0.045378,0.046718,0.046252,0.04571,0.044378,0.047062,0.043706,0.0
44602,0.044484,0.046257,0.046405,0.046612,0.04716,0.046039,0.046534,0.
046689,0.044312,0.046843,0.043349,0.047741,0.045696,0.045778,0.048301,
0.044067,0.049056
0.19698,0.19841,0.1976,0.19972,0.19987,0.20204,0.19887,0.19734,0.20082
,0.20013,0.19851,0.19933,0.19893,0.19839,0.20182,0.19922,0.19966,0.203
44,0.20088,0.1957,0.31124,0.2801,0.33199,0.26573,0.19972,0.19997,0.202
72,0.19835,0.19612,0.20188,0.19886,0.19914,0.19808,0.19764,0.20415,0.1
9916,0.1963,0.1975,0.199,0.35796,0.26729,0.29963,0.27306,0.28918,0.278
41,0.28245,0.1976,0.1962,0.20123,0.20069,0.19667,0.20034,0.19789,0.199
39,0.19688,0.20057,0.20074,0.20014,0.19683,0.2013,0.1992,0.38811,0.358
61,0.27118,0.30764,0.27033,0.29129,0.29282,0.27792,0.27834,0.27599,0.1
9784,0.19966,0.20244,0.19904,0.19701,0.19904,0.20058,0.19947,0.20117,0
.19926,0.19886,0.19837,0.19948,0.2,0.20304,0.35215,0.35002,0.38039,0.2
4501,0.3267,0.28964,0.28744,0.29094,0.28991,0.30249,0.29133,0.29283,0.
20312,0.2019,0.19868,0.19899,0.2016,0.19972,0.19861,0.19752,0.20049,0.
20163,0.20043,0.2007,0.20109,0.20174,0.19704,0.20146,0.3556,0.37724,0.
38605,0.38271,0.29287,0.29532,0.25933,0.29177,0.27831,0.27968,0.27735,
0.29152,0.29577,0.30127,0.20034,0.20044,0.20212,0.20131,0.19798,0.1980
9,0.19974,0.19871,0.20132,0.2004,0.19915,0.20069,0.19874,0.20179,0.197
28,0.19804,0.33253,0.36596,0.39458,0.3715,0.36604,0.38562,0.28111,0.27
519,0.27089,0.29226,0.28023,0.30344,0.26592,0.28336,0.27428,0.28626,0.
19751,0.19942,0.2012,0.19758,0.19889,0.20049,0.1982,0.20135,0.19882,0.
19929,0.20069,0.2032,0.20097,0.19803,0.20064,0.19937,0.2012,0.38054,0.
39045,0.36695,0.39014,0.36443,0.40645,0.29073,0.26208,0.28963,0.31011,
0.29699,0.30078,0.29285,0.28663,0.28907,0.27334,0.29112,0.19858,0.2007
4,0.19804,0.19616,0.19892,0.198,0.19576,0.19965,0.19716,0.20213,0.2000
7,0.1956,0.20074,0.12748,0.10738,0.12721,0.19969,0.34669,0.38112,0.352
44,0.39207,0.38219,0.3762,0.3592,0.29227,0.27218,0.30236,0.27612,0.311
53,0.27413,0.28556,0.29065,0.28403,0.27994,0.27657,0.19766,0.2005,0.20
129,0.20129,0.19877,0.19914,0.20056,0.19553,0.19771,0.2018,0.2001,0.10
088,0.12129,0.12192,0.12205,0.12135,0.12602,0.20068,0.34469,0.38144,0.
381,0.36627,0.36849,0.3592,0.36577,0.28585,0.29557,0.27453,0.30399,0.2
8008,0.29458,0.29245,0.29546,0.29709,0.27956,0.25792,0.19727,0.20344,0
.20044,0.19895,0.19611,0.20061,0.20134,0.19937,0.20261,0.12033,0.12419
,0.13398,0.12135,0.12092,0.12061,0.12167,0.1289,0.12055,0.19809,0.3725
,0.36523,0.40431,0.38763,0.36619,0.37028,0.37509,0.27969,0.28231,0.292
22,0.29717,0.29293,0.28017,0.2977,0.26818,0.28523,0.27855,0.28585,0.26
34,0.19727,0.19793,0.20088,0.20091,0.19979,0.2025,0.11313,0.123,0.1258
7,0.12242,0.13878,0.12329,0.11557,0.12228,0.1233,0.14002,0.12337,0.122
42,0.38324,0.39064,0.36651,0.40225,0.37185,0.37789,0.37188,0.28077,0.2
7116,0.28467,0.29694,0.2937,0.3078,0.28864,0.29284,0.29195,0.29461,0.2
9256,0.27328,0.20103,0.19772,0.20154,0.11736,0.14046,0.11035,0.10986,0

.12575,0.11867,0.12301,0.12211,0.12559,0.12413,0.12192,0.13179,0.12704
,0.14688,0.1236,0.36076,0.35376,0.37412,0.35211,0.36567,0.37588,0.4220
5,0.35715,0.37323,0.28163,0.29037,0.2778,0.28883,0.27132,0.27417,0.287
96,0.28729,0.28612,0.29109,0.30375,0.12093,0.12327,0.12045,0.1201,0.12
331,0.12203,0.11732,0.12355,0.12739,0.11972,0.10759,0.12088,0.12169,0.
12125,0.12183,0.10386,0.11616,0.12727,0.36244,0.37992,0.42104,0.38403,
0.36767,0.37177,0.40096,0.35518,0.36199,0.30559,0.27292,0.22553,0.1943
4,0.21008,0.20932,0.20608,0.21456,0.22627,0.18943,0.20691,0.12493,0.13
577,0.11295,0.1079,0.12045,0.13893,0.12535,0.12019,0.12791,0.10222,0.1
1946,0.12296,0.11947,0.12085,0.12683,0.11736,0.12149,0.11825,0.3605,0.
36732,0.38175,0.40963,0.38568,0.3561,0.3878,0.35379,0.36831,0.1976,0.2
125,0.2012,0.22139,0.19829,0.23573,0.19299,0.23494,0.21301,0.2093,0.18
938,0.12159,0.12423,0.13427,0.12179,0.11825,0.14198,0.14228,0.12096,0.
1357,0.1249,0.12078,0.12684,0.13531,0.11314,0.11129,0.12192,0.11316,0.
12238,0.38677,0.38417,0.36746,0.37834,0.37751,0.38737,0.37926,0.2057,0
.20768,0.25824,0.1954,0.20151,0.20999,0.20726,0.19562,0.19715,0.20573,
0.21101,0.21327,0.20419,0.12122,0.1222,0.13119,0.11386,0.12247,0.12002
,0.1228,0.14032,0.12225,0.12222,0.11302,0.12425,0.11755,0.12177,0.1144
4,0.1146,0.11727,0.13474,0.37178,0.37724,0.35708,0.40487,0.37527,0.365
78,0.21083,0.23265,0.22954,0.1849,0.20405,0.21436,0.19682,0.20567,0.20
292,0.22404,0.20894,0.21485,0.2083,0.23718,0.11957,0.12263,0.11962,0.1
2001,0.11308,0.12113,0.11657,0.11862,0.12657,0.1263,0.14194,0.1223,0.1
209,0.11528,0.1346,0.12853,0.10988,0.12203,0.36941,0.37925,0.38324,0.3
7027,0.37883,0.24286,0.21891,0.20565,0.22015,0.19538,0.16315,0.2014,0.
22204,0.21464,0.20648,0.19713,0.2131,0.20266,0.20843,0.21138,0.1367,0.
1091,0.13753,0.11775,0.10431,0.12087,0.11698,0.13681,0.1104,0.12155,0.
11381,0.12166,0.12207,0.1195,0.12471,0.13258,0.11233,0.12194,0.38825,0
.36412,0.37262,0.20428,0.21905,0.20555,0.22801,0.19755,0.22771,0.18278
,0.22452,0.22832,0.20557,0.19757,0.23399,0.19301,0.20995,0.21801,0.186
11,0.18587,0.11741,0.12147,0.11028,0.12245,0.11316,0.12971,0.13092,0.1
2274,0.12063,0.10806,0.12166,0.12254,0.12479,0.1366,0.1128,0.12264,0.1
2082,0.1208,0.36125,0.21911,0.22772,0.20702,0.18234,0.21762,0.20107,0.
20783,0.1924,0.20287,0.21189,0.21082,0.20831,0.20135,0.23267,0.20081,0
.21315,0.20942,0.20684,0.12127,0.12202,0.12223,0.12054,0.11847,0.11415
,0.11731,0.12251,0.12644,0.12474,0.12098,0.1208,0.11878,0.12981,0.1461
2,0.11984,0.10685,0.042734,0.20269,0.20942,0.20517,0.20277,0.20219,0.1
799,0.2037,0.24555,0.19857,0.26821,0.23574,0.17542,0.18078,0.21034,0.2
0961,0.20311,0.19538,0.19113,0.20134,0.1206,0.12218,0.12398,0.12178,0.
11944,0.0974,0.12206,0.11282,0.12638,0.11795,0.13208,0.11846,0.11965,0
.12459,0.12145,0.12093,0.12063,0.044499,0.22783,0.2045,0.20998,0.24329
,0.20809,0.18843,0.18707,0.17989,0.16719,0.21255,0.1988,0.23749,0.1677
,0.20374,0.20191,0.21432,0.22103,0.23064,0.1185,0.12093,0.12141,0.1246
8,0.12285,0.10582,0.1408,0.12564,0.12651,0.12209,0.12797,0.12162,0.120
79,0.12373,0.11287,0.12314,0.11734,0.11992,0.044002,0.21755,0.24889,0.
20038,0.21963,0.23027,0.20586,0.1955,0.20967,0.19896,0.21147,0.20044,0
.1978,0.19243,0.211,0.21107,0.20702,0.19191,0.19778,0.13455,0.12241,0.
12047,0.12132,0.12046,0.14583,0.11487,0.11995,0.11805,0.12302,0.1219,0
.12033,0.10953,0.11627,0.1143,0.12218,0.13376,0.042793,0.04286,0.04528
2,0.20794,0.2152,0.22258,0.20383,0.22512,0.1992,0.19774,0.20011,0.2182
5,0.18378,0.18793,0.20775,0.18704,0.21093,0.22497,0.10301,0.12513,0.12
908,0.1223,0.12179,0.12164,0.11473,0.12645,0.10882,0.12795,0.12221,0.1
1252,0.11866,0.11881,0.11416,0.11398,0.12133,0.11957,0.04286,0.044579,

0.043388,0.046234,0.1481,0.21871,0.19538,0.17864,0.21851,0.20439,0.244
51,0.25907,0.23366,0.21253,0.19584,0.20445,0.13843,0.11605,0.1048,0.11
375,0.13585,0.13479,0.1393,0.1125,0.12588,0.12328,0.13525,0.13227,0.12
148,0.13634,0.1212,0.12361,0.1257,0.12806,0.10771,0.12241,0.041077,0.0
43996,0.04474,0.044666,0.043713,0.043589,0.21773,0.21347,0.26306,0.211
84,0.18898,0.19971,0.19804,0.21274,0.11099,0.10328,0.093679,0.12231,0.
12124,0.11934,0.11882,0.12563,0.09451,0.12183,0.131,0.15905,0.12005,0.
11903,0.14662,0.12539,0.1218,0.09589,0.12245,0.11825,0.11805,0.11455,0
.12582,0.044468,0.045379,0.041502,0.046308,0.042734,0.044783,0.2321,0.
2128,0.23176,0.20999,0.21749,0.044571,0.043,0.12196,0.1204,0.12163,0.1
2133,0.12137,0.12139,0.11925,0.1302,0.11835,0.11375,0.11863,0.11694,0.
1267,0.12144,0.11466,0.12118,0.12384,0.11725,0.12383,0.12628,0.12494,0
.044262,0.043034,0.04126,0.043932,0.043436,0.045273,0.044966,0.043948,
0.04271,0.042104,0.2095,0.18312,0.041973,0.042758,0.043256,0.044144,0.
13244,0.097638,0.12113,0.12311,0.12399,0.1339,0.12349,0.11846,0.12323,
0.11509,0.1123,0.13674,0.12773,0.11922,0.12434,0.12187,0.1122,0.13155,
0.1282,0.043445,0.043691,0.044451,0.045458,0.043521,0.043391,0.044905,
0.044023,0.044093,0.043306,0.043582,0.04686,0.042944,0.044399,0.045204
,0.041287,0.042232,0.1185,0.12846,0.1107,0.10218,0.1206,0.11609,0.1242
,0.1103,0.11768,0.1219,0.11873,0.12654,0.11258,0.1214,0.12857,0.12149,
0.043095,0.044587,0.044226,0.044569,0.045266,0.045003,0.04296,0.04634,
0.04482,0.044102,0.046677,0.043416,0.04317,0.043277,0.04741,0.043367,0
.043984,0.042059,0.046368,0.1128,0.1145,0.1422,0.12194,0.11112,0.12123
,0.12156,0.1272,0.11603,0.12567,0.1273,0.12974,0.10942,0.044126,0.0448
45,0.044272,0.044316,0.044873,0.043956,0.042994,0.045466,0.042424,0.04
3323,0.042804,0.047091,0.043491,0.042842,0.043114,0.042944,0.044953,0.
044653,0.042905,0.044615,0.042573,0.11542,0.12272,0.13057,0.1226,0.131
17,0.10858,0.11522,0.12109,0.1203,0.044224,0.042943,0.043653,0.043959,
0.042664,0.043561,0.042835,0.043836,0.045236,0.04415,0.044502,0.043089
,0.046923,0.043261,0.043115,0.043114,0.044265,0.041513,0.042491,0.0437
96,0.043839,0.044762,0.044968,0.042809,0.04494,0.11405,0.13237,0.12133
,0.10517,0.042964,0.043819,0.043271,0.044275,0.041647,0.043052,0.04389
8,0.044141,0.044476,0.045765,0.045119,0.043786,0.044142,0.042779,0.046
077,0.043262,0.04371,0.042936,0.042798,0.042638,0.045249,0.042679,0.04
2623,0.043224,0.045636,0.043653,0.04668,0.045618,0.042034,0.047219,0.0
45451,0.04163,0.046697,0.044756,0.043301,0.04309,0.043256,0.042755,0.0
42798,0.044319,0.045029,0.04574,0.04589,0.043821,0.043068,0.043955,0.0
45531,0.042456,0.04515,0.044841,0.040206,0.043891,0.04212,0.044487,0.0
44754,0.04537,0.04402,0.043769,0.044149,0.042989,0.04432,0.044266,0.04
4347,0.044225,0.044692,0.043744,0.044065,0.043398,0.044539,0.044521,0.
044867,0.047449,0.043507,0.045126,0.041295,0.044253,0.046532,0.045066,
0.042499,0.044357,0.042786,0.045829,0.044915,0.042905,0.044457,0.04239
8,0.042856,0.043997,0.042916,0.042735,0.043304,0.043612,0.043748,0.042
635,0.046315,0.04342,0.04387,0.044926,0.044638,0.04565,0.044575,0.0457
69,0.044044,0.041066,0.043586,0.046106,0.043425,0.042747,0.045868,0.04
3445,0.042386,0.043687,0.042753,0.046129,0.04528,0.042413,0.043193,0.0
4395,0.0439,0.045276,0.042737,0.043829,0.044148,0.045427,0.043148,0.04
471,0.044902,0.044043,0.044821,0.042828,0.040778,0.044746,0.043148,0.0
44862,0.044356,0.044082,0.045279,0.042038,0.045832,0.04296,0.043432,0.
042785,0.044739,0.041768,0.046065,0.044642,0.044515,0.043437,0.045628,
0.044131,0.042666,0.044037,0.043658,0.042528,0.044822,0.04537,0.043263
,0.044604,0.042925,0.042081,0.044696,0.043708,0.045779,0.043286,0.0435

42,0.044389,0.043781,0.045268,0.044698,0.045817,0.04388,0.043934,0.044933,0.043744,0.042922,0.045067,0.04258,0.041823,0.042776,0.0443,0.044965,0.04382,0.045533,0.044033,0.043474,0.043992,0.04219,0.044915,0.04224,0.045593,0.04322,0.043271,0.045443,0.043038,0.045657
0.198,0.19836,0.19858,0.20222,0.20114,0.20396,0.20064,0.19887,0.20298,0.20049,0.20077,0.19985,0.20031,0.20006,0.20456,0.19941,0.20137,0.20457,0.20301,0.19781,0.3043,0.27653,0.32678,0.26858,0.20147,0.20139,0.2042,0.19948,0.19845,0.20231,0.20022,0.2009,0.1996,0.19846,0.20492,0.20059,0.1975,0.1983,0.20185,0.34319,0.27595,0.29719,0.27495,0.29221,0.27234,0.27785,0.2003,0.19939,0.20217,0.20076,0.19826,0.20393,0.19952,0.20072,0.19819,0.20213,0.20181,0.20032,0.19785,0.20237,0.20096,0.38558,0.35173,0.27363,0.30985,0.26728,0.29273,0.29042,0.27407,0.27898,0.27162,0.19945,0.20059,0.20406,0.20016,0.19976,0.20058,0.20198,0.20118,0.20176,0.20103,0.20035,0.19962,0.20034,0.2001,0.20366,0.34934,0.34168,0.3915,0.24467,0.32159,0.28583,0.28935,0.28881,0.28763,0.29764,0.29099,0.28794,0.20264,0.20301,0.20022,0.20114,0.20361,0.20161,0.20049,0.19793,0.20217,0.20313,0.2014,0.2014,0.20232,0.20168,0.20024,0.20209,0.35887,0.36404,0.38798,0.3822,0.29503,0.28916,0.26046,0.28821,0.27856,0.28317,0.27536,0.2858,0.29248,0.29444,0.20272,0.2027,0.20275,0.2023,0.19889,0.2012,0.20018,0.2,0.20064,0.20142,0.2001,0.20073,0.19807,0.20259,0.19969,0.20015,0.32214,0.3702,0.38866,0.36026,0.35039,0.37627,0.28064,0.27097,0.27222,0.29432,0.27629,0.30533,0.26695,0.27526,0.27217,0.28392,0.19929,0.2011,0.20196,0.19896,0.2001,0.20263,0.20013,0.20284,0.20003,0.2013,0.20273,0.20501,0.2019,0.19958,0.20161,0.19992,0.20301,0.37301,0.375,0.36133,0.37441,0.36802,0.39972,0.28282,0.25835,0.28894,0.30535,0.29445,0.29573,0.29181,0.28525,0.29335,0.27256,0.28963,0.1997,0.20195,0.19959,0.19729,0.19944,0.19955,0.19751,0.20114,0.19735,0.20427,0.20092,0.1967,0.20229,0.12698,0.10686,0.12764,0.20224,0.34191,0.38533,0.34732,0.38662,0.37129,0.37411,0.35256,0.29409,0.27626,0.30056,0.27952,0.30849,0.26906,0.28194,0.28562,0.2813,0.2768,0.2784,0.19807,0.20048,0.20264,0.20144,0.20016,0.20087,0.20224,0.19755,0.19901,0.20273,0.20001,0.099865,0.12061,0.12177,0.12247,0.12131,0.12693,0.20202,0.33723,0.37304,0.36085,0.36794,0.36086,0.35991,0.35333,0.28692,0.29417,0.27191,0.30552,0.27322,0.29878,0.29121,0.29335,0.29937,0.27594,0.25837,0.19946,0.20373,0.20066,0.20067,0.19763,0.20096,0.20246,0.20079,0.20273,0.12168,0.12387,0.13469,0.12121,0.12131,0.12159,0.12174,0.12911,0.12033,0.1998,0.3628,0.35286,0.38632,0.37506,0.37339,0.37448,0.38283,0.2803,0.27996,0.28968,0.29537,0.29104,0.27431,0.29372,0.26711,0.28812,0.27621,0.28468,0.26038,0.19869,0.19851,0.20161,0.20258,0.20146,0.20276,0.1134,0.12252,0.12646,0.12207,0.13926,0.12404,0.1152,0.12252,0.12291,0.13989,0.12343,0.12249,0.36742,0.3894,0.35694,0.38957,0.3696,0.36438,0.37056,0.27988,0.27731,0.28823,0.2869,0.2879,0.30281,0.29202,0.28705,0.29058,0.29292,0.29259,0.27199,0.20219,0.19932,0.20287,0.11655,0.14084,0.1102,0.10989,0.12602,0.11847,0.12268,0.12259,0.12498,0.12453,0.12137,0.13255,0.1273,0.14736,0.12313,0.36293,0.35785,0.37704,0.35472,0.37002,0.37171,0.41268,0.3539,0.36448,0.28038,0.28103,0.28096,0.28339,0.26472,0.27378,0.28165,0.29009,0.28211,0.28992,0.2982,0.12086,0.12349,0.12013,0.12065,0.12394,0.12158,0.11685,0.12368,0.12652,0.1196,0.1077,0.12072,0.12143,0.12177,0.12179,0.10395,0.11637,0.12653,0.35172,0.37366,0.42027,0.37204,0.34984,0.36603,0.38864,0.34921,0.36687,0.30868,0.27698,0.22268,0.19102,0.21303,0.20128,0.20462,0.21314,0.21959,0.18505,0.2055,0.12559,0.13657,0.11325,0.10759,0.12069,0.13981,0.12517,0.12019,0.12824,0.10167,0.

11941,0.12234,0.11988,0.12119,0.12674,0.11681,0.12138,0.11849,0.35848,
0.36253,0.3719,0.40291,0.3763,0.35052,0.39526,0.34313,0.36106,0.18823,
0.20687,0.19991,0.22149,0.19917,0.23865,0.19048,0.23119,0.21091,0.2070
9,0.18832,0.12129,0.12526,0.13476,0.12268,0.1181,0.14175,0.14278,0.121
78,0.13632,0.125,0.12158,0.12642,0.13681,0.11275,0.11141,0.12214,0.112
93,0.12218,0.37296,0.38708,0.36271,0.37228,0.38313,0.37987,0.39009,0.1
957,0.21304,0.24813,0.18934,0.20046,0.20929,0.20509,0.19742,0.19879,0.
20727,0.20735,0.20965,0.2028,0.12097,0.12162,0.13113,0.11404,0.12263,0
.12013,0.12232,0.14111,0.12245,0.12272,0.1135,0.12504,0.11801,0.12147,
0.11486,0.11418,0.11775,0.13489,0.37258,0.37711,0.35445,0.40346,0.3868
7,0.3661,0.21752,0.23063,0.21947,0.18567,0.19902,0.21622,0.19261,0.207
17,0.20253,0.2196,0.20312,0.20958,0.20694,0.23674,0.11963,0.12274,0.12
018,0.12083,0.11289,0.12103,0.11751,0.11859,0.12618,0.12663,0.14296,0.
12248,0.12112,0.1152,0.13498,0.12881,0.10913,0.12249,0.36226,0.37693,0
.39441,0.34622,0.36233,0.23802,0.21563,0.20798,0.21061,0.1831,0.16635,
0.19579,0.21657,0.21639,0.20106,0.19653,0.21285,0.19863,0.20584,0.2100
6,0.13706,0.10887,0.13762,0.11731,0.10344,0.12103,0.11672,0.13675,0.11
027,0.12152,0.11366,0.12197,0.12198,0.11905,0.12497,0.13288,0.11305,0.
12171,0.37255,0.35964,0.36265,0.20412,0.22195,0.19594,0.21783,0.19349,
0.22066,0.18215,0.21093,0.22487,0.19743,0.19521,0.2313,0.19685,0.20793
,0.20819,0.18544,0.1844,0.11768,0.12174,0.10957,0.12175,0.11337,0.1299
,0.13137,0.12344,0.12072,0.10849,0.12183,0.12316,0.12438,0.13684,0.113
66,0.12282,0.12123,0.1209,0.35589,0.21534,0.22071,0.20378,0.18234,0.22
133,0.1932,0.20501,0.189,0.20319,0.21466,0.20735,0.20778,0.19831,0.228
29,0.19922,0.20963,0.20651,0.20459,0.12145,0.12177,0.12259,0.12014,0.1
1743,0.11405,0.11711,0.12264,0.12681,0.12559,0.12063,0.12145,0.11843,0
.12934,0.14618,0.11949,0.10629,0.041406,0.19553,0.20633,0.20411,0.1975
1,0.19606,0.17826,0.1982,0.24289,0.19778,0.26527,0.23109,0.17235,0.180
31,0.2098,0.21218,0.19908,0.19064,0.18726,0.20141,0.12057,0.12237,0.12
437,0.12186,0.12014,0.097217,0.12223,0.11298,0.12638,0.11792,0.13224,0
.118,0.12018,0.12487,0.12165,0.12008,0.12048,0.042802,0.21747,0.20589,
0.20623,0.23868,0.19948,0.19385,0.18521,0.17325,0.15911,0.21201,0.1993
3,0.23305,0.16671,0.19719,0.19635,0.2108,0.21426,0.22737,0.11873,0.121
1,0.1214,0.12433,0.12265,0.10532,0.14065,0.12501,0.12671,0.12183,0.128
19,0.12207,0.12175,0.12342,0.11355,0.12302,0.11709,0.1199,0.042546,0.2
1189,0.25042,0.19731,0.22173,0.22146,0.20114,0.18794,0.21299,0.19068,0
.20332,0.19859,0.19749,0.1893,0.2155,0.21714,0.20568,0.19532,0.19784,0
.13466,0.12213,0.12089,0.12155,0.12035,0.14549,0.11498,0.11999,0.1185,
0.12309,0.12157,0.12031,0.109,0.11593,0.11423,0.12191,0.13336,0.041883
,0.041762,0.042817,0.20098,0.20742,0.21581,0.19967,0.21447,0.20042,0.1
9156,0.19097,0.21476,0.18177,0.18632,0.21301,0.18374,0.20459,0.22169,0
.10254,0.12472,0.12867,0.12232,0.12234,0.12227,0.11437,0.12582,0.10887
,0.12827,0.12205,0.1123,0.11862,0.11867,0.11411,0.11404,0.12139,0.1192
1,0.041971,0.043239,0.041783,0.045793,0.14856,0.21386,0.18295,0.169,0.
20945,0.20495,0.24638,0.24624,0.22098,0.2115,0.19563,0.20182,0.13795,0
.11587,0.10437,0.11366,0.13523,0.13512,0.13952,0.11219,0.12566,0.12277
,0.13549,0.13132,0.1221,0.13716,0.12072,0.1232,0.12553,0.12875,0.10747
,0.12193,0.040522,0.043763,0.042732,0.042713,0.042577,0.042584,0.21787
,0.209,0.25919,0.21151,0.18639,0.19444,0.19176,0.20637,0.11155,0.10318
,0.093695,0.12186,0.12117,0.11916,0.11887,0.12543,0.093901,0.12267,0.1
3042,0.15949,0.12004,0.11917,0.14754,0.12584,0.12184,0.0956,0.12277,0.
11795,0.11793,0.11417,0.12573,0.043385,0.044449,0.040031,0.044096,0.04

1399,0.043355,0.23415,0.21046,0.22473,0.20434,0.21306,0.043343,0.04147
8,0.12195,0.11927,0.12119,0.12145,0.12173,0.12145,0.11893,0.13083,0.11
872,0.11303,0.11869,0.11716,0.12705,0.12147,0.11406,0.12157,0.1235,0.1
1695,0.12319,0.12677,0.12498,0.042773,0.042999,0.040108,0.043564,0.042
527,0.044006,0.044594,0.042378,0.041245,0.041875,0.20957,0.18091,0.039
902,0.041386,0.042079,0.042741,0.13204,0.097276,0.12216,0.12335,0.1241
,0.13353,0.12357,0.11849,0.12296,0.11522,0.11207,0.13708,0.12776,0.119
07,0.12512,0.12268,0.11231,0.13156,0.12843,0.041624,0.042284,0.043212,
0.043762,0.042861,0.042707,0.0425,0.042724,0.043071,0.042471,0.042332,
0.045509,0.042633,0.042482,0.043897,0.040442,0.040589,0.1186,0.12802,0
.1101,0.1021,0.12062,0.11497,0.12454,0.11014,0.11789,0.12214,0.11818,0
.12568,0.1125,0.12107,0.12814,0.122,0.041288,0.043078,0.043708,0.04369
8,0.043828,0.043414,0.041564,0.044477,0.043744,0.041853,0.04541,0.0432
65,0.042148,0.04159,0.045026,0.041385,0.043417,0.041102,0.0449,0.11181
,0.11448,0.14309,0.12227,0.11145,0.12175,0.12175,0.12771,0.11553,0.126
13,0.12672,0.12955,0.10879,0.042309,0.042982,0.043731,0.043537,0.04259
1,0.042987,0.042315,0.043622,0.041661,0.041702,0.04212,0.045468,0.0424
5,0.041964,0.041699,0.041169,0.043984,0.042787,0.042036,0.042426,0.041
771,0.11588,0.12276,0.1303,0.1228,0.13164,0.1088,0.11542,0.12139,0.121
14,0.041831,0.04209,0.042833,0.042495,0.0415,0.042436,0.04171,0.043003
,0.043184,0.043261,0.043468,0.04105,0.044668,0.041934,0.042,0.042532,0
.042796,0.039925,0.04145,0.042949,0.04253,0.043398,0.044462,0.040943,0
.044201,0.11414,0.13273,0.1212,0.10522,0.040728,0.042329,0.041855,0.04
3525,0.041765,0.041171,0.042967,0.042483,0.043624,0.044665,0.044073,0.
042387,0.042175,0.041567,0.044228,0.042034,0.04221,0.042277,0.042079,0
.04163,0.044784,0.041007,0.04108,0.041523,0.044198,0.041482,0.044684,0
.043154,0.042274,0.045217,0.044191,0.04082,0.044732,0.043088,0.041982,
0.040856,0.0415,0.041572,0.041338,0.04339,0.043653,0.04427,0.044426,0.
042511,0.041302,0.042378,0.044751,0.041038,0.043879,0.043763,0.039985,
0.042707,0.04074,0.04353,0.043673,0.044499,0.043333,0.042942,0.043261,
0.042033,0.042866,0.043326,0.044195,0.043366,0.043871,0.041487,0.04316
8,0.042319,0.043926,0.043593,0.043719,0.046022,0.04258,0.044359,0.0405
52,0.043131,0.045195,0.043351,0.04118,0.043787,0.041328,0.043206,0.043
435,0.040949,0.043517,0.040128,0.042366,0.042781,0.042128,0.041471,0.0
42425,0.04281,0.042842,0.040539,0.044481,0.041962,0.042697,0.043625,0.
042735,0.045057,0.043375,0.044698,0.042407,0.039877,0.043021,0.044357,
0.042028,0.04099,0.044743,0.042289,0.040901,0.040615,0.042386,0.044848
,0.043001,0.040824,0.040813,0.042478,0.041673,0.043562,0.041575,0.0427
96,0.042125,0.043332,0.042695,0.042294,0.04434,0.042634,0.04322,0.0416
34,0.039921,0.044467,0.04219,0.043315,0.042442,0.043219,0.043974,0.041
811,0.044799,0.041458,0.042555,0.041437,0.043991,0.039993,0.044374,0.0
43172,0.0429,0.041045,0.043958,0.042422,0.040961,0.043196,0.041854,0.0
41757,0.0432,0.044342,0.042094,0.043152,0.040589,0.040271,0.04326,0.04
1883,0.043433,0.042376,0.041787,0.0434,0.042658,0.043739,0.043117,0.04
4582,0.042006,0.043503,0.043721,0.041459,0.041721,0.044405,0.040922,0.
040523,0.04176,0.043201,0.043666,0.042248,0.043881,0.042138,0.04255,0.
041804,0.041756,0.043973,0.041327,0.044201,0.04341,0.041834,0.043528,0
.041852,0.044942
0.19846,0.19923,0.19861,0.20259,0.20233,0.20386,0.20174,0.20102,0.2046
1,0.20213,0.20244,0.20039,0.20219,0.20098,0.20376,0.20132,0.2029,0.204
79,0.20416,0.1995,0.30266,0.27408,0.33251,0.26601,0.20246,0.20382,0.20
605,0.20088,0.19889,0.20408,0.20103,0.20281,0.20108,0.20088,0.20578,0.

20015,0.20011,0.19937,0.20229,0.33941,0.26925,0.29122,0.28239,0.28845,
0.26789,0.27266,0.20127,0.20035,0.2053,0.2025,0.19955,0.20463,0.20115,
0.20199,0.19877,0.20194,0.20243,0.20178,0.20008,0.20361,0.20346,0.3871
7,0.35475,0.27099,0.3112,0.26802,0.29197,0.28349,0.27868,0.27383,0.264
73,0.20218,0.20258,0.20479,0.20152,0.20161,0.2028,0.20227,0.20082,0.20
229,0.20227,0.2026,0.20083,0.20241,0.20121,0.20383,0.34426,0.3418,0.38
497,0.24583,0.32203,0.28221,0.28932,0.28648,0.28658,0.30043,0.29295,0.
28591,0.20342,0.2036,0.20174,0.20241,0.2042,0.20354,0.20173,0.19866,0.
2039,0.20412,0.20244,0.20267,0.20249,0.20384,0.20174,0.20339,0.34769,0
.3693,0.37323,0.37888,0.29481,0.28205,0.26133,0.2825,0.27587,0.28269,0
.2719,0.28637,0.28983,0.29334,0.20357,0.20389,0.20328,0.20373,0.2012,0
.20241,0.20108,0.20082,0.20218,0.20175,0.20227,0.20214,0.19962,0.20464
,0.20035,0.20164,0.32029,0.36895,0.37791,0.35815,0.35164,0.37442,0.278
09,0.27145,0.27151,0.29282,0.27764,0.29242,0.26692,0.2809,0.26815,0.27
82,0.20026,0.20237,0.20196,0.20078,0.20179,0.2029,0.20215,0.20382,0.20
082,0.20306,0.20367,0.20617,0.20373,0.20115,0.20362,0.2016,0.20536,0.3
6805,0.36753,0.35523,0.37118,0.37074,0.40469,0.28505,0.25478,0.28635,0
.30152,0.29913,0.29469,0.2986,0.28199,0.2815,0.27074,0.29098,0.20087,0
.20263,0.20077,0.19883,0.20031,0.19994,0.19963,0.2038,0.19918,0.20426,
0.20161,0.19709,0.20407,0.12758,0.10654,0.12754,0.20303,0.32586,0.3801
4,0.3288,0.38372,0.36048,0.36528,0.34384,0.29259,0.27927,0.29748,0.282
66,0.30612,0.26998,0.27935,0.28107,0.28266,0.26938,0.27585,0.19998,0.2
0036,0.20377,0.20221,0.20171,0.20241,0.20277,0.19905,0.19993,0.2032,0.
20173,0.10037,0.12015,0.12165,0.12237,0.12068,0.12745,0.20273,0.33882,
0.3692,0.35523,0.37094,0.34926,0.36412,0.36061,0.28175,0.29708,0.26464
,0.30479,0.27127,0.29615,0.28923,0.2924,0.29511,0.28117,0.25622,0.2010
7,0.2045,0.20133,0.20146,0.19817,0.202,0.20322,0.20225,0.2026,0.12218,
0.1235,0.13493,0.12169,0.12129,0.12135,0.12147,0.12983,0.12043,0.19999
,0.34812,0.34776,0.38316,0.35997,0.37661,0.37974,0.37766,0.27543,0.281
37,0.28616,0.29424,0.28808,0.27635,0.28387,0.2664,0.28474,0.27471,0.27
38,0.26349,0.20028,0.19956,0.20292,0.20451,0.20256,0.20404,0.11342,0.1
2297,0.12708,0.1214,0.1394,0.12492,0.11462,0.12311,0.12314,0.14059,0.1
2371,0.12239,0.37126,0.39015,0.36166,0.38484,0.36309,0.36877,0.3581,0.
27956,0.28371,0.28774,0.28138,0.28782,0.30151,0.28908,0.28308,0.29256,
0.2971,0.29199,0.26735,0.20288,0.20046,0.20357,0.11673,0.14053,0.10994
,0.11006,0.12606,0.11895,0.12281,0.12219,0.12437,0.12465,0.12088,0.132
73,0.12741,0.14822,0.12351,0.35167,0.34341,0.37877,0.36021,0.36573,0.3
8065,0.38596,0.34008,0.35324,0.28105,0.27991,0.27572,0.28673,0.26884,0
.27387,0.27338,0.28104,0.28004,0.2862,0.30106,0.12125,0.12359,0.11966,
0.12069,0.12345,0.12224,0.11733,0.12381,0.12677,0.11932,0.10776,0.1208
5,0.12189,0.1214,0.12146,0.10384,0.11634,0.12631,0.36268,0.36646,0.403
97,0.35869,0.35036,0.36217,0.38236,0.34186,0.36223,0.30935,0.27468,0.2
2419,0.19002,0.21331,0.19972,0.20539,0.21111,0.22182,0.17956,0.20615,0
.12452,0.13707,0.11265,0.1077,0.12041,0.13942,0.1259,0.12119,0.12871,0
.1021,0.11978,0.12215,0.12031,0.12085,0.12699,0.11702,0.12111,0.11879,
0.36839,0.36874,0.37759,0.41249,0.36145,0.34437,0.38938,0.3415,0.35451
,0.1876,0.20492,0.2027,0.2166,0.19579,0.23698,0.19038,0.22985,0.20856,
0.2026,0.19046,0.12191,0.12559,0.1351,0.12274,0.11767,0.14207,0.14406,
0.12177,0.13685,0.12507,0.12186,0.12669,0.13671,0.11254,0.11104,0.1227
7,0.11355,0.12176,0.35546,0.38066,0.37065,0.37872,0.36535,0.37328,0.37
899,0.19847,0.20385,0.24439,0.1885,0.20594,0.2065,0.20627,0.19571,0.20
134,0.20371,0.20473,0.21191,0.20334,0.12049,0.12186,0.13208,0.11439,0.

12267,0.11933,0.12291,0.14083,0.12217,0.12267,0.11362,0.12488,0.11936,
0.12191,0.11513,0.11434,0.11744,0.13527,0.37377,0.3754,0.36058,0.39156
,0.3872,0.3567,0.21708,0.22129,0.21023,0.18485,0.19849,0.22077,0.19517
,0.20435,0.20165,0.21737,0.20386,0.209,0.20635,0.23682,0.11884,0.12315
,0.12054,0.12124,0.1128,0.12088,0.11752,0.11926,0.12644,0.12686,0.1438
3,0.12281,0.12117,0.11507,0.13513,0.12861,0.10846,0.12243,0.35262,0.36
766,0.39202,0.35765,0.34555,0.23306,0.2086,0.20953,0.20977,0.1824,0.16
634,0.1922,0.22029,0.21535,0.2014,0.19975,0.21493,0.19953,0.2072,0.210
47,0.13744,0.10804,0.13796,0.1167,0.10269,0.12099,0.11726,0.13621,0.11
073,0.12138,0.1133,0.12266,0.12271,0.11951,0.12519,0.13386,0.11318,0.1
2216,0.36041,0.35332,0.36268,0.20665,0.22037,0.19415,0.21611,0.19551,0
.21623,0.18238,0.20122,0.22323,0.19283,0.19097,0.22853,0.19673,0.21216
,0.20415,0.18577,0.18579,0.11832,0.1218,0.10884,0.12138,0.11421,0.1304
3,0.13061,0.12425,0.11997,0.10872,0.12264,0.12383,0.12478,0.13741,0.11
285,0.1224,0.12147,0.12165,0.35874,0.20673,0.21668,0.20233,0.1785,0.21
411,0.19032,0.20158,0.18891,0.19788,0.21198,0.20243,0.20877,0.19484,0.
22762,0.19896,0.20937,0.20271,0.20465,0.12173,0.12061,0.12212,0.12066,
0.11706,0.11466,0.1167,0.12301,0.1264,0.12621,0.12074,0.12132,0.11832,
0.12988,0.14639,0.11951,0.10643,0.040848,0.19584,0.19918,0.19957,0.196
91,0.19479,0.17378,0.19219,0.24416,0.19566,0.25801,0.23127,0.17289,0.1
8061,0.2059,0.20909,0.19208,0.19454,0.19151,0.19822,0.12024,0.12262,0.
12481,0.12223,0.11962,0.096194,0.12277,0.1123,0.12671,0.11839,0.13211,
0.11837,0.11974,0.12596,0.12245,0.11944,0.12035,0.041868,0.22532,0.199
75,0.20497,0.23971,0.20107,0.18799,0.18706,0.16885,0.15763,0.21296,0.1
9966,0.23166,0.16737,0.19168,0.19657,0.21163,0.21153,0.23145,0.11905,0
.12109,0.12132,0.12434,0.12318,0.10495,0.14127,0.12504,0.12696,0.12242
,0.12848,0.12234,0.12098,0.12276,0.11384,0.12199,0.11719,0.12001,0.041
487,0.22184,0.24644,0.20224,0.22548,0.21964,0.19909,0.18625,0.20988,0.
19207,0.21006,0.19248,0.19218,0.19209,0.21573,0.21587,0.20218,0.1885,0
.19824,0.13426,0.12166,0.12033,0.12206,0.12036,0.14628,0.11479,0.11983
,0.11805,0.12332,0.12175,0.12082,0.10912,0.11583,0.11389,0.1217,0.1336
,0.040544,0.040583,0.042366,0.20123,0.20592,0.21474,0.19816,0.21103,0.
19639,0.19282,0.19544,0.21201,0.18139,0.18397,0.21185,0.18256,0.19867,
0.21641,0.10229,0.12448,0.12909,0.12253,0.12245,0.12218,0.1137,0.12599
,0.10874,0.12818,0.12243,0.11178,0.11822,0.1189,0.11465,0.11436,0.1210
5,0.11901,0.04126,0.041743,0.04189,0.044652,0.15248,0.21406,0.18377,0.
16258,0.20953,0.20578,0.2361,0.23836,0.22563,0.2102,0.19346,0.20168,0.
13873,0.11617,0.10404,0.11314,0.13579,0.13557,0.13942,0.1117,0.1268,0.
12303,0.13555,0.13184,0.12242,0.13744,0.12083,0.12279,0.12585,0.12987,
0.10815,0.12216,0.039863,0.043077,0.041994,0.04104,0.041086,0.041294,0
.21989,0.20381,0.25944,0.20793,0.18674,0.19268,0.18426,0.20037,0.11168
,0.10338,0.092841,0.12209,0.12127,0.11979,0.11959,0.12507,0.093652,0.1
2343,0.13018,0.15979,0.11997,0.11922,0.14781,0.12624,0.12232,0.095393,
0.12305,0.11763,0.11856,0.11347,0.12625,0.041611,0.042936,0.039196,0.0
42575,0.040683,0.042347,0.2223,0.20774,0.22903,0.19561,0.21025,0.04226
3,0.040227,0.1219,0.11995,0.1215,0.12133,0.12154,0.1227,0.11949,0.1313
3,0.11987,0.11262,0.11889,0.11671,0.1265,0.12208,0.11366,0.12241,0.123
36,0.11754,0.12477,0.12613,0.12475,0.041406,0.042274,0.038858,0.042967
,0.041324,0.043183,0.042791,0.041655,0.039886,0.040595,0.20364,0.17991
,0.040134,0.039408,0.040835,0.041617,0.13173,0.096802,0.12237,0.12371,
0.12457,0.13291,0.12315,0.11893,0.12338,0.11584,0.1118,0.13773,0.12883
,0.11898,0.12559,0.12275,0.11209,0.13139,0.12854,0.04053,0.041652,0.04

3643,0.042233,0.042607,0.041781,0.042078,0.041099,0.041705,0.041625,0.
042298,0.044298,0.040511,0.041314,0.043329,0.039145,0.039952,0.11824,0
.12807,0.11065,0.10235,0.12113,0.11535,0.12478,0.11018,0.11765,0.12219
,0.1179,0.12596,0.11253,0.12102,0.12913,0.12245,0.040564,0.040972,0.04
3013,0.042077,0.042414,0.042809,0.040427,0.043607,0.042977,0.040005,0.
044466,0.042445,0.041834,0.040153,0.043321,0.040503,0.042468,0.040136,
0.043884,0.1121,0.11492,0.14343,0.12251,0.11111,0.1222,0.12174,0.12781
,0.11539,0.1264,0.12655,0.13003,0.10813,0.040775,0.04279,0.042725,0.04
259,0.041938,0.041592,0.041682,0.042505,0.041063,0.040467,0.041973,0.0
4511,0.042228,0.04123,0.040032,0.041839,0.04223,0.041268,0.039933,0.04
1378,0.041348,0.11583,0.1229,0.13097,0.12226,0.13178,0.10849,0.11618,0
.12121,0.12159,0.041351,0.041407,0.041199,0.041768,0.041877,0.040533,0
.041024,0.04268,0.041851,0.042413,0.041446,0.040105,0.042757,0.040835,
0.041421,0.041814,0.0412,0.039717,0.040453,0.042556,0.042562,0.042757,
0.042457,0.039091,0.042876,0.11386,0.13295,0.12149,0.10539,0.040052,0.
040312,0.040914,0.043464,0.041098,0.04078,0.042131,0.042205,0.04296,0.
043387,0.043171,0.041117,0.040867,0.041368,0.043047,0.041029,0.042915,
0.040966,0.041152,0.040646,0.043247,0.039417,0.040365,0.040662,0.04371
2,0.041163,0.0437,0.042795,0.042078,0.044481,0.043433,0.040095,0.04301
6,0.042093,0.040543,0.040506,0.040768,0.0416,0.042022,0.04276,0.042301
,0.042438,0.043252,0.041238,0.040589,0.042484,0.043509,0.0404,0.043426
,0.043037,0.039264,0.041851,0.040036,0.041314,0.043357,0.0427,0.041348
,0.041738,0.041926,0.040336,0.042086,0.042436,0.042732,0.043165,0.0427
37,0.040635,0.042326,0.041176,0.041813,0.042197,0.043281,0.044444,0.04
1371,0.04302,0.040288,0.041993,0.043783,0.043208,0.040143,0.043048,0.0
4111,0.042533,0.0428,0.040724,0.042818,0.039695,0.041498,0.041689,0.04
1343,0.041044,0.041952,0.041706,0.042527,0.040298,0.043122,0.04019,0.0
41448,0.043416,0.042012,0.043863,0.042189,0.042674,0.041439,0.039492,0
.04203,0.042408,0.040617,0.040001,0.04399,0.041395,0.039978,0.040233,0
.041453,0.044484,0.042577,0.040127,0.039106,0.041185,0.041507,0.042097
,0.040864,0.041835,0.040538,0.041664,0.041818,0.041934,0.042931,0.0413
52,0.04206,0.041487,0.039636,0.042336,0.04104,0.041817,0.042074,0.0424
3,0.042631,0.041319,0.043794,0.041381,0.041488,0.041028,0.042954,0.039
052,0.04267,0.041568,0.042335,0.041609,0.042956,0.041696,0.039912,0.04
175,0.040894,0.040603,0.042017,0.042707,0.041717,0.041324,0.04084,0.04
0264,0.042985,0.040392,0.042018,0.041782,0.040867,0.042785,0.041989,0.
043215,0.041657,0.043265,0.040608,0.04167,0.042306,0.040341,0.040426,0
.042501,0.039538,0.040031,0.040778,0.042256,0.043201,0.042393,0.042643
,0.040916,0.041952,0.040024,0.040357,0.042991,0.039367,0.043421,0.0424
87,0.041533,0.041979,0.041768,0.044271
0.19866,0.20036,0.20016,0.20462,0.20155,0.20449,0.20326,0.20233,0.2053
1,0.20265,0.20299,0.20295,0.20367,0.2012,0.20449,0.20125,0.20458,0.205
78,0.2048,0.20157,0.29409,0.27187,0.33503,0.267,0.20387,0.2053,0.20558
,0.2013,0.19979,0.2048,0.20281,0.20365,0.20188,0.20279,0.2075,0.20039,
0.20081,0.20018,0.20269,0.34604,0.2731,0.28825,0.28396,0.28812,0.27186
,0.27489,0.20165,0.20175,0.20427,0.20267,0.20073,0.20547,0.20223,0.201
68,0.20098,0.20189,0.20422,0.20283,0.20263,0.20396,0.20444,0.38283,0.3
6443,0.2704,0.31054,0.26598,0.28784,0.27995,0.28023,0.27203,0.26245,0.
20344,0.20352,0.20697,0.20343,0.20176,0.20342,0.20435,0.20132,0.20465,
0.20411,0.20368,0.20222,0.20422,0.20213,0.20475,0.34005,0.345,0.3807,0
.24585,0.3167,0.28754,0.29131,0.2836,0.29143,0.29952,0.29594,0.28117,0
.20353,0.20346,0.20229,0.20151,0.20664,0.20429,0.20289,0.2006,0.20401,

0.2047,0.20378,0.20371,0.20241,0.20547,0.20277,0.20391,0.35082,0.36798
,0.36387,0.37674,0.29537,0.28721,0.26135,0.2817,0.27447,0.27339,0.2705
3,0.28687,0.29079,0.28705,0.20419,0.20606,0.20376,0.20544,0.20324,0.20
302,0.20144,0.20038,0.20303,0.20335,0.20332,0.2026,0.19991,0.20455,0.2
0235,0.20221,0.33207,0.36704,0.36043,0.33424,0.3661,0.37019,0.27601,0.
26738,0.27813,0.29642,0.27614,0.29101,0.2681,0.27528,0.27015,0.278,0.2
0143,0.20282,0.20283,0.20067,0.20259,0.20443,0.20239,0.20474,0.20242,0
.20406,0.20513,0.20565,0.20562,0.20185,0.20457,0.20328,0.20631,0.3599,
0.38168,0.36074,0.37481,0.37364,0.39356,0.27834,0.26034,0.28253,0.3070
1,0.29225,0.2955,0.29367,0.28051,0.27769,0.27343,0.29072,0.20158,0.203
69,0.2025,0.19909,0.20154,0.20131,0.20016,0.20478,0.20053,0.2044,0.202
06,0.19834,0.20424,0.12797,0.10735,0.12811,0.20447,0.32564,0.36997,0.3
2449,0.38527,0.34719,0.36203,0.34522,0.29593,0.28126,0.29497,0.27823,0
.30797,0.26912,0.27758,0.27633,0.28138,0.26926,0.2728,0.20131,0.20187,
0.20418,0.20293,0.20389,0.20421,0.20457,0.2,0.20175,0.20457,0.20349,0.
1003,0.11986,0.12189,0.12269,0.12115,0.12756,0.2041,0.33596,0.36187,0.
3627,0.37377,0.34637,0.35324,0.35374,0.288,0.3024,0.2709,0.29679,0.266
76,0.29736,0.28308,0.29184,0.29128,0.27366,0.25424,0.20249,0.20373,0.2
0327,0.20239,0.19989,0.20374,0.20377,0.20377,0.20549,0.12226,0.12334,0
.13566,0.12267,0.12026,0.12165,0.12164,0.12986,0.1203,0.20152,0.345,0.
34334,0.38251,0.35104,0.36958,0.38209,0.3713,0.26924,0.27813,0.28618,0
.29427,0.28471,0.27284,0.28098,0.264,0.27937,0.26919,0.27179,0.26307,0
.20172,0.20075,0.20363,0.20587,0.20217,0.20609,0.11318,0.12336,0.12717
,0.12156,0.13991,0.12556,0.11466,0.12275,0.1232,0.14037,0.12527,0.1222
,0.35431,0.38849,0.35669,0.36461,0.35705,0.35687,0.36477,0.27473,0.281
57,0.28949,0.27623,0.28404,0.29548,0.28956,0.28007,0.29053,0.29136,0.2
8889,0.27017,0.20421,0.2014,0.20458,0.11679,0.14183,0.11022,0.11069,0.
12625,0.11862,0.12297,0.12249,0.12443,0.12476,0.12129,0.13321,0.12763,
0.14869,0.12393,0.34384,0.34459,0.3763,0.36172,0.36235,0.37201,0.38152
,0.34007,0.35152,0.28889,0.28357,0.27523,0.28864,0.2655,0.27831,0.2755
4,0.28148,0.27588,0.28842,0.29765,0.12092,0.12323,0.12031,0.1206,0.123
48,0.12219,0.11768,0.12471,0.12666,0.11977,0.10806,0.12076,0.12169,0.1
2111,0.12171,0.10411,0.11639,0.12693,0.3712,0.3664,0.40578,0.36751,0.3
4588,0.36386,0.37864,0.34573,0.37436,0.29765,0.27193,0.22509,0.1881,0.
21313,0.19561,0.20619,0.21198,0.2183,0.18092,0.20588,0.12483,0.13729,0
.11312,0.10782,0.1214,0.14002,0.12601,0.1209,0.12824,0.10227,0.11955,0
.12275,0.12027,0.12106,0.12739,0.11732,0.12124,0.11889,0.35936,0.35899
,0.38188,0.40812,0.3583,0.34816,0.37614,0.34838,0.34677,0.18552,0.2032
8,0.20664,0.21437,0.19477,0.23447,0.19224,0.22712,0.20967,0.20265,0.18
941,0.12157,0.12673,0.13555,0.12253,0.11752,0.14259,0.1441,0.12203,0.1
3602,0.12484,0.12236,0.12731,0.13734,0.11295,0.11102,0.12248,0.11387,0
.12159,0.36462,0.36219,0.36939,0.38107,0.36108,0.36941,0.38023,0.19797
,0.20706,0.24184,0.18435,0.21122,0.20756,0.20181,0.19359,0.1975,0.2082
9,0.20545,0.21214,0.19835,0.12105,0.12209,0.13203,0.11496,0.12283,0.11
942,0.12286,0.14124,0.12273,0.12257,0.11342,0.12473,0.11961,0.12203,0.
11538,0.11463,0.11805,0.13556,0.37438,0.37947,0.35045,0.38677,0.37463,
0.34537,0.22224,0.22458,0.19921,0.18477,0.18638,0.21924,0.19724,0.2074
4,0.2025,0.21714,0.20301,0.20976,0.20784,0.23402,0.11936,0.12345,0.120
9,0.12233,0.11274,0.12134,0.11704,0.11963,0.127,0.12711,0.14319,0.1223
9,0.1213,0.11576,0.13528,0.1289,0.10819,0.12185,0.35579,0.35795,0.3801
5,0.36516,0.35263,0.21918,0.20366,0.20772,0.20977,0.17767,0.16403,0.19
578,0.22007,0.21049,0.1954,0.19856,0.21467,0.199,0.20721,0.21246,0.137

54,0.10796,0.13802,0.11703,0.10233,0.12162,0.11726,0.13625,0.11017,0.1
2124,0.11303,0.12266,0.12283,0.12007,0.12546,0.13441,0.11353,0.12184,0
.36029,0.34184,0.37823,0.20219,0.22108,0.18964,0.20911,0.18987,0.21011
,0.18446,0.20353,0.21724,0.19658,0.18971,0.22844,0.19301,0.21108,0.202
54,0.18689,0.18307,0.11864,0.12228,0.10894,0.12156,0.11507,0.12993,0.1
3117,0.12377,0.12029,0.10851,0.12317,0.12392,0.12535,0.13819,0.11286,0
.12269,0.12162,0.12124,0.34942,0.20009,0.21185,0.20138,0.17816,0.209,0
.19396,0.20019,0.19176,0.2029,0.21484,0.20192,0.20092,0.1933,0.22382,0
.19928,0.20711,0.1991,0.20379,0.12262,0.12057,0.1217,0.12061,0.11627,0
.11501,0.11731,0.12332,0.12729,0.12615,0.12175,0.12198,0.11886,0.12991
,0.14686,0.11938,0.10691,0.040488,0.19102,0.19126,0.20102,0.18903,0.19
007,0.1748,0.19604,0.24146,0.19641,0.26099,0.22456,0.17156,0.18074,0.2
0639,0.21039,0.193,0.19461,0.19321,0.19998,0.12034,0.12307,0.12489,0.1
2248,0.11969,0.09622,0.12324,0.11281,0.12712,0.11882,0.13237,0.11822,0
.11979,0.12634,0.12311,0.12013,0.12028,0.041212,0.22107,0.20487,0.2074
1,0.23662,0.20773,0.18619,0.18611,0.17009,0.14988,0.20369,0.19604,0.22
866,0.16771,0.19214,0.1928,0.20947,0.20929,0.2256,0.11871,0.12048,0.12
178,0.12475,0.12359,0.10515,0.14253,0.12506,0.1275,0.12302,0.12851,0.1
227,0.12165,0.1227,0.1142,0.12217,0.11771,0.12035,0.040464,0.22052,0.2
5172,0.20476,0.21651,0.21451,0.19351,0.18274,0.20247,0.19209,0.20723,0
.19433,0.19169,0.19225,0.21404,0.21408,0.1934,0.19252,0.19716,0.13497,
0.12147,0.12203,0.12187,0.12127,0.14602,0.11518,0.11972,0.11871,0.1230
4,0.12165,0.12126,0.10941,0.11588,0.11354,0.12233,0.1341,0.039831,0.04
0381,0.041444,0.20022,0.20341,0.20434,0.19209,0.20189,0.1953,0.1885,0.
19077,0.20983,0.18498,0.18175,0.20881,0.18254,0.19799,0.21236,0.10236,
0.12448,0.12974,0.12258,0.12232,0.12238,0.11393,0.12657,0.10862,0.1285
5,0.12214,0.11135,0.11913,0.11935,0.11428,0.1146,0.12163,0.11938,0.041
304,0.040706,0.040837,0.044103,0.14999,0.21228,0.1834,0.16698,0.21027,
0.20399,0.22546,0.2299,0.21964,0.20937,0.19684,0.20498,0.13886,0.11617
,0.10403,0.11325,0.13625,0.13552,0.14012,0.11154,0.12689,0.12362,0.135
03,0.13215,0.12254,0.13784,0.12071,0.1226,0.12599,0.13018,0.10785,0.12
266,0.039433,0.042274,0.040316,0.039594,0.040893,0.040094,0.20694,0.19
896,0.25798,0.20567,0.18821,0.19888,0.18083,0.19801,0.11235,0.1029,0.0
93145,0.1222,0.12114,0.12024,0.12012,0.12611,0.093452,0.12426,0.13096,
0.1598,0.12063,0.11954,0.1486,0.12596,0.12268,0.096076,0.12365,0.11767
,0.11859,0.11359,0.12687,0.040263,0.043023,0.039106,0.041483,0.039817,
0.041413,0.21606,0.20751,0.2298,0.18949,0.20446,0.042201,0.040527,0.12
317,0.12062,0.12246,0.12146,0.12192,0.12261,0.11898,0.13092,0.1209,0.1
1269,0.11866,0.1174,0.12677,0.1227,0.11378,0.12207,0.12343,0.11778,0.1
2475,0.12685,0.12496,0.040969,0.040662,0.038631,0.04194,0.04104,0.0418
9,0.042457,0.04019,0.03982,0.039448,0.19916,0.1831,0.038943,0.039657,0
.039802,0.04051,0.13166,0.097061,0.12264,0.12463,0.12556,0.13347,0.123
98,0.11872,0.12358,0.11532,0.11253,0.13855,0.1293,0.1193,0.12608,0.122
54,0.11198,0.13128,0.12931,0.040461,0.040488,0.042792,0.040874,0.04120
5,0.041269,0.040968,0.040726,0.041113,0.040135,0.041816,0.044009,0.040
571,0.040423,0.042514,0.038465,0.039565,0.11854,0.12866,0.11118,0.1026
8,0.12111,0.11632,0.12526,0.10947,0.11704,0.12246,0.11881,0.12602,0.11
298,0.12146,0.12879,0.12291,0.039959,0.040044,0.041813,0.042212,0.0414
59,0.042669,0.040182,0.042837,0.042603,0.038951,0.043505,0.041575,0.04
1836,0.040946,0.042853,0.040011,0.041988,0.039046,0.043115,0.11264,0.1
1442,0.14329,0.12308,0.11083,0.12279,0.12234,0.12863,0.11534,0.1264,0.
12662,0.1305,0.10825,0.03958,0.042538,0.042257,0.041757,0.041454,0.040

479,0.040555,0.041277,0.040158,0.040157,0.04081,0.043599,0.041515,0.04
0368,0.038834,0.040891,0.04184,0.041244,0.039,0.041868,0.040993,0.1150
6,0.12344,0.1304,0.12263,0.13122,0.10815,0.11613,0.12134,0.12117,0.040
46,0.040052,0.040329,0.040606,0.04177,0.040196,0.041196,0.040704,0.041
174,0.041551,0.041291,0.039175,0.042118,0.040443,0.040254,0.040531,0.0
39802,0.038589,0.040515,0.041834,0.042196,0.041672,0.041719,0.039057,0
.042569,0.11408,0.13356,0.12231,0.10464,0.039743,0.039843,0.039707,0.0
42908,0.039716,0.039985,0.041545,0.040439,0.041995,0.04278,0.04361,0.0
40344,0.040699,0.041064,0.041299,0.041223,0.043101,0.040903,0.039485,0
.040223,0.042248,0.040157,0.039719,0.039776,0.042696,0.039918,0.043352
,0.041735,0.040477,0.043439,0.041932,0.03965,0.04188,0.041014,0.04032,
0.040461,0.040707,0.041729,0.041437,0.041936,0.040729,0.041684,0.04356
1,0.040364,0.040129,0.041558,0.043433,0.038912,0.042995,0.041946,0.037
884,0.041163,0.039122,0.04016,0.042424,0.04138,0.040804,0.041176,0.041
196,0.040497,0.042098,0.041192,0.041301,0.04176,0.041666,0.039208,0.04
2352,0.040262,0.040654,0.041688,0.042379,0.043686,0.040145,0.042834,0.
040216,0.041088,0.043378,0.042244,0.039707,0.042391,0.040673,0.041426,
0.042152,0.040305,0.041839,0.038993,0.040494,0.04004,0.040957,0.039873
,0.041259,0.042153,0.04189,0.04016,0.042517,0.0394,0.041357,0.041927,0
.04043,0.042424,0.041689,0.041335,0.041144,0.038981,0.04055,0.041383,0
.040719,0.039046,0.043213,0.041287,0.039461,0.039864,0.040152,0.043229
,0.040362,0.040317,0.039012,0.040674,0.040647,0.041768,0.039126,0.0418
42,0.040162,0.041116,0.041066,0.040912,0.04149,0.040325,0.041646,0.039
899,0.039164,0.041173,0.041006,0.041329,0.04126,0.041983,0.041165,0.04
1466,0.043665,0.040113,0.040776,0.03988,0.042419,0.037903,0.042209,0.0
4144,0.041248,0.040804,0.042475,0.040067,0.039915,0.041015,0.040287,0.
04144,0.042324,0.041956,0.041399,0.041045,0.040948,0.039604,0.043164,0
.039945,0.040229,0.041841,0.040323,0.042392,0.042082,0.042602,0.040275
,0.042471,0.039621,0.041214,0.041968,0.039505,0.040728,0.042236,0.0392
06,0.038757,0.041251,0.041414,0.042654,0.04141,0.042413,0.039487,0.040
523,0.0397,0.03892,0.042013,0.038653,0.043674,0.040559,0.041226,0.0415
03,0.040632,0.043131
0.20069,0.20173,0.1996,0.20534,0.20292,0.205,0.20316,0.20247,0.20578,0
.20319,0.20424,0.20283,0.20644,0.20188,0.20625,0.20276,0.20618,0.20706
,0.20665,0.20363,0.30359,0.26635,0.32695,0.26309,0.20433,0.2042,0.2058
9,0.20321,0.20004,0.20376,0.20315,0.20376,0.20149,0.20285,0.20882,0.20
168,0.20278,0.20154,0.20376,0.34404,0.27132,0.29051,0.28646,0.29305,0.
26532,0.27434,0.20345,0.20129,0.20397,0.20276,0.20206,0.20525,0.20321,
0.20108,0.20277,0.2035,0.20462,0.20366,0.20352,0.20536,0.20458,0.37947
,0.35777,0.27433,0.3114,0.26848,0.28723,0.28242,0.27705,0.27058,0.2662
9,0.20459,0.20446,0.20672,0.20475,0.20315,0.20365,0.20498,0.20263,0.20
52,0.20399,0.20475,0.20224,0.2055,0.2022,0.20651,0.33246,0.33904,0.378
93,0.24426,0.30496,0.29645,0.28467,0.27499,0.2882,0.30069,0.2921,0.279
46,0.20396,0.20404,0.20345,0.20305,0.20654,0.2054,0.20327,0.20151,0.20
537,0.20585,0.20496,0.20432,0.20231,0.20643,0.20295,0.20409,0.33307,0.
35709,0.35301,0.3641,0.28761,0.28561,0.2602,0.28727,0.27731,0.26717,0.
27222,0.28929,0.29106,0.28357,0.20512,0.20667,0.20312,0.20682,0.20261,
0.20474,0.2028,0.2008,0.20357,0.20453,0.20408,0.2047,0.20166,0.20448,0
.20371,0.20302,0.3302,0.35651,0.34984,0.34154,0.3617,0.36664,0.27234,0
.27425,0.27765,0.29518,0.28208,0.29389,0.27439,0.27337,0.27161,0.28363
,0.20189,0.20419,0.20426,0.20162,0.20449,0.20294,0.20411,0.20559,0.203
11,0.20407,0.20389,0.2067,0.20686,0.20229,0.20469,0.20417,0.20878,0.37

872,0.38195,0.35197,0.36466,0.36769,0.38823,0.27611,0.26498,0.27953,0.
3067,0.28061,0.29386,0.28437,0.2787,0.27563,0.27567,0.28701,0.20227,0.
20708,0.20425,0.20128,0.20207,0.20068,0.20099,0.20597,0.20187,0.20472,
0.20391,0.19802,0.20626,0.1282,0.10695,0.12797,0.20556,0.3396,0.37612,
0.3211,0.37765,0.34819,0.36728,0.35117,0.29759,0.28094,0.29632,0.27687
,0.29664,0.26685,0.27636,0.27496,0.27778,0.26675,0.26822,0.20156,0.203
05,0.20403,0.20347,0.20458,0.20562,0.20553,0.20041,0.20189,0.20536,0.2
0451,0.10093,0.12003,0.1214,0.12248,0.1205,0.12663,0.20368,0.33066,0.3
8118,0.36737,0.37092,0.3387,0.36767,0.36363,0.28738,0.30057,0.27089,0.
29767,0.26192,0.29131,0.28035,0.29792,0.288,0.2733,0.25553,0.20279,0.2
0448,0.20448,0.20309,0.20081,0.20436,0.20456,0.20594,0.20613,0.12322,0
.12359,0.13598,0.12274,0.12027,0.12237,0.12195,0.13022,0.12018,0.20178
,0.34495,0.33988,0.39072,0.34453,0.37442,0.38134,0.37211,0.26813,0.284
76,0.28341,0.29328,0.2815,0.27424,0.27713,0.26043,0.28013,0.26813,0.26
699,0.26349,0.20215,0.20274,0.20424,0.20749,0.2029,0.20725,0.11411,0.1
234,0.12723,0.12166,0.13988,0.12508,0.11482,0.12325,0.12298,0.14082,0.
1261,0.12288,0.33531,0.39648,0.36509,0.36767,0.36792,0.35174,0.37005,0
.27125,0.27303,0.28666,0.27805,0.27694,0.2931,0.28347,0.28484,0.29948,
0.28863,0.28691,0.27531,0.20405,0.20256,0.2034,0.11633,0.14247,0.11076
,0.11076,0.12594,0.1192,0.12286,0.122,0.1248,0.1244,0.12167,0.13379,0.
12802,0.14954,0.12385,0.33455,0.34382,0.38521,0.35696,0.3696,0.38394,0
.35494,0.33114,0.33695,0.2886,0.27675,0.27317,0.28912,0.26826,0.27548,
0.27046,0.27572,0.27075,0.28924,0.29848,0.12167,0.12343,0.12133,0.1199
8,0.12272,0.12227,0.11737,0.12519,0.12723,0.11952,0.10815,0.12015,0.12
177,0.12099,0.12198,0.10447,0.11623,0.12799,0.35374,0.38098,0.40236,0.
36135,0.33687,0.38727,0.37312,0.33545,0.36979,0.30001,0.27033,0.22758,
0.1851,0.21497,0.19474,0.19934,0.21265,0.2194,0.18018,0.20741,0.12486,
0.13678,0.11298,0.10818,0.12199,0.14083,0.12635,0.12113,0.12851,0.1018
7,0.11959,0.12347,0.11969,0.12165,0.12795,0.11838,0.12096,0.11884,0.35
362,0.35204,0.37452,0.40205,0.35915,0.35355,0.36752,0.34905,0.34869,0.
18053,0.20308,0.20815,0.21291,0.19538,0.23881,0.18658,0.22363,0.21022,
0.20145,0.19176,0.12153,0.12602,0.13594,0.12232,0.117,0.14227,0.14469,
0.12262,0.13652,0.12491,0.12246,0.12623,0.13765,0.11354,0.1104,0.1227,
0.11424,0.12241,0.35873,0.36418,0.36488,0.37322,0.3564,0.37271,0.37249
,0.19497,0.20932,0.2388,0.18205,0.21314,0.20473,0.20297,0.19158,0.1977
3,0.20996,0.20328,0.21399,0.19934,0.12149,0.12255,0.13274,0.11507,0.12
236,0.11913,0.12305,0.14159,0.12309,0.12301,0.11443,0.12478,0.12019,0.
12164,0.11532,0.11429,0.11784,0.13567,0.36861,0.37398,0.33814,0.37485,
0.35646,0.34821,0.22408,0.21714,0.19154,0.1824,0.18902,0.21863,0.19819
,0.20756,0.20029,0.21295,0.20167,0.20507,0.20897,0.22753,0.12001,0.123
15,0.1204,0.12258,0.1126,0.12108,0.11644,0.11932,0.127,0.12667,0.14338
,0.12182,0.12174,0.11563,0.13533,0.12904,0.1088,0.12196,0.35432,0.3581
1,0.3842,0.35766,0.34328,0.21666,0.20403,0.20077,0.21014,0.17873,0.166
01,0.19739,0.22276,0.21301,0.19157,0.19348,0.21038,0.19399,0.20335,0.2
1145,0.13798,0.10832,0.13865,0.11732,0.10227,0.12225,0.11679,0.13631,0
.10994,0.12178,0.11329,0.12317,0.12277,0.11951,0.12545,0.13383,0.11341
,0.12149,0.36407,0.3387,0.37228,0.19703,0.21988,0.18192,0.20632,0.1899
6,0.21016,0.18342,0.20849,0.21512,0.19663,0.18918,0.22733,0.19077,0.20
923,0.2006,0.19169,0.18784,0.11879,0.12193,0.10917,0.12239,0.1156,0.13
012,0.13189,0.12363,0.12072,0.10863,0.12273,0.12349,0.12476,0.13943,0.
11341,0.12322,0.12164,0.12183,0.32728,0.20349,0.22261,0.19955,0.17526,
0.2086,0.19394,0.19639,0.19423,0.20218,0.20919,0.19915,0.19805,0.19204

,0.22456,0.19898,0.20369,0.19429,0.20785,0.12362,0.12098,0.1222,0.1205
9,0.11661,0.11489,0.11761,0.12312,0.12685,0.12721,0.12143,0.12281,0.11
947,0.13073,0.14672,0.11954,0.10667,0.040862,0.18963,0.19032,0.19864,0
.18906,0.19282,0.17228,0.19621,0.24708,0.19112,0.26119,0.22245,0.17027
,0.17921,0.20359,0.21026,0.19127,0.1916,0.19095,0.20422,0.12066,0.1232
7,0.12484,0.12305,0.11935,0.095267,0.12298,0.11253,0.12726,0.1196,0.13
315,0.11892,0.12072,0.12725,0.12289,0.12071,0.12086,0.040626,0.22737,0
.20016,0.20226,0.22427,0.2007,0.18332,0.18464,0.16983,0.15657,0.2095,0
.19894,0.22921,0.16747,0.19236,0.18948,0.20664,0.2062,0.22891,0.1186,0
.12104,0.12178,0.12503,0.12302,0.10514,0.14211,0.12469,0.12733,0.12284
,0.12769,0.12253,0.12131,0.12304,0.11412,0.12208,0.11762,0.1197,0.0397
27,0.21611,0.25796,0.2052,0.20735,0.20844,0.18888,0.17971,0.21004,0.18
518,0.20376,0.1958,0.18936,0.19402,0.2155,0.21093,0.19482,0.18892,0.19
863,0.13493,0.12186,0.12246,0.12191,0.12135,0.14659,0.11622,0.12036,0.
11844,0.12343,0.12152,0.12012,0.10943,0.11509,0.11396,0.1227,0.13362,0
.039574,0.040131,0.040599,0.19273,0.20198,0.2081,0.19129,0.214,0.19354
,0.18798,0.19242,0.21088,0.18716,0.18538,0.20147,0.18457,0.19426,0.213
61,0.10204,0.12543,0.12982,0.12255,0.12291,0.12239,0.11445,0.12675,0.1
0837,0.1289,0.1222,0.1109,0.11934,0.1193,0.11361,0.11462,0.12153,0.119
79,0.041196,0.039731,0.040363,0.043428,0.14731,0.20788,0.18367,0.15637
,0.21142,0.19888,0.22703,0.22703,0.2142,0.20447,0.19566,0.20754,0.1395
1,0.11666,0.10456,0.11236,0.13639,0.13628,0.14094,0.1115,0.12704,0.124
56,0.13527,0.132,0.12293,0.13822,0.12087,0.12312,0.12663,0.13026,0.108
08,0.12278,0.039359,0.042111,0.039918,0.040219,0.040258,0.03939,0.208,
0.20011,0.24961,0.21186,0.18584,0.19785,0.17365,0.20091,0.11258,0.1031
6,0.093243,0.12263,0.12126,0.12028,0.1202,0.12668,0.093204,0.12444,0.1
3104,0.16052,0.12005,0.11972,0.14913,0.12605,0.12221,0.09594,0.12395,0
.11736,0.11839,0.11359,0.12695,0.040281,0.042488,0.038987,0.042034,0.0
39124,0.041212,0.21855,0.20161,0.23243,0.19234,0.20291,0.041416,0.0403
23,0.12324,0.12092,0.12253,0.12229,0.12224,0.12254,0.1199,0.13245,0.12
159,0.11253,0.11877,0.11714,0.12706,0.12234,0.11364,0.12194,0.12355,0.
1178,0.12494,0.12668,0.12601,0.040312,0.039911,0.038375,0.04115,0.0409
07,0.041349,0.041221,0.040058,0.040319,0.039152,0.19191,0.18221,0.0382
64,0.038618,0.040155,0.040648,0.13225,0.096492,0.1234,0.12548,0.12643,
0.13424,0.12378,0.11845,0.12416,0.1157,0.1133,0.13886,0.13071,0.11983,
0.1263,0.12271,0.11221,0.13256,0.12923,0.040101,0.039785,0.04212,0.039
973,0.039324,0.040916,0.04007,0.040679,0.040822,0.040209,0.041807,0.04
2815,0.040247,0.039975,0.041641,0.039199,0.038502,0.11844,0.12939,0.11
072,0.10238,0.12119,0.11589,0.12567,0.10828,0.11683,0.12325,0.11919,0.
12591,0.11252,0.122,0.12918,0.12235,0.039321,0.039862,0.041239,0.04161
5,0.040964,0.042325,0.04069,0.041284,0.041944,0.039505,0.042856,0.0403
57,0.040328,0.039795,0.041766,0.039779,0.041979,0.038751,0.041375,0.11
282,0.11481,0.14356,0.12272,0.11077,0.12336,0.12298,0.12919,0.11544,0.
12668,0.12683,0.13036,0.10872,0.039356,0.041615,0.04229,0.040636,0.040
772,0.039713,0.040827,0.040152,0.040445,0.039474,0.04019,0.041569,0.04
0372,0.040763,0.038482,0.040719,0.042082,0.039904,0.038872,0.041406,0.
040369,0.11501,0.12369,0.13013,0.12324,0.13127,0.10866,0.11584,0.1207,
0.12084,0.039925,0.039902,0.041042,0.040464,0.04105,0.039186,0.041025,
0.0396,0.040492,0.042401,0.040486,0.039027,0.042309,0.040466,0.040187,
0.040627,0.039938,0.037979,0.040475,0.040435,0.041619,0.04052,0.041707
,0.040789,0.042242,0.11427,0.13376,0.12302,0.10463,0.037474,0.039507,0
.039243,0.042576,0.038896,0.038359,0.040669,0.040512,0.041088,0.042127

,0.042,0.040077,0.040361,0.040559,0.040298,0.040491,0.041973,0.040362,
0.039115,0.03988,0.042012,0.03949,0.038481,0.039348,0.042104,0.039563,
0.04239,0.041095,0.039463,0.042412,0.041623,0.040008,0.041743,0.040488
,0.039248,0.040138,0.040001,0.04031,0.040322,0.042182,0.040611,0.04078
3,0.042288,0.039077,0.039627,0.041928,0.042736,0.03798,0.04249,0.04203
9,0.037886,0.041639,0.038721,0.039944,0.041846,0.040862,0.040926,0.040
423,0.040993,0.040112,0.041428,0.040414,0.040424,0.040019,0.041104,0.0
39716,0.042267,0.040926,0.040313,0.04042,0.04116,0.044005,0.039842,0.0
41902,0.040391,0.040276,0.043552,0.042308,0.039207,0.04199,0.040219,0.
040869,0.041254,0.039427,0.040523,0.038481,0.040375,0.039194,0.040485,
0.039999,0.039779,0.041379,0.0414,0.039616,0.041519,0.038752,0.040373,
0.041153,0.039749,0.041396,0.041098,0.04028,0.040157,0.037985,0.039863
,0.040533,0.040613,0.039297,0.042786,0.041396,0.038958,0.039844,0.0398
56,0.041866,0.039697,0.039951,0.03858,0.040169,0.039606,0.041362,0.039
204,0.041101,0.039423,0.04041,0.040789,0.040549,0.040426,0.039708,0.04
1223,0.040752,0.039258,0.042001,0.040701,0.040916,0.041033,0.040593,0.
040966,0.040409,0.042906,0.039234,0.040558,0.039122,0.041334,0.038137,
0.041541,0.040265,0.040151,0.039771,0.041941,0.03931,0.039065,0.040589
,0.039977,0.040579,0.041492,0.041624,0.04103,0.039768,0.040539,0.03921
5,0.042191,0.04016,0.040128,0.041887,0.040144,0.041825,0.042112,0.0416
21,0.039313,0.042419,0.038983,0.039387,0.041354,0.038953,0.040788,0.04
2043,0.038722,0.038205,0.039121,0.041399,0.042519,0.040629,0.042359,0.
039132,0.040597,0.039438,0.039431,0.041715,0.03812,0.042653,0.041299,0
.04006,0.040811,0.039654,0.041809
0.2022,0.20233,0.20151,0.20515,0.20436,0.20453,0.20338,0.20369,0.20639
,0.2031,0.20485,0.20458,0.20705,0.20352,0.20599,0.2025,0.20701,0.20795
,0.20759,0.20544,0.30689,0.2656,0.32679,0.2678,0.20515,0.20611,0.20757
,0.20241,0.20075,0.20536,0.20474,0.20363,0.20232,0.20266,0.21001,0.202
52,0.20395,0.20162,0.20452,0.33586,0.26566,0.28555,0.28502,0.29029,0.2
6028,0.27707,0.20403,0.20102,0.20493,0.20366,0.20286,0.20669,0.20456,0
.20012,0.20255,0.20424,0.20588,0.20364,0.205,0.20563,0.20545,0.3884,0.
35974,0.27818,0.30449,0.2662,0.27809,0.28527,0.27752,0.27419,0.2705,0.
2043,0.20455,0.20754,0.20486,0.20415,0.20409,0.20482,0.20402,0.20591,0
.20473,0.20493,0.20306,0.20689,0.2026,0.20694,0.33858,0.34571,0.37611,
0.24508,0.30131,0.28961,0.27674,0.27899,0.28735,0.30209,0.28424,0.2760
3,0.20448,0.20384,0.20531,0.20454,0.20684,0.20503,0.20394,0.20368,0.20
581,0.20537,0.20537,0.20583,0.20397,0.20741,0.20374,0.20454,0.3255,0.3
3834,0.35428,0.37215,0.28591,0.28583,0.25921,0.28532,0.27363,0.26695,0
.27173,0.28612,0.28851,0.27584,0.20565,0.20826,0.2048,0.20743,0.20396,
0.20477,0.20549,0.2024,0.20455,0.20536,0.20706,0.20603,0.20321,0.20452
,0.20456,0.20351,0.32401,0.34751,0.35268,0.34398,0.35463,0.3561,0.2700
4,0.2724,0.27802,0.28858,0.28047,0.2959,0.27435,0.26875,0.27063,0.2807
3,0.20289,0.20474,0.20525,0.20271,0.20715,0.20297,0.20617,0.20622,0.20
408,0.20446,0.20604,0.20724,0.20647,0.20273,0.20497,0.20542,0.20898,0.
37174,0.38373,0.35028,0.35245,0.36816,0.38943,0.27199,0.26509,0.27749,
0.30423,0.27923,0.28816,0.27901,0.27791,0.28122,0.27278,0.28429,0.2023
,0.20877,0.20454,0.20156,0.20323,0.20203,0.20212,0.20536,0.20457,0.205
86,0.20479,0.19914,0.20635,0.12896,0.10705,0.12891,0.20678,0.34604,0.3
7496,0.32448,0.37,0.34608,0.35572,0.35485,0.29687,0.27734,0.2986,0.281
41,0.29203,0.26921,0.27694,0.27425,0.2765,0.26693,0.26581,0.20317,0.20
401,0.20536,0.20476,0.20523,0.2063,0.20637,0.20052,0.20361,0.20652,0.2
0453,0.10104,0.12047,0.12213,0.12248,0.12198,0.12736,0.20392,0.33581,0

.36633,0.3648,0.36802,0.34042,0.34944,0.3758,0.28887,0.29405,0.26841,0
.29506,0.2647,0.28829,0.27569,0.29353,0.28666,0.27572,0.25812,0.20396,
0.20413,0.20535,0.20462,0.20177,0.20562,0.20676,0.20585,0.20645,0.1235
9,0.12398,0.13606,0.12323,0.12054,0.12208,0.12237,0.12967,0.1212,0.202
04,0.34861,0.35987,0.38642,0.34662,0.3704,0.38334,0.36393,0.26325,0.28
375,0.27648,0.28904,0.28162,0.27505,0.27474,0.26274,0.27526,0.268,0.27
206,0.26196,0.20346,0.20276,0.20612,0.20713,0.20266,0.20817,0.11414,0.
12307,0.12718,0.1218,0.1393,0.12546,0.11511,0.12301,0.12353,0.14072,0.
12633,0.12243,0.33719,0.39694,0.36619,0.3516,0.354,0.35191,0.36883,0.2
7035,0.27762,0.28992,0.27717,0.27997,0.2862,0.29148,0.28446,0.29971,0.
28464,0.28558,0.27544,0.2047,0.20421,0.20494,0.1161,0.14335,0.11019,0.
1109,0.12614,0.11945,0.12279,0.12224,0.126,0.12457,0.122,0.1336,0.1275
4,0.15013,0.12472,0.33584,0.32994,0.38433,0.35505,0.36255,0.3787,0.348
7,0.33801,0.3328,0.28531,0.27906,0.27489,0.2886,0.26577,0.27651,0.2690
9,0.28168,0.2686,0.28508,0.29927,0.12144,0.12267,0.12147,0.11976,0.122
58,0.12256,0.11755,0.12492,0.12849,0.12028,0.10801,0.12059,0.12184,0.1
2106,0.12208,0.10499,0.11621,0.12718,0.35636,0.394,0.40594,0.35674,0.3
3023,0.38258,0.36267,0.3379,0.36823,0.29737,0.26919,0.22416,0.18562,0.
21579,0.19507,0.19658,0.21239,0.22246,0.18054,0.20681,0.12528,0.13681,
0.11291,0.10846,0.12287,0.14176,0.12714,0.12119,0.12914,0.10272,0.1198
3,0.12359,0.12013,0.12146,0.12754,0.11907,0.12107,0.1189,0.36069,0.349
55,0.3688,0.40089,0.34821,0.35328,0.37235,0.34336,0.35519,0.17444,0.20
073,0.20317,0.21725,0.19356,0.23621,0.18694,0.22107,0.20654,0.19957,0.
19395,0.12188,0.12646,0.13655,0.1225,0.11741,0.14262,0.14558,0.12298,0
.13684,0.12516,0.12242,0.12596,0.13791,0.11354,0.11097,0.12334,0.11391
,0.12259,0.36092,0.37468,0.36825,0.35971,0.35297,0.37186,0.3846,0.1922
6,0.21183,0.24049,0.17749,0.20945,0.20261,0.2037,0.19141,0.1985,0.2126
3,0.2016,0.20952,0.19448,0.12138,0.12243,0.13392,0.11493,0.12245,0.119
87,0.12412,0.14191,0.12233,0.12299,0.11482,0.12465,0.1214,0.12355,0.11
529,0.11352,0.11822,0.13534,0.35633,0.37002,0.34624,0.36775,0.34043,0.
34498,0.22009,0.21067,0.19236,0.18601,0.20027,0.22112,0.19602,0.20824,
0.20398,0.20981,0.19911,0.20651,0.20637,0.22948,0.12065,0.1232,0.12061
,0.12214,0.11254,0.12095,0.11685,0.11949,0.12719,0.12677,0.14373,0.121
31,0.12227,0.1154,0.13586,0.12922,0.10856,0.12197,0.34945,0.36964,0.37
791,0.35188,0.34394,0.22529,0.19821,0.19859,0.21015,0.18154,0.1641,0.1
9645,0.21923,0.20799,0.19045,0.19495,0.21286,0.19666,0.20403,0.20963,0
.13837,0.10843,0.13837,0.11813,0.10227,0.12261,0.11638,0.1361,0.11049,
0.1211,0.11394,0.12354,0.12397,0.11969,0.12523,0.13439,0.11376,0.12185
,0.35081,0.35199,0.37568,0.19305,0.21395,0.18064,0.20621,0.18879,0.209
23,0.18585,0.20838,0.21216,0.19747,0.1883,0.22186,0.19014,0.20517,0.19
964,0.18984,0.1904,0.1185,0.12231,0.10885,0.12234,0.11543,0.13006,0.13
205,0.12376,0.12054,0.10872,0.12262,0.12412,0.1255,0.13959,0.11347,0.1
2366,0.12199,0.12191,0.3196,0.19942,0.22185,0.19592,0.17214,0.20739,0.
18622,0.19792,0.19609,0.20102,0.20755,0.19868,0.19856,0.19084,0.22112,
0.19318,0.20287,0.19741,0.20547,0.12371,0.12047,0.12267,0.12121,0.1165
7,0.11437,0.11822,0.12245,0.12691,0.12643,0.12161,0.12333,0.11949,0.13
115,0.14809,0.11984,0.10679,0.040591,0.1923,0.19018,0.19629,0.18511,0.
18849,0.16899,0.19094,0.24344,0.19084,0.26292,0.21884,0.17192,0.17761,
0.20014,0.20823,0.19302,0.1952,0.18753,0.20218,0.12025,0.1234,0.12444,
0.12232,0.11986,0.095838,0.12195,0.11249,0.12761,0.11973,0.13321,0.119
07,0.12064,0.12715,0.12282,0.12053,0.1213,0.04101,0.22654,0.19772,0.20
433,0.22157,0.1989,0.18348,0.18152,0.16258,0.15018,0.21333,0.19749,0.2

2854,0.16862,0.1925,0.19348,0.20422,0.20954,0.23222,0.11918,0.1205,0.1
2282,0.12508,0.12289,0.10499,0.14301,0.12494,0.12754,0.12296,0.12814,0
.12283,0.12244,0.12238,0.11454,0.12251,0.11832,0.11979,0.039476,0.2103
7,0.25318,0.19664,0.21038,0.20152,0.19838,0.17974,0.20811,0.18713,0.20
093,0.19549,0.19314,0.19397,0.21511,0.21027,0.19575,0.19348,0.19941,0.
13482,0.1229,0.12197,0.12282,0.12245,0.14705,0.1169,0.12088,0.11885,0.
12375,0.12203,0.11994,0.10876,0.11565,0.11374,0.12287,0.13437,0.038956
,0.039365,0.039767,0.19427,0.19407,0.20144,0.19254,0.22048,0.19405,0.1
8163,0.19585,0.20207,0.18667,0.18258,0.19654,0.18371,0.19422,0.21233,0
.10161,0.12609,0.13053,0.12239,0.12321,0.12309,0.11471,0.12767,0.10913
,0.12862,0.12233,0.11058,0.11922,0.11945,0.11366,0.11428,0.12183,0.119
82,0.040242,0.039737,0.040574,0.042851,0.14491,0.20816,0.1813,0.15398,
0.20493,0.19588,0.22455,0.22263,0.21369,0.20593,0.19354,0.20661,0.1397
1,0.11643,0.10394,0.11272,0.13669,0.1362,0.14168,0.1117,0.12723,0.1242
9,0.13539,0.13252,0.12338,0.13805,0.12032,0.1239,0.12661,0.13073,0.108
55,0.12296,0.039678,0.041917,0.03949,0.039755,0.0409,0.038449,0.20618,
0.19703,0.24588,0.20989,0.18406,0.19601,0.17267,0.19746,0.11242,0.1033
2,0.092781,0.12261,0.12119,0.1211,0.12076,0.1266,0.092864,0.12378,0.13
157,0.1608,0.12055,0.12002,0.14942,0.1262,0.12265,0.095777,0.12386,0.1
1744,0.11836,0.11418,0.12715,0.040185,0.042445,0.039045,0.041164,0.040
101,0.041361,0.21793,0.20562,0.2273,0.18689,0.20217,0.040006,0.040381,
0.12399,0.1212,0.12274,0.12192,0.12259,0.12283,0.12064,0.13189,0.12223
,0.11283,0.11873,0.11735,0.12708,0.12249,0.11409,0.12233,0.12395,0.117
78,0.12531,0.12708,0.12653,0.039755,0.039543,0.038952,0.040481,0.04190
8,0.041467,0.038942,0.040188,0.039389,0.038559,0.18587,0.18446,0.03737
7,0.0382,0.039826,0.040307,0.13263,0.096755,0.12303,0.1257,0.12629,0.1
342,0.12405,0.11793,0.1239,0.11609,0.11323,0.13919,0.13113,0.11967,0.1
2678,0.12195,0.11295,0.13309,0.12949,0.039965,0.039998,0.041502,0.0395
13,0.040036,0.041262,0.03955,0.04024,0.040292,0.03964,0.041606,0.04165
,0.039971,0.039428,0.042141,0.038935,0.038186,0.1184,0.12931,0.11089,0
.1022,0.1206,0.11578,0.12539,0.10889,0.11685,0.12276,0.11905,0.12649,0
.11272,0.12245,0.12907,0.12214,0.039052,0.039325,0.041705,0.04175,0.03
9319,0.04157,0.03944,0.040857,0.041566,0.039454,0.04216,0.040595,0.039
717,0.039239,0.041026,0.039549,0.04118,0.03963,0.040137,0.11247,0.1147
6,0.14426,0.12338,0.1112,0.12396,0.12368,0.12942,0.11541,0.12722,0.126
83,0.13088,0.10877,0.039569,0.041487,0.04096,0.041123,0.040314,0.03945
4,0.039865,0.040252,0.040441,0.039536,0.03978,0.041468,0.039445,0.0396
96,0.037374,0.040069,0.041319,0.039726,0.038993,0.040627,0.040475,0.11
56,0.12409,0.13005,0.12374,0.13194,0.10921,0.11592,0.12141,0.12118,0.0
39837,0.039841,0.040373,0.03927,0.040514,0.03882,0.039536,0.039314,0.0
40303,0.041976,0.039519,0.037615,0.042919,0.040178,0.040664,0.040081,0
.039992,0.037544,0.04027,0.039454,0.041237,0.040258,0.04211,0.040502,0
.041904,0.11434,0.13415,0.12302,0.10443,0.037655,0.037764,0.038529,0.0
40648,0.038174,0.038589,0.040632,0.040174,0.040776,0.041175,0.041364,0
.040331,0.03896,0.040381,0.040029,0.040187,0.041427,0.040214,0.039264,
0.040196,0.040979,0.039695,0.0394,0.039191,0.041267,0.038746,0.040607,
0.040347,0.039578,0.041864,0.042188,0.039786,0.041025,0.039873,0.03832
2,0.039252,0.039818,0.039509,0.041014,0.04216,0.040637,0.040767,0.0405
93,0.038494,0.039387,0.041466,0.04249,0.037852,0.041315,0.040695,0.037
846,0.040908,0.038061,0.039761,0.041811,0.039758,0.040498,0.040097,0.0
4099,0.039497,0.040623,0.040054,0.04008,0.039387,0.039944,0.040201,0.0
41386,0.040305,0.040669,0.039923,0.040642,0.043681,0.039689,0.040917,0

.039221,0.03986,0.042705,0.041811,0.039201,0.04214,0.03937,0.040559,0.
040771,0.039956,0.039562,0.038019,0.03987,0.039305,0.04014,0.039956,0.
039501,0.041391,0.040293,0.038544,0.041322,0.038278,0.040459,0.040584,
0.03935,0.041017,0.040242,0.040011,0.040332,0.038949,0.038407,0.040954
,0.04088,0.038882,0.042276,0.041817,0.03919,0.03943,0.038773,0.040611,
0.039485,0.038813,0.038479,0.039091,0.040025,0.041305,0.03928,0.039517
,0.039347,0.041029,0.040637,0.040342,0.040478,0.038546,0.040683,0.0396
51,0.038205,0.041757,0.039761,0.040694,0.040533,0.039978,0.040681,0.03
9015,0.043288,0.038467,0.040239,0.039002,0.040747,0.038223,0.041834,0.
039658,0.039681,0.039438,0.042151,0.038468,0.038835,0.040382,0.039831,
0.040777,0.040282,0.040454,0.040697,0.039501,0.039353,0.039143,0.04077
,0.038339,0.039545,0.041005,0.039517,0.041662,0.041461,0.041225,0.0390
66,0.041422,0.038138,0.039279,0.041173,0.039408,0.040525,0.039865,0.03
8596,0.037578,0.038936,0.040831,0.041585,0.040574,0.042095,0.039367,0.
040992,0.038789,0.039938,0.041533,0.037542,0.041702,0.041372,0.039618,
0.040184,0.03991,0.040958

#simdataS2SNR20:

Kep_f=1.2
Kep_f=1.2
Kep_s=0.1
Ktrans_f=0.05
Ktrans_s=0.03
del_t=0.5
gtSf=0 0 0 0 0 0
0 0 0 0 0 0
0 0 0 0 0 0
0 0 0 0 0 0
0 0 0 0 0 0
0 0 0 0 0 0
0 0 0 0 0 0
0 0 0 0 0 0
0 0 0 0 0 0
0 0 0 0 0 0
0 0 0 0 0 0
0 0 0 0 0 0
0 0 0 0 0 0
0 0 0 0 0 0
0 0 0 0 0 0
0 0 0 0 0 0
0 0 0 0 0 0
0 0 0 0 0 0
0 0 0 0 0 0
0 0 0 0 0 0
0 0 0 0 0 0
0 0 0 0 0 0
0 0 0 0 0 0
0 0 0 0 0 0
0 0 0 0 0 0
0 0 0 0 0 0

0	0	0	0	0	0
0	0	0	0	0	0
0	0	0	0	0	0
0	0	0	0	0	0
0	0	0	0	0	0
0	0	0	0	0	0
0	0	0	0	0	0
0	0	0	0	0	0
0	0	0	0.5	0.42948	0.47589
0	0	0	0	0	0
0	0	0	0	0	0
0	0	0	0	0	0
0	0	0	0	0	0
0	0	0	0	0	0
0.50893	0.57408	0.56937	0.5	0.54437	0.56822
0	0	0	0	0	0
0	0	0	0	0	0
0	0	0	0	0	0
0	0	0	0	0	0
0	0	0	0	0.46439	0.56132
0.44634	0.50375	0.45543	0.48206	0.49612	0.63481
0.5	0	0	0	0	0
0	0	0	0	0	0
0	0	0	0	0	0
0	0	0	0	0	0
0	0	0	0.4967	0.50346	0.51369
0.5	0.47364	0.5	0.4923	0.40215	0.50108
0.5	0.54287	0.51665	0	0	0
0	0	0	0	0	0
0	0	0	0	0	0
0	0	0	0	0	0
0	0.52637	0.42059	0.5	0.36333	0.5
0.4806	0.39421	0.70845	0.5	0.5	0.44952
0.5267	0.58888	0.45405	0.5	0	0
0	0	0	0	0	0
0	0	0	0	0	0
0	0	0	0	0	0
0.5	0.41024	0.41458	0.49602	0.5	0.51901
0.53488	0.5	0.28932	0.49837	0.54152	0.52118
0.49335	0.49797	0.5	0.45678	0.35821	0.45026
0	0	0	0	0	0
0	0	0	0	0	0.33816
0.33333	0.33032	0.36411	0.30647	0.3604	0.33333
0.30854	0.30406	0.58516	0.51386	0.5	0.51275
0.5	0.67466	0.5146	0.47486	0.55755	0.50427
0.42436	0.56689	0.48447	0.50312	0.49317	0.55896
0.53459	0.53887	0	0	0	0
0	0	0	0	0	0.50883
0.33333	0.22966	0.33766	0.33333	0.32337	0.33333
0.37886	0.34025	0.27578	0.29143	0.5	0.65593
0.49317	0.5	0.47684	0.5	0.61135	0.50619
0.45504	0.50792	0.58653	0.50166	0.43765	0.48616

0.45617	0.44038	0.52251	0.42041	0	0
0	0	0	0	0	0.48991
0.41992	0.43988	0.36074	0.25769	0.33333	0.29158
0.27887	0.33487	0.32928	0.27594	0.3151	0.29991
0.5	0.38696	0.5	0.56591	0.64994	0.61097
0.5863	0.5	0.5	0.5	0.49126	0.5
0.49479	0.49939	0.5	0.52983	0.68846	0.45611
0	0	0	0	0	0
0.44654	0.47469	0.52232	0.62194	0.54682	0.33513
0.40367	0.27559	0.38141	0.36679	0.35923	0.3962
0.29812	0.30816	0.49388	0.52456	0.5	0.52923
0.58974	0.46036	0.5308	0.65638	0.4821	0.53082
0.44777	0.59991	0.54212	0.48175	0.53405	0.50091
0.53088	0.6105	0	0	0	0
0	0.53253	0.49648	0.5	0.52639	0.53215
0.5	0.39598	0.36792	0.231	0.38238	0.37418
0.33333	0.33333	0.33333	0.33333	0.50924	0.54465
0.45978	0.5	0.5	0.47912	0.39255	0.48636
0.54043	0.5	0.51297	0.55295	0.5	0.52037
0.51258	0.5	0.43606	0.46274	0	0
0	0.5	0.50435	0.49315	0.50115	0.48662
0.5	0.4836	0.59566	0.33333	0.34484	0.33703
0.49279	0.36836	0.3191	0.33333	0.38827	0.33333
0.28064	0.47816	0.49875	0.5	0.5	0.5
0.53636	0.4976	0.40315	0.51506	0.47097	0.57398
0.5	0.57569	0.69115	0.48011	0.45986	0.50646
0	0.47599	0.47518	0.52554	0.5	0.4303
0.50062	0.39966	0.5	0.51905	0.41504	0.33333
0.29697	0.34894	0.3542	0.33333	0.37883	0.37949
0.34571	0.46766	0.50837	0.5342	0.5719	0.5036
0.5	0.52167	0.56705	0.50675	0.47253	0.50482
0.5	0.5	0.55927	0.60388	0.47862	0.49358
1	0.56256	0.5	0.54112	0.68511	0.45469
0.68527	0.58665	0.47266	0.4605	0.5	0.33333
0.22474	0.34502	0.39564	0.35722	0.20926	0.33955
0.31024	0.33948	0.46622	0.48846	0.49033	0.46577
0.48505	0.40128	0.48578	0.59445	0.5	0.62579
0.5	0.5	0.44728	0.53158	0.5	0.47672
0.5	1	0.44037	0.40578	0.4923	0.5
0.62162	0.5	0.5	0.48783	0.5	0.48531
0.24649	0.36635	0.32687	0.34406	0.33333	0.31349
0.33807	0.34969	0.5	0.54578	0.55432	0.40152
0.48514	0.40657	0.4998	0.45037	0.49339	0.5011
0.5428	0.5	0.48452	0.5914	0.5	0.50684
0.54446	0.53368	1	0.45298	0.49175	0.68035
0.55204	0.46149	0.5145	0.5	0.49082	0.49486
0.5	0.36962	0.41833	0.33739	0.33123	0.29173
0.36771	0.35539	0.28468	0.55152	0.5	0.33017
0.50322	0.43859	0.5	0.48883	0.50533	0.57043
0.43734	0.5	0.55439	0.47226	0.51319	0.5
0.49487	0.34592	1	1	1	0.29306
0.53356	0.5	0.5	0.39678	0.46648	0.5

0.5	0.5859	0.33333	0.37576	0.36445	0.26749
0.32324	0.33302	0.55358	0.5	0.5	0.39672
0.66508	0.58341	0.44134	0.46887	0.6049	0.5183
0.48837	0.48022	0.57197	0.42116	0.84552	0.58125
0.40797	0.47638	1	1	1	1
0.5	0.43146	0.40579	0.53264	0.5	0.60062
0.53724	0.5488	0.5	0.40505	0.33233	0.36897
0.55989	0.5	0.17878	0.53281	0.55079	0.51616
0.52447	0.5	0.3883	0.48858	0.5	0.42339
0.53726	0.5	0.2491	0.46429	0.49975	0.46733
0.5	0.46659	1	1	1	1
1	1	0.43311	0.41273	0.49576	0.5
0.2845	0.58527	0.48233	0.5	0.37921	0.4856
0.42865	0.47795	0.48621	0.47362	0.36488	0.5
0.51487	0.47686	0.5	0.43052	0.49028	0.52918
0.57505	0.54034	0.56387	0.5	0.5	0.54566
0.5	0.5	0.56163	1	1	1
1	1	1	0.48773	0.5	0.43754
0.47181	0.47187	1	1	0.51844	0.48257
0.59348	0.5	0.5	0.5	0.5	0.50357
0.5	0.46689	0.5	0.47933	0.5	0.29551
0.34368	0.53185	0.47263	0.5	0.47393	0.5
0.5	1	1	1	1	1
1	1	1	1	1	0.51812
0.44656	1	1	1	1	0.4636
0.61496	0.45225	0.53577	0.53863	0.41003	0.5
0.5	0.5	0.5	0.50893	0.44322	0.5
0.5	0.44441	0.48334	0.50681	0.4752	0.55039
1	1	1	1	1	1
1	1	1	1	1	1
1	1	1	1	1	0.49539
0.5	0.5012	0.57671	0.46642	0.57995	0.53386
0.5	0.49254	0.42423	0.48485	0.5015	0.43297
0.45164	0.46959	0.51973	1	1	1
1	1	1	1	1	1
1	1	1	1	1	1
1	1	1	1	0.5858	0.49489
0.38925	0.49756	0.57401	0.5	0.46803	0.48146
0.5	0.49343	0.45415	0.50071	0.35089	1
1	1	1	1	1	1
1	1	1	1	1	1
1	1	1	1	1	1
1	1	0.5	0.59043	0.51695	0.51554
0.51211	0.69906	0.5	0.5	0.51134	1
1	1	1	1	1	1
1	1	1	1	1	1
1	1	1	1	1	1
1	1	1	1	1	1
0.59734	0.48259	0.44464	0.64073	1	1
1	1	1	1	1	1
1	1	1	1	1	1
1	1	1	1	1	1

1 1 1 1 1 1
1 1 1 1 1 1
1 1 1 1 1 1
1 1 1 1 1 1
1 1 1 1 1 1
1 1 1 1 1 1
1 1 1 1 1 1
1 1 1 1 1 1
1 1 1 1 1 1
1 1 1 1 1 1
1 1 1 1 1 1
1 1 1 1 1 1
1 1 1 1 1 1
1 1 1 1 1 1
1 1 1 1 1 1
1 1 1 1 1 1
1 1 1 1 1 1
1 1 1 1 1 1
1 1 1 1 1 1
1 1 1 1 1 1
1 1 1 1 1 1
1 1 1 1 1 1
1 1 1 1 1 1
1 1 1 1 1 1
1 1 1 1 1 1
1 1 1 1 1 1
1 1 1 1 1 1
1 1 1 1 1 1
1 1 1 1 1 1
1
gtSs=1 1 1 1 1 1
1 1 1 1 1 1
1 1 1 1 1 1
1 1 0.46269 0.53764 0.5 0.50089
1 1 1 1 1 1
1 1 1 1 1 1
1 1 1 0 0.50644 0.51017
0.5 0.47263 0.67583 0.5 1 1
1 1 1 1 1 1
1 1 1 1 1 1
1 0 0 0.63001 0.55085 0.5
0.5 0.51349 0.44699 0.44649 0.57038 1
1 1 1 1 1 1
1 1 1 1 1 1
1 1 0 0 0 0.48856
0.45439 0.38819 0.53725 0.43592 0.60057 0.30122
0.48825 0.5 1 1 1 1
1 1 1 1 1 1
1 1 1 1 1 1
0 0 0 0 0.57601 0.45928
0.49717 0.47196 0.50705 0.5 0.47534 0.5
0.5 0.5 1 1 1 1

1	1	1	1	1	1
1	1	1	1	1	1
0	0	0	0	0	0
0.5	0.37727	0.5	0.5	0.50117	0.46685
0.49124	0.5	0.5	0.4927	1	1
1	1	1	1	1	1
1	1	1	1	1	1
1	1	1	0	0	0
0	0	0	0.46587	0.5	0.42892
0.51268	0.48168	0.40297	0.5	0.5	0.5
0.52445	0.46583	1	1	1	1
1	1	1	1	1	1
1	1	1	0.5	0.57052	0.52411
1	0	0	0	0	0
0	0	0.48546	0.44021	0.5	0.5
0.45615	0.52676	0.61455	0.5	0.49639	0.51694
0.5	1	1	1	1	1
1	1	1	1	1	1
0.49107	0.42592	0.43063	0.5	0.45563	0.43178
1	0	0	0	0	0
0	0	0.55542	0.5	0.5	0.48744
0.53957	0.44117	0.5	0.41995	0.5	0.50021
0.39389	1	1	1	1	1
1	1	1	1	0.53561	0.43868
0.55366	0.49625	0.54457	0.51794	0.50388	0.36519
0.5	1	0	0	0	0
0	0	0	0.46194	0.45163	0.46575
0.5	0.56155	0.5	0.48928	0.54939	0.5
0.40228	0.51784	0.5	1	1	1
1	1	1	0.5033	0.49654	0.48631
0.5	0.52636	0.5	0.5077	0.59785	0.49892
0.5	0.45713	0.48335	0	0	0
0	0	0	0	0.50917	0.52604
0.4529	0.5	0.5	0.47842	0.53235	0.43198
0.45641	0.4759	0.51822	0.54398	1	1
1	0.47363	0.57941	0.5	0.63667	0.5
0.5194	0.60579	0.29155	0.5	0.5	0.55048
0.4733	0.41112	0.54595	0.5	0	0
0	0	0	0	0	0
0	0.5	0.53618	0.59769	0.49764	0.49186
0.65255	0.5	0.51506	0.51758	0.47199	0.49891
0.5	0.58976	0.58542	0.50398	0.5	0.48099
0.46512	0.5	0.71068	0.50163	0.45848	0.47882
0.50665	0.50203	0.5	0.54322	0.64179	0.54974
0	0	0	0	0	0
0	0	0	0.46758	0.46897	0.32368
0.33333	0.31158	0.27178	0.34677	0.2792	0.33333
0.34573	0.27462	0.41484	0.48614	0.5	0.48725
0.5	0.32534	0.4854	0.52514	0.44245	0.49573
0.57564	0.43311	0.51553	0.49688	0.50683	0.44104
0.46541	0.46113	0	0	0	0
0	0	0	0	0	0

0.33333 0.38517 0.33766 0.33333 0.33831 0.33333
0.37886 0.36768 0.36211 0.38585 0.5 0.34407
0.50683 0.5 0.52316 0.5 0.38865 0.49381
0.54496 0.49208 0.41347 0.49834 0.56235 0.51384
0.54383 0.55962 0.47749 0.57959 0 0
0 0 0 0 0 0
0 0 0.27851 0.38436 0.33333 0.33202
0.34232 0.36289 0.3296 0.36604 0.39338 0.35004
0.5 0.61304 0.5 0.43409 0.35006 0.38903
0.4137 0.5 0.5 0.5 0.50874 0.5
0.50521 0.50061 0.5 0.47017 0.31154 0.54389
0 0 0 0 0 0
0 0 0 0 0 0.33513
0.28515 0.3622 0.38141 0.3449 0.33835 0.14564
0.35094 0.2939 0.50612 0.47544 0.5 0.47077
0.41026 0.53964 0.4692 0.34362 0.5179 0.46918
0.55223 0.40009 0.45788 0.51825 0.46595 0.49909
0.46912 0.3895 0 0 0 0
0 0 0 0 0 0
0 0.34249 0.39452 0.42285 0.32544 0.25164
0.33333 0.33333 0.33333 0.33333 0.49076 0.45535
0.54022 0.5 0.5 0.52088 0.60745 0.51364
0.45957 0.5 0.48703 0.44705 0.5 0.47963
0.48742 0.5 0.56394 0.53726 0 0
0 0 0 0 0 0
0 0 0 0.33333 0.34484 0.32593
0.22155 0.36743 0.33708 0.33333 0.37892 0.33333
0.71936 0.52184 0.50125 0.5 0.5 0.5
0.46364 0.5024 0.59685 0.48494 0.52903 0.42602
0.5 0.42431 0.30885 0.51989 0.54014 0.49354
0 0 0 0 0 0
0 0 0 0 0.37046 0.33333
0.35152 0.33273 0.37505 0.33333 0.24235 0.30211
0.34571 0.53234 0.49163 0.4658 0.4281 0.4964
0.5 0.47833 0.43295 0.49325 0.52747 0.49518
0.5 0.5 0.44073 0.39612 0.52138 0.50642
0 0 0 0 0 0
0 0 0 0 0 0.33333
0.43734 0.22295 0.36552 0.32785 0.3612 0.33955
0.34071 0.37449 0.53378 0.51154 0.50967 0.53423
0.51495 0.59872 0.51422 0.40555 0.5 0.37421
0.5 0.5 0.55272 0.46842 0.5 0.52328
0.5 0 0 0 0 0
0 0 0 0 0 0
0.31057 0.30954 0.33523 0.3184 0.33333 0.30171
0.33807 0.34969 0.5 0.45422 0.44568 0.59848
0.51486 0.59343 0.5002 0.54963 0.50661 0.4989
0.4572 0.5 0.51548 0.4086 0.5 0.49316
0.45554 0.46632 0 0 0 0
0 0 0 0 0 0
0 0.28567 0.32168 0.33739 0.33439 0.38921
0.33726 0.35539 0.32714 0.44848 0.5 0.66983

0.49678	0.56141	0.5	0.51117	0.49467	0.42957
0.56266	0.5	0.44561	0.52774	0.48681	0.5
0.50513	0.65408	0	0	0	0
0	0	0	0	0	0
0	0	0.33333	0.37576	0.36445	0.38692
0.33838	0.3316	0.44642	0.5	0.5	0.60328
0.33492	0.41659	0.55866	0.53113	0.3951	0.4817
0.51163	0.51978	0.42803	0.57884	0.15448	0.41875
0.59203	0.52362	0	0	0	0
0	0	0	0	0	0
0	0	0	0.40505	0.33384	0.36408
0.44011	0.5	0.82122	0.46719	0.44921	0.48384
0.47553	0.5	0.6117	0.51142	0.5	0.57661
0.46274	0.5	0.7509	0.53571	0.50025	0.53267
0.5	0.53341	0	0	0	0
0	0	0	0	0	0
0	0	0	0	0.62079	0.5144
0.57135	0.52205	0.51379	0.52638	0.63512	0.5
0.48513	0.52314	0.5	0.56948	0.50972	0.47082
0.42495	0.45966	0.43613	0.5	0.5	0.45434
0.5	0.5	0.43837	0	0	0
0	0	0	0	0	0
0	0	0	0	0.48156	0.51743
0.40652	0.5	0.5	0.5	0.5	0.49643
0.5	0.53311	0.5	0.52067	0.5	0.70449
0.65632	0.46815	0.52737	0.5	0.52607	0.5
0.5	0	0	0	0	0
0	0	0	0	0	0
0	0	0	0	0	0.5364
0.38504	0.54775	0.46423	0.46137	0.58997	0.5
0.5	0.5	0.5	0.49107	0.55678	0.5
0.5	0.55559	0.51666	0.49319	0.5248	0.44961
0	0	0	0	0	0
0	0	0	0	0	0
0	0	0	0	0	0.50461
0.5	0.4988	0.42329	0.53358	0.42005	0.46614
0.5	0.50746	0.57577	0.51515	0.4985	0.56703
0.54836	0.53041	0.48027	0	0	0
0	0	0	0	0	0
0	0	0	0	0	0
0	0	0	0	0.4142	0.50511
0.61075	0.50244	0.42599	0.5	0.53197	0.51854
0.5	0.50657	0.54585	0.49929	0.64911	0
0	0	0	0	0	0
0	0	0	0	0	0
0	0	0	0	0	0
0	0	0.5	0.40957	0.48305	0.48446
0.48789	0.30094	0.5	0.5	0.48866	0
0	0	0	0	0	0
0	0	0	0	0	0
0	0	0	0	0	0
0	0	0	0	0	0

```
0.40266 0.51741 0.55536 0.35927 0 0
0 0 0 0 0 0
0 0 0 0 0 0
0 0 0 0 0 0
0 0 0 0 0 0
0 0 0 0 0 0
0 0 0 0 0 0
0 0 0 0 0 0
0 0 0 0 0 0
0 0 0 0 0 0
0 0 0 0 0 0
0 0 0 0 0 0
0 0 0 0 0 0
0 0 0 0 0 0
0 0 0 0 0 0
0 0 0 0 0 0
0 0 0 0 0 0
0 0 0 0 0 0
0 0 0 0 0 0
0 0 0 0 0 0
0 0 0 0 0 0
0 0 0 0 0 0
0 0 0 0 0 0
0 0 0 0 0 0
0 0 0 0 0 0
0 0 0 0 0 0
0 0 0 0 0 0
0 0 0 0 0 0
0 0 0 0 0 0
0 0 0 0 0 0
0 0 0 0 0 0
0 0 0 0 0 0
0 0 0 0 0 0
0
gtSp=0 0 0 0 0 0
0 0 0 0 0 0
0 0 0 0 0 0
0 0 0.53731 0.46236 0.5 0.49911
0 0 0 0 0 0
0 0 0 0 0 0
0 0 0 1 0.49356 0.48983
0.5 0.52737 0.32417 0.5 0 0
0 0 0 0 0 0
0 0 0 0 0 0
0 1 1 0.36999 0.44915 0.5
0.5 0.48651 0.55301 0.55351 0.42962 0
0 0 0 0 0 0
0 0 0 0 0 0
0 0 1 1 1 0.51144
0.54561 0.61181 0.46275 0.56408 0.39943 0.69878
0.51175 0.5 0 0 0 0
0 0 0 0 0 0
```

0	0	0	0	0	0
1	1	1	1	0.42399	0.54072
0.50283	0.52804	0.49295	0.5	0.52466	0.5
0.5	0.5	0	0	0	0
0	0	0	0	0	0
0	0	0	0	0	0
1	1	1	1	1	1
0.5	0.62273	0.5	0.5	0.49883	0.53315
0.50876	0.5	0.5	0.5073	0	0
0	0	0	0	0	0
0	0	0	0	0	0
0	0	0	1	1	1
1	1	1	0.53413	0.5	0.57108
0.48732	0.51832	0.59703	0.5	0.5	0.5
0.47555	0.53417	0	0	0	0
0	0	0	0	0	0
0	0	0	0	0	0
0	1	1	1	1	1
1	1	0.51454	0.55979	0.5	0.5
0.54385	0.47324	0.38545	0.5	0.50361	0.48306
0.5	0	0	0	0	0
0	0	0	0	0	0
0	0	0	0	0	0
0	1	1	1	1	1
1	1	0.44458	0.5	0.5	0.51256
0.46043	0.55883	0.5	0.58005	0.5	0.49979
0.60611	0	0	0	0	0
0	0	0	0	0	0
0	0	0	0	0	0
0	0	1	1	1	1
1	1	1	0.53806	0.54837	0.53425
0.5	0.43845	0.5	0.51072	0.45061	0.5
0.59772	0.48216	0.5	0	0	0
0	0	0	0	0	0
0	0	0	0	0	0
0	0	0	1	1	1
1	1	1	1	0.49083	0.47396
0.5471	0.5	0.5	0.52158	0.46765	0.56802
0.54359	0.5241	0.48178	0.45602	0	0
0	0	0	0	0	0
0	0	0	0	0	0
0	0	0	0	1	1
1	1	1	1	1	1
1	0.5	0.46382	0.40231	0.50236	0.50814
0.34745	0.5	0.48494	0.48242	0.52801	0.50109
0	0	0	0	0	0
0	0	0	0	0	0
0	0	0	0	0	0
1	1	1	1	1	1
1	1	1	0.53242	0.53103	0.33816
0.33333	0.3581	0.36411	0.34677	0.3604	0.33333
0.34573	0.42132	0	0	0	0

0	0	0	0	0	0
0	0	0	0	0	0
0	0	1	1	1	1
1	1	1	1	1	0.49117
0.33333	0.38517	0.32467	0.33333	0.33831	0.33333
0.24228	0.29207	0.36211	0.32272	0	0
0	0	0	0	0	0
0	0	0	0	0	0
0	0	0	0	1	1
1	1	1	1	1	0.51009
0.58008	0.56012	0.36074	0.35796	0.33333	0.3764
0.3788	0.30224	0.34112	0.35802	0.29152	0.35004
0	0	0	0	0	0
0	0	0	0	0	0
0	0	0	0	0	0
1	1	1	1	1	1
0.55346	0.52531	0.47768	0.37806	0.45318	0.32975
0.31118	0.3622	0.23719	0.2883	0.30241	0.45817
0.35094	0.39794	0	0	0	0
0	0	0	0	0	0
0	0	0	0	0	0
0	0	1	1	1	1
1	0.46747	0.50352	0.5	0.47361	0.46785
0.5	0.26153	0.23756	0.34615	0.29218	0.37418
0.33333	0.33333	0.33333	0.33333	0	0
0	0	0	0	0	0
0	0	0	0	0	0
0	0	0	0	1	1
1	0.5	0.49565	0.50685	0.49885	0.51338
0.5	0.5164	0.40434	0.33333	0.31031	0.33703
0.28566	0.2642	0.34383	0.33333	0.23281	0.33333
0	0	0	0	0	0
0	0	0	0	0	0
0	0	0	0	0	0
1	0.52401	0.52482	0.47446	0.5	0.5697
0.49938	0.60034	0.5	0.48095	0.21451	0.33333
0.35152	0.31834	0.27075	0.33333	0.37883	0.3184
0.30858	0	0	0	0	0
0	0	0	0	0	0
0	0	0	0	0	0
0	0.43744	0.5	0.45888	0.31489	0.54531
0.31473	0.41335	0.52734	0.5395	0.5	0.33333
0.33792	0.43203	0.23884	0.31493	0.42954	0.3209
0.34905	0.28603	0	0	0	0
0	0	0	0	0	0
0	0	0	0	0	0
0	0	0.55963	0.59422	0.5077	0.5
0.37838	0.5	0.5	0.51217	0.5	0.51469
0.44295	0.32411	0.33791	0.33754	0.33333	0.3848
0.32387	0.30061	0	0	0	0
0	0	0	0	0	0
0	0	0	0	0	0

0	0	0	0.54702	0.50825	0.31965
0.44796	0.53851	0.4855	0.5	0.50918	0.50514
0.5	0.34471	0.25999	0.32523	0.33439	0.31906
0.29503	0.28922	0.38817	0	0	0
0	0	0	0	0	0
0	0	0	0	0	0
0	0	0	0	0	0.70694
0.46644	0.5	0.5	0.60322	0.53352	0.5
0.5	0.4141	0.33333	0.24849	0.2711	0.34559
0.33838	0.33538	0	0	0	0
0	0	0	0	0	0
0	0	0	0	0	0
0	0	0	0	0	0
0.5	0.56854	0.59421	0.46736	0.5	0.39938
0.46276	0.4512	0.5	0.18991	0.33384	0.26696
0	0	0	0	0	0
0	0	0	0	0	0
0	0	0	0	0	0
0	0	0	0	0	0
0	0	0.56689	0.58727	0.50424	0.5
0.7155	0.41473	0.51767	0.5	0	0
0	0	0	0	0	0
0	0	0	0	0	0
0	0	0	0	0	0
0	0	0	0	0	0
0	0	0	0.51227	0.5	0.56246
0.52819	0.52813	0	0	0	0
0	0	0	0	0	0
0	0	0	0	0	0
0	0	0	0	0	0
0	0	0	0	0	0
0	0	0	0	0	0.48188
0.55344	0	0	0	0	0
0	0	0	0	0	0
0	0	0	0	0	0
0	0	0	0	0	0
0	0	0	0	0	0
0	0	0	0	0	0
0	0	0	0	0	0
0	0	0	0	0	0
0	0	0	0	0	0
0	0	0	0	0	0
0	0	0	0	0	0
0	0	0	0	0	0
0	0	0	0	0	0
0	0	0	0	0	0
0	0	0	0	0	0
0	0	0	0	0	0
0	0	0	0	0	0
0	0	0	0	0	0
0	0	0	0	0	0
0	0	0	0	0	0

0	0	0	0	0	0
0	0	0	0	0	0
0	0	0	0	0	0
0	0	0	0	0	0
0	0	0	0	0	0
0	0	0	0	0	0
0	0	0	0	0	0
0	0	0	0	0	0
0	0	0	0	0	0
0	0	0	0	0	0
0	0	0	0	0	0
0	0	0	0	0	0
0	0	0	0	0	0
0	0	0	0	0	0
0	0	0	0	0	0
0	0	0	0	0	0
0	0	0	0	0	0
0	0	0	0	0	0
0	0	0	0	0	0
0	0	0	0	0	0
0	0	0	0	0	0
0	0	0	0	0	0
0	0	0	0	0	0
0	0	0	0	0	0
0	0	0	0	0	0
0	0	0	0	0	0
0	0	0	0	0	0
0	0	0	0	0	0
0	0	0	0	0	0
0	0	0	0	0	0
0	0	0	0	0	0
0	0	0	0	0	0
0	0	0	0	0	0
0	0	0	0	0	0
0	0	0	0	0	0
0	0	0	0	0	0
0	0	0	0	0	0

0

gtTCf=
0
0.11925
0.14534
0.13574
0.11593
0.096197
0.079948
0.067704
0.058926
0.052819
0.048646
0.045817

0.043898
0.042582
0.041662
0.040998
0.040498
0.040102

gtTCs=
0
0.07155
0.116
0.14393
0.16177
0.17342
0.18126
0.18671
0.19067
0.19366
0.19601
0.19792
0.19952
0.20088
0.20206
0.20309
0.20398
0.20476

gtTCp=
0
2.385
1.5979
1.1196
0.8287
0.65143
0.54309
0.47655
0.43538
0.4096
0.39316
0.38239
0.37506
0.36982
0.36585
0.36266
0.35995
0.35753

X_mask=
2.2204e-016,2.2204e-016,2.2204e-016,5.1623e-005,2.2204e-016,2.2204e-016,0.00027668,0.00075049,0.00068662,0.00035063,0.00094933,2.2204e-016,0.00016621,0.00012421,0.00021209,2.2204e-016,0.00021865,0.00039997,0.00030607,2.2204e-016,2.2204e-016,2.2204e-

016,0.0064036,2.2204e-016,2.2204e-
016,0.00032067,0.0011019,0.00040821,0.00010781,0.00023074,0.00013328,0
.00086696,2.2204e-016,0.00042177,0.00055025,0.00041074,6.7833e-
005,2.2204e-016,0.0004003,0.0037053,7.6902e-005,0.0016312,2.2204e-
016,2.2204e-016,2.2204e-016,0.0023092,0.00064577,2.2204e-016,2.2204e-
016,0.00013906,0.00012353,2.2204e-016,0.00066459,2.2204e-
016,0.00034791,2.2204e-016,0.00056458,1.9363e-005,2.2204e-
016,0.00048524,9.8262e-005,2.2204e-016,2.2204e-
016,0.0023501,0.00156,0.0027362,2.2204e-
016,0.001347,0.0025262,0.0037056,0.00081782,0.00042785,5.9706e-
005,0.00016284,0.00048887,8.5383e-005,0.00044502,0.00033369,2.2204e-
016,2.2204e-016,0.0007048,0.00026871,0.00091467,2.2204e-016,2.2204e-
016,0.00010884,2.2204e-016,2.2204e-016,2.2204e-016,2.2204e-
016,0.0025795,0.0007665,2.2204e-
016,0.0030216,0.0022421,0.0016647,0.0008328,2.2204e-016,2.2204e-
016,2.2204e-016,2.2204e-016,2.2204e-016,2.2204e-
016,0.00024277,2.2204e-016,8.647e-005,2.2204e-016,2.2204e-
016,0.00048101,0.00011617,0.00033921,2.2204e-016,2.2204e-016,2.2204e-
016,0.0057756,0.0090529,0.0032114,0.0011313,2.2204e-016,2.2204e-
016,0.0010511,0.0042319,0.0025354,2.2204e-016,2.2204e-
016,0.0025465,2.2204e-016,0.001929,0.00042155,2.2204e-016,2.2204e-
016,0.0010545,2.2204e-016,4.3914e-
005,0.00065252,0.00069383,0.0001924,2.2204e-016,0.00091482,2.2204e-
016,0.00072326,2.2204e-016,5.3144e-
005,0.00046,0.0078504,0.010407,2.2204e-016,2.2204e-016,2.2204e-
016,0.0020773,2.2204e-016,2.2204e-016,2.2204e-016,2.2204e-
016,0.0011699,2.2204e-016,0.00032578,0.0066833,2.2204e-
016,0.0033828,2.2204e-016,2.2204e-016,2.2204e-016,2.2204e-016,7.4978e-
005,0.00055378,2.2204e-016,0.00025663,0.00028416,2.2204e-
016,0.00022903,2.2204e-016,0.00053638,2.2204e-
016,0.00031616,0.00063417,2.2204e-016,0.0015932,0.010833,2.2204e-
016,2.2204e-016,2.2204e-016,0.0010527,0.00027198,2.2204e-
016,0.0050069,0.0010038,0.001163,2.2204e-016,0.00042157,2.2204e-
016,0.0033497,2.2204e-016,2.2204e-016,2.2204e-
016,0.0011293,0.00018675,2.2204e-
016,0.00021625,0.00057062,0.00047586,2.2204e-016,0.00067801,9.9527e-
005,0.00015424,2.2204e-016,2.2204e-016,2.2204e-016,2.2204e-
016,5.3866e-005,2.2204e-016,0.0042168,0.0013903,2.2204e-
016,0.0045759,2.2204e-016,0.00011304,0.014152,0.0028594,2.2204e-
016,0.0022805,2.2204e-016,2.2204e-016,2.2204e-016,2.2204e-
016,0.0022524,0.0032078,2.2204e-016,2.2204e-016,7.0906e-
005,0.00036558,2.2204e-016,2.2204e-016,2.2204e-016,2.2204e-
016,2.2204e-016,2.2204e-016,0.00046208,2.2204e-016,2.2204e-
016,2.2204e-016,2.2204e-016,2.2204e-016,0.00060918,2.2204e-
016,0.0004466,0.00171,0.0014373,0.0045642,2.2204e-016,2.2204e-
016,0.00048161,2.2204e-016,2.2204e-016,0.00021602,6.4993e-
005,0.00085894,2.2204e-016,0.0043531,2.2204e-016,2.2204e-016,2.2204e-
016,2.2204e-016,0.0014211,0.0010674,2.2204e-016,2.2204e-
016,0.00074974,0.00097061,2.2204e-016,2.2204e-016,2.2204e-016,2.2204e-
016,2.2204e-016,2.2204e-016,0.00030821,0.00014477,2.2204e-016,2.2204e-
016,2.2204e-016,0.00029623,2.2204e-016,2.2204e-
016,0.00076889,0.00491,2.2204e-016,2.2204e-

016,0.0092434,0.003312,2.2204e-016,2.2204e-
016,0.0015457,0.0014477,2.2204e-016,2.2204e-016,0.00083297,2.2204e-
016,2.2204e-016,2.2204e-016,2.2204e-016,2.2204e-
016,0.002396,0.00074063,2.2204e-016,2.2204e-016,2.2204e-
016,0.00054858,2.2204e-016,4.7883e-005,0.00046754,2.2204e-016,2.2204e-
016,0.00010218,2.2204e-016,2.2204e-016,0.00036163,2.2204e-
016,0.00033785,2.2204e-016,2.2204e-016,0.00023344,0.0026037,2.2204e-
016,0.002602,2.2204e-016,0.00024518,0.0066988,2.2204e-
016,0.00020513,2.2204e-016,2.2204e-
016,0.0035711,0.00168,0.0025765,0.0012604,2.2204e-
016,0.0040794,0.0025297,2.2204e-016,0.0010219,2.2204e-
016,0.00050531,2.2204e-016,2.2204e-016,2.2204e-016,2.2204e-
016,2.2204e-016,2.2204e-016,0.00010723,2.2204e-016,2.2204e-
016,0.00075836,0.00018907,2.2204e-016,2.2204e-016,0.00036172,2.2204e-
016,2.2204e-016,0.0065963,0.0074173,0.001636,2.2204e-
016,0.0073395,0.011687,2.2204e-016,0.0030989,2.2204e-016,2.2204e-
016,0.0011509,0.00034056,2.2204e-016,2.2204e-016,0.00055302,2.2204e-
016,2.2204e-
016,0.0042755,0.0010912,0.0051182,0.00013043,0.00032144,2.2204e-
016,2.2204e-016,2.2204e-016,2.2204e-016,2.0043e-005,5.5381e-
005,0.00035245,2.2204e-016,0.00024997,2.2204e-016,0.00028554,6.7484e-
005,0.00073585,8.8942e-005,2.2204e-016,2.2204e-
016,0.0027771,0.005344,2.2204e-
016,0.0027754,0.0072965,0.0014759,2.2204e-
016,0.012965,0.0059087,2.2204e-016,2.2204e-016,2.2204e-
016,0.0022333,2.2204e-016,2.2204e-016,2.2204e-016,2.2204e-016,2.2204e-
016,0.00055856,2.2204e-016,2.2204e-016,2.2204e-016,2.2204e-
016,2.2204e-016,2.2204e-016,5.0893e-
005,0.00045025,0.00059649,0.00014085,2.2204e-016,2.2204e-016,2.2204e-
016,9.6648e-005,0.00035727,6.5111e-007,2.2204e-016,2.2204e-
016,2.0197e-005,0.0042917,0.0071391,2.2204e-
016,0.0038206,0.0032837,2.2204e-016,2.2204e-016,0.0012758,2.2204e-
016,2.2204e-016,0.00167,0.0014863,2.2204e-016,0.00064788,2.2204e-
016,0.0022635,2.2204e-016,0.0015501,0.0017671,0.00048321,2.2204e-
016,0.0001515,0.00012478,2.2204e-016,3.0021e-
005,0.00063898,0.00084187,2.2204e-016,0.00032239,0.00013965,2.2204e-
016,0.00022366,2.2204e-016,2.2204e-016,0.00029736,5.7447e-005,2.2204e-
016,2.2204e-016,0.010134,2.2204e-016,0.0027364,0.0117,2.2204e-
016,2.2204e-016,0.00077859,2.2204e-016,2.2204e-016,0.0031812,2.2204e-
016,2.2204e-016,2.2204e-016,2.2204e-
016,0.00082615,0.003298,0.0017596,2.2204e-016,3.2839e-005,2.2204e-
016,0.00020948,2.2204e-016,2.2204e-016,0.00047871,8.7114e-
005,0.000647,0.00012021,0.00049973,2.2204e-016,2.2204e-016,2.2204e-
016,2.2204e-016,2.2204e-016,0.0005681,8.5151e-005,2.2204e-
016,0.00059534,2.2204e-016,2.2204e-016,0.0037951,2.2204e-016,2.2204e-
016,2.2204e-016,0.0016882,2.2204e-016,0.0029034,2.2204e-016,2.2204e-
016,2.2204e-016,0.00015954,0.00072443,0.0028419,2.2204e-
016,0.00079082,0.00046105,2.2204e-016,0.00065284,0.0026546,2.2204e-
016,2.2204e-016,2.2204e-016,0.00073596,3.7675e-
005,0.00011363,0.00024228,6.0761e-005,2.2204e-016,2.2204e-
016,0.00012237,0.00044646,0.00011983,2.2204e-016,2.2204e-016,2.2204e-
016,2.2204e-016,8.3404e-005,2.2204e-016,0.0016714,2.2204e-016,2.2204e-

016,0.0083293,0.0024968,2.2204e-016,2.2204e-016,0.0056312,2.2204e-
016,2.2204e-016,2.2204e-016,2.2204e-016,2.2204e-016,2.2204e-
016,0.00096505,2.2204e-016,2.2204e-016,0.00031312,2.2204e-016,2.2204e-
016,2.2204e-016,2.2204e-016,2.2204e-016,0.0002649,2.2204e-016,4.862e-
006,2.2204e-016,2.2204e-016,0.00016326,0.00048637,0.00016851,2.2204e-
016,0.00026753,6.4778e-005,2.2204e-016,0.00069448,5.8082e-
005,0.0068018,0.003355,0.01259,0.0047748,2.2204e-016,2.2204e-
016,0.0016455,2.2204e-016,2.2204e-016,2.2204e-
016,0.0016135,0.0010885,0.0023724,0.0013194,2.2204e-
016,0.00073859,0.001934,2.2204e-016,0.00066759,0.00087351,2.2204e-
016,2.2204e-016,2.2204e-016,2.2204e-016,2.2204e-016,5.3244e-
006,2.2204e-016,5.2117e-005,2.2204e-016,2.2204e-016,2.2204e-
016,2.2204e-016,7.235e-005,2.2204e-016,2.2204e-
016,0.00023518,0.00074094,0.0005667,0.0007922,2.2204e-
016,0.0013582,2.2204e-016,2.2204e-016,0.0047456,0.0024258,2.2204e-
016,0.0038944,2.2204e-016,2.2204e-016,2.2204e-016,0.001134,2.2204e-
016,2.2204e-016,0.0010158,0.00061582,0.0015529,0.0017018,2.2204e-
016,0.00082659,2.2204e-016,6.1585e-005,0.00045533,2.2204e-016,8.2225e-
005,0.00012233,2.2204e-016,0.00047323,2.2204e-016,2.2204e-016,2.2204e-
016,9.2658e-005,2.2204e-016,2.2204e-016,0.00010638,0.00022799,2.2204e-
016,2.2204e-016,2.2204e-016,2.2204e-
016,0.0023326,0.00062971,0.0029726,2.2204e-
016,0.0061914,0.0022686,2.2204e-
016,0.0012581,0.011094,0.0003559,2.2204e-016,2.2204e-016,2.2204e-
016,2.2204e-016,0.0050093,0.00012559,0.00019481,0.00031327,2.2204e-
016,2.2204e-016,0.00032588,2.2204e-016,8.5067e-005,2.2204e-
016,0.00010688,0.00016251,2.2204e-016,2.2204e-016,0.00023905,2.2204e-
016,0.00022043,0.00050873,0.00048095,2.2204e-
016,0.0030728,0.0022537,2.2204e-016,2.2204e-016,2.2204e-
016,0.0026553,2.2204e-016,0.0015245,0.0002998,2.2204e-016,2.2204e-
016,2.2204e-016,2.2204e-016,0.0022164,2.2204e-
016,0.003087,0.00092101,2.2204e-016,2.2204e-
016,0.00024823,0.00045633,3.0765e-
005,0.00021527,0.00012637,0.00015934,2.2204e-016,2.2204e-016,2.2204e-
016,2.2204e-016,2.2204e-016,0.00015629,0.0001823,2.2204e-016,2.2204e-
016,2.2204e-016,2.2204e-
016,0.00083505,0.0024282,0.00047908,0.0012823,2.2204e-016,2.2204e-
016,0.0010445,2.2204e-016,2.2204e-016,2.2204e-
016,0.00092733,0.00098556,0.0011401,0.00051396,0.0029868,2.2204e-
016,8.2812e-006,2.2204e-016,2.2204e-016,7.3602e-005,2.2204e-
016,2.2204e-016,4.1825e-005,8.2772e-005,2.2204e-016,2.2204e-
016,2.2204e-016,2.2204e-016,4.4009e-005,0.00043474,7.3104e-
005,2.2204e-016,0.00026338,2.2204e-016,2.2204e-016,1.6755e-
006,2.2204e-016,2.2204e-016,2.2204e-016,2.2204e-016,0.0033496,2.2204e-
016,0.0020938,2.2204e-016,0.0020014,0.00019899,0.0013393,2.2204e-
016,2.2204e-016,2.2204e-016,0.00077177,2.2204e-
016,0.0025826,0.00021757,2.2204e-016,2.5868e-006,2.2204e-
016,0.00016543,0.00021722,0.00020217,0.00014661,3.8932e-005,2.2204e-
016,2.2204e-016,2.2204e-016,0.00012628,2.2204e-016,2.2204e-
016,2.7057e-005,0.00039763,0.00042493,0.00062253,0.00040936,2.2204e-
016,2.2204e-016,2.2204e-016,0.0047284,2.2204e-
016,0.0039183,0.0031035,0.003545,0.0049727,2.2204e-

016,0.00040064,0.00015934,0.0013278,0.0015402,2.2204e-
016,0.00017509,0.00023485,0.00014345,2.2204e-016,0.00016972,2.2204e-
016,2.2204e-016,2.2204e-016,0.00040219,8.1617e-005,2.2204e-
016,2.2204e-016,2.2204e-016,0.00014918,0.00021611,2.2204e-
016,0.0002224,0.00016427,2.2204e-016,2.2204e-016,2.2204e-016,2.2204e-
016,2.2204e-016,2.2204e-016,2.2204e-016,2.2204e-016,2.2204e-
016,2.2204e-016,2.2204e-016,0.001167,2.2204e-016,0.011183,2.2204e-
016,0.00025333,6.549e-005,2.2204e-016,2.2204e-016,0.00010657,2.2204e-
016,0.00012064,2.2204e-016,0.00031624,0.00036097,0.00012907,2.2204e-
016,5.4086e-005,0.00019601,0.00010111,6.4567e-005,2.2204e-016,2.2204e-
016,0.00021367,2.2204e-016,2.2204e-016,3.7043e-005,0.00025134,9.5469e-
005,2.2204e-016,0.0003307,0.00026567,0.00036709,0.00028273,2.2204e-
016,0.00034661,2.2204e-016,2.2204e-016,2.2204e-016,2.2204e-
016,2.2204e-016,2.2204e-016,2.2204e-016,0.00035461,2.2204e-
016,2.2204e-016,5.618e-005,4.8203e-005,2.2204e-
016,0.00016403,0.0001578,0.00047542,0.00033055,2.2204e-
016,0.00043145,0.00026865,2.2204e-
016,0.00041234,0.00011592,0.00016893,2.2204e-016,2.2204e-016,2.2204e-
016,2.2204e-016,2.2204e-016,2.2204e-016,0.00050961,2.2204e-
016,2.2204e-016,0.0004592,2.2204e-016,2.2204e-
016,0.0049341,0.0046752,2.2204e-016,0.00033663,2.2204e-016,2.2204e-
016,2.2204e-016,2.4495e-005,2.2204e-016,0.00014583,8.0077e-
005,0.00044726,0.00013214,0.00011317,2.2204e-016,0.00017002,2.2204e-
016,2.2204e-016,2.2204e-
016,0.00027487,0.00033503,0.00043715,0.00012552,2.0069e-005,2.2204e-
016,2.2204e-016,0.00016721,9.9366e-005,2.2204e-016,2.2204e-
016,2.2204e-016,2.2204e-016,2.2204e-016,0.00065949,2.2204e-
016,0.00038683,0.00032419,2.2204e-016,2.2204e-016,0.00026223,2.2204e-
016,2.2204e-016,2.2204e-016,2.2204e-016,2.2204e-016,3.8334e-
005,0.00029098,0.00016354,0.00029217,0.00032794,2.2204e-
016,0.00028177,5.5759e-006,2.2204e-016,6.9708e-005,2.2204e-
016,2.2204e-016,0.00029525,1.4146e-005,2.2204e-016,2.2204e-
016,6.4195e-005,9.319e-005,0.00022123,2.2204e-016,0.00057248,2.2204e-
016,0.00035829,0.00066802,0.0003141,2.2204e-016,0.00011194,2.2204e-
016,0.00039944,2.2204e-016,2.2204e-016,0.00030854,2.2204e-
016,0.00012372,0.00011749,0.00057616,2.2204e-016,2.2204e-
016,0.0003457,7.226e-005,2.2204e-016,2.2204e-016,4.9801e-006,2.2204e-
016,0.00035828,0.00029479,0.00084969,0.00026792,2.2204e-
016,0.00046775,5.8107e-005,6.2519e-005,2.2204e-016,2.2204e-
016,2.2204e-016,2.2204e-016,2.2204e-016,2.2204e-
016,0.00045964,0.00028791,2.2204e-016,4.768e-005,2.2204e-016,2.2204e-
016,2.2204e-016,2.2204e-016,0.00013269,5.3533e-005,2.2204e-
016,0.00060201,2.2204e-016,2.2204e-016,2.2204e-016,0.00020988,2.2204e-
016,0.00020189,0.00023329,2.3669e-005,0.00041125,2.2204e-
016,0.00045814,2.2204e-016,0.00011429,0.00056002,0.00028314,2.2204e-
016,0.00049097,0.00020015,2.2204e-016,1.5183e-005,2.2204e-
016,0.00062935,2.2204e-016,2.2204e-016,0.0002617,2.2204e-016,2.2204e-
016,0.00042088,0.0001179,2.2204e-016,2.2204e-016,0.00051377,2.2204e-
016,0.00047862,0.0005078,2.2204e-016,0.0001206,2.2204e-016,2.2204e-
016,0.00038701,2.2204e-016,2.2204e-016,0.00019772,0.00010044,2.2204e-
016,0.00010489,2.2204e-016,2.2204e-016,0.00025834,2.2204e-
016,0.00050362,0.00044668,2.2204e-016,0.00029026,2.2204e-

016,0.00072417,2.2204e-016,2.2204e-016,2.2204e-016,2.2204e-
016,0.00027067,2.2204e-016,0.00034568,2.2204e-016,2.2204e-
016,0.00014974,1.3377e-006,2.2204e-
016,0.00054288,0.00038998,0.00032963,2.2204e-016,2.2204e-016,2.2204e-
016,2.2204e-016,2.2204e-016,0.00041676,0.00055252,2.2204e-
016,0.00052616,0.00024448,8.6205e-005,2.2204e-016,2.2204e-
016,0.00012308,2.2204e-016,0.0001333,2.2204e-016,0.00017441,2.2204e-
016,2.2204e-016,8.9472e-005,0.00016428,0.00019738,2.2204e-
016,0.00035376,6.7724e-006,2.2204e-016,0.00031722,2.2204e-
016,0.0002818,2.7015e-005,0.00058906,2.2204e-016,2.2204e-
016,0.00017472,2.2204e-016,0.00023866,0.00018727,2.2204e-016,2.2204e-
016,2.2204e-016,3.4101e-005,2.2204e-016,2.2204e-016,1.8177e-
006,0.00022332,2.2227e-005,0.00034053,0.00035807,2.2204e-016,2.2204e-
016,5.6887e-005,9.5369e-005,2.2204e-016,0.00079265,2.2204e-
016,7.7626e-005,2.2204e-016,0.00038667,0.0005317,6.5277e-005,2.2204e-
016,0.00027923,2.2204e-016,2.2204e-016,8.6367e-005,2.2204e-
016,2.2204e-016,0.00026504,2.2204e-016,2.2204e-016,0.00033713,2.2204e-
016,2.2204e-016,0.00014535,0.00012331,2.2204e-016,2.2204e-
016,0.00010224,2.2204e-016,2.2204e-016,2.2204e-016,2.2204e-
016,2.2204e-016,2.2204e-016,0.00053203,2.2204e-016,8.5882e-
005,0.00044347,2.2204e-016,2.2204e-016,0.00049462,2.2204e-
016,0.00025404,0.00018659,0.00033854,2.2204e-016,2.2204e-016,2.2204e-
016,5.3655e-005,2.2204e-016,0.00050063,0.00021763,2.2204e-016,2.2204e-
016,2.2204e-016,2.2204e-016,0.00020515,2.2204e-016,2.2204e-
016,2.2204e-016,2.2204e-016,0.00043786,8.1501e-005,2.2204e-
016,2.2204e-016,2.2204e-016,2.2204e-016,0.00031799,2.2204e-
016,0.00064798,0.00034852,0.00035898,0.00015928,8.246e-
005,0.00032725,0.00074255,2.2204e-016,2.2204e-016,4.7078e-005,2.2204e-
016,2.2204e-016,2.2204e-016,4.405e-006,3.8136e-005,0.00052604,2.2204e-
016,2.2204e-016,2.2204e-016,2.2204e-016,1.5349e-005,2.2204e-
016,2.2204e-016,2.2204e-016,0.00052826,2.2204e-016,2.2204e-
016,0.00018348,0.0006861,7.7899e-007,2.2204e-016,2.2204e-016,2.2204e-
016,5.9994e-005,2.2204e-016,0.00021254,2.2204e-016,0.00015111
0.070816,0.071326,0.071067,0.071711,0.071716,0.071574,0.071595,0.07174
7,0.072179,0.071564,0.072601,0.071808,0.07168,0.071274,0.071569,0.0715
61,0.072333,0.071694,0.071563,0.072002,1.3131,1.1369,1.2318,1.2256,0.0
7023,0.071865,0.072883,0.071683,0.072018,0.071626,0.071739,0.072443,0.
071215,0.071865,0.072043,0.071428,0.071492,0.071018,0.07153,2.3874,1.2
122,1.2064,1.2248,1.2859,0.81986,1.2329,0.072184,0.071127,0.071446,0.0
71898,0.071822,0.070363,0.072167,0.071625,0.072431,0.070916,0.07213,0.
071891,0.070995,0.071848,0.07151,2.3783,2.3788,0.93041,1.1114,1.2299,1
.2264,1.1987,1.3526,1.355,1.0654,0.071971,0.071553,0.071485,0.072176,0
.071645,0.071923,0.071767,0.071456,0.071021,0.072402,0.072045,0.072193
,0.070766,0.070955,0.071457,2.388,2.3768,2.3722,1.2522,1.335,1.4887,1.
1368,1.3769,0.99796,1.6888,1.2544,1.2262,0.071801,0.071602,0.071059,0.
071888,0.071254,0.07169,0.071692,0.071679,0.071086,0.071021,0.071894,0
.072003,0.071525,0.070533,0.071311,0.070665,2.393,2.392,2.3856,2.3842,
1.0515,1.3197,1.2362,1.2976,1.2147,1.2292,1.287,1.2293,1.2272,1.2301,0
.071694,0.072053,0.071527,0.072716,0.070596,0.071599,0.07252,0.072424,
0.071579,0.071718,0.072653,0.071197,0.072354,0.07028,0.070985,0.072401
,2.3887,2.3923,2.3735,2.3895,2.3848,2.3899,1.2282,1.5091,1.2286,1.2267
,1.2267,1.3065,1.2477,1.2341,1.2234,1.2481,0.071006,0.071392,0.071015,

0.071876,0.071322,0.072329,0.071185,0.07144,0.07163,0.071299,0.072006,
0.070987,0.071948,0.07121,0.071938,0.072136,0.071036,2.3857,2.3985,2.3
78,2.3833,2.3817,2.384,1.3082,1.2204,1.3999,1.2004,1.2722,1.451,1.2308
,1.2279,1.2318,1.1704,1.3073,0.070785,0.072724,0.071794,0.070909,0.071
652,0.072146,0.071453,0.071336,0.072345,0.071349,0.07172,0.070612,0.07
0533,0.095104,0.091419,0.094108,0.071008,2.3878,2.3865,2.3806,2.3873,2
.3849,2.3849,2.3969,1.2652,1.3662,1.2305,1.2252,1.3285,1.1653,0.96291,
1.2306,1.2396,1.1886,1.2293,0.070994,0.071797,0.070742,0.071219,0.0709
42,0.071664,0.070375,0.070051,0.072306,0.07085,0.071148,0.095853,0.098
504,0.098387,0.096025,0.097736,0.099024,0.072869,2.3857,2.3904,2.3801,
2.3842,2.3846,2.3848,2.3808,1.1012,1.2285,1.2293,1.2561,1.1401,1.3582,
1.2272,1.4097,1.2269,1.2289,1.4754,0.071406,0.071547,0.072243,0.0726,0
.071491,0.070697,0.070787,0.071219,0.071883,0.093561,0.098822,0.093061
,0.09536,0.093054,0.094299,0.095399,0.10173,0.095128,0.071831,2.3895,2
.3775,2.3848,2.3913,2.3869,2.3824,2.3803,1.3196,1.3427,1.3052,1.2236,1
.0884,1.2245,1.2498,1.1128,1.2237,1.4524,1.1883,1.2291,0.070775,0.0717
18,0.071568,0.072016,0.070924,0.071219,0.095552,0.095782,0.095559,0.09
5349,0.094155,0.095229,0.095207,0.090586,0.095614,0.09529,0.097171,0.0
96276,2.3852,2.3828,2.3859,2.3877,2.3866,2.3958,2.3853,1.2069,1.1645,1
.3353,1.2302,1.2287,1.2796,1.1519,1.3857,1.332,1.285,1.1853,1.1253,0.0
70829,0.072212,0.071203,0.096736,0.09113,0.095174,0.0885,0.095255,0.09
4635,0.089643,0.10511,0.095965,0.095599,0.09269,0.096349,0.099867,0.09
3334,0.095468,2.3947,2.3926,2.3861,2.3854,2.395,2.3965,2.3845,2.387,2.
3798,1.2268,1.1463,1.0032,1.2312,1.2459,0.87491,1.2265,1.1887,1.1924,1
.2966,1.2364,0.095685,0.091234,0.090909,0.095191,0.095299,0.095831,0.0
97144,0.095518,0.085847,0.094534,0.09765,0.096201,0.095134,0.095223,0.
096069,0.093308,0.08829,0.092886,2.3836,2.3884,2.3851,2.3819,2.3915,2.
3863,2.3772,2.3983,2.3929,1.3035,1.2995,0.8683,0.86133,0.91669,0.9306,
0.88798,0.92311,0.85858,0.88643,1.0584,0.099284,0.096067,0.095489,0.09
5752,0.095169,0.10388,0.096525,0.094571,0.098184,0.095565,0.091684,0.0
98602,0.094746,0.095954,0.09519,0.098276,0.096449,0.097587,2.3863,2.38
99,2.3832,2.39,2.3911,2.3809,2.3802,2.3865,2.3824,1.2287,0.85978,0.976
7,0.83821,0.8592,0.87022,0.85872,0.65004,0.76515,0.92489,0.83226,0.095
331,0.10293,0.095589,0.095244,0.094367,0.095972,0.10154,0.095391,0.093
646,0.096129,0.099457,0.095694,0.092433,0.094433,0.093654,0.092911,0.0
95934,0.091236,2.4008,2.3824,2.3908,2.3947,2.3819,2.3727,2.3877,1.2679
,1.4317,1.3904,0.92233,0.91154,0.85642,0.95539,0.96255,0.78989,0.87827
,0.91143,0.76117,0.88959,0.095539,0.089813,0.095439,0.099075,0.10269,0
.1011,0.099755,0.09592,0.09521,0.095313,0.094483,0.095164,0.095079,0.0
95936,0.095509,0.096524,0.10532,0.093069,2.3811,2.3899,2.3822,2.3838,2
.3764,2.391,1.3692,1.31,1.2022,0.97441,1.1445,0.85121,0.81088,0.92678,
0.6376,0.75742,0.78901,1.1454,0.90052,1.0083,0.094756,0.096451,0.09496
1,0.097558,0.099652,0.093682,0.09749,0.10297,0.094622,0.096665,0.09297
4,0.10071,0.097556,0.093998,0.097259,0.095422,0.096797,0.10087,2.3859,
2.3877,2.3853,2.3854,2.3907,1.1792,1.258,1.2487,1.1983,1.1788,1.2493,0
.69331,0.63764,0.88371,0.76219,0.95662,0.85789,0.85452,0.85903,0.85597
,0.09581,0.096994,0.093176,0.094689,0.095456,0.093786,0.09028,0.094578
,0.097259,0.095693,0.096329,0.098299,0.095262,0.096657,0.096221,0.0953
11,0.092942,0.093609,2.3886,2.3889,2.3964,1.2566,1.2365,1.2643,1.2514,
1.2805,1.2513,1.2891,1.0385,0.8614,0.80781,0.86874,0.75541,0.7008,0.88
564,0.85778,0.62891,0.85912,0.08416,0.094156,0.094788,0.094938,0.09514
9,0.095099,0.096613,0.09519,0.09042,0.095801,0.094132,0.098685,0.09543

6,0.098944,0.10407,0.094527,0.094268,0.096088,2.3852,1.3032,1.3102,1.1
944,1.2502,1.4132,1.2547,1.4764,1.2537,1.2047,0.58668,0.85614,0.90006,
0.82338,0.71486,0.85928,0.96605,0.82815,0.805,0.093507,0.096533,0.0968
85,0.098868,0.095949,0.095296,0.096443,0.098724,0.095583,0.094648,0.09
5395,0.095426,0.095222,0.098422,0.10011,0.094333,0.09522,0.11922,1.111
2,1.2473,1.158,0.83279,1.3595,0.83269,1.0593,1.3135,1.3463,1.2539,0.85
79,0.86492,1.0988,0.64322,0.81647,1.0761,0.8307,0.89024,0.75348,0.0938
69,0.094868,0.095182,0.093742,0.094385,0.091007,0.09412,0.099874,0.095
128,0.10148,0.095683,0.094929,0.092447,0.09733,0.095547,0.094575,0.095
981,0.11939,1.3849,1.469,1.2716,1.2476,0.97592,1.2496,1.2545,1.2746,1.
2536,1.2867,1.1045,0.83605,0.868,0.86714,0.85983,0.97721,0.83897,0.785
06,0.095263,0.097541,0.098111,0.09123,0.094602,0.091098,0.095465,0.092
957,0.094863,0.095144,0.097379,0.095077,0.094443,0.099707,0.095696,0.0
95207,0.097221,0.096949,0.1183,1.3601,1.2737,0.8445,1.1359,1.336,1.215
7,1.2532,1.2701,1.2615,1.2501,0.8876,0.69352,0.84004,0.86186,0.82668,0
.77209,0.75788,0.98172,0.097722,0.0955,0.087324,0.095409,0.092461,0.09
5481,0.094933,0.095309,0.098536,0.092277,0.095267,0.098437,0.094265,0.
095795,0.095732,0.095264,0.088027,0.11957,0.11879,0.11933,1.7167,1.173
9,1.2525,1.2498,1.4861,1.3254,1.2533,1.2527,1.059,0.85626,0.66422,0.71
666,0.88441,0.8678,0.8648,0.097979,0.094768,0.095329,0.090336,0.10338,
0.099545,0.092699,0.094016,0.10038,0.096212,0.094728,0.094155,0.098953
,0.091415,0.1113,0.099322,0.091146,0.094672,0.11984,0.11974,0.1191,0.1
1905,1.2468,1.413,1.4649,1.1817,1.2563,1.0277,1.174,1.1388,1.2528,0.52
994,0.86053,0.70703,0.098153,0.095218,0.080172,0.097297,0.097775,0.096
284,0.096441,0.095468,0.089905,0.095299,0.095433,0.091543,0.097071,0.0
95066,0.083311,0.093777,0.094895,0.093837,0.095465,0.093859,0.11824,0.
11913,0.11899,0.11896,0.11919,0.11923,1.3993,1.4475,1.2557,1.2489,1.74
24,1.0569,1.3025,1.2499,0.089905,0.094827,0.091528,0.094288,0.094869,0
.093655,0.089356,0.095243,0.096312,0.094739,0.095588,0.091808,0.094809
,0.09712,0.098829,0.097212,0.098497,0.095182,0.095531,0.097545,0.09511
5,0.095619,0.09867,0.11935,0.1191,0.1194,0.11956,0.11937,0.11967,1.275
7,1.2512,1.3912,1.3136,1.3038,0.11938,0.11909,0.096156,0.094239,0.0999
26,0.095176,0.095308,0.09566,0.095521,0.094889,0.095665,0.094164,0.095
79,0.094651,0.095463,0.085816,0.088375,0.096998,0.094631,0.095544,0.09
4524,0.095138,0.095215,0.11846,0.11902,0.11889,0.11893,0.1196,0.11881,
0.11881,0.11973,0.11906,0.11892,1.2147,1.3775,0.11906,0.11941,0.1189,0
.11928,0.093672,0.10096,0.092889,0.096978,0.097422,0.091659,0.095775,0
.095631,0.095419,0.09559,0.095673,0.092277,0.095252,0.095874,0.09316,0
.095111,0.095694,0.094068,0.097883,0.1187,0.11948,0.11928,0.11915,0.11
91,0.11937,0.11931,0.11876,0.12,0.11893,0.11933,0.11971,0.11909,0.1187
,0.11938,0.11898,0.11895,0.094975,0.095238,0.095587,0.098885,0.093874,
0.099268,0.097273,0.09582,0.094736,0.09211,0.094605,0.095556,0.092266,
0.093105,0.093828,0.096726,0.11942,0.11921,0.11927,0.11932,0.11899,0.1
1961,0.11911,0.11969,0.11897,0.11968,0.12026,0.11912,0.11887,0.11883,0
.11912,0.1197,0.11916,0.11888,0.11939,0.09948,0.095178,0.090183,0.0957
43,0.098773,0.095224,0.094001,0.094557,0.09521,0.094906,0.093266,0.095
486,0.08864,0.11936,0.12016,0.11962,0.11861,0.11929,0.11918,0.1195,0.1
1903,0.11913,0.11886,0.11876,0.119,0.11933,0.1198,0.11963,0.11927,0.11
939,0.11874,0.11874,0.11923,0.11858,0.095513,0.099817,0.096115,0.09667
2,0.09567,0.10474,0.095325,0.095781,0.095524,0.11948,0.11963,0.1193,0.
11976,0.11879,0.11961,0.11881,0.11917,0.11991,0.11958,0.119,0.11962,0.
11948,0.11868,0.1195,0.11901,0.11995,0.11878,0.11886,0.11933,0.11931,0

.11896,0.11958,0.11934,0.11883,0.099772,0.095023,0.092713,0.10267,0.11
968,0.11858,0.11896,0.11947,0.11875,0.11937,0.11932,0.11885,0.1195,0.1
1932,0.11884,0.11936,0.11872,0.11915,0.11933,0.11884,0.11989,0.1195,0.
11862,0.11942,0.11929,0.11962,0.11882,0.11896,0.11889,0.11874,0.11942,
0.11904,0.11981,0.11911,0.11901,0.11909,0.11898,0.11876,0.11953,0.1193
7,0.11971,0.11934,0.11862,0.11908,0.11883,0.11831,0.11959,0.11983,0.11
866,0.11974,0.11934,0.11918,0.11874,0.11916,0.1196,0.11932,0.1194,0.11
918,0.11919,0.11914,0.11944,0.11963,0.11923,0.11927,0.11844,0.11964,0.
11934,0.11936,0.12025,0.11909,0.11935,0.11952,0.11993,0.11944,0.1191,0
.11938,0.11913,0.11953,0.11934,0.119,0.11897,0.11855,0.11942,0.11845,0
.1187,0.1194,0.11933,0.11948,0.11921,0.1198,0.11911,0.11902,0.11939,0.
11921,0.11887,0.11987,0.1191,0.1191,0.11961,0.11954,0.1195,0.11951,0.1
1915,0.11976,0.1192,0.1195,0.11927,0.11906,0.1188,0.11945,0.11919,0.11
91,0.11999,0.11881,0.11874,0.11912,0.1198,0.11913,0.11896,0.11944,0.11
923,0.11888,0.11886,0.11927,0.11874,0.11897,0.11948,0.11912,0.11939,0.
11935,0.11915,0.11896,0.11977,0.11918,0.11955,0.11947,0.1195,0.11898,0
.11866,0.11928,0.1196,0.11868,0.11983,0.11986,0.1193,0.11932,0.1191,0.
11919,0.11976,0.11878,0.11846,0.11902,0.11901,0.11959,0.11949,0.11916,
0.11895,0.11906,0.11818,0.11933,0.11862,0.11972,0.11973,0.11955,0.1192
9,0.11913,0.11956,0.11975,0.11905,0.11836,0.11927,0.1193,0.11922,0.119
11,0.11946,0.11925,0.11975,0.11903,0.1189,0.11907,0.11871,0.11946,0.11
939,0.11822,0.11934,0.11989,0.11907,0.11915,0.1193,0.11994,0.11927,0.1
1907,0.11943,0.11846,0.11917,0.11933,0.11954,0.11879,0.11936
0.11513,0.11584,0.11532,0.11606,0.11604,0.11578,0.11568,0.11608,0.1165
3,0.11609,0.11733,0.11639,0.11608,0.1157,0.11595,0.11591,0.11668,0.115
88,0.11687,0.11678,0.9119,0.79649,0.86064,0.85531,0.11441,0.1159,0.117
09,0.11625,0.11659,0.11587,0.11672,0.11655,0.11568,0.11624,0.11626,0.1
1595,0.11586,0.11545,0.11624,1.5969,0.84757,0.84662,0.85469,0.89247,0.
59506,0.86293,0.1163,0.11549,0.11621,0.11629,0.11609,0.11448,0.11647,0
.11636,0.11672,0.1151,0.1167,0.11622,0.11553,0.11646,0.11625,1.5879,1.
5911,0.66618,0.78365,0.85905,0.85488,0.83712,0.93614,0.93829,0.7522,0.
11621,0.11602,0.11605,0.11678,0.11609,0.11606,0.11612,0.1156,0.11552,0
.11665,0.11686,0.11626,0.11536,0.11611,0.11604,1.5988,1.5903,1.5865,0.
87244,0.92435,1.0255,0.79601,0.95123,0.711,1.1519,0.8747,0.85604,0.115
93,0.11614,0.11583,0.11639,0.11596,0.11597,0.11605,0.1164,0.11587,0.11
558,0.11623,0.11638,0.11607,0.1145,0.11553,0.11536,1.6098,1.6022,1.597
3,1.6023,0.74426,0.91537,0.86193,0.90359,0.8484,0.85743,0.89388,0.8570
3,0.857,0.85866,0.11633,0.11617,0.11631,0.11667,0.11495,0.11635,0.1173
3,0.1169,0.11621,0.11606,0.11731,0.11555,0.11669,0.11492,0.11555,0.117
03,1.5956,1.6069,1.587,1.6013,1.5994,1.6012,0.8549,1.0366,0.85645,0.85
628,0.85576,0.9074,0.87128,0.86343,0.85227,0.87207,0.11549,0.11575,0.1
1558,0.11608,0.11562,0.11708,0.11599,0.11586,0.11614,0.11588,0.11654,0
.11579,0.11658,0.1155,0.11627,0.11637,0.11502,1.5984,1.6044,1.5903,1.6
015,1.5967,1.5975,0.90851,0.84826,0.9692,0.84038,0.88591,0.99683,0.859
,0.85525,0.85869,0.81812,0.90808,0.11531,0.11714,0.11598,0.11556,0.115
89,0.11585,0.1159,0.11555,0.11689,0.11551,0.11641,0.11532,0.11511,0.13
042,0.12805,0.1297,0.11538,1.6007,1.5964,1.5906,1.5987,1.5956,1.5966,1
.6119,0.88046,0.94755,0.86062,0.85375,0.91983,0.816,0.68638,0.85983,0.
86609,0.82951,0.85957,0.1158,0.11634,0.11521,0.11571,0.11494,0.11611,0
.11471,0.11489,0.11663,0.11521,0.11598,0.13086,0.13248,0.13245,0.13125
,0.13244,0.13281,0.11728,1.5983,1.6038,1.5928,1.5977,1.596,1.5955,1.59
38,0.77375,0.85691,0.85881,0.87285,0.80172,0.9381,0.85554,0.97003,0.85

557,0.85771,1.0153,0.11564,0.11588,0.11666,0.11735,0.11581,0.11522,0.1
1503,0.11543,0.11601,0.12941,0.13274,0.12919,0.13045,0.12904,0.12995,0
.13057,0.13434,0.13036,0.11596,1.6038,1.589,1.596,1.5997,1.6,1.5953,1.
5967,0.91669,0.92947,0.90666,0.85356,0.76909,0.85428,0.86976,0.78191,0
.8551,1.0018,0.83136,0.85984,0.11532,0.11609,0.11612,0.11616,0.11549,0
.11592,0.13106,0.13096,0.13056,0.13068,0.12994,0.13053,0.1305,0.1276,0
.13051,0.13087,0.13168,0.13143,1.5994,1.5966,1.5953,1.6025,1.6013,1.60
84,1.5998,0.84238,0.8169,0.92517,0.85845,0.85782,0.88802,0.808,0.95827
,0.9241,0.89299,0.8291,0.78918,0.11497,0.11677,0.11592,0.13147,0.12774
,0.13046,0.12646,0.1305,0.13038,0.12703,0.13655,0.13124,0.13075,0.1291
,0.13121,0.13357,0.12947,0.13055,1.6055,1.6043,1.5998,1.5985,1.6067,1.
605,1.5988,1.598,1.5924,0.85477,0.80503,0.71453,0.86038,0.86869,0.6307
4,0.85706,0.8278,0.83465,0.90108,0.86254,0.13111,0.12814,0.12788,0.130
72,0.13054,0.1309,0.13174,0.13099,0.12497,0.13015,0.13249,0.13098,0.13
065,0.13071,0.13138,0.12951,0.12606,0.12929,1.596,1.6003,1.5946,1.5962
,1.606,1.5966,1.5909,1.6114,1.6051,0.90783,0.90231,0.62501,0.62233,0.6
5749,0.66547,0.63829,0.66228,0.61896,0.63848,0.74864,0.13281,0.13108,0
.13058,0.13075,0.13044,0.13609,0.13155,0.13034,0.13235,0.13081,0.12812
,0.1328,0.13054,0.13106,0.13056,0.13243,0.13098,0.13204,1.5954,1.6081,
1.5979,1.6036,1.6019,1.5968,1.5969,1.6044,1.5961,0.85663,0.62043,0.696
57,0.6072,0.62117,0.62778,0.6189,0.48575,0.55983,0.66448,0.60312,0.130
54,0.13527,0.13085,0.13049,0.13005,0.13125,0.13463,0.13056,0.12952,0.1
3118,0.13314,0.13083,0.12874,0.12998,0.12978,0.1291,0.13098,0.1282,1.6
108,1.596,1.6034,1.6081,1.5967,1.5882,1.5979,0.88091,0.987,0.95888,0.6
5879,0.65265,0.6174,0.68231,0.68578,0.57622,0.63447,0.65456,0.55611,0.
63807,0.1309,0.12716,0.13076,0.13305,0.13546,0.13433,0.13341,0.13109,0
.13041,0.13038,0.1299,0.13033,0.1305,0.1311,0.13072,0.13127,0.13725,0.
12908,1.5982,1.6028,1.5931,1.5968,1.5918,1.6083,0.94448,0.90715,0.8403
3,0.69297,0.80228,0.61505,0.58968,0.6638,0.47761,0.55649,0.57437,0.802
32,0.64712,0.71601,0.13015,0.1313,0.13037,0.13225,0.13316,0.12973,0.13
215,0.13525,0.1304,0.13151,0.12897,0.13396,0.13207,0.1295,0.13211,0.13
064,0.1313,0.13403,1.5967,1.6008,1.6007,1.5997,1.6069,0.82552,0.87459,
0.86984,0.83797,0.82496,0.86865,0.51299,0.47797,0.63703,0.55809,0.6838
1,0.61917,0.61734,0.62093,0.6197,0.13103,0.13128,0.12932,0.13015,0.130
67,0.1296,0.12752,0.13029,0.13177,0.1309,0.13127,0.13259,0.1307,0.1315
6,0.13105,0.13051,0.12923,0.12927,1.6002,1.6067,1.6087,0.8752,0.86088,
0.87687,0.87402,0.88794,0.86961,0.89388,0.73585,0.62392,0.58927,0.6262
9,0.55336,0.51905,0.6366,0.62056,0.47262,0.61924,0.12337,0.13008,0.130
31,0.13021,0.13077,0.13034,0.13125,0.13058,0.12741,0.13064,0.12987,0.1
3275,0.13083,0.13279,0.1359,0.1302,0.13002,0.13116,1.5992,0.90418,0.90
943,0.83481,0.8698,0.97557,0.87585,1.0148,0.87347,0.84042,0.44489,0.61
754,0.6466,0.59652,0.52698,0.62177,0.68849,0.6008,0.58763,0.1293,0.131
65,0.13146,0.13281,0.13132,0.13062,0.13139,0.13281,0.13079,0.13024,0.1
3081,0.13071,0.1306,0.13256,0.13372,0.1301,0.13044,0.14544,0.78008,0.8
6447,0.81208,0.60321,0.9428,0.60303,0.74953,0.9104,0.93244,0.87233,0.6
1732,0.62363,0.7779,0.48097,0.59347,0.75962,0.60042,0.63937,0.55232,0.
1297,0.13035,0.13053,0.12971,0.12993,0.12791,0.12954,0.13341,0.13019,0
.13439,0.13084,0.13023,0.12863,0.13215,0.13081,0.13016,0.13108,0.1455,
0.95774,1.0102,0.88386,0.86746,0.69355,0.86989,0.87501,0.88502,0.87211
,0.89149,0.7782,0.60406,0.62632,0.62425,0.62038,0.69595,0.60928,0.5726
3,0.13047,0.13194,0.13229,0.12832,0.13024,0.12787,0.13061,0.12886,0.13
041,0.13039,0.13179,0.13062,0.13009,0.13309,0.1306,0.13035,0.1318,0.13

156,0.14475,0.93999,0.88511,0.60971,0.79847,0.92532,0.84976,0.87326,0.
88135,0.87642,0.86927,0.63785,0.51467,0.60986,0.62284,0.59983,0.56471,
0.55559,0.69909,0.13206,0.13076,0.12576,0.13045,0.12899,0.13081,0.1305
5,0.13046,0.13266,0.12893,0.13034,0.13278,0.1303,0.13066,0.13086,0.130
52,0.12604,0.14573,0.1447,0.14508,1.1688,0.82242,0.87015,0.8684,1.0237
,0.91946,0.87193,0.87076,0.74886,0.61924,0.49452,0.52875,0.63701,0.626
13,0.62376,0.13228,0.13012,0.13049,0.12743,0.13546,0.13323,0.12899,0.1
2985,0.13368,0.13111,0.13034,0.1296,0.13285,0.12831,0.14019,0.13304,0.
12797,0.13023,0.14556,0.14559,0.1451,0.14521,0.86646,0.97537,1.0061,0.
82606,0.87661,0.72994,0.82502,0.79842,0.87357,0.40871,0.62181,0.52272,
0.13235,0.13059,0.12097,0.1321,0.13205,0.13102,0.13147,0.13051,0.12745
,0.13065,0.13074,0.12833,0.13144,0.13057,0.12338,0.1296,0.13026,0.1297
9,0.13095,0.12999,0.14436,0.14519,0.14532,0.14503,0.1452,0.14529,0.965
46,0.99675,0.87117,0.86776,1.1843,0.74545,0.90776,0.87102,0.1273,0.130
34,0.12785,0.12997,0.13067,0.1295,0.12695,0.13048,0.13111,0.1302,0.130
8,0.12845,0.13018,0.13196,0.13256,0.13198,0.13261,0.13037,0.13058,0.13
178,0.13047,0.1308,0.13271,0.14527,0.14529,0.1456,0.14562,0.14553,0.14
571,0.88645,0.87107,0.96137,0.91149,0.89867,0.14536,0.14505,0.13114,0.
13,0.13341,0.13041,0.13058,0.13097,0.13092,0.13017,0.13092,0.13015,0.1
3119,0.13019,0.13063,0.12502,0.12657,0.13165,0.13017,0.13064,0.13035,0
.1305,0.13042,0.14447,0.14501,0.14476,0.14524,0.1459,0.14494,0.14508,0
.14582,0.14537,0.14513,0.84839,0.95345,0.14511,0.14553,0.14514,0.14537
,0.12966,0.13401,0.12891,0.13153,0.13191,0.12858,0.13101,0.13101,0.130
43,0.13087,0.13086,0.12876,0.13055,0.13116,0.12927,0.13066,0.13068,0.1
2972,0.13209,0.1447,0.1455,0.14541,0.14496,0.1451,0.14557,0.1455,0.144
71,0.14594,0.14507,0.14537,0.14564,0.1452,0.14482,0.14538,0.14492,0.14
491,0.13036,0.13013,0.13092,0.13293,0.12993,0.13287,0.13204,0.13106,0.
13025,0.12852,0.13011,0.13088,0.12884,0.12937,0.12964,0.13167,0.1453,0
.14535,0.14558,0.14558,0.14495,0.14574,0.14537,0.14566,0.14515,0.14604
,0.14576,0.14507,0.14507,0.14494,0.14488,0.14625,0.14537,0.14489,0.145
59,0.13315,0.13072,0.12727,0.13096,0.13268,0.13045,0.12998,0.13014,0.1
3065,0.13045,0.12927,0.13083,0.1266,0.14536,0.1461,0.14563,0.14475,0.1
452,0.1452,0.14534,0.14492,0.14544,0.14537,0.14494,0.14501,0.14572,0.1
4588,0.14576,0.14543,0.14523,0.145,0.14468,0.14519,0.14454,0.13107,0.1
336,0.13125,0.13162,0.1306,0.13631,0.13061,0.131,0.13073,0.14579,0.145
75,0.14566,0.14552,0.14521,0.14558,0.14492,0.14512,0.14602,0.14548,0.1
4504,0.14583,0.1454,0.14454,0.1456,0.14504,0.14566,0.1446,0.14497,0.14
553,0.14551,0.14505,0.14554,0.14541,0.14492,0.13334,0.13047,0.12932,0.
13525,0.14591,0.14506,0.14504,0.14529,0.14476,0.14541,0.14576,0.14502,
0.14566,0.14548,0.14487,0.14526,0.14457,0.14545,0.14556,0.14503,0.1458
,0.14567,0.14454,0.14553,0.1454,0.14586,0.14493,0.14475,0.14507,0.1448
8,0.14511,0.14507,0.14573,0.14488,0.14539,0.14526,0.14521,0.14493,0.14
56,0.14531,0.14532,0.14563,0.14487,0.14541,0.14539,0.14462,0.1455,0.14
592,0.14476,0.14573,0.14518,0.14526,0.14501,0.14518,0.14554,0.14578,0.
1455,0.1452,0.14545,0.14536,0.1454,0.14573,0.14544,0.14545,0.14484,0.1
4566,0.14543,0.14525,0.14633,0.14494,0.14553,0.14551,0.1461,0.14547,0.
14523,0.14545,0.14548,0.1456,0.14527,0.14513,0.14531,0.14457,0.14557,0
.14474,0.14538,0.14533,0.14533,0.14538,0.14505,0.14541,0.14522,0.14524
,0.14527,0.14538,0.14495,0.14575,0.14519,0.14509,0.14588,0.14586,0.145
49,0.14521,0.14511,0.1458,0.1456,0.1456,0.14576,0.14534,0.14512,0.1457
6,0.14548,0.14528,0.14584,0.14494,0.14479,0.14507,0.14572,0.14534,0.14
482,0.14543,0.14503,0.14482,0.14485,0.14528,0.14487,0.14527,0.14568,0.

14541,0.14549,0.14564,0.14534,0.1449,0.14593,0.14516,0.14567,0.14553,0
.14582,0.14521,0.14475,0.14533,0.14569,0.14478,0.14589,0.14599,0.14552
,0.14542,0.14534,0.14497,0.14563,0.14505,0.14469,0.14504,0.14514,0.145
71,0.14535,0.14528,0.14526,0.14501,0.14469,0.14545,0.14503,0.14588,0.1
4574,0.14553,0.14538,0.1452,0.1456,0.14577,0.14506,0.14458,0.1454,0.14
567,0.14532,0.14542,0.14549,0.1455,0.14566,0.14519,0.14481,0.14528,0.1
4456,0.14542,0.14531,0.14446,0.14534,0.14577,0.14528,0.14533,0.14537,0
.14589,0.14555,0.14516,0.14541,0.14466,0.14519,0.1457,0.14577,0.14488,
0.14549
0.14332,0.14401,0.14364,0.14407,0.14397,0.14361,0.14348,0.14404,0.1443
3,0.14417,0.14482,0.14437,0.14397,0.1436,0.14371,0.14353,0.14471,0.143
4,0.14474,0.14502,0.66776,0.59274,0.63486,0.63023,0.14241,0.14397,0.14
501,0.14398,0.14463,0.14371,0.14502,0.14443,0.14375,0.14437,0.14481,0.
14389,0.14421,0.14327,0.14409,1.1228,0.62405,0.62402,0.63098,0.65245,0
.45959,0.63743,0.14444,0.14352,0.14414,0.14436,0.14396,0.14198,0.14403
,0.14411,0.14471,0.14341,0.14436,0.1439,0.14362,0.14392,0.14448,1.1099
,1.1111,0.50643,0.58186,0.63416,0.63058,0.61779,0.68347,0.68489,0.5613
6,0.14408,0.14434,0.1437,0.14491,0.14424,0.14448,0.14415,0.1437,0.1431
6,0.14443,0.14473,0.14439,0.14375,0.14425,0.14372,1.1194,1.1157,1.1071
,0.63897,0.6741,0.74265,0.59052,0.69637,0.53601,0.82474,0.64221,0.6306
5,0.14365,0.14423,0.14389,0.14433,0.14403,0.14382,0.14428,0.14425,0.14
372,0.14331,0.1443,0.14418,0.14418,0.14284,0.14308,0.14317,1.1293,1.12
38,1.122,1.1256,0.55667,0.66924,0.63635,0.66442,0.6265,0.63321,0.65546
,0.63149,0.63104,0.63482,0.14456,0.14444,0.14418,0.14453,0.14287,0.144
26,0.145,0.14442,0.14399,0.14355,0.14485,0.14323,0.14455,0.14311,0.143
84,0.14492,1.1181,1.129,1.1058,1.1261,1.1206,1.122,0.63008,0.75004,0.6
3097,0.63174,0.63187,0.66626,0.64169,0.63739,0.62738,0.64182,0.14369,0
.14401,0.14405,0.14407,0.14338,0.14484,0.1439,0.14412,0.144,0.14388,0.
14445,0.14333,0.14472,0.14293,0.14422,0.14413,0.14274,1.1158,1.1224,1.
1145,1.1267,1.117,1.1172,0.66583,0.62439,0.70727,0.62167,0.65418,0.724
04,0.63259,0.62953,0.63472,0.60378,0.66686,0.14356,0.14503,0.14354,0.1
4339,0.1439,0.14376,0.14414,0.14317,0.14474,0.14338,0.14436,0.14337,0.
14321,0.13953,0.13996,0.13974,0.14334,1.1214,1.1201,1.1129,1.1202,1.11
73,1.1253,1.135,0.64715,0.69286,0.63423,0.62724,0.67269,0.60532,0.5185
9,0.63504,0.63989,0.61418,0.6326,0.14376,0.14425,0.14331,0.14378,0.142
95,0.14375,0.1425,0.14278,0.14475,0.14323,0.1438,0.13969,0.13898,0.139
01,0.14012,0.13973,0.13925,0.14509,1.1178,1.1267,1.1159,1.122,1.1183,1
.1121,1.1181,0.57811,0.63178,0.63276,0.63927,0.59646,0.68087,0.62845,0
.70635,0.63249,0.63327,0.73615,0.14368,0.14386,0.14479,0.1448,0.14353,
0.14342,0.14303,0.14322,0.14341,0.13984,0.13981,0.14033,0.13944,0.1399
5,0.13983,0.13998,0.13853,0.13945,0.14391,1.1228,1.1124,1.1234,1.1224,
1.1223,1.1158,1.1231,0.67157,0.67967,0.66563,0.62711,0.57586,0.62996,0
.64082,0.5821,0.62803,0.73093,0.61457,0.63439,0.14333,0.1441,0.14418,0
.1441,0.14352,0.14383,0.14021,0.14004,0.13925,0.13979,0.13998,0.13995,
0.13977,0.14056,0.14001,0.13981,0.13926,0.13996,1.1225,1.1207,1.1173,1
.1166,1.1246,1.1251,1.1208,0.62362,0.60476,0.6763,0.62971,0.63215,0.65
161,0.60153,0.69805,0.67697,0.65843,0.61292,0.58538,0.14266,0.14494,0.
14441,0.13972,0.13987,0.13982,0.14057,0.13971,0.14021,0.14031,0.13791,
0.14019,0.14005,0.14008,0.13944,0.13941,0.14027,0.13972,1.1259,1.1254,
1.1219,1.1159,1.1292,1.1237,1.118,1.1195,1.1149,0.62916,0.59759,0.5387
,0.63398,0.6393,0.48283,0.63144,0.61095,0.61783,0.65914,0.63823,0.1403
6,0.14033,0.14048,0.14006,0.1396,0.13924,0.13976,0.14005,0.14171,0.139

45,0.14012,0.13935,0.13995,0.13998,0.14024,0.14004,0.14109,0.14043,1.1
19,1.1182,1.1185,1.1168,1.1246,1.1208,1.1149,1.1327,1.1267,0.66654,0.6
609,0.46979,0.46795,0.49215,0.49662,0.47846,0.49468,0.46521,0.47975,0.
55153,0.13903,0.1396,0.13983,0.1395,0.1397,0.13881,0.14018,0.14054,0.1
3933,0.13993,0.13999,0.13962,0.14016,0.14018,0.13986,0.13938,0.139,0.1
397,1.1156,1.1335,1.1162,1.1218,1.1234,1.1215,1.1218,1.1238,1.113,0.61
64,0.46662,0.52083,0.4585,0.46695,0.47388,0.46611,0.37713,0.42635,0.49
962,0.45782,0.1394,0.13877,0.14018,0.13976,0.13988,0.14041,0.13949,0.1
3939,0.14041,0.14013,0.13895,0.1397,0.14027,0.13972,0.1406,0.14055,0.1
3951,0.14049,1.1285,1.1179,1.1271,1.1304,1.1168,1.11,1.1201,0.63202,0.
70504,0.68565,0.48994,0.4897,0.46558,0.50904,0.51181,0.43811,0.47684,0
.49037,0.42512,0.47962,0.14016,0.14068,0.13999,0.13963,0.13876,0.13945
,0.1394,0.14025,0.13962,0.13968,0.13974,0.13928,0.1399,0.14039,0.14008
,0.13919,0.13923,0.13998,1.1171,1.1242,1.1156,1.1148,1.1118,1.1325,0.6
7653,0.6501,0.60673,0.50602,0.5803,0.46308,0.44478,0.49772,0.37113,0.4
2393,0.43637,0.58386,0.48588,0.53085,0.1396,0.13943,0.13963,0.14017,0.
13905,0.14022,0.14023,0.13853,0.14036,0.13961,0.14002,0.13949,0.13958,
0.13934,0.13984,0.13979,0.13939,0.13931,1.1165,1.1162,1.1247,1.1261,1.
1259,0.59625,0.62633,0.62548,0.60692,0.59664,0.62548,0.39385,0.3713,0.
48261,0.42319,0.50714,0.46657,0.4664,0.46771,0.46753,0.13988,0.13861,0
.13993,0.13947,0.14005,0.1395,0.14062,0.14007,0.13946,0.14006,0.13973,
0.13967,0.13981,0.14002,0.13994,0.13986,0.14073,0.13995,1.121,1.1284,1
.1309,0.63091,0.61888,0.63103,0.63162,0.63912,0.625,0.64373,0.53577,0.
46876,0.44612,0.47065,0.41872,0.3991,0.47797,0.46723,0.36729,0.46522,0
.14104,0.13997,0.13948,0.13945,0.13993,0.13945,0.13902,0.13996,0.14023
,0.13933,0.14003,0.13911,0.1399,0.13914,0.138,0.14015,0.14091,0.14007,
1.1228,0.64798,0.65646,0.60285,0.62519,0.69807,0.63193,0.7241,0.62979,
0.60404,0.34734,0.46534,0.48478,0.45148,0.40573,0.46839,0.51263,0.4526
2,0.44551,0.13985,0.14032,0.13947,0.13918,0.14017,0.1399,0.13978,0.139
28,0.13986,0.1402,0.1398,0.13986,0.13965,0.13957,0.13866,0.14004,0.139
72,0.13593,0.56854,0.62021,0.58721,0.44533,0.67716,0.4447,0.54558,0.65
26,0.67041,0.62993,0.46454,0.47223,0.57024,0.3722,0.44846,0.5618,0.454
4,0.4781,0.42101,0.14027,0.14,0.14028,0.14024,0.13965,0.14088,0.13938,
0.13913,0.13942,0.13902,0.13978,0.13933,0.13991,0.14005,0.13985,0.1404
7,0.14006,0.13587,0.6882,0.72258,0.63801,0.62458,0.50703,0.62698,0.629
77,0.63384,0.62762,0.6418,0.57335,0.45474,0.47003,0.46754,0.46767,0.51
796,0.46044,0.43615,0.13966,0.13938,0.13941,0.14117,0.13978,0.14074,0.
13981,0.13996,0.13979,0.13967,0.13944,0.13979,0.13995,0.13912,0.1397,0
.13926,0.13935,0.13975,0.13542,0.67259,0.63775,0.44912,0.57989,0.66097
,0.61148,0.63289,0.63448,0.63009,0.62648,0.47751,0.39467,0.46065,0.469
22,0.45362,0.4297,0.42552,0.5198,0.13931,0.14006,0.1412,0.13936,0.1403
8,0.13981,0.14011,0.13965,0.13946,0.14048,0.13957,0.13984,0.1407,0.139
16,0.13997,0.13991,0.14128,0.13606,0.13521,0.13565,0.83061,0.59674,0.6
2616,0.62452,0.73274,0.66034,0.62706,0.62709,0.54504,0.46525,0.38319,0
.40615,0.48102,0.47073,0.46894,0.13952,0.1393,0.13944,0.1403,0.13846,0
.13913,0.14028,0.13999,0.1388,0.13956,0.14004,0.13949,0.13917,0.14047,
0.13665,0.13922,0.14044,0.1404,0.13595,0.13623,0.13568,0.13555,0.62227
,0.69854,0.71585,0.59711,0.63298,0.53505,0.5974,0.57829,0.62984,0.3261
1,0.46994,0.4013,0.13916,0.13971,0.14216,0.14004,0.13929,0.13937,0.139
59,0.13963,0.1408,0.14021,0.13994,0.14018,0.13913,0.13982,0.14194,0.14
,0.13942,0.14031,0.14001,0.14029,0.13473,0.13554,0.13578,0.13541,0.135
73,0.13591,0.6894,0.71276,0.62515,0.62359,0.83979,0.5419,0.65424,0.626

75,0.14085,0.1402,0.13975,0.13997,0.14037,0.13932,0.1411,0.13978,0.139
55,0.14034,0.13987,0.14013,0.13985,0.14016,0.13878,0.13954,0.13955,0.1
3965,0.13973,0.13928,0.1398,0.14001,0.1397,0.13578,0.1359,0.13622,0.13
579,0.13602,0.13603,0.63858,0.6271,0.6902,0.65443,0.63975,0.13583,0.13
563,0.13955,0.13972,0.13904,0.13952,0.1396,0.14018,0.14026,0.13939,0.1
3987,0.14039,0.1403,0.14004,0.13989,0.14204,0.14144,0.13962,0.14035,0.
13968,0.14068,0.13968,0.1397,0.13497,0.1356,0.13526,0.13594,0.13646,0.
13505,0.1355,0.13617,0.13607,0.13571,0.61202,0.6853,0.13573,0.13599,0.
13582,0.13572,0.14031,0.13892,0.1402,0.13933,0.13961,0.14095,0.14005,0
.14007,0.13963,0.14014,0.13978,0.14,0.13962,0.14034,0.14039,0.14027,0.
13968,0.13979,0.13971,0.13516,0.1359,0.13597,0.13489,0.13536,0.13617,0
.13568,0.13536,0.13616,0.1355,0.13565,0.1361,0.13584,0.1354,0.13617,0.
1355,0.13543,0.13985,0.13932,0.14003,0.13913,0.14046,0.13932,0.13967,0
.14041,0.13939,0.14028,0.13974,0.14015,0.14059,0.14062,0.13997,0.14017
,0.13555,0.13569,0.13585,0.13609,0.13561,0.13614,0.13573,0.13584,0.135
84,0.13627,0.13608,0.13564,0.13573,0.1354,0.13536,0.13609,0.13571,0.13
533,0.13568,0.13917,0.1399,0.14061,0.14027,0.13925,0.13976,0.14038,0.1
3994,0.1399,0.13992,0.14037,0.13986,0.14145,0.13567,0.13635,0.13616,0.
13536,0.13557,0.13535,0.13576,0.13532,0.13571,0.13548,0.13543,0.13544,
0.136,0.13604,0.1361,0.13619,0.13564,0.13537,0.13535,0.13543,0.13534,0
.14012,0.1391,0.13984,0.13987,0.13937,0.13791,0.13984,0.13998,0.13956,
0.13588,0.136,0.13621,0.13596,0.13552,0.13578,0.13547,0.13583,0.13663,
0.13579,0.13551,0.13624,0.13592,0.13514,0.13624,0.1353,0.13614,0.13517
,0.13541,0.13576,0.13608,0.13537,0.13589,0.13557,0.13562,0.13883,0.140
3,0.14072,0.13905,0.1366,0.13523,0.13541,0.13576,0.13537,0.13607,0.136
14,0.13541,0.13608,0.13584,0.1354,0.13572,0.13476,0.13554,0.13581,0.13
533,0.13639,0.13549,0.13519,0.13559,0.13583,0.1361,0.13579,0.13528,0.1
3542,0.13538,0.13546,0.1356,0.13613,0.13536,0.13572,0.13551,0.13573,0.
13565,0.13591,0.13567,0.13605,0.13565,0.13541,0.13591,0.136,0.13514,0.
13589,0.13659,0.13506,0.136,0.13564,0.1356,0.1356,0.13564,0.13595,0.13
617,0.13571,0.13547,0.13599,0.13575,0.13556,0.13607,0.13602,0.13577,0.
13546,0.13599,0.1356,0.13569,0.1367,0.13531,0.13609,0.13612,0.13647,0.
13569,0.13556,0.1358,0.1359,0.13597,0.13558,0.1353,0.13556,0.13518,0.1
3575,0.13513,0.1361,0.13595,0.13562,0.13551,0.1355,0.13561,0.13575,0.1
3566,0.13534,0.13618,0.13525,0.13653,0.13535,0.13554,0.136,0.13642,0.1
3604,0.13572,0.13547,0.13614,0.13609,0.13581,0.13614,0.13575,0.13545,0
.13614,0.13575,0.13563,0.1361,0.13548,0.13535,0.13565,0.13605,0.13578,
0.13542,0.1358,0.13549,0.1353,0.1354,0.1357,0.1351,0.1356,0.13599,0.13
557,0.13578,0.13593,0.13552,0.13536,0.13621,0.13563,0.13582,0.13613,0.
13605,0.13567,0.13513,0.13584,0.13634,0.13529,0.13606,0.13629,0.13575,
0.13543,0.1358,0.1356,0.13602,0.13567,0.13493,0.13534,0.13585,0.13603,
0.13581,0.13539,0.13543,0.13534,0.13524,0.13587,0.13549,0.13646,0.1359
3,0.13597,0.13551,0.13569,0.13583,0.13618,0.13563,0.13496,0.13602,0.13
611,0.13576,0.13584,0.13577,0.13606,0.13594,0.13569,0.13532,0.13588,0.
13484,0.13572,0.13581,0.13488,0.13582,0.13644,0.13572,0.13549,0.13605,
0.13637,0.13552,0.13551,0.13556,0.13475,0.13569,0.13619,0.13619,0.1355
8,0.13598
0.16157,0.16203,0.16144,0.16186,0.16109,0.16098,0.1623,0.16187,0.16186
,0.16194,0.16256,0.16242,0.16167,0.16123,0.16189,0.16119,0.1625,0.1611
4,0.16231,0.16237,0.51982,0.46951,0.49694,0.49445,0.16056,0.16225,0.16
267,0.16162,0.16222,0.16151,0.16289,0.16203,0.16173,0.16208,0.16249,0.
16197,0.16277,0.16097,0.16177,0.8341,0.49069,0.49001,0.4944,0.50675,0.

37728,0.49872,0.16216,0.16174,0.16219,0.16202,0.16154,0.15968,0.1624,0
.16176,0.1624,0.16109,0.16192,0.16144,0.16136,0.16168,0.16231,0.82395,
0.82012,0.41025,0.46123,0.49715,0.49514,0.48597,0.52803,0.53277,0.4472
5,0.16168,0.16234,0.16141,0.16278,0.16224,0.16193,0.16174,0.16116,0.16
129,0.16217,0.16269,0.16205,0.16143,0.16172,0.16167,0.82613,0.82963,0.
81875,0.49626,0.52489,0.56852,0.4656,0.53977,0.43096,0.62955,0.49999,0
.49707,0.16148,0.16197,0.16198,0.16237,0.16178,0.16126,0.16169,0.16181
,0.16189,0.16135,0.16187,0.16216,0.16204,0.16056,0.16097,0.16165,0.837
87,0.83088,0.8298,0.8329,0.44259,0.51988,0.49907,0.51677,0.49033,0.497
59,0.51237,0.49636,0.49417,0.49769,0.16259,0.16227,0.16246,0.16197,0.1
6036,0.16156,0.16305,0.16233,0.1618,0.16171,0.16312,0.16133,0.16274,0.
16065,0.16193,0.1625,0.82416,0.83829,0.8213,0.83518,0.82889,0.83021,0.
49528,0.5769,0.49473,0.49171,0.49466,0.51962,0.50238,0.50276,0.49263,0
.50468,0.16145,0.16169,0.16187,0.16226,0.16108,0.16264,0.16184,0.16172
,0.16188,0.16221,0.16205,0.16154,0.16217,0.16087,0.1624,0.16191,0.1611
4,0.8248,0.82825,0.82295,0.83914,0.82875,0.82629,0.52123,0.48824,0.547
16,0.4873,0.51103,0.55981,0.49517,0.49205,0.49858,0.47374,0.51867,0.16
135,0.16302,0.16127,0.16149,0.16161,0.16186,0.16189,0.16091,0.16277,0.
16153,0.16188,0.16104,0.16109,0.13833,0.14173,0.13978,0.16095,0.8284,0
.82973,0.82387,0.82592,0.82499,0.82841,0.84847,0.50423,0.53812,0.49714
,0.4919,0.52313,0.4781,0.41723,0.49852,0.50288,0.48328,0.49512,0.16145
,0.16225,0.16135,0.16172,0.16104,0.16212,0.16065,0.16109,0.16274,0.161
16,0.16153,0.13799,0.13534,0.13548,0.1391,0.1371,0.13579,0.16307,0.825
07,0.83313,0.82955,0.83061,0.83111,0.82202,0.82815,0.45807,0.49559,0.4
9432,0.49851,0.47096,0.52676,0.49365,0.54801,0.49723,0.49543,0.56395,0
.16171,0.16194,0.16287,0.16254,0.16127,0.16145,0.16054,0.16143,0.16117
,0.14024,0.13644,0.14151,0.13838,0.14069,0.1396,0.13928,0.13281,0.1384
2,0.16174,0.83109,0.81949,0.83114,0.8299,0.8298,0.82531,0.83008,0.5237
5,0.52828,0.51699,0.49021,0.45911,0.49468,0.50342,0.46173,0.49345,0.56
405,0.4849,0.49714,0.16123,0.16153,0.16204,0.16179,0.1613,0.16129,0.13
922,0.1389,0.13789,0.13881,0.14002,0.13913,0.13926,0.14346,0.13903,0.1
3852,0.13669,0.13837,0.8318,0.83278,0.82999,0.82729,0.83256,0.83107,0.
83164,0.48839,0.47542,0.52648,0.49475,0.49586,0.50806,0.47321,0.54009,
0.52736,0.51592,0.48208,0.46354,0.16025,0.16235,0.16247,0.13793,0.1416
,0.13887,0.14485,0.13904,0.13965,0.1435,0.12913,0.13878,0.13887,0.1409
9,0.1376,0.13487,0.14088,0.1388,0.83104,0.83702,0.83528,0.82999,0.8385
,0.83033,0.82843,0.83164,0.8268,0.49312,0.47231,0.43151,0.49616,0.5004
4,0.39418,0.49479,0.47998,0.48618,0.51407,0.50131,0.1393,0.14289,0.142
62,0.13935,0.13867,0.13764,0.1374,0.13916,0.14869,0.1389,0.13772,0.137
59,0.13912,0.13911,0.13936,0.14069,0.14525,0.14135,0.83139,0.82449,0.8
2492,0.82492,0.83603,0.8296,0.82684,0.84656,0.8363,0.52052,0.51517,0.3
6933,0.36998,0.38703,0.3876,0.37804,0.38679,0.3685,0.37899,0.42814,0.1
3473,0.13814,0.13895,0.13831,0.13873,0.13141,0.13857,0.14028,0.13607,0
.13897,0.14178,0.13602,0.13995,0.13908,0.13916,0.13636,0.13684,0.1373,
0.8252,0.8428,0.82453,0.82838,0.83483,0.8321,0.83159,0.82858,0.82301,0
.46412,0.36724,0.41086,0.36396,0.36979,0.37529,0.36837,0.30645,0.34132
,0.39462,0.36423,0.13855,0.1318,0.13959,0.139,0.13974,0.1395,0.13439,0
.13813,0.14132,0.1388,0.13487,0.13881,0.14181,0.13921,0.14127,0.14192,
0.13809,0.14229,0.83601,0.82622,0.83648,0.83958,0.82252,0.81614,0.8264
,0.47534,0.52876,0.51538,0.38371,0.38758,0.36846,0.39795,0.40215,0.349
49,0.37765,0.38693,0.34153,0.37943,0.13932,0.14361,0.1388,0.13609,0.13
193,0.13414,0.13532,0.13892,0.1386,0.13895,0.13896,0.13832,0.13919,0.1

3946,0.13926,0.13705,0.13095,0.14082,0.82587,0.83249,0.82689,0.8304,0.
82272,0.844,0.506,0.48674,0.45777,0.38411,0.43841,0.36714,0.35089,0.39
273,0.30143,0.33703,0.34643,0.44729,0.38477,0.41416,0.13886,0.13776,0.
13855,0.13799,0.13467,0.14073,0.13818,0.13173,0.13973,0.13744,0.14116,
0.1346,0.13699,0.13947,0.13761,0.13904,0.13711,0.13404,0.82914,0.82806
,0.83027,0.83587,0.83875,0.45038,0.46885,0.47071,0.45885,0.44728,0.467
95,0.31753,0.30343,0.38573,0.33633,0.39416,0.36856,0.36996,0.36987,0.3
6954,0.13853,0.13623,0.14062,0.13855,0.13888,0.13924,0.14365,0.13984,0
.13703,0.13901,0.13829,0.13665,0.13882,0.13809,0.1384,0.13885,0.14219,
0.1406,0.83228,0.83867,0.83754,0.47456,0.46616,0.47203,0.47627,0.47896
,0.47012,0.48454,0.40713,0.37042,0.35508,0.37154,0.32996,0.32186,0.379
53,0.36852,0.29867,0.36816,0.14781,0.13976,0.13857,0.13846,0.1392,0.13
831,0.13676,0.139,0.14289,0.13788,0.13998,0.13525,0.13889,0.13523,0.12
981,0.13968,0.14155,0.13871,0.83089,0.4869,0.49297,0.45308,0.47068,0.5
2321,0.47692,0.5425,0.47258,0.45451,0.28342,0.36781,0.38249,0.35715,0.
32707,0.37261,0.40011,0.3571,0.35472,0.14009,0.13908,0.13717,0.13542,0
.1391,0.13903,0.13829,0.13563,0.13863,0.1403,0.13877,0.13872,0.13854,0
.1364,0.13389,0.13983,0.139,0.11627,0.43086,0.46387,0.44408,0.34158,0.
51059,0.34005,0.41599,0.49224,0.50309,0.47626,0.36814,0.37689,0.44075,
0.30268,0.35634,0.43869,0.36121,0.37649,0.33879,0.14043,0.13944,0.1394
8,0.14039,0.13917,0.14331,0.13885,0.1346,0.13836,0.13314,0.13874,0.138
24,0.14089,0.13798,0.13866,0.14046,0.1393,0.11638,0.5163,0.54155,0.481
11,0.47013,0.38302,0.47212,0.4748,0.47515,0.47217,0.48173,0.44429,0.35
875,0.37089,0.36791,0.36982,0.4043,0.36468,0.34802,0.13891,0.13637,0.1
3624,0.14396,0.13915,0.14302,0.13905,0.14107,0.13888,0.13861,0.13693,0
.13876,0.13947,0.13457,0.13856,0.13814,0.13666,0.13766,0.11567,0.50606
,0.48027,0.34324,0.43564,0.4959,0.45743,0.47919,0.47756,0.47481,0.4698
6,0.37373,0.31605,0.36586,0.36959,0.36119,0.34276,0.33999,0.40643,0.13
635,0.13909,0.1468,0.13823,0.14156,0.13869,0.13937,0.13858,0.13594,0.1
4173,0.13871,0.13687,0.14077,0.13761,0.13893,0.13892,0.14615,0.11596,0
.11533,0.11588,0.6212,0.45135,0.47162,0.46948,0.5487,0.49634,0.47336,0
.47325,0.41371,0.36805,0.3112,0.32684,0.37949,0.37186,0.37084,0.13661,
0.13849,0.13847,0.14301,0.13132,0.135,0.14149,0.1401,0.13385,0.13797,0
.13946,0.13903,0.13548,0.14258,0.12256,0.13527,0.14296,0.14011,0.11613
,0.11616,0.11607,0.11564,0.46825,0.52287,0.53591,0.44913,0.4772,0.4056
4,0.44987,0.43716,0.47174,0.2697,0.37331,0.32292,0.13599,0.13851,0.152
95,0.13767,0.13671,0.13778,0.13765,0.13872,0.14419,0.13961,0.13901,0.1
4194,0.13685,0.13878,0.15048,0.1406,0.13853,0.14082,0.13914,0.1404,0.1
1503,0.11569,0.11599,0.1162,0.11607,0.11617,0.51692,0.53439,0.47116,0.
46644,0.62235,0.40948,0.49179,0.47251,0.14438,0.13984,0.14151,0.14003,
0.13985,0.1393,0.14522,0.13885,0.13802,0.13997,0.13913,0.14179,0.13944
,0.13779,0.13486,0.13705,0.13608,0.13849,0.13878,0.13655,0.13882,0.139
04,0.13617,0.11606,0.1163,0.11633,0.11608,0.11625,0.11624,0.48025,0.47
348,0.51517,0.49148,0.4793,0.11588,0.11588,0.13802,0.13952,0.1345,0.13
841,0.13877,0.13953,0.13939,0.13824,0.13899,0.14074,0.13921,0.14002,0.
13886,0.14857,0.14632,0.13747,0.14019,0.13865,0.14043,0.13869,0.13873,
0.11522,0.11602,0.11565,0.11631,0.11674,0.11535,0.11569,0.11616,0.1162
5,0.11568,0.46206,0.51546,0.11598,0.11592,0.11608,0.11589,0.1408,0.133
63,0.14089,0.13724,0.13741,0.14301,0.13908,0.13912,0.13841,0.13918,0.1
3843,0.14114,0.13874,0.13929,0.14156,0.14002,0.13834,0.13977,0.13659,0
.11536,0.11633,0.11622,0.11499,0.1157,0.11617,0.1161,0.11557,0.11604,0
.11572,0.11607,0.11626,0.11627,0.11541,0.11637,0.11574,0.11579,0.1389,

0.13836,0.13907,0.13528,0.14067,0.13536,0.13742,0.13921,0.13899,0.1422
1,0.13917,0.13889,0.14187,0.14134,0.14001,0.13827,0.11584,0.1159,0.115
93,0.1163,0.1158,0.11611,0.11621,0.11599,0.11616,0.1163,0.11608,0.1156
1,0.1162,0.1159,0.11588,0.11617,0.11558,0.11553,0.1157,0.13494,0.13919
,0.14399,0.13946,0.13544,0.13882,0.14051,0.13978,0.13883,0.13918,0.140
9,0.13872,0.14628,0.11565,0.11637,0.11644,0.11536,0.1157,0.11586,0.116
32,0.11535,0.11596,0.11586,0.11561,0.11561,0.11634,0.1161,0.11626,0.11
605,0.11575,0.11561,0.11572,0.11578,0.116,0.13889,0.13469,0.13803,0.13
813,0.13784,0.12947,0.1388,0.13909,0.13822,0.11612,0.11595,0.11645,0.1
1618,0.11569,0.11594,0.11553,0.1162,0.11668,0.11604,0.11578,0.11658,0.
1159,0.11542,0.11674,0.11553,0.11631,0.11569,0.11557,0.11594,0.11643,0
.11544,0.11595,0.11604,0.11599,0.13406,0.13993,0.14187,0.13255,0.11678
,0.11537,0.11553,0.1161,0.11583,0.11623,0.11618,0.11539,0.11619,0.1162
5,0.11535,0.11568,0.11485,0.11554,0.11605,0.11537,0.11659,0.11559,0.11
519,0.11555,0.11606,0.11648,0.11566,0.11582,0.11543,0.11553,0.11554,0.
11563,0.11651,0.11565,0.11587,0.11573,0.11601,0.11608,0.11599,0.11583,
0.11628,0.11587,0.11567,0.11596,0.11644,0.11546,0.11621,0.11629,0.1154
9,0.11614,0.11574,0.11589,0.11605,0.11579,0.11633,0.11649,0.11578,0.11
565,0.11634,0.11586,0.11571,0.11605,0.11611,0.11581,0.11552,0.11602,0.
11587,0.11593,0.11681,0.11539,0.11628,0.1166,0.1166,0.11578,0.11564,0.
1162,0.11609,0.11605,0.11573,0.11553,0.11596,0.11561,0.11616,0.11544,0
.11591,0.11619,0.11605,0.11549,0.11594,0.11566,0.11594,0.11599,0.11538
,0.11655,0.11541,0.11655,0.11563,0.11576,0.11603,0.11656,0.11623,0.115
81,0.11564,0.11643,0.11611,0.11587,0.11634,0.11586,0.11568,0.11616,0.1
1581,0.1159,0.11636,0.11588,0.11582,0.11592,0.11606,0.11587,0.11529,0.
11589,0.11556,0.11561,0.11545,0.11568,0.11552,0.11573,0.11627,0.11574,
0.11587,0.11611,0.11613,0.11557,0.11621,0.1155,0.11626,0.11642,0.11592
,0.11544,0.11511,0.11592,0.11631,0.11517,0.11618,0.11648,0.11605,0.115
71,0.11587,0.11588,0.11623,0.1158,0.11519,0.11544,0.11593,0.11627,0.11
589,0.11546,0.11573,0.11556,0.11554,0.11571,0.11569,0.11653,0.11596,0.
1161,0.11556,0.11625,0.11601,0.11671,0.11612,0.11522,0.11638,0.11651,0
.11573,0.11644,0.11572,0.11613,0.11581,0.11602,0.11586,0.11623,0.11508
,0.11623,0.11589,0.1152,0.11603,0.11672,0.11595,0.11547,0.11607,0.1165
4,0.11596,0.11557,0.11618,0.11515,0.11525,0.11617,0.11636,0.11576,0.11
615
0.17337,0.17355,0.17302,0.17314,0.17306,0.17281,0.1739,0.17324,0.17281
,0.17358,0.17391,0.17418,0.17364,0.17301,0.1736,0.17301,0.17397,0.1729
7,0.17398,0.17416,0.42871,0.39542,0.41373,0.41028,0.17205,0.17395,0.17
374,0.17324,0.1737,0.17295,0.17434,0.17365,0.17327,0.17354,0.1737,0.17
375,0.17429,0.17248,0.17368,0.65386,0.40938,0.40835,0.41115,0.41981,0.
32815,0.41519,0.17385,0.17341,0.17396,0.17336,0.17309,0.17161,0.17431,
0.17331,0.17414,0.17297,0.17349,0.17338,0.17294,0.17367,0.17405,0.6487
7,0.64453,0.35229,0.38881,0.41357,0.41593,0.40601,0.43559,0.43862,0.37
64,0.17314,0.17414,0.17254,0.17438,0.1739,0.1741,0.17308,0.17282,0.173
42,0.174,0.17461,0.17392,0.17303,0.17322,0.17307,0.64611,0.65114,0.645
19,0.41363,0.43462,0.46184,0.38985,0.44611,0.36667,0.50872,0.41775,0.4
1376,0.17312,0.17372,0.17321,0.1738,0.17331,0.17327,0.17329,0.17348,0.
17338,0.17295,0.1732,0.1742,0.17343,0.17272,0.17275,0.17304,0.65993,0.
65395,0.65322,0.6566,0.37468,0.42901,0.4156,0.42887,0.40994,0.41504,0.
42499,0.41308,0.4127,0.41531,0.17432,0.17364,0.17406,0.17368,0.17152,0
.17302,0.17467,0.17376,0.17374,0.17332,0.17452,0.17285,0.17413,0.17225
,0.1735,0.17464,0.64692,0.6583,0.644,0.66068,0.65011,0.64849,0.41244,0

.47019,0.41178,0.40814,0.41276,0.42909,0.41804,0.42143,0.41025,0.42241
,0.17331,0.17371,0.17338,0.174,0.17283,0.17416,0.17353,0.17348,0.17368
,0.17365,0.17349,0.17352,0.17349,0.17217,0.17403,0.17357,0.17307,0.649
96,0.65208,0.64446,0.65966,0.6497,0.6463,0.43127,0.40691,0.44872,0.404
78,0.42612,0.45957,0.41189,0.41,0.41525,0.39526,0.42842,0.17292,0.1747
2,0.17275,0.17309,0.17312,0.17376,0.17323,0.17252,0.17438,0.17341,0.17
321,0.17277,0.17274,0.13436,0.13979,0.13655,0.17289,0.64921,0.65009,0.
6443,0.6511,0.64661,0.65116,0.66831,0.41911,0.44333,0.41403,0.40797,0.
43029,0.39989,0.35593,0.41543,0.41814,0.40158,0.41306,0.17348,0.17387,
0.17341,0.17368,0.17289,0.17364,0.17252,0.17295,0.17432,0.17284,0.1733
,0.13362,0.12893,0.12932,0.13517,0.13178,0.12956,0.17495,0.64903,0.652
8,0.65252,0.65563,0.6501,0.64375,0.65133,0.38439,0.41413,0.41036,0.413
31,0.39675,0.43438,0.40982,0.45001,0.41431,0.4121,0.46068,0.17357,0.17
36,0.17468,0.17444,0.17299,0.17295,0.17229,0.17307,0.17235,0.1374,0.13
037,0.13901,0.13435,0.13824,0.13615,0.1353,0.12438,0.13435,0.17325,0.6
5575,0.6461,0.64925,0.65242,0.65436,0.6471,0.64746,0.4319,0.43494,0.42
721,0.40607,0.38658,0.41069,0.41884,0.39063,0.40934,0.46155,0.40669,0.
41568,0.17338,0.17283,0.17406,0.17366,0.17295,0.17316,0.13538,0.13477,
0.1336,0.13474,0.13672,0.13506,0.13536,0.14235,0.13509,0.13442,0.1315,
0.13353,0.65735,0.65418,0.65297,0.65295,0.65046,0.65211,0.6536,0.40683
,0.3976,0.43525,0.41096,0.41234,0.41931,0.39775,0.44493,0.43503,0.4280
4,0.40286,0.39155,0.17192,0.17396,0.17386,0.1333,0.14035,0.13473,0.145
05,0.13502,0.13634,0.14262,0.11861,0.13463,0.13497,0.13848,0.13271,0.1
2823,0.13839,0.13488,0.65217,0.6589,0.65579,0.65055,0.66131,0.65055,0.
65185,0.65823,0.64495,0.41159,0.39452,0.3668,0.41286,0.41691,0.33965,0
.41241,0.39925,0.40725,0.42478,0.41768,0.1351,0.14162,0.14135,0.13526,
0.13462,0.13311,0.13229,0.13514,0.15141,0.13498,0.13228,0.13304,0.1350
8,0.13525,0.13537,0.1381,0.14562,0.13868,0.65468,0.64677,0.64937,0.648
89,0.66008,0.64924,0.65343,0.66883,0.66044,0.43,0.42752,0.30692,0.3079
1,0.31892,0.31993,0.31482,0.31919,0.30349,0.31657,0.35099,0.12813,0.13
375,0.13499,0.13379,0.1348,0.12189,0.13431,0.13702,0.13013,0.13474,0.1
4022,0.12974,0.13626,0.13502,0.13533,0.13039,0.13174,0.13198,0.65032,0
.66447,0.64884,0.64758,0.65686,0.65611,0.6565,0.65491,0.64811,0.36845,
0.30469,0.34141,0.30356,0.30671,0.31038,0.30809,0.25944,0.28767,0.3278
2,0.30623,0.1344,0.12303,0.13566,0.13475,0.13668,0.13531,0.12677,0.134
,0.1386,0.13472,0.1279,0.13455,0.13991,0.13581,0.13866,0.13976,0.13324
,0.14077,0.65826,0.64959,0.66467,0.66205,0.64953,0.63773,0.6511,0.3751
3,0.42059,0.40683,0.31607,0.32312,0.30687,0.32934,0.33557,0.29367,0.31
485,0.32252,0.28871,0.31588,0.1352,0.14325,0.1347,0.13002,0.12285,0.12
658,0.12855,0.135,0.13456,0.13512,0.13512,0.13452,0.13527,0.13554,0.13
542,0.13234,0.12117,0.13817,0.6518,0.65362,0.64975,0.64965,0.64431,0.6
6692,0.39793,0.38392,0.36114,0.30341,0.34856,0.3064,0.29128,0.32655,0.
25662,0.28083,0.28843,0.35957,0.32143,0.3415,0.13502,0.13323,0.13455,0
.13303,0.12783,0.13805,0.13292,0.12268,0.13607,0.13246,0.13918,0.12729
,0.13159,0.13593,0.13265,0.13507,0.13232,0.12649,0.65212,0.65206,0.653
84,0.6571,0.65706,0.35849,0.36887,0.37352,0.36429,0.35318,0.37058,0.26
724,0.25909,0.32273,0.28211,0.32423,0.30622,0.30802,0.30618,0.309,0.13
434,0.13098,0.13788,0.13459,0.13473,0.13599,0.14296,0.13629,0.13182,0.
13522,0.13389,0.13107,0.13489,0.13339,0.13404,0.13449,0.14004,0.13788,
0.64967,0.65885,0.66123,0.37617,0.36996,0.37127,0.37649,0.37958,0.3714
7,0.38461,0.32356,0.30971,0.2979,0.3099,0.27146,0.27138,0.31669,0.3056
6,0.25357,0.30653,0.15077,0.13641,0.13465,0.13441,0.13519,0.1345,0.131

65,0.13509,0.14181,0.13333,0.13704,0.1288,0.13492,0.12875,0.1196,0.135
98,0.13859,0.13455,0.65543,0.38368,0.38915,0.35979,0.37235,0.41129,0.3
7747,0.43003,0.37277,0.35828,0.24186,0.30599,0.31928,0.29767,0.27707,0
.31069,0.32826,0.29778,0.29781,0.13715,0.13462,0.13192,0.12908,0.13495
,0.13496,0.13372,0.12955,0.13439,0.13726,0.13462,0.13471,0.13424,0.130
36,0.12659,0.13658,0.13519,0.096478,0.34308,0.36703,0.35128,0.27254,0.
40601,0.26942,0.33123,0.38888,0.39797,0.37582,0.30604,0.3177,0.36007,0
.25613,0.29781,0.36209,0.30103,0.31125,0.28644,0.13736,0.13592,0.13564
,0.13734,0.13564,0.14241,0.13519,0.12744,0.13415,0.12527,0.13468,0.134
29,0.13847,0.13312,0.13492,0.13708,0.13521,0.096545,0.40894,0.42753,0.
38156,0.3727,0.30352,0.37063,0.37592,0.37714,0.37384,0.38453,0.36493,0
.29845,0.3083,0.30489,0.3071,0.33288,0.3049,0.2922,0.13449,0.13087,0.1
3049,0.14301,0.13553,0.14204,0.13503,0.13886,0.13514,0.13448,0.13156,0
.13451,0.13594,0.1277,0.13448,0.13404,0.13122,0.13242,0.095973,0.39832
,0.37922,0.27292,0.34551,0.39362,0.36151,0.37904,0.3796,0.37613,0.3702
4,0.30903,0.2643,0.30505,0.30844,0.30398,0.2858,0.28501,0.33682,0.1308
2,0.13501,0.14818,0.1339,0.13963,0.13478,0.13592,0.13455,0.12949,0.139
7,0.13438,0.13095,0.13751,0.13339,0.13494,0.13486,0.14684,0.096286,0.0
95557,0.096288,0.49063,0.35758,0.37475,0.37074,0.43146,0.39452,0.3756,
0.37664,0.32809,0.3068,0.2645,0.27581,0.31773,0.3093,0.30865,0.13086,0
.13479,0.13441,0.14237,0.12205,0.12825,0.13929,0.13697,0.12652,0.13332
,0.13578,0.13576,0.12912,0.14107,0.1076,0.12873,0.14199,0.13694,0.0963
6,0.096195,0.096376,0.09592,0.37138,0.41396,0.42387,0.35473,0.37782,0.
32396,0.35642,0.34629,0.3746,0.23338,0.3122,0.27364,0.13015,0.1343,0.1
593,0.1327,0.13109,0.13315,0.13271,0.13448,0.14357,0.1358,0.13528,0.14
037,0.1317,0.13489,0.15431,0.13769,0.13446,0.13769,0.13494,0.13738,0.0
95562,0.09617,0.095988,0.096244,0.096118,0.096541,0.40727,0.42193,0.37
211,0.36776,0.49559,0.3247,0.3899,0.37309,0.14394,0.13594,0.13978,0.13
666,0.13597,0.13597,0.14548,0.13493,0.13379,0.13669,0.13493,0.13991,0.
1359,0.13282,0.12851,0.13175,0.12996,0.13441,0.13482,0.13113,0.13481,0
.13508,0.13011,0.095979,0.096863,0.096528,0.096361,0.096399,0.09656,0.
38034,0.37274,0.4063,0.38977,0.37618,0.096121,0.096201,0.13336,0.13617
,0.12758,0.13427,0.13479,0.13548,0.13519,0.13411,0.13504,0.13754,0.135
3,0.13664,0.13497,0.15105,0.14732,0.13221,0.137,0.13448,0.13727,0.1346
5,0.13458,0.095474,0.096304,0.096154,0.096829,0.096905,0.09577,0.09583
2,0.096234,0.096638,0.095918,0.36807,0.40704,0.09636,0.096179,0.096024
,0.096243,0.1381,0.12613,0.13826,0.13191,0.13184,0.14176,0.1349,0.1351
6,0.13436,0.13492,0.13422,0.13883,0.13465,0.13522,0.1392,0.13643,0.134
03,0.13644,0.13105,0.09586,0.096392,0.096525,0.095573,0.096051,0.09645
3,0.096666,0.095877,0.096167,0.095727,0.096057,0.096518,0.096739,0.095
576,0.09638,0.095899,0.096276,0.13494,0.13399,0.13484,0.12872,0.13788,
0.1286,0.13232,0.13508,0.13548,0.14055,0.13577,0.1345,0.14005,0.13859,
0.13689,0.13349,0.095885,0.096179,0.096033,0.096676,0.095862,0.096191,
0.096774,0.096297,0.096299,0.096615,0.096217,0.095744,0.096531,0.09607
5,0.096162,0.096127,0.095825,0.095809,0.096089,0.12825,0.13547,0.14335
,0.13538,0.12916,0.1349,0.13742,0.13638,0.13478,0.13548,0.13823,0.1348
2,0.14689,0.096111,0.096711,0.096553,0.0956,0.095925,0.096049,0.096316
,0.0956,0.096271,0.096053,0.095803,0.096049,0.096751,0.096223,0.096775
,0.096352,0.096076,0.095826,0.096266,0.096319,0.096047,0.13478,0.12774
,0.13343,0.13355,0.13347,0.11919,0.13464,0.13516,0.13383,0.096414,0.09
6437,0.096553,0.096427,0.096177,0.096015,0.095916,0.096175,0.097005,0.
096204,0.095936,0.096593,0.096388,0.095766,0.096753,0.095779,0.096594,

0.096131,0.095649,0.096045,0.096684,0.095583,0.096158,0.09627,0.096295
,0.12695,0.13648,0.13949,0.12377,0.096924,0.095622,0.095793,0.096563,0
.096063,0.096834,0.096622,0.095297,0.096315,0.096338,0.095703,0.095907
,0.095218,0.095944,0.096242,0.095959,0.096526,0.095609,0.095393,0.0957
17,0.096241,0.096619,0.096092,0.096162,0.096092,0.095615,0.095654,0.09
603,0.096842,0.096197,0.095988,0.09599,0.096356,0.096559,0.096218,0.09
6374,0.096597,0.095828,0.096012,0.096316,0.097022,0.095914,0.096748,0.
096387,0.095778,0.096491,0.096139,0.096081,0.096513,0.096121,0.096259,
0.096642,0.095738,0.096126,0.096612,0.096124,0.096,0.096046,0.096144,0
.095867,0.095928,0.096291,0.096186,0.096473,0.097106,0.095743,0.096488
,0.096692,0.096917,0.095872,0.095691,0.0962,0.096203,0.096236,0.095712
,0.095544,0.096164,0.096106,0.096153,0.095613,0.095986,0.096414,0.0961
46,0.096063,0.096123,0.096141,0.096195,0.096723,0.095408,0.097005,0.09
5642,0.096802,0.096033,0.096074,0.09614,0.096781,0.096115,0.096015,0.0
95902,0.096712,0.096324,0.096096,0.096443,0.096095,0.095928,0.096304,0
.096192,0.095845,0.096631,0.096172,0.095923,0.096378,0.096507,0.096076
,0.095783,0.096042,0.095765,0.095656,0.095785,0.096154,0.095841,0.0958
84,0.096773,0.095993,0.096161,0.096376,0.096602,0.095507,0.096455,0.09
6027,0.096267,0.09677,0.096553,0.095927,0.095406,0.096248,0.096577,0.0
95648,0.096621,0.096391,0.096245,0.09568,0.095766,0.096251,0.096765,0.
096068,0.095811,0.09574,0.096276,0.096178,0.095996,0.095872,0.095999,0
.095955,0.09587,0.096002,0.096096,0.096801,0.096222,0.096411,0.096147,
0.096864,0.096297,0.097019,0.095863,0.095583,0.096852,0.096999,0.09606
9,0.096428,0.095717,0.096333,0.095891,0.096238,0.096488,0.096572,0.095
489,0.096732,0.096375,0.095542,0.096434,0.096933,0.096464,0.095606,0.0
95983,0.096944,0.096227,0.095941,0.096439,0.095508,0.095439,0.096356,0
.096597,0.09625,0.09615
0.18109,0.18128,0.18119,0.18037,0.18123,0.18044,0.18187,0.18107,0.1803
4,0.1816,0.18148,0.18201,0.1815,0.18122,0.18132,0.18105,0.1812,0.18158
,0.18187,0.18186,0.37495,0.3482,0.36177,0.3611,0.17989,0.1819,0.18163,
0.1811,0.18137,0.18096,0.182,0.18138,0.18103,0.1813,0.18154,0.18142,0.
18209,0.18036,0.18136,0.54485,0.35935,0.35992,0.36057,0.36587,0.29857,
0.36473,0.18167,0.18204,0.18198,0.18106,0.18108,0.17966,0.18209,0.1814
5,0.18132,0.18114,0.18071,0.18086,0.18083,0.1817,0.18206,0.53916,0.534
77,0.31469,0.34536,0.36146,0.36334,0.35639,0.38045,0.37992,0.33463,0.1
8124,0.1821,0.18017,0.18229,0.18202,0.18184,0.18124,0.18116,0.18109,0.
18155,0.18201,0.18174,0.1813,0.18126,0.18132,0.54009,0.54315,0.53416,0
.36102,0.3777,0.39826,0.3435,0.38881,0.32793,0.43572,0.36696,0.36264,0
.18091,0.18164,0.18089,0.18202,0.18125,0.18113,0.18093,0.18169,0.18104
,0.18084,0.18168,0.18223,0.18108,0.18107,0.18083,0.18059,0.54934,0.550
48,0.54713,0.54555,0.33442,0.37399,0.3657,0.37494,0.36176,0.36355,0.36
964,0.36334,0.36217,0.36397,0.1823,0.18133,0.18155,0.18141,0.17966,0.1
8097,0.18234,0.18139,0.1814,0.18097,0.18233,0.18087,0.18205,0.18053,0.
18142,0.18224,0.54289,0.55071,0.538,0.54879,0.54561,0.53864,0.36254,0.
40617,0.36263,0.35857,0.36354,0.37459,0.3682,0.37158,0.36243,0.371,0.1
8146,0.18198,0.18143,0.18189,0.18044,0.18172,0.18161,0.18179,0.18131,0
.18148,0.18145,0.18162,0.1813,0.18005,0.18202,0.18147,0.18081,0.54134,
0.54965,0.53603,0.54489,0.54036,0.53482,0.37482,0.35591,0.39148,0.3566
5,0.37536,0.39677,0.36273,0.36143,0.36541,0.34842,0.37438,0.18085,0.18
27,0.18071,0.18084,0.18091,0.18142,0.18077,0.18054,0.18215,0.18121,0.1
8165,0.18037,0.18086,0.13026,0.1374,0.13289,0.18045,0.54149,0.54598,0.
53333,0.54041,0.53954,0.54196,0.5625,0.36816,0.38678,0.3631,0.35947,0.

37256,0.35238,0.31842,0.36487,0.36632,0.35382,0.36142,0.18132,0.18177,
0.1811,0.18137,0.18076,0.18162,0.18046,0.1808,0.18228,0.18094,0.18118,
0.12929,0.1229,0.12347,0.13108,0.12656,0.12365,0.18281,0.54003,0.55002
,0.54732,0.54407,0.54364,0.53562,0.54182,0.34028,0.36272,0.36009,0.363
1,0.35158,0.37804,0.36209,0.39016,0.36326,0.36064,0.3977,0.18181,0.181
66,0.18214,0.18244,0.18082,0.18104,0.1807,0.18082,0.18011,0.1339,0.124
71,0.13621,0.13017,0.13514,0.13247,0.13119,0.11676,0.13019,0.18122,0.5
4665,0.5418,0.53903,0.54406,0.54523,0.53682,0.54033,0.37811,0.38035,0.
37365,0.35684,0.34527,0.36082,0.36891,0.34466,0.35819,0.40028,0.35841,
0.3649,0.18092,0.18062,0.1819,0.18158,0.18084,0.18121,0.13111,0.1306,0
.12941,0.13068,0.13322,0.13074,0.13161,0.14047,0.13081,0.13019,0.12625
,0.12904,0.55119,0.55129,0.54621,0.54737,0.54273,0.54408,0.54428,0.357
88,0.35146,0.37968,0.36242,0.36373,0.36784,0.35103,0.38807,0.37892,0.3
7504,0.3577,0.34646,0.18009,0.18187,0.1824,0.1285,0.13805,0.13045,0.14
419,0.13069,0.13248,0.14082,0.10956,0.13074,0.13109,0.13555,0.12786,0.
12193,0.13522,0.13069,0.54096,0.5482,0.54858,0.54261,0.55167,0.54278,0
.55,0.54641,0.53821,0.36135,0.34977,0.32751,0.36479,0.36673,0.30626,0.
362,0.35253,0.35993,0.36977,0.36752,0.13102,0.13957,0.13913,0.13123,0.
13045,0.12849,0.12734,0.13104,0.1522,0.131,0.12663,0.12827,0.13119,0.1
3087,0.13102,0.13476,0.14488,0.13566,0.54558,0.53724,0.54359,0.53713,0
.54749,0.54146,0.54229,0.56068,0.55129,0.37776,0.37288,0.2687,0.26921,
0.27585,0.27704,0.27647,0.27487,0.26448,0.27611,0.30333,0.12179,0.1293
4,0.13069,0.12933,0.13042,0.11342,0.12976,0.13335,0.12461,0.13042,0.13
794,0.12392,0.13237,0.13075,0.13122,0.12485,0.12673,0.12685,0.54253,0.
55882,0.53798,0.53503,0.54914,0.5529,0.54441,0.54437,0.54212,0.30659,0
.2669,0.2981,0.26543,0.26583,0.27023,0.26916,0.22965,0.25166,0.28723,0
.26876,0.13037,0.11498,0.13145,0.13048,0.13296,0.13091,0.11989,0.12963
,0.13548,0.13038,0.12168,0.13036,0.13711,0.13194,0.13536,0.13698,0.128
4,0.13881,0.55162,0.5412,0.55318,0.55346,0.53695,0.52798,0.54016,0.311
59,0.35395,0.34065,0.27283,0.28365,0.2683,0.2865,0.29198,0.25944,0.274
46,0.28135,0.25353,0.27486,0.13099,0.14202,0.13047,0.12414,0.11512,0.1
1971,0.12229,0.13099,0.13038,0.13096,0.1312,0.13045,0.13094,0.13128,0.
13123,0.12768,0.11248,0.13499,0.54308,0.54732,0.54014,0.54148,0.53707,
0.56168,0.32959,0.32238,0.29956,0.25282,0.29103,0.26719,0.25258,0.2848
1,0.22745,0.24574,0.25371,0.30702,0.28106,0.29557,0.13096,0.12817,0.13
042,0.12804,0.12148,0.1348,0.1278,0.11459,0.13228,0.12751,0.13635,0.12
053,0.12623,0.13203,0.12758,0.13093,0.12744,0.11963,0.54489,0.54266,0.
54711,0.54958,0.54912,0.29901,0.30492,0.30993,0.30253,0.29349,0.30889,
0.23535,0.23069,0.28527,0.24802,0.2803,0.2683,0.2696,0.26621,0.26854,0
.13008,0.12528,0.1347,0.1302,0.13063,0.13244,0.14127,0.13228,0.12684,0
.13093,0.12937,0.12549,0.13068,0.12885,0.1294,0.13052,0.13725,0.13455,
0.54639,0.55042,0.54842,0.31565,0.30963,0.30806,0.31264,0.3154,0.30948
,0.32283,0.26987,0.27127,0.26145,0.27143,0.23624,0.24009,0.27663,0.267
03,0.2247,0.26725,0.15185,0.13279,0.13043,0.1303,0.13102,0.1304,0.1265
8,0.13092,0.1399,0.12882,0.13365,0.12266,0.13059,0.12254,0.11086,0.132
22,0.13535,0.13033,0.55137,0.31832,0.32444,0.30164,0.30996,0.34315,0.3
1425,0.35881,0.31311,0.29814,0.2148,0.26906,0.27924,0.26087,0.24483,0.
2719,0.28182,0.25909,0.26055,0.13352,0.13015,0.12683,0.1233,0.13046,0.
13069,0.12885,0.12363,0.12998,0.13356,0.13042,0.13045,0.13028,0.12477,
0.11969,0.13281,0.13126,0.079909,0.28724,0.30436,0.2928,0.22661,0.3399
2,0.22384,0.27532,0.3237,0.33089,0.31379,0.2692,0.28037,0.30782,0.2267
4,0.26119,0.31511,0.26381,0.27196,0.25309,0.13421,0.13181,0.13189,0.13

426,0.13196,0.14071,0.13128,0.12101,0.12995,0.11822,0.13037,0.13021,0.
13569,0.1283,0.1308,0.13344,0.13084,0.080264,0.34036,0.35908,0.32054,0
.3107,0.25188,0.3095,0.31284,0.31358,0.31228,0.31808,0.31572,0.26092,0
.26741,0.26583,0.26707,0.28748,0.26667,0.25519,0.13042,0.12563,0.12505
,0.14128,0.13166,0.14014,0.1307,0.13601,0.13131,0.13018,0.12629,0.1301
9,0.13215,0.12135,0.13005,0.12964,0.12584,0.12745,0.079645,0.33263,0.3
1696,0.22641,0.28939,0.32744,0.30083,0.31663,0.31744,0.31275,0.30864,0
.26893,0.23087,0.26759,0.26885,0.26777,0.24971,0.25056,0.2919,0.12549,
0.13069,0.14808,0.12995,0.13682,0.13077,0.13193,0.13012,0.12366,0.1368
5,0.13016,0.12524,0.13406,0.12876,0.1305,0.13079,0.14632,0.080113,0.07
9653,0.079902,0.40934,0.29939,0.31332,0.31079,0.35994,0.32927,0.31444,
0.31438,0.27511,0.2681,0.23526,0.24347,0.27842,0.2693,0.26934,0.12533,
0.13062,0.13023,0.14054,0.11381,0.1219,0.13647,0.13329,0.11985,0.12855
,0.13193,0.13193,0.12315,0.13883,0.095447,0.12243,0.14018,0.1331,0.079
992,0.079959,0.079975,0.079245,0.30839,0.34378,0.35293,0.29638,0.31293
,0.27065,0.29747,0.28881,0.31348,0.20939,0.27138,0.24246,0.12473,0.130
15,0.16307,0.12786,0.12573,0.12852,0.12781,0.13004,0.14212,0.13179,0.1
3103,0.13809,0.12678,0.13066,0.15612,0.13438,0.13009,0.13429,0.1306,0.
13394,0.079699,0.080085,0.079824,0.079795,0.080019,0.0803,0.33935,0.35
048,0.30996,0.30773,0.41024,0.26802,0.32553,0.31169,0.14279,0.13202,0.
13723,0.13283,0.13224,0.13228,0.14417,0.13087,0.12928,0.13311,0.13079,
0.1372,0.13177,0.12801,0.12256,0.12661,0.12407,0.13006,0.13037,0.12584
,0.13039,0.13073,0.12443,0.079892,0.08051,0.080284,0.080073,0.080363,0
.080039,0.31429,0.3119,0.33936,0.32453,0.31207,0.07967,0.079985,0.1284
4,0.13232,0.1209,0.12999,0.1306,0.13129,0.13104,0.12984,0.13091,0.1342
3,0.13117,0.13281,0.13068,0.15163,0.14678,0.12733,0.13345,0.13012,0.13
348,0.13047,0.13055,0.079492,0.079717,0.080079,0.08042,0.080724,0.0792
49,0.079655,0.080296,0.080146,0.079843,0.30693,0.33739,0.080014,0.0798
46,0.079828,0.080004,0.13484,0.1191,0.13526,0.12679,0.12677,0.14,0.130
89,0.13087,0.13021,0.13066,0.1297,0.13581,0.13055,0.13107,0.13644,0.13
282,0.1298,0.13275,0.12568,0.079231,0.080487,0.079729,0.07949,0.079941
,0.080244,0.08059,0.079676,0.079857,0.079559,0.079798,0.08045,0.080233
,0.079157,0.080147,0.079465,0.079949,0.13082,0.12983,0.13053,0.12274,0
.13434,0.12263,0.12724,0.13105,0.13176,0.13806,0.13186,0.13028,0.13764
,0.13578,0.1333,0.12871,0.079508,0.079894,0.079719,0.080199,0.079773,0
.079864,0.080202,0.079973,0.080041,0.080305,0.080011,0.079596,0.08015,
0.079778,0.080015,0.079779,0.079642,0.079393,0.080047,0.12183,0.13143,
0.14185,0.13124,0.12283,0.1306,0.1339,0.13247,0.13083,0.13126,0.13543,
0.13068,0.1462,0.079925,0.08051,0.080341,0.07936,0.079636,0.080051,0.0
79996,0.079028,0.079573,0.079739,0.079653,0.07967,0.080408,0.080245,0.
080454,0.080002,0.079833,0.079612,0.079893,0.0802,0.08023,0.13057,0.12
133,0.12886,0.12887,0.12876,0.11011,0.13072,0.13113,0.12922,0.080433,0
.080385,0.080457,0.080329,0.079879,0.079365,0.079775,0.079676,0.080705
,0.08016,0.07991,0.080327,0.079824,0.079848,0.080486,0.079402,0.0804,0
.080088,0.079376,0.079781,0.080515,0.079335,0.079987,0.079925,0.079998
,0.12046,0.13273,0.13681,0.11614,0.08069,0.079445,0.079632,0.080508,0.
079932,0.080834,0.080411,0.07886,0.079851,0.079764,0.079416,0.080108,0
.079121,0.079678,0.080219,0.079982,0.080193,0.079175,0.078956,0.079498
,0.079819,0.080085,0.079659,0.080012,0.08002,0.079508,0.079624,0.07994
2,0.080429,0.080131,0.079587,0.079793,0.079896,0.080197,0.080051,0.080
212,0.080075,0.079607,0.079849,0.079832,0.080344,0.079655,0.080317,0.0
79872,0.079833,0.080205,0.079831,0.080117,0.080243,0.079689,0.079991,0

.080169,0.079516,0.079602,0.079949,0.079744,0.079711,0.079947,0.07994,
0.079539,0.079987,0.079973,0.079789,0.080274,0.080832,0.079539,0.08044
1,0.080262,0.080614,0.079689,0.07956,0.079859,0.079983,0.080181,0.0793
92,0.079747,0.080139,0.080075,0.079993,0.079354,0.079909,0.080316,0.08
0203,0.080253,0.080029,0.080028,0.079817,0.080538,0.079311,0.080614,0.
079358,0.080579,0.079946,0.079994,0.079872,0.080769,0.0797,0.079855,0.
079787,0.080087,0.079963,0.079804,0.08036,0.079947,0.079828,0.08017,0.
079943,0.079574,0.080593,0.079526,0.079905,0.080033,0.079955,0.079712,
0.079578,0.080069,0.079582,0.079502,0.079497,0.080035,0.079681,0.07983
2,0.080564,0.079718,0.079771,0.080118,0.080454,0.079206,0.079963,0.079
76,0.080078,0.080403,0.080391,0.079651,0.079238,0.079793,0.080279,0.07
9538,0.080336,0.079974,0.079827,0.079442,0.079467,0.079906,0.080456,0.
079881,0.079407,0.079403,0.080138,0.079924,0.079589,0.079875,0.079869,
0.079828,0.079652,0.079842,0.080131,0.080179,0.079958,0.080199,0.08011
2,0.080647,0.080116,0.080843,0.079746,0.079141,0.080222,0.080816,0.079
851,0.08009,0.079442,0.079885,0.079476,0.079838,0.080612,0.080345,0.07
911,0.080325,0.079922,0.079324,0.079995,0.080592,0.08007,0.0794,0.0798
91,0.080761,0.080026,0.079921,0.080194,0.079249,0.079332,0.080061,0.08
0394,0.080084,0.079965
0.18673,0.18667,0.18696,0.18581,0.1866,0.1859,0.18736,0.18669,0.18551,
0.18666,0.18716,0.18701,0.18695,0.1864,0.18634,0.18669,0.18657,0.18677
,0.18752,0.18712,0.34113,0.31785,0.33169,0.3301,0.1852,0.18702,0.18749
,0.18652,0.18661,0.18679,0.18709,0.18652,0.18622,0.18687,0.1871,0.1868
1,0.18774,0.18594,0.18704,0.47407,0.32897,0.32974,0.3304,0.33533,0.280
49,0.33378,0.18728,0.18768,0.18746,0.18613,0.18645,0.18509,0.18788,0.1
8698,0.18661,0.18677,0.18632,0.186,0.18628,0.1874,0.18753,0.47446,0.47
171,0.29394,0.3192,0.33258,0.33101,0.32688,0.34631,0.34589,0.30914,0.1
8701,0.18743,0.18596,0.18763,0.1876,0.18704,0.1868,0.18673,0.18707,0.1
8726,0.18752,0.18712,0.18662,0.18648,0.18673,0.47456,0.48085,0.46901,0
.33032,0.3438,0.35685,0.31534,0.35493,0.303,0.39393,0.33528,0.33274,0.
18637,0.18689,0.18642,0.18768,0.18624,0.18648,0.1862,0.18742,0.18663,0
.18619,0.18718,0.18768,0.18635,0.18673,0.18685,0.18615,0.48325,0.48646
,0.47842,0.47942,0.31011,0.33983,0.33387,0.34067,0.33169,0.33352,0.339
44,0.33022,0.33073,0.33368,0.18772,0.18666,0.18704,0.18705,0.18575,0.1
8685,0.18768,0.18712,0.18682,0.18646,0.18755,0.18613,0.18761,0.18623,0
.18657,0.18753,0.46976,0.4825,0.47413,0.47545,0.48002,0.47054,0.33081,
0.36625,0.33347,0.3291,0.33412,0.34083,0.33692,0.34219,0.33283,0.33965
,0.18711,0.18699,0.18648,0.18751,0.18604,0.18763,0.18694,0.18726,0.186
51,0.18665,0.18705,0.18758,0.18707,0.18574,0.18718,0.18703,0.18633,0.4
757,0.48424,0.47264,0.47777,0.47989,0.46702,0.34333,0.3267,0.35405,0.3
2854,0.3424,0.35832,0.33121,0.33086,0.33527,0.31916,0.3423,0.18658,0.1
8809,0.18634,0.18651,0.18613,0.18677,0.18613,0.18594,0.1876,0.18663,0.
18674,0.18597,0.18658,0.12679,0.1351,0.12998,0.18566,0.47616,0.47969,0
.46544,0.46962,0.46989,0.47448,0.49101,0.33457,0.35187,0.33252,0.32999
,0.3396,0.32162,0.29574,0.33297,0.33474,0.3264,0.33102,0.1871,0.18693,
0.18693,0.18683,0.18638,0.18684,0.18626,0.18668,0.18787,0.18609,0.1860
9,0.12575,0.11825,0.11881,0.12763,0.12235,0.11893,0.18803,0.47255,0.48
466,0.4853,0.47792,0.47571,0.47314,0.47914,0.31342,0.3335,0.33012,0.33
232,0.32256,0.34433,0.3301,0.35311,0.3315,0.33097,0.35997,0.1871,0.187
03,0.18809,0.18768,0.18602,0.18694,0.18637,0.18659,0.18619,0.13119,0.1
2035,0.13401,0.12653,0.13296,0.12941,0.12788,0.11083,0.12683,0.18672,0
.47935,0.4784,0.47043,0.4808,0.47838,0.47008,0.47594,0.34526,0.34693,0

.34264,0.32819,0.31882,0.33329,0.33646,0.31837,0.32818,0.36166,0.32832
,0.33541,0.18653,0.18643,0.18705,0.18711,0.18618,0.1867,0.12793,0.127,
0.12585,0.12741,0.13019,0.12736,0.12831,0.13872,0.12744,0.12694,0.1221
3,0.12543,0.48307,0.48078,0.47846,0.48035,0.47744,0.47438,0.47625,0.32
794,0.32343,0.34508,0.33343,0.33622,0.33525,0.32372,0.35263,0.34546,0.
3425,0.32973,0.31913,0.1857,0.18714,0.18791,0.12467,0.13611,0.12701,0.
14306,0.12738,0.1294,0.13929,0.10237,0.12732,0.12774,0.13308,0.12408,0
.11694,0.13264,0.12729,0.47695,0.48338,0.47526,0.47547,0.48354,0.48086
,0.47859,0.48006,0.47039,0.33153,0.32277,0.30417,0.33375,0.33634,0.286
36,0.33046,0.32433,0.33055,0.3392,0.33614,0.12756,0.13778,0.13705,0.12
779,0.12706,0.12461,0.12355,0.12756,0.15245,0.1275,0.1224,0.12449,0.12
793,0.12744,0.12737,0.13204,0.14412,0.13302,0.47663,0.47214,0.48032,0.
47027,0.48097,0.47464,0.47317,0.4946,0.48441,0.34656,0.33936,0.24317,0
.24548,0.24876,0.24977,0.25089,0.24774,0.24011,0.25233,0.27372,0.11712
,0.12584,0.12736,0.12558,0.12714,0.10683,0.12603,0.13064,0.12019,0.126
9,0.13579,0.11914,0.12927,0.12727,0.12802,0.12047,0.12274,0.1228,0.473
73,0.49283,0.47323,0.47022,0.48382,0.48502,0.47878,0.48058,0.47852,0.2
6695,0.2413,0.27157,0.24181,0.24166,0.2464,0.24412,0.20997,0.2314,0.26
201,0.24433,0.12711,0.10883,0.12816,0.12724,0.12995,0.12758,0.1147,0.1
2613,0.13292,0.1267,0.11673,0.1269,0.13498,0.12885,0.13272,0.13453,0.1
2455,0.13656,0.4811,0.47455,0.48458,0.48623,0.47131,0.46439,0.47336,0.
27228,0.31094,0.29812,0.24698,0.25814,0.24356,0.2607,0.26458,0.23832,0
.24982,0.25562,0.23162,0.24984,0.12785,0.14058,0.127,0.11956,0.10926,0
.11434,0.1174,0.1275,0.12714,0.12762,0.12804,0.12703,0.12748,0.12778,0
.12782,0.12385,0.10578,0.13223,0.47465,0.47578,0.4789,0.47908,0.46734,
0.48945,0.28961,0.2805,0.26274,0.22148,0.2542,0.24518,0.2276,0.2607,0.
20881,0.22409,0.23119,0.27299,0.25588,0.26768,0.12778,0.12412,0.12668,
0.12433,0.11642,0.132,0.12392,0.10851,0.12926,0.12351,0.1339,0.11539,0
.12206,0.12902,0.12347,0.1274,0.12388,0.11414,0.47894,0.47705,0.48345,
0.48186,0.48153,0.25883,0.26896,0.27037,0.26406,0.2557,0.27006,0.21475
,0.21256,0.26167,0.226,0.25306,0.24494,0.24316,0.24137,0.24608,0.12651
,0.12131,0.13214,0.12681,0.12723,0.12952,0.13965,0.12922,0.12269,0.127
5,0.12546,0.12101,0.12735,0.12496,0.12572,0.12716,0.13502,0.13161,0.47
875,0.48525,0.47934,0.27637,0.27136,0.26988,0.27415,0.27703,0.26726,0.
2815,0.23614,0.24572,0.23726,0.24608,0.21211,0.21959,0.25146,0.242,0.2
06,0.2426,0.15234,0.12973,0.1271,0.12703,0.1273,0.12722,0.12262,0.1273
9,0.13827,0.12516,0.1307,0.11821,0.12707,0.11769,0.10409,0.12914,0.132
63,0.12668,0.48376,0.27847,0.28451,0.26359,0.27284,0.29816,0.27248,0.3
1074,0.27307,0.26121,0.19761,0.24513,0.25314,0.23746,0.22376,0.24728,0
.25361,0.23408,0.23677,0.13039,0.12662,0.12299,0.11877,0.127,0.12729,0
.12509,0.11924,0.12655,0.13077,0.12685,0.12711,0.12701,0.12026,0.11454
,0.13003,0.128,0.067486,0.25009,0.26546,0.25387,0.19695,0.29731,0.1958
8,0.24094,0.28386,0.28883,0.2742,0.24468,0.25813,0.27596,0.20797,0.237
9,0.28623,0.24141,0.24677,0.23282,0.13165,0.1285,0.12829,0.13148,0.128
73,0.13881,0.12812,0.11606,0.12658,0.11261,0.12704,0.12723,0.13329,0.1
2429,0.12743,0.13024,0.12719,0.067877,0.29607,0.3152,0.28039,0.26949,0
.21766,0.27054,0.27605,0.27336,0.27248,0.27688,0.28429,0.23644,0.24233
,0.24121,0.24341,0.26005,0.24195,0.23237,0.12728,0.12152,0.12041,0.139
8,0.1286,0.13871,0.12732,0.13345,0.12796,0.12689,0.12217,0.127,0.12895
,0.11621,0.12662,0.12619,0.12169,0.12354,0.067156,0.28785,0.27715,0.19
501,0.25192,0.28691,0.26129,0.27726,0.27556,0.27205,0.26901,0.24183,0.
21035,0.24294,0.24406,0.24545,0.22715,0.22901,0.26421,0.12128,0.1272,0

.14783,0.12663,0.13463,0.12732,0.12874,0.12691,0.11909,0.13439,0.12723
,0.12091,0.13116,0.12497,0.12704,0.1275,0.14583,0.067927,0.067421,0.06
7595,0.35609,0.26184,0.27277,0.27012,0.31361,0.28637,0.27545,0.27638,0
.24087,0.24294,0.21641,0.22338,0.25371,0.24509,0.24593,0.12081,0.12715
,0.12698,0.13909,0.10756,0.11707,0.13408,0.13044,0.11456,0.12506,0.128
56,0.12877,0.11837,0.13677,0.085932,0.1176,0.13821,0.13011,0.067667,0.
067792,0.06805,0.067249,0.26832,0.30454,0.30907,0.25892,0.27451,0.2352
3,0.26092,0.25083,0.27363,0.19412,0.24644,0.22233,0.12059,0.12673,0.16
518,0.12371,0.12131,0.12501,0.12393,0.12659,0.14052,0.1287,0.12754,0.1
3602,0.12262,0.12738,0.15706,0.13169,0.12684,0.13131,0.12721,0.13088,0
.067573,0.067808,0.067694,0.067506,0.068023,0.06826,0.29961,0.30694,0.
27157,0.26879,0.36212,0.23418,0.28547,0.27096,0.1411,0.12872,0.13524,0
.12986,0.1291,0.12978,0.14303,0.12747,0.12535,0.13023,0.12739,0.13505,
0.1282,0.12415,0.11776,0.12253,0.11974,0.12689,0.12711,0.12156,0.12715
,0.12717,0.11987,0.067424,0.068052,0.06796,0.067695,0.067962,0.067976,
0.27322,0.27396,0.29602,0.28666,0.27448,0.067329,0.067641,0.12465,0.12
922,0.11593,0.12642,0.12719,0.12776,0.12768,0.12654,0.12757,0.13125,0.
12772,0.12962,0.12713,0.15205,0.14598,0.12344,0.13062,0.12672,0.13063,
0.12699,0.12688,0.067214,0.067493,0.068044,0.068151,0.068598,0.06714,0
.06732,0.067977,0.067975,0.067873,0.26663,0.29551,0.067547,0.067531,0.
067596,0.067821,0.13231,0.11355,0.13275,0.12275,0.12268,0.13832,0.1274
5,0.12739,0.12679,0.12684,0.12609,0.1335,0.12712,0.12766,0.13398,0.129
7,0.12645,0.12986,0.12128,0.067179,0.068036,0.06742,0.067395,0.067774,
0.067913,0.068385,0.067127,0.067793,0.067468,0.067643,0.068348,0.06776
2,0.066873,0.067943,0.067278,0.067623,0.12744,0.12663,0.12724,0.11807,
0.13147,0.11766,0.12331,0.12775,0.12804,0.13596,0.12869,0.12697,0.1352
9,0.13312,0.13035,0.12497,0.067629,0.067675,0.067734,0.067929,0.06768,
0.067631,0.067964,0.06769,0.067996,0.067576,0.067698,0.067177,0.067686
,0.067439,0.067902,0.067456,0.067258,0.067178,0.067676,0.11681,0.1281,
0.1403,0.12762,0.11807,0.12754,0.13127,0.12939,0.12741,0.12818,0.13267
,0.12712,0.14531,0.067731,0.068312,0.068054,0.067015,0.067577,0.067805
,0.067731,0.066724,0.067313,0.067585,0.067583,0.06761,0.067893,0.06802
5,0.068122,0.067424,0.067572,0.067231,0.067813,0.067719,0.068081,0.127
41,0.11653,0.12529,0.12521,0.12537,0.10326,0.12733,0.12756,0.12562,0.0
67633,0.067953,0.067899,0.068012,0.067533,0.067269,0.067667,0.067493,0
.068194,0.06798,0.067772,0.068136,0.067511,0.067498,0.068164,0.06721,0
.067803,0.067808,0.067115,0.06774,0.068251,0.066816,0.067719,0.067698,
0.067514,0.1156,0.12976,0.13444,0.11028,0.067982,0.067329,0.067438,0.0
68336,0.067836,0.068483,0.068073,0.066821,0.067859,0.067897,0.066996,0
.068151,0.067013,0.067615,0.068013,0.068026,0.068103,0.067215,0.066912
,0.067447,0.067502,0.067628,0.067409,0.067783,0.067632,0.067254,0.0671
88,0.067574,0.068118,0.068435,0.067674,0.06768,0.067688,0.067917,0.067
928,0.067705,0.068073,0.067516,0.067866,0.067477,0.068016,0.067245,0.0
6809,0.067581,0.067389,0.067907,0.067637,0.067782,0.067904,0.067674,0.
067656,0.067797,0.06704,0.067454,0.067643,0.067683,0.06747,0.067817,0.
067546,0.067524,0.067826,0.067841,0.067842,0.067889,0.068266,0.067561,
0.068393,0.068276,0.068624,0.067253,0.067369,0.067343,0.067978,0.06789
8,0.067187,0.067607,0.067686,0.067885,0.068211,0.067243,0.067629,0.067
766,0.067697,0.067871,0.067947,0.067693,0.067862,0.068502,0.067175,0.0
68435,0.066854,0.068272,0.06784,0.067635,0.067526,0.068254,0.067239,0.
067437,0.067579,0.067805,0.067881,0.067641,0.068021,0.067833,0.067722,
0.067974,0.067714,0.067368,0.068305,0.067071,0.06771,0.067952,0.06769,

0.067448,0.067578,0.067639,0.067641,0.067611,0.067535,0.067639,0.06722
7,0.067244,0.068228,0.067256,0.067647,0.067589,0.06829,0.067306,0.0676
64,0.067245,0.067616,0.067896,0.068199,0.067596,0.066897,0.067757,0.06
7941,0.067065,0.068296,0.067661,0.067575,0.067327,0.067295,0.067751,0.
068167,0.067739,0.067203,0.067259,0.068127,0.067608,0.06753,0.067614,0
.067789,0.067692,0.067651,0.067693,0.067845,0.067491,0.067525,0.068002
,0.067881,0.068279,0.068194,0.068296,0.06755,0.066904,0.068094,0.06869
1,0.067545,0.067801,0.067509,0.06767,0.067227,0.067691,0.06829,0.06794
1,0.066974,0.068363,0.06774,0.067458,0.06771,0.068394,0.067623,0.06732
5,0.067714,0.068449,0.067683,0.067785,0.067808,0.067222,0.067026,0.068
029,0.068201,0.067696,0.067826
0.19048,0.18992,0.19088,0.19008,0.19062,0.19002,0.19138,0.19081,0.1895
5,0.1909,0.19109,0.19088,0.19112,0.19078,0.18996,0.19058,0.19046,0.190
95,0.19123,0.19115,0.31915,0.30053,0.31346,0.31195,0.18893,0.19125,0.1
9125,0.19029,0.19041,0.19062,0.19069,0.19059,0.19023,0.19117,0.19069,0
.19056,0.19163,0.18966,0.19099,0.43611,0.31062,0.31066,0.31202,0.31617
,0.26958,0.31459,0.19081,0.19148,0.19105,0.19032,0.19004,0.18947,0.191
47,0.19122,0.19044,0.19053,0.19062,0.19025,0.19022,0.19155,0.19115,0.4
3244,0.42832,0.28212,0.30261,0.31567,0.31387,0.30881,0.32668,0.32625,0
.29111,0.191,0.19141,0.19013,0.19144,0.19152,0.19097,0.19076,0.19101,0
.19091,0.19115,0.19158,0.19068,0.19071,0.19035,0.19063,0.43013,0.43588
,0.42833,0.31188,0.32142,0.33168,0.29791,0.33184,0.29035,0.36307,0.315
7,0.31536,0.19043,0.19111,0.19045,0.19148,0.1903,0.19047,0.19024,0.191
52,0.19109,0.19006,0.19081,0.19163,0.18979,0.19047,0.19072,0.18976,0.4
4191,0.44574,0.44101,0.43511,0.29661,0.32061,0.31448,0.31987,0.31079,0
.31387,0.31829,0.31257,0.31355,0.31578,0.19165,0.19086,0.1913,0.19093,
0.18996,0.19093,0.19154,0.19089,0.19039,0.19049,0.19165,0.1901,0.19202
,0.19009,0.19028,0.19133,0.42765,0.44598,0.43333,0.43447,0.44167,0.433
37,0.31328,0.3411,0.314,0.312,0.3143,0.32201,0.31652,0.32216,0.31435,0
.32097,0.19091,0.19096,0.19044,0.19137,0.19006,0.19181,0.19111,0.19119
,0.1905,0.19072,0.19098,0.19125,0.19087,0.18955,0.19108,0.19099,0.1899
7,0.43316,0.44321,0.42938,0.43624,0.43967,0.42949,0.32314,0.30814,0.33
247,0.31031,0.32109,0.33632,0.31117,0.31154,0.31616,0.30302,0.32427,0.
19084,0.19265,0.19043,0.19042,0.19031,0.19054,0.18978,0.18975,0.19161,
0.19105,0.1908,0.18943,0.19024,0.12442,0.13368,0.1278,0.18926,0.43269,
0.43821,0.42259,0.42809,0.43243,0.43593,0.44089,0.31854,0.33174,0.3129
,0.30981,0.32012,0.30328,0.28274,0.31567,0.31677,0.30799,0.31401,0.191
14,0.19129,0.19139,0.19098,0.19009,0.19091,0.19024,0.1908,0.19154,0.19
024,0.19021,0.12347,0.11515,0.11566,0.12518,0.11942,0.11583,0.19166,0.
43184,0.44115,0.44865,0.4371,0.43472,0.43014,0.43796,0.29603,0.31493,0
.31091,0.31375,0.30713,0.32259,0.31393,0.33046,0.31332,0.31145,0.33705
,0.19082,0.19097,0.19204,0.19195,0.18998,0.19078,0.19044,0.19083,0.190
07,0.12936,0.11696,0.1323,0.12403,0.13113,0.12755,0.1255,0.1068,0.1244
4,0.19067,0.43619,0.43936,0.43056,0.44356,0.43499,0.43095,0.43324,0.32
573,0.32799,0.32039,0.30941,0.30319,0.31562,0.31792,0.30358,0.30956,0.
33823,0.3098,0.31752,0.19028,0.19002,0.19081,0.19078,0.18995,0.19078,0
.1255,0.12456,0.12294,0.12503,0.12812,0.12499,0.12607,0.13747,0.12494,
0.12453,0.11914,0.12277,0.44014,0.43796,0.43727,0.44202,0.43321,0.4353
4,0.43487,0.31153,0.30552,0.3239,0.31331,0.31457,0.31454,0.30643,0.330
05,0.32552,0.32213,0.31208,0.30266,0.18986,0.19087,0.19226,0.12195,0.1
3478,0.12462,0.14271,0.12489,0.12731,0.13859,0.097291,0.12471,0.12572,
0.13144,0.12123,0.11348,0.13075,0.12508,0.4349,0.43851,0.43446,0.43109

,0.43764,0.44058,0.43385,0.43966,0.43338,0.31385,0.30388,0.29119,0.312
77,0.31911,0.27506,0.31157,0.3068,0.31235,0.31887,0.31748,0.12491,0.13
635,0.13574,0.12565,0.12471,0.12177,0.12059,0.12525,0.1527,0.12504,0.1
1954,0.1221,0.12575,0.12534,0.12515,0.13029,0.14345,0.13119,0.43573,0.
43238,0.4412,0.42609,0.44031,0.43867,0.43088,0.45187,0.44005,0.32694,0
.32157,0.22934,0.23087,0.23377,0.23136,0.23535,0.23072,0.22526,0.23741
,0.25449,0.11355,0.12289,0.1247,0.12301,0.12466,0.10212,0.12347,0.1283
9,0.117,0.12446,0.13436,0.1158,0.12685,0.12487,0.12571,0.11708,0.11975
,0.11988,0.42945,0.45144,0.42899,0.43102,0.43974,0.44407,0.43985,0.440
7,0.43878,0.24243,0.22509,0.25557,0.22672,0.2249,0.23143,0.22807,0.197
77,0.21753,0.24641,0.22954,0.12486,0.10441,0.12567,0.12504,0.12775,0.1
2509,0.11101,0.12353,0.13106,0.12383,0.11331,0.12445,0.13343,0.12667,0
.13087,0.13276,0.12188,0.13526,0.43204,0.4365,0.44451,0.44402,0.42935,
0.42507,0.43757,0.24671,0.28084,0.26958,0.23125,0.24337,0.22731,0.2447
7,0.24752,0.22507,0.23357,0.24036,0.21809,0.23344,0.12539,0.13956,0.12
478,0.11635,0.10499,0.1105,0.11389,0.12509,0.12475,0.12517,0.12579,0.1
247,0.12504,0.12543,0.12549,0.12091,0.10091,0.13043,0.43442,0.43452,0.
43555,0.43698,0.42587,0.44421,0.261,0.25437,0.23924,0.1988,0.23075,0.2
3049,0.21171,0.24529,0.1981,0.21111,0.21681,0.25201,0.23881,0.2501,0.1
2529,0.12119,0.12436,0.12165,0.1127,0.13002,0.12108,0.10404,0.12732,0.
12062,0.13204,0.11167,0.11918,0.12687,0.12054,0.12481,0.12141,0.11029,
0.43841,0.44162,0.44297,0.43913,0.43975,0.23275,0.24679,0.24622,0.2418
1,0.23137,0.24406,0.20159,0.20178,0.24673,0.2106,0.23555,0.22906,0.227
17,0.22732,0.23106,0.12412,0.11819,0.13017,0.12443,0.12468,0.1273,0.13
867,0.12678,0.11959,0.12498,0.12284,0.11784,0.12504,0.12241,0.12343,0.
12464,0.13348,0.12978,0.43653,0.44109,0.43691,0.25102,0.24411,0.24486,
0.24688,0.25205,0.2405,0.2532,0.21414,0.22943,0.22339,0.23047,0.19649,
0.20666,0.23609,0.22554,0.19391,0.22696,0.15292,0.12758,0.12444,0.1246
4,0.12468,0.12488,0.11988,0.12495,0.13705,0.12274,0.12845,0.11478,0.12
469,0.11424,0.099144,0.12725,0.13088,0.1241,0.44023,0.25319,0.25895,0.
23762,0.24653,0.27228,0.2486,0.28305,0.24703,0.23669,0.1863,0.22971,0.
23842,0.22137,0.21017,0.23167,0.2356,0.21855,0.22169,0.12844,0.12406,0
.12029,0.11547,0.12462,0.12481,0.12222,0.11588,0.12428,0.1287,0.12453,
0.12474,0.1245,0.11707,0.111,0.12799,0.12563,0.058855,0.2289,0.24357,0
.22913,0.1786,0.27001,0.1775,0.21724,0.26,0.26172,0.24997,0.22862,0.24
308,0.25593,0.19615,0.22259,0.26785,0.2278,0.23216,0.21885,0.12974,0.1
261,0.1258,0.12944,0.12633,0.13755,0.12596,0.11246,0.12415,0.10868,0.1
2463,0.12469,0.1315,0.12158,0.12485,0.12818,0.1249,0.058983,0.26881,0.
28708,0.25365,0.24547,0.19796,0.24437,0.25055,0.24829,0.24812,0.25018,
0.26349,0.22112,0.22718,0.2266,0.22666,0.24216,0.22735,0.21871,0.12485
,0.11832,0.11746,0.13866,0.12657,0.13775,0.12502,0.13144,0.1258,0.1243
4,0.1193,0.12447,0.12701,0.11249,0.12431,0.12371,0.11886,0.12079,0.058
347,0.26097,0.25111,0.17548,0.22672,0.26241,0.23705,0.25189,0.24979,0.
24617,0.24477,0.22561,0.19696,0.22624,0.22855,0.23046,0.21216,0.2146,0
.24572,0.11806,0.12479,0.14729,0.12437,0.13312,0.12489,0.12647,0.12421
,0.11587,0.13249,0.12489,0.11775,0.12917,0.12252,0.12448,0.12519,0.145
13,0.059277,0.05887,0.058892,0.32682,0.2361,0.2474,0.24467,0.28577,0.2
5994,0.249,0.24845,0.21855,0.22738,0.20483,0.20965,0.23902,0.23008,0.2
3006,0.11757,0.12471,0.12471,0.13786,0.10336,0.11339,0.13232,0.12885,0
.11071,0.12249,0.12615,0.12652,0.1153,0.13516,0.078794,0.11415,0.13722
,0.1283,0.058932,0.058818,0.059106,0.058666,0.24429,0.27754,0.28046,0.
23418,0.24978,0.21426,0.23851,0.22714,0.2493,0.18356,0.22966,0.2097,0.

11731,0.12453,0.16736,0.12079,0.11828,0.12251,0.12126,0.12424,0.13964,
0.1267,0.12525,0.1347,0.11964,0.12508,0.15785,0.12957,0.12448,0.12935,
0.12453,0.12896,0.058888,0.059044,0.058888,0.059032,0.059117,0.059536,
0.27441,0.27888,0.24752,0.24312,0.33219,0.21467,0.26033,0.24619,0.1402
,0.12657,0.1336,0.12761,0.12675,0.12781,0.14219,0.1252,0.12288,0.12775
,0.1251,0.1335,0.12623,0.12133,0.1146,0.11959,0.11662,0.1245,0.1247,0.
1186,0.12472,0.12473,0.11679,0.058723,0.059183,0.059266,0.058981,0.059
103,0.059145,0.24956,0.24784,0.27058,0.2589,0.24985,0.05872,0.058729,0
.12201,0.12684,0.11212,0.12419,0.12474,0.12527,0.12522,0.12409,0.12492
,0.12926,0.12515,0.12762,0.12468,0.15249,0.14512,0.12059,0.12835,0.124
21,0.12843,0.12466,0.12438,0.058355,0.05871,0.059259,0.059578,0.059684
,0.058186,0.058371,0.05901,0.059324,0.059296,0.24107,0.26804,0.058577,
0.058741,0.058895,0.058989,0.13022,0.10991,0.13103,0.11971,0.11966,0.1
3715,0.12518,0.12499,0.12435,0.12436,0.12363,0.1318,0.12459,0.12525,0.
13213,0.1274,0.12391,0.12795,0.1183,0.058467,0.059094,0.058791,0.05853
1,0.058518,0.059116,0.059241,0.058562,0.059298,0.058934,0.058652,0.059
321,0.059164,0.058342,0.059116,0.058689,0.058959,0.12517,0.12417,0.125
03,0.11454,0.12963,0.11404,0.12041,0.12531,0.12599,0.13462,0.12653,0.1
2452,0.13363,0.13141,0.12852,0.12232,0.059013,0.058913,0.058532,0.0591
67,0.05891,0.058935,0.059228,0.058709,0.059195,0.058675,0.059011,0.058
52,0.058757,0.058684,0.059043,0.059015,0.058299,0.058416,0.058705,0.11
329,0.12549,0.13946,0.12518,0.11457,0.12515,0.12933,0.12724,0.12498,0.
12574,0.13106,0.12461,0.14468,0.058972,0.05931,0.059224,0.058256,0.058
956,0.059272,0.05884,0.058209,0.058533,0.058693,0.058716,0.058704,0.05
9121,0.058957,0.05958,0.058695,0.059129,0.058277,0.058693,0.058751,0.0
59125,0.12483,0.11286,0.12268,0.12268,0.1228,0.098342,0.12487,0.12546,
0.12322,0.058889,0.058983,0.059018,0.059014,0.058964,0.058593,0.058945
,0.058944,0.059241,0.059383,0.058989,0.059529,0.058915,0.058588,0.0594
13,0.058614,0.058975,0.059062,0.058422,0.059284,0.059499,0.058088,0.05
8769,0.059096,0.058623,0.11193,0.12759,0.13269,0.10615,0.059297,0.0585
09,0.058866,0.059442,0.059104,0.059779,0.059307,0.05817,0.059131,0.058
874,0.058496,0.059324,0.05831,0.058693,0.058994,0.059223,0.059227,0.05
8455,0.058091,0.058689,0.058742,0.058912,0.058334,0.058922,0.059123,0.
058437,0.058286,0.058755,0.059347,0.059655,0.058864,0.058737,0.058904,
0.05903,0.059232,0.058804,0.05907,0.058905,0.059317,0.059161,0.059567,
0.058702,0.059351,0.05908,0.058805,0.05901,0.058622,0.059135,0.058894,
0.058538,0.059112,0.058678,0.058381,0.058676,0.058941,0.058789,0.05854
3,0.059322,0.058674,0.058632,0.059025,0.059036,0.059111,0.058699,0.059
44,0.05845,0.059318,0.059591,0.059474,0.058456,0.058949,0.058785,0.059
437,0.059005,0.058574,0.058744,0.059009,0.059016,0.059391,0.058342,0.0
5885,0.058901,0.058892,0.058817,0.059215,0.058788,0.059306,0.059615,0.
058437,0.059778,0.058234,0.059459,0.05938,0.058826,0.058729,0.059284,0
.058766,0.058647,0.058609,0.059117,0.059131,0.058833,0.059304,0.059323
,0.058478,0.058843,0.058877,0.058661,0.059674,0.058361,0.058769,0.0589
92,0.058757,0.058288,0.058939,0.059,0.058936,0.058836,0.058794,0.05874
7,0.058559,0.058732,0.059467,0.058848,0.058427,0.058805,0.059309,0.058
333,0.0589,0.058439,0.059158,0.05929,0.059376,0.058944,0.058199,0.0589
5,0.059169,0.058587,0.059592,0.058811,0.059146,0.058551,0.058334,0.059
304,0.059301,0.059103,0.058375,0.05868,0.05923,0.058553,0.058517,0.058
928,0.05896,0.059026,0.058755,0.05856,0.059159,0.058513,0.058886,0.059
361,0.059079,0.05995,0.059377,0.059393,0.058683,0.058259,0.059328,0.06
0133,0.059069,0.058929,0.058719,0.059133,0.058423,0.058822,0.059287,0.

059099,0.058195,0.05958,0.058792,0.058592,0.058872,0.059271,0.059034,0
.058712,0.059044,0.059556,0.058862,0.058992,0.059117,0.058472,0.058403
,0.05927,0.059513,0.05883,0.058922
0.1939,0.19291,0.19388,0.19342,0.19392,0.19323,0.19423,0.19363,0.19232
,0.1939,0.19421,0.19405,0.19386,0.19372,0.19287,0.1933,0.19335,0.19397
,0.1944,0.19428,0.30623,0.29111,0.30155,0.30322,0.19249,0.19414,0.1939
5,0.19337,0.19351,0.19339,0.19392,0.19337,0.19317,0.19436,0.19405,0.19
347,0.19446,0.19283,0.19392,0.41419,0.30014,0.29927,0.3016,0.30623,0.2
6339,0.30304,0.19394,0.19409,0.19437,0.19278,0.19302,0.19275,0.19428,0
.19433,0.19335,0.19345,0.19327,0.19359,0.193,0.19413,0.19394,0.40658,0
.399,0.27263,0.29224,0.3041,0.30557,0.29846,0.31479,0.31263,0.28294,0.
19398,0.19444,0.19293,0.19393,0.19418,0.19449,0.19348,0.19391,0.19367,
0.19374,0.19438,0.19349,0.19393,0.19335,0.1937,0.40826,0.40864,0.40014
,0.29943,0.31027,0.31747,0.28798,0.31765,0.28122,0.34557,0.30379,0.301
57,0.19289,0.19421,0.19308,0.1945,0.19301,0.19367,0.19272,0.19452,0.19
416,0.19307,0.1938,0.19453,0.19268,0.19384,0.19396,0.19292,0.41408,0.4
2096,0.41657,0.40799,0.28617,0.30795,0.30266,0.30763,0.29781,0.30246,0
.30445,0.30206,0.30359,0.30396,0.19439,0.194,0.19451,0.19386,0.19285,0
.19355,0.19474,0.19368,0.19367,0.19363,0.19394,0.19328,0.19492,0.193,0
.19328,0.19447,0.39941,0.41984,0.40738,0.40484,0.4158,0.40229,0.30171,
0.32638,0.30226,0.2997,0.30302,0.30676,0.30234,0.31255,0.30376,0.30876
,0.19376,0.19402,0.19348,0.19454,0.1931,0.19478,0.19436,0.19406,0.1934
1,0.19366,0.19392,0.19368,0.194,0.19265,0.19408,0.19362,0.19362,0.4085
3,0.41354,0.40179,0.4085,0.41541,0.40476,0.30938,0.29533,0.31982,0.299
35,0.3094,0.3212,0.29994,0.3003,0.30497,0.29124,0.31451,0.19319,0.1957
4,0.19285,0.19369,0.19367,0.19292,0.19309,0.19278,0.19456,0.19447,0.19
403,0.19255,0.19351,0.12299,0.13285,0.12672,0.19265,0.40899,0.4121,0.3
9691,0.40451,0.40664,0.40797,0.41354,0.30662,0.31682,0.30168,0.29844,0
.30784,0.29168,0.27351,0.3041,0.30451,0.29749,0.30048,0.19409,0.19399,
0.19463,0.19373,0.19316,0.19384,0.19353,0.19414,0.19485,0.1936,0.19391
,0.12175,0.11291,0.1136,0.12371,0.11724,0.1136,0.19436,0.40375,0.41248
,0.42362,0.4084,0.41267,0.40482,0.41164,0.28578,0.30285,0.29936,0.3013
3,0.29667,0.30983,0.30238,0.31849,0.29944,0.29969,0.32386,0.19389,0.19
381,0.19464,0.19467,0.1929,0.19359,0.19351,0.19381,0.19268,0.12802,0.1
1477,0.13083,0.12246,0.12966,0.12618,0.12386,0.10419,0.12272,0.19358,0
.41204,0.41235,0.40696,0.41551,0.4136,0.40769,0.40938,0.3123,0.31485,0
.30558,0.29922,0.29342,0.30432,0.30595,0.29326,0.2991,0.324,0.29867,0.
30705,0.19334,0.19315,0.19427,0.1937,0.19285,0.19362,0.12399,0.12289,0
.12132,0.12332,0.12651,0.12325,0.12453,0.13663,0.12318,0.12293,0.11718
,0.12099,0.4166,0.41251,0.41282,0.41367,0.41074,0.41017,0.41423,0.3011
7,0.29415,0.31077,0.3005,0.302,0.30425,0.29554,0.31717,0.31121,0.30818
,0.3012,0.29158,0.19291,0.19392,0.19482,0.11993,0.13384,0.12288,0.1425
4,0.12355,0.12563,0.13812,0.093828,0.12322,0.1242,0.13043,0.11948,0.11
094,0.12942,0.1237,0.4111,0.41376,0.40488,0.40897,0.41132,0.41415,0.41
069,0.41273,0.40513,0.30289,0.29366,0.28338,0.30158,0.30657,0.26786,0.
30002,0.2978,0.30096,0.30497,0.3044,0.12327,0.13579,0.13493,0.12421,0.
12308,0.11999,0.11859,0.12365,0.15296,0.12323,0.11746,0.12069,0.12431,
0.124,0.12373,0.12903,0.14313,0.13035,0.41153,0.41199,0.41036,0.4017,0
.41304,0.41206,0.40229,0.42469,0.41065,0.3135,0.31006,0.22,0.22264,0.2
2505,0.22272,0.22505,0.21961,0.21616,0.22821,0.24559,0.11121,0.12115,0
.12311,0.12146,0.1231,0.098854,0.12182,0.12722,0.11503,0.1228,0.13343,
0.11361,0.12552,0.12314,0.12419,0.11502,0.11797,0.11775,0.4046,0.42423

,0.40638,0.40613,0.41098,0.42034,0.41266,0.41578,0.41435,0.22498,0.217
41,0.24361,0.21773,0.21647,0.22106,0.21898,0.19098,0.20927,0.23652,0.2
2123,0.12308,0.10137,0.12411,0.12343,0.12621,0.12354,0.10838,0.1218,0.
12981,0.1223,0.11094,0.12279,0.13234,0.12511,0.12977,0.13163,0.12006,0
.13454,0.40754,0.41195,0.41874,0.4193,0.4028,0.40191,0.41068,0.23099,0
.26441,0.25345,0.21904,0.2332,0.21743,0.23353,0.23611,0.21595,0.22403,
0.23079,0.21,0.22438,0.12385,0.13892,0.12306,0.11433,0.10235,0.10786,0
.11142,0.12347,0.12295,0.12384,0.12417,0.12314,0.12346,0.12391,0.12404
,0.11914,0.09733,0.12933,0.4124,0.40698,0.41219,0.40873,0.39712,0.4185
,0.24444,0.2388,0.22658,0.18705,0.2151,0.2193,0.20063,0.23437,0.19107,
0.20337,0.20805,0.23957,0.23031,0.23929,0.12389,0.11933,0.1228,0.11972
,0.1103,0.12884,0.11926,0.10098,0.12601,0.11876,0.13086,0.10919,0.1174
5,0.12552,0.11874,0.1233,0.11949,0.10755,0.4111,0.4172,0.41685,0.41057
,0.41516,0.21901,0.23128,0.23073,0.22844,0.21402,0.22946,0.1947,0.1935
5,0.23792,0.20126,0.22334,0.21912,0.21911,0.21897,0.22095,0.12226,0.11
636,0.12909,0.12306,0.12326,0.12596,0.138,0.12521,0.11777,0.12354,0.12
117,0.11578,0.12338,0.12064,0.12167,0.12334,0.13236,0.12882,0.41024,0.
41499,0.40938,0.23333,0.22872,0.2294,0.23251,0.2355,0.22469,0.23656,0.
19885,0.21958,0.21573,0.21901,0.18714,0.19931,0.22451,0.21547,0.18754,
0.2183,0.15316,0.12629,0.12289,0.12312,0.12319,0.12346,0.11797,0.12331
,0.13624,0.12087,0.12713,0.11238,0.12289,0.11206,0.095805,0.126,0.1296
9,0.12254,0.41148,0.23475,0.24297,0.22197,0.22967,0.25339,0.23335,0.26
557,0.23114,0.22125,0.17969,0.21986,0.22954,0.21353,0.20335,0.22113,0.
22366,0.20828,0.2119,0.12745,0.12257,0.11849,0.11343,0.12312,0.12324,0
.12069,0.1139,0.12278,0.12726,0.1227,0.12307,0.12283,0.11527,0.10832,0
.12674,0.12423,0.052719,0.2131,0.22747,0.21417,0.16542,0.25342,0.16544
,0.20452,0.24184,0.24541,0.23259,0.21866,0.23336,0.24285,0.18927,0.212
05,0.2574,0.21952,0.2228,0.20897,0.12875,0.1248,0.12431,0.12815,0.1249
5,0.13662,0.12448,0.11006,0.12265,0.10598,0.12316,0.12335,0.13032,0.11
96,0.12332,0.12691,0.1231,0.053059,0.25363,0.26999,0.23783,0.22798,0.1
8525,0.22905,0.23415,0.23127,0.2337,0.23517,0.25095,0.21106,0.21871,0.
21674,0.21699,0.23208,0.21801,0.20962,0.12323,0.11637,0.11552,0.13787,
0.12515,0.13709,0.12354,0.13013,0.12435,0.12279,0.11737,0.12302,0.1255
7,0.11026,0.1227,0.12232,0.11694,0.11886,0.052607,0.24615,0.2368,0.162
18,0.21326,0.24639,0.22028,0.23479,0.23091,0.23163,0.2281,0.21541,0.18
916,0.2172,0.21744,0.22202,0.20385,0.2043,0.23382,0.11591,0.12327,0.14
732,0.12266,0.13208,0.1235,0.12481,0.12268,0.1138,0.13129,0.12338,0.11
569,0.12751,0.12103,0.12297,0.12388,0.14512,0.05307,0.052888,0.052682,
0.31091,0.22105,0.2316,0.23002,0.26745,0.2425,0.23113,0.23242,0.20576,
0.2179,0.19722,0.20152,0.22828,0.21926,0.21955,0.11557,0.1232,0.12307,
0.13739,0.10044,0.11134,0.13129,0.12765,0.10809,0.12089,0.1245,0.1253,
0.11309,0.13442,0.074302,0.11202,0.13655,0.12683,0.05275,0.052594,0.05
3184,0.052309,0.22856,0.26063,0.26028,0.2206,0.23092,0.19832,0.22164,0
.21248,0.23383,0.17712,0.21902,0.2021,0.11508,0.12288,0.16867,0.11912,
0.11643,0.12095,0.11954,0.12291,0.1389,0.12515,0.12366,0.13368,0.11766
,0.12337,0.15896,0.12843,0.12305,0.12817,0.12269,0.12805,0.052896,0.05
3149,0.052781,0.053134,0.053071,0.0535,0.25864,0.26123,0.22987,0.22803
,0.31127,0.20064,0.24186,0.23138,0.13987,0.12502,0.13305,0.12633,0.125
55,0.12661,0.1419,0.12357,0.12125,0.12641,0.12339,0.13259,0.12464,0.11
973,0.11241,0.11761,0.11453,0.12313,0.12292,0.11665,0.12299,0.12326,0.
11462,0.052595,0.052879,0.05317,0.052899,0.05301,0.052976,0.23449,0.23
28,0.25327,0.23987,0.23515,0.052105,0.052663,0.12022,0.12541,0.10993,0

.12254,0.1231,0.12349,0.12346,0.12274,0.12336,0.12793,0.12351,0.1263,0
.12311,0.15253,0.14522,0.11869,0.12721,0.12281,0.12719,0.12306,0.1227,
0.052155,0.052461,0.053056,0.053407,0.053441,0.052155,0.052136,0.05283
6,0.052985,0.052903,0.22535,0.2488,0.052311,0.052694,0.052952,0.052881
,0.12891,0.10714,0.13003,0.11789,0.11772,0.13629,0.12362,0.12341,0.122
57,0.12255,0.12205,0.13096,0.1232,0.12359,0.13108,0.12593,0.12247,0.12
661,0.11618,0.052265,0.05281,0.052745,0.052499,0.052541,0.053072,0.052
95,0.052475,0.053149,0.052765,0.052659,0.05326,0.053123,0.052537,0.052
802,0.052641,0.052895,0.12349,0.12272,0.12338,0.11226,0.12854,0.1117,0
.11861,0.12369,0.12448,0.13365,0.12534,0.12311,0.13288,0.13036,0.12728
,0.12078,0.052967,0.053007,0.052539,0.052807,0.052717,0.052945,0.05314
5,0.052347,0.053126,0.052672,0.052903,0.052434,0.052833,0.052494,0.052
851,0.052729,0.052327,0.052416,0.052483,0.11073,0.12395,0.13883,0.1236
9,0.11245,0.12369,0.12809,0.12585,0.12355,0.12416,0.1297,0.12292,0.144
48,0.052952,0.05311,0.053208,0.052275,0.052714,0.05295,0.052662,0.0524
24,0.052663,0.052638,0.052824,0.052391,0.053112,0.052793,0.053269,0.05
264,0.053119,0.052316,0.052868,0.052481,0.052879,0.12327,0.11052,0.120
99,0.12102,0.12113,0.094957,0.12344,0.12378,0.1216,0.052795,0.05277,0.
052809,0.052976,0.052685,0.052711,0.052834,0.052905,0.053087,0.052999,
0.052639,0.053206,0.052901,0.052261,0.053259,0.052559,0.052867,0.05311
,0.052626,0.0528,0.053503,0.052283,0.052819,0.053062,0.052553,0.1095,0
.12605,0.13158,0.10324,0.053395,0.052484,0.052778,0.053474,0.052919,0.
05336,0.053213,0.052341,0.052898,0.052642,0.052369,0.053247,0.05229,0.
052824,0.052967,0.052978,0.053078,0.052442,0.051992,0.052616,0.052598,
0.052707,0.052143,0.052614,0.053155,0.052207,0.0521,0.052857,0.053384,
0.053448,0.052866,0.052449,0.052482,0.052827,0.053058,0.052873,0.05287
5,0.052851,0.05308,0.05307,0.053607,0.052419,0.05289,0.053023,0.052721
,0.052841,0.052395,0.053621,0.052785,0.052626,0.053029,0.052567,0.0523
97,0.052585,0.052825,0.05278,0.052263,0.053019,0.05238,0.052463,0.0532
23,0.052719,0.052907,0.052554,0.053408,0.052599,0.053454,0.053258,0.05
3449,0.052328,0.052442,0.052865,0.053341,0.052957,0.052462,0.052425,0.
052465,0.053116,0.053173,0.052393,0.052529,0.052809,0.052765,0.052726,
0.053098,0.052722,0.053055,0.05339,0.052258,0.053727,0.052325,0.053172
,0.053018,0.05261,0.052616,0.053185,0.052725,0.052643,0.052743,0.05313
7,0.053107,0.052375,0.052932,0.05326,0.052188,0.052918,0.052626,0.0525
35,0.053616,0.052292,0.052905,0.053163,0.052757,0.052019,0.053049,0.05
2737,0.05279,0.052558,0.052567,0.052761,0.052593,0.052698,0.053246,0.0
52466,0.052302,0.052689,0.053247,0.05226,0.052909,0.05232,0.053064,0.0
53316,0.053405,0.052959,0.052384,0.052631,0.053451,0.05283,0.053502,0.
052809,0.052746,0.05268,0.052518,0.05329,0.053021,0.052787,0.052094,0.
052565,0.052488,0.052452,0.052591,0.052413,0.05279,0.052768,0.052745,0
.05281,0.052623,0.052625,0.052688,0.053414,0.052866,0.053509,0.053013,
0.0532,0.052469,0.052348,0.053115,0.054041,0.052965,0.052856,0.052395,
0.052996,0.052445,0.052531,0.053119,0.052839,0.051845,0.053458,0.05245
9,0.052397,0.052717,0.053231,0.052836,0.052732,0.052756,0.05361,0.0526
17,0.05301,0.053022,0.052764,0.052376,0.053316,0.053221,0.052543,0.053
083
0.19657,0.19569,0.19621,0.19634,0.19621,0.19531,0.1965,0.19629,0.19444
,0.19643,0.19643,0.1964,0.19607,0.19611,0.1949,0.1958,0.19543,0.19603,
0.19666,0.19684,0.29695,0.28453,0.2939,0.29383,0.19488,0.19644,0.19608
,0.19597,0.19588,0.19551,0.19655,0.19568,0.19567,0.19655,0.19685,0.196
02,0.19686,0.19503,0.19641,0.39522,0.29303,0.29411,0.29533,0.29857,0.2

5879,0.2952,0.19652,0.19626,0.19684,0.19482,0.19538,0.19539,0.19718,0.
19669,0.1957,0.19595,0.19526,0.19599,0.19541,0.19655,0.19651,0.38912,0
.38065,0.26742,0.28605,0.29443,0.29865,0.29179,0.30732,0.30506,0.27623
,0.1965,0.19679,0.19535,0.19632,0.19649,0.19678,0.19582,0.19591,0.1958
2,0.19585,0.19663,0.19542,0.19598,0.19611,0.19608,0.38954,0.3905,0.384
16,0.29394,0.30338,0.30976,0.2825,0.31075,0.27549,0.33773,0.29737,0.29
413,0.19538,0.19613,0.19523,0.197,0.19559,0.19603,0.19516,0.19674,0.19
628,0.1955,0.19623,0.1966,0.1952,0.19593,0.19629,0.19572,0.39766,0.398
98,0.39771,0.39417,0.27953,0.30022,0.29464,0.29942,0.29114,0.29457,0.2
9653,0.29413,0.29668,0.29738,0.19659,0.19651,0.19653,0.19662,0.19481,0
.19585,0.19653,0.19588,0.19595,0.19612,0.19625,0.19576,0.1974,0.19511,
0.19562,0.19648,0.38506,0.40233,0.39165,0.38634,0.39647,0.3852,0.29554
,0.31752,0.29597,0.29182,0.29417,0.29788,0.29496,0.30454,0.29808,0.299
62,0.19562,0.19628,0.19583,0.19645,0.19567,0.19693,0.19661,0.19651,0.1
9587,0.19609,0.19678,0.19603,0.19604,0.19492,0.19633,0.19592,0.19616,0
.3897,0.39702,0.38506,0.39198,0.39295,0.3906,0.3008,0.28896,0.31406,0.
29322,0.30192,0.31203,0.2944,0.29406,0.29746,0.28558,0.30835,0.19538,0
.19788,0.19516,0.1962,0.19585,0.19526,0.19565,0.19549,0.19701,0.19662,
0.19656,0.19505,0.19597,0.12205,0.13216,0.12611,0.19514,0.39433,0.3953
9,0.38041,0.38924,0.39132,0.38841,0.39585,0.2998,0.31127,0.29634,0.291
9,0.29897,0.28706,0.26882,0.29928,0.29748,0.29154,0.2947,0.19689,0.196
31,0.197,0.19566,0.19519,0.19615,0.19604,0.19622,0.1968,0.19625,0.1966
2,0.12107,0.11162,0.11209,0.12276,0.11599,0.11234,0.19678,0.38462,0.39
587,0.4056,0.39268,0.39155,0.38612,0.3931,0.27994,0.29855,0.29176,0.29
387,0.29147,0.30222,0.29626,0.30793,0.29117,0.29254,0.31419,0.19577,0.
19599,0.1967,0.1971,0.19514,0.19553,0.1964,0.19621,0.19489,0.1273,0.11
328,0.13001,0.12134,0.12892,0.12538,0.12314,0.10246,0.12175,0.19604,0.
39359,0.39635,0.38804,0.39942,0.39807,0.39079,0.39058,0.30232,0.30799,
0.29861,0.29385,0.28784,0.29813,0.29933,0.28756,0.29347,0.31533,0.2918
7,0.29954,0.19544,0.19556,0.19652,0.19591,0.19522,0.19581,0.12305,0.12
2,0.12047,0.1222,0.12586,0.12246,0.12373,0.13629,0.12216,0.12209,0.115
88,0.1201,0.39399,0.40087,0.39845,0.40037,0.39426,0.39384,0.40027,0.29
502,0.28906,0.30246,0.29269,0.29287,0.29599,0.2887,0.30533,0.30232,0.2
9962,0.29256,0.2865,0.19514,0.19657,0.19731,0.11865,0.13355,0.12205,0.
14256,0.1226,0.12495,0.13762,0.091574,0.12232,0.12311,0.12992,0.1187,0
.10949,0.12883,0.12251,0.39728,0.39296,0.38545,0.39115,0.39819,0.39588
,0.39107,0.39698,0.38495,0.29592,0.28634,0.27824,0.29302,0.29931,0.264
23,0.29133,0.29067,0.29592,0.29657,0.29766,0.1223,0.13594,0.13466,0.12
316,0.12198,0.1191,0.11728,0.12278,0.15346,0.12233,0.11618,0.11958,0.1
2349,0.12322,0.12294,0.12861,0.14313,0.12962,0.3968,0.39443,0.39728,0.
38299,0.39817,0.39597,0.38916,0.40692,0.39316,0.3068,0.30297,0.21465,0
.21589,0.21822,0.21663,0.21884,0.21341,0.20981,0.22168,0.23871,0.10984
,0.12016,0.12223,0.1203,0.12224,0.096893,0.12059,0.12642,0.11381,0.121
71,0.1332,0.1122,0.12452,0.12206,0.1231,0.11378,0.11696,0.11651,0.3892
,0.40762,0.39266,0.3872,0.39386,0.40071,0.39567,0.40009,0.39924,0.2150
6,0.21163,0.23643,0.21097,0.21037,0.21418,0.21028,0.1875,0.20427,0.228
29,0.21721,0.12204,0.099386,0.12333,0.12248,0.1254,0.12282,0.10662,0.1
209,0.12898,0.1212,0.10953,0.12182,0.13178,0.12428,0.12913,0.13107,0.1
1884,0.134,0.3917,0.39507,0.40184,0.40481,0.38653,0.38846,0.3993,0.223
03,0.2499,0.24301,0.21203,0.2267,0.21068,0.22688,0.23056,0.21088,0.217
29,0.22386,0.20374,0.21859,0.12279,0.13893,0.1221,0.11326,0.10046,0.10
642,0.1098,0.12258,0.12222,0.12298,0.1235,0.12203,0.12248,0.12315,0.12

31,0.11807,0.09523,0.12869,0.39308,0.3912,0.39743,0.39389,0.38387,0.40
331,0.23111,0.22934,0.2132,0.17683,0.20555,0.21287,0.19376,0.22733,0.1
8634,0.19811,0.20326,0.22974,0.22199,0.23293,0.12298,0.11832,0.12202,0
.11862,0.10881,0.12823,0.11802,0.099167,0.12517,0.11754,0.13008,0.1072
1,0.11636,0.12476,0.11763,0.12238,0.11859,0.10598,0.39629,0.39996,0.39
799,0.40023,0.40114,0.20939,0.22383,0.22038,0.21869,0.20334,0.21977,0.
18997,0.18859,0.23155,0.19573,0.21638,0.21203,0.21228,0.21332,0.21531,
0.12119,0.11514,0.12834,0.1221,0.12229,0.12544,0.13796,0.12436,0.11648
,0.12281,0.12021,0.11456,0.12238,0.11951,0.12077,0.12262,0.13189,0.128
18,0.39464,0.40096,0.39089,0.22206,0.21829,0.21877,0.22107,0.2246,0.21
483,0.22764,0.18871,0.21272,0.20966,0.21177,0.18131,0.19506,0.21846,0.
21039,0.18341,0.21224,0.15376,0.12557,0.12221,0.12209,0.12239,0.12246,
0.11668,0.12262,0.13598,0.11981,0.12673,0.11099,0.12203,0.11076,0.0935
09,0.12525,0.12899,0.12153,0.39507,0.22428,0.23191,0.21127,0.22054,0.2
461,0.22173,0.25511,0.2218,0.21262,0.17525,0.21349,0.22166,0.20721,0.1
9705,0.21514,0.21564,0.20247,0.20579,0.12677,0.12144,0.11734,0.11183,0
.122,0.1224,0.11943,0.11241,0.1218,0.12636,0.12187,0.12203,0.12184,0.1
1409,0.10675,0.12587,0.12332,0.048602,0.20363,0.21774,0.20474,0.15822,
0.24103,0.1569,0.19584,0.23047,0.23288,0.22225,0.21223,0.22842,0.23394
,0.18551,0.20703,0.25012,0.21298,0.21559,0.20334,0.12808,0.12375,0.123
41,0.12749,0.12421,0.13658,0.12354,0.10849,0.12183,0.10413,0.12229,0.1
2213,0.12965,0.11816,0.12248,0.12609,0.12227,0.048963,0.24071,0.25917,
0.22847,0.21683,0.17448,0.22187,0.22385,0.22169,0.2251,0.22309,0.24443
,0.20593,0.21241,0.21027,0.2112,0.22604,0.21181,0.20436,0.1224,0.1151,
0.11435,0.13732,0.1245,0.13681,0.12261,0.12967,0.12336,0.1217,0.11603,
0.12215,0.12458,0.10855,0.12179,0.12136,0.11563,0.11771,0.04845,0.2357
1,0.22467,0.15548,0.20344,0.23428,0.21099,0.22781,0.22146,0.22249,0.21
821,0.20849,0.1832,0.21185,0.21081,0.21578,0.19817,0.19882,0.22794,0.1
1475,0.12218,0.14738,0.12141,0.13166,0.12254,0.12383,0.12182,0.11232,0
.13096,0.12228,0.11439,0.12674,0.12003,0.12205,0.12312,0.14504,0.04882
6,0.048549,0.048665,0.29432,0.20913,0.22002,0.22181,0.25607,0.23275,0.
22035,0.22107,0.19565,0.2121,0.19225,0.19792,0.22226,0.21331,0.2144,0.
11433,0.12252,0.12225,0.1371,0.098597,0.10981,0.13071,0.12682,0.10667,
0.1198,0.1236,0.12485,0.11148,0.13389,0.071396,0.11069,0.13606,0.12601
,0.048398,0.048785,0.048898,0.048358,0.21825,0.24869,0.24699,0.21085,0
.22152,0.18679,0.21042,0.20279,0.22528,0.17313,0.21249,0.19718,0.11365
,0.122,0.16978,0.11775,0.11517,0.11986,0.11842,0.12203,0.13859,0.12416
,0.1227,0.13351,0.1165,0.12232,0.1594,0.128,0.12231,0.12708,0.12172,0.
12759,0.048918,0.048947,0.048892,0.048938,0.049088,0.049189,0.24833,0.
25224,0.2187,0.21774,0.29788,0.18995,0.23067,0.22108,0.1398,0.12413,0.
13249,0.12528,0.12481,0.12611,0.1418,0.12258,0.12012,0.12561,0.12269,0
.13223,0.12382,0.11847,0.11113,0.11634,0.11308,0.12229,0.12196,0.11545
,0.12214,0.12226,0.1132,0.048416,0.048797,0.049083,0.048869,0.048386,0
.04881,0.22406,0.22071,0.24383,0.23025,0.22594,0.047929,0.048513,0.119
25,0.12455,0.10855,0.12173,0.12229,0.12256,0.12233,0.12199,0.12257,0.1
2728,0.12242,0.12549,0.12216,0.15276,0.14543,0.11755,0.12656,0.12221,0
.1267,0.12206,0.12182,0.048171,0.048264,0.048301,0.049191,0.049253,0.0
47789,0.048012,0.04862,0.048915,0.048744,0.21627,0.23968,0.048153,0.04
8654,0.048592,0.048788,0.12848,0.10527,0.12956,0.11682,0.11631,0.13566
,0.12264,0.12234,0.1214,0.12187,0.12108,0.13033,0.12234,0.12257,0.1305
4,0.12503,0.12152,0.12591,0.1149,0.048339,0.048631,0.048529,0.048455,0
.048185,0.048936,0.048636,0.048463,0.048907,0.048528,0.048632,0.049116

,0.04909,0.048428,0.048742,0.048417,0.048753,0.12278,0.12172,0.12224,0
.11074,0.12781,0.11038,0.11765,0.12271,0.12364,0.13353,0.12461,0.12246
,0.13242,0.13005,0.12648,0.11975,0.048701,0.049203,0.048645,0.048344,0
.048467,0.049041,0.048803,0.048156,0.048796,0.04883,0.048283,0.048579,
0.048647,0.048329,0.048782,0.048512,0.048288,0.048365,0.048228,0.10945
,0.12292,0.13874,0.12271,0.11085,0.12269,0.12718,0.1249,0.12247,0.1234
3,0.12905,0.12214,0.14444,0.048432,0.048874,0.048839,0.048082,0.048543
,0.048698,0.048516,0.048155,0.048698,0.048362,0.048492,0.048256,0.0489
19,0.048811,0.048782,0.048786,0.048683,0.04828,0.048612,0.048531,0.048
744,0.12224,0.10889,0.11968,0.12013,0.12003,0.092802,0.12264,0.12273,0
.12053,0.048751,0.048558,0.048671,0.048969,0.048549,0.048299,0.048496,
0.048678,0.04888,0.048661,0.0488,0.049044,0.048648,0.048221,0.048996,0
.048702,0.048967,0.048829,0.048418,0.048736,0.048855,0.048278,0.048705
,0.049049,0.048144,0.1079,0.12502,0.13081,0.10146,0.049408,0.048275,0.
048709,0.049249,0.048993,0.048979,0.04853,0.047852,0.048718,0.048463,0
.048351,0.049101,0.048181,0.048673,0.048839,0.048984,0.04913,0.048488,
0.048012,0.048357,0.048938,0.048438,0.047936,0.048891,0.048875,0.04792
5,0.048,0.048629,0.049076,0.049388,0.048745,0.048362,0.048368,0.04876,
0.049091,0.048824,0.048661,0.048458,0.048822,0.048976,0.04925,0.048151
,0.048687,0.048689,0.048831,0.048544,0.0482,0.049469,0.048318,0.048446
,0.048871,0.048608,0.048316,0.048724,0.048647,0.04844,0.048366,0.04887
8,0.048314,0.04841,0.048895,0.048446,0.048875,0.048474,0.049087,0.0482
72,0.049004,0.04924,0.049012,0.048455,0.048108,0.048614,0.049361,0.049
14,0.048385,0.047876,0.048548,0.049036,0.049005,0.048085,0.048385,0.04
8788,0.048685,0.048762,0.048744,0.0486,0.048687,0.049097,0.048432,0.04
9743,0.048175,0.048849,0.048946,0.048469,0.04856,0.048981,0.048732,0.0
48586,0.04883,0.04912,0.048792,0.048049,0.049132,0.049095,0.04778,0.04
9153,0.048304,0.048524,0.049623,0.048402,0.048486,0.049054,0.048605,0.
047633,0.049059,0.0483,0.048779,0.048442,0.048246,0.04867,0.048152,0.0
48273,0.049006,0.048703,0.048361,0.048367,0.049057,0.04826,0.048655,0.
048328,0.048697,0.049098,0.049132,0.048679,0.048382,0.04829,0.049209,0
.048342,0.049265,0.048956,0.048833,0.048537,0.048544,0.049155,0.048824
,0.04827,0.047813,0.048763,0.048229,0.048396,0.048528,0.04836,0.048747
,0.048737,0.048631,0.04853,0.048209,0.04858,0.048702,0.049073,0.048744
,0.049429,0.049062,0.049378,0.048473,0.048352,0.048647,0.049748,0.0482
8,0.048519,0.048291,0.048796,0.048157,0.048199,0.049077,0.048625,0.047
681,0.049112,0.048361,0.048296,0.048759,0.04911,0.048766,0.048535,0.04
8556,0.049191,0.048057,0.048706,0.048623,0.048574,0.04841,0.0491,0.049
081,0.048497,0.048605
0.19844,0.19767,0.19822,0.19838,0.1978,0.19739,0.19817,0.19872,0.19646
,0.19833,0.1987,0.19782,0.19768,0.19817,0.19673,0.19791,0.19736,0.1979
7,0.19839,0.19863,0.29308,0.28164,0.29011,0.28944,0.19699,0.19812,0.19
856,0.19786,0.1975,0.19746,0.19811,0.19733,0.19754,0.19827,0.1983,0.19
809,0.19869,0.19685,0.19863,0.38429,0.28892,0.28928,0.28863,0.29614,0.
25704,0.29,0.19861,0.19843,0.19863,0.19697,0.19765,0.19764,0.1987,0.19
831,0.19747,0.19772,0.19755,0.19791,0.1976,0.19837,0.19877,0.38196,0.3
702,0.26617,0.28093,0.29117,0.29327,0.28829,0.30389,0.30285,0.27347,0.
19888,0.19786,0.19707,0.19825,0.19817,0.19865,0.19767,0.19757,0.19791,
0.19799,0.19845,0.19683,0.19821,0.1983,0.19776,0.37921,0.37895,0.37449
,0.28792,0.29599,0.30401,0.27983,0.30522,0.27087,0.32991,0.2931,0.2909
2,0.19738,0.19831,0.19739,0.19875,0.19759,0.19789,0.19705,0.19844,0.19
838,0.19728,0.19746,0.19852,0.19757,0.19785,0.19827,0.19755,0.38478,0.

38901,0.38961,0.38434,0.27695,0.29411,0.28896,0.29307,0.28578,0.2897,0
.2921,0.29047,0.29138,0.29265,0.19824,0.19857,0.19841,0.1979,0.19637,0
.19772,0.19807,0.19773,0.19814,0.19829,0.1975,0.1978,0.19917,0.19731,0
.19788,0.19812,0.37288,0.39054,0.38219,0.37468,0.38314,0.3731,0.29124,
0.31086,0.29148,0.28968,0.28836,0.29365,0.29001,0.29842,0.29165,0.2961
5,0.19745,0.19814,0.19767,0.19787,0.19757,0.19862,0.19881,0.19801,0.19
8,0.19826,0.19848,0.19759,0.19811,0.19725,0.19803,0.19797,0.19799,0.37
672,0.39062,0.37394,0.38181,0.38332,0.38,0.29595,0.28528,0.30892,0.287
57,0.29667,0.30717,0.28898,0.29108,0.29235,0.28244,0.30242,0.19741,0.1
9946,0.19732,0.1977,0.19775,0.19714,0.19792,0.19743,0.19859,0.19852,0.
1984,0.19743,0.19788,0.12141,0.13205,0.12563,0.19698,0.38277,0.38328,0
.37149,0.37627,0.38214,0.378,0.38397,0.29529,0.30576,0.29003,0.28686,0
.29551,0.28246,0.2669,0.29333,0.29419,0.28786,0.2914,0.19883,0.19823,0
.19866,0.19733,0.19718,0.19791,0.19804,0.1985,0.19897,0.19837,0.19853,
0.12057,0.1108,0.11121,0.12218,0.11521,0.11171,0.19889,0.37541,0.38709
,0.39417,0.38197,0.38264,0.37381,0.38358,0.27516,0.29287,0.28924,0.290
69,0.28989,0.29833,0.29121,0.30129,0.28763,0.28812,0.3088,0.19769,0.19
831,0.1985,0.1992,0.19702,0.19738,0.19809,0.19836,0.19717,0.12696,0.11
265,0.12984,0.12093,0.12885,0.12472,0.12272,0.10127,0.12144,0.1979,0.3
8523,0.38519,0.37498,0.38777,0.38695,0.37964,0.3766,0.29681,0.30277,0.
29152,0.28961,0.28107,0.29446,0.29508,0.28331,0.2908,0.30696,0.28716,0
.29338,0.1973,0.19787,0.19853,0.1978,0.19725,0.19783,0.12261,0.12163,0
.1197,0.12191,0.12563,0.12199,0.1233,0.13638,0.12153,0.12168,0.11524,0
.11939,0.38265,0.38685,0.38444,0.38559,0.37976,0.38127,0.38652,0.28921
,0.28534,0.29615,0.29011,0.28794,0.29065,0.28578,0.30089,0.29695,0.292
5,0.28856,0.28319,0.19726,0.19868,0.19894,0.11803,0.13347,0.12167,0.14
261,0.12209,0.12473,0.13784,0.089965,0.12191,0.12249,0.12963,0.11776,0
.10855,0.12864,0.12208,0.39346,0.38499,0.3777,0.37998,0.38723,0.38479,
0.38568,0.38544,0.37597,0.29235,0.28211,0.27438,0.29079,0.29407,0.2614
3,0.28795,0.28563,0.28896,0.29153,0.29079,0.12171,0.13594,0.13443,0.12
281,0.12142,0.11857,0.11666,0.12221,0.15407,0.12187,0.11555,0.11875,0.
12328,0.1227,0.12225,0.12857,0.14349,0.12942,0.38494,0.38515,0.38695,0
.37222,0.38856,0.38547,0.37806,0.39826,0.37985,0.29825,0.29864,0.20981
,0.21176,0.21417,0.21134,0.21478,0.20951,0.20807,0.21674,0.23254,0.108
78,0.11958,0.12173,0.1199,0.12177,0.095468,0.11997,0.12601,0.11325,0.1
2115,0.13295,0.11135,0.12428,0.12168,0.12254,0.11315,0.11623,0.11586,0
.38033,0.39663,0.37637,0.37486,0.38049,0.39183,0.38423,0.3876,0.38739,
0.20759,0.20901,0.2332,0.20691,0.2066,0.21051,0.20622,0.185,0.20017,0.
22478,0.21425,0.12135,0.098083,0.12301,0.12222,0.12518,0.12227,0.10542
,0.12035,0.12858,0.12072,0.10872,0.12145,0.13187,0.12377,0.12894,0.130
87,0.11837,0.13404,0.37882,0.38285,0.38845,0.3906,0.37598,0.37375,0.38
895,0.21632,0.24119,0.2331,0.2088,0.22365,0.20719,0.22178,0.22745,0.20
735,0.21261,0.22043,0.20046,0.21646,0.12229,0.13908,0.12179,0.11249,0.
099454,0.10535,0.10897,0.12191,0.12193,0.12251,0.12303,0.1216,0.12196,
0.12275,0.12226,0.11755,0.093938,0.12843,0.38347,0.37828,0.38627,0.379
85,0.37506,0.39224,0.22634,0.22319,0.20803,0.1716,0.199,0.20948,0.1903
4,0.22382,0.18311,0.19587,0.19958,0.22391,0.2178,0.22869,0.12266,0.117
96,0.1218,0.11811,0.10794,0.12817,0.11737,0.097896,0.1248,0.11691,0.12
986,0.10615,0.11571,0.12412,0.11675,0.12183,0.11793,0.10495,0.38487,0.
39314,0.3839,0.38654,0.38743,0.2016,0.21527,0.21441,0.20994,0.19773,0.
21246,0.18603,0.18644,0.22853,0.19311,0.21184,0.20934,0.20956,0.20802,
0.21101,0.12071,0.11448,0.12803,0.12169,0.1221,0.12487,0.13787,0.12388

,0.11576,0.12267,0.11977,0.11401,0.12164,0.11894,0.11995,0.12219,0.131
73,0.12798,0.38247,0.38749,0.37577,0.21499,0.21236,0.21169,0.21148,0.2
1785,0.20601,0.22123,0.18313,0.20963,0.20378,0.20873,0.17789,0.19117,0
.21553,0.20665,0.18082,0.20812,0.15465,0.1252,0.12189,0.1218,0.1219,0.
12183,0.11595,0.12204,0.13607,0.11908,0.12642,0.11022,0.12181,0.10992,
0.092165,0.12476,0.12861,0.12091,0.38797,0.21859,0.22307,0.20295,0.215
67,0.24057,0.2143,0.24561,0.2153,0.20652,0.17272,0.20962,0.21735,0.203
53,0.19311,0.21126,0.20959,0.19848,0.20139,0.12626,0.12098,0.11658,0.1
1097,0.1218,0.12211,0.11894,0.11172,0.12117,0.12613,0.12127,0.12145,0.
12126,0.11325,0.10574,0.12538,0.12283,0.045866,0.19765,0.21279,0.19758
,0.15186,0.23304,0.15255,0.18704,0.22313,0.22806,0.21401,0.20818,0.224
7,0.22852,0.1825,0.20355,0.24454,0.20854,0.21247,0.19964,0.12766,0.123
32,0.12301,0.12733,0.12369,0.13663,0.12329,0.10748,0.1213,0.10294,0.12
174,0.1216,0.1296,0.1175,0.12208,0.1256,0.12182,0.046163,0.23189,0.250
62,0.22138,0.2106,0.16961,0.21661,0.21588,0.21522,0.22072,0.217,0.2389
9,0.20169,0.20981,0.20481,0.20773,0.22227,0.20805,0.20097,0.12188,0.11
449,0.11367,0.1371,0.12419,0.13669,0.12211,0.12937,0.12291,0.12132,0.1
1543,0.12187,0.12438,0.10802,0.12153,0.12073,0.11474,0.11714,0.045536,
0.2272,0.22004,0.15197,0.19832,0.2255,0.2047,0.22127,0.21372,0.21603,0
.21282,0.20513,0.17978,0.20756,0.20678,0.21217,0.19397,0.19516,0.22418
,0.11404,0.1215,0.14754,0.12091,0.13158,0.12203,0.12366,0.12127,0.1115
4,0.13076,0.12176,0.11379,0.12639,0.1197,0.12152,0.12274,0.1454,0.0459
69,0.045578,0.046106,0.2843,0.20229,0.21425,0.21682,0.24967,0.22659,0.
21313,0.21069,0.19014,0.20913,0.18835,0.19527,0.21745,0.20988,0.20898,
0.11361,0.12163,0.12179,0.1372,0.097121,0.109,0.13042,0.1266,0.1057,0.
11911,0.12342,0.12436,0.11064,0.13389,0.069344,0.10984,0.13603,0.12552
,0.045883,0.046355,0.046278,0.045731,0.21034,0.24199,0.24054,0.20213,0
.2134,0.17932,0.20332,0.19749,0.21944,0.17089,0.20877,0.1945,0.11282,0
.12166,0.17073,0.117,0.11464,0.11916,0.11764,0.12134,0.13889,0.12369,0
.12229,0.13347,0.11587,0.12178,0.1601,0.12751,0.12197,0.12657,0.12145,
0.12732,0.046296,0.045928,0.046017,0.046382,0.046327,0.046483,0.24071,
0.24351,0.21374,0.21214,0.28963,0.18402,0.22225,0.2148,0.13999,0.12382
,0.13251,0.1247,0.12454,0.12589,0.1422,0.12218,0.11948,0.12505,0.12235
,0.13196,0.12342,0.11782,0.11011,0.11565,0.11241,0.12198,0.1215,0.1148
5,0.12183,0.12172,0.11246,0.045362,0.046009,0.046361,0.046345,0.045768
,0.046012,0.21566,0.21281,0.2352,0.22394,0.21935,0.045087,0.046094,0.1
1898,0.12417,0.10759,0.12147,0.1218,0.12194,0.12185,0.12162,0.12196,0.
12702,0.1218,0.12504,0.12172,0.15319,0.14572,0.11685,0.12634,0.1217,0.
12637,0.12163,0.12127,0.045418,0.045338,0.045297,0.046571,0.046556,0.0
44759,0.045248,0.046137,0.046206,0.045804,0.20964,0.23456,0.045572,0.0
45947,0.045595,0.046068,0.12831,0.1045,0.12911,0.11599,0.11581,0.13556
,0.12222,0.12178,0.12098,0.12178,0.12055,0.13019,0.12185,0.1218,0.1302
8,0.12466,0.12119,0.12553,0.11408,0.045603,0.045741,0.045861,0.045541,
0.045201,0.046024,0.045847,0.045585,0.046099,0.045959,0.045934,0.04652
6,0.04596,0.045684,0.045751,0.045426,0.045754,0.12244,0.12132,0.1216,0
.10987,0.12744,0.1093,0.11706,0.1223,0.1231,0.13331,0.12422,0.12201,0.
13235,0.12975,0.12598,0.11909,0.045799,0.046142,0.045923,0.045419,0.04
5633,0.04623,0.045791,0.045459,0.045974,0.046111,0.045255,0.045692,0.0
45898,0.04565,0.046121,0.045394,0.045681,0.045389,0.045328,0.10868,0.1
225,0.1385,0.12246,0.1102,0.12232,0.12683,0.12457,0.12169,0.12298,0.12
846,0.12159,0.1447,0.045679,0.045678,0.046025,0.045419,0.045703,0.0458
27,0.045416,0.045425,0.045716,0.045762,0.045881,0.045632,0.045976,0.04

6129,0.04583,0.04614,0.045549,0.045571,0.045907,0.045662,0.045786,0.12
185,0.10817,0.11925,0.11962,0.11969,0.09147,0.1221,0.12202,0.12016,0.0
45865,0.045821,0.045897,0.046077,0.045794,0.045579,0.045743,0.045729,0
.046158,0.045731,0.045844,0.046207,0.045467,0.045626,0.046568,0.04582,
0.045889,0.046167,0.045543,0.045909,0.046013,0.045446,0.045988,0.04589
4,0.045326,0.10686,0.12475,0.13066,0.10025,0.046254,0.045709,0.045803,
0.046639,0.046119,0.04638,0.045503,0.045209,0.04579,0.045803,0.045422,
0.046453,0.04523,0.046031,0.045921,0.046234,0.046257,0.045865,0.045278
,0.045308,0.046256,0.045662,0.045156,0.046297,0.046251,0.045126,0.0454
3,0.045849,0.045746,0.046575,0.046101,0.0458,0.045683,0.046059,0.04631
4,0.046029,0.045901,0.045589,0.046171,0.046268,0.046719,0.045269,0.045
699,0.045872,0.045879,0.045556,0.045385,0.046451,0.045869,0.045836,0.0
45678,0.045797,0.045546,0.045821,0.046067,0.045877,0.045412,0.045818,0
.045213,0.04566,0.04587,0.045481,0.046124,0.045414,0.046354,0.045364,0
.046108,0.046332,0.045853,0.045608,0.045633,0.045944,0.046428,0.04617,
0.045572,0.045406,0.045719,0.046055,0.046267,0.045237,0.045674,0.04586
6,0.045759,0.046055,0.045814,0.045574,0.045918,0.046276,0.045636,0.046
722,0.04548,0.045713,0.045921,0.045815,0.045441,0.04625,0.045805,0.045
878,0.045687,0.046305,0.046094,0.045201,0.046131,0.046249,0.045174,0.0
46492,0.045437,0.045591,0.046595,0.045368,0.045913,0.046312,0.046121,0
.044958,0.046297,0.045288,0.046057,0.045878,0.045429,0.045719,0.045459
,0.045557,0.046394,0.046162,0.045295,0.045793,0.046329,0.045829,0.0461
94,0.045757,0.045863,0.04645,0.046379,0.045904,0.045714,0.045134,0.046
339,0.045652,0.046647,0.045925,0.046003,0.045499,0.045743,0.046499,0.0
45973,0.045659,0.044698,0.046131,0.045263,0.045838,0.045724,0.045644,0
.045835,0.04596,0.045906,0.04564,0.045061,0.04558,0.045847,0.045985,0.
045971,0.046646,0.046238,0.046542,0.045584,0.045771,0.045751,0.046959,
0.045501,0.045749,0.045796,0.045879,0.045566,0.045068,0.046156,0.04586
,0.045006,0.046025,0.045494,0.045391,0.045894,0.046465,0.046049,0.0458
34,0.045664,0.046268,0.045072,0.045803,0.046153,0.045534,0.04551,0.046
301,0.046324,0.045784,0.045693
0.19979,0.19929,0.19991,0.19961,0.19963,0.19923,0.19977,0.20066,0.1978
6,0.19966,0.20008,0.19962,0.19903,0.1996,0.19812,0.19971,0.19921,0.199
46,0.19994,0.20022,0.29417,0.27747,0.28727,0.28544,0.19925,0.19949,0.2
002,0.19972,0.19908,0.19886,0.19988,0.19888,0.19902,0.20016,0.2,0.1994
2,0.20012,0.19863,0.20044,0.3778,0.2857,0.28813,0.28526,0.29307,0.2565
4,0.28796,0.19965,0.19993,0.20012,0.19845,0.19931,0.19889,0.20014,0.19
96,0.19873,0.19949,0.19874,0.19977,0.19896,0.19974,0.20013,0.37244,0.3
6816,0.26547,0.27824,0.28825,0.29058,0.28651,0.30162,0.30038,0.27118,0
.2006,0.19945,0.19852,0.20007,0.1995,0.20053,0.19913,0.19949,0.19935,0
.19955,0.19979,0.19838,0.19962,0.1996,0.19932,0.37171,0.37275,0.36802,
0.2861,0.29291,0.29876,0.27684,0.29921,0.2676,0.32241,0.29103,0.28928,
0.19923,0.20006,0.19969,0.20056,0.19947,0.19969,0.19863,0.20032,0.2000
3,0.19887,0.19899,0.19999,0.19933,0.19945,0.19942,0.19913,0.37717,0.38
587,0.38594,0.37327,0.27477,0.28946,0.28704,0.2914,0.28418,0.28677,0.2
8791,0.28677,0.2873,0.28936,0.19981,0.19932,0.20016,0.19907,0.19781,0.
19958,0.19945,0.19951,0.19947,0.20016,0.19899,0.19962,0.20069,0.19906,
0.19941,0.19972,0.36271,0.38359,0.37057,0.37095,0.37745,0.36261,0.2869
,0.30476,0.28756,0.28691,0.28596,0.29171,0.28614,0.29571,0.28965,0.292
16,0.19978,0.19964,0.19972,0.19989,0.1991,0.20024,0.19982,0.19987,0.19
968,0.19942,0.19981,0.19982,0.19962,0.19859,0.19931,0.19951,0.1992,0.3
6626,0.38408,0.36878,0.37418,0.37607,0.37424,0.29143,0.28302,0.30439,0

.28411,0.29455,0.30386,0.2844,0.28829,0.28894,0.27904,0.29786,0.19927,
0.20061,0.1991,0.19956,0.19944,0.19866,0.19917,0.19898,0.20028,0.2,0.1
9962,0.19907,0.19969,0.121,0.13181,0.1256,0.19867,0.37512,0.37927,0.36
73,0.37174,0.37224,0.37099,0.37572,0.291,0.30105,0.28584,0.28398,0.293
49,0.27806,0.2662,0.2904,0.29267,0.28715,0.2887,0.20028,0.1999,0.19994
,0.19878,0.1986,0.19925,0.1995,0.20022,0.20073,0.20008,0.2,0.12014,0.1
1052,0.11059,0.12182,0.11481,0.11129,0.20013,0.36839,0.38218,0.38164,0
.36909,0.37218,0.36493,0.37258,0.27393,0.28755,0.28749,0.28654,0.28874
,0.29383,0.28777,0.29756,0.28714,0.28383,0.30413,0.19908,0.19966,0.200
04,0.20098,0.19882,0.19929,0.20005,0.20012,0.19888,0.12706,0.11211,0.1
2988,0.12099,0.12867,0.12466,0.12242,0.10058,0.12133,0.19972,0.37642,0
.37841,0.36889,0.381,0.37498,0.37077,0.36963,0.29295,0.29927,0.28692,0
.28677,0.27935,0.29008,0.29278,0.2809,0.28607,0.30249,0.28545,0.29185,
0.19884,0.19953,0.20008,0.1997,0.19904,0.19924,0.1224,0.1216,0.11956,0
.12154,0.12539,0.12189,0.12315,0.13641,0.12149,0.1216,0.115,0.11928,0.
37425,0.38063,0.37421,0.37691,0.36747,0.37173,0.37796,0.28762,0.28223,
0.2943,0.28472,0.28357,0.28745,0.28328,0.29861,0.29355,0.28701,0.28635
,0.28035,0.19874,0.19998,0.2005,0.11774,0.13393,0.12155,0.14302,0.1219
7,0.12473,0.13804,0.088994,0.12178,0.12214,0.12956,0.11766,0.10796,0.1
2869,0.12195,0.38513,0.37985,0.37177,0.3717,0.38046,0.37958,0.37495,0.
37613,0.36654,0.28883,0.2791,0.27253,0.28721,0.29279,0.26047,0.28638,0
.28273,0.28767,0.28885,0.28634,0.12157,0.13624,0.13462,0.12259,0.12131
,0.11842,0.11627,0.12187,0.15459,0.1214,0.1151,0.11853,0.12295,0.12275
,0.12223,0.12867,0.14377,0.12943,0.37788,0.37957,0.3761,0.36935,0.3831
,0.38034,0.36958,0.38534,0.37594,0.29624,0.29778,0.20768,0.20878,0.210
69,0.20698,0.21206,0.20613,0.20497,0.21364,0.22688,0.10817,0.11932,0.1
2161,0.11974,0.12158,0.094485,0.11966,0.12618,0.11287,0.121,0.13314,0.
1109,0.12415,0.12164,0.12255,0.11286,0.1158,0.1155,0.37247,0.39093,0.3
7286,0.36743,0.37411,0.38528,0.3799,0.38249,0.38057,0.2027,0.20694,0.2
2977,0.2022,0.20458,0.20778,0.20519,0.18314,0.19728,0.22245,0.2109,0.1
2135,0.097427,0.12315,0.12205,0.12484,0.12198,0.1049,0.12029,0.12884,0
.12019,0.10824,0.12114,0.13168,0.12348,0.12912,0.13113,0.11842,0.13439
,0.36804,0.37206,0.37878,0.38506,0.37188,0.36534,0.38523,0.21225,0.235
74,0.22991,0.20603,0.22158,0.20469,0.21886,0.2247,0.20409,0.21023,0.21
808,0.19708,0.21526,0.12206,0.13966,0.12155,0.11222,0.098995,0.10478,0
.1086,0.12173,0.12184,0.12206,0.12288,0.12154,0.12194,0.12261,0.12197,
0.11735,0.093019,0.12823,0.3769,0.37278,0.37986,0.37386,0.36894,0.3839
7,0.22234,0.2194,0.20274,0.16776,0.19347,0.20747,0.18965,0.22039,0.181
47,0.19331,0.19685,0.22159,0.21538,0.22473,0.1225,0.11791,0.12154,0.11
774,0.1075,0.12785,0.1169,0.097272,0.1248,0.11659,0.12993,0.10587,0.11
513,0.12418,0.11654,0.12152,0.11763,0.10438,0.38055,0.38241,0.37903,0.
37822,0.38005,0.19665,0.21139,0.21026,0.20585,0.19333,0.20766,0.18465,
0.18438,0.22525,0.19239,0.20804,0.20685,0.20483,0.20606,0.20933,0.1205
3,0.11417,0.12797,0.12173,0.12212,0.12475,0.13802,0.12359,0.11556,0.12
257,0.11953,0.11376,0.12143,0.1185,0.11959,0.12198,0.13172,0.12787,0.3
736,0.3785,0.37068,0.20688,0.20606,0.20855,0.20665,0.21363,0.20223,0.2
1659,0.17795,0.20785,0.20168,0.20624,0.1758,0.18978,0.21322,0.20569,0.
1795,0.20603,0.15522,0.12519,0.12167,0.12166,0.12192,0.12172,0.1159,0.
12171,0.13621,0.11899,0.12651,0.10985,0.12162,0.10946,0.091146,0.12482
,0.12866,0.12067,0.37684,0.21399,0.21847,0.19917,0.21106,0.23589,0.210
39,0.23994,0.21157,0.20054,0.17087,0.20713,0.21412,0.20129,0.18961,0.2
0778,0.20528,0.19581,0.19775,0.12637,0.12076,0.11613,0.11051,0.12152,0

.12188,0.11866,0.11154,0.1208,0.12627,0.12121,0.1212,0.12116,0.11277,0
.10528,0.12532,0.12262,0.043784,0.19136,0.20846,0.19297,0.14836,0.2270
3,0.14919,0.18237,0.21937,0.22402,0.21233,0.20507,0.223,0.22311,0.1794
7,0.20043,0.23991,0.20585,0.20925,0.19621,0.12748,0.12321,0.12283,0.12
717,0.12345,0.13671,0.12346,0.10684,0.12114,0.10243,0.12164,0.12144,0.
1298,0.11696,0.12175,0.12564,0.12165,0.044239,0.22939,0.24654,0.2177,0
.20549,0.16801,0.20982,0.21214,0.20805,0.21585,0.21111,0.23506,0.1997,
0.20839,0.20403,0.20539,0.21954,0.20447,0.19957,0.12166,0.11411,0.1134
6,0.13729,0.12419,0.13672,0.12199,0.12943,0.1227,0.12107,0.11505,0.121
55,0.12415,0.10769,0.12158,0.12062,0.11446,0.11698,0.043905,0.22417,0.
21738,0.14935,0.19307,0.22033,0.1999,0.21771,0.20927,0.21344,0.20898,0
.20075,0.17769,0.20413,0.20365,0.20873,0.19125,0.19383,0.22232,0.11383
,0.1214,0.14802,0.12049,0.13157,0.12205,0.12353,0.12102,0.11103,0.1308
2,0.12172,0.1131,0.12608,0.11939,0.12134,0.12261,0.1458,0.043807,0.043
55,0.044339,0.28184,0.19733,0.20788,0.21397,0.24593,0.22303,0.20835,0.
20717,0.18651,0.20594,0.18598,0.19335,0.216,0.20765,0.20705,0.11347,0.
12162,0.12153,0.13744,0.096515,0.10858,0.13058,0.1262,0.10503,0.11904,
0.12336,0.12433,0.11046,0.13403,0.068131,0.10922,0.13609,0.1257,0.0437
72,0.04446,0.044331,0.043908,0.20511,0.23909,0.23681,0.19704,0.20757,0
.17591,0.19851,0.19242,0.21358,0.16946,0.20644,0.19227,0.11243,0.12137
,0.1718,0.11688,0.1142,0.11866,0.11738,0.12137,0.13946,0.12357,0.12206
,0.13378,0.11568,0.12165,0.16082,0.12741,0.12172,0.1266,0.12121,0.1272
6,0.044388,0.044033,0.04397,0.044449,0.044262,0.044595,0.23819,0.24019
,0.20934,0.20847,0.28328,0.18194,0.219,0.21061,0.14046,0.12363,0.13301
,0.12475,0.12434,0.12575,0.14255,0.1221,0.11932,0.12513,0.12209,0.1321
6,0.1233,0.11753,0.10988,0.11527,0.11217,0.12182,0.12162,0.11457,0.121
62,0.12161,0.11188,0.043753,0.044076,0.044053,0.044483,0.043945,0.0441
62,0.20979,0.20968,0.23065,0.21933,0.21299,0.043196,0.043933,0.11872,0
.12414,0.10702,0.12151,0.12166,0.12161,0.12173,0.12148,0.12182,0.12705
,0.12154,0.1248,0.12142,0.15365,0.1461,0.11664,0.12625,0.12138,0.12604
,0.12134,0.12111,0.04324,0.043424,0.043565,0.044646,0.044431,0.042644,
0.043504,0.044111,0.044381,0.043927,0.20503,0.22965,0.043773,0.044107,
0.043659,0.044338,0.1281,0.10408,0.12915,0.11568,0.11548,0.13572,0.121
92,0.12153,0.12085,0.12182,0.12065,0.13034,0.12165,0.12156,0.13032,0.1
2464,0.12093,0.12535,0.11379,0.043829,0.04411,0.043498,0.043413,0.0433
61,0.044258,0.044098,0.043471,0.044179,0.044024,0.044176,0.044497,0.04
4102,0.043882,0.043798,0.043329,0.043915,0.12254,0.12118,0.12134,0.109
58,0.12747,0.10875,0.11704,0.12199,0.1229,0.13324,0.1239,0.12182,0.132
25,0.12956,0.12595,0.11879,0.043941,0.044222,0.043944,0.043439,0.04361
7,0.044353,0.044075,0.04339,0.044119,0.044403,0.043416,0.043951,0.0438
02,0.044048,0.043988,0.043764,0.043902,0.043555,0.043983,0.10806,0.122
21,0.13889,0.12225,0.10971,0.12198,0.12681,0.12466,0.12146,0.12283,0.1
2846,0.12125,0.14479,0.044191,0.043465,0.043893,0.043523,0.043898,0.04
3675,0.04337,0.043539,0.04383,0.043845,0.044217,0.043585,0.044221,0.04
4176,0.043858,0.044081,0.043492,0.043451,0.043964,0.043733,0.044054,0.
12204,0.1077,0.11913,0.11937,0.11955,0.09048,0.12196,0.12179,0.12005,0
.044056,0.043798,0.043808,0.044221,0.043666,0.043604,0.043701,0.04361,
0.04416,0.043914,0.043967,0.044031,0.043177,0.043568,0.044651,0.044116
,0.043858,0.044122,0.043165,0.043872,0.043899,0.043563,0.043968,0.0438
9,0.043489,0.10624,0.12465,0.13052,0.099692,0.044081,0.04354,0.043611,
0.044596,0.044424,0.044284,0.04368,0.043442,0.043979,0.043729,0.043283
,0.044681,0.043674,0.044043,0.044225,0.044253,0.044226,0.043826,0.0432

84,0.04335,0.044261,0.043959,0.043057,0.044442,0.044402,0.043254,0.043
554,0.043669,0.043751,0.044743,0.044098,0.043841,0.0439,0.044225,0.044
187,0.043958,0.044161,0.043596,0.044162,0.043851,0.044598,0.043271,0.0
44024,0.043803,0.043687,0.043648,0.043438,0.044602,0.043929,0.044008,0
.043583,0.043721,0.043598,0.044126,0.044313,0.043817,0.043416,0.043862
,0.043297,0.043856,0.043942,0.043649,0.044345,0.043457,0.0444,0.043444
,0.044197,0.044863,0.043786,0.04371,0.04397,0.044132,0.044543,0.04415,
0.043818,0.043748,0.044115,0.044008,0.044346,0.043076,0.043834,0.04382
6,0.044118,0.043925,0.043913,0.043711,0.043892,0.044336,0.043903,0.044
623,0.043566,0.043938,0.043998,0.043644,0.043684,0.043639,0.043875,0.0
43866,0.043899,0.044547,0.044228,0.043355,0.043967,0.044541,0.043635,0
.044632,0.043556,0.043815,0.044756,0.043699,0.043993,0.044607,0.044256
,0.043255,0.044143,0.043447,0.044115,0.043767,0.043669,0.04378,0.04358
4,0.043439,0.044472,0.044193,0.043384,0.0442,0.044136,0.04379,0.043968
,0.043845,0.043626,0.044512,0.044347,0.044181,0.043797,0.042933,0.0443
19,0.043848,0.04463,0.044218,0.04408,0.043552,0.044025,0.044468,0.0441
28,0.043827,0.042887,0.044326,0.043209,0.043837,0.043981,0.043471,0.04
4016,0.044033,0.04388,0.043686,0.04314,0.043455,0.043764,0.044054,0.04
4046,0.044677,0.044094,0.044967,0.043552,0.043788,0.043933,0.045107,0.
043617,0.044065,0.043809,0.044015,0.043655,0.043177,0.044258,0.043789,
0.043003,0.044155,0.043355,0.043238,0.044078,0.044565,0.044246,0.04385
3,0.043571,0.044319,0.043021,0.043886,0.044232,0.043698,0.043487,0.044
304,0.044462,0.043903,0.043514
0.20123,0.20053,0.20146,0.20066,0.20156,0.20084,0.20097,0.20207,0.1998
3,0.20055,0.20112,0.20074,0.20047,0.20093,0.19995,0.20094,0.20077,0.20
084,0.20131,0.20139,0.29185,0.2756,0.28621,0.28268,0.20093,0.20109,0.2
017,0.20113,0.20079,0.20024,0.20136,0.2003,0.20024,0.20156,0.2012,0.20
092,0.2012,0.19987,0.20157,0.37261,0.28505,0.28662,0.28443,0.29197,0.2
5534,0.28543,0.20134,0.20087,0.20127,0.19975,0.20079,0.19982,0.20167,0
.20081,0.1999,0.20098,0.2001,0.2009,0.20036,0.20127,0.20148,0.36753,0.
36305,0.26531,0.27764,0.28463,0.28781,0.28366,0.30047,0.29719,0.26994,
0.20194,0.20074,0.20026,0.20135,0.20081,0.20177,0.20059,0.2011,0.20076
,0.20088,0.20114,0.20019,0.20079,0.20075,0.20092,0.36225,0.37057,0.366
84,0.28352,0.29099,0.29719,0.27537,0.2977,0.26631,0.3142,0.28915,0.286
86,0.20103,0.20129,0.20087,0.20162,0.20082,0.20106,0.20013,0.20141,0.2
0127,0.20044,0.20102,0.20112,0.20066,0.20093,0.20089,0.20092,0.37231,0
.37591,0.37681,0.36513,0.27111,0.28736,0.2845,0.28907,0.28146,0.28437,
0.28521,0.28695,0.28206,0.28699,0.20112,0.20084,0.20149,0.20053,0.1996
,0.20104,0.20083,0.20091,0.20091,0.20152,0.20036,0.20112,0.2019,0.2002
8,0.20058,0.2009,0.35696,0.38339,0.36682,0.36438,0.37175,0.35695,0.285
16,0.30372,0.28444,0.28394,0.2852,0.2921,0.28365,0.29228,0.28819,0.288
25,0.20094,0.20142,0.20107,0.20166,0.20035,0.20141,0.20064,0.20129,0.2
0121,0.20055,0.20157,0.20094,0.2007,0.20023,0.20083,0.20094,0.20073,0.
3571,0.37602,0.36237,0.37403,0.372,0.37147,0.29007,0.2824,0.30055,0.28
338,0.29358,0.3021,0.28174,0.28543,0.28842,0.27734,0.29492,0.2007,0.20
177,0.20059,0.20067,0.20093,0.20038,0.20063,0.20012,0.20142,0.20135,0.
20073,0.20061,0.20106,0.12086,0.13204,0.12555,0.20025,0.37161,0.37198,
0.36304,0.36851,0.36619,0.36469,0.36915,0.28865,0.29891,0.2826,0.28437
,0.29227,0.27651,0.26505,0.28717,0.29187,0.28415,0.28661,0.20185,0.201
29,0.20116,0.2006,0.20005,0.20059,0.20126,0.20139,0.20169,0.20114,0.20
115,0.12029,0.1104,0.11042,0.12176,0.11486,0.1111,0.20127,0.36353,0.37
368,0.37477,0.36141,0.37017,0.35753,0.36761,0.27297,0.28485,0.28689,0.

28686,0.28686,0.29309,0.28487,0.29582,0.28556,0.28288,0.3009,0.19991,0
.20118,0.20131,0.20223,0.2006,0.20063,0.20152,0.20148,0.20075,0.12733,
0.11192,0.13019,0.12084,0.12888,0.12499,0.12231,0.10033,0.12124,0.2011
,0.37046,0.3698,0.36145,0.37788,0.37282,0.36093,0.36488,0.29061,0.2967
7,0.28555,0.28441,0.27859,0.28845,0.28936,0.28068,0.2828,0.30106,0.283
91,0.28791,0.20072,0.20119,0.20161,0.20108,0.20023,0.20042,0.12247,0.1
2125,0.11945,0.12167,0.12552,0.12215,0.12331,0.13677,0.12168,0.12164,0
.11495,0.11932,0.36941,0.37648,0.36808,0.37222,0.36314,0.37021,0.37633
,0.28438,0.28122,0.29436,0.28369,0.28225,0.28787,0.28183,0.29906,0.292
9,0.28255,0.2863,0.27736,0.2,0.20121,0.20187,0.11765,0.13432,0.12142,0
.14326,0.12176,0.12463,0.13835,0.088494,0.12182,0.12235,0.12962,0.1176
1,0.10751,0.1288,0.12177,0.376,0.36765,0.36926,0.36459,0.3759,0.37091,
0.36947,0.37472,0.36317,0.28474,0.27814,0.27194,0.28623,0.29003,0.2595
9,0.28535,0.27967,0.2865,0.28617,0.28416,0.12171,0.13626,0.1349,0.1226
1,0.12164,0.11831,0.11601,0.12229,0.15503,0.1215,0.11513,0.11846,0.122
87,0.1227,0.12199,0.12893,0.14389,0.12964,0.37462,0.37646,0.36541,0.36
269,0.37325,0.37936,0.36502,0.37851,0.36954,0.29446,0.29442,0.20551,0.
20634,0.20788,0.20551,0.21089,0.20466,0.20237,0.21206,0.225,0.10797,0.
11927,0.12153,0.11956,0.12169,0.09405,0.11949,0.12629,0.11281,0.12087,
0.13332,0.11076,0.12421,0.12161,0.1229,0.1128,0.11575,0.11527,0.36773,
0.38296,0.36767,0.3643,0.36942,0.38274,0.37254,0.3797,0.37757,0.19882,
0.20381,0.22724,0.20112,0.20232,0.20508,0.20346,0.18104,0.19726,0.2219
5,0.21051,0.12132,0.096873,0.12316,0.12222,0.12497,0.12181,0.10434,0.1
1998,0.12903,0.12026,0.10799,0.12127,0.1319,0.12361,0.12931,0.13149,0.
11845,0.13481,0.36069,0.37082,0.37636,0.38215,0.36646,0.35885,0.37813,
0.20645,0.23203,0.22816,0.20271,0.21988,0.20016,0.21719,0.22343,0.2014
7,0.20789,0.21681,0.19676,0.21289,0.12209,0.14001,0.12169,0.11193,0.09
8456,0.10457,0.10831,0.12201,0.12201,0.1221,0.12299,0.12141,0.122,0.12
249,0.12211,0.11734,0.09237,0.12854,0.37214,0.3663,0.37183,0.36675,0.3
6289,0.37585,0.21932,0.21459,0.19951,0.16421,0.19063,0.20532,0.18777,0
.21787,0.18097,0.19253,0.19579,0.2199,0.21247,0.22257,0.12258,0.11802,
0.12173,0.11755,0.10715,0.12802,0.11686,0.096928,0.12479,0.11642,0.130
26,0.10562,0.11526,0.1243,0.11618,0.12146,0.11752,0.10395,0.37624,0.37
523,0.37296,0.37621,0.37873,0.19458,0.20949,0.20764,0.20149,0.19431,0.
20528,0.18283,0.18352,0.22495,0.19045,0.20692,0.20377,0.20293,0.20348,
0.20734,0.12042,0.11411,0.12816,0.12149,0.12207,0.12483,0.13824,0.1235
9,0.1156,0.12255,0.11954,0.11354,0.12139,0.1186,0.11968,0.12198,0.1317
4,0.12801,0.36515,0.37029,0.36578,0.20432,0.20324,0.20361,0.20272,0.21
034,0.19964,0.21546,0.17639,0.20725,0.19947,0.20412,0.173,0.18828,0.21
082,0.20508,0.17799,0.20327,0.15591,0.12553,0.12166,0.12179,0.12193,0.
12166,0.11577,0.12166,0.13666,0.11887,0.12697,0.10984,0.12172,0.10932,
0.090849,0.12459,0.12888,0.12065,0.37284,0.21161,0.21349,0.19822,0.206
38,0.23343,0.20662,0.23651,0.20696,0.1948,0.16999,0.20434,0.21245,0.20
086,0.18905,0.20561,0.20264,0.19418,0.19621,0.12635,0.12058,0.11628,0.
11031,0.1215,0.12188,0.11857,0.1114,0.12103,0.12617,0.12113,0.12126,0.
12132,0.11285,0.10525,0.1254,0.12255,0.042629,0.18896,0.20653,0.19146,
0.14545,0.22217,0.14612,0.17772,0.21798,0.22239,0.2072,0.20227,0.22237
,0.2201,0.17845,0.19782,0.23734,0.20331,0.20682,0.19527,0.12746,0.1235
4,0.12276,0.12725,0.12357,0.13733,0.12378,0.1066,0.12137,0.10202,0.121
52,0.12151,0.1298,0.11699,0.12194,0.12566,0.12162,0.042881,0.22629,0.2
4295,0.21412,0.20112,0.16533,0.20739,0.20721,0.20399,0.21077,0.20919,0
.23275,0.19857,0.20824,0.20188,0.20365,0.21721,0.20226,0.1977,0.12149,

0.11395,0.11323,0.13761,0.12421,0.13696,0.12184,0.12955,0.12291,0.1212
1,0.11498,0.12148,0.12416,0.10755,0.12171,0.12061,0.11457,0.1168,0.042
618,0.22268,0.21423,0.14609,0.18836,0.217,0.1974,0.21464,0.20455,0.206
53,0.20624,0.19871,0.17678,0.2019,0.20243,0.20801,0.18985,0.19218,0.21
915,0.11363,0.12154,0.14845,0.12067,0.13182,0.12205,0.12322,0.12098,0.
11089,0.13089,0.12174,0.11307,0.12609,0.11932,0.1215,0.1226,0.14636,0.
042736,0.042289,0.043168,0.277,0.19353,0.20434,0.20927,0.24403,0.22267
,0.20595,0.20448,0.18325,0.20382,0.18477,0.19074,0.21471,0.20668,0.204
11,0.11325,0.12164,0.12191,0.13786,0.095915,0.10818,0.13074,0.1265,0.1
0468,0.11895,0.12352,0.12451,0.11037,0.13422,0.067227,0.10908,0.13628,
0.12573,0.042202,0.043051,0.042951,0.042509,0.2019,0.23545,0.23147,0.1
9321,0.20406,0.17384,0.19234,0.18677,0.20839,0.16737,0.2045,0.19131,0.
11223,0.1215,0.17264,0.11679,0.11392,0.11873,0.11746,0.12151,0.13979,0
.12364,0.12177,0.13377,0.11583,0.12181,0.16145,0.1272,0.12183,0.12664,
0.12125,0.12743,0.04321,0.042421,0.042757,0.043043,0.043032,0.043217,0
.23336,0.23651,0.20579,0.20357,0.27908,0.18046,0.21391,0.2075,0.14077,
0.12379,0.13324,0.12486,0.12414,0.12615,0.14305,0.12217,0.11944,0.1254
5,0.12218,0.13259,0.1233,0.11741,0.10965,0.11528,0.11212,0.12189,0.121
8,0.11446,0.12188,0.12142,0.11174,0.042522,0.042529,0.042573,0.043159,
0.042676,0.042458,0.20592,0.20594,0.22614,0.21742,0.2112,0.04228,0.042
847,0.11894,0.12449,0.10705,0.12156,0.12165,0.1216,0.12158,0.12145,0.1
2201,0.12706,0.12141,0.12504,0.12124,0.15439,0.14635,0.11661,0.12651,0
.12123,0.12606,0.12158,0.12103,0.041865,0.042427,0.042122,0.043455,0.0
43545,0.041331,0.042425,0.042822,0.043252,0.042743,0.19976,0.22745,0.0
42226,0.042713,0.041791,0.04303,0.12823,0.10393,0.12917,0.11571,0.1153
5,0.13589,0.12182,0.12144,0.12097,0.12182,0.12062,0.13052,0.12166,0.12
149,0.13044,0.12478,0.12112,0.12553,0.11376,0.042477,0.042672,0.042324
,0.042379,0.042125,0.042754,0.042632,0.042318,0.043113,0.043089,0.0427
84,0.043133,0.042932,0.042673,0.042543,0.04175,0.042579,0.12259,0.1212
4,0.1214,0.10953,0.12742,0.10844,0.11688,0.12205,0.12304,0.13348,0.123
85,0.12169,0.13272,0.12986,0.12609,0.11894,0.042713,0.042706,0.042603,
0.042169,0.042566,0.043163,0.04292,0.042212,0.043106,0.042775,0.041498
,0.042705,0.042263,0.042405,0.042789,0.042389,0.042728,0.042315,0.0424
78,0.10802,0.12219,0.13937,0.12246,0.10957,0.12187,0.12672,0.12466,0.1
2157,0.12281,0.1285,0.1215,0.14541,0.04285,0.042147,0.042663,0.042002,
0.042693,0.042153,0.042044,0.042345,0.042759,0.042337,0.042894,0.04241
9,0.042907,0.042939,0.042736,0.042771,0.042348,0.042295,0.042764,0.042
635,0.042647,0.12204,0.10747,0.11889,0.11932,0.11942,0.089973,0.12198,
0.12188,0.12023,0.042408,0.042478,0.042492,0.042774,0.042347,0.042327,
0.042324,0.042164,0.042683,0.042935,0.042658,0.042566,0.041773,0.04216
8,0.043044,0.042778,0.042549,0.042644,0.042027,0.04279,0.042718,0.0423
97,0.042681,0.042513,0.042345,0.106,0.12458,0.13069,0.099107,0.042808,
0.042292,0.042185,0.043127,0.04302,0.043022,0.042474,0.04231,0.042845,
0.042351,0.041999,0.04326,0.042383,0.042698,0.042832,0.042878,0.043025
,0.042649,0.042392,0.042178,0.042884,0.042532,0.042217,0.043255,0.0431
69,0.042081,0.042106,0.042474,0.042306,0.043275,0.042588,0.042358,0.04
2503,0.043067,0.042742,0.04275,0.042464,0.042818,0.043069,0.042461,0.0
42941,0.042179,0.042715,0.042427,0.042359,0.042448,0.042138,0.04315,0.
042642,0.042782,0.042243,0.042529,0.04216,0.04268,0.042879,0.042366,0.
042264,0.042466,0.042041,0.04226,0.042829,0.04242,0.043008,0.042382,0.
043304,0.042139,0.042756,0.043304,0.04243,0.042465,0.042428,0.042639,0
.043321,0.042982,0.042243,0.042689,0.043249,0.042811,0.043012,0.042183

,0.042568,0.042473,0.042432,0.042527,0.042288,0.04278,0.04276,0.043087
,0.042761,0.043176,0.041972,0.042494,0.042752,0.042325,0.042232,0.0421
04,0.042391,0.04222,0.042948,0.042924,0.042798,0.041935,0.042463,0.042
789,0.042694,0.04354,0.042203,0.042654,0.043184,0.042403,0.042544,0.04
3271,0.042936,0.041766,0.042988,0.042004,0.042477,0.042306,0.042318,0.
042538,0.042552,0.041952,0.042884,0.042847,0.042083,0.043032,0.042628,
0.042625,0.042548,0.042385,0.04267,0.043265,0.042669,0.042627,0.042957
,0.041667,0.042852,0.04243,0.043336,0.042739,0.042901,0.042458,0.04256
,0.043355,0.0429,0.042371,0.041427,0.042538,0.042184,0.04264,0.042601,
0.042251,0.042722,0.042824,0.04236,0.042359,0.041902,0.042136,0.04252,
0.042353,0.042692,0.043245,0.042757,0.043359,0.042188,0.042556,0.04277
9,0.043683,0.042343,0.042662,0.04288,0.042807,0.041849,0.042073,0.0428
13,0.042822,0.041898,0.042828,0.042011,0.042127,0.042908,0.043154,0.04
2915,0.042406,0.042228,0.043015,0.041781,0.042489,0.043029,0.042535,0.
041862,0.043277,0.042919,0.042519,0.0421
0.20254,0.20174,0.20283,0.2015,0.20274,0.202,0.20212,0.2031,0.20107,0.
20166,0.20232,0.20206,0.20139,0.20225,0.20133,0.20214,0.20207,0.20188,
0.20222,0.20243,0.28832,0.27526,0.28333,0.28176,0.20207,0.20248,0.2027
7,0.20197,0.20218,0.20191,0.20233,0.20126,0.2014,0.20237,0.20252,0.202
02,0.20277,0.20122,0.20271,0.36881,0.28363,0.28435,0.28316,0.28905,0.2
5347,0.28337,0.2024,0.20197,0.20284,0.20111,0.20189,0.201,0.20309,0.20
259,0.20116,0.20193,0.20117,0.20218,0.20151,0.20241,0.20278,0.35735,0.
36229,0.2653,0.27635,0.28361,0.28459,0.28297,0.29738,0.29273,0.27081,0
.2034,0.20186,0.20158,0.20271,0.20174,0.20292,0.20203,0.2021,0.20186,0
.20206,0.20232,0.20177,0.202,0.2017,0.20183,0.36415,0.36378,0.36159,0.
28193,0.29187,0.29775,0.27624,0.29626,0.26546,0.31233,0.28727,0.2859,0
.20267,0.20253,0.20222,0.20294,0.20192,0.20194,0.20141,0.20263,0.20243
,0.20135,0.20215,0.202,0.20186,0.20227,0.20177,0.20167,0.36727,0.37275
,0.37476,0.3613,0.26986,0.2864,0.28148,0.28561,0.2812,0.28246,0.28393,
0.2842,0.28161,0.28646,0.20227,0.20213,0.20256,0.20167,0.20108,0.20171
,0.20212,0.20232,0.20219,0.20262,0.20136,0.20277,0.20278,0.20178,0.201
64,0.20208,0.3553,0.37522,0.36094,0.36122,0.36456,0.35345,0.28494,0.30
176,0.28202,0.28302,0.28412,0.29127,0.28135,0.28739,0.28617,0.28553,0.
20216,0.20234,0.20241,0.20245,0.20145,0.20235,0.20188,0.20282,0.20238,
0.20169,0.20275,0.20191,0.2019,0.2013,0.20183,0.20195,0.20178,0.35102,
0.36775,0.36096,0.36817,0.3668,0.36467,0.28918,0.28182,0.29971,0.28101
,0.29195,0.29848,0.28183,0.28367,0.28752,0.27736,0.29162,0.20151,0.202
78,0.20192,0.20167,0.20188,0.20166,0.20171,0.20084,0.20205,0.20276,0.2
0168,0.20144,0.20241,0.12104,0.13242,0.12564,0.20177,0.36446,0.3667,0.
36042,0.36763,0.36479,0.36278,0.36594,0.28467,0.29692,0.27993,0.28213,
0.28839,0.27605,0.26543,0.28591,0.2904,0.28137,0.28714,0.20277,0.20219
,0.20235,0.20221,0.20148,0.20181,0.20227,0.20243,0.20285,0.20272,0.202
29,0.12044,0.11023,0.11023,0.12199,0.11488,0.11098,0.20228,0.35938,0.3
7163,0.36953,0.35517,0.36305,0.35336,0.36587,0.27108,0.28538,0.28336,0
.28453,0.28616,0.29098,0.28215,0.2929,0.28477,0.28385,0.29859,0.2015,0
.20215,0.20213,0.2031,0.20158,0.20212,0.20282,0.20262,0.20189,0.12739,
0.11203,0.13048,0.12096,0.12894,0.12526,0.12246,0.10032,0.12149,0.2016
8,0.36869,0.36766,0.35584,0.37109,0.37059,0.35605,0.36213,0.28862,0.29
526,0.28574,0.28275,0.27702,0.28617,0.28918,0.27783,0.27874,0.30011,0.
28457,0.28682,0.20214,0.20208,0.20282,0.20232,0.20144,0.20147,0.12255,
0.12125,0.11961,0.12186,0.12571,0.1221,0.12343,0.13709,0.12188,0.12182
,0.11484,0.11933,0.36412,0.36967,0.36586,0.36673,0.3623,0.36585,0.3700

1,0.28141,0.2822,0.29346,0.28348,0.28208,0.28598,0.28049,0.29621,0.290
27,0.28266,0.28423,0.27802,0.2015,0.20216,0.20276,0.11779,0.13452,0.12
158,0.14352,0.12185,0.12466,0.13895,0.088138,0.12189,0.1226,0.12984,0.
11761,0.10768,0.12898,0.12179,0.37161,0.362,0.36705,0.36378,0.37504,0.
36394,0.36024,0.36732,0.3589,0.28375,0.27778,0.27065,0.28654,0.28947,0
.26026,0.28333,0.27968,0.28494,0.2844,0.28235,0.1216,0.1366,0.13529,0.
12272,0.12177,0.11834,0.11623,0.12225,0.15558,0.12166,0.11499,0.11859,
0.12298,0.12263,0.12196,0.12911,0.1447,0.12978,0.37145,0.37353,0.36386
,0.35967,0.36889,0.37279,0.36282,0.37515,0.36443,0.29333,0.2944,0.2060
4,0.20529,0.20821,0.20387,0.21095,0.20403,0.20223,0.21153,0.2232,0.107
98,0.11916,0.12168,0.11951,0.12197,0.093952,0.11967,0.12633,0.11267,0.
12135,0.13328,0.1108,0.12441,0.12203,0.12315,0.11283,0.1158,0.11547,0.
36675,0.37788,0.3628,0.36112,0.36488,0.38139,0.36726,0.37716,0.37309,0
.19635,0.20202,0.22489,0.20093,0.19978,0.20387,0.19995,0.17993,0.19569
,0.22052,0.20865,0.1216,0.096746,0.12316,0.12232,0.12512,0.12194,0.104
23,0.12019,0.12898,0.12049,0.10797,0.12135,0.13206,0.12384,0.12947,0.1
3162,0.11852,0.13541,0.35675,0.36055,0.3744,0.37578,0.36614,0.35293,0.
37235,0.20407,0.23033,0.22416,0.20169,0.22082,0.19907,0.21543,0.22356,
0.1997,0.20597,0.21541,0.19505,0.21114,0.12214,0.14033,0.12158,0.1117,
0.098256,0.10439,0.10822,0.12193,0.12235,0.12225,0.12309,0.12183,0.122
31,0.12272,0.12215,0.11756,0.092051,0.12896,0.37329,0.36241,0.36715,0.
36433,0.35721,0.37595,0.21768,0.2108,0.19688,0.16297,0.18721,0.20368,0
.18771,0.21729,0.18024,0.19113,0.19512,0.21782,0.21203,0.22063,0.12297
,0.11802,0.12173,0.1176,0.10711,0.12834,0.11696,0.097072,0.12501,0.116
64,0.13042,0.10563,0.11527,0.12452,0.1162,0.12149,0.11764,0.10398,0.36
943,0.3709,0.37098,0.3756,0.37616,0.19348,0.20522,0.20491,0.19862,0.19
247,0.20805,0.18111,0.1827,0.2241,0.18864,0.20555,0.20253,0.20219,0.20
184,0.20525,0.12035,0.11419,0.1286,0.1216,0.1221,0.12468,0.13892,0.123
83,0.11565,0.12274,0.11952,0.11347,0.12149,0.11827,0.11964,0.12216,0.1
3191,0.12818,0.36289,0.36686,0.35876,0.20065,0.19952,0.20063,0.20275,0
.2082,0.19674,0.21134,0.1732,0.2065,0.19963,0.2031,0.17183,0.18623,0.2
0882,0.20344,0.17641,0.20265,0.15676,0.12563,0.12188,0.12184,0.1222,0.
1216,0.11598,0.12198,0.13677,0.11885,0.12727,0.10995,0.12217,0.10916,0
.090431,0.12467,0.12901,0.12088,0.36892,0.2092,0.21104,0.19599,0.2016,
0.23183,0.20416,0.23155,0.20329,0.19407,0.16949,0.20244,0.21008,0.1994
5,0.18789,0.20352,0.20031,0.19314,0.19657,0.12658,0.12061,0.11655,0.11
031,0.12165,0.12188,0.11862,0.11132,0.12131,0.12619,0.12136,0.12137,0.
12159,0.1128,0.10483,0.12569,0.12248,0.041695,0.18623,0.20386,0.18937,
0.14248,0.22035,0.14354,0.17695,0.21338,0.21742,0.20456,0.20049,0.2225
5,0.21699,0.17717,0.19629,0.23504,0.20285,0.20784,0.1941,0.12738,0.123
83,0.12304,0.12742,0.12358,0.13777,0.1238,0.10652,0.12151,0.10193,0.12
164,0.12163,0.12984,0.11722,0.12205,0.12576,0.12166,0.041867,0.21949,0
.2407,0.21059,0.20043,0.16342,0.20299,0.20534,0.20119,0.20734,0.20339,
0.23105,0.19673,0.20748,0.20148,0.20313,0.2148,0.2006,0.19717,0.12162,
0.11388,0.11326,0.13785,0.12419,0.13715,0.12185,0.12989,0.12305,0.1212
,0.115,0.12142,0.12439,0.10765,0.12196,0.12075,0.11443,0.1169,0.041431
,0.2186,0.21107,0.14382,0.18738,0.21313,0.19583,0.21101,0.20166,0.2023
9,0.20228,0.19802,0.17665,0.20076,0.20158,0.20723,0.18918,0.19127,0.21
794,0.11378,0.12153,0.14902,0.12076,0.13199,0.12226,0.12328,0.12116,0.
11105,0.1315,0.12181,0.11304,0.12646,0.11926,0.12154,0.12273,0.14682,0
.041835,0.041307,0.041919,0.27357,0.19348,0.20309,0.20663,0.24,0.22141
,0.2028,0.20246,0.18071,0.20409,0.1838,0.18958,0.21378,0.20589,0.2037,

0.11328,0.1217,0.12209,0.13794,0.095753,0.1083,0.1309,0.12653,0.10446,
0.11935,0.12365,0.12473,0.11049,0.13444,0.066714,0.109,0.13636,0.12595
,0.041214,0.042344,0.042097,0.041493,0.19978,0.2336,0.22898,0.19129,0.
20135,0.16996,0.18857,0.18346,0.20614,0.16731,0.20408,0.19031,0.11225,
0.12147,0.17365,0.11664,0.11403,0.1186,0.11758,0.12159,0.14031,0.12387
,0.12192,0.13421,0.11578,0.12224,0.16211,0.12739,0.12226,0.1271,0.1214
2,0.12758,0.041926,0.041395,0.04173,0.042158,0.04233,0.04225,0.22957,0
.23693,0.20371,0.20107,0.27578,0.17825,0.20979,0.20421,0.14114,0.12377
,0.13336,0.12536,0.12411,0.12642,0.14334,0.1221,0.11944,0.12554,0.1222
8,0.13293,0.1233,0.1177,0.10988,0.11564,0.11213,0.122,0.12176,0.11448,
0.12179,0.12162,0.11167,0.041764,0.041793,0.041531,0.042314,0.041737,0
.04154,0.20315,0.20122,0.22152,0.21268,0.21025,0.041385,0.041834,0.119
06,0.12435,0.10709,0.12177,0.12185,0.12174,0.12191,0.12162,0.12207,0.1
2745,0.12144,0.12524,0.12139,0.1549,0.14682,0.11672,0.1266,0.12137,0.1
2637,0.12148,0.12127,0.041163,0.041766,0.041281,0.04246,0.042929,0.040
703,0.041279,0.041889,0.042269,0.041837,0.19953,0.22167,0.041247,0.041
553,0.040827,0.042136,0.12843,0.10354,0.12952,0.11577,0.11543,0.13583,
0.12207,0.12159,0.1211,0.12203,0.12085,0.13066,0.1217,0.12169,0.13069,
0.12494,0.12161,0.12562,0.11383,0.041359,0.041864,0.041616,0.041487,0.
041262,0.041962,0.041875,0.041426,0.041998,0.042269,0.041654,0.041843,
0.041801,0.041925,0.041743,0.040946,0.041737,0.12248,0.12136,0.12154,0
.10952,0.12753,0.10832,0.11706,0.12207,0.12335,0.13378,0.12407,0.12189
,0.13285,0.12995,0.12647,0.11909,0.041659,0.041887,0.041589,0.041117,0
.041312,0.041973,0.041863,0.041499,0.042216,0.04217,0.040721,0.041938,
0.040978,0.041381,0.041912,0.041488,0.041994,0.041442,0.041853,0.10789
,0.12253,0.13989,0.12237,0.10974,0.122,0.12684,0.12489,0.12178,0.12296
,0.12881,0.12157,0.14596,0.04203,0.041299,0.041853,0.041045,0.041743,0
.041193,0.041272,0.04163,0.041545,0.04116,0.041572,0.041558,0.041693,0
.041955,0.041812,0.041892,0.041333,0.041394,0.041792,0.04187,0.041903,
0.1221,0.10717,0.11911,0.11946,0.1196,0.089864,0.12208,0.12209,0.1202,
0.041708,0.04172,0.041543,0.042241,0.041201,0.041464,0.041249,0.041366
,0.041669,0.041922,0.041687,0.04166,0.040999,0.041105,0.042215,0.04183
,0.041777,0.041518,0.040937,0.04187,0.041981,0.041386,0.041559,0.04144
1,0.041186,0.10587,0.12481,0.13092,0.09893,0.041586,0.041284,0.04131,0
.041968,0.041951,0.042108,0.041561,0.041228,0.041777,0.041314,0.041005
,0.04219,0.041623,0.04195,0.041729,0.041748,0.042103,0.041776,0.041618
,0.041023,0.041894,0.041501,0.041148,0.041931,0.042448,0.041158,0.0412
25,0.041797,0.041338,0.042123,0.041344,0.041386,0.041642,0.041963,0.04
175,0.041478,0.041619,0.041831,0.041698,0.041823,0.041957,0.04136,0.04
1872,0.041587,0.041309,0.041378,0.041184,0.041978,0.041497,0.041786,0.
041586,0.041808,0.041121,0.041689,0.041918,0.041215,0.041347,0.041523,
0.04092,0.041265,0.042036,0.041265,0.04213,0.041225,0.04251,0.041536,0
.041808,0.042186,0.041398,0.041128,0.041803,0.04175,0.042142,0.041997,
0.041285,0.041715,0.042408,0.041728,0.042019,0.04158,0.041546,0.04167,
0.041356,0.041728,0.041553,0.042201,0.042012,0.04198,0.041812,0.042322
,0.040892,0.041542,0.04195,0.041536,0.041687,0.04073,0.041589,0.04125,
0.042023,0.042079,0.041623,0.041018,0.041617,0.042059,0.04188,0.042564
,0.041385,0.041874,0.042172,0.041538,0.04145,0.042094,0.041858,0.04098
4,0.041903,0.041296,0.041767,0.041648,0.041714,0.041807,0.041341,0.040
877,0.042232,0.041889,0.040913,0.042311,0.041838,0.041752,0.041496,0.0
41551,0.041666,0.042394,0.041754,0.041709,0.041884,0.040775,0.041768,0
.041573,0.042528,0.041631,0.042133,0.041652,0.041589,0.042489,0.04186,

0.041585,0.040462,0.041729,0.041079,0.041619,0.041493,0.041504,0.04142
,0.041799,0.041623,0.041205,0.040985,0.041212,0.041553,0.041195,0.0419
18,0.042008,0.04196,0.042256,0.041424,0.041726,0.04197,0.042854,0.0415
65,0.041744,0.041602,0.041759,0.041201,0.041428,0.04218,0.042111,0.041
299,0.041842,0.040837,0.041081,0.04185,0.042055,0.042173,0.041195,0.04
1235,0.042043,0.040717,0.041837,0.042176,0.041495,0.041107,0.042425,0.
042064,0.041658,0.041076
0.2035,0.20285,0.20404,0.20214,0.20364,0.20302,0.20311,0.20406,0.20215
,0.20277,0.20339,0.20313,0.20265,0.20295,0.20251,0.20318,0.20301,0.203
08,0.20349,0.20311,0.28905,0.27619,0.28213,0.27903,0.20286,0.20316,0.2
0398,0.20265,0.20323,0.20263,0.2031,0.20266,0.20282,0.20343,0.20343,0.
20285,0.20322,0.20196,0.20351,0.36675,0.2815,0.28436,0.2823,0.28723,0.
25438,0.28031,0.20369,0.20307,0.20405,0.20238,0.20284,0.20205,0.20385,
0.2036,0.20182,0.20261,0.20267,0.20271,0.20292,0.20341,0.20399,0.35086
,0.35878,0.26485,0.27303,0.28129,0.28221,0.28091,0.2961,0.29376,0.2705
6,0.20437,0.20274,0.20247,0.20325,0.20281,0.20414,0.20289,0.20343,0.20
282,0.20366,0.20321,0.20276,0.20308,0.20276,0.20269,0.35968,0.36019,0.
36269,0.28183,0.28955,0.29692,0.27502,0.29598,0.26532,0.30911,0.28858,
0.28369,0.20362,0.20375,0.20366,0.20366,0.20317,0.2029,0.20203,0.20343
,0.20323,0.20248,0.20328,0.20332,0.20306,0.20289,0.2025,0.20265,0.3625
8,0.36702,0.37631,0.35834,0.26789,0.28955,0.28005,0.28432,0.28096,0.27
939,0.28381,0.28298,0.28168,0.28506,0.20368,0.20303,0.20324,0.20286,0.
20218,0.2028,0.20344,0.20334,0.20337,0.20348,0.20207,0.20374,0.20342,0
.20265,0.20283,0.20277,0.35341,0.36992,0.36227,0.35547,0.36121,0.3508,
0.28268,0.29991,0.28085,0.28298,0.28068,0.28828,0.28317,0.28779,0.2847
8,0.28268,0.20341,0.20359,0.20358,0.20371,0.20277,0.20346,0.20295,0.20
407,0.2028,0.2026,0.20381,0.20307,0.20283,0.20215,0.20287,0.20291,0.20
261,0.35156,0.36453,0.36011,0.36308,0.3649,0.35936,0.28938,0.28003,0.2
99,0.27793,0.28884,0.29615,0.2831,0.28252,0.28471,0.27588,0.29214,0.20
251,0.20341,0.20272,0.20271,0.2025,0.20278,0.20267,0.20208,0.20331,0.2
0342,0.20275,0.20236,0.20365,0.12113,0.13292,0.1258,0.20302,0.35929,0.
36267,0.35628,0.36711,0.3665,0.35776,0.36303,0.28392,0.29557,0.27805,0
.28029,0.28775,0.27598,0.26519,0.28349,0.28903,0.28037,0.28628,0.20393
,0.20316,0.20323,0.20348,0.20287,0.20314,0.20339,0.20371,0.2038,0.2033
1,0.20287,0.12053,0.11041,0.11015,0.12218,0.11512,0.11116,0.20345,0.36
101,0.36687,0.36598,0.3511,0.35841,0.3509,0.36279,0.27363,0.2853,0.281
22,0.28533,0.28373,0.28836,0.28082,0.29224,0.28441,0.2824,0.29832,0.20
252,0.2031,0.20341,0.20463,0.20244,0.20312,0.20354,0.20384,0.20292,0.1
2763,0.11218,0.13057,0.12123,0.12912,0.12545,0.12279,0.10015,0.12147,0
.2025,0.36515,0.3641,0.35377,0.36172,0.36929,0.35374,0.35576,0.28868,0
.29283,0.28432,0.28176,0.27564,0.28609,0.28841,0.2749,0.27642,0.29683,
0.28321,0.28602,0.20291,0.20281,0.2036,0.20298,0.20252,0.20241,0.12266
,0.12106,0.11976,0.12206,0.126,0.12226,0.12383,0.13732,0.12217,0.12207
,0.11492,0.11963,0.35948,0.37138,0.36367,0.36128,0.36405,0.36816,0.367
08,0.2816,0.28133,0.29142,0.28153,0.28252,0.28635,0.27906,0.29534,0.29
167,0.28205,0.28299,0.27767,0.20242,0.20328,0.20335,0.11788,0.1345,0.1
2166,0.14393,0.12217,0.12465,0.13945,0.088135,0.12207,0.12258,0.13021,
0.11771,0.10781,0.12927,0.12181,0.3684,0.35503,0.36364,0.36538,0.37211
,0.35839,0.35577,0.36819,0.35502,0.28333,0.2774,0.27072,0.2862,0.28926
,0.26002,0.28093,0.2803,0.28416,0.28161,0.28288,0.12176,0.13685,0.1354
9,0.12279,0.12177,0.11846,0.11643,0.12224,0.15645,0.12199,0.1151,0.118
75,0.1233,0.12261,0.12225,0.12939,0.14509,0.12984,0.37127,0.37172,0.35

987,0.36066,0.3663,0.36665,0.3603,0.3711,0.36177,0.29111,0.29141,0.204
66,0.20524,0.20592,0.20358,0.20884,0.20224,0.20183,0.21062,0.22335,0.1
08,0.11918,0.12185,0.11956,0.12221,0.093878,0.1198,0.12661,0.11268,0.1
2164,0.1337,0.11108,0.12471,0.12196,0.12349,0.11294,0.11582,0.11557,0.
36124,0.37354,0.3594,0.35974,0.36248,0.38006,0.35942,0.3739,0.37005,0.
19493,0.2023,0.22403,0.19973,0.19797,0.20297,0.19913,0.17875,0.19635,0
.21924,0.20792,0.12206,0.096652,0.12335,0.12225,0.12536,0.1219,0.10427
,0.12044,0.12927,0.12069,0.108,0.1217,0.13217,0.12418,0.12953,0.13176,
0.11851,0.13599,0.34848,0.3597,0.36792,0.37383,0.36132,0.34856,0.36734
,0.20028,0.22748,0.22227,0.20064,0.22007,0.19867,0.21675,0.22205,0.198
89,0.20419,0.21458,0.19414,0.20955,0.12224,0.14059,0.1217,0.11174,0.09
7972,0.10432,0.10826,0.12202,0.12248,0.12222,0.12331,0.12177,0.1224,0.
12291,0.1221,0.11781,0.092061,0.12913,0.36822,0.36143,0.3616,0.36162,0
.35106,0.36991,0.21368,0.20917,0.19479,0.16062,0.18477,0.20351,0.18741
,0.21351,0.17906,0.18865,0.19569,0.21383,0.2104,0.22001,0.12338,0.1178
9,0.12185,0.1178,0.10731,0.12845,0.11709,0.096648,0.12517,0.11688,0.13
083,0.10567,0.11537,0.1245,0.11622,0.12159,0.11764,0.10397,0.36331,0.3
6646,0.36723,0.3707,0.37335,0.19279,0.20342,0.20321,0.19551,0.19013,0.
20675,0.18162,0.18219,0.22241,0.18703,0.2042,0.20116,0.20215,0.20149,0
.20447,0.12023,0.11425,0.12892,0.12173,0.12226,0.12489,0.13926,0.12398
,0.11584,0.12285,0.11991,0.11341,0.12152,0.11846,0.11988,0.12246,0.132
23,0.12848,0.35905,0.36346,0.35077,0.19848,0.19662,0.19836,0.20292,0.2
0863,0.19406,0.20777,0.17183,0.20563,0.19713,0.20282,0.16983,0.18538,0
.20798,0.20178,0.17595,0.20225,0.15738,0.12579,0.122,0.12221,0.12243,0
.12185,0.11587,0.1224,0.13739,0.11922,0.12729,0.11008,0.12245,0.10926,
0.090445,0.12512,0.12929,0.12104,0.36425,0.21126,0.21123,0.19284,0.199
16,0.23128,0.20113,0.22735,0.19957,0.19225,0.1687,0.20166,0.20806,0.19
79,0.18581,0.20154,0.19873,0.19298,0.196,0.12658,0.12044,0.11651,0.110
29,0.12168,0.12225,0.11866,0.11136,0.1214,0.12648,0.12172,0.12176,0.12
182,0.11291,0.10486,0.12584,0.12279,0.041163,0.18506,0.20116,0.18733,0
.14207,0.21943,0.14067,0.17554,0.21003,0.2122,0.20066,0.19988,0.2215,0
.21332,0.17708,0.19449,0.23588,0.20362,0.20681,0.19438,0.12755,0.12415
,0.12336,0.12735,0.12388,0.13807,0.1243,0.10667,0.12181,0.10194,0.1220
6,0.12201,0.12999,0.11707,0.12215,0.12621,0.12191,0.04104,0.21784,0.24
074,0.20948,0.2006,0.1612,0.20226,0.20367,0.20064,0.20557,0.20368,0.23
146,0.19543,0.20601,0.20223,0.20137,0.21456,0.19919,0.19594,0.12187,0.
11409,0.11343,0.13822,0.12461,0.13747,0.12196,0.13004,0.12334,0.12138,
0.11515,0.12159,0.12489,0.10773,0.12218,0.12092,0.11446,0.1171,0.04065
9,0.21484,0.20782,0.14289,0.18502,0.21171,0.19482,0.20872,0.19897,0.20
027,0.20127,0.19851,0.17535,0.20097,0.20227,0.20665,0.18881,0.18978,0.
21753,0.1138,0.12179,0.1496,0.12132,0.13204,0.12216,0.12357,0.12112,0.
11123,0.13197,0.1219,0.11294,0.12664,0.11981,0.12192,0.12309,0.14741,0
.041052,0.040648,0.041261,0.26917,0.19109,0.20068,0.20343,0.23531,0.22
06,0.20317,0.20115,0.18109,0.20311,0.18299,0.18838,0.21249,0.2062,0.20
308,0.11359,0.12184,0.12217,0.13838,0.09561,0.10837,0.13116,0.127,0.10
449,0.11947,0.12362,0.12476,0.11064,0.13474,0.066083,0.10902,0.13681,0
.12621,0.040816,0.041429,0.041284,0.040719,0.19876,0.23282,0.22598,0.1
8943,0.20101,0.16774,0.18723,0.18215,0.2051,0.16709,0.20264,0.1895,0.1
1225,0.12161,0.17429,0.1166,0.11374,0.11893,0.11782,0.12186,0.14056,0.
12409,0.12206,0.13461,0.11586,0.12238,0.16253,0.1276,0.12241,0.12747,0
.12162,0.12765,0.041234,0.040756,0.041229,0.041241,0.041512,0.04152,0.
22412,0.23524,0.20256,0.199,0.27244,0.17612,0.2077,0.20404,0.14138,0.1

2399,0.13356,0.12573,0.12438,0.12665,0.14364,0.12231,0.11966,0.12558,0
.12235,0.1333,0.12378,0.11794,0.10993,0.11585,0.11231,0.12207,0.1219,0
.11469,0.12209,0.12204,0.11191,0.041121,0.041298,0.040786,0.041421,0.0
4114,0.040728,0.1998,0.19821,0.21907,0.21329,0.20978,0.041101,0.041283
,0.11914,0.12465,0.1072,0.12203,0.12196,0.12232,0.12223,0.1217,0.12213
,0.12752,0.12138,0.12565,0.1217,0.15554,0.14723,0.11686,0.12674,0.1215
4,0.12674,0.12194,0.12148,0.04056,0.041169,0.040719,0.041816,0.042061,
0.040496,0.040437,0.040814,0.041538,0.041184,0.19912,0.21793,0.04105,0
.040728,0.040167,0.041436,0.12854,0.10346,0.12963,0.1159,0.11566,0.136
27,0.12221,0.12158,0.12156,0.12208,0.12106,0.13105,0.12194,0.12205,0.1
3077,0.12515,0.1218,0.12592,0.1139,0.040652,0.040943,0.041024,0.04055,
0.040568,0.040969,0.04133,0.040843,0.041422,0.041795,0.040954,0.040988
,0.04083,0.041509,0.040982,0.040078,0.040998,0.12271,0.12165,0.12166,0
.10939,0.12762,0.10834,0.11732,0.12204,0.12357,0.13412,0.12453,0.12206
,0.13316,0.13039,0.12673,0.1193,0.040989,0.041367,0.041071,0.040886,0.
040778,0.041178,0.041024,0.040735,0.04118,0.041473,0.040039,0.041307,0
.040354,0.040577,0.041103,0.040918,0.041326,0.040847,0.04116,0.1078,0.
12271,0.14031,0.12257,0.10995,0.12224,0.127,0.12503,0.12219,0.12311,0.
12913,0.12187,0.14656,0.041393,0.040202,0.041404,0.040239,0.041182,0.0
40466,0.04071,0.041278,0.041043,0.040452,0.041144,0.041062,0.040787,0.
041145,0.040873,0.041062,0.040835,0.040566,0.040936,0.041258,0.041129,
0.12219,0.1073,0.11928,0.11945,0.11975,0.089799,0.12233,0.12215,0.1200
1,0.041062,0.041169,0.040809,0.041489,0.040484,0.040934,0.040784,0.040
846,0.04068,0.041115,0.040827,0.041271,0.040618,0.040568,0.04156,0.041
187,0.041131,0.041053,0.040196,0.04135,0.041201,0.040567,0.040882,0.04
0853,0.040735,0.10601,0.12497,0.13097,0.098953,0.041127,0.04065,0.0407
26,0.041423,0.041409,0.041619,0.04114,0.040394,0.041071,0.040146,0.040
525,0.041471,0.040925,0.041177,0.041219,0.041185,0.041553,0.041058,0.0
40727,0.040523,0.041157,0.041303,0.040643,0.041354,0.041354,0.040611,0
.040542,0.041141,0.040828,0.041827,0.040825,0.040724,0.041049,0.041326
,0.041079,0.040823,0.041295,0.040939,0.040895,0.041268,0.041532,0.0407
01,0.041102,0.040716,0.040737,0.041193,0.040684,0.041395,0.040779,0.04
093,0.040789,0.04124,0.040616,0.041159,0.041072,0.040569,0.040568,0.04
0879,0.040235,0.04063,0.04161,0.040583,0.041168,0.040542,0.041755,0.04
0723,0.041321,0.041377,0.040653,0.04058,0.040869,0.041157,0.040948,0.0
41213,0.040859,0.040971,0.041516,0.041058,0.041359,0.040906,0.040871,0
.040782,0.040521,0.041173,0.0407,0.041771,0.041223,0.041487,0.04123,0.
041519,0.040325,0.041021,0.040885,0.040976,0.040989,0.040434,0.040973,
0.040571,0.041266,0.04152,0.040754,0.040585,0.040998,0.041548,0.041285
,0.041547,0.040863,0.041275,0.041421,0.040712,0.04071,0.041356,0.04123
5,0.040364,0.041278,0.040613,0.041039,0.041236,0.041193,0.041049,0.040
69,0.040164,0.041214,0.041403,0.04048,0.041645,0.041081,0.041031,0.040
916,0.040945,0.040987,0.041786,0.041054,0.041088,0.041024,0.04,0.04101
7,0.040986,0.041458,0.040967,0.041055,0.040691,0.04089,0.041936,0.0410
28,0.041069,0.039728,0.041127,0.040533,0.04083,0.040719,0.04087,0.0408
12,0.041117,0.040909,0.040432,0.040238,0.040634,0.040674,0.040348,0.04
1188,0.041367,0.041071,0.041517,0.040622,0.040963,0.041414,0.042155,0.
040631,0.040894,0.040995,0.040879,0.040419,0.040779,0.04142,0.041241,0
.040651,0.041253,0.040196,0.040054,0.041026,0.041496,0.041354,0.040306
,0.040932,0.04133,0.039971,0.041265,0.04158,0.041076,0.040515,0.041707
,0.041299,0.041217,0.040493

0.20429,0.20314,0.20427,0.20339,0.20469,0.20368,0.20418,0.20521,0.2031
3,0.20386,0.20421,0.20404,0.20306,0.20377,0.20348,0.20397,0.20378,0.20
398,0.20431,0.20393,0.28721,0.27272,0.28316,0.27824,0.20422,0.20423,0.
20492,0.20348,0.20402,0.20332,0.20387,0.20389,0.20381,0.2042,0.20469,0
.20394,0.20424,0.20342,0.20434,0.36646,0.28129,0.283,0.28085,0.2865,0.
25399,0.27981,0.20433,0.20384,0.2048,0.20299,0.20384,0.20305,0.20463,0
.20453,0.20292,0.2034,0.20364,0.20348,0.20406,0.20441,0.20481,0.3503,0
.35034,0.26399,0.2726,0.28152,0.28101,0.2804,0.29423,0.2946,0.26999,0.
20511,0.20365,0.20349,0.20388,0.20359,0.20519,0.20403,0.20463,0.20395,
0.20481,0.20381,0.20374,0.20391,0.20325,0.20385,0.35721,0.35815,0.3568
,0.28009,0.29046,0.29468,0.27497,0.29397,0.26347,0.3068,0.2868,0.28321
,0.20405,0.20459,0.2045,0.20413,0.20416,0.20422,0.20264,0.20406,0.2037
4,0.20316,0.20412,0.20399,0.20431,0.20346,0.20337,0.20366,0.36,0.36765
,0.37155,0.35725,0.26893,0.28924,0.27917,0.28382,0.28075,0.27739,0.284
67,0.28408,0.28146,0.28471,0.20456,0.20388,0.20415,0.20393,0.20279,0.2
0412,0.20451,0.20405,0.20394,0.20457,0.20299,0.20492,0.20473,0.20322,0
.20359,0.20317,0.34786,0.36975,0.35568,0.35562,0.36508,0.34741,0.28224
,0.2956,0.28008,0.28048,0.27986,0.28746,0.28304,0.28736,0.28383,0.2823
6,0.20408,0.20443,0.20443,0.20445,0.20364,0.20462,0.20405,0.20501,0.20
416,0.20275,0.20455,0.20386,0.20351,0.20325,0.20367,0.20386,0.20384,0.
34786,0.36127,0.35598,0.3588,0.36324,0.3548,0.28577,0.28031,0.29626,0.
2766,0.28568,0.29469,0.2838,0.28132,0.28187,0.27321,0.29191,0.20341,0.
2043,0.20354,0.20343,0.20346,0.20411,0.20341,0.20229,0.20441,0.2048,0.
20329,0.20305,0.20464,0.12149,0.13332,0.1261,0.20428,0.35563,0.3583,0.
35799,0.3599,0.35873,0.3571,0.36435,0.28223,0.29261,0.27865,0.27732,0.
28945,0.2775,0.26573,0.2811,0.28767,0.27983,0.28405,0.20467,0.20421,0.
20453,0.20399,0.20397,0.20445,0.20429,0.20457,0.20465,0.20454,0.20412,
0.12108,0.11017,0.11031,0.12236,0.11501,0.11123,0.20452,0.35713,0.3669
2,0.36594,0.3523,0.3592,0.34597,0.36037,0.27254,0.28721,0.27977,0.2839
3,0.28255,0.28828,0.2796,0.29135,0.2837,0.28242,0.29861,0.20352,0.2043
,0.20395,0.20517,0.2032,0.20401,0.20453,0.2049,0.20402,0.12759,0.11213
,0.13083,0.12117,0.12943,0.12564,0.12302,0.10011,0.12171,0.20349,0.363
51,0.3599,0.35371,0.35919,0.36698,0.3491,0.35061,0.28813,0.29093,0.283
42,0.28067,0.27366,0.28591,0.28886,0.27476,0.27525,0.29645,0.28198,0.2
8442,0.20371,0.20322,0.20423,0.20402,0.20337,0.20326,0.12281,0.12133,0
.12006,0.12227,0.12599,0.12241,0.12391,0.13762,0.12225,0.12224,0.115,0
.11974,0.35866,0.36998,0.3607,0.361,0.36589,0.36482,0.36359,0.28046,0.
27983,0.29125,0.2812,0.28313,0.28514,0.27847,0.2933,0.28815,0.28311,0.
28249,0.27583,0.20338,0.20433,0.2041,0.11795,0.1348,0.12188,0.14428,0.
12243,0.12505,0.14004,0.087861,0.12259,0.12284,0.13062,0.11781,0.10792
,0.12953,0.12208,0.36492,0.35226,0.3618,0.36048,0.37201,0.35559,0.3540
6,0.36399,0.35338,0.28053,0.277,0.26969,0.28422,0.28638,0.25819,0.2814
8,0.27717,0.28411,0.27889,0.27933,0.12207,0.13704,0.1359,0.12321,0.121
92,0.11869,0.11654,0.12234,0.15699,0.12216,0.11522,0.11902,0.12324,0.1
2272,0.12242,0.12962,0.14538,0.13001,0.36845,0.36737,0.35544,0.35735,0
.36506,0.36528,0.35525,0.37538,0.359,0.28792,0.28952,0.20452,0.2043,0.
20407,0.20305,0.20697,0.20081,0.20079,0.21015,0.22285,0.10822,0.11945,
0.12198,0.11963,0.12225,0.093709,0.11987,0.1266,0.1128,0.1217,0.13395,
0.11141,0.12502,0.12205,0.12358,0.11314,0.11592,0.11593,0.35755,0.3735
3,0.35666,0.3588,0.36229,0.37461,0.35928,0.36802,0.36673,0.19306,0.201
94,0.22281,0.20006,0.19691,0.2019,0.19789,0.17778,0.19504,0.21911,0.20
734,0.12215,0.096557,0.12324,0.12242,0.12562,0.12211,0.10423,0.12073,0

.12941,0.12082,0.1082,0.12184,0.1326,0.12423,0.12978,0.13208,0.11862,0
.13639,0.34515,0.35628,0.36208,0.36674,0.35831,0.35041,0.36662,0.19867
,0.22605,0.22241,0.19862,0.21979,0.19916,0.21536,0.22108,0.19776,0.204
12,0.21366,0.19554,0.20962,0.12261,0.14076,0.122,0.11169,0.097825,0.10
44,0.10826,0.12198,0.12266,0.12222,0.12365,0.12171,0.12273,0.12272,0.1
2223,0.11785,0.091769,0.12932,0.36217,0.35808,0.35742,0.36021,0.34881,
0.36487,0.2125,0.20729,0.19412,0.15832,0.18311,0.20247,0.187,0.212,0.1
781,0.18849,0.19508,0.21027,0.20967,0.21775,0.12369,0.11829,0.12214,0.
11773,0.10747,0.1286,0.11733,0.09654,0.12526,0.11697,0.13105,0.10588,0
.11565,0.12491,0.11641,0.12167,0.11776,0.10387,0.35976,0.36385,0.36143
,0.36632,0.36835,0.19021,0.20218,0.19961,0.19445,0.18935,0.20541,0.181
4,0.18086,0.222,0.18662,0.20373,0.20022,0.20076,0.20126,0.20464,0.1204
6,0.11424,0.12914,0.1221,0.12252,0.1252,0.13944,0.12412,0.11586,0.1230
4,0.12025,0.11354,0.12182,0.11893,0.12026,0.12267,0.13256,0.12852,0.35
788,0.36021,0.34645,0.19382,0.19574,0.19545,0.20126,0.20774,0.19264,0.
20829,0.17145,0.20408,0.19592,0.20079,0.16931,0.18355,0.20702,0.20231,
0.17523,0.20091,0.15777,0.12597,0.12246,0.12243,0.12262,0.12215,0.1161
,0.12254,0.138,0.11931,0.12744,0.11018,0.12263,0.10941,0.090388,0.1254
6,0.12951,0.12109,0.3641,0.21114,0.20813,0.19064,0.19747,0.22842,0.198
56,0.22354,0.19928,0.19238,0.16836,0.20012,0.20656,0.19567,0.18624,0.2
0151,0.19817,0.19232,0.19473,0.12693,0.12083,0.11664,0.1104,0.12181,0.
12244,0.11876,0.11152,0.12187,0.12693,0.12188,0.12212,0.12216,0.11281,
0.10488,0.12599,0.12288,0.040635,0.18228,0.19931,0.18711,0.14062,0.218
02,0.14023,0.17574,0.21016,0.20895,0.1982,0.19848,0.22123,0.21042,0.17
66,0.19458,0.23583,0.20242,0.20536,0.19308,0.12768,0.1242,0.12367,0.12
734,0.12405,0.13828,0.12468,0.10695,0.12201,0.10189,0.12207,0.12206,0.
13016,0.11713,0.12217,0.12647,0.12215,0.040578,0.21675,0.24022,0.2079,
0.19863,0.15978,0.1996,0.20001,0.1983,0.20316,0.20186,0.23088,0.19525,
0.20443,0.20221,0.20169,0.21237,0.19771,0.19444,0.1219,0.1142,0.11383,
0.13856,0.12464,0.13769,0.12206,0.13019,0.1234,0.1218,0.11532,0.12196,
0.1252,0.10797,0.12234,0.12106,0.11449,0.11733,0.040378,0.21308,0.2051
2,0.14158,0.18245,0.2102,0.1942,0.20673,0.19664,0.19853,0.19951,0.1975
5,0.17581,0.19992,0.2012,0.20518,0.18884,0.18918,0.21703,0.11382,0.121
86,0.15,0.1217,0.13244,0.12249,0.12376,0.1212,0.11117,0.13227,0.12219,
0.11299,0.12694,0.1202,0.12212,0.12317,0.1478,0.040813,0.040234,0.0406
12,0.26135,0.18868,0.19839,0.20259,0.23288,0.21736,0.19985,0.20093,0.1
8015,0.20405,0.18266,0.1885,0.21179,0.20515,0.2026,0.11378,0.1221,0.12
252,0.13876,0.09575,0.1084,0.13145,0.12742,0.10488,0.11987,0.12373,0.1
2514,0.11064,0.13492,0.065654,0.10917,0.13724,0.12618,0.040255,0.04089
,0.040713,0.040281,0.19658,0.23019,0.2229,0.18991,0.20083,0.16666,0.18
534,0.18045,0.20412,0.16632,0.20166,0.18919,0.11242,0.12195,0.17522,0.
1165,0.11391,0.11924,0.11784,0.12207,0.14101,0.12449,0.12219,0.13478,0
.11623,0.12276,0.16323,0.12801,0.12255,0.1278,0.12197,0.12806,0.04085,
0.040116,0.04049,0.040668,0.041125,0.041156,0.22257,0.23456,0.20036,0.
19761,0.27047,0.17474,0.20619,0.20287,0.14175,0.12439,0.13405,0.12627,
0.12436,0.1268,0.14372,0.12238,0.12015,0.12578,0.12256,0.13358,0.12423
,0.11797,0.10972,0.11615,0.11241,0.12237,0.12191,0.11453,0.12236,0.122
29,0.11214,0.04058,0.04078,0.040666,0.040731,0.040611,0.04006,0.20018,
0.19741,0.21706,0.20893,0.20791,0.040597,0.040662,0.11929,0.12501,0.10
743,0.12209,0.12233,0.12249,0.12233,0.12175,0.122,0.12785,0.12147,0.12
575,0.12192,0.15624,0.14747,0.11702,0.12682,0.12165,0.12682,0.12219,0.
12203,0.040209,0.040482,0.040289,0.041135,0.041741,0.03996,0.039764,0.

040393,0.041104,0.040401,0.19825,0.21666,0.040424,0.040008,0.039697,0.
040706,0.12882,0.10341,0.12999,0.11597,0.11585,0.13672,0.12243,0.12172
,0.1218,0.12205,0.12101,0.13132,0.12214,0.12245,0.13087,0.12545,0.1221
,0.12612,0.11396,0.040149,0.040175,0.040656,0.040061,0.040167,0.040364
,0.041018,0.040031,0.040976,0.041515,0.040362,0.040123,0.040426,0.0410
43,0.040531,0.039636,0.040254,0.12299,0.12194,0.12196,0.10979,0.12777,
0.10841,0.11733,0.12226,0.12354,0.1341,0.12477,0.12234,0.13341,0.13069
,0.12717,0.11929,0.040508,0.040893,0.040424,0.040154,0.040197,0.040616
,0.040476,0.040451,0.040491,0.040824,0.039745,0.040773,0.040044,0.0402
42,0.040449,0.040524,0.040841,0.040138,0.040543,0.1079,0.12289,0.14067
,0.12273,0.10999,0.12254,0.12722,0.1253,0.12226,0.1233,0.12937,0.12194
,0.14674,0.040878,0.039627,0.040957,0.039908,0.040574,0.040085,0.04026
9,0.040334,0.040492,0.04025,0.040651,0.040519,0.040406,0.040408,0.0403
45,0.040314,0.040115,0.04021,0.040429,0.040762,0.040573,0.12252,0.1074
7,0.11943,0.11955,0.11973,0.089618,0.12253,0.12241,0.12003,0.040541,0.
040755,0.04057,0.040808,0.040122,0.040419,0.040308,0.040207,0.04032,0.
04057,0.040206,0.040728,0.040065,0.040186,0.041366,0.040912,0.040619,0
.040476,0.040014,0.040711,0.040846,0.040015,0.040487,0.040506,0.040223
,0.10605,0.12511,0.13113,0.099032,0.040009,0.040088,0.040294,0.040727,
0.040498,0.041432,0.040619,0.039986,0.040686,0.039589,0.040149,0.04110
1,0.040802,0.040626,0.040685,0.040496,0.040979,0.040822,0.040433,0.040
114,0.040453,0.040959,0.040264,0.040812,0.040869,0.040088,0.040218,0.0
40315,0.040318,0.041185,0.040549,0.040239,0.040416,0.04098,0.040629,0.
040268,0.040544,0.040427,0.040646,0.04106,0.040913,0.040277,0.040651,0
.040182,0.040734,0.040607,0.040135,0.040953,0.040724,0.040641,0.040397
,0.040694,0.040223,0.040733,0.04074,0.040151,0.040173,0.040562,0.03991
,0.040055,0.041279,0.040134,0.040665,0.040109,0.041378,0.040415,0.0411
,0.040659,0.040137,0.040407,0.040287,0.040618,0.040508,0.040619,0.0404
78,0.040475,0.040963,0.040814,0.040703,0.040211,0.040313,0.040209,0.04
024,0.03991,0.040262,0.041138,0.040479,0.040946,0.040588,0.040943,0.03
9991,0.040831,0.040779,0.040427,0.040372,0.039655,0.040508,0.039798,0.
041175,0.040995,0.040263,0.040316,0.040601,0.040996,0.040424,0.041419,
0.040786,0.040712,0.040889,0.040416,0.040295,0.040835,0.040698,0.03996
6,0.040732,0.040108,0.040615,0.040711,0.040723,0.040543,0.040148,0.039
841,0.041119,0.041102,0.04046,0.041194,0.040444,0.040377,0.04056,0.040
182,0.040481,0.041266,0.040343,0.040569,0.040506,0.039468,0.040798,0.0
40245,0.041045,0.040347,0.040767,0.040275,0.0404,0.04119,0.040245,0.04
0644,0.039545,0.04066,0.039885,0.040317,0.040415,0.040437,0.040179,0.0
4076,0.040389,0.039749,0.039733,0.039954,0.040263,0.039776,0.040691,0.
040904,0.040609,0.041505,0.039853,0.040296,0.040893,0.041845,0.040031,
0.040604,0.040577,0.040296,0.040065,0.040005,0.040736,0.041005,0.04004
7,0.040704,0.039785,0.03949,0.04049,0.040775,0.040835,0.040009,0.04036
1,0.040785,0.039877,0.040672,0.040719,0.040799,0.039976,0.041073,0.040
853,0.040848,0.039902
0.20486,0.20362,0.20492,0.20443,0.20527,0.20437,0.20473,0.2057,0.20389
,0.20483,0.20492,0.20517,0.20449,0.20424,0.20444,0.20492,0.20463,0.204
72,0.20485,0.20476,0.28536,0.27195,0.28465,0.27409,0.20551,0.20497,0.2
056,0.20456,0.2046,0.20447,0.2046,0.20462,0.20497,0.20515,0.20498,0.20
434,0.20505,0.20461,0.20535,0.36364,0.28016,0.27948,0.28131,0.28567,0.
25424,0.2786,0.20525,0.20467,0.20584,0.20375,0.20482,0.20355,0.20582,0
.20511,0.20363,0.20409,0.20513,0.20395,0.20487,0.20463,0.20559,0.35018
,0.34805,0.26243,0.2714,0.28104,0.2809,0.2769,0.29379,0.29274,0.26961,

0.20579,0.20462,0.20465,0.20481,0.20429,0.20605,0.20496,0.20525,0.2049
9,0.2058,0.20439,0.2045,0.20502,0.20402,0.2045,0.35222,0.35682,0.35011
,0.27875,0.28797,0.2928,0.27233,0.29188,0.26442,0.30604,0.28583,0.2821
7,0.20496,0.20541,0.2051,0.20522,0.20459,0.20491,0.20387,0.20455,0.204
93,0.20365,0.20484,0.2053,0.20496,0.20426,0.20361,0.20434,0.35882,0.36
504,0.36873,0.35386,0.26906,0.28698,0.27876,0.28537,0.28078,0.27697,0.
28296,0.28299,0.28031,0.28438,0.20518,0.20505,0.20499,0.2048,0.20397,0
.20475,0.20586,0.20461,0.20442,0.20562,0.20386,0.20514,0.20589,0.20438
,0.2044,0.20426,0.34787,0.36461,0.35112,0.35073,0.36609,0.34211,0.2807
1,0.29489,0.278,0.27973,0.28064,0.2858,0.27971,0.28645,0.28214,0.2815,
0.20506,0.20498,0.205,0.20542,0.20442,0.20474,0.20486,0.20567,0.20477,
0.20376,0.20519,0.20483,0.20386,0.20402,0.20476,0.20493,0.20447,0.3471
1,0.35675,0.35293,0.35627,0.36216,0.35338,0.28651,0.28028,0.29533,0.27
596,0.28551,0.29344,0.28306,0.28167,0.28331,0.27224,0.29095,0.20436,0.
20526,0.20413,0.20446,0.20497,0.20497,0.20448,0.20381,0.20574,0.20525,
0.20447,0.20408,0.20523,0.12173,0.13358,0.12634,0.20475,0.35663,0.3541
8,0.35619,0.35843,0.35581,0.35138,0.36491,0.28238,0.29132,0.27922,0.27
713,0.2856,0.27745,0.26398,0.27999,0.28498,0.27944,0.28256,0.20549,0.2
0485,0.20509,0.20479,0.20501,0.20466,0.20524,0.20506,0.20572,0.20502,0
.2047,0.12122,0.11039,0.11041,0.12242,0.11521,0.11125,0.2056,0.35613,0
.36147,0.36327,0.35109,0.3574,0.34712,0.35996,0.27219,0.28432,0.27888,
0.28277,0.28116,0.28851,0.27716,0.29334,0.28512,0.2813,0.29656,0.20386
,0.20525,0.20483,0.20601,0.20364,0.2045,0.20555,0.20546,0.2047,0.1277,
0.11229,0.1311,0.12128,0.12959,0.12554,0.12326,0.10005,0.12194,0.2039,
0.36224,0.35597,0.35357,0.35669,0.36135,0.35152,0.35272,0.28675,0.2905
,0.28305,0.27875,0.27163,0.28471,0.28591,0.27669,0.27553,0.29428,0.281
33,0.28471,0.20443,0.20383,0.20482,0.20486,0.20429,0.20412,0.12324,0.1
2165,0.12033,0.12241,0.12642,0.12263,0.12411,0.13798,0.1223,0.12248,0.
1152,0.11997,0.35408,0.36672,0.36168,0.35716,0.36342,0.3591,0.3581,0.2
8231,0.27838,0.28886,0.28082,0.2813,0.28187,0.27705,0.29455,0.28978,0.
2823,0.28244,0.2745,0.2045,0.20521,0.20515,0.11805,0.13543,0.12202,0.1
4475,0.12271,0.12515,0.14009,0.087833,0.12285,0.12275,0.1311,0.11815,0
.10795,0.12968,0.12208,0.3627,0.35495,0.36086,0.35882,0.3635,0.35255,0
.35214,0.36521,0.35353,0.28042,0.2768,0.26961,0.28387,0.28346,0.25707,
0.27929,0.27709,0.28441,0.27824,0.28093,0.122,0.13727,0.13606,0.12335,
0.12225,0.1188,0.11669,0.12241,0.15715,0.12248,0.11528,0.1191,0.12356,
0.12302,0.12232,0.12972,0.14576,0.13027,0.36954,0.36608,0.3538,0.35633
,0.36482,0.36486,0.35978,0.36846,0.35517,0.28699,0.28888,0.20291,0.203
46,0.20212,0.2026,0.20687,0.20119,0.1993,0.20998,0.2228,0.10806,0.1197
8,0.12202,0.11994,0.1228,0.093769,0.12008,0.12688,0.11293,0.12195,0.13
44,0.11164,0.12507,0.12228,0.12374,0.11308,0.11603,0.11623,0.35811,0.3
6797,0.35669,0.36008,0.35918,0.36732,0.36197,0.36507,0.36204,0.19184,0
.20167,0.22205,0.20028,0.19758,0.20111,0.19696,0.17784,0.195,0.21843,0
.20498,0.12213,0.096411,0.12317,0.12286,0.12592,0.12225,0.10419,0.1210
9,0.12978,0.1209,0.10813,0.12185,0.13284,0.12447,0.12999,0.13246,0.118
91,0.13658,0.34137,0.35732,0.35408,0.36347,0.3516,0.34838,0.36413,0.19
481,0.2245,0.22056,0.19732,0.21895,0.19804,0.21502,0.2192,0.19567,0.20
231,0.2136,0.19528,0.20919,0.12267,0.14128,0.12194,0.11178,0.098099,0.
10438,0.10854,0.12223,0.12285,0.12231,0.12391,0.12193,0.12325,0.12309,
0.12258,0.11776,0.091587,0.12913,0.36052,0.35981,0.35599,0.36015,0.347
84,0.36237,0.21058,0.20513,0.1945,0.1581,0.18016,0.2027,0.18618,0.2112
8,0.17696,0.18791,0.19467,0.2093,0.20904,0.21727,0.12387,0.11828,0.122

27,0.11811,0.1076,0.12862,0.11756,0.096496,0.12547,0.11691,0.1315,0.10
599,0.1158,0.12517,0.11652,0.12184,0.11759,0.10381,0.35881,0.36295,0.3
5984,0.36477,0.36744,0.18953,0.20154,0.19685,0.19334,0.18915,0.20349,0
.18066,0.18087,0.22216,0.18653,0.20262,0.20056,0.19951,0.20247,0.20412
,0.12074,0.11448,0.12935,0.12231,0.12252,0.12552,0.13977,0.12444,0.115
85,0.12329,0.12053,0.11364,0.12193,0.11906,0.12052,0.12277,0.13296,0.1
2862,0.35375,0.35827,0.34721,0.19259,0.19427,0.19479,0.19855,0.20718,0
.19094,0.20682,0.17098,0.20387,0.19578,0.19912,0.16898,0.18466,0.20594
,0.20018,0.17481,0.19977,0.1581,0.12613,0.12276,0.12265,0.12265,0.1223
6,0.11639,0.12287,0.13829,0.11942,0.12749,0.11021,0.12305,0.10953,0.09
059,0.12551,0.12965,0.12139,0.35929,0.21192,0.20803,0.19029,0.1953,0.2
2698,0.19644,0.2209,0.20122,0.1888,0.16932,0.19913,0.20692,0.19435,0.1
8607,0.19989,0.19804,0.19103,0.1942,0.12726,0.12093,0.11705,0.11052,0.
12185,0.12292,0.11882,0.11141,0.12204,0.12713,0.12204,0.12214,0.12254,
0.11292,0.10496,0.12608,0.12298,0.04047,0.18104,0.19966,0.18465,0.1394
5,0.21849,0.14,0.17463,0.2063,0.20818,0.19584,0.19686,0.21906,0.20913,
0.17573,0.19423,0.23591,0.20116,0.20378,0.19324,0.12786,0.12456,0.1237
9,0.12749,0.12436,0.13842,0.12504,0.10705,0.12214,0.10199,0.12243,0.12
218,0.13052,0.11716,0.1225,0.12678,0.12248,0.039814,0.21693,0.23649,0.
20594,0.19725,0.15994,0.19752,0.19905,0.19777,0.20131,0.20235,0.23122,
0.19513,0.20323,0.201,0.20183,0.21188,0.19753,0.19331,0.12219,0.11425,
0.11375,0.13864,0.12496,0.13803,0.12223,0.13043,0.12343,0.12204,0.1155
5,0.12195,0.12531,0.10808,0.1226,0.1215,0.11465,0.11761,0.039653,0.214
86,0.20232,0.14102,0.18059,0.20861,0.19326,0.20337,0.19732,0.19626,0.1
9786,0.19652,0.17513,0.20056,0.20055,0.20312,0.18783,0.18817,0.21402,0
.11405,0.12212,0.15038,0.12188,0.1326,0.12243,0.12408,0.12105,0.11109,
0.13272,0.12234,0.11317,0.12696,0.12034,0.12225,0.12319,0.14807,0.0403
73,0.039945,0.040023,0.26049,0.18748,0.19652,0.20096,0.23202,0.21481,0
.19941,0.19861,0.17773,0.20402,0.1814,0.18772,0.2111,0.20488,0.20285,0
.11377,0.12245,0.12274,0.13911,0.095725,0.10835,0.13172,0.1274,0.105,0
.11987,0.12392,0.12542,0.11084,0.13504,0.065592,0.10914,0.13769,0.1263
2,0.040022,0.040423,0.040468,0.039954,0.19256,0.23072,0.2198,0.18871,0
.19832,0.16694,0.18465,0.17981,0.20281,0.16588,0.20274,0.18845,0.11246
,0.12195,0.17578,0.11676,0.11416,0.11939,0.11808,0.12219,0.14101,0.124
72,0.12243,0.13509,0.11635,0.12284,0.16389,0.12835,0.1226,0.12798,0.12
234,0.12811,0.040406,0.039612,0.039993,0.040309,0.040434,0.040687,0.22
153,0.23462,0.20102,0.19589,0.26918,0.1732,0.20444,0.20337,0.14222,0.1
2475,0.13438,0.12657,0.12467,0.12706,0.14411,0.12239,0.12045,0.12566,0
.12284,0.1338,0.12431,0.1182,0.10987,0.11637,0.11249,0.12219,0.12182,0
.11476,0.12253,0.1224,0.11226,0.040244,0.040619,0.040332,0.040497,0.04
0445,0.039658,0.20024,0.19557,0.21764,0.20797,0.20866,0.040077,0.04036
3,0.11959,0.12522,0.10751,0.12237,0.12249,0.12248,0.12231,0.12184,0.12
239,0.12805,0.12154,0.12619,0.12218,0.15674,0.14793,0.11738,0.12727,0.
12182,0.1268,0.12221,0.1222,0.040242,0.040424,0.039985,0.040638,0.0415
21,0.039452,0.039413,0.03967,0.040332,0.039937,0.19645,0.21844,0.03991
8,0.039397,0.039538,0.040356,0.12894,0.1035,0.13037,0.11613,0.11612,0.
13718,0.12273,0.12195,0.12197,0.12222,0.12123,0.1315,0.12231,0.12245,0
.13128,0.12552,0.12192,0.12659,0.11407,0.039738,0.039565,0.040344,0.03
9695,0.039866,0.040248,0.040307,0.039773,0.04051,0.041026,0.039921,0.0
40005,0.040038,0.040448,0.040026,0.039365,0.039709,0.12319,0.1221,0.12
218,0.10972,0.12789,0.10849,0.11741,0.12223,0.12383,0.13421,0.12514,0.
12265,0.13363,0.13107,0.12727,0.11956,0.040022,0.040355,0.040088,0.039

72,0.039903,0.040783,0.03988,0.04022,0.039994,0.04044,0.03959,0.040301
,0.039587,0.039793,0.040419,0.040232,0.040388,0.039944,0.040237,0.1080
2,0.12317,0.14096,0.12323,0.11013,0.12285,0.12738,0.12554,0.12277,0.12
35,0.12982,0.12238,0.14699,0.040628,0.03931,0.040649,0.039395,0.040213
,0.039988,0.039683,0.039839,0.040315,0.039984,0.040156,0.040514,0.0401
69,0.040386,0.039711,0.04009,0.039758,0.039807,0.0401,0.040338,0.04029
7,0.12253,0.10752,0.11967,0.11969,0.12015,0.089472,0.12293,0.12268,0.1
2035,0.040179,0.040558,0.03995,0.040321,0.039543,0.039917,0.04011,0.03
9797,0.040089,0.040288,0.039615,0.040424,0.039827,0.039996,0.040759,0.
040773,0.040229,0.040082,0.039733,0.040301,0.040286,0.039867,0.040176,
0.040179,0.039707,0.10624,0.1254,0.13149,0.099172,0.039489,0.03999,0.0
39843,0.040704,0.040192,0.040867,0.040217,0.03956,0.040418,0.039262,0.
039895,0.040911,0.040052,0.040233,0.040124,0.039943,0.040564,0.040379,
0.040005,0.039921,0.039903,0.040252,0.039978,0.040378,0.040499,0.03971
5,0.039718,0.040099,0.04019,0.040836,0.040149,0.03963,0.03968,0.040516
,0.039758,0.039765,0.040051,0.040313,0.040047,0.040765,0.04052,0.03985
4,0.040338,0.039772,0.039923,0.040228,0.039866,0.040285,0.040143,0.039
916,0.040093,0.040271,0.039527,0.040242,0.040539,0.039745,0.039751,0.0
40113,0.039317,0.039372,0.040925,0.039454,0.040379,0.039125,0.04075,0.
039835,0.040345,0.040192,0.039745,0.040145,0.039625,0.040171,0.04033,0
.040178,0.039755,0.040024,0.040432,0.040263,0.0401,0.039753,0.039884,0
.039676,0.039923,0.039665,0.040003,0.040783,0.040475,0.040372,0.040391
,0.040338,0.039775,0.040375,0.040385,0.039792,0.040065,0.03927,0.04017
4,0.039325,0.040678,0.040487,0.040098,0.039794,0.040263,0.040738,0.040
188,0.041005,0.040101,0.040245,0.04054,0.040178,0.040023,0.040384,0.04
0169,0.039539,0.040577,0.039806,0.039942,0.040283,0.040147,0.040397,0.
039723,0.039722,0.040623,0.04052,0.040119,0.040443,0.040238,0.039875,0
.040126,0.039947,0.040112,0.040916,0.039908,0.040328,0.040122,0.039207
,0.040452,0.039714,0.040333,0.040089,0.040378,0.040043,0.039893,0.0405
94,0.039723,0.040314,0.039208,0.040539,0.039533,0.039837,0.040257,0.03
997,0.039994,0.040474,0.040156,0.039276,0.039583,0.039758,0.039717,0.0
39694,0.040337,0.040596,0.039982,0.04104,0.039289,0.039894,0.040455,0.
041312,0.039772,0.040183,0.040377,0.039788,0.03954,0.039437,0.040171,0
.04068,0.03986,0.040372,0.039536,0.039078,0.040157,0.04041,0.040421,0.
039548,0.040085,0.040283,0.039437,0.040213,0.040011,0.040333,0.04005,0
.040432,0.040214,0.040387,0.039746

#simdataS3SNR10:

Kep_f=1.2
Kep_f=1.2
Kep_s=0.5
Ktrans_f=0.05
Ktrans_s=0.06
del_t=0.5
gtSf=0 0 0 0 0 0
0 0 0 0 0 0
0 0 0 0 0 0
0 0 0 0 0 0
0 0 0 0 0 0
0 0 0 0 0 0

0	0	0	0	0	0
0	0	0	0	0	0
0	0	0	0	0	0
0	0	0	0	0	0
0	0	0	0	0	0
0	0	0	0	0	0
0	0	0	0	0	0
0	0	0	0	0	0
0	0	0	0	0	0
0	0	0	0	0	0
0	0	0	0	0	0
0	0	0	0	0	0
0	0	0	0	0	0
0	0	0	0	0	0
0	0	0	0	0	0
0	0	0	0	0	0
0	0	0	0	0	0
0	0	0	0	0	0
0	0	0	0	0	0
0	0	0	0	0	0
0	0	0	0	0	0
0	0	0	0	0	0
0	0	0	0	0	0
0	0	0	0	0	0
0	0	0	0	0	0
0	0	0	0	0	0
0	0	0	0	0	0
0	0	0	0	0	0
0	0	0	0.54646	0.5	0.39276
0	0	0	0	0	0
0	0	0	0	0	0
0	0	0	0	0	0
0	0	0	0	0	0
0	0	0	0	0	0
0.44584	0.46383	0.5	0.51113	0.56762	0.53348
0	0	0	0	0	0
0	0	0	0	0	0
0	0	0	0	0	0
0	0	0	0	0	0
0	0	0	0	0.5	0.38325
0.54478	0.5	0.53371	0.28144	0.5	0.49852
0.49729	0	0	0	0	0
0	0	0	0	0	0
0	0	0	0	0	0
0	0	0	0	0	0
0	0	0	0.28893	0.48799	0.4683
0.52162	0.5	0.5	0.49537	0.5	0.45915
0.52311	0.75961	0.5	0	0	0
0	0	0	0	0	0
0	0	0	0	0	0
0	0	0	0	0	0
0	0.6344	0.50947	0.50073	0.47209	0.61584

0.46036	0.57387	0.57631	0.61434	0.34181	0.5886
0.48045	0.47081	0.55011	0.51746	0	0
0	0	0	0	0	0
0	0	0	0	0	0
0	0	0	0	0	0
0.5	0.53447	0.45907	0.51233	0.53238	0.5
0.45961	0.5	0.42994	0.54163	0.51158	0.48692
0.53489	0.49568	0.45304	0.37631	0.50009	0.43575
0	0	0	0	0	0
0	0	0	0	0	0.31154
0.36777	0.358	0.38928	0.32821	0.3642	0.23551
0.32054	0.37227	0.42075	0.5	0.47413	0.50949
0.67391	0.53633	0.35166	0.616	0.47204	0.48637
0.55731	0.5	0.53167	0.5	0.5	0.5
0.61154	0.44864	0	0	0	0
0	0	0	0	0	0.50979
0.30608	0.30325	0.30824	0.37791	0.31566	0.32955
0.33333	0.35523	0.24284	0.37714	0.5	0.57556
0.50384	0.48481	0.49398	0.49919	0.5	0.58813
0.56114	0.5	0.5	0.52499	0.48749	0.5
0.41353	0.50683	0.47339	0.51589	0	0
0	0	0	0	0	0.5852
0.50861	0.5	0.38093	0.35364	0.31605	0.33333
0.34399	0.31247	0.23573	0.30671	0.33788	0.38032
0.48765	0.46539	0.45214	0.4719	0.5	0.5
0.57871	0.43472	0.50256	0.56993	0.49462	0.47866
0.46084	0.5681	0.5	0.45497	0.52053	0.5
0	0	0	0	0	0
0.5454	0.50724	0.51469	0.39959	0.5	0.22765
0.33333	0.37247	0.36712	0.39398	0.36588	0.35049
0.3562	0.33333	0.37927	0.60311	0.63661	0.50067
0.5	0.53067	0.48416	0.37404	0.4479	0.41038
0.49375	0.5	0.5	0.5	0.53668	0.49848
0.50949	0.51956	0	0	0	0
0	0.51737	0.57798	0.5	0.49148	0.54838
0.55727	0.34781	0.36684	0.3323	0.36789	0.35807
0.32029	0.40501	0.29761	0.39126	0.4442	0.5
0.50459	0.31002	0.54145	0.61553	0.49278	0.60572
0.43713	0.48576	0.56039	0.52535	0.57678	0.5
0.43683	0.5	0.50791	0.58356	0	0
0	0.47972	0.64854	0.5	0.58805	0.5
0.51733	0.49843	0.405	0.33333	0.34441	0.4117
0.23495	0.34315	0.34384	0.36471	0.33333	0.30441
0.64163	0.52829	0.5219	0.5	0.53472	0.42198
0.54419	0.40118	0.43383	0.5	0.5	0.5
0.5	0.48653	0.59409	0.53148	0.56004	0.54895
0	0.49933	0.4737	0.53923	0.37901	0.55225
0.5	0.47206	0.39465	0.43667	0.29272	0.33892
0.34251	0.33333	0.39124	0.30691	0.37163	0.33333
0.33333	0.53194	0.5	0.41976	0.49013	0.50595
0.61588	0.5	0.53589	0.55175	0.5	0.46672
0.47552	0.52234	0.5	0.37917	0.5	0.5

1	0.4919	0.47418	0.49955	0.48775	0.5
0.57915	0.5	0.59622	0.49846	0.45356	0.33333
0.34074	0.3101	0.37456	0.31798	0.35905	0.28081
0.33333	0.3388	0.5	0.52492	0.59945	0.5
0.44122	0.38061	0.51907	0.46979	0.5	0.53191
0.55503	0.50234	0.5	0.42755	0.56282	0.59359
0.5	1	0.54385	0.53798	0.4377	0.56751
0.57555	0.5	0.5	0.44848	0.45216	0.52551
0.34103	0.33439	0.30016	0.34136	0.37616	0.33333
0.36788	0.37327	0.5069	0.6826	0.5	0.49818
0.48358	0.5	0.56082	0.49615	0.5	0.51084
0.5337	0.31577	0.41859	0.43801	0.5	0.40923
0.5	0.52971	1	0.41618	0.60028	0.52998
0.5	0.52612	0.71134	0.52067	0.52062	0.56603
0.39399	0.3426	0.33865	0.37501	0.33871	0.33864
0.31682	0.391	0.338	0.5	0.44441	0.49258
0.53031	0.5	0.48962	0.498	0.44874	0.4956
0.5	0.4101	0.52035	0.5	0.54431	0.5
0.5	0.57103	1	1	1	0.49563
0.5	0.43756	0.5	0.42468	0.54259	0.52437
0.61232	0.5	0.39147	0.34681	0.33333	0.32584
0.30845	0.3468	0.46293	0.5	0.5	0.49354
0.5	0.56043	0.51789	0.55738	0.28035	0.52908
0.50909	0.5	0.52628	0.33948	0.5	0.5
0.50732	0.44732	1	1	1	1
0.52103	0.5	0.60017	0.54755	0.5	0.5
0.5	0.5	0.5	0.36817	0.282	0.34995
0.48826	0.5	0.4601	0.54027	0.4705	0.62429
0.54084	0.47939	0.5	0.5	0.5	0.5
0.52109	0.35258	0.5003	0.40036	0.5	0.42149
0.5	0.55536	1	1	1	1
1	1	0.5	0.49192	0.38241	0.5105
0.45211	0.5	0.38901	0.4831	0.5	0.55473
0.53201	0.6056	0.59459	0.53767	0.45708	0.45828
0.4588	0.52007	0.32922	0.67042	0.37071	0.47436
0.5	0.53644	0.5	0.44708	0.49797	0.52102
0.52157	0.40909	0.5	1	1	1
1	1	1	0.49348	0.5	0.62486
0.4567	0.54933	1	1	0.449	0.47658
0.43828	0.5	0.5	0.46881	0.55145	0.52092
0.52486	0.454	0.56766	0.42881	0.45759	0.46864
0.652	0.56728	0.44835	0.46177	0.54074	0.5755
0.5139	1	1	1	1	1
1	1	1	1	1	0.50684
0.5	1	1	1	1	0.43978
0.51421	0.54258	0.48775	0.5	0.50285	0.6063
0.40998	0.45427	0.46203	0.42881	0.55452	0.5
0.47741	0.5	0.48833	0.53565	0.53573	0.55342
1	1	1	1	1	1
1	1	1	1	1	1
1	1	1	1	1	0.44529
0.5	0.42246	0.51976	0.5	0.52067	0.5

```
0.5      0.52684     0.42909     0.51105         0.5         0.5
0.47214          0.5     0.51327           1           1           1
1            1           1           1           1           1
1            1           1           1           1           1
1            1           1           1      0.4317     0.52018
0.35525       0.4645     0.53527         0.5     0.47983     0.58869
0.49872      0.56236      0.4656     0.35588      0.4578           1
1            1           1           1           1           1
1            1           1           1           1           1
1            1           1           1           1           1
1            1      0.4599     0.36941     0.54261     0.62052
0.5      0.42437     0.47259         0.5     0.59144           1
1            1           1           1           1           1
1            1           1           1           1           1
1            1           1           1           1           1
1            1           1           1           1           1
0.5      0.43581     0.58909     0.39208           1           1
1            1           1           1           1           1
1            1           1           1           1           1
1            1           1           1           1           1
1            1           1           1           1           1
1            1           1           1           1           1
1            1           1           1           1           1
1            1           1           1           1           1
1            1           1           1           1           1
1            1           1           1           1           1
1            1           1           1           1           1
1            1           1           1           1           1
1            1           1           1           1           1
1            1           1           1           1           1
1            1           1           1           1           1
1            1           1           1           1           1
1            1           1           1           1           1
1            1           1           1           1           1
1            1           1           1           1           1
1            1           1           1           1           1
1            1           1           1           1           1
1            1           1           1           1           1
1            1           1           1           1           1
1            1           1           1           1           1
1            1           1           1           1           1
1            1           1           1           1           1
1            1           1           1           1           1
1            1           1           1           1           1
1            1           1           1           1           1
1            1           1           1           1           1
1            1           1           1           1           1
1            1           1           1           1           1
1            1           1           1           1           1
1
gtSs=1           1           1           1           1           1
1            1           1           1           1           1
```

1 1 1 1 1 1
1 1 0.50207 0.40829 0.38328 0.54994
1 1 1 1 1 1
1 1 1 1 1 1
1 1 1 0 0.45469 0.48074
0.51895 0.54355 0.46843 0.42607 1 1
1 1 1 1 1 1
1 1 1 1 1 1
1 0 0 0.46656 0.5 0.47207
0.37308 0.44404 0.39438 0.39568 0.42568 1
1 1 1 1 1 1
1 1 1 1 1 1
1 1 0 0 0 0.5
0.5 0.48752 0.5 0.5 0.56048 0.43555
0.50089 0.5 1 1 1 1
1 1 1 1 1 1
1 1 1 1 1 1
0 0 0 0 0.52099 0.5
0.51519 0.46038 0.5 0.51167 0.43167 0.47268
0.54606 0.54996 1 1 1 1
1 1 1 1 1 1
1 1 1 1 1 1
0 0 0 0 0 0
0.54309 0.47991 0.48613 0.49144 0.5 0.48666
0.51563 0.43449 0.50499 0.50461 1 1
1 1 1 1 1 1
1 1 1 1 1 1
1 1 1 0 0 0
0 0 0 0.44225 0.50346 0.54333
0.5 0.44084 0.68603 0.56744 0.43661 0.50877
0.47318 0.60787 1 1 1 1
1 1 1 1 1 1
1 1 1 0.45354 0.5 0.60724
1 0 0 0 0
0 0 0.49005 0.5 0.5 0.56446
0.51202 0.5 0.43494 0.5 0.54847 0.64762
0.54 1 1 1 1 1
1 1 1 1 1 1
0.55416 0.53617 0.5 0.48887 0.43238 0.46652
1 0 0 0 0 0
0 0 0.48918 0.6528 0.5042 0.39564
0.5 0.52547 0.46367 0.49272 0.39506 0.53249
0.36445 1 1 1 1 1
1 1 1 1 0.5 0.61675
0.45522 0.5 0.46629 0.71856 0.5 0.50148
0.50271 1 0 0 0 0
0 0 0 0.38981 0.63274 0.48456
0.5 0.63986 0.50189 0.55684 0.5 0.63469
0.49983 0.58084 0.5 1 1 1
1 1 1 0.71107 0.51201 0.5317
0.47838 0.5 0.5 0.50463 0.5 0.54085
0.47689 0.24039 0.5 0 0 0

0	0	0	0	0.53018	0.49793
0.49869	0.52717	0.30603	0.48818	0.524	0.5599
0.5	0.49519	0.5	0.52211	1	1
1	0.3656	0.49053	0.49927	0.52791	0.38416
0.53964	0.42613	0.42369	0.38566	0.65819	0.4114
0.51955	0.52919	0.44989	0.48254	0	0
0	0	0	0	0	0
0	0.5	0.48915	0.53103	0.50502	0.39447
0.5	0.5	0.5	0.46865	0.42711	0.44138
0.5	0.46553	0.54093	0.48767	0.46762	0.5
0.54039	0.5	0.57006	0.45837	0.48842	0.51308
0.46511	0.50432	0.54696	0.62369	0.49991	0.56425
0	0	0	0	0	0
0	0	0	0.54867	0.43191	0.34423
0.36777	0.284	0.3782	0.34098	0.40604	0.36274
0.35455	0.37227	0.57925	0.5	0.52587	0.49051
0.32609	0.46367	0.64834	0.384	0.52796	0.51363
0.44269	0.5	0.46833	0.5	0.5	0.5
0.38846	0.55136	0	0	0	0
0	0	0	0	0	0
0.34696	0.33686	0.34588	0.31156	0.41223	0.37962
0.33333	0.22085	0.46623	0.37714	0.5	0.42444
0.49616	0.51519	0.50602	0.50081	0.5	0.41187
0.43886	0.5	0.5	0.47501	0.51251	0.5
0.58647	0.49317	0.52661	0.48411	0	0
0	0	0	0	0	0
0	0	0.23814	0.35364	0.34197	0.33333
0.31203	0.35148	0.37652	0.34664	0.32423	0.38032
0.51235	0.53461	0.54786	0.5281	0.5	0.5
0.42129	0.56528	0.49744	0.43007	0.50538	0.52134
0.53916	0.4319	0.5	0.54503	0.47947	0.5
0	0	0	0	0	0
0	0	0	0	0	0.38617
0.33333	0.33783	0.32102	0.21205	0.25706	0.25428
0.2876	0.33333	0.62073	0.39689	0.36339	0.49933
0.5	0.46933	0.51584	0.62596	0.5521	0.58962
0.50625	0.5	0.5	0.5	0.46332	0.50152
0.49051	0.48044	0	0	0	0
0	0	0	0	0	0
0	0.34781	0.31593	0.3511	0.33867	0.27313
0.33462	0.20273	0.3512	0.20192	0.5558	0.5
0.49541	0.68998	0.45855	0.38447	0.50722	0.39428
0.56287	0.51424	0.43961	0.47465	0.42322	0.5
0.56317	0.5	0.49209	0.41644	0	0
0	0	0	0	0	0
0	0	0	0.33333	0.31893	0.34402
0.38253	0.3598	0.30878	0.27058	0.33333	0.3863
0.35837	0.47171	0.4781	0.5	0.46528	0.57802
0.45581	0.59882	0.56617	0.5	0.5	0.5
0.5	0.51347	0.40591	0.46852	0.43996	0.45105
0	0	0	0	0	0
0	0	0	0	0.35364	0.33892

0.31498	0.33333	0.38541	0.39357	0.25673	0.33333
0.33333	0.46806	0.5	0.58024	0.50987	0.49405
0.38412	0.5	0.46411	0.44825	0.5	0.53328
0.52448	0.47766	0.5	0.62083	0.5	0.5
0	0	0	0	0	0
0	0	0	0	0	0.33333
0.34599	0.34495	0.28314	0.2987	0.38411	0.35959
0.33333	0.3388	0.5	0.47508	0.40055	0.5
0.55878	0.61939	0.48093	0.53021	0.5	0.46809
0.44497	0.49766	0.5	0.57245	0.43718	0.40641
0.5	0	0	0	0	0
0	0	0	0	0	0
0.41469	0.33439	0.38592	0.34311	0.27178	0.33333
0.26424	0.32092	0.4931	0.3174	0.5	0.50182
0.51642	0.5	0.43918	0.50385	0.5	0.48916
0.4663	0.68423	0.58141	0.56199	0.5	0.59077
0.5	0.47029	0	0	0	0
0	0	0	0	0	0
0	0.31479	0.33865	0.24998	0.33871	0.28763
0.32337	0.31475	0.338	0.5	0.55559	0.50742
0.46969	0.5	0.51038	0.502	0.55126	0.5044
0.5	0.5899	0.47965	0.5	0.45569	0.5
0.5	0.42897	0	0	0	0
0	0	0	0	0	0
0	0	0.29025	0.30637	0.33333	0.30787
0.23827	0.30641	0.53707	0.5	0.5	0.50646
0.5	0.43957	0.48211	0.44262	0.71965	0.47092
0.49091	0.5	0.47372	0.66052	0.5	0.5
0.49268	0.55268	0	0	0	0
0	0	0	0	0	0
0	0	0	0.26367	0.359	0.30011
0.51174	0.5	0.5399	0.45973	0.5295	0.37571
0.45916	0.52061	0.5	0.5	0.5	0.5
0.47891	0.64742	0.4997	0.59964	0.5	0.57851
0.5	0.44464	0	0	0	0
0	0	0	0	0	0
0	0	0	0	0.5	0.44527
0.46799	0.3944	0.40541	0.46233	0.54292	0.54172
0.5412	0.47993	0.67078	0.32958	0.62929	0.52564
0.5	0.46356	0.5	0.55292	0.50203	0.47898
0.47843	0.59091	0.5	0	0	0
0	0	0	0	0	0
0	0	0	0	0.551	0.52342
0.56172	0.5	0.5	0.53119	0.44855	0.47908
0.47514	0.546	0.43234	0.57119	0.54241	0.53136
0.348	0.43272	0.55165	0.53823	0.45926	0.4245
0.4861	0	0	0	0	0
0	0	0	0	0	0
0	0	0	0	0	0.56022
0.48579	0.45742	0.51225	0.5	0.49715	0.3937
0.59002	0.54573	0.53797	0.57119	0.44548	0.5
0.52259	0.5	0.51167	0.46435	0.46427	0.44658

0	0	0	0	0	0
0	0	0	0	0	0
0	0	0	0	0	0.55471
0.5	0.57754	0.48024	0.5	0.47933	0.5
0.5	0.47316	0.57091	0.48895	0.5	0.5
0.52786	0.5	0.48673	0	0	0
0	0	0	0	0	0
0	0	0	0	0	0
0	0	0	0	0.5683	0.47982
0.64475	0.5355	0.46473	0.5	0.52017	0.41131
0.50128	0.43764	0.5344	0.64412	0.5422	0
0	0	0	0	0	0
0	0	0	0	0	0
0	0	0	0	0	0
0	0	0.5401	0.63059	0.45739	0.37948
0.5	0.57563	0.52741	0.5	0.40856	0
0	0	0	0	0	0
0	0	0	0	0	0
0	0	0	0	0	0
0	0	0	0	0	0
0.5	0.56419	0.41091	0.60792	0	0
0	0	0	0	0	0
0	0	0	0	0	0
0	0	0	0	0	0
0	0	0	0	0	0
0	0	0	0	0	0
0	0	0	0	0	0
0	0	0	0	0	0
0	0	0	0	0	0
0	0	0	0	0	0
0	0	0	0	0	0
0	0	0	0	0	0
0	0	0	0	0	0
0	0	0	0	0	0
0	0	0	0	0	0
0	0	0	0	0	0
0	0	0	0	0	0
0	0	0	0	0	0
0	0	0	0	0	0
0	0	0	0	0	0
0	0	0	0	0	0
0	0	0	0	0	0
0	0	0	0	0	0
0	0	0	0	0	0
0	0	0	0	0	0
0	0	0	0	0	0
0	0	0	0	0	0
0	0	0	0	0	0
0	0	0	0	0	0
0	0	0	0	0	0
0	0	0	0	0	0
0	0	0	0	0	0
0	0	0	0	0	0

0 0 0 0 0 0
0
gtSp=0 0 0 0 0 0
0 0 0 0 0 0
0 0 0 0 0 0
0 0 0.49793 0.59171 0.61672 0.45006
0 0 0 0 0 0
0 0 0 0 0 0
0 0 0 1 0.54531 0.51926
0.48105 0.45645 0.53157 0.57393 0 0
0 0 0 0 0 0
0 0 0 0 0 0
0 1 1 0.53344 0.5 0.52793
0.62692 0.55596 0.60562 0.60432 0.57432 0
0 0 0 0 0 0
0 0 0 0 0 0
0 0 1 1 1 0.5
0.5 0.51248 0.5 0.5 0.43952 0.56445
0.49911 0.5 0 0 0 0
0 0 0 0 0 0
0 0 0 0 0 0
1 1 1 1 0.47901 0.5
0.48481 0.53962 0.5 0.48833 0.56833 0.52732
0.45394 0.45004 0 0 0 0
0 0 0 0 0 0
0 0 0 0 0 0
1 1 1 1 1 1
0.45691 0.52009 0.51387 0.50856 0.5 0.51334
0.48437 0.56551 0.49501 0.49539 0 0
0 0 0 0 0 0
0 0 0 0 0 0
0 0 0 1 1 1
1 1 1 0.55775 0.49654 0.45667
0.5 0.55916 0.31397 0.43256 0.56339 0.49123
0.52682 0.39213 0 0 0 0
0 0 0 0 0 0
0 0 0 0 0 0
0 1 1 1 1 1
1 1 0.50995 0.5 0.5 0.43554
0.48798 0.5 0.56506 0.5 0.45153 0.35238
0.46 0 0 0 0 0
0 0 0 0 0 0
0 0 0 0 0 0
0 1 1 1 1 1
1 1 0.51082 0.3472 0.4958 0.60436
0.5 0.47453 0.53633 0.50728 0.60494 0.46751
0.63555 0 0 0 0 0
0 0 0 0 0 0
0 0 0 0 0 0
0 0 1 1 1 1
1 1 1 0.61019 0.36726 0.51544
0.5 0.36014 0.49811 0.44316 0.5 0.36531

0.50017	0.41916	0.5	0	0	0
0	0	0	0	0	0
0	0	0	0	0	0
0	0	0	1	1	1
1	1	1	1	0.46982	0.50207
0.50131	0.47283	0.69397	0.51182	0.476	0.4401
0.5	0.50481	0.5	0.47789	0	0
0	0	0	0	0	0
0	0	0	0	0	0
0	0	0	0	1	1
1	1	1	1	1	1
1	0.5	0.51085	0.46897	0.49498	0.60553
0.5	0.5	0.5	0.53135	0.57289	0.55862
0	0	0	0	0	0
0	0	0	0	0	0
0	0	0	0	0	0
1	1	1	1	1	1
1	1	1	0.45133	0.56809	0.34423
0.26447	0.358	0.23253	0.33081	0.22976	0.40174
0.32491	0.25546	0	0	0	0
0	0	0	0	0	0
0	0	0	0	0	0
0	0	1	1	1	1
1	1	1	1	1	0.49021
0.34696	0.35989	0.34588	0.31053	0.27211	0.29083
0.33333	0.42392	0.29093	0.24572	0	0
0	0	0	0	0	0
0	0	0	0	0	0
0	0	0	0	1	1
1	1	1	1	1	0.4148
0.49139	0.5	0.38093	0.29273	0.34197	0.33333
0.34399	0.33605	0.38775	0.34664	0.33788	0.23935
0	0	0	0	0	0
0	0	0	0	0	0
0	0	0	0	0	0
1	1	1	1	1	1
0.4546	0.49276	0.48531	0.60041	0.5	0.38617
0.33333	0.2897	0.31186	0.39398	0.37707	0.39523
0.3562	0.33333	0	0	0	0
0	0	0	0	0	0
0	0	0	0	0	0
0	0	1	1	1	1
1	0.48263	0.42202	0.5	0.50852	0.45162
0.44273	0.30439	0.31724	0.3166	0.29344	0.36881
0.34509	0.39226	0.3512	0.40682	0	0
0	0	0	0	0	0
0	0	0	0	0	0
0	0	0	0	1	1
1	0.52028	0.35146	0.5	0.41195	0.5
0.48267	0.50157	0.595	0.33333	0.33667	0.24428
0.38253	0.29705	0.34737	0.36471	0.33333	0.30929
0	0	0	0	0	0

0	0	0	0	0	0
0	0	0	0	0	0
1	0.50067	0.5263	0.46077	0.62099	0.44775
0.5	0.52794	0.60535	0.56333	0.35364	0.32217
0.34251	0.33333	0.22335	0.29952	0.37163	0.33333
0.33333	0	0	0	0	0
0	0	0	0	0	0
0	0	0	0	0	0
0	0.5081	0.52582	0.50045	0.51225	0.5
0.42085	0.5	0.40378	0.50154	0.54644	0.33333
0.31327	0.34495	0.3423	0.38332	0.25684	0.35959
0.33333	0.3224	0	0	0	0
0	0	0	0	0	0
0	0	0	0	0	0
0	0	0.45615	0.46202	0.5623	0.43249
0.42445	0.5	0.5	0.55152	0.54784	0.47449
0.24428	0.33123	0.31391	0.31553	0.35206	0.33333
0.36788	0.30581	0	0	0	0
0	0	0	0	0	0
0	0	0	0	0	0
0	0	0	0.58382	0.39972	0.47002
0.5	0.47388	0.28866	0.47933	0.47938	0.43397
0.60601	0.3426	0.3227	0.37501	0.32259	0.37373
0.35981	0.29425	0.324	0	0	0
0	0	0	0	0	0
0	0	0	0	0	0
0	0	0	0	0	0.50437
0.5	0.56244	0.5	0.57532	0.45741	0.47563
0.38768	0.5	0.31828	0.34681	0.33333	0.36629
0.45328	0.3468	0	0	0	0
0	0	0	0	0	0
0	0	0	0	0	0
0	0	0	0	0	0
0.47897	0.5	0.39983	0.45245	0.5	0.5
0.5	0.5	0.5	0.36817	0.359	0.34995
0	0	0	0	0	0
0	0	0	0	0	0
0	0	0	0	0	0
0	0	0	0	0	0
0	0	0.5	0.50808	0.61759	0.4895
0.54789	0.5	0.61099	0.5169	0	0
0	0	0	0	0	0
0	0	0	0	0	0
0	0	0	0	0	0
0	0	0	0	0	0
0	0	0	0.50652	0.5	0.37514
0.5433	0.45067	0	0	0	0
0	0	0	0	0	0
0	0	0	0	0	0
0	0	0	0	0	0
0	0	0	0	0	0
0	0	0	0	0	0.49316

0.5	0	0	0	0	0
0	0	0	0	0	0
0	0	0	0	0	0
0	0	0	0	0	0
0	0	0	0	0	0
0	0	0	0	0	0
0	0	0	0	0	0
0	0	0	0	0	0
0	0	0	0	0	0
0	0	0	0	0	0
0	0	0	0	0	0
0	0	0	0	0	0
0	0	0	0	0	0
0	0	0	0	0	0
0	0	0	0	0	0
0	0	0	0	0	0
0	0	0	0	0	0
0	0	0	0	0	0
0	0	0	0	0	0
0	0	0	0	0	0
0	0	0	0	0	0
0	0	0	0	0	0
0	0	0	0	0	0
0	0	0	0	0	0
0	0	0	0	0	0
0	0	0	0	0	0
0	0	0	0	0	0
0	0	0	0	0	0
0	0	0	0	0	0
0	0	0	0	0	0
0	0	0	0	0	0
0	0	0	0	0	0
0	0	0	0	0	0
0	0	0	0	0	0
0	0	0	0	0	0
0	0	0	0	0	0
0	0	0	0	0	0
0	0	0	0	0	0
0	0	0	0	0	0
0	0	0	0	0	0
0	0	0	0	0	0
0	0	0	0	0	0
0	0	0	0	0	0
0	0	0	0	0	0
0	0	0	0	0	0
0	0	0	0	0	0
0	0	0	0	0	0
0	0	0	0	0	0
0	0	0	0	0	0
0	0	0	0	0	0
0	0	0	0	0	0
0	0	0	0	0	0

0	0	0	0	0	0
0	0	0	0	0	0
0	0	0	0	0	0
0	0	0	0	0	0
0	0	0	0	0	0
0					

gtTCf=
0
0.11925
0.14534
0.13574
0.11593
0.096197
0.079948
0.067704
0.058926
0.052819
0.048646
0.045817
0.043898
0.042582
0.041662
0.040998
0.040498
0.040102

gtTCs=
0
0.1431
0.20732
0.22864
0.22778
0.21648
0.20118
0.18528
0.17042
0.1573
0.14609
0.13672
0.12898
0.12264
0.11746
0.11324
0.10979
0.10696

gtTCp=
0
2.385
1.5979
1.1196

0.8287
0.65143
0.54309
0.47655
0.43538
0.4096
0.39316
0.38239
0.37506
0.36982
0.36585
0.36266
0.35995
0.35753

X_mask=
2.2204e-016,0.0026852,2.2204e-016,2.2204e-
016,0.006109,0.0023182,2.2204e-016,2.2204e-016,2.2204e-016,2.2204e-
016,2.2204e-016,0.0064992,0.003362,0.0027694,2.2204e-
016,0.0014256,0.011033,0.0037964,2.2204e-016,2.2204e-016,2.2204e-
016,2.2204e-016,2.2204e-016,2.2204e-016,2.2204e-016,2.2204e-
016,2.2204e-016,0.009111,2.2204e-016,0.0047121,2.2204e-016,2.2204e-
016,0.0027031,0.0089883,0.0010095,2.2204e-
016,0.0025831,0.0043025,0.00038202,2.2204e-016,2.2204e-016,2.2204e-
016,2.2204e-016,0.062313,2.2204e-016,2.2204e-016,2.2204e-
016,0.013631,2.2204e-016,2.2204e-016,2.2204e-016,2.2204e-016,2.2204e-
016,0.0014797,2.2204e-
016,0.00012774,0.0059836,0.0078167,0.0073677,0.0059423,0.0027517,2.220
4e-016,2.2204e-016,2.2204e-016,0.013964,0.032107,0.034258,2.2204e-
016,0.029295,2.2204e-016,2.2204e-016,2.2204e-016,0.0011289,2.2204e-
016,8.453e-005,0.003534,2.2204e-
016,0.010721,0.0010298,0.003641,2.2204e-
016,0.0029991,0.00073983,2.2204e-016,0.0070749,0.0046709,2.2204e-
016,0.1188,2.2204e-016,2.2204e-
016,0.04476,0.015483,0.014815,0.0089749,2.2204e-
016,0.010682,0.008789,2.2204e-016,2.2204e-
016,0.0065183,0.0083011,2.2204e-016,2.2204e-016,0.0056229,2.2204e-
016,2.2204e-016,0.0042418,2.2204e-016,0.0008288,2.2204e-
016,0.0033849,2.2204e-016,0.0012617,2.2204e-016,2.2204e-
016,0.036564,2.2204e-016,2.2204e-016,0.018267,2.2204e-016,2.2204e-
016,0.010532,0.024585,2.2204e-016,2.2204e-016,0.068388,2.2204e-
016,2.2204e-016,2.2204e-016,2.2204e-016,2.2204e-016,0.0027828,2.2204e-
016,2.2204e-016,0.00015563,0.0033751,2.2204e-016,2.2204e-
016,0.0087735,0.0038179,2.2204e-016,2.2204e-016,2.2204e-
016,0.0014693,2.2204e-016,2.2204e-016,0.09652,2.2204e-
016,0.064601,2.2204e-016,2.2204e-
016,0.049097,0.01279,0.075246,0.0088654,0.020399,0.053199,0.028434,0.0
37023,2.2204e-016,0.0068384,0.00049006,2.2204e-016,2.2204e-
016,2.2204e-016,0.0035251,0.012821,2.2204e-
016,0.00024772,0.0033755,0.010971,0.0053976,2.2204e-016,2.2204e-
016,0.010683,2.2204e-016,2.2204e-016,2.2204e-016,2.2204e-016,2.2204e-
016,0.028328,0.048445,2.2204e-016,2.2204e-

016,0.0026874,0.027864,0.026831,0.036319,0.06113,2.2204e-016,2.2204e-016,2.2204e-016,2.2204e-016,0.046753,2.2204e-016,2.2204e-016,2.2204e-016,0.00016332,0.004623,2.2204e-016,2.2204e-016,0.0045427,2.2204e-016,2.2204e-016,0.0093047,0.0053244,0.0048977,0.0026653,0.0011354,0.0019828,0.0021463,2.2204e-016,2.2204e-016,2.2204e-016,0.0080972,2.2204e-016,0.19536,2.2204e-016,2.2204e-016,0.0028997,2.2204e-016,0.023231,2.2204e-016,0.0080536,2.2204e-016,0.012667,2.2204e-016,0.0092869,2.2204e-016,0.0030467,0.0066165,2.2204e-016,2.2204e-016,0.004169,2.2204e-016,2.2204e-016,0.006044,0.0031125,0.0007654,0.0031251,0.0046006,2.2204e-016,2.2204e-016,2.2204e-016,2.2204e-016,2.2204e-016,2.2204e-016,0.030166,2.2204e-016,0.033233,2.2204e-016,0.018008,2.2204e-016,0.037698,2.2204e-016,0.0088143,2.2204e-016,2.2204e-016,2.2204e-016,2.2204e-016,0.039295,2.2204e-016,2.2204e-016,0.013584,2.2204e-016,0.00040164,2.2204e-016,0.0071523,0.0012305,0.003069,2.2204e-016,2.2204e-016,2.2204e-016,2.2204e-016,0.0061888,0.0012227,0.0035317,2.2204e-016,2.2204e-016,0.014046,0.0052109,2.2204e-016,0.0084062,2.2204e-016,0.012377,2.2204e-016,2.2204e-016,2.2204e-016,0.060566,0.0019166,2.2204e-016,2.2204e-016,2.2204e-016,0.0033125,0.019823,2.2204e-016,2.2204e-016,2.2204e-016,2.2204e-016,0.020036,2.2204e-016,0.0076816,2.2204e-016,2.2204e-016,2.2204e-016,0.0036326,2.2204e-016,0.0061664,0.0026993,2.2204e-016,0.0018327,0.0048524,0.00132,2.2204e-016,2.2204e-016,0.0084597,0.0084116,0.0065861,0.0029232,2.2204e-016,2.2204e-016,2.2204e-016,2.2204e-016,2.2204e-016,0.071509,2.2204e-016,2.2204e-016,0.045431,2.2204e-016,0.063582,0.03452,0.032955,0.024354,2.2204e-016,0.01976,0.026034,2.2204e-016,0.024877,2.2204e-016,2.2204e-016,0.0067833,2.2204e-016,0.0012861,0.0017061,0.0032986,2.2204e-016,0.0057374,2.2204e-016,2.2204e-016,2.2204e-016,0.0037604,2.2204e-016,2.2204e-016,2.2204e-016,0.00075347,2.2204e-016,0.0029014,2.2204e-016,2.2204e-016,2.2204e-016,0.058884,0.0035083,2.2204e-016,0.043852,0.01929,2.2204e-016,0.016579,0.0033809,0.018444,2.2204e-016,0.013608,2.2204e-016,0.0061394,0.016384,0.0020986,2.2204e-016,0.01248,0.029553,0.0060137,0.0037968,0.012551,2.2204e-016,0.010287,0.0030562,2.2204e-016,0.0019878,0.0039754,2.2204e-016,0.0023504,2.2204e-016,0.001335,2.2204e-016,0.00209,0.0050911,0.0056302,0.00089458,2.2204e-016,2.2204e-016,2.2204e-016,2.2204e-016,0.095551,0.13159,0.02273,0.012892,2.2204e-016,2.2204e-016,2.2204e-016,0.004119,2.2204e-016,0.017519,2.2204e-016,2.2204e-016,2.2204e-016,0.009904,0.038845,0.017344,0.0042845,2.2204e-016,0.00032897,2.2204e-016,0.0018784,2.2204e-016,0.0011143,2.2204e-016,0.00069502,0.0021074,2.2204e-016,2.2204e-016,2.2204e-016,0.006005,2.2204e-016,0.0037412,2.2204e-016,0.006262,0.00025832,0.052258,2.2204e-016,0.012158,2.2204e-016,0.07409,2.2204e-016,2.2204e-016,2.2204e-016,2.2204e-016,2.2204e-016,2.2204e-016,7.3566e-007,2.2204e-016,2.2204e-016,0.0045046,0.0047851,0.0052197,2.2204e-016,2.2204e-016,0.002086,2.2204e-016,0.005775,0.0061261,2.2204e-016,2.2204e-016,0.0019593,0.0068597,0.00011498,0.0024915,0.00045786,2.2204e-

016,0.0055221,2.2204e-016,0.00016048,2.2204e-
016,0.0015437,0.0053077,2.2204e-016,2.2204e-016,0.1108,2.2204e-
016,2.2204e-016,2.2204e-016,2.2204e-016,0.024914,2.2204e-
016,0.036426,0.0028406,2.2204e-
016,0.008621,0.012733,0.013187,0.01585,0.0030142,0.0072482,2.2204e-
016,0.016262,0.00062595,0.00042572,0.0063674,0.0045742,2.2204e-
016,0.00036534,2.2204e-016,0.0015788,2.2204e-016,0.0026818,2.2204e-
016,2.2204e-016,0.00028971,0.0051449,0.0021321,0.00089462,2.2204e-
016,2.2204e-016,2.2204e-016,0.045023,2.2204e-016,0.030609,2.2204e-
016,2.2204e-016,0.019425,0.0027982,2.2204e-
016,0.01591,0.052726,2.2204e-016,0.012595,2.2204e-016,2.2204e-
016,2.2204e-016,2.2204e-016,2.2204e-016,0.012194,2.2204e-016,2.2204e-
016,0.0016567,0.0037034,0.0043153,0.010311,0.0093822,0.0023563,0.00086
278,2.2204e-016,8.8602e-005,0.0035128,0.0043452,2.2204e-
016,0.00039285,2.2204e-016,2.2204e-016,2.2204e-016,2.2204e-
016,2.2204e-016,0.022804,0.011667,0.05656,2.2204e-016,2.2204e-
016,0.013378,0.035265,2.2204e-016,2.2204e-
016,0.028246,0.0071908,2.2204e-016,2.2204e-
016,0.0056872,0.0034131,0.0025217,2.2204e-016,2.2204e-016,2.2204e-
016,2.2204e-016,0.0073404,0.0074789,2.2204e-016,2.2204e-016,2.2204e-
016,0.0034795,0.0056403,0.006748,0.0045233,2.2204e-016,2.2204e-
016,0.0042223,0.0026395,0.00066244,0.0047602,2.2204e-016,2.2204e-
016,0.026441,2.2204e-016,2.2204e-016,2.2204e-016,2.2204e-016,2.2204e-
016,0.029059,2.2204e-016,2.2204e-016,0.019841,2.2204e-
016,0.018536,0.00024926,0.012784,2.2204e-
016,0.010579,0.021116,0.012288,0.0033673,0.0024488,0.0034458,0.0049915
,2.2204e-016,0.0041352,0.0012489,2.2204e-016,2.2204e-
016,0.0015066,2.2204e-016,0.0087836,2.2204e-016,2.2204e-016,2.2204e-
016,2.2204e-016,0.0027465,2.2204e-016,2.2204e-016,2.2204e-
016,0.055661,0.016301,0.066617,0.031706,0.010388,0.02471,2.2204e-
016,2.2204e-016,2.2204e-016,0.010188,2.2204e-016,0.021875,2.2204e-
016,2.2204e-016,2.2204e-016,0.009693,2.2204e-016,2.2204e-016,2.2204e-
016,0.0047602,0.0048704,2.2204e-
016,0.0033747,0.0013218,0.00071772,2.2204e-
016,0.0036148,0.0061308,2.2204e-
016,0.001213,0.001454,0.0040663,2.2204e-016,2.2204e-016,2.2204e-
016,0.0019466,2.2204e-016,2.2204e-
016,0.0063853,0.023207,0.011697,0.0051191,0.040445,0.015967,0.023789,0
.014906,2.2204e-016,0.011359,2.2204e-
016,0.0055037,0.016295,0.0098338,0.022192,2.2204e-016,2.2204e-
016,0.02555,0.0053505,2.2204e-016,2.2204e-016,2.2204e-
016,0.0039296,2.2204e-016,0.00090423,2.2204e-016,2.2204e-
016,0.0061209,2.2204e-016,0.0021083,2.2204e-016,2.2204e-016,2.2204e-
016,2.2204e-016,0.0053123,2.2204e-016,0.032248,2.2204e-
016,0.012702,0.0029873,2.2204e-
016,0.024161,0.016256,0.0050789,0.015243,2.2204e-016,0.011949,2.2204e-
016,0.020289,2.2204e-016,2.2204e-
016,0.0062372,0.04562,0.018161,0.0018783,2.2204e-016,2.2204e-
016,2.2204e-016,0.013113,0.0045346,0.0031808,2.2204e-
016,0.00026989,0.005273,0.0029846,2.2204e-
016,0.0011411,0.0025102,0.0073037,2.2204e-016,2.2204e-
016,0.0015273,1.2335e-005,0.037897,0.017268,2.2204e-016,2.2204e-

016,0.017859,2.2204e-016,2.2204e-016,2.2204e-016,2.2204e-016,2.2204e-
016,2.2204e-016,2.2204e-016,0.0021682,2.2204e-016,0.0055221,2.2204e-
016,0.022249,2.2204e-016,0.003506,2.2204e-016,2.2204e-016,2.2204e-
016,2.2204e-016,2.2204e-016,0.0013697,2.2204e-016,2.2204e-
016,0.0018585,2.2204e-016,0.0066269,0.004972,0.0022195,2.2204e-
016,0.0063554,0.0015381,0.0023918,0.003321,2.2204e-
016,0.0037145,0.0020038,0.043465,2.2204e-016,2.2204e-
016,0.048487,2.2204e-016,0.041281,2.2204e-016,2.2204e-
016,0.0023264,2.2204e-016,2.2204e-016,2.2204e-016,2.2204e-
016,0.0045818,2.2204e-016,2.2204e-016,2.2204e-016,2.2204e-
016,0.0032957,2.2204e-016,0.00048378,2.2204e-016,0.0039872,2.2204e-
016,2.2204e-016,2.2204e-016,0.010053,2.2204e-
016,0.0014818,0.0052915,0.0029725,2.2204e-016,2.2204e-016,2.2204e-
016,0.00093454,0.0028957,2.2204e-016,0.016863,0.0048088,2.2204e-
016,2.2204e-016,0.014027,0.060571,2.2204e-016,0.021571,2.2204e-
016,2.2204e-016,2.2204e-016,2.2204e-016,2.2204e-016,2.2204e-
016,2.2204e-016,0.0023587,2.2204e-016,0.0077406,0.00093288,2.2204e-
016,0.0037663,0.00091983,2.2204e-016,2.2204e-016,2.2204e-
016,0.0075444,2.2204e-016,0.0010214,2.2204e-
016,0.00048939,0.0003996,0.0031519,0.0070164,0.0024688,0.0046621,0.000
10253,0.019989,2.2204e-016,0.048186,2.2204e-016,2.2204e-
016,0.0053586,2.2204e-016,0.011547,2.2204e-016,2.2204e-
016,0.005725,2.2204e-016,2.2204e-
016,0.0064463,0.00046218,0.0067936,0.0059684,2.2204e-
016,0.0007985,2.2204e-016,0.005262,2.2204e-016,2.2204e-
016,0.00088655,2.2204e-016,0.00064473,2.2204e-016,0.011545,2.2204e-
016,2.2204e-016,0.0033336,2.2204e-016,0.0017747,2.2204e-016,2.2204e-
016,2.2204e-016,0.00037547,2.2204e-016,2.2204e-016,0.0089738,2.2204e-
016,0.017534,2.2204e-
016,0.00056827,0.00162,0.0017137,0.00087294,2.2204e-
016,0.0058472,0.0014242,2.2204e-016,2.2204e-016,2.2204e-
016,0.0024834,2.2204e-016,0.0042757,2.2204e-
016,0.0027145,0.0062118,0.00926,0.0020558,2.2204e-
016,0.008465,2.2204e-016,0.01114,0.0072247,2.2204e-
016,0.0014993,0.0037333,2.2204e-016,0.0010556,0.0068433,2.2204e-
016,2.2204e-016,0.0031326,0.05097,5.511e-005,2.2204e-016,2.2204e-
016,0.0037727,2.2204e-016,2.2204e-016,0.0051536,2.2204e-
016,0.001861,2.2204e-016,2.2204e-016,2.2204e-016,2.2204e-
016,0.0078458,0.00086781,2.2204e-016,0.0022805,2.2204e-
016,0.0068829,2.2204e-016,0.0023867,2.2204e-016,0.00055427,2.2204e-
016,0.0027288,0.0069271,0.0069163,0.00097238,2.2204e-
016,0.0033544,2.2204e-016,2.2204e-016,0.0031575,2.2204e-
016,0.0010984,2.2204e-016,2.2204e-016,2.2204e-
016,0.0038821,0.0021704,2.2204e-016,0.0016219,2.6736e-005,2.2204e-
016,2.2204e-016,2.2204e-
016,0.0041336,0.0031284,0.0049387,0.00779,2.2204e-
016,0.0046724,0.0027041,0.0041657,0.010348,2.2204e-
016,0.0014994,0.0030985,2.2204e-
016,0.0031174,0.0028558,0.0016244,2.2204e-
016,0.0011758,0.0047518,2.2204e-016,2.2204e-016,2.2204e-
016,0.00131,2.2204e-016,2.2204e-016,2.2204e-016,2.2204e-
016,0.0025618,0.0028984,0.0027614,0.0034749,0.00065652,0.0022891,2.220

4e-016,2.2204e-016,0.0038401,0.0026812,0.0017001,2.2204e-016,2.2204e-016,2.2204e-016,2.2204e-016,0.0039502,0.0021564,2.2204e-016,2.2204e-016,2.2204e-016,2.2204e-016,0.0011876,0.0016371,2.2204e-016,2.2204e-016,2.2204e-016,0.010395,0.0022901,0.0044436,2.2204e-016,2.2204e-016,0.003034,2.2204e-016,0.0001718,0.0012868,0.0016704,2.2204e-016,2.2204e-016,2.2204e-016,0.0035178,0.0020966,2.2204e-016,2.2204e-016,0.001255,2.2204e-016,2.2204e-016,2.2204e-016,0.0031702,0.0031805,6.8479e-005,0.0011149,0.0031513,2.2204e-016,0.00058146,2.2204e-016,2.2204e-016,0.0020596,0.0019949,2.2204e-016,0.0037129,2.2204e-016,2.2204e-016,0.0024529,0.0047649,0.00033075,0.00099198,0.0020126,2.2204e-016,0.0028149,0.0055516,0.0011149,2.2204e-016,0.0037274,0.0019124,0.0030325,2.2204e-016,2.2204e-016,2.2204e-016,0.00013288,0.00054822,2.2204e-016,2.2204e-016,0.0042001,2.2204e-016,2.2204e-016,2.2204e-016,2.2204e-016,2.2204e-016,0.0041967,0.0021805,2.2204e-016,2.2204e-016,0.002498,0.0045371,2.2204e-016,0.0017278,2.2204e-016,2.2204e-016,0.0020913,0.0028802,0.0047027,0.0015042,2.2204e-016,0.0006151,2.2204e-016,2.2204e-016,2.2204e-016,0.0095487,2.2204e-016,2.2204e-016,2.2204e-016,2.2204e-016,2.2204e-016,2.2204e-016,0.0044925,0.0027589,2.2204e-016,2.2204e-016,0.0061967,0.0021884,0.0031822,0.00087666,0.0081129,2.2204e-016,2.2204e-016,2.2204e-016,0.0034451,0.0011569,0.0059076,2.2204e-016,2.2204e-016,0.0079858,2.2204e-016,2.2204e-016,2.2204e-016,2.2204e-016,0.0097059,2.2204e-016,0.0018163,2.2204e-016,0.0044964,2.2204e-016,2.2204e-016,0.0038598,0.0037216,2.2204e-016,2.2204e-016,2.2204e-016,2.2204e-016,2.2204e-016,0.0086144,2.2204e-016,0.0055393,2.2204e-016,2.2204e-016,0.0026241,2.2204e-016,0.0039483,0.0030288,2.2204e-016,0.002681,0.009755,2.2204e-016,0.0018983,0.0076755,9.0164e-005,2.2204e-016,0.0016828,2.2204e-016,2.2204e-016,0.00075103,4.0476e-005,2.2204e-016,2.2204e-016,2.2204e-016,0.0031434,0.0059064,2.2204e-016,2.2204e-016,0.0038917,2.2204e-016,2.2204e-016,2.2204e-016,0.00072383,2.2204e-016,0.0058786,0.0013127,2.2204e-016,2.2204e-016,2.2204e-016,0.0052973,2.2204e-016,2.2204e-016,0.0048091,2.2204e-016,2.2204e-016,2.2204e-016,0.0046254,2.2204e-016,2.2204e-016,2.2204e-016,0.0060034,2.2204e-016,2.2204e-016,0.0053713,0.0037723,0.0013226,2.2204e-016,0.0004972,0.0032645,0.0020576,0.0044915,0.0055613,2.2204e-016,0.00042246,2.2204e-016,2.2204e-016,0.00071051,2.2204e-016,2.2204e-016,2.2204e-016,2.2204e-016,2.2204e-016,2.2204e-016,2.2204e-016,0.0078258,2.2204e-016,0.001683,0.00056837,0.0017884,2.2204e-016,0.0046844,2.2204e-016,0.0024135,0.003376,0.00079617,2.2204e-016,2.2204e-016,0.00080239,2.2204e-016,0.0016664,2.2204e-016,0.0016042,2.2204e-016,0.0012544,0.0051365,0.0057025,0.0025973,2.2204e-016,0.0012579,2.2204e-016,2.2204e-016,0.0015793,2.2204e-016,2.2204e-016,2.2204e-016,0.0052698,0.0010924,0.00042686,2.2204e-016,2.2204e-016,2.2204e-016,0.00063559,2.2204e-016,2.2204e-016,2.2204e-016,2.2204e-016,2.2204e-016,0.001058,2.2204e-016
0.14108,0.14684,0.13495,0.1302,0.1496,0.1491,0.13919,0.13917,0.14184,0.14504,0.13138,0.15132,0.14183,0.14377,0.14341,0.14783,0.15212,0.14739,0.13739,0.13272,1.2279,1.4454,1.4928,1.1166,0.14088,0.14193,0.14077,0

.15259,0.14204,0.14648,0.13894,0.13853,0.1417,0.15366,0.14178,0.13928,
0.14662,0.14553,0.14332,2.3649,1.3705,1.2701,1.2484,1.2111,1.3051,1.43
09,0.14253,0.15719,0.1403,0.13862,0.13629,0.13245,0.13881,0.13887,0.13
938,0.14589,0.14894,0.14868,0.14686,0.14889,0.14467,2.3458,2.3684,1.33
97,1.2786,1.34,1.5994,1.3215,1.5388,1.445,1.3969,0.13851,0.14403,0.140
69,0.143,0.14934,0.1421,0.15508,0.14387,0.14753,0.12944,0.14722,0.1414
5,0.12992,0.14922,0.14448,2.364,2.4805,2.3574,1.2096,1.306,1.3122,1.26
94,1.2848,1.0959,1.4075,1.2648,1.2707,0.13845,0.149,0.15111,0.13863,0.
13747,0.14463,0.13798,0.1388,0.14676,0.14394,0.14428,0.1407,0.14428,0.
14127,0.14279,0.13628,2.3343,2.4317,2.2793,2.3778,1.2546,1.2743,1.1688
,1.3335,1.2949,1.2092,1.3751,1.3977,1.1617,1.158,0.14183,0.13507,0.142
17,0.14843,0.14232,0.13757,0.14385,0.14504,0.13083,0.1303,0.15121,0.14
598,0.137,0.14582,0.13893,0.14355,2.4095,2.3554,2.4884,2.3058,2.4231,2
.343,1.1713,1.3472,1.3323,1.3355,1.266,1.319,1.2958,1.4367,1.2913,1.21
,0.148,0.14567,0.14174,0.14323,0.14366,0.15051,0.15057,0.14033,0.14436
,0.14553,0.15823,0.1465,0.12982,0.14101,0.15391,0.14163,0.13776,2.3031
,2.3354,2.3621,2.4265,2.4146,2.2489,1.4061,1.2759,1.1803,1.2923,1.4407
,0.91988,1.081,1.3662,1.2353,1.3104,1.0767,0.13829,0.13621,0.13655,0.1
4337,0.14672,0.14473,0.13805,0.14346,0.14007,0.13886,0.15369,0.15097,0
.14563,0.13365,0.1311,0.1337,0.14655,2.2766,2.3333,2.2982,2.3845,2.347
1,2.5819,2.3669,1.2626,1.2475,1.2275,1.1128,1.2179,1.2796,1.3786,1.278
5,1.1234,0.94989,1.1512,0.14704,0.14683,0.1403,0.14047,0.14531,0.1422,
0.14444,0.14349,0.14781,0.14193,0.1444,0.13724,0.12901,0.12639,0.12878
,0.12686,0.12724,0.1432,2.382,2.2987,2.4185,2.3158,2.4352,2.3724,2.414
9,1.2712,0.94201,1.2447,1.5042,1.2732,1.1851,1.3853,1.2378,1.4771,1.19
05,1.5531,0.14188,0.14597,0.14753,0.14522,0.14327,0.14671,0.14079,0.13
745,0.14443,0.13825,0.13549,0.13558,0.12975,0.12846,0.14976,0.13615,0.
12756,0.1366,0.13958,2.4148,2.3547,2.3676,2.2654,2.4807,2.3707,2.3128,
1.5224,0.96824,1.3062,1.2775,0.92821,1.2571,1.1111,1.2215,0.99181,1.23
4,1.0952,1.2396,0.1415,0.14013,0.14577,0.1331,0.14815,0.145,0.1332,0.1
3416,0.13778,0.13119,0.12987,0.13405,0.14123,0.13814,0.14008,0.134,0.1
1745,0.12697,2.3095,2.2512,2.3312,2.4591,2.3622,2.3682,2.3801,1.153,1.
3171,1.3021,1.2352,1.7746,1.2773,1.2212,1.1599,1.225,1.2927,1.2155,1.1
927,0.15254,0.14074,0.14372,0.12985,0.13231,0.12651,0.14121,0.12664,0.
13207,0.12713,0.13465,0.12474,0.125,0.12235,0.13465,0.13121,0.13533,0.
12807,2.2881,2.3393,2.4803,2.4189,2.3329,2.4225,2.356,2.3012,2.4029,1.
2439,1.3163,1.1585,1.2576,1.4597,1.2611,1.2821,1.2684,1.3092,1.4474,1.
4341,0.13975,0.13256,0.14793,0.1249,0.13918,0.13498,0.12716,0.13172,0.
13552,0.12365,0.13399,0.12617,0.13153,0.13313,0.13693,0.13705,0.13571,
0.13653,2.3863,2.323,2.3086,2.3868,2.4651,2.5134,2.397,2.4089,2.3687,1
.1768,1.3915,0.92456,0.69609,0.96083,0.638,0.86109,0.63195,1.042,0.889
43,0.72705,0.13667,0.1307,0.13358,0.12431,0.12691,0.12654,0.1385,0.124
68,0.12991,0.13549,0.12882,0.1254,0.13071,0.13643,0.12694,0.13222,0.11
936,0.13692,2.3933,2.3933,2.3489,2.3747,2.3533,2.4442,2.3345,2.3434,2.
3032,1.1956,0.89531,0.91507,0.91069,0.81136,0.71476,0.77751,0.88587,1.
1003,0.78149,0.66039,0.13734,0.1268,0.13678,0.13839,0.13102,0.12607,0.
13625,0.13655,0.12977,0.13337,0.12833,0.12804,0.1391,0.13092,0.13386,0
.12678,0.13655,0.13634,2.3061,2.3549,2.447,2.3655,2.3868,2.3477,2.3551
,1.0712,1.2113,1.2443,0.986,0.75608,0.9012,0.89812,0.93557,0.90377,1.0
05,0.91534,0.87382,0.66169,0.13251,0.13016,0.14018,0.13492,0.12797,0.1
3445,0.13013,0.13283,0.12775,0.13227,0.12901,0.12672,0.13452,0.13002,0
.13064,0.13486,0.13197,0.12602,2.3355,2.4144,2.3193,2.4552,2.3125,2.28

28,1.154,1.246,1.2118,1.487,1.2941,0.99051,0.91363,0.75844,0.83285,1.0
05,0.96308,1.0015,0.94377,0.85792,0.13462,0.12838,0.13059,0.13475,0.13
945,0.14163,0.1337,0.13545,0.13138,0.12917,0.13166,0.13214,0.12361,0.1
3195,0.13065,0.12698,0.128,0.12867,2.3696,2.3763,2.423,2.4503,2.3018,1
.2097,1.0872,1.2921,1.2571,1.1272,1.1589,0.81961,0.8277,0.84044,0.7936
8,0.94668,0.91028,0.95395,0.92253,1.0246,0.13064,0.1383,0.1353,0.13439
,0.12185,0.12464,0.13496,0.13486,0.13987,0.13767,0.1256,0.11871,0.1312
3,0.13013,0.13418,0.13528,0.12635,0.12982,2.4054,2.3449,2.3476,1.271,0
.90738,1.2082,1.0822,1.1853,1.1998,1.2725,1.4663,0.89148,0.89425,0.682
31,0.97169,0.8244,0.9296,0.95478,0.8751,0.83007,0.13251,0.13579,0.1276
1,0.13399,0.13063,0.13104,0.13009,0.13244,0.13002,0.13813,0.13012,0.13
101,0.12791,0.12738,0.13221,0.12467,0.12737,0.12884,2.4616,1.255,1.350
9,1.2011,1.5383,1.1563,1.2465,1.2935,1.4691,1.4209,0.91818,0.89019,0.8
5235,0.88983,0.62876,0.82559,0.93388,0.85356,0.84361,0.13554,0.13531,0
.12993,0.13441,0.12902,0.12724,0.12596,0.13614,0.13158,0.13162,0.13501
,0.13467,0.13522,0.12662,0.12936,0.12366,0.13373,0.11285,1.2582,1.3199
,1.2653,1.2633,1.2325,1.118,1.2594,1.0446,1.2442,1.322,0.88373,0.83189
,0.92685,0.91163,0.99018,0.72495,0.9223,0.86672,0.87079,0.13671,0.1295
8,0.12258,0.12521,0.13356,0.12852,0.13196,0.13068,0.12677,0.13342,0.12
283,0.1328,0.12678,0.13003,0.11787,0.12282,0.13581,0.11723,1.1931,1.14
17,1.393,1.0878,1.0734,1.2847,1.2899,1.3671,1.3565,1.1507,0.69849,0.83
969,0.87065,0.83455,0.90665,0.87228,0.99897,0.83467,0.13515,0.12802,0.
13213,0.13101,0.14202,0.13295,0.12933,0.12439,0.12888,0.13658,0.13454,
0.13432,0.1321,0.13776,0.14064,0.1347,0.12546,0.13274,0.1206,1.4654,1.
0452,1.1467,1.2206,1.212,0.76874,1.1862,1.1883,1.0634,1.4742,0.9002,0.
85809,0.99354,0.83577,0.96974,0.93387,0.81679,0.86025,0.13421,0.13346,
0.12867,0.12501,0.129,0.12406,0.13076,0.12725,0.12087,0.12996,0.12749,
0.13738,0.13492,0.13165,0.13012,0.1394,0.12955,0.12212,0.1223,0.11895,
1.2684,1.2767,1.4249,1.1958,1.3989,1.198,1.1766,1.0243,1.2263,0.84627,
0.91741,0.85331,0.95549,1.1464,0.90288,0.13183,0.1275,0.13384,0.12888,
0.13055,0.13137,0.1282,0.12942,0.13104,0.13467,0.12461,0.12698,0.13018
,0.14342,0.13001,0.12834,0.13467,0.13671,0.12142,0.11418,0.11762,0.120
49,1.2132,1.1707,1.0702,1.1456,1.2431,1.234,1.2535,1.3094,1.2116,0.972
74,0.92897,0.91405,0.12593,0.1241,0.13145,0.12549,0.13677,0.13032,0.13
27,0.13924,0.13287,0.12721,0.13132,0.13674,0.13063,0.1369,0.12702,0.14
243,0.12982,0.13272,0.12366,0.1293,0.11867,0.123,0.12367,0.12566,0.124
49,0.11895,1.2757,1.2661,1.5601,1.1838,1.3534,1.2657,1.4741,1.2871,0.1
3262,0.12381,0.1372,0.12737,0.12277,0.13905,0.13238,0.13751,0.13772,0.
12752,0.13263,0.1263,0.1406,0.11921,0.13183,0.12972,0.12408,0.13491,0.
12396,0.14054,0.12855,0.13541,0.13509,0.11185,0.12149,0.12047,0.12013,
0.12027,0.11749,1.2068,1.2428,0.98601,1.2901,1.15,0.11821,0.11941,0.13
381,0.13104,0.13507,0.12721,0.1377,0.1368,0.12547,0.12851,0.11879,0.13
942,0.12842,0.13485,0.12927,0.13229,0.13433,0.1377,0.13572,0.13482,0.1
366,0.12558,0.14153,0.12696,0.11883,0.12163,0.11832,0.11724,0.12161,0.
12676,0.11198,0.11849,0.11993,1.2705,1.2376,0.11513,0.11616,0.11989,0.
11592,0.12689,0.13712,0.13185,0.13388,0.13324,0.12802,0.12931,0.13174,
0.13927,0.13369,0.12958,0.1319,0.13251,0.13693,0.13145,0.13426,0.12439
,0.13186,0.12222,0.11927,0.12897,0.12689,0.12006,0.11148,0.12287,0.115
64,0.12094,0.12275,0.11708,0.11894,0.12094,0.11584,0.11441,0.1246,0.12
039,0.1167,0.13394,0.13142,0.12679,0.12173,0.12569,0.13422,0.13485,0.1
3483,0.14094,0.13223,0.13819,0.13046,0.13545,0.14177,0.12972,0.13299,0
.1192,0.11636,0.12074,0.12089,0.12079,0.12176,0.12236,0.1231,0.11737,0

.11344,0.11662,0.12155,0.11926,0.11614,0.11768,0.11759,0.11999,0.12236
,0.11939,0.13348,0.12964,0.13669,0.12669,0.12791,0.13313,0.13203,0.131
33,0.12418,0.12905,0.12416,0.13203,0.13378,0.12344,0.1141,0.11615,0.11
683,0.11535,0.11949,0.12509,0.11923,0.11742,0.12119,0.1286,0.1183,0.11
938,0.11631,0.11769,0.1212,0.1151,0.119,0.11973,0.12371,0.10678,0.1295
6,0.12961,0.13296,0.13042,0.12839,0.13165,0.13796,0.12576,0.12734,0.11
501,0.12286,0.12127,0.12075,0.12005,0.12414,0.11563,0.12114,0.11934,0.
11719,0.12126,0.12033,0.11694,0.12199,0.11527,0.12048,0.12032,0.1226,0
.11878,0.12013,0.11862,0.11555,0.12399,0.12409,0.11996,0.12594,0.13247
,0.12666,0.13414,0.11649,0.11628,0.11756,0.11782,0.12204,0.1152,0.1114
8,0.12249,0.11982,0.11429,0.11364,0.11819,0.11514,0.12112,0.12116,0.11
676,0.11872,0.1235,0.12271,0.11607,0.12002,0.12053,0.11912,0.12385,0.1
1895,0.12328,0.12025,0.11336,0.12072,0.11994,0.11487,0.11528,0.12538,0
.12009,0.11882,0.12156,0.11779,0.12013,0.11295,0.12446,0.12299,0.11801
,0.11502,0.1257,0.1227,0.12275,0.12046,0.12766,0.1183,0.11768,0.11842,
0.12048,0.12279,0.12427,0.11625,0.11969,0.12834,0.1157,0.11757,0.11438
,0.12155,0.12656,0.11703,0.12246,0.11941,0.12121,0.11714,0.11471,0.119
49,0.12307,0.11513,0.11186,0.12049,0.11414,0.11637,0.12838,0.11478,0.1
2282,0.1136,0.1153,0.12303,0.11471,0.12333,0.11831,0.11789,0.11822,0.1
2778,0.10917,0.12422,0.12725,0.12066,0.10652,0.12382,0.11007,0.11148,0
.12014,0.11706,0.11291,0.11345,0.11605,0.12002,0.12723,0.11196,0.11738
,0.12314,0.11525,0.10954,0.11764,0.12089,0.11908,0.12476,0.11788,0.115
65,0.11716,0.11624,0.12559,0.11634,0.11692,0.12429,0.11529,0.11562,0.1
1207,0.12456,0.11622,0.1149,0.11895,0.1235,0.11783,0.1156,0.12488,0.12
252,0.12084,0.11786,0.12024,0.1209,0.12191,0.12178,0.12529,0.11546,0.1
2057,0.11344,0.11365,0.11755,0.11623,0.11637,0.11437,0.11582,0.11188,0
.11831,0.11587,0.1287,0.11549,0.1197,0.1233,0.12037,0.11661,0.12538,0.
11929,0.12351,0.12176,0.12081,0.11737,0.11802,0.11998,0.11832,0.1202,0
.11705,0.12391,0.11443,0.12109,0.12322,0.12845,0.12156,0.11705,0.11923
,0.11116,0.12023,0.1245,0.11744,0.11143,0.11676,0.12886,0.11814,0.1208
3,0.11467,0.11827,0.11721,0.12011,0.1198,0.11751,0.11402,0.11709,0.117
49,0.12364,0.12023
0.20449,0.21414,0.20102,0.19366,0.21154,0.21631,0.20097,0.20367,0.2065
6,0.20868,0.19668,0.21658,0.20288,0.20585,0.20732,0.21357,0.21641,0.21
23,0.20352,0.20145,0.86832,1.0138,1.0361,0.80205,0.20142,0.20666,0.207
53,0.21751,0.20416,0.21191,0.20613,0.20205,0.20512,0.21164,0.20464,0.2
0104,0.21449,0.21408,0.20613,1.5897,0.95871,0.89934,0.90523,0.88666,0.
93464,0.9993,0.20558,0.21822,0.20223,0.20204,0.20582,0.19516,0.20498,0
.20247,0.20345,0.20456,0.21382,0.20945,0.2086,0.21084,0.2067,1.5501,1.
581,0.9392,0.92366,0.96793,1.1147,0.92013,1.0354,1.0066,0.96102,0.2018
8,0.20678,0.20182,0.2028,0.21422,0.20461,0.21431,0.20695,0.21175,0.198
07,0.21,0.20649,0.19256,0.21413,0.21,1.5717,1.6844,1.5637,0.8485,0.942
26,0.93807,0.90139,0.93512,0.78424,0.98234,0.90406,0.91059,0.20639,0.2
1059,0.21681,0.20056,0.20463,0.21108,0.19958,0.2069,0.20799,0.20735,0.
20914,0.20186,0.20828,0.20419,0.20691,0.20251,1.5703,1.6165,1.4555,1.5
601,0.92659,0.92349,0.84648,0.91348,0.92691,0.88341,0.97383,1.0245,0.8
392,0.82564,0.20669,0.20052,0.20055,0.21244,0.2063,0.20366,0.20905,0.2
0509,0.19625,0.19577,0.21382,0.21041,0.19721,0.21231,0.20775,0.20223,1
.6496,1.5823,1.7643,1.4554,1.6202,1.604,0.83532,0.97939,0.94442,0.9584
,0.90468,0.92852,0.91405,1.0189,0.93174,0.86532,0.21293,0.21103,0.2058
5,0.20692,0.2097,0.21313,0.21195,0.20604,0.207,0.21101,0.22091,0.2134,
0.19526,0.20486,0.21658,0.20178,0.20155,1.5519,1.5366,1.5305,1.6299,1.

636,1.4383,1.0056,0.89922,0.85687,0.93117,1.0272,0.70327,0.77371,0.929
42,0.88589,0.93313,0.8037,0.20152,0.20168,0.2044,0.20673,0.21143,0.205
94,0.20306,0.20888,0.20177,0.20291,0.21759,0.21,0.21303,0.17443,0.1768
1,0.18093,0.2126,1.504,1.5361,1.5143,1.5766,1.5934,1.7493,1.591,0.8676
9,0.90509,0.85639,0.79259,0.86901,0.90757,0.98143,0.91989,0.8108,0.703
59,0.82828,0.20777,0.20833,0.20754,0.20972,0.21083,0.2075,0.21095,0.20
742,0.21399,0.20533,0.20608,0.18553,0.17616,0.16957,0.17503,0.16816,0.
17011,0.20816,1.6166,1.486,1.6523,1.5681,1.6651,1.627,1.6289,0.89671,0
.71393,0.87155,1.0628,0.88568,0.84549,0.97052,0.86384,1.0434,0.8577,1.
101,0.20651,0.20627,0.21026,0.21068,0.21103,0.21408,0.20522,0.20103,0.
20958,0.17789,0.18829,0.17844,0.17333,0.17259,0.20262,0.18341,0.17444,
0.18356,0.20514,1.6236,1.5567,1.5724,1.4731,1.7173,1.5785,1.5214,1.042
7,0.70816,0.93262,0.89974,0.71228,0.90278,0.79762,0.88011,0.72734,0.87
22,0.78875,0.88671,0.20623,0.20683,0.21048,0.20202,0.20977,0.20735,0.1
8557,0.18087,0.18614,0.17463,0.17287,0.17648,0.1849,0.18265,0.18576,0.
18068,0.15244,0.1707,1.5603,1.4661,1.5515,1.6646,1.5762,1.596,1.622,0.
803,0.94857,0.92889,0.87953,1.2372,0.90361,0.89209,0.8377,0.87625,0.92
877,0.86949,0.84899,0.21375,0.20254,0.20808,0.17152,0.17981,0.16877,0.
18522,0.16832,0.17875,0.16883,0.17636,0.16362,0.17909,0.16091,0.18051,
0.17915,0.17698,0.17337,1.4999,1.5371,1.6863,1.6274,1.5268,1.6009,1.57
23,1.5487,1.5606,0.87046,0.93212,0.82564,0.88336,1.0063,0.91459,0.9238
2,0.90533,0.91501,0.99883,1.0255,0.18221,0.17331,0.18804,0.17161,0.182
34,0.17905,0.17263,0.17734,0.18448,0.16836,0.17816,0.1756,0.17682,0.17
801,0.18408,0.18869,0.18211,0.18489,1.5886,1.5485,1.5065,1.5902,1.6251
,1.7263,1.6385,1.6195,1.5703,0.87486,0.97348,0.6875,0.52489,0.69783,0.
48993,0.61395,0.48881,0.7522,0.66403,0.55819,0.18348,0.17753,0.18184,0
.1681,0.16523,0.17384,0.18874,0.16412,0.17548,0.18198,0.17464,0.17273,
0.17418,0.17764,0.17128,0.17316,0.15963,0.1882,1.6293,1.5765,1.5742,1.
6543,1.5677,1.6611,1.5757,1.5703,1.4927,0.83819,0.65861,0.65262,0.6619
2,0.58937,0.53295,0.59332,0.65197,0.79083,0.59476,0.50993,0.18561,0.17
122,0.18355,0.18492,0.17502,0.17173,0.17979,0.18053,0.17228,0.17962,0.
17287,0.16921,0.18595,0.17608,0.18382,0.17007,0.18213,0.17545,1.5292,1
.6061,1.6707,1.5821,1.6236,1.554,1.5698,0.76169,0.86129,0.87976,0.7047
4,0.56272,0.66274,0.64717,0.69366,0.66673,0.73855,0.66813,0.6254,0.515
08,0.18037,0.17722,0.18405,0.17799,0.17525,0.18193,0.17099,0.18067,0.1
7122,0.17569,0.17439,0.17629,0.18203,0.17474,0.17809,0.18045,0.17427,0
.16907,1.5212,1.5893,1.5243,1.6581,1.5499,1.5045,0.81057,0.8784,0.8574
3,1.0122,0.90866,0.70665,0.67783,0.56161,0.61551,0.70412,0.69255,0.718
17,0.71205,0.61543,0.18633,0.17043,0.17009,0.18211,0.18332,0.18821,0.1
7913,0.18895,0.1789,0.17482,0.17852,0.17822,0.16988,0.17701,0.17478,0.
17441,0.17179,0.17462,1.535,1.5875,1.6737,1.6133,1.5256,0.8288,0.76327
,0.9126,0.84183,0.78046,0.81825,0.60658,0.61437,0.6241,0.60323,0.6847,
0.66795,0.6674,0.6736,0.69907,0.179,0.18421,0.18169,0.18668,0.16862,0.
16492,0.18081,0.17293,0.1871,0.18109,0.16591,0.16363,0.17052,0.17605,0
.18089,0.18068,0.17148,0.17706,1.6525,1.5458,1.5265,0.90482,0.6276,0.8
3842,0.75544,0.81247,0.82461,0.8931,0.99199,0.64836,0.64976,0.51707,0.
70539,0.62825,0.68433,0.69692,0.64274,0.60964,0.17367,0.17935,0.1723,0
.1799,0.18017,0.17884,0.17215,0.18154,0.17998,0.18231,0.17714,0.17392,
0.17399,0.17161,0.16959,0.17086,0.17466,0.17012,1.6191,0.8776,0.94554,
0.85547,1.052,0.81367,0.8504,0.90759,1.0018,0.9929,0.66697,0.66412,0.6
2616,0.666,0.48138,0.62933,0.66018,0.63394,0.61623,0.17663,0.17941,0.1
7698,0.18118,0.1752,0.16758,0.17213,0.17987,0.17533,0.1729,0.18196,0.1

7902,0.17626,0.1729,0.17874,0.16978,0.17741,0.13894,0.87178,0.95622,0.
88585,0.87368,0.8648,0.80856,0.87481,0.72539,0.84342,0.90281,0.65013,0
.62686,0.69574,0.67747,0.69832,0.55852,0.67202,0.65582,0.64727,0.1849,
0.17336,0.16507,0.17265,0.17975,0.1769,0.17814,0.18006,0.17473,0.17543
,0.16489,0.17669,0.1721,0.18064,0.16347,0.16831,0.17892,0.14508,0.8432
3,0.80057,0.94543,0.75658,0.7587,0.89455,0.91569,0.94132,0.92702,0.778
57,0.53232,0.6288,0.64398,0.6014,0.64459,0.64681,0.73976,0.63546,0.181
68,0.16684,0.17561,0.17665,0.18761,0.17944,0.17337,0.17462,0.17591,0.1
8259,0.17849,0.18648,0.18614,0.18672,0.18538,0.18534,0.1724,0.1746,0.1
4602,0.98718,0.7507,0.80836,0.8511,0.85018,0.55181,0.82282,0.8259,0.73
866,1.0089,0.66433,0.64903,0.71374,0.61483,0.6866,0.67676,0.60669,0.64
217,0.17804,0.17929,0.17247,0.16527,0.17339,0.16913,0.17499,0.17639,0.
16891,0.17449,0.17352,0.17867,0.18046,0.17466,0.17404,0.18212,0.17201,
0.14726,0.14936,0.14628,0.87215,0.91707,0.9923,0.8069,0.94225,0.84658,
0.81595,0.75942,0.85702,0.62049,0.67618,0.63089,0.70416,0.79982,0.6496
6,0.17784,0.17079,0.17658,0.17749,0.17512,0.17375,0.16757,0.17358,0.18
304,0.17701,0.16954,0.17198,0.1745,0.19094,0.17428,0.17269,0.18013,0.1
8584,0.14881,0.14138,0.14393,0.14532,0.86199,0.79762,0.76823,0.79846,0
.86997,0.85434,0.86485,0.91594,0.85447,0.71617,0.70176,0.66375,0.17177
,0.17063,0.17364,0.17131,0.18457,0.17019,0.17326,0.18497,0.18001,0.169
68,0.17318,0.18468,0.17418,0.188,0.1717,0.19259,0.17469,0.18481,0.1658
,0.17311,0.14451,0.14874,0.15115,0.15133,0.14873,0.14538,0.93139,0.864
62,1.0513,0.79791,0.9548,0.87793,0.98772,0.89155,0.17696,0.16522,0.185
45,0.16758,0.16174,0.18104,0.18226,0.18303,0.18155,0.1738,0.18473,0.16
375,0.19102,0.16601,0.17996,0.17283,0.17447,0.18151,0.1663,0.18405,0.1
7437,0.18689,0.17969,0.13703,0.14776,0.14684,0.14724,0.14523,0.14268,0
.81298,0.85361,0.69828,0.87727,0.80067,0.14814,0.14844,0.17902,0.17649
,0.18408,0.17123,0.18462,0.18529,0.17097,0.16814,0.16402,0.18666,0.170
34,0.18276,0.17579,0.17873,0.17321,0.17888,0.18272,0.18242,0.17986,0.1
6592,0.18466,0.15377,0.14408,0.14597,0.14425,0.14227,0.14475,0.15307,0
.13562,0.14707,0.146,0.89837,0.88534,0.1413,0.14538,0.14683,0.14352,0.
17412,0.18159,0.17131,0.18055,0.17715,0.17163,0.16782,0.18124,0.18473,
0.17978,0.18004,0.17536,0.17625,0.17883,0.17738,0.17758,0.16953,0.1762
9,0.16235,0.14354,0.15562,0.15362,0.14675,0.1383,0.15174,0.14209,0.147
03,0.14802,0.14316,0.14649,0.1447,0.1417,0.14147,0.14902,0.14592,0.141
23,0.17859,0.17659,0.17482,0.16795,0.17115,0.17997,0.18056,0.17814,0.1
824,0.18235,0.18351,0.17492,0.18401,0.18465,0.1726,0.17854,0.14599,0.1
4153,0.14665,0.14542,0.14908,0.14514,0.15144,0.15159,0.14251,0.14405,0
.14636,0.14744,0.14652,0.14117,0.14317,0.14367,0.14534,0.15049,0.14501
,0.18315,0.17379,0.18477,0.17104,0.17358,0.17936,0.17878,0.17529,0.170
81,0.16969,0.1711,0.17917,0.17923,0.15072,0.14045,0.14291,0.14217,0.14
252,0.14115,0.14678,0.14668,0.145,0.14617,0.15247,0.14153,0.14589,0.14
137,0.14459,0.14683,0.14535,0.1442,0.14315,0.1503,0.13235,0.17387,0.18
105,0.17644,0.16898,0.17624,0.17455,0.18539,0.16849,0.16711,0.14257,0.
15148,0.14758,0.14812,0.14836,0.15067,0.14389,0.14842,0.14675,0.14393,
0.14808,0.1478,0.14442,0.14666,0.14409,0.14956,0.14391,0.14527,0.14616
,0.14547,0.14461,0.14242,0.15065,0.14852,0.14795,0.17156,0.17855,0.164
58,0.18031,0.14463,0.14201,0.146,0.14462,0.14597,0.14181,0.13949,0.146
93,0.15034,0.14197,0.1396,0.1456,0.14149,0.14606,0.1462,0.13965,0.1450
5,0.14991,0.14825,0.14103,0.14805,0.146,0.14482,0.15362,0.14548,0.1497
4,0.14631,0.13784,0.15012,0.1473,0.14069,0.14212,0.14956,0.14609,0.145
24,0.14619,0.14069,0.14565,0.13903,0.14881,0.14902,0.14357,0.14193,0.1

4574,0.14758,0.14819,0.14784,0.15029,0.14627,0.14605,0.14614,0.14298,0
.14908,0.15048,0.14471,0.14803,0.15035,0.1422,0.14624,0.1411,0.14543,0
.15167,0.14396,0.14851,0.14632,0.14799,0.13854,0.14145,0.14679,0.14712
,0.14325,0.13877,0.14593,0.14205,0.14165,0.15704,0.13769,0.14969,0.140
76,0.14207,0.14729,0.13996,0.14989,0.14593,0.14444,0.14763,0.1511,0.13
584,0.14737,0.15558,0.14434,0.13423,0.14893,0.13714,0.13908,0.14753,0.
143,0.13789,0.14117,0.14402,0.14749,0.15221,0.13968,0.14222,0.14777,0.
14213,0.13534,0.14247,0.14401,0.14348,0.15068,0.1463,0.14088,0.14438,0
.14323,0.15524,0.14117,0.14147,0.15013,0.14164,0.14238,0.13654,0.15119
,0.14373,0.1433,0.14311,0.1479,0.14355,0.14123,0.15298,0.14772,0.14574
,0.14555,0.14879,0.14498,0.14502,0.14535,0.15056,0.13871,0.14557,0.139
12,0.13928,0.14269,0.14245,0.14091,0.13914,0.14075,0.13717,0.14276,0.1
4068,0.1528,0.14127,0.14464,0.15002,0.14925,0.14301,0.14985,0.14481,0.
1492,0.14879,0.14634,0.14169,0.14443,0.14894,0.14472,0.14985,0.14249,0
.14792,0.14063,0.14798,0.14659,0.15215,0.15083,0.14752,0.14393,0.14116
,0.14694,0.15145,0.1445,0.14132,0.14412,0.15413,0.14509,0.14399,0.1395
4,0.14108,0.14402,0.14712,0.1469,0.14562,0.14262,0.145,0.1416,0.1465,0
.15125
0.22584,0.23443,0.22019,0.21275,0.23513,0.23235,0.22218,0.22407,0.2318
4,0.22822,0.22183,0.23503,0.22471,0.22762,0.23085,0.23243,0.24049,0.23
53,0.22835,0.22146,0.64519,0.74299,0.78719,0.59768,0.22407,0.22638,0.2
2401,0.23781,0.22343,0.23351,0.22566,0.22619,0.22807,0.23386,0.22443,0
.2251,0.23262,0.2339,0.22924,1.1299,0.70588,0.67825,0.67089,0.68073,0.
676,0.72581,0.22543,0.24052,0.22335,0.22321,0.22931,0.21408,0.22721,0.
22309,0.22273,0.22628,0.2334,0.23161,0.22833,0.22803,0.22705,1.0851,1.
1316,0.68488,0.68951,0.72992,0.80713,0.66282,0.73801,0.71287,0.70907,0
.22763,0.22555,0.22524,0.22742,0.2343,0.22409,0.23879,0.2294,0.23355,0
.21888,0.23122,0.22582,0.2168,0.23956,0.22498,1.0988,1.1878,1.0681,0.6
1348,0.70077,0.69508,0.68106,0.72045,0.60081,0.69425,0.66127,0.68216,0
.22603,0.23596,0.2369,0.22257,0.22574,0.23162,0.22028,0.22836,0.23101,
0.22746,0.23382,0.22332,0.23098,0.22851,0.22842,0.22329,1.1084,1.1477,
1.0103,1.063,0.71129,0.6841,0.63337,0.68436,0.66924,0.66414,0.70259,0.
76361,0.64177,0.61522,0.22808,0.22265,0.2238,0.22724,0.22752,0.22564,0
.23737,0.22382,0.21533,0.21522,0.23627,0.22816,0.22322,0.23392,0.22595
,0.22194,1.1614,1.0946,1.2395,1.0211,1.1377,1.0787,0.63712,0.72552,0.7
0094,0.72334,0.66994,0.68539,0.68183,0.74749,0.71061,0.64058,0.23552,0
.22929,0.22663,0.22558,0.23157,0.23832,0.23454,0.22398,0.22415,0.23863
,0.24298,0.23514,0.21582,0.22641,0.2359,0.22191,0.22531,1.091,1.0578,1
.0595,1.1724,1.1246,0.88934,0.75227,0.67247,0.6526,0.70654,0.73797,0.5
6202,0.59355,0.67836,0.65347,0.68233,0.62431,0.22028,0.22013,0.22963,0
.22757,0.23322,0.22908,0.2243,0.22746,0.22568,0.22536,0.23532,0.23624,
0.23165,0.17708,0.18227,0.19305,0.23471,1.0695,1.1016,1.0281,1.0897,1.
0739,1.2199,1.096,0.62734,0.67379,0.63266,0.59777,0.66088,0.6653,0.696
05,0.70236,0.59187,0.54652,0.6144,0.23234,0.23232,0.22706,0.23089,0.23
046,0.22697,0.23,0.23134,0.23435,0.22966,0.2308,0.19438,0.18321,0.1780
1,0.18074,0.17069,0.17688,0.22882,1.1251,1.013,1.1634,1.1018,1.1648,1.
1003,1.1474,0.66459,0.56164,0.64721,0.81607,0.64166,0.63461,0.74261,0.
65645,0.77703,0.67757,0.81292,0.22797,0.22849,0.23093,0.22896,0.23258,
0.23427,0.22681,0.22502,0.23477,0.18741,0.20125,0.18173,0.18176,0.1790
6,0.21527,0.18805,0.17898,0.18781,0.2297,1.1463,1.0617,1.0769,1.0082,1
.252,1.1158,1.034,0.77137,0.55646,0.68488,0.64523,0.54365,0.67273,0.60
692,0.65753,0.56519,0.64545,0.60337,0.67369,0.23035,0.2263,0.23106,0.2

2064,0.23192,0.23048,0.19785,0.18763,0.1919,0.17622,0.18227,0.18233,0.
19059,0.18922,0.19308,0.18632,0.15143,0.17543,1.0563,0.99758,1.0799,1.
1791,1.0875,1.1106,1.1073,0.59066,0.68612,0.70642,0.67106,0.89234,0.66
275,0.65687,0.62268,0.66554,0.70243,0.65033,0.65624,0.23698,0.22522,0.
23323,0.17332,0.18295,0.17406,0.19669,0.17027,0.18549,0.1726,0.18096,0
.16866,0.19266,0.16631,0.18726,0.18487,0.18236,0.18056,1.0476,1.0447,1
.1666,1.1347,1.0727,1.0989,1.083,1.0952,1.1128,0.65637,0.68956,0.60153
,0.63192,0.7223,0.67936,0.72512,0.67477,0.68468,0.7205,0.74485,0.18527
,0.17659,0.19534,0.1815,0.18441,0.1823,0.17905,0.18155,0.19128,0.17061
,0.18358,0.18162,0.17881,0.18481,0.18899,0.19748,0.18877,0.19308,1.097
6,1.0775,1.0173,1.0782,1.1106,1.2426,1.1285,1.2011,1.0881,0.66827,0.70
353,0.53112,0.40655,0.5323,0.38228,0.46525,0.3798,0.56279,0.53072,0.43
962,0.19338,0.18666,0.19055,0.17216,0.1674,0.1741,0.1957,0.16818,0.179
95,0.19031,0.17645,0.18029,0.17949,0.18481,0.17774,0.17862,0.16635,0.1
9458,1.1673,1.0989,1.0827,1.1576,1.0956,1.2013,1.0758,1.1004,1.0164,0.
61945,0.49831,0.48793,0.49198,0.43598,0.42559,0.45999,0.49898,0.57554,
0.4654,0.40812,0.19274,0.17155,0.18625,0.1883,0.18243,0.17706,0.1872,0
.18384,0.17467,0.18428,0.18158,0.17738,0.18965,0.18248,0.19294,0.17073
,0.1894,0.18306,1.0276,1.1505,1.1408,1.0831,1.161,1.0671,1.0872,0.5655
1,0.60632,0.63195,0.52278,0.44297,0.50943,0.47868,0.53214,0.5087,0.551
75,0.50832,0.46912,0.40983,0.18397,0.1799,0.18977,0.18506,0.18332,0.18
691,0.17417,0.18788,0.17874,0.1805,0.17925,0.18688,0.1891,0.17592,0.18
164,0.18635,0.18134,0.17397,1.0519,1.1107,1.0474,1.1449,1.0897,1.0575,
0.56808,0.62724,0.6033,0.71745,0.66687,0.52637,0.50338,0.4252,0.46243,
0.5191,0.49746,0.53451,0.54547,0.45369,0.19663,0.1719,0.16961,0.18467,
0.18808,0.19489,0.18368,0.19634,0.18778,0.18537,0.18391,0.18178,0.1777
6,0.18318,0.17823,0.18074,0.18166,0.17939,1.0403,1.1249,1.1935,1.1045,
1.105,0.60288,0.55857,0.66627,0.61259,0.55582,0.58143,0.4577,0.45929,0
.48722,0.46456,0.51064,0.509,0.46399,0.51236,0.50242,0.18834,0.19094,0
.18388,0.1993,0.17681,0.16907,0.18029,0.17494,0.1946,0.18936,0.17262,0
.16968,0.17837,0.18461,0.18852,0.18668,0.17859,0.18,1.1523,1.0328,1.02
99,0.65778,0.4424,0.59535,0.56784,0.57094,0.59153,0.63429,0.72245,0.47
267,0.49606,0.40042,0.53319,0.49439,0.50794,0.52061,0.50718,0.47279,0.
17627,0.18526,0.17655,0.18834,0.18208,0.18867,0.17601,0.19057,0.18991,
0.18641,0.18446,0.18083,0.18447,0.17736,0.17151,0.17626,0.17571,0.1771
8,1.1621,0.65328,0.68368,0.62837,0.73726,0.58076,0.5893,0.65935,0.7089
2,0.69456,0.50339,0.50629,0.46013,0.52035,0.3787,0.49931,0.48642,0.472
9,0.45965,0.18291,0.18476,0.18445,0.18563,0.18121,0.16594,0.17602,0.18
32,0.17974,0.17721,0.18659,0.18614,0.18005,0.17916,0.18864,0.1765,0.18
577,0.13019,0.63031,0.67548,0.64565,0.63643,0.62684,0.58966,0.62502,0.
52716,0.59133,0.66222,0.4932,0.48215,0.53582,0.51623,0.52493,0.43426,0
.49252,0.49375,0.4857,0.19196,0.17595,0.17078,0.18078,0.18577,0.1881,0
.18267,0.18611,0.18034,0.17981,0.16559,0.18123,0.17716,0.18861,0.16863
,0.16803,0.18478,0.13592,0.61662,0.58564,0.65157,0.53856,0.5597,0.6206
4,0.65973,0.70262,0.65271,0.56618,0.41744,0.48732,0.49237,0.46945,0.47
725,0.48922,0.55719,0.48902,0.18607,0.16366,0.18132,0.17958,0.19125,0.
18676,0.17719,0.17878,0.18092,0.1887,0.18294,0.19643,0.19181,0.19383,0
.18911,0.19726,0.18138,0.17896,0.1348,0.70653,0.56237,0.57953,0.60441,
0.60085,0.40175,0.59746,0.58335,0.51869,0.65884,0.49826,0.49752,0.5346
5,0.45486,0.509,0.4951,0.45736,0.49414,0.18559,0.18628,0.1819,0.16984,
0.17564,0.1736,0.18016,0.18001,0.17393,0.18336,0.18274,0.18358,0.18713
,0.18144,0.1817,0.18589,0.17407,0.13622,0.13935,0.13596,0.63157,0.6620

6,0.70664,0.55944,0.69131,0.63948,0.59411,0.54977,0.61362,0.46828,0.50
851,0.4749,0.53273,0.56965,0.48781,0.18462,0.17693,0.18405,0.18274,0.1
7679,0.17746,0.1753,0.17869,0.19138,0.18355,0.17794,0.17772,0.17996,0.
20033,0.17924,0.1805,0.18468,0.19501,0.14134,0.13074,0.13522,0.13531,0
.60664,0.55875,0.55886,0.5903,0.62863,0.61541,0.62911,0.67619,0.59716,
0.54979,0.55596,0.50972,0.17812,0.17576,0.18107,0.17462,0.19175,0.1741
6,0.17603,0.18859,0.18552,0.17471,0.17812,0.19157,0.17905,0.20076,0.18
033,0.19999,0.1796,0.19123,0.17139,0.17566,0.13235,0.13733,0.14285,0.1
4071,0.13752,0.13744,0.67424,0.60733,0.76976,0.55448,0.69398,0.61978,0
.66883,0.66084,0.18285,0.17276,0.18828,0.17334,0.16724,0.18297,0.187,0
.18938,0.18898,0.18095,0.19623,0.16417,0.19802,0.17531,0.18637,0.17684
,0.17954,0.18877,0.17586,0.18142,0.18264,0.19481,0.18578,0.12733,0.136
81,0.13971,0.13929,0.13659,0.13218,0.56403,0.63277,0.53487,0.61377,0.5
9187,0.1393,0.14051,0.18738,0.18502,0.19007,0.1787,0.18914,0.19568,0.1
7841,0.17553,0.16638,0.19426,0.17457,0.18925,0.1849,0.18687,0.17249,0.
1838,0.19088,0.18791,0.18267,0.16666,0.19131,0.14431,0.13518,0.1338,0.
13401,0.13262,0.13234,0.1452,0.1268,0.1376,0.13763,0.64453,0.63911,0.1
3179,0.13605,0.13692,0.13711,0.18368,0.18822,0.18122,0.18976,0.18144,0
.18046,0.16861,0.18948,0.1954,0.18645,0.19053,0.17779,0.18549,0.18592,
0.18497,0.18108,0.1722,0.17669,0.16423,0.13336,0.14508,0.14479,0.1397,
0.13145,0.14221,0.1363,0.13672,0.13956,0.13088,0.13909,0.13409,0.12889
,0.13205,0.13793,0.13666,0.13062,0.18773,0.18203,0.18184,0.17188,0.182
09,0.18294,0.18375,0.18769,0.18816,0.18618,0.18823,0.18032,0.18663,0.1
8885,0.17831,0.18454,0.13808,0.13165,0.1385,0.13388,0.14,0.13364,0.139
8,0.14147,0.1337,0.13667,0.13719,0.13987,0.13716,0.12876,0.13445,0.135
12,0.1368,0.14222,0.13602,0.19213,0.17866,0.19402,0.18096,0.17805,0.18
392,0.18585,0.17898,0.17726,0.17276,0.18274,0.1877,0.18762,0.14033,0.1
3165,0.13199,0.13401,0.13513,0.13202,0.14085,0.13766,0.13455,0.136,0.1
4122,0.13433,0.136,0.13328,0.13677,0.13684,0.1347,0.13528,0.13584,0.14
032,0.12393,0.18319,0.19293,0.18076,0.17015,0.17949,0.18555,0.19051,0.
17229,0.17138,0.13403,0.14259,0.13906,0.13767,0.13605,0.1432,0.13495,0
.14125,0.13412,0.13409,0.14095,0.13819,0.13942,0.1388,0.13534,0.13676,
0.13301,0.13421,0.13549,0.13313,0.13588,0.13318,0.14201,0.13864,0.1363
4,0.17717,0.18578,0.16821,0.19132,0.13571,0.12842,0.13592,0.1346,0.139
07,0.1311,0.13261,0.13739,0.14097,0.13027,0.13192,0.13603,0.13543,0.13
367,0.13553,0.13081,0.13689,0.13798,0.13987,0.13202,0.13509,0.14125,0.
13776,0.14255,0.13457,0.13788,0.13711,0.12635,0.14029,0.13326,0.13225,
0.13369,0.13776,0.13394,0.13472,0.13779,0.1311,0.13679,0.12988,0.13862
,0.1361,0.13531,0.13244,0.13463,0.13818,0.13989,0.14287,0.14284,0.1369
,0.13565,0.13428,0.1331,0.13897,0.14089,0.13409,0.14042,0.13992,0.1339
1,0.14058,0.13071,0.13513,0.14316,0.13347,0.13881,0.13377,0.13696,0.13
058,0.1325,0.13742,0.13871,0.1335,0.1303,0.13386,0.13485,0.13578,0.143
8,0.13114,0.13927,0.13329,0.13142,0.13598,0.13094,0.1407,0.13843,0.135
64,0.13481,0.14011,0.12823,0.13764,0.14758,0.13523,0.12555,0.13861,0.1
2907,0.13216,0.13824,0.13279,0.13038,0.13246,0.13475,0.13649,0.14243,0
.13104,0.13092,0.14057,0.13531,0.1248,0.13338,0.13578,0.13425,0.14181,
0.13595,0.13003,0.13517,0.13314,0.14368,0.13107,0.13101,0.13983,0.1341
6,0.13403,0.12782,0.14062,0.1337,0.13402,0.13335,0.13906,0.13366,0.134
12,0.14425,0.13706,0.13583,0.13438,0.14026,0.13551,0.13449,0.13672,0.1
3965,0.13088,0.13766,0.13064,0.1292,0.1337,0.1314,0.13321,0.12781,0.13
267,0.13021,0.1346,0.13009,0.14289,0.13133,0.13344,0.13803,0.14233,0.1
3535,0.14167,0.13711,0.13716,0.13658,0.13588,0.13277,0.13287,0.14101,0

.13666,0.1404,0.13519,0.13921,0.13356,0.13817,0.13458,0.14239,0.14255,
0.13694,0.13228,0.13384,0.13735,0.1421,0.13782,0.13441,0.135,0.14284,0
.13669,0.13354,0.13329,0.13256,0.13535,0.13647,0.13789,0.13891,0.13458
,0.13467,0.13363,0.13648,0.14064
0.21961,0.2295,0.21972,0.21552,0.23649,0.23121,0.22496,0.22116,0.22656
,0.22453,0.22495,0.23627,0.22482,0.22835,0.23157,0.23623,0.24133,0.232
29,0.2254,0.21865,0.51906,0.55275,0.5884,0.46733,0.2229,0.226,0.22312,
0.23336,0.22123,0.23056,0.22124,0.2233,0.22931,0.23818,0.22267,0.2269,
0.23393,0.23219,0.22613,0.84291,0.56068,0.53637,0.53321,0.55157,0.5024
,0.55831,0.22167,0.23896,0.22267,0.22156,0.22962,0.21149,0.22393,0.222
36,0.22622,0.2228,0.23529,0.23645,0.22631,0.22587,0.22728,0.78841,0.86
585,0.52906,0.54781,0.5827,0.59662,0.50863,0.55813,0.57654,0.52929,0.2
3126,0.22932,0.22577,0.22779,0.23578,0.22518,0.23438,0.2257,0.23702,0.
22117,0.23144,0.22808,0.21702,0.23759,0.22446,0.81086,0.88341,0.80619,
0.47451,0.54822,0.53888,0.54248,0.57468,0.48229,0.5444,0.50817,0.54758
,0.227,0.23507,0.23401,0.22438,0.22646,0.23302,0.22092,0.22975,0.22737
,0.22572,0.23177,0.22845,0.22574,0.2285,0.22793,0.22854,0.81774,0.8069
6,0.7309,0.71355,0.55631,0.5251,0.49704,0.54017,0.5157,0.50963,0.52379
,0.61516,0.5156,0.49582,0.22462,0.22157,0.22632,0.22842,0.2279,0.22396
,0.23315,0.22129,0.21795,0.21368,0.23695,0.22787,0.22201,0.22913,0.224
08,0.22143,0.8673,0.80987,0.96522,0.70091,0.86648,0.75097,0.51333,0.56
256,0.56992,0.57938,0.51292,0.5502,0.56167,0.5947,0.56505,0.48319,0.23
292,0.22819,0.22514,0.2303,0.22951,0.24189,0.23074,0.22211,0.22272,0.2
3455,0.24063,0.23543,0.21751,0.2267,0.23497,0.21977,0.22029,0.79229,0.
8024,0.79996,0.87139,0.84351,0.63512,0.5645,0.54045,0.50333,0.53951,0.
58184,0.45814,0.46606,0.50989,0.5084,0.53277,0.49482,0.22567,0.22153,0
.22938,0.22725,0.23317,0.22733,0.22285,0.22335,0.22549,0.22506,0.23295
,0.23322,0.2302,0.16889,0.16918,0.18631,0.23279,0.81592,0.80773,0.7654
5,0.79899,0.80885,0.87208,0.77729,0.47527,0.54756,0.4898,0.47681,0.510
13,0.50288,0.54567,0.54636,0.46455,0.44199,0.48613,0.2259,0.23294,0.22
331,0.22663,0.22737,0.22577,0.23127,0.2306,0.23061,0.22689,0.2322,0.18
335,0.17053,0.16841,0.17027,0.16083,0.16839,0.22745,0.83217,0.70639,0.
84189,0.83832,0.85193,0.81337,0.83709,0.51873,0.45588,0.48655,0.66222,
0.48358,0.50175,0.56549,0.51469,0.58008,0.53274,0.60977,0.22836,0.228,
0.23089,0.22628,0.22987,0.23352,0.22321,0.22308,0.23415,0.17504,0.1910
7,0.172,0.17429,0.16992,0.20488,0.17772,0.16942,0.17712,0.2284,0.86073
,0.77001,0.76869,0.72182,0.97009,0.86772,0.77818,0.5738,0.46026,0.5400
5,0.48485,0.44647,0.52367,0.46669,0.5296,0.46415,0.48604,0.47788,0.536
65,0.2295,0.22585,0.22782,0.22044,0.23281,0.22779,0.19555,0.17749,0.18
229,0.16897,0.16954,0.17546,0.18049,0.17728,0.18513,0.1754,0.13992,0.1
653,0.747,0.71424,0.79492,0.89421,0.82494,0.81049,0.8194,0.46125,0.529
06,0.57673,0.52112,0.69236,0.51529,0.52397,0.48082,0.52975,0.54427,0.5
0988,0.50925,0.23441,0.22689,0.23324,0.15823,0.16752,0.16046,0.18627,0
.16134,0.1732,0.15608,0.17011,0.15858,0.184,0.15444,0.17441,0.17265,0.
16941,0.16686,0.78883,0.78775,0.87759,0.81911,0.83128,0.81423,0.79948,
0.79313,0.83816,0.5204,0.53258,0.47717,0.49031,0.55869,0.55571,0.57338
,0.49949,0.53645,0.54224,0.59798,0.17403,0.1697,0.18363,0.16943,0.1753
4,0.17011,0.17063,0.17134,0.18228,0.16325,0.17062,0.17643,0.16782,0.17
306,0.18294,0.18916,0.17722,0.17854,0.79576,0.77121,0.74068,0.77528,0.
81623,0.90328,0.78283,0.94225,0.81176,0.52813,0.53387,0.40758,0.31575,
0.41012,0.29893,0.37174,0.30976,0.44986,0.42294,0.35263,0.18275,0.1738
9,0.1791,0.16368,0.15417,0.16437,0.18942,0.15557,0.1706,0.1791,0.16171

,0.1711,0.16841,0.17583,0.17057,0.16994,0.15246,0.17974,0.83209,0.7962
8,0.79901,0.86654,0.76201,0.90024,0.81727,0.82311,0.72378,0.45579,0.37
306,0.37756,0.39107,0.35237,0.34282,0.36871,0.39723,0.43314,0.38881,0.
33563,0.18417,0.16169,0.17437,0.1733,0.17481,0.16768,0.17833,0.17194,0
.16187,0.17475,0.17193,0.16632,0.17929,0.17414,0.18213,0.16113,0.17971
,0.17236,0.7751,0.86954,0.83871,0.80063,0.88067,0.77056,0.77231,0.4182
2,0.4392,0.44421,0.41347,0.34672,0.3963,0.37944,0.42599,0.41123,0.4224
4,0.4165,0.36695,0.344,0.17521,0.16875,0.17729,0.17794,0.17475,0.17936
,0.16316,0.1775,0.17019,0.17028,0.1719,0.17372,0.17681,0.1615,0.17059,
0.17496,0.16856,0.16753,0.78862,0.82273,0.80317,0.81979,0.84562,0.7607
3,0.4506,0.48343,0.4457,0.51834,0.51154,0.40867,0.39952,0.32512,0.3450
3,0.41677,0.38904,0.42573,0.44456,0.35463,0.18736,0.15924,0.15537,0.17
249,0.18001,0.18094,0.17638,0.18922,0.1748,0.17543,0.17404,0.17267,0.1
682,0.17401,0.17077,0.1741,0.17321,0.16649,0.75007,0.89231,0.8939,0.82
769,0.76793,0.45159,0.42237,0.49886,0.45306,0.41836,0.45862,0.38179,0.
36322,0.38305,0.369,0.38014,0.41798,0.35675,0.40254,0.37252,0.17656,0.
18089,0.17483,0.1927,0.16363,0.15422,0.1695,0.15991,0.18648,0.18156,0.
1626,0.15907,0.16574,0.17752,0.17994,0.17776,0.17042,0.17089,0.8416,0.
75904,0.73424,0.49163,0.35316,0.42946,0.43551,0.41901,0.46075,0.47293,
0.52511,0.36228,0.39423,0.33071,0.41066,0.38933,0.38326,0.39714,0.4036
6,0.35834,0.1636,0.17819,0.16516,0.17562,0.17503,0.17852,0.16485,0.181
07,0.1822,0.17021,0.17339,0.17039,0.17832,0.16486,0.15743,0.16599,0.16
663,0.16364,0.82276,0.4807,0.52509,0.48586,0.55251,0.42497,0.43214,0.4
8887,0.54151,0.51611,0.3986,0.38354,0.34195,0.39308,0.31682,0.40077,0.
35074,0.35696,0.3554,0.17196,0.1736,0.17548,0.17558,0.16753,0.156,0.16
243,0.17486,0.16728,0.16878,0.17982,0.17964,0.16815,0.16717,0.18253,0.
16741,0.17525,0.10955,0.48548,0.51191,0.50372,0.46739,0.4718,0.45554,0
.46117,0.3896,0.4416,0.4824,0.39657,0.36627,0.43361,0.39517,0.38423,0.
34766,0.38318,0.39385,0.38502,0.18065,0.16659,0.15919,0.17045,0.18013,
0.18073,0.17231,0.1741,0.17126,0.16977,0.15735,0.17122,0.16677,0.17742
,0.16006,0.15668,0.17555,0.11564,0.46144,0.44009,0.49889,0.4081,0.4160
2,0.45552,0.49721,0.53459,0.50325,0.416,0.34885,0.3883,0.37879,0.37289
,0.37909,0.38478,0.4395,0.398,0.17392,0.14909,0.17013,0.17099,0.17842,
0.17605,0.16541,0.16885,0.17208,0.17553,0.16852,0.19222,0.18147,0.1828
7,0.17786,0.18711,0.1743,0.16322,0.11685,0.55882,0.44561,0.43481,0.434
02,0.45578,0.30522,0.4454,0.43171,0.39726,0.47985,0.40169,0.38438,0.41
565,0.35486,0.38152,0.36533,0.33894,0.38905,0.17401,0.17839,0.1744,0.1
5615,0.16825,0.16516,0.17157,0.1699,0.16611,0.17133,0.17803,0.1745,0.1
7653,0.17348,0.17148,0.17221,0.16336,0.11709,0.11877,0.1158,0.46725,0.
49252,0.5538,0.42461,0.5058,0.47859,0.47151,0.4259,0.47802,0.37137,0.3
8907,0.37369,0.40997,0.44733,0.38232,0.17307,0.16995,0.17554,0.1738,0.
16576,0.16593,0.16827,0.16605,0.18647,0.17375,0.16909,0.1675,0.17015,0
.19375,0.16875,0.16818,0.17275,0.18432,0.1189,0.11214,0.11772,0.11466,
0.46642,0.42241,0.43697,0.46196,0.46022,0.45976,0.49519,0.52006,0.4406
1,0.44356,0.45918,0.39503,0.17021,0.16596,0.17073,0.16746,0.18102,0.15
965,0.16422,0.17522,0.17766,0.16945,0.16707,0.18021,0.16997,0.19251,0.
17143,0.19084,0.16836,0.18583,0.16426,0.16284,0.11249,0.11663,0.12291,
0.12087,0.11574,0.11804,0.53824,0.45893,0.59106,0.39112,0.54484,0.4459
4,0.45996,0.49105,0.17244,0.16046,0.17662,0.16068,0.15711,0.17242,0.17
535,0.1825,0.18058,0.16906,0.18695,0.1515,0.1926,0.16934,0.1761,0.1679
3,0.17155,0.17889,0.16751,0.17249,0.17421,0.18091,0.17626,0.11086,0.11
439,0.11787,0.11797,0.11391,0.11418,0.41356,0.46696,0.40474,0.44709,0.

45648,0.1189,0.12479,0.18124,0.17052,0.18226,0.16819,0.17896,0.18583,0
.1662,0.16571,0.15655,0.18355,0.16407,0.18032,0.17716,0.17651,0.15869,
0.17108,0.18179,0.17715,0.16931,0.15952,0.18102,0.12475,0.11381,0.1151
4,0.11476,0.11351,0.11457,0.123,0.1092,0.11781,0.11428,0.50895,0.49605
,0.11233,0.1155,0.11509,0.11846,0.17642,0.17354,0.16636,0.17994,0.1738
3,0.17258,0.15682,0.18016,0.18516,0.1768,0.18384,0.16229,0.17563,0.173
,0.17487,0.17218,0.16223,0.16643,0.15504,0.11533,0.12259,0.12634,0.121
15,0.11049,0.12075,0.1192,0.11761,0.11863,0.11046,0.11782,0.11654,0.10
976,0.11456,0.11827,0.11341,0.11202,0.17855,0.1716,0.17453,0.1626,0.17
092,0.16866,0.17219,0.17401,0.17524,0.17756,0.17781,0.16628,0.17597,0.
17815,0.17181,0.1713,0.11656,0.11093,0.11411,0.11504,0.1198,0.11398,0.
11876,0.12181,0.11583,0.11406,0.11623,0.12191,0.11741,0.10848,0.11434,
0.11546,0.11797,0.11885,0.1162,0.18562,0.16769,0.18682,0.16809,0.16787
,0.17466,0.1744,0.16435,0.16787,0.16126,0.17406,0.17703,0.17829,0.1189
5,0.11377,0.11167,0.11182,0.11414,0.11263,0.12019,0.11863,0.11661,0.11
701,0.12374,0.11483,0.11361,0.11272,0.11904,0.11701,0.11816,0.1172,0.1
1611,0.11975,0.10259,0.17409,0.1885,0.16993,0.15959,0.16931,0.18046,0.
17752,0.16265,0.1577,0.1112,0.11971,0.11854,0.11788,0.11387,0.12103,0.
11514,0.121,0.11925,0.11472,0.12123,0.11792,0.12028,0.12038,0.11409,0.
11525,0.11489,0.11528,0.11364,0.11269,0.11613,0.11182,0.1239,0.11701,0
.11482,0.16396,0.17937,0.15653,0.18454,0.11577,0.11032,0.1145,0.11348,
0.12066,0.11102,0.11568,0.12056,0.11941,0.11186,0.111,0.11645,0.11556,
0.1154,0.11587,0.11452,0.11507,0.11951,0.1214,0.11261,0.11385,0.1179,0
.1148,0.11968,0.11492,0.11668,0.11696,0.1064,0.11357,0.11602,0.11251,0
.11436,0.11877,0.11483,0.11586,0.11832,0.11034,0.11568,0.11233,0.12168
,0.11638,0.11181,0.1153,0.11425,0.11788,0.11903,0.128,0.12199,0.11571,
0.1161,0.11176,0.11004,0.11777,0.11945,0.11131,0.11854,0.11752,0.11558
,0.12021,0.11093,0.11269,0.12218,0.1178,0.12012,0.11146,0.11934,0.1149
3,0.11536,0.11752,0.11851,0.11081,0.10797,0.11237,0.11408,0.11614,0.12
604,0.1133,0.11687,0.11605,0.11073,0.11283,0.11143,0.12233,0.11708,0.1
1739,0.11584,0.12096,0.10742,0.11976,0.12967,0.11451,0.10449,0.1175,0.
10963,0.11392,0.12224,0.11097,0.11194,0.11173,0.11655,0.11898,0.12317,
0.10837,0.11446,0.1212,0.11276,0.10466,0.11573,0.11936,0.11281,0.1208,
0.11561,0.11119,0.11532,0.11664,0.12292,0.11278,0.111,0.12137,0.11249,
0.11719,0.11296,0.11926,0.11553,0.11149,0.11373,0.11837,0.11502,0.1153
9,0.12643,0.11764,0.11593,0.11362,0.11967,0.11324,0.11309,0.11663,0.12
091,0.11098,0.11896,0.11129,0.11217,0.11548,0.11326,0.11306,0.10979,0.
11432,0.10881,0.11481,0.10902,0.12301,0.11015,0.11347,0.11626,0.12505,
0.11725,0.12363,0.1164,0.1166,0.1152,0.11738,0.11007,0.11287,0.12296,0
.11608,0.11927,0.11409,0.11595,0.11488,0.12063,0.11481,0.12201,0.12348
,0.11666,0.11213,0.11728,0.11757,0.12327,0.12428,0.11329,0.11423,0.124
73,0.11678,0.11407,0.11341,0.1113,0.11501,0.11313,0.11618,0.11848,0.11
659,0.1138,0.11472,0.11587,0.12273
0.21052,0.21793,0.20602,0.20722,0.22592,0.21851,0.21626,0.20937,0.2119
2,0.21324,0.2159,0.22921,0.21375,0.21649,0.21714,0.22451,0.22697,0.220
31,0.21361,0.20703,0.43051,0.45579,0.46742,0.37825,0.21431,0.21173,0.2
117,0.22236,0.21168,0.21686,0.21366,0.21105,0.21903,0.22266,0.21113,0.
21838,0.22249,0.22343,0.21872,0.65386,0.4562,0.4162,0.43782,0.45767,0.
39986,0.4271,0.21806,0.22689,0.21249,0.21149,0.21724,0.20248,0.21247,0
.2129,0.21738,0.20906,0.22695,0.22164,0.21749,0.21474,0.21548,0.61572,
0.68224,0.41364,0.4457,0.48355,0.47915,0.39619,0.45012,0.46666,0.44455
,0.2181,0.21773,0.21344,0.21633,0.22256,0.2172,0.22335,0.21585,0.2264,

0.20639,0.2222,0.21765,0.20698,0.22336,0.21624,0.59642,0.69416,0.65911
,0.36846,0.45581,0.44464,0.43718,0.49294,0.38994,0.44144,0.40305,0.451
14,0.21406,0.2216,0.22,0.21532,0.21672,0.21658,0.21403,0.21618,0.21885
,0.21254,0.2247,0.22113,0.21418,0.21403,0.22091,0.2192,0.68067,0.62708
,0.56997,0.51779,0.45129,0.44392,0.40978,0.44253,0.43286,0.43873,0.435
97,0.50216,0.43807,0.41663,0.21397,0.21246,0.21455,0.22175,0.21465,0.2
1344,0.2198,0.20861,0.206,0.20506,0.22366,0.21954,0.21259,0.2183,0.212
16,0.20653,0.70386,0.64908,0.7782,0.56106,0.71584,0.60031,0.42498,0.48
031,0.48209,0.4758,0.42245,0.44849,0.47259,0.49539,0.47972,0.38154,0.2
1738,0.21832,0.20945,0.22188,0.21671,0.22863,0.21568,0.21311,0.20907,0
.21987,0.22818,0.22503,0.20232,0.21637,0.22493,0.21449,0.20991,0.64924
,0.60512,0.60564,0.65192,0.72135,0.49976,0.46247,0.43555,0.40126,0.421
25,0.48958,0.38185,0.37308,0.42861,0.40607,0.44158,0.41044,0.21722,0.2
1289,0.21635,0.21939,0.22246,0.21384,0.2121,0.21167,0.20965,0.21204,0.
22427,0.21946,0.21839,0.15563,0.1531,0.17388,0.2211,0.59687,0.60485,0.
62269,0.62609,0.62585,0.67554,0.63108,0.40889,0.47059,0.40831,0.38825,
0.43217,0.40228,0.44261,0.44392,0.39683,0.35547,0.40537,0.21393,0.2191
5,0.21129,0.21575,0.21791,0.21628,0.2159,0.22137,0.21741,0.2146,0.2203
,0.16816,0.15186,0.15004,0.15442,0.14619,0.15145,0.21205,0.64264,0.555
12,0.68814,0.676,0.68225,0.62161,0.66203,0.4411,0.39181,0.39326,0.5580
9,0.39745,0.4122,0.4488,0.44706,0.46313,0.44611,0.48913,0.21875,0.2178
9,0.22285,0.21205,0.2192,0.219,0.20995,0.21486,0.22132,0.15742,0.17588
,0.15683,0.15926,0.1548,0.19068,0.15989,0.15509,0.16274,0.21634,0.6687
6,0.6312,0.56039,0.58019,0.74759,0.69323,0.61415,0.46146,0.39127,0.440
82,0.38309,0.37728,0.41014,0.36994,0.46339,0.39337,0.40231,0.4042,0.46
169,0.22075,0.21237,0.21073,0.20873,0.22064,0.22199,0.17865,0.1603,0.1
6657,0.15408,0.15343,0.15427,0.17045,0.16185,0.16871,0.15957,0.12118,0
.15417,0.5512,0.47951,0.65407,0.75397,0.66158,0.62101,0.65135,0.38314,
0.4411,0.49573,0.42042,0.58142,0.43315,0.4211,0.40556,0.42999,0.43081,
0.44053,0.4245,0.22203,0.21516,0.2181,0.14274,0.1516,0.14677,0.17301,0
.14458,0.15999,0.1431,0.15635,0.14189,0.16948,0.13934,0.15535,0.15589,
0.15589,0.15126,0.60994,0.60213,0.68719,0.62415,0.68883,0.6558,0.66708
,0.65123,0.70479,0.43072,0.43739,0.38362,0.40012,0.45516,0.48737,0.471
26,0.43867,0.41191,0.43283,0.49484,0.15785,0.15433,0.16768,0.15475,0.1
5885,0.15242,0.15696,0.1559,0.16616,0.14783,0.15408,0.15911,0.15093,0.
15087,0.166,0.17747,0.16014,0.16175,0.63795,0.58103,0.56971,0.58149,0.
62376,0.71235,0.62926,0.80624,0.62662,0.43704,0.43056,0.33978,0.2589,0
.32421,0.24704,0.32629,0.27253,0.35755,0.34053,0.30923,0.16539,0.15557
,0.16007,0.14541,0.13386,0.14855,0.17375,0.1375,0.15913,0.16481,0.1455
,0.15879,0.15548,0.16234,0.15569,0.15379,0.13865,0.16675,0.6598,0.5784
6,0.62002,0.66136,0.6258,0.71509,0.63782,0.64267,0.56785,0.36718,0.322
37,0.30307,0.32325,0.26286,0.29654,0.30131,0.33747,0.34164,0.33693,0.2
8642,0.16655,0.1432,0.15844,0.1593,0.15809,0.15598,0.16512,0.15601,0.1
4754,0.16058,0.15613,0.14951,0.16263,0.16181,0.16737,0.1502,0.16423,0.
15763,0.61069,0.65214,0.659,0.5967,0.67262,0.6107,0.60643,0.33761,0.36
944,0.34559,0.34208,0.28194,0.33263,0.31534,0.34446,0.33293,0.33355,0.
33415,0.30171,0.29648,0.16009,0.15614,0.16204,0.16252,0.16173,0.16612,
0.14898,0.16263,0.15578,0.15269,0.15823,0.15935,0.1613,0.14693,0.15274
,0.15891,0.15303,0.14895,0.65253,0.66,0.62645,0.6429,0.6879,0.57631,0.
35891,0.38643,0.33828,0.39835,0.40751,0.34675,0.3214,0.25736,0.28287,0
.32901,0.30916,0.34725,0.36797,0.27543,0.17363,0.14537,0.13922,0.15925
,0.16286,0.16414,0.16073,0.17425,0.16153,0.1612,0.16271,0.15681,0.1534

2,0.15691,0.15608,0.15785,0.15379,0.15147,0.60714,0.67509,0.7304,0.622
97,0.58213,0.35829,0.34201,0.38461,0.31988,0.32643,0.38682,0.30203,0.3
2158,0.33118,0.29822,0.30507,0.34984,0.28079,0.33476,0.30207,0.16244,0
.16704,0.16006,0.17685,0.14642,0.13577,0.15318,0.14447,0.17153,0.16322
,0.14518,0.14567,0.15464,0.16382,0.16621,0.16083,0.15754,0.15273,0.662
61,0.59467,0.56123,0.38794,0.28572,0.35976,0.35863,0.33059,0.3629,0.39
739,0.41426,0.29652,0.31526,0.28249,0.32774,0.32902,0.31715,0.32239,0.
33828,0.29481,0.14542,0.16235,0.15155,0.15757,0.15625,0.16827,0.1489,0
.16606,0.16629,0.15259,0.15794,0.15468,0.16164,0.14835,0.13932,0.15145
,0.15167,0.14916,0.62697,0.3744,0.41987,0.40805,0.41477,0.32659,0.3356
5,0.36482,0.44617,0.41302,0.31662,0.30618,0.27963,0.30904,0.27072,0.34
413,0.2576,0.28934,0.2952,0.15794,0.16008,0.16136,0.16114,0.15164,0.13
938,0.14734,0.15896,0.15074,0.1553,0.16716,0.16449,0.14763,0.15273,0.1
6578,0.15308,0.16011,0.089622,0.37996,0.39585,0.38175,0.36447,0.38742,
0.35864,0.35817,0.32921,0.31865,0.37434,0.31593,0.29294,0.35514,0.3236
,0.31306,0.28869,0.30314,0.30608,0.32874,0.16512,0.14921,0.14292,0.154
1,0.16837,0.1652,0.15489,0.15938,0.1593,0.15143,0.14056,0.1528,0.14666
,0.16023,0.1424,0.1395,0.15828,0.094178,0.34988,0.3341,0.39128,0.31926
,0.31249,0.35466,0.39474,0.43044,0.40312,0.3247,0.29215,0.32585,0.3094
6,0.30779,0.29369,0.32838,0.37601,0.33775,0.1579,0.13172,0.15499,0.154
9,0.16536,0.16231,0.14828,0.15776,0.15432,0.16167,0.14919,0.18123,0.16
953,0.16905,0.16046,0.17143,0.15753,0.15067,0.094648,0.45359,0.36644,0
.33367,0.3352,0.34564,0.2469,0.34909,0.33105,0.3195,0.37503,0.32519,0.
30433,0.35501,0.29658,0.31387,0.27282,0.27789,0.31973,0.15492,0.16002,
0.15864,0.14184,0.15416,0.14843,0.15723,0.15557,0.15361,0.15631,0.1648
,0.15999,0.15993,0.15771,0.15395,0.15846,0.14856,0.099016,0.097001,0.0
95475,0.37181,0.3785,0.44568,0.34197,0.41672,0.37284,0.38657,0.3319,0.
39085,0.30678,0.31309,0.30675,0.33149,0.36899,0.32111,0.1588,0.15367,0
.16487,0.15608,0.15273,0.14887,0.1537,0.15043,0.17561,0.15681,0.15374,
0.15287,0.15529,0.17646,0.15187,0.15569,0.15595,0.1702,0.098958,0.0938
05,0.095737,0.096584,0.36547,0.32265,0.35704,0.35494,0.38043,0.38562,0
.38758,0.42644,0.33812,0.35728,0.3839,0.31457,0.15423,0.15103,0.15654,
0.15232,0.16375,0.1423,0.15009,0.15931,0.16192,0.15297,0.1523,0.16464,
0.15439,0.1767,0.15779,0.17571,0.15276,0.17004,0.14319,0.14623,0.09235
4,0.098818,0.10283,0.10175,0.097847,0.097606,0.42274,0.36703,0.49139,0
.2996,0.45772,0.34314,0.34822,0.38002,0.15796,0.14443,0.15925,0.14804,
0.1407,0.1561,0.15667,0.16707,0.16432,0.15505,0.17272,0.13666,0.17893,
0.15322,0.1625,0.14663,0.1536,0.16311,0.15079,0.15824,0.15639,0.16344,
0.15779,0.090266,0.093414,0.094969,0.0982,0.093346,0.096821,0.31075,0.
36315,0.33274,0.345,0.36824,0.10074,0.10675,0.1665,0.15643,0.16749,0.1
534,0.16683,0.16783,0.14951,0.14987,0.14312,0.16661,0.14836,0.16448,0.
16332,0.15964,0.13824,0.15661,0.16303,0.16108,0.15504,0.14735,0.16621,
0.10239,0.096999,0.095428,0.096499,0.090527,0.094071,0.1024,0.091494,0
.098054,0.094788,0.41636,0.3929,0.095521,0.093534,0.095372,0.09696,0.1
6294,0.15804,0.15213,0.16445,0.15691,0.15415,0.14043,0.16456,0.16656,0
.16067,0.17177,0.14778,0.16144,0.15883,0.16122,0.158,0.14829,0.15022,0
.14045,0.10067,0.10165,0.10343,0.10092,0.092,0.096973,0.096855,0.09636
1,0.097086,0.088554,0.098647,0.099074,0.090193,0.094633,0.098075,0.096
031,0.090866,0.16292,0.1565,0.1576,0.14732,0.15679,0.15112,0.15468,0.1
5697,0.15903,0.16546,0.16296,0.14983,0.16176,0.15841,0.15495,0.15963,0
.095889,0.093005,0.091458,0.092958,0.10136,0.094533,0.10051,0.10331,0.
095473,0.094837,0.093792,0.10031,0.098407,0.089272,0.095598,0.097235,0

.10093,0.099634,0.098127,0.16903,0.15294,0.17559,0.15561,0.15409,0.160
83,0.15992,0.15005,0.15111,0.14679,0.15702,0.16352,0.16106,0.099207,0.
094561,0.089301,0.091862,0.09674,0.090545,0.099651,0.098989,0.097376,0
.097753,0.10629,0.095815,0.097022,0.09546,0.098316,0.09718,0.10028,0.0
9669,0.093948,0.099535,0.084482,0.16105,0.17318,0.15516,0.14324,0.1536
2,0.1668,0.15987,0.14945,0.14323,0.093541,0.098538,0.099061,0.099844,0
.092783,0.10261,0.09464,0.10134,0.097763,0.095003,0.09883,0.099404,0.0
98271,0.1023,0.09572,0.096775,0.096519,0.095307,0.094482,0.09609,0.095
806,0.090445,0.10497,0.096514,0.094635,0.14586,0.1625,0.13996,0.17115,
0.09734,0.090551,0.096466,0.093001,0.10188,0.093021,0.097699,0.10255,0
.098869,0.094946,0.090494,0.096729,0.09262,0.096766,0.094508,0.094189,
0.094375,0.10127,0.10446,0.092737,0.093712,0.095128,0.095487,0.099788,
0.094586,0.095905,0.097231,0.090571,0.094274,0.095325,0.095468,0.09441
9,0.098023,0.09285,0.097145,0.10049,0.091735,0.095873,0.090402,0.10561
,0.098857,0.092804,0.099418,0.093469,0.099916,0.10037,0.10583,0.10329,
0.092788,0.098704,0.094076,0.092593,0.098269,0.095372,0.094929,0.09693
7,0.10013,0.094332,0.10069,0.09236,0.093535,0.10357,0.095596,0.097606,
0.092713,0.098753,0.094627,0.093774,0.098433,0.099774,0.088744,0.08652
,0.093668,0.094483,0.096768,0.10759,0.094121,0.097881,0.098115,0.09181
,0.091576,0.089888,0.10549,0.098953,0.09607,0.098065,0.10203,0.088832,
0.099612,0.10952,0.094644,0.08686,0.095155,0.087878,0.097347,0.10075,0
.089755,0.091423,0.090499,0.096419,0.10118,0.10118,0.090517,0.094946,0
.099349,0.093734,0.087694,0.095738,0.098673,0.093315,0.1016,0.096761,0
.091821,0.095767,0.094597,0.10002,0.092725,0.090946,0.098608,0.092097,
0.099231,0.093608,0.098777,0.095569,0.093098,0.0936,0.095624,0.096515,
0.097594,0.10631,0.094818,0.093672,0.094632,0.097966,0.093595,0.09433,
0.098032,0.097919,0.0907,0.096906,0.094247,0.092233,0.094673,0.094803,
0.094077,0.090942,0.095076,0.08665,0.096894,0.091167,0.10079,0.094181,
0.097763,0.098921,0.10612,0.096553,0.1062,0.095455,0.097563,0.095955,0
.096934,0.089437,0.09548,0.10467,0.094845,0.097853,0.094406,0.098412,0
.09406,0.099068,0.094515,0.099212,0.10305,0.099494,0.092837,0.098361,0
.095419,0.09979,0.10138,0.094856,0.094201,0.099532,0.095794,0.095742,0
.093629,0.093787,0.095429,0.093277,0.094898,0.09689,0.094235,0.094975,
0.096082,0.095576,0.099875
0.19344,0.20471,0.19204,0.19654,0.21028,0.20112,0.20165,0.19347,0.1947
1,0.19458,0.20057,0.21457,0.19724,0.20246,0.20214,0.2119,0.21145,0.202
83,0.1991,0.19456,0.37252,0.37825,0.38591,0.31692,0.20334,0.19603,0.19
727,0.2083,0.198,0.20208,0.19736,0.19504,0.20423,0.20676,0.19922,0.200
12,0.20775,0.204,0.20263,0.54343,0.40676,0.36557,0.39257,0.37908,0.342
4,0.34636,0.2015,0.21283,0.19678,0.19804,0.19627,0.19113,0.19218,0.199
09,0.20243,0.19091,0.21152,0.20439,0.20556,0.1987,0.2015,0.53349,0.597
57,0.36265,0.37869,0.3648,0.41778,0.36413,0.39267,0.39217,0.38208,0.19
899,0.19985,0.19886,0.20808,0.20821,0.20221,0.20628,0.20241,0.21263,0.
19569,0.20616,0.20506,0.19034,0.20688,0.20513,0.52418,0.55014,0.57823,
0.31571,0.39192,0.38302,0.3926,0.41205,0.33836,0.38956,0.36647,0.38356
,0.19284,0.20425,0.20262,0.20276,0.20319,0.20195,0.20219,0.1998,0.2027
3,0.20007,0.20537,0.20428,0.20275,0.19753,0.20434,0.2069,0.60006,0.509
16,0.46065,0.4347,0.38873,0.36724,0.34929,0.38294,0.36175,0.3557,0.358
06,0.4167,0.39686,0.38343,0.20075,0.19715,0.1984,0.20528,0.20215,0.197
47,0.20294,0.20049,0.18976,0.18817,0.20544,0.20297,0.19744,0.20428,0.2
0035,0.1913,0.5858,0.54996,0.66691,0.44025,0.60255,0.47732,0.35955,0.3
9627,0.43485,0.41897,0.33565,0.39441,0.41053,0.43419,0.40599,0.31093,0

.20392,0.20105,0.19534,0.2044,0.20149,0.2097,0.20067,0.19905,0.19943,0
.20645,0.21038,0.21081,0.19042,0.20352,0.2079,0.20044,0.1978,0.53821,0
.49195,0.45794,0.54808,0.60914,0.46742,0.40937,0.36912,0.35312,0.32851
,0.41353,0.33816,0.3071,0.38498,0.3499,0.39908,0.34783,0.20021,0.19824
,0.20111,0.20419,0.20828,0.20143,0.19886,0.19855,0.19568,0.19514,0.212
09,0.20566,0.20414,0.13876,0.13724,0.1581,0.20735,0.48779,0.49118,0.53
994,0.5463,0.49315,0.53706,0.55296,0.36156,0.41244,0.33431,0.31828,0.3
3356,0.34755,0.38053,0.38284,0.33225,0.30987,0.35912,0.19455,0.20157,0
.19595,0.19728,0.20701,0.19923,0.19848,0.20366,0.20476,0.19636,0.20548
,0.15562,0.13726,0.13355,0.13677,0.13119,0.13713,0.19754,0.56351,0.421
75,0.49319,0.57535,0.57903,0.51942,0.52802,0.3676,0.33261,0.32552,0.47
966,0.34373,0.35903,0.39295,0.34733,0.40137,0.39384,0.40646,0.20371,0.
20325,0.20492,0.19708,0.19981,0.20128,0.19681,0.1963,0.20371,0.14001,0
.15963,0.13898,0.14314,0.1408,0.17412,0.1441,0.13938,0.14841,0.20241,0
.58094,0.4882,0.48934,0.4693,0.61672,0.55937,0.51238,0.39937,0.33621,0
.3672,0.3222,0.32092,0.34861,0.32389,0.39714,0.35134,0.36238,0.34141,0
.39932,0.20777,0.19581,0.19589,0.19246,0.20406,0.20113,0.15917,0.14625
,0.15033,0.13809,0.13851,0.1372,0.15104,0.14733,0.15134,0.14452,0.1069
4,0.13778,0.43787,0.41408,0.49954,0.64143,0.56966,0.52979,0.56911,0.32
197,0.36702,0.43318,0.36232,0.51359,0.38474,0.38444,0.34632,0.36541,0.
35978,0.36975,0.35288,0.20861,0.20156,0.20097,0.12458,0.13938,0.13265,
0.15427,0.12921,0.14374,0.12758,0.13716,0.1287,0.15691,0.12552,0.13784
,0.14037,0.14089,0.13694,0.50562,0.46821,0.56762,0.54295,0.57888,0.527
91,0.5164,0.55527,0.56523,0.37698,0.3806,0.33658,0.34785,0.39558,0.428
28,0.39755,0.37312,0.33631,0.36271,0.41749,0.13852,0.13801,0.15473,0.1
4147,0.14308,0.13948,0.14009,0.13931,0.15321,0.13362,0.13731,0.14208,0
.13463,0.13812,0.14938,0.15937,0.14604,0.148,0.50949,0.49558,0.44639,0
.46593,0.52403,0.61471,0.51384,0.66302,0.52467,0.38889,0.36358,0.289,0
.22896,0.27974,0.21231,0.28905,0.2273,0.29307,0.28994,0.26165,0.14857,
0.14237,0.14204,0.12908,0.11957,0.13375,0.15396,0.1232,0.14509,0.14491
,0.13178,0.14581,0.13758,0.14756,0.135,0.13638,0.12491,0.1487,0.59622,
0.51948,0.50976,0.55785,0.53025,0.5848,0.53254,0.50086,0.45186,0.28888
,0.28122,0.2445,0.28228,0.22752,0.25386,0.25398,0.28923,0.28887,0.3078
4,0.23991,0.14712,0.12623,0.13963,0.14082,0.13952,0.13857,0.14978,0.13
96,0.13066,0.14693,0.14206,0.13247,0.14353,0.14733,0.1497,0.13331,0.14
772,0.14328,0.4839,0.54959,0.51255,0.50238,0.57812,0.4972,0.53301,0.29
442,0.31307,0.28251,0.31218,0.25,0.29759,0.26592,0.30169,0.28813,0.282
91,0.26428,0.26126,0.24225,0.14278,0.13995,0.14646,0.14516,0.14769,0.1
49,0.13174,0.14811,0.1404,0.13566,0.14128,0.14633,0.1443,0.13406,0.138
44,0.14101,0.1374,0.1342,0.52439,0.54797,0.54223,0.52439,0.59285,0.459
5,0.29316,0.35028,0.28511,0.36321,0.3379,0.28954,0.28148,0.22476,0.242
51,0.2795,0.25653,0.28836,0.31342,0.24282,0.16063,0.12762,0.12338,0.14
227,0.14781,0.14703,0.146,0.1567,0.14564,0.14584,0.14462,0.14028,0.138
14,0.13853,0.13675,0.14053,0.13917,0.13339,0.49135,0.55775,0.59506,0.5
0861,0.47673,0.31098,0.29702,0.33048,0.27126,0.24865,0.33646,0.25994,0
.26564,0.27823,0.25874,0.25402,0.28199,0.22459,0.28621,0.24758,0.15096
,0.15262,0.14033,0.15932,0.13079,0.12158,0.1397,0.12927,0.15769,0.1483
9,0.12837,0.13161,0.13866,0.14657,0.14596,0.14716,0.14316,0.13467,0.54
778,0.45076,0.46409,0.31955,0.2321,0.286,0.30938,0.2569,0.28463,0.3184
1,0.33738,0.26162,0.25491,0.24119,0.27367,0.27947,0.28069,0.27971,0.28
972,0.26339,0.13054,0.14476,0.13419,0.14095,0.14423,0.15293,0.13267,0.
14948,0.15107,0.13913,0.14001,0.13579,0.14588,0.13136,0.12139,0.13679,

0.13704,0.13478,0.50601,0.32384,0.34246,0.35276,0.36418,0.27949,0.2941
1,0.29939,0.37469,0.35454,0.25852,0.25705,0.24015,0.27042,0.23612,0.30
51,0.20306,0.24476,0.24629,0.14356,0.14598,0.14466,0.14134,0.1364,0.12
281,0.13322,0.14217,0.13219,0.14167,0.14917,0.14826,0.13192,0.13816,0.
15277,0.14062,0.14663,0.074003,0.32002,0.32509,0.32648,0.31552,0.32019
,0.30838,0.30872,0.26918,0.25778,0.33201,0.26808,0.26454,0.31304,0.264
69,0.25316,0.25346,0.24251,0.2751,0.28642,0.15071,0.13521,0.12814,0.14
012,0.15275,0.14771,0.14004,0.1447,0.14373,0.13369,0.12767,0.13733,0.1
3259,0.14766,0.12657,0.12519,0.14645,0.07828,0.27241,0.29007,0.32264,0
.2739,0.27176,0.28731,0.35947,0.36012,0.34409,0.26637,0.24647,0.28411,
0.26649,0.25318,0.2431,0.28778,0.32926,0.27473,0.14351,0.12026,0.14047
,0.139,0.1479,0.14378,0.12877,0.142,0.14011,0.14457,0.13529,0.16196,0.
15655,0.15475,0.14132,0.15801,0.13834,0.13469,0.07884,0.36475,0.31811,
0.25607,0.27092,0.29091,0.19989,0.28657,0.25829,0.26435,0.30283,0.2850
1,0.25378,0.30338,0.26477,0.25442,0.23361,0.2442,0.28341,0.13964,0.145
45,0.14037,0.1259,0.13844,0.13085,0.1409,0.14233,0.13905,0.1377,0.1499
7,0.14788,0.14409,0.14211,0.14136,0.14109,0.13169,0.083975,0.081964,0.
07577,0.31207,0.31669,0.38746,0.28554,0.36832,0.31644,0.31721,0.27189,
0.33691,0.26244,0.25869,0.24796,0.28396,0.31905,0.27301,0.14423,0.1390
3,0.15125,0.13908,0.13401,0.13362,0.13704,0.13971,0.16181,0.14277,0.13
619,0.13662,0.14041,0.16032,0.13879,0.14323,0.14583,0.15156,0.08502,0.
079448,0.079068,0.077028,0.32173,0.26428,0.29553,0.28348,0.29654,0.334
29,0.3132,0.36834,0.28057,0.31493,0.33346,0.2696,0.14047,0.13684,0.142
5,0.13407,0.14799,0.12435,0.13344,0.1402,0.14533,0.13402,0.13653,0.147
7,0.14148,0.15917,0.14079,0.16004,0.13482,0.1529,0.13011,0.1282,0.0787
59,0.079917,0.086628,0.083688,0.079483,0.080323,0.37379,0.30359,0.4172
2,0.22778,0.39417,0.31134,0.27189,0.31175,0.13842,0.12792,0.14686,0.13
069,0.12923,0.1375,0.14281,0.15456,0.14751,0.13962,0.16212,0.12026,0.1
6668,0.13773,0.14616,0.1315,0.14199,0.1473,0.13474,0.14334,0.13943,0.1
5156,0.14438,0.070395,0.077161,0.07966,0.079977,0.078813,0.082333,0.25
946,0.3053,0.27883,0.26706,0.3053,0.08499,0.090616,0.14896,0.14115,0.1
5377,0.13701,0.15005,0.14906,0.13286,0.13629,0.12742,0.14907,0.13381,0
.15139,0.15215,0.14519,0.12545,0.14146,0.1471,0.1434,0.14023,0.1321,0.
14869,0.085074,0.079591,0.082685,0.079684,0.073797,0.07741,0.087362,0.
079818,0.081284,0.078415,0.34294,0.33045,0.078852,0.079751,0.078412,0.
082551,0.14862,0.13992,0.13647,0.15096,0.13952,0.13901,0.12785,0.14892
,0.15031,0.14267,0.15308,0.13425,0.1447,0.14612,0.14161,0.13787,0.1366
6,0.13301,0.12472,0.082169,0.086303,0.086666,0.08353,0.074694,0.081939
,0.078062,0.079921,0.082307,0.070484,0.085513,0.081776,0.075169,0.0788
04,0.083113,0.077977,0.073049,0.14611,0.13711,0.14495,0.12945,0.13999,
0.13052,0.13805,0.13819,0.14064,0.14999,0.14684,0.1311,0.14728,0.13941
,0.13997,0.14088,0.077643,0.077298,0.073771,0.07673,0.083867,0.076932,
0.082027,0.088484,0.07772,0.076565,0.076691,0.085378,0.081758,0.072714
,0.079743,0.080168,0.084356,0.081947,0.081956,0.15229,0.13974,0.16092,
0.14379,0.13839,0.14757,0.14268,0.13625,0.13621,0.12647,0.14234,0.1484
5,0.14281,0.082524,0.077853,0.074016,0.078909,0.080297,0.078015,0.0831
17,0.085351,0.078457,0.086331,0.091532,0.083614,0.081373,0.081074,0.07
983,0.078835,0.084758,0.080402,0.075996,0.082107,0.07034,0.14861,0.154
94,0.13763,0.12923,0.14198,0.15482,0.14527,0.13437,0.12728,0.079377,0.
081066,0.081862,0.083608,0.076455,0.084175,0.079617,0.08591,0.081097,0
.079164,0.081853,0.082511,0.084222,0.083078,0.079631,0.078071,0.081363
,0.078401,0.078793,0.080062,0.08173,0.077415,0.088305,0.078941,0.07841

7,0.12954,0.14466,0.12387,0.15406,0.08093,0.075338,0.080979,0.075555,0
.086018,0.077564,0.081895,0.087936,0.08065,0.079326,0.074302,0.080642,
0.077405,0.079517,0.080221,0.07903,0.080727,0.085911,0.089154,0.078214
,0.078442,0.078776,0.078594,0.080922,0.080377,0.07885,0.082105,0.07513
8,0.077565,0.079402,0.078549,0.076925,0.0781,0.080381,0.080166,0.08571
8,0.075383,0.078628,0.074725,0.085977,0.081335,0.078556,0.082828,0.076
467,0.08603,0.084142,0.089535,0.087835,0.075439,0.08149,0.080235,0.075
873,0.081778,0.078468,0.080401,0.079926,0.08543,0.078041,0.083829,0.07
6822,0.077182,0.087022,0.076554,0.083605,0.074725,0.082279,0.075286,0.
076306,0.080107,0.081481,0.073188,0.07238,0.078125,0.081382,0.080199,0
.091384,0.077387,0.080263,0.081016,0.075807,0.074335,0.073251,0.086066
,0.080993,0.081388,0.078692,0.08626,0.074986,0.084928,0.092167,0.07897
8,0.068719,0.077934,0.071473,0.078521,0.082448,0.074085,0.077377,0.072
121,0.07868,0.084942,0.082905,0.072223,0.076694,0.079361,0.079186,0.07
3283,0.079007,0.080418,0.076599,0.085797,0.077444,0.076738,0.079232,0.
082209,0.085179,0.07517,0.074794,0.081447,0.076318,0.083612,0.07398,0.
084779,0.079838,0.078695,0.078425,0.076797,0.083841,0.081093,0.087768,
0.080672,0.0783,0.077879,0.077476,0.076255,0.076568,0.081929,0.082801,
0.075383,0.079053,0.075835,0.076482,0.081869,0.076049,0.079778,0.07620
6,0.079988,0.07103,0.080341,0.073846,0.085903,0.077285,0.080908,0.0823
8,0.090079,0.078182,0.087721,0.077208,0.082254,0.080044,0.080516,0.073
701,0.079827,0.086856,0.076872,0.084452,0.082373,0.083981,0.07876,0.08
5718,0.081756,0.08244,0.085242,0.079579,0.074258,0.083354,0.080791,0.0
83519,0.085117,0.076549,0.075419,0.08193,0.078932,0.080934,0.080421,0.
077227,0.077924,0.076811,0.077452,0.081339,0.077106,0.077633,0.077546,
0.080202,0.08399
0.1766,0.18788,0.17852,0.18138,0.19244,0.18507,0.18488,0.17292,0.17968
,0.17955,0.18018,0.19477,0.18455,0.18496,0.18343,0.19462,0.19445,0.188
87,0.18664,0.17911,0.32699,0.32823,0.34338,0.28213,0.18702,0.18174,0.1
8557,0.19258,0.18069,0.18494,0.17865,0.18596,0.18269,0.19034,0.18487,0
.18575,0.19221,0.19097,0.18677,0.4936,0.3516,0.34601,0.3464,0.34595,0.
29211,0.31154,0.18747,0.19606,0.18013,0.18136,0.18051,0.18114,0.17311,
0.17995,0.18629,0.18135,0.19903,0.18603,0.19106,0.18378,0.18235,0.4369
8,0.48164,0.29855,0.3494,0.28506,0.35116,0.3088,0.3297,0.3606,0.34343,
0.18348,0.18224,0.17776,0.18809,0.19025,0.18696,0.18791,0.18335,0.1971
3,0.17957,0.19292,0.191,0.17368,0.19068,0.19244,0.4592,0.47862,0.52457
,0.2738,0.36148,0.33415,0.34465,0.36542,0.28939,0.29983,0.31636,0.3544
3,0.17744,0.18744,0.19166,0.18316,0.1857,0.17939,0.18031,0.18024,0.187
08,0.18192,0.19076,0.18675,0.18929,0.18745,0.18889,0.18803,0.49963,0.4
4285,0.38219,0.35755,0.32461,0.29907,0.31645,0.32866,0.31557,0.31954,0
.32039,0.39787,0.35644,0.33879,0.18822,0.18154,0.18278,0.18767,0.18579
,0.17944,0.19093,0.18479,0.17624,0.17372,0.19137,0.18543,0.18269,0.185
33,0.1827,0.1781,0.50589,0.47981,0.58898,0.41118,0.57459,0.43559,0.317
82,0.34981,0.40001,0.37798,0.29509,0.36431,0.36798,0.40916,0.35935,0.2
713,0.19065,0.18589,0.18361,0.18893,0.18566,0.18903,0.18663,0.18587,0.
18662,0.18987,0.19339,0.19372,0.17424,0.1926,0.18927,0.18754,0.18347,0
.49223,0.44158,0.34503,0.47954,0.4922,0.41169,0.35989,0.32647,0.30971,
0.29675,0.38608,0.29499,0.27092,0.31175,0.32065,0.33924,0.3142,0.18349
,0.1798,0.18469,0.18825,0.18886,0.18374,0.18208,0.18677,0.17681,0.1785
,0.19255,0.1918,0.18777,0.12271,0.1216,0.14349,0.18886,0.40219,0.4465,
0.49664,0.48398,0.42628,0.45894,0.48766,0.32206,0.35903,0.27861,0.2967
4,0.30353,0.29279,0.35944,0.32727,0.31956,0.29074,0.32322,0.17968,0.18

032,0.17984,0.18601,0.19556,0.18448,0.18524,0.18739,0.18875,0.18413,0.
18598,0.14103,0.12939,0.12075,0.11849,0.11941,0.12291,0.1804,0.49299,0
.34703,0.45627,0.49945,0.4723,0.46743,0.49407,0.3278,0.29061,0.28784,0
.40861,0.29376,0.31242,0.36266,0.30589,0.35946,0.34715,0.36566,0.18799
,0.18738,0.19111,0.18584,0.18735,0.18793,0.18255,0.17911,0.19093,0.126
49,0.13982,0.12294,0.13064,0.12694,0.15966,0.12703,0.12828,0.13656,0.1
8521,0.53738,0.41703,0.43513,0.43785,0.5474,0.46744,0.43601,0.30799,0.
29714,0.33962,0.26295,0.2761,0.30967,0.29879,0.32843,0.29072,0.32162,0
.31425,0.3734,0.18902,0.1817,0.17867,0.17716,0.19448,0.18353,0.14226,0
.13208,0.13385,0.1244,0.1238,0.12362,0.13559,0.13244,0.13738,0.12899,0
.09588,0.12344,0.43939,0.33474,0.41462,0.56966,0.47758,0.45094,0.50725
,0.28854,0.34249,0.40309,0.31688,0.46497,0.35324,0.34068,0.28387,0.318
63,0.33021,0.33813,0.31406,0.1853,0.18468,0.18591,0.1103,0.12424,0.115
1,0.13954,0.11531,0.12967,0.1138,0.1205,0.1133,0.14556,0.11094,0.12648
,0.12482,0.12337,0.12164,0.4244,0.38712,0.51243,0.4333,0.49509,0.44755
,0.45546,0.48141,0.47171,0.33614,0.33121,0.30277,0.31459,0.34631,0.363
22,0.36019,0.30988,0.2849,0.31733,0.37215,0.12616,0.12546,0.14003,0.12
413,0.12797,0.12687,0.12855,0.12355,0.13903,0.11915,0.12117,0.12566,0.
1218,0.12134,0.13417,0.14668,0.13067,0.13373,0.43889,0.47429,0.39593,0
.47667,0.41726,0.5724,0.45168,0.60613,0.48563,0.34558,0.32742,0.24788,
0.20147,0.24096,0.17469,0.25546,0.21226,0.26985,0.24574,0.24819,0.1314
2,0.12707,0.12705,0.11366,0.10954,0.12109,0.13984,0.10911,0.13069,0.12
765,0.12284,0.12907,0.12406,0.13536,0.12113,0.12371,0.11063,0.13165,0.
50449,0.50707,0.42446,0.5223,0.45928,0.50939,0.46273,0.40034,0.3603,0.
24548,0.25998,0.22095,0.24613,0.1881,0.22471,0.23091,0.26412,0.2592,0.
27912,0.21467,0.1346,0.11614,0.12705,0.13046,0.12671,0.12305,0.13407,0
.12325,0.11357,0.13129,0.12858,0.11796,0.13094,0.13469,0.13906,0.12163
,0.13319,0.1271,0.42493,0.44302,0.45587,0.42649,0.55369,0.42349,0.4718
4,0.25938,0.28558,0.25259,0.26316,0.21529,0.25996,0.23456,0.24818,0.25
599,0.25779,0.22456,0.21725,0.21518,0.126,0.12796,0.13188,0.13165,0.13
402,0.13289,0.11896,0.13264,0.12546,0.12129,0.12832,0.13297,0.13065,0.
1213,0.12487,0.12634,0.12631,0.12425,0.47599,0.47011,0.45115,0.41595,0
.5178,0.40652,0.26539,0.28265,0.25703,0.3253,0.29717,0.25183,0.24912,0
.18899,0.21246,0.25191,0.22909,0.24188,0.27892,0.2127,0.1453,0.11405,0
.10816,0.12767,0.13132,0.1306,0.13286,0.14263,0.13081,0.12926,0.12793,
0.13124,0.12003,0.12774,0.12006,0.12993,0.12527,0.11864,0.41774,0.4919
1,0.52246,0.48456,0.36595,0.2563,0.26127,0.27873,0.21439,0.22861,0.304
58,0.22791,0.2215,0.2537,0.23576,0.22401,0.23902,0.20795,0.25345,0.205
47,0.13569,0.13785,0.1258,0.14887,0.11695,0.10286,0.12296,0.1155,0.138
88,0.13683,0.11407,0.11719,0.12411,0.13075,0.13457,0.13184,0.12945,0.1
2396,0.48551,0.43095,0.41103,0.28322,0.20939,0.24715,0.2833,0.2279,0.2
6304,0.26995,0.31433,0.23985,0.2316,0.20928,0.25221,0.24003,0.26841,0.
24796,0.25148,0.23404,0.12038,0.12988,0.12182,0.12588,0.13284,0.13732,
0.12017,0.13521,0.13776,0.1265,0.12529,0.1192,0.133,0.12135,0.10545,0.
12376,0.1227,0.12118,0.45613,0.25511,0.30368,0.30856,0.35143,0.24056,0
.21164,0.2632,0.34515,0.3067,0.22058,0.23868,0.22686,0.23411,0.20581,0
.26756,0.17785,0.208,0.22047,0.13082,0.13276,0.12773,0.13002,0.12063,0
.10792,0.11907,0.12746,0.11806,0.12653,0.1349,0.13715,0.1196,0.12121,0
.13702,0.12519,0.13178,0.066821,0.27702,0.29884,0.30398,0.2796,0.28962
,0.2465,0.26614,0.24406,0.2263,0.27127,0.25987,0.23826,0.2764,0.23162,
0.22196,0.21566,0.22372,0.23351,0.25578,0.13514,0.12051,0.11431,0.1229
2,0.13959,0.13454,0.12478,0.12995,0.12853,0.12268,0.11542,0.12786,0.11

706,0.13306,0.11547,0.11212,0.13382,0.066066,0.23738,0.262,0.29444,0.2
1801,0.25796,0.2387,0.31859,0.32053,0.26878,0.21124,0.21984,0.2565,0.2
342,0.22118,0.21477,0.25544,0.30082,0.24601,0.1276,0.10445,0.1253,0.12
657,0.13187,0.12849,0.11116,0.12587,0.12717,0.12782,0.11969,0.14564,0.
14186,0.14151,0.12607,0.14113,0.12274,0.12122,0.066733,0.30411,0.28217
,0.20095,0.2437,0.26404,0.17346,0.26653,0.21006,0.20077,0.25928,0.2575
,0.22504,0.25892,0.22698,0.22768,0.21001,0.21361,0.24869,0.12592,0.134
19,0.12559,0.11212,0.1227,0.12039,0.12631,0.13015,0.12803,0.12247,0.13
731,0.13251,0.12929,0.12701,0.1257,0.12802,0.11957,0.072632,0.071605,0
.064939,0.28017,0.28722,0.33945,0.24357,0.31418,0.30242,0.26314,0.2543
3,0.29431,0.23523,0.22863,0.21766,0.25127,0.28347,0.23701,0.12834,0.12
496,0.13529,0.12756,0.11728,0.11818,0.12314,0.12233,0.14513,0.12977,0.
12393,0.12363,0.12771,0.14582,0.12399,0.13071,0.13023,0.14022,0.072999
,0.068047,0.068082,0.060836,0.28457,0.24041,0.24381,0.25272,0.27734,0.
28279,0.28352,0.31989,0.25674,0.2934,0.29219,0.2576,0.12395,0.12614,0.
13089,0.11609,0.13696,0.1125,0.12366,0.12714,0.13193,0.12083,0.123,0.1
3349,0.12765,0.14548,0.12865,0.14926,0.11761,0.13859,0.1146,0.11722,0.
063937,0.069191,0.074069,0.071853,0.067523,0.069193,0.33389,0.27624,0.
36783,0.18478,0.3487,0.27912,0.23,0.29662,0.12259,0.11425,0.13263,0.11
547,0.11648,0.12259,0.13178,0.14011,0.13829,0.12291,0.14622,0.1079,0.1
4931,0.12654,0.13361,0.11693,0.12775,0.1314,0.12026,0.12856,0.12439,0.
13807,0.12979,0.0588,0.064644,0.068198,0.070194,0.064778,0.070906,0.20
585,0.28182,0.23318,0.22146,0.26165,0.070259,0.080107,0.1347,0.13113,0
.13773,0.1214,0.13371,0.1335,0.11807,0.12057,0.11572,0.13465,0.11738,0
.13524,0.13552,0.12995,0.11666,0.12708,0.13233,0.12944,0.12535,0.11893
,0.13396,0.067807,0.066388,0.072827,0.068315,0.06039,0.066039,0.075688
,0.063951,0.06799,0.065329,0.30267,0.28109,0.065558,0.067952,0.066629,
0.06659,0.13467,0.12382,0.12231,0.13611,0.12857,0.12659,0.11441,0.1380
2,0.13437,0.12705,0.139,0.12398,0.13012,0.12948,0.13154,0.12399,0.1238
3,0.1192,0.11299,0.071421,0.075858,0.074844,0.07152,0.066558,0.068086,
0.066118,0.067597,0.068009,0.060728,0.071701,0.070533,0.064411,0.06641
2,0.072247,0.066135,0.061716,0.13297,0.1223,0.13032,0.11733,0.12731,0.
11704,0.12234,0.123,0.12409,0.13918,0.12984,0.11822,0.13287,0.12636,0.
12841,0.1254,0.06663,0.062711,0.062771,0.064831,0.071612,0.065979,0.06
6366,0.076532,0.067195,0.062811,0.065949,0.073944,0.072139,0.061551,0.
066271,0.066964,0.071056,0.069761,0.070088,0.13527,0.12514,0.14671,0.1
3082,0.12689,0.13053,0.13016,0.12172,0.12258,0.11613,0.12632,0.13597,0
.12511,0.067226,0.063997,0.06231,0.069719,0.065791,0.066436,0.073484,0
.071767,0.06905,0.073324,0.078012,0.073504,0.070038,0.069396,0.064325,
0.067263,0.069935,0.070121,0.063452,0.068113,0.058143,0.12939,0.13922,
0.12561,0.11462,0.12906,0.14079,0.13138,0.12291,0.11447,0.065997,0.068
622,0.069558,0.074047,0.066299,0.072962,0.067492,0.074654,0.066328,0.0
65266,0.072263,0.069499,0.0702,0.070699,0.068399,0.065384,0.066727,0.0
68622,0.067374,0.066517,0.068342,0.064133,0.074829,0.068826,0.06594,0.
11725,0.13181,0.11319,0.14117,0.069572,0.06109,0.070472,0.064408,0.071
811,0.064257,0.070189,0.075567,0.069741,0.066078,0.061792,0.06872,0.06
631,0.067936,0.067208,0.070078,0.06791,0.072958,0.07718,0.067013,0.066
8,0.06768,0.065507,0.06674,0.066865,0.067098,0.067397,0.064884,0.06309
5,0.065062,0.065747,0.066229,0.06531,0.067177,0.070564,0.070023,0.0643
87,0.064012,0.066669,0.07181,0.068146,0.06535,0.068414,0.066219,0.0710
78,0.071795,0.079942,0.074317,0.06308,0.069053,0.06638,0.062395,0.0715
54,0.065396,0.068866,0.067527,0.071935,0.063775,0.06953,0.063837,0.065

104,0.075445,0.064022,0.072047,0.062903,0.070063,0.0645,0.063391,0.068
355,0.068281,0.058908,0.059617,0.066346,0.072117,0.066849,0.07768,0.06
5792,0.068635,0.069718,0.064654,0.061081,0.061206,0.074789,0.068983,0.
073603,0.06795,0.071947,0.060718,0.072253,0.077141,0.066977,0.057426,0
.062945,0.062139,0.065913,0.073985,0.064327,0.064778,0.059204,0.063199
,0.074875,0.070859,0.058656,0.066142,0.069648,0.067472,0.059843,0.0679
55,0.067787,0.066513,0.069358,0.066661,0.063383,0.066549,0.069151,0.07
1495,0.061371,0.064093,0.071114,0.066674,0.071011,0.063053,0.07474,0.0
69869,0.069062,0.066964,0.062658,0.072742,0.069683,0.076262,0.067238,0
.065778,0.066554,0.064877,0.065301,0.066016,0.068307,0.068807,0.066008
,0.066545,0.063045,0.062611,0.070424,0.062839,0.068881,0.062954,0.0710
38,0.060441,0.068165,0.061773,0.069826,0.066263,0.068645,0.071936,0.07
5679,0.067176,0.072212,0.066275,0.069295,0.067079,0.06843,0.059321,0.0
67222,0.074355,0.066809,0.070883,0.069489,0.071572,0.066444,0.072625,0
.06838,0.07135,0.071706,0.064888,0.064369,0.068636,0.066864,0.068313,0
.071458,0.066625,0.063394,0.071208,0.070234,0.069543,0.070685,0.063882
,0.066715,0.063883,0.065582,0.068578,0.066575,0.062833,0.06457,0.06846
3,0.0711
0.16314,0.17074,0.1652,0.1645,0.18221,0.17201,0.17014,0.15868,0.16577,
0.16452,0.16644,0.18094,0.16827,0.17219,0.16522,0.17691,0.17204,0.1775
5,0.17485,0.16079,0.29685,0.30332,0.34657,0.27332,0.17308,0.1696,0.169
58,0.17407,0.1703,0.17349,0.16419,0.17154,0.17293,0.17045,0.1709,0.172
83,0.17614,0.17511,0.17088,0.46871,0.33786,0.32598,0.32351,0.33243,0.2
9607,0.30971,0.16981,0.18085,0.1612,0.16389,0.16513,0.16354,0.15561,0.
16838,0.17293,0.16652,0.18208,0.17202,0.17422,0.16873,0.16554,0.46458,
0.44964,0.28193,0.30194,0.27017,0.33036,0.28759,0.31297,0.31986,0.3396
1,0.16943,0.1679,0.15958,0.17424,0.1773,0.16933,0.17479,0.16918,0.1817
7,0.16667,0.17646,0.17779,0.16185,0.17704,0.17981,0.43551,0.4208,0.461
32,0.26025,0.35314,0.32286,0.31089,0.32964,0.26194,0.2799,0.28846,0.31
059,0.1634,0.17133,0.17939,0.16678,0.1705,0.16634,0.16699,0.16831,0.17
503,0.1671,0.1796,0.1737,0.17369,0.17031,0.17227,0.17099,0.4374,0.3998
2,0.34828,0.34881,0.30727,0.25623,0.31078,0.28015,0.29528,0.30056,0.31
2,0.36568,0.31791,0.32176,0.17266,0.16436,0.17034,0.17152,0.17175,0.17
056,0.17564,0.17418,0.16623,0.15788,0.17399,0.1681,0.1683,0.16674,0.16
547,0.16567,0.4531,0.40292,0.48737,0.37821,0.51783,0.38873,0.30334,0.3
3613,0.37981,0.34537,0.28762,0.33703,0.3283,0.39259,0.34679,0.26123,0.
18073,0.17545,0.16951,0.17524,0.16726,0.17525,0.17232,0.16572,0.17567,
0.17203,0.17511,0.17475,0.15896,0.17983,0.17113,0.17408,0.16703,0.4477
,0.39815,0.2824,0.42194,0.46633,0.36073,0.32413,0.29352,0.27042,0.2680
8,0.37447,0.27248,0.24076,0.26941,0.29651,0.30892,0.27314,0.16616,0.16
592,0.16294,0.17293,0.17362,0.16901,0.16464,0.17154,0.16141,0.16612,0.
1764,0.17581,0.17059,0.11304,0.11405,0.13213,0.16798,0.36094,0.41914,0
.47057,0.43242,0.39225,0.39874,0.47513,0.28157,0.32593,0.2236,0.27068,
0.27831,0.25374,0.34644,0.31368,0.30222,0.26448,0.2886,0.1663,0.17154,
0.1667,0.1686,0.18074,0.16911,0.16899,0.17089,0.17274,0.16923,0.17188,
0.12881,0.11885,0.1091,0.10966,0.10366,0.11046,0.16756,0.45799,0.3033,
0.42634,0.43503,0.41751,0.40722,0.44914,0.31636,0.2698,0.2683,0.35429,
0.26811,0.29036,0.33875,0.30244,0.34477,0.31457,0.34627,0.17566,0.1718
5,0.17824,0.17315,0.17398,0.17404,0.16639,0.16215,0.17298,0.11306,0.12
532,0.11274,0.11889,0.11779,0.14909,0.11355,0.11685,0.12212,0.1737,0.4
9037,0.33181,0.37006,0.39166,0.53329,0.4588,0.39692,0.26637,0.26494,0.
31419,0.23854,0.24427,0.26892,0.25073,0.32178,0.26232,0.27148,0.27862,

0.34439,0.17564,0.16782,0.16241,0.16739,0.18016,0.1687,0.13312,0.12051
,0.1201,0.11164,0.11097,0.11259,0.12259,0.11827,0.12596,0.11954,0.0868
15,0.11129,0.42235,0.31523,0.4249,0.49865,0.41716,0.43629,0.50297,0.28
207,0.32608,0.35033,0.2993,0.42717,0.31189,0.30817,0.25626,0.29799,0.2
7196,0.32176,0.29731,0.17188,0.17215,0.17112,0.097996,0.11288,0.10528,
0.1215,0.10326,0.12011,0.10396,0.11016,0.10813,0.13434,0.10181,0.1139,
0.11354,0.10928,0.11175,0.42504,0.38031,0.46983,0.38625,0.46113,0.4179
5,0.42796,0.45592,0.45629,0.31022,0.31488,0.25054,0.29604,0.34404,0.34
037,0.33781,0.27565,0.26543,0.30347,0.34574,0.11807,0.1124,0.12743,0.1
1335,0.11296,0.11706,0.11769,0.11263,0.12464,0.11232,0.10942,0.11064,0
.11008,0.1087,0.12132,0.13031,0.11614,0.12115,0.39107,0.43147,0.35695,
0.42858,0.3757,0.52836,0.42988,0.55077,0.46429,0.31476,0.28039,0.24877
,0.18513,0.22648,0.16045,0.24213,0.17774,0.23886,0.2338,0.22119,0.1180
7,0.11484,0.1139,0.10015,0.098168,0.10967,0.12548,0.097947,0.11571,0.1
1773,0.10917,0.11733,0.11578,0.1199,0.10931,0.11005,0.10195,0.12006,0.
40033,0.46783,0.40394,0.44365,0.46677,0.44454,0.42221,0.38243,0.39066,
0.21701,0.24466,0.19085,0.22466,0.15415,0.21503,0.21679,0.24325,0.2204
1,0.26302,0.20645,0.12361,0.10632,0.11488,0.1189,0.11492,0.11083,0.122
15,0.10622,0.10165,0.11567,0.11354,0.10773,0.11743,0.12238,0.127,0.109
82,0.1179,0.11476,0.40329,0.37225,0.36524,0.45153,0.53627,0.38388,0.40
01,0.24973,0.24454,0.23302,0.2177,0.2024,0.25095,0.22066,0.24017,0.238
18,0.23706,0.19465,0.20844,0.18965,0.11593,0.11646,0.11831,0.11792,0.1
1825,0.11763,0.10832,0.11997,0.11431,0.10915,0.11329,0.12017,0.12069,0
.10652,0.11506,0.11655,0.10983,0.11339,0.41978,0.4454,0.4052,0.37184,0
.45324,0.37595,0.23253,0.25079,0.21854,0.33959,0.2632,0.23907,0.22407,
0.16445,0.20184,0.2126,0.19841,0.21974,0.26848,0.20016,0.13155,0.10321
,0.095874,0.11394,0.1206,0.11726,0.11965,0.12993,0.11924,0.11906,0.113
5,0.11718,0.10901,0.11626,0.10663,0.11877,0.11429,0.10307,0.36579,0.44
813,0.44663,0.3999,0.30099,0.24108,0.23869,0.2383,0.18127,0.20297,0.25
708,0.20284,0.20065,0.23423,0.21266,0.19656,0.21218,0.19945,0.23611,0.
16613,0.11976,0.12478,0.11229,0.13403,0.10877,0.093548,0.11024,0.10534
,0.12539,0.12519,0.10433,0.10575,0.11446,0.1176,0.1243,0.12009,0.11484
,0.11445,0.46257,0.38053,0.38159,0.2546,0.17156,0.22878,0.25701,0.2143
2,0.21894,0.25237,0.2989,0.2157,0.21278,0.18986,0.22701,0.21931,0.2429
4,0.22413,0.22478,0.22449,0.10429,0.11807,0.10945,0.11542,0.11889,0.12
697,0.11132,0.12113,0.1266,0.11336,0.11445,0.10624,0.12087,0.10802,0.0
9585,0.11021,0.11188,0.10819,0.39755,0.21519,0.26542,0.28486,0.31902,0
.22611,0.20991,0.21646,0.3328,0.26732,0.20535,0.21188,0.21064,0.21141,
0.19422,0.23083,0.1516,0.17765,0.19603,0.11782,0.12316,0.11862,0.11484
,0.11104,0.09663,0.11168,0.11417,0.10464,0.11973,0.12038,0.12251,0.106
5,0.10951,0.1261,0.11197,0.1189,0.058761,0.24664,0.28425,0.28846,0.250
03,0.23709,0.23527,0.23239,0.21188,0.21063,0.28104,0.25126,0.22051,0.2
4569,0.20065,0.20465,0.19548,0.20498,0.20684,0.24097,0.12394,0.10868,0
.10182,0.11108,0.12804,0.12124,0.11264,0.11683,0.11761,0.11029,0.10512
,0.11197,0.10892,0.12019,0.10345,0.099011,0.1228,0.055139,0.2211,0.237
59,0.24838,0.22168,0.21423,0.22324,0.29796,0.28153,0.29145,0.21242,0.1
9527,0.23506,0.20598,0.19851,0.19976,0.22765,0.27174,0.22169,0.11643,0
.092279,0.11462,0.11247,0.11765,0.11919,0.096614,0.1112,0.11498,0.1113
4,0.10633,0.13155,0.12883,0.12858,0.11445,0.12834,0.11083,0.11019,0.05
9386,0.31013,0.24258,0.18072,0.22178,0.23443,0.16141,0.23355,0.19089,0
.18274,0.26236,0.22167,0.20322,0.22551,0.20394,0.20825,0.19259,0.19049
,0.23606,0.11356,0.12046,0.11298,0.099826,0.10834,0.10565,0.11669,0.11

977,0.1139,0.11435,0.12279,0.12017,0.1146,0.11466,0.11527,0.11813,0.10
965,0.062218,0.064536,0.052813,0.27195,0.25954,0.31908,0.23233,0.29291
,0.26494,0.22917,0.22909,0.26949,0.20505,0.21604,0.21625,0.22663,0.271
63,0.22388,0.11607,0.11391,0.12462,0.1123,0.1069,0.10961,0.11761,0.108
98,0.13347,0.1151,0.11108,0.11317,0.11076,0.1315,0.11364,0.11454,0.117
87,0.13057,0.064425,0.061148,0.059585,0.050992,0.2767,0.23293,0.21677,
0.21388,0.26342,0.25276,0.25955,0.28728,0.21652,0.25962,0.26363,0.2292
3,0.10813,0.11293,0.11868,0.10775,0.12579,0.098007,0.11102,0.11761,0.1
2024,0.11207,0.11307,0.12035,0.11711,0.1292,0.11496,0.13729,0.10825,0.
12548,0.10137,0.10642,0.055284,0.062549,0.065156,0.062096,0.057883,0.0
60895,0.30866,0.25529,0.34367,0.16766,0.34416,0.25104,0.22519,0.27083,
0.11198,0.10346,0.11947,0.10281,0.10309,0.11278,0.1201,0.12302,0.1273,
0.10835,0.13649,0.096196,0.1352,0.11455,0.11757,0.10728,0.11809,0.1207
1,0.10962,0.12011,0.11495,0.12744,0.11785,0.050601,0.054563,0.057418,0
.062044,0.058402,0.061669,0.18647,0.27398,0.202,0.21262,0.2411,0.06179
3,0.072416,0.12436,0.11936,0.1222,0.11102,0.12362,0.12437,0.10401,0.10
954,0.10562,0.1247,0.10505,0.12579,0.12208,0.11709,0.10659,0.11405,0.1
2033,0.12221,0.11077,0.10629,0.11852,0.058581,0.057223,0.065431,0.0601
13,0.05376,0.056652,0.064311,0.05641,0.060216,0.059118,0.27196,0.28281
,0.059638,0.056769,0.05773,0.055846,0.12092,0.11058,0.11164,0.12316,0.
1184,0.11535,0.10207,0.12614,0.1202,0.1153,0.12575,0.11277,0.1151,0.11
794,0.11772,0.11387,0.1119,0.10916,0.10603,0.061168,0.068531,0.067516,
0.064715,0.056985,0.058707,0.059632,0.055547,0.059965,0.051984,0.06286
,0.060261,0.055974,0.054524,0.065274,0.059054,0.053084,0.11963,0.10746
,0.11852,0.10545,0.11309,0.10564,0.10763,0.11108,0.11345,0.13015,0.118
33,0.10996,0.12166,0.1133,0.117,0.11478,0.059948,0.053578,0.052932,0.0
56939,0.061864,0.055969,0.060056,0.067405,0.058519,0.053591,0.053741,0
.064111,0.061819,0.052899,0.060272,0.05828,0.065413,0.063556,0.062346,
0.12314,0.11479,0.13233,0.11797,0.11382,0.11571,0.11562,0.10912,0.1112
5,0.10663,0.11486,0.12464,0.11397,0.057512,0.051514,0.058808,0.061382,
0.061544,0.057714,0.064631,0.063631,0.061562,0.064155,0.065555,0.06464
2,0.062447,0.059243,0.058705,0.057384,0.065496,0.062427,0.052999,0.059
777,0.052679,0.11453,0.13002,0.11245,0.10385,0.11795,0.12624,0.12236,0
.11109,0.10146,0.054438,0.061207,0.061174,0.061483,0.057041,0.061978,0
.060502,0.063402,0.055847,0.056641,0.063561,0.059867,0.062348,0.060504
,0.061187,0.057288,0.057292,0.058804,0.061394,0.057662,0.060864,0.0542
2,0.065162,0.058451,0.058791,0.1108,0.12016,0.10082,0.12414,0.059684,0
.051407,0.060684,0.058872,0.059989,0.055413,0.060554,0.064915,0.062092
,0.056698,0.050808,0.058964,0.055076,0.060192,0.057833,0.060962,0.0579
14,0.060199,0.066511,0.059634,0.060495,0.057819,0.058053,0.060055,0.05
6567,0.060394,0.057428,0.053016,0.055318,0.055217,0.057102,0.05615,0.0
59575,0.059359,0.060977,0.063234,0.054675,0.054247,0.058592,0.061748,0
.062261,0.053653,0.059596,0.057435,0.062775,0.064017,0.072861,0.064291
,0.055572,0.060317,0.058116,0.052722,0.064184,0.05698,0.059506,0.05781
3,0.065339,0.053707,0.059184,0.057159,0.059378,0.065998,0.057054,0.064
239,0.054389,0.059796,0.052586,0.055902,0.059607,0.057931,0.05169,0.05
5067,0.055257,0.062525,0.055298,0.067358,0.053992,0.05882,0.060648,0.0
52415,0.054193,0.051327,0.066007,0.058528,0.065536,0.05687,0.062925,0.
049481,0.066382,0.068785,0.055987,0.050422,0.05467,0.052601,0.059508,0
.063934,0.060096,0.056671,0.049534,0.053956,0.065203,0.063456,0.051594
,0.057158,0.062276,0.057946,0.049516,0.062653,0.056628,0.059554,0.0595
3,0.05632,0.059365,0.058027,0.058892,0.063048,0.050916,0.056903,0.0642

44,0.058498,0.062556,0.054035,0.064566,0.061973,0.06037,0.056368,0.055
231,0.063306,0.058794,0.066425,0.060206,0.056343,0.058978,0.056613,0.0
55425,0.058464,0.059139,0.061823,0.060515,0.057125,0.054611,0.055076,0
.061022,0.055749,0.059627,0.055277,0.05878,0.049908,0.0582,0.053916,0.
060233,0.057601,0.058158,0.062169,0.066659,0.055922,0.063675,0.058572,
0.057248,0.059328,0.05994,0.047486,0.059751,0.065991,0.059672,0.063917
,0.062997,0.063568,0.056796,0.062056,0.059261,0.06069,0.062183,0.05643
4,0.054377,0.059238,0.057366,0.05804,0.061516,0.058591,0.055026,0.0611
69,0.058216,0.059812,0.062002,0.05587,0.057569,0.056861,0.054692,0.060
104,0.053604,0.05612,0.057183,0.058943,0.065311
0.14912,0.15656,0.15335,0.14766,0.16976,0.15892,0.15769,0.14994,0.1546
7,0.15305,0.15241,0.1706,0.15662,0.16042,0.15218,0.16248,0.16227,0.167
55,0.15957,0.15188,0.29791,0.27958,0.29484,0.24412,0.1574,0.15395,0.15
441,0.15864,0.15302,0.16196,0.15246,0.15711,0.16134,0.15863,0.15637,0.
16147,0.16649,0.16301,0.15731,0.41848,0.31618,0.29897,0.30805,0.30934,
0.27499,0.29103,0.15483,0.16275,0.15543,0.14837,0.15233,0.14983,0.1474
5,0.15635,0.15581,0.15345,0.169,0.158,0.16288,0.15169,0.15667,0.46817,
0.37087,0.26656,0.28749,0.26201,0.32808,0.26651,0.26561,0.32057,0.3225
9,0.15229,0.15446,0.1485,0.16157,0.16366,0.15876,0.16219,0.15509,0.168
32,0.15496,0.16508,0.16079,0.1534,0.15836,0.16906,0.45223,0.37248,0.43
352,0.25017,0.31875,0.293,0.32422,0.27761,0.241,0.24791,0.28027,0.2875
6,0.1511,0.16216,0.16043,0.15739,0.15457,0.15526,0.15812,0.15432,0.161
23,0.15302,0.16669,0.15647,0.1626,0.15901,0.16169,0.15652,0.41008,0.31
518,0.3302,0.29855,0.28854,0.24576,0.2846,0.29146,0.27822,0.30167,0.28
38,0.33341,0.30682,0.29831,0.15875,0.15389,0.15995,0.15506,0.16006,0.1
5856,0.15961,0.15739,0.15108,0.14973,0.16155,0.1574,0.15801,0.15405,0.
15672,0.15124,0.40312,0.41806,0.45691,0.37431,0.45839,0.37662,0.27527,
0.34057,0.34629,0.32963,0.29064,0.32565,0.295,0.38801,0.30272,0.24807,
0.16719,0.1634,0.15672,0.16008,0.14894,0.16263,0.15921,0.15286,0.1587,
0.15904,0.16105,0.16408,0.14801,0.16629,0.15893,0.16293,0.15268,0.4286
2,0.37786,0.3146,0.37012,0.45134,0.35314,0.29048,0.26104,0.25506,0.257
89,0.34956,0.26138,0.25646,0.26756,0.27753,0.29204,0.2523,0.15743,0.15
817,0.15204,0.15976,0.16305,0.15189,0.14962,0.16265,0.14941,0.15249,0.
16364,0.16346,0.15908,0.10355,0.1052,0.12194,0.15381,0.34841,0.39884,0
.47144,0.44534,0.34859,0.37868,0.43527,0.26767,0.32015,0.2255,0.25825,
0.25613,0.24269,0.34021,0.28905,0.27746,0.24952,0.27698,0.15157,0.1611
8,0.15076,0.15701,0.16752,0.15407,0.15367,0.1555,0.15861,0.15555,0.161
06,0.11452,0.10963,0.098022,0.10323,0.097386,0.10366,0.15598,0.4084,0.
3363,0.41357,0.39179,0.3978,0.38548,0.46349,0.29164,0.26044,0.24117,0.
33311,0.26103,0.27465,0.34411,0.28256,0.32015,0.29064,0.29718,0.16592,
0.15574,0.16377,0.15852,0.15921,0.16046,0.15215,0.14916,0.15881,0.1033
5,0.11964,0.10418,0.11161,0.10817,0.13653,0.10213,0.10608,0.11044,0.16
27,0.47176,0.35718,0.34963,0.3698,0.50675,0.42984,0.33952,0.24068,0.23
372,0.30859,0.22243,0.23018,0.26918,0.23445,0.29722,0.24223,0.27288,0.
24308,0.32264,0.16153,0.15234,0.15082,0.15629,0.16671,0.15722,0.12412,
0.10815,0.10758,0.10173,0.10413,0.10198,0.1138,0.10682,0.11379,0.10995
,0.080526,0.098415,0.35487,0.3688,0.41881,0.48283,0.36043,0.40053,0.49
596,0.24882,0.31381,0.32995,0.26675,0.41441,0.27747,0.29369,0.24067,0.
28563,0.24409,0.31445,0.28363,0.15967,0.16139,0.15351,0.089529,0.10032
,0.094406,0.1111,0.095861,0.10817,0.093312,0.10332,0.099245,0.1246,0.0
97451,0.10005,0.10545,0.10177,0.10332,0.36789,0.33442,0.47632,0.36299,
0.44795,0.40106,0.41263,0.42003,0.44564,0.27927,0.29049,0.2521,0.30068

,0.32424,0.32731,0.31317,0.27859,0.25553,0.29552,0.29431,0.10418,0.103
84,0.11753,0.10426,0.10039,0.10657,0.10703,0.1077,0.11744,0.10365,0.09
9851,0.10226,0.10213,0.099259,0.11099,0.11934,0.10396,0.11095,0.35979,
0.41627,0.32949,0.39044,0.34764,0.50369,0.39857,0.51448,0.44132,0.3025
8,0.28895,0.23428,0.17419,0.228,0.14475,0.21715,0.16729,0.22804,0.217,
0.21218,0.11107,0.10684,0.1068,0.092392,0.087326,0.10016,0.11623,0.089
48,0.10499,0.11148,0.099697,0.11015,0.10414,0.10839,0.1021,0.10096,0.0
89994,0.10837,0.38858,0.42896,0.35355,0.4433,0.42827,0.40527,0.39082,0
.32687,0.3899,0.19078,0.2327,0.17275,0.2098,0.14678,0.18418,0.20777,0.
22754,0.19258,0.23881,0.17984,0.11315,0.096049,0.10717,0.10788,0.10741
,0.10059,0.10854,0.09822,0.093124,0.10723,0.10283,0.10017,0.10798,0.11
118,0.11676,0.09942,0.10773,0.10124,0.36799,0.37092,0.36009,0.41482,0.
52218,0.33364,0.36522,0.23244,0.2287,0.23956,0.19719,0.18067,0.21532,0
.22166,0.21416,0.21806,0.21571,0.16048,0.18889,0.17806,0.10581,0.10953
,0.10987,0.10804,0.11098,0.10592,0.098529,0.10815,0.10938,0.099991,0.1
0585,0.10985,0.10598,0.097255,0.10533,0.10707,0.1001,0.10386,0.39684,0
.40642,0.38307,0.31747,0.43203,0.32785,0.19837,0.25175,0.2017,0.28068,
0.25318,0.21534,0.19945,0.15429,0.16906,0.19836,0.19581,0.21107,0.2472
6,0.18526,0.11874,0.095201,0.088462,0.10363,0.10799,0.10733,0.11135,0.
1218,0.11515,0.11564,0.10042,0.10499,0.098732,0.10643,0.09567,0.11177,
0.10254,0.091407,0.36744,0.39755,0.41977,0.40318,0.29493,0.20887,0.206
18,0.23965,0.17205,0.2004,0.24085,0.17597,0.18629,0.21393,0.18797,0.18
179,0.19268,0.18482,0.23013,0.16677,0.11018,0.11308,0.10325,0.12128,0.
10138,0.085002,0.10154,0.097581,0.11688,0.11534,0.09294,0.098038,0.100
68,0.1096,0.11445,0.10626,0.10402,0.10719,0.41727,0.38131,0.39369,0.23
01,0.15593,0.20698,0.25142,0.20075,0.1934,0.24152,0.27568,0.21995,0.20
324,0.17271,0.2128,0.19943,0.22911,0.20944,0.21244,0.21092,0.095638,0.
10974,0.10127,0.10442,0.10914,0.11595,0.10257,0.10959,0.11759,0.10257,
0.10516,0.095171,0.10974,0.099456,0.085527,0.099263,0.10231,0.0989,0.3
933,0.1927,0.23789,0.24348,0.27885,0.21459,0.2061,0.19839,0.29331,0.23
925,0.20971,0.20449,0.19513,0.20986,0.17388,0.209,0.14324,0.16762,0.19
56,0.10726,0.11358,0.11126,0.10611,0.099397,0.086537,0.1011,0.105,0.09
8393,0.11424,0.10791,0.11887,0.09686,0.1012,0.11491,0.10328,0.10587,0.
049967,0.22262,0.26797,0.28264,0.24085,0.21951,0.20063,0.21018,0.18591
,0.21521,0.26989,0.23479,0.20891,0.23094,0.1813,0.19398,0.18856,0.2097
6,0.19249,0.22949,0.11172,0.098329,0.090891,0.09943,0.11971,0.11157,0.
099888,0.10701,0.11017,0.099417,0.099785,0.10059,0.10336,0.11069,0.096
776,0.087658,0.11074,0.04714,0.20916,0.22474,0.20981,0.19697,0.22294,0
.21668,0.26443,0.2276,0.25516,0.21513,0.17911,0.22442,0.19611,0.18712,
0.18044,0.2048,0.24894,0.19868,0.10563,0.086636,0.10715,0.10112,0.1095
1,0.107,0.088247,0.09866,0.10838,0.10111,0.096918,0.12183,0.11558,0.11
565,0.10282,0.11881,0.10234,0.10301,0.051053,0.30252,0.21835,0.17919,0
.20277,0.22522,0.13432,0.22687,0.19893,0.15209,0.2426,0.19311,0.20347,
0.19696,0.18757,0.19547,0.17736,0.18982,0.23103,0.10376,0.10803,0.1058
3,0.089834,0.10184,0.097567,0.106,0.11392,0.10369,0.10235,0.11072,0.10
814,0.10626,0.10528,0.10577,0.10817,0.09954,0.054133,0.058241,0.050156
,0.25926,0.25206,0.2849,0.2242,0.26274,0.2419,0.21463,0.22381,0.25548,
0.18864,0.19975,0.20292,0.20613,0.25059,0.20708,0.10694,0.10607,0.1139
6,0.10452,0.096138,0.1026,0.10664,0.097313,0.11989,0.10516,0.10037,0.1
0271,0.10337,0.12082,0.10005,0.10478,0.10815,0.12049,0.057701,0.056774
,0.056039,0.046452,0.25396,0.23149,0.22228,0.19657,0.26094,0.24271,0.2
5858,0.24882,0.21789,0.26444,0.24071,0.19333,0.10071,0.10382,0.10643,0

.098923,0.1153,0.091086,0.10124,0.1086,0.10637,0.10728,0.10114,0.1123,
0.10774,0.1157,0.10443,0.12561,0.10064,0.11584,0.095421,0.097051,0.048
876,0.053923,0.05871,0.059174,0.050398,0.053213,0.29633,0.22752,0.3100
2,0.16462,0.32391,0.26714,0.22562,0.25049,0.10601,0.097225,0.10906,0.0
92029,0.090679,0.1007,0.10971,0.11199,0.11768,0.10101,0.12384,0.083941
,0.12521,0.10544,0.10853,0.10289,0.1097,0.11142,0.098906,0.10436,0.104
85,0.11576,0.10927,0.041646,0.04715,0.050829,0.057524,0.049278,0.05514
2,0.16753,0.24538,0.19198,0.21727,0.23364,0.059568,0.064634,0.1169,0.1
095,0.1118,0.10291,0.11212,0.1125,0.095153,0.099606,0.095473,0.113,0.0
94648,0.11651,0.11143,0.10649,0.099948,0.1026,0.10766,0.11671,0.10377,
0.095899,0.10786,0.050151,0.05129,0.059594,0.052368,0.049472,0.05241,0
.057694,0.051783,0.052788,0.050847,0.2387,0.25891,0.052612,0.050993,0.
049388,0.050239,0.11227,0.10089,0.10362,0.11539,0.11014,0.10707,0.0915
4,0.11996,0.1106,0.10565,0.11478,0.106,0.10671,0.10727,0.10738,0.10371
,0.10357,0.099338,0.097726,0.055889,0.05887,0.058479,0.06083,0.051584,
0.053042,0.052752,0.051517,0.054193,0.046814,0.056897,0.054136,0.04835
7,0.049489,0.057574,0.055625,0.04886,0.10854,0.099129,0.11176,0.096988
,0.10229,0.0974,0.098303,0.10409,0.10065,0.12081,0.10518,0.096168,0.11
344,0.10794,0.10633,0.10687,0.048853,0.049027,0.048082,0.054317,0.0584
93,0.046477,0.056565,0.063128,0.050451,0.044485,0.046088,0.056437,0.05
567,0.047082,0.054886,0.052312,0.057364,0.054921,0.054378,0.11341,0.10
688,0.12215,0.10838,0.10585,0.10116,0.10511,0.10243,0.098211,0.099724,
0.1093,0.11251,0.10202,0.051541,0.04653,0.053653,0.060502,0.056222,0.0
53777,0.055832,0.056207,0.054109,0.06058,0.059639,0.060126,0.054068,0.
053362,0.054832,0.052497,0.056734,0.059316,0.04781,0.051392,0.048549,0
.10265,0.12365,0.10491,0.096335,0.10656,0.11796,0.11654,0.10372,0.0947
28,0.047784,0.055357,0.053084,0.056388,0.052509,0.054872,0.054164,0.05
7393,0.052649,0.052243,0.054931,0.053269,0.057783,0.054708,0.054472,0.
051596,0.052125,0.052104,0.056708,0.052198,0.049727,0.048573,0.058718,
0.05041,0.053243,0.10255,0.10953,0.09038,0.11236,0.055645,0.048514,0.0
57472,0.052798,0.053297,0.051144,0.05275,0.059863,0.055675,0.050536,0.
041341,0.053198,0.050449,0.053817,0.050837,0.055165,0.050912,0.052485,
0.061402,0.054445,0.052394,0.050314,0.04996,0.053332,0.050597,0.055371
,0.051409,0.049686,0.04794,0.04749,0.051547,0.048873,0.051779,0.052007
,0.052501,0.056874,0.047371,0.045019,0.050702,0.055101,0.055348,0.0476
41,0.052867,0.05082,0.05655,0.056178,0.065816,0.058407,0.050147,0.0490
14,0.050432,0.04774,0.05855,0.049058,0.05239,0.048434,0.061806,0.04856
8,0.048879,0.049514,0.053163,0.058513,0.052773,0.057111,0.04868,0.0539
43,0.048908,0.050581,0.055854,0.052479,0.044179,0.049584,0.047687,0.05
7497,0.052542,0.062467,0.049344,0.054954,0.051344,0.049478,0.046993,0.
048541,0.060373,0.053844,0.060583,0.054428,0.056549,0.043036,0.058536,
0.062184,0.047136,0.046237,0.048268,0.046237,0.053108,0.056192,0.05258
2,0.048954,0.043324,0.046256,0.057357,0.056333,0.045009,0.054606,0.054
71,0.054087,0.043523,0.056644,0.05032,0.052249,0.056002,0.051062,0.055
966,0.051536,0.052738,0.057476,0.044613,0.048186,0.055243,0.051459,0.0
57864,0.048188,0.058992,0.055687,0.056909,0.052679,0.049702,0.05525,0.
053708,0.058676,0.057046,0.050769,0.054299,0.048762,0.049519,0.053102,
0.053122,0.053758,0.052702,0.050494,0.047011,0.049855,0.056099,0.05183
6,0.052305,0.046736,0.051803,0.045697,0.054283,0.049096,0.053038,0.052
493,0.053735,0.054437,0.057458,0.04876,0.05742,0.052375,0.050147,0.054
181,0.054375,0.043846,0.050363,0.061544,0.053584,0.058164,0.0553,0.055
694,0.049494,0.053491,0.051698,0.053625,0.054703,0.049024,0.049657,0.0

52433,0.050413,0.053351,0.053236,0.052356,0.051956,0.052249,0.051948,0.054707,0.053817,0.050897,0.053055,0.050574,0.053055,0.053704,0.049093,0.050597,0.053188,0.05376,0.059995
0.13427,0.14145,0.13952,0.13269,0.15898,0.14744,0.14752,0.1358,0.14327,0.14291,0.1395,0.1577,0.14223,0.14739,0.14118,0.15129,0.15117,0.15629,0.14538,0.14303,0.26557,0.24246,0.31641,0.20825,0.14888,0.13982,0.14313,0.15276,0.14077,0.15192,0.14626,0.14322,0.14806,0.14782,0.14609,0.15222,0.15262,0.14977,0.14579,0.43049,0.31397,0.29059,0.30719,0.27278,0.27095,0.27944,0.14534,0.14925,0.14374,0.14003,0.14355,0.1347,0.13998,0.14505,0.14408,0.14494,0.15695,0.14737,0.14769,0.143,0.14325,0.45121,0.39103,0.25778,0.26418,0.23881,0.31516,0.25543,0.26996,0.31725,0.30762,0.14101,0.14678,0.1374,0.15073,0.15365,0.14879,0.14655,0.14464,0.15106,0.14329,0.15464,0.15173,0.14362,0.14242,0.15688,0.4282,0.39904,0.40619,0.22634,0.30757,0.29276,0.33006,0.26827,0.2282,0.24402,0.27189,0.26754,0.14164,0.15423,0.1471,0.14673,0.14545,0.14639,0.1477,0.14411,0.15034,0.14428,0.15341,0.14399,0.15369,0.14887,0.14968,0.14191,0.37739,0.34184,0.31928,0.30082,0.26761,0.23511,0.26901,0.27006,0.23147,0.28831,0.27709,0.31802,0.29194,0.28795,0.14395,0.14555,0.14873,0.14344,0.14664,0.15073,0.14724,0.14536,0.14426,0.14061,0.14459,0.14707,0.14626,0.14188,0.14977,0.14043,0.39417,0.41379,0.40615,0.37325,0.43495,0.33005,0.27155,0.29838,0.30718,0.30283,0.25222,0.32793,0.29538,0.35759,0.30218,0.23523,0.15656,0.15438,0.14541,0.15048,0.13825,0.15083,0.14798,0.14208,0.14664,0.14226,0.14896,0.15377,0.13195,0.15009,0.149,0.15356,0.1424,0.40572,0.37802,0.33399,0.35764,0.42908,0.31611,0.26629,0.23909,0.2516,0.26537,0.33367,0.23748,0.22963,0.26146,0.25023,0.28139,0.23904,0.14683,0.14845,0.13849,0.14946,0.15223,0.14126,0.13838,0.15431,0.1398,0.14512,0.14989,0.15226,0.14654,0.094926,0.10121,0.11794,0.1421,0.34651,0.37589,0.47267,0.37238,0.31369,0.34906,0.36739,0.26097,0.29332,0.22491,0.25231,0.2437,0.22953,0.33748,0.27181,0.25633,0.23349,0.26958,0.14041,0.15057,0.13839,0.15005,0.15655,0.14578,0.14294,0.14086,0.14987,0.14203,0.15313,0.10661,0.10015,0.090819,0.092224,0.09028,0.09674,0.14211,0.38421,0.30971,0.39513,0.36735,0.39259,0.35165,0.44334,0.28845,0.24208,0.22515,0.32389,0.24819,0.26715,0.32109,0.25091,0.29658,0.2847,0.26262,0.15363,0.14289,0.14839,0.15232,0.14803,0.14823,0.14611,0.13704,0.147,0.096239,0.11088,0.096809,0.10502,0.097006,0.12887,0.098575,0.097522,0.097584,0.15177,0.45927,0.37181,0.34556,0.38898,0.46424,0.37037,0.32496,0.25099,0.23381,0.29428,0.22619,0.22246,0.25929,0.22312,0.28338,0.22734,0.25971,0.24593,0.31075,0.15477,0.14142,0.13984,0.1473,0.15568,0.14667,0.11556,0.095891,0.099291,0.094868,0.10045,0.094817,0.10458,0.097989,0.10234,0.099477,0.077775,0.091981,0.30349,0.31221,0.44843,0.48976,0.34286,0.37676,0.46088,0.24305,0.30498,0.32101,0.23389,0.4258,0.24088,0.28252,0.22369,0.26408,0.23793,0.29024,0.28654,0.14663,0.15271,0.14065,0.081436,0.093636,0.088083,0.10094,0.089562,0.10034,0.088545,0.094594,0.094398,0.11308,0.091375,0.09379,0.097373,0.093188,0.094762,0.37906,0.34704,0.3882,0.35461,0.46238,0.43377,0.39824,0.42105,0.45528,0.264,0.25392,0.25734,0.28645,0.32234,0.34303,0.29765,0.24688,0.24753,0.27487,0.28769,0.093088,0.09287,0.10531,0.096269,0.093508,0.09952,0.099768,0.10218,0.10999,0.095603,0.093464,0.096383,0.090198,0.091995,0.10334,0.10845,0.098478,0.10265,0.32741,0.36347,0.31654,0.3268,0.33804,0.47755,0.41779,0.4666,0.43753,0.30069,0.27366,0.22358,0.16439,0.20646,0.12377,0.21311,0.1598,0.1995,0.20406,0.18428,0.10251,0.097412,0.096561,0.081714,0.080637,0.090223,0.10617,0.086448,0.097617,0.10293,0.09325

8,0.10092,0.098606,0.098095,0.095189,0.095744,0.087516,0.099797,0.3383
9,0.40982,0.35208,0.41746,0.39737,0.35668,0.39164,0.33012,0.32173,0.17
946,0.22468,0.16248,0.21322,0.14205,0.15988,0.2005,0.21617,0.18812,0.2
2816,0.15718,0.10461,0.088933,0.10059,0.097114,0.098383,0.094543,0.099
369,0.088228,0.085049,0.097369,0.092229,0.096384,0.098268,0.1048,0.110
06,0.091709,0.10497,0.096684,0.33166,0.32205,0.33477,0.36699,0.48423,0
.32726,0.36082,0.21822,0.20343,0.214,0.17964,0.18138,0.20136,0.2146,0.
21012,0.21054,0.21148,0.13986,0.18118,0.16571,0.10019,0.10235,0.106,0.
10233,0.10324,0.098828,0.08963,0.099848,0.098202,0.091909,0.098617,0.1
0353,0.099958,0.092452,0.097671,0.097283,0.092825,0.095558,0.37573,0.3
8621,0.34559,0.32799,0.42759,0.32503,0.17915,0.25026,0.19448,0.26155,0
.22525,0.19986,0.19688,0.13964,0.16049,0.20216,0.19496,0.19621,0.23214
,0.1862,0.11067,0.087363,0.081774,0.095739,0.10302,0.096451,0.10453,0.
11081,0.10722,0.10599,0.09128,0.095417,0.092449,0.098044,0.092641,0.10
415,0.096898,0.084507,0.33095,0.36835,0.39892,0.36623,0.26009,0.20151,
0.19267,0.24367,0.15665,0.18974,0.21904,0.16184,0.18772,0.19262,0.1791
9,0.17209,0.18906,0.17263,0.22682,0.1579,0.10395,0.10383,0.095692,0.10
883,0.094433,0.080574,0.089959,0.091731,0.10698,0.10652,0.084755,0.091
66,0.092277,0.10387,0.10362,0.097396,0.095331,0.10104,0.40077,0.38734,
0.41852,0.22167,0.13993,0.18118,0.22776,0.17824,0.19677,0.2075,0.28302
,0.21492,0.19845,0.16494,0.21561,0.1806,0.22394,0.18922,0.20894,0.2031
5,0.089182,0.10034,0.09149,0.096993,0.098336,0.10967,0.092199,0.10273,
0.10721,0.092413,0.099152,0.089136,0.098491,0.089683,0.078187,0.092618
,0.093238,0.093225,0.35835,0.17774,0.23192,0.21651,0.26419,0.19067,0.1
8937,0.19539,0.2823,0.2093,0.20869,0.18896,0.18075,0.19681,0.1653,0.21
568,0.12987,0.16468,0.19171,0.10292,0.10522,0.10014,0.097815,0.089175,
0.079365,0.093203,0.096647,0.088114,0.10156,0.099648,0.10865,0.094608,
0.092356,0.10586,0.096947,0.098169,0.045802,0.20909,0.26477,0.26437,0.
24572,0.22417,0.19224,0.20331,0.18629,0.20058,0.23864,0.23844,0.2125,0
.24316,0.18742,0.19236,0.18369,0.19234,0.17152,0.2253,0.10195,0.088377
,0.08308,0.091273,0.10806,0.10239,0.09208,0.10222,0.1018,0.093487,0.09
5572,0.093011,0.094945,0.10185,0.090427,0.080305,0.10212,0.041384,0.18
628,0.21379,0.19542,0.19284,0.19945,0.22244,0.25684,0.22181,0.23169,0.
2164,0.1545,0.21214,0.19977,0.17777,0.16063,0.17767,0.2277,0.18031,0.0
94742,0.082108,0.096374,0.095019,0.098246,0.10187,0.083909,0.09412,0.0
99745,0.093873,0.087839,0.1108,0.10323,0.1072,0.093645,0.11126,0.09541
6,0.093357,0.051416,0.28566,0.20395,0.18694,0.19569,0.25147,0.12206,0.
20968,0.17584,0.13986,0.25555,0.18549,0.1964,0.20758,0.17624,0.18603,0
.15928,0.17626,0.20339,0.094172,0.098364,0.10073,0.084099,0.092785,0.0
89044,0.10206,0.10181,0.095724,0.096402,0.10302,0.098094,0.099066,0.09
7026,0.097342,0.10263,0.094211,0.048852,0.053039,0.042803,0.27922,0.26
236,0.27709,0.23755,0.2493,0.23003,0.19498,0.21288,0.23335,0.17926,0.1
9958,0.18447,0.20789,0.22451,0.19788,0.10131,0.098945,0.10561,0.095498
,0.089648,0.093831,0.10039,0.085356,0.11037,0.099103,0.096048,0.095015
,0.094098,0.11348,0.092034,0.10024,0.10294,0.11556,0.055309,0.051121,0
.054486,0.04184,0.24719,0.22026,0.20228,0.1774,0.24946,0.2123,0.24364,
0.22749,0.22205,0.25301,0.21389,0.17767,0.097478,0.09569,0.09495,0.092
607,0.10256,0.084544,0.094786,0.1013,0.10096,0.10231,0.094058,0.10236,
0.10175,0.10573,0.1003,0.11615,0.092222,0.10943,0.085852,0.093918,0.04
3622,0.047686,0.054051,0.054114,0.048549,0.050816,0.28529,0.21704,0.31
013,0.17042,0.31742,0.25247,0.23754,0.23341,0.099765,0.090578,0.1021,0
.08652,0.083036,0.089785,0.099839,0.10507,0.10757,0.095009,0.11596,0.0

78633,0.11811,0.097477,0.10151,0.094987,0.098397,0.10232,0.092554,0.09
8942,0.097647,0.10734,0.099319,0.04046,0.042754,0.046914,0.054521,0.04
5189,0.051619,0.1862,0.22656,0.18683,0.23519,0.2385,0.052678,0.059263,
0.10557,0.10625,0.10293,0.095155,0.10305,0.10543,0.088977,0.095635,0.0
92319,0.10619,0.088074,0.10914,0.10523,0.097638,0.092796,0.094582,0.10
132,0.10803,0.095354,0.090593,0.098036,0.044803,0.04731,0.055648,0.049
528,0.042776,0.048693,0.051178,0.048494,0.048723,0.05033,0.22593,0.238
8,0.048429,0.047695,0.047322,0.043107,0.10453,0.094544,0.095858,0.1104
9,0.098118,0.097939,0.086045,0.10769,0.10832,0.099586,0.10844,0.10026,
0.10087,0.098158,0.10292,0.097993,0.098139,0.088317,0.091437,0.052375,
0.055,0.057166,0.058909,0.04301,0.049722,0.047399,0.044834,0.050286,0.
040756,0.051724,0.050533,0.044266,0.045465,0.051148,0.050484,0.043155,
0.10208,0.092603,0.10498,0.08696,0.093627,0.09124,0.094772,0.095414,0.
094986,0.11268,0.097327,0.089819,0.104,0.10021,0.096628,0.096313,0.041
392,0.042896,0.043173,0.051048,0.055317,0.045056,0.053786,0.056282,0.0
41701,0.042465,0.043096,0.054399,0.051656,0.037984,0.047263,0.04864,0.
053261,0.051317,0.050384,0.10796,0.0978,0.10964,0.10205,0.097689,0.092
019,0.095622,0.094278,0.09154,0.092201,0.099674,0.10207,0.093365,0.048
888,0.042438,0.046525,0.055743,0.051604,0.049529,0.047257,0.050143,0.0
49824,0.055797,0.051429,0.055939,0.050103,0.050306,0.051598,0.046998,0
.053125,0.052864,0.041686,0.047425,0.044975,0.095908,0.11091,0.097871,
0.088866,0.09921,0.10781,0.11056,0.096925,0.08908,0.046295,0.049149,0.
048643,0.050521,0.049385,0.049704,0.052384,0.049918,0.045691,0.047024,
0.049839,0.049285,0.054789,0.05003,0.051494,0.049853,0.052191,0.050359
,0.051632,0.047195,0.045197,0.043783,0.050437,0.045259,0.048853,0.0949
,0.10236,0.08265,0.10423,0.049319,0.047167,0.055161,0.048267,0.051293,
0.045087,0.045625,0.057249,0.052772,0.045205,0.035035,0.051126,0.04771
7,0.051594,0.043745,0.049359,0.046887,0.04871,0.058328,0.050563,0.0479
64,0.045576,0.047283,0.051701,0.04413,0.050564,0.048963,0.050124,0.042
633,0.042203,0.045425,0.045783,0.045702,0.046669,0.046855,0.05453,0.04
1935,0.040306,0.046449,0.050513,0.050955,0.045113,0.045824,0.044062,0.
053298,0.04858,0.060054,0.054388,0.043462,0.047582,0.047488,0.044473,0
.053653,0.045108,0.047016,0.046075,0.057371,0.043525,0.047419,0.047278
,0.048207,0.054419,0.049965,0.050814,0.045091,0.050907,0.045359,0.0486
47,0.048365,0.047254,0.04143,0.047324,0.043593,0.054383,0.051662,0.056
331,0.045733,0.050332,0.045916,0.044098,0.040738,0.046674,0.057878,0.0
51404,0.056548,0.049252,0.051312,0.038081,0.056191,0.053807,0.048531,0
.038739,0.042956,0.04294,0.046604,0.054035,0.050098,0.044943,0.041087,
0.044103,0.050258,0.049781,0.043037,0.049371,0.052836,0.048592,0.04081
5,0.055379,0.048552,0.049275,0.055465,0.046113,0.050219,0.049698,0.047
047,0.051378,0.040379,0.042803,0.050609,0.047164,0.051454,0.048533,0.0
56144,0.050595,0.051448,0.050408,0.047309,0.049031,0.049377,0.054451,0
.05157,0.047881,0.050289,0.041912,0.046363,0.051878,0.051225,0.04751,0
.048855,0.049801,0.042492,0.044296,0.052782,0.04629,0.04914,0.039694,0
.051262,0.039364,0.052839,0.045358,0.048752,0.045213,0.050351,0.04937,
0.05404,0.045745,0.0535,0.048033,0.044657,0.049495,0.046244,0.040202,0
.043021,0.05739,0.048779,0.056141,0.053759,0.05103,0.044191,0.049303,0
.047903,0.051726,0.048246,0.049404,0.04502,0.047648,0.045655,0.049892,
0.046911,0.049377,0.046291,0.047899,0.046337,0.047593,0.048973,0.05042
8,0.051275,0.045977,0.046279,0.050778,0.044047,0.044875,0.052665,0.053
213,0.053802

0.12243,0.13946,0.13332,0.12471,0.14823,0.13441,0.1386,0.13057,0.1372,
0.13414,0.12842,0.14823,0.12997,0.13923,0.13397,0.14546,0.14003,0.1413
8,0.14061,0.1361,0.25104,0.23327,0.29145,0.20576,0.13909,0.13519,0.132
27,0.14656,0.13465,0.14164,0.13465,0.13085,0.13702,0.13778,0.1364,0.14
384,0.14186,0.14042,0.13701,0.38785,0.27585,0.28186,0.27439,0.26507,0.
26096,0.28937,0.13631,0.13714,0.13601,0.13106,0.13503,0.12422,0.13367,
0.1348,0.13176,0.12924,0.14499,0.13843,0.13605,0.13546,0.13446,0.41233
,0.3285,0.24036,0.25668,0.23264,0.30077,0.24077,0.2531,0.30853,0.31755
,0.13725,0.13847,0.12831,0.14054,0.14163,0.14365,0.13446,0.13142,0.143
4,0.13503,0.14185,0.14718,0.13603,0.13203,0.14857,0.41108,0.36755,0.36
846,0.19875,0.29524,0.30402,0.30126,0.23867,0.21509,0.23079,0.27275,0.
22704,0.12954,0.14173,0.13545,0.13753,0.13413,0.13642,0.13865,0.12803,
0.13786,0.13784,0.14471,0.12956,0.13851,0.13891,0.13902,0.13326,0.3645
7,0.32188,0.33192,0.30083,0.28161,0.22039,0.25287,0.27319,0.23115,0.28
397,0.24528,0.30014,0.29543,0.29111,0.13414,0.13783,0.13498,0.13344,0.
14071,0.14285,0.13375,0.14048,0.14079,0.13625,0.13676,0.13502,0.13338,
0.13145,0.14123,0.13071,0.40206,0.42508,0.40225,0.33403,0.39411,0.3112
8,0.24309,0.25211,0.29917,0.29142,0.25741,0.3042,0.26524,0.33653,0.274
65,0.2482,0.14309,0.14105,0.13759,0.13793,0.13136,0.14321,0.14013,0.13
034,0.13844,0.13212,0.13935,0.14347,0.1186,0.14296,0.14286,0.13882,0.1
3703,0.45269,0.34527,0.27309,0.34092,0.41406,0.28355,0.24643,0.24243,0
.24913,0.25347,0.31852,0.2404,0.22278,0.2571,0.23557,0.28668,0.2182,0.
13988,0.14167,0.13008,0.13412,0.14401,0.13561,0.12816,0.14401,0.13292,
0.13852,0.13641,0.14072,0.13897,0.090275,0.09178,0.11356,0.13514,0.327
04,0.36734,0.46073,0.36784,0.30607,0.35022,0.3558,0.24571,0.29787,0.22
049,0.22959,0.25242,0.21119,0.29015,0.27592,0.26136,0.22578,0.24766,0.
13124,0.14426,0.12648,0.14195,0.14801,0.14069,0.13444,0.13053,0.14212,
0.13432,0.14302,0.097798,0.093663,0.088967,0.084113,0.081265,0.090215,
0.13543,0.35494,0.3325,0.32237,0.37517,0.40114,0.38249,0.43848,0.27891
,0.23742,0.21057,0.3014,0.22528,0.25758,0.31345,0.21768,0.2848,0.2657,
0.26295,0.14471,0.13435,0.14316,0.14409,0.13754,0.13795,0.13926,0.1312
,0.1368,0.087905,0.099147,0.08837,0.098467,0.088933,0.12034,0.092402,0
.09041,0.088696,0.13985,0.41145,0.37295,0.35939,0.35542,0.41221,0.3529
1,0.28206,0.25947,0.21559,0.28788,0.23048,0.21009,0.24811,0.21333,0.26
613,0.22222,0.24596,0.24951,0.27759,0.14743,0.13255,0.12555,0.13763,0.
1435,0.13415,0.10976,0.090639,0.093941,0.089702,0.091423,0.089348,0.09
7873,0.094192,0.096644,0.094459,0.075254,0.086328,0.28921,0.31113,0.47
742,0.43417,0.28182,0.38721,0.48573,0.22771,0.28436,0.30701,0.23788,0.
41993,0.2259,0.27575,0.22435,0.25898,0.20256,0.27599,0.25495,0.13883,0
.13951,0.13499,0.078527,0.087164,0.080827,0.095502,0.085921,0.09467,0.
079034,0.086888,0.08807,0.10632,0.086024,0.089168,0.089146,0.081694,0.
089399,0.32727,0.36193,0.35931,0.35943,0.39103,0.41258,0.41889,0.45904
,0.40946,0.23637,0.2426,0.2445,0.28617,0.32013,0.33275,0.27344,0.25334
,0.24877,0.25108,0.29278,0.086212,0.08692,0.10098,0.089409,0.085458,0.
097174,0.093076,0.096307,0.10284,0.090311,0.087185,0.091872,0.085117,0
.084869,0.099879,0.10584,0.093024,0.096991,0.32357,0.36833,0.32646,0.3
3479,0.30244,0.45828,0.34631,0.49361,0.41876,0.28765,0.26701,0.20441,0
.14292,0.20504,0.1289,0.19026,0.15051,0.17188,0.20081,0.17853,0.09773,
0.088073,0.090289,0.078763,0.074796,0.08307,0.098329,0.08362,0.093439,
0.09769,0.08826,0.09504,0.093628,0.090589,0.09207,0.091072,0.083732,0.
094001,0.31467,0.39969,0.3579,0.41972,0.38392,0.36172,0.40267,0.31202,
0.33236,0.2035,0.22421,0.15382,0.20137,0.13123,0.14602,0.18829,0.22668

,0.20792,0.21495,0.14827,0.097864,0.085871,0.095584,0.092506,0.090019,
0.088723,0.091035,0.083507,0.080633,0.092607,0.086987,0.086963,0.08749
1,0.099961,0.10447,0.087532,0.097755,0.088199,0.2866,0.2932,0.33278,0.
33839,0.50858,0.3188,0.35843,0.20495,0.17986,0.20083,0.19113,0.17839,0
.20108,0.20396,0.2023,0.21675,0.20601,0.14013,0.16655,0.1657,0.09427,0
.094772,0.10062,0.096527,0.099285,0.089922,0.085221,0.093798,0.092013,
0.083282,0.095118,0.094525,0.092093,0.085167,0.090928,0.094426,0.08659
,0.086153,0.37236,0.42866,0.36974,0.36542,0.40344,0.32732,0.16046,0.22
161,0.18799,0.26818,0.2006,0.19804,0.1902,0.1345,0.16743,0.19398,0.184
63,0.19351,0.21556,0.19183,0.10369,0.083061,0.076533,0.088377,0.091011
,0.091394,0.09468,0.10046,0.10031,0.097807,0.086515,0.086381,0.085013,
0.09067,0.084127,0.099239,0.087412,0.07724,0.31506,0.36681,0.38906,0.3
5136,0.2363,0.19147,0.18698,0.22282,0.15627,0.18243,0.22045,0.14709,0.
17145,0.18513,0.17964,0.17057,0.19776,0.14903,0.22669,0.15542,0.096569
,0.094402,0.091911,0.10557,0.086799,0.074042,0.085353,0.081799,0.10365
,0.09925,0.081467,0.087673,0.087099,0.096295,0.10004,0.090846,0.090527
,0.093946,0.39247,0.35706,0.38611,0.21908,0.14496,0.19776,0.23521,0.17
161,0.20364,0.19826,0.2538,0.20582,0.19532,0.16271,0.19311,0.17455,0.2
1175,0.19172,0.20443,0.20974,0.083351,0.091233,0.086681,0.094793,0.091
495,0.10454,0.087045,0.094966,0.10055,0.0879,0.094786,0.083133,0.09284
6,0.080331,0.073849,0.087212,0.086582,0.085023,0.30956,0.17944,0.22683
,0.21216,0.25764,0.17056,0.19083,0.18579,0.26413,0.2473,0.18886,0.2006
2,0.16976,0.19426,0.15254,0.22038,0.12926,0.16253,0.19664,0.095354,0.0
96083,0.093328,0.092995,0.082545,0.074889,0.089545,0.092203,0.083909,0
.096261,0.095388,0.10147,0.087595,0.087967,0.099331,0.087404,0.092332,
0.044822,0.18411,0.22612,0.25757,0.25525,0.20814,0.18862,0.18627,0.174
05,0.19437,0.24963,0.21939,0.20442,0.24104,0.18175,0.19712,0.18303,0.1
8544,0.15859,0.21089,0.0974,0.084952,0.079641,0.087475,0.10017,0.09492
6,0.08431,0.098134,0.090449,0.086166,0.089153,0.085525,0.085905,0.0969
82,0.083445,0.074535,0.094976,0.040089,0.17134,0.2161,0.16515,0.19837,
0.18806,0.22477,0.25139,0.2077,0.21808,0.19876,0.15,0.21083,0.19642,0.
17322,0.16695,0.17061,0.2106,0.16249,0.088359,0.07661,0.092826,0.08899
6,0.090695,0.095549,0.076991,0.084658,0.092398,0.087311,0.080436,0.103
49,0.098389,0.09753,0.084898,0.10321,0.085767,0.0937,0.050953,0.27041,
0.19353,0.19324,0.20973,0.21646,0.1068,0.2106,0.17047,0.13399,0.24823,
0.18598,0.18632,0.20481,0.1835,0.18374,0.16818,0.17611,0.2017,0.086161
,0.091989,0.096186,0.080364,0.088342,0.086255,0.095247,0.093034,0.0937
07,0.089378,0.097866,0.093753,0.092987,0.091564,0.090951,0.094368,0.09
0077,0.050163,0.046698,0.042267,0.28358,0.25975,0.25362,0.23303,0.2215
6,0.23149,0.18956,0.19287,0.20973,0.17642,0.19939,0.18274,0.2144,0.217
92,0.19928,0.091529,0.09336,0.097514,0.090929,0.084788,0.088035,0.0941
39,0.077284,0.10176,0.094752,0.089088,0.085978,0.091976,0.10792,0.0840
24,0.092687,0.098117,0.10267,0.05074,0.047861,0.054997,0.040434,0.2477
5,0.18971,0.20014,0.1911,0.21392,0.23766,0.2425,0.2103,0.23278,0.23513
,0.20397,0.16413,0.09052,0.090988,0.092442,0.085522,0.096386,0.078402,
0.088081,0.097659,0.092764,0.093471,0.087034,0.099164,0.096064,0.09795
4,0.095228,0.10639,0.088556,0.10121,0.080571,0.091119,0.040087,0.04283
5,0.048374,0.052604,0.047939,0.045726,0.28869,0.20426,0.31253,0.17548,
0.29289,0.28814,0.25334,0.22329,0.091848,0.085025,0.094454,0.080208,0.
079202,0.085129,0.09569,0.096391,0.097482,0.08644,0.11169,0.072172,0.1
1149,0.093067,0.096946,0.087712,0.091066,0.095317,0.086511,0.090901,0.
094208,0.099214,0.0946,0.038245,0.041226,0.046656,0.050399,0.043226,0.

048642,0.17974,0.2309,0.18553,0.2092,0.24973,0.051279,0.054509,0.09482
4,0.094926,0.094347,0.087559,0.096131,0.09849,0.084481,0.091547,0.0868
5,0.099082,0.085128,0.10045,0.10324,0.092092,0.090581,0.09096,0.093925
,0.10153,0.088443,0.083398,0.09489,0.04316,0.044377,0.052916,0.046199,
0.040003,0.0468,0.050304,0.045339,0.047638,0.048291,0.22956,0.23219,0.
044634,0.047798,0.045395,0.037372,0.095914,0.085463,0.091546,0.10103,0
.092628,0.091565,0.081249,0.10447,0.09855,0.094836,0.10103,0.090613,0.
093039,0.094015,0.099819,0.092834,0.090138,0.088267,0.083188,0.048691,
0.050815,0.05102,0.055663,0.040074,0.046457,0.044485,0.042828,0.049157
,0.043119,0.049737,0.045841,0.042539,0.044442,0.051599,0.045984,0.0436
09,0.095714,0.091095,0.098348,0.08367,0.086932,0.085223,0.089585,0.090
528,0.089415,0.10345,0.089944,0.086625,0.094873,0.092331,0.091107,0.08
8548,0.040136,0.04216,0.042509,0.048187,0.052308,0.041305,0.050084,0.0
52204,0.041589,0.040753,0.04113,0.053365,0.052078,0.036603,0.046177,0.
046526,0.049657,0.045867,0.047079,0.098093,0.092508,0.10528,0.097602,0
.0909,0.088478,0.092165,0.086758,0.087141,0.087729,0.093219,0.098978,0
.089223,0.048343,0.036588,0.045566,0.052835,0.048401,0.048108,0.044948
,0.047251,0.048347,0.049797,0.050209,0.05329,0.049476,0.049787,0.04795
1,0.046071,0.052374,0.051121,0.039158,0.044074,0.041717,0.088932,0.103
71,0.09034,0.083921,0.090246,0.10628,0.10403,0.088924,0.080653,0.04774
9,0.047017,0.044823,0.049689,0.045293,0.046342,0.050868,0.046057,0.042
695,0.045192,0.049921,0.045431,0.052147,0.045151,0.049456,0.049923,0.0
49988,0.049436,0.044866,0.04531,0.041641,0.042489,0.048377,0.040427,0.
046059,0.088709,0.094635,0.080129,0.10024,0.047936,0.046205,0.052241,0
.046519,0.048092,0.041387,0.040217,0.05495,0.049497,0.040657,0.032874,
0.04899,0.043242,0.049972,0.040205,0.042345,0.04584,0.046257,0.053216,
0.046394,0.045173,0.041703,0.046238,0.050381,0.039013,0.04712,0.045609
,0.047117,0.042124,0.039232,0.046457,0.0443,0.044089,0.045882,0.04674,
0.050289,0.040817,0.036947,0.042952,0.049805,0.047294,0.040994,0.04307
1,0.043527,0.051518,0.046027,0.05456,0.053155,0.041437,0.04472,0.04438
1,0.04153,0.05032,0.041757,0.043673,0.044382,0.053488,0.042667,0.04477
5,0.042385,0.04246,0.05217,0.047835,0.046256,0.042988,0.04675,0.040705
,0.044863,0.047651,0.044336,0.038788,0.043103,0.039989,0.051807,0.0514
91,0.054273,0.042397,0.046704,0.044934,0.041949,0.035554,0.042639,0.05
5278,0.04853,0.054485,0.044339,0.051511,0.035199,0.057042,0.051899,0.0
46761,0.036815,0.039755,0.039502,0.045672,0.04879,0.049853,0.043018,0.
041292,0.042711,0.045516,0.045072,0.037085,0.047817,0.051432,0.044806,
0.038453,0.053527,0.043991,0.047999,0.048191,0.042991,0.048612,0.04643
6,0.045856,0.049092,0.037205,0.040572,0.049509,0.044699,0.049322,0.042
05,0.050883,0.048008,0.048803,0.049127,0.044514,0.045785,0.043451,0.04
8724,0.046174,0.045036,0.046626,0.041714,0.043823,0.05284,0.047638,0.0
45301,0.047751,0.04702,0.039326,0.039835,0.047312,0.044399,0.045581,0.
036687,0.048881,0.032903,0.047047,0.042589,0.046537,0.04569,0.050818,0
.045869,0.049509,0.043712,0.050525,0.043532,0.041929,0.046587,0.045732
,0.037479,0.040546,0.053271,0.045866,0.054621,0.051107,0.047313,0.0422
34,0.04832,0.044073,0.048358,0.047531,0.047527,0.039989,0.043312,0.040
478,0.046941,0.04146,0.045902,0.046103,0.044882,0.04531,0.044107,0.047
956,0.04697,0.052424,0.044818,0.045106,0.048234,0.043781,0.044233,0.04
8166,0.05008,0.048243
0.1201,0.12747,0.13267,0.11658,0.13725,0.13108,0.13083,0.12061,0.12931
,0.1324,0.1221,0.1402,0.12198,0.135,0.12295,0.13589,0.13102,0.13341,0.
13401,0.12882,0.24276,0.23977,0.26495,0.20237,0.13487,0.12907,0.1273,0

.13667,0.12843,0.13681,0.12999,0.12364,0.13047,0.12904,0.12665,0.13525
,0.13695,0.12871,0.12841,0.39996,0.27106,0.26175,0.26285,0.27982,0.255
27,0.2685,0.13075,0.12949,0.12539,0.12161,0.12919,0.11464,0.12522,0.12
729,0.13005,0.12336,0.13882,0.13276,0.12719,0.13126,0.12968,0.38864,0.
33326,0.21466,0.25903,0.2355,0.29731,0.22449,0.25056,0.30915,0.29235,0
.13381,0.12669,0.12005,0.13063,0.13702,0.13528,0.12609,0.12605,0.13802
,0.1312,0.12969,0.13814,0.12922,0.12519,0.13816,0.43489,0.3575,0.36466
,0.2148,0.27691,0.30111,0.30663,0.21876,0.19354,0.21513,0.24943,0.218,
0.12159,0.13488,0.13073,0.13362,0.12529,0.12933,0.12874,0.12239,0.1338
1,0.12946,0.13676,0.12622,0.12592,0.1289,0.13419,0.12661,0.36859,0.298
77,0.34668,0.30256,0.26284,0.20248,0.2519,0.27124,0.21304,0.26897,0.24
311,0.28698,0.30033,0.27186,0.1291,0.13119,0.12617,0.12328,0.13034,0.1
3659,0.12839,0.13188,0.13305,0.12792,0.12978,0.13036,0.131,0.12595,0.1
3249,0.12593,0.35708,0.43224,0.34216,0.32331,0.4021,0.35833,0.25584,0.
24038,0.29937,0.29743,0.22077,0.2803,0.26268,0.33337,0.24987,0.22653,0
.13374,0.13159,0.13162,0.12434,0.12135,0.13678,0.13173,0.12582,0.13068
,0.12426,0.13171,0.13361,0.11049,0.1343,0.13459,0.12936,0.13003,0.4392
3,0.32207,0.28207,0.3333,0.44075,0.33162,0.23478,0.25521,0.2342,0.2451
1,0.29451,0.24387,0.21951,0.22862,0.23782,0.27966,0.21241,0.1372,0.133
74,0.12549,0.12767,0.13425,0.12674,0.12312,0.13873,0.12453,0.12471,0.1
2885,0.13266,0.13294,0.084438,0.089668,0.10306,0.12611,0.32148,0.37407
,0.44026,0.35998,0.3177,0.36338,0.3464,0.25402,0.26965,0.23591,0.20322
,0.23286,0.20491,0.27673,0.27828,0.2552,0.23269,0.23309,0.12683,0.1366
4,0.12112,0.13382,0.14016,0.13553,0.12137,0.12293,0.13331,0.12764,0.13
356,0.094978,0.088446,0.086831,0.079766,0.077857,0.091306,0.13037,0.35
21,0.29478,0.29482,0.37322,0.40414,0.35247,0.41615,0.29844,0.24547,0.2
0538,0.28916,0.22651,0.24416,0.28995,0.21347,0.29044,0.25078,0.24377,0
.13582,0.12804,0.13556,0.14027,0.13109,0.12608,0.13273,0.1236,0.12988,
0.082632,0.094765,0.08252,0.092574,0.081349,0.11006,0.092153,0.088562,
0.082341,0.12846,0.34295,0.39144,0.36361,0.3285,0.36533,0.36856,0.3086
9,0.21702,0.22088,0.299,0.22125,0.20643,0.23926,0.22098,0.24764,0.2255
4,0.24625,0.25661,0.27786,0.13577,0.12655,0.12118,0.13173,0.13564,0.12
409,0.10173,0.084478,0.093613,0.08466,0.086206,0.084214,0.091974,0.088
637,0.094334,0.08829,0.07392,0.083784,0.31299,0.34749,0.42381,0.42877,
0.28242,0.38123,0.4587,0.21074,0.28583,0.31947,0.19998,0.37855,0.23818
,0.25614,0.2294,0.25005,0.20152,0.25464,0.2454,0.13321,0.1301,0.12429,
0.076858,0.080577,0.076438,0.086739,0.077689,0.091168,0.070704,0.08396
7,0.083576,0.099961,0.083058,0.085163,0.084415,0.074071,0.084429,0.352
17,0.32848,0.33346,0.3453,0.43734,0.41697,0.40311,0.433,0.40327,0.2226
3,0.24161,0.25142,0.27063,0.31799,0.30886,0.25972,0.2516,0.24742,0.224
23,0.31496,0.078168,0.078571,0.093606,0.085876,0.082087,0.094199,0.091
435,0.090864,0.096824,0.087624,0.080332,0.088187,0.080177,0.082557,0.0
9592,0.10074,0.089315,0.090904,0.33967,0.37882,0.30753,0.31161,0.27788
,0.45086,0.35593,0.51069,0.41391,0.26459,0.2557,0.19473,0.13699,0.2051
3,0.12774,0.19241,0.14097,0.14023,0.195,0.17948,0.087106,0.083746,0.08
5721,0.076924,0.073564,0.0741,0.09428,0.079656,0.08743,0.091961,0.0834
59,0.089736,0.086992,0.08394,0.088958,0.083875,0.077375,0.086854,0.346
41,0.39414,0.40469,0.39728,0.39921,0.36143,0.41258,0.33178,0.35303,0.2
1994,0.22305,0.12811,0.19277,0.1366,0.14124,0.19077,0.20898,0.21226,0.
204,0.14245,0.092984,0.082144,0.087852,0.088212,0.087017,0.08402,0.088
079,0.082335,0.074635,0.090041,0.082795,0.082186,0.083614,0.094758,0.1
008,0.083449,0.093214,0.082978,0.28116,0.29823,0.3264,0.33421,0.488,0.

30553,0.29452,0.20593,0.17537,0.21782,0.1937,0.17849,0.18375,0.18303,0
.18842,0.21452,0.1946,0.14431,0.16278,0.15267,0.088083,0.093224,0.0964
34,0.090812,0.089765,0.08617,0.079386,0.087767,0.085933,0.08161,0.0893
51,0.090989,0.085185,0.081541,0.083319,0.089794,0.081851,0.081036,0.38
643,0.35226,0.36857,0.41331,0.37479,0.30777,0.17335,0.21692,0.18388,0.
23094,0.21445,0.18641,0.16895,0.13449,0.1669,0.18485,0.19125,0.19637,0
.21064,0.1718,0.10148,0.080058,0.07333,0.081911,0.082421,0.0874,0.0918
54,0.091739,0.092314,0.093874,0.086616,0.08002,0.084112,0.088603,0.076
639,0.096323,0.081798,0.070769,0.304,0.34151,0.34224,0.33892,0.22853,0
.19577,0.1936,0.23581,0.16299,0.17976,0.21736,0.14797,0.17166,0.17872,
0.17641,0.18267,0.18377,0.16769,0.20512,0.15331,0.087758,0.089547,0.08
7627,0.09778,0.080948,0.068499,0.08523,0.075492,0.098679,0.09545,0.076
399,0.084841,0.082416,0.090382,0.09631,0.084683,0.085178,0.086329,0.41
348,0.31144,0.35651,0.20997,0.15759,0.22326,0.22061,0.15165,0.19984,0.
1742,0.23385,0.19989,0.18365,0.16478,0.17534,0.16903,0.198,0.16374,0.2
0451,0.20589,0.076315,0.088153,0.079242,0.089131,0.086798,0.09844,0.08
0612,0.084025,0.09666,0.086291,0.089826,0.079584,0.090604,0.074988,0.0
69259,0.082535,0.079768,0.081365,0.2762,0.14976,0.2152,0.19518,0.26665
,0.15475,0.19115,0.17056,0.26537,0.22787,0.17746,0.2009,0.17455,0.1765
2,0.13964,0.22493,0.14191,0.15336,0.18677,0.089715,0.093929,0.087754,0
.086185,0.076625,0.070712,0.08511,0.086851,0.08459,0.091967,0.091029,0
.093893,0.084074,0.083666,0.096602,0.080018,0.088922,0.044732,0.17976,
0.21368,0.25255,0.23295,0.1961,0.198,0.18554,0.15818,0.18094,0.26603,0
.19573,0.20228,0.23606,0.17437,0.17004,0.17301,0.18114,0.17115,0.20612
,0.090857,0.077832,0.076081,0.083156,0.094108,0.089927,0.080688,0.0925
05,0.089264,0.081949,0.08458,0.081876,0.081944,0.092468,0.073199,0.070
18,0.090303,0.039438,0.14714,0.21143,0.17086,0.20467,0.20971,0.20386,0
.2345,0.22091,0.20322,0.21863,0.14447,0.19174,0.19908,0.16531,0.16541,
0.16037,0.1985,0.16946,0.082067,0.074139,0.086458,0.085902,0.084736,0.
09103,0.075755,0.080126,0.086194,0.083289,0.079657,0.099059,0.092001,0
.092497,0.084008,0.096179,0.083741,0.090279,0.051248,0.25435,0.1655,0.
19412,0.22516,0.20826,0.11424,0.18254,0.18645,0.12397,0.22976,0.17957,
0.18766,0.19777,0.16334,0.17782,0.17775,0.16752,0.18167,0.084106,0.088
397,0.090513,0.076218,0.084978,0.082422,0.086976,0.089563,0.092977,0.0
84545,0.09264,0.088339,0.086973,0.083981,0.084714,0.08799,0.087143,0.0
46818,0.043545,0.036874,0.28474,0.27335,0.23452,0.22378,0.20913,0.2329
7,0.18509,0.18995,0.19716,0.18296,0.19063,0.16613,0.20528,0.20726,0.19
431,0.085011,0.092177,0.097572,0.087406,0.081329,0.082443,0.086858,0.0
76383,0.098335,0.091104,0.085161,0.082217,0.08952,0.10107,0.080023,0.0
88511,0.091287,0.098916,0.049168,0.046238,0.048903,0.038588,0.22842,0.
16838,0.21565,0.18898,0.22649,0.23358,0.23249,0.20003,0.22926,0.2258,0
.17386,0.15361,0.08702,0.086053,0.087115,0.079202,0.089754,0.075655,0.
083265,0.092909,0.089207,0.08943,0.083537,0.09573,0.093167,0.093217,0.
088069,0.10123,0.089225,0.096481,0.077502,0.090054,0.038895,0.041917,0
.043158,0.050368,0.044518,0.046123,0.27892,0.16356,0.28078,0.17963,0.2
8608,0.28754,0.23447,0.19422,0.086179,0.079944,0.08792,0.080591,0.0729
36,0.083636,0.088675,0.090485,0.092943,0.082433,0.10786,0.068365,0.107
17,0.088397,0.092176,0.085308,0.084354,0.092561,0.082072,0.086028,0.08
9667,0.094112,0.089285,0.03708,0.039954,0.042758,0.049959,0.040354,0.0
46363,0.17003,0.22287,0.17318,0.19871,0.26209,0.049639,0.051422,0.0875
59,0.088485,0.08922,0.082437,0.090621,0.093787,0.078289,0.085995,0.080
171,0.095343,0.079283,0.093534,0.094618,0.08977,0.086393,0.08342,0.091

453,0.096478,0.083711,0.078206,0.088088,0.040502,0.042529,0.049497,0.0
42817,0.037668,0.046651,0.049813,0.042478,0.043343,0.046137,0.22934,0.
22205,0.044005,0.046154,0.042089,0.036902,0.089734,0.082644,0.088517,0
.094842,0.085639,0.083622,0.076279,0.099862,0.092524,0.087389,0.096435
,0.087727,0.088563,0.087296,0.095518,0.087701,0.08247,0.081258,0.07941
3,0.044232,0.048545,0.049477,0.051104,0.042037,0.045351,0.04231,0.0396
21,0.046866,0.040649,0.050208,0.044195,0.03922,0.041937,0.050142,0.043
967,0.043197,0.086885,0.085726,0.093526,0.078948,0.080438,0.0824,0.083
537,0.084467,0.083225,0.096177,0.085339,0.079918,0.088679,0.086963,0.0
85837,0.085396,0.041507,0.042156,0.03546,0.046658,0.04966,0.039237,0.0
47028,0.047285,0.040273,0.043232,0.040837,0.052943,0.04946,0.036126,0.
041015,0.044558,0.046706,0.043378,0.042033,0.095633,0.086922,0.10096,0
.093613,0.091346,0.084514,0.083451,0.084691,0.086955,0.086468,0.08728,
0.089057,0.085175,0.046148,0.0363,0.044439,0.0489,0.044885,0.044958,0.
041499,0.045956,0.044463,0.045602,0.046647,0.050266,0.045945,0.045519,
0.049391,0.044748,0.04898,0.048245,0.037945,0.041933,0.041464,0.081203
,0.098832,0.085405,0.078561,0.08369,0.099584,0.097241,0.084031,0.07493
9,0.046918,0.044197,0.043493,0.048468,0.042071,0.045217,0.047921,0.043
312,0.042857,0.040257,0.047171,0.044429,0.050958,0.043061,0.048002,0.0
46587,0.046134,0.046809,0.043083,0.044171,0.040366,0.043502,0.044132,0
.040569,0.042632,0.084581,0.091137,0.079275,0.10048,0.042603,0.045933,
0.050716,0.045303,0.045878,0.038472,0.037689,0.051682,0.04531,0.039662
,0.032442,0.047127,0.04257,0.04575,0.039788,0.040259,0.0433,0.042396,0
.052221,0.043936,0.042234,0.040208,0.044596,0.047686,0.039399,0.044932
,0.043656,0.045411,0.041914,0.036776,0.043844,0.042595,0.043017,0.0405
59,0.04378,0.049119,0.041227,0.039726,0.0425,0.047745,0.042564,0.03934
5,0.040082,0.040658,0.049428,0.047286,0.053502,0.050605,0.039222,0.041
065,0.043174,0.039483,0.048484,0.042307,0.042408,0.043121,0.051196,0.0
41284,0.044568,0.038458,0.03888,0.049541,0.044632,0.043519,0.041621,0.
044053,0.039278,0.043176,0.043563,0.04662,0.037909,0.040758,0.039114,0
.050461,0.049459,0.050553,0.041113,0.046483,0.041594,0.040577,0.036011
,0.042139,0.053315,0.042886,0.053629,0.045441,0.049008,0.033619,0.0544
45,0.051371,0.047405,0.034897,0.037701,0.035768,0.041207,0.047453,0.04
965,0.04499,0.039045,0.037811,0.045717,0.045253,0.035988,0.046419,0.04
8737,0.0396,0.034381,0.053384,0.043584,0.047032,0.04894,0.037929,0.046
258,0.042961,0.045305,0.046014,0.039254,0.040084,0.04584,0.04266,0.043
835,0.038003,0.048768,0.044512,0.043762,0.043265,0.041859,0.041942,0.0
41407,0.047041,0.04656,0.043951,0.046208,0.041868,0.04141,0.047582,0.0
45403,0.045625,0.044013,0.047595,0.038209,0.040059,0.04761,0.041652,0.
045273,0.035517,0.044462,0.035162,0.048019,0.041581,0.048903,0.041333,
0.046991,0.042519,0.047434,0.041435,0.050811,0.041485,0.037451,0.04419
5,0.044537,0.034517,0.037422,0.05022,0.042971,0.050869,0.050824,0.0428
42,0.037493,0.049032,0.044608,0.044682,0.04699,0.046188,0.037951,0.042
977,0.036365,0.045537,0.039952,0.042855,0.044,0.042035,0.041955,0.0440
64,0.051075,0.046578,0.051449,0.044234,0.04269,0.048311,0.040884,0.040
288,0.045375,0.045996,0.042946
0.11644,0.12025,0.12816,0.11247,0.13006,0.12436,0.1254,0.1127,0.12615,
0.12739,0.1096,0.13489,0.1176,0.12677,0.11542,0.12548,0.12645,0.12602,
0.12233,0.12162,0.23072,0.24559,0.26897,0.18875,0.12783,0.11798,0.1183
8,0.12956,0.12205,0.13202,0.12816,0.11972,0.12343,0.12222,0.11752,0.13
394,0.13234,0.12285,0.12236,0.36586,0.26146,0.26193,0.26443,0.28085,0.
25332,0.27083,0.12501,0.1232,0.12343,0.11925,0.12424,0.10696,0.11457,0

.12059,0.12789,0.11855,0.13448,0.12591,0.12265,0.12558,0.12445,0.367,0
.29784,0.19191,0.25777,0.19118,0.2629,0.22315,0.24448,0.2939,0.30545,0
.12382,0.12031,0.11222,0.12401,0.13005,0.12575,0.11552,0.12151,0.13014
,0.12395,0.12084,0.13317,0.12276,0.1194,0.12809,0.4174,0.37891,0.31652
,0.22912,0.24644,0.27502,0.31502,0.21975,0.21001,0.21242,0.25633,0.237
98,0.11841,0.12712,0.12557,0.12239,0.12062,0.11765,0.12389,0.1189,0.12
414,0.12716,0.12812,0.11945,0.12084,0.12162,0.1315,0.11715,0.36096,0.2
9008,0.35216,0.2964,0.25493,0.20002,0.25271,0.26717,0.22916,0.2617,0.2
387,0.26272,0.29974,0.2597,0.12437,0.12391,0.11882,0.12207,0.12442,0.1
2625,0.12109,0.12569,0.12709,0.12117,0.12335,0.1272,0.12994,0.11933,0.
12775,0.11977,0.37572,0.43752,0.34321,0.38323,0.41988,0.35737,0.25814,
0.21011,0.27357,0.27138,0.19394,0.27604,0.24585,0.32648,0.25056,0.2295
5,0.13142,0.12339,0.12547,0.11871,0.11628,0.12912,0.12614,0.12265,0.12
537,0.11839,0.12333,0.13162,0.10811,0.12854,0.13221,0.12715,0.12526,0.
42449,0.35258,0.29835,0.34193,0.42518,0.37238,0.22883,0.24263,0.20447,
0.2274,0.27415,0.24113,0.20931,0.21686,0.23733,0.27988,0.21677,0.12986
,0.12324,0.11743,0.12387,0.12918,0.1202,0.12038,0.12897,0.12171,0.1198
3,0.12093,0.12654,0.12587,0.082387,0.086936,0.099954,0.12435,0.33348,0
.36852,0.42998,0.38101,0.2992,0.32585,0.29541,0.26392,0.27303,0.22731,
0.20632,0.22879,0.21108,0.26106,0.27264,0.24398,0.21881,0.24322,0.1273
7,0.12929,0.1142,0.1263,0.12993,0.12915,0.11628,0.11739,0.12545,0.1193
3,0.12628,0.090284,0.085248,0.078117,0.072311,0.077965,0.087799,0.1220
7,0.38561,0.3187,0.29597,0.35138,0.44424,0.37371,0.43082,0.31543,0.241
12,0.21658,0.2661,0.23074,0.24565,0.28283,0.19862,0.27894,0.22914,0.22
975,0.131,0.12405,0.12865,0.13287,0.11707,0.12149,0.12652,0.12466,0.12
354,0.077385,0.086806,0.080305,0.088983,0.077248,0.10024,0.086896,0.08
2222,0.078713,0.12376,0.36064,0.42118,0.33556,0.33837,0.37152,0.34665,
0.34561,0.21448,0.20639,0.31364,0.21698,0.20311,0.23303,0.21779,0.2197
1,0.22095,0.25276,0.22709,0.28565,0.12631,0.1197,0.11485,0.12425,0.128
3,0.11695,0.09883,0.077853,0.090837,0.080465,0.085518,0.082299,0.08959
,0.081088,0.091554,0.085256,0.071351,0.08207,0.29543,0.35583,0.40044,0
.44559,0.31389,0.42476,0.44464,0.21991,0.27393,0.30852,0.18897,0.37335
,0.246,0.23631,0.22348,0.2602,0.21327,0.24796,0.23724,0.13103,0.1229,0
.12074,0.075268,0.078259,0.073094,0.083035,0.076806,0.085228,0.071056,
0.080902,0.08087,0.093935,0.079893,0.082453,0.080118,0.071974,0.082528
,0.39807,0.29183,0.34637,0.37614,0.42922,0.41621,0.41125,0.45264,0.392
51,0.2079,0.25581,0.22751,0.29549,0.27573,0.32729,0.24939,0.23355,0.27
568,0.22499,0.27977,0.073418,0.075022,0.086748,0.081793,0.079859,0.089
932,0.088946,0.088537,0.089173,0.084703,0.074805,0.085633,0.078664,0.0
75883,0.092776,0.099407,0.084646,0.086244,0.33039,0.38724,0.31896,0.30
133,0.28348,0.41859,0.31059,0.53268,0.41774,0.24575,0.25855,0.20156,0.
13909,0.2092,0.1383,0.188,0.14795,0.15799,0.19981,0.17901,0.084059,0.0
78896,0.082067,0.077718,0.070054,0.072114,0.085459,0.076727,0.079229,0
.089245,0.077406,0.088624,0.082799,0.082817,0.079671,0.077455,0.073119
,0.08369,0.35866,0.43984,0.38289,0.34341,0.3975,0.32093,0.41878,0.3565
,0.36451,0.22024,0.23067,0.12722,0.18864,0.13626,0.14198,0.18454,0.196
44,0.21961,0.19221,0.13813,0.088734,0.076886,0.085463,0.087383,0.08308
5,0.079407,0.085641,0.074242,0.07546,0.083706,0.078225,0.0815,0.080118
,0.091372,0.095903,0.082177,0.088279,0.077438,0.31239,0.26915,0.33639,
0.30017,0.4357,0.32646,0.26569,0.19973,0.1799,0.20303,0.18467,0.16854,
0.2085,0.17553,0.19775,0.19943,0.19067,0.15775,0.17239,0.14582,0.08589
1,0.08914,0.091137,0.084111,0.088081,0.083262,0.074584,0.086712,0.0846

69,0.080847,0.084203,0.087051,0.084913,0.079326,0.076377,0.089492,0.07
7173,0.079263,0.40433,0.35255,0.38021,0.43649,0.36175,0.28043,0.17953,
0.18963,0.17784,0.23991,0.21271,0.18247,0.1695,0.13786,0.16522,0.16463
,0.18154,0.19081,0.2067,0.16556,0.096727,0.075935,0.070455,0.082674,0.
079336,0.083775,0.087857,0.088376,0.086675,0.092976,0.081049,0.073375,
0.083033,0.085307,0.069996,0.08974,0.078422,0.06793,0.30342,0.33253,0.
29407,0.33268,0.24704,0.18557,0.21055,0.23009,0.17032,0.19025,0.22805,
0.14568,0.16213,0.16809,0.16964,0.17827,0.1907,0.15892,0.19903,0.14435
,0.083586,0.084177,0.083189,0.09148,0.077965,0.061414,0.077903,0.07004
7,0.092979,0.092391,0.073709,0.08131,0.079548,0.083988,0.08966,0.08201
,0.079193,0.080834,0.39563,0.33268,0.37607,0.19423,0.15191,0.23013,0.2
1668,0.18082,0.19821,0.1639,0.22802,0.20304,0.18458,0.16964,0.15522,0.
16923,0.20288,0.14766,0.19235,0.20553,0.070283,0.082901,0.077397,0.086
342,0.082352,0.09154,0.076297,0.078859,0.096815,0.083294,0.086354,0.07
9093,0.086632,0.073609,0.067707,0.081272,0.079036,0.077035,0.23314,0.1
5848,0.2039,0.20561,0.25594,0.16862,0.19488,0.18024,0.26088,0.25283,0.
18846,0.19511,0.17999,0.17298,0.12428,0.22437,0.15251,0.1529,0.17389,0
.085655,0.092038,0.085913,0.078617,0.074908,0.066448,0.083715,0.082547
,0.08152,0.087581,0.088546,0.087917,0.076001,0.081845,0.093542,0.07695
9,0.087461,0.04627,0.1803,0.21236,0.22754,0.22673,0.20981,0.19528,0.17
594,0.14764,0.20073,0.27796,0.1872,0.18121,0.23212,0.14888,0.1686,0.17
706,0.16963,0.16449,0.22031,0.084374,0.074958,0.074758,0.081612,0.0867
15,0.089747,0.075492,0.085214,0.081955,0.079547,0.079328,0.080475,0.08
1795,0.087726,0.074275,0.06956,0.085064,0.035948,0.15245,0.19645,0.175
68,0.19492,0.2249,0.20622,0.24677,0.2215,0.20968,0.20387,0.1497,0.1785
1,0.20412,0.16408,0.15871,0.15459,0.20185,0.15953,0.078353,0.067744,0.
08163,0.08282,0.080581,0.086677,0.074794,0.073874,0.080618,0.076748,0.
073155,0.091583,0.086893,0.085562,0.081088,0.09021,0.079376,0.08641,0.
052042,0.26644,0.15254,0.1833,0.22847,0.21941,0.11452,0.20153,0.18082,
0.1197,0.23854,0.15552,0.18339,0.20163,0.16909,0.18207,0.17068,0.16843
,0.16258,0.079335,0.085779,0.086113,0.075106,0.083738,0.081212,0.08524
4,0.085321,0.089631,0.081984,0.08568,0.086708,0.081814,0.081561,0.0814
78,0.084231,0.081562,0.047227,0.041478,0.03617,0.28185,0.25007,0.22806
,0.21307,0.20654,0.22351,0.15449,0.15992,0.16254,0.18169,0.17402,0.154
32,0.21681,0.19094,0.19267,0.083663,0.085808,0.08984,0.084771,0.076558
,0.078935,0.084537,0.071759,0.093549,0.087071,0.08392,0.080307,0.08889
5,0.09833,0.08141,0.087044,0.08786,0.092372,0.046279,0.044364,0.049362
,0.036912,0.22307,0.1832,0.21224,0.16367,0.21563,0.21128,0.22426,0.211
89,0.22373,0.21679,0.18534,0.14378,0.080159,0.082844,0.084939,0.076215
,0.086777,0.07283,0.078562,0.088184,0.083799,0.084408,0.085012,0.08975
1,0.091333,0.086146,0.085653,0.096053,0.084663,0.092593,0.071067,0.084
118,0.035249,0.04194,0.043034,0.051092,0.042848,0.046005,0.27465,0.166
4,0.27694,0.18111,0.2627,0.27717,0.22837,0.17997,0.08278,0.076687,0.08
0226,0.080596,0.067062,0.082904,0.086195,0.084098,0.092548,0.080268,0.
10519,0.06419,0.10822,0.083303,0.084691,0.078758,0.082044,0.09099,0.07
9125,0.082438,0.083378,0.092505,0.0877,0.03486,0.037559,0.043281,0.048
187,0.039295,0.047991,0.18906,0.23379,0.17401,0.19378,0.24845,0.047599
,0.051307,0.083107,0.085903,0.084113,0.079268,0.090943,0.087468,0.0757
15,0.08262,0.078923,0.090371,0.073876,0.090215,0.088357,0.083836,0.081
916,0.077426,0.090648,0.094266,0.079391,0.075034,0.085342,0.04432,0.04
4569,0.050363,0.041564,0.040409,0.044018,0.044922,0.040182,0.041737,0.
044143,0.22288,0.23797,0.040325,0.042206,0.037843,0.03965,0.088348,0.0

80042,0.082462,0.089547,0.078301,0.080236,0.075742,0.091568,0.090045,0
.083423,0.091534,0.081372,0.08378,0.083333,0.090847,0.081244,0.080107,
0.080791,0.075517,0.043744,0.047612,0.048266,0.047173,0.039397,0.04141
,0.040751,0.039874,0.046092,0.038291,0.049002,0.042344,0.037358,0.0425
36,0.050802,0.046737,0.040887,0.081827,0.079288,0.0908,0.074668,0.0763
52,0.074716,0.082948,0.079532,0.076824,0.092673,0.083212,0.077594,0.08
7236,0.083038,0.087655,0.080723,0.037163,0.041899,0.0324,0.043241,0.04
5415,0.040215,0.04426,0.045762,0.037785,0.039002,0.041613,0.051377,0.0
49171,0.03436,0.040344,0.041218,0.046623,0.04421,0.041112,0.092351,0.0
80127,0.099288,0.089576,0.087728,0.081684,0.080334,0.080386,0.079511,0
.081372,0.084041,0.081631,0.07868,0.045516,0.03391,0.040476,0.046011,0
.041087,0.04403,0.042351,0.04242,0.045695,0.042323,0.044266,0.047906,0
.042756,0.045361,0.048782,0.043653,0.046696,0.044414,0.037241,0.040577
,0.039406,0.075856,0.095827,0.081446,0.071906,0.075811,0.094799,0.0916
32,0.080797,0.072713,0.046603,0.044998,0.042377,0.049048,0.04446,0.044
423,0.048049,0.042483,0.040327,0.035799,0.043484,0.042493,0.05057,0.04
1055,0.046658,0.046125,0.043513,0.045222,0.040449,0.044695,0.037577,0.
04635,0.044476,0.042692,0.043598,0.080409,0.085868,0.073302,0.095439,0
.039767,0.045603,0.052515,0.04389,0.042031,0.039373,0.039241,0.049392,
0.042136,0.039793,0.034256,0.046717,0.045052,0.043094,0.039492,0.04108
6,0.043136,0.043913,0.049756,0.044875,0.04331,0.038468,0.044464,0.0428
42,0.037013,0.04351,0.042935,0.042288,0.039619,0.035232,0.041854,0.043
598,0.043777,0.038795,0.040984,0.048692,0.038256,0.036981,0.039341,0.0
46127,0.039381,0.039779,0.039858,0.04131,0.048715,0.04672,0.05079,0.04
8487,0.038767,0.043974,0.041543,0.038952,0.046187,0.041753,0.045358,0.
044193,0.047774,0.038508,0.042692,0.035631,0.038515,0.046838,0.044354,
0.041604,0.040714,0.039621,0.036503,0.041789,0.042845,0.045746,0.03957
,0.038289,0.038469,0.046702,0.049762,0.046851,0.040858,0.046052,0.0397
16,0.039599,0.033317,0.040508,0.049784,0.039577,0.051489,0.043511,0.04
7699,0.031431,0.051819,0.047754,0.045197,0.034063,0.037973,0.037797,0.
040461,0.04486,0.048245,0.042132,0.038754,0.037591,0.042321,0.043489,0
.036269,0.046597,0.043596,0.039035,0.035014,0.052247,0.039849,0.0486,0
.048226,0.033995,0.041368,0.042931,0.046382,0.041779,0.041708,0.038524
,0.042772,0.044558,0.044772,0.03366,0.045038,0.044497,0.042942,0.04073
8,0.04112,0.041819,0.039654,0.045863,0.046244,0.041543,0.044365,0.0401
52,0.04095,0.0445,0.042624,0.042739,0.040326,0.04666,0.035673,0.036381
,0.047085,0.041025,0.044887,0.035815,0.043113,0.036932,0.045089,0.0417
82,0.049658,0.038431,0.044943,0.039726,0.045705,0.038073,0.049267,0.04
0905,0.037403,0.042585,0.040083,0.031998,0.036839,0.04894,0.039562,0.0
48443,0.046978,0.042551,0.037135,0.045391,0.046707,0.0447,0.04284,0.04
5576,0.037788,0.040624,0.037436,0.044715,0.036928,0.041945,0.044228,0.
038501,0.042347,0.042595,0.043737,0.042127,0.049534,0.041322,0.0394,0.
047306,0.038763,0.041675,0.04332,0.045556,0.040765
0.11195,0.11283,0.1249,0.10957,0.12332,0.11876,0.12379,0.10853,0.11971
,0.12326,0.10187,0.12778,0.11009,0.12337,0.11286,0.11667,0.12158,0.121
05,0.11889,0.12253,0.23392,0.24409,0.30324,0.18251,0.12407,0.11538,0.1
1451,0.12299,0.12053,0.12123,0.12595,0.11464,0.1208,0.11527,0.11196,0.
12467,0.12696,0.11421,0.11478,0.35018,0.2762,0.25355,0.28163,0.28182,0
.2484,0.2644,0.11984,0.11919,0.11975,0.11577,0.11873,0.1043,0.11219,0.
11642,0.12549,0.11327,0.12721,0.12069,0.11777,0.1163,0.12073,0.3658,0.
29858,0.20342,0.24868,0.20068,0.25234,0.24476,0.23906,0.25841,0.31685,
0.11714,0.11372,0.10979,0.12087,0.12553,0.12118,0.10958,0.11669,0.1219

8,0.11808,0.11629,0.12589,0.11522,0.11587,0.12437,0.41875,0.407,0.3090
4,0.24654,0.21228,0.24741,0.30865,0.18834,0.19312,0.19854,0.26115,0.23
168,0.11477,0.12002,0.11914,0.11326,0.11123,0.11362,0.12209,0.11168,0.
11735,0.11963,0.12199,0.11482,0.11549,0.11331,0.1241,0.11668,0.36392,0
.29845,0.33128,0.31448,0.2565,0.21496,0.27699,0.27782,0.20109,0.2616,0
.21965,0.24298,0.27312,0.23101,0.11772,0.11983,0.11755,0.12132,0.11748
,0.11916,0.11609,0.12228,0.12076,0.11653,0.12057,0.12184,0.12014,0.114
36,0.11856,0.11407,0.36103,0.42039,0.37267,0.37503,0.43971,0.34936,0.2
4796,0.19088,0.2784,0.26959,0.18762,0.23337,0.24805,0.30514,0.23954,0.
22369,0.12635,0.12245,0.12402,0.11269,0.1095,0.12378,0.12169,0.11744,0
.11259,0.11215,0.11731,0.12673,0.10473,0.1213,0.12433,0.11904,0.1172,0
.36598,0.35051,0.3026,0.35857,0.40358,0.34367,0.20168,0.24726,0.21299,
0.20114,0.27343,0.24159,0.19831,0.21926,0.21957,0.29374,0.21901,0.1230
9,0.11685,0.1127,0.11973,0.12182,0.11365,0.11671,0.12565,0.11415,0.115
37,0.1156,0.12437,0.12059,0.082037,0.083198,0.094283,0.11439,0.33606,0
.35457,0.39661,0.39496,0.31902,0.32897,0.32092,0.26291,0.25052,0.23564
,0.20284,0.23084,0.1985,0.25799,0.25614,0.2352,0.2031,0.23794,0.12202,
0.12362,0.11324,0.11946,0.12487,0.12264,0.11066,0.11815,0.1191,0.11534
,0.11418,0.087805,0.080513,0.072449,0.073782,0.076007,0.086336,0.1187,
0.33942,0.33728,0.33976,0.36274,0.44145,0.37348,0.43461,0.30218,0.2297
2,0.23377,0.2626,0.19911,0.23171,0.26909,0.18313,0.27908,0.21941,0.226
47,0.12125,0.11488,0.1248,0.12485,0.11271,0.11556,0.12348,0.11574,0.12
05,0.072783,0.081504,0.080399,0.079341,0.075574,0.095501,0.0824,0.0781
03,0.078626,0.11611,0.33547,0.40481,0.29985,0.33447,0.35666,0.36305,0.
32409,0.20893,0.20088,0.29194,0.22317,0.19392,0.22685,0.20096,0.22045,
0.23589,0.24047,0.21262,0.27767,0.12051,0.11301,0.10783,0.11744,0.1215
8,0.11506,0.090037,0.075635,0.090802,0.079085,0.081431,0.079583,0.0834
65,0.077963,0.088497,0.080162,0.067493,0.076452,0.28198,0.38153,0.4429
3,0.43997,0.33921,0.43406,0.44626,0.20924,0.25806,0.29343,0.18727,0.34
768,0.24687,0.21396,0.23714,0.22357,0.20311,0.24546,0.25064,0.1301,0.1
2088,0.11585,0.076704,0.07302,0.075386,0.083067,0.072017,0.079701,0.06
6172,0.079456,0.07776,0.093777,0.076403,0.080272,0.076281,0.067559,0.0
81258,0.39621,0.27205,0.34185,0.34855,0.39524,0.39692,0.36377,0.4627,0
.40888,0.21535,0.2725,0.23604,0.27662,0.25666,0.29837,0.24587,0.22092,
0.2724,0.20287,0.26334,0.070753,0.073995,0.081252,0.080033,0.080056,0.
085193,0.083218,0.082283,0.084214,0.077945,0.069298,0.077687,0.074302,
0.071669,0.087872,0.096717,0.07926,0.079397,0.33581,0.46691,0.31473,0.
29665,0.25088,0.38829,0.33366,0.52942,0.41483,0.23928,0.26954,0.19531,
0.13084,0.20662,0.13199,0.18148,0.12791,0.15114,0.20426,0.16504,0.0826
17,0.078122,0.078655,0.076783,0.067006,0.069144,0.085384,0.073227,0.08
1,0.082419,0.075599,0.083784,0.080115,0.079166,0.076837,0.078169,0.069
373,0.07893,0.38784,0.41123,0.46757,0.35362,0.38129,0.28983,0.46133,0.
39297,0.33454,0.22405,0.22869,0.11965,0.18249,0.133,0.14294,0.18455,0.
19314,0.21166,0.18742,0.13548,0.082974,0.07199,0.08666,0.084172,0.0771
27,0.075773,0.081645,0.072105,0.070458,0.080833,0.079808,0.079286,0.07
9182,0.087164,0.087805,0.078306,0.084811,0.075119,0.32516,0.26947,0.30
793,0.3645,0.42863,0.3324,0.24535,0.19461,0.18084,0.18935,0.16971,0.16
57,0.19777,0.17419,0.1826,0.18891,0.16332,0.14957,0.16264,0.13997,0.08
2298,0.083565,0.091459,0.082049,0.083158,0.081731,0.070382,0.083232,0.
0806,0.078319,0.077683,0.080948,0.081339,0.078641,0.075183,0.088058,0.
073369,0.071984,0.42304,0.3365,0.39389,0.4373,0.35332,0.28458,0.17502,
0.18663,0.18554,0.22158,0.23269,0.1802,0.17278,0.13484,0.16372,0.18002

,0.16419,0.17273,0.20422,0.15728,0.094534,0.074857,0.068086,0.080559,0
.077533,0.079888,0.086061,0.088063,0.08456,0.087198,0.076706,0.071621,
0.078365,0.08131,0.066716,0.083777,0.074936,0.066813,0.29826,0.30734,0
.31548,0.36221,0.287,0.17069,0.20857,0.23693,0.16549,0.19709,0.21652,0
.14387,0.16178,0.15146,0.16268,0.16848,0.20256,0.17191,0.17897,0.14773
,0.079337,0.080405,0.083099,0.085499,0.076964,0.057729,0.072698,0.0671
83,0.088915,0.092415,0.067133,0.079776,0.080373,0.076727,0.092006,0.08
1304,0.077799,0.077005,0.37854,0.30496,0.38935,0.20424,0.15185,0.22773
,0.20765,0.16674,0.19521,0.14347,0.24383,0.21211,0.18731,0.15341,0.128
86,0.16936,0.18861,0.15449,0.18882,0.19583,0.06871,0.082022,0.07179,0.
07984,0.078197,0.090124,0.072368,0.079044,0.095306,0.076886,0.081406,0
.074209,0.081479,0.069581,0.06606,0.081106,0.078405,0.078224,0.25489,0
.15882,0.20784,0.19347,0.25321,0.16354,0.16953,0.19467,0.26576,0.276,0
.16777,0.18393,0.16111,0.17603,0.12201,0.22736,0.15461,0.15532,0.18919
,0.083088,0.091018,0.083708,0.078226,0.071767,0.063448,0.081949,0.0783
95,0.08021,0.083429,0.087072,0.083824,0.076286,0.077552,0.089806,0.078
697,0.086292,0.046227,0.1987,0.2117,0.21894,0.20027,0.19436,0.19052,0.
18111,0.16118,0.18556,0.25971,0.17632,0.17622,0.22016,0.14138,0.15996,
0.16324,0.17133,0.1521,0.21703,0.081399,0.075153,0.07368,0.080299,0.08
136,0.088641,0.074308,0.083674,0.079731,0.075445,0.079913,0.078583,0.0
79718,0.089887,0.069732,0.069651,0.081273,0.036322,0.15475,0.20469,0.1
904,0.18901,0.21135,0.19686,0.23285,0.21617,0.19817,0.19367,0.13862,0.
18111,0.20147,0.1816,0.1629,0.13549,0.20388,0.1626,0.075722,0.066331,0
.080799,0.077806,0.080446,0.081929,0.073371,0.07127,0.079798,0.075772,
0.070154,0.088374,0.087284,0.086356,0.07955,0.084014,0.077888,0.081234
,0.052045,0.2781,0.16813,0.18702,0.25455,0.23022,0.1067,0.20972,0.1823
9,0.11114,0.24451,0.15792,0.16612,0.19561,0.17272,0.17761,0.1627,0.170
37,0.15466,0.076621,0.082152,0.084318,0.07174,0.079526,0.081122,0.0843
3,0.082706,0.085814,0.079191,0.082053,0.080346,0.079388,0.078797,0.079
315,0.077225,0.078074,0.048115,0.041465,0.032823,0.28087,0.24568,0.212
14,0.21824,0.20766,0.23,0.16471,0.16286,0.17454,0.18672,0.1631,0.16228
,0.21733,0.16078,0.1907,0.082365,0.080942,0.086584,0.083047,0.076541,0
.077972,0.079305,0.07385,0.090364,0.082111,0.078334,0.073293,0.08447,0
.096928,0.079747,0.087639,0.084985,0.089431,0.046988,0.044422,0.04694,
0.035113,0.23201,0.18695,0.20809,0.16718,0.21173,0.19607,0.20179,0.201
59,0.21645,0.20287,0.19224,0.14566,0.079829,0.080605,0.082206,0.069413
,0.080465,0.069478,0.075215,0.084178,0.07992,0.079656,0.084372,0.08889
6,0.086765,0.079432,0.083867,0.093733,0.080487,0.089933,0.066657,0.083
379,0.035151,0.041946,0.041984,0.048187,0.039145,0.044459,0.25279,0.17
279,0.26819,0.19931,0.26335,0.25779,0.23215,0.17594,0.079177,0.070591,
0.0775,0.078968,0.067459,0.081098,0.08209,0.082446,0.091599,0.077681,0
.10229,0.06298,0.10387,0.08083,0.082795,0.075741,0.07939,0.085282,0.07
608,0.078771,0.078562,0.086149,0.082351,0.033164,0.036474,0.038249,0.0
45054,0.040626,0.046205,0.18988,0.22367,0.17214,0.17674,0.25014,0.0459
69,0.048442,0.081331,0.077819,0.078176,0.077214,0.086619,0.082729,0.07
5871,0.081742,0.074739,0.087867,0.075425,0.082129,0.08734,0.082226,0.0
79073,0.078012,0.090019,0.088128,0.077008,0.075093,0.082157,0.040365,0
.043744,0.048973,0.042921,0.039032,0.041492,0.044558,0.040655,0.038481
,0.042925,0.22741,0.24406,0.039626,0.039364,0.038565,0.041026,0.087071
,0.076449,0.07669,0.081955,0.079282,0.076394,0.070203,0.089266,0.08298
,0.079598,0.087728,0.079086,0.081125,0.078465,0.089162,0.077846,0.0755
88,0.07498,0.077749,0.043491,0.04839,0.048591,0.045445,0.041029,0.0409

19,0.038089,0.037154,0.044122,0.036636,0.047706,0.039861,0.036297,0.04
1794,0.047335,0.045674,0.040326,0.079903,0.076541,0.087179,0.069887,0.
073227,0.071645,0.08162,0.07831,0.07519,0.087868,0.080843,0.072745,0.0
79646,0.083726,0.082443,0.077211,0.034413,0.038286,0.031866,0.043372,0
.043181,0.038502,0.041351,0.040715,0.03939,0.039507,0.040082,0.049856,
0.049166,0.034251,0.038961,0.040567,0.046438,0.04142,0.041424,0.091201
,0.077217,0.097335,0.087311,0.081758,0.075984,0.078064,0.080059,0.0806
99,0.077094,0.08164,0.078487,0.076529,0.0442,0.034763,0.041638,0.04567
8,0.042375,0.040261,0.043167,0.042605,0.042744,0.037256,0.044553,0.048
405,0.041955,0.044758,0.045341,0.04174,0.047179,0.041967,0.03373,0.038
854,0.036811,0.074332,0.090256,0.080817,0.069411,0.071936,0.089682,0.0
86091,0.080077,0.070447,0.044838,0.045275,0.039271,0.048508,0.042775,0
.041378,0.047291,0.040546,0.038982,0.033311,0.045364,0.041267,0.047339
,0.041692,0.044789,0.048125,0.043761,0.043524,0.040528,0.04582,0.03382
3,0.043145,0.044087,0.041203,0.044756,0.076339,0.079958,0.072201,0.090
62,0.038505,0.045047,0.053345,0.042934,0.038483,0.040829,0.036127,0.04
5752,0.039138,0.037485,0.035966,0.04245,0.044377,0.042878,0.040313,0.0
42371,0.041633,0.040419,0.046699,0.044747,0.042319,0.037999,0.045159,0
.040441,0.035427,0.042601,0.042258,0.039873,0.0415,0.036627,0.041899,0
.043335,0.042728,0.036521,0.040255,0.050357,0.037645,0.035936,0.040287
,0.047696,0.038706,0.039025,0.040969,0.040432,0.045646,0.043081,0.0462
53,0.046267,0.037664,0.04461,0.042319,0.036689,0.043028,0.040699,0.046
494,0.045412,0.046139,0.038343,0.039145,0.035953,0.035734,0.045119,0.0
4335,0.041272,0.039058,0.039521,0.037134,0.040471,0.040568,0.046636,0.
038993,0.036579,0.035799,0.042499,0.046884,0.044058,0.042232,0.043105,
0.038606,0.039461,0.034309,0.039746,0.046337,0.038017,0.047787,0.04351
8,0.04623,0.03519,0.04866,0.046014,0.044993,0.033742,0.03858,0.036426,
0.042966,0.043381,0.047914,0.045024,0.039229,0.035521,0.043943,0.04224
4,0.034515,0.043763,0.0414,0.038021,0.03374,0.051899,0.037641,0.046109
,0.044047,0.034032,0.04133,0.041626,0.043945,0.042594,0.040115,0.03652
7,0.042735,0.045501,0.046095,0.033671,0.042276,0.042957,0.042011,0.039
366,0.037023,0.041633,0.037893,0.04582,0.046725,0.040483,0.043748,0.03
8991,0.040442,0.043439,0.04081,0.040265,0.039253,0.046592,0.031416,0.0
35134,0.044313,0.038339,0.046097,0.036342,0.040334,0.035893,0.047229,0
.04098,0.048766,0.035183,0.046243,0.038283,0.046693,0.038215,0.046455,
0.035248,0.038088,0.042309,0.038587,0.033785,0.034557,0.044128,0.03761
2,0.045323,0.044637,0.040951,0.036463,0.043935,0.045785,0.042775,0.041
018,0.042668,0.033271,0.038759,0.0368,0.042271,0.037307,0.040952,0.042
208,0.038889,0.043038,0.040536,0.044156,0.039591,0.046194,0.040634,0.0
35954,0.046637,0.038207,0.042637,0.041062,0.041682,0.040958
0.10724,0.11126,0.117,0.10595,0.11283,0.11649,0.12,0.10762,0.11869,0.1
1273,0.098998,0.12397,0.10715,0.11969,0.10983,0.11258,0.11743,0.12043,
0.11358,0.12005,0.21735,0.2361,0.31431,0.17865,0.11811,0.11334,0.1102,
0.12079,0.11869,0.11458,0.12203,0.10954,0.1171,0.11264,0.10572,0.12046
,0.11802,0.10928,0.11022,0.34263,0.27796,0.25509,0.26512,0.28985,0.256
31,0.25698,0.11749,0.11365,0.11727,0.11006,0.11229,0.10176,0.10972,0.1
1699,0.11568,0.11185,0.12301,0.11542,0.11795,0.10908,0.11823,0.40155,0
.28563,0.23026,0.24543,0.21252,0.26301,0.24083,0.21677,0.26644,0.30563
,0.11268,0.11185,0.1025,0.11568,0.12367,0.11368,0.10588,0.11395,0.1206
8,0.11845,0.1134,0.11918,0.1125,0.11096,0.12203,0.37809,0.43084,0.3156
,0.21897,0.22841,0.22042,0.29744,0.19427,0.20603,0.20053,0.24499,0.243
9,0.11031,0.11531,0.11666,0.10326,0.10995,0.10591,0.11682,0.10912,0.11

241,0.11998,0.11808,0.1113,0.11055,0.1144,0.11936,0.11542,0.36854,0.30
711,0.37935,0.3008,0.23199,0.21585,0.26393,0.25996,0.18642,0.25832,0.2
4396,0.24981,0.2575,0.24481,0.11239,0.11325,0.11246,0.11581,0.11325,0.
10921,0.11279,0.11507,0.1151,0.1104,0.1133,0.11623,0.11799,0.11141,0.1
1754,0.11078,0.35581,0.40969,0.34793,0.36737,0.44198,0.36297,0.25139,0
.20016,0.28096,0.26454,0.1919,0.22869,0.23739,0.33152,0.22516,0.21511,
0.12138,0.11827,0.12116,0.11273,0.10638,0.11779,0.1175,0.11476,0.11216
,0.10877,0.11715,0.11599,0.098749,0.11566,0.11734,0.11778,0.11227,0.39
806,0.3791,0.33254,0.33699,0.37047,0.35727,0.21419,0.24463,0.21478,0.1
9955,0.27108,0.23368,0.20529,0.24327,0.20279,0.30074,0.22219,0.11302,0
.11097,0.10582,0.11156,0.11653,0.10819,0.1137,0.11967,0.106,0.11157,0.
1107,0.12209,0.1137,0.077572,0.083827,0.089306,0.11115,0.28722,0.37282
,0.36097,0.36655,0.31897,0.2998,0.34167,0.24417,0.25883,0.22709,0.2193
6,0.21596,0.21454,0.24632,0.24236,0.23299,0.20206,0.22106,0.11665,0.11
791,0.107,0.11398,0.12165,0.11838,0.11076,0.11638,0.11343,0.11031,0.11
066,0.083854,0.079424,0.072141,0.070845,0.072391,0.083966,0.11768,0.32
957,0.36376,0.3316,0.36345,0.40111,0.36748,0.39776,0.29989,0.20844,0.2
3055,0.25387,0.20709,0.21762,0.26103,0.19092,0.28225,0.22521,0.23459,0
.12125,0.11143,0.12591,0.11842,0.10867,0.111,0.11785,0.11176,0.11156,0
.069094,0.080564,0.081135,0.077282,0.075562,0.089829,0.079418,0.074409
,0.077272,0.11535,0.34439,0.39601,0.27727,0.33763,0.33808,0.40374,0.30
343,0.21519,0.19192,0.29299,0.21591,0.19638,0.21994,0.18267,0.21613,0.
23217,0.23643,0.20038,0.26081,0.11619,0.10807,0.10327,0.11382,0.11907,
0.10941,0.088418,0.072781,0.086087,0.078813,0.079574,0.074827,0.081834
,0.073385,0.087946,0.07684,0.067,0.07533,0.26841,0.38154,0.40692,0.415
06,0.35953,0.41201,0.42552,0.18887,0.27587,0.2865,0.21254,0.33674,0.23
864,0.22105,0.2346,0.21955,0.23208,0.23605,0.23612,0.12513,0.11619,0.1
1545,0.076058,0.071494,0.073168,0.079578,0.068783,0.078052,0.067881,0.
075508,0.075818,0.08993,0.071608,0.076003,0.0749,0.069517,0.079792,0.4
1266,0.29955,0.32347,0.36484,0.39887,0.37263,0.36854,0.46093,0.41784,0
.20212,0.262,0.23836,0.29098,0.24279,0.28098,0.24711,0.22192,0.27004,0
.20493,0.2568,0.068667,0.073689,0.079348,0.07403,0.076001,0.081756,0.0
79859,0.078008,0.080852,0.075309,0.067895,0.075057,0.07291,0.067327,0.
085183,0.091798,0.077841,0.076275,0.36458,0.42405,0.35242,0.30366,0.30
406,0.37711,0.32344,0.52358,0.4039,0.22459,0.26758,0.19828,0.14434,0.1
9524,0.14242,0.17109,0.12758,0.15922,0.2184,0.15836,0.079954,0.078698,
0.079062,0.073962,0.064314,0.069955,0.082268,0.07116,0.077967,0.07858,
0.072774,0.082033,0.075192,0.079006,0.072033,0.076192,0.0669,0.078459,
0.35318,0.4442,0.44313,0.38099,0.39685,0.32062,0.44677,0.3887,0.34102,
0.22074,0.21199,0.1147,0.17662,0.14268,0.13911,0.18513,0.17646,0.22242
,0.17254,0.12541,0.079238,0.069631,0.084845,0.081441,0.074707,0.07962,
0.080771,0.067207,0.068384,0.076547,0.07954,0.076065,0.078435,0.087463
,0.081047,0.074758,0.083001,0.075518,0.34227,0.30088,0.33798,0.35736,0
.4312,0.33502,0.26448,0.19012,0.17376,0.18507,0.17486,0.1619,0.18507,0
.17366,0.17965,0.19275,0.15928,0.13947,0.1751,0.13452,0.079177,0.07883
8,0.090181,0.076196,0.079522,0.079613,0.071039,0.080857,0.082004,0.076
586,0.072106,0.081798,0.080361,0.077544,0.074862,0.08446,0.071804,0.07
005,0.4073,0.34509,0.38672,0.39629,0.35148,0.27817,0.16383,0.18143,0.1
8208,0.23053,0.22575,0.1816,0.16837,0.13735,0.15788,0.18033,0.16484,0.
18526,0.19912,0.16112,0.087751,0.073147,0.066191,0.078243,0.069545,0.0
76381,0.07957,0.086834,0.081172,0.08827,0.074038,0.070662,0.072965,0.0
75331,0.065255,0.082286,0.069572,0.066214,0.30652,0.32245,0.27704,0.38

405,0.30546,0.1682,0.19882,0.22742,0.1376,0.22413,0.22438,0.123,0.1500
5,0.15926,0.15819,0.17663,0.21303,0.16868,0.17912,0.1454,0.079576,0.07
9589,0.077411,0.082078,0.076272,0.058076,0.067706,0.066123,0.083532,0.
086825,0.064495,0.084875,0.076371,0.073296,0.08878,0.079669,0.073178,0
.074267,0.38163,0.28803,0.40995,0.21717,0.15744,0.22503,0.18899,0.1541
7,0.18364,0.16176,0.23246,0.21051,0.17378,0.15389,0.13668,0.16701,0.18
757,0.14517,0.18265,0.18387,0.063654,0.075807,0.071433,0.077916,0.0735
08,0.085061,0.069958,0.075237,0.091675,0.075742,0.080958,0.072406,0.07
8655,0.066773,0.064996,0.077227,0.075878,0.076802,0.24737,0.16464,0.18
204,0.19059,0.23865,0.19057,0.1743,0.19044,0.27113,0.25963,0.16541,0.1
7896,0.15545,0.17424,0.12242,0.22158,0.15102,0.15381,0.19997,0.080098,
0.089612,0.082984,0.076649,0.071014,0.063442,0.078474,0.075956,0.08030
2,0.081416,0.084075,0.07884,0.071758,0.073885,0.085796,0.077492,0.0846
71,0.045655,0.194,0.21601,0.19481,0.21672,0.19124,0.17264,0.1627,0.156
96,0.18836,0.25641,0.16686,0.18128,0.20188,0.12365,0.168,0.15478,0.175
6,0.14287,0.21391,0.077959,0.073144,0.070492,0.080304,0.080347,0.08354
3,0.075409,0.081627,0.07784,0.071192,0.077554,0.07507,0.07633,0.086312
,0.068803,0.066606,0.078339,0.034394,0.15226,0.20149,0.19804,0.19188,0
.21333,0.19403,0.21553,0.19807,0.19858,0.20537,0.14002,0.16689,0.21487
,0.19099,0.17906,0.11771,0.17844,0.16282,0.073523,0.061308,0.07983,0.0
77772,0.07914,0.082218,0.06859,0.07256,0.078172,0.074998,0.070759,0.08
388,0.083718,0.083984,0.075201,0.08434,0.07296,0.076226,0.049107,0.301
09,0.15699,0.19815,0.26432,0.21712,0.10936,0.20095,0.18674,0.10645,0.2
3528,0.16308,0.16542,0.19699,0.16442,0.19193,0.16028,0.15818,0.13459,0
.073728,0.077011,0.084223,0.072828,0.078468,0.076976,0.080779,0.080485
,0.083482,0.077552,0.079079,0.075812,0.078901,0.076694,0.073152,0.0759
18,0.080812,0.046743,0.037582,0.03353,0.27214,0.21697,0.18188,0.22018,
0.21458,0.22967,0.18058,0.15246,0.17738,0.17481,0.15983,0.14657,0.2030
5,0.17355,0.19035,0.078964,0.075776,0.086046,0.077559,0.07832,0.072213
,0.077632,0.071138,0.086393,0.079526,0.075388,0.06937,0.078179,0.09346
3,0.079053,0.084369,0.082814,0.082896,0.046616,0.046725,0.044918,0.037
36,0.2289,0.17968,0.2032,0.16482,0.19192,0.1905,0.19256,0.19873,0.2097
9,0.1852,0.18373,0.12252,0.077465,0.078925,0.079817,0.068679,0.077998,
0.067235,0.074279,0.0802,0.079468,0.077887,0.08315,0.087926,0.083914,0
.073738,0.078086,0.094125,0.079299,0.087312,0.064876,0.079867,0.033537
,0.042442,0.039572,0.04485,0.039406,0.041168,0.22809,0.16834,0.25473,0
.21432,0.25726,0.26293,0.23918,0.17325,0.075003,0.072598,0.077186,0.07
593,0.065876,0.078286,0.080729,0.078305,0.087215,0.074211,0.096666,0.0
6077,0.098583,0.07653,0.080007,0.071683,0.078487,0.081074,0.073569,0.0
77067,0.079328,0.083406,0.08182,0.034801,0.038139,0.036171,0.045488,0.
039874,0.044242,0.1974,0.21765,0.17037,0.16766,0.2596,0.045519,0.04883
7,0.077063,0.075481,0.078033,0.077087,0.083926,0.081558,0.072659,0.078
514,0.06899,0.08534,0.074991,0.079875,0.082914,0.078414,0.075586,0.071
239,0.09017,0.083892,0.07124,0.074874,0.07636,0.041329,0.04669,0.04781
4,0.044926,0.038683,0.042257,0.044338,0.038262,0.037298,0.039372,0.223
71,0.23133,0.040058,0.036979,0.040653,0.038914,0.083296,0.074547,0.075
173,0.08085,0.079802,0.071252,0.067079,0.08625,0.079316,0.079462,0.087
276,0.0782,0.073678,0.077394,0.088655,0.075582,0.077335,0.073183,0.071
281,0.042078,0.046159,0.048927,0.04358,0.037781,0.040024,0.035559,0.03
6959,0.043446,0.036939,0.046979,0.041612,0.038464,0.042379,0.044398,0.
045343,0.039254,0.077668,0.070514,0.083145,0.06775,0.073632,0.072022,0
.077015,0.073495,0.074214,0.087082,0.080989,0.07022,0.07753,0.082061,0

.078253,0.075773,0.034577,0.038691,0.036325,0.041893,0.041072,0.040177
,0.037311,0.040886,0.037362,0.0415,0.038517,0.048705,0.046401,0.035318
,0.040694,0.038028,0.045262,0.04217,0.043068,0.090863,0.074289,0.09518
5,0.085938,0.076651,0.074411,0.079674,0.078132,0.079724,0.073554,0.079
802,0.07909,0.075217,0.041707,0.037016,0.045012,0.04597,0.041453,0.038
557,0.039517,0.041266,0.041112,0.034766,0.044692,0.045765,0.040629,0.0
42777,0.040867,0.040749,0.047515,0.041666,0.032034,0.039214,0.035685,0
.074026,0.084202,0.074402,0.067106,0.070974,0.082181,0.082456,0.080701
,0.072061,0.042247,0.043401,0.035073,0.045538,0.044075,0.042952,0.0453
52,0.041624,0.03559,0.034003,0.043678,0.040316,0.046573,0.042179,0.044
204,0.043842,0.045982,0.040031,0.037881,0.045174,0.035384,0.044509,0.0
4545,0.040717,0.046625,0.077862,0.079783,0.067169,0.085074,0.039069,0.
041844,0.052408,0.041181,0.039767,0.039471,0.037766,0.04255,0.040021,0
.036654,0.032216,0.038982,0.043609,0.043021,0.042698,0.041774,0.040946
,0.041831,0.046624,0.041231,0.03968,0.036816,0.04113,0.039564,0.035875
,0.040944,0.0409,0.040542,0.038445,0.037927,0.043141,0.042693,0.04222,
0.03551,0.040922,0.049067,0.037373,0.034793,0.040593,0.043984,0.036721
,0.04008,0.039437,0.040103,0.046376,0.043634,0.044633,0.044456,0.04013
5,0.043955,0.04078,0.036178,0.04225,0.040935,0.044401,0.044332,0.04880
4,0.039079,0.038053,0.034165,0.034633,0.042702,0.041543,0.040982,0.039
369,0.035334,0.036839,0.042065,0.038327,0.044931,0.039154,0.035053,0.0
34059,0.042714,0.044962,0.043636,0.03882,0.042715,0.038989,0.038133,0.
033797,0.039712,0.044771,0.03924,0.043408,0.041825,0.046555,0.035761,0
.046095,0.043423,0.042706,0.033047,0.040866,0.03502,0.04361,0.042635,0
.045172,0.044869,0.035312,0.03429,0.045469,0.044576,0.033984,0.042456,
0.040046,0.038467,0.02963,0.052883,0.036912,0.046147,0.042118,0.031984
,0.039556,0.04368,0.044366,0.041155,0.042029,0.03525,0.039239,0.047815
,0.043622,0.033501,0.04089,0.046274,0.040064,0.039594,0.036407,0.03731
1,0.034804,0.043735,0.045962,0.041202,0.044876,0.038837,0.039879,0.042
502,0.038319,0.038158,0.041923,0.044687,0.031095,0.034305,0.043865,0.0
38807,0.044829,0.034985,0.038421,0.036962,0.043778,0.043365,0.047235,0
.035669,0.045098,0.03679,0.046353,0.039954,0.04644,0.036383,0.038114,0
.039711,0.039198,0.036,0.036532,0.04236,0.035529,0.045071,0.046623,0.0
41388,0.03535,0.041708,0.04096,0.042766,0.04104,0.043634,0.033928,0.03
3562,0.037767,0.041376,0.036163,0.038682,0.040487,0.038328,0.041334,0.
042391,0.040654,0.037755,0.046804,0.04149,0.03729,0.046483,0.036653,0.
038964,0.03938,0.03767,0.037229
0.10626,0.1083,0.11208,0.10245,0.11028,0.11269,0.11567,0.1023,0.11725,
0.108,0.09382,0.11817,0.10344,0.11668,0.10905,0.11174,0.11349,0.11621,
0.10726,0.11684,0.20777,0.22416,0.2926,0.20445,0.1117,0.10936,0.10654,
0.1164,0.1127,0.11439,0.11437,0.10037,0.11606,0.10697,0.1055,0.11603,0
.10902,0.10362,0.11002,0.33283,0.27075,0.25727,0.26537,0.28358,0.24202
,0.22197,0.11186,0.11124,0.11536,0.1052,0.1074,0.098591,0.11126,0.1133
,0.11159,0.10992,0.11664,0.11029,0.11344,0.10432,0.11582,0.38378,0.319
07,0.24297,0.22267,0.21942,0.26429,0.26769,0.18781,0.22937,0.28687,0.1
0914,0.10725,0.10121,0.11344,0.11706,0.11263,0.10285,0.11172,0.11602,0
.11536,0.1112,0.11382,0.11003,0.10827,0.11534,0.3872,0.40719,0.36749,0
.22986,0.19893,0.23088,0.28983,0.19576,0.18906,0.20399,0.23373,0.24896
,0.106,0.10941,0.11502,0.10327,0.10356,0.10057,0.10897,0.10797,0.11134
,0.11308,0.11265,0.10829,0.10602,0.11192,0.11338,0.1112,0.39329,0.2881
2,0.37867,0.30391,0.2307,0.2008,0.27917,0.26012,0.1953,0.2456,0.22504,
0.25154,0.25412,0.24628,0.11079,0.11188,0.11088,0.11208,0.11072,0.1059

4,0.11189,0.11557,0.10698,0.10917,0.1034,0.10807,0.11757,0.11387,0.116
01,0.11134,0.3771,0.40033,0.37045,0.32721,0.46723,0.3255,0.25464,0.222
99,0.26989,0.24544,0.20303,0.25528,0.23731,0.30073,0.22251,0.21592,0.1
1886,0.11158,0.12094,0.10742,0.10164,0.11139,0.11227,0.11227,0.11129,0
.11175,0.11665,0.11184,0.095446,0.10808,0.11531,0.11555,0.1076,0.427,0
.3767,0.31225,0.35284,0.36241,0.32354,0.23095,0.23151,0.19155,0.19946,
0.26774,0.21264,0.20416,0.25588,0.19476,0.29214,0.21561,0.11039,0.1073
6,0.10444,0.11078,0.10603,0.10317,0.11067,0.11583,0.10597,0.10652,0.10
583,0.11839,0.11159,0.074632,0.080807,0.088031,0.10758,0.26769,0.37345
,0.35157,0.34517,0.31978,0.29444,0.37205,0.26504,0.2688,0.20632,0.1973
3,0.21719,0.20643,0.24442,0.2501,0.24513,0.19807,0.20691,0.10989,0.114
92,0.10524,0.11309,0.11596,0.11476,0.10494,0.10944,0.10708,0.10728,0.1
1135,0.079514,0.07329,0.070636,0.069678,0.071445,0.081625,0.11509,0.38
191,0.3716,0.32202,0.3737,0.42377,0.3535,0.35996,0.31914,0.20301,0.240
09,0.25461,0.22151,0.23333,0.24087,0.20501,0.27418,0.22745,0.23603,0.1
1659,0.10435,0.12262,0.11506,0.10579,0.10282,0.11296,0.10289,0.10328,0
.068807,0.079995,0.07748,0.076007,0.073046,0.090806,0.080092,0.072603,
0.077063,0.10811,0.33296,0.41297,0.273,0.37147,0.29653,0.38853,0.29839
,0.21295,0.17381,0.30207,0.19878,0.19021,0.22286,0.18453,0.21358,0.229
63,0.24165,0.19364,0.25679,0.1094,0.10217,0.1013,0.11334,0.11521,0.105
08,0.087849,0.069807,0.083397,0.079593,0.074559,0.073371,0.079889,0.07
3501,0.088185,0.073302,0.063694,0.074024,0.24781,0.37086,0.38886,0.439
51,0.34489,0.38639,0.40481,0.18984,0.26139,0.27942,0.19598,0.32958,0.2
4257,0.2026,0.2254,0.21585,0.24538,0.23034,0.25133,0.12126,0.1124,0.11
122,0.074836,0.068024,0.071338,0.076398,0.067108,0.072455,0.068822,0.0
71192,0.075537,0.088136,0.069407,0.072045,0.077168,0.065926,0.076552,0
.42009,0.28891,0.35837,0.38891,0.35977,0.37891,0.32871,0.45618,0.44382
,0.18539,0.2431,0.23158,0.27016,0.22583,0.25099,0.24456,0.21029,0.2564
3,0.20825,0.2531,0.067769,0.072567,0.075244,0.073606,0.073847,0.080329
,0.07938,0.073614,0.083465,0.072468,0.066868,0.070931,0.073141,0.06524
7,0.080924,0.088365,0.077702,0.074448,0.35777,0.42101,0.33395,0.34834,
0.31685,0.36562,0.30099,0.50345,0.37155,0.20864,0.27551,0.20683,0.1492
3,0.17658,0.1449,0.16959,0.11862,0.16888,0.21439,0.13714,0.077206,0.07
6518,0.079928,0.071657,0.061242,0.069951,0.080113,0.070722,0.077664,0.
074291,0.07074,0.078652,0.074215,0.078094,0.071432,0.072255,0.063947,0
.077427,0.34008,0.46962,0.44998,0.37938,0.39367,0.32007,0.46123,0.3755
,0.3014,0.19975,0.19578,0.11985,0.18247,0.13588,0.14307,0.17646,0.1716
6,0.22208,0.1835,0.11949,0.074056,0.067841,0.083578,0.081098,0.07333,0
.080471,0.07894,0.063081,0.064919,0.070829,0.076517,0.074048,0.079039,
0.085417,0.077306,0.073244,0.079528,0.075651,0.31708,0.29459,0.31264,0
.36253,0.41602,0.35332,0.25869,0.19708,0.17978,0.18782,0.18496,0.16382
,0.17953,0.18439,0.18806,0.18591,0.17006,0.13181,0.16585,0.1419,0.0769
64,0.076198,0.085476,0.07228,0.07233,0.078071,0.067585,0.080654,0.0780
43,0.073282,0.070208,0.081435,0.075933,0.077048,0.071536,0.081963,0.07
2281,0.068076,0.42272,0.33267,0.365,0.39116,0.32633,0.31799,0.16312,0.
16605,0.18178,0.21806,0.23319,0.16387,0.16513,0.13655,0.15051,0.18702,
0.15332,0.19417,0.19337,0.15471,0.08707,0.073229,0.067233,0.076555,0.0
68174,0.073969,0.077595,0.082935,0.078322,0.08716,0.071304,0.069356,0.
069817,0.074401,0.064943,0.077857,0.069602,0.064373,0.31392,0.32447,0.
30417,0.3874,0.31916,0.17194,0.21968,0.23078,0.13268,0.24267,0.21365,0
.13411,0.1427,0.14928,0.14516,0.1856,0.20404,0.18022,0.16802,0.16339,0
.076675,0.076421,0.076382,0.081668,0.074794,0.058889,0.063702,0.063591

,0.082136,0.087788,0.064023,0.080321,0.073127,0.073667,0.083572,0.0761
54,0.072797,0.075073,0.34079,0.26175,0.36539,0.21965,0.1606,0.22514,0.
17691,0.16757,0.18019,0.15952,0.21713,0.21429,0.17593,0.15513,0.13785,
0.1538,0.20246,0.14732,0.17684,0.18012,0.062958,0.073949,0.068439,0.07
8465,0.07319,0.081155,0.067493,0.073164,0.08968,0.07512,0.08005,0.0692
22,0.077461,0.067527,0.066382,0.075033,0.070785,0.074645,0.29588,0.158
01,0.17759,0.1603,0.26136,0.1862,0.14603,0.19546,0.25865,0.26004,0.167
94,0.1538,0.15721,0.16544,0.11291,0.21621,0.14575,0.16103,0.18466,0.07
5274,0.088876,0.077683,0.075974,0.067576,0.055025,0.074444,0.071646,0.
076639,0.07904,0.082107,0.077367,0.073957,0.071969,0.084985,0.072412,0
.083501,0.044923,0.21311,0.22505,0.18869,0.21688,0.18285,0.15966,0.154
83,0.16816,0.19212,0.22563,0.15829,0.1778,0.20286,0.12747,0.17995,0.14
564,0.15987,0.15194,0.20888,0.073559,0.073651,0.069969,0.080258,0.0780
69,0.081757,0.073484,0.078924,0.075361,0.068828,0.07044,0.073202,0.074
993,0.081899,0.069025,0.068188,0.074413,0.03507,0.16717,0.19679,0.1919
1,0.18345,0.22279,0.19497,0.2134,0.18504,0.216,0.19617,0.13775,0.17759
,0.21459,0.18924,0.16838,0.12282,0.17705,0.16534,0.070557,0.061159,0.0
78462,0.07461,0.075236,0.080951,0.067314,0.070963,0.076936,0.072276,0.
067016,0.081584,0.080778,0.085676,0.073724,0.08375,0.07277,0.073796,0.
049943,0.30384,0.16719,0.18922,0.25195,0.21439,0.10954,0.20775,0.18974
,0.11798,0.21167,0.15431,0.17348,0.18891,0.15435,0.20058,0.16554,0.160
56,0.14661,0.073592,0.077626,0.081877,0.073663,0.082982,0.074755,0.076
339,0.078307,0.080026,0.073561,0.079399,0.07544,0.081104,0.074228,0.06
771,0.075982,0.078808,0.04725,0.038545,0.032932,0.27065,0.19473,0.1885
7,0.22055,0.20352,0.20478,0.20352,0.14885,0.17112,0.16523,0.16238,0.14
875,0.21106,0.1624,0.17659,0.077405,0.075809,0.080455,0.075745,0.07910
3,0.066557,0.07471,0.069123,0.079464,0.075492,0.07435,0.068579,0.07311
7,0.089507,0.078006,0.081349,0.083593,0.082529,0.043873,0.042221,0.042
865,0.037405,0.23858,0.19807,0.18424,0.16165,0.17823,0.18816,0.20909,0
.18472,0.23933,0.18326,0.18256,0.1252,0.07663,0.075573,0.077446,0.0665
77,0.080535,0.063666,0.073764,0.074947,0.079971,0.073991,0.077603,0.08
4404,0.08362,0.070499,0.075831,0.092988,0.076587,0.083132,0.064453,0.0
74413,0.031678,0.043189,0.038445,0.043964,0.039723,0.038246,0.22995,0.
17343,0.26234,0.2076,0.23698,0.2427,0.23051,0.19288,0.074751,0.068162,
0.075207,0.07676,0.064312,0.076918,0.077943,0.075196,0.08448,0.070396,
0.096197,0.054606,0.098843,0.072494,0.07815,0.071337,0.074731,0.078924
,0.073143,0.07216,0.076627,0.080949,0.078192,0.034269,0.04036,0.035358
,0.044658,0.03971,0.044391,0.20377,0.2343,0.15546,0.1776,0.23298,0.045
972,0.045866,0.074495,0.074674,0.077291,0.077852,0.079145,0.078824,0.0
72009,0.07529,0.06584,0.083627,0.074327,0.077732,0.080021,0.078985,0.0
72845,0.068954,0.087872,0.080511,0.069975,0.073318,0.074429,0.042756,0
.048188,0.046918,0.044064,0.038739,0.04238,0.043761,0.037123,0.037025,
0.039126,0.19504,0.23314,0.042411,0.038341,0.042649,0.039191,0.081542,
0.074707,0.074677,0.079565,0.079379,0.06831,0.063783,0.086023,0.078222
,0.078357,0.083618,0.07278,0.067289,0.076678,0.087291,0.075372,0.07591
3,0.069939,0.069243,0.040296,0.048114,0.04492,0.041444,0.036534,0.0408
6,0.034649,0.036277,0.041244,0.038899,0.047568,0.039598,0.03767,0.0420
38,0.044075,0.045921,0.035906,0.074565,0.068197,0.079219,0.067171,0.07
0742,0.069879,0.075125,0.069952,0.072663,0.086599,0.078477,0.070696,0.
076607,0.080039,0.076907,0.073463,0.035638,0.036934,0.034504,0.041146,
0.040016,0.04089,0.035997,0.043723,0.034791,0.040565,0.03872,0.047507,
0.04144,0.034739,0.041767,0.037131,0.045001,0.043343,0.041205,0.08503,

0.069229,0.091285,0.084199,0.072852,0.0736,0.076778,0.073005,0.080879,
0.072411,0.079137,0.07964,0.074007,0.042514,0.034178,0.046524,0.043999
,0.041577,0.038955,0.03849,0.036927,0.039049,0.036006,0.041554,0.04267
4,0.040661,0.044611,0.039637,0.045668,0.045458,0.041716,0.03049,0.0368
68,0.038866,0.073784,0.083138,0.077634,0.066373,0.066893,0.083504,0.08
0782,0.079155,0.069738,0.043115,0.041757,0.034828,0.046462,0.042179,0.
040713,0.044546,0.044213,0.035064,0.035488,0.044504,0.04168,0.047951,0
.042225,0.04312,0.042409,0.043341,0.040579,0.03509,0.045223,0.036682,0
.042105,0.043251,0.037772,0.045001,0.074864,0.078358,0.066984,0.086133
,0.035577,0.037774,0.050858,0.042133,0.040152,0.036492,0.037512,0.0413
38,0.038289,0.03521,0.032489,0.04203,0.042889,0.039809,0.043436,0.0412
04,0.037683,0.044258,0.047523,0.037057,0.038695,0.036351,0.041107,0.03
8734,0.032521,0.043215,0.041024,0.037517,0.040009,0.039634,0.041179,0.
041181,0.041878,0.037476,0.041249,0.048434,0.039016,0.035818,0.042685,
0.043892,0.036133,0.040271,0.039631,0.039019,0.045311,0.042102,0.04150
3,0.041163,0.041106,0.045073,0.040779,0.036325,0.040051,0.04304,0.0449
57,0.041452,0.047546,0.038732,0.041122,0.033872,0.036747,0.041603,0.03
9511,0.03894,0.042692,0.033713,0.037222,0.041063,0.038751,0.045363,0.0
39003,0.037939,0.035201,0.039865,0.041767,0.038824,0.037684,0.040983,0
.037976,0.042731,0.032815,0.037665,0.044128,0.038469,0.04325,0.039378,
0.04456,0.035297,0.044185,0.043802,0.042457,0.031208,0.042887,0.033574
,0.043774,0.039419,0.043919,0.047694,0.033188,0.032567,0.043449,0.0464
62,0.037033,0.040323,0.042665,0.038675,0.030579,0.050035,0.037404,0.04
5842,0.04074,0.032792,0.041126,0.041478,0.048013,0.042682,0.041246,0.0
36423,0.04001,0.048555,0.042407,0.034497,0.041169,0.045843,0.041609,0.
039975,0.036814,0.03843,0.035319,0.042722,0.043854,0.039089,0.044802,0
.038528,0.042729,0.042605,0.038112,0.040333,0.046451,0.043505,0.0293,0
.031682,0.043624,0.03794,0.043582,0.032082,0.037652,0.03602,0.043751,0
.0441,0.044485,0.036042,0.045658,0.03826,0.043646,0.038056,0.045768,0.
036355,0.038268,0.041755,0.040837,0.035702,0.032518,0.046676,0.035442,
0.045148,0.045799,0.039888,0.039377,0.041266,0.039116,0.045692,0.04318
3,0.045399,0.032603,0.033404,0.038479,0.03973,0.036583,0.037736,0.0392
,0.034797,0.042195,0.042463,0.035947,0.037825,0.048749,0.042459,0.0378
6,0.047071,0.038246,0.04064,0.042392,0.037643,0.032513
0.10431,0.10361,0.10564,0.10383,0.10333,0.1125,0.11218,0.099439,0.1213
9,0.10876,0.092078,0.11031,0.10315,0.11549,0.10762,0.10704,0.10976,0.1
1557,0.09987,0.11428,0.2091,0.2473,0.2848,0.20677,0.11062,0.10979,0.10
287,0.10985,0.10895,0.11139,0.11306,0.097617,0.11156,0.10565,0.10554,0
.11375,0.10735,0.10058,0.10509,0.30648,0.28376,0.24354,0.25527,0.27848
,0.22867,0.21502,0.10899,0.11052,0.1128,0.096031,0.10783,0.099624,0.10
237,0.11262,0.10781,0.10877,0.10973,0.10879,0.10991,0.10338,0.11097,0.
35469,0.33292,0.24512,0.23561,0.22998,0.24963,0.23194,0.16013,0.2171,0
.31448,0.11047,0.10496,0.099771,0.11297,0.11615,0.11068,0.10376,0.1085
1,0.11194,0.11048,0.10883,0.1126,0.10558,0.10906,0.11107,0.40751,0.383
39,0.30853,0.19778,0.20074,0.22028,0.29533,0.20295,0.17449,0.20047,0.2
3042,0.25591,0.10143,0.1099,0.11076,0.10178,0.10097,0.098126,0.10215,0
.10252,0.10891,0.11473,0.10895,0.10285,0.10638,0.1134,0.10807,0.10298,
0.39311,0.34428,0.39148,0.28972,0.24019,0.18983,0.26819,0.25934,0.2258
1,0.21735,0.21148,0.23293,0.25375,0.24632,0.10613,0.10291,0.10908,0.10
837,0.11008,0.10261,0.10915,0.1102,0.10704,0.10952,0.098171,0.11085,0.
1186,0.11018,0.11308,0.10491,0.4045,0.39262,0.39533,0.35752,0.38764,0.
29237,0.24368,0.24328,0.25229,0.25477,0.17825,0.24168,0.23653,0.26195,

0.23028,0.21533,0.11398,0.1103,0.1175,0.10616,0.098998,0.10533,0.11174
,0.10648,0.10731,0.11023,0.11381,0.10811,0.093093,0.10735,0.1137,0.110
47,0.1053,0.41224,0.39418,0.29819,0.38544,0.36226,0.3097,0.22503,0.223
44,0.20981,0.18164,0.24088,0.21549,0.20762,0.26806,0.19321,0.28083,0.2
2072,0.10633,0.10513,0.099659,0.10539,0.10374,0.10157,0.10923,0.10956,
0.103,0.10297,0.10373,0.11713,0.10983,0.075133,0.080075,0.085998,0.105
61,0.28499,0.42083,0.34918,0.30853,0.3511,0.2502,0.37197,0.25227,0.251
42,0.19034,0.19198,0.22075,0.21153,0.25606,0.22749,0.23218,0.18366,0.2
1874,0.10504,0.11054,0.098664,0.10473,0.11305,0.10751,0.09689,0.10285,
0.10282,0.10432,0.10629,0.078387,0.072505,0.069403,0.066286,0.074099,0
.078563,0.11222,0.34144,0.40442,0.32805,0.38719,0.39211,0.32253,0.3090
7,0.3006,0.19351,0.22543,0.2476,0.23284,0.22181,0.2205,0.20005,0.25481
,0.21885,0.24401,0.11333,0.10296,0.11774,0.11076,0.10281,0.1018,0.1128
7,0.10065,0.10288,0.067412,0.078833,0.074084,0.074331,0.073372,0.08552
,0.079336,0.073704,0.077774,0.10417,0.32732,0.38114,0.29432,0.3823,0.3
3283,0.41036,0.28416,0.21318,0.17906,0.27939,0.2242,0.19036,0.22553,0.
19909,0.22912,0.22113,0.22483,0.18591,0.25827,0.10734,0.10152,0.10448,
0.10835,0.10816,0.10091,0.088021,0.069373,0.081211,0.074736,0.074081,0
.07333,0.07615,0.071604,0.084951,0.071675,0.062966,0.073805,0.25481,0.
38996,0.4004,0.43586,0.33249,0.38546,0.36999,0.21514,0.27429,0.27465,0
.19138,0.34383,0.22837,0.18963,0.21638,0.22474,0.22991,0.22174,0.23446
,0.11669,0.11319,0.11048,0.072651,0.069288,0.072105,0.076006,0.066689,
0.073551,0.065288,0.070128,0.07516,0.08572,0.064653,0.072804,0.076432,
0.06406,0.073523,0.40225,0.29974,0.36505,0.36435,0.29566,0.35729,0.331
21,0.45019,0.40803,0.19492,0.21255,0.22369,0.25681,0.24942,0.25914,0.2
2548,0.21086,0.24938,0.20651,0.22829,0.067683,0.06963,0.070269,0.07026
4,0.071601,0.076794,0.07938,0.070467,0.081652,0.072948,0.072859,0.0690
99,0.07362,0.065823,0.078578,0.084962,0.076167,0.075812,0.35709,0.4416
1,0.30419,0.338,0.33472,0.35375,0.27244,0.45392,0.37452,0.19422,0.2767
7,0.19514,0.14765,0.18501,0.13952,0.17469,0.11109,0.18858,0.20439,0.12
428,0.074895,0.077779,0.076361,0.070518,0.061602,0.068114,0.075201,0.0
69119,0.073623,0.073316,0.070461,0.080067,0.072543,0.075662,0.069666,0
.071814,0.0625,0.077393,0.34166,0.48039,0.42332,0.39017,0.39108,0.3460
2,0.41315,0.42271,0.3009,0.19978,0.19535,0.13036,0.18386,0.14742,0.140
32,0.1758,0.19411,0.21282,0.18063,0.11647,0.075094,0.06408,0.086019,0.
078679,0.070482,0.079569,0.074844,0.063231,0.063587,0.06702,0.074706,0
.072885,0.076321,0.085748,0.077153,0.077113,0.079255,0.070602,0.2891,0
.29614,0.34441,0.35689,0.41492,0.34314,0.24333,0.18934,0.17783,0.19837
,0.17119,0.14752,0.17568,0.18165,0.18314,0.18899,0.1613,0.12962,0.1569
4,0.15026,0.078055,0.073193,0.082188,0.071065,0.072935,0.077366,0.0619
05,0.078014,0.079461,0.071882,0.069291,0.078614,0.071461,0.074034,0.07
4558,0.08095,0.069871,0.068144,0.41785,0.33223,0.34437,0.38348,0.33386
,0.32271,0.14386,0.18136,0.17229,0.22958,0.21995,0.16913,0.1692,0.1337
5,0.15528,0.18669,0.1659,0.19492,0.19387,0.16323,0.081818,0.071218,0.0
67698,0.074159,0.067531,0.070598,0.078352,0.080395,0.075692,0.083902,0
.067079,0.070108,0.068114,0.076011,0.063687,0.075991,0.069736,0.062302
,0.315,0.32282,0.29663,0.37062,0.32749,0.16817,0.21798,0.21756,0.1406,
0.23362,0.19783,0.13162,0.15371,0.16391,0.13113,0.17572,0.21765,0.1652
2,0.16941,0.18783,0.075742,0.073743,0.074568,0.080394,0.071637,0.05786
6,0.063004,0.060919,0.079961,0.082863,0.064966,0.071996,0.071296,0.070
736,0.082169,0.074832,0.070559,0.075615,0.3806,0.29011,0.33458,0.21008
,0.15409,0.22593,0.18652,0.1592,0.17057,0.15913,0.21864,0.1995,0.16218

,0.16666,0.14492,0.14884,0.18738,0.15724,0.16279,0.16217,0.064239,0.07
1584,0.066089,0.07513,0.073409,0.079883,0.063851,0.071452,0.089044,0.0
72813,0.075909,0.071369,0.076554,0.065469,0.066271,0.075227,0.071021,0
.077125,0.34911,0.15357,0.18383,0.1803,0.26161,0.2031,0.14231,0.18698,
0.25333,0.23466,0.17438,0.14957,0.16366,0.16582,0.11164,0.22719,0.1508
3,0.15002,0.18485,0.07309,0.085793,0.076956,0.075615,0.068762,0.052204
,0.072432,0.067998,0.075296,0.077372,0.079817,0.073796,0.074503,0.0725
5,0.087627,0.0715,0.078443,0.046795,0.21504,0.21725,0.19326,0.19786,0.
17887,0.16132,0.12624,0.16135,0.20472,0.18946,0.16237,0.16786,0.19346,
0.13136,0.17998,0.13397,0.16853,0.13963,0.19147,0.072441,0.075635,0.06
6802,0.078244,0.075641,0.078779,0.073099,0.079704,0.074866,0.068797,0.
068346,0.075171,0.071901,0.078619,0.069491,0.068107,0.076035,0.033741,
0.17203,0.19124,0.17685,0.1825,0.21208,0.21351,0.20147,0.20035,0.19588
,0.18858,0.13772,0.17678,0.20428,0.1858,0.16277,0.12895,0.18775,0.1623
,0.071241,0.058604,0.074603,0.075138,0.07419,0.079314,0.067675,0.06689
7,0.074008,0.069274,0.064191,0.080889,0.080357,0.08593,0.07575,0.07969
1,0.073788,0.072767,0.046797,0.2887,0.17593,0.1893,0.22517,0.21908,0.1
1814,0.2116,0.17691,0.11795,0.19609,0.15825,0.16675,0.16985,0.16206,0.
19468,0.15861,0.17622,0.14314,0.074188,0.07553,0.077246,0.072574,0.080
156,0.073101,0.077283,0.073795,0.080201,0.071452,0.077263,0.071544,0.0
82604,0.072372,0.06451,0.072518,0.077184,0.046102,0.035422,0.031701,0.
27672,0.1996,0.19983,0.21588,0.1916,0.20147,0.20966,0.1421,0.17047,0.1
6546,0.15333,0.14725,0.20064,0.16681,0.19115,0.073859,0.074686,0.07922
4,0.075283,0.078813,0.062048,0.070091,0.069618,0.080277,0.072793,0.073
819,0.0673,0.072884,0.085293,0.075345,0.07832,0.078603,0.079633,0.0435
65,0.042485,0.040752,0.037472,0.24003,0.19281,0.17607,0.15515,0.17818,
0.19177,0.21212,0.18126,0.24257,0.19601,0.16898,0.1313,0.077826,0.0719
13,0.072132,0.06888,0.076201,0.063452,0.072102,0.076288,0.074651,0.072
901,0.076066,0.082399,0.081923,0.073408,0.07414,0.089904,0.072062,0.08
2227,0.061683,0.070924,0.03111,0.045591,0.039211,0.040672,0.038094,0.0
3725,0.23818,0.15986,0.25939,0.21853,0.19937,0.22274,0.24843,0.18937,0
.069978,0.069033,0.072035,0.071655,0.060054,0.073975,0.076615,0.07206,
0.08068,0.067783,0.094062,0.054247,0.093228,0.072409,0.075926,0.068565
,0.073726,0.075683,0.076164,0.06857,0.078115,0.082408,0.076458,0.03425
9,0.041037,0.037,0.045811,0.039626,0.042068,0.19975,0.24435,0.15931,0.
17399,0.22804,0.044551,0.040987,0.074294,0.075087,0.077327,0.073626,0.
076072,0.074682,0.068298,0.074112,0.06375,0.083799,0.073371,0.075764,0
.076054,0.075947,0.068595,0.069062,0.084929,0.077366,0.06812,0.070655,
0.074071,0.041839,0.049486,0.04542,0.040468,0.036526,0.041384,0.044166
,0.032565,0.040774,0.038008,0.19517,0.2276,0.045237,0.035517,0.044285,
0.039714,0.079448,0.072321,0.071228,0.075789,0.076598,0.069339,0.06488
4,0.085993,0.077118,0.073022,0.079158,0.071118,0.066537,0.075383,0.083
251,0.070451,0.072423,0.067572,0.068453,0.039106,0.049223,0.043298,0.0
37347,0.03734,0.04256,0.039406,0.035654,0.040832,0.037487,0.047488,0.0
37747,0.038153,0.041843,0.044181,0.044501,0.036103,0.072831,0.06651,0.
079336,0.063272,0.068344,0.069062,0.073575,0.072758,0.072307,0.085135,
0.07882,0.068519,0.073421,0.080617,0.074895,0.070305,0.038249,0.033268
,0.039148,0.040757,0.040579,0.041653,0.037527,0.042009,0.036972,0.0380
03,0.040728,0.044931,0.041239,0.033636,0.04261,0.036538,0.043225,0.042
343,0.040033,0.082296,0.069922,0.0933,0.082398,0.069395,0.074899,0.074
485,0.072433,0.07403,0.072838,0.075382,0.08022,0.067722,0.040966,0.033
652,0.046595,0.042329,0.043089,0.039776,0.036868,0.037154,0.037558,0.0

34904,0.04319,0.039452,0.038936,0.044154,0.039642,0.044097,0.04349,0.0
40415,0.031544,0.038497,0.035462,0.06899,0.080702,0.078142,0.06442,0.0
62156,0.082118,0.08034,0.078403,0.068876,0.042097,0.042015,0.034344,0.
046956,0.040852,0.044402,0.042432,0.044804,0.03272,0.037458,0.044389,0
.041059,0.046455,0.043181,0.043432,0.041342,0.044625,0.039386,0.035853
,0.047721,0.037309,0.042132,0.045225,0.03709,0.044947,0.075788,0.07597
5,0.065415,0.081146,0.036758,0.038966,0.049441,0.043967,0.03883,0.0378
01,0.037535,0.03982,0.038161,0.033908,0.037348,0.040658,0.041421,0.039
748,0.044756,0.040592,0.034629,0.042946,0.046068,0.036701,0.037524,0.0
37068,0.041741,0.039897,0.030771,0.04677,0.043742,0.036615,0.038935,0.
038519,0.039881,0.039888,0.040517,0.037691,0.038156,0.046245,0.03915,0
.039966,0.046653,0.04284,0.036063,0.042849,0.037391,0.039059,0.043077,
0.040052,0.040963,0.039348,0.044972,0.04701,0.041515,0.036184,0.040266
,0.039972,0.043164,0.039324,0.045722,0.040831,0.040524,0.033676,0.0377
47,0.040417,0.038118,0.035973,0.042955,0.032739,0.036871,0.042204,0.03
495,0.042878,0.037669,0.035409,0.033622,0.042008,0.04053,0.03918,0.039
38,0.041205,0.038497,0.041619,0.032494,0.038069,0.045366,0.041708,0.04
3441,0.039528,0.043906,0.035931,0.041441,0.043511,0.04173,0.031681,0.0
42882,0.031264,0.045369,0.040173,0.041829,0.046233,0.032907,0.033743,0
.044409,0.048563,0.03712,0.041669,0.043235,0.038178,0.031423,0.050504,
0.037887,0.044752,0.039828,0.034019,0.040217,0.041433,0.048983,0.04254
9,0.041471,0.039082,0.04055,0.049348,0.042694,0.034086,0.042262,0.0440
01,0.04333,0.035578,0.037957,0.038537,0.030783,0.040086,0.042931,0.038
456,0.048247,0.038369,0.038784,0.040988,0.038377,0.039166,0.045287,0.0
42066,0.032795,0.029373,0.043146,0.039296,0.042395,0.032699,0.038056,0
.032676,0.04555,0.044989,0.044384,0.036981,0.045237,0.037793,0.040819,
0.038252,0.046329,0.035176,0.041399,0.042113,0.040451,0.036592,0.03473
1,0.048434,0.035126,0.041171,0.04446,0.041893,0.038961,0.038626,0.0373
12,0.046003,0.042376,0.043448,0.033747,0.032108,0.038947,0.03973,0.039
182,0.037442,0.040135,0.034209,0.042779,0.043389,0.034494,0.037021,0.0
50151,0.044705,0.037294,0.0444,0.038199,0.04295,0.039374,0.034344,0.03
2588

#simdataS3SNR15:

Kep_f=1.2
Kep_f=1.2
Kep_s=0.5
Ktrans_f=0.05
Ktrans_s=0.06
del_t=0.5
gtSf=0 0 0 0 0 0
0 0 0 0 0 0
0 0 0 0 0 0
0 0 0 0 0 0
0 0 0 0 0 0
0 0 0 0 0 0
0 0 0 0 0 0
0 0 0 0 0 0
0 0 0 0 0 0
0 0 0 0 0 0

0	0	0	0	0	0
0	0	0	0	0	0
0	0	0	0	0	0
0	0	0	0	0	0
0	0	0	0	0	0
0	0	0	0	0	0
0	0	0	0	0	0
0	0	0	0	0	0
0	0	0	0	0	0
0	0	0	0	0	0
0	0	0	0	0	0
0	0	0	0	0	0
0	0	0	0	0	0
0	0	0	0	0	0
0	0	0	0	0	0
0	0	0	0	0	0
0	0	0	0	0	0
0	0	0	0	0	0
0	0	0	0	0	0
0	0	0	0	0	0
0	0	0	0	0	0
0	0	0	0	0	0
0	0	0	0	0	0
0	0	0	0	0	0
0	0	0	0.54548	0.5	0.44643
0	0	0	0	0	0
0	0	0	0	0	0
0	0	0	0	0	0
0	0	0	0	0	0
0	0	0	0	0	0
0.5	0.38922	0.5	0.55132	0.5	0.54227
0	0	0	0	0	0
0	0	0	0	0	0
0	0	0	0	0	0
0	0	0	0	0	0
0	0	0	0	0.5	0.46169
0.55073	0.55826	0.5	0.54574	0.45762	0.51325
0.55336	0	0	0	0	0
0	0	0	0	0	0
0	0	0	0	0	0
0	0	0	0	0	0
0	0	0	0.49285	0.5	0.41147
0.56106	0.5	0.49428	0.56929	0.60297	0.55256
0.5	0.56646	0.46739	0	0	0
0	0	0	0	0	0
0	0	0	0	0	0
0	0	0	0	0	0
0	0.5	0.67658	0.5059	0.61793	0.5
0.46619	0.48164	0.5	0.52469	0.59386	0.50275
0.46235	0.53217	0.5	0.5	0	0
0	0	0	0	0	0
0	0	0	0	0	0

0	0	0	0	0	0
0.5	0.56077	0.5	0.51916	0.41945	0.5
0.56346	0.4984	0.4745	0.5	0.48817	0.40112
0.53358	0.45307	0.54768	0.5	0.56591	0.5
0	0	0	0	0	0
0	0	0	0	0	0.33333
0.34312	0.41239	0.40185	0.37256	0.33333	0.33333
0.32543	0.34223	0.5	0.5	0.43466	0.5
0.5	0.51304	0.5	0.47701	0.5	0.5
0.52911	0.5085	0.46177	0.58669	0.5	0.50578
0.5	0.5	0	0	0	0
0	0	0	0	0	0.49678
0.32109	0.32889	0.33333	0.33333	0.31331	0.4012
0.35532	0.39086	0.33333	0.34013	0.49453	0.44607
0.4576	0.51205	0.58655	0.46215	0.43727	0.5
0.50393	0.44209	0.72355	0.5	0.51928	0.5
0.5085	0.5	0.49485	0.50109	0	0
0	0	0	0	0	0.45709
0.44155	0.49547	0.33368	0.20421	0.33333	0.32285
0.34023	0.33333	0.35688	0.33333	0.34106	0.334
0.51837	0.47494	0.41169	0.46412	0.4547	0.45301
0.5	0.54423	0.5	0.51163	0.5	0.49248
0.5	0.38134	0.55939	0.48	0.47031	0.36482
0	0	0	0	0	0
0.52953	0.36997	0.56768	0.44662	0.58043	0.34926
0.30557	0.33421	0.39704	0.33333	0.33333	0.33333
0.33333	0.37179	0.54213	0.5	0.40649	0.52814
0.5	0.44097	0.52811	0.5	0.57753	0.26904
0.48449	0.42114	0.5	0.53425	0.56298	0.54892
0.5	0.48934	0	0	0	0
0	0.5	0.49661	0.41099	0.5	0.5
0.51895	0.36987	0.35887	0.34565	0.36668	0.35383
0.42321	0.37171	0.4275	0.34455	0.51186	0.43933
0.34814	0.54843	0.46076	0.50516	0.5	0.5
0.46312	0.5	0.46461	0.48117	0.4924	0.52776
0.4758	0.49701	0.5	0.30982	0	0
0	0.59245	0.5	0.4859	0.59057	0.57812
0.52755	0.54905	0.49172	0.42233	0.34732	0.41013
0.33333	0.40804	0.35021	0.29987	0.25994	0.32908
0.40758	0.49425	0.5146	0.5	0.49193	0.50445
0.5	0.56119	0.5	0.47277	0.5	0.59057
0.50156	0.5	0.49687	0.5	0.49799	0.5
0	0.5	0.5	0.5	0.48781	0.4903
0.52232	0.5	0.5	0.44102	0.33333	0.38624
0.28912	0.35349	0.36859	0.3648	0.36002	0.29751
0.37987	0.51352	0.5	0.5714	0.56488	0.50889
0.50957	0.52838	0.4017	0.43994	0.48414	0.54581
0.47845	0.4792	0.48134	0.5059	0.51375	0.5
1	0.5	0.602	0.5	0.5	0.5
0.52471	0.5	0.5	0.68298	0.40834	0.33333
0.34108	0.38319	0.33114	0.42063	0.31796	0.36057
0.39263	0.33712	0.53903	0.63533	0.54114	0.5

0.27834	0.72481	0.44075	0.53851	0.5	0.43603
0.52089	0.49034	0.53421	0.5	0.44167	0.49612
0.5	1	0.35469	0.50572	0.50811	0.50206
0.32304	0.45054	0.51344	0.5	0.65888	0.46354
0.33943	0.33333	0.34332	0.37494	0.35637	0.23791
0.32661	0.35446	0.5	0.5	0.53758	0.40866
0.46541	0.5518	0.5	0.46847	0.64698	0.41459
0.48082	0.48569	0.49016	0.5	0.5	0.5
0.49363	0.5	1	0.45315	0.62298	0.45103
0.48568	0.51879	0.42046	0.54665	0.54559	0.53502
0.5	0.30893	0.28839	0.25134	0.36095	0.29809
0.35704	0.32409	0.29519	0.5	0.51347	0.46075
0.47447	0.5	0.54933	0.55252	0.52873	0.43168
0.5	0.5	0.5	0.57698	0.52232	0.5
0.49896	0.45838	1	1	1	0.41542
0.62006	0.55163	0.5	0.56516	0.51746	0.5
0.55789	0.40883	0.40081	0.36725	0.33712	0.35179
0.25369	0.33333	0.5	0.42285	0.51337	0.47575
0.49441	0.55031	0.5	0.56988	0.5207	0.56612
0.49974	0.49746	0.40533	0.4375	0.47588	0.47239
0.5428	0.51602	1	1	1	1
0.50965	0.52398	0.5	0.45233	0.46973	0.51068
0.50147	0.53965	0.5	0.33333	0.32182	0.30535
0.45766	0.50384	0.27248	0.41311	0.45682	0.4849
0.53313	0.5232	0.43761	0.5	0.51541	0.4559
0.5	0.47607	0.52751	0.46156	0.5	0.43667
0.5082	0.63033	1	1	1	1
1	1	0.5	0.62614	0.5	0.5186
0.5556	0.35099	0.41326	0.70483	0.48093	0.60187
0.51511	0.50504	0.5	0.4454	0.31764	0.55781
0.50796	0.5	0.54975	0.4749	0.5	0.51394
0.54949	0.5	0.51271	0.5	0.54501	0.5
0.4336	0.36308	0.42623	1	1	1
1	1	1	0.45185	0.65688	0.5
0.4882	0.5451	1	1	0.5	0.4239
0.5	0.47396	0.51951	0.53265	0.5	0.53706
0.53941	0.49994	0.43814	0.5	0.50961	0.44677
0.50146	0.35275	0.65424	0.5	0.47766	0.37264
0.52242	1	1	1	1	1
1	1	1	1	1	0.5
0.5	1	1	1	1	0.52273
0.5	0.68822	0.41371	0.57763	0.48173	0.49569
0.52357	0.45454	0.42211	0.5	0.53706	0.37305
0.5	0.34926	0.57272	0.52737	0.48282	0.48539
1	1	1	1	1	1
1	1	1	1	1	1
1	1	1	1	1	0.52661
0.3896	0.42122	0.58583	0.38299	0.5	0.5
0.42629	0.55766	0.42362	0.5	0.5	0.48097
0.40798	0.35838	0.47839	1	1	1
1	1	1	1	1	1
1	1	1	1	1	1

1 1 1 1 0.53035 0.5
0.48754 0.54496 0.5 0.5 0.50222 0.51095
0.5 0.57958 0.56542 0.51951 0.52955 1
1 1 1 1 1 1
1 1 1 1 1 1
1 1 1 1 1 1
1 1 0.5 0.32088 0.45069 0.52802
0.52017 0.50395 0.45856 0.41988 0.5 1
1 1 1 1 1 1
1 1 1 1 1 1
1 1 1 1 1 1
1 1 1 1 1 1
0.57817 0.45705 0.56151 0.5 1 1
1 1 1 1 1 1
1 1 1 1 1 1
1 1 1 1 1 1
1 1 1 1 1 1
1 1 1 1 1 1
1 1 1 1 1 1
1 1 1 1 1 1
1 1 1 1 1 1
1 1 1 1 1 1
1 1 1 1 1 1
1 1 1 1 1 1
1 1 1 1 1 1
1 1 1 1 1 1
1 1 1 1 1 1
1 1 1 1 1 1
1 1 1 1 1 1
1 1 1 1 1 1
1 1 1 1 1 1
1 1 1 1 1 1
1 1 1 1 1 1
1 1 1 1 1 1
1 1 1 1 1 1
1 1 1 1 1 1
1 1 1 1 1 1
1 1 1 1 1 1
1 1 1 1 1 1
1 1 1 1 1 1
1 1 1 1 1 1
1 1 1 1 1 1
1 1 1 1 1 1
1 1 1 1 1 1
1 1 1 1 1 1
1
gtSs=1 1 1 1 1 1
1 1 1 1 1 1
1 1 1 1 1 1
1 1 0.58718 0.60442 0.4756 0.46726
1 1 1 1 1 1
1 1 1 1 1 1

1	1	1	0	0.49985	0.54023
0.5	0.47075	0.4906	0.4873	1	1
1	1	1	1	1	1
1	1	1	1	1	1
1	0	0	0.56415	0.5	0.56422
0.5	0.59727	0.51184	0.52698	0.5	1
1	1	1	1	1	1
1	1	1	1	1	1
1	1	0	0	0	0.55576
0.46039	0.33538	0.51971	0.5	0.5	0.55644
0.51015	0.5295	1	1	1	1
1	1	1	1	1	1
1	1	1	1	1	1
0	0	0	0	0.5	0.5
0.5	0.50364	0.41142	0.52453	0.53883	0.57751
0.48451	0.56244	1	1	1	1
1	1	1	1	1	1
1	1	1	1	1	1
0	0	0	0	0	0
0.45162	0.46919	0.47308	0.52782	0.58663	0.43053
0.5	0.54112	0.47976	0.37899	1	1
1	1	1	1	1	1
1	1	1	1	1	1
1	1	1	0	0	0
0	0	0	0.38132	0.53967	0.56398
0.43463	0.5	0.48589	0.46783	0.50957	0.57875
0.42412	0.51065	1	1	1	1
1	1	1	1	1	1
1	1	1	0.45452	0.5	0.55357
1	0	0	0	0	0
0	0	0.5747	0.36124	0.50328	0.38784
0.50437	0.50183	0.51581	0.5	0.5	0.5
0.47753	1	1	1	1	1
1	1	1	1	1	1
0.5	0.61078	0.5	0.44868	0.5	0.45773
1	0	0	0	0	0
0	0	0.48395	0.40553	0.5	0.48388
0.5	0.36406	0.53974	0.55147	0.48836	0.52247
0.5	1	1	1	1	1
1	1	1	1	0.5	0.53831
0.44927	0.44174	0.5	0.45426	0.54238	0.48675
0.44664	1	0	0	0	0
0	0	0	0.48334	0.51611	0.48007
0.42078	0.56869	0.45463	0.56262	0.51199	0.5715
0.5708	0.47177	0.5	1	1	1
1	1	1	0.50715	0.5	0.58853
0.43894	0.5	0.50572	0.43071	0.39703	0.44744
0.5	0.43354	0.53261	0	0	0
0	0	0	0	0.44313	0.48312
0.5	0.5	0.53759	0.45775	0.38338	0.49915
0.4128	0.4674	0.45806	0.42736	1	1
1	0.5	0.32342	0.4941	0.38207	0.5

0.53381	0.51836	0.5	0.47531	0.40614	0.49725
0.53765	0.46783	0.5	0.5	0	0
0	0	0	0	0	0
0	0.50767	0.44373	0.45444	0.41638	0.51335
0.45597	0.5	0.5	0.37438	0.5	0.53195
0.5	0.43923	0.5	0.48084	0.58055	0.5
0.43654	0.5016	0.5255	0.5	0.51183	0.59888
0.46642	0.54693	0.45232	0.5	0.43409	0.5
0	0	0	0	0	0
0	0	0	0.56164	0.49506	0.33333
0.34312	0.224	0.10316	0.29468	0.33333	0.33333
0.33729	0.31554	0.5	0.5	0.56534	0.5
0.5	0.48696	0.5	0.52299	0.5	0.5
0.47089	0.4915	0.53823	0.41331	0.5	0.49422
0.5	0.5	0	0	0	0
0	0	0	0	0	0
0.35344	0.42515	0.33333	0.33333	0.34335	0.1976
0.36984	0.42831	0.33333	0.34389	0.50547	0.55393
0.5424	0.48795	0.41345	0.53785	0.56273	0.5
0.49607	0.55791	0.27645	0.5	0.48072	0.5
0.4915	0.5	0.50515	0.49891	0	0
0	0	0	0	0	0
0	0	0.4122	0.3716	0.33333	0.39744
0.34023	0.33333	0.28624	0.33333	0.31787	0.334
0.48163	0.52506	0.58831	0.53588	0.5453	0.54699
0.5	0.45577	0.5	0.48837	0.5	0.50752
0.5	0.61866	0.44061	0.52	0.52969	0.63518
0	0	0	0	0	0
0	0	0	0	0	0.30147
0.37186	0.33159	0.40958	0.33333	0.33333	0.33333
0.33333	0.26531	0.45787	0.5	0.59351	0.47186
0.5	0.55903	0.47189	0.5	0.42247	0.73096
0.51551	0.57886	0.5	0.46575	0.43702	0.45108
0.5	0.51066	0	0	0	0
0	0	0	0	0	0
0	0.26026	0.31739	0.30264	0.29147	0.35383
0.15359	0.30433	0.26797	0.45818	0.48814	0.56067
0.65186	0.45157	0.53924	0.49484	0.5	0.5
0.53688	0.5	0.53539	0.51883	0.5076	0.47224
0.5242	0.50299	0.5	0.69018	0	0
0	0	0	0	0	0
0	0	0	0.20541	0.31964	0.36389
0.33333	0.29258	0.28469	0.35006	0.36097	0.33633
0.59242	0.50575	0.4854	0.5	0.50807	0.49555
0.5	0.43881	0.5	0.52723	0.5	0.40943
0.49844	0.5	0.50313	0.5	0.50201	0.5
0	0	0	0	0	0
0	0	0	0	0.33333	0.29074
0.35544	0.35349	0.23387	0.27795	0.27844	0.35125
0.33947	0.48648	0.5	0.4286	0.43512	0.49111
0.49043	0.47162	0.5983	0.56006	0.51586	0.45419
0.52155	0.5208	0.51866	0.4941	0.48625	0.5

0	0	0	0	0	0
0	0	0	0	0	0.33333
0.30656	0.23362	0.33443	0.3523	0.34116	0.33626
0.25367	0.2659	0.46097	0.36467	0.45886	0.5
0.72166	0.27519	0.55925	0.46149	0.5	0.56397
0.47911	0.50966	0.46579	0.5	0.55833	0.50388
0.5	0	0	0	0	0
0	0	0	0	0	0
0.31164	0.33333	0.34332	0.33302	0.28726	0.38105
0.33464	0.32967	0.5	0.5	0.46242	0.59134
0.53459	0.4482	0.5	0.53153	0.35302	0.58541
0.51918	0.51431	0.50984	0.5	0.5	0.5
0.50637	0.5	0	0	0	0
0	0	0	0	0	0
0	0.36939	0.3558	0.40602	0.36095	0.35095
0.41012	0.35959	0.36792	0.5	0.48653	0.53925
0.52553	0.5	0.45067	0.44748	0.47127	0.56832
0.5	0.5	0.5	0.42302	0.47768	0.5
0.50104	0.54162	0	0	0	0
0	0	0	0	0	0
0	0	0.19837	0.34846	0.33712	0.35179
0.42577	0.33333	0.5	0.57715	0.48663	0.52425
0.50559	0.44969	0.5	0.43012	0.4793	0.43388
0.50026	0.50254	0.59467	0.5625	0.52412	0.52761
0.4572	0.48398	0	0	0	0
0	0	0	0	0	0
0	0	0	0.33333	0.33909	0.33139
0.54234	0.49616	0.72752	0.58689	0.54318	0.5151
0.46687	0.4768	0.56239	0.5	0.48459	0.5441
0.5	0.52393	0.47249	0.53844	0.5	0.56333
0.4918	0.36967	0	0	0	0
0	0	0	0	0	0
0	0	0	0	0.51907	0.39813
0.48489	0.49496	0.5	0.5546	0.68236	0.44219
0.49204	0.5	0.45025	0.5251	0.5	0.48606
0.45051	0.5	0.48729	0.5	0.45499	0.5
0.5664	0.63692	0.57377	0	0	0
0	0	0	0	0	0
0	0	0	0	0.5	0.5761
0.5	0.52604	0.48049	0.46735	0.5	0.46294
0.46059	0.50006	0.56186	0.5	0.49039	0.55323
0.49854	0.64725	0.34576	0.5	0.52234	0.62736
0.47758	0	0	0	0	0
0	0	0	0	0	0
0	0	0	0	0	0.47727
0.5	0.31178	0.58629	0.42237	0.51827	0.50431
0.47643	0.54546	0.57789	0.5	0.46294	0.62695
0.5	0.65074	0.42728	0.47263	0.51718	0.51461
0	0	0	0	0	0
0	0	0	0	0	0
0	0	0	0	0	0.47339
0.6104	0.57878	0.41417	0.61701	0.5	0.5

```
0.57371    0.44234    0.57638    0.5    0.5    0.51903
0.59202    0.64162    0.52161    0    0    0
0    0    0    0    0    0
0    0    0    0    0    0
0    0    0    0    0.46965    0.5
0.51246    0.45504    0.5    0.5    0.49778    0.48905
0.5    0.42042    0.43458    0.48049    0.47045    0
0    0    0    0    0    0
0    0    0    0    0    0
0    0    0    0    0    0
0    0    0.5    0.67912    0.54931    0.47198
0.47983    0.49605    0.54144    0.58012    0.5    0
0    0    0    0    0    0
0    0    0    0    0    0
0    0    0    0    0    0
0    0    0    0    0    0
0.42183    0.54295    0.43849    0.5    0    0
0    0    0    0    0    0
0    0    0    0    0    0
0    0    0    0    0    0
0    0    0    0    0    0
0    0    0    0    0    0
0    0    0    0    0    0
0    0    0    0    0    0
0    0    0    0    0    0
0    0    0    0    0    0
0    0    0    0    0    0
0    0    0    0    0    0
0    0    0    0    0    0
0    0    0    0    0    0
0    0    0    0    0    0
0    0    0    0    0    0
0    0    0    0    0    0
0    0    0    0    0    0
0    0    0    0    0    0
0    0    0    0    0    0
0    0    0    0    0    0
0    0    0    0    0    0
0    0    0    0    0    0
0    0    0    0    0    0
0    0    0    0    0    0
0    0    0    0    0    0
0    0    0    0    0    0
0    0    0    0    0    0
0    0    0    0    0    0
0    0    0    0    0    0
0    0    0    0    0    0
0    0    0    0    0    0
0    0    0    0    0    0
0
gtSp=0    0    0    0    0    0
0    0    0    0    0    0
```

0	0	0	0	0	0	
0	0	0.41282	0.39558	0.5244	0.53274	
0	0	0	0	0	0	
0	0	0	0	0	0	
0	0	0	1	0.50015	0.45977	
0.5	0.52925	0.5094	0.5127	0	0	
0	0	0	0	0	0	
0	0	0	0	0	0	
0	1	1	0.43585	0.5	0.43578	
0.5	0.40273	0.48816	0.47302	0.5	0	
0	0	0	0	0	0	
0	0	0	0	0	0	
0	0	1	1	1	0.44424	
0.53961	0.66462	0.48029	0.5	0.5	0.44356	
0.48985	0.4705	0	0	0	0	
0	0	0	0	0	0	
0	0	0	0	0	0	
1	1	1	1	0.5	0.5	
0.5	0.49636	0.58858	0.47547	0.46117	0.42249	
0.51549	0.43756	0	0	0	0	
0	0	0	0	0	0	
0	0	0	0	0	0	
1	1	1	1	1	1	
0.54838	0.53081	0.52692	0.47218	0.41337	0.56947	
0.5	0.45888	0.52024	0.62101	0	0	
0	0	0	0	0	0	
0	0	0	0	0	0	
0	0	0	1	1	1	
1	1	1	0.61868	0.46033	0.43602	
0.56537	0.5	0.51411	0.53217	0.49043	0.42125	
0.57588	0.48935	0	0	0	0	
0	0	0	0	0	0	
0	0	0	0	0	0	
0	1	1	1	1	1	
1	1	0.4253	0.63876	0.49672	0.61216	
0.49563	0.49817	0.48419	0.5	0.5	0.5	
0.52247	0	0	0	0	0	
0	0	0	0	0	0	
0	0	0	0	0	0	
0	1	1	1	1	1	
1	1	0.51605	0.59447	0.5	0.51612	
0.5	0.63594	0.46026	0.44853	0.51164	0.47753	
0.5	0	0	0	0	0	
0	0	0	0	0	0	
0	0	0	0	0	0	
0	0	1	1	1	1	
1	1	1	0.51666	0.48389	0.51993	
0.57922	0.43131	0.54537	0.43738	0.48801	0.4285	
0.4292	0.52823	0.5	0	0	0	
0	0	0	0	0	0	
0	0	0	0	0	0	
0	0	0	1	1	1	

1	1	1	1	0.55687	0.51688
0.5	0.5	0.46241	0.54225	0.61662	0.50085
0.5872	0.5326	0.54194	0.57264	0	0
0	0	0	0	0	0
0	0	0	0	0	0
0	0	0	0	1	1
1	1	1	1	1	1
1	0.49233	0.55627	0.54556	0.58362	0.48665
0.54403	0.5	0.5	0.62562	0.5	0.46805
0	0	0	0	0	0
0	0	0	0	0	0
0	0	0	0	0	0
1	1	1	1	1	1
1	1	1	0.43836	0.50494	0.33333
0.31375	0.36362	0.49499	0.33276	0.33333	0.33333
0.33729	0.34223	0	0	0	0
0	0	0	0	0	0
0	0	0	0	0	0
0	0	1	1	1	1
1	1	1	1	1	0.50322
0.32547	0.24596	0.33333	0.33333	0.34335	0.4012
0.27484	0.18083	0.33333	0.31598	0	0
0	0	0	0	0	0
0	0	0	0	0	0
0	0	0	0	1	1
1	1	1	1	1	0.54291
0.55845	0.50453	0.25412	0.42419	0.33333	0.27971
0.31954	0.33333	0.35688	0.33333	0.34106	0.33201
0	0	0	0	0	0
0	0	0	0	0	0
0	0	0	0	0	0
1	1	1	1	1	1
0.47047	0.63003	0.43232	0.55338	0.41957	0.34926
0.32257	0.33421	0.19337	0.33333	0.33333	0.33333
0.33333	0.36291	0	0	0	0
0	0	0	0	0	0
0	0	0	0	0	0
0	0	1	1	1	1
1	0.5	0.50339	0.58901	0.5	0.5
0.48105	0.36987	0.32374	0.35171	0.34184	0.29234
0.42321	0.32396	0.30453	0.19727	0	0
0	0	0	0	0	0
0	0	0	0	0	0
0	0	0	0	1	1
1	0.40755	0.5	0.5141	0.40943	0.42188
0.47245	0.45095	0.50828	0.37226	0.33304	0.22598
0.33333	0.29938	0.3651	0.35006	0.37908	0.33459
0	0	0	0	0	0
0	0	0	0	0	0
0	0	0	0	0	0
1	0.5	0.5	0.5	0.51219	0.5097
0.47768	0.5	0.5	0.55898	0.33333	0.32302

0.35544	0.29301	0.39754	0.35725	0.36154	0.35125
0.28066	0	0	0	0	0
0	0	0	0	0	0
0	0	0	0	0	0
0	0.5	0.398	0.5	0.5	0.5
0.47529	0.5	0.5	0.31702	0.59166	0.33333
0.35236	0.38319	0.33443	0.22708	0.34088	0.30316
0.3537	0.39698	0	0	0	0
0	0	0	0	0	0
0	0	0	0	0	0
0	0	0.64531	0.49428	0.49189	0.49794
0.67696	0.54946	0.48656	0.5	0.34112	0.53646
0.34893	0.33333	0.31335	0.29204	0.35637	0.38105
0.33875	0.31587	0	0	0	0
0	0	0	0	0	0
0	0	0	0	0	0
0	0	0	0.54685	0.37702	0.54897
0.51432	0.48121	0.57954	0.45335	0.45441	0.46498
0.5	0.32168	0.3558	0.34265	0.2781	0.35095
0.23284	0.31632	0.33689	0	0	0
0	0	0	0	0	0
0	0	0	0	0	0
0	0	0	0	0	0.58458
0.37994	0.44837	0.5	0.43484	0.48254	0.5
0.44211	0.59117	0.40081	0.28429	0.32577	0.29642
0.32054	0.33333	0	0	0	0
0	0	0	0	0	0
0	0	0	0	0	0
0	0	0	0	0	0
0.49035	0.47602	0.5	0.54767	0.53027	0.48932
0.49853	0.46035	0.5	0.33333	0.33909	0.36325
0	0	0	0	0	0
0	0	0	0	0	0
0	0	0	0	0	0
0	0	0	0	0	0
0	0	0.5	0.37386	0.5	0.4814
0.4444	0.64901	0.58674	0.29517	0	0
0	0	0	0	0	0
0	0	0	0	0	0
0	0	0	0	0	0
0	0	0	0	0	0
0	0	0	0.54815	0.34312	0.5
0.5118	0.4549	0	0	0	0
0	0	0	0	0	0
0	0	0	0	0	0
0	0	0	0	0	0
0	0	0	0	0	0
0	0	0	0	0	0.5
0.5	0	0	0	0	0
0	0	0	0	0	0
0	0	0	0	0	0
0	0	0	0	0	0

0	0	0	0	0	0
0	0	0	0	0	0
0	0	0	0	0	0
0	0	0	0	0	0
0	0	0	0	0	0
0	0	0	0	0	0
0	0	0	0	0	0
0	0	0	0	0	0
0	0	0	0	0	0
0	0	0	0	0	0
0	0	0	0	0	0
0	0	0	0	0	0
0	0	0	0	0	0
0	0	0	0	0	0
0	0	0	0	0	0
0	0	0	0	0	0
0	0	0	0	0	0
0	0	0	0	0	0
0	0	0	0	0	0
0	0	0	0	0	0
0	0	0	0	0	0
0	0	0	0	0	0
0	0	0	0	0	0
0	0	0	0	0	0
0	0	0	0	0	0
0	0	0	0	0	0
0	0	0	0	0	0
0	0	0	0	0	0
0	0	0	0	0	0
0	0	0	0	0	0
0	0	0	0	0	0
0	0	0	0	0	0
0	0	0	0	0	0
0	0	0	0	0	0
0	0	0	0	0	0
0	0	0	0	0	0
0	0	0	0	0	0
0	0	0	0	0	0
0	0	0	0	0	0
0	0	0	0	0	0
0	0	0	0	0	0
0	0	0	0	0	0
0	0	0	0	0	0
0	0	0	0	0	0
0	0	0	0	0	0
0	0	0	0	0	0
0	0	0	0	0	0
0	0	0	0	0	0
0	0	0	0	0	0
0	0	0	0	0	0
0	0	0	0	0	0
0	0	0	0	0	0

0	0	0	0	0	0
0					

gtTCf=
0
0.11925
0.14534
0.13574
0.11593
0.096197
0.079948
0.067704
0.058926
0.052819
0.048646
0.045817
0.043898
0.042582
0.041662
0.040998
0.040498
0.040102

gtTCs=
0
0.1431
0.20732
0.22864
0.22778
0.21648
0.20118
0.18528
0.17042
0.1573
0.14609
0.13672
0.12898
0.12264
0.11746
0.11324
0.10979
0.10696

gtTCp=
0
2.385
1.5979
1.1196
0.8287
0.65143
0.54309
0.47655

0.43538
0.4096
0.39316
0.38239
0.37506
0.36982
0.36585
0.36266
0.35995
0.35753

X_mask=
2.2204e-016,2.2204e-016,2.2204e-016,0.00051671,0.0003141,2.2204e-016,0.00020454,0.0016283,2.2204e-016,0.0002678,8.4213e-005,0.00021968,0.0032482,0.0045197,2.2204e-016,0.0011855,2.2204e-016,0.0019774,2.2204e-016,2.2204e-016,0.004405,0.0069356,0.0062076,2.2204e-016,0.00035969,0.00045389,0.0012096,0.00037244,0.00060047,0.001562,0.0010299,0.0015948,2.2204e-016,2.2204e-016,0.00096143,0.002141,2.2204e-016,0.0012562,0.00052372,2.2204e-016,2.2204e-016,2.2204e-016,2.2204e-016,0.011048,0.0011994,0.020024,0.0014159,2.2204e-016,0.00044991,2.2204e-016,2.2204e-016,2.2204e-016,0.0028047,2.2204e-016,0.00083912,2.2204e-016,0.0030664,2.2204e-016,0.001533,0.0019084,2.2204e-016,2.2204e-016,0.018467,0.0096016,2.2204e-016,2.2204e-016,2.2204e-016,0.0017811,0.0028746,2.2204e-016,0.0073668,2.2204e-016,0.002956,2.2204e-016,2.2204e-016,2.2204e-016,0.0014451,0.00087104,2.2204e-016,2.2204e-016,0.0013974,0.00039639,6.2174e-005,2.2204e-016,0.00098209,2.2204e-016,0.026559,0.0091723,2.2204e-016,0.0068396,0.0022733,2.2204e-016,2.2204e-016,0.0026838,2.2204e-016,2.2204e-016,2.2204e-016,0.011663,2.2204e-016,0.00041559,0.0022909,4.4461e-005,0.00020385,2.2204e-016,0.0017171,2.2204e-016,0.0016671,2.2204e-016,3.7616e-005,0.00022179,0.00092531,0.00090166,2.2204e-016,0.0011275,0.022733,0.0033475,2.2204e-016,0.004034,0.0063121,2.2204e-016,0.0064056,0.011996,0.01888,0.0048235,2.2204e-016,2.2204e-016,2.2204e-016,0.0046123,0.00053984,2.2204e-016,2.2204e-016,0.00020818,2.2204e-016,0.0032261,0.00017491,0.0015175,0.0034247,2.2204e-016,0.0020931,2.2204e-016,2.2204e-016,0.0010657,2.2204e-016,2.2204e-016,0.0042052,2.2204e-016,2.2204e-016,2.2204e-016,2.2204e-016,0.014355,0.007624,2.2204e-016,2.2204e-016,2.2204e-016,0.0029145,0.0026345,2.2204e-016,0.012163,0.0052289,2.2204e-016,2.2204e-016,9.1036e-005,2.2204e-016,2.2204e-016,2.2204e-016,0.0013782,0.00013622,0.0024655,0.00033919,2.2204e-016,0.00037332,2.2204e-016,2.2204e-016,0.00053628,2.2204e-016,0.0011444,2.2204e-016,0.021004,2.2204e-016,2.2204e-016,2.2204e-016,0.0062771,2.2204e-016,0.014331,0.0039946,0.01436,0.0020554,0.005452,2.2204e-016,2.2204e-016,2.2204e-016,2.2204e-016,2.2204e-016,2.2204e-016,0.0025663,0.00075181,2.2204e-016,2.2204e-016,0.0010332,2.2204e-

016,2.2204e-016,2.2204e-016,0.00052307,2.2204e-016,2.2204e-
016,0.00033015,2.2204e-016,2.2204e-016,0.0002323,2.2204e-016,2.2204e-
016,2.2204e-016,2.2204e-016,0.00051587,2.2204e-016,0.0013243,2.2204e-
016,2.2204e-016,0.0079149,2.2204e-016,2.2204e-016,2.2204e-
016,0.0026268,0.0062338,0.0012893,2.2204e-016,2.2204e-
016,0.00026509,0.00024477,2.2204e-016,2.2204e-016,2.2204e-
016,0.00189,2.2204e-016,2.2204e-016,2.2204e-
016,0.0017767,0.00082054,0.00049591,2.2204e-016,2.2204e-016,2.2204e-
016,2.2204e-016,2.2204e-
016,0.00011756,0.0018004,0.0019584,0.019552,2.2204e-
016,0.0013379,2.2204e-016,2.2204e-
016,0.018198,0.0050892,0.0059996,0.002184,2.2204e-
016,0.015014,0.0060088,0.0091089,0.00076189,0.0014109,0.0081696,0.0162
05,2.2204e-016,0.00021396,0.0013947,2.2204e-016,2.2204e-
016,0.0028881,0.0017443,0.0028953,2.2204e-016,2.2204e-016,2.2204e-
016,2.2204e-016,0.00025304,0.0003497,2.2204e-016,2.2204e-
016,0.00062507,2.2204e-016,2.2204e-016,2.2204e-016,0.0022379,2.2204e-
016,0.031274,0.013318,0.0047576,2.2204e-
016,0.0020044,0.0003809,2.2204e-016,2.2204e-016,0.018081,2.2204e-
016,2.2204e-016,0.0019741,0.0089569,0.0099673,2.2204e-016,2.2204e-
016,0.00094139,2.2204e-016,2.2204e-016,2.2204e-
016,0.00014043,0.00054061,2.2204e-016,2.2204e-016,2.2204e-016,2.2204e-
016,2.2204e-016,0.00012511,0.00075049,2.2204e-016,2.2204e-016,2.2204e-
016,2.2204e-016,0.0011225,0.0013636,0.023431,2.2204e-
016,0.0029379,2.2204e-016,0.0028744,0.0022071,2.2204e-
016,0.00013686,0.012835,0.0079659,0.0021581,0.0030393,2.2204e-
016,0.011839,2.2204e-016,2.2204e-
016,0.00040309,0.0098072,0.0039579,2.2204e-016,0.00014946,2.2204e-
016,0.0015055,2.2204e-
016,0.00046427,0.001168,0.0002478,0.0023886,0.0009271,2.2204e-
016,1.0898e-005,2.2204e-016,2.2204e-016,2.2204e-
016,0.0016177,0.00037293,0.0011638,2.2204e-016,2.2204e-
016,0.010231,2.2204e-016,0.019386,0.0035105,0.0099908,2.2204e-
016,2.2204e-016,0.0017232,0.0054723,2.2204e-016,2.2204e-
016,0.015324,0.008908,0.0031012,0.001948,2.2204e-016,2.2204e-
016,0.010546,0.00032736,0.00024866,0.0011102,0.0014153,0.00064573,0.00
11887,0.0012034,2.2204e-016,2.2204e-016,9.1825e-
005,0.00020587,2.2204e-016,0.00053944,2.2204e-
016,0.00037642,0.00092321,2.2204e-016,0.0017817,2.2204e-
016,0.035993,2.2204e-016,2.2204e-016,0.00084128,2.2204e-016,2.2204e-
016,0.013944,2.2204e-016,2.2204e-016,0.023493,2.2204e-
016,0.010589,0.0065774,2.2204e-016,0.0045141,2.2204e-016,2.2204e-
016,0.0047993,0.0040461,0.00053901,1.3303e-
005,0.0012111,0.00068201,0.0011032,0.001234,2.2204e-016,2.2204e-
016,2.2204e-016,8.2398e-
005,0.0014466,0.00029498,0.00093004,0.0013711,0.00094123,2.2204e-
016,2.2204e-016,2.2204e-016,2.2204e-016,2.2204e-
016,0.026009,0.020522,0.0055209,0.031739,0.015435,2.2204e-
016,0.020708,2.2204e-016,2.2204e-016,2.2204e-016,0.0003719,2.2204e-
016,2.2204e-016,0.0020175,0.0062523,2.2204e-016,2.2204e-
016,0.0042974,0.0013473,0.00036074,0.0010581,2.2204e-016,2.2204e-
016,2.2204e-016,0.0017254,2.2204e-016,0.0023545,2.2204e-

016,0.00099679,2.2204e-016,0.00072633,0.00091845,2.2204e-
016,0.00093541,0.0012129,0.0014126,0.026462,2.2204e-016,2.2204e-
016,2.2204e-016,2.2204e-
016,0.035234,0.022313,0.00054314,0.0030644,2.2204e-016,2.2204e-
016,0.0088107,0.0033107,2.2204e-016,2.2204e-
016,0.014491,0.0054831,2.2204e-016,2.2204e-016,0.0056761,2.2204e-
016,2.2204e-016,2.2204e-016,0.0002435,2.2204e-016,0.0028299,2.2204e-
016,0.0018134,2.2204e-016,0.00059367,0.00041431,0.00049682,2.2204e-
016,0.0018726,2.2204e-016,2.2204e-016,2.2204e-016,2.2204e-
016,0.0065483,2.2204e-016,2.2204e-016,2.2204e-016,0.012521,2.2204e-
016,2.2204e-016,0.0047367,2.2204e-
016,0.0040927,0.013832,0.010721,0.00068952,0.013688,2.2204e-
016,2.2204e-016,0.0034096,2.2204e-016,0.0059422,0.0018641,2.2204e-
016,2.2204e-016,0.00045904,0.00071033,2.2204e-
016,0.00067306,0.00061978,0.00035625,2.2204e-016,0.00023729,2.2204e-
016,0.0015403,2.2204e-016,0.0027463,2.2204e-016,0.00030726,2.2204e-
016,0.0018759,2.2204e-016,2.2204e-
016,0.0020997,0.0020315,0.0094509,2.2204e-016,0.015278,4.8284e-
005,0.0054622,0.0047919,0.010024,0.0024723,2.2204e-
016,0.0010529,2.2204e-016,0.0047634,0.014206,0.0029291,6.5703e-
005,0.0013316,2.2204e-016,0.00019877,2.2204e-016,0.00069064,2.2204e-
016,2.2204e-016,2.2204e-016,2.2204e-016,2.2204e-016,2.2204e-
016,0.0010086,2.2204e-016,0.0021016,2.2204e-
016,0.00054799,0.002387,2.2204e-
016,0.00258,0.012486,0.0022559,0.0026075,0.0081287,0.01029,0.019605,0.
011532,0.0021805,0.021547,2.2204e-016,2.2204e-
016,0.014216,0.0055701,2.2204e-016,2.2204e-016,2.2204e-016,2.2204e-
016,0.0018955,0.00098283,2.2204e-016,0.0018467,2.2204e-
016,0.0020058,2.2204e-016,9.6447e-005,2.2204e-016,2.2204e-
016,0.0005109,2.2204e-016,0.0015544,0.0013035,2.2204e-016,2.2204e-
016,2.2204e-
016,0.00084176,0.0015854,0.0014681,0.0013939,0.026447,2.2204e-
016,2.2204e-016,2.2204e-016,0.011455,2.2204e-016,0.0049703,2.2204e-
016,0.005593,0.0066293,2.2204e-
016,0.00082752,0.0037466,0.0040158,0.012632,0.0042657,0.00045893,2.220
4e-016,2.2204e-016,2.2204e-016,2.2204e-016,2.2204e-016,2.2204e-
016,2.2204e-016,2.2204e-016,2.2204e-
016,0.00095553,0.00060411,0.0012327,0.00063439,2.2204e-
016,0.00036616,2.2204e-016,0.00048234,2.2204e-016,2.2204e-016,2.2204e-
016,0.027648,0.0026124,0.0043083,0.0075243,2.2204e-
016,0.00021927,0.013017,2.2204e-016,2.2204e-016,2.2204e-016,2.2204e-
016,0.0026992,2.2204e-016,2.2204e-016,2.2204e-016,2.2204e-016,2.2204e-
016,2.2204e-016,0.003764,0.00044585,2.2204e-016,2.2204e-016,2.2204e-
016,0.00044162,2.2204e-016,8.9902e-005,2.2204e-
016,0.00070782,0.00053517,9.6209e-005,0.0013147,0.0010597,2.2204e-
016,0.001918,2.2204e-016,0.00059402,0.00050298,2.2204e-016,2.2204e-
016,2.2204e-016,0.00031023,2.2204e-016,2.2204e-016,0.0053735,2.2204e-
016,0.011115,0.010272,0.0071295,2.2204e-016,2.2204e-
016,0.0076386,2.2204e-016,0.0078787,0.0080695,0.0023507,2.2204e-
016,2.2204e-016,2.2204e-016,0.0010966,0.0043936,2.2204e-016,2.2204e-
016,2.2204e-016,2.2204e-016,3.9533e-005,2.2204e-
016,0.00028555,2.2204e-016,2.2204e-016,2.2204e-016,2.2204e-

016,0.00012965,0.00094759,2.2204e-016,2.2204e-016,2.2204e-
016,0.013126,2.2204e-016,0.0076889,2.2204e-016,2.2204e-
016,0.010963,2.2204e-016,0.010534,2.2204e-016,2.2204e-016,2.2204e-
016,2.2204e-016,2.2204e-016,2.2204e-016,2.2204e-016,2.2204e-
016,0.00069155,2.2204e-016,0.0013716,2.2204e-016,2.2204e-
016,0.00017181,2.2204e-016,0.0016867,2.2204e-
016,0.00024586,0.0023821,0.002382,0.00073564,2.2204e-016,2.2204e-
016,2.2204e-016,0.0013266,2.2204e-016,2.2204e-
016,0.00023007,0.012811,5.4216e-
005,0.0012831,0.010278,0.0010389,2.2204e-
016,0.0024512,0.0062807,2.2204e-
016,0.0045922,0.0035801,0.0026682,0.0034427,0.0088698,2.2204e-
016,0.0013828,2.2204e-016,0.0003726,2.2204e-016,2.2204e-016,2.2204e-
016,0.0020876,0.00057999,0.00012159,0.0021885,2.2204e-
016,0.0017211,0.0007229,2.2204e-
016,0.0024311,0.0026075,0.00063139,0.00027216,2.2204e-
016,0.00064972,0.0008911,2.2204e-016,0.003874,0.00037371,2.2204e-
016,2.2204e-016,2.2204e-016,2.2204e-016,0.010119,0.0030326,2.2204e-
016,2.2204e-016,2.2204e-016,0.0040868,2.2204e-
016,0.00028963,0.0036683,2.2204e-016,0.00074124,2.2204e-016,2.2204e-
016,2.2204e-016,0.00011935,0.0002032,2.2204e-016,2.2204e-016,2.2204e-
016,2.2204e-016,2.2204e-016,2.2204e-016,2.2204e-016,0.0012942,1.3013e-
006,0.0006977,2.2204e-016,2.2204e-016,0.0015519,2.2204e-016,2.2204e-
016,2.2204e-016,2.2204e-016,2.2204e-016,2.2204e-
016,0.0048894,0.0060432,2.2204e-016,0.010028,2.2204e-
016,0.0014373,2.2204e-016,0.00069995,2.2204e-016,0.00097302,8.8239e-
005,0.0015073,0.0011401,0.0010664,2.2204e-016,2.2204e-
016,0.0017946,2.2204e-016,2.2204e-016,0.0017561,2.2204e-016,2.2204e-
016,0.00099699,2.2204e-016,2.2204e-016,2.2204e-016,0.000179,2.2204e-
016,2.2204e-016,0.00070792,2.2204e-016,4.414e-
005,0.00053718,0.00067448,2.2204e-
016,0.0023277,0.0034201,0.0056896,0.011628,0.0002116,0.0010477,3.1393e
-005,0.00046937,0.00025863,2.2204e-016,2.2204e-
016,0.00057848,0.00052462,0.0017001,0.00015544,0.0015186,2.2204e-
016,0.00077038,0.0021794,2.2204e-016,0.0014743,0.00010837,2.2204e-
016,2.2204e-016,2.2204e-016,2.2204e-016,2.2204e-016,2.2204e-
016,0.0013008,0.0007472,2.2204e-016,0.0011882,0.00033795,2.2204e-
016,5.9193e-005,2.2204e-016,2.2204e-016,8.2962e-005,0.022254,2.2204e-
016,2.2204e-016,2.2204e-016,2.2204e-016,2.2204e-
016,0.0021888,0.00033817,0.0021434,2.2204e-016,2.2204e-
016,0.0012318,0.0032418,0.0014983,2.2204e-016,0.001785,2.2204e-
016,0.00045206,2.2204e-016,2.2204e-016,0.0010127,2.2204e-
016,0.0032074,2.2204e-016,2.2204e-016,2.2204e-016,2.2204e-016,2.2204e-
016,2.2204e-
016,0.001504,0.00096606,0.001094,0.00022228,0.00029246,2.2204e-
016,2.2204e-016,0.00081296,0.00081927,0.00053954,2.2204e-016,2.2204e-
016,0.00064269,0.0015287,0.00023742,2.2204e-016,2.2204e-
016,0.0005918,0.001376,2.2204e-016,2.2204e-016,2.2204e-016,2.2204e-
016,2.2204e-016,2.2204e-016,2.2204e-016,0.0014931,0.00054125,2.2204e-
016,2.2204e-016,2.2204e-016,2.2204e-
016,0.00093979,0.00062147,0.0014747,2.2204e-
016,0.0016359,0.0020714,2.2204e-016,0.0020138,0.00077335,2.2204e-

016,0.0016942,2.2204e-016,2.2204e-016,0.00033419,2.2204e-016,2.2204e-
016,2.2204e-016,2.2204e-016,2.2204e-016,2.2204e-016,2.2204e-
016,0.0025662,2.2204e-016,2.2204e-016,0.0021332,0.00015705,2.2204e-
016,2.2204e-016,0.00036561,2.2204e-016,2.2204e-
016,0.00033141,0.0020482,0.001164,0.0025125,2.2204e-
016,0.0023422,2.2204e-016,0.0012138,0.0023053,2.2204e-
016,0.00072222,0.0017805,0.00046197,0.00057021,0.0007284,0.00062458,0.
0021096,2.2204e-016,2.2204e-016,2.2204e-016,0.00054815,2.2204e-
016,2.2204e-016,0.00038096,0.0011122,0.0018369,0.0014177,2.2204e-
016,2.2204e-016,2.2204e-016,2.2204e-016,2.2204e-016,2.2204e-
016,2.2204e-016,2.2204e-016,2.2204e-016,0.00063791,0.0013679,2.2204e-
016,2.2204e-016,2.2204e-016,0.0011791,2.2204e-016,0.00066753,2.2204e-
016,2.2204e-016,2.2204e-016,2.2204e-016,0.0010101,2.2204e-016,2.2204e-
016,2.2204e-016,0.00055189,0.0011704,2.2204e-
016,0.0019247,0.00071739,2.2204e-016,0.002632,0.0011017,2.2204e-
016,9.5125e-005,2.2204e-016,2.2204e-016,2.2204e-016,2.2204e-
016,0.00047851,2.2204e-016,0.00050061,2.2204e-016,2.2204e-
016,0.0011408,0.0019003,2.2204e-016,0.002453,2.2204e-
016,0.002733,2.2204e-016,1.7831e-006,0.00063782,0.0017773,2.2204e-
016,0.00031583,2.2204e-016,0.00022689,0.0010074,2.2204e-016,2.2204e-
016,0.00084171,0.00023669,2.2204e-016,0.00011176,2.2204e-
016,0.0021256,2.2204e-016,0.0012978,2.2204e-016,2.2204e-
016,0.00082602,2.2204e-016,0.00040183,2.2204e-016,2.2204e-
016,0.0026616,3.6176e-005,2.2204e-016,2.2204e-
016,0.0013835,0.0004792,2.2204e-016,2.2204e-016,2.2204e-
016,0.0020428,0.00026012,2.2204e-016,2.2204e-
016,0.00067804,0.00010434,2.2204e-016,0.0019243,2.2204e-
016,0.0010921,0.00018752,2.2204e-016,0.0013363,0.00079758,3.3805e-
005,0.00014607,2.2204e-016,2.2204e-016,2.2204e-016,0.00096258,2.2204e-
016,0.00064239,0.001962,2.2204e-016,2.2204e-016,0.00088445,2.2204e-
016,2.2204e-016,0.00025924,8.4057e-005,2.2204e-016,2.2204e-
016,0.00062127,0.00023458,0.0002896,2.2204e-
016,0.0024875,0.00061491,0.00015776,2.2204e-016,0.00058154,2.2204e-
016,2.2204e-016,0.00014907,2.2204e-016,0.0014638,0.000243,2.2204e-
016,0.00048778,0.0016949,0.0011273,2.2204e-016,2.2204e-
016,0.0015676,2.2204e-016,0.00017979,2.2204e-
016,0.0014074,0.0015464,0.0010906,2.2204e-
016,0.00088741,0.00048552,0.0022507,0.00034765,0.00088268,0.0027742,4.
2218e-005,0.0017667,2.2204e-016,2.2204e-
016,0.00031067,0.00069044,0.0002172,2.2204e-016,2.2204e-016,2.2204e-
016,0.00079795,0.00023623,2.2204e-016,2.2204e-016,0.002464,2.2204e-
016,5.7644e-005,2.2204e-
016,0.0019655,0.0022966,0.00015528,0.0028992,2.2204e-
016,0.00093048,2.2204e-016,0.0028541,2.2204e-016,2.2204e-016,2.2204e-
016,2.2204e-016,2.2204e-016,2.2204e-016,2.2204e-016,2.2204e-
016,2.2204e-016,2.2204e-016,0.00057071,2.2204e-016,2.2204e-
016,0.0011252,2.2204e-016,0.00023694,2.2204e-
016,0.0018977,0.0017066,0.0008456,0.00038782,2.2204e-016,2.2204e-
016,0.0034671,0.00013268,7.3592e-005,2.2204e-016,0.0014058,9.8779e-
005,0.0019838,0.001234,0.001502,2.2204e-016,0.000467,2.2204e-
016,0.0011357,2.2204e-016,2.2204e-016,2.2204e-016,1.0963e-005,2.2204e-
016,0.00027605,0.00021926,2.2204e-016,2.2204e-016,2.2204e-016

0.14066,0.13855,0.1434,0.14395,0.14312,0.14281,0.14309,0.14374,0.14374
,0.14305,0.14277,0.14297,0.14583,0.14629,0.14122,0.14429,0.14062,0.144
85,0.14042,0.14285,1.073,1.0349,1.3227,1.3343,0.1438,0.14259,0.14248,0
.14316,0.14512,0.1445,0.14337,0.14534,0.14258,0.14123,0.14312,0.14634,
0.14313,0.14454,0.14391,2.3941,1.2595,1.1663,1.2524,1.3468,1.2796,1.30
44,0.14384,0.14325,0.14399,0.14015,0.13949,0.14361,0.14536,0.14229,0.1
4434,0.14077,0.14498,0.14245,0.1438,0.14547,0.14135,2.3273,2.3959,1.12
48,1.2482,1.1249,1.2599,1.0474,1.2389,1.1984,1.2707,0.1433,0.14476,0.1
4191,0.14305,0.14253,0.14445,0.14335,0.14042,0.14086,0.14496,0.14402,0
.14433,0.1431,0.14455,0.14168,2.4029,2.3858,2.3633,1.1505,1.3531,1.610
6,1.2157,1.2604,1.249,1.1253,1.239,1.2093,0.14208,0.14341,0.14546,0.14
31,0.14484,0.14384,0.14323,0.14135,0.14528,0.13913,0.14261,0.14361,0.1
4308,0.14509,0.14273,0.14495,2.4042,2.4015,2.3627,2.3895,1.2676,1.2444
,1.2675,1.2657,1.472,1.2088,1.1607,1.0825,1.2969,1.128,0.14299,0.14152
,0.14372,0.14268,0.1431,0.1458,0.14204,0.1448,0.14784,0.14119,0.14556,
0.143,0.14137,0.14238,0.14122,0.14092,2.3745,2.3992,2.383,2.3376,2.380
2,2.3988,1.3705,1.3363,1.3215,1.2011,1.0735,1.4262,1.2551,1.1872,1.310
7,1.5364,0.14152,0.14445,0.14121,0.13995,0.14069,0.14404,0.14342,0.145
73,0.14337,0.13846,0.14323,0.14249,0.1419,0.1441,0.14135,0.14452,0.140
1,2.4069,2.358,2.3792,2.3783,2.4012,2.3511,1.5461,1.185,1.1406,1.4113,
1.2707,1.2858,1.3396,1.2328,1.0729,1.4378,1.2261,0.14677,0.14373,0.142
21,0.14304,0.14271,0.1431,0.14368,0.14263,0.14397,0.14304,0.14394,0.14
36,0.13983,0.12963,0.13121,0.13077,0.14108,2.374,2.3829,2.3934,2.3785,
2.3933,2.3602,2.3478,1.1101,1.5625,1.2519,1.5088,1.2599,1.2611,1.226,1
.2622,1.2524,1.2683,1.3124,0.14168,0.1423,0.1415,0.14399,0.14029,0.141
52,0.14185,0.1454,0.14377,0.14375,0.14339,0.13069,0.13106,0.13012,0.12
854,0.13146,0.13383,0.14537,2.4081,2.3629,2.3889,2.3667,2.3654,2.403,2
.3967,1.3081,1.4877,1.2645,1.311,1.2747,1.5861,1.1746,1.1503,1.3033,1.
236,1.2612,0.14179,0.14594,0.14136,0.14202,0.14505,0.14297,0.14486,0.1
4051,0.14135,0.12847,0.1306,0.13103,0.13027,0.13027,0.12991,0.13257,0.
12978,0.12794,0.14102,2.3833,2.3634,2.4034,2.3899,2.3884,2.3826,2.3849
,1.3093,1.2223,1.304,1.4574,1.107,1.3536,1.1253,1.2367,1.1111,1.1058,1
.3282,1.2694,0.14436,0.1415,0.14304,0.1431,0.14342,0.14195,0.13133,0.1
312,0.13241,0.12858,0.13167,0.13123,0.12612,0.12745,0.12826,0.12869,0.
12993,0.13429,2.4026,2.3391,2.3893,2.3825,2.3843,2.3736,2.3731,1.3902,
1.3109,1.2691,1.2611,1.1828,1.3481,1.5423,1.2569,1.4486,1.3388,1.3799,
1.4259,0.14206,0.14382,0.14133,0.13272,0.12507,0.13174,0.13054,0.13061
,0.1348,0.13243,0.12977,0.13137,0.12542,0.13121,0.1309,0.13264,0.13081
,0.13192,2.3622,2.3661,2.3981,2.3759,2.4099,2.384,2.3818,2.3718,2.3801
,1.2511,1.3982,1.3521,1.4499,1.2453,1.3659,1.2696,1.2728,1.5437,1.261,
1.2043,0.13261,0.131,0.13058,0.13195,0.13405,0.13223,0.13165,0.13052,0
.12935,0.13115,0.13136,0.13299,0.132,0.13065,0.12996,0.13331,0.12979,0
.13317,2.3422,2.4263,2.3715,2.3696,2.3664,2.3691,2.375,2.4047,2.3659,1
.1295,1.2967,0.87974,0.84876,0.95879,1.2281,0.8855,0.86891,0.8767,0.89
227,0.90337,0.13122,0.13112,0.13445,0.1325,0.1315,0.13223,0.13065,0.13
075,0.13092,0.13011,0.13083,0.1302,0.13288,0.13048,0.13248,0.13033,0.1
2983,0.12843,2.3594,2.3816,2.4079,2.3903,2.367,2.4097,2.4032,2.3673,2.
4056,1.2516,0.86321,0.68811,0.88018,0.87842,0.8927,1.0348,0.75806,0.53
711,0.87571,0.84866,0.13359,0.13401,0.13283,0.12861,0.12831,0.13182,0.
13521,0.13032,0.13375,0.13253,0.12723,0.12894,0.1306,0.13102,0.12876,0
.13124,0.13123,0.13229,2.4111,2.3717,2.3797,2.3697,2.36,2.4158,2.4035,
1.3504,1.3905,1.2501,0.6991,1.0932,0.8892,0.75924,0.83992,0.89514,0.94

124,0.88206,0.90215,0.88842,0.12914,0.12953,0.13254,0.13056,0.1316,0.1
3345,0.12866,0.13128,0.13065,0.13115,0.13132,0.1322,0.12926,0.13716,0.
12937,0.13109,0.12942,0.13292,2.3988,2.3404,2.3623,2.3967,2.4089,2.371
1,1.1743,1.5525,1.0951,1.3861,1.0885,0.9274,0.86021,0.90145,0.56501,0.
88485,0.88847,0.88015,0.88993,0.94695,0.1271,0.12984,0.13418,0.13017,0
.13067,0.13348,0.13062,0.1325,0.12942,0.13766,0.1319,0.13598,0.12941,0
.13232,0.12546,0.13149,0.12809,0.13165,2.383,2.378,2.3834,2.3853,2.402
9,1.2458,1.2745,1.4514,1.2535,1.2583,1.2132,0.9643,0.85187,0.92659,0.9
0158,0.79111,1.1007,0.86163,0.81446,0.57608,0.12928,0.13338,0.1333,0.1
312,0.1306,0.12917,0.12967,0.13039,0.12953,0.12985,0.13278,0.13055,0.1
3376,0.12785,0.13255,0.133,0.13056,0.13797,2.4,2.3913,2.3775,1.0525,1.
265,1.3046,1.0632,1.0793,1.2125,1.1385,1.2662,0.97982,0.88246,0.63754,
0.88186,0.79849,0.95085,0.92396,0.99014,0.89005,0.13562,0.13129,0.1332
,0.13119,0.13277,0.12969,0.13032,0.13111,0.13215,0.13361,0.13327,0.126
52,0.12986,0.13027,0.13026,0.1325,0.1328,0.13173,2.4135,1.2363,1.2454,
1.2521,1.2832,1.2736,1.1956,1.2308,1.2492,1.3867,0.87713,0.85671,0.935
26,0.79142,1.0366,0.93878,0.94444,0.91591,0.76141,0.13001,0.13128,0.12
879,0.12828,0.13013,0.13027,0.12882,0.13499,0.13392,0.13278,0.13133,0.
1299,0.13282,0.13155,0.13108,0.12987,0.13111,0.1194,1.2764,1.0235,1.25
37,1.2583,1.2388,1.1887,1.2633,1.2374,0.82692,1.4451,0.88089,0.92157,0
.99029,0.88118,0.63978,0.90071,0.81667,0.93129,1.0256,0.1314,0.12651,0
.13104,0.13044,0.1369,0.12589,0.13264,0.13026,0.1318,0.13364,0.13119,0
.13339,0.13266,0.13159,0.13481,0.1297,0.13196,0.12033,1.5885,1.2364,1.
2222,1.2435,1.6476,1.3521,1.2194,1.2521,0.90349,1.3453,0.92553,0.87702
,0.83379,0.79502,0.92818,0.99546,0.90428,0.84013,0.13111,0.1312,0.1300
3,0.13487,0.13677,0.12753,0.12892,0.12994,0.12758,0.13405,0.12952,0.13
213,0.13111,0.1296,0.13112,0.12996,0.1299,0.13112,0.11907,1.3533,0.970
04,1.3738,1.2801,1.2194,1.422,1.1474,1.1595,1.1734,1.2601,0.8538,0.925
85,0.9113,0.74652,0.92035,0.65501,0.84065,0.88825,0.13121,0.12985,0.13
313,0.13091,0.13044,0.1303,0.12864,0.13137,0.12994,0.13131,0.13321,0.1
3435,0.12888,0.13017,0.13036,0.12984,0.13393,0.11903,0.11872,0.11978,1
.4536,0.97795,1.1397,1.2686,1.1008,1.2061,1.2482,1.1267,1.4606,1.037,0
.77559,0.86392,0.80157,0.86307,0.87841,0.13174,0.13247,0.13006,0.13111
,0.13038,0.12892,0.13376,0.12949,0.13042,0.131,0.13061,0.13268,0.13412
,0.13053,0.13499,0.13407,0.13108,0.13232,0.11754,0.11974,0.11985,0.119
24,1.2257,1.1953,1.2537,1.3599,1.3099,1.2257,1.2579,1.1605,1.2336,0.88
107,0.89703,0.94882,0.13172,0.13114,0.13941,0.13244,0.13272,0.13083,0.
12998,0.12984,0.13241,0.13202,0.12898,0.12936,0.1296,0.13081,0.13053,0
.13085,0.1316,0.13525,0.13115,0.1292,0.11692,0.11855,0.12093,0.11954,0
.11837,0.11778,1.2535,0.94711,1.2406,1.2057,1.1297,1.5818,1.46,0.78289
,0.13312,0.12533,0.13058,0.13063,0.13217,0.13262,0.13769,0.13036,0.131
7,0.1295,0.12804,0.13363,0.12872,0.12843,0.1305,0.12892,0.12926,0.1328
2,0.12927,0.12958,0.13063,0.13482,0.13322,0.11807,0.11945,0.11857,0.11
892,0.11952,0.11904,1.3545,0.89564,1.2548,1.2786,1.1585,0.11886,0.1203
9,0.13059,0.13394,0.1318,0.13097,0.12895,0.13139,0.13189,0.13097,0.131
17,0.1326,0.13188,0.13143,0.13335,0.13107,0.1319,0.13481,0.12561,0.129
22,0.13202,0.13272,0.12973,0.11721,0.12068,0.12023,0.11772,0.12026,0.1
1902,0.11864,0.11929,0.11617,0.1186,1.2545,1.2742,0.1173,0.118,0.11861
,0.11844,0.12952,0.13263,0.12717,0.13484,0.12848,0.13123,0.13192,0.133
17,0.13297,0.13222,0.13235,0.13036,0.13476,0.13053,0.13378,0.12874,0.1
2828,0.13428,0.12881,0.11902,0.11812,0.11846,0.11883,0.11778,0.12032,0
.12027,0.12004,0.11975,0.11931,0.11698,0.11872,0.12031,0.11923,0.11928

,0.11885,0.11836,0.13069,0.13615,0.13286,0.12709,0.13308,0.13169,0.131
85,0.13117,0.12872,0.13245,0.12805,0.13048,0.13108,0.13118,0.13555,0.1
3232,0.11847,0.11899,0.11605,0.1193,0.11964,0.11945,0.12038,0.119,0.12
05,0.12116,0.11874,0.12189,0.11892,0.11818,0.12114,0.11811,0.11714,0.1
1951,0.11722,0.13009,0.13096,0.1296,0.12865,0.12949,0.13125,0.13326,0.
1304,0.13068,0.13163,0.12974,0.1296,0.13053,0.11935,0.11668,0.11993,0.
12027,0.12132,0.12065,0.12099,0.11904,0.1213,0.11988,0.11998,0.12033,0
.11752,0.11955,0.121,0.12053,0.11945,0.12074,0.11923,0.12061,0.11862,0
.13096,0.13336,0.13351,0.12861,0.12974,0.13251,0.13315,0.13416,0.13289
,0.1179,0.11784,0.11861,0.11762,0.11834,0.11879,0.1195,0.1185,0.1195,0
.11944,0.12079,0.11848,0.11792,0.11799,0.12063,0.11891,0.12148,0.1173,
0.11939,0.11931,0.1178,0.11955,0.11885,0.11913,0.11806,0.12916,0.13378
,0.12865,0.13373,0.1197,0.11822,0.12151,0.12031,0.118,0.11957,0.11937,
0.11885,0.11927,0.11861,0.12119,0.11836,0.12014,0.11709,0.11869,0.1202
1,0.12017,0.11818,0.12136,0.11825,0.12212,0.11891,0.12015,0.11963,0.12
126,0.11672,0.11942,0.11704,0.11933,0.12053,0.11776,0.11987,0.11979,0.
1196,0.1181,0.11796,0.11774,0.12155,0.11732,0.12066,0.11899,0.11834,0.
12053,0.1187,0.11984,0.11768,0.11886,0.12152,0.11935,0.11762,0.11761,0
.12072,0.12002,0.11704,0.11912,0.11769,0.1211,0.11943,0.11783,0.11929,
0.12023,0.12011,0.11622,0.12102,0.11872,0.11926,0.11953,0.11833,0.1198
8,0.1205,0.11857,0.11853,0.11919,0.11812,0.11973,0.12008,0.11877,0.120
29,0.12014,0.11767,0.11735,0.12121,0.11873,0.11788,0.12046,0.11989,0.1
1774,0.11849,0.11972,0.11938,0.11864,0.11917,0.12141,0.11937,0.11897,0
.11723,0.12034,0.1193,0.1174,0.11845,0.11848,0.12027,0.11923,0.11777,0
.11941,0.12104,0.11949,0.11847,0.1192,0.12006,0.11877,0.11893,0.11685,
0.12037,0.12053,0.12027,0.11907,0.11999,0.12007,0.12161,0.1197,0.12039
,0.12225,0.11986,0.12077,0.11892,0.11755,0.11928,0.11919,0.11781,0.118
06,0.11867,0.11806,0.12007,0.11979,0.1182,0.11987,0.12228,0.11823,0.11
976,0.11862,0.12148,0.12137,0.11965,0.12147,0.11788,0.11996,0.11953,0.
12098,0.11926,0.11824,0.11782,0.11736,0.11919,0.11831,0.11859,0.1196,0
.11839,0.11756,0.12032,0.11804,0.11826,0.12065,0.11935,0.11897,0.11949
,0.12027,0.12154,0.11994,0.12012,0.12008,0.11885,0.1234,0.11924,0.1186
7,0.11677,0.1206,0.11901,0.12135,0.12119,0.12129,0.11853,0.11929,0.118
23,0.12022,0.12004,0.11761,0.11733,0.11914,0.12073,0.11876,0.11886,0.1
1894,0.11904,0.1176
0.20581,0.20174,0.2079,0.20857,0.20758,0.208,0.20774,0.20869,0.20696,0
.20713,0.20799,0.20853,0.20966,0.2104,0.20495,0.20877,0.20547,0.20971,
0.20367,0.20726,0.78322,0.76457,0.93876,0.94392,0.20844,0.20774,0.2051
6,0.20668,0.21006,0.20917,0.20876,0.21046,0.20627,0.20568,0.20839,0.20
975,0.20746,0.2098,0.20689,1.5987,0.89771,0.84305,0.89121,0.95934,0.91
58,0.92018,0.20739,0.20761,0.20739,0.20386,0.20379,0.20751,0.20893,0.2
0698,0.20923,0.20577,0.20926,0.20742,0.20914,0.20969,0.20696,1.5515,1.
611,0.82027,0.88922,0.82279,0.89686,0.7732,0.88503,0.86392,0.90681,0.2
0708,0.20976,0.20718,0.20635,0.20711,0.20912,0.20788,0.20489,0.20432,0
.20983,0.20818,0.20806,0.20704,0.20899,0.20534,1.6168,1.6011,1.5852,0.
84254,0.96137,1.1179,0.86822,0.89398,0.89233,0.80826,0.89328,0.87498,0
.20868,0.20782,0.20908,0.20634,0.20762,0.20885,0.20756,0.20655,0.20863
,0.20309,0.20766,0.20736,0.20768,0.21029,0.20503,0.21019,1.6114,1.6089
,1.5765,1.5965,0.90095,0.88515,0.90704,0.91026,1.0291,0.86232,0.83527,
0.79143,0.9236,0.81873,0.20654,0.20648,0.20943,0.20754,0.20838,0.21036
,0.20739,0.20804,0.21106,0.20595,0.20997,0.20699,0.20565,0.20678,0.205
16,0.20581,1.6006,1.6134,1.5948,1.5559,1.59,1.611,0.96222,0.94191,0.94

1,0.86943,0.78799,1.0065,0.89618,0.8659,0.93595,1.0685,0.20614,0.20784
,0.20627,0.20407,0.20536,0.2098,0.2069,0.2095,0.20696,0.20429,0.20865,
0.2065,0.20689,0.20805,0.20492,0.20807,0.20497,1.62,1.5781,1.5879,1.59
55,1.6082,1.5557,1.091,0.85431,0.83284,0.9913,0.90565,0.91325,0.95275,
0.88241,0.7799,1.0104,0.87432,0.21008,0.20734,0.20741,0.20745,0.20663,
0.20578,0.20853,0.20852,0.20861,0.20738,0.20833,0.20866,0.20369,0.1724
2,0.1767,0.17802,0.20587,1.5895,1.5895,1.5958,1.5938,1.6128,1.5834,1.5
654,0.81283,1.0866,0.89873,1.0494,0.90669,0.90356,0.87569,0.89565,0.89
102,0.90517,0.93453,0.20539,0.20733,0.20574,0.20873,0.20559,0.20585,0.
20649,0.20954,0.20697,0.20931,0.20848,0.17508,0.1802,0.17591,0.1727,0.
17665,0.17609,0.20955,1.6287,1.5696,1.5917,1.5878,1.5976,1.6123,1.618,
0.93419,1.047,0.91085,0.93125,0.91223,1.107,0.8496,0.83795,0.92704,0.8
9412,0.89769,0.20671,0.21039,0.20585,0.20533,0.20966,0.20598,0.20824,0
.20462,0.20609,0.1735,0.17813,0.17404,0.17339,0.17554,0.17379,0.179,0.
17437,0.17225,0.20649,1.5875,1.5767,1.6094,1.6044,1.61,1.6009,1.5852,0
.93137,0.87614,0.93096,1.0148,0.80642,0.95263,0.81596,0.89319,0.81108,
0.80313,0.93518,0.90491,0.20769,0.20617,0.20732,0.20792,0.20671,0.2055
8,0.17587,0.17741,0.18182,0.17176,0.17694,0.17646,0.16817,0.16791,0.17
128,0.17422,0.17269,0.1794,1.6213,1.551,1.6059,1.5981,1.5958,1.5914,1.
5941,0.98474,0.93246,0.90169,0.90482,0.85348,0.94358,1.0736,0.89318,1.
0276,0.95549,0.9846,1.0109,0.20596,0.2083,0.20705,0.17861,0.16379,0.17
648,0.17032,0.17635,0.18129,0.17848,0.17488,0.17599,0.16883,0.17504,0.
17649,0.17615,0.1756,0.17642,1.5802,1.5823,1.6097,1.5819,1.6276,1.5964
,1.5909,1.5782,1.5901,0.89704,0.98867,0.94466,1.0235,0.88934,0.96303,0
.91208,0.91287,1.0798,0.90444,0.87122,0.1776,0.17364,0.17537,0.17587,0
.18126,0.17714,0.17436,0.17554,0.17549,0.17576,0.17786,0.18283,0.17514
,0.17683,0.17251,0.17868,0.17182,0.17893,1.5605,1.64,1.577,1.5882,1.59
5,1.5789,1.5735,1.6283,1.5598,0.8234,0.92738,0.64844,0.62941,0.69191,0
.85606,0.64615,0.63984,0.64396,0.65972,0.66347,0.17669,0.17601,0.1813,
0.17656,0.17648,0.17709,0.17634,0.17726,0.17607,0.17589,0.17509,0.1745
6,0.18118,0.17253,0.17735,0.17479,0.17455,0.17369,1.5648,1.5876,1.6323
,1.6158,1.5759,1.6241,1.6119,1.5753,1.608,0.87239,0.63918,0.53002,0.65
032,0.64175,0.65579,0.73886,0.56962,0.42986,0.64094,0.63377,0.17844,0.
18089,0.1804,0.17262,0.17011,0.17849,0.18383,0.17532,0.17916,0.1792,0.
16363,0.17473,0.17592,0.17717,0.17395,0.17583,0.17679,0.17737,1.6368,1
.5899,1.6101,1.5843,1.5748,1.6349,1.6067,0.93443,0.96235,0.86939,0.538
02,0.79116,0.65035,0.57161,0.62527,0.66078,0.68944,0.6481,0.6654,0.662
21,0.17392,0.17579,0.18241,0.17697,0.1782,0.17942,0.17476,0.17442,0.17
497,0.17593,0.17661,0.17816,0.17368,0.18692,0.17209,0.17685,0.17661,0.
18311,1.6054,1.5556,1.5756,1.5928,1.611,1.5742,0.81588,1.0736,0.7728,0
.95855,0.77446,0.68291,0.63437,0.66642,0.45064,0.65142,0.65751,0.64429
,0.6634,0.68466,0.17113,0.17387,0.18227,0.17477,0.17609,0.18216,0.1743
4,0.17822,0.17013,0.19101,0.17827,0.18457,0.17546,0.17614,0.16833,0.17
467,0.17389,0.17752,1.6081,1.6014,1.5907,1.6046,1.6214,0.86918,0.89737
,0.98655,0.86847,0.88256,0.84613,0.69949,0.63058,0.67668,0.65793,0.589
51,0.78948,0.63402,0.60643,0.45756,0.17313,0.17998,0.18424,0.17371,0.1
7684,0.17414,0.17368,0.17556,0.17708,0.17577,0.17867,0.17655,0.17847,0
.17146,0.1793,0.17753,0.17673,0.19134,1.5949,1.6074,1.5857,0.74004,0.8
9194,0.91447,0.75375,0.76566,0.85746,0.79992,0.87393,0.7099,0.65317,0.
49098,0.65337,0.58868,0.68939,0.68218,0.7206,0.65479,0.18363,0.17703,0
.17845,0.17619,0.17754,0.17612,0.17505,0.17461,0.17719,0.17987,0.17834
,0.16834,0.17554,0.17627,0.17577,0.17705,0.17937,0.17761,1.6271,0.8607

6,0.85929,0.8814,0.89617,0.88284,0.83416,0.84334,0.86886,0.95947,0.647
33,0.63408,0.68483,0.58908,0.74603,0.67876,0.68763,0.66689,0.58026,0.1
7394,0.17596,0.17082,0.1714,0.17627,0.17627,0.17249,0.18327,0.18036,0.
17893,0.1737,0.17634,0.17888,0.17628,0.17586,0.17531,0.17567,0.14571,0
.8926,0.72693,0.86921,0.88156,0.85901,0.82854,0.88556,0.85498,0.59642,
0.98399,0.6471,0.6722,0.71861,0.64676,0.49417,0.66461,0.61209,0.67797,
0.74161,0.17551,0.16678,0.17516,0.17671,0.1901,0.16315,0.18159,0.17445
,0.17698,0.1804,0.17627,0.17832,0.1761,0.17787,0.18213,0.1758,0.17769,
0.14647,1.0884,0.8635,0.85257,0.8665,1.1274,0.93315,0.85389,0.87103,0.
65325,0.93902,0.67866,0.64838,0.6176,0.59872,0.67621,0.73109,0.6666,0.
61841,0.17684,0.1756,0.17308,0.18407,0.18346,0.17138,0.17499,0.17758,0
.16733,0.18156,0.17486,0.17696,0.17636,0.17592,0.177,0.17464,0.17463,0
.17606,0.14552,0.934,0.69098,0.95178,0.88369,0.85058,0.97568,0.7979,0.
811,0.8208,0.87693,0.63063,0.68059,0.6735,0.55699,0.67651,0.51033,0.62
089,0.65275,0.17557,0.17409,0.17934,0.17749,0.17572,0.17395,0.17082,0.
17518,0.17792,0.17668,0.17824,0.17945,0.17153,0.1743,0.17648,0.17491,0
.18032,0.14559,0.14468,0.14555,1.0051,0.69725,0.79518,0.88597,0.77552,
0.83767,0.8679,0.7932,1.0134,0.74844,0.5828,0.63699,0.59902,0.64582,0.
64216,0.17642,0.18112,0.17507,0.17736,0.17545,0.17136,0.17803,0.17357,
0.17587,0.17426,0.17591,0.17886,0.18312,0.1788,0.18264,0.17872,0.17407
,0.17695,0.1446,0.14569,0.14553,0.14559,0.85112,0.8357,0.87463,0.94762
,0.90443,0.85698,0.87032,0.81324,0.85107,0.64532,0.66058,0.69622,0.178
78,0.17592,0.19306,0.18264,0.18026,0.17612,0.17406,0.17512,0.17941,0.1
7691,0.17399,0.17663,0.17341,0.17662,0.17409,0.17644,0.17726,0.18332,0
.17699,0.16981,0.14196,0.14542,0.14738,0.14668,0.14528,0.14445,0.87684
,0.66909,0.86334,0.83998,0.79567,1.0823,1.0106,0.57257,0.1784,0.16679,
0.17545,0.17685,0.17689,0.17998,0.18848,0.17216,0.17648,0.17499,0.1711
,0.18066,0.17502,0.17324,0.17396,0.1739,0.17409,0.17801,0.17248,0.1754
5,0.17845,0.18542,0.18218,0.14407,0.14574,0.14464,0.1441,0.14626,0.145
03,0.93083,0.64672,0.87823,0.88332,0.81027,0.14472,0.14586,0.1758,0.18
309,0.17612,0.17692,0.17314,0.17539,0.17601,0.17586,0.17481,0.17882,0.
17898,0.17672,0.17732,0.17804,0.17696,0.18613,0.16556,0.17339,0.17791,
0.18201,0.17402,0.14381,0.14659,0.14522,0.14352,0.14608,0.14529,0.1456
2,0.14568,0.14269,0.14453,0.87427,0.89156,0.14335,0.1439,0.14539,0.144
26,0.17358,0.17695,0.16508,0.18375,0.16988,0.17629,0.1775,0.17744,0.17
995,0.17936,0.17736,0.17518,0.18551,0.17462,0.18467,0.17131,0.17218,0.
17957,0.17454,0.14505,0.14488,0.14495,0.14473,0.14399,0.14745,0.14683,
0.1461,0.1451,0.14469,0.14296,0.14424,0.14678,0.14494,0.14515,0.14537,
0.14437,0.1747,0.18545,0.18167,0.16996,0.18277,0.17706,0.17699,0.17937
,0.17277,0.1806,0.17299,0.17604,0.17736,0.17971,0.1859,0.17778,0.14399
,0.14633,0.1436,0.14533,0.14586,0.1453,0.14666,0.14587,0.14581,0.14737
,0.14508,0.14772,0.14503,0.14446,0.14715,0.14425,0.14272,0.14585,0.143
28,0.17475,0.17614,0.17527,0.1727,0.17633,0.17611,0.17831,0.17581,0.17
626,0.17435,0.17158,0.17401,0.17409,0.14546,0.14382,0.14584,0.14671,0.
14769,0.14704,0.14704,0.14556,0.1471,0.14655,0.14513,0.14651,0.1433,0.
14576,0.14663,0.14597,0.14579,0.1459,0.14592,0.14686,0.14459,0.17538,0
.18542,0.18011,0.17316,0.17219,0.17746,0.17866,0.18225,0.17727,0.14421
,0.14493,0.14505,0.14432,0.1443,0.14553,0.14499,0.14397,0.14551,0.1448
8,0.14733,0.14355,0.14355,0.14374,0.14679,0.14552,0.14736,0.1433,0.145
29,0.14508,0.14531,0.14517,0.14402,0.14539,0.14295,0.17016,0.18095,0.1
7138,0.17847,0.14492,0.14452,0.14783,0.14647,0.14418,0.14623,0.14466,0
.14451,0.14632,0.14475,0.14737,0.1451,0.14625,0.143,0.14487,0.14653,0.

14685,0.14401,0.14788,0.1451,0.14769,0.14533,0.14522,0.14648,0.14744,0
.14362,0.14586,0.14273,0.14553,0.14649,0.14496,0.14548,0.14494,0.14569
,0.14425,0.14392,0.14452,0.14738,0.14383,0.14658,0.144,0.14436,0.14647
,0.14541,0.14462,0.14384,0.14505,0.14801,0.14524,0.14423,0.14285,0.145
91,0.1456,0.14317,0.14421,0.14416,0.14768,0.14541,0.14408,0.14551,0.14
652,0.14591,0.14162,0.14699,0.14552,0.14533,0.14457,0.14508,0.14576,0.
14687,0.14464,0.14465,0.14571,0.14397,0.14687,0.1457,0.14603,0.14716,0
.14634,0.14469,0.14334,0.14632,0.14504,0.1449,0.14719,0.14622,0.14407,
0.14472,0.14602,0.14612,0.14555,0.14476,0.1473,0.14565,0.14464,0.14342
,0.14583,0.14594,0.14328,0.14519,0.14381,0.14674,0.14569,0.1438,0.1454
2,0.14746,0.14491,0.14474,0.14495,0.14699,0.14529,0.14498,0.14335,0.14
702,0.14606,0.14675,0.1447,0.14642,0.14554,0.14739,0.14517,0.14651,0.1
4815,0.14591,0.14706,0.14505,0.1434,0.14462,0.14502,0.14377,0.14366,0.
1442,0.14387,0.14643,0.14522,0.14382,0.14617,0.14791,0.14448,0.14586,0
.14435,0.14777,0.14611,0.14595,0.14836,0.14449,0.1463,0.14659,0.14736,
0.14535,0.14421,0.14354,0.14369,0.14547,0.14455,0.14498,0.14617,0.1439
,0.14393,0.14504,0.14471,0.14393,0.14541,0.14465,0.14476,0.14512,0.145
9,0.14727,0.14565,0.14613,0.14577,0.14471,0.14904,0.14572,0.14506,0.14
273,0.14727,0.1447,0.14736,0.14632,0.14843,0.14509,0.14563,0.14382,0.1
4519,0.14522,0.14409,0.14395,0.14557,0.14715,0.14441,0.14497,0.14525,0
.1447,0.14508
0.22623,0.22364,0.22712,0.22811,0.22937,0.22939,0.22953,0.22931,0.2288
6,0.22914,0.22965,0.22887,0.23089,0.23172,0.22613,0.22938,0.2266,0.230
02,0.22403,0.22797,0.6007,0.58991,0.68351,0.6977,0.23011,0.22883,0.226
2,0.22694,0.22962,0.23093,0.23046,0.23019,0.22752,0.22638,0.22989,0.23
053,0.22907,0.23333,0.22774,1.1176,0.67213,0.6363,0.65938,0.7169,0.679
29,0.68487,0.22935,0.22801,0.22817,0.22665,0.22602,0.22796,0.22966,0.2
2858,0.23103,0.22684,0.2307,0.22875,0.23109,0.23124,0.22686,1.0673,1.1
385,0.62477,0.66145,0.62988,0.66746,0.594,0.66247,0.64551,0.68235,0.22
968,0.22967,0.22812,0.22783,0.22739,0.22962,0.22963,0.22535,0.22596,0.
22944,0.2286,0.22864,0.2279,0.2313,0.22591,1.1305,1.1089,1.1094,0.6367
6,0.70941,0.80607,0.65899,0.66641,0.66641,0.60912,0.67217,0.66231,0.22
839,0.22897,0.23138,0.22777,0.22842,0.22945,0.22883,0.2286,0.22881,0.2
2492,0.22968,0.22817,0.22926,0.23038,0.22716,0.23104,1.1226,1.1261,1.0
982,1.106,0.67565,0.6576,0.67963,0.67965,0.75623,0.64332,0.62871,0.601
23,0.68355,0.61615,0.22873,0.22828,0.23035,0.22775,0.22916,0.23023,0.2
2894,0.22998,0.23148,0.22768,0.22954,0.22849,0.22699,0.22776,0.22687,0
.22717,1.1314,1.1155,1.1283,1.0754,1.114,1.1337,0.70973,0.69096,0.6996
9,0.66397,0.60182,0.74425,0.68212,0.65539,0.69449,0.77611,0.22727,0.22
83,0.22813,0.22699,0.22713,0.23022,0.22954,0.23084,0.22768,0.2264,0.22
922,0.22984,0.22941,0.22947,0.22589,0.22858,0.22616,1.1492,1.0956,1.10
34,1.1235,1.1314,1.0701,0.79534,0.64707,0.63307,0.72936,0.68344,0.6781
7,0.70547,0.65349,0.59407,0.74256,0.65417,0.23041,0.22979,0.22795,0.22
78,0.2291,0.22765,0.2306,0.22904,0.23029,0.22879,0.22947,0.22955,0.224
37,0.17746,0.18291,0.18645,0.22823,1.1132,1.1104,1.111,1.1128,1.1403,1
.1115,1.0835,0.62421,0.79177,0.67567,0.76854,0.67993,0.67287,0.65709,0
.6627,0.65967,0.67242,0.69498,0.22588,0.22945,0.22814,0.22978,0.22589,
0.22673,0.22847,0.22928,0.22795,0.23143,0.22941,0.1806,0.18925,0.18179
,0.17765,0.18177,0.17953,0.23172,1.1336,1.087,1.114,1.107,1.1232,1.128
8,1.1354,0.69573,0.77467,0.67375,0.68899,0.67598,0.80752,0.63771,0.637
43,0.68536,0.67183,0.67341,0.22813,0.23198,0.22806,0.22613,0.23031,0.2
2717,0.23015,0.22582,0.22711,0.17921,0.18477,0.17822,0.1769,0.18212,0.

17825,0.18606,0.1801,0.17718,0.22902,1.1194,1.1059,1.1293,1.1151,1.134
,1.1278,1.1054,0.69656,0.65501,0.69449,0.74342,0.6085,0.70689,0.62028,
0.67453,0.61424,0.60975,0.69277,0.67825,0.23007,0.22787,0.2284,0.22792
,0.22785,0.22586,0.18215,0.18248,0.18894,0.17611,0.18257,0.18219,0.172
74,0.17141,0.17607,0.18013,0.17594,0.18545,1.1456,1.0714,1.1292,1.1197
,1.1099,1.1209,1.1123,0.7227,0.69282,0.67362,0.67812,0.64135,0.70073,0
.78659,0.66464,0.75842,0.70452,0.73445,0.75869,0.22805,0.23007,0.23024
,0.18462,0.16335,0.1819,0.17203,0.18124,0.18779,0.18435,0.1814,0.18126
,0.17266,0.18021,0.18368,0.1799,0.18245,0.18132,1.1141,1.0981,1.1287,1
.104,1.1545,1.1214,1.0993,1.0897,1.115,0.6704,0.73018,0.69697,0.75369,
0.67015,0.72277,0.68392,0.68746,0.78452,0.67863,0.65819,0.18388,0.1781
1,0.18132,0.18106,0.19069,0.18262,0.17633,0.18343,0.18114,0.18097,0.18
432,0.19135,0.18004,0.18366,0.17747,0.18448,0.17576,0.18481,1.0858,1.1
591,1.1049,1.1132,1.1184,1.1014,1.0998,1.1335,1.0851,0.62071,0.6953,0.
49227,0.48361,0.52,0.61961,0.49391,0.4818,0.48555,0.49767,0.50883,0.18
217,0.18182,0.18928,0.18243,0.18234,0.18317,0.18181,0.18479,0.18295,0.
18279,0.18039,0.18016,0.18688,0.17439,0.18344,0.18104,0.18063,0.17926,
1.0852,1.1052,1.1549,1.1295,1.0978,1.1421,1.1464,1.0993,1.1244,0.6347,
0.48744,0.42269,0.49738,0.48326,0.4961,0.54899,0.44173,0.34821,0.48219
,0.48739,0.18316,0.18901,0.18811,0.17771,0.17343,0.18556,0.19209,0.180
6,0.18448,0.18691,0.16221,0.18073,0.18124,0.18113,0.17975,0.18063,0.18
208,0.1843,1.146,1.1108,1.1405,1.0996,1.0958,1.1631,1.1121,0.67006,0.6
9221,0.6218,0.42663,0.59205,0.49463,0.44552,0.47403,0.49896,0.52211,0.
49318,0.50362,0.5037,0.17866,0.18267,0.18933,0.1855,0.18515,0.1868,0.1
8085,0.17944,0.18007,0.18207,0.18226,0.18418,0.17912,0.19658,0.17647,0
.1836,0.18385,0.1941,1.1217,1.0781,1.09,1.1117,1.1424,1.0949,0.5916,0.
76459,0.56511,0.68852,0.56597,0.52032,0.48505,0.50855,0.36048,0.49319,
0.49755,0.49091,0.50492,0.51063,0.17432,0.18066,0.19109,0.17904,0.1825
5,0.18977,0.17883,0.18432,0.17433,0.20358,0.18417,0.19308,0.18147,0.18
156,0.17266,0.17946,0.18076,0.18353,1.1209,1.1315,1.112,1.1276,1.15,0.
6288,0.65101,0.70184,0.62598,0.63864,0.61289,0.52706,0.47909,0.5118,0.
49969,0.45408,0.58615,0.48792,0.46261,0.37169,0.17858,0.18713,0.19499,
0.17787,0.18291,0.17931,0.18022,0.18075,0.18505,0.18198,0.18562,0.1831
,0.18438,0.17656,0.18519,0.18473,0.18057,0.20236,1.1207,1.1228,1.1038,
0.53865,0.64767,0.66162,0.55122,0.55551,0.62272,0.57693,0.6238,0.52992
,0.50189,0.38917,0.49213,0.4517,0.51878,0.51916,0.53992,0.50238,0.1926
7,0.183,0.18242,0.18318,0.18352,0.18245,0.18071,0.17819,0.18299,0.1879
2,0.18407,0.17202,0.18092,0.18263,0.18169,0.18341,0.18602,0.18415,1.14
08,0.62521,0.61277,0.63931,0.64578,0.63745,0.60298,0.61081,0.63029,0.6
9076,0.49484,0.48423,0.52072,0.45274,0.55865,0.50564,0.51576,0.5031,0.
45016,0.17919,0.18213,0.1742,0.17466,0.18174,0.18136,0.1782,0.19233,0.
18734,0.18485,0.17774,0.18123,0.18514,0.18305,0.18106,0.18176,0.18119,
0.13619,0.65195,0.53269,0.6307,0.63352,0.62042,0.60191,0.63833,0.59933
,0.44081,0.70887,0.48897,0.51073,0.52816,0.49699,0.3924,0.4994,0.47208
,0.50267,0.56034,0.18075,0.16892,0.17919,0.18269,0.20291,0.16296,0.189
84,0.17991,0.18169,0.188,0.18219,0.18464,0.18054,0.18441,0.18953,0.181
05,0.18336,0.13675,0.76414,0.62502,0.613,0.62764,0.79284,0.66725,0.619
71,0.62736,0.4835,0.6753,0.5141,0.49157,0.47502,0.46317,0.50913,0.5499
6,0.51178,0.47095,0.18263,0.18067,0.1778,0.19175,0.19022,0.17567,0.181
57,0.18507,0.1686,0.1902,0.18269,0.18451,0.18187,0.18223,0.18169,0.180
62,0.18184,0.18189,0.13599,0.67892,0.50685,0.69163,0.6336,0.61706,0.69
38,0.569,0.58318,0.5925,0.63115,0.48216,0.51616,0.51526,0.42598,0.5126

9,0.40302,0.47568,0.50008,0.18083,0.17957,0.18654,0.18309,0.18126,0.17
786,0.17508,0.18068,0.18596,0.18274,0.18402,0.18598,0.17554,0.17748,0.
18286,0.18096,0.18783,0.13638,0.13454,0.1348,0.71326,0.51363,0.57697,0
.63526,0.55788,0.60588,0.62944,0.57423,0.72585,0.55684,0.44593,0.48693
,0.46422,0.49952,0.49137,0.18356,0.18979,0.18003,0.18289,0.18268,0.175
97,0.18485,0.17629,0.18104,0.17773,0.18183,0.18434,0.19247,0.18717,0.1
8875,0.18753,0.17888,0.18191,0.13568,0.13642,0.13512,0.13541,0.60804,0
.60426,0.62848,0.67634,0.65138,0.62024,0.62427,0.58983,0.60149,0.49009
,0.50425,0.52365,0.18673,0.18177,0.20691,0.19215,0.18757,0.18196,0.177
96,0.18034,0.18739,0.18326,0.18076,0.18338,0.18001,0.18322,0.17985,0.1
8423,0.18246,0.19102,0.18134,0.17211,0.13291,0.13543,0.13812,0.137,0.1
3595,0.13452,0.62428,0.48483,0.62719,0.6042,0.57757,0.77221,0.7213,0.4
2196,0.18572,0.16963,0.18063,0.18252,0.18217,0.18694,0.19939,0.17575,0
.182,0.18089,0.17466,0.18854,0.18048,0.1794,0.17853,0.18026,0.17941,0.
18391,0.17646,0.18045,0.18684,0.19572,0.19009,0.1348,0.13658,0.1354,0.
13428,0.13641,0.13569,0.66782,0.47682,0.6265,0.63255,0.5913,0.13478,0.
13675,0.18128,0.19256,0.18225,0.18313,0.17941,0.17978,0.18233,0.18015,
0.17946,0.18424,0.1869,0.1836,0.18206,0.18607,0.1826,0.19643,0.1669,0.
18006,0.18452,0.19195,0.17963,0.13473,0.13782,0.13595,0.13332,0.13709,
0.13585,0.13573,0.13561,0.1337,0.13539,0.62745,0.65445,0.13405,0.13481
,0.13499,0.13452,0.17817,0.18251,0.16537,0.19154,0.17311,0.18264,0.183
8,0.18292,0.1873,0.18845,0.18297,0.18032,0.19384,0.18139,0.19451,0.175
24,0.17851,0.18478,0.18233,0.13556,0.13598,0.13488,0.13488,0.13444,0.1
3849,0.13593,0.13702,0.13508,0.13544,0.13368,0.13505,0.13619,0.13581,0
.13522,0.13587,0.13475,0.18036,0.19492,0.19012,0.17271,0.19315,0.18261
,0.18246,0.18802,0.17706,0.18927,0.17978,0.18162,0.18418,0.18921,0.195
5,0.18403,0.13456,0.1366,0.13449,0.13523,0.13669,0.13572,0.13661,0.136
19,0.1372,0.13719,0.13469,0.13735,0.13538,0.13475,0.1378,0.13493,0.132
77,0.13626,0.13357,0.18059,0.18275,0.18187,0.17653,0.18242,0.18212,0.1
8476,0.18108,0.18228,0.17697,0.17442,0.17954,0.17997,0.13531,0.13482,0
.13604,0.13587,0.13721,0.13746,0.13768,0.13541,0.1368,0.13729,0.13532,
0.13673,0.13441,0.13626,0.13698,0.13718,0.13604,0.13631,0.13681,0.1375
9,0.13451,0.18193,0.19813,0.18734,0.17771,0.17846,0.18377,0.185,0.1907
8,0.18338,0.13503,0.13488,0.136,0.13458,0.13418,0.13594,0.13603,0.1345
2,0.13665,0.13551,0.13706,0.13366,0.13439,0.13517,0.13824,0.13649,0.13
87,0.13344,0.13542,0.13514,0.13574,0.13606,0.13373,0.13603,0.13272,0.1
739,0.18851,0.17708,0.18483,0.13502,0.13485,0.13784,0.13565,0.13547,0.
13596,0.13547,0.13398,0.13641,0.1356,0.13679,0.13608,0.13649,0.13461,0
.13541,0.13685,0.13715,0.13331,0.1381,0.13581,0.13759,0.13451,0.1361,0
.13761,0.13784,0.13393,0.13553,0.13331,0.13549,0.13719,0.13514,0.13573
,0.13586,0.13536,0.13413,0.13505,0.13474,0.13701,0.13329,0.13639,0.134
45,0.13601,0.1374,0.13566,0.13458,0.13455,0.13466,0.13829,0.13533,0.13
482,0.13299,0.13641,0.13626,0.13377,0.13473,0.13491,0.13841,0.13576,0.
13435,0.1357,0.13652,0.13571,0.13176,0.13644,0.1358,0.13507,0.13499,0.
13558,0.13487,0.13593,0.13509,0.13534,0.13671,0.1342,0.13684,0.13589,0
.13569,0.13798,0.13608,0.13528,0.13319,0.13614,0.1346,0.13519,0.13775,
0.13691,0.13485,0.13539,0.13687,0.13664,0.13607,0.13542,0.13739,0.1364
7,0.13506,0.13396,0.13623,0.1364,0.13419,0.13564,0.13444,0.13741,0.136
57,0.13487,0.1352,0.1383,0.13478,0.13543,0.13499,0.13757,0.13487,0.134
76,0.13464,0.13688,0.13631,0.13774,0.13492,0.13675,0.13528,0.13829,0.1
3614,0.13628,0.13831,0.13649,0.13707,0.1355,0.13468,0.13547,0.13524,0.
13502,0.1348,0.13427,0.13358,0.13725,0.13656,0.13473,0.13614,0.13798,0

.13433,0.13574,0.1349,0.13756,0.13689,0.1365,0.13798,0.1349,0.13714,0.
1364,0.13707,0.13533,0.13499,0.13497,0.1341,0.13609,0.13538,0.13583,0.
1359,0.13394,0.13523,0.13611,0.13505,0.1345,0.1353,0.13476,0.13484,0.1
3567,0.13682,0.13738,0.136,0.1359,0.13553,0.13529,0.13848,0.13666,0.13
58,0.13312,0.13722,0.13471,0.13806,0.13696,0.13907,0.13548,0.1351,0.13
336,0.13639,0.13514,0.13505,0.13481,0.13605,0.13668,0.13516,0.13551,0.
13613,0.13663,0.13421
0.22644,0.22379,0.22801,0.22685,0.22819,0.2288,0.22767,0.23005,0.22832
,0.22771,0.22869,0.22917,0.2311,0.2298,0.22659,0.22921,0.22588,0.22788
,0.22332,0.22651,0.48363,0.47558,0.5259,0.5451,0.22859,0.22813,0.22699
,0.22548,0.22764,0.22893,0.23074,0.22929,0.22684,0.22729,0.22909,0.229
4,0.22741,0.23274,0.22668,0.82606,0.52389,0.50159,0.52021,0.55475,0.53
196,0.5402,0.22825,0.22624,0.22599,0.22538,0.22592,0.22709,0.22936,0.2
2657,0.23017,0.2264,0.22844,0.22746,0.23071,0.22995,0.2262,0.78842,0.8
4516,0.49937,0.51473,0.50348,0.51984,0.47875,0.52224,0.51261,0.53192,0
.22977,0.22824,0.22785,0.22675,0.22589,0.22933,0.22914,0.2245,0.22566,
0.22913,0.22768,0.22747,0.22744,0.23139,0.22663,0.83641,0.82844,0.8211
9,0.5064,0.55062,0.60662,0.51631,0.52234,0.52177,0.47943,0.5348,0.5257
5,0.22725,0.22757,0.22844,0.22751,0.22822,0.22883,0.2277,0.22906,0.227
99,0.22406,0.228,0.22606,0.22967,0.23,0.22606,0.23042,0.8352,0.82809,0
.8038,0.83198,0.52801,0.50995,0.52885,0.53273,0.58769,0.51063,0.49787,
0.47791,0.53835,0.48935,0.22928,0.227,0.22841,0.22608,0.22828,0.22648,
0.22862,0.22943,0.22956,0.22699,0.22815,0.22713,0.22714,0.22591,0.2268
,0.22634,0.83388,0.82251,0.84174,0.80284,0.82985,0.83248,0.55393,0.541
34,0.5492,0.52489,0.48479,0.57696,0.53936,0.52,0.54209,0.59753,0.22798
,0.22817,0.22814,0.22575,0.22621,0.22913,0.22755,0.22988,0.22541,0.226
78,0.2278,0.22858,0.22868,0.22752,0.22568,0.2269,0.22538,0.85087,0.802
8,0.81432,0.82934,0.84573,0.78699,0.60839,0.5071,0.50267,0.55725,0.532
22,0.52869,0.55673,0.50958,0.47226,0.58319,0.5083,0.22947,0.22912,0.22
66,0.22766,0.22808,0.22829,0.22938,0.22819,0.22809,0.22814,0.22848,0.2
2744,0.22372,0.16587,0.17348,0.17753,0.2263,0.82334,0.83238,0.81521,0.
83473,0.85185,0.82388,0.7933,0.49407,0.60573,0.53591,0.59618,0.54077,0
.5287,0.51458,0.51318,0.51384,0.52855,0.53676,0.22461,0.22926,0.22776,
0.23104,0.22574,0.2272,0.22901,0.22757,0.22689,0.22991,0.22787,0.17075
,0.18156,0.1708,0.16706,0.17022,0.16824,0.23054,0.8318,0.80822,0.82988
,0.79753,0.83781,0.83017,0.85079,0.54185,0.59721,0.52814,0.53034,0.531
58,0.62271,0.49979,0.50698,0.53682,0.5294,0.52386,0.22732,0.2304,0.228
75,0.22613,0.22784,0.22437,0.22978,0.22503,0.22639,0.16838,0.17572,0.1
6677,0.16587,0.17037,0.16729,0.17689,0.17003,0.16492,0.22833,0.82684,0
.79817,0.85031,0.81832,0.84728,0.83431,0.81186,0.54635,0.5119,0.54577,
0.578,0.48276,0.55622,0.49324,0.53351,0.49079,0.48617,0.53655,0.53089,
0.22976,0.22652,0.22846,0.22911,0.22634,0.2254,0.17149,0.17285,0.18106
,0.16479,0.17292,0.17092,0.16097,0.15854,0.16604,0.17058,0.16449,0.176
35,0.85843,0.79211,0.83346,0.81598,0.82444,0.82871,0.81748,0.55456,0.5
4812,0.52666,0.53306,0.50596,0.54744,0.60021,0.51495,0.58661,0.54929,0
.58175,0.57971,0.22747,0.22786,0.22947,0.17533,0.14999,0.17232,0.15953
,0.17057,0.17698,0.17565,0.17077,0.17014,0.16066,0.17049,0.17544,0.169
81,0.17225,0.17007,0.82359,0.79706,0.83525,0.82202,0.85687,0.82275,0.8
0584,0.80157,0.83403,0.53179,0.5716,0.53373,0.58117,0.52181,0.56724,0.
53528,0.5372,0.60219,0.52952,0.5234,0.17279,0.16604,0.17007,0.16957,0.
18117,0.17283,0.16433,0.17251,0.17149,0.17024,0.17378,0.1819,0.16886,0
.17378,0.16567,0.17433,0.16305,0.17415,0.79475,0.86092,0.81784,0.82823

,0.84735,0.81687,0.80719,0.84512,0.79596,0.48796,0.55142,0.39003,0.386
41,0.39924,0.47536,0.38773,0.3791,0.38208,0.39071,0.4104,0.17331,0.172
57,0.18028,0.1703,0.17178,0.1732,0.17243,0.17448,0.17282,0.17045,0.169
21,0.16966,0.17755,0.1627,0.17378,0.17182,0.17045,0.16982,0.80604,0.81
636,0.85141,0.84052,0.80227,0.85348,0.85978,0.80005,0.83192,0.47117,0.
38534,0.34259,0.39059,0.38221,0.38778,0.42157,0.35951,0.28693,0.37904,
0.38719,0.1726,0.17897,0.17822,0.16817,0.16126,0.1749,0.18217,0.17043,
0.17352,0.17763,0.14772,0.17152,0.16956,0.17186,0.16927,0.16837,0.1713
6,0.17334,0.86509,0.8092,0.85876,0.81593,0.80224,0.86121,0.81765,0.501
64,0.5235,0.47255,0.34577,0.46628,0.38372,0.36238,0.37437,0.39354,0.41
205,0.39276,0.39979,0.40193,0.16828,0.17296,0.18108,0.17634,0.17544,0.
17859,0.17037,0.16774,0.16955,0.17233,0.17102,0.17334,0.16861,0.18815,
0.16564,0.17317,0.1739,0.1859,0.8376,0.80006,0.80913,0.8116,0.84674,0.
80915,0.44916,0.57241,0.42751,0.50944,0.43079,0.41058,0.38472,0.40208,
0.29659,0.38929,0.39533,0.39037,0.39944,0.40075,0.16232,0.17044,0.1826
1,0.16793,0.17214,0.17994,0.16842,0.17423,0.16238,0.19755,0.17351,0.18
34,0.17183,0.16962,0.16118,0.16767,0.17005,0.17353,0.83287,0.83707,0.8
253,0.82451,0.85729,0.47796,0.48807,0.52855,0.46879,0.4783,0.46135,0.4
1225,0.38156,0.39984,0.39216,0.3627,0.45424,0.38516,0.36394,0.30395,0.
16856,0.17731,0.18831,0.16667,0.17287,0.17042,0.17026,0.17078,0.17503,
0.17299,0.17583,0.17404,0.1744,0.16608,0.17449,0.17352,0.16998,0.19436
,0.82517,0.83457,0.82153,0.40393,0.48911,0.49737,0.4171,0.42622,0.4756
5,0.4292,0.46046,0.41358,0.39621,0.31625,0.38856,0.35708,0.40316,0.416
92,0.42462,0.39981,0.18313,0.17054,0.17115,0.17423,0.17413,0.17121,0.1
7003,0.16712,0.17328,0.17786,0.17323,0.15939,0.17105,0.17126,0.17144,0
.17313,0.17454,0.17358,0.85112,0.46732,0.45558,0.47803,0.48416,0.48093
,0.45371,0.45022,0.48005,0.5205,0.38902,0.38257,0.40818,0.36042,0.4369
6,0.39318,0.40291,0.39775,0.35842,0.16935,0.17313,0.16264,0.1638,0.171
32,0.17096,0.16703,0.18315,0.17723,0.17466,0.16552,0.17171,0.17465,0.1
7256,0.17057,0.17121,0.17092,0.11638,0.49288,0.40562,0.46992,0.47994,0
.47528,0.45477,0.48489,0.44796,0.33837,0.53764,0.38958,0.39899,0.40432
,0.39609,0.31709,0.4006,0.37248,0.39089,0.44139,0.16979,0.15489,0.1672
4,0.17261,0.19614,0.14856,0.18054,0.1697,0.17115,0.17951,0.17128,0.174
37,0.17048,0.17358,0.18045,0.1701,0.17339,0.11759,0.56983,0.47096,0.45
786,0.46409,0.58862,0.50167,0.46942,0.45987,0.3744,0.50151,0.40971,0.3
8607,0.37814,0.3663,0.39586,0.43392,0.40478,0.37052,0.17239,0.17004,0.
16685,0.18246,0.17996,0.16547,0.16956,0.17555,0.15655,0.18116,0.17399,
0.1742,0.1728,0.1712,0.17137,0.17118,0.17163,0.17167,0.11625,0.51357,0
.38073,0.51495,0.47468,0.46287,0.50913,0.42667,0.44291,0.44646,0.48125
,0.37883,0.40624,0.41331,0.34329,0.40501,0.32956,0.37317,0.3966,0.1691
3,0.16848,0.17619,0.17179,0.17082,0.16629,0.16464,0.17001,0.17792,0.17
246,0.17417,0.1756,0.16286,0.16717,0.17329,0.16994,0.17929,0.11688,0.1
1538,0.1145,0.53358,0.39647,0.44519,0.4764,0.42,0.45474,0.47208,0.4311
9,0.5471,0.43416,0.36026,0.38485,0.37228,0.39684,0.38472,0.17165,0.179
88,0.16899,0.1723,0.17375,0.16368,0.17361,0.16405,0.17026,0.16587,0.17
224,0.17411,0.18516,0.17879,0.17812,0.17751,0.16789,0.17182,0.11643,0.
11728,0.11541,0.11636,0.45722,0.4588,0.47175,0.50343,0.4914,0.46532,0.
46679,0.44773,0.44161,0.39132,0.40341,0.40993,0.17673,0.17073,0.20128,
0.18368,0.17725,0.17226,0.16618,0.16919,0.17781,0.17353,0.17015,0.1751
7,0.16945,0.17387,0.16835,0.17449,0.17169,0.18206,0.17122,0.15981,0.11
291,0.11542,0.11859,0.11818,0.11669,0.11496,0.4596,0.36558,0.47993,0.4
5687,0.43737,0.57263,0.54623,0.32385,0.17506,0.15806,0.16926,0.17164,0

.17258,0.17842,0.19284,0.16406,0.17163,0.17174,0.1644,0.17873,0.17,0.1
6885,0.16715,0.16976,0.16891,0.17371,0.16541,0.16968,0.1779,0.18838,0.
18004,0.1161,0.11649,0.11507,0.11351,0.11716,0.11567,0.50463,0.36432,0
.47515,0.47875,0.44886,0.11495,0.1161,0.17166,0.18266,0.17089,0.17379,
0.16926,0.16919,0.1713,0.16881,0.1686,0.17403,0.1775,0.17349,0.17171,0
.17677,0.17175,0.18878,0.15333,0.17044,0.1742,0.18424,0.16848,0.11516,
0.1182,0.11539,0.11459,0.11714,0.11546,0.11543,0.11524,0.1143,0.11536,
0.47317,0.49167,0.11466,0.11576,0.11537,0.11516,0.16828,0.17261,0.1518
1,0.18199,0.16162,0.17207,0.1739,0.17226,0.17732,0.18064,0.17257,0.169
23,0.18457,0.17095,0.18807,0.16261,0.16646,0.17395,0.17261,0.11621,0.1
1668,0.11526,0.11499,0.1155,0.11776,0.11617,0.11662,0.11462,0.11648,0.
1143,0.11514,0.11675,0.11525,0.1155,0.11677,0.11544,0.1695,0.18609,0.1
8068,0.16202,0.18551,0.17123,0.17266,0.17825,0.1654,0.18139,0.17,0.170
59,0.17312,0.18188,0.18838,0.1749,0.11418,0.11811,0.11494,0.11532,0.11
626,0.11529,0.11722,0.11562,0.11725,0.11776,0.11569,0.11727,0.1159,0.1
1509,0.11741,0.11611,0.11281,0.11666,0.11393,0.17053,0.17149,0.17218,0
.1644,0.17219,0.17169,0.17505,0.17094,0.1729,0.16463,0.16296,0.16887,0
.16844,0.11573,0.11507,0.1162,0.11571,0.11687,0.11765,0.11759,0.11554,
0.11763,0.11671,0.11521,0.11661,0.11521,0.11739,0.11735,0.11664,0.1152
,0.11568,0.11799,0.11824,0.11588,0.17153,0.19089,0.17816,0.16872,0.167
57,0.1729,0.17514,0.18132,0.17276,0.11481,0.11486,0.11545,0.11454,0.11
424,0.11602,0.11643,0.11582,0.11748,0.11625,0.11735,0.11343,0.1144,0.1
1601,0.11958,0.11605,0.11767,0.11424,0.1159,0.11447,0.1162,0.11646,0.1
1373,0.11588,0.11286,0.16268,0.17906,0.16477,0.17479,0.11547,0.11536,0
.11782,0.11595,0.11503,0.1157,0.11534,0.11444,0.11666,0.11525,0.11727,
0.11603,0.11591,0.11476,0.11498,0.11777,0.11706,0.11404,0.11797,0.1147
4,0.11813,0.11528,0.1154,0.1188,0.11782,0.11363,0.11528,0.11389,0.1171
,0.11799,0.11525,0.11558,0.11628,0.11535,0.11438,0.11578,0.11478,0.117
35,0.11279,0.11689,0.11553,0.11659,0.11773,0.11691,0.11443,0.11483,0.1
1452,0.11757,0.11591,0.11494,0.11275,0.11625,0.11595,0.11442,0.11498,0
.11701,0.11799,0.11534,0.11393,0.11507,0.11698,0.11687,0.11258,0.11623
,0.11609,0.1145,0.11472,0.11632,0.11575,0.11621,0.11569,0.11595,0.1165
4,0.1149,0.11684,0.11638,0.11568,0.11851,0.11737,0.11552,0.113,0.11623
,0.11523,0.11491,0.11733,0.11672,0.11515,0.11542,0.11656,0.11655,0.116
21,0.11642,0.11746,0.11728,0.11566,0.1141,0.11638,0.1162,0.11429,0.115
48,0.11542,0.11801,0.11636,0.1145,0.11572,0.11795,0.11484,0.11587,0.11
564,0.11787,0.11471,0.11519,0.11464,0.11667,0.11664,0.1179,0.11635,0.1
1659,0.11584,0.11802,0.11629,0.11701,0.11934,0.1158,0.1173,0.11597,0.1
1595,0.11582,0.11545,0.11607,0.11533,0.11469,0.11345,0.11847,0.11668,0
.1151,0.11686,0.11797,0.11453,0.11604,0.11615,0.117,0.11677,0.11633,0.
11725,0.11577,0.11655,0.11593,0.11629,0.11507,0.11579,0.11502,0.11462,
0.11608,0.1165,0.1158,0.11579,0.11392,0.11517,0.11706,0.1148,0.11603,0
.11508,0.11481,0.11435,0.1161,0.11678,0.11801,0.11563,0.1159,0.11457,0
.11509,0.1173,0.11653,0.11554,0.11318,0.11804,0.11449,0.11848,0.11666,
0.11876,0.11597,0.11579,0.1139,0.11644,0.11497,0.11439,0.11456,0.1151,
0.11569,0.11474,0.11491,0.11743,0.11629,0.11525
0.21566,0.21316,0.217,0.21536,0.21665,0.21824,0.21607,0.21921,0.21549,
0.21717,0.21728,0.21647,0.21918,0.21877,0.21508,0.21799,0.21497,0.2175
2,0.21233,0.21307,0.39838,0.39962,0.43148,0.45163,0.2163,0.21692,0.215
68,0.21518,0.21494,0.21681,0.21875,0.21836,0.21613,0.21562,0.21765,0.2
1906,0.2148,0.22134,0.21526,0.65891,0.42675,0.42056,0.42871,0.45598,0.
42954,0.44093,0.21754,0.21574,0.21513,0.2141,0.21362,0.21708,0.21866,0

.21625,0.21803,0.21401,0.21729,0.21762,0.21877,0.21852,0.21444,0.62223
,0.6459,0.41416,0.42036,0.41934,0.42727,0.40659,0.43128,0.41894,0.4356
3,0.21903,0.21628,0.21709,0.21513,0.21488,0.2179,0.21763,0.2145,0.2151
3,0.21566,0.21669,0.21676,0.21423,0.21921,0.21605,0.66835,0.65348,0.65
57,0.42371,0.4459,0.49622,0.41806,0.43198,0.42334,0.39919,0.44013,0.43
233,0.21585,0.21653,0.21634,0.21718,0.21719,0.21749,0.21645,0.21735,0.
21611,0.21336,0.21403,0.21377,0.21897,0.21906,0.21595,0.21963,0.65904,
0.64907,0.61228,0.65456,0.43863,0.41712,0.43144,0.43768,0.47057,0.4191
9,0.41018,0.39428,0.4426,0.40807,0.21789,0.2165,0.21693,0.21462,0.2164
2,0.21524,0.21755,0.21754,0.2175,0.21754,0.21639,0.21619,0.21548,0.214
68,0.21546,0.21472,0.66037,0.66441,0.66795,0.62568,0.65442,0.66341,0.4
4768,0.44648,0.44821,0.4299,0.40796,0.46236,0.44793,0.43181,0.44644,0.
48639,0.21683,0.21539,0.21729,0.2141,0.21583,0.21862,0.2167,0.21785,0.
21373,0.21446,0.21666,0.21789,0.21713,0.21592,0.21527,0.21685,0.21552,
0.67104,0.61047,0.64674,0.65553,0.66943,0.62246,0.48639,0.42073,0.4178
7,0.45933,0.43027,0.43673,0.46097,0.41655,0.39425,0.4725,0.41759,0.217
69,0.21719,0.21391,0.21569,0.21601,0.2175,0.2175,0.21745,0.21694,0.216
01,0.21818,0.21699,0.21141,0.14889,0.1579,0.16357,0.21527,0.65614,0.64
728,0.61748,0.66804,0.66419,0.65716,0.62417,0.41252,0.49623,0.43711,0.
48845,0.44603,0.43324,0.41358,0.41865,0.4234,0.43491,0.44122,0.21497,0
.21885,0.21689,0.22009,0.2146,0.21651,0.21884,0.2177,0.21653,0.2184,0.
2156,0.15382,0.16716,0.1566,0.15166,0.1547,0.15301,0.21915,0.6429,0.63
866,0.6547,0.62622,0.65325,0.65839,0.68572,0.44494,0.48404,0.43233,0.4
3214,0.43331,0.5047,0.41706,0.42003,0.4448,0.43784,0.43024,0.21554,0.2
1796,0.21772,0.21547,0.2172,0.2144,0.21835,0.21407,0.21441,0.15361,0.1
6035,0.15015,0.14987,0.1555,0.15222,0.16121,0.15497,0.14842,0.21584,0.
64323,0.61992,0.66513,0.64693,0.67745,0.64716,0.62527,0.45359,0.42557,
0.45223,0.46683,0.40099,0.45629,0.40459,0.43762,0.40848,0.39807,0.4366
,0.43382,0.21826,0.21578,0.21726,0.21678,0.21545,0.21356,0.15767,0.157
46,0.16545,0.1485,0.15762,0.15661,0.14603,0.14108,0.15063,0.156,0.1496
8,0.16086,0.658,0.60789,0.6563,0.64583,0.63888,0.64921,0.62311,0.45411
,0.4497,0.42979,0.43521,0.42368,0.44389,0.48806,0.42135,0.47654,0.4570
7,0.47233,0.47815,0.21656,0.21467,0.21787,0.15997,0.13298,0.15623,0.14
255,0.15535,0.16111,0.1603,0.15507,0.15378,0.14395,0.15473,0.1607,0.15
429,0.15691,0.15553,0.64934,0.61782,0.6435,0.63934,0.6785,0.65338,0.63
859,0.61943,0.65921,0.43765,0.4686,0.44156,0.47134,0.42848,0.46325,0.4
4284,0.44331,0.49366,0.43935,0.43496,0.15766,0.14901,0.1551,0.1543,0.1
6699,0.15756,0.14852,0.15753,0.1568,0.15599,0.15866,0.16826,0.15286,0.
15789,0.15075,0.15881,0.14735,0.15877,0.63416,0.68916,0.63518,0.6528,0
.67659,0.64915,0.63127,0.65326,0.62126,0.40424,0.45826,0.31615,0.31881
,0.31831,0.38158,0.31585,0.31268,0.31148,0.31577,0.33712,0.15715,0.157
92,0.16589,0.15501,0.15599,0.15668,0.15647,0.15821,0.1585,0.15431,0.15
387,0.15447,0.16159,0.14666,0.15907,0.15582,0.15505,0.15431,0.62844,0.
63875,0.67864,0.6636,0.6208,0.66051,0.68638,0.63249,0.66216,0.37164,0.
3141,0.28726,0.32486,0.3119,0.31215,0.35087,0.30291,0.24276,0.31257,0.
31489,0.15584,0.16284,0.16286,0.153,0.14542,0.15934,0.16815,0.15562,0.
15702,0.16187,0.12909,0.1558,0.15335,0.15685,0.15481,0.15392,0.15555,0
.1573,0.68224,0.63375,0.67847,0.63965,0.63636,0.66461,0.64273,0.40068,
0.41908,0.37641,0.29205,0.38398,0.31459,0.30334,0.30482,0.32688,0.3398
5,0.3215,0.32846,0.33157,0.15215,0.15765,0.16707,0.16103,0.15999,0.163
68,0.15521,0.15215,0.15454,0.15693,0.15541,0.15793,0.15297,0.17272,0.1
4997,0.15704,0.15928,0.17115,0.65139,0.63278,0.64272,0.63496,0.66603,0

.64373,0.35651,0.45022,0.33803,0.3961,0.33665,0.33233,0.31543,0.33202,
0.25069,0.32441,0.32793,0.32165,0.32429,0.32609,0.1471,0.15434,0.16849
,0.15245,0.15656,0.1649,0.15358,0.15965,0.14629,0.18426,0.15758,0.1676
9,0.15637,0.15426,0.14644,0.15111,0.15418,0.15789,0.65645,0.64451,0.64
911,0.6402,0.67044,0.37723,0.3853,0.4247,0.37602,0.38381,0.36647,0.329
45,0.31321,0.32738,0.32168,0.29718,0.36976,0.31395,0.30001,0.25758,0.1
5227,0.16311,0.17519,0.15007,0.15786,0.15471,0.1547,0.15524,0.16024,0.
15668,0.16007,0.15837,0.15762,0.15108,0.15974,0.15747,0.1544,0.18111,0
.63313,0.65182,0.64167,0.31964,0.3864,0.39586,0.3273,0.33807,0.38087,0
.33975,0.35137,0.33935,0.32637,0.26307,0.31984,0.29587,0.32626,0.34676
,0.3518,0.32988,0.16844,0.15565,0.15534,0.15851,0.15772,0.15568,0.1546
7,0.15125,0.15764,0.1609,0.15801,0.14308,0.15666,0.15489,0.15567,0.156
47,0.15818,0.15776,0.66312,0.37563,0.3612,0.38134,0.39142,0.37677,0.35
579,0.34548,0.38431,0.41703,0.32301,0.31173,0.33451,0.29706,0.35751,0.
32262,0.33419,0.32341,0.29707,0.15407,0.15782,0.14635,0.14775,0.15563,
0.15461,0.15098,0.16914,0.16222,0.15822,0.14911,0.15573,0.1591,0.15733
,0.15515,0.15512,0.15676,0.096315,0.38814,0.32285,0.36495,0.37867,0.37
1,0.35799,0.37917,0.34938,0.27083,0.42852,0.3229,0.3259,0.32661,0.3279
1,0.26029,0.32369,0.30911,0.31602,0.35676,0.15419,0.1383,0.1514,0.1569
3,0.18222,0.13135,0.16696,0.15408,0.15539,0.16427,0.15661,0.15831,0.15
469,0.15708,0.16544,0.15453,0.15696,0.097255,0.44902,0.37124,0.36418,0
.36636,0.45265,0.40154,0.37236,0.36335,0.29507,0.40096,0.3367,0.31704,
0.31287,0.30064,0.32271,0.35592,0.33175,0.30814,0.15666,0.15576,0.1512
4,0.16678,0.16478,0.15022,0.15404,0.15931,0.13981,0.1663,0.15885,0.159
19,0.15719,0.15585,0.15576,0.15459,0.15606,0.15681,0.096117,0.40925,0.
29862,0.40835,0.38136,0.3602,0.40605,0.3383,0.34789,0.34519,0.38235,0.
31223,0.33853,0.34832,0.28636,0.33121,0.27837,0.30867,0.32538,0.15595,
0.15319,0.161,0.15616,0.15483,0.15081,0.14982,0.1537,0.16367,0.15659,0
.15823,0.15958,0.14607,0.15173,0.15772,0.15457,0.16279,0.096875,0.0955
77,0.094609,0.42168,0.31601,0.35672,0.3808,0.32892,0.36018,0.37906,0.3
394,0.44359,0.3501,0.29651,0.31282,0.30955,0.32986,0.3163,0.15579,0.16
505,0.15413,0.15679,0.15824,0.14833,0.15843,0.1477,0.15523,0.14836,0.1
5604,0.15957,0.17,0.16385,0.16249,0.16138,0.15293,0.15545,0.096639,0.0
96699,0.095843,0.097253,0.35297,0.36291,0.3699,0.39327,0.39413,0.36346
,0.36564,0.36203,0.34366,0.32185,0.33379,0.34272,0.16193,0.15565,0.187
21,0.1686,0.16168,0.15676,0.1511,0.15336,0.16297,0.15798,0.15441,0.160
34,0.15399,0.15818,0.15336,0.15998,0.15482,0.16748,0.15476,0.14267,0.0
945,0.095774,0.09726,0.096933,0.097408,0.095121,0.35999,0.28965,0.3812
3,0.36199,0.34932,0.45124,0.42517,0.25845,0.15971,0.14208,0.15433,0.15
611,0.1564,0.16298,0.17693,0.14827,0.15636,0.1568,0.14872,0.16378,0.15
375,0.15367,0.15199,0.15446,0.15287,0.15853,0.15062,0.15405,0.1632,0.1
7334,0.16482,0.09522,0.096841,0.095246,0.094388,0.096353,0.096589,0.39
277,0.28873,0.36218,0.38176,0.35597,0.095527,0.097281,0.15654,0.16655,
0.15504,0.15982,0.15382,0.15486,0.15518,0.15374,0.15245,0.158,0.16331,
0.15817,0.1555,0.16161,0.15675,0.17464,0.13726,0.15473,0.15841,0.17071
,0.15317,0.096127,0.097747,0.095928,0.095943,0.097134,0.095452,0.09648
9,0.094126,0.094112,0.095902,0.37464,0.38763,0.094349,0.096154,0.09522
5,0.094922,0.15352,0.15741,0.13403,0.1675,0.1456,0.15677,0.15708,0.156
78,0.16272,0.16556,0.15703,0.15315,0.16958,0.15549,0.17451,0.14647,0.1
5088,0.15756,0.1569,0.095504,0.097447,0.095871,0.096122,0.09553,0.0979
03,0.096234,0.096343,0.095718,0.096756,0.094035,0.096053,0.097472,0.09
6455,0.096662,0.097721,0.095499,0.15274,0.17174,0.16693,0.14596,0.1702

,0.15538,0.15608,0.1632,0.15121,0.16758,0.15439,0.15425,0.15824,0.1670
2,0.1746,0.15932,0.095087,0.098351,0.095316,0.096615,0.096136,0.095442
,0.097323,0.095806,0.097731,0.098485,0.095599,0.09689,0.096634,0.09474
4,0.097216,0.096712,0.093186,0.096011,0.094983,0.15451,0.15579,0.15673
,0.14841,0.15623,0.15567,0.1587,0.15432,0.1582,0.1479,0.14794,0.1537,0
.15323,0.09601,0.095729,0.095714,0.095043,0.097166,0.098659,0.097532,0
.095363,0.097208,0.096423,0.094955,0.096816,0.095495,0.097787,0.096798
,0.096132,0.09562,0.095832,0.09774,0.097356,0.095193,0.15612,0.17614,0
.16321,0.15242,0.1526,0.15593,0.16059,0.16588,0.15719,0.094603,0.09554
9,0.096409,0.095394,0.094597,0.095214,0.098253,0.096845,0.097762,0.096
231,0.098051,0.094199,0.093748,0.096989,0.099553,0.096231,0.097787,0.0
94959,0.096114,0.094095,0.09645,0.097091,0.09483,0.09611,0.093016,0.14
625,0.16341,0.14807,0.15906,0.095754,0.095868,0.0981,0.096356,0.095259
,0.097282,0.095726,0.095142,0.096471,0.095675,0.09748,0.094796,0.09567
4,0.095083,0.094235,0.097464,0.096794,0.094413,0.097897,0.095597,0.098
405,0.095662,0.094974,0.098386,0.097848,0.093875,0.095299,0.095327,0.0
97222,0.098538,0.095014,0.095967,0.096525,0.096437,0.09588,0.097489,0.
095917,0.097004,0.093708,0.097308,0.095029,0.097462,0.09793,0.096531,0
.09612,0.094985,0.095246,0.097179,0.096226,0.094692,0.094134,0.096794,
0.096258,0.094649,0.096341,0.09783,0.097159,0.096074,0.093939,0.095191
,0.097777,0.097176,0.092188,0.09609,0.096275,0.094988,0.09516,0.095936
,0.095742,0.096482,0.096301,0.097098,0.097024,0.095859,0.096298,0.0964
87,0.096094,0.098749,0.097142,0.096148,0.092778,0.096697,0.095574,0.09
5186,0.097268,0.096549,0.095823,0.096565,0.096102,0.096593,0.096171,0.
09694,0.096945,0.098443,0.096535,0.094346,0.095818,0.096547,0.095188,0
.095595,0.095648,0.09755,0.096143,0.094756,0.096919,0.098729,0.095091,
0.096379,0.095808,0.097749,0.094424,0.096219,0.0946,0.096333,0.096646,
0.097098,0.09703,0.096108,0.09662,0.098175,0.096425,0.097442,0.099544,
0.096571,0.098024,0.097164,0.09628,0.096559,0.095787,0.096597,0.095869
,0.094658,0.092783,0.099341,0.097442,0.0955,0.097117,0.098684,0.095097
,0.096518,0.096434,0.096817,0.096421,0.096886,0.096588,0.095763,0.0961
69,0.095633,0.096557,0.094948,0.096198,0.095547,0.095325,0.096347,0.09
6183,0.095903,0.095892,0.0944,0.096092,0.096759,0.095033,0.09617,0.095
071,0.095233,0.094535,0.096524,0.095334,0.097947,0.09626,0.096281,0.09
4015,0.095479,0.096782,0.0969,0.095201,0.093702,0.097991,0.095167,0.09
8696,0.096664,0.098119,0.095756,0.095865,0.093691,0.096889,0.095324,0.
093926,0.094653,0.095339,0.096171,0.094232,0.095009,0.09813,0.096128,0
.095244
0.19993,0.19822,0.20176,0.20188,0.19989,0.20495,0.20009,0.20426,0.2012
5,0.20179,0.2016,0.20104,0.20401,0.20315,0.2008,0.20164,0.2005,0.20242
,0.19698,0.1979,0.34344,0.34429,0.36721,0.38769,0.20041,0.20124,0.2018
1,0.20024,0.20003,0.20217,0.20267,0.20376,0.20144,0.20025,0.2026,0.203
96,0.20116,0.20483,0.19987,0.5484,0.36916,0.3664,0.37202,0.39158,0.361
2,0.37498,0.20043,0.1999,0.19938,0.19961,0.19829,0.20239,0.20212,0.200
62,0.20206,0.1999,0.20122,0.19973,0.20377,0.20224,0.19919,0.5225,0.546
63,0.35172,0.35948,0.36068,0.36266,0.34725,0.37334,0.36201,0.38153,0.2
0315,0.20099,0.20304,0.19922,0.20107,0.20298,0.20297,0.19968,0.19908,0
.19972,0.20181,0.20076,0.19912,0.20405,0.20255,0.55254,0.54235,0.55726
,0.36743,0.37512,0.4201,0.36028,0.37198,0.35858,0.34877,0.3758,0.37448
,0.20012,0.20261,0.2014,0.2015,0.20145,0.20203,0.20122,0.20125,0.20143
,0.19836,0.1993,0.1994,0.20289,0.20255,0.20061,0.2046,0.55232,0.5322,0
.50634,0.54891,0.37735,0.35969,0.36779,0.37302,0.40546,0.36367,0.35205

,0.34442,0.38134,0.35835,0.20074,0.20063,0.20108,0.19925,0.20085,0.198
75,0.20136,0.20067,0.20109,0.20255,0.20053,0.19948,0.20044,0.19956,0.2
001,0.20005,0.54726,0.54707,0.55157,0.52509,0.54105,0.55596,0.39045,0.
38161,0.38414,0.36728,0.35288,0.39446,0.38899,0.36648,0.37805,0.40647,
0.20152,0.20082,0.20113,0.19835,0.19952,0.20269,0.19997,0.20185,0.1988
6,0.19947,0.20047,0.20281,0.2013,0.20231,0.20078,0.20131,0.201,0.56308
,0.51101,0.54753,0.5385,0.55973,0.51374,0.41283,0.35924,0.35743,0.3999
3,0.3657,0.3792,0.38738,0.35696,0.34686,0.39394,0.35619,0.20298,0.2022
7,0.19963,0.20022,0.20034,0.20095,0.20302,0.2022,0.2035,0.19944,0.2023
,0.20311,0.19742,0.13283,0.14253,0.14721,0.19918,0.54861,0.54473,0.506
74,0.57127,0.55603,0.54814,0.51933,0.36089,0.41934,0.38018,0.41206,0.3
8079,0.37172,0.36047,0.36226,0.36085,0.37055,0.36655,0.19967,0.20255,0
.20212,0.20498,0.19854,0.20151,0.20453,0.20305,0.20183,0.20158,0.19929
,0.13893,0.15144,0.14094,0.1363,0.13954,0.13798,0.20375,0.54251,0.5339
1,0.54988,0.52868,0.54488,0.54837,0.57032,0.37651,0.41496,0.35735,0.36
36,0.37158,0.43589,0.35858,0.36397,0.3822,0.381,0.36832,0.19975,0.2029
3,0.20161,0.20089,0.201,0.19969,0.20258,0.19816,0.19745,0.13784,0.1440
4,0.13307,0.13485,0.14093,0.13647,0.14581,0.13893,0.13333,0.20176,0.52
073,0.50128,0.54531,0.55584,0.57496,0.53662,0.51873,0.37976,0.36773,0.
38848,0.39389,0.34692,0.38243,0.35027,0.37796,0.35529,0.34445,0.36831,
0.36961,0.20354,0.20002,0.20205,0.2028,0.20149,0.19891,0.14015,0.14096
,0.14967,0.13348,0.14189,0.14189,0.13086,0.12563,0.13456,0.14038,0.133
96,0.14493,0.55101,0.52245,0.53541,0.55301,0.51941,0.52484,0.51812,0.3
8449,0.38189,0.37608,0.37182,0.36422,0.37399,0.41759,0.36313,0.39767,0
.38923,0.41152,0.40818,0.20295,0.19905,0.20196,0.14372,0.11704,0.14049
,0.12696,0.13901,0.14489,0.14392,0.1388,0.13803,0.12814,0.13924,0.1444
7,0.1389,0.14104,0.13995,0.54159,0.50007,0.53444,0.52586,0.57869,0.537
79,0.54304,0.51899,0.54793,0.37133,0.40286,0.3824,0.40004,0.37114,0.39
842,0.38212,0.3808,0.41893,0.38129,0.37564,0.1423,0.13298,0.13986,0.13
827,0.15064,0.14149,0.13202,0.14189,0.14204,0.14084,0.14367,0.15276,0.
13679,0.14242,0.1352,0.14316,0.13224,0.14383,0.54125,0.58488,0.53743,0
.53589,0.55502,0.5378,0.53127,0.54448,0.5055,0.34797,0.3956,0.26919,0.
27249,0.2706,0.3166,0.27441,0.26454,0.26179,0.26718,0.28666,0.14228,0.
14234,0.15111,0.13944,0.14099,0.14128,0.14192,0.14236,0.14166,0.13856,
0.13806,0.13845,0.1459,0.13122,0.14366,0.1409,0.13879,0.13942,0.5245,0
.52289,0.56719,0.54915,0.52629,0.55673,0.57047,0.53064,0.55733,0.30906
,0.26557,0.24564,0.27736,0.26358,0.26678,0.30036,0.26391,0.213,0.26471
,0.26516,0.13906,0.14676,0.14619,0.13744,0.12877,0.14434,0.15184,0.140
27,0.14078,0.14658,0.11256,0.14003,0.13667,0.14122,0.14045,0.13965,0.1
3977,0.1413,0.56816,0.52824,0.5681,0.52738,0.52903,0.55692,0.53389,0.3
3571,0.35603,0.31006,0.25118,0.32071,0.2638,0.25927,0.26162,0.27692,0.
29403,0.27563,0.28023,0.281,0.1369,0.14122,0.15159,0.14556,0.14387,0.1
4795,0.14033,0.13689,0.13758,0.1404,0.13971,0.14195,0.13782,0.15656,0.
13391,0.14158,0.14258,0.15594,0.53904,0.53296,0.53009,0.52395,0.55181,
0.53713,0.30282,0.37238,0.28077,0.33805,0.27852,0.27951,0.27015,0.2889
,0.21967,0.2772,0.27534,0.2816,0.27851,0.2824,0.13225,0.13989,0.15291,
0.13646,0.14139,0.14892,0.13655,0.14366,0.12978,0.16708,0.14195,0.1510
3,0.14114,0.13818,0.13066,0.13458,0.13704,0.14126,0.54078,0.54199,0.54
662,0.5217,0.55439,0.31228,0.31921,0.35987,0.30747,0.31272,0.30414,0.2
8382,0.26119,0.28177,0.27824,0.2589,0.31537,0.26915,0.25452,0.22459,0.
13581,0.14657,0.1592,0.13372,0.1425,0.13993,0.1401,0.13967,0.1458,0.14
111,0.14355,0.14281,0.14223,0.13475,0.14396,0.14131,0.13956,0.16428,0.

52747,0.54073,0.53782,0.26577,0.32076,0.33601,0.27188,0.28248,0.32297,
0.2804,0.28683,0.28919,0.28269,0.22926,0.27643,0.25611,0.27597,0.29615
,0.30374,0.28368,0.15246,0.14121,0.13919,0.14226,0.14244,0.13979,0.139
53,0.13564,0.14142,0.14409,0.14289,0.12753,0.1416,0.1386,0.14043,0.141
48,0.14302,0.14175,0.54599,0.3121,0.30347,0.31722,0.32886,0.31376,0.30
199,0.28294,0.31743,0.35015,0.27298,0.26615,0.28489,0.25421,0.29934,0.
27175,0.2872,0.28265,0.25626,0.1393,0.14232,0.13019,0.13177,0.14006,0.
13952,0.13613,0.15372,0.14667,0.14253,0.13334,0.14051,0.14348,0.14245,
0.14018,0.13968,0.14031,0.079229,0.32212,0.26012,0.30259,0.31451,0.314
18,0.30481,0.3161,0.29272,0.22488,0.35081,0.28006,0.27704,0.27414,0.27
969,0.22075,0.27654,0.26716,0.26741,0.30145,0.13873,0.12262,0.13563,0.
14168,0.16724,0.11467,0.15111,0.13775,0.13974,0.14821,0.14067,0.1418,0
.13794,0.14085,0.14964,0.13853,0.14144,0.080999,0.3691,0.30827,0.30707
,0.30121,0.38353,0.33924,0.30592,0.30422,0.24117,0.33327,0.29262,0.270
54,0.26406,0.25454,0.27324,0.30833,0.28247,0.26394,0.14094,0.1397,0.13
573,0.15124,0.14831,0.13429,0.13799,0.14334,0.12334,0.14971,0.14371,0.
14267,0.142,0.14129,0.1401,0.13857,0.14074,0.14044,0.080069,0.34561,0.
24252,0.34162,0.31485,0.30096,0.33401,0.27872,0.28827,0.28352,0.31942,
0.26812,0.28754,0.29571,0.24594,0.28029,0.24237,0.26564,0.27969,0.1403
5,0.13661,0.14502,0.14076,0.13974,0.13499,0.13406,0.13629,0.14829,0.14
182,0.14188,0.14426,0.12989,0.13734,0.14126,0.14074,0.14762,0.079056,0
.079114,0.078366,0.35043,0.26494,0.29861,0.31545,0.27423,0.30708,0.313
22,0.27638,0.36632,0.30158,0.25485,0.27362,0.26684,0.28012,0.27626,0.1
3893,0.14914,0.1389,0.1411,0.14221,0.13333,0.14403,0.13092,0.13855,0.1
3164,0.14128,0.14294,0.15394,0.14836,0.14516,0.14458,0.13627,0.13924,0
.079954,0.08132,0.078215,0.080155,0.28898,0.30545,0.30501,0.32518,0.33
423,0.29878,0.30227,0.2996,0.27943,0.27445,0.28597,0.29559,0.14664,0.1
397,0.17217,0.15273,0.14554,0.14017,0.13529,0.13814,0.1478,0.14209,0.1
3834,0.1454,0.13882,0.14281,0.13597,0.14425,0.13918,0.15045,0.13898,0.
1268,0.077636,0.079511,0.08091,0.080365,0.080351,0.079227,0.3007,0.243
99,0.32287,0.30087,0.29441,0.37426,0.3594,0.21652,0.1437,0.12664,0.139
61,0.13965,0.14076,0.14766,0.16107,0.13325,0.14025,0.14116,0.1326,0.14
67,0.13906,0.13802,0.13477,0.13809,0.13671,0.14269,0.1342,0.13933,0.14
743,0.15833,0.14898,0.078312,0.080737,0.079733,0.078002,0.079893,0.079
77,0.31445,0.24257,0.29652,0.32209,0.3009,0.079001,0.080656,0.14032,0.
15159,0.13913,0.14368,0.13855,0.13861,0.14016,0.13714,0.13723,0.14284,
0.14778,0.14349,0.1403,0.14596,0.14056,0.15883,0.12069,0.13877,0.14214
,0.15493,0.13721,0.079542,0.081645,0.079702,0.079884,0.08076,0.078523,
0.080348,0.077651,0.077862,0.080094,0.31475,0.31726,0.078232,0.080293,
0.078234,0.078681,0.13763,0.14042,0.1186,0.15162,0.13104,0.14108,0.141
19,0.13998,0.14704,0.14996,0.14077,0.13754,0.15499,0.13928,0.16021,0.1
3141,0.13419,0.14153,0.14235,0.08012,0.080555,0.079447,0.079767,0.0783
92,0.081718,0.080009,0.080156,0.079586,0.080064,0.077051,0.078944,0.08
0484,0.079775,0.080413,0.081703,0.079467,0.1366,0.15583,0.1514,0.13028
,0.15473,0.14005,0.13914,0.1494,0.13404,0.15076,0.13924,0.13854,0.1425
9,0.15157,0.15828,0.14417,0.078854,0.082696,0.078033,0.080501,0.079515
,0.079842,0.080348,0.079703,0.080611,0.082234,0.077922,0.079834,0.0804
01,0.079003,0.080751,0.080753,0.076956,0.079993,0.078906,0.13907,0.140
24,0.13994,0.13348,0.14228,0.14079,0.14194,0.13917,0.14154,0.13209,0.1
3119,0.13769,0.13748,0.080784,0.079427,0.079828,0.079047,0.080527,0.08
2173,0.081226,0.079329,0.080086,0.080745,0.079441,0.079714,0.079739,0.
081201,0.08017,0.080169,0.079235,0.079269,0.081162,0.080803,0.078475,0

.13976,0.16065,0.14748,0.13731,0.1367,0.14015,0.14467,0.14902,0.14072,
0.078277,0.078593,0.080662,0.078515,0.078675,0.079788,0.080759,0.08001
8,0.081219,0.080473,0.081806,0.078679,0.077447,0.080681,0.082569,0.079
777,0.081018,0.0787,0.079339,0.078079,0.080464,0.080742,0.077503,0.080
222,0.077714,0.13106,0.14628,0.1314,0.14297,0.079266,0.079153,0.081645
,0.079462,0.078835,0.080059,0.079352,0.079489,0.080546,0.079192,0.0798
54,0.07912,0.080045,0.078034,0.077748,0.081226,0.081516,0.078285,0.081
191,0.079689,0.082015,0.079595,0.07838,0.081365,0.080226,0.077686,0.07
879,0.077518,0.080838,0.082719,0.078793,0.079135,0.079821,0.079761,0.0
78903,0.079678,0.08005,0.080874,0.077002,0.079815,0.078987,0.080677,0.
081898,0.080844,0.079963,0.077736,0.079124,0.080726,0.080484,0.079589,
0.078022,0.080149,0.079979,0.079096,0.079311,0.081562,0.081021,0.07944
5,0.078508,0.079432,0.08167,0.080176,0.076469,0.079589,0.079763,0.0786
24,0.078887,0.079908,0.079713,0.080018,0.079768,0.080362,0.079712,0.07
9966,0.079953,0.079694,0.079376,0.082467,0.081367,0.080324,0.077073,0.
08003,0.079967,0.079123,0.080656,0.080238,0.079119,0.080581,0.080331,0
.07956,0.08044,0.079854,0.079845,0.081696,0.080399,0.078463,0.079883,0
.080107,0.078676,0.079137,0.079212,0.079691,0.080306,0.078337,0.080802
,0.082095,0.079114,0.079565,0.079484,0.082534,0.078363,0.079642,0.0781
15,0.080642,0.079037,0.081267,0.081053,0.078977,0.080738,0.081414,0.08
1091,0.082041,0.083887,0.080543,0.08191,0.080934,0.080193,0.080689,0.0
79374,0.080386,0.079183,0.077107,0.077167,0.083272,0.081022,0.079468,0
.080811,0.082213,0.079579,0.080858,0.079563,0.081543,0.079962,0.081059
,0.080792,0.079537,0.08018,0.079057,0.080403,0.07912,0.07964,0.079671,
0.079713,0.07989,0.080292,0.079328,0.080025,0.078831,0.080678,0.079524
,0.079776,0.079111,0.079255,0.079058,0.079113,0.080046,0.080155,0.0818
76,0.080335,0.080116,0.078519,0.079719,0.081661,0.080155,0.079143,0.07
8199,0.081821,0.079103,0.082349,0.08041,0.081521,0.079479,0.079703,0.0
78116,0.080397,0.078809,0.077339,0.078501,0.079632,0.079517,0.077705,0
.078624,0.081127,0.079555,0.079535
0.18534,0.18215,0.1851,0.186,0.18451,0.18841,0.18481,0.18732,0.18463,0
.18658,0.18615,0.18414,0.1884,0.18713,0.18591,0.18535,0.18417,0.18592,
0.1815,0.18131,0.3069,0.31078,0.33721,0.34188,0.18426,0.18516,0.18599,
0.18492,0.18364,0.18692,0.18627,0.18721,0.18665,0.18465,0.18581,0.1883
1,0.1848,0.18872,0.18335,0.46899,0.32459,0.33048,0.33539,0.35135,0.317
52,0.32482,0.18379,0.18346,0.18475,0.18199,0.18394,0.18647,0.18684,0.1
8321,0.18607,0.18392,0.18491,0.18499,0.18904,0.18526,0.18256,0.44963,0
.48961,0.3086,0.32175,0.32548,0.32183,0.31355,0.32947,0.32545,0.33629,
0.1863,0.18429,0.18617,0.18435,0.18534,0.18715,0.18666,0.1836,0.18282,
0.1848,0.18454,0.18436,0.18377,0.18959,0.18676,0.47392,0.49007,0.467,0
.32908,0.33162,0.37365,0.31805,0.3303,0.31528,0.31035,0.34497,0.33358,
0.18399,0.18684,0.1857,0.18549,0.18636,0.18509,0.18689,0.18436,0.18625
,0.18375,0.1837,0.18361,0.18764,0.1855,0.18472,0.18866,0.49429,0.4642,
0.43348,0.47166,0.33769,0.3211,0.31295,0.33093,0.36036,0.31639,0.31474
,0.31367,0.33998,0.32183,0.18504,0.18482,0.18482,0.18388,0.18495,0.183
29,0.18564,0.18526,0.18485,0.18749,0.18344,0.18526,0.18491,0.18411,0.1
8295,0.18371,0.48596,0.49005,0.48038,0.44497,0.48228,0.49992,0.35177,0
.33359,0.34306,0.32999,0.31368,0.34593,0.34144,0.3232,0.32836,0.36051,
0.18564,0.18401,0.18501,0.18254,0.1842,0.18595,0.18248,0.18675,0.18242
,0.18477,0.18558,0.18695,0.18591,0.18496,0.18546,0.18565,0.1854,0.4902
6,0.45003,0.49397,0.47665,0.47684,0.44029,0.36152,0.32199,0.32102,0.35
565,0.32413,0.33177,0.34453,0.31762,0.30697,0.35184,0.30866,0.18623,0.

18597,0.18309,0.18455,0.18611,0.18422,0.18758,0.18519,0.18644,0.18315,
0.1867,0.18724,0.1813,0.11918,0.12905,0.13215,0.1842,0.47107,0.47808,0
.44621,0.49414,0.48979,0.50209,0.45567,0.31513,0.37411,0.33565,0.36722
,0.3357,0.33895,0.32176,0.32103,0.32685,0.33469,0.32518,0.18462,0.1863
5,0.18385,0.1884,0.18311,0.18521,0.18919,0.18742,0.18574,0.18561,0.182
97,0.12514,0.1376,0.12721,0.12141,0.12608,0.1239,0.18785,0.4799,0.4671
2,0.48245,0.45455,0.47279,0.48355,0.50227,0.33648,0.37062,0.31319,0.32
322,0.33059,0.38107,0.31954,0.32186,0.34493,0.34545,0.3271,0.18443,0.1
8793,0.18626,0.1854,0.18396,0.18369,0.18568,0.18346,0.18108,0.12402,0.
12958,0.11944,0.12061,0.1271,0.12259,0.13105,0.12442,0.11888,0.18564,0
.45898,0.4345,0.47451,0.4932,0.50748,0.45643,0.47955,0.34183,0.31881,0
.34405,0.34926,0.3096,0.33479,0.30944,0.33337,0.31992,0.30986,0.33598,
0.33287,0.1874,0.18335,0.1866,0.18767,0.18399,0.18351,0.12692,0.12715,
0.13593,0.11974,0.12808,0.12849,0.11649,0.11187,0.12104,0.12729,0.1206
7,0.13135,0.48763,0.45678,0.47191,0.48057,0.44893,0.44739,0.45157,0.34
491,0.34402,0.33661,0.32674,0.32291,0.33255,0.37206,0.32538,0.35227,0.
35479,0.36808,0.3635,0.18753,0.18347,0.18516,0.12918,0.10317,0.12587,0
.11414,0.1248,0.13025,0.13041,0.12332,0.12405,0.11506,0.12532,0.1301,0
.12502,0.12802,0.12604,0.48165,0.43191,0.46687,0.44086,0.51771,0.46044
,0.47276,0.44069,0.47577,0.33195,0.35779,0.33864,0.35684,0.33504,0.351
98,0.34367,0.33779,0.37396,0.34006,0.33712,0.12897,0.11914,0.12556,0.1
2562,0.13608,0.12705,0.11769,0.12835,0.12811,0.126,0.12864,0.13809,0.1
2423,0.129,0.12148,0.12928,0.11841,0.12991,0.46397,0.51177,0.47547,0.4
7345,0.47576,0.46567,0.45835,0.48204,0.44091,0.31049,0.35058,0.24076,0
.24363,0.23347,0.27743,0.24268,0.23605,0.23265,0.23609,0.2543,0.12823,
0.12637,0.13558,0.12535,0.12646,0.12622,0.12731,0.12845,0.12745,0.1236
9,0.12409,0.12429,0.13216,0.11718,0.12968,0.12666,0.12471,0.12577,0.46
258,0.44569,0.49679,0.48925,0.45976,0.4913,0.50559,0.4728,0.46935,0.26
812,0.23879,0.21651,0.24651,0.22648,0.23902,0.26777,0.23952,0.18612,0.
22875,0.23435,0.12582,0.1326,0.13053,0.1242,0.11461,0.13057,0.13611,0.
12673,0.1267,0.13344,0.099244,0.12693,0.12334,0.12632,0.12737,0.12641,
0.12403,0.12679,0.49816,0.45567,0.51248,0.46631,0.45899,0.48146,0.4692
6,0.28983,0.31671,0.27052,0.21916,0.28591,0.23727,0.23198,0.2289,0.243
86,0.26187,0.24795,0.25478,0.24577,0.12416,0.12647,0.13889,0.13151,0.1
3086,0.13352,0.126,0.12235,0.12444,0.1267,0.12588,0.1283,0.12473,0.142
28,0.11931,0.12747,0.12943,0.14149,0.46873,0.46188,0.45664,0.46081,0.4
7537,0.46656,0.26532,0.32316,0.24267,0.30235,0.23562,0.24843,0.23894,0
.25248,0.19301,0.24149,0.24327,0.24455,0.25149,0.24989,0.11871,0.125,0
.13856,0.12197,0.12741,0.13372,0.12307,0.12968,0.11621,0.15169,0.12864
,0.13686,0.12746,0.124,0.11732,0.12153,0.12334,0.12722,0.48905,0.46263
,0.47873,0.45859,0.49561,0.27494,0.27934,0.31659,0.26635,0.27041,0.265
16,0.24889,0.2288,0.25129,0.24548,0.23436,0.27492,0.23517,0.22926,0.19
993,0.12257,0.13382,0.1434,0.11907,0.12714,0.12649,0.12522,0.12554,0.1
3171,0.12585,0.12973,0.12806,0.12825,0.12056,0.13047,0.12849,0.1253,0.
14982,0.46321,0.48025,0.45717,0.23628,0.27539,0.29537,0.23798,0.25208,
0.28508,0.24954,0.24589,0.25398,0.25655,0.2052,0.24271,0.22336,0.24252
,0.26094,0.27114,0.25226,0.13796,0.12659,0.1239,0.12903,0.12785,0.1259
6,0.12603,0.12183,0.12714,0.12929,0.12828,0.11508,0.12768,0.12543,0.12
656,0.12828,0.12871,0.12914,0.49173,0.27179,0.26332,0.27598,0.28377,0.
27784,0.26795,0.24454,0.27772,0.31173,0.24659,0.23383,0.25141,0.22425,
0.26576,0.24073,0.25927,0.24769,0.22726,0.12486,0.12769,0.11641,0.1183
1,0.12585,0.12553,0.12191,0.13848,0.13357,0.12786,0.11971,0.12642,0.13

059,0.12856,0.12738,0.12448,0.12533,0.066642,0.28023,0.22366,0.26447,0
.27645,0.27327,0.27624,0.2771,0.25501,0.19904,0.30087,0.25049,0.24627,
0.24063,0.24634,0.19684,0.24254,0.23605,0.23661,0.2615,0.12432,0.10834
,0.12068,0.12792,0.15361,0.10053,0.13618,0.1233,0.12642,0.13315,0.1271
8,0.12766,0.12347,0.12606,0.13417,0.12453,0.12667,0.068616,0.31464,0.2
7407,0.2699,0.26343,0.33659,0.30228,0.27159,0.25887,0.20883,0.29718,0.
2602,0.23417,0.23831,0.23037,0.24161,0.26899,0.25616,0.23611,0.12589,0
.12495,0.1219,0.13636,0.13387,0.12143,0.12407,0.12937,0.10987,0.13565,
0.12933,0.12895,0.12776,0.12778,0.12507,0.12391,0.12722,0.1256,0.06720
2,0.30205,0.20754,0.30494,0.27454,0.26636,0.29546,0.24859,0.25402,0.24
635,0.28752,0.23553,0.25247,0.26328,0.21046,0.24636,0.2167,0.2344,0.24
79,0.12578,0.12323,0.13077,0.12592,0.12592,0.12148,0.12121,0.12233,0.1
3398,0.12755,0.12676,0.12979,0.11519,0.12233,0.12832,0.12718,0.133,0.0
66926,0.068117,0.066346,0.31357,0.23356,0.25886,0.27457,0.24204,0.2642
6,0.2702,0.22723,0.31357,0.27081,0.22624,0.24071,0.23413,0.25089,0.246
76,0.12615,0.13429,0.12442,0.12755,0.12733,0.11961,0.12956,0.11715,0.1
2411,0.11783,0.12752,0.12751,0.13946,0.13318,0.13186,0.13182,0.12243,0
.12561,0.067411,0.068766,0.065921,0.068681,0.24942,0.26371,0.26397,0.2
8465,0.29963,0.26475,0.26287,0.25728,0.23711,0.23749,0.25743,0.25484,0
.1315,0.12409,0.1565,0.13961,0.1303,0.12612,0.12218,0.12292,0.13347,0.
1271,0.1246,0.13157,0.12413,0.12948,0.12144,0.12942,0.12668,0.1356,0.1
2385,0.1139,0.065816,0.06626,0.068016,0.067887,0.068699,0.068043,0.256
27,0.21351,0.28607,0.26168,0.2567,0.32066,0.31316,0.19009,0.12992,0.11
48,0.12573,0.12515,0.12642,0.13285,0.14537,0.11845,0.12604,0.12763,0.1
1839,0.13284,0.12486,0.12473,0.11971,0.1242,0.12328,0.12845,0.12017,0.
12485,0.13305,0.14355,0.13372,0.065415,0.068036,0.067413,0.06563,0.066
313,0.068817,0.27244,0.20987,0.2522,0.27733,0.2683,0.067728,0.06915,0.
12592,0.13701,0.12516,0.12951,0.12459,0.12298,0.12659,0.12317,0.1226,0
.12854,0.13466,0.12953,0.1267,0.13319,0.12686,0.14357,0.10725,0.12513,
0.12765,0.14041,0.12367,0.06699,0.068692,0.066947,0.068055,0.067174,0.
066043,0.068213,0.065696,0.066047,0.066894,0.27829,0.26881,0.067562,0.
068202,0.065684,0.067054,0.12289,0.1263,0.10529,0.13814,0.11827,0.1262
4,0.12743,0.12542,0.13313,0.13652,0.12666,0.12284,0.13968,0.12573,0.14
555,0.11696,0.12028,0.12813,0.12756,0.067664,0.068392,0.067163,0.06768
7,0.06683,0.069539,0.067771,0.068129,0.067763,0.067004,0.066128,0.0665
87,0.067425,0.067914,0.067845,0.069677,0.06608,0.1233,0.14089,0.1372,0
.11674,0.14011,0.12673,0.12534,0.13557,0.12022,0.13648,0.12473,0.12432
,0.12917,0.13774,0.14377,0.12978,0.067032,0.069908,0.066146,0.069625,0
.067685,0.067717,0.068658,0.067664,0.067176,0.069963,0.065398,0.068168
,0.068284,0.067648,0.067185,0.069308,0.065176,0.06719,0.066592,0.12446
,0.12723,0.12582,0.11985,0.12799,0.12797,0.12784,0.12459,0.1274,0.1187
2,0.11717,0.1231,0.12363,0.067188,0.067937,0.067875,0.06699,0.068785,0
.068866,0.070377,0.067901,0.068017,0.068286,0.066821,0.067404,0.066792
,0.068337,0.067755,0.066855,0.067229,0.067901,0.069328,0.068895,0.0672
41,0.12552,0.14739,0.13328,0.12371,0.1226,0.1258,0.13068,0.13547,0.126
29,0.066678,0.067097,0.067624,0.065915,0.065935,0.067354,0.068489,0.06
8029,0.068487,0.068595,0.07004,0.066738,0.065287,0.069131,0.070202,0.0
67712,0.068394,0.066301,0.067447,0.065901,0.068751,0.069204,0.06471,0.
068238,0.066694,0.11713,0.13207,0.11778,0.12805,0.067392,0.066571,0.07
0146,0.067145,0.06556,0.068988,0.067518,0.067448,0.068753,0.06707,0.06
813,0.067249,0.067848,0.065933,0.066022,0.068829,0.069288,0.067293,0.0
69997,0.067067,0.069306,0.067962,0.066325,0.068689,0.067672,0.065156,0

.067645,0.065511,0.068488,0.070177,0.066855,0.066506,0.068396,0.068141
,0.06624,0.066763,0.068326,0.069397,0.064012,0.067838,0.066753,0.06744
9,0.068491,0.068997,0.068487,0.065811,0.066515,0.06795,0.068096,0.0674
93,0.065118,0.067408,0.068036,0.067064,0.06799,0.069145,0.068752,0.067
73,0.067193,0.066802,0.069021,0.067948,0.06535,0.067091,0.066933,0.066
968,0.065666,0.067246,0.066438,0.067443,0.067323,0.067294,0.066116,0.0
67602,0.068013,0.068299,0.067736,0.070713,0.069023,0.069423,0.065003,0
.068315,0.067685,0.067168,0.069084,0.068388,0.067365,0.068519,0.067436
,0.067317,0.068163,0.069097,0.06767,0.068105,0.068262,0.065987,0.06806
6,0.067143,0.066567,0.067628,0.066375,0.06705,0.068359,0.066239,0.0693
32,0.069416,0.067992,0.066984,0.068,0.069728,0.066423,0.067113,0.06589
3,0.068043,0.067458,0.069194,0.067445,0.067498,0.068013,0.06985,0.0688
19,0.070271,0.071766,0.067581,0.069576,0.067829,0.067909,0.068686,0.06
7387,0.067285,0.06656,0.065164,0.06548,0.071314,0.068037,0.067352,0.06
8447,0.071222,0.067056,0.068564,0.069117,0.068964,0.067073,0.069512,0.
068593,0.06825,0.067876,0.066658,0.067709,0.066649,0.066287,0.067894,0
.067761,0.06781,0.068363,0.067237,0.068234,0.066837,0.068906,0.067736,
0.067059,0.06744,0.065826,0.066631,0.067737,0.06787,0.067091,0.069523,
0.067819,0.068195,0.06598,0.067079,0.06862,0.06863,0.067196,0.066608,0
.069168,0.066938,0.069527,0.068197,0.068224,0.067627,0.067062,0.065938
,0.068329,0.066859,0.06518,0.067264,0.06676,0.066582,0.065825,0.066114
,0.068494,0.067281,0.067157
0.16847,0.16813,0.17009,0.1715,0.17094,0.17299,0.16858,0.17235,0.17177
,0.17222,0.17168,0.16886,0.17428,0.17301,0.17136,0.17136,0.16987,0.171
5,0.16673,0.16542,0.28014,0.28631,0.32069,0.31401,0.17057,0.17033,0.17
,0.1692,0.17018,0.17274,0.17152,0.17111,0.17145,0.16976,0.1706,0.17467
,0.16834,0.17205,0.16793,0.42278,0.2952,0.30482,0.31214,0.32467,0.2853
9,0.29686,0.16978,0.16916,0.16915,0.16745,0.16955,0.17197,0.17073,0.16
873,0.17125,0.16979,0.16917,0.17016,0.17273,0.17038,0.16851,0.41983,0.
43693,0.27722,0.29651,0.30017,0.28732,0.28911,0.29982,0.30331,0.30967,
0.17241,0.17024,0.17008,0.16906,0.17029,0.1726,0.17022,0.16862,0.16792
,0.1693,0.16982,0.17078,0.16949,0.1726,0.17231,0.42952,0.44131,0.42454
,0.29761,0.30164,0.34153,0.29077,0.30195,0.29432,0.28349,0.30918,0.304
19,0.16913,0.17259,0.17228,0.17146,0.17179,0.17026,0.17126,0.16935,0.1
7115,0.16879,0.16974,0.16912,0.17154,0.17107,0.17012,0.17446,0.46599,0
.42429,0.39029,0.43415,0.31117,0.29787,0.29534,0.29886,0.32722,0.29067
,0.28846,0.28265,0.31735,0.29763,0.16914,0.1703,0.16983,0.16924,0.1711
4,0.16989,0.17096,0.17041,0.17046,0.17279,0.16897,0.17011,0.17071,0.16
869,0.16745,0.1702,0.44328,0.45352,0.43597,0.39976,0.44842,0.45493,0.3
2023,0.30705,0.31367,0.30308,0.2881,0.32083,0.3144,0.29268,0.30464,0.3
3204,0.17062,0.17002,0.17054,0.16805,0.16834,0.17091,0.16753,0.17139,0
.16849,0.16963,0.17081,0.17304,0.17044,0.17001,0.17016,0.17138,0.17108
,0.45503,0.40443,0.45551,0.42839,0.43599,0.41502,0.32287,0.29132,0.287
09,0.32655,0.28993,0.2939,0.3139,0.28397,0.27728,0.32328,0.27453,0.171
66,0.16873,0.16853,0.16942,0.1717,0.16882,0.17261,0.17015,0.17175,0.16
909,0.17305,0.17205,0.16648,0.10763,0.1174,0.12143,0.17055,0.44606,0.4
441,0.41623,0.4566,0.45288,0.45957,0.42065,0.28782,0.34126,0.30024,0.3
3871,0.3097,0.31056,0.29729,0.29396,0.30421,0.30392,0.3029,0.17064,0.1
717,0.16859,0.17258,0.16895,0.1695,0.17392,0.17322,0.17051,0.16996,0.1
6876,0.11254,0.12554,0.11472,0.10961,0.11479,0.11247,0.17305,0.42847,0
.43217,0.44111,0.40741,0.43788,0.44305,0.45882,0.30459,0.34141,0.28797
,0.28971,0.30256,0.34303,0.28121,0.28406,0.31358,0.32061,0.30288,0.169

97,0.17339,0.17198,0.17107,0.16977,0.16789,0.17178,0.16879,0.16786,0.1
135,0.11897,0.10833,0.10957,0.11573,0.11116,0.11918,0.11414,0.10806,0.
16971,0.42355,0.40018,0.43687,0.44964,0.46175,0.40259,0.43739,0.31695,
0.29237,0.31596,0.32314,0.28339,0.31037,0.27925,0.30315,0.29329,0.2843
1,0.30092,0.29841,0.17283,0.16969,0.17051,0.17162,0.16962,0.16921,0.11
435,0.1157,0.12363,0.10827,0.11641,0.11705,0.1058,0.10142,0.11036,0.11
471,0.10963,0.1183,0.43974,0.41142,0.42534,0.43509,0.42268,0.42705,0.4
167,0.30911,0.31762,0.30757,0.29855,0.30106,0.30834,0.34255,0.30074,0.
32085,0.32452,0.32641,0.33883,0.17086,0.16777,0.16955,0.1167,0.093045,
0.11288,0.10142,0.11321,0.11878,0.11788,0.11236,0.11278,0.10421,0.1128
8,0.11902,0.11252,0.11452,0.11488,0.43489,0.39477,0.42905,0.41564,0.47
026,0.42513,0.44067,0.40264,0.44304,0.30322,0.34065,0.31968,0.32271,0.
30322,0.32574,0.31681,0.30996,0.34375,0.30932,0.30641,0.117,0.10799,0.
11477,0.11355,0.12421,0.11495,0.10685,0.11564,0.11499,0.11354,0.11597,
0.12607,0.11201,0.11807,0.11028,0.11674,0.10702,0.11771,0.41643,0.4521
,0.43236,0.44049,0.42021,0.42585,0.41563,0.4478,0.40321,0.28398,0.3143
9,0.21823,0.22157,0.21694,0.25679,0.21802,0.21383,0.21615,0.21039,0.23
014,0.11653,0.11351,0.12261,0.11398,0.11464,0.11476,0.11592,0.11587,0.
11558,0.11206,0.11341,0.11281,0.12108,0.10514,0.11729,0.11437,0.11281,
0.11386,0.42947,0.39202,0.45776,0.44368,0.42215,0.43857,0.47236,0.4267
3,0.44696,0.23866,0.21595,0.19804,0.22598,0.2046,0.21736,0.24385,0.216
97,0.17025,0.20747,0.21152,0.11441,0.12123,0.11902,0.11273,0.10488,0.1
1764,0.12267,0.11394,0.11695,0.12176,0.089795,0.11486,0.11199,0.11511,
0.11504,0.11407,0.1125,0.11534,0.46064,0.41861,0.47094,0.42989,0.41837
,0.44047,0.41313,0.25755,0.29007,0.25592,0.2027,0.26399,0.21472,0.2092
3,0.20845,0.21912,0.23927,0.22162,0.23267,0.22567,0.11183,0.11491,0.12
592,0.11971,0.118,0.12055,0.11382,0.11101,0.11344,0.1154,0.11362,0.116
57,0.11316,0.1301,0.10811,0.11545,0.11786,0.12847,0.41543,0.41956,0.42
808,0.43388,0.43623,0.42493,0.24639,0.30194,0.21724,0.27166,0.21503,0.
22631,0.21469,0.22757,0.17455,0.22209,0.22481,0.2215,0.23129,0.22659,0
.107,0.11276,0.12606,0.11004,0.11632,0.12073,0.11169,0.118,0.10514,0.1
3935,0.11686,0.12385,0.11521,0.11235,0.10563,0.10944,0.11214,0.11546,0
.44493,0.42244,0.43399,0.42575,0.45622,0.25395,0.25605,0.28088,0.24312
,0.24157,0.24469,0.23397,0.21207,0.22478,0.21959,0.2149,0.24422,0.2176
,0.20339,0.18091,0.11221,0.12173,0.13148,0.10773,0.1168,0.114,0.11352,
0.11385,0.11935,0.11397,0.11735,0.1158,0.11591,0.10992,0.11792,0.1152,
0.11406,0.13662,0.41385,0.4251,0.41064,0.20887,0.24731,0.26683,0.21791
,0.23347,0.25998,0.22538,0.21575,0.22383,0.23857,0.18725,0.22068,0.204
65,0.21555,0.2353,0.2432,0.23089,0.12477,0.11487,0.11225,0.11614,0.116
36,0.11409,0.1137,0.11092,0.11512,0.11646,0.11604,0.10263,0.11593,0.11
413,0.11451,0.11717,0.11707,0.11758,0.44997,0.25462,0.23249,0.25139,0.
25417,0.25657,0.24414,0.22818,0.25505,0.29033,0.22246,0.20993,0.22552,
0.20155,0.23825,0.22398,0.23471,0.22109,0.20958,0.11303,0.11585,0.1040
4,0.10717,0.1142,0.11358,0.10985,0.1273,0.12081,0.11633,0.10857,0.1147
3,0.11897,0.11822,0.11522,0.11251,0.11397,0.058316,0.25668,0.20179,0.2
3971,0.2575,0.2434,0.25129,0.25039,0.22849,0.17759,0.27433,0.22935,0.2
2031,0.21914,0.22227,0.17722,0.22038,0.2155,0.21389,0.24184,0.11167,0.
097469,0.10945,0.11516,0.14089,0.08944,0.12391,0.1106,0.11477,0.12067,
0.11517,0.11519,0.11206,0.11299,0.12314,0.11301,0.1137,0.059364,0.2832
,0.24615,0.24757,0.24506,0.31237,0.27857,0.25353,0.23968,0.18714,0.265
52,0.23941,0.21402,0.21607,0.21262,0.22247,0.24645,0.23097,0.21342,0.1
1399,0.11424,0.11031,0.12552,0.12118,0.10993,0.11308,0.11652,0.10001,0

.1239,0.11712,0.1174,0.11542,0.11648,0.11348,0.11212,0.11572,0.1144,0.
058834,0.2722,0.18766,0.28793,0.25633,0.23583,0.26689,0.22494,0.23346,
0.2241,0.26425,0.21411,0.229,0.24258,0.18919,0.21784,0.1984,0.21523,0.
22169,0.11397,0.11321,0.12031,0.11376,0.11347,0.10984,0.10875,0.11092,
0.12228,0.11608,0.11415,0.11716,0.10422,0.1098,0.11588,0.11549,0.12029
,0.05837,0.059347,0.057641,0.27824,0.21219,0.23725,0.26175,0.22007,0.2
4464,0.25253,0.21234,0.28761,0.24677,0.20444,0.21834,0.21562,0.23083,0
.22835,0.11417,0.12267,0.11295,0.11517,0.11517,0.10817,0.11793,0.10559
,0.11256,0.10642,0.11643,0.11486,0.12674,0.12143,0.11981,0.11876,0.110
45,0.11351,0.058589,0.059814,0.058529,0.058706,0.2273,0.24087,0.23908,
0.24717,0.27201,0.24136,0.23793,0.23522,0.21426,0.21884,0.23059,0.2326
2,0.12027,0.11347,0.14342,0.12674,0.11882,0.11388,0.11071,0.11194,0.12
069,0.11543,0.11314,0.1191,0.11237,0.11792,0.1107,0.11698,0.11439,0.12
318,0.11227,0.10205,0.057387,0.058578,0.058511,0.058637,0.060278,0.057
701,0.22427,0.18936,0.25103,0.23702,0.23071,0.2856,0.28438,0.17404,0.1
1758,0.10333,0.11314,0.11306,0.11541,0.12022,0.13232,0.10811,0.11388,0
.11559,0.10635,0.12002,0.11349,0.11288,0.10817,0.11233,0.11155,0.1157,
0.10905,0.11425,0.12136,0.13069,0.12093,0.057236,0.059632,0.058787,0.0
57644,0.057103,0.060361,0.24491,0.18545,0.22363,0.25646,0.24227,0.0588
13,0.060174,0.113,0.12348,0.11235,0.11639,0.11306,0.11163,0.11385,0.11
148,0.11024,0.11547,0.12287,0.11706,0.11447,0.12038,0.11637,0.13093,0.
097105,0.11464,0.11528,0.1276,0.11076,0.058315,0.059169,0.058241,0.059
27,0.058958,0.055944,0.059387,0.056425,0.057952,0.057475,0.24989,0.244
78,0.059379,0.059656,0.056614,0.057825,0.11125,0.11488,0.094814,0.1254
4,0.10719,0.11463,0.11576,0.11423,0.12198,0.1234,0.11438,0.11213,0.126
91,0.11342,0.13213,0.10563,0.10961,0.1147,0.11572,0.058947,0.060093,0.
059022,0.058938,0.057959,0.061119,0.058754,0.059926,0.059103,0.05768,0
.058137,0.058781,0.058332,0.059366,0.059375,0.060157,0.057429,0.11186,
0.12853,0.12444,0.10535,0.12744,0.11674,0.11306,0.1232,0.10876,0.12412
,0.11441,0.11286,0.11683,0.12457,0.13094,0.11801,0.058715,0.061062,0.0
57181,0.060431,0.058767,0.05778,0.060323,0.058153,0.058775,0.05985,0.0
56905,0.060018,0.05905,0.058958,0.058895,0.060072,0.056187,0.058036,0.
058364,0.1125,0.11557,0.11403,0.10866,0.11613,0.1164,0.11522,0.1126,0.
11485,0.1071,0.10662,0.11144,0.11195,0.057613,0.058509,0.057842,0.0573
44,0.059409,0.059353,0.061892,0.059803,0.059317,0.059314,0.057971,0.05
8168,0.057912,0.059792,0.059481,0.05745,0.057735,0.058304,0.060293,0.0
59953,0.057423,0.11299,0.13492,0.12201,0.11243,0.11119,0.11514,0.11786
,0.12353,0.11321,0.057854,0.058225,0.05982,0.057052,0.056443,0.059485,
0.059521,0.057945,0.0606,0.059406,0.06102,0.058472,0.056882,0.06052,0.
061024,0.05853,0.059192,0.058398,0.058188,0.05726,0.059073,0.059888,0.
056589,0.058944,0.057425,0.1064,0.11966,0.10657,0.11686,0.058324,0.058
002,0.062791,0.058836,0.056656,0.060397,0.059092,0.058958,0.059977,0.0
58546,0.058823,0.057084,0.059362,0.057084,0.057701,0.060277,0.059645,0
.059386,0.060713,0.058216,0.060461,0.059051,0.058041,0.05956,0.058557,
0.057395,0.05858,0.057087,0.059518,0.06127,0.058048,0.058081,0.059456,
0.059313,0.056893,0.057272,0.05995,0.05962,0.05477,0.058972,0.058315,0
.059038,0.059447,0.058786,0.058438,0.056746,0.058394,0.059828,0.058936
,0.058411,0.057636,0.059053,0.0583,0.058919,0.059705,0.059829,0.060012
,0.058808,0.059111,0.058273,0.060049,0.058711,0.056433,0.058305,0.0576
05,0.058209,0.057157,0.057801,0.057493,0.05888,0.059618,0.059038,0.057
537,0.058671,0.059216,0.059983,0.058783,0.06192,0.059554,0.060948,0.05
7004,0.059794,0.059481,0.058204,0.061218,0.060307,0.05917,0.059058,0.0

58242,0.057556,0.059409,0.060509,0.058566,0.059627,0.05959,0.057876,0.
058706,0.05811,0.05869,0.058835,0.058375,0.05784,0.060339,0.058347,0.0
60062,0.060323,0.058575,0.057979,0.059193,0.062119,0.057071,0.058445,0
.057078,0.059479,0.057987,0.060117,0.058832,0.058898,0.059435,0.060296
,0.060048,0.061268,0.06159,0.058956,0.060344,0.059171,0.059498,0.05975
,0.059046,0.059728,0.057232,0.057143,0.056464,0.061478,0.059366,0.0584
75,0.059562,0.062396,0.058248,0.059206,0.060529,0.060914,0.05791,0.060
976,0.059342,0.059343,0.060373,0.058269,0.059813,0.057669,0.057696,0.0
58976,0.058819,0.059156,0.05986,0.057879,0.059886,0.05703,0.060831,0.0
59174,0.058036,0.05754,0.057272,0.058311,0.058854,0.058954,0.058152,0.
061929,0.05803,0.05897,0.058079,0.057938,0.059618,0.059534,0.058345,0.
058014,0.059784,0.058486,0.060656,0.059535,0.059735,0.058928,0.058471,
0.057048,0.059394,0.057766,0.056075,0.058142,0.057719,0.057861,0.05807
8,0.058542,0.058806,0.058189,0.05903
0.15459,0.1543,0.15638,0.15849,0.15754,0.1604,0.15576,0.15996,0.15853,
0.15932,0.15786,0.15648,0.15987,0.15876,0.15674,0.15872,0.15606,0.1593
,0.15368,0.15202,0.25932,0.26565,0.30167,0.29219,0.1568,0.15698,0.1569
2,0.15652,0.15729,0.15995,0.15861,0.15854,0.15848,0.15649,0.15724,0.16
07,0.1539,0.15808,0.15444,0.37984,0.27098,0.28775,0.29217,0.30029,0.26
854,0.27967,0.15821,0.15559,0.1562,0.15393,0.15687,0.15876,0.15652,0.1
5685,0.15884,0.15619,0.15703,0.15815,0.15847,0.15756,0.15549,0.40416,0
.40461,0.25977,0.27901,0.27731,0.26899,0.26912,0.2807,0.27877,0.28411,
0.15954,0.15628,0.15734,0.1559,0.15761,0.15981,0.15699,0.15518,0.15449
,0.15623,0.15662,0.15787,0.15807,0.15819,0.15905,0.38146,0.40707,0.385
69,0.27301,0.28494,0.31468,0.26733,0.28281,0.27015,0.26991,0.29056,0.2
8508,0.15658,0.15909,0.15925,0.15907,0.15751,0.15706,0.1582,0.15717,0.
15727,0.15554,0.15617,0.15648,0.15874,0.15798,0.15796,0.16006,0.42885,
0.38506,0.37785,0.39773,0.2913,0.27591,0.28407,0.27019,0.3045,0.27159,
0.26794,0.26064,0.29716,0.27951,0.15598,0.15777,0.15604,0.15711,0.1580
4,0.15798,0.15722,0.15773,0.15718,0.1597,0.15483,0.15784,0.1574,0.1559
,0.15463,0.15796,0.40733,0.42133,0.42294,0.37089,0.4195,0.44045,0.294,
0.29148,0.28853,0.28579,0.26554,0.3065,0.29917,0.26937,0.28362,0.30874
,0.15656,0.15661,0.15788,0.15518,0.15659,0.15722,0.15411,0.15793,0.155
88,0.15608,0.15718,0.16019,0.1575,0.1578,0.15619,0.15974,0.15754,0.423
62,0.39001,0.40724,0.40698,0.40171,0.40203,0.30305,0.27493,0.26879,0.2
9714,0.26625,0.2744,0.29948,0.25956,0.25542,0.29355,0.25314,0.15891,0.
15719,0.15532,0.15809,0.15978,0.15593,0.15839,0.15775,0.15882,0.15646,
0.16004,0.15924,0.15434,0.099404,0.10722,0.11178,0.15672,0.40942,0.415
43,0.40316,0.44147,0.43546,0.43561,0.39932,0.27247,0.32674,0.27995,0.3
123,0.291,0.29023,0.27812,0.27155,0.27588,0.28414,0.28458,0.1577,0.159
26,0.15529,0.15807,0.1557,0.15638,0.16121,0.15873,0.1572,0.15783,0.156
92,0.10285,0.1156,0.10518,0.10117,0.10588,0.1029,0.1602,0.40724,0.4031
8,0.40917,0.38143,0.39456,0.42679,0.41141,0.29238,0.31415,0.26908,0.26
821,0.28542,0.31559,0.26637,0.26568,0.28905,0.30306,0.28353,0.15607,0.
15987,0.15782,0.15775,0.1566,0.15632,0.15792,0.15446,0.1548,0.10395,0.
10945,0.099397,0.10009,0.10625,0.1022,0.10909,0.10466,0.09795,0.15598,
0.40087,0.4027,0.40898,0.44885,0.43182,0.38415,0.40754,0.30069,0.27417
,0.29576,0.30258,0.26307,0.29037,0.25936,0.28104,0.26911,0.26858,0.278
33,0.27348,0.15844,0.15619,0.15759,0.15833,0.15727,0.15655,0.10439,0.1
0628,0.11354,0.098694,0.10677,0.10638,0.096271,0.091648,0.10157,0.1049
3,0.1002,0.10832,0.41467,0.37087,0.40691,0.40418,0.39181,0.41102,0.381
11,0.28365,0.29538,0.28764,0.27822,0.27933,0.28791,0.31748,0.2847,0.29

349,0.30212,0.30274,0.31058,0.15842,0.15583,0.15748,0.10689,0.085268,0
.10313,0.09293,0.10273,0.10888,0.10815,0.10223,0.10325,0.094537,0.1036
3,0.10939,0.10332,0.10422,0.10591,0.41412,0.379,0.41872,0.39482,0.4298
5,0.40079,0.42049,0.38635,0.42208,0.28194,0.32505,0.2952,0.30037,0.278
26,0.29339,0.30628,0.28008,0.32083,0.28396,0.28981,0.10665,0.099696,0.
10583,0.10436,0.11394,0.10482,0.098139,0.10548,0.10505,0.104,0.10669,0
.11558,0.10197,0.10809,0.10111,0.10611,0.097565,0.10657,0.39369,0.4319
3,0.39682,0.40062,0.39465,0.39193,0.38712,0.42145,0.37145,0.26749,0.29
411,0.20122,0.20849,0.20339,0.2376,0.20665,0.20134,0.20565,0.19603,0.2
1412,0.10753,0.10458,0.11424,0.10442,0.10495,0.10469,0.10597,0.10665,0
.10566,0.10154,0.10471,0.10317,0.11129,0.096748,0.10701,0.10523,0.1040
1,0.10425,0.40841,0.38472,0.42393,0.4277,0.40083,0.42183,0.42905,0.407
49,0.41632,0.22439,0.20063,0.18249,0.21595,0.19279,0.20128,0.22656,0.1
9934,0.15886,0.19189,0.20356,0.10481,0.11124,0.10832,0.10306,0.096624,
0.10711,0.1131,0.10394,0.10741,0.11104,0.082065,0.10423,0.10357,0.1047
8,0.10591,0.10499,0.10275,0.10525,0.43767,0.40727,0.44379,0.39806,0.39
159,0.42469,0.39055,0.24113,0.27434,0.24007,0.1877,0.24344,0.20308,0.1
9506,0.19534,0.20511,0.22251,0.21001,0.21489,0.21315,0.10312,0.10557,0
.11619,0.10967,0.10793,0.10956,0.10447,0.1011,0.10343,0.10519,0.10425,
0.10697,0.10411,0.11958,0.099241,0.10477,0.10832,0.11899,0.40464,0.401
81,0.39923,0.41431,0.41119,0.40644,0.23148,0.29046,0.20089,0.24671,0.2
0134,0.21086,0.20259,0.21715,0.16203,0.20921,0.20984,0.20599,0.21476,0
.21043,0.097298,0.1026,0.11558,0.10057,0.10707,0.11164,0.10263,0.10778
,0.095608,0.12826,0.10781,0.11356,0.10543,0.10317,0.095889,0.099536,0.
10256,0.10605,0.41191,0.40174,0.39923,0.39989,0.43385,0.23417,0.24109,
0.26792,0.22777,0.23144,0.23221,0.21923,0.19502,0.21209,0.20544,0.2049
9,0.22975,0.20305,0.1928,0.168,0.10276,0.11085,0.12083,0.099194,0.1080
2,0.10406,0.10275,0.10375,0.10984,0.10487,0.10767,0.10574,0.10636,0.10
06,0.1079,0.10571,0.10478,0.12483,0.39062,0.39707,0.393,0.19195,0.2363
1,0.24982,0.20032,0.21435,0.24603,0.21524,0.19703,0.20677,0.221,0.1757
3,0.20583,0.1916,0.20033,0.21834,0.22869,0.21727,0.1155,0.10459,0.1021
3,0.10581,0.10683,0.10534,0.10417,0.10074,0.10601,0.10587,0.10519,0.09
359,0.10635,0.10688,0.10448,0.10619,0.10606,0.1068,0.41284,0.2483,0.21
269,0.23325,0.24158,0.23699,0.23005,0.21931,0.235,0.26804,0.20473,0.19
37,0.21277,0.18665,0.22291,0.2054,0.22148,0.20573,0.19917,0.10405,0.10
592,0.096084,0.097758,0.10424,0.10344,0.10056,0.11605,0.11098,0.10528,
0.098844,0.10563,0.10896,0.10941,0.10588,0.10372,0.1036,0.052129,0.239
56,0.18944,0.23109,0.24617,0.22917,0.23852,0.23464,0.21902,0.16437,0.2
4951,0.21566,0.20505,0.2041,0.20618,0.16418,0.20734,0.20249,0.20039,0.
22978,0.10111,0.087973,0.099367,0.10541,0.12922,0.081405,0.11353,0.101
89,0.10559,0.11069,0.10446,0.10709,0.10237,0.10395,0.11316,0.10318,0.1
0334,0.052378,0.2574,0.229,0.23189,0.22803,0.29816,0.26595,0.23482,0.2
2196,0.17362,0.253,0.22146,0.19582,0.19884,0.196,0.20983,0.22812,0.218
23,0.20328,0.10499,0.10481,0.10135,0.11506,0.11112,0.10181,0.10371,0.1
0583,0.091287,0.11412,0.10723,0.10648,0.10575,0.10631,0.10443,0.1024,0
.10563,0.1055,0.052508,0.25885,0.1757,0.26663,0.23467,0.22344,0.24069,
0.20701,0.21369,0.20949,0.24233,0.204,0.2137,0.22633,0.18066,0.2078,0.
18182,0.20319,0.21449,0.10493,0.10447,0.10935,0.10401,0.10346,0.099413
,0.10045,0.10185,0.11179,0.10551,0.10455,0.10697,0.094961,0.10152,0.10
641,0.10539,0.11037,0.051765,0.052634,0.05113,0.25966,0.19913,0.22245,
0.24211,0.20945,0.22942,0.23732,0.19684,0.27211,0.22985,0.19264,0.2017
,0.20004,0.21396,0.20968,0.1046,0.114,0.10479,0.10584,0.10491,0.10046,

0.10708,0.097502,0.10273,0.097016,0.10723,0.10552,0.11634,0.11217,0.11
105,0.10881,0.10067,0.10483,0.052822,0.053843,0.052455,0.052097,0.2111
,0.22317,0.22035,0.22608,0.25537,0.22972,0.223,0.21816,0.2031,0.20167,
0.21088,0.21678,0.10989,0.10412,0.13137,0.11653,0.10749,0.1031,0.10158
,0.1022,0.11035,0.10488,0.1038,0.11035,0.10239,0.10916,0.10004,0.10784
,0.10555,0.11205,0.10254,0.093105,0.051437,0.05269,0.053296,0.052324,0
.053732,0.050649,0.20759,0.17947,0.24055,0.22443,0.22033,0.26455,0.273
55,0.15945,0.10868,0.094301,0.10368,0.10371,0.10542,0.11103,0.12191,0.
097855,0.10414,0.10649,0.098017,0.10942,0.10352,0.10324,0.099501,0.101
52,0.10257,0.10626,0.098941,0.1038,0.11047,0.12063,0.11132,0.051025,0.
053376,0.052254,0.051502,0.051028,0.053972,0.23265,0.17789,0.20648,0.2
4263,0.22483,0.053475,0.053692,0.10371,0.1124,0.10314,0.10599,0.10381,
0.10287,0.10404,0.10306,0.1008,0.10521,0.11196,0.10716,0.10412,0.11134
,0.10618,0.11998,0.087718,0.10654,0.10515,0.118,0.10213,0.052902,0.052
777,0.051241,0.053762,0.05242,0.050298,0.053496,0.051272,0.051868,0.05
1694,0.23524,0.22722,0.053435,0.052823,0.051291,0.052334,0.10309,0.104
41,0.086759,0.11417,0.098914,0.10432,0.10707,0.10456,0.1112,0.11295,0.
10516,0.10252,0.11542,0.10362,0.12139,0.096595,0.099965,0.10497,0.1055
6,0.053883,0.054014,0.052982,0.052559,0.051654,0.055049,0.052219,0.052
695,0.053292,0.051794,0.052929,0.051244,0.051715,0.053657,0.052857,0.0
54343,0.051581,0.10215,0.11813,0.11394,0.096189,0.11607,0.1071,0.10414
,0.11373,0.098929,0.1133,0.10535,0.10336,0.10735,0.11499,0.12109,0.107
9,0.05317,0.054666,0.051217,0.053994,0.05346,0.052392,0.05478,0.049975
,0.052317,0.05376,0.051008,0.05362,0.053575,0.053685,0.053586,0.053732
,0.050569,0.052021,0.052977,0.103,0.1056,0.10426,0.099503,0.10587,0.10
697,0.10461,0.10224,0.10509,0.097371,0.09739,0.10311,0.10279,0.051964,
0.052727,0.051762,0.051473,0.053309,0.053507,0.054191,0.053866,0.05332
2,0.053844,0.051312,0.052022,0.052201,0.053171,0.053148,0.0507,0.05204
2,0.051365,0.053848,0.054152,0.052038,0.10394,0.12337,0.11223,0.1036,0
.10125,0.10471,0.10763,0.11402,0.10395,0.052347,0.052064,0.053645,0.05
1391,0.050831,0.053909,0.053632,0.051194,0.053797,0.052596,0.054597,0.
052771,0.05083,0.053681,0.054223,0.052753,0.052769,0.051947,0.051847,0
.051966,0.053316,0.053351,0.052327,0.052746,0.051276,0.096153,0.10888,
0.098329,0.10809,0.053147,0.051879,0.056502,0.052558,0.050881,0.05368,
0.054225,0.052215,0.054251,0.052037,0.052458,0.050548,0.052861,0.05021
5,0.05164,0.053819,0.053726,0.052599,0.053953,0.050608,0.0535,0.053087
,0.052822,0.052595,0.05314,0.051143,0.051698,0.051642,0.053575,0.05443
1,0.051888,0.052139,0.052546,0.053393,0.050234,0.051211,0.053365,0.053
174,0.048599,0.053274,0.051221,0.053265,0.052812,0.052382,0.05161,0.05
1274,0.053013,0.053813,0.05372,0.05238,0.051791,0.053315,0.051955,0.05
299,0.053072,0.054162,0.054193,0.053124,0.053551,0.052438,0.053719,0.0
53297,0.049227,0.051007,0.052308,0.051643,0.051641,0.051952,0.051115,0
.052707,0.053722,0.052465,0.051221,0.052027,0.052557,0.053632,0.053161
,0.055507,0.053137,0.05423,0.051923,0.053954,0.053128,0.051947,0.05464
7,0.052988,0.052922,0.052618,0.052543,0.051559,0.052338,0.055173,0.053
544,0.053061,0.053252,0.052041,0.052039,0.05169,0.053142,0.05137,0.051
076,0.051668,0.054121,0.052361,0.054192,0.054148,0.051525,0.051749,0.0
52788,0.055287,0.051221,0.05277,0.052054,0.05336,0.052196,0.052556,0.0
53142,0.05222,0.05373,0.055078,0.054433,0.055141,0.054608,0.053491,0.0
53903,0.053783,0.052813,0.052772,0.051855,0.053432,0.050277,0.050956,0
.049715,0.055148,0.054234,0.052345,0.054106,0.05474,0.052161,0.052886,
0.054267,0.055124,0.051857,0.054388,0.052623,0.053644,0.053433,0.05136

1,0.0532,0.051179,0.051641,0.052609,0.052212,0.052352,0.054131,0.05272
8,0.053407,0.051836,0.055109,0.052291,0.052504,0.052139,0.052383,0.052
308,0.053102,0.052431,0.051783,0.055803,0.051484,0.053199,0.052419,0.0
51931,0.053019,0.053262,0.052594,0.052577,0.053386,0.053339,0.053384,0
.052711,0.054184,0.052765,0.053159,0.051393,0.053064,0.051414,0.050643
,0.051425,0.052286,0.051949,0.052283,0.05161,0.05393,0.052174,0.052953
0.14413,0.14304,0.14558,0.14827,0.14574,0.14963,0.14351,0.14846,0.1471
9,0.14877,0.14613,0.14614,0.14761,0.14743,0.14499,0.14676,0.1438,0.147
68,0.14235,0.1412,0.24879,0.248,0.28964,0.28564,0.1451,0.14721,0.1439,
0.14465,0.14624,0.14918,0.14573,0.14692,0.14799,0.14498,0.14751,0.1497
5,0.14417,0.14796,0.14399,0.36637,0.26489,0.27062,0.27356,0.28646,0.25
388,0.27499,0.14707,0.14492,0.14568,0.14212,0.14473,0.1468,0.14577,0.1
4573,0.14849,0.1463,0.14504,0.14843,0.14727,0.14653,0.14408,0.38947,0.
38883,0.2437,0.26673,0.25668,0.26082,0.25534,0.26694,0.26153,0.26219,0
.14696,0.1451,0.14483,0.14492,0.14629,0.14882,0.14643,0.14257,0.14309,
0.14495,0.14521,0.14719,0.1472,0.14632,0.1471,0.35687,0.40024,0.37495,
0.25915,0.27478,0.30358,0.25344,0.26658,0.25898,0.26133,0.27511,0.2704
6,0.14372,0.14838,0.14839,0.14825,0.14501,0.14577,0.14659,0.14563,0.14
453,0.14581,0.1456,0.14593,0.14674,0.14749,0.14683,0.14836,0.40889,0.3
7279,0.36874,0.38923,0.27984,0.26052,0.26965,0.26028,0.2915,0.25819,0.
25544,0.24441,0.278,0.26431,0.14524,0.14563,0.1466,0.14518,0.14659,0.1
4646,0.14655,0.14662,0.14542,0.14899,0.14424,0.14628,0.14741,0.146,0.1
4472,0.14594,0.39538,0.40996,0.40812,0.3617,0.38934,0.42433,0.29001,0.
28163,0.28026,0.27306,0.25377,0.28965,0.2846,0.24966,0.26937,0.28931,0
.14584,0.14485,0.14646,0.1441,0.14627,0.14513,0.14252,0.14529,0.14541,
0.1433,0.14655,0.14924,0.14656,0.14609,0.14485,0.14846,0.14558,0.40838
,0.38796,0.39571,0.39083,0.36894,0.37857,0.28981,0.25644,0.2562,0.2854
6,0.25232,0.25963,0.28862,0.24701,0.24202,0.28567,0.24442,0.14841,0.14
69,0.143,0.1474,0.14742,0.14418,0.1461,0.14658,0.14783,0.14539,0.15016
,0.14899,0.14394,0.091241,0.099106,0.10367,0.14451,0.39121,0.39465,0.3
8913,0.42716,0.40744,0.40787,0.39383,0.25426,0.31813,0.26268,0.30257,0
.27688,0.28228,0.26321,0.2608,0.26159,0.27218,0.27161,0.14679,0.14791,
0.14348,0.14785,0.14539,0.14525,0.14996,0.14767,0.14474,0.14577,0.1456
7,0.09495,0.10744,0.097953,0.094437,0.097053,0.095502,0.15003,0.39517,
0.38515,0.38839,0.36913,0.38249,0.40696,0.40244,0.2754,0.29691,0.25514
,0.24952,0.26586,0.30275,0.25617,0.25704,0.2728,0.28523,0.27079,0.1445
6,0.14762,0.1461,0.14604,0.14571,0.14535,0.147,0.14366,0.14425,0.09721
2,0.10113,0.092901,0.092669,0.098249,0.095157,0.10047,0.096192,0.09174
6,0.14561,0.38444,0.38632,0.40503,0.42446,0.40885,0.37492,0.39254,0.27
709,0.27016,0.28064,0.29093,0.25392,0.2822,0.24335,0.26893,0.25852,0.2
5023,0.27349,0.25831,0.14662,0.14471,0.14524,0.14643,0.14571,0.14662,0
.096422,0.099434,0.10465,0.090771,0.099681,0.099801,0.088841,0.083895,
0.093998,0.097034,0.093101,0.10074,0.39812,0.35354,0.40259,0.39108,0.3
8182,0.39399,0.3673,0.2714,0.27946,0.27553,0.26038,0.26841,0.2768,0.30
317,0.27686,0.27482,0.28362,0.29109,0.30472,0.14817,0.14395,0.14662,0.
099298,0.07973,0.09645,0.085686,0.095832,0.10135,0.099433,0.095647,0.0
9539,0.087873,0.096974,0.10141,0.09578,0.097483,0.097639,0.39193,0.372
51,0.39706,0.37405,0.40716,0.38212,0.39673,0.38473,0.39892,0.26588,0.3
05,0.2839,0.28914,0.26332,0.28206,0.29344,0.26817,0.29981,0.27045,0.27
332,0.098598,0.092985,0.097617,0.095532,0.10582,0.096996,0.09105,0.096
697,0.09696,0.096829,0.098641,0.10835,0.094568,0.099889,0.094322,0.098
102,0.090539,0.0994,0.38134,0.41063,0.3934,0.37846,0.39719,0.38146,0.3

8183,0.40661,0.36379,0.25382,0.2798,0.1901,0.20018,0.19276,0.22408,0.1
9373,0.19562,0.19586,0.18669,0.20479,0.099449,0.097375,0.10657,0.09799
1,0.097898,0.096398,0.098455,0.099303,0.097592,0.094216,0.097466,0.096
108,0.10286,0.090235,0.098604,0.098281,0.096846,0.096053,0.38015,0.374
64,0.40822,0.41041,0.38309,0.40535,0.4065,0.41211,0.39128,0.21292,0.18
638,0.17176,0.19826,0.18045,0.19561,0.21552,0.18826,0.15182,0.18204,0.
19227,0.098056,0.10366,0.099948,0.095303,0.08998,0.099501,0.10476,0.09
523,0.099229,0.10264,0.075191,0.097031,0.096483,0.098081,0.099064,0.09
9147,0.095172,0.097297,0.42561,0.39316,0.42601,0.39082,0.36504,0.41153
,0.37654,0.23032,0.25827,0.22395,0.1772,0.22697,0.19521,0.18014,0.1853
2,0.19564,0.20845,0.20148,0.20237,0.19727,0.096104,0.098585,0.10777,0.
10218,0.1002,0.10167,0.095199,0.093535,0.095968,0.097629,0.096031,0.09
9197,0.096542,0.11254,0.093422,0.098146,0.1002,0.11052,0.38788,0.37084
,0.38512,0.40147,0.39559,0.37965,0.22336,0.28376,0.18511,0.23489,0.188
29,0.20429,0.19057,0.20346,0.14922,0.19663,0.20026,0.19641,0.20537,0.1
9916,0.091457,0.094724,0.10833,0.093111,0.09855,0.10449,0.09401,0.0997
65,0.088736,0.11929,0.09925,0.10492,0.097483,0.095414,0.088369,0.09219
,0.095537,0.099551,0.39216,0.39558,0.39837,0.38488,0.41907,0.2212,0.22
991,0.25091,0.22207,0.21839,0.22049,0.2083,0.18623,0.20221,0.19707,0.1
9089,0.22071,0.19369,0.18086,0.15919,0.095755,0.1033,0.1116,0.091865,0
.1001,0.096784,0.094258,0.097056,0.10236,0.097043,0.099557,0.099407,0.
09819,0.092846,0.099709,0.098397,0.096908,0.11596,0.37321,0.37666,0.36
855,0.18615,0.22624,0.24391,0.19282,0.20358,0.23299,0.19989,0.183,0.19
601,0.20608,0.16604,0.20135,0.17973,0.18889,0.20016,0.21893,0.20444,0.
10761,0.096726,0.094453,0.097106,0.098321,0.097276,0.095733,0.093949,0
.097322,0.098826,0.097003,0.087681,0.098507,0.098566,0.097119,0.098057
,0.098634,0.098778,0.38315,0.22815,0.20284,0.23349,0.23707,0.22185,0.2
1314,0.2058,0.22322,0.25582,0.19137,0.18073,0.20415,0.18133,0.20808,0.
19765,0.21068,0.20152,0.18912,0.096944,0.097331,0.089451,0.091005,0.09
7751,0.095909,0.093763,0.10878,0.10291,0.097553,0.092152,0.097702,0.10
208,0.10122,0.098582,0.095515,0.095881,0.048361,0.23035,0.17645,0.2215
4,0.23433,0.21755,0.22287,0.22272,0.20999,0.15384,0.23723,0.20138,0.20
186,0.19753,0.19931,0.15639,0.1977,0.19217,0.19195,0.22375,0.094359,0.
08102,0.092533,0.097267,0.11854,0.075263,0.10527,0.094398,0.099594,0.1
0178,0.097543,0.099618,0.095502,0.096607,0.10503,0.094872,0.094307,0.0
48622,0.24337,0.22139,0.21617,0.20491,0.28844,0.25615,0.22613,0.21225,
0.16724,0.24247,0.20405,0.18137,0.19055,0.1834,0.19315,0.2133,0.20619,
0.19614,0.097919,0.097258,0.094312,0.10578,0.10276,0.09423,0.096407,0.
097518,0.084828,0.10564,0.10054,0.09954,0.098757,0.097738,0.097065,0.0
96391,0.098478,0.096644,0.04829,0.25002,0.17191,0.25902,0.21914,0.2123
4,0.23421,0.19475,0.20501,0.19792,0.22881,0.1927,0.20834,0.21732,0.170
89,0.19688,0.17335,0.1917,0.20548,0.097468,0.096257,0.1016,0.095744,0.
095024,0.092671,0.092435,0.094994,0.10314,0.097756,0.097824,0.099413,0
.087581,0.093122,0.098985,0.09743,0.10217,0.048182,0.048678,0.046715,0
.25195,0.19011,0.2223,0.22433,0.19891,0.22055,0.22448,0.18632,0.25334,
0.21448,0.18203,0.18347,0.19017,0.20439,0.19966,0.097019,0.10725,0.097
053,0.098231,0.096999,0.092949,0.099431,0.090318,0.095247,0.089283,0.1
0023,0.097478,0.10776,0.10338,0.10368,0.10152,0.093538,0.097169,0.0488
68,0.049324,0.048643,0.046769,0.21092,0.21434,0.21272,0.21928,0.2461,0
.22386,0.2137,0.20559,0.19096,0.19733,0.20469,0.20456,0.10157,0.096194
,0.12186,0.10798,0.099958,0.095996,0.094775,0.09477,0.10221,0.098358,0
.096303,0.10135,0.09398,0.10043,0.093346,0.10025,0.097599,0.10271,0.09

4776,0.085867,0.047821,0.04898,0.048529,0.047801,0.049141,0.046524,0.2
0857,0.17462,0.23275,0.20863,0.2032,0.26503,0.25595,0.15562,0.099655,0
.08693,0.09694,0.095604,0.098755,0.10223,0.11372,0.091712,0.0973,0.097
982,0.091769,0.10086,0.096075,0.096291,0.092416,0.0928,0.095006,0.0987
06,0.091922,0.096623,0.10148,0.11173,0.10311,0.046815,0.048961,0.04758
1,0.046731,0.046898,0.048979,0.2236,0.17178,0.19361,0.22239,0.21338,0.
048905,0.049313,0.09586,0.10386,0.096031,0.098785,0.096153,0.09506,0.0
97223,0.094822,0.093011,0.095654,0.10445,0.098824,0.097037,0.10359,0.0
97387,0.11041,0.081146,0.09845,0.098133,0.109,0.094874,0.04898,0.04853
6,0.046938,0.04965,0.047735,0.046263,0.050076,0.047199,0.047939,0.0475
17,0.22799,0.21528,0.048963,0.049539,0.04751,0.049443,0.095762,0.09816
2,0.07975,0.10693,0.092978,0.097742,0.099004,0.097244,0.10336,0.10518,
0.096743,0.095415,0.1077,0.096391,0.11268,0.088995,0.091734,0.097169,0
.098268,0.050145,0.049823,0.049083,0.048089,0.047293,0.050329,0.048154
,0.048876,0.049046,0.047675,0.048801,0.048226,0.047826,0.049769,0.0480
95,0.049518,0.047443,0.094085,0.10957,0.1054,0.089028,0.10646,0.098922
,0.097029,0.10486,0.0909,0.10553,0.097559,0.095697,0.1002,0.10643,0.11
167,0.10009,0.049321,0.050062,0.047563,0.050086,0.048691,0.048321,0.05
0795,0.04678,0.048487,0.049603,0.047059,0.049544,0.049741,0.048813,0.0
48052,0.048744,0.04669,0.048223,0.048041,0.09522,0.098577,0.096746,0.0
92639,0.098952,0.098808,0.096504,0.095067,0.097474,0.090545,0.091119,0
.095113,0.095148,0.047386,0.047867,0.047425,0.047218,0.049307,0.049683
,0.049691,0.049588,0.048705,0.049627,0.047511,0.048348,0.047208,0.0486
75,0.049295,0.047058,0.047276,0.048517,0.050196,0.050049,0.048471,0.09
6857,0.11432,0.10285,0.096011,0.092719,0.096839,0.099477,0.10691,0.096
282,0.048118,0.047576,0.049413,0.04694,0.046916,0.049709,0.050264,0.04
7752,0.049117,0.049042,0.049522,0.048737,0.046834,0.050367,0.049634,0.
048087,0.048221,0.047525,0.047935,0.048055,0.049787,0.048591,0.048576,
0.048188,0.047416,0.090128,0.10107,0.091254,0.099012,0.048329,0.048112
,0.053232,0.048967,0.046918,0.048812,0.049527,0.048342,0.05044,0.04792
5,0.048579,0.047162,0.0479,0.045174,0.047223,0.049015,0.049552,0.04789
5,0.049252,0.0462,0.049597,0.048766,0.047038,0.049039,0.048865,0.04705
5,0.048187,0.047483,0.048671,0.050201,0.04717,0.04883,0.047804,0.04930
3,0.046472,0.046862,0.048347,0.04865,0.044289,0.048,0.047713,0.048728,
0.049099,0.047544,0.046794,0.047442,0.049919,0.049623,0.049578,0.04903
7,0.048234,0.048786,0.048392,0.049167,0.048423,0.049722,0.049775,0.048
788,0.04984,0.048543,0.049511,0.04852,0.046468,0.047044,0.047191,0.048
245,0.047809,0.047182,0.046551,0.049258,0.049702,0.049163,0.047897,0.0
48541,0.048139,0.050467,0.049826,0.050569,0.050002,0.050776,0.047697,0
.048783,0.049578,0.048527,0.050261,0.048505,0.048477,0.049081,0.048269
,0.047029,0.047821,0.049853,0.04941,0.049598,0.048958,0.047998,0.04703
2,0.047874,0.04929,0.047175,0.046911,0.047909,0.050003,0.047157,0.0501
03,0.050469,0.046836,0.04659,0.047836,0.050856,0.046753,0.048747,0.048
938,0.049133,0.04872,0.04927,0.048634,0.047845,0.048905,0.050747,0.049
366,0.050828,0.049721,0.049353,0.050315,0.049728,0.048184,0.048523,0.0
46816,0.049536,0.046215,0.04647,0.045452,0.050423,0.049222,0.048026,0.
049442,0.050876,0.047175,0.047561,0.05011,0.050426,0.047535,0.049146,0
.048904,0.047927,0.050511,0.047606,0.048509,0.046841,0.047593,0.048295
,0.048342,0.048029,0.050695,0.048448,0.050035,0.046963,0.050649,0.0476
65,0.049139,0.047948,0.047713,0.048254,0.049362,0.047075,0.047491,0.05
0254,0.047263,0.049162,0.04839,0.04845,0.048827,0.049138,0.049037,0.04
9273,0.049051,0.049231,0.049631,0.050302,0.050056,0.048275,0.048125,0.

047685,0.048498,0.047287,0.047425,0.047568,0.048213,0.047925,0.047169,
0.047486,0.048787,0.046979,0.04961
0.1333,0.13334,0.13551,0.13881,0.13734,0.14047,0.1346,0.13948,0.13903,
0.13906,0.13614,0.13717,0.13965,0.13779,0.13556,0.13649,0.13431,0.1372
1,0.13522,0.13223,0.24029,0.2376,0.2838,0.27487,0.13676,0.13788,0.1341
3,0.13567,0.13627,0.13813,0.1363,0.13617,0.1385,0.13518,0.13732,0.1405
8,0.13444,0.13812,0.13314,0.34968,0.26319,0.26038,0.26501,0.27233,0.24
324,0.25708,0.13719,0.13651,0.13471,0.13354,0.13417,0.13781,0.1361,0.1
3587,0.1389,0.13604,0.13683,0.13803,0.13808,0.13635,0.13523,0.38042,0.
38072,0.23159,0.25616,0.2448,0.26345,0.24571,0.25358,0.25327,0.251,0.1
3619,0.13656,0.13648,0.13654,0.13706,0.13986,0.13683,0.13385,0.13471,0
.13615,0.13591,0.13654,0.13674,0.13731,0.13773,0.34883,0.38427,0.36242
,0.25049,0.2657,0.29706,0.2352,0.25159,0.24752,0.25346,0.26625,0.26523
,0.13417,0.13765,0.13849,0.13854,0.13604,0.13669,0.13704,0.13664,0.135
8,0.137,0.13651,0.13678,0.13719,0.13879,0.13737,0.13795,0.4002,0.36697
,0.36633,0.38182,0.27195,0.25008,0.26024,0.25056,0.28032,0.24628,0.243
35,0.229,0.27136,0.24889,0.13596,0.13584,0.13708,0.13646,0.13681,0.137
18,0.13771,0.13728,0.13619,0.13849,0.13474,0.13782,0.13854,0.13678,0.1
3361,0.1353,0.38841,0.41162,0.40794,0.34842,0.38531,0.4068,0.27245,0.2
7686,0.26142,0.26177,0.24288,0.28471,0.27577,0.23471,0.26096,0.28045,0
.13647,0.13513,0.13672,0.1345,0.13775,0.13522,0.13342,0.13517,0.13497,
0.13485,0.13626,0.1399,0.13765,0.13568,0.13728,0.13964,0.13812,0.39266
,0.36865,0.38415,0.37274,0.3595,0.36182,0.28299,0.23998,0.25443,0.2714
7,0.24279,0.25367,0.27941,0.24223,0.23365,0.27554,0.23352,0.13772,0.13
622,0.13414,0.13887,0.13813,0.13432,0.1363,0.13575,0.13873,0.13715,0.1
3938,0.14027,0.13506,0.086839,0.093344,0.09803,0.13513,0.37548,0.3757,
0.38416,0.41021,0.38913,0.38983,0.3786,0.24365,0.31452,0.25221,0.29408
,0.27173,0.27344,0.24769,0.25625,0.25364,0.27203,0.25989,0.13693,0.138
24,0.13524,0.13727,0.13775,0.13582,0.14091,0.13818,0.13493,0.13588,0.1
3721,0.089181,0.10009,0.091972,0.08807,0.091224,0.088907,0.14047,0.388
38,0.37549,0.37265,0.36831,0.37947,0.39633,0.38293,0.26204,0.28622,0.2
4399,0.23417,0.24755,0.29086,0.24198,0.2443,0.25817,0.26791,0.25709,0.
13436,0.13935,0.13783,0.13693,0.13642,0.13654,0.13753,0.1349,0.13492,0
.090758,0.094493,0.086105,0.087375,0.091131,0.089153,0.094463,0.08942,
0.086364,0.13584,0.37228,0.3912,0.39645,0.42529,0.39698,0.35279,0.3866
3,0.26784,0.2604,0.26379,0.27963,0.24281,0.27558,0.2362,0.25867,0.2476
7,0.2392,0.26138,0.25013,0.1372,0.13548,0.13602,0.1368,0.13654,0.13639
,0.090095,0.093513,0.097846,0.084223,0.093114,0.093531,0.084073,0.0793
12,0.087389,0.092033,0.085414,0.094339,0.37733,0.34165,0.39248,0.37938
,0.37373,0.37483,0.34688,0.24702,0.27035,0.26934,0.25746,0.25762,0.271
65,0.29064,0.26059,0.26615,0.27757,0.27909,0.29508,0.13806,0.13452,0.1
3685,0.092791,0.075532,0.089952,0.080862,0.08942,0.09502,0.092501,0.09
052,0.090137,0.082268,0.091629,0.09559,0.088859,0.091849,0.091705,0.38
052,0.3481,0.38674,0.37117,0.39966,0.36906,0.38715,0.37288,0.38385,0.2
576,0.29793,0.2764,0.27612,0.24919,0.27682,0.28243,0.26432,0.29089,0.2
6305,0.26526,0.091707,0.086508,0.091472,0.089953,0.099154,0.090358,0.0
85215,0.090282,0.09121,0.091428,0.092341,0.10101,0.088534,0.0936,0.089
031,0.091923,0.085493,0.092898,0.36075,0.39594,0.3877,0.36857,0.3883,0
.39013,0.38309,0.39435,0.35157,0.2342,0.27259,0.18079,0.18934,0.18327,
0.21549,0.18368,0.18962,0.18404,0.18604,0.19542,0.092129,0.091681,0.09
8854,0.090481,0.090752,0.089973,0.091881,0.093705,0.091584,0.088989,0.
091195,0.089155,0.096955,0.085371,0.092941,0.092356,0.091068,0.089967,

0.36384,0.37246,0.39292,0.39715,0.37041,0.40784,0.40515,0.40694,0.3833
2,0.2054,0.18093,0.16425,0.19608,0.17786,0.19064,0.19989,0.18263,0.144
53,0.17645,0.1811,0.092312,0.096896,0.093369,0.089633,0.083985,0.09360
8,0.09789,0.089681,0.093063,0.095404,0.069914,0.09039,0.091632,0.09024
8,0.093414,0.093711,0.088433,0.090788,0.42272,0.39173,0.40747,0.37253,
0.34182,0.38662,0.36939,0.23225,0.24342,0.22049,0.1707,0.22029,0.18281
,0.17541,0.17509,0.18866,0.19384,0.19867,0.19029,0.19169,0.089596,0.09
2653,0.10095,0.094598,0.09336,0.094997,0.090461,0.087513,0.08908,0.090
749,0.09008,0.092306,0.090187,0.10567,0.087254,0.092972,0.094861,0.105
66,0.37509,0.37129,0.3802,0.39464,0.38233,0.37363,0.21362,0.27479,0.17
814,0.23285,0.18068,0.19554,0.18175,0.19426,0.14054,0.1953,0.19641,0.1
8662,0.1923,0.18944,0.085583,0.088945,0.10147,0.087298,0.092945,0.0975
12,0.088057,0.093303,0.08427,0.11181,0.093024,0.098384,0.090274,0.0887
49,0.0821,0.086274,0.090363,0.093966,0.3897,0.38954,0.38484,0.38082,0.
406,0.21129,0.22258,0.249,0.21102,0.20279,0.21384,0.20104,0.18399,0.19
462,0.19314,0.18621,0.21853,0.18984,0.17149,0.1497,0.089943,0.096387,0
.10433,0.08706,0.092793,0.089763,0.08882,0.091294,0.095378,0.091079,0.
093464,0.094173,0.091513,0.085557,0.093747,0.09201,0.091739,0.1086,0.3
568,0.3644,0.36381,0.18351,0.21878,0.23894,0.17994,0.19794,0.23137,0.1
9319,0.18553,0.18759,0.19326,0.16221,0.18883,0.168,0.18106,0.19038,0.2
0991,0.19334,0.10107,0.092287,0.087476,0.091169,0.092245,0.091428,0.09
0722,0.086472,0.090912,0.092765,0.091382,0.081855,0.092037,0.091146,0.
091858,0.090516,0.093755,0.092642,0.38084,0.2202,0.19548,0.22999,0.231
69,0.2146,0.20782,0.19456,0.21636,0.25108,0.17997,0.17242,0.1946,0.169
46,0.2016,0.19408,0.20544,0.19241,0.17806,0.091622,0.091449,0.085215,0
.085175,0.092014,0.090308,0.08848,0.10175,0.095671,0.091066,0.08714,0.
09283,0.095703,0.095248,0.092078,0.089542,0.089736,0.046101,0.21919,0.
17152,0.21762,0.22151,0.20744,0.21497,0.21366,0.20443,0.1506,0.23552,0
.19546,0.19356,0.18858,0.19055,0.14782,0.19269,0.18008,0.18246,0.21122
,0.088311,0.075862,0.085927,0.090068,0.11114,0.071196,0.097677,0.08892
4,0.092942,0.096118,0.091493,0.092826,0.089211,0.090587,0.098499,0.087
867,0.089338,0.045747,0.24129,0.22159,0.20668,0.19762,0.27553,0.23951,
0.22547,0.20732,0.16036,0.22712,0.20087,0.17708,0.18487,0.17534,0.1894
1,0.20494,0.19735,0.18942,0.090837,0.089889,0.087921,0.097583,0.09597,
0.088422,0.090561,0.091348,0.080362,0.099011,0.094415,0.093284,0.09220
2,0.091385,0.090614,0.089968,0.092166,0.090941,0.045529,0.25271,0.1674
2,0.24337,0.2156,0.20273,0.22548,0.19453,0.20149,0.19335,0.2236,0.1815
7,0.19858,0.20926,0.16203,0.19186,0.16489,0.18101,0.19773,0.090743,0.0
90861,0.094608,0.089561,0.089083,0.08877,0.086998,0.088949,0.09744,0.0
91825,0.092773,0.093917,0.082391,0.088302,0.092926,0.091046,0.096193,0
.044633,0.046154,0.044081,0.23726,0.1839,0.2121,0.21379,0.18947,0.2139
4,0.22461,0.18241,0.2478,0.21246,0.17617,0.17441,0.18026,0.19498,0.190
02,0.091557,0.1,0.091274,0.092397,0.090538,0.086637,0.092904,0.083717,
0.090057,0.084071,0.094267,0.091948,0.10107,0.096192,0.09674,0.095581,
0.08687,0.091446,0.045599,0.046019,0.047161,0.043427,0.20502,0.20824,0
.21382,0.21017,0.2396,0.22302,0.20153,0.20547,0.18985,0.18829,0.194,0.
19347,0.095111,0.090397,0.11565,0.10238,0.093244,0.091223,0.089646,0.0
89485,0.096077,0.09241,0.089259,0.095478,0.089209,0.094146,0.086507,0.
092866,0.090805,0.097897,0.088824,0.08019,0.045617,0.045548,0.045415,0
.045187,0.046107,0.043659,0.20433,0.17374,0.22275,0.2005,0.19223,0.258
08,0.25531,0.14785,0.093984,0.082112,0.090483,0.088131,0.092669,0.0961
95,0.10753,0.086032,0.091931,0.092597,0.086786,0.094019,0.089946,0.090

389,0.086057,0.087337,0.089633,0.092513,0.08656,0.091541,0.095007,0.10
54,0.096525,0.04435,0.046185,0.04461,0.044308,0.042985,0.047541,0.2138
8,0.15828,0.18864,0.21189,0.19836,0.045701,0.047372,0.090279,0.096497,
0.089338,0.093057,0.089076,0.088866,0.091844,0.090251,0.086828,0.08996
3,0.098561,0.091803,0.090496,0.096423,0.090391,0.10279,0.076127,0.0913
74,0.092969,0.10333,0.089036,0.046947,0.045413,0.044596,0.046152,0.044
553,0.0435,0.047353,0.044915,0.044872,0.04525,0.22075,0.20756,0.04579,
0.047111,0.044594,0.045849,0.089178,0.092936,0.074034,0.099737,0.08714
2,0.092221,0.093309,0.091,0.097612,0.098677,0.090506,0.089861,0.1005,0
.09095,0.1055,0.083014,0.086192,0.088889,0.092413,0.047727,0.047733,0.
045353,0.045597,0.044048,0.047581,0.044862,0.045608,0.046117,0.045263,
0.046514,0.045695,0.044744,0.046621,0.044644,0.046528,0.044551,0.08819
5,0.10267,0.098191,0.082531,0.099098,0.091655,0.090443,0.098379,0.0855
92,0.099492,0.091297,0.090902,0.094918,0.10003,0.10378,0.093847,0.0468
93,0.046463,0.04414,0.046831,0.046288,0.046027,0.047395,0.045462,0.045
425,0.047003,0.044589,0.046882,0.047078,0.045705,0.045226,0.045348,0.0
43953,0.045174,0.045103,0.089526,0.091445,0.090773,0.087685,0.093122,0
.092504,0.089891,0.089669,0.090977,0.084796,0.086072,0.087885,0.088875
,0.044401,0.045489,0.044962,0.044325,0.045901,0.046368,0.047005,0.0470
44,0.044741,0.047342,0.044622,0.045142,0.044475,0.045508,0.04691,0.044
561,0.044952,0.04721,0.047125,0.047557,0.045409,0.089779,0.10693,0.096
726,0.090309,0.086831,0.092202,0.093733,0.1011,0.089047,0.045725,0.044
8,0.047124,0.044543,0.044127,0.046758,0.04705,0.044789,0.04529,0.04714
4,0.046096,0.044804,0.042849,0.047285,0.046664,0.045994,0.045943,0.044
54,0.045518,0.044891,0.0467,0.046619,0.046406,0.044701,0.044754,0.0843
57,0.09533,0.085508,0.092538,0.045459,0.046603,0.049738,0.046661,0.044
5,0.044802,0.047022,0.045153,0.047879,0.044781,0.046605,0.043623,0.045
178,0.042024,0.044644,0.046443,0.047136,0.044814,0.046838,0.04396,0.04
687,0.04579,0.044561,0.044787,0.046406,0.045189,0.046125,0.044681,0.04
6436,0.047064,0.044488,0.045739,0.044354,0.046139,0.043698,0.045222,0.
045762,0.046069,0.040601,0.04455,0.045548,0.045969,0.046373,0.044478,0
.043756,0.043911,0.046256,0.046461,0.046821,0.045711,0.045016,0.045776
,0.045616,0.045402,0.044967,0.046609,0.046796,0.045602,0.047238,0.0456
78,0.047027,0.04519,0.043719,0.044604,0.044265,0.045135,0.045663,0.045
543,0.04368,0.046285,0.04765,0.045686,0.044877,0.045964,0.045436,0.046
673,0.04693,0.047866,0.047511,0.048314,0.044885,0.045459,0.047023,0.04
5099,0.048114,0.045823,0.04565,0.046402,0.045084,0.044665,0.044969,0.0
46422,0.045701,0.046344,0.045667,0.045952,0.044605,0.045193,0.04576,0.
044514,0.044042,0.045229,0.047155,0.043798,0.046554,0.046911,0.044196,
0.045173,0.04492,0.047749,0.044113,0.045866,0.046481,0.045614,0.045462
,0.045984,0.045412,0.046336,0.04657,0.047168,0.046807,0.04773,0.046577
,0.046073,0.048023,0.045987,0.046304,0.045438,0.043782,0.046957,0.0437
29,0.043219,0.04356,0.047958,0.045926,0.046151,0.046481,0.047336,0.044
769,0.044905,0.046373,0.04777,0.044721,0.045581,0.046169,0.045106,0.04
654,0.043934,0.045408,0.044035,0.044835,0.045235,0.046,0.044926,0.0474
5,0.04679,0.047136,0.044208,0.046994,0.044556,0.04539,0.045412,0.04445
8,0.045504,0.047074,0.044223,0.044507,0.048102,0.044215,0.047064,0.045
7,0.045121,0.046221,0.045726,0.045865,0.0458,0.046107,0.045541,0.04636
9,0.047211,0.047213,0.045284,0.045015,0.045247,0.045353,0.044667,0.044
137,0.045484,0.045374,0.045613,0.04497,0.044771,0.045666,0.044069,0.04
7113

0.12696,0.12564,0.12796,0.13261,0.1294,0.13112,0.12605,0.13164,0.13032
,0.13107,0.12933,0.12933,0.13284,0.13155,0.12883,0.13024,0.12733,0.130
37,0.128,0.12388,0.2361,0.22504,0.27292,0.26699,0.12996,0.13094,0.1265
3,0.12743,0.12846,0.13077,0.1277,0.12899,0.13045,0.12707,0.12972,0.133
07,0.12765,0.13025,0.12462,0.35122,0.25656,0.25676,0.25907,0.268,0.234
56,0.25534,0.12739,0.12856,0.12795,0.12697,0.12799,0.12963,0.12702,0.1
282,0.13032,0.12709,0.12887,0.12977,0.12923,0.12768,0.12934,0.36566,0.
38335,0.22417,0.24906,0.23913,0.24552,0.2444,0.24821,0.24551,0.24736,0
.12802,0.12791,0.12835,0.12801,0.12919,0.13168,0.12934,0.12522,0.12799
,0.12894,0.1271,0.1267,0.12916,0.12939,0.1298,0.35381,0.37401,0.35387,
0.2391,0.25254,0.29257,0.22286,0.25065,0.24011,0.23762,0.25586,0.25485
,0.12741,0.12912,0.13061,0.13065,0.1287,0.12854,0.13038,0.12934,0.1277
5,0.12814,0.12832,0.12963,0.12931,0.13192,0.12901,0.1302,0.38981,0.370
77,0.36198,0.36841,0.26383,0.24702,0.25408,0.24705,0.26973,0.23958,0.2
3508,0.21618,0.26633,0.22908,0.12956,0.1273,0.12984,0.12843,0.12987,0.
12987,0.13022,0.12982,0.12909,0.13085,0.12676,0.13188,0.12976,0.12861,
0.12693,0.12875,0.3747,0.39944,0.39435,0.33818,0.38258,0.39801,0.26283
,0.26484,0.25782,0.24945,0.23247,0.28476,0.26092,0.23792,0.25331,0.274
93,0.12998,0.12746,0.12828,0.12604,0.12856,0.1266,0.12497,0.12736,0.12
857,0.12672,0.12895,0.13174,0.12933,0.12752,0.12921,0.13249,0.12983,0.
39178,0.34928,0.37932,0.35903,0.35144,0.35162,0.27559,0.23065,0.23742,
0.25986,0.2388,0.25216,0.27654,0.23115,0.22341,0.26757,0.22871,0.1296,
0.12994,0.12558,0.12998,0.12911,0.12701,0.12752,0.12941,0.13,0.13002,0
.1316,0.13175,0.12699,0.082785,0.08857,0.091552,0.12789,0.38164,0.3774
7,0.36641,0.40596,0.38866,0.38467,0.37539,0.23247,0.30584,0.24381,0.28
847,0.26029,0.26663,0.24563,0.25632,0.24007,0.26133,0.25143,0.1286,0.1
3034,0.12664,0.12966,0.13045,0.12812,0.13202,0.13092,0.12758,0.12834,0
.12901,0.084116,0.095065,0.087265,0.083304,0.086248,0.085444,0.13211,0
.38896,0.36905,0.37421,0.36249,0.3784,0.38987,0.38213,0.25187,0.27719,
0.23784,0.22968,0.24142,0.27768,0.23758,0.2346,0.24868,0.25872,0.2525,
0.12592,0.13051,0.13012,0.1314,0.12836,0.12842,0.12955,0.12595,0.12664
,0.086706,0.090048,0.080976,0.08245,0.086973,0.084614,0.089436,0.08541
2,0.082297,0.12854,0.36698,0.3742,0.37336,0.41899,0.39399,0.34497,0.37
696,0.26497,0.25175,0.24947,0.27236,0.23646,0.26977,0.23559,0.25635,0.
24486,0.22843,0.25521,0.24015,0.1305,0.12814,0.12756,0.12893,0.12807,0
.12841,0.085922,0.088986,0.09259,0.079631,0.087264,0.088731,0.079778,0
.074808,0.081404,0.087649,0.081505,0.089207,0.38459,0.34941,0.38051,0.
37726,0.37243,0.36467,0.34865,0.23777,0.26629,0.26117,0.25104,0.25124,
0.25986,0.28878,0.2606,0.25769,0.26941,0.26741,0.28773,0.13121,0.12611
,0.12883,0.087583,0.070919,0.085499,0.076836,0.085797,0.089856,0.08822
6,0.085461,0.084851,0.076873,0.086182,0.090403,0.084512,0.087039,0.088
046,0.35774,0.34416,0.3814,0.35942,0.40057,0.36259,0.39589,0.35776,0.3
8971,0.24286,0.28688,0.27032,0.27137,0.24958,0.26458,0.28042,0.25587,0
.28664,0.25503,0.25395,0.086941,0.081398,0.086574,0.085703,0.09398,0.0
86545,0.081408,0.085291,0.086231,0.087034,0.087241,0.095786,0.084941,0
.089067,0.083387,0.086469,0.080826,0.088416,0.34575,0.37628,0.37052,0.
35855,0.37639,0.38059,0.36722,0.38806,0.3444,0.22608,0.25566,0.17117,0
.18808,0.17872,0.2088,0.18339,0.18286,0.18181,0.18157,0.19135,0.086425
,0.086436,0.093163,0.084379,0.085545,0.086274,0.086937,0.087845,0.0870
72,0.084161,0.086195,0.084102,0.090681,0.08081,0.087254,0.0868,0.08676
4,0.085871,0.36016,0.3562,0.3723,0.40123,0.36336,0.40312,0.39858,0.384
79,0.37204,0.19341,0.177,0.15711,0.19456,0.17022,0.18509,0.18591,0.175

18,0.1397,0.16714,0.17442,0.086783,0.091941,0.087345,0.085488,0.079779
,0.087898,0.092788,0.085065,0.088305,0.090324,0.065961,0.085615,0.0872
91,0.085953,0.088748,0.089631,0.084281,0.086474,0.41148,0.38356,0.3950
2,0.35972,0.34491,0.38966,0.37281,0.22142,0.23506,0.21707,0.16433,0.21
761,0.18051,0.16955,0.1712,0.18363,0.18713,0.18863,0.18121,0.18865,0.0
8512,0.087405,0.094823,0.089765,0.088028,0.091384,0.085475,0.082285,0.
084061,0.086599,0.084976,0.086642,0.086785,0.10024,0.083063,0.087884,0
.089506,0.099925,0.37978,0.36757,0.3796,0.39982,0.36363,0.38529,0.2010
1,0.28003,0.18142,0.22541,0.18294,0.18526,0.17412,0.19401,0.13655,0.18
968,0.19372,0.17408,0.18868,0.18272,0.080683,0.084516,0.095137,0.08265
4,0.088895,0.09247,0.083096,0.087307,0.080764,0.10653,0.088151,0.09337
3,0.085883,0.083513,0.077499,0.081108,0.084711,0.088266,0.38507,0.3769
,0.37494,0.3775,0.39862,0.21206,0.21435,0.24691,0.20515,0.20024,0.2059
9,0.19297,0.18014,0.19335,0.18489,0.17943,0.21011,0.18364,0.16734,0.14
474,0.086005,0.091029,0.097278,0.082571,0.089701,0.086279,0.085216,0.0
86527,0.08976,0.086015,0.087659,0.08885,0.08703,0.082113,0.087533,0.08
7235,0.08619,0.10248,0.34068,0.35629,0.37659,0.18067,0.2075,0.23705,0.
17847,0.19424,0.22263,0.18518,0.18651,0.18311,0.18733,0.15474,0.17951,
0.16242,0.17788,0.18686,0.19383,0.18777,0.096468,0.088091,0.082754,0.0
85614,0.087468,0.086224,0.0858,0.081408,0.086727,0.087562,0.085834,0.0
77385,0.087316,0.086195,0.086672,0.084588,0.088708,0.088369,0.37663,0.
22055,0.19573,0.21754,0.22419,0.2141,0.20569,0.19076,0.21206,0.24068,0
.17302,0.16822,0.1904,0.16468,0.19487,0.18822,0.20015,0.18661,0.16879,
0.086774,0.087208,0.081502,0.079899,0.086054,0.085886,0.084846,0.09576
7,0.090868,0.086047,0.081938,0.087424,0.090596,0.090023,0.087784,0.084
219,0.08493,0.04292,0.21501,0.17032,0.20934,0.22019,0.20527,0.20628,0.
20412,0.20551,0.14569,0.23631,0.18901,0.18683,0.18459,0.18524,0.14326,
0.18668,0.17283,0.18072,0.20297,0.083018,0.072532,0.081906,0.086124,0.
1044,0.066744,0.092902,0.084105,0.087605,0.091216,0.086136,0.088291,0.
08374,0.085992,0.093856,0.08401,0.084968,0.044323,0.23521,0.2168,0.205
96,0.19376,0.28067,0.23403,0.21506,0.19868,0.15736,0.22896,0.19657,0.1
7334,0.17697,0.16805,0.18388,0.19925,0.18603,0.18349,0.08585,0.085294,
0.084213,0.092955,0.091121,0.083732,0.086003,0.086553,0.075022,0.09251
,0.088836,0.088129,0.087091,0.085828,0.085565,0.085352,0.087919,0.0860
28,0.043278,0.24245,0.16167,0.23695,0.21079,0.19908,0.21754,0.18922,0.
19847,0.19449,0.21384,0.17196,0.18889,0.20028,0.15239,0.1877,0.1599,0.
17612,0.19449,0.086048,0.085341,0.089359,0.085535,0.082836,0.083551,0.
0836,0.084634,0.092718,0.086628,0.087747,0.088908,0.078542,0.084125,0.
087546,0.086634,0.09065,0.043282,0.043604,0.042128,0.24048,0.18192,0.2
0866,0.20618,0.17908,0.20689,0.2136,0.17927,0.24528,0.20597,0.16812,0.
16925,0.16936,0.18618,0.18482,0.086402,0.094961,0.086573,0.087886,0.08
5088,0.081408,0.087613,0.079387,0.085909,0.08038,0.088964,0.086744,0.0
95531,0.090598,0.092916,0.089201,0.082411,0.086292,0.043403,0.044571,0
.045002,0.040996,0.19938,0.20322,0.20694,0.20993,0.23026,0.21896,0.196
79,0.2007,0.1885,0.18518,0.19014,0.19196,0.090535,0.085072,0.10959,0.0
97818,0.089059,0.08496,0.085524,0.085584,0.091119,0.088352,0.084618,0.
089073,0.084595,0.088827,0.082714,0.087825,0.086237,0.093291,0.084381,
0.0764,0.043977,0.043798,0.044834,0.044342,0.043528,0.04237,0.2032,0.1
7183,0.20823,0.19765,0.19112,0.25259,0.24937,0.14419,0.089105,0.078417
,0.085222,0.084351,0.086794,0.091144,0.10219,0.082133,0.087351,0.08887
8,0.082141,0.088293,0.084234,0.085622,0.081983,0.083266,0.085277,0.088
422,0.082758,0.087052,0.090117,0.099524,0.091814,0.042268,0.044491,0.0

43138,0.041618,0.041071,0.044099,0.21448,0.14898,0.18439,0.21187,0.198
9,0.043873,0.044872,0.085965,0.09174,0.085543,0.087966,0.085224,0.0845
27,0.086802,0.086239,0.081162,0.085669,0.094698,0.086963,0.086291,0.09
0928,0.087461,0.097121,0.072081,0.087284,0.086665,0.098092,0.084714,0.
044677,0.043544,0.042839,0.043659,0.042823,0.042779,0.044667,0.044126,
0.043337,0.043086,0.21526,0.20382,0.043855,0.045099,0.043085,0.044416,
0.084464,0.089703,0.069042,0.094402,0.083443,0.08862,0.088625,0.085497
,0.091901,0.09274,0.085958,0.084069,0.095053,0.086457,0.1003,0.079893,
0.08176,0.085517,0.086268,0.045583,0.045729,0.044177,0.043311,0.041843
,0.045341,0.042745,0.043498,0.044464,0.042583,0.044582,0.042976,0.0426
2,0.04517,0.042346,0.043337,0.04247,0.084081,0.096314,0.09312,0.077658
,0.093559,0.086882,0.085142,0.093835,0.081094,0.094082,0.08641,0.08690
1,0.088489,0.095153,0.098319,0.088411,0.044922,0.044023,0.041703,0.045
534,0.043804,0.045067,0.046229,0.042664,0.043412,0.044952,0.043006,0.0
4494,0.044855,0.043721,0.043278,0.043588,0.042137,0.043516,0.043731,0.
082797,0.087435,0.086362,0.083403,0.089297,0.087944,0.08663,0.084237,0
.086224,0.07957,0.081602,0.084109,0.084741,0.042038,0.042828,0.043251,
0.042558,0.043552,0.044017,0.044957,0.044604,0.042819,0.045274,0.04288
8,0.043469,0.04365,0.043249,0.044208,0.043161,0.043542,0.045065,0.0446
04,0.04586,0.044743,0.084151,0.099071,0.091735,0.086291,0.083718,0.086
973,0.088949,0.095201,0.08448,0.043881,0.043006,0.044744,0.04298,0.042
278,0.044988,0.045103,0.043047,0.042786,0.044405,0.044372,0.043262,0.0
41897,0.045159,0.04474,0.044194,0.04491,0.042615,0.042589,0.043098,0.0
44711,0.04285,0.043896,0.042966,0.042855,0.080682,0.089386,0.081594,0.
087551,0.042586,0.045934,0.046521,0.04453,0.043145,0.042034,0.04435,0.
042964,0.045394,0.042693,0.044817,0.04207,0.044186,0.039862,0.043801,0
.044244,0.045055,0.042713,0.04427,0.041757,0.045126,0.0437,0.041562,0.
042518,0.044727,0.042992,0.043971,0.043388,0.044069,0.045801,0.042359,
0.04428,0.04314,0.043707,0.041379,0.043492,0.043598,0.045309,0.039444,
0.042245,0.043261,0.044247,0.044627,0.042614,0.041399,0.041769,0.04568
6,0.044095,0.045363,0.0444,0.042701,0.043723,0.042952,0.043138,0.04275
,0.04536,0.045688,0.043138,0.046834,0.043767,0.044543,0.042836,0.04215
1,0.042945,0.042432,0.043218,0.043995,0.043409,0.041418,0.044119,0.045
995,0.043322,0.041866,0.043087,0.043829,0.044903,0.044464,0.044838,0.0
45087,0.046157,0.043235,0.045032,0.045378,0.042283,0.045871,0.044345,0
.044247,0.043261,0.042897,0.042624,0.043334,0.044299,0.042535,0.044992
,0.04381,0.044768,0.04286,0.043076,0.044424,0.043044,0.041796,0.043755
,0.045657,0.04262,0.045716,0.044667,0.042626,0.044502,0.04281,0.045326
,0.042563,0.045072,0.045176,0.042597,0.043779,0.043848,0.04344,0.04465
3,0.044137,0.045003,0.044899,0.045756,0.044543,0.043108,0.046136,0.044
934,0.044091,0.043801,0.041697,0.044813,0.04261,0.041899,0.042438,0.04
5486,0.043761,0.044252,0.044943,0.044982,0.043829,0.043682,0.044205,0.
046194,0.041861,0.043302,0.044223,0.043628,0.045042,0.043064,0.043254,
0.042207,0.042317,0.043224,0.044188,0.043273,0.04438,0.043666,0.044976
,0.041729,0.04465,0.041928,0.043485,0.043762,0.04202,0.043808,0.044467
,0.042596,0.041848,0.047083,0.042129,0.044782,0.043128,0.043283,0.0439
48,0.043842,0.044107,0.044045,0.04394,0.043141,0.044137,0.045034,0.044
963,0.044456,0.043347,0.043313,0.044954,0.043589,0.042298,0.044256,0.0
43402,0.04289,0.043182,0.042339,0.043978,0.041353,0.045615
0.11924,0.11888,0.12206,0.12464,0.12397,0.12515,0.12181,0.12523,0.1243
9,0.12478,0.12414,0.12305,0.12563,0.12503,0.12241,0.12311,0.12165,0.12
41,0.12359,0.11751,0.2394,0.21279,0.26821,0.25804,0.1237,0.12509,0.120

9,0.12266,0.12228,0.12462,0.12189,0.12269,0.1254,0.12128,0.12249,0.125
1,0.11989,0.12281,0.11869,0.34377,0.24276,0.24153,0.24926,0.2555,0.229
79,0.24998,0.12131,0.12202,0.12299,0.12073,0.12107,0.12254,0.12215,0.1
2212,0.12242,0.11969,0.12262,0.12477,0.12313,0.12164,0.12131,0.36047,0
.38036,0.22285,0.24272,0.23573,0.24045,0.23682,0.24346,0.24439,0.24042
,0.121,0.12183,0.12274,0.12215,0.12296,0.12528,0.12404,0.1196,0.12234,
0.12141,0.11984,0.12142,0.12255,0.12226,0.12297,0.34343,0.38278,0.3428
9,0.23673,0.24007,0.28593,0.22237,0.24354,0.23793,0.23562,0.26238,0.24
996,0.12118,0.12168,0.12537,0.12495,0.12157,0.12206,0.12334,0.12227,0.
12226,0.12232,0.12139,0.12336,0.12274,0.12613,0.12119,0.12413,0.37263,
0.36213,0.35744,0.3706,0.25283,0.24523,0.24823,0.2463,0.25929,0.24388,
0.22673,0.21662,0.26838,0.22542,0.12321,0.12199,0.12298,0.1218,0.12303
,0.1234,0.12315,0.12339,0.12287,0.12484,0.11978,0.12535,0.1237,0.12116
,0.12013,0.12413,0.37702,0.37927,0.37878,0.33541,0.37357,0.37856,0.263
31,0.25629,0.24911,0.23661,0.23031,0.27158,0.2599,0.23129,0.24043,0.27
947,0.12326,0.12019,0.12285,0.1197,0.12198,0.12019,0.11876,0.12173,0.1
2395,0.11905,0.12273,0.12432,0.12174,0.12087,0.12332,0.12661,0.12326,0
.39245,0.34162,0.37772,0.36929,0.35531,0.34363,0.26704,0.23078,0.23248
,0.25686,0.23378,0.24896,0.26279,0.2304,0.22367,0.26174,0.22529,0.1218
5,0.12343,0.12083,0.12364,0.12262,0.12033,0.12028,0.12234,0.12384,0.12
263,0.12504,0.12467,0.12038,0.078411,0.085356,0.087129,0.12141,0.37585
,0.36809,0.35975,0.39452,0.39802,0.37996,0.37541,0.22274,0.30322,0.243
19,0.27645,0.25523,0.25914,0.23928,0.2506,0.23908,0.25414,0.25204,0.12
308,0.12389,0.11915,0.12412,0.12368,0.12254,0.12684,0.1246,0.11991,0.1
2094,0.12298,0.080218,0.091167,0.083539,0.079171,0.0826,0.082312,0.125
26,0.39357,0.37176,0.3645,0.36882,0.3872,0.39031,0.3813,0.24662,0.2705
8,0.23769,0.232,0.24044,0.27444,0.23365,0.23096,0.23797,0.25811,0.2490
4,0.12059,0.12514,0.12519,0.12458,0.12153,0.12226,0.12282,0.11814,0.12
028,0.081016,0.085674,0.077328,0.079397,0.081965,0.08098,0.085327,0.08
1397,0.077522,0.12269,0.37176,0.37435,0.37446,0.40995,0.40022,0.34043,
0.37053,0.2538,0.2402,0.2433,0.26362,0.22838,0.2616,0.2313,0.25425,0.2
3842,0.22681,0.24996,0.23468,0.12561,0.12247,0.12228,0.1224,0.12275,0.
12199,0.082633,0.084439,0.088723,0.077517,0.082993,0.084611,0.075972,0
.071472,0.077739,0.083966,0.078196,0.084684,0.38298,0.35049,0.37281,0.
3681,0.36212,0.36542,0.35707,0.22958,0.26023,0.25302,0.24685,0.24723,0
.25412,0.28231,0.25647,0.25269,0.25493,0.26103,0.28391,0.12516,0.11889
,0.12187,0.082804,0.067357,0.082628,0.074411,0.082073,0.086195,0.08366
6,0.081866,0.0813,0.073997,0.081579,0.086439,0.080813,0.082488,0.08358
6,0.36581,0.34911,0.37284,0.35296,0.39422,0.35151,0.38015,0.36168,0.38
687,0.24009,0.27575,0.26332,0.26938,0.24575,0.25371,0.27361,0.24451,0.
2827,0.2466,0.25028,0.08356,0.078378,0.08107,0.082306,0.089751,0.08214
7,0.077663,0.081473,0.082368,0.083062,0.084622,0.091714,0.080638,0.085
969,0.079611,0.082235,0.077072,0.084373,0.3499,0.37155,0.35676,0.34831
,0.35673,0.38219,0.36471,0.37399,0.34267,0.22105,0.25729,0.16902,0.185
19,0.17142,0.20614,0.17025,0.17884,0.17938,0.17892,0.18667,0.082727,0.
08355,0.089126,0.08255,0.081893,0.083338,0.082898,0.082908,0.082635,0.
081435,0.082486,0.08013,0.086613,0.076479,0.084005,0.083338,0.083238,0
.082637,0.34967,0.34352,0.35714,0.39585,0.35838,0.40376,0.38395,0.3800
1,0.36447,0.19329,0.18062,0.15208,0.18888,0.16955,0.17733,0.18638,0.16
684,0.13477,0.16368,0.16921,0.082192,0.086178,0.084891,0.081702,0.0767
71,0.083226,0.089327,0.081513,0.085217,0.085768,0.063542,0.082119,0.08
2966,0.082113,0.08514,0.085685,0.0806,0.08144,0.40485,0.38424,0.37659,

0.36341,0.34437,0.38017,0.37239,0.21574,0.23897,0.21341,0.1657,0.20631
,0.17634,0.16572,0.16416,0.1821,0.18351,0.18992,0.18101,0.18215,0.0815
05,0.083089,0.090225,0.085366,0.08475,0.088103,0.081948,0.078554,0.080
337,0.083312,0.082057,0.083925,0.083107,0.09651,0.079793,0.08383,0.086
664,0.095693,0.35642,0.36456,0.373,0.39784,0.36942,0.39522,0.19645,0.2
7691,0.17378,0.21772,0.17843,0.18086,0.16711,0.18914,0.13452,0.18072,0
.18772,0.16966,0.18401,0.17695,0.075912,0.080719,0.089943,0.07937,0.08
4744,0.087183,0.079734,0.083228,0.077249,0.10162,0.084591,0.088872,0.0
81771,0.079364,0.0747,0.077408,0.080315,0.084578,0.38195,0.36996,0.364
27,0.36466,0.38105,0.21146,0.21866,0.24605,0.20505,0.19568,0.20374,0.1
8652,0.17518,0.18976,0.18222,0.17783,0.20494,0.17751,0.16382,0.14505,0
.083222,0.086996,0.092502,0.078966,0.085912,0.084224,0.081708,0.081536
,0.085289,0.081792,0.084335,0.083829,0.084125,0.078519,0.084383,0.0834
72,0.082409,0.097395,0.33829,0.35339,0.37104,0.18367,0.20583,0.23806,0
.16878,0.1891,0.22451,0.18542,0.1836,0.18061,0.17987,0.14871,0.17513,0
.16186,0.17192,0.18369,0.19342,0.17967,0.091752,0.082732,0.079505,0.08
2116,0.083627,0.081934,0.081225,0.077343,0.082455,0.084127,0.082706,0.
074556,0.083577,0.083202,0.083197,0.081071,0.085618,0.085547,0.38479,0
.21911,0.19414,0.21286,0.22361,0.21505,0.20418,0.18829,0.21222,0.2384,
0.1698,0.16423,0.18585,0.16573,0.19119,0.17968,0.19522,0.18104,0.16499
,0.082362,0.082041,0.077518,0.075319,0.082636,0.082004,0.081446,0.0929
15,0.086642,0.082745,0.077618,0.08318,0.086113,0.085933,0.084007,0.081
183,0.080844,0.041467,0.20559,0.16575,0.20586,0.21471,0.20403,0.20616,
0.20019,0.2046,0.14414,0.23684,0.18397,0.18538,0.18048,0.18234,0.13803
,0.18556,0.17108,0.17442,0.198,0.08023,0.069611,0.077638,0.081528,0.09
8784,0.06345,0.087591,0.081442,0.08281,0.08741,0.08262,0.08541,0.07968
9,0.083447,0.088886,0.080667,0.081028,0.042554,0.22899,0.20847,0.20324
,0.19576,0.2744,0.22352,0.21694,0.19796,0.15313,0.22658,0.19243,0.1672
3,0.17641,0.16529,0.18256,0.19199,0.17974,0.18142,0.081531,0.082286,0.
07992,0.08965,0.087847,0.07962,0.082905,0.081599,0.071781,0.088115,0.0
84484,0.082156,0.082026,0.080605,0.080993,0.081905,0.083446,0.082599,0
.041836,0.22944,0.15645,0.24183,0.20924,0.20493,0.21488,0.188,0.18784,
0.18843,0.21165,0.16946,0.18345,0.19339,0.14851,0.1821,0.15584,0.17147
,0.18253,0.081848,0.081686,0.08481,0.08248,0.078986,0.0798,0.079022,0.
080573,0.08792,0.08508,0.084638,0.084895,0.075487,0.080017,0.083507,0.
082077,0.086849,0.041562,0.042044,0.04215,0.23647,0.17865,0.20384,0.20
428,0.17445,0.20314,0.21071,0.17836,0.23976,0.19675,0.1632,0.16539,0.1
6253,0.18249,0.17574,0.083078,0.090803,0.082734,0.08368,0.081702,0.078
03,0.084561,0.076167,0.081673,0.077107,0.085086,0.083379,0.090843,0.08
6669,0.088364,0.084725,0.078722,0.08229,0.042049,0.044123,0.043957,0.0
40766,0.19781,0.20111,0.20909,0.19869,0.22616,0.21955,0.19647,0.20185,
0.18786,0.18543,0.19065,0.18857,0.08672,0.080828,0.10347,0.094275,0.08
6054,0.080553,0.082571,0.080788,0.086295,0.083485,0.080913,0.084844,0.
080211,0.083339,0.078579,0.08388,0.082881,0.089251,0.080266,0.073281,0
.04315,0.043207,0.043628,0.043203,0.042396,0.04122,0.20024,0.17083,0.2
0645,0.1978,0.19344,0.24295,0.24425,0.14333,0.084545,0.074868,0.08127,
0.080492,0.083815,0.0863,0.098173,0.078714,0.083654,0.085731,0.079308,
0.083687,0.078916,0.081195,0.078757,0.07924,0.08108,0.083674,0.078995,
0.08271,0.085654,0.094827,0.087452,0.042113,0.041783,0.041634,0.040267
,0.039898,0.043205,0.21088,0.14839,0.18722,0.21092,0.19406,0.042199,0.
043427,0.082821,0.087082,0.081104,0.083209,0.081079,0.081473,0.08319,0
.08185,0.078136,0.082636,0.089251,0.083335,0.082172,0.08702,0.083132,0

.093951,0.069122,0.083415,0.082013,0.094852,0.081542,0.043696,0.041778
,0.041975,0.042876,0.041636,0.041182,0.042817,0.043595,0.041552,0.0421
22,0.21305,0.20196,0.042955,0.043872,0.042459,0.043058,0.080799,0.0860
9,0.066064,0.090662,0.07936,0.085232,0.084026,0.08253,0.088031,0.08838
8,0.081412,0.080206,0.091194,0.083502,0.094912,0.076513,0.078253,0.083
754,0.082071,0.043828,0.043867,0.042548,0.041252,0.040585,0.043783,0.0
41538,0.042307,0.043819,0.040819,0.04342,0.041178,0.042051,0.044512,0.
041303,0.041644,0.040946,0.081685,0.092215,0.08941,0.074712,0.089458,0
.082649,0.081005,0.090559,0.07824,0.089957,0.082014,0.082933,0.084368,
0.091114,0.093595,0.083705,0.043918,0.043153,0.040502,0.043451,0.04240
1,0.043726,0.045932,0.042693,0.042061,0.042983,0.042325,0.044209,0.042
984,0.042009,0.042518,0.042596,0.041772,0.043316,0.042314,0.07944,0.08
3904,0.08313,0.079678,0.084921,0.084864,0.08253,0.080921,0.082368,0.07
6413,0.07791,0.079844,0.080682,0.040843,0.042094,0.042924,0.041932,0.0
4252,0.042271,0.042828,0.043241,0.040526,0.043726,0.04156,0.0424,0.042
29,0.042223,0.042393,0.041807,0.042538,0.043403,0.042676,0.045059,0.04
3731,0.08017,0.095432,0.087832,0.081772,0.080833,0.083605,0.085507,0.0
91258,0.080023,0.042725,0.041925,0.043392,0.04234,0.040939,0.044486,0.
043519,0.041971,0.04171,0.043043,0.042478,0.042622,0.040027,0.043343,0
.043132,0.043373,0.043411,0.042425,0.040872,0.041639,0.043857,0.040865
,0.043194,0.042402,0.042326,0.076732,0.083947,0.077769,0.082681,0.0413
62,0.044191,0.044193,0.044138,0.042015,0.040708,0.04245,0.041889,0.043
498,0.040674,0.043665,0.040347,0.043026,0.038755,0.043928,0.043823,0.0
43459,0.04099,0.043468,0.040355,0.043868,0.042052,0.041147,0.04228,0.0
43643,0.041297,0.042773,0.042487,0.042949,0.044316,0.040288,0.042828,0
.041375,0.043081,0.040703,0.042581,0.042546,0.043635,0.038357,0.040836
,0.041965,0.043024,0.042709,0.040993,0.040813,0.040893,0.043386,0.0425
12,0.043909,0.043384,0.041257,0.043002,0.041658,0.042392,0.04175,0.043
82,0.044148,0.042733,0.045246,0.043009,0.044334,0.042431,0.040557,0.04
1526,0.041038,0.042304,0.042961,0.041816,0.039827,0.042033,0.044759,0.
042379,0.041265,0.042067,0.042006,0.043032,0.042549,0.042846,0.04461,0
.044645,0.042437,0.043253,0.043577,0.041307,0.044104,0.042848,0.042601
,0.042057,0.040826,0.040728,0.041876,0.042203,0.041723,0.043159,0.0424
07,0.041691,0.041539,0.041361,0.042905,0.04175,0.041442,0.042147,0.043
96,0.040887,0.042985,0.043456,0.040837,0.043644,0.041852,0.043555,0.04
1827,0.043888,0.04409,0.041439,0.042435,0.041754,0.041369,0.043653,0.0
41442,0.044491,0.042928,0.043791,0.04316,0.042541,0.044401,0.043607,0.
04242,0.04202,0.041246,0.042622,0.041187,0.041471,0.041458,0.044715,0.
042706,0.042996,0.043217,0.043907,0.042809,0.042588,0.042877,0.045826,
0.04161,0.042085,0.042435,0.041714,0.043022,0.042521,0.042024,0.041301
,0.040257,0.041579,0.04189,0.042333,0.042549,0.043075,0.044225,0.04027
3,0.043005,0.040732,0.043216,0.042623,0.041031,0.042948,0.042628,0.042
533,0.041211,0.045361,0.041504,0.043826,0.042765,0.041461,0.041608,0.0
4203,0.041693,0.043314,0.043074,0.042421,0.043099,0.043401,0.042561,0.
042903,0.042479,0.041534,0.043414,0.04227,0.041483,0.043664,0.042136,0
.04236,0.042134,0.04224,0.042653,0.040404,0.044419
0.11375,0.11441,0.11763,0.11867,0.11827,0.11979,0.11745,0.12048,0.1188
8,0.1183,0.12028,0.11834,0.12027,0.11966,0.11756,0.11833,0.11643,0.119
41,0.11824,0.11239,0.23904,0.20039,0.26598,0.25779,0.11898,0.12074,0.1
1487,0.11671,0.11906,0.11877,0.11759,0.11794,0.11878,0.11719,0.11695,0
.11901,0.11467,0.11672,0.11389,0.33527,0.22698,0.23316,0.24492,0.25334
,0.23333,0.24592,0.11804,0.11635,0.11822,0.11548,0.11506,0.11812,0.116

93,0.11645,0.11771,0.11313,0.11728,0.12033,0.11817,0.11769,0.11556,0.3
6958,0.37915,0.21841,0.23732,0.23308,0.23768,0.22558,0.23592,0.24118,0
.23221,0.11528,0.11617,0.1198,0.11713,0.11699,0.12014,0.11756,0.11428,
0.11675,0.11594,0.11577,0.11677,0.11782,0.11721,0.1175,0.33705,0.36808
,0.34226,0.22351,0.24412,0.27707,0.22163,0.23897,0.22996,0.23948,0.250
89,0.24616,0.11565,0.11718,0.1178,0.11924,0.1171,0.11648,0.11733,0.117
27,0.11564,0.11841,0.11759,0.12026,0.11786,0.12095,0.11635,0.11914,0.3
6616,0.36842,0.33397,0.36891,0.25127,0.24504,0.24226,0.24288,0.25271,0
.23815,0.22054,0.20594,0.25583,0.22473,0.11839,0.11575,0.11786,0.11734
,0.11871,0.1167,0.11852,0.11861,0.11724,0.11928,0.11474,0.12024,0.1171
,0.11676,0.11491,0.11995,0.37459,0.37901,0.35621,0.34086,0.36359,0.373
77,0.26477,0.2459,0.24164,0.23496,0.22877,0.26813,0.25145,0.22113,0.23
758,0.27122,0.11817,0.11701,0.11778,0.11526,0.11684,0.11514,0.11463,0.
11654,0.11918,0.11449,0.11754,0.12004,0.1166,0.11595,0.11819,0.12033,0
.11811,0.3864,0.34076,0.38145,0.37175,0.34694,0.34707,0.26258,0.2307,0
.23253,0.25025,0.23067,0.24583,0.25325,0.23061,0.22085,0.25793,0.21958
,0.11817,0.11703,0.11547,0.11637,0.11804,0.1138,0.1152,0.11815,0.11792
,0.11894,0.11926,0.11891,0.11485,0.075929,0.082167,0.083516,0.11609,0.
38275,0.36399,0.37307,0.39202,0.38851,0.36677,0.38169,0.2215,0.29841,0
.23406,0.28436,0.25177,0.2524,0.2355,0.25469,0.23204,0.24718,0.24829,0
.1179,0.11829,0.11442,0.1186,0.11864,0.11691,0.12106,0.11851,0.11534,0
.11665,0.11833,0.07757,0.086957,0.081351,0.076933,0.078953,0.078793,0.
11925,0.38511,0.36748,0.35583,0.36304,0.38162,0.38144,0.37571,0.2335,0
.26334,0.23242,0.2232,0.22894,0.27586,0.22843,0.22547,0.23421,0.25101,
0.24581,0.11568,0.11931,0.11935,0.12019,0.11648,0.11805,0.11704,0.1131
7,0.11523,0.078076,0.083092,0.074339,0.076453,0.078524,0.077842,0.0832
97,0.079578,0.074371,0.11709,0.3755,0.36559,0.37397,0.40582,0.39926,0.
35059,0.3578,0.25274,0.23617,0.24365,0.26972,0.22637,0.25498,0.22355,0
.24891,0.23432,0.22576,0.24817,0.23074,0.11998,0.11637,0.11745,0.11825
,0.11694,0.11635,0.079384,0.080535,0.08478,0.074911,0.079035,0.080971,
0.072109,0.069293,0.074168,0.080914,0.075407,0.080827,0.37591,0.35329,
0.3775,0.36607,0.34779,0.36715,0.34511,0.22733,0.25506,0.24891,0.24181
,0.23618,0.25473,0.27602,0.24921,0.23832,0.25396,0.25044,0.27661,0.120
52,0.11388,0.11765,0.07837,0.065668,0.079301,0.071375,0.079608,0.08285
6,0.081409,0.077868,0.078196,0.071278,0.078379,0.083116,0.077212,0.079
105,0.079803,0.37031,0.34051,0.37326,0.34019,0.39994,0.34767,0.3759,0.
37417,0.38383,0.23815,0.26587,0.24902,0.26322,0.23787,0.24609,0.26772,
0.23661,0.2819,0.23348,0.24475,0.079999,0.076739,0.079312,0.078628,0.0
86553,0.079965,0.075284,0.079172,0.079459,0.080031,0.080912,0.087338,0
.078053,0.082844,0.07674,0.078993,0.073939,0.081405,0.35001,0.37461,0.
36047,0.35183,0.34574,0.37836,0.36317,0.38557,0.35057,0.21915,0.24701,
0.16827,0.17621,0.16957,0.20173,0.16392,0.17269,0.17292,0.17706,0.1790
3,0.078594,0.080773,0.08611,0.079801,0.078652,0.078846,0.080349,0.0787
12,0.079387,0.077198,0.079868,0.077882,0.083195,0.072747,0.080828,0.08
0288,0.080604,0.079654,0.3498,0.33237,0.33933,0.39399,0.37062,0.39235,
0.37375,0.38126,0.35939,0.19253,0.17564,0.14942,0.1816,0.16581,0.17318
,0.19009,0.16552,0.13239,0.16209,0.16421,0.079707,0.0827,0.081155,0.07
7372,0.075257,0.080875,0.085835,0.078993,0.082349,0.08225,0.061011,0.0
80059,0.079717,0.079084,0.081915,0.082697,0.078293,0.078419,0.38394,0.
35765,0.37541,0.35245,0.34422,0.38297,0.36719,0.21113,0.2301,0.20498,0
.16131,0.20269,0.1728,0.16118,0.16185,0.18086,0.18156,0.18776,0.17512,
0.17769,0.078217,0.079709,0.086127,0.082459,0.081263,0.084311,0.080208

,0.074476,0.077487,0.08061,0.078494,0.081042,0.079052,0.092722,0.07720
8,0.080913,0.08368,0.092187,0.3539,0.36954,0.35892,0.38609,0.36121,0.3
8946,0.1971,0.27697,0.17486,0.22579,0.17493,0.17449,0.16653,0.17833,0.
1291,0.17552,0.18291,0.16701,0.17895,0.17169,0.074625,0.078018,0.08704
6,0.075995,0.082098,0.08354,0.076866,0.080142,0.073855,0.097872,0.0827
67,0.085117,0.078801,0.076573,0.072155,0.074834,0.077047,0.08141,0.363
45,0.35731,0.36265,0.35908,0.37151,0.2131,0.21083,0.24223,0.19924,0.19
292,0.19186,0.18093,0.17378,0.1902,0.17655,0.17554,0.19981,0.17214,0.1
5942,0.13814,0.080085,0.083614,0.087973,0.075648,0.083929,0.08057,0.07
8335,0.078198,0.081841,0.07923,0.082042,0.081338,0.081263,0.074954,0.0
81726,0.080166,0.078697,0.094353,0.32996,0.35278,0.35754,0.1786,0.1991
5,0.22631,0.17175,0.18824,0.21295,0.1842,0.18087,0.18094,0.17625,0.146
57,0.17164,0.16219,0.16551,0.17762,0.19536,0.17406,0.088439,0.080108,0
.076663,0.079149,0.081491,0.078177,0.078307,0.074531,0.079789,0.081743
,0.079818,0.071767,0.080877,0.0794,0.07891,0.078049,0.083458,0.082532,
0.3944,0.21699,0.19712,0.20647,0.22651,0.20823,0.19965,0.19004,0.21369
,0.23931,0.16644,0.16237,0.18066,0.16414,0.18801,0.17819,0.18735,0.178
15,0.16179,0.078834,0.07786,0.074386,0.073778,0.079209,0.079834,0.0783
99,0.0886,0.083827,0.078882,0.074426,0.079267,0.083024,0.081514,0.0805
98,0.078227,0.077711,0.040588,0.20446,0.16567,0.20643,0.20561,0.19952,
0.19572,0.19536,0.20797,0.14196,0.23776,0.18176,0.18689,0.18076,0.1780
7,0.13534,0.18162,0.16665,0.17731,0.19304,0.077371,0.068428,0.075745,0
.078209,0.094631,0.061322,0.083608,0.079414,0.07951,0.08366,0.079077,0
.08212,0.076127,0.081347,0.085299,0.078464,0.078023,0.042197,0.22566,0
.2102,0.20139,0.20037,0.27452,0.22162,0.21759,0.19675,0.15898,0.22336,
0.19072,0.1624,0.17488,0.16558,0.17967,0.19139,0.17583,0.17345,0.07775
9,0.079289,0.076366,0.086682,0.08489,0.077091,0.079216,0.078805,0.0686
71,0.085089,0.081235,0.079043,0.079286,0.078133,0.078815,0.079202,0.08
2232,0.080443,0.041212,0.21861,0.15666,0.23554,0.20776,0.20561,0.21193
,0.187,0.18479,0.1811,0.21195,0.16682,0.17997,0.19252,0.14571,0.17526,
0.14988,0.1673,0.18295,0.078918,0.079192,0.081921,0.0793,0.077759,0.07
6719,0.07656,0.077324,0.085,0.081773,0.081956,0.081179,0.072092,0.0774
36,0.080017,0.079476,0.083653,0.041128,0.040934,0.041019,0.24173,0.175
78,0.19627,0.20709,0.17121,0.20175,0.20698,0.18129,0.23538,0.18668,0.1
5725,0.15973,0.15591,0.1831,0.17126,0.079649,0.087571,0.078449,0.08156
6,0.079566,0.074709,0.080495,0.074061,0.077581,0.075346,0.081992,0.080
707,0.088068,0.083497,0.084798,0.081969,0.075519,0.080131,0.041753,0.0
42999,0.042868,0.039963,0.19584,0.20813,0.20838,0.1998,0.23093,0.2186,
0.19253,0.19612,0.18464,0.18237,0.18532,0.18598,0.082432,0.078577,0.10
03,0.091465,0.082674,0.077485,0.079446,0.077665,0.081987,0.080327,0.07
8461,0.082198,0.077875,0.080099,0.075181,0.081167,0.079738,0.085716,0.
077736,0.07107,0.041126,0.042546,0.042912,0.043229,0.041407,0.040803,0
.19895,0.16656,0.206,0.20366,0.19355,0.24282,0.23518,0.14389,0.080889,
0.072578,0.0783,0.078715,0.080425,0.082563,0.095034,0.075638,0.081353,
0.082791,0.076748,0.080186,0.075911,0.07799,0.075671,0.078133,0.077767
,0.080501,0.077024,0.078811,0.081782,0.091545,0.082639,0.040831,0.0418
84,0.041013,0.039506,0.039141,0.041825,0.21184,0.14754,0.18758,0.20755
,0.18014,0.041064,0.043371,0.078351,0.08532,0.078168,0.081507,0.077911
,0.079418,0.080068,0.078699,0.074895,0.080178,0.084379,0.079801,0.0804
91,0.083879,0.080806,0.089955,0.066771,0.080164,0.078734,0.090867,0.07
8334,0.042232,0.040715,0.041449,0.041471,0.041054,0.040945,0.041711,0.
042655,0.040719,0.041263,0.21067,0.20312,0.042394,0.04244,0.041534,0.0

41035,0.078876,0.083427,0.063579,0.087317,0.077118,0.081125,0.080798,0
.078351,0.083665,0.085217,0.078463,0.076723,0.08678,0.07988,0.091231,0
.073515,0.075568,0.080898,0.079766,0.042296,0.042739,0.041181,0.039374
,0.039407,0.042484,0.040527,0.040666,0.042316,0.040312,0.042732,0.0400
95,0.040289,0.043121,0.040153,0.040902,0.039238,0.07804,0.089054,0.085
514,0.072438,0.085708,0.07933,0.079166,0.086835,0.075085,0.087283,0.07
8568,0.078988,0.080626,0.088557,0.089224,0.081109,0.043142,0.042583,0.
0391,0.04187,0.042227,0.041957,0.044619,0.041794,0.040684,0.042287,0.0
40979,0.043276,0.041644,0.041428,0.040903,0.040836,0.040858,0.041591,0
.0416,0.075825,0.079681,0.078704,0.076456,0.082549,0.08222,0.079507,0.
077653,0.079446,0.073726,0.075365,0.077079,0.077996,0.039671,0.041481,
0.042235,0.041048,0.0412,0.042096,0.042071,0.042312,0.039894,0.041586,
0.040894,0.041584,0.041689,0.0411,0.041019,0.040326,0.041517,0.0419,0.
042774,0.043858,0.043269,0.078353,0.091813,0.084272,0.077644,0.077599,
0.080555,0.082579,0.089571,0.077298,0.042718,0.040911,0.043069,0.04111
6,0.03972,0.043794,0.042554,0.04156,0.040945,0.042384,0.041414,0.04197
4,0.038953,0.043324,0.042175,0.041472,0.042376,0.042013,0.040229,0.041
622,0.042354,0.040391,0.040991,0.040907,0.041827,0.073754,0.081716,0.0
75442,0.080102,0.039903,0.043244,0.042668,0.042754,0.041537,0.039032,0
.042087,0.040084,0.042728,0.039546,0.042393,0.039649,0.042538,0.039345
,0.043443,0.043302,0.042806,0.040122,0.041294,0.039075,0.043061,0.0409
36,0.040299,0.040728,0.042752,0.041143,0.041345,0.041377,0.042122,0.04
3148,0.039561,0.043017,0.040562,0.042245,0.040161,0.04288,0.041928,0.0
41201,0.038079,0.040418,0.040187,0.042994,0.042171,0.040596,0.040756,0
.039616,0.042741,0.041633,0.042957,0.041833,0.040538,0.042259,0.040938
,0.041083,0.041284,0.04238,0.042397,0.041176,0.04412,0.042418,0.043542
,0.04208,0.039879,0.041145,0.039267,0.041231,0.042818,0.040112,0.03881
5,0.040223,0.043766,0.041634,0.041755,0.041178,0.041331,0.042265,0.041
37,0.042581,0.04415,0.043458,0.041469,0.041948,0.044351,0.040388,0.043
024,0.041321,0.043125,0.041117,0.03937,0.039545,0.040937,0.042427,0.04
0931,0.041995,0.041557,0.040969,0.041079,0.040048,0.04186,0.041494,0.0
40645,0.040512,0.043427,0.040092,0.042504,0.042162,0.040127,0.043432,0
.040633,0.042292,0.040786,0.042766,0.043789,0.041311,0.04165,0.041305,
0.040499,0.042867,0.040953,0.042894,0.041638,0.042623,0.042519,0.04264
8,0.042605,0.042135,0.041629,0.040404,0.040685,0.042022,0.040021,0.040
324,0.039506,0.044165,0.041986,0.042635,0.042463,0.042841,0.042054,0.0
42222,0.041681,0.044244,0.04017,0.040811,0.041902,0.040297,0.04152,0.0
41017,0.041448,0.041857,0.039927,0.041841,0.040921,0.040335,0.040788,0
.042296,0.043758,0.039849,0.041827,0.039994,0.042164,0.042037,0.040133
,0.041808,0.04163,0.040894,0.041068,0.043821,0.039825,0.043435,0.04258
3,0.040835,0.040478,0.041386,0.041743,0.043418,0.041677,0.041265,0.041
704,0.042308,0.04223,0.041628,0.042249,0.040693,0.043469,0.040886,0.04
1539,0.043305,0.041584,0.041014,0.041581,0.041771,0.042642,0.040063,0.
043144
0.10895,0.11097,0.11356,0.11309,0.11369,0.11451,0.11178,0.11616,0.1147
5,0.11375,0.11451,0.11325,0.11507,0.11439,0.11321,0.11465,0.11104,0.11
503,0.11323,0.10936,0.23467,0.20108,0.25331,0.2583,0.11434,0.1153,0.11
155,0.11142,0.11409,0.11585,0.11402,0.1146,0.11399,0.1136,0.11213,0.11
454,0.10977,0.11362,0.11004,0.33123,0.22358,0.23422,0.23854,0.25189,0.
23179,0.24524,0.11362,0.11219,0.11569,0.11161,0.11164,0.11314,0.11275,
0.11187,0.11278,0.10912,0.11331,0.11613,0.11369,0.11329,0.11192,0.3727
7,0.36111,0.21963,0.23283,0.22974,0.23279,0.22312,0.23026,0.2361,0.224

15,0.11279,0.11182,0.11446,0.11286,0.11271,0.11518,0.11217,0.10901,0.1
1195,0.1135,0.11343,0.11262,0.11383,0.11254,0.11321,0.33276,0.3673,0.3
3975,0.2188,0.23993,0.27098,0.22438,0.22881,0.22188,0.23363,0.24223,0.
23631,0.11268,0.1128,0.11382,0.11531,0.11198,0.11267,0.11204,0.11355,0
.11203,0.11359,0.11321,0.11483,0.11334,0.11739,0.1121,0.11579,0.37023,
0.37221,0.32797,0.36204,0.25046,0.24007,0.23341,0.24236,0.24718,0.2304
2,0.21841,0.20865,0.25528,0.21433,0.11523,0.11209,0.11242,0.11291,0.11
446,0.11259,0.11312,0.11326,0.11317,0.11536,0.1112,0.11565,0.1129,0.11
429,0.11088,0.11557,0.36256,0.38002,0.35976,0.33734,0.35607,0.36152,0.
25699,0.24498,0.24263,0.23536,0.22391,0.26276,0.24605,0.21844,0.22932,
0.27041,0.11405,0.11398,0.11411,0.11153,0.11219,0.11147,0.11048,0.1123
5,0.11459,0.11077,0.11328,0.11513,0.11248,0.11188,0.11208,0.11636,0.11
327,0.37969,0.35751,0.37852,0.34789,0.34167,0.3507,0.25866,0.22742,0.2
3754,0.25225,0.2225,0.24657,0.23867,0.23476,0.21687,0.25588,0.21745,0.
11506,0.11317,0.11074,0.11272,0.11256,0.10959,0.11113,0.1139,0.1151,0.
11566,0.11616,0.11447,0.1115,0.073465,0.079112,0.080637,0.11088,0.3654
9,0.37425,0.36536,0.39304,0.38199,0.36255,0.37872,0.21272,0.29347,0.23
063,0.27386,0.25144,0.26012,0.23149,0.25282,0.2291,0.24613,0.24382,0.1
1517,0.11458,0.11017,0.11514,0.11457,0.11366,0.11647,0.11463,0.11184,0
.1119,0.11403,0.075755,0.083609,0.078338,0.075508,0.076302,0.0769,0.11
554,0.39061,0.38005,0.33249,0.36388,0.38073,0.36326,0.38856,0.23215,0.
25349,0.23288,0.22084,0.23026,0.27474,0.22456,0.22087,0.23291,0.2393,0
.24035,0.11137,0.11408,0.11467,0.11549,0.11133,0.11309,0.11398,0.10965
,0.11107,0.07521,0.079377,0.072727,0.073382,0.075335,0.07585,0.08054,0
.077631,0.071665,0.11167,0.37564,0.37813,0.37701,0.40157,0.39835,0.345
58,0.34281,0.25051,0.23021,0.24851,0.27289,0.22545,0.25456,0.22192,0.2
4443,0.22942,0.22524,0.24102,0.22761,0.11489,0.11187,0.11305,0.1137,0.
11237,0.11246,0.0771,0.078112,0.081243,0.072139,0.076458,0.078015,0.07
0446,0.068457,0.071742,0.079548,0.073961,0.078576,0.35665,0.35573,0.37
801,0.37063,0.33785,0.37679,0.34586,0.23418,0.2504,0.24961,0.24083,0.2
2927,0.25467,0.26705,0.23989,0.23832,0.25488,0.25005,0.27064,0.11479,0
.10994,0.11417,0.07682,0.063775,0.077512,0.069699,0.077659,0.080321,0.
079429,0.074825,0.076287,0.068309,0.07535,0.081345,0.075681,0.076862,0
.077574,0.37389,0.34591,0.36986,0.34863,0.39296,0.3553,0.36807,0.37509
,0.3759,0.23815,0.25487,0.24445,0.25768,0.23874,0.24346,0.26669,0.2289
5,0.27114,0.2378,0.24344,0.077912,0.074954,0.076409,0.077119,0.083853,
0.078081,0.07244,0.077297,0.07807,0.077637,0.077583,0.083864,0.075493,
0.080914,0.074541,0.076979,0.071807,0.079252,0.35337,0.38756,0.35752,0
.35359,0.33958,0.38468,0.36079,0.3841,0.34197,0.21629,0.24437,0.16018,
0.1717,0.16542,0.20047,0.16182,0.17158,0.16529,0.17797,0.18255,0.07628
,0.077204,0.082581,0.076677,0.075394,0.07595,0.078602,0.075917,0.07766
6,0.075901,0.077057,0.075631,0.080525,0.071144,0.078421,0.078512,0.078
091,0.077786,0.35716,0.33324,0.34805,0.3787,0.37652,0.3898,0.38922,0.3
8469,0.35533,0.1952,0.17132,0.14732,0.17821,0.16533,0.17305,0.18254,0.
15856,0.13006,0.15847,0.16354,0.077267,0.07945,0.079502,0.075611,0.073
465,0.078597,0.083309,0.077045,0.081263,0.079363,0.058879,0.078232,0.0
7668,0.077071,0.078443,0.079047,0.074169,0.075547,0.37376,0.35866,0.39
22,0.35271,0.33094,0.36696,0.3758,0.21003,0.22813,0.20399,0.15772,0.19
843,0.16615,0.15948,0.15726,0.17746,0.1804,0.1827,0.17322,0.17597,0.07
6273,0.077412,0.082541,0.080192,0.078719,0.08138,0.077666,0.072709,0.0
75629,0.078064,0.075727,0.078118,0.076174,0.08978,0.074439,0.078319,0.
080879,0.089283,0.35266,0.37527,0.36099,0.3808,0.35826,0.37233,0.19361

,0.27417,0.1754,0.22812,0.16906,0.17654,0.16862,0.16742,0.12389,0.1718
3,0.18088,0.16549,0.17506,0.16818,0.072925,0.076093,0.084008,0.073559,
0.078845,0.08157,0.07448,0.077157,0.071501,0.093898,0.080215,0.082304,
0.076947,0.075267,0.070336,0.072696,0.075628,0.079687,0.36403,0.34819,
0.3602,0.35713,0.37639,0.20817,0.20743,0.23928,0.20054,0.19256,0.18805
,0.17906,0.16893,0.18182,0.17113,0.17183,0.19692,0.16924,0.15579,0.135
83,0.07827,0.081778,0.084166,0.073767,0.081338,0.078078,0.075822,0.075
792,0.078774,0.076537,0.079281,0.078046,0.07825,0.072026,0.079525,0.07
6617,0.075238,0.090894,0.33633,0.35906,0.35402,0.1793,0.19227,0.21892,
0.16883,0.18076,0.20583,0.18771,0.18167,0.17755,0.17393,0.14135,0.1779
8,0.15683,0.16623,0.17728,0.1911,0.16905,0.085355,0.077705,0.073833,0.
077229,0.079612,0.076739,0.074174,0.072547,0.076439,0.080278,0.077565,
0.069693,0.077997,0.077526,0.075799,0.076161,0.081029,0.079672,0.37833
,0.21852,0.20236,0.20843,0.22442,0.21076,0.20137,0.19105,0.20989,0.236
32,0.16339,0.15396,0.17969,0.16318,0.18752,0.16856,0.18221,0.17025,0.1
601,0.076988,0.07628,0.072774,0.07191,0.077006,0.075829,0.075223,0.087
127,0.080518,0.077132,0.071308,0.077641,0.078856,0.079346,0.07691,0.07
6492,0.075708,0.039949,0.20249,0.16205,0.20692,0.20227,0.19656,0.1985,
0.19249,0.19835,0.14336,0.23552,0.17868,0.18102,0.183,0.17149,0.12945,
0.17957,0.16406,0.17341,0.19311,0.075316,0.066649,0.073164,0.075799,0.
091924,0.059025,0.079615,0.076059,0.077329,0.080917,0.077366,0.079437,
0.073261,0.079259,0.082647,0.075913,0.075786,0.041783,0.22024,0.20396,
0.2002,0.207,0.27005,0.22744,0.21241,0.19696,0.15348,0.2125,0.1897,0.1
5898,0.17018,0.1652,0.17923,0.18944,0.171,0.17395,0.07548,0.078063,0.0
7371,0.085108,0.082386,0.074316,0.077516,0.076641,0.067538,0.080809,0.
078544,0.076911,0.076212,0.075164,0.076673,0.076667,0.07923,0.077405,0
.040641,0.21925,0.15334,0.23061,0.21346,0.19533,0.21231,0.18592,0.1842
5,0.17556,0.21159,0.16651,0.18034,0.18489,0.14466,0.17433,0.14778,0.16
662,0.17941,0.077446,0.077742,0.078947,0.076127,0.075458,0.075261,0.07
3717,0.074401,0.082002,0.078181,0.078836,0.078805,0.070868,0.076509,0.
076906,0.077307,0.081555,0.039742,0.040193,0.040417,0.23794,0.17397,0.
19239,0.2099,0.16998,0.2031,0.20673,0.18255,0.23512,0.18727,0.15514,0.
15629,0.15313,0.1778,0.17426,0.078659,0.085023,0.07521,0.07994,0.07651
5,0.073089,0.077344,0.07144,0.075976,0.07337,0.07958,0.078089,0.085465
,0.081006,0.083187,0.07842,0.074376,0.079065,0.040591,0.042049,0.04179
5,0.039712,0.19039,0.21099,0.21298,0.19969,0.22832,0.20899,0.19155,0.1
9364,0.1841,0.17742,0.17786,0.189,0.080334,0.076303,0.096326,0.088208,
0.081186,0.075165,0.077457,0.075995,0.080114,0.077879,0.076471,0.08031
8,0.075893,0.07753,0.074139,0.07928,0.075839,0.082152,0.076551,0.06848
1,0.040733,0.041547,0.042173,0.042531,0.040009,0.040119,0.19067,0.1634
3,0.20459,0.19943,0.19349,0.23865,0.2269,0.14081,0.078538,0.070602,0.0
75396,0.076457,0.07743,0.080539,0.091214,0.073662,0.078662,0.079773,0.
075193,0.077606,0.073875,0.074974,0.073901,0.075386,0.076436,0.078742,
0.074742,0.0761,0.07892,0.088284,0.080351,0.040128,0.040895,0.041094,0
.038625,0.038861,0.041741,0.20169,0.14895,0.19472,0.20688,0.17687,0.03
993,0.043665,0.076386,0.083658,0.075022,0.078487,0.075726,0.077493,0.0
77949,0.075347,0.072739,0.077919,0.082417,0.077766,0.076887,0.082154,0
.077178,0.084826,0.065497,0.077245,0.076245,0.087808,0.075434,0.042566
,0.040758,0.041046,0.040772,0.039754,0.039757,0.040892,0.042564,0.0394
4,0.040502,0.20193,0.19718,0.041997,0.041215,0.041247,0.041404,0.07819
1,0.080531,0.060955,0.084453,0.075504,0.078502,0.078699,0.074457,0.080
462,0.081582,0.075557,0.072437,0.084811,0.07805,0.088971,0.070755,0.07

3891,0.077305,0.076605,0.042154,0.041194,0.041204,0.038644,0.039476,0.
042362,0.04004,0.040155,0.041316,0.039892,0.041638,0.040311,0.040395,0
.042372,0.039682,0.040387,0.038965,0.075729,0.086059,0.082997,0.071436
,0.08384,0.076606,0.076726,0.084138,0.073251,0.084782,0.076835,0.07603
7,0.076986,0.085505,0.087542,0.07863,0.042456,0.042542,0.039168,0.0418
06,0.041839,0.040715,0.043775,0.041826,0.040945,0.041325,0.040695,0.04
274,0.040656,0.041138,0.040328,0.04033,0.040979,0.04097,0.04131,0.0738
82,0.078142,0.076649,0.074325,0.080187,0.07902,0.076904,0.076405,0.076
707,0.071166,0.072767,0.075224,0.075534,0.039919,0.040666,0.041164,0.0
40013,0.040834,0.041257,0.040693,0.041634,0.039437,0.041784,0.040299,0
.040525,0.04182,0.039663,0.040305,0.039848,0.041783,0.041504,0.041505,
0.042963,0.042876,0.076208,0.088753,0.081938,0.075901,0.075618,0.07777
8,0.080558,0.087256,0.073971,0.041934,0.040663,0.042182,0.040112,0.038
337,0.042507,0.041683,0.041339,0.040829,0.042026,0.041275,0.04083,0.03
8627,0.042614,0.04184,0.040525,0.040971,0.04143,0.039914,0.040645,0.04
2263,0.039421,0.040815,0.039733,0.042274,0.070973,0.078645,0.073258,0.
077235,0.040725,0.042065,0.041554,0.042139,0.040847,0.039261,0.040967,
0.039953,0.042268,0.038605,0.041806,0.039511,0.04216,0.039301,0.042172
,0.041859,0.041758,0.03929,0.04041,0.038397,0.042034,0.040621,0.040098
,0.040049,0.041583,0.040923,0.040969,0.040456,0.04086,0.043178,0.03843
8,0.042426,0.03914,0.040907,0.039161,0.041554,0.041476,0.04086,0.03727
1,0.040102,0.04036,0.042103,0.04135,0.04035,0.040458,0.039386,0.041956
,0.040613,0.042846,0.041277,0.040198,0.041815,0.040772,0.040781,0.0410
29,0.041505,0.041054,0.041267,0.043401,0.042145,0.043659,0.040949,0.03
9146,0.04039,0.038781,0.040602,0.041823,0.039899,0.038185,0.040608,0.0
42408,0.040955,0.041549,0.040357,0.041612,0.043247,0.040722,0.042482,0
.043167,0.042471,0.04128,0.041516,0.044024,0.040959,0.042278,0.04127,0
.042285,0.040568,0.039011,0.039653,0.039913,0.041777,0.039941,0.041591
,0.041323,0.041172,0.039786,0.039114,0.041296,0.041372,0.040344,0.0407
65,0.042597,0.039517,0.041116,0.041687,0.039692,0.042807,0.040397,0.04
1834,0.039832,0.04246,0.042948,0.040824,0.041508,0.04202,0.040237,0.04
26,0.040811,0.041781,0.041711,0.04199,0.041554,0.041966,0.042092,0.041
811,0.040986,0.039686,0.039515,0.040985,0.039734,0.040334,0.037842,0.0
44533,0.042363,0.041566,0.041306,0.042024,0.041629,0.040432,0.041395,0
.042949,0.038357,0.03972,0.041884,0.04014,0.041769,0.040657,0.040515,0
.041555,0.039396,0.042022,0.039457,0.040643,0.039982,0.04216,0.043091,
0.038485,0.041125,0.039443,0.041022,0.041356,0.040026,0.040822,0.04072
6,0.041862,0.040758,0.043027,0.039985,0.042692,0.042062,0.039686,0.039
585,0.041024,0.040871,0.041887,0.041671,0.040839,0.041155,0.04106,0.04
0938,0.041663,0.041242,0.039771,0.042725,0.039698,0.041286,0.04319,0.0
40767,0.040141,0.04091,0.041091,0.041872,0.039045,0.041803
0.10571,0.10778,0.1098,0.10829,0.10948,0.10987,0.10966,0.1125,0.1095,0
.11185,0.11126,0.10863,0.11243,0.11045,0.11021,0.11169,0.10855,0.11146
,0.1087,0.10544,0.23203,0.20092,0.25394,0.25466,0.11106,0.11069,0.1078
7,0.10845,0.11068,0.10986,0.11121,0.11088,0.1121,0.11004,0.10921,0.111
45,0.10594,0.11149,0.10588,0.32451,0.22267,0.23602,0.23583,0.25288,0.2
3063,0.23854,0.10948,0.10943,0.11243,0.10858,0.10786,0.10964,0.10844,0
.10777,0.10893,0.10408,0.10959,0.11219,0.10962,0.11114,0.10796,0.37963
,0.35306,0.22001,0.23616,0.22678,0.23043,0.22474,0.22578,0.23493,0.228
57,0.10907,0.10726,0.11098,0.10937,0.10934,0.11194,0.10874,0.10577,0.1
0857,0.11091,0.1102,0.11013,0.11068,0.10954,0.10997,0.32445,0.36333,0.
3326,0.2167,0.23889,0.27044,0.21729,0.22353,0.21967,0.23252,0.23279,0.

22884,0.10922,0.11019,0.11072,0.11,0.10819,0.10925,0.10713,0.10926,0.1
0968,0.1092,0.10924,0.10985,0.11015,0.11404,0.10846,0.11177,0.35533,0.
36805,0.32795,0.35242,0.24482,0.23002,0.2285,0.2411,0.24403,0.22248,0.
2116,0.20879,0.24629,0.21336,0.11291,0.10755,0.11067,0.10899,0.11161,0
.10784,0.108,0.10959,0.1093,0.11055,0.1094,0.11238,0.10934,0.11155,0.1
085,0.11188,0.35831,0.37747,0.33761,0.33725,0.34632,0.35361,0.25553,0.
23928,0.23788,0.23555,0.22506,0.25024,0.24292,0.21891,0.22541,0.27133,
0.10966,0.10944,0.11004,0.10684,0.10873,0.10749,0.10863,0.10892,0.1108
4,0.1079,0.10995,0.11217,0.10898,0.10887,0.10933,0.11343,0.11026,0.376
48,0.35646,0.36636,0.34728,0.3422,0.34617,0.25807,0.21865,0.23181,0.24
524,0.22405,0.24445,0.23114,0.22869,0.2174,0.2493,0.21688,0.11059,0.10
83,0.10698,0.11078,0.10924,0.10619,0.10829,0.10946,0.11277,0.11048,0.1
1258,0.11146,0.10725,0.070509,0.077602,0.079343,0.10685,0.36478,0.3754
3,0.36813,0.37721,0.39149,0.36362,0.37493,0.20725,0.28791,0.23391,0.27
033,0.24297,0.25899,0.22761,0.24093,0.22744,0.2475,0.23779,0.11126,0.1
1143,0.10665,0.11117,0.11146,0.10901,0.11196,0.11136,0.10746,0.10916,0
.11036,0.074969,0.082569,0.076181,0.074028,0.074071,0.074478,0.11186,0
.38349,0.37704,0.32468,0.34546,0.3735,0.37064,0.38604,0.23658,0.25074,
0.22938,0.22579,0.22782,0.27593,0.22232,0.22205,0.22481,0.23487,0.2415
4,0.1086,0.11081,0.11158,0.1113,0.10919,0.10984,0.11124,0.1067,0.10805
,0.073426,0.077057,0.071892,0.070384,0.074558,0.073222,0.078547,0.0758
79,0.069829,0.10823,0.37269,0.39738,0.38213,0.40116,0.39478,0.34955,0.
33892,0.25113,0.2311,0.24695,0.27393,0.22042,0.24721,0.21428,0.2424,0.
2257,0.2285,0.24373,0.22338,0.1118,0.10799,0.10994,0.10997,0.10773,0.1
0928,0.076179,0.075372,0.080093,0.070109,0.074477,0.075322,0.069239,0.
066633,0.071434,0.07695,0.071669,0.077274,0.34226,0.35176,0.37169,0.36
22,0.34112,0.37364,0.35352,0.22857,0.24563,0.24548,0.23357,0.22576,0.2
5477,0.26588,0.23784,0.22583,0.24614,0.23962,0.26914,0.11139,0.10738,0
.11038,0.074942,0.062853,0.07571,0.067247,0.076463,0.078426,0.077121,0
.073344,0.073217,0.067465,0.074222,0.080238,0.074005,0.075044,0.074751
,0.36291,0.35177,0.37385,0.35115,0.37909,0.36349,0.35363,0.3687,0.3945
7,0.23268,0.25839,0.24346,0.25989,0.23624,0.24275,0.26617,0.23609,0.26
676,0.22895,0.2405,0.076519,0.073343,0.074239,0.074698,0.082436,0.0759
42,0.07082,0.075221,0.076051,0.075003,0.076286,0.08124,0.074656,0.0786
32,0.071291,0.074533,0.070194,0.077084,0.34942,0.37795,0.36302,0.35097
,0.34673,0.37454,0.35759,0.37019,0.34241,0.21611,0.24081,0.16049,0.171
03,0.16326,0.19092,0.1604,0.17216,0.16488,0.17092,0.1799,0.073799,0.07
5608,0.0804,0.07609,0.073437,0.073164,0.076657,0.072648,0.075728,0.073
882,0.075829,0.073984,0.078968,0.067943,0.076455,0.076473,0.075255,0.0
77146,0.35666,0.31955,0.34737,0.38313,0.38031,0.38816,0.3855,0.37439,0
.3505,0.20371,0.16512,0.14608,0.17845,0.16611,0.17091,0.18253,0.15711,
0.12796,0.15845,0.16376,0.074821,0.07756,0.077182,0.073064,0.071615,0.
077179,0.081997,0.075709,0.078329,0.076561,0.057261,0.075028,0.075234,
0.075353,0.075688,0.07696,0.073362,0.073749,0.36837,0.35967,0.38051,0.
36326,0.33377,0.37243,0.35693,0.21159,0.22901,0.19753,0.1505,0.19601,0
.1625,0.15473,0.15792,0.18025,0.17498,0.18543,0.16777,0.17804,0.074624
,0.075684,0.079171,0.078699,0.075679,0.07942,0.075822,0.07011,0.074013
,0.076211,0.074089,0.07589,0.074808,0.088814,0.073457,0.076095,0.07750
9,0.088334,0.35847,0.36908,0.36368,0.38083,0.35441,0.3648,0.18983,0.26
977,0.17355,0.22836,0.16742,0.17265,0.16677,0.16561,0.11958,0.167,0.17
407,0.16726,0.16737,0.17275,0.07142,0.075247,0.0818,0.07173,0.077933,0
.079436,0.073074,0.074695,0.068654,0.090431,0.078354,0.079854,0.075983

,0.073134,0.069538,0.070427,0.073739,0.078188,0.36944,0.3471,0.353,0.3
529,0.36235,0.20347,0.20708,0.23449,0.19031,0.19878,0.1901,0.17977,0.1
6756,0.18347,0.16944,0.16609,0.19597,0.16148,0.15791,0.13037,0.076289,
0.079674,0.082337,0.072688,0.079226,0.075636,0.073686,0.072426,0.07752
8,0.074749,0.077005,0.074242,0.075916,0.070216,0.078043,0.075155,0.073
707,0.089289,0.32683,0.35165,0.33295,0.17556,0.19576,0.21854,0.16942,0
.18072,0.20035,0.18587,0.18261,0.17533,0.17105,0.13981,0.17855,0.15682
,0.16538,0.17629,0.18502,0.17249,0.082634,0.076326,0.072578,0.07536,0.
077367,0.073463,0.070936,0.071135,0.074273,0.076913,0.074452,0.068218,
0.07654,0.076957,0.073176,0.073335,0.079321,0.077836,0.36733,0.21761,0
.19703,0.20667,0.22893,0.20988,0.20175,0.18662,0.21227,0.23468,0.15735
,0.15401,0.1817,0.1645,0.19067,0.16526,0.17827,0.17338,0.15905,0.07442
6,0.074884,0.070079,0.069895,0.074952,0.072957,0.07311,0.084947,0.0790
29,0.074974,0.068357,0.076807,0.076279,0.077498,0.0761,0.073936,0.0735
2,0.039447,0.20307,0.16182,0.20139,0.19782,0.19671,0.19946,0.19381,0.1
9314,0.13824,0.22901,0.17711,0.17993,0.18481,0.17016,0.12941,0.17419,0
.16817,0.17066,0.19228,0.073297,0.065393,0.071259,0.073404,0.089863,0.
057656,0.078061,0.073198,0.075445,0.078282,0.07494,0.076519,0.071023,0
.077212,0.079868,0.074939,0.07292,0.040467,0.217,0.20672,0.19792,0.201
59,0.26044,0.22863,0.21312,0.19203,0.15563,0.21192,0.18765,0.15401,0.1
7193,0.15937,0.17394,0.18685,0.16618,0.1717,0.073557,0.076405,0.071062
,0.082026,0.079575,0.072456,0.075243,0.073974,0.065241,0.078235,0.0768
08,0.075551,0.075837,0.073013,0.075173,0.075974,0.077847,0.076263,0.03
9655,0.21908,0.15336,0.22922,0.2096,0.19758,0.20132,0.18285,0.17997,0.
17546,0.20694,0.16335,0.168,0.18062,0.14343,0.17056,0.1459,0.16727,0.1
7756,0.074436,0.075917,0.076807,0.074212,0.074137,0.073707,0.072242,0.
071746,0.079301,0.076067,0.077349,0.076299,0.071048,0.075312,0.074691,
0.075292,0.079229,0.038966,0.039766,0.039587,0.2334,0.1775,0.18872,0.2
0948,0.17117,0.1925,0.21076,0.18031,0.2305,0.18271,0.1505,0.15464,0.15
467,0.17338,0.1697,0.076229,0.083359,0.07348,0.076438,0.074892,0.07154
,0.075104,0.068757,0.073466,0.070669,0.077899,0.076373,0.083664,0.0779
45,0.080344,0.076341,0.071923,0.077101,0.040499,0.041117,0.041576,0.04
0507,0.18788,0.2096,0.20479,0.19532,0.22735,0.21047,0.19533,0.19177,0.
18931,0.17675,0.17452,0.18461,0.079207,0.074325,0.092937,0.085243,0.07
9384,0.073743,0.07457,0.074551,0.078159,0.075802,0.073658,0.078635,0.0
75426,0.075909,0.071596,0.077657,0.074624,0.080704,0.074536,0.066566,0
.040267,0.040589,0.041414,0.041365,0.038766,0.039583,0.19448,0.16152,0
.19711,0.20186,0.18549,0.23841,0.22869,0.13953,0.076459,0.068971,0.073
023,0.073956,0.075929,0.079099,0.088776,0.073063,0.076559,0.079344,0.0
73875,0.075952,0.072175,0.073918,0.071773,0.074243,0.075374,0.077931,0
.07411,0.073163,0.077035,0.086039,0.077317,0.040251,0.041094,0.040813,
0.038602,0.038361,0.04165,0.19946,0.14281,0.19678,0.19805,0.18251,0.03
922,0.041676,0.073743,0.082,0.073406,0.076555,0.074776,0.076338,0.0756
08,0.073219,0.071095,0.076252,0.080192,0.075653,0.074737,0.080504,0.07
5078,0.082752,0.06401,0.07537,0.075168,0.08457,0.074046,0.041357,0.040
905,0.040289,0.04039,0.039534,0.038719,0.03959,0.041698,0.039498,0.039
509,0.2014,0.20018,0.040635,0.041276,0.039029,0.039997,0.076078,0.0789
06,0.059312,0.082681,0.074069,0.07794,0.076595,0.072926,0.078974,0.080
833,0.073655,0.069908,0.082136,0.074747,0.086603,0.069401,0.072075,0.0
73947,0.076363,0.041409,0.040845,0.040929,0.038194,0.039349,0.041295,0
.038981,0.039809,0.041122,0.038943,0.041226,0.039642,0.040176,0.04189,
0.039462,0.039277,0.038483,0.071912,0.082791,0.081216,0.069689,0.08189

1,0.074465,0.075257,0.082106,0.070898,0.082793,0.075348,0.074691,0.074416,0.083637,0.084679,0.07422,0.041077,0.042511,0.038416,0.040357,0.042089,0.040886,0.043412,0.041674,0.040478,0.041076,0.039672,0.042308,0.04037,0.041296,0.038716,0.039661,0.040929,0.040987,0.04047,0.073284,0.075145,0.074847,0.072119,0.077216,0.077169,0.074335,0.07419,0.074982,0.070088,0.070673,0.073331,0.073454,0.03974,0.039831,0.040682,0.040109,0.040646,0.040611,0.039614,0.040847,0.039069,0.041726,0.039613,0.039565,0.041723,0.039519,0.039078,0.03964,0.041852,0.041294,0.040468,0.042463,0.043538,0.074423,0.086399,0.078299,0.07379,0.073096,0.075552,0.079214,0.084805,0.071518,0.041291,0.040265,0.041522,0.039978,0.03898,0.042198,0.040807,0.040754,0.039896,0.041899,0.0404,0.040399,0.0389,0.041892,0.040696,0.040382,0.040747,0.041032,0.039638,0.040542,0.040882,0.039023,0.039865,0.038949,0.041764,0.069691,0.076877,0.071349,0.075612,0.039901,0.04211,0.041454,0.042159,0.040839,0.039097,0.040976,0.039397,0.042015,0.038836,0.040372,0.038145,0.041503,0.039044,0.041635,0.040982,0.041402,0.038983,0.040493,0.038673,0.041135,0.041334,0.040064,0.039867,0.041574,0.039712,0.040893,0.040491,0.040448,0.042795,0.038635,0.041561,0.038277,0.040574,0.039047,0.040745,0.042561,0.039524,0.037934,0.040373,0.039985,0.042097,0.040953,0.039579,0.04045,0.037785,0.040797,0.040281,0.042508,0.039786,0.038095,0.03981,0.040122,0.040284,0.040373,0.041167,0.040884,0.04017,0.043499,0.041551,0.043301,0.039812,0.038585,0.03993,0.037546,0.039336,0.041304,0.040973,0.03827,0.041016,0.041798,0.039673,0.041156,0.040154,0.041308,0.042577,0.040151,0.042155,0.041823,0.042381,0.040451,0.040188,0.043726,0.039348,0.041683,0.040646,0.041734,0.039237,0.038047,0.038629,0.039471,0.04164,0.040171,0.040883,0.040649,0.039999,0.03881,0.039787,0.041341,0.040987,0.039856,0.039811,0.041561,0.038944,0.039654,0.041141,0.03941,0.041621,0.039544,0.040899,0.039281,0.041665,0.042105,0.040771,0.041142,0.04159,0.040559,0.042155,0.041231,0.04056,0.039866,0.041194,0.040783,0.042319,0.041652,0.041097,0.040732,0.038948,0.038985,0.040742,0.039958,0.0395,0.037549,0.0432,0.041211,0.040696,0.039859,0.04139,0.041058,0.040285,0.039902,0.041816,0.038296,0.039943,0.04133,0.039162,0.04195,0.0402,0.040492,0.041082,0.039875,0.040324,0.038543,0.040496,0.039092,0.041868,0.042239,0.037824,0.040893,0.037992,0.039818,0.040017,0.039505,0.040396,0.039649,0.040593,0.040794,0.041952,0.039477,0.042327,0.042248,0.039227,0.040303,0.040211,0.040227,0.040942,0.04163,0.040556,0.04009,0.04079,0.040148,0.041054,0.041685,0.039693,0.042105,0.039416,0.03896,0.042472,0.0401,0.040313,0.040958,0.040644,0.041252,0.038757,0.041078
0.10423,0.10627,0.10546,0.10475,0.10666,0.10749,0.10741,0.10844,0.10654,0.1079,0.10786,0.10632,0.10916,0.10632,0.10717,0.11003,0.10592,0.10813,0.1062,0.10437,0.22417,0.19248,0.24636,0.25074,0.10924,0.10802,0.10534,0.10581,0.10916,0.10687,0.10839,0.10798,0.10956,0.10622,0.1059,0.10876,0.10312,0.10882,0.10339,0.32503,0.21926,0.23548,0.22653,0.24612,0.22285,0.23499,0.10667,0.10705,0.10973,0.10684,0.10549,0.10747,0.1054,0.1062,0.10733,0.10171,0.10738,0.10885,0.10607,0.10747,0.10406,0.3834,0.34883,0.22203,0.24041,0.22428,0.23337,0.221,0.22147,0.23015,0.22899,0.10635,0.10415,0.10825,0.10747,0.10521,0.10778,0.10804,0.10317,0.10585,0.10699,0.10881,0.10844,0.10671,0.10692,0.10783,0.33937,0.36149,0.33908,0.21141,0.23735,0.26877,0.21765,0.21424,0.21961,0.23051,0.23115,0.22974,0.10614,0.10781,0.10849,0.10622,0.1053,0.1045,0.10453,0.10515,0.10707,0.10778,0.10721,0.10741,0.10847,0.1098,0.10643,0.10883,0.34165,0.36477,0.33125,0.35091,0.23908,0.2345,0.22835,0.24797,0.24542,0.22297,

0.21566,0.20515,0.2352,0.20866,0.10932,0.10344,0.10829,0.1058,0.10841,
0.10507,0.10538,0.10746,0.10596,0.10673,0.1073,0.10936,0.10648,0.10917
,0.10583,0.10809,0.36045,0.37293,0.34987,0.34658,0.3397,0.35475,0.2532
,0.23485,0.23854,0.22807,0.22559,0.24047,0.2405,0.21615,0.22629,0.2692
8,0.10722,0.10786,0.10844,0.10334,0.10591,0.10412,0.10543,0.10577,0.10
772,0.10492,0.10625,0.1093,0.10602,0.10678,0.10659,0.10961,0.10717,0.3
6189,0.36346,0.35814,0.34649,0.3484,0.35504,0.25859,0.21402,0.21871,0.
24804,0.21888,0.24059,0.23601,0.2353,0.21461,0.24687,0.21486,0.10638,0
.10469,0.10375,0.10745,0.10745,0.10319,0.10647,0.10817,0.1102,0.10664,
0.10978,0.10836,0.10537,0.069183,0.075277,0.077966,0.10326,0.37156,0.3
8142,0.36428,0.37123,0.40574,0.38386,0.38622,0.20377,0.2879,0.24109,0.
27362,0.24252,0.25059,0.22166,0.23624,0.22617,0.24104,0.23256,0.10856,
0.1071,0.104,0.10809,0.10984,0.10696,0.10937,0.1083,0.10453,0.10594,0.
10716,0.072275,0.080609,0.07495,0.071804,0.073545,0.073123,0.10828,0.3
6755,0.36605,0.33281,0.34949,0.37082,0.37447,0.38651,0.23883,0.25185,0
.22898,0.2226,0.23123,0.27017,0.22001,0.22151,0.22882,0.22468,0.23347,
0.10611,0.10731,0.10963,0.10745,0.10668,0.10517,0.10813,0.10439,0.1045
4,0.071868,0.075052,0.070317,0.068693,0.073766,0.07245,0.077222,0.0735
92,0.067491,0.10659,0.37915,0.39279,0.36967,0.3964,0.39254,0.35482,0.3
3344,0.24629,0.23493,0.24296,0.27045,0.21422,0.24689,0.20928,0.2355,0.
22473,0.22044,0.23884,0.22328,0.10935,0.10511,0.10656,0.1084,0.10552,0
.10598,0.073991,0.073065,0.077828,0.067913,0.073328,0.074154,0.067303,
0.065077,0.069677,0.075366,0.071872,0.074513,0.33512,0.35885,0.36779,0
.36263,0.36157,0.37337,0.34652,0.22564,0.24161,0.24199,0.22472,0.22452
,0.24973,0.25985,0.23317,0.22633,0.24534,0.23622,0.26117,0.10868,0.104
31,0.10673,0.072922,0.060975,0.073515,0.064891,0.074803,0.07623,0.0755
84,0.071984,0.072527,0.066465,0.0726,0.078153,0.071846,0.073433,0.0734
28,0.37861,0.35288,0.35982,0.34362,0.37141,0.3605,0.36661,0.36633,0.38
265,0.23112,0.25002,0.24267,0.2578,0.23047,0.2428,0.2619,0.23236,0.260
87,0.22295,0.23728,0.07463,0.071529,0.07175,0.072338,0.080659,0.07468,
0.068949,0.073034,0.073574,0.074038,0.075299,0.08008,0.073518,0.077325
,0.070046,0.071328,0.068859,0.075626,0.3375,0.36113,0.36292,0.36051,0.
33277,0.36962,0.3625,0.35966,0.33905,0.2132,0.23732,0.15782,0.16159,0.
167,0.18696,0.15621,0.17202,0.1606,0.17146,0.17046,0.071817,0.075619,0
.077687,0.073786,0.072466,0.070494,0.074347,0.071642,0.075166,0.07245,
0.073828,0.071387,0.077805,0.066358,0.07506,0.073191,0.074407,0.075475
,0.34774,0.33952,0.34027,0.37202,0.37354,0.39664,0.38209,0.38452,0.337
87,0.20653,0.16309,0.14155,0.17702,0.16382,0.17221,0.17954,0.15435,0.1
2861,0.16046,0.16272,0.07238,0.075888,0.075327,0.072286,0.070773,0.075
565,0.080138,0.075036,0.075593,0.075928,0.056392,0.073964,0.074555,0.0
73528,0.073427,0.074934,0.071665,0.072645,0.35807,0.36581,0.36608,0.35
3,0.33212,0.36044,0.34863,0.21206,0.22539,0.18724,0.15009,0.19676,0.15
736,0.15167,0.15705,0.17796,0.17392,0.18148,0.1637,0.17525,0.072455,0.
073536,0.078643,0.075614,0.073731,0.077085,0.073888,0.069394,0.072101,
0.074822,0.073146,0.072982,0.073957,0.086253,0.070983,0.07456,0.075197
,0.084971,0.35981,0.36246,0.35025,0.3843,0.3504,0.36493,0.19251,0.2706
5,0.17215,0.23021,0.17133,0.16821,0.16446,0.16656,0.11975,0.15951,0.17
018,0.16306,0.16904,0.1739,0.070758,0.073242,0.080373,0.069285,0.07570
2,0.077662,0.072414,0.073736,0.067838,0.087727,0.077192,0.077416,0.074
746,0.071569,0.068673,0.068227,0.071985,0.077948,0.38555,0.35604,0.358
6,0.35238,0.3667,0.20101,0.20971,0.23532,0.18554,0.19624,0.18902,0.172
47,0.16564,0.18357,0.16874,0.16554,0.1989,0.15843,0.15426,0.12655,0.07

4954,0.078014,0.080554,0.071272,0.077221,0.073587,0.07247,0.070636,0.0
7674,0.074317,0.074788,0.072917,0.074685,0.06933,0.076475,0.073754,0.0
72307,0.086782,0.32844,0.34972,0.33454,0.17279,0.20856,0.21367,0.16949
,0.16956,0.20354,0.1859,0.18406,0.16871,0.16925,0.1389,0.17578,0.15325
,0.16255,0.17912,0.18428,0.17293,0.08048,0.074366,0.071175,0.074307,0.
075389,0.071759,0.069827,0.069588,0.073966,0.076077,0.073292,0.066607,
0.076454,0.073571,0.072897,0.071282,0.078696,0.075864,0.3682,0.21474,0
.18508,0.20064,0.23464,0.20743,0.2022,0.1911,0.20956,0.23348,0.15729,0
.15076,0.17455,0.16307,0.19457,0.16444,0.17811,0.16779,0.16087,0.07306
8,0.074405,0.067572,0.068613,0.072718,0.071496,0.071313,0.082633,0.077
686,0.072414,0.067003,0.075351,0.074608,0.075423,0.073807,0.073037,0.0
71481,0.039499,0.20611,0.1594,0.19789,0.1937,0.19526,0.20163,0.18961,0
.18926,0.13833,0.22659,0.17874,0.17837,0.18285,0.1706,0.12921,0.17452,
0.16135,0.17079,0.18773,0.072337,0.063543,0.070395,0.072268,0.087718,0
.057651,0.07629,0.072518,0.073559,0.077187,0.074453,0.074832,0.070154,
0.075731,0.078457,0.073495,0.070862,0.040136,0.21642,0.19419,0.19313,0
.20369,0.26051,0.23102,0.21091,0.1903,0.15117,0.2078,0.18526,0.15186,0
.16555,0.15664,0.17327,0.18444,0.17043,0.17498,0.072206,0.07385,0.0698
17,0.079151,0.077072,0.070314,0.074146,0.07185,0.063563,0.07627,0.0753
53,0.073384,0.074385,0.072667,0.073073,0.074932,0.075187,0.073789,0.03
903,0.21758,0.14861,0.22933,0.20524,0.20212,0.19766,0.18875,0.18423,0.
16946,0.20055,0.16347,0.16758,0.18015,0.14061,0.16804,0.1431,0.16714,0
.17614,0.072713,0.074257,0.076535,0.071866,0.072336,0.071667,0.070706,
0.070105,0.078158,0.074944,0.075456,0.073811,0.06937,0.073075,0.073411
,0.074786,0.077664,0.038377,0.040198,0.03904,0.23112,0.17585,0.18363,0
.20478,0.17295,0.19177,0.20928,0.18009,0.22992,0.17422,0.14503,0.15176
,0.15422,0.16826,0.16424,0.075221,0.08022,0.071687,0.075249,0.073641,0
.070134,0.072397,0.067374,0.071986,0.068351,0.076446,0.075137,0.082282
,0.076833,0.078627,0.074899,0.069528,0.074083,0.040837,0.04111,0.04121
,0.040779,0.19197,0.2046,0.20785,0.19602,0.2289,0.21277,0.18458,0.1875
2,0.1864,0.17561,0.17337,0.18032,0.077819,0.072575,0.091542,0.082774,0
.077797,0.071414,0.073983,0.07283,0.076269,0.073802,0.072473,0.076568,
0.074296,0.074272,0.069788,0.076007,0.07274,0.07862,0.072562,0.064843,
0.040216,0.040322,0.041273,0.04237,0.037649,0.039711,0.19897,0.1602,0.
20177,0.19485,0.18395,0.23754,0.22822,0.13665,0.075177,0.06677,0.07156
1,0.072862,0.074516,0.077113,0.086503,0.070898,0.075622,0.077229,0.072
017,0.074761,0.070491,0.0717,0.069281,0.073364,0.072877,0.075881,0.072
835,0.072551,0.07611,0.083907,0.075375,0.0404,0.039761,0.040521,0.0386
68,0.03748,0.041376,0.19281,0.13981,0.19857,0.19348,0.18121,0.038828,0
.041324,0.073879,0.080505,0.071808,0.073965,0.073569,0.074782,0.073831
,0.071972,0.069349,0.074851,0.077862,0.07438,0.072417,0.077861,0.07505
1,0.080809,0.062192,0.073953,0.073652,0.082705,0.071966,0.041171,0.039
533,0.040025,0.039813,0.040231,0.037657,0.039539,0.040546,0.039321,0.0
39124,0.19831,0.1984,0.041452,0.041808,0.038114,0.039607,0.075287,0.07
6602,0.058013,0.080084,0.071507,0.075938,0.075313,0.071821,0.077821,0.
077763,0.072341,0.068284,0.079647,0.074117,0.084756,0.067456,0.070583,
0.072642,0.075287,0.041949,0.040049,0.041142,0.037231,0.040133,0.04130
3,0.038773,0.038796,0.039321,0.038791,0.040112,0.039283,0.040065,0.041
488,0.039386,0.040663,0.037657,0.070954,0.080765,0.078707,0.068545,0.0
7993,0.07248,0.074551,0.080138,0.068478,0.081128,0.074005,0.073323,0.0
73485,0.08249,0.082273,0.073573,0.039436,0.04313,0.03752,0.038978,0.04
1337,0.041382,0.042336,0.041321,0.041198,0.041085,0.039205,0.041802,0.

040572,0.040897,0.038907,0.039343,0.039804,0.042248,0.039516,0.072806,
0.073445,0.073259,0.070064,0.074842,0.074874,0.073004,0.07297,0.074231
,0.069027,0.069826,0.071173,0.072095,0.039075,0.040246,0.040201,0.0395
3,0.040403,0.039921,0.038753,0.040557,0.039566,0.040083,0.039352,0.038
806,0.040551,0.03913,0.03876,0.039451,0.041965,0.040944,0.039129,0.042
217,0.042663,0.072576,0.08413,0.07608,0.071789,0.072042,0.074209,0.076
981,0.08262,0.068827,0.040793,0.040207,0.040153,0.039827,0.038887,0.04
1849,0.040194,0.039745,0.040187,0.040645,0.040066,0.039526,0.039638,0.
040563,0.039969,0.039759,0.039168,0.040036,0.039397,0.040892,0.040986,
0.038603,0.039914,0.039549,0.040991,0.069294,0.075858,0.069543,0.07497
5,0.038879,0.042161,0.040876,0.042076,0.040293,0.03883,0.040486,0.0389
34,0.04149,0.038246,0.039647,0.038003,0.041692,0.038707,0.040538,0.039
491,0.040431,0.038697,0.040191,0.03846,0.040876,0.041696,0.040323,0.03
9172,0.04092,0.039243,0.040461,0.04021,0.039669,0.042169,0.038178,0.04
1426,0.038779,0.040575,0.038335,0.04007,0.042754,0.039028,0.03734,0.03
9491,0.040122,0.042569,0.040884,0.039432,0.039537,0.038306,0.039616,0.
040293,0.041555,0.03866,0.037799,0.038884,0.039739,0.039509,0.039884,0
.040639,0.041021,0.03923,0.042178,0.040848,0.043781,0.039323,0.038515,
0.039632,0.036949,0.038804,0.04055,0.040684,0.038956,0.040553,0.040615
,0.039438,0.040555,0.039894,0.039716,0.04229,0.038994,0.041701,0.04009
5,0.042203,0.040372,0.038992,0.043299,0.038613,0.042219,0.040482,0.040
574,0.039248,0.037413,0.037953,0.039172,0.042311,0.03935,0.040573,0.04
0318,0.039727,0.037043,0.039285,0.041158,0.041161,0.039662,0.039409,0.
041615,0.038302,0.040175,0.040509,0.038386,0.041317,0.038646,0.039748,
0.039363,0.041454,0.040699,0.040252,0.040676,0.041698,0.040841,0.04093
8,0.041127,0.039823,0.039862,0.040769,0.040524,0.042593,0.041019,0.040
303,0.040163,0.039912,0.03811,0.040183,0.039619,0.040609,0.03674,0.044
237,0.041127,0.038988,0.039029,0.040696,0.040595,0.040826,0.039943,0.0
41686,0.038839,0.040084,0.040497,0.03956,0.041159,0.038933,0.039868,0.
040358,0.038983,0.040751,0.03819,0.040878,0.038081,0.041857,0.041692,0
.037738,0.039775,0.037707,0.039631,0.038779,0.03813,0.039962,0.038713,
0.039676,0.040739,0.04159,0.038405,0.041933,0.041743,0.039552,0.040065
,0.040644,0.040151,0.040931,0.041523,0.039855,0.039484,0.040007,0.0406
09,0.040352,0.041083,0.038796,0.041217,0.039432,0.038707,0.042097,0.03
9097,0.04047,0.041183,0.040487,0.040451,0.037417,0.040234

#simdataS3SNR20:

Kep_f=1.2
Kep_f=1.2
Kep_s=0.5
Ktrans_f=0.05
Ktrans_s=0.06
del_t=0.5
gtSf=0 0 0 0 0 0
0 0 0 0 0 0
0 0 0 0 0 0
0 0 0 0 0 0
0 0 0 0 0 0
0 0 0 0 0 0
0 0 0 0 0 0

0	0	0	0	0	0
0	0	0	0	0	0
0	0	0	0	0	0
0	0	0	0	0	0
0	0	0	0	0	0
0	0	0	0	0	0
0	0	0	0	0	0
0	0	0	0	0	0
0	0	0	0	0	0
0	0	0	0	0	0
0	0	0	0	0	0
0	0	0	0	0	0
0	0	0	0	0	0
0	0	0	0	0	0
0	0	0	0	0	0
0	0	0	0	0	0
0	0	0	0	0	0
0	0	0	0	0	0
0	0	0	0	0	0
0	0	0	0	0	0
0	0	0	0	0	0
0	0	0	0	0	0
0	0	0	0	0	0
0	0	0	0	0	0
0	0	0	0	0	0
0	0	0	0	0	0
0	0	0	0	0	0
0	0	0	0.5	0.66771	0.39998
0	0	0	0	0	0
0	0	0	0	0	0
0	0	0	0	0	0
0	0	0	0	0	0
0	0	0	0	0	0
0.51942	0.56773	0.4262	0.71832	0.57041	0.48816
0	0	0	0	0	0
0	0	0	0	0	0
0	0	0	0	0	0
0	0	0	0	0	0
0	0	0	0	0.60091	0.49167
0.51161	0.58172	0.37807	0.55977	0.5	0.5
0.35561	0	0	0	0	0
0	0	0	0	0	0
0	0	0	0	0	0
0	0	0	0	0	0
0	0	0	0.42832	0.51597	0.51807
0.4694	0.49592	0.54335	0.46458	0.45468	0.47077
0.5	0.50561	0.5	0	0	0
0	0	0	0	0	0
0	0	0	0	0	0
0	0	0	0	0	0
0	0.42913	0.48998	0.46229	0.50989	0.61649
0.37922	0.55869	0.57222	0.5	0.53449	0.5

0.5	0.47625	0.59491	0.4675	0	0
0	0	0	0	0	0
0	0	0	0	0	0
0	0	0	0	0	0
0.44539	0.63258	0.54562	0.46147	0.52795	0.53033
0.51434	0.5	0.53699	0.50162	0.55101	0.50102
0.4872	0.5	0.5024	0.49392	0.54823	0.56679
0	0	0	0	0	0
0	0	0	0	0	0.33333
0.36045	0.33254	0.33333	0.31483	0.33333	0.35352
0.2958	0.33457	0.5	0.52409	0.47566	0.4918
0.47211	0.56883	0.5	0.63945	0.44411	0.57223
0.42274	0.47471	0.5	0.53171	0.5	0.42856
0.46292	0.51891	0	0	0	0
0	0	0	0	0	0.47639
0.36443	0.34415	0.38149	0.34935	0.33999	0.34695
0.27662	0.29544	0.33731	0.31203	0.41929	0.64051
0.5	0.58852	0.45171	0.49619	0.5	0.63773
0.51358	0.50312	0.31188	0.52043	0.5	0.52639
0.38596	0.43564	0.5	0.52439	0	0
0	0	0	0	0	0.58291
0.5529	0.5	0.34739	0.41405	0.18561	0.42715
0.33767	0.35601	0.2842	0.34627	0.30608	0.35819
0.49305	0.5	0.52165	0.52585	0.54942	0.56703
0.41442	0.45258	0.50427	0.50578	0.53239	0.5
0.5	0.5	0.44702	0.5	0.62406	0.52734
0	0	0	0	0	0
0.51448	0.47413	0.53609	0.49051	0.49635	0.35303
0.39567	0.37013	0.38022	0.36257	0.28305	0.38464
0.39266	0.28355	0.36739	0.51942	0.5	0.40079
0.59	0.44178	0.5	0.50851	0.54461	0.52521
0.5	0.49998	0.41896	0.51886	0.55148	0.43023
0.39862	0.5	0	0	0	0
0	0.35644	0.5	0.65417	0.52498	0.40666
0.45882	0.32158	0.39046	0.32688	0.35308	0.33333
0.33333	0.31141	0.31773	0.26491	0.51537	0.5
0.5	0.42774	0.53229	0.5	0.5	0.5917
0.38462	0.49609	0.44422	0.57905	0.5415	0.35254
0.55072	0.55507	0.5	0.56795	0	0
0	0.61725	0.48066	0.52411	0.58943	0.5
0.49686	0.53775	0.5	0.35994	0.34149	0.32952
0.29964	0.33949	0.34958	0.35264	0.37488	0.29503
0.60149	0.59889	0.4867	0.41857	0.5	0.5
0.46238	0.5	0.60468	0.48365	0.55041	0.4847
0.40507	0.49617	0.48159	0.49893	0.50041	0.5
0	0.54821	0.5	0.47587	0.49856	0.36174
0.62139	0.49741	0.52998	0.39218	0.33333	0.33081
0.35209	0.3541	0.29478	0.34211	0.30152	0.36252
0.33455	0.5	0.50514	0.58108	0.5	0.5
0.54697	0.56266	0.40991	0.39531	0.58902	0.5
0.5	0.55949	0.46914	0.58549	0.43427	0.34358
1	0.50491	0.5	0.5	0.4949	0.5

0.46648	0.52504	0.40941	0.60995	0.42063	0.41417
0.36835	0.33507	0.33333	0.36138	0.35261	0.29451
0.34444	0.39094	0.5	0.53616	0.45665	0.5
0.41156	0.5259	0.51761	0.5	0.47277	0.5
0.5	0.60625	0.658	0.5	0.5	0.56607
0.56615	1	0.38582	0.5	0.5	0.45139
0.48751	0.48194	0.51921	0.49176	0.52542	0.51179
0.33333	0.2181	0.30592	0.33418	0.26427	0.33333
0.40431	0.3728	0.56999	0.53641	0.55726	0.55579
0.5	0.5	0.45132	0.5	0.46311	0.55202
0.17697	0.5576	0.43777	0.4486	0.48786	0.62278
0.5	0.5	1	0.37772	0.5	0.59003
0.39599	0.55476	0.5	0.51728	0.53677	0.46786
0.46271	0.33333	0.37163	0.3142	0.28273	0.31418
0.23489	0.36846	0.34616	0.57936	0.5372	0.40531
0.5	0.71277	0.45083	0.5	0.40184	0.54253
0.40152	0.44906	0.38569	0.46265	0.54369	0.5
0.56468	0.57708	1	1	1	0.54053
0.53013	0.51679	0.48076	0.38498	0.5319	0.5
0.48592	0.5	0.34602	0.24772	0.33004	0.29655
0.32256	0.3732	0.48384	0.5	0.5	0.573
0.49375	0.44304	0.61127	0.5	0.34187	0.5
0.51194	0.50282	0.5	0.5	0.30612	0.41072
0.5338	0.52113	1	1	1	1
0.6046	0.62804	0.35479	0.4524	0.43578	0.48426
0.49099	0.77729	0.49809	0.25233	0.39275	0.33333
0.50779	0.5	0.56327	0.55475	0.54629	0.50744
0.49754	0.47935	0.55074	0.57974	0.5	0.432
0.57592	0.51053	0.49131	0.5	0.44881	0.55814
0.4096	0.49993	1	1	1	1
1	1	0.39444	0.47807	0.5	0.47352
0.45017	0.33081	0.49428	0.52105	0.5	0.41036
0.60726	0.41241	0.59118	0.5	0.5	0.55047
0.45859	0.49929	0.48164	0.46153	0.51583	0.50318
0.50251	0.5	0.55028	0.47691	0.51884	0.31824
0.57687	0.49392	0.5	1	1	1
1	1	1	0.48931	0.51802	0.5
0.43278	0.5	1	1	0.56676	0.53575
0.53182	0.50779	0.53679	0.5	0.49251	0.5
0.52155	0.47282	0.48458	0.5	0.53857	0.60452
0.43809	0.436	0.38469	0.5	0.44838	0.5
0.5	1	1	1	1	1
1	1	1	1	1	0.44974
0.47747	1	1	1	1	0.5087
0.5	0.54631	0.44851	0.55075	0.40081	0.48689
0.54337	0.5	0.49638	0.48036	0.39925	0.45674
0.5	0.54328	0.55434	0.5	0.46298	0.52429
1	1	1	1	1	1
1	1	1	1	1	1
1	1	1	1	1	0.5
0.53601	0.5	0.5	0.52829	0.5	0.47724
0.52247	0.5	0.38797	0.56495	0.44273	0.49164

```
0.44278     0.44379         0.5           1          1          1
1           1           1           1           1           1
1           1           1           1           1           1
1           1           1           1     0.44076         0.5
0.57302     0.57081     0.50978     0.59587     0.48268     0.57101
0.5      0.6081     0.55143     0.48138         0.5           1
1           1           1           1           1           1
1           1           1           1           1           1
1           1           1           1           1           1
1           1     0.54058         0.5     0.54283     0.51726
0.46698     0.50217         0.5      0.4333     0.45727           1
1           1           1           1           1           1
1           1           1           1           1           1
1           1           1           1           1           1
1           1           1           1           1           1
0.44386         0.5     0.49596         0.5           1           1
1           1           1           1           1           1
1           1           1           1           1           1
1           1           1           1           1           1
1           1           1           1           1           1
1           1           1           1           1           1
1           1           1           1           1           1
1           1           1           1           1           1
1           1           1           1           1           1
1           1           1           1           1           1
1           1           1           1           1           1
1           1           1           1           1           1
1           1           1           1           1           1
1           1           1           1           1           1
1           1           1           1           1           1
1           1           1           1           1           1
1           1           1           1           1           1
1           1           1           1           1           1
1           1           1           1           1           1
1           1           1           1           1           1
1           1           1           1           1           1
1           1           1           1           1           1
1           1           1           1           1           1
1           1           1           1           1           1
1           1           1           1           1           1
1           1           1           1           1           1
1           1           1           1           1           1
1           1           1           1           1           1
1           1           1           1           1           1
1           1           1           1           1           1
1           1           1           1           1           1
1           1           1           1           1           1
1           1           1           1           1           1
1
gtSs=1          1           1           1           1           1
1           1           1           1           1           1
1           1           1           1           1           1
```

1 1 0.5 0.64461 0.57444 0.55629
1 1 1 1 1 1
1 1 1 1 1 1
1 1 1 0 0.41079 0.55446
0.44996 0.55644 0.5 0.49659 1 1
1 1 1 1 1 1
1 1 1 1 1 1
1 0 0 0.53247 0.34808 0.49529
0.48233 0.39892 0.34138 0.53793 0.44239 1
1 1 1 1 1 1
1 1 1 1 1 1
1 1 0 0 0 0.47124
0.55792 0.45959 0.4019 0.60403 0.5 0.52563
0.44276 0.5 1 1 1 1
1 1 1 1 1 1
1 1 1 1 1 1
0 0 0 0 0.5 0.5
0.49095 0.51536 0.4116 0.5 0.51936 0.5
0.44619 0.47563 1 1 1 1
1 1 1 1 1 1
1 1 1 1 1 1
0 0 0 0 0 0
0.5 0.52101 0.55424 0.50117 0.51471 0.5
0.51297 0.5 0.49215 0.43785 1 1
1 1 1 1 1 1
1 1 1 1 1 1
1 1 1 0 0 0
0 0 0 0.53762 0.456 0.50768
0.5 0.5675 0.5 0.54921 0.72656 0.54783
0.44906 0.5 1 1 1 1
1 1 1 1 1 1
1 1 1 0.5 0.33229 0.60002
1 0 0 0 0 0
0 0 0.5 0.50049 0.50129 0.55446
0.51426 0.5 0.58112 0.5 0.45363 0.45385
0.46534 1 1 1 1 1
1 1 1 1 1 1
0.48058 0.43227 0.5738 0.28168 0.42959 0.51184
1 0 0 0 0 0
0 0 0.47146 0.46266 0.5 0.5
0.58736 0.54818 0.56718 0.52179 0.4409 0.35145
0.55305 1 1 1 1 1
1 1 1 1 0.39909 0.50833
0.48839 0.41828 0.62193 0.44023 0.5 0.5
0.64439 1 0 0 0 0
0 0 0 0.33206 0.51218 0.49852
0.5 0.47755 0.45008 0.47003 0.47987 0.49462
0.55612 0.5207 0.5 1 1 1
1 1 1 0.57168 0.48403 0.48193
0.5306 0.50408 0.45665 0.53542 0.54532 0.52923
0.5 0.49439 0.5 0 0 0
0 0 0 0 0.6163 0.60815

0.64255	0.48212	0.5	0.5	0.53129	0.5
0.51981	0.55202	0.51183	0.39454	1	1
1	0.57087	0.51002	0.53771	0.49011	0.38351
0.62078	0.44131	0.42778	0.5	0.46551	0.5
0.5	0.52375	0.40509	0.5325	0	0
0	0	0	0	0	0
0	0.50281	0.54925	0.5145	0.52108	0.59692
0.5	0.46089	0.34493	0.49287	0.55843	0.57496
0.55461	0.36742	0.45438	0.53853	0.47205	0.46967
0.48566	0.5	0.46301	0.49838	0.44899	0.49898
0.5128	0.5	0.4976	0.50608	0.45177	0.43321
0	0	0	0	0	0
0	0	0	0.52496	0.5	0.33333
0.3156	0.28396	0.33333	0.34259	0.33333	0.29296
0.3521	0.33087	0.5	0.47591	0.52434	0.5082
0.52789	0.43117	0.5	0.36055	0.55589	0.42777
0.57726	0.52529	0.5	0.46829	0.5	0.57144
0.53708	0.48109	0	0	0	0
0	0	0	0	0	0
0.25117	0.32075	0.2973	0.27548	0.32002	0.34695
0.34078	0.35228	0.32538	0.34398	0.58071	0.35949
0.5	0.41148	0.54829	0.50381	0.5	0.36227
0.48642	0.49688	0.68812	0.47957	0.5	0.47361
0.61404	0.56436	0.5	0.47561	0	0
0	0	0	0	0	0
0	0	0.30521	0.21024	0.42836	0.30778
0.33767	0.28799	0.38832	0.34627	0.34696	0.35819
0.50695	0.5	0.47835	0.47415	0.45058	0.43297
0.58558	0.54742	0.49573	0.49422	0.46761	0.5
0.5	0.5	0.55298	0.5	0.37594	0.47266
0	0	0	0	0	0
0	0	0	0	0	0.34251
0.29369	0.25974	0.28997	0.32313	0.35847	0.23071
0.33325	0.33967	0.63261	0.48058	0.5	0.59921
0.41	0.55822	0.5	0.49149	0.45539	0.47479
0.5	0.50002	0.58104	0.48114	0.44852	0.56977
0.60138	0.5	0	0	0	0
0	0	0	0	0	0
0	0.32386	0.39046	0.31989	0.29384	0.33333
0.33333	0.37236	0.349	0.36755	0.48463	0.5
0.5	0.57226	0.46771	0.5	0.5	0.4083
0.61538	0.50391	0.55578	0.42095	0.4585	0.64746
0.44928	0.44493	0.5	0.43205	0	0
0	0	0	0	0	0
0	0	0	0.35994	0.34149	0.30779
0.35018	0.32103	0.41327	0.29472	0.34855	0.36429
0.39851	0.40111	0.5133	0.58143	0.5	0.5
0.53762	0.5	0.39532	0.51635	0.44959	0.5153
0.59493	0.50383	0.51841	0.50107	0.49959	0.5
0	0	0	0	0	0
0	0	0	0	0.33333	0.48091
0.26926	0.34607	0.33743	0.34211	0.34326	0.41113

0.33455	0.5	0.49486	0.41892	0.5	0.5
0.45303	0.43734	0.59009	0.60469	0.41098	0.5
0.5	0.44051	0.53086	0.41451	0.56573	0.65642
0	0	0	0	0	0
0	0	0	0	0	0.17167
0.26329	0.32473	0.33333	0.37913	0.33296	0.35275
0.33206	0.39094	0.5	0.46384	0.54335	0.5
0.58844	0.4741	0.48239	0.5	0.52723	0.5
0.5	0.39375	0.342	0.5	0.5	0.43393
0.43385	0	0	0	0	0
0	0	0	0	0	0
0.33333	0.39095	0.29092	0.33164	0.36787	0.33333
0.35004	0.25441	0.43001	0.46359	0.44274	0.44421
0.5	0.5	0.54868	0.5	0.53689	0.44798
0.82303	0.4424	0.56223	0.5514	0.51214	0.37722
0.5	0.5	0	0	0	0
0	0	0	0	0	0
0	0.33333	0.2698	0.33791	0.37564	0.34291
0.38965	0.22085	0.30768	0.42064	0.4628	0.59469
0.5	0.28723	0.54917	0.5	0.59816	0.45747
0.59848	0.55094	0.61431	0.53735	0.45631	0.5
0.43532	0.42292	0	0	0	0
0	0	0	0	0	0
0	0	0.34602	0.37614	0.33498	0.26036
0.30202	0.32677	0.51616	0.5	0.5	0.427
0.50625	0.55696	0.38873	0.5	0.65813	0.5
0.48806	0.49718	0.5	0.5	0.69388	0.58928
0.4662	0.47887	0	0	0	0
0	0	0	0	0	0
0	0	0	0.34251	0.38419	0.33333
0.49221	0.5	0.43673	0.44525	0.45371	0.49256
0.50246	0.52065	0.44926	0.42026	0.5	0.568
0.42408	0.48947	0.50869	0.5	0.55119	0.44186
0.5904	0.50007	0	0	0	0
0	0	0	0	0	0
0	0	0	0	0.5	0.58964
0.39274	0.58759	0.40882	0.5	0.5	0.44953
0.54141	0.50071	0.51836	0.53847	0.48417	0.49682
0.49749	0.5	0.44972	0.52309	0.48116	0.68176
0.42313	0.50608	0.5	0	0	0
0	0	0	0	0	0
0	0	0	0	0.43324	0.46425
0.46818	0.49221	0.46321	0.5	0.50749	0.5
0.47845	0.52718	0.51542	0.5	0.46143	0.39548
0.56191	0.564	0.61531	0.5	0.55162	0.5
0.5	0	0	0	0	0
0	0	0	0	0	0
0	0	0	0	0	0.4913
0.5	0.45369	0.55149	0.44925	0.59919	0.51311
0.45663	0.5	0.50362	0.51964	0.60075	0.54326
0.5	0.45672	0.44566	0.5	0.53702	0.47571
0	0	0	0	0	0

0	0	0	0	0	0
0	0	0	0	0	0.5
0.46399	0.5	0.5	0.47171	0.5	0.52276
0.47753	0.5	0.61203	0.43505	0.55727	0.50836
0.55722	0.55621	0.5	0	0	0
0	0	0	0	0	0
0	0	0	0	0	0
0	0	0	0	0.55924	0.5
0.42698	0.42919	0.49022	0.40413	0.51732	0.42899
0.5	0.3919	0.44857	0.51862	0.5	0
0	0	0	0	0	0
0	0	0	0	0	0
0	0	0	0	0	0
0	0	0.45942	0.5	0.45717	0.48274
0.53302	0.49783	0.5	0.5667	0.54273	0
0	0	0	0	0	0
0	0	0	0	0	0
0	0	0	0	0	0
0	0	0	0	0	0
0.55614	0.5	0.50404	0.5	0	0
0	0	0	0	0	0
0	0	0	0	0	0
0	0	0	0	0	0
0	0	0	0	0	0
0	0	0	0	0	0
0	0	0	0	0	0
0	0	0	0	0	0
0	0	0	0	0	0
0	0	0	0	0	0
0	0	0	0	0	0
0	0	0	0	0	0
0	0	0	0	0	0
0	0	0	0	0	0
0	0	0	0	0	0
0	0	0	0	0	0
0	0	0	0	0	0
0	0	0	0	0	0
0	0	0	0	0	0
0	0	0	0	0	0
0	0	0	0	0	0
0	0	0	0	0	0
0	0	0	0	0	0
0	0	0	0	0	0
0	0	0	0	0	0
0	0	0	0	0	0
0	0	0	0	0	0
0	0	0	0	0	0
0	0	0	0	0	0
0	0	0	0	0	0
0	0	0	0	0	0
0	0	0	0	0	0

0 0 0 0 0 0
0
gtSp=0 0 0 0 0 0
0 0 0 0 0 0
0 0 0 0 0 0
0 0 0.5 0.35539 0.42556 0.44371
0 0 0 0 0 0
0 0 0 0 0 0
0 0 0 1 0.58921 0.44554
0.55004 0.44356 0.5 0.50341 0 0
0 0 0 0 0 0
0 0 0 0 0 0
0 1 1 0.46753 0.65192 0.50471
0.51767 0.60108 0.65862 0.46207 0.55761 0
0 0 0 0 0 0
0 0 0 0 0 0
0 0 1 1 1 0.52876
0.44208 0.54041 0.5981 0.39597 0.5 0.47437
0.55724 0.5 0 0 0 0
0 0 0 0 0 0
0 0 0 0 0 0
1 1 1 1 0.5 0.5
0.50905 0.48464 0.5884 0.5 0.48064 0.5
0.55381 0.52437 0 0 0 0
0 0 0 0 0 0
0 0 0 0 0 0
1 1 1 1 1 1
0.5 0.47899 0.44576 0.49883 0.48529 0.5
0.48703 0.5 0.50785 0.56215 0 0
0 0 0 0 0 0
0 0 0 0 0 0
0 0 0 1 1 1
1 1 1 0.46238 0.544 0.49232
0.5 0.4325 0.5 0.45079 0.27344 0.45217
0.55094 0.5 0 0 0 0
0 0 0 0 0 0
0 0 0 0 0 0
0 1 1 1 1 1
1 1 0.5 0.49951 0.49871 0.44554
0.48574 0.5 0.41888 0.5 0.54637 0.54615
0.53466 0 0 0 0 0
0 0 0 0 0 0
0 0 0 0 0 0
0 1 1 1 1 1
1 1 0.52854 0.53734 0.5 0.5
0.41264 0.45182 0.43282 0.47821 0.5591 0.64855
0.44695 0 0 0 0 0
0 0 0 0 0 0
0 0 0 0 0 0
0 0 1 1 1 1
1 1 1 0.66794 0.48782 0.50148
0.5 0.52245 0.54992 0.52997 0.52013 0.50538

0.44388	0.4793	0.5	0	0	0
0	0	0	0	0	0
0	0	0	0	0	0
0	0	0	1	1	1
1	1	1	1	0.3837	0.39185
0.35745	0.51788	0.5	0.5	0.46871	0.5
0.48019	0.44798	0.48817	0.60546	0	0
0	0	0	0	0	0
0	0	0	0	0	0
0	0	0	0	1	1
1	1	1	1	1	1
1	0.49719	0.45075	0.4855	0.47892	0.40308
0.5	0.53911	0.65507	0.50713	0.44157	0.42504
0	0	0	0	0	0
0	0	0	0	0	0
0	0	0	0	0	0
1	1	1	1	1	1
1	1	1	0.47504	0.5	0.33333
0.32395	0.3835	0.33333	0.34259	0.33333	0.35352
0.3521	0.33457	0	0	0	0
0	0	0	0	0	0
0	0	0	0	0	0
0	0	1	1	1	1
1	1	1	1	1	0.52361
0.3844	0.3351	0.32121	0.37518	0.33999	0.30611
0.3826	0.35228	0.33731	0.34398	0	0
0	0	0	0	0	0
0	0	0	0	0	0
0	0	0	0	1	1
1	1	1	1	1	0.41709
0.4471	0.5	0.34739	0.3757	0.38603	0.26507
0.32466	0.35601	0.32749	0.30747	0.34696	0.28361
0	0	0	0	0	0
0	0	0	0	0	0
0	0	0	0	0	0
1	1	1	1	1	1
0.48552	0.52587	0.46391	0.50949	0.50365	0.30446
0.31064	0.37013	0.32981	0.3143	0.35847	0.38464
0.27408	0.37679	0	0	0	0
0	0	0	0	0	0
0	0	0	0	0	0
0	0	1	1	1	1
1	0.64356	0.5	0.34583	0.47502	0.59334
0.54118	0.35456	0.21908	0.35323	0.35308	0.33333
0.33333	0.31623	0.33327	0.36755	0	0
0	0	0	0	0	0
0	0	0	0	0	0
0	0	0	0	1	1
1	0.38275	0.51934	0.47589	0.41057	0.5
0.50314	0.46225	0.5	0.28012	0.31702	0.36269
0.35018	0.33949	0.23716	0.35264	0.27657	0.34068
0	0	0	0	0	0

0	0	0	0	0	0
0	0	0	0	0	0
1	0.45179	0.5	0.52413	0.50144	0.63826
0.37861	0.50259	0.47002	0.60782	0.33333	0.18828
0.37865	0.29983	0.36778	0.31578	0.35522	0.22635
0.33091	0	0	0	0	0
0	0	0	0	0	0
0	0	0	0	0	0
0	0.49509	0.5	0.5	0.5051	0.5
0.53352	0.47496	0.59059	0.39005	0.57937	0.41417
0.36835	0.3402	0.33333	0.2595	0.31443	0.35275
0.3235	0.21812	0	0	0	0
0	0	0	0	0	0
0	0	0	0	0	0
0	0	0.61418	0.5	0.5	0.54861
0.51249	0.51806	0.48079	0.50824	0.47458	0.48821
0.33333	0.39095	0.40316	0.33418	0.36787	0.33333
0.24565	0.3728	0	0	0	0
0	0	0	0	0	0
0	0	0	0	0	0
0	0	0	0.62228	0.5	0.40997
0.60401	0.44524	0.5	0.48272	0.46323	0.53214
0.53729	0.33333	0.35857	0.34789	0.34163	0.34291
0.37546	0.41069	0.34616	0	0	0
0	0	0	0	0	0
0	0	0	0	0	0
0	0	0	0	0	0.45947
0.46987	0.48321	0.51924	0.61502	0.4681	0.5
0.51408	0.5	0.30795	0.37614	0.33498	0.44309
0.37542	0.30002	0	0	0	0
0	0	0	0	0	0
0	0	0	0	0	0
0	0	0	0	0	0
0.3954	0.37196	0.64521	0.5476	0.56422	0.51574
0.50901	0.22271	0.50191	0.40516	0.22306	0.33333
0	0	0	0	0	0
0	0	0	0	0	0
0	0	0	0	0	0
0	0	0	0	0	0
0	0	0.60556	0.52193	0.5	0.52648
0.54983	0.66919	0.50572	0.47895	0	0
0	0	0	0	0	0
0	0	0	0	0	0
0	0	0	0	0	0
0	0	0	0	0	0
0	0	0	0.51069	0.48198	0.5
0.56722	0.5	0	0	0	0
0	0	0	0	0	0
0	0	0	0	0	0
0	0	0	0	0	0
0	0	0	0	0	0
0	0	0	0	0	0.55026

0.52253	0	0	0	0	0
0	0	0	0	0	0
0	0	0	0	0	0
0	0	0	0	0	0
0	0	0	0	0	0
0	0	0	0	0	0
0	0	0	0	0	0
0	0	0	0	0	0
0	0	0	0	0	0
0	0	0	0	0	0
0	0	0	0	0	0
0	0	0	0	0	0
0	0	0	0	0	0
0	0	0	0	0	0
0	0	0	0	0	0
0	0	0	0	0	0
0	0	0	0	0	0
0	0	0	0	0	0
0	0	0	0	0	0
0	0	0	0	0	0
0	0	0	0	0	0
0	0	0	0	0	0
0	0	0	0	0	0
0	0	0	0	0	0
0	0	0	0	0	0
0	0	0	0	0	0
0	0	0	0	0	0
0	0	0	0	0	0
0	0	0	0	0	0
0	0	0	0	0	0
0	0	0	0	0	0
0	0	0	0	0	0
0	0	0	0	0	0
0	0	0	0	0	0
0	0	0	0	0	0
0	0	0	0	0	0
0	0	0	0	0	0
0	0	0	0	0	0
0	0	0	0	0	0
0	0	0	0	0	0
0	0	0	0	0	0
0	0	0	0	0	0
0	0	0	0	0	0
0	0	0	0	0	0
0	0	0	0	0	0
0	0	0	0	0	0
0	0	0	0	0	0
0	0	0	0	0	0
0	0	0	0	0	0
0	0	0	0	0	0
0	0	0	0	0	0
0	0	0	0	0	0

0	0	0	0	0	0
0	0	0	0	0	0
0	0	0	0	0	0
0	0	0	0	0	0
0	0	0	0	0	0
0					

gtTCf=
0
0.11925
0.14534
0.13574
0.11593
0.096197
0.079948
0.067704
0.058926
0.052819
0.048646
0.045817
0.043898
0.042582
0.041662
0.040998
0.040498
0.040102

gtTCs=
0
0.1431
0.20732
0.22864
0.22778
0.21648
0.20118
0.18528
0.17042
0.1573
0.14609
0.13672
0.12898
0.12264
0.11746
0.11324
0.10979
0.10696

gtTCp=
0
2.385
1.5979
1.1196

0.8287
0.65143
0.54309
0.47655
0.43538
0.4096
0.39316
0.38239
0.37506
0.36982
0.36585
0.36266
0.35995
0.35753

X_mask=
0.00028396,0.00066969,0.0006532,2.2204e-016,2.2204e-016,0.0011166,2.2204e-016,2.2204e-016,0.00015458,0.00059642,2.2204e-016,0.00059249,2.2204e-016,2.2204e-016,0.00042049,2.2204e-016,2.2204e-016,6.359e-005,2.2204e-016,0.00040351,2.2204e-016,0.0036027,2.2204e-016,0.0030861,2.2204e-016,2.2204e-016,3.3448e-005,0.0002973,0.00045526,0.00030435,4.9327e-005,2.2204e-016,2.2204e-016,2.2204e-016,2.2204e-016,2.2204e-016,2.2204e-016,2.2204e-016,0.0013024,0.0061308,2.2204e-016,2.2204e-016,2.2204e-016,2.2204e-016,0.00029764,2.2204e-016,2.2204e-016,2.2204e-016,0.0011139,0.00040569,2.2204e-016,0.00022834,0.00051618,2.2204e-016,2.7282e-005,2.2204e-016,9.123e-005,0.0010531,2.2204e-016,5.9963e-006,2.2204e-016,2.2204e-016,2.2204e-016,2.2204e-016,2.2204e-016,0.00079612,2.2204e-016,0.001502,2.2204e-016,0.0029536,0.0041353,2.2204e-016,2.2204e-016,2.2204e-016,2.2204e-016,0.00043096,2.2204e-016,2.2204e-016,0.00028273,0.00045107,2.2204e-016,2.2204e-016,6.6831e-005,0.0004035,2.2204e-016,9.6321e-005,2.2204e-016,0.0035856,2.2204e-016,2.2204e-016,2.2204e-016,0.00221,2.2204e-016,0.0035844,0.0045755,0.0026372,2.2204e-016,2.2204e-016,0.0001115,2.2204e-016,2.2204e-016,2.2204e-016,0.00015359,0.00035554,0.00059934,2.2204e-016,0.0004894,7.5216e-006,2.2204e-016,2.2204e-016,2.2204e-016,6.8112e-005,0.000811,2.2204e-016,0.0052523,2.2204e-016,0.0023573,2.2204e-016,0.00060672,2.2204e-016,2.2204e-016,2.2204e-016,0.0011494,0.00042219,0.0027165,0.00046436,0.00022458,0.0062751,0.00074145,2.2204e-016,2.5702e-005,2.2204e-016,9.3119e-007,2.2204e-016,2.2204e-016,2.2204e-016,0.00042018,2.2204e-016,2.2204e-016,0.00022014,0.00058844,2.2204e-016,0.00041771,0.00021081,2.2204e-016,0.0016898,2.2204e-016,0.0083956,2.2204e-016,2.2204e-016,0.00039296,0.00086773,2.2204e-016,0.0074736,2.2204e-016,0.00044324,0.0022361,0.0056073,2.2204e-016,2.2204e-016,8.7787e-005,2.2204e-016,0.00026786,0.00030626,2.2204e-016,2.2204e-016,0.00042717,2.2204e-016,0.00088885,2.2204e-016,2.2204e-016,0.0003941,2.2204e-016,0.00030038,0.00024326,0.00091321,0.00067224,0.0019785,0.0063653,2.2204e-016,0.0063757,2.2204e-016,0.00070235,0.0045972,2.2204e-016,2.2204e-016,2.2204e-016,2.2204e-016,2.2204e-016,2.2204e-

016,0.00036041,2.2204e-016,0.0040226,0.0015042,2.2204e-
016,0.00016226,0.00068178,2.2204e-016,3.6901e-005,8.8054e-
005,0.00035219,0.00016842,0.00036549,2.2204e-016,2.2204e-016,4.027e-
005,2.2204e-016,0.00074908,2.2204e-016,0.00011357,2.2204e-016,2.2204e-
016,0.0042963,2.2204e-016,0.0069291,2.2204e-016,2.2204e-016,2.2204e-
016,0.0024482,2.2204e-016,2.2204e-016,2.2204e-016,0.0016274,2.2204e-
016,2.2204e-016,0.0025608,2.2204e-016,4.1568e-005,2.2204e-016,2.2204e-
016,0.00025326,0.00047311,2.2204e-016,2.2204e-016,2.2204e-016,5.816e-
005,2.2204e-016,0.00027923,2.2204e-016,2.2204e-
016,0.00034846,0.00013613,0.00067005,3.7251e-005,0.00046313,2.2204e-
016,2.2204e-
016,0.0091897,0.0062153,0.0068384,0.0043153,0.0032636,2.2204e-
016,0.0036518,2.2204e-016,2.2204e-016,0.0027795,0.0032941,2.2204e-
016,2.2204e-016,0.00042966,2.2204e-016,2.2204e-016,2.2204e-
016,0.0032413,0.00033919,2.2204e-016,2.2204e-016,0.00015849,2.2204e-
016,0.001416,2.2204e-016,2.2204e-016,0.00056288,2.2204e-016,2.2204e-
016,8.521e-005,0.00029092,0.0008708,2.2204e-
016,0.00065175,0.000211,0.00037666,0.00042584,2.2204e-
016,0.0091055,0.0018725,2.2204e-016,0.00021177,0.0057699,2.2204e-
016,2.2204e-016,2.2204e-016,2.2204e-016,0.0015806,2.2204e-
016,0.0099107,2.2204e-016,2.2204e-016,0.0016368,2.2204e-016,2.2204e-
016,2.2204e-016,2.2204e-016,0.00085741,2.2204e-
016,0.00038466,0.00057305,2.2204e-016,0.00042391,2.2204e-
016,0.00017185,5.813e-005,0.00059527,0.00037902,2.2204e-016,2.2204e-
016,2.2204e-016,0.00058305,6.743e-005,0.00012464,2.2204e-
016,0.0016702,2.2204e-016,2.2204e-016,2.2204e-016,2.2204e-016,2.2204e-
016,0.0057621,0.00047919,2.6464e-005,2.2204e-016,2.2204e-016,2.2204e-
016,0.0029559,2.2204e-016,2.2204e-
016,0.0036028,0.00029814,0.0027685,1.8938e-005,2.2204e-016,2.2204e-
016,2.2204e-016,0.00010806,2.2015e-005,0.0001605,2.2204e-016,2.2204e-
016,2.2204e-016,2.2204e-016,2.2204e-016,2.2204e-016,2.2204e-
016,2.2204e-016,2.2204e-016,0.00055621,2.2204e-016,0.0035032,2.2204e-
016,0.002168,0.0023125,0.00058323,2.2204e-016,2.2204e-
016,0.0069888,0.00051003,0.0072517,0.00053282,0.0018519,0.0005705,2.22
04e-016,2.2204e-016,0.0033486,9.6777e-005,2.2204e-
016,0.0010511,0.0005168,0.00047529,2.2204e-016,2.2204e-016,2.2204e-
016,3.415e-005,5.4655e-005,0.00065181,2.2204e-016,2.2204e-
016,0.0003603,0.00055982,0.00013869,0.000185,0.00028388,2.2204e-
016,2.2204e-016,2.2204e-016,2.2204e-016,0.0013063,2.2204e-016,2.2204e-
016,0.0087699,0.0062071,0.012091,2.2204e-016,2.2204e-
016,0.0038082,0.0043899,2.2204e-016,0.00018811,2.2204e-016,2.2204e-
016,2.2204e-016,2.2204e-016,2.2204e-016,2.2204e-016,0.0027441,2.2204e-
016,4.6282e-006,0.0011451,5.697e-
005,0.00042475,0.00017713,0.00078221,0.00011821,2.2204e-016,2.2204e-
016,0.00097848,0.00081205,2.2204e-016,2.2204e-
016,0.00026678,0.00057812,2.2204e-016,2.2204e-016,2.2204e-
016,0.010451,0.0014684,0.0017184,2.2204e-016,2.2204e-016,2.2204e-
016,2.2204e-016,0.0017825,2.2204e-016,2.2204e-016,2.2204e-016,2.2204e-
016,0.00075695,2.2204e-016,0.0029697,2.2204e-016,2.2204e-
016,0.00076171,0.000647,0.00085599,2.2204e-016,2.2204e-016,2.2204e-
016,2.2204e-016,0.00013031,0.0010303,2.2204e-016,0.00010056,2.2204e-
016,2.2204e-016,0.00043934,0.00031295,2.2204e-016,2.2204e-

016,0.00045183,0.00069021,0.00062799,0.00069212,0.0026859,2.2204e-
016,0.0085091,0.005301,0.002156,2.2204e-016,0.0086363,2.2204e-
016,2.2204e-016,0.0025414,2.2204e-016,0.00089585,2.2204e-016,2.2204e-
016,2.2204e-016,2.2204e-016,2.2204e-016,2.2204e-016,2.2204e-
016,2.2204e-016,2.2204e-016,0.00099357,0.00019541,0.00024954,2.2204e-
016,2.2204e-016,0.00051169,2.2204e-016,2.2204e-016,2.2204e-
016,2.2204e-016,2.2204e-016,0.0005917,0.00020643,2.2204e-
016,0.00032727,0.0003192,2.2204e-016,2.2204e-016,2.2204e-016,2.2204e-
016,2.2204e-016,0.0019393,2.2204e-
016,0.0002662,0.0004624,0.0042571,0.0032986,2.2204e-
016,0.00020066,2.2204e-016,2.2204e-016,2.2204e-016,2.2204e-
016,2.2204e-016,2.2204e-
016,0.0013438,0.00094165,0.00070461,0.00049562,0.00031263,0.00042579,0
.00029459,0.00033601,2.2204e-016,2.2204e-016,1.1834e-
005,0.00022872,2.2204e-016,0.00018021,2.2204e-016,2.2204e-016,2.2204e-
016,0.00026114,2.2204e-016,2.2204e-016,2.2204e-016,2.2204e-
016,0.0058367,0.0026791,0.010569,0.0031542,0.0024274,0.00014652,2.2204
e-016,2.2204e-016,2.2204e-016,0.0028238,2.2204e-016,2.2204e-
016,2.2204e-016,0.00079061,0.0025239,0.0017253,2.2204e-016,2.2204e-
016,2.0308e-006,2.2204e-016,0.0014053,0.00022069,2.2204e-
016,0.00023184,2.2204e-016,0.00030717,2.5798e-005,8.5391e-
005,0.00048616,0.00021921,2.2204e-016,2.2204e-016,0.00032932,2.2204e-
016,0.00026715,2.2204e-016,2.2204e-016,1.488e-005,2.2204e-016,2.2204e-
016,2.2204e-016,0.0016782,0.0021172,2.2204e-
016,0.0022691,0.00089428,2.2204e-016,0.00018914,0.0014531,2.2204e-
016,2.2204e-016,2.2204e-016,2.2204e-016,2.2204e-016,2.2204e-
016,0.00020514,0.00015851,0.00087695,2.2204e-016,6.2527e-005,2.2204e-
016,2.2204e-016,0.00021032,0.00031221,2.2204e-016,2.2204e-
016,0.00045351,0.00060481,2.2204e-016,0.00040122,4.3424e-
005,0.00021909,0.00018247,2.2204e-016,0.00096499,2.2204e-016,2.2204e-
016,2.2204e-016,2.2204e-016,0.00050941,0.0028604,2.2204e-
016,0.003208,0.0031433,0.00067544,2.2204e-016,0.00095526,2.2204e-
016,0.0016211,0.00048894,2.2204e-016,2.2204e-016,0.00055341,2.2204e-
016,2.2204e-016,0.00024719,2.2204e-016,0.00016613,2.2204e-
016,0.00024033,2.2204e-
016,0.00057127,0.00040566,0.00049277,0.00027731,0.00025671,0.0002584,2
.2204e-016,2.2204e-016,3.0781e-
005,0.00041362,0.0002783,0.00097409,2.2204e-016,2.2204e-
016,0.0024489,2.2204e-016,2.2204e-016,2.2204e-016,2.2204e-
016,0.00124,2.2204e-016,2.2204e-016,2.2204e-016,0.0014585,2.2204e-
016,2.2204e-016,0.002152,2.2204e-016,2.2204e-016,2.2204e-016,2.2204e-
016,0.00048595,8.6403e-005,0.0002238,2.2204e-
016,0.00046999,0.00020585,0.001113,2.2204e-016,2.2204e-016,2.2204e-
016,2.2204e-016,2.2204e-016,2.2204e-016,2.2204e-016,2.2204e-
016,2.2204e-016,8.7996e-005,2.2204e-016,0.0024146,2.2204e-
016,0.0027861,0.0014037,1.58e-005,0.0038328,2.2204e-016,2.2204e-
016,6.8915e-005,2.2204e-016,2.2204e-016,2.2204e-016,0.0023606,2.2204e-
016,2.2204e-016,0.002226,2.2204e-016,5.4483e-
005,0.0001633,0.00021663,2.2204e-016,2.2204e-016,2.2204e-
016,0.00022879,2.2204e-016,2.2204e-016,2.2204e-
016,0.00015773,0.00082992,0.00026488,2.2204e-016,2.2204e-
016,0.00023272,2.2204e-016,2.2204e-016,2.2204e-016,2.2204e-

016,2.2204e-016,2.2204e-016,0.0024368,0.0040304,2.2204e-016,2.2204e-
016,0.0037022,0.00010991,0.00021769,2.2204e-016,0.00053238,2.2204e-
016,0.0018691,0.0019935,2.2204e-016,2.2204e-
016,0.00030062,0.00045527,0.00040539,2.2204e-016,2.2204e-016,7.5796e-
005,2.2204e-016,2.2204e-016,0.00036053,2.2204e-016,2.2204e-
016,2.2204e-016,2.2204e-016,0.00057691,2.2204e-016,2.2204e-
016,0.00027745,2.2204e-016,2.2204e-016,2.2204e-
016,0.00023451,0.0015036,2.2204e-016,0.00018314,2.2204e-
016,0.0011074,0.0030761,0.0043912,2.2204e-
016,0.0018498,0.0016903,0.0024303,0.0011191,0.0010169,0.00068381,2.220
4e-016,0.00014654,2.7179e-006,0.00056236,4.6441e-
005,0.00038017,2.2204e-016,0.00024517,0.00037605,3.4475e-
005,0.00046638,2.2204e-016,2.2204e-016,2.2204e-016,2.2204e-
016,2.2204e-016,0.00034136,0.00021089,2.2204e-016,2.2204e-
016,0.00011321,2.2204e-016,2.2204e-016,0.0040237,2.2204e-016,2.2204e-
016,2.2204e-016,2.2204e-016,0.003786,0.00043917,0.00036002,2.2204e-
016,7.2304e-005,2.2204e-016,2.2204e-016,0.0008792,0.00055729,2.2204e-
016,2.2204e-016,2.2204e-016,2.2204e-016,2.2204e-
016,0.00048973,2.2204e-016,2.2204e-016,3.404e-005,2.2204e-
016,0.0004163,2.2204e-016,8.8754e-005,0.0001287,2.2204e-016,2.2204e-
016,5.8886e-005,0.00031872,0.00041186,2.2204e-016,2.2204e-016,4.079e-
006,0.00070603,2.2204e-016,0.0033597,0.0026468,2.2204e-016,2.2204e-
016,2.2204e-016,0.0027335,2.2204e-016,0.00035683,2.2204e-
016,0.00029036,2.2204e-016,2.2204e-016,0.00068161,2.2204e-
016,0.00094145,2.2204e-016,2.2204e-016,2.2204e-016,2.2204e-
016,2.2204e-016,2.2204e-016,0.00041155,2.2204e-016,1.8636e-
005,2.2204e-016,0.00034458,0.00013323,2.2204e-016,2.2204e-016,2.2204e-
016,0.00011958,0.00012576,2.2204e-016,2.2204e-016,0.0001105,7.5483e-
005,0.004127,2.2204e-016,2.2204e-016,2.2204e-016,0.0047699,2.2204e-
016,2.2204e-016,0.00040913,2.2204e-016,2.2204e-016,0.00076199,2.2204e-
016,2.2204e-016,0.00014855,0.00027966,7.5484e-005,2.2204e-016,2.2204e-
016,0.00023325,2.2204e-016,0.00016417,2.2204e-016,5.6286e-007,2.2204e-
016,0.0002421,2.2204e-016,2.2204e-016,6.2284e-006,2.2204e-016,2.2204e-
016,2.2204e-016,0.00017567,0.00073313,2.2204e-
016,0.00037508,0.0006148,0.00040777,2.2204e-016,2.2204e-
016,0.0052258,2.2204e-016,2.2204e-016,0.00020063,0.00021247,2.2204e-
016,2.2204e-016,2.2204e-016,5.2675e-005,2.2204e-
016,0.00037493,2.2204e-016,2.2204e-016,2.2204e-016,2.2204e-
016,0.00035227,2.2204e-016,2.2204e-016,2.2204e-016,2.2204e-
016,2.2204e-016,0.00037646,0.00033262,2.2204e-
016,0.00033738,0.00030412,0.00025227,8.805e-005,2.2204e-
016,0.0004879,0.0004524,0.00056666,2.2204e-016,0.000307,7.3218e-
005,0.00068631,0.00017288,0.00023857,0.00042078,2.2204e-016,2.2204e-
016,2.2204e-016,2.2204e-016,0.00012435,2.2204e-016,2.2204e-
016,2.2204e-016,2.2204e-016,2.2204e-016,0.00025317,2.2204e-
016,2.2204e-016,0.000396,0.0001525,0.00013115,0.0001669,9.2136e-
005,2.2204e-016,0.00014339,0.00010741,1.4027e-005,2.2204e-
016,0.00043114,2.2204e-016,2.2204e-016,2.2204e-016,2.2204e-
016,0.00048874,2.2204e-016,2.2204e-016,0.00043323,2.2204e-
016,0.00052,2.2204e-016,0.00018993,0.00032542,0.0012307,2.2204e-
016,2.2204e-016,2.2204e-016,0.00023121,0.00088968,2.2204e-016,2.2204e-
016,0.00016001,0.0001641,1.8053e-005,0.00025602,2.2204e-016,2.2204e-

016,1.8214e-005,0.00077506,2.2204e-016,0.00053836,0.00012047,2.2204e-016,7.527e-005,2.2204e-016,2.2204e-016,2.2204e-016,5.4478e-005,2.2204e-016,2.2204e-016,2.2204e-016,2.2204e-016,2.2204e-016,0.00024049,0.00048807,0.00067744,5.6779e-005,2.2204e-016,2.2204e-016,2.2204e-016,8.8608e-005,2.2204e-016,2.2204e-016,2.2204e-016,0.00045726,4.7902e-005,5.5002e-005,0.00047988,2.2204e-016,2.2204e-016,0.00034643,2.2204e-016,2.2204e-016,2.2204e-016,0.00049934,0.00047976,2.2204e-016,2.2204e-016,0.00020648,0.00040476,0.00018533,2.2204e-016,2.2204e-016,2.2204e-016,0.00018619,2.2204e-016,0.00023046,0.00042908,0.00025786,2.2204e-016,1.0294e-005,0.00036692,2.2204e-016,2.2204e-016,2.2204e-016,0.0003494,2.2204e-016,0.0010035,2.2204e-016,2.2204e-016,2.2204e-016,0.00024124,2.2204e-016,0.00011761,3.373e-005,7.0961e-005,2.2204e-016,2.2204e-016,2.2204e-016,2.2204e-016,2.2204e-016,0.00048719,0.0001078,2.2204e-016,2.2204e-016,2.2204e-016,2.2204e-016,0.00012242,2.2204e-016,0.00022403,2.2204e-016,0.00017432,0.00066449,0.0001002,2.2204e-016,6.9782e-005,0.00016568,4.4988e-005,2.2204e-016,5.2805e-005,0.00074416,2.2204e-016,2.2204e-016,0.0010616,8.4368e-005,2.2204e-016,0.00011858,0.00030167,4.9899e-005,6.3057e-005,0.00018374,0.00011961,6.1694e-006,2.2204e-016,2.2204e-016,2.2204e-016,2.2204e-016,2.2204e-016,2.2204e-016,2.2204e-016,0.0003143,2.2204e-016,2.2204e-016,0.00019234,2.2204e-016,0.00076093,0.00043969,2.2204e-016,0.00021389,2.2204e-016,0.00044587,2.2204e-016,2.2204e-016,0.00018143,0.00031653,2.2204e-016,2.2204e-016,2.2204e-016,0.0005127,6.9899e-005,1.885e-005,2.2204e-016,2.2204e-016,0.0002105,0.00044979,0.00037448,2.2204e-016,0.00047847,5.2838e-005,7.2112e-005,0.00028233,0.00041486,0.00023321,2.2204e-016,0.00043695,0.00022839,0.0001155,0.00020039,2.2204e-016,0.00043012,2.0544e-005,2.2204e-016,2.2204e-016,2.2204e-016,2.2204e-016,0.00068794,2.2204e-016,2.2204e-016,0.00030062,2.2204e-016,2.2204e-016,2.2204e-016,2.2204e-016,2.2204e-016,2.2204e-016,0.00046768,0.00013195,2.2204e-016,0.00074775,2.2204e-016,2.2204e-016,0.00022911,2.2204e-016,0.00024954,2.2204e-016,2.2204e-016,0.00020708,2.2204e-016,3.6e-005,0.00050773,0.00044622,0.00051512,0.00023768,2.2204e-016,0.00014973,2.2204e-016,0.00042626,2.2204e-016,0.00038698,0.00019526,2.2204e-016,2.2204e-016,0.0002787,0.00023767,2.2204e-016,2.2204e-016,0.00033728,0.00048306,0.00050184,0.00010716,0.00023308,2.2204e-016,2.2204e-016,2.2204e-016,0.00038818,9.795e-005,2.2204e-016,2.2204e-016,2.2204e-016,2.2204e-016,2.2204e-016,2.2204e-016,2.2204e-016,0.00039802,2.2204e-016,0.00065199,0.0005676,0.0007201,2.2204e-016,3.0461e-005,2.2204e-016,0.00090361,0.00017101,2.2204e-016,2.2204e-016,2.2204e-016,2.2204e-016,2.2204e-016,2.2204e-016,0.0005391,0.00035025,0.00043528,9.6232e-005,2.2204e-016,0.00020423,2.2204e-016,0.00031866,2.2204e-016,2.2204e-016,0.00035796,2.2204e-016,2.2204e-016,8.4788e-005,2.2204e-016,2.2204e-016,2.2204e-016,2.2204e-016,0.00010411,2.2204e-016,0.00014716

0.14353,0.1443,0.1438,0.14257,0.14291,0.14419,0.14266,0.14225,0.14309,0.14358,0.14223,0.14373,0.14256,0.14283,0.14375,0.14209,0.14206,0.1437

,0.14308,0.14283,1.2571,0.94267,1.0965,1.1411,0.14314,0.14305,0.14301,
0.14333,0.14342,0.14315,0.14351,0.14268,0.14274,0.14334,0.14298,0.1427
5,0.14194,0.14314,0.14452,2.3922,1.4663,1.1323,1.3766,1.1348,1.2645,1.
2711,0.14248,0.14244,0.14448,0.14309,0.14252,0.14323,0.14342,0.14263,0
.14301,0.14258,0.14333,0.14389,0.14271,0.14297,0.14277,2.3752,2.3806,1
.1883,1.5966,1.2754,1.3043,1.493,1.6164,1.1809,1.3957,0.14248,0.14214,
0.1425,0.14244,0.14351,0.14252,0.14283,0.14337,0.1437,0.14218,0.14318,
0.14331,0.14332,0.14269,0.14344,2.3779,2.3881,2.3792,1.3255,1.1334,1.3
558,1.479,1.034,1.2685,1.2107,1.3924,1.2597,0.14321,0.14268,0.14211,0.
14266,0.14305,0.14406,0.14389,0.14319,0.14317,0.14314,0.14256,0.14233,
0.14221,0.14307,0.14389,0.14239,2.3859,2.3807,2.3922,2.3841,1.2682,1.2
575,1.2841,1.2288,1.4647,1.2616,1.2245,1.2669,1.3855,1.3252,0.14379,0.
1436,0.14339,0.14303,0.14252,0.14329,0.1434,0.14302,0.14334,0.1431,0.1
4242,0.14377,0.14446,0.14297,0.14347,0.14292,2.3743,2.39,2.3827,2.3936
,2.3823,2.3778,1.2622,1.2165,1.1422,1.2674,1.2291,1.2667,1.2366,1.2683
,1.2812,1.4038,0.14302,0.14227,0.14366,0.14346,0.14298,0.14216,0.14344
,0.14297,0.14381,0.14245,0.14311,0.14348,0.1425,0.14337,0.14307,0.1441
5,0.14385,2.3863,2.3909,2.3869,2.3873,2.3816,2.3863,1.1839,1.3596,1.24
65,1.2644,1.1091,1.2595,1.1534,0.75563,1.1571,1.381,1.2651,0.14221,0.1
4341,0.14379,0.14253,0.14354,0.14355,0.14347,0.14313,0.14336,0.1424,0.
14243,0.14283,0.14278,0.13204,0.12634,0.13355,0.14307,2.384,2.3905,2.3
816,2.3903,2.3888,2.3799,2.382,1.2655,1.259,1.2613,1.1403,1.2345,1.262
5,1.0817,1.268,1.3687,1.3663,1.3392,0.1433,0.14372,0.14368,0.14286,0.1
4207,0.14274,0.14275,0.14297,0.1432,0.14246,0.14239,0.13074,0.12952,0.
13367,0.12607,0.12972,0.13114,0.14291,2.3979,2.3898,2.3874,2.3888,2.38
55,2.3809,2.3886,1.3257,1.344,1.2661,1.2694,1.0652,1.1548,1.1143,1.208
2,1.3989,1.595,1.1478,0.14348,0.14324,0.14206,0.14336,0.14256,0.14425,
0.14253,0.14287,0.14381,0.12859,0.1308,0.13091,0.1297,0.13491,0.12898,
0.13206,0.13162,0.13495,0.14342,2.3769,2.3961,2.3846,2.3775,2.384,2.39
07,2.3825,1.6389,1.2358,1.2624,1.2643,1.313,1.3852,1.3249,1.3087,1.278
9,1.1367,1.2188,1.2609,0.14286,0.14436,0.14214,0.14381,0.14348,0.14244
,0.13315,0.13027,0.13101,0.13208,0.13164,0.13046,0.13184,0.1315,0.1314
6,0.13157,0.13117,0.13156,2.3779,2.3874,2.3756,2.3797,2.3763,2.3828,2.
3777,1.0096,1.0226,0.94437,1.3043,1.2565,1.2648,1.1955,1.2611,1.2184,1
.1507,1.2377,1.5031,0.14322,0.14314,0.14312,0.13267,0.13154,0.13215,0.
13121,0.12824,0.13324,0.12925,0.12932,0.13097,0.13008,0.13059,0.1312,0
.13153,0.1295,0.13208,2.3894,2.3871,2.3879,2.3849,2.3891,2.3797,2.3843
,2.3899,2.385,1.2652,1.1531,1.2345,1.2176,1.0464,1.2598,1.3541,1.6128,
1.2756,1.1336,1.0954,0.13313,0.12793,0.12973,0.13111,0.13066,0.13044,0
.13135,0.13067,0.12957,0.13137,0.13075,0.13148,0.1316,0.13159,0.13085,
0.13147,0.12964,0.12881,2.3898,2.383,2.3782,2.3915,2.3895,2.3945,2.379
7,2.3703,2.3876,1.2129,1.262,0.88218,0.85887,0.99109,0.87912,0.90167,0
.88281,0.92781,0.92682,0.88319,0.13115,0.13196,0.13194,0.1319,0.13172,
0.13023,0.13125,0.12772,0.13238,0.13041,0.13413,0.13105,0.1312,0.13066
,0.13178,0.13296,0.13194,0.1299,2.3967,2.3824,2.3858,2.3768,2.3799,2.3
821,2.3817,2.3846,2.3832,1.3011,0.99537,0.88486,0.85607,0.97465,0.8990
2,0.81912,0.99484,0.92535,0.89088,0.90737,0.1321,0.12714,0.13102,0.128
87,0.13202,0.13208,0.13099,0.12779,0.13013,0.13063,0.13601,0.13092,0.1
3113,0.12979,0.13393,0.13367,0.13176,0.13124,2.3859,2.3719,2.3984,2.39
04,2.3857,2.3793,2.3928,1.0576,1.1331,1.2524,0.91338,0.97675,1.0029,0.
72761,0.86192,0.92883,0.87141,0.82289,0.91251,0.76842,0.13124,0.1323,0
.13073,0.13077,0.12956,0.12913,0.13369,0.13235,0.131,0.13058,0.13015,0

.13076,0.13176,0.13153,0.13217,0.13176,0.12839,0.13053,2.3817,2.3819,2
.382,2.3819,2.3941,2.3707,1.2179,1.3089,1.1742,1.279,1.2581,0.81843,0.
82896,0.96464,0.8741,0.83645,0.93982,0.99501,0.74896,0.9829,0.13483,0.
13113,0.13149,0.13388,0.12942,0.1328,0.13103,0.1303,0.13037,0.13067,0.
13045,0.13121,0.13304,0.13036,0.12976,0.13306,0.13268,0.13131,2.3796,2
.3787,2.3883,2.3901,2.3897,1.5811,1.254,0.90337,1.1923,1.4577,1.3437,0
.93245,0.62444,0.92678,0.92342,0.88246,0.88567,0.84523,0.88112,0.95979
,0.131,0.13082,0.13245,0.13329,0.13003,0.13144,0.13063,0.12966,0.13399
,0.13159,0.13276,0.12957,0.13006,0.13447,0.13049,0.12964,0.13165,0.129
49,2.3894,2.3866,2.3815,0.98599,1.2916,1.2012,1.0519,1.2511,1.2605,1.1
688,1.2515,0.76325,0.84794,0.94591,0.91903,0.89541,0.66498,0.92395,0.7
533,0.89898,0.12907,0.12954,0.13159,0.1335,0.131,0.13094,0.13199,0.131
63,0.12784,0.13098,0.13057,0.13184,0.13287,0.13136,0.13158,0.13169,0.1
3137,0.13073,2.3887,1.1399,1.2527,1.3015,1.2566,1.5638,0.97866,1.2587,
1.1872,1.4986,0.88246,0.55625,0.9854,0.80601,0.96099,0.84278,0.92952,0
.64089,0.87807,0.13051,0.13019,0.12946,0.13084,0.13172,0.12969,0.12949
,0.13346,0.1339,0.12914,0.1318,0.13124,0.12982,0.13234,0.12931,0.13234
,0.13482,0.11979,1.2412,1.2551,1.2487,1.262,1.2557,1.3258,1.1948,1.456
3,1.0024,1.4319,1.0605,0.95877,0.89795,0.88336,0.71787,0.84039,0.92939
,0.85742,0.6223,0.13109,0.13036,0.1324,0.13069,0.13352,0.13022,0.13113
,0.13149,0.13282,0.13021,0.13135,0.12866,0.12708,0.13121,0.1308,0.1293
3,0.12906,0.11882,1.5116,1.2481,1.2548,1.3631,1.2832,1.2934,1.2087,1.2
754,1.1952,1.2248,0.88293,1.0093,1.038,0.88006,0.96584,0.88085,0.6849,
0.97175,0.12936,0.13029,0.12983,0.13017,0.1305,0.13097,0.13159,0.13146
,0.13166,0.12984,0.13826,0.1298,0.13364,0.13261,0.13141,0.12781,0.1310
3,0.13067,0.11907,1.5252,1.2495,1.0472,1.483,1.1315,1.2549,1.2052,1.16
89,1.3284,1.3406,0.88501,0.93702,0.91508,0.90008,0.90606,0.97889,1.054
6,0.90741,0.12934,0.13066,0.13361,0.13013,0.12596,0.13227,0.13054,0.13
305,0.13066,0.1334,0.13242,0.13381,0.13126,0.13064,0.13099,0.12942,0.1
2947,0.1192,0.11929,0.11924,1.1605,1.1845,1.2102,1.2935,1.5083,1.182,1
.2552,1.2904,1.2508,0.82751,0.98109,0.88851,1.13,0.97818,0.80699,0.131
2,0.13102,0.13129,0.13015,0.13152,0.13251,0.12776,0.13164,0.13524,0.13
109,0.13112,0.13031,0.131,0.13122,0.13584,0.1331,0.13061,0.13057,0.119
2,0.11912,0.11946,0.11864,1.0131,0.96503,1.5769,1.3552,1.3968,1.2885,1
.2773,0.62314,1.2577,1.0447,0.63383,0.87985,0.13097,0.13167,0.12997,0.
12937,0.12963,0.13076,0.13142,0.13158,0.13032,0.12853,0.13096,0.13253,
0.12821,0.13146,0.13066,0.13129,0.13206,0.12983,0.13339,0.1317,0.11968
,0.11979,0.11895,0.11882,0.11906,0.11983,1.4893,1.305,1.2544,1.3059,1.
3619,1.6324,1.2689,1.2039,0.13147,0.13335,0.12913,0.13304,0.12867,0.13
185,0.1307,0.13093,0.13206,0.13082,0.13152,0.13151,0.13092,0.13021,0.1
3127,0.13093,0.13029,0.13147,0.13093,0.13582,0.12908,0.1308,0.13053,0.
11941,0.11933,0.11892,0.1192,0.11927,0.11937,1.2795,1.212,1.2484,1.403
6,1.2564,0.11916,0.11938,0.13002,0.13001,0.12995,0.13161,0.12972,0.131
03,0.13153,0.13153,0.13065,0.13161,0.13112,0.13134,0.13057,0.12861,0.1
3274,0.13276,0.13316,0.1318,0.13204,0.13094,0.13098,0.11905,0.1191,0.1
1927,0.11953,0.11987,0.11877,0.11966,0.11977,0.11943,0.11875,1.3657,1.
3076,0.11881,0.11922,0.11936,0.11968,0.13057,0.13091,0.1302,0.13222,0.
12943,0.13384,0.13117,0.12972,0.1302,0.13101,0.1321,0.13318,0.13216,0.
13075,0.12935,0.12941,0.13179,0.13246,0.13003,0.11939,0.11983,0.11939,
0.11948,0.119,0.11965,0.11945,0.11965,0.11888,0.11952,0.11902,0.11979,
0.11946,0.11932,0.11971,0.1195,0.11931,0.13128,0.12993,0.13145,0.13049
,0.13043,0.13076,0.13141,0.1306,0.13116,0.1329,0.12955,0.133,0.13135,0

.13274,0.13293,0.13143,0.11907,0.11931,0.11943,0.11926,0.11888,0.11977
,0.11931,0.11888,0.1186,0.11896,0.11948,0.11899,0.1192,0.11977,0.11862
,0.11995,0.11922,0.11934,0.11945,0.13362,0.13105,0.12909,0.12979,0.131
09,0.13001,0.13173,0.12934,0.13117,0.12895,0.13,0.13158,0.13065,0.1191
2,0.11924,0.12001,0.11877,0.11998,0.11924,0.11891,0.11923,0.11853,0.11
919,0.1186,0.11908,0.11949,0.11903,0.11929,0.11873,0.11915,0.11941,0.1
1968,0.11996,0.11927,0.13004,0.13055,0.13004,0.13082,0.13177,0.1306,0.
13109,0.13301,0.13207,0.11931,0.11951,0.11899,0.11904,0.11957,0.119,0.
11914,0.11891,0.11988,0.11987,0.11909,0.11864,0.11967,0.11956,0.11947,
0.11906,0.11913,0.11895,0.11923,0.11817,0.11948,0.11927,0.11951,0.1182
6,0.1194,0.13291,0.13075,0.13129,0.13092,0.11976,0.11863,0.12027,0.119
62,0.11854,0.11905,0.11929,0.11859,0.11924,0.11931,0.11925,0.11901,0.1
1917,0.11896,0.11894,0.11882,0.11958,0.11931,0.11894,0.11882,0.11879,0
.11851,0.11951,0.11881,0.11965,0.11893,0.1194,0.11983,0.11896,0.11884,
0.11929,0.11943,0.11971,0.11922,0.1195,0.12014,0.11921,0.11941,0.12002
,0.11947,0.11916,0.11935,0.11957,0.11908,0.11923,0.11944,0.11956,0.119
34,0.11892,0.11883,0.11892,0.11911,0.11891,0.11915,0.11896,0.11923,0.1
1877,0.11914,0.11951,0.11879,0.11977,0.11944,0.11862,0.11948,0.11915,0
.11979,0.11937,0.11917,0.11941,0.11965,0.1192,0.11904,0.1192,0.1201,0.
11907,0.11917,0.11927,0.11792,0.1196,0.11986,0.11989,0.11872,0.11966,0
.11933,0.11947,0.11949,0.11996,0.11945,0.11917,0.11948,0.11942,0.11947
,0.11938,0.11909,0.11967,0.11925,0.11856,0.1189,0.11844,0.11876,0.1200
6,0.119,0.11874,0.11977,0.11924,0.11909,0.11914,0.11879,0.11917,0.1187
4,0.11959,0.11944,0.11879,0.11997,0.1194,0.1186,0.11944,0.11896,0.1194
4,0.11907,0.11902,0.11933,0.11907,0.1192,0.1197,0.11955,0.11973,0.1191
3,0.11851,0.11942,0.11903,0.11981,0.11922,0.11955,0.11926,0.11882,0.11
92,0.11966,0.11959,0.11879,0.11892,0.11973,0.11966,0.11982,0.1195,0.11
917,0.11919,0.11861,0.11911,0.11954,0.11952,0.11938,0.11876,0.11911,0.
11892,0.11886,0.11898,0.11927,0.11948,0.11931,0.11981,0.11983,0.11958,
0.11896,0.11936,0.11828,0.11997,0.11925,0.11896,0.11877,0.11928,0.1193
,0.11871,0.1189,0.11986,0.11938,0.11977,0.1195,0.11917,0.11932,0.11911
,0.11956,0.1194,0.11912,0.11931,0.11894,0.11897,0.11943,0.11841,0.1191
8,0.11911,0.11886,0.11898,0.11885,0.1193
0.20782,0.2083,0.20786,0.20676,0.20749,0.20885,0.20705,0.2067,0.20671,
0.20743,0.20625,0.20764,0.20648,0.20746,0.20779,0.20616,0.20663,0.2084
8,0.20725,0.20771,0.89778,0.70606,0.79746,0.82698,0.20721,0.2072,0.207
65,0.20697,0.20761,0.20727,0.20777,0.20709,0.20698,0.20758,0.20749,0.2
0725,0.20622,0.20708,0.2085,1.6039,1.0296,0.81729,0.97277,0.82492,0.90
444,0.90849,0.20654,0.20686,0.20884,0.2071,0.20677,0.20759,0.20765,0.2
0687,0.20701,0.2071,0.20741,0.20808,0.20724,0.20756,0.20718,1.592,1.59
7,0.85555,1.1062,0.90689,0.92866,1.0454,1.1217,0.8518,0.98547,0.20718,
0.20621,0.2071,0.20674,0.20785,0.20711,0.20717,0.20756,0.20777,0.20664
,0.20774,0.20764,0.20779,0.20709,0.20801,1.5888,1.6014,1.5955,0.93821,
0.8212,0.95974,1.0368,0.76057,0.90605,0.87268,0.98508,0.89799,0.20764,
0.20686,0.20647,0.20667,0.20716,0.20799,0.20807,0.20744,0.20762,0.2074
6,0.20689,0.20649,0.20665,0.20744,0.2082,0.20686,1.6017,1.5981,1.6032,
1.5935,0.90699,0.89758,0.91778,0.88152,1.0275,0.89881,0.88103,0.90565,
0.97937,0.94297,0.20806,0.20778,0.2075,0.20754,0.20686,0.20744,0.20733
,0.20721,0.20769,0.20755,0.20629,0.20768,0.20843,0.20711,0.2078,0.2069
1,1.5853,1.6018,1.5943,1.6061,1.596,1.5954,0.90069,0.87388,0.82829,0.9
0817,0.87718,0.9039,0.88526,0.90558,0.91239,0.99051,0.20679,0.2066,0.2
0768,0.20782,0.2075,0.20648,0.20761,0.20705,0.20765,0.20676,0.20764,0.

2074,0.20694,0.20729,0.20746,0.20826,0.20819,1.5945,1.6067,1.5999,1.59
78,1.5944,1.6002,0.85596,0.96188,0.89166,0.90271,0.80685,0.89727,0.833
51,0.58833,0.83671,0.97771,0.90379,0.20633,0.20758,0.20818,0.20664,0.2
0792,0.20744,0.2078,0.20732,0.2072,0.20688,0.20672,0.20716,0.20717,0.1
7712,0.16505,0.18265,0.20765,1.5978,1.605,1.5913,1.6014,1.6036,1.5923,
1.5934,0.90771,0.89917,0.90121,0.82567,0.88851,0.90366,0.78968,0.90675
,0.96657,0.96622,0.94718,0.20746,0.20831,0.20798,0.20671,0.20676,0.207
38,0.20736,0.20701,0.2078,0.2067,0.20698,0.17543,0.17221,0.18125,0.162
95,0.17226,0.17705,0.20719,1.6146,1.6079,1.5994,1.6019,1.5994,1.5907,1
.5993,0.9369,0.95254,0.90371,0.90754,0.77849,0.83397,0.81011,0.86628,0
.98964,1.107,0.83182,0.20762,0.20731,0.20604,0.20755,0.20695,0.20874,0
.20636,0.20725,0.20791,0.16998,0.17636,0.17521,0.17171,0.18444,0.17239
,0.17728,0.17702,0.18526,0.20787,1.5885,1.6062,1.5988,1.5902,1.5952,1.
6053,1.5966,1.1316,0.88566,0.89779,0.90172,0.93209,0.97894,0.93811,0.9
3134,0.91203,0.82439,0.87599,0.90014,0.20709,0.20843,0.20674,0.20809,0
.20742,0.20672,0.18075,0.17511,0.17528,0.17854,0.17664,0.17385,0.1782,
0.17843,0.17837,0.17686,0.17596,0.17708,1.5939,1.6025,1.5848,1.5877,1.
5859,1.5976,1.5922,0.74552,0.75434,0.70467,0.92731,0.89659,0.90495,0.8
5977,0.9006,0.8749,0.83384,0.88591,1.0525,0.20756,0.20728,0.20779,0.18
062,0.17699,0.17851,0.17636,0.16905,0.18339,0.17247,0.17149,0.17635,0.
17386,0.17594,0.17651,0.17764,0.17121,0.17857,1.6018,1.5965,1.5983,1.5
994,1.602,1.5899,1.5925,1.6012,1.6001,0.9042,0.83274,0.88489,0.87425,0
.76564,0.89992,0.95807,1.1203,0.90696,0.82112,0.79925,0.18047,0.16769,
0.17292,0.17787,0.1744,0.17438,0.17568,0.17596,0.17346,0.17599,0.17351
,0.17665,0.17702,0.17684,0.17569,0.17677,0.17276,0.17136,1.6024,1.5967
,1.5904,1.6036,1.6045,1.6074,1.5953,1.5844,1.6001,0.87155,0.90062,0.64
961,0.63187,0.71669,0.64685,0.66055,0.65074,0.67668,0.67991,0.64853,0.
17621,0.17614,0.17775,0.17735,0.17761,0.1724,0.17645,0.1678,0.17953,0.
17264,0.18227,0.17706,0.17642,0.17469,0.1768,0.1806,0.17822,0.17441,1.
6041,1.5962,1.6012,1.5891,1.5923,1.5935,1.5962,1.5962,1.5974,0.90131,0
.71811,0.6509,0.63171,0.70727,0.66186,0.61002,0.72324,0.67646,0.65468,
0.66571,0.18031,0.16716,0.17629,0.1706,0.17893,0.17692,0.17589,0.1677,
0.17468,0.17573,0.18799,0.17548,0.17615,0.1739,0.18389,0.18123,0.17705
,0.1752,1.5947,1.5831,1.6064,1.606,1.6015,1.5901,1.6096,0.74505,0.7954
9,0.87141,0.66899,0.70545,0.73124,0.54947,0.63639,0.67694,0.64735,0.61
118,0.6712,0.57728,0.17663,0.17725,0.17497,0.17495,0.17278,0.17125,0.1
8206,0.1793,0.17575,0.17576,0.17433,0.17611,0.17672,0.17693,0.17934,0.
17705,0.16855,0.17452,1.5966,1.593,1.5943,1.5933,1.6057,1.5855,0.84879
,0.90477,0.82397,0.89139,0.87528,0.61019,0.61607,0.69944,0.64267,0.618
03,0.68922,0.717,0.5645,0.71461,0.185,0.1755,0.17661,0.18269,0.17127,0
.18012,0.17615,0.17522,0.17381,0.17467,0.17547,0.17629,0.18137,0.1753,
0.17314,0.18096,0.18171,0.17625,1.599,1.5952,1.6024,1.6034,1.6019,1.08
26,0.87147,0.64926,0.83194,1.0005,0.92941,0.6825,0.48769,0.67813,0.674
61,0.6514,0.65144,0.6288,0.64953,0.70264,0.1753,0.17638,0.17724,0.1816
8,0.174,0.17655,0.17587,0.17114,0.18337,0.1771,0.1799,0.17169,0.17325,
0.18514,0.17381,0.17265,0.17675,0.17201,1.6023,1.6001,1.5923,0.70274,0
.89663,0.8409,0.74308,0.86934,0.87674,0.8176,0.87219,0.57517,0.62823,0
.68844,0.67243,0.65721,0.51471,0.67495,0.56829,0.6622,0.17018,0.1708,0
.17747,0.1816,0.17632,0.17615,0.17864,0.17646,0.16949,0.177,0.17366,0.
17726,0.18173,0.17674,0.17758,0.17677,0.17645,0.17615,1.6036,0.80013,0
.87291,0.90005,0.87424,1.0722,0.69652,0.87484,0.83145,1.0284,0.65015,0
.44759,0.71276,0.60093,0.69933,0.62539,0.68009,0.49864,0.6481,0.17571,

0.17544,0.17173,0.17632,0.177,0.17304,0.17239,0.18218,0.18313,0.17075,
0.177,0.17657,0.17264,0.17881,0.17088,0.18033,0.186,0.14606,0.86571,0.
87311,0.8677,0.8751,0.87584,0.91928,0.83487,1.0003,0.71144,0.98783,0.7
5634,0.69597,0.66043,0.65054,0.54721,0.62438,0.68231,0.6325,0.48692,0.
17637,0.17444,0.17911,0.17559,0.18184,0.17438,0.176,0.1767,0.17908,0.1
7577,0.17681,0.16986,0.16634,0.17642,0.17586,0.17215,0.17168,0.14497,1
.0352,0.86841,0.8735,0.94415,0.89211,0.89807,0.84317,0.88521,0.83442,0
.85451,0.65099,0.73333,0.74618,0.64788,0.70644,0.65009,0.52382,0.7036,
0.17175,0.17404,0.1726,0.17316,0.1757,0.17571,0.17863,0.17665,0.17835,
0.17298,0.19539,0.17286,0.18069,0.17944,0.1771,0.16842,0.1762,0.17636,
0.14521,1.0478,0.86829,0.7394,1.0191,0.79544,0.87376,0.84017,0.81893,0
.92159,0.92908,0.65189,0.68206,0.67136,0.6619,0.66559,0.71462,0.75527,
0.66298,0.17153,0.17448,0.18245,0.17548,0.16292,0.17915,0.17616,0.1816
6,0.17403,0.18239,0.17944,0.18357,0.17782,0.17431,0.17598,0.17216,0.17
209,0.14528,0.14554,0.14544,0.81433,0.82781,0.8446,0.89526,1.0344,0.82
779,0.87488,0.89707,0.86971,0.61607,0.71766,0.65504,0.80634,0.71105,0.
60183,0.17689,0.17617,0.1769,0.17227,0.17655,0.1803,0.16859,0.17723,0.
18677,0.17618,0.17569,0.17561,0.17626,0.17611,0.18832,0.18175,0.17459,
0.17491,0.14559,0.14515,0.14559,0.14512,0.71855,0.68908,1.0777,0.93704
,0.96308,0.89462,0.88859,0.46777,0.87694,0.75486,0.49279,0.64825,0.176
26,0.17663,0.17262,0.17241,0.1726,0.17561,0.17663,0.17748,0.17343,0.17
107,0.17617,0.18022,0.1709,0.17648,0.17608,0.17647,0.1793,0.17247,0.18
19,0.17675,0.14588,0.146,0.14474,0.1451,0.14543,0.14597,1.0222,0.90618
,0.87422,0.90253,0.94236,1.1145,0.8834,0.83983,0.17658,0.18147,0.17037
,0.18187,0.17054,0.17707,0.17585,0.17401,0.17912,0.17595,0.17736,0.177
68,0.17539,0.17534,0.17647,0.17608,0.17382,0.17755,0.17512,0.18774,0.1
7102,0.17612,0.1754,0.14535,0.14548,0.14499,0.14516,0.14537,0.1453,0.8
9124,0.84739,0.86711,0.96881,0.87474,0.14517,0.14546,0.17267,0.17378,0
.1741,0.1763,0.17355,0.17608,0.17726,0.17672,0.17495,0.17811,0.17694,0
.17624,0.17407,0.17014,0.18047,0.18023,0.18265,0.1768,0.17943,0.17597,
0.17636,0.14533,0.14529,0.14548,0.14558,0.14576,0.14479,0.14557,0.1458
5,0.14571,0.14488,0.94243,0.90792,0.14508,0.14545,0.14543,0.14557,0.17
527,0.17616,0.17346,0.17947,0.17281,0.18297,0.17668,0.17343,0.17509,0.
17657,0.17844,0.18233,0.17886,0.17606,0.17272,0.17266,0.17686,0.17911,
0.17428,0.14577,0.14602,0.14533,0.14614,0.1452,0.14579,0.14533,0.14557
,0.14508,0.14568,0.14535,0.14607,0.14573,0.14552,0.14565,0.14534,0.145
12,0.17654,0.17409,0.17631,0.17582,0.17425,0.17598,0.17783,0.17503,0.1
7678,0.18265,0.17255,0.18038,0.17685,0.18013,0.18027,0.17636,0.14508,0
.1455,0.1454,0.14499,0.14481,0.14569,0.14538,0.14518,0.14455,0.14503,0
.14564,0.14486,0.1449,0.14599,0.14487,0.14619,0.14527,0.14543,0.14558,
0.18088,0.17612,0.17129,0.17214,0.17585,0.17134,0.17762,0.17162,0.1762
2,0.17004,0.17314,0.17756,0.17576,0.14492,0.1456,0.14601,0.14504,0.146
26,0.14539,0.14476,0.14533,0.14468,0.1457,0.1447,0.14509,0.14551,0.145
11,0.14528,0.14499,0.14517,0.14545,0.14571,0.14592,0.14535,0.17375,0.1
7578,0.17357,0.17533,0.17813,0.17579,0.17611,0.18085,0.17893,0.14545,0
.1453,0.14514,0.14505,0.14546,0.14503,0.14527,0.14503,0.14584,0.14579,
0.14513,0.14483,0.14578,0.14579,0.14547,0.14486,0.14528,0.14514,0.1452
2,0.14448,0.14564,0.14553,0.14583,0.14443,0.14571,0.18,0.17588,0.1764,
0.17633,0.1459,0.14497,0.14636,0.14565,0.14489,0.14543,0.14529,0.14488
,0.14542,0.14558,0.14523,0.14491,0.1454,0.14524,0.14495,0.14492,0.1459
1,0.1453,0.14518,0.14518,0.14495,0.14474,0.14547,0.14501,0.14548,0.144
98,0.14556,0.1458,0.14505,0.14494,0.14542,0.14546,0.14586,0.14554,0.14

534,0.14614,0.14542,0.14548,0.1461,0.14536,0.14516,0.14529,0.14538,0.1
4524,0.1449,0.14558,0.14561,0.14546,0.1452,0.14492,0.1452,0.14495,0.14
503,0.14538,0.14499,0.14497,0.14508,0.14507,0.1456,0.14475,0.14567,0.1
4533,0.14498,0.14538,0.14514,0.14588,0.14519,0.1453,0.14562,0.14594,0.
14527,0.14516,0.14547,0.14599,0.14531,0.14493,0.14514,0.14431,0.14569,
0.14553,0.14605,0.1451,0.14555,0.14519,0.14543,0.1456,0.14595,0.14575,
0.14541,0.14555,0.14556,0.1454,0.14531,0.14508,0.14551,0.14514,0.14452
,0.14484,0.14438,0.14488,0.14629,0.14507,0.14502,0.14566,0.14507,0.144
88,0.14537,0.14529,0.14545,0.14487,0.14525,0.14559,0.1451,0.14591,0.14
523,0.14467,0.14537,0.14512,0.14567,0.14503,0.14509,0.14543,0.14525,0.
14522,0.14555,0.14559,0.14565,0.14505,0.14442,0.14527,0.14525,0.14594,
0.14536,0.14564,0.14525,0.14518,0.14519,0.14556,0.14565,0.14518,0.1447
8,0.14631,0.14585,0.14616,0.14546,0.14527,0.14566,0.14459,0.14493,0.14
546,0.14546,0.14504,0.14513,0.14525,0.14522,0.1452,0.145,0.14553,0.145
74,0.14538,0.14584,0.14629,0.1456,0.14501,0.1455,0.14432,0.14584,0.145
23,0.14479,0.14483,0.14543,0.14529,0.14465,0.14493,0.14584,0.1454,0.14
573,0.14575,0.14535,0.14486,0.14512,0.14556,0.14538,0.14524,0.1453,0.1
4456,0.14503,0.14527,0.14481,0.14531,0.14524,0.14484,0.14531,0.14494,0
.14519
0.22868,0.2296,0.22889,0.22824,0.22847,0.22974,0.22838,0.22807,0.22827
,0.22875,0.22753,0.22915,0.2278,0.22868,0.22908,0.22756,0.22787,0.2294
7,0.22856,0.22884,0.67081,0.55015,0.60452,0.62795,0.22842,0.22876,0.22
904,0.22851,0.22911,0.22874,0.22897,0.22795,0.22852,0.22894,0.2287,0.2
2909,0.22783,0.22864,0.22984,1.1267,0.75459,0.61816,0.71971,0.62583,0.
67461,0.67892,0.22774,0.22832,0.22955,0.2278,0.22762,0.22885,0.22871,0
.22813,0.2282,0.22841,0.22846,0.22982,0.22835,0.22895,0.22829,1.1106,1
.1179,0.64187,0.8038,0.67683,0.69,0.76796,0.81296,0.64213,0.72787,0.22
813,0.22743,0.22828,0.22829,0.22925,0.2282,0.22822,0.22879,0.22911,0.2
2794,0.22907,0.22881,0.2291,0.22879,0.22894,1.1116,1.1208,1.1183,0.696
66,0.62065,0.71094,0.75948,0.58284,0.67662,0.65573,0.72809,0.67029,0.2
2921,0.22782,0.22768,0.22827,0.22831,0.22902,0.22928,0.22854,0.22923,0
.2286,0.22858,0.22792,0.228,0.22889,0.22974,0.22793,1.1235,1.1237,1.12
8,1.115,0.67828,0.67253,0.68442,0.66081,0.75502,0.67088,0.66208,0.6794
9,0.72202,0.7024,0.22916,0.2286,0.22869,0.22894,0.22844,0.22927,0.2290
1,0.22847,0.22901,0.22886,0.22796,0.22935,0.2296,0.22848,0.22941,0.228
64,1.1082,1.1252,1.1118,1.1241,1.1134,1.1177,0.67384,0.65629,0.62857,0
.67989,0.65719,0.6768,0.66371,0.67801,0.68092,0.73042,0.22855,0.2279,0
.22892,0.22939,0.22869,0.22814,0.22878,0.22835,0.22887,0.22823,0.229,0
.22882,0.22843,0.22827,0.22855,0.22968,0.22919,1.1192,1.1223,1.1229,1.
1153,1.1172,1.1219,0.64668,0.71213,0.66501,0.67476,0.61345,0.66935,0.6
2783,0.4736,0.63131,0.72091,0.67576,0.22821,0.22879,0.22948,0.22804,0.
22895,0.22888,0.22895,0.22853,0.22871,0.22826,0.22817,0.22854,0.2283,0
.18299,0.16597,0.19163,0.22923,1.1178,1.1302,1.113,1.1221,1.1257,1.112
6,1.1139,0.67842,0.67143,0.67191,0.62624,0.66732,0.67587,0.601,0.67979
,0.71355,0.71477,0.70143,0.22867,0.22973,0.22923,0.22834,0.22786,0.228
19,0.22869,0.22881,0.2294,0.22826,0.22815,0.18063,0.1759,0.18929,0.161
95,0.17599,0.18321,0.2285,1.1338,1.1297,1.1205,1.1244,1.1202,1.1171,1.
1193,0.6941,0.70559,0.6739,0.67813,0.59283,0.62942,0.61626,0.64934,0.7
3126,0.80341,0.63017,0.22867,0.22867,0.22796,0.22876,0.2283,0.23,0.227
54,0.22856,0.22918,0.173,0.18271,0.18082,0.17504,0.19381,0.17695,0.182
89,0.18296,0.19538,0.22934,1.1108,1.1289,1.1235,1.1118,1.1198,1.1231,1
.1181,0.82105,0.66138,0.66974,0.67319,0.69399,0.72434,0.69408,0.69168,

0.68235,0.62382,0.65833,0.67392,0.22864,0.2298,0.22763,0.22939,0.22914
,0.22793,0.1888,0.18057,0.18037,0.18529,0.18253,0.17834,0.18499,0.1857
9,0.18526,0.18266,0.18192,0.18299,1.1143,1.119,1.1059,1.1113,1.1095,1.
1202,1.113,0.57456,0.57942,0.54774,0.68795,0.6672,0.67411,0.64611,0.67
531,0.65646,0.63124,0.6634,0.76992,0.22915,0.22895,0.22944,0.18908,0.1
8327,0.18596,0.18212,0.17133,0.19332,0.17602,0.1751,0.18211,0.17837,0.
18174,0.18259,0.18431,0.17421,0.18571,1.1198,1.1153,1.1215,1.1234,1.12
51,1.1121,1.1146,1.1221,1.1213,0.67778,0.62637,0.66501,0.65813,0.58511
,0.66969,0.71103,0.8131,0.67317,0.62418,0.60844,0.18807,0.16918,0.1774
4,0.18514,0.1793,0.17942,0.18103,0.18188,0.17792,0.18189,0.17787,0.182
64,0.18344,0.18251,0.18145,0.18268,0.1773,0.17538,1.1203,1.1205,1.116,
1.1247,1.1242,1.1284,1.1162,1.1089,1.1186,0.6542,0.67299,0.49433,0.480
59,0.53505,0.49142,0.50066,0.49453,0.50911,0.51629,0.49181,0.1822,0.18
111,0.18439,0.18325,0.18457,0.17608,0.18227,0.16964,0.18704,0.17635,0.
19042,0.1834,0.18224,0.17987,0.18265,0.18849,0.18552,0.18023,1.1217,1.
1184,1.1191,1.1096,1.1164,1.1139,1.1174,1.1188,1.118,0.64662,0.53728,0
.49401,0.47882,0.52973,0.50255,0.46734,0.54374,0.51029,0.49746,0.50562
,0.18861,0.16877,0.18197,0.17393,0.18602,0.1829,0.18215,0.16926,0.1802
5,0.18125,0.1995,0.1807,0.18198,0.1787,0.19291,0.18836,0.1832,0.18033,
1.1186,1.1038,1.1284,1.1284,1.1258,1.1095,1.1317,0.53937,0.57586,0.627
47,0.50688,0.52695,0.55483,0.42452,0.48396,0.50861,0.49687,0.46805,0.5
0875,0.44422,0.18284,0.18274,0.18001,0.18019,0.17715,0.17511,0.19095,0
.18663,0.18149,0.18161,0.17904,0.18178,0.18285,0.18281,0.18687,0.18296
,0.1707,0.17966,1.1179,1.1157,1.1164,1.1161,1.1248,1.1053,0.61294,0.64
876,0.59614,0.64186,0.63054,0.46791,0.46905,0.5243,0.48728,0.47351,0.5
2353,0.53399,0.43749,0.53832,0.19473,0.18056,0.1824,0.19162,0.17433,0.
18796,0.18194,0.18072,0.17847,0.17942,0.18142,0.18199,0.18954,0.18036,
0.1773,0.18883,0.19093,0.18208,1.1177,1.1179,1.127,1.1227,1.1276,0.771
53,0.62614,0.47571,0.60045,0.71459,0.66583,0.51668,0.38832,0.51349,0.5
097,0.49594,0.49467,0.48198,0.49502,0.53158,0.18066,0.1829,0.18283,0.1
8956,0.17908,0.18192,0.18155,0.17415,0.19258,0.18271,0.18776,0.17514,0
.17822,0.19551,0.17788,0.1766,0.18271,0.17622,1.1241,1.1223,1.1135,0.5
1246,0.6446,0.60868,0.54073,0.62562,0.6298,0.59089,0.6291,0.44527,0.48
128,0.51842,0.50964,0.49917,0.40651,0.51018,0.44066,0.50377,0.17298,0.
17382,0.18367,0.19028,0.18246,0.18209,0.18616,0.18245,0.17197,0.18313,
0.17837,0.18363,0.19086,0.18259,0.18379,0.18247,0.1827,0.18136,1.1283,
0.57944,0.62833,0.64649,0.63059,0.76554,0.50781,0.62984,0.60138,0.7325
3,0.49599,0.36541,0.53486,0.46096,0.52863,0.47861,0.51495,0.39522,0.49
481,0.18179,0.1811,0.17479,0.1822,0.18284,0.17783,0.17634,0.19098,0.19
224,0.17397,0.18267,0.18234,0.17667,0.18564,0.17421,0.18843,0.19689,0.
13649,0.62451,0.63086,0.62432,0.62999,0.63067,0.66103,0.60126,0.71468,
0.51916,0.70434,0.55831,0.5222,0.50347,0.49464,0.42698,0.477,0.51809,0
.4837,0.38779,0.18231,0.17901,0.18627,0.1814,0.19065,0.18001,0.18126,0
.18211,0.18547,0.1815,0.18274,0.17273,0.16725,0.18189,0.18186,0.17587,
0.17572,0.13541,0.73764,0.62482,0.63073,0.67927,0.64184,0.64331,0.6083
8,0.63663,0.60132,0.61707,0.49494,0.55205,0.55549,0.4918,0.53627,0.495
23,0.41089,0.52582,0.17523,0.17901,0.17656,0.17695,0.18164,0.18177,0.1
8601,0.18238,0.18534,0.17718,0.21131,0.17701,0.18871,0.18701,0.1833,0.
17078,0.18209,0.18233,0.13567,0.74476,0.62395,0.53804,0.72761,0.57752,
0.62944,0.60399,0.59014,0.66212,0.66536,0.49833,0.51349,0.50988,0.5048
3,0.50556,0.54044,0.56199,0.50052,0.17513,0.18,0.19125,0.18144,0.16203
,0.18662,0.1819,0.19074,0.17845,0.19138,0.18705,0.19263,0.18516,0.1787

4,0.18177,0.17599,0.17553,0.13538,0.13603,0.1358,0.58748,0.59776,0.607
44,0.64549,0.73659,0.59614,0.62775,0.64397,0.62784,0.47125,0.54252,0.4
9951,0.59688,0.53343,0.46144,0.18351,0.18171,0.18255,0.17606,0.18275,0
.18759,0.17147,0.18303,0.19744,0.18214,0.18105,0.18103,0.18217,0.18198
,0.20001,0.19099,0.17939,0.18031,0.13568,0.13548,0.13562,0.13564,0.524
76,0.50429,0.76705,0.67054,0.69008,0.64208,0.63889,0.35375,0.63112,0.5
6731,0.39106,0.49301,0.18196,0.18235,0.17642,0.17701,0.17705,0.18132,0
.18239,0.18433,0.17778,0.17462,0.18191,0.18872,0.17462,0.18211,0.18207
,0.18209,0.18668,0.17685,0.19038,0.18239,0.13635,0.13632,0.1354,0.1353
3,0.13589,0.13628,0.72861,0.65159,0.63066,0.64807,0.67295,0.79394,0.63
625,0.60593,0.18284,0.19007,0.1728,0.19032,0.17374,0.18242,0.18166,0.1
7841,0.18614,0.18214,0.18355,0.18496,0.18067,0.18138,0.18247,0.18178,0
.17814,0.18407,0.18041,0.19966,0.1747,0.18253,0.1815,0.13544,0.13615,0
.13533,0.13563,0.13572,0.13564,0.64119,0.61335,0.62376,0.69499,0.63166
,0.13515,0.13599,0.17667,0.17852,0.17915,0.18211,0.17852,0.18214,0.183
06,0.18254,0.18004,0.18484,0.18319,0.18206,0.179,0.17222,0.18832,0.188
24,0.19187,0.18251,0.18704,0.18183,0.18201,0.13594,0.13557,0.13593,0.1
355,0.13604,0.13515,0.13615,0.13611,0.13593,0.13516,0.67427,0.65418,0.
13588,0.13599,0.13599,0.13597,0.18095,0.1822,0.17773,0.18729,0.17661,0
.19204,0.18338,0.17779,0.1809,0.18247,0.18486,0.19154,0.18616,0.18208,
0.17757,0.17657,0.18261,0.1858,0.17941,0.13594,0.13635,0.13565,0.13637
,0.13552,0.13633,0.1358,0.13594,0.1353,0.13624,0.1358,0.13635,0.13598,
0.13605,0.13606,0.13577,0.13588,0.18234,0.17892,0.18165,0.18192,0.1791
1,0.18202,0.18478,0.18001,0.1825,0.19184,0.1766,0.18775,0.18305,0.1872
1,0.18757,0.18216,0.13532,0.13608,0.13589,0.13574,0.13528,0.13625,0.13
587,0.13578,0.13485,0.13563,0.13589,0.13528,0.13526,0.13614,0.13522,0.
1364,0.13598,0.13604,0.13593,0.18803,0.18228,0.17488,0.17575,0.18127,0
.17418,0.18395,0.17533,0.18239,0.17264,0.1778,0.18441,0.18181,0.13552,
0.13596,0.13605,0.13531,0.13668,0.13557,0.13535,0.13596,0.1354,0.13596
,0.13527,0.13544,0.13593,0.13569,0.13556,0.13554,0.13581,0.13566,0.135
57,0.13633,0.13587,0.17843,0.18179,0.17786,0.18124,0.18528,0.18173,0.1
8205,0.189,0.18618,0.13575,0.13551,0.13582,0.13569,0.13593,0.1357,0.13
539,0.13524,0.1363,0.13614,0.13578,0.13508,0.13621,0.13617,0.13556,0.1
3522,0.13561,0.13566,0.13567,0.13476,0.13615,0.13604,0.13616,0.13448,0
.13637,0.18759,0.18195,0.1823,0.18203,0.13611,0.13506,0.13659,0.13576,
0.13533,0.13593,0.13588,0.1353,0.13613,0.13573,0.13557,0.1355,0.1355,0
.13591,0.13523,0.13553,0.13615,0.13532,0.13549,0.13551,0.13536,0.13529
,0.13582,0.13546,0.13585,0.13557,0.13559,0.13623,0.13527,0.13534,0.135
64,0.13574,0.13644,0.13607,0.1356,0.13656,0.13563,0.13562,0.13641,0.13
557,0.13571,0.13577,0.13573,0.13561,0.13512,0.13581,0.13589,0.13572,0.
13573,0.13547,0.13571,0.13533,0.1354,0.13575,0.13577,0.1355,0.13566,0.
13568,0.13614,0.13514,0.13599,0.13576,0.13547,0.13584,0.13565,0.13634,
0.13569,0.13566,0.13604,0.13639,0.13582,0.13565,0.13583,0.13647,0.1356
4,0.13555,0.13557,0.13494,0.13641,0.13583,0.13611,0.13551,0.13589,0.13
545,0.13601,0.13622,0.13639,0.13621,0.13585,0.13604,0.1359,0.13576,0.1
3584,0.13525,0.13622,0.13571,0.13519,0.13513,0.13469,0.13541,0.13638,0
.13536,0.13561,0.13625,0.13568,0.13554,0.13561,0.13556,0.13606,0.13535
,0.13546,0.13615,0.13564,0.1365,0.1357,0.1353,0.13559,0.13548,0.13613,
0.13576,0.13545,0.13565,0.13557,0.13573,0.13613,0.13604,0.13597,0.1355
2,0.13495,0.13574,0.13592,0.13655,0.13564,0.13592,0.13542,0.13553,0.13
548,0.13608,0.13617,0.13578,0.13512,0.13677,0.13622,0.13622,0.13597,0.
13594,0.1362,0.13538,0.13536,0.13581,0.13575,0.13529,0.1355,0.13584,0.

13602,0.13586,0.1353,0.13586,0.1361,0.13583,0.13613,0.13664,0.13601,0.
1356,0.13604,0.13459,0.13603,0.13552,0.13537,0.13512,0.1358,0.13555,0.
13517,0.13535,0.13642,0.13577,0.13628,0.136,0.13563,0.13519,0.1355,0.1
3595,0.13575,0.13568,0.13565,0.13492,0.13577,0.13547,0.13548,0.13588,0
.13581,0.1353,0.1357,0.13535,0.13583
0.22802,0.22844,0.22791,0.22756,0.2281,0.2289,0.22708,0.2273,0.2275,0.
2281,0.22681,0.22834,0.22704,0.2281,0.22813,0.22701,0.22684,0.22834,0.
22796,0.22818,0.52584,0.44562,0.48069,0.4988,0.22755,0.22788,0.22848,0
.2274,0.22802,0.22782,0.22804,0.22712,0.22782,0.22769,0.22801,0.22832,
0.22694,0.22796,0.22876,0.84006,0.58322,0.48687,0.55857,0.4961,0.52947
,0.52935,0.22714,0.22745,0.22841,0.22711,0.2266,0.22822,0.22786,0.2273
9,0.22707,0.22743,0.22781,0.22846,0.22786,0.22787,0.22729,0.82208,0.82
477,0.50553,0.61412,0.5285,0.53923,0.59165,0.62195,0.5064,0.56498,0.22
763,0.22657,0.22764,0.22733,0.22834,0.22755,0.22746,0.22772,0.22827,0.
22721,0.22849,0.22783,0.2287,0.22771,0.22804,0.8226,0.82727,0.82757,0.
54238,0.49151,0.55148,0.58607,0.46723,0.529,0.51379,0.56594,0.5249,0.2
2797,0.22714,0.22721,0.22755,0.22742,0.22808,0.22863,0.22745,0.22853,0
.22786,0.22773,0.22749,0.2273,0.22784,0.22869,0.22726,0.83587,0.82984,
0.83567,0.82412,0.53138,0.52607,0.53463,0.51931,0.58377,0.52327,0.5200
2,0.5336,0.56185,0.54937,0.2281,0.22824,0.22785,0.2281,0.22763,0.22802
,0.22813,0.22747,0.22838,0.22805,0.22703,0.22849,0.22895,0.22769,0.227
89,0.22726,0.81377,0.83514,0.82148,0.82944,0.82529,0.82595,0.52846,0.5
1538,0.49651,0.53586,0.51557,0.53008,0.52359,0.53235,0.53147,0.56402,0
.22751,0.22698,0.22843,0.22899,0.22819,0.22756,0.22764,0.22727,0.22783
,0.22735,0.22813,0.2282,0.22771,0.22748,0.22739,0.22865,0.22816,0.8276
2,0.83197,0.82709,0.82321,0.82495,0.83457,0.51003,0.55164,0.52285,0.52
895,0.4865,0.52318,0.49594,0.39281,0.49854,0.56065,0.53051,0.22698,0.2
2789,0.22903,0.22731,0.22755,0.22776,0.22777,0.22821,0.22792,0.22726,0
.22708,0.22759,0.22731,0.17239,0.15258,0.18354,0.22791,0.8272,0.84163,
0.81944,0.83065,0.83495,0.82657,0.82226,0.53194,0.52748,0.52735,0.4947
6,0.52368,0.52787,0.47626,0.53215,0.55335,0.55725,0.54664,0.22802,0.22
883,0.22842,0.22785,0.22719,0.22724,0.22814,0.22807,0.22842,0.22792,0.
22721,0.17055,0.16409,0.18044,0.14746,0.16436,0.1732,0.22732,0.84282,0
.83995,0.83132,0.83354,0.83021,0.82697,0.82966,0.53913,0.54815,0.52848
,0.53241,0.47096,0.49829,0.49124,0.5102,0.56819,0.61402,0.49795,0.2275
9,0.2279,0.22724,0.22783,0.22766,0.2295,0.22676,0.22757,0.22781,0.1605
,0.17229,0.17054,0.16323,0.18597,0.16544,0.17221,0.17209,0.18772,0.228
19,0.82245,0.8383,0.83211,0.82424,0.82714,0.83114,0.82432,0.6241,0.519
19,0.52434,0.52886,0.53876,0.56127,0.53875,0.53957,0.53265,0.49404,0.5
186,0.52806,0.22764,0.22875,0.22642,0.22814,0.22835,0.22698,0.17996,0.
17011,0.16949,0.17562,0.17212,0.16756,0.17521,0.17625,0.17567,0.17239,
0.17108,0.17241,0.82439,0.82458,0.81522,0.82061,0.82333,0.83524,0.8261
2,0.46153,0.46534,0.44406,0.53831,0.52326,0.52798,0.51171,0.52919,0.51
559,0.50041,0.52462,0.59184,0.22838,0.22795,0.22797,0.18012,0.17337,0.
17664,0.17123,0.1587,0.18557,0.16487,0.16326,0.17195,0.16758,0.17154,0
.17229,0.17454,0.16193,0.17615,0.83174,0.82767,0.83329,0.8311,0.83398,
0.81977,0.82211,0.83374,0.82765,0.53267,0.49513,0.52351,0.51789,0.4657
9,0.52433,0.55266,0.6226,0.52775,0.49486,0.48564,0.17853,0.15624,0.166
64,0.17562,0.16822,0.16879,0.17036,0.17136,0.16654,0.17155,0.16645,0.1
7177,0.17343,0.17195,0.17118,0.17276,0.16627,0.16392,0.83285,0.8331,0.
82667,0.83349,0.83313,0.83579,0.8257,0.81866,0.82759,0.51549,0.52752,0
.39318,0.37896,0.41744,0.38904,0.39607,0.39031,0.39835,0.40858,0.38833

,0.17134,0.17001,0.17446,0.17265,0.17531,0.16436,0.17174,0.15651,0.177
77,0.16476,0.18143,0.17354,0.17157,0.16904,0.17239,0.17982,0.17558,0.1
7016,0.83075,0.83002,0.8262,0.81795,0.83166,0.82287,0.8263,0.82843,0.8
2817,0.48622,0.4167,0.39054,0.37546,0.41255,0.39673,0.36983,0.42682,0.
4004,0.39256,0.39828,0.17979,0.15592,0.1717,0.16179,0.17669,0.1724,0.1
7185,0.15611,0.16962,0.17123,0.19281,0.16966,0.17206,0.1683,0.18473,0.
17941,0.17279,0.16971,0.8295,0.81714,0.83607,0.83709,0.83583,0.82094,0
.84169,0.40615,0.43464,0.473,0.39876,0.40847,0.43683,0.33976,0.38326,0
.39884,0.3958,0.37099,0.40406,0.3542,0.17243,0.17215,0.16917,0.16916,0
.16596,0.16376,0.18215,0.17696,0.1715,0.17104,0.16773,0.17141,0.17239,
0.17246,0.17746,0.17257,0.15833,0.1687,0.82541,0.82597,0.82416,0.82445
,0.83874,0.81421,0.46001,0.48489,0.45135,0.4845,0.47259,0.3728,0.37185
,0.40977,0.38416,0.37387,0.41254,0.41349,0.35109,0.42238,0.18671,0.170
12,0.17199,0.18295,0.16236,0.17881,0.17173,0.17033,0.16753,0.16883,0.1
7119,0.17157,0.18051,0.16987,0.166,0.17981,0.18239,0.17173,0.82651,0.8
253,0.83188,0.83155,0.83552,0.57789,0.47288,0.36044,0.45377,0.53295,0.
49782,0.40722,0.31666,0.40256,0.4001,0.39106,0.3895,0.38355,0.39106,0.
42104,0.17017,0.17246,0.17245,0.18069,0.16804,0.17154,0.17135,0.16215,
0.18448,0.17212,0.1782,0.16338,0.16747,0.18818,0.1665,0.16506,0.17214,
0.16481,0.83005,0.83005,0.82608,0.38819,0.48277,0.45919,0.41,0.47173,0
.47171,0.44602,0.47467,0.35612,0.38149,0.40638,0.40052,0.39223,0.32987
,0.40076,0.35245,0.39911,0.16058,0.16163,0.17328,0.18163,0.1721,0.1720
4,0.17633,0.17188,0.15995,0.1731,0.1673,0.17404,0.18234,0.17222,0.1738
7,0.17271,0.17253,0.17071,0.83839,0.43772,0.47096,0.48489,0.47619,0.57
156,0.38457,0.47318,0.45417,0.54967,0.39146,0.30385,0.416,0.36767,0.41
661,0.37995,0.40601,0.3214,0.39012,0.17133,0.17102,0.16291,0.17199,0.1
7251,0.16634,0.16494,0.18199,0.18345,0.1623,0.17238,0.17232,0.16532,0.
17579,0.16222,0.17923,0.18968,0.1167,0.47158,0.47568,0.47182,0.47227,0
.47534,0.49656,0.45348,0.53406,0.39401,0.52431,0.42933,0.40815,0.39653
,0.38963,0.34356,0.3795,0.41025,0.38311,0.31704,0.17185,0.16807,0.1771
4,0.17113,0.18202,0.16908,0.17064,0.17193,0.17533,0.17116,0.17237,0.16
066,0.15392,0.17139,0.17183,0.16433,0.16441,0.11571,0.5522,0.46854,0.4
7614,0.50975,0.48119,0.48298,0.45629,0.47775,0.4545,0.46499,0.39205,0.
43175,0.43255,0.38938,0.42365,0.39193,0.33096,0.40944,0.16364,0.16805,
0.16536,0.16527,0.17117,0.17159,0.17679,0.17206,0.17554,0.16606,0.2072
1,0.16539,0.17938,0.1775,0.17344,0.15809,0.17147,0.172,0.11607,0.55818
,0.46983,0.40658,0.54405,0.43989,0.47522,0.45273,0.44466,0.49838,0.500
75,0.39402,0.40224,0.4013,0.3993,0.39778,0.42552,0.43524,0.39215,0.163
09,0.16893,0.18264,0.17115,0.14749,0.1774,0.17145,0.18234,0.16755,0.18
279,0.17748,0.18403,0.17553,0.16766,0.1717,0.16441,0.16359,0.11573,0.1
1657,0.11604,0.44332,0.44982,0.45917,0.4843,0.55039,0.44831,0.47114,0.
48645,0.47145,0.37476,0.42548,0.39575,0.46388,0.41789,0.36751,0.17372,
0.17148,0.17214,0.16455,0.17281,0.17837,0.15922,0.17262,0.18997,0.1717
1,0.17043,0.17114,0.17197,0.17153,0.19304,0.18247,0.16819,0.16965,0.11
607,0.11585,0.11594,0.11568,0.39958,0.38297,0.5725,0.50523,0.51709,0.4
8163,0.47959,0.27352,0.47401,0.4442,0.31783,0.39011,0.17167,0.17176,0.
16511,0.1656,0.16594,0.17109,0.17198,0.17439,0.16667,0.16299,0.17147,0
.17963,0.16288,0.17113,0.17195,0.17183,0.17713,0.16591,0.18185,0.17224
,0.11647,0.11639,0.11583,0.11564,0.11596,0.11612,0.54729,0.49094,0.474
22,0.48664,0.50322,0.59089,0.47812,0.45742,0.17239,0.18142,0.16013,0.1
814,0.1618,0.17194,0.17186,0.16686,0.17673,0.17171,0.17348,0.17548,0.1
7003,0.17117,0.17218,0.17148,0.16682,0.17424,0.16971,0.19264,0.16281,0

.17246,0.17106,0.11581,0.11657,0.11553,0.11558,0.11553,0.11597,0.4834,
0.46417,0.46945,0.5227,0.47472,0.11516,0.11583,0.165,0.16745,0.16839,0
.17138,0.16768,0.17196,0.17231,0.17223,0.1692,0.17523,0.1729,0.17155,0
.16777,0.15988,0.17876,0.17923,0.18388,0.1722,0.1773,0.17143,0.1719,0.
11627,0.11595,0.1162,0.11601,0.11614,0.11543,0.11622,0.11626,0.11613,0
.11531,0.50579,0.49087,0.11602,0.11629,0.11626,0.1159,0.17046,0.1717,0
.16654,0.17802,0.16505,0.18339,0.17281,0.16661,0.17065,0.17217,0.17494
,0.18303,0.17643,0.17175,0.16636,0.16518,0.17206,0.17589,0.16886,0.116
23,0.11661,0.11574,0.11628,0.11588,0.11654,0.11569,0.11636,0.11535,0.1
1649,0.11595,0.1165,0.11609,0.11606,0.11621,0.1162,0.1159,0.17206,0.16
774,0.17139,0.17173,0.16812,0.17212,0.17491,0.16931,0.1722,0.18357,0.1
6473,0.17847,0.17317,0.1783,0.17788,0.1718,0.11546,0.11627,0.11622,0.1
1575,0.11554,0.11639,0.11607,0.11619,0.11485,0.11564,0.11632,0.11573,0
.1156,0.11643,0.11552,0.1164,0.11578,0.11589,0.11632,0.17905,0.17157,0
.16351,0.16423,0.1706,0.16195,0.17375,0.16365,0.17204,0.16049,0.16652,
0.17455,0.17127,0.11557,0.11604,0.11634,0.11543,0.11697,0.11568,0.1155
4,0.11597,0.11573,0.11626,0.11582,0.11575,0.11659,0.11603,0.11593,0.11
566,0.11606,0.11587,0.11582,0.11605,0.11619,0.16742,0.17142,0.1666,0.1
7057,0.17581,0.17133,0.17168,0.1802,0.17662,0.11598,0.11581,0.11606,0.
11587,0.1159,0.11599,0.11558,0.1156,0.11666,0.11638,0.11608,0.11524,0.
11652,0.11644,0.11595,0.11549,0.1158,0.11619,0.11561,0.11525,0.11644,0
.11627,0.11629,0.11476,0.1164,0.17856,0.17142,0.17178,0.17153,0.11633,
0.11547,0.11676,0.1157,0.1155,0.11598,0.11577,0.11551,0.11632,0.11595,
0.1157,0.1159,0.11562,0.11622,0.11522,0.1156,0.11615,0.1155,0.11566,0.
11594,0.11568,0.1155,0.1159,0.11559,0.11596,0.11567,0.11573,0.11627,0.
11555,0.11543,0.11547,0.11599,0.11623,0.11644,0.11581,0.11682,0.11555,
0.11566,0.1166,0.11574,0.11576,0.11587,0.1156,0.11596,0.1152,0.11607,0
.11621,0.1159,0.11574,0.11574,0.11602,0.11561,0.11541,0.11584,0.11573,
0.11555,0.11554,0.11565,0.11601,0.11558,0.11591,0.11553,0.11568,0.1162
,0.11574,0.1165,0.11599,0.11597,0.11607,0.11634,0.11586,0.11632,0.1158
7,0.11677,0.11577,0.11579,0.1157,0.11539,0.11684,0.11637,0.11641,0.115
74,0.11619,0.11583,0.11611,0.11607,0.11659,0.11626,0.11594,0.11645,0.1
1638,0.11598,0.11583,0.1155,0.11609,0.116,0.11503,0.11529,0.11471,0.11
558,0.11657,0.11589,0.11597,0.11643,0.11604,0.11564,0.11575,0.11581,0.
11616,0.11564,0.11562,0.11613,0.11593,0.11661,0.11578,0.11541,0.11611,
0.11579,0.11637,0.11563,0.11566,0.11579,0.11601,0.11597,0.11599,0.1161
3,0.11605,0.11555,0.11511,0.11592,0.11614,0.11678,0.11587,0.11607,0.11
601,0.11536,0.1159,0.11616,0.11647,0.11606,0.11546,0.11688,0.11644,0.1
1642,0.11603,0.11597,0.11656,0.11585,0.11531,0.116,0.11598,0.11543,0.1
1586,0.11613,0.11612,0.11589,0.11563,0.11617,0.11628,0.11637,0.1161,0.
11666,0.11611,0.11555,0.11624,0.11493,0.11629,0.11549,0.11518,0.11518,
0.11593,0.11555,0.11531,0.11555,0.11642,0.11613,0.11624,0.11593,0.1156
4,0.11558,0.11576,0.1163,0.1157,0.11571,0.11577,0.11511,0.11564,0.1158
3,0.11566,0.11641,0.11596,0.11592,0.11588,0.11572,0.11599
0.21691,0.21694,0.21628,0.21622,0.2167,0.21773,0.21589,0.21576,0.21604
,0.21709,0.21534,0.21714,0.21577,0.21667,0.21716,0.21589,0.21573,0.216
73,0.21688,0.21712,0.43245,0.37609,0.39737,0.41363,0.21668,0.21671,0.2
1718,0.21597,0.21662,0.21664,0.217,0.21588,0.2164,0.21642,0.21682,0.21
665,0.21539,0.21654,0.21768,0.65925,0.47216,0.40178,0.45199,0.41111,0.
43792,0.43324,0.21592,0.21631,0.21688,0.21577,0.21542,0.21697,0.21712,
0.21623,0.21572,0.21573,0.21661,0.21708,0.2174,0.21618,0.21599,0.65039
,0.64888,0.41796,0.49126,0.43611,0.43995,0.48068,0.50075,0.41914,0.462

18,0.21589,0.21543,0.21603,0.21637,0.21728,0.21681,0.2162,0.21676,0.21
707,0.21581,0.21703,0.21649,0.21743,0.21659,0.2168,0.64668,0.64617,0.6
5132,0.44527,0.40638,0.45348,0.47385,0.38844,0.43562,0.42286,0.45888,0
.43173,0.21697,0.21594,0.21605,0.21644,0.21601,0.21678,0.21733,0.21596
,0.21645,0.21692,0.2166,0.21612,0.21626,0.21637,0.21774,0.21575,0.6586
5,0.64941,0.65911,0.65143,0.43945,0.43226,0.43866,0.42509,0.47407,0.43
084,0.42801,0.43767,0.45867,0.44981,0.21678,0.21705,0.21694,0.21714,0.
21633,0.2165,0.21673,0.21645,0.21725,0.21678,0.21595,0.21741,0.21765,0
.21642,0.21684,0.21592,0.63833,0.65912,0.64209,0.6526,0.6476,0.65344,0
.4343,0.42294,0.41146,0.44035,0.42615,0.43846,0.43092,0.43797,0.43696,
0.46041,0.21624,0.21559,0.21717,0.21779,0.21695,0.2161,0.21637,0.21597
,0.21644,0.21618,0.21646,0.21697,0.21675,0.21645,0.21635,0.21808,0.216
73,0.64748,0.6557,0.65464,0.64572,0.64838,0.65758,0.4214,0.44948,0.429
21,0.43366,0.40433,0.42983,0.41012,0.33613,0.41387,0.45917,0.43736,0.2
1517,0.2162,0.21798,0.21594,0.21638,0.2161,0.2163,0.21686,0.2166,0.215
96,0.21562,0.21624,0.21617,0.15644,0.13566,0.16873,0.21662,0.65114,0.6
5731,0.64078,0.65303,0.66004,0.65335,0.64431,0.43843,0.43369,0.43338,0
.40909,0.43119,0.43334,0.39563,0.43762,0.45288,0.45486,0.44548,0.21644
,0.21787,0.21749,0.21643,0.21608,0.21624,0.21678,0.21682,0.21712,0.216
92,0.21595,0.15478,0.14814,0.16602,0.12982,0.14822,0.15775,0.21631,0.6
654,0.65934,0.65746,0.65784,0.65089,0.65002,0.65178,0.44227,0.44758,0.
43447,0.43965,0.39033,0.4133,0.40802,0.42078,0.46712,0.4954,0.41295,0.
2161,0.21653,0.2158,0.21701,0.21677,0.21779,0.21523,0.21643,0.21654,0.
14357,0.15639,0.15529,0.14716,0.17146,0.14941,0.15675,0.15635,0.17312,
0.21651,0.64122,0.6612,0.65242,0.64799,0.65267,0.6561,0.65043,0.50184,
0.4288,0.43018,0.43366,0.44177,0.45929,0.437,0.44138,0.43793,0.40838,0
.42599,0.43485,0.21661,0.21759,0.21488,0.2166,0.21692,0.21646,0.16532,
0.15444,0.15378,0.16025,0.15683,0.15154,0.16017,0.16108,0.16014,0.1566
4,0.15539,0.15682,0.64991,0.64137,0.63926,0.64541,0.64242,0.65617,0.64
662,0.3859,0.38909,0.37396,0.44088,0.43082,0.43507,0.42248,0.43426,0.4
245,0.4149,0.43194,0.47992,0.21714,0.21685,0.21679,0.16534,0.15814,0.1
6146,0.15579,0.14209,0.17116,0.14901,0.14737,0.15609,0.15162,0.15625,0
.15665,0.15924,0.14547,0.16097,0.65295,0.65475,0.65643,0.65825,0.65182
,0.64587,0.64331,0.65235,0.65063,0.4391,0.40918,0.43053,0.42732,0.3873
2,0.428,0.45318,0.5045,0.43391,0.40982,0.40337,0.1634,0.13981,0.15117,
0.16012,0.1526,0.15319,0.15495,0.15606,0.15085,0.15567,0.15064,0.15607
,0.15797,0.15664,0.15604,0.15731,0.15036,0.14817,0.65048,0.66002,0.647
92,0.652,0.65487,0.65552,0.64702,0.63651,0.64709,0.42504,0.43343,0.324
57,0.30989,0.33924,0.31961,0.32507,0.32188,0.32565,0.33575,0.31807,0.1
5591,0.15447,0.15913,0.15728,0.16001,0.14811,0.15628,0.13995,0.16257,0
.1487,0.16649,0.15871,0.1559,0.15317,0.157,0.16511,0.16068,0.15475,0.6
556,0.65334,0.64684,0.64304,0.64714,0.64525,0.64955,0.65448,0.65288,0.
38208,0.33877,0.32023,0.30662,0.33666,0.32597,0.3052,0.34803,0.32689,0
.32316,0.32686,0.1646,0.1394,0.15646,0.14529,0.16164,0.15677,0.15629,0
.13977,0.15389,0.15606,0.17834,0.1537,0.1564,0.15251,0.17039,0.16427,0
.15713,0.15375,0.65645,0.6378,0.65666,0.65988,0.66007,0.64526,0.66033,
0.321,0.34333,0.37336,0.32646,0.33141,0.36031,0.28117,0.31646,0.32521,
0.32829,0.30679,0.3302,0.29334,0.15715,0.15636,0.15369,0.1536,0.15012,
0.1477,0.16707,0.16217,0.1558,0.15509,0.15222,0.15582,0.15635,0.15689,
0.1622,0.15722,0.14171,0.15303,0.64404,0.65018,0.64563,0.64771,0.6603,
0.64184,0.36491,0.38191,0.35853,0.38338,0.37124,0.30788,0.30708,0.3344
3,0.31547,0.3084,0.33888,0.33591,0.29093,0.34652,0.17214,0.15441,0.156

35,0.16846,0.14541,0.16385,0.1561,0.15481,0.15148,0.15289,0.15549,0.15
589,0.16574,0.15439,0.14985,0.16439,0.168,0.15644,0.65305,0.65035,0.65
627,0.65032,0.66145,0.45644,0.3745,0.28551,0.35996,0.41959,0.39316,0.3
3639,0.2656,0.32975,0.32876,0.32114,0.31904,0.31671,0.32167,0.34825,0.
15459,0.15685,0.157,0.16595,0.15217,0.15581,0.15592,0.14584,0.16954,0.
15679,0.16321,0.14714,0.15147,0.17379,0.15066,0.14925,0.15635,0.14838,
0.64713,0.65179,0.64883,0.30665,0.38207,0.3642,0.32567,0.37202,0.37302
,0.35233,0.3787,0.29643,0.31687,0.33208,0.32869,0.32173,0.27686,0.3263
3,0.29341,0.32907,0.14391,0.14537,0.15804,0.16637,0.15654,0.15642,0.16
107,0.15642,0.14352,0.15787,0.15147,0.15853,0.16785,0.15664,0.1587,0.1
5728,0.157,0.15561,0.65777,0.34737,0.3734,0.38234,0.37505,0.45227,0.30
559,0.37545,0.3599,0.43578,0.32075,0.25817,0.33967,0.30414,0.34102,0.3
1198,0.3343,0.26958,0.32107,0.15574,0.1552,0.14662,0.15651,0.15705,0.1
5023,0.14909,0.16689,0.1692,0.146,0.15692,0.15706,0.14934,0.16045,0.14
591,0.16424,0.17556,0.096984,0.3726,0.37685,0.3726,0.37387,0.37635,0.3
9211,0.35853,0.41958,0.31243,0.41312,0.34845,0.3327,0.32617,0.32041,0.
28615,0.31238,0.33723,0.3147,0.26602,0.15632,0.15216,0.16212,0.15565,0
.16749,0.15351,0.155,0.15664,0.16025,0.15549,0.15655,0.14419,0.13735,0
.1559,0.15639,0.14809,0.14823,0.096017,0.43672,0.37087,0.37566,0.40335
,0.38107,0.38186,0.36281,0.37965,0.36088,0.36832,0.32297,0.35457,0.353
47,0.32136,0.34904,0.32173,0.27683,0.33277,0.14781,0.15204,0.14921,0.1
4943,0.15582,0.15615,0.16199,0.15637,0.16047,0.15031,0.19446,0.14955,0
.16421,0.16263,0.15793,0.14157,0.15602,0.15657,0.096195,0.44005,0.3741
4,0.32246,0.43202,0.34742,0.37577,0.3556,0.35162,0.39601,0.39647,0.323
52,0.32939,0.3293,0.32964,0.32629,0.351,0.35305,0.32269,0.14686,0.1529
1,0.16795,0.15582,0.13057,0.1623,0.15585,0.16751,0.15178,0.16848,0.162
38,0.16958,0.16039,0.1515,0.15618,0.14815,0.14741,0.096333,0.096441,0.
096015,0.35002,0.35648,0.36093,0.38169,0.4318,0.3545,0.37305,0.38622,0
.37383,0.30951,0.34847,0.32531,0.37946,0.34098,0.3032,0.15834,0.15566,
0.15633,0.14838,0.15742,0.16354,0.14257,0.15705,0.17574,0.15633,0.1549
,0.15526,0.15668,0.15612,0.17928,0.16818,0.1522,0.15409,0.096167,0.095
838,0.096137,0.095809,0.31585,0.30343,0.45221,0.4012,0.41019,0.37854,0
.38088,0.21863,0.37348,0.3631,0.26624,0.32037,0.15616,0.15608,0.14887,
0.14999,0.14982,0.15556,0.15598,0.1588,0.1508,0.14678,0.15577,0.16452,
0.14668,0.15576,0.15651,0.15637,0.16237,0.14985,0.16692,0.15651,0.0965
93,0.096575,0.096272,0.096041,0.096425,0.096,0.43497,0.38929,0.37549,0
.38426,0.39748,0.46679,0.37705,0.36196,0.15704,0.16672,0.14385,0.16666
,0.1452,0.15651,0.15638,0.15029,0.1615,0.15603,0.15828,0.1602,0.15433,
0.15561,0.15615,0.156,0.15092,0.15888,0.15401,0.17848,0.14705,0.15679,
0.15612,0.096154,0.096774,0.095998,0.095983,0.095957,0.096046,0.38391,
0.36892,0.36817,0.41418,0.37627,0.095443,0.096036,0.14896,0.15135,0.15
257,0.15584,0.15219,0.15681,0.15671,0.15706,0.15375,0.15981,0.15751,0.
15591,0.15199,0.14333,0.16396,0.16425,0.16966,0.15682,0.16245,0.15589,
0.15629,0.096555,0.096402,0.096414,0.096256,0.096525,0.095766,0.096629
,0.096563,0.096254,0.095584,0.40092,0.39059,0.096205,0.096603,0.096494
,0.096209,0.15505,0.15642,0.15063,0.16303,0.14918,0.16829,0.15755,0.15
053,0.15497,0.15699,0.15926,0.16842,0.16146,0.15616,0.15011,0.14917,0.
15638,0.16063,0.15318,0.096478,0.096628,0.096059,0.096348,0.095973,0.0
96495,0.095917,0.096766,0.095541,0.096647,0.096357,0.096771,0.096534,0
.096273,0.096684,0.096469,0.096402,0.15636,0.15153,0.15586,0.15596,0.1
5267,0.15635,0.15954,0.15363,0.1568,0.16907,0.1486,0.16337,0.1576,0.16
295,0.16268,0.15611,0.095978,0.09662,0.096385,0.095742,0.095679,0.0968

08,0.096391,0.09646,0.095072,0.096056,0.096685,0.096227,0.095592,0.096
328,0.095821,0.096609,0.096526,0.09608,0.096617,0.16394,0.15582,0.1474
2,0.14809,0.15527,0.14566,0.15843,0.14747,0.15656,0.14421,0.15067,0.15
915,0.15593,0.096126,0.096348,0.096301,0.095722,0.096811,0.095948,0.09
5804,0.096045,0.095968,0.096624,0.096052,0.095928,0.096647,0.096175,0.
096354,0.096006,0.09643,0.096088,0.096237,0.096084,0.096289,0.15124,0.
15616,0.15069,0.1548,0.16064,0.156,0.15618,0.16535,0.16122,0.096047,0.
096232,0.09632,0.095978,0.096348,0.096364,0.095758,0.096005,0.096665,0
.096719,0.096411,0.095438,0.096648,0.096549,0.096094,0.095778,0.096304
,0.096593,0.095622,0.095755,0.0964,0.096407,0.096538,0.095046,0.096603
,0.16378,0.15607,0.15631,0.15578,0.096462,0.095513,0.096795,0.096073,0
.095559,0.09619,0.095968,0.095948,0.096591,0.096689,0.096157,0.096027,
0.096126,0.096601,0.095266,0.096101,0.096478,0.0958,0.095955,0.096328,
0.095777,0.095791,0.096195,0.096149,0.09631,0.09617,0.095923,0.096621,
0.095541,0.095687,0.095661,0.09608,0.096472,0.096634,0.096361,0.097003
,0.095896,0.096065,0.096887,0.095764,0.095881,0.096242,0.095768,0.0960
14,0.095465,0.096209,0.096174,0.096393,0.095736,0.09595,0.096403,0.096
166,0.09577,0.096177,0.096039,0.095501,0.095893,0.096202,0.096154,0.09
5627,0.0965,0.095688,0.095775,0.096009,0.095967,0.096532,0.096225,0.09
6552,0.096147,0.096647,0.096116,0.096563,0.09582,0.096827,0.096044,0.0
96077,0.095633,0.095803,0.09691,0.096666,0.096833,0.096196,0.096608,0.
095985,0.096253,0.096179,0.096528,0.096374,0.096234,0.096701,0.096508,
0.096305,0.096129,0.095646,0.096482,0.09631,0.095415,0.095456,0.09521,
0.095984,0.096913,0.095891,0.096399,0.097019,0.096298,0.095943,0.09592
6,0.096125,0.09656,0.095965,0.095447,0.096376,0.096484,0.096764,0.0961
4,0.095807,0.096437,0.095871,0.096655,0.095829,0.095866,0.095915,0.096
089,0.096072,0.096226,0.096489,0.096296,0.096192,0.095649,0.096168,0.0
96289,0.097343,0.096313,0.096277,0.096324,0.095501,0.096228,0.096687,0
.096792,0.096154,0.096024,0.097065,0.096556,0.096543,0.096211,0.096023
,0.096857,0.096273,0.095521,0.096155,0.096247,0.095485,0.096385,0.0963
82,0.096164,0.096266,0.095757,0.096478,0.096094,0.096216,0.096221,0.09
7161,0.096404,0.095796,0.096183,0.094996,0.096415,0.095764,0.095642,0.
095581,0.09604,0.095909,0.095398,0.095639,0.096836,0.096377,0.096423,0
.096105,0.095769,0.09563,0.095696,0.096549,0.096066,0.095669,0.095725,
0.095663,0.096256,0.095818,0.09589,0.096798,0.096253,0.096301,0.096114
,0.096185,0.096083
0.20197,0.20141,0.20088,0.20096,0.20165,0.20229,0.2006,0.20083,0.20094
,0.20163,0.20047,0.20197,0.20005,0.20136,0.20154,0.20073,0.20066,0.201
44,0.20113,0.20188,0.3696,0.3278,0.34444,0.35788,0.20167,0.20122,0.201
55,0.20095,0.20166,0.20147,0.20143,0.20029,0.20142,0.20162,0.20172,0.2
0124,0.20038,0.20087,0.20177,0.54726,0.40583,0.34508,0.38553,0.35466,0
.37685,0.37116,0.20076,0.20109,0.20112,0.20084,0.19981,0.20189,0.20182
,0.20144,0.2001,0.20046,0.20128,0.20159,0.2022,0.20065,0.20034,0.53869
,0.54277,0.35953,0.41385,0.3751,0.37734,0.41006,0.42399,0.3594,0.39669
,0.20047,0.19996,0.20114,0.20118,0.20191,0.2016,0.20068,0.20157,0.2019
7,0.20062,0.20151,0.2015,0.20222,0.20138,0.20162,0.54056,0.54137,0.544
89,0.3819,0.35173,0.38833,0.40614,0.33622,0.37242,0.36269,0.39259,0.37
066,0.20146,0.20073,0.20079,0.20112,0.20075,0.20174,0.20158,0.20085,0.
20136,0.20199,0.20124,0.20095,0.20094,0.20091,0.20223,0.20062,0.54732,
0.54101,0.55039,0.54605,0.37552,0.37026,0.37628,0.36729,0.40608,0.3703
6,0.36959,0.37066,0.39307,0.38404,0.20177,0.20141,0.2017,0.20187,0.201
08,0.20111,0.20144,0.20079,0.20179,0.20108,0.20073,0.20162,0.20196,0.2

0112,0.20141,0.20066,0.53039,0.54698,0.53447,0.54772,0.53976,0.54678,0
.37428,0.36079,0.3557,0.37876,0.3663,0.37299,0.37081,0.37602,0.37525,0
.39129,0.20105,0.20046,0.20194,0.2025,0.20155,0.20073,0.20101,0.20093,
0.20141,0.20066,0.2011,0.20154,0.20142,0.20093,0.20097,0.20258,0.20166
,0.53426,0.54548,0.5454,0.54024,0.543,0.54829,0.36324,0.38309,0.36714,
0.37313,0.34725,0.36922,0.3535,0.29567,0.35747,0.39189,0.37507,0.20044
,0.20118,0.2027,0.2008,0.20121,0.20087,0.20128,0.20165,0.20159,0.20068
,0.20024,0.20085,0.20062,0.1406,0.11968,0.15313,0.20137,0.54026,0.5469
1,0.5327,0.54887,0.55211,0.54354,0.53448,0.37551,0.37282,0.36907,0.349
61,0.37021,0.37446,0.34153,0.37658,0.38898,0.39042,0.382,0.20104,0.202
07,0.20208,0.20148,0.20046,0.20095,0.20119,0.20157,0.20203,0.20155,0.2
0048,0.13864,0.13232,0.15018,0.1139,0.13262,0.14218,0.20116,0.55683,0.
54713,0.55096,0.54976,0.53748,0.54322,0.5432,0.37805,0.38206,0.3756,0.
37783,0.33543,0.35469,0.35117,0.36131,0.39901,0.4226,0.35717,0.20138,0
.20097,0.20059,0.20142,0.2014,0.20252,0.20015,0.2012,0.20121,0.12759,0
.14099,0.14001,0.13145,0.15572,0.13349,0.14081,0.14044,0.15705,0.20114
,0.53371,0.5506,0.5434,0.53774,0.54512,0.54627,0.54059,0.425,0.36865,0
.36776,0.37232,0.37768,0.39242,0.37343,0.37694,0.37419,0.35309,0.36521
,0.37296,0.20104,0.2018,0.20011,0.20109,0.20138,0.20139,0.14964,0.1384
5,0.13801,0.14424,0.14121,0.13601,0.14414,0.14531,0.14428,0.14059,0.13
977,0.14072,0.5392,0.5396,0.52902,0.53497,0.53663,0.54294,0.5409,0.334
95,0.33714,0.32553,0.37791,0.36811,0.37446,0.36458,0.37183,0.36221,0.3
5866,0.37153,0.41051,0.20188,0.20149,0.20114,0.14926,0.14215,0.14587,0
.13978,0.12621,0.15552,0.13344,0.13129,0.1401,0.13577,0.14064,0.14095,
0.14348,0.12982,0.14509,0.54549,0.54468,0.54424,0.54827,0.54621,0.5340
2,0.53925,0.54463,0.54204,0.37706,0.35338,0.37257,0.36631,0.33485,0.36
844,0.38999,0.42574,0.37262,0.35178,0.34759,0.14749,0.12413,0.13491,0.
14402,0.13672,0.13715,0.13916,0.14039,0.13486,0.14002,0.13456,0.14014,
0.14192,0.14076,0.14024,0.14141,0.13441,0.13261,0.54278,0.55299,0.5420
1,0.54268,0.54769,0.55218,0.53992,0.53025,0.53887,0.3664,0.37197,0.277
02,0.26515,0.28936,0.2714,0.27818,0.27498,0.27586,0.28656,0.26983,0.14
031,0.13873,0.14316,0.14124,0.14417,0.13221,0.14059,0.12385,0.14664,0.
1328,0.15075,0.14298,0.14014,0.13726,0.14128,0.1491,0.14478,0.13913,0.
54596,0.54368,0.5369,0.53272,0.54303,0.53787,0.54278,0.5446,0.54751,0.
31879,0.28838,0.27438,0.26103,0.28491,0.27783,0.26291,0.2961,0.27922,0
.27439,0.28015,0.14895,0.12324,0.14027,0.12938,0.14617,0.14115,0.14072
,0.12407,0.13794,0.14022,0.16282,0.13748,0.14082,0.13664,0.1547,0.1487
2,0.14162,0.1379,0.55135,0.52677,0.5459,0.55432,0.55052,0.54126,0.5491
6,0.2655,0.28492,0.31093,0.27886,0.2803,0.311,0.23923,0.27108,0.27622,
0.28195,0.26286,0.28201,0.25144,0.1414,0.14063,0.13785,0.13777,0.13447
,0.13157,0.15123,0.14626,0.13976,0.13926,0.13638,0.14059,0.14034,0.140
92,0.14652,0.14101,0.1262,0.13729,0.53801,0.54457,0.53803,0.54065,0.54
981,0.53727,0.30756,0.31643,0.29997,0.32156,0.30963,0.26498,0.2634,0.2
8559,0.26985,0.26328,0.28958,0.28367,0.25047,0.29393,0.15652,0.13836,0
.14078,0.15276,0.12946,0.14819,0.14074,0.13923,0.13564,0.13721,0.13979
,0.14025,0.14989,0.13858,0.13443,0.14869,0.15257,0.14061,0.54499,0.544
17,0.5493,0.54382,0.54758,0.37958,0.31334,0.23661,0.29897,0.34698,0.32
681,0.28751,0.22958,0.2815,0.28004,0.2753,0.27435,0.27143,0.27453,0.29
9,0.13868,0.14105,0.14123,0.14984,0.13657,0.14035,0.1402,0.13013,0.153
75,0.14137,0.14772,0.13135,0.1356,0.15806,0.1346,0.13337,0.14047,0.132
37,0.54328,0.53589,0.5373,0.25459,0.31798,0.3045,0.26918,0.30966,0.310
49,0.29346,0.31718,0.2546,0.2717,0.28373,0.2805,0.27496,0.23969,0.2777

7,0.25248,0.28163,0.12841,0.12916,0.14228,0.15045,0.14056,0.14053,0.14
521,0.14058,0.12751,0.14213,0.1354,0.14281,0.15255,0.14074,0.14303,0.1
4157,0.14104,0.13968,0.54383,0.28777,0.31082,0.32022,0.31253,0.37901,0
.25463,0.31322,0.29872,0.36035,0.27376,0.22605,0.28821,0.26141,0.28969
,0.26546,0.28604,0.2308,0.27605,0.14016,0.13953,0.13071,0.14079,0.1411
6,0.13431,0.13287,0.15132,0.15386,0.13006,0.14088,0.14165,0.1334,0.144
44,0.13017,0.14844,0.16015,0.08063,0.30967,0.31438,0.3103,0.31182,0.31
375,0.32729,0.29795,0.3514,0.26175,0.34384,0.29468,0.28409,0.27864,0.2
7464,0.24594,0.26845,0.28885,0.26891,0.23006,0.14048,0.1363,0.14618,0.
13995,0.15157,0.13754,0.1386,0.14061,0.1444,0.13968,0.1406,0.12831,0.1
2138,0.14039,0.14057,0.13235,0.13258,0.079772,0.36694,0.30916,0.31226,
0.33593,0.31743,0.31903,0.30305,0.31728,0.30173,0.30727,0.27664,0.3027
3,0.29991,0.27486,0.29952,0.27675,0.2382,0.28275,0.13215,0.13635,0.133
52,0.13352,0.14017,0.14024,0.1463,0.14059,0.14453,0.13442,0.17878,0.13
331,0.14851,0.14695,0.14265,0.12612,0.13996,0.14057,0.079919,0.36796,0
.31072,0.26781,0.36244,0.29092,0.31331,0.2962,0.29571,0.3309,0.33069,0
.27617,0.2806,0.2814,0.28209,0.27974,0.30014,0.29839,0.27471,0.13108,0
.13716,0.15208,0.14004,0.11464,0.14642,0.14012,0.15186,0.13566,0.15307
,0.14648,0.15383,0.14432,0.13567,0.14009,0.13237,0.13153,0.080172,0.08
0086,0.079663,0.28929,0.29721,0.30027,0.31819,0.35989,0.29676,0.30778,
0.32276,0.31312,0.26361,0.29726,0.27834,0.3227,0.2902,0.25978,0.14296,
0.14017,0.14065,0.13244,0.14161,0.14815,0.12712,0.14138,0.15997,0.1406
2,0.13889,0.1396,0.14085,0.14013,0.1641,0.15224,0.13641,0.13794,0.0797
52,0.079502,0.080016,0.079654,0.26351,0.2535,0.37648,0.33459,0.34147,0
.31533,0.31719,0.18168,0.31279,0.30952,0.2297,0.27409,0.14034,0.14034,
0.13338,0.13424,0.13411,0.13972,0.14031,0.14311,0.13502,0.13054,0.1396
1,0.14853,0.1307,0.13959,0.14052,0.14081,0.14661,0.13408,0.15144,0.140
78,0.08029,0.080334,0.080122,0.079816,0.080338,0.080008,0.36186,0.3250
8,0.31257,0.31985,0.33142,0.38763,0.3122,0.2998,0.14119,0.15108,0.1283
5,0.15121,0.12931,0.14091,0.14084,0.13438,0.14541,0.14028,0.1425,0.144
66,0.13858,0.14028,0.14081,0.14027,0.13529,0.14302,0.13833,0.16307,0.1
3111,0.1409,0.14002,0.079919,0.080932,0.079811,0.079392,0.079597,0.079
856,0.32074,0.30721,0.30346,0.34422,0.31171,0.079299,0.079796,0.13266,
0.13586,0.13689,0.14007,0.13629,0.14126,0.14098,0.14086,0.13785,0.1441
4,0.1424,0.14003,0.1362,0.12772,0.14821,0.14851,0.15434,0.14134,0.1471
5,0.14044,0.14094,0.080244,0.080013,0.080195,0.079714,0.080455,0.07994
1,0.080447,0.080432,0.079996,0.079164,0.3348,0.32815,0.079803,0.080454
,0.080569,0.079822,0.13918,0.14065,0.13453,0.14726,0.13323,0.15227,0.1
4193,0.13439,0.13934,0.14124,0.14306,0.15257,0.146,0.14058,0.13443,0.1
3326,0.14067,0.14498,0.13734,0.080006,0.080498,0.079751,0.080126,0.079
951,0.080236,0.079811,0.080516,0.07949,0.080384,0.079809,0.080686,0.08
0224,0.080261,0.080424,0.080076,0.08009,0.14046,0.13587,0.14017,0.1403
,0.1365,0.14061,0.14374,0.13787,0.14084,0.15371,0.13226,0.1475,0.14154
,0.14725,0.14693,0.14033,0.079808,0.080585,0.080025,0.079574,0.079565,
0.080298,0.080405,0.080064,0.079079,0.079618,0.080428,0.080176,0.07953
5,0.080163,0.079496,0.080467,0.080243,0.079915,0.080176,0.14825,0.1399
7,0.13127,0.13226,0.13958,0.12948,0.14273,0.13144,0.14088,0.12812,0.13
489,0.14364,0.14013,0.079791,0.080216,0.079972,0.079671,0.080633,0.079
902,0.079719,0.0797,0.079593,0.080791,0.07941,0.079262,0.080347,0.0799
54,0.079808,0.079682,0.079834,0.079759,0.080227,0.079861,0.080257,0.13
513,0.14019,0.13482,0.13889,0.14464,0.13994,0.14078,0.14917,0.14539,0.
079559,0.080102,0.0802,0.079689,0.080021,0.080379,0.079471,0.07984,0.0

80455,0.08041,0.079985,0.079537,0.080319,0.079975,0.079778,0.079784,0.
080185,0.080354,0.079527,0.079962,0.079958,0.079958,0.080139,0.078852,
0.080193,0.14807,0.14003,0.14018,0.13999,0.080095,0.079566,0.080235,0.
079837,0.079626,0.079888,0.079903,0.079701,0.080405,0.080665,0.080078,
0.079805,0.0801,0.080509,0.079201,0.079794,0.080146,0.079515,0.079772,
0.080211,0.079611,0.079379,0.079834,0.079475,0.079945,0.080005,0.07981
9,0.080196,0.079438,0.079698,0.079212,0.080106,0.080015,0.080388,0.079
898,0.080982,0.079767,0.079607,0.080328,0.079556,0.079564,0.080191,0.0
79798,0.079781,0.079356,0.079724,0.079745,0.080073,0.079403,0.079731,0
.080198,0.079804,0.079533,0.079934,0.079811,0.079214,0.079533,0.080059
,0.079929,0.079509,0.080275,0.079597,0.07955,0.079616,0.079752,0.07972
2,0.079861,0.08038,0.079784,0.080341,0.079794,0.080228,0.079614,0.0807
14,0.079585,0.079944,0.079614,0.079486,0.080541,0.080482,0.080789,0.08
0122,0.08026,0.079736,0.080072,0.079525,0.080066,0.080045,0.07987,0.08
0419,0.080032,0.080429,0.079775,0.079336,0.07997,0.080007,0.079348,0.0
79397,0.079132,0.079702,0.080583,0.079459,0.080297,0.080708,0.08023,0.
079844,0.079828,0.079787,0.080332,0.07957,0.079339,0.080156,0.080259,0
.080518,0.079865,0.079489,0.079977,0.079527,0.080421,0.079326,0.079627
,0.079578,0.079921,0.079866,0.079987,0.080389,0.079976,0.079928,0.0796
83,0.080034,0.080339,0.081088,0.080077,0.0804,0.080209,0.079575,0.0799
35,0.080393,0.080505,0.079943,0.079814,0.080683,0.080462,0.080109,0.07
9971,0.079817,0.080884,0.079764,0.07903,0.079921,0.080037,0.079132,0.0
80171,0.079906,0.079998,0.07996,0.079342,0.080277,0.07982,0.080005,0.0
80014,0.080602,0.080371,0.079409,0.079843,0.078843,0.080084,0.079665,0
.079242,0.079262,0.079682,0.079595,0.079557,0.079415,0.080605,0.080283
,0.080183,0.080183,0.079523,0.079546,0.079365,0.080324,0.079813,0.0795
66,0.07903,0.079689,0.079741,0.079699,0.07911,0.080191,0.080045,0.0799
94,0.080149,0.079965,0.080252
0.18572,0.18554,0.18501,0.18506,0.18559,0.18605,0.1852,0.18486,0.18551
,0.18553,0.18484,0.18584,0.18409,0.18548,0.18527,0.18544,0.18466,0.185
54,0.18537,0.18518,0.32826,0.29245,0.30725,0.31969,0.1855,0.18542,0.18
55,0.18527,0.18559,0.18558,0.18581,0.18466,0.18548,0.18588,0.18637,0.1
854,0.18439,0.18498,0.18557,0.4828,0.35912,0.30698,0.34293,0.31435,0.3
3426,0.32875,0.18513,0.18553,0.18488,0.18507,0.18388,0.18577,0.18573,0
.18555,0.18421,0.18428,0.18565,0.18597,0.18592,0.18509,0.18445,0.47417
,0.47952,0.31964,0.36976,0.33468,0.33665,0.36379,0.37599,0.31726,0.350
01,0.18485,0.18411,0.1855,0.18474,0.18625,0.18571,0.18508,0.18564,0.18
611,0.18483,0.18536,0.18571,0.18638,0.1855,0.18576,0.47216,0.47628,0.4
7797,0.33812,0.31295,0.34466,0.35855,0.30138,0.33194,0.32105,0.34931,0
.32792,0.18534,0.18491,0.18503,0.18508,0.18478,0.18561,0.18562,0.18502
,0.18536,0.18565,0.18524,0.18465,0.18488,0.18498,0.18618,0.18481,0.479
27,0.47478,0.48111,0.48374,0.33325,0.33011,0.33456,0.32597,0.35545,0.3
284,0.32941,0.32882,0.35034,0.34239,0.18621,0.18574,0.18559,0.18612,0.
18531,0.18468,0.18563,0.18504,0.18543,0.18495,0.18499,0.18557,0.18591,
0.18487,0.18522,0.18455,0.46121,0.48081,0.47203,0.48434,0.47646,0.4843
,0.33393,0.31956,0.31543,0.3363,0.32709,0.33138,0.32872,0.3354,0.33306
,0.34768,0.18538,0.18449,0.18656,0.18679,0.18592,0.18515,0.18482,0.184
99,0.18509,0.18478,0.18518,0.18552,0.18547,0.18475,0.18488,0.18666,0.1
8555,0.46707,0.47879,0.4804,0.47175,0.47335,0.48576,0.32545,0.33987,0.
32565,0.33066,0.30916,0.3283,0.31565,0.26603,0.31781,0.34742,0.33281,0
.18453,0.1849,0.18681,0.18472,0.18518,0.18473,0.18537,0.18599,0.18564,
0.18495,0.18473,0.18533,0.18511,0.12639,0.10624,0.13848,0.18527,0.4741

2,0.48301,0.4631,0.48069,0.48566,0.47838,0.46791,0.3353,0.33342,0.3271
5,0.31021,0.32717,0.33507,0.30496,0.3356,0.34464,0.3475,0.3381,0.18522
,0.18589,0.18592,0.18581,0.18524,0.18524,0.18522,0.18567,0.18591,0.185
94,0.18496,0.12459,0.1181,0.13577,0.10062,0.11848,0.12807,0.18535,0.48
59,0.48045,0.48222,0.48727,0.46756,0.47758,0.47909,0.33572,0.34148,0.3
376,0.33681,0.29768,0.31617,0.31287,0.32081,0.3543,0.37311,0.3179,0.18
596,0.18525,0.18501,0.18521,0.18504,0.18618,0.18436,0.1852,0.18516,0.1
1395,0.12728,0.12573,0.11761,0.14111,0.11951,0.12674,0.12625,0.14264,0
.18525,0.47329,0.47795,0.47288,0.47295,0.47652,0.47817,0.47934,0.3771,
0.3278,0.32747,0.32725,0.33436,0.34757,0.33146,0.3339,0.33288,0.31433,
0.32512,0.33188,0.18535,0.18583,0.18409,0.18524,0.18518,0.18504,0.1352
1,0.12427,0.12392,0.12998,0.12706,0.12209,0.13018,0.13103,0.13032,0.12
633,0.12585,0.12649,0.47608,0.47497,0.46148,0.47636,0.46951,0.47331,0.
47466,0.29828,0.2995,0.2917,0.33414,0.32633,0.33229,0.3257,0.33093,0.3
2359,0.3187,0.32932,0.36417,0.18555,0.18594,0.18498,0.13527,0.12795,0.
1317,0.12558,0.11285,0.14125,0.11954,0.11761,0.12646,0.12176,0.12664,0
.12685,0.12956,0.11629,0.13093,0.48032,0.47833,0.4761,0.48264,0.48216,
0.4677,0.47943,0.47843,0.47497,0.33427,0.31562,0.33127,0.32642,0.29786
,0.32872,0.34683,0.37655,0.33244,0.31419,0.30921,0.13298,0.11024,0.121
16,0.13003,0.12303,0.12309,0.12481,0.12639,0.12095,0.12587,0.12081,0.1
2581,0.12804,0.12655,0.12655,0.12728,0.12029,0.11864,0.47669,0.48482,0
.47967,0.47368,0.47997,0.48473,0.47807,0.46475,0.47008,0.32799,0.33292
,0.24401,0.2349,0.25667,0.23866,0.24774,0.24417,0.24391,0.2538,0.23809
,0.12625,0.12436,0.12902,0.12727,0.13006,0.11804,0.12663,0.11041,0.132
21,0.11909,0.13619,0.12843,0.12623,0.12326,0.12716,0.13499,0.13074,0.1
2514,0.47696,0.47201,0.47209,0.47075,0.4722,0.47434,0.47506,0.47952,0.
47953,0.28028,0.2545,0.24299,0.23221,0.25111,0.24497,0.23288,0.26331,0
.24732,0.24298,0.24686,0.13437,0.10994,0.12626,0.11526,0.1319,0.12744,
0.12682,0.11076,0.12414,0.12606,0.14804,0.12328,0.12682,0.12259,0.1403
9,0.13421,0.12735,0.12369,0.4823,0.46359,0.48066,0.48943,0.48326,0.477
05,0.47986,0.23359,0.24991,0.27301,0.24569,0.24827,0.27784,0.21087,0.2
394,0.24473,0.24887,0.2329,0.25072,0.22272,0.12724,0.12642,0.1239,0.12
382,0.12081,0.11787,0.1367,0.13211,0.12577,0.12528,0.12253,0.12645,0.1
2579,0.1268,0.1322,0.12687,0.1124,0.1238,0.47256,0.47746,0.4705,0.4717
2,0.48227,0.46983,0.27052,0.27587,0.25932,0.28074,0.27043,0.23692,0.23
229,0.25254,0.2373,0.23244,0.25538,0.25042,0.22149,0.26038,0.14237,0.1
243,0.12686,0.13835,0.11588,0.13439,0.12674,0.12548,0.12138,0.12324,0.
12594,0.12615,0.13579,0.12441,0.12058,0.13456,0.13785,0.12664,0.4788,0
.47829,0.47552,0.47577,0.4772,0.33238,0.27387,0.2064,0.26143,0.30333,0
.2814,0.25504,0.2039,0.24894,0.24821,0.24304,0.24512,0.24067,0.2422,0.
26645,0.12447,0.12715,0.12691,0.13566,0.12247,0.12669,0.12601,0.1168,0
.13941,0.12738,0.133,0.11774,0.12126,0.14337,0.12087,0.11962,0.12638,0
.11822,0.4775,0.46861,0.46807,0.22423,0.27943,0.26527,0.23515,0.26871,
0.27146,0.25574,0.27739,0.22627,0.23944,0.25004,0.24785,0.24275,0.2132
,0.24521,0.22556,0.25013,0.11439,0.11511,0.12831,0.13635,0.12653,0.126
47,0.1308,0.12642,0.11413,0.12831,0.12147,0.12855,0.13784,0.12658,0.12
881,0.12753,0.12702,0.12557,0.47752,0.25096,0.27216,0.27921,0.27265,0.
33238,0.22154,0.27356,0.26144,0.31514,0.24305,0.20231,0.25625,0.23195,
0.25657,0.23626,0.25444,0.20548,0.24399,0.126,0.12531,0.11699,0.12646,
0.1274,0.1206,0.11901,0.13688,0.13972,0.11619,0.12677,0.12711,0.11964,
0.13037,0.11631,0.13378,0.14547,0.068287,0.26917,0.2757,0.2709,0.27211
,0.27125,0.28439,0.26131,0.30857,0.22816,0.30186,0.25662,0.25108,0.246

08,0.2439,0.21821,0.23905,0.25455,0.23936,0.2043,0.12607,0.12219,0.132
12,0.12576,0.1374,0.12341,0.12445,0.1263,0.13005,0.12569,0.12645,0.114
42,0.10809,0.12589,0.12674,0.11848,0.11851,0.067407,0.32336,0.27217,0.
27372,0.2933,0.2795,0.28048,0.26415,0.27789,0.26465,0.26923,0.2451,0.2
6762,0.26364,0.2427,0.2646,0.24521,0.21037,0.24872,0.11838,0.12217,0.1
1965,0.11974,0.12613,0.12665,0.13208,0.12666,0.13039,0.12016,0.16345,0
.11906,0.13458,0.13267,0.12794,0.11237,0.12558,0.12663,0.06807,0.32088
,0.27074,0.23348,0.31828,0.25554,0.2753,0.25813,0.25861,0.28937,0.2882
3,0.24388,0.24677,0.25069,0.24876,0.24908,0.26816,0.26287,0.24326,0.11
768,0.12309,0.13751,0.12583,0.1011,0.13218,0.12589,0.13712,0.12156,0.1
3843,0.1324,0.13958,0.13008,0.12163,0.1261,0.11854,0.11736,0.067751,0.
068098,0.067505,0.25236,0.25891,0.26453,0.2789,0.31223,0.25942,0.27002
,0.28226,0.27312,0.23288,0.26166,0.24664,0.2854,0.25627,0.22995,0.1283
,0.12626,0.12648,0.11867,0.12741,0.13362,0.11335,0.12744,0.14543,0.126
21,0.12449,0.12575,0.12672,0.12599,0.1495,0.1375,0.12252,0.12404,0.067
402,0.0673,0.067691,0.067232,0.22974,0.22121,0.32891,0.29293,0.29603,0
.27374,0.27817,0.15789,0.2731,0.27452,0.20287,0.24296,0.12628,0.12626,
0.11964,0.12053,0.12027,0.12568,0.12625,0.12893,0.12124,0.11688,0.1259
2,0.13374,0.11681,0.12549,0.12673,0.1265,0.13243,0.11996,0.13728,0.126
75,0.067708,0.068038,0.067972,0.067574,0.067863,0.06776,0.31513,0.2826
4,0.27456,0.28008,0.29012,0.33775,0.27167,0.26221,0.12681,0.13711,0.11
444,0.13668,0.11584,0.1269,0.1268,0.12053,0.13137,0.1258,0.12831,0.130
5,0.12414,0.12615,0.127,0.12652,0.12098,0.12902,0.12435,0.14826,0.1177
4,0.12676,0.12651,0.067706,0.068664,0.06768,0.06703,0.067342,0.067754,
0.2814,0.26647,0.2654,0.30128,0.27065,0.067204,0.067582,0.11895,0.1220
4,0.1225,0.1261,0.12265,0.12702,0.12702,0.12664,0.12394,0.12992,0.1283
4,0.12607,0.12229,0.11398,0.13381,0.13398,0.13985,0.12731,0.13307,0.12
661,0.12668,0.068085,0.067841,0.067808,0.067605,0.068225,0.067492,0.06
8466,0.068014,0.067653,0.067122,0.29428,0.28533,0.067592,0.06835,0.068
121,0.067552,0.12526,0.12661,0.12017,0.13288,0.11925,0.13824,0.12769,0
.12089,0.12563,0.12735,0.12854,0.13801,0.13197,0.12658,0.12062,0.1193,
0.12653,0.13086,0.12368,0.06785,0.068219,0.067393,0.067995,0.067818,0.
067979,0.06748,0.067983,0.067385,0.068238,0.067563,0.068195,0.067879,0
.06821,0.068173,0.067755,0.06782,0.12626,0.12177,0.12618,0.1261,0.1224
8,0.12663,0.12955,0.12389,0.12718,0.13905,0.1183,0.13318,0.12738,0.133
07,0.13284,0.12614,0.067457,0.068336,0.067796,0.067034,0.067345,0.0680
4,0.068044,0.067991,0.067147,0.067309,0.068148,0.067895,0.067246,0.067
783,0.067283,0.068096,0.067904,0.067669,0.067789,0.13392,0.12609,0.117
62,0.11859,0.1254,0.11563,0.1289,0.11749,0.12662,0.11443,0.12092,0.129
41,0.12576,0.067726,0.068155,0.067723,0.067621,0.068186,0.067686,0.067
596,0.067477,0.066998,0.068441,0.067195,0.067115,0.068058,0.06784,0.06
729,0.067365,0.067512,0.067642,0.067846,0.067421,0.068083,0.12085,0.12
622,0.12119,0.12481,0.13023,0.12571,0.12674,0.13495,0.13106,0.067457,0
.068065,0.068131,0.067279,0.06799,0.068316,0.067269,0.06734,0.068098,0
.068004,0.067893,0.067235,0.068062,0.067703,0.067522,0.0674,0.067695,0
.068173,0.067494,0.067934,0.068051,0.067928,0.068036,0.066692,0.067633
,0.13396,0.12603,0.12605,0.1262,0.067993,0.067153,0.067963,0.067444,0.
067164,0.067664,0.067609,0.067687,0.067826,0.068234,0.067867,0.067466,
0.067933,0.06808,0.06704,0.06769,0.067835,0.06755,0.067423,0.06772,0.0
67302,0.067626,0.067717,0.067295,0.067523,0.067923,0.067545,0.06833,0.
06688,0.067445,0.067124,0.067458,0.067763,0.068179,0.067471,0.068407,0
.06759,0.067354,0.068064,0.06742,0.067385,0.068171,0.067548,0.06756,0.

06719,0.067511,0.067378,0.067632,0.067421,0.067552,0.067743,0.067522,0
.067131,0.067651,0.067633,0.066748,0.067108,0.067723,0.067841,0.067516
,0.068171,0.067522,0.067527,0.068038,0.067487,0.067739,0.067749,0.0680
62,0.067715,0.06818,0.067242,0.068017,0.067106,0.068311,0.067338,0.067
742,0.067339,0.067268,0.068426,0.06827,0.068302,0.067867,0.067745,0.06
7534,0.06773,0.067464,0.06768,0.067728,0.067792,0.067959,0.067875,0.06
829,0.06741,0.067101,0.067545,0.067868,0.067148,0.067154,0.06685,0.067
453,0.068173,0.06728,0.068253,0.068047,0.06801,0.067457,0.067391,0.067
373,0.067713,0.067475,0.06695,0.067922,0.068075,0.068517,0.067825,0.06
7423,0.067572,0.067233,0.068304,0.067214,0.067617,0.067474,0.067835,0.
067859,0.067758,0.068027,0.067899,0.067567,0.067431,0.067783,0.068065,
0.0691,0.067733,0.068237,0.068053,0.06735,0.067597,0.068074,0.068241,0
.067463,0.067978,0.068682,0.068163,0.067804,0.067361,0.067411,0.068598
,0.067602,0.066943,0.067977,0.067643,0.067214,0.067578,0.067933,0.0674
2,0.067654,0.066827,0.067955,0.067989,0.067733,0.067512,0.067941,0.068
039,0.067402,0.067165,0.067029,0.067823,0.06743,0.067091,0.067065,0.06
7259,0.067377,0.066985,0.06696,0.068543,0.067867,0.067849,0.067668,0.0
67302,0.067394,0.067337,0.068294,0.067725,0.067402,0.066892,0.067525,0
.067561,0.067469,0.067005,0.068153,0.068041,0.067792,0.067535,0.067524
,0.067863
0.17088,0.17047,0.17044,0.17001,0.17113,0.17118,0.17008,0.16977,0.1705
8,0.17113,0.17041,0.17148,0.16931,0.1707,0.17032,0.17066,0.16975,0.170
85,0.17042,0.17056,0.30137,0.26722,0.28184,0.29214,0.1705,0.17091,0.17
081,0.17019,0.17078,0.17107,0.17121,0.16978,0.17045,0.17074,0.17103,0.
17054,0.16959,0.17027,0.17046,0.43404,0.32848,0.28214,0.31519,0.2909,0
.30604,0.30135,0.17034,0.17034,0.1701,0.17005,0.16935,0.1709,0.171,0.1
7029,0.16976,0.16942,0.17066,0.17161,0.17106,0.17019,0.16953,0.43146,0
.43512,0.29106,0.3375,0.30524,0.30936,0.33214,0.34612,0.29206,0.3206,0
.17005,0.16946,0.17037,0.17034,0.1711,0.17087,0.16976,0.17028,0.17112,
0.17,0.17023,0.17089,0.17185,0.17044,0.17112,0.42784,0.43337,0.43681,0
.3085,0.28656,0.31708,0.32861,0.27609,0.30219,0.29582,0.31907,0.30005,
0.17067,0.16986,0.17016,0.17049,0.16983,0.17069,0.17079,0.1699,0.17026
,0.17097,0.17026,0.16957,0.17044,0.16995,0.17137,0.17005,0.4379,0.4328
7,0.44,0.44145,0.30568,0.30386,0.30643,0.29826,0.32556,0.30062,0.30102
,0.30205,0.32083,0.31168,0.17129,0.1709,0.17058,0.17127,0.17052,0.1701
1,0.17097,0.17035,0.17067,0.17015,0.17057,0.17049,0.17088,0.1698,0.170
49,0.1699,0.41932,0.43969,0.43207,0.44673,0.43239,0.44476,0.3067,0.293
64,0.28943,0.30778,0.29954,0.30275,0.30081,0.30567,0.30498,0.31808,0.1
7053,0.16952,0.1718,0.17178,0.1706,0.17019,0.17032,0.17029,0.17006,0.1
7001,0.1703,0.17071,0.17079,0.16971,0.17022,0.17171,0.1703,0.42315,0.4
3675,0.43507,0.4307,0.4359,0.43929,0.29936,0.31043,0.29789,0.30159,0.2
824,0.3027,0.28863,0.24359,0.28914,0.31694,0.30251,0.16962,0.1703,0.17
193,0.16983,0.17073,0.17022,0.17084,0.17116,0.17051,0.17019,0.16978,0.
17008,0.17027,0.1144,0.09545,0.12636,0.17032,0.43134,0.44279,0.42522,0
.44061,0.44702,0.43578,0.42742,0.31,0.30485,0.29971,0.28445,0.30105,0.
30677,0.27938,0.30826,0.31675,0.31745,0.30831,0.17007,0.17077,0.17086,
0.17114,0.17059,0.17009,0.17098,0.17101,0.17055,0.17115,0.17002,0.1128
1,0.10657,0.12355,0.09058,0.10731,0.11676,0.17053,0.44704,0.43367,0.43
827,0.44304,0.43158,0.43215,0.43726,0.31058,0.31442,0.30788,0.30823,0.
27405,0.28848,0.286,0.29452,0.32485,0.3391,0.29182,0.17121,0.17047,0.1
6994,0.17057,0.17029,0.17136,0.1692,0.17097,0.17051,0.10292,0.11564,0.
11369,0.10632,0.12871,0.10795,0.11452,0.11453,0.12998,0.17024,0.4352,0

.43608,0.42927,0.43295,0.43481,0.43944,0.43923,0.34249,0.29918,0.30092
,0.30037,0.3055,0.3185,0.30473,0.30426,0.30559,0.28743,0.29427,0.30373
,0.17012,0.17075,0.16964,0.17052,0.17062,0.1702,0.12305,0.11238,0.1122
4,0.11797,0.11515,0.11053,0.11801,0.11916,0.11822,0.11472,0.11388,0.11
469,0.43509,0.43005,0.41978,0.43665,0.42339,0.43491,0.42586,0.2721,0.2
7346,0.2687,0.30567,0.29873,0.30387,0.29857,0.30121,0.29784,0.29224,0.
3012,0.33326,0.17075,0.1708,0.16985,0.12276,0.11626,0.11969,0.11394,0.
10203,0.12871,0.10804,0.10641,0.11498,0.10996,0.11495,0.1151,0.11787,0
.10524,0.11878,0.44071,0.4353,0.43711,0.43606,0.44046,0.42918,0.43648,
0.43804,0.43185,0.30707,0.28883,0.30415,0.29707,0.27252,0.30168,0.3171
,0.34333,0.30577,0.28792,0.28303,0.12101,0.099175,0.10966,0.11796,0.11
137,0.11151,0.11318,0.11445,0.10941,0.11425,0.10927,0.11434,0.11625,0.
11459,0.1148,0.11547,0.10885,0.10712,0.43235,0.4378,0.43435,0.43282,0.
43861,0.44501,0.43373,0.42811,0.42949,0.29961,0.30383,0.22194,0.21458,
0.23579,0.21789,0.22538,0.2225,0.22162,0.23126,0.21607,0.11437,0.11265
,0.11706,0.11542,0.1184,0.10673,0.11484,0.099522,0.1202,0.10743,0.1237
2,0.1164,0.11448,0.11153,0.11518,0.12291,0.11866,0.1132,0.43137,0.4300
8,0.43069,0.42653,0.43052,0.43384,0.4319,0.43855,0.43736,0.25294,0.231
17,0.22132,0.21359,0.22839,0.22229,0.21221,0.24014,0.22529,0.22076,0.2
2487,0.12232,0.09905,0.11443,0.10362,0.12003,0.11579,0.11523,0.1,0.112
59,0.11424,0.13541,0.1115,0.11485,0.11088,0.12754,0.1221,0.1155,0.1123
9,0.44211,0.42765,0.43941,0.4447,0.4413,0.43472,0.4401,0.21043,0.22638
,0.2471,0.22442,0.22584,0.25388,0.19196,0.21871,0.22383,0.22673,0.2121
4,0.23064,0.20404,0.11546,0.11471,0.11244,0.11199,0.10926,0.1063,0.124
,0.11983,0.11373,0.11371,0.11068,0.11483,0.11408,0.11499,0.12032,0.114
76,0.10102,0.11233,0.43342,0.43974,0.43246,0.43257,0.44224,0.43623,0.2
4779,0.25015,0.23499,0.25497,0.24484,0.21607,0.21156,0.2294,0.21489,0.
21229,0.23296,0.22708,0.20117,0.23816,0.12984,0.11287,0.11511,0.12582,
0.10478,0.12219,0.11486,0.11391,0.10979,0.11165,0.11411,0.11467,0.1235
6,0.11293,0.1086,0.12262,0.12532,0.11498,0.43757,0.43524,0.43716,0.435
81,0.43415,0.30157,0.24674,0.18786,0.23686,0.27525,0.25594,0.23173,0.1
8611,0.22762,0.22698,0.22138,0.22521,0.22215,0.2199,0.245,0.11335,0.11
56,0.1154,0.12308,0.11107,0.11502,0.11418,0.10544,0.12683,0.11571,0.12
069,0.10646,0.10993,0.13074,0.1096,0.10774,0.11441,0.10681,0.43902,0.4
2634,0.42115,0.20262,0.2546,0.24104,0.2144,0.24373,0.24694,0.23387,0.2
5407,0.20635,0.21824,0.22774,0.22536,0.21956,0.19451,0.22305,0.20618,0
.22989,0.10341,0.1037,0.11618,0.12399,0.11474,0.1146,0.11891,0.11474,0
.10271,0.11641,0.10967,0.11661,0.1249,0.11464,0.11642,0.11552,0.11511,
0.11393,0.43857,0.22818,0.24654,0.25354,0.24438,0.30232,0.19769,0.2482
2,0.23883,0.28555,0.22146,0.18488,0.23464,0.20985,0.23331,0.21581,0.23
141,0.18771,0.22107,0.11423,0.11358,0.10573,0.11422,0.11537,0.10904,0.
10753,0.12455,0.127,0.10503,0.11497,0.11517,0.10812,0.11826,0.10482,0.
12156,0.13302,0.05955,0.24415,0.2504,0.24772,0.24643,0.24822,0.25828,0
.23778,0.28091,0.2074,0.27182,0.23314,0.22792,0.22458,0.22342,0.19806,
0.21575,0.23327,0.21855,0.18626,0.11425,0.1109,0.12003,0.11382,0.12474
,0.11198,0.11272,0.11446,0.11774,0.11417,0.1147,0.10346,0.097089,0.114
39,0.11507,0.10729,0.1075,0.058589,0.29314,0.24988,0.24698,0.26558,0.2
5442,0.25314,0.24131,0.25325,0.23918,0.24487,0.22324,0.24498,0.24023,0
.221,0.24044,0.22367,0.19153,0.22572,0.10709,0.11081,0.10821,0.10812,0
.1141,0.11479,0.11997,0.11489,0.11852,0.10865,0.14941,0.10787,0.12196,
0.12037,0.11588,0.10134,0.11371,0.11492,0.059243,0.29029,0.2444,0.2130
3,0.28893,0.2319,0.24978,0.23744,0.2354,0.26315,0.2603,0.22278,0.22517

,0.22768,0.2261,0.2277,0.24281,0.23813,0.22195,0.10638,0.1115,0.1251,0
.11407,0.090451,0.12034,0.11445,0.12478,0.11008,0.12617,0.12038,0.1273
9,0.11816,0.10996,0.11426,0.10724,0.10597,0.059137,0.059261,0.058782,0
.22895,0.23616,0.24009,0.25274,0.28213,0.23412,0.2438,0.25606,0.2502,0
.21179,0.23852,0.22583,0.26107,0.23423,0.20938,0.11619,0.11442,0.11439
,0.10707,0.1153,0.12109,0.10205,0.11566,0.13261,0.11435,0.11295,0.1139
9,0.11482,0.11437,0.13661,0.12543,0.11147,0.11262,0.058671,0.058695,0.
058879,0.058466,0.20746,0.19948,0.3,0.26478,0.27031,0.24663,0.25199,0.
14198,0.24756,0.24912,0.18582,0.22087,0.1143,0.11427,0.10807,0.10915,0
.10922,0.11404,0.11461,0.11682,0.10961,0.10532,0.11371,0.12158,0.1053,
0.11354,0.11475,0.11473,0.12008,0.10833,0.12482,0.11526,0.059,0.059218
,0.05945,0.058722,0.059139,0.05881,0.28567,0.25657,0.24853,0.25631,0.2
6375,0.30763,0.24749,0.23632,0.115,0.12478,0.10339,0.12399,0.10473,0.1
1536,0.11512,0.10901,0.11941,0.11414,0.11638,0.11897,0.11248,0.11428,0
.11519,0.11453,0.10961,0.11722,0.11246,0.13579,0.10641,0.11469,0.11471
,0.058866,0.059853,0.05882,0.058704,0.058721,0.0587,0.25522,0.24218,0.
23743,0.2747,0.24598,0.058367,0.058755,0.10742,0.11018,0.11069,0.11421
,0.11078,0.11494,0.11508,0.11463,0.11227,0.11701,0.11638,0.11431,0.111
02,0.103,0.12146,0.12199,0.12734,0.11528,0.12084,0.11462,0.11484,0.059
112,0.059087,0.058954,0.059122,0.059156,0.05901,0.059756,0.059142,0.05
8928,0.058226,0.26981,0.2598,0.059039,0.059797,0.059145,0.058995,0.113
04,0.11475,0.10887,0.12067,0.10769,0.12553,0.11566,0.1095,0.11386,0.11
568,0.11644,0.1252,0.11982,0.11493,0.10926,0.10782,0.11505,0.11854,0.1
1204,0.059046,0.059025,0.059038,0.059302,0.05891,0.059034,0.05904,0.05
9177,0.058575,0.059275,0.058784,0.059452,0.05906,0.059119,0.059284,0.0
5904,0.059031,0.11443,0.11051,0.11432,0.11443,0.11084,0.115,0.11779,0.
11258,0.11539,0.1267,0.10703,0.12103,0.11543,0.12087,0.12096,0.11437,0
.058549,0.059542,0.05896,0.058301,0.058797,0.059153,0.059169,0.059372,
0.058476,0.058693,0.059322,0.059029,0.058631,0.058803,0.05833,0.059215
,0.059255,0.058907,0.058935,0.12162,0.11422,0.10607,0.10737,0.11369,0.
10427,0.11694,0.10627,0.11466,0.10302,0.10895,0.11735,0.11394,0.058962
,0.059105,0.058822,0.058689,0.059207,0.058742,0.058685,0.058712,0.0584
93,0.059551,0.058081,0.058456,0.059274,0.05921,0.058557,0.058554,0.058
694,0.058946,0.059024,0.058958,0.05898,0.10954,0.11433,0.10954,0.11315
,0.11832,0.11392,0.1149,0.1224,0.11908,0.058994,0.05947,0.0592,0.05864
8,0.05925,0.059479,0.058488,0.05862,0.059619,0.059289,0.05906,0.058398
,0.059229,0.058931,0.058733,0.058529,0.058803,0.059636,0.058598,0.0591
16,0.059251,0.059119,0.059291,0.057888,0.058939,0.12209,0.11416,0.1139
3,0.11446,0.059032,0.058486,0.05906,0.05862,0.058396,0.059008,0.058823
,0.058821,0.05923,0.059544,0.059155,0.058484,0.059542,0.059394,0.05816
1,0.059059,0.058959,0.058716,0.058778,0.059011,0.058736,0.059034,0.058
912,0.058759,0.05873,0.059155,0.058756,0.059434,0.058153,0.058717,0.05
8453,0.058381,0.05899,0.059343,0.058856,0.059586,0.058735,0.058522,0.0
59353,0.058497,0.058418,0.059228,0.058715,0.058791,0.058433,0.058694,0
.058907,0.058803,0.058728,0.058751,0.05894,0.058864,0.058589,0.058809,
0.05887,0.05811,0.058487,0.058931,0.059107,0.058765,0.05906,0.059012,0
.058895,0.059062,0.058519,0.059148,0.059067,0.059375,0.058978,0.059311
,0.058494,0.059538,0.058246,0.059299,0.058623,0.058667,0.058865,0.0584
9,0.059381,0.059486,0.059429,0.059029,0.058944,0.058684,0.058703,0.058
898,0.059105,0.059013,0.058983,0.05933,0.058757,0.059237,0.058675,0.05
847,0.059005,0.059334,0.058364,0.058494,0.058286,0.058684,0.059346,0.0
58581,0.059641,0.059062,0.059199,0.058517,0.058794,0.058603,0.058878,0

.058603,0.058653,0.059132,0.058952,0.059301,0.059167,0.058625,0.058843
,0.058261,0.059425,0.058516,0.059191,0.058872,0.058922,0.059102,0.0591
49,0.059162,0.059387,0.059038,0.05873,0.058932,0.059166,0.059987,0.059
054,0.060061,0.059105,0.058691,0.058965,0.059068,0.059206,0.058664,0.0
58931,0.059369,0.059325,0.058466,0.058852,0.058719,0.059548,0.058952,0
.058308,0.059058,0.059003,0.058102,0.058877,0.059033,0.058889,0.058829
,0.057945,0.059171,0.059197,0.058688,0.05857,0.059107,0.059433,0.05871
2,0.058135,0.05836,0.058898,0.058933,0.058278,0.058223,0.058421,0.0585
18,0.058206,0.058397,0.059494,0.058901,0.058988,0.058973,0.058531,0.05
8813,0.05848,0.059583,0.058854,0.05877,0.058238,0.058847,0.05923,0.058
511,0.058299,0.05941,0.058861,0.058923,0.058647,0.058731,0.059037
0.15778,0.15717,0.1576,0.15719,0.15817,0.15806,0.15761,0.15682,0.15702
,0.15758,0.15719,0.15815,0.15643,0.1575,0.15711,0.15729,0.15674,0.1581
8,0.15758,0.1568,0.28271,0.24954,0.26388,0.27139,0.15712,0.15814,0.157
57,0.1572,0.15781,0.15772,0.15766,0.15676,0.15711,0.1577,0.15766,0.157
4,0.15693,0.15713,0.15702,0.41497,0.30528,0.26351,0.2977,0.27108,0.287
76,0.28172,0.15695,0.15708,0.15704,0.15674,0.15662,0.1581,0.15766,0.15
721,0.15675,0.15596,0.1571,0.15827,0.15825,0.15736,0.15668,0.40678,0.4
0207,0.27056,0.3152,0.28359,0.28895,0.31118,0.32515,0.27369,0.2989,0.1
5701,0.1561,0.15746,0.15693,0.15806,0.15757,0.15676,0.15729,0.15826,0.
15701,0.1573,0.15798,0.15869,0.15714,0.15772,0.4011,0.40731,0.41465,0.
28933,0.26956,0.29567,0.30749,0.2568,0.28295,0.2756,0.29937,0.27974,0.
15785,0.15695,0.15735,0.15672,0.15698,0.15738,0.15768,0.15731,0.1568,0
.15756,0.157,0.15693,0.15757,0.15707,0.15778,0.15715,0.41178,0.41051,0
.41613,0.4168,0.28625,0.2843,0.28809,0.27975,0.30604,0.28177,0.28062,0
.28192,0.3004,0.29492,0.15811,0.15754,0.15743,0.15789,0.15755,0.15689,
0.15817,0.15721,0.1577,0.157,0.15753,0.15729,0.15792,0.15669,0.15743,0
.15698,0.39215,0.41814,0.40679,0.42188,0.4103,0.42023,0.28615,0.27306,
0.27114,0.28712,0.28073,0.28383,0.2814,0.2848,0.28328,0.2981,0.15742,0
.1564,0.1588,0.15844,0.15706,0.15727,0.1575,0.15722,0.15734,0.15683,0.
15689,0.15771,0.15784,0.15645,0.15661,0.15862,0.15753,0.40023,0.41788,
0.40759,0.41032,0.41737,0.41576,0.27933,0.28956,0.27919,0.28352,0.2640
1,0.28247,0.26934,0.227,0.26965,0.29721,0.28205,0.15657,0.1574,0.15891
,0.15681,0.15716,0.15684,0.15748,0.15771,0.15722,0.15703,0.15678,0.157
34,0.15709,0.10476,0.087269,0.11618,0.1571,0.40519,0.41678,0.40175,0.4
1186,0.41976,0.41099,0.40199,0.28849,0.28537,0.27994,0.26565,0.28093,0
.28849,0.26102,0.28901,0.29711,0.29755,0.28968,0.15688,0.1575,0.15772,
0.15798,0.15755,0.15671,0.15787,0.15776,0.15758,0.15787,0.15677,0.1034
,0.09756,0.11341,0.082649,0.097998,0.10679,0.15743,0.42014,0.41119,0.4
0988,0.41402,0.40423,0.40826,0.41115,0.28769,0.29378,0.29129,0.28678,0
.25478,0.2685,0.26762,0.27459,0.30312,0.32126,0.27374,0.1576,0.15698,0
.15735,0.15733,0.15699,0.15816,0.15634,0.15832,0.15719,0.094137,0.1059
5,0.10434,0.097167,0.11826,0.098794,0.10495,0.10505,0.11951,0.15711,0.
41075,0.41169,0.40163,0.40548,0.41293,0.41315,0.4116,0.32039,0.27869,0
.28223,0.27958,0.28549,0.29641,0.28626,0.28505,0.28515,0.26855,0.27371
,0.28337,0.15732,0.15771,0.15667,0.15734,0.15752,0.15736,0.11291,0.103
12,0.10273,0.10824,0.10533,0.10116,0.10821,0.10931,0.10846,0.10484,0.1
0424,0.1052,0.40655,0.40854,0.39645,0.41061,0.39859,0.41161,0.4033,0.2
5285,0.25486,0.24881,0.28701,0.27757,0.28474,0.27914,0.28188,0.28081,0
.27367,0.28167,0.31362,0.1579,0.15753,0.15683,0.11271,0.10687,0.10948,
0.10462,0.093184,0.11833,0.098731,0.097549,0.10547,0.10078,0.10548,0.1
0531,0.10817,0.096437,0.1091,0.41299,0.40918,0.40876,0.41539,0.41362,0

.40775,0.409,0.41407,0.40807,0.28523,0.26989,0.28523,0.27857,0.25256,0
.28185,0.29817,0.32241,0.28437,0.26881,0.26376,0.11091,0.090429,0.1001
4,0.10801,0.10239,0.1023,0.10327,0.10494,0.10018,0.10444,0.099718,0.10
449,0.10655,0.10458,0.10519,0.10594,0.099598,0.098243,0.40647,0.4117,0
.41083,0.40898,0.41346,0.41824,0.40765,0.40364,0.40338,0.28145,0.28332
,0.20536,0.19921,0.22069,0.20394,0.2112,0.20639,0.20741,0.21498,0.2021
9,0.10445,0.1028,0.10737,0.10583,0.10841,0.097792,0.10507,0.09104,0.11
016,0.098275,0.11362,0.10676,0.10501,0.10224,0.10531,0.11252,0.10904,0
.1037,0.40377,0.39935,0.40184,0.40469,0.40153,0.40807,0.40552,0.41542,
0.40637,0.23802,0.2154,0.20724,0.19818,0.21399,0.20713,0.1978,0.22363,
0.20952,0.20609,0.20871,0.11206,0.090722,0.10478,0.094395,0.10967,0.10
619,0.10566,0.09115,0.10334,0.10468,0.12489,0.10228,0.1052,0.10171,0.1
1713,0.11225,0.10578,0.10283,0.41245,0.40562,0.41301,0.41397,0.4137,0.
40825,0.41109,0.19643,0.21233,0.22982,0.20946,0.20932,0.2362,0.17781,0
.20456,0.20831,0.20989,0.19706,0.21331,0.18939,0.10544,0.10535,0.103,0
.10285,0.10017,0.097384,0.1138,0.10972,0.10431,0.10421,0.10119,0.1053,
0.10413,0.10521,0.11045,0.10499,0.092147,0.10274,0.40935,0.4126,0.4077
,0.40985,0.41469,0.40971,0.23145,0.23383,0.21895,0.23695,0.2284,0.2017
,0.19906,0.21495,0.20109,0.19778,0.21805,0.21242,0.18759,0.22192,0.119
03,0.10334,0.10538,0.11523,0.095892,0.11205,0.10491,0.10441,0.10067,0.
10249,0.10444,0.10496,0.11367,0.10351,0.099582,0.11275,0.11465,0.10542
,0.40839,0.40719,0.41348,0.41209,0.41014,0.28259,0.23119,0.17545,0.221
43,0.25562,0.24017,0.21372,0.17236,0.21167,0.21294,0.20631,0.21094,0.2
0619,0.20615,0.22976,0.10387,0.10589,0.1058,0.11286,0.10184,0.10515,0.
10462,0.096327,0.11641,0.10581,0.11134,0.0975,0.1004,0.12015,0.10021,0
.098553,0.10498,0.097646,0.41102,0.4026,0.3965,0.18797,0.23738,0.22695
,0.19941,0.23048,0.23056,0.21836,0.23875,0.19213,0.20282,0.21204,0.210
09,0.20461,0.18122,0.20782,0.19046,0.21623,0.094123,0.095132,0.10671,0
.11363,0.10552,0.1045,0.10921,0.10554,0.093723,0.10664,0.10075,0.10683
,0.11447,0.10498,0.10691,0.10602,0.10558,0.10424,0.41658,0.2142,0.2312
,0.23635,0.22935,0.28209,0.18452,0.23215,0.22321,0.26985,0.20603,0.170
69,0.21939,0.1955,0.21629,0.20229,0.21604,0.17476,0.20611,0.10426,0.10
418,0.096917,0.10469,0.10564,0.099421,0.098273,0.11442,0.11669,0.09628
6,0.10544,0.10565,0.09891,0.10831,0.096074,0.11161,0.12218,0.053269,0.
22928,0.23358,0.23094,0.22946,0.23197,0.24225,0.223,0.26518,0.19678,0.
25799,0.21789,0.21288,0.20907,0.20725,0.18406,0.20268,0.21748,0.20375,
0.17283,0.10451,0.10151,0.11008,0.10376,0.11469,0.10253,0.10355,0.1048
7,0.10784,0.10469,0.10495,0.094521,0.088734,0.10511,0.10532,0.098132,0
.098274,0.052551,0.27426,0.23749,0.22996,0.24672,0.23657,0.23915,0.225
2,0.23666,0.22325,0.22784,0.20903,0.22816,0.22471,0.20694,0.22362,0.20
75,0.17785,0.20936,0.098079,0.10129,0.099273,0.098339,0.10463,0.10514,
0.10974,0.10503,0.10868,0.099441,0.13822,0.098474,0.11153,0.11045,0.10
607,0.092575,0.1042,0.10496,0.052876,0.27326,0.22787,0.20022,0.27083,0
.21705,0.23434,0.22256,0.21949,0.24733,0.24317,0.20802,0.20981,0.21197
,0.21034,0.21336,0.2272,0.22183,0.20723,0.097077,0.10217,0.11469,0.104
73,0.082102,0.11005,0.1049,0.11488,0.10077,0.11589,0.11063,0.11664,0.1
0845,0.10063,0.1047,0.098372,0.096951,0.052922,0.053013,0.052636,0.212
55,0.21992,0.22425,0.23685,0.2644,0.22036,0.2315,0.24113,0.2342,0.1989
6,0.22205,0.21161,0.24176,0.21887,0.19481,0.10659,0.10475,0.10497,0.09
8059,0.10571,0.11156,0.093067,0.10577,0.122,0.10504,0.1034,0.10487,0.1
0499,0.10516,0.12591,0.11528,0.10192,0.10312,0.052664,0.05255,0.052776
,0.051936,0.19262,0.18428,0.28191,0.24827,0.25252,0.23118,0.23764,0.13

081,0.23134,0.23376,0.17226,0.20546,0.10474,0.10438,0.098941,0.099673,
0.10032,0.10443,0.10489,0.10726,0.10054,0.095912,0.1044,0.11153,0.0963
23,0.10399,0.10481,0.10531,0.10999,0.099315,0.1143,0.10565,0.052811,0.
052751,0.053275,0.052852,0.052714,0.052926,0.26941,0.23806,0.23201,0.2
3828,0.24897,0.28931,0.23065,0.22174,0.10514,0.11443,0.094934,0.11382,
0.095869,0.10551,0.10576,0.099614,0.10947,0.10465,0.10668,0.10927,0.10
301,0.10438,0.1056,0.1053,0.10007,0.10707,0.10286,0.12474,0.097197,0.1
0489,0.10495,0.052721,0.053892,0.052999,0.052414,0.052495,0.052677,0.2
3865,0.2254,0.2225,0.25709,0.23159,0.052,0.052549,0.098277,0.10071,0.1
0128,0.10435,0.10151,0.10532,0.10547,0.1051,0.10286,0.10698,0.10673,0.
10456,0.10148,0.093895,0.11161,0.112,0.11696,0.10565,0.11086,0.10482,0
.10536,0.052883,0.052908,0.05294,0.053015,0.053088,0.052803,0.05335,0.
053168,0.052791,0.052388,0.25428,0.2426,0.053,0.053418,0.053014,0.0530
13,0.10361,0.10547,0.099813,0.1105,0.098694,0.11522,0.10607,0.10009,0.
10424,0.10618,0.1066,0.11491,0.11016,0.10503,0.1001,0.098738,0.1055,0.
10862,0.10289,0.052738,0.053017,0.052814,0.053316,0.05315,0.053034,0.0
53014,0.052972,0.052498,0.052989,0.05237,0.053249,0.052774,0.052816,0.
053284,0.052909,0.052879,0.1048,0.10077,0.10466,0.10475,0.10148,0.1057
6,0.10766,0.10334,0.10553,0.11627,0.097924,0.11071,0.10579,0.11083,0.1
1081,0.10469,0.052374,0.053433,0.052987,0.052245,0.052565,0.053136,0.0
53102,0.053372,0.052302,0.052691,0.053032,0.052996,0.052591,0.053012,0
.05228,0.053145,0.052861,0.052669,0.052833,0.11145,0.10465,0.097074,0.
098225,0.10394,0.095272,0.10763,0.097085,0.10492,0.094124,0.09933,0.10
794,0.10444,0.053024,0.053018,0.052905,0.052549,0.053029,0.052538,0.05
2755,0.052752,0.052398,0.05321,0.052209,0.052244,0.053266,0.053023,0.0
52327,0.052532,0.052449,0.052878,0.052766,0.052697,0.05289,0.10041,0.1
0502,0.10031,0.10352,0.10827,0.10474,0.10533,0.11226,0.10946,0.052842,
0.053339,0.053225,0.052643,0.053431,0.053262,0.052557,0.052563,0.05340
8,0.053027,0.05307,0.052602,0.053049,0.052641,0.052488,0.052444,0.0526
53,0.053376,0.052374,0.053115,0.052962,0.053031,0.053046,0.051818,0.05
3145,0.11207,0.10408,0.10457,0.10504,0.052938,0.052529,0.053044,0.0523
1,0.052336,0.053189,0.052635,0.052714,0.052967,0.053463,0.052721,0.052
651,0.053485,0.05328,0.052071,0.052999,0.052598,0.052767,0.052514,0.05
3099,0.052486,0.052944,0.052903,0.052858,0.052332,0.052991,0.052467,0.
053289,0.052199,0.052509,0.052643,0.052209,0.052859,0.053199,0.05288,0
.053463,0.052787,0.052446,0.053049,0.052263,0.052285,0.052947,0.052623
,0.052683,0.052129,0.052626,0.05281,0.052764,0.052606,0.052552,0.05265
8,0.052686,0.052563,0.052957,0.052981,0.052124,0.052447,0.052652,0.052
909,0.052558,0.052844,0.052894,0.052708,0.052858,0.052451,0.053082,0.0
53144,0.053533,0.052652,0.053042,0.052487,0.053383,0.052262,0.053061,0
.052711,0.052664,0.052921,0.052595,0.053321,0.05327,0.053266,0.052837,
0.052726,0.052845,0.052573,0.052849,0.053011,0.053064,0.052764,0.05329
3,0.052637,0.053007,0.052713,0.052213,0.052595,0.053297,0.052354,0.052
464,0.051921,0.052746,0.053105,0.052589,0.05369,0.053023,0.053261,0.05
2235,0.052626,0.052506,0.052959,0.052642,0.052934,0.053025,0.052775,0.
052926,0.053247,0.052714,0.052508,0.05222,0.053154,0.052569,0.053029,0
.052685,0.052868,0.052846,0.052889,0.05286,0.053173,0.053144,0.052726,
0.052761,0.052924,0.053888,0.053202,0.053736,0.05287,0.052842,0.05295,
0.052908,0.053001,0.052702,0.052555,0.052964,0.052911,0.052598,0.05273
5,0.052606,0.053177,0.052702,0.052192,0.05304,0.052848,0.051859,0.0526
93,0.052658,0.052722,0.052471,0.051614,0.053178,0.053206,0.052632,0.05
2307,0.053023,0.053154,0.052581,0.052182,0.052189,0.052692,0.052553,0.

05211,0.052196,0.05223,0.052812,0.052155,0.051954,0.053172,0.052981,0.052961,0.053136,0.052224,0.0528,0.052423,0.053452,0.05291,0.052705,0.05238,0.052788,0.052821,0.052533,0.05235,0.053108,0.052629,0.052738,0.052642,0.052565,0.052876
0.14644,0.14594,0.14613,0.14589,0.14719,0.14681,0.14617,0.14587,0.14596,0.1461,0.14615,0.14678,0.1456,0.14634,0.14591,0.14629,0.14559,0.14711,0.14612,0.14576,0.26807,0.2358,0.25023,0.25648,0.14605,0.14703,0.14649,0.14605,0.14633,0.14626,0.14589,0.14565,0.14577,0.14634,0.14628,0.14589,0.14583,0.14623,0.14595,0.39963,0.29358,0.24833,0.28409,0.25718,0.27332,0.268,0.14589,0.14598,0.14644,0.14565,0.14541,0.14653,0.14642,0.14623,0.14564,0.14485,0.14615,0.14679,0.14687,0.14567,0.14566,0.38707,0.38904,0.25658,0.30508,0.27044,0.27519,0.29609,0.30752,0.25939,0.28522,0.14543,0.14494,0.14624,0.14549,0.1468,0.14606,0.14557,0.14655,0.1474,0.14607,0.14598,0.14626,0.14733,0.14616,0.14665,0.38224,0.39303,0.39248,0.27849,0.2566,0.28206,0.2948,0.24458,0.26822,0.26195,0.28495,0.26604,0.14628,0.14594,0.14608,0.1454,0.14588,0.14617,0.14657,0.1459,0.14584,0.14617,0.14583,0.14587,0.14632,0.14601,0.14628,0.14566,0.39215,0.39635,0.39984,0.3978,0.27212,0.27015,0.27335,0.26368,0.29178,0.26733,0.2639,0.27015,0.28584,0.2783,0.14675,0.14638,0.14596,0.14656,0.14628,0.1455,0.14698,0.1458,0.14662,0.14616,0.1465,0.14666,0.14614,0.14565,0.14609,0.1462,0.36971,0.39645,0.38856,0.40695,0.40177,0.40327,0.27241,0.26098,0.25907,0.27334,0.26689,0.26958,0.26609,0.2688,0.26854,0.28563,0.1462,0.14548,0.14761,0.14695,0.14619,0.14617,0.14632,0.14634,0.14614,0.14534,0.14579,0.14643,0.14672,0.14553,0.14561,0.1472,0.14617,0.38319,0.40166,0.39137,0.39003,0.39964,0.39706,0.26345,0.27442,0.26526,0.26792,0.24947,0.26725,0.25669,0.21337,0.25462,0.28352,0.26778,0.14521,0.14619,0.14745,0.1456,0.14579,0.1454,0.14646,0.14587,0.14602,0.14611,0.14592,0.14575,0.14603,0.097107,0.080933,0.10727,0.14642,0.38866,0.40135,0.38673,0.39974,0.40319,0.3935,0.38402,0.27339,0.27117,0.26763,0.25188,0.26571,0.27557,0.24901,0.27521,0.28266,0.28357,0.27633,0.14558,0.146,0.14682,0.14638,0.14627,0.14578,0.14694,0.14612,0.14614,0.14663,0.14596,0.095669,0.090495,0.10541,0.076538,0.090519,0.09913,0.14605,0.40688,0.39109,0.39251,0.39949,0.38864,0.39554,0.39465,0.2739,0.27835,0.27571,0.27359,0.2405,0.25493,0.25491,0.2593,0.28743,0.31061,0.26005,0.14625,0.1458,0.14613,0.14573,0.14594,0.14673,0.14537,0.14703,0.14612,0.086922,0.098106,0.096659,0.089855,0.10973,0.091536,0.096787,0.097053,0.11101,0.14614,0.39096,0.39473,0.38641,0.38873,0.39927,0.39631,0.3949,0.30788,0.26596,0.26674,0.26582,0.27009,0.2848,0.27077,0.27216,0.27344,0.25672,0.26017,0.26718,0.14646,0.1467,0.14528,0.14617,0.14625,0.14605,0.10468,0.095406,0.095075,0.10036,0.097604,0.093638,0.10013,0.10137,0.10009,0.097232,0.096714,0.097552,0.39395,0.39556,0.38444,0.39703,0.38141,0.39339,0.38926,0.23865,0.24122,0.23571,0.275,0.26251,0.27048,0.26549,0.26685,0.26514,0.25709,0.26507,0.29777,0.14683,0.1461,0.14532,0.10428,0.099105,0.10153,0.096899,0.086459,0.10988,0.091559,0.090342,0.097745,0.093477,0.097958,0.09766,0.10023,0.089301,0.10086,0.39891,0.3889,0.39249,0.39442,0.39704,0.39377,0.39615,0.39728,0.39327,0.27175,0.25489,0.27243,0.26459,0.24155,0.26969,0.28226,0.30607,0.27184,0.25485,0.24907,0.10286,0.08384,0.0929,0.10052,0.094912,0.095228,0.095427,0.097278,0.093138,0.09675,0.092346,0.096946,0.098869,0.096997,0.097159,0.098264,0.092196,0.090782,0.38778,0.39436,0.39165,0.39437,0.39579,0.40094,0.39616,0.38462,0.39101,0.2651,0.27186,0.19538,0.18819,0.20982,0.19409,0.19884,0.19466,0.19655,0.20372,0.1922,0.096679,0.095325,0.10011,0.09

7849,0.10048,0.090706,0.096973,0.084127,0.10166,0.091011,0.1054,0.0986
88,0.097612,0.0945,0.097033,0.10402,0.101,0.096134,0.38869,0.38761,0.3
8934,0.38971,0.3828,0.38949,0.38808,0.40054,0.39309,0.22726,0.20615,0.
19502,0.18819,0.20261,0.19676,0.18707,0.21195,0.19799,0.1955,0.19926,0
.10411,0.083911,0.096818,0.087164,0.10159,0.098202,0.097908,0.084409,0
.095758,0.097107,0.11608,0.09468,0.097359,0.094458,0.10829,0.10367,0.0
98188,0.095239,0.39419,0.39069,0.39776,0.39891,0.39514,0.39031,0.3944,
0.18733,0.20355,0.22053,0.19907,0.20044,0.22341,0.16901,0.19382,0.1965
1,0.20082,0.18574,0.20356,0.17805,0.097416,0.097394,0.095656,0.095298,
0.092848,0.090049,0.10565,0.10176,0.096773,0.096462,0.093661,0.097481,
0.096616,0.097686,0.10276,0.097145,0.085423,0.094739,0.3947,0.39495,0.
38952,0.39141,0.39299,0.39187,0.22364,0.22529,0.20725,0.22618,0.21801,
0.19055,0.18979,0.20363,0.18935,0.18693,0.20558,0.20166,0.17886,0.2117
2,0.11037,0.096035,0.097721,0.1064,0.08868,0.10367,0.097561,0.096544,0
.093348,0.095019,0.097364,0.09742,0.10563,0.095714,0.092136,0.10465,0.
1063,0.097752,0.39323,0.39236,0.39508,0.40032,0.39008,0.27043,0.22049,
0.16713,0.20908,0.2426,0.23086,0.20292,0.16229,0.20049,0.20204,0.19682
,0.19993,0.19645,0.19683,0.21677,0.096003,0.098225,0.098227,0.10462,0.
094376,0.097256,0.097013,0.089207,0.10766,0.098136,0.10314,0.090332,0.
092943,0.11131,0.092579,0.091726,0.09708,0.090352,0.39461,0.38341,0.38
511,0.17923,0.23054,0.21534,0.19044,0.22108,0.22186,0.20763,0.22715,0.
18153,0.1925,0.20119,0.19961,0.19349,0.1718,0.19762,0.18153,0.20491,0.
087152,0.088116,0.098841,0.10526,0.098088,0.09682,0.10112,0.097818,0.0
86998,0.098806,0.093368,0.099242,0.10626,0.097204,0.099131,0.098283,0.
09772,0.096421,0.40241,0.20276,0.22478,0.22501,0.21963,0.27028,0.17783
,0.22386,0.21259,0.25592,0.19508,0.16118,0.20993,0.18571,0.20586,0.192
38,0.20742,0.1645,0.19656,0.096604,0.096847,0.089607,0.097029,0.097712
,0.092135,0.091113,0.10604,0.10846,0.089322,0.098164,0.097999,0.091571
,0.10024,0.08892,0.1031,0.11309,0.048813,0.22136,0.22392,0.22359,0.215
84,0.21993,0.23348,0.21335,0.25662,0.18804,0.25049,0.20543,0.2031,0.19
912,0.19561,0.17298,0.19343,0.20839,0.19409,0.16179,0.096663,0.093961,
0.10184,0.0961,0.10618,0.095238,0.096072,0.097131,0.099583,0.097014,0.
096849,0.08771,0.08194,0.097405,0.097828,0.091016,0.091033,0.04837,0.2
5953,0.22622,0.21944,0.23817,0.22691,0.22637,0.21427,0.22624,0.21297,0
.21731,0.1979,0.21726,0.21332,0.19625,0.21229,0.19649,0.16838,0.19942,
0.090666,0.093789,0.091975,0.09142,0.097225,0.09713,0.10224,0.097294,0
.1011,0.09214,0.12832,0.091557,0.10328,0.10241,0.098151,0.085482,0.096
89,0.097489,0.048503,0.26102,0.21764,0.18916,0.25929,0.20772,0.22274,0
.211,0.20803,0.23386,0.23247,0.19854,0.20001,0.20221,0.19899,0.20135,0
.21579,0.21132,0.1976,0.090107,0.094583,0.1062,0.097144,0.076041,0.102
01,0.097148,0.10664,0.093076,0.10745,0.10283,0.10808,0.10047,0.09345,0
.096893,0.090998,0.089799,0.048846,0.048809,0.048188,0.2054,0.20991,0.
21222,0.22639,0.25217,0.21259,0.21952,0.23038,0.22558,0.18835,0.21162,
0.20071,0.22867,0.20706,0.18465,0.098663,0.09709,0.09733,0.090691,0.09
8135,0.10316,0.086365,0.097898,0.11335,0.097385,0.095631,0.097402,0.09
7377,0.097299,0.11693,0.10704,0.09458,0.095571,0.048422,0.048427,0.048
564,0.047787,0.18324,0.17671,0.26631,0.23713,0.24416,0.21939,0.22559,0
.12399,0.22238,0.22191,0.16238,0.19568,0.096969,0.096754,0.091535,0.09
2106,0.09282,0.096471,0.09721,0.099324,0.092964,0.088989,0.096914,0.10
317,0.089308,0.096359,0.097305,0.097692,0.10163,0.092217,0.10607,0.098
22,0.04857,0.048563,0.049005,0.048576,0.048766,0.048629,0.25723,0.2294
1,0.21948,0.2265,0.23818,0.27847,0.22101,0.21195,0.097505,0.10638,0.08

7697,0.10543,0.088727,0.097553,0.098319,0.092568,0.10134,0.097109,0.09
9162,0.10109,0.095617,0.097174,0.097942,0.097667,0.092667,0.099189,0.0
95347,0.11612,0.09006,0.097148,0.09731,0.048751,0.049562,0.048844,0.04
8271,0.048201,0.048775,0.22831,0.21489,0.21276,0.24375,0.22097,0.04770
7,0.048465,0.09106,0.092858,0.093942,0.096655,0.094354,0.097804,0.0977
04,0.097546,0.095543,0.099394,0.098537,0.097209,0.094013,0.086932,0.10
335,0.10363,0.10839,0.097792,0.10262,0.097242,0.097816,0.048386,0.0486
82,0.048609,0.048769,0.049139,0.048483,0.049138,0.048931,0.048567,0.04
8359,0.24412,0.23191,0.048868,0.049328,0.048866,0.048718,0.096219,0.09
7563,0.092389,0.10237,0.091428,0.10708,0.098057,0.092885,0.096284,0.09
8869,0.09871,0.10651,0.10207,0.097399,0.092282,0.091357,0.097941,0.100
61,0.095463,0.048618,0.048832,0.048595,0.048988,0.048959,0.048686,0.04
8637,0.048908,0.048473,0.048952,0.048245,0.048949,0.048176,0.048658,0.
048923,0.049041,0.048533,0.097024,0.093278,0.097154,0.097346,0.093837,
0.097982,0.099686,0.09585,0.097779,0.10796,0.090506,0.1028,0.09817,0.1
0265,0.10307,0.096964,0.048323,0.049318,0.048789,0.048261,0.048255,0.0
48821,0.049066,0.048968,0.048213,0.048571,0.049071,0.048811,0.048307,0
.048595,0.047924,0.048974,0.048743,0.048447,0.048472,0.1035,0.096865,0
.089905,0.090747,0.096299,0.088323,0.099752,0.0902,0.097401,0.087404,0
.091823,0.10024,0.096572,0.048856,0.048597,0.048942,0.048347,0.048757,
0.048577,0.048442,0.048323,0.048043,0.048993,0.047927,0.047798,0.04898
3,0.048848,0.04825,0.048413,0.048377,0.048723,0.048923,0.048855,0.0488
2,0.093243,0.097285,0.092907,0.095972,0.10051,0.09701,0.097465,0.104,0
.10137,0.048766,0.049057,0.049283,0.048439,0.049245,0.049085,0.04845,0
.048309,0.049038,0.049082,0.048774,0.048649,0.048917,0.048554,0.048368
,0.048177,0.048545,0.049169,0.048475,0.048862,0.048971,0.048652,0.0487
65,0.04774,0.048659,0.10355,0.096167,0.097011,0.097306,0.048705,0.0483
48,0.049136,0.048053,0.048374,0.048763,0.048656,0.048452,0.048621,0.04
9665,0.048761,0.048696,0.049218,0.048941,0.048067,0.048753,0.048679,0.
048838,0.048228,0.048641,0.048612,0.048627,0.048693,0.048954,0.047852,
0.048524,0.048002,0.048958,0.048195,0.048145,0.04822,0.048022,0.048621
,0.049209,0.048624,0.04929,0.048445,0.048469,0.048748,0.048081,0.04821
7,0.048771,0.048415,0.048541,0.047999,0.048478,0.048545,0.048572,0.048
294,0.048511,0.048509,0.048519,0.048235,0.048652,0.048685,0.048404,0.0
4822,0.048641,0.048808,0.0484,0.048965,0.048694,0.048647,0.048684,0.04
8443,0.048939,0.048921,0.049406,0.048347,0.048758,0.048264,0.049388,0.
047796,0.048773,0.048698,0.048519,0.049045,0.048286,0.049183,0.049089,
0.049156,0.048896,0.048483,0.048477,0.0486,0.048822,0.048682,0.04929,0
.048594,0.048951,0.048503,0.048999,0.048591,0.048219,0.048648,0.049069
,0.048434,0.048447,0.047711,0.048625,0.048764,0.048408,0.049517,0.0486
28,0.049373,0.048188,0.048323,0.048508,0.049326,0.04849,0.048982,0.048
872,0.048636,0.048745,0.049001,0.04879,0.048658,0.04824,0.048882,0.048
733,0.048667,0.048902,0.048938,0.048891,0.048749,0.048815,0.049057,0.0
49309,0.048692,0.048432,0.048648,0.0494,0.049203,0.049343,0.048562,0.0
48714,0.048552,0.048591,0.048884,0.048173,0.048277,0.048966,0.049317,0
.048583,0.048753,0.04835,0.048581,0.048739,0.048362,0.048849,0.048743,
0.047682,0.04879,0.048656,0.048498,0.048039,0.047401,0.049171,0.048878
,0.048784,0.047887,0.04887,0.048867,0.048361,0.048042,0.048239,0.04822
3,0.048387,0.048078,0.048092,0.048013,0.048354,0.048215,0.048002,0.048
836,0.048798,0.04837,0.049114,0.047849,0.048539,0.04828,0.048932,0.048
623,0.048704,0.048177,0.048797,0.04849,0.048332,0.04837,0.04892,0.0487
18,0.048325,0.048426,0.048292,0.049057

0.13726,0.13663,0.13643,0.13633,0.13806,0.13748,0.13712,0.13661,0.1368
2,0.13692,0.13685,0.13749,0.13624,0.13721,0.13647,0.1373,0.13639,0.137
45,0.13653,0.13658,0.25984,0.22659,0.24021,0.24695,0.13658,0.13745,0.1
3727,0.13689,0.13727,0.13668,0.13666,0.13616,0.1367,0.13665,0.13702,0.
1363,0.13645,0.13684,0.13653,0.38695,0.28008,0.23882,0.27511,0.24819,0
.26167,0.25996,0.13603,0.13675,0.13694,0.13601,0.13595,0.13672,0.13709
,0.13663,0.13638,0.13548,0.13676,0.13693,0.13772,0.13638,0.1361,0.3706
3,0.38151,0.2464,0.29444,0.2575,0.26382,0.28555,0.29747,0.25028,0.2751
5,0.13645,0.13577,0.13654,0.13624,0.13733,0.13686,0.13618,0.13749,0.13
769,0.13713,0.13649,0.13717,0.13831,0.13702,0.13733,0.371,0.37966,0.37
965,0.26828,0.24792,0.27134,0.28354,0.23598,0.25796,0.25407,0.27466,0.
25501,0.13628,0.13663,0.13677,0.13613,0.13674,0.1366,0.13737,0.13637,0
.13686,0.13688,0.13636,0.13663,0.13642,0.13674,0.13647,0.13626,0.37935
,0.38867,0.39082,0.38623,0.25895,0.26016,0.26285,0.25172,0.27867,0.260
17,0.25192,0.26126,0.27416,0.26887,0.13687,0.13754,0.13652,0.13674,0.1
3615,0.13587,0.13758,0.13651,0.13741,0.13639,0.13759,0.13706,0.13702,0
.13666,0.13704,0.13667,0.36165,0.38224,0.37597,0.39306,0.39132,0.39197
,0.26327,0.25134,0.24815,0.26231,0.25384,0.26094,0.25589,0.25769,0.260
57,0.27601,0.13658,0.13623,0.13787,0.1375,0.13656,0.13687,0.13696,0.13
694,0.13698,0.13613,0.13628,0.13734,0.13772,0.1361,0.13614,0.13774,0.1
3685,0.37307,0.38941,0.37664,0.3819,0.39299,0.38258,0.25254,0.26652,0.
25669,0.25786,0.24029,0.25591,0.24889,0.20284,0.24518,0.27171,0.25909,
0.13576,0.13662,0.13816,0.1362,0.13654,0.13599,0.13681,0.13637,0.13653
,0.13693,0.13685,0.13639,0.13694,0.091284,0.075972,0.10032,0.13682,0.3
8013,0.38977,0.37108,0.38763,0.394,0.38527,0.36875,0.26155,0.26294,0.2
5792,0.24089,0.25735,0.26632,0.23764,0.26391,0.27217,0.2721,0.26608,0.
13646,0.13668,0.13711,0.13654,0.13676,0.13679,0.13776,0.13663,0.13669,
0.13726,0.1366,0.089579,0.084347,0.098723,0.071707,0.085283,0.092808,0
.13683,0.39152,0.3763,0.3796,0.38619,0.37373,0.38658,0.38468,0.26585,0
.26773,0.26294,0.26174,0.23045,0.24458,0.2448,0.25072,0.27768,0.29713,
0.24838,0.13672,0.13622,0.13671,0.13607,0.13667,0.1374,0.13632,0.13731
,0.1367,0.081672,0.091938,0.090654,0.084221,0.10254,0.085969,0.090576,
0.090735,0.10415,0.13688,0.38588,0.38212,0.37616,0.3809,0.38724,0.3865
6,0.38418,0.29701,0.25833,0.25518,0.25463,0.2612,0.27658,0.26091,0.262
67,0.26205,0.24647,0.25113,0.25665,0.137,0.13751,0.13653,0.13729,0.136
9,0.13653,0.098162,0.089104,0.089106,0.093758,0.091641,0.087549,0.0941
43,0.095111,0.093586,0.091308,0.090586,0.091408,0.38104,0.38166,0.3786
7,0.38586,0.37256,0.38607,0.38138,0.22801,0.23284,0.22705,0.26421,0.25
218,0.25971,0.25394,0.25544,0.25599,0.24759,0.25511,0.2873,0.13737,0.1
3646,0.13568,0.097792,0.092504,0.095239,0.09085,0.081119,0.10304,0.086
199,0.084345,0.091975,0.087686,0.091972,0.091521,0.093928,0.083747,0.0
94455,0.38612,0.37629,0.38507,0.3802,0.38642,0.38543,0.38882,0.38377,0
.38507,0.26121,0.24557,0.26064,0.25534,0.23125,0.25936,0.27034,0.29567
,0.26024,0.24655,0.24012,0.096392,0.078559,0.087212,0.094135,0.089045,
0.089269,0.089267,0.091197,0.08737,0.090589,0.08617,0.090806,0.09305,0
.090806,0.09101,0.092286,0.086615,0.085169,0.37831,0.38102,0.38157,0.3
854,0.38204,0.39177,0.38697,0.37906,0.38341,0.25467,0.26062,0.18687,0.
18103,0.20257,0.18585,0.18966,0.18713,0.18937,0.19596,0.18393,0.090928
,0.089469,0.093806,0.091507,0.093942,0.085124,0.090712,0.079029,0.0953
81,0.085728,0.098691,0.092547,0.091744,0.088645,0.091067,0.097325,0.09
4919,0.089755,0.37872,0.37755,0.37547,0.37989,0.37161,0.37801,0.38036,
0.3861,0.38302,0.22075,0.19853,0.18673,0.18106,0.19674,0.1881,0.1789,0

.20416,0.18985,0.18842,0.19142,0.097616,0.07895,0.090934,0.081518,0.09
5148,0.092025,0.091836,0.079164,0.089918,0.091367,0.10878,0.088435,0.0
9124,0.08832,0.10116,0.097326,0.091961,0.089222,0.3855,0.37778,0.38693
,0.38721,0.38776,0.38237,0.38353,0.18083,0.19843,0.21327,0.1909,0.1899
3,0.21522,0.16164,0.18745,0.19043,0.1932,0.17805,0.19653,0.17045,0.091
433,0.091193,0.089511,0.089858,0.087539,0.084399,0.098773,0.094876,0.0
90528,0.090531,0.087693,0.09129,0.090296,0.091374,0.096131,0.090834,0.
079822,0.088549,0.38586,0.38152,0.37958,0.37833,0.37805,0.38243,0.2173
,0.21819,0.20113,0.22099,0.21228,0.18412,0.18208,0.19584,0.18155,0.181
01,0.19716,0.1947,0.17084,0.20268,0.10326,0.089784,0.091639,0.10011,0.
082962,0.097303,0.091287,0.090372,0.087864,0.089343,0.091171,0.091022,
0.099163,0.089577,0.086118,0.098274,0.099878,0.091702,0.38273,0.38285,
0.38199,0.39039,0.38432,0.26249,0.21133,0.16081,0.20316,0.23426,0.2219
8,0.19576,0.15499,0.19373,0.1936,0.18927,0.19121,0.18759,0.18921,0.208
88,0.089511,0.091896,0.092043,0.098021,0.088688,0.090987,0.091213,0.08
3821,0.10093,0.092258,0.096684,0.084724,0.086897,0.10455,0.0869,0.0860
14,0.091053,0.084725,0.38055,0.37482,0.37594,0.17419,0.22342,0.21119,0
.18426,0.21414,0.21708,0.202,0.2213,0.17442,0.18292,0.1938,0.19242,0.1
8576,0.16504,0.18857,0.17447,0.19766,0.081798,0.082885,0.092602,0.0985
58,0.091973,0.090836,0.094924,0.091716,0.081757,0.092525,0.08747,0.093
072,0.099744,0.091189,0.093179,0.091965,0.091639,0.090525,0.39281,0.19
581,0.21675,0.21806,0.21221,0.26096,0.17339,0.2171,0.2037,0.24749,0.18
686,0.15359,0.20053,0.17728,0.19883,0.1845,0.19934,0.1576,0.18935,0.09
0442,0.090905,0.084043,0.090845,0.091556,0.086586,0.085263,0.099394,0.
10159,0.083697,0.091986,0.092059,0.085733,0.093861,0.083301,0.096533,0
.10584,0.046114,0.2167,0.21721,0.21775,0.20965,0.21627,0.22496,0.20684
,0.25076,0.18116,0.24448,0.19792,0.19508,0.19035,0.18833,0.16585,0.186
75,0.20012,0.18651,0.1546,0.090673,0.08841,0.095735,0.090249,0.099603,
0.089429,0.090127,0.091142,0.093042,0.090906,0.09065,0.082093,0.077103
,0.091083,0.091502,0.085461,0.085349,0.045813,0.2519,0.22043,0.21401,0
.23119,0.22317,0.21931,0.20529,0.21979,0.20474,0.21187,0.18923,0.20808
,0.20654,0.18872,0.20493,0.18804,0.16115,0.1934,0.084868,0.08807,0.086
016,0.085619,0.091165,0.090956,0.095662,0.091099,0.094745,0.086414,0.1
2026,0.085934,0.096784,0.0962,0.091772,0.080295,0.091001,0.091322,0.04
5777,0.25331,0.20846,0.18238,0.24955,0.20095,0.21578,0.20392,0.20087,0
.22556,0.22711,0.19163,0.19329,0.19455,0.19136,0.19356,0.20733,0.20192
,0.19072,0.084431,0.088572,0.099406,0.091322,0.071702,0.095109,0.09147
,0.10012,0.087093,0.10069,0.096543,0.10112,0.093916,0.087593,0.090955,
0.085223,0.083885,0.046092,0.046011,0.045364,0.19734,0.20479,0.20495,0
.21895,0.24363,0.20746,0.21309,0.22179,0.21753,0.1804,0.20406,0.19225,
0.22034,0.19957,0.17851,0.092383,0.090962,0.091257,0.08487,0.092173,0.
096551,0.080794,0.091687,0.10619,0.091487,0.089803,0.091177,0.091345,0
.091249,0.10895,0.099962,0.088816,0.089724,0.045437,0.045638,0.045804,
0.045315,0.17653,0.16995,0.25934,0.23109,0.23669,0.21266,0.21908,0.119
06,0.21542,0.21423,0.15508,0.18707,0.090854,0.090736,0.085724,0.086093
,0.086811,0.090479,0.091198,0.09309,0.087008,0.083676,0.09089,0.096485
,0.08369,0.090368,0.091159,0.091862,0.095112,0.086247,0.099379,0.09191
1,0.045746,0.045881,0.046063,0.045583,0.045931,0.045955,0.24957,0.2223
,0.21369,0.22305,0.22964,0.27221,0.21277,0.20743,0.091599,0.09992,0.08
2391,0.098408,0.08299,0.091745,0.092104,0.086997,0.095121,0.091153,0.0
93055,0.094557,0.089299,0.091307,0.091574,0.091445,0.08679,0.09262,0.0
89335,0.10852,0.084715,0.090785,0.091358,0.045887,0.046312,0.046171,0.

045475,0.045647,0.045806,0.22148,0.20991,0.2039,0.23658,0.21428,0.0449
32,0.045538,0.08556,0.087066,0.088041,0.090452,0.088594,0.091822,0.091
545,0.09128,0.089841,0.093239,0.092563,0.090999,0.08777,0.081486,0.096
855,0.097094,0.10189,0.091873,0.095771,0.090986,0.091848,0.045372,0.04
5661,0.045807,0.045992,0.046199,0.045749,0.046086,0.046212,0.045706,0.
045397,0.23415,0.22536,0.045938,0.046379,0.046019,0.045883,0.090147,0.
091408,0.086805,0.09581,0.085574,0.10029,0.092253,0.08711,0.090102,0.0
92313,0.092224,0.099851,0.095704,0.091254,0.086503,0.086051,0.091696,0
.094286,0.089683,0.045708,0.045938,0.045579,0.046147,0.045963,0.045879
,0.046049,0.0461,0.045604,0.045853,0.045131,0.046332,0.045255,0.045548
,0.04577,0.046304,0.045558,0.091176,0.087307,0.09112,0.091199,0.087834
,0.091433,0.093569,0.090205,0.091709,0.10101,0.084885,0.096703,0.09177
7,0.09633,0.096534,0.091268,0.045557,0.046201,0.045831,0.045576,0.0455
68,0.046063,0.046278,0.045924,0.045499,0.045271,0.046262,0.045971,0.04
5655,0.045763,0.045165,0.046081,0.045843,0.045417,0.045461,0.097161,0.
090875,0.084215,0.084918,0.090321,0.082623,0.093126,0.084922,0.091554,
0.082173,0.086199,0.093925,0.090516,0.045957,0.045606,0.046308,0.04541
4,0.046018,0.045641,0.045298,0.045529,0.045204,0.045815,0.045288,0.045
084,0.046027,0.045855,0.045406,0.045702,0.045629,0.046105,0.046236,0.0
46182,0.046151,0.087502,0.091364,0.087019,0.090066,0.094049,0.091303,0
.091567,0.097593,0.095186,0.046076,0.046219,0.046033,0.045571,0.046318
,0.046178,0.045459,0.045695,0.046246,0.046062,0.04576,0.045601,0.04593
2,0.045737,0.045465,0.045355,0.045371,0.04629,0.045499,0.045977,0.0462
36,0.045831,0.045661,0.044989,0.045654,0.097062,0.0902,0.091291,0.0911
53,0.045782,0.045284,0.046382,0.045293,0.045459,0.045853,0.045572,0.04
5615,0.045773,0.046832,0.045848,0.045975,0.046568,0.045703,0.045368,0.
045476,0.04575,0.046084,0.045087,0.045517,0.045952,0.045841,0.045979,0
.046396,0.045131,0.045562,0.04537,0.045975,0.045471,0.045325,0.045531,
0.045059,0.045815,0.046225,0.045859,0.046068,0.045936,0.045733,0.04590
2,0.045238,0.04537,0.045907,0.045707,0.045422,0.045097,0.04571,0.04577
7,0.045963,0.04535,0.045452,0.04572,0.045805,0.045481,0.046237,0.04589
3,0.045427,0.045557,0.046142,0.045803,0.045678,0.046257,0.045913,0.045
388,0.045811,0.045416,0.04613,0.046004,0.046926,0.04541,0.045837,0.045
474,0.046368,0.045063,0.045492,0.046035,0.045805,0.0461,0.045824,0.046
151,0.046087,0.046133,0.046221,0.045614,0.045435,0.045806,0.046023,0.0
45673,0.046141,0.046036,0.04611,0.045776,0.046241,0.046141,0.045541,0.
045777,0.046112,0.045668,0.045924,0.045058,0.045887,0.04574,0.045809,0
.046874,0.045636,0.046591,0.045169,0.045564,0.045649,0.046268,0.045964
,0.046179,0.046081,0.045717,0.045668,0.046335,0.04595,0.045667,0.04544
6,0.045794,0.045948,0.045758,0.046085,0.046164,0.045927,0.045695,0.045
85,0.045962,0.046435,0.045749,0.046007,0.045917,0.046377,0.046369,0.04
6245,0.045943,0.045852,0.045617,0.045733,0.045852,0.045513,0.045472,0.
046078,0.046012,0.045794,0.046232,0.045415,0.045797,0.045971,0.045553,
0.046221,0.045616,0.045062,0.045808,0.045804,0.045833,0.045279,0.04493
,0.046256,0.045909,0.045839,0.045289,0.046027,0.046062,0.045635,0.0449
62,0.045526,0.045375,0.045579,0.045624,0.045202,0.04545,0.045294,0.045
348,0.045375,0.046283,0.046023,0.045558,0.046227,0.045232,0.045508,0.0
45112,0.046269,0.045755,0.045583,0.045797,0.04587,0.045484,0.045516,0.
045451,0.046078,0.045863,0.045631,0.045675,0.045373,0.046262
0.12948,0.12892,0.12842,0.12857,0.13061,0.12993,0.12901,0.12918,0.1289
7,0.12918,0.12912,0.13005,0.12867,0.12914,0.12864,0.12909,0.1287,0.130
33,0.12877,0.12853,0.25315,0.21783,0.23328,0.23725,0.12929,0.1302,0.12

983,0.12889,0.12933,0.12885,0.1288,0.12824,0.12901,0.12875,0.12933,0.1
2866,0.12866,0.12845,0.12858,0.37688,0.27299,0.23197,0.26855,0.23851,0
.25473,0.2517,0.12883,0.12875,0.12931,0.12862,0.12868,0.1284,0.12967,0
.12887,0.12871,0.12812,0.1291,0.12921,0.12993,0.12904,0.12842,0.36453,
0.37538,0.23936,0.28453,0.25222,0.25821,0.27784,0.28995,0.24212,0.2683
6,0.12889,0.12833,0.12889,0.1287,0.12938,0.12919,0.12843,0.12956,0.129
39,0.12977,0.12894,0.12933,0.13048,0.1295,0.12948,0.36295,0.37094,0.36
844,0.25786,0.23928,0.26287,0.27376,0.22617,0.25201,0.2463,0.2673,0.24
741,0.12849,0.12891,0.12873,0.12903,0.12897,0.12872,0.13028,0.1289,0.1
2942,0.12916,0.12891,0.12926,0.12852,0.12897,0.12888,0.12886,0.37334,0
.38268,0.38659,0.37838,0.24959,0.25362,0.25367,0.24353,0.27045,0.25301
,0.24448,0.25461,0.26863,0.26031,0.12886,0.12974,0.12905,0.12889,0.128
53,0.12795,0.12965,0.12868,0.12946,0.1286,0.12972,0.12915,0.12908,0.12
871,0.12899,0.12894,0.35396,0.37669,0.3683,0.38564,0.38479,0.385,0.254
78,0.24468,0.24074,0.25334,0.24629,0.2541,0.24984,0.24987,0.25307,0.26
67,0.12897,0.12847,0.13024,0.12966,0.12866,0.1296,0.12971,0.12935,0.12
954,0.12862,0.12884,0.12921,0.12959,0.1285,0.12847,0.12999,0.12883,0.3
6262,0.3821,0.37181,0.37855,0.38352,0.37832,0.2452,0.25792,0.25041,0.2
5066,0.23367,0.24686,0.2403,0.19524,0.23877,0.26377,0.25,0.12765,0.128
46,0.13051,0.12845,0.12897,0.12784,0.12909,0.12837,0.1287,0.12868,0.12
894,0.12859,0.12925,0.086468,0.072143,0.094812,0.1293,0.36848,0.38325,
0.3671,0.37728,0.38737,0.37549,0.36138,0.25537,0.25677,0.24911,0.23404
,0.24814,0.25538,0.22991,0.25565,0.26181,0.26656,0.25772,0.12886,0.128
3,0.12895,0.12879,0.129,0.12914,0.13002,0.12891,0.12943,0.12967,0.1286
4,0.084864,0.079923,0.093141,0.068033,0.080835,0.087972,0.12886,0.3859
6,0.37062,0.37297,0.3785,0.37028,0.37643,0.37693,0.25673,0.26106,0.256
58,0.25183,0.223,0.23895,0.23777,0.24413,0.27061,0.2903,0.24107,0.1289
3,0.12834,0.12899,0.1284,0.12932,0.12941,0.12875,0.12999,0.12859,0.077
743,0.08712,0.085599,0.079839,0.097024,0.08129,0.086027,0.086021,0.098
542,0.12963,0.37641,0.37202,0.36877,0.3748,0.38078,0.38176,0.38064,0.2
8928,0.25045,0.24659,0.24688,0.25426,0.26923,0.25386,0.25522,0.25349,0
.24194,0.24381,0.24878,0.12927,0.12972,0.12918,0.12952,0.1288,0.12908,
0.093002,0.084345,0.084654,0.088535,0.086525,0.082752,0.088998,0.09003
1,0.088608,0.086496,0.085926,0.086503,0.37207,0.37392,0.37196,0.37796,
0.36408,0.38007,0.37928,0.22078,0.2235,0.21843,0.2553,0.24343,0.25317,
0.24473,0.24629,0.24871,0.24027,0.24504,0.28171,0.12942,0.12848,0.1280
5,0.092657,0.087614,0.089808,0.086151,0.076586,0.097279,0.081528,0.079
707,0.087091,0.083215,0.086927,0.086662,0.08853,0.079709,0.089522,0.38
205,0.37248,0.37645,0.37125,0.38186,0.38013,0.38295,0.3739,0.37582,0.2
5615,0.23758,0.25222,0.24717,0.22505,0.25108,0.26315,0.28945,0.25299,0
.23924,0.23427,0.091096,0.074549,0.082525,0.088946,0.084798,0.084648,0
.084572,0.086492,0.082799,0.085817,0.082009,0.085838,0.087715,0.08622,
0.086293,0.087554,0.082362,0.080869,0.37465,0.37515,0.37564,0.37772,0.
37549,0.38277,0.37794,0.3717,0.37329,0.24639,0.25198,0.18048,0.17516,0
.19582,0.18083,0.18483,0.18084,0.18244,0.18931,0.17807,0.086291,0.0849
74,0.088488,0.08659,0.088555,0.080494,0.086239,0.075508,0.090127,0.081
286,0.093285,0.08732,0.086812,0.083813,0.08611,0.091843,0.089623,0.084
963,0.37117,0.36923,0.36836,0.37555,0.36627,0.37163,0.37147,0.37991,0.
37557,0.2179,0.19211,0.18163,0.17631,0.19117,0.18339,0.17406,0.19731,0
.18572,0.18315,0.18585,0.092602,0.074527,0.086361,0.077558,0.090329,0.
087057,0.087143,0.074978,0.085333,0.086415,0.10258,0.083948,0.086278,0
.083847,0.095515,0.092577,0.086894,0.08477,0.37575,0.37196,0.37616,0.3

8024,0.37717,0.37338,0.37548,0.17805,0.194,0.20769,0.18704,0.18545,0.2
0938,0.15734,0.1817,0.18613,0.18896,0.17448,0.19156,0.16467,0.086575,0
.086287,0.084645,0.085381,0.082885,0.080054,0.093234,0.090046,0.085515
,0.085408,0.082839,0.086412,0.085348,0.086524,0.090923,0.086023,0.0759
07,0.084066,0.37351,0.36954,0.37251,0.3728,0.37176,0.37458,0.21048,0.2
1511,0.19853,0.21582,0.20873,0.17877,0.17699,0.1891,0.17732,0.17803,0.
19079,0.19236,0.16559,0.19505,0.097631,0.085103,0.086781,0.094668,0.07
8552,0.091717,0.086846,0.085659,0.083615,0.084767,0.086628,0.086403,0.
093811,0.084842,0.081683,0.093022,0.094388,0.086811,0.37466,0.37681,0.
37201,0.38378,0.38049,0.25607,0.20658,0.15726,0.20033,0.23018,0.21737,
0.18987,0.15024,0.18757,0.18953,0.18338,0.18363,0.18036,0.18341,0.2026
1,0.08471,0.086817,0.08693,0.092789,0.08365,0.086069,0.086348,0.079465
,0.095672,0.087128,0.091714,0.080462,0.081991,0.098796,0.082554,0.0816
19,0.086028,0.079811,0.37142,0.37142,0.37254,0.16947,0.21928,0.20455,0
.17925,0.20942,0.21453,0.19664,0.21678,0.16888,0.17741,0.1892,0.18544,
0.18048,0.16043,0.18411,0.16812,0.19157,0.077677,0.078663,0.087764,0.0
93217,0.087183,0.08616,0.089995,0.086667,0.077548,0.087712,0.082603,0.
087677,0.094357,0.086038,0.088419,0.087081,0.086789,0.085863,0.38393,0
.19112,0.21117,0.2127,0.20722,0.2556,0.17045,0.21398,0.20246,0.24393,0
.17899,0.1478,0.19385,0.17135,0.19273,0.17981,0.19234,0.15188,0.18367,
0.085435,0.08625,0.079872,0.085745,0.086609,0.08193,0.080824,0.094019,
0.096327,0.079671,0.087206,0.087063,0.081288,0.088771,0.079242,0.09128
2,0.099485,0.044296,0.21157,0.21107,0.21374,0.20708,0.21369,0.21984,0.
20268,0.24809,0.17724,0.23681,0.19342,0.18959,0.18516,0.18281,0.16031,
0.17981,0.19489,0.18055,0.14947,0.085839,0.083625,0.090341,0.085278,0.
094395,0.084428,0.085644,0.086593,0.088023,0.085916,0.085951,0.077637,
0.073066,0.086033,0.086491,0.081011,0.080757,0.043782,0.2458,0.21643,0
.20835,0.22721,0.21668,0.21602,0.20037,0.21464,0.20018,0.20731,0.1841,
0.20271,0.20068,0.18253,0.19743,0.18234,0.15612,0.18862,0.080732,0.083
4,0.081216,0.081517,0.086517,0.086307,0.090606,0.086339,0.089531,0.082
141,0.11391,0.081589,0.091527,0.090921,0.086961,0.076308,0.086187,0.08
5938,0.043787,0.24793,0.2045,0.17906,0.24263,0.19759,0.21079,0.19916,0
.19538,0.21946,0.22257,0.18501,0.18816,0.18774,0.18567,0.18725,0.202,0
.1967,0.1842,0.07996,0.084057,0.094003,0.086298,0.068496,0.090121,0.08
6658,0.0946,0.08239,0.095589,0.091356,0.095322,0.08884,0.083084,0.0861
22,0.081169,0.079708,0.043809,0.044436,0.043536,0.19173,0.19806,0.2003
2,0.21239,0.23714,0.20479,0.2128,0.21831,0.21399,0.17367,0.19778,0.186
22,0.21472,0.1956,0.17168,0.087173,0.086055,0.086135,0.0801,0.087103,0
.091652,0.076751,0.086628,0.10047,0.086199,0.084995,0.08658,0.086777,0
.086204,0.10283,0.09477,0.083993,0.085037,0.043592,0.043851,0.044019,0
.043348,0.17154,0.1664,0.25185,0.22431,0.23043,0.20737,0.21339,0.1161,
0.20973,0.20859,0.1505,0.18159,0.086146,0.086223,0.080938,0.081371,0.0
82543,0.085287,0.086359,0.088451,0.082961,0.07944,0.085876,0.091346,0.
079117,0.085655,0.086228,0.086916,0.089733,0.081735,0.094014,0.086872,
0.043833,0.044174,0.044177,0.04385,0.043993,0.044048,0.24601,0.21542,0
.20847,0.21638,0.22657,0.2641,0.20891,0.20138,0.086758,0.094568,0.0782
16,0.093655,0.078402,0.086631,0.087288,0.082509,0.089966,0.086271,0.08
8063,0.08956,0.084669,0.086046,0.086622,0.08639,0.082438,0.08768,0.084
371,0.10217,0.080221,0.086146,0.086513,0.043949,0.044291,0.044163,0.04
3746,0.043763,0.044038,0.21899,0.20434,0.20132,0.23082,0.21179,0.04321
,0.043824,0.081053,0.0828,0.083317,0.085462,0.083756,0.08679,0.086557,
0.086288,0.084959,0.088384,0.088006,0.086144,0.083308,0.077203,0.09140

4,0.092163,0.096074,0.087327,0.09079,0.085932,0.087429,0.043442,0.0432
58,0.043902,0.0443,0.044336,0.043861,0.044031,0.044409,0.043726,0.0432
74,0.22865,0.22159,0.043896,0.04433,0.044119,0.043645,0.085456,0.08663
5,0.082432,0.090738,0.081365,0.094609,0.087176,0.082386,0.085544,0.086
991,0.087215,0.09434,0.090787,0.086475,0.081607,0.081582,0.086955,0.08
9107,0.084855,0.043996,0.044043,0.043568,0.044305,0.044206,0.044177,0.
044058,0.044356,0.043663,0.044026,0.043706,0.044424,0.043475,0.043755,
0.043846,0.044178,0.043651,0.086114,0.082699,0.086081,0.086428,0.08318
8,0.086472,0.088486,0.085505,0.086863,0.095746,0.080362,0.091408,0.087
112,0.090988,0.091241,0.086327,0.043739,0.044401,0.04395,0.043527,0.04
3689,0.044037,0.044273,0.043909,0.043728,0.043306,0.044574,0.043925,0.
043765,0.04361,0.043325,0.043962,0.043765,0.043383,0.04361,0.092007,0.
086157,0.079717,0.080527,0.085321,0.07823,0.088318,0.080286,0.086878,0
.077934,0.081783,0.089067,0.085823,0.04404,0.043656,0.044274,0.043643,
0.044159,0.043819,0.043115,0.043917,0.043072,0.043871,0.043544,0.04328
8,0.044069,0.043948,0.043377,0.043532,0.043611,0.043887,0.044403,0.044
118,0.043841,0.082994,0.08657,0.082738,0.085658,0.08917,0.086366,0.086
762,0.092264,0.09039,0.043872,0.044434,0.043713,0.043894,0.044311,0.04
4615,0.043717,0.043841,0.044458,0.044212,0.043879,0.043672,0.044504,0.
043962,0.04369,0.043419,0.043208,0.044242,0.043549,0.04425,0.044239,0.
043809,0.043728,0.042941,0.04391,0.092198,0.085077,0.086282,0.086672,0
.044154,0.043538,0.044453,0.043576,0.043572,0.043781,0.043301,0.043665
,0.044031,0.044655,0.044005,0.044052,0.044609,0.04382,0.043331,0.04379
,0.043697,0.04396,0.043264,0.043801,0.044169,0.044105,0.044236,0.04441
2,0.043592,0.043918,0.04349,0.044243,0.043554,0.043299,0.043703,0.0429
71,0.043728,0.044773,0.04386,0.043931,0.043876,0.044202,0.043915,0.043
295,0.043362,0.044021,0.043891,0.043459,0.043435,0.043549,0.043676,0.0
43826,0.043519,0.043249,0.043885,0.043943,0.043436,0.044102,0.044162,0
.043866,0.043615,0.043969,0.044097,0.043834,0.044272,0.044099,0.043286
,0.043843,0.043665,0.04393,0.043948,0.044998,0.043513,0.044084,0.04371
2,0.044173,0.043156,0.04385,0.0439,0.043717,0.044174,0.044016,0.044161
,0.043958,0.044176,0.044453,0.043836,0.043366,0.043744,0.044408,0.0438
69,0.044187,0.043727,0.044126,0.043746,0.04446,0.044237,0.043367,0.044
01,0.044216,0.043461,0.044171,0.043373,0.044002,0.04378,0.043715,0.044
677,0.043536,0.04437,0.043273,0.043793,0.043464,0.044329,0.043845,0.04
4321,0.044169,0.043756,0.04364,0.044334,0.043964,0.043665,0.043651,0.0
4417,0.044073,0.043855,0.044017,0.044048,0.044265,0.043907,0.043937,0.
044279,0.044552,0.043912,0.04391,0.043747,0.044469,0.044469,0.043927,0
.043755,0.043927,0.043671,0.043892,0.044016,0.043714,0.043604,0.043899
,0.044095,0.044112,0.044223,0.043529,0.043879,0.043888,0.044057,0.0443
35,0.043652,0.043125,0.043952,0.043902,0.044,0.043496,0.043092,0.04441
1,0.044066,0.043925,0.043453,0.044241,0.044155,0.043487,0.043392,0.043
651,0.043456,0.043425,0.043826,0.043407,0.043661,0.043264,0.043692,0.0
43262,0.044623,0.044074,0.043775,0.0443,0.043049,0.043503,0.043237,0.0
44437,0.043729,0.043688,0.043936,0.044327,0.043546,0.043867,0.043298,0
.043678,0.044051,0.043781,0.043901,0.043593,0.044258
0.12322,0.1225,0.12196,0.12268,0.12468,0.12318,0.12271,0.12267,0.1226,
0.12268,0.12271,0.12323,0.1222,0.12275,0.12242,0.12311,0.12232,0.12413
,0.12292,0.12257,0.2474,0.21165,0.22677,0.23082,0.12319,0.12363,0.1232
,0.12227,0.12306,0.12266,0.12218,0.12186,0.12271,0.12245,0.12286,0.122
3,0.12242,0.12183,0.12215,0.37566,0.26718,0.22617,0.26212,0.23343,0.24
797,0.24584,0.12276,0.12217,0.12298,0.12269,0.12195,0.12241,0.12371,0.

12216,0.12252,0.12162,0.12267,0.12281,0.12384,0.12256,0.12219,0.35582,
0.37136,0.23481,0.2791,0.24464,0.25145,0.27317,0.28404,0.23686,0.26261
,0.1227,0.12228,0.12284,0.12261,0.12345,0.12264,0.12232,0.1229,0.12293
,0.1234,0.12254,0.12268,0.12373,0.12277,0.12323,0.35999,0.36528,0.3603
6,0.25252,0.23173,0.25768,0.27116,0.21965,0.24531,0.24047,0.26073,0.24
273,0.12211,0.1228,0.12247,0.1228,0.12274,0.12202,0.124,0.12259,0.123,
0.12267,0.122,0.12303,0.12203,0.12278,0.12304,0.12251,0.36889,0.37546,
0.38404,0.37414,0.24365,0.24849,0.24881,0.23891,0.26685,0.24575,0.2376
6,0.24881,0.26498,0.25377,0.12258,0.12321,0.12278,0.12219,0.12255,0.12
144,0.12298,0.12233,0.12294,0.12199,0.12348,0.12253,0.12286,0.12254,0.
12298,0.12283,0.35332,0.37149,0.36567,0.37776,0.3762,0.37823,0.24829,0
.23637,0.23569,0.24737,0.23967,0.25054,0.24375,0.24633,0.24914,0.26355
,0.12222,0.12255,0.1237,0.12344,0.12263,0.12284,0.12314,0.12293,0.1232
5,0.12236,0.12241,0.12273,0.12359,0.12238,0.12223,0.124,0.12241,0.3559
7,0.3764,0.36488,0.37156,0.37591,0.37279,0.23828,0.25252,0.24545,0.243
08,0.22903,0.24089,0.23309,0.18935,0.23607,0.25866,0.2448,0.12154,0.12
23,0.12384,0.1217,0.12226,0.12133,0.12271,0.12214,0.12265,0.12242,0.12
319,0.12224,0.12278,0.082936,0.06925,0.090573,0.12299,0.36512,0.38046,
0.36553,0.37067,0.37749,0.37402,0.35446,0.25023,0.24969,0.24274,0.2301
4,0.24303,0.24968,0.22356,0.24791,0.25582,0.26132,0.25302,0.12213,0.12
234,0.12214,0.12237,0.12264,0.12253,0.12369,0.12267,0.12282,0.12348,0.
12203,0.081192,0.076483,0.089042,0.065176,0.077501,0.083707,0.1227,0.3
8079,0.36859,0.37162,0.37214,0.36831,0.36915,0.37339,0.25199,0.25597,0
.24985,0.24561,0.21717,0.23526,0.23106,0.23879,0.26506,0.28717,0.23532
,0.12237,0.12224,0.12288,0.12178,0.12298,0.12337,0.12212,0.1235,0.1221
4,0.074446,0.08341,0.081682,0.076389,0.092261,0.078111,0.0823,0.082344
,0.094053,0.1232,0.36712,0.36874,0.36314,0.36908,0.37477,0.37801,0.375
24,0.28727,0.24498,0.23988,0.24243,0.24621,0.26305,0.24788,0.25052,0.2
4865,0.23599,0.23982,0.24496,0.12262,0.12353,0.12297,0.12301,0.12274,0
.12292,0.088762,0.080441,0.080854,0.084476,0.082626,0.078974,0.085148,
0.086473,0.084423,0.082702,0.081973,0.082858,0.36517,0.36469,0.36585,0
.37326,0.35574,0.37151,0.37342,0.21433,0.21696,0.21294,0.25101,0.23823
,0.24624,0.23917,0.23959,0.24205,0.23428,0.24078,0.27209,0.12272,0.122
14,0.12169,0.088453,0.083751,0.08617,0.082251,0.073296,0.092657,0.0779
06,0.076297,0.083344,0.079549,0.083365,0.082879,0.084712,0.076166,0.08
5859,0.37612,0.36209,0.37163,0.36278,0.3779,0.37601,0.37923,0.36955,0.
36948,0.25067,0.23147,0.24405,0.24051,0.22144,0.24471,0.25927,0.28443,
0.2467,0.23336,0.22875,0.087565,0.071238,0.078815,0.085268,0.081432,0.
081105,0.080449,0.082816,0.079255,0.082024,0.078498,0.082122,0.083674,
0.082731,0.082728,0.083758,0.078792,0.077269,0.36941,0.37109,0.37214,0
.37261,0.37045,0.38098,0.37563,0.37205,0.37097,0.24172,0.24286,0.17635
,0.17036,0.19075,0.17923,0.18047,0.1758,0.17838,0.18477,0.17482,0.0825
19,0.081409,0.084439,0.082804,0.084665,0.077203,0.082453,0.072162,0.08
5957,0.077628,0.088982,0.083354,0.082904,0.080054,0.082277,0.08774,0.0
85306,0.081237,0.35996,0.36176,0.36574,0.36931,0.36581,0.36688,0.36894
,0.3733,0.37105,0.21324,0.18844,0.17789,0.17346,0.18585,0.18014,0.1711
7,0.19238,0.18237,0.17866,0.18247,0.088568,0.071743,0.08279,0.074275,0
.086221,0.083077,0.083281,0.071794,0.081457,0.08273,0.098004,0.080574,
0.08281,0.079678,0.091172,0.088262,0.083168,0.081007,0.3683,0.36962,0.
37048,0.3772,0.37151,0.37073,0.36788,0.17544,0.19012,0.20504,0.18214,0
.18139,0.2038,0.15327,0.17648,0.18313,0.18364,0.17066,0.18558,0.16137,
0.082613,0.082013,0.080658,0.081723,0.079455,0.076303,0.089083,0.08581

7,0.081521,0.081614,0.079483,0.082687,0.081328,0.08265,0.08675,0.08213
9,0.072574,0.080108,0.36522,0.36689,0.36616,0.37187,0.36622,0.36884,0.
20755,0.21132,0.19626,0.21201,0.20652,0.17245,0.17332,0.18481,0.17293,
0.17421,0.18673,0.18908,0.162,0.19083,0.09332,0.081411,0.083051,0.0901
42,0.075016,0.08746,0.082974,0.082089,0.079896,0.081111,0.082871,0.082
506,0.089749,0.080943,0.078316,0.08861,0.089717,0.082965,0.36816,0.373
55,0.36187,0.3754,0.37572,0.25099,0.20511,0.15555,0.19796,0.22491,0.21
291,0.1857,0.14594,0.18385,0.18372,0.17893,0.17949,0.17648,0.17845,0.1
9799,0.080934,0.082699,0.083144,0.088632,0.07974,0.082479,0.082512,0.0
76069,0.091196,0.083164,0.087728,0.076501,0.078468,0.094049,0.079079,0
.078005,0.0823,0.076171,0.36556,0.36727,0.36871,0.16509,0.21407,0.2021
2,0.17542,0.2069,0.20832,0.19424,0.21116,0.16403,0.17167,0.18593,0.180
68,0.17642,0.15603,0.18102,0.16393,0.18792,0.074251,0.07551,0.083953,0
.089141,0.083124,0.082935,0.085844,0.082579,0.074557,0.083837,0.07923,
0.084175,0.090106,0.082162,0.084224,0.083351,0.082887,0.08215,0.37879,
0.18898,0.20786,0.21089,0.20368,0.25175,0.16714,0.21113,0.20015,0.2393
3,0.17306,0.14331,0.18739,0.16658,0.18979,0.17355,0.18683,0.14836,0.17
897,0.081874,0.082207,0.076673,0.081931,0.082686,0.078556,0.077258,0.0
89701,0.091719,0.075968,0.083478,0.083093,0.077962,0.084935,0.075705,0
.08707,0.095238,0.042635,0.20752,0.20743,0.21114,0.204,0.20863,0.2135,
0.19892,0.24377,0.17573,0.23524,0.19151,0.18652,0.18107,0.17858,0.1560
6,0.17682,0.18899,0.17692,0.14563,0.08212,0.079933,0.085906,0.081282,0
.090584,0.081086,0.082052,0.083138,0.084283,0.082425,0.082167,0.0744,0
.070015,0.082139,0.082538,0.077458,0.077253,0.042545,0.24368,0.21209,0
.20728,0.22342,0.21397,0.2095,0.19651,0.21245,0.19757,0.20587,0.1809,0
.19796,0.1972,0.17872,0.19409,0.17772,0.15334,0.18623,0.07739,0.079316
,0.077595,0.077579,0.082686,0.082146,0.08678,0.082642,0.085572,0.07868
9,0.10866,0.077366,0.086787,0.087122,0.083181,0.072939,0.08248,0.08199
2,0.043053,0.24381,0.20032,0.17457,0.24167,0.1938,0.2081,0.19652,0.193
33,0.21643,0.22,0.18005,0.1842,0.18373,0.18226,0.18288,0.196,0.19196,0
.18076,0.07606,0.080245,0.089673,0.082669,0.065401,0.085774,0.082894,0
.090502,0.07876,0.09134,0.087009,0.091002,0.085279,0.079672,0.082472,0
.077512,0.076204,0.04229,0.043196,0.042128,0.18921,0.19632,0.19814,0.2
1163,0.23543,0.20233,0.20799,0.21772,0.21241,0.17039,0.19464,0.18194,0
.20891,0.18996,0.16694,0.083203,0.082376,0.082792,0.076734,0.083108,0.
087728,0.073518,0.082639,0.095717,0.082692,0.081495,0.083207,0.082652,
0.081915,0.097951,0.090249,0.080315,0.081402,0.042019,0.042627,0.04267
8,0.042476,0.16777,0.16324,0.25066,0.22302,0.22466,0.2052,0.21,0.11479
,0.20316,0.20396,0.14511,0.17795,0.082691,0.082272,0.077446,0.077917,0
.078796,0.081411,0.082508,0.08461,0.079652,0.076045,0.082022,0.087239,
0.075697,0.081985,0.082766,0.083159,0.085834,0.077995,0.089834,0.08303
2,0.04252,0.042528,0.042742,0.042578,0.042466,0.04276,0.24321,0.21107,
0.20693,0.21497,0.2216,0.26055,0.20728,0.20075,0.082936,0.090223,0.074
831,0.089471,0.075305,0.082968,0.083454,0.079208,0.086021,0.082252,0.0
84239,0.085385,0.080721,0.082312,0.082739,0.082668,0.078604,0.083744,0
.080536,0.097424,0.07687,0.08231,0.082624,0.042718,0.042945,0.042836,0
.042171,0.042657,0.042315,0.21485,0.20094,0.19887,0.22632,0.20905,0.04
2063,0.042142,0.077284,0.079382,0.079801,0.08179,0.080195,0.083225,0.0
82701,0.081938,0.081065,0.084643,0.084118,0.08236,0.079509,0.074136,0.
087514,0.087923,0.091665,0.083429,0.086894,0.082186,0.083692,0.041874,
0.041818,0.042474,0.043308,0.043154,0.042529,0.04284,0.04305,0.042328,
0.04253,0.22375,0.21944,0.04241,0.042813,0.042787,0.042457,0.081413,0.

08275,0.078496,0.086433,0.077887,0.090277,0.083187,0.078767,0.081873,0
.083155,0.083263,0.089841,0.08653,0.082568,0.078548,0.078129,0.082896,
0.085361,0.081339,0.042659,0.042764,0.042339,0.043018,0.042632,0.04285
5,0.042991,0.043109,0.042427,0.042639,0.04255,0.043174,0.04206,0.04246
8,0.042381,0.042864,0.042549,0.082045,0.079111,0.08196,0.082721,0.0795
49,0.082317,0.084142,0.081848,0.082709,0.09148,0.077356,0.087242,0.083
235,0.086996,0.086938,0.082469,0.042303,0.0434,0.04241,0.042649,0.0422
86,0.042832,0.042982,0.042629,0.042414,0.042128,0.04311,0.042535,0.042
511,0.042314,0.042135,0.042453,0.042302,0.042036,0.042606,0.088011,0.0
82242,0.076608,0.07737,0.081716,0.074913,0.084458,0.076787,0.082646,0.
074609,0.07808,0.085072,0.081993,0.042795,0.042257,0.042979,0.042166,0
.042643,0.042632,0.041768,0.042453,0.041903,0.042526,0.042447,0.041666
,0.042831,0.042114,0.042045,0.042211,0.042493,0.04254,0.042712,0.04271
2,0.042477,0.079509,0.082935,0.079309,0.081726,0.085303,0.082603,0.082
765,0.088045,0.08641,0.042421,0.042988,0.042264,0.042401,0.042746,0.04
3257,0.042442,0.042779,0.042916,0.042792,0.04248,0.042447,0.043075,0.0
42421,0.042395,0.042248,0.041833,0.042885,0.042466,0.043066,0.043014,0
.042699,0.041967,0.041357,0.042359,0.087895,0.08105,0.082275,0.082888,
0.042654,0.042125,0.04306,0.042364,0.042296,0.042568,0.042096,0.042482
,0.042937,0.043544,0.042878,0.042889,0.043541,0.042627,0.042207,0.0425
14,0.042561,0.042769,0.041899,0.042386,0.04307,0.042978,0.042788,0.042
821,0.042437,0.042442,0.042025,0.042798,0.042027,0.041724,0.041961,0.0
416,0.042406,0.043497,0.042472,0.042933,0.042499,0.042867,0.0425,0.041
985,0.041944,0.042698,0.04236,0.042279,0.042194,0.042202,0.042411,0.04
2599,0.04221,0.041859,0.042454,0.042999,0.042369,0.043044,0.04274,0.04
246,0.042357,0.042451,0.042501,0.042671,0.042856,0.042458,0.042132,0.0
42541,0.042345,0.042509,0.0427,0.043361,0.042225,0.042788,0.042082,0.0
42921,0.042176,0.042432,0.042705,0.042461,0.04304,0.042406,0.042615,0.
042577,0.042731,0.042964,0.042238,0.042091,0.042484,0.043076,0.042708,
0.042885,0.042118,0.042678,0.042394,0.043024,0.043134,0.042201,0.04256
4,0.042727,0.042204,0.042795,0.042082,0.042585,0.042697,0.042685,0.043
214,0.042203,0.043028,0.042085,0.042312,0.042087,0.04291,0.042363,0.04
3026,0.042685,0.042515,0.042348,0.042861,0.042629,0.042213,0.042481,0.
042915,0.04252,0.042675,0.042567,0.042944,0.042804,0.042869,0.04286,0.
04262,0.043276,0.042458,0.042466,0.042341,0.043034,0.04302,0.042624,0.
042644,0.042549,0.042364,0.042661,0.042762,0.042472,0.042556,0.042702,
0.042814,0.042881,0.042993,0.042231,0.042487,0.042681,0.042862,0.04307
1,0.042425,0.041874,0.04255,0.042479,0.042749,0.042072,0.041459,0.0431
89,0.042747,0.042718,0.042021,0.043007,0.04281,0.042294,0.041937,0.042
173,0.042321,0.04212,0.042659,0.042171,0.042525,0.042297,0.042448,0.04
215,0.043012,0.042647,0.042424,0.042819,0.041722,0.042076,0.041937,0.0
42843,0.042179,0.042352,0.042511,0.042966,0.042148,0.042523,0.041979,0
.042573,0.042821,0.042439,0.042231,0.042486,0.042729
0.11825,0.11726,0.11699,0.11718,0.11884,0.11766,0.11842,0.11746,0.1175
2,0.11671,0.11757,0.11812,0.11721,0.11749,0.11697,0.11766,0.11751,0.11
896,0.11796,0.11708,0.24234,0.20732,0.22243,0.22648,0.11749,0.11857,0.
11785,0.11735,0.11825,0.11753,0.11716,0.11622,0.11724,0.11757,0.11773,
0.11738,0.11691,0.11705,0.11669,0.37181,0.26458,0.22316,0.2578,0.2296,
0.24498,0.24125,0.11737,0.11674,0.1173,0.11732,0.1168,0.11712,0.11874,
0.1173,0.11717,0.11637,0.11717,0.11807,0.11861,0.11748,0.11648,0.35288
,0.37494,0.23043,0.27255,0.24154,0.24835,0.26808,0.27661,0.23079,0.257
32,0.11779,0.11734,0.11776,0.11777,0.11796,0.11727,0.11674,0.11743,0.1

1755,0.11824,0.11702,0.11812,0.11871,0.11765,0.11783,0.35736,0.36335,0
.35696,0.24753,0.22721,0.25003,0.26873,0.21343,0.24164,0.23514,0.25585
,0.23713,0.11703,0.11797,0.11707,0.1176,0.1179,0.11724,0.11891,0.11744
,0.118,0.1171,0.11717,0.11842,0.11729,0.11768,0.11804,0.11743,0.3647,0
.36712,0.37694,0.37166,0.23854,0.24306,0.24516,0.23416,0.2641,0.2378,0
.23382,0.24495,0.26158,0.2487,0.11751,0.11807,0.1175,0.11705,0.11721,0
.11651,0.11784,0.11725,0.11781,0.11712,0.11822,0.11789,0.1179,0.11741,
0.11756,0.11784,0.34892,0.36648,0.36475,0.37649,0.36819,0.37561,0.2430
1,0.23144,0.23026,0.24076,0.23495,0.24624,0.23652,0.24394,0.24335,0.25
853,0.11719,0.11784,0.11816,0.11832,0.11765,0.11774,0.11808,0.11748,0.
11808,0.11735,0.1173,0.11732,0.11882,0.11708,0.11674,0.11879,0.11681,0
.35599,0.37277,0.35841,0.37181,0.36935,0.36853,0.2326,0.24809,0.24071,
0.23995,0.22607,0.23725,0.22739,0.18301,0.22967,0.25515,0.23827,0.1167
7,0.117,0.1183,0.11639,0.11686,0.11594,0.11705,0.11667,0.1177,0.11747,
0.11813,0.11705,0.11719,0.079806,0.066731,0.087053,0.11739,0.36047,0.3
7846,0.3641,0.36369,0.37364,0.36628,0.34865,0.24382,0.24696,0.2371,0.2
2619,0.2411,0.2436,0.21808,0.24463,0.2508,0.25675,0.25002,0.11737,0.11
763,0.11713,0.11735,0.11756,0.11768,0.11868,0.11766,0.11747,0.11787,0.
11672,0.078326,0.073847,0.085463,0.062668,0.075024,0.080604,0.11719,0.
374,0.36388,0.36683,0.36664,0.36122,0.3659,0.37228,0.24705,0.24921,0.2
4475,0.2418,0.21343,0.23046,0.22657,0.23456,0.26209,0.28474,0.22862,0.
11776,0.11728,0.11787,0.11671,0.1181,0.11871,0.11707,0.11794,0.11685,0
.071811,0.080347,0.078443,0.073686,0.088511,0.074918,0.079181,0.079459
,0.090238,0.11782,0.3663,0.35994,0.35814,0.36457,0.37361,0.37737,0.367
65,0.28419,0.23748,0.23379,0.23936,0.24195,0.25797,0.24521,0.24625,0.2
4303,0.22974,0.23539,0.24036,0.1175,0.1187,0.11814,0.11808,0.11753,0.1
1799,0.085577,0.07746,0.077843,0.081704,0.079518,0.076052,0.081854,0.0
83406,0.081454,0.079823,0.078764,0.079504,0.35883,0.36064,0.35947,0.36
99,0.35038,0.36532,0.36908,0.20905,0.21146,0.20793,0.24824,0.23588,0.2
4113,0.23454,0.23747,0.23893,0.23142,0.23468,0.26713,0.11741,0.117,0.1
1676,0.085399,0.080893,0.082929,0.078768,0.070547,0.08916,0.07503,0.07
3562,0.080292,0.076985,0.080504,0.079638,0.081138,0.073414,0.082556,0.
36963,0.35692,0.37131,0.3587,0.3727,0.37151,0.37435,0.3656,0.3634,0.24
728,0.22738,0.23879,0.23681,0.21664,0.24299,0.25332,0.28218,0.24231,0.
22927,0.22129,0.083923,0.069019,0.075952,0.082059,0.07832,0.077858,0.0
77204,0.0796,0.076482,0.078753,0.07577,0.079127,0.080588,0.079824,0.07
9579,0.080737,0.076293,0.074308,0.36751,0.37135,0.37188,0.37198,0.3634
5,0.37943,0.37034,0.36951,0.3627,0.23669,0.23829,0.17295,0.16605,0.187
28,0.17568,0.1784,0.17214,0.17299,0.18055,0.17119,0.079526,0.0784,0.08
1451,0.079952,0.081769,0.074789,0.079153,0.069353,0.083004,0.075102,0.
085572,0.080211,0.07973,0.07739,0.079422,0.084538,0.082368,0.077861,0.
36119,0.35657,0.35824,0.36683,0.36041,0.3637,0.36461,0.36829,0.36137,0
.20884,0.18545,0.17582,0.17015,0.18458,0.17636,0.16758,0.18847,0.17848
,0.17623,0.1792,0.085194,0.069348,0.079533,0.071941,0.083141,0.080122,
0.08004,0.069296,0.078846,0.079509,0.094047,0.077694,0.079586,0.076795
,0.087906,0.08492,0.079721,0.078197,0.36606,0.36479,0.36433,0.37377,0.
36906,0.37096,0.36431,0.17539,0.18776,0.20299,0.17927,0.17835,0.19953,
0.14981,0.17546,0.17896,0.17999,0.16842,0.1822,0.15768,0.079404,0.0789
7,0.077839,0.07884,0.076396,0.073512,0.08595,0.082617,0.077847,0.07865
8,0.076784,0.079703,0.078366,0.079338,0.083389,0.07904,0.070205,0.0773
14,0.35945,0.35946,0.36401,0.36454,0.36167,0.36577,0.20474,0.21147,0.1
9507,0.20915,0.20295,0.1696,0.17069,0.18213,0.17011,0.17091,0.18418,0.

18741,0.15886,0.18655,0.089566,0.078098,0.079939,0.086903,0.072232,0.0
8442,0.079829,0.079181,0.076711,0.078286,0.079893,0.079366,0.086289,0.
078267,0.075514,0.08542,0.086335,0.079718,0.36794,0.3678,0.36313,0.370
57,0.36789,0.24562,0.20652,0.15457,0.19672,0.22169,0.21344,0.18127,0.1
426,0.18092,0.18004,0.17657,0.17761,0.17293,0.17543,0.19362,0.078003,0
.079462,0.08004,0.085647,0.076942,0.079537,0.079633,0.07317,0.087458,0
.080108,0.084722,0.073583,0.075982,0.090448,0.076143,0.075144,0.079024
,0.073572,0.36696,0.36449,0.36647,0.16479,0.21041,0.19909,0.17217,0.20
451,0.20645,0.19285,0.20752,0.16017,0.16789,0.18228,0.17919,0.17285,0.
15293,0.17876,0.15868,0.18461,0.071664,0.072883,0.080815,0.086067,0.07
9909,0.080088,0.082583,0.079303,0.072102,0.080948,0.076425,0.081043,0.
087157,0.079306,0.08105,0.080436,0.07981,0.079106,0.36989,0.18765,0.20
244,0.20634,0.2019,0.24865,0.16462,0.2078,0.19732,0.23683,0.16991,0.13
953,0.18354,0.16276,0.18751,0.17003,0.18469,0.14469,0.17603,0.078848,0
.079499,0.073999,0.078592,0.079812,0.075973,0.074837,0.085888,0.087886
,0.073124,0.080346,0.079982,0.075177,0.081472,0.072931,0.08379,0.09178
8,0.041747,0.20364,0.2053,0.20729,0.20273,0.20732,0.21077,0.19516,0.23
914,0.17211,0.23199,0.18928,0.18151,0.18031,0.17583,0.15279,0.17223,0.
18492,0.17438,0.14256,0.079411,0.076991,0.082453,0.078201,0.087075,0.0
77951,0.078862,0.079985,0.081117,0.079375,0.078949,0.071747,0.067813,0
.079323,0.079291,0.074346,0.074478,0.041772,0.24343,0.21005,0.20558,0.
21979,0.21194,0.20683,0.19277,0.21019,0.19715,0.20172,0.17845,0.19347,
0.19371,0.17492,0.18946,0.17449,0.14904,0.18426,0.074419,0.075897,0.07
4563,0.075044,0.079461,0.0794,0.083364,0.079611,0.082464,0.075928,0.10
447,0.074427,0.083549,0.083862,0.080265,0.069927,0.079327,0.078912,0.0
42124,0.24095,0.197,0.17205,0.23939,0.19081,0.2081,0.19374,0.19187,0.2
1265,0.21743,0.17849,0.18028,0.18079,0.17895,0.17992,0.19161,0.18901,0
.17646,0.073388,0.077465,0.086348,0.079704,0.063254,0.082539,0.07992,0
.086741,0.075584,0.087544,0.084055,0.087941,0.081948,0.076314,0.07962,
0.074779,0.07352,0.041474,0.042094,0.041238,0.18606,0.19266,0.19587,0.
21032,0.22828,0.19761,0.20387,0.21749,0.20968,0.16665,0.19021,0.17769,
0.20497,0.18656,0.16305,0.079987,0.079212,0.079699,0.073954,0.080527,0
.084322,0.070948,0.079554,0.091868,0.079541,0.078499,0.079956,0.079938
,0.078793,0.094511,0.086816,0.077292,0.078293,0.041449,0.041528,0.0418
47,0.041464,0.16642,0.16094,0.24491,0.22173,0.22164,0.2028,0.20659,0.1
1356,0.20346,0.20101,0.14137,0.17541,0.079762,0.07908,0.074809,0.07554
8,0.075652,0.078526,0.079522,0.08126,0.07682,0.073313,0.079128,0.08377
1,0.073003,0.079027,0.080026,0.079869,0.082833,0.075347,0.086223,0.079
943,0.041383,0.041721,0.041698,0.041777,0.041639,0.041651,0.24102,0.20
783,0.20533,0.21489,0.21736,0.25654,0.20379,0.19855,0.079946,0.086615,
0.071905,0.085825,0.072783,0.079989,0.080398,0.076194,0.082711,0.07892
2,0.08111,0.082038,0.077964,0.079372,0.079958,0.079615,0.075514,0.0805
62,0.077522,0.0938,0.074172,0.079348,0.079658,0.041882,0.042075,0.0417
14,0.041463,0.041457,0.041549,0.20987,0.19663,0.19594,0.22241,0.20622,
0.041076,0.040973,0.074559,0.076626,0.076835,0.079132,0.077403,0.08021
7,0.079788,0.078813,0.077992,0.081735,0.080922,0.079451,0.076552,0.071
531,0.084193,0.084897,0.088274,0.080547,0.083593,0.079045,0.080181,0.0
41175,0.040899,0.041598,0.042406,0.042044,0.041695,0.041832,0.042109,0
.041374,0.041602,0.21904,0.21556,0.041205,0.04212,0.041869,0.041818,0.
078418,0.079506,0.07581,0.083103,0.075186,0.086799,0.080038,0.075692,0
.078621,0.079988,0.080242,0.086508,0.083096,0.079403,0.075758,0.075427
,0.079573,0.082229,0.078291,0.041948,0.041532,0.041945,0.041912,0.0417

23,0.041948,0.04194,0.042025,0.041395,0.041483,0.042042,0.041933,0.040
785,0.04165,0.04151,0.041696,0.041776,0.079136,0.07635,0.079235,0.0793
6,0.077023,0.079262,0.081048,0.078791,0.079444,0.088023,0.074882,0.083
927,0.080343,0.083582,0.083716,0.079365,0.041493,0.042315,0.041491,0.0
42064,0.041385,0.041473,0.041941,0.041723,0.041135,0.041348,0.04217,0.
041706,0.041325,0.04128,0.041228,0.041329,0.041404,0.041156,0.041604,0
.084642,0.079171,0.073894,0.074224,0.078613,0.072289,0.081415,0.074089
,0.079521,0.071816,0.075093,0.081536,0.078789,0.04201,0.041159,0.04189
7,0.041166,0.041738,0.041705,0.041163,0.041478,0.040718,0.041678,0.041
642,0.040872,0.042209,0.041267,0.041149,0.041465,0.041495,0.041605,0.0
41706,0.041731,0.041294,0.0765,0.079975,0.076493,0.078523,0.082109,0.0
79282,0.079616,0.084631,0.083246,0.041359,0.04187,0.041198,0.041609,0.
041343,0.042132,0.04157,0.041804,0.041992,0.041439,0.041613,0.041609,0
.042422,0.041314,0.041459,0.041356,0.04109,0.041939,0.041551,0.041946,
0.042437,0.042172,0.041302,0.040871,0.04144,0.084648,0.078088,0.079114
,0.079795,0.04157,0.041392,0.041956,0.041471,0.041234,0.041861,0.04117
,0.041566,0.041791,0.042699,0.041924,0.041884,0.042675,0.041719,0.0412
31,0.041354,0.041313,0.041909,0.041173,0.041405,0.042069,0.041992,0.04
189,0.041945,0.041473,0.041271,0.040953,0.041628,0.041138,0.041073,0.0
41199,0.04064,0.041536,0.042569,0.041678,0.041936,0.041684,0.041961,0.
04165,0.041064,0.041217,0.041852,0.041258,0.041331,0.041345,0.041302,0
.041666,0.041759,0.041471,0.041043,0.0417,0.042045,0.041235,0.041827,0
.041706,0.041565,0.041162,0.04158,0.041553,0.042086,0.041672,0.04142,0
.041246,0.041583,0.041489,0.041771,0.041919,0.042491,0.041309,0.041734
,0.041189,0.041924,0.041275,0.041619,0.041882,0.04167,0.04185,0.041539
,0.041747,0.041629,0.041736,0.042115,0.041425,0.040811,0.04088,0.04254
8,0.041878,0.041967,0.041257,0.041593,0.041322,0.041997,0.042205,0.041
345,0.041225,0.041411,0.041502,0.041689,0.041511,0.041611,0.041833,0.0
41629,0.042362,0.041201,0.04231,0.041205,0.041389,0.041137,0.042242,0.
041424,0.042331,0.04177,0.041761,0.041388,0.041967,0.041969,0.041344,0
.041555,0.041931,0.041645,0.041639,0.041354,0.04187,0.041924,0.042039,
0.04195,0.041702,0.042039,0.041574,0.041436,0.041696,0.0418,0.042094,0
.041717,0.041898,0.041663,0.041388,0.041757,0.041606,0.04184,0.041642,
0.041384,0.042225,0.042019,0.042314,0.041472,0.041678,0.041674,0.04214
9,0.041631,0.041728,0.041088,0.041907,0.041397,0.042147,0.041382,0.040
546,0.041947,0.041744,0.041731,0.041246,0.042223,0.041882,0.041275,0.0
41066,0.04139,0.041313,0.041245,0.041758,0.04127,0.041817,0.041163,0.0
41708,0.041314,0.041964,0.041625,0.041485,0.04185,0.041321,0.041365,0.
041194,0.041457,0.04153,0.041498,0.041285,0.042084,0.041349,0.041727,0
.040788,0.041509,0.042392,0.041346,0.041192,0.041352,0.041645
0.11417,0.11314,0.11273,0.11345,0.11475,0.11395,0.11377,0.11341,0.1131
3,0.11254,0.11299,0.11329,0.11315,0.11369,0.11258,0.11327,0.11357,0.11
456,0.11362,0.11302,0.23833,0.2029,0.21868,0.22444,0.11335,0.11427,0.1
1352,0.11297,0.11406,0.1136,0.113,0.11224,0.113,0.11345,0.11339,0.1129
3,0.11284,0.11249,0.11213,0.37423,0.26068,0.21696,0.25477,0.22432,0.24
158,0.23813,0.11331,0.11197,0.11275,0.11352,0.11238,0.11279,0.11457,0.
11328,0.11274,0.11198,0.1129,0.11384,0.1142,0.11304,0.1128,0.35246,0.3
7138,0.22823,0.27138,0.23706,0.24471,0.26313,0.27316,0.22679,0.25378,0
.11328,0.11334,0.11338,0.11357,0.11361,0.11275,0.11275,0.11345,0.11319
,0.11373,0.11269,0.114,0.11427,0.11375,0.11378,0.35841,0.364,0.35571,0
.24469,0.22274,0.24872,0.26479,0.21053,0.23925,0.23104,0.25306,0.23341
,0.11311,0.11337,0.11308,0.1133,0.11382,0.11253,0.11405,0.11296,0.1135

9,0.1129,0.11289,0.1137,0.11308,0.11357,0.11401,0.11311,0.36339,0.3625
2,0.36917,0.36678,0.23469,0.23897,0.24254,0.23066,0.25851,0.23787,0.23
117,0.24002,0.25756,0.24598,0.11329,0.1135,0.11318,0.11318,0.11291,0.1
1228,0.1133,0.11268,0.11372,0.11243,0.11383,0.11359,0.1141,0.1133,0.11
357,0.11317,0.34919,0.36099,0.36019,0.36762,0.36523,0.37101,0.23928,0.
227,0.22566,0.23835,0.23289,0.2419,0.23276,0.24152,0.24009,0.25385,0.1
1298,0.11363,0.11408,0.11405,0.11379,0.11368,0.1141,0.11316,0.11365,0.
1133,0.11334,0.11305,0.1149,0.11276,0.11257,0.11434,0.11264,0.3541,0.3
6456,0.35456,0.3723,0.36268,0.36389,0.22894,0.24416,0.238,0.23784,0.22
16,0.23281,0.22292,0.17959,0.22449,0.252,0.23676,0.11208,0.1131,0.1142
2,0.11236,0.11306,0.11169,0.11293,0.11289,0.11379,0.11296,0.11392,0.11
234,0.1125,0.077518,0.064952,0.08476,0.11281,0.35772,0.37473,0.3616,0.
35916,0.37084,0.3627,0.34599,0.24133,0.24156,0.23616,0.22252,0.2371,0.
24038,0.2172,0.24257,0.24485,0.25287,0.24331,0.11305,0.1132,0.11322,0.
11318,0.11314,0.11329,0.11404,0.11349,0.11292,0.11389,0.11251,0.076091
,0.07157,0.082598,0.060878,0.072684,0.078095,0.11303,0.36591,0.35829,0
.36126,0.36502,0.35859,0.36069,0.36957,0.24279,0.24232,0.24087,0.23908
,0.20962,0.2267,0.22113,0.22841,0.25907,0.2833,0.22384,0.1136,0.11299,
0.11368,0.11264,0.11363,0.11465,0.11303,0.1135,0.11309,0.069851,0.0780
74,0.076116,0.071596,0.085909,0.072918,0.076686,0.077104,0.087234,0.11
369,0.36446,0.36043,0.35587,0.36688,0.36841,0.37753,0.36271,0.27806,0.
23424,0.23226,0.2359,0.23899,0.25219,0.23962,0.24351,0.24015,0.22466,0
.23258,0.23744,0.11312,0.11411,0.11435,0.11383,0.11337,0.11369,0.08267
6,0.075151,0.075449,0.079376,0.077403,0.074035,0.079443,0.080954,0.079
008,0.077223,0.076129,0.077281,0.35622,0.35696,0.35394,0.36862,0.34657
,0.36371,0.3675,0.20549,0.2079,0.20286,0.24746,0.23177,0.23691,0.23068
,0.23192,0.23313,0.22924,0.23012,0.26292,0.11285,0.1133,0.11283,0.0824
36,0.078752,0.080197,0.076234,0.068528,0.086367,0.07295,0.07118,0.0777
31,0.074728,0.077945,0.076894,0.078502,0.070967,0.079811,0.3644,0.3529
6,0.36862,0.35644,0.36894,0.36781,0.3725,0.36302,0.35523,0.24182,0.224
36,0.23385,0.23165,0.21411,0.23779,0.25183,0.27787,0.23685,0.22638,0.2
1673,0.081353,0.067359,0.073732,0.079433,0.075545,0.075465,0.074904,0.
077148,0.074167,0.076682,0.073716,0.076604,0.077934,0.077505,0.07723,0
.078215,0.073848,0.072031,0.36794,0.36491,0.36479,0.3677,0.35915,0.377
83,0.36257,0.36141,0.35625,0.23224,0.23374,0.17185,0.163,0.18521,0.173
05,0.17635,0.16873,0.17073,0.17901,0.16931,0.077351,0.076079,0.079089,
0.077682,0.079545,0.072828,0.076619,0.067519,0.080536,0.072743,0.08273
6,0.077811,0.077249,0.074851,0.076873,0.082072,0.079991,0.075475,0.358
79,0.35788,0.35572,0.36215,0.35907,0.36406,0.35362,0.36808,0.35972,0.2
0637,0.18168,0.17298,0.16619,0.18111,0.17411,0.16301,0.18624,0.17571,0
.17446,0.17772,0.082505,0.067164,0.077241,0.069928,0.080497,0.078082,0
.07744,0.067515,0.076229,0.07711,0.091381,0.075463,0.076841,0.074893,0
.085292,0.082112,0.077407,0.075558,0.36683,0.35991,0.36379,0.37,0.3691
,0.36702,0.36218,0.17535,0.18406,0.20109,0.17743,0.17459,0.1962,0.1480
7,0.17345,0.17802,0.17639,0.16725,0.17852,0.15564,0.077011,0.076504,0.
074887,0.076707,0.074287,0.071544,0.083724,0.079651,0.075883,0.076126,
0.074724,0.077581,0.076253,0.077342,0.081068,0.07665,0.068119,0.074891
,0.35687,0.35819,0.35949,0.36345,0.35995,0.3618,0.20188,0.20904,0.1931
7,0.20541,0.20078,0.16748,0.16668,0.18031,0.16803,0.16721,0.17849,0.18
581,0.1561,0.1846,0.086849,0.075957,0.077492,0.084273,0.070032,0.08146
2,0.077544,0.076498,0.073998,0.075825,0.077345,0.07697,0.084017,0.0760
03,0.073313,0.082505,0.083501,0.077342,0.36279,0.36467,0.36404,0.36872

,0.36614,0.2449,0.2022,0.15186,0.19716,0.22139,0.21251,0.18026,0.13992
,0.17699,0.17767,0.17377,0.17431,0.16923,0.1729,0.1911,0.075481,0.0767
42,0.077264,0.082745,0.074863,0.077013,0.077178,0.070709,0.084939,0.07
7671,0.08188,0.071368,0.07388,0.087899,0.073888,0.072503,0.076789,0.07
1268,0.36483,0.35901,0.36475,0.1633,0.20795,0.19744,0.17095,0.20316,0.
20308,0.19069,0.20439,0.15837,0.16531,0.17961,0.17688,0.17061,0.14994,
0.17696,0.15494,0.18163,0.069605,0.070849,0.078357,0.083318,0.077733,0
.077577,0.07965,0.076791,0.06998,0.078554,0.073835,0.078301,0.084588,0
.076508,0.078581,0.07796,0.077113,0.076529,0.36718,0.18637,0.19881,0.2
0525,0.19933,0.24618,0.16272,0.20311,0.19629,0.23597,0.16756,0.13703,0
.18087,0.15957,0.18669,0.16777,0.18211,0.14226,0.17284,0.076477,0.0769
44,0.071505,0.076183,0.077377,0.073477,0.072712,0.082955,0.084897,0.07
1239,0.078095,0.077489,0.07313,0.078844,0.070564,0.081242,0.088722,0.0
41098,0.2026,0.20315,0.20489,0.20145,0.2042,0.20906,0.19465,0.23493,0.
16794,0.23001,0.18905,0.18029,0.17804,0.17263,0.15067,0.17084,0.1811,0
.17285,0.13946,0.076673,0.074688,0.079988,0.075821,0.084521,0.075597,0
.076501,0.077659,0.078665,0.077317,0.076835,0.069413,0.065554,0.07651,
0.077431,0.072515,0.072511,0.040897,0.2403,0.2088,0.20435,0.21908,0.20
969,0.20549,0.19049,0.20793,0.19556,0.19935,0.17508,0.19019,0.19322,0.
17233,0.18418,0.17348,0.14585,0.18164,0.072547,0.073357,0.072165,0.072
705,0.077198,0.077085,0.081104,0.076859,0.079921,0.073676,0.10062,0.07
245,0.081054,0.080977,0.077884,0.067759,0.076975,0.076463,0.04144,0.24
011,0.19731,0.17018,0.23718,0.18793,0.20693,0.19252,0.18827,0.20975,0.
21437,0.17586,0.17797,0.17905,0.17614,0.17628,0.1876,0.18735,0.17319,0
.071227,0.074934,0.083647,0.077319,0.06155,0.079763,0.077191,0.083832,
0.073383,0.084813,0.081376,0.085121,0.079886,0.074083,0.077376,0.07249
7,0.070814,0.040739,0.041202,0.040825,0.18321,0.18842,0.19418,0.20709,
0.22782,0.19428,0.20002,0.21425,0.20762,0.16431,0.18583,0.17574,0.2016
6,0.18306,0.16044,0.077524,0.076916,0.077249,0.071995,0.077955,0.08125
6,0.068755,0.076926,0.088702,0.077079,0.07606,0.077435,0.077533,0.0760
55,0.091538,0.084147,0.074967,0.076185,0.04099,0.040947,0.041252,0.040
893,0.16547,0.16006,0.23959,0.21683,0.21817,0.20184,0.20411,0.11291,0.
20103,0.19866,0.14007,0.17227,0.077087,0.076752,0.072648,0.073492,0.07
329,0.076125,0.077311,0.078742,0.074335,0.071137,0.0767,0.081013,0.070
871,0.076745,0.077795,0.077495,0.080419,0.073249,0.083442,0.077259,0.0
40545,0.040967,0.040806,0.041223,0.040957,0.040838,0.23779,0.20843,0.2
0295,0.21197,0.21531,0.25376,0.19906,0.19795,0.077586,0.083855,0.06956
9,0.083039,0.070611,0.07726,0.078012,0.073947,0.079992,0.07688,0.07845
5,0.07938,0.075653,0.076744,0.077094,0.077393,0.073451,0.078137,0.0754
77,0.090638,0.071922,0.076979,0.077232,0.041454,0.041526,0.041031,0.04
0798,0.041053,0.040842,0.20816,0.19491,0.19259,0.21934,0.20299,0.04056
,0.040316,0.072545,0.074438,0.074555,0.076705,0.074721,0.077631,0.0774
1,0.076508,0.075787,0.07924,0.078603,0.077219,0.074071,0.069113,0.0815
16,0.08239,0.085333,0.077785,0.080651,0.076303,0.0779,0.04061,0.04013,
0.0409,0.041566,0.041336,0.041056,0.040964,0.041301,0.040879,0.04115,0
.21641,0.21436,0.040576,0.041267,0.041147,0.041048,0.075638,0.077219,0
.073528,0.08044,0.072445,0.084142,0.077766,0.073291,0.076446,0.077638,
0.077921,0.084001,0.080629,0.076914,0.073444,0.073265,0.077494,0.07994
1,0.076131,0.041099,0.040799,0.041073,0.041257,0.041186,0.041189,0.041
077,0.04101,0.040865,0.040998,0.041678,0.041602,0.040176,0.040936,0.04
0783,0.040747,0.041135,0.076771,0.074004,0.076764,0.076827,0.07455,0.0
77217,0.07837,0.07659,0.076973,0.085123,0.072628,0.081253,0.077705,0.0

80895,0.080832,0.076818,0.040617,0.041821,0.040787,0.041348,0.040609,0
.040762,0.041104,0.040882,0.040501,0.040897,0.041525,0.04078,0.040615,
0.04044,0.040851,0.040633,0.040735,0.040563,0.041155,0.082102,0.076929
,0.071657,0.072246,0.076237,0.070059,0.078584,0.072031,0.076935,0.0697
95,0.0728,0.079276,0.076523,0.041331,0.040519,0.040969,0.040716,0.0413
64,0.040837,0.04034,0.040753,0.039891,0.041146,0.040924,0.04048,0.0417
74,0.040571,0.04061,0.040649,0.040926,0.040941,0.041189,0.040785,0.040
838,0.074051,0.077686,0.074254,0.076323,0.079577,0.077216,0.077445,0.0
8174,0.080645,0.041042,0.041409,0.040474,0.041168,0.040855,0.041588,0.
041033,0.041342,0.04149,0.040668,0.040586,0.040979,0.041522,0.040817,0
.041005,0.040821,0.040443,0.04115,0.040891,0.041196,0.041564,0.041556,
0.040636,0.040316,0.040492,0.081828,0.076017,0.076772,0.077546,0.04089
3,0.041041,0.041359,0.041049,0.040375,0.041388,0.041162,0.040659,0.041
304,0.042004,0.041132,0.041425,0.042024,0.04096,0.040431,0.040677,0.04
0713,0.041333,0.040817,0.040721,0.041066,0.041171,0.041261,0.041539,0.
040783,0.040423,0.040497,0.040988,0.040652,0.040475,0.040507,0.040337,
0.040891,0.041913,0.041201,0.041424,0.040912,0.041339,0.041326,0.04013
4,0.04072,0.040911,0.040579,0.04078,0.04069,0.040506,0.041344,0.040955
,0.041375,0.040598,0.041031,0.041583,0.040657,0.041415,0.041243,0.0409
61,0.040644,0.040944,0.040786,0.041406,0.041303,0.040758,0.040554,0.04
1094,0.040461,0.041106,0.041451,0.041664,0.040879,0.04087,0.040669,0.0
41271,0.040515,0.040983,0.041274,0.041099,0.040808,0.040798,0.041132,0
.041169,0.040768,0.04163,0.040787,0.04053,0.040561,0.041563,0.040978,0
.041515,0.040634,0.041025,0.04086,0.041412,0.041376,0.041042,0.040665,
0.040734,0.040732,0.040903,0.040975,0.040656,0.040948,0.041118,0.04162
9,0.04054,0.041628,0.040619,0.040722,0.040653,0.041592,0.041019,0.0416
35,0.0409,0.041193,0.040505,0.04132,0.041442,0.041005,0.040669,0.04129
6,0.041137,0.041002,0.040522,0.040963,0.041181,0.041599,0.041309,0.040
715,0.04083,0.040983,0.040556,0.041161,0.040957,0.041263,0.040944,0.04
1332,0.04065,0.040888,0.041009,0.040774,0.04106,0.041164,0.040644,0.04
1556,0.041132,0.041792,0.040924,0.040721,0.041163,0.041311,0.04069,0.0
41223,0.040305,0.041424,0.040734,0.041243,0.0409,0.040032,0.04133,0.04
0998,0.041104,0.040727,0.041727,0.040938,0.0407,0.040577,0.040748,0.04
0314,0.040821,0.04138,0.040639,0.041264,0.040611,0.040696,0.040846,0.0
41382,0.040933,0.040916,0.041055,0.040745,0.040831,0.040398,0.040756,0
.040767,0.040883,0.040956,0.041347,0.040552,0.040985,0.039915,0.0408,0
.041574,0.040847,0.040619,0.040711,0.040986
0.11001,0.10951,0.10936,0.11029,0.11154,0.11038,0.11043,0.11022,0.1100
1,0.10923,0.1097,0.10984,0.10982,0.10992,0.10948,0.10958,0.11017,0.111
17,0.11037,0.10959,0.23529,0.19919,0.21669,0.22175,0.10975,0.11083,0.1
1,0.10898,0.11065,0.10998,0.1094,0.1088,0.10971,0.10979,0.11016,0.1095
6,0.10971,0.109,0.10855,0.37156,0.25513,0.215,0.25284,0.22351,0.24122,
0.23549,0.10963,0.10856,0.10974,0.11044,0.10892,0.10901,0.11101,0.1097
3,0.1089,0.10896,0.10939,0.11065,0.11025,0.10972,0.10961,0.34926,0.367
31,0.22492,0.26702,0.23266,0.24057,0.26008,0.26915,0.22385,0.25094,0.1
1018,0.1099,0.11012,0.11018,0.11054,0.10922,0.10945,0.10959,0.11024,0.
11045,0.10938,0.11053,0.11045,0.11,0.11044,0.35459,0.36645,0.35434,0.2
4153,0.22,0.2416,0.26071,0.20642,0.23428,0.22744,0.25008,0.23281,0.109
24,0.10967,0.10969,0.10969,0.11001,0.10922,0.11032,0.10885,0.10984,0.1
0996,0.10907,0.1104,0.10938,0.10975,0.11026,0.10954,0.3622,0.36346,0.3
6404,0.3594,0.23542,0.23521,0.23921,0.22644,0.25375,0.23452,0.22991,0.
23692,0.25448,0.24241,0.10959,0.11001,0.10958,0.1096,0.10933,0.10918,0

.10991,0.10882,0.11006,0.10886,0.1111,0.11012,0.11069,0.10962,0.11029,
0.10997,0.34828,0.35917,0.36217,0.36246,0.36534,0.3706,0.23745,0.22388
,0.22365,0.23492,0.23047,0.24011,0.22888,0.23895,0.23767,0.25227,0.109
21,0.11025,0.11009,0.11077,0.10994,0.11031,0.11039,0.10933,0.11016,0.1
0978,0.10923,0.10954,0.11149,0.10974,0.10887,0.11063,0.10942,0.35358,0
.35914,0.35004,0.37123,0.36212,0.36729,0.22669,0.2425,0.23314,0.23467,
0.21844,0.23013,0.22085,0.17699,0.22012,0.24879,0.23489,0.10863,0.1099
1,0.11074,0.10887,0.1096,0.10803,0.10947,0.10976,0.1103,0.10943,0.1103
,0.10901,0.1093,0.075221,0.063188,0.082129,0.1092,0.35333,0.36881,0.35
626,0.36085,0.36556,0.35845,0.33978,0.23629,0.23762,0.23238,0.22164,0.
23518,0.23753,0.21501,0.24032,0.24271,0.24661,0.23783,0.10923,0.10939,
0.10911,0.10967,0.11001,0.10961,0.11093,0.11026,0.10952,0.10997,0.1095
7,0.074061,0.069836,0.080179,0.059482,0.070819,0.076311,0.10966,0.3619
7,0.35729,0.35495,0.36332,0.35967,0.35943,0.36384,0.24081,0.24023,0.23
734,0.23626,0.20672,0.22426,0.21876,0.22466,0.25427,0.27944,0.22079,0.
11014,0.10968,0.10996,0.10928,0.11061,0.1109,0.10912,0.1106,0.10979,0.
0682,0.076345,0.074282,0.069809,0.083803,0.070979,0.074415,0.075052,0.
085157,0.11036,0.36142,0.35994,0.34778,0.36441,0.36574,0.37382,0.35543
,0.27701,0.23066,0.22874,0.23282,0.23649,0.24901,0.23834,0.2402,0.2373
6,0.22034,0.23089,0.23451,0.10934,0.11082,0.11062,0.11048,0.1101,0.110
36,0.080369,0.072956,0.073533,0.077395,0.075132,0.071861,0.07715,0.078
731,0.076875,0.075362,0.074139,0.07546,0.35346,0.35534,0.3539,0.36043,
0.34406,0.3616,0.36153,0.20091,0.20382,0.2013,0.243,0.22863,0.23275,0.
22771,0.22834,0.22911,0.22782,0.22699,0.2603,0.10952,0.11004,0.10999,0
.080125,0.076507,0.077907,0.074161,0.066995,0.083943,0.071053,0.069543
,0.075802,0.072892,0.076005,0.074991,0.076383,0.06906,0.077936,0.35968
,0.35314,0.36525,0.3564,0.36676,0.364,0.36943,0.35413,0.35268,0.23502,
0.22293,0.23107,0.22862,0.21069,0.23309,0.2492,0.27659,0.23287,0.22328
,0.21247,0.079157,0.065552,0.071796,0.077233,0.073536,0.073382,0.07287
5,0.074889,0.071957,0.074919,0.072362,0.07478,0.076113,0.075403,0.0755
67,0.075951,0.071786,0.070281,0.36473,0.36217,0.35928,0.36391,0.35159,
0.37345,0.36037,0.3554,0.35291,0.23026,0.23358,0.16997,0.16077,0.1834,
0.16985,0.17508,0.16583,0.16851,0.17768,0.16708,0.075355,0.074029,0.07
6961,0.075868,0.077248,0.071,0.074889,0.065639,0.078723,0.070918,0.080
606,0.075856,0.07532,0.07289,0.07495,0.079923,0.07776,0.073533,0.35588
,0.35745,0.3554,0.3579,0.35349,0.3641,0.35391,0.36375,0.35823,0.20443,
0.18005,0.17044,0.16405,0.17943,0.17278,0.16113,0.18234,0.17433,0.1719
1,0.17555,0.080333,0.065551,0.074851,0.068199,0.07836,0.075884,0.07530
2,0.065773,0.074266,0.075274,0.089009,0.073611,0.07475,0.07298,0.08306
2,0.080205,0.075428,0.073631,0.3666,0.35748,0.36479,0.36745,0.35867,0.
36263,0.36102,0.17379,0.18492,0.20027,0.17615,0.17198,0.19326,0.14635,
0.16962,0.17489,0.17391,0.16562,0.17844,0.15367,0.074947,0.074533,0.07
2994,0.074536,0.072306,0.070026,0.081448,0.077393,0.074017,0.074232,0.
073044,0.075588,0.074018,0.07539,0.078803,0.074735,0.066601,0.072922,0
.35604,0.35441,0.35933,0.35672,0.36012,0.36077,0.19714,0.20754,0.19111
,0.20224,0.19971,0.16394,0.16373,0.17754,0.16544,0.16383,0.17573,0.182
88,0.1548,0.18327,0.084275,0.07396,0.07546,0.081935,0.068149,0.079187,
0.075919,0.0745,0.072059,0.073943,0.075393,0.075063,0.08182,0.074218,0
.071208,0.080416,0.080823,0.075366,0.35849,0.365,0.36227,0.36664,0.365
46,0.24534,0.20138,0.1512,0.19654,0.22096,0.20749,0.17684,0.13727,0.17
485,0.17472,0.17023,0.17074,0.16768,0.17192,0.18793,0.073542,0.074912,
0.075326,0.080362,0.073049,0.075104,0.075513,0.069237,0.083092,0.07571

2,0.079368,0.069776,0.072321,0.085437,0.072145,0.070753,0.074987,0.069
377,0.36014,0.35439,0.36501,0.16363,0.20654,0.19561,0.1689,0.20328,0.2
0215,0.19011,0.20245,0.1569,0.16386,0.17823,0.17473,0.16898,0.1478,0.1
7637,0.15266,0.18041,0.067615,0.068863,0.076268,0.081268,0.075741,0.07
5635,0.077451,0.074718,0.068421,0.076145,0.07209,0.076142,0.082335,0.0
74767,0.076578,0.075867,0.075454,0.074753,0.36291,0.1856,0.19994,0.203
85,0.19885,0.24579,0.16165,0.20132,0.19406,0.2329,0.16703,0.13474,0.17
841,0.15771,0.18274,0.16489,0.17932,0.1395,0.1706,0.07464,0.075174,0.0
69849,0.074269,0.075295,0.071713,0.07085,0.080613,0.082422,0.069785,0.
076167,0.075569,0.070946,0.076824,0.068996,0.079018,0.086344,0.040389,
0.20141,0.20097,0.20399,0.20288,0.20073,0.20802,0.19293,0.23498,0.1637
6,0.229,0.18757,0.17697,0.17487,0.1712,0.14805,0.16816,0.17998,0.17127
,0.13692,0.074465,0.072752,0.077858,0.074008,0.082151,0.073698,0.07464
1,0.075522,0.076897,0.075185,0.074879,0.067873,0.064203,0.074581,0.075
264,0.071101,0.070834,0.04062,0.23704,0.20684,0.20233,0.21658,0.20828,
0.20641,0.18822,0.2042,0.19338,0.19771,0.17303,0.18747,0.19009,0.16927
,0.18408,0.16992,0.14285,0.17962,0.070624,0.071878,0.07055,0.070894,0.
074909,0.075216,0.078875,0.075289,0.077787,0.071844,0.09712,0.070061,0
.078946,0.078362,0.07604,0.066075,0.07529,0.074608,0.040949,0.23844,0.
19506,0.17043,0.23475,0.18718,0.20365,0.19081,0.18759,0.20742,0.21277,
0.17388,0.17614,0.17689,0.17318,0.17358,0.18381,0.18545,0.17159,0.0696
46,0.073035,0.081564,0.07548,0.060217,0.077651,0.07535,0.081541,0.0716
73,0.082427,0.07898,0.082831,0.07761,0.072094,0.075604,0.070916,0.0693
5,0.040239,0.040843,0.040294,0.17999,0.18591,0.19301,0.20648,0.22663,0
.19231,0.19908,0.21341,0.20365,0.16235,0.18524,0.17326,0.1991,0.18114,
0.15883,0.075823,0.074797,0.075254,0.070408,0.076098,0.079087,0.066746
,0.074913,0.086703,0.075244,0.074124,0.075563,0.075348,0.073994,0.0893
91,0.081673,0.073061,0.074155,0.040255,0.040397,0.04081,0.040258,0.165
73,0.15903,0.23964,0.21513,0.21597,0.20064,0.2045,0.11141,0.19977,0.19
553,0.13817,0.16994,0.075211,0.074611,0.070808,0.072038,0.071368,0.074
214,0.075427,0.076618,0.071942,0.069846,0.0747,0.078803,0.069301,0.074
767,0.075921,0.075499,0.078363,0.071335,0.080986,0.075577,0.039918,0.0
40645,0.040268,0.040818,0.040939,0.040364,0.23507,0.20635,0.20323,0.20
887,0.21145,0.25086,0.19769,0.19699,0.07573,0.081062,0.067831,0.080849
,0.068906,0.07537,0.076004,0.071945,0.077832,0.074938,0.076419,0.07763
8,0.074145,0.074921,0.075342,0.075613,0.071693,0.076345,0.073496,0.088
222,0.070055,0.074649,0.075165,0.040806,0.041075,0.04049,0.040488,0.04
0629,0.040311,0.20515,0.19505,0.19426,0.21731,0.20218,0.039877,0.04015
8,0.070379,0.072704,0.072906,0.074682,0.072667,0.075679,0.07549,0.0745
04,0.073945,0.077572,0.07629,0.075093,0.072069,0.06765,0.079785,0.0797
79,0.083024,0.075551,0.078592,0.074437,0.075779,0.040122,0.039806,0.04
0478,0.041056,0.040819,0.040719,0.040449,0.040925,0.040287,0.040532,0.
21522,0.21259,0.040344,0.040701,0.040654,0.040768,0.073768,0.074804,0.
07168,0.078572,0.070668,0.081867,0.075597,0.071477,0.074489,0.07545,0.
07626,0.081822,0.078293,0.074853,0.071441,0.071306,0.075426,0.07788,0.
074512,0.040421,0.040506,0.040533,0.040468,0.040391,0.040667,0.040593,
0.040562,0.040527,0.040462,0.041156,0.0411,0.039777,0.040347,0.040493,
0.040196,0.040746,0.074689,0.072221,0.074931,0.074982,0.072378,0.07539
1,0.076264,0.074534,0.075186,0.08269,0.070875,0.079008,0.075341,0.0789
78,0.079001,0.074384,0.03999,0.041317,0.040269,0.04097,0.040323,0.0405
07,0.040389,0.040574,0.040191,0.040634,0.040975,0.039857,0.040393,0.04
0016,0.040317,0.039978,0.040048,0.040354,0.040328,0.079789,0.075068,0.

070196,0.070623,0.074192,0.068141,0.076598,0.070269,0.074935,0.068199,
0.071241,0.077667,0.074874,0.041144,0.040268,0.04025,0.040193,0.04074,
0.040339,0.039706,0.039951,0.039612,0.040408,0.040368,0.040092,0.04123
9,0.040212,0.040005,0.039882,0.040302,0.040319,0.041,0.04027,0.040388,
0.072263,0.075438,0.072517,0.074275,0.07718,0.074875,0.075581,0.079262
,0.078432,0.040328,0.040656,0.040004,0.040662,0.040271,0.040983,0.0401
85,0.040956,0.040843,0.040354,0.040118,0.040726,0.0412,0.040155,0.0403
56,0.040564,0.039944,0.040621,0.040485,0.040663,0.041401,0.041072,0.03
9891,0.039723,0.040181,0.079225,0.074487,0.074918,0.075172,0.040453,0.
040681,0.040719,0.040956,0.039804,0.040957,0.040611,0.04059,0.040938,0
.041097,0.040534,0.040934,0.041432,0.040401,0.040103,0.039945,0.039959
,0.040703,0.04031,0.040687,0.040456,0.040631,0.040741,0.040981,0.03989
3,0.040197,0.039847,0.040562,0.039918,0.039531,0.039855,0.039758,0.040
298,0.041478,0.040562,0.04081,0.0403,0.040786,0.040993,0.03963,0.04028
8,0.040547,0.040318,0.040138,0.040065,0.040183,0.041027,0.040505,0.040
738,0.039964,0.040378,0.040737,0.040075,0.041027,0.040792,0.040157,0.0
40142,0.040202,0.040511,0.04097,0.040941,0.040244,0.040011,0.040743,0.
039882,0.040477,0.040945,0.041256,0.040226,0.040469,0.040188,0.040448,
0.040282,0.040313,0.040766,0.04029,0.040094,0.040601,0.040465,0.041032
,0.040126,0.041294,0.040499,0.040014,0.040146,0.040978,0.040494,0.0407
89,0.040222,0.040316,0.040557,0.040825,0.041107,0.040831,0.040053,0.04
0316,0.040282,0.040529,0.040723,0.03983,0.040465,0.040768,0.041274,0.0
39909,0.040831,0.040154,0.040369,0.040181,0.040984,0.040622,0.04091,0.
04058,0.040488,0.040259,0.040755,0.040822,0.040382,0.039968,0.040671,0
.040524,0.040247,0.040221,0.040387,0.040953,0.040846,0.040732,0.040279
,0.040258,0.040559,0.040075,0.040398,0.040316,0.040864,0.040187,0.0408
36,0.040282,0.040471,0.040536,0.040134,0.040605,0.040728,0.040407,0.04
0943,0.040746,0.041174,0.040454,0.040577,0.041234,0.040575,0.039957,0.
040709,0.039963,0.040968,0.039973,0.040869,0.040615,0.039606,0.040395,
0.040531,0.040764,0.040635,0.041082,0.040628,0.040368,0.040333,0.04011
4,0.039894,0.040198,0.041101,0.040077,0.040869,0.040079,0.040049,0.040
636,0.040933,0.040692,0.040537,0.040519,0.040271,0.04019,0.040055,0.04
0479,0.040277,0.040449,0.04076,0.040916,0.039933,0.040469,0.039338,0.0
40139,0.041116,0.040428,0.040232,0.04031,0.040717
0.10735,0.10621,0.1064,0.10755,0.10925,0.10728,0.10804,0.1074,0.10769,
0.10601,0.10707,0.1067,0.10711,0.1067,0.10656,0.10668,0.10766,0.10843,
0.10737,0.10711,0.233,0.19557,0.21421,0.21833,0.10723,0.10788,0.10714,
0.10637,0.1073,0.10704,0.10672,0.1062,0.10696,0.10685,0.10742,0.10641,
0.10701,0.10597,0.106,0.37247,0.25241,0.21527,0.24742,0.22113,0.23923,
0.23467,0.10681,0.10572,0.10675,0.1074,0.1065,0.10639,0.10803,0.10662,
0.10658,0.10602,0.10651,0.10806,0.10657,0.10662,0.1069,0.3506,0.36321,
0.22197,0.2659,0.22865,0.2386,0.25666,0.26601,0.22267,0.24665,0.10763,
0.10682,0.10709,0.10724,0.1074,0.10644,0.10663,0.10721,0.10744,0.10727
,0.10633,0.10758,0.10806,0.10704,0.10763,0.3564,0.36187,0.35176,0.2374
3,0.22041,0.23966,0.25775,0.20341,0.22955,0.2276,0.24852,0.23028,0.106
04,0.10669,0.1072,0.1068,0.10725,0.10662,0.10733,0.10577,0.10674,0.107
24,0.10662,0.1077,0.10645,0.10699,0.10704,0.10631,0.36202,0.35878,0.35
678,0.3538,0.23274,0.23258,0.23874,0.22504,0.25149,0.23096,0.22859,0.2
3046,0.25492,0.24156,0.10667,0.10735,0.1068,0.10698,0.10687,0.10616,0.
10714,0.10596,0.10765,0.10641,0.10857,0.10774,0.10749,0.10689,0.10751,
0.10728,0.34631,0.35582,0.36107,0.3592,0.36263,0.36537,0.23277,0.22257
,0.22055,0.23064,0.22872,0.23645,0.22662,0.23351,0.23649,0.24781,0.106

32,0.10715,0.10714,0.10771,0.10708,0.10722,0.10774,0.107,0.10692,0.107
05,0.10624,0.10643,0.10851,0.10662,0.10596,0.10758,0.1069,0.34814,0.35
454,0.3495,0.36836,0.35952,0.36897,0.22522,0.24223,0.23172,0.23145,0.2
1683,0.22966,0.21928,0.17385,0.21943,0.2451,0.2334,0.10601,0.10704,0.1
0789,0.10623,0.10701,0.10498,0.10686,0.10711,0.10746,0.10629,0.10723,0
.10637,0.10661,0.073925,0.061779,0.080117,0.10635,0.35534,0.3618,0.354
75,0.35881,0.36233,0.35738,0.33288,0.23226,0.23298,0.22929,0.21887,0.2
3269,0.23286,0.21111,0.2393,0.23989,0.24479,0.23703,0.10655,0.10649,0.
10641,0.10729,0.10692,0.10647,0.10811,0.10712,0.10694,0.1075,0.10684,0
.07262,0.068469,0.078316,0.058307,0.069396,0.075108,0.10696,0.35913,0.
3581,0.35566,0.35814,0.35431,0.35502,0.36317,0.23699,0.23929,0.23281,0
.23305,0.20263,0.22064,0.21404,0.2231,0.25093,0.27272,0.21719,0.10712,
0.10714,0.10686,0.10642,0.10761,0.10777,0.10625,0.10743,0.10662,0.0671
36,0.074562,0.072711,0.068838,0.0819,0.069636,0.073109,0.073264,0.0832
76,0.10729,0.35957,0.35595,0.34514,0.36072,0.36447,0.36653,0.35366,0.2
7086,0.22885,0.226,0.23027,0.23303,0.24736,0.23623,0.23825,0.23361,0.2
1697,0.22857,0.23105,0.10643,0.10819,0.10782,0.10717,0.10748,0.10796,0
.078515,0.071363,0.071808,0.075719,0.073366,0.070044,0.075464,0.077155
,0.075322,0.073663,0.072993,0.07402,0.35215,0.35551,0.35504,0.35734,0.
34363,0.36071,0.35966,0.19848,0.2005,0.19942,0.23857,0.22696,0.22677,0
.22633,0.22698,0.22579,0.22418,0.22342,0.25641,0.10691,0.10735,0.10685
,0.078347,0.075156,0.076124,0.072766,0.065714,0.082142,0.069837,0.0680
3,0.074434,0.071622,0.074274,0.073053,0.074694,0.067619,0.075943,0.354
01,0.35107,0.36058,0.3516,0.36537,0.35508,0.36621,0.35647,0.3519,0.231
11,0.2191,0.22913,0.22616,0.20784,0.22761,0.24586,0.27231,0.23135,0.21
929,0.21052,0.077489,0.0645,0.070212,0.075918,0.072201,0.071619,0.0714
56,0.073503,0.070639,0.073301,0.070675,0.073482,0.07469,0.073686,0.073
873,0.074189,0.070318,0.069293,0.36142,0.35823,0.35675,0.36354,0.35123
,0.37018,0.35802,0.35382,0.35233,0.22766,0.23127,0.16892,0.15977,0.179
09,0.16814,0.1738,0.16412,0.16769,0.17522,0.1643,0.074082,0.072344,0.0
75492,0.074377,0.075868,0.069293,0.073303,0.064534,0.076936,0.069425,0
.078903,0.074367,0.073674,0.071518,0.073313,0.078303,0.076294,0.071874
,0.35336,0.35763,0.35036,0.35895,0.34996,0.36253,0.35347,0.36312,0.355
63,0.20152,0.17766,0.16778,0.16225,0.17812,0.17011,0.1611,0.18085,0.17
285,0.17076,0.17396,0.078152,0.064263,0.073187,0.066662,0.07705,0.0741
92,0.074014,0.064461,0.072616,0.073974,0.086911,0.072239,0.073274,0.07
1265,0.081287,0.078573,0.073669,0.071771,0.36165,0.35252,0.36234,0.365
9,0.35596,0.36064,0.36323,0.17247,0.18364,0.19961,0.17435,0.17034,0.19
162,0.14577,0.1691,0.17301,0.17309,0.16329,0.17713,0.15203,0.073307,0.
072961,0.071385,0.072668,0.070529,0.068867,0.079807,0.075194,0.07304,0
.072537,0.071371,0.073856,0.072695,0.073819,0.076932,0.072866,0.065237
,0.071559,0.35909,0.35279,0.3554,0.35565,0.35759,0.35926,0.19622,0.206
12,0.19131,0.20166,0.1991,0.16308,0.16199,0.17614,0.16484,0.16236,0.17
365,0.17919,0.15246,0.18144,0.082277,0.07266,0.073619,0.079967,0.06656
3,0.077486,0.074212,0.073217,0.070767,0.072242,0.073642,0.073372,0.079
773,0.072425,0.069937,0.078281,0.079283,0.073486,0.35572,0.35999,0.361
8,0.36344,0.36342,0.24521,0.20194,0.14859,0.19347,0.21924,0.20728,0.17
498,0.13483,0.17237,0.17275,0.16798,0.16971,0.16517,0.16992,0.18643,0.
071962,0.073332,0.074039,0.078465,0.071098,0.0739,0.073939,0.067907,0.
081096,0.074516,0.077769,0.068061,0.071115,0.083586,0.070424,0.069806,
0.072814,0.068124,0.356,0.35483,0.36009,0.16168,0.20471,0.19287,0.1683
8,0.20309,0.20006,0.18825,0.19986,0.15472,0.16224,0.1755,0.1717,0.1668

8,0.14595,0.17465,0.15022,0.17969,0.066128,0.06774,0.074509,0.079132,0
.074038,0.074121,0.075695,0.072911,0.067056,0.074593,0.07067,0.07467,0
.080561,0.073124,0.075026,0.074414,0.074004,0.073341,0.36435,0.18283,0
.19943,0.20363,0.19892,0.24374,0.15994,0.20019,0.19294,0.23123,0.16356
,0.13219,0.17775,0.1568,0.18016,0.16267,0.17826,0.13817,0.16792,0.0731
48,0.073526,0.068428,0.072589,0.07328,0.07023,0.069359,0.078632,0.0806
69,0.068364,0.0749,0.074177,0.069572,0.075078,0.068003,0.076882,0.0844
71,0.04013,0.19776,0.20086,0.20237,0.20307,0.20055,0.2066,0.19066,0.23
259,0.16396,0.22641,0.18637,0.17527,0.17261,0.16846,0.14612,0.1672,0.1
7708,0.1679,0.1366,0.072959,0.071142,0.076092,0.072522,0.080392,0.0721
75,0.072795,0.073905,0.075096,0.073823,0.073236,0.066435,0.063176,0.07
3083,0.07387,0.069861,0.069048,0.040219,0.23551,0.20334,0.20029,0.2148
3,0.20425,0.20297,0.19022,0.20186,0.19171,0.19974,0.17134,0.18665,0.18
733,0.16833,0.18405,0.1694,0.14141,0.17644,0.069365,0.070308,0.068958,
0.069355,0.073568,0.073608,0.076834,0.073469,0.075885,0.07047,0.094707
,0.068676,0.077112,0.076399,0.074512,0.064764,0.073718,0.073131,0.0404
45,0.23562,0.1921,0.16923,0.23354,0.1849,0.20366,0.18847,0.18747,0.203
69,0.21235,0.17254,0.17469,0.17488,0.17158,0.17245,0.1825,0.18087,0.16
848,0.068066,0.071529,0.079843,0.074053,0.059079,0.075988,0.073799,0.0
79557,0.070291,0.080433,0.077286,0.080924,0.075758,0.070664,0.073869,0
.069916,0.067953,0.039891,0.040334,0.039882,0.17941,0.18512,0.19214,0.
20625,0.22649,0.19198,0.20022,0.21136,0.1992,0.161,0.18146,0.17032,0.1
9976,0.17884,0.15768,0.074128,0.073266,0.073919,0.06889,0.074156,0.077
004,0.065659,0.073119,0.084896,0.073575,0.072776,0.074137,0.073534,0.0
72485,0.087264,0.079644,0.071639,0.072396,0.039973,0.039784,0.040257,0
.04016,0.16381,0.15794,0.23795,0.2109,0.2141,0.2001,0.20231,0.11061,0.
19923,0.19356,0.13733,0.16667,0.073531,0.073236,0.06927,0.070541,0.070
143,0.072808,0.073619,0.075056,0.070228,0.068214,0.073011,0.077221,0.0
68053,0.072821,0.074112,0.073955,0.076521,0.070217,0.078991,0.074011,0
.039625,0.040555,0.039955,0.040607,0.040368,0.039681,0.23332,0.20537,0
.20279,0.20663,0.21025,0.25023,0.1963,0.19479,0.073877,0.078771,0.0665
71,0.07919,0.067628,0.073743,0.074161,0.070487,0.076099,0.073251,0.074
702,0.076268,0.072307,0.073296,0.073761,0.073657,0.070672,0.074687,0.0
71932,0.086232,0.069023,0.073186,0.073605,0.040471,0.040758,0.040078,0
.040111,0.040142,0.039832,0.20373,0.19394,0.1957,0.21642,0.20108,0.039
48,0.039615,0.068951,0.071134,0.071188,0.072943,0.071156,0.074054,0.07
3864,0.073031,0.072636,0.075446,0.074868,0.073507,0.070502,0.066765,0.
077945,0.078042,0.081129,0.073829,0.07686,0.072792,0.074353,0.039673,0
.039732,0.040072,0.040453,0.040432,0.040256,0.040212,0.040486,0.039798
,0.04027,0.21339,0.21198,0.039842,0.040289,0.040402,0.040396,0.072656,
0.073442,0.070285,0.076889,0.069313,0.080085,0.074048,0.070051,0.07283
4,0.073572,0.074651,0.079991,0.076598,0.073271,0.069872,0.070026,0.074
066,0.076651,0.072644,0.040146,0.039907,0.039884,0.039872,0.040062,0.0
40246,0.040491,0.040288,0.04006,0.039927,0.040853,0.040363,0.039453,0.
039767,0.040044,0.039823,0.040263,0.072905,0.070766,0.073657,0.073287,
0.070875,0.073946,0.074581,0.073453,0.073754,0.081056,0.069445,0.07743
2,0.073655,0.077556,0.077388,0.073107,0.039455,0.040623,0.039908,0.040
396,0.040158,0.040117,0.039967,0.040553,0.039749,0.04041,0.040358,0.03
9585,0.039653,0.039558,0.04016,0.039446,0.039332,0.039928,0.040005,0.0
77751,0.073438,0.068871,0.069158,0.072493,0.067013,0.074831,0.068754,0
.073535,0.066699,0.069903,0.07569,0.073529,0.040591,0.03975,0.03964,0.
039942,0.040501,0.04016,0.039355,0.03963,0.039455,0.03986,0.040258,0.0

39656,0.040697,0.040147,0.039558,0.039361,0.039811,0.040052,0.040446,0
.04007,0.039852,0.070704,0.073506,0.070834,0.072627,0.075648,0.073145,
0.073489,0.077753,0.076922,0.040008,0.040355,0.039638,0.040132,0.04019
8,0.040661,0.039764,0.040534,0.040295,0.040145,0.039705,0.040321,0.041
032,0.039851,0.039853,0.040284,0.039509,0.039866,0.039984,0.039963,0.0
41239,0.040704,0.039719,0.039203,0.039793,0.077295,0.073045,0.073199,0
.073619,0.040148,0.040443,0.04043,0.040435,0.039489,0.040662,0.040302,
0.039933,0.040581,0.040719,0.040215,0.040557,0.041181,0.040066,0.03964
3,0.039451,0.039744,0.039966,0.039883,0.040138,0.040104,0.040181,0.040
088,0.040368,0.039833,0.03988,0.039108,0.040305,0.039633,0.039194,0.03
9493,0.039426,0.039966,0.041078,0.039762,0.040592,0.039904,0.040294,0.
040616,0.039221,0.040045,0.040287,0.040215,0.039678,0.039671,0.03979,0
.040651,0.039855,0.040466,0.039448,0.03994,0.04049,0.039831,0.040795,0
.04042,0.039814,0.039484,0.040027,0.04029,0.040474,0.040681,0.040271,0
.039509,0.040421,0.039273,0.039939,0.040498,0.040524,0.040072,0.039704
,0.039615,0.039973,0.039789,0.039693,0.040428,0.039996,0.039774,0.0401
46,0.040016,0.040385,0.039795,0.040947,0.04011,0.039381,0.039672,0.040
416,0.040094,0.040378,0.040101,0.039936,0.040274,0.040481,0.040433,0.0
40392,0.039714,0.04014,0.039923,0.040231,0.040432,0.03918,0.039989,0.0
40293,0.040604,0.039596,0.040395,0.040184,0.04001,0.040095,0.04063,0.0
40335,0.040666,0.039714,0.039953,0.040039,0.040256,0.040508,0.039954,0
.039431,0.039951,0.040123,0.039887,0.039758,0.039835,0.040299,0.040521
,0.040458,0.040151,0.039882,0.040268,0.039603,0.039896,0.039733,0.0404
48,0.039571,0.04037,0.039753,0.040038,0.040302,0.040035,0.040153,0.040
377,0.039849,0.040325,0.040336,0.040824,0.039852,0.039881,0.040787,0.0
40149,0.039606,0.040181,0.039358,0.04062,0.039822,0.040313,0.040281,0.
039321,0.040137,0.040036,0.040605,0.04018,0.04073,0.039923,0.039789,0.
039974,0.039767,0.039537,0.039885,0.040708,0.039784,0.040548,0.039622,
0.039903,0.040396,0.040589,0.039895,0.040199,0.040083,0.039513,0.04014
,0.039588,0.040177,0.039846,0.040126,0.040365,0.040663,0.03989,0.04010
2,0.03926,0.039583,0.040697,0.040245,0.03953,0.040052,0.040166

#simdataS4SNR10:

Kep_f=1.5
Kep_f=1.5
Kep_s=0.6
Ktrans_f=0.08
Ktrans_s=0.05
del_t=0.5
gtSf=0 0 0 0 0 0
0 0 0 0 0 0
0 0 0 0 0 0
0 0 0 0 0 0
0 0 0 0 0 0
0 0 0 0 0 0
0 0 0 0 0 0
0 0 0 0 0 0
0 0 0 0 0 0
0 0 0 0 0 0
0 0 0 0 0 0

0	0	0	0	0	0
0	0	0	0	0	0
0	0	0	0	0	0
0	0	0	0	0	0
0	0	0	0	0	0
0	0	0	0	0	0
0	0	0	0	0	0
0	0	0	0	0	0
0	0	0	0	0	0
0	0	0	0	0	0
0	0	0	0	0	0
0	0	0	0	0	0
0	0	0	0	0	0
0	0	0	0	0	0
0	0	0	0	0	0
0	0	0	0	0	0
0	0	0	0	0	0
0	0	0	0	0	0
0	0	0	0	0	0
0	0	0	0	0	0
0	0	0	0	0	0
0	0	0	0	0	0
0	0	0	0	0	0
0	0	0	0.57309	0.5	0.5437
0	0	0	0	0	0
0	0	0	0	0	0
0	0	0	0	0	0
0	0	0	0	0	0
0	0	0	0	0	0
0.5	0.5	0.5	0.47929	0.5	0.5
0	0	0	0	0	0
0	0	0	0	0	0
0	0	0	0	0	0
0	0	0	0	0	0
0	0	0	0	0.55796	0.54039
0.5	0.5	0.5	0.5	0.48976	0.45797
0.47316	0	0	0	0	0
0	0	0	0	0	0
0	0	0	0	0	0
0	0	0	0	0	0
0	0	0	0.5	0.55684	0.43185
0.62667	0.54691	0.58628	0.47268	0.47257	0.40902
0.58969	0.4253	0.5	0	0	0
0	0	0	0	0	0
0	0	0	0	0	0
0	0	0	0	0	0
0	0.47858	0.5	0.5856	0.5358	0.5
0.51957	0.5	0.53643	0.49211	0.41849	0.57557
0.49312	0.4656	0.43812	0.50533	0	0
0	0	0	0	0	0
0	0	0	0	0	0
0	0	0	0	0	0

0.48075	0.5	0.57615	0.41005	0.5	0.5
0.5	0.38919	0.5	0.5	0.42231	0.5
0.5	0.51853	0.5	0.51547	0.3845	0.6068
0	0	0	0	0	0
0	0	0	0	0	0.31193
0.31341	0.23375	0.33333	0.34245	0.33333	0.32196
0.33401	0.28525	0.45917	0.4597	0.53833	0.47219
0.52066	0.68409	0.49286	0.46065	0.43755	0.5
0.5	0.48506	0.52889	0.4974	0.55339	0.4916
0.49778	0.59002	0	0	0	0
0	0	0	0	0	0.5
0.33477	0.37636	0.35105	0.41553	0.3182	0.29294
0.24197	0.33464	0.30527	0.40255	0.60097	0.49014
0.52532	0.62556	0.50284	0.5	0.44976	0.56975
0.5	0.50604	0.56368	0.49971	0.43735	0.60807
0.47839	0.59968	0.54566	0.50721	0	0
0	0	0	0	0	0.46389
0.5	0.58454	0.23784	0.45355	0.33186	0.3646
0.36342	0.40095	0.297	0.30265	0.37709	0.33333
0.5	0.48562	0.5	0.5	0.47599	0.5
0.57724	0.5	0.48333	0.532	0.63766	0.5
0.42342	0.50944	0.46412	0.48804	0.48943	0.62222
0	0	0	0	0	0
0.5	0.5	0.52303	0.52393	0.49839	0.29243
0.40815	0.42961	0.23506	0.37422	0.37117	0.37409
0.36466	0.33333	0.47886	0.4281	0.44523	0.46261
0.5	0.5	0.52906	0.4711	0.45403	0.39615
0.53187	0.46065	0.5	0.50717	0.68404	0.56553
0.51956	0.49663	0	0	0	0
0	0.50581	0.5	0.53897	0.5	0.5
0.5	0.33333	0.29497	0.30915	0.36841	0.33333
0.35477	0.38493	0.24377	0.34225	0.47117	0.51826
0.56488	0.46099	0.6328	0.41002	0.53219	0.58474
0.51586	0.5	0.51588	0.55394	0.5	0.5316
0.57274	0.50184	0.61406	0.5	0	0
0	0.5	0.39518	0.5	0.49456	0.49037
0.379	0.56709	0.55431	0.27876	0.31422	0.35253
0.33333	0.33333	0.33333	0.29319	0.33581	0.31359
0.5	0.5	0.5	0.54497	0.48532	0.36745
0.50019	0.5	0.5	0.5	0.58126	0.5
0.52342	0.4465	0.7282	0.52064	0.5	0.36135
0	0.5	0.32919	0.49481	0.42068	0.54187
0.45977	0.49813	0.50525	0.54	0.24076	0.44077
0.32478	0.33333	0.30564	0.26969	0.40034	0.20922
0.33333	0.53121	0.56946	0.35462	0.5	0.45457
0.66764	0.5	0.48693	0.5	0.43547	0.50383
0.45941	0.52445	0.52148	0.5	0.49112	0.49249
1	0.40879	0.54051	0.39967	0.52708	0.4875
0.5	0.4586	0.52086	0.5	0.44125	0.33742
0.35132	0.3488	0.38838	0.39724	0.34419	0.31482
0.35521	0.33333	0.49699	0.45716	0.5	0.47489
0.51021	0.4397	0.5	0.55703	0.5	0.52404

0.5	0.37582	0.41332	0.43064	0.42498	0.5
0.5	1	0.55875	0.49976	0.5	0.50031
0.45782	0.44472	0.34579	0.6079	0.43768	0.5
0.35994	0.34617	0.3467	0.35237	0.37464	0.23054
0.37966	0.36532	0.44201	0.65711	0.52027	0.4696
0.48389	0.5	0.48574	0.5	0.36631	0.54556
0.5	0.5	0.5	0.36022	0.52904	0.6306
0.5	0.50644	1	0.49014	0.5	0.53605
0.50676	0.5	0.40395	0.65269	0.56702	0.54463
0.50496	0.33831	0.28527	0.28449	0.33363	0.35287
0.335	0.32158	0.34822	0.53612	0.59599	0.51917
0.62705	0.53978	0.54749	0.51401	0.51931	0.43857
0.4989	0.43488	0.3594	0.5	0.51356	0.47763
0.53883	0.45325	1	1	1	0.48915
0.43805	0.59325	0.5	0.51134	0.5	0.5
0.5	0.5	0.35484	0.31175	0.33333	0.41077
0.33333	0.38544	0.48934	0.52847	0.56456	0.43661
0.56219	0.44638	0.5	0.56016	0.5	0.3962
0.60563	0.54267	0.5	0.47302	0.5	0.47452
0.53467	0.44638	1	1	1	1
0.5	0.46414	0.52367	0.57376	0.49107	0.43014
0.57294	0.46136	0.50245	0.29941	0.33333	0.3523
0.4457	0.5	0.5	0.53992	0.5	0.55524
0.42329	0.5	0.50339	0.5774	0.5	0.57857
0.44455	0.3624	0.5568	0.5	0.52927	0.48581
0.45448	0.37352	1	1	1	1
1	1	0.48264	0.48685	0.45258	0.40954
0.51632	0.55164	0.5	0.37636	0.45842	0.5
0.5	0.5	0.51933	0.5	0.59292	0.46527
0.64568	0.53718	0.56705	0.55396	0.50646	0.5
0.52251	0.52016	0.5	0.43607	0.5	0.5342
0.5	0.5	0.46887	1	1	1
1	1	1	0.5	0.52542	0.56691
0.5	0.49	1	1	0.44825	0.38806
0.5	0.47746	0.57261	0.48428	0.5	0.5
0.45324	0.48089	0.41926	0.49552	0.6145	0.46794
0.42079	0.5	0.5	0.51737	0.47415	0.38507
0.45478	1	1	1	1	1
1	1	1	1	1	0.50355
0.46963	1	1	1	1	0.4956
0.5	0.59328	0.58868	0.72512	0.50914	0.40882
0.6184	0.60852	0.51956	0.56269	0.49008	0.49118
0.49883	0.44935	0.36651	0.53247	0.54966	0.5
1	1	1	1	1	1
1	1	1	1	1	1
1	1	1	1	1	0.54563
0.52684	0.5	0.5	0.5374	0.5	0.42135
0.48462	0.46569	0.47647	0.59543	0.5	0.36757
0.43166	0.5	0.54165	1	1	1
1	1	1	1	1	1
1	1	1	1	1	1
1	1	1	1	0.5	0.51918

```
0.49798     0.49299        0.5        0.5     0.51074     0.49964
0.5     0.63192     0.44607     0.41324     0.51034           1
1           1           1           1           1           1
1           1           1           1           1           1
1           1           1           1           1           1
1           1     0.43963        0.5     0.45256     0.45882
0.52834        0.5     0.53721     0.54825        0.5           1
1           1           1           1           1           1
1           1           1           1           1           1
1           1           1           1           1           1
1           1           1           1           1           1
0.55243      0.4737     0.48653     0.38454           1           1
1           1           1           1           1           1
1           1           1           1           1           1
1           1           1           1           1           1
1           1           1           1           1           1
1           1           1           1           1           1
1           1           1           1           1           1
1           1           1           1           1           1
1           1           1           1           1           1
1           1           1           1           1           1
1           1           1           1           1           1
1           1           1           1           1           1
1           1           1           1           1           1
1           1           1           1           1           1
1           1           1           1           1           1
1           1           1           1           1           1
1           1           1           1           1           1
1           1           1           1           1           1
1           1           1           1           1           1
1           1           1           1           1           1
1           1           1           1           1           1
1           1           1           1           1           1
1           1           1           1           1           1
1           1           1           1           1           1
1           1           1           1           1           1
1           1           1           1           1           1
1           1           1           1           1           1
1           1           1           1           1           1
1           1           1           1           1           1
1           1           1           1           1           1
1           1           1           1           1           1
1           1           1           1           1           1
1           1           1           1           1           1
1
gtSs=1           1           1           1           1           1
1           1           1           1           1           1
1           1           1           1           1           1
1           1      0.4616     0.51649     0.33955     0.50826
1           1           1           1           1           1
1           1           1           1           1           1
1           1           1           0         0.5     0.53118
```

0.61844 0.5 0.5 0.59796 1 1
1 1 1 1 1 1
1 1 1 1 1 1
1 0 0 0.51302 0.54543 0.52012
0.50093 0.5 0.52728 0.5 0.53167 1
1 1 1 1 1 1
1 1 1 1 1 1
1 1 0 0 0 0.47601
0.43084 0.50959 0.54618 0.5 0.5 0.53609
0.66279 0.53931 1 1 1 1
1 1 1 1 1 1
1 1 1 1 1 1
0 0 0 0 0.5 0.54893
0.43573 0.58022 0.5 0.49127 0.43026 0.5
0.5 0.49612 1 1 1 1
1 1 1 1 1 1
1 1 1 1 1 1
0 0 0 0 0 0
0.56814 0.45089 0.5 0.51139 0.47999 0.49097
0.61675 0.60157 0.44065 0.50968 1 1
1 1 1 1 1 1
1 1 1 1 1 1
1 1 1 0 0 0
0 0 0 0.5351 0.5 0.4834
0.5 0.44524 0.5 0.5 0.5 0.5
0.44526 0.45735 1 1 1 1
1 1 1 1 1 1
1 1 1 0.42691 0.5 0.4563
1 0 0 0 0 0
0 0 0.49542 0.35381 0.55607 0.27262
0.5 0.47287 0.49911 0.49741 0.5 0.5
0.63289 1 1 1 1 1
1 1 1 1 1 1
0.5 0.5 0.5 0.52071 0.5 0.5
1 0 0 0 0 0
0 0 0.51418 0.51621 0.48077 0.45714
0.5869 0.49745 0.5547 0.47658 0.5 0.52007
0.52723 1 1 1 1 1
1 1 1 1 0.44204 0.45961
0.5 0.5 0.5 0.5 0.51024 0.54203
0.52684 1 0 0 0 0
0 0 0 0.42066 0.51138 0.52247
0.57637 0.53637 0.52626 0.57205 0.48017 0.5
0.50563 0.5696 0.48368 1 1 1
1 1 1 0.5 0.44316 0.56815
0.37333 0.45309 0.41372 0.52732 0.52743 0.59098
0.41031 0.5747 0.5 0 0 0
0 0 0 0 0.49674 0.4963
0.48347 0.5 0.48195 0.61877 0.44825 0.53472
0.4474 0.5 0.5 0.47239 1 1
1 0.52142 0.5 0.4144 0.4642 0.5
0.48043 0.5 0.46357 0.50789 0.58151 0.42443

0.50688	0.5344	0.56188	0.49467	0	0
0	0	0	0	0	0
0	0.57577	0.48629	0.37874	0.5	0.60449
0.49499	0.49113	0.51812	0.5	0.52504	0.49417
0.51925	0.5	0.42385	0.58995	0.5	0.5
0.5	0.61081	0.5	0.5	0.57769	0.5
0.5	0.48147	0.5	0.48453	0.6155	0.3932
0	0	0	0	0	0
0	0	0	0.5	0.50726	0.34404
0.35557	0.38312	0.33333	0.34245	0.33333	0.33902
0.33197	0.35737	0.54083	0.5403	0.46167	0.52781
0.47934	0.31591	0.50714	0.53935	0.56245	0.5
0.5	0.51494	0.47111	0.5026	0.44661	0.5084
0.50222	0.40998	0	0	0	0
0	0	0	0	0	0
0.33477	0.37636	0.35105	0.41553	0.3409	0.35353
0.3798	0.35006	0.34737	0.30681	0.39903	0.50986
0.47468	0.37444	0.49716	0.5	0.55024	0.43025
0.5	0.49396	0.43632	0.50029	0.56265	0.39193
0.52161	0.40032	0.45434	0.49279	0	0
0	0	0	0	0	0
0	0	0.38108	0.32315	0.31889	0.35497
0.33517	0.28396	0.36387	0.31529	0.24581	0.33333
0.5	0.51438	0.5	0.5	0.52401	0.5
0.42276	0.5	0.51667	0.468	0.36234	0.5
0.57658	0.49056	0.53588	0.51196	0.51057	0.37778
0	0	0	0	0	0
0	0	0	0	0	0.25162
0.28169	0.27211	0.42535	0.25157	0.25767	0.31334
0.27068	0.33333	0.52114	0.5719	0.55477	0.53739
0.5	0.5	0.47094	0.5289	0.54597	0.60385
0.46813	0.53935	0.5	0.49283	0.31596	0.43447
0.48044	0.50337	0	0	0	0
0	0	0	0	0	0
0	0.33333	0.40567	0.34543	0.32073	0.33333
0.30723	0.38493	0.48507	0.34225	0.52883	0.48174
0.43512	0.53901	0.3672	0.58998	0.46781	0.41526
0.48414	0.5	0.48412	0.44606	0.5	0.4684
0.42726	0.49816	0.38594	0.5	0	0
0	0	0	0	0	0
0	0	0	0.36474	0.36954	0.43021
0.33333	0.33333	0.33333	0.33642	0.33581	0.32851
0.5	0.5	0.5	0.45503	0.51468	0.63255
0.49981	0.5	0.5	0.5	0.41874	0.5
0.47658	0.5535	0.2718	0.47936	0.5	0.63865
0	0	0	0	0	0
0	0	0	0	0.37962	0.36464
0.35595	0.33333	0.42592	0.40603	0.40034	0.39539
0.33333	0.46879	0.43054	0.64538	0.5	0.54543
0.33236	0.5	0.51307	0.5	0.56453	0.49617
0.54059	0.47555	0.47852	0.5	0.50888	0.50751
0	0	0	0	0	0

0	0	0	0	0	0.33742
0.29737	0.3488	0.26236	0.44425	0.3991	0.35748
0.32847	0.33333	0.50301	0.54284	0.5	0.52511
0.48979	0.5603	0.5	0.44297	0.5	0.47596
0.5	0.62418	0.58668	0.56936	0.57502	0.5
0.5	0	0	0	0	0
0	0	0	0	0	0
0.28013	0.30766	0.3179	0.34676	0.25073	0.38473
0.32283	0.36532	0.55799	0.34289	0.47973	0.5304
0.51611	0.5	0.51426	0.5	0.63369	0.45444
0.5	0.5	0.5	0.63978	0.47096	0.3694
0.5	0.49356	0	0	0	0
0	0	0	0	0	0
0	0.33983	0.35609	0.34074	0.34934	0.31977
0.30423	0.353	0.34822	0.46388	0.40401	0.48083
0.37295	0.46022	0.45251	0.48599	0.48069	0.56143
0.5011	0.56512	0.6406	0.5	0.48644	0.52237
0.46117	0.54675	0	0	0	0
0	0	0	0	0	0
0	0	0.38961	0.34413	0.33333	0.24129
0.33333	0.28238	0.51066	0.47153	0.43544	0.56339
0.43781	0.55362	0.5	0.43984	0.5	0.6038
0.39437	0.45733	0.5	0.52698	0.5	0.52548
0.46533	0.55362	0	0	0	0
0	0	0	0	0	0
0	0	0	0.3503	0.33333	0.3325
0.5543	0.5	0.5	0.46008	0.5	0.44476
0.57671	0.5	0.49661	0.4226	0.5	0.42143
0.55545	0.6376	0.4432	0.5	0.47073	0.51419
0.54552	0.62648	0	0	0	0
0	0	0	0	0	0
0	0	0	0	0.54158	0.5
0.5	0.5	0.48067	0.5	0.40708	0.53473
0.35432	0.46282	0.43295	0.44604	0.49354	0.5
0.47749	0.47984	0.5	0.56393	0.5	0.4658
0.5	0.5	0.53113	0	0	0
0	0	0	0	0	0
0	0	0	0	0.55175	0.61194
0.5	0.52254	0.42739	0.51572	0.5	0.5
0.54676	0.51911	0.58074	0.50448	0.3855	0.53206
0.57921	0.5	0.5	0.48263	0.52585	0.61493
0.54522	0	0	0	0	0
0	0	0	0	0	0
0	0	0	0	0	0.5044
0.5	0.40672	0.41132	0.27488	0.49086	0.59118
0.3816	0.39148	0.48044	0.43731	0.50992	0.50882
0.50117	0.55065	0.63349	0.46753	0.45034	0.5
0	0	0	0	0	0
0	0	0	0	0	0
0	0	0	0	0	0.45437
0.47316	0.5	0.5	0.4626	0.5	0.57865
0.51538	0.53431	0.52353	0.40457	0.5	0.63243

```
0.56834 0.5 0.45835 0 0 0
0 0 0 0 0 0
0 0 0 0 0 0
0 0 0 0 0.5 0.48082
0.50202 0.50701 0.5 0.5 0.48926 0.50036
0.5 0.36808 0.55393 0.58676 0.48966 0
0 0 0 0 0 0
0 0 0 0 0 0
0 0 0 0 0 0
0 0 0.56037 0.5 0.54744 0.54118
0.47166 0.5 0.46279 0.45175 0.5 0
0 0 0 0 0 0
0 0 0 0 0 0
0 0 0 0 0 0
0 0 0 0 0 0
0.44757 0.5263 0.51347 0.61546 0 0
0 0 0 0 0 0
0 0 0 0 0 0
0 0 0 0 0 0
0 0 0 0 0 0
0 0 0 0 0 0
0 0 0 0 0 0
0 0 0 0 0 0
0 0 0 0 0 0
0 0 0 0 0 0
0 0 0 0 0 0
0 0 0 0 0 0
0 0 0 0 0 0
0 0 0 0 0 0
0 0 0 0 0 0
0 0 0 0 0 0
0 0 0 0 0 0
0 0 0 0 0 0
0 0 0 0 0 0
0 0 0 0 0 0
0 0 0 0 0 0
0 0 0 0 0 0
0 0 0 0 0 0
0 0 0 0 0 0
0 0 0 0 0 0
0 0 0 0 0 0
0 0 0 0 0 0
0 0 0 0 0 0
0 0 0 0 0 0
0 0 0 0 0 0
0 0 0 0 0 0
0 0 0 0 0 0
0 0 0 0 0 0
0
gtSp=0 0 0 0 0 0
0 0 0 0 0 0
0 0 0 0 0 0
```

0 0 0.5384 0.48351 0.66045 0.49174
0 0 0 0 0 0
0 0 0 0 0 0
0 0 0 1 0.5 0.46882
0.38156 0.5 0.5 0.40204 0 0
0 0 0 0 0 0
0 0 0 0 0 0
0 1 1 0.48698 0.45457 0.47988
0.49907 0.5 0.47272 0.5 0.46833 0
0 0 0 0 0 0
0 0 0 0 0 0
0 0 1 1 1 0.52399
0.56916 0.49041 0.45382 0.5 0.5 0.46391
0.33721 0.46069 0 0 0 0
0 0 0 0 0 0
0 0 0 0 0 0
1 1 1 1 0.5 0.45107
0.56427 0.41978 0.5 0.50873 0.56974 0.5
0.5 0.50388 0 0 0 0
0 0 0 0 0 0
0 0 0 0 0 0
1 1 1 1 1 1
0.43186 0.54911 0.5 0.48861 0.52001 0.50903
0.38325 0.39843 0.55935 0.49032 0 0
0 0 0 0 0 0
0 0 0 0 0 0
0 0 0 1 1 1
1 1 1 0.4649 0.5 0.5166
0.5 0.55476 0.5 0.5 0.5 0.5
0.55474 0.54265 0 0 0 0
0 0 0 0 0 0
0 0 0 0 0 0
0 1 1 1 1 1
1 1 0.50458 0.64619 0.44393 0.72738
0.5 0.52713 0.50089 0.50259 0.5 0.5
0.36711 0 0 0 0 0
0 0 0 0 0 0
0 0 0 0 0 0
0 1 1 1 1 1
1 1 0.48582 0.48379 0.51923 0.54286
0.4131 0.50255 0.4453 0.52342 0.5 0.47993
0.47277 0 0 0 0 0
0 0 0 0 0 0
0 0 0 0 0 0
0 0 1 1 1 1
1 1 1 0.57934 0.48862 0.47753
0.42363 0.46363 0.47374 0.42795 0.51983 0.5
0.49437 0.4304 0.51632 0 0 0
0 0 0 0 0 0
0 0 0 0 0 0
0 0 0 1 1 1
1 1 1 1 0.50326 0.5037

0.51653	0.5	0.51805	0.38123	0.55175	0.46528
0.5526	0.5	0.5	0.52761	0	0
0	0	0	0	0	0
0	0	0	0	0	0
0	0	0	0	1	1
1	1	1	1	1	1
1	0.42423	0.51371	0.62126	0.5	0.39551
0.50501	0.50887	0.48188	0.5	0.47496	0.50583
0	0	0	0	0	0
0	0	0	0	0	0
0	0	0	0	0	0
1	1	1	1	1	1
1	1	1	0.5	0.49274	0.34404
0.33102	0.38312	0.33333	0.31511	0.33333	0.33902
0.33401	0.35737	0	0	0	0
0	0	0	0	0	0
0	0	0	0	0	0
0	0	1	1	1	1
1	1	1	1	1	0.5
0.33047	0.24728	0.2979	0.16894	0.3409	0.35353
0.37823	0.3153	0.34737	0.29064	0	0
0	0	0	0	0	0
0	0	0	0	0	0
0	0	0	0	1	1
1	1	1	1	1	0.53611
0.5	0.41546	0.38108	0.22331	0.34925	0.28043
0.30141	0.3151	0.33913	0.38206	0.37709	0.33333
0	0	0	0	0	0
0	0	0	0	0	0
0	0	0	0	0	0
1	1	1	1	1	1
0.5	0.5	0.47697	0.47607	0.50161	0.45595
0.31015	0.29828	0.3396	0.37422	0.37117	0.31256
0.36466	0.33333	0	0	0	0
0	0	0	0	0	0
0	0	0	0	0	0
0	0	1	1	1	1
1	0.49419	0.5	0.46103	0.5	0.5
0.5	0.33333	0.29937	0.34543	0.31086	0.33333
0.338	0.23014	0.27116	0.31549	0	0
0	0	0	0	0	0
0	0	0	0	0	0
0	0	0	0	1	1
1	0.5	0.60482	0.5	0.50544	0.50963
0.621	0.43291	0.44569	0.3565	0.31624	0.21725
0.33333	0.33333	0.33333	0.3704	0.32838	0.35789
0	0	0	0	0	0
0	0	0	0	0	0
0	0	0	0	0	0
1	0.5	0.67081	0.50519	0.57932	0.45813
0.54023	0.50187	0.49475	0.46	0.37962	0.19459
0.31927	0.33333	0.26844	0.32428	0.19931	0.39539

0.33333	0	0	0	0	0
0	0	0	0	0	0
0	0	0	0	0	0
0	0.59121	0.45949	0.60033	0.47292	0.5125
0.5	0.5414	0.47914	0.5	0.55875	0.32515
0.35132	0.30239	0.34927	0.1585	0.25671	0.3277
0.31632	0.33333	0	0	0	0
0	0	0	0	0	0
0	0	0	0	0	0
0	0	0.44125	0.50024	0.5	0.49969
0.54218	0.55528	0.65421	0.3921	0.56232	0.5
0.35994	0.34617	0.33539	0.30087	0.37464	0.38473
0.29752	0.26936	0	0	0	0
0	0	0	0	0	0
0	0	0	0	0	0
0	0	0	0.50986	0.5	0.46395
0.49324	0.5	0.59605	0.34731	0.43298	0.45537
0.49504	0.32186	0.35864	0.37478	0.31704	0.32736
0.36077	0.32542	0.30356	0	0	0
0	0	0	0	0	0
0	0	0	0	0	0
0	0	0	0	0	0.51085
0.56195	0.40675	0.5	0.48866	0.5	0.5
0.5	0.5	0.25555	0.34413	0.33333	0.34794
0.33333	0.33218	0	0	0	0
0	0	0	0	0	0
0	0	0	0	0	0
0	0	0	0	0	0
0.5	0.53586	0.47633	0.42624	0.50893	0.56986
0.42706	0.53864	0.49755	0.3503	0.33333	0.3152
0	0	0	0	0	0
0	0	0	0	0	0
0	0	0	0	0	0
0	0	0	0	0	0
0	0	0.51736	0.51315	0.54742	0.59046
0.48368	0.44836	0.5	0.62364	0	0
0	0	0	0	0	0
0	0	0	0	0	0
0	0	0	0	0	0
0	0	0	0	0	0
0	0	0	0.5	0.47458	0.43309
0.5	0.51	0	0	0	0
0	0	0	0	0	0
0	0	0	0	0	0
0	0	0	0	0	0
0	0	0	0	0	0
0	0	0	0	0	0.49645
0.53037	0	0	0	0	0
0	0	0	0	0	0
0	0	0	0	0	0
0	0	0	0	0	0
0	0	0	0	0	0

0	0	0	0	0	0
0	0	0	0	0	0
0	0	0	0	0	0
0	0	0	0	0	0
0	0	0	0	0	0
0	0	0	0	0	0
0	0	0	0	0	0
0	0	0	0	0	0
0	0	0	0	0	0
0	0	0	0	0	0
0	0	0	0	0	0
0	0	0	0	0	0
0	0	0	0	0	0
0	0	0	0	0	0
0	0	0	0	0	0
0	0	0	0	0	0
0	0	0	0	0	0
0	0	0	0	0	0
0	0	0	0	0	0
0	0	0	0	0	0
0	0	0	0	0	0
0	0	0	0	0	0
0	0	0	0	0	0
0	0	0	0	0	0
0	0	0	0	0	0
0	0	0	0	0	0
0	0	0	0	0	0
0	0	0	0	0	0
0	0	0	0	0	0
0	0	0	0	0	0
0	0	0	0	0	0
0	0	0	0	0	0
0	0	0	0	0	0
0	0	0	0	0	0
0	0	0	0	0	0
0	0	0	0	0	0
0	0	0	0	0	0
0	0	0	0	0	0
0	0	0	0	0	0
0	0	0	0	0	0
0	0	0	0	0	0
0	0	0	0	0	0
0	0	0	0	0	0
0	0	0	0	0	0
0	0	0	0	0	0
0	0	0	0	0	0
0	0	0	0	0	0
0	0	0	0	0	0
0	0	0	0	0	0
0	0	0	0	0	0
0	0	0	0	0	0

0	0	0	0	0	0
0					

gtTCf=
0
0.1908
0.21796
0.19252
0.15724
0.12639
0.10315
0.086848
0.075855
0.068599
0.063857
0.060755
0.058703
0.057315
0.056342
0.055627
0.055072
0.054617

gtTCs=
0
0.11925
0.16824
0.18061
0.17524
0.16239
0.14746
0.13307
0.12035
0.10964
0.10088
0.093851
0.08828
0.08389
0.08044
0.077724
0.075577
0.073866

gtTCp=
0
2.385
1.5979
1.1196
0.8287
0.65143
0.54309
0.47655

0.43538
0.4096
0.39316
0.38239
0.37506
0.36982
0.36585
0.36266
0.35995
0.35753

X_mask=
0.00051182,0.0057893,0.0010368,0.002745,0.0016492,2.2204e-016,2.2204e-016,0.00245,2.2204e-016,2.2204e-016,0.0076532,0.0057538,0.0086345,0.0053954,2.2204e-016,2.2204e-016,0.0020597,0.0066204,0.00017473,2.2204e-016,2.2204e-016,0.012847,2.2204e-016,2.2204e-016,2.2204e-016,2.2204e-016,2.2204e-016,2.2204e-016,7.4076e-005,2.2204e-016,2.2204e-016,2.2204e-016,2.2204e-016,0.0048005,0.0031499,2.2204e-016,2.2204e-016,2.2204e-016,2.2204e-016,0.038588,2.2204e-016,2.2204e-016,2.2204e-016,2.2204e-016,2.2204e-016,0.0091653,2.2204e-016,2.2204e-016,0.0056399,0.0029274,2.2204e-016,2.2204e-016,0.0018306,2.2204e-016,0.00017228,2.2204e-016,0.0083356,0.0059593,0.002827,2.2204e-016,2.2204e-016,2.2204e-016,2.2204e-016,2.2204e-016,2.2204e-016,2.2204e-016,0.0061041,2.2204e-016,2.2204e-016,0.019809,0.03622,9.3346e-005,0.0017843,0.0025289,2.2204e-016,2.2204e-016,0.0003574,0.0043425,2.2204e-016,2.2204e-016,2.2204e-016,2.2204e-016,0.0029597,2.2204e-016,2.2204e-016,2.2204e-016,0.022541,0.064638,2.2204e-016,2.2204e-016,2.2204e-016,2.2204e-016,2.2204e-016,2.2204e-016,0.063066,2.2204e-016,0.010967,0.053742,2.2204e-016,0.0049675,0.0031125,0.0040832,0.0011104,2.2204e-016,2.2204e-016,0.0012051,2.2204e-016,2.2204e-016,2.2204e-016,2.2204e-016,0.0070013,0.0001927,2.2204e-016,0.0062557,2.2204e-016,0.00035953,0.12871,0.013784,0.017614,2.2204e-016,2.2204e-016,0.014627,2.2204e-016,2.2204e-016,0.014233,0.032089,0.0025512,0.030301,0.0012709,2.2204e-016,2.2204e-016,0.000967,0.0055692,2.2204e-016,0.0034603,0.00092651,0.0083948,0.0040807,2.2204e-016,2.2204e-016,2.2204e-016,0.0010841,0.0065919,0.0029231,2.2204e-016,2.2204e-016,0.064709,0.0119,2.2204e-016,2.2204e-016,2.2204e-016,2.2204e-016,0.022295,0.056038,2.2204e-016,2.2204e-016,2.2204e-016,0.029987,0.01716,0.013,2.2204e-016,0.0087363,0.0084331,0.0081118,0.0022282,0.0024898,0.0054299,0.00635,2.2204e-016,2.2204e-016,0.0028548,0.0013943,0.010778,0.0013615,0.0045581,2.2204e-016,2.2204e-016,2.2204e-016,2.2204e-016,2.2204e-016,0.044885,0.0091959,2.2204e-016,2.2204e-016,0.020531,0.024089,0.005094,0.021558,2.2204e-016,0.027775,2.2204e-016,0.016344,0.055712,2.2204e-016,0.0026298,0.00052897,2.2204e-016,2.2204e-016,2.2204e-016,0.0027381,0.010185,0.00014101,2.2204e-016,0.0013329,0.0025451,0.0019133,0.0054114,0.0038581,0.010923,0.00885

25,2.2204e-016,0.0055012,0.0060672,0.040679,2.2204e-
016,0.035173,0.093466,0.086574,0.00089131,0.037413,2.2204e-
016,2.2204e-016,0.00035764,0.024999,2.2204e-
016,0.02549,0.014988,2.2204e-016,0.026361,2.2204e-016,2.2204e-
016,0.0056202,2.2204e-016,2.2204e-016,2.2204e-016,2.2204e-016,2.2204e-
016,0.0042723,0.0018911,0.001417,2.2204e-016,2.2204e-016,2.2204e-
016,0.014812,2.2204e-016,2.2204e-016,2.2204e-016,2.2204e-
016,0.053561,0.0088361,2.2204e-016,2.2204e-016,0.093473,2.2204e-
016,2.2204e-016,2.2204e-016,0.0054894,0.060933,2.2204e-
016,0.034508,0.011073,2.2204e-016,0.032675,2.2204e-016,2.2204e-
016,0.0039538,2.2204e-016,2.2204e-016,2.2204e-016,2.2204e-016,2.2204e-
016,2.2204e-016,0.0057225,2.2204e-016,2.2204e-016,0.0048681,2.2204e-
016,0.0012572,0.00036195,0.0091744,2.2204e-016,2.2204e-016,2.2204e-
016,2.2204e-016,0.04134,0.027898,2.2204e-016,2.2204e-016,2.2204e-
016,2.2204e-016,0.022311,2.2204e-016,0.011492,2.2204e-016,2.2204e-
016,0.023228,0.0067259,0.0083871,0.0020116,2.2204e-016,2.2204e-
016,0.0079844,0.042418,0.00066951,2.2204e-016,0.00062487,2.2204e-
016,0.0011582,2.2204e-016,2.2204e-016,0.00658,2.2204e-016,2.2204e-
016,0.0060524,2.2204e-016,2.2204e-016,0.0076588,2.2204e-016,2.2204e-
016,0.0036794,0.0047894,0.033117,2.2204e-016,2.2204e-016,2.2204e-
016,0.066159,0.040513,2.2204e-016,0.025094,2.2204e-016,2.2204e-
016,2.2204e-016,2.2204e-016,2.2204e-016,0.032516,2.2204e-
016,0.038267,0.018412,0.030093,0.011116,0.0017761,2.2204e-
016,0.00091309,0.0085591,2.2204e-016,2.2204e-
016,0.011375,0.00043833,2.2204e-016,2.2204e-
016,0.00081712,0.0027279,2.2204e-016,0.0035612,2.2204e-
016,0.00072276,2.2204e-016,2.2204e-
016,0.053581,0.063144,0.0052366,2.2204e-
016,0.0053287,0.083668,2.2204e-016,2.2204e-
016,0.038917,0.017044,2.2204e-016,2.2204e-016,2.2204e-
016,0.022677,0.014096,2.2204e-016,2.2204e-
016,0.0052696,0.023494,2.2204e-016,2.2204e-016,2.2204e-
016,0.0045115,2.2204e-016,2.2204e-016,2.2204e-
016,0.0012848,0.0038078,0.0015428,2.2204e-016,2.2204e-
016,0.0021377,2.2204e-016,0.0039975,2.2204e-
016,0.0039636,0.0016592,0.0032354,2.2204e-016,2.2204e-016,2.2204e-
016,0.045144,0.009118,0.056926,2.2204e-016,0.055029,2.2204e-
016,0.0070516,0.016428,0.031394,2.2204e-016,0.00051216,2.2204e-
016,2.2204e-016,2.2204e-016,2.2204e-016,2.2204e-016,0.022865,2.2204e-
016,0.0035998,2.2204e-016,0.0020383,2.2204e-016,0.0032699,2.2204e-
016,0.0086448,0.0072671,2.2204e-016,2.2204e-016,2.2204e-
016,0.0038694,0.0084239,0.0035202,2.2204e-016,2.2204e-016,2.2204e-
016,0.012326,2.2204e-016,2.2204e-016,2.2204e-016,2.2204e-
016,0.0042311,2.2204e-016,2.2204e-
016,0.12343,0.020703,0.0063286,2.2204e-016,0.012865,2.2204e-
016,0.017023,2.2204e-016,0.012323,0.013945,0.0049736,2.2204e-
016,0.0014433,0.0061001,0.0079065,2.2204e-016,2.2204e-016,2.2204e-
016,0.0033007,0.0045708,2.2204e-
016,0.00090837,0.0059775,0.0024464,0.0013804,0.0062184,0.010706,2.2204
e-016,0.0032679,2.2204e-016,0.047387,2.2204e-016,0.080658,2.2204e-
016,0.17918,0.068664,2.2204e-016,2.2204e-
016,0.061653,0.0027848,2.2204e-016,2.2204e-016,2.2204e-016,2.2204e-

016,2.2204e-016,2.2204e-016,2.2204e-016,2.2204e-016,0.048255,2.2204e-
016,0.0018287,0.0020406,0.0008532,2.2204e-016,0.0051834,2.2204e-
016,0.0019588,2.2204e-016,2.2204e-016,2.2204e-016,2.2204e-016,2.2204e-
016,2.2204e-016,2.2204e-016,0.0072374,2.2204e-
016,0.0151,0.0018351,0.01466,2.2204e-016,0.053116,2.2204e-
016,0.0067763,0.0036409,0.00044313,0.011416,2.2204e-016,2.2204e-
016,2.2204e-016,2.2204e-016,2.2204e-016,0.044472,2.2204e-
016,0.017127,2.2204e-016,0.013023,2.2204e-016,2.2204e-
016,0.0051903,0.0077643,2.2204e-016,2.2204e-016,0.0044511,2.2204e-
016,2.2204e-016,0.0037929,2.2204e-016,0.0014878,0.0026315,2.2204e-
016,0.0030077,2.2204e-016,0.005705,0.0033283,2.2204e-016,2.2204e-
016,0.076346,0.048717,0.11567,0.0054923,2.2204e-016,0.015601,2.2204e-
016,2.2204e-016,2.2204e-016,0.053351,0.0030073,2.2204e-016,2.2204e-
016,0.0034767,2.2204e-016,0.015652,2.2204e-016,2.2204e-
016,0.0027029,2.2204e-016,2.2204e-016,2.2204e-016,2.2204e-016,2.2204e-
016,2.2204e-016,2.2204e-016,0.0088728,2.2204e-016,2.2204e-016,2.2204e-
016,0.0047741,0.0038014,0.0018153,0.0099815,2.2204e-016,2.2204e-
016,2.2204e-016,0.010183,0.0079972,0.059805,2.2204e-
016,0.0039304,0.015311,0.023932,0.064589,0.033039,0.050619,2.2204e-
016,0.028746,0.033308,0.015729,0.01435,2.2204e-016,2.2204e-
016,2.2204e-016,2.2204e-016,2.2204e-016,0.0077029,2.2204e-
016,0.0042691,2.2204e-016,0.0011984,2.2204e-
016,0.0075133,0.0026887,0.020666,2.2204e-
016,0.00050512,0.009069,0.0087755,0.008121,0.0049001,2.2204e-
016,0.0050091,0.0081091,2.2204e-016,2.2204e-
016,0.024629,0.037129,2.2204e-016,2.2204e-016,0.043003,2.2204e-
016,0.060503,2.2204e-016,0.031849,2.2204e-
016,0.0044012,0.0020221,2.2204e-016,0.021445,2.2204e-
016,0.010972,2.2204e-016,0.018646,2.2204e-
016,0.0069697,0.0053531,0.0024631,0.0021124,0.0076271,0.0025416,2.2204
e-016,2.2204e-016,2.2204e-016,0.0062818,2.2204e-016,2.2204e-
016,0.0042814,0.0017202,0.0024007,0.0036262,0.00051941,2.2204e-
016,2.2204e-016,0.029534,2.2204e-
016,0.024763,0.021499,0.037013,2.2204e-016,0.041429,2.2204e-
016,0.023086,2.2204e-016,2.2204e-016,0.043493,2.2204e-
016,0.0016939,2.2204e-016,2.2204e-016,2.2204e-016,2.2204e-016,2.2204e-
016,2.2204e-016,2.2204e-016,2.2204e-016,2.2204e-016,2.2204e-
016,2.2204e-016,0.003613,0.0016163,0.0014905,2.2204e-
016,0.0048668,2.2204e-016,2.2204e-016,0.010843,8.8353e-005,2.2204e-
016,2.2204e-016,2.2204e-016,2.2204e-016,2.2204e-016,2.2204e-
016,2.2204e-016,0.0014546,0.0084644,2.2204e-016,2.2204e-016,2.2204e-
016,0.0062137,2.2204e-016,0.036135,0.017157,2.2204e-016,7.7749e-
006,0.013879,0.0028972,2.2204e-016,0.0081885,0.002477,2.2204e-
016,0.0025248,0.0038682,2.2204e-016,2.2204e-
016,0.009371,0.0027689,0.0024798,2.2204e-
016,0.012068,0.0081482,0.00093867,2.2204e-016,2.2204e-
016,0.0037345,2.2204e-016,2.2204e-
016,0.0061193,0.032983,0.038656,0.0050599,0.0015702,0.0091095,0.007063
7,0.013209,0.026019,2.2204e-016,2.2204e-016,2.2204e-016,2.2204e-
016,2.2204e-016,0.024887,2.2204e-016,2.2204e-016,0.017359,2.2204e-
016,2.2204e-016,0.006768,0.0058793,0.0028243,0.0060436,2.2204e-
016,2.2204e-016,2.2204e-016,0.0018328,2.2204e-016,2.2204e-016,2.2204e-

016,0.0035138,0.0038313,0.0046357,0.0082295,2.2204e-016,2.2204e-
016,0.045162,2.2204e-016,2.2204e-016,0.021519,2.2204e-016,2.2204e-
016,2.2204e-016,0.097076,2.2204e-016,0.002341,2.2204e-016,2.2204e-
016,0.036555,2.2204e-016,2.2204e-016,0.0015874,2.2204e-016,2.2204e-
016,0.0031712,0.0030536,0.011329,2.2204e-016,2.2204e-
016,0.0030241,2.2204e-016,2.2204e-016,0.0014224,2.2204e-016,2.2204e-
016,0.012728,0.00038344,2.2204e-016,6.5112e-
005,0.0044045,0.0029157,0.0023029,2.2204e-016,2.2204e-016,2.2204e-
016,0.017209,0.0625,2.2204e-016,2.2204e-016,2.2204e-
016,0.041872,2.2204e-016,0.037754,2.2204e-016,0.0039871,2.2204e-
016,0.0025666,2.2204e-016,0.0010317,2.2204e-016,2.2204e-
016,0.012935,0.0040968,2.2204e-016,2.2204e-016,0.00020133,2.2204e-
016,2.2204e-016,2.2204e-016,0.00084536,0.0097112,2.2204e-016,2.2204e-
016,2.2204e-016,0.013519,2.2204e-016,0.0041876,0.00090246,2.2204e-
016,0.013066,2.2204e-016,0.024746,0.023711,2.2204e-016,2.2204e-
016,2.2204e-016,2.2204e-
016,0.021951,0.00097581,0.0073783,0.0012894,0.0055043,2.2204e-
016,2.2204e-016,2.2204e-016,0.0021037,0.0045579,0.0069129,2.2204e-
016,0.00114,2.2204e-016,0.0083622,0.00052441,2.2204e-
016,0.0016691,2.2204e-016,2.2204e-
016,0.0054561,0.0061972,0.0021001,0.0052735,0.005118,0.00909,2.2204e-
016,2.2204e-016,0.0027992,0.0042772,2.2204e-016,0.034404,2.2204e-
016,0.0017376,0.031538,2.2204e-016,2.2204e-016,0.0033293,2.2204e-
016,2.2204e-016,0.0045764,2.2204e-016,0.0024094,2.2204e-016,2.2204e-
016,2.2204e-016,2.2204e-016,0.0057156,0.0094432,0.0014135,2.2204e-
016,2.2204e-016,2.2204e-016,0.0011865,0.0021031,2.2204e-
016,0.00027124,2.2204e-016,0.0053491,0.012303,2.2204e-016,2.2204e-
016,0.01364,2.2204e-016,2.2204e-016,2.2204e-016,2.2204e-016,2.2204e-
016,2.2204e-016,0.016823,0.0065477,0.0032918,2.2204e-016,2.2204e-
016,2.2204e-016,0.0020883,2.2204e-016,2.2204e-016,2.2204e-
016,0.002984,0.0024483,6.7963e-005,0.007524,2.2204e-016,3.9734e-
005,0.016343,0.0024276,0.004389,0.0030029,0.00028613,0.0014725,2.2204e
-016,2.2204e-016,2.2204e-016,2.2204e-016,0.0071444,2.2204e-
016,0.0032283,0.0028901,0.0031131,2.2204e-016,0.00839,2.2204e-
016,2.2204e-016,0.0055174,0.0062967,2.2204e-016,2.2204e-
016,0.0011603,0.0099963,2.2204e-016,2.2204e-016,2.2204e-
016,0.0019911,2.2204e-016,2.2204e-
016,0.0022153,0.0011992,0.00048573,0.003766,2.2204e-
016,0.002802,2.2204e-016,2.2204e-016,2.2204e-
016,0.0027536,0.0021715,2.2204e-016,2.2204e-
016,0.0029206,0.0018042,0.0082631,0.0042399,2.2204e-
016,0.0052417,0.0068635,0.00090516,0.0063451,0.0012886,2.2204e-
016,0.0011023,0.00034447,2.2204e-016,2.2204e-016,0.0050238,2.2204e-
016,0.0081643,2.2204e-016,2.2204e-016,2.2204e-
016,0.0099433,0.0027142,2.2204e-016,2.2204e-016,2.2204e-
016,0.0054858,2.2204e-016,0.0019694,2.2204e-016,2.2204e-
016,0.0044971,2.2204e-016,2.2204e-016,2.2204e-016,2.2204e-
016,0.0076362,2.2204e-016,2.2204e-016,2.2204e-016,0.0040856,2.2204e-
016,0.0063888,2.2204e-016,0.0021816,0.001868,0.008007,2.2204e-
016,2.2204e-016,0.0050784,2.2204e-016,0.0004798,0.0047581,2.2204e-
016,2.2204e-016,0.0024575,0.0045642,2.2204e-016,2.2204e-
016,0.009339,2.2204e-016,0.0044824,2.2204e-

016,0.0028879,0.00070899,0.0021566,2.2204e-
016,0.0024673,0.010706,2.2204e-016,0.003393,0.0010404,2.2204e-
016,2.2204e-016,2.2204e-016,2.2204e-016,0.0007042,2.2204e-
016,0.0012609,2.2204e-016,2.2204e-016,2.2204e-016,2.2204e-
016,0.0016257,2.2204e-016,2.2204e-016,0.0011951,2.2204e-016,2.2204e-
016,0.0013563,0.0042788,0.0045672,2.2204e-016,2.2204e-016,2.2204e-
016,2.2204e-016,2.2204e-016,0.0044461,2.2204e-
016,0.0072766,0.0060434,0.004161,2.2204e-
016,0.016632,0.0040503,2.2204e-016,2.2204e-016,2.2204e-
016,0.0068252,0.0066281,2.2204e-016,2.2204e-
016,0.0011539,0.0085837,0.0083677,2.2204e-016,0.0021201,2.2204e-
016,2.2204e-016,2.2204e-016,0.0089387,0.0075247,2.2204e-016,2.2204e-
016,2.2204e-016,2.2204e-016,2.2204e-
016,0.0014718,0.0024809,0.0061528,0.00089319,2.2204e-
016,0.0014362,2.2204e-016,2.2204e-016,0.0085439,2.2204e-
016,0.0017433,2.2204e-016,2.2204e-016,0.0091543,0.0047669,2.2204e-
016,2.2204e-016,0.005711,0.0014066,2.2204e-
016,0.0022366,0.002004,2.2204e-016,0.0045148,0.0055542,2.2204e-
016,2.2204e-016,0.0035587,0.0076008,2.2204e-
016,0.0043902,0.00025602,0.0055244,2.2204e-
016,0.0016398,0.002096,0.00018857,2.2204e-016,2.2204e-016,2.2204e-
016,0.00223,2.2204e-016,2.2204e-016,2.2204e-016,2.2204e-
016,0.0005343,0.0057738,0.0035241,2.2204e-016,0.0071085,2.2204e-
016,2.2204e-016,2.2204e-016,2.2204e-016,2.2204e-
016,0.0030255,0.0066191,0.0036552,0.0021036,0.00077199,2.2204e-
016,2.2204e-016,0.0044031,0.0053177,2.2204e-016,0.014915,6.2832e-
006,2.2204e-016,2.2204e-016,0.0058157,2.2204e-016,2.2204e-016,2.2204e-
016,0.0036536,0.0071687,2.2204e-016,2.2204e-016,2.2204e-016,2.2204e-
016,2.2204e-016,2.2204e-016,2.2204e-016,0.0042189,2.2204e-
016,0.0050406,2.2204e-016,2.2204e-016,0.0082348,2.2204e-
016,0.0019622,2.2204e-016,0.0023213,2.2204e-016,2.2204e-016,2.2204e-
016,0.0018981,2.2204e-016,2.2204e-016,2.2204e-016,2.2204e-
016,0.0074838,0.004641,0.0038208,0.0027993,0.0055504,2.2204e-
016,2.2204e-016,0.00050908,0.011063,2.2204e-016,2.2204e-
016,0.0041418,2.2204e-016,0.012959,0.0085577,2.2204e-
016,0.0026213,0.0047878,2.2204e-016,2.2204e-016,2.2204e-016,2.2204e-
016,0.012632,0.0040341,2.2204e-016,0.0059356,0.0032947,2.2204e-
016,2.2204e-016,2.2204e-016,0.0074304,2.2204e-016,0.0062014,2.2204e-
016,0.016964,0.008443,2.2204e-016,0.00010251,0.0051525,2.2204e-
016,2.2204e-016,0.0047883,2.2204e-016,0.001307,2.2204e-016,2.2204e-
016,2.2204e-016,0.0034425,2.2204e-016,0.010813,2.2204e-016,2.2204e-
016,2.2204e-016,0.002153,0.0091371,2.2204e-016
0.12025,0.1243,0.12093,0.1219,0.1183,0.11339,0.11198,0.12236,0.12201,0
.11157,0.13024,0.12337,0.12737,0.12269,0.11283,0.1159,0.12462,0.11924,
0.11709,0.11371,1.3299,1.2437,1.5864,1.2131,0.11607,0.109,0.11419,0.11
632,0.11406,0.12025,0.11576,0.11931,0.11804,0.11994,0.12236,0.11363,0.
11508,0.1223,0.11485,2.4319,1.2424,1.1755,0.97242,1.2506,1.2483,1.0207
,0.11475,0.11859,0.12123,0.12183,0.11893,0.11046,0.11781,0.11332,0.119
12,0.11496,0.13066,0.1254,0.12085,0.11728,0.11682,2.3101,2.3161,1.2013
,1.1256,1.2016,1.2651,1.2369,1.2034,1.2781,1.216,0.11398,0.1196,0.1199
5,0.11396,0.11677,0.12069,0.12309,0.12227,0.11301,0.11966,0.11318,0.12
148,0.11585,0.11927,0.11547,2.4004,2.4222,2.3639,1.3071,1.3482,1.2038,

1.1166,1.2105,1.3036,1.1271,0.88935,1.2263,0.11902,0.12169,0.12304,0.1
2207,0.12036,0.11381,0.11852,0.12019,0.11574,0.11447,0.11757,0.11204,0
.12962,0.11747,0.11979,0.12784,2.3462,2.3521,2.4731,2.3935,1.2929,1.14
13,1.3974,1.0999,1.248,1.2532,1.4176,1.2956,1.2503,1.2858,0.12312,0.10
769,0.11894,0.12099,0.12777,0.11708,0.12364,0.11834,0.12641,0.12421,0.
11191,0.11727,0.11742,0.11961,0.12508,0.12167,2.2794,2.3172,2.4409,2.3
809,2.3502,2.3372,1.0577,1.3333,1.2954,1.3041,1.2825,1.2843,0.99069,1.
0488,1.4059,1.2593,0.11323,0.12881,0.12623,0.12614,0.11873,0.11983,0.1
2079,0.12691,0.11603,0.11711,0.1191,0.12044,0.13358,0.12102,0.1236,0.1
1327,0.11475,2.3701,2.3729,2.3623,2.4264,2.3993,2.3065,1.1789,1.2986,1
.3195,1.2473,1.3945,1.2251,1.2605,1.2575,1.2789,1.424,1.3101,0.12029,0
.11996,0.11779,0.11536,0.11935,0.11733,0.12678,0.12115,0.12135,0.11865
,0.12451,0.122,0.12179,0.15952,0.16482,0.16332,0.11981,2.3945,2.3765,2
.4282,2.3685,2.4423,2.5066,2.4732,1.2583,1.6205,1.1134,1.7713,1.2546,1
.3228,1.218,1.2977,1.2834,1.2155,0.98274,0.11878,0.11065,0.1255,0.1213
,0.1184,0.12088,0.11674,0.11125,0.12508,0.12354,0.12156,0.15,0.15191,0
.15186,0.16697,0.15105,0.14682,0.1187,2.2804,2.4391,2.4014,2.3668,2.38
38,2.4825,2.3263,1.2211,1.1803,1.3007,1.4139,1.0605,1.3026,1.1342,1.29
78,1.2916,1.2086,1.2126,0.12371,0.11165,0.12176,0.1178,0.12007,0.11261
,0.11787,0.1229,0.11521,0.15674,0.1622,0.15362,0.15909,0.15432,0.16617
,0.15854,0.14314,0.14611,0.11554,2.4172,2.3839,2.3079,2.3344,2.3367,2.
3214,2.4146,1.3796,1.2424,1.1799,1.0673,1.1917,1.2067,1.085,1.3187,1.2
119,1.245,1.0963,1.3446,0.12269,0.11365,0.12037,0.11449,0.12225,0.1147
3,0.15366,0.16641,0.14344,0.15598,0.16519,0.1551,0.14992,0.15848,0.145
45,0.15942,0.15336,0.15749,2.4306,2.2912,2.3571,2.3155,2.4452,2.4452,2
.3735,1.2769,1.2663,1.2887,1.1872,1.2995,0.96572,1.4159,1.1694,1.4087,
1.2537,1.2559,1.3219,0.1234,0.11651,0.1224,0.16312,0.15381,0.16161,0.1
665,0.15539,0.1561,0.1528,0.15899,0.15886,0.14882,0.16277,0.15281,0.15
059,0.14145,0.15572,2.4409,2.4066,2.4164,2.3087,2.3955,2.5094,2.3466,2
.3325,2.4194,1.0879,1.294,1.5209,1.2549,1.0428,1.2863,1.2507,1.2257,1.
2508,1.2092,1.2526,0.14377,0.15202,0.16526,0.14825,0.14627,0.15075,0.1
5079,0.15108,0.15607,0.15241,0.14445,0.15929,0.15268,0.15974,0.15133,0
.16019,0.14908,0.16804,2.3678,2.3248,2.3804,2.4608,2.3293,2.4696,2.350
9,2.4251,2.4032,1.2604,1.2624,0.95289,0.8868,1.0008,0.88228,0.83036,0.
87732,0.90541,0.89085,0.95147,0.15102,0.15893,0.15701,0.15369,0.14955,
0.16976,0.15069,0.1571,0.15543,0.15216,0.14731,0.14853,0.16253,0.16154
,0.16328,0.15363,0.14166,0.15352,2.4432,2.3287,2.3536,2.3438,2.3837,2.
3901,2.3196,2.3407,2.4939,1.312,0.89263,0.69437,0.84246,0.52877,0.9333
7,0.91396,1.0113,0.87663,0.9431,0.80251,0.16148,0.15942,0.16057,0.1613
1,0.1501,0.1573,0.15405,0.16322,0.1539,0.1578,0.16601,0.15566,0.15252,
0.16754,0.16376,0.15665,0.16164,0.15768,2.4358,2.368,2.4506,2.3523,2.5
333,2.4488,2.3728,1.3426,1.3636,1.0893,0.98386,0.64866,0.93361,0.76654
,0.80153,0.8638,0.89064,0.98554,1.0336,0.86107,0.16044,0.15546,0.16023
,0.14496,0.15966,0.14825,0.16036,0.15262,0.14851,0.15891,0.16391,0.152
74,0.14484,0.15593,0.16018,0.15192,0.17022,0.16351,2.4009,2.3702,2.418
4,2.3343,2.3764,2.3894,1.2934,1.2939,1.238,1.1804,1.195,1.165,0.84187,
0.87311,0.90008,1.0091,0.96712,0.88511,0.97426,0.88353,0.15668,0.15597
,0.14943,0.14878,0.16027,0.15641,0.15539,0.15602,0.14909,0.14713,0.159
83,0.1478,0.15775,0.15216,0.17196,0.16591,0.15656,0.14992,2.4514,2.436
3,2.4473,2.3887,2.3434,1.2863,1.2503,1.1655,1.2801,1.3282,1.3055,0.897
69,0.8228,0.92308,0.81483,0.91652,0.88124,0.65798,0.74995,0.84869,0.14
329,0.15612,0.15063,0.15029,0.16219,0.14023,0.16364,0.15444,0.15282,0.

14682,0.15862,0.16527,0.15977,0.16322,0.16148,0.14487,0.15705,0.16177,
2.4051,2.4195,2.3572,1.284,1.5457,1.3092,1.3485,1.3282,1.593,1.1164,1.
2034,0.97992,0.86804,0.65633,0.89561,0.89847,0.8939,0.96799,0.8706,0.9
8566,0.15407,0.15782,0.14693,0.16068,0.15179,0.15094,0.15694,0.17724,0
.15086,0.1541,0.17034,0.16076,0.16182,0.16096,0.17001,0.1603,0.16377,0
.1413,2.3454,1.3148,1.6493,1.2914,1.4184,1.2164,1.3233,1.3462,1.2589,1
.2063,0.98524,0.5909,0.88137,0.88745,0.76685,0.87222,0.60376,0.99975,0
.9083,0.15606,0.16767,0.14961,0.15909,0.15147,0.17289,0.16101,0.15195,
0.15581,0.14821,0.16174,0.149,0.15089,0.16194,0.16343,0.15329,0.15765,
0.19023,1.4581,1.165,1.5197,1.1848,1.3432,1.307,1.4294,1.2117,1.3358,1
.4106,0.88811,0.92429,0.81289,0.97459,0.48857,0.72084,0.88503,0.84172,
0.86887,0.15433,0.15205,0.15789,0.15131,0.15632,0.14207,0.15223,0.1570
5,0.15849,0.1572,0.15751,0.14418,0.15275,0.14987,0.14219,0.16294,0.155
58,0.1813,1.1384,1.2267,1.2521,1.2836,1.3501,1.3594,1.6358,1.0631,1.36
73,1.2988,0.9319,0.92045,0.88607,0.86066,1.0023,0.98627,0.82431,0.7686
8,0.15606,0.15999,0.16217,0.153,0.14678,0.15649,0.15812,0.1523,0.1434,
0.17181,0.15514,0.1597,0.14806,0.15527,0.1635,0.16109,0.14299,0.15087,
0.19879,1.2536,1.3081,1.215,1.3149,1.3266,1.5037,0.94859,1.1431,1.2016
,1.2829,0.90527,0.939,0.98048,0.83563,0.8749,0.95572,0.89919,0.81657,0
.15231,0.17157,0.14984,0.15491,0.16514,0.16549,0.1631,0.16378,0.1498,0
.14682,0.14824,0.14827,0.15606,0.1527,0.14998,0.16282,0.15574,0.19743,
0.19686,0.18568,1.2534,1.4614,1.0183,1.2942,1.2766,1.2834,1.281,1.2737
,1.3859,0.71452,0.93201,0.90129,0.92317,0.93106,0.89233,0.15465,0.1549
,0.15373,0.14481,0.16284,0.1578,0.16323,0.1563,0.14766,0.15174,0.15672
,0.15402,0.15227,0.1522,0.15278,0.16872,0.16068,0.14593,0.18747,0.1967
5,0.19544,0.19446,1.2687,1.3609,1.1929,1.1387,1.3937,1.4553,1.1127,1.3
619,1.3241,0.94306,0.94373,0.86067,0.15326,0.15333,0.15595,0.15348,0.1
5496,0.16111,0.15458,0.17023,0.1584,0.15519,0.15385,0.16177,0.15333,0.
14092,0.15333,0.15663,0.16207,0.15225,0.14216,0.14281,0.20232,0.1857,0
.19752,0.19094,0.18393,0.20162,1.3339,1.3557,1.4182,1.3925,1.2153,1.15
66,1.2879,1.5705,0.15309,0.16007,0.15715,0.16095,0.15309,0.14434,0.151
73,0.15053,0.16944,0.16736,0.15625,0.15425,0.15095,0.15903,0.15424,0.1
5286,0.15413,0.14881,0.14554,0.15829,0.16324,0.15464,0.15439,0.19449,0
.20167,0.1878,0.18155,0.19499,0.19205,1.2185,1.2726,1.1305,1.2899,1.34
93,0.18456,0.19335,0.15194,0.14252,0.14675,0.1567,0.15615,0.15325,0.15
61,0.15486,0.14873,0.14801,0.15348,0.16315,0.1655,0.14973,0.14465,0.14
873,0.15914,0.15772,0.15188,0.14325,0.14439,0.19971,0.20318,0.19187,0.
19305,0.20066,0.18249,0.18116,0.18567,0.18952,0.18469,1.2776,1.3629,0.
19983,0.19758,0.18596,0.18878,0.15342,0.16057,0.1591,0.15675,0.16715,0
.15827,0.15282,0.16484,0.16643,0.15184,0.16065,0.16833,0.15543,0.16114
,0.15139,0.14203,0.15706,0.15437,0.14957,0.19034,0.18584,0.19318,0.186
15,0.19566,0.1941,0.19052,0.18832,0.19974,0.19399,0.18865,0.19668,0.19
561,0.18882,0.18848,0.18967,0.20166,0.15542,0.15096,0.15298,0.15273,0.
15488,0.1539,0.15376,0.15448,0.1498,0.15769,0.15855,0.15724,0.13677,0.
14503,0.15055,0.1619,0.1949,0.18333,0.18756,0.195,0.1898,0.19628,0.192
16,0.18373,0.20193,0.19857,0.19176,0.19642,0.1951,0.19032,0.19036,0.19
301,0.17889,0.18123,0.19527,0.15247,0.16362,0.14426,0.1536,0.14543,0.1
6352,0.1558,0.15402,0.15418,0.15547,0.15739,0.1487,0.15878,0.18359,0.1
8124,0.19148,0.17898,0.1854,0.18177,0.18708,0.19911,0.18571,0.1814,0.1
813,0.19313,0.18847,0.19789,0.1847,0.19118,0.19237,0.20003,0.18749,0.1
8096,0.19739,0.1488,0.15212,0.15952,0.14993,0.15791,0.15679,0.15832,0.
15677,0.14669,0.19606,0.18536,0.19443,0.18091,0.19746,0.19096,0.19493,

0.19279,0.199,0.20258,0.18224,0.19566,0.19079,0.18976,0.18851,0.18681,
0.18465,0.19378,0.19267,0.19166,0.17548,0.19237,0.18913,0.18854,0.1928
3,0.15781,0.15262,0.15238,0.14687,0.18872,0.19346,0.19375,0.19265,0.17
719,0.18876,0.17217,0.18708,0.18472,0.19208,0.19124,0.19946,0.19084,0.
1964,0.17794,0.20739,0.19794,0.1885,0.18316,0.18882,0.19825,0.19961,0.
18807,0.1828,0.19388,0.20043,0.19586,0.18679,0.19241,0.1853,0.18701,0.
19208,0.19971,0.19894,0.18301,0.18836,0.18028,0.18435,0.18146,0.19715,
0.1914,0.19227,0.19456,0.1862,0.19282,0.18547,0.18767,0.19843,0.18605,
0.18789,0.18959,0.19201,0.20193,0.19529,0.18978,0.17856,0.20054,0.1919
4,0.18679,0.19293,0.19462,0.17954,0.19004,0.19875,0.18618,0.17891,0.19
545,0.19831,0.18942,0.19323,0.1917,0.19418,0.17957,0.19348,0.19433,0.1
9264,0.18904,0.18702,0.18885,0.19554,0.18424,0.18516,0.19132,0.18357,0
.19355,0.19658,0.1963,0.18346,0.19789,0.18953,0.18414,0.18612,0.18576,
0.18669,0.19536,0.19196,0.19069,0.19742,0.19048,0.17122,0.1866,0.19202
,0.19883,0.18118,0.205,0.18906,0.18599,0.18217,0.19991,0.18682,0.18785
,0.18838,0.19651,0.20069,0.18255,0.18819,0.18082,0.18821,0.18062,0.185
75,0.18817,0.19694,0.18819,0.1984,0.18669,0.18491,0.19643,0.18796,0.19
107,0.18838,0.19308,0.18686,0.18675,0.18247,0.19235,0.18604,0.18566,0.
1858,0.18441,0.20395,0.19667,0.19543,0.19806,0.19194,0.18647,0.18633,0
.19017,0.19721,0.18463,0.19113,0.1945,0.19021,0.20414,0.19428,0.18009,
0.19271,0.1979,0.18435,0.18808,0.18884,0.19146,0.20446,0.19697,0.1909,
0.1962,0.19412,0.19245,0.19337,0.18914,0.20114,0.18448,0.19451,0.19412
,0.20693,0.19603,0.18175,0.19666,0.19952,0.18893,0.18403,0.19649,0.187
34,0.19339,0.18772,0.18699,0.18539,0.19327,0.18852,0.19944,0.17627,0.1
8583,0.18937,0.19223,0.19935,0.173
0.16961,0.17401,0.17218,0.16998,0.1669,0.1643,0.16085,0.16893,0.17018,
0.16061,0.18059,0.1716,0.17463,0.17077,0.1647,0.16175,0.16838,0.1671,0
.16803,0.15947,0.9525,0.90623,1.086,0.88431,0.16664,0.16005,0.15983,0.
17062,0.16649,0.16956,0.16622,0.16869,0.16354,0.16702,0.17208,0.16377,
0.16489,0.1684,0.16235,1.5953,0.87511,0.82762,0.69172,0.88309,0.87861,
0.7316,0.16182,0.16695,0.17292,0.17233,0.16739,0.15537,0.16501,0.16372
,0.16879,0.16595,0.17542,0.17146,0.16873,0.16737,0.16685,1.5397,1.5578
,0.85312,0.78684,0.85347,0.9161,0.88601,0.85026,0.90372,0.89408,0.1615
4,0.16774,0.16763,0.16408,0.16907,0.17053,0.17385,0.16901,0.16129,0.16
986,0.16467,0.16853,0.16292,0.16731,0.1643,1.5876,1.5899,1.6173,0.9421
2,0.91898,0.84139,0.80259,0.85169,0.93305,0.78714,0.66808,0.912,0.1636
4,0.17061,0.17312,0.17201,0.17104,0.16442,0.16807,0.16862,0.16718,0.16
52,0.16532,0.15714,0.1776,0.16676,0.16895,0.17794,1.5571,1.5766,1.6781
,1.6048,0.92262,0.81338,0.98373,0.80145,0.87605,0.90428,0.99652,0.9034
3,0.87503,0.91613,0.17161,0.16009,0.17046,0.17209,0.17645,0.16958,0.17
277,0.16522,0.17583,0.17573,0.15965,0.1692,0.16689,0.16748,0.17338,0.1
6977,1.5166,1.568,1.64,1.5769,1.6041,1.5111,0.7509,0.92057,0.92014,0.9
2556,0.90658,0.90863,0.70278,0.77356,0.99623,0.87009,0.16452,0.17531,0
.17421,0.1745,0.16892,0.1695,0.17097,0.17718,0.16449,0.16774,0.16751,0
.17199,0.18152,0.16643,0.16988,0.16131,0.16195,1.6032,1.555,1.5671,1.6
438,1.6262,1.4976,0.82443,0.93994,0.91836,0.88527,0.97826,0.8507,0.882
68,0.89589,0.91613,1.0328,0.92189,0.16856,0.16809,0.16754,0.16593,0.16
665,0.16833,0.17529,0.17089,0.16955,0.16789,0.1761,0.17196,0.17314,0.2
0015,0.20115,0.19931,0.16686,1.5958,1.6064,1.6685,1.5892,1.6705,1.7185
,1.6709,0.88162,1.136,0.78425,1.1831,0.88269,0.92523,0.85232,0.92225,0
.89669,0.8577,0.71744,0.16683,0.16024,0.17273,0.16978,0.16635,0.16909,
0.16544,0.15834,0.17238,0.17311,0.17041,0.19103,0.18579,0.18614,0.2061

7,0.19093,0.18636,0.17003,1.5125,1.6536,1.613,1.5576,1.6464,1.7214,1.5
371,0.86353,0.85341,0.91226,1.0016,0.76173,0.92267,0.79701,0.92626,0.9
2003,0.85139,0.86175,0.17243,0.1613,0.16795,0.16784,0.17084,0.16306,0.
16635,0.17179,0.16783,0.19466,0.1975,0.19328,0.19631,0.19107,0.20305,0
.19752,0.18233,0.18612,0.16481,1.6244,1.6126,1.508,1.5901,1.5639,1.570
1,1.6441,0.95157,0.90646,0.83254,0.76597,0.8609,0.86025,0.77757,0.9392
8,0.84477,0.87755,0.77306,0.95849,0.17024,0.16246,0.17111,0.16672,0.17
372,0.1668,0.19055,0.2026,0.18419,0.19237,0.19693,0.18947,0.19136,0.19
445,0.18367,0.19687,0.19312,0.1976,1.666,1.5224,1.5975,1.5721,1.6669,1
.6865,1.5807,0.89552,0.86086,0.90413,0.8342,0.91326,0.70104,1.0122,0.8
229,0.97553,0.88507,0.90925,0.93276,0.17257,0.16869,0.17106,0.20282,0.
19173,0.19744,0.20531,0.19047,0.19349,0.19189,0.19964,0.19922,0.18692,
0.19955,0.19173,0.19167,0.1811,0.19564,1.675,1.6357,1.622,1.5038,1.590
6,1.6873,1.5397,1.5704,1.6144,0.78753,0.93077,1.0359,0.87854,0.75629,0
.88508,0.87436,0.85673,0.89776,0.87614,0.89808,0.18135,0.18739,0.20136
,0.18705,0.18818,0.18809,0.19021,0.19095,0.19422,0.1895,0.1836,0.19572
,0.19137,0.1955,0.19231,0.19689,0.19245,0.20003,1.568,1.5727,1.5874,1.
63,1.5822,1.6683,1.6227,1.6677,1.6264,0.89245,0.89609,0.70347,0.65409,
0.73974,0.65116,0.61582,0.628,0.65713,0.64858,0.69978,0.18828,0.19982,
0.19489,0.19403,0.18608,0.20279,0.18868,0.19549,0.19605,0.19166,0.1879
6,0.18892,0.20043,0.19695,0.20263,0.18994,0.18202,0.19007,1.6407,1.529
8,1.5413,1.5276,1.6149,1.6402,1.526,1.5403,1.6964,0.94464,0.65204,0.53
342,0.63566,0.43884,0.70256,0.65233,0.73545,0.64836,0.70294,0.60594,0.
19819,0.19724,0.19648,0.19985,0.18821,0.19769,0.19423,0.20002,0.19317,
0.20006,0.20404,0.19248,0.18971,0.20638,0.20058,0.19245,0.2001,0.19097
,1.6542,1.6179,1.651,1.5352,1.7079,1.6675,1.5904,0.93914,0.99851,0.792
09,0.70522,0.50408,0.68058,0.55703,0.58969,0.61611,0.64267,0.68812,0.7
42,0.62962,0.1981,0.19377,0.19663,0.18521,0.19873,0.18732,0.19926,0.19
36,0.1884,0.19945,0.1984,0.19233,0.18442,0.19234,0.19808,0.19031,0.204
46,0.20057,1.5825,1.5756,1.6194,1.4875,1.5917,1.6256,0.90865,0.91269,0
.8652,0.834,0.82419,0.81854,0.62663,0.64425,0.66146,0.73982,0.69931,0.
68093,0.70078,0.65398,0.19462,0.19313,0.18538,0.18757,0.19576,0.19381,
0.191,0.18867,0.18824,0.18688,0.19895,0.18588,0.19601,0.19335,0.20646,
0.20354,0.1909,0.18911,1.6613,1.6568,1.6719,1.6267,1.5531,0.90496,0.87
057,0.84411,0.88274,0.94002,0.91589,0.65707,0.62675,0.67715,0.59055,0.
66014,0.6558,0.51663,0.5668,0.62699,0.18553,0.19067,0.18854,0.18898,0.
19799,0.18001,0.20237,0.19286,0.18992,0.18604,0.19546,0.20241,0.20332,
0.20016,0.20177,0.18393,0.19442,0.19744,1.6456,1.6273,1.5675,0.91806,1
.0853,0.93427,0.94311,0.92435,1.0951,0.77875,0.86793,0.71607,0.64056,0
[truncated: 602,130 more chars]
