# Supplementary material for: Unsupervised Deconvolution of Dynamic Imaging Reveals Intratumor Vascular Heterogeneity and Repopulation Dynamics
Source: PLoS One. 2014 Nov 7;9(11):e112143. doi: 10.1371/journal.pone.0112143 (PMC4224420; doi:10.1371/journal.pone.0112143)
Supplement: Data S3 — DCE-MRI data sets arising from a longitudinal study of tumor response to anti-angiogenic therapy. (PDF) [file pone.0112143.s013.pdf]

Data S3

Data 3-1 Before Treatment

Baseline	0	0	0	0	0	0	0	0
0	0	0	0	0	0	0	0	0
0	0	0	0	0	0	0	0	0
0	0	0	0	0	0	0	0	0
0	0	0	0	0	0	0	0	0
0	0	0	0	0	0	0	0	0
0	0	0	0	0	0	0	0	0
0	0	0	0	0	0	0	0	0
0	0	0	0	0	0	0	0	0
0	0	0	0	0	0	0	0	0
0	0	0	0	0	0	0	0	0
0	0	0	0	0	0	0	0	0
0	0	0	0	0	0	0	0	0
0	0	0	0	0	0	0	0	0
0	0	0	0	0	0	0	0	0
0	0	0	0	0	0	0	0	0
0	0	0	0	0	0	0	0	0
0	0	0	0	0	0	0	0	0
0	0	0	0	0	0	0	0	0
0	0	0	0	0	0	0	0	0
0	0	0	0	0	0	0	0	0
0	0	0	0	0	0	0	0	0
0	0	0	0	0	0	0	0	0
0	0	0	0	0	0	0	0	0
0.0014	0.0015	0.0019	0.0022	0.0022	0.0027	0.003	0.0032	0.0033
0.0436	0.0261	0.0217	0.0222	0.0227	0.0232	0.0229	0.0236	0.0232
0.0171	0.0099	0.0072	0.0071	0.0073	0.0076	0.0071	0.0081	0.0077
0	0	0	0	0	0	0	0	0
0.0415	0.0249	0.022	0.0217	0.022	0.0228	0.0212	0.0224	0.0224
0.0961	0.057	0.0468	0.0433	0.0432	0.0452	0.043	0.0443	0.0452
0	0	0	0	0	0	0	0	0
0.0092	0.0056	0.0048	0.0043	0.0043	0.0046	0.0043	0.0042	0.0044
0.0088	0.0052	0.004	0.0039	0.0041	0.004	0.0037	0.0037	0.0037
0.0341	0.0201	0.017	0.0168	0.0169	0.0162	0.016	0.0152	0.0158
0.0422	0.0235	0.0172	0.0172	0.0167	0.0174	0.0165	0.0164	0.0164
0.0163	0.0095	0.0074	0.0064	0.0065	0.0063	0.0065	0.0065	0.0064
0	0	0	0	0	0	0	0	0
0	0	0	0	0	0	0	0	0
0	0	0	0	0	0	0	0	0
0	0	0	0	0	0	0	0	0
0.0178	0.0116	0.0095	0.009	0.0087	0.0086	0.0082	0.0085	0.008
0.008	0.0046	0.0036	0.0035	0.0033	0.0033	0.003	0.0032	0.0035
0	0	0	0	0	0	0	0	0
0	0	0	0	0	0	0	0	0

0.0179	0.0114	0.0092	0.0088	0.0087	0.0083	0.0076	0.008	0.0084
0.0248	0.0141	0.0121	0.0116	0.0118	0.012	0.0125	0.0118	0.0115
0.0251	0.0138	0.0113	0.0103	0.0099	0.0098	0.009	0.0091	0.0095
0.0182	0.0108	0.0091	0.0085	0.009	0.0092	0.0094	0.0089	0.0083
0	0	0	0	0	0	0	0	0
0	0	0	0	0	0	0	0	0
0	0	0	0	0	0	0	0	0
0	0	0	0	0	0	0	0	0
0	0	0	0	0	0	0	0	0
0	0	0	0	0	0	0	0	0
0	0	0	0	0	0	0	0	0
0	0	0	0	0	0	0	0	0
0	0	0	0	0	0	0	0	0
0	0	0	0	0	0	0	0	0
0	0	0	0	0	0	0	0	0
0	0	0	0	0	0	0	0	0
0	0	0	0	0	0	0	0	0
0	0	0	0	0	0	0	0	0
0	0	0	0	0	0	0	0	0
0	0	0	0	0	0	0	0	0
0	0	0	0	0	0	0	0	0
0	0	0	0	0	0	0	0	0
0	0	0	0	0	0	0	0	0
0	0	0	0	0	0	0	0	0
0	0	0	0	0	0	0	0	0
0	0	0	0	0	0	0	0	0
0	0	0	0	0	0	0	0	0
0	0	0	0	0	0	0	0	0
0	0	0	0	0	0	0	0	0
0	0	0	0	0	0	0	0	0
0.0427	0.0258	0.0211	0.0195	0.0196	0.0198	0.0202	0.0208	0.0199
0.0008	0.0009	0.0011	0.0012	0.0014	0.0016	0.0018	0.0019	0.0018
0.04	0.0235	0.0183	0.0187	0.0194	0.02	0.0184	0.019	0.0192
0.0531	0.0315	0.0254	0.0242	0.0215	0.023	0.0223	0.0232	0.0245
0.0004	0.0004	0.0005	0.0006	0.0007	0.0007	0.0008	0.0009	0.0009
0.0003	0.0004	0.0005	0.0006	0.0006	0.0007	0.0008	0.0009	0.0009
0	0	0	0	0	0	0	0	0
0.052	0.0325	0.0255	0.026	0.0265	0.0268	0.0273	0.0268	0.0278
0	0	0	0	0	0	0	0	0
0	0	0	0	0	0	0	0	0
0	0	0	0	0	0	0	0	0
0	0	0	0	0	0	0	0	0
0	0	0	0	0	0	0	0	0
0.0167	0.0093	0.0076	0.0069	0.0071	0.0078	0.0078	0.0079	0.0083

0.0078	0.0046	0.0036	0.0035	0.0034	0.0035	0.0035	0.0038	0.0041
0	0	0	0	0	0	0	0	0
0	0	0	0	0	0	0	0	0
0	0	0	0	0	0	0	0	0
0	0	0	0	0	0	0	0	0
0.0258	0.0152	0.0124	0.0116	0.0128	0.0136	0.0124	0.0122	0.0128
0.0418	0.0255	0.0184	0.0174	0.0178	0.018	0.016	0.018	0.0177
0.0326	0.0178	0.0147	0.0146	0.0144	0.0136	0.0135	0.013	0.0126
0.0086	0.0049	0.0038	0.0037	0.0036	0.0036	0.0033	0.003	0.0032
0	0	0	0	0	0	0	0	0
0	0	0	0	0	0	0	0	0
0	0	0	0	0	0	0	0	0
0	0	0	0	0	0	0	0	0
0	0	0	0	0	0	0	0	0
0	0	0	0	0	0	0	0	0
0	0	0	0	0	0	0	0	0
0	0	0	0	0	0	0	0	0
0	0	0	0	0	0	0	0	0
0	0	0	0	0	0	0	0	0
0	0	0	0	0	0	0	0	0
0	0	0	0	0	0	0	0	0
0	0	0	0	0	0	0	0	0
0	0	0	0	0	0	0	0	0
0	0	0	0	0	0	0	0	0
0	0	0	0	0	0	0	0	0
0	0	0	0	0	0	0	0	0
0	0	0	0	0	0	0	0	0
0	0	0	0	0	0	0	0	0
0	0	0	0	0	0	0	0	0
0	0	0	0	0	0	0	0	0
0	0	0	0	0	0	0	0	0
0	0	0	0	0	0	0	0	0
0	0	0	0	0	0	0	0	0
0	0	0	0	0	0	0	0	0
0	0	0	0	0	0	0	0	0
0	0	0	0	0	0	0	0	0
0.0565	0.0348	0.027	0.0253	0.0259	0.0246	0.0232	0.0269	0.0289
0.0311	0.0176	0.0131	0.0135	0.0145	0.0142	0.0129	0.0135	0.0125
0.0688	0.0429	0.0368	0.0356	0.0349	0.0348	0.0349	0.0351	0.0352
0.081	0.0479	0.0375	0.0335	0.0319	0.0339	0.0337	0.0374	0.0375
0.0266	0.0161	0.0131	0.0114	0.0115	0.0114	0.0109	0.0115	0.012
0.0165	0.0094	0.0076	0.007	0.0074	0.0077	0.0076	0.0076	0.0071
0.0542	0.0309	0.0239	0.0251	0.0259	0.0272	0.027	0.0277	0.0276
0.0646	0.0386	0.0315	0.0329	0.0324	0.0335	0.0327	0.0331	0.0298

0.0996	0.0609	0.051	0.0507	0.0526	0.0496	0.0476	0.0458	0.0485
0.0843	0.0484	0.0381	0.0361	0.0362	0.0362	0.0341	0.0355	0.0388
0.0749	0.0457	0.0378	0.0374	0.0366	0.0387	0.0366	0.036	0.0392
0.0755	0.046	0.0383	0.0379	0.0349	0.0344	0.0341	0.035	0.0342
0.0756	0.0478	0.0369	0.0368	0.0376	0.0347	0.0327	0.0318	0.0334
0.052	0.0331	0.0269	0.0253	0.024	0.0233	0.0252	0.0257	0.0253
0.0086	0.005	0.004	0.0037	0.0037	0.0037	0.0033	0.0035	0.0035
0	0	0	0	0	0	0	0	0
0	0	0	0	0	0	0	0	0
0	0	0	0	0	0	0	0	0
0.0092	0.0057	0.0047	0.0044	0.0043	0.0043	0.0043	0.0042	0.0044
0.0258	0.0156	0.0121	0.0119	0.0115	0.0118	0.0111	0.0108	0.0111
0.0418	0.0239	0.0179	0.0174	0.0183	0.0197	0.0176	0.0165	0.0173
0.035	0.022	0.0192	0.0189	0.0188	0.0179	0.0163	0.0168	0.0185
0.0174	0.0109	0.0089	0.0083	0.0083	0.0084	0.0077	0.0075	0.0084
0	0	0	0	0	0	0	0	0
0	0	0	0	0	0	0	0	0
0	0	0	0	0	0	0	0	0
0	0	0	0	0	0	0	0	0
0	0	0	0	0	0	0	0	0
0	0	0	0	0	0	0	0	0
0	0	0	0	0	0	0	0	0
0	0	0	0	0	0	0	0	0
0	0	0	0	0	0	0	0	0
0	0	0	0	0	0	0	0	0
0	0	0	0	0	0	0	0	0
0	0	0	0	0	0	0	0	0
0	0	0	0	0	0	0	0	0
0	0	0	0	0	0	0	0	0
0	0	0	0	0	0	0	0	0
0	0	0	0	0	0	0	0	0
0	0	0	0	0	0	0	0	0
0	0	0	0	0	0	0	0	0
0	0	0	0	0	0	0	0	0
0	0	0	0	0	0	0	0	0
0	0	0	0	0	0	0	0	0
0	0	0	0	0	0	0	0	0
0	0	0	0	0	0	0	0	0
0	0	0	0	0	0	0	0	0
0	0	0	0	0	0	0	0	0
0	0	0	0	0	0	0	0	0
0	0	0	0	0	0	0	0	0
0.0011	0.0013	0.0017	0.0019	0.002	0.0022	0.0025	0.0029	0.0029
0.127	0.0709	0.0574	0.0593	0.0604	0.0613	0.0602	0.0639	0.064

0.0003	0.0003	0.0004	0.0005	0.0005	0.0006	0.0007	0.0008	0.0008
0.0008	0.0011	0.0014	0.0016	0.0019	0.0021	0.0022	0.0022	0.0023
0.0495	0.0277	0.022	0.0216	0.0244	0.0243	0.0248	0.0251	0.0235
0.0732	0.044	0.035	0.0331	0.0341	0.0347	0.034	0.0341	0.0343
0.0178	0.0113	0.0098	0.0095	0.0097	0.0102	0.0102	0.0106	0.0107
0.0014	0.0016	0.0019	0.0021	0.0022	0.0025	0.0028	0.0033	0.0033
0.0436	0.0275	0.023	0.0223	0.0222	0.0225	0.0216	0.0235	0.0229
0.0079	0.0043	0.0035	0.0034	0.0034	0.0034	0.0036	0.0039	0.0036
0	0	0	0	0	0	0	0	0
0	0	0	0	0	0	0	0	0
0	0	0	0	0	0	0	0	0
0	0	0	0	0	0	0	0	0
0.0083	0.0049	0.004	0.004	0.0039	0.0039	0.0036	0.0037	0.0036
0.0342	0.0197	0.0163	0.0159	0.0158	0.0163	0.0155	0.0171	0.0153
0.0873	0.0519	0.0416	0.037	0.0396	0.0402	0.0401	0.0401	0.0369
0.0532	0.0326	0.0257	0.0255	0.0245	0.0246	0.0222	0.0229	0.0251
0.0153	0.0086	0.0066	0.0064	0.0063	0.0068	0.0067	0.0068	0.007
0	0	0	0	0	0	0	0	0
0.0085	0.0047	0.0037	0.0035	0.0032	0.003	0.0028	0.0027	0.003
0.0247	0.0148	0.0109	0.0107	0.0108	0.0114	0.011	0.0114	0.0119
0.0415	0.0237	0.0195	0.0175	0.0185	0.0189	0.0175	0.0164	0.0167
0.0417	0.0238	0.0198	0.0183	0.0175	0.017	0.0185	0.0197	0.0197
0	0	0	0	0	0	0	0	0
0	0	0	0	0	0	0	0	0
0	0	0	0	0	0	0	0	0
0	0	0	0	0	0	0	0	0
0	0	0	0	0	0	0	0	0
0	0	0	0	0	0	0	0	0
0	0	0	0	0	0	0	0	0
0	0	0	0	0	0	0	0	0
0	0	0	0	0	0	0	0	0
0	0	0	0	0	0	0	0	0
0	0	0	0	0	0	0	0	0
0	0	0	0	0	0	0	0	0
0	0	0	0	0	0	0	0	0
0	0	0	0	0	0	0	0	0
0	0	0	0	0	0	0	0	0
0	0	0	0	0	0	0	0	0
0	0	0	0	0	0	0	0	0
0	0	0	0	0	0	0	0	0
0	0	0	0	0	0	0	0	0
0	0	0	0	0	0	0	0	0
0	0	0	0	0	0	0	0	0
0	0	0	0	0	0	0	0	0

0	0	0	0	0	0	0	0	0
0	0	0	0	0	0	0	0	0
0	0	0	0	0	0	0	0	0
0	0	0	0	0	0	0	0	0
0.0107	0.0074	0.0073	0.008	0.0083	0.0088	0.0089	0.0095	0.0092
0.0168	0.01	0.0077	0.0067	0.0068	0.0069	0.0066	0.007	0.0069
0	0	0	0	0	0	0	0	0
0.0175	0.0108	0.0089	0.0089	0.009	0.0095	0.0097	0.0094	0.0091
0	0	0	0	0	0	0	0	0
0	0	0	0	0	0	0	0	0
0.1883	0.1124	0.0912	0.0859	0.0898	0.0927	0.0895	0.0909	0.0885
0.1198	0.0714	0.0533	0.048	0.0491	0.0514	0.0509	0.0504	0.052
0	0	0	0	0	0	0	0	0
0	0	0	0	0	0	0	0	0
0	0	0	0	0	0	0	0	0
0.0256	0.0156	0.0125	0.0126	0.0121	0.0125	0.012	0.0122	0.0123
0.0179	0.0112	0.0091	0.0087	0.0088	0.0086	0.008	0.0081	0.0078
0	0	0	0	0	0	0	0	0
0	0	0	0	0	0	0	0	0
0.0086	0.0052	0.0044	0.0044	0.0043	0.0045	0.0041	0.0043	0.0045
0.0243	0.0139	0.0101	0.0101	0.0106	0.0105	0.0098	0.0095	0.0097
0.0086	0.0049	0.0041	0.0039	0.004	0.0041	0.0038	0.0041	0.004
0	0	0	0	0	0	0	0	0
0	0	0	0	0	0	0	0	0
0.0173	0.0103	0.008	0.0076	0.0075	0.0069	0.0061	0.0059	0.0058
0.0366	0.022	0.0176	0.0156	0.0143	0.0148	0.0141	0.0145	0.0145
0.0431	0.0257	0.0204	0.0198	0.0189	0.02	0.0191	0.0209	0.0218
0.0472	0.0299	0.0242	0.0238	0.0233	0.0219	0.0213	0.021	0.0207
0	0	0	0	0	0	0	0	0
0	0	0	0	0	0	0	0	0
0	0	0	0	0	0	0	0	0
0	0	0	0	0	0	0	0	0
0	0	0	0	0	0	0	0	0
0	0	0	0	0	0	0	0	0
0	0	0	0	0	0	0	0	0
0	0	0	0	0	0	0	0	0
0	0	0	0	0	0	0	0	0
0	0	0	0	0	0	0	0	0
0	0	0	0	0	0	0	0	0
0	0	0	0	0	0	0	0	0
0	0	0	0	0	0	0	0	0
0	0	0	0	0	0	0	0	0
0	0	0	0	0	0	0	0	0
0	0	0	0	0	0	0	0	0

0	0	0	0	0	0	0	0	0
0	0	0	0	0	0	0	0	0
0	0	0	0	0	0	0	0	0
0	0	0	0	0	0	0	0	0
0	0	0	0	0	0	0	0	0
0	0	0	0	0	0	0	0	0
0	0	0	0	0	0	0	0	0
0	0	0	0	0	0	0	0	0
0	0	0	0	0	0	0	0	0
0	0	0	0	0	0	0	0	0
0.1181	0.0718	0.0562	0.0566	0.0571	0.0591	0.0565	0.0564	0.0544
0	0	0	0	0	0	0	0	0
0.1684	0.1011	0.0791	0.0748	0.0783	0.0841	0.0788	0.0791	0.0797
0.4888	0.273	0.223	0.1943	0.2133	0.2158	0.1991	0.2336	0.241
0.1707	0.1013	0.082	0.0738	0.0751	0.0791	0.0747	0.0781	0.0742
0.1517	0.0927	0.075	0.0723	0.0761	0.0763	0.0739	0.0748	0.0641
0.8202	0.4872	0.3622	0.3662	0.3451	0.3343	0.338	0.3201	0.3181
0.9192	0.553	0.4425	0.4091	0.4045	0.4157	0.4025	0.4343	0.4362
0.2126	0.1329	0.1042	0.0945	0.0994	0.098	0.0997	0.1028	0.0945
0.114	0.0699	0.0583	0.0549	0.0525	0.0529	0.0478	0.0478	0.049
0.059	0.0344	0.0285	0.0272	0.0276	0.0295	0.0266	0.0266	0.0275
0.0949	0.0591	0.0467	0.045	0.0452	0.0433	0.0427	0.0408	0.0423
0.1211	0.0723	0.0546	0.0584	0.055	0.0591	0.0589	0.062	0.0559
0.0821	0.0467	0.0389	0.0377	0.0408	0.0431	0.0403	0.0405	0.0411
0.0699	0.042	0.03	0.0277	0.0273	0.0283	0.0271	0.0281	0.0282
0.0685	0.0414	0.0327	0.032	0.0308	0.0302	0.0302	0.0324	0.0338
0	0	0	0	0	0	0	0	0
0	0	0	0	0	0	0	0	0
0	0	0	0	0	0	0	0	0
0.0245	0.0141	0.0115	0.0105	0.0116	0.0118	0.0112	0.0122	0.0118
0.0477	0.0302	0.0256	0.024	0.0229	0.0224	0.0227	0.0231	0.0234
0.0488	0.0282	0.0216	0.0198	0.0194	0.0201	0.02	0.0194	0.02
0.024	0.0143	0.0108	0.0108	0.0117	0.0117	0.0102	0.0102	0.0107
0	0	0	0	0	0	0	0	0
0	0	0	0	0	0	0	0	0
0	0	0	0	0	0	0	0	0
0	0	0	0	0	0	0	0	0
0	0	0	0	0	0	0	0	0
0	0	0	0	0	0	0	0	0
0	0	0	0	0	0	0	0	0
0	0	0	0	0	0	0	0	0
0	0	0	0	0	0	0	0	0
0	0	0	0	0	0	0	0	0
0	0	0	0	0	0	0	0	0

0	0	0	0	0	0	0	0	0
0	0	0	0	0	0	0	0	0
0	0	0	0	0	0	0	0	0
0	0	0	0	0	0	0	0	0
0	0	0	0	0	0	0	0	0
0	0	0	0	0	0	0	0	0
0	0	0	0	0	0	0	0	0
0	0	0	0	0	0	0	0	0
0	0	0	0	0	0	0	0	0
0	0	0	0	0	0	0	0	0
0	0	0	0	0	0	0	0	0
0	0	0	0	0	0	0	0	0
0	0	0	0	0	0	0	0	0
0	0	0	0	0	0	0	0	0
0	0	0	0	0	0	0	0	0
0	0	0	0	0	0	0	0	0
0.1507	0.0815	0.0666	0.0618	0.0635	0.0645	0.0636	0.0685	0.0676
0.4321	0.2514	0.2171	0.2204	0.23	0.2282	0.1995	0.1962	0.2124
0.5955	0.3673	0.297	0.2622	0.2616	0.2691	0.2674	0.2554	0.267
0.7139	0.4518	0.3724	0.3776	0.3815	0.3849	0.3629	0.3676	0.3483
0.3971	0.2426	0.1946	0.164	0.1609	0.1586	0.154	0.1525	0.1552
0.2606	0.1508	0.1207	0.1125	0.1114	0.1122	0.1118	0.1166	0.1299
0.8164	0.5174	0.4152	0.3993	0.3736	0.3973	0.3805	0.4044	0.3812
0.9585	0.5693	0.4372	0.4502	0.4409	0.4713	0.4505	0.4553	0.4698
0.7258	0.4378	0.3642	0.3656	0.3695	0.379	0.357	0.3798	0.3745
0.693	0.4201	0.3669	0.3587	0.3558	0.3754	0.3584	0.3576	0.35
0.9247	0.5819	0.4595	0.4015	0.3703	0.3857	0.3851	0.3841	0.389
1.0229	0.5695	0.432	0.433	0.4527	0.4679	0.495	0.4837	0.4701
0.979	0.5705	0.4455	0.4414	0.457	0.4673	0.4005	0.3796	0.3742
0.8025	0.4799	0.3956	0.3816	0.3574	0.3454	0.3626	0.356	0.3476
0.3112	0.1739	0.1441	0.1412	0.134	0.136	0.1427	0.1396	0.1447
0	0	0	0	0	0	0	0	0
0.1151	0.0649	0.0496	0.049	0.0504	0.0497	0.0517	0.0503	0.0464
0.0954	0.0547	0.0505	0.0469	0.0497	0.0522	0.0475	0.049	0.0493
0.0709	0.0428	0.035	0.0335	0.0332	0.032	0.0335	0.0335	0.033
0.0509	0.0291	0.0245	0.023	0.0243	0.0236	0.0227	0.0225	0.0224
0.0441	0.0257	0.0212	0.0199	0.0206	0.0204	0.0175	0.018	0.0185
0.0325	0.0198	0.0156	0.0143	0.0149	0.0155	0.0145	0.016	0.0164
0.0167	0.0102	0.0081	0.0077	0.0074	0.0078	0.0068	0.0071	0.0072
0	0	0	0	0	0	0	0	0
0	0	0	0	0	0	0	0	0
0	0	0	0	0	0	0	0	0
0	0	0	0	0	0	0	0	0
0	0	0	0	0	0	0	0	0

0	0	0	0	0	0	0	0	0
0	0	0	0	0	0	0	0	0
0	0	0	0	0	0	0	0	0
0	0	0	0	0	0	0	0	0
0	0	0	0	0	0	0	0	0
0	0	0	0	0	0	0	0	0
0	0	0	0	0	0	0	0	0
0	0	0	0	0	0	0	0	0
0	0	0	0	0	0	0	0	0
0	0	0	0	0	0	0	0	0
0	0	0	0	0	0	0	0	0
0	0	0	0	0	0	0	0	0
0	0	0	0	0	0	0	0	0
0	0	0	0	0	0	0	0	0
0	0	0	0	0	0	0	0	0
0	0	0	0	0	0	0	0	0
0	0	0	0	0	0	0	0	0
0	0	0	0	0	0	0	0	0
0	0	0	0	0	0	0	0	0
0	0	0	0	0	0	0	0	0
0	0	0	0	0	0	0	0	0
0	0	0	0	0	0	0	0	0
0.1411	0.083	0.0713	0.0728	0.0753	0.0755	0.0702	0.0712	0.0702
1.1663	0.7458	0.6	0.6118	0.5947	0.5643	0.5385	0.5271	0.5264
0.8173	0.4933	0.391	0.3979	0.4004	0.4028	0.4134	0.4389	0.4353
0.4354	0.27	0.2204	0.2173	0.2198	0.2292	0.2181	0.2143	0.2054
0.3236	0.1926	0.1558	0.1473	0.145	0.1541	0.1509	0.1572	0.1551
0.0602	0.0375	0.0308	0.0291	0.0301	0.0312	0.0301	0.0299	0.0298
0.1106	0.065	0.0528	0.0494	0.0512	0.0541	0.0513	0.0557	0.0546
0.1517	0.0931	0.0773	0.0805	0.0808	0.0763	0.0795	0.0771	0.0779
0.5096	0.2884	0.2244	0.2151	0.2133	0.2149	0.2154	0.2295	0.2603
0.4353	0.2726	0.2172	0.2002	0.2067	0.213	0.2067	0.2044	0.1951
0.4886	0.2938	0.2277	0.208	0.2242	0.203	0.1962	0.218	0.2147
0.757	0.4184	0.301	0.302	0.3144	0.3462	0.3304	0.3613	0.3549
1.093	0.5973	0.4854	0.4417	0.467	0.4669	0.4434	0.4623	0.4632
1.1614	0.6396	0.5172	0.5156	0.5106	0.5289	0.5141	0.5195	0.5153
1.0195	0.5916	0.4541	0.4341	0.4347	0.4767	0.4807	0.4784	0.4898
0.8665	0.5356	0.4488	0.4308	0.4216	0.415	0.3793	0.3601	0.3681
0.3561	0.2023	0.1588	0.1576	0.1636	0.1678	0.1648	0.1662	0.178
0.3047	0.1863	0.1496	0.1464	0.1511	0.1576	0.1485	0.1414	0.1505
0.1713	0.0997	0.083	0.0795	0.0789	0.0828	0.0823	0.0794	0.0759
0.0566	0.0354	0.0281	0.0256	0.0253	0.0234	0.0221	0.0224	0.0218
0	0	0	0	0	0	0	0	0
0	0	0	0	0	0	0	0	0

0.0274	0.0174	0.0149	0.014	0.0143	0.015	0.0153	0.0151	0.0149
0.0516	0.0297	0.0252	0.0242	0.0236	0.024	0.0228	0.0231	0.0215
0	0	0	0	0	0	0	0	0
0	0	0	0	0	0	0	0	0
0	0	0	0	0	0	0	0	0
0	0	0	0	0	0	0	0	0
0	0	0	0	0	0	0	0	0
0	0	0	0	0	0	0	0	0
0	0	0	0	0	0	0	0	0
0	0	0	0	0	0	0	0	0
0	0	0	0	0	0	0	0	0
0	0	0	0	0	0	0	0	0
0	0	0	0	0	0	0	0	0
0.002	0.0039	0.0043	0.0044	0.0045	0.0043	0.0044	0.0043	0.0042
0.0028	0.0088	0.0081	0.0084	0.0078	0.0071	0.0067	0.005	0.0054
0.0175	0.0177	0.0188	0.0207	0.0212	0.0222	0.0227	0.0234	0.0229
0.0083	0.0049	0.004	0.0038	0.0042	0.0044	0.0043	0.004	0.0038
0.0118	0.0093	0.0092	0.0097	0.0104	0.0113	0.0118	0.0121	0.0122
0.0181	0.0127	0.0106	0.0102	0.0107	0.0107	0.0104	0.01	0.0095
0.0167	0.0096	0.0078	0.0069	0.007	0.0075	0.0075	0.0078	0.0076
0	0	0	0	0	0	0	0	0
0.031	0.0232	0.0216	0.0212	0.0214	0.0221	0.022	0.0223	0.0223
0.025	0.017	0.0139	0.0141	0.0153	0.0158	0.0159	0.0158	0.0157
0.0802	0.055	0.0443	0.0413	0.0414	0.039	0.0367	0.039	0.0392
0.0274	0.0193	0.0164	0.0158	0.0155	0.0152	0.0152	0.0145	0.0142
0.0489	0.0325	0.0295	0.0298	0.0297	0.0311	0.0333	0.0344	0.0338
0.1531	0.0997	0.0853	0.0814	0.0859	0.0885	0.0889	0.091	0.0903
0.0038	0.0041	0.0054	0.0063	0.0073	0.0078	0.0084	0.0099	0.01
0.1228	0.0781	0.0646	0.0628	0.0614	0.064	0.0639	0.0615	0.0619
0.3536	0.2041	0.1558	0.1496	0.1386	0.1458	0.1464	0.1489	0.1479
0.3594	0.204	0.1655	0.1639	0.1594	0.1595	0.1497	0.1587	0.1496
0.1043	0.0635	0.0502	0.0449	0.0428	0.0434	0.0431	0.0429	0.0432
0.0017	0.002	0.0025	0.0031	0.003	0.0035	0.0039	0.0045	0.0045
0.0737	0.0454	0.0368	0.0313	0.0321	0.0319	0.0327	0.0297	0.028
0.0767	0.0442	0.0349	0.0353	0.034	0.0357	0.0345	0.0364	0.0351
0	0	0	0	0	0	0	0	0
0.3556	0.2208	0.1785	0.1566	0.1513	0.1484	0.1458	0.1598	0.1574
0.4494	0.2676	0.2144	0.2082	0.1983	0.2106	0.195	0.1909	0.1993
0.6973	0.4205	0.3458	0.3384	0.3459	0.3458	0.3404	0.3576	0.3439
0.3434	0.2132	0.1771	0.1705	0.1666	0.1638	0.1626	0.1548	0.1424
0.3043	0.1691	0.1339	0.1195	0.1235	0.1268	0.1198	0.1166	0.1183
1.3057	0.8192	0.6427	0.5974	0.5687	0.5572	0.5492	0.5635	0.5639
1.2305	0.7371	0.5578	0.531	0.5201	0.5297	0.4878	0.5114	0.4715
0.4082	0.2536	0.1999	0.192	0.19	0.1934	0.1826	0.1784	0.1805

0.8097	0.4741	0.3983	0.3836	0.3886	0.371	0.3452	0.3391	0.3428
0.9234	0.5867	0.482	0.4075	0.3874	0.3798	0.3689	0.3895	0.3967
0.9739	0.5777	0.5072	0.4595	0.4695	0.4375	0.4082	0.41	0.4122
0.5299	0.32	0.2618	0.2626	0.2802	0.2788	0.2625	0.278	0.2651
0.1719	0.106	0.0899	0.0814	0.0868	0.0904	0.0922	0.0967	0.0945
0.0012	0.0015	0.0019	0.0025	0.0028	0.0033	0.0037	0.0036	0.0032
0	0	0	0	0	0	0	0	0
0.0089	0.0054	0.0043	0.0042	0.0044	0.0046	0.0043	0.0042	0.0042
0	0	0	0	0	0	0	0	0
0	0	0	0	0	0	0	0	0
0	0	0	0	0	0	0	0	0
0	0	0	0	0	0	0	0	0
0	0	0	0	0	0	0	0	0
0	0	0	0	0	0	0	0	0
0	0	0	0	0	0	0	0	0
0	0	0	0	0	0	0	0	0
0	0	0	0	0	0	0	0	0
0	0	0	0	0	0	0	0	0
0.0074	0.0136	0.0145	0.0154	0.0168	0.0162	0.0174	0.0175	0.0168
0.0047	0.0048	0.0058	0.0066	0.0071	0.0084	0.009	0.0095	0.0094
0	0	0	0	0	0	0	0	0
0	0	0	0	0	0	0	0	0
0.0089	0.0055	0.0046	0.0045	0.0045	0.0046	0.004	0.004	0.0037
0.015	0.0167	0.0167	0.017	0.0173	0.0179	0.019	0.0192	0.0188
0.0161	0.0193	0.0194	0.0207	0.0219	0.0224	0.024	0.0243	0.0245
0.0119	0.0091	0.0094	0.0103	0.011	0.0114	0.0124	0.0131	0.0131
0.0602	0.0468	0.0424	0.0424	0.0435	0.0448	0.0454	0.0462	0.0453
0.1085	0.0624	0.0502	0.0482	0.0518	0.05	0.0492	0.0499	0.0475
0.0558	0.0359	0.0298	0.0296	0.0287	0.0297	0.0289	0.0283	0.0268
0.0045	0.0131	0.0127	0.0131	0.013	0.0124	0.0126	0.0112	0.011
0.0181	0.0251	0.0289	0.0327	0.0374	0.0376	0.044	0.0458	0.047
0.0576	0.0782	0.0927	0.099	0.1106	0.1282	0.1511	0.1606	0.164
0.2232	0.191	0.1836	0.1934	0.1995	0.2087	0.2263	0.2477	0.2523
0.1151	0.1111	0.1292	0.1444	0.1565	0.1711	0.1738	0.1964	0.1861
0.0155	0.0177	0.0215	0.0245	0.0271	0.0292	0.0302	0.0347	0.0343
0.0281	0.0196	0.0159	0.0158	0.0156	0.015	0.0147	0.0144	0.0141
0.0435	0.0271	0.0228	0.021	0.0215	0.0227	0.0214	0.0225	0.0224
0.0347	0.023	0.0203	0.0198	0.0198	0.0191	0.019	0.0195	0.0186
0.0286	0.0191	0.017	0.0173	0.0176	0.0179	0.0181	0.0178	0.0179
0	0	0	0	0	0	0	0	0
0.051	0.0316	0.0262	0.0263	0.0267	0.0261	0.0264	0.0261	0.0252
0.1886	0.126	0.1035	0.103	0.1055	0.0999	0.0939	0.0878	0.0856
0.3179	0.1963	0.1558	0.1531	0.142	0.1468	0.1396	0.1502	0.1447
0.4446	0.25	0.2185	0.2089	0.2049	0.2059	0.2118	0.2181	0.1985

0.4794	0.282	0.223	0.2198	0.207	0.2183	0.2063	0.2199	0.2322
0.279	0.1692	0.1323	0.1222	0.1279	0.1287	0.1238	0.1308	0.1245
0.4046	0.2467	0.1935	0.1899	0.1739	0.1772	0.1717	0.1673	0.1695
0.6225	0.3857	0.3246	0.3085	0.3009	0.2972	0.288	0.2769	0.2796
0.4205	0.2566	0.214	0.2057	0.2062	0.2045	0.1964	0.1906	0.1971
0.4881	0.3025	0.2402	0.2262	0.2099	0.2131	0.2067	0.2157	0.2249
0.7804	0.4783	0.3941	0.3831	0.3875	0.4138	0.3816	0.383	0.4092
1.0053	0.559	0.4434	0.4169	0.4059	0.4183	0.4117	0.4229	0.4654
1.0946	0.6643	0.5629	0.5517	0.5503	0.5078	0.4844	0.4818	0.4953
0.8205	0.493	0.4012	0.3822	0.3825	0.3893	0.3803	0.4081	0.4037
0.4665	0.2874	0.24	0.2301	0.2366	0.2326	0.2213	0.2278	0.224
0.1142	0.0705	0.0578	0.0534	0.0519	0.051	0.0518	0.0529	0.0542
0.0003	0.0003	0.0004	0.0004	0.0005	0.0006	0.0006	0.0007	0.0007
0.0464	0.03	0.0255	0.0243	0.0245	0.0267	0.0269	0.028	0.0273
0	0	0	0	0	0	0	0	0
0	0	0	0	0	0	0	0	0
0	0	0	0	0	0	0	0	0
0	0	0	0	0	0	0	0	0
0	0	0	0	0	0	0	0	0
0	0	0	0	0	0	0	0	0
0	0	0	0	0	0	0	0	0
0	0	0	0	0	0	0	0	0
0	0	0	0	0	0	0	0	0
0	0	0	0	0	0	0	0	0
0.0106	0.0086	0.0073	0.0068	0.0068	0.0063	0.0061	0.0059	0.0058
0.0038	0.0044	0.0053	0.0064	0.0071	0.0078	0.0093	0.0102	0.0099
0.0084	0.012	0.017	0.02	0.0204	0.024	0.0271	0.0276	0.0271
0.0055	0.0084	0.0092	0.0098	0.0104	0.0109	0.0118	0.0121	0.0118
0.0013	0.0023	0.0024	0.0026	0.0028	0.003	0.0034	0.0033	0.0033
0.0005	0.0014	0.0014	0.0013	0.0012	0.001	0.001	0.0009	0.0009
0.0103	0.0132	0.0148	0.0167	0.0179	0.0186	0.0193	0.0199	0.0196
0.0052	0.0096	0.0109	0.0115	0.0124	0.0125	0.0142	0.014	0.0142
0.053	0.0345	0.0297	0.0281	0.0288	0.0293	0.0277	0.028	0.0288
0.0141	0.0147	0.0162	0.0188	0.0207	0.0202	0.0244	0.0261	0.0267
0.0581	0.1126	0.1173	0.1192	0.1226	0.122	0.1271	0.1226	0.1245
0.2584	0.266	0.2421	0.2492	0.2535	0.2385	0.2382	0.2224	0.2235
0.2261	0.2165	0.2007	0.2025	0.2069	0.202	0.2045	0.2011	0.1979
0.0694	0.0972	0.1214	0.1427	0.1655	0.1809	0.1928	0.2071	0.1981
0.1548	0.1398	0.1636	0.1937	0.2036	0.2351	0.2511	0.2493	0.2431
0.1459	0.1485	0.1529	0.1591	0.1839	0.2132	0.2331	0.2499	0.2537
0.1774	0.1419	0.1512	0.1631	0.1718	0.1879	0.1992	0.2075	0.2018
0.0319	0.0235	0.0223	0.0233	0.0247	0.0269	0.0285	0.0299	0.0306
0.0022	0.0026	0.0028	0.0034	0.0038	0.0041	0.0044	0.0048	0.0045
0.0429	0.0242	0.0197	0.0178	0.0169	0.0177	0.0173	0.0173	0.0183

0.12	0.0819	0.067	0.0631	0.0644	0.0645	0.0614	0.0614	0.0581
0.0644	0.0467	0.0385	0.0379	0.0353	0.0347	0.0364	0.0365	0.035
0.0497	0.028	0.0224	0.0224	0.0227	0.0221	0.0202	0.0196	0.0192
0.1405	0.0801	0.0682	0.0625	0.0637	0.0659	0.0618	0.0642	0.0612
0.1209	0.0681	0.0564	0.0556	0.0568	0.0574	0.0547	0.0529	0.0525
0.112	0.067	0.0552	0.0484	0.0491	0.0493	0.0443	0.0436	0.0435
0.0044	0.0106	0.0103	0.0106	0.0103	0.0097	0.01	0.0089	0.0084
0.0717	0.0447	0.039	0.0362	0.0368	0.0383	0.0374	0.036	0.0343
0.4636	0.2702	0.2094	0.2003	0.1995	0.2064	0.2027	0.197	0.2091
0.3316	0.1929	0.1483	0.1473	0.1398	0.1396	0.1315	0.1444	0.1418
0.1708	0.1056	0.0835	0.08	0.078	0.0798	0.0792	0.0827	0.084
0.6874	0.4199	0.3324	0.3114	0.334	0.3366	0.3301	0.3256	0.3375
0.8424	0.5346	0.4454	0.4408	0.4275	0.41	0.3943	0.4157	0.4156
0.8053	0.4691	0.3942	0.3427	0.3263	0.3214	0.3192	0.3217	0.3383
0.5117	0.2929	0.2476	0.2378	0.2306	0.2335	0.2342	0.2311	0.2302
0.3557	0.2142	0.1642	0.1593	0.157	0.1606	0.1567	0.1604	0.1761
0.4007	0.2364	0.1817	0.1647	0.1689	0.1725	0.155	0.1678	0.1608
0.2589	0.158	0.1223	0.12	0.119	0.1191	0.1178	0.1225	0.128
0	0	0	0	0	0	0	0	0
0.0279	0.0173	0.015	0.0148	0.0148	0.0153	0.0155	0.0162	0.0167
0	0	0	0	0	0	0	0	0
0	0	0	0	0	0	0	0	0
0	0	0	0	0	0	0	0	0
0	0	0	0	0	0	0	0	0
0	0	0	0	0	0	0	0	0
0	0	0	0	0	0	0	0	0
0	0	0	0	0	0	0	0	0
0	0	0	0	0	0	0	0	0
0	0	0	0	0	0	0	0	0
0	0	0	0	0	0	0	0	0
0.0085	0.0054	0.0043	0.004	0.0044	0.0048	0.0044	0.0045	0.0045
0.0245	0.0233	0.0236	0.0269	0.0286	0.0301	0.032	0.0329	0.0324
0.0209	0.0348	0.0396	0.049	0.0501	0.054	0.061	0.0608	0.0686
0.0173	0.0249	0.0282	0.0295	0.0299	0.0324	0.0355	0.036	0.035
0.004	0.0051	0.0061	0.0065	0.0066	0.0076	0.0088	0.0098	0.0097
0.0054	0.0141	0.0139	0.0143	0.0146	0.0129	0.0135	0.0122	0.0121
0.0429	0.0872	0.0919	0.0928	0.0979	0.096	0.1029	0.0986	0.0981
0.0701	0.1395	0.1492	0.1569	0.1626	0.1609	0.1663	0.1583	0.1512
0.0669	0.131	0.138	0.1417	0.1464	0.1483	0.1524	0.1473	0.1445
0.1303	0.1423	0.1432	0.1439	0.1505	0.154	0.1629	0.1642	0.1637
0.6496	0.4937	0.4322	0.42	0.4141	0.414	0.4107	0.4053	0.4128
1.302	0.9256	0.7897	0.7668	0.7842	0.7597	0.7314	0.6911	0.6922
1.0739	0.7391	0.6271	0.5939	0.602	0.5714	0.5708	0.5285	0.5163
0.224	0.2087	0.1987	0.2014	0.2057	0.21	0.2183	0.2182	0.2173

0.0803	0.1088	0.1356	0.1464	0.1525	0.1688	0.1952	0.1969	0.1943
0.1568	0.163	0.1774	0.2004	0.2198	0.2361	0.2612	0.2715	0.2565
0.1392	0.1251	0.1351	0.1487	0.1623	0.1801	0.1931	0.2021	0.2027
0.0764	0.0633	0.06	0.0615	0.0639	0.0653	0.0677	0.0685	0.0675
0.0121	0.0305	0.0296	0.0303	0.0296	0.0281	0.0287	0.0255	0.0231
0.0073	0.02	0.0182	0.0178	0.0168	0.0136	0.0129	0.0104	0.0095
0.0607	0.0404	0.0362	0.0338	0.0335	0.0323	0.0318	0.0313	0.0284
0.1004	0.0852	0.0722	0.0683	0.0669	0.0664	0.0647	0.0625	0.0603
0.0195	0.0416	0.0395	0.0397	0.0395	0.0345	0.0328	0.0283	0.0274
0.0079	0.0212	0.0205	0.0205	0.0203	0.018	0.0186	0.0173	0.0167
0.0774	0.0615	0.0551	0.054	0.0542	0.0533	0.0547	0.0541	0.0521
0	0	0	0	0	0	0	0	0
0	0	0	0	0	0	0	0	0
0.0841	0.0536	0.0433	0.0428	0.0419	0.04	0.0381	0.0399	0.04
0.2072	0.1154	0.091	0.0849	0.0891	0.0913	0.0851	0.095	0.098
0.1087	0.0697	0.0563	0.0539	0.0544	0.0535	0.0487	0.051	0.0501
0.0587	0.0324	0.0252	0.0254	0.026	0.0259	0.0233	0.0246	0.0239
0.3002	0.1881	0.1508	0.1499	0.1629	0.1661	0.1635	0.1573	0.1553
0.7775	0.4622	0.3593	0.3702	0.3736	0.3777	0.3796	0.391	0.4044
0.7832	0.4625	0.3687	0.3582	0.3518	0.3608	0.348	0.3567	0.3695
0.574	0.3334	0.2769	0.2553	0.2624	0.2581	0.2441	0.2417	0.233
0.6351	0.3679	0.2892	0.2954	0.2875	0.2749	0.2787	0.2974	0.2855
1.0308	0.6335	0.5264	0.5073	0.5271	0.5266	0.5165	0.5195	0.5021
0.6958	0.416	0.3366	0.3032	0.2894	0.3368	0.3368	0.335	0.3372
0.1685	0.0967	0.0751	0.0737	0.073	0.0687	0.0669	0.0661	0.0722
0	0	0	0	0	0	0	0	0
0	0	0	0	0	0	0	0	0
0	0	0	0	0	0	0	0	0
0	0	0	0	0	0	0	0	0
0	0	0	0	0	0	0	0	0
0	0	0	0	0	0	0	0	0
0	0	0	0	0	0	0	0	0
0	0	0	0	0	0	0	0	0
0	0	0	0	0	0	0	0	0
0	0	0	0	0	0	0	0	0
0	0	0	0	0	0	0	0	0
0.0106	0.0079	0.0078	0.0079	0.0086	0.0093	0.01	0.0107	0.0102
0.0101	0.0066	0.006	0.0061	0.0062	0.0065	0.0065	0.0067	0.0064
0.0207	0.02	0.0225	0.0233	0.0247	0.0258	0.0283	0.0309	0.0307
0	0	0	0	0	0	0	0	0
0.0017	0.0023	0.0033	0.0041	0.0049	0.0058	0.0065	0.0069	0.0069
0.0205	0.0508	0.0525	0.0546	0.0547	0.051	0.0531	0.0487	0.0474
0.0532	0.1419	0.1424	0.145	0.1422	0.1413	0.1406	0.1332	0.1275
0.085	0.2196	0.2148	0.2145	0.2106	0.1933	0.197	0.1659	0.1665

0.1964	0.2976	0.2929	0.2879	0.2842	0.2631	0.2657	0.2373	0.2284
0.087	0.1812	0.1873	0.1946	0.2006	0.1882	0.1989	0.1811	0.1794
0.1798	0.1899	0.1815	0.1857	0.1914	0.1915	0.1991	0.1978	0.1956
0.8317	0.5588	0.4724	0.4775	0.4794	0.4774	0.4561	0.4399	0.4427
0.9611	0.7108	0.5922	0.5537	0.5628	0.5624	0.5454	0.5399	0.5135
0.3293	0.3125	0.2877	0.2844	0.2755	0.2661	0.2678	0.257	0.2481
0.0649	0.1145	0.1281	0.1296	0.1343	0.1361	0.1459	0.1482	0.1462
0.1151	0.1289	0.1487	0.1641	0.1781	0.1887	0.1998	0.1989	0.1989
0.0682	0.1352	0.1437	0.1515	0.1576	0.1576	0.1629	0.1567	0.1516
0.1235	0.2156	0.1977	0.2033	0.2004	0.1823	0.1782	0.1618	0.1666
0.2251	0.3507	0.3171	0.3142	0.3076	0.2883	0.2952	0.2588	0.2576
0.2757	0.384	0.3426	0.3455	0.3414	0.3101	0.3192	0.2811	0.2634
0.2239	0.3269	0.2952	0.2891	0.2843	0.2638	0.2556	0.2122	0.2013
0.2139	0.3253	0.3018	0.2997	0.2926	0.2695	0.2707	0.238	0.2459
0.2182	0.3509	0.3039	0.2892	0.2801	0.2673	0.2637	0.2335	0.2321
0.2183	0.2958	0.2549	0.2544	0.258	0.2297	0.2165	0.1881	0.1992
0.0133	0.036	0.0335	0.033	0.0315	0.0288	0.026	0.0221	0.0209
0.0251	0.0456	0.0451	0.0455	0.0466	0.0423	0.0446	0.041	0.0391
0.0946	0.0899	0.0786	0.0795	0.0822	0.0801	0.0797	0.0751	0.0731
0.105	0.0761	0.0651	0.0652	0.0667	0.0671	0.0664	0.0646	0.064
0.0114	0.0191	0.0211	0.0215	0.0222	0.0228	0.0252	0.0257	0.0239
0.0181	0.0247	0.0246	0.0254	0.0259	0.025	0.0266	0.0257	0.0251
0.189	0.1064	0.0898	0.0774	0.0809	0.0784	0.0795	0.0843	0.0841
0.3181	0.2092	0.1753	0.1548	0.1525	0.1496	0.1502	0.1562	0.1575
0.5706	0.3415	0.2597	0.2482	0.2421	0.2523	0.2245	0.25	0.2528
0.7363	0.4613	0.3829	0.3618	0.3457	0.36	0.3428	0.3431	0.3285
0.8398	0.4929	0.4235	0.4088	0.4087	0.4207	0.4038	0.4032	0.4029
1.0925	0.69	0.6008	0.5958	0.5587	0.5002	0.4994	0.5065	0.4722
1.2823	0.7609	0.6013	0.5703	0.5486	0.5121	0.4547	0.5142	0.4983
0.7164	0.4181	0.3382	0.2955	0.3149	0.3313	0.309	0.3083	0.3307
0.1298	0.0788	0.0664	0.0658	0.0684	0.0714	0.0712	0.0701	0.0665
0	0	0	0	0	0	0	0	0
0	0	0	0	0	0	0	0	0
0	0	0	0	0	0	0	0	0
0	0	0	0	0	0	0	0	0
0	0	0	0	0	0	0	0	0
0	0	0	0	0	0	0	0	0
0	0	0	0	0	0	0	0	0
0	0	0	0	0	0	0	0	0
0	0	0	0	0	0	0	0	0
0	0	0	0	0	0	0	0	0
0	0	0	0	0	0	0	0	0
0.0015	0.0042	0.004	0.0041	0.0039	0.0035	0.0037	0.0029	0.0029
0	0	0	0	0	0	0	0	0

0.0167	0.0149	0.0161	0.019	0.0212	0.0237	0.0245	0.0256	0.0268
0.0086	0.005	0.004	0.0038	0.0041	0.0044	0.0041	0.0042	0.004
0.0248	0.0241	0.0233	0.0239	0.0245	0.0247	0.0259	0.0257	0.0255
0.051	0.0808	0.0827	0.0855	0.087	0.0852	0.0885	0.0841	0.0824
0.0748	0.1398	0.1419	0.1405	0.1464	0.1417	0.1461	0.1366	0.1312
0.0862	0.1764	0.1737	0.171	0.1719	0.1621	0.1691	0.1549	0.1547
0.2317	0.3149	0.2892	0.2874	0.2863	0.258	0.2453	0.2185	0.1892
0.14	0.2336	0.2235	0.222	0.2177	0.2037	0.2051	0.18	0.1843
0.0792	0.1483	0.1556	0.1579	0.1615	0.1605	0.1704	0.1658	0.1596
0.1542	0.159	0.1586	0.1633	0.1762	0.1854	0.1977	0.2025	0.1999
0.2626	0.2494	0.2358	0.2383	0.2443	0.2432	0.25	0.2421	0.239
0.1077	0.1799	0.1768	0.1825	0.1817	0.1762	0.1792	0.1657	0.1598
0.0838	0.1579	0.164	0.1711	0.1767	0.1749	0.1817	0.167	0.1677
0.0765	0.1499	0.1584	0.1659	0.1703	0.1641	0.1746	0.1667	0.1617
0.0552	0.2179	0.2242	0.2327	0.2331	0.2056	0.2053	0.164	0.1622
0.2522	0.3784	0.3491	0.333	0.321	0.2658	0.2615	0.2276	0.2393
0.4241	0.4784	0.4143	0.425	0.4104	0.385	0.36	0.3241	0.3306
0.5392	0.5608	0.5159	0.4973	0.4723	0.4221	0.41	0.3581	0.3708
0.4294	0.5061	0.4574	0.4528	0.4509	0.4129	0.4032	0.3425	0.3529
0.1573	0.323	0.3208	0.318	0.3254	0.2926	0.2824	0.2448	0.2326
0.2604	0.3703	0.3483	0.332	0.3239	0.2943	0.2896	0.253	0.2337
0.5248	0.4885	0.4376	0.4404	0.4201	0.3922	0.387	0.3633	0.3714
0.6602	0.5334	0.4315	0.4268	0.4226	0.4168	0.3945	0.3637	0.3607
0.4933	0.4905	0.4281	0.3901	0.3757	0.357	0.3656	0.3418	0.3584
0.0882	0.2397	0.2394	0.2385	0.2429	0.2184	0.2177	0.1649	0.1585
0.0726	0.1881	0.1791	0.178	0.174	0.1562	0.1563	0.1342	0.1322
0.0961	0.1182	0.1181	0.1229	0.1259	0.1247	0.1304	0.1267	0.1244
0.1381	0.0911	0.0812	0.0804	0.0841	0.088	0.0899	0.0933	0.0921
0.336	0.2065	0.1667	0.1743	0.1684	0.1643	0.1601	0.1665	0.1647
0.4443	0.2581	0.2173	0.1938	0.1915	0.2006	0.1907	0.2023	0.1902
0.3376	0.2062	0.1714	0.1657	0.165	0.1656	0.1562	0.1557	0.1562
0.281	0.1661	0.1415	0.1321	0.1262	0.1255	0.1199	0.1217	0.1277
0.3299	0.204	0.1708	0.166	0.1625	0.1631	0.1593	0.1628	0.1642
0.537	0.3278	0.2701	0.2528	0.2344	0.2479	0.2517	0.2624	0.2711
0.7326	0.4462	0.3819	0.3719	0.3492	0.3482	0.3429	0.366	0.3644
0.5177	0.314	0.256	0.2327	0.2266	0.2269	0.206	0.216	0.2255
0.1445	0.0874	0.0698	0.0665	0.0648	0.067	0.0616	0.062	0.0593
0.0169	0.0104	0.0086	0.008	0.0079	0.008	0.0077	0.0076	0.0076
0	0	0	0	0	0	0	0	0
0	0	0	0	0	0	0	0	0
0	0	0	0	0	0	0	0	0
0	0	0	0	0	0	0	0	0
0	0	0	0	0	0	0	0	0
0	0	0	0	0	0	0	0	0

0	0	0	0	0	0	0	0	0
0	0	0	0	0	0	0	0	0
0	0	0	0	0	0	0	0	0
0	0	0	0	0	0	0	0	0
0	0	0	0	0	0	0	0	0
0	0	0	0	0	0	0	0	0
0.0087	0.0051	0.0042	0.004	0.0039	0.0038	0.0037	0.0037	0.0035
0.0188	0.0131	0.0107	0.0105	0.0103	0.0105	0.0104	0.01	0.0096
0.0347	0.0367	0.0345	0.0342	0.0345	0.033	0.0337	0.0326	0.0319
0.0808	0.1036	0.1081	0.1088	0.1152	0.1186	0.1276	0.1279	0.1279
0.0919	0.1108	0.1121	0.1207	0.1314	0.1383	0.1477	0.1488	0.144
0.0937	0.0954	0.1113	0.1234	0.1279	0.139	0.1548	0.1684	0.1644
0.9332	0.6129	0.5263	0.5207	0.5606	0.5407	0.5376	0.5314	0.5364
0.7378	0.5891	0.4951	0.4838	0.4624	0.4749	0.4864	0.4847	0.4714
0.1854	0.2459	0.2304	0.2299	0.2316	0.2149	0.2182	0.2071	0.1946
0.0766	0.1549	0.1665	0.1685	0.1734	0.1713	0.186	0.1762	0.1658
0.0986	0.159	0.1651	0.1724	0.1775	0.1751	0.1848	0.1821	0.1777
0.1816	0.2009	0.1964	0.2023	0.2086	0.2094	0.2189	0.2157	0.2132
0.2143	0.2308	0.2207	0.2234	0.2283	0.2262	0.2296	0.2227	0.22
0.1129	0.1921	0.1867	0.1882	0.1922	0.1874	0.1881	0.17	0.1697
0.1242	0.3072	0.2951	0.2995	0.2904	0.2765	0.2783	0.2376	0.2224
0.2152	0.3729	0.3561	0.3443	0.3377	0.313	0.3086	0.2679	0.2603
0.3437	0.4591	0.4232	0.4133	0.4103	0.3574	0.3562	0.3126	0.3197
0.3539	0.4316	0.3905	0.3761	0.3853	0.3526	0.3346	0.3053	0.297
0.2841	0.3943	0.353	0.3486	0.3459	0.3203	0.3093	0.2626	0.274
0.1174	0.2538	0.24	0.2491	0.2492	0.2281	0.224	0.1847	0.1765
0.0957	0.171	0.1728	0.1723	0.1745	0.1679	0.1719	0.1575	0.1523
0.2272	0.2371	0.2146	0.2062	0.2068	0.2017	0.2064	0.19	0.1901
1.2633	0.8855	0.7454	0.7383	0.6976	0.6504	0.6378	0.6307	0.5972
0.9057	0.7567	0.6666	0.638	0.6334	0.6098	0.6123	0.6073	0.612
0.3577	0.4447	0.4102	0.3917	0.3952	0.3447	0.338	0.301	0.2879
0.1926	0.3634	0.3462	0.3366	0.3414	0.3184	0.3043	0.2511	0.2461
0.2508	0.3163	0.2987	0.2972	0.2899	0.2802	0.285	0.2643	0.2536
0.0466	0.0715	0.0782	0.0856	0.0992	0.1109	0.1299	0.1339	0.1327
0.0804	0.0868	0.0939	0.1115	0.119	0.1195	0.1337	0.1364	0.1277
0.0555	0.044	0.0426	0.044	0.0454	0.0473	0.0499	0.051	0.0503
0.2202	0.1352	0.1174	0.1136	0.1205	0.1267	0.1304	0.1367	0.1278
0.008	0.0048	0.0042	0.0039	0.004	0.0042	0.0041	0.0043	0.0041
0.0276	0.0169	0.0132	0.0115	0.0121	0.0128	0.0116	0.0127	0.0122
0.3396	0.2101	0.1722	0.151	0.1465	0.1435	0.1363	0.1427	0.145
0.5387	0.3266	0.2877	0.256	0.2502	0.2853	0.2992	0.2781	0.2556
0.5254	0.3072	0.2389	0.2238	0.2377	0.2265	0.2124	0.2029	0.1938
0.272	0.165	0.1309	0.1234	0.1316	0.1346	0.1311	0.1337	0.143
0.042	0.0258	0.0205	0.0187	0.0184	0.0182	0.0167	0.018	0.0186

0	0	0	0	0	0	0	0	0
0	0	0	0	0	0	0	0	0
0	0	0	0	0	0	0	0	0
0	0	0	0	0	0	0	0	0
0	0	0	0	0	0	0	0	0
0	0	0	0	0	0	0	0	0
0	0	0	0	0	0	0	0	0
0	0	0	0	0	0	0	0	0
0	0	0	0	0	0	0	0	0
0	0	0	0	0	0	0	0	0
0.0031	0.0046	0.0059	0.0064	0.0072	0.0075	0.0081	0.0083	0.008
0.0007	0.0025	0.0024	0.0024	0.0023	0.0022	0.0022	0.0017	0.0016
0.0097	0.0068	0.006	0.0055	0.0055	0.0053	0.0051	0.0051	0.005
0.02	0.0138	0.013	0.013	0.0138	0.0142	0.0147	0.0157	0.0155
0.0418	0.038	0.0342	0.034	0.0344	0.0333	0.0347	0.034	0.0344
0.0975	0.1116	0.1132	0.1177	0.1256	0.1318	0.1388	0.1392	0.1377
0.1053	0.1195	0.122	0.1292	0.1359	0.135	0.1458	0.1478	0.1489
0.1084	0.1162	0.1239	0.1308	0.1334	0.1512	0.168	0.1736	0.1742
0.2893	0.2479	0.2258	0.2189	0.2269	0.2209	0.2244	0.2183	0.2165
0.3999	0.3397	0.3071	0.2988	0.2794	0.2662	0.261	0.246	0.2368
0.2483	0.319	0.2981	0.3097	0.3013	0.2811	0.2677	0.2357	0.2243
0.1201	0.234	0.2356	0.2348	0.2416	0.2255	0.2365	0.2123	0.2006
0.1194	0.1762	0.1779	0.1829	0.1837	0.1776	0.185	0.172	0.1724
0.0612	0.0872	0.0929	0.1036	0.1137	0.1213	0.1265	0.1303	0.126
0.0843	0.0968	0.1029	0.1069	0.1146	0.1173	0.1212	0.1265	0.1257
0.105	0.1488	0.1521	0.1614	0.1693	0.1713	0.1802	0.1793	0.1746
0.0673	0.1374	0.1417	0.1462	0.1522	0.1457	0.152	0.1443	0.143
0.1579	0.2968	0.2835	0.2679	0.2695	0.2213	0.2288	0.1818	0.1776
0.0806	0.2605	0.2563	0.2522	0.2394	0.2159	0.2178	0.1888	0.197
0.165	0.3458	0.3254	0.3209	0.3161	0.2779	0.2593	0.2234	0.2179
0.3148	0.4419	0.418	0.4016	0.3808	0.3502	0.3424	0.298	0.2778
0.3685	0.3718	0.3241	0.3254	0.3068	0.2812	0.2741	0.2432	0.2377
0.2591	0.2629	0.2441	0.2447	0.2403	0.233	0.2389	0.2259	0.2214
0.0507	0.2131	0.2112	0.2243	0.2274	0.2057	0.2123	0.1934	0.1909
0.2959	0.3881	0.3578	0.3625	0.3677	0.3153	0.3125	0.2637	0.2625
0.4622	0.4626	0.4006	0.3802	0.3777	0.3546	0.3617	0.3355	0.3309
0.4683	0.4044	0.3642	0.3554	0.354	0.3392	0.3194	0.3068	0.3004
0.6119	0.5953	0.5255	0.5012	0.5023	0.459	0.4201	0.3981	0.3719
0.336	0.4114	0.3836	0.3766	0.3773	0.3402	0.3355	0.2987	0.3024
0.0582	0.1122	0.1272	0.1366	0.145	0.1484	0.1649	0.1561	0.1544
0.1535	0.1458	0.1528	0.1708	0.1845	0.2057	0.2198	0.2266	0.228
0.0738	0.0825	0.0941	0.1029	0.1155	0.1202	0.1304	0.1468	0.1463
0.1142	0.0865	0.0865	0.0888	0.0915	0.1024	0.1096	0.1172	0.1164
0.0067	0.0094	0.0126	0.0142	0.0145	0.0166	0.0176	0.0187	0.0188

0.0443	0.0257	0.0219	0.0212	0.021	0.0214	0.022	0.0212	0.0193
0.1988	0.1164	0.0935	0.0923	0.0909	0.0951	0.0975	0.0985	0.0984
0.1738	0.1058	0.0829	0.084	0.0844	0.0863	0.0817	0.0835	0.0818
0.1273	0.0726	0.0571	0.0554	0.0556	0.0557	0.058	0.0594	0.0598
0.1051	0.0655	0.0523	0.0493	0.0465	0.0487	0.047	0.0497	0.0464
0	0	0	0	0	0	0	0	0
0	0	0	0	0	0	0	0	0
0	0	0	0	0	0	0	0	0
0	0	0	0	0	0	0	0	0
0	0	0	0	0	0	0	0	0
0	0	0	0	0	0	0	0	0
0	0	0	0	0	0	0	0	0
0	0	0	0	0	0	0	0	0
0	0	0	0	0	0	0	0	0
0	0	0	0	0	0	0	0	0
0	0	0	0	0	0	0	0	0
0.0286	0.0259	0.0225	0.0221	0.0215	0.0205	0.0196	0.0195	0.0186
0.0392	0.0263	0.0235	0.0231	0.0246	0.0257	0.0263	0.0273	0.0275
0.0384	0.031	0.0255	0.0241	0.0228	0.0216	0.0213	0.0204	0.0188
0.0018	0.003	0.0032	0.0034	0.0035	0.0035	0.0038	0.0039	0.0036
0.0145	0.0232	0.0242	0.0276	0.0298	0.03	0.0317	0.032	0.0317
0.1237	0.1416	0.1447	0.1519	0.1591	0.1588	0.1648	0.1655	0.1672
0.1295	0.1643	0.1623	0.1686	0.176	0.1733	0.18	0.176	0.1712
0.0638	0.1543	0.1581	0.1665	0.166	0.1549	0.1665	0.1534	0.1576
0.1414	0.241	0.2401	0.238	0.2356	0.2164	0.2177	0.1954	0.1977
0.219	0.2905	0.2555	0.2567	0.2374	0.2252	0.229	0.2146	0.2138
0.2894	0.341	0.3055	0.3074	0.3129	0.2846	0.2888	0.2619	0.2515
0.1048	0.2055	0.209	0.2072	0.2059	0.1924	0.1954	0.1789	0.1779
0.0717	0.1336	0.1409	0.1534	0.1603	0.1647	0.1733	0.1701	0.1724
0.334	0.2887	0.2606	0.2631	0.2706	0.2742	0.2776	0.2736	0.2693
0.3532	0.3062	0.2699	0.2587	0.2596	0.2501	0.2504	0.248	0.2504
0.0526	0.111	0.1135	0.1109	0.1151	0.1133	0.1234	0.1165	0.1144
0.0596	0.1305	0.1368	0.1482	0.1539	0.1521	0.1637	0.1573	0.1597
0.107	0.2143	0.2045	0.2073	0.1999	0.1787	0.1792	0.1676	0.1627
0.1323	0.2994	0.2797	0.2795	0.2708	0.2468	0.2369	0.2056	0.1996
0.1519	0.3081	0.2823	0.3004	0.2933	0.267	0.2501	0.2188	0.2195
0.2408	0.3544	0.3275	0.3263	0.3257	0.3078	0.308	0.251	0.2597
0.2041	0.3134	0.2893	0.287	0.2967	0.2705	0.2743	0.226	0.2252
0.1446	0.2586	0.2363	0.2296	0.2348	0.2167	0.2251	0.1991	0.1913
0.2154	0.358	0.335	0.3276	0.3289	0.2869	0.2864	0.2592	0.2546
0.1248	0.3396	0.3303	0.3202	0.3312	0.2812	0.266	0.2189	0.211
0.1087	0.2761	0.2413	0.2403	0.2278	0.1888	0.1918	0.1533	0.1514
0.2972	0.3537	0.3209	0.3194	0.3204	0.3044	0.3003	0.2662	0.2505
0.4184	0.447	0.4126	0.4069	0.4001	0.3557	0.3532	0.307	0.3132

0.1611	0.3092	0.283	0.2677	0.294	0.257	0.2532	0.2102	0.1894
0.0708	0.2139	0.2143	0.2116	0.2148	0.1941	0.1907	0.1545	0.1554
0.1092	0.1877	0.1912	0.1971	0.1961	0.1871	0.1933	0.1788	0.1738
0.077	0.1975	0.2018	0.21	0.2069	0.1969	0.2109	0.1974	0.1905
0.0599	0.1216	0.1294	0.1357	0.137	0.1357	0.1461	0.1478	0.1477
0.0425	0.031	0.0286	0.03	0.0317	0.0336	0.0365	0.0386	0.0385
0.1808	0.1101	0.0872	0.0833	0.0839	0.0874	0.092	0.0939	0.0933
0.2514	0.1605	0.1274	0.1246	0.1307	0.135	0.1379	0.1411	0.1361
0.1869	0.1134	0.0939	0.0871	0.0919	0.095	0.0962	0.0958	0.0959
0.0664	0.0395	0.0313	0.029	0.0302	0.0309	0.0296	0.0315	0.0302
0.0147	0.0209	0.019	0.0194	0.0188	0.0176	0.0182	0.0154	0.0142
0.0339	0.0212	0.0173	0.0167	0.0173	0.0173	0.0166	0.0176	0.0165
0.0087	0.0053	0.0044	0.0039	0.0037	0.0038	0.0039	0.0039	0.0041
0.0084	0.0051	0.0041	0.0039	0.0039	0.004	0.0039	0.0041	0.004
0	0	0	0	0	0	0	0	0
0	0	0	0	0	0	0	0	0
0	0	0	0	0	0	0	0	0
0	0	0	0	0	0	0	0	0
0	0	0	0	0	0	0	0	0
0	0	0	0	0	0	0	0	0
0	0	0	0	0	0	0	0	0
0	0	0	0	0	0	0	0	0
0.0553	0.037	0.0307	0.0279	0.0283	0.0281	0.0271	0.0262	0.0261
0.0281	0.0182	0.0147	0.0142	0.014	0.0144	0.0137	0.0133	0.013
0.0515	0.0342	0.0303	0.0289	0.0285	0.0279	0.0271	0.0276	0.0264
0.0833	0.0609	0.0553	0.0559	0.0579	0.059	0.0608	0.0627	0.063
0.0698	0.0888	0.0949	0.1012	0.1036	0.1092	0.1226	0.1232	0.1239
0.0843	0.1607	0.1608	0.1666	0.1693	0.169	0.1784	0.1628	0.1604
0.1323	0.2399	0.2292	0.225	0.2249	0.2052	0.2077	0.1876	0.1835
0.1504	0.2587	0.2501	0.2459	0.2438	0.2259	0.2288	0.1967	0.198
0.0755	0.2269	0.2208	0.2184	0.2101	0.1927	0.1915	0.1653	0.1713
0.2776	0.379	0.3466	0.343	0.3372	0.324	0.3202	0.2802	0.2811
0.1962	0.3319	0.3063	0.2821	0.2874	0.249	0.2482	0.2129	0.2212
0.1524	0.2466	0.2411	0.2409	0.2468	0.2272	0.2279	0.1935	0.1861
0.0938	0.1482	0.1489	0.1551	0.1545	0.1538	0.1599	0.1497	0.1488
0.3944	0.3973	0.3541	0.3524	0.3656	0.3428	0.3501	0.3311	0.3231
0.8658	0.7348	0.6527	0.6548	0.6448	0.5835	0.5584	0.5276	0.5299
0.7133	0.642	0.5659	0.517	0.5184	0.4927	0.5011	0.4625	0.4445
0.3318	0.2785	0.2514	0.2512	0.2567	0.2561	0.2582	0.2561	0.2536
0.0734	0.2269	0.2205	0.2212	0.2255	0.2101	0.2194	0.1844	0.1714
0.12	0.3133	0.2854	0.2813	0.275	0.2388	0.253	0.2157	0.1957
0.3294	0.45	0.416	0.4118	0.4074	0.3597	0.3481	0.297	0.2743
0.1978	0.3435	0.313	0.3056	0.3147	0.2918	0.283	0.2342	0.2278
0.1102	0.2803	0.2764	0.2568	0.2615	0.2337	0.2225	0.1801	0.1782

0.1571	0.3144	0.294	0.3147	0.3173	0.2866	0.2682	0.2286	0.2236
0.0943	0.282	0.2729	0.2801	0.2719	0.2502	0.2446	0.2013	0.1788
0.0806	0.2811	0.2657	0.2559	0.2447	0.2222	0.217	0.1642	0.1516
0.1054	0.2914	0.2619	0.2521	0.2553	0.2275	0.2384	0.1969	0.1903
0.1975	0.3285	0.3109	0.3055	0.3127	0.2831	0.2683	0.2408	0.2344
0.2554	0.3321	0.3022	0.2973	0.297	0.2786	0.2829	0.2565	0.2657
0.1193	0.2595	0.2375	0.2402	0.2413	0.231	0.2138	0.1804	0.171
0.1142	0.2858	0.2698	0.2704	0.288	0.248	0.2412	0.2044	0.2053
0.282	0.3998	0.3679	0.3308	0.3338	0.3144	0.3163	0.2758	0.2673
0.2835	0.4022	0.3677	0.3711	0.3922	0.3391	0.351	0.3057	0.2919
0.1288	0.2451	0.2359	0.2309	0.2368	0.2117	0.2161	0.1854	0.187
0.079	0.1648	0.1722	0.1731	0.1767	0.1667	0.1738	0.16	0.1533
0.0819	0.1137	0.1271	0.1388	0.1477	0.1539	0.1586	0.1687	0.1727
0.0893	0.0812	0.0816	0.0858	0.0919	0.1029	0.1135	0.1249	0.124
0.0856	0.0688	0.0648	0.0662	0.0697	0.0725	0.075	0.0768	0.0758
0.0507	0.0453	0.0396	0.039	0.0388	0.038	0.038	0.0359	0.0357
0.0595	0.0404	0.0361	0.0355	0.0367	0.0387	0.0395	0.0417	0.0409
0.0837	0.0453	0.0412	0.0392	0.0393	0.0378	0.0357	0.036	0.0396
0.0086	0.0053	0.0043	0.004	0.0039	0.0039	0.0037	0.0041	0.004
0.0079	0.0047	0.0035	0.0031	0.0031	0.0033	0.0032	0.0035	0.0033
0	0	0	0	0	0	0	0	0
0	0	0	0	0	0	0	0	0
0	0	0	0	0	0	0	0	0
0	0	0	0	0	0	0	0	0
0	0	0	0	0	0	0	0	0
0	0	0	0	0	0	0	0	0
0	0	0	0	0	0	0	0	0
0	0	0	0	0	0	0	0	0
0.0428	0.0232	0.0195	0.0186	0.0204	0.0207	0.0207	0.0216	0.0219
0	0	0	0	0	0	0	0	0
0.0428	0.0267	0.0217	0.0218	0.023	0.0232	0.0228	0.0228	0.0242
0.127	0.0979	0.0892	0.0883	0.0901	0.0919	0.0954	0.0931	0.0903
0.1173	0.1485	0.1557	0.1638	0.1706	0.1733	0.1869	0.1882	0.1851
0.0714	0.2019	0.2031	0.204	0.203	0.1829	0.1975	0.1679	0.165
0.0681	0.2549	0.2448	0.2557	0.241	0.2054	0.2085	0.172	0.1815
0.2583	0.3799	0.3478	0.34	0.3441	0.3159	0.305	0.2748	0.2659
0.2906	0.3917	0.3523	0.3276	0.3304	0.2832	0.2845	0.2539	0.2481
0.3899	0.4061	0.3261	0.3208	0.3124	0.3067	0.2913	0.2531	0.2477
0.1488	0.3378	0.3252	0.3179	0.3036	0.284	0.2624	0.2347	0.2306
0.0896	0.261	0.2622	0.261	0.2631	0.229	0.2321	0.2055	0.1976
0.0614	0.1604	0.1565	0.1643	0.1655	0.1472	0.1493	0.1377	0.1346
0.2468	0.3068	0.279	0.2788	0.2856	0.2664	0.2648	0.2382	0.2361
1.1118	0.8889	0.7497	0.7359	0.6971	0.6934	0.6765	0.656	0.677
1.2006	0.8746	0.6842	0.6498	0.6544	0.6245	0.6735	0.6637	0.6865

0.492	0.3966	0.3312	0.3335	0.3421	0.3362	0.3214	0.3071	0.2849
0.0782	0.2367	0.2247	0.2199	0.214	0.1794	0.1765	0.1389	0.1332
0.2899	0.4049	0.3658	0.3405	0.3516	0.3186	0.3007	0.2736	0.2651
0.5554	0.5502	0.4746	0.4416	0.4342	0.4216	0.4205	0.3852	0.3858
0.2742	0.3998	0.3752	0.3453	0.3442	0.3116	0.3099	0.2605	0.2546
0.1105	0.3082	0.2723	0.2767	0.2812	0.2423	0.2472	0.1927	0.1827
0.2468	0.3672	0.3337	0.3298	0.3238	0.2976	0.3033	0.263	0.2662
0.1694	0.3168	0.2981	0.2951	0.2962	0.2623	0.2548	0.2115	0.2035
0.2389	0.3832	0.3529	0.3425	0.3277	0.2891	0.2945	0.246	0.2218
0.1009	0.3082	0.2774	0.2629	0.2581	0.2338	0.2222	0.173	0.1753
0.1872	0.3739	0.3646	0.3467	0.3305	0.2941	0.279	0.2315	0.2197
0.1451	0.2418	0.2334	0.2238	0.2272	0.2095	0.2112	0.1866	0.1827
0.0734	0.1612	0.1643	0.1696	0.1724	0.1647	0.1725	0.1603	0.1639
0.2119	0.3188	0.2963	0.2923	0.2977	0.2626	0.2629	0.2214	0.2303
0.4434	0.5311	0.4754	0.4396	0.4364	0.3964	0.384	0.3623	0.3503
0.5195	0.5312	0.4608	0.4479	0.4472	0.4302	0.4181	0.3987	0.3712
0.2243	0.3675	0.3517	0.3497	0.3555	0.3059	0.2965	0.2767	0.2629
0.0755	0.2242	0.2323	0.2233	0.2294	0.2063	0.2052	0.1686	0.1643
0.0714	0.169	0.1717	0.1748	0.1794	0.1714	0.1797	0.1614	0.1602
0.0698	0.1135	0.1231	0.1385	0.143	0.1518	0.1664	0.1613	0.1515
0.0952	0.0824	0.1037	0.1136	0.1202	0.1358	0.1502	0.154	0.149
0.0397	0.0361	0.0368	0.0376	0.0392	0.0424	0.0441	0.0457	0.0455
0.0175	0.0104	0.0082	0.0077	0.0077	0.0077	0.0078	0.0083	0.0084
0.0284	0.0217	0.0192	0.0178	0.0176	0.0171	0.0171	0.0168	0.0168
0.0081	0.0045	0.0036	0.0035	0.0034	0.0035	0.0036	0.0036	0.0039
0.0083	0.005	0.0041	0.0038	0.0037	0.0035	0.0036	0.0039	0.0036
0	0	0	0	0	0	0	0	0
0	0	0	0	0	0	0	0	0
0	0	0	0	0	0	0	0	0
0	0	0	0	0	0	0	0	0
0	0	0	0	0	0	0	0	0
0	0	0	0	0	0	0	0	0
0	0	0	0	0	0	0	0	0
0	0	0	0	0	0	0	0	0
0	0	0	0	0	0	0	0	0
0.003	0.0073	0.007	0.0073	0.0074	0.0063	0.0063	0.0053	0.0055
0.0161	0.0277	0.027	0.0278	0.0269	0.0256	0.0258	0.0236	0.0223
0.0969	0.1308	0.1219	0.1242	0.1302	0.1239	0.1239	0.1143	0.1127
0.135	0.206	0.2057	0.2086	0.2058	0.2064	0.2088	0.1932	0.1923
0.0808	0.2288	0.2194	0.2177	0.2114	0.1866	0.1893	0.1612	0.1595
0.1289	0.3238	0.3073	0.3124	0.3029	0.2885	0.2859	0.2447	0.2484
0.2104	0.3598	0.3348	0.3222	0.3155	0.2846	0.2873	0.2599	0.2623
0.2655	0.361	0.3381	0.3292	0.3317	0.2978	0.3053	0.2771	0.2818
0.3063	0.3604	0.3312	0.3236	0.2939	0.2715	0.2775	0.2524	0.2498

0.201	0.3297	0.3228	0.3149	0.2973	0.2813	0.2994	0.2549	0.237
0.1887	0.3238	0.3055	0.2933	0.3062	0.2645	0.2587	0.2261	0.2213
0.103	0.2248	0.2182	0.2343	0.2227	0.2023	0.1978	0.1769	0.1739
0.1363	0.2245	0.2089	0.2136	0.2069	0.1918	0.1981	0.1768	0.1731
0.5647	0.4774	0.4135	0.4281	0.4185	0.3874	0.3607	0.3453	0.3517
0.6912	0.5038	0.4287	0.4066	0.3947	0.3762	0.3652	0.381	0.3687
0.2768	0.2651	0.239	0.2438	0.2539	0.243	0.2396	0.226	0.2201
0.1009	0.2663	0.2565	0.244	0.2486	0.231	0.2335	0.1917	0.1887
0.427	0.4718	0.426	0.4088	0.3928	0.3809	0.3612	0.3123	0.3134
0.6716	0.5868	0.5361	0.5134	0.5099	0.4802	0.4505	0.4293	0.4263
0.2844	0.4082	0.3783	0.3485	0.3339	0.2888	0.3093	0.2709	0.2703
0.0816	0.2681	0.2563	0.2579	0.2497	0.2263	0.2353	0.1994	0.1904
0.2725	0.373	0.3377	0.3247	0.3211	0.2843	0.2583	0.2191	0.2158
0.2836	0.3572	0.3235	0.3242	0.3303	0.3029	0.305	0.2839	0.2817
0.2781	0.3889	0.3595	0.3387	0.3473	0.3364	0.3162	0.2666	0.2786
0.1909	0.3445	0.324	0.3235	0.3073	0.2813	0.2814	0.2403	0.2254
0.1349	0.3363	0.3227	0.3135	0.3183	0.2761	0.276	0.2384	0.211
0.0987	0.2019	0.199	0.1974	0.1921	0.1792	0.184	0.162	0.1599
0.081	0.117	0.1229	0.1299	0.1336	0.1343	0.1414	0.1504	0.1525
0.1885	0.2664	0.2476	0.2472	0.2466	0.2296	0.2255	0.2045	0.1996
0.3403	0.4174	0.3751	0.3583	0.3584	0.311	0.2968	0.2526	0.2731
0.4235	0.4306	0.3764	0.387	0.3895	0.3652	0.367	0.3432	0.3332
0.1423	0.2581	0.248	0.2462	0.2425	0.2234	0.2213	0.1939	0.1934
0.0829	0.1969	0.182	0.179	0.1763	0.153	0.1577	0.1386	0.134
0.0982	0.2465	0.235	0.2208	0.2197	0.1856	0.1876	0.1518	0.1526
0.1465	0.2522	0.2449	0.2431	0.2486	0.2325	0.2364	0.2174	0.2068
0.1144	0.1307	0.1347	0.1432	0.1488	0.1539	0.1712	0.1778	0.1776
0.0239	0.0168	0.0194	0.0223	0.0246	0.0282	0.03	0.0318	0.0304
0	0	0	0	0	0	0	0	0
0.002	0.0027	0.004	0.0054	0.0068	0.008	0.0091	0.0102	0.0106
0.0086	0.0048	0.0038	0.0037	0.0036	0.0038	0.0036	0.0036	0.0036
0.008	0.0045	0.0033	0.0033	0.0032	0.0032	0.0031	0.0032	0.0032
0	0	0	0	0	0	0	0	0
0	0	0	0	0	0	0	0	0
0	0	0	0	0	0	0	0	0
0	0	0	0	0	0	0	0	0
0	0	0	0	0	0	0	0	0
0	0	0	0	0	0	0	0	0
0	0	0	0	0	0	0	0	0
0	0	0	0	0	0	0	0	0
0.0023	0.0035	0.0039	0.0042	0.0042	0.0042	0.0045	0.0045	0.0043
0.0424	0.0269	0.0223	0.023	0.0226	0.0223	0.0224	0.0223	0.0233
0.0269	0.0419	0.0396	0.0395	0.04	0.0369	0.0383	0.0341	0.0332
0.0615	0.1589	0.1528	0.1603	0.1638	0.1547	0.1555	0.1387	0.133

0.1704	0.2855	0.2687	0.2654	0.2723	0.2576	0.247	0.2209	0.2189
0.2584	0.3839	0.3537	0.3461	0.3393	0.3197	0.3211	0.2726	0.2495
0.1701	0.3179	0.2997	0.2986	0.3002	0.2732	0.2712	0.221	0.2285
0.0879	0.2058	0.203	0.2039	0.2012	0.1825	0.178	0.155	0.1609
0.1682	0.2848	0.264	0.2572	0.2593	0.2403	0.2437	0.2098	0.2042
0.0728	0.2239	0.219	0.2057	0.2029	0.1741	0.1848	0.154	0.1461
0.2107	0.3313	0.3011	0.3028	0.3109	0.2802	0.2775	0.2455	0.2325
0.1406	0.2798	0.2703	0.2682	0.2756	0.2513	0.2404	0.2059	0.2031
0.115	0.2748	0.256	0.2549	0.2598	0.2336	0.2393	0.2038	0.1897
0.1853	0.3064	0.2656	0.2459	0.2475	0.2216	0.2199	0.1919	0.2062
0.0579	0.1636	0.1507	0.15	0.1619	0.1387	0.1416	0.1181	0.1076
0.1045	0.1396	0.1367	0.1378	0.1389	0.1373	0.1416	0.1341	0.1313
0.0639	0.1552	0.1565	0.1598	0.1644	0.1647	0.1671	0.149	0.142
0.0551	0.2152	0.2026	0.1999	0.193	0.1761	0.1713	0.129	0.1284
0.313	0.3794	0.3611	0.3454	0.339	0.3224	0.3082	0.2744	0.2611
0.3971	0.4285	0.3931	0.3962	0.3935	0.3639	0.3695	0.3508	0.3426
0.2114	0.3838	0.3571	0.3621	0.3601	0.3253	0.3202	0.2602	0.2614
0.0802	0.2186	0.2177	0.2218	0.216	0.1954	0.1994	0.1678	0.1615
0.1372	0.2195	0.203	0.2094	0.2085	0.1859	0.1824	0.165	0.1566
0.2284	0.3301	0.3122	0.3124	0.3068	0.2765	0.2606	0.2184	0.2297
0.1728	0.3193	0.3094	0.3095	0.2993	0.2757	0.2804	0.2417	0.2364
0.0699	0.2062	0.2085	0.199	0.1997	0.1765	0.1854	0.1473	0.1521
0.092	0.2648	0.2763	0.2679	0.2616	0.2194	0.2294	0.1989	0.2027
0.0984	0.2306	0.2299	0.2268	0.2262	0.1978	0.1992	0.1785	0.1771
0.0683	0.1388	0.1488	0.1516	0.1526	0.1523	0.1636	0.1565	0.1539
0.1228	0.2611	0.2491	0.2425	0.2367	0.2049	0.2115	0.1843	0.1778
0.1805	0.3498	0.3452	0.3367	0.3215	0.3022	0.3134	0.2613	0.2562
0.1974	0.3544	0.3354	0.3206	0.3128	0.2795	0.2745	0.2458	0.2391
0.0746	0.1406	0.1423	0.144	0.1436	0.1345	0.1398	0.1254	0.1241
0.0974	0.1695	0.1706	0.1762	0.1823	0.1808	0.1877	0.179	0.1723
0.1603	0.3116	0.2841	0.2791	0.2747	0.2472	0.2478	0.2047	0.199
0.1969	0.38	0.3466	0.3327	0.3209	0.2648	0.2559	0.2101	0.2087
0.0993	0.1952	0.192	0.1965	0.2014	0.1748	0.1804	0.1556	0.1549
0.04	0.0567	0.0547	0.0553	0.0554	0.0508	0.0526	0.0492	0.0487
0.1106	0.0693	0.0542	0.05	0.0482	0.0492	0.0448	0.0472	0.0488
0.2347	0.1463	0.1212	0.1104	0.1139	0.1128	0.1146	0.1179	0.1182
0.0087	0.0053	0.0045	0.0043	0.0041	0.0043	0.004	0.004	0.004
0.0092	0.0056	0.0046	0.0044	0.0041	0.004	0.004	0.0039	0.0037
0	0	0	0	0	0	0	0	0
0	0	0	0	0	0	0	0	0
0	0	0	0	0	0	0	0	0
0	0	0	0	0	0	0	0	0
0	0	0	0	0	0	0	0	0
0	0	0	0	0	0	0	0	0

0	0	0	0	0	0	0	0	0
0	0	0	0	0	0	0	0	0
0.0041	0.0086	0.0086	0.0089	0.0092	0.0088	0.0095	0.0089	0.0087
0.0847	0.0487	0.0386	0.0343	0.0338	0.0342	0.0309	0.0352	0.0333
0.0477	0.0346	0.0297	0.0284	0.0278	0.0274	0.0277	0.0282	0.029
0.0892	0.1563	0.1544	0.1554	0.1537	0.1421	0.1435	0.1342	0.1343
0.293	0.3635	0.3239	0.3096	0.2953	0.2695	0.2622	0.2356	0.2196
0.4347	0.5113	0.4471	0.4405	0.4395	0.4133	0.3957	0.3527	0.3475
0.2349	0.3604	0.3339	0.3183	0.3297	0.291	0.2994	0.2554	0.2531
0.0545	0.1697	0.1731	0.1777	0.1828	0.1731	0.1808	0.1591	0.1582
0.2043	0.3007	0.2866	0.2888	0.2943	0.2695	0.2594	0.216	0.2093
0.0852	0.2589	0.2484	0.2437	0.2452	0.2081	0.2105	0.1806	0.1732
0.1755	0.3175	0.3043	0.2706	0.261	0.2268	0.2336	0.1994	0.1995
0.097	0.2763	0.2565	0.2603	0.2581	0.2328	0.2276	0.1855	0.19
0.211	0.3659	0.342	0.3362	0.338	0.3086	0.2968	0.2606	0.2472
0.4032	0.4077	0.3627	0.3517	0.3452	0.3114	0.3127	0.2783	0.2865
0.0866	0.175	0.1654	0.1543	0.1558	0.1414	0.1323	0.1102	0.1145
0.1158	0.1806	0.1827	0.1858	0.1882	0.1789	0.185	0.1723	0.1695
0.0785	0.1759	0.1772	0.1807	0.1806	0.1714	0.1876	0.1744	0.1677
0.1099	0.2526	0.2428	0.234	0.2367	0.2192	0.2258	0.1875	0.1779
0.105	0.2539	0.2445	0.2468	0.2435	0.2136	0.2183	0.1824	0.1858
0.1102	0.2898	0.2763	0.2718	0.2585	0.2185	0.2236	0.1763	0.1571
0.0881	0.2613	0.2639	0.2443	0.252	0.2308	0.234	0.1895	0.1744
0.117	0.202	0.1903	0.1938	0.2011	0.1807	0.1862	0.1659	0.1615
0.1135	0.1962	0.1929	0.1895	0.1913	0.1738	0.1806	0.1562	0.1498
0.137	0.3214	0.3029	0.2954	0.291	0.2511	0.2542	0.1999	0.2153
0.1005	0.2792	0.2489	0.256	0.2597	0.2441	0.2287	0.2045	0.1926
0.094	0.2226	0.2189	0.2198	0.2131	0.1904	0.1972	0.1743	0.1625
0.089	0.2757	0.282	0.274	0.2842	0.2433	0.2476	0.1975	0.193
0.1557	0.324	0.3058	0.2945	0.2787	0.235	0.2279	0.1899	0.1891
0.1329	0.252	0.2357	0.2396	0.2472	0.2202	0.2141	0.1885	0.1803
0.1162	0.2539	0.2484	0.2418	0.2405	0.2156	0.2034	0.1761	0.1834
0.0812	0.2125	0.212	0.2163	0.2051	0.1727	0.1832	0.1468	0.1404
0.0746	0.2295	0.2172	0.2143	0.2202	0.2091	0.2093	0.1695	0.1657
0.1039	0.2425	0.2291	0.2322	0.2462	0.2237	0.2119	0.1823	0.1902
0.0717	0.1878	0.1813	0.1768	0.1824	0.1685	0.1727	0.154	0.1527
0.0738	0.2447	0.232	0.2357	0.2378	0.2067	0.1949	0.16	0.1624
0.148	0.3714	0.3656	0.3574	0.3435	0.3025	0.3078	0.257	0.2446
0.1067	0.2819	0.2659	0.2734	0.2758	0.2419	0.2377	0.2035	0.1958
0.0766	0.1336	0.1314	0.1353	0.1343	0.1296	0.1365	0.1256	0.1226
0.2955	0.1844	0.1557	0.1481	0.1504	0.153	0.1453	0.1453	0.1486
0.5233	0.3062	0.2569	0.2294	0.2393	0.238	0.2352	0.2464	0.2416
0.0087	0.005	0.0042	0.0041	0.004	0.004	0.0037	0.0041	0.004
0.0094	0.0058	0.0047	0.0044	0.0042	0.0042	0.0041	0.004	0.0041

0	0	0	0	0	0	0	0	0
0	0	0	0	0	0	0	0	0
0	0	0	0	0	0	0	0	0
0	0	0	0	0	0	0	0	0
0	0	0	0	0	0	0	0	0
0	0	0	0	0	0	0	0	0
0	0	0	0	0	0	0	0	0
0	0	0	0	0	0	0	0	0
0.0105	0.01	0.0088	0.0084	0.0083	0.0077	0.0078	0.0068	0.0067
0.0651	0.0377	0.0286	0.0286	0.0284	0.0294	0.0268	0.0255	0.0291
0.0356	0.0328	0.0314	0.0322	0.033	0.0335	0.0346	0.0347	0.0342
0.1364	0.1435	0.135	0.1354	0.135	0.1317	0.1342	0.1284	0.1242
0.387	0.3711	0.3283	0.3176	0.3255	0.3101	0.3114	0.284	0.2877
0.3899	0.4733	0.4298	0.4118	0.3925	0.3659	0.3609	0.3285	0.329
0.1811	0.3463	0.3045	0.2932	0.3019	0.2561	0.2524	0.2216	0.213
0.116	0.2818	0.2567	0.2715	0.2644	0.2324	0.2289	0.1979	0.184
0.1085	0.2682	0.253	0.2401	0.2444	0.2178	0.2124	0.1732	0.1663
0.1097	0.2708	0.2584	0.2617	0.2499	0.2169	0.2235	0.1881	0.1927
0.1936	0.3159	0.2881	0.2831	0.2789	0.2501	0.2407	0.2168	0.225
0.1801	0.3502	0.3239	0.3183	0.3202	0.2758	0.275	0.2269	0.2188
0.3769	0.431	0.3956	0.3763	0.3633	0.3394	0.3441	0.3073	0.2678
0.534	0.43	0.349	0.3339	0.353	0.3321	0.3508	0.34	0.3397
0.2174	0.1943	0.1762	0.1694	0.1653	0.1605	0.1568	0.148	0.1514
0.0746	0.1538	0.1553	0.1582	0.1622	0.155	0.164	0.1511	0.1453
0.1796	0.2468	0.2358	0.2367	0.2359	0.2272	0.2336	0.2186	0.2172
0.1355	0.2579	0.2381	0.2431	0.2448	0.2147	0.2133	0.185	0.1796
0.1126	0.2568	0.2436	0.2484	0.2437	0.2242	0.2318	0.1999	0.202
0.0597	0.2227	0.2215	0.2383	0.2452	0.2113	0.2061	0.1722	0.1661
0.174	0.3114	0.3003	0.2939	0.2953	0.268	0.2681	0.2244	0.2052
0.1845	0.2622	0.2385	0.2419	0.2423	0.2291	0.2342	0.2091	0.2132
0.1876	0.3027	0.2854	0.2742	0.2682	0.2434	0.2519	0.223	0.2135
0.1796	0.3497	0.325	0.3162	0.3073	0.2844	0.2798	0.2308	0.2182
0.1115	0.2657	0.2553	0.2544	0.2516	0.2216	0.2246	0.1734	0.1745
0.1369	0.2371	0.2334	0.2284	0.2207	0.2073	0.2112	0.1849	0.1885
0.1232	0.2884	0.2671	0.2591	0.2606	0.2269	0.2212	0.1851	0.1878
0.1576	0.3401	0.3129	0.2913	0.2908	0.25	0.2593	0.2022	0.1815
0.1908	0.328	0.3059	0.302	0.2952	0.2785	0.2638	0.2254	0.2164
0.1379	0.2424	0.2204	0.2133	0.2111	0.1917	0.1922	0.1754	0.1737
0.1129	0.1737	0.1727	0.1788	0.1818	0.1728	0.1776	0.1671	0.1647
0.1515	0.2282	0.2179	0.2169	0.2162	0.2025	0.2095	0.2014	0.1999
0.1225	0.2968	0.2772	0.2522	0.2532	0.2113	0.2263	0.1934	0.1923
0.1024	0.275	0.2619	0.2659	0.2408	0.2027	0.2127	0.1744	0.1676
0.1233	0.3379	0.3282	0.3279	0.3091	0.2663	0.2694	0.2298	0.2301
0.1158	0.3075	0.2907	0.2797	0.2622	0.2174	0.2246	0.1937	0.1993

0.124	0.2789	0.2619	0.2651	0.2522	0.2114	0.2033	0.1554	0.1518
0.1154	0.1709	0.1626	0.1651	0.1676	0.1607	0.167	0.1536	0.1498
0.2275	0.1449	0.1285	0.1199	0.1181	0.1251	0.1184	0.1203	0.1192
0.3993	0.2487	0.1997	0.1904	0.1915	0.1999	0.1979	0.2042	0.208
0.0093	0.0058	0.0046	0.0041	0.0042	0.0043	0.0044	0.0043	0.0042
0.0092	0.0058	0.0047	0.0045	0.0044	0.0041	0.0041	0.0043	0.0046
0	0	0	0	0	0	0	0	0
0	0	0	0	0	0	0	0	0
0	0	0	0	0	0	0	0	0
0	0	0	0	0	0	0	0	0
0	0	0	0	0	0	0	0	0
0	0	0	0	0	0	0	0	0
0	0	0	0	0	0	0	0	0
0	0	0	0	0	0	0	0	0
0.0104	0.0088	0.0077	0.0076	0.0076	0.0077	0.0078	0.0078	0.0076
0.0278	0.049	0.0479	0.0494	0.05	0.0476	0.0492	0.0467	0.0459
0.0447	0.0895	0.0937	0.0944	0.0937	0.0917	0.0984	0.0918	0.0924
0.1674	0.1503	0.1334	0.1296	0.1238	0.1187	0.1201	0.1116	0.1129
0.391	0.3455	0.2952	0.2933	0.2964	0.2705	0.2735	0.268	0.2548
0.3234	0.4175	0.3868	0.3695	0.3519	0.3224	0.3117	0.2736	0.2603
0.1456	0.3217	0.321	0.3165	0.3094	0.2763	0.2778	0.2401	0.2334
0.1604	0.3345	0.3262	0.3166	0.3124	0.2731	0.2795	0.2278	0.227
0.0963	0.238	0.2144	0.2035	0.2079	0.1863	0.1874	0.1641	0.1639
0.2228	0.37	0.3529	0.3571	0.3491	0.3232	0.3147	0.2761	0.2882
0.1119	0.3176	0.3117	0.2946	0.2855	0.2493	0.235	0.1786	0.1694
0.0521	0.2463	0.2335	0.2334	0.2321	0.2021	0.2198	0.1838	0.1682
0.2349	0.3602	0.322	0.3093	0.2966	0.2607	0.2528	0.2167	0.201
0.3691	0.3401	0.3067	0.2992	0.2963	0.2894	0.2842	0.2528	0.2471
0.2918	0.2449	0.213	0.206	0.2053	0.2006	0.2021	0.1965	0.1888
0.0569	0.1231	0.1243	0.1271	0.1274	0.1193	0.1256	0.1132	0.1111
0.106	0.1365	0.132	0.1333	0.1377	0.1341	0.1355	0.1261	0.1248
0.0542	0.134	0.1407	0.1415	0.1414	0.1333	0.1336	0.1207	0.1201
0.0867	0.1621	0.1613	0.1681	0.1692	0.1644	0.1701	0.1585	0.155
0.1124	0.2039	0.1964	0.2007	0.2027	0.192	0.1963	0.1857	0.1851
0.1349	0.2713	0.2538	0.251	0.2591	0.2375	0.2302	0.2123	0.2029
0.1431	0.2814	0.2717	0.2772	0.2756	0.2377	0.2439	0.212	0.2159
0.1581	0.316	0.2941	0.293	0.2957	0.2431	0.2431	0.2095	0.2146
0.1811	0.3042	0.2811	0.2897	0.2756	0.2564	0.2427	0.2054	0.2143
0.1432	0.2666	0.2566	0.2473	0.2374	0.2111	0.1989	0.1775	0.1763
0.0982	0.1771	0.1729	0.1811	0.1792	0.1671	0.1679	0.1505	0.1496
0.0855	0.2208	0.2151	0.2233	0.2257	0.2048	0.2103	0.1853	0.1799
0.092	0.2662	0.2543	0.2489	0.2422	0.1965	0.1964	0.1527	0.1519
0.1388	0.3	0.2859	0.2771	0.2557	0.2356	0.2325	0.1909	0.1836
0.1205	0.2369	0.2203	0.2197	0.2136	0.1995	0.2005	0.1857	0.1759

0.1128	0.1717	0.1669	0.1697	0.171	0.1629	0.1675	0.1537	0.1514
0.2104	0.2758	0.2457	0.2483	0.2399	0.213	0.2111	0.191	0.1949
0.1478	0.2797	0.2792	0.2723	0.2763	0.2333	0.2225	0.189	0.1842
0.1264	0.3392	0.3241	0.3084	0.3017	0.2565	0.2524	0.1953	0.2113
0.1529	0.322	0.3047	0.3131	0.2867	0.2491	0.2571	0.2271	0.2234
0.1825	0.2451	0.2274	0.2326	0.2376	0.2181	0.2112	0.1935	0.1853
0.1791	0.1951	0.1837	0.1873	0.1919	0.1823	0.1794	0.1662	0.1668
0.1236	0.1493	0.1475	0.1492	0.1525	0.152	0.1589	0.1542	0.152
0.0251	0.031	0.0286	0.0288	0.0284	0.0272	0.0279	0.0253	0.0245
0.0241	0.0236	0.022	0.0223	0.0225	0.0223	0.0228	0.0225	0.0223
0.009	0.0056	0.0047	0.0045	0.0045	0.0045	0.0045	0.0048	0.0046
0.0085	0.0049	0.004	0.004	0.0041	0.004	0.004	0.0043	0.0046
0.0007	0.0026	0.0024	0.0023	0.0022	0.0021	0.002	0.0016	0.0015
0.0007	0.0008	0.001	0.0013	0.0014	0.0016	0.0018	0.0019	0.0019
0.0096	0.0063	0.0053	0.0053	0.0053	0.0056	0.0055	0.0057	0.0055
0.01	0.0107	0.0097	0.0093	0.0094	0.0095	0.0095	0.0087	0.0086
0.0027	0.0039	0.0041	0.0046	0.0051	0.0055	0.0062	0.0062	0.0064
0.0118	0.0143	0.0129	0.0127	0.0127	0.0122	0.0122	0.0114	0.0117
0.1104	0.0771	0.067	0.0667	0.0656	0.0655	0.0625	0.0609	0.0604
0.1855	0.1129	0.0993	0.093	0.0953	0.0993	0.1025	0.109	0.1102
0.0309	0.0616	0.065	0.0694	0.0727	0.0734	0.0779	0.0747	0.0731
0.1112	0.1706	0.1687	0.1682	0.1695	0.1655	0.1702	0.1586	0.154
0.1389	0.1886	0.1817	0.1863	0.1881	0.1773	0.1771	0.162	0.1577
0.0926	0.1528	0.1464	0.1518	0.1505	0.1405	0.1431	0.1293	0.1265
0.0879	0.2111	0.1938	0.206	0.1995	0.1784	0.1707	0.1379	0.1332
0.1636	0.3269	0.2986	0.3083	0.3035	0.2731	0.2715	0.2431	0.2239
0.1729	0.3251	0.3028	0.2944	0.2887	0.2583	0.258	0.2231	0.2139
0.1246	0.2687	0.2608	0.2531	0.2547	0.2345	0.2297	0.2008	0.1924
0.1023	0.2027	0.1995	0.1987	0.1983	0.1872	0.1886	0.17	0.1609
0.2832	0.3798	0.358	0.3381	0.3345	0.2936	0.3032	0.2654	0.2543
0.1887	0.368	0.3368	0.3338	0.3375	0.2934	0.2758	0.2438	0.2389
0.0759	0.2752	0.2751	0.2784	0.2803	0.2361	0.223	0.1964	0.1832
0.1049	0.2782	0.2599	0.2666	0.2718	0.2553	0.244	0.1937	0.1977
0.2983	0.2892	0.2628	0.2473	0.2492	0.2407	0.2252	0.2151	0.2079
0.591	0.3803	0.3035	0.2693	0.2733	0.2712	0.2669	0.251	0.26
0.2796	0.2416	0.2018	0.2046	0.2017	0.1923	0.1904	0.1783	0.1827
0.4847	0.2993	0.2373	0.239	0.2368	0.2366	0.2233	0.2315	0.2142
0.1739	0.1999	0.1911	0.1898	0.1896	0.1869	0.192	0.1798	0.1742
0.0955	0.1983	0.1937	0.1952	0.1889	0.1796	0.1813	0.1611	0.1617
0.1364	0.2202	0.2177	0.2116	0.2103	0.1936	0.1993	0.1802	0.1736
0.0973	0.229	0.2206	0.2245	0.2194	0.2064	0.2156	0.181	0.1823
0.0772	0.2528	0.2357	0.2379	0.2494	0.2174	0.2157	0.1846	0.1915
0.1267	0.295	0.2972	0.2997	0.2941	0.2698	0.257	0.2145	0.2048
0.109	0.2394	0.221	0.2216	0.2255	0.2085	0.2048	0.182	0.1753

0.1669	0.2542	0.2406	0.2347	0.2269	0.2025	0.2051	0.1881	0.1901
0.1176	0.2284	0.2268	0.2271	0.2343	0.2148	0.21	0.1828	0.1773
0.0927	0.2398	0.2223	0.2118	0.2108	0.1914	0.2055	0.1778	0.1805
0.1531	0.3156	0.2796	0.2764	0.2603	0.2327	0.2323	0.213	0.1912
0.1445	0.2511	0.2351	0.2295	0.2372	0.2118	0.2119	0.1825	0.176
0.2209	0.2696	0.2465	0.2335	0.2262	0.1916	0.197	0.173	0.174
0.2583	0.3333	0.2986	0.2962	0.2998	0.2664	0.2641	0.2201	0.2328
0.1941	0.306	0.2859	0.2785	0.268	0.2437	0.2422	0.2079	0.1959
0.0467	0.2277	0.224	0.2292	0.237	0.2038	0.208	0.1723	0.1566
0.08	0.255	0.2313	0.2292	0.2282	0.1988	0.1921	0.151	0.1502
0.145	0.3027	0.2883	0.282	0.2747	0.237	0.2334	0.1963	0.1687
0.2953	0.285	0.2501	0.2467	0.2524	0.246	0.2492	0.2312	0.2246
0.3639	0.2694	0.2305	0.2271	0.2322	0.2295	0.2227	0.2284	0.2235
0.2332	0.2431	0.2155	0.2142	0.206	0.187	0.1865	0.1755	0.1769
0.0146	0.017	0.0165	0.0169	0.0172	0.0171	0.0178	0.0173	0.0169
0.0111	0.0116	0.0102	0.0097	0.0096	0.0095	0.0098	0.0087	0.0087
0	0	0	0	0	0	0	0	0
0	0	0	0	0	0	0	0	0
0.0005	0.0015	0.0015	0.0016	0.0017	0.0016	0.0017	0.0016	0.0016
0.0084	0.0048	0.004	0.004	0.004	0.0042	0.004	0.0038	0.0037
0.0246	0.0144	0.0113	0.0117	0.0121	0.0129	0.0125	0.0135	0.0137
0.0444	0.028	0.022	0.0218	0.0222	0.0218	0.0208	0.021	0.0218
0.0361	0.0233	0.0196	0.0185	0.0183	0.0187	0.0183	0.0188	0.018
0.0399	0.0232	0.0165	0.0143	0.0154	0.0166	0.016	0.0152	0.0159
0.106	0.0689	0.0574	0.055	0.055	0.0586	0.0571	0.0568	0.0579
0.212	0.152	0.134	0.1352	0.1395	0.1433	0.144	0.1473	0.1482
0.1998	0.185	0.1812	0.1861	0.1916	0.1959	0.203	0.2069	0.2063
0.0603	0.1135	0.1261	0.1475	0.158	0.1557	0.1676	0.166	0.1641
0.0569	0.1271	0.1312	0.1387	0.1437	0.1425	0.1514	0.1399	0.1352
0.1334	0.268	0.2563	0.2564	0.2429	0.2179	0.2227	0.1919	0.1999
0.1435	0.3212	0.3084	0.2801	0.27	0.2345	0.2449	0.2	0.1933
0.0804	0.2348	0.2203	0.2295	0.2234	0.2042	0.2016	0.1705	0.1744
0.0723	0.2101	0.2023	0.1984	0.1989	0.1705	0.1751	0.1463	0.1349
0.119	0.2386	0.2339	0.2332	0.2475	0.2312	0.2336	0.2064	0.1952
0.0719	0.2154	0.2212	0.2243	0.2117	0.1778	0.1787	0.1556	0.1522
0.1384	0.2939	0.292	0.2799	0.2686	0.2434	0.2364	0.1913	0.1918
0.1054	0.3066	0.2713	0.2735	0.2762	0.2364	0.2325	0.1878	0.1896
0.1488	0.2945	0.2997	0.2828	0.2726	0.2494	0.2501	0.232	0.2106
0.3549	0.3333	0.2898	0.2945	0.2857	0.2707	0.2702	0.2429	0.2506
0.653	0.4035	0.3254	0.3012	0.2986	0.2982	0.2566	0.2713	0.2726
0.9749	0.585	0.4969	0.453	0.4196	0.4427	0.4539	0.432	0.4319
0.6115	0.3837	0.3107	0.2989	0.2868	0.2695	0.2656	0.2661	0.2492
0.4331	0.2934	0.2405	0.2311	0.2312	0.2457	0.2442	0.2383	0.2346
0.223	0.2656	0.2422	0.2396	0.2321	0.2118	0.2141	0.1979	0.195

0.1293	0.2643	0.236	0.2448	0.2368	0.2095	0.2161	0.187	0.1889
0.1366	0.2787	0.258	0.232	0.238	0.2184	0.2158	0.1729	0.161
0.0869	0.2484	0.2435	0.2541	0.2631	0.2338	0.2393	0.2082	0.2076
0.1213	0.2897	0.2609	0.2811	0.2796	0.2381	0.249	0.197	0.1779
0.1135	0.3007	0.2923	0.2842	0.2698	0.2165	0.2225	0.1959	0.1957
0.154	0.3115	0.2753	0.277	0.2659	0.2374	0.2339	0.1855	0.1697
0.1308	0.3042	0.2847	0.2851	0.2757	0.237	0.2415	0.1909	0.1765
0.1159	0.273	0.2633	0.2417	0.2467	0.2148	0.2134	0.1849	0.1738
0.0887	0.2446	0.2293	0.2071	0.2119	0.1835	0.1907	0.1675	0.1559
0.1998	0.3543	0.3327	0.317	0.3118	0.2824	0.2769	0.2282	0.2197
0.1519	0.3071	0.2842	0.2794	0.298	0.263	0.2652	0.2304	0.2369
0.2038	0.3409	0.3085	0.2872	0.2891	0.2508	0.2501	0.2215	0.2294
0.1249	0.2873	0.275	0.274	0.2719	0.2535	0.2572	0.2157	0.206
0.0664	0.1863	0.1835	0.1851	0.1833	0.1607	0.1649	0.136	0.1411
0.202	0.3021	0.28	0.2733	0.2783	0.2515	0.2467	0.2171	0.211
0.1383	0.2883	0.2679	0.2646	0.2641	0.2386	0.2453	0.2022	0.2125
0.1382	0.282	0.243	0.2514	0.2416	0.2101	0.2095	0.1719	0.1743
0.278	0.3445	0.3095	0.3002	0.2994	0.2649	0.268	0.224	0.2219
0.3121	0.3696	0.3165	0.3185	0.3164	0.2945	0.293	0.2703	0.271
0.2242	0.3087	0.2754	0.2738	0.2598	0.2346	0.2268	0.1977	0.1975
0.0248	0.0293	0.0275	0.027	0.0263	0.0247	0.0242	0.0219	0.0207
0.0562	0.0368	0.0301	0.0277	0.0265	0.0282	0.0291	0.0294	0.0282
0	0	0	0	0	0	0	0	0
0	0	0	0	0	0	0	0	0
0.0169	0.0103	0.0085	0.0083	0.0085	0.0086	0.0087	0.0088	0.0084
0.0097	0.0088	0.0079	0.0073	0.0073	0.0067	0.0064	0.006	0.0062
0.0185	0.0115	0.0096	0.0097	0.0104	0.0107	0.0107	0.0109	0.011
0.0434	0.0259	0.022	0.0205	0.0195	0.0186	0.018	0.018	0.0177
0.0546	0.0343	0.0277	0.0268	0.026	0.027	0.0258	0.0259	0.0264
0.0548	0.0338	0.0282	0.0259	0.0245	0.0266	0.0263	0.0259	0.0265
0.0914	0.0672	0.0608	0.0593	0.0601	0.0592	0.0601	0.0602	0.0609
0.1607	0.1487	0.1439	0.1478	0.1505	0.1529	0.1587	0.1562	0.1557
0.1826	0.2382	0.2313	0.2296	0.2351	0.2258	0.2325	0.2217	0.2147
0.1451	0.1919	0.1878	0.1948	0.199	0.1944	0.2051	0.1973	0.1947
0.1296	0.1814	0.1737	0.1746	0.1763	0.1686	0.1682	0.1567	0.1545
0.1536	0.3059	0.3003	0.3015	0.3021	0.2699	0.2636	0.2376	0.2379
0.1303	0.3149	0.3046	0.2778	0.2704	0.2208	0.2343	0.1962	0.183
0.0975	0.2189	0.209	0.2062	0.2073	0.1824	0.1763	0.1479	0.1524
0.0933	0.2224	0.2072	0.2074	0.2072	0.1837	0.1867	0.1694	0.1645
0.1259	0.2521	0.2353	0.2264	0.2288	0.214	0.2054	0.182	0.1851
0.0987	0.251	0.2446	0.2435	0.2474	0.2237	0.2303	0.2021	0.1875
0.0938	0.2323	0.2245	0.2171	0.2044	0.1754	0.1956	0.1579	0.1579
0.1093	0.2987	0.2954	0.292	0.291	0.2491	0.2516	0.2145	0.2168
0.1974	0.3232	0.3112	0.2992	0.2996	0.2552	0.252	0.2143	0.2251

0.3712	0.292	0.2455	0.2276	0.2356	0.2337	0.2382	0.2342	0.2366
0.556	0.3217	0.2512	0.2378	0.2374	0.2419	0.255	0.262	0.261
0.6827	0.4212	0.318	0.2919	0.3186	0.3456	0.3359	0.3622	0.3689
0.4875	0.325	0.2662	0.2529	0.2414	0.2531	0.2476	0.2667	0.2728
0.3059	0.2029	0.1765	0.1733	0.174	0.1833	0.1843	0.1923	0.186
0.2627	0.2658	0.2374	0.2338	0.2277	0.2192	0.2104	0.1989	0.1984
0.1515	0.2925	0.2683	0.2488	0.2537	0.215	0.2295	0.206	0.2037
0.1267	0.3242	0.3127	0.3174	0.319	0.2816	0.2756	0.2275	0.229
0.1467	0.3088	0.3081	0.3101	0.2996	0.26	0.2727	0.224	0.2264
0.1391	0.2619	0.2391	0.2368	0.2194	0.1961	0.1831	0.1505	0.1571
0.1024	0.2521	0.2419	0.2408	0.2363	0.2241	0.2305	0.2019	0.1926
0.1432	0.3004	0.2875	0.2824	0.2881	0.2499	0.251	0.2175	0.2182
0.2808	0.3781	0.3387	0.3251	0.3241	0.2981	0.2748	0.2387	0.2421
0.1951	0.3489	0.3159	0.312	0.3078	0.2702	0.2604	0.2177	0.2034
0.14	0.3137	0.2878	0.2965	0.2896	0.2736	0.288	0.2428	0.234
0.1693	0.3512	0.3113	0.2988	0.2902	0.2404	0.2566	0.2184	0.2256
0.1027	0.272	0.2601	0.2575	0.2515	0.2366	0.213	0.171	0.1597
0.1443	0.295	0.2752	0.2635	0.2455	0.2166	0.2216	0.1907	0.1884
0.2495	0.3664	0.3385	0.3273	0.3195	0.3018	0.2999	0.2462	0.2314
0.2978	0.3316	0.3042	0.2936	0.2909	0.2664	0.2679	0.2328	0.2265
0.2771	0.3122	0.2814	0.2786	0.2784	0.2788	0.2698	0.2613	0.2546
0.2379	0.3601	0.333	0.3282	0.3171	0.2853	0.2702	0.2361	0.2299
0.174	0.3046	0.2809	0.2715	0.2616	0.2465	0.2384	0.2105	0.2044
0.187	0.3195	0.2931	0.2796	0.2754	0.2368	0.2298	0.1948	0.199
0.2595	0.3891	0.366	0.358	0.3454	0.3248	0.3099	0.253	0.2556
0.1126	0.2111	0.1882	0.1931	0.1978	0.177	0.1755	0.1582	0.1601
0.0153	0.0229	0.0207	0.0194	0.02	0.0187	0.0187	0.0168	0.0161
0.0796	0.0478	0.0379	0.0359	0.0362	0.0359	0.033	0.0312	0.0296
0	0	0	0	0	0	0	0	0
0	0	0	0	0	0	0	0	0
0.0014	0.0016	0.0021	0.0024	0.0025	0.0027	0.0031	0.0035	0.0036
0.0029	0.0069	0.0066	0.0062	0.0062	0.0053	0.0053	0.0044	0.004
0.0005	0.0006	0.0008	0.001	0.0011	0.0013	0.0015	0.0015	0.0016
0.026	0.0151	0.0123	0.0123	0.0122	0.0132	0.0129	0.0127	0.0127
0.0389	0.03	0.025	0.0245	0.024	0.0247	0.0237	0.0222	0.0215
0.0261	0.0157	0.0126	0.0124	0.0118	0.0119	0.0112	0.0114	0.0109
0.0388	0.0422	0.0408	0.0413	0.0422	0.0418	0.0431	0.042	0.0412
0.1073	0.145	0.1441	0.1459	0.1477	0.1436	0.1499	0.1421	0.1385
0.1057	0.2246	0.2193	0.2232	0.221	0.2081	0.206	0.1729	0.1614
0.2877	0.3048	0.2758	0.2813	0.2814	0.2632	0.2598	0.2336	0.2402
0.4674	0.4291	0.3916	0.3833	0.3766	0.356	0.3597	0.3348	0.3109
0.2464	0.3199	0.2903	0.2887	0.2876	0.2755	0.2518	0.2158	0.2137
0.0867	0.2252	0.2164	0.2284	0.227	0.2035	0.2149	0.1921	0.1788
0.0974	0.2141	0.2078	0.209	0.2137	0.193	0.1853	0.1539	0.1527

0.1495	0.2704	0.2645	0.2535	0.2507	0.228	0.2312	0.1995	0.2002
0.0889	0.2221	0.211	0.2169	0.2104	0.1885	0.1888	0.1605	0.1606
0.1186	0.2069	0.1998	0.2039	0.2014	0.1915	0.1927	0.1745	0.178
0.1419	0.2572	0.241	0.2476	0.2366	0.2048	0.2022	0.1826	0.1777
0.1434	0.3239	0.3079	0.3033	0.2915	0.2669	0.2544	0.2014	0.1783
0.1974	0.3513	0.324	0.3103	0.3188	0.2824	0.2787	0.2287	0.2191
0.1203	0.2188	0.2145	0.2167	0.207	0.2004	0.1931	0.1742	0.1734
0.1473	0.178	0.1729	0.1733	0.1747	0.1681	0.1724	0.1611	0.1581
0.2204	0.2344	0.2199	0.2199	0.2238	0.2232	0.2303	0.2234	0.2182
0.1232	0.1473	0.1426	0.1436	0.1474	0.1452	0.151	0.1455	0.1436
0.1819	0.2131	0.1961	0.1951	0.1934	0.1805	0.1838	0.1725	0.1717
0.2725	0.3277	0.2995	0.289	0.2866	0.2598	0.2494	0.2272	0.2178
0.1815	0.3285	0.2902	0.2856	0.3021	0.2573	0.268	0.2311	0.2263
0.1291	0.3388	0.3193	0.3088	0.3068	0.2829	0.2715	0.2274	0.2296
0.1684	0.303	0.2934	0.2769	0.2669	0.2396	0.2467	0.2077	0.2082
0.1332	0.1883	0.1862	0.1895	0.1938	0.1884	0.1928	0.1847	0.1821
0.0791	0.1559	0.1644	0.1719	0.174	0.1687	0.1823	0.1802	0.1749
0.1463	0.2728	0.2688	0.2674	0.2677	0.2416	0.2511	0.2201	0.2184
0.4051	0.4186	0.3871	0.3887	0.3831	0.3622	0.3484	0.3165	0.3166
0.268	0.3682	0.3415	0.3195	0.2934	0.262	0.245	0.208	0.2101
0.1156	0.2706	0.2486	0.2491	0.2556	0.2232	0.2117	0.1824	0.1971
0.1014	0.2446	0.2323	0.2308	0.238	0.2165	0.2165	0.1899	0.1877
0.071	0.2189	0.2161	0.2165	0.2144	0.1965	0.2049	0.1822	0.1773
0.1515	0.2794	0.2613	0.2669	0.2669	0.2502	0.2555	0.228	0.2202
0.3413	0.3751	0.3253	0.3211	0.3206	0.3064	0.2942	0.2583	0.2647
0.4622	0.4194	0.3595	0.3756	0.3773	0.357	0.3449	0.329	0.3137
0.3187	0.3796	0.3365	0.3044	0.2982	0.2753	0.28	0.2524	0.2523
0.221	0.3514	0.3302	0.331	0.3212	0.2792	0.273	0.2257	0.2174
0.13	0.2881	0.2716	0.2581	0.2522	0.2076	0.1993	0.1688	0.1767
0.128	0.2712	0.2554	0.2633	0.2697	0.2302	0.2334	0.1995	0.1957
0.147	0.2519	0.2296	0.2153	0.2117	0.1876	0.1893	0.1687	0.1604
0.0464	0.126	0.1228	0.1237	0.1294	0.1102	0.1168	0.1035	0.0999
0.0073	0.0234	0.0221	0.0211	0.0208	0.0179	0.017	0.0128	0.0131
0.0683	0.0426	0.0358	0.0357	0.0348	0.0367	0.0342	0.0339	0.0318
0	0	0	0	0	0	0	0	0
0	0	0	0	0	0	0	0	0
0.0019	0.0024	0.0029	0.0032	0.0035	0.0036	0.0039	0.0042	0.0043
0.0032	0.0086	0.0081	0.0081	0.0079	0.0068	0.007	0.0058	0.0052
0	0	0	0	0	0	0	0	0
0.0256	0.0153	0.0115	0.0107	0.0114	0.0114	0.0114	0.0113	0.0108
0.036	0.0271	0.0238	0.0228	0.0225	0.0231	0.0225	0.0217	0.0217
0.0149	0.0084	0.0065	0.0063	0.0064	0.0064	0.0064	0.0059	0.006
0.0163	0.0252	0.0237	0.0247	0.0241	0.0226	0.0229	0.0212	0.0209
0.0644	0.1067	0.1069	0.1117	0.1157	0.1108	0.1143	0.105	0.103

0.0571	0.1259	0.1312	0.1352	0.1378	0.1307	0.1356	0.1238	0.1251
0.3218	0.2997	0.2549	0.2513	0.254	0.2331	0.2363	0.2199	0.2171
0.4207	0.4279	0.3763	0.3804	0.3876	0.3668	0.3546	0.314	0.2891
0.2646	0.3081	0.2839	0.2791	0.2827	0.2716	0.2664	0.2422	0.2346
0.078	0.1726	0.1755	0.1738	0.1803	0.1681	0.1735	0.1516	0.1501
0.1154	0.2363	0.2316	0.2289	0.2288	0.2094	0.2147	0.1934	0.1924
0.15	0.223	0.2148	0.2139	0.2197	0.1946	0.2008	0.1796	0.1804
0.0875	0.1581	0.1611	0.1694	0.1752	0.1729	0.1778	0.1711	0.1661
0.0878	0.1753	0.1743	0.1808	0.1822	0.1772	0.1855	0.1663	0.1617
0.1366	0.2924	0.2756	0.2816	0.2804	0.2515	0.2416	0.2162	0.2182
0.2195	0.3919	0.3571	0.344	0.3423	0.3135	0.3158	0.266	0.265
0.1141	0.2864	0.2819	0.262	0.2544	0.2256	0.2265	0.2087	0.2011
0.0813	0.2332	0.218	0.2151	0.2177	0.2025	0.1999	0.1782	0.1631
0.0799	0.2212	0.229	0.2263	0.2189	0.1991	0.2028	0.1811	0.1774
0.0776	0.1989	0.2	0.2007	0.2061	0.1925	0.2048	0.1914	0.189
0.0878	0.1908	0.1871	0.1876	0.1831	0.1731	0.1793	0.1574	0.1457
0.0943	0.2233	0.2204	0.2238	0.2301	0.2026	0.1986	0.1726	0.1734
0.2611	0.3737	0.3538	0.3486	0.3415	0.2927	0.315	0.285	0.2808
0.1602	0.3142	0.2823	0.2854	0.2897	0.2768	0.2677	0.2304	0.2212
0.0997	0.2906	0.2917	0.3061	0.284	0.2451	0.2372	0.1903	0.1927
0.1572	0.2976	0.2784	0.2707	0.2663	0.2354	0.228	0.1892	0.1871
0.115	0.148	0.148	0.1565	0.1638	0.1679	0.1771	0.1705	0.1696
0.0458	0.0684	0.0888	0.1067	0.1187	0.1287	0.146	0.1529	0.1482
0.13	0.2171	0.2128	0.2133	0.2169	0.2024	0.2046	0.1889	0.1871
0.1535	0.3036	0.2823	0.2641	0.2728	0.2456	0.2483	0.2042	0.2143
0.09	0.2244	0.2245	0.2232	0.2184	0.1942	0.1933	0.1562	0.1619
0.1065	0.1827	0.1784	0.1804	0.1819	0.1727	0.1741	0.1546	0.1528
0.1954	0.2426	0.2284	0.222	0.2203	0.1983	0.1978	0.184	0.1832
0.164	0.2714	0.2584	0.2567	0.2514	0.232	0.2345	0.2118	0.208
0.1067	0.2421	0.227	0.2163	0.2172	0.183	0.1833	0.1461	0.1487
0.2304	0.3473	0.3257	0.3075	0.3085	0.2669	0.2556	0.2253	0.2161
0.3274	0.3708	0.3276	0.3051	0.2907	0.2626	0.2659	0.2449	0.2518
0.1768	0.2879	0.2561	0.2494	0.2443	0.2093	0.2156	0.1777	0.1738
0.1216	0.269	0.2455	0.2487	0.233	0.2111	0.2152	0.177	0.1702
0.1256	0.3119	0.3032	0.3004	0.3084	0.2553	0.2517	0.2006	0.1941
0.1641	0.3164	0.2999	0.2913	0.2967	0.2553	0.267	0.2377	0.2163
0.1779	0.2871	0.2714	0.2752	0.2792	0.2399	0.2345	0.1998	0.1929
0.1076	0.1987	0.1913	0.1907	0.1985	0.1722	0.1696	0.1465	0.1486
0.0219	0.0434	0.0364	0.0355	0.0359	0.0303	0.0291	0.0251	0.0252
0.0741	0.0415	0.0329	0.0298	0.0305	0.032	0.0296	0.0312	0.0315
0	0	0	0	0	0	0	0	0
0	0	0	0	0	0	0	0	0
0.01	0.0069	0.0061	0.0063	0.0064	0.0067	0.007	0.0073	0.0074
0.0093	0.0094	0.0086	0.0085	0.0086	0.0079	0.0076	0.0065	0.0066

0.0266	0.0169	0.0142	0.014	0.0139	0.0134	0.0122	0.0126	0.0131
0.042	0.0241	0.0198	0.0181	0.0185	0.0176	0.0149	0.0164	0.0159
0.0352	0.022	0.0172	0.0176	0.0173	0.0197	0.0186	0.0191	0.0184
0.017	0.0108	0.0088	0.0085	0.0086	0.0086	0.0084	0.0083	0.0085
0.027	0.0201	0.0168	0.0166	0.0169	0.0158	0.0144	0.0133	0.0137
0.082	0.0872	0.0804	0.0802	0.08	0.077	0.0763	0.0695	0.0677
0.2206	0.143	0.1269	0.1312	0.1301	0.1355	0.1373	0.1452	0.1439
0.1775	0.1709	0.1534	0.1494	0.1501	0.1412	0.1365	0.1299	0.1236
0.1578	0.2643	0.2353	0.2284	0.2355	0.213	0.2166	0.1806	0.1804
0.138	0.255	0.2349	0.2311	0.2307	0.2039	0.2056	0.1796	0.1751
0.1155	0.2186	0.223	0.2253	0.239	0.2145	0.2078	0.1834	0.1863
0.0899	0.2337	0.2314	0.2249	0.2216	0.1895	0.1906	0.161	0.148
0.0982	0.1971	0.1917	0.196	0.1919	0.1791	0.1873	0.1672	0.1635
0.1191	0.1607	0.1608	0.162	0.1699	0.1709	0.1807	0.1753	0.1749
0.1121	0.2416	0.2308	0.2258	0.2263	0.206	0.2103	0.1932	0.1881
0.1002	0.3022	0.2799	0.2837	0.2883	0.2762	0.2685	0.2003	0.19
0.1192	0.2712	0.2588	0.2585	0.2581	0.2309	0.2284	0.1964	0.1899
0.0745	0.1891	0.1856	0.1832	0.1847	0.167	0.1767	0.1577	0.1505
0.1211	0.234	0.2228	0.2308	0.2302	0.2037	0.1995	0.18	0.1728
0.2332	0.3164	0.2862	0.2855	0.276	0.2559	0.251	0.2239	0.2115
0.1178	0.2398	0.2353	0.236	0.2447	0.2101	0.216	0.1891	0.1897
0.1673	0.2986	0.2729	0.2693	0.2641	0.2337	0.2269	0.1957	0.1869
0.0726	0.2264	0.2124	0.2125	0.2077	0.2042	0.2065	0.1722	0.171
0.173	0.3155	0.305	0.3068	0.3033	0.2747	0.2795	0.2351	0.2152
0.1443	0.2936	0.2779	0.2641	0.2699	0.2283	0.2311	0.1851	0.1882
0.0885	0.2632	0.2454	0.2319	0.2418	0.2106	0.2062	0.1771	0.1834
0.1088	0.2866	0.2611	0.2543	0.2524	0.2249	0.221	0.1766	0.1788
0.1066	0.1848	0.1807	0.187	0.1904	0.1831	0.1936	0.1769	0.174
0.0729	0.1048	0.1121	0.1297	0.1407	0.1476	0.1646	0.1638	0.1589
0.0844	0.1509	0.1486	0.1518	0.1553	0.1496	0.1541	0.1363	0.136
0.0895	0.2438	0.2424	0.236	0.2291	0.2075	0.2197	0.1865	0.1909
0.0614	0.2002	0.2023	0.2117	0.2019	0.175	0.1746	0.16	0.1535
0.1185	0.2162	0.2009	0.2012	0.2082	0.1912	0.1979	0.177	0.1729
0.194	0.3013	0.2739	0.2856	0.2793	0.2451	0.2425	0.2056	0.2087
0.1633	0.3216	0.3077	0.2881	0.2958	0.2629	0.2518	0.2199	0.2115
0.1033	0.2756	0.2796	0.27	0.2824	0.2459	0.2397	0.1944	0.185
0.0624	0.2309	0.2348	0.2379	0.2391	0.2068	0.2093	0.1889	0.194
0.1041	0.244	0.2223	0.2168	0.2131	0.1846	0.1746	0.1537	0.1382
0.1123	0.2229	0.2204	0.2139	0.206	0.1806	0.1743	0.1395	0.1317
0.0645	0.1664	0.1662	0.1671	0.175	0.1567	0.164	0.1436	0.1385
0.1319	0.2601	0.2448	0.2421	0.249	0.2312	0.2379	0.2057	0.2011
0.1726	0.296	0.2849	0.2733	0.2707	0.2415	0.2379	0.2181	0.2123
0.2205	0.3715	0.3463	0.3325	0.34	0.3009	0.2863	0.2551	0.2653
0.1816	0.298	0.2717	0.2578	0.2635	0.2464	0.2553	0.227	0.2228

0.056	0.0526	0.0448	0.0438	0.0431	0.0411	0.0406	0.0373	0.0395
0.1046	0.0618	0.0501	0.0457	0.0446	0.0439	0.0433	0.0452	0.0464
0	0	0	0	0	0	0	0	0
0	0	0	0	0	0	0	0	0
0.0018	0.0037	0.004	0.0042	0.0043	0.0044	0.0046	0.0042	0.0041
0.0088	0.0055	0.0046	0.0042	0.0043	0.0046	0.0047	0.0045	0.0044
0.0367	0.022	0.0183	0.0173	0.017	0.0183	0.0176	0.0174	0.0175
0.0449	0.0277	0.0224	0.0221	0.0209	0.0205	0.0195	0.021	0.0197
0.018	0.0105	0.0086	0.0086	0.0089	0.0088	0.0082	0.0079	0.0078
0.0018	0.002	0.0026	0.0031	0.0032	0.0037	0.004	0.0044	0.0046
0.0261	0.0166	0.014	0.0123	0.013	0.0133	0.0126	0.0137	0.0141
0.0902	0.0603	0.0491	0.0489	0.0469	0.0443	0.0423	0.0444	0.0434
0.2794	0.1934	0.1661	0.1598	0.1543	0.1535	0.1567	0.1474	0.1399
0.114	0.1501	0.1443	0.1503	0.139	0.1244	0.1277	0.1124	0.1129
0.0677	0.2141	0.2066	0.206	0.1962	0.1816	0.1826	0.166	0.165
0.0853	0.25	0.2442	0.2427	0.2515	0.2231	0.2407	0.1985	0.1998
0.1085	0.2645	0.2654	0.2628	0.2559	0.2275	0.2401	0.2084	0.2088
0.1196	0.2928	0.2785	0.2592	0.2798	0.2477	0.2435	0.2012	0.1977
0.0905	0.2382	0.2238	0.2383	0.2371	0.2093	0.2104	0.1905	0.186
0.1147	0.2007	0.1935	0.1917	0.1875	0.1809	0.1866	0.1686	0.167
0.1809	0.318	0.2873	0.2829	0.2847	0.249	0.2547	0.2129	0.2102
0.0961	0.2668	0.2411	0.2214	0.2336	0.205	0.2026	0.1674	0.1668
0.1332	0.228	0.2161	0.2105	0.2087	0.1925	0.1924	0.1748	0.1767
0.0596	0.1253	0.1321	0.1434	0.148	0.1433	0.1509	0.1384	0.1416
0.1539	0.2013	0.1946	0.196	0.2004	0.1919	0.1982	0.1875	0.1861
0.3239	0.3497	0.319	0.3148	0.3136	0.2927	0.2895	0.2593	0.2519
0.1616	0.3221	0.3005	0.3043	0.3023	0.2695	0.2513	0.2072	0.2155
0.1063	0.2943	0.268	0.2755	0.2628	0.2289	0.2477	0.1943	0.1916
0.1362	0.293	0.2857	0.3075	0.2897	0.2463	0.2502	0.2105	0.2145
0.1254	0.3118	0.2981	0.2824	0.2842	0.2492	0.2515	0.1954	0.1959
0.1275	0.2813	0.2636	0.2648	0.2555	0.2315	0.2293	0.1802	0.1855
0.1561	0.3056	0.2907	0.2914	0.2953	0.2602	0.2573	0.2125	0.207
0.0964	0.2803	0.2547	0.244	0.2338	0.2072	0.1981	0.1668	0.1676
0.1245	0.2887	0.2802	0.2788	0.2568	0.2251	0.2152	0.1851	0.1746
0.1242	0.2325	0.2267	0.229	0.231	0.2089	0.2053	0.1864	0.1828
0.1036	0.2296	0.2255	0.2341	0.2278	0.2086	0.2141	0.1903	0.1822
0.1526	0.2524	0.2354	0.2314	0.2242	0.2061	0.2161	0.2017	0.1996
0.0987	0.2147	0.1969	0.1925	0.1985	0.1808	0.1868	0.1697	0.1586
0.1291	0.2465	0.2268	0.2311	0.2264	0.2064	0.208	0.1837	0.185
0.1401	0.2928	0.2895	0.2718	0.2621	0.2294	0.232	0.201	0.1963
0.0709	0.2512	0.2483	0.2599	0.2564	0.2074	0.2019	0.1447	0.1586
0.1339	0.2991	0.2618	0.2592	0.2501	0.2163	0.2105	0.1668	0.1493
0.1443	0.3045	0.2882	0.2774	0.2799	0.2509	0.2539	0.2086	0.2059
0.1066	0.2622	0.2529	0.2474	0.2399	0.204	0.1983	0.1648	0.1544

0.1024	0.2334	0.2251	0.2169	0.2163	0.1918	0.1888	0.1591	0.1664
0.1062	0.2049	0.1988	0.1912	0.1927	0.1819	0.1816	0.1581	0.1597
0.0922	0.2073	0.1961	0.196	0.1905	0.1676	0.1631	0.1443	0.1394
0.1842	0.3177	0.2995	0.2927	0.286	0.2545	0.2486	0.2041	0.2008
0.2142	0.3668	0.3465	0.339	0.3418	0.3074	0.3123	0.2787	0.2957
0.143	0.2725	0.2629	0.2636	0.2488	0.2255	0.2204	0.1778	0.1897
0.0547	0.0364	0.0311	0.0313	0.0312	0.0299	0.0288	0.0291	0.0296
0.0987	0.0609	0.0498	0.0485	0.0474	0.0482	0.0428	0.0437	0.0415
0	0	0	0	0	0	0	0	0
0	0	0	0	0	0	0	0	0
0.0017	0.0043	0.0042	0.004	0.0041	0.0037	0.0037	0.0032	0.0031
0	0	0	0	0	0	0	0	0
0.0106	0.0076	0.0072	0.0075	0.0079	0.0085	0.0087	0.009	0.0089
0.0193	0.0152	0.0135	0.0128	0.0126	0.0119	0.0118	0.0111	0.0108
0	0	0	0	0	0	0	0	0
0.0046	0.0089	0.0097	0.0104	0.0109	0.0106	0.0114	0.0106	0.0105
0.0032	0.0083	0.0083	0.0081	0.0081	0.0071	0.0068	0.006	0.0056
0.0741	0.0429	0.0356	0.0325	0.0345	0.0351	0.0378	0.0359	0.0371
0.1335	0.089	0.0763	0.0707	0.074	0.0747	0.0727	0.0711	0.072
0.1492	0.1899	0.1752	0.1738	0.1754	0.1621	0.1596	0.1512	0.1504
0.1389	0.2623	0.2535	0.2553	0.26	0.2446	0.2442	0.2124	0.2067
0.0827	0.2413	0.2339	0.2375	0.2366	0.2079	0.2057	0.1811	0.179
0.0993	0.2641	0.2691	0.2655	0.2557	0.2331	0.2253	0.1931	0.1936
0.0991	0.2864	0.2794	0.2774	0.2799	0.2524	0.2465	0.2166	0.2198
0.133	0.291	0.2754	0.2628	0.2531	0.2296	0.2285	0.2019	0.1914
0.0757	0.1759	0.1711	0.1822	0.1815	0.1706	0.1714	0.1574	0.1518
0.0908	0.1948	0.1921	0.1897	0.1904	0.1816	0.1868	0.1632	0.1616
0.0629	0.149	0.1429	0.1433	0.1453	0.131	0.1311	0.1116	0.1058
0.1774	0.2069	0.1976	0.1929	0.1929	0.1866	0.1897	0.1767	0.1697
0.0811	0.146	0.1476	0.1562	0.1559	0.1549	0.1638	0.1535	0.151
0.1613	0.1927	0.1871	0.1867	0.1878	0.1834	0.1909	0.1824	0.1821
0.3291	0.3543	0.3256	0.3117	0.315	0.2875	0.2809	0.2518	0.2527
0.205	0.3591	0.3458	0.3442	0.3267	0.2908	0.2682	0.2233	0.2119
0.1377	0.3097	0.2877	0.2726	0.255	0.2287	0.2031	0.1512	0.1323
0.1911	0.3212	0.299	0.2854	0.287	0.2522	0.251	0.2181	0.2222
0.0631	0.1998	0.2019	0.1969	0.1951	0.1741	0.1834	0.1589	0.161
0.0919	0.2144	0.2035	0.2034	0.2058	0.1816	0.1821	0.1597	0.1615
0.2134	0.328	0.2984	0.2874	0.2858	0.2611	0.268	0.2447	0.2427
0.1323	0.313	0.3012	0.3041	0.2979	0.2517	0.237	0.1902	0.1727
0.1058	0.2819	0.2649	0.2482	0.257	0.2229	0.2028	0.174	0.1714
0.1684	0.3327	0.3078	0.2945	0.2975	0.2616	0.2581	0.2224	0.2047
0.1098	0.2679	0.2517	0.2536	0.2402	0.2116	0.1949	0.1702	0.1672
0.1147	0.2691	0.248	0.2401	0.2418	0.2067	0.2081	0.17	0.1598
0.0906	0.1896	0.191	0.1968	0.195	0.1869	0.1937	0.178	0.177

0.0906	0.128	0.1385	0.1463	0.1505	0.1504	0.1637	0.1612	0.1632
0.0803	0.159	0.1639	0.1671	0.1739	0.1666	0.1745	0.1665	0.1608
0.0841	0.2285	0.2211	0.2193	0.215	0.1828	0.1846	0.1493	0.1506
0.1215	0.2751	0.2595	0.2766	0.2827	0.2427	0.2451	0.2153	0.1955
0.2196	0.346	0.3199	0.3174	0.312	0.2817	0.2619	0.2091	0.1869
0.1186	0.2784	0.2491	0.2576	0.2648	0.222	0.2309	0.1958	0.1867
0.1808	0.3297	0.3079	0.2916	0.2794	0.2464	0.2537	0.1865	0.1764
0.1125	0.2481	0.2289	0.2266	0.2248	0.202	0.1868	0.1624	0.1613
0.0947	0.256	0.2406	0.2516	0.2581	0.2221	0.2331	0.1933	0.1991
0.101	0.2835	0.271	0.2656	0.2516	0.2203	0.2104	0.1838	0.1828
0.1643	0.3363	0.3181	0.3145	0.315	0.2775	0.2609	0.2099	0.1917
0.0816	0.2106	0.207	0.2034	0.1994	0.1837	0.1879	0.1575	0.1522
0.0124	0.0156	0.0142	0.0136	0.0131	0.0118	0.0115	0.0099	0.01
0.0777	0.0573	0.0467	0.0466	0.0466	0.0474	0.0463	0.0451	0.0456
0	0	0	0	0	0	0	0	0
0	0	0	0	0	0	0	0	0
0	0	0	0	0	0	0	0	0
0	0	0	0	0	0	0	0	0
0	0	0	0	0	0	0	0	0
0	0	0	0	0	0	0	0	0
0	0	0	0	0	0	0	0	0
0	0	0	0	0	0	0	0	0
0	0	0	0	0	0	0	0	0
0	0	0	0	0	0	0	0	0
0.0006	0.0009	0.0011	0.0013	0.0013	0.0014	0.0015	0.0015	0.0015
0.0252	0.0542	0.0523	0.0521	0.0528	0.049	0.0488	0.0425	0.0424
0.0892	0.1554	0.151	0.1508	0.1497	0.1387	0.1394	0.1241	0.1187
0.4809	0.4436	0.4019	0.3798	0.3782	0.3698	0.3596	0.3382	0.3475
0.2882	0.3387	0.3011	0.2934	0.2918	0.2637	0.2583	0.2326	0.2342
0.0886	0.2104	0.2	0.1905	0.1915	0.1592	0.1638	0.1344	0.1328
0.2625	0.2909	0.2568	0.2491	0.258	0.2412	0.2335	0.2004	0.1992
0.1064	0.2232	0.2201	0.2184	0.2167	0.1963	0.2022	0.1729	0.1658
0.0736	0.1685	0.1707	0.179	0.183	0.175	0.1762	0.1544	0.1568
0.0601	0.1286	0.1285	0.1312	0.1424	0.1371	0.1459	0.1332	0.1321
0.0694	0.118	0.1251	0.1297	0.1378	0.1374	0.1507	0.1435	0.1402
0.0932	0.1387	0.1422	0.1458	0.1513	0.1473	0.1561	0.1571	0.1544
0.142	0.2114	0.2053	0.1964	0.196	0.1776	0.183	0.1584	0.1547
0.2094	0.3664	0.3218	0.3039	0.295	0.2503	0.2593	0.2162	0.2225
0.1531	0.311	0.3067	0.3038	0.3199	0.29	0.2796	0.2407	0.2407
0.1432	0.216	0.2107	0.2069	0.2052	0.1968	0.2004	0.1835	0.1772
0.148	0.1406	0.1353	0.1379	0.1409	0.1429	0.1489	0.1474	0.1447
0.1016	0.0996	0.1004	0.108	0.1173	0.1278	0.1321	0.1418	0.1439
0.1307	0.2003	0.19	0.1899	0.1887	0.1802	0.1772	0.1622	0.1556
0.1778	0.3206	0.2935	0.2825	0.2882	0.2589	0.2583	0.2159	0.2183

0.1236	0.303	0.3047	0.2998	0.3066	0.2681	0.2653	0.2439	0.2468
0.1393	0.307	0.2888	0.2874	0.2867	0.2444	0.2617	0.2072	0.1916
0.1477	0.3234	0.2992	0.2752	0.2679	0.2305	0.2384	0.2098	0.2008
0.1584	0.3377	0.2931	0.2799	0.2716	0.2433	0.248	0.21	0.2104
0.1304	0.2929	0.2724	0.2683	0.2672	0.2316	0.236	0.214	0.2154
0.101	0.1937	0.1968	0.2007	0.2015	0.1899	0.1964	0.1807	0.1815
0.1109	0.1283	0.1421	0.1514	0.1623	0.1695	0.1776	0.1831	0.1873
0.1042	0.1542	0.1555	0.1578	0.1633	0.1595	0.1701	0.1584	0.1602
0.0789	0.2276	0.2237	0.2169	0.2202	0.1956	0.2045	0.1719	0.1617
0.1237	0.3092	0.2904	0.2825	0.2852	0.2414	0.2274	0.1718	0.1752
0.1692	0.3035	0.2962	0.2957	0.2808	0.2464	0.2433	0.202	0.2073
0.0714	0.233	0.2214	0.2141	0.2215	0.1886	0.1823	0.143	0.1245
0.1995	0.2934	0.2669	0.2636	0.2756	0.2522	0.2394	0.2132	0.2027
0.0961	0.2425	0.2245	0.2244	0.2182	0.1893	0.1853	0.1521	0.145
0.1161	0.2827	0.2745	0.2653	0.26	0.2366	0.2484	0.2195	0.2051
0.2462	0.4077	0.3863	0.3709	0.3652	0.3198	0.3367	0.2841	0.2936
0.2232	0.3264	0.2958	0.2853	0.2902	0.2624	0.2455	0.2038	0.199
0.0442	0.0633	0.0592	0.057	0.0568	0.052	0.0498	0.0434	0.0407
0.2236	0.1315	0.1084	0.1038	0.1107	0.1114	0.1038	0.1143	0.1019
0.5872	0.3701	0.2988	0.2806	0.2941	0.3095	0.2786	0.2774	0.2614
0.1063	0.0643	0.0536	0.0515	0.0488	0.0496	0.0496	0.0535	0.053
0.0558	0.0349	0.0271	0.026	0.0249	0.0255	0.024	0.0246	0.024
0	0	0	0	0	0	0	0	0
0	0	0	0	0	0	0	0	0
0	0	0	0	0	0	0	0	0
0	0	0	0	0	0	0	0	0
0	0	0	0	0	0	0	0	0
0	0	0	0	0	0	0	0	0
0	0	0	0	0	0	0	0	0
0	0	0	0	0	0	0	0	0
0	0	0	0	0	0	0	0	0
0.046	0.029	0.0234	0.0236	0.0234	0.0228	0.0226	0.0236	0.024
0.173	0.1188	0.1012	0.0975	0.0986	0.0991	0.0888	0.0852	0.0876
0.8627	0.5762	0.4585	0.4622	0.4659	0.4714	0.4645	0.4607	0.4562
0.8992	0.6089	0.5123	0.4989	0.495	0.477	0.4463	0.4574	0.4266
0.2925	0.2897	0.2567	0.2515	0.2495	0.2346	0.2289	0.2112	0.2063
0.1617	0.2743	0.2507	0.251	0.2528	0.2239	0.2234	0.2018	0.2025
0.0844	0.2547	0.2493	0.2641	0.2672	0.2369	0.2276	0.1919	0.188
0.1618	0.2968	0.2758	0.2729	0.2748	0.2621	0.2661	0.2306	0.217
0.1729	0.2454	0.239	0.2467	0.249	0.2362	0.2413	0.2249	0.2202
0.1077	0.1523	0.1552	0.1575	0.1648	0.1651	0.1763	0.1737	0.1737
0.0595	0.1578	0.1628	0.1672	0.166	0.1548	0.1527	0.136	0.1319
0.118	0.2872	0.2527	0.2524	0.2535	0.2205	0.22	0.1873	0.1964
0.1245	0.2727	0.25	0.245	0.2547	0.2228	0.2321	0.1851	0.1828

0.0654	0.1747	0.1608	0.1618	0.1609	0.1492	0.1515	0.1301	0.1193
0.0819	0.1739	0.1797	0.1822	0.1834	0.1766	0.1846	0.1716	0.1663
0.1225	0.1461	0.1508	0.1597	0.1657	0.17	0.1809	0.1814	0.1793
0.0934	0.0935	0.1131	0.1259	0.1266	0.1482	0.1629	0.1731	0.1752
0.1226	0.1894	0.1842	0.1861	0.1967	0.1828	0.1914	0.1747	0.1733
0.1273	0.2821	0.2641	0.2518	0.2415	0.2092	0.2116	0.159	0.1593
0.1019	0.2977	0.2992	0.2965	0.2782	0.2513	0.2652	0.2152	0.2033
0.1646	0.3412	0.3265	0.3206	0.325	0.2844	0.2624	0.2268	0.2227
0.1103	0.2825	0.28	0.281	0.2898	0.2562	0.24	0.1861	0.1835
0.1175	0.3128	0.2897	0.2908	0.2846	0.248	0.2466	0.2112	0.1963
0.1373	0.2863	0.268	0.2447	0.2437	0.2092	0.2008	0.1576	0.1533
0.0835	0.1809	0.1791	0.1764	0.1761	0.1651	0.1688	0.1567	0.162
0.1146	0.1806	0.1812	0.1862	0.1918	0.1881	0.1932	0.1801	0.1778
0.1267	0.2258	0.2187	0.2202	0.2232	0.2081	0.2126	0.1888	0.1923
0.1287	0.2992	0.3001	0.2912	0.2769	0.2433	0.2427	0.2042	0.2024
0.1634	0.3406	0.3257	0.2876	0.2936	0.2503	0.2622	0.2287	0.2216
0.1366	0.3002	0.2732	0.2697	0.2774	0.2467	0.2453	0.2055	0.2039
0.1467	0.3454	0.3409	0.3383	0.3117	0.2764	0.2765	0.2349	0.2437
0.0969	0.2542	0.2422	0.2428	0.2373	0.2041	0.197	0.1571	0.138
0.192	0.2909	0.2648	0.2602	0.2541	0.2312	0.2346	0.212	0.2201
0.3157	0.3357	0.2946	0.2762	0.2701	0.2504	0.2367	0.2156	0.205
0.2477	0.3884	0.3812	0.3679	0.3492	0.3078	0.3184	0.3011	0.2848
0.1199	0.2968	0.2723	0.2593	0.2655	0.2282	0.2314	0.1833	0.174
0.1396	0.1099	0.099	0.1019	0.1029	0.0939	0.0888	0.0808	0.0796
0.607	0.3645	0.3176	0.3031	0.2879	0.289	0.2902	0.3002	0.2949
1.0665	0.6279	0.512	0.5228	0.5259	0.5101	0.5089	0.5384	0.5316
0.0718	0.0409	0.0337	0.0312	0.0313	0.0325	0.0296	0.0319	0.0341
0.0324	0.0197	0.0155	0.0143	0.0149	0.0149	0.0136	0.014	0.0144
0	0	0	0	0	0	0	0	0
0	0	0	0	0	0	0	0	0
0	0	0	0	0	0	0	0	0
0	0	0	0	0	0	0	0	0
0	0	0	0	0	0	0	0	0
0	0	0	0	0	0	0	0	0
0	0	0	0	0	0	0	0	0
0	0	0	0	0	0	0	0	0
0.0464	0.0377	0.0312	0.0301	0.0287	0.0281	0.0278	0.028	0.0285
0.0987	0.0583	0.0463	0.0446	0.0472	0.0517	0.0467	0.0465	0.045
0.0432	0.0267	0.0229	0.0227	0.0212	0.0207	0.0193	0.0196	0.0196
0.6202	0.3848	0.3224	0.3209	0.3172	0.3096	0.3054	0.2971	0.276
0.8682	0.5482	0.4283	0.4263	0.428	0.4559	0.4295	0.4206	0.4179
0.2868	0.27	0.2388	0.229	0.2206	0.2098	0.2069	0.1832	0.1778
0.1156	0.2734	0.2588	0.2636	0.2665	0.2321	0.236	0.2009	0.1866
0.2095	0.3405	0.3208	0.3234	0.3183	0.2955	0.2835	0.2402	0.2326

0.0698	0.2195	0.2172	0.215	0.2112	0.1953	0.1911	0.1705	0.1636
0.1271	0.2712	0.2556	0.2501	0.2536	0.2155	0.2141	0.1813	0.1776
0.1402	0.3119	0.3034	0.283	0.2861	0.2663	0.2619	0.2218	0.2156
0.1346	0.3041	0.2824	0.2867	0.2745	0.2671	0.2637	0.2209	0.2211
0.1904	0.2775	0.2699	0.2519	0.2395	0.2105	0.2119	0.1887	0.1946
0.1716	0.1676	0.1552	0.1567	0.1596	0.1606	0.1645	0.1605	0.1604
0.1229	0.1003	0.1053	0.114	0.1198	0.1303	0.1411	0.146	0.1472
0.1431	0.1747	0.1794	0.1855	0.1935	0.1962	0.2055	0.2028	0.201
0.0862	0.1325	0.1451	0.1572	0.1698	0.1736	0.1874	0.1839	0.1815
0.0899	0.1293	0.1388	0.144	0.1474	0.1491	0.1634	0.1709	0.1686
0.0956	0.208	0.2065	0.2115	0.2112	0.1927	0.1963	0.1742	0.1642
0.0918	0.2708	0.2508	0.2419	0.2504	0.22	0.2294	0.2084	0.2134
0.0822	0.2657	0.2574	0.2459	0.2377	0.2121	0.2063	0.1574	0.1551
0.1763	0.3224	0.3002	0.2802	0.2842	0.2542	0.2516	0.2154	0.2256
0.2508	0.3648	0.3434	0.3382	0.3311	0.304	0.2994	0.2628	0.2503
0.1086	0.3104	0.2971	0.2894	0.2928	0.2448	0.2362	0.218	0.2087
0.1147	0.2836	0.2522	0.2519	0.2492	0.2109	0.2057	0.1768	0.1657
0.1049	0.2066	0.2017	0.2037	0.2046	0.1917	0.193	0.1786	0.1738
0.1134	0.2197	0.2052	0.2074	0.2119	0.1889	0.1898	0.1665	0.161
0.1663	0.2951	0.2744	0.2758	0.2772	0.2692	0.2712	0.2383	0.2462
0.1075	0.2734	0.2536	0.2548	0.2579	0.2363	0.2361	0.1845	0.1834
0.1054	0.2771	0.2686	0.2638	0.2522	0.2243	0.23	0.1847	0.1818
0.0967	0.2628	0.2405	0.2293	0.2324	0.1902	0.1928	0.1489	0.1449
0.1321	0.3017	0.281	0.2715	0.2699	0.2534	0.2468	0.199	0.1943
0.1032	0.2749	0.2597	0.2587	0.2492	0.2193	0.2172	0.1895	0.1806
0.2434	0.3192	0.3012	0.2998	0.2991	0.2849	0.2729	0.2419	0.2341
0.3275	0.3715	0.3283	0.3205	0.3163	0.2996	0.2849	0.2572	0.2586
0.129	0.3293	0.3016	0.2913	0.2916	0.2432	0.2393	0.203	0.2061
0.0796	0.2236	0.2183	0.2136	0.2116	0.1837	0.186	0.1528	0.146
0.2035	0.1467	0.1207	0.119	0.1177	0.1188	0.1189	0.1122	0.1113
0.3387	0.2003	0.1488	0.1443	0.1516	0.1654	0.1703	0.176	0.1701
0.3185	0.1917	0.1465	0.1357	0.1312	0.1186	0.1082	0.1149	0.1201
0.0521	0.0328	0.0251	0.0246	0.0246	0.0256	0.0262	0.0264	0.0253
0.0175	0.0111	0.0089	0.0086	0.0089	0.0096	0.0092	0.009	0.0091
0	0	0	0	0	0	0	0	0
0	0	0	0	0	0	0	0	0
0	0	0	0	0	0	0	0	0
0	0	0	0	0	0	0	0	0
0	0	0	0	0	0	0	0	0
0	0	0	0	0	0	0	0	0
0	0	0	0	0	0	0	0	0
0	0	0	0	0	0	0	0	0
0.0037	0.007	0.0069	0.0072	0.0074	0.0071	0.0072	0.0067	0.0065
0.1103	0.065	0.0512	0.0476	0.0517	0.056	0.0577	0.0565	0.0602

0.0055	0.0161	0.0147	0.0147	0.0143	0.0124	0.0121	0.0095	0.0088
0.0707	0.0486	0.0396	0.0361	0.0365	0.0389	0.039	0.0401	0.04
0.3412	0.229	0.1914	0.1786	0.1809	0.1819	0.1757	0.1789	0.1735
0.1303	0.1874	0.1705	0.171	0.1708	0.1593	0.1625	0.1491	0.1466
0.0962	0.2222	0.2145	0.2133	0.2091	0.1905	0.2007	0.1791	0.1704
0.1129	0.1971	0.1931	0.2003	0.2048	0.1887	0.1924	0.1769	0.1701
0.0799	0.1906	0.1932	0.1895	0.1927	0.1819	0.1858	0.1707	0.1672
0.0917	0.2422	0.2321	0.2275	0.2308	0.2028	0.1957	0.1665	0.1557
0.099	0.2996	0.2995	0.2708	0.2656	0.2305	0.2276	0.1847	0.1815
0.2297	0.3369	0.3074	0.2988	0.2974	0.2713	0.272	0.2473	0.2432
0.288	0.2366	0.212	0.2126	0.2178	0.213	0.2109	0.2095	0.2053
0.2263	0.1607	0.1527	0.1572	0.1653	0.178	0.1893	0.1971	0.1934
0.132	0.1164	0.1292	0.1348	0.1478	0.1624	0.1804	0.1922	0.195
0.1469	0.147	0.1747	0.1842	0.1896	0.2126	0.2291	0.2472	0.242
0.0627	0.0844	0.112	0.1313	0.146	0.1552	0.1745	0.1729	0.1699
0.0868	0.1564	0.1611	0.168	0.1683	0.1695	0.1797	0.1716	0.1688
0.1544	0.287	0.2755	0.2656	0.27	0.241	0.2357	0.1951	0.1949
0.0944	0.257	0.2649	0.2555	0.2536	0.2337	0.2179	0.1917	0.1941
0.1482	0.311	0.3039	0.308	0.3114	0.2937	0.2938	0.2505	0.2477
0.2962	0.3971	0.3859	0.3575	0.3612	0.3063	0.3019	0.2651	0.2719
0.3394	0.4082	0.3633	0.3667	0.3685	0.3391	0.3235	0.278	0.2803
0.2024	0.373	0.3478	0.3299	0.3178	0.2841	0.2772	0.2492	0.2393
0.1199	0.3129	0.3019	0.3052	0.3044	0.2787	0.2596	0.208	0.1853
0.0973	0.2383	0.2259	0.2226	0.2131	0.1874	0.1976	0.1674	0.1779
0.1191	0.2786	0.2771	0.2806	0.2746	0.2454	0.2495	0.2117	0.2093
0.1379	0.3133	0.2961	0.2941	0.2837	0.2514	0.2457	0.1954	0.1977
0.1384	0.3237	0.2997	0.2904	0.2807	0.2524	0.2447	0.1986	0.1962
0.095	0.2796	0.2788	0.2626	0.2634	0.222	0.2368	0.1845	0.17
0.094	0.286	0.2828	0.2719	0.2742	0.2424	0.2448	0.2171	0.2316
0.148	0.2657	0.2541	0.2633	0.2577	0.2351	0.2209	0.1973	0.1915
0.1668	0.3107	0.2975	0.2953	0.2795	0.2434	0.2349	0.2143	0.1969
0.1971	0.3018	0.2792	0.2811	0.2848	0.2545	0.2509	0.2151	0.1973
0.2117	0.3743	0.3468	0.3477	0.3259	0.2808	0.2667	0.2394	0.2343
0.09	0.2059	0.2033	0.2084	0.1951	0.165	0.1644	0.1269	0.1294
0.2359	0.1971	0.1795	0.1621	0.1669	0.1619	0.1575	0.147	0.146
0.4525	0.3154	0.2781	0.248	0.2389	0.2228	0.2156	0.2159	0.2045
0.2347	0.153	0.1304	0.1302	0.1296	0.1226	0.1108	0.1105	0.1123
0.0004	0.0005	0.0005	0.0006	0.0007	0.0008	0.0009	0.0009	0.0009
0.018	0.0108	0.009	0.0087	0.0089	0.0091	0.0092	0.0092	0.0093
0	0	0	0	0	0	0	0	0
0	0	0	0	0	0	0	0	0
0	0	0	0	0	0	0	0	0
0	0	0	0	0	0	0	0	0
0	0	0	0	0	0	0	0	0

0	0	0	0	0	0	0	0	0
0	0	0	0	0	0	0	0	0
0	0	0	0	0	0	0	0	0
0	0	0	0	0	0	0	0	0
0.0003	0.0004	0.0005	0.0006	0.0006	0.0007	0.0008	0.0008	0.0008
0.081	0.0512	0.0454	0.0443	0.0466	0.0483	0.0465	0.0471	0.0471
0.0089	0.0055	0.0045	0.0042	0.0042	0.0043	0.0039	0.0041	0.004
0.0038	0.0045	0.0057	0.0076	0.0077	0.0093	0.0105	0.0127	0.0133
0.1052	0.1275	0.1252	0.1281	0.1305	0.1293	0.1338	0.1301	0.1276
0.1138	0.1918	0.1885	0.1852	0.1892	0.1756	0.186	0.172	0.1706
0.1013	0.1848	0.181	0.1825	0.1891	0.1787	0.1843	0.1746	0.1733
0.0627	0.1018	0.1144	0.1233	0.1286	0.1371	0.1638	0.1581	0.1557
0.1829	0.2444	0.2374	0.2382	0.2359	0.2215	0.2226	0.199	0.1971
0.0743	0.1839	0.1788	0.1889	0.1925	0.1781	0.1792	0.1616	0.1623
0.0776	0.1607	0.1687	0.1732	0.1742	0.1651	0.1711	0.1613	0.1633
0.1547	0.1375	0.1363	0.1424	0.1452	0.1508	0.1595	0.1613	0.1596
0.218	0.1559	0.1507	0.1541	0.1626	0.1715	0.1834	0.19	0.1845
0.1177	0.1429	0.1501	0.1555	0.1584	0.1696	0.1806	0.1786	0.1766
0.0586	0.0999	0.1204	0.1319	0.1472	0.1454	0.1529	0.1562	0.1561
0.0848	0.1037	0.1123	0.1355	0.1487	0.1578	0.1877	0.1946	0.1918
0.0836	0.1044	0.1248	0.1423	0.1263	0.146	0.1728	0.1749	0.1626
0.0976	0.1409	0.1509	0.1491	0.1559	0.1639	0.1812	0.1868	0.1806
0.11	0.2235	0.2192	0.2217	0.2267	0.1991	0.2085	0.1831	0.1795
0.1063	0.2799	0.2656	0.2585	0.2511	0.2175	0.2123	0.1756	0.1772
0.1364	0.3109	0.2992	0.2953	0.2932	0.2671	0.2841	0.2518	0.2392
0.3238	0.4089	0.3667	0.3665	0.3779	0.3464	0.3275	0.2951	0.2828
0.3469	0.3878	0.3563	0.354	0.3569	0.3213	0.3169	0.2946	0.2939
0.1645	0.3016	0.269	0.2569	0.2546	0.2302	0.2302	0.1952	0.2004
0.1109	0.2587	0.2448	0.2315	0.2186	0.1888	0.1895	0.1548	0.1542
0.1896	0.3492	0.3137	0.3073	0.2962	0.2692	0.2559	0.2167	0.22
0.1321	0.3009	0.2974	0.2881	0.2837	0.2461	0.2544	0.2085	0.196
0.1022	0.2821	0.2536	0.248	0.2422	0.223	0.214	0.1526	0.1457
0.1279	0.3276	0.3106	0.2981	0.2973	0.2622	0.275	0.2182	0.2175
0.1135	0.3083	0.2771	0.2757	0.2777	0.2378	0.2266	0.1952	0.2053
0.0926	0.2574	0.245	0.2523	0.2505	0.2169	0.2121	0.1813	0.1787
0.1911	0.3226	0.2883	0.2723	0.2604	0.2259	0.2261	0.1771	0.162
0.1053	0.2619	0.251	0.2507	0.2541	0.2168	0.2284	0.1848	0.1841
0.2116	0.3425	0.3088	0.2919	0.2771	0.2532	0.2502	0.2007	0.2082
0.2018	0.2965	0.2726	0.2718	0.2652	0.239	0.2419	0.208	0.2023
0.0691	0.0671	0.0618	0.0606	0.0592	0.054	0.0522	0.0476	0.0472
0.1045	0.0601	0.0467	0.0436	0.0424	0.0472	0.0422	0.0451	0.0432
0.2343	0.1567	0.1272	0.1303	0.1272	0.1281	0.1165	0.1197	0.1154
0.2296	0.1419	0.1101	0.1078	0.1091	0.1066	0.0968	0.1006	0.1047
0.1106	0.0813	0.0705	0.0657	0.0659	0.0589	0.0601	0.0564	0.0524

0	0	0	0	0	0	0	0	0
0	0	0	0	0	0	0	0	0
0	0	0	0	0	0	0	0	0
0	0	0	0	0	0	0	0	0
0	0	0	0	0	0	0	0	0
0	0	0	0	0	0	0	0	0
0	0	0	0	0	0	0	0	0
0	0	0	0	0	0	0	0	0
0	0	0	0	0	0	0	0	0
0	0	0	0	0	0	0	0	0
0.025	0.0139	0.0105	0.0101	0.01	0.0101	0.0097	0.0104	0.0101
0.013	0.0129	0.0124	0.0128	0.0133	0.0136	0.0143	0.0143	0.0142
0.08	0.0497	0.0399	0.0374	0.0376	0.0394	0.0388	0.0383	0.0357
0.1276	0.0904	0.0822	0.0813	0.0822	0.0837	0.0831	0.083	0.0801
0.1034	0.1701	0.1683	0.1736	0.1778	0.1716	0.1733	0.1627	0.1635
0.1144	0.2318	0.2344	0.2371	0.2335	0.2176	0.2302	0.2052	0.1909
0.1218	0.1952	0.1909	0.1976	0.2044	0.1948	0.2029	0.1897	0.1883
0.1222	0.1428	0.1473	0.1563	0.1692	0.1808	0.2036	0.2059	0.1985
0.1172	0.1838	0.1899	0.1963	0.2001	0.1944	0.2024	0.1925	0.1911
0.0966	0.2308	0.2279	0.2233	0.229	0.2099	0.2091	0.1795	0.1743
0.0692	0.1673	0.1581	0.1689	0.1704	0.1589	0.1641	0.1492	0.1508
0.0513	0.0987	0.1048	0.1088	0.1186	0.1213	0.1323	0.1268	0.1252
0.0784	0.138	0.1401	0.1471	0.1497	0.1466	0.1579	0.156	0.1516
0.0731	0.1289	0.141	0.1574	0.1675	0.1684	0.1775	0.1719	0.1662
0.0718	0.0826	0.1047	0.125	0.1396	0.1477	0.1579	0.1614	0.1748
0.1057	0.1019	0.126	0.1491	0.1565	0.1755	0.2039	0.2085	0.2014
0.0882	0.1211	0.1473	0.1564	0.1624	0.1683	0.1864	0.1897	0.193
0.1021	0.1003	0.1235	0.1302	0.1374	0.1537	0.1628	0.1828	0.1778
0.1126	0.164	0.1707	0.1786	0.1849	0.1863	0.1981	0.1909	0.1861
0.0814	0.261	0.2513	0.2527	0.2419	0.2108	0.2176	0.1828	0.1788
0.0974	0.3043	0.2707	0.2855	0.2828	0.2458	0.2582	0.2145	0.193
0.2059	0.3444	0.314	0.3072	0.3039	0.2754	0.2555	0.2187	0.2025
0.2559	0.388	0.3498	0.3292	0.3353	0.2985	0.2946	0.2784	0.2778
0.1043	0.2415	0.2336	0.2186	0.2155	0.1799	0.1858	0.1534	0.152
0.0742	0.203	0.2063	0.2079	0.2013	0.1799	0.1883	0.1642	0.1521
0.1728	0.3575	0.3164	0.2966	0.3052	0.2643	0.2497	0.2207	0.208
0.1439	0.3125	0.2975	0.3018	0.3101	0.2749	0.2662	0.2312	0.2296
0.1128	0.2808	0.2676	0.2529	0.253	0.2381	0.2417	0.2147	0.217
0.1527	0.3056	0.2994	0.2891	0.2886	0.2587	0.2402	0.2045	0.1921
0.179	0.2756	0.2443	0.252	0.2517	0.2357	0.2375	0.2005	0.1883
0.1523	0.2599	0.2473	0.2423	0.2441	0.2156	0.2029	0.1737	0.1645
0.1584	0.3343	0.3296	0.3316	0.3142	0.2898	0.2868	0.2443	0.2548
0.185	0.3523	0.3253	0.3245	0.3445	0.2845	0.281	0.2406	0.2495
0.1759	0.2645	0.2406	0.2486	0.2393	0.2146	0.2137	0.1712	0.1731

0.0937	0.0894	0.0812	0.079	0.0789	0.072	0.0694	0.0632	0.0625
0.0278	0.0169	0.0138	0.0136	0.014	0.0147	0.0143	0.0146	0.0139
0.0014	0.0041	0.0041	0.0044	0.0043	0.0039	0.0041	0.0037	0.0037
0.0092	0.0055	0.0044	0.0042	0.0045	0.0044	0.0044	0.0043	0.0044
0	0	0	0	0	0	0	0	0
0.001	0.0026	0.0026	0.0026	0.0026	0.0022	0.0022	0.0019	0.0019
0	0	0	0	0	0	0	0	0
0	0	0	0	0	0	0	0	0
0	0	0	0	0	0	0	0	0
0	0	0	0	0	0	0	0	0
0	0	0	0	0	0	0	0	0
0	0	0	0	0	0	0	0	0
0	0	0	0	0	0	0	0	0
0	0	0	0	0	0	0	0	0
0	0	0	0	0	0	0	0	0
0	0	0	0	0	0	0	0	0
0.0684	0.0459	0.0362	0.0344	0.0341	0.0321	0.0294	0.0283	0.0295
0.0052	0.0136	0.0133	0.0132	0.0132	0.0116	0.0121	0.011	0.0107
0.0421	0.0246	0.0198	0.0186	0.0199	0.0196	0.0187	0.0178	0.0186
0.0687	0.0667	0.0619	0.0621	0.0626	0.0607	0.0597	0.0573	0.0555
0.051	0.1271	0.1246	0.1218	0.123	0.1104	0.1191	0.1095	0.1099
0.0705	0.1708	0.1711	0.1749	0.1721	0.1574	0.1622	0.1456	0.1371
0.0669	0.1359	0.1336	0.1343	0.1394	0.1328	0.139	0.1254	0.1234
0.1395	0.15	0.1513	0.1577	0.1623	0.1614	0.1724	0.1747	0.1731
0.0585	0.0865	0.1092	0.1272	0.1296	0.1404	0.1555	0.1587	0.1517
0.2765	0.3435	0.3171	0.3164	0.3228	0.2978	0.2944	0.2733	0.2718
0.2968	0.4069	0.3695	0.3443	0.3423	0.3025	0.2953	0.2514	0.2388
0.1512	0.3025	0.2852	0.2801	0.2947	0.2645	0.2725	0.234	0.2182
0.06	0.2178	0.2182	0.2111	0.2178	0.2104	0.2136	0.1821	0.178
0.179	0.2048	0.2013	0.2057	0.2113	0.2128	0.221	0.2154	0.2122
0.2117	0.157	0.1582	0.1728	0.1888	0.2074	0.2257	0.2365	0.2318
0.2113	0.1978	0.1969	0.207	0.2151	0.2285	0.2452	0.2518	0.2453
0.1039	0.1682	0.1741	0.1789	0.1814	0.1794	0.1908	0.1799	0.1775
0.0724	0.0935	0.1171	0.1456	0.1532	0.1645	0.1809	0.1949	0.192
0.0852	0.1111	0.1222	0.1363	0.146	0.1602	0.1685	0.1771	0.1701
0.1144	0.2662	0.2571	0.251	0.2512	0.2103	0.199	0.1722	0.1605
0.0997	0.2812	0.2737	0.2655	0.267	0.228	0.2434	0.1944	0.1937
0.1288	0.2917	0.2812	0.2778	0.2611	0.239	0.2278	0.1934	0.1894
0.1696	0.3414	0.3168	0.3142	0.3009	0.2641	0.2573	0.2157	0.2085
0.0899	0.2426	0.2378	0.2418	0.2359	0.2125	0.2109	0.1763	0.1737
0.1009	0.2493	0.2384	0.2274	0.2273	0.1995	0.1946	0.1598	0.1559
0.1935	0.3492	0.3246	0.3094	0.3011	0.273	0.2729	0.2374	0.2363
0.0859	0.25	0.2358	0.2418	0.2441	0.2113	0.2222	0.1808	0.175
0.0786	0.2289	0.2189	0.2055	0.1952	0.1777	0.1869	0.1585	0.1492

0.3092	0.3145	0.2857	0.2877	0.2856	0.263	0.2522	0.2198	0.2117
0.448	0.3809	0.329	0.313	0.3108	0.2864	0.2722	0.2538	0.2603
0.2741	0.3003	0.2731	0.2718	0.2701	0.2546	0.2554	0.2382	0.2428
0.1475	0.2662	0.2399	0.2325	0.2229	0.1953	0.1904	0.1533	0.1496
0.1729	0.2742	0.2669	0.2549	0.2515	0.222	0.2281	0.191	0.1887
0.0683	0.1015	0.0917	0.0875	0.0855	0.0725	0.0716	0.0574	0.059
0.0003	0.0003	0.0004	0.0005	0.0006	0.0007	0.0008	0.0008	0.0008
0.0388	0.0301	0.026	0.026	0.0267	0.0254	0.0247	0.0227	0.0229
0.1375	0.0861	0.0689	0.0661	0.0684	0.0698	0.0635	0.0575	0.0546
0.1501	0.0918	0.074	0.0719	0.0736	0.0696	0.0705	0.0735	0.0756
0.0757	0.0577	0.052	0.0486	0.0472	0.0455	0.0461	0.042	0.0406
0	0	0	0	0	0	0	0	0
0	0	0	0	0	0	0	0	0
0	0	0	0	0	0	0	0	0
0	0	0	0	0	0	0	0	0
0	0	0	0	0	0	0	0	0
0	0	0	0	0	0	0	0	0
0	0	0	0	0	0	0	0	0
0	0	0	0	0	0	0	0	0
0	0	0	0	0	0	0	0	0
0	0	0	0	0	0	0	0	0
0	0	0	0	0	0	0	0	0
0.0017	0.0021	0.0024	0.0029	0.0032	0.0036	0.0041	0.0044	0.0045
0.0167	0.0099	0.0085	0.0083	0.008	0.0083	0.0083	0.0085	0.0083
0.0618	0.0369	0.0306	0.0286	0.0275	0.0274	0.026	0.0277	0.0277
0.0008	0.001	0.0013	0.0014	0.0015	0.0016	0.0018	0.002	0.0019
0.0173	0.0272	0.026	0.0257	0.0255	0.0239	0.0243	0.0211	0.0207
0.0699	0.0678	0.0583	0.0579	0.0584	0.057	0.0558	0.0525	0.0508
0.0087	0.0221	0.0226	0.0233	0.0244	0.0235	0.0235	0.022	0.022
0.0071	0.009	0.0108	0.0153	0.0172	0.0199	0.022	0.0228	0.0225
0.0686	0.0463	0.0419	0.0424	0.0419	0.0439	0.0457	0.0482	0.0474
0.6128	0.4595	0.3981	0.3865	0.3971	0.3822	0.3759	0.3631	0.3785
0.7415	0.6742	0.6033	0.5595	0.5399	0.5093	0.5141	0.5005	0.5004
0.4249	0.5293	0.4821	0.4669	0.4497	0.4099	0.38	0.3483	0.324
0.1088	0.2993	0.2963	0.2778	0.2759	0.2535	0.2618	0.2318	0.2157
0.2622	0.3643	0.3401	0.3324	0.3277	0.2978	0.2904	0.2499	0.2459
0.2685	0.3135	0.2844	0.2724	0.2762	0.2586	0.2558	0.2343	0.2296
0.0685	0.1452	0.1457	0.1506	0.1467	0.142	0.147	0.1357	0.1316
0.1091	0.1918	0.1883	0.1817	0.1845	0.175	0.1786	0.1661	0.1618
0.0818	0.1432	0.1538	0.1553	0.1614	0.1595	0.1705	0.175	0.1697
0.0903	0.0995	0.116	0.1232	0.1254	0.1395	0.1562	0.1634	0.1671
0.1297	0.2431	0.2278	0.2245	0.2196	0.2002	0.2006	0.1807	0.1691
0.0773	0.2574	0.2397	0.2524	0.2462	0.2391	0.2374	0.1875	0.1662
0.0785	0.2148	0.2032	0.1931	0.1923	0.1815	0.1843	0.1596	0.1585

0.1078	0.2662	0.2505	0.2485	0.2616	0.2331	0.246	0.2096	0.2111
0.1567	0.3144	0.2861	0.2842	0.2807	0.2455	0.247	0.2106	0.2138
0.0832	0.2266	0.2084	0.2086	0.2158	0.1984	0.2052	0.1771	0.1719
0.1625	0.244	0.2393	0.2398	0.2365	0.2244	0.2289	0.2026	0.1958
0.089	0.2345	0.2367	0.2336	0.2387	0.2047	0.2105	0.1709	0.1614
0.0684	0.196	0.188	0.1835	0.1907	0.1744	0.1754	0.147	0.1515
0.3914	0.2813	0.2304	0.2265	0.2202	0.2211	0.2116	0.2095	0.2052
0.6505	0.4631	0.3909	0.3814	0.3788	0.368	0.3471	0.3241	0.3392
0.4078	0.3859	0.3356	0.3345	0.3338	0.3168	0.3197	0.2725	0.257
0.0508	0.0822	0.0759	0.0738	0.0717	0.0657	0.063	0.0547	0.0552
0.0541	0.0367	0.0311	0.0301	0.0314	0.0337	0.0319	0.0327	0.0333
0.0656	0.0386	0.033	0.0302	0.0305	0.0311	0.0279	0.0272	0.0279
0.0458	0.0298	0.0247	0.0237	0.0232	0.0223	0.0201	0.019	0.0197
0	0	0	0	0	0	0	0	0
0	0	0	0	0	0	0	0	0
0.0268	0.02	0.0175	0.0161	0.0175	0.017	0.0164	0.0154	0.0156
0.0543	0.0342	0.0289	0.0266	0.0272	0.0272	0.0254	0.0257	0.0258
0.0444	0.0333	0.0265	0.0267	0.0266	0.0257	0.0251	0.0236	0.0227
0	0	0	0	0	0	0	0	0
0.0078	0.0044	0.0037	0.0034	0.0036	0.0037	0.0037	0.0038	0.0039
0	0	0	0	0	0	0	0	0
0	0	0	0	0	0	0	0	0
0	0	0	0	0	0	0	0	0
0	0	0	0	0	0	0	0	0
0	0	0	0	0	0	0	0	0
0	0	0	0	0	0	0	0	0
0	0	0	0	0	0	0	0	0
0	0	0	0	0	0	0	0	0
0	0	0	0	0	0	0	0	0
0	0	0	0	0	0	0	0	0
0	0	0	0	0	0	0	0	0
0	0	0	0	0	0	0	0	0
0	0	0	0	0	0	0	0	0
0	0	0	0	0	0	0	0	0
0	0	0	0	0	0	0	0	0
0	0	0	0	0	0	0	0	0
0.1165	0.067	0.0514	0.0507	0.0528	0.0554	0.0536	0.0559	0.0542
0.591	0.3703	0.3149	0.3068	0.3149	0.3491	0.3307	0.3326	0.3261
0.7315	0.5762	0.4859	0.4715	0.4636	0.4402	0.4506	0.4382	0.4427
0.2765	0.3599	0.3352	0.3228	0.3208	0.2933	0.2921	0.2583	0.2448
0.1353	0.3061	0.2835	0.2845	0.2944	0.2526	0.2532	0.2004	0.1999
0.1444	0.3063	0.3124	0.2891	0.2979	0.2618	0.2474	0.1995	0.1998
0.135	0.256	0.2338	0.2357	0.2297	0.199	0.195	0.1758	0.1662
0.1679	0.2614	0.2292	0.2199	0.2216	0.1931	0.1931	0.1688	0.1689

0.2904	0.2879	0.2627	0.2578	0.2564	0.2458	0.2455	0.2244	0.2169
0.1736	0.1928	0.1875	0.1901	0.1945	0.191	0.1983	0.1934	0.1926
0.0938	0.1199	0.1336	0.1448	0.1528	0.1627	0.1749	0.1782	0.1744
0.0468	0.0723	0.0806	0.0907	0.0937	0.1094	0.1233	0.1393	0.1298
0.0511	0.1001	0.1086	0.1134	0.1174	0.1177	0.1251	0.1239	0.1247
0.1244	0.1939	0.1932	0.1948	0.1969	0.1914	0.1947	0.1763	0.1721
0.1508	0.2386	0.2213	0.2203	0.2188	0.1931	0.1876	0.1665	0.1626
0.0752	0.2011	0.1964	0.1931	0.1914	0.1619	0.1657	0.1407	0.1444
0.0872	0.1762	0.1835	0.178	0.177	0.1654	0.1718	0.1455	0.1479
0.0953	0.1604	0.1601	0.1695	0.1709	0.1686	0.1748	0.1685	0.1654
0.0957	0.1716	0.1734	0.1823	0.186	0.1766	0.1878	0.1775	0.1714
0.0913	0.1563	0.1476	0.1497	0.153	0.142	0.1452	0.1323	0.1306
0.0744	0.0688	0.0617	0.0613	0.0631	0.0597	0.0584	0.0555	0.0539
0.0725	0.0619	0.0557	0.056	0.0565	0.0573	0.0575	0.057	0.0559
0.0624	0.0742	0.0693	0.0668	0.0664	0.0614	0.0616	0.0555	0.0564
0.0264	0.017	0.014	0.0144	0.0147	0.0139	0.0131	0.0134	0.0137
0	0	0	0	0	0	0	0	0
0	0	0	0	0	0	0	0	0
0	0	0	0	0	0	0	0	0
0	0	0	0	0	0	0	0	0
0	0	0	0	0	0	0	0	0
0	0	0	0	0	0	0	0	0
0	0	0	0	0	0	0	0	0
0	0	0	0	0	0	0	0	0
0	0	0	0	0	0	0	0	0
0	0	0	0	0	0	0	0	0
0	0	0	0	0	0	0	0	0
0	0	0	0	0	0	0	0	0
0	0	0	0	0	0	0	0	0
0	0	0	0	0	0	0	0	0
0	0	0	0	0	0	0	0	0
0	0	0	0	0	0	0	0	0
0	0	0	0	0	0	0	0	0
0	0	0	0	0	0	0	0	0
0	0	0	0	0	0	0	0	0
0	0	0	0	0	0	0	0	0
0	0	0	0	0	0	0	0	0
0	0	0	0	0	0	0	0	0
0	0	0	0	0	0	0	0	0
0	0	0	0	0	0	0	0	0
0	0	0	0	0	0	0	0	0
0	0	0	0	0	0	0	0	0
0.0063	0.0174	0.0171	0.0174	0.0162	0.0145	0.0148	0.0132	0.0122
0.1176	0.0773	0.0685	0.0664	0.0678	0.0681	0.0675	0.0658	0.063

0.1575	0.1298	0.1171	0.12	0.1228	0.1233	0.1264	0.1268	0.125
0.129	0.1547	0.1587	0.1655	0.1769	0.1772	0.1909	0.1912	0.1888
0.1126	0.1524	0.1622	0.1751	0.1834	0.1904	0.206	0.2059	0.2034
0.0826	0.1526	0.1583	0.1653	0.1754	0.1671	0.1833	0.1704	0.1617
0.2635	0.3012	0.2792	0.28	0.2744	0.2514	0.2443	0.2227	0.2088
0.6524	0.5556	0.4859	0.4692	0.4545	0.438	0.4221	0.3787	0.3646
0.914	0.6437	0.5402	0.5194	0.5315	0.5184	0.5205	0.4905	0.5097
0.4203	0.3176	0.281	0.2775	0.2683	0.2691	0.2527	0.2493	0.2653
0.0828	0.1165	0.1169	0.124	0.1282	0.1358	0.1406	0.1391	0.1378
0.0627	0.0841	0.0966	0.106	0.1144	0.125	0.1387	0.1497	0.1483
0.0973	0.1072	0.1201	0.1337	0.1464	0.1625	0.1877	0.1935	0.1957
0.0582	0.0972	0.1172	0.1327	0.1468	0.155	0.169	0.1779	0.1696
0.0535	0.1088	0.1106	0.118	0.1251	0.1225	0.1321	0.1267	0.1234
0.0865	0.1394	0.1407	0.1454	0.1524	0.1518	0.1591	0.1505	0.1463
0.066	0.1674	0.166	0.1766	0.1759	0.161	0.1688	0.1544	0.1441
0.0784	0.16	0.1665	0.1693	0.1752	0.1682	0.177	0.1703	0.1677
0.0527	0.1013	0.1054	0.1107	0.1151	0.1153	0.1211	0.113	0.1121
0.0336	0.0505	0.0488	0.0493	0.0501	0.0484	0.0506	0.047	0.0451
0.0481	0.0501	0.0452	0.0448	0.0446	0.0432	0.0442	0.0424	0.0421
0.0512	0.0535	0.0522	0.0536	0.0548	0.0552	0.0575	0.0572	0.0565
0.0374	0.038	0.0367	0.0374	0.0382	0.0385	0.0402	0.0397	0.0394
0.01	0.0109	0.0096	0.0096	0.0097	0.0089	0.0085	0.0077	0.0076
0	0	0	0	0	0	0	0	0
0	0	0	0	0	0	0	0	0
0	0	0	0	0	0	0	0	0
0	0	0	0	0	0	0	0	0
0	0	0	0	0	0	0	0	0
0	0	0	0	0	0	0	0	0
0	0	0	0	0	0	0	0	0
0	0	0	0	0	0	0	0	0
0	0	0	0	0	0	0	0	0
0	0	0	0	0	0	0	0	0
0	0	0	0	0	0	0	0	0
0	0	0	0	0	0	0	0	0
0	0	0	0	0	0	0	0	0
0	0	0	0	0	0	0	0	0
0	0	0	0	0	0	0	0	0
0	0	0	0	0	0	0	0	0
0	0	0	0	0	0	0	0	0
0	0	0	0	0	0	0	0	0
0	0	0	0	0	0	0	0	0
0	0	0	0	0	0	0	0	0
0	0	0	0	0	0	0	0	0

0	0	0	0	0	0	0	0	0
0	0	0	0	0	0	0	0	0
0	0	0	0	0	0	0	0	0
0	0	0	0	0	0	0	0	0
0.0445	0.0289	0.0234	0.0221	0.0221	0.0227	0.0199	0.0201	0.0213
0	0	0	0	0	0	0	0	0
0.0151	0.0254	0.0302	0.034	0.0374	0.0402	0.0443	0.0455	0.0458
0.0677	0.0751	0.0859	0.0931	0.1066	0.113	0.1285	0.1332	0.127
0.1282	0.1179	0.131	0.1458	0.1599	0.175	0.1916	0.2019	0.1964
0.043	0.0722	0.0849	0.0934	0.104	0.1138	0.128	0.1339	0.1285
0.2237	0.2256	0.2086	0.2045	0.2038	0.1948	0.1953	0.185	0.1809
0.6521	0.5062	0.4372	0.4495	0.4624	0.4509	0.4383	0.4115	0.4025
0.7198	0.519	0.4284	0.4224	0.4051	0.3856	0.4006	0.3924	0.403
0.2597	0.2256	0.2021	0.1888	0.195	0.1928	0.1928	0.1884	0.1867
0.058	0.1221	0.1268	0.1323	0.1352	0.129	0.1326	0.1211	0.1195
0.1121	0.1602	0.1617	0.1666	0.1686	0.1655	0.1717	0.1674	0.1631
0.1526	0.1816	0.1851	0.1926	0.1991	0.2019	0.2153	0.2172	0.2157
0.0886	0.1301	0.138	0.1506	0.1498	0.1528	0.1711	0.1694	0.1723
0.0508	0.0912	0.0958	0.0981	0.1053	0.1048	0.1139	0.1125	0.1088
0.0384	0.075	0.0853	0.0906	0.0964	0.1001	0.1091	0.1105	0.1126
0.0631	0.1413	0.1406	0.1433	0.1415	0.1331	0.1418	0.1284	0.1272
0.0617	0.1207	0.1266	0.1256	0.1292	0.1253	0.1317	0.1225	0.123
0.0155	0.0244	0.0265	0.0282	0.0294	0.0316	0.0353	0.0358	0.0346
0	0	0	0	0	0	0	0	0
0.0227	0.0238	0.0217	0.0218	0.0225	0.0217	0.022	0.0205	0.0201
0.0275	0.0315	0.0313	0.0317	0.0324	0.0324	0.0337	0.033	0.0327
0.0029	0.003	0.0042	0.0053	0.0058	0.0069	0.0087	0.0098	0.0095
0.0044	0.011	0.0106	0.011	0.0109	0.0095	0.01	0.0089	0.0088
0	0	0	0	0	0	0	0	0
0	0	0	0	0	0	0	0	0
0	0	0	0	0	0	0	0	0
0	0	0	0	0	0	0	0	0
0	0	0	0	0	0	0	0	0
0	0	0	0	0	0	0	0	0
0	0	0	0	0	0	0	0	0
0	0	0	0	0	0	0	0	0
0	0	0	0	0	0	0	0	0
0	0	0	0	0	0	0	0	0
0	0	0	0	0	0	0	0	0
0	0	0	0	0	0	0	0	0
0	0	0	0	0	0	0	0	0
0	0	0	0	0	0	0	0	0
0	0	0	0	0	0	0	0	0
0	0	0	0	0	0	0	0	0

0	0	0	0	0	0	0	0	0
0	0	0	0	0	0	0	0	0
0	0	0	0	0	0	0	0	0
0	0	0	0	0	0	0	0	0
0	0	0	0	0	0	0	0	0
0	0	0	0	0	0	0	0	0
0	0	0	0	0	0	0	0	0
0	0	0	0	0	0	0	0	0
0	0	0	0	0	0	0	0	0
0	0	0	0	0	0	0	0	0
0.104	0.0773	0.0647	0.0641	0.063	0.0612	0.0577	0.0602	0.0581
0.1147	0.0772	0.0604	0.0553	0.0561	0.0572	0.0566	0.058	0.0574
0.0562	0.0415	0.0348	0.032	0.0316	0.0308	0.0309	0.0315	0.0322
0.0133	0.0113	0.0118	0.013	0.0138	0.0148	0.0164	0.0174	0.0171
0.0417	0.0365	0.0376	0.0414	0.0434	0.0458	0.0504	0.054	0.0521
0.0398	0.0518	0.0543	0.0567	0.0582	0.0595	0.0627	0.061	0.06
0.0918	0.1115	0.1022	0.101	0.104	0.0997	0.1009	0.0939	0.0898
0.2169	0.1951	0.1682	0.165	0.1662	0.1653	0.1633	0.1516	0.1464
0.0492	0.1157	0.1115	0.1102	0.1023	0.0963	0.0994	0.0879	0.0817
0.0367	0.1022	0.0988	0.0992	0.0993	0.0892	0.0929	0.0852	0.0794
0.0495	0.1248	0.1184	0.1189	0.1199	0.1122	0.1072	0.0892	0.09
0.0775	0.1484	0.1474	0.1477	0.1428	0.1327	0.135	0.1178	0.1173
0.0575	0.1242	0.1271	0.1312	0.1323	0.1264	0.1261	0.1155	0.1129
0.105	0.1256	0.123	0.127	0.1292	0.1289	0.1344	0.1313	0.1294
0.0919	0.0777	0.0678	0.0682	0.0685	0.0663	0.0675	0.0676	0.0663
0.0083	0.0046	0.0035	0.0033	0.0034	0.0037	0.0036	0.0037	0.0036
0.0144	0.038	0.0376	0.0387	0.0388	0.0372	0.0386	0.0344	0.0334
0.0121	0.0269	0.0278	0.0278	0.0284	0.027	0.0291	0.0269	0.0268
0.001	0.0028	0.0027	0.0028	0.0029	0.0027	0.0027	0.0025	0.0024
0	0	0	0	0	0	0	0	0
0.0089	0.0057	0.0048	0.0046	0.0047	0.0047	0.0044	0.0044	0.0042
0.0091	0.0067	0.0056	0.0054	0.0054	0.0056	0.0055	0.0054	0.0052
0	0	0	0	0	0	0	0	0
0.0006	0.0007	0.0009	0.0011	0.0013	0.0015	0.0017	0.0018	0.0016
0	0	0	0	0	0	0	0	0
0	0	0	0	0	0	0	0	0
0	0	0	0	0	0	0	0	0
0	0	0	0	0	0	0	0	0
0	0	0	0	0	0	0	0	0
0	0	0	0	0	0	0	0	0
0	0	0	0	0	0	0	0	0
0	0	0	0	0	0	0	0	0
0	0	0	0	0	0	0	0	0
0	0	0	0	0	0	0	0	0

0	0	0	0	0	0	0	0	0
0	0	0	0	0	0	0	0	0
0	0	0	0	0	0	0	0	0
0	0	0	0	0	0	0	0	0
0	0	0	0	0	0	0	0	0
0	0	0	0	0	0	0	0	0
0	0	0	0	0	0	0	0	0
0	0	0	0	0	0	0	0	0
0	0	0	0	0	0	0	0	0
0	0	0	0	0	0	0	0	0
0	0	0	0	0	0	0	0	0
0	0	0	0	0	0	0	0	0
0	0	0	0	0	0	0	0	0
0	0	0	0	0	0	0	0	0
0	0	0	0	0	0	0	0	0
0	0	0	0	0	0	0	0	0
0	0	0	0	0	0	0	0	0
0.0435	0.0254	0.021	0.0196	0.0191	0.0209	0.0218	0.0217	0.0205
0.0268	0.0183	0.0148	0.0145	0.0138	0.0138	0.0132	0.0128	0.0121
0.0018	0.0051	0.005	0.005	0.0049	0.0042	0.0043	0.0035	0.0035
0.0025	0.0068	0.0066	0.0063	0.006	0.0054	0.0053	0.0045	0.004
0.052	0.0461	0.0434	0.0432	0.0424	0.0424	0.042	0.0395	0.0388
0.0163	0.0253	0.0229	0.0224	0.0224	0.0203	0.0195	0.0158	0.0152
0.0003	0.0011	0.0012	0.0011	0.0011	0.001	0.0011	0.0009	0.0009
0.0083	0.0245	0.0227	0.0223	0.0218	0.0202	0.0196	0.0164	0.0168
0.0377	0.0314	0.0266	0.0259	0.0257	0.0271	0.0267	0.0264	0.0252
0.0721	0.0467	0.0369	0.0338	0.035	0.0342	0.0314	0.0334	0.0342
0.0038	0.01	0.0097	0.0093	0.0095	0.0086	0.008	0.0067	0.0067
0.0082	0.0186	0.0188	0.0186	0.0185	0.0182	0.0196	0.0177	0.0177
0.01	0.0193	0.0202	0.0213	0.0219	0.0214	0.0224	0.0222	0.0218
0.082	0.0526	0.0452	0.0442	0.0449	0.0471	0.0473	0.0481	0.0448
0.091	0.0591	0.0533	0.0541	0.0539	0.0574	0.057	0.0616	0.0625
0.0163	0.0095	0.0078	0.0078	0.0079	0.008	0.008	0.0075	0.0078
0.009	0.0051	0.0044	0.0042	0.004	0.0042	0.0038	0.0039	0.0039
0.0024	0.0075	0.0071	0.0074	0.0073	0.0064	0.0062	0.0052	0.0052
0.0073	0.0183	0.0172	0.0152	0.0149	0.0135	0.0137	0.0122	0.0117
0.0103	0.0071	0.0063	0.0062	0.0061	0.0064	0.0064	0.0066	0.0064
0.0178	0.0112	0.0092	0.0091	0.0084	0.0085	0.0084	0.0084	0.0085
0.0095	0.0093	0.0085	0.0079	0.0079	0.0073	0.0077	0.0072	0.007
0.0022	0.0025	0.0029	0.0031	0.0039	0.0043	0.0052	0.0055	0.0053
0	0	0	0	0	0	0	0	0
0	0	0	0	0	0	0	0	0
0	0	0	0	0	0	0	0	0
0	0	0	0	0	0	0	0	0

0	0	0	0	0	0	0	0	0
0	0	0	0	0	0	0	0	0
0	0	0	0	0	0	0	0	0
0	0	0	0	0	0	0	0	0
0	0	0	0	0	0	0	0	0
0	0	0	0	0	0	0	0	0
0	0	0	0	0	0	0	0	0
0	0	0	0	0	0	0	0	0
0	0	0	0	0	0	0	0	0
0	0	0	0	0	0	0	0	0
0	0	0	0	0	0	0	0	0
0	0	0	0	0	0	0	0	0
0	0	0	0	0	0	0	0	0
0	0	0	0	0	0	0	0	0
0	0	0	0	0	0	0	0	0
0	0	0	0	0	0	0	0	0
0	0	0	0	0	0	0	0	0
0	0	0	0	0	0	0	0	0
0	0	0	0	0	0	0	0	0
0	0	0	0	0	0	0	0	0
0	0	0	0	0	0	0	0	0
0	0	0	0	0	0	0	0	0
0.0543	0.0341	0.0295	0.028	0.0261	0.0259	0.0231	0.0243	0.0252
0.0023	0.0027	0.0034	0.004	0.0048	0.0052	0.0059	0.0062	0.0064
0.001	0.0012	0.0014	0.0016	0.0018	0.0019	0.0022	0.0024	0.0025
0.0265	0.0158	0.0131	0.0127	0.0125	0.0128	0.0133	0.0132	0.0126
0.0422	0.0247	0.0197	0.0178	0.0192	0.0174	0.0165	0.0175	0.017
0.025	0.0155	0.0128	0.0112	0.0111	0.0115	0.0116	0.0111	0.011
0.0175	0.0114	0.0099	0.0098	0.0104	0.0101	0.0095	0.0096	0.0097
0.0289	0.0184	0.0159	0.0158	0.0161	0.0162	0.016	0.0163	0.016
0.1546	0.0985	0.0829	0.0744	0.0775	0.0779	0.0736	0.0746	0.0747
0.0034	0.0062	0.0065	0.0066	0.0069	0.007	0.0072	0.0071	0.0069
0.0036	0.0061	0.0071	0.0079	0.0081	0.0087	0.0098	0.0102	0.0099
0.0589	0.0367	0.0308	0.0299	0.0313	0.0339	0.0345	0.0364	0.0371
0.0367	0.0222	0.0184	0.0162	0.016	0.0155	0.0153	0.0162	0.0161
0.0009	0.0026	0.0025	0.0024	0.0023	0.002	0.002	0.0016	0.0015
0.0169	0.0274	0.03	0.0315	0.0322	0.034	0.036	0.0363	0.0349
0.1113	0.1073	0.1076	0.1112	0.1178	0.1245	0.1318	0.1347	0.1359
0.0422	0.0301	0.0282	0.0286	0.0288	0.0298	0.0311	0.0323	0.032
0.0201	0.0162	0.0147	0.0144	0.0142	0.0137	0.0137	0.0135	0.0131
0	0	0	0	0	0	0	0	0
0.0017	0.002	0.0027	0.0028	0.0032	0.0036	0.0041	0.0043	0.0043
0.0107	0.0078	0.0075	0.0079	0.0083	0.0091	0.0098	0.0104	0.01
0.0173	0.0098	0.008	0.0078	0.0075	0.0075	0.0073	0.0076	0.0078

0.0082	0.0049	0.004	0.0036	0.0038	0.004	0.0039	0.0041	0.0038
0.0098	0.0081	0.0071	0.0068	0.0065	0.0062	0.0059	0.0055	0.0058
0	0	0	0	0	0	0	0	0
0	0	0	0	0	0	0	0	0
0	0	0	0	0	0	0	0	0
0	0	0	0	0	0	0	0	0
0	0	0	0	0	0	0	0	0
0	0	0	0	0	0	0	0	0
0	0	0	0	0	0	0	0	0
0	0	0	0	0	0	0	0	0
0	0	0	0	0	0	0	0	0
0	0	0	0	0	0	0	0	0
0	0	0	0	0	0	0	0	0
0	0	0	0	0	0	0	0	0
0	0	0	0	0	0	0	0	0
0	0	0	0	0	0	0	0	0
0	0	0	0	0	0	0	0	0
0	0	0	0	0	0	0	0	0
0	0	0	0	0	0	0	0	0
0	0	0	0	0	0	0	0	0
0	0	0	0	0	0	0	0	0
0	0	0	0	0	0	0	0	0
0	0	0	0	0	0	0	0	0
0	0	0	0	0	0	0	0	0
0	0	0	0	0	0	0	0	0
0	0	0	0	0	0	0	0	0
0	0	0	0	0	0	0	0	0
0	0	0	0	0	0	0	0	0
0.1456	0.0947	0.0804	0.0756	0.0741	0.0703	0.0722	0.0704	0.0703
0.034	0.0232	0.0197	0.0187	0.0181	0.0186	0.0199	0.0183	0.0172
0.0278	0.0201	0.0167	0.0156	0.016	0.0157	0.0141	0.0143	0.0148
0.0851	0.0508	0.0428	0.0435	0.0401	0.0411	0.0425	0.0444	0.0424
0.0672	0.0428	0.0368	0.035	0.0372	0.038	0.0368	0.0344	0.0353
0.0173	0.0107	0.0087	0.0085	0.0075	0.0077	0.0075	0.0076	0.007
0.0354	0.0239	0.0195	0.0187	0.0194	0.0179	0.0163	0.0171	0.0179
0.0796	0.0596	0.0527	0.0515	0.051	0.0493	0.0476	0.0446	0.0431
0.0525	0.0313	0.0258	0.0265	0.0267	0.0287	0.0282	0.0293	0.0279
0	0	0	0	0	0	0	0	0
0.0008	0.0024	0.0023	0.0023	0.0021	0.0019	0.0019	0.0017	0.0015
0.0742	0.0488	0.0412	0.0399	0.0398	0.0398	0.0377	0.0386	0.0378
0.0934	0.0606	0.0514	0.0506	0.0508	0.0504	0.0509	0.0475	0.0483
0.0275	0.0183	0.0151	0.0146	0.0139	0.0135	0.0128	0.0139	0.0136
0.0005	0.0007	0.0009	0.001	0.0011	0.0011	0.0014	0.0015	0.0017
0.0158	0.0148	0.0161	0.0178	0.0192	0.0199	0.0211	0.0215	0.0211

0.0428	0.036	0.0334	0.0338	0.0346	0.0344	0.0352	0.0348	0.0347
0.0231	0.0235	0.0212	0.0212	0.0214	0.0211	0.0215	0.0204	0.0204
0	0	0	0	0	0	0	0	0
0	0	0	0	0	0	0	0	0
0	0	0	0	0	0	0	0	0
0.0084	0.0048	0.004	0.0039	0.0038	0.0038	0.0036	0.0041	0.0042
0.0083	0.005	0.004	0.0038	0.0035	0.0036	0.0032	0.0029	0.003
0.0009	0.0026	0.0024	0.0022	0.0022	0.002	0.0019	0.0016	0.0017
0	0	0	0	0	0	0	0	0
0	0	0	0	0	0	0	0	0
0	0	0	0	0	0	0	0	0
0	0	0	0	0	0	0	0	0
0	0	0	0	0	0	0	0	0
0	0	0	0	0	0	0	0	0
0	0	0	0	0	0	0	0	0
0	0	0	0	0	0	0	0	0
0	0	0	0	0	0	0	0	0
0	0	0	0	0	0	0	0	0
0	0	0	0	0	0	0	0	0
0	0	0	0	0	0	0	0	0
0	0	0	0	0	0	0	0	0
0	0	0	0	0	0	0	0	0
0	0	0	0	0	0	0	0	0
0	0	0	0	0	0	0	0	0
0	0	0	0	0	0	0	0	0
0	0	0	0	0	0	0	0	0
0	0	0	0	0	0	0	0	0
0	0	0	0	0	0	0	0	0
0	0	0	0	0	0	0	0	0
0	0	0	0	0	0	0	0	0
0	0	0	0	0	0	0	0	0
0	0	0	0	0	0	0	0	0
0	0	0	0	0	0	0	0	0
0	0	0	0	0	0	0	0	0
0	0	0	0	0	0	0	0	0
0.0006	0.0008	0.0011	0.0013	0.0013	0.0015	0.0017	0.0018	0.0018
0.0092	0.0061	0.0055	0.0054	0.0055	0.0059	0.006	0.0063	0.0063
0.0189	0.0167	0.0145	0.014	0.014	0.0131	0.0126	0.0122	0.0127
0.0046	0.0106	0.01	0.0098	0.0095	0.0083	0.0078	0.0061	0.0061
0.0396	0.0239	0.0179	0.0157	0.0171	0.0165	0.0161	0.0164	0.0172
0.0813	0.0502	0.0411	0.0369	0.0414	0.043	0.0401	0.04	0.0419
0.0264	0.0165	0.0133	0.0127	0.0124	0.0123	0.012	0.0123	0.0125
0.0366	0.0272	0.0227	0.0207	0.0218	0.0209	0.0216	0.0207	0.0192
0.0599	0.0378	0.0331	0.0327	0.0331	0.0346	0.0343	0.0356	0.0347

0.0649	0.0419	0.0339	0.0299	0.0294	0.0291	0.0296	0.0273	0.0266
0	0	0	0	0	0	0	0	0
0.0012	0.0038	0.0037	0.0036	0.0034	0.0029	0.0029	0.0024	0.0024
0.0439	0.0294	0.0258	0.0246	0.0241	0.0259	0.0238	0.0244	0.0231
0.0934	0.0587	0.0471	0.0512	0.0531	0.0533	0.0515	0.0519	0.0501
0.0192	0.0135	0.0119	0.0115	0.0115	0.011	0.0108	0.0107	0.0109
0.0336	0.0189	0.0142	0.0137	0.0144	0.0146	0.0143	0.0151	0.0154
0.0085	0.0049	0.004	0.004	0.004	0.0042	0.0041	0.0041	0.004
0.003	0.0051	0.0055	0.006	0.0063	0.0065	0.0071	0.0072	0.0067
0.0038	0.0091	0.0091	0.0092	0.0094	0.0079	0.0081	0.0074	0.0066
0	0	0	0	0	0	0	0	0
0	0	0	0	0	0	0	0	0
0.0028	0.0055	0.0058	0.0059	0.0059	0.0057	0.006	0.0057	0.0058
0.0006	0.0009	0.0013	0.0016	0.0019	0.0021	0.0022	0.0023	0.0022
0	0	0	0	0	0	0	0	0
0	0	0	0	0	0	0	0	0
0	0	0	0	0	0	0	0	0
0	0	0	0	0	0	0	0	0
0	0	0	0	0	0	0	0	0
0	0	0	0	0	0	0	0	0
0	0	0	0	0	0	0	0	0
0	0	0	0	0	0	0	0	0
0	0	0	0	0	0	0	0	0
0	0	0	0	0	0	0	0	0
0	0	0	0	0	0	0	0	0
0	0	0	0	0	0	0	0	0
0	0	0	0	0	0	0	0	0
0	0	0	0	0	0	0	0	0
0	0	0	0	0	0	0	0	0
0	0	0	0	0	0	0	0	0
0	0	0	0	0	0	0	0	0
0	0	0	0	0	0	0	0	0
0	0	0	0	0	0	0	0	0
0	0	0	0	0	0	0	0	0
0	0	0	0	0	0	0	0	0
0	0	0	0	0	0	0	0	0
0	0	0	0	0	0	0	0	0
0	0	0	0	0	0	0	0	0
0	0	0	0	0	0	0	0	0
0	0	0	0	0	0	0	0	0
0	0	0	0	0	0	0	0	0
0	0	0	0	0	0	0	0	0
0	0	0	0	0	0	0	0	0
0	0	0	0	0	0	0	0	0

0	0	0	0	0	0	0	0	0
0	0	0	0	0	0	0	0	0
0	0	0	0	0	0	0	0	0
0	0	0	0	0	0	0	0	0
0	0	0	0	0	0	0	0	0
0	0	0	0	0	0	0	0	0
0	0	0	0	0	0	0	0	0
0	0	0	0	0	0	0	0	0
0	0	0	0	0	0	0	0	0
0	0	0	0	0	0	0	0	0
0	0	0	0	0	0	0	0	0
0	0	0	0	0	0	0	0	0
0	0	0	0	0	0	0	0	0
0	0	0	0	0	0	0	0	0
0	0	0	0	0	0	0	0	0
0	0	0	0	0	0	0	0	0
0	0	0	0	0	0	0	0	0
0	0	0	0	0	0	0	0	0
0	0	0	0	0	0	0	0	0
0	0	0	0	0	0	0	0	0
0	0	0	0	0	0	0	0	0
0	0	0	0	0	0	0	0	0
0	0	0	0	0	0	0	0	0
0	0	0	0	0	0	0	0	0
0	0	0	0	0	0	0	0	0
0	0	0	0	0	0	0	0	0
0	0	0	0	0	0	0	0	0
0	0	0	0	0	0	0	0	0
0	0	0	0	0	0	0	0	0
0	0	0	0	0	0	0	0	0
0	0	0	0	0	0	0	0	0
0	0	0	0	0	0	0	0	0
0	0	0	0	0	0	0	0	0
0	0	0	0	0	0	0	0	0
0	0	0	0	0	0	0	0	0
0	0	0	0	0	0	0	0	0
0	0	0	0	0	0	0	0	0
0	0	0	0	0	0	0	0	0
0	0	0	0	0	0	0	0	0
0	0	0	0	0	0	0	0	0
0	0	0	0	0	0	0	0	0
0	0	0	0	0	0	0	0	0
0	0	0	0	0	0	0	0	0
0	0	0	0	0	0	0	0	0

0	0	0	0	0	0	0	0	0
0	0	0	0	0	0	0	0	0
0	0	0	0	0	0	0	0	0
0	0	0	0	0	0	0	0	0
0	0	0	0	0	0	0	0	0
0	0	0	0	0	0	0	0	0
0	0	0	0	0	0	0	0	0
0	0	0	0	0	0	0	0	0
0	0	0	0	0	0	0	0	0
0	0	0	0	0	0	0	0	0
0	0	0	0	0	0	0	0	0
0	0	0	0	0	0	0	0	0
0	0	0	0	0	0	0	0	0
0	0	0	0	0	0	0	0	0
0	0	0	0	0	0	0	0	0
0	0	0	0	0	0	0	0	0
0	0	0	0	0	0	0	0	0
0	0	0	0	0	0	0	0	0
0	0	0	0	0	0	0	0	0
0	0	0	0	0	0	0	0	0
0	0	0	0	0	0	0	0	0
0	0	0	0	0	0	0	0	0
0	0	0	0	0	0	0	0	0
0	0	0	0	0	0	0	0	0
0	0	0	0	0	0	0	0	0
0	0	0	0	0	0	0	0	0
0	0	0	0	0	0	0	0	0
0	0	0	0	0	0	0	0	0
0	0	0	0	0	0	0	0	0
0	0	0	0	0	0	0	0	0
0	0	0	0	0	0	0	0	0
0	0	0	0	0	0	0	0	0
0	0	0	0	0	0	0	0	0
0	0	0	0	0	0	0	0	0
0	0	0	0	0	0	0	0	0
0	0	0	0	0	0	0	0	0

0	0	0	0	0	0	0	0	0
0	0	0	0	0	0	0	0	0
0	0	0	0	0	0	0	0	0
0	0	0	0	0	0	0	0	0
0	0	0	0	0	0	0	0	0
0	0	0	0	0	0	0	0	0
0	0	0	0	0	0	0	0	0
0	0	0	0	0	0	0	0	0
0	0	0	0	0	0	0	0	0
0	0	0	0	0	0	0	0	0
0	0	0	0	0	0	0	0	0
0	0	0	0	0	0	0	0	0
0	0	0	0	0	0	0	0	0
0	0	0	0	0	0	0	0	0
0	0	0	0	0	0	0	0	0
0	0	0	0	0	0	0	0	0
0	0	0	0	0	0	0	0	0
0	0	0	0	0	0	0	0	0
0	0	0	0	0	0	0	0	0
0	0	0	0	0	0	0	0	0
0	0	0	0	0	0	0	0	0
0	0	0	0	0	0	0	0	0
0	0	0	0	0	0	0	0	0
0	0	0	0	0	0	0	0	0
0.0034	0.0036	0.0036	0.0038	0.0041	0.0044	0.0046	0.0044	0.0044
0.0213	0.0208	0.0204	0.0199	0.0201	0.0207	0.0199	0.0185	0.0194
0.007	0.0067	0.0067	0.0067	0.0065	0.007	0.0068	0.0058	0.0059
0	0	0	0	0	0	0	0	0
0.0198	0.0207	0.0198	0.0213	0.0195	0.0202	0.0212	0.0208	0.0221
0.043	0.0448	0.0449	0.0451	0.0435	0.0461	0.0459	0.0436	0.0456
0	0	0	0	0	0	0	0	0
0.0038	0.004	0.0038	0.0041	0.0037	0.0036	0.0039	0.0037	0.0037
0.0034	0.0034	0.0033	0.0033	0.0033	0.0031	0.003	0.0029	0.003
0.0141	0.0154	0.0149	0.0148	0.0133	0.0136	0.0138	0.0138	0.0147
0.015	0.0159	0.0156	0.0163	0.0154	0.0164	0.0157	0.0154	0.016
0.0057	0.0066	0.0068	0.0067	0.0067	0.0069	0.0066	0.0071	0.0073
0	0	0	0	0	0	0	0	0
0	0	0	0	0	0	0	0	0
0	0	0	0	0	0	0	0	0
0	0	0	0	0	0	0	0	0
0.0068	0.0073	0.007	0.0073	0.0069	0.0071	0.0073	0.0075	0.0083
0.0031	0.0031	0.0033	0.0033	0.0031	0.003	0.0033	0.0031	0.0033
0	0	0	0	0	0	0	0	0
0	0	0	0	0	0	0	0	0

0.008	0.008	0.0077	0.0074	0.0066	0.0066	0.0067	0.0069	0.007
0.01	0.01	0.0097	0.0103	0.0105	0.0105	0.0108	0.0111	0.0108
0.0094	0.0096	0.0088	0.0091	0.0082	0.0077	0.008	0.0082	0.0085
0.0077	0.0084	0.0085	0.0084	0.0073	0.0071	0.0078	0.0083	0.0088
0	0	0	0	0	0	0	0	0
0	0	0	0	0	0	0	0	0
0	0	0	0	0	0	0	0	0
0	0	0	0	0	0	0	0	0
0	0	0	0	0	0	0	0	0
0	0	0	0	0	0	0	0	0
0	0	0	0	0	0	0	0	0
0	0	0	0	0	0	0	0	0
0	0	0	0	0	0	0	0	0
0	0	0	0	0	0	0	0	0
0	0	0	0	0	0	0	0	0
0	0	0	0	0	0	0	0	0
0	0	0	0	0	0	0	0	0
0	0	0	0	0	0	0	0	0
0	0	0	0	0	0	0	0	0
0	0	0	0	0	0	0	0	0
0	0	0	0	0	0	0	0	0
0	0	0	0	0	0	0	0	0
0	0	0	0	0	0	0	0	0
0	0	0	0	0	0	0	0	0
0	0	0	0	0	0	0	0	0
0	0	0	0	0	0	0	0	0
0	0	0	0	0	0	0	0	0
0	0	0	0	0	0	0	0	0
0	0	0	0	0	0	0	0	0
0	0	0	0	0	0	0	0	0
0.0174	0.0173	0.0157	0.0163	0.0145	0.0153	0.0154	0.0156	0.0153
0.0018	0.0017	0.0018	0.0019	0.0019	0.0019	0.0021	0.0022	0.0023
0.0163	0.0162	0.016	0.0155	0.0139	0.016	0.0154	0.0155	0.0156
0.0231	0.0231	0.023	0.0225	0.0206	0.0212	0.0197	0.0194	0.0191
0.001	0.001	0.001	0.001	0.001	0.0011	0.0011	0.0011	0.0012
0.0009	0.0009	0.0009	0.001	0.001	0.001	0.001	0.001	0.0011
0	0	0	0	0	0	0	0	0
0.0248	0.0249	0.0248	0.0236	0.0215	0.0209	0.0197	0.0183	0.0187
0	0	0	0	0	0	0	0	0
0	0	0	0	0	0	0	0	0
0	0	0	0	0	0	0	0	0
0	0	0	0	0	0	0	0	0
0	0	0	0	0	0	0	0	0
0.0078	0.0081	0.0079	0.0077	0.0075	0.0079	0.0081	0.0077	0.0078

0.0038	0.0038	0.0039	0.0038	0.0035	0.0035	0.0034	0.0035	0.0037
0	0	0	0	0	0	0	0	0
0	0	0	0	0	0	0	0	0
0	0	0	0	0	0	0	0	0
0	0	0	0	0	0	0	0	0
0.0111	0.0113	0.0104	0.0105	0.0098	0.0089	0.0097	0.0091	0.0095
0.0159	0.0161	0.0134	0.0145	0.0131	0.0138	0.0148	0.0153	0.0171
0.0105	0.0115	0.0114	0.0116	0.0111	0.0122	0.0113	0.0112	0.0117
0.003	0.0031	0.0031	0.0033	0.0032	0.0035	0.0033	0.0031	0.0029
0	0	0	0	0	0	0	0	0
0	0	0	0	0	0	0	0	0
0	0	0	0	0	0	0	0	0
0	0	0	0	0	0	0	0	0
0	0	0	0	0	0	0	0	0
0	0	0	0	0	0	0	0	0
0	0	0	0	0	0	0	0	0
0	0	0	0	0	0	0	0	0
0	0	0	0	0	0	0	0	0
0	0	0	0	0	0	0	0	0
0	0	0	0	0	0	0	0	0
0	0	0	0	0	0	0	0	0
0	0	0	0	0	0	0	0	0
0	0	0	0	0	0	0	0	0
0	0	0	0	0	0	0	0	0
0	0	0	0	0	0	0	0	0
0	0	0	0	0	0	0	0	0
0	0	0	0	0	0	0	0	0
0	0	0	0	0	0	0	0	0
0	0	0	0	0	0	0	0	0
0	0	0	0	0	0	0	0	0
0	0	0	0	0	0	0	0	0
0	0	0	0	0	0	0	0	0
0	0	0	0	0	0	0	0	0
0	0	0	0	0	0	0	0	0
0	0	0	0	0	0	0	0	0
0	0	0	0	0	0	0	0	0
0.0278	0.0293	0.0284	0.0307	0.0292	0.0303	0.0317	0.0298	0.0267
0.0104	0.0125	0.0133	0.0134	0.013	0.014	0.0141	0.0146	0.0146
0.0308	0.0313	0.032	0.0294	0.0296	0.0313	0.0303	0.0301	0.0294
0.0331	0.0342	0.0292	0.031	0.0326	0.0337	0.0329	0.0321	0.0341
0.0099	0.0097	0.0099	0.0106	0.0093	0.0099	0.0094	0.0099	0.0099
0.0063	0.007	0.007	0.0072	0.0064	0.0071	0.0072	0.007	0.0071
0.0246	0.0242	0.0243	0.025	0.0208	0.021	0.0199	0.0179	0.0204
0.0275	0.0316	0.0297	0.0292	0.0287	0.0303	0.0284	0.0264	0.0261

0.0451	0.0406	0.0398	0.0397	0.0354	0.0377	0.0391	0.039	0.0368
0.0383	0.0365	0.0323	0.0324	0.0322	0.0315	0.0314	0.0316	0.0301
0.0361	0.0338	0.0334	0.0345	0.032	0.0335	0.033	0.0337	0.0354
0.0316	0.0334	0.0317	0.0316	0.0291	0.0295	0.0291	0.027	0.0297
0.0297	0.0314	0.0279	0.0287	0.0293	0.0286	0.0296	0.026	0.0308
0.0227	0.0222	0.0214	0.023	0.0202	0.0218	0.0231	0.0229	0.0229
0.0032	0.0035	0.0033	0.0035	0.0034	0.0038	0.0037	0.0037	0.0037
0	0	0	0	0	0	0	0	0
0	0	0	0	0	0	0	0	0
0	0	0	0	0	0	0	0	0
0.0039	0.0036	0.0034	0.0035	0.0031	0.003	0.003	0.003	0.0031
0.0103	0.0109	0.0107	0.0103	0.0101	0.01	0.0089	0.009	0.0092
0.0157	0.0168	0.0166	0.0179	0.0177	0.0175	0.0175	0.0169	0.0168
0.0173	0.0167	0.0153	0.0145	0.0131	0.0128	0.0126	0.0131	0.0134
0.0076	0.0072	0.0072	0.0078	0.0072	0.0069	0.0072	0.0071	0.0077
0	0	0	0	0	0	0	0	0
0	0	0	0	0	0	0	0	0
0	0	0	0	0	0	0	0	0
0	0	0	0	0	0	0	0	0
0	0	0	0	0	0	0	0	0
0	0	0	0	0	0	0	0	0
0	0	0	0	0	0	0	0	0
0	0	0	0	0	0	0	0	0
0	0	0	0	0	0	0	0	0
0	0	0	0	0	0	0	0	0
0	0	0	0	0	0	0	0	0
0	0	0	0	0	0	0	0	0
0	0	0	0	0	0	0	0	0
0	0	0	0	0	0	0	0	0
0	0	0	0	0	0	0	0	0
0	0	0	0	0	0	0	0	0
0	0	0	0	0	0	0	0	0
0	0	0	0	0	0	0	0	0
0	0	0	0	0	0	0	0	0
0	0	0	0	0	0	0	0	0
0	0	0	0	0	0	0	0	0
0	0	0	0	0	0	0	0	0
0	0	0	0	0	0	0	0	0
0	0	0	0	0	0	0	0	0
0	0	0	0	0	0	0	0	0
0	0	0	0	0	0	0	0	0
0	0	0	0	0	0	0	0	0
0	0	0	0	0	0	0	0	0
0.0031	0.0032	0.0032	0.0031	0.0033	0.0034	0.0034	0.0034	0.0035
0.0622	0.0651	0.063	0.0627	0.0551	0.0577	0.0557	0.0566	0.0607

0.0008	0.0008	0.0009	0.001	0.001	0.0011	0.0012	0.0012	0.0012
0.0024	0.0024	0.0027	0.0028	0.0028	0.0027	0.0029	0.003	0.0031
0.0217	0.0211	0.0218	0.0216	0.019	0.0197	0.0171	0.0181	0.0175
0.0334	0.0353	0.0334	0.033	0.0282	0.0279	0.0273	0.0269	0.0265
0.01	0.0102	0.0102	0.0105	0.0101	0.0108	0.0105	0.0107	0.011
0.0032	0.003	0.0033	0.0033	0.0034	0.0035	0.0035	0.0035	0.0036
0.0209	0.0209	0.0201	0.0198	0.0174	0.017	0.0181	0.019	0.0191
0.0033	0.0037	0.0039	0.0035	0.0032	0.0035	0.0037	0.0036	0.0035
0	0	0	0	0	0	0	0	0
0	0	0	0	0	0	0	0	0
0	0	0	0	0	0	0	0	0
0	0	0	0	0	0	0	0	0
0.0033	0.0033	0.0032	0.0033	0.0032	0.0034	0.0034	0.0033	0.0036
0.0146	0.0143	0.0152	0.0154	0.015	0.0144	0.0127	0.013	0.0131
0.0324	0.0344	0.0336	0.0331	0.0345	0.0335	0.0328	0.0309	0.0323
0.0243	0.0237	0.0244	0.0241	0.0226	0.0216	0.0219	0.0212	0.0208
0.0057	0.0053	0.0042	0.0044	0.004	0.0048	0.0051	0.0057	0.0053
0	0	0	0	0	0	0	0	0
0.0025	0.0027	0.0031	0.0034	0.0032	0.0034	0.003	0.003	0.0034
0.0111	0.0111	0.0104	0.0106	0.0097	0.0107	0.0102	0.0104	0.0101
0.0149	0.0168	0.0173	0.0169	0.0152	0.0151	0.0153	0.0155	0.0164
0.0171	0.0182	0.0165	0.0164	0.0144	0.0149	0.0157	0.0164	0.0178
0	0	0	0	0	0	0	0	0
0	0	0	0	0	0	0	0	0
0	0	0	0	0	0	0	0	0
0	0	0	0	0	0	0	0	0
0	0	0	0	0	0	0	0	0
0	0	0	0	0	0	0	0	0
0	0	0	0	0	0	0	0	0
0	0	0	0	0	0	0	0	0
0	0	0	0	0	0	0	0	0
0	0	0	0	0	0	0	0	0
0	0	0	0	0	0	0	0	0
0	0	0	0	0	0	0	0	0
0	0	0	0	0	0	0	0	0
0	0	0	0	0	0	0	0	0
0	0	0	0	0	0	0	0	0
0	0	0	0	0	0	0	0	0
0	0	0	0	0	0	0	0	0
0	0	0	0	0	0	0	0	0
0	0	0	0	0	0	0	0	0
0	0	0	0	0	0	0	0	0
0	0	0	0	0	0	0	0	0
0	0	0	0	0	0	0	0	0

0	0	0	0	0	0	0	0	0
0	0	0	0	0	0	0	0	0
0	0	0	0	0	0	0	0	0
0	0	0	0	0	0	0	0	0
0.0094	0.0096	0.0096	0.0099	0.01	0.0103	0.0105	0.0106	0.0108
0.006	0.0062	0.006	0.0058	0.0059	0.0057	0.0056	0.0057	0.0058
0	0	0	0	0	0	0	0	0
0.0088	0.0087	0.0087	0.0087	0.0079	0.008	0.0077	0.0075	0.0078
0	0	0	0	0	0	0	0	0
0	0	0	0	0	0	0	0	0
0.0791	0.0831	0.0729	0.0709	0.0626	0.0728	0.0707	0.0775	0.0777
0.0497	0.05	0.0491	0.05	0.0458	0.0464	0.0408	0.0372	0.0398
0	0	0	0	0	0	0	0	0
0	0	0	0	0	0	0	0	0
0	0	0	0	0	0	0	0	0
0.0117	0.0116	0.0116	0.0123	0.0113	0.0117	0.0112	0.0109	0.0114
0.0072	0.0075	0.0069	0.0061	0.0061	0.0062	0.0059	0.0064	0.007
0	0	0	0	0	0	0	0	0
0	0	0	0	0	0	0	0	0
0.0042	0.0041	0.004	0.004	0.0039	0.0036	0.0037	0.0036	0.0038
0.0084	0.0078	0.008	0.0087	0.0084	0.0091	0.0103	0.0111	0.0111
0.0038	0.0039	0.0038	0.0036	0.0034	0.0034	0.0036	0.0035	0.0036
0	0	0	0	0	0	0	0	0
0	0	0	0	0	0	0	0	0
0.0054	0.0055	0.0054	0.0057	0.0058	0.0056	0.0053	0.0054	0.0057
0.0128	0.0133	0.0124	0.0124	0.0113	0.0122	0.0122	0.0127	0.0136
0.019	0.0194	0.0192	0.0187	0.0179	0.0176	0.0179	0.0175	0.0176
0.0192	0.0211	0.0196	0.0208	0.02	0.0206	0.0193	0.0183	0.0182
0	0	0	0	0	0	0	0	0
0	0	0	0	0	0	0	0	0
0	0	0	0	0	0	0	0	0
0	0	0	0	0	0	0	0	0
0	0	0	0	0	0	0	0	0
0	0	0	0	0	0	0	0	0
0	0	0	0	0	0	0	0	0
0	0	0	0	0	0	0	0	0
0	0	0	0	0	0	0	0	0
0	0	0	0	0	0	0	0	0
0	0	0	0	0	0	0	0	0
0	0	0	0	0	0	0	0	0
0	0	0	0	0	0	0	0	0
0	0	0	0	0	0	0	0	0
0	0	0	0	0	0	0	0	0
0	0	0	0	0	0	0	0	0

0	0	0	0	0	0	0	0	0
0	0	0	0	0	0	0	0	0
0	0	0	0	0	0	0	0	0
0	0	0	0	0	0	0	0	0
0	0	0	0	0	0	0	0	0
0	0	0	0	0	0	0	0	0
0	0	0	0	0	0	0	0	0
0	0	0	0	0	0	0	0	0
0	0	0	0	0	0	0	0	0
0	0	0	0	0	0	0	0	0
0.0523	0.0518	0.0522	0.0524	0.0523	0.0558	0.0549	0.0555	0.0572
0	0	0	0	0	0	0	0	0
0.0699	0.0789	0.0732	0.0704	0.0632	0.063	0.0615	0.0621	0.0671
0.2214	0.2261	0.2013	0.2024	0.1883	0.1957	0.1881	0.187	0.1911
0.0708	0.0741	0.0762	0.0737	0.0727	0.0749	0.0773	0.0767	0.0709
0.0564	0.0644	0.0669	0.0656	0.0632	0.0611	0.0625	0.0562	0.0504
0.2789	0.2979	0.2888	0.3245	0.3058	0.3258	0.3124	0.2863	0.3057
0.3754	0.3775	0.3556	0.3617	0.3281	0.3653	0.3563	0.3677	0.3698
0.0794	0.0774	0.075	0.0729	0.072	0.0776	0.0804	0.0829	0.0788
0.0497	0.0488	0.0502	0.0514	0.0454	0.0493	0.0475	0.0472	0.0471
0.0251	0.0248	0.0216	0.0205	0.0203	0.0211	0.0211	0.0206	0.0221
0.0392	0.0401	0.0416	0.0427	0.04	0.0415	0.0391	0.0401	0.0397
0.0528	0.0539	0.0517	0.0477	0.0468	0.0516	0.0507	0.0499	0.0498
0.0359	0.0354	0.0329	0.0327	0.0294	0.0301	0.0292	0.0295	0.0296
0.0262	0.026	0.0258	0.0274	0.026	0.0265	0.0246	0.0225	0.024
0.0309	0.0312	0.0302	0.0299	0.0282	0.0276	0.025	0.0246	0.0257
0	0	0	0	0	0	0	0	0
0	0	0	0	0	0	0	0	0
0	0	0	0	0	0	0	0	0
0.01	0.0106	0.0096	0.0101	0.0088	0.0099	0.0105	0.0101	0.0111
0.0227	0.0212	0.0193	0.0193	0.0188	0.0186	0.0174	0.0164	0.0161
0.0179	0.0182	0.0183	0.0189	0.0176	0.0168	0.0155	0.0161	0.0153
0.0092	0.0095	0.0091	0.0089	0.0087	0.0093	0.0096	0.0104	0.0103
0	0	0	0	0	0	0	0	0
0	0	0	0	0	0	0	0	0
0	0	0	0	0	0	0	0	0
0	0	0	0	0	0	0	0	0
0	0	0	0	0	0	0	0	0
0	0	0	0	0	0	0	0	0
0	0	0	0	0	0	0	0	0
0	0	0	0	0	0	0	0	0
0	0	0	0	0	0	0	0	0
0	0	0	0	0	0	0	0	0
0	0	0	0	0	0	0	0	0

0	0	0	0	0	0	0	0	0
0	0	0	0	0	0	0	0	0
0	0	0	0	0	0	0	0	0
0	0	0	0	0	0	0	0	0
0	0	0	0	0	0	0	0	0
0	0	0	0	0	0	0	0	0
0	0	0	0	0	0	0	0	0
0	0	0	0	0	0	0	0	0
0	0	0	0	0	0	0	0	0
0	0	0	0	0	0	0	0	0
0	0	0	0	0	0	0	0	0
0	0	0	0	0	0	0	0	0
0	0	0	0	0	0	0	0	0
0	0	0	0	0	0	0	0	0
0	0	0	0	0	0	0	0	0
0	0	0	0	0	0	0	0	0
0.0565	0.0604	0.0591	0.0579	0.0579	0.0569	0.0601	0.0608	0.0618
0.2004	0.225	0.2095	0.2139	0.1944	0.2059	0.184	0.1976	0.1925
0.2297	0.2296	0.2179	0.2237	0.2045	0.2098	0.2065	0.1905	0.1849
0.3126	0.3225	0.3226	0.3255	0.2911	0.3084	0.3089	0.3089	0.3223
0.1487	0.1386	0.1313	0.1288	0.1213	0.1287	0.1302	0.1353	0.1322
0.1205	0.1218	0.1049	0.1076	0.0982	0.0955	0.097	0.1018	0.1037
0.3544	0.3607	0.3516	0.3281	0.3159	0.3716	0.3418	0.3766	0.3831
0.4512	0.4437	0.4482	0.4227	0.3812	0.3976	0.3856	0.3421	0.3537
0.3418	0.334	0.2856	0.3027	0.2513	0.259	0.2527	0.2503	0.2722
0.3204	0.3478	0.3378	0.3281	0.3078	0.3272	0.3187	0.3074	0.3093
0.3297	0.3445	0.3306	0.3368	0.2917	0.296	0.3473	0.3423	0.3641
0.4396	0.4678	0.4831	0.4827	0.4691	0.4953	0.5039	0.5005	0.4955
0.3387	0.3529	0.3697	0.3689	0.3522	0.3996	0.42	0.4137	0.3919
0.3146	0.314	0.3445	0.3141	0.2818	0.2954	0.2874	0.2771	0.2786
0.1333	0.1444	0.1382	0.1372	0.1378	0.127	0.1247	0.1261	0.1321
0	0	0	0	0	0	0	0	0
0.0421	0.0401	0.0424	0.0426	0.0426	0.0445	0.0462	0.0485	0.0528
0.0438	0.0452	0.0454	0.0463	0.0439	0.0433	0.0411	0.0382	0.0393
0.0304	0.032	0.0315	0.0337	0.0321	0.0333	0.0349	0.0319	0.033
0.0206	0.0223	0.0221	0.0218	0.0216	0.0221	0.0222	0.0225	0.0222
0.0163	0.0177	0.0176	0.016	0.014	0.0149	0.0151	0.0153	0.0163
0.0149	0.016	0.0157	0.0153	0.0148	0.0143	0.0156	0.0158	0.0168
0.0068	0.0072	0.0075	0.0072	0.0073	0.0078	0.0075	0.0071	0.0074
0	0	0	0	0	0	0	0	0
0	0	0	0	0	0	0	0	0
0	0	0	0	0	0	0	0	0
0	0	0	0	0	0	0	0	0
0	0	0	0	0	0	0	0	0

0	0	0	0	0	0	0	0	0
0	0	0	0	0	0	0	0	0
0	0	0	0	0	0	0	0	0
0	0	0	0	0	0	0	0	0
0	0	0	0	0	0	0	0	0
0	0	0	0	0	0	0	0	0
0	0	0	0	0	0	0	0	0
0	0	0	0	0	0	0	0	0
0	0	0	0	0	0	0	0	0
0	0	0	0	0	0	0	0	0
0	0	0	0	0	0	0	0	0
0	0	0	0	0	0	0	0	0
0	0	0	0	0	0	0	0	0
0	0	0	0	0	0	0	0	0
0	0	0	0	0	0	0	0	0
0	0	0	0	0	0	0	0	0
0	0	0	0	0	0	0	0	0
0	0	0	0	0	0	0	0	0
0	0	0	0	0	0	0	0	0
0	0	0	0	0	0	0	0	0
0	0	0	0	0	0	0	0	0
0	0	0	0	0	0	0	0	0
0.0639	0.0635	0.0616	0.0669	0.0636	0.0643	0.0631	0.063	0.0622
0.418	0.408	0.4179	0.4306	0.4331	0.4287	0.4286	0.4501	0.4653
0.3968	0.391	0.4014	0.3787	0.3765	0.3617	0.3386	0.3565	0.3684
0.1892	0.1924	0.2006	0.2187	0.2165	0.2186	0.2185	0.2139	0.2131
0.1384	0.1402	0.1378	0.1429	0.1375	0.1456	0.1531	0.1437	0.1484
0.0281	0.0304	0.0281	0.0271	0.0247	0.0259	0.0274	0.0279	0.0272
0.0485	0.0483	0.048	0.0501	0.0507	0.0495	0.0479	0.0492	0.0489
0.0698	0.0709	0.0732	0.0727	0.0666	0.0683	0.0691	0.0648	0.0672
0.2312	0.2307	0.2151	0.2122	0.1922	0.1744	0.1844	0.1869	0.2021
0.1831	0.1894	0.1776	0.1734	0.174	0.179	0.1776	0.1732	0.1725
0.201	0.1961	0.1928	0.177	0.1649	0.1751	0.1861	0.2001	0.1893
0.3293	0.3371	0.3188	0.333	0.2991	0.3391	0.3109	0.2901	0.3067
0.3953	0.4318	0.3963	0.4213	0.4493	0.4607	0.4607	0.4214	0.4181
0.4739	0.5042	0.4999	0.4824	0.4516	0.4637	0.4183	0.4001	0.4311
0.433	0.4356	0.4265	0.4418	0.4114	0.404	0.4086	0.3996	0.3979
0.3412	0.3592	0.3561	0.3551	0.3374	0.367	0.3785	0.3715	0.3711
0.1536	0.148	0.1581	0.159	0.1426	0.1538	0.1576	0.1546	0.1628
0.1421	0.1376	0.1406	0.1469	0.1423	0.1436	0.1395	0.1406	0.1351
0.0701	0.0674	0.0651	0.0679	0.0666	0.0705	0.0695	0.0688	0.0695
0.0198	0.0215	0.0233	0.0221	0.0209	0.0223	0.022	0.0221	0.0227
0	0	0	0	0	0	0	0	0
0	0	0	0	0	0	0	0	0

0.0133	0.0124	0.0118	0.0125	0.0126	0.014	0.0139	0.0129	0.0124
0.0198	0.0192	0.0205	0.0222	0.0202	0.0223	0.0207	0.0183	0.0191
0	0	0	0	0	0	0	0	0
0	0	0	0	0	0	0	0	0
0	0	0	0	0	0	0	0	0
0	0	0	0	0	0	0	0	0
0	0	0	0	0	0	0	0	0
0	0	0	0	0	0	0	0	0
0	0	0	0	0	0	0	0	0
0	0	0	0	0	0	0	0	0
0	0	0	0	0	0	0	0	0
0	0	0	0	0	0	0	0	0
0	0	0	0	0	0	0	0	0
0.0041	0.0039	0.004	0.0041	0.0044	0.0045	0.0045	0.0044	0.0042
0.005	0.0043	0.0041	0.0036	0.0038	0.0041	0.0046	0.0046	0.0035
0.023	0.0225	0.0229	0.0233	0.0232	0.0246	0.0257	0.0257	0.0262
0.0034	0.0035	0.0032	0.0032	0.0029	0.0034	0.0036	0.0037	0.0036
0.0124	0.0121	0.0127	0.0129	0.0128	0.0137	0.0141	0.0141	0.0145
0.009	0.0092	0.009	0.0087	0.0087	0.0093	0.0094	0.0094	0.0097
0.0074	0.0072	0.0066	0.0074	0.0069	0.0073	0.0069	0.0072	0.0077
0	0	0	0	0	0	0	0	0
0.0213	0.0215	0.0215	0.0214	0.0219	0.0226	0.0235	0.0235	0.0239
0.0143	0.0146	0.0143	0.0136	0.013	0.0134	0.0143	0.0145	0.014
0.0349	0.0352	0.0348	0.0359	0.0347	0.0372	0.033	0.0353	0.0336
0.0135	0.0141	0.0136	0.0137	0.0141	0.0142	0.0148	0.015	0.0156
0.0313	0.0314	0.0316	0.0326	0.0321	0.033	0.0339	0.0353	0.0357
0.0843	0.0857	0.0845	0.0814	0.0805	0.0846	0.0886	0.0907	0.0893
0.0112	0.0113	0.0113	0.0115	0.0119	0.0118	0.0116	0.0126	0.0122
0.0551	0.0556	0.0569	0.0563	0.0538	0.0579	0.0573	0.0532	0.055
0.1359	0.1406	0.127	0.1301	0.122	0.1272	0.1201	0.1102	0.1147
0.1418	0.1453	0.134	0.1401	0.1424	0.1591	0.1566	0.1487	0.1565
0.0402	0.0454	0.0451	0.0444	0.0405	0.0451	0.0404	0.0388	0.0389
0.0048	0.0051	0.0056	0.0057	0.0063	0.006	0.006	0.0061	0.006
0.024	0.0247	0.025	0.0266	0.0257	0.0252	0.0248	0.0241	0.0236
0.0312	0.0319	0.0282	0.0287	0.0249	0.0263	0.0266	0.0278	0.0282
0	0	0	0	0	0	0	0	0
0.1434	0.1534	0.146	0.1393	0.1347	0.1473	0.1474	0.1327	0.1305
0.1885	0.1904	0.1788	0.1842	0.1758	0.1777	0.1633	0.1597	0.1584
0.3031	0.3	0.2644	0.2931	0.2821	0.2543	0.2593	0.256	0.2602
0.1269	0.1272	0.1209	0.1168	0.1165	0.1053	0.1091	0.117	0.1183
0.0986	0.1056	0.1069	0.1126	0.1112	0.1094	0.1135	0.1196	0.119
0.5157	0.5933	0.5519	0.5599	0.5137	0.5485	0.5187	0.485	0.4488
0.3847	0.4156	0.4088	0.4375	0.4237	0.4467	0.413	0.3796	0.4265
0.156	0.1564	0.1519	0.1513	0.1467	0.1548	0.1405	0.1366	0.1454

0.2993	0.3441	0.3299	0.3251	0.3105	0.3505	0.3372	0.3374	0.3226
0.3844	0.425	0.3987	0.4279	0.3963	0.3906	0.373	0.3997	0.3928
0.3619	0.3681	0.3568	0.3469	0.2956	0.3437	0.339	0.334	0.3486
0.237	0.2485	0.2325	0.2334	0.2124	0.2216	0.2168	0.2013	0.1958
0.0873	0.0836	0.079	0.0756	0.0731	0.0735	0.0694	0.065	0.0646
0.0034	0.0032	0.0037	0.0037	0.004	0.0041	0.0042	0.004	0.0039
0	0	0	0	0	0	0	0	0
0.0038	0.0038	0.0037	0.0037	0.0038	0.0039	0.0041	0.0039	0.0037
0	0	0	0	0	0	0	0	0
0	0	0	0	0	0	0	0	0
0	0	0	0	0	0	0	0	0
0	0	0	0	0	0	0	0	0
0	0	0	0	0	0	0	0	0
0	0	0	0	0	0	0	0	0
0	0	0	0	0	0	0	0	0
0	0	0	0	0	0	0	0	0
0	0	0	0	0	0	0	0	0
0	0	0	0	0	0	0	0	0
0.017	0.0161	0.0167	0.0168	0.018	0.0188	0.0195	0.0197	0.0195
0.0096	0.0088	0.0095	0.0096	0.0104	0.0109	0.0116	0.0115	0.0123
0	0	0	0	0	0	0	0	0
0	0	0	0	0	0	0	0	0
0.0035	0.0036	0.0035	0.0034	0.003	0.0032	0.0036	0.0035	0.0033
0.0185	0.018	0.018	0.018	0.0184	0.0189	0.0191	0.0189	0.0189
0.0237	0.0229	0.0236	0.0233	0.0241	0.0254	0.0263	0.0265	0.0263
0.013	0.0131	0.0133	0.0134	0.0138	0.0145	0.015	0.0148	0.015
0.0427	0.0428	0.043	0.0435	0.0444	0.0455	0.0463	0.0456	0.0456
0.0408	0.0404	0.0365	0.0381	0.034	0.036	0.0329	0.0369	0.0373
0.0255	0.026	0.0259	0.0261	0.0264	0.028	0.0291	0.0292	0.0287
0.0099	0.0089	0.0091	0.0086	0.0089	0.0094	0.01	0.0098	0.0091
0.0491	0.0449	0.044	0.045	0.0495	0.052	0.0533	0.0523	0.0559
0.1638	0.1529	0.1575	0.1566	0.1614	0.1538	0.1606	0.1617	0.1712
0.2563	0.2553	0.2543	0.2639	0.2772	0.2779	0.2855	0.2883	0.2983
0.1842	0.1814	0.1929	0.1943	0.2036	0.2195	0.2269	0.2375	0.2367
0.0353	0.0346	0.0364	0.0357	0.0393	0.0413	0.0441	0.0444	0.0446
0.0137	0.0144	0.0138	0.014	0.0134	0.0134	0.0135	0.0126	0.0123
0.0216	0.0232	0.0222	0.0224	0.0222	0.0219	0.0224	0.0219	0.0228
0.0171	0.0173	0.017	0.0177	0.0156	0.0168	0.0165	0.0167	0.0166
0.0171	0.0173	0.017	0.0175	0.0168	0.018	0.0179	0.0179	0.0181
0	0	0	0	0	0	0	0	0
0.0239	0.0252	0.0256	0.0269	0.0255	0.0249	0.0236	0.0245	0.0236
0.0749	0.0769	0.074	0.0739	0.0723	0.0717	0.0721	0.0726	0.0736
0.128	0.1333	0.1291	0.1267	0.1192	0.129	0.1255	0.1195	0.1312
0.182	0.1899	0.2003	0.1903	0.1763	0.1837	0.1776	0.176	0.1841

0.1941	0.2082	0.187	0.1779	0.1678	0.1829	0.1827	0.1872	0.2027
0.1103	0.1173	0.1123	0.115	0.1032	0.1111	0.1039	0.0948	0.0947
0.1533	0.158	0.1522	0.1581	0.1659	0.168	0.1534	0.1543	0.1666
0.2588	0.2582	0.2559	0.2784	0.2886	0.292	0.2737	0.2792	0.2822
0.1803	0.1755	0.1837	0.1823	0.1854	0.1802	0.1844	0.1875	0.1939
0.204	0.225	0.2178	0.2311	0.2015	0.2126	0.189	0.1859	0.184
0.3423	0.3647	0.349	0.3721	0.3425	0.3442	0.334	0.3296	0.3244
0.4286	0.4568	0.4661	0.4495	0.4286	0.4633	0.4807	0.4868	0.5043
0.4614	0.4588	0.4902	0.4783	0.4703	0.4613	0.4738	0.4444	0.4134
0.3389	0.3374	0.3005	0.3175	0.2992	0.3322	0.3349	0.3238	0.3203
0.198	0.1967	0.1933	0.1954	0.1791	0.1812	0.1788	0.1733	0.1797
0.0528	0.0527	0.0493	0.0513	0.0486	0.0514	0.0505	0.0484	0.0455
0.0007	0.0007	0.0008	0.0008	0.0009	0.001	0.001	0.0011	0.0011
0.0255	0.0252	0.0242	0.0226	0.0228	0.0241	0.0248	0.0236	0.0237
0	0	0	0	0	0	0	0	0
0	0	0	0	0	0	0	0	0
0	0	0	0	0	0	0	0	0
0	0	0	0	0	0	0	0	0
0	0	0	0	0	0	0	0	0
0	0	0	0	0	0	0	0	0
0	0	0	0	0	0	0	0	0
0	0	0	0	0	0	0	0	0
0	0	0	0	0	0	0	0	0
0	0	0	0	0	0	0	0	0
0.0049	0.0045	0.0045	0.0046	0.0045	0.004	0.0044	0.0044	0.0045
0.0101	0.0103	0.0108	0.0108	0.0117	0.0127	0.0133	0.0137	0.0139
0.0288	0.0287	0.0299	0.0327	0.0348	0.0345	0.0357	0.0341	0.0369
0.0122	0.0115	0.012	0.0124	0.0128	0.0134	0.0141	0.0138	0.014
0.0034	0.0033	0.0035	0.0035	0.0036	0.0035	0.0036	0.0036	0.0036
0.0008	0.0007	0.0007	0.0006	0.0006	0.0006	0.0007	0.0007	0.0006
0.0192	0.018	0.019	0.0196	0.0207	0.022	0.0227	0.0229	0.0224
0.014	0.0131	0.0142	0.0143	0.0152	0.0164	0.017	0.0174	0.0178
0.0273	0.0271	0.0264	0.0267	0.0256	0.0276	0.029	0.029	0.0297
0.0273	0.0273	0.0279	0.0295	0.0303	0.0332	0.0351	0.0346	0.0342
0.1226	0.1139	0.1097	0.1084	0.1136	0.1157	0.1227	0.1209	0.1214
0.201	0.1948	0.1949	0.1911	0.1908	0.1905	0.1961	0.1922	0.1928
0.188	0.1828	0.1809	0.1801	0.1806	0.1834	0.1877	0.1858	0.182
0.2	0.1978	0.2151	0.2218	0.2314	0.2362	0.2467	0.2468	0.2524
0.2618	0.2571	0.248	0.2494	0.2664	0.2759	0.2803	0.279	0.2809
0.2563	0.2519	0.2603	0.2581	0.2768	0.2878	0.2955	0.2901	0.2881
0.1976	0.2015	0.2113	0.2136	0.2225	0.235	0.2394	0.2407	0.2445
0.03	0.0301	0.0302	0.0298	0.0299	0.0307	0.0313	0.031	0.0314
0.0046	0.0048	0.0051	0.0054	0.0058	0.0065	0.0073	0.0074	0.0076
0.0169	0.0172	0.0153	0.0162	0.0154	0.0152	0.0159	0.0151	0.0133

0.0524	0.0531	0.0531	0.0513	0.0501	0.0524	0.0495	0.0534	0.0508
0.03	0.0313	0.0312	0.0296	0.0288	0.0295	0.0299	0.0288	0.0282
0.0162	0.0186	0.0177	0.0177	0.0168	0.0175	0.0192	0.019	0.019
0.0606	0.0615	0.0629	0.06	0.0598	0.0553	0.0591	0.0521	0.0487
0.0462	0.0438	0.046	0.0457	0.0463	0.0475	0.045	0.0419	0.0442
0.0417	0.0437	0.0429	0.0453	0.0427	0.0453	0.042	0.0383	0.0337
0.008	0.0075	0.0073	0.0072	0.0078	0.0076	0.008	0.0076	0.0069
0.0325	0.0325	0.0299	0.0309	0.0301	0.0324	0.0321	0.0333	0.034
0.1864	0.1944	0.1798	0.1938	0.1973	0.1919	0.2026	0.2045	0.2002
0.1341	0.1412	0.1317	0.1318	0.1256	0.1364	0.1329	0.1393	0.1363
0.0767	0.0786	0.0776	0.0725	0.0677	0.072	0.0709	0.0662	0.071
0.3226	0.3294	0.3127	0.3238	0.3119	0.3349	0.3426	0.335	0.3193
0.3814	0.4049	0.3887	0.4045	0.359	0.3846	0.3948	0.3819	0.3935
0.2466	0.2631	0.244	0.2543	0.2432	0.2509	0.2913	0.3008	0.3176
0.2209	0.2256	0.2046	0.1931	0.174	0.1796	0.1704	0.1658	0.1849
0.1632	0.1708	0.1794	0.1855	0.179	0.1902	0.1757	0.172	0.176
0.1328	0.1376	0.1448	0.1391	0.1299	0.1399	0.1471	0.1494	0.1655
0.1129	0.1124	0.1021	0.1043	0.091	0.0946	0.0916	0.0984	0.0963
0	0	0	0	0	0	0	0	0
0.0159	0.0163	0.0161	0.0167	0.0164	0.0171	0.0169	0.0167	0.0173
0	0	0	0	0	0	0	0	0
0	0	0	0	0	0	0	0	0
0	0	0	0	0	0	0	0	0
0	0	0	0	0	0	0	0	0
0	0	0	0	0	0	0	0	0
0	0	0	0	0	0	0	0	0
0	0	0	0	0	0	0	0	0
0	0	0	0	0	0	0	0	0
0	0	0	0	0	0	0	0	0
0	0	0	0	0	0	0	0	0
0.0041	0.0043	0.0041	0.0037	0.0037	0.0038	0.0036	0.0036	0.0036
0.0323	0.0318	0.0335	0.0337	0.0377	0.0382	0.0389	0.0389	0.04
0.0752	0.0739	0.0796	0.0849	0.094	0.1023	0.1037	0.106	0.1081
0.0347	0.0331	0.0344	0.0354	0.0367	0.0384	0.0389	0.0397	0.041
0.0105	0.0103	0.0108	0.0107	0.0109	0.011	0.0112	0.0118	0.012
0.0117	0.0112	0.0104	0.0095	0.0106	0.0104	0.011	0.0108	0.0106
0.0975	0.0905	0.0927	0.0924	0.095	0.0965	0.1032	0.1022	0.0997
0.1452	0.1377	0.1433	0.1414	0.1558	0.1567	0.1625	0.1595	0.1542
0.1446	0.1408	0.1361	0.1329	0.1378	0.143	0.1468	0.1457	0.1446
0.1611	0.1588	0.1618	0.1617	0.1655	0.1713	0.1774	0.178	0.1756
0.3799	0.3852	0.3657	0.3735	0.3624	0.3637	0.3789	0.3827	0.3849
0.6561	0.6533	0.655	0.6393	0.6011	0.6267	0.6345	0.6176	0.5906
0.4873	0.4898	0.4667	0.4836	0.4735	0.4882	0.4933	0.4488	0.4295
0.2079	0.2048	0.2072	0.2087	0.2111	0.2176	0.2224	0.2207	0.2194

0.1864	0.1754	0.1787	0.1834	0.1912	0.1979	0.2026	0.2162	0.2296
0.2608	0.2576	0.2655	0.2735	0.2864	0.3171	0.323	0.3197	0.3263
0.2041	0.2035	0.2137	0.2215	0.2275	0.2346	0.2273	0.2231	0.238
0.0656	0.0656	0.0662	0.0668	0.0682	0.0712	0.0734	0.0735	0.0728
0.0201	0.0183	0.0195	0.0202	0.0214	0.0209	0.022	0.0234	0.0214
0.0089	0.0081	0.0075	0.0074	0.0076	0.0069	0.0073	0.0069	0.0056
0.0261	0.0268	0.0272	0.03	0.0278	0.0257	0.0265	0.0253	0.0266
0.0549	0.0563	0.0533	0.0527	0.051	0.0525	0.0513	0.052	0.0541
0.0222	0.0199	0.0189	0.0165	0.0182	0.0186	0.0187	0.0177	0.0166
0.0152	0.0142	0.0149	0.0134	0.0137	0.0128	0.0131	0.0119	0.011
0.0496	0.0492	0.0486	0.0481	0.048	0.051	0.0523	0.0519	0.0535
0	0	0	0	0	0	0	0	0
0	0	0	0	0	0	0	0	0
0.0359	0.0373	0.0367	0.0373	0.0348	0.0375	0.036	0.0354	0.0374
0.0759	0.0858	0.084	0.084	0.0767	0.0777	0.0822	0.0845	0.0893
0.0446	0.0444	0.044	0.0434	0.0387	0.0417	0.0397	0.0375	0.0396
0.0219	0.0236	0.023	0.023	0.0199	0.0213	0.0219	0.0218	0.0227
0.1367	0.1393	0.1456	0.1481	0.1448	0.1454	0.1433	0.1417	0.1397
0.3581	0.3686	0.3531	0.3683	0.3194	0.3437	0.3539	0.3485	0.3844
0.3304	0.3694	0.3417	0.3226	0.3051	0.311	0.3008	0.2901	0.2734
0.2016	0.2009	0.1939	0.1963	0.1944	0.2118	0.2027	0.2187	0.2113
0.2638	0.2828	0.2772	0.2687	0.2645	0.2635	0.2823	0.28	0.2628
0.449	0.4288	0.388	0.4141	0.4067	0.4312	0.4282	0.439	0.4325
0.3135	0.3256	0.3241	0.3181	0.2917	0.3213	0.2931	0.2846	0.3046
0.0635	0.0648	0.0655	0.0664	0.0654	0.0714	0.0736	0.0728	0.0726
0	0	0	0	0	0	0	0	0
0	0	0	0	0	0	0	0	0
0	0	0	0	0	0	0	0	0
0	0	0	0	0	0	0	0	0
0	0	0	0	0	0	0	0	0
0	0	0	0	0	0	0	0	0
0	0	0	0	0	0	0	0	0
0	0	0	0	0	0	0	0	0
0	0	0	0	0	0	0	0	0
0	0	0	0	0	0	0	0	0
0	0	0	0	0	0	0	0	0
0.01	0.0102	0.0105	0.0108	0.0115	0.012	0.0122	0.0119	0.0122
0.0062	0.0063	0.0064	0.0064	0.0062	0.0064	0.0068	0.0067	0.0067
0.0299	0.0303	0.032	0.031	0.0314	0.0332	0.0351	0.0355	0.0357
0	0	0	0	0	0	0	0	0
0.0076	0.0078	0.0076	0.0082	0.0081	0.0081	0.0086	0.0085	0.0084
0.0453	0.043	0.0421	0.0425	0.0431	0.0444	0.0463	0.0432	0.0418
0.1159	0.107	0.105	0.1078	0.1112	0.1104	0.1064	0.105	0.0975
0.1516	0.1389	0.1372	0.1448	0.1507	0.1405	0.1299	0.1243	0.1144

0.2005	0.1818	0.181	0.1902	0.1924	0.1825	0.1811	0.1811	0.1735
0.1752	0.1555	0.1607	0.1572	0.1694	0.1701	0.1844	0.1833	0.1829
0.1862	0.182	0.1832	0.1836	0.187	0.1918	0.196	0.1947	0.1924
0.4013	0.4029	0.3859	0.3779	0.3528	0.3656	0.3844	0.3729	0.3648
0.46	0.4621	0.4675	0.4726	0.4776	0.4976	0.4912	0.492	0.4701
0.2315	0.2213	0.219	0.2109	0.2085	0.2061	0.2067	0.2054	0.2017
0.1598	0.151	0.1528	0.1529	0.1581	0.161	0.169	0.1699	0.1685
0.2041	0.195	0.2003	0.2089	0.2198	0.2324	0.2525	0.2561	0.2521
0.1444	0.1392	0.1409	0.1402	0.1498	0.151	0.1528	0.149	0.146
0.1487	0.1392	0.134	0.1237	0.1223	0.1165	0.1219	0.1123	0.0917
0.2304	0.2128	0.2119	0.2072	0.2112	0.2155	0.2114	0.2117	0.187
0.2381	0.2306	0.2285	0.2032	0.2043	0.1904	0.1822	0.1646	0.1623
0.1937	0.1871	0.1843	0.1816	0.1946	0.1816	0.1681	0.1598	0.1436
0.2243	0.2149	0.2116	0.2008	0.1923	0.1711	0.1723	0.1617	0.1402
0.2043	0.1968	0.1941	0.1747	0.183	0.1679	0.1585	0.1426	0.1318
0.1722	0.1713	0.1629	0.1544	0.1468	0.1409	0.135	0.1303	0.1147
0.0183	0.0158	0.0151	0.017	0.0178	0.0155	0.0159	0.0139	0.0101
0.0348	0.0321	0.0322	0.0309	0.0312	0.0295	0.0297	0.0299	0.0285
0.067	0.0666	0.066	0.0655	0.063	0.0628	0.0623	0.0611	0.0583
0.0583	0.0589	0.0586	0.0596	0.0571	0.0593	0.0595	0.0599	0.0593
0.0238	0.0229	0.0227	0.0229	0.024	0.0253	0.0264	0.0259	0.0252
0.0239	0.0227	0.0223	0.0226	0.0232	0.023	0.0238	0.0236	0.0231
0.0791	0.0783	0.0754	0.0823	0.0792	0.0874	0.0864	0.0834	0.084
0.1358	0.1429	0.1374	0.135	0.1191	0.1156	0.1167	0.1131	0.1243
0.2369	0.239	0.2261	0.2258	0.2222	0.236	0.2397	0.2386	0.2268
0.3035	0.3167	0.3337	0.3179	0.302	0.3101	0.2991	0.2737	0.306
0.3634	0.3727	0.3442	0.354	0.323	0.3296	0.3303	0.3018	0.32
0.3849	0.3694	0.372	0.3287	0.31	0.3656	0.3529	0.3762	0.3991
0.502	0.5046	0.4648	0.4821	0.4228	0.4736	0.4742	0.4653	0.4674
0.2733	0.2769	0.2734	0.2816	0.2592	0.2735	0.2998	0.2716	0.2736
0.0617	0.0641	0.0631	0.0631	0.0598	0.0597	0.0633	0.0613	0.0644
0	0	0	0	0	0	0	0	0
0	0	0	0	0	0	0	0	0
0	0	0	0	0	0	0	0	0
0	0	0	0	0	0	0	0	0
0	0	0	0	0	0	0	0	0
0	0	0	0	0	0	0	0	0
0	0	0	0	0	0	0	0	0
0	0	0	0	0	0	0	0	0
0	0	0	0	0	0	0	0	0
0	0	0	0	0	0	0	0	0
0	0	0	0	0	0	0	0	0
0.0025	0.0021	0.0019	0.0016	0.0017	0.0013	0.0013	0.0013	0.0009
0	0	0	0	0	0	0	0	0

0.0271	0.025	0.0249	0.0259	0.0274	0.0294	0.0311	0.0313	0.0324
0.0034	0.0037	0.0035	0.0036	0.0035	0.004	0.0036	0.0034	0.0032
0.0246	0.0238	0.0241	0.0243	0.0249	0.0257	0.0263	0.0263	0.026
0.0786	0.0761	0.0773	0.0762	0.077	0.0774	0.0791	0.0784	0.0758
0.122	0.1121	0.1106	0.1068	0.1122	0.1177	0.1239	0.1192	0.1136
0.1426	0.1325	0.1394	0.1366	0.1386	0.1371	0.1405	0.1363	0.1285
0.168	0.152	0.1418	0.1527	0.15	0.1376	0.1531	0.1516	0.1351
0.1689	0.1605	0.1573	0.1558	0.1655	0.1543	0.1591	0.1576	0.1413
0.1567	0.1503	0.1521	0.1519	0.1557	0.1574	0.164	0.1623	0.1541
0.1954	0.1953	0.204	0.2051	0.2128	0.2167	0.2235	0.218	0.2174
0.2285	0.2224	0.2198	0.2204	0.2252	0.2278	0.2302	0.228	0.2281
0.1563	0.1491	0.1507	0.1489	0.1589	0.1584	0.1632	0.1565	0.1486
0.1647	0.1582	0.1607	0.1661	0.1712	0.1734	0.1788	0.1764	0.171
0.1572	0.1483	0.1528	0.1473	0.1547	0.1527	0.1587	0.1593	0.1574
0.1428	0.1284	0.1247	0.1326	0.1429	0.131	0.1454	0.1395	0.1301
0.2104	0.1935	0.1761	0.1878	0.1953	0.1885	0.1893	0.192	0.1804
0.2871	0.2733	0.2587	0.2577	0.2578	0.244	0.2789	0.252	0.2349
0.3218	0.3274	0.3122	0.3131	0.3038	0.2987	0.3122	0.2741	0.2567
0.3213	0.3078	0.3069	0.2941	0.2833	0.2635	0.2673	0.2623	0.2674
0.1985	0.1908	0.1898	0.1727	0.1653	0.1569	0.1722	0.1692	0.1319
0.2086	0.2011	0.1853	0.1651	0.1761	0.1608	0.1586	0.1621	0.1468
0.3351	0.3185	0.3145	0.3038	0.2936	0.2829	0.2718	0.2692	0.2439
0.2969	0.2875	0.2978	0.3152	0.3069	0.3163	0.2917	0.2682	0.2509
0.3151	0.3051	0.2892	0.2953	0.2964	0.2864	0.2736	0.2816	0.2578
0.1433	0.1331	0.122	0.1157	0.1208	0.1168	0.1158	0.1153	0.1094
0.1162	0.1068	0.1044	0.0976	0.1007	0.0886	0.1053	0.106	0.0945
0.1207	0.1168	0.1167	0.1185	0.1216	0.1232	0.1266	0.1269	0.1246
0.0868	0.0893	0.0904	0.0906	0.0909	0.0952	0.0948	0.0953	0.0953
0.1374	0.1462	0.1422	0.1473	0.138	0.1402	0.1355	0.145	0.1346
0.1795	0.1858	0.1882	0.1941	0.1807	0.1853	0.1953	0.199	0.1955
0.1382	0.1487	0.1516	0.1413	0.1147	0.1276	0.1315	0.1291	0.1247
0.1179	0.1197	0.1096	0.1086	0.1046	0.1151	0.111	0.1182	0.1135
0.1481	0.1517	0.1487	0.1469	0.1407	0.1437	0.1435	0.1373	0.1402
0.27	0.251	0.2261	0.2256	0.218	0.2297	0.2088	0.2293	0.2436
0.3077	0.3023	0.3231	0.3038	0.2935	0.2874	0.2665	0.2717	0.288
0.2143	0.2266	0.2185	0.1975	0.1862	0.1962	0.2065	0.2062	0.2049
0.0549	0.0594	0.0576	0.0637	0.0636	0.0675	0.0674	0.0678	0.0701
0.0075	0.0068	0.0072	0.0072	0.0068	0.0074	0.0077	0.0079	0.0076
0	0	0	0	0	0	0	0	0
0	0	0	0	0	0	0	0	0
0	0	0	0	0	0	0	0	0
0	0	0	0	0	0	0	0	0
0	0	0	0	0	0	0	0	0
0	0	0	0	0	0	0	0	0

0	0	0	0	0	0	0	0	0
0	0	0	0	0	0	0	0	0
0	0	0	0	0	0	0	0	0
0	0	0	0	0	0	0	0	0
0	0	0	0	0	0	0	0	0
0	0	0	0	0	0	0	0	0
0.0032	0.0032	0.0029	0.0032	0.0028	0.003	0.0031	0.0032	0.0034
0.0089	0.0084	0.0076	0.0072	0.007	0.0074	0.0073	0.0071	0.0068
0.0295	0.0287	0.0286	0.0289	0.0293	0.0292	0.0304	0.0297	0.0291
0.1236	0.1189	0.122	0.1244	0.1284	0.1336	0.1368	0.1316	0.1353
0.1437	0.1396	0.1445	0.1458	0.1505	0.1536	0.1565	0.1613	0.1561
0.1632	0.1569	0.1689	0.1654	0.1725	0.1806	0.1872	0.1881	0.1878
0.4695	0.4453	0.4273	0.413	0.4053	0.4393	0.4348	0.4202	0.3997
0.4341	0.424	0.3938	0.4006	0.3884	0.385	0.3863	0.3816	0.3908
0.1774	0.1731	0.172	0.1639	0.1613	0.1571	0.1626	0.159	0.151
0.165	0.1516	0.1546	0.1531	0.1581	0.1594	0.1604	0.1646	0.1573
0.1762	0.1658	0.1691	0.1702	0.1765	0.178	0.1804	0.1775	0.1785
0.204	0.197	0.1998	0.2012	0.2023	0.2092	0.2162	0.2158	0.213
0.2084	0.2037	0.2023	0.204	0.2081	0.2113	0.2125	0.2099	0.2071
0.1521	0.1438	0.144	0.1393	0.1469	0.1456	0.1485	0.1482	0.1403
0.1974	0.1882	0.1816	0.1677	0.1799	0.172	0.1652	0.1529	0.1371
0.2185	0.1938	0.1833	0.1908	0.1974	0.2042	0.2008	0.1759	0.1509
0.2838	0.2659	0.2372	0.2315	0.2516	0.2257	0.2313	0.2357	0.2019
0.2644	0.2549	0.2294	0.21	0.2096	0.1965	0.2058	0.1885	0.1735
0.2487	0.2412	0.222	0.2152	0.2181	0.2172	0.2114	0.2089	0.204
0.1567	0.1515	0.1477	0.1407	0.1309	0.1164	0.1144	0.1151	0.1082
0.1431	0.1346	0.1319	0.1282	0.1375	0.1375	0.1469	0.1423	0.1378
0.1724	0.1659	0.1623	0.1608	0.1632	0.1641	0.1633	0.1624	0.1595
0.519	0.511	0.4485	0.4786	0.4312	0.4213	0.4145	0.398	0.4069
0.5422	0.5304	0.5161	0.5115	0.5015	0.5133	0.5008	0.4734	0.4508
0.2621	0.2249	0.224	0.2074	0.1963	0.1929	0.1797	0.1746	0.1736
0.2162	0.197	0.2031	0.1893	0.1797	0.1675	0.1787	0.1789	0.1713
0.2303	0.2236	0.2192	0.2158	0.223	0.2254	0.2213	0.2156	0.2049
0.137	0.1328	0.1343	0.1421	0.1638	0.1707	0.1824	0.1798	0.1781
0.1301	0.1277	0.1336	0.1364	0.1441	0.1503	0.162	0.1558	0.1628
0.0494	0.0487	0.0492	0.0491	0.0495	0.0511	0.0523	0.0523	0.0523
0.1229	0.1234	0.1255	0.1246	0.1136	0.1233	0.1201	0.1156	0.1133
0.0037	0.0035	0.0031	0.0029	0.0027	0.0029	0.003	0.0031	0.0031
0.0113	0.0122	0.0124	0.0129	0.0127	0.0134	0.0128	0.012	0.0118
0.1241	0.1306	0.1201	0.1313	0.116	0.1358	0.1394	0.1306	0.1307
0.2349	0.2413	0.2098	0.2176	0.202	0.2079	0.2188	0.231	0.2333
0.1737	0.1687	0.1558	0.1501	0.128	0.137	0.1358	0.1419	0.1354
0.1249	0.1226	0.1144	0.1114	0.1102	0.1056	0.0986	0.1005	0.1036
0.0169	0.0167	0.0172	0.0189	0.017	0.0177	0.0182	0.0175	0.0167

0	0	0	0	0	0	0	0	0
0	0	0	0	0	0	0	0	0
0	0	0	0	0	0	0	0	0
0	0	0	0	0	0	0	0	0
0	0	0	0	0	0	0	0	0
0	0	0	0	0	0	0	0	0
0	0	0	0	0	0	0	0	0
0	0	0	0	0	0	0	0	0
0	0	0	0	0	0	0	0	0
0	0	0	0	0	0	0	0	0
0.0082	0.0079	0.0081	0.0083	0.0086	0.0092	0.0097	0.0098	0.0105
0.0016	0.0014	0.0013	0.0013	0.0012	0.0011	0.001	0.0012	0.0011
0.0046	0.0043	0.0039	0.0039	0.0035	0.0036	0.0035	0.0037	0.0035
0.0151	0.015	0.0155	0.0159	0.0155	0.0161	0.0165	0.0163	0.0166
0.0319	0.0315	0.0318	0.032	0.0322	0.0331	0.0338	0.033	0.0325
0.1376	0.1306	0.134	0.1343	0.137	0.1429	0.1466	0.1479	0.1489
0.1441	0.1405	0.143	0.1456	0.1489	0.1566	0.1625	0.1606	0.159
0.1823	0.1814	0.1874	0.19	0.1983	0.2025	0.2002	0.2038	0.2089
0.2062	0.2009	0.2062	0.2032	0.2012	0.205	0.2055	0.2034	0.2029
0.2013	0.2041	0.2049	0.1905	0.1945	0.1987	0.2009	0.1994	0.1947
0.2057	0.1929	0.198	0.1862	0.1823	0.1768	0.1914	0.1858	0.1779
0.1828	0.1659	0.1608	0.1524	0.1588	0.157	0.1612	0.164	0.1582
0.1608	0.1534	0.1534	0.1529	0.1582	0.1658	0.1741	0.1795	0.1727
0.1297	0.1237	0.133	0.1354	0.1377	0.1408	0.1486	0.1512	0.1562
0.1258	0.1222	0.1257	0.1296	0.1346	0.1374	0.1386	0.1413	0.1386
0.1713	0.1682	0.171	0.1756	0.182	0.1918	0.1965	0.1951	0.1942
0.1365	0.1291	0.1302	0.1315	0.133	0.1345	0.1399	0.1422	0.14
0.1545	0.1352	0.1333	0.1167	0.1239	0.1212	0.1288	0.1266	0.1092
0.1603	0.1378	0.1214	0.1058	0.1187	0.1107	0.1132	0.1194	0.1092
0.2009	0.1975	0.1801	0.156	0.1635	0.1393	0.1416	0.1485	0.1079
0.2518	0.2247	0.22	0.215	0.2238	0.2171	0.2204	0.2152	0.2224
0.2026	0.202	0.2084	0.1998	0.1898	0.1897	0.1898	0.1838	0.1839
0.2083	0.1999	0.1983	0.1986	0.1972	0.2012	0.2057	0.2034	0.1996
0.1807	0.1743	0.1796	0.1739	0.173	0.1623	0.1643	0.159	0.1383
0.2281	0.2191	0.2163	0.2028	0.1964	0.197	0.2029	0.1897	0.1875
0.274	0.2674	0.2753	0.2652	0.251	0.2531	0.26	0.2566	0.2228
0.2708	0.2727	0.263	0.2661	0.2667	0.2745	0.285	0.2843	0.2745
0.3264	0.3201	0.2833	0.2721	0.2782	0.2861	0.3003	0.2861	0.2783
0.2807	0.2655	0.2356	0.2327	0.2398	0.2302	0.2281	0.2269	0.2151
0.1529	0.1483	0.1482	0.1478	0.1552	0.1606	0.1674	0.1672	0.1721
0.2362	0.2264	0.2318	0.2366	0.2463	0.255	0.2674	0.2719	0.2738
0.1563	0.1576	0.1627	0.1564	0.1633	0.1719	0.1756	0.1765	0.1821
0.1207	0.1184	0.1228	0.12	0.1184	0.1259	0.1302	0.1323	0.1379
0.0189	0.0202	0.0204	0.0229	0.0248	0.0271	0.0282	0.0274	0.0277

0.0173	0.0176	0.0158	0.0152	0.0136	0.0137	0.0141	0.014	0.0142
0.0875	0.0956	0.0949	0.0973	0.0969	0.1008	0.0989	0.0954	0.0908
0.0707	0.07	0.069	0.0625	0.0602	0.0623	0.0661	0.0643	0.0686
0.0531	0.0554	0.0551	0.0577	0.0529	0.055	0.0527	0.0524	0.0536
0.0424	0.0475	0.0442	0.0426	0.0388	0.0373	0.0368	0.0338	0.0334
0	0	0	0	0	0	0	0	0
0	0	0	0	0	0	0	0	0
0	0	0	0	0	0	0	0	0
0	0	0	0	0	0	0	0	0
0	0	0	0	0	0	0	0	0
0	0	0	0	0	0	0	0	0
0	0	0	0	0	0	0	0	0
0	0	0	0	0	0	0	0	0
0	0	0	0	0	0	0	0	0
0	0	0	0	0	0	0	0	0
0	0	0	0	0	0	0	0	0
0.0166	0.0153	0.0141	0.0141	0.0141	0.0149	0.0144	0.0143	0.0139
0.0269	0.0273	0.0271	0.0273	0.0261	0.0271	0.0273	0.0274	0.0277
0.0185	0.0186	0.0174	0.0175	0.0161	0.0166	0.0153	0.0145	0.0135
0.0037	0.0036	0.0037	0.0035	0.0039	0.004	0.0041	0.0041	0.0041
0.0314	0.029	0.0315	0.0322	0.033	0.0339	0.035	0.0356	0.0352
0.1624	0.158	0.1609	0.1633	0.1651	0.1723	0.1785	0.1789	0.178
0.1644	0.1615	0.1638	0.1636	0.1684	0.1721	0.1797	0.1784	0.1776
0.1538	0.1489	0.1539	0.1542	0.158	0.1573	0.1624	0.1616	0.1546
0.1725	0.1561	0.1474	0.1436	0.1526	0.1465	0.1552	0.156	0.1418
0.1778	0.1633	0.1633	0.1705	0.1701	0.1718	0.1714	0.1566	0.1429
0.2142	0.1995	0.1943	0.1901	0.1899	0.1764	0.1835	0.1683	0.1538
0.1695	0.1569	0.1526	0.1498	0.1561	0.1526	0.1621	0.1643	0.1529
0.1663	0.1563	0.1593	0.1633	0.1724	0.1724	0.1816	0.1801	0.1826
0.2531	0.2496	0.2435	0.2461	0.2496	0.2524	0.2568	0.2538	0.2533
0.2278	0.2208	0.2143	0.2111	0.2016	0.205	0.2093	0.213	0.2124
0.1119	0.1074	0.1096	0.1105	0.113	0.117	0.1165	0.1116	0.1082
0.1539	0.1423	0.141	0.1378	0.1513	0.1567	0.1636	0.1637	0.1622
0.1434	0.1307	0.1222	0.1127	0.1173	0.1247	0.1326	0.1287	0.1139
0.1675	0.1631	0.1627	0.1567	0.1679	0.1686	0.1669	0.1588	0.1444
0.1848	0.1757	0.1598	0.1582	0.1739	0.142	0.1547	0.164	0.1432
0.2047	0.1806	0.1766	0.1834	0.2064	0.1976	0.2185	0.2143	0.2087
0.1894	0.1771	0.1725	0.1636	0.165	0.162	0.1646	0.156	0.1399
0.1732	0.1577	0.1437	0.1477	0.1508	0.1493	0.1438	0.1459	0.1349
0.2042	0.1936	0.1885	0.1768	0.1761	0.1799	0.1806	0.1704	0.1484
0.1765	0.1473	0.1381	0.1348	0.1442	0.1252	0.1296	0.0902	0.0884
0.1307	0.1149	0.1068	0.101	0.0951	0.1134	0.1197	0.0978	0.0875
0.2139	0.2094	0.2097	0.2069	0.2074	0.2238	0.2275	0.2069	0.1897
0.2783	0.2609	0.2438	0.2428	0.2248	0.2277	0.2393	0.2338	0.22

0.1678	0.1444	0.1638	0.1391	0.1361	0.1245	0.1418	0.1443	0.1229
0.1404	0.1274	0.1329	0.1253	0.1239	0.1201	0.1285	0.1276	0.1105
0.1596	0.1543	0.1537	0.1523	0.1617	0.1589	0.1692	0.1632	0.1517
0.1762	0.1669	0.1674	0.1645	0.1735	0.172	0.1814	0.182	0.1721
0.1437	0.1359	0.1348	0.1319	0.1392	0.1381	0.1417	0.1406	0.1376
0.0382	0.0384	0.0409	0.0403	0.0407	0.0431	0.0432	0.0445	0.0449
0.0854	0.0838	0.0846	0.0878	0.0851	0.0921	0.0938	0.094	0.099
0.1331	0.1298	0.1319	0.1385	0.1285	0.1269	0.1278	0.1264	0.1273
0.0824	0.0816	0.0848	0.0861	0.0833	0.0866	0.089	0.0789	0.0805
0.027	0.0281	0.0288	0.0278	0.0249	0.0256	0.0247	0.0227	0.023
0.0127	0.0119	0.0121	0.0114	0.0113	0.0102	0.0113	0.0105	0.0097
0.0148	0.0157	0.0155	0.0145	0.0134	0.0141	0.0131	0.0132	0.014
0.0034	0.0036	0.0033	0.0032	0.003	0.0033	0.0033	0.0031	0.0031
0.0036	0.0038	0.0035	0.0033	0.0031	0.0032	0.0032	0.0029	0.003
0	0	0	0	0	0	0	0	0
0	0	0	0	0	0	0	0	0
0	0	0	0	0	0	0	0	0
0	0	0	0	0	0	0	0	0
0	0	0	0	0	0	0	0	0
0	0	0	0	0	0	0	0	0
0	0	0	0	0	0	0	0	0
0	0	0	0	0	0	0	0	0
0.0236	0.0237	0.0232	0.0251	0.0239	0.0258	0.0246	0.0241	0.0228
0.0124	0.0135	0.013	0.013	0.012	0.0125	0.0132	0.0126	0.0125
0.0234	0.0235	0.0232	0.0252	0.0248	0.0234	0.023	0.024	0.0236
0.0592	0.0585	0.0593	0.0601	0.0594	0.0619	0.0628	0.0614	0.0608
0.1283	0.1184	0.1222	0.1248	0.1299	0.1293	0.1362	0.1387	0.1382
0.1556	0.1464	0.1528	0.1481	0.1551	0.1589	0.1656	0.163	0.1585
0.1694	0.1586	0.1544	0.1464	0.1556	0.1534	0.1537	0.1598	0.1568
0.1805	0.1633	0.1707	0.1678	0.1731	0.1649	0.1665	0.1693	0.151
0.1543	0.1407	0.1383	0.1228	0.1262	0.1275	0.1301	0.1258	0.1027
0.2516	0.2312	0.2299	0.2282	0.2247	0.2306	0.2229	0.2192	0.1833
0.1926	0.186	0.1763	0.1733	0.1656	0.1506	0.1444	0.1485	0.1247
0.173	0.163	0.1619	0.1501	0.1562	0.1538	0.1619	0.1632	0.1498
0.1396	0.1336	0.1335	0.1339	0.1428	0.1446	0.1491	0.1472	0.1419
0.2791	0.2611	0.2405	0.2377	0.2461	0.25	0.2615	0.2475	0.2514
0.4591	0.464	0.4526	0.4671	0.4039	0.4135	0.4068	0.3846	0.3772
0.4039	0.4209	0.4011	0.3966	0.3929	0.399	0.3951	0.371	0.3429
0.24	0.2382	0.2339	0.2351	0.2301	0.2412	0.2391	0.2349	0.2331
0.1559	0.1418	0.1355	0.1346	0.1475	0.1409	0.1421	0.1397	0.1312
0.1604	0.1265	0.1191	0.1156	0.1108	0.1003	0.1103	0.1005	0.0808
0.245	0.2256	0.2128	0.196	0.1969	0.1837	0.196	0.2122	0.1984
0.2049	0.1885	0.1977	0.1978	0.2019	0.1978	0.186	0.1861	0.1315
0.1582	0.1459	0.1434	0.1187	0.1089	0.0846	0.0996	0.1076	0.0858

0.1998	0.1885	0.1743	0.1725	0.1721	0.1574	0.1619	0.1462	0.1122
0.1699	0.1511	0.1657	0.1456	0.1599	0.1418	0.1559	0.1432	0.1259
0.1341	0.1129	0.1089	0.0972	0.0886	0.0972	0.1004	0.1134	0.1022
0.1622	0.1426	0.1518	0.1467	0.1625	0.1464	0.1467	0.1412	0.1173
0.1914	0.1742	0.1564	0.1463	0.1429	0.1467	0.1478	0.1415	0.1406
0.2278	0.2142	0.2073	0.1808	0.1812	0.1622	0.1753	0.1724	0.1573
0.1478	0.1243	0.1167	0.1022	0.1069	0.1081	0.1336	0.1266	0.1183
0.1761	0.1595	0.1445	0.1382	0.142	0.132	0.1375	0.1256	0.1275
0.219	0.2106	0.1912	0.2075	0.2052	0.1875	0.1872	0.1881	0.1781
0.2632	0.2581	0.2302	0.2167	0.219	0.1848	0.1887	0.1774	0.1674
0.1572	0.1376	0.1326	0.1362	0.1392	0.1392	0.1476	0.1419	0.1317
0.1433	0.1357	0.1367	0.1332	0.1434	0.143	0.1526	0.1543	0.1465
0.1777	0.1699	0.1783	0.1744	0.1852	0.1798	0.1868	0.1937	0.1923
0.1264	0.131	0.1365	0.1397	0.1408	0.1486	0.1526	0.1532	0.1614
0.0732	0.072	0.073	0.0727	0.0727	0.0756	0.0765	0.0766	0.0778
0.0315	0.0302	0.0281	0.0275	0.0287	0.0297	0.0297	0.029	0.0295
0.0397	0.04	0.0407	0.0417	0.0426	0.0444	0.0445	0.0469	0.047
0.0362	0.0391	0.0376	0.0373	0.0361	0.038	0.0383	0.0374	0.0397
0.0037	0.0036	0.0033	0.0034	0.0031	0.0031	0.0031	0.0033	0.0033
0.003	0.0031	0.0032	0.0033	0.0031	0.0033	0.0033	0.0033	0.0033
0	0	0	0	0	0	0	0	0
0	0	0	0	0	0	0	0	0
0	0	0	0	0	0	0	0	0
0	0	0	0	0	0	0	0	0
0	0	0	0	0	0	0	0	0
0	0	0	0	0	0	0	0	0
0	0	0	0	0	0	0	0	0
0	0	0	0	0	0	0	0	0
0.0215	0.0234	0.0223	0.0221	0.021	0.0219	0.0212	0.0214	0.0213
0	0	0	0	0	0	0	0	0
0.0225	0.0229	0.0229	0.0228	0.0219	0.0213	0.0201	0.0203	0.0207
0.0851	0.0872	0.0888	0.089	0.0872	0.0913	0.0929	0.0926	0.0917
0.1838	0.1832	0.185	0.1869	0.1962	0.2023	0.2076	0.2058	0.2016
0.1517	0.1324	0.1348	0.135	0.1423	0.1426	0.1537	0.154	0.1503
0.1533	0.1273	0.1205	0.1272	0.138	0.1159	0.1244	0.1447	0.1361
0.2188	0.2024	0.1874	0.1822	0.1797	0.1803	0.1926	0.1738	0.1486
0.2138	0.2069	0.2008	0.1887	0.1976	0.1876	0.2009	0.197	0.1587
0.2194	0.224	0.2179	0.2031	0.2128	0.2095	0.2174	0.2149	0.2162
0.1879	0.1815	0.1794	0.177	0.1846	0.1591	0.1599	0.1389	0.1267
0.1798	0.1722	0.173	0.1675	0.1887	0.1672	0.1587	0.1533	0.1236
0.127	0.1138	0.1105	0.1006	0.1115	0.1121	0.1189	0.1143	0.1108
0.2193	0.2134	0.2069	0.2057	0.2002	0.1913	0.1915	0.1936	0.1764
0.5945	0.5718	0.5232	0.5191	0.4974	0.4993	0.5051	0.4961	0.4971
0.64	0.6408	0.6001	0.5898	0.5653	0.5687	0.5278	0.5494	0.5365

0.2495	0.2343	0.2288	0.2377	0.2443	0.2409	0.2359	0.238	0.2365
0.1102	0.0943	0.0941	0.0871	0.1038	0.0818	0.0929	0.0952	0.0711
0.2058	0.1812	0.1546	0.1367	0.1309	0.129	0.1362	0.1326	0.1135
0.3466	0.3293	0.3205	0.3089	0.2912	0.2831	0.2737	0.2835	0.2623
0.2243	0.2028	0.1844	0.1707	0.1747	0.1749	0.171	0.174	0.1639
0.1612	0.1424	0.127	0.1202	0.1367	0.1285	0.143	0.1449	0.1069
0.2377	0.2335	0.2191	0.2029	0.1996	0.1795	0.1991	0.1867	0.1678
0.1735	0.1578	0.1483	0.1405	0.1423	0.1306	0.1222	0.1184	0.0979
0.191	0.1618	0.1496	0.1453	0.1439	0.1479	0.1697	0.1572	0.1298
0.1343	0.1175	0.1093	0.1204	0.1392	0.1235	0.1327	0.1328	0.1075
0.1924	0.1794	0.1823	0.1865	0.1945	0.1881	0.1862	0.1772	0.157
0.1561	0.1386	0.1349	0.1291	0.13	0.1248	0.1339	0.1327	0.1207
0.1543	0.1426	0.1389	0.1319	0.1355	0.1352	0.1444	0.1488	0.1411
0.1978	0.1864	0.1788	0.1701	0.1747	0.1738	0.1729	0.1654	0.1624
0.2946	0.2592	0.2459	0.242	0.2551	0.2771	0.2649	0.2508	0.244
0.3249	0.3227	0.3081	0.2791	0.276	0.2593	0.2727	0.279	0.2667
0.226	0.1994	0.1972	0.1698	0.1598	0.1472	0.1668	0.1626	0.1557
0.1557	0.142	0.1244	0.1155	0.1267	0.1226	0.116	0.1061	0.0931
0.1518	0.1457	0.1509	0.1504	0.1519	0.1519	0.1617	0.1605	0.1523
0.1624	0.1595	0.1584	0.1585	0.1658	0.1776	0.1817	0.1787	0.1956
0.1529	0.1535	0.1657	0.1763	0.1894	0.1911	0.203	0.1991	0.1978
0.0454	0.0457	0.0454	0.0456	0.0465	0.0477	0.0498	0.0493	0.0504
0.0078	0.0083	0.0075	0.0076	0.0073	0.0074	0.0071	0.0073	0.0079
0.0143	0.0145	0.0144	0.0128	0.0118	0.0123	0.012	0.0115	0.011
0.0035	0.0034	0.0031	0.0032	0.003	0.0033	0.0033	0.0033	0.003
0.0032	0.0034	0.0033	0.0034	0.0031	0.003	0.003	0.0029	0.0033
0	0	0	0	0	0	0	0	0
0	0	0	0	0	0	0	0	0
0	0	0	0	0	0	0	0	0
0	0	0	0	0	0	0	0	0
0	0	0	0	0	0	0	0	0
0	0	0	0	0	0	0	0	0
0	0	0	0	0	0	0	0	0
0	0	0	0	0	0	0	0	0
0	0	0	0	0	0	0	0	0
0.0051	0.0043	0.0043	0.0044	0.0045	0.0041	0.0043	0.0041	0.0038
0.0194	0.0183	0.0188	0.0187	0.0186	0.0181	0.0179	0.0183	0.0175
0.1052	0.0994	0.0993	0.0991	0.1001	0.0963	0.0975	0.0959	0.0893
0.1824	0.1741	0.173	0.1731	0.1773	0.1774	0.18	0.1817	0.1764
0.1409	0.1259	0.1266	0.1044	0.1094	0.0969	0.1032	0.0905	0.0851
0.2225	0.2019	0.191	0.1671	0.189	0.1716	0.159	0.154	0.1306
0.219	0.2124	0.1976	0.1675	0.1695	0.1757	0.1702	0.1636	0.1431
0.2523	0.2316	0.2197	0.2124	0.2177	0.2199	0.2175	0.2145	0.1886
0.2081	0.2107	0.1972	0.1849	0.1742	0.1816	0.1955	0.1854	0.1673

0.199	0.1926	0.18	0.1734	0.1675	0.1574	0.151	0.1377	0.1416
0.2035	0.1942	0.1803	0.1806	0.1979	0.1792	0.1695	0.1701	0.1357
0.1522	0.1402	0.149	0.1319	0.1323	0.1339	0.1407	0.1343	0.1224
0.1477	0.1465	0.1394	0.1381	0.1389	0.1372	0.1525	0.1483	0.1359
0.3168	0.3286	0.3183	0.3147	0.3232	0.3305	0.3376	0.3099	0.2977
0.343	0.3272	0.3271	0.3128	0.2836	0.2928	0.2952	0.2879	0.3131
0.1949	0.1843	0.1844	0.187	0.1905	0.1896	0.1821	0.1847	0.1898
0.1472	0.1256	0.1293	0.1128	0.1134	0.1131	0.0996	0.0936	0.0842
0.2472	0.2339	0.2279	0.2029	0.188	0.188	0.202	0.2004	0.1917
0.3718	0.3526	0.3301	0.3244	0.3037	0.3148	0.314	0.3339	0.3281
0.2317	0.2113	0.2141	0.2024	0.2005	0.1932	0.195	0.1679	0.1405
0.1728	0.1541	0.1437	0.147	0.1471	0.1302	0.1307	0.1249	0.0979
0.1848	0.1835	0.1756	0.1606	0.1504	0.1631	0.1591	0.1523	0.1464
0.2515	0.231	0.2229	0.2294	0.2211	0.2172	0.217	0.2169	0.1928
0.2395	0.2075	0.2038	0.1878	0.188	0.1867	0.2019	0.1922	0.1763
0.1983	0.1909	0.1937	0.1981	0.1957	0.1816	0.1753	0.181	0.1546
0.1853	0.1592	0.1344	0.1394	0.1307	0.1324	0.1419	0.1371	0.1183
0.1513	0.141	0.1402	0.1387	0.1432	0.142	0.1435	0.135	0.1244
0.1519	0.1468	0.1507	0.1527	0.1563	0.1599	0.1676	0.1677	0.1606
0.1858	0.1687	0.1672	0.1596	0.1666	0.1634	0.1672	0.1766	0.1739
0.2217	0.2036	0.19	0.2021	0.1844	0.1887	0.197	0.2	0.2012
0.3082	0.2916	0.2727	0.2776	0.2727	0.273	0.2889	0.2956	0.2578
0.1674	0.1537	0.1465	0.1511	0.1535	0.1526	0.1565	0.1404	0.1211
0.1214	0.1101	0.1075	0.1026	0.1109	0.1111	0.1223	0.1259	0.1226
0.1431	0.1325	0.1253	0.1274	0.1361	0.1382	0.1394	0.1263	0.1137
0.1911	0.1785	0.1811	0.1769	0.1794	0.1808	0.1929	0.1943	0.1804
0.1717	0.1657	0.1706	0.1713	0.1728	0.1842	0.1896	0.1902	0.1903
0.0298	0.0302	0.0305	0.0301	0.0301	0.0313	0.0323	0.0326	0.0335
0	0	0	0	0	0	0	0	0
0.011	0.0113	0.0118	0.0124	0.0129	0.013	0.0135	0.0138	0.0143
0.0036	0.0033	0.0031	0.0032	0.0031	0.0036	0.0033	0.0034	0.0033
0.0029	0.003	0.0029	0.003	0.0028	0.0033	0.0035	0.0036	0.0038
0	0	0	0	0	0	0	0	0
0	0	0	0	0	0	0	0	0
0	0	0	0	0	0	0	0	0
0	0	0	0	0	0	0	0	0
0	0	0	0	0	0	0	0	0
0	0	0	0	0	0	0	0	0
0	0	0	0	0	0	0	0	0
0	0	0	0	0	0	0	0	0
0.0044	0.0042	0.0044	0.0045	0.0047	0.005	0.0052	0.0051	0.0053
0.0209	0.021	0.02	0.0211	0.0223	0.022	0.0244	0.0239	0.0233
0.029	0.0254	0.0262	0.0258	0.0257	0.025	0.0246	0.0249	0.0235
0.1275	0.117	0.1166	0.1109	0.1214	0.1254	0.1252	0.12	0.1085

0.1989	0.1832	0.1749	0.1693	0.1684	0.1662	0.1701	0.1634	0.1591
0.2123	0.1959	0.1847	0.1746	0.1757	0.1696	0.1851	0.1867	0.1632
0.2024	0.1827	0.1672	0.1639	0.153	0.1473	0.1471	0.1416	0.1122
0.1529	0.1427	0.1422	0.1398	0.1461	0.1434	0.1483	0.1414	0.1353
0.1861	0.1736	0.1685	0.1732	0.1757	0.1645	0.1672	0.1642	0.1463
0.1215	0.1072	0.1009	0.0961	0.1019	0.0998	0.1084	0.1148	0.0958
0.1924	0.1645	0.1644	0.1535	0.1544	0.1373	0.1499	0.1496	0.1456
0.1836	0.1734	0.1522	0.1305	0.1228	0.1195	0.1274	0.1179	0.0992
0.157	0.1434	0.1383	0.1324	0.1427	0.133	0.1346	0.119	0.1127
0.1808	0.1674	0.1578	0.1606	0.1646	0.1605	0.162	0.156	0.1462
0.0954	0.0867	0.0853	0.0806	0.089	0.0811	0.0855	0.0811	0.0687
0.1225	0.1142	0.1109	0.1117	0.1157	0.1147	0.1154	0.1163	0.1129
0.1397	0.1287	0.1267	0.1259	0.1336	0.133	0.137	0.1364	0.1317
0.0949	0.0975	0.094	0.0919	0.1056	0.1128	0.1211	0.1235	0.1088
0.2306	0.2165	0.2114	0.2108	0.1958	0.1844	0.1863	0.1819	0.1748
0.2942	0.2904	0.2878	0.2601	0.2641	0.2472	0.2477	0.2175	0.1899
0.2212	0.1968	0.1723	0.1531	0.1678	0.1469	0.1649	0.1527	0.1385
0.14	0.1326	0.136	0.1338	0.1436	0.1352	0.143	0.1489	0.1429
0.1454	0.1423	0.1329	0.1276	0.1383	0.1395	0.1464	0.1332	0.1258
0.2059	0.1918	0.1819	0.1704	0.1824	0.1748	0.168	0.1798	0.1671
0.2094	0.1756	0.1697	0.1647	0.1439	0.1301	0.1232	0.1164	0.0964
0.1474	0.1416	0.1343	0.1226	0.1367	0.146	0.1482	0.1488	0.1349
0.1881	0.1509	0.1397	0.1213	0.1156	0.1001	0.1094	0.1066	0.0995
0.1571	0.1371	0.1373	0.1363	0.1344	0.1286	0.1339	0.1292	0.1256
0.1496	0.1452	0.1448	0.1419	0.1452	0.1475	0.154	0.1502	0.1488
0.1627	0.1441	0.1482	0.1478	0.1517	0.1466	0.1513	0.1533	0.1469
0.2287	0.2064	0.194	0.1794	0.1843	0.1816	0.1924	0.1966	0.1708
0.2107	0.1911	0.1718	0.163	0.1563	0.1374	0.1211	0.126	0.0966
0.1211	0.1143	0.1135	0.1151	0.1228	0.1217	0.1243	0.1281	0.1214
0.166	0.1598	0.1632	0.1651	0.1745	0.1715	0.1764	0.1752	0.1669
0.172	0.1635	0.163	0.157	0.167	0.1641	0.164	0.1626	0.1461
0.1724	0.1723	0.1534	0.1519	0.1561	0.1418	0.1503	0.153	0.1217
0.1321	0.1138	0.1139	0.1124	0.1081	0.1075	0.1149	0.1056	0.1056
0.0447	0.0425	0.0435	0.0434	0.046	0.0445	0.0451	0.045	0.0432
0.045	0.0485	0.0433	0.0439	0.0394	0.0402	0.0425	0.0411	0.0387
0.1077	0.1092	0.109	0.103	0.0918	0.0928	0.0934	0.0991	0.0989
0.0037	0.0038	0.0036	0.0038	0.0036	0.0037	0.0037	0.0037	0.0035
0.0031	0.003	0.0027	0.003	0.0028	0.0029	0.0027	0.0026	0.0029
0	0	0	0	0	0	0	0	0
0	0	0	0	0	0	0	0	0
0	0	0	0	0	0	0	0	0
0	0	0	0	0	0	0	0	0
0	0	0	0	0	0	0	0	0
0	0	0	0	0	0	0	0	0

0	0	0	0	0	0	0	0	0
0	0	0	0	0	0	0	0	0
0.0083	0.0077	0.0076	0.0073	0.0076	0.0075	0.0078	0.0077	0.0071
0.0325	0.0308	0.0299	0.0289	0.0266	0.0283	0.0293	0.0293	0.0307
0.0264	0.0256	0.0253	0.0252	0.0235	0.0238	0.0232	0.0222	0.021
0.1288	0.1225	0.1201	0.1194	0.1278	0.129	0.1373	0.1325	0.1227
0.2073	0.1979	0.1912	0.186	0.1775	0.1766	0.1707	0.1674	0.1783
0.2858	0.2415	0.2311	0.2098	0.215	0.221	0.2233	0.2169	0.2138
0.2267	0.2207	0.2238	0.2171	0.2076	0.1911	0.2017	0.1957	0.1811
0.1504	0.1388	0.1387	0.1308	0.134	0.1246	0.13	0.1252	0.1201
0.186	0.1617	0.1629	0.1622	0.1707	0.1748	0.1806	0.1687	0.1476
0.1533	0.1251	0.1382	0.1326	0.1338	0.1191	0.1229	0.1329	0.1169
0.1667	0.1333	0.1285	0.1311	0.1352	0.1366	0.1511	0.1494	0.1381
0.1735	0.1541	0.1606	0.1689	0.1609	0.1455	0.1634	0.1642	0.1492
0.2172	0.2042	0.1842	0.1646	0.1594	0.1432	0.1484	0.1431	0.1172
0.26	0.2586	0.2421	0.2277	0.2256	0.2189	0.2079	0.2037	0.2088
0.1028	0.09	0.084	0.0788	0.0922	0.0904	0.0934	0.0839	0.0818
0.1614	0.1531	0.1556	0.1562	0.1594	0.1625	0.1673	0.1628	0.1603
0.1563	0.1463	0.1416	0.1381	0.1488	0.1551	0.1617	0.1599	0.1475
0.1567	0.1321	0.1316	0.1285	0.1259	0.1304	0.142	0.1489	0.1302
0.1625	0.1435	0.1323	0.1225	0.1273	0.1312	0.1422	0.1382	0.1155
0.1216	0.1052	0.1062	0.1027	0.1031	0.0982	0.1253	0.1235	0.116
0.1336	0.1349	0.1108	0.1041	0.1029	0.096	0.1143	0.1059	0.0956
0.1472	0.1411	0.1315	0.1305	0.1336	0.1287	0.1348	0.1304	0.1171
0.1304	0.1244	0.1227	0.1285	0.1359	0.1368	0.1394	0.1391	0.1331
0.1813	0.1615	0.1551	0.1516	0.151	0.1457	0.15	0.1411	0.1212
0.1641	0.1406	0.1191	0.1202	0.1277	0.1221	0.1306	0.1372	0.1346
0.1394	0.1245	0.1318	0.1248	0.1235	0.1181	0.1244	0.1341	0.128
0.1549	0.15	0.1443	0.1328	0.1242	0.1168	0.1301	0.1244	0.0982
0.1565	0.15	0.1435	0.1362	0.1332	0.144	0.1406	0.1402	0.1301
0.147	0.1363	0.1274	0.1295	0.1369	0.1412	0.1393	0.1326	0.1203
0.1786	0.1577	0.1505	0.1411	0.1442	0.149	0.1518	0.1529	0.1395
0.1141	0.0981	0.0942	0.0969	0.1062	0.1022	0.1132	0.1048	0.0913
0.1377	0.1362	0.1183	0.107	0.1128	0.1115	0.1049	0.1122	0.0892
0.1758	0.1576	0.1531	0.1544	0.1559	0.1539	0.1548	0.1502	0.1391
0.1349	0.1233	0.1256	0.1274	0.1339	0.1328	0.1351	0.1289	0.11
0.1355	0.1391	0.127	0.1168	0.1256	0.1138	0.1115	0.0935	0.0843
0.2049	0.1721	0.1607	0.1523	0.1515	0.1559	0.1332	0.119	0.0988
0.166	0.1544	0.1413	0.1342	0.1453	0.141	0.1412	0.149	0.1387
0.1172	0.1114	0.1113	0.1103	0.1151	0.1182	0.1209	0.1186	0.1107
0.1394	0.1415	0.1281	0.1311	0.1314	0.136	0.1357	0.1435	0.1386
0.2116	0.2223	0.2187	0.2346	0.2143	0.2227	0.2091	0.2147	0.2384
0.0034	0.0035	0.0031	0.0031	0.0031	0.0034	0.0034	0.0031	0.003
0.0037	0.0037	0.0036	0.0036	0.0033	0.0034	0.0036	0.0034	0.0033

0	0	0	0	0	0	0	0	0
0	0	0	0	0	0	0	0	0
0	0	0	0	0	0	0	0	0
0	0	0	0	0	0	0	0	0
0	0	0	0	0	0	0	0	0
0	0	0	0	0	0	0	0	0
0	0	0	0	0	0	0	0	0
0	0	0	0	0	0	0	0	0
0.0058	0.0058	0.0058	0.0056	0.0053	0.0049	0.0051	0.0049	0.0045
0.0275	0.0267	0.0254	0.028	0.0247	0.0282	0.0265	0.0271	0.0268
0.0332	0.0324	0.0329	0.0333	0.0337	0.0347	0.0358	0.0355	0.0352
0.1158	0.1102	0.1069	0.1052	0.107	0.1091	0.1096	0.1099	0.1055
0.2584	0.2472	0.2381	0.2291	0.2194	0.2095	0.2091	0.196	0.1962
0.275	0.2719	0.2507	0.245	0.2244	0.2551	0.2419	0.2456	0.2143
0.1731	0.1505	0.1494	0.1376	0.1389	0.1315	0.1363	0.1265	0.1112
0.1652	0.1502	0.1508	0.1354	0.1458	0.1444	0.1447	0.1399	0.1207
0.1493	0.1373	0.1356	0.1312	0.1369	0.1167	0.1162	0.0998	0.1011
0.1643	0.144	0.1385	0.1275	0.1302	0.1177	0.1196	0.1065	0.0805
0.1911	0.1757	0.1663	0.1532	0.1626	0.1617	0.1572	0.1615	0.1546
0.1884	0.1777	0.1656	0.1567	0.1436	0.1432	0.1627	0.165	0.1471
0.2309	0.2116	0.2009	0.1989	0.1908	0.1766	0.1698	0.1651	0.1435
0.309	0.3161	0.3064	0.2954	0.2855	0.2958	0.2843	0.2706	0.2519
0.1361	0.1318	0.1269	0.1272	0.1229	0.127	0.1305	0.1268	0.1274
0.1432	0.1391	0.1385	0.1342	0.147	0.1489	0.1521	0.1522	0.1422
0.1995	0.1881	0.18	0.1756	0.1825	0.1815	0.1815	0.1782	0.1715
0.154	0.1333	0.1265	0.1261	0.1174	0.114	0.1277	0.1327	0.1244
0.1906	0.1692	0.1579	0.1424	0.1473	0.1423	0.1424	0.1261	0.119
0.1455	0.139	0.1336	0.1186	0.1264	0.1158	0.1124	0.1058	0.0816
0.1841	0.1703	0.1683	0.1633	0.1604	0.1419	0.1473	0.1388	0.1192
0.1939	0.1899	0.1849	0.1849	0.1805	0.1868	0.1914	0.1886	0.1834
0.1993	0.1892	0.19	0.1875	0.1917	0.1822	0.1844	0.1674	0.1576
0.186	0.165	0.1613	0.1655	0.1733	0.1677	0.1784	0.1593	0.1356
0.1441	0.1304	0.1201	0.1214	0.123	0.1207	0.1352	0.1315	0.11
0.1647	0.1571	0.1558	0.1505	0.156	0.1581	0.1657	0.1548	0.1474
0.1555	0.1421	0.156	0.1368	0.1416	0.1564	0.1603	0.1558	0.1455
0.1458	0.1212	0.1158	0.0973	0.1124	0.107	0.132	0.1351	0.1047
0.1958	0.1889	0.1772	0.1688	0.1852	0.1774	0.1783	0.1659	0.147
0.1566	0.1519	0.1543	0.1547	0.1769	0.177	0.1752	0.1729	0.164
0.1537	0.1471	0.1497	0.147	0.1537	0.154	0.1572	0.1552	0.1519
0.1808	0.1768	0.1778	0.1748	0.1807	0.1841	0.1875	0.1855	0.1745
0.1624	0.1379	0.1131	0.0989	0.1011	0.0997	0.1108	0.0959	0.0995
0.1515	0.1434	0.1334	0.1126	0.1248	0.1258	0.1239	0.122	0.1074
0.1845	0.1627	0.1544	0.1518	0.1606	0.1318	0.1214	0.1206	0.1163
0.1767	0.1768	0.1859	0.1683	0.1612	0.137	0.1447	0.1419	0.1324

0.1384	0.1218	0.1087	0.1024	0.1023	0.1012	0.0961	0.0792	0.0773
0.1413	0.135	0.1333	0.1354	0.1356	0.1362	0.1394	0.1348	0.1294
0.1113	0.1147	0.1109	0.1228	0.1184	0.1225	0.1193	0.1182	0.1116
0.1843	0.1891	0.1821	0.1807	0.1833	0.1822	0.182	0.1791	0.1798
0.0037	0.0042	0.0039	0.0038	0.0035	0.0036	0.0037	0.0035	0.0032
0.0043	0.0043	0.0043	0.0047	0.0041	0.0039	0.004	0.0037	0.0038
0	0	0	0	0	0	0	0	0
0	0	0	0	0	0	0	0	0
0	0	0	0	0	0	0	0	0
0	0	0	0	0	0	0	0	0
0	0	0	0	0	0	0	0	0
0	0	0	0	0	0	0	0	0
0	0	0	0	0	0	0	0	0
0	0	0	0	0	0	0	0	0
0.007	0.0068	0.0069	0.0067	0.0068	0.0068	0.0065	0.0063	0.0062
0.0438	0.0408	0.0399	0.0397	0.0411	0.0398	0.0415	0.0412	0.0397
0.0906	0.0835	0.0867	0.0852	0.0899	0.0927	0.0967	0.0927	0.0854
0.1034	0.1039	0.1022	0.1023	0.0987	0.1002	0.1009	0.0983	0.094
0.2314	0.2272	0.2204	0.2191	0.1997	0.1906	0.1994	0.1896	0.1776
0.2384	0.2202	0.2242	0.2517	0.2468	0.248	0.247	0.2429	0.2112
0.1991	0.1849	0.1861	0.1782	0.1853	0.1788	0.1853	0.1821	0.1608
0.1971	0.1809	0.1853	0.1762	0.1638	0.1648	0.1645	0.16	0.1472
0.1372	0.1275	0.1111	0.1028	0.1156	0.1211	0.1213	0.1158	0.0962
0.2368	0.2139	0.2013	0.1897	0.2112	0.1994	0.202	0.2028	0.1822
0.1253	0.1196	0.1114	0.1092	0.1115	0.103	0.1085	0.1229	0.1033
0.1455	0.1198	0.1319	0.1466	0.1781	0.1657	0.1689	0.1626	0.1254
0.1876	0.1609	0.1384	0.1383	0.1328	0.1112	0.1178	0.1254	0.1013
0.2182	0.224	0.2124	0.2277	0.2277	0.2404	0.2208	0.2151	0.1975
0.1738	0.1682	0.1665	0.1648	0.1619	0.1645	0.1619	0.1665	0.1664
0.106	0.0977	0.1023	0.0996	0.1026	0.1012	0.1055	0.1078	0.1036
0.1168	0.1118	0.1089	0.1103	0.1116	0.1126	0.1169	0.1113	0.1069
0.1123	0.1043	0.1034	0.1082	0.115	0.1172	0.1207	0.1149	0.1084
0.1495	0.1409	0.1411	0.1387	0.1426	0.1433	0.1459	0.1427	0.1351
0.1715	0.161	0.1535	0.1508	0.1538	0.156	0.1617	0.1595	0.1478
0.1706	0.1488	0.1489	0.1375	0.1539	0.1555	0.1498	0.1532	0.1371
0.1949	0.195	0.2056	0.1905	0.1978	0.2036	0.2062	0.2112	0.181
0.1768	0.1631	0.1564	0.1537	0.1731	0.1553	0.1593	0.1629	0.1429
0.1823	0.172	0.1631	0.1509	0.1549	0.1494	0.1518	0.1396	0.1294
0.1549	0.1502	0.1461	0.1373	0.1439	0.1424	0.1437	0.1433	0.1153
0.1499	0.1457	0.1384	0.1331	0.1435	0.1417	0.1476	0.1467	0.139
0.1599	0.1588	0.1489	0.1464	0.1475	0.1346	0.1393	0.1418	0.1315
0.1394	0.1268	0.1155	0.1023	0.0983	0.0939	0.1105	0.0865	0.0755
0.1577	0.1563	0.1521	0.1376	0.1491	0.1365	0.1463	0.1238	0.1111
0.1499	0.1381	0.1414	0.129	0.1401	0.1224	0.1339	0.1386	0.1174

0.1412	0.1346	0.1334	0.1262	0.1304	0.1265	0.1315	0.1261	0.1197
0.1787	0.1768	0.1724	0.168	0.1774	0.1733	0.1693	0.1636	0.1548
0.1436	0.1368	0.1358	0.1478	0.1515	0.1435	0.1501	0.1538	0.1425
0.1887	0.164	0.1511	0.1303	0.1376	0.1366	0.1583	0.125	0.1067
0.1799	0.15	0.1454	0.1424	0.156	0.1586	0.1616	0.1553	0.1307
0.1712	0.1569	0.1517	0.1417	0.1401	0.1502	0.1569	0.158	0.1534
0.15	0.1456	0.1411	0.136	0.1348	0.1321	0.138	0.1296	0.128
0.1437	0.1389	0.1385	0.1381	0.1424	0.1459	0.1505	0.1482	0.1458
0.0226	0.0213	0.0215	0.0218	0.0217	0.0214	0.0222	0.0208	0.0206
0.0213	0.0205	0.0203	0.0202	0.0205	0.0211	0.0216	0.0215	0.0211
0.0042	0.0044	0.004	0.004	0.0038	0.0038	0.0037	0.0035	0.0034
0.004	0.0042	0.004	0.004	0.004	0.0037	0.0037	0.0036	0.0036
0.0012	0.0011	0.001	0.0011	0.001	0.0009	0.0011	0.0011	0.0009
0.002	0.0021	0.0021	0.0021	0.0022	0.0022	0.0024	0.0024	0.0024
0.0051	0.0051	0.005	0.0051	0.0049	0.0052	0.0053	0.0053	0.0055
0.0076	0.0073	0.0067	0.0061	0.0058	0.0056	0.0057	0.0056	0.0053
0.0067	0.0065	0.0071	0.007	0.0073	0.0078	0.0085	0.009	0.0088
0.0109	0.0103	0.01	0.0096	0.0091	0.0087	0.0085	0.0081	0.0074
0.0525	0.0532	0.0495	0.0452	0.0449	0.0467	0.0489	0.0444	0.0445
0.1036	0.1031	0.0996	0.0934	0.0874	0.0926	0.094	0.0963	0.0991
0.0716	0.0704	0.0706	0.0703	0.0752	0.076	0.0831	0.0788	0.0796
0.1479	0.1392	0.1394	0.1339	0.1383	0.1402	0.1456	0.1448	0.142
0.1455	0.1365	0.1383	0.1377	0.1407	0.1371	0.1376	0.1378	0.1318
0.1183	0.1106	0.1007	0.0991	0.1031	0.1061	0.1026	0.1036	0.0903
0.1069	0.0877	0.0775	0.0801	0.0808	0.0707	0.0735	0.069	0.0605
0.1853	0.1834	0.1636	0.1676	0.1784	0.1808	0.1858	0.1663	0.1498
0.1898	0.1614	0.1539	0.1464	0.156	0.1382	0.1425	0.1319	0.1248
0.17	0.1514	0.1328	0.1251	0.1288	0.1167	0.131	0.1239	0.119
0.1471	0.1389	0.1337	0.1302	0.125	0.1203	0.1281	0.1259	0.1115
0.2252	0.2173	0.2185	0.2048	0.183	0.1908	0.2082	0.1896	0.168
0.2157	0.2139	0.2133	0.2035	0.2025	0.2049	0.2063	0.1921	0.1761
0.1526	0.1281	0.1073	0.0863	0.098	0.109	0.0827	0.0917	0.077
0.1671	0.1298	0.1304	0.1256	0.1258	0.1145	0.0984	0.0877	0.0751
0.1774	0.1774	0.1773	0.1691	0.1694	0.1638	0.1676	0.1701	0.1553
0.2547	0.2639	0.2568	0.2641	0.2613	0.2674	0.2448	0.2325	0.2384
0.1695	0.1623	0.1461	0.1486	0.1385	0.1368	0.1384	0.1384	0.1217
0.1992	0.2156	0.1956	0.2015	0.1879	0.1934	0.2021	0.2011	0.1867
0.1677	0.1599	0.1599	0.1593	0.1622	0.163	0.1671	0.162	0.1558
0.1536	0.1404	0.1384	0.1431	0.142	0.1401	0.1425	0.1426	0.1364
0.1572	0.1479	0.1475	0.1438	0.1467	0.149	0.159	0.1576	0.1523
0.1653	0.1494	0.1571	0.1462	0.1556	0.1446	0.1486	0.1467	0.1302
0.1731	0.1453	0.1446	0.1352	0.1349	0.1305	0.1339	0.1223	0.105
0.1735	0.144	0.1383	0.1367	0.1441	0.1403	0.1406	0.1332	0.1053
0.165	0.1441	0.1518	0.1544	0.1516	0.1469	0.1502	0.1379	0.1249

0.1609	0.1456	0.1445	0.1405	0.1504	0.1492	0.1518	0.1508	0.1366
0.1567	0.1483	0.1482	0.1458	0.1514	0.1483	0.1507	0.1419	0.1348
0.1516	0.1237	0.109	0.1172	0.1273	0.1248	0.123	0.1213	0.1119
0.161	0.1388	0.1234	0.1187	0.1349	0.129	0.1205	0.1138	0.1067
0.1552	0.1529	0.1444	0.1427	0.1527	0.1448	0.1409	0.133	0.1213
0.1642	0.1678	0.1604	0.1625	0.1678	0.1671	0.172	0.1701	0.1583
0.1928	0.1797	0.1666	0.1516	0.1531	0.141	0.1301	0.1318	0.123
0.1697	0.1704	0.1633	0.1598	0.1739	0.1729	0.1806	0.165	0.1531
0.1337	0.1162	0.1047	0.1032	0.1137	0.1047	0.1123	0.1054	0.0981
0.1229	0.1018	0.1041	0.109	0.107	0.0891	0.0915	0.0908	0.0779
0.1511	0.1483	0.15	0.1557	0.1608	0.1496	0.1474	0.1375	0.128
0.2092	0.1967	0.1875	0.1767	0.1742	0.1735	0.1759	0.1847	0.1791
0.2048	0.2062	0.1936	0.1883	0.1867	0.1955	0.1949	0.1947	0.1981
0.1658	0.1634	0.161	0.1612	0.1598	0.1626	0.1598	0.1523	0.1429
0.0162	0.0156	0.0156	0.0154	0.0158	0.016	0.0166	0.0162	0.016
0.0076	0.0071	0.0061	0.006	0.0057	0.0061	0.0059	0.0056	0.0053
0	0	0	0	0	0	0	0	0
0	0	0	0	0	0	0	0	0
0.0016	0.0015	0.0015	0.0015	0.0015	0.0015	0.0016	0.0016	0.0016
0.0035	0.0034	0.0034	0.0035	0.0035	0.0037	0.004	0.0039	0.0044
0.0129	0.0138	0.014	0.0141	0.0135	0.0139	0.014	0.0141	0.0145
0.0201	0.018	0.0173	0.0191	0.0179	0.019	0.0182	0.018	0.0171
0.0177	0.0172	0.0172	0.0178	0.017	0.017	0.0166	0.0159	0.0163
0.0146	0.016	0.0181	0.0185	0.0182	0.0177	0.0172	0.0171	0.0173
0.0519	0.0519	0.0516	0.0506	0.0498	0.0512	0.0535	0.0516	0.0495
0.1391	0.1383	0.1388	0.1397	0.1371	0.1415	0.1434	0.1413	0.1465
0.2002	0.1962	0.1994	0.2007	0.2038	0.2073	0.2118	0.2109	0.2117
0.1685	0.162	0.167	0.1696	0.1774	0.1864	0.194	0.197	0.1976
0.133	0.1263	0.1289	0.1251	0.1374	0.14	0.1454	0.1459	0.145
0.1745	0.1611	0.1491	0.1306	0.1377	0.1253	0.1306	0.1262	0.1048
0.1523	0.1404	0.1309	0.1271	0.1271	0.1353	0.1346	0.1179	0.1204
0.1558	0.134	0.1261	0.1135	0.1205	0.1059	0.1132	0.1099	0.1018
0.1274	0.1049	0.1059	0.1079	0.1205	0.1186	0.1232	0.1208	0.1135
0.1773	0.1718	0.1515	0.1508	0.1665	0.1485	0.1544	0.1462	0.1351
0.1253	0.11	0.1068	0.1129	0.1134	0.1105	0.1233	0.1161	0.1078
0.1764	0.1612	0.1563	0.1664	0.1697	0.1575	0.16	0.1547	0.142
0.1576	0.129	0.1254	0.1244	0.1337	0.1241	0.124	0.1019	0.1048
0.1835	0.1649	0.1612	0.1539	0.1596	0.1378	0.1329	0.1306	0.1032
0.2251	0.222	0.2064	0.2015	0.1959	0.188	0.1815	0.1689	0.1511
0.2368	0.252	0.2513	0.2758	0.2451	0.2523	0.2494	0.2382	0.2363
0.3938	0.4239	0.3927	0.4235	0.4018	0.4118	0.3914	0.397	0.4042
0.2269	0.2532	0.2409	0.257	0.2203	0.2217	0.2324	0.2225	0.2114
0.2117	0.223	0.2198	0.2164	0.1949	0.2065	0.2004	0.1925	0.184
0.171	0.1666	0.1603	0.1537	0.1415	0.1457	0.1477	0.1418	0.1445

0.1703	0.1579	0.1437	0.1395	0.1295	0.1216	0.1137	0.1214	0.1017
0.1447	0.1308	0.1247	0.1121	0.106	0.1081	0.1077	0.1078	0.0995
0.1834	0.1641	0.1532	0.1478	0.1494	0.1536	0.1584	0.1326	0.1163
0.1536	0.1544	0.157	0.1381	0.1421	0.1289	0.1272	0.1233	0.1152
0.163	0.148	0.1288	0.1029	0.0993	0.1064	0.11	0.1072	0.0879
0.1513	0.1443	0.1433	0.1394	0.1418	0.1407	0.1491	0.1482	0.1269
0.1479	0.1479	0.1557	0.1501	0.1583	0.1547	0.1518	0.1587	0.1412
0.1535	0.1538	0.1516	0.1503	0.1496	0.1426	0.1561	0.1463	0.1424
0.1332	0.1262	0.1196	0.1278	0.1354	0.1402	0.1468	0.1539	0.138
0.1804	0.1744	0.1642	0.154	0.1625	0.1482	0.1586	0.1472	0.1163
0.2195	0.2057	0.1803	0.1561	0.1573	0.1365	0.1415	0.1421	0.1385
0.2054	0.189	0.1816	0.1793	0.1862	0.159	0.1727	0.1679	0.1531
0.1698	0.1701	0.16	0.1549	0.1592	0.1442	0.1401	0.1391	0.1275
0.1258	0.1111	0.1057	0.1126	0.1211	0.1242	0.1247	0.1243	0.1034
0.1876	0.1807	0.1707	0.1717	0.1736	0.1844	0.1833	0.1671	0.153
0.1872	0.1767	0.1712	0.1468	0.1452	0.1363	0.1463	0.1578	0.1314
0.1394	0.1436	0.1147	0.1058	0.1286	0.121	0.1223	0.116	0.0931
0.192	0.1918	0.1962	0.1701	0.1656	0.1647	0.1774	0.1756	0.1681
0.2439	0.2287	0.2156	0.2097	0.2023	0.2004	0.2032	0.1991	0.1871
0.176	0.1626	0.152	0.149	0.1445	0.1403	0.15	0.1429	0.1177
0.0184	0.017	0.0163	0.0157	0.0156	0.0151	0.0155	0.0146	0.014
0.0258	0.0263	0.0261	0.0275	0.0272	0.0268	0.0255	0.027	0.0273
0	0	0	0	0	0	0	0	0
0	0	0	0	0	0	0	0	0
0.0081	0.0085	0.0078	0.0077	0.0075	0.0081	0.008	0.0081	0.0082
0.0055	0.0051	0.0049	0.0045	0.0045	0.0044	0.0043	0.0039	0.0037
0.0103	0.0104	0.0101	0.01	0.0097	0.0098	0.0101	0.01	0.0101
0.0181	0.0185	0.0199	0.0204	0.0185	0.0188	0.0179	0.0191	0.0184
0.0251	0.0264	0.026	0.0257	0.0233	0.0229	0.0223	0.0233	0.0224
0.024	0.0227	0.0213	0.0204	0.0209	0.0204	0.0209	0.0186	0.018
0.0578	0.0576	0.0555	0.0546	0.0541	0.0572	0.0584	0.0593	0.0583
0.1512	0.1483	0.1497	0.1509	0.1527	0.1571	0.1619	0.1618	0.1614
0.2027	0.1933	0.1937	0.1939	0.1998	0.199	0.2031	0.1994	0.1988
0.1872	0.1783	0.1759	0.1747	0.1782	0.1812	0.1856	0.1873	0.1865
0.1419	0.1358	0.1351	0.1345	0.1446	0.15	0.1576	0.1579	0.1494
0.2093	0.1838	0.1816	0.1791	0.1811	0.1631	0.1717	0.1635	0.1594
0.1598	0.1366	0.1338	0.1334	0.1439	0.1445	0.1584	0.1625	0.1528
0.1391	0.1289	0.1196	0.1282	0.1278	0.1104	0.1055	0.1131	0.1066
0.1502	0.1354	0.1372	0.1392	0.1487	0.1438	0.1473	0.1502	0.1457
0.1667	0.1608	0.1543	0.1471	0.1446	0.1343	0.1413	0.1391	0.1318
0.1603	0.1436	0.1414	0.1402	0.1485	0.1366	0.1514	0.1506	0.1334
0.1241	0.1108	0.0998	0.0918	0.1041	0.0946	0.0936	0.0961	0.0935
0.1877	0.1722	0.1588	0.1664	0.1714	0.1545	0.1527	0.1459	0.1315
0.1949	0.1777	0.1786	0.1843	0.1801	0.1559	0.1602	0.154	0.1315

0.2198	0.2048	0.196	0.1895	0.1838	0.1931	0.1857	0.1931	0.1948
0.2521	0.2487	0.2424	0.2384	0.2341	0.2311	0.2171	0.2152	0.2375
0.3235	0.3291	0.3073	0.3204	0.2994	0.3075	0.321	0.2984	0.2787
0.2483	0.248	0.2458	0.2382	0.2349	0.2369	0.232	0.2141	0.2206
0.1731	0.1678	0.1713	0.1743	0.1716	0.1709	0.175	0.178	0.1774
0.176	0.1805	0.1751	0.1645	0.1637	0.1679	0.1655	0.1767	0.1719
0.1581	0.1463	0.1436	0.1345	0.1388	0.1264	0.1291	0.1352	0.1214
0.1976	0.1703	0.1617	0.1602	0.1755	0.1585	0.1717	0.1771	0.1652
0.1914	0.1834	0.1667	0.1731	0.1663	0.1686	0.1674	0.1506	0.14
0.1387	0.1294	0.1216	0.1166	0.1243	0.1095	0.1218	0.1182	0.107
0.1692	0.1428	0.1355	0.1279	0.1402	0.1266	0.1423	0.1375	0.1187
0.1938	0.1747	0.1737	0.1643	0.1606	0.1493	0.1346	0.1208	0.1027
0.2198	0.2166	0.1989	0.1832	0.1823	0.1733	0.1831	0.1731	0.1593
0.1806	0.1688	0.167	0.1592	0.1599	0.1394	0.1549	0.1544	0.1332
0.2026	0.1817	0.1844	0.192	0.1886	0.1489	0.1579	0.1508	0.125
0.2015	0.1833	0.1811	0.1663	0.1614	0.1487	0.1464	0.1529	0.1383
0.1451	0.1339	0.1267	0.1208	0.1233	0.122	0.1181	0.1185	0.103
0.1477	0.1276	0.1261	0.1092	0.1259	0.125	0.1172	0.1244	0.1075
0.2122	0.1989	0.194	0.1781	0.184	0.188	0.1824	0.1656	0.1493
0.1997	0.1964	0.1857	0.1876	0.183	0.1788	0.1852	0.1797	0.1775
0.2223	0.2145	0.2032	0.2003	0.1975	0.193	0.2041	0.2034	0.2029
0.1981	0.1894	0.1834	0.1718	0.1784	0.1559	0.1517	0.1508	0.1428
0.1748	0.1593	0.1666	0.16	0.1359	0.1309	0.1316	0.1222	0.1209
0.1771	0.1605	0.1464	0.1237	0.1238	0.1141	0.124	0.1133	0.1105
0.2415	0.2463	0.233	0.1988	0.1916	0.1857	0.2037	0.2008	0.1885
0.1449	0.1361	0.1335	0.1242	0.1216	0.1117	0.1085	0.1024	0.0896
0.0143	0.0141	0.013	0.0129	0.013	0.0124	0.0126	0.0128	0.0112
0.0262	0.0275	0.0263	0.0259	0.0246	0.0239	0.0252	0.0261	0.0257
0	0	0	0	0	0	0	0	0
0	0	0	0	0	0	0	0	0
0.0039	0.0039	0.0039	0.004	0.004	0.004	0.0042	0.0042	0.0043
0.0034	0.003	0.003	0.0027	0.0031	0.0027	0.003	0.0031	0.0023
0.0018	0.0017	0.0019	0.002	0.0019	0.002	0.0022	0.0022	0.0023
0.0117	0.0114	0.0118	0.0114	0.01	0.0104	0.011	0.0105	0.0111
0.0197	0.0198	0.0191	0.0194	0.0184	0.0193	0.0195	0.02	0.0199
0.0105	0.0106	0.0106	0.0114	0.0105	0.0103	0.0109	0.0096	0.0102
0.0392	0.038	0.0382	0.0379	0.0388	0.0395	0.0406	0.0406	0.0402
0.134	0.1279	0.1313	0.1313	0.1366	0.1388	0.1433	0.1422	0.1418
0.1485	0.1371	0.1239	0.1262	0.1316	0.1275	0.1312	0.1232	0.1192
0.2078	0.1892	0.183	0.1829	0.1843	0.1901	0.1875	0.1869	0.1737
0.2746	0.2552	0.2469	0.2496	0.2399	0.2443	0.236	0.234	0.2367
0.1968	0.1845	0.1882	0.1875	0.1871	0.1849	0.1837	0.1787	0.1557
0.1506	0.1348	0.1354	0.123	0.127	0.1262	0.1318	0.1252	0.113
0.1311	0.1232	0.1135	0.1164	0.1286	0.1309	0.1339	0.1394	0.1314

0.1789	0.1719	0.169	0.1494	0.1564	0.1464	0.1446	0.148	0.1337
0.1484	0.1344	0.1366	0.1183	0.1165	0.1154	0.1238	0.1237	0.1152
0.1628	0.1479	0.1437	0.1515	0.1598	0.1615	0.1617	0.1534	0.1418
0.1583	0.1365	0.1373	0.1283	0.1319	0.1094	0.1185	0.1196	0.1016
0.1569	0.1717	0.1609	0.1607	0.159	0.1521	0.1505	0.1378	0.1217
0.1799	0.151	0.1404	0.1333	0.1277	0.141	0.1299	0.1276	0.1094
0.147	0.1327	0.1271	0.1366	0.1361	0.1329	0.139	0.135	0.1263
0.1497	0.1438	0.1405	0.1407	0.1423	0.1469	0.1491	0.1453	0.1407
0.2038	0.1992	0.195	0.1974	0.1996	0.2001	0.1996	0.197	0.1929
0.1381	0.134	0.1336	0.1326	0.1363	0.1385	0.1433	0.1415	0.139
0.1573	0.1534	0.1521	0.1532	0.152	0.1519	0.1564	0.1592	0.147
0.1907	0.1792	0.1598	0.1529	0.1592	0.1581	0.1629	0.1616	0.149
0.1983	0.1899	0.1785	0.1661	0.1611	0.1461	0.1463	0.1447	0.1232
0.2048	0.1907	0.1755	0.1687	0.1662	0.1583	0.1481	0.1352	0.1143
0.1693	0.1486	0.1508	0.1474	0.1439	0.1386	0.1484	0.143	0.1342
0.1721	0.164	0.1658	0.1622	0.1693	0.1704	0.1776	0.1783	0.1748
0.1715	0.1648	0.1626	0.1611	0.1716	0.173	0.1788	0.1837	0.1831
0.193	0.1805	0.1839	0.1744	0.167	0.1685	0.18	0.193	0.1702
0.2591	0.2313	0.2218	0.2141	0.2119	0.2182	0.2205	0.2361	0.2255
0.179	0.1624	0.154	0.1423	0.1485	0.1372	0.1478	0.1413	0.1372
0.1712	0.1567	0.1532	0.1414	0.1403	0.1297	0.1407	0.1394	0.1287
0.1572	0.1471	0.1296	0.1222	0.1186	0.1285	0.1388	0.1297	0.1079
0.1533	0.1382	0.146	0.1272	0.1389	0.1398	0.1556	0.1482	0.1423
0.1929	0.18	0.166	0.1578	0.1682	0.1591	0.1682	0.1567	0.1541
0.2304	0.2282	0.2269	0.2097	0.2136	0.2155	0.2228	0.2224	0.1846
0.2824	0.2739	0.2644	0.2586	0.2359	0.2302	0.2426	0.2397	0.2131
0.2238	0.2029	0.1862	0.1899	0.1838	0.171	0.1876	0.1881	0.1802
0.1932	0.1832	0.1701	0.1653	0.1531	0.1485	0.1542	0.1454	0.1287
0.1564	0.1492	0.1432	0.1333	0.13	0.1334	0.1567	0.1409	0.1369
0.1726	0.1573	0.154	0.1441	0.1406	0.1225	0.1087	0.1065	0.0931
0.1326	0.1325	0.1283	0.1317	0.1242	0.1141	0.1169	0.1231	0.1076
0.0876	0.0871	0.0818	0.0708	0.0743	0.0691	0.072	0.064	0.0533
0.0124	0.0111	0.0106	0.01	0.0105	0.0103	0.0116	0.0108	0.0076
0.0306	0.0304	0.0268	0.0287	0.0285	0.0279	0.0292	0.0272	0.0247
0	0	0	0	0	0	0	0	0
0	0	0	0	0	0	0	0	0
0.0044	0.0041	0.0044	0.0046	0.0049	0.0053	0.0055	0.0058	0.0061
0.0043	0.004	0.0032	0.003	0.0035	0.003	0.0029	0.0033	0.0026
0	0	0	0	0	0	0	0	0
0.0103	0.0112	0.0116	0.011	0.0088	0.0094	0.0093	0.0083	0.009
0.0199	0.0191	0.0179	0.0184	0.0179	0.0183	0.0183	0.0174	0.0185
0.005	0.0055	0.0056	0.0057	0.0054	0.0056	0.0059	0.0059	0.0058
0.0198	0.0189	0.0191	0.0192	0.0197	0.0197	0.019	0.0187	0.0176
0.0985	0.0898	0.0915	0.0888	0.0933	0.0942	0.0985	0.096	0.0924

0.1188	0.1125	0.111	0.1056	0.11	0.1111	0.114	0.112	0.1071
0.1943	0.1871	0.1824	0.1811	0.1758	0.1766	0.1788	0.1646	0.1635
0.2561	0.2391	0.2429	0.2372	0.2161	0.2268	0.2408	0.24	0.253
0.2091	0.1916	0.1963	0.1919	0.1963	0.1938	0.1894	0.1926	0.1825
0.1379	0.132	0.1331	0.134	0.1399	0.1414	0.1456	0.1436	0.1344
0.1749	0.1516	0.1478	0.1437	0.1595	0.1495	0.1556	0.147	0.1318
0.1599	0.1522	0.147	0.1411	0.1423	0.1482	0.1548	0.1523	0.1507
0.1566	0.1477	0.1484	0.1495	0.1578	0.1659	0.1708	0.1679	0.1644
0.1472	0.1372	0.1365	0.1343	0.1423	0.1449	0.1566	0.1544	0.1497
0.1882	0.1751	0.1717	0.1686	0.1809	0.1675	0.1792	0.1594	0.1443
0.2104	0.2136	0.1949	0.196	0.1881	0.1975	0.2153	0.1999	0.1875
0.1777	0.1626	0.156	0.1537	0.159	0.14	0.1389	0.1411	0.1306
0.1427	0.1309	0.1329	0.1329	0.1331	0.1319	0.1389	0.1429	0.132
0.1635	0.148	0.1515	0.1535	0.1613	0.1556	0.1616	0.1649	0.1509
0.1694	0.1593	0.1557	0.1632	0.168	0.1734	0.1755	0.1749	0.1675
0.1366	0.1317	0.1227	0.1165	0.1256	0.1256	0.1285	0.1309	0.1264
0.1486	0.1287	0.1304	0.1174	0.12	0.1291	0.1325	0.133	0.1301
0.2374	0.2096	0.2079	0.2148	0.2229	0.2071	0.2081	0.1936	0.1742
0.1927	0.1845	0.1622	0.1539	0.1686	0.1613	0.1667	0.1566	0.1487
0.1644	0.1508	0.135	0.1186	0.1112	0.098	0.0967	0.0926	0.0736
0.1509	0.1504	0.143	0.133	0.1377	0.1352	0.1368	0.1503	0.1458
0.1692	0.1647	0.1674	0.1682	0.1733	0.1799	0.1868	0.1866	0.19
0.1639	0.1621	0.1705	0.1717	0.1886	0.1934	0.204	0.1915	0.203
0.168	0.1551	0.1489	0.1418	0.1537	0.1459	0.1479	0.1452	0.1394
0.193	0.1922	0.1779	0.172	0.19	0.1768	0.1705	0.1542	0.1392
0.1487	0.134	0.1316	0.139	0.1482	0.1467	0.1498	0.1376	0.1248
0.1351	0.1291	0.1262	0.1262	0.1318	0.1291	0.1275	0.1235	0.1149
0.1622	0.1551	0.1502	0.1475	0.1499	0.1529	0.1546	0.1547	0.1495
0.181	0.168	0.1638	0.1602	0.1628	0.158	0.1597	0.159	0.1504
0.1231	0.1045	0.1034	0.1047	0.1192	0.1052	0.116	0.1103	0.1052
0.1881	0.1842	0.1802	0.1635	0.1678	0.1698	0.1677	0.1599	0.1419
0.2171	0.1947	0.1943	0.1858	0.1822	0.1698	0.1718	0.1587	0.1537
0.1451	0.1341	0.1397	0.147	0.137	0.1238	0.1339	0.1283	0.1161
0.15	0.1393	0.119	0.1136	0.1191	0.1206	0.1339	0.1304	0.1102
0.1733	0.1507	0.1393	0.1293	0.1302	0.1261	0.1429	0.1368	0.1136
0.1769	0.1603	0.1525	0.1558	0.1521	0.1389	0.1555	0.1374	0.1212
0.1711	0.1509	0.1517	0.139	0.1393	0.1303	0.1301	0.1223	0.1114
0.1146	0.1058	0.106	0.0932	0.1009	0.0979	0.1059	0.0935	0.0785
0.021	0.0194	0.0195	0.0185	0.0196	0.0165	0.0178	0.0172	0.0129
0.0297	0.0299	0.0285	0.0287	0.0291	0.0313	0.0287	0.0272	0.0271
0	0	0	0	0	0	0	0	0
0	0	0	0	0	0	0	0	0
0.0072	0.0071	0.0073	0.0075	0.0076	0.008	0.008	0.0079	0.008
0.0059	0.0059	0.006	0.006	0.006	0.0057	0.0056	0.0054	0.0049

0.0118	0.0121	0.0109	0.0111	0.0112	0.0117	0.0115	0.0119	0.0122
0.016	0.0164	0.016	0.0158	0.0157	0.0165	0.0172	0.0167	0.015
0.0179	0.0189	0.0177	0.018	0.0178	0.019	0.0185	0.0177	0.017
0.0078	0.0082	0.0078	0.0075	0.0069	0.0072	0.0072	0.0069	0.0068
0.0129	0.0127	0.0122	0.0112	0.0108	0.0102	0.0108	0.0106	0.0111
0.0612	0.0582	0.0565	0.0556	0.0535	0.0542	0.0552	0.0536	0.0561
0.1312	0.1315	0.1278	0.1338	0.1306	0.1344	0.1346	0.1348	0.1377
0.11	0.1105	0.108	0.1113	0.1103	0.1094	0.112	0.1086	0.1057
0.1612	0.1564	0.1369	0.1227	0.1157	0.1016	0.1055	0.0964	0.0835
0.1588	0.1562	0.1584	0.1548	0.1608	0.155	0.1473	0.1437	0.1378
0.1595	0.1483	0.1435	0.1335	0.1413	0.1379	0.1404	0.1383	0.1291
0.1368	0.1247	0.1268	0.1271	0.1259	0.124	0.1325	0.1328	0.1224
0.154	0.1425	0.1392	0.1373	0.1415	0.1366	0.1457	0.1415	0.134
0.1741	0.1673	0.1684	0.168	0.1751	0.1784	0.1848	0.184	0.1796
0.161	0.153	0.1389	0.1325	0.149	0.1494	0.163	0.151	0.1406
0.1722	0.1531	0.1527	0.1412	0.1533	0.1417	0.1393	0.1313	0.1114
0.1779	0.1518	0.1565	0.1654	0.1594	0.152	0.1649	0.1632	0.1296
0.1454	0.1398	0.1409	0.1369	0.1445	0.1474	0.1571	0.1505	0.1368
0.1557	0.1516	0.144	0.1421	0.1503	0.1426	0.1391	0.1396	0.1322
0.2028	0.1872	0.1792	0.1798	0.1739	0.1621	0.1589	0.144	0.1357
0.1739	0.162	0.1545	0.1364	0.1457	0.1479	0.1551	0.1525	0.1431
0.1614	0.1439	0.14	0.1362	0.1462	0.1344	0.1357	0.1306	0.1164
0.1471	0.1412	0.1393	0.145	0.1568	0.1497	0.1592	0.1441	0.1245
0.1911	0.1779	0.1633	0.1516	0.1477	0.1534	0.1431	0.1316	0.118
0.1676	0.168	0.177	0.1577	0.1778	0.1742	0.1711	0.1603	0.1483
0.1546	0.1416	0.1221	0.1131	0.1306	0.1025	0.1135	0.114	0.0971
0.1471	0.1399	0.1277	0.115	0.1194	0.1135	0.1212	0.1227	0.105
0.1648	0.1598	0.1567	0.1522	0.1554	0.1544	0.1621	0.1617	0.153
0.1588	0.1572	0.1539	0.1599	0.1691	0.1826	0.1922	0.1865	0.1931
0.1275	0.122	0.124	0.1236	0.1255	0.1224	0.1258	0.1231	0.1164
0.1822	0.1593	0.1581	0.155	0.1581	0.1546	0.1693	0.1732	0.1555
0.1293	0.1155	0.1097	0.1195	0.126	0.1306	0.1354	0.129	0.1211
0.1531	0.1407	0.1358	0.125	0.1326	0.1244	0.1256	0.1151	0.1086
0.1837	0.1741	0.17	0.1639	0.1768	0.1716	0.1686	0.1602	0.1373
0.1862	0.1572	0.1405	0.1528	0.1603	0.1507	0.1654	0.1634	0.1335
0.15	0.1423	0.1317	0.1173	0.1298	0.1113	0.115	0.1186	0.1044
0.1634	0.1468	0.1509	0.1342	0.1193	0.096	0.1036	0.1046	0.0861
0.1216	0.0962	0.0895	0.0803	0.0812	0.0756	0.0724	0.071	0.0538
0.1249	0.1116	0.1138	0.1061	0.1114	0.1082	0.1086	0.1134	0.0895
0.1195	0.1073	0.1059	0.0888	0.0982	0.0971	0.112	0.1002	0.0863
0.1771	0.1578	0.1603	0.1504	0.1486	0.1379	0.1376	0.1353	0.1116
0.1963	0.1685	0.1615	0.1373	0.1372	0.1247	0.1286	0.1253	0.1142
0.2355	0.2211	0.2183	0.2119	0.2272	0.2083	0.2183	0.2052	0.173
0.1876	0.1794	0.1675	0.1529	0.1495	0.1344	0.143	0.1445	0.1303

0.0371	0.0346	0.0309	0.0313	0.0297	0.0322	0.0323	0.0302	0.0283
0.0396	0.0405	0.0402	0.0421	0.0428	0.0447	0.046	0.0464	0.0457
0	0	0	0	0	0	0	0	0
0	0	0	0	0	0	0	0	0
0.0039	0.0038	0.0038	0.0037	0.0039	0.004	0.0042	0.0041	0.0039
0.0038	0.0038	0.0038	0.0038	0.004	0.0041	0.004	0.0041	0.0042
0.016	0.0161	0.0154	0.0158	0.0155	0.0162	0.015	0.0144	0.0151
0.0175	0.0182	0.0187	0.0178	0.0176	0.018	0.0176	0.018	0.0164
0.0068	0.0069	0.0069	0.0066	0.006	0.0061	0.0056	0.0055	0.0059
0.0047	0.0049	0.0051	0.0055	0.0057	0.0059	0.0058	0.0059	0.0061
0.0128	0.0132	0.0126	0.0124	0.0118	0.0118	0.0119	0.0112	0.0115
0.0424	0.0445	0.0439	0.0446	0.0435	0.0428	0.041	0.0387	0.0351
0.1294	0.1331	0.1251	0.1281	0.118	0.1241	0.1239	0.1205	0.1188
0.0988	0.0922	0.0866	0.0827	0.0837	0.0758	0.0798	0.0738	0.0678
0.1461	0.1394	0.1567	0.1494	0.1432	0.1367	0.1392	0.1377	0.1204
0.1873	0.1478	0.1283	0.1129	0.1224	0.1319	0.122	0.1069	0.0937
0.194	0.1819	0.1643	0.1536	0.1701	0.167	0.165	0.1646	0.1404
0.166	0.1424	0.1272	0.1083	0.1142	0.104	0.0986	0.0931	0.0964
0.1577	0.1489	0.1436	0.1357	0.1464	0.1495	0.1436	0.1413	0.1354
0.1532	0.1411	0.1403	0.1431	0.1495	0.1495	0.158	0.1541	0.1481
0.1902	0.1761	0.1597	0.1556	0.163	0.1703	0.1809	0.1885	0.1744
0.1397	0.1323	0.1382	0.1306	0.1217	0.1103	0.1201	0.1122	0.0994
0.1587	0.1431	0.1308	0.1344	0.1432	0.1482	0.1474	0.1381	0.1279
0.1399	0.1341	0.1329	0.1303	0.1422	0.1477	0.1575	0.1561	0.1517
0.1739	0.1682	0.168	0.1638	0.1647	0.1691	0.173	0.1671	0.1652
0.2295	0.2244	0.2081	0.202	0.1827	0.1864	0.1851	0.1909	0.184
0.1825	0.1697	0.1786	0.179	0.1692	0.1666	0.182	0.1845	0.1702
0.1656	0.1477	0.1418	0.1428	0.1436	0.1454	0.1487	0.1496	0.1278
0.1869	0.1667	0.1647	0.1648	0.1524	0.1391	0.1384	0.1497	0.1373
0.1739	0.1672	0.1592	0.1523	0.1526	0.1428	0.1329	0.1404	0.1097
0.167	0.1329	0.1226	0.1304	0.1388	0.1294	0.1277	0.1027	0.0962
0.1737	0.1511	0.1492	0.1372	0.1348	0.1099	0.0976	0.0796	0.079
0.1355	0.1119	0.1161	0.1059	0.1164	0.1036	0.1091	0.1061	0.0891
0.1556	0.1349	0.1333	0.1286	0.1393	0.1377	0.1422	0.1388	0.119
0.1705	0.1612	0.1632	0.1484	0.1476	0.1495	0.1581	0.1532	0.1388
0.154	0.1332	0.1311	0.1293	0.1391	0.1235	0.1297	0.1409	0.1341
0.1725	0.1605	0.1528	0.1439	0.1529	0.1509	0.1549	0.1514	0.1378
0.148	0.1401	0.1349	0.1316	0.1465	0.1426	0.1459	0.153	0.1439
0.1693	0.1591	0.1589	0.1588	0.1716	0.1735	0.1816	0.1721	0.1596
0.184	0.1745	0.1655	0.1532	0.1616	0.1567	0.1592	0.1468	0.1225
0.1089	0.0861	0.0815	0.0709	0.0857	0.092	0.108	0.1102	0.1057
0.1299	0.1105	0.1187	0.1147	0.1204	0.1126	0.1205	0.1168	0.1062
0.174	0.1509	0.1514	0.1446	0.1401	0.1347	0.1461	0.1291	0.1219
0.1222	0.1221	0.1171	0.1194	0.1228	0.1295	0.1293	0.1218	0.1084

0.1377	0.1341	0.125	0.1217	0.1302	0.1179	0.1263	0.1215	0.1106
0.1428	0.1312	0.1295	0.1232	0.1235	0.1185	0.1207	0.1266	0.1116
0.1175	0.1129	0.1054	0.1009	0.1085	0.1031	0.1081	0.1069	0.1001
0.1751	0.1631	0.148	0.1343	0.132	0.1314	0.1253	0.1253	0.107
0.2446	0.22	0.2021	0.192	0.2048	0.2084	0.2071	0.2038	0.184
0.1607	0.157	0.1648	0.1514	0.1548	0.1536	0.1627	0.1538	0.1398
0.0279	0.0279	0.0273	0.0275	0.0268	0.0279	0.0271	0.0265	0.0251
0.037	0.0389	0.0372	0.0387	0.0378	0.0381	0.0391	0.0386	0.0351
0	0	0	0	0	0	0	0	0
0	0	0	0	0	0	0	0	0
0.0027	0.0025	0.0026	0.0024	0.0027	0.0027	0.0029	0.0029	0.0026
0	0	0	0	0	0	0	0	0
0.0087	0.0087	0.0091	0.0094	0.0095	0.01	0.0101	0.0101	0.0104
0.0096	0.0096	0.0092	0.0085	0.0085	0.0081	0.0079	0.0074	0.0072
0	0	0	0	0	0	0	0	0
0.0104	0.0102	0.0104	0.0102	0.0108	0.0106	0.0112	0.011	0.0108
0.0053	0.0051	0.0049	0.0047	0.0047	0.0043	0.0045	0.0044	0.0041
0.0322	0.0339	0.0328	0.0335	0.0352	0.0349	0.034	0.0315	0.0332
0.0671	0.0666	0.064	0.0634	0.0592	0.0612	0.0586	0.0589	0.0581
0.1387	0.1375	0.1323	0.1329	0.1324	0.1335	0.135	0.1309	0.129
0.1969	0.1821	0.1783	0.17	0.1731	0.1705	0.1799	0.1679	0.145
0.168	0.1585	0.1364	0.1426	0.1477	0.1291	0.1369	0.1268	0.1248
0.1714	0.1552	0.1507	0.139	0.1381	0.1322	0.1315	0.1138	0.09
0.1726	0.1451	0.141	0.1328	0.1383	0.1153	0.1225	0.1247	0.1018
0.1625	0.1541	0.1502	0.1368	0.1219	0.1152	0.12	0.1287	0.1054
0.1416	0.1389	0.1358	0.1326	0.1316	0.1273	0.132	0.1266	0.1169
0.1518	0.1398	0.1408	0.132	0.135	0.1391	0.1411	0.1374	0.1346
0.0965	0.086	0.085	0.078	0.0847	0.0886	0.094	0.0915	0.0839
0.1539	0.1509	0.1453	0.1426	0.1411	0.1434	0.1509	0.1465	0.1373
0.1508	0.1461	0.1522	0.1516	0.156	0.1611	0.1676	0.1673	0.163
0.1702	0.1633	0.1639	0.1645	0.1675	0.1677	0.1722	0.1722	0.1676
0.2329	0.2128	0.2054	0.1956	0.1917	0.1918	0.1754	0.1804	0.1662
0.2006	0.1884	0.1805	0.1611	0.1533	0.1357	0.1234	0.1116	0.0885
0.116	0.1134	0.1103	0.1076	0.1069	0.1001	0.115	0.1239	0.1035
0.1979	0.1831	0.1643	0.1427	0.1305	0.1296	0.1261	0.1231	0.0931
0.1474	0.1283	0.1236	0.1259	0.1353	0.1313	0.1308	0.1318	0.1209
0.1434	0.1333	0.1338	0.1305	0.1352	0.1303	0.1381	0.1324	0.1175
0.1999	0.1864	0.185	0.178	0.1806	0.1824	0.1723	0.1635	0.1542
0.132	0.136	0.1312	0.1303	0.1461	0.1449	0.1522	0.1578	0.1202
0.1439	0.1375	0.1444	0.1316	0.1394	0.1273	0.1307	0.1419	0.1226
0.1805	0.1587	0.1585	0.1525	0.1652	0.1467	0.1626	0.1508	0.1387
0.1366	0.1319	0.1332	0.123	0.112	0.1033	0.1029	0.0976	0.0947
0.14	0.1266	0.1116	0.0927	0.1074	0.0961	0.1031	0.1005	0.09
0.1676	0.1613	0.1562	0.1523	0.1582	0.1527	0.1577	0.1515	0.1446

0.1639	0.1515	0.1539	0.154	0.1604	0.1652	0.1734	0.174	0.1746
0.1556	0.1458	0.1495	0.1467	0.1531	0.1562	0.1635	0.1574	0.1516
0.1377	0.1225	0.1021	0.0941	0.1043	0.1077	0.1268	0.1175	0.111
0.1766	0.1564	0.1446	0.1377	0.1449	0.1497	0.1519	0.1392	0.1285
0.175	0.1802	0.1643	0.163	0.1632	0.1438	0.1514	0.1542	0.1468
0.1655	0.1483	0.1269	0.1176	0.1167	0.1101	0.1217	0.1205	0.1227
0.1451	0.1208	0.1293	0.1216	0.127	0.1081	0.1148	0.1233	0.1064
0.1363	0.1378	0.1347	0.1304	0.1335	0.1338	0.1336	0.1305	0.1179
0.1749	0.1546	0.1424	0.1261	0.134	0.1185	0.116	0.1057	0.0957
0.1583	0.1375	0.1399	0.133	0.1269	0.1217	0.1164	0.1098	0.1048
0.1685	0.1395	0.1277	0.1182	0.1273	0.117	0.1242	0.138	0.1244
0.1315	0.1147	0.1074	0.0995	0.097	0.0905	0.0911	0.0859	0.0683
0.0089	0.0083	0.0079	0.007	0.0069	0.0071	0.0069	0.0069	0.0062
0.0423	0.0409	0.0417	0.0417	0.042	0.0418	0.0425	0.0408	0.0428
0	0	0	0	0	0	0	0	0
0	0	0	0	0	0	0	0	0
0	0	0	0	0	0	0	0	0
0	0	0	0	0	0	0	0	0
0	0	0	0	0	0	0	0	0
0	0	0	0	0	0	0	0	0
0	0	0	0	0	0	0	0	0
0	0	0	0	0	0	0	0	0
0	0	0	0	0	0	0	0	0
0	0	0	0	0	0	0	0	0
0.0016	0.0016	0.0017	0.0018	0.002	0.0022	0.0023	0.0024	0.0025
0.0409	0.0392	0.038	0.037	0.0379	0.0367	0.0368	0.0349	0.0315
0.1047	0.1004	0.0924	0.0864	0.0898	0.0888	0.0938	0.0896	0.083
0.309	0.3053	0.295	0.2957	0.2908	0.2809	0.2885	0.2734	0.2604
0.2104	0.2027	0.1871	0.1642	0.1655	0.1639	0.1697	0.1699	0.1665
0.1128	0.1027	0.1006	0.0989	0.091	0.0865	0.0899	0.1014	0.0788
0.1654	0.1652	0.154	0.1598	0.1557	0.1439	0.1461	0.1416	0.1296
0.1502	0.1455	0.1474	0.1421	0.1496	0.1285	0.132	0.1264	0.1173
0.1492	0.1368	0.1382	0.1371	0.1405	0.1427	0.1468	0.1481	0.1401
0.125	0.1148	0.1149	0.1111	0.1211	0.1217	0.127	0.1272	0.1206
0.1344	0.1283	0.1327	0.1331	0.141	0.1443	0.1481	0.1519	0.1495
0.1512	0.146	0.1449	0.1485	0.1549	0.1586	0.1622	0.1598	0.1553
0.1341	0.1184	0.1133	0.1121	0.1239	0.1229	0.1218	0.1201	0.1127
0.2005	0.1799	0.1658	0.1685	0.1804	0.1777	0.185	0.1693	0.1612
0.2146	0.1962	0.1799	0.1689	0.1711	0.1741	0.1742	0.1592	0.1342
0.1637	0.1548	0.1499	0.1463	0.1472	0.1484	0.161	0.153	0.1454
0.1407	0.1372	0.1387	0.1382	0.1402	0.1442	0.1484	0.148	0.1468
0.1476	0.1427	0.1463	0.1468	0.1524	0.1568	0.1628	0.1723	0.1754
0.1409	0.137	0.1355	0.1326	0.1336	0.1347	0.1424	0.1385	0.1323
0.1774	0.1751	0.1663	0.1602	0.1728	0.158	0.1632	0.1581	0.1399

0.2181	0.2068	0.195	0.1746	0.1788	0.1568	0.1325	0.1208	0.116
0.1763	0.1484	0.1511	0.1409	0.1385	0.1331	0.1388	0.152	0.1356
0.1791	0.1692	0.1435	0.1401	0.1395	0.1247	0.1212	0.1253	0.1083
0.1793	0.1465	0.1445	0.1324	0.1612	0.1446	0.1273	0.1267	0.1119
0.1869	0.1661	0.144	0.1339	0.1421	0.1563	0.146	0.1333	0.1046
0.1684	0.1558	0.1552	0.1536	0.1578	0.1592	0.1583	0.1531	0.1512
0.1813	0.1745	0.1794	0.1855	0.194	0.1964	0.2005	0.2067	0.2076
0.1524	0.15	0.1521	0.1493	0.1566	0.1608	0.1663	0.1662	0.1637
0.1484	0.1279	0.1141	0.0989	0.1025	0.1026	0.1139	0.1076	0.0983
0.1354	0.1216	0.1218	0.1239	0.1202	0.1112	0.0894	0.083	0.0691
0.1865	0.185	0.1704	0.1727	0.1645	0.1589	0.1536	0.1329	0.116
0.0941	0.0795	0.0783	0.0692	0.07	0.0726	0.0911	0.0966	0.0793
0.1801	0.1695	0.1693	0.1635	0.1639	0.1485	0.149	0.14	0.1198
0.1263	0.1047	0.0995	0.0956	0.1017	0.0955	0.1015	0.0942	0.0846
0.1742	0.1555	0.1569	0.1631	0.1553	0.1387	0.1413	0.133	0.1054
0.2385	0.211	0.1941	0.1752	0.1916	0.1884	0.206	0.2202	0.1933
0.1788	0.1759	0.1769	0.164	0.1693	0.1613	0.1609	0.1482	0.1344
0.0355	0.0324	0.0305	0.0302	0.0288	0.0283	0.0292	0.0295	0.0249
0.0863	0.0897	0.0861	0.0866	0.0803	0.0815	0.0829	0.08	0.0832
0.2488	0.2452	0.2379	0.2186	0.2081	0.2326	0.2263	0.2096	0.2184
0.0482	0.0512	0.0459	0.0445	0.0404	0.0406	0.0392	0.037	0.04
0.0218	0.0225	0.0206	0.022	0.0199	0.0198	0.0186	0.0185	0.0191
0	0	0	0	0	0	0	0	0
0	0	0	0	0	0	0	0	0
0	0	0	0	0	0	0	0	0
0	0	0	0	0	0	0	0	0
0	0	0	0	0	0	0	0	0
0	0	0	0	0	0	0	0	0
0	0	0	0	0	0	0	0	0
0	0	0	0	0	0	0	0	0
0	0	0	0	0	0	0	0	0
0.0228	0.0228	0.0232	0.0238	0.0217	0.0224	0.0236	0.0224	0.0233
0.0846	0.0837	0.0813	0.0802	0.0754	0.0776	0.079	0.0808	0.0765
0.3985	0.4085	0.3936	0.3772	0.3564	0.3803	0.3587	0.3555	0.3472
0.4238	0.417	0.3799	0.3739	0.3489	0.3655	0.3633	0.3819	0.3785
0.1777	0.1772	0.1704	0.1577	0.1576	0.1452	0.1537	0.149	0.1403
0.1764	0.155	0.1424	0.14	0.1473	0.1443	0.1617	0.1608	0.1447
0.1689	0.149	0.1559	0.1389	0.1359	0.1404	0.1505	0.14	0.1229
0.1993	0.1777	0.1755	0.1619	0.1605	0.1557	0.1568	0.1527	0.1405
0.2037	0.1946	0.1917	0.183	0.1782	0.1842	0.1895	0.1837	0.1708
0.1704	0.16	0.1652	0.1625	0.1667	0.1693	0.1758	0.1757	0.176
0.1233	0.1141	0.1136	0.1082	0.1159	0.1138	0.1211	0.1188	0.1108
0.1777	0.1421	0.1315	0.1244	0.121	0.1237	0.135	0.1291	0.1157
0.1592	0.1378	0.1457	0.1418	0.1497	0.1409	0.1324	0.14	0.1049

0.1046	0.0905	0.0916	0.0898	0.0985	0.0973	0.1131	0.1146	0.0963
0.1541	0.144	0.1446	0.1431	0.1514	0.1549	0.1658	0.1576	0.147
0.1733	0.1679	0.1709	0.1719	0.1805	0.1846	0.1898	0.1867	0.1881
0.1755	0.1695	0.1823	0.1843	0.1945	0.2052	0.2135	0.2069	0.213
0.1627	0.1523	0.1541	0.1508	0.161	0.1619	0.1664	0.1615	0.1567
0.1461	0.1339	0.1373	0.1383	0.1395	0.1296	0.1224	0.1106	0.0969
0.1645	0.1504	0.1441	0.1455	0.1452	0.1459	0.1546	0.1491	0.1312
0.1815	0.1488	0.152	0.1442	0.1385	0.1441	0.1342	0.1229	0.1117
0.1657	0.1467	0.1462	0.1293	0.1443	0.1256	0.1209	0.1379	0.1289
0.1813	0.1563	0.1332	0.1133	0.1102	0.1015	0.1126	0.0943	0.087
0.1394	0.1149	0.1139	0.1082	0.1166	0.1212	0.1155	0.1067	0.0917
0.1488	0.1436	0.1363	0.1326	0.1368	0.1387	0.142	0.1452	0.1355
0.1669	0.1621	0.1619	0.1597	0.1613	0.1606	0.1687	0.1669	0.1632
0.1751	0.1651	0.1612	0.1558	0.17	0.1698	0.1771	0.1764	0.1686
0.1934	0.1762	0.1732	0.1728	0.171	0.1623	0.1918	0.1715	0.1343
0.178	0.162	0.1575	0.1573	0.1484	0.1454	0.1573	0.1476	0.1421
0.1719	0.1733	0.1424	0.1466	0.1389	0.1354	0.1456	0.1508	0.1333
0.2097	0.193	0.186	0.1744	0.1746	0.1705	0.1741	0.167	0.1696
0.1168	0.0935	0.0941	0.0938	0.1084	0.0982	0.0925	0.0853	0.0711
0.1982	0.1856	0.1747	0.1617	0.1449	0.1178	0.1178	0.1232	0.1143
0.1782	0.1669	0.1752	0.1718	0.1675	0.1791	0.1771	0.1636	0.1549
0.2458	0.2364	0.2306	0.2046	0.2139	0.1954	0.1853	0.1625	0.1454
0.1458	0.1258	0.1151	0.1196	0.1163	0.1075	0.1117	0.1033	0.0853
0.0706	0.0706	0.0702	0.0689	0.0677	0.0642	0.0601	0.0618	0.0612
0.2754	0.285	0.2763	0.2726	0.2354	0.2318	0.2227	0.2226	0.2211
0.4968	0.4744	0.4536	0.4559	0.4543	0.4778	0.4668	0.4345	0.4203
0.0326	0.0301	0.0294	0.0304	0.0279	0.0274	0.0269	0.0274	0.0281
0.0119	0.0123	0.013	0.0131	0.0122	0.0132	0.0124	0.0123	0.0139
0	0	0	0	0	0	0	0	0
0	0	0	0	0	0	0	0	0
0	0	0	0	0	0	0	0	0
0	0	0	0	0	0	0	0	0
0	0	0	0	0	0	0	0	0
0	0	0	0	0	0	0	0	0
0	0	0	0	0	0	0	0	0
0	0	0	0	0	0	0	0	0
0.0252	0.0252	0.0244	0.0242	0.0225	0.0225	0.0215	0.0223	0.022
0.0422	0.0461	0.0457	0.0422	0.0358	0.0406	0.0414	0.0423	0.0435
0.0176	0.0174	0.0176	0.0165	0.0165	0.0165	0.0156	0.015	0.0153
0.2782	0.2814	0.27	0.2925	0.2836	0.2644	0.2686	0.2607	0.2578
0.3818	0.4057	0.3774	0.4199	0.4118	0.4065	0.3975	0.3782	0.3876
0.1707	0.1636	0.1412	0.1432	0.1305	0.1272	0.1264	0.1264	0.1283
0.1736	0.1618	0.1671	0.1627	0.1713	0.1628	0.1586	0.1482	0.1296
0.1923	0.1755	0.1583	0.1674	0.1695	0.159	0.16	0.1536	0.1518

0.1424	0.1443	0.1319	0.1205	0.126	0.1109	0.1161	0.1084	0.0975
0.1533	0.1367	0.1413	0.1425	0.1343	0.1232	0.1306	0.1202	0.1059
0.1949	0.1782	0.1668	0.1591	0.1648	0.1538	0.1718	0.1532	0.1381
0.1928	0.1748	0.1644	0.1559	0.151	0.14	0.1353	0.1248	0.1089
0.1741	0.1424	0.1361	0.1443	0.1526	0.1415	0.1349	0.1187	0.1098
0.1512	0.1461	0.147	0.1473	0.1493	0.1527	0.1554	0.1532	0.1504
0.1478	0.1546	0.1591	0.1604	0.1639	0.1683	0.1744	0.1788	0.177
0.1956	0.1908	0.1934	0.1927	0.1973	0.2015	0.208	0.2051	0.2081
0.1837	0.1729	0.1802	0.1846	0.1869	0.1972	0.1981	0.2008	0.1947
0.1697	0.1628	0.1719	0.1725	0.1772	0.1866	0.1896	0.1859	0.1861
0.1467	0.1393	0.1365	0.1277	0.1317	0.1389	0.1356	0.1278	0.1171
0.1753	0.1626	0.1598	0.1545	0.1539	0.1529	0.1412	0.1527	0.1378
0.1409	0.1262	0.1273	0.1213	0.11	0.1124	0.1248	0.1102	0.095
0.2068	0.1816	0.1899	0.1745	0.1758	0.161	0.1841	0.1903	0.1683
0.2216	0.2015	0.2075	0.1887	0.1813	0.1604	0.1662	0.1481	0.135
0.1746	0.1572	0.1689	0.1529	0.1601	0.1572	0.1647	0.1559	0.1413
0.1501	0.1477	0.1381	0.1376	0.1273	0.1234	0.1357	0.1396	0.1243
0.1565	0.1485	0.1488	0.145	0.146	0.1427	0.1502	0.1453	0.127
0.1447	0.1266	0.1275	0.1293	0.1382	0.1323	0.1377	0.1406	0.1222
0.2194	0.2161	0.1959	0.1966	0.1951	0.1879	0.2033	0.1897	0.1778
0.1506	0.1495	0.1413	0.1239	0.1405	0.1273	0.1351	0.1277	0.1272
0.1555	0.1429	0.1587	0.153	0.1571	0.1411	0.1436	0.131	0.1085
0.1398	0.1199	0.1143	0.0869	0.1063	0.1064	0.1086	0.1085	0.0965
0.1692	0.1645	0.1628	0.1497	0.1551	0.1386	0.1427	0.1419	0.1267
0.1586	0.1307	0.1306	0.1193	0.1125	0.1002	0.0982	0.1031	0.0989
0.2215	0.2045	0.2034	0.1999	0.1912	0.1741	0.171	0.1801	0.1561
0.2005	0.193	0.1835	0.1839	0.1889	0.1935	0.1767	0.1671	0.1475
0.1731	0.1384	0.1314	0.1154	0.1254	0.135	0.1414	0.1264	0.1032
0.1289	0.1098	0.1153	0.1051	0.101	0.0984	0.1022	0.1032	0.0912
0.097	0.0963	0.0982	0.0938	0.0893	0.0932	0.0885	0.0879	0.0812
0.1565	0.1578	0.1502	0.1519	0.1355	0.1335	0.1219	0.1108	0.1208
0.101	0.0968	0.099	0.1058	0.1064	0.1208	0.1301	0.1296	0.1273
0.0234	0.025	0.0254	0.0257	0.0248	0.0256	0.024	0.0232	0.0223
0.0081	0.0075	0.0069	0.0073	0.0074	0.0075	0.0079	0.0077	0.0073
0	0	0	0	0	0	0	0	0
0	0	0	0	0	0	0	0	0
0	0	0	0	0	0	0	0	0
0	0	0	0	0	0	0	0	0
0	0	0	0	0	0	0	0	0
0	0	0	0	0	0	0	0	0
0	0	0	0	0	0	0	0	0
0	0	0	0	0	0	0	0	0
0.0063	0.0058	0.0059	0.0059	0.0062	0.0063	0.0067	0.0064	0.0063
0.0545	0.0572	0.0571	0.0571	0.0555	0.0562	0.0503	0.046	0.0444

0.0071	0.0053	0.0042	0.0039	0.0053	0.0051	0.0053	0.0044	0.0036
0.0352	0.0348	0.0342	0.0344	0.0344	0.0319	0.0323	0.0332	0.0323
0.1548	0.1594	0.1547	0.1579	0.1437	0.1534	0.1508	0.1376	0.1358
0.1331	0.1216	0.118	0.1147	0.123	0.1214	0.1289	0.1209	0.1149
0.1456	0.1431	0.1492	0.1481	0.1566	0.1417	0.1381	0.1266	0.1174
0.1581	0.1586	0.1555	0.1464	0.1564	0.1488	0.1575	0.1568	0.1527
0.1499	0.1448	0.1364	0.1339	0.1473	0.1435	0.1508	0.1444	0.1327
0.1397	0.124	0.1123	0.0979	0.1003	0.0894	0.1019	0.0955	0.0767
0.1708	0.1597	0.1529	0.1457	0.1577	0.1401	0.1419	0.1437	0.1171
0.2172	0.2022	0.1973	0.1911	0.188	0.1663	0.1903	0.189	0.159
0.1849	0.1824	0.1825	0.1837	0.1849	0.1861	0.195	0.1921	0.195
0.1882	0.1881	0.1894	0.1906	0.1929	0.2002	0.2065	0.2086	0.2164
0.2046	0.19	0.1931	0.2067	0.2135	0.232	0.2427	0.2446	0.2512
0.2483	0.2363	0.247	0.2612	0.2682	0.2673	0.2775	0.2766	0.288
0.159	0.1534	0.1568	0.1671	0.1916	0.1975	0.2083	0.2135	0.228
0.1586	0.1509	0.153	0.1546	0.1626	0.1641	0.1649	0.1624	0.1617
0.1703	0.1485	0.1467	0.145	0.14	0.1226	0.1281	0.1251	0.1047
0.1684	0.143	0.1232	0.1209	0.1225	0.1185	0.1104	0.0973	0.0758
0.214	0.1885	0.1996	0.1875	0.1768	0.168	0.1603	0.1407	0.1142
0.2244	0.2149	0.2099	0.1882	0.1936	0.189	0.1959	0.176	0.157
0.2483	0.2221	0.2102	0.1945	0.2091	0.2061	0.2077	0.2024	0.2009
0.1932	0.1754	0.1541	0.1413	0.1447	0.1312	0.143	0.1327	0.1381
0.1719	0.15	0.1412	0.1334	0.1308	0.1303	0.1244	0.1143	0.0894
0.1574	0.1325	0.13	0.1279	0.1188	0.1165	0.1305	0.1303	0.1274
0.1947	0.18	0.1649	0.167	0.1721	0.1696	0.1697	0.1583	0.1456
0.1517	0.1398	0.1266	0.1401	0.1341	0.1358	0.1379	0.1396	0.1169
0.1585	0.1257	0.1242	0.1139	0.1186	0.0958	0.1	0.1074	0.0975
0.1432	0.1296	0.1203	0.1214	0.1412	0.1376	0.142	0.1324	0.1334
0.2163	0.189	0.1827	0.1695	0.1689	0.1616	0.1727	0.1568	0.1556
0.1694	0.1489	0.1471	0.148	0.1448	0.1356	0.149	0.1371	0.1336
0.1596	0.1449	0.1314	0.1132	0.1266	0.1222	0.1166	0.11	0.1033
0.1621	0.1609	0.1572	0.1671	0.176	0.1751	0.1813	0.1864	0.1609
0.2023	0.2007	0.2013	0.1875	0.1807	0.163	0.1486	0.1553	0.1169
0.1042	0.0913	0.0788	0.0754	0.0743	0.0792	0.0832	0.0926	0.0855
0.1324	0.1209	0.1111	0.1051	0.1027	0.1057	0.1145	0.1117	0.1059
0.1917	0.1975	0.1972	0.1853	0.1699	0.1807	0.1759	0.1678	0.17
0.1061	0.1014	0.1035	0.1039	0.1036	0.1028	0.0989	0.0965	0.0968
0.0009	0.0009	0.001	0.001	0.0011	0.0012	0.0012	0.0011	0.0012
0.0081	0.0079	0.0074	0.0074	0.0063	0.0065	0.0067	0.0068	0.0071
0	0	0	0	0	0	0	0	0
0	0	0	0	0	0	0	0	0
0	0	0	0	0	0	0	0	0
0	0	0	0	0	0	0	0	0
0	0	0	0	0	0	0	0	0

0	0	0	0	0	0	0	0	0
0	0	0	0	0	0	0	0	0
0	0	0	0	0	0	0	0	0
0	0	0	0	0	0	0	0	0
0.0008	0.0009	0.0009	0.0009	0.001	0.0011	0.0012	0.0011	0.0011
0.0423	0.0441	0.0454	0.0449	0.0428	0.0432	0.0421	0.0409	0.0411
0.0034	0.0033	0.0033	0.0031	0.0028	0.003	0.0029	0.0032	0.0031
0.0143	0.0145	0.0148	0.0154	0.0163	0.0159	0.0167	0.0166	0.0172
0.1232	0.119	0.1175	0.1188	0.1222	0.1249	0.1298	0.1292	0.1271
0.1617	0.1495	0.1482	0.1422	0.1467	0.151	0.1567	0.1545	0.1527
0.1661	0.1571	0.1545	0.1511	0.1564	0.1587	0.1602	0.1598	0.1565
0.158	0.1518	0.1521	0.1562	0.1652	0.1712	0.1806	0.18	0.1854
0.1847	0.1733	0.1675	0.1682	0.1722	0.1776	0.18	0.1741	0.1668
0.1464	0.1381	0.1427	0.1351	0.1403	0.1263	0.1279	0.1308	0.1234
0.1497	0.1352	0.1347	0.1307	0.1344	0.1287	0.1333	0.1363	0.1287
0.1568	0.153	0.1562	0.1566	0.1628	0.1674	0.1724	0.1725	0.1743
0.184	0.1819	0.1839	0.1843	0.1865	0.1932	0.1968	0.1937	0.2012
0.1747	0.1685	0.169	0.1736	0.1795	0.1852	0.1925	0.1937	0.1917
0.1546	0.1436	0.1561	0.1571	0.1678	0.1652	0.1754	0.1795	0.1721
0.1925	0.1982	0.2132	0.205	0.2226	0.2248	0.2234	0.2225	0.2414
0.1837	0.1723	0.1816	0.1956	0.2054	0.2333	0.2519	0.2678	0.2726
0.1815	0.1769	0.1745	0.1711	0.1823	0.1888	0.1967	0.1957	0.1988
0.1672	0.1441	0.1387	0.1344	0.1432	0.1381	0.1374	0.1347	0.1342
0.1613	0.1601	0.1575	0.1351	0.1341	0.1295	0.1348	0.1255	0.1149
0.2134	0.2033	0.2023	0.194	0.1999	0.1689	0.1681	0.1577	0.1249
0.2351	0.2066	0.19	0.1858	0.198	0.2137	0.214	0.1939	0.1662
0.2538	0.2468	0.2544	0.2401	0.2285	0.2224	0.2112	0.1969	0.1807
0.1704	0.1591	0.1552	0.1524	0.1674	0.1677	0.1809	0.1666	0.1614
0.1356	0.1382	0.131	0.121	0.1329	0.1162	0.1232	0.1101	0.0957
0.1969	0.1619	0.1562	0.1556	0.1593	0.1611	0.1605	0.1576	0.1289
0.171	0.1466	0.1351	0.111	0.122	0.1159	0.1145	0.1294	0.1009
0.1205	0.1117	0.0878	0.0739	0.0689	0.074	0.0933	0.1152	0.1026
0.1901	0.171	0.1578	0.154	0.1488	0.1311	0.1288	0.1226	0.1012
0.1853	0.1617	0.1483	0.1322	0.1415	0.1425	0.1469	0.1249	0.1233
0.1686	0.1472	0.1254	0.1174	0.1237	0.1134	0.1328	0.1305	0.1037
0.1474	0.1442	0.1502	0.1387	0.1392	0.1509	0.1728	0.1721	0.1606
0.1705	0.1398	0.1252	0.1184	0.126	0.1115	0.1154	0.1095	0.0898
0.1752	0.1662	0.1434	0.1405	0.1403	0.1386	0.1447	0.1299	0.1243
0.192	0.1722	0.1557	0.1445	0.1381	0.1403	0.1458	0.1385	0.1261
0.0436	0.0433	0.04	0.0402	0.0408	0.04	0.0442	0.0421	0.0377
0.035	0.0375	0.0383	0.0428	0.0444	0.0497	0.0462	0.0465	0.0475
0.1067	0.1125	0.1014	0.0978	0.0901	0.1004	0.0906	0.0896	0.0945
0.0984	0.1028	0.1056	0.0945	0.0853	0.0821	0.0857	0.0862	0.0858
0.0502	0.0502	0.0487	0.0466	0.0442	0.0422	0.0414	0.0382	0.039

0	0	0	0	0	0	0	0	0
0	0	0	0	0	0	0	0	0
0	0	0	0	0	0	0	0	0
0	0	0	0	0	0	0	0	0
0	0	0	0	0	0	0	0	0
0	0	0	0	0	0	0	0	0
0	0	0	0	0	0	0	0	0
0	0	0	0	0	0	0	0	0
0	0	0	0	0	0	0	0	0
0	0	0	0	0	0	0	0	0
0.0092	0.0097	0.0093	0.0096	0.0094	0.0097	0.0096	0.0097	0.0099
0.0139	0.0135	0.0137	0.0136	0.0139	0.0144	0.0149	0.015	0.0151
0.032	0.0341	0.0351	0.0359	0.0334	0.033	0.0319	0.0329	0.0318
0.0765	0.0763	0.0758	0.077	0.0752	0.0778	0.0819	0.0815	0.0827
0.1574	0.1469	0.1482	0.1458	0.1517	0.1525	0.1544	0.1528	0.1448
0.1796	0.168	0.1661	0.1588	0.1706	0.156	0.1658	0.1675	0.1525
0.1786	0.1743	0.1746	0.1707	0.1786	0.1791	0.1877	0.188	0.1818
0.1939	0.189	0.1963	0.2011	0.202	0.2105	0.2207	0.23	0.2266
0.184	0.1735	0.1756	0.1738	0.1762	0.1791	0.1864	0.1869	0.1818
0.1405	0.1309	0.1358	0.1425	0.1493	0.1388	0.1384	0.1412	0.1325
0.1429	0.1332	0.1334	0.1395	0.1441	0.147	0.1464	0.1453	0.1358
0.1215	0.119	0.1213	0.121	0.1306	0.1299	0.1346	0.1332	0.1352
0.149	0.1478	0.1544	0.153	0.1583	0.1571	0.1611	0.1596	0.1557
0.1703	0.1618	0.1663	0.1711	0.1729	0.1831	0.1854	0.191	0.1935
0.1898	0.1907	0.2014	0.1996	0.2047	0.2248	0.2255	0.229	0.2358
0.2042	0.209	0.2227	0.2302	0.2405	0.2438	0.2479	0.2553	0.2539
0.1913	0.2003	0.205	0.1983	0.2065	0.2147	0.2289	0.2292	0.2426
0.1871	0.1836	0.1991	0.2029	0.2156	0.2415	0.2458	0.243	0.2363
0.1837	0.1733	0.1767	0.1782	0.1856	0.1859	0.1914	0.1859	0.1812
0.1742	0.164	0.1438	0.135	0.1483	0.1492	0.1477	0.1383	0.1234
0.1612	0.1365	0.1399	0.1496	0.1675	0.155	0.1526	0.1534	0.1158
0.1915	0.1762	0.1664	0.1724	0.1788	0.1522	0.1611	0.1585	0.1353
0.2333	0.2193	0.2026	0.1883	0.1968	0.1942	0.2118	0.2064	0.1896
0.1441	0.1202	0.1252	0.1191	0.1251	0.1224	0.1178	0.1083	0.0992
0.1372	0.126	0.1145	0.1112	0.1244	0.1218	0.1241	0.111	0.1002
0.1732	0.1548	0.1418	0.1484	0.1418	0.1427	0.1392	0.1293	0.1103
0.2038	0.1849	0.1658	0.1489	0.1399	0.1271	0.1376	0.1208	0.1069
0.1823	0.1679	0.1589	0.1447	0.152	0.1421	0.1378	0.1298	0.1246
0.1712	0.1418	0.1468	0.1313	0.148	0.1273	0.1384	0.1308	0.1177
0.1604	0.1498	0.1598	0.1565	0.1378	0.1305	0.1343	0.1235	0.1157
0.1403	0.1317	0.1286	0.1248	0.1254	0.119	0.1156	0.1156	0.1089
0.2313	0.1925	0.172	0.1411	0.1473	0.1291	0.1389	0.1321	0.1161
0.2234	0.1999	0.2133	0.1958	0.1989	0.1792	0.1838	0.1796	0.147
0.154	0.1411	0.1297	0.1244	0.1243	0.1183	0.1226	0.1201	0.1113

0.0534	0.0514	0.0464	0.0498	0.046	0.0503	0.0507	0.0516	0.0481
0.0121	0.0131	0.0133	0.0127	0.0124	0.0125	0.0129	0.0134	0.0137
0.0033	0.003	0.0029	0.003	0.0031	0.0031	0.0031	0.0029	0.0028
0.004	0.0039	0.0037	0.0038	0.0036	0.0038	0.0037	0.0039	0.0039
0	0	0	0	0	0	0	0	0
0.0017	0.0015	0.0015	0.0015	0.0015	0.0013	0.0014	0.0015	0.0013
0	0	0	0	0	0	0	0	0
0	0	0	0	0	0	0	0	0
0	0	0	0	0	0	0	0	0
0	0	0	0	0	0	0	0	0
0	0	0	0	0	0	0	0	0
0	0	0	0	0	0	0	0	0
0	0	0	0	0	0	0	0	0
0	0	0	0	0	0	0	0	0
0	0	0	0	0	0	0	0	0
0	0	0	0	0	0	0	0	0
0.0263	0.0304	0.0283	0.0297	0.0282	0.0279	0.0274	0.028	0.0297
0.01	0.0089	0.0087	0.0085	0.0085	0.0086	0.0086	0.0089	0.0087
0.0178	0.0173	0.0175	0.0168	0.0167	0.0166	0.0161	0.0159	0.0168
0.0505	0.0495	0.0494	0.0481	0.0482	0.049	0.0512	0.0504	0.0484
0.0978	0.093	0.0917	0.0869	0.0931	0.093	0.0995	0.1033	0.1005
0.1287	0.1193	0.1186	0.1118	0.1135	0.1081	0.117	0.1186	0.1101
0.1163	0.1072	0.1058	0.1028	0.1124	0.1116	0.1151	0.1129	0.1147
0.1694	0.1599	0.1658	0.1658	0.1733	0.1768	0.1769	0.1783	0.1746
0.1425	0.1417	0.1437	0.1443	0.15	0.1555	0.1692	0.1649	0.1692
0.2463	0.2309	0.215	0.2067	0.2154	0.209	0.2114	0.2095	0.2036
0.2076	0.1882	0.1711	0.1697	0.1828	0.1628	0.1609	0.1571	0.1429
0.1933	0.1689	0.1487	0.1354	0.1515	0.1362	0.1518	0.1394	0.1199
0.1711	0.1538	0.153	0.1519	0.1579	0.1626	0.1557	0.1407	0.1182
0.2051	0.1964	0.1973	0.1968	0.203	0.2078	0.2125	0.2118	0.2119
0.2387	0.2429	0.2546	0.2635	0.2724	0.2869	0.2973	0.2942	0.2951
0.2409	0.2335	0.2357	0.2409	0.2492	0.2586	0.263	0.2651	0.2722
0.1689	0.1613	0.1623	0.1601	0.1684	0.1708	0.1793	0.178	0.1708
0.1888	0.1838	0.1953	0.2086	0.202	0.216	0.2164	0.2266	0.2369
0.1805	0.1836	0.1917	0.1961	0.2007	0.2136	0.2179	0.2124	0.2161
0.135	0.1184	0.1223	0.1202	0.1254	0.129	0.115	0.1137	0.1126
0.1736	0.1709	0.1807	0.1703	0.1896	0.1864	0.1812	0.1731	0.1479
0.1589	0.1455	0.1293	0.1329	0.144	0.1237	0.1268	0.1163	0.1031
0.1744	0.1599	0.1609	0.155	0.1603	0.1691	0.1875	0.1745	0.1633
0.1454	0.1387	0.1397	0.1316	0.1401	0.1371	0.1313	0.1241	0.1103
0.1383	0.1262	0.1279	0.1185	0.125	0.1131	0.1132	0.115	0.106
0.2101	0.1871	0.1834	0.1827	0.1877	0.1817	0.1821	0.1698	0.1448
0.1513	0.147	0.1363	0.1351	0.1242	0.1206	0.13	0.1496	0.1413
0.1204	0.1036	0.099	0.0997	0.0989	0.094	0.0911	0.0898	0.082

0.1863	0.172	0.1629	0.1617	0.1626	0.1699	0.1742	0.1742	0.168
0.2465	0.24	0.2405	0.2317	0.2283	0.2294	0.2285	0.2258	0.2215
0.2123	0.2018	0.1957	0.203	0.1962	0.2021	0.2116	0.2065	0.1869
0.1228	0.1239	0.1166	0.1131	0.118	0.1177	0.1216	0.1237	0.1097
0.1652	0.1423	0.1419	0.1429	0.1275	0.1207	0.1344	0.1304	0.1132
0.0474	0.0462	0.044	0.0439	0.047	0.047	0.0528	0.0532	0.0517
0.0008	0.0009	0.0009	0.0009	0.0009	0.001	0.0011	0.001	0.0011
0.0203	0.0208	0.0206	0.0211	0.0194	0.0206	0.0213	0.021	0.019
0.0492	0.0479	0.0452	0.0452	0.0441	0.0453	0.0518	0.0554	0.0552
0.0693	0.0719	0.0681	0.0659	0.0602	0.0604	0.063	0.0572	0.0586
0.0381	0.0394	0.0378	0.0354	0.0351	0.034	0.0351	0.0324	0.0294
0	0	0	0	0	0	0	0	0
0	0	0	0	0	0	0	0	0
0	0	0	0	0	0	0	0	0
0	0	0	0	0	0	0	0	0
0	0	0	0	0	0	0	0	0
0	0	0	0	0	0	0	0	0
0	0	0	0	0	0	0	0	0
0	0	0	0	0	0	0	0	0
0	0	0	0	0	0	0	0	0
0	0	0	0	0	0	0	0	0
0	0	0	0	0	0	0	0	0
0.0049	0.0048	0.0048	0.0048	0.0049	0.0051	0.0055	0.0058	0.0055
0.0075	0.0077	0.0073	0.0068	0.0066	0.0065	0.0069	0.0076	0.008
0.0251	0.0269	0.0262	0.0252	0.026	0.024	0.0261	0.0241	0.0228
0.0021	0.0021	0.0022	0.0022	0.0022	0.0022	0.0023	0.0024	0.0024
0.0174	0.017	0.0178	0.0177	0.0187	0.0183	0.019	0.0185	0.0171
0.0449	0.0437	0.0424	0.0428	0.0432	0.0439	0.0461	0.045	0.0441
0.021	0.019	0.0195	0.0195	0.0201	0.02	0.02	0.0206	0.0196
0.0219	0.02	0.0214	0.0226	0.0236	0.0248	0.0262	0.0294	0.0318
0.0457	0.0472	0.0472	0.0484	0.0479	0.0503	0.0498	0.0492	0.0496
0.3321	0.3291	0.32	0.3179	0.3186	0.3054	0.2879	0.2809	0.2818
0.4341	0.4178	0.4213	0.4234	0.4326	0.4335	0.4051	0.3933	0.3853
0.2749	0.2522	0.2179	0.203	0.2048	0.2096	0.2166	0.2016	0.1902
0.1836	0.1645	0.1658	0.1645	0.1735	0.1529	0.1773	0.1687	0.1496
0.2069	0.1901	0.185	0.1653	0.1677	0.1596	0.1827	0.1707	0.1586
0.2099	0.2094	0.2106	0.2067	0.2049	0.2025	0.1951	0.2017	0.1777
0.1228	0.1096	0.1116	0.1135	0.1185	0.1207	0.1279	0.1252	0.1237
0.1525	0.133	0.1349	0.1342	0.139	0.1377	0.1488	0.1431	0.1373
0.1705	0.1645	0.1668	0.1747	0.1883	0.1869	0.1944	0.1937	0.1933
0.176	0.1735	0.1783	0.1734	0.1788	0.1803	0.1799	0.1868	0.1843
0.1537	0.1407	0.1353	0.1309	0.134	0.1373	0.142	0.1402	0.1371
0.1458	0.13	0.1077	0.0795	0.1001	0.09	0.104	0.1022	0.0878
0.1439	0.1277	0.1262	0.1206	0.1292	0.1313	0.1335	0.128	0.123

0.1824	0.1544	0.15	0.134	0.159	0.1555	0.1589	0.156	0.1524
0.1774	0.156	0.1578	0.1435	0.143	0.1223	0.1227	0.1079	0.1003
0.1424	0.1398	0.1332	0.1388	0.1477	0.1519	0.1588	0.1546	0.142
0.1816	0.1769	0.1803	0.1675	0.1661	0.1635	0.1628	0.1658	0.1523
0.142	0.1298	0.1124	0.1165	0.1273	0.1173	0.1268	0.1188	0.1003
0.1472	0.1332	0.1301	0.1234	0.1279	0.1299	0.1291	0.1235	0.1075
0.193	0.1868	0.1914	0.1859	0.1687	0.1638	0.1614	0.1677	0.1648
0.3187	0.3315	0.3252	0.3239	0.3182	0.3237	0.3447	0.3394	0.3156
0.2249	0.2133	0.1988	0.1946	0.1984	0.1989	0.2161	0.2183	0.2043
0.0484	0.0467	0.0462	0.0447	0.0495	0.0478	0.0486	0.0464	0.045
0.0301	0.03	0.0289	0.0287	0.0275	0.0278	0.0252	0.0248	0.0247
0.0223	0.0245	0.0269	0.0266	0.0255	0.0287	0.0279	0.0286	0.0285
0.019	0.0184	0.0175	0.0164	0.0166	0.0169	0.0176	0.017	0.0153
0	0	0	0	0	0	0	0	0
0	0	0	0	0	0	0	0	0
0.0141	0.0141	0.0143	0.0129	0.0121	0.0116	0.0119	0.0114	0.0118
0.0229	0.0213	0.0203	0.0191	0.0186	0.0197	0.0189	0.0192	0.0191
0.0221	0.0225	0.0212	0.0208	0.0191	0.0209	0.021	0.0203	0.0212
0	0	0	0	0	0	0	0	0
0.0035	0.0035	0.0035	0.0037	0.0036	0.0039	0.0039	0.0041	0.0041
0	0	0	0	0	0	0	0	0
0	0	0	0	0	0	0	0	0
0	0	0	0	0	0	0	0	0
0	0	0	0	0	0	0	0	0
0	0	0	0	0	0	0	0	0
0	0	0	0	0	0	0	0	0
0	0	0	0	0	0	0	0	0
0	0	0	0	0	0	0	0	0
0	0	0	0	0	0	0	0	0
0	0	0	0	0	0	0	0	0
0	0	0	0	0	0	0	0	0
0	0	0	0	0	0	0	0	0
0	0	0	0	0	0	0	0	0
0	0	0	0	0	0	0	0	0
0	0	0	0	0	0	0	0	0
0	0	0	0	0	0	0	0	0
0.0496	0.0523	0.0522	0.0528	0.0526	0.0545	0.0526	0.0569	0.0586
0.2909	0.3174	0.3229	0.3204	0.2993	0.2803	0.2663	0.2524	0.28
0.3835	0.3691	0.3368	0.3203	0.3017	0.2995	0.301	0.2918	0.2574
0.2054	0.1849	0.1832	0.1841	0.1912	0.1747	0.1759	0.1748	0.176
0.1707	0.1555	0.1304	0.1395	0.143	0.1423	0.1579	0.1562	0.13
0.1683	0.1662	0.1588	0.1628	0.1713	0.155	0.1476	0.14	0.1317
0.1516	0.1467	0.1408	0.1278	0.1255	0.122	0.1179	0.1035	0.091
0.1575	0.1471	0.1499	0.1402	0.1266	0.1204	0.1337	0.1167	0.1098

0.1898	0.1852	0.1801	0.1725	0.1725	0.1761	0.1796	0.173	0.176
0.183	0.1763	0.1769	0.1773	0.1829	0.1856	0.1914	0.1873	0.1838
0.1799	0.1739	0.1819	0.1798	0.1976	0.2031	0.2198	0.2175	0.2225
0.1297	0.1304	0.1398	0.1425	0.1521	0.1588	0.1621	0.1677	0.1619
0.1192	0.1117	0.1158	0.1176	0.1203	0.1286	0.1314	0.1288	0.1272
0.1582	0.1479	0.1488	0.1479	0.1544	0.1554	0.1648	0.1598	0.1498
0.1413	0.1243	0.1139	0.1068	0.1079	0.1092	0.1162	0.1143	0.1055
0.1392	0.1269	0.1238	0.1131	0.1128	0.1116	0.1126	0.119	0.1098
0.1341	0.1242	0.1203	0.1196	0.1235	0.1247	0.1325	0.1264	0.1144
0.1589	0.1542	0.1594	0.1585	0.1658	0.1668	0.1728	0.1697	0.1648
0.1659	0.1556	0.1571	0.1571	0.1664	0.1734	0.1758	0.1713	0.1654
0.1176	0.1109	0.1089	0.113	0.1206	0.1212	0.127	0.1241	0.118
0.0476	0.0454	0.0426	0.0441	0.0428	0.0428	0.0441	0.0431	0.0415
0.0537	0.0536	0.0531	0.0532	0.0528	0.0551	0.0569	0.0569	0.056
0.053	0.0516	0.0517	0.0508	0.0501	0.0519	0.0543	0.0533	0.0498
0.0122	0.013	0.0129	0.013	0.0124	0.012	0.0114	0.0119	0.0122
0	0	0	0	0	0	0	0	0
0	0	0	0	0	0	0	0	0
0	0	0	0	0	0	0	0	0
0	0	0	0	0	0	0	0	0
0	0	0	0	0	0	0	0	0
0	0	0	0	0	0	0	0	0
0	0	0	0	0	0	0	0	0
0	0	0	0	0	0	0	0	0
0	0	0	0	0	0	0	0	0
0	0	0	0	0	0	0	0	0
0	0	0	0	0	0	0	0	0
0	0	0	0	0	0	0	0	0
0	0	0	0	0	0	0	0	0
0	0	0	0	0	0	0	0	0
0	0	0	0	0	0	0	0	0
0	0	0	0	0	0	0	0	0
0	0	0	0	0	0	0	0	0
0	0	0	0	0	0	0	0	0
0	0	0	0	0	0	0	0	0
0	0	0	0	0	0	0	0	0
0	0	0	0	0	0	0	0	0
0	0	0	0	0	0	0	0	0
0	0	0	0	0	0	0	0	0
0	0	0	0	0	0	0	0	0
0	0	0	0	0	0	0	0	0
0	0	0	0	0	0	0	0	0
0.0109	0.01	0.0098	0.0102	0.0103	0.0093	0.0093	0.0094	0.0079
0.0584	0.0559	0.0552	0.0576	0.0562	0.0599	0.0592	0.0579	0.0591

0.12	0.1197	0.1161	0.115	0.1164	0.12	0.1224	0.1248	0.126
0.1856	0.1773	0.1831	0.186	0.1862	0.1934	0.2047	0.2057	0.2018
0.2033	0.1999	0.2015	0.2011	0.2122	0.2207	0.2289	0.2271	0.2284
0.1574	0.1525	0.1558	0.1594	0.1655	0.1669	0.1745	0.1743	0.1625
0.1803	0.1697	0.1609	0.1625	0.1625	0.1728	0.1701	0.1682	0.1653
0.3461	0.3161	0.3165	0.3262	0.3227	0.3351	0.3285	0.343	0.3573
0.4523	0.4467	0.4184	0.3821	0.3646	0.3936	0.3692	0.3651	0.3881
0.2533	0.2524	0.2656	0.2507	0.2312	0.2402	0.2349	0.2387	0.2341
0.1367	0.1312	0.1306	0.137	0.1431	0.145	0.1487	0.1508	0.1511
0.156	0.1519	0.1529	0.1472	0.153	0.1534	0.1644	0.1749	0.1728
0.1977	0.1939	0.197	0.1989	0.2104	0.2185	0.2251	0.2318	0.2358
0.1696	0.1674	0.1697	0.1697	0.1854	0.1951	0.2084	0.2188	0.2125
0.1254	0.1198	0.1263	0.1314	0.1373	0.14	0.1427	0.1397	0.1358
0.1422	0.1356	0.1353	0.1346	0.1406	0.1442	0.153	0.1516	0.1487
0.1337	0.1286	0.1324	0.1285	0.1366	0.1376	0.146	0.1495	0.1348
0.161	0.1507	0.1479	0.1463	0.1532	0.1553	0.1638	0.161	0.156
0.1118	0.1064	0.1042	0.1057	0.1121	0.1132	0.1169	0.1155	0.1101
0.0415	0.0401	0.0413	0.0407	0.0415	0.0412	0.0425	0.0426	0.04
0.0391	0.0381	0.0374	0.0369	0.0369	0.037	0.038	0.0376	0.0371
0.0548	0.0533	0.0538	0.054	0.0552	0.0566	0.0576	0.0574	0.0572
0.0383	0.0372	0.0376	0.0375	0.0385	0.0393	0.0404	0.0403	0.0401
0.0066	0.0065	0.0064	0.0069	0.0071	0.0068	0.0066	0.0063	0.0059
0	0	0	0	0	0	0	0	0
0	0	0	0	0	0	0	0	0
0	0	0	0	0	0	0	0	0
0	0	0	0	0	0	0	0	0
0	0	0	0	0	0	0	0	0
0	0	0	0	0	0	0	0	0
0	0	0	0	0	0	0	0	0
0	0	0	0	0	0	0	0	0
0	0	0	0	0	0	0	0	0
0	0	0	0	0	0	0	0	0
0	0	0	0	0	0	0	0	0
0	0	0	0	0	0	0	0	0
0	0	0	0	0	0	0	0	0
0	0	0	0	0	0	0	0	0
0	0	0	0	0	0	0	0	0
0	0	0	0	0	0	0	0	0
0	0	0	0	0	0	0	0	0
0	0	0	0	0	0	0	0	0
0	0	0	0	0	0	0	0	0
0	0	0	0	0	0	0	0	0
0	0	0	0	0	0	0	0	0
0	0	0	0	0	0	0	0	0

0	0	0	0	0	0	0	0	0
0	0	0	0	0	0	0	0	0
0	0	0	0	0	0	0	0	0
0	0	0	0	0	0	0	0	0
0.0197	0.0196	0.0178	0.0176	0.0172	0.0183	0.0168	0.0161	0.0154
0	0	0	0	0	0	0	0	0
0.0464	0.0454	0.0466	0.0462	0.0481	0.0493	0.0497	0.0503	0.049
0.1277	0.1231	0.1306	0.1315	0.139	0.1485	0.1542	0.1567	0.1641
0.2022	0.2007	0.2072	0.2136	0.2228	0.224	0.232	0.2391	0.2486
0.1286	0.1234	0.1248	0.1288	0.1386	0.1411	0.1413	0.1445	0.1482
0.1637	0.1561	0.149	0.149	0.1411	0.1406	0.1376	0.1388	0.134
0.3656	0.3614	0.3179	0.338	0.3031	0.2984	0.2924	0.2928	0.2971
0.3425	0.3556	0.3398	0.3174	0.308	0.3225	0.3254	0.3144	0.2917
0.1657	0.1535	0.1507	0.1456	0.1436	0.147	0.1429	0.1426	0.1347
0.1125	0.1089	0.1087	0.1066	0.1138	0.1128	0.1169	0.1111	0.108
0.1577	0.1531	0.1569	0.1547	0.1606	0.162	0.1679	0.1683	0.1659
0.2096	0.2018	0.2039	0.2071	0.2111	0.2111	0.2169	0.2165	0.2158
0.1709	0.1632	0.1651	0.1658	0.1701	0.1795	0.1905	0.1949	0.1936
0.1098	0.1018	0.1108	0.1141	0.1202	0.1248	0.1308	0.1338	0.131
0.1113	0.1083	0.1112	0.1112	0.1156	0.1171	0.1211	0.1206	0.1202
0.1252	0.1165	0.1158	0.1185	0.1257	0.1295	0.1315	0.1323	0.1311
0.1179	0.1091	0.1105	0.1082	0.1161	0.1153	0.1147	0.1155	0.1165
0.0386	0.0381	0.0402	0.0402	0.0414	0.0441	0.0456	0.0463	0.0456
0	0	0	0	0	0	0	0	0
0.0178	0.0175	0.0167	0.0169	0.0167	0.0172	0.0175	0.0168	0.016
0.0317	0.0305	0.0309	0.0308	0.0315	0.0323	0.0332	0.0333	0.0327
0.0095	0.0101	0.0104	0.0109	0.0118	0.0124	0.0123	0.0129	0.0131
0.0076	0.0067	0.0067	0.0067	0.0062	0.0051	0.0054	0.006	0.0053
0	0	0	0	0	0	0	0	0
0	0	0	0	0	0	0	0	0
0	0	0	0	0	0	0	0	0
0	0	0	0	0	0	0	0	0
0	0	0	0	0	0	0	0	0
0	0	0	0	0	0	0	0	0
0	0	0	0	0	0	0	0	0
0	0	0	0	0	0	0	0	0
0	0	0	0	0	0	0	0	0
0	0	0	0	0	0	0	0	0
0	0	0	0	0	0	0	0	0
0	0	0	0	0	0	0	0	0
0	0	0	0	0	0	0	0	0
0	0	0	0	0	0	0	0	0
0	0	0	0	0	0	0	0	0
0	0	0	0	0	0	0	0	0

0	0	0	0	0	0	0	0	0
0	0	0	0	0	0	0	0	0
0	0	0	0	0	0	0	0	0
0	0	0	0	0	0	0	0	0
0	0	0	0	0	0	0	0	0
0	0	0	0	0	0	0	0	0
0	0	0	0	0	0	0	0	0
0	0	0	0	0	0	0	0	0
0	0	0	0	0	0	0	0	0
0	0	0	0	0	0	0	0	0
0.0544	0.0533	0.05	0.0494	0.0513	0.0542	0.0554	0.054	0.0541
0.0546	0.0549	0.0507	0.0509	0.0491	0.0483	0.0454	0.0459	0.0463
0.0274	0.0273	0.0265	0.0283	0.028	0.0286	0.0285	0.0287	0.0281
0.0185	0.0173	0.0177	0.0182	0.0191	0.02	0.0201	0.0201	0.0204
0.0519	0.0531	0.0534	0.0544	0.056	0.0605	0.0629	0.0645	0.0653
0.0593	0.0584	0.0603	0.0618	0.0641	0.0662	0.0662	0.0663	0.0665
0.0825	0.0778	0.0771	0.0743	0.0721	0.0721	0.0753	0.0775	0.0752
0.1342	0.1295	0.1213	0.12	0.1158	0.1131	0.1073	0.1051	0.1016
0.0689	0.0601	0.0641	0.0635	0.0683	0.058	0.0611	0.0619	0.0579
0.0709	0.0646	0.0621	0.0606	0.0613	0.0584	0.059	0.0547	0.0456
0.0769	0.0742	0.0738	0.0676	0.076	0.0789	0.0827	0.0761	0.0699
0.104	0.0955	0.0931	0.0911	0.0874	0.0834	0.0878	0.0872	0.0853
0.1086	0.0996	0.1016	0.102	0.1068	0.1046	0.11	0.1057	0.0995
0.1253	0.1208	0.1205	0.1207	0.1251	0.127	0.1284	0.1279	0.1257
0.0616	0.0616	0.0619	0.0622	0.0624	0.0639	0.0643	0.0639	0.0635
0.0034	0.0034	0.0031	0.0033	0.0031	0.0032	0.0033	0.0032	0.0032
0.0307	0.0286	0.0284	0.0294	0.0317	0.0313	0.0326	0.0326	0.0303
0.0258	0.0239	0.0237	0.0225	0.0236	0.0238	0.025	0.0249	0.0245
0.0023	0.002	0.002	0.002	0.002	0.002	0.0022	0.0022	0.0022
0	0	0	0	0	0	0	0	0
0.0038	0.0038	0.0035	0.0036	0.0033	0.0036	0.0037	0.0037	0.0036
0.0048	0.005	0.0049	0.0047	0.0041	0.0041	0.004	0.004	0.0039
0	0	0	0	0	0	0	0	0
0.0017	0.0015	0.0015	0.0016	0.0017	0.0019	0.002	0.0022	0.0022
0	0	0	0	0	0	0	0	0
0	0	0	0	0	0	0	0	0
0	0	0	0	0	0	0	0	0
0	0	0	0	0	0	0	0	0
0	0	0	0	0	0	0	0	0
0	0	0	0	0	0	0	0	0
0	0	0	0	0	0	0	0	0
0	0	0	0	0	0	0	0	0
0	0	0	0	0	0	0	0	0
0	0	0	0	0	0	0	0	0

0	0	0	0	0	0	0	0	0
0	0	0	0	0	0	0	0	0
0	0	0	0	0	0	0	0	0
0	0	0	0	0	0	0	0	0
0	0	0	0	0	0	0	0	0
0	0	0	0	0	0	0	0	0
0	0	0	0	0	0	0	0	0
0	0	0	0	0	0	0	0	0
0	0	0	0	0	0	0	0	0
0	0	0	0	0	0	0	0	0
0	0	0	0	0	0	0	0	0
0	0	0	0	0	0	0	0	0
0	0	0	0	0	0	0	0	0
0	0	0	0	0	0	0	0	0
0	0	0	0	0	0	0	0	0
0	0	0	0	0	0	0	0	0
0	0	0	0	0	0	0	0	0
0.0191	0.0195	0.0189	0.019	0.0181	0.019	0.0192	0.0179	0.0181
0.0109	0.0115	0.0112	0.0113	0.0112	0.0123	0.0122	0.0119	0.0119
0.0032	0.0031	0.003	0.0026	0.0026	0.0025	0.0026	0.0026	0.0024
0.0034	0.0029	0.0027	0.003	0.0033	0.0034	0.0033	0.0031	0.0026
0.0359	0.0342	0.0329	0.0333	0.0333	0.0326	0.0317	0.0302	0.0302
0.0125	0.0123	0.012	0.0126	0.011	0.0103	0.012	0.0118	0.0097
0.0008	0.0007	0.0008	0.0007	0.0007	0.0006	0.0006	0.0006	0.0005
0.015	0.0123	0.0115	0.0112	0.0119	0.0113	0.0108	0.0104	0.0089
0.0218	0.0228	0.0215	0.0208	0.0198	0.0206	0.0202	0.0194	0.019
0.0311	0.0317	0.0312	0.0326	0.0322	0.0334	0.0336	0.032	0.032
0.0058	0.0054	0.0053	0.005	0.0052	0.0048	0.0047	0.0047	0.004
0.0167	0.0153	0.0151	0.0148	0.0152	0.0154	0.0159	0.0163	0.0154
0.0211	0.0198	0.0201	0.0208	0.0212	0.0219	0.0232	0.0229	0.023
0.0414	0.0429	0.0435	0.0461	0.0437	0.047	0.047	0.048	0.0472
0.0602	0.059	0.0599	0.0614	0.0591	0.0642	0.0644	0.064	0.0635
0.007	0.0067	0.0063	0.0064	0.0062	0.0063	0.0067	0.007	0.0068
0.0038	0.0036	0.0036	0.0038	0.0036	0.0038	0.0035	0.0034	0.0034
0.0041	0.0038	0.0034	0.0033	0.0034	0.0032	0.0034	0.0031	0.0025
0.0101	0.0094	0.0092	0.0097	0.0098	0.0085	0.0096	0.0088	0.0086
0.0063	0.0064	0.0063	0.0063	0.0063	0.0066	0.0066	0.0068	0.0068
0.008	0.0082	0.0074	0.0078	0.0066	0.0069	0.007	0.0072	0.0071
0.0062	0.0059	0.0057	0.0051	0.0049	0.0049	0.0053	0.0053	0.0052
0.0056	0.0056	0.0053	0.0054	0.0058	0.0064	0.0065	0.0066	0.007
0	0	0	0	0	0	0	0	0
0	0	0	0	0	0	0	0	0
0	0	0	0	0	0	0	0	0
0	0	0	0	0	0	0	0	0

0	0	0	0	0	0	0	0	0
0	0	0	0	0	0	0	0	0
0	0	0	0	0	0	0	0	0
0	0	0	0	0	0	0	0	0
0	0	0	0	0	0	0	0	0
0	0	0	0	0	0	0	0	0
0	0	0	0	0	0	0	0	0
0	0	0	0	0	0	0	0	0
0	0	0	0	0	0	0	0	0
0	0	0	0	0	0	0	0	0
0	0	0	0	0	0	0	0	0
0	0	0	0	0	0	0	0	0
0	0	0	0	0	0	0	0	0
0	0	0	0	0	0	0	0	0
0	0	0	0	0	0	0	0	0
0	0	0	0	0	0	0	0	0
0	0	0	0	0	0	0	0	0
0	0	0	0	0	0	0	0	0
0	0	0	0	0	0	0	0	0
0	0	0	0	0	0	0	0	0
0	0	0	0	0	0	0	0	0
0	0	0	0	0	0	0	0	0
0.0235	0.0243	0.023	0.0217	0.022	0.0223	0.0226	0.022	0.0237
0.0071	0.0066	0.0069	0.0071	0.0073	0.0075	0.0077	0.0074	0.0076
0.0026	0.0026	0.0027	0.0027	0.0027	0.0029	0.003	0.0032	0.0033
0.0106	0.0105	0.01	0.0094	0.0086	0.0081	0.0091	0.0096	0.009
0.015	0.0154	0.0142	0.0143	0.0124	0.0125	0.013	0.011	0.0101
0.0105	0.0111	0.0098	0.0108	0.0095	0.0098	0.0101	0.0104	0.0108
0.0084	0.0084	0.0084	0.0087	0.0083	0.0089	0.0089	0.0088	0.0091
0.0151	0.0151	0.0151	0.0151	0.0143	0.0151	0.0149	0.0157	0.0165
0.0721	0.072	0.0679	0.0708	0.0644	0.0659	0.064	0.0605	0.0592
0.0068	0.0065	0.0068	0.0069	0.0072	0.0071	0.0075	0.0073	0.0072
0.0096	0.0095	0.0102	0.0104	0.0103	0.0109	0.0113	0.0118	0.0119
0.0349	0.0364	0.0352	0.0353	0.0332	0.0343	0.0342	0.0334	0.0334
0.0157	0.0154	0.015	0.0149	0.014	0.014	0.0141	0.012	0.0125
0.0012	0.0011	0.001	0.001	0.0011	0.001	0.0011	0.0011	0.001
0.0351	0.0328	0.0343	0.0346	0.0371	0.0381	0.0381	0.0381	0.0373
0.1339	0.1293	0.1328	0.1355	0.1421	0.1498	0.1506	0.1501	0.1516
0.0321	0.032	0.0324	0.0326	0.0319	0.0339	0.0335	0.0328	0.0329
0.0121	0.0123	0.0122	0.0125	0.0122	0.0124	0.0128	0.0131	0.0129
0	0	0	0	0	0	0	0	0
0.0044	0.0044	0.0047	0.0047	0.005	0.0053	0.0056	0.0057	0.0059
0.0097	0.0096	0.0098	0.01	0.0103	0.0108	0.0108	0.0107	0.0109
0.0073	0.0074	0.0071	0.0071	0.0069	0.0073	0.0079	0.0076	0.0077

0.0031	0.0031	0.0032	0.0033	0.0031	0.0034	0.0035	0.0034	0.0035
0.0052	0.0053	0.0051	0.0054	0.0052	0.0049	0.0049	0.0046	0.0044
0	0	0	0	0	0	0	0	0
0	0	0	0	0	0	0	0	0
0	0	0	0	0	0	0	0	0
0	0	0	0	0	0	0	0	0
0	0	0	0	0	0	0	0	0
0	0	0	0	0	0	0	0	0
0	0	0	0	0	0	0	0	0
0	0	0	0	0	0	0	0	0
0	0	0	0	0	0	0	0	0
0	0	0	0	0	0	0	0	0
0	0	0	0	0	0	0	0	0
0	0	0	0	0	0	0	0	0
0	0	0	0	0	0	0	0	0
0	0	0	0	0	0	0	0	0
0	0	0	0	0	0	0	0	0
0	0	0	0	0	0	0	0	0
0	0	0	0	0	0	0	0	0
0	0	0	0	0	0	0	0	0
0	0	0	0	0	0	0	0	0
0	0	0	0	0	0	0	0	0
0	0	0	0	0	0	0	0	0
0	0	0	0	0	0	0	0	0
0	0	0	0	0	0	0	0	0
0	0	0	0	0	0	0	0	0
0	0	0	0	0	0	0	0	0
0	0	0	0	0	0	0	0	0
0.0605	0.0577	0.0602	0.0622	0.0618	0.0613	0.0607	0.0623	0.0633
0.0157	0.0163	0.0161	0.016	0.0159	0.0165	0.0177	0.0175	0.0167
0.0129	0.0136	0.0132	0.0137	0.013	0.0137	0.0129	0.0124	0.0126
0.0392	0.039	0.0358	0.036	0.0337	0.0369	0.0366	0.0375	0.0384
0.0339	0.0345	0.0343	0.0358	0.034	0.0336	0.0331	0.031	0.0306
0.0064	0.0067	0.0065	0.007	0.007	0.0076	0.0076	0.0073	0.0072
0.0148	0.0152	0.0137	0.0139	0.0137	0.0149	0.0147	0.0154	0.0152
0.0386	0.0377	0.0375	0.0351	0.0348	0.0361	0.0328	0.0314	0.0298
0.0271	0.0272	0.0277	0.0281	0.0267	0.0277	0.0279	0.0272	0.0283
0	0	0	0	0	0	0	0	0
0.0012	0.0009	0.0009	0.0009	0.0011	0.0011	0.0011	0.0011	0.001
0.0349	0.0342	0.0343	0.0353	0.0343	0.0352	0.0368	0.0358	0.0353
0.0449	0.0452	0.0451	0.0431	0.0416	0.044	0.0438	0.0435	0.0469
0.0135	0.0141	0.0128	0.0133	0.0125	0.0118	0.0111	0.0112	0.012
0.0016	0.0016	0.0016	0.0016	0.0018	0.0019	0.002	0.002	0.002
0.0219	0.0227	0.0229	0.0236	0.0247	0.025	0.0254	0.0266	0.0272

0.0334	0.0331	0.0333	0.034	0.034	0.0346	0.0349	0.0353	0.035
0.0193	0.0187	0.0184	0.0179	0.0169	0.0171	0.0167	0.0163	0.016
0	0	0	0	0	0	0	0	0
0	0	0	0	0	0	0	0	0
0	0	0	0	0	0	0	0	0
0.0036	0.0036	0.0035	0.0033	0.0031	0.0033	0.0036	0.0035	0.0036
0.0028	0.0026	0.0024	0.0024	0.0025	0.0024	0.0022	0.0025	0.0028
0.0015	0.0012	0.0011	0.001	0.0012	0.001	0.001	0.0009	0.0009
0	0	0	0	0	0	0	0	0
0	0	0	0	0	0	0	0	0
0	0	0	0	0	0	0	0	0
0	0	0	0	0	0	0	0	0
0	0	0	0	0	0	0	0	0
0	0	0	0	0	0	0	0	0
0	0	0	0	0	0	0	0	0
0	0	0	0	0	0	0	0	0
0	0	0	0	0	0	0	0	0
0	0	0	0	0	0	0	0	0
0	0	0	0	0	0	0	0	0
0	0	0	0	0	0	0	0	0
0	0	0	0	0	0	0	0	0
0	0	0	0	0	0	0	0	0
0	0	0	0	0	0	0	0	0
0	0	0	0	0	0	0	0	0
0	0	0	0	0	0	0	0	0
0	0	0	0	0	0	0	0	0
0	0	0	0	0	0	0	0	0
0	0	0	0	0	0	0	0	0
0	0	0	0	0	0	0	0	0
0	0	0	0	0	0	0	0	0
0	0	0	0	0	0	0	0	0
0	0	0	0	0	0	0	0	0
0	0	0	0	0	0	0	0	0
0	0	0	0	0	0	0	0	0
0	0	0	0	0	0	0	0	0
0.002	0.0021	0.0022	0.0022	0.0022	0.0022	0.0023	0.0023	0.0025
0.0062	0.0062	0.0062	0.0064	0.0062	0.0064	0.0064	0.0063	0.0064
0.0113	0.011	0.0114	0.0112	0.0102	0.0097	0.0105	0.0107	0.0107
0.0048	0.0044	0.0041	0.004	0.0044	0.0041	0.0038	0.0035	0.003
0.0138	0.0146	0.0153	0.0145	0.0128	0.0139	0.014	0.0154	0.0161
0.0383	0.0379	0.036	0.0373	0.0348	0.0368	0.0382	0.0374	0.0382
0.0115	0.0126	0.0125	0.0126	0.0118	0.0122	0.0114	0.0117	0.0124
0.017	0.0169	0.0171	0.0178	0.0165	0.0161	0.0165	0.0171	0.0176
0.0326	0.0311	0.0309	0.0312	0.0283	0.0274	0.028	0.0284	0.0282

0.024	0.025	0.0225	0.0205	0.0192	0.0206	0.0197	0.0218	0.0242
0	0	0	0	0	0	0	0	0
0.0021	0.0016	0.0016	0.0016	0.0017	0.0017	0.0017	0.0017	0.0012
0.021	0.0205	0.0197	0.0184	0.0176	0.0184	0.0197	0.0193	0.0178
0.0471	0.0447	0.045	0.0441	0.0423	0.043	0.043	0.0426	0.045
0.0101	0.0102	0.0103	0.0099	0.0098	0.0095	0.0092	0.0093	0.0095
0.0146	0.0149	0.0134	0.0145	0.0145	0.0141	0.0139	0.0145	0.0155
0.0036	0.0036	0.0035	0.0036	0.0036	0.0035	0.0037	0.0034	0.0035
0.0067	0.0064	0.0067	0.0067	0.0071	0.0072	0.0075	0.0078	0.0081
0.0059	0.0054	0.0057	0.0053	0.0058	0.0057	0.0063	0.0063	0.006
0	0	0	0	0	0	0	0	0
0	0	0	0	0	0	0	0	0
0.0056	0.0052	0.0054	0.0052	0.0055	0.0057	0.0061	0.0061	0.006
0.0023	0.0024	0.0027	0.0027	0.0028	0.0029	0.003	0.0031	0.0033
0	0	0	0	0	0	0	0	0
0	0	0	0	0	0	0	0	0
0	0	0	0	0	0	0	0	0
0	0	0	0	0	0	0	0	0
0	0	0	0	0	0	0	0	0
0	0	0	0	0	0	0	0	0
0	0	0	0	0	0	0	0	0
0	0	0	0	0	0	0	0	0
0	0	0	0	0	0	0	0	0
0	0	0	0	0	0	0	0	0
0	0	0	0	0	0	0	0	0
0	0	0	0	0	0	0	0	0
0	0	0	0	0	0	0	0	0
0	0	0	0	0	0	0	0	0
0	0	0	0	0	0	0	0	0
0	0	0	0	0	0	0	0	0
0	0	0	0	0	0	0	0	0
0	0	0	0	0	0	0	0	0
0	0	0	0	0	0	0	0	0
0	0	0	0	0	0	0	0	0
0	0	0	0	0	0	0	0	0
0	0	0	0	0	0	0	0	0
0	0	0	0	0	0	0	0	0
0	0	0	0	0	0	0	0	0
0	0	0	0	0	0	0	0	0
0	0	0	0	0	0	0	0	0
0	0	0	0	0	0	0	0	0
0	0	0	0	0	0	0	0	0
0	0	0	0	0	0	0	0	0
0	0	0	0	0	0	0	0	0

0	0	0	0	0	0	0	0	0
0	0	0	0	0	0	0	0	0
0	0	0	0	0	0	0	0	0
0	0	0	0	0	0	0	0	0
0	0	0	0	0	0	0	0	0
0	0	0	0	0	0	0	0	0
0	0	0	0	0	0	0	0	0
0	0	0	0	0	0	0	0	0
0	0	0	0	0	0	0	0	0
0	0	0	0	0	0	0	0	0
0	0	0	0	0	0	0	0	0
0	0	0	0	0	0	0	0	0
0	0	0	0	0	0	0	0	0
0	0	0	0	0	0	0	0	0
0	0	0	0	0	0	0	0	0
0	0	0	0	0	0	0	0	0
0	0	0	0	0	0	0	0	0
0	0	0	0	0	0	0	0	0
0	0	0	0	0	0	0	0	0
0	0	0	0	0	0	0	0	0
0	0	0	0	0	0	0	0	0
0	0	0	0	0	0	0	0	0
0	0	0	0	0	0	0	0	0
0	0	0	0	0	0	0	0	0
0	0	0	0	0	0	0	0	0
0	0	0	0	0	0	0	0	0
0	0	0	0	0	0	0	0	0
0	0	0	0	0	0	0	0	0
0	0	0	0	0	0	0	0	0
0	0	0	0	0	0	0	0	0
0	0	0	0	0	0	0	0	0
0	0	0	0	0	0	0	0	0
0	0	0	0	0	0	0	0	0
0	0	0	0	0	0	0	0	0
0	0	0	0	0	0	0	0	0
0	0	0	0	0	0	0	0	0
0	0	0	0	0	0	0	0	0
0	0	0	0	0	0	0	0	0
0	0	0	0	0	0	0	0	0
0	0	0	0	0	0	0	0	0
0	0	0	0	0	0	0	0	0
0	0	0	0	0	0	0	0	0
0	0	0	0	0	0	0	0	0
0	0	0	0	0	0	0	0	0

0	0	0	0	0	0	0	0	0
0	0	0	0	0	0	0	0	0
0	0	0	0	0	0	0	0	0
0	0	0	0	0	0	0	0	0
0	0	0	0	0	0	0	0	0
0	0	0	0	0	0	0	0	0
0	0	0	0	0	0	0	0	0
0	0	0	0	0	0	0	0	0
0	0	0	0	0	0	0	0	0
0	0	0	0	0	0	0	0	0
0	0	0	0	0	0	0	0	0
0	0	0	0	0	0	0	0	0
0	0	0	0	0	0	0	0	0
0	0	0	0	0	0	0	0	0
0	0	0	0	0	0	0	0	0
0	0	0	0	0	0	0	0	0
0	0	0	0	0	0	0	0	0
0	0	0	0	0	0	0	0	0
0	0	0	0	0	0	0	0	0
0	0	0	0	0	0	0	0	0
0	0	0	0	0	0	0	0	0
0	0	0	0	0	0	0	0	0
0	0	0	0	0	0	0	0	0
0	0	0	0	0	0	0	0	0
0	0	0	0	0	0	0	0	0
0	0	0	0	0	0	0	0	0
0	0	0	0	0	0	0	0	0
0	0	0	0	0	0	0	0	0
0	0	0	0	0	0	0	0	0
0	0	0	0	0	0	0	0	0
0	0	0	0	0	0	0	0	0
0	0	0	0	0	0	0	0	0
0	0	0	0	0	0	0	0	0
0	0	0	0	0	0	0	0	0
0	0	0	0	0	0	0	0	0
0	0	0	0	0	0	0	0	0

Data 3-2 During Treatment (interim)

During	0	0	0	0	0	0	0	0
0	0	0	0	0	0	0	0	0
0	0	0	0	0	0	0	0	0
0	0	0	0	0	0	0	0	0
0	0	0	0	0	0	0	0	0
0	0	0	0	0	0	0	0	0
0	0	0	0	0	0	0	0	0
0	0	0	0	0	0	0	0	0
0	0	0	0	0	0	0	0	0
0	0	0	0	0	0	0	0	0
0	0	0	0	0	0	0	0	0
0	0	0	0	0	0	0	0	0
0	0	0	0	0	0	0	0	0
0	0	0	0	0	0	0	0	0
0	0	0	0	0	0	0	0	0
0	0	0	0	0	0	0	0	0
0.0233	0.0114	0.0093	0.0099	0.0095	0.0109	0.012	0.0102	0.0098
0.0005	0.0015	0.0024	0.0029	0.0027	0.0026	0.0027	0.0021	0.0024
0.0003	0.0012	0.0023	0.0023	0.0021	0.0021	0.0021	0.0016	0.0016
0.0412	0.0152	0.0146	0.0117	0.0108	0.0133	0.0146	0.0129	0.0136
0.0407	0.0204	0.0164	0.0148	0.0147	0.0152	0.0141	0.0117	0.0086
0	0	0	0	0	0	0	0	0
0	0	0	0	0	0	0	0	0
0.0078	0.0045	0.0042	0.0049	0.0045	0.0054	0.0057	0.005	0.005
0.0156	0.007	0.006	0.0047	0.0055	0.0057	0.0055	0.0044	0.0033
0.0372	0.0158	0.0116	0.0106	0.0112	0.0122	0.0132	0.0118	0.0111
0.0072	0.0068	0.0082	0.0105	0.0095	0.0111	0.0126	0.011	0.0114
0.0003	0.0006	0.0012	0.0012	0.0011	0.0011	0.0011	0.0008	0.0007
0.0073	0.0026	0.0018	0.0015	0.0018	0.0018	0.0017	0.0013	0.0011
-0.0004	0.0037	0.0045	0.0066	0.0061	0.0065	0.0078	0.007	0.0068
-0.0017	0.001	0.0012	0.0026	0.0023	0.0034	0.0047	0.004	0.0042
0.0396	0.0185	0.0156	0.0143	0.0149	0.0152	0.0142	0.0129	0.0134
0	0	0	0	0	0	0	0	0
0.0086	0.0057	0.0072	0.0069	0.0065	0.0065	0.0061	0.0046	0.0041
0.0148	0.0086	0.0095	0.0092	0.009	0.01	0.0104	0.0084	0.0079
0.0003	0.0008	0.0011	0.0017	0.0014	0.0017	0.002	0.0018	0.0019
0	0	0	0	0	0	0	0	0
-0.0001	0.0002	0.0002	0.0005	0.0005	0.0006	0.0008	0.0007	0.0009
0	0.0007	0.001	0.0017	0.0017	0.002	0.0025	0.0023	0.0023
0	0	0	0	0	0	0	0	0
0	0	0	0	0	0	0	0	0
0	0	0	0	0	0	0	0	0
0	0	0	0	0	0	0	0	0
0	0	0	0	0	0	0	0	0

0	0	0	0	0	0	0	0	0
0	0	0	0	0	0	0	0	0
0	0	0	0	0	0	0	0	0
0	0	0	0	0	0	0	0	0
0	0	0	0	0	0	0	0	0
0	0	0	0	0	0	0	0	0
0	0	0	0	0	0	0	0	0
0	0	0	0	0	0	0	0	0
0	0	0	0	0	0	0	0	0
0	0	0	0	0	0	0	0	0
0	0	0	0	0	0	0	0	0
0	0	0	0	0	0	0	0	0
0	0	0	0	0	0	0	0	0
0	0	0	0	0	0	0	0	0
0	0	0	0	0	0	0	0	0
0	0	0	0	0	0	0	0	0
0	0	0	0	0	0	0	0	0
0	0	0	0	0	0	0	0	0
0	0	0	0	0	0	0	0	0
0	0	0	0	0	0	0	0	0
0	0	0	0	0	0	0	0	0
0	0	0	0	0	0	0	0	0
0.0004	0.0013	0.0025	0.0026	0.0024	0.0026	0.0027	0.0023	0.0022
0	0	0	0	0	0	0	0	0
-0.0006	0.0007	0.0009	0.0016	0.0015	0.0021	0.0024	0.0023	0.0026
0	0.0006	0.0007	0.001	0.001	0.0014	0.0017	0.0016	0.0014
0.0004	0.0011	0.0021	0.0023	0.0021	0.002	0.002	0.0015	0.0014
0.0438	0.0189	0.0157	0.015	0.0139	0.0133	0.0111	0.0078	0.0054
0.0639	0.0285	0.0197	0.0162	0.0142	0.0183	0.0187	0.0175	0.0157
0.0236	0.0099	0.0077	0.0058	0.0057	0.0061	0.0061	0.0055	0.0053
0.0001	0.0006	0.0009	0.0014	0.0013	0.0015	0.0018	0.0016	0.0016
0	0.0015	0.0018	0.0025	0.0023	0.0027	0.0033	0.0032	0.0031
-0.001	0.0012	0.0018	0.0035	0.0028	0.0036	0.004	0.0038	0.0042
-0.0003	0.0006	0.0013	0.0019	0.002	0.0023	0.0027	0.0022	0.0022
0.0075	0.0043	0.0045	0.005	0.0044	0.0051	0.0051	0.0043	0.0044
0	0	0	0	0	0	0	0	0
0.0002	0.0006	0.0012	0.0011	0.0011	0.001	0.001	0.0008	0.0008
0.0011	0.0057	0.0079	0.0101	0.0094	0.01	0.0119	0.0109	0.0108
0.0002	0.0008	0.0015	0.0016	0.0014	0.0016	0.0018	0.0015	0.0015
0.0005	0.0013	0.0024	0.0024	0.0022	0.0021	0.0021	0.0015	0.0014
-0.0001	0.0011	0.0018	0.0022	0.0021	0.0022	0.0025	0.0023	0.0025
-0.0001	0.0003	0.0003	0.0005	0.0004	0.0005	0.0007	0.0006	0.0007
0	0	0	0	0	0	0	0	0
0.0084	0.0048	0.0056	0.0054	0.0051	0.0053	0.005	0.0041	0.0037

0.0081	0.0055	0.0067	0.0067	0.0063	0.0068	0.0069	0.0057	0.0054
0.0002	0.0005	0.0004	0.0007	0.0008	0.0009	0.0011	0.0009	0.0009
0	0	0	0	0	0	0	0	0
0	0	0	0	0	0	0	0	0
0	0	0	0	0	0	0	0	0
0	0	0	0	0	0	0	0	0
0	0	0	0	0	0	0	0	0
0	0	0	0	0	0	0	0	0
0	0	0	0	0	0	0	0	0
0	0	0	0	0	0	0	0	0
0	0	0	0	0	0	0	0	0
0	0	0	0	0	0	0	0	0
0	0	0	0	0	0	0	0	0
0	0	0	0	0	0	0	0	0
0	0	0	0	0	0	0	0	0
0	0	0	0	0	0	0	0	0
0	0	0	0	0	0	0	0	0
0	0	0	0	0	0	0	0	0
0	0	0	0	0	0	0	0	0
0	0	0	0	0	0	0	0	0
0	0	0	0	0	0	0	0	0
0	0	0	0	0	0	0	0	0
0	0	0	0	0	0	0	0	0
0	0	0	0	0	0	0	0	0
0	0	0	0	0	0	0	0	0
0	0	0	0	0	0	0	0	0
0	0	0	0	0	0	0	0	0
0	0	0	0	0	0	0	0	0
0.0221	0.0094	0.0084	0.0082	0.0088	0.009	0.0085	0.0079	0.0081
0.0218	0.0087	0.007	0.0059	0.0062	0.0058	0.0051	0.0051	0.0049
0.0345	0.0139	0.0114	0.012	0.0108	0.0138	0.0145	0.0132	0.0126
0.0214	0.0074	0.0075	0.0058	0.0063	0.0071	0.0078	0.0065	0.0066
0.0003	0.0006	0.0012	0.0012	0.0011	0.001	0.0011	0.0008	0.0009
-0.0004	0.0026	0.0043	0.0062	0.0052	0.0061	0.0075	0.0056	0.0057
-0.0008	0.0042	0.0073	0.0099	0.0093	0.0111	0.0129	0.0121	0.0127
0	0	0	0	0	0	0	0	0
0	0	0	0	0	0	0	0	0
0.0224	0.0094	0.0068	0.0072	0.0077	0.0082	0.0071	0.0056	0.0043
0.0072	0.0029	0.0022	0.0018	0.0018	0.0022	0.0021	0.0019	0.0014
0.0002	0.0013	0.0021	0.0025	0.0024	0.0027	0.0029	0.0025	0.0023
0.0319	0.0173	0.0158	0.0144	0.0138	0.0151	0.0155	0.0125	0.0123
0.0161	0.0086	0.0087	0.0076	0.0069	0.0073	0.0071	0.0056	0.0046
0.0003	0.0005	0.0011	0.0011	0.001	0.0009	0.001	0.0008	0.0008
0.0052	0.0063	0.0064	0.01	0.0093	0.0122	0.015	0.0128	0.0132

0.0076	0.0044	0.0039	0.0048	0.0046	0.0054	0.0059	0.0051	0.0049
0	0	0	0	0	0	0	0	0
0	0	0	0	0	0	0	0	0
0	0	0	0	0	0	0	0	0
0.0147	0.0072	0.0066	0.0053	0.0053	0.0061	0.0064	0.0053	0.0045
0.0098	0.0078	0.0099	0.0097	0.0093	0.0095	0.0092	0.0071	0.0064
0.0015	0.0031	0.0052	0.0052	0.0045	0.0041	0.0041	0.0033	0.003
0.0147	0.0076	0.0063	0.0065	0.0063	0.007	0.0072	0.0061	0.0066
0	0	0	0	0	0	0	0	0
0	0	0	0	0	0	0	0	0
0	0	0	0	0	0	0	0	0
0	0	0	0	0	0	0	0	0
0	0	0	0	0	0	0	0	0
0	0	0	0	0	0	0	0	0
0	0	0	0	0	0	0	0	0
0	0	0	0	0	0	0	0	0
0	0	0	0	0	0	0	0	0
0	0	0	0	0	0	0	0	0
0	0	0	0	0	0	0	0	0
0	0	0	0	0	0	0	0	0
0	0	0	0	0	0	0	0	0
0	0	0	0	0	0	0	0	0
0	0	0	0	0	0	0	0	0
0	0	0	0	0	0	0	0	0
0	0	0	0	0	0	0	0	0
0	0	0	0	0	0	0	0	0
0	0	0	0	0	0	0	0	0
0	0	0	0	0	0	0	0	0
0	0	0	0	0	0	0	0	0
0	0	0	0	0	0	0	0	0
0	0	0	0	0	0	0	0	0
0	0	0	0	0	0	0	0	0
0	0	0	0	0	0	0	0	0
0	0	0	0	0	0	0	0	0
0.0063	0.0045	0.0046	0.0061	0.0061	0.0078	0.0088	0.008	0.0079
-0.0002	0.0025	0.0024	0.0047	0.004	0.0057	0.007	0.0067	0.0067
0.0218	0.0103	0.0084	0.0082	0.0085	0.0096	0.0107	0.0097	0.0098
0.0435	0.007	0	-0.0006	-0.0002	0.003	0.0021	0.001	0.0017
0	0	0	0	0	0	0	0	0
0.0001	0.0006	0.0012	0.0011	0.0011	0.001	0.001	0.0008	0.0007
0.0002	0.0005	0.0011	0.0011	0.001	0.001	0.001	0.0008	0.0007
0.0356	0.0135	0.0071	0.0065	0.0067	0.007	0.0067	0.0032	0.0042
0.0291	0.0166	0.0177	0.0179	0.0168	0.0192	0.0218	0.0187	0.0175
0.0364	0.0157	0.0118	0.0099	0.0102	0.0117	0.0102	0.0081	0.0082

0	0	0	0	0	0	0	0	0
0.0002	0.0009	0.0015	0.0018	0.0017	0.0018	0.002	0.0017	0.0016
0.0079	0.0041	0.0037	0.0031	0.0032	0.0034	0.003	0.0027	0.0023
0.0152	0.0065	0.0055	0.005	0.0047	0.0057	0.0052	0.0049	0.0055
0.0146	0.0088	0.0083	0.0093	0.0091	0.0109	0.0105	0.0089	0.0085
0.0475	0.0223	0.0199	0.0169	0.0157	0.0171	0.0162	0.0135	0.0092
0.0071	0.0031	0.0027	0.0029	0.0027	0.0033	0.0033	0.003	0.0027
0	0	0	0	0	0	0	0	0
0	0	0	0	0	0	0	0	0
0	0	0	0	0	0	0	0	0
0.0141	0.0049	0.003	0.0021	0.0028	0.0038	0.0039	0.0042	0.0038
0.0002	0.0009	0.0021	0.0021	0.0019	0.0019	0.0018	0.0014	0.0013
0	0	0	0	0	0	0	0	0
0.0156	0.0075	0.006	0.0065	0.0067	0.0078	0.0078	0.0074	0.0078
0	0	0	0	0	0	0	0	0
0	0	0	0	0	0	0	0	0
0	0	0	0	0	0	0	0	0
0	0	0	0	0	0	0	0	0
0	0	0	0	0	0	0	0	0
0	0	0	0	0	0	0	0	0
0	0	0	0	0	0	0	0	0
0	0	0	0	0	0	0	0	0
0	0	0	0	0	0	0	0	0
0	0	0	0	0	0	0	0	0
0	0	0	0	0	0	0	0	0
0	0	0	0	0	0	0	0	0
0	0	0	0	0	0	0	0	0
0	0	0	0	0	0	0	0	0
0	0	0	0	0	0	0	0	0
0	0	0	0	0	0	0	0	0
0	0	0	0	0	0	0	0	0
0	0	0	0	0	0	0	0	0
0	0	0	0	0	0	0	0	0
0	0	0	0	0	0	0	0	0
0	0	0	0	0	0	0	0	0
0	0	0	0	0	0	0	0	0
0	0	0	0	0	0	0	0	0
0	0	0	0	0	0	0	0	0
0	0	0	0	0	0	0	0	0
0	0	0	0	0	0	0	0	0
0.0147	0.0064	0.0045	0.0034	0.003	0.0037	0.0037	0.0038	0.004
0.001	0.003	0.0052	0.0057	0.0054	0.0056	0.0057	0.005	0.0045
0.0246	0.0141	0.0148	0.0138	0.013	0.0128	0.0129	0.0108	0.0103
0.0075	0.004	0.0042	0.004	0.0039	0.0036	0.0033	0.0025	0.0024

0.0005	0.0035	0.0041	0.0051	0.0051	0.0064	0.0073	0.0064	0.0066
0.0483	0.0184	0.0143	0.0125	0.0125	0.0126	0.0142	0.0111	0.0065
0.0905	0.0435	0.0328	0.0261	0.0229	0.0304	0.028	0.0245	0.0222
0.0017	0.0068	0.0082	0.0102	0.0091	0.0112	0.0116	0.0105	0.0114
0.0004	0.0019	0.0043	0.0041	0.0039	0.0043	0.0042	0.0032	0.003
0.001	0.0038	0.0066	0.0081	0.0072	0.0078	0.0088	0.0074	0.0078
0.0006	0.0017	0.0034	0.0036	0.0032	0.0032	0.0034	0.0027	0.0025
-0.0003	0.0027	0.004	0.0054	0.0051	0.0062	0.0073	0.0056	0.0057
0.0216	0.0118	0.0104	0.0116	0.0122	0.0143	0.0154	0.0128	0.0131
0.0217	0.0104	0.0086	0.0099	0.009	0.0105	0.0103	0.0099	0.0091
-0.0004	0.0034	0.0051	0.0103	0.0103	0.0107	0.013	0.0121	0.0117
0.0012	0.0037	0.0074	0.0077	0.0069	0.006	0.0058	0.0042	0.004
0.0006	0.0014	0.0024	0.0025	0.0022	0.0023	0.0023	0.0019	0.0017
0.0093	0.0082	0.011	0.0119	0.011	0.0118	0.0123	0.01	0.0096
0.0089	0.0071	0.0095	0.0098	0.0094	0.0095	0.0096	0.0074	0.007
0.0002	0.0011	0.0022	0.0022	0.0019	0.0019	0.0019	0.0015	0.0013
-0.0001	0.0008	0.001	0.0015	0.0013	0.0017	0.0017	0.0016	0.0015
0	0	0	0	0	0	0	0	0
0	0	0	0	0	0	0	0	0
0	0.0006	0.0007	0.0012	0.001	0.0014	0.0018	0.0018	0.0018
0	0	0	0	0	0	0	0	0
0	0	0	0	0	0	0	0	0
0	0	0	0	0	0	0	0	0
0	0	0	0	0	0	0	0	0
0	0	0	0	0	0	0	0	0
0	0	0	0	0	0	0	0	0
0	0	0	0	0	0	0	0	0
0	0	0	0	0	0	0	0	0
0	0	0	0	0	0	0	0	0
0	0	0	0	0	0	0	0	0
0	0	0	0	0	0	0	0	0
0	0	0	0	0	0	0	0	0
0	0	0	0	0	0	0	0	0
0	0	0	0	0	0	0	0	0
0	0	0	0	0	0	0	0	0
0	0	0	0	0	0	0	0	0
0	0	0	0	0	0	0	0	0
0	0	0	0	0	0	0	0	0
0	0	0	0	0	0	0	0	0
0	0	0	0	0	0	0	0	0
0	0	0	0	0	0	0	0	0
0	0	0	0	0	0	0	0	0
0	0	0	0	0	0	0	0	0
0	0	0	0	0	0	0	0	0
0	0	0	0	0	0	0	0	0
0	0	0	0	0	0	0	0	0

0	0	0	0	0	0	0	0	0
0	0	0	0	0	0	0	0	0
0.0138	0.0049	0.0035	0.0027	0.0036	0.0032	0.0037	0.0031	0.0022
0.0089	0.0069	0.0088	0.0087	0.0081	0.0079	0.0082	0.0069	0.0064
0.0013	0.0045	0.0102	0.0107	0.0098	0.0094	0.0094	0.0072	0.0065
0.0012	0.0034	0.0069	0.0068	0.0061	0.0061	0.0062	0.0045	0.0041
-0.0009	0.0054	0.0087	0.0134	0.0112	0.0134	0.0157	0.0141	0.0149
0.3372	0.1556	0.1237	0.109	0.0935	0.1066	0.1188	0.1185	0.1054
0.4325	0.201	0.1555	0.1323	0.1463	0.1598	0.1538	0.1375	0.1258
0.1107	0.0513	0.0398	0.0359	0.029	0.0335	0.0397	0.0356	0.032
0.0003	0.0006	0.0011	0.0011	0.0009	0.001	0.0009	0.0007	0.0007
0.0177	0.0172	0.0208	0.0289	0.028	0.032	0.0363	0.0324	0.033
0.0515	0.0296	0.029	0.0304	0.0292	0.0343	0.0347	0.0295	0.0264
0.1052	0.0623	0.0603	0.0607	0.0562	0.0625	0.0657	0.0557	0.0546
0.1729	0.0955	0.0859	0.0865	0.0844	0.0895	0.092	0.0749	0.0759
0.1303	0.0648	0.0517	0.0387	0.038	0.0375	0.0388	0.039	0.0339
0.0258	0.0174	0.0209	0.0212	0.02	0.0207	0.0208	0.0171	0.016
0.0061	0.0241	0.0403	0.0494	0.0454	0.048	0.0556	0.0433	0.0441
0.0098	0.0111	0.0162	0.0168	0.0153	0.0147	0.0153	0.012	0.0108
0.0717	0.0375	0.0386	0.042	0.0419	0.0488	0.0468	0.038	0.0385
0.0807	0.0412	0.0442	0.0407	0.0364	0.0399	0.0407	0.0344	0.031
0.0145	0.0084	0.0111	0.0108	0.0102	0.0106	0.0102	0.0078	0.0076
-0.0001	0.0003	0.0004	0.0006	0.0006	0.0007	0.0009	0.0009	0.0009
0.0002	0.0009	0.0011	0.0014	0.0013	0.0015	0.0019	0.0018	0.0019
0.0004	0.0007	0.0013	0.0013	0.0012	0.0011	0.001	0.0007	0.0007
0.0006	0.0014	0.0027	0.0027	0.0025	0.0023	0.0024	0.0018	0.0017
0	0	0	0	0	0	0	0	0
0	0	0	0	0	0	0	0	0
0	0	0	0	0	0	0	0	0
0	0	0	0	0	0	0	0	0
0	0	0	0	0	0	0	0	0
0	0	0	0	0	0	0	0	0
0	0	0	0	0	0	0	0	0
0	0	0	0	0	0	0	0	0
0	0	0	0	0	0	0	0	0
0	0	0	0	0	0	0	0	0
0	0	0	0	0	0	0	0	0
0	0	0	0	0	0	0	0	0
0	0	0	0	0	0	0	0	0
0	0	0	0	0	0	0	0	0
0	0	0	0	0	0	0	0	0
0	0	0	0	0	0	0	0	0
0	0	0	0	0	0	0	0	0
0	0	0	0	0	0	0	0	0

0	0	0	0	0	0	0	0	0
0	0	0	0	0	0	0	0	0
0	0	0	0	0	0	0	0	0
0	0	0	0	0	0	0	0	0
0	0	0	0	0	0	0	0	0
0	0	0	0	0	0	0	0	0
0	0	0	0	0	0	0	0	0
0	0	0	0	0	0	0	0	0
-0.001	0.0051	0.0077	0.0098	0.0115	0.0133	0.0144	0.0116	0.0114
0.0701	0.0345	0.0304	0.0348	0.0333	0.0358	0.0351	0.0279	0.0263
0.4018	0.1684	0.1335	0.1239	0.1329	0.1332	0.141	0.1224	0.1248
0.7827	0.4134	0.3083	0.2739	0.2546	0.2408	0.2153	0.188	0.1552
0.7659	0.3514	0.2935	0.2578	0.2471	0.2822	0.2892	0.2425	0.2367
0.799	0.3172	0.2349	0.2277	0.2296	0.2544	0.2365	0.142	0.132
0.7052	0.3463	0.2408	0.2051	0.2404	0.243	0.2179	0.2276	0.1925
0.3433	0.1599	0.0993	0.0835	0.0975	0.1075	0.1291	0.1115	0.0993
0.0196	0.042	0.0428	0.0688	0.059	0.0748	0.0869	0.0789	0.0841
0.0748	0.0787	0.0798	0.1038	0.0931	0.1056	0.123	0.1073	0.1154
0.0876	0.0721	0.0814	0.1043	0.1031	0.1181	0.1337	0.1119	0.1125
0.136	0.1003	0.0962	0.1249	0.1188	0.1373	0.1526	0.1393	0.1405
0.1885	0.1078	0.1035	0.1236	0.1141	0.1339	0.1403	0.1202	0.1139
0.1535	0.0808	0.0795	0.0818	0.0796	0.0856	0.0871	0.0746	0.0719
0.069	0.0546	0.06	0.0723	0.0656	0.0766	0.0858	0.0739	0.0749
0.0325	0.0435	0.0646	0.0815	0.0726	0.0932	0.1059	0.0954	0.0976
0.0246	0.0241	0.0318	0.0353	0.0319	0.0357	0.0374	0.032	0.0306
0.1571	0.0792	0.0754	0.0795	0.0802	0.0875	0.0929	0.0769	0.0697
0.1873	0.0983	0.0848	0.0729	0.0701	0.0837	0.0768	0.0643	0.0592
0.038	0.0192	0.017	0.0132	0.0149	0.0154	0.0144	0.0124	0.011
0	0	0	0	0	0	0	0	0
0.0076	0.0052	0.0053	0.0057	0.0052	0.0065	0.0068	0.0054	0.0053
0.0262	0.0097	0.0076	0.0068	0.0077	0.0088	0.0083	0.0051	0.0053
0.0005	0.0013	0.0024	0.0023	0.002	0.002	0.0022	0.0017	0.0017
0	0	0	0	0	0	0	0	0
0	0	0	0	0	0	0	0	0
0	0	0	0	0	0	0	0	0
0	0	0	0	0	0	0	0	0
0	0	0	0	0	0	0	0	0
0	0	0	0	0	0	0	0	0
0	0	0	0	0	0	0	0	0
0	0	0	0	0	0	0	0	0
0	0	0	0	0	0	0	0	0
0	0	0	0	0	0	0	0	0
0	0	0	0	0	0	0	0	0
0	0	0	0	0	0	0	0	0

0	0	0	0	0	0	0	0	0
0	0	0	0	0	0	0	0	0
0	0	0	0	0	0	0	0	0
0	0	0	0	0	0	0	0	0
0	0	0	0	0	0	0	0	0
0	0	0	0	0	0	0	0	0
0	0	0	0	0	0	0	0	0
0	0	0	0	0	0	0	0	0
0	0	0	0	0	0	0	0	0
0	0	0	0	0	0	0	0	0
0	0	0	0	0	0	0	0	0
0	0	0	0	0	0	0	0	0
0	0	0	0	0	0	0	0	0
0	0	0	0	0	0	0	0	0
0.0585	0.0263	0.0231	0.0205	0.018	0.0209	0.0231	0.0173	0.0138
0.0466	0.0228	0.0207	0.0173	0.0155	0.0152	0.0154	0.0116	0.0082
0.3616	0.1686	0.1412	0.1423	0.1235	0.1536	0.1502	0.1285	0.1293
0.6012	0.3064	0.2402	0.2492	0.2664	0.272	0.2538	0.2175	0.1984
0.3121	0.1422	0.1292	0.1122	0.1124	0.1261	0.1219	0.1039	0.0861
0.0471	0.0571	0.0733	0.0882	0.078	0.0902	0.0978	0.0799	0.0813
0.0313	0.0548	0.0693	0.0847	0.0734	0.0887	0.1063	0.0902	0.0962
0.0217	0.0221	0.0246	0.0342	0.0314	0.0366	0.0419	0.0355	0.0363
0.0334	0.0374	0.0425	0.0518	0.0465	0.0515	0.0604	0.0509	0.0501
0.0156	0.0211	0.0287	0.0371	0.0329	0.04	0.0448	0.038	0.0382
0.0043	0.0314	0.0452	0.0637	0.0583	0.0682	0.0759	0.0612	0.0617
-0.0061	0.0363	0.0656	0.0946	0.0848	0.0891	0.1	0.0809	0.085
0.0565	0.046	0.0593	0.0658	0.0614	0.0643	0.0667	0.0557	0.0558
0.0663	0.0462	0.0543	0.0573	0.0536	0.0576	0.0612	0.0506	0.0477
0.0279	0.0479	0.0605	0.0798	0.0699	0.077	0.0816	0.0662	0.0678
0.0316	0.0326	0.0434	0.0494	0.0439	0.0476	0.0554	0.0476	0.0471
0.0415	0.0355	0.0437	0.0504	0.046	0.051	0.0549	0.0464	0.0462
0.2149	0.1033	0.1005	0.1107	0.108	0.1191	0.1121	0.0991	0.0921
0.2377	0.0959	0.0753	0.0774	0.0669	0.0778	0.0841	0.0695	0.0609
0.0624	0.0296	0.0225	0.0169	0.0148	0.0178	0.0201	0.0153	0.0129
0	0	0	0	0	0	0	0	0
0.0227	0.0122	0.0111	0.0115	0.0111	0.0129	0.014	0.0129	0.0123
0.0522	0.0211	0.0157	0.013	0.012	0.0131	0.0122	0.0068	0.0083
0	0	0	0	0	0	0	0	0
0	0	0	0	0	0	0	0	0
0	0	0	0	0	0	0	0	0
0	0	0	0	0	0	0	0	0
0	0	0	0	0	0	0	0	0
0	0	0	0	0	0	0	0	0
0	0	0	0	0	0	0	0	0

0	0	0	0	0	0	0	0	0
0	0	0	0	0	0	0	0	0
0	0	0	0	0	0	0	0	0
0	0	0	0	0	0	0	0	0
0	0	0	0	0	0	0	0	0
0	0	0	0	0	0	0	0	0
0	0	0	0	0	0	0	0	0
0	0	0	0	0	0	0	0	0
0	0	0	0	0	0	0	0	0
0	0	0	0	0	0	0	0	0
0	0	0	0	0	0	0	0	0
0	0	0	0	0	0	0	0	0
0.0017	0.0059	0.0102	0.0106	0.0093	0.0092	0.0092	0.0072	0.0064
-0.001	0.0003	0.0004	0.0016	0.0017	0.0025	0.0034	0.0029	0.0028
0.0039	0.0093	0.0175	0.017	0.0157	0.0142	0.0145	0.0122	0.0125
0.004	0.0091	0.0174	0.0173	0.0155	0.0157	0.0161	0.0125	0.0131
0.0217	0.0097	0.0073	0.0076	0.0087	0.0097	0.01	0.0097	0.0083
0.03	0.0135	0.0102	0.0097	0.0102	0.0111	0.0106	0.0083	0.0077
0.003	0.0064	0.011	0.011	0.0103	0.0101	0.0098	0.008	0.007
0	0	0	0	0	0	0	0	0
-0.0002	0.0025	0.003	0.0047	0.0044	0.0048	0.0061	0.0054	0.0057
0.0542	0.0445	0.0618	0.0635	0.0591	0.0616	0.0628	0.0506	0.0485
0.1256	0.0665	0.0576	0.0561	0.0554	0.0593	0.0593	0.0474	0.0468
0.1227	0.0659	0.0692	0.0718	0.0726	0.0821	0.0865	0.0703	0.0624
0.0538	0.086	0.1293	0.1472	0.1303	0.1337	0.1454	0.1178	0.1103
0.0268	0.0819	0.1098	0.1371	0.1225	0.1327	0.157	0.1253	0.1309
-0.002	0.0542	0.0958	0.1286	0.1072	0.113	0.1225	0.0889	0.116
0.0523	0.0857	0.1155	0.1421	0.1172	0.1615	0.1826	0.1547	0.1587
0.0081	0.0597	0.1101	0.1369	0.1189	0.1229	0.1418	0.1231	0.1202
0.0392	0.099	0.1473	0.1792	0.1569	0.1728	0.1856	0.1584	0.1613
0.0425	0.0812	0.1243	0.1536	0.1311	0.1416	0.151	0.123	0.1271
0.0455	0.1008	0.171	0.1873	0.1697	0.1735	0.1816	0.1553	0.1493
0.0252	0.0779	0.1514	0.148	0.1414	0.1289	0.1409	0.107	0.1009
0.0293	0.0864	0.1522	0.1641	0.1464	0.1496	0.1613	0.1366	0.1292
0.0332	0.0606	0.096	0.1024	0.0899	0.0988	0.1028	0.0791	0.076
0.0104	0.0118	0.0195	0.0188	0.0167	0.0164	0.0159	0.0125	0.0111
0.0036	0.0144	0.0193	0.0331	0.0317	0.0358	0.0466	0.0424	0.0432
0.1394	0.0862	0.0863	0.0995	0.0916	0.1033	0.1107	0.0943	0.0927
0.1401	0.0527	0.0344	0.0358	0.0379	0.0426	0.0432	0.035	0.0353
0.015	0.009	0.0117	0.0121	0.0111	0.0107	0.0106	0.0082	0.0077
0.0008	0.0028	0.0058	0.006	0.0055	0.0054	0.0055	0.0045	0.0043
0.0145	0.0068	0.0051	0.0045	0.0048	0.0051	0.0051	0.0048	0.0045
0.0044	0.0115	0.0191	0.0222	0.0194	0.0186	0.0205	0.0159	0.0164
0.0079	0.0035	0.0028	0.0024	0.0029	0.0028	0.0028	0.0023	0.0021

0.001	0.0026	0.005	0.0053	0.005	0.0052	0.0054	0.0044	0.004
0.0005	0.0017	0.0027	0.003	0.0026	0.0026	0.0028	0.002	0.0021
0.0075	0.0036	0.0027	0.0027	0.0026	0.0026	0.0029	0.0022	0.0019
0	0	0	0	0	0	0	0	0
0	0	0	0	0	0	0	0	0
0.0003	0.0007	0.0013	0.0013	0.0012	0.0012	0.0012	0.0009	0.0009
0.0286	0.0124	0.0106	0.0087	0.0097	0.0108	0.0113	0.0105	0.0089
0.0084	0.0042	0.0031	0.0028	0.0027	0.0028	0.0026	0.0023	0.0024
0	0	0	0	0	0	0	0	0
0	0	0	0	0	0	0	0	0
0	0	0	0	0	0	0	0	0
0	0	0	0	0	0	0	0	0
0	0	0	0	0	0	0	0	0
0	0	0	0	0	0	0	0	0
0	0	0	0	0	0	0	0	0
0	0	0	0	0	0	0	0	0
0	0	0	0	0	0	0	0	0
0	0	0	0	0	0	0	0	0
0.0018	0.0047	0.0069	0.0093	0.009	0.0097	0.0114	0.0098	0.01
0.0083	0.0041	0.0032	0.0028	0.0029	0.0029	0.0026	0.0023	0.002
0.0295	0.0132	0.0124	0.0141	0.0125	0.0156	0.0163	0.0135	0.0126
0.0012	0.0046	0.0049	0.0061	0.0058	0.0074	0.0094	0.0086	0.0091
0.0006	0.0013	0.0024	0.0025	0.0023	0.002	0.0021	0.0016	0.0014
0.0002	0.0009	0.0019	0.0019	0.0017	0.0016	0.0018	0.0015	0.0014
0.0443	0.0204	0.0175	0.0194	0.0194	0.0231	0.0238	0.0223	0.023
0.0929	0.041	0.0407	0.0388	0.0341	0.037	0.041	0.0337	0.0286
0.0013	0.0047	0.0084	0.0083	0.0076	0.007	0.0073	0.0061	0.0061
0.0074	0.0499	0.0723	0.0895	0.0736	0.0851	0.0945	0.0717	0.0757
0.0255	0.04	0.0544	0.0722	0.0598	0.0673	0.074	0.0684	0.0717
0.0526	0.0413	0.056	0.0551	0.0517	0.0519	0.052	0.0419	0.0406
0.0751	0.1086	0.1815	0.1985	0.184	0.1881	0.1859	0.1466	0.1399
0.0686	0.1104	0.1848	0.2055	0.1852	0.1774	0.1814	0.1478	0.1419
0.0536	0.1062	0.1931	0.2033	0.1915	0.1929	0.2038	0.1637	0.163
-0.0011	0.0745	0.1588	0.1679	0.1481	0.1694	0.1874	0.1455	0.1379
0.0984	0.1481	0.2558	0.2594	0.2327	0.2262	0.2335	0.1754	0.1627
0.0392	0.0772	0.1388	0.1538	0.1391	0.1382	0.14	0.1131	0.1056
0.0322	0.0857	0.1324	0.1527	0.139	0.1499	0.1693	0.1416	0.141
-0.0002	0.041	0.0855	0.1037	0.0901	0.0887	0.1075	0.0898	0.0952
0.0096	0.0618	0.0888	0.1165	0.1057	0.1159	0.1379	0.1244	0.1229
0.0297	0.0536	0.0743	0.0944	0.0801	0.0955	0.1119	0.0958	0.1005
0.093	0.0949	0.1347	0.1426	0.1324	0.135	0.1326	0.1062	0.1015
0.1031	0.1257	0.2021	0.1933	0.1739	0.1751	0.1714	0.1222	0.1205
0.0779	0.0681	0.0986	0.0999	0.0906	0.0939	0.0945	0.0759	0.0706
0.0456	0.0338	0.0385	0.0448	0.0424	0.0465	0.0492	0.0411	0.0416

0.0119	0.0159	0.0227	0.0248	0.0228	0.0233	0.024	0.0199	0.0195
0.0003	0.0029	0.003	0.0055	0.0048	0.0058	0.0075	0.0073	0.0079
−0.0028	0.0027	0.0047	0.0074	0.0069	0.0089	0.0119	0.0101	0.0115
0.0038	0.0087	0.0164	0.0174	0.0152	0.0149	0.0148	0.0107	0.0102
0.0003	0.0007	0.0013	0.0013	0.0012	0.0011	0.0011	0.0008	0.0007
−0.0005	0.0006	0.0006	0.0013	0.0013	0.0019	0.0021	0.0018	0.0021
0.0142	0.0073	0.0087	0.0084	0.0077	0.0083	0.0082	0.006	0.0047
0.0081	0.0049	0.0052	0.0051	0.0048	0.0048	0.0047	0.004	0.0037
0.0311	0.0145	0.0111	0.0107	0.0107	0.0113	0.0103	0.0091	0.008
0.0319	0.0147	0.0097	0.0093	0.0088	0.0112	0.0101	0.0099	0.0094
0	0	0	0	0	0	0	0	0
0.0001	0.0005	0.0011	0.0011	0.001	0.001	0.001	0.0008	0.0007
0.015	0.0062	0.0048	0.0038	0.0045	0.0048	0.0045	0.0038	0.0036
0	0	0	0	0	0	0	0	0
0	0	0	0	0	0	0	0	0
0	0	0	0	0	0	0	0	0
0	0	0	0	0	0	0	0	0
0	0	0	0	0	0	0	0	0
0	0	0	0	0	0	0	0	0
0	0	0	0	0	0	0	0	0
0	0	0	0	0	0	0	0	0
0	0	0	0	0	0	0	0	0
0	0	0	0	0	0	0	0	0
0	0	0	0	0	0	0	0	0
0	0	0	0	0	0	0	0	0
0.0011	0.0061	0.0092	0.0112	0.0102	0.0111	0.0131	0.0108	0.0113
0	0	0	0	0	0	0	0	0
0	0	0	0	0	0	0	0	0
0.0169	0.0171	0.0235	0.0258	0.0237	0.0256	0.0279	0.0226	0.0225
0.0219	0.0135	0.0144	0.0155	0.0142	0.0159	0.0166	0.0143	0.014
0	0	0	0	0	0	0	0	0
0	0	0	0	0	0	0	0	0
0.0395	0.0256	0.0267	0.0296	0.0282	0.0318	0.0329	0.028	0.0275
0.0236	0.058	0.0873	0.1026	0.0923	0.0979	0.1077	0.0895	0.0866
0.0186	0.0796	0.1355	0.1538	0.1509	0.1497	0.1554	0.1291	0.1339
0.0383	0.0706	0.1281	0.1404	0.1232	0.1234	0.1231	0.0922	0.0881
0.0645	0.1128	0.2161	0.2227	0.2068	0.2108	0.205	0.1398	0.1309
0.1092	0.1643	0.2793	0.277	0.2435	0.2393	0.2291	0.1826	0.1778
0.0563	0.115	0.2233	0.2198	0.1766	0.1647	0.1736	0.1394	0.1306
0.0134	0.0864	0.2076	0.1982	0.1821	0.1668	0.1731	0.1377	0.128
0.0865	0.1456	0.2425	0.2474	0.226	0.2163	0.2064	0.1621	0.153
0.0385	0.0837	0.1156	0.1301	0.112	0.1184	0.1263	0.1067	0.1095
−0.0136	0.0591	0.1004	0.1437	0.1169	0.1349	0.1634	0.1437	0.1429
0.0272	0.0832	0.128	0.1526	0.13	0.1458	0.1624	0.1259	0.1339

-0.0112	0.0401	0.0724	0.1048	0.0989	0.1155	0.1393	0.118	0.1281
0.0884	0.0986	0.1363	0.1585	0.1413	0.1579	0.1744	0.1457	0.1461
0.1011	0.0982	0.1418	0.1531	0.1431	0.1492	0.1545	0.1289	0.1216
0.1302	0.1565	0.2736	0.2581	0.2387	0.2273	0.2361	0.1834	0.1797
0.2539	0.1862	0.2699	0.2673	0.2458	0.2476	0.2555	0.2045	0.1789
0.0824	0.0692	0.0961	0.0955	0.0872	0.0856	0.0871	0.0686	0.0655
-0.0015	0.0068	0.009	0.0146	0.0127	0.0172	0.022	0.0187	0.0188
0.0382	0.0148	0.0109	0.0077	0.0074	0.0096	0.0101	0.0085	0.008
0.0741	0.0353	0.0314	0.0298	0.0282	0.0334	0.0356	0.0316	0.0249
0.0373	0.0209	0.0187	0.0205	0.0184	0.02	0.0216	0.0185	0.0193
0	0	0	0	0	0	0	0	0
0.0004	0.0013	0.0026	0.0026	0.0023	0.0022	0.0023	0.0018	0.0016
0.0007	0.0026	0.0048	0.0048	0.0043	0.0042	0.0043	0.0037	0.0037
0.0003	0.0014	0.0026	0.0028	0.0027	0.0028	0.0029	0.0024	0.0021
0.015	0.0061	0.0046	0.0046	0.0043	0.0043	0.0052	0.004	0.0038
0.0365	0.0152	0.0108	0.0091	0.0098	0.0114	0.0122	0.0103	0.0083
0.0076	0.0034	0.0023	0.0021	0.0024	0.0026	0.0027	0.0022	0.0018
0.0076	0.0038	0.0036	0.0033	0.0031	0.0033	0.0033	0.0028	0.0026
0.0088	0.0046	0.0043	0.0035	0.0029	0.0032	0.0028	0.0024	0.0022
0	0	0	0	0	0	0	0	0
0	0	0	0	0	0	0	0	0
0	0	0	0	0	0	0	0	0
0	0	0	0	0	0	0	0	0
0	0	0	0	0	0	0	0	0
0	0	0	0	0	0	0	0	0
0	0	0	0	0	0	0	0	0
0	0	0	0	0	0	0	0	0
0	0	0	0	0	0	0	0	0
0	0	0	0	0	0	0	0	0
0	0	0	0	0	0	0	0	0
0.0002	0.0011	0.0023	0.0022	0.002	0.0019	0.0019	0.0015	0.0015
0.0217	0.0107	0.0105	0.0107	0.0104	0.0123	0.0129	0.0117	0.0109
0.0921	0.0415	0.0412	0.0313	0.0301	0.0251	0.0249	0.0192	0.0152
0.0652	0.0352	0.0366	0.0344	0.0332	0.036	0.0357	0.0274	0.0281
-0.0036	0.012	0.0224	0.0362	0.0335	0.0391	0.0409	0.0336	0.0334
0.0065	0.015	0.025	0.0243	0.0224	0.0215	0.022	0.0174	0.0174
0.025	0.0195	0.0281	0.0284	0.0258	0.0258	0.0255	0.0209	0.0194
0.0708	0.0586	0.0565	0.0735	0.0672	0.0812	0.0889	0.0819	0.0803
0.0398	0.0275	0.027	0.0327	0.0296	0.0332	0.0453	0.0371	0.0396
0.0591	0.0522	0.0793	0.0838	0.0766	0.0797	0.0831	0.0667	0.0655
0.0852	0.1189	0.1929	0.195	0.1749	0.1707	0.1837	0.1496	0.141
0.0332	0.087	0.1592	0.1761	0.1591	0.1663	0.1765	0.1308	0.1355
0.0546	0.1229	0.2384	0.2466	0.2249	0.2057	0.2137	0.1658	0.1468
0.0429	0.1237	0.2249	0.2314	0.2009	0.1981	0.2046	0.1564	0.1414

0.0664	0.1113	0.1862	0.1896	0.1798	0.1773	0.1768	0.1399	0.1332
0.0316	0.0613	0.1115	0.1206	0.1101	0.1107	0.1148	0.0924	0.0938
0.0373	0.0964	0.16	0.1572	0.1418	0.1419	0.1451	0.1211	0.1049
0.0236	0.0876	0.1356	0.1612	0.1492	0.1519	0.1595	0.1431	0.1424
0.0422	0.0871	0.1507	0.1682	0.1419	0.1503	0.154	0.1234	0.1336
0.0349	0.0799	0.1444	0.1633	0.1411	0.1426	0.1431	0.1057	0.1042
0.0588	0.0869	0.1402	0.1564	0.1443	0.1504	0.1601	0.1336	0.1307
0.0896	0.1141	0.1889	0.1897	0.1719	0.1674	0.1703	0.1347	0.1344
0.0637	0.0828	0.123	0.1364	0.1215	0.1272	0.1364	0.1101	0.1092
0.0377	0.0681	0.1267	0.1378	0.1227	0.1308	0.134	0.1121	0.1063
0.1803	0.1474	0.2192	0.2272	0.2171	0.207	0.211	0.1659	0.1622
0.1851	0.1581	0.2123	0.2141	0.1881	0.1807	0.1686	0.1259	0.1167
0.0418	0.0375	0.0527	0.0571	0.0517	0.0552	0.0562	0.0468	0.0453
0.0634	0.0365	0.0329	0.0378	0.0346	0.0411	0.0471	0.0399	0.039
0.2096	0.1072	0.104	0.1026	0.1002	0.1152	0.1134	0.0945	0.095
0.1913	0.0891	0.0681	0.0614	0.0585	0.0729	0.0756	0.075	0.0752
0.0418	0.0141	0.0089	0.0064	0.0052	0.0058	0.0054	0.0059	0.0081
0.0436	0.0409	0.0603	0.065	0.0602	0.0613	0.0624	0.0515	0.0503
0.0007	0.0027	0.0054	0.0058	0.0052	0.0053	0.0056	0.0046	0.0042
0.0006	0.0017	0.0028	0.0029	0.0025	0.0026	0.0028	0.0023	0.0022
0.0002	0.0006	0.0005	0.0008	0.0008	0.0009	0.001	0.0009	0.0011
0	0	0	0	0	0	0	0	0
0	0	0	0	0	0	0	0	0
0.0003	0.0007	0.0012	0.0012	0.001	0.001	0.0009	0.0007	0.0006
0.0139	0.0058	0.0037	0.0035	0.003	0.0037	0.0035	0.0029	0.0021
0	0	0	0	0	0	0	0	0
0	0	0	0	0	0	0	0	0
0	0	0	0	0	0	0	0	0
0	0	0	0	0	0	0	0	0
0	0	0	0	0	0	0	0	0
0	0	0	0	0	0	0	0	0
0	0	0	0	0	0	0	0	0
0	0	0	0	0	0	0	0	0
0	0	0	0	0	0	0	0	0
0	0	0	0	0	0	0	0	0
0	0	0	0	0	0	0	0	0
0.0877	0.0386	0.0337	0.0329	0.0321	0.0348	0.0359	0.0306	0.0301
0	0	0	0	0	0	0	0	0
-0.0008	0.0015	0.0018	0.0037	0.0036	0.0044	0.0053	0.0045	0.0048
0.0002	0.0044	0.005	0.0074	0.0072	0.0077	0.0096	0.0082	0.0086
0.0676	0.0412	0.0509	0.0505	0.0496	0.0515	0.0537	0.0459	0.0448
0.1854	0.1104	0.1242	0.1317	0.1289	0.1332	0.137	0.1148	0.1159
0.1976	0.1252	0.1357	0.1448	0.1347	0.1509	0.1602	0.1334	0.1292
0.1273	0.0764	0.0851	0.0853	0.0811	0.0929	0.096	0.0802	0.0777

0.0608	0.0729	0.1141	0.1194	0.1105	0.1096	0.1136	0.1007	0.1006
0.0427	0.0649	0.1208	0.1321	0.1276	0.1178	0.121	0.0938	0.0817
0.0525	0.1077	0.1791	0.1851	0.166	0.1737	0.1816	0.1316	0.1309
0.029	0.0937	0.1625	0.1804	0.1618	0.1759	0.1961	0.1628	0.149
0.0391	0.1025	0.2045	0.2116	0.1787	0.1832	0.1936	0.1568	0.1458
0.0898	0.1374	0.2458	0.2514	0.2276	0.2248	0.2283	0.1861	0.1608
0.067	0.1039	0.1835	0.1888	0.1839	0.1835	0.1846	0.1546	0.1477
-0.0272	0.0315	0.0776	0.1086	0.1025	0.1159	0.127	0.1053	0.1107
0.019	0.0656	0.1198	0.1371	0.1248	0.1249	0.1281	0.0988	0.0977
0.0181	0.0769	0.1335	0.1599	0.14	0.1472	0.1579	0.1253	0.1414
0.0465	0.0877	0.1491	0.1615	0.1414	0.1513	0.1594	0.131	0.1276
0.0386	0.0799	0.1295	0.1432	0.1277	0.13	0.1393	0.1185	0.1201
0.0192	0.0916	0.1325	0.1508	0.1341	0.1396	0.1539	0.1251	0.1284
0.0059	0.0519	0.0937	0.1165	0.1082	0.1167	0.1302	0.1082	0.1107
0.0327	0.0699	0.0798	0.1225	0.1047	0.135	0.152	0.1235	0.1175
0.0571	0.0864	0.0854	0.121	0.1136	0.142	0.1599	0.1491	0.163
0.0085	0.0446	0.081	0.09	0.0775	0.0863	0.0979	0.0893	0.0902
0.1783	0.1636	0.2407	0.2343	0.2071	0.2064	0.2096	0.1609	0.1561
0.0168	0.076	0.1546	0.1644	0.1425	0.1395	0.1495	0.1216	0.1135
0.0018	0.0305	0.0417	0.057	0.0554	0.0629	0.0796	0.0719	0.0709
0.2421	0.1074	0.0864	0.0941	0.0895	0.1008	0.1083	0.0886	0.0948
0.2806	0.1293	0.0933	0.0986	0.0913	0.097	0.0981	0.0709	0.0543
0.1482	0.0768	0.0714	0.0715	0.0744	0.0813	0.0827	0.0724	0.0677
0.2099	0.1024	0.0996	0.1043	0.097	0.1082	0.1021	0.0846	0.0861
0.0824	0.0355	0.0364	0.0315	0.0292	0.0328	0.0325	0.0252	0.0258
0.0003	0.0014	0.0028	0.003	0.0027	0.0029	0.0029	0.0023	0.0024
0	0.0003	0.0004	0.0006	0.0006	0.0008	0.0009	0.0009	0.0009
0	0	0	0	0	0	0	0	0
0	0	0	0	0	0	0	0	0
0.0002	0.0006	0.0012	0.0012	0.0011	0.0011	0.001	0.0009	0.0009
0.0158	0.0071	0.0061	0.0058	0.0059	0.0058	0.0058	0.0048	0.0041
0.0166	0.007	0.0052	0.0049	0.0046	0.0053	0.0047	0.0042	0.0041
0	0	0	0	0	0	0	0	0
0	0	0	0	0	0	0	0	0
0	0	0	0	0	0	0	0	0
0	0	0	0	0	0	0	0	0
0	0	0	0	0	0	0	0	0
0	0	0	0	0	0	0	0	0
0	0	0	0	0	0	0	0	0
0	0	0	0	0	0	0	0	0
0	0	0	0	0	0	0	0	0
0	0	0	0	0	0	0	0	0
0	0	0	0	0	0	0	0	0
0.0097	0.0115	0.0181	0.0186	0.0172	0.0175	0.0177	0.0143	0.0137

0.0266	0.0102	0.0078	0.0061	0.0065	0.0089	0.0091	0.0084	0.0081
0.0295	0.014	0.0115	0.0104	0.0104	0.0121	0.0127	0.01	0.0085
0.0565	0.0605	0.0906	0.0934	0.0866	0.0805	0.0797	0.0656	0.067
0.0704	0.0759	0.1142	0.1187	0.1055	0.1095	0.1139	0.0918	0.0928
0.0393	0.0407	0.0566	0.0676	0.0607	0.0697	0.0789	0.0664	0.0661
0.0305	0.0439	0.0605	0.0702	0.066	0.0691	0.0754	0.0666	0.0651
0.0249	0.0728	0.1399	0.1568	0.142	0.1377	0.1518	0.1187	0.114
0.0334	0.0902	0.1647	0.1715	0.1559	0.1648	0.1849	0.147	0.1412
0.0276	0.0836	0.1466	0.1502	0.1405	0.1442	0.1515	0.1209	0.1145
0.0081	0.0442	0.0981	0.1207	0.1053	0.1133	0.1287	0.1036	0.0996
0.1067	0.1001	0.143	0.1523	0.1364	0.1433	0.15	0.1263	0.122
0.1477	0.1303	0.1918	0.2023	0.1906	0.1961	0.2039	0.1669	0.1669
0.0826	0.1078	0.1697	0.1662	0.1522	0.1473	0.1533	0.1219	0.1237
0.0502	0.1176	0.1734	0.1885	0.1692	0.1864	0.1915	0.1613	0.1646
0.0388	0.0921	0.1479	0.1722	0.1637	0.1794	0.1828	0.1456	0.1455
0.0376	0.067	0.1037	0.1227	0.114	0.1261	0.1305	0.1146	0.112
0.0546	0.0963	0.1524	0.1648	0.156	0.1662	0.1659	0.1299	0.1267
0.0138	0.0512	0.0857	0.1111	0.0967	0.1139	0.1248	0.1038	0.106
0.0098	0.0803	0.1668	0.1903	0.1759	0.182	0.1866	0.1435	0.1426
0.0193	0.0826	0.1268	0.1457	0.1279	0.1369	0.1457	0.1184	0.1254
0.077	0.1233	0.1825	0.2103	0.1927	0.2157	0.2281	0.1876	0.1768
0.0606	0.0917	0.1274	0.158	0.1378	0.1695	0.1909	0.1622	0.1662
0.201	0.1237	0.1329	0.1407	0.1266	0.1494	0.1559	0.131	0.1358
0.3915	0.2004	0.2017	0.2138	0.2037	0.2089	0.2139	0.1708	0.1621
0.1715	0.142	0.1807	0.2015	0.1851	0.2006	0.2105	0.1802	0.1767
0.035	0.1021	0.123	0.1719	0.1507	0.1742	0.2084	0.1757	0.1831
0.1189	0.0936	0.0993	0.1339	0.1273	0.1556	0.1569	0.1352	0.1295
0.1733	0.092	0.0967	0.1051	0.1071	0.1202	0.1196	0.1025	0.0936
0.0715	0.0346	0.0353	0.0359	0.0345	0.0385	0.0367	0.0315	0.0274
0.1415	0.0505	0.0462	0.0491	0.0484	0.0443	0.0453	0.0388	0.0364
0.1994	0.085	0.0639	0.0653	0.0584	0.067	0.0643	0.064	0.0545
0.0768	0.0354	0.0329	0.0294	0.0306	0.0338	0.0325	0.0293	0.0288
0.0139	0.005	0.003	0.003	0.0026	0.0035	0.0031	0.0024	0.0022
0.016	0.0069	0.0041	0.0032	0.0024	0.0032	0.0023	0.0013	0.0012
0	0	0	0	0	0	0	0	0
0.0004	0.0007	0.0013	0.0013	0.0012	0.0011	0.0011	0.001	0.0009
0.0077	0.0038	0.0035	0.0033	0.0033	0.0033	0.0033	0.0027	0.0027
0.0137	0.0048	0.0035	0.0019	0.0023	0.0033	0.0027	0.0023	0.0024
0	0	0	0	0	0	0	0	0
0	0	0	0	0	0	0	0	0
0	0	0	0	0	0	0	0	0
0	0	0	0	0	0	0	0	0
0	0	0	0	0	0	0	0	0
0	0	0	0	0	0	0	0	0

0	0	0	0	0	0	0	0	0
0	0	0	0	0	0	0	0	0
0	0	0	0	0	0	0	0	0
0	0	0	0	0	0	0	0	0
0	0	0	0	0	0	0	0	0
0.0038	0.0082	0.0145	0.0144	0.0125	0.0122	0.0124	0.0099	0.009
0	0	0	0	0	0	0	0	0
0.0351	0.0311	0.0411	0.0431	0.0398	0.042	0.0442	0.0361	0.0352
0.1189	0.0947	0.1256	0.1322	0.1204	0.1255	0.1321	0.1078	0.1036
0.1494	0.0807	0.0844	0.0708	0.069	0.072	0.0691	0.0546	0.0535
0.0701	0.0363	0.0341	0.0376	0.0351	0.0409	0.0456	0.04	0.0376
0.0581	0.1226	0.1611	0.2027	0.1838	0.184	0.2024	0.1696	0.1639
0.013	0.0743	0.1225	0.1512	0.137	0.1525	0.1599	0.1532	0.1623
0.1237	0.1147	0.1682	0.1803	0.1674	0.1764	0.1856	0.1507	0.1485
0.175	0.1203	0.1423	0.1572	0.1452	0.1576	0.1639	0.1346	0.1276
0.1752	0.137	0.1757	0.1848	0.1661	0.1755	0.1802	0.1488	0.1435
0.142	0.1016	0.1128	0.1231	0.1141	0.1262	0.1345	0.1103	0.1108
0.1407	0.1217	0.1567	0.1685	0.153	0.1651	0.1708	0.1403	0.1372
0.1027	0.1235	0.1901	0.2009	0.1941	0.1936	0.1985	0.1608	0.1611
0.0186	0.0785	0.1519	0.1676	0.1565	0.158	0.1619	0.1335	0.1446
0.0397	0.0764	0.1312	0.1512	0.1354	0.1423	0.1456	0.1113	0.1098
0.0259	0.064	0.1081	0.1314	0.1157	0.1233	0.1367	0.1163	0.1189
0.0059	0.0689	0.1282	0.1427	0.1287	0.1426	0.1472	0.1298	0.125
0.0258	0.0818	0.1439	0.1661	0.1517	0.16	0.1687	0.1428	0.1389
0.094	0.1552	0.2397	0.2477	0.2209	0.2093	0.1968	0.1497	0.1319
0.1215	0.143	0.24	0.2477	0.2297	0.2204	0.2214	0.176	0.1709
0.1272	0.1587	0.2639	0.25	0.2345	0.2236	0.2247	0.1731	0.1532
0.0745	0.1119	0.1955	0.207	0.188	0.1838	0.1841	0.1375	0.1312
0.2027	0.1736	0.2455	0.2486	0.2323	0.2364	0.2327	0.1842	0.1747
0.1743	0.1379	0.1827	0.1849	0.1691	0.175	0.1787	0.1485	0.141
0.0761	0.1075	0.156	0.1884	0.1636	0.1741	0.1864	0.1617	0.1591
-0.0108	0.06	0.0641	0.1264	0.1008	0.1389	0.1675	0.1374	0.1756
0.0742	0.0754	0.0814	0.1221	0.1033	0.1385	0.1616	0.1342	0.1475
0.2169	0.1258	0.1264	0.1419	0.1295	0.1464	0.16	0.1427	0.1399
0.2437	0.1066	0.0878	0.0962	0.0813	0.1025	0.1008	0.0884	0.0771
0.3545	0.1404	0.1088	0.0859	0.0889	0.1047	0.1005	0.0761	0.0718
0.1606	0.064	0.0418	0.0391	0.0393	0.0522	0.06	0.0466	0.0438
0.0506	0.0211	0.0165	0.0151	0.0131	0.0134	0.0154	0.0112	0.0104
0	0.0004	0.0004	0.0006	0.0006	0.0007	0.0009	0.0008	0.0008
0.016	0.0069	0.0057	0.0051	0.0049	0.0052	0.0058	0.0055	0.005
0	0	0	0	0	0	0	0	0
0.0003	0.0007	0.0012	0.0012	0.0012	0.0012	0.0011	0.0008	0.0008
0.0001	0.0005	0.001	0.0011	0.001	0.001	0.001	0.0007	0.0007
0.0076	0.0033	0.0024	0.0018	0.0019	0.0021	0.002	0.0015	0.0013

0	0	0	0	0	0	0	0	0
0	0	0	0	0	0	0	0	0
0	0	0	0	0	0	0	0	0
0	0	0	0	0	0	0	0	0
0	0	0	0	0	0	0	0	0
0	0	0	0	0	0	0	0	0
0	0	0	0	0	0	0	0	0
0	0	0	0	0	0	0	0	0
0	0	0	0	0	0	0	0	0
0	0	0	0	0	0	0	0	0
0.0084	0.008	0.0109	0.0127	0.0117	0.013	0.0146	0.0123	0.0118
-0.0005	0.001	0.0015	0.0021	0.002	0.0024	0.003	0.0027	0.0031
0	0	0	0	0	0	0	0	0
0.0037	0.0237	0.0385	0.0467	0.0431	0.0476	0.0504	0.0396	0.039
0.0976	0.079	0.091	0.1055	0.1019	0.1165	0.1218	0.1041	0.1035
0.2571	0.1213	0.0988	0.0978	0.1038	0.1241	0.1399	0.1215	0.1193
0.4663	0.2209	0.1829	0.1853	0.1905	0.207	0.2061	0.1627	0.1299
0.5069	0.2287	0.2362	0.2388	0.2261	0.2684	0.2657	0.2061	0.2008
0.0101	0.0718	0.1188	0.1499	0.1426	0.159	0.1635	0.1395	0.1463
0.2529	0.1562	0.1806	0.1788	0.1713	0.1798	0.1861	0.1486	0.1374
0.4391	0.2019	0.2045	0.1933	0.1924	0.2104	0.2088	0.1765	0.1528
0.4001	0.2717	0.3494	0.3412	0.3161	0.3182	0.3156	0.2459	0.2314
0.1853	0.1682	0.2428	0.2471	0.2305	0.2385	0.2468	0.2027	0.1987
0.0581	0.1224	0.2194	0.2181	0.1982	0.1985	0.2096	0.1509	0.1365
0.1062	0.1479	0.2426	0.2446	0.2227	0.2254	0.2352	0.1748	0.1549
0.0785	0.1175	0.2077	0.2128	0.2004	0.1973	0.2033	0.1697	0.1572
0.0455	0.101	0.1802	0.1843	0.1603	0.1498	0.1591	0.1222	0.1112
0.0398	0.1157	0.1891	0.2095	0.1839	0.1795	0.1856	0.1488	0.1393
0.049	0.0913	0.1706	0.1682	0.1619	0.1611	0.1712	0.1443	0.1402
0.143	0.1538	0.2213	0.2314	0.2075	0.2001	0.204	0.1688	0.1607
0.2218	0.1563	0.2057	0.1997	0.1856	0.1907	0.1857	0.1484	0.1325
0.2568	0.1972	0.277	0.2674	0.2579	0.2531	0.2495	0.2005	0.1871
0.1393	0.1546	0.2481	0.243	0.2114	0.2086	0.2124	0.1662	0.1597
0.0268	0.0987	0.205	0.2071	0.1868	0.1798	0.1691	0.1352	0.1294
0.2583	0.1624	0.24	0.2321	0.2148	0.2175	0.2088	0.1741	0.1612
0.0996	0.151	0.2666	0.2807	0.2492	0.2372	0.2342	0.1865	0.1642
0.0422	0.095	0.1572	0.1814	0.1677	0.1798	0.188	0.1521	0.153
0.0879	0.1273	0.1425	0.1896	0.1674	0.179	0.2088	0.1741	0.1784
-0.004	0.0621	0.0817	0.1233	0.1187	0.1543	0.1707	0.151	0.1517
0.0486	0.0697	0.0929	0.1406	0.138	0.1786	0.2046	0.1822	0.1788
0.1635	0.1103	0.1064	0.1413	0.1372	0.1745	0.1867	0.163	0.1644
0.1441	0.0857	0.0764	0.0809	0.0753	0.0816	0.0926	0.0791	0.0752
0.0749	0.0306	0.0272	0.022	0.0209	0.0228	0.0251	0.0227	0.0222
0.0004	0.0014	0.0027	0.0029	0.0026	0.0027	0.0031	0.0025	0.0023

-0.0001	0.0002	0.0003	0.0006	0.0005	0.0007	0.0008	0.0007	0.0008
0	0	0	0	0	0	0	0	0
0	0	0	0	0	0	0	0	0
0.0002	0.0005	0.0011	0.0011	0.001	0.0009	0.0009	0.0007	0.0007
0.0001	0.0005	0.0011	0.0011	0.0009	0.0009	0.0009	0.0007	0.0006
0.0072	0.0026	0.0021	0.0019	0.0018	0.0016	0.0014	0.0008	0.0007
0	0	0	0	0	0	0	0	0
0	0	0	0	0	0	0	0	0
0.0002	0.0018	0.0023	0.0032	0.003	0.0031	0.0038	0.0031	0.0031
0	0	0	0	0	0	0	0	0
0	0	0	0	0	0	0	0	0
0.0002	0.0005	0.0011	0.001	0.0009	0.0009	0.0009	0.0006	0.0005
-0.0001	0.0029	0.005	0.0067	0.0061	0.0076	0.0086	0.0072	0.0079
0.0004	0.0022	0.0034	0.0042	0.0038	0.0042	0.0047	0.0038	0.0037
0	0.0003	0.0004	0.0007	0.0007	0.0009	0.0011	0.001	0.001
0.0004	0.0021	0.0025	0.0043	0.0034	0.0042	0.0057	0.0049	0.0052
0	0	0	0	0	0	0	0	0
0.0813	0.0349	0.0321	0.029	0.0283	0.0367	0.0375	0.0339	0.0324
0.0001	0.0006	0.0006	0.0013	0.0011	0.0015	0.0018	0.0016	0.0019
0.1476	0.0755	0.0726	0.0791	0.0723	0.0791	0.0837	0.0757	0.0735
0.0673	0.036	0.0294	0.0371	0.035	0.0405	0.0452	0.0397	0.04
0.1654	0.0712	0.0625	0.0591	0.0632	0.0616	0.0709	0.0588	0.0526
0.204	0.0969	0.0823	0.085	0.0928	0.11	0.1258	0.115	0.1069
0.8579	0.4117	0.3042	0.2822	0.2464	0.2525	0.249	0.2154	0.1943
0.9002	0.3916	0.3889	0.3482	0.3567	0.3989	0.3699	0.2847	0.2464
0.4544	0.264	0.2582	0.2534	0.2487	0.2809	0.2709	0.2107	0.1845
0.0971	0.1124	0.1629	0.1718	0.1601	0.1718	0.1758	0.1494	0.1367
0.1352	0.1512	0.236	0.2244	0.2053	0.2009	0.1949	0.1494	0.142
0.134	0.119	0.2056	0.2058	0.1863	0.1791	0.1803	0.1513	0.1409
0.0791	0.1442	0.2575	0.2447	0.2278	0.2239	0.2113	0.1791	0.1684
0.0385	0.1067	0.219	0.2299	0.2054	0.2024	0.2043	0.1594	0.1591
0.122	0.1765	0.2762	0.2655	0.2366	0.2339	0.2298	0.1694	0.161
0.0502	0.1389	0.2505	0.255	0.2264	0.2109	0.2239	0.1801	0.164
0.1182	0.1363	0.2369	0.2432	0.2293	0.2177	0.219	0.1707	0.1603
0.0314	0.0948	0.1953	0.1862	0.1619	0.1599	0.1807	0.1319	0.1292
0.0691	0.1067	0.1797	0.1853	0.1592	0.1414	0.1544	0.1195	0.1175
0.5641	0.3075	0.298	0.2651	0.2609	0.2594	0.2755	0.2309	0.2101
0.3503	0.2151	0.2823	0.2709	0.2534	0.2456	0.2525	0.2036	0.1957
0.0278	0.0822	0.1558	0.159	0.1491	0.1462	0.1543	0.1136	0.1144
0.2353	0.1654	0.2298	0.2234	0.2089	0.21	0.2041	0.1636	0.1595
0.274	0.1994	0.2696	0.2546	0.2409	0.2481	0.2509	0.1979	0.1752
0.0333	0.0993	0.1919	0.193	0.1711	0.1688	0.1593	0.1193	0.115
0.1401	0.1505	0.2226	0.2398	0.2228	0.2255	0.2336	0.1876	0.1789
0.055	0.098	0.1301	0.1493	0.1319	0.1467	0.1488	0.118	0.1248

-0.0206	0.0502	0.0597	0.0931	0.0856	0.1209	0.1428	0.1386	0.1239
0.0887	0.1013	0.1247	0.157	0.1427	0.1669	0.1778	0.1624	0.161
-0.0042	0.05	0.087	0.1402	0.1324	0.1551	0.1856	0.1604	0.1616
0.0914	0.0852	0.1	0.1252	0.1154	0.1291	0.141	0.1243	0.119
0.1189	0.0654	0.0757	0.0724	0.0672	0.0725	0.0738	0.0643	0.0542
0.0496	0.0239	0.0239	0.0204	0.0189	0.0225	0.0224	0.0204	0.0165
-0.0003	0.0004	0.0006	0.001	0.0009	0.0012	0.0015	0.0011	0.0011
0.0001	0.0015	0.0034	0.0036	0.0033	0.0035	0.0036	0.0029	0.0029
0.0591	0.0251	0.0198	0.0186	0.0202	0.0227	0.0239	0.0241	0.0208
0.0332	0.0205	0.0225	0.0213	0.0211	0.0194	0.0206	0.0172	0.016
0.0015	0.0046	0.0101	0.0105	0.0094	0.0093	0.0087	0.0068	0.0066
0.0811	0.0319	0.0236	0.0208	0.0209	0.0222	0.0249	0.0201	0.0231
0	0	0	0	0	0	0	0	0
0	0	0	0	0	0	0	0	0
0	0	0	0	0	0	0	0	0
-0.0002	0.0001	0.0002	0.0004	0.0005	0.0006	0.0006	0.0006	0.0007
-0.0001	0.0003	0.0004	0.0006	0.0004	0.0006	0.0007	0.0006	0.0007
0	0.0006	0.0006	0.0012	0.0011	0.0012	0.0013	0.0012	0.0012
0.0005	0.0023	0.0033	0.0042	0.0042	0.0047	0.005	0.0041	0.0038
0.0006	0.0014	0.0026	0.0023	0.0021	0.002	0.0019	0.0013	0.0013
0.0003	0.0007	0.0012	0.0012	0.001	0.001	0.0011	0.0008	0.0008
0.0003	0.0007	0.0013	0.0012	0.0011	0.0011	0.0011	0.0009	0.0008
0.003	0.0076	0.0133	0.0128	0.0118	0.0103	0.0104	0.0084	0.0072
0	0	0	0	0	0	0	0	0
0.0356	0.0129	0.0097	0.0099	0.0117	0.0123	0.0113	0.0096	0.0095
0.0029	0.0138	0.029	0.0287	0.0259	0.0246	0.0234	0.0181	0.0158
0.6565	0.2717	0.2035	0.1989	0.2343	0.233	0.2102	0.1934	0.1666
0.2433	0.124	0.1164	0.1159	0.1087	0.1121	0.108	0.0897	0.0756
0.0087	0.0518	0.0856	0.103	0.0939	0.0942	0.1037	0.0866	0.0885
-0.0044	0.0455	0.0577	0.0894	0.0609	0.0856	0.1047	0.0946	0.1006
0.3996	0.2267	0.2325	0.2451	0.2382	0.2592	0.2568	0.2195	0.1985
0.1604	0.1294	0.1671	0.1722	0.1575	0.1623	0.1706	0.138	0.1318
0.0087	0.0516	0.071	0.1019	0.081	0.0942	0.118	0.1109	0.1057
0.1024	0.1385	0.2144	0.2232	0.2069	0.2096	0.2101	0.1725	0.1597
0.1465	0.1433	0.2285	0.2293	0.2099	0.2217	0.2165	0.177	0.1646
0.0528	0.1117	0.1928	0.2057	0.188	0.1808	0.1859	0.1539	0.1428
0.03	0.0856	0.1614	0.1564	0.1471	0.1418	0.1467	0.109	0.1062
0.0227	0.0836	0.1845	0.2009	0.1693	0.162	0.1544	0.1089	0.0948
0.1125	0.1536	0.2465	0.2383	0.2182	0.2101	0.2212	0.1772	0.1573
0.1178	0.1654	0.2595	0.2558	0.2344	0.2266	0.2198	0.184	0.1749
0.1351	0.1371	0.2061	0.2132	0.1953	0.1955	0.202	0.1746	0.1626
0.2011	0.1654	0.2402	0.2386	0.219	0.2158	0.2208	0.1821	0.1677
0.1563	0.1511	0.2248	0.224	0.21	0.2134	0.2164	0.1757	0.1637
0.0929	0.1233	0.2408	0.2408	0.2151	0.2066	0.2145	0.1592	0.1529

0.3202	0.2151	0.2601	0.2467	0.2292	0.236	0.2349	0.1871	0.1714
0.7454	0.4016	0.3893	0.3231	0.2954	0.3051	0.2784	0.2322	0.2234
0.2449	0.1791	0.2382	0.2271	0.209	0.1998	0.2015	0.1549	0.1421
0.101	0.1157	0.1887	0.1922	0.1815	0.1838	0.1825	0.137	0.1294
0.0173	0.0954	0.1806	0.1852	0.164	0.168	0.1678	0.1332	0.1304
0.0529	0.0717	0.1064	0.1276	0.1239	0.1432	0.1622	0.1343	0.1482
0.0609	0.0769	0.0969	0.1384	0.1238	0.1498	0.159	0.1244	0.1217
0.0111	0.0839	0.1414	0.1832	0.1568	0.1778	0.1918	0.1733	0.1721
0.0138	0.0575	0.0755	0.111	0.0913	0.1239	0.1475	0.12	0.1403
0.0353	0.0469	0.0688	0.0966	0.1031	0.1253	0.1454	0.1299	0.1503
-0.0027	0.0417	0.0712	0.0849	0.0791	0.0855	0.0933	0.0753	0.0725
-0.0083	0.0008	0.006	0.0138	0.0126	0.0162	0.0207	0.018	0.0201
0.263	0.1165	0.107	0.1054	0.0908	0.1009	0.0942	0.0835	0.06
0.2565	0.0964	0.0702	0.0584	0.0663	0.0786	0.0815	0.0774	0.0661
0.1787	0.0927	0.0866	0.0921	0.0895	0.0986	0.0964	0.0838	0.0863
0.2379	0.1295	0.1329	0.1373	0.1266	0.1327	0.1347	0.1117	0.1043
0.1174	0.0597	0.0583	0.0534	0.0555	0.0591	0.0637	0.0491	0.0489
0.0085	0.0157	0.0253	0.0243	0.0226	0.0208	0.0215	0.0162	0.0154
0	0	0	0	0	0	0	0	0
0	0	0	0	0	0	0	0	0
0	0	0	0	0	0	0	0	0
-0.0002	0.0006	0.0008	0.0017	0.0014	0.0024	0.0027	0.0025	0.003
-0.0004	0.0015	0.0013	0.0028	0.0028	0.0035	0.0042	0.0039	0.0043
0	0	0	0	0	0	0	0	0
0	0	0	0	0	0	0	0	0
0.0002	0.0006	0.0012	0.0011	0.0009	0.0009	0.0009	0.0006	0.0006
0.0006	0.0014	0.0025	0.0025	0.0022	0.002	0.002	0.0015	0.0014
0.0007	0.0019	0.0039	0.0038	0.0036	0.0035	0.0035	0.0028	0.0026
0.1616	0.0785	0.0558	0.0447	0.0556	0.0555	0.0577	0.0556	0.0469
0.0003	0.0015	0.0031	0.0032	0.0029	0.0028	0.0029	0.0022	0.002
0.055	0.0259	0.0214	0.0229	0.0233	0.0258	0.0277	0.022	0.0204
0	0	0	0	0	0	0	0	0
0.9721	0.3825	0.3231	0.3581	0.3496	0.3339	0.3552	0.2676	0.2186
0.5676	0.2341	0.1717	0.1324	0.1437	0.1631	0.158	0.1373	0.1334
0.403	0.2087	0.2027	0.1952	0.1929	0.205	0.2142	0.178	0.1725
0.029	0.075	0.1095	0.1303	0.1139	0.1501	0.1832	0.1602	0.1592
0.046	0.0978	0.1265	0.1565	0.1402	0.1605	0.1826	0.1535	0.1437
0.0284	0.0965	0.1684	0.179	0.1625	0.1672	0.1721	0.14	0.1469
-0.021	0.0336	0.0617	0.0912	0.075	0.0978	0.1114	0.0951	0.094
0.0197	0.0476	0.0854	0.1083	0.1041	0.1079	0.1239	0.1052	0.1171
0.0739	0.0955	0.1268	0.1428	0.1351	0.1469	0.1619	0.1375	0.1325
0.0026	0.0594	0.1043	0.1335	0.1096	0.1327	0.1417	0.1202	0.1169
0.0509	0.0956	0.1493	0.1599	0.1436	0.138	0.1484	0.1136	0.114
0.0693	0.1258	0.221	0.2279	0.1904	0.1933	0.1952	0.151	0.1357

0.0548	0.1422	0.2593	0.2477	0.2174	0.2092	0.1909	0.1396	0.1262
0.1874	0.1739	0.2579	0.2487	0.2274	0.2197	0.2218	0.1845	0.171
0.642	0.3166	0.3013	0.2757	0.2551	0.2976	0.2601	0.2477	0.2308
0.5767	0.3021	0.331	0.289	0.2627	0.2785	0.2706	0.2036	0.1976
0.0364	0.0888	0.183	0.1809	0.151	0.1448	0.1604	0.1185	0.1215
0.0658	0.1247	0.2304	0.2235	0.1963	0.191	0.205	0.1573	0.1489
0.1524	0.1475	0.2122	0.2196	0.2072	0.208	0.2116	0.167	0.1507
0.1221	0.1353	0.2029	0.2026	0.1853	0.189	0.1963	0.1569	0.1438
0.0758	0.0987	0.145	0.1595	0.1474	0.1562	0.1712	0.1397	0.1377
0.1145	0.1139	0.1609	0.1774	0.1577	0.1664	0.18	0.1543	0.1497
0.0264	0.0905	0.1652	0.1863	0.1593	0.1597	0.1594	0.1307	0.1281
0.0639	0.0827	0.0963	0.1259	0.1105	0.139	0.1543	0.1342	0.1363
0.1269	0.0886	0.0978	0.1192	0.1134	0.1298	0.1386	0.1223	0.1219
0.0398	0.096	0.1618	0.1783	0.1707	0.1762	0.1841	0.1558	0.1555
0.0121	0.0695	0.1356	0.1718	0.156	0.1797	0.2045	0.1849	0.1774
0.0236	0.0721	0.0742	0.1152	0.1193	0.1617	0.1863	0.1615	0.1556
0.2397	0.1493	0.153	0.1739	0.1549	0.1808	0.1923	0.165	0.1658
0.0536	0.035	0.0319	0.0432	0.0391	0.0519	0.0675	0.0627	0.0633
0.1786	0.1026	0.1092	0.1111	0.1077	0.1176	0.124	0.1007	0.0941
0.2226	0.1297	0.1178	0.1275	0.1237	0.1373	0.1463	0.1204	0.1102
0.2104	0.1028	0.0933	0.1103	0.1101	0.1199	0.132	0.1163	0.1161
0.4066	0.1911	0.172	0.1473	0.1422	0.1584	0.1484	0.1279	0.1192
0.2821	0.1256	0.0947	0.065	0.073	0.0788	0.0698	0.0535	0.0476
0	0	0	0	0	0	0	0	0
0	0	0	0	0	0	0	0	0
0	0	0	0	0	0	0	0	0
0	0	0	0	0	0	0	0	0
-0.0006	0.0013	0.002	0.0033	0.0032	0.0041	0.005	0.0048	0.0048
-0.0001	0.0012	0.002	0.0026	0.0026	0.0028	0.0032	0.0027	0.003
0	0	0	0	0	0	0	0	0
0	0	0	0	0	0	0	0	0
-0.0001	0.0003	0.0007	0.0011	0.0011	0.0016	0.0018	0.0017	0.0018
0.0002	0.0015	0.0014	0.0023	0.0019	0.0024	0.0027	0.0024	0.0025
-0.0001	0.0016	0.002	0.0027	0.0023	0.0029	0.0038	0.0036	0.0037
0.0002	0.0038	0.0083	0.009	0.0078	0.0074	0.0075	0.0055	0.0055
0	0	0	0	0	0	0	0	0
0.0393	0.0144	0.0099	0.0089	0.0111	0.0104	0.0093	0.0081	0.008
0.0112	0.0266	0.0487	0.052	0.0464	0.0441	0.0453	0.0335	0.0341
0.3078	0.161	0.182	0.1629	0.1504	0.167	0.1779	0.1423	0.1239
0.7844	0.3304	0.2715	0.2371	0.1991	0.2159	0.2142	0.1529	0.1152
0.7857	0.3576	0.2689	0.2446	0.2392	0.2874	0.3164	0.2701	0.2851
0.0826	0.1056	0.1121	0.1618	0.1591	0.191	0.2298	0.1947	0.1977
0.0066	0.0757	0.116	0.1563	0.1426	0.1748	0.1869	0.1657	0.1664
0.0397	0.1031	0.1618	0.1791	0.1583	0.1606	0.1658	0.1399	0.1312

0.0248	0.0689	0.1123	0.1342	0.1211	0.1357	0.1488	0.1178	0.1175
0.0217	0.0608	0.0937	0.1255	0.1197	0.1459	0.159	0.1326	0.1316
-0.0008	0.0414	0.0531	0.0924	0.0762	0.1076	0.1377	0.1261	0.1232
0.0102	0.0679	0.0869	0.111	0.1008	0.1207	0.1357	0.1198	0.1165
0.0218	0.0715	0.1391	0.146	0.1345	0.1417	0.144	0.1158	0.1204
0.0787	0.1232	0.2294	0.2385	0.222	0.2237	0.2257	0.1709	0.1555
0.1885	0.1775	0.281	0.2741	0.2581	0.2633	0.266	0.2095	0.1876
0.0984	0.1129	0.2123	0.2081	0.1993	0.1903	0.2014	0.1486	0.1445
0.5195	0.2291	0.2405	0.2364	0.2202	0.2374	0.2505	0.2094	0.2015
0.858	0.4182	0.3833	0.3417	0.3277	0.3384	0.355	0.3039	0.2765
0.3976	0.2449	0.2993	0.2732	0.2718	0.2806	0.2781	0.2497	0.2335
0.0437	0.1029	0.1877	0.192	0.1822	0.1835	0.1897	0.1503	0.1314
0.0604	0.0976	0.1649	0.1708	0.1531	0.1517	0.1525	0.1285	0.1265
0.1376	0.1228	0.2021	0.2062	0.1899	0.1955	0.192	0.1413	0.1297
0.1015	0.1267	0.2207	0.2255	0.2044	0.1893	0.1941	0.1443	0.1408
0.0115	0.0689	0.1325	0.1439	0.1276	0.1307	0.1395	0.1016	0.102
0.1797	0.1386	0.1708	0.182	0.1672	0.1732	0.1799	0.1518	0.1453
-0.0026	0.0562	0.1342	0.1694	0.1527	0.157	0.1784	0.1373	0.1321
0.0094	0.0614	0.1059	0.1407	0.1231	0.1335	0.1472	0.1264	0.1243
0.1812	0.1237	0.1645	0.1689	0.1563	0.158	0.1534	0.1232	0.1176
0.0152	0.0972	0.1867	0.2125	0.1771	0.1972	0.2115	0.1735	0.1599
0.0308	0.0775	0.1024	0.1264	0.1107	0.1457	0.1739	0.1612	0.17
0.0867	0.0781	0.0913	0.1081	0.0988	0.1135	0.1182	0.0934	0.0938
0.048	0.0388	0.05	0.0537	0.0488	0.0518	0.0529	0.0436	0.0419
0.1621	0.1023	0.1197	0.1147	0.1072	0.1149	0.1162	0.0974	0.0888
0.2311	0.1373	0.1337	0.1428	0.1377	0.152	0.1576	0.1376	0.1329
0.3136	0.1517	0.1257	0.1313	0.1317	0.1504	0.1559	0.1338	0.125
0.2199	0.099	0.0903	0.076	0.0759	0.09	0.0783	0.0722	0.0755
0	0	0	0	0	0	0	0	0
0.0396	0.0222	0.0239	0.0223	0.0199	0.0205	0.0215	0.0172	0.0148
0	0	0	0	0	0	0	0	0
0	0	0	0	0	0	0	0	0
0	0	0	0	0	0	0	0	0
-0.0013	0.0003	0.0009	0.002	0.0018	0.0025	0.0028	0.0026	0.0024
0.0005	0.0011	0.0022	0.0021	0.002	0.0018	0.0019	0.0014	0.0014
0.0005	0.0012	0.0023	0.0024	0.0022	0.0021	0.002	0.0017	0.0016
0	0	0	0	0	0	0	0	0
0.0001	0.0015	0.0017	0.0026	0.0024	0.0029	0.0037	0.0035	0.0037
-0.0002	0.0003	0.0006	0.001	0.001	0.0015	0.0016	0.0014	0.0014
-0.0001	0.0002	0.0003	0.0006	0.0006	0.0008	0.001	0.0009	0.0009
0	0.0003	0.0004	0.0006	0.0005	0.0008	0.0009	0.0009	0.001
-0.0002	0.0009	0.0017	0.0025	0.0023	0.0029	0.0032	0.0029	0.0029
0.0018	0.0064	0.0133	0.014	0.0119	0.0121	0.0125	0.0103	0.0107
0.1664	0.0607	0.0417	0.0481	0.0506	0.0608	0.0609	0.0553	0.0586

-0.0071	0.0262	0.0268	0.0463	0.0436	0.0592	0.0662	0.0571	0.0624
0.2131	0.149	0.1991	0.1899	0.181	0.1761	0.1723	0.1338	0.1194
0.1284	0.1194	0.1724	0.1828	0.1648	0.1768	0.1832	0.1535	0.1435
0.0121	0.0644	0.0937	0.1207	0.114	0.119	0.1341	0.1171	0.1161
0.1372	0.1492	0.2183	0.2295	0.1993	0.2053	0.2089	0.1728	0.1698
0.1369	0.1547	0.2526	0.2519	0.234	0.2283	0.2271	0.1856	0.1807
0.0589	0.0819	0.1448	0.1467	0.1343	0.1383	0.1467	0.1196	0.1131
0.0066	0.0725	0.0911	0.1345	0.1402	0.1514	0.1619	0.1348	0.1387
-0.022	0.0305	0.034	0.0727	0.0775	0.1077	0.1357	0.1096	0.1092
0.0009	0.0439	0.0695	0.0993	0.0825	0.0945	0.117	0.0962	0.1051
0.029	0.0651	0.1072	0.1276	0.1206	0.1338	0.1452	0.1141	0.1088
0.0542	0.0847	0.1424	0.1533	0.1319	0.1364	0.1458	0.1223	0.115
0.0323	0.1006	0.1696	0.1766	0.161	0.1574	0.168	0.1297	0.1342
0.0418	0.0797	0.149	0.1536	0.1383	0.1365	0.1536	0.1232	0.1176
0.171	0.1445	0.2134	0.2149	0.1949	0.1909	0.1972	0.1575	0.1427
0.3817	0.2076	0.2349	0.2334	0.212	0.2223	0.1987	0.1555	0.1517
0.4019	0.1715	0.2076	0.2086	0.1983	0.2064	0.2053	0.1572	0.1224
0.2657	0.1945	0.2498	0.2471	0.2268	0.2309	0.2304	0.1787	0.167
0.0607	0.1151	0.2155	0.2284	0.2105	0.2023	0.2021	0.1683	0.1652
0.0888	0.1044	0.1772	0.1827	0.1708	0.1765	0.1769	0.1378	0.1217
0.2438	0.1859	0.2554	0.2546	0.2289	0.2318	0.2255	0.172	0.1508
0.0203	0.0835	0.161	0.1867	0.1762	0.1827	0.1901	0.151	0.1436
0.0958	0.0991	0.1414	0.1606	0.1454	0.1558	0.1645	0.1403	0.1397
0.0057	0.0553	0.1177	0.1449	0.1261	0.1323	0.1402	0.109	0.1055
-0.0012	0.0629	0.1039	0.1324	0.1199	0.1445	0.1493	0.1234	0.1341
0.1108	0.1223	0.189	0.1933	0.18	0.1762	0.1822	0.1479	0.1374
0.0339	0.1097	0.2157	0.2292	0.2059	0.2125	0.2099	0.1714	0.1688
0.0965	0.0795	0.1007	0.1252	0.112	0.136	0.1623	0.1428	0.1415
-0.0042	0.0578	0.0738	0.1022	0.0969	0.1271	0.1545	0.1535	0.1591
-0.0007	0.023	0.0372	0.0572	0.0464	0.0584	0.0643	0.0505	0.046
0.1123	0.0589	0.0581	0.0583	0.0547	0.0571	0.055	0.0436	0.0369
0.2389	0.1167	0.1132	0.096	0.1001	0.1123	0.1099	0.0786	0.0714
0.3743	0.1842	0.1529	0.1423	0.1409	0.1563	0.1623	0.1422	0.126
0.2475	0.109	0.0843	0.0733	0.0755	0.0688	0.0811	0.0673	0.0669
0.01	0.0106	0.0162	0.0165	0.0149	0.0147	0.0156	0.0122	0.0115
0.0142	0.007	0.0072	0.0073	0.0069	0.0078	0.0078	0.007	0.007
0	0	0	0	0	0	0	0	0
0	0	0	0	0	0	0	0	0
0	0	0	0	0	0	0	0	0
0	0	0	0	0	0	0	0	0
0.001	0.0023	0.0037	0.0038	0.0036	0.0035	0.0034	0.0029	0.0029
0.0005	0.002	0.0032	0.0035	0.0031	0.0032	0.0035	0.0028	0.0029
-0.0006	0.001	0.0018	0.0029	0.0024	0.0033	0.0042	0.0039	0.0041
0	0	0	0	0	0	0	0	0

0	0	0	0	0	0	0	0	0
0.0005	0.0012	0.0023	0.0023	0.002	0.0018	0.0019	0.0013	0.0013
0.0012	0.0029	0.0062	0.0062	0.0058	0.0057	0.0057	0.0044	0.004
0.061	0.0207	0.0145	0.0125	0.0118	0.0158	0.0164	0.0122	0.0094
0.1098	0.0458	0.039	0.0303	0.0274	0.0324	0.0373	0.0318	0.0282
0.0294	0.025	0.0295	0.0359	0.0343	0.0383	0.0439	0.0352	0.0348
0.1149	0.0644	0.0552	0.0653	0.0668	0.0782	0.0877	0.0787	0.0774
0.0391	0.0616	0.0985	0.1072	0.0993	0.098	0.0963	0.077	0.0784
0.0113	0.0507	0.0772	0.1029	0.0818	0.0918	0.1102	0.0945	0.0937
0.0064	0.0641	0.0974	0.1251	0.1123	0.1373	0.1529	0.1275	0.1427
0.0986	0.1118	0.1502	0.1645	0.1448	0.1621	0.1653	0.1427	0.1434
0.0944	0.1278	0.1981	0.2028	0.1906	0.189	0.1869	0.1537	0.1375
0.0279	0.0813	0.1149	0.1365	0.1291	0.1453	0.151	0.1332	0.1294
-0.0147	0.0417	0.0626	0.0975	0.0759	0.1125	0.1311	0.135	0.1533
-0.0633	0.0177	0.0591	0.1175	0.118	0.1443	0.1654	0.1442	0.1488
0.0195	0.0577	0.0862	0.1281	0.1032	0.1301	0.1426	0.1239	0.1382
-0.0074	0.0432	0.0832	0.1199	0.1102	0.1222	0.1345	0.115	0.1171
0.0314	0.0721	0.0871	0.1114	0.1036	0.1092	0.1163	0.1009	0.1114
0.0569	0.0777	0.0925	0.1326	0.1096	0.1324	0.1594	0.1309	0.1307
0.0277	0.064	0.0828	0.1199	0.1061	0.1357	0.1588	0.1385	0.1352
0.0405	0.0954	0.1258	0.1623	0.1406	0.1524	0.1648	0.1417	0.1463
0.4012	0.2091	0.1871	0.1676	0.1612	0.1693	0.163	0.1363	0.1329
0.6895	0.3323	0.2924	0.304	0.3229	0.3222	0.3104	0.2505	0.2159
0.2233	0.1526	0.1998	0.1956	0.1809	0.1899	0.189	0.1477	0.1421
-0.0076	0.0633	0.1666	0.177	0.169	0.1666	0.1775	0.1387	0.1358
0.1508	0.153	0.2412	0.2368	0.2234	0.2155	0.2152	0.189	0.1753
0.1542	0.134	0.1781	0.1888	0.1731	0.1742	0.1905	0.1543	0.1473
0.0199	0.0818	0.1396	0.1511	0.1327	0.1339	0.1527	0.1254	0.1304
0.0197	0.0717	0.1247	0.1392	0.1244	0.1286	0.1386	0.1073	0.1141
0.1993	0.1568	0.1964	0.2036	0.1902	0.1977	0.2033	0.1696	0.1608
0.2821	0.1702	0.2341	0.2257	0.2005	0.214	0.2137	0.1699	0.1718
0.0686	0.1366	0.2401	0.2321	0.1818	0.1767	0.174	0.1329	0.1029
0.0546	0.1169	0.2051	0.199	0.1824	0.173	0.1763	0.1487	0.1493
0.0291	0.0963	0.1426	0.1733	0.1547	0.1753	0.2137	0.1872	0.1827
0.082	0.0848	0.1074	0.1395	0.1369	0.1674	0.1928	0.1596	0.1609
0.2319	0.1433	0.1529	0.1619	0.1525	0.1734	0.1778	0.1514	0.1441
0.7271	0.32	0.249	0.233	0.2656	0.2781	0.2581	0.2279	0.2082
0.6468	0.2842	0.2638	0.2196	0.2004	0.2202	0.2148	0.1789	0.1485
0.1753	0.1101	0.1298	0.1312	0.1234	0.1319	0.1288	0.0996	0.0892
0.0804	0.0458	0.0491	0.0478	0.0465	0.0513	0.0509	0.0422	0.0419
0.0712	0.0374	0.0418	0.0376	0.0318	0.0335	0.0352	0.0291	0.0279
0	0	0	0	0	0	0	0	0
0	0	0	0	0	0	0	0	0
0	0	0	0	0	0	0	0	0

0	0	0	0	0	0	0	0	0
0.0003	0.0008	0.0013	0.0012	0.0011	0.001	0.001	0.0008	0.0008
0.0005	0.0012	0.0024	0.0024	0.0021	0.0021	0.0022	0.0016	0.0014
-0.0003	0.0013	0.0015	0.0026	0.0024	0.0028	0.0034	0.0029	0.0034
0.0006	0.0013	0.0017	0.003	0.0028	0.0037	0.0046	0.004	0.0038
0.0003	0.0006	0.0012	0.0012	0.0011	0.001	0.0011	0.0009	0.0008
0.0013	0.0052	0.0108	0.0118	0.0113	0.0122	0.0133	0.0109	0.0101
-0.0007	0.0147	0.0276	0.0361	0.0326	0.0338	0.0415	0.0332	0.0344
0.0359	0.0173	0.0155	0.0159	0.016	0.0176	0.0172	0.0165	0.0144
0.0005	0.0008	0.0013	0.0014	0.0012	0.0011	0.0012	0.0009	0.0008
-0.0001	0.0013	0.0014	0.0024	0.0018	0.0024	0.003	0.0026	0.0028
0.0032	0.0142	0.0178	0.0232	0.0195	0.0232	0.027	0.0246	0.025
0.2399	0.1429	0.1491	0.16	0.152	0.1716	0.1751	0.147	0.1388
-0.0006	0.0488	0.0775	0.1018	0.105	0.1086	0.1201	0.1003	0.0964
0.0421	0.0538	0.0742	0.1021	0.0971	0.1349	0.1445	0.122	0.1307
0.1995	0.1465	0.123	0.1607	0.153	0.1855	0.2277	0.1939	0.2092
0.0581	0.0863	0.0947	0.1352	0.1262	0.1496	0.1564	0.1496	0.1529
0.0239	0.0752	0.1135	0.1519	0.1356	0.1402	0.1643	0.139	0.1379
0.0042	0.0745	0.1138	0.186	0.1726	0.1815	0.1956	0.1769	0.1829
0.0044	0.0538	0.0716	0.1128	0.1148	0.1226	0.1487	0.1224	0.1435
-0.0018	0.046	0.0738	0.0968	0.0901	0.1111	0.1204	0.1053	0.1156
0.0032	0.0586	0.0787	0.1121	0.1028	0.142	0.1591	0.1379	0.1452
0.0352	0.0989	0.1527	0.1667	0.1577	0.1685	0.1811	0.1484	0.1531
0.0509	0.104	0.1784	0.1814	0.1631	0.1668	0.1612	0.1274	0.1283
0.0419	0.0972	0.1501	0.164	0.1438	0.1578	0.1643	0.1238	0.1282
0.0487	0.0907	0.1375	0.1479	0.1344	0.1319	0.1475	0.1309	0.1263
0.0116	0.0739	0.1333	0.1633	0.1523	0.1613	0.1768	0.1496	0.1464
0.0355	0.0816	0.1292	0.1434	0.1304	0.137	0.1557	0.1316	0.1241
0.2084	0.1749	0.2397	0.2468	0.2249	0.2224	0.2256	0.1818	0.1705
0.1252	0.115	0.1677	0.1756	0.1661	0.171	0.1769	0.1427	0.1328
0.0433	0.0995	0.1739	0.1677	0.152	0.1503	0.1539	0.1235	0.1266
0.0918	0.1291	0.2278	0.2335	0.2089	0.2124	0.2144	0.1656	0.1525
0.0001	0.0583	0.1198	0.1403	0.1306	0.1365	0.1534	0.1276	0.1337
0.111	0.1079	0.1484	0.1655	0.1457	0.1611	0.1748	0.1484	0.1452
-0.0048	0.0513	0.0957	0.1131	0.0893	0.106	0.1336	0.1193	0.1218
0.2584	0.1395	0.1348	0.149	0.1434	0.168	0.1851	0.1483	0.1515
0.7448	0.3594	0.3295	0.2595	0.2211	0.2545	0.214	0.1783	0.1622
0.4309	0.2657	0.3196	0.3053	0.2751	0.2747	0.2495	0.2333	0.2148
0.0583	0.1585	0.2548	0.2682	0.2338	0.2273	0.2349	0.1786	0.1722
0.0191	0.0918	0.1842	0.1977	0.177	0.1763	0.1813	0.1333	0.1271
-0.0083	0.0571	0.1128	0.1349	0.1169	0.1249	0.1339	0.1027	0.1127
0.0247	0.0807	0.1137	0.1283	0.1144	0.1256	0.1449	0.1177	0.1228
0.4988	0.2459	0.2185	0.2142	0.1919	0.2341	0.2513	0.2049	0.1998
0.6791	0.326	0.2557	0.2467	0.2428	0.2698	0.2691	0.2363	0.2554

0.3576	0.1572	0.1369	0.1306	0.1336	0.1542	0.1472	0.123	0.1104
0.3679	0.1504	0.1095	0.0972	0.0933	0.0966	0.1088	0.1021	0.0796
0.2618	0.1211	0.0992	0.0838	0.0887	0.11	0.1027	0.0951	0.0833
0.0003	0.0064	0.0064	0.0096	0.0078	0.0094	0.0133	0.0125	0.0127
0	0	0	0	0	0	0	0	0
0	0	0	0	0	0	0	0	0
0.0008	0.0017	0.0021	0.0029	0.0024	0.003	0.0034	0.0032	0.0036
0.0003	0.0006	0.0012	0.0012	0.0011	0.0011	0.0011	0.0009	0.0008
0	0	0	0	0	0	0	0	0
0.0003	0.0011	0.0012	0.0018	0.0016	0.0019	0.0022	0.002	0.0021
0	0	0	0	0	0	0	0	0
0.0013	0.0032	0.0063	0.0065	0.0062	0.0061	0.0063	0.005	0.0047
0.0041	0.0167	0.0277	0.0328	0.0307	0.0327	0.0346	0.0295	0.0306
0.02	0.0488	0.0651	0.0778	0.0742	0.0745	0.0837	0.0665	0.0724
0.0408	0.0211	0.018	0.0189	0.0177	0.0189	0.0194	0.0186	0.0161
0.0358	0.0137	0.0113	0.0097	0.0108	0.0116	0.0129	0.0128	0.0131
0.1313	0.0757	0.078	0.0858	0.0818	0.0957	0.1028	0.0891	0.0898
-0.0055	0.0307	0.0464	0.0728	0.0687	0.0829	0.0969	0.0828	0.0857
0.0197	0.0797	0.1038	0.1529	0.1372	0.1665	0.178	0.1466	0.1437
0.0006	0.0393	0.0451	0.102	0.0792	0.0914	0.1277	0.0982	0.0998
0.0912	0.0889	0.1082	0.1487	0.143	0.1991	0.2379	0.1891	0.2064
-0.0732	-0.0081	0.0154	0.0686	0.0608	0.0912	0.1294	0.1115	0.1226
0.0334	0.0668	0.1012	0.1412	0.1273	0.1523	0.1715	0.1475	0.1547
0.0287	0.0747	0.0924	0.1206	0.1193	0.136	0.1649	0.1464	0.155
0.0157	0.0713	0.0983	0.1233	0.1057	0.1259	0.1315	0.1145	0.1265
0.0134	0.0623	0.0575	0.105	0.0886	0.127	0.163	0.1397	0.1677
0.0631	0.1155	0.1265	0.1554	0.1482	0.1618	0.187	0.1513	0.1572
0.0286	0.0786	0.1205	0.1346	0.1157	0.1263	0.1512	0.1321	0.125
0.0646	0.1227	0.2163	0.2254	0.2088	0.21	0.2122	0.1711	0.1688
0.144	0.1651	0.2652	0.2592	0.2565	0.2481	0.2424	0.1941	0.1732
0.1746	0.1645	0.2582	0.2505	0.2301	0.2218	0.2241	0.1756	0.168
0.0407	0.1006	0.2095	0.221	0.19	0.1911	0.1992	0.1551	0.1428
0.0551	0.1161	0.2252	0.2311	0.2	0.2004	0.2138	0.1677	0.1491
0.0793	0.1059	0.2061	0.2061	0.1881	0.1892	0.1841	0.143	0.1343
0.0171	0.0786	0.1518	0.152	0.1365	0.1348	0.1478	0.103	0.0953
0.0273	0.089	0.1545	0.1659	0.1484	0.1538	0.1589	0.1321	0.1356
0.0752	0.1327	0.2322	0.2392	0.2122	0.2155	0.2125	0.1582	0.1446
0.0206	0.078	0.1485	0.1511	0.1499	0.1499	0.1475	0.1116	0.107
0.0207	0.0813	0.137	0.1487	0.1317	0.1394	0.1502	0.1267	0.132
0.1872	0.1327	0.143	0.1691	0.1643	0.1837	0.1992	0.17	0.1694
0.1093	0.0944	0.1134	0.1464	0.1327	0.155	0.173	0.141	0.1385
0.0512	0.0536	0.0724	0.0798	0.0735	0.0794	0.0851	0.0687	0.072
0.7913	0.3719	0.2947	0.2676	0.2876	0.311	0.3314	0.2997	0.2657
0.8969	0.4653	0.4277	0.4012	0.4183	0.4344	0.4511	0.4071	0.3668

0.1256	0.1372	0.2296	0.2199	0.197	0.1953	0.1967	0.1479	0.1333
0.2595	0.1983	0.2658	0.2491	0.2294	0.2406	0.2473	0.2009	0.187
0.1405	0.139	0.1945	0.2039	0.1848	0.1879	0.1941	0.156	0.1531
0.004	0.0877	0.1285	0.1635	0.1563	0.1821	0.1933	0.1728	0.1792
0.0477	0.0602	0.0796	0.1063	0.0967	0.119	0.1335	0.116	0.1213
0.1639	0.104	0.1097	0.1179	0.1067	0.121	0.131	0.1133	0.1147
0.177	0.0808	0.0735	0.0755	0.0738	0.0831	0.0794	0.0652	0.0669
0.1408	0.0619	0.05	0.0463	0.05	0.0556	0.0597	0.0605	0.0536
0.0074	0.0037	0.0036	0.003	0.0024	0.0027	0.0024	0.0022	0.0016
0.009	0.0072	0.0104	0.0101	0.0089	0.0094	0.0092	0.0074	0.0072
0	0	0	0	0	0	0	0	0
0	0	0	0	0	0	0	0	0
0.0009	0.0041	0.0063	0.0082	0.0074	0.0078	0.0082	0.0067	0.0071
0.0002	0.0006	0.0011	0.0011	0.001	0.001	0.0009	0.0008	0.0007
0.0072	0.0029	0.0022	0.0023	0.002	0.0018	0.0013	0.0011	0.0012
0.0145	0.0078	0.0072	0.0086	0.0084	0.0092	0.0097	0.0085	0.0085
0.0156	0.0117	0.0145	0.0153	0.014	0.0148	0.0156	0.0131	0.0126
0.0545	0.0394	0.0474	0.0504	0.0477	0.0529	0.0564	0.0476	0.0448
0.0394	0.0348	0.044	0.0497	0.0447	0.0491	0.0539	0.0446	0.0449
0.0008	0.0076	0.0123	0.018	0.0152	0.0176	0.0205	0.0166	0.0168
0.0646	0.0426	0.0395	0.0465	0.0468	0.0547	0.0609	0.0514	0.0533
0.0176	0.0187	0.022	0.0317	0.0289	0.0367	0.04	0.0366	0.0394
0.142	0.092	0.1154	0.1141	0.1057	0.1094	0.1152	0.0928	0.0876
0.0248	0.0885	0.1342	0.1656	0.1465	0.1549	0.1649	0.1271	0.1322
0.1579	0.1241	0.1465	0.1591	0.1469	0.1651	0.1748	0.1442	0.1351
0.0593	0.0752	0.0945	0.1226	0.1099	0.139	0.16	0.1304	0.1403
-0.0023	0.0586	0.0993	0.1337	0.1238	0.1334	0.1507	0.128	0.1287
-0.0069	0.0535	0.0925	0.1258	0.1157	0.1408	0.15	0.1325	0.129
-0.0051	0.0464	0.1113	0.1368	0.1317	0.1363	0.1466	0.1366	0.1287
0.1937	0.1588	0.2372	0.2387	0.2202	0.2063	0.2092	0.1615	0.1497
0.1054	0.136	0.221	0.2134	0.1992	0.1996	0.1912	0.1507	0.1404
0.0449	0.0763	0.1159	0.1395	0.1353	0.1423	0.1511	0.1215	0.1172
0.0481	0.0705	0.0988	0.1281	0.132	0.1402	0.1555	0.1348	0.1313
-0.0009	0.0795	0.1423	0.1677	0.1462	0.1493	0.163	0.1355	0.134
0.0841	0.1169	0.2147	0.2137	0.1902	0.1853	0.1903	0.1535	0.1518
0.1547	0.1422	0.2178	0.209	0.1932	0.1928	0.1894	0.1456	0.1238
0.2624	0.2042	0.2785	0.2779	0.2605	0.259	0.2443	0.1867	0.1744
0.0205	0.1023	0.211	0.2152	0.1934	0.1879	0.1902	0.1437	0.1219
0.1557	0.1818	0.2743	0.2673	0.2424	0.2301	0.2275	0.1806	0.1771
0.1838	0.1404	0.2008	0.1997	0.1871	0.193	0.1916	0.1524	0.1435
0.7326	0.4099	0.3843	0.3604	0.3824	0.3481	0.3263	0.2699	0.2352
0.1441	0.1613	0.2402	0.2549	0.2361	0.2313	0.2291	0.1919	0.1823
0.0194	0.0801	0.1389	0.1556	0.1371	0.1459	0.1561	0.1144	0.1169
0.0994	0.0968	0.1286	0.1497	0.1378	0.159	0.1693	0.1427	0.1501

0.0649	0.1171	0.1899	0.1974	0.1757	0.1688	0.1712	0.1324	0.1242
0.0326	0.0838	0.1503	0.1737	0.1601	0.1694	0.1866	0.1519	0.1472
0.1599	0.1265	0.1645	0.1803	0.1688	0.1826	0.1938	0.1573	0.1444
0.1318	0.1054	0.1427	0.1414	0.1327	0.1352	0.1322	0.1066	0.1039
0.2808	0.1822	0.236	0.233	0.2213	0.2293	0.2279	0.1806	0.1568
0.4961	0.2743	0.3063	0.2842	0.2976	0.3003	0.2813	0.2454	0.2446
0.1788	0.1324	0.2006	0.1964	0.1742	0.1823	0.1782	0.1425	0.1342
0.1166	0.1571	0.2586	0.2658	0.2379	0.2335	0.2365	0.1934	0.1903
0.1227	0.1512	0.238	0.2403	0.2259	0.2282	0.2292	0.1841	0.1836
0.0971	0.0887	0.1233	0.1384	0.1296	0.1427	0.1525	0.1276	0.1227
0.2127	0.1479	0.1724	0.2062	0.195	0.2253	0.2457	0.2067	0.2001
-0.0258	0.0373	0.0536	0.0874	0.0644	0.0775	0.1158	0.095	0.0906
0.0079	0.0348	0.0633	0.0701	0.0674	0.0676	0.0709	0.0579	0.0514
0.4688	0.2136	0.1627	0.1536	0.1531	0.1734	0.1952	0.163	0.1442
0.1317	0.0566	0.041	0.0351	0.0398	0.0389	0.0405	0.0361	0.0288
0.0483	0.0218	0.0185	0.0163	0.0152	0.0182	0.0212	0.0178	0.0163
0	0	0	0	0	0	0	0	0
0	0	0	0	0	0	0	0	0
0.0443	0.0184	0.0099	0.0079	0.0083	0.0074	0.0076	0.0056	0.006
0.0323	0.015	0.0133	0.0116	0.0107	0.0109	0.0102	0.0097	0.0086
-0.0007	0.0012	0.0015	0.0028	0.0025	0.0031	0.0041	0.0041	0.0041
0	0	0	0	0	0	0	0	0
0.001	0.0063	0.0119	0.0117	0.0103	0.0092	0.0104	0.0079	0.0076
0.1664	0.0894	0.091	0.0886	0.0862	0.0926	0.0911	0.0757	0.0735
0.199	0.0987	0.0842	0.0905	0.0915	0.1006	0.1127	0.0982	0.0947
0.0974	0.0449	0.0413	0.037	0.037	0.0365	0.0352	0.0341	0.0283
0.0041	0.0296	0.0628	0.0722	0.0649	0.0681	0.0741	0.0552	0.0543
0.0009	0.0286	0.0624	0.0686	0.0582	0.0608	0.0639	0.0523	0.0515
0.0443	0.0675	0.0975	0.1182	0.1066	0.1188	0.1305	0.1181	0.1166
0.1211	0.0868	0.0859	0.1062	0.1	0.1233	0.1385	0.1246	0.1195
0.1612	0.1368	0.1777	0.1908	0.1759	0.1819	0.1904	0.1584	0.1526
0.0298	0.0974	0.1922	0.21	0.1837	0.1808	0.1924	0.1521	0.1481
0.1327	0.139	0.2292	0.2237	0.1996	0.1941	0.1937	0.1532	0.1392
0.0743	0.1009	0.16	0.1753	0.1592	0.1638	0.1729	0.1445	0.1347
0.3633	0.2001	0.2356	0.2141	0.2022	0.2069	0.2077	0.1595	0.145
0.5058	0.3018	0.3422	0.3451	0.331	0.3317	0.3428	0.2868	0.2729
0.1554	0.1779	0.2752	0.2784	0.2455	0.2457	0.2471	0.1988	0.197
0.0338	0.1043	0.2185	0.2157	0.1932	0.1887	0.1793	0.1374	0.132
0.0575	0.1053	0.185	0.1907	0.1718	0.1729	0.1624	0.1313	0.1262
0.0064	0.0576	0.1084	0.1329	0.1219	0.1298	0.1302	0.1133	0.0991
0.033	0.0819	0.1511	0.1739	0.1571	0.1508	0.1538	0.1233	0.1253
0.1243	0.1469	0.2395	0.2397	0.2227	0.2194	0.2229	0.174	0.1738
0.0365	0.1124	0.2122	0.2245	0.2018	0.1985	0.2003	0.1544	0.1464
0.0484	0.1056	0.185	0.1995	0.1725	0.1786	0.1768	0.1446	0.1341

0.2256	0.1723	0.234	0.2216	0.211	0.2122	0.2033	0.164	0.1508
0.0381	0.0772	0.1617	0.1612	0.1521	0.1493	0.1503	0.116	0.1036
0.1558	0.1362	0.2217	0.2364	0.2133	0.2161	0.2311	0.1915	0.1824
0.0389	0.1012	0.1812	0.204	0.1882	0.1798	0.1867	0.146	0.1483
0.1014	0.0879	0.1121	0.1296	0.1162	0.1294	0.1419	0.1185	0.1147
0.2671	0.1529	0.1664	0.1699	0.1591	0.1755	0.1808	0.1606	0.1496
0.187	0.1432	0.2179	0.2169	0.2091	0.203	0.1936	0.1491	0.1342
0.0499	0.1164	0.1972	0.2019	0.1772	0.175	0.1975	0.1498	0.1429
0.1679	0.1207	0.1406	0.1564	0.146	0.161	0.1727	0.1434	0.1421
0.1994	0.1302	0.1463	0.1486	0.1378	0.1459	0.1475	0.1241	0.1223
0.0799	0.1096	0.202	0.2091	0.1897	0.1921	0.1918	0.1409	0.1271
0.2614	0.1997	0.268	0.2592	0.2526	0.2518	0.2443	0.1997	0.184
0.2394	0.18	0.2451	0.2551	0.2379	0.2393	0.2352	0.1889	0.1905
0.0239	0.0667	0.1607	0.1767	0.1542	0.1575	0.1593	0.131	0.1184
0.0544	0.1184	0.2155	0.2209	0.2074	0.2022	0.1995	0.1505	0.1424
0.4551	0.2359	0.2495	0.2363	0.2226	0.2384	0.2413	0.2054	0.1807
0.4587	0.2294	0.234	0.201	0.1826	0.2195	0.2162	0.1807	0.1724
0.2274	0.1567	0.167	0.1809	0.1609	0.1856	0.1982	0.1727	0.1722
0.021	0.0718	0.1269	0.1409	0.1358	0.1379	0.1425	0.1169	0.1016
0.3145	0.1577	0.1496	0.1471	0.1538	0.1789	0.1684	0.1399	0.1263
0.1987	0.0916	0.0731	0.0674	0.0676	0.0787	0.078	0.067	0.0603
0.0013	0.0031	0.0058	0.0061	0.0057	0.0054	0.0052	0.0044	0.0044
0	0	0	0	0	0	0	0	0
0	0	0	0	0	0	0	0	0
0	0	0	0	0	0	0	0	0
0.0005	0.0019	0.0028	0.0033	0.0029	0.0034	0.0039	0.0031	0.0029
0.0078	0.0054	0.0059	0.0066	0.0064	0.0071	0.0075	0.0064	0.0065
0.0203	0.0085	0.0074	0.0051	0.0045	0.0053	0.0051	0.0045	0.0043
0.0086	0.0069	0.0103	0.01	0.0092	0.0094	0.0091	0.0073	0.0064
0.0251	0.0211	0.0273	0.0296	0.0269	0.0287	0.0296	0.0249	0.024
0.0459	0.0306	0.0336	0.038	0.0352	0.0393	0.0435	0.0368	0.0351
0.0084	0.0055	0.0065	0.0063	0.0058	0.0057	0.0059	0.0044	0.0044
0.0974	0.068	0.0728	0.0896	0.0799	0.0927	0.1019	0.0888	0.0899
0.0105	0.022	0.0254	0.0397	0.037	0.039	0.0406	0.0327	0.0331
0.0473	0.0474	0.0535	0.0698	0.0646	0.0764	0.0886	0.0801	0.0768
0.0825	0.1007	0.1143	0.1482	0.1369	0.1485	0.1659	0.1381	0.1363
0.2023	0.1781	0.2389	0.2464	0.2285	0.2381	0.2495	0.2036	0.1906
0.0334	0.0965	0.1728	0.174	0.1633	0.1537	0.1617	0.1225	0.1113
0.0993	0.1324	0.2073	0.2141	0.1885	0.1948	0.1931	0.1541	0.1441
0.0521	0.1322	0.2364	0.2479	0.2275	0.2182	0.2203	0.1663	0.1324
0.2505	0.1825	0.2378	0.2283	0.2135	0.2215	0.2216	0.1707	0.1579
0.322	0.2266	0.282	0.278	0.2563	0.2555	0.264	0.208	0.1953
0.0576	0.1084	0.2026	0.2219	0.1991	0.1952	0.192	0.142	0.128
0.0754	0.1492	0.2691	0.2712	0.2504	0.2267	0.2347	0.1774	0.166

0.1501	0.1628	0.2611	0.2576	0.2274	0.2203	0.2239	0.1816	0.1763
0.0606	0.0851	0.1367	0.1502	0.1377	0.1513	0.152	0.1266	0.1235
0.0217	0.0763	0.1154	0.1434	0.1254	0.1344	0.1438	0.1216	0.128
0.043	0.0984	0.1683	0.1776	0.1591	0.1604	0.1644	0.1304	0.1306
0.1155	0.125	0.2104	0.2094	0.1908	0.2009	0.2027	0.1612	0.158
0.0138	0.0791	0.1491	0.1569	0.14	0.1466	0.157	0.1256	0.1196
0.0213	0.1006	0.2031	0.2113	0.182	0.1791	0.1908	0.1519	0.1391
0.1251	0.1631	0.2739	0.2868	0.2576	0.2496	0.247	0.1916	0.1757
0.1058	0.1369	0.2293	0.2167	0.1911	0.1875	0.1953	0.1443	0.1334
-0.0038	0.0585	0.1083	0.1304	0.107	0.1218	0.1363	0.0985	0.0989
0.1671	0.117	0.125	0.1579	0.1487	0.1747	0.185	0.1583	0.1512
0.422	0.2408	0.2584	0.2395	0.2215	0.2262	0.2301	0.1966	0.1884
0.1308	0.136	0.1748	0.199	0.1834	0.1959	0.2064	0.1732	0.1771
0.2038	0.1423	0.1465	0.1647	0.1541	0.1755	0.1868	0.1568	0.1571
0.0459	0.07	0.0766	0.1089	0.0917	0.114	0.1363	0.1173	0.1176
0.0853	0.1077	0.1696	0.1767	0.1612	0.1686	0.1822	0.1537	0.1423
0.242	0.166	0.2378	0.2329	0.2061	0.1915	0.1771	0.1407	0.1327
0.1873	0.1582	0.2357	0.2278	0.2027	0.2008	0.2042	0.1638	0.1537
0.0256	0.0786	0.1884	0.2002	0.1803	0.1716	0.1714	0.1262	0.1286
0.0386	0.094	0.1607	0.1709	0.1573	0.1496	0.156	0.1218	0.125
0.0132	0.0718	0.159	0.1633	0.1504	0.1525	0.1597	0.1176	0.1099
0.1486	0.1527	0.2509	0.2512	0.227	0.2085	0.2042	0.1594	0.1443
0.1178	0.1175	0.1778	0.1784	0.1615	0.1619	0.1681	0.1341	0.13
0.6194	0.2442	0.2058	0.1976	0.2055	0.1915	0.2176	0.1747	0.1687
0.4533	0.2328	0.2036	0.2038	0.2048	0.2313	0.2235	0.1884	0.1983
0.0019	0.0055	0.0114	0.0115	0.0102	0.0105	0.01	0.0077	0.0071
0.0027	0.0072	0.0118	0.0126	0.0108	0.0118	0.0126	0.0106	0.0105
0.0502	0.0216	0.017	0.0181	0.0189	0.0214	0.0226	0.018	0.0161
0	0	0	0	0	0	0	0	0
0	0	0	0	0	0	0	0	0
0.0436	0.0166	0.0104	0.0099	0.0092	0.0132	0.014	0.0136	0.0117
0.0006	0.0015	0.0027	0.0027	0.0024	0.0023	0.0023	0.0018	0.0016
0.0004	0.0023	0.0035	0.0046	0.0043	0.0046	0.0049	0.004	0.0042
0.0147	0.0078	0.0067	0.0078	0.0074	0.0086	0.0095	0.0083	0.0076
-0.0001	0.0002	0.0003	0.0006	0.0006	0.0007	0.0007	0.0007	0.0007
0.0018	0.0085	0.0103	0.0139	0.0134	0.0158	0.0182	0.0167	0.0184
0.2524	0.1176	0.0954	0.099	0.0931	0.1036	0.1084	0.0929	0.0995
0.5441	0.2538	0.1961	0.2036	0.1987	0.2187	0.2429	0.1896	0.1722
0.0393	0.0316	0.0306	0.0451	0.045	0.0557	0.0646	0.0567	0.0595
0.0007	0.0238	0.0349	0.0458	0.037	0.0441	0.0553	0.0433	0.0475
0.2012	0.1005	0.0898	0.0986	0.0925	0.1047	0.1108	0.0948	0.0903
0.0713	0.096	0.0999	0.1419	0.1244	0.1507	0.177	0.161	0.153
0.6299	0.3216	0.2925	0.2818	0.265	0.2749	0.2763	0.2483	0.2123
0.6182	0.3473	0.4288	0.4007	0.3764	0.3842	0.3841	0.3216	0.2701

0.0269	0.0954	0.1999	0.202	0.1843	0.1767	0.1934	0.1424	0.1367
0.0317	0.0962	0.1904	0.1955	0.1703	0.1599	0.179	0.1365	0.1204
0.1378	0.1376	0.2306	0.2215	0.1949	0.1894	0.1873	0.1407	0.1275
0.0438	0.1311	0.2319	0.2448	0.2131	0.2083	0.2203	0.1795	0.1642
0.0432	0.1119	0.1995	0.199	0.1769	0.1737	0.1665	0.1305	0.1331
0.0929	0.1426	0.2364	0.2329	0.2106	0.2013	0.2032	0.1619	0.1566
0.1423	0.1847	0.2957	0.2945	0.2628	0.2588	0.2584	0.1948	0.1867
0.0369	0.0937	0.165	0.1759	0.1581	0.1495	0.1513	0.1177	0.1247
0.0543	0.0903	0.1146	0.154	0.1339	0.1611	0.1812	0.1542	0.1509
0.0129	0.048	0.0784	0.1084	0.0862	0.1174	0.1304	0.1133	0.1197
0.0318	0.0898	0.1589	0.1708	0.1481	0.1508	0.1548	0.1342	0.1404
0.0935	0.121	0.2039	0.2081	0.1864	0.1882	0.1875	0.1473	0.1378
0.0384	0.1014	0.1938	0.2016	0.1725	0.175	0.1744	0.1357	0.127
0.0633	0.1107	0.1858	0.1941	0.1794	0.1797	0.1904	0.1356	0.1304
0.0267	0.0806	0.1127	0.1497	0.1353	0.1427	0.1674	0.1529	0.1595
0.1904	0.1233	0.1091	0.1427	0.1306	0.1527	0.181	0.1522	0.1532
0.188	0.1456	0.1786	0.1979	0.1814	0.1944	0.2037	0.169	0.1628
0.0365	0.0867	0.1575	0.1679	0.1424	0.1456	0.1591	0.1285	0.1202
0.0202	0.042	0.046	0.0856	0.0778	0.099	0.1256	0.1077	0.1079
0.3294	0.1923	0.1765	0.2029	0.1902	0.2136	0.22	0.1966	0.1895
0.0387	0.0569	0.0731	0.0986	0.0855	0.0981	0.1195	0.0997	0.0994
0.2599	0.187	0.2482	0.233	0.2116	0.2137	0.2149	0.1741	0.1678
0.6489	0.3045	0.3099	0.2855	0.2752	0.3189	0.3105	0.2794	0.2272
0.4399	0.253	0.2998	0.2747	0.2606	0.2523	0.255	0.2156	0.202
0.098	0.1401	0.2314	0.2393	0.2156	0.2115	0.2101	0.1574	0.1492
0.0603	0.1075	0.1806	0.1968	0.1706	0.1745	0.1843	0.1433	0.1368
0.0272	0.0763	0.1363	0.1561	0.1459	0.1483	0.1547	0.1263	0.1278
0.1535	0.1763	0.26	0.2638	0.2395	0.2399	0.2482	0.1988	0.1851
0.0526	0.0939	0.1396	0.1499	0.1333	0.1479	0.1571	0.1316	0.1378
0.6287	0.2513	0.2107	0.1935	0.1837	0.1949	0.2074	0.1646	0.169
0.5914	0.2627	0.2263	0.1753	0.1599	0.2046	0.2342	0.1996	0.1654
0.0708	0.0523	0.0581	0.0697	0.0625	0.0722	0.0768	0.0688	0.0655
-0.001	0.0021	0.0026	0.0057	0.0055	0.0068	0.0087	0.0077	0.0085
0	0	0	0	0	0	0	0	0
0	0	0	0	0	0	0	0	0
0	0	0	0	0	0	0	0	0
0.0307	0.0154	0.0129	0.0124	0.0116	0.0129	0.0139	0.0125	0.0107
0.0004	0.0012	0.0024	0.0023	0.0021	0.0018	0.0019	0.0015	0.0014
0.0003	0.0007	0.0012	0.0013	0.0012	0.0011	0.0011	0.0009	0.0009
0	0	0	0	0	0	0	0	0
0	0	0	0	0	0	0	0	0
0.0959	0.0475	0.038	0.0374	0.0409	0.0424	0.0395	0.034	0.0339
0.3785	0.1925	0.173	0.1611	0.1542	0.1626	0.1723	0.1412	0.1269
0.5001	0.2371	0.1917	0.197	0.1926	0.2188	0.2486	0.2066	0.1882

0.34	0.1802	0.1901	0.1882	0.1725	0.1859	0.1952	0.1659	0.1651
0.1013	0.0527	0.0489	0.0536	0.0541	0.0609	0.0659	0.0595	0.0598
0.2335	0.1058	0.0763	0.0777	0.076	0.0933	0.0992	0.0808	0.0735
0.2796	0.1583	0.152	0.1678	0.1633	0.1889	0.226	0.198	0.2001
0.9332	0.4474	0.3792	0.317	0.3251	0.3762	0.352	0.2746	0.2653
1.0719	0.5176	0.4154	0.3672	0.3182	0.3548	0.3385	0.2798	0.2277
0.4774	0.2635	0.2721	0.2584	0.2483	0.2573	0.271	0.2229	0.2347
0.5349	0.3019	0.3292	0.2938	0.2841	0.2905	0.3005	0.2633	0.244
0.1538	0.1452	0.2359	0.2309	0.2134	0.2013	0.2031	0.1668	0.1512
0.0546	0.1379	0.249	0.2619	0.2387	0.2256	0.2331	0.1865	0.1819
0.1356	0.1405	0.2161	0.2146	0.1932	0.1939	0.1898	0.1523	0.137
0.478	0.3021	0.3424	0.3201	0.3056	0.3081	0.3126	0.2426	0.233
0.1924	0.1665	0.2464	0.2437	0.228	0.2253	0.2285	0.1804	0.1723
0.0053	0.0601	0.1044	0.1286	0.1154	0.1145	0.1293	0.1113	0.1194
0.0465	0.0634	0.0903	0.1229	0.1001	0.1338	0.1681	0.1599	0.1753
-0.0414	0.0256	0.075	0.1233	0.0998	0.1339	0.1577	0.1342	0.1388
0.0038	0.0645	0.0828	0.112	0.0975	0.1076	0.137	0.1241	0.1341
0.0771	0.0995	0.1222	0.1439	0.1266	0.1451	0.1528	0.1325	0.1316
0.0272	0.0852	0.1125	0.1378	0.1211	0.1341	0.1402	0.1246	0.1264
0.2918	0.1697	0.1685	0.1811	0.1811	0.2026	0.2168	0.1795	0.1686
0.6515	0.3265	0.2711	0.2778	0.2847	0.3179	0.3152	0.2776	0.2879
0.7206	0.3774	0.3323	0.3307	0.3195	0.343	0.3568	0.3447	0.3178
0.4285	0.2437	0.2336	0.2203	0.2132	0.2308	0.2331	0.1907	0.1916
0.2125	0.1511	0.1752	0.193	0.1794	0.1962	0.2078	0.1707	0.1667
0.0443	0.0862	0.1223	0.1471	0.1343	0.1507	0.1662	0.1358	0.1355
0.0936	0.0928	0.1307	0.1568	0.1465	0.1691	0.1887	0.1546	0.1533
0.0784	0.0968	0.1377	0.1552	0.1378	0.1528	0.1636	0.1383	0.136
0.4501	0.2547	0.2654	0.2384	0.2224	0.2208	0.2169	0.1597	0.1543
0.6757	0.3053	0.2791	0.2633	0.2573	0.2534	0.2625	0.2077	0.1846
0.441	0.2489	0.2687	0.243	0.2409	0.2531	0.2564	0.211	0.1735
0.2615	0.1931	0.2608	0.252	0.2423	0.2434	0.2442	0.1888	0.1758
0.052	0.1101	0.1799	0.1948	0.1621	0.1577	0.1632	0.1375	0.1343
0.0301	0.0893	0.155	0.1701	0.1547	0.1565	0.1606	0.1357	0.1282
0.0128	0.0704	0.132	0.1542	0.1355	0.1388	0.152	0.1191	0.1052
0.1072	0.0917	0.1255	0.1435	0.1293	0.145	0.1601	0.1345	0.1325
0.5845	0.253	0.2196	0.2177	0.2245	0.2759	0.2913	0.2652	0.2483
0.5392	0.2344	0.1868	0.1847	0.2001	0.234	0.225	0.2029	0.1886
0.3222	0.1604	0.1488	0.1337	0.1184	0.1363	0.1533	0.1302	0.1226
0.0579	0.0297	0.0364	0.0354	0.0349	0.036	0.0348	0.0295	0.0249
0.0549	0.0218	0.0178	0.0145	0.0126	0.0151	0.014	0.0139	0.0139
0	0	0	0	0	0	0	0	0
0	0	0	0	0	0	0	0	0
0.0001	0.0005	0.0011	0.0012	0.0011	0.0011	0.0011	0.0009	0.0009
0.0365	0.0169	0.0142	0.0163	0.0149	0.0156	0.0163	0.0138	0.0119

0.0652	0.0239	0.0167	0.0169	0.0193	0.0222	0.0236	0.0184	0.0171
0	0	0	0	0	0	0	0	0
0.0232	0.0135	0.0113	0.0135	0.0141	0.0157	0.0175	0.0157	0.0154
0.2025	0.0955	0.0821	0.081	0.0782	0.0925	0.0891	0.0722	0.0654
0.2493	0.1268	0.1187	0.1216	0.1199	0.1289	0.1364	0.1229	0.1236
0.0922	0.0672	0.075	0.0866	0.0838	0.0964	0.1025	0.0899	0.0892
0.3141	0.1549	0.1491	0.1517	0.1532	0.1696	0.1745	0.1503	0.1515
0.083	0.0372	0.028	0.0207	0.015	0.0172	0.0168	0.0189	0.0172
0.0984	0.0564	0.0541	0.0619	0.0587	0.0713	0.0815	0.0717	0.071
0.5353	0.2592	0.2407	0.2165	0.2199	0.2481	0.2648	0.2287	0.2016
0.9173	0.448	0.3729	0.2894	0.2892	0.2975	0.2804	0.2438	0.2024
0.5958	0.2737	0.2246	0.2116	0.1942	0.2133	0.2221	0.2007	0.2073
0.7607	0.4266	0.4013	0.3456	0.3186	0.3376	0.3208	0.2897	0.2661
0.487	0.2765	0.2921	0.2885	0.2694	0.2962	0.2833	0.2242	0.2091
0.0636	0.1355	0.2298	0.2114	0.1968	0.1923	0.1989	0.1695	0.1721
0.027	0.1091	0.1994	0.2018	0.1854	0.189	0.1937	0.1505	0.1354
0.2621	0.1745	0.219	0.2064	0.1887	0.1871	0.1805	0.1463	0.1333
0.6545	0.3473	0.3473	0.3199	0.3199	0.3105	0.3077	0.2212	0.1956
0.2754	0.1764	0.1994	0.207	0.1982	0.2188	0.2258	0.1804	0.1671
0.0033	0.0464	0.0783	0.1116	0.1137	0.1392	0.1677	0.1498	0.1538
0.0774	0.0954	0.1119	0.1591	0.1482	0.1612	0.1934	0.1608	0.1791
-0.0032	0.0578	0.0739	0.1282	0.1241	0.1499	0.1678	0.1579	0.1706
0.0985	0.0906	0.0997	0.147	0.1334	0.1661	0.1854	0.1668	0.1642
0.0411	0.0687	0.0628	0.102	0.0847	0.1072	0.1276	0.1144	0.1285
0.0353	0.0636	0.0942	0.1187	0.1101	0.1219	0.1335	0.1048	0.113
0.5361	0.2539	0.2233	0.2072	0.2143	0.2258	0.2281	0.1894	0.1722
0.6165	0.2824	0.2432	0.2277	0.2077	0.2348	0.259	0.2315	0.2031
0.0771	0.1019	0.1361	0.1628	0.1586	0.1695	0.1866	0.1642	0.1687
-0.0207	0.0424	0.04	0.0892	0.0798	0.1168	0.1516	0.1243	0.1335
0.2906	0.1593	0.1461	0.1562	0.1451	0.1589	0.1709	0.139	0.1447
0.2672	0.1683	0.1788	0.1859	0.1707	0.1904	0.1901	0.1642	0.1618
0.2195	0.1313	0.1336	0.145	0.1395	0.1581	0.1727	0.1472	0.1437
0.0118	0.0582	0.0935	0.1081	0.0943	0.1136	0.1286	0.1097	0.1084
0.0195	0.0853	0.1837	0.1754	0.1524	0.1411	0.1443	0.1102	0.0936
0.236	0.1829	0.2517	0.2444	0.2282	0.2288	0.2329	0.1927	0.1777
0.2522	0.202	0.2919	0.2856	0.2685	0.2588	0.2502	0.2081	0.1989
0.234	0.1611	0.2299	0.2234	0.2075	0.2057	0.1981	0.166	0.1522
0.3578	0.2347	0.2971	0.2961	0.2748	0.286	0.2799	0.2403	0.2298
0.0056	0.0858	0.1382	0.175	0.1653	0.1708	0.1889	0.1507	0.1523
-0.012	0.0285	0.0623	0.0816	0.0675	0.0905	0.1029	0.0863	0.0875
0.2619	0.165	0.1922	0.1931	0.1756	0.1936	0.1969	0.1579	0.139
0.6238	0.2316	0.174	0.1956	0.2168	0.1724	0.1766	0.1368	0.1199
0.9568	0.3932	0.3718	0.3331	0.3442	0.3357	0.3361	0.3139	0.3087
1.0858	0.4676	0.3685	0.2892	0.2972	0.3265	0.3264	0.3677	0.324

0.3172	0.146	0.1195	0.0967	0.1029	0.1077	0.1093	0.106	0.0853
0	0	0	0	0	0	0	0	0
0	0	0	0	0	0	0	0	0
0	0	0	0	0	0	0	0	0
0.0533	0.0235	0.0187	0.0167	0.0151	0.0149	0.0155	0.0146	0.0144
0.0375	0.0164	0.0129	0.0112	0.011	0.0129	0.0136	0.0105	0.0116
0	0.0003	0.0005	0.0007	0.0006	0.0008	0.001	0.0009	0.0009
0	0	0	0	0	0	0	0	0
0.0002	0.0005	0.0011	0.0011	0.001	0.0009	0.0009	0.0007	0.0006
0.1884	0.0809	0.0632	0.0608	0.0637	0.0642	0.0731	0.0622	0.0625
0.4128	0.2118	0.1885	0.1727	0.1562	0.1655	0.1525	0.1284	0.109
0.3706	0.158	0.1097	0.0883	0.0938	0.122	0.1265	0.1226	0.1053
0.1849	0.0909	0.0842	0.0926	0.0938	0.1043	0.0951	0.0734	0.0703
0.0689	0.0418	0.0464	0.0443	0.043	0.0459	0.0442	0.0376	0.0383
-0.0081	0.013	0.0173	0.034	0.0226	0.0369	0.049	0.0467	0.0459
0.1118	0.0912	0.1166	0.1353	0.1298	0.1496	0.1646	0.1467	0.1493
0.1634	0.1229	0.155	0.1605	0.1478	0.1564	0.1629	0.131	0.1218
0.0356	0.09	0.1776	0.1743	0.1534	0.1481	0.1386	0.1093	0.1065
0.4889	0.279	0.2992	0.2714	0.2492	0.2882	0.2893	0.2333	0.203
0.2104	0.1615	0.2331	0.2382	0.2202	0.2238	0.2218	0.1735	0.162
0.0526	0.1358	0.2326	0.235	0.2105	0.1983	0.21	0.1582	0.144
0.0361	0.1096	0.2074	0.2108	0.1872	0.1849	0.1943	0.1406	0.1244
0.1078	0.1193	0.2031	0.21	0.2024	0.1972	0.1903	0.1489	0.1293
0.2222	0.1553	0.22	0.2176	0.2089	0.2036	0.204	0.1478	0.1444
0.0525	0.0813	0.1409	0.1484	0.1439	0.1479	0.1577	0.1274	0.1207
0.0059	0.0576	0.1237	0.1339	0.13	0.1393	0.1457	0.1083	0.1102
0.1687	0.1235	0.171	0.1739	0.155	0.1608	0.1664	0.1352	0.1328
0.131	0.0999	0.1133	0.1276	0.1169	0.1318	0.1457	0.1238	0.1264
0.0994	0.0729	0.0706	0.1013	0.0867	0.1099	0.1323	0.1099	0.1158
0.2	0.1469	0.148	0.1809	0.1656	0.2048	0.2208	0.2023	0.2057
0.2279	0.1621	0.1915	0.201	0.1893	0.2035	0.2191	0.1835	0.176
0.2477	0.1475	0.1537	0.1486	0.1386	0.1466	0.143	0.1163	0.1172
0.5119	0.2944	0.2868	0.277	0.2469	0.2458	0.242	0.2122	0.1869
0.2654	0.1862	0.2424	0.236	0.2174	0.2201	0.2236	0.1928	0.1866
0.4465	0.2521	0.2495	0.2478	0.2323	0.2363	0.2348	0.182	0.1857
0.1122	0.1049	0.157	0.164	0.1524	0.1591	0.1641	0.1326	0.1264
0.1192	0.1054	0.16	0.1529	0.1372	0.1351	0.1302	0.1082	0.0998
0.2743	0.1902	0.2489	0.2514	0.227	0.2261	0.2373	0.1938	0.177
0.1663	0.1346	0.1799	0.1874	0.1715	0.178	0.1811	0.1449	0.136
0.1048	0.1044	0.1624	0.1579	0.1421	0.145	0.1499	0.1237	0.1138
0.4936	0.2674	0.3171	0.2725	0.2563	0.2862	0.2783	0.2406	0.2259
0.3893	0.2379	0.3029	0.2889	0.2733	0.2759	0.2811	0.2378	0.2133
0.0941	0.1059	0.1783	0.1832	0.1722	0.171	0.1819	0.1444	0.1312
0.2998	0.2029	0.2666	0.2553	0.2315	0.2276	0.2198	0.1864	0.1704

0.1163	0.1291	0.1935	0.2099	0.1893	0.1996	0.2118	0.1633	0.1566
-0.0418	0.0256	0.0545	0.0802	0.067	0.0913	0.1083	0.0946	0.1018
0.0805	0.1006	0.152	0.1697	0.1568	0.1681	0.1779	0.1471	0.1406
0.4349	0.2377	0.215	0.2115	0.1973	0.2106	0.2033	0.1572	0.1433
0.2975	0.1396	0.15	0.1373	0.1347	0.1404	0.1372	0.1221	0.1145
0.1222	0.055	0.0417	0.0332	0.0307	0.032	0.0347	0.0288	0.0283
0	0	0	0	0	0	0	0	0
0.1459	0.0703	0.053	0.0518	0.052	0.0571	0.0526	0.0485	0.0482
0	0	0	0	0	0	0	0	0
0	0	0	0	0	0	0	0	0
0.0546	0.0232	0.0193	0.0169	0.017	0.0152	0.0152	0.0148	0.0135
0	0	0	0	0	0	0	0	0
-0.0004	0.0004	0.0005	0.0013	0.001	0.0013	0.0016	0.0014	0.0014
0.0639	0.0223	0.017	0.0137	0.0131	0.0141	0.0147	0.013	0.0086
0	0	0	0	0	0	0	0	0
0	0	0	0	0	0	0	0	0
0.1517	0.0684	0.0581	0.0605	0.0544	0.0639	0.074	0.0635	0.0491
0.4477	0.2121	0.1944	0.1812	0.1925	0.2223	0.232	0.1857	0.182
0.103	0.0493	0.0409	0.0336	0.0355	0.0408	0.04	0.0377	0.0336
0.2343	0.1233	0.1146	0.1181	0.1198	0.1281	0.1376	0.1154	0.1056
0.1488	0.0755	0.0667	0.0686	0.0665	0.0753	0.0796	0.0686	0.0701
0.0516	0.1167	0.1563	0.2008	0.173	0.1987	0.217	0.1894	0.1807
0.0351	0.0939	0.1543	0.1815	0.1677	0.1778	0.1813	0.1428	0.1475
0.1434	0.1193	0.1637	0.1708	0.1593	0.1619	0.1675	0.1376	0.1314
0.3814	0.1789	0.1759	0.1721	0.1548	0.1778	0.1862	0.17	0.1515
0.2338	0.171	0.2591	0.2457	0.2197	0.2223	0.2326	0.1777	0.1586
0.0434	0.131	0.274	0.2675	0.2386	0.2359	0.2452	0.1943	0.1842
0.181	0.169	0.2547	0.2418	0.2296	0.2253	0.2271	0.1848	0.1697
0.1149	0.1443	0.2281	0.2276	0.1966	0.1812	0.1888	0.1583	0.1395
0.0622	0.1265	0.2315	0.226	0.2056	0.1944	0.205	0.1529	0.1381
0.0527	0.1087	0.1855	0.1863	0.1683	0.173	0.1712	0.1317	0.1243
0.0918	0.1303	0.2048	0.219	0.2004	0.2069	0.2058	0.1588	0.1529
0.1333	0.1306	0.2242	0.2212	0.2058	0.1955	0.1958	0.1512	0.1425
0.0832	0.112	0.1767	0.1795	0.1588	0.1569	0.1672	0.1348	0.1229
0.1282	0.1143	0.1427	0.1663	0.1534	0.1698	0.1816	0.1596	0.1631
0.0959	0.0768	0.0844	0.1187	0.111	0.1343	0.1402	0.1227	0.12
0.1656	0.1184	0.1324	0.1522	0.1462	0.1767	0.187	0.1648	0.159
0.0782	0.089	0.1319	0.1413	0.135	0.1433	0.151	0.1205	0.1156
0.455	0.279	0.2908	0.2696	0.2609	0.2692	0.2532	0.2128	0.2006
0.3602	0.1909	0.21	0.1914	0.1866	0.2035	0.2058	0.1798	0.177
0.7507	0.3909	0.3843	0.3142	0.2684	0.2323	0.2318	0.1922	0.1781
0.1113	0.1417	0.2328	0.232	0.203	0.2017	0.2116	0.1605	0.1552
0.1339	0.1624	0.2559	0.2567	0.2385	0.2384	0.2455	0.2	0.1953
0.0125	0.0888	0.1693	0.1723	0.1579	0.1578	0.1437	0.1012	0.1152

0.0762	0.1129	0.1853	0.1894	0.1698	0.1763	0.1828	0.1534	0.1531
0.1311	0.1622	0.2689	0.2657	0.2396	0.2364	0.2304	0.186	0.1757
0.2692	0.1702	0.2018	0.1892	0.1752	0.178	0.179	0.1484	0.1321
0.3746	0.2043	0.2239	0.2131	0.1923	0.2012	0.2068	0.1575	0.1214
0.173	0.1617	0.253	0.2512	0.2258	0.227	0.2272	0.1759	0.1657
0.0447	0.115	0.2279	0.237	0.2243	0.2059	0.2056	0.1595	0.1579
0.0203	0.1001	0.1978	0.2158	0.2031	0.1938	0.2003	0.1613	0.1567
0.0625	0.0851	0.0941	0.137	0.1257	0.1722	0.1974	0.1757	0.1798
-0.0094	0.0408	0.0558	0.0999	0.0924	0.1158	0.1332	0.1274	0.136
0.0041	0.0296	0.0596	0.0672	0.0616	0.0674	0.0729	0.057	0.0595
-0.004	0.0107	0.0141	0.0227	0.0173	0.0246	0.0302	0.0271	0.0272
0.1065	0.0775	0.0921	0.0971	0.0916	0.0955	0.0998	0.0831	0.0823
0.023	0.0137	0.0181	0.0176	0.0164	0.0169	0.0164	0.0135	0.0122
0.0006	0.0014	0.0025	0.0027	0.0022	0.0021	0.002	0.0014	0.0013
0	0	0	0	0	0	0	0	0
0	0	0	0	0	0	0	0	0
0.0003	0.0007	0.0012	0.0012	0.0012	0.0011	0.0011	0.0009	0.0008
0.0081	0.0036	0.0022	0.0019	0.002	0.002	0.0019	0.0016	0.0012
0	0	0	0	0	0	0	0	0
0	0	0	0	0	0	0	0	0
0	0	0	0	0	0	0	0	0
0.0416	0.0213	0.0197	0.0179	0.0163	0.0181	0.0191	0.0167	0.016
0.057	0.0278	0.0259	0.0252	0.0259	0.03	0.0294	0.0252	0.0231
0.0629	0.0342	0.0328	0.0337	0.0325	0.0359	0.0376	0.0324	0.0303
-0.0019	0.0118	0.0255	0.0329	0.0289	0.0394	0.048	0.0373	0.0395
0.0853	0.0673	0.0736	0.0819	0.078	0.0847	0.092	0.0808	0.0773
0.0921	0.0654	0.0719	0.0839	0.0792	0.0851	0.0927	0.0829	0.0826
0.0152	0.0446	0.0683	0.0874	0.0774	0.0949	0.1088	0.0906	0.0869
-0.0033	0.0681	0.107	0.1449	0.1392	0.1685	0.1878	0.1502	0.1577
0.0126	0.0681	0.0871	0.1197	0.105	0.1193	0.1391	0.1177	0.1271
0.0931	0.0886	0.1143	0.1336	0.1202	0.1312	0.1407	0.1224	0.1188
0.1236	0.13	0.1864	0.2068	0.189	0.1971	0.2125	0.1808	0.1787
0.1033	0.1283	0.229	0.245	0.2216	0.218	0.2278	0.1874	0.1696
0.4175	0.2335	0.2595	0.2491	0.2261	0.2292	0.2308	0.1966	0.1847
0.6881	0.3316	0.338	0.2973	0.2682	0.2819	0.2811	0.2209	0.1734
0.4013	0.2421	0.3099	0.295	0.2829	0.2963	0.2828	0.2239	0.1966
0.0135	0.081	0.1828	0.1916	0.1684	0.1599	0.1684	0.1314	0.1182
0.1618	0.1452	0.2045	0.2099	0.1938	0.1996	0.1975	0.1624	0.1554
0.012	0.075	0.1709	0.1886	0.1715	0.1724	0.1794	0.1418	0.1351
0.1952	0.1709	0.2476	0.2527	0.2298	0.2232	0.2147	0.1769	0.1701
0.1702	0.1574	0.2397	0.2332	0.2213	0.2259	0.2204	0.1708	0.1595
0.008	0.0577	0.0749	0.093	0.0748	0.0919	0.1211	0.0937	0.1118
0.0179	0.0778	0.1061	0.1196	0.1117	0.1237	0.1347	0.105	0.0972
0.1093	0.1102	0.1664	0.1689	0.154	0.1532	0.1535	0.1201	0.1097

0.0903	0.1285	0.2348	0.2301	0.2082	0.2155	0.209	0.157	0.1411
0.1119	0.1501	0.2428	0.2456	0.2247	0.2224	0.2123	0.1687	0.1657
0.1656	0.1561	0.2367	0.2249	0.2075	0.2073	0.2184	0.173	0.1697
0.1179	0.1345	0.2173	0.2277	0.203	0.2066	0.207	0.1783	0.1736
0.0312	0.0774	0.137	0.1486	0.1322	0.1309	0.1405	0.1185	0.1153
0.025	0.0848	0.1593	0.1579	0.1517	0.1459	0.1442	0.1146	0.1004
0.1615	0.1745	0.2616	0.2528	0.233	0.2332	0.2293	0.1851	0.1752
0.2196	0.176	0.2577	0.2578	0.2449	0.2383	0.2457	0.2019	0.1829
0.1992	0.1691	0.272	0.2659	0.2415	0.242	0.2366	0.1871	0.1768
0.0369	0.1049	0.1961	0.1948	0.1759	0.172	0.1788	0.1392	0.1315
0.175	0.1658	0.2607	0.2509	0.2333	0.2314	0.2468	0.2038	0.1842
0.2931	0.1907	0.2659	0.2532	0.2381	0.238	0.2491	0.1943	0.1872
0.1046	0.1351	0.2338	0.2456	0.2067	0.2111	0.2302	0.1798	0.173
0.0042	0.0871	0.0959	0.1383	0.1304	0.1541	0.184	0.1683	0.1847
0.0284	0.0281	0.0271	0.0384	0.036	0.0437	0.0463	0.0403	0.0428
0.161	0.0824	0.0848	0.0931	0.0924	0.1034	0.1076	0.0954	0.0937
0.1291	0.0812	0.0868	0.0903	0.0821	0.0874	0.0867	0.0733	0.0697
0.014	0.0518	0.0932	0.1003	0.0911	0.0933	0.0952	0.0775	0.074
0.0032	0.012	0.0214	0.0232	0.023	0.0229	0.0244	0.0199	0.0202
0.0074	0.0033	0.0022	0.0019	0.0021	0.0024	0.0024	0.0022	0.002
0	0	0	0	0	0	0	0	0
0	0	0	0	0	0	0	0	0
0.0004	0.0016	0.0024	0.0031	0.0029	0.0032	0.0036	0.003	0.0035
0.0004	0.001	0.001	0.0015	0.0013	0.0016	0.0021	0.0019	0.002
0	0	0	0	0	0	0	0	0
0	0	0	0	0	0	0	0	0
0.0148	0.0057	0.004	0.0038	0.0043	0.0049	0.0053	0.0055	0.0046
0.0703	0.0329	0.0275	0.0257	0.025	0.0255	0.0269	0.0222	0.0175
0.0942	0.0436	0.04	0.0447	0.0429	0.0469	0.0463	0.0414	0.0399
0.098	0.0458	0.0384	0.0434	0.041	0.0461	0.0449	0.044	0.0409
0.3806	0.1906	0.1956	0.2042	0.1891	0.2094	0.2137	0.1838	0.1841
0.1152	0.0598	0.055	0.0541	0.0502	0.0593	0.0615	0.0489	0.0486
0	0	0	0	0	0	0	0	0
0.0199	0.0435	0.0513	0.0598	0.0519	0.0542	0.0549	0.0484	0.0532
0.086	0.0862	0.1191	0.1557	0.1376	0.1612	0.1739	0.1539	0.1625
0.039	0.0779	0.0943	0.1682	0.1698	0.1833	0.1957	0.1729	0.181
0.0059	0.0816	0.1014	0.1525	0.1255	0.1318	0.151	0.1172	0.1321
0.0036	0.0667	0.084	0.122	0.1044	0.121	0.1323	0.1129	0.1189
0.0426	0.0927	0.1605	0.172	0.1626	0.1538	0.1572	0.1311	0.1333
0.3042	0.1871	0.2285	0.214	0.2059	0.2107	0.2282	0.1775	0.1577
0.2879	0.1927	0.2415	0.2286	0.2184	0.2213	0.2277	0.1828	0.1643
0.2933	0.2172	0.2648	0.2554	0.2416	0.2437	0.2419	0.2084	0.2066
0.0761	0.1411	0.252	0.2618	0.2352	0.2407	0.2212	0.1817	0.155
0.0486	0.0932	0.1648	0.1881	0.1735	0.1806	0.1889	0.1627	0.1618

0.0673	0.0869	0.0953	0.1265	0.1096	0.1301	0.1417	0.1151	0.1194
0.0029	0.0835	0.1436	0.1771	0.1714	0.1769	0.1957	0.1548	0.1604
0.122	0.1392	0.1946	0.2106	0.1866	0.1982	0.2054	0.17	0.1606
0.0557	0.0798	0.0941	0.1314	0.1227	0.1549	0.1773	0.1584	0.1799
-0.0093	0.0484	0.0909	0.1265	0.1157	0.1332	0.1488	0.1212	0.1407
0.1313	0.1378	0.217	0.2181	0.1983	0.1911	0.1962	0.1437	0.1352
0.495	0.3117	0.3332	0.2933	0.2897	0.2793	0.2895	0.2293	0.1816
0.2706	0.194	0.2538	0.2515	0.2341	0.231	0.2295	0.1782	0.1629
0.0305	0.0968	0.2001	0.2058	0.1768	0.1618	0.1586	0.1271	0.1204
0.11	0.1227	0.1883	0.1976	0.1811	0.186	0.1861	0.1482	0.1469
0.0361	0.0917	0.1707	0.19	0.1675	0.1636	0.1769	0.1342	0.1319
0.2236	0.1604	0.2082	0.2099	0.1969	0.1967	0.191	0.1557	0.1556
0.191	0.1398	0.2095	0.2066	0.1887	0.1835	0.1867	0.1565	0.137
0.2476	0.1907	0.2603	0.2464	0.2215	0.2264	0.2246	0.1861	0.1737
0.5152	0.2486	0.2473	0.216	0.2049	0.212	0.2091	0.1689	0.1348
0.3711	0.26	0.345	0.3089	0.2881	0.2997	0.3046	0.256	0.2209
0.2605	0.1733	0.2054	0.1969	0.1805	0.1823	0.1805	0.133	0.1211
0.326	0.1865	0.2323	0.2264	0.2192	0.2348	0.2326	0.1939	0.1946
0.0254	0.108	0.1578	0.1914	0.1779	0.2018	0.2135	0.1772	0.1845
0.057	0.0592	0.0881	0.0925	0.0832	0.0892	0.0931	0.0697	0.0673
0.1256	0.0713	0.0746	0.073	0.0715	0.0766	0.0795	0.0675	0.0618
0.1175	0.0625	0.0625	0.068	0.0671	0.0744	0.078	0.0675	0.0673
0.0578	0.0543	0.0687	0.0803	0.0741	0.0843	0.0885	0.0728	0.0736
0.0579	0.0397	0.0479	0.0493	0.0471	0.0494	0.051	0.0416	0.0389
0.0604	0.0272	0.0198	0.0199	0.0213	0.022	0.0253	0.0203	0.0165
0.0065	0.0067	0.0075	0.0089	0.0074	0.0097	0.0104	0.009	0.0091
0	0	0	0	0	0	0	0	0
0	0	0	0	0	0	0	0	0
0	0.0011	0.0018	0.0024	0.0022	0.0026	0.0029	0.0025	0.0027
0.0075	0.0033	0.0022	0.0021	0.002	0.0024	0.0026	0.0021	0.0019
0.0068	0.0029	0.0023	0.002	0.0018	0.0023	0.0024	0.0023	0.0018
-0.0001	0.0002	0.0003	0.0006	0.0006	0.0007	0.0009	0.0008	0.0008
0.0079	0.0046	0.0041	0.0045	0.0046	0.005	0.0055	0.005	0.0049
0.0527	0.0239	0.0202	0.0182	0.0185	0.0199	0.0191	0.018	0.017
0.0782	0.0387	0.0355	0.033	0.028	0.0322	0.0323	0.0225	0.0209
0.0808	0.0414	0.0365	0.0365	0.0357	0.0374	0.0395	0.0334	0.0311
0.2211	0.1132	0.0966	0.096	0.0897	0.0986	0.0912	0.0798	0.0663
0.0667	0.0326	0.0298	0.0338	0.0335	0.039	0.0408	0.037	0.0357
0	0	0	0	0	0	0	0	0
0.0039	0.0228	0.0326	0.0408	0.0362	0.0389	0.0418	0.0314	0.033
0.0253	0.051	0.0602	0.0785	0.0753	0.0842	0.1108	0.1064	0.1069
-0.007	0.0472	0.0731	0.0913	0.0904	0.109	0.1186	0.0997	0.1064
0.0776	0.0912	0.1137	0.1337	0.1182	0.1401	0.1563	0.1279	0.123
0.1595	0.1008	0.1104	0.1446	0.1354	0.1607	0.1787	0.1406	0.1475

0.1151	0.1289	0.1889	0.2072	0.1874	0.1971	0.2104	0.1793	0.1818
0.0861	0.1241	0.2165	0.2172	0.1863	0.1852	0.1773	0.1321	0.1341
0.0537	0.1036	0.1871	0.193	0.18	0.1694	0.17	0.1428	0.1346
0.022	0.0837	0.1632	0.1728	0.1542	0.1479	0.1537	0.1064	0.1066
0.3572	0.2356	0.3224	0.3109	0.2837	0.2793	0.2664	0.209	0.2047
0.024	0.0762	0.1523	0.1501	0.1355	0.1335	0.1355	0.1009	0.0891
0.0045	0.0617	0.0812	0.1066	0.1024	0.1229	0.1312	0.1204	0.1239
0.0944	0.087	0.1032	0.1593	0.1596	0.2002	0.2177	0.1813	0.1856
-0.0226	0.032	0.0445	0.0806	0.0644	0.0856	0.1033	0.0849	0.0961
-0.0006	0.0694	0.1033	0.1315	0.1269	0.1549	0.1769	0.1392	0.163
0.0259	0.0561	0.0593	0.0918	0.0918	0.1214	0.1418	0.1164	0.1373
0.0674	0.0988	0.1496	0.174	0.1569	0.175	0.1886	0.1587	0.1699
0.1241	0.1042	0.1398	0.1486	0.1385	0.1469	0.1514	0.1253	0.121
0.1044	0.1106	0.1726	0.1825	0.1645	0.1731	0.1772	0.1456	0.1416
0.1127	0.1457	0.2244	0.2271	0.2154	0.2123	0.2169	0.1733	0.1646
0.1546	0.1332	0.2069	0.2026	0.1852	0.1819	0.1831	0.1488	0.1339
0.3863	0.2409	0.2926	0.2848	0.2659	0.2535	0.2532	0.1995	0.1815
0.0516	0.12	0.2326	0.2213	0.2068	0.2087	0.2137	0.1724	0.1711
0.0537	0.1299	0.2414	0.2415	0.2182	0.2117	0.1958	0.1405	0.1455
0.3242	0.2141	0.2583	0.2445	0.2256	0.2231	0.2079	0.1637	0.1564
0.4686	0.2602	0.2653	0.2333	0.2632	0.2859	0.2674	0.2128	0.207
0.2768	0.1721	0.2259	0.2052	0.2014	0.2061	0.2079	0.1723	0.1602
0.3303	0.2172	0.2745	0.2524	0.2441	0.231	0.2396	0.1905	0.1824
0.1408	0.1434	0.204	0.2138	0.2018	0.2091	0.2153	0.1793	0.1666
0.0257	0.0844	0.1142	0.1514	0.138	0.1546	0.1572	0.1381	0.1318
0.0591	0.072	0.1126	0.1142	0.1033	0.1052	0.1089	0.0847	0.081
0.1271	0.0757	0.0895	0.0857	0.085	0.0798	0.0795	0.066	0.0594
0.0779	0.0703	0.0764	0.0971	0.0909	0.102	0.1213	0.1065	0.1093
-0.0086	0.0504	0.0701	0.1303	0.1469	0.1762	0.1855	0.161	0.1719
0.0133	0.0167	0.02	0.0261	0.0259	0.0304	0.038	0.0337	0.0347
0.0444	0.0154	0.0123	0.0104	0.0092	0.0116	0.0091	0.0074	0.0088
0.0026	0.0095	0.0184	0.0196	0.0176	0.0184	0.0185	0.0154	0.015
0	0	0	0	0	0	0	0	0
0	0	0	0	0	0	0	0	0
0.0073	0.0032	0.003	0.0027	0.0023	0.0025	0.0025	0.0023	0.0019
0.0156	0.0061	0.0048	0.0029	0.0029	0.0032	0.0037	0.0031	0.0033
0	0	0	0	0	0	0	0	0
0	0	0	0	0	0	0	0	0
0	0	0	0	0	0	0	0	0
0.0006	0.0012	0.0024	0.0024	0.0021	0.002	0.0019	0.0014	0.0015
0.0004	0.0017	0.0032	0.0033	0.0029	0.0026	0.0024	0.0017	0.0017
0.0011	0.0027	0.0052	0.0054	0.005	0.0047	0.0048	0.0038	0.0038
0.0011	0.005	0.0063	0.0103	0.0088	0.0106	0.0124	0.0105	0.0109
0	0.0026	0.0043	0.0056	0.0051	0.0058	0.0069	0.0059	0.0062

0.0571	0.029	0.0304	0.0251	0.0281	0.0298	0.0325	0.0275	0.0268
-0.004	0.0099	0.0152	0.0223	0.0207	0.0251	0.0272	0.0199	0.021
0.0016	0.0155	0.0317	0.0362	0.0337	0.0376	0.0378	0.0309	0.0317
0.0559	0.0649	0.0984	0.1036	0.0939	0.0972	0.0987	0.0806	0.074
0.1763	0.1403	0.1739	0.1847	0.1706	0.1794	0.1862	0.1542	0.1459
0.2304	0.1456	0.1716	0.1803	0.1711	0.1836	0.1853	0.1514	0.1464
0.053	0.0885	0.1437	0.1588	0.1507	0.1558	0.1659	0.1344	0.1325
0.0207	0.085	0.1539	0.1674	0.1493	0.146	0.1642	0.134	0.1237
0.2816	0.1864	0.2282	0.2307	0.216	0.2176	0.2206	0.1754	0.1585
0.4486	0.2224	0.2508	0.2421	0.234	0.2249	0.2462	0.1978	0.1667
0.2928	0.1878	0.2555	0.2459	0.2379	0.2357	0.2156	0.1639	0.1429
0.137	0.1441	0.2395	0.2409	0.2197	0.2166	0.2125	0.1771	0.16
0.0409	0.0781	0.132	0.1349	0.1216	0.1177	0.1231	0.0958	0.0904
0.847	0.4262	0.4048	0.3591	0.3406	0.3861	0.3339	0.283	0.2386
0.508	0.278	0.3062	0.2895	0.2807	0.3108	0.3137	0.2632	0.2098
0.2565	0.1727	0.2251	0.2242	0.2115	0.2105	0.2147	0.1747	0.1613
-0.0168	0.034	0.0552	0.0794	0.0751	0.0976	0.1108	0.0939	0.0919
-0.0056	0.039	0.0538	0.093	0.0697	0.1102	0.1365	0.1269	0.1209
0.0566	0.0888	0.1244	0.1373	0.1224	0.1452	0.1547	0.1303	0.1237
0.0317	0.0875	0.1723	0.1998	0.1742	0.1752	0.1865	0.1512	0.1478
0.1038	0.1068	0.1703	0.1755	0.1607	0.1658	0.1742	0.1475	0.1369
0.4794	0.2232	0.2313	0.2402	0.2313	0.2377	0.2542	0.2011	0.1605
0.6699	0.3486	0.3214	0.3168	0.3309	0.3433	0.3062	0.2611	0.2285
0.3818	0.2341	0.2903	0.2813	0.2623	0.2757	0.2758	0.2292	0.2254
0.0334	0.106	0.2057	0.2156	0.1984	0.1938	0.1938	0.1562	0.1366
0.0339	0.1126	0.2342	0.2439	0.2117	0.1961	0.1921	0.1496	0.143
0.3587	0.1984	0.2356	0.2206	0.2115	0.2244	0.2166	0.1769	0.1622
0.4622	0.2468	0.262	0.2467	0.2345	0.2402	0.2241	0.1988	0.1795
0.6516	0.347	0.3571	0.3435	0.34	0.3679	0.3595	0.2832	0.275
0.4949	0.234	0.2096	0.2202	0.2198	0.2483	0.2654	0.2354	0.2245
0.0472	0.0683	0.095	0.11	0.0929	0.104	0.1089	0.0949	0.0927
0.007	0.0383	0.0645	0.0755	0.0721	0.0705	0.0802	0.0641	0.0591
-0.0001	0.0068	0.0125	0.0154	0.0144	0.0155	0.0184	0.0142	0.0148
0.01	0.0488	0.0684	0.116	0.1132	0.1454	0.174	0.1589	0.1653
-0.0091	0.0466	0.0576	0.0888	0.0767	0.1073	0.1242	0.1166	0.1225
0.0021	0.0074	0.0107	0.0154	0.0144	0.0163	0.0193	0.0172	0.0156
0.0018	0.0069	0.0105	0.012	0.0108	0.0114	0.0126	0.0112	0.0115
0	0	0	0	0	0	0	0	0
0	0	0	0	0	0	0	0	0
0	0	0	0	0	0	0	0	0
0.0003	0.0007	0.0012	0.0012	0.0011	0.001	0.0009	0.0007	0.0006
0.0158	0.0088	0.0078	0.007	0.0061	0.0067	0.0063	0.0052	0.0049
0	0	0	0	0	0	0	0	0
0	0	0	0	0	0	0	0	0

0	0	0	0	0	0	0	0	0
0.0003	0.0007	0.0012	0.0012	0.001	0.001	0.001	0.0008	0.0007
0.0001	0.0011	0.0022	0.0022	0.002	0.0019	0.0019	0.0015	0.0015
0.0004	0.0012	0.0024	0.0023	0.0021	0.0019	0.0018	0.0013	0.0013
0.0079	0.0044	0.0043	0.004	0.0039	0.004	0.0035	0.0023	0.0019
0.002	0.0069	0.0137	0.0145	0.0123	0.0132	0.0137	0.0107	0.0101
0.0017	0.0035	0.0064	0.0061	0.0053	0.0049	0.0048	0.0039	0.0037
0.0228	0.0133	0.0118	0.0143	0.0129	0.0159	0.0178	0.0156	0.0158
0.1583	0.0704	0.0682	0.0632	0.0592	0.0654	0.071	0.0533	0.048
0.2461	0.1354	0.137	0.1376	0.1305	0.1333	0.1299	0.1069	0.1109
0.148	0.1106	0.135	0.1536	0.1435	0.1627	0.1796	0.1547	0.156
-0.0055	0.0546	0.0983	0.1284	0.1192	0.1338	0.1495	0.1161	0.1161
0.0022	0.065	0.0927	0.1112	0.0837	0.0951	0.1162	0.0862	0.0808
0.056	0.0916	0.1425	0.158	0.146	0.1578	0.1713	0.1398	0.1346
0.4305	0.2336	0.2386	0.2144	0.2025	0.2249	0.2372	0.1839	0.1867
0.8791	0.4184	0.3632	0.2994	0.2856	0.3465	0.3532	0.2306	0.2003
0.1892	0.1646	0.2523	0.244	0.2271	0.2229	0.2197	0.1649	0.1623
0.511	0.28	0.316	0.2861	0.2533	0.2535	0.2564	0.1949	0.1829
0.3308	0.1835	0.2225	0.2172	0.2148	0.2078	0.1989	0.158	0.1578
0.9061	0.3861	0.3756	0.363	0.3293	0.3781	0.379	0.3264	0.2413
1.2706	0.4662	0.3238	0.2817	0.2256	0.292	0.3152	0.2519	0.2392
0.7791	0.3492	0.335	0.344	0.32	0.3458	0.3461	0.2701	0.2277
0.099	0.1355	0.2212	0.2166	0.1994	0.1917	0.1922	0.151	0.1409
0.431	0.2364	0.2546	0.2391	0.2411	0.2513	0.2283	0.1911	0.1774
1.103	0.4275	0.331	0.2715	0.3494	0.3567	0.3649	0.3491	0.3128
0.6766	0.3607	0.376	0.3649	0.3766	0.3926	0.3939	0.3083	0.2858
0.142	0.129	0.1896	0.1905	0.1742	0.1761	0.1808	0.1385	0.1297
0.8066	0.3832	0.3111	0.2597	0.2628	0.2827	0.2829	0.2309	0.2515
0.8144	0.3621	0.3041	0.2946	0.3074	0.3613	0.3276	0.2604	0.2596
0.851	0.4404	0.4196	0.364	0.329	0.327	0.32	0.2441	0.2281
0.7862	0.3386	0.3017	0.2036	0.2043	0.2446	0.2283	0.1501	0.1506
0.5672	0.2847	0.2914	0.2782	0.2669	0.2663	0.2694	0.2222	0.2283
0.6655	0.3672	0.3759	0.3679	0.3296	0.3375	0.3144	0.2789	0.2771
0.8614	0.4512	0.4171	0.4029	0.3609	0.3988	0.3994	0.3654	0.3194
0.8598	0.407	0.3192	0.2611	0.2555	0.2552	0.264	0.1873	0.1691
0.111	0.101	0.1497	0.1471	0.1354	0.1323	0.1353	0.113	0.1062
0.0767	0.0453	0.0498	0.0466	0.0444	0.0479	0.0475	0.0398	0.0353
0	0	0	0	0	0	0	0	0
-0.0012	0.0183	0.027	0.0369	0.0373	0.0409	0.0494	0.0469	0.0475
0.0353	0.0618	0.0749	0.1149	0.1059	0.1227	0.1353	0.1184	0.1132
0.0461	0.0217	0.0171	0.0192	0.0195	0.0214	0.0219	0.0178	0.018
0.0445	0.0228	0.0245	0.0224	0.0216	0.0227	0.0223	0.0182	0.0161
0.03	0.0168	0.0167	0.0165	0.0174	0.0182	0.0187	0.0144	0.0145
0	0	0	0	0	0	0	0	0

0	0	0	0	0	0	0	0	0
0	0	0	0	0	0	0	0	0
-0.0002	0.0005	0.0011	0.0014	0.0012	0.0015	0.0018	0.0015	0.0017
0	0.0006	0.0013	0.0015	0.0014	0.0017	0.0018	0.0015	0.0015
0.0075	0.0039	0.0036	0.0035	0.0034	0.0036	0.0038	0.0034	0.0034
-0.0001	0.0002	0.0002	0.0004	0.0004	0.0005	0.0007	0.0006	0.0007
0.0001	0.0004	0.0005	0.0008	0.0007	0.0008	0.0009	0.0009	0.001
0.0224	0.0102	0.0082	0.0069	0.0069	0.007	0.0064	0.0072	0.0065
0.033	0.0137	0.0098	0.0097	0.0103	0.0132	0.0118	0.0086	0.0096
0.0161	0.0072	0.0056	0.0048	0.0041	0.005	0.0043	0.0035	0.0034
0.0808	0.0277	0.0225	0.0234	0.0263	0.025	0.0221	0.0215	0.0207
-0.0019	0.0044	0.0049	0.01	0.0082	0.0112	0.0141	0.0125	0.0123
0.0002	0.0024	0.0019	0.0038	0.0038	0.0047	0.0048	0.0049	0.0048
0.1698	0.0973	0.0939	0.0786	0.0761	0.0883	0.081	0.0683	0.0631
0.3489	0.1824	0.1866	0.191	0.1778	0.1904	0.18	0.1448	0.1276
0.2466	0.1182	0.1185	0.1107	0.0945	0.1154	0.124	0.1031	0.0899
0.1155	0.0707	0.0613	0.0645	0.059	0.0679	0.0673	0.0591	0.0591
0.0159	0.0334	0.0519	0.0701	0.0629	0.0749	0.0814	0.072	0.0747
0.1184	0.1081	0.1258	0.1552	0.1375	0.1702	0.1884	0.1539	0.1545
0.0715	0.0938	0.1344	0.1543	0.1304	0.1427	0.164	0.1323	0.1344
0.0785	0.0915	0.1393	0.1459	0.1319	0.1307	0.137	0.1076	0.1025
0.6074	0.282	0.2772	0.238	0.2489	0.2332	0.2318	0.2188	0.1894
0.9425	0.3957	0.3263	0.2923	0.3063	0.3181	0.3745	0.3416	0.315
0.4986	0.2492	0.2664	0.2415	0.2246	0.2555	0.2585	0.2051	0.1958
0.3996	0.2532	0.2929	0.2674	0.2499	0.2546	0.2361	0.1959	0.193
0.5826	0.2837	0.2515	0.2361	0.2087	0.2229	0.2355	0.2124	0.2182
1.2472	0.5434	0.4625	0.4472	0.4649	0.4463	0.441	0.3509	0.3801
0.8707	0.4305	0.3682	0.3354	0.2934	0.322	0.2946	0.2515	0.2274
0.2371	0.1899	0.2619	0.2435	0.2292	0.2284	0.2204	0.169	0.1544
0.5869	0.2924	0.2394	0.2194	0.2277	0.2294	0.2369	0.2059	0.1675
1.3776	0.6259	0.5156	0.4291	0.464	0.4621	0.4742	0.3906	0.3202
1.0522	0.3811	0.3214	0.2366	0.2509	0.3133	0.2995	0.2933	0.2808
0.6214	0.2995	0.3224	0.3062	0.2922	0.2706	0.2389	0.1865	0.1617
0.943	0.4288	0.4035	0.3328	0.3048	0.3282	0.3367	0.2879	0.3058
0.8886	0.4263	0.3535	0.3148	0.2951	0.3275	0.325	0.2487	0.2179
1.1757	0.5259	0.4643	0.4029	0.3662	0.4191	0.4139	0.3728	0.3866
1.2832	0.5762	0.4599	0.3737	0.4654	0.4785	0.4699	0.3861	0.3715
1.1076	0.5102	0.449	0.3742	0.3356	0.3227	0.3195	0.3214	0.2833
1.3821	0.5295	0.4378	0.4052	0.4329	0.4428	0.4195	0.298	0.2613
0.7638	0.3649	0.3405	0.3043	0.2625	0.2602	0.2614	0.2235	0.2124
0.1392	0.1204	0.1629	0.1652	0.1521	0.1492	0.1532	0.1203	0.1103
0.0117	0.0419	0.0529	0.0629	0.0568	0.0639	0.0643	0.0554	0.0585
0.0259	0.0288	0.0252	0.036	0.0302	0.0462	0.0523	0.0464	0.0529
0.0336	0.0442	0.057	0.0914	0.0917	0.1195	0.1542	0.1339	0.1451

0.0375	0.0636	0.0761	0.1022	0.09	0.1051	0.1224	0.1146	0.1252
0.0003	0.0014	0.0023	0.003	0.0028	0.0035	0.0038	0.0032	0.0033
0.0536	0.0203	0.0152	0.0169	0.0183	0.0225	0.0228	0.02	0.0201
0.228	0.121	0.1091	0.1206	0.1083	0.1176	0.1326	0.1125	0.1095
0.2317	0.1128	0.0842	0.0761	0.0809	0.0905	0.0961	0.0811	0.068
0.016	0.0122	0.0148	0.0166	0.0159	0.0171	0.018	0.0148	0.0148
0	0	0	0	0	0	0	0	0
0	0	0	0	0	0	0	0	0
0.0003	0.001	0.0018	0.0019	0.0017	0.0017	0.0018	0.0015	0.0014
0.0005	0.0013	0.0018	0.0021	0.002	0.0021	0.002	0.0017	0.0016
0.0073	0.0035	0.0035	0.0034	0.0032	0.0036	0.0035	0.0028	0.0029
0.0001	0.0008	0.0012	0.0014	0.0013	0.0014	0.0016	0.0011	0.0011
0.0081	0.0042	0.0037	0.0034	0.0036	0.0038	0.0036	0.0031	0.0032
0.0302	0.0129	0.0087	0.0097	0.0113	0.0118	0.0113	0.0095	0.008
0.028	0.0111	0.0085	0.0082	0.0087	0.0099	0.0082	0.0066	0.0057
0.0139	0.0056	0.0048	0.0051	0.005	0.0052	0.0046	0.0037	0.003
0.0012	0.0111	0.0207	0.0236	0.0218	0.0235	0.0252	0.0198	0.0203
0	0	0	0	0	0	0	0	0
0.0295	0.0148	0.0119	0.0116	0.0123	0.0144	0.0147	0.0125	0.0128
0.1734	0.0766	0.0692	0.0638	0.0606	0.0677	0.0716	0.0651	0.0638
0.1952	0.1071	0.1038	0.1024	0.1002	0.1034	0.0989	0.0883	0.0796
0.0882	0.0646	0.073	0.0854	0.0814	0.0932	0.0989	0.088	0.0862
0.1053	0.0757	0.0796	0.0894	0.0819	0.0908	0.1007	0.0862	0.0835
0.1788	0.1097	0.1183	0.124	0.1197	0.1341	0.1431	0.1246	0.1166
0.0599	0.1159	0.1737	0.1921	0.1717	0.1792	0.1957	0.1641	0.1564
0.0639	0.0874	0.0955	0.1291	0.1117	0.1335	0.1545	0.1372	0.1414
0.0394	0.1026	0.1693	0.2096	0.1781	0.1833	0.2058	0.1736	0.1544
0.1789	0.1104	0.1266	0.1269	0.1193	0.1314	0.1335	0.113	0.1098
1.0118	0.4257	0.3291	0.3008	0.2902	0.2579	0.2138	0.1925	0.2002
0.5137	0.3127	0.3827	0.3612	0.3339	0.3548	0.3497	0.2815	0.2701
0.3258	0.2044	0.2507	0.2511	0.2357	0.2298	0.2474	0.2139	0.1943
0.6131	0.2665	0.2222	0.175	0.1668	0.1867	0.1857	0.1433	0.1243
0.6973	0.3247	0.2744	0.2167	0.216	0.1985	0.2119	0.1789	0.194
0.3549	0.2129	0.255	0.2322	0.2204	0.2243	0.2149	0.1654	0.1456
0.2031	0.1667	0.2349	0.2294	0.2063	0.199	0.2053	0.166	0.1498
0.2374	0.14	0.1734	0.1721	0.1586	0.1662	0.1749	0.1456	0.1311
0.3762	0.1846	0.1898	0.1659	0.1583	0.1623	0.1695	0.1341	0.1261
0.6184	0.292	0.2913	0.2432	0.1983	0.2051	0.1956	0.1646	0.1506
0.9243	0.4228	0.3401	0.3203	0.2943	0.2989	0.3024	0.2725	0.2561
0.9435	0.3155	0.2667	0.2547	0.2713	0.2758	0.2717	0.2274	0.2155
0.7776	0.3585	0.3209	0.2844	0.2844	0.2766	0.2476	0.2034	0.1896
0.8423	0.4176	0.369	0.3231	0.3531	0.3487	0.3185	0.2383	0.2096
0.8996	0.329	0.2133	0.1885	0.1603	0.1984	0.1819	0.1978	0.1465
1.3007	0.5699	0.475	0.3523	0.3923	0.4365	0.401	0.3934	0.3093

1.4182	0.6373	0.4826	0.5328	0.4926	0.5309	0.5534	0.5409	0.5019
0.4075	0.2341	0.2632	0.2445	0.2372	0.2444	0.2446	0.1974	0.1691
0.044	0.0612	0.0907	0.1023	0.0951	0.1053	0.1144	0.0876	0.0835
0.0991	0.0807	0.0698	0.0924	0.0881	0.1069	0.1204	0.1048	0.1027
-0.0401	0.023	0.038	0.0946	0.0738	0.1031	0.1505	0.147	0.1571
0.0778	0.1005	0.1042	0.1465	0.1341	0.1423	0.1631	0.1546	0.1598
0.0465	0.0309	0.0301	0.0367	0.0331	0.0376	0.0416	0.0393	0.0421
0.001	0.0024	0.0048	0.0048	0.0045	0.0042	0.0041	0.0032	0.0031
0.0463	0.0264	0.0286	0.0276	0.0269	0.0287	0.0305	0.0254	0.0255
0.2133	0.0832	0.0549	0.0519	0.0604	0.0679	0.069	0.0598	0.0612
0.1733	0.0369	0.0193	0.0182	0.0219	0.0418	0.039	0.036	0.0334
0.0015	0.004	0.0073	0.008	0.0069	0.0062	0.0059	0.0047	0.0042
0	0	0	0	0	0	0	0	0
0	0	0	0	0	0	0	0	0
0.0001	0.0006	0.0011	0.0011	0.0011	0.0011	0.001	0.0008	0.0007
0.0001	0.0005	0.001	0.001	0.0009	0.0009	0.0009	0.0007	0.0006
0.0003	0.0007	0.0012	0.0013	0.0012	0.0011	0.0011	0.0008	0.0008
0.0001	0.0005	0.0011	0.0011	0.001	0.0009	0.0009	0.0007	0.0007
0	0	0	0	0	0	0	0	0
0	0	0	0	0	0	0	0	0
0	0	0	0	0	0	0	0	0
0	0	0	0	0	0	0	0	0
0.0001	0.0004	0.0004	0.0007	0.0006	0.0007	0.0008	0.0007	0.0008
0.0289	0.0111	0.0068	0.0047	0.0052	0.0066	0.0065	0.0048	0.0036
0.0248	0.0179	0.0221	0.0218	0.0203	0.0211	0.0215	0.018	0.017
0.0007	0.0073	0.0151	0.016	0.0144	0.015	0.0156	0.0131	0.0117
0.0001	0.0177	0.0249	0.0443	0.04	0.0527	0.0627	0.0553	0.0612
0.0428	0.069	0.0945	0.1152	0.0975	0.1114	0.1265	0.112	0.1167
0.0549	0.0551	0.076	0.0827	0.0776	0.0822	0.0848	0.0717	0.0692
0.014	0.039	0.0604	0.0721	0.0688	0.0737	0.0797	0.0706	0.067
0.0005	0.0101	0.0181	0.0228	0.0224	0.0236	0.0278	0.0226	0.0226
0.0622	0.0707	0.1001	0.1109	0.105	0.1141	0.1206	0.0997	0.0917
0.0146	0.0483	0.0752	0.1022	0.1074	0.1219	0.1444	0.1311	0.1404
0.1227	0.1105	0.1473	0.169	0.1537	0.1729	0.1838	0.1605	0.1614
0.1278	0.0946	0.1309	0.1287	0.1201	0.1232	0.1253	0.0996	0.0946
1.0609	0.4478	0.3866	0.3239	0.3149	0.2844	0.2375	0.2194	0.2478
0.717	0.3924	0.4159	0.4022	0.3637	0.3989	0.3944	0.331	0.3102
0.3398	0.2384	0.299	0.2885	0.2889	0.2762	0.2795	0.2248	0.2154
0.4638	0.262	0.3002	0.2736	0.2683	0.2706	0.3003	0.2371	0.2118
0.2324	0.2084	0.3076	0.3102	0.289	0.2737	0.2673	0.2127	0.1867
0.3261	0.236	0.2986	0.2913	0.2776	0.2739	0.2743	0.2161	0.1927
0.709	0.3879	0.3971	0.3523	0.3405	0.3644	0.3332	0.2708	0.2412
0.5702	0.2898	0.3171	0.3152	0.3367	0.3542	0.3757	0.3177	0.2652
0.5174	0.2988	0.3143	0.297	0.2851	0.2825	0.2749	0.2249	0.2093

0.8055	0.4259	0.3964	0.3432	0.349	0.3733	0.3277	0.2888	0.2386
1.1731	0.5377	0.4377	0.3299	0.3172	0.3611	0.3244	0.2455	0.2105
0.7616	0.3903	0.3568	0.2835	0.2634	0.2707	0.2553	0.1715	0.1421
0.6424	0.3468	0.3572	0.3441	0.3342	0.3432	0.3428	0.2667	0.2317
0.6943	0.3731	0.3201	0.2941	0.2843	0.3405	0.3505	0.2969	0.2838
0.6314	0.2919	0.2497	0.2401	0.2679	0.3079	0.2726	0.2182	0.2254
0.3631	0.215	0.2516	0.2514	0.2334	0.2512	0.2536	0.221	0.218
-0.0022	0.0536	0.0839	0.1114	0.0959	0.1082	0.1369	0.1151	0.1064
0.1756	0.0977	0.0846	0.0974	0.0981	0.1183	0.1273	0.1166	0.1157
0.2427	0.122	0.1006	0.1074	0.1024	0.1154	0.1164	0.1054	0.1001
0.048	0.0396	0.0419	0.0513	0.0468	0.0554	0.0602	0.0528	0.0555
0.004	0.0144	0.0222	0.0258	0.025	0.0253	0.0262	0.0224	0.022
0	0	0	0	0	0	0	0	0
0.0255	0.0443	0.0737	0.0778	0.0738	0.0735	0.0764	0.0649	0.0632
0.0416	0.0498	0.087	0.0854	0.0765	0.0779	0.0796	0.0642	0.0606
0.0665	0.0304	0.0273	0.0271	0.0257	0.0263	0.0243	0.0201	0.0171
0.0551	0.0306	0.0309	0.0294	0.0324	0.0346	0.033	0.0251	0.0236
-0.0004	0.0014	0.001	0.0031	0.0028	0.0035	0.0041	0.0043	0.0048
0	0	0	0	0	0	0	0	0
0	0	0	0	0	0	0	0	0
0	0	0	0	0	0	0	0	0
0	0	0	0	0	0	0	0	0
0	0	0	0	0	0	0	0	0
0	0	0	0	0	0	0	0	0
0	0	0	0	0	0	0	0	0
0	0	0	0	0	0	0	0	0
0	0	0	0	0	0	0	0	0
0	0	0	0	0	0	0	0	0
0.0005	0.0013	0.0018	0.0019	0.0018	0.002	0.0022	0.0017	0.0017
0.0003	0.0015	0.0026	0.0029	0.0024	0.0024	0.0023	0.0018	0.0019
0.001	0.0032	0.0058	0.0067	0.0055	0.0055	0.0054	0.0044	0.0042
0	0.0027	0.0059	0.0069	0.0062	0.0064	0.0067	0.0054	0.0053
0	0.0039	0.0084	0.0098	0.0088	0.0091	0.0105	0.0079	0.0079
0.0018	0.0061	0.0094	0.0117	0.0102	0.0096	0.01	0.0075	0.0081
0.0015	0.0056	0.0088	0.0099	0.0086	0.0103	0.0112	0.0089	0.008
-0.0005	0.004	0.0071	0.0092	0.0077	0.009	0.0097	0.0089	0.0088
0.0149	0.0083	0.0076	0.008	0.0072	0.0085	0.0094	0.0088	0.0087
0.0631	0.0802	0.1142	0.1213	0.1064	0.1062	0.1086	0.0912	0.0878
0.1018	0.1087	0.1518	0.1731	0.1566	0.1709	0.1824	0.1522	0.1478
0.0379	0.0952	0.1689	0.1815	0.1651	0.1703	0.1801	0.1428	0.1448
0.0314	0.0843	0.1532	0.1697	0.1555	0.1634	0.1619	0.1279	0.1321
0.3048	0.1699	0.1597	0.1616	0.1589	0.1646	0.1695	0.1447	0.1385
0.6069	0.3086	0.294	0.29	0.2905	0.3307	0.3164	0.2633	0.243
0.6244	0.3238	0.3094	0.2926	0.2794	0.2734	0.27	0.2173	0.2145

1.0408	0.4779	0.4121	0.4109	0.4164	0.3917	0.4092	0.4195	0.4033
0.4687	0.2832	0.3049	0.2823	0.2735	0.2728	0.2635	0.2055	0.1812
0.3392	0.1634	0.1874	0.1665	0.1773	0.1957	0.1826	0.166	0.1414
0.8545	0.3809	0.302	0.2542	0.2287	0.2667	0.2487	0.1933	0.1727
0.6469	0.2946	0.2618	0.2364	0.2161	0.2242	0.2448	0.1998	0.1948
0.1092	0.1301	0.204	0.2162	0.1954	0.1915	0.2067	0.1695	0.1541
0.1817	0.1399	0.1805	0.1821	0.1642	0.1644	0.1666	0.1381	0.1298
0.5206	0.2379	0.2081	0.2004	0.2083	0.2396	0.2389	0.219	0.1892
0.5991	0.3216	0.3137	0.2874	0.273	0.2893	0.2802	0.235	0.2408
0.0836	0.1094	0.1628	0.1771	0.1549	0.1628	0.1733	0.1409	0.1323
0.0198	0.0861	0.1496	0.1683	0.1489	0.1596	0.1762	0.1457	0.1537
0.1141	0.1479	0.2014	0.2269	0.2101	0.2205	0.2328	0.1988	0.1948
0.0524	0.0944	0.1226	0.1485	0.1339	0.1481	0.1487	0.1247	0.1255
0.0069	0.0234	0.0297	0.0438	0.0429	0.0486	0.0526	0.0449	0.0442
0.3346	0.1342	0.1115	0.1119	0.0992	0.121	0.135	0.1153	0.1025
0.4751	0.1836	0.1182	0.1103	0.1129	0.1053	0.1105	0.1102	0.1036
0.1409	0.0547	0.0374	0.0248	0.0356	0.046	0.0347	0.0291	0.0229
0.0143	0.0059	0.0049	0.0052	0.0053	0.0052	0.0053	0.0048	0.0044
0.0036	0.0097	0.0159	0.0165	0.0154	0.0148	0.0153	0.0108	0.0111
0.0288	0.0326	0.0529	0.0517	0.0487	0.0482	0.0464	0.0361	0.0327
0.0126	0.022	0.0414	0.042	0.0364	0.0364	0.0363	0.0294	0.0291
0.0014	0.0076	0.0147	0.0148	0.0139	0.0143	0.0157	0.0122	0.0107
0	0.0007	0.0013	0.0016	0.0014	0.0015	0.0016	0.0014	0.0015
0	0	0	0	0	0	0	0	0
0	0	0	0	0	0	0	0	0
0	0	0	0	0	0	0	0	0
0	0	0	0	0	0	0	0	0
0	0	0	0	0	0	0	0	0
0	0	0	0	0	0	0	0	0
0	0	0	0	0	0	0	0	0
0	0	0	0	0	0	0	0	0
0	0	0	0	0	0	0	0	0
0	0	0	0	0	0	0	0	0
0	0	0	0	0	0	0	0	0
0	0	0	0	0	0	0	0	0
0	0	0	0	0	0	0	0	0
0.0005	0.0018	0.0029	0.0031	0.0027	0.003	0.0032	0.0028	0.0028
0.0004	0.0024	0.0044	0.005	0.0046	0.0049	0.0052	0.0042	0.0039
0.0009	0.0029	0.0053	0.0058	0.0053	0.0054	0.0057	0.0043	0.0041
-0.0002	0.002	0.0039	0.0045	0.0041	0.0045	0.0051	0.0041	0.004
0.0005	0.0026	0.0039	0.0052	0.0052	0.0056	0.0065	0.0052	0.0046
0	0.0021	0.0033	0.0045	0.0041	0.0044	0.0048	0.0039	0.0039
-0.0022	0.0073	0.0138	0.0172	0.0155	0.0158	0.0188	0.0136	0.014
0.0255	0.058	0.0748	0.0943	0.0862	0.0969	0.1133	0.1009	0.1007

0.0368	0.0496	0.0628	0.0896	0.0775	0.0961	0.1104	0.0946	0.1071
0.019	0.0761	0.1136	0.1498	0.1242	0.1317	0.146	0.1246	0.125
-0.0031	0.0724	0.1245	0.1501	0.1463	0.1722	0.1831	0.1515	0.1594
0.0786	0.0846	0.1202	0.1365	0.1293	0.1424	0.1538	0.1259	0.1307
0.0532	0.0794	0.1037	0.1307	0.128	0.1504	0.1626	0.1369	0.1302
0.0107	0.0634	0.1027	0.1209	0.1042	0.1194	0.132	0.1113	0.1109
0.0196	0.0752	0.11	0.1358	0.1169	0.1185	0.1285	0.1211	0.1191
0.0477	0.1075	0.1928	0.1951	0.1756	0.1847	0.1884	0.1516	0.1497
0.0464	0.11	0.1897	0.2077	0.1879	0.1975	0.1943	0.1552	0.1659
0.0363	0.0737	0.1333	0.1582	0.1433	0.1524	0.1636	0.1415	0.1364
0.0596	0.0896	0.1283	0.1511	0.1354	0.1348	0.1348	0.1198	0.1071
0.021	0.0833	0.1476	0.1608	0.1463	0.15	0.1622	0.139	0.1364
0.0508	0.0881	0.1482	0.1692	0.1456	0.1548	0.1591	0.1308	0.1188
0.2297	0.1709	0.217	0.2234	0.2086	0.2219	0.2298	0.192	0.1865
-0.0127	0.0678	0.1124	0.1554	0.1359	0.1589	0.1804	0.1566	0.1543
0.0007	0.0496	0.0859	0.1152	0.0947	0.1226	0.1335	0.1031	0.1106
0.2014	0.1399	0.1331	0.1588	0.1588	0.1927	0.2214	0.186	0.1856
0.1754	0.1483	0.1425	0.1762	0.1603	0.1837	0.2012	0.1802	0.1793
0.0296	0.0357	0.0488	0.0581	0.0523	0.0592	0.0666	0.061	0.059
0.0221	0.0575	0.0913	0.117	0.1017	0.1121	0.1261	0.1155	0.1153
0.0036	0.0693	0.0947	0.121	0.1159	0.1209	0.1299	0.1009	0.1182
0.5081	0.2497	0.2124	0.1783	0.1702	0.1605	0.1647	0.1577	0.1567
0.2048	0.087	0.083	0.0813	0.0859	0.0883	0.0889	0.0697	0.0638
0.0724	0.0354	0.0319	0.0266	0.0274	0.0262	0.0261	0.0226	0.0228
0	0	0	0	0	0	0	0	0
0.0024	0.0053	0.0102	0.0103	0.0088	0.0085	0.0089	0.0068	0.0068
0.0012	0.0039	0.0068	0.0068	0.0059	0.0055	0.0062	0.0047	0.0044
0	0.0004	0.0003	0.0005	0.0004	0.0005	0.0007	0.0006	0.0006
0.0139	0.0059	0.0046	0.005	0.0043	0.005	0.0042	0.0029	0.0024
0.0315	0.0152	0.0132	0.0117	0.0117	0.012	0.0109	0.0094	0.007
0	0	0	0	0	0	0	0	0
0	0	0	0	0	0	0	0	0
0	0	0	0	0	0	0	0	0
0	0	0	0	0	0	0	0	0
0	0	0	0	0	0	0	0	0
0	0	0	0	0	0	0	0	0
0	0	0	0	0	0	0	0	0
0	0	0	0	0	0	0	0	0
0	0	0	0	0	0	0	0	0
0	0	0	0	0	0	0	0	0
0	0	0	0	0	0	0	0	0
0	0	0	0	0	0	0	0	0
0	0	0	0	0	0	0	0	0
0.0001	0.0004	0.0011	0.0011	0.0009	0.0009	0.001	0.0008	0.0008

0.0001	0.0005	0.001	0.001	0.001	0.0009	0.0009	0.0007	0.0007
0.0003	0.0007	0.0012	0.0012	0.001	0.001	0.001	0.0007	0.0007
0	0	0	0	0	0	0	0	0
0	0	0	0	0	0	0	0	0
0.0022	0.0049	0.0039	0.0061	0.0057	0.0071	0.0077	0.0072	0.0074
0.0635	0.0382	0.0348	0.0389	0.0344	0.0417	0.0453	0.0409	0.0404
0.0742	0.0492	0.0467	0.0504	0.0455	0.052	0.0554	0.0493	0.0491
0.0277	0.0648	0.0781	0.1162	0.1053	0.1353	0.157	0.1357	0.1432
0.0195	0.037	0.0409	0.068	0.0524	0.0718	0.0909	0.0778	0.0919
0.0279	0.031	0.046	0.0497	0.0456	0.0479	0.0495	0.0395	0.0374
0.0419	0.0767	0.1127	0.1293	0.1145	0.1279	0.1356	0.1106	0.1142
0.0762	0.089	0.1298	0.1481	0.1343	0.1523	0.1641	0.1321	0.1324
0.0091	0.054	0.1156	0.1303	0.1184	0.1228	0.1272	0.1026	0.0968
0.0446	0.0405	0.0564	0.0592	0.0539	0.0573	0.0591	0.0495	0.047
0.24	0.1126	0.1155	0.106	0.094	0.108	0.1106	0.0856	0.081
0.0171	0.0507	0.0914	0.0975	0.0875	0.0867	0.0892	0.0673	0.0655
0.0197	0.0611	0.1127	0.108	0.091	0.0803	0.0813	0.0583	0.0576
0.1452	0.0935	0.1239	0.1292	0.1189	0.1185	0.1176	0.0942	0.0888
0.425	0.2097	0.2117	0.1939	0.2096	0.2179	0.225	0.1858	0.1766
0.8319	0.3848	0.3605	0.3257	0.3462	0.357	0.3677	0.3098	0.2506
0.3392	0.1655	0.153	0.1289	0.1197	0.1419	0.1374	0.1033	0.0918
0.0746	0.0472	0.0456	0.0544	0.0504	0.0604	0.0685	0.0614	0.0607
0.4144	0.1701	0.1259	0.1198	0.1122	0.1496	0.1457	0.1368	0.1331
0.3406	0.161	0.1348	0.13	0.1342	0.1395	0.1385	0.1338	0.1259
0.0267	0.0259	0.0397	0.0406	0.0361	0.0365	0.0371	0.0295	0.0282
0.4286	0.2022	0.1842	0.1822	0.1745	0.1881	0.1677	0.1389	0.1113
0.0603	0.0539	0.0598	0.0735	0.0649	0.0783	0.0887	0.0742	0.0767
0.4792	0.211	0.2071	0.1857	0.174	0.1787	0.1577	0.1384	0.109
0.1672	0.0808	0.0809	0.0778	0.0773	0.0866	0.0765	0.0704	0.0653
0.0445	0.0181	0.014	0.0147	0.0136	0.0142	0.0146	0.0127	0.0132
0	0	0	0	0	0	0	0	0
0	0	0	0	0	0	0	0	0
0	0	0	0	0	0	0	0	0
0	0.0003	0.0003	0.0005	0.0004	0.0004	0.0006	0.0005	0.0006
0.0151	0.0074	0.0075	0.0069	0.007	0.008	0.0082	0.0072	0.0072
0.0223	0.0125	0.015	0.0147	0.0146	0.0139	0.0137	0.0115	0.0101
0	0	0	0	0	0	0	0	0
0	0	0	0	0	0	0	0	0
0	0	0	0	0	0	0	0	0
0	0	0	0	0	0	0	0	0
0	0	0	0	0	0	0	0	0
0	0	0	0	0	0	0	0	0
0	0	0	0	0	0	0	0	0
0	0	0	0	0	0	0	0	0

0	0	0	0	0	0	0	0	0
0	0	0	0	0	0	0	0	0
0	0.0003	0.0004	0.0007	0.0006	0.0007	0.0008	0.0007	0.0008
-0.0001	0.0002	0.0003	0.0007	0.0007	0.0008	0.0009	0.0008	0.0009
0	0	0	0	0	0	0	0	0
0	0	0	0	0	0	0	0	0
0	0	0	0	0	0	0	0	0
0	0	0	0	0	0	0	0	0
0	0	0	0	0	0	0	0	0
0	0	0	0	0	0	0	0	0
0.0408	0.026	0.032	0.0295	0.0289	0.03	0.0314	0.024	0.0217
0.0366	0.0632	0.1119	0.1185	0.1111	0.1112	0.1141	0.095	0.094
0.0023	0.0166	0.0239	0.0339	0.0246	0.0306	0.0397	0.0316	0.0345
0.0168	0.0227	0.0281	0.0389	0.0351	0.0437	0.0484	0.0421	0.044
0.2263	0.137	0.164	0.1577	0.1418	0.1377	0.1414	0.1093	0.1042
0.223	0.1089	0.0986	0.0944	0.0979	0.1118	0.1161	0.1016	0.0865
0.18	0.0864	0.0711	0.0677	0.0704	0.0776	0.0827	0.075	0.0713
0.235	0.119	0.1189	0.1093	0.0987	0.1054	0.1032	0.0936	0.0926
0.0974	0.0582	0.0694	0.0677	0.0598	0.0613	0.0592	0.0468	0.0456
0.3265	0.1251	0.1009	0.0902	0.087	0.0909	0.0966	0.0868	0.0967
1.0578	0.4901	0.3817	0.3251	0.3034	0.3354	0.3236	0.3584	0.3993
0.7299	0.2871	0.2541	0.2403	0.2183	0.2405	0.2958	0.2544	0.2122
0.0444	0.0334	0.037	0.0426	0.0386	0.042	0.046	0.0388	0.0392
0.1146	0.071	0.0716	0.0693	0.0635	0.0704	0.071	0.0587	0.053
0.0829	0.044	0.0472	0.0467	0.0462	0.0473	0.0477	0.039	0.0409
0	0.0003	0.0003	0.0006	0.0004	0.0006	0.0007	0.0007	0.0008
0.7528	0.3346	0.2398	0.2027	0.1961	0.25	0.2593	0.2699	0.213
0.4796	0.1829	0.1492	0.0974	0.1314	0.1482	0.1256	0.0837	0.0762
0.399	0.1937	0.1752	0.1616	0.1761	0.1913	0.2014	0.1783	0.1687
0.4924	0.2057	0.1661	0.1686	0.177	0.1913	0.1669	0.1468	0.1314
0.7361	0.3161	0.2709	0.2658	0.2661	0.312	0.3028	0.2657	0.2422
0.893	0.3669	0.3255	0.2976	0.2807	0.2963	0.2993	0.3063	0.2891
0.0186	0.0304	0.0485	0.0495	0.0449	0.0443	0.0468	0.0378	0.0348
0.1994	0.1185	0.1123	0.1124	0.0994	0.1045	0.1076	0.0889	0.0889
0.0109	0.0205	0.0342	0.0355	0.0309	0.032	0.0318	0.0257	0.0247
0.0011	0.0063	0.0109	0.0118	0.0105	0.0112	0.0123	0.0107	0.0098
-0.0002	0.0002	0.0002	0.0005	0.0005	0.0006	0.0008	0.0007	0.0007
0.0244	0.0136	0.0132	0.011	0.0111	0.0121	0.0108	0.0082	0.0076
0.0254	0.0138	0.012	0.0108	0.0103	0.0105	0.0103	0.0084	0.0071
0.0163	0.008	0.0075	0.007	0.0067	0.0076	0.0076	0.0062	0.0057
-0.0007	0.0014	0.0045	0.0049	0.005	0.0048	0.0049	0.0042	0.004
0.0013	0.0035	0.0063	0.0068	0.0059	0.0058	0.0061	0.0046	0.0044
0	0	0	0	0	0	0	0	0
0	0	0	0	0	0	0	0	0

0	0	0	0	0	0	0	0	0
0	0	0	0	0	0	0	0	0
0	0	0	0	0	0	0	0	0
0	0	0	0	0	0	0	0	0
0	0	0	0	0	0	0	0	0
0	0	0	0	0	0	0	0	0
0	0	0	0	0	0	0	0	0
0	0	0	0	0	0	0	0	0
0.0003	0.0017	0.0025	0.0031	0.0027	0.003	0.0034	0.003	0.0029
0.0002	0.0014	0.0019	0.0024	0.0022	0.0024	0.0028	0.0023	0.0026
0.0001	0.0012	0.002	0.0024	0.0021	0.0024	0.0025	0.0019	0.0021
0.0002	0.0009	0.0016	0.0018	0.0016	0.0017	0.0018	0.0015	0.0014
0	0	0	0	0	0	0	0	0
0	0	0	0	0	0	0	0	0
0.0003	0.0007	0.0012	0.0012	0.0011	0.0011	0.0011	0.0009	0.0009
0.0003	0.0007	0.0012	0.0012	0.001	0.001	0.001	0.0008	0.0008
0.0088	0.0062	0.0079	0.0073	0.0067	0.0069	0.0066	0.0053	0.0049
0.0751	0.0455	0.048	0.0506	0.0476	0.0547	0.0559	0.049	0.0471
0.0646	0.0642	0.0825	0.0933	0.0853	0.0979	0.1066	0.0888	0.0875
0.0883	0.0691	0.0928	0.0924	0.0864	0.0857	0.0842	0.0702	0.0642
0.1891	0.0866	0.0837	0.0721	0.0677	0.0782	0.0826	0.0738	0.0823
0.2607	0.1367	0.1223	0.1133	0.1095	0.1203	0.1212	0.0914	0.0867
0.4646	0.2273	0.2241	0.2145	0.1992	0.2057	0.2109	0.1711	0.1567
0.6961	0.3216	0.2226	0.2025	0.2017	0.255	0.2431	0.2166	0.2244
0.4875	0.1826	0.1367	0.1428	0.1519	0.1507	0.1492	0.1229	0.0925
0.0012	0.0042	0.0092	0.0092	0.0079	0.0072	0.0071	0.0053	0.0056
0.181	0.0769	0.0632	0.0516	0.0541	0.0629	0.0532	0.0454	0.0401
0.3205	0.149	0.1217	0.1241	0.1283	0.1412	0.1465	0.1261	0.1209
0.1713	0.0949	0.0975	0.1045	0.1003	0.1158	0.1254	0.1003	0.0938
0.3638	0.1663	0.1539	0.1484	0.1317	0.146	0.1498	0.1189	0.113
0.3276	0.1644	0.1687	0.1504	0.1452	0.1575	0.1556	0.1291	0.1117
0.3283	0.1745	0.1531	0.1496	0.1526	0.1734	0.18	0.1635	0.1434
0.5249	0.247	0.2201	0.2053	0.1745	0.2053	0.1995	0.1872	0.1655
0.1485	0.1022	0.0915	0.1154	0.114	0.1346	0.1464	0.1302	0.1357
0.2264	0.119	0.0976	0.1083	0.1158	0.126	0.1273	0.1074	0.1039
0.5574	0.2602	0.2383	0.2394	0.2321	0.2455	0.2389	0.1765	0.1681
0.1627	0.1306	0.1621	0.1799	0.1709	0.1861	0.1974	0.1579	0.1575
0.5296	0.205	0.2104	0.2057	0.2212	0.2178	0.2144	0.212	0.1953
0.4276	0.1831	0.1393	0.1055	0.0893	0.0824	0.0874	0.066	0.0628
0.5679	0.2798	0.2394	0.2359	0.2089	0.2269	0.2467	0.2166	0.2156
0.0026	0.0091	0.0181	0.0205	0.0186	0.0197	0.0213	0.0164	0.0161
0.0001	0.0024	0.0031	0.0044	0.0044	0.0052	0.006	0.0052	0.0052
0	0.0004	0.0004	0.0007	0.0007	0.0008	0.0009	0.0009	0.0009
0.0239	0.0106	0.0092	0.0082	0.0089	0.009	0.0085	0.0082	0.0074

0.0152	0.0073	0.0059	0.0056	0.0055	0.0065	0.0062	0.0053	0.0044
0.014	0.0052	0.004	0.0026	0.0023	0.0022	0.0019	0.001	0.0005
0.0075	0.0033	0.0027	0.0026	0.0024	0.0025	0.0027	0.0022	0.002
-0.0002	0.0001	0.0003	0.0006	0.0004	0.0006	0.0008	0.0006	0.0007
0	0	0	0	0	0	0	0	0
0	0	0	0	0	0	0	0	0
0	0	0	0	0	0	0	0	0
0	0	0	0	0	0	0	0	0
0	0	0	0	0	0	0	0	0
0	0	0	0	0	0	0	0	0
0	0	0	0	0	0	0	0	0
0	0	0	0	0	0	0	0	0
0	0	0	0	0	0	0	0	0
0	0	0	0	0	0	0	0	0
0.0014	0.0035	0.0054	0.0062	0.0058	0.0064	0.0072	0.0064	0.0061
0.0002	0.0026	0.0048	0.0053	0.0046	0.005	0.0052	0.0043	0.0043
0	0.0016	0.0031	0.0037	0.0034	0.0037	0.0041	0.0033	0.0033
-0.0003	0.0009	0.0015	0.0022	0.002	0.0023	0.0026	0.0022	0.002
0.0001	0.0005	0.0006	0.0009	0.0009	0.0011	0.0011	0.0011	0.001
0	0.0007	0.0013	0.0015	0.0014	0.0015	0.0017	0.0013	0.0013
0.0002	0.0007	0.0012	0.0012	0.001	0.0009	0.001	0.0008	0.0007
0.0005	0.0012	0.0023	0.0022	0.002	0.002	0.0019	0.0015	0.0014
0.0027	0.0079	0.0119	0.0131	0.0124	0.0129	0.0136	0.0117	0.012
0	0	0	0	0	0	0	0	0
0.0032	0.0307	0.0723	0.0748	0.0654	0.0637	0.0663	0.05	0.0488
0.0943	0.0801	0.1082	0.1093	0.1017	0.1033	0.1057	0.0842	0.0815
0.1739	0.0917	0.081	0.0738	0.069	0.066	0.0659	0.0536	0.0416
0.0945	0.0773	0.1087	0.104	0.0979	0.0998	0.1035	0.0826	0.0813
0.0563	0.0779	0.1234	0.1199	0.1101	0.1049	0.1019	0.078	0.0704
0.0741	0.0371	0.0373	0.0388	0.0345	0.0372	0.0365	0.0287	0.0273
0.0697	0.0326	0.0329	0.032	0.0308	0.0343	0.0347	0.0277	0.0291
-0.0001	0.0003	0.0004	0.0007	0.0006	0.0007	0.0008	0.0008	0.0009
0.0692	0.0244	0.0123	0.0108	0.0119	0.0171	0.0187	0.0163	0.0164
0.0028	0.009	0.0141	0.0172	0.015	0.0169	0.0188	0.0155	0.0157
-0.0009	0.016	0.0289	0.0412	0.0384	0.0417	0.0461	0.0415	0.0444
0.1614	0.063	0.0588	0.068	0.0658	0.0736	0.0691	0.0499	0.046
0.0548	0.0241	0.0203	0.0189	0.0163	0.0214	0.0241	0.0213	0.0199
0.0218	0.0302	0.0384	0.0487	0.0437	0.0551	0.0607	0.0505	0.0497
0.0347	0.0451	0.0609	0.0746	0.07	0.0828	0.0888	0.0724	0.0696
0.0397	0.0317	0.0386	0.0448	0.0413	0.046	0.0498	0.0417	0.0416
-0.0077	0.0101	0.0098	0.0206	0.0174	0.03	0.0436	0.0357	0.0349
0.3552	0.1347	0.1071	0.1214	0.1321	0.1641	0.1518	0.1311	0.1082
0.2238	0.0764	0.0559	0.056	0.055	0.0724	0.0794	0.079	0.063
0.1592	0.0636	0.0466	0.0358	0.0403	0.052	0.0493	0.0452	0.0444

0.007	0.0188	0.0351	0.036	0.03	0.03	0.0305	0.0227	0.0227
0.0526	0.0259	0.0246	0.0241	0.0222	0.0234	0.0231	0.019	0.0155
0.0608	0.0304	0.0303	0.0316	0.0287	0.0332	0.0338	0.0285	0.0263
0.015	0.0077	0.0064	0.0058	0.0055	0.0061	0.0073	0.0065	0.0065
0	0	0	0	0	0	0	0	0
0	0	0	0	0	0	0	0	0
0	0	0	0	0	0	0	0	0
0.0001	0.0003	0.0005	0.0008	0.0006	0.0007	0.0008	0.0007	0.0008
0.0225	0.0107	0.0095	0.0089	0.0076	0.0083	0.01	0.0091	0.009
0	0	0	0	0	0	0	0	0
0	0	0	0	0	0	0	0	0
0	0	0	0	0	0	0	0	0
0	0	0	0	0	0	0	0	0
0	0	0	0	0	0	0	0	0
0	0	0	0	0	0	0	0	0
0	0	0	0	0	0	0	0	0
0	0	0	0	0	0	0	0	0
0	0	0	0	0	0	0	0	0
0	0	0	0	0	0	0	0	0
0	0	0	0	0	0	0	0	0
0.0013	0.0027	0.0046	0.0049	0.0043	0.0041	0.0043	0.0035	0.0031
0.0006	0.0015	0.0027	0.0029	0.0026	0.0027	0.003	0.0024	0.0026
0.0001	0.0009	0.0016	0.0018	0.0016	0.0016	0.0017	0.0015	0.0015
0	0.0004	0.0005	0.0006	0.0007	0.0008	0.0008	0.0008	0.0008
0.0002	0.0006	0.0006	0.0008	0.0007	0.0008	0.001	0.0009	0.0008
0	0	0	0	0	0	0	0	0
0	0	0	0	0	0	0	0	0
0.0001	0.0005	0.0011	0.0012	0.001	0.0009	0.0009	0.0008	0.0007
0	0	0	0	0	0	0	0	0
0.0002	0.0026	0.0035	0.0063	0.0055	0.0065	0.0074	0.0066	0.0064
0.0634	0.0345	0.0368	0.0331	0.0296	0.032	0.0319	0.0259	0.0229
0.0862	0.0333	0.0231	0.0226	0.0253	0.0279	0.028	0.0218	0.0199
0	0.0004	0.0005	0.0007	0.0006	0.0006	0.0008	0.0007	0.0008
0.0042	0.0167	0.0359	0.0383	0.0335	0.0306	0.0313	0.0236	0.0248
0.0023	0.0054	0.009	0.0096	0.0089	0.0088	0.0091	0.0074	0.007
0.0297	0.0125	0.0095	0.0084	0.0088	0.0101	0.0096	0.0083	0.0078
0.0303	0.0135	0.0111	0.009	0.0093	0.0099	0.0105	0.0086	0.0068
0.0319	0.0238	0.0295	0.0318	0.0293	0.0317	0.0338	0.0276	0.0272
0.011	0.0153	0.0226	0.0253	0.0221	0.0234	0.0243	0.0196	0.0182
0.0072	0.0026	0.002	0.0019	0.0019	0.0024	0.0026	0.0023	0.002
0.0372	0.0179	0.014	0.0129	0.0127	0.0156	0.0161	0.0161	0.0144
0.0026	0.0107	0.0198	0.0212	0.0198	0.0209	0.0225	0.019	0.0183
0.0004	0.0046	0.0102	0.0094	0.0083	0.0089	0.008	0.0058	0.0047
0.0143	0.0063	0.0047	0.0039	0.0037	0.0037	0.0037	0.0032	0.0036

0.0362	0.0167	0.0156	0.0152	0.0172	0.0152	0.0126	0.009	0.0094
0.003	0.0086	0.0145	0.0152	0.0133	0.0124	0.0126	0.0101	0.0098
0.0003	0.0024	0.0056	0.0053	0.0046	0.0044	0.0044	0.0033	0.0029
0.0883	0.04	0.0343	0.0311	0.0307	0.0336	0.0297	0.0225	0.0222
0.0032	0.007	0.0124	0.0129	0.0116	0.0109	0.0109	0.0091	0.0083
0	0	0	0	0	0	0	0	0
0.0544	0.0226	0.0182	0.0198	0.0197	0.019	0.0218	0.0192	0.0184
0.0021	0.0083	0.0162	0.0162	0.0148	0.0141	0.0145	0.0108	0.0106
0.0684	0.0384	0.0328	0.0331	0.0293	0.0307	0.0302	0.0248	0.0199
0.024	0.0123	0.0127	0.0118	0.0099	0.0098	0.0095	0.0077	0.0076
0.0076	0.0041	0.0054	0.005	0.0051	0.0052	0.0052	0.0046	0.0041
0	0.0004	0.001	0.0011	0.001	0.0009	0.001	0.0008	0.0008
-0.0001	0.0001	0.0002	0.0005	0.0005	0.0006	0.0008	0.0007	0.0008
0.0005	0.0025	0.0049	0.0056	0.0048	0.0049	0.0052	0.0042	0.0038
0.0229	0.0121	0.0139	0.0133	0.0124	0.0133	0.0122	0.0103	0.0096
0.0003	0.0008	0.0014	0.0014	0.0012	0.0012	0.0011	0.0009	0.0009
0	0	0	0	0	0	0	0	0
0	0	0	0	0	0	0	0	0
0	0	0	0	0	0	0	0	0
0	0	0	0	0	0	0	0	0
0	0	0	0	0	0	0	0	0
0	0	0	0	0	0	0	0	0
0	0	0	0	0	0	0	0	0
0	0	0	0	0	0	0	0	0
0	0	0	0	0	0	0	0	0
0	0	0	0	0	0	0	0	0
0.0008	0.002	0.0039	0.004	0.0037	0.0032	0.0035	0.0027	0.0025
0.0004	0.0007	0.0012	0.0012	0.0011	0.001	0.0011	0.0008	0.0007
0	0	0	0	0	0	0	0	0
0	0	0	0	0	0	0	0	0
0	0	0	0	0	0	0	0	0
0	0	0	0	0	0	0	0	0
0	0	0	0	0	0	0	0	0
0	0	0	0	0	0	0	0	0
0.0521	0.0233	0.0196	0.0173	0.0168	0.0176	0.0169	0.0179	0.0153
0	0	0	0	0	0	0	0	0
0.0001	0.0007	0.0008	0.0011	0.0011	0.0013	0.0016	0.0016	0.0018
0.0398	0.0208	0.0207	0.0184	0.0192	0.0192	0.0183	0.017	0.015
0.0025	0.005	0.0085	0.0083	0.0075	0.0074	0.0072	0.0056	0.0055
0.0159	0.0072	0.0065	0.006	0.0055	0.0058	0.0062	0.0059	0.0042
0.047	0.0212	0.0179	0.0149	0.0139	0.0158	0.0149	0.0119	0.0117
0.0028	0.0059	0.0083	0.0098	0.0085	0.0098	0.0108	0.0088	0.0094
-0.0002	0.0022	0.0026	0.0044	0.0036	0.0045	0.0057	0.0056	0.0062
0	0	0	0	0	0	0	0	0

0	0	0	0	0	0	0	0	0
0.0443	0.0199	0.0188	0.0175	0.0158	0.015	0.0156	0.0119	0.0116
0.0001	0.0005	0.0005	0.0007	0.0007	0.0008	0.0009	0.0008	0.0009
-0.0002	0.0006	0.0006	0.0011	0.0009	0.0012	0.0014	0.0012	0.0014
0.1079	0.0466	0.0327	0.0333	0.0255	0.0249	0.0237	0.0163	0.0164
0	0	0	0	0	0	0	0	0
0	0	0	0	0	0	0	0	0
0.0386	0.0137	0.011	0.0093	0.0102	0.0113	0.0126	0.0097	0.009
0.0677	0.0353	0.0324	0.0301	0.0282	0.0317	0.0297	0.0254	0.0255
0	0	0	0	0	0	0	0	0
0.0753	0.0259	0.0178	0.011	0.0116	0.0126	0.0117	0.0075	0.0073
0.0184	0.0178	0.0289	0.0282	0.0251	0.0253	0.0262	0.0219	0.02
0.0003	0.0011	0.0024	0.0023	0.002	0.0021	0.0019	0.0016	0.0016
0	0.0011	0.0014	0.0022	0.002	0.0027	0.0033	0.0028	0.0027
0	0	0	0	0	0	0	0	0
0	0	0	0	0	0	0	0	0
0.0003	0.0008	0.0013	0.0013	0.0012	0.0012	0.0011	0.0009	0.0008
0.0069	0.0031	0.0035	0.0034	0.0028	0.0033	0.0033	0.0027	0.0022
-0.0006	0	0.0001	0.0006	0.0007	0.0011	0.0015	0.0013	0.0014
0.0072	0.0041	0.0048	0.0051	0.0048	0.0052	0.0055	0.0043	0.0043
0.0084	0.0056	0.0065	0.0065	0.0061	0.0066	0.0069	0.0057	0.0056
0	0	0	0	0	0	0	0	0
0	0	0	0	0	0	0	0	0
0	0	0	0	0	0	0	0	0
0	0	0	0	0	0	0	0	0
0	0	0	0	0	0	0	0	0
0	0	0	0	0	0	0	0	0
0	0	0	0	0	0	0	0	0
0	0	0	0	0	0	0	0	0
0	0	0	0	0	0	0	0	0
0	0	0	0	0	0	0	0	0
0	0	0	0	0	0	0	0	0
0	0	0	0	0	0	0	0	0
0	0	0	0	0	0	0	0	0
0	0	0	0	0	0	0	0	0
0	0	0	0	0	0	0	0	0
0	0	0	0	0	0	0	0	0
0	0	0	0	0	0	0	0	0
0	0	0	0	0	0	0	0	0
0	0	0	0	0	0	0	0	0
0	0	0	0	0	0	0	0	0
0	0	0	0	0	0	0	0	0
0	0	0	0	0	0	0	0	0

0	0	0	0	0	0	0	0	0
0	0	0	0	0	0	0	0	0
0	0	0	0	0	0	0	0	0
0	0	0	0	0	0	0	0	0
0	0	0	0	0	0	0	0	0
0	0	0	0	0	0	0	0	0
0	0	0	0	0	0	0	0	0
0	0	0	0	0	0	0	0	0
0	0	0	0	0	0	0	0	0
0	0	0	0	0	0	0	0	0
0	0	0	0	0	0	0	0	0
0	0	0	0	0	0	0	0	0
0	0	0	0	0	0	0	0	0
0	0	0	0	0	0	0	0	0
0	0	0	0	0	0	0	0	0
0	0	0	0	0	0	0	0	0
0	0	0	0	0	0	0	0	0
0	0	0	0	0	0	0	0	0
0	0	0	0	0	0	0	0	0
0	0	0	0	0	0	0	0	0
0	0	0	0	0	0	0	0	0
0	0	0	0	0	0	0	0	0
0	0	0	0	0	0	0	0	0
0	0	0	0	0	0	0	0	0
0	0	0	0	0	0	0	0	0
0	0	0	0	0	0	0	0	0
0	0	0	0	0	0	0	0	0
0	0	0	0	0	0	0	0	0
0	0	0	0	0	0	0	0	0
0	0	0	0	0	0	0	0	0
0	0	0	0	0	0	0	0	0
0	0	0	0	0	0	0	0	0
0	0	0	0	0	0	0	0	0
0	0	0	0	0	0	0	0	0
0	0	0	0	0	0	0	0	0
0	0	0	0	0	0	0	0	0
0	0	0	0	0	0	0	0	0
0	0	0	0	0	0	0	0	0
0	0	0	0	0	0	0	0	0
0	0	0	0	0	0	0	0	0
0	0	0	0	0	0	0	0	0
0	0	0	0	0	0	0	0	0
0	0	0	0	0	0	0	0	0
0	0	0	0	0	0	0	0	0

0	0	0	0	0	0	0	0	0
0	0	0	0	0	0	0	0	0
0	0	0	0	0	0	0	0	0
0	0	0	0	0	0	0	0	0
0	0	0	0	0	0	0	0	0
0	0	0	0	0	0	0	0	0
0	0	0	0	0	0	0	0	0
0	0	0	0	0	0	0	0	0
0	0	0	0	0	0	0	0	0
0	0	0	0	0	0	0	0	0
0	0	0	0	0	0	0	0	0
0	0	0	0	0	0	0	0	0
0	0	0	0	0	0	0	0	0
0	0	0	0	0	0	0	0	0
0	0	0	0	0	0	0	0	0
0	0	0	0	0	0	0	0	0
0	0	0	0	0	0	0	0	0
0	0	0	0	0	0	0	0	0
0	0	0	0	0	0	0	0	0
0	0	0	0	0	0	0	0	0
0	0	0	0	0	0	0	0	0
0	0	0	0	0	0	0	0	0
0	0	0	0	0	0	0	0	0
0	0	0	0	0	0	0	0	0
0	0	0	0	0	0	0	0	0
0	0	0	0	0	0	0	0	0
0	0	0	0	0	0	0	0	0
0	0	0	0	0	0	0	0	0
0	0	0	0	0	0	0	0	0
0	0	0	0	0	0	0	0	0
0	0	0	0	0	0	0	0	0
0	0	0	0	0	0	0	0	0
0	0	0	0	0	0	0	0	0
0	0	0	0	0	0	0	0	0
0	0	0	0	0	0	0	0	0
0	0	0	0	0	0	0	0	0

0	0	0	0	0	0	0	0	0
0	0	0	0	0	0	0	0	0
0	0	0	0	0	0	0	0	0
0	0	0	0	0	0	0	0	0
0	0	0	0	0	0	0	0	0
0	0	0	0	0	0	0	0	0
0	0	0	0	0	0	0	0	0
0	0	0	0	0	0	0	0	0
0	0	0	0	0	0	0	0	0
0	0	0	0	0	0	0	0	0
0	0	0	0	0	0	0	0	0
0	0	0	0	0	0	0	0	0
0	0	0	0	0	0	0	0	0
0	0	0	0	0	0	0	0	0
0	0	0	0	0	0	0	0	0
0	0	0	0	0	0	0	0	0
0.0102	0.0113	0.0107	0.0116	0.01	0.0111	0.011	0.0128	0.011
0.0025	0.0027	0.0027	0.0024	0.0024	0.0026	0.0026	0.0031	0.0025
0.0018	0.0017	0.0017	0.0016	0.0015	0.0014	0.0014	0.0014	0.0013
0.0134	0.0146	0.0142	0.0144	0.0131	0.0122	0.0112	0.0163	0.0164
0.0097	0.01	0.0102	0.0134	0.0131	0.0139	0.0128	0.0158	0.0163
0	0	0	0	0	0	0	0	0
0	0	0	0	0	0	0	0	0
0.0051	0.0052	0.005	0.0054	0.0052	0.0054	0.0056	0.0072	0.0057
0.0032	0.0032	0.002	0.0027	0.0016	0.0018	0.0014	0.0039	0.004
0.0141	0.0143	0.0127	0.0135	0.0114	0.0132	0.0122	0.0146	0.0132
0.012	0.0128	0.0124	0.0132	0.0126	0.0138	0.0142	0.0166	0.0131
0.0007	0.0007	0.0007	0.0007	0.0007	0.0007	0.0007	0.0007	0.0007
0.0009	0.0008	0.0009	0.0012	0.001	0.001	0.0012	0.0016	0.0017
0.0075	0.0079	0.0086	0.0094	0.0079	0.0089	0.0089	0.0118	0.0095
0.0048	0.0056	0.0063	0.0073	0.0075	0.0071	0.0076	0.0094	0.0069
0.0154	0.0163	0.0158	0.0191	0.0151	0.014	0.012	0.0137	0.0135
0	0	0	0	0	0	0	0	0
0.0039	0.0042	0.0042	0.0042	0.0039	0.004	0.0041	0.0047	0.0047
0.0083	0.0088	0.0087	0.0093	0.009	0.0092	0.0096	0.0114	0.0102
0.0021	0.002	0.0021	0.0022	0.0023	0.0026	0.0026	0.0029	0.0023
0	0	0	0	0	0	0	0	0
0.001	0.0011	0.001	0.0011	0.0011	0.0011	0.0012	0.0013	0.001
0.0025	0.0025	0.0025	0.0025	0.0027	0.0031	0.003	0.0037	0.0028
0	0	0	0	0	0	0	0	0
0	0	0	0	0	0	0	0	0
0	0	0	0	0	0	0	0	0
0	0	0	0	0	0	0	0	0
0	0	0	0	0	0	0	0	0

0	0	0	0	0	0	0	0	0
0	0	0	0	0	0	0	0	0
0	0	0	0	0	0	0	0	0
0	0	0	0	0	0	0	0	0
0	0	0	0	0	0	0	0	0
0	0	0	0	0	0	0	0	0
0	0	0	0	0	0	0	0	0
0	0	0	0	0	0	0	0	0
0	0	0	0	0	0	0	0	0
0	0	0	0	0	0	0	0	0
0	0	0	0	0	0	0	0	0
0	0	0	0	0	0	0	0	0
0	0	0	0	0	0	0	0	0
0	0	0	0	0	0	0	0	0
0	0	0	0	0	0	0	0	0
0	0	0	0	0	0	0	0	0
0	0	0	0	0	0	0	0	0
0	0	0	0	0	0	0	0	0
0	0	0	0	0	0	0	0	0
0	0	0	0	0	0	0	0	0
0	0	0	0	0	0	0	0	0
0	0	0	0	0	0	0	0	0
0.0023	0.0023	0.0023	0.0021	0.0021	0.0019	0.0022	0.0024	0.0021
0	0	0	0	0	0	0	0	0
0.0028	0.0029	0.0027	0.003	0.0028	0.0031	0.0032	0.004	0.003
0.0016	0.0016	0.0014	0.0014	0.0014	0.0015	0.0016	0.0023	0.0018
0.0014	0.0012	0.0013	0.0012	0.0012	0.0012	0.0013	0.0013	0.0012
0.0042	0.0073	0.0057	0.0088	0.0077	0.0081	0.0087	0.0111	0.0115
0.0173	0.0172	0.0152	0.0151	0.0115	0.011	0.0138	0.0181	0.0149
0.0044	0.0059	0.0054	0.0062	0.0057	0.0051	0.0048	0.0059	0.0063
0.0017	0.0018	0.0018	0.0018	0.0018	0.002	0.0019	0.0024	0.0017
0.0032	0.0036	0.0038	0.0038	0.0039	0.0039	0.0039	0.0048	0.0037
0.0042	0.0042	0.0042	0.0042	0.0042	0.0039	0.004	0.0053	0.0035
0.0025	0.0025	0.0025	0.0023	0.0024	0.0025	0.0026	0.003	0.0024
0.0047	0.0051	0.005	0.0051	0.0046	0.0052	0.0053	0.0064	0.0052
0	0	0	0	0	0	0	0	0
0.0008	0.0007	0.0006	0.0006	0.0005	0.0005	0.0005	0.0005	0.0004
0.0117	0.0118	0.0123	0.0128	0.0128	0.0136	0.0143	0.016	0.012
0.0015	0.0017	0.0016	0.0017	0.0017	0.0018	0.0019	0.002	0.0016
0.0013	0.0013	0.0013	0.0011	0.0012	0.0011	0.0011	0.0012	0.0012
0.0024	0.0023	0.0023	0.0025	0.0025	0.0027	0.0028	0.0033	0.0027
0.0007	0.0008	0.0008	0.001	0.001	0.0011	0.001	0.0013	0.001
0	0	0	0	0	0	0	0	0
0.0039	0.004	0.0039	0.0036	0.0033	0.0033	0.0035	0.004	0.004

0.0058	0.0059	0.0059	0.0061	0.0058	0.0059	0.006	0.0064	0.0057
0.001	0.0011	0.0011	0.001	0.001	0.0012	0.001	0.0013	0.0009
0	0	0	0	0	0	0	0	0
0	0	0	0	0	0	0	0	0
0	0	0	0	0	0	0	0	0
0	0	0	0	0	0	0	0	0
0	0	0	0	0	0	0	0	0
0	0	0	0	0	0	0	0	0
0	0	0	0	0	0	0	0	0
0	0	0	0	0	0	0	0	0
0	0	0	0	0	0	0	0	0
0	0	0	0	0	0	0	0	0
0	0	0	0	0	0	0	0	0
0	0	0	0	0	0	0	0	0
0	0	0	0	0	0	0	0	0
0	0	0	0	0	0	0	0	0
0	0	0	0	0	0	0	0	0
0	0	0	0	0	0	0	0	0
0	0	0	0	0	0	0	0	0
0	0	0	0	0	0	0	0	0
0	0	0	0	0	0	0	0	0
0	0	0	0	0	0	0	0	0
0	0	0	0	0	0	0	0	0
0	0	0	0	0	0	0	0	0
0	0	0	0	0	0	0	0	0
0	0	0	0	0	0	0	0	0
0	0	0	0	0	0	0	0	0
0	0	0	0	0	0	0	0	0
0.0082	0.0096	0.0093	0.0081	0.0076	0.0066	0.0067	0.0055	0.0053
0.006	0.0062	0.006	0.0073	0.0064	0.008	0.007	0.0083	0.0071
0.0113	0.0118	0.0114	0.0112	0.0104	0.01	0.0088	0.0117	0.012
0.0059	0.0065	0.0066	0.0076	0.0074	0.0082	0.0076	0.0096	0.0076
0.0008	0.0008	0.0008	0.0008	0.0007	0.0006	0.0006	0.0007	0.0007
0.0065	0.0072	0.0076	0.0076	0.0079	0.0081	0.0085	0.0101	0.0078
0.0135	0.0142	0.0152	0.0157	0.0143	0.0149	0.015	0.0185	0.0145
0	0	0	0	0	0	0	0	0
0	0	0	0	0	0	0	0	0
0.0062	0.0071	0.0066	0.0071	0.0055	0.0066	0.0074	0.007	0.0061
0.0013	0.0013	0.0012	0.0014	0.0012	0.0018	0.0022	0.0026	0.0022
0.0025	0.0025	0.0026	0.0025	0.0026	0.0028	0.003	0.0036	0.0028
0.0118	0.0128	0.0126	0.0145	0.0137	0.0141	0.0148	0.0163	0.0141
0.0042	0.0046	0.0045	0.0054	0.005	0.0054	0.006	0.0072	0.007
0.0008	0.0008	0.0008	0.0008	0.0007	0.0007	0.0007	0.0007	0.0006
0.0144	0.0151	0.0153	0.0157	0.0163	0.0183	0.019	0.023	0.0172

0.0051	0.0054	0.0053	0.0058	0.0053	0.006	0.0059	0.0075	0.0062
0	0	0	0	0	0	0	0	0
0	0	0	0	0	0	0	0	0
0	0	0	0	0	0	0	0	0
0.0041	0.005	0.0047	0.0048	0.0044	0.0042	0.004	0.0051	0.0045
0.0068	0.0066	0.006	0.0061	0.0058	0.0056	0.0059	0.0063	0.0061
0.003	0.0034	0.0035	0.0028	0.0029	0.0028	0.0029	0.0028	0.0025
0.0064	0.0072	0.007	0.0075	0.0072	0.0077	0.0075	0.0094	0.0075
0	0	0	0	0	0	0	0	0
0	0	0	0	0	0	0	0	0
0	0	0	0	0	0	0	0	0
0	0	0	0	0	0	0	0	0
0	0	0	0	0	0	0	0	0
0	0	0	0	0	0	0	0	0
0	0	0	0	0	0	0	0	0
0	0	0	0	0	0	0	0	0
0	0	0	0	0	0	0	0	0
0	0	0	0	0	0	0	0	0
0	0	0	0	0	0	0	0	0
0	0	0	0	0	0	0	0	0
0	0	0	0	0	0	0	0	0
0	0	0	0	0	0	0	0	0
0	0	0	0	0	0	0	0	0
0	0	0	0	0	0	0	0	0
0	0	0	0	0	0	0	0	0
0	0	0	0	0	0	0	0	0
0	0	0	0	0	0	0	0	0
0	0	0	0	0	0	0	0	0
0	0	0	0	0	0	0	0	0
0	0	0	0	0	0	0	0	0
0	0	0	0	0	0	0	0	0
0	0	0	0	0	0	0	0	0
0	0	0	0	0	0	0	0	0
0	0	0	0	0	0	0	0	0
0.0078	0.0087	0.0089	0.0091	0.0093	0.0095	0.0096	0.0117	0.0096
0.0072	0.0089	0.0092	0.0085	0.0075	0.0081	0.0088	0.0114	0.0087
0.01	0.0104	0.011	0.012	0.0117	0.0126	0.0127	0.015	0.013
0.0059	0.0074	0.0062	0.0072	0.0046	0.0093	0.0141	0.0153	0.0162
0	0	0	0	0	0	0	0	0
0.0007	0.0007	0.0006	0.0006	0.0007	0.0007	0.0007	0.0006	0.0006
0.0007	0.0007	0.0007	0.0007	0.0007	0.0007	0.0006	0.0006	0.0006
0.0044	0.005	0.0044	0.0075	0.007	0.01	0.0106	0.0142	0.0125
0.0184	0.0196	0.0193	0.0198	0.0191	0.0195	0.0195	0.0233	0.0198
0.011	0.0125	0.0124	0.0156	0.0138	0.0125	0.0131	0.0159	0.0135

0	0	0	0	0	0	0	0	0
0.0017	0.0017	0.0017	0.0016	0.0016	0.0015	0.0017	0.002	0.0016
0.0025	0.0025	0.0022	0.0029	0.0028	0.003	0.003	0.0033	0.0027
0.0054	0.0043	0.0043	0.0045	0.0041	0.0049	0.0046	0.005	0.0047
0.009	0.01	0.0102	0.0105	0.0095	0.0103	0.0106	0.0131	0.0108
0.0095	0.0125	0.0114	0.0146	0.0127	0.0131	0.0124	0.015	0.0162
0.003	0.0034	0.003	0.0033	0.003	0.0033	0.0036	0.0042	0.0037
0	0	0	0	0	0	0	0	0
0	0	0	0	0	0	0	0	0
0	0	0	0	0	0	0	0	0
0.0042	0.0046	0.0048	0.0051	0.005	0.0052	0.0047	0.0053	0.0052
0.0014	0.0015	0.0015	0.0014	0.0014	0.0013	0.0012	0.0012	0.0013
0	0	0	0	0	0	0	0	0
0.0079	0.008	0.0079	0.0087	0.0082	0.0081	0.0077	0.0095	0.008
0	0	0	0	0	0	0	0	0
0	0	0	0	0	0	0	0	0
0	0	0	0	0	0	0	0	0
0	0	0	0	0	0	0	0	0
0	0	0	0	0	0	0	0	0
0	0	0	0	0	0	0	0	0
0	0	0	0	0	0	0	0	0
0	0	0	0	0	0	0	0	0
0	0	0	0	0	0	0	0	0
0	0	0	0	0	0	0	0	0
0	0	0	0	0	0	0	0	0
0	0	0	0	0	0	0	0	0
0	0	0	0	0	0	0	0	0
0	0	0	0	0	0	0	0	0
0	0	0	0	0	0	0	0	0
0	0	0	0	0	0	0	0	0
0	0	0	0	0	0	0	0	0
0	0	0	0	0	0	0	0	0
0	0	0	0	0	0	0	0	0
0	0	0	0	0	0	0	0	0
0	0	0	0	0	0	0	0	0
0	0	0	0	0	0	0	0	0
0	0	0	0	0	0	0	0	0
0	0	0	0	0	0	0	0	0
0	0	0	0	0	0	0	0	0
0	0	0	0	0	0	0	0	0
0.0032	0.0037	0.0043	0.0049	0.0037	0.0038	0.0035	0.0041	0.0037
0.0048	0.0052	0.0055	0.005	0.005	0.0054	0.0056	0.0064	0.0051
0.0098	0.0102	0.0104	0.0106	0.0098	0.0097	0.0098	0.0107	0.0107
0.0023	0.0023	0.0025	0.0025	0.0021	0.0021	0.0021	0.0024	0.0023

0.0067	0.0068	0.0064	0.0065	0.007	0.0076	0.0075	0.0088	0.0073
0.0067	0.009	0.0086	0.0111	0.0078	0.0111	0.0113	0.0135	0.0119
0.0214	0.0206	0.0217	0.0259	0.0243	0.0286	0.0263	0.0319	0.0324
0.0129	0.0138	0.0138	0.013	0.0125	0.0141	0.015	0.0179	0.013
0.0029	0.0029	0.0029	0.0027	0.0028	0.0027	0.0025	0.0024	0.002
0.0077	0.0081	0.0081	0.0078	0.0079	0.0077	0.008	0.0085	0.0064
0.0025	0.0025	0.0025	0.0026	0.0023	0.0021	0.0021	0.0022	0.0022
0.0062	0.0063	0.0064	0.0063	0.0065	0.0068	0.0072	0.0088	0.0066
0.0137	0.0148	0.0143	0.015	0.0149	0.0161	0.0166	0.0201	0.0165
0.0095	0.0097	0.0104	0.0108	0.0102	0.0113	0.0118	0.0137	0.0113
0.0112	0.0123	0.0132	0.0129	0.0124	0.0129	0.0124	0.0146	0.0109
0.0044	0.0043	0.0044	0.0041	0.0035	0.0035	0.0035	0.0038	0.004
0.0018	0.0017	0.0016	0.0015	0.0016	0.0015	0.0015	0.0014	0.0015
0.0098	0.0101	0.0099	0.0101	0.0098	0.01	0.0104	0.0119	0.01
0.0071	0.0071	0.0072	0.0074	0.0067	0.0067	0.007	0.0078	0.007
0.0014	0.0012	0.0011	0.0011	0.0009	0.001	0.0011	0.001	0.001
0.0016	0.0018	0.0017	0.0019	0.0021	0.0021	0.0022	0.0027	0.0022
0	0	0	0	0	0	0	0	0
0	0	0	0	0	0	0	0	0
0.0017	0.0019	0.0019	0.002	0.0017	0.0018	0.0019	0.0024	0.0016
0	0	0	0	0	0	0	0	0
0	0	0	0	0	0	0	0	0
0	0	0	0	0	0	0	0	0
0	0	0	0	0	0	0	0	0
0	0	0	0	0	0	0	0	0
0	0	0	0	0	0	0	0	0
0	0	0	0	0	0	0	0	0
0	0	0	0	0	0	0	0	0
0	0	0	0	0	0	0	0	0
0	0	0	0	0	0	0	0	0
0	0	0	0	0	0	0	0	0
0	0	0	0	0	0	0	0	0
0	0	0	0	0	0	0	0	0
0	0	0	0	0	0	0	0	0
0	0	0	0	0	0	0	0	0
0	0	0	0	0	0	0	0	0
0	0	0	0	0	0	0	0	0
0	0	0	0	0	0	0	0	0
0	0	0	0	0	0	0	0	0
0	0	0	0	0	0	0	0	0
0	0	0	0	0	0	0	0	0
0	0	0	0	0	0	0	0	0
0	0	0	0	0	0	0	0	0

0	0	0	0	0	0	0	0	0
0	0	0	0	0	0	0	0	0
0.003	0.0033	0.0026	0.0042	0.0039	0.0037	0.0034	0.0046	0.0043
0.0064	0.0065	0.0067	0.0063	0.0059	0.0062	0.0063	0.0069	0.0062
0.0066	0.0058	0.0054	0.0052	0.0046	0.0048	0.0049	0.0044	0.0043
0.004	0.0043	0.0047	0.0045	0.004	0.0042	0.0041	0.0037	0.0038
0.0162	0.0167	0.0164	0.0157	0.0144	0.0145	0.0155	0.0175	0.0132
0.1015	0.1079	0.0936	0.1119	0.0877	0.0941	0.0729	0.0925	0.0761
0.1157	0.1299	0.1269	0.1499	0.121	0.1221	0.1085	0.146	0.1438
0.0389	0.0446	0.0442	0.0491	0.0414	0.0471	0.0462	0.0574	0.0468
0.0007	0.0007	0.0007	0.0007	0.0007	0.0006	0.0006	0.0005	0.0005
0.0344	0.038	0.0392	0.0408	0.0404	0.0442	0.0447	0.0539	0.042
0.0288	0.0314	0.0318	0.0339	0.0319	0.0349	0.0359	0.0426	0.036
0.0578	0.0573	0.0545	0.0555	0.0543	0.0612	0.0632	0.0766	0.0628
0.0791	0.088	0.0884	0.0903	0.0851	0.0874	0.0887	0.1128	0.0961
0.0337	0.0321	0.0343	0.0362	0.0284	0.0291	0.0263	0.0323	0.0286
0.0164	0.017	0.0166	0.0176	0.0155	0.0167	0.0173	0.0198	0.0178
0.0496	0.0531	0.0509	0.0489	0.0503	0.0491	0.0522	0.0619	0.0506
0.0111	0.0116	0.0116	0.0116	0.0113	0.0122	0.012	0.0132	0.0115
0.0389	0.0402	0.0395	0.0414	0.0407	0.0433	0.0434	0.0537	0.0464
0.0339	0.0341	0.0317	0.0352	0.0349	0.0363	0.0369	0.0464	0.042
0.0077	0.0087	0.0086	0.009	0.0081	0.0092	0.0094	0.0104	0.0097
0.0009	0.0009	0.0009	0.0009	0.001	0.0012	0.0011	0.0015	0.0012
0.0019	0.002	0.0021	0.0021	0.0021	0.0022	0.0024	0.0027	0.002
0.0007	0.0007	0.0007	0.0007	0.0007	0.0006	0.0007	0.0007	0.0007
0.0017	0.0016	0.0017	0.0015	0.0015	0.0015	0.0015	0.0015	0.0013
0	0	0	0	0	0	0	0	0
0	0	0	0	0	0	0	0	0
0	0	0	0	0	0	0	0	0
0	0	0	0	0	0	0	0	0
0	0	0	0	0	0	0	0	0
0	0	0	0	0	0	0	0	0
0	0	0	0	0	0	0	0	0
0	0	0	0	0	0	0	0	0
0	0	0	0	0	0	0	0	0
0	0	0	0	0	0	0	0	0
0	0	0	0	0	0	0	0	0
0	0	0	0	0	0	0	0	0
0	0	0	0	0	0	0	0	0
0	0	0	0	0	0	0	0	0
0	0	0	0	0	0	0	0	0
0	0	0	0	0	0	0	0	0
0	0	0	0	0	0	0	0	0
0	0	0	0	0	0	0	0	0

0	0	0	0	0	0	0	0	0
0	0	0	0	0	0	0	0	0
0	0	0	0	0	0	0	0	0
0	0	0	0	0	0	0	0	0
0	0	0	0	0	0	0	0	0
0	0	0	0	0	0	0	0	0
0	0	0	0	0	0	0	0	0
0	0	0	0	0	0	0	0	0
0.0143	0.0149	0.0143	0.0155	0.0142	0.0147	0.0165	0.0204	0.0148
0.0261	0.0295	0.0291	0.0321	0.0313	0.0339	0.0375	0.0465	0.0379
0.1146	0.1216	0.109	0.1328	0.1225	0.1258	0.1294	0.1511	0.152
0.1605	0.1668	0.1862	0.2188	0.1691	0.173	0.1657	0.2089	0.1814
0.2242	0.1865	0.1963	0.2129	0.1833	0.1888	0.2127	0.2412	0.267
0.1353	0.1588	0.1418	0.1785	0.1323	0.1777	0.1877	0.2022	0.1766
0.2007	0.2163	0.1856	0.2303	0.2043	0.2247	0.241	0.274	0.2219
0.0952	0.1057	0.1021	0.1104	0.088	0.1113	0.1195	0.1292	0.1186
0.0819	0.0849	0.0887	0.0868	0.0865	0.0973	0.0885	0.1163	0.0813
0.1173	0.1283	0.1227	0.1273	0.1218	0.125	0.118	0.1514	0.1169
0.1185	0.1217	0.126	0.1257	0.1246	0.1297	0.1407	0.1611	0.1323
0.1427	0.151	0.1596	0.1607	0.1622	0.1651	0.1663	0.2057	0.1679
0.1198	0.1307	0.1261	0.1364	0.1328	0.1402	0.1415	0.1699	0.1364
0.0703	0.0772	0.0733	0.0789	0.0767	0.0798	0.0804	0.0951	0.085
0.0778	0.082	0.0816	0.0851	0.0798	0.0853	0.0855	0.1046	0.0866
0.1037	0.1096	0.1106	0.1092	0.1054	0.1133	0.118	0.1423	0.118
0.0319	0.0318	0.0308	0.0322	0.0315	0.0338	0.0346	0.0408	0.0352
0.0716	0.0754	0.0727	0.0774	0.0732	0.0826	0.0869	0.1045	0.0921
0.0588	0.0589	0.0621	0.0645	0.061	0.0704	0.0791	0.09	0.083
0.0102	0.012	0.0109	0.013	0.0126	0.0139	0.0138	0.0169	0.017
0	0	0	0	0	0	0	0	0
0.0057	0.006	0.006	0.0062	0.0063	0.0073	0.0075	0.0087	0.0069
0.0055	0.0069	0.0065	0.009	0.0074	0.0085	0.0085	0.0115	0.0111
0.0016	0.0014	0.0014	0.0013	0.0012	0.0013	0.0013	0.0011	0.0011
0	0	0	0	0	0	0	0	0
0	0	0	0	0	0	0	0	0
0	0	0	0	0	0	0	0	0
0	0	0	0	0	0	0	0	0
0	0	0	0	0	0	0	0	0
0	0	0	0	0	0	0	0	0
0	0	0	0	0	0	0	0	0
0	0	0	0	0	0	0	0	0
0	0	0	0	0	0	0	0	0
0	0	0	0	0	0	0	0	0
0	0	0	0	0	0	0	0	0
0	0	0	0	0	0	0	0	0

0	0	0	0	0	0	0	0	0
0	0	0	0	0	0	0	0	0
0	0	0	0	0	0	0	0	0
0	0	0	0	0	0	0	0	0
0	0	0	0	0	0	0	0	0
0	0	0	0	0	0	0	0	0
0	0	0	0	0	0	0	0	0
0	0	0	0	0	0	0	0	0
0	0	0	0	0	0	0	0	0
0	0	0	0	0	0	0	0	0
0	0	0	0	0	0	0	0	0
0	0	0	0	0	0	0	0	0
0	0	0	0	0	0	0	0	0
0	0	0	0	0	0	0	0	0
0.0146	0.0169	0.017	0.0183	0.015	0.0138	0.0111	0.0152	0.0142
0.0115	0.0134	0.0131	0.0131	0.0123	0.0126	0.0142	0.015	0.0141
0.1293	0.1388	0.1418	0.1432	0.1462	0.1458	0.1422	0.1641	0.1565
0.1761	0.1882	0.1769	0.2174	0.1584	0.184	0.1805	0.2169	0.1901
0.0912	0.1073	0.109	0.129	0.102	0.1097	0.1044	0.1305	0.1145
0.0837	0.0955	0.0927	0.096	0.0955	0.1006	0.1082	0.1265	0.1003
0.0904	0.0969	0.0962	0.1036	0.1008	0.1036	0.0991	0.1296	0.1026
0.0394	0.0417	0.042	0.0438	0.0418	0.044	0.0462	0.056	0.0436
0.0553	0.0581	0.0573	0.0617	0.0617	0.0722	0.0728	0.0893	0.0691
0.0427	0.0432	0.0415	0.0458	0.0455	0.0469	0.0495	0.0607	0.0506
0.0611	0.0635	0.0636	0.0602	0.0659	0.0692	0.0759	0.0807	0.0588
0.0816	0.095	0.0969	0.108	0.1147	0.1195	0.124	0.1415	0.1117
0.0609	0.0622	0.0632	0.0637	0.0591	0.0629	0.0646	0.0756	0.0639
0.0493	0.0511	0.0511	0.0521	0.0491	0.0501	0.0499	0.0582	0.0498
0.0763	0.0795	0.0806	0.0799	0.0798	0.0853	0.0911	0.1111	0.0868
0.05	0.0536	0.0569	0.056	0.0566	0.0613	0.0621	0.0753	0.0621
0.0477	0.0501	0.0485	0.0495	0.0475	0.0499	0.0511	0.0595	0.0496
0.1036	0.1112	0.1111	0.1192	0.1125	0.1219	0.1204	0.1366	0.1175
0.0669	0.0835	0.0924	0.1005	0.0817	0.0899	0.1018	0.1228	0.1072
0.0132	0.0119	0.0104	0.0119	0.0116	0.0105	0.0115	0.0142	0.0164
0	0	0	0	0	0	0	0	0
0.0125	0.0125	0.0119	0.0128	0.0121	0.0134	0.0142	0.0162	0.0141
0.01	0.0116	0.0087	0.0095	0.0081	0.0093	0.008	0.0124	0.0136
0	0	0	0	0	0	0	0	0
0	0	0	0	0	0	0	0	0
0	0	0	0	0	0	0	0	0
0	0	0	0	0	0	0	0	0
0	0	0	0	0	0	0	0	0
0	0	0	0	0	0	0	0	0
0	0	0	0	0	0	0	0	0

0	0	0	0	0	0	0	0	0
0	0	0	0	0	0	0	0	0
0	0	0	0	0	0	0	0	0
0	0	0	0	0	0	0	0	0
0	0	0	0	0	0	0	0	0
0	0	0	0	0	0	0	0	0
0	0	0	0	0	0	0	0	0
0	0	0	0	0	0	0	0	0
0	0	0	0	0	0	0	0	0
0	0	0	0	0	0	0	0	0
0	0	0	0	0	0	0	0	0
0	0	0	0	0	0	0	0	0
0.0068	0.0069	0.0071	0.0069	0.008	0.0082	0.0076	0.0078	0.0074
0.0035	0.0037	0.0036	0.0034	0.0035	0.0035	0.0038	0.0051	0.0036
0.0133	0.0134	0.0129	0.0121	0.0105	0.0101	0.0099	0.0101	0.0098
0.0121	0.0127	0.0126	0.0113	0.0117	0.0113	0.0111	0.0099	0.0088
0.0089	0.0096	0.0087	0.0098	0.0082	0.0088	0.0084	0.0093	0.0084
0.0094	0.0097	0.0094	0.012	0.0106	0.0101	0.011	0.0124	0.0127
0.0074	0.007	0.0065	0.0064	0.0061	0.006	0.0063	0.0064	0.0057
0	0	0	0	0	0	0	0	0
0.0059	0.0068	0.0069	0.0071	0.0068	0.0071	0.0083	0.0105	0.0082
0.052	0.0539	0.0537	0.0521	0.0481	0.0492	0.0493	0.0564	0.0506
0.0519	0.0545	0.0545	0.0624	0.0605	0.0673	0.0688	0.088	0.075
0.0647	0.069	0.0638	0.0664	0.0629	0.0688	0.0675	0.0795	0.0766
0.1147	0.1217	0.12	0.1195	0.1156	0.1255	0.131	0.1439	0.1216
0.1411	0.139	0.1528	0.1494	0.1438	0.1442	0.1491	0.172	0.1321
0.144	0.158	0.1649	0.1577	0.1614	0.1742	0.1829	0.212	0.1796
0.1676	0.1788	0.1587	0.139	0.1531	0.1651	0.169	0.206	0.142
0.128	0.1314	0.1401	0.1373	0.1397	0.1521	0.1572	0.1903	0.1504
0.1719	0.1898	0.1799	0.1818	0.1704	0.1788	0.2003	0.2185	0.1801
0.1253	0.1307	0.1358	0.1434	0.1462	0.1589	0.1649	0.1947	0.1642
0.1518	0.147	0.1541	0.138	0.1316	0.1411	0.134	0.1492	0.1289
0.1064	0.1065	0.1021	0.0958	0.0972	0.0951	0.0956	0.1011	0.088
0.1355	0.1263	0.133	0.1264	0.1236	0.1311	0.132	0.139	0.1206
0.0767	0.078	0.0816	0.081	0.0729	0.077	0.0778	0.0933	0.0831
0.0114	0.0115	0.011	0.0113	0.0106	0.011	0.0126	0.0134	0.0138
0.0461	0.0447	0.0469	0.0483	0.045	0.0441	0.0462	0.055	0.0434
0.0965	0.1049	0.109	0.115	0.1054	0.1106	0.114	0.1349	0.108
0.0402	0.0453	0.0474	0.0488	0.0536	0.0623	0.0549	0.0621	0.0544
0.0079	0.0081	0.0081	0.0087	0.008	0.0085	0.0082	0.0089	0.0086
0.0044	0.0046	0.0046	0.0042	0.0038	0.0041	0.0042	0.0047	0.0043
0.0045	0.005	0.0054	0.0064	0.0058	0.0063	0.0065	0.0075	0.0066
0.0172	0.0182	0.0172	0.0156	0.0151	0.0161	0.0161	0.018	0.0152
0.0018	0.0015	0.0015	0.0012	0.0008	0.0009	0.0009	0.0011	0.0008

0.0041	0.0043	0.0046	0.0044	0.0042	0.0039	0.004	0.0042	0.0036
0.0022	0.0023	0.0023	0.0022	0.0022	0.0022	0.0021	0.0025	0.002
0.002	0.0027	0.0027	0.0031	0.0029	0.003	0.0033	0.0039	0.0033
0	0	0	0	0	0	0	0	0
0	0	0	0	0	0	0	0	0
0.001	0.001	0.001	0.001	0.001	0.001	0.001	0.0009	0.0009
0.0092	0.0097	0.0098	0.0121	0.0103	0.0111	0.0107	0.0115	0.0107
0.0029	0.0031	0.0028	0.0029	0.0021	0.0021	0.0021	0.002	0.0018
0	0	0	0	0	0	0	0	0
0	0	0	0	0	0	0	0	0
0	0	0	0	0	0	0	0	0
0	0	0	0	0	0	0	0	0
0	0	0	0	0	0	0	0	0
0	0	0	0	0	0	0	0	0
0	0	0	0	0	0	0	0	0
0	0	0	0	0	0	0	0	0
0	0	0	0	0	0	0	0	0
0	0	0	0	0	0	0	0	0
0.0108	0.0113	0.0117	0.0111	0.0115	0.0118	0.011	0.013	0.0107
0.0022	0.0022	0.0016	0.0022	0.0019	0.0023	0.0024	0.003	0.0026
0.0123	0.0126	0.0133	0.0146	0.0117	0.0134	0.0131	0.0152	0.0128
0.0094	0.0098	0.0104	0.0106	0.0104	0.0116	0.0123	0.0144	0.0108
0.0012	0.0013	0.0013	0.0012	0.0012	0.001	0.0012	0.0012	0.0012
0.0015	0.0013	0.0015	0.0014	0.0014	0.0014	0.0013	0.0013	0.0012
0.0216	0.0211	0.0224	0.023	0.0196	0.0203	0.0205	0.0268	0.0233
0.0297	0.0314	0.0304	0.0284	0.0233	0.0278	0.0255	0.0308	0.0271
0.006	0.0061	0.0064	0.0057	0.0053	0.0051	0.0044	0.0045	0.0045
0.0817	0.0902	0.0867	0.0935	0.0896	0.0961	0.0971	0.1104	0.0818
0.084	0.0899	0.0887	0.0894	0.0857	0.0942	0.098	0.1154	0.0928
0.0405	0.0419	0.0428	0.0453	0.0417	0.0431	0.0432	0.0469	0.042
0.1404	0.1345	0.1361	0.1261	0.1155	0.118	0.1286	0.1449	0.1246
0.1523	0.1497	0.1525	0.1375	0.1335	0.1371	0.1501	0.1546	0.1318
0.1704	0.1632	0.1665	0.1459	0.1436	0.1368	0.1479	0.1628	0.1509
0.1452	0.1434	0.1556	0.1613	0.1589	0.1556	0.1644	0.1755	0.1388
0.162	0.1681	0.1663	0.1408	0.1342	0.1396	0.1451	0.1511	0.1393
0.1057	0.1105	0.1135	0.1068	0.1055	0.1145	0.1165	0.1257	0.11
0.1419	0.1441	0.1473	0.1436	0.1326	0.139	0.1324	0.1468	0.1166
0.0935	0.0945	0.1006	0.0954	0.0977	0.0973	0.0974	0.1047	0.0843
0.1209	0.1295	0.1222	0.1099	0.1049	0.1066	0.1104	0.1392	0.1154
0.1057	0.1213	0.1184	0.1219	0.1358	0.1403	0.1301	0.1527	0.1159
0.107	0.1132	0.1114	0.1134	0.1073	0.1118	0.1123	0.1281	0.1106
0.1254	0.1203	0.1151	0.1039	0.1028	0.1022	0.1092	0.1131	0.1055
0.0738	0.075	0.0747	0.0759	0.0763	0.0735	0.0749	0.0821	0.0746
0.0435	0.0451	0.0453	0.0465	0.0441	0.0465	0.0459	0.0548	0.0446

0.0196	0.0206	0.0203	0.0207	0.0201	0.0208	0.022	0.0251	0.0218
0.0081	0.008	0.0083	0.0085	0.0083	0.0091	0.0097	0.0119	0.0098
0.0121	0.0132	0.0134	0.0151	0.0138	0.015	0.0163	0.0194	0.0142
0.0102	0.0108	0.0112	0.0111	0.0107	0.0107	0.0106	0.0111	0.0113
0.0008	0.0006	0.0006	0.0006	0.0006	0.0006	0.0006	0.0006	0.0005
0.0023	0.0026	0.0027	0.0023	0.0022	0.0025	0.0026	0.0033	0.0023
0.0051	0.0056	0.0062	0.0073	0.007	0.0071	0.0068	0.0079	0.0077
0.0037	0.0039	0.0037	0.0037	0.0033	0.0038	0.004	0.005	0.0048
0.0091	0.01	0.0099	0.0104	0.0072	0.0072	0.0082	0.0106	0.0099
0.0093	0.0092	0.0071	0.0097	0.0072	0.0078	0.0079	0.0095	0.0087
0	0	0	0	0	0	0	0	0
0.0007	0.0008	0.0008	0.0008	0.0007	0.0008	0.0007	0.0007	0.0006
0.0041	0.0049	0.004	0.0044	0.0033	0.0035	0.0039	0.0048	0.0041
0	0	0	0	0	0	0	0	0
0	0	0	0	0	0	0	0	0
0	0	0	0	0	0	0	0	0
0	0	0	0	0	0	0	0	0
0	0	0	0	0	0	0	0	0
0	0	0	0	0	0	0	0	0
0	0	0	0	0	0	0	0	0
0	0	0	0	0	0	0	0	0
0	0	0	0	0	0	0	0	0
0	0	0	0	0	0	0	0	0
0	0	0	0	0	0	0	0	0
0	0	0	0	0	0	0	0	0
0.0113	0.012	0.013	0.0124	0.0139	0.0148	0.0144	0.018	0.0145
0	0	0	0	0	0	0	0	0
0	0	0	0	0	0	0	0	0
0.0236	0.0232	0.0236	0.0239	0.0237	0.025	0.0261	0.0293	0.0245
0.014	0.0142	0.014	0.0145	0.0142	0.0151	0.0158	0.0186	0.0155
0	0	0	0	0	0	0	0	0
0	0	0	0	0	0	0	0	0
0.0289	0.0302	0.0298	0.03	0.0288	0.0307	0.0309	0.0367	0.0306
0.0784	0.085	0.088	0.0957	0.0968	0.1012	0.1091	0.1183	0.0934
0.1381	0.1306	0.1285	0.1229	0.1235	0.1251	0.1267	0.1341	0.1117
0.0973	0.0995	0.1121	0.0979	0.0958	0.0965	0.1016	0.1036	0.0884
0.1405	0.1284	0.1256	0.1133	0.0995	0.1081	0.1109	0.111	0.1
0.1878	0.1843	0.1841	0.1724	0.1587	0.1485	0.1537	0.1485	0.1457
0.1203	0.1177	0.1179	0.1111	0.109	0.1048	0.1147	0.11	0.1075
0.1253	0.139	0.1415	0.1336	0.1092	0.1088	0.1061	0.1041	0.0901
0.1599	0.1677	0.1683	0.1654	0.1605	0.1699	0.1764	0.1761	0.1551
0.1107	0.1138	0.1127	0.1149	0.1146	0.1183	0.1264	0.1448	0.1191
0.1445	0.1559	0.1666	0.1681	0.1896	0.1986	0.2091	0.2378	0.1888
0.152	0.1492	0.1443	0.1435	0.1434	0.1472	0.1575	0.1841	0.1557

0.1304	0.1488	0.1485	0.138	0.1432	0.1467	0.155	0.1959	0.1507
0.1621	0.1678	0.1671	0.1719	0.1706	0.1858	0.1857	0.2164	0.1725
0.1209	0.1239	0.1238	0.1271	0.12	0.1241	0.1255	0.1414	0.1164
0.1827	0.1718	0.1558	0.1433	0.1331	0.135	0.1399	0.1453	0.1267
0.1736	0.1683	0.1614	0.1627	0.1484	0.1543	0.169	0.1846	0.1716
0.0673	0.0697	0.0672	0.0649	0.0639	0.0666	0.0662	0.0716	0.0654
0.0199	0.0231	0.0234	0.0244	0.0233	0.0241	0.0249	0.0289	0.0217
0.0076	0.0082	0.0094	0.0099	0.0086	0.0075	0.0067	0.0098	0.0078
0.0224	0.0231	0.0228	0.0266	0.0229	0.026	0.025	0.0304	0.0272
0.0197	0.0207	0.0223	0.0236	0.0223	0.0239	0.0232	0.027	0.022
0	0	0	0	0	0	0	0	0
0.0016	0.0016	0.0015	0.0014	0.0013	0.0013	0.0012	0.0012	0.0013
0.0038	0.0039	0.004	0.0037	0.0035	0.0037	0.0038	0.0039	0.0034
0.0022	0.0022	0.0022	0.0022	0.0021	0.0022	0.0023	0.0026	0.0021
0.0046	0.005	0.0044	0.005	0.0043	0.0047	0.0052	0.0062	0.0049
0.0092	0.0098	0.0079	0.0071	0.006	0.0068	0.0075	0.0099	0.0087
0.0021	0.0028	0.0028	0.0031	0.0029	0.0026	0.0025	0.0028	0.0024
0.0028	0.0029	0.0029	0.003	0.0025	0.0025	0.0021	0.0026	0.0024
0.002	0.0021	0.002	0.0019	0.0013	0.0011	0.0015	0.002	0.0018
0	0	0	0	0	0	0	0	0
0	0	0	0	0	0	0	0	0
0	0	0	0	0	0	0	0	0
0	0	0	0	0	0	0	0	0
0	0	0	0	0	0	0	0	0
0	0	0	0	0	0	0	0	0
0	0	0	0	0	0	0	0	0
0	0	0	0	0	0	0	0	0
0	0	0	0	0	0	0	0	0
0	0	0	0	0	0	0	0	0
0	0	0	0	0	0	0	0	0
0.0015	0.0014	0.0014	0.0013	0.0013	0.0012	0.0013	0.0014	0.0013
0.0116	0.013	0.0129	0.013	0.012	0.0128	0.0129	0.0154	0.0131
0.0148	0.0191	0.0154	0.0203	0.019	0.0219	0.0264	0.029	0.0302
0.0301	0.0315	0.0317	0.0328	0.0308	0.0322	0.0296	0.0319	0.0337
0.0369	0.0398	0.0411	0.0412	0.0389	0.0444	0.0445	0.0519	0.0423
0.0176	0.0185	0.0163	0.0151	0.0144	0.0152	0.0166	0.0179	0.0161
0.0196	0.0207	0.0216	0.022	0.0206	0.0207	0.0195	0.0209	0.0191
0.0842	0.0872	0.0847	0.0924	0.0872	0.1026	0.1057	0.1259	0.0939
0.0419	0.0448	0.046	0.048	0.045	0.0491	0.0497	0.0617	0.0475
0.0648	0.0672	0.0683	0.0647	0.0609	0.0609	0.0619	0.0678	0.0603
0.1425	0.1391	0.1395	0.1324	0.1334	0.137	0.1416	0.1472	0.1326
0.1364	0.1419	0.1379	0.1263	0.1267	0.1252	0.1305	0.144	0.1211
0.1494	0.1404	0.1415	0.1311	0.1269	0.1277	0.1267	0.1133	0.1229
0.1426	0.1401	0.147	0.1453	0.1408	0.1549	0.163	0.1549	0.1434

0.1422	0.1405	0.1427	0.1389	0.1245	0.129	0.1317	0.1373	0.1229
0.0983	0.1047	0.1093	0.1084	0.095	0.0985	0.1018	0.1055	0.0877
0.0971	0.0955	0.0874	0.087	0.0825	0.0826	0.0836	0.0798	0.066
0.1471	0.1419	0.1556	0.1545	0.1644	0.1684	0.1672	0.1819	0.1473
0.1398	0.1395	0.1455	0.1347	0.1357	0.1429	0.1569	0.1695	0.1461
0.1053	0.1087	0.1055	0.0999	0.1038	0.1107	0.1086	0.1222	0.1053
0.1384	0.1348	0.1429	0.1425	0.1341	0.1444	0.1434	0.1597	0.1343
0.1327	0.1366	0.1266	0.1206	0.1105	0.103	0.1083	0.116	0.1006
0.1179	0.121	0.1152	0.1077	0.1055	0.1152	0.1203	0.1311	0.1055
0.115	0.1066	0.1083	0.1137	0.1111	0.1128	0.1162	0.1247	0.1077
0.1642	0.1728	0.1748	0.1722	0.1552	0.1456	0.1495	0.1654	0.15
0.1216	0.1237	0.1151	0.1102	0.0985	0.0925	0.0906	0.0926	0.09
0.0476	0.0485	0.0485	0.0476	0.0452	0.0469	0.049	0.0534	0.0471
0.0438	0.0476	0.0467	0.0499	0.0498	0.0581	0.0586	0.0717	0.0596
0.099	0.0977	0.1004	0.1079	0.1023	0.1051	0.1126	0.1319	0.1177
0.079	0.0845	0.0811	0.0859	0.0742	0.0754	0.0788	0.0857	0.0742
0.0102	0.0119	0.0111	0.0132	0.0115	0.0133	0.0139	0.0154	0.0165
0.0533	0.0569	0.056	0.0551	0.052	0.0518	0.0543	0.0593	0.051
0.0042	0.0041	0.0043	0.0041	0.0039	0.0039	0.0039	0.004	0.0038
0.0024	0.0023	0.0022	0.0022	0.0021	0.0021	0.0021	0.0022	0.002
0.0011	0.0011	0.0011	0.0011	0.0011	0.0012	0.0012	0.0014	0.0011
0	0	0	0	0	0	0	0	0
0	0	0	0	0	0	0	0	0
0.0006	0.0007	0.0006	0.0006	0.0006	0.0006	0.0007	0.0006	0.0006
0.0017	0.0022	0.0021	0.0033	0.0034	0.0038	0.0036	0.0046	0.0052
0	0	0	0	0	0	0	0	0
0	0	0	0	0	0	0	0	0
0	0	0	0	0	0	0	0	0
0	0	0	0	0	0	0	0	0
0	0	0	0	0	0	0	0	0
0	0	0	0	0	0	0	0	0
0	0	0	0	0	0	0	0	0
0	0	0	0	0	0	0	0	0
0	0	0	0	0	0	0	0	0
0	0	0	0	0	0	0	0	0
0	0	0	0	0	0	0	0	0
0.036	0.0377	0.0387	0.0406	0.038	0.0435	0.0436	0.0531	0.0419
0	0	0	0	0	0	0	0	0
0.0048	0.0055	0.0059	0.0062	0.0053	0.0056	0.0062	0.0081	0.0061
0.0093	0.0103	0.0094	0.0092	0.0096	0.01	0.0096	0.0115	0.0088
0.043	0.0438	0.0415	0.0423	0.0398	0.0418	0.043	0.0493	0.0441
0.1203	0.1263	0.1239	0.1244	0.1137	0.1225	0.1271	0.1509	0.1278
0.1331	0.1396	0.1366	0.1402	0.1337	0.1422	0.1429	0.1664	0.1413
0.0814	0.0871	0.0848	0.0915	0.0832	0.0917	0.0928	0.1112	0.0947

0.0964	0.0948	0.1011	0.0948	0.09	0.0843	0.09	0.0928	0.0837
0.0801	0.0796	0.0732	0.0758	0.0731	0.0728	0.0762	0.0824	0.0743
0.1357	0.136	0.1496	0.1429	0.1388	0.1328	0.136	0.1319	0.123
0.1565	0.1502	0.1466	0.134	0.1407	0.1338	0.1299	0.1428	0.126
0.1481	0.1529	0.1544	0.145	0.1386	0.1357	0.1408	0.1448	0.1317
0.1647	0.1646	0.1718	0.1559	0.1453	0.1518	0.1537	0.1586	0.151
0.1384	0.1421	0.1359	0.1375	0.1329	0.1486	0.1539	0.1489	0.131
0.1186	0.1211	0.1208	0.1217	0.1247	0.136	0.1511	0.1704	0.1371
0.1027	0.1101	0.1165	0.118	0.1207	0.1202	0.13	0.1474	0.1246
0.1567	0.1617	0.1508	0.1593	0.16	0.1593	0.1661	0.1927	0.1509
0.1315	0.1379	0.139	0.1253	0.1167	0.1241	0.1263	0.1471	0.1281
0.1248	0.1312	0.137	0.1381	0.1332	0.1326	0.1304	0.1511	0.1275
0.1284	0.1325	0.1247	0.1294	0.1236	0.1379	0.1329	0.1594	0.1178
0.1141	0.1139	0.1145	0.117	0.1162	0.1345	0.1413	0.163	0.1337
0.1317	0.1409	0.1497	0.1515	0.1554	0.1571	0.1621	0.2067	0.1546
0.1806	0.1859	0.1732	0.1802	0.1719	0.185	0.1967	0.2388	0.1791
0.0958	0.0942	0.0914	0.0917	0.0846	0.09	0.0874	0.1089	0.0914
0.1633	0.1612	0.165	0.1573	0.1445	0.1524	0.1551	0.1733	0.1629
0.1135	0.1213	0.1231	0.1163	0.1107	0.1188	0.1229	0.1353	0.1248
0.0747	0.0785	0.0823	0.0784	0.074	0.0744	0.0753	0.0862	0.0598
0.1011	0.1084	0.1122	0.1153	0.1019	0.1048	0.1047	0.1193	0.1053
0.0753	0.0932	0.0983	0.1102	0.1008	0.1165	0.1089	0.1466	0.1404
0.0681	0.0752	0.0722	0.0713	0.0658	0.0705	0.0747	0.088	0.0755
0.091	0.1044	0.1126	0.1261	0.1193	0.1238	0.1271	0.147	0.1237
0.0254	0.0296	0.0302	0.0332	0.0274	0.0299	0.0306	0.0352	0.0302
0.0024	0.0024	0.0026	0.0024	0.0025	0.0025	0.0027	0.0029	0.0025
0.0008	0.0009	0.001	0.0009	0.0009	0.0009	0.001	0.0012	0.0009
0	0	0	0	0	0	0	0	0
0	0	0	0	0	0	0	0	0
0.0009	0.0009	0.0008	0.0008	0.0007	0.0007	0.0007	0.0007	0.0007
0.0042	0.0042	0.0038	0.0045	0.0046	0.0045	0.0048	0.006	0.0055
0.0049	0.0054	0.0052	0.0053	0.0051	0.0056	0.0054	0.0062	0.0069
0	0	0	0	0	0	0	0	0
0	0	0	0	0	0	0	0	0
0	0	0	0	0	0	0	0	0
0	0	0	0	0	0	0	0	0
0	0	0	0	0	0	0	0	0
0	0	0	0	0	0	0	0	0
0	0	0	0	0	0	0	0	0
0	0	0	0	0	0	0	0	0
0	0	0	0	0	0	0	0	0
0	0	0	0	0	0	0	0	0
0	0	0	0	0	0	0	0	0
0.0143	0.0145	0.0145	0.0152	0.015	0.0147	0.0144	0.0162	0.0136

0.0084	0.0092	0.0098	0.0097	0.0078	0.0076	0.0057	0.0078	0.0051
0.0084	0.0106	0.0096	0.0098	0.0079	0.0081	0.0097	0.0121	0.0104
0.0664	0.0669	0.0656	0.0624	0.0549	0.0561	0.0544	0.0549	0.0519
0.0994	0.1022	0.0978	0.0976	0.0973	0.1002	0.1021	0.1114	0.0958
0.0709	0.0777	0.0821	0.0842	0.0811	0.0856	0.0838	0.0995	0.0819
0.0665	0.0662	0.0664	0.0639	0.0609	0.0628	0.0626	0.0765	0.0624
0.1146	0.1205	0.1291	0.1316	0.1354	0.1303	0.1319	0.1341	0.1246
0.1477	0.1505	0.137	0.1401	0.1286	0.1295	0.1336	0.1476	0.1284
0.1195	0.1292	0.1205	0.1158	0.1138	0.1168	0.1172	0.1399	0.1121
0.1063	0.116	0.1247	0.1201	0.1239	0.1307	0.1293	0.1471	0.1216
0.1271	0.1297	0.1289	0.1311	0.1281	0.1295	0.1356	0.1499	0.1263
0.17	0.1688	0.1709	0.1643	0.1539	0.1621	0.1678	0.185	0.1618
0.126	0.1285	0.133	0.1261	0.1149	0.1118	0.1173	0.1186	0.1127
0.1649	0.1808	0.1798	0.182	0.175	0.1816	0.1778	0.2013	0.1726
0.1438	0.1581	0.1577	0.1533	0.1435	0.1526	0.1544	0.1822	0.1591
0.1261	0.1362	0.1292	0.1268	0.1285	0.1289	0.1315	0.1467	0.1195
0.1304	0.1359	0.1296	0.136	0.1302	0.1286	0.1267	0.1521	0.1263
0.1062	0.1008	0.1004	0.0986	0.1016	0.1012	0.1024	0.1165	0.0976
0.134	0.1422	0.1505	0.1501	0.1491	0.1482	0.1602	0.1759	0.147
0.1365	0.1353	0.1347	0.1248	0.1292	0.1272	0.1336	0.1492	0.1242
0.1899	0.2025	0.2018	0.2034	0.2054	0.2008	0.2083	0.2282	0.1845
0.172	0.1892	0.1897	0.1975	0.191	0.2045	0.1967	0.2344	0.1967
0.1468	0.1572	0.1592	0.165	0.159	0.1665	0.1621	0.1901	0.1571
0.1704	0.1737	0.1784	0.1838	0.163	0.17	0.1659	0.1832	0.1497
0.1799	0.1922	0.1879	0.191	0.1832	0.1936	0.1965	0.2288	0.1928
0.2036	0.1835	0.1902	0.1938	0.1996	0.2062	0.219	0.2708	0.2025
0.1384	0.1488	0.1563	0.1677	0.1697	0.1826	0.1924	0.2339	0.2005
0.0962	0.0984	0.0945	0.1022	0.0908	0.0976	0.097	0.116	0.1012
0.0262	0.0259	0.0268	0.0248	0.0242	0.0232	0.0223	0.0287	0.0255
0.0356	0.0427	0.0474	0.0558	0.0513	0.0615	0.0634	0.0762	0.0671
0.0557	0.0502	0.0429	0.0534	0.0528	0.0572	0.0615	0.068	0.058
0.0322	0.0273	0.0234	0.0261	0.0258	0.0283	0.0222	0.022	0.0235
0.0024	0.0035	0.0032	0.0032	0.003	0.004	0.0041	0.0048	0.0051
0.0011	0.0012	0.0013	0.0014	0.001	0.0018	0.0017	0.0033	0.0029
0	0	0	0	0	0	0	0	0
0.0009	0.0009	0.0009	0.0008	0.0008	0.0008	0.0008	0.0008	0.0007
0.0024	0.0022	0.0022	0.0028	0.0024	0.0023	0.0024	0.0026	0.0026
0.0021	0.0022	0.0025	0.0024	0.0026	0.0037	0.0035	0.0048	0.0042
0	0	0	0	0	0	0	0	0
0	0	0	0	0	0	0	0	0
0	0	0	0	0	0	0	0	0
0	0	0	0	0	0	0	0	0
0	0	0	0	0	0	0	0	0
0	0	0	0	0	0	0	0	0

0	0	0	0	0	0	0	0	0
0	0	0	0	0	0	0	0	0
0	0	0	0	0	0	0	0	0
0	0	0	0	0	0	0	0	0
0	0	0	0	0	0	0	0	0
0.0091	0.0083	0.0079	0.0068	0.0061	0.0057	0.0065	0.0055	0.0059
0	0	0	0	0	0	0	0	0
0.0363	0.0374	0.0371	0.0381	0.0359	0.0381	0.0384	0.0432	0.0365
0.1102	0.1141	0.1156	0.1173	0.1115	0.115	0.1155	0.127	0.1073
0.0545	0.0607	0.0656	0.0692	0.0541	0.0603	0.0565	0.0602	0.0544
0.0425	0.0462	0.044	0.0455	0.0433	0.0481	0.0501	0.0604	0.0502
0.1736	0.1928	0.2093	0.2005	0.2136	0.23	0.2307	0.2696	0.2067
0.1677	0.1725	0.1718	0.1693	0.1822	0.1759	0.1808	0.2125	0.1782
0.1483	0.1491	0.152	0.1521	0.1411	0.147	0.151	0.1677	0.1427
0.14	0.1474	0.1502	0.1527	0.1455	0.1541	0.1505	0.1774	0.1495
0.1534	0.1622	0.1613	0.1666	0.1526	0.1622	0.1693	0.1899	0.1668
0.1164	0.1275	0.1258	0.126	0.1182	0.1249	0.1278	0.1516	0.1288
0.1401	0.1467	0.1446	0.1466	0.1367	0.1434	0.1448	0.1687	0.1491
0.1611	0.1578	0.1569	0.1521	0.1449	0.1523	0.1539	0.1735	0.143
0.1469	0.1456	0.147	0.1358	0.132	0.1282	0.1302	0.14	0.1271
0.1141	0.1158	0.1179	0.1171	0.1155	0.1191	0.1139	0.1271	0.1099
0.1169	0.1176	0.1199	0.1195	0.113	0.1136	0.1163	0.1361	0.1113
0.1308	0.1224	0.1218	0.119	0.1348	0.1411	0.1493	0.171	0.1499
0.1466	0.1431	0.1497	0.1411	0.1354	0.1472	0.1459	0.1559	0.1288
0.1349	0.135	0.134	0.1179	0.1124	0.1359	0.1425	0.1464	0.1283
0.1778	0.1786	0.1727	0.1643	0.1562	0.1566	0.1693	0.172	0.161
0.1724	0.165	0.16	0.1631	0.1537	0.15	0.1541	0.1564	0.1482
0.1383	0.1444	0.1585	0.1492	0.151	0.1506	0.1498	0.161	0.1466
0.1816	0.1961	0.1958	0.1901	0.175	0.1825	0.1911	0.2074	0.1863
0.1443	0.1473	0.1441	0.1474	0.1371	0.1407	0.1399	0.158	0.1374
0.1587	0.1624	0.1676	0.1744	0.1743	0.1828	0.1893	0.2214	0.1891
0.1809	0.1974	0.1846	0.1862	0.2007	0.2199	0.2197	0.2625	0.1845
0.1552	0.1629	0.1589	0.1636	0.1611	0.1793	0.1894	0.2385	0.1948
0.1471	0.1609	0.1566	0.1661	0.158	0.1686	0.1758	0.2089	0.1823
0.0833	0.1028	0.1007	0.1195	0.1176	0.1348	0.1328	0.166	0.1492
0.0686	0.0906	0.0927	0.0979	0.0894	0.096	0.0949	0.118	0.0871
0.0463	0.0477	0.0528	0.0554	0.0507	0.0484	0.0514	0.0617	0.0545
0.012	0.0122	0.0106	0.0131	0.0096	0.0095	0.0103	0.0147	0.0154
0.0008	0.0008	0.0008	0.0008	0.0008	0.0008	0.0009	0.0012	0.0009
0.0057	0.0058	0.0056	0.0061	0.0054	0.0053	0.0047	0.0056	0.0049
0	0	0	0	0	0	0	0	0
0.0008	0.0008	0.0008	0.0007	0.0006	0.0005	0.0006	0.0007	0.0006
0.0006	0.0006	0.0007	0.0007	0.0007	0.0006	0.0007	0.0007	0.0007
0.0012	0.0011	0.0009	0.0011	0.0008	0.0007	0.0008	0.0009	0.001

0	0	0	0	0	0	0	0	0
0	0	0	0	0	0	0	0	0
0	0	0	0	0	0	0	0	0
0	0	0	0	0	0	0	0	0
0	0	0	0	0	0	0	0	0
0	0	0	0	0	0	0	0	0
0	0	0	0	0	0	0	0	0
0	0	0	0	0	0	0	0	0
0	0	0	0	0	0	0	0	0
0	0	0	0	0	0	0	0	0
0.0119	0.0127	0.0124	0.0128	0.0126	0.0138	0.0143	0.0165	0.0137
0.0031	0.0033	0.0033	0.0032	0.0033	0.0038	0.0039	0.0051	0.0038
0	0	0	0	0	0	0	0	0
0.0428	0.0436	0.0472	0.0438	0.0427	0.0434	0.0457	0.0518	0.0444
0.1003	0.1098	0.1111	0.1124	0.1086	0.1151	0.1172	0.1341	0.1085
0.1241	0.1283	0.1147	0.1322	0.127	0.1371	0.1402	0.1633	0.143
0.1512	0.1448	0.144	0.1676	0.1646	0.1864	0.1749	0.1967	0.1924
0.2229	0.2178	0.2331	0.2428	0.2241	0.2229	0.2496	0.2983	0.2622
0.1483	0.1539	0.1484	0.1512	0.154	0.1533	0.1497	0.1633	0.1416
0.1408	0.158	0.1615	0.1569	0.145	0.1542	0.1636	0.1791	0.1636
0.1638	0.1692	0.1532	0.1916	0.1577	0.1654	0.1643	0.1909	0.1835
0.2407	0.2304	0.2083	0.2107	0.1811	0.1927	0.1854	0.2093	0.2065
0.1929	0.1927	0.1882	0.1808	0.1719	0.1705	0.1801	0.1927	0.1784
0.1422	0.1512	0.1597	0.1503	0.1365	0.1275	0.1306	0.1322	0.1287
0.152	0.1473	0.1411	0.139	0.1352	0.1262	0.1393	0.1543	0.148
0.1465	0.1512	0.1574	0.1435	0.131	0.1303	0.1337	0.1462	0.1303
0.1087	0.1033	0.1109	0.1059	0.0971	0.0977	0.1088	0.1097	0.0972
0.1592	0.158	0.1544	0.1632	0.1561	0.1487	0.1569	0.1678	0.1323
0.1431	0.1352	0.1398	0.1272	0.1197	0.1114	0.1147	0.1298	0.1235
0.1688	0.1703	0.1677	0.1622	0.1569	0.157	0.1615	0.1698	0.1514
0.1223	0.1326	0.1269	0.1368	0.1137	0.1175	0.1245	0.132	0.125
0.1827	0.1827	0.1875	0.1805	0.1652	0.1663	0.1675	0.1824	0.1719
0.1497	0.1469	0.1447	0.1478	0.1253	0.1296	0.147	0.1528	0.1483
0.138	0.1341	0.138	0.118	0.1121	0.1027	0.109	0.1176	0.1135
0.1716	0.1677	0.1627	0.1619	0.1601	0.171	0.1951	0.1927	0.1735
0.1747	0.1694	0.1632	0.1452	0.156	0.1424	0.1486	0.1597	0.1502
0.1526	0.1665	0.1726	0.1685	0.163	0.1703	0.1816	0.1988	0.1566
0.1941	0.2026	0.197	0.1983	0.1978	0.1985	0.2232	0.2644	0.206
0.1876	0.1877	0.197	0.1997	0.1907	0.2017	0.2044	0.2489	0.1764
0.179	0.1932	0.202	0.2046	0.1937	0.2101	0.2099	0.2623	0.2093
0.1688	0.1704	0.169	0.1787	0.1806	0.1942	0.212	0.2574	0.2045
0.0773	0.0788	0.0778	0.0835	0.0751	0.0819	0.0886	0.1075	0.0882
0.0226	0.0225	0.0207	0.0258	0.0196	0.0173	0.0177	0.0217	0.0234
0.0023	0.0024	0.0025	0.0024	0.0024	0.0025	0.0024	0.0026	0.0021

0.0008	0.0009	0.0009	0.001	0.001	0.0011	0.001	0.0012	0.0009
0	0	0	0	0	0	0	0	0
0	0	0	0	0	0	0	0	0
0.0008	0.0007	0.0008	0.0007	0.0007	0.0007	0.0007	0.0006	0.0005
0.0006	0.0006	0.0005	0.0004	0.0004	0.0004	0.0004	0.0005	0.0005
0.0011	0.0014	0.0012	0.0014	0.0015	0.0016	0.0016	0.0019	0.0018
0	0	0	0	0	0	0	0	0
0	0	0	0	0	0	0	0	0
0.003	0.0031	0.0027	0.0027	0.0029	0.0031	0.0031	0.0046	0.0037
0	0	0	0	0	0	0	0	0
0	0	0	0	0	0	0	0	0
0.0006	0.0006	0.0006	0.0006	0.0006	0.0006	0.0006	0.0006	0.0005
0.0082	0.0086	0.0087	0.0078	0.0077	0.0076	0.0076	0.0084	0.0062
0.004	0.0041	0.0043	0.0043	0.0043	0.0043	0.0045	0.0052	0.0041
0.001	0.0009	0.001	0.001	0.0009	0.0009	0.001	0.0013	0.0009
0.0052	0.0053	0.0055	0.0053	0.0054	0.0062	0.0065	0.0072	0.0054
0	0	0	0	0	0	0	0	0
0.0333	0.032	0.0326	0.039	0.0348	0.0383	0.0352	0.0426	0.0409
0.002	0.002	0.0023	0.0023	0.0022	0.0024	0.0026	0.0031	0.0025
0.0731	0.0741	0.0717	0.0711	0.0595	0.0629	0.0651	0.0747	0.0685
0.0406	0.0428	0.0459	0.0483	0.0456	0.0509	0.0511	0.0659	0.0539
0.0561	0.0647	0.0602	0.0674	0.0593	0.0666	0.0672	0.0749	0.0696
0.1124	0.1141	0.1144	0.1162	0.0984	0.1112	0.1145	0.141	0.1267
0.2302	0.2515	0.2342	0.2112	0.1889	0.1944	0.2359	0.2949	0.2407
0.2891	0.2687	0.2535	0.319	0.2579	0.2647	0.2569	0.3146	0.2766
0.1855	0.1832	0.1671	0.1663	0.1553	0.1848	0.1924	0.2347	0.22
0.1462	0.1597	0.1604	0.1624	0.1582	0.1615	0.164	0.1821	0.1523
0.1396	0.1379	0.1382	0.1402	0.1303	0.1157	0.112	0.1186	0.1184
0.1454	0.1531	0.1567	0.1472	0.1337	0.1335	0.1256	0.1385	0.1292
0.1816	0.1614	0.1538	0.1418	0.1216	0.126	0.1217	0.1205	0.1052
0.1697	0.1619	0.1542	0.1501	0.1519	0.1365	0.1436	0.1558	0.1458
0.1668	0.1567	0.1495	0.1423	0.1285	0.1248	0.1242	0.1303	0.1289
0.164	0.1546	0.136	0.1379	0.1346	0.1309	0.1276	0.1213	0.1108
0.1755	0.1825	0.1802	0.1817	0.1644	0.1721	0.1876	0.1977	0.1956
0.1337	0.1249	0.1261	0.1171	0.1248	0.114	0.1125	0.1214	0.1154
0.1238	0.1181	0.1246	0.1147	0.109	0.1104	0.1192	0.1302	0.1045
0.201	0.2072	0.2156	0.2283	0.199	0.2003	0.1907	0.2172	0.1892
0.1835	0.1808	0.1822	0.1804	0.1666	0.1841	0.1929	0.2147	0.2014
0.1141	0.1103	0.1148	0.1035	0.1019	0.0962	0.101	0.0978	0.0971
0.1622	0.1585	0.1594	0.1731	0.159	0.1667	0.1732	0.171	0.1551
0.1801	0.1861	0.1882	0.1931	0.1756	0.1804	0.1717	0.1789	0.1688
0.1176	0.1197	0.1241	0.1206	0.1108	0.1183	0.1295	0.1239	0.1124
0.179	0.1843	0.1866	0.183	0.1907	0.1903	0.1933	0.214	0.1855
0.1305	0.1404	0.1461	0.1451	0.1273	0.1409	0.1474	0.1776	0.1443

0.1341	0.1549	0.1548	0.1486	0.1536	0.1659	0.1793	0.208	0.1547
0.1722	0.181	0.1779	0.1767	0.1784	0.1821	0.1954	0.2309	0.1672
0.1685	0.1767	0.1873	0.2073	0.1958	0.2146	0.2223	0.257	0.179
0.1201	0.1251	0.1358	0.1348	0.1246	0.1323	0.1381	0.1652	0.1334
0.0553	0.0544	0.0515	0.051	0.0414	0.0405	0.0414	0.0431	0.0411
0.0189	0.0198	0.021	0.0195	0.017	0.0192	0.0162	0.0202	0.0208
0.0013	0.0016	0.0015	0.0018	0.0018	0.002	0.0022	0.0027	0.002
0.0031	0.0032	0.0031	0.0034	0.0031	0.0031	0.003	0.0033	0.0028
0.0186	0.0175	0.017	0.0169	0.0171	0.0171	0.0171	0.019	0.0177
0.0161	0.0168	0.0174	0.0186	0.0171	0.0182	0.0167	0.0185	0.0179
0.0067	0.0064	0.0067	0.0059	0.006	0.0057	0.0054	0.0054	0.0051
0.0245	0.0208	0.0192	0.0268	0.0178	0.0208	0.0199	0.0239	0.0203
0	0	0	0	0	0	0	0	0
0	0	0	0	0	0	0	0	0
0	0	0	0	0	0	0	0	0
0.0008	0.0008	0.0008	0.0008	0.0008	0.001	0.001	0.0013	0.0011
0.0006	0.0008	0.0008	0.0009	0.0009	0.0011	0.001	0.0012	0.0009
0.0013	0.0015	0.0015	0.0017	0.0017	0.0018	0.0019	0.0023	0.0017
0.004	0.0045	0.0043	0.0048	0.0047	0.0047	0.0048	0.0055	0.0046
0.0014	0.0013	0.0012	0.0013	0.0011	0.0011	0.0011	0.0011	0.0011
0.0008	0.0008	0.0008	0.0007	0.0007	0.0006	0.0006	0.0006	0.0006
0.0008	0.0007	0.0008	0.0007	0.0007	0.0007	0.0007	0.0007	0.0007
0.0073	0.0079	0.0084	0.0079	0.0074	0.0067	0.007	0.0068	0.0064
0	0	0	0	0	0	0	0	0
0.0107	0.0112	0.011	0.0102	0.0071	0.0086	0.0113	0.0117	0.0108
0.0172	0.0174	0.0184	0.0168	0.0144	0.0135	0.0121	0.0137	0.0143
0.1481	0.1254	0.1363	0.164	0.1222	0.1408	0.148	0.204	0.2006
0.0779	0.0892	0.081	0.0865	0.0757	0.0846	0.0885	0.1036	0.096
0.0965	0.1051	0.0989	0.0964	0.0863	0.0866	0.0834	0.1093	0.0857
0.1094	0.1106	0.1158	0.1285	0.1457	0.1577	0.1613	0.2037	0.1621
0.1994	0.2177	0.2196	0.229	0.2217	0.239	0.2417	0.2886	0.2381
0.1362	0.1428	0.14	0.1458	0.1343	0.1408	0.1412	0.1615	0.1422
0.1203	0.116	0.1248	0.1231	0.1177	0.1312	0.1329	0.1599	0.1207
0.1623	0.1647	0.167	0.164	0.1583	0.1538	0.1522	0.1594	0.1343
0.161	0.1629	0.1558	0.1479	0.1378	0.1378	0.1324	0.1312	0.1257
0.1348	0.1348	0.1311	0.1223	0.1196	0.131	0.1349	0.1443	0.1228
0.1106	0.1039	0.1136	0.1172	0.1113	0.109	0.11	0.1099	0.1084
0.1056	0.114	0.1132	0.1144	0.0963	0.1052	0.107	0.1133	0.1123
0.1565	0.1555	0.1508	0.1532	0.1413	0.129	0.1319	0.1348	0.1271
0.1788	0.1628	0.1635	0.1604	0.142	0.1446	0.1485	0.158	0.1473
0.1664	0.1625	0.1592	0.1589	0.1499	0.1484	0.1374	0.1501	0.1339
0.1744	0.1793	0.18	0.1755	0.1629	0.1627	0.1597	0.1741	0.1693
0.1591	0.1565	0.1569	0.1516	0.1378	0.1427	0.1431	0.1474	0.1426
0.1605	0.1731	0.1842	0.1776	0.1628	0.1683	0.16	0.1586	0.1402

0.1757	0.1993	0.1996	0.1849	0.1537	0.1493	0.1575	0.1725	0.1755
0.2281	0.2488	0.2716	0.2807	0.2565	0.2687	0.248	0.2768	0.2913
0.1469	0.1451	0.139	0.1437	0.132	0.1345	0.1395	0.1476	0.1368
0.1398	0.1413	0.1423	0.1411	0.1392	0.1354	0.1301	0.1346	0.1159
0.1266	0.1207	0.1208	0.1202	0.1244	0.1097	0.1196	0.1458	0.1354
0.1574	0.167	0.1745	0.1779	0.1777	0.1802	0.1761	0.1947	0.1574
0.1327	0.1439	0.1511	0.1492	0.1529	0.1591	0.1592	0.1862	0.1484
0.1784	0.1754	0.178	0.1831	0.1833	0.1955	0.198	0.2299	0.1875
0.1439	0.1634	0.1594	0.1749	0.17	0.1887	0.2026	0.2245	0.1747
0.1678	0.194	0.1955	0.2041	0.1952	0.1996	0.2022	0.2276	0.174
0.0745	0.0743	0.0705	0.0762	0.0783	0.0806	0.086	0.0948	0.0755
0.0235	0.0261	0.0258	0.0248	0.0251	0.0264	0.0265	0.0325	0.0248
0.0542	0.065	0.0599	0.0705	0.0418	0.0526	0.0578	0.0643	0.059
0.0688	0.0908	0.0919	0.1028	0.1025	0.0973	0.0884	0.1005	0.0862
0.0887	0.0921	0.0858	0.0921	0.0887	0.0944	0.0989	0.1182	0.0977
0.1087	0.1152	0.1105	0.1136	0.1006	0.1172	0.1154	0.1395	0.1222
0.0564	0.0579	0.0545	0.058	0.0529	0.0561	0.05	0.0589	0.0532
0.0133	0.0119	0.0124	0.0113	0.0107	0.0102	0.01	0.0091	0.0101
0	0	0	0	0	0	0	0	0
0	0	0	0	0	0	0	0	0
0	0	0	0	0	0	0	0	0
0.0029	0.0029	0.0032	0.0033	0.0037	0.0042	0.0039	0.0048	0.0035
0.0048	0.0051	0.0052	0.0051	0.0051	0.0049	0.0052	0.0066	0.0051
0	0	0	0	0	0	0	0	0
0	0	0	0	0	0	0	0	0
0.0006	0.0006	0.0006	0.0006	0.0006	0.0006	0.0007	0.0006	0.0006
0.0015	0.0016	0.0015	0.0013	0.0013	0.0012	0.0013	0.0011	0.001
0.0027	0.0026	0.0023	0.0022	0.002	0.0021	0.0021	0.0022	0.0023
0.0482	0.0555	0.048	0.0532	0.044	0.0494	0.0483	0.0508	0.0526
0.0021	0.0022	0.0024	0.0021	0.0018	0.0018	0.0017	0.0019	0.0018
0.0205	0.0217	0.0233	0.0273	0.022	0.024	0.0228	0.0267	0.0198
0	0	0	0	0	0	0	0	0
0.2048	0.2755	0.329	0.4048	0.2986	0.3417	0.3433	0.385	0.3717
0.1509	0.1819	0.1653	0.1978	0.1452	0.1407	0.1065	0.1189	0.1121
0.1817	0.1802	0.188	0.1935	0.1785	0.1843	0.1766	0.2132	0.191
0.1546	0.1721	0.174	0.1697	0.1749	0.1867	0.1922	0.2336	0.1699
0.1491	0.1634	0.1566	0.162	0.1636	0.1718	0.1868	0.2164	0.1729
0.162	0.1608	0.1498	0.1521	0.1503	0.1439	0.1399	0.1499	0.1224
0.1112	0.1138	0.1156	0.1176	0.1185	0.1148	0.13	0.1504	0.116
0.1143	0.131	0.1416	0.1319	0.125	0.1404	0.1362	0.1603	0.1375
0.1365	0.1322	0.1366	0.1353	0.1338	0.1362	0.139	0.1573	0.1241
0.1107	0.1221	0.124	0.1176	0.1045	0.1036	0.1049	0.1303	0.098
0.1228	0.1209	0.1296	0.1249	0.1202	0.1215	0.1205	0.1361	0.1138
0.1255	0.1154	0.1099	0.1073	0.0906	0.0939	0.1031	0.1134	0.1026

0.1262	0.1509	0.1483	0.1433	0.1427	0.134	0.1407	0.1453	0.1307
0.1689	0.1651	0.1587	0.1627	0.1622	0.1633	0.16	0.1671	0.1642
0.2167	0.2217	0.2125	0.221	0.1756	0.2013	0.2074	0.2362	0.2111
0.1947	0.2033	0.1945	0.2411	0.2148	0.1998	0.2158	0.229	0.2218
0.1151	0.119	0.1188	0.1111	0.0934	0.0976	0.1039	0.1078	0.0992
0.1462	0.1409	0.1405	0.1426	0.1398	0.1303	0.1318	0.1322	0.116
0.1525	0.16	0.1577	0.162	0.1515	0.1553	0.1605	0.1701	0.1607
0.1373	0.141	0.1406	0.1357	0.1264	0.1369	0.1349	0.1485	0.1303
0.1425	0.1505	0.1504	0.1483	0.1417	0.1438	0.1497	0.1711	0.1453
0.1501	0.1548	0.1552	0.1573	0.1484	0.1544	0.1556	0.1702	0.1458
0.1391	0.147	0.1439	0.1325	0.1328	0.1316	0.1402	0.1624	0.1445
0.1429	0.146	0.1441	0.1477	0.1377	0.1522	0.1658	0.1968	0.1497
0.1263	0.1334	0.1326	0.1392	0.1288	0.1338	0.1379	0.1662	0.1363
0.1598	0.1529	0.1587	0.1531	0.1531	0.1605	0.1651	0.1804	0.1488
0.1796	0.1952	0.1877	0.1807	0.1864	0.2053	0.2042	0.2248	0.1858
0.1662	0.1801	0.2136	0.2144	0.1901	0.2046	0.2151	0.2641	0.1952
0.1677	0.1675	0.1587	0.1729	0.1604	0.1695	0.1783	0.2179	0.1714
0.0639	0.0712	0.0691	0.0743	0.0745	0.0755	0.076	0.0971	0.079
0.1002	0.1034	0.1043	0.1113	0.1111	0.1178	0.1255	0.1427	0.1226
0.1155	0.1289	0.126	0.1415	0.1368	0.1452	0.1443	0.1727	0.1441
0.1314	0.1411	0.1391	0.147	0.133	0.1432	0.1473	0.1699	0.144
0.1399	0.1375	0.141	0.1581	0.1502	0.1576	0.1667	0.1766	0.1608
0.0517	0.0585	0.0671	0.079	0.0575	0.0675	0.0696	0.0764	0.0615
0	0	0	0	0	0	0	0	0
0	0	0	0	0	0	0	0	0
0	0	0	0	0	0	0	0	0
0	0	0	0	0	0	0	0	0
0.0052	0.0053	0.0056	0.0062	0.0064	0.0068	0.0076	0.0088	0.0069
0.0032	0.0031	0.0033	0.0032	0.0029	0.0034	0.0034	0.0039	0.0028
0	0	0	0	0	0	0	0	0
0	0	0	0	0	0	0	0	0
0.0019	0.0021	0.002	0.0021	0.0021	0.0024	0.0025	0.0029	0.0023
0.003	0.0029	0.003	0.0032	0.0032	0.0038	0.004	0.0047	0.0037
0.0039	0.0042	0.0044	0.0047	0.0047	0.005	0.0053	0.0065	0.0056
0.0059	0.0051	0.0052	0.0044	0.0046	0.0049	0.0045	0.0044	0.0041
0	0	0	0	0	0	0	0	0
0.0088	0.0091	0.0091	0.0137	0.0143	0.0156	0.0143	0.017	0.0166
0.0314	0.0315	0.0297	0.0284	0.0259	0.0276	0.0275	0.0268	0.0229
0.1328	0.1373	0.1306	0.1407	0.1288	0.1241	0.1317	0.153	0.1462
0.1051	0.1149	0.1401	0.1234	0.0983	0.1016	0.082	0.1361	0.1169
0.2556	0.2708	0.2521	0.2625	0.2654	0.2563	0.2598	0.3231	0.2976
0.2107	0.2343	0.2297	0.2309	0.24	0.2561	0.2601	0.3117	0.2389
0.1742	0.18	0.1683	0.1615	0.1539	0.1725	0.1696	0.2049	0.1768
0.141	0.1508	0.1418	0.1373	0.1337	0.1442	0.1441	0.1656	0.1424

0.1147	0.1196	0.1239	0.1229	0.1319	0.1297	0.1224	0.137	0.1074
0.154	0.1523	0.1498	0.1458	0.1464	0.1539	0.1556	0.1865	0.1593
0.1275	0.1558	0.159	0.1648	0.1617	0.1751	0.1748	0.1995	0.1591
0.124	0.1327	0.1364	0.1313	0.1398	0.1503	0.1632	0.177	0.1428
0.1234	0.1297	0.1299	0.1259	0.1226	0.1287	0.1346	0.1472	0.1238
0.1557	0.1516	0.1395	0.1425	0.1383	0.1382	0.1373	0.1415	0.1477
0.1892	0.1969	0.1909	0.1848	0.1701	0.1733	0.1802	0.1967	0.1845
0.1539	0.1505	0.1477	0.1472	0.1334	0.1428	0.1565	0.1518	0.1546
0.2024	0.2187	0.2109	0.233	0.1963	0.2296	0.2297	0.2417	0.2441
0.2753	0.2915	0.3255	0.3704	0.3185	0.3173	0.2953	0.2984	0.3378
0.2406	0.236	0.2405	0.2318	0.2049	0.2122	0.2162	0.2496	0.2249
0.1369	0.1375	0.1404	0.1318	0.134	0.1331	0.1308	0.1483	0.1379
0.1283	0.1272	0.1332	0.1252	0.1243	0.1215	0.1235	0.1229	0.1153
0.1374	0.1351	0.1242	0.1238	0.1126	0.1257	0.1367	0.1528	0.1408
0.1496	0.1564	0.158	0.1517	0.1479	0.1557	0.1589	0.1722	0.1565
0.1055	0.1046	0.1035	0.1038	0.1084	0.1033	0.107	0.1169	0.1007
0.1526	0.1583	0.1577	0.1612	0.1537	0.1604	0.1649	0.1839	0.16
0.1341	0.1273	0.1294	0.1177	0.1172	0.1332	0.1404	0.1537	0.1237
0.1282	0.1403	0.1383	0.1378	0.1499	0.162	0.1711	0.192	0.1718
0.127	0.1246	0.133	0.1337	0.1256	0.1232	0.1192	0.1295	0.1206
0.1538	0.1703	0.1756	0.1872	0.1817	0.1904	0.1817	0.1866	0.1454
0.1769	0.1882	0.1961	0.1832	0.1706	0.1736	0.1842	0.2222	0.1635
0.1005	0.1153	0.1154	0.1173	0.1177	0.1186	0.1225	0.1432	0.1154
0.0432	0.0453	0.0455	0.0452	0.0428	0.0444	0.0461	0.0536	0.0454
0.0925	0.0959	0.0948	0.0991	0.0934	0.1001	0.0954	0.1084	0.0972
0.1394	0.1495	0.1452	0.1504	0.1382	0.1513	0.1458	0.1678	0.1419
0.1365	0.1431	0.1386	0.1374	0.1191	0.1242	0.1203	0.1555	0.1275
0.0788	0.0846	0.0856	0.0822	0.0773	0.0768	0.0691	0.0855	0.0891
0	0	0	0	0	0	0	0	0
0.0157	0.0159	0.0159	0.0169	0.0163	0.0167	0.0165	0.0187	0.0162
0	0	0	0	0	0	0	0	0
0	0	0	0	0	0	0	0	0
0	0	0	0	0	0	0	0	0
0.0025	0.0035	0.0036	0.0039	0.0039	0.0041	0.0047	0.0054	0.0039
0.0014	0.0013	0.0012	0.0012	0.0012	0.0011	0.0012	0.0011	0.0011
0.0017	0.0018	0.0019	0.0017	0.0015	0.0014	0.0013	0.0011	0.0012
0	0	0	0	0	0	0	0	0
0.0035	0.004	0.0041	0.004	0.0038	0.0042	0.0046	0.0048	0.0036
0.0015	0.0015	0.0017	0.0018	0.002	0.0023	0.0026	0.0028	0.0023
0.001	0.0011	0.0011	0.0012	0.0013	0.0012	0.0013	0.0014	0.0011
0.001	0.0011	0.0011	0.0011	0.001	0.0013	0.0013	0.0016	0.0012
0.0028	0.0028	0.0028	0.0027	0.0025	0.0026	0.0026	0.003	0.0024
0.0106	0.0105	0.011	0.0096	0.0098	0.01	0.0094	0.0089	0.0088
0.0633	0.0684	0.0624	0.0629	0.0532	0.0624	0.0707	0.0904	0.0786

0.0669	0.0675	0.0674	0.076	0.0744	0.078	0.078	0.096	0.069
0.1332	0.1439	0.1454	0.1437	0.1257	0.13	0.1351	0.1505	0.1413
0.1504	0.1574	0.1615	0.166	0.1577	0.1637	0.1643	0.1847	0.1635
0.1243	0.1359	0.1391	0.1361	0.135	0.1454	0.1472	0.1727	0.1409
0.1716	0.1723	0.1739	0.1665	0.1666	0.1765	0.1749	0.1877	0.1612
0.1796	0.1824	0.1692	0.1709	0.1727	0.1748	0.1765	0.1784	0.1549
0.1133	0.1137	0.1129	0.1078	0.1076	0.1072	0.1175	0.1242	0.1124
0.137	0.1453	0.1522	0.156	0.171	0.1704	0.1767	0.1945	0.1452
0.1278	0.1527	0.1559	0.1553	0.1473	0.1833	0.1856	0.2216	0.1783
0.113	0.1311	0.1425	0.1433	0.1493	0.1632	0.1684	0.1984	0.1503
0.1083	0.1122	0.1168	0.1171	0.1118	0.1172	0.1212	0.1447	0.1135
0.1143	0.1248	0.1249	0.1279	0.1208	0.1181	0.118	0.1316	0.11
0.129	0.1315	0.1381	0.1285	0.1306	0.1293	0.1274	0.1272	0.1109
0.1099	0.1225	0.1283	0.1202	0.1068	0.1062	0.0958	0.0959	0.0807
0.1465	0.1423	0.1411	0.1419	0.1334	0.1338	0.135	0.1404	0.1322
0.1579	0.1548	0.1465	0.1587	0.1354	0.1444	0.1624	0.1846	0.179
0.1433	0.1439	0.1258	0.14	0.1096	0.1131	0.1176	0.1482	0.1517
0.1773	0.1772	0.1826	0.1821	0.1757	0.1808	0.178	0.196	0.1734
0.1771	0.1871	0.1922	0.1769	0.161	0.1549	0.1629	0.1533	0.1371
0.1274	0.1248	0.1255	0.1221	0.118	0.1172	0.1124	0.117	0.1103
0.1561	0.1572	0.1579	0.1554	0.1527	0.1504	0.1525	0.164	0.1543
0.1544	0.1586	0.1538	0.1363	0.1333	0.1213	0.1264	0.1288	0.1074
0.1394	0.1484	0.1504	0.1445	0.1401	0.1504	0.1491	0.1722	0.1443
0.1115	0.1101	0.1165	0.1108	0.1055	0.1075	0.1139	0.124	0.1071
0.1421	0.1591	0.156	0.1318	0.1149	0.1246	0.1458	0.1767	0.126
0.1346	0.1424	0.1376	0.1334	0.1219	0.1102	0.1023	0.1048	0.1015
0.1815	0.1689	0.1547	0.1592	0.1646	0.159	0.1602	0.1648	0.1304
0.1519	0.1695	0.1705	0.1823	0.1809	0.1867	0.1922	0.2417	0.2025
0.1719	0.168	0.1695	0.1492	0.1579	0.1677	0.1565	0.1997	0.1545
0.0526	0.0584	0.0591	0.0618	0.0669	0.0747	0.0798	0.0928	0.0771
0.0393	0.0369	0.0313	0.0364	0.0329	0.0328	0.032	0.0395	0.0355
0.0767	0.0825	0.0829	0.0919	0.0741	0.0757	0.0832	0.0872	0.0718
0.1266	0.1383	0.1499	0.159	0.137	0.15	0.1379	0.1641	0.1593
0.061	0.0671	0.0736	0.0719	0.0777	0.088	0.0876	0.1067	0.0891
0.0122	0.0124	0.0123	0.0121	0.0112	0.0118	0.0122	0.0132	0.012
0.008	0.0085	0.0081	0.0087	0.0077	0.0078	0.0087	0.01	0.0088
0	0	0	0	0	0	0	0	0
0	0	0	0	0	0	0	0	0
0	0	0	0	0	0	0	0	0
0	0	0	0	0	0	0	0	0
0.0027	0.0028	0.0027	0.0025	0.0024	0.0025	0.0025	0.0025	0.0022
0.0029	0.0029	0.003	0.0031	0.0029	0.0033	0.0035	0.0039	0.0034
0.0042	0.0045	0.005	0.0045	0.0043	0.0046	0.0049	0.0063	0.0054
0	0	0	0	0	0	0	0	0

0	0	0	0	0	0	0	0	0
0.0015	0.0015	0.0015	0.0014	0.0014	0.0014	0.0014	0.0014	0.0011
0.0039	0.0035	0.0036	0.0029	0.0022	0.0021	0.002	0.0024	0.0023
0.0123	0.0135	0.0157	0.0196	0.0208	0.0189	0.0219	0.028	0.0251
0.0333	0.0381	0.037	0.0398	0.032	0.035	0.0423	0.0453	0.0466
0.0364	0.0391	0.0404	0.0407	0.0405	0.0436	0.0443	0.0537	0.0432
0.0814	0.0862	0.0822	0.0882	0.0852	0.0898	0.0917	0.1081	0.0886
0.0791	0.0791	0.0811	0.0766	0.0787	0.074	0.0796	0.0869	0.0743
0.1017	0.1093	0.1109	0.1144	0.1218	0.1268	0.1417	0.1568	0.1297
0.1571	0.1663	0.1685	0.1867	0.19	0.1927	0.1961	0.2258	0.1763
0.1508	0.1546	0.1592	0.1633	0.1532	0.1503	0.152	0.1692	0.1423
0.1346	0.128	0.1291	0.1223	0.109	0.1127	0.1132	0.1117	0.1019
0.1291	0.1291	0.1297	0.128	0.1245	0.1282	0.1314	0.1529	0.1338
0.1804	0.2034	0.2187	0.2081	0.1764	0.1957	0.2101	0.2482	0.1873
0.1595	0.1714	0.1697	0.1712	0.1594	0.1843	0.1959	0.2347	0.1729
0.1509	0.1549	0.1528	0.1536	0.1578	0.1661	0.1762	0.2285	0.1783
0.1191	0.1207	0.1277	0.1296	0.132	0.1454	0.1673	0.1931	0.1563
0.1176	0.1261	0.136	0.1353	0.1324	0.1458	0.1646	0.2207	0.1811
0.1382	0.1465	0.1439	0.1453	0.14	0.1531	0.1676	0.2028	0.1554
0.1427	0.1487	0.1505	0.1453	0.1431	0.1566	0.1749	0.206	0.173
0.1468	0.1518	0.1606	0.1661	0.1728	0.1712	0.1769	0.2019	0.1653
0.1309	0.1402	0.1407	0.1378	0.1252	0.1455	0.152	0.1867	0.1657
0.2455	0.2608	0.2524	0.2852	0.2632	0.2643	0.2834	0.3297	0.3171
0.1453	0.1499	0.148	0.1466	0.1415	0.1485	0.1571	0.1759	0.1574
0.1418	0.1344	0.1344	0.1332	0.1231	0.1133	0.1234	0.1306	0.1133
0.1761	0.1762	0.1707	0.1562	0.1475	0.1452	0.1481	0.1598	0.1604
0.156	0.1608	0.1581	0.1597	0.147	0.15	0.1469	0.1618	0.1415
0.1353	0.1411	0.139	0.1253	0.1246	0.1256	0.1382	0.1583	0.1331
0.1229	0.1258	0.1239	0.125	0.1181	0.1155	0.1204	0.1298	0.1085
0.1664	0.1706	0.1726	0.1736	0.1598	0.1617	0.1651	0.1846	0.1597
0.1772	0.175	0.1685	0.169	0.1383	0.1471	0.1439	0.1596	0.1408
0.0849	0.0989	0.1215	0.121	0.1149	0.1075	0.1278	0.1335	0.1298
0.1436	0.1402	0.1422	0.1362	0.121	0.1272	0.1427	0.1319	0.1177
0.1909	0.208	0.2014	0.2034	0.1929	0.19	0.1962	0.2485	0.2065
0.1577	0.1669	0.1684	0.1755	0.1741	0.18	0.1869	0.2227	0.178
0.1495	0.1581	0.153	0.1535	0.1413	0.1505	0.1467	0.182	0.1564
0.1651	0.1682	0.1314	0.1342	0.099	0.1097	0.1348	0.2175	0.2214
0.1765	0.2062	0.2125	0.2319	0.2256	0.2312	0.2406	0.2775	0.2473
0.0879	0.0925	0.0917	0.1012	0.0921	0.0933	0.0967	0.1095	0.1017
0.0473	0.0479	0.0467	0.0446	0.0409	0.0426	0.0418	0.0483	0.0447
0.0303	0.0288	0.0265	0.0281	0.0241	0.0249	0.0252	0.0282	0.0256
0	0	0	0	0	0	0	0	0
0	0	0	0	0	0	0	0	0
0	0	0	0	0	0	0	0	0

0	0	0	0	0	0	0	0	0
0.0008	0.0008	0.0008	0.0007	0.0006	0.0005	0.0006	0.0006	0.0005
0.0015	0.0015	0.0016	0.0014	0.0014	0.0012	0.0013	0.0014	0.0015
0.0037	0.0038	0.004	0.0043	0.0043	0.0047	0.0044	0.0049	0.0038
0.0041	0.0043	0.0047	0.005	0.0046	0.0049	0.0052	0.0061	0.0048
0.0007	0.0007	0.0007	0.0007	0.0007	0.0007	0.0008	0.0008	0.0007
0.0109	0.0107	0.011	0.0105	0.0106	0.0106	0.0108	0.0121	0.0105
0.0399	0.037	0.0412	0.039	0.0373	0.0395	0.0392	0.045	0.0371
0.0173	0.0178	0.0171	0.0192	0.0185	0.018	0.0176	0.0205	0.0184
0.0007	0.0007	0.0007	0.0007	0.0007	0.0007	0.0007	0.0007	0.0006
0.0034	0.0036	0.0035	0.0037	0.0037	0.0042	0.0042	0.0051	0.0038
0.0261	0.0264	0.0272	0.0289	0.0284	0.0299	0.0336	0.0385	0.0291
0.1455	0.156	0.1577	0.1653	0.1601	0.1717	0.1748	0.2003	0.1657
0.1017	0.1057	0.1076	0.1183	0.1227	0.1311	0.1257	0.1591	0.1257
0.1476	0.1634	0.1766	0.1768	0.1706	0.181	0.1884	0.221	0.1649
0.2138	0.2324	0.2387	0.2488	0.2363	0.254	0.2596	0.313	0.2381
0.1448	0.149	0.1503	0.14	0.1468	0.1662	0.1696	0.1987	0.154
0.1484	0.162	0.1638	0.1699	0.17	0.1859	0.1783	0.226	0.1753
0.2065	0.2215	0.2112	0.1938	0.2036	0.2126	0.2196	0.2769	0.204
0.1397	0.1502	0.1564	0.1625	0.1668	0.1815	0.2008	0.2348	0.1826
0.1112	0.1159	0.1166	0.1059	0.1121	0.1081	0.1117	0.1379	0.1064
0.1459	0.1391	0.1458	0.1416	0.1536	0.174	0.1995	0.2382	0.188
0.1581	0.1658	0.1776	0.1735	0.1683	0.164	0.1677	0.1831	0.1358
0.1306	0.1322	0.1359	0.1361	0.1315	0.1337	0.1312	0.1467	0.1319
0.1293	0.1345	0.1423	0.134	0.1324	0.1388	0.1384	0.1637	0.1298
0.1366	0.1388	0.1461	0.1362	0.1409	0.1431	0.1425	0.1605	0.1307
0.1432	0.1459	0.152	0.151	0.152	0.1605	0.1715	0.182	0.1484
0.1322	0.1341	0.1394	0.1348	0.1332	0.1421	0.1473	0.1607	0.1344
0.1748	0.181	0.1792	0.1783	0.1719	0.1804	0.1842	0.1999	0.1791
0.1394	0.1439	0.139	0.1464	0.1316	0.1391	0.1462	0.1664	0.141
0.1309	0.1226	0.1207	0.1075	0.0943	0.0949	0.0975	0.092	0.0905
0.1515	0.1588	0.1518	0.1468	0.1392	0.133	0.1355	0.1495	0.1364
0.147	0.1528	0.1552	0.1515	0.1461	0.1361	0.1334	0.1422	0.1129
0.1505	0.1608	0.1593	0.1606	0.1525	0.1576	0.1655	0.1867	0.1572
0.1239	0.1397	0.1466	0.1544	0.157	0.1601	0.1681	0.1913	0.1658
0.153	0.1658	0.1617	0.1718	0.1615	0.174	0.1726	0.2142	0.1775
0.1715	0.1885	0.1817	0.2209	0.2146	0.201	0.2126	0.2363	0.1902
0.2162	0.2128	0.2227	0.2265	0.2021	0.1967	0.1972	0.2251	0.1971
0.1742	0.1644	0.1668	0.1506	0.1449	0.1325	0.138	0.1216	0.1199
0.1357	0.131	0.1308	0.1217	0.1234	0.1267	0.1132	0.1245	0.0986
0.1204	0.1243	0.1259	0.123	0.1252	0.1329	0.1477	0.1671	0.1434
0.1418	0.1454	0.143	0.1425	0.1533	0.1544	0.1627	0.1796	0.1529
0.2123	0.2238	0.209	0.2215	0.1951	0.212	0.2309	0.275	0.2615
0.2291	0.2379	0.2153	0.2298	0.2039	0.2381	0.2552	0.2936	0.275

0.1235	0.115	0.1095	0.1346	0.1236	0.1253	0.1357	0.1609	0.134
0.0609	0.0946	0.0884	0.1236	0.1168	0.1281	0.1253	0.1359	0.1183
0.0828	0.0879	0.0851	0.0967	0.0917	0.1029	0.0851	0.1108	0.1054
0.0132	0.0129	0.0117	0.0127	0.0126	0.0149	0.0161	0.0196	0.0142
0	0	0	0	0	0	0	0	0
0	0	0	0	0	0	0	0	0
0.0037	0.0038	0.0038	0.0038	0.0041	0.0042	0.0042	0.005	0.0038
0.0007	0.0007	0.0007	0.0006	0.0006	0.0005	0.0005	0.0005	0.0005
0	0	0	0	0	0	0	0	0
0.0021	0.0022	0.0023	0.0023	0.0022	0.0024	0.0026	0.0032	0.0026
0	0	0	0	0	0	0	0	0
0.0046	0.0046	0.0047	0.0044	0.0041	0.0038	0.004	0.0037	0.0034
0.0309	0.032	0.0312	0.0318	0.0314	0.0328	0.036	0.0421	0.0341
0.0724	0.0761	0.077	0.0772	0.0813	0.0852	0.0878	0.0969	0.0763
0.0158	0.017	0.0171	0.0186	0.0173	0.0179	0.017	0.0198	0.0162
0.0142	0.016	0.014	0.0153	0.0147	0.0161	0.0166	0.0185	0.017
0.096	0.1074	0.1046	0.1092	0.1044	0.1097	0.1145	0.1335	0.1066
0.0815	0.0885	0.0996	0.1063	0.106	0.1064	0.1078	0.1244	0.0978
0.1495	0.1401	0.1584	0.1573	0.1591	0.1549	0.1616	0.1954	0.1399
0.1231	0.1381	0.1731	0.1778	0.2005	0.2044	0.1987	0.2497	0.1873
0.2271	0.2379	0.2399	0.2343	0.2264	0.2479	0.2604	0.31	0.2321
0.1376	0.1411	0.1467	0.1522	0.152	0.1461	0.1415	0.1907	0.1362
0.1655	0.1739	0.1722	0.1668	0.1644	0.18	0.1845	0.2343	0.179
0.1607	0.168	0.1652	0.1554	0.1603	0.168	0.1725	0.1977	0.1561
0.1238	0.1266	0.1358	0.1382	0.1379	0.1516	0.1563	0.1794	0.1372
0.1848	0.1891	0.189	0.1855	0.2033	0.2221	0.2358	0.2823	0.2183
0.1567	0.1523	0.1696	0.1674	0.1563	0.1808	0.1717	0.227	0.1768
0.1344	0.1347	0.1349	0.1374	0.1253	0.1349	0.1326	0.1547	0.1338
0.1654	0.165	0.1588	0.1546	0.156	0.153	0.1465	0.1433	0.1358
0.1857	0.1886	0.1848	0.184	0.1628	0.1598	0.1663	0.1631	0.1484
0.1644	0.1599	0.1631	0.1578	0.1456	0.1507	0.1478	0.1516	0.1462
0.1451	0.139	0.1283	0.1159	0.1141	0.1157	0.1303	0.1293	0.1274
0.1724	0.1723	0.1757	0.166	0.1645	0.1627	0.163	0.1711	0.1609
0.1401	0.1406	0.1472	0.1433	0.1449	0.1387	0.1436	0.1474	0.1367
0.1063	0.1089	0.1011	0.1016	0.0916	0.0961	0.1055	0.1187	0.115
0.1319	0.1338	0.1252	0.12	0.1176	0.1163	0.1217	0.1388	0.1198
0.1461	0.1432	0.1496	0.1448	0.1487	0.1542	0.1588	0.157	0.1317
0.1068	0.1038	0.103	0.0997	0.1021	0.1068	0.1052	0.1181	0.0988
0.1346	0.1332	0.1348	0.126	0.1146	0.1202	0.131	0.1542	0.1186
0.1776	0.1803	0.1863	0.1928	0.1883	0.1982	0.1968	0.2283	0.1838
0.1467	0.1589	0.1648	0.1753	0.1748	0.1969	0.198	0.2231	0.181
0.0748	0.0798	0.079	0.0799	0.0747	0.0764	0.081	0.0938	0.0814
0.2701	0.3055	0.2611	0.2472	0.2507	0.2709	0.2792	0.3543	0.3012
0.3385	0.3357	0.3137	0.3184	0.278	0.2946	0.2771	0.3793	0.3296

0.1299	0.1325	0.1287	0.1204	0.1149	0.1157	0.1224	0.123	0.1164
0.1904	0.1851	0.1775	0.1767	0.1627	0.1735	0.17	0.1804	0.172
0.162	0.1686	0.1672	0.1652	0.1547	0.1542	0.1604	0.1845	0.1615
0.1824	0.1877	0.1785	0.1675	0.1681	0.1807	0.1836	0.2184	0.1827
0.1279	0.1435	0.1478	0.1589	0.1534	0.1684	0.1676	0.2056	0.1664
0.1187	0.1241	0.1186	0.1257	0.1195	0.1311	0.1335	0.1658	0.1387
0.0655	0.0727	0.0708	0.0798	0.0699	0.077	0.0831	0.1057	0.0985
0.0508	0.053	0.0577	0.0659	0.0589	0.0622	0.0556	0.0648	0.0611
0.0017	0.002	0.0018	0.0021	0.0022	0.0024	0.0026	0.0032	0.0032
0.0072	0.0072	0.007	0.007	0.0064	0.0063	0.0062	0.0064	0.0063
0	0	0	0	0	0	0	0	0
0	0	0	0	0	0	0	0	0
0.007	0.0065	0.0073	0.0071	0.0069	0.0069	0.0066	0.008	0.0068
0.0007	0.0007	0.0007	0.0007	0.0006	0.0006	0.0006	0.0007	0.0006
0.0011	0.0014	0.0013	0.0015	0.0009	0.0008	0.001	0.0013	0.0014
0.0087	0.0094	0.0089	0.0092	0.009	0.0103	0.0105	0.0128	0.0111
0.0131	0.0136	0.0139	0.0142	0.0135	0.0136	0.014	0.0163	0.014
0.0469	0.0487	0.0476	0.0495	0.0473	0.0506	0.0514	0.0596	0.0515
0.0465	0.0479	0.049	0.0483	0.0469	0.0509	0.0534	0.0647	0.054
0.0157	0.0169	0.0178	0.0164	0.0155	0.017	0.0197	0.0238	0.0158
0.0559	0.0594	0.0593	0.0574	0.051	0.0526	0.0549	0.0689	0.0559
0.0418	0.0482	0.0484	0.049	0.0465	0.0507	0.0489	0.0637	0.0475
0.0876	0.0886	0.091	0.096	0.0879	0.0911	0.093	0.1055	0.09
0.1454	0.1556	0.1634	0.1505	0.1617	0.1781	0.1811	0.198	0.1629
0.1473	0.1524	0.1489	0.1547	0.1456	0.1565	0.1634	0.196	0.1661
0.1497	0.1741	0.1691	0.1698	0.1602	0.1746	0.1804	0.2179	0.1791
0.1498	0.1539	0.1567	0.1669	0.1638	0.171	0.1861	0.2169	0.1725
0.1342	0.1431	0.1341	0.1406	0.1354	0.1567	0.1586	0.2058	0.1698
0.142	0.1549	0.1467	0.1352	0.1403	0.1579	0.1691	0.1868	0.1572
0.1466	0.1516	0.1459	0.1405	0.1278	0.1338	0.1343	0.1472	0.1343
0.1401	0.1384	0.144	0.1347	0.1226	0.1219	0.1268	0.1294	0.1171
0.1264	0.1259	0.127	0.1247	0.1189	0.1352	0.1405	0.1606	0.1316
0.149	0.163	0.1671	0.1723	0.183	0.1844	0.192	0.2231	0.1777
0.1442	0.1528	0.1636	0.153	0.1479	0.1588	0.1698	0.1846	0.1542
0.1555	0.1511	0.1574	0.1459	0.1432	0.14	0.1434	0.1385	0.1339
0.117	0.1195	0.1269	0.1184	0.1138	0.1098	0.1068	0.1129	0.1041
0.1794	0.1824	0.1766	0.1811	0.1718	0.1774	0.188	0.1958	0.1708
0.1218	0.1216	0.1267	0.1227	0.1206	0.143	0.1436	0.1418	0.1294
0.1802	0.1822	0.1667	0.1581	0.1532	0.1553	0.1647	0.1776	0.1693
0.1452	0.1467	0.1373	0.1318	0.1184	0.1182	0.12	0.1265	0.1187
0.2189	0.2386	0.2358	0.255	0.2172	0.2292	0.2549	0.304	0.2835
0.1808	0.1807	0.1799	0.1693	0.1564	0.1513	0.1496	0.1582	0.1469
0.1143	0.1214	0.127	0.1277	0.1264	0.1313	0.1349	0.1542	0.1293
0.1544	0.1555	0.1512	0.1502	0.1449	0.1519	0.1546	0.1806	0.1512

0.1244	0.1272	0.124	0.1211	0.1124	0.122	0.1273	0.1368	0.1212
0.1474	0.1419	0.1465	0.1479	0.1415	0.1455	0.1389	0.1535	0.1367
0.1458	0.1499	0.1505	0.1553	0.1508	0.1594	0.1659	0.1892	0.1631
0.1048	0.1044	0.102	0.1007	0.0942	0.0963	0.0993	0.1057	0.0977
0.1642	0.1778	0.1686	0.1605	0.1405	0.1473	0.1539	0.1639	0.1558
0.2192	0.2266	0.2085	0.2288	0.2004	0.2182	0.2087	0.2215	0.2203
0.139	0.1412	0.1395	0.1418	0.1322	0.1389	0.1393	0.1469	0.1304
0.174	0.1747	0.1817	0.1821	0.1681	0.1581	0.1556	0.1563	0.1402
0.1895	0.1852	0.1876	0.1858	0.1768	0.1894	0.1859	0.186	0.1676
0.1253	0.131	0.1274	0.1266	0.119	0.1315	0.131	0.1545	0.1292
0.2051	0.2205	0.2212	0.2335	0.2226	0.2219	0.2356	0.2781	0.2169
0.0945	0.0928	0.0911	0.0893	0.0986	0.1183	0.1193	0.1572	0.1113
0.0515	0.0515	0.0529	0.0549	0.0529	0.0522	0.0481	0.055	0.0491
0.1465	0.1553	0.1501	0.1778	0.1704	0.1748	0.1575	0.1968	0.1848
0.0258	0.0277	0.0306	0.0346	0.0284	0.0312	0.0259	0.0386	0.0307
0.0159	0.0174	0.0182	0.0209	0.0183	0.0173	0.0177	0.0235	0.0221
0	0	0	0	0	0	0	0	0
0	0	0	0	0	0	0	0	0
0.0065	0.0085	0.0064	0.0075	0.0055	0.0087	0.0104	0.0119	0.0112
0.0078	0.0078	0.0078	0.0084	0.0081	0.0106	0.0114	0.0133	0.0125
0.0045	0.005	0.0051	0.0048	0.0048	0.0055	0.0061	0.0068	0.0054
0	0	0	0	0	0	0	0	0
0.0081	0.0081	0.008	0.0064	0.0066	0.0066	0.0065	0.0066	0.0057
0.0728	0.0784	0.0772	0.0827	0.0786	0.0867	0.0851	0.1057	0.0892
0.0957	0.1008	0.1055	0.1155	0.1081	0.1157	0.1158	0.1324	0.1226
0.033	0.036	0.0313	0.037	0.0314	0.0318	0.0295	0.0377	0.0348
0.0587	0.0655	0.0636	0.0673	0.0686	0.0686	0.0773	0.0885	0.0724
0.0574	0.0602	0.0589	0.0539	0.0542	0.0564	0.0571	0.0694	0.0585
0.1229	0.13	0.1287	0.1197	0.1136	0.1216	0.1242	0.1412	0.1091
0.1277	0.1406	0.1462	0.1531	0.1442	0.1572	0.1519	0.1912	0.1544
0.1599	0.1675	0.165	0.1703	0.1654	0.1714	0.1734	0.1901	0.1607
0.1546	0.1486	0.169	0.1677	0.1593	0.1605	0.162	0.1682	0.146
0.1453	0.1499	0.1585	0.1468	0.1434	0.1446	0.1548	0.1568	0.1508
0.1331	0.132	0.1378	0.1296	0.1259	0.1305	0.1394	0.139	0.1258
0.1457	0.1543	0.1524	0.1655	0.1578	0.1701	0.159	0.1662	0.1496
0.2606	0.2671	0.2712	0.277	0.23	0.2444	0.2478	0.2775	0.258
0.2005	0.1928	0.1864	0.1796	0.1718	0.1685	0.1629	0.1657	0.1468
0.1343	0.1281	0.1444	0.1273	0.1233	0.1131	0.1173	0.1224	0.1156
0.1437	0.1543	0.1508	0.1339	0.1225	0.1273	0.1315	0.1412	0.1239
0.1082	0.1152	0.1257	0.1201	0.1199	0.1211	0.1386	0.1565	0.1395
0.1327	0.1325	0.1346	0.1347	0.1387	0.1427	0.1597	0.1699	0.1367
0.1828	0.1894	0.1886	0.169	0.1578	0.1531	0.1569	0.1628	0.1553
0.1509	0.1576	0.1461	0.142	0.1363	0.1372	0.1461	0.1384	0.1371
0.1431	0.1326	0.1271	0.1226	0.115	0.1195	0.1181	0.1405	0.1294

0.1566	0.1644	0.156	0.1589	0.1476	0.1506	0.1567	0.1752	0.1622
0.0988	0.0979	0.1103	0.1016	0.0974	0.0942	0.1062	0.1121	0.1032
0.1826	0.1785	0.1773	0.1677	0.1528	0.1581	0.1658	0.1733	0.1558
0.1515	0.1509	0.1402	0.1351	0.1329	0.1331	0.1393	0.1363	0.1059
0.1204	0.1279	0.1292	0.131	0.1312	0.1401	0.1416	0.1698	0.1383
0.1542	0.1593	0.1546	0.1666	0.1511	0.1642	0.1643	0.176	0.1503
0.1467	0.1557	0.1497	0.1516	0.1423	0.1477	0.1383	0.1499	0.1356
0.1479	0.15	0.1505	0.1369	0.129	0.1292	0.1331	0.1384	0.1226
0.1516	0.1597	0.1585	0.1576	0.1515	0.1622	0.1672	0.1938	0.1637
0.1233	0.1325	0.1313	0.1348	0.1246	0.1303	0.1312	0.1515	0.1329
0.1314	0.1359	0.1381	0.1397	0.134	0.1395	0.134	0.1433	0.1256
0.1805	0.183	0.1879	0.1811	0.1727	0.1695	0.1723	0.1853	0.1644
0.1862	0.1885	0.1824	0.1829	0.1711	0.1829	0.1724	0.185	0.162
0.1224	0.1259	0.1186	0.1111	0.1061	0.1005	0.0942	0.0987	0.0928
0.1471	0.1406	0.1491	0.1368	0.1377	0.1356	0.1315	0.1241	0.119
0.1934	0.2158	0.2207	0.221	0.1974	0.2015	0.194	0.2246	0.2214
0.1829	0.2044	0.2187	0.2283	0.21	0.2312	0.226	0.2656	0.2288
0.1766	0.1863	0.1791	0.1851	0.1775	0.1902	0.1928	0.2325	0.1918
0.1069	0.1117	0.1078	0.1118	0.114	0.1266	0.1254	0.1329	0.1161
0.1274	0.1302	0.1365	0.1633	0.1419	0.143	0.1398	0.1598	0.141
0.0636	0.0721	0.0682	0.0712	0.0662	0.0738	0.0679	0.0774	0.072
0.0044	0.0039	0.0037	0.0035	0.0033	0.0033	0.0031	0.0031	0.0029
0	0	0	0	0	0	0	0	0
0	0	0	0	0	0	0	0	0
0	0	0	0	0	0	0	0	0
0.0031	0.0035	0.0036	0.0035	0.0034	0.0037	0.0038	0.0047	0.0037
0.0066	0.0072	0.0071	0.0071	0.0068	0.0072	0.0071	0.0086	0.0073
0.0044	0.0044	0.0035	0.0053	0.004	0.0054	0.0045	0.0059	0.0055
0.0067	0.0065	0.0062	0.0062	0.0063	0.0061	0.0061	0.0067	0.0063
0.0255	0.0269	0.0263	0.026	0.0247	0.0267	0.0267	0.0305	0.0254
0.0372	0.0387	0.0387	0.0402	0.0363	0.0394	0.0411	0.0491	0.0417
0.0046	0.0046	0.0043	0.0045	0.0041	0.0044	0.0044	0.0052	0.0044
0.0977	0.1056	0.1067	0.1128	0.1101	0.1163	0.1178	0.1382	0.1142
0.0332	0.037	0.0415	0.0435	0.0461	0.0479	0.0522	0.0603	0.0488
0.0819	0.0857	0.0892	0.0929	0.0943	0.1013	0.1016	0.1194	0.0926
0.1541	0.1578	0.1545	0.1738	0.1613	0.1785	0.1856	0.2173	0.1811
0.2003	0.2039	0.2016	0.1965	0.1939	0.1963	0.1942	0.2294	0.1996
0.111	0.1244	0.1477	0.1392	0.1334	0.1289	0.1298	0.139	0.1266
0.1489	0.1513	0.1426	0.1367	0.1307	0.1252	0.1314	0.148	0.1438
0.1434	0.1422	0.1575	0.1473	0.1344	0.1316	0.1359	0.1561	0.1619
0.1562	0.1565	0.1471	0.1393	0.1305	0.1302	0.1338	0.1431	0.1418
0.1964	0.196	0.1971	0.1935	0.1897	0.1835	0.1787	0.181	0.1862
0.1296	0.1338	0.1448	0.1307	0.1239	0.1234	0.1328	0.1356	0.1311
0.1661	0.1603	0.152	0.1432	0.145	0.1325	0.1392	0.1406	0.1223

0.1744	0.1894	0.1944	0.1837	0.1734	0.1692	0.1675	0.1654	0.162
0.1283	0.1293	0.1365	0.1318	0.1172	0.1186	0.1289	0.1429	0.1253
0.1349	0.1454	0.1489	0.1496	0.1357	0.1397	0.1454	0.1665	0.1386
0.1338	0.1302	0.1257	0.122	0.1144	0.1121	0.1185	0.1218	0.1036
0.1625	0.1592	0.1593	0.1388	0.1325	0.135	0.1386	0.1315	0.1221
0.1189	0.1152	0.119	0.1076	0.101	0.1009	0.1129	0.1191	0.1083
0.1404	0.1404	0.1569	0.1696	0.1558	0.1481	0.1574	0.1531	0.1578
0.1646	0.1614	0.153	0.1439	0.1373	0.1403	0.1497	0.1502	0.1398
0.1329	0.1378	0.1412	0.1345	0.1323	0.1313	0.1367	0.1395	0.1306
0.1066	0.1143	0.119	0.1214	0.1198	0.1267	0.1336	0.1621	0.1409
0.1577	0.1727	0.1788	0.1802	0.1726	0.1944	0.2012	0.2349	0.1917
0.1919	0.211	0.2138	0.2219	0.2047	0.2242	0.2151	0.2397	0.2108
0.1888	0.188	0.1901	0.1977	0.1871	0.193	0.1937	0.2272	0.1819
0.1674	0.1801	0.1769	0.182	0.1696	0.1736	0.1819	0.2154	0.1778
0.1321	0.1338	0.1377	0.1358	0.1312	0.1366	0.1344	0.1646	0.1324
0.1464	0.1489	0.1498	0.1455	0.1407	0.1474	0.1551	0.1647	0.1471
0.1323	0.1431	0.1474	0.1515	0.1448	0.1531	0.1594	0.1694	0.1688
0.1628	0.1609	0.1667	0.1589	0.1524	0.1477	0.1434	0.1417	0.1396
0.1293	0.1356	0.1455	0.1378	0.1372	0.117	0.1295	0.1356	0.1277
0.1249	0.1209	0.122	0.1221	0.124	0.1273	0.1208	0.1391	0.1266
0.1147	0.1145	0.1156	0.1048	0.0997	0.1073	0.1172	0.1062	0.101
0.1447	0.1525	0.1651	0.161	0.1471	0.1416	0.159	0.1778	0.1653
0.1295	0.1276	0.1205	0.1161	0.1092	0.1152	0.1181	0.1261	0.1098
0.1697	0.168	0.1511	0.183	0.1809	0.1959	0.1803	0.2572	0.2317
0.1899	0.1929	0.1924	0.2042	0.1777	0.186	0.1631	0.2005	0.1873
0.0073	0.0077	0.0074	0.0073	0.0069	0.0072	0.0074	0.0077	0.0078
0.0109	0.011	0.0109	0.0111	0.0105	0.0097	0.011	0.0123	0.0107
0.0162	0.0174	0.016	0.0203	0.0174	0.02	0.021	0.027	0.0239
0	0	0	0	0	0	0	0	0
0	0	0	0	0	0	0	0	0
0.0114	0.0129	0.0155	0.0171	0.0156	0.018	0.0164	0.0207	0.0184
0.0017	0.0016	0.0014	0.0013	0.0013	0.0013	0.0012	0.0012	0.0009
0.0046	0.0053	0.0058	0.0056	0.0054	0.006	0.0061	0.0073	0.006
0.0086	0.0098	0.0095	0.0098	0.0086	0.0088	0.0087	0.0112	0.0093
0.0008	0.0009	0.0009	0.001	0.001	0.0011	0.001	0.0012	0.001
0.0184	0.0187	0.0192	0.0174	0.0178	0.018	0.0216	0.0254	0.0195
0.1069	0.1263	0.1227	0.1328	0.1197	0.1204	0.1182	0.1491	0.1253
0.1738	0.1999	0.2216	0.2401	0.2192	0.2371	0.2247	0.252	0.2411
0.0636	0.064	0.0668	0.0692	0.0712	0.0766	0.078	0.0928	0.0762
0.0503	0.058	0.0633	0.0661	0.066	0.0677	0.0759	0.0935	0.0684
0.1014	0.1107	0.1113	0.1259	0.115	0.1216	0.1172	0.1446	0.12
0.1589	0.1679	0.1558	0.1574	0.1685	0.1986	0.2028	0.2524	0.1988
0.2258	0.2374	0.2473	0.2687	0.2098	0.2327	0.2482	0.308	0.2764
0.2658	0.2926	0.2789	0.2846	0.2696	0.2946	0.3091	0.3169	0.2862

0.1389	0.139	0.1407	0.1273	0.1248	0.1141	0.1309	0.13	0.1226
0.1252	0.1243	0.1326	0.1391	0.1294	0.1419	0.1295	0.1317	0.118
0.1339	0.1375	0.1391	0.1353	0.1203	0.1265	0.1219	0.1289	0.1245
0.1627	0.154	0.1563	0.1598	0.1593	0.1596	0.1466	0.1509	0.1481
0.1362	0.1334	0.1378	0.1272	0.1187	0.1221	0.1369	0.1336	0.1286
0.1564	0.1505	0.145	0.152	0.1431	0.1443	0.147	0.1375	0.1245
0.1875	0.1896	0.1772	0.1671	0.1521	0.1546	0.1683	0.1629	0.1521
0.1326	0.1299	0.1419	0.1423	0.1483	0.1523	0.1479	0.1568	0.1335
0.161	0.1678	0.1828	0.178	0.1717	0.1926	0.1967	0.2305	0.1812
0.1432	0.1437	0.1512	0.1489	0.1503	0.1587	0.1657	0.205	0.1526
0.1429	0.1361	0.1397	0.1305	0.1247	0.1285	0.1292	0.145	0.1285
0.1449	0.1405	0.1364	0.1366	0.1191	0.1182	0.1156	0.1283	0.1126
0.1393	0.1292	0.1321	0.12	0.1223	0.1163	0.1281	0.1369	0.1256
0.1277	0.123	0.1175	0.1217	0.1165	0.1194	0.1302	0.1487	0.1333
0.1766	0.1883	0.1934	0.222	0.2124	0.2213	0.2277	0.2514	0.2052
0.1571	0.1688	0.1819	0.1902	0.1933	0.2136	0.2265	0.2626	0.2103
0.1708	0.1809	0.1822	0.1818	0.174	0.1837	0.1881	0.215	0.1871
0.1249	0.1299	0.131	0.123	0.1273	0.1189	0.1258	0.1456	0.1219
0.1181	0.1344	0.1338	0.143	0.1285	0.1382	0.1472	0.1861	0.14
0.1994	0.2213	0.2205	0.2287	0.2089	0.2144	0.2136	0.2588	0.2073
0.1117	0.1184	0.1176	0.1195	0.117	0.119	0.124	0.1521	0.1178
0.1738	0.1672	0.1656	0.1603	0.148	0.1503	0.1484	0.1673	0.1565
0.258	0.2318	0.2626	0.255	0.2296	0.2144	0.2319	0.2562	0.2586
0.2029	0.2146	0.1908	0.2037	0.1834	0.1864	0.1779	0.2022	0.1952
0.1531	0.15	0.1501	0.1472	0.1438	0.1528	0.1597	0.1739	0.1649
0.1406	0.1551	0.1684	0.1487	0.1484	0.1445	0.154	0.1689	0.1373
0.1245	0.1273	0.1267	0.1167	0.1107	0.112	0.1147	0.1295	0.1108
0.1958	0.1933	0.1984	0.1861	0.174	0.1664	0.1703	0.1804	0.1681
0.141	0.1422	0.1393	0.1408	0.1409	0.1479	0.1407	0.1665	0.1338
0.1901	0.1964	0.1848	0.2389	0.2176	0.2318	0.2419	0.3275	0.2623
0.1897	0.2107	0.1982	0.2039	0.1737	0.1797	0.1609	0.2035	0.1621
0.0674	0.0751	0.0756	0.0783	0.0751	0.0792	0.0803	0.096	0.0785
0.0093	0.0106	0.0095	0.0102	0.011	0.0119	0.0119	0.0141	0.0108
0	0	0	0	0	0	0	0	0
0	0	0	0	0	0	0	0	0
0	0	0	0	0	0	0	0	0
0.0099	0.0107	0.0095	0.0115	0.0104	0.0114	0.0114	0.0152	0.0129
0.0014	0.0013	0.0014	0.0013	0.0012	0.0011	0.0011	0.0011	0.0011
0.0009	0.0009	0.0008	0.0008	0.0007	0.0007	0.0008	0.0008	0.0007
0	0	0	0	0	0	0	0	0
0	0	0	0	0	0	0	0	0
0.0313	0.0338	0.0366	0.0366	0.0278	0.0302	0.0316	0.0354	0.029
0.1256	0.1217	0.1226	0.124	0.1059	0.1104	0.1265	0.1533	0.1409
0.1727	0.1945	0.1868	0.2013	0.1515	0.1671	0.1503	0.1854	0.1584

0.17	0.1702	0.1631	0.1708	0.1703	0.1855	0.1799	0.2132	0.1972
0.0642	0.0708	0.0684	0.0722	0.0683	0.0748	0.0764	0.0958	0.0783
0.0989	0.1082	0.104	0.1234	0.1	0.1058	0.1035	0.1376	0.1117
0.1994	0.215	0.197	0.2076	0.1973	0.2082	0.2251	0.2654	0.2127
0.2731	0.3068	0.2705	0.3225	0.2951	0.2965	0.2903	0.4101	0.3969
0.2745	0.3249	0.3551	0.3742	0.3541	0.3933	0.3576	0.4331	0.3865
0.2081	0.2195	0.2296	0.223	0.2082	0.2255	0.2261	0.2433	0.2366
0.2354	0.2449	0.2293	0.2417	0.2006	0.2003	0.2008	0.2146	0.2011
0.1616	0.1646	0.1645	0.1457	0.1378	0.1346	0.1369	0.1496	0.1447
0.182	0.179	0.1972	0.1733	0.1631	0.1578	0.1563	0.1622	0.1455
0.1454	0.1449	0.1327	0.1269	0.1285	0.1241	0.1199	0.1266	0.1161
0.255	0.2567	0.2531	0.2548	0.2271	0.2208	0.2067	0.242	0.2269
0.1781	0.1741	0.1722	0.1661	0.154	0.1537	0.1547	0.1707	0.1631
0.1161	0.1275	0.1311	0.13	0.1367	0.1374	0.1465	0.1542	0.1254
0.1788	0.1693	0.1724	0.1745	0.1769	0.1747	0.1695	0.21	0.1592
0.1726	0.187	0.1831	0.2009	0.1892	0.2187	0.2203	0.257	0.1888
0.131	0.1323	0.135	0.1396	0.159	0.1654	0.175	0.2046	0.1692
0.1479	0.1622	0.1673	0.1722	0.1687	0.1775	0.1859	0.2129	0.1699
0.128	0.1218	0.1215	0.1253	0.1207	0.1252	0.1449	0.1702	0.1307
0.181	0.1989	0.2019	0.2129	0.1972	0.2014	0.208	0.242	0.2071
0.277	0.3188	0.318	0.3045	0.2938	0.3099	0.3353	0.3842	0.3316
0.3007	0.2734	0.256	0.2939	0.2699	0.2925	0.2933	0.3462	0.3078
0.1901	0.2053	0.1937	0.2004	0.1893	0.2131	0.2177	0.2475	0.2262
0.1751	0.1825	0.1848	0.1919	0.1804	0.1877	0.1952	0.2251	0.1908
0.1455	0.1489	0.1381	0.1412	0.132	0.1446	0.1431	0.1627	0.1302
0.1587	0.1745	0.171	0.1807	0.1761	0.1817	0.1949	0.2217	0.1821
0.1376	0.1458	0.1484	0.1363	0.1333	0.1433	0.1393	0.1588	0.1317
0.153	0.1835	0.1762	0.1952	0.1731	0.1674	0.1677	0.1899	0.1866
0.2064	0.1922	0.1751	0.1931	0.165	0.206	0.1963	0.2297	0.2396
0.1949	0.2078	0.1978	0.2058	0.1876	0.207	0.2041	0.2036	0.1889
0.1896	0.1934	0.1934	0.1895	0.1786	0.1769	0.1675	0.1728	0.1559
0.1358	0.141	0.1424	0.1407	0.1383	0.1386	0.1427	0.1502	0.1444
0.1299	0.1331	0.1266	0.1234	0.1207	0.1217	0.119	0.1343	0.1155
0.1213	0.1372	0.142	0.1323	0.129	0.1365	0.1281	0.157	0.132
0.1459	0.1586	0.1551	0.1612	0.1593	0.171	0.1798	0.2172	0.1813
0.2624	0.2597	0.2661	0.2613	0.239	0.3057	0.283	0.3391	0.2758
0.208	0.2096	0.1892	0.209	0.1558	0.1715	0.1786	0.2227	0.1941
0.1289	0.121	0.1118	0.1165	0.1045	0.1073	0.1075	0.1365	0.1234
0.0227	0.0245	0.0224	0.0236	0.0225	0.0227	0.0246	0.0276	0.0258
0.0136	0.0148	0.014	0.016	0.0137	0.0144	0.0118	0.0164	0.0138
0	0	0	0	0	0	0	0	0
0	0	0	0	0	0	0	0	0
0.0008	0.0008	0.0008	0.0008	0.0008	0.0007	0.0007	0.0007	0.0007
0.0127	0.0122	0.0125	0.0134	0.0121	0.0133	0.0133	0.0155	0.0146

0.018	0.0184	0.015	0.016	0.0163	0.0217	0.0186	0.0217	0.0191
0	0	0	0	0	0	0	0	0
0.016	0.0167	0.0173	0.0177	0.0175	0.0193	0.0195	0.0226	0.0183
0.0721	0.0817	0.0816	0.0889	0.086	0.0956	0.098	0.1155	0.0931
0.1235	0.1299	0.1232	0.1296	0.122	0.1351	0.1309	0.1547	0.1375
0.0911	0.0968	0.0988	0.1004	0.095	0.1002	0.0975	0.1188	0.1
0.1547	0.1637	0.1562	0.1684	0.1613	0.174	0.1684	0.203	0.1683
0.0177	0.0172	0.0118	0.0139	0.011	0.0152	0.0135	0.0212	0.02
0.0741	0.078	0.0723	0.0739	0.0717	0.079	0.0776	0.0957	0.08
0.2267	0.2411	0.2467	0.2592	0.2379	0.267	0.2665	0.3196	0.2898
0.1896	0.2184	0.2386	0.2874	0.2301	0.2551	0.2533	0.3393	0.3141
0.2044	0.2142	0.203	0.1992	0.1609	0.1956	0.2068	0.233	0.2086
0.2427	0.2667	0.2564	0.295	0.267	0.2691	0.2841	0.3358	0.3022
0.2205	0.2377	0.2239	0.2288	0.1823	0.2097	0.1951	0.2286	0.2015
0.1692	0.1745	0.1771	0.146	0.1299	0.136	0.1364	0.1352	0.137
0.1366	0.1376	0.1404	0.1206	0.1116	0.1106	0.118	0.1242	0.1125
0.131	0.1379	0.1398	0.1304	0.1159	0.1243	0.1275	0.1375	0.1326
0.2261	0.2169	0.2332	0.2289	0.1915	0.202	0.2012	0.245	0.2365
0.1758	0.1878	0.1905	0.2027	0.1847	0.1923	0.1961	0.2296	0.1918
0.1542	0.1583	0.1691	0.1752	0.1803	0.1848	0.1732	0.2062	0.1637
0.2155	0.2132	0.2088	0.2128	0.2073	0.2103	0.2187	0.2539	0.1943
0.1794	0.1935	0.1747	0.1569	0.1346	0.1555	0.1846	0.228	0.1651
0.1753	0.1887	0.1895	0.2025	0.1936	0.2147	0.2184	0.2781	0.2182
0.1537	0.1523	0.1531	0.1481	0.1506	0.1687	0.1847	0.2221	0.1777
0.1247	0.1203	0.1252	0.1235	0.1145	0.1213	0.1192	0.1435	0.1141
0.2015	0.2157	0.1865	0.2115	0.1942	0.2101	0.2397	0.257	0.228
0.1911	0.1908	0.165	0.1954	0.1738	0.1731	0.1835	0.2398	0.2046
0.1801	0.1921	0.1903	0.1845	0.1793	0.1829	0.1838	0.2009	0.1667
0.1305	0.1295	0.12	0.1412	0.146	0.1688	0.178	0.2278	0.1606
0.15	0.1629	0.1592	0.1692	0.1555	0.1809	0.1907	0.2367	0.2066
0.1645	0.1782	0.1799	0.1808	0.1671	0.1796	0.1773	0.2104	0.1799
0.1557	0.1686	0.1785	0.1903	0.1772	0.1889	0.1929	0.2278	0.1885
0.1242	0.1297	0.124	0.1146	0.1296	0.1353	0.1403	0.1598	0.1324
0.1034	0.1084	0.095	0.0937	0.0954	0.0863	0.0918	0.0946	0.0894
0.1778	0.1836	0.1781	0.1827	0.1693	0.1672	0.1663	0.1745	0.1705
0.1949	0.2018	0.2016	0.1999	0.1876	0.1812	0.1827	0.1804	0.1749
0.1541	0.1564	0.1555	0.1556	0.1435	0.1388	0.1336	0.153	0.1545
0.2283	0.2261	0.2085	0.2138	0.1919	0.1875	0.1819	0.2052	0.1894
0.1614	0.1657	0.1701	0.17	0.1626	0.1663	0.1701	0.1962	0.1632
0.0851	0.1023	0.1005	0.0925	0.0931	0.0999	0.094	0.119	0.0907
0.1442	0.1514	0.154	0.1518	0.1352	0.1363	0.1442	0.1543	0.1495
0.1626	0.1693	0.1916	0.22	0.2234	0.2369	0.2305	0.2773	0.2395
0.317	0.3366	0.3239	0.3837	0.3544	0.3091	0.23	0.2904	0.2656
0.2349	0.2722	0.2821	0.3236	0.2852	0.3114	0.2711	0.3101	0.2721

0.0922	0.0978	0.0793	0.0848	0.0646	0.0785	0.0704	0.092	0.0921
0	0	0	0	0	0	0	0	0
0	0	0	0	0	0	0	0	0
0	0	0	0	0	0	0	0	0
0.0161	0.0165	0.0159	0.0188	0.0156	0.015	0.0165	0.0211	0.0188
0.0115	0.0111	0.0121	0.0135	0.0108	0.0115	0.013	0.0179	0.0145
0.0009	0.001	0.001	0.0009	0.001	0.0011	0.0011	0.0014	0.001
0	0	0	0	0	0	0	0	0
0.0006	0.0006	0.0007	0.0007	0.0006	0.0006	0.0006	0.0005	0.0005
0.0567	0.0625	0.0582	0.0681	0.0428	0.0552	0.0643	0.0732	0.061
0.1004	0.0823	0.0809	0.089	0.0924	0.0925	0.1074	0.1387	0.1213
0.0998	0.1146	0.1061	0.1285	0.1164	0.131	0.1194	0.139	0.1417
0.0751	0.0772	0.08	0.0825	0.0781	0.0859	0.0901	0.1074	0.0957
0.0383	0.0427	0.0407	0.0431	0.0408	0.0409	0.0374	0.0427	0.0395
0.0542	0.0596	0.0679	0.0675	0.0684	0.0798	0.0835	0.0985	0.0695
0.1563	0.158	0.1594	0.1656	0.1593	0.1693	0.1721	0.2082	0.1768
0.132	0.136	0.1302	0.1332	0.1267	0.135	0.1374	0.1566	0.1352
0.107	0.1062	0.098	0.1032	0.1024	0.1056	0.1091	0.1177	0.109
0.2199	0.2265	0.2009	0.2056	0.1934	0.1985	0.1974	0.2376	0.2226
0.162	0.1627	0.1681	0.1641	0.1578	0.1713	0.1721	0.1809	0.1744
0.1657	0.1727	0.1739	0.1472	0.1389	0.1363	0.1331	0.1194	0.1076
0.1301	0.1243	0.122	0.1233	0.1121	0.1209	0.1317	0.1425	0.129
0.1429	0.1406	0.1369	0.123	0.1159	0.1146	0.115	0.122	0.1173
0.1388	0.1375	0.1431	0.1467	0.1391	0.1413	0.1507	0.1594	0.1502
0.1241	0.121	0.1235	0.1179	0.1086	0.1082	0.1107	0.1228	0.1009
0.1131	0.1111	0.1086	0.1066	0.1044	0.1155	0.117	0.1313	0.1115
0.1413	0.1504	0.1508	0.1511	0.1464	0.1494	0.1492	0.173	0.1533
0.1346	0.1443	0.1424	0.1449	0.1439	0.1588	0.16	0.1883	0.1552
0.1304	0.146	0.1362	0.1429	0.143	0.1615	0.1641	0.2081	0.162
0.2163	0.229	0.232	0.2378	0.2244	0.2337	0.2446	0.2913	0.2376
0.1809	0.1883	0.1886	0.1891	0.1839	0.1924	0.198	0.2267	0.1987
0.1178	0.1288	0.1362	0.1504	0.1385	0.1406	0.1448	0.1627	0.1473
0.2	0.2049	0.1935	0.2095	0.1787	0.2098	0.2255	0.2661	0.2351
0.1877	0.1948	0.1971	0.1917	0.1856	0.1885	0.1913	0.2122	0.2019
0.1854	0.1923	0.177	0.1927	0.1754	0.1995	0.2043	0.2428	0.2055
0.1348	0.1362	0.1366	0.1334	0.1315	0.1361	0.1368	0.1458	0.1286
0.0978	0.0954	0.0931	0.0894	0.079	0.0814	0.0864	0.0929	0.087
0.1902	0.1828	0.1866	0.1725	0.1563	0.1645	0.1667	0.1918	0.1792
0.1391	0.1418	0.1402	0.1362	0.1284	0.1343	0.1415	0.1578	0.1398
0.1195	0.1272	0.122	0.1077	0.0986	0.1006	0.1045	0.1045	0.0941
0.2126	0.2282	0.2315	0.2494	0.2109	0.2011	0.2001	0.2334	0.2243
0.2162	0.2294	0.2225	0.2241	0.2115	0.2003	0.1815	0.2023	0.1919
0.1372	0.1372	0.1294	0.1277	0.1199	0.1198	0.1233	0.1223	0.1144
0.1752	0.1792	0.1791	0.171	0.1557	0.1701	0.166	0.1919	0.1655

0.1689	0.1757	0.1817	0.1854	0.1799	0.1853	0.1906	0.2135	0.1839
0.1072	0.1144	0.1185	0.1277	0.1326	0.1418	0.1509	0.1867	0.1543
0.1515	0.1458	0.1431	0.1351	0.1367	0.1365	0.1416	0.1544	0.126
0.1449	0.1591	0.1517	0.1615	0.1371	0.1402	0.1481	0.1771	0.1525
0.1028	0.1033	0.1037	0.1012	0.0855	0.102	0.1137	0.129	0.1151
0.0292	0.0228	0.0232	0.0302	0.0262	0.0206	0.0302	0.0326	0.0313
0	0	0	0	0	0	0	0	0
0.0554	0.0583	0.0617	0.0625	0.0582	0.0609	0.0517	0.0663	0.0597
0	0	0	0	0	0	0	0	0
0	0	0	0	0	0	0	0	0
0.0147	0.0161	0.0132	0.0126	0.0111	0.0124	0.0116	0.0143	0.0155
0	0	0	0	0	0	0	0	0
0.0016	0.0016	0.0018	0.0019	0.0022	0.0026	0.0027	0.0034	0.0028
0.0116	0.0145	0.0127	0.0189	0.017	0.0188	0.0224	0.0268	0.0245
0	0	0	0	0	0	0	0	0
0	0	0	0	0	0	0	0	0
0.0478	0.057	0.0571	0.0619	0.0546	0.0575	0.0608	0.0696	0.0581
0.1699	0.1851	0.1969	0.2008	0.1729	0.1884	0.1892	0.2415	0.2275
0.0343	0.0343	0.0371	0.0426	0.0374	0.0368	0.0368	0.0421	0.0385
0.113	0.1227	0.1264	0.1425	0.1482	0.1492	0.144	0.1724	0.1475
0.0686	0.0749	0.0716	0.0773	0.0716	0.0738	0.0731	0.0884	0.0679
0.1904	0.1899	0.1816	0.1791	0.1727	0.193	0.189	0.2202	0.1882
0.1632	0.1617	0.1584	0.1509	0.1512	0.158	0.1672	0.1925	0.1563
0.1341	0.1388	0.1387	0.1416	0.136	0.1419	0.1473	0.1693	0.1474
0.1631	0.1695	0.1652	0.1747	0.1469	0.1723	0.1881	0.2307	0.2169
0.1737	0.1863	0.1806	0.1784	0.1613	0.1741	0.173	0.1939	0.1717
0.1863	0.1683	0.1736	0.176	0.165	0.1566	0.162	0.1719	0.1603
0.1657	0.1671	0.1647	0.1614	0.1443	0.1509	0.151	0.1606	0.1556
0.1468	0.1431	0.1513	0.1387	0.1326	0.1312	0.1328	0.1372	0.1204
0.1494	0.1451	0.1477	0.1414	0.14	0.1508	0.1484	0.1492	0.1341
0.138	0.141	0.1459	0.1415	0.1377	0.1301	0.1232	0.1273	0.1182
0.1453	0.1436	0.1423	0.1335	0.1307	0.1249	0.1258	0.1342	0.1297
0.1458	0.1388	0.135	0.1373	0.132	0.1357	0.1395	0.1395	0.1289
0.1289	0.1282	0.1284	0.1154	0.1157	0.1139	0.1154	0.1208	0.1002
0.1673	0.1777	0.172	0.1693	0.1644	0.1689	0.1693	0.1988	0.1651
0.1331	0.1461	0.1394	0.1502	0.1497	0.1634	0.1584	0.1916	0.152
0.172	0.1763	0.181	0.1774	0.1646	0.1849	0.1887	0.2253	0.1711
0.1168	0.1227	0.122	0.1196	0.1151	0.1244	0.1264	0.1406	0.1227
0.2069	0.2282	0.2089	0.2348	0.2097	0.2107	0.2167	0.2414	0.2249
0.1749	0.1822	0.1808	0.1667	0.1655	0.1628	0.1755	0.1903	0.1772
0.2008	0.1877	0.1948	0.1567	0.1147	0.1366	0.1496	0.1919	0.1741
0.1627	0.174	0.1682	0.1605	0.1417	0.1437	0.1537	0.156	0.1436
0.2066	0.2085	0.1986	0.1906	0.1762	0.1803	0.1878	0.1925	0.1781
0.1284	0.1162	0.1266	0.1188	0.1071	0.103	0.1084	0.0951	0.0912

0.1535	0.159	0.1585	0.1565	0.1515	0.1525	0.1511	0.1546	0.1346
0.1814	0.1862	0.1862	0.1773	0.1649	0.1559	0.1653	0.1693	0.1532
0.1325	0.134	0.1376	0.1359	0.1143	0.1271	0.134	0.1445	0.1247
0.127	0.1485	0.1644	0.176	0.1588	0.1554	0.1728	0.1968	0.2012
0.1667	0.1665	0.1622	0.1593	0.1476	0.1575	0.166	0.1725	0.1635
0.1764	0.1871	0.1954	0.1767	0.1673	0.1543	0.1517	0.1387	0.1334
0.1594	0.1654	0.1698	0.1608	0.1622	0.1601	0.1644	0.1728	0.1477
0.1823	0.1956	0.2063	0.2161	0.2192	0.2423	0.2547	0.2902	0.2335
0.1436	0.1591	0.1663	0.1643	0.1608	0.1765	0.1897	0.2411	0.1711
0.0604	0.0581	0.0592	0.0631	0.063	0.0668	0.0706	0.0692	0.0563
0.03	0.0329	0.0342	0.0323	0.0325	0.0354	0.0347	0.0389	0.0306
0.0859	0.0883	0.0866	0.0861	0.082	0.0843	0.0822	0.0951	0.081
0.0122	0.0132	0.0135	0.0132	0.0119	0.0124	0.0121	0.0134	0.0136
0.0015	0.0015	0.0014	0.0012	0.0011	0.0011	0.0011	0.0012	0.0011
0	0	0	0	0	0	0	0	0
0	0	0	0	0	0	0	0	0
0.0008	0.0007	0.0007	0.0007	0.0005	0.0006	0.0005	0.0005	0.0004
0.0013	0.0017	0.0012	0.0014	0.0016	0.0017	0.0017	0.0022	0.0019
0	0	0	0	0	0	0	0	0
0	0	0	0	0	0	0	0	0
0	0	0	0	0	0	0	0	0
0.0173	0.0192	0.0178	0.0158	0.0141	0.0143	0.0158	0.0184	0.0166
0.023	0.0256	0.0242	0.0245	0.0227	0.0236	0.0247	0.0297	0.0266
0.0331	0.0349	0.0357	0.0379	0.034	0.0346	0.0351	0.0416	0.036
0.0402	0.0394	0.0372	0.038	0.0364	0.0367	0.035	0.0424	0.0326
0.0803	0.0823	0.0819	0.0845	0.0822	0.0892	0.0945	0.1085	0.0918
0.0892	0.0923	0.0903	0.0963	0.0897	0.0955	0.1002	0.1161	0.0953
0.093	0.0949	0.0927	0.091	0.0859	0.0863	0.0951	0.1198	0.0905
0.1519	0.1563	0.1475	0.1338	0.1302	0.1461	0.1518	0.1948	0.1546
0.131	0.1346	0.1359	0.1447	0.1446	0.1609	0.1633	0.1887	0.146
0.1292	0.1411	0.1398	0.1451	0.1408	0.1467	0.1508	0.1713	0.1383
0.1891	0.2016	0.1973	0.1969	0.1913	0.1957	0.1975	0.2274	0.1898
0.1734	0.1674	0.1747	0.1637	0.1638	0.1701	0.1678	0.1764	0.1502
0.2092	0.2141	0.2252	0.2176	0.1827	0.2021	0.1941	0.2177	0.2022
0.178	0.1809	0.1829	0.1968	0.1815	0.2039	0.2316	0.2816	0.2643
0.1923	0.2069	0.2056	0.211	0.1959	0.1937	0.2093	0.2254	0.2035
0.1148	0.1177	0.1274	0.125	0.1217	0.1147	0.1078	0.108	0.1027
0.1574	0.1614	0.1551	0.153	0.1471	0.1569	0.1629	0.1809	0.1599
0.1446	0.1457	0.1491	0.1474	0.1377	0.1433	0.1484	0.1634	0.1476
0.1696	0.1743	0.1658	0.1715	0.1587	0.1646	0.1696	0.1846	0.1794
0.1626	0.1655	0.1605	0.1516	0.1488	0.1415	0.1375	0.1483	0.1294
0.1167	0.1445	0.1474	0.1396	0.1367	0.1457	0.1576	0.1995	0.156
0.1144	0.1238	0.1196	0.1217	0.1269	0.1279	0.1296	0.1545	0.1245
0.1132	0.1078	0.102	0.1035	0.0978	0.0981	0.1044	0.1099	0.1014

0.1493	0.1548	0.16	0.158	0.1398	0.1316	0.1422	0.1344	0.1183
0.1711	0.1667	0.1702	0.1633	0.1496	0.1581	0.1546	0.1562	0.1503
0.1764	0.1747	0.1606	0.1512	0.1449	0.1466	0.1429	0.1508	0.1446
0.1719	0.1733	0.1725	0.166	0.1519	0.1524	0.1506	0.1606	0.1528
0.1095	0.113	0.1093	0.1051	0.1019	0.1041	0.1086	0.1206	0.0949
0.0975	0.1076	0.1098	0.101	0.0953	0.0884	0.0958	0.0992	0.0832
0.1769	0.1775	0.1724	0.1645	0.1554	0.1497	0.1579	0.1617	0.154
0.1812	0.1864	0.1868	0.2013	0.1751	0.1644	0.1655	0.1763	0.1703
0.1727	0.1584	0.164	0.1497	0.1356	0.1417	0.1434	0.1488	0.14
0.1324	0.1366	0.1256	0.1195	0.1262	0.1178	0.1283	0.1184	0.1256
0.1819	0.1754	0.17	0.1665	0.1525	0.145	0.1571	0.1645	0.1489
0.2025	0.2185	0.2189	0.2026	0.1901	0.2001	0.1938	0.2083	0.2051
0.1821	0.1873	0.1909	0.1854	0.179	0.1727	0.1687	0.1907	0.169
0.1916	0.2021	0.2086	0.2086	0.2053	0.2266	0.2251	0.2714	0.2153
0.0468	0.049	0.0483	0.052	0.0492	0.0566	0.0588	0.071	0.0575
0.0966	0.1014	0.1038	0.1084	0.0995	0.1075	0.1057	0.1294	0.1149
0.07	0.0737	0.074	0.0783	0.0731	0.0781	0.0798	0.0925	0.0836
0.0742	0.0744	0.0694	0.066	0.0654	0.0662	0.0679	0.0773	0.0654
0.0219	0.0221	0.0226	0.0225	0.021	0.0222	0.0233	0.0243	0.0216
0.0027	0.0028	0.0023	0.0023	0.0019	0.0021	0.0021	0.0028	0.0025
0	0	0	0	0	0	0	0	0
0	0	0	0	0	0	0	0	0
0.0039	0.004	0.004	0.004	0.004	0.0043	0.0043	0.0049	0.0042
0.0018	0.002	0.002	0.002	0.0019	0.0022	0.0023	0.0026	0.0019
0	0	0	0	0	0	0	0	0
0	0	0	0	0	0	0	0	0
0.0048	0.0055	0.0056	0.0063	0.0056	0.0068	0.0063	0.0071	0.0057
0.0186	0.021	0.0219	0.0222	0.0163	0.0186	0.0168	0.0217	0.0194
0.043	0.0465	0.043	0.0457	0.0445	0.0468	0.0498	0.057	0.0564
0.0427	0.0452	0.0441	0.0473	0.0404	0.0418	0.0405	0.0472	0.0409
0.1913	0.2041	0.1901	0.2157	0.2011	0.2213	0.2147	0.2585	0.2098
0.0521	0.0532	0.0525	0.0565	0.053	0.0546	0.0525	0.0629	0.0569
0	0	0	0	0	0	0	0	0
0.0582	0.0589	0.0586	0.0594	0.0565	0.0569	0.062	0.0766	0.0627
0.1648	0.1722	0.1806	0.1886	0.184	0.1893	0.1879	0.2308	0.1826
0.2034	0.2171	0.208	0.1998	0.1917	0.2098	0.2231	0.2701	0.2242
0.1534	0.1783	0.1836	0.2001	0.198	0.199	0.2103	0.2687	0.2044
0.1393	0.1515	0.1498	0.1576	0.1632	0.1811	0.1832	0.2105	0.1743
0.1316	0.1263	0.1188	0.1056	0.1089	0.1139	0.1172	0.1205	0.1038
0.1639	0.1727	0.162	0.161	0.1442	0.1496	0.1511	0.1837	0.1868
0.1824	0.1915	0.1872	0.1877	0.1691	0.1789	0.1861	0.2148	0.1878
0.207	0.2125	0.2111	0.2043	0.1947	0.1958	0.192	0.1963	0.1764
0.15	0.1467	0.1492	0.1414	0.1428	0.1346	0.1199	0.1156	0.1086
0.1554	0.1643	0.1667	0.1693	0.1539	0.1514	0.166	0.1818	0.1504

0.1242	0.1383	0.1425	0.1387	0.1417	0.1596	0.1678	0.1922	0.1569
0.1679	0.1773	0.1882	0.17	0.1669	0.1705	0.173	0.1953	0.1599
0.1707	0.1778	0.1773	0.1803	0.1713	0.178	0.1741	0.2018	0.1682
0.1772	0.1785	0.1754	0.1764	0.1649	0.1942	0.1935	0.2348	0.179
0.1464	0.1553	0.1527	0.1624	0.165	0.1702	0.1705	0.2092	0.1715
0.1396	0.1353	0.1382	0.1384	0.1236	0.1236	0.1262	0.1349	0.1269
0.1808	0.1917	0.1897	0.196	0.1844	0.199	0.2135	0.2526	0.2256
0.1707	0.1815	0.1806	0.1874	0.1767	0.1702	0.1738	0.1913	0.1691
0.1132	0.1167	0.118	0.1102	0.1088	0.1158	0.1169	0.1192	0.1031
0.1436	0.1425	0.1422	0.14	0.1316	0.1331	0.1411	0.1528	0.1289
0.1309	0.1279	0.132	0.1247	0.1247	0.1202	0.1279	0.1541	0.1323
0.1649	0.1588	0.1649	0.1699	0.1575	0.1493	0.1574	0.1663	0.1499
0.137	0.1358	0.1238	0.1257	0.1138	0.1191	0.123	0.1373	0.1235
0.1736	0.1698	0.1773	0.1683	0.1487	0.1516	0.1652	0.1725	0.1582
0.1687	0.1793	0.1902	0.1984	0.1917	0.2052	0.2138	0.246	0.2492
0.2203	0.2132	0.1998	0.1899	0.1688	0.1714	0.1669	0.1848	0.1801
0.1229	0.1361	0.1336	0.131	0.1194	0.1271	0.1333	0.1466	0.1427
0.2077	0.2108	0.212	0.2162	0.1952	0.2039	0.2188	0.2418	0.2184
0.1815	0.1825	0.1888	0.1742	0.1845	0.1883	0.1973	0.223	0.1849
0.0713	0.0738	0.0745	0.0708	0.0716	0.0756	0.0751	0.0829	0.0723
0.0618	0.0676	0.0663	0.0725	0.0661	0.0676	0.0703	0.0814	0.0748
0.067	0.0698	0.0675	0.0699	0.0638	0.0718	0.0699	0.0806	0.0676
0.0754	0.0776	0.0773	0.0792	0.0783	0.0855	0.0855	0.0997	0.0805
0.04	0.042	0.0423	0.0428	0.0384	0.0393	0.0392	0.0452	0.0398
0.0123	0.0128	0.0109	0.012	0.0108	0.0115	0.0132	0.0141	0.0147
0.0099	0.011	0.0112	0.0121	0.0121	0.0128	0.0131	0.0158	0.0112
0	0	0	0	0	0	0	0	0
0	0	0	0	0	0	0	0	0
0.0028	0.003	0.0028	0.0028	0.0028	0.0028	0.0029	0.0033	0.0025
0.002	0.0027	0.0029	0.0034	0.0029	0.0028	0.0029	0.0035	0.0027
0.0021	0.0025	0.0023	0.0027	0.0029	0.0031	0.0031	0.0039	0.0032
0.0007	0.0008	0.0008	0.0008	0.0009	0.001	0.001	0.0013	0.001
0.0051	0.0053	0.0053	0.0053	0.0045	0.0048	0.0047	0.0058	0.0048
0.0172	0.019	0.0186	0.0191	0.0174	0.0196	0.0213	0.0236	0.0218
0.0232	0.0248	0.0243	0.0264	0.0228	0.0279	0.0254	0.0307	0.0317
0.031	0.0325	0.0293	0.0307	0.0255	0.0292	0.027	0.0338	0.0301
0.07	0.0639	0.0692	0.0857	0.0851	0.0862	0.0862	0.1117	0.1082
0.0388	0.0396	0.0377	0.043	0.0425	0.0431	0.0425	0.0535	0.047
0	0	0	0	0	0	0	0	0
0.0369	0.0375	0.0397	0.0395	0.0398	0.0451	0.0464	0.0532	0.0427
0.1164	0.1307	0.1338	0.1396	0.1391	0.149	0.151	0.1757	0.1438
0.1225	0.1277	0.1282	0.1408	0.1421	0.1456	0.1485	0.1678	0.1432
0.1316	0.1354	0.1358	0.1406	0.1408	0.1509	0.1469	0.1758	0.1381
0.1587	0.1646	0.1681	0.173	0.1636	0.1794	0.1821	0.2134	0.1805

0.1893	0.1908	0.193	0.19	0.183	0.1948	0.2026	0.2321	0.1918
0.1382	0.1436	0.1424	0.1437	0.1349	0.1242	0.1388	0.1424	0.1268
0.1397	0.1411	0.1342	0.1147	0.1214	0.1119	0.1097	0.1126	0.1073
0.1053	0.1113	0.1126	0.1037	0.1118	0.1199	0.1188	0.1278	0.1212
0.2188	0.2158	0.2037	0.2034	0.179	0.1904	0.1929	0.1991	0.1865
0.0903	0.0924	0.0965	0.0909	0.0893	0.0968	0.1051	0.104	0.0969
0.1442	0.1625	0.1588	0.1607	0.1436	0.165	0.158	0.1872	0.1408
0.1875	0.1964	0.1979	0.2111	0.2125	0.2168	0.2161	0.2755	0.2058
0.0996	0.1344	0.1323	0.145	0.1492	0.167	0.1775	0.2191	0.1757
0.1776	0.1878	0.1772	0.1699	0.1714	0.1573	0.1739	0.2124	0.1673
0.1585	0.1607	0.1493	0.1465	0.1406	0.1621	0.1627	0.2166	0.1716
0.1749	0.1832	0.1833	0.1854	0.1805	0.1832	0.1934	0.2191	0.1633
0.128	0.1334	0.1325	0.1326	0.1259	0.131	0.1374	0.1542	0.1334
0.1465	0.1489	0.1456	0.139	0.1304	0.1347	0.1337	0.1447	0.1292
0.159	0.1559	0.1545	0.1569	0.1482	0.1504	0.1598	0.1658	0.1516
0.1391	0.143	0.1423	0.1406	0.1358	0.1333	0.1373	0.149	0.1417
0.1855	0.1847	0.1899	0.1901	0.1659	0.174	0.1597	0.1937	0.1768
0.169	0.1654	0.1513	0.1372	0.1288	0.1332	0.1327	0.1336	0.1293
0.1508	0.1515	0.1599	0.1451	0.1419	0.1348	0.1338	0.1434	0.1337
0.1718	0.1793	0.1651	0.1765	0.1571	0.1559	0.1553	0.1749	0.1495
0.217	0.2054	0.2021	0.1944	0.1763	0.195	0.1949	0.2241	0.2024
0.1583	0.1586	0.1616	0.1646	0.1499	0.1589	0.166	0.1811	0.1647
0.183	0.1819	0.18	0.1792	0.1751	0.1889	0.1984	0.219	0.1976
0.1764	0.1815	0.1848	0.1843	0.1798	0.1893	0.1916	0.2088	0.1869
0.1597	0.1695	0.1807	0.1844	0.1906	0.1861	0.1877	0.2096	0.1533
0.0834	0.0867	0.0901	0.0849	0.085	0.0858	0.0869	0.0974	0.0884
0.0574	0.0554	0.0541	0.0546	0.0485	0.0483	0.0521	0.0636	0.0615
0.1135	0.1158	0.1186	0.118	0.1092	0.1171	0.1158	0.1443	0.1136
0.166	0.1839	0.1801	0.1868	0.1945	0.1922	0.2007	0.2421	0.1789
0.0382	0.0398	0.0386	0.0385	0.0397	0.0432	0.0446	0.0538	0.0418
0.0061	0.0069	0.0047	0.0067	0.0029	0.0076	0.0085	0.0122	0.0113
0.0162	0.0163	0.0154	0.0145	0.0138	0.0139	0.0139	0.0148	0.0122
0	0	0	0	0	0	0	0	0
0	0	0	0	0	0	0	0	0
0.0015	0.0017	0.0015	0.0016	0.0015	0.0015	0.0017	0.0019	0.0018
0.0038	0.0044	0.0042	0.005	0.004	0.0043	0.0042	0.0053	0.0054
0	0	0	0	0	0	0	0	0
0	0	0	0	0	0	0	0	0
0	0	0	0	0	0	0	0	0
0.0016	0.0016	0.0016	0.0014	0.0014	0.0012	0.0013	0.0013	0.0011
0.0018	0.0018	0.0021	0.0018	0.0019	0.0017	0.0017	0.0017	0.0013
0.0036	0.0033	0.0034	0.0033	0.003	0.003	0.0032	0.0029	0.0029
0.0115	0.0123	0.0123	0.0129	0.0137	0.0156	0.0153	0.0189	0.014
0.0066	0.0064	0.006	0.0057	0.0058	0.0055	0.0062	0.0075	0.0063

0.0285	0.0308	0.0283	0.0264	0.0252	0.0236	0.0241	0.0271	0.0242
0.0231	0.0258	0.0266	0.025	0.0264	0.0279	0.0298	0.0353	0.0287
0.0349	0.0376	0.0379	0.0386	0.0383	0.039	0.0399	0.043	0.0349
0.0776	0.0781	0.0792	0.0759	0.0735	0.0773	0.0761	0.0856	0.0738
0.1467	0.154	0.1575	0.1596	0.154	0.157	0.1611	0.1818	0.1544
0.1458	0.1506	0.1473	0.1466	0.1375	0.1498	0.1498	0.1743	0.1551
0.1363	0.1317	0.1401	0.1376	0.1327	0.1391	0.141	0.1578	0.1368
0.1262	0.1206	0.1166	0.1165	0.1098	0.1115	0.1169	0.1198	0.114
0.1633	0.1652	0.1634	0.1678	0.1549	0.1597	0.1707	0.185	0.1696
0.1834	0.2005	0.1908	0.2032	0.1939	0.1955	0.2043	0.2347	0.2203
0.1534	0.1571	0.1599	0.1642	0.1544	0.1575	0.1493	0.1678	0.1533
0.1633	0.1682	0.1723	0.1605	0.1481	0.1511	0.159	0.1684	0.1606
0.0932	0.1033	0.1105	0.1029	0.1007	0.1122	0.1139	0.1118	0.0939
0.2262	0.2427	0.2711	0.2715	0.2458	0.2495	0.241	0.2642	0.2014
0.204	0.2323	0.2222	0.2284	0.2103	0.2241	0.2451	0.2799	0.2575
0.1717	0.1861	0.1869	0.1883	0.1706	0.1741	0.1653	0.186	0.1724
0.0932	0.1057	0.1112	0.1055	0.1198	0.1287	0.1209	0.1421	0.0932
0.1294	0.1377	0.1472	0.1569	0.1488	0.1492	0.1465	0.1824	0.1404
0.1359	0.1454	0.1508	0.1537	0.1563	0.1622	0.1685	0.1915	0.1653
0.1499	0.1413	0.1394	0.1464	0.1448	0.1392	0.1427	0.1502	0.1314
0.1481	0.1457	0.1492	0.146	0.1363	0.1372	0.1447	0.1574	0.1362
0.1643	0.1869	0.1863	0.208	0.1851	0.1888	0.1847	0.2009	0.1754
0.2057	0.2127	0.2253	0.2516	0.2152	0.1998	0.2046	0.2411	0.2396
0.2443	0.2388	0.2293	0.2248	0.2106	0.2255	0.2459	0.275	0.2392
0.1297	0.1299	0.1441	0.1323	0.1163	0.1202	0.1262	0.1234	0.1077
0.1679	0.1628	0.1685	0.17	0.1675	0.1694	0.1641	0.1762	0.1575
0.1766	0.1828	0.1763	0.1797	0.1689	0.1563	0.1673	0.1836	0.1689
0.1929	0.2008	0.2008	0.2097	0.1852	0.1748	0.1791	0.1911	0.1745
0.2842	0.2942	0.2667	0.2798	0.2705	0.2771	0.2803	0.3313	0.3348
0.2449	0.2524	0.2542	0.2664	0.2467	0.2463	0.2635	0.2984	0.2749
0.1058	0.1134	0.1124	0.1142	0.1097	0.1166	0.1239	0.1382	0.1102
0.0684	0.0703	0.0754	0.0769	0.0809	0.0854	0.0908	0.1052	0.0824
0.0156	0.0166	0.0161	0.0173	0.0175	0.0182	0.0181	0.0206	0.0175
0.1845	0.1926	0.1801	0.1855	0.189	0.198	0.2094	0.2599	0.2153
0.1249	0.1354	0.1173	0.1308	0.1395	0.1494	0.1616	0.2124	0.1615
0.0147	0.0167	0.0164	0.0176	0.0155	0.0161	0.017	0.021	0.0159
0.012	0.0123	0.0125	0.0126	0.013	0.0126	0.0135	0.0148	0.0117
0	0	0	0	0	0	0	0	0
0	0	0	0	0	0	0	0	0
0	0	0	0	0	0	0	0	0
0.0005	0.0005	0.0005	0.0005	0.0004	0.0004	0.0004	0.0004	0.0004
0.0048	0.0043	0.0042	0.0047	0.0047	0.0052	0.0062	0.0074	0.0067
0	0	0	0	0	0	0	0	0
0	0	0	0	0	0	0	0	0

0	0	0	0	0	0	0	0	0
0.0006	0.0006	0.0007	0.0007	0.0007	0.0007	0.0007	0.0007	0.0007
0.0016	0.0016	0.0015	0.0013	0.0013	0.0014	0.0013	0.0014	0.0013
0.0013	0.0014	0.0013	0.0012	0.0012	0.0013	0.0012	0.0013	0.0013
0.0022	0.0024	0.0022	0.002	0.002	0.0021	0.0019	0.0023	0.002
0.0104	0.0099	0.0099	0.0095	0.0095	0.0093	0.0087	0.0082	0.0075
0.0037	0.0038	0.0038	0.0035	0.0033	0.0031	0.0031	0.0034	0.0034
0.0169	0.0179	0.0187	0.0203	0.0184	0.0199	0.0212	0.0274	0.0227
0.046	0.0574	0.0564	0.0646	0.0588	0.0617	0.0645	0.072	0.0595
0.1096	0.1013	0.1028	0.1172	0.1189	0.1282	0.1274	0.1464	0.1381
0.1665	0.1768	0.1753	0.1785	0.1706	0.1822	0.1845	0.2109	0.1761
0.1313	0.1276	0.1307	0.1288	0.1276	0.1384	0.1489	0.1841	0.155
0.0877	0.1019	0.1118	0.1123	0.1102	0.1253	0.1291	0.1532	0.1256
0.1419	0.1533	0.1589	0.1608	0.1537	0.1522	0.1496	0.1681	0.1394
0.2158	0.2369	0.2188	0.212	0.2004	0.2079	0.1871	0.1869	0.1462
0.2211	0.2732	0.256	0.2634	0.2221	0.2317	0.2374	0.3149	0.3394
0.1671	0.1648	0.1662	0.1593	0.1565	0.1545	0.1465	0.148	0.1326
0.1772	0.1822	0.1704	0.1698	0.1571	0.1705	0.176	0.196	0.1967
0.1414	0.1395	0.1414	0.1364	0.111	0.1131	0.1136	0.1424	0.1092
0.2666	0.2339	0.2286	0.2116	0.1649	0.1348	0.1265	0.1954	0.1789
0.3102	0.3532	0.3548	0.3944	0.3652	0.414	0.3734	0.3945	0.3919
0.2315	0.2782	0.2752	0.3081	0.2593	0.2799	0.2674	0.3125	0.2841
0.1504	0.1482	0.1586	0.1549	0.1535	0.1408	0.1372	0.1393	0.1263
0.1745	0.1787	0.1654	0.1753	0.1576	0.1727	0.1869	0.2041	0.1886
0.2787	0.2441	0.2302	0.2401	0.2097	0.2782	0.3048	0.4183	0.4115
0.2889	0.278	0.2578	0.2861	0.2469	0.245	0.2592	0.2864	0.2599
0.1289	0.1254	0.1184	0.11	0.1102	0.114	0.1212	0.123	0.1126
0.2822	0.2871	0.2816	0.2726	0.2014	0.2325	0.2878	0.338	0.2839
0.2602	0.276	0.2607	0.2538	0.2575	0.2519	0.2312	0.296	0.2846
0.2646	0.2846	0.278	0.2536	0.2148	0.2277	0.2233	0.3297	0.3021
0.1479	0.1963	0.1867	0.2214	0.1451	0.1562	0.1624	0.2125	0.1685
0.2184	0.2255	0.2374	0.2668	0.2357	0.2822	0.2846	0.3375	0.3052
0.2708	0.2949	0.2685	0.2849	0.2506	0.2232	0.2228	0.2494	0.2319
0.2971	0.2979	0.2455	0.3105	0.2439	0.3025	0.2731	0.3184	0.308
0.1428	0.1583	0.1554	0.2182	0.2067	0.2302	0.2396	0.3266	0.298
0.1124	0.114	0.1138	0.1128	0.1031	0.1039	0.1051	0.1115	0.1029
0.0377	0.04	0.0392	0.0387	0.033	0.033	0.0375	0.042	0.0417
0	0	0	0	0	0	0	0	0
0.0498	0.0532	0.0544	0.0531	0.0547	0.0571	0.0574	0.0646	0.0516
0.1172	0.1219	0.1226	0.1262	0.1361	0.1507	0.1515	0.1975	0.1572
0.0174	0.0172	0.0172	0.0176	0.0141	0.0156	0.0173	0.0213	0.0195
0.016	0.0165	0.0173	0.0171	0.0141	0.0156	0.0164	0.0195	0.0192
0.0157	0.0171	0.0166	0.0176	0.0164	0.0168	0.0172	0.021	0.0191
0	0	0	0	0	0	0	0	0

0	0	0	0	0	0	0	0	0
0	0	0	0	0	0	0	0	0
0.0018	0.002	0.0018	0.0018	0.0018	0.0018	0.0017	0.0019	0.0015
0.0016	0.0017	0.0018	0.0018	0.0018	0.0017	0.0018	0.002	0.0018
0.0038	0.0039	0.0037	0.0036	0.0037	0.0038	0.0037	0.0043	0.0037
0.0008	0.0009	0.001	0.001	0.001	0.0011	0.0011	0.0014	0.0011
0.001	0.001	0.001	0.0009	0.0008	0.0009	0.001	0.0012	0.0008
0.0064	0.0072	0.0082	0.0094	0.0087	0.0091	0.0087	0.0101	0.0109
0.0089	0.0087	0.0103	0.0095	0.0086	0.0085	0.01	0.0116	0.0106
0.0034	0.0045	0.0038	0.0055	0.0047	0.0045	0.0045	0.0053	0.0056
0.019	0.0164	0.0172	0.0175	0.0136	0.0193	0.0131	0.0182	0.0146
0.0136	0.0153	0.0181	0.0172	0.016	0.0178	0.0193	0.0219	0.0151
0.0055	0.0064	0.0069	0.0075	0.0072	0.007	0.0074	0.009	0.0073
0.0571	0.063	0.06	0.0641	0.0601	0.0634	0.0766	0.0845	0.0815
0.1391	0.154	0.1691	0.1647	0.1538	0.1688	0.1691	0.1811	0.1544
0.0811	0.0864	0.0917	0.102	0.0823	0.0843	0.0834	0.0951	0.0847
0.0647	0.0656	0.0627	0.0642	0.0576	0.0651	0.067	0.0761	0.0663
0.0757	0.0874	0.084	0.0771	0.0799	0.0917	0.0972	0.122	0.0889
0.1709	0.1811	0.1793	0.1817	0.1788	0.1921	0.1904	0.2285	0.1843
0.1414	0.142	0.138	0.1409	0.1398	0.1483	0.1503	0.1832	0.1516
0.1085	0.1099	0.1101	0.1076	0.1017	0.1028	0.1118	0.1229	0.1066
0.2124	0.2121	0.2113	0.2191	0.1904	0.1865	0.2013	0.2275	0.2303
0.321	0.3059	0.2456	0.2616	0.2413	0.2538	0.2666	0.3035	0.2586
0.1902	0.1944	0.1819	0.2035	0.1794	0.1936	0.1748	0.2078	0.1877
0.178	0.1785	0.1682	0.173	0.1497	0.1511	0.1623	0.1868	0.1681
0.2179	0.2362	0.2193	0.2502	0.1904	0.1917	0.2023	0.2436	0.2439
0.4062	0.3783	0.3415	0.3094	0.189	0.2449	0.2199	0.2568	0.2745
0.2326	0.2433	0.2545	0.2882	0.2174	0.2128	0.2352	0.2656	0.2214
0.1592	0.1638	0.1686	0.1589	0.1468	0.1529	0.158	0.1704	0.1564
0.1948	0.1929	0.2038	0.2335	0.2003	0.2086	0.1655	0.2098	0.2151
0.3489	0.3781	0.3727	0.4237	0.4162	0.4287	0.4072	0.4175	0.3873
0.2666	0.2664	0.2795	0.3678	0.2381	0.2338	0.2267	0.3093	0.2835
0.1908	0.1921	0.2002	0.2207	0.1962	0.1735	0.1946	0.2376	0.2127
0.326	0.3449	0.3547	0.3764	0.3449	0.3501	0.3351	0.344	0.3024
0.2267	0.2327	0.2398	0.2584	0.2857	0.3048	0.2954	0.3319	0.3513
0.372	0.3934	0.3295	0.4207	0.3247	0.2812	0.2821	0.4043	0.3819
0.4101	0.422	0.4193	0.4798	0.3654	0.3973	0.3666	0.5098	0.4567
0.2887	0.278	0.2825	0.3239	0.3144	0.3912	0.3807	0.4648	0.3998
0.2982	0.4256	0.3695	0.3928	0.3436	0.333	0.2731	0.3239	0.2861
0.2202	0.2597	0.2301	0.2259	0.1888	0.2181	0.2108	0.2343	0.2365
0.1184	0.12	0.1202	0.1155	0.1101	0.1106	0.1112	0.1276	0.113
0.0646	0.0658	0.0653	0.0613	0.0605	0.0649	0.0641	0.0719	0.0553
0.0567	0.0584	0.0645	0.0682	0.0632	0.0673	0.0692	0.0896	0.0646
0.1536	0.147	0.1391	0.1511	0.1655	0.1652	0.1489	0.1854	0.1371

0.1241	0.1369	0.1315	0.1408	0.1439	0.1517	0.1587	0.1796	0.1415
0.0034	0.0037	0.0039	0.0037	0.0035	0.0035	0.0036	0.0041	0.0033
0.0212	0.0219	0.0222	0.0226	0.0204	0.0212	0.0217	0.0281	0.0245
0.1162	0.1268	0.1264	0.1356	0.1267	0.1361	0.1452	0.1741	0.1459
0.0608	0.0612	0.0619	0.0643	0.0572	0.0488	0.0538	0.0758	0.0647
0.0158	0.0167	0.0168	0.0173	0.0164	0.017	0.0178	0.0209	0.0173
0	0	0	0	0	0	0	0	0
0	0	0	0	0	0	0	0	0
0.0016	0.0016	0.0016	0.0015	0.0015	0.0015	0.0014	0.0017	0.0014
0.0017	0.0018	0.0018	0.0017	0.0018	0.0018	0.0019	0.0022	0.0018
0.0029	0.0031	0.0031	0.0037	0.0035	0.004	0.0038	0.0047	0.0042
0.0013	0.0014	0.0013	0.0014	0.0014	0.0015	0.0016	0.0018	0.0014
0.0031	0.0034	0.0034	0.0037	0.0034	0.0035	0.0033	0.004	0.0034
0.0076	0.0071	0.0051	0.0057	0.0038	0.0043	0.0042	0.006	0.0051
0.0044	0.0047	0.006	0.007	0.0062	0.0078	0.0075	0.0099	0.0081
0.0033	0.0045	0.0042	0.0046	0.0046	0.0059	0.0068	0.0077	0.0068
0.0201	0.0198	0.0202	0.0201	0.0201	0.0235	0.0238	0.0257	0.0205
0	0	0	0	0	0	0	0	0
0.0147	0.0156	0.0155	0.0174	0.0158	0.0152	0.0151	0.0171	0.0142
0.0619	0.0665	0.0594	0.0676	0.0702	0.0684	0.068	0.0767	0.068
0.0851	0.0924	0.0957	0.1002	0.0937	0.0941	0.1034	0.1149	0.0993
0.0878	0.0958	0.0927	0.0951	0.092	0.1021	0.1066	0.1253	0.1017
0.0864	0.0887	0.0897	0.0918	0.0858	0.0922	0.0941	0.1151	0.0977
0.1273	0.1337	0.1367	0.1402	0.132	0.1345	0.1358	0.1597	0.1344
0.158	0.1551	0.1618	0.1652	0.1529	0.1705	0.1711	0.1986	0.164
0.1492	0.157	0.1605	0.1723	0.1627	0.1732	0.1761	0.2073	0.164
0.1722	0.1718	0.1761	0.1603	0.1646	0.1635	0.1805	0.2139	0.1703
0.1094	0.1155	0.1138	0.1179	0.1147	0.1181	0.1159	0.1362	0.1185
0.2383	0.2643	0.2589	0.2836	0.2454	0.2881	0.3094	0.3528	0.3188
0.2521	0.2599	0.2822	0.2865	0.2634	0.2741	0.2679	0.282	0.2595
0.2013	0.2141	0.2319	0.2305	0.2034	0.2068	0.1987	0.2215	0.2078
0.1437	0.1587	0.1482	0.1524	0.1398	0.1348	0.1309	0.1611	0.169
0.1668	0.2189	0.2045	0.2182	0.165	0.2039	0.229	0.2641	0.2362
0.1507	0.171	0.1685	0.1642	0.1486	0.133	0.1393	0.1584	0.1403
0.1606	0.16	0.1619	0.1646	0.1499	0.1505	0.1574	0.1634	0.1578
0.1365	0.1347	0.1366	0.1349	0.1212	0.1195	0.1191	0.1326	0.1197
0.1283	0.1435	0.1315	0.1352	0.1339	0.139	0.1538	0.1496	0.1295
0.138	0.1663	0.1641	0.1753	0.1558	0.1837	0.1728	0.2205	0.1787
0.226	0.2585	0.2624	0.2873	0.2172	0.2677	0.2165	0.2906	0.2726
0.2176	0.1557	0.1578	0.1351	0.0557	0.0889	0.0945	0.1718	0.1609
0.1926	0.2053	0.233	0.2452	0.1901	0.2095	0.2357	0.2864	0.2986
0.2011	0.1887	0.1982	0.2501	0.2135	0.1945	0.203	0.2696	0.2319
0.1642	0.1637	0.1947	0.2571	0.2359	0.2365	0.2577	0.3014	0.2357
0.3464	0.4089	0.3815	0.4247	0.3103	0.3604	0.3465	0.3983	0.3918

0.4368	0.3663	0.3712	0.3657	0.2599	0.2906	0.2529	0.3025	0.3002
0.1818	0.1792	0.1775	0.1763	0.1516	0.161	0.1706	0.194	0.164
0.0909	0.094	0.0983	0.0967	0.0975	0.0996	0.1072	0.1192	0.0981
0.1032	0.1199	0.1185	0.1239	0.1137	0.1219	0.125	0.1495	0.1119
0.133	0.1659	0.1616	0.1757	0.1935	0.1904	0.2117	0.2585	0.1896
0.159	0.1746	0.1773	0.1855	0.1726	0.1953	0.1911	0.234	0.1799
0.0442	0.0475	0.0449	0.047	0.046	0.0491	0.0497	0.0592	0.047
0.0033	0.0035	0.0037	0.0031	0.0029	0.003	0.0029	0.0027	0.0025
0.0267	0.0286	0.029	0.0303	0.0293	0.0288	0.0315	0.0349	0.0305
0.0632	0.0644	0.0603	0.0657	0.0541	0.0577	0.0494	0.0599	0.0555
0.0292	0.0285	0.0347	0.0366	0.0274	0.0332	0.043	0.0672	0.0692
0.004	0.0038	0.0039	0.0039	0.004	0.004	0.0041	0.0039	0.0039
0	0	0	0	0	0	0	0	0
0	0	0	0	0	0	0	0	0
0.0008	0.0007	0.0008	0.0007	0.0007	0.0006	0.0006	0.0007	0.0006
0.0007	0.0007	0.0007	0.0007	0.0007	0.0007	0.0008	0.0007	0.0007
0.0008	0.0008	0.0008	0.0008	0.0008	0.0007	0.0007	0.0007	0.0006
0.0007	0.0008	0.0007	0.0006	0.0006	0.0006	0.0006	0.0007	0.0006
0	0	0	0	0	0	0	0	0
0	0	0	0	0	0	0	0	0
0	0	0	0	0	0	0	0	0
0	0	0	0	0	0	0	0	0
0.0008	0.0009	0.001	0.001	0.0011	0.0011	0.0012	0.0015	0.0013
0.0035	0.0062	0.0072	0.0069	0.0065	0.0077	0.0084	0.0115	0.012
0.0172	0.0179	0.0175	0.0178	0.0168	0.018	0.0183	0.0207	0.019
0.0128	0.0132	0.0131	0.0124	0.0114	0.0116	0.0117	0.0126	0.0108
0.0765	0.0777	0.0756	0.0786	0.0839	0.0835	0.0841	0.1055	0.0803
0.1197	0.125	0.1236	0.1247	0.1287	0.1435	0.1379	0.1636	0.1357
0.0741	0.0758	0.0746	0.0764	0.073	0.0757	0.0751	0.0841	0.0699
0.0684	0.074	0.0756	0.076	0.0826	0.0811	0.0842	0.0977	0.0766
0.0242	0.0259	0.0264	0.0246	0.0242	0.0258	0.0275	0.0323	0.0252
0.0945	0.0975	0.1	0.0971	0.094	0.0997	0.1041	0.1142	0.0946
0.1343	0.1266	0.1323	0.1211	0.1192	0.1327	0.1264	0.1533	0.1122
0.1704	0.182	0.1764	0.1731	0.1686	0.1804	0.1821	0.2119	0.1712
0.0984	0.0995	0.0976	0.1013	0.0937	0.1006	0.1014	0.1102	0.0965
0.259	0.27	0.2549	0.2688	0.1883	0.2456	0.2361	0.3374	0.2983
0.33	0.3485	0.3285	0.327	0.3106	0.2732	0.2765	0.3062	0.3035
0.2132	0.2189	0.2046	0.2087	0.1887	0.1915	0.2033	0.2238	0.2147
0.2243	0.2298	0.2366	0.2383	0.1911	0.2028	0.1972	0.2294	0.2284
0.1952	0.1954	0.1899	0.1867	0.1738	0.1892	0.181	0.1902	0.1738
0.1932	0.1994	0.2017	0.1853	0.1762	0.1799	0.1913	0.2183	0.2
0.2264	0.2514	0.2536	0.2594	0.2314	0.2391	0.2382	0.2618	0.2341
0.2616	0.2816	0.2503	0.2284	0.199	0.2206	0.1919	0.2128	0.1934
0.2184	0.2321	0.2066	0.2309	0.2017	0.1951	0.1955	0.2255	0.2407

0.208	0.2154	0.2049	0.2092	0.2123	0.2408	0.2545	0.2795	0.2617
0.2188	0.2755	0.2432	0.2564	0.2197	0.2485	0.2457	0.2669	0.251
0.16	0.1887	0.1939	0.2243	0.1851	0.2008	0.1895	0.2191	0.1831
0.2339	0.2363	0.2023	0.1864	0.1664	0.1574	0.1632	0.1797	0.1599
0.2767	0.2832	0.2515	0.2381	0.2136	0.2499	0.205	0.2269	0.2392
0.1981	0.2234	0.2298	0.2334	0.2094	0.2131	0.217	0.2706	0.2487
0.2205	0.2348	0.2262	0.2447	0.2262	0.2303	0.2342	0.2649	0.2395
0.1081	0.1154	0.1206	0.1181	0.1125	0.1227	0.1268	0.1515	0.1242
0.1235	0.1332	0.1371	0.147	0.1298	0.1382	0.1431	0.1697	0.1418
0.0987	0.099	0.0866	0.0916	0.0832	0.0859	0.0833	0.1075	0.0869
0.0562	0.0618	0.0618	0.0632	0.0636	0.0663	0.0682	0.083	0.0673
0.0243	0.0252	0.0245	0.0242	0.0242	0.027	0.0267	0.0303	0.0255
0	0	0	0	0	0	0	0	0
0.0636	0.0645	0.0662	0.0613	0.0578	0.0497	0.0511	0.056	0.0484
0.0588	0.0579	0.0532	0.0501	0.0468	0.0502	0.0533	0.0523	0.0539
0.0171	0.0183	0.0184	0.0217	0.0185	0.0222	0.021	0.023	0.0194
0.023	0.0216	0.0197	0.0212	0.0193	0.023	0.0237	0.0258	0.0242
0.0049	0.0053	0.0052	0.0052	0.0048	0.0051	0.0059	0.0079	0.0056
0	0	0	0	0	0	0	0	0
0	0	0	0	0	0	0	0	0
0	0	0	0	0	0	0	0	0
0	0	0	0	0	0	0	0	0
0	0	0	0	0	0	0	0	0
0	0	0	0	0	0	0	0	0
0	0	0	0	0	0	0	0	0
0	0	0	0	0	0	0	0	0
0	0	0	0	0	0	0	0	0
0	0	0	0	0	0	0	0	0
0.0019	0.0019	0.0018	0.0017	0.0017	0.0016	0.0017	0.0019	0.0016
0.0021	0.0021	0.0021	0.0019	0.0018	0.0018	0.0018	0.0022	0.0019
0.0048	0.0049	0.0049	0.0048	0.0044	0.0044	0.0044	0.0049	0.0044
0.0052	0.0055	0.0054	0.0055	0.0056	0.0057	0.0065	0.0069	0.006
0.0078	0.0082	0.0085	0.0083	0.0075	0.0071	0.0082	0.0091	0.0078
0.0086	0.0093	0.0097	0.0097	0.0098	0.0096	0.0103	0.0119	0.0096
0.008	0.009	0.0096	0.0097	0.0107	0.0111	0.0105	0.0123	0.01
0.009	0.0096	0.009	0.0091	0.0095	0.0094	0.0102	0.0121	0.0097
0.009	0.0094	0.0093	0.0096	0.009	0.0102	0.0101	0.0123	0.0103
0.0934	0.1016	0.0997	0.0984	0.0917	0.0977	0.1009	0.1161	0.1007
0.1504	0.1519	0.1451	0.1454	0.1394	0.1467	0.1428	0.1703	0.1365
0.1538	0.1608	0.1536	0.154	0.1571	0.1665	0.1684	0.1874	0.1766
0.1286	0.1292	0.1197	0.1185	0.1185	0.1173	0.1275	0.141	0.1277
0.1416	0.1476	0.1404	0.146	0.1339	0.1437	0.1347	0.1607	0.137
0.2558	0.2561	0.247	0.238	0.1967	0.1992	0.1875	0.2262	0.2033
0.224	0.2227	0.2057	0.2378	0.22	0.2538	0.2394	0.2943	0.2473

0.3347	0.2856	0.2846	0.3253	0.2236	0.2584	0.239	0.2423	0.209
0.1926	0.1934	0.1798	0.1814	0.1849	0.2034	0.2163	0.2393	0.208
0.1344	0.1223	0.1195	0.1241	0.1132	0.1095	0.1168	0.125	0.1111
0.1904	0.1997	0.2347	0.2276	0.1941	0.1936	0.2153	0.2659	0.217
0.2051	0.2467	0.2446	0.2668	0.263	0.2695	0.2601	0.286	0.2719
0.1612	0.166	0.1651	0.1571	0.1524	0.1514	0.1466	0.1578	0.139
0.1316	0.1319	0.126	0.1199	0.11	0.1135	0.1204	0.133	0.1189
0.1898	0.2058	0.1993	0.2378	0.2182	0.217	0.1991	0.2311	0.1946
0.2405	0.2565	0.2382	0.2582	0.2185	0.233	0.2293	0.2819	0.2815
0.1405	0.1405	0.1423	0.1417	0.1406	0.1383	0.1418	0.157	0.1268
0.1637	0.1712	0.1834	0.1819	0.1762	0.174	0.1746	0.1869	0.1498
0.2039	0.213	0.2149	0.2131	0.2058	0.2103	0.213	0.2425	0.1944
0.1333	0.1385	0.1336	0.1266	0.1314	0.1396	0.1411	0.1587	0.1276
0.0522	0.0554	0.0559	0.0612	0.0592	0.0558	0.0603	0.0719	0.0572
0.1041	0.1233	0.1074	0.0981	0.0866	0.1137	0.1297	0.1533	0.1541
0.1091	0.1374	0.1469	0.1514	0.1286	0.1599	0.142	0.1506	0.1592
0.0258	0.03	0.0284	0.0246	0.0181	0.0206	0.0241	0.0327	0.0284
0.0045	0.0044	0.0044	0.0046	0.0034	0.0038	0.0044	0.006	0.0058
0.0119	0.0116	0.0113	0.0105	0.01	0.0101	0.0102	0.0104	0.0101
0.0323	0.0304	0.0321	0.0305	0.0275	0.0254	0.026	0.0257	0.0258
0.029	0.0298	0.0291	0.0287	0.0293	0.0284	0.026	0.0264	0.0232
0.0112	0.0115	0.0115	0.0107	0.0117	0.0114	0.0118	0.0116	0.01
0.0017	0.0017	0.0018	0.0019	0.0018	0.0019	0.0019	0.002	0.0017
0	0	0	0	0	0	0	0	0
0	0	0	0	0	0	0	0	0
0	0	0	0	0	0	0	0	0
0	0	0	0	0	0	0	0	0
0	0	0	0	0	0	0	0	0
0	0	0	0	0	0	0	0	0
0	0	0	0	0	0	0	0	0
0	0	0	0	0	0	0	0	0
0	0	0	0	0	0	0	0	0
0	0	0	0	0	0	0	0	0
0	0	0	0	0	0	0	0	0
0	0	0	0	0	0	0	0	0
0	0	0	0	0	0	0	0	0
0.0028	0.0026	0.0025	0.0024	0.0022	0.0023	0.0024	0.0027	0.0023
0.0041	0.0043	0.0043	0.0041	0.0037	0.0039	0.0041	0.0045	0.0039
0.0042	0.0045	0.0045	0.0045	0.0043	0.004	0.0039	0.0045	0.004
0.0041	0.0045	0.0049	0.0048	0.0047	0.0049	0.0052	0.0062	0.005
0.0046	0.0047	0.0051	0.0056	0.0057	0.0054	0.006	0.0072	0.0057
0.0049	0.0052	0.0053	0.0049	0.005	0.0053	0.0059	0.0071	0.0055
0.0152	0.0156	0.0163	0.0178	0.0184	0.0192	0.0213	0.0249	0.0202
0.111	0.1131	0.1191	0.1219	0.1165	0.119	0.1142	0.1394	0.113

0.1119	0.1145	0.1072	0.106	0.104	0.1079	0.1162	0.1373	0.1159
0.1366	0.1519	0.1672	0.1527	0.1592	0.1717	0.183	0.221	0.1727
0.1727	0.1878	0.197	0.2041	0.1951	0.211	0.2106	0.2361	0.1787
0.1329	0.1351	0.1343	0.1389	0.1346	0.1519	0.1539	0.1751	0.1439
0.1398	0.1437	0.1474	0.1433	0.1466	0.1644	0.1597	0.1959	0.1648
0.1211	0.1238	0.1248	0.1246	0.1202	0.1365	0.1379	0.1483	0.1247
0.1239	0.1222	0.1195	0.1138	0.1123	0.1124	0.1223	0.1446	0.1126
0.1472	0.1393	0.1461	0.1383	0.1368	0.1374	0.1402	0.1545	0.1365
0.1768	0.166	0.1617	0.1541	0.1488	0.1532	0.1543	0.1718	0.149
0.1381	0.1443	0.1376	0.1372	0.1276	0.1325	0.1419	0.1575	0.1257
0.1081	0.1214	0.1265	0.1265	0.1201	0.1183	0.1161	0.1356	0.1085
0.1402	0.141	0.1333	0.125	0.1172	0.1183	0.1236	0.1402	0.1177
0.1286	0.124	0.129	0.1256	0.1233	0.126	0.1244	0.1346	0.1292
0.197	0.2023	0.2045	0.2049	0.194	0.1976	0.1991	0.2308	0.2028
0.1658	0.1761	0.1961	0.1884	0.1848	0.1787	0.1871	0.2242	0.1754
0.114	0.1162	0.1179	0.1115	0.1046	0.1076	0.1044	0.1288	0.0868
0.1951	0.2047	0.2145	0.2252	0.213	0.2303	0.2363	0.286	0.2314
0.1841	0.1968	0.1928	0.1915	0.1796	0.1876	0.1912	0.2178	0.1659
0.063	0.0663	0.0686	0.0672	0.0669	0.0704	0.0756	0.0899	0.069
0.1224	0.1225	0.1239	0.1273	0.1354	0.1483	0.1513	0.168	0.1355
0.1276	0.1284	0.1286	0.1282	0.1241	0.1416	0.1433	0.1599	0.1254
0.1828	0.2009	0.2038	0.2175	0.1977	0.2021	0.1896	0.2431	0.211
0.0685	0.0745	0.0674	0.0768	0.0708	0.0715	0.0745	0.0878	0.0785
0.0233	0.0248	0.0228	0.0233	0.0228	0.0222	0.0216	0.0231	0.0214
0	0	0	0	0	0	0	0	0
0.0065	0.0062	0.0068	0.0066	0.0063	0.0061	0.0065	0.0065	0.0064
0.0048	0.005	0.0047	0.0042	0.004	0.0043	0.0046	0.0046	0.0041
0.0007	0.0008	0.0008	0.0008	0.0009	0.0009	0.001	0.0013	0.0009
0.0035	0.0037	0.0032	0.0033	0.0028	0.0028	0.0028	0.0047	0.0045
0.0069	0.0078	0.0061	0.0057	0.0057	0.0066	0.0075	0.0073	0.0049
0	0	0	0	0	0	0	0	0
0	0	0	0	0	0	0	0	0
0	0	0	0	0	0	0	0	0
0	0	0	0	0	0	0	0	0
0	0	0	0	0	0	0	0	0
0	0	0	0	0	0	0	0	0
0	0	0	0	0	0	0	0	0
0	0	0	0	0	0	0	0	0
0	0	0	0	0	0	0	0	0
0	0	0	0	0	0	0	0	0
0	0	0	0	0	0	0	0	0
0	0	0	0	0	0	0	0	0
0	0	0	0	0	0	0	0	0
0.0008	0.0008	0.0007	0.0007	0.0006	0.0006	0.0006	0.0006	0.0006

0.0007	0.0006	0.0006	0.0006	0.0007	0.0006	0.0007	0.0007	0.0006
0.0008	0.0008	0.0008	0.0008	0.0007	0.0006	0.0006	0.0006	0.0006
0	0	0	0	0	0	0	0	0
0	0	0	0	0	0	0	0	0
0.0088	0.0096	0.0097	0.0092	0.0094	0.0093	0.0091	0.0107	0.0076
0.0403	0.0426	0.0426	0.0431	0.042	0.0459	0.0466	0.0566	0.045
0.0514	0.0552	0.0554	0.0574	0.0549	0.0582	0.0586	0.069	0.0554
0.1345	0.1486	0.1473	0.1425	0.1398	0.1611	0.1542	0.1845	0.1448
0.0938	0.1005	0.1052	0.1206	0.1099	0.1222	0.1345	0.1645	0.1313
0.0382	0.0401	0.0406	0.0404	0.0393	0.0406	0.0411	0.0445	0.0375
0.116	0.1192	0.1195	0.1159	0.1074	0.1091	0.1161	0.128	0.1028
0.1443	0.1504	0.1537	0.1579	0.1515	0.1579	0.1689	0.1878	0.1586
0.0987	0.0973	0.0974	0.0928	0.084	0.087	0.0885	0.1008	0.0815
0.0482	0.0508	0.0513	0.0506	0.0482	0.0508	0.0523	0.0587	0.0511
0.0887	0.1015	0.1067	0.1084	0.1015	0.1129	0.1145	0.1277	0.1093
0.0622	0.0629	0.0608	0.0579	0.0512	0.0456	0.049	0.0535	0.0444
0.0606	0.0671	0.0688	0.0668	0.0615	0.0547	0.0564	0.0528	0.0452
0.0904	0.0851	0.092	0.0947	0.0865	0.088	0.0862	0.0927	0.0832
0.1593	0.1755	0.1749	0.1861	0.1553	0.1658	0.171	0.195	0.1834
0.2521	0.2461	0.2234	0.2354	0.2113	0.2604	0.3045	0.3732	0.3336
0.1018	0.1239	0.1137	0.128	0.0953	0.1126	0.1154	0.1379	0.1356
0.0672	0.0717	0.0726	0.076	0.0745	0.0783	0.077	0.0954	0.0761
0.1368	0.1449	0.1289	0.1586	0.1398	0.1496	0.1593	0.1935	0.1494
0.1224	0.1287	0.1365	0.1446	0.1307	0.1345	0.1393	0.1634	0.1497
0.0293	0.0305	0.0302	0.0299	0.0302	0.0313	0.0333	0.0362	0.0308
0.1154	0.1252	0.1392	0.1537	0.1322	0.1473	0.158	0.1921	0.1731
0.0865	0.0917	0.0944	0.0979	0.0965	0.104	0.1077	0.1281	0.1014
0.1442	0.1352	0.1163	0.1348	0.1203	0.1403	0.137	0.1915	0.1757
0.0723	0.0685	0.0661	0.0697	0.0584	0.0608	0.0613	0.068	0.0628
0.0155	0.0159	0.0159	0.0182	0.0169	0.0172	0.0186	0.0213	0.02
0	0	0	0	0	0	0	0	0
0	0	0	0	0	0	0	0	0
0	0	0	0	0	0	0	0	0
0.0006	0.0007	0.0007	0.0007	0.0008	0.0007	0.0007	0.001	0.0007
0.0072	0.0073	0.0069	0.0068	0.0054	0.0055	0.0058	0.0067	0.0062
0.0111	0.0105	0.0114	0.0115	0.0104	0.0116	0.0124	0.0138	0.0125
0	0	0	0	0	0	0	0	0
0	0	0	0	0	0	0	0	0
0	0	0	0	0	0	0	0	0
0	0	0	0	0	0	0	0	0
0	0	0	0	0	0	0	0	0
0	0	0	0	0	0	0	0	0
0	0	0	0	0	0	0	0	0
0	0	0	0	0	0	0	0	0

0	0	0	0	0	0	0	0	0
0	0	0	0	0	0	0	0	0
0.0009	0.0009	0.0009	0.0009	0.0008	0.0008	0.0009	0.0011	0.0008
0.0009	0.0009	0.0009	0.0009	0.0009	0.001	0.001	0.0013	0.0009
0	0	0	0	0	0	0	0	0
0	0	0	0	0	0	0	0	0
0	0	0	0	0	0	0	0	0
0	0	0	0	0	0	0	0	0
0	0	0	0	0	0	0	0	0
0	0	0	0	0	0	0	0	0
0.0216	0.0222	0.0218	0.0225	0.0201	0.0205	0.0193	0.0199	0.0187
0.0946	0.1017	0.1013	0.0958	0.0938	0.0981	0.0956	0.1009	0.0967
0.0375	0.0427	0.0411	0.0422	0.0432	0.0473	0.0509	0.0622	0.0429
0.0456	0.0473	0.0446	0.0451	0.0465	0.051	0.0566	0.0659	0.0527
0.1124	0.1141	0.1185	0.125	0.1167	0.1304	0.1306	0.1429	0.1274
0.0982	0.0987	0.103	0.1024	0.0911	0.0882	0.0943	0.1115	0.1034
0.0794	0.0792	0.0748	0.0763	0.0652	0.0663	0.0718	0.0912	0.0867
0.0878	0.1027	0.0982	0.1	0.0966	0.096	0.0995	0.1173	0.1116
0.0469	0.0463	0.0463	0.0468	0.0407	0.0404	0.0444	0.0519	0.0463
0.1054	0.123	0.1162	0.1266	0.102	0.1205	0.1057	0.1269	0.1195
0.374	0.3702	0.4189	0.4329	0.3757	0.3745	0.3651	0.4821	0.4667
0.213	0.1738	0.1681	0.1962	0.1732	0.2003	0.2205	0.2668	0.2124
0.0401	0.0427	0.0427	0.0442	0.0422	0.0449	0.0458	0.0559	0.046
0.0566	0.0584	0.0578	0.064	0.061	0.0629	0.0638	0.0731	0.0636
0.0439	0.0461	0.0459	0.0464	0.0447	0.0469	0.0481	0.0586	0.0517
0.0008	0.0009	0.001	0.001	0.001	0.0011	0.001	0.0013	0.0011
0.2392	0.2696	0.233	0.243	0.1818	0.2294	0.2005	0.2558	0.2952
0.0946	0.0984	0.0967	0.112	0.0944	0.0924	0.0772	0.1186	0.1274
0.1536	0.1476	0.15	0.1627	0.1497	0.15	0.1342	0.1725	0.1875
0.1306	0.1432	0.1365	0.1626	0.1425	0.1634	0.1602	0.1757	0.152
0.2224	0.2642	0.2489	0.234	0.2275	0.2072	0.2151	0.2372	0.2184
0.3065	0.3064	0.3176	0.3153	0.3073	0.2734	0.2685	0.3117	0.3596
0.035	0.0357	0.0335	0.0321	0.0341	0.0335	0.034	0.0376	0.0328
0.0961	0.1044	0.1032	0.111	0.1072	0.1172	0.1201	0.1358	0.1161
0.0266	0.0263	0.0267	0.0264	0.0249	0.026	0.0271	0.0292	0.0265
0.0104	0.0111	0.011	0.0097	0.0095	0.01	0.0098	0.0112	0.0095
0.0008	0.0009	0.001	0.001	0.0008	0.0008	0.0008	0.001	0.0007
0.0091	0.0099	0.0091	0.011	0.0099	0.0097	0.0098	0.0114	0.0099
0.0079	0.0086	0.0104	0.0104	0.0103	0.0091	0.0077	0.0096	0.0083
0.0056	0.0055	0.0052	0.0056	0.0041	0.0049	0.0054	0.0057	0.0055
0.0042	0.0041	0.004	0.0037	0.0038	0.0042	0.0039	0.0043	0.0039
0.0042	0.004	0.004	0.0038	0.0037	0.0036	0.0038	0.0042	0.0037
0	0	0	0	0	0	0	0	0
0	0	0	0	0	0	0	0	0

0	0	0	0	0	0	0	0	0
0	0	0	0	0	0	0	0	0
0	0	0	0	0	0	0	0	0
0	0	0	0	0	0	0	0	0
0	0	0	0	0	0	0	0	0
0	0	0	0	0	0	0	0	0
0	0	0	0	0	0	0	0	0
0	0	0	0	0	0	0	0	0
0.0032	0.0036	0.0035	0.0034	0.0036	0.004	0.004	0.0048	0.0039
0.0029	0.0029	0.0029	0.0028	0.0029	0.003	0.003	0.0034	0.0027
0.0024	0.0026	0.0026	0.0025	0.0025	0.0028	0.0028	0.0032	0.0025
0.0014	0.0014	0.0016	0.0015	0.0014	0.0015	0.0015	0.0018	0.0015
0	0	0	0	0	0	0	0	0
0	0	0	0	0	0	0	0	0
0.0009	0.0008	0.0008	0.0008	0.0008	0.0007	0.0007	0.0006	0.0006
0.0008	0.0008	0.0008	0.0007	0.0006	0.0006	0.0006	0.0007	0.0006
0.005	0.0051	0.0052	0.0054	0.0052	0.0052	0.0049	0.0053	0.0051
0.0481	0.0505	0.0497	0.0516	0.0477	0.0519	0.0538	0.0615	0.0539
0.0886	0.0962	0.0924	0.0892	0.0802	0.0807	0.0843	0.1016	0.0843
0.0642	0.0598	0.0588	0.0602	0.0533	0.0523	0.0517	0.0589	0.0561
0.0785	0.079	0.075	0.0761	0.0669	0.0691	0.066	0.0747	0.0672
0.0814	0.1025	0.0946	0.0935	0.0879	0.1011	0.1132	0.1418	0.1225
0.1676	0.1836	0.1765	0.1731	0.1315	0.1478	0.1651	0.1926	0.1845
0.2463	0.2949	0.2829	0.2892	0.284	0.2678	0.2629	0.3478	0.3131
0.1294	0.1459	0.136	0.1472	0.1198	0.137	0.1229	0.1672	0.1461
0.0058	0.0059	0.0058	0.0049	0.0048	0.0047	0.0047	0.0044	0.0042
0.0377	0.0438	0.0386	0.0465	0.0427	0.0465	0.0551	0.0608	0.0545
0.1299	0.1405	0.1461	0.1578	0.1465	0.1699	0.1694	0.1988	0.1746
0.0974	0.1023	0.1016	0.1057	0.0985	0.1131	0.116	0.1494	0.1239
0.1339	0.1292	0.1217	0.1267	0.0992	0.1091	0.1181	0.1378	0.1381
0.1313	0.1338	0.1218	0.1382	0.1146	0.1091	0.1166	0.1283	0.1204
0.1459	0.1638	0.1533	0.1463	0.13	0.1331	0.1229	0.1483	0.124
0.1835	0.1883	0.1738	0.1833	0.1606	0.179	0.1692	0.2194	0.1947
0.1404	0.1479	0.1542	0.161	0.1556	0.1663	0.1723	0.2101	0.1739
0.113	0.1165	0.1075	0.1122	0.1039	0.1045	0.1145	0.1346	0.1179
0.2085	0.2432	0.2184	0.2269	0.209	0.246	0.242	0.2749	0.2525
0.1731	0.1817	0.1773	0.1887	0.1811	0.1931	0.1935	0.2273	0.1935
0.2151	0.2166	0.2111	0.236	0.2108	0.2485	0.2279	0.2691	0.2637
0.091	0.1128	0.0976	0.0975	0.1073	0.0825	0.0751	0.0793	0.0666
0.2238	0.2121	0.1935	0.2052	0.1803	0.2058	0.1895	0.2452	0.2307
0.017	0.0173	0.0164	0.0154	0.0155	0.016	0.0173	0.0198	0.0164
0.0057	0.0063	0.0066	0.0061	0.0057	0.0058	0.006	0.007	0.0056
0.001	0.001	0.0009	0.0009	0.0009	0.001	0.001	0.0011	0.0008
0.0068	0.0069	0.0064	0.0071	0.005	0.0048	0.0054	0.0062	0.0062

0.0043	0.0042	0.0047	0.0045	0.0044	0.004	0.0039	0.0048	0.0039
0.0016	0.002	0.0012	0.0012	0.0013	0.0021	0.0024	0.0032	0.0028
0.002	0.0022	0.002	0.0025	0.0022	0.0025	0.0021	0.003	0.0028
0.0007	0.0008	0.0009	0.001	0.0009	0.0011	0.001	0.0013	0.001
0	0	0	0	0	0	0	0	0
0	0	0	0	0	0	0	0	0
0	0	0	0	0	0	0	0	0
0	0	0	0	0	0	0	0	0
0	0	0	0	0	0	0	0	0
0	0	0	0	0	0	0	0	0
0	0	0	0	0	0	0	0	0
0	0	0	0	0	0	0	0	0
0	0	0	0	0	0	0	0	0
0	0	0	0	0	0	0	0	0
0.0064	0.0066	0.0064	0.0062	0.0058	0.0059	0.0063	0.007	0.0058
0.0047	0.0054	0.0054	0.0052	0.005	0.0047	0.0046	0.0053	0.0046
0.0033	0.0036	0.0037	0.0036	0.0034	0.0036	0.0036	0.0041	0.0036
0.002	0.0019	0.0019	0.002	0.0021	0.0021	0.0023	0.0029	0.0023
0.0011	0.0012	0.0011	0.0011	0.0011	0.0013	0.0013	0.0015	0.0011
0.0014	0.0015	0.0015	0.0015	0.0015	0.0015	0.0015	0.0018	0.0015
0.0007	0.0007	0.0007	0.0007	0.0007	0.0007	0.0008	0.0008	0.0007
0.0015	0.0015	0.0013	0.001	0.001	0.001	0.0011	0.0011	0.0011
0.0118	0.0125	0.0121	0.0128	0.0126	0.0129	0.0133	0.0146	0.0121
0	0	0	0	0	0	0	0	0
0.0497	0.0514	0.0519	0.0529	0.0494	0.045	0.0427	0.0443	0.0417
0.0826	0.0873	0.0887	0.0883	0.0837	0.0861	0.0876	0.0986	0.0859
0.0447	0.0485	0.0523	0.0549	0.0499	0.0575	0.0619	0.0752	0.0693
0.0809	0.0809	0.0794	0.0795	0.0744	0.0716	0.072	0.0748	0.0724
0.0708	0.0687	0.0676	0.0666	0.0585	0.0556	0.0613	0.0639	0.0652
0.0278	0.0338	0.0335	0.0388	0.0344	0.0367	0.0349	0.0421	0.042
0.0324	0.0368	0.037	0.0359	0.0307	0.0319	0.029	0.0325	0.0304
0.0008	0.001	0.001	0.001	0.0011	0.0012	0.0012	0.0014	0.0011
0.0183	0.0195	0.0195	0.0235	0.0228	0.0212	0.0218	0.023	0.0243
0.0158	0.0146	0.0155	0.0167	0.0171	0.0161	0.0161	0.0185	0.0155
0.0453	0.0441	0.0408	0.0453	0.0478	0.0508	0.0477	0.0592	0.0439
0.0548	0.06	0.0599	0.0692	0.0587	0.0588	0.0667	0.0792	0.0669
0.0202	0.0234	0.0238	0.0261	0.0233	0.0237	0.0247	0.0291	0.026
0.0569	0.0556	0.0578	0.062	0.0627	0.0633	0.0648	0.079	0.0577
0.0679	0.0717	0.0724	0.0715	0.0733	0.0753	0.0792	0.0963	0.0773
0.0418	0.0425	0.0431	0.0432	0.0419	0.0461	0.048	0.0581	0.0469
0.0431	0.049	0.0463	0.0468	0.0485	0.0553	0.0563	0.0711	0.0528
0.1065	0.1154	0.1197	0.1222	0.1094	0.1168	0.1205	0.149	0.1339
0.0726	0.0819	0.0845	0.0828	0.0883	0.0952	0.0903	0.11	0.0914
0.0479	0.0463	0.0548	0.0595	0.0542	0.073	0.0656	0.0817	0.07

0.0225	0.0216	0.0208	0.0204	0.0193	0.0194	0.019	0.0184	0.0166
0.0178	0.0164	0.0166	0.0201	0.0166	0.0194	0.019	0.0207	0.0203
0.0275	0.0295	0.029	0.0295	0.0284	0.0301	0.0288	0.0322	0.0288
0.007	0.0074	0.0071	0.0075	0.0069	0.0071	0.0073	0.0089	0.0079
0	0	0	0	0	0	0	0	0
0	0	0	0	0	0	0	0	0
0	0	0	0	0	0	0	0	0
0.0008	0.0008	0.0008	0.0009	0.001	0.001	0.0011	0.0013	0.0011
0.0078	0.0076	0.0077	0.0082	0.0073	0.0077	0.0068	0.008	0.0075
0	0	0	0	0	0	0	0	0
0	0	0	0	0	0	0	0	0
0	0	0	0	0	0	0	0	0
0	0	0	0	0	0	0	0	0
0	0	0	0	0	0	0	0	0
0	0	0	0	0	0	0	0	0
0	0	0	0	0	0	0	0	0
0	0	0	0	0	0	0	0	0
0	0	0	0	0	0	0	0	0
0	0	0	0	0	0	0	0	0
0	0	0	0	0	0	0	0	0
0.0034	0.0035	0.0035	0.0034	0.0031	0.0029	0.003	0.0031	0.0024
0.0027	0.0026	0.0025	0.0025	0.0024	0.0023	0.0024	0.0026	0.0022
0.0015	0.0017	0.0017	0.0016	0.0015	0.0015	0.0015	0.0018	0.0013
0.0008	0.0008	0.0009	0.0009	0.0009	0.0009	0.0009	0.0013	0.0008
0.0009	0.001	0.001	0.0011	0.0011	0.001	0.0011	0.0015	0.0012
0	0	0	0	0	0	0	0	0
0	0	0	0	0	0	0	0	0
0.0007	0.0006	0.0006	0.0005	0.0005	0.0005	0.0005	0.0004	0.0005
0	0	0	0	0	0	0	0	0
0.0067	0.007	0.0075	0.007	0.0076	0.0076	0.0085	0.0098	0.0075
0.0248	0.0269	0.026	0.0265	0.0255	0.0273	0.0294	0.0326	0.0295
0.0181	0.0225	0.019	0.0221	0.0177	0.0257	0.0255	0.0309	0.0258
0.0008	0.0009	0.001	0.001	0.0009	0.001	0.0011	0.0013	0.0009
0.0241	0.0226	0.0231	0.0227	0.0229	0.0224	0.0224	0.0236	0.0212
0.0069	0.0071	0.0071	0.0066	0.0066	0.0065	0.0062	0.0066	0.0056
0.0099	0.0115	0.0104	0.0109	0.0087	0.0084	0.009	0.0099	0.0096
0.0076	0.0079	0.0088	0.0088	0.0084	0.009	0.0082	0.0092	0.01
0.0288	0.0297	0.0288	0.0299	0.0291	0.0308	0.0318	0.0366	0.0311
0.0194	0.0201	0.0203	0.0209	0.0196	0.0205	0.0213	0.0248	0.0217
0.0023	0.0024	0.0023	0.0029	0.0024	0.0027	0.0026	0.0028	0.0026
0.0134	0.0131	0.0129	0.0149	0.0133	0.0141	0.0123	0.0138	0.0138
0.0174	0.0171	0.0185	0.0192	0.0184	0.0187	0.0194	0.0213	0.0181
0.005	0.0049	0.0048	0.0048	0.0044	0.0044	0.0051	0.0045	0.0051
0.003	0.0036	0.0039	0.004	0.0038	0.0047	0.0054	0.0061	0.006

0.0089	0.0102	0.0095	0.011	0.0111	0.0116	0.0107	0.011	0.0118
0.01	0.01	0.0096	0.0089	0.0089	0.0083	0.0084	0.0091	0.0073
0.0027	0.003	0.0034	0.0033	0.0036	0.0034	0.0035	0.0034	0.0031
0.0209	0.023	0.029	0.0279	0.0278	0.0308	0.0324	0.0418	0.04
0.0084	0.0081	0.0087	0.0085	0.008	0.0083	0.0078	0.0071	0.0068
0	0	0	0	0	0	0	0	0
0.0193	0.0222	0.026	0.0311	0.0273	0.0258	0.0269	0.0326	0.029
0.0115	0.0113	0.0111	0.0106	0.0092	0.0095	0.0096	0.0095	0.0089
0.0203	0.0226	0.0191	0.0199	0.0178	0.0166	0.0184	0.0209	0.0196
0.0084	0.0082	0.0065	0.007	0.0059	0.0072	0.0074	0.0084	0.0077
0.0038	0.0043	0.004	0.0038	0.0035	0.0034	0.0032	0.0037	0.0037
0.0007	0.0007	0.0007	0.0006	0.0006	0.0005	0.0005	0.0006	0.0006
0.0008	0.0009	0.001	0.001	0.001	0.001	0.001	0.0011	0.0008
0.0038	0.0038	0.0041	0.0041	0.0041	0.004	0.0042	0.0044	0.0038
0.0098	0.0105	0.01	0.0107	0.0101	0.01	0.0107	0.012	0.0104
0.001	0.0009	0.0009	0.0009	0.0008	0.0009	0.0009	0.0009	0.0009
0	0	0	0	0	0	0	0	0
0	0	0	0	0	0	0	0	0
0	0	0	0	0	0	0	0	0
0	0	0	0	0	0	0	0	0
0	0	0	0	0	0	0	0	0
0	0	0	0	0	0	0	0	0
0	0	0	0	0	0	0	0	0
0	0	0	0	0	0	0	0	0
0	0	0	0	0	0	0	0	0
0	0	0	0	0	0	0	0	0
0.0024	0.0021	0.0021	0.0019	0.0019	0.0018	0.002	0.002	0.002
0.0007	0.0007	0.0007	0.0007	0.0006	0.0006	0.0007	0.0007	0.0006
0	0	0	0	0	0	0	0	0
0	0	0	0	0	0	0	0	0
0	0	0	0	0	0	0	0	0
0	0	0	0	0	0	0	0	0
0	0	0	0	0	0	0	0	0
0	0	0	0	0	0	0	0	0
0.0176	0.0185	0.0166	0.0211	0.0193	0.0217	0.0214	0.0235	0.022
0	0	0	0	0	0	0	0	0
0.0018	0.0018	0.0018	0.0019	0.0019	0.0021	0.0022	0.0026	0.0019
0.0162	0.0178	0.0184	0.0192	0.017	0.0173	0.0165	0.0185	0.0188
0.0057	0.0056	0.0059	0.0058	0.0055	0.0049	0.0045	0.0044	0.0043
0.0045	0.0036	0.0034	0.0032	0.0015	0.0012	0.0014	0.0026	0.0029
0.0128	0.0139	0.0129	0.0166	0.0153	0.0163	0.0165	0.0206	0.0185
0.0094	0.0098	0.0102	0.0099	0.0105	0.0104	0.0106	0.0128	0.0098
0.0067	0.0072	0.0074	0.0076	0.0074	0.008	0.0084	0.0102	0.0076
0	0	0	0	0	0	0	0	0

0	0	0	0	0	0	0	0	0
0.0114	0.0148	0.0122	0.0137	0.0119	0.0128	0.016	0.0203	0.0196
0.001	0.001	0.0011	0.0011	0.001	0.001	0.0012	0.0013	0.0009
0.0016	0.0017	0.0019	0.002	0.0019	0.0021	0.0021	0.0026	0.002
0.0217	0.024	0.0192	0.0257	0.017	0.02	0.0209	0.0288	0.029
0	0	0	0	0	0	0	0	0
0	0	0	0	0	0	0	0	0
0.0105	0.0134	0.0131	0.0135	0.012	0.0121	0.0092	0.0125	0.0097
0.025	0.0266	0.0268	0.0289	0.0257	0.0269	0.0269	0.031	0.0309
0	0	0	0	0	0	0	0	0
0.011	0.0104	0.0093	0.0138	0.0035	0.0063	0.0065	0.0155	0.0166
0.0205	0.0211	0.0214	0.0202	0.0187	0.0189	0.0188	0.0194	0.0177
0.0017	0.0016	0.0017	0.0015	0.0014	0.0013	0.0014	0.0013	0.0013
0.003	0.0032	0.0031	0.0031	0.0031	0.0034	0.0033	0.0041	0.003
0	0	0	0	0	0	0	0	0
0	0	0	0	0	0	0	0	0
0.0008	0.0008	0.0008	0.0007	0.0007	0.0007	0.0008	0.0008	0.0007
0.0023	0.0021	0.002	0.002	0.0018	0.0019	0.0016	0.0019	0.0015
0.0015	0.0015	0.0016	0.0017	0.0017	0.0017	0.0017	0.0021	0.0015
0.0046	0.0049	0.005	0.0054	0.0052	0.0054	0.0054	0.006	0.0052
0.0056	0.0055	0.0055	0.0056	0.0053	0.0055	0.0055	0.0061	0.0053
0	0	0	0	0	0	0	0	0
0	0	0	0	0	0	0	0	0
0	0	0	0	0	0	0	0	0
0	0	0	0	0	0	0	0	0
0	0	0	0	0	0	0	0	0
0	0	0	0	0	0	0	0	0
0	0	0	0	0	0	0	0	0
0	0	0	0	0	0	0	0	0
0	0	0	0	0	0	0	0	0
0	0	0	0	0	0	0	0	0
0	0	0	0	0	0	0	0	0
0	0	0	0	0	0	0	0	0
0	0	0	0	0	0	0	0	0
0	0	0	0	0	0	0	0	0
0	0	0	0	0	0	0	0	0
0	0	0	0	0	0	0	0	0
0	0	0	0	0	0	0	0	0
0	0	0	0	0	0	0	0	0
0	0	0	0	0	0	0	0	0
0	0	0	0	0	0	0	0	0
0	0	0	0	0	0	0	0	0
0	0	0	0	0	0	0	0	0
0	0	0	0	0	0	0	0	0

0	0	0	0	0	0	0	0	0
0	0	0	0	0	0	0	0	0
0	0	0	0	0	0	0	0	0
0	0	0	0	0	0	0	0	0
0	0	0	0	0	0	0	0	0
0	0	0	0	0	0	0	0	0
0	0	0	0	0	0	0	0	0
0	0	0	0	0	0	0	0	0
0	0	0	0	0	0	0	0	0
0	0	0	0	0	0	0	0	0
0	0	0	0	0	0	0	0	0
0	0	0	0	0	0	0	0	0
0	0	0	0	0	0	0	0	0
0	0	0	0	0	0	0	0	0
0	0	0	0	0	0	0	0	0
0	0	0	0	0	0	0	0	0
0	0	0	0	0	0	0	0	0
0	0	0	0	0	0	0	0	0
0	0	0	0	0	0	0	0	0
0	0	0	0	0	0	0	0	0
0	0	0	0	0	0	0	0	0
0	0	0	0	0	0	0	0	0
0	0	0	0	0	0	0	0	0
0	0	0	0	0	0	0	0	0
0	0	0	0	0	0	0	0	0
0	0	0	0	0	0	0	0	0
0	0	0	0	0	0	0	0	0
0	0	0	0	0	0	0	0	0
0	0	0	0	0	0	0	0	0
0	0	0	0	0	0	0	0	0
0	0	0	0	0	0	0	0	0
0	0	0	0	0	0	0	0	0
0	0	0	0	0	0	0	0	0
0	0	0	0	0	0	0	0	0
0	0	0	0	0	0	0	0	0
0	0	0	0	0	0	0	0	0
0	0	0	0	0	0	0	0	0
0	0	0	0	0	0	0	0	0
0	0	0	0	0	0	0	0	0
0	0	0	0	0	0	0	0	0
0	0	0	0	0	0	0	0	0
0	0	0	0	0	0	0	0	0
0	0	0	0	0	0	0	0	0
0	0	0	0	0	0	0	0	0

0	0	0	0	0	0	0	0	0
0	0	0	0	0	0	0	0	0
0	0	0	0	0	0	0	0	0
0	0	0	0	0	0	0	0	0
0	0	0	0	0	0	0	0	0
0	0	0	0	0	0	0	0	0
0	0	0	0	0	0	0	0	0
0	0	0	0	0	0	0	0	0
0	0	0	0	0	0	0	0	0
0	0	0	0	0	0	0	0	0
0	0	0	0	0	0	0	0	0
0	0	0	0	0	0	0	0	0
0	0	0	0	0	0	0	0	0
0	0	0	0	0	0	0	0	0
0	0	0	0	0	0	0	0	0
0	0	0	0	0	0	0	0	0
0	0	0	0	0	0	0	0	0
0	0	0	0	0	0	0	0	0
0	0	0	0	0	0	0	0	0
0	0	0	0	0	0	0	0	0
0	0	0	0	0	0	0	0	0
0	0	0	0	0	0	0	0	0
0	0	0	0	0	0	0	0	0
0	0	0	0	0	0	0	0	0
0	0	0	0	0	0	0	0	0
0	0	0	0	0	0	0	0	0
0	0	0	0	0	0	0	0	0
0	0	0	0	0	0	0	0	0
0	0	0	0	0	0	0	0	0
0	0	0	0	0	0	0	0	0
0	0	0	0	0	0	0	0	0
0	0	0	0	0	0	0	0	0
0	0	0	0	0	0	0	0	0
0	0	0	0	0	0	0	0	0
0	0	0	0	0	0	0	0	0
0	0	0	0	0	0	0	0	0

Data 3-3 After Treatment

After	0	0	0	0	0	0	0	0
0	0	0	0	0	0	0	0	0
0	0	0	0	0	0	0	0	0
0	0	0	0	0	0	0	0	0
0	0	0	0	0	0	0	0	0
0	0	0	0	0	0	0	0	0
0	0	0	0	0	0	0	0	0
0	0	0	0	0	0	0	0	0
0	0	0	0	0	0	0	0	0
0	0	0	0	0	0	0	0	0
0	0	0	0	0	0	0	0	0
0	0	0	0	0	0	0	0	0
0	0	0	0	0	0	0	0	0
0	0	0	0	0	0	0	0	0
0	0	0	0	0	0	0	0	0
0	0	0	0	0	0	0	0	0
0	0	0	0	0	0	0	0	0
0	0	0	0	0	0	0	0	0
0	0	0	0	0	0	0	0	0
0	0	0	0	0	0	0	0	0
0	0	0	0	0	0	0	0	0
0	0	0	0	0	0	0	0	0
0	0	0	0	0	0	0	0	0
0	0	0	0	0	0	0	0	0
0	0	0	0	0	0	0	0	0
0	0	0	0	0	0	0	0	0
0	0	0	0	0	0	0	0	0
0	0	0	0	0	0	0	0	0
0	0	0	0	0	0	0	0	0
0	0	0	0	0	0	0	0	0
0	0	0	0	0	0	0	0	0
0	0	0	0	0	0	0	0	0
0	0	0	0	0	0	0	0	0
0	0	0	0	0	0	0	0	0
0	0	0	0	0	0	0	0	0
0	0	0	0	0	0	0	0	0
0	0	0	0	0	0	0	0	0
0	0	0	0	0	0	0	0	0
0	0	0	0	0	0	0	0	0
0	0	0	0	0	0	0	0	0
0	0	0	0	0	0	0	0	0
0	0	0	0	0	0	0	0	0
0	0	0	0	0	0	0	0	0
0	0	0	0	0	0	0	0	0

0	0	0	0	0	0	0	0	0
0	0	0	0	0	0	0	0	0
0	0	0	0	0	0	0	0	0
0	0	0	0	0	0	0	0	0
0	0	0	0	0	0	0	0	0
0	0	0	0	0	0	0	0	0
0	0	0	0	0	0	0	0	0
0	0	0	0	0	0	0	0	0
0	0	0	0	0	0	0	0	0
0	0	0	0	0	0	0	0	0
0	0	0	0	0	0	0	0	0
0	0	0	0	0	0	0	0	0
0	0	0	0	0	0	0	0	0
0	0	0	0	0	0	0	0	0
0	0	0	0	0	0	0	0	0
0	0	0	0	0	0	0	0	0
0	0	0	0	0	0	0	0	0
0	0	0	0	0	0	0	0	0
0	0	0	0	0	0	0	0	0
0	0	0	0	0	0	0	0	0
0	0	0	0	0	0	0	0	0
0	0	0	0	0	0	0	0	0
0	0	0	0	0	0	0	0	0
0	0	0	0	0	0	0	0	0
0	0	0	0	0	0	0	0	0
0	0	0	0	0	0	0	0	0
0	0	0	0	0	0	0	0	0
0	0	0	0	0	0	0	0	0
0	0	0	0	0	0	0	0	0
0	0	0	0	0	0	0	0	0
0	0	0	0	0	0	0	0	0
0	0	0	0	0	0	0	0	0
0	0	0	0	0	0	0	0	0
0	0	0	0	0	0	0	0	0
0	0	0	0	0	0	0	0	0
0	0	0	0	0	0	0	0	0
0	0	0	0	0	0	0	0	0
0	0	0	0	0	0	0	0	0
0	0	0	0	0	0	0	0	0
0	0	0	0	0	0	0	0	0
0	0	0	0	0	0	0	0	0
0	0	0	0	0	0	0	0	0
0	0	0	0	0	0	0	0	0
0	0	0	0	0	0	0	0	0
0	0	0	0	0	0	0	0	0

0	0	0	0	0	0	0	0	0
0	0	0	0	0	0	0	0	0
0	0	0	0	0	0	0	0	0
0	0	0	0	0	0	0	0	0
0	0	0	0	0	0	0	0	0
0	0	0	0	0	0	0	0	0
0	0	0	0	0	0	0	0	0
0	0	0	0	0	0	0	0	0
0	0	0	0	0	0	0	0	0
0	0	0	0	0	0	0	0	0
0	0	0	0	0	0	0	0	0
0	0	0	0	0	0	0	0	0
0	0	0	0	0	0	0	0	0
0	0	0	0	0	0	0	0	0
0	0	0	0	0	0	0	0	0
0	0	0	0	0	0	0	0	0
0	0	0	0	0	0	0	0	0
0	0	0	0	0	0	0	0	0
0	0	0	0	0	0	0	0	0
0	0	0	0	0	0	0	0	0
0	0	0	0	0	0	0	0	0
0	0	0	0	0	0	0	0	0
0	0	0	0	0	0	0	0	0
0	0	0	0	0	0	0	0	0
0	0	0	0	0	0	0	0	0
0	0	0	0	0	0	0	0	0
0	0	0	0	0	0	0	0	0
0	0	0	0	0	0	0	0	0
0	0	0	0	0	0	0	0	0
0	0	0	0	0	0	0	0	0
0	0	0	0	0	0	0	0	0
0	0	0	0	0	0	0	0	0
0	0	0	0	0	0	0	0	0
0	0	0	0	0	0	0	0	0
0	0	0	0	0	0	0	0	0
0	0	0	0	0	0	0	0	0
0	0	0	0	0	0	0	0	0
0	0	0	0	0	0	0	0	0
0	0	0	0	0	0	0	0	0
0	0	0	0	0	0	0	0	0
0	0	0	0	0	0	0	0	0
0	0	0	0	0	0	0	0	0
0	0	0	0	0	0	0	0	0
0	0	0	0	0	0	0	0	0

0	0	0	0	0	0	0	0	0
0	0	0	0	0	0	0	0	0
0	0	0	0	0	0	0	0	0
0	0	0	0	0	0	0	0	0
0	0	0	0	0	0	0	0	0
0	0	0	0	0	0	0	0	0
0	0	0	0	0	0	0	0	0
0	0	0	0	0	0	0	0	0
0	0	0	0	0	0	0	0	0
0	0	0	0	0	0	0	0	0
0	0	0	0	0	0	0	0	0
0	0	0	0	0	0	0	0	0
0	0	0	0	0	0	0	0	0
0	0	0	0	0	0	0	0	0
0	0	0	0	0	0	0	0	0
0	0	0	0	0	0	0	0	0
0	0	0	0	0	0	0	0	0
0	0	0	0	0	0	0	0	0
0	0	0	0	0	0	0	0	0
0	0	0	0	0	0	0	0	0
0	0	0	0	0	0	0	0	0
0	0	0	0	0	0	0	0	0
0	0	0	0	0	0	0	0	0
0	0	0	0	0	0	0	0	0
0	0	0	0	0	0	0	0	0
0	0	0	0	0	0	0	0	0
0	0	0	0	0	0	0	0	0
0	0	0	0	0	0	0	0	0
0	0	0	0	0	0	0	0	0
0	0	0	0	0	0	0	0	0
0	0	0	0	0	0	0	0	0
0	0	0	0	0	0	0	0	0
0	0	0	0	0	0	0	0	0
0	0	0	0	0	0	0	0	0
0	0	0	0	0	0	0	0	0
0	0	0	0	0	0	0	0	0
0	0	0	0	0	0	0	0	0
0	0	0	0	0	0	0	0	0
0	0	0	0	0	0	0	0	0
0	0	0	0	0	0	0	0	0
0	0	0	0	0	0	0	0	0
0	0	0	0	0	0	0	0	0
0	0	0	0	0	0	0	0	0
0	0	0	0	0	0	0	0	0

0	0	0	0	0	0	0	0	0
0	0	0	0	0	0	0	0	0
0	0	0	0	0	0	0	0	0
0	0	0	0	0	0	0	0	0
0	0	0	0	0	0	0	0	0
0	0	0	0	0	0	0	0	0
0	0	0	0	0	0	0	0	0
0	0	0	0	0	0	0	0	0
0	0	0	0	0	0	0	0	0
0	0	0	0	0	0	0	0	0
0	0	0	0	0	0	0	0	0
0	0	0	0	0	0	0	0	0
0	0	0	0	0	0	0	0	0
0	0	0	0	0	0	0	0	0
0	0	0	0	0	0	0	0	0
0	0	0	0	0	0	0	0	0
0	0	0	0	0	0	0	0	0
0	0	0	0	0	0	0	0	0
0	0	0	0	0	0	0	0	0
0	0	0	0	0	0	0	0	0
0	0	0	0	0	0	0	0	0
0	0	0	0	0	0	0	0	0
0	0	0	0	0	0	0	0	0
0	0	0	0	0	0	0	0	0
0	0	0	0	0	0	0	0	0
0	0	0	0	0	0	0	0	0
0	0	0	0	0	0	0	0	0
0	0	0	0	0	0	0	0	0
0	0	0	0	0	0	0	0	0
0	0	0	0	0	0	0	0	0
0	0	0	0	0	0	0	0	0
0	0	0	0	0	0	0	0	0
0	0	0	0	0	0	0	0	0
0	0	0	0	0	0	0	0	0
0	0	0	0	0	0	0	0	0
0	0	0	0	0	0	0	0	0
0	0	0	0	0	0	0	0	0
0	0	0	0	0	0	0	0	0
0	0	0	0	0	0	0	0	0
0	0	0	0	0	0	0	0	0
0	0	0	0	0	0	0	0	0
0	0	0	0	0	0	0	0	0
0	0	0	0	0	0	0	0	0
0	0	0	0	0	0	0	0	0

0	0	0	0	0	0	0	0	0
0	0	0	0	0	0	0	0	0
0	0	0	0	0	0	0	0	0
0	0	0	0	0	0	0	0	0
0	0	0	0	0	0	0	0	0
0	0	0	0	0	0	0	0	0
0	0	0	0	0	0	0	0	0
0	0	0	0	0	0	0	0	0
0	0	0	0	0	0	0	0	0
0	0	0	0	0	0	0	0	0
0	0	0	0	0	0	0	0	0
0	0	0	0	0	0	0	0	0
0	0	0	0	0	0	0	0	0
0	0	0	0	0	0	0	0	0
0	0	0	0	0	0	0	0	0
0	0	0	0	0	0	0	0	0
0	0	0	0	0	0	0	0	0
0	0	0	0	0	0	0	0	0
0	0	0	0	0	0	0	0	0
0	0	0	0	0	0	0	0	0
0	0	0	0	0	0	0	0	0
0	0	0	0	0	0	0	0	0
0	0	0	0	0	0	0	0	0
0	0	0	0	0	0	0	0	0
0	0	0	0	0	0	0	0	0
0	0	0	0	0	0	0	0	0
0	0	0	0	0	0	0	0	0
0	0	0	0	0	0	0	0	0
0	0	0	0	0	0	0	0	0
0	0	0	0	0	0	0	0	0
0	0	0	0	0	0	0	0	0
0	0	0	0	0	0	0	0	0
0	0	0	0	0	0	0	0	0
0	0	0	0	0	0	0	0	0
0	0	0	0	0	0	0	0	0
0	0	0	0	0	0	0	0	0
0	0	0	0	0	0	0	0	0
0	0	0	0	0	0	0	0	0
0	0	0	0	0	0	0	0	0
0	0	0	0	0	0	0	0	0
0	0	0	0	0	0	0	0	0
0	0	0	0	0	0	0	0	0
0	0	0	0	0	0	0	0	0
0	0	0	0	0	0	0	0	0

0	0	0	0	0	0	0	0	0
0	0	0	0	0	0	0	0	0
0	0	0	0	0	0	0	0	0
0	0	0	0	0	0	0	0	0
0	0	0	0	0	0	0	0	0
0	0	0	0	0	0	0	0	0
0	0	0	0	0	0	0	0	0
0	0	0	0	0	0	0	0	0
0	0	0	0	0	0	0	0	0
0	0	0	0	0	0	0	0	0
0	0	0	0	0	0	0	0	0
0	0	0	0	0	0	0	0	0
0	0	0	0	0	0	0	0	0
0	0	0	0	0	0	0	0	0
0	0	0	0	0	0	0	0	0
0	0	0	0	0	0	0	0	0
0	0	0	0	0	0	0	0	0
0	0	0	0	0	0	0	0	0
0	0	0	0	0	0	0	0	0
0	0	0	0	0	0	0	0	0
0	0	0	0	0	0	0	0	0
0	0	0	0	0	0	0	0	0
0	0	0	0	0	0	0	0	0
0	0	0	0	0	0	0	0	0
0	0	0	0	0	0	0	0	0
0	0	0	0	0	0	0	0	0
0	0	0	0	0	0	0	0	0
0	0	0	0	0	0	0	0	0
0	0	0	0	0	0	0	0	0
0	0	0	0	0	0	0	0	0
0	0	0	0	0	0	0	0	0
0	0	0	0	0	0	0	0	0
0	0	0	0	0	0	0	0	0
0	0	0	0	0	0	0	0	0
0	0	0	0	0	0	0	0	0
0	0	0	0	0	0	0	0	0
0	0	0	0	0	0	0	0	0
0	0	0	0	0	0	0	0	0
0	0	0	0	0	0	0	0	0
0	0	0	0	0	0	0	0	0
0	0	0	0	0	0	0	0	0
0	0	0	0	0	0	0	0	0
0	0	0	0	0	0	0	0	0
0	0	0	0	0	0	0	0	0

0	0	0	0	0	0	0	0	0
0	0	0	0	0	0	0	0	0
0	0	0	0	0	0	0	0	0
0	0	0	0	0	0	0	0	0
0	0	0	0	0	0	0	0	0
0	0	0	0	0	0	0	0	0
0	0	0	0	0	0	0	0	0
0	0	0	0	0	0	0	0	0
0	0	0	0	0	0	0	0	0
0	0	0	0	0	0	0	0	0
0	0	0	0	0	0	0	0	0
0	0	0	0	0	0	0	0	0
0	0	0	0	0	0	0	0	0
0	0	0	0	0	0	0	0	0
0	0	0	0	0	0	0	0	0
0	0	0	0	0	0	0	0	0
0	0	0	0	0	0	0	0	0
0	0	0	0	0	0	0	0	0
0	0	0	0	0	0	0	0	0
0	0	0	0	0	0	0	0	0
0	0	0	0	0	0	0	0	0
0	0	0	0	0	0	0	0	0
0	0	0	0	0	0	0	0	0
0	0	0	0	0	0	0	0	0
0	0	0	0	0	0	0	0	0
0	0	0	0	0	0	0	0	0
0	0	0	0	0	0	0	0	0
0	0	0	0	0	0	0	0	0
0	0	0	0	0	0	0	0	0
0	0	0	0	0	0	0	0	0
0	0	0	0	0	0	0	0	0
0	0	0	0	0	0	0	0	0
0	0	0	0	0	0	0	0	0
0	0	0	0	0	0	0	0	0
0	0	0	0	0	0	0	0	0
0	0	0	0	0	0	0	0	0
0	0	0	0	0	0	0	0	0
0	0	0	0	0	0	0	0	0
0	0	0	0	0	0	0	0	0
0	0	0	0	0	0	0	0	0
0	0	0	0	0	0	0	0	0
0	0	0	0	0	0	0	0	0
0	0	0	0	0	0	0	0	0
0	0	0	0	0	0	0	0	0

0	0	0	0	0	0	0	0	0
0	0	0	0	0	0	0	0	0
0	0	0	0	0	0	0	0	0
0	0	0	0	0	0	0	0	0
0	0	0	0	0	0	0	0	0
0	0	0	0	0	0	0	0	0
0	0	0	0	0	0	0	0	0
0	0	0	0	0	0	0	0	0
0	0	0	0	0	0	0	0	0
0	0	0	0	0	0	0	0	0
0	0	0	0	0	0	0	0	0
0	0	0	0	0	0	0	0	0
0	0	0	0	0	0	0	0	0
0	0	0	0	0	0	0	0	0
0	0	0	0	0	0	0	0	0
0	0	0	0	0	0	0	0	0
0	0	0	0	0	0	0	0	0
0	0	0	0	0	0	0	0	0
0	0	0	0	0	0	0	0	0
0	0	0	0	0	0	0	0	0
0	0	0	0	0	0	0	0	0
0	0	0	0	0	0	0	0	0
0	0	0	0	0	0	0	0	0
0	0	0	0	0	0	0	0	0
0	0	0	0	0	0	0	0	0
0	0	0	0	0	0	0	0	0
0	0	0	0	0	0	0	0	0
0	0	0	0	0	0	0	0	0
0	0	0	0	0	0	0	0	0
0	0	0	0	0	0	0	0	0
0	0	0	0	0	0	0	0	0
0	0	0	0	0	0	0	0	0
0	0	0	0	0	0	0	0	0
0	0	0	0	0	0	0	0	0
0	0	0	0	0	0	0	0	0
0	0	0	0	0	0	0	0	0
0	0	0	0	0	0	0	0	0
0	0	0	0	0	0	0	0	0
0	0	0	0	0	0	0	0	0
0	0	0	0	0	0	0	0	0
0	0	0	0	0	0	0	0	0
0	0	0	0	0	0	0	0	0
0	0	0	0	0	0	0	0	0
0	0	0	0	0	0	0	0	0

0	0	0	0	0	0	0	0	0
0	0	0	0	0	0	0	0	0
0	0	0	0	0	0	0	0	0
0	0	0	0	0	0	0	0	0
0	0	0	0	0	0	0	0	0
0	0	0	0	0	0	0	0	0
0	0	0	0	0	0	0	0	0
0	0	0	0	0	0	0	0	0
0	0	0	0	0	0	0	0	0
0	0	0	0	0	0	0	0	0
0	0	0	0	0	0	0	0	0
0	0	0	0	0	0	0	0	0
0	0	0	0	0	0	0	0	0
0	0	0	0	0	0	0	0	0
0	0	0	0	0	0	0	0	0
0	0	0	0	0	0	0	0	0
0	0	0	0	0	0	0	0	0
0	0	0	0	0	0	0	0	0
0	0	0	0	0	0	0	0	0
0	0	0	0	0	0	0	0	0
0.0011	0.0016	0.0013	0.0014	0.0012	0.0014	0.0015	0.0016	0.0015
0	0	0	0	0	0	0	0	0
0	0	0	0	0	0	0	0	0
0.0015	0.0024	0.0022	0.0025	0.002	0.0023	0.0025	0.0027	0.0028
0.0017	0.0027	0.0022	0.003	0.0026	0.0028	0.0031	0.0034	0.0033
0	0	0	0	0	0	0	0	0
0	0	0	0	0	0	0	0	0
0.0002	0.0004	0.0004	0.0005	0.0004	0.0005	0.0006	0.0007	0.0007
0	0	0	0	0	0	0	0	0
0.0006	0.0011	0.001	0.0011	0.0008	0.0009	0.0012	0.0014	0.0014
0.0005	0.001	0.0009	0.001	0.0008	0.001	0.0011	0.0012	0.0012
0	0	0	0	0	0	0	0	0
0	0	0	0	0	0	0	0	0
0.0007	0.0012	0.0011	0.0013	0.001	0.0012	0.0013	0.0014	0.0014
0.0005	0.0009	0.0008	0.001	0.0008	0.001	0.0011	0.0012	0.0013
0	0	0	0	0	0	0	0	0
0	0	0	0	0	0	0	0	0
0	0	0	0	0	0	0	0	0
0	0	0	0	0	0	0	0	0
0	0	0	0	0	0	0	0	0
0	0	0	0	0	0	0	0	0
0	0	0	0	0	0	0	0	0
0	0	0	0	0	0	0	0	0
0	0	0	0	0	0	0	0	0

0	0	0	0	0	0	0	0	0
0	0	0	0	0	0	0	0	0
0	0	0	0	0	0	0	0	0
0	0	0	0	0	0	0	0	0
0	0	0	0	0	0	0	0	0
0	0	0	0	0	0	0	0	0
0	0	0	0	0	0	0	0	0
0	0	0	0	0	0	0	0	0
0	0	0	0	0	0	0	0	0
0	0	0	0	0	0	0	0	0
0	0	0	0	0	0	0	0	0
0	0	0	0	0	0	0	0	0
0	0	0	0	0	0	0	0	0
0	0	0	0	0	0	0	0	0
0	0	0	0	0	0	0	0	0
0	0	0	0	0	0	0	0	0
0	0	0	0	0	0	0	0	0
0	0	0	0	0	0	0	0	0
0	0	0	0	0	0	0	0	0
0	0	0	0	0	0	0	0	0
0	0	0	0	0	0	0	0	0
0	0	0	0	0	0	0	0	0
0	0	0	0	0	0	0	0	0
0	0	0	0	0	0	0	0	0
0	0	0	0	0	0	0	0	0
0	0	0	0	0	0	0	0	0
0.007	0.0095	0.007	0.0077	0.0047	0.0061	0.0067	0.0048	0.0035
0.0102	0.0125	0.0095	0.0103	0.0076	0.0092	0.0089	0.0076	0.0056
0.0086	0.0104	0.0071	0.0073	0.0046	0.0063	0.0061	0.0047	0.0031
0.0097	0.0122	0.0094	0.01	0.0063	0.0078	0.0078	0.0071	0.0051
0.0094	0.0119	0.0085	0.0095	0.0061	0.0078	0.0077	0.0065	0.0051
0.0062	0.0089	0.0059	0.0075	0.0049	0.0066	0.0063	0.0049	0.0045
0.0064	0.0089	0.0054	0.0062	0.0041	0.0059	0.0062	0.0046	0.0026
0.0087	0.0108	0.0072	0.008	0.0053	0.0076	0.0077	0.0059	0.0048
0	0	0	0	0	0	0	0	0
0	0	0	0	0	0	0	0	0
0	0	0	0	0	0	0	0	0
0	0	0	0	0	0	0	0	0
0	0	0	0	0	0	0	0	0
0	0	0	0	0	0	0	0	0
0	0	0	0	0	0	0	0	0
0	0	0	0	0	0	0	0	0
0	0	0	0	0	0	0	0	0
0	0	0	0	0	0	0	0	0

0	0	0	0	0	0	0	0	0
0	0	0	0	0	0	0	0	0
0	0	0	0	0	0	0	0	0
0	0	0	0	0	0	0	0	0
0	0	0	0	0	0	0	0	0
0	0	0	0	0	0	0	0	0
0	0	0	0	0	0	0	0	0
0	0	0	0	0	0	0	0	0
0	0	0	0	0	0	0	0	0
0	0	0	0	0	0	0	0	0
0	0	0	0	0	0	0	0	0
0	0	0	0	0	0	0	0	0
0	0	0	0	0	0	0	0	0
0	0	0	0	0	0	0	0	0
0	0	0	0	0	0	0	0	0
0	0	0	0	0	0	0	0	0
0	0	0	0	0	0	0	0	0
0	0	0	0	0	0	0	0	0
0	0	0	0	0	0	0	0	0
0	0	0	0	0	0	0	0	0
0	0	0	0	0	0	0	0	0
0	0	0	0	0	0	0	0	0
0	0	0	0	0	0	0	0	0
0	0	0	0	0	0	0	0	0
0	0	0	0	0	0	0	0	0
0	0	0	0	0	0	0	0	0
0	0	0	0	0	0	0	0	0
0	0	0	0	0	0	0	0	0
0	0	0	0	0	0	0	0	0
0	0	0	0	0	0	0	0	0
0	0	0	0	0	0	0	0	0
0	0	0	0	0	0	0	0	0
0.0094	0.0124	0.0085	0.009	0.0061	0.0072	0.0067	0.0052	0.0036
0.0073	0.0104	0.006	0.0074	0.0043	0.0062	0.0064	0.0056	0.004
0.0074	0.0097	0.0062	0.0063	0.0034	0.0054	0.0058	0.0053	0.0047
0.0043	0.0068	0.0039	0.005	0.0025	0.0034	0.0033	0.0024	0.0012
0.0088	0.0102	0.0064	0.0072	0.0035	0.0056	0.0055	0.005	0.0045
0.0078	0.0109	0.0077	0.0094	0.0057	0.007	0.0071	0.0059	0.005
0.0089	0.012	0.0089	0.01	0.0074	0.009	0.0099	0.0087	0.0065
0.0074	0.0097	0.0061	0.0083	0.0052	0.0066	0.0064	0.0045	0.0036
0.0004	0.0006	0.0005	0.0005	0.0004	0.0004	0.0005	0.0006	0.0006
0	0	0	0	0	0	0	0	0
0	0	0	0	0	0	0	0	0
0	0	0	0	0	0	0	0	0

0	0	0	0	0	0	0	0	0
0	0	0	0	0	0	0	0	0
0	0	0	0	0	0	0	0	0
0	0	0	0	0	0	0	0	0
0	0	0	0	0	0	0	0	0
0	0	0	0	0	0	0	0	0
0	0	0	0	0	0	0	0	0
0	0	0	0	0	0	0	0	0
0	0	0	0	0	0	0	0	0
0	0	0	0	0	0	0	0	0
0	0	0	0	0	0	0	0	0
0	0	0	0	0	0	0	0	0
0	0	0	0	0	0	0	0	0
0	0	0	0	0	0	0	0	0
0	0	0	0	0	0	0	0	0
0	0	0	0	0	0	0	0	0
0	0	0	0	0	0	0	0	0
0	0	0	0	0	0	0	0	0
0	0	0	0	0	0	0	0	0
0	0	0	0	0	0	0	0	0
0	0	0	0	0	0	0	0	0
0	0	0	0	0	0	0	0	0
0	0	0	0	0	0	0	0	0
0	0	0	0	0	0	0	0	0
0	0	0	0	0	0	0	0	0
0	0	0	0	0	0	0	0	0
0	0	0	0	0	0	0	0	0
0	0	0	0	0	0	0	0	0
0	0	0	0	0	0	0	0	0
0	0	0	0	0	0	0	0	0
0	0	0	0	0	0	0	0	0
0	0	0	0	0	0	0	0	0
0	0	0	0	0	0	0	0	0
0	0	0	0	0	0	0	0	0
0	0	0	0	0	0	0	0	0
0	0	0	0	0	0	0	0	0
0	0	0	0	0	0	0	0	0
0	0	0	0	0	0	0	0	0
0.009	0.0113	0.0074	0.0085	0.0059	0.0076	0.0074	0.0057	0.0051
0.0094	0.0129	0.0092	0.01	0.0061	0.0081	0.0082	0.0074	0.0062
0.0093	0.0121	0.0092	0.0093	0.0064	0.0082	0.0082	0.0073	0.0072
0.0076	0.0102	0.0072	0.0077	0.0047	0.0058	0.0069	0.0062	0.0042
0.0075	0.0104	0.007	0.0087	0.0062	0.0075	0.007	0.0054	0.0053
0.0089	0.0123	0.0086	0.0101	0.0066	0.0091	0.0098	0.0077	0.0064

0.0111	0.0147	0.0114	0.0126	0.0088	0.0111	0.0122	0.0118	0.0101
0.0068	0.0097	0.0075	0.0085	0.0052	0.0065	0.0065	0.0056	0.0039
0.0013	0.0022	0.0018	0.0025	0.0021	0.0021	0.0027	0.0029	0.0027
0.0007	0.0011	0.001	0.0012	0.0009	0.001	0.0013	0.0015	0.0015
0.0009	0.0015	0.0014	0.0016	0.0014	0.0014	0.0015	0.0016	0.0016
0.0008	0.0012	0.001	0.0012	0.0011	0.0012	0.0014	0.0014	0.0013
0.0008	0.0012	0.001	0.0013	0.0009	0.001	0.0012	0.0013	0.0013
0	0	0	0	0	0	0	0	0
0	0	0	0	0	0	0	0	0
0	0	0	0	0	0	0	0	0
0	0	0	0	0	0	0	0	0
0	0	0	0	0	0	0	0	0
0	0	0	0	0	0	0	0	0
0	0	0	0	0	0	0	0	0
0	0	0	0	0	0	0	0	0
0	0	0	0	0	0	0	0	0
0	0	0	0	0	0	0	0	0
0	0	0	0	0	0	0	0	0
0	0	0	0	0	0	0	0	0
0	0	0	0	0	0	0	0	0
0	0	0	0	0	0	0	0	0
0	0	0	0	0	0	0	0	0
0	0	0	0	0	0	0	0	0
0	0	0	0	0	0	0	0	0
0	0	0	0	0	0	0	0	0
0	0	0	0	0	0	0	0	0
0	0	0	0	0	0	0	0	0
0	0	0	0	0	0	0	0	0
0	0	0	0	0	0	0	0	0
0	0	0	0	0	0	0	0	0
0	0	0	0	0	0	0	0	0
0	0	0	0	0	0	0	0	0
0	0	0	0	0	0	0	0	0
0	0	0	0	0	0	0	0	0
0	0	0	0	0	0	0	0	0
0	0	0	0	0	0	0	0	0
0	0	0	0	0	0	0	0	0
0	0	0	0	0	0	0	0	0
0	0	0	0	0	0	0	0	0
0	0	0	0	0	0	0	0	0
0	0	0	0	0	0	0	0	0
0	0	0	0	0	0	0	0	0
0	0	0	0	0	0	0	0	0
0	0	0	0	0	0	0	0	0
0	0	0	0	0	0	0	0	0

0	0	0	0	0	0	0	0	0
0.0004	0.0006	0.0006	0.0007	0.0006	0.0006	0.0007	0.0007	0.0008
0.0003	0.0006	0.0004	0.0006	0.0005	0.0005	0.0006	0.0007	0.0007
0	0	0	0	0	0	0	0	0
0	0	0	0	0	0	0	0	0
0.0005	0.001	0.0008	0.001	0.0008	0.0009	0.001	0.0012	0.0011
0.0008	0.0015	0.0012	0.0016	0.0014	0.0016	0.0018	0.002	0.002
0	0	0	0	0	0	0	0	0
0.001	0.0017	0.0015	0.0017	0.0014	0.0016	0.0017	0.0019	0.0019
0	0	0	0	0	0	0	0	0
0	0	0	0	0	0	0	0	0
0.001	0.0015	0.0012	0.0014	0.0012	0.0012	0.0013	0.0016	0.0017
0.0008	0.0012	0.001	0.0013	0.0011	0.0012	0.0015	0.0016	0.0015
0	0	0	0	0	0	0	0	0
0	0	0	0	0	0	0	0	0
0.0003	0.0005	0.0004	0.0005	0.0004	0.0005	0.0005	0.0006	0.0006
0	0	0	0	0	0	0	0	0
0	0	0	0	0	0	0	0	0
0	0	0	0	0	0	0	0	0
0	0	0	0	0	0	0	0	0
0	0	0	0	0	0	0	0	0
0	0	0	0	0	0	0	0	0
0	0	0	0	0	0	0	0	0
0	0	0	0	0	0	0	0	0
0	0	0	0	0	0	0	0	0
0	0	0	0	0	0	0	0	0
0	0	0	0	0	0	0	0	0
0	0	0	0	0	0	0	0	0
0	0	0	0	0	0	0	0	0
0	0	0	0	0	0	0	0	0
0	0	0	0	0	0	0	0	0
0	0	0	0	0	0	0	0	0
0	0	0	0	0	0	0	0	0
0	0	0	0	0	0	0	0	0
0	0	0	0	0	0	0	0	0
0	0	0	0	0	0	0	0	0
0	0	0	0	0	0	0	0	0
0	0	0	0	0	0	0	0	0
0	0	0	0	0	0	0	0	0
0	0	0	0	0	0	0	0	0
0	0	0	0	0	0	0	0	0
0	0	0	0	0	0	0	0	0
0	0	0	0	0	0	0	0	0

0	0	0	0	0	0	0	0	0
0	0	0	0	0	0	0	0	0
0	0	0	0	0	0	0	0	0
0	0	0	0	0	0	0	0	0
0	0	0	0	0	0	0	0	0
0	0	0	0	0	0	0	0	0
0	0	0	0	0	0	0	0	0
0	0	0	0	0	0	0	0	0
0	0	0	0	0	0	0	0	0
0.0003	0.0006	0.0005	0.0006	0.0005	0.0005	0.0006	0.0006	0.0006
0.0002	0.0004	0.0003	0.0004	0.0003	0.0004	0.0005	0.0006	0.0006
0	0	0	0	0	0	0	0	0
0	0	0	0	0	0	0	0	0
0	0	0	0	0	0	0	0	0
0.0004	0.0006	0.0005	0.0006	0.0005	0.0006	0.0006	0.0007	0.0007
0	0	0	0	0	0	0	0	0
0	0	0	0	0	0	0	0	0
0.0005	0.0007	0.0006	0.0007	0.0005	0.0005	0.0006	0.0007	0.0007
0.0003	0.0006	0.0005	0.0006	0.0005	0.0005	0.0006	0.0007	0.0007
0	0	0	0	0	0	0	0	0
0	0	0	0	0	0	0	0	0
0.0003	0.0005	0.0005	0.0006	0.0005	0.0006	0.0006	0.0008	0.0007
0	0	0	0	0	0	0	0	0
0	0	0	0	0	0	0	0	0
0	0	0	0	0	0	0	0	0
0	0	0	0	0	0	0	0	0
0	0	0	0	0	0	0	0	0
0	0	0	0	0	0	0	0	0
0	0	0	0	0	0	0	0	0
0	0	0	0	0	0	0	0	0
0	0	0	0	0	0	0	0	0
0	0	0	0	0	0	0	0	0
0	0	0	0	0	0	0	0	0
0	0	0	0	0	0	0	0	0
0	0	0	0	0	0	0	0	0
0	0	0	0	0	0	0	0	0
0	0	0	0	0	0	0	0	0
0	0	0	0	0	0	0	0	0
0	0	0	0	0	0	0	0	0
0	0	0	0	0	0	0	0	0
0	0	0	0	0	0	0	0	0
0	0	0	0	0	0	0	0	0
0	0	0	0	0	0	0	0	0
0	0	0	0	0	0	0	0	0

0	0	0	0	0	0	0	0	0
0	0	0	0	0	0	0	0	0
0	0	0	0	0	0	0	0	0
0	0	0	0	0	0	0	0	0
0	0	0	0	0	0	0	0	0
0	0	0	0	0	0	0	0	0
0	0	0	0	0	0	0	0	0
0	0	0	0	0	0	0	0	0
0	0	0	0	0	0	0	0	0
0	0	0	0	0	0	0	0	0
0	0	0	0	0	0	0	0	0
0	0	0	0	0	0	0	0	0
0.0166	0.0221	0.0161	0.0182	0.0109	0.0129	0.0126	0.0121	0.0099
0.0199	0.0243	0.018	0.0195	0.0134	0.0152	0.0151	0.0134	0.0097
0.0157	0.0207	0.0135	0.013	0.0064	0.0109	0.0121	0.0104	0.0089
0.0205	0.0292	0.0199	0.0241	0.0173	0.0204	0.0205	0.0185	0.0168
0.0139	0.0243	0.0184	0.0214	0.0149	0.0186	0.0192	0.0171	0.015
0.0168	0.0225	0.0147	0.0185	0.0108	0.0144	0.0136	0.0113	0.0102
0.015	0.0193	0.0142	0.0168	0.0102	0.0138	0.013	0.0115	0.0087
0.0148	0.0202	0.0151	0.0174	0.0111	0.0147	0.0126	0.0112	0.0088
0.0013	0.0019	0.0017	0.0019	0.0016	0.0016	0.0018	0.0019	0.0018
0	0	0	0	0	0	0	0	0
0	0	0	0	0	0	0	0	0
0.0014	0.0024	0.002	0.0023	0.0021	0.0023	0.0024	0.0027	0.0026
0.0018	0.003	0.0025	0.003	0.0025	0.0027	0.0029	0.0032	0.0032
0	0	0	0	0	0	0	0	0
0.0004	0.0006	0.0005	0.0006	0.0005	0.0006	0.0007	0.0007	0.0008
0.0018	0.0034	0.0029	0.0034	0.0025	0.003	0.0034	0.0041	0.0041
0	0	0	0	0	0	0	0	0
0	0	0	0	0	0	0	0	0
0	0	0	0	0	0	0	0	0
0	0	0	0	0	0	0	0	0
0	0	0	0	0	0	0	0	0
0	0	0	0	0	0	0	0	0
0	0	0	0	0	0	0	0	0
0	0	0	0	0	0	0	0	0
0	0	0	0	0	0	0	0	0
0	0	0	0	0	0	0	0	0
0	0	0	0	0	0	0	0	0
0	0	0	0	0	0	0	0	0
0	0	0	0	0	0	0	0	0
0	0	0	0	0	0	0	0	0
0	0	0	0	0	0	0	0	0

0	0	0	0	0	0	0	0	0
0	0	0	0	0	0	0	0	0
0	0	0	0	0	0	0	0	0
0	0	0	0	0	0	0	0	0
0	0	0	0	0	0	0	0	0
0	0	0	0	0	0	0	0	0
0	0	0	0	0	0	0	0	0
0	0	0	0	0	0	0	0	0
0	0	0	0	0	0	0	0	0
0	0	0	0	0	0	0	0	0
0	0	0	0	0	0	0	0	0
0	0	0	0	0	0	0	0	0
0	0	0	0	0	0	0	0	0
0	0	0	0	0	0	0	0	0
0	0	0	0	0	0	0	0	0
0	0	0	0	0	0	0	0	0
0	0	0	0	0	0	0	0	0
0	0	0	0	0	0	0	0	0
0.044	0.059	0.043	0.0455	0.0309	0.0368	0.039	0.0356	0.0315
0.0312	0.0442	0.0326	0.0357	0.0256	0.0376	0.0369	0.032	0.0293
0.0368	0.0486	0.0343	0.0365	0.022	0.0277	0.0264	0.0233	0.0212
0.0365	0.0516	0.0365	0.043	0.0293	0.0372	0.036	0.0347	0.0314
0.0409	0.0562	0.0404	0.0419	0.0303	0.039	0.0422	0.036	0.0308
0.0324	0.0451	0.0305	0.0339	0.0194	0.0271	0.0251	0.0244	0.0205
0.032	0.0387	0.0263	0.0297	0.0165	0.0254	0.023	0.0187	0.015
0.0424	0.0538	0.041	0.044	0.0319	0.0384	0.039	0.0328	0.0271
0.0018	0.0035	0.0028	0.0034	0.0027	0.0029	0.0036	0.0041	0.0043
0.0005	0.0011	0.0009	0.0011	0.001	0.0011	0.0012	0.0014	0.0012
0.001	0.0018	0.0015	0.0017	0.0015	0.0017	0.0018	0.002	0.002
0.0037	0.0059	0.0053	0.0064	0.0058	0.0066	0.0073	0.0082	0.0079
0.0026	0.0048	0.0036	0.0045	0.0036	0.0044	0.0057	0.0063	0.007
0.0018	0.0032	0.003	0.0035	0.0028	0.0035	0.0037	0.0041	0.0041
0.0029	0.0047	0.004	0.0045	0.0035	0.004	0.0045	0.0051	0.0049
0.0031	0.0058	0.005	0.0072	0.0061	0.007	0.0076	0.0081	0.008
0	0	0	0	0	0	0	0	0
0	0	0	0	0	0	0	0	0
0	0	0	0	0	0	0	0	0
0	0	0	0	0	0	0	0	0
0	0	0	0	0	0	0	0	0
0	0	0	0	0	0	0	0	0
0	0	0	0	0	0	0	0	0
0	0	0	0	0	0	0	0	0
0	0	0	0	0	0	0	0	0
0	0	0	0	0	0	0	0	0

0	0	0	0	0	0	0	0	0
0	0	0	0	0	0	0	0	0
0	0	0	0	0	0	0	0	0
0	0	0	0	0	0	0	0	0
0	0	0	0	0	0	0	0	0
0	0	0	0	0	0	0	0	0
0	0	0	0	0	0	0	0	0
0	0	0	0	0	0	0	0	0
0	0	0	0	0	0	0	0	0
0	0	0	0	0	0	0	0	0
0	0	0	0	0	0	0	0	0
0	0	0	0	0	0	0	0	0
0	0	0	0	0	0	0	0	0
0	0	0	0	0	0	0	0	0
0	0	0	0	0	0	0	0	0
0	0	0	0	0	0	0	0	0
0	0	0	0	0	0	0	0	0
0	0	0	0	0	0	0	0	0
0.0002	0.0012	0.0012	0.0013	0.0011	0.0012	0.0009	0.001	0.0008
0.0004	0.0006	0.0005	0.0006	0.0005	0.0005	0.0005	0.0006	0.0006
0	0	0	0	0	0	0	0	0
0	0	0	0	0	0	0	0	0
0.0018	0.0036	0.0031	0.0035	0.003	0.0035	0.0042	0.0049	0.0046
0.004	0.0096	0.009	0.0115	0.0092	0.0101	0.0109	0.0121	0.0121
0.1679	0.2329	0.1508	0.1926	0.1065	0.1498	0.1694	0.1523	0.1202
0.3774	0.5297	0.3366	0.3724	0.2668	0.2917	0.2737	0.2815	0.2626
0.2271	0.3006	0.2198	0.2389	0.1743	0.1999	0.1934	0.1733	0.1578
0.0573	0.0869	0.0676	0.0839	0.06	0.071	0.0789	0.0821	0.0779
0.1681	0.2438	0.1752	0.2041	0.1358	0.1676	0.1847	0.1637	0.1457
0.0674	0.1173	0.0958	0.0986	0.0753	0.0915	0.0821	0.0724	0.0614
0.0226	0.1229	0.1133	0.1031	0.0811	0.1046	0.067	0.0593	0.0288
0.4176	0.5535	0.3652	0.441	0.2854	0.3995	0.3514	0.3348	0.2993
0.1077	0.1588	0.1054	0.1299	0.0772	0.1076	0.1062	0.0896	0.0721
0.0468	0.0779	0.0627	0.0731	0.0507	0.063	0.0543	0.0392	0.0331
0.0203	0.0447	0.0355	0.042	0.0278	0.0382	0.0323	0.0232	0.0162
0.1216	0.1851	0.1368	0.1659	0.1175	0.1485	0.1628	0.1513	0.1335
0.168	0.2418	0.1803	0.2051	0.1594	0.1862	0.1836	0.1793	0.1663
0.0165	0.0309	0.027	0.0294	0.0217	0.0268	0.0266	0.0257	0.0226
0.0044	0.017	0.0154	0.0147	0.0108	0.0124	0.0108	0.0134	0.0108
0.0916	0.1366	0.1	0.1173	0.0858	0.1057	0.1052	0.0986	0.0872
0.0021	0.0039	0.0031	0.004	0.0033	0.0034	0.004	0.0049	0.0048
0.0003	0.0005	0.0004	0.0005	0.0003	0.0004	0.0005	0.0006	0.0006
0	0	0	0	0	0	0	0	0
0.0005	0.0007	0.0007	0.0007	0.0006	0.0007	0.0007	0.0008	0.0007

0	0	0	0	0	0	0	0	0
0	0	0	0	0	0	0	0	0
0.0003	0.0005	0.0004	0.0005	0.0004	0.0005	0.0005	0.0006	0.0007
0	0	0	0	0	0	0	0	0
0	0	0	0	0	0	0	0	0
0	0	0	0	0	0	0	0	0
0	0	0	0	0	0	0	0	0
0	0	0	0	0	0	0	0	0
0	0	0	0	0	0	0	0	0
0	0	0	0	0	0	0	0	0
0	0	0	0	0	0	0	0	0
0	0	0	0	0	0	0	0	0
0	0	0	0	0	0	0	0	0
0	0	0	0	0	0	0	0	0
0	0	0	0	0	0	0	0	0
0	0	0	0	0	0	0	0	0
0	0	0	0	0	0	0	0	0
0	0	0	0	0	0	0	0	0
0	0	0	0	0	0	0	0	0
0	0	0	0	0	0	0	0	0
0	0	0	0	0	0	0	0	0
0	0	0	0	0	0	0	0	0
0.0006	0.0017	0.0014	0.0015	0.0012	0.0014	0.0011	0.0014	0.0011
0	0	0	0	0	0	0	0	0
0	0	0	0	0	0	0	0	0
0.0003	0.0006	0.0005	0.0006	0.0005	0.0005	0.0006	0.0007	0.0007
0.0003	0.0006	0.0005	0.0006	0.0005	0.0005	0.0006	0.0007	0.0007
0	0	0	0	0	0	0	0	0
0.0007	0.0049	0.004	0.0045	0.0036	0.0045	0.0033	0.0036	0.0025
0.0009	0.0087	0.0088	0.0082	0.0057	0.0073	0.0061	0.0069	0.0051
0.0454	0.0846	0.0678	0.0728	0.0581	0.0703	0.0654	0.0609	0.0508
0.1389	0.1713	0.1319	0.1408	0.0864	0.1299	0.1361	0.1144	0.0931
0.0707	0.1101	0.0763	0.0894	0.0617	0.0837	0.0787	0.0718	0.0577
0.0137	0.0391	0.0314	0.0354	0.0278	0.0337	0.0282	0.0323	0.0298
0.0191	0.0477	0.0394	0.0464	0.0378	0.0484	0.0489	0.0526	0.0532
0.0268	0.072	0.0607	0.0666	0.0503	0.0575	0.0505	0.0602	0.0598
0.0404	0.1924	0.1776	0.1737	0.1422	0.1887	0.1564	0.1455	0.1113
0.1862	0.3521	0.2743	0.2789	0.1751	0.2391	0.2103	0.1499	0.114
0.119	0.2083	0.153	0.1635	0.1045	0.1373	0.1355	0.1168	0.0858
0.0487	0.1779	0.1529	0.1724	0.1305	0.1616	0.1288	0.1269	0.0817
0.105	0.2325	0.2163	0.2131	0.1642	0.203	0.1701	0.1565	0.1159
0.114	0.2133	0.1732	0.191	0.1329	0.1704	0.1528	0.1479	0.1234
0.1667	0.2308	0.1712	0.1981	0.1419	0.1833	0.1919	0.1808	0.17
0.1032	0.1549	0.1154	0.1403	0.1029	0.1288	0.1409	0.1361	0.1281

0.0593	0.0867	0.0621	0.0656	0.0386	0.0486	0.0492	0.0355	0.0264
0.0774	0.1144	0.086	0.0895	0.0576	0.0776	0.0627	0.0524	0.0451
0.0025	0.0048	0.0046	0.0054	0.0044	0.0048	0.0056	0.0061	0.0057
0.0003	0.0005	0.0004	0.0005	0.0004	0.0005	0.0006	0.0006	0.0006
0	0	0	0	0	0	0	0	0
0.0004	0.0006	0.0006	0.0007	0.0006	0.0006	0.0007	0.0008	0.0008
0	0	0	0	0	0	0	0	0
0	0	0	0	0	0	0	0	0
0.0004	0.0006	0.0005	0.0006	0.0006	0.0006	0.0007	0.0007	0.0008
0	0	0	0	0	0	0	0	0
0	0	0	0	0	0	0	0	0
0	0	0	0	0	0	0	0	0
0	0	0	0	0	0	0	0	0
0	0	0	0	0	0	0	0	0
0	0	0	0	0	0	0	0	0
0	0	0	0	0	0	0	0	0
0	0	0	0	0	0	0	0	0
0	0	0	0	0	0	0	0	0
0	0	0	0	0	0	0	0	0
0	0	0	0	0	0	0	0	0
0	0	0	0	0	0	0	0	0
0	0	0	0	0	0	0	0	0
0	0	0	0	0	0	0	0	0
0	0	0	0	0	0	0	0	0
0	0	0	0	0	0	0	0	0
0	0	0	0	0	0	0	0	0
0	0	0	0	0	0	0	0	0
0	0	0	0	0	0	0	0	0
0.0012	0.0035	0.0031	0.0035	0.0028	0.0036	0.0033	0.0032	0.003
0.0002	0.001	0.0009	0.001	0.0007	0.001	0.0007	0.0007	0.0005
0	0	0	0	0	0	0	0	0
0.0004	0.0005	0.0004	0.0005	0.0004	0.0005	0.0006	0.0007	0.0007
0.001	0.0017	0.0014	0.0017	0.0013	0.0015	0.0018	0.002	0.002
0.0006	0.0022	0.002	0.002	0.0016	0.0018	0.0013	0.0017	0.0012
0.0015	0.0076	0.0072	0.0079	0.0062	0.0081	0.006	0.0058	0.0042
0.0019	0.0106	0.0105	0.0108	0.0099	0.0111	0.0094	0.0104	0.0087
0.0027	0.0787	0.0853	0.0891	0.077	0.0978	0.0742	0.0759	0.0602
0.0184	0.0486	0.0415	0.0403	0.0326	0.0413	0.0315	0.0312	0.0265
0.1217	0.1717	0.1246	0.1459	0.1026	0.132	0.1396	0.1323	0.1249
0.1574	0.2248	0.1761	0.1999	0.1492	0.182	0.1874	0.1874	0.1639
0.0307	0.1365	0.1245	0.1263	0.0987	0.1233	0.0806	0.0941	0.0774
0.0312	0.1677	0.1553	0.1581	0.1285	0.1766	0.1386	0.1505	0.1143
0.0282	0.16	0.132	0.1425	0.1084	0.1467	0.1131	0.113	0.0832
0.0497	0.1389	0.1137	0.1207	0.0883	0.1188	0.103	0.1037	0.0886

0.0466	0.1634	0.1336	0.1399	0.09	0.1218	0.1138	0.1194	0.0968
0.1156	0.2522	0.2164	0.2451	0.1936	0.2406	0.1922	0.1881	0.1422
0.1049	0.2439	0.2206	0.2198	0.1498	0.1941	0.1626	0.1544	0.1367
0.0978	0.1678	0.141	0.1567	0.1223	0.1453	0.146	0.1376	0.1149
0.1054	0.1558	0.1344	0.1488	0.1208	0.138	0.15	0.1581	0.1512
0.0836	0.1542	0.1246	0.1504	0.1212	0.1412	0.1574	0.1578	0.1482
0.0999	0.1365	0.1051	0.1161	0.0863	0.1058	0.1018	0.0993	0.0853
0.0531	0.0639	0.0449	0.0497	0.0321	0.0406	0.0432	0.0381	0.0262
0.0045	0.0082	0.0071	0.008	0.0071	0.0078	0.009	0.0096	0.009
0.0005	0.0011	0.0008	0.0011	0.0008	0.0009	0.0011	0.0012	0.0013
0	0	0	0	0	0	0	0	0
0.0003	0.0006	0.0004	0.0006	0.0005	0.0005	0.0006	0.0007	0.0007
0	0	0	0	0	0	0	0	0
0	0	0	0	0	0	0	0	0
0.0003	0.0006	0.0004	0.0005	0.0005	0.0005	0.0005	0.0006	0.0006
0	0	0	0	0	0	0	0	0
0	0	0	0	0	0	0	0	0
0	0	0	0	0	0	0	0	0
0	0	0	0	0	0	0	0	0
0	0	0	0	0	0	0	0	0
0	0	0	0	0	0	0	0	0
0	0	0	0	0	0	0	0	0
0	0	0	0	0	0	0	0	0
0	0	0	0	0	0	0	0	0
0	0	0	0	0	0	0	0	0
0	0	0	0	0	0	0	0	0
0	0	0	0	0	0	0	0	0
0	0	0	0	0	0	0	0	0
0	0	0	0	0	0	0	0	0
0	0	0	0	0	0	0	0	0
0	0	0	0	0	0	0	0	0
0	0	0	0	0	0	0	0	0
0	0	0	0	0	0	0	0	0
0.0011	0.0029	0.0023	0.0025	0.002	0.0023	0.0023	0.0026	0.0023
0.0003	0.001	0.0009	0.0009	0.0007	0.0009	0.0007	0.0008	0.0005
0	0	0	0	0	0	0	0	0
0.0007	0.0013	0.0011	0.0013	0.0011	0.0011	0.0013	0.0013	0.0013
0.0007	0.0011	0.0009	0.0012	0.0009	0.0011	0.0012	0.0014	0.0013
0.0001	0.0009	0.0008	0.0007	0.0006	0.0009	0.0006	0.0007	0.0007
0.0001	0.0046	0.0044	0.0043	0.0032	0.0052	0.0037	0.0039	0.0035
0	0.0073	0.0063	0.0064	0.0043	0.0073	0.0053	0.0056	0.0046
0.0193	0.0544	0.0445	0.0486	0.0365	0.0469	0.0409	0.0419	0.0295
0.1093	0.1833	0.1464	0.1649	0.1173	0.1398	0.1475	0.1454	0.1161

0.2275	0.3356	0.2629	0.2951	0.2009	0.254	0.2481	0.2369	0.2065
0.2718	0.3713	0.2688	0.2919	0.2051	0.2626	0.2539	0.2067	0.1598
0.0869	0.2435	0.1893	0.2363	0.175	0.228	0.1609	0.1274	0.0748
0.1064	0.261	0.2006	0.186	0.1095	0.1742	0.1438	0.1401	0.0874
0.0477	0.1508	0.1338	0.1421	0.1056	0.1389	0.1266	0.125	0.1134
0.0344	0.1188	0.0983	0.1116	0.0681	0.0966	0.0743	0.0792	0.0515
-0.0148	0.1197	0.1017	0.1149	0.0854	0.1184	0.0941	0.0852	0.0603
0.2082	0.3379	0.2691	0.2798	0.2165	0.2724	0.2606	0.2449	0.2007
0.1956	0.2895	0.2116	0.2367	0.1686	0.1899	0.1822	0.165	0.1485
0.0682	0.1459	0.1157	0.1306	0.0949	0.1228	0.1093	0.1065	0.0944
0.053	0.1362	0.1053	0.1234	0.093	0.1255	0.1013	0.1025	0.0936
0.075	0.1987	0.1422	0.1505	0.1121	0.164	0.1345	0.1204	0.0918
0.0771	0.1645	0.1206	0.1497	0.1147	0.1439	0.1426	0.1465	0.1265
0.0353	0.07	0.0594	0.0756	0.0554	0.0616	0.0758	0.0812	0.0802
0.0055	0.0098	0.0086	0.0097	0.0084	0.0088	0.01	0.0108	0.0109
0.0008	0.0012	0.0011	0.0014	0.0012	0.0012	0.0014	0.0014	0.0014
0	0	0	0	0	0	0	0	0
0.0003	0.0006	0.0005	0.0006	0.0005	0.0005	0.0006	0.0007	0.0007
0	0	0	0	0	0	0	0	0
0	0	0	0	0	0	0	0	0
0.0003	0.0006	0.0005	0.0006	0.0005	0.0006	0.0007	0.0007	0.0007
0	0	0	0	0	0	0	0	0
0	0	0	0	0	0	0	0	0
0	0	0	0	0	0	0	0	0
0	0	0	0	0	0	0	0	0
0	0	0	0	0	0	0	0	0
0	0	0	0	0	0	0	0	0
0	0	0	0	0	0	0	0	0
0	0	0	0	0	0	0	0	0
0	0	0	0	0	0	0	0	0
0	0	0	0	0	0	0	0	0
0	0	0	0	0	0	0	0	0
0	0	0	0	0	0	0	0	0
0	0	0	0	0	0	0	0	0
0	0	0	0	0	0	0	0	0
0	0	0	0	0	0	0	0	0
0	0	0	0	0	0	0	0	0
0	0	0	0	0	0	0	0	0
0	0	0	0	0	0	0	0	0
0	0	0	0	0	0	0	0	0
0.0006	0.001	0.0008	0.0011	0.0009	0.001	0.0011	0.0012	0.0011
0.0005	0.0019	0.0018	0.0019	0.0016	0.002	0.0018	0.002	0.0018
0.0007	0.0029	0.0023	0.0025	0.0018	0.0026	0.0019	0.0021	0.0019
0.0005	0.0026	0.0023	0.0026	0.002	0.0024	0.0021	0.0022	0.0018

0	0	0	0	0	0	0	0	0
0	0	0	0	0	0	0	0	0
0.0009	0.0014	0.0011	0.0013	0.0011	0.0012	0.0014	0.0015	0.0015
0.003	0.0052	0.0047	0.0055	0.0047	0.0051	0.0055	0.0062	0.0065
0.0117	0.0225	0.0201	0.0259	0.0196	0.0234	0.0255	0.0274	0.0282
0.1071	0.1597	0.1215	0.1397	0.098	0.1283	0.1354	0.1292	0.127
0.1568	0.2313	0.1785	0.2032	0.1486	0.1809	0.1855	0.1676	0.1457
0.1413	0.2078	0.1609	0.1831	0.1379	0.1705	0.1916	0.188	0.173
0.1587	0.2282	0.1628	0.1931	0.1338	0.1666	0.1737	0.1704	0.1492
0.097	0.1884	0.1399	0.1623	0.111	0.1402	0.1365	0.14	0.1257
0.0815	0.1696	0.1322	0.1502	0.1087	0.1414	0.1208	0.121	0.0999
0.0663	0.2016	0.1731	0.1806	0.126	0.1735	0.1419	0.1382	0.1289
0.1443	0.2855	0.2118	0.2201	0.1601	0.2233	0.1955	0.1451	0.1118
0.2048	0.3439	0.2756	0.2983	0.219	0.2794	0.2691	0.2309	0.1954
0.11	0.2258	0.1554	0.176	0.1095	0.1507	0.133	0.121	0.089
0.0997	0.228	0.2036	0.2189	0.1758	0.2231	0.1848	0.1814	0.1348
0.2299	0.3867	0.2998	0.3318	0.2462	0.3355	0.2582	0.2185	0.1148
0.393	0.6077	0.457	0.4951	0.3301	0.4196	0.4188	0.3569	0.2954
0.3618	0.5041	0.3518	0.3886	0.2563	0.3457	0.3639	0.3264	0.3108
0.0522	0.0989	0.0884	0.1132	0.0962	0.1064	0.1234	0.1349	0.1353
0.0036	0.0087	0.0071	0.0082	0.0077	0.0077	0.0098	0.0106	0.0113
0.0008	0.0011	0.0009	0.0011	0.0008	0.001	0.0011	0.0013	0.0013
0	0	0	0	0	0	0	0	0
0.0005	0.0008	0.0007	0.0007	0.0006	0.0006	0.0007	0.0008	0.0007
0.0003	0.0006	0.0005	0.0006	0.0005	0.0005	0.0006	0.0006	0.0006
0	0	0	0	0	0	0	0	0
0	0	0	0	0	0	0	0	0
0	0	0	0	0	0	0	0	0
0	0	0	0	0	0	0	0	0
0	0	0	0	0	0	0	0	0
0	0	0	0	0	0	0	0	0
0	0	0	0	0	0	0	0	0
0	0	0	0	0	0	0	0	0
0	0	0	0	0	0	0	0	0
0	0	0	0	0	0	0	0	0
0	0	0	0	0	0	0	0	0
0	0	0	0	0	0	0	0	0
0	0	0	0	0	0	0	0	0
0	0	0	0	0	0	0	0	0
0	0	0	0	0	0	0	0	0
0	0	0	0	0	0	0	0	0
0	0	0	0	0	0	0	0	0
0	0	0	0	0	0	0	0	0

0	0	0	0	0	0	0	0	0
0	0	0	0	0	0	0	0	0
0.0002	0.0005	0.0004	0.0005	0.0004	0.0004	0.0005	0.0006	0.0006
0.0003	0.0013	0.0012	0.0013	0.001	0.0013	0.0012	0.0013	0.001
0.0021	0.0051	0.0046	0.0048	0.0037	0.0046	0.004	0.0042	0.0037
0.0006	0.0036	0.0034	0.0034	0.0028	0.0035	0.0028	0.003	0.0023
-0.0001	0.0008	0.0006	0.0007	0.0005	0.0006	0.0004	0.0005	0.0004
0	0	0	0	0	0	0	0	0
0.0013	0.0021	0.0017	0.0021	0.0018	0.002	0.0021	0.0024	0.0024
0.0059	0.0085	0.0068	0.0086	0.0076	0.0081	0.0095	0.01	0.0096
0.0415	0.0758	0.0665	0.0817	0.0657	0.0728	0.0729	0.0878	0.0856
0.06	0.0845	0.0544	0.0654	0.0477	0.0626	0.0661	0.0622	0.0481
0.0338	0.0494	0.0366	0.0423	0.0304	0.0368	0.0413	0.0389	0.0335
0.0454	0.0943	0.0803	0.0978	0.0798	0.0894	0.0956	0.1092	0.1098
0.0963	0.1666	0.1317	0.1453	0.1088	0.139	0.149	0.1539	0.1444
0.1358	0.2406	0.1839	0.2046	0.1358	0.162	0.159	0.1417	0.1194
0.2519	0.3643	0.2576	0.3008	0.2157	0.2771	0.3067	0.2737	0.2245
0.3565	0.5459	0.3967	0.4431	0.3056	0.3881	0.334	0.2764	0.2366
0.2906	0.4665	0.3732	0.3813	0.2555	0.3193	0.2951	0.2117	0.1895
0.2907	0.4942	0.2981	0.3341	0.2211	0.3055	0.286	0.1782	0.1263
0.1203	0.2777	0.1941	0.2171	0.1703	0.2353	0.2221	0.1911	0.1278
0.0681	0.2092	0.2021	0.2127	0.1789	0.2269	0.1649	0.1452	0.1128
0.4035	0.5701	0.4176	0.4924	0.3328	0.4085	0.3528	0.3095	0.2379
0.9157	1.0936	0.7178	0.6863	0.4303	0.5808	0.5411	0.3746	0.3329
0.5952	0.7744	0.5221	0.6021	0.4388	0.6154	0.5587	0.5144	0.4057
0.0675	0.1273	0.1046	0.1315	0.105	0.1226	0.1325	0.1344	0.1286
0.0071	0.0119	0.0095	0.0118	0.01	0.0105	0.0112	0.0129	0.0122
0.0007	0.0012	0.0009	0.0012	0.001	0.001	0.0011	0.0013	0.0013
0	0	0	0	0	0	0	0	0
0.0009	0.0014	0.0013	0.0014	0.0013	0.0014	0.0015	0.0016	0.0016
0.0006	0.001	0.0008	0.0011	0.0009	0.001	0.0011	0.0012	0.0012
0	0	0	0	0	0	0	0	0
0	0	0	0	0	0	0	0	0
0	0	0	0	0	0	0	0	0
0	0	0	0	0	0	0	0	0
0	0	0	0	0	0	0	0	0
0	0	0	0	0	0	0	0	0
0	0	0	0	0	0	0	0	0
0	0	0	0	0	0	0	0	0
0	0	0	0	0	0	0	0	0
0	0	0	0	0	0	0	0	0
0	0	0	0	0	0	0	0	0
0	0	0	0	0	0	0	0	0
0	0	0	0	0	0	0	0	0

0	0	0	0	0	0	0	0	0
0	0	0	0	0	0	0	0	0
0	0	0	0	0	0	0	0	0
0	0	0	0	0	0	0	0	0
0	0	0	0	0	0	0	0	0
0	0	0	0	0	0	0	0	0
0	0	0	0	0	0	0	0	0
0	0	0	0	0	0	0	0	0
0	0	0	0	0	0	0	0	0
0.0002	0.0009	0.0007	0.0007	0.0006	0.0008	0.0006	0.0007	0.0005
0.0006	0.0028	0.0025	0.0025	0.0018	0.0024	0.0015	0.0016	0.0012
0.0002	0.0019	0.0018	0.002	0.0017	0.0022	0.002	0.002	0.0016
0.0001	0.0009	0.0009	0.0009	0.0007	0.0008	0.0005	0.0006	0.0005
0	0	0	0	0	0	0	0	0
0.0007	0.0012	0.0009	0.0012	0.0009	0.0011	0.0013	0.0013	0.0011
0.0026	0.0047	0.0042	0.005	0.0039	0.0044	0.0049	0.0058	0.0061
0.0448	0.0572	0.0357	0.039	0.0244	0.0334	0.0289	0.0253	0.0196
0.0173	0.0346	0.0291	0.0323	0.0252	0.0305	0.0287	0.0319	0.0282
0.0489	0.0828	0.0554	0.0656	0.0465	0.0673	0.0627	0.0551	0.0406
0.0482	0.184	0.1645	0.1715	0.1338	0.1808	0.1385	0.1493	0.1174
0.0279	0.1973	0.1645	0.1474	0.1201	0.1641	0.1232	0.1476	0.1044
0.1902	0.3495	0.2506	0.2906	0.2081	0.2795	0.2631	0.2396	0.1923
0.4058	0.5477	0.3877	0.4333	0.2497	0.3157	0.3172	0.2501	0.1893
0.3638	0.5241	0.3364	0.3955	0.2432	0.3686	0.3933	0.3242	0.2197
0.4574	0.6674	0.4967	0.5111	0.3344	0.4272	0.396	0.3462	0.2582
0.5279	0.6821	0.4285	0.4957	0.3298	0.4982	0.472	0.4083	0.3296
0.3297	0.4838	0.3648	0.4213	0.2741	0.3293	0.3206	0.2538	0.1814
0.0756	0.1683	0.1414	0.1409	0.1047	0.1331	0.1066	0.1078	0.0857
0.3073	0.4375	0.329	0.3297	0.2002	0.2816	0.2981	0.2644	0.2134
0.6848	0.9146	0.6274	0.7158	0.4202	0.5622	0.5928	0.4763	0.3725
0.5176	0.7353	0.4076	0.5229	0.3512	0.5068	0.5306	0.4383	0.3315
0.1344	0.2103	0.1613	0.1948	0.1414	0.1686	0.1751	0.1718	0.1588
0.0111	0.016	0.0129	0.016	0.0135	0.0141	0.0151	0.0169	0.0164
0.0007	0.0012	0.001	0.0012	0.0009	0.001	0.0012	0.0013	0.0013
0	0	0	0	0	0	0	0	0
0.0007	0.001	0.0009	0.0011	0.0008	0.001	0.0013	0.0013	0.0013
0.0005	0.001	0.0008	0.0011	0.0009	0.0011	0.0012	0.0014	0.0015
0	0	0	0	0	0	0	0	0
0	0	0	0	0	0	0	0	0
0	0	0	0	0	0	0	0	0
0	0	0	0	0	0	0	0	0
0	0	0	0	0	0	0	0	0
0	0	0	0	0	0	0	0	0
0	0	0	0	0	0	0	0	0

0	0	0	0	0	0	0	0	0
0	0	0	0	0	0	0	0	0
0	0	0	0	0	0	0	0	0
0	0	0	0	0	0	0	0	0
0	0	0	0	0	0	0	0	0
0	0	0	0	0	0	0	0	0
0	0	0	0	0	0	0	0	0
0	0	0	0	0	0	0	0	0
0	0	0	0	0	0	0	0	0
0	0	0	0	0	0	0	0	0
0	0	0	0	0	0	0	0	0
0	0	0	0	0	0	0	0	0
0	0	0	0	0	0	0	0	0
0	0	0	0	0	0	0	0	0
0.0004	0.0016	0.0014	0.0015	0.0011	0.0014	0.0012	0.0014	0.0012
-0.0001	0.0015	0.0015	0.0018	0.0015	0.0018	0.0012	0.0013	0.0011
0.0002	0.0017	0.0014	0.0014	0.0009	0.0014	0.001	0.001	0.0008
0.0002	0.0013	0.0011	0.0013	0.001	0.0012	0.0011	0.0011	0.0009
0.0012	0.0021	0.0018	0.0021	0.0017	0.0019	0.002	0.002	0.002
0.0002	0.0013	0.001	0.0012	0.0011	0.0014	0.0012	0.0013	0.001
0.0008	0.0043	0.0037	0.0036	0.0026	0.0033	0.0027	0.0026	0.0018
0.0004	0.0058	0.0054	0.0057	0.0045	0.0062	0.004	0.0038	0.0023
0.0335	0.0489	0.0305	0.0355	0.021	0.0305	0.0276	0.019	0.0171
0.0038	0.0073	0.006	0.008	0.006	0.008	0.0093	0.011	0.0115
0.1311	0.2096	0.1559	0.1741	0.1278	0.1522	0.1272	0.1285	0.1042
0.1929	0.396	0.3348	0.3374	0.2435	0.3051	0.2719	0.2466	0.1882
0.0136	0.1634	0.1668	0.1387	0.1158	0.1733	0.1247	0.1121	0.0656
0.1148	0.2708	0.2086	0.2177	0.1433	0.1971	0.1497	0.1285	0.0775
0.4197	0.5792	0.4396	0.4839	0.3183	0.4248	0.4054	0.3341	0.2387
0.2366	0.3614	0.2945	0.2924	0.2048	0.2781	0.2712	0.2503	0.2129
0.4351	0.6415	0.4424	0.4578	0.2884	0.4294	0.4174	0.3181	0.2492
0.7036	0.9896	0.7393	0.7578	0.4742	0.6388	0.545	0.4258	0.3338
0.5783	0.8061	0.5809	0.6397	0.4358	0.5216	0.5119	0.3672	0.2823
0.1108	0.1881	0.1423	0.1555	0.128	0.1652	0.1578	0.1425	0.1245
0.0748	0.1615	0.1309	0.1479	0.1109	0.1361	0.1298	0.1271	0.1123
0.512	0.6886	0.5106	0.5744	0.3978	0.5288	0.5654	0.5552	0.4486
0.4543	0.6698	0.4608	0.523	0.3047	0.3957	0.3375	0.3513	0.3072
0.1221	0.1746	0.1267	0.1453	0.1084	0.1388	0.146	0.1483	0.1374
0.0069	0.0123	0.0117	0.0147	0.0113	0.0138	0.0148	0.0155	0.0161
0.0006	0.0012	0.001	0.0012	0.0009	0.0009	0.001	0.0012	0.0012
0	0	0	0	0	0	0	0	0
0.0008	0.0012	0.0011	0.0013	0.001	0.0011	0.0012	0.0013	0.0014
0.0011	0.0018	0.0016	0.002	0.0017	0.0018	0.002	0.0023	0.0023
0	0	0	0	0	0	0	0	0

0	0	0	0	0	0	0	0	0
0.0004	0.0007	0.0006	0.0006	0.0006	0.0006	0.0007	0.0008	0.0008
0	0	0	0	0	0	0	0	0
0	0	0	0	0	0	0	0	0
0	0	0	0	0	0	0	0	0
0	0	0	0	0	0	0	0	0
0	0	0	0	0	0	0	0	0
0	0	0	0	0	0	0	0	0
0	0	0	0	0	0	0	0	0
0	0	0	0	0	0	0	0	0
0	0	0	0	0	0	0	0	0
0	0	0	0	0	0	0	0	0
0	0	0	0	0	0	0	0	0
0	0	0	0	0	0	0	0	0
0	0	0	0	0	0	0	0	0
0	0	0	0	0	0	0	0	0
0	0	0	0	0	0	0	0	0
0	0	0	0	0	0	0	0	0
0	0	0	0	0	0	0	0	0
0	0	0	0	0	0	0	0	0
0	0	0	0	0	0	0	0	0
0	0	0	0	0	0	0	0	0
0	0	0	0	0	0	0	0	0
0	0	0	0	0	0	0	0	0
0	0	0	0	0	0	0	0	0
0	0	0	0	0	0	0	0	0
0	0	0	0	0	0	0	0	0
0	0	0	0	0	0	0	0	0
0.0005	0.0033	0.0032	0.0032	0.0026	0.0033	0.0027	0.0029	0.0023
0.1299	0.1945	0.1304	0.1428	0.0915	0.1156	0.1173	0.0954	0.0743
0.4222	0.5996	0.4282	0.4642	0.3132	0.3837	0.3321	0.3363	0.3046
0.1506	0.317	0.2485	0.2555	0.1811	0.2383	0.186	0.18	0.1235
0.0618	0.2163	0.1865	0.1986	0.1795	0.2203	0.18	0.1819	0.137
0.0569	0.2162	0.1805	0.183	0.1251	0.1457	0.1099	0.1158	0.0951
0.0152	0.1472	0.1338	0.1335	0.1076	0.1422	0.111	0.1095	0.0997
0.0404	0.1833	0.1599	0.1618	0.1148	0.1613	0.1326	0.1259	0.0885
0.2055	0.3285	0.249	0.2748	0.1873	0.2253	0.2226	0.1843	0.1332
0.4854	0.7128	0.4472	0.4897	0.3434	0.5415	0.5453	0.5105	0.4116
0.1804	0.3463	0.2327	0.2627	0.1437	0.1985	0.2137	0.176	0.1451
0.0339	0.1254	0.1098	0.1195	0.085	0.1048	0.0883	0.1028	0.094
0.3021	0.409	0.2947	0.3287	0.2379	0.2699	0.2804	0.2313	0.2098
0.4845	0.6759	0.4618	0.5141	0.3381	0.4368	0.4324	0.3649	0.2839
0.3172	0.4864	0.3544	0.4075	0.2857	0.3352	0.3552	0.3063	0.2608
0.1261	0.1899	0.1416	0.1647	0.1221	0.1479	0.1591	0.1502	0.1503

0.0061	0.0138	0.0129	0.0155	0.0112	0.0112	0.0105	0.0118	0.0101
0.0029	0.0043	0.0037	0.0038	0.0034	0.0038	0.004	0.0044	0.0043
0	0	0	0	0	0	0	0	0
0	0.003	0.0027	0.0025	0.0021	0.0026	0.0018	0.002	0.0017
0.0004	0.001	0.0008	0.0011	0.0008	0.001	0.001	0.0011	0.0013
0	0	0	0	0	0	0	0	0
0	0	0	0	0	0	0	0	0
0.0008	0.0012	0.0011	0.0013	0.001	0.0011	0.0012	0.0014	0.0014
0	0	0	0	0	0	0	0	0
0	0	0	0	0	0	0	0	0
0	0	0	0	0	0	0	0	0
0	0	0	0	0	0	0	0	0
0	0	0	0	0	0	0	0	0
0	0	0	0	0	0	0	0	0
0	0	0	0	0	0	0	0	0
0	0	0	0	0	0	0	0	0
0	0	0	0	0	0	0	0	0
0	0	0	0	0	0	0	0	0
0	0	0	0	0	0	0	0	0
0	0	0	0	0	0	0	0	0
0	0	0	0	0	0	0	0	0
0	0	0	0	0	0	0	0	0
0	0	0	0	0	0	0	0	0
0	0	0	0	0	0	0	0	0
0	0	0	0	0	0	0	0	0
0	0	0	0	0	0	0	0	0
0	0	0	0	0	0	0	0	0
0	0	0	0	0	0	0	0	0
0	0	0	0	0	0	0	0	0
0	0	0	0	0	0	0	0	0
0	0	0	0	0	0	0	0	0
0	0	0	0	0	0	0	0	0
0	0	0	0	0	0	0	0	0
0	0	0	0	0	0	0	0	0
0.1335	0.1659	0.1153	0.1104	0.0721	0.0959	0.0914	0.0762	0.0615
0.052	0.0726	0.0452	0.0493	0.0294	0.0429	0.04	0.0368	0.0281
0.2389	0.3439	0.2278	0.2786	0.1942	0.2739	0.2679	0.2063	0.1799
0.1509	0.3128	0.2598	0.2685	0.1996	0.2651	0.2414	0.1932	0.1112
0.0032	0.1965	0.1663	0.1816	0.1523	0.2003	0.147	0.1403	0.1084
0.0806	0.2435	0.1808	0.1868	0.1403	0.1708	0.1282	0.1308	0.0836
0.0046	0.1434	0.1156	0.1258	0.0739	0.1058	0.0811	0.0916	0.0691
0.1232	0.2599	0.2042	0.2126	0.1537	0.2123	0.1881	0.1746	0.1417
0.2216	0.3827	0.2822	0.3	0.2261	0.2751	0.2486	0.2061	0.145
0.254	0.4635	0.3142	0.3522	0.2228	0.3279	0.2718	0.2087	0.1553

0.0572	0.2009	0.1931	0.1964	0.1351	0.1609	0.1008	0.1127	0.0755
0.2097	0.3568	0.2864	0.3049	0.2028	0.2586	0.2504	0.223	0.1946
0.4939	0.7063	0.4725	0.5681	0.3193	0.4859	0.5203	0.3725	0.3088
0.5324	0.6745	0.4603	0.5394	0.3191	0.4517	0.4519	0.4285	0.4172
0.123	0.1869	0.1458	0.1747	0.131	0.1558	0.1748	0.1727	0.1605
0.0695	0.0985	0.0873	0.1	0.0828	0.0895	0.093	0.0962	0.0967
0.0083	0.0152	0.0128	0.0144	0.0107	0.0126	0.0127	0.0138	0.0133
0.0011	0.0019	0.0017	0.0023	0.0018	0.0019	0.0023	0.0025	0.0026
0	0	0	0	0	0	0	0	0
0.0005	0.0031	0.0031	0.0034	0.0027	0.0035	0.0026	0.003	0.0022
0.001	0.0016	0.0014	0.0017	0.0015	0.0015	0.0017	0.0019	0.002
0	0	0	0	0	0	0	0	0
-0.0002	0.0006	0.0006	0.0006	0.0005	0.0006	0.0004	0.0005	0.0003
0.0003	0.0005	0.0004	0.0006	0.0005	0.0005	0.0007	0.0008	0.0008
0	0	0	0	0	0	0	0	0
0	0	0	0	0	0	0	0	0
0	0	0	0	0	0	0	0	0
0	0	0	0	0	0	0	0	0
0	0	0	0	0	0	0	0	0
0	0	0	0	0	0	0	0	0
0	0	0	0	0	0	0	0	0
0	0	0	0	0	0	0	0	0
0	0	0	0	0	0	0	0	0
0	0	0	0	0	0	0	0	0
0	0	0	0	0	0	0	0	0
0	0	0	0	0	0	0	0	0
0	0	0	0	0	0	0	0	0
0	0	0	0	0	0	0	0	0
0	0	0	0	0	0	0	0	0
0	0	0	0	0	0	0	0	0
0	0	0	0	0	0	0	0	0
0	0	0	0	0	0	0	0	0
0	0	0	0	0	0	0	0	0
0	0	0	0	0	0	0	0	0
0	0	0	0	0	0	0	0	0
0	0	0	0	0	0	0	0	0
0	0	0	0	0	0	0	0	0
0	0	0	0	0	0	0	0	0
0	0	0	0	0	0	0	0	0
0	0	0	0	0	0	0	0	0
0.0007	0.0087	0.0085	0.0089	0.0078	0.0112	0.0092	0.0085	0.0067
0.0099	0.0158	0.014	0.0178	0.0142	0.017	0.0189	0.0219	0.0216
0.0248	0.0504	0.0386	0.0457	0.0319	0.0402	0.0406	0.0363	0.0335
0.0222	0.1314	0.1086	0.1289	0.0894	0.1161	0.0984	0.0998	0.0678

0.0037	0.1647	0.1521	0.1449	0.1237	0.1746	0.1397	0.1514	0.1397
0.1381	0.3427	0.2864	0.2798	0.203	0.2564	0.2142	0.2076	0.1581
0.1928	0.3594	0.271	0.29	0.2125	0.3056	0.2373	0.2152	0.1596
0.0958	0.2403	0.183	0.1841	0.1314	0.1779	0.1173	0.1182	0.0996
0.9186	1.2033	0.9229	1.14	0.78	0.8477	0.8381	0.7115	0.7532
0.6724	0.9555	0.6456	0.7188	0.5194	0.6798	0.6511	0.4921	0.3508
0.1543	0.2766	0.2218	0.2392	0.1813	0.2205	0.2045	0.1967	0.1528
0.0906	0.2265	0.1789	0.195	0.1427	0.179	0.15	0.1511	0.1094
0.3838	0.6008	0.4422	0.4854	0.3002	0.3723	0.3805	0.33	0.2768
0.2358	0.3223	0.2361	0.2707	0.1942	0.2479	0.2492	0.2176	0.1789
0.0227	0.0323	0.0272	0.0324	0.0246	0.0273	0.0334	0.0366	0.038
0.0648	0.1109	0.0762	0.0831	0.0516	0.0797	0.0846	0.0687	0.0525
0.0051	0.0116	0.0107	0.0111	0.0086	0.0096	0.0095	0.0098	0.0095
0.0004	0.0007	0.0006	0.0007	0.0006	0.0007	0.0008	0.0008	0.0008
0	0	0	0	0	0	0	0	0
0.0007	0.0033	0.0026	0.0027	0.0022	0.003	0.0025	0.0026	0.002
0.0008	0.0015	0.0013	0.0017	0.0015	0.0017	0.0019	0.0023	0.0021
0	0	0	0	0	0	0	0	0
0.0001	0.001	0.0009	0.0009	0.0006	0.0008	0.0006	0.0006	0.0005
0.0002	0.0005	0.0005	0.0006	0.0005	0.0006	0.0007	0.0008	0.0008
0	0	0	0	0	0	0	0	0
0	0	0	0	0	0	0	0	0
0	0	0	0	0	0	0	0	0
0	0	0	0	0	0	0	0	0
0	0	0	0	0	0	0	0	0
0	0	0	0	0	0	0	0	0
0	0	0	0	0	0	0	0	0
0	0	0	0	0	0	0	0	0
0	0	0	0	0	0	0	0	0
0	0	0	0	0	0	0	0	0
0	0	0	0	0	0	0	0	0
0	0	0	0	0	0	0	0	0
0	0	0	0	0	0	0	0	0
0	0	0	0	0	0	0	0	0
0	0	0	0	0	0	0	0	0
0	0	0	0	0	0	0	0	0
0	0	0	0	0	0	0	0	0
0	0	0	0	0	0	0	0	0
0	0	0	0	0	0	0	0	0
0	0	0	0	0	0	0	0	0
0	0	0	0	0	0	0	0	0
0	0	0	0	0	0	0	0	0
0	0	0	0	0	0	0	0	0
0	0	0	0	0	0	0	0	0

0	0	0	0	0	0	0	0	0
0	0	0	0	0	0	0	0	0
0.1095	0.1386	0.1004	0.1093	0.0725	0.0847	0.0859	0.0612	0.0464
0.1413	0.1855	0.1433	0.1627	0.124	0.1454	0.154	0.147	0.1361
0.1582	0.1998	0.1223	0.15	0.0925	0.1417	0.1344	0.1031	0.0675
0.0879	0.1568	0.1224	0.131	0.0919	0.1214	0.1169	0.1024	0.0873
0.0917	0.1909	0.1489	0.1611	0.127	0.1598	0.1435	0.1505	0.1389
0.4898	0.7256	0.5005	0.5499	0.3568	0.4909	0.4298	0.3552	0.271
0.5305	0.8213	0.5768	0.6302	0.4118	0.5655	0.6013	0.4685	0.3908
0.3418	0.5181	0.3655	0.3815	0.2709	0.3427	0.3513	0.2945	0.2169
0.7906	1.0465	0.732	0.8278	0.5652	0.6813	0.6487	0.5058	0.3889
0.6932	1.0287	0.7615	0.8681	0.6183	0.7733	0.7579	0.619	0.5991
0.326	0.4791	0.2831	0.3199	0.1438	0.2329	0.2629	0.1841	0.1717
0.034	0.1213	0.0979	0.102	0.0804	0.1078	0.0939	0.1122	0.1012
0.0558	0.1222	0.108	0.1203	0.0953	0.1128	0.1153	0.1194	0.1099
0.0808	0.1406	0.1146	0.1231	0.0877	0.1164	0.1117	0.1112	0.1005
0.0157	0.079	0.0679	0.073	0.0488	0.075	0.0553	0.0496	0.0385
0.0542	0.1474	0.119	0.1274	0.0759	0.1135	0.0932	0.0829	0.0542
0.0005	0.0066	0.0061	0.0065	0.0057	0.007	0.0059	0.0058	0.0046
0	0	0	0	0	0	0	0	0
0	0	0	0	0	0	0	0	0
0.0001	0.0026	0.0026	0.0026	0.0019	0.0027	0.0021	0.0023	0.0019
0.0008	0.0016	0.0016	0.0019	0.0015	0.0017	0.0018	0.002	0.002
0.0005	0.0007	0.0007	0.0007	0.0006	0.0006	0.0006	0.0007	0.0006
0	0.0008	0.0007	0.0008	0.0006	0.0007	0.0005	0.0005	0.0002
0	0	0	0	0	0	0	0	0
0	0	0	0	0	0	0	0	0
0	0	0	0	0	0	0	0	0
0	0	0	0	0	0	0	0	0
0	0	0	0	0	0	0	0	0
0	0	0	0	0	0	0	0	0
0	0	0	0	0	0	0	0	0
0	0	0	0	0	0	0	0	0
0	0	0	0	0	0	0	0	0
0	0	0	0	0	0	0	0	0
0	0	0	0	0	0	0	0	0
0	0	0	0	0	0	0	0	0
0	0	0	0	0	0	0	0	0
0	0	0	0	0	0	0	0	0
0	0	0	0	0	0	0	0	0
0	0	0	0	0	0	0	0	0
0	0	0	0	0	0	0	0	0
0	0	0	0	0	0	0	0	0
0	0	0	0	0	0	0	0	0

0	0	0	0	0	0	0	0	0
0	0	0	0	0	0	0	0	0
0	0	0	0	0	0	0	0	0
0	0	0	0	0	0	0	0	0
0	0	0	0	0	0	0	0	0
0	0	0	0	0	0	0	0	0
0	0	0	0	0	0	0	0	0
0	0	0	0	0	0	0	0	0
0.014	0.0217	0.0186	0.0206	0.017	0.0184	0.021	0.0242	0.0261
0.0835	0.1405	0.111	0.1314	0.1018	0.1252	0.1347	0.1356	0.1218
0.0779	0.1153	0.0835	0.0937	0.0674	0.0822	0.0792	0.0691	0.0563
0.0151	0.0232	0.0176	0.0204	0.0152	0.0179	0.0195	0.0196	0.0185
0.1044	0.1489	0.0952	0.1045	0.0686	0.097	0.1041	0.0906	0.0799
0.6387	0.8083	0.4834	0.5595	0.3872	0.5075	0.4703	0.4253	0.3168
0.8927	1.1028	0.7683	0.8554	0.5973	0.865	0.8307	0.639	0.5347
0.8078	1.1029	0.7712	0.8843	0.615	0.8057	0.7403	0.5721	0.4632
0.5691	0.7319	0.4454	0.4843	0.2237	0.2921	0.3216	0.2501	0.2121
0.9056	1.0763	0.7838	0.8244	0.5552	0.6948	0.7189	0.6328	0.5199
0.4083	0.6492	0.493	0.4903	0.3039	0.4237	0.3483	0.2723	0.1632
0.0119	0.1208	0.103	0.1145	0.0844	0.1232	0.0956	0.0964	0.0621
0.0257	0.0368	0.0287	0.0328	0.0234	0.0282	0.0313	0.0295	0.027
0.047	0.0952	0.0696	0.0801	0.0577	0.0744	0.0634	0.0559	0.0467
0.0071	0.0925	0.0654	0.0736	0.0544	0.1014	0.0777	0.0823	0.0664
0.0075	0.0444	0.0357	0.0388	0.0294	0.0354	0.0272	0.0236	0.0096
-0.0009	0.0053	0.0048	0.0058	0.0043	0.0061	0.0038	0.0039	0.0022
0	0	0	0	0	0	0	0	0
0	0	0	0	0	0	0	0	0
0.0006	0.0025	0.0022	0.0023	0.002	0.0024	0.002	0.0023	0.0019
0.0005	0.001	0.0009	0.0011	0.001	0.001	0.0012	0.0014	0.0014
0.0002	0.0005	0.0004	0.0006	0.0005	0.0006	0.0007	0.0007	0.0006
0.0001	0.0009	0.0007	0.0007	0.0004	0.0006	0.0003	0.0004	0.0002
0	0	0	0	0	0	0	0	0
0	0	0	0	0	0	0	0	0
0	0	0	0	0	0	0	0	0
0	0	0	0	0	0	0	0	0
0	0	0	0	0	0	0	0	0
0	0	0	0	0	0	0	0	0
0	0	0	0	0	0	0	0	0
0	0	0	0	0	0	0	0	0
0	0	0	0	0	0	0	0	0
0	0	0	0	0	0	0	0	0
0	0	0	0	0	0	0	0	0
0	0	0	0	0	0	0	0	0
0	0	0	0	0	0	0	0	0

0	0	0	0	0	0	0	0	0
0	0	0	0	0	0	0	0	0
0	0	0	0	0	0	0	0	0
0	0	0	0	0	0	0	0	0
0	0	0	0	0	0	0	0	0
0	0	0	0	0	0	0	0	0
0	0	0	0	0	0	0	0	0
0	0	0	0	0	0	0	0	0
0	0	0	0	0	0	0	0	0
0	0	0	0	0	0	0	0	0
0	0	0	0	0	0	0	0	0
0	0	0	0	0	0	0	0	0
0	0	0	0	0	0	0	0	0
0	0	0	0	0	0	0	0	0
0.0408	0.0696	0.0524	0.0622	0.0421	0.0554	0.0509	0.0483	0.0467
0.1136	0.1995	0.1483	0.1658	0.126	0.1656	0.1641	0.1582	0.1406
0.0602	0.106	0.086	0.1015	0.0753	0.0882	0.0925	0.1019	0.0918
0.1093	0.1563	0.1175	0.139	0.1079	0.1257	0.1347	0.13	0.1208
0.045	0.0687	0.0506	0.0593	0.0442	0.0589	0.0631	0.0575	0.0536
0.1059	0.1365	0.1035	0.1189	0.0876	0.1047	0.107	0.0833	0.0756
0.3078	0.4519	0.3079	0.3426	0.2187	0.2868	0.3132	0.2983	0.2294
0.3405	0.4502	0.3354	0.3736	0.2565	0.2884	0.2679	0.2047	0.1516
0.6543	0.9622	0.7441	0.773	0.4992	0.6913	0.7036	0.5943	0.477
0.3053	0.4843	0.309	0.3771	0.2444	0.3383	0.3468	0.2885	0.24
0.0324	0.1529	0.1511	0.1556	0.1131	0.1309	0.0942	0.1031	0.083
0.0948	0.2056	0.1529	0.164	0.1201	0.146	0.1157	0.1133	0.076
0.0796	0.1414	0.1193	0.1235	0.0812	0.097	0.08	0.0565	0.0319
-0.0032	0.0177	0.0132	0.0164	0.0109	0.0174	0.0119	0.012	0.0092
0.0431	0.079	0.0506	0.0597	0.0332	0.0513	0.0467	0.0426	0.0345
0.0165	0.0437	0.0284	0.031	0.0184	0.0312	0.0285	0.0284	0.0258
-0.0001	0.0082	0.0082	0.0081	0.0061	0.0088	0.0063	0.0058	0.0036
-0.0001	0.0006	0.0005	0.0005	0.0003	0.0004	0.0002	0.0003	0.0001
0	0	0	0	0	0	0	0	0
-0.0003	0.0013	0.0013	0.0014	0.0011	0.0016	0.001	0.001	0.0006
0.0002	0.0005	0.0004	0.0004	0.0003	0.0004	0.0005	0.0006	0.0006
0.0003	0.0005	0.0005	0.0006	0.0005	0.0005	0.0006	0.0006	0.0006
0.0004	0.0014	0.0013	0.0012	0.001	0.0013	0.0012	0.0012	0.0011
0.0004	0.0006	0.0005	0.0006	0.0005	0.0006	0.0007	0.0007	0.0007
0	0	0	0	0	0	0	0	0
0	0	0	0	0	0	0	0	0
0	0	0	0	0	0	0	0	0
0	0	0	0	0	0	0	0	0
0	0	0	0	0	0	0	0	0
0	0	0	0	0	0	0	0	0

0	0	0	0	0	0	0	0	0
0	0	0	0	0	0	0	0	0
0	0	0	0	0	0	0	0	0
0	0	0	0	0	0	0	0	0
0	0	0	0	0	0	0	0	0
0	0	0	0	0	0	0	0	0
0	0	0	0	0	0	0	0	0
0	0	0	0	0	0	0	0	0
0	0	0	0	0	0	0	0	0
0	0	0	0	0	0	0	0	0
0	0	0	0	0	0	0	0	0
0	0	0	0	0	0	0	0	0
0	0	0	0	0	0	0	0	0
0	0	0	0	0	0	0	0	0
0	0	0	0	0	0	0	0	0
0	0	0	0	0	0	0	0	0
0	0	0	0	0	0	0	0	0
0	0	0	0	0	0	0	0	0
0	0	0	0	0	0	0	0	0
0	0	0	0	0	0	0	0	0
0.0417	0.0682	0.0427	0.05	0.0307	0.0466	0.0451	0.0366	0.0297
0.1768	0.2715	0.1993	0.2351	0.1529	0.2104	0.2038	0.183	0.1494
0.1057	0.153	0.1119	0.1258	0.0962	0.1147	0.1217	0.1162	0.0982
0.2346	0.3086	0.2346	0.2566	0.1812	0.2148	0.2164	0.2166	0.1917
0.1672	0.2301	0.1691	0.1758	0.1216	0.1634	0.1594	0.1519	0.1392
0.0045	0.019	0.017	0.0185	0.0156	0.0196	0.017	0.0185	0.0181
0.0344	0.0864	0.0733	0.0839	0.0705	0.0782	0.0743	0.075	0.0733
0.0485	0.0916	0.0697	0.0759	0.0583	0.0762	0.0725	0.0682	0.0566
0.2029	0.3285	0.2236	0.2185	0.1542	0.1976	0.1881	0.1664	0.1271
0.003	0.02	0.0179	0.0196	0.015	0.019	0.0162	0.018	0.0148
0.0022	0.0039	0.0034	0.0043	0.0037	0.0038	0.0043	0.0046	0.0045
0.1858	0.2642	0.1839	0.2165	0.1428	0.1802	0.1688	0.1456	0.1175
0.0894	0.2408	0.1939	0.1977	0.1372	0.169	0.1502	0.1255	0.0896
0.0403	0.1545	0.1243	0.1298	0.0801	0.1194	0.0862	0.0753	0.0587
0.2288	0.3528	0.2504	0.2599	0.1606	0.2187	0.2298	0.1816	0.1707
0.0438	0.1373	0.11	0.1121	0.0731	0.111	0.093	0.0804	0.053
0.0024	0.0102	0.0104	0.0094	0.0082	0.0103	0.0087	0.009	0.0064
0.0009	0.0026	0.0023	0.0023	0.0018	0.0022	0.0018	0.0019	0.0014
0.0006	0.0015	0.0014	0.0014	0.0011	0.0015	0.0013	0.0014	0.0013
-0.0002	0.0013	0.0011	0.0011	0.0008	0.0013	0.0009	0.0008	0.0007
0	0	0	0	0	0	0	0	0
0.0004	0.0006	0.0005	0.0006	0.0005	0.0006	0.0007	0.0007	0.0007
0.0008	0.002	0.0017	0.0019	0.0014	0.0019	0.0016	0.0016	0.0014
0.0005	0.001	0.001	0.0013	0.001	0.0011	0.0013	0.0013	0.0014

0	0	0	0	0	0	0	0	0
0	0	0	0	0	0	0	0	0
0	0	0	0	0	0	0	0	0
0	0	0	0	0	0	0	0	0
0	0	0	0	0	0	0	0	0
0	0	0	0	0	0	0	0	0
0	0	0	0	0	0	0	0	0
0	0	0	0	0	0	0	0	0
0	0	0	0	0	0	0	0	0
0	0	0	0	0	0	0	0	0
0	0	0	0	0	0	0	0	0
0	0	0	0	0	0	0	0	0
0	0	0	0	0	0	0	0	0
0	0	0	0	0	0	0	0	0
0	0	0	0	0	0	0	0	0
0	0	0	0	0	0	0	0	0
0	0	0	0	0	0	0	0	0
0	0	0	0	0	0	0	0	0
0	0	0	0	0	0	0	0	0
0	0	0	0	0	0	0	0	0
0	0	0	0	0	0	0	0	0
0	0	0	0	0	0	0	0	0
0	0	0	0	0	0	0	0	0
0	0	0	0	0	0	0	0	0
0	0	0	0	0	0	0	0	0
0	0	0	0	0	0	0	0	0
0.0913	0.1448	0.0923	0.1167	0.0597	0.0891	0.0957	0.0734	0.0491
0.3931	0.5002	0.347	0.3835	0.2287	0.3022	0.3034	0.2128	0.1448
0.1402	0.1967	0.1237	0.1358	0.0691	0.1108	0.121	0.1108	0.1063
0.0317	0.0491	0.0407	0.0403	0.0279	0.0336	0.0322	0.0279	0.0233
0.0655	0.0995	0.0699	0.0746	0.0464	0.066	0.0651	0.0595	0.0562
0.0077	0.0145	0.0138	0.0149	0.0133	0.0147	0.0159	0.018	0.0168
0.1434	0.2213	0.1621	0.1822	0.122	0.1558	0.1643	0.1532	0.1313
0.1797	0.2912	0.2059	0.2115	0.1316	0.167	0.1767	0.146	0.1139
0.0995	0.1515	0.113	0.1309	0.0952	0.121	0.127	0.1153	0.1022
0.256	0.3383	0.2144	0.2493	0.1626	0.2381	0.2348	0.2108	0.169
0.5032	0.6757	0.5081	0.5398	0.3829	0.4719	0.4388	0.3448	0.2759
0.4019	0.5262	0.307	0.3824	0.2313	0.3472	0.3393	0.2515	0.1729
0.0075	0.0347	0.0318	0.0336	0.0274	0.0347	0.0285	0.0312	0.0274
0.2454	0.3379	0.2477	0.2415	0.1622	0.2029	0.1942	0.1637	0.1395
0.5205	0.6793	0.4654	0.46	0.292	0.3978	0.4346	0.3429	0.2345
0.1934	0.2724	0.1943	0.1802	0.1246	0.1602	0.1611	0.1443	0.1226
0.0032	0.0136	0.0122	0.0113	0.0088	0.0105	0.0087	0.0092	0.0074
0.0003	0.0031	0.0029	0.0032	0.0024	0.0031	0.0023	0.0025	0.0022

0.0008	0.0019	0.0017	0.0019	0.0014	0.0018	0.0017	0.0018	0.0016
0.0003	0.0018	0.0019	0.0019	0.0013	0.0018	0.0014	0.0015	0.0012
0	0	0	0	0	0	0	0	0
0	0	0	0	0	0	0	0	0
0.0008	0.0021	0.0019	0.0021	0.0018	0.0021	0.0021	0.0022	0.0019
0.0005	0.001	0.0009	0.0011	0.0009	0.0009	0.0012	0.0013	0.0012
0	0	0	0	0	0	0	0	0
0	0	0	0	0	0	0	0	0
0	0	0	0	0	0	0	0	0
0	0	0	0	0	0	0	0	0
0	0	0	0	0	0	0	0	0
0	0	0	0	0	0	0	0	0
0	0	0	0	0	0	0	0	0
0	0	0	0	0	0	0	0	0
0	0	0	0	0	0	0	0	0
0	0	0	0	0	0	0	0	0
0	0	0	0	0	0	0	0	0
0	0	0	0	0	0	0	0	0
0	0	0	0	0	0	0	0	0
0	0	0	0	0	0	0	0	0
0	0	0	0	0	0	0	0	0
0	0	0	0	0	0	0	0	0
0	0	0	0	0	0	0	0	0
0	0	0	0	0	0	0	0	0
0	0	0	0	0	0	0	0	0
0	0	0	0	0	0	0	0	0
0	0	0	0	0	0	0	0	0
0	0	0	0	0	0	0	0	0
0	0	0	0	0	0	0	0	0
0	0	0	0	0	0	0	0	0
0	0	0	0	0	0	0	0	0
0	0	0	0	0	0	0	0	0
0.0317	0.0395	0.0263	0.0312	0.0199	0.0288	0.0318	0.0294	0.0225
0.0366	0.0443	0.0311	0.0343	0.0224	0.0306	0.0322	0.024	0.0195
0.0288	0.0398	0.0232	0.0257	0.016	0.0215	0.0234	0.0195	0.018
0.0265	0.036	0.0222	0.0239	0.0132	0.0192	0.0192	0.0136	0.0076
0.0397	0.0506	0.037	0.044	0.0278	0.0337	0.0307	0.023	0.0213
0.0336	0.0464	0.0336	0.039	0.0274	0.0341	0.0339	0.0276	0.023
0.0324	0.0456	0.0319	0.037	0.0245	0.0331	0.0346	0.0292	0.0246
0.0352	0.0501	0.037	0.0412	0.0292	0.0356	0.0379	0.0331	0.0306
0.0609	0.0767	0.0571	0.0621	0.0415	0.0494	0.0449	0.0414	0.0309
0.0481	0.0728	0.0514	0.0616	0.0412	0.0503	0.0495	0.0427	0.0329
0.0561	0.0756	0.0518	0.0593	0.0392	0.0453	0.0496	0.0393	0.0278
0.0487	0.0629	0.0434	0.0489	0.0254	0.0382	0.0392	0.0324	0.0238

0.0605	0.079	0.0559	0.0591	0.0352	0.0487	0.0459	0.0376	0.028
0.0527	0.0693	0.0445	0.0527	0.0291	0.0366	0.0386	0.0328	0.0311
0.0545	0.0704	0.0492	0.0558	0.0403	0.0493	0.0476	0.0396	0.0306
0.0583	0.0689	0.0399	0.0436	0.0291	0.0432	0.0427	0.0329	0.0214
0	0	0	0	0	0	0	0	0
0	0	0	0	0	0	0	0	0
0	0	0	0	0	0	0	0	0
0	0	0	0	0	0	0	0	0
0	0	0	0	0	0	0	0	0
0	0	0	0	0	0	0	0	0
0	0	0	0	0	0	0	0	0
0	0	0	0	0	0	0	0	0
0	0	0	0	0	0	0	0	0
0	0	0	0	0	0	0	0	0
0	0	0	0	0	0	0	0	0
0	0	0	0	0	0	0	0	0
0	0	0	0	0	0	0	0	0
0	0	0	0	0	0	0	0	0
0	0	0	0	0	0	0	0	0
0	0	0	0	0	0	0	0	0
0	0	0	0	0	0	0	0	0
0	0	0	0	0	0	0	0	0
0	0	0	0	0	0	0	0	0
0	0	0	0	0	0	0	0	0
0	0	0	0	0	0	0	0	0
0	0	0	0	0	0	0	0	0
0	0	0	0	0	0	0	0	0
0	0	0	0	0	0	0	0	0
0	0	0	0	0	0	0	0	0
0	0	0	0	0	0	0	0	0
0	0	0	0	0	0	0	0	0
0	0	0	0	0	0	0	0	0
0	0	0	0	0	0	0	0	0
0	0	0	0	0	0	0	0	0
0	0	0	0	0	0	0	0	0
0	0	0	0	0	0	0	0	0
0	0	0	0	0	0	0	0	0
0.0191	0.0241	0.0177	0.0175	0.0114	0.0163	0.0157	0.0134	0.013
0.0182	0.0229	0.0161	0.0179	0.0115	0.015	0.0158	0.0123	0.008
0.016	0.0207	0.0138	0.0145	0.009	0.0125	0.0119	0.0099	0.0074
0.0165	0.0211	0.0142	0.0163	0.009	0.0134	0.0122	0.0082	0.0062
0.0215	0.025	0.0171	0.0179	0.0101	0.014	0.0147	0.0103	0.0072
0.0165	0.0217	0.015	0.016	0.0094	0.0133	0.013	0.0102	0.0077

0.0187	0.0239	0.017	0.0194	0.0121	0.0155	0.0154	0.0141	0.0108
0.0195	0.0263	0.0188	0.0219	0.0158	0.0187	0.0176	0.015	0.0135
0.0144	0.0209	0.0127	0.0159	0.0112	0.0146	0.0137	0.0115	0.0103
0.0197	0.0241	0.018	0.0196	0.0121	0.0156	0.0162	0.0131	0.009
0.0171	0.0217	0.0157	0.0164	0.0108	0.0151	0.0136	0.0106	0.0098
0.0166	0.0228	0.0154	0.0171	0.0132	0.0169	0.0159	0.0138	0.0092
0.0164	0.0213	0.0153	0.0183	0.0119	0.0153	0.0155	0.0143	0.0112
0.0137	0.0195	0.0136	0.0159	0.0121	0.0148	0.0158	0.0134	0.0118
0.0184	0.0228	0.0161	0.0169	0.0116	0.0152	0.0156	0.0113	0.0074
0.0141	0.0198	0.0134	0.0144	0.0084	0.0099	0.0097	0.0075	0.0067
0	0	0	0	0	0	0	0	0
0	0	0	0	0	0	0	0	0
0	0	0	0	0	0	0	0	0
0	0	0	0	0	0	0	0	0
0	0	0	0	0	0	0	0	0
0	0	0	0	0	0	0	0	0
0	0	0	0	0	0	0	0	0
0	0	0	0	0	0	0	0	0
0	0	0	0	0	0	0	0	0
0	0	0	0	0	0	0	0	0
0	0	0	0	0	0	0	0	0
0	0	0	0	0	0	0	0	0
0	0	0	0	0	0	0	0	0
0	0	0	0	0	0	0	0	0
0	0	0	0	0	0	0	0	0
0	0	0	0	0	0	0	0	0
0	0	0	0	0	0	0	0	0
0	0	0	0	0	0	0	0	0
0	0	0	0	0	0	0	0	0
0	0	0	0	0	0	0	0	0
0	0	0	0	0	0	0	0	0
0	0	0	0	0	0	0	0	0
0	0	0	0	0	0	0	0	0
0	0	0	0	0	0	0	0	0
0	0	0	0	0	0	0	0	0
0	0	0	0	0	0	0	0	0
0	0	0	0	0	0	0	0	0
0	0	0	0	0	0	0	0	0
0	0	0	0	0	0	0	0	0
0	0	0	0	0	0	0	0	0
0	0	0	0	0	0	0	0	0
0	0	0	0	0	0	0	0	0
0	0	0	0	0	0	0	0	0
0	0	0	0	0	0	0	0	0

0.0003	0.0005	0.0004	0.0005	0.0004	0.0005	0.0006	0.0006	0.0007
0	0	0	0	0	0	0	0	0
0	0	0	0	0	0	0	0	0
0	0	0	0	0	0	0	0	0
0	0	0	0	0	0	0	0	0
0	0	0	0	0	0	0	0	0
0	0	0	0	0	0	0	0	0
0	0	0	0	0	0	0	0	0
0	0	0	0	0	0	0	0	0
0	0	0	0	0	0	0	0	0
0	0	0	0	0	0	0	0	0
0	0	0	0	0	0	0	0	0
0	0	0	0	0	0	0	0	0
0.0002	0.0005	0.0004	0.0005	0.0004	0.0005	0.0005	0.0006	0.0006
0.0004	0.0006	0.0006	0.0007	0.0005	0.0005	0.0006	0.0006	0.0006
0.0004	0.0006	0.0005	0.0005	0.0004	0.0004	0.0005	0.0006	0.0006
0	0	0	0	0	0	0	0	0
0	0	0	0	0	0	0	0	0
0	0	0	0	0	0	0	0	0
0	0	0	0	0	0	0	0	0
0	0	0	0	0	0	0	0	0
0	0	0	0	0	0	0	0	0
0	0	0	0	0	0	0	0	0
0	0	0	0	0	0	0	0	0
0	0	0	0	0	0	0	0	0
0	0	0	0	0	0	0	0	0
0	0	0	0	0	0	0	0	0
0	0	0	0	0	0	0	0	0
0	0	0	0	0	0	0	0	0
0	0	0	0	0	0	0	0	0
0	0	0	0	0	0	0	0	0
0	0	0	0	0	0	0	0	0
0	0	0	0	0	0	0	0	0
0	0	0	0	0	0	0	0	0
0	0	0	0	0	0	0	0	0
0	0	0	0	0	0	0	0	0
0	0	0	0	0	0	0	0	0
0	0	0	0	0	0	0	0	0
0	0	0	0	0	0	0	0	0
0	0	0	0	0	0	0	0	0
0	0	0	0	0	0	0	0	0
0	0	0	0	0	0	0	0	0
0	0	0	0	0	0	0	0	0
0	0	0	0	0	0	0	0	0

0	0	0	0	0	0	0	0	0
0	0	0	0	0	0	0	0	0
0	0	0	0	0	0	0	0	0
0	0	0	0	0	0	0	0	0
0	0	0	0	0	0	0	0	0
0	0	0	0	0	0	0	0	0
0.0006	0.001	0.0008	0.001	0.0009	0.001	0.001	0.0012	0.0012
0.0008	0.0012	0.0009	0.0012	0.0011	0.0012	0.0013	0.0015	0.0015
0.0005	0.0007	0.0006	0.0006	0.0005	0.0005	0.0006	0.0007	0.0007
0.0004	0.0006	0.0005	0.0006	0.0005	0.0006	0.0007	0.0007	0.0007
0	0	0	0	0	0	0	0	0
0	0	0	0	0	0	0	0	0
0	0	0	0	0	0	0	0	0
0	0	0	0	0	0	0	0	0
0	0	0	0	0	0	0	0	0
0	0	0	0	0	0	0	0	0
0	0	0	0	0	0	0	0	0
0	0	0	0	0	0	0	0	0
0	0	0	0	0	0	0	0	0
0.0003	0.0006	0.0005	0.0006	0.0005	0.0005	0.0006	0.0006	0.0006
0.0003	0.0006	0.0005	0.0006	0.0005	0.0005	0.0006	0.0007	0.0007
0.0002	0.0004	0.0004	0.0004	0.0003	0.0004	0.0004	0.0006	0.0006
0	0	0	0	0	0	0	0	0
0	0	0	0	0	0	0	0	0
0	0	0	0	0	0	0	0	0
0	0	0	0	0	0	0	0	0
0	0	0	0	0	0	0	0	0
0	0	0	0	0	0	0	0	0
0	0	0	0	0	0	0	0	0
0	0	0	0	0	0	0	0	0
0	0	0	0	0	0	0	0	0
0	0	0	0	0	0	0	0	0
0	0	0	0	0	0	0	0	0
0	0	0	0	0	0	0	0	0
0	0	0	0	0	0	0	0	0
0	0	0	0	0	0	0	0	0
0	0	0	0	0	0	0	0	0
0	0	0	0	0	0	0	0	0
0	0	0	0	0	0	0	0	0
0	0	0	0	0	0	0	0	0
0	0	0	0	0	0	0	0	0
0	0	0	0	0	0	0	0	0
0	0	0	0	0	0	0	0	0
0	0	0	0	0	0	0	0	0

0	0	0	0	0	0	0	0	0
0	0	0	0	0	0	0	0	0
0	0	0	0	0	0	0	0	0
0	0	0	0	0	0	0	0	0
0	0	0	0	0	0	0	0	0
0	0	0	0	0	0	0	0	0
0	0	0	0	0	0	0	0	0
0	0	0	0	0	0	0	0	0
0	0	0	0	0	0	0	0	0
0	0	0	0	0	0	0	0	0
0	0	0	0	0	0	0	0	0
0	0	0	0	0	0	0	0	0
0.0066	0.0095	0.0068	0.0075	0.005	0.0065	0.0068	0.0054	0.0047
0.0077	0.0092	0.0068	0.0087	0.0059	0.0078	0.0083	0.0065	0.0051
0.0082	0.011	0.0075	0.0087	0.0068	0.0079	0.0077	0.0062	0.0053
0.0099	0.0121	0.0096	0.0094	0.0058	0.0072	0.0063	0.0057	0.0051
0.0083	0.011	0.0075	0.0081	0.0056	0.0069	0.0073	0.0063	0.0049
0.0099	0.0126	0.0087	0.0094	0.0063	0.0088	0.0086	0.0075	0.0061
0.009	0.0114	0.008	0.0094	0.0063	0.0086	0.0086	0.0072	0.0056
0.0085	0.0123	0.0081	0.0085	0.0061	0.0078	0.0079	0.0068	0.0055
0.007	0.01	0.0067	0.0076	0.0037	0.0048	0.0049	0.0034	0.0033
0.0065	0.0091	0.0052	0.0071	0.0046	0.0062	0.0063	0.0053	0.0042
0.0093	0.0123	0.0089	0.0097	0.0069	0.0092	0.0085	0.0074	0.0063
0.0075	0.0103	0.0073	0.0081	0.0058	0.0073	0.0081	0.0069	0.0062
0.0082	0.0117	0.0082	0.0091	0.0053	0.0068	0.0069	0.006	0.0046
0.0064	0.0086	0.0055	0.0069	0.0033	0.0059	0.0058	0.0042	0.0028
0.0091	0.0109	0.0074	0.0089	0.0058	0.0071	0.007	0.0049	0.0038
0.0078	0.0101	0.0074	0.008	0.0052	0.0068	0.0072	0.0055	0.0042
0	0	0	0	0	0	0	0	0
0	0	0	0	0	0	0	0	0
0	0	0	0	0	0	0	0	0
0	0	0	0	0	0	0	0	0
0	0	0	0	0	0	0	0	0
0	0	0	0	0	0	0	0	0
0	0	0	0	0	0	0	0	0
0	0	0	0	0	0	0	0	0
0	0	0	0	0	0	0	0	0
0	0	0	0	0	0	0	0	0
0	0	0	0	0	0	0	0	0
0	0	0	0	0	0	0	0	0
0	0	0	0	0	0	0	0	0
0	0	0	0	0	0	0	0	0
0	0	0	0	0	0	0	0	0
0	0	0	0	0	0	0	0	0

0	0	0	0	0	0	0	0	0
0	0	0	0	0	0	0	0	0
0	0	0	0	0	0	0	0	0
0	0	0	0	0	0	0	0	0
0	0	0	0	0	0	0	0	0
0	0	0	0	0	0	0	0	0
0	0	0	0	0	0	0	0	0
0	0	0	0	0	0	0	0	0
0	0	0	0	0	0	0	0	0
0	0	0	0	0	0	0	0	0
0	0	0	0	0	0	0	0	0
0	0	0	0	0	0	0	0	0
0	0	0	0	0	0	0	0	0
0	0	0	0	0	0	0	0	0
0	0	0	0	0	0	0	0	0
0	0	0	0	0	0	0	0	0
0	0	0	0	0	0	0	0	0
0	0	0	0	0	0	0	0	0
0.0077	0.0098	0.0069	0.008	0.005	0.0062	0.0062	0.0053	0.0033
0.0083	0.0101	0.008	0.0079	0.0047	0.0061	0.0062	0.0053	0.0034
0.0058	0.0084	0.0053	0.0055	0.0031	0.0048	0.0056	0.004	0.0029
0.0079	0.0109	0.0078	0.0089	0.0063	0.0071	0.0069	0.006	0.0039
0.0073	0.0093	0.0058	0.0069	0.004	0.0053	0.0057	0.0049	0.0043
0.0083	0.0106	0.0073	0.0085	0.0053	0.0068	0.0072	0.0058	0.0047
0.0081	0.011	0.0074	0.009	0.0064	0.0075	0.0072	0.0057	0.0036
0.0098	0.0123	0.0088	0.0102	0.0068	0.0083	0.0089	0.0075	0.0056
0.001	0.0017	0.0014	0.0018	0.0015	0.0016	0.0019	0.0021	0.0021
0.0005	0.0014	0.0012	0.0014	0.0011	0.0012	0.0014	0.0016	0.0017
0.0009	0.0018	0.0015	0.0017	0.0014	0.0015	0.0018	0.0019	0.0019
0.0005	0.001	0.0008	0.0011	0.001	0.001	0.0012	0.0015	0.0015
0.0003	0.0006	0.0005	0.0006	0.0005	0.0006	0.0006	0.0007	0.0007
0	0	0	0	0	0	0	0	0
0	0	0	0	0	0	0	0	0
0	0	0	0	0	0	0	0	0
0	0	0	0	0	0	0	0	0
0	0	0	0	0	0	0	0	0
0	0	0	0	0	0	0	0	0
0	0	0	0	0	0	0	0	0
0	0	0	0	0	0	0	0	0
0	0	0	0	0	0	0	0	0
0	0	0	0	0	0	0	0	0
0	0	0	0	0	0	0	0	0
0	0	0	0	0	0	0	0	0
0	0	0	0	0	0	0	0	0

0	0	0	0	0	0	0	0	0
0	0	0	0	0	0	0	0	0
0	0	0	0	0	0	0	0	0
0	0	0	0	0	0	0	0	0
0	0	0	0	0	0	0	0	0
0	0	0	0	0	0	0	0	0
0	0	0	0	0	0	0	0	0
0	0	0	0	0	0	0	0	0
0	0	0	0	0	0	0	0	0
0	0	0	0	0	0	0	0	0
0	0	0	0	0	0	0	0	0
0	0	0	0	0	0	0	0	0
0	0	0	0	0	0	0	0	0
0	0	0	0	0	0	0	0	0
0	0	0	0	0	0	0	0	0
0	0	0	0	0	0	0	0	0
0	0	0	0	0	0	0	0	0
0	0	0	0	0	0	0	0	0
0	0	0	0	0	0	0	0	0
0	0	0	0	0	0	0	0	0
0	0	0	0	0	0	0	0	0
0	0	0	0	0	0	0	0	0
0	0	0	0	0	0	0	0	0
0	0	0	0	0	0	0	0	0
0.0085	0.0115	0.008	0.0094	0.0063	0.0086	0.0077	0.0063	0.0043
0.0065	0.0099	0.0068	0.0073	0.0048	0.0072	0.0079	0.0065	0.0052
0.0072	0.0096	0.0059	0.0065	0.0044	0.0061	0.0064	0.0059	0.0055
0.0068	0.0094	0.0063	0.007	0.0053	0.0059	0.0064	0.0047	0.003
0.0087	0.0115	0.0087	0.0092	0.0059	0.0068	0.0065	0.0048	0.0038
0.0077	0.0103	0.008	0.0095	0.0067	0.008	0.0082	0.0068	0.0046
0.0088	0.0108	0.0075	0.0089	0.0067	0.0088	0.0087	0.0067	0.005
0.0066	0.0097	0.0069	0.0083	0.0055	0.007	0.0073	0.0054	0.0044
0.0008	0.0012	0.001	0.0012	0.001	0.001	0.0012	0.0012	0.0013
0.001	0.0015	0.0012	0.0013	0.001	0.001	0.0011	0.0012	0.0012
0.0002	0.0005	0.0004	0.0005	0.0004	0.0005	0.0006	0.0007	0.0007
0.0004	0.0006	0.0005	0.0006	0.0006	0.0006	0.0007	0.0007	0.0007
0.0004	0.0006	0.0005	0.0007	0.0005	0.0006	0.0007	0.0008	0.0008
0	0	0	0	0	0	0	0	0
0	0	0	0	0	0	0	0	0
0	0	0	0	0	0	0	0	0
0	0	0	0	0	0	0	0	0
0	0	0	0	0	0	0	0	0
0	0	0	0	0	0	0	0	0
0	0	0	0	0	0	0	0	0

0	0	0	0	0	0	0	0	0
0	0	0	0	0	0	0	0	0
0	0	0	0	0	0	0	0	0
0	0	0	0	0	0	0	0	0
0	0	0	0	0	0	0	0	0
0	0	0	0	0	0	0	0	0
0	0	0	0	0	0	0	0	0
0	0	0	0	0	0	0	0	0
0	0	0	0	0	0	0	0	0
0	0	0	0	0	0	0	0	0
0	0	0	0	0	0	0	0	0
0	0	0	0	0	0	0	0	0
0	0	0	0	0	0	0	0	0
0	0	0	0	0	0	0	0	0
0	0	0	0	0	0	0	0	0
0	0	0	0	0	0	0	0	0
0	0	0	0	0	0	0	0	0
0	0	0	0	0	0	0	0	0
0	0	0	0	0	0	0	0	0
0	0	0	0	0	0	0	0	0
0	0	0	0	0	0	0	0	0
0	0	0	0	0	0	0	0	0
0	0	0	0	0	0	0	0	0
0	0	0	0	0	0	0	0	0
0	0	0	0	0	0	0	0	0
0	0	0	0	0	0	0	0	0
0	0	0	0	0	0	0	0	0
0	0	0	0	0	0	0	0	0
0	0	0	0	0	0	0	0	0
0	0	0	0	0	0	0	0	0
0.0009	0.0017	0.0013	0.0016	0.0012	0.0015	0.0016	0.0018	0.0018
0.001	0.0014	0.0012	0.0014	0.0011	0.0012	0.0014	0.0016	0.0015
0.0003	0.0005	0.0004	0.0005	0.0003	0.0004	0.0005	0.0006	0.0006
0	0	0	0	0	0	0	0	0
0	0	0	0	0	0	0	0	0
0	0	0	0	0	0	0	0	0
0.0004	0.0006	0.0005	0.0006	0.0006	0.0006	0.0006	0.0007	0.0006
0.0003	0.0005	0.0004	0.0005	0.0004	0.0004	0.0005	0.0006	0.0006
0.008	0.0097	0.0068	0.0073	0.0042	0.0056	0.0055	0.0036	0.0031
0.0076	0.0095	0.0069	0.0071	0.0033	0.0045	0.0043	0.0027	0.0013
0.0081	0.0109	0.0072	0.008	0.0059	0.0072	0.0064	0.004	0.0027
0.0075	0.0109	0.0073	0.0085	0.0057	0.0074	0.0069	0.006	0.0043
0.0072	0.0089	0.0055	0.0062	0.0038	0.0055	0.0061	0.0048	0.0042
0.0068	0.0091	0.0062	0.0066	0.0042	0.0058	0.0057	0.0049	0.0033

0.0075	0.0105	0.0074	0.0079	0.006	0.0075	0.0073	0.0062	0.0047
0.0054	0.0079	0.0043	0.0059	0.0039	0.0053	0.006	0.005	0.003
0	0	0	0	0	0	0	0	0
0	0	0	0	0	0	0	0	0
0	0	0	0	0	0	0	0	0
0	0	0	0	0	0	0	0	0
0	0	0	0	0	0	0	0	0
0	0	0	0	0	0	0	0	0
0	0	0	0	0	0	0	0	0
0	0	0	0	0	0	0	0	0
0	0	0	0	0	0	0	0	0
0	0	0	0	0	0	0	0	0
0	0	0	0	0	0	0	0	0
0	0	0	0	0	0	0	0	0
0	0	0	0	0	0	0	0	0
0	0	0	0	0	0	0	0	0
0	0	0	0	0	0	0	0	0
0	0	0	0	0	0	0	0	0
0	0	0	0	0	0	0	0	0
0	0	0	0	0	0	0	0	0
0	0	0	0	0	0	0	0	0
0	0	0	0	0	0	0	0	0
0	0	0	0	0	0	0	0	0
0	0	0	0	0	0	0	0	0
0	0	0	0	0	0	0	0	0
0	0	0	0	0	0	0	0	0
0	0	0	0	0	0	0	0	0
0	0	0	0	0	0	0	0	0
0	0	0	0	0	0	0	0	0
0	0	0	0	0	0	0	0	0
0	0	0	0	0	0	0	0	0
0	0	0	0	0	0	0	0	0
0	0	0	0	0	0	0	0	0
0	0	0	0	0	0	0	0	0
0	0	0	0	0	0	0	0	0
0	0	0	0	0	0	0	0	0
0	0	0	0	0	0	0	0	0
0	0	0	0	0	0	0	0	0
0	0	0	0	0	0	0	0	0
0	0	0	0	0	0	0	0	0
0	0	0	0	0	0	0	0	0
0	0	0	0	0	0	0	0	0
0	0	0	0	0	0	0	0	0
0	0	0	0	0	0	0	0	0

0	0	0	0	0	0	0	0	0
0	0	0	0	0	0	0	0	0
0	0	0	0	0	0	0	0	0
0	0	0	0	0	0	0	0	0
0	0	0	0	0	0	0	0	0
0	0	0	0	0	0	0	0	0
0	0	0	0	0	0	0	0	0
0	0	0	0	0	0	0	0	0
0	0	0	0	0	0	0	0	0
0	0	0	0	0	0	0	0	0
0	0	0	0	0	0	0	0	0
0	0	0	0	0	0	0	0	0
0	0	0	0	0	0	0	0	0
0	0	0	0	0	0	0	0	0
0	0	0	0	0	0	0	0	0
0	0	0	0	0	0	0	0	0
0	0	0	0	0	0	0	0	0
0	0	0	0	0	0	0	0	0
0	0	0	0	0	0	0	0	0
0	0	0	0	0	0	0	0	0
0	0	0	0	0	0	0	0	0
0	0	0	0	0	0	0	0	0
0	0	0	0	0	0	0	0	0
0	0	0	0	0	0	0	0	0
0	0	0	0	0	0	0	0	0
0	0	0	0	0	0	0	0	0
0	0	0	0	0	0	0	0	0
0	0	0	0	0	0	0	0	0
0	0	0	0	0	0	0	0	0
0	0	0	0	0	0	0	0	0
0	0	0	0	0	0	0	0	0
0	0	0	0	0	0	0	0	0
0	0	0	0	0	0	0	0	0
0	0	0	0	0	0	0	0	0
0	0	0	0	0	0	0	0	0
0	0	0	0	0	0	0	0	0
0	0	0	0	0	0	0	0	0
0	0	0	0	0	0	0	0	0
0	0	0	0	0	0	0	0	0
0	0	0	0	0	0	0	0	0
0	0	0	0	0	0	0	0	0
0	0	0	0	0	0	0	0	0
0	0	0	0	0	0	0	0	0
0	0	0	0	0	0	0	0	0

0	0	0	0	0	0	0	0	0
0	0	0	0	0	0	0	0	0
0	0	0	0	0	0	0	0	0
0	0	0	0	0	0	0	0	0
0	0	0	0	0	0	0	0	0
0	0	0	0	0	0	0	0	0
0	0	0	0	0	0	0	0	0
0	0	0	0	0	0	0	0	0
0	0	0	0	0	0	0	0	0
0	0	0	0	0	0	0	0	0
0	0	0	0	0	0	0	0	0
0	0	0	0	0	0	0	0	0
0	0	0	0	0	0	0	0	0
0	0	0	0	0	0	0	0	0
0	0	0	0	0	0	0	0	0
0	0	0	0	0	0	0	0	0
0	0	0	0	0	0	0	0	0
0	0	0	0	0	0	0	0	0
0	0	0	0	0	0	0	0	0
0	0	0	0	0	0	0	0	0
0	0	0	0	0	0	0	0	0
0	0	0	0	0	0	0	0	0
0	0	0	0	0	0	0	0	0
0	0	0	0	0	0	0	0	0
0	0	0	0	0	0	0	0	0
0	0	0	0	0	0	0	0	0
0	0	0	0	0	0	0	0	0
0	0	0	0	0	0	0	0	0
0	0	0	0	0	0	0	0	0
0	0	0	0	0	0	0	0	0
0	0	0	0	0	0	0	0	0
0	0	0	0	0	0	0	0	0
0	0	0	0	0	0	0	0	0
0	0	0	0	0	0	0	0	0
0	0	0	0	0	0	0	0	0
0	0	0	0	0	0	0	0	0
0	0	0	0	0	0	0	0	0
0	0	0	0	0	0	0	0	0
0	0	0	0	0	0	0	0	0
0	0	0	0	0	0	0	0	0
0	0	0	0	0	0	0	0	0
0	0	0	0	0	0	0	0	0
0	0	0	0	0	0	0	0	0
0	0	0	0	0	0	0	0	0
0	0	0	0	0	0	0	0	0

0	0	0	0	0	0	0	0	0
0	0	0	0	0	0	0	0	0
0	0	0	0	0	0	0	0	0
0	0	0	0	0	0	0	0	0
0	0	0	0	0	0	0	0	0
0	0	0	0	0	0	0	0	0
0	0	0	0	0	0	0	0	0
0	0	0	0	0	0	0	0	0
0	0	0	0	0	0	0	0	0
0	0	0	0	0	0	0	0	0
0	0	0	0	0	0	0	0	0
0	0	0	0	0	0	0	0	0
0	0	0	0	0	0	0	0	0
0	0	0	0	0	0	0	0	0
0	0	0	0	0	0	0	0	0
0	0	0	0	0	0	0	0	0
0	0	0	0	0	0	0	0	0
0	0	0	0	0	0	0	0	0
0	0	0	0	0	0	0	0	0
0	0	0	0	0	0	0	0	0
0	0	0	0	0	0	0	0	0
0	0	0	0	0	0	0	0	0
0	0	0	0	0	0	0	0	0
0	0	0	0	0	0	0	0	0
0	0	0	0	0	0	0	0	0
0	0	0	0	0	0	0	0	0
0	0	0	0	0	0	0	0	0
0	0	0	0	0	0	0	0	0
0	0	0	0	0	0	0	0	0
0	0	0	0	0	0	0	0	0
0	0	0	0	0	0	0	0	0
0	0	0	0	0	0	0	0	0
0	0	0	0	0	0	0	0	0
0	0	0	0	0	0	0	0	0
0	0	0	0	0	0	0	0	0
0	0	0	0	0	0	0	0	0
0	0	0	0	0	0	0	0	0
0	0	0	0	0	0	0	0	0
0	0	0	0	0	0	0	0	0
0	0	0	0	0	0	0	0	0
0	0	0	0	0	0	0	0	0
0	0	0	0	0	0	0	0	0
0	0	0	0	0	0	0	0	0
0	0	0	0	0	0	0	0	0

0	0	0	0	0	0	0	0	0
0	0	0	0	0	0	0	0	0
0	0	0	0	0	0	0	0	0
0	0	0	0	0	0	0	0	0
0	0	0	0	0	0	0	0	0
0	0	0	0	0	0	0	0	0
0	0	0	0	0	0	0	0	0
0	0	0	0	0	0	0	0	0
0	0	0	0	0	0	0	0	0
0	0	0	0	0	0	0	0	0
0	0	0	0	0	0	0	0	0
0	0	0	0	0	0	0	0	0
0	0	0	0	0	0	0	0	0
0	0	0	0	0	0	0	0	0
0	0	0	0	0	0	0	0	0
0	0	0	0	0	0	0	0	0
0	0	0	0	0	0	0	0	0
0	0	0	0	0	0	0	0	0
0	0	0	0	0	0	0	0	0
0	0	0	0	0	0	0	0	0
0	0	0	0	0	0	0	0	0
0	0	0	0	0	0	0	0	0
0	0	0	0	0	0	0	0	0
0	0	0	0	0	0	0	0	0
0	0	0	0	0	0	0	0	0
0	0	0	0	0	0	0	0	0
0	0	0	0	0	0	0	0	0
0	0	0	0	0	0	0	0	0
0	0	0	0	0	0	0	0	0
0	0	0	0	0	0	0	0	0
0	0	0	0	0	0	0	0	0
0	0	0	0	0	0	0	0	0
0	0	0	0	0	0	0	0	0
0	0	0	0	0	0	0	0	0
0	0	0	0	0	0	0	0	0
0	0	0	0	0	0	0	0	0
0	0	0	0	0	0	0	0	0
0	0	0	0	0	0	0	0	0
0	0	0	0	0	0	0	0	0
0	0	0	0	0	0	0	0	0
0	0	0	0	0	0	0	0	0
0	0	0	0	0	0	0	0	0
0	0	0	0	0	0	0	0	0
0	0	0	0	0	0	0	0	0

0	0	0	0	0	0	0	0	0
0	0	0	0	0	0	0	0	0
0	0	0	0	0	0	0	0	0
0	0	0	0	0	0	0	0	0
0	0	0	0	0	0	0	0	0
0	0	0	0	0	0	0	0	0
0	0	0	0	0	0	0	0	0
0	0	0	0	0	0	0	0	0
0	0	0	0	0	0	0	0	0
0	0	0	0	0	0	0	0	0
0	0	0	0	0	0	0	0	0
0	0	0	0	0	0	0	0	0
0	0	0	0	0	0	0	0	0
0	0	0	0	0	0	0	0	0
0	0	0	0	0	0	0	0	0
0	0	0	0	0	0	0	0	0
0	0	0	0	0	0	0	0	0
0	0	0	0	0	0	0	0	0
0	0	0	0	0	0	0	0	0
0	0	0	0	0	0	0	0	0
0	0	0	0	0	0	0	0	0
0	0	0	0	0	0	0	0	0
0	0	0	0	0	0	0	0	0
0	0	0	0	0	0	0	0	0
0	0	0	0	0	0	0	0	0
0	0	0	0	0	0	0	0	0
0	0	0	0	0	0	0	0	0
0	0	0	0	0	0	0	0	0
0	0	0	0	0	0	0	0	0
0	0	0	0	0	0	0	0	0
0	0	0	0	0	0	0	0	0
0	0	0	0	0	0	0	0	0
0	0	0	0	0	0	0	0	0
0	0	0	0	0	0	0	0	0
0	0	0	0	0	0	0	0	0
0	0	0	0	0	0	0	0	0
0	0	0	0	0	0	0	0	0
0	0	0	0	0	0	0	0	0
0	0	0	0	0	0	0	0	0
0	0	0	0	0	0	0	0	0
0	0	0	0	0	0	0	0	0
0	0	0	0	0	0	0	0	0
0	0	0	0	0	0	0	0	0
0	0	0	0	0	0	0	0	0

0	0	0	0	0	0	0	0	0
0	0	0	0	0	0	0	0	0
0	0	0	0	0	0	0	0	0
0	0	0	0	0	0	0	0	0
0	0	0	0	0	0	0	0	0
0	0	0	0	0	0	0	0	0
0	0	0	0	0	0	0	0	0
0	0	0	0	0	0	0	0	0
0	0	0	0	0	0	0	0	0
0	0	0	0	0	0	0	0	0
0	0	0	0	0	0	0	0	0
0	0	0	0	0	0	0	0	0
0	0	0	0	0	0	0	0	0
0	0	0	0	0	0	0	0	0
0	0	0	0	0	0	0	0	0
0	0	0	0	0	0	0	0	0
0	0	0	0	0	0	0	0	0
0	0	0	0	0	0	0	0	0
0	0	0	0	0	0	0	0	0
0	0	0	0	0	0	0	0	0
0	0	0	0	0	0	0	0	0
0	0	0	0	0	0	0	0	0
0	0	0	0	0	0	0	0	0
0	0	0	0	0	0	0	0	0
0	0	0	0	0	0	0	0	0
0	0	0	0	0	0	0	0	0
0	0	0	0	0	0	0	0	0
0	0	0	0	0	0	0	0	0
0	0	0	0	0	0	0	0	0
0	0	0	0	0	0	0	0	0
0	0	0	0	0	0	0	0	0
0	0	0	0	0	0	0	0	0
0	0	0	0	0	0	0	0	0
0	0	0	0	0	0	0	0	0
0	0	0	0	0	0	0	0	0
0	0	0	0	0	0	0	0	0
0	0	0	0	0	0	0	0	0
0	0	0	0	0	0	0	0	0
0	0	0	0	0	0	0	0	0
0	0	0	0	0	0	0	0	0
0	0	0	0	0	0	0	0	0
0	0	0	0	0	0	0	0	0
0	0	0	0	0	0	0	0	0
0	0	0	0	0	0	0	0	0

0	0	0	0	0	0	0	0	0
0	0	0	0	0	0	0	0	0
0	0	0	0	0	0	0	0	0
0	0	0	0	0	0	0	0	0
0	0	0	0	0	0	0	0	0
0	0	0	0	0	0	0	0	0
0	0	0	0	0	0	0	0	0
0	0	0	0	0	0	0	0	0
0	0	0	0	0	0	0	0	0
0	0	0	0	0	0	0	0	0
0	0	0	0	0	0	0	0	0
0	0	0	0	0	0	0	0	0
0	0	0	0	0	0	0	0	0
0	0	0	0	0	0	0	0	0
0	0	0	0	0	0	0	0	0
0	0	0	0	0	0	0	0	0
0	0	0	0	0	0	0	0	0
0	0	0	0	0	0	0	0	0
0	0	0	0	0	0	0	0	0
0	0	0	0	0	0	0	0	0
0	0	0	0	0	0	0	0	0
0	0	0	0	0	0	0	0	0
0	0	0	0	0	0	0	0	0
0	0	0	0	0	0	0	0	0
0	0	0	0	0	0	0	0	0
0	0	0	0	0	0	0	0	0
0	0	0	0	0	0	0	0	0
0	0	0	0	0	0	0	0	0
0	0	0	0	0	0	0	0	0
0	0	0	0	0	0	0	0	0
0	0	0	0	0	0	0	0	0
0	0	0	0	0	0	0	0	0
0	0	0	0	0	0	0	0	0
0	0	0	0	0	0	0	0	0
0	0	0	0	0	0	0	0	0
0	0	0	0	0	0	0	0	0
0	0	0	0	0	0	0	0	0
0	0	0	0	0	0	0	0	0
0	0	0	0	0	0	0	0	0
0	0	0	0	0	0	0	0	0
0	0	0	0	0	0	0	0	0
0	0	0	0	0	0	0	0	0
0	0	0	0	0	0	0	0	0
0	0	0	0	0	0	0	0	0

0	0	0	0	0	0	0	0	0
0	0	0	0	0	0	0	0	0
0	0	0	0	0	0	0	0	0
0	0	0	0	0	0	0	0	0
0	0	0	0	0	0	0	0	0
0	0	0	0	0	0	0	0	0
0	0	0	0	0	0	0	0	0
0	0	0	0	0	0	0	0	0
0	0	0	0	0	0	0	0	0
0	0	0	0	0	0	0	0	0
0	0	0	0	0	0	0	0	0
0	0	0	0	0	0	0	0	0
0	0	0	0	0	0	0	0	0
0	0	0	0	0	0	0	0	0
0	0	0	0	0	0	0	0	0
0	0	0	0	0	0	0	0	0
0	0	0	0	0	0	0	0	0
0	0	0	0	0	0	0	0	0
0	0	0	0	0	0	0	0	0
0	0	0	0	0	0	0	0	0
0	0	0	0	0	0	0	0	0
0	0	0	0	0	0	0	0	0
0	0	0	0	0	0	0	0	0
0	0	0	0	0	0	0	0	0
0	0	0	0	0	0	0	0	0
0	0	0	0	0	0	0	0	0
0	0	0	0	0	0	0	0	0
0	0	0	0	0	0	0	0	0
0	0	0	0	0	0	0	0	0
0	0	0	0	0	0	0	0	0
0	0	0	0	0	0	0	0	0
0	0	0	0	0	0	0	0	0
0	0	0	0	0	0	0	0	0
0	0	0	0	0	0	0	0	0
0	0	0	0	0	0	0	0	0
0	0	0	0	0	0	0	0	0
0	0	0	0	0	0	0	0	0
0	0	0	0	0	0	0	0	0
0	0	0	0	0	0	0	0	0
0	0	0	0	0	0	0	0	0
0	0	0	0	0	0	0	0	0
0	0	0	0	0	0	0	0	0
0	0	0	0	0	0	0	0	0
0	0	0	0	0	0	0	0	0

0	0	0	0	0	0	0	0	0
0	0	0	0	0	0	0	0	0
0	0	0	0	0	0	0	0	0
0	0	0	0	0	0	0	0	0
0	0	0	0	0	0	0	0	0
0	0	0	0	0	0	0	0	0
0	0	0	0	0	0	0	0	0
0	0	0	0	0	0	0	0	0
0	0	0	0	0	0	0	0	0
0	0	0	0	0	0	0	0	0
0	0	0	0	0	0	0	0	0
0	0	0	0	0	0	0	0	0
0	0	0	0	0	0	0	0	0
0	0	0	0	0	0	0	0	0
0	0	0	0	0	0	0	0	0
0	0	0	0	0	0	0	0	0
0	0	0	0	0	0	0	0	0
0	0	0	0	0	0	0	0	0
0	0	0	0	0	0	0	0	0
0	0	0	0	0	0	0	0	0
0	0	0	0	0	0	0	0	0
0	0	0	0	0	0	0	0	0
0	0	0	0	0	0	0	0	0
0	0	0	0	0	0	0	0	0
0	0	0	0	0	0	0	0	0
0	0	0	0	0	0	0	0	0
0	0	0	0	0	0	0	0	0
0	0	0	0	0	0	0	0	0
0	0	0	0	0	0	0	0	0
0	0	0	0	0	0	0	0	0
0	0	0	0	0	0	0	0	0
0	0	0	0	0	0	0	0	0
0	0	0	0	0	0	0	0	0
0	0	0	0	0	0	0	0	0
0	0	0	0	0	0	0	0	0
0	0	0	0	0	0	0	0	0
0	0	0	0	0	0	0	0	0
0	0	0	0	0	0	0	0	0
0	0	0	0	0	0	0	0	0
0	0	0	0	0	0	0	0	0
0	0	0	0	0	0	0	0	0
0	0	0	0	0	0	0	0	0
0	0	0	0	0	0	0	0	0
0	0	0	0	0	0	0	0	0

0	0	0	0	0	0	0	0	0
0	0	0	0	0	0	0	0	0
0	0	0	0	0	0	0	0	0
0	0	0	0	0	0	0	0	0
0	0	0	0	0	0	0	0	0
0	0	0	0	0	0	0	0	0
0	0	0	0	0	0	0	0	0
0	0	0	0	0	0	0	0	0
0	0	0	0	0	0	0	0	0
0	0	0	0	0	0	0	0	0
0	0	0	0	0	0	0	0	0
0	0	0	0	0	0	0	0	0
0	0	0	0	0	0	0	0	0
0	0	0	0	0	0	0	0	0
0	0	0	0	0	0	0	0	0
0	0	0	0	0	0	0	0	0
0	0	0	0	0	0	0	0	0
0	0	0	0	0	0	0	0	0
0	0	0	0	0	0	0	0	0
0	0	0	0	0	0	0	0	0
0	0	0	0	0	0	0	0	0
0	0	0	0	0	0	0	0	0
0	0	0	0	0	0	0	0	0
0	0	0	0	0	0	0	0	0
0	0	0	0	0	0	0	0	0
0	0	0	0	0	0	0	0	0
0	0	0	0	0	0	0	0	0
0	0	0	0	0	0	0	0	0
0	0	0	0	0	0	0	0	0
0	0	0	0	0	0	0	0	0
0	0	0	0	0	0	0	0	0
0	0	0	0	0	0	0	0	0
0	0	0	0	0	0	0	0	0
0	0	0	0	0	0	0	0	0
0	0	0	0	0	0	0	0	0
0	0	0	0	0	0	0	0	0
0	0	0	0	0	0	0	0	0
0	0	0	0	0	0	0	0	0
0	0	0	0	0	0	0	0	0
0	0	0	0	0	0	0	0	0
0	0	0	0	0	0	0	0	0
0	0	0	0	0	0	0	0	0
0	0	0	0	0	0	0	0	0
0	0	0	0	0	0	0	0	0

0	0	0	0	0	0	0	0	0
0	0	0	0	0	0	0	0	0
0	0	0	0	0	0	0	0	0
0	0	0	0	0	0	0	0	0
0	0	0	0	0	0	0	0	0
0	0	0	0	0	0	0	0	0
0	0	0	0	0	0	0	0	0
0	0	0	0	0	0	0	0	0
0	0	0	0	0	0	0	0	0
0	0	0	0	0	0	0	0	0
0	0	0	0	0	0	0	0	0
0	0	0	0	0	0	0	0	0
0	0	0	0	0	0	0	0	0
0	0	0	0	0	0	0	0	0
0	0	0	0	0	0	0	0	0
0	0	0	0	0	0	0	0	0
0	0	0	0	0	0	0	0	0
0	0	0	0	0	0	0	0	0
0	0	0	0	0	0	0	0	0
0	0	0	0	0	0	0	0	0
0	0	0	0	0	0	0	0	0
0	0	0	0	0	0	0	0	0
0	0	0	0	0	0	0	0	0
0	0	0	0	0	0	0	0	0
0	0	0	0	0	0	0	0	0
0	0	0	0	0	0	0	0	0
0	0	0	0	0	0	0	0	0
0	0	0	0	0	0	0	0	0
0	0	0	0	0	0	0	0	0
0	0	0	0	0	0	0	0	0
0	0	0	0	0	0	0	0	0
0	0	0	0	0	0	0	0	0
0	0	0	0	0	0	0	0	0
0	0	0	0	0	0	0	0	0
0	0	0	0	0	0	0	0	0
0	0	0	0	0	0	0	0	0

0	0	0	0	0
0	0	0	0	0
0	0	0	0	0
0	0	0	0	0
0	0	0	0	0
0	0	0	0	0
0	0	0	0	0
0	0	0	0	0
0	0	0	0	0
0	0	0	0	0
0	0	0	0	0
0	0	0	0	0
0	0	0	0	0
0	0	0	0	0
0	0	0	0	0
0	0	0	0	0
0	0	0	0	0
0	0	0	0	0
0	0	0	0	0
0	0	0	0	0
0	0	0	0	0
0	0	0	0	0
0	0	0	0	0
0	0	0	0	0
0	0	0	0	0
0	0	0	0	0
0	0	0	0	0
0	0	0	0	0
0	0	0	0	0
0	0	0	0	0
0	0	0	0	0
0	0	0	0	0
0	0	0	0	0
0	0	0	0	0
0	0	0	0	0
0	0	0	0	0
0	0	0	0	0
0	0	0	0	0
0	0	0	0	0
0	0	0	0	0
0	0	0	0	0
0	0	0	0	0
0	0	0	0	0
0	0	0	0	0

0	0	0	0	0
0	0	0	0	0
0	0	0	0	0
0	0	0	0	0
0	0	0	0	0
0	0	0	0	0
0	0	0	0	0
0	0	0	0	0
0	0	0	0	0
0	0	0	0	0
0	0	0	0	0
0	0	0	0	0
0	0	0	0	0
0	0	0	0	0
0	0	0	0	0
0	0	0	0	0
0	0	0	0	0
0	0	0	0	0
0	0	0	0	0
0	0	0	0	0
0	0	0	0	0
0	0	0	0	0
0	0	0	0	0
0	0	0	0	0
0	0	0	0	0
0	0	0	0	0
0	0	0	0	0
0	0	0	0	0
0	0	0	0	0
0	0	0	0	0
0	0	0	0	0
0	0	0	0	0
0	0	0	0	0
0	0	0	0	0
0	0	0	0	0
0	0	0	0	0
0	0	0	0	0
0	0	0	0	0
0	0	0	0	0
0	0	0	0	0
0	0	0	0	0
0	0	0	0	0
0	0	0	0	0
0	0	0	0	0

0	0	0	0	0
0	0	0	0	0
0	0	0	0	0
0	0	0	0	0
0	0	0	0	0
0	0	0	0	0
0	0	0	0	0
0	0	0	0	0
0	0	0	0	0
0	0	0	0	0
0	0	0	0	0
0	0	0	0	0
0	0	0	0	0
0	0	0	0	0
0	0	0	0	0
0	0	0	0	0
0	0	0	0	0
0	0	0	0	0
0	0	0	0	0
0	0	0	0	0
0	0	0	0	0
0	0	0	0	0
0	0	0	0	0
0	0	0	0	0
0	0	0	0	0
0	0	0	0	0
0	0	0	0	0
0	0	0	0	0
0	0	0	0	0
0	0	0	0	0
0	0	0	0	0
0	0	0	0	0
0	0	0	0	0
0	0	0	0	0
0	0	0	0	0
0	0	0	0	0
0	0	0	0	0
0	0	0	0	0
0	0	0	0	0
0	0	0	0	0
0	0	0	0	0
0	0	0	0	0
0	0	0	0	0
0	0	0	0	0

0	0	0	0	0
0	0	0	0	0
0	0	0	0	0
0	0	0	0	0
0	0	0	0	0
0	0	0	0	0
0	0	0	0	0
0	0	0	0	0
0	0	0	0	0
0	0	0	0	0
0	0	0	0	0
0	0	0	0	0
0	0	0	0	0
0	0	0	0	0
0	0	0	0	0
0	0	0	0	0
0	0	0	0	0
0	0	0	0	0
0	0	0	0	0
0	0	0	0	0
0	0	0	0	0
0	0	0	0	0
0	0	0	0	0
0	0	0	0	0
0	0	0	0	0
0	0	0	0	0
0	0	0	0	0
0	0	0	0	0
0	0	0	0	0
0	0	0	0	0
0	0	0	0	0
0	0	0	0	0
0	0	0	0	0
0	0	0	0	0
0	0	0	0	0
0	0	0	0	0
0	0	0	0	0
0	0	0	0	0
0	0	0	0	0
0	0	0	0	0
0	0	0	0	0
0	0	0	0	0
0	0	0	0	0
0	0	0	0	0

0	0	0	0	0
0	0	0	0	0
0	0	0	0	0
0	0	0	0	0
0	0	0	0	0
0	0	0	0	0
0	0	0	0	0
0	0	0	0	0
0	0	0	0	0
0	0	0	0	0
0	0	0	0	0
0	0	0	0	0
0	0	0	0	0
0	0	0	0	0
0	0	0	0	0
0	0	0	0	0
0	0	0	0	0
0	0	0	0	0
0	0	0	0	0
0	0	0	0	0
0	0	0	0	0
0	0	0	0	0
0	0	0	0	0
0	0	0	0	0
0	0	0	0	0
0	0	0	0	0
0	0	0	0	0
0	0	0	0	0
0	0	0	0	0
0	0	0	0	0
0	0	0	0	0
0	0	0	0	0
0	0	0	0	0
0	0	0	0	0
0	0	0	0	0
0	0	0	0	0
0	0	0	0	0
0	0	0	0	0
0	0	0	0	0
0	0	0	0	0
0	0	0	0	0
0	0	0	0	0
0	0	0	0	0
0	0	0	0	0

0	0	0	0	0
0	0	0	0	0
0	0	0	0	0
0	0	0	0	0
0	0	0	0	0
0	0	0	0	0
0	0	0	0	0
0	0	0	0	0
0	0	0	0	0
0	0	0	0	0
0	0	0	0	0
0	0	0	0	0
0	0	0	0	0
0	0	0	0	0
0	0	0	0	0
0	0	0	0	0
0	0	0	0	0
0	0	0	0	0
0	0	0	0	0
0	0	0	0	0
0	0	0	0	0
0	0	0	0	0
0	0	0	0	0
0	0	0	0	0
0	0	0	0	0
0	0	0	0	0
0	0	0	0	0
0	0	0	0	0
0	0	0	0	0
0	0	0	0	0
0	0	0	0	0
0	0	0	0	0
0	0	0	0	0
0	0	0	0	0
0	0	0	0	0
0	0	0	0	0
0	0	0	0	0
0	0	0	0	0
0	0	0	0	0
0	0	0	0	0
0	0	0	0	0
0	0	0	0	0
0	0	0	0	0
0	0	0	0	0

0	0	0	0	0
0	0	0	0	0
0	0	0	0	0
0	0	0	0	0
0	0	0	0	0
0	0	0	0	0
0	0	0	0	0
0	0	0	0	0
0	0	0	0	0
0	0	0	0	0
0	0	0	0	0
0	0	0	0	0
0	0	0	0	0
0	0	0	0	0
0	0	0	0	0
0	0	0	0	0
0	0	0	0	0
0	0	0	0	0
0	0	0	0	0
0	0	0	0	0
0	0	0	0	0
0	0	0	0	0
0	0	0	0	0
0	0	0	0	0
0	0	0	0	0
0	0	0	0	0
0	0	0	0	0
0	0	0	0	0
0	0	0	0	0
0	0	0	0	0
0	0	0	0	0
0	0	0	0	0
0	0	0	0	0
0	0	0	0	0
0	0	0	0	0
0	0	0	0	0
0	0	0	0	0
0	0	0	0	0
0	0	0	0	0
0	0	0	0	0
0	0	0	0	0
0	0	0	0	0
0	0	0	0	0
0	0	0	0	0

0	0	0	0	0
0	0	0	0	0
0	0	0	0	0
0	0	0	0	0
0	0	0	0	0
0	0	0	0	0
0	0	0	0	0
0	0	0	0	0
0	0	0	0	0
0	0	0	0	0
0	0	0	0	0
0	0	0	0	0
0	0	0	0	0
0	0	0	0	0
0	0	0	0	0
0	0	0	0	0
0	0	0	0	0
0	0	0	0	0
0	0	0	0	0
0	0	0	0	0
0	0	0	0	0
0	0	0	0	0
0	0	0	0	0
0	0	0	0	0
0	0	0	0	0
0	0	0	0	0
0	0	0	0	0
0	0	0	0	0
0	0	0	0	0
0	0	0	0	0
0	0	0	0	0
0	0	0	0	0
0	0	0	0	0
0	0	0	0	0
0	0	0	0	0
0	0	0	0	0
0	0	0	0	0
0	0	0	0	0
0	0	0	0	0
0	0	0	0	0
0	0	0	0	0
0	0	0	0	0
0	0	0	0	0
0	0	0	0	0

0	0	0	0	0
0	0	0	0	0
0	0	0	0	0
0	0	0	0	0
0	0	0	0	0
0	0	0	0	0
0	0	0	0	0
0	0	0	0	0
0	0	0	0	0
0	0	0	0	0
0	0	0	0	0
0	0	0	0	0
0	0	0	0	0
0	0	0	0	0
0	0	0	0	0
0	0	0	0	0
0	0	0	0	0
0	0	0	0	0
0	0	0	0	0
0	0	0	0	0
0	0	0	0	0
0	0	0	0	0
0	0	0	0	0
0	0	0	0	0
0	0	0	0	0
0	0	0	0	0
0	0	0	0	0
0	0	0	0	0
0	0	0	0	0
0	0	0	0	0
0	0	0	0	0
0	0	0	0	0
0	0	0	0	0
0	0	0	0	0
0	0	0	0	0
0	0	0	0	0
0	0	0	0	0
0	0	0	0	0
0	0	0	0	0
0	0	0	0	0
0	0	0	0	0
0	0	0	0	0
0	0	0	0	0
0	0	0	0	0

0	0	0	0	0
0	0	0	0	0
0	0	0	0	0
0	0	0	0	0
0	0	0	0	0
0	0	0	0	0
0	0	0	0	0
0	0	0	0	0
0	0	0	0	0
0	0	0	0	0
0	0	0	0	0
0	0	0	0	0
0	0	0	0	0
0	0	0	0	0
0	0	0	0	0
0	0	0	0	0
0	0	0	0	0
0	0	0	0	0
0	0	0	0	0
0	0	0	0	0
0.0015	0.0016	0.0016	0.0015	0.0013
0	0	0	0	0
0	0	0	0	0
0.0027	0.0027	0.0028	0.0026	0.0022
0.0032	0.0033	0.0033	0.0032	0.0031
0	0	0	0	0
0	0	0	0	0
0.0007	0.0008	0.0008	0.0007	0.0006
0	0	0	0	0
0.0014	0.0016	0.0016	0.0015	0.0014
0.0013	0.0013	0.0013	0.0013	0.0013
0	0	0	0	0
0	0	0	0	0
0.0015	0.0014	0.0015	0.0013	0.0012
0.0014	0.0015	0.0015	0.0013	0.0012
0	0	0	0	0
0	0	0	0	0
0	0	0	0	0
0	0	0	0	0
0	0	0	0	0
0	0	0	0	0
0	0	0	0	0
0	0	0	0	0
0	0	0	0	0

0	0	0	0	0
0	0	0	0	0
0	0	0	0	0
0	0	0	0	0
0	0	0	0	0
0	0	0	0	0
0	0	0	0	0
0	0	0	0	0
0	0	0	0	0
0	0	0	0	0
0	0	0	0	0
0	0	0	0	0
0	0	0	0	0
0	0	0	0	0
0	0	0	0	0
0	0	0	0	0
0	0	0	0	0
0	0	0	0	0
0	0	0	0	0
0	0	0	0	0
0	0	0	0	0
0	0	0	0	0
0	0	0	0	0
0	0	0	0	0
0	0	0	0	0
0	0	0	0	0
0.0037	0.0046	0.0041	0.0024	0.0041
0.0068	0.0078	0.0066	0.005	0.0066
0.0039	0.0051	0.0046	0.0032	0.0048
0.0062	0.0065	0.006	0.0049	0.006
0.006	0.0066	0.0051	0.0033	0.0047
0.0052	0.0055	0.0041	0.0026	0.0045
0.0036	0.0047	0.0047	0.0019	0.0041
0.0063	0.0074	0.0062	0.0046	0.0064
0	0	0	0	0
0	0	0	0	0
0	0	0	0	0
0	0	0	0	0
0	0	0	0	0
0	0	0	0	0
0	0	0	0	0
0	0	0	0	0
0	0	0	0	0
0	0	0	0	0

0	0	0	0	0
0	0	0	0	0
0	0	0	0	0
0	0	0	0	0
0	0	0	0	0
0	0	0	0	0
0	0	0	0	0
0	0	0	0	0
0	0	0	0	0
0	0	0	0	0
0	0	0	0	0
0	0	0	0	0
0	0	0	0	0
0	0	0	0	0
0	0	0	0	0
0	0	0	0	0
0	0	0	0	0
0	0	0	0	0
0	0	0	0	0
0	0	0	0	0
0	0	0	0	0
0	0	0	0	0
0	0	0	0	0
0	0	0	0	0
0	0	0	0	0
0	0	0	0	0
0	0	0	0	0
0	0	0	0	0
0	0	0	0	0
0	0	0	0	0
0	0	0	0	0
0	0	0	0	0
0.0044	0.0056	0.005	0.0036	0.0056
0.0037	0.005	0.0052	0.0028	0.0051
0.0062	0.0076	0.0072	0.0053	0.0054
0.0021	0.0032	0.0025	0.0012	0.0035
0.0046	0.0054	0.0051	0.0038	0.0054
0.0059	0.0077	0.0058	0.0042	0.0063
0.0077	0.0088	0.0082	0.0062	0.008
0.0052	0.0063	0.0055	0.0037	0.0058
0.0006	0.0006	0.0006	0.0006	0.0005
0	0	0	0	0
0	0	0	0	0
0	0	0	0	0

0	0	0	0	0
0	0	0	0	0
0	0	0	0	0
0	0	0	0	0
0	0	0	0	0
0	0	0	0	0
0	0	0	0	0
0	0	0	0	0
0	0	0	0	0
0	0	0	0	0
0	0	0	0	0
0	0	0	0	0
0	0	0	0	0
0	0	0	0	0
0	0	0	0	0
0	0	0	0	0
0	0	0	0	0
0	0	0	0	0
0	0	0	0	0
0	0	0	0	0
0	0	0	0	0
0	0	0	0	0
0	0	0	0	0
0	0	0	0	0
0	0	0	0	0
0	0	0	0	0
0	0	0	0	0
0	0	0	0	0
0	0	0	0	0
0	0	0	0	0
0	0	0	0	0
0	0	0	0	0
0	0	0	0	0
0	0	0	0	0
0	0	0	0	0
0	0	0	0	0
0	0	0	0	0
0	0	0	0	0
0.0059	0.0067	0.0064	0.0052	0.0069
0.007	0.0089	0.0067	0.0048	0.0064
0.0083	0.0091	0.0084	0.0075	0.0089
0.0052	0.0062	0.0053	0.0038	0.006
0.0061	0.0071	0.0062	0.0045	0.0065
0.0083	0.0092	0.0081	0.0063	0.0076

0.0109	0.0111	0.0104	0.0078	0.0092
0.0052	0.0058	0.0052	0.0036	0.0049
0.0029	0.0031	0.0032	0.003	0.0028
0.0015	0.0015	0.0015	0.0014	0.0013
0.0016	0.0015	0.0016	0.0014	0.0012
0.0013	0.0014	0.0014	0.0014	0.0012
0.0012	0.0014	0.0015	0.0013	0.0013
0	0	0	0	0
0	0	0	0	0
0	0	0	0	0
0	0	0	0	0
0	0	0	0	0
0	0	0	0	0
0	0	0	0	0
0	0	0	0	0
0	0	0	0	0
0	0	0	0	0
0	0	0	0	0
0	0	0	0	0
0	0	0	0	0
0	0	0	0	0
0	0	0	0	0
0	0	0	0	0
0	0	0	0	0
0	0	0	0	0
0	0	0	0	0
0	0	0	0	0
0	0	0	0	0
0	0	0	0	0
0	0	0	0	0
0	0	0	0	0
0	0	0	0	0
0	0	0	0	0
0	0	0	0	0
0	0	0	0	0
0	0	0	0	0
0	0	0	0	0
0	0	0	0	0
0	0	0	0	0
0	0	0	0	0
0	0	0	0	0
0	0	0	0	0
0	0	0	0	0
0	0	0	0	0
0	0	0	0	0

0	0	0	0	0
0.0008	0.0008	0.0009	0.0008	0.0007
0.0007	0.0008	0.0008	0.0008	0.0006
0	0	0	0	0
0	0	0	0	0
0.001	0.0012	0.0013	0.0012	0.0011
0.0019	0.0021	0.0019	0.0018	0.0017
0	0	0	0	0
0.002	0.0022	0.0021	0.0021	0.0018
0	0	0	0	0
0	0	0	0	0
0.0016	0.0018	0.0018	0.0015	0.0013
0.0014	0.0016	0.0016	0.0015	0.0014
0	0	0	0	0
0	0	0	0	0
0.0006	0.0007	0.0007	0.0006	0.0006
0	0	0	0	0
0	0	0	0	0
0	0	0	0	0
0	0	0	0	0
0	0	0	0	0
0	0	0	0	0
0	0	0	0	0
0	0	0	0	0
0	0	0	0	0
0	0	0	0	0
0	0	0	0	0
0	0	0	0	0
0	0	0	0	0
0	0	0	0	0
0	0	0	0	0
0	0	0	0	0
0	0	0	0	0
0	0	0	0	0
0	0	0	0	0
0	0	0	0	0
0	0	0	0	0
0	0	0	0	0
0	0	0	0	0
0	0	0	0	0
0	0	0	0	0
0	0	0	0	0
0	0	0	0	0
0	0	0	0	0
0	0	0	0	0

0	0	0	0	0
0	0	0	0	0
0	0	0	0	0
0	0	0	0	0
0	0	0	0	0
0	0	0	0	0
0	0	0	0	0
0	0	0	0	0
0	0	0	0	0
0.0006	0.0007	0.0008	0.0007	0.0007
0.0006	0.0007	0.0007	0.0007	0.0007
0	0	0	0	0
0	0	0	0	0
0	0	0	0	0
0.0007	0.0008	0.0008	0.0008	0.0007
0	0	0	0	0
0	0	0	0	0
0.0007	0.0008	0.0008	0.0007	0.0007
0.0007	0.0008	0.0008	0.0007	0.0007
0	0	0	0	0
0	0	0	0	0
0.0006	0.0007	0.0007	0.0007	0.0006
0	0	0	0	0
0	0	0	0	0
0	0	0	0	0
0	0	0	0	0
0	0	0	0	0
0	0	0	0	0
0	0	0	0	0
0	0	0	0	0
0	0	0	0	0
0	0	0	0	0
0	0	0	0	0
0	0	0	0	0
0	0	0	0	0
0	0	0	0	0
0	0	0	0	0
0	0	0	0	0
0	0	0	0	0
0	0	0	0	0
0	0	0	0	0
0	0	0	0	0
0	0	0	0	0
0	0	0	0	0

0	0	0	0	0
0	0	0	0	0
0	0	0	0	0
0	0	0	0	0
0	0	0	0	0
0	0	0	0	0
0	0	0	0	0
0	0	0	0	0
0	0	0	0	0
0	0	0	0	0
0	0	0	0	0
0	0	0	0	0
0.0127	0.0155	0.0134	0.0116	0.0148
0.011	0.0118	0.011	0.0088	0.0119
0.011	0.0122	0.0102	0.0088	0.0131
0.0177	0.0207	0.0201	0.0163	0.0191
0.016	0.0194	0.0184	0.0137	0.0176
0.0111	0.0128	0.0116	0.0078	0.0139
0.0082	0.0098	0.0071	0.003	0.0074
0.0107	0.0128	0.0111	0.0085	0.0112
0.0018	0.0021	0.0021	0.002	0.0017
0	0	0	0	0
0	0	0	0	0
0.0026	0.0028	0.0029	0.0025	0.0023
0.0032	0.0036	0.0038	0.0035	0.003
0	0	0	0	0
0.0008	0.0009	0.0009	0.0008	0.0007
0.0039	0.004	0.0043	0.0043	0.0041
0	0	0	0	0
0	0	0	0	0
0	0	0	0	0
0	0	0	0	0
0	0	0	0	0
0	0	0	0	0
0	0	0	0	0
0	0	0	0	0
0	0	0	0	0
0	0	0	0	0
0	0	0	0	0
0	0	0	0	0
0	0	0	0	0
0	0	0	0	0
0	0	0	0	0
0	0	0	0	0

0	0	0	0	0
0	0	0	0	0
0	0	0	0	0
0	0	0	0	0
0	0	0	0	0
0	0	0	0	0
0	0	0	0	0
0	0	0	0	0
0	0	0	0	0
0	0	0	0	0
0	0	0	0	0
0	0	0	0	0
0	0	0	0	0
0	0	0	0	0
0	0	0	0	0
0	0	0	0	0
0	0	0	0	0
0	0	0	0	0
0.0357	0.0418	0.0332	0.0257	0.0338
0.0333	0.0399	0.0415	0.0337	0.0404
0.0261	0.0307	0.0273	0.0221	0.0294
0.0368	0.0403	0.0383	0.029	0.0387
0.0346	0.0418	0.042	0.0358	0.0453
0.0277	0.0307	0.0251	0.0156	0.0237
0.02	0.0224	0.0215	0.0165	0.0219
0.0306	0.0395	0.0328	0.0268	0.0341
0.0044	0.0047	0.005	0.0048	0.0046
0.0012	0.0013	0.0012	0.0011	0.0009
0.002	0.0021	0.002	0.0019	0.0017
0.0076	0.0076	0.0083	0.007	0.0062
0.0067	0.0075	0.008	0.0075	0.007
0.004	0.0042	0.0043	0.0037	0.0032
0.0051	0.0059	0.0058	0.0051	0.0048
0.0078	0.0087	0.0086	0.0082	0.0068
0	0	0	0	0
0	0	0	0	0
0	0	0	0	0
0	0	0	0	0
0	0	0	0	0
0	0	0	0	0
0	0	0	0	0
0	0	0	0	0
0	0	0	0	0
0	0	0	0	0

0	0	0	0	0
0	0	0	0	0
0	0	0	0	0
0	0	0	0	0
0	0	0	0	0
0	0	0	0	0
0	0	0	0	0
0	0	0	0	0
0	0	0	0	0
0	0	0	0	0
0	0	0	0	0
0	0	0	0	0
0	0	0	0	0
0	0	0	0	0
0	0	0	0	0
0	0	0	0	0
0	0	0	0	0
0	0	0	0	0
0.0009	0.0012	0.0013	0.001	0.001
0.0006	0.0006	0.0007	0.0007	0.0006
0	0	0	0	0
0	0	0	0	0
0.0044	0.0045	0.0049	0.0046	0.0045
0.0114	0.0123	0.0128	0.0116	0.0105
0.1469	0.1766	0.1681	0.1197	0.1567
0.3027	0.3514	0.296	0.2215	0.3141
0.1808	0.1885	0.1531	0.099	0.1425
0.0792	0.0888	0.0895	0.0776	0.0817
0.1643	0.1792	0.1728	0.1409	0.1565
0.0628	0.0753	0.0663	0.0545	0.0602
0.0243	0.0479	0.0341	0.033	0.0486
0.3403	0.3654	0.2923	0.2245	0.2814
0.0953	0.1162	0.0816	0.0527	0.0883
0.0412	0.0478	0.042	0.0314	0.0468
0.0177	0.0232	0.0175	0.0146	0.0251
0.1425	0.1636	0.1546	0.1253	0.1365
0.1752	0.192	0.1899	0.1523	0.1674
0.023	0.0265	0.0258	0.0222	0.0243
0.012	0.014	0.013	0.0124	0.0137
0.0901	0.1011	0.096	0.0854	0.0945
0.0051	0.0056	0.0057	0.0055	0.0049
0.0006	0.0007	0.0008	0.0007	0.0007
0	0	0	0	0
0.0007	0.0007	0.0007	0.0006	0.0006

0	0	0	0	0
0	0	0	0	0
0.0006	0.0007	0.0007	0.0007	0.0007
0	0	0	0	0
0	0	0	0	0
0	0	0	0	0
0	0	0	0	0
0	0	0	0	0
0	0	0	0	0
0	0	0	0	0
0	0	0	0	0
0	0	0	0	0
0	0	0	0	0
0	0	0	0	0
0	0	0	0	0
0	0	0	0	0
0	0	0	0	0
0	0	0	0	0
0	0	0	0	0
0	0	0	0	0
0	0	0	0	0
0	0	0	0	0
0.0009	0.0011	0.0012	0.0012	0.0012
0	0	0	0	0
0	0	0	0	0
0.0007	0.0007	0.0008	0.0008	0.0008
0.0007	0.0007	0.0007	0.0006	0.0005
0	0	0	0	0
0.0023	0.0029	0.0027	0.0026	0.0026
0.0046	0.0064	0.0062	0.0054	0.0066
0.0551	0.0652	0.0642	0.051	0.0626
0.1061	0.1201	0.0963	0.0628	0.0801
0.0592	0.0739	0.0655	0.0529	0.0711
0.0253	0.0327	0.0325	0.0313	0.0314
0.052	0.0541	0.0552	0.0517	0.0528
0.0583	0.0697	0.0659	0.0604	0.0578
0.0989	0.1303	0.1273	0.1092	0.1133
0.1094	0.1513	0.1306	0.0927	0.1743
0.0948	0.1304	0.1118	0.0886	0.12
0.0799	0.0984	0.0948	0.0811	0.1012
0.1141	0.1359	0.1287	0.1057	0.1444
0.1332	0.1543	0.1433	0.1243	0.1434
0.1845	0.2075	0.2033	0.1808	0.1835
0.133	0.1488	0.1391	0.1241	0.1294

0.0381	0.0476	0.0369	0.0274	0.0424
0.0557	0.0771	0.0612	0.0427	0.0619
0.0057	0.0058	0.0061	0.0059	0.0054
0.0007	0.0007	0.0007	0.0007	0.0007
0	0	0	0	0
0.0008	0.0008	0.0007	0.0007	0.0006
0	0	0	0	0
0	0	0	0	0
0.0007	0.0008	0.0008	0.0008	0.0007
0	0	0	0	0
0	0	0	0	0
0	0	0	0	0
0	0	0	0	0
0	0	0	0	0
0	0	0	0	0
0	0	0	0	0
0	0	0	0	0
0	0	0	0	0
0	0	0	0	0
0	0	0	0	0
0	0	0	0	0
0	0	0	0	0
0	0	0	0	0
0	0	0	0	0
0	0	0	0	0
0	0	0	0	0
0	0	0	0	0
0	0	0	0	0
0.0029	0.0033	0.0032	0.0031	0.003
0.0005	0.0006	0.0005	0.0005	0.0005
0	0	0	0	0
0.0006	0.0007	0.0007	0.0006	0.0006
0.0018	0.0019	0.002	0.0017	0.0014
0.0011	0.0012	0.0011	0.0009	0.0014
0.0038	0.0059	0.0049	0.0053	0.0062
0.0081	0.0082	0.0087	0.0088	0.0075
0.0594	0.0687	0.0609	0.064	0.0606
0.0257	0.0331	0.0367	0.0369	0.0383
0.1352	0.1533	0.1474	0.1277	0.141
0.1731	0.1869	0.1793	0.1542	0.1678
0.0697	0.0898	0.0839	0.0827	0.0943
0.0901	0.1036	0.0904	0.0854	0.1049
0.0949	0.1057	0.1005	0.0985	0.1172
0.0771	0.105	0.0977	0.0944	0.0956

0.0888	0.1128	0.1026	0.0823	0.0972
0.1289	0.1646	0.1468	0.1395	0.1689
0.1317	0.1642	0.1503	0.1332	0.1639
0.1219	0.1462	0.1406	0.125	0.1325
0.1543	0.1605	0.1614	0.1437	0.1388
0.1613	0.178	0.1716	0.1444	0.1451
0.0922	0.1045	0.096	0.081	0.0903
0.0337	0.0367	0.0322	0.0233	0.0328
0.009	0.0096	0.0094	0.0088	0.008
0.0013	0.0014	0.0014	0.0013	0.0012
0	0	0	0	0
0.0006	0.0007	0.0007	0.0007	0.0006
0	0	0	0	0
0	0	0	0	0
0.0005	0.0006	0.0006	0.0006	0.0005
0	0	0	0	0
0	0	0	0	0
0	0	0	0	0
0	0	0	0	0
0	0	0	0	0
0	0	0	0	0
0	0	0	0	0
0	0	0	0	0
0	0	0	0	0
0	0	0	0	0
0	0	0	0	0
0	0	0	0	0
0	0	0	0	0
0	0	0	0	0
0	0	0	0	0
0	0	0	0	0
0	0	0	0	0
0	0	0	0	0
0	0	0	0	0
0.0023	0.0026	0.0029	0.0029	0.0024
0.0005	0.0008	0.0006	0.0005	0.0006
0	0	0	0	0
0.0014	0.0016	0.0016	0.0015	0.0013
0.0013	0.0014	0.0015	0.0014	0.0013
0.0007	0.0008	0.0008	0.0008	0.0009
0.0031	0.0035	0.003	0.0029	0.0031
0.0046	0.0063	0.006	0.0047	0.0055
0.0297	0.034	0.0326	0.0275	0.0366
0.1267	0.16	0.1543	0.1408	0.1534

0.2212	0.2743	0.2505	0.2133	0.2499
0.1646	0.208	0.2028	0.1423	0.1843
0.0883	0.1471	0.141	0.1154	0.1737
0.0695	0.1025	0.1082	0.1028	0.1318
0.109	0.1371	0.1257	0.116	0.128
0.0481	0.0696	0.062	0.0606	0.0651
0.0518	0.0677	0.0561	0.065	0.0748
0.2026	0.2409	0.2331	0.1909	0.2369
0.1759	0.2138	0.2046	0.1622	0.1871
0.0931	0.1183	0.1136	0.0975	0.1083
0.0762	0.0996	0.1006	0.0886	0.0967
0.0907	0.1241	0.112	0.0999	0.1191
0.128	0.1438	0.135	0.1127	0.1334
0.0827	0.094	0.0965	0.0972	0.0845
0.0101	0.0116	0.0112	0.0099	0.0093
0.0014	0.0015	0.0015	0.0015	0.0013
0	0	0	0	0
0.0006	0.0007	0.0007	0.0007	0.0006
0	0	0	0	0
0	0	0	0	0
0.0007	0.0008	0.0008	0.0008	0.0008
0	0	0	0	0
0	0	0	0	0
0	0	0	0	0
0	0	0	0	0
0	0	0	0	0
0	0	0	0	0
0	0	0	0	0
0	0	0	0	0
0	0	0	0	0
0	0	0	0	0
0	0	0	0	0
0	0	0	0	0
0	0	0	0	0
0	0	0	0	0
0	0	0	0	0
0	0	0	0	0
0	0	0	0	0
0	0	0	0	0
0	0	0	0	0
0.0012	0.0013	0.0014	0.0012	0.0012
0.0017	0.002	0.0022	0.0023	0.0021
0.0019	0.0021	0.0024	0.0022	0.0021
0.0014	0.0019	0.0019	0.0018	0.0018

0	0	0	0	0
0	0	0	0	0
0.0015	0.0015	0.0014	0.0014	0.0013
0.0067	0.007	0.0069	0.0059	0.006
0.0313	0.036	0.0358	0.0316	0.0241
0.1411	0.1617	0.1573	0.1311	0.1383
0.1529	0.1776	0.1672	0.1384	0.158
0.1767	0.1976	0.1966	0.1712	0.1758
0.1492	0.1739	0.1601	0.132	0.1526
0.1284	0.1626	0.1594	0.1409	0.1534
0.1049	0.1316	0.1269	0.1191	0.1383
0.1278	0.1624	0.1531	0.1356	0.1501
0.1276	0.1758	0.1732	0.1228	0.1319
0.2294	0.2843	0.2569	0.2163	0.2741
0.1194	0.1592	0.1446	0.1219	0.1721
0.1266	0.1566	0.1521	0.1406	0.1459
0.1173	0.155	0.133	0.0714	0.1081
0.3516	0.4321	0.4098	0.287	0.4286
0.3482	0.3694	0.3361	0.2685	0.3446
0.1347	0.139	0.1404	0.1188	0.1184
0.0112	0.0131	0.0139	0.0132	0.0114
0.0013	0.0013	0.0015	0.0014	0.0014
0	0	0	0	0
0.0008	0.0008	0.0009	0.0008	0.0007
0.0006	0.0006	0.0006	0.0005	0.0005
0	0	0	0	0
0	0	0	0	0
0	0	0	0	0
0	0	0	0	0
0	0	0	0	0
0	0	0	0	0
0	0	0	0	0
0	0	0	0	0
0	0	0	0	0
0	0	0	0	0
0	0	0	0	0
0	0	0	0	0
0	0	0	0	0
0	0	0	0	0
0	0	0	0	0
0	0	0	0	0
0	0	0	0	0
0	0	0	0	0
0	0	0	0	0

0	0	0	0	0
0	0	0	0	0
0.0006	0.0007	0.0007	0.0007	0.0006
0.0008	0.0011	0.0011	0.0011	0.0012
0.0039	0.0048	0.0048	0.0046	0.0045
0.0019	0.0024	0.0028	0.0026	0.0029
0.0003	0.0004	0.0005	0.0004	0.0006
0	0	0	0	0
0.0025	0.0026	0.0026	0.0024	0.0021
0.0091	0.0092	0.0094	0.0093	0.0081
0.0872	0.094	0.0958	0.0922	0.0864
0.0552	0.0724	0.065	0.0451	0.0569
0.0342	0.0414	0.041	0.0346	0.0363
0.1099	0.1197	0.1208	0.1057	0.0994
0.1447	0.1594	0.1587	0.1379	0.1385
0.1311	0.1702	0.1501	0.1165	0.1566
0.2551	0.2932	0.2732	0.1968	0.265
0.2345	0.2995	0.2794	0.2031	0.2897
0.22	0.2717	0.2257	0.1881	0.2682
0.1748	0.232	0.2054	0.1421	0.2351
0.1407	0.1431	0.0974	0.0714	0.1322
0.112	0.1446	0.1307	0.1067	0.1139
0.2899	0.3296	0.2876	0.23	0.3159
0.3854	0.4914	0.4075	0.2431	0.4319
0.4948	0.5985	0.5114	0.3407	0.4784
0.1287	0.1411	0.141	0.1241	0.1303
0.0116	0.0117	0.0134	0.0118	0.0112
0.0013	0.0015	0.0015	0.0014	0.0012
0	0	0	0	0
0.0016	0.0017	0.0017	0.0015	0.0015
0.0012	0.0011	0.0013	0.0012	0.001
0	0	0	0	0
0	0	0	0	0
0	0	0	0	0
0	0	0	0	0
0	0	0	0	0
0	0	0	0	0
0	0	0	0	0
0	0	0	0	0
0	0	0	0	0
0	0	0	0	0
0	0	0	0	0
0	0	0	0	0
0	0	0	0	0

0	0	0	0	0
0	0	0	0	0
0	0	0	0	0
0	0	0	0	0
0	0	0	0	0
0	0	0	0	0
0	0	0	0	0
0	0	0	0	0
0	0	0	0	0
0.0004	0.0005	0.0005	0.0007	0.0007
0.0009	0.0012	0.0011	0.0009	0.0011
0.0013	0.0017	0.0016	0.0015	0.0018
0.0004	0.0006	0.0006	0.0006	0.0006
0	0	0	0	0
0.0012	0.0013	0.0013	0.0011	0.001
0.0057	0.0064	0.0063	0.0056	0.005
0.0207	0.0215	0.0147	0.0143	0.0211
0.0291	0.0315	0.0289	0.0263	0.028
0.0505	0.0664	0.0571	0.0458	0.0617
0.0988	0.1164	0.1019	0.0923	0.1242
0.087	0.1217	0.1099	0.0902	0.1065
0.2014	0.2382	0.2318	0.1825	0.2549
0.2756	0.3174	0.2842	0.1766	0.294
0.2824	0.3363	0.3047	0.204	0.303
0.2764	0.3292	0.2789	0.2391	0.3669
0.3683	0.4405	0.367	0.2534	0.4296
0.2293	0.2516	0.2123	0.1557	0.276
0.0776	0.1029	0.0963	0.0816	0.0879
0.2292	0.2656	0.2311	0.1816	0.2324
0.508	0.5875	0.5137	0.3545	0.5698
0.4475	0.5054	0.4341	0.2891	0.4698
0.18	0.2071	0.2067	0.1645	0.1757
0.0159	0.018	0.0185	0.0173	0.0157
0.0013	0.0013	0.0013	0.0013	0.0011
0	0	0	0	0
0.0013	0.0013	0.0014	0.0013	0.0013
0.0015	0.0016	0.0016	0.0014	0.0014
0	0	0	0	0
0	0	0	0	0
0	0	0	0	0
0	0	0	0	0
0	0	0	0	0
0	0	0	0	0
0	0	0	0	0

0	0	0	0	0
0	0	0	0	0
0	0	0	0	0
0	0	0	0	0
0	0	0	0	0
0	0	0	0	0
0	0	0	0	0
0	0	0	0	0
0	0	0	0	0
0	0	0	0	0
0	0	0	0	0
0	0	0	0	0
0	0	0	0	0
0	0	0	0	0
0.0011	0.0014	0.0013	0.0011	0.0012
0.0009	0.0011	0.0011	0.0009	0.0008
0.0004	0.0009	0.0008	0.0011	0.0011
0.0008	0.0009	0.0009	0.0008	0.0008
0.002	0.0022	0.0024	0.0021	0.0022
0.0009	0.0011	0.0011	0.001	0.001
0.0014	0.0019	0.0019	0.0017	0.0018
0.0019	0.0025	0.0024	0.0019	0.0023
0.0235	0.0331	0.0289	0.0168	0.0273
0.0113	0.0126	0.0127	0.0119	0.0112
0.1106	0.1218	0.1165	0.0914	0.1201
0.1804	0.2389	0.2132	0.1665	0.1997
0.0614	0.1106	0.1207	0.0893	0.1155
0.0871	0.1356	0.1081	0.1046	0.1331
0.3096	0.3524	0.3183	0.1787	0.2784
0.2251	0.2415	0.228	0.1872	0.2228
0.3171	0.3455	0.3136	0.2562	0.3415
0.4544	0.5586	0.4742	0.4087	0.6312
0.334	0.4175	0.3432	0.2929	0.4519
0.1185	0.1443	0.1412	0.1245	0.1432
0.1093	0.1322	0.119	0.1044	0.1123
0.4655	0.5981	0.5069	0.3981	0.4901
0.4179	0.4398	0.3207	0.1979	0.3647
0.1482	0.1658	0.1721	0.1475	0.1624
0.0173	0.0186	0.0183	0.0169	0.0162
0.0013	0.0014	0.0014	0.0013	0.0012
0	0	0	0	0
0.0015	0.0016	0.0016	0.0015	0.0013
0.0024	0.0024	0.0023	0.002	0.0017
0	0	0	0	0

0	0	0	0	0
0.0008	0.0007	0.0008	0.0007	0.0006
0	0	0	0	0
0	0	0	0	0
0	0	0	0	0
0	0	0	0	0
0	0	0	0	0
0	0	0	0	0
0	0	0	0	0
0	0	0	0	0
0	0	0	0	0
0	0	0	0	0
0	0	0	0	0
0	0	0	0	0
0	0	0	0	0
0	0	0	0	0
0	0	0	0	0
0	0	0	0	0
0	0	0	0	0
0	0	0	0	0
0	0	0	0	0
0	0	0	0	0
0	0	0	0	0
0	0	0	0	0
0	0	0	0	0
0	0	0	0	0
0	0	0	0	0
0	0	0	0	0
0.0021	0.0027	0.0027	0.0026	0.0026
0.0815	0.113	0.1051	0.083	0.1097
0.3161	0.4622	0.3932	0.3325	0.4447
0.1383	0.1804	0.1546	0.1236	0.1635
0.127	0.1615	0.1558	0.14	0.1655
0.0866	0.1131	0.0983	0.0801	0.113
0.0635	0.0997	0.0925	0.0716	0.0725
0.0636	0.1052	0.1084	0.1056	0.1225
0.1661	0.1912	0.1792	0.1683	0.19
0.4186	0.5574	0.4539	0.3828	0.4956
0.1676	0.1405	0.1074	0.0755	0.1239
0.089	0.1152	0.1142	0.1136	0.1231
0.2026	0.2385	0.2181	0.1712	0.2375
0.2918	0.3571	0.3206	0.2018	0.2858
0.2961	0.3284	0.2932	0.2295	0.2878
0.1696	0.1946	0.1869	0.1611	0.1708

0.0106	0.0125	0.0127	0.0116	0.0116
0.0043	0.0046	0.0049	0.0044	0.004
0	0	0	0	0
0.0016	0.002	0.0019	0.0018	0.002
0.0012	0.0015	0.0016	0.0016	0.0014
0	0	0	0	0
0	0	0	0	0
0.0015	0.0016	0.0017	0.0016	0.0014
0	0	0	0	0
0	0	0	0	0
0	0	0	0	0
0	0	0	0	0
0	0	0	0	0
0	0	0	0	0
0	0	0	0	0
0	0	0	0	0
0	0	0	0	0
0	0	0	0	0
0	0	0	0	0
0	0	0	0	0
0	0	0	0	0
0	0	0	0	0
0	0	0	0	0
0	0	0	0	0
0	0	0	0	0
0	0	0	0	0
0	0	0	0	0
0	0	0	0	0
0	0	0	0	0
0	0	0	0	0
0	0	0	0	0
0	0	0	0	0
0	0	0	0	0
0	0	0	0	0
0.072	0.0804	0.0602	0.0468	0.0732
0.0351	0.0455	0.0373	0.0186	0.0336
0.1944	0.2417	0.1993	0.1317	0.1693
0.1313	0.1656	0.1641	0.1316	0.1752
0.076	0.1084	0.1136	0.1003	0.1159
0.0911	0.1333	0.1237	0.119	0.133
0.0465	0.0811	0.0801	0.0861	0.0847
0.1377	0.1647	0.1493	0.1301	0.1562
0.1887	0.248	0.1856	0.1163	0.1723
0.2002	0.2816	0.2472	0.1251	0.2016

0.0632	0.0961	0.0924	0.0712	0.0917
0.2071	0.212	0.1769	0.1613	0.1944
0.3819	0.4238	0.2859	0.123	0.2732
0.4616	0.477	0.4292	0.3355	0.4369
0.1621	0.1753	0.1702	0.1454	0.163
0.0945	0.1016	0.1021	0.0945	0.0838
0.0131	0.0147	0.0145	0.0129	0.012
0.0027	0.003	0.003	0.0026	0.0025
0	0	0	0	0
0.002	0.0025	0.0026	0.0024	0.0027
0.0018	0.002	0.0022	0.0022	0.002
0	0	0	0	0
0.0003	0.0005	0.0005	0.0005	0.0007
0.0008	0.0008	0.0008	0.0008	0.0007
0	0	0	0	0
0	0	0	0	0
0	0	0	0	0
0	0	0	0	0
0	0	0	0	0
0	0	0	0	0
0	0	0	0	0
0	0	0	0	0
0	0	0	0	0
0	0	0	0	0
0	0	0	0	0
0	0	0	0	0
0	0	0	0	0
0	0	0	0	0
0	0	0	0	0
0	0	0	0	0
0	0	0	0	0
0	0	0	0	0
0	0	0	0	0
0	0	0	0	0
0	0	0	0	0
0	0	0	0	0
0	0	0	0	0
0	0	0	0	0
0	0	0	0	0
0	0	0	0	0
0	0	0	0	0
0.0054	0.0065	0.0065	0.007	0.0078
0.0206	0.0218	0.0223	0.0192	0.0183
0.0336	0.0397	0.038	0.034	0.0367
0.0639	0.0854	0.0959	0.0974	0.0989

0.1286	0.1698	0.1493	0.1564	0.1601
0.1306	0.1964	0.1645	0.1568	0.2071
0.1698	0.1926	0.2011	0.1922	0.2295
0.0814	0.1156	0.1167	0.1013	0.1363
0.8145	0.9145	0.7297	0.5609	0.8039
0.3972	0.4536	0.3143	0.263	0.4031
0.1679	0.1799	0.1288	0.0985	0.1277
0.1197	0.157	0.1505	0.1132	0.1489
0.3282	0.353	0.2666	0.132	0.2713
0.1844	0.247	0.2118	0.1611	0.1976
0.0367	0.0407	0.043	0.0404	0.0369
0.072	0.08	0.0726	0.0577	0.0738
0.0089	0.0101	0.0096	0.0088	0.009
0.0008	0.0009	0.0009	0.0008	0.0007
0	0	0	0	0
0.0019	0.0023	0.0024	0.0021	0.0022
0.0021	0.0022	0.0022	0.0021	0.002
0	0	0	0	0
0.0005	0.0006	0.0006	0.0006	0.0006
0.0008	0.0008	0.0008	0.0008	0.0007
0	0	0	0	0
0	0	0	0	0
0	0	0	0	0
0	0	0	0	0
0	0	0	0	0
0	0	0	0	0
0	0	0	0	0
0	0	0	0	0
0	0	0	0	0
0	0	0	0	0
0	0	0	0	0
0	0	0	0	0
0	0	0	0	0
0	0	0	0	0
0	0	0	0	0
0	0	0	0	0
0	0	0	0	0
0	0	0	0	0
0	0	0	0	0
0	0	0	0	0
0	0	0	0	0
0	0	0	0	0
0	0	0	0	0
0	0	0	0	0

0	0	0	0	0
0	0	0	0	0
0.0552	0.0731	0.0645	0.0513	0.0729
0.1517	0.1677	0.1558	0.1274	0.1351
0.0557	0.0766	0.0731	0.054	0.0955
0.0912	0.099	0.0856	0.07	0.0862
0.1459	0.1601	0.1578	0.1506	0.1684
0.2959	0.4228	0.413	0.2786	0.382
0.461	0.5881	0.4926	0.3958	0.4869
0.2465	0.3427	0.2947	0.2503	0.3659
0.4337	0.5369	0.4463	0.2818	0.4594
0.787	0.8598	0.7024	0.4691	0.6851
0.2435	0.3303	0.2676	0.2167	0.319
0.0855	0.1024	0.1121	0.1049	0.089
0.1062	0.1148	0.1112	0.1025	0.1057
0.0998	0.1221	0.1144	0.1011	0.1156
0.0411	0.0491	0.0458	0.0348	0.0469
0.0521	0.0838	0.0677	0.0518	0.0622
0.0034	0.0042	0.0046	0.0037	0.0042
0	0	0	0	0
0	0	0	0	0
0.0017	0.002	0.0019	0.0021	0.0018
0.002	0.0023	0.0024	0.0022	0.0019
0.0007	0.0008	0.0008	0.0007	0.0007
0.0002	0.0004	0.0004	0.0004	0.0004
0	0	0	0	0
0	0	0	0	0
0	0	0	0	0
0	0	0	0	0
0	0	0	0	0
0	0	0	0	0
0	0	0	0	0
0	0	0	0	0
0	0	0	0	0
0	0	0	0	0
0	0	0	0	0
0	0	0	0	0
0	0	0	0	0
0	0	0	0	0
0	0	0	0	0
0	0	0	0	0
0	0	0	0	0
0	0	0	0	0
0	0	0	0	0

0	0	0	0	0
0	0	0	0	0
0	0	0	0	0
0	0	0	0	0
0	0	0	0	0
0	0	0	0	0
0	0	0	0	0
0	0	0	0	0
0.0258	0.0286	0.0278	0.0267	0.0237
0.1147	0.1236	0.1171	0.0959	0.1025
0.0603	0.0731	0.0608	0.0462	0.0613
0.0187	0.0209	0.0202	0.0174	0.0186
0.0782	0.1028	0.0915	0.0691	0.0915
0.385	0.4418	0.4158	0.2903	0.4436
0.6875	0.7543	0.6135	0.4226	0.6468
0.5289	0.5796	0.4751	0.2995	0.5465
0.3123	0.3576	0.3006	0.19	0.3458
0.5998	0.6302	0.5335	0.3939	0.6395
0.1905	0.2869	0.2539	0.2138	0.3225
0.0511	0.0777	0.0698	0.056	0.0651
0.0295	0.032	0.0301	0.0269	0.0287
0.0425	0.0533	0.0419	0.0305	0.0443
0.0753	0.0924	0.0898	0.0646	0.0664
0.0066	0.0164	0.0189	0.0199	0.0229
0.0013	0.0029	0.0031	0.0037	0.0033
0	0	0	0	0
0	0	0	0	0
0.0015	0.0019	0.0018	0.0015	0.0015
0.0014	0.0015	0.0015	0.0014	0.0013
0.0007	0.0007	0.0007	0.0006	0.0005
0.0003	0.0004	0.0003	0.0004	0.0005
0	0	0	0	0
0	0	0	0	0
0	0	0	0	0
0	0	0	0	0
0	0	0	0	0
0	0	0	0	0
0	0	0	0	0
0	0	0	0	0
0	0	0	0	0
0	0	0	0	0
0	0	0	0	0
0	0	0	0	0
0	0	0	0	0

0	0	0	0	0
0	0	0	0	0
0	0	0	0	0
0	0	0	0	0
0	0	0	0	0
0	0	0	0	0
0	0	0	0	0
0	0	0	0	0
0	0	0	0	0
0	0	0	0	0
0	0	0	0	0
0	0	0	0	0
0	0	0	0	0
0	0	0	0	0
0.049	0.0574	0.0551	0.0437	0.0498
0.1384	0.1585	0.144	0.126	0.1382
0.0983	0.1128	0.1135	0.1061	0.1071
0.1241	0.1356	0.1304	0.1131	0.1235
0.0607	0.0649	0.0609	0.0481	0.0527
0.0852	0.1005	0.0866	0.0678	0.0898
0.2496	0.3107	0.2679	0.219	0.2816
0.195	0.2368	0.2023	0.1652	0.2229
0.5671	0.6473	0.5302	0.4583	0.5816
0.3075	0.3661	0.3141	0.227	0.3241
0.0677	0.0786	0.0538	0.047	0.0698
0.0852	0.0986	0.0889	0.0651	0.0848
0.0413	0.0543	0.0526	0.0404	0.0666
0.0073	0.0126	0.0128	0.0163	0.0182
0.0354	0.0396	0.0345	0.0259	0.0388
0.0273	0.0298	0.0239	0.0193	0.0261
0.0029	0.004	0.003	0.0037	0.0033
0.0002	0.0003	0.0003	0.0004	0.0004
0	0	0	0	0
0.0006	0.0009	0.0009	0.0009	0.001
0.0006	0.0007	0.0007	0.0007	0.0007
0.0007	0.0007	0.0007	0.0007	0.0005
0.0011	0.0012	0.0011	0.0009	0.0011
0.0006	0.0006	0.0007	0.0007	0.0006
0	0	0	0	0
0	0	0	0	0
0	0	0	0	0
0	0	0	0	0
0	0	0	0	0
0	0	0	0	0

0	0	0	0	0
0	0	0	0	0
0	0	0	0	0
0	0	0	0	0
0	0	0	0	0
0	0	0	0	0
0	0	0	0	0
0	0	0	0	0
0	0	0	0	0
0	0	0	0	0
0	0	0	0	0
0	0	0	0	0
0	0	0	0	0
0	0	0	0	0
0	0	0	0	0
0	0	0	0	0
0	0	0	0	0
0	0	0	0	0
0	0	0	0	0
0	0	0	0	0
0.0406	0.0485	0.0442	0.027	0.0379
0.1581	0.1879	0.1814	0.1429	0.1871
0.1048	0.1182	0.1068	0.0855	0.1076
0.2125	0.234	0.2131	0.1692	0.2057
0.1461	0.1589	0.1487	0.1013	0.1371
0.0199	0.0235	0.0224	0.0204	0.0208
0.0728	0.0915	0.0875	0.0714	0.073
0.0541	0.0701	0.064	0.0534	0.0516
0.1301	0.1571	0.1461	0.0902	0.1386
0.0143	0.0174	0.0167	0.0168	0.0205
0.0048	0.0055	0.0051	0.0049	0.0045
0.1224	0.1333	0.0997	0.0758	0.1488
0.0951	0.1195	0.1213	0.119	0.1759
0.0376	0.0646	0.0578	0.0508	0.082
0.1813	0.2069	0.2038	0.1465	0.2248
0.0536	0.0855	0.0637	0.0476	0.0667
0.0051	0.0078	0.0058	0.0053	0.0063
0.0012	0.0016	0.0016	0.0014	0.0017
0.0012	0.0013	0.0013	0.0011	0.0011
0.0006	0.001	0.0008	0.0009	0.0011
0	0	0	0	0
0.0007	0.0007	0.0007	0.0006	0.0006
0.0014	0.0018	0.0017	0.0016	0.0016
0.0014	0.0016	0.0016	0.0015	0.0013

0	0	0	0	0
0	0	0	0	0
0	0	0	0	0
0	0	0	0	0
0	0	0	0	0
0	0	0	0	0
0	0	0	0	0
0	0	0	0	0
0	0	0	0	0
0	0	0	0	0
0	0	0	0	0
0	0	0	0	0
0	0	0	0	0
0	0	0	0	0
0	0	0	0	0
0	0	0	0	0
0	0	0	0	0
0	0	0	0	0
0	0	0	0	0
0	0	0	0	0
0	0	0	0	0
0	0	0	0	0
0	0	0	0	0
0	0	0	0	0
0	0	0	0	0
0	0	0	0	0
0.0689	0.0851	0.0601	0.0425	0.079
0.2112	0.2556	0.2173	0.1343	0.2181
0.1303	0.1435	0.1295	0.1001	0.1356
0.0236	0.0317	0.0263	0.0198	0.0224
0.0695	0.0747	0.0687	0.0514	0.0693
0.0159	0.0171	0.0171	0.0139	0.0117
0.1365	0.1531	0.156	0.1336	0.1478
0.1307	0.1609	0.1478	0.115	0.162
0.1044	0.1156	0.1129	0.0971	0.1103
0.1824	0.2074	0.1879	0.1216	0.1637
0.3644	0.4012	0.2938	0.1767	0.2972
0.2288	0.2953	0.3099	0.2406	0.3304
0.0253	0.0305	0.0289	0.0248	0.025
0.1449	0.1794	0.1365	0.1075	0.1495
0.26	0.3294	0.2652	0.1352	0.2155
0.1371	0.1635	0.14	0.1044	0.1353
0.0063	0.0085	0.008	0.0075	0.0081
0.002	0.0026	0.0027	0.0028	0.0026

0.0015	0.0018	0.0019	0.0017	0.0016
0.001	0.0012	0.0011	0.0011	0.0011
0	0	0	0	0
0	0	0	0	0
0.0017	0.002	0.0019	0.002	0.0019
0.001	0.0012	0.0013	0.0012	0.0011
0	0	0	0	0
0	0	0	0	0
0	0	0	0	0
0	0	0	0	0
0	0	0	0	0
0	0	0	0	0
0	0	0	0	0
0	0	0	0	0
0	0	0	0	0
0	0	0	0	0
0	0	0	0	0
0	0	0	0	0
0	0	0	0	0
0	0	0	0	0
0	0	0	0	0
0	0	0	0	0
0	0	0	0	0
0	0	0	0	0
0	0	0	0	0
0	0	0	0	0
0	0	0	0	0
0	0	0	0	0
0	0	0	0	0
0	0	0	0	0
0	0	0	0	0
0	0	0	0	0
0.0291	0.0324	0.0262	0.0178	0.0247
0.0235	0.0255	0.0209	0.011	0.0193
0.0233	0.0276	0.0244	0.0198	0.0263
0.0128	0.0157	0.0146	0.0067	0.0126
0.0267	0.0313	0.0284	0.0227	0.0272
0.0236	0.0314	0.0277	0.0223	0.0305
0.0275	0.0327	0.0273	0.0196	0.0286
0.0364	0.0414	0.0371	0.0314	0.038
0.0398	0.0447	0.0382	0.0279	0.0424
0.0328	0.0401	0.0357	0.0244	0.0448
0.0302	0.0323	0.0288	0.0158	0.0261
0.0317	0.0365	0.0291	0.0211	0.0366

0.032	0.0401	0.0287	0.0184	0.0275
0.041	0.0454	0.0444	0.0333	0.0461
0.0425	0.0521	0.0436	0.0316	0.0502
0.0273	0.0353	0.0316	0.0177	0.037
0	0	0	0	0
0	0	0	0	0
0	0	0	0	0
0	0	0	0	0
0	0	0	0	0
0	0	0	0	0
0	0	0	0	0
0	0	0	0	0
0	0	0	0	0
0	0	0	0	0
0	0	0	0	0
0	0	0	0	0
0	0	0	0	0
0	0	0	0	0
0	0	0	0	0
0	0	0	0	0
0	0	0	0	0
0	0	0	0	0
0	0	0	0	0
0	0	0	0	0
0	0	0	0	0
0	0	0	0	0
0	0	0	0	0
0	0	0	0	0
0	0	0	0	0
0	0	0	0	0
0	0	0	0	0
0	0	0	0	0
0	0	0	0	0
0	0	0	0	0
0	0	0	0	0
0	0	0	0	0
0	0	0	0	0
0	0	0	0	0
0.0158	0.0186	0.0159	0.0128	0.0141
0.01	0.0129	0.0098	0.0051	0.0102
0.0098	0.011	0.0087	0.0059	0.0107
0.0079	0.0107	0.0099	0.0057	0.0117
0.01	0.012	0.0094	0.008	0.0094
0.0095	0.0119	0.0098	0.0061	0.0093

0.0139	0.0152	0.0143	0.0119	0.0168
0.0163	0.0196	0.0173	0.0142	0.0191
0.0109	0.0122	0.0111	0.0077	0.0114
0.0114	0.0134	0.0119	0.0086	0.013
0.0127	0.0144	0.0125	0.0072	0.0121
0.0119	0.0138	0.0129	0.0103	0.0135
0.0136	0.016	0.0128	0.009	0.0124
0.0131	0.0164	0.0143	0.0112	0.0155
0.0107	0.0125	0.0094	0.0064	0.0088
0.0091	0.0105	0.0088	0.0042	0.0079
0	0	0	0	0
0	0	0	0	0
0	0	0	0	0
0	0	0	0	0
0	0	0	0	0
0	0	0	0	0
0	0	0	0	0
0	0	0	0	0
0	0	0	0	0
0	0	0	0	0
0	0	0	0	0
0	0	0	0	0
0	0	0	0	0
0	0	0	0	0
0	0	0	0	0
0	0	0	0	0
0	0	0	0	0
0	0	0	0	0
0	0	0	0	0
0	0	0	0	0
0	0	0	0	0
0	0	0	0	0
0	0	0	0	0
0	0	0	0	0
0	0	0	0	0
0	0	0	0	0
0	0	0	0	0
0	0	0	0	0
0	0	0	0	0
0	0	0	0	0
0	0	0	0	0
0	0	0	0	0
0	0	0	0	0
0	0	0	0	0

0.0006	0.0006	0.0007	0.0006	0.0006
0	0	0	0	0
0	0	0	0	0
0	0	0	0	0
0	0	0	0	0
0	0	0	0	0
0	0	0	0	0
0	0	0	0	0
0	0	0	0	0
0	0	0	0	0
0	0	0	0	0
0	0	0	0	0
0	0	0	0	0
0.0006	0.0007	0.0007	0.0007	0.0006
0.0006	0.0007	0.0007	0.0006	0.0005
0.0006	0.0006	0.0007	0.0007	0.0006
0	0	0	0	0
0	0	0	0	0
0	0	0	0	0
0	0	0	0	0
0	0	0	0	0
0	0	0	0	0
0	0	0	0	0
0	0	0	0	0
0	0	0	0	0
0	0	0	0	0
0	0	0	0	0
0	0	0	0	0
0	0	0	0	0
0	0	0	0	0
0	0	0	0	0
0	0	0	0	0
0	0	0	0	0
0	0	0	0	0
0	0	0	0	0
0	0	0	0	0
0	0	0	0	0
0	0	0	0	0
0	0	0	0	0
0	0	0	0	0
0	0	0	0	0
0	0	0	0	0
0	0	0	0	0
0	0	0	0	0

0	0	0	0	0
0	0	0	0	0
0	0	0	0	0
0	0	0	0	0
0	0	0	0	0
0	0	0	0	0
0.0012	0.0013	0.0014	0.0013	0.0012
0.0015	0.0015	0.0015	0.0015	0.0013
0.0007	0.0007	0.0008	0.0007	0.0007
0.0007	0.0008	0.0008	0.0008	0.0007
0	0	0	0	0
0	0	0	0	0
0	0	0	0	0
0	0	0	0	0
0	0	0	0	0
0	0	0	0	0
0	0	0	0	0
0	0	0	0	0
0	0	0	0	0
0.0006	0.0007	0.0007	0.0007	0.0006
0.0006	0.0007	0.0007	0.0006	0.0006
0.0006	0.0006	0.0006	0.0005	0.0005
0	0	0	0	0
0	0	0	0	0
0	0	0	0	0
0	0	0	0	0
0	0	0	0	0
0	0	0	0	0
0	0	0	0	0
0	0	0	0	0
0	0	0	0	0
0	0	0	0	0
0	0	0	0	0
0	0	0	0	0
0	0	0	0	0
0	0	0	0	0
0	0	0	0	0
0	0	0	0	0
0	0	0	0	0
0	0	0	0	0
0	0	0	0	0
0	0	0	0	0
0	0	0	0	0
0	0	0	0	0

0	0	0	0	0
0	0	0	0	0
0	0	0	0	0
0	0	0	0	0
0	0	0	0	0
0	0	0	0	0
0	0	0	0	0
0	0	0	0	0
0	0	0	0	0
0	0	0	0	0
0	0	0	0	0
0	0	0	0	0
0.0061	0.0076	0.0064	0.0047	0.0065
0.0068	0.0074	0.0066	0.0047	0.0054
0.0057	0.0065	0.006	0.0052	0.0072
0.0063	0.0076	0.0068	0.0055	0.0069
0.0059	0.0074	0.0068	0.0052	0.0068
0.0072	0.0091	0.0077	0.0056	0.0084
0.0066	0.0079	0.0061	0.0044	0.0058
0.0064	0.0068	0.0059	0.0049	0.0064
0.0036	0.0047	0.0037	0.0023	0.0037
0.0057	0.0068	0.0064	0.005	0.0072
0.0077	0.0088	0.0076	0.0057	0.0078
0.0069	0.0082	0.0079	0.0066	0.0083
0.0053	0.0066	0.0062	0.0053	0.0077
0.0037	0.005	0.004	0.0019	0.0039
0.0049	0.0053	0.004	0.0025	0.0042
0.0054	0.0064	0.0054	0.0041	0.0061
0	0	0	0	0
0	0	0	0	0
0	0	0	0	0
0	0	0	0	0
0	0	0	0	0
0	0	0	0	0
0	0	0	0	0
0	0	0	0	0
0	0	0	0	0
0	0	0	0	0
0	0	0	0	0
0	0	0	0	0
0	0	0	0	0
0	0	0	0	0
0	0	0	0	0
0	0	0	0	0

0	0	0	0	0
0	0	0	0	0
0	0	0	0	0
0	0	0	0	0
0	0	0	0	0
0	0	0	0	0
0	0	0	0	0
0	0	0	0	0
0	0	0	0	0
0	0	0	0	0
0	0	0	0	0
0	0	0	0	0
0	0	0	0	0
0	0	0	0	0
0	0	0	0	0
0	0	0	0	0
0	0	0	0	0
0	0	0	0	0
0.0048	0.0057	0.0047	0.0018	0.0046
0.0046	0.0049	0.0041	0.0023	0.0045
0.0036	0.0049	0.0041	0.0023	0.0047
0.0039	0.0051	0.0049	0.0029	0.0052
0.0049	0.0047	0.0036	0.0023	0.0037
0.0051	0.006	0.0053	0.0027	0.005
0.0047	0.0058	0.0048	0.0033	0.0054
0.0066	0.0076	0.0077	0.0058	0.007
0.0021	0.0022	0.0022	0.0021	0.0018
0.0018	0.002	0.0021	0.0019	0.0016
0.0018	0.002	0.0022	0.0021	0.002
0.0014	0.0015	0.0015	0.0013	0.0012
0.0007	0.0008	0.0008	0.0007	0.0007
0	0	0	0	0
0	0	0	0	0
0	0	0	0	0
0	0	0	0	0
0	0	0	0	0
0	0	0	0	0
0	0	0	0	0
0	0	0	0	0
0	0	0	0	0
0	0	0	0	0
0	0	0	0	0
0	0	0	0	0
0	0	0	0	0

0	0	0	0	0
0	0	0	0	0
0	0	0	0	0
0	0	0	0	0
0	0	0	0	0
0	0	0	0	0
0	0	0	0	0
0	0	0	0	0
0	0	0	0	0
0	0	0	0	0
0	0	0	0	0
0	0	0	0	0
0	0	0	0	0
0	0	0	0	0
0	0	0	0	0
0	0	0	0	0
0	0	0	0	0
0	0	0	0	0
0	0	0	0	0
0	0	0	0	0
0	0	0	0	0
0	0	0	0	0
0	0	0	0	0
0	0	0	0	0
0.0052	0.0056	0.0049	0.0036	0.006
0.0051	0.0051	0.0043	0.0027	0.0046
0.0057	0.0063	0.0053	0.0038	0.0059
0.0036	0.0051	0.0042	0.003	0.0047
0.0044	0.006	0.0047	0.0029	0.0045
0.0065	0.0082	0.007	0.0053	0.0061
0.0058	0.0069	0.0053	0.0037	0.0058
0.0055	0.0067	0.0058	0.0042	0.0068
0.0013	0.0014	0.0014	0.0014	0.0013
0.0013	0.0014	0.0014	0.0013	0.0012
0.0007	0.0008	0.0008	0.0007	0.0007
0.0007	0.0007	0.0007	0.0007	0.0007
0.0008	0.0009	0.0009	0.0008	0.0007
0	0	0	0	0
0	0	0	0	0
0	0	0	0	0
0	0	0	0	0
0	0	0	0	0
0	0	0	0	0
0	0	0	0	0

0	0	0	0	0
0	0	0	0	0
0	0	0	0	0
0	0	0	0	0
0	0	0	0	0
0	0	0	0	0
0	0	0	0	0
0	0	0	0	0
0	0	0	0	0
0	0	0	0	0
0	0	0	0	0
0	0	0	0	0
0	0	0	0	0
0	0	0	0	0
0	0	0	0	0
0	0	0	0	0
0	0	0	0	0
0	0	0	0	0
0	0	0	0	0
0	0	0	0	0
0	0	0	0	0
0	0	0	0	0
0	0	0	0	0
0	0	0	0	0
0	0	0	0	0
0	0	0	0	0
0	0	0	0	0
0	0	0	0	0
0	0	0	0	0
0	0	0	0	0
0.0018	0.002	0.002	0.0019	0.0017
0.0015	0.0015	0.0015	0.0015	0.0013
0.0006	0.0006	0.0007	0.0006	0.0005
0	0	0	0	0
0	0	0	0	0
0	0	0	0	0
0.0007	0.0007	0.0007	0.0007	0.0006
0.0006	0.0006	0.0007	0.0006	0.0005
0.0037	0.0044	0.004	0.0022	0.0041
0.0028	0.0042	0.003	0.0017	0.0037
0.0041	0.0066	0.0056	0.0043	0.0062
0.0048	0.0065	0.0054	0.0048	0.0067
0.0051	0.0068	0.0064	0.0052	0.0065
0.0042	0.005	0.0039	0.0016	0.0033

0.0048	0.0065	0.0047	0.004	0.0062
0.0051	0.0062	0.0051	0.0041	0.0054
0	0	0	0	0
0	0	0	0	0
0	0	0	0	0
0	0	0	0	0
0	0	0	0	0
0	0	0	0	0
0	0	0	0	0
0	0	0	0	0
0	0	0	0	0
0	0	0	0	0
0	0	0	0	0
0	0	0	0	0
0	0	0	0	0
0	0	0	0	0
0	0	0	0	0
0	0	0	0	0
0	0	0	0	0
0	0	0	0	0
0	0	0	0	0
0	0	0	0	0
0	0	0	0	0
0	0	0	0	0
0	0	0	0	0
0	0	0	0	0
0	0	0	0	0
0	0	0	0	0
0	0	0	0	0
0	0	0	0	0
0	0	0	0	0
0	0	0	0	0
0	0	0	0	0
0	0	0	0	0
0	0	0	0	0
0	0	0	0	0
0	0	0	0	0
0	0	0	0	0
0	0	0	0	0
0	0	0	0	0
0	0	0	0	0
0	0	0	0	0
0	0	0	0	0
0	0	0	0	0

0	0	0	0	0
0	0	0	0	0
0	0	0	0	0
0	0	0	0	0
0	0	0	0	0
0	0	0	0	0
0	0	0	0	0
0	0	0	0	0
0	0	0	0	0
0	0	0	0	0
0	0	0	0	0
0	0	0	0	0
0	0	0	0	0
0	0	0	0	0
0	0	0	0	0
0	0	0	0	0
0	0	0	0	0
0	0	0	0	0
0	0	0	0	0
0	0	0	0	0
0	0	0	0	0
0	0	0	0	0
0	0	0	0	0
0	0	0	0	0
0	0	0	0	0
0	0	0	0	0
0	0	0	0	0
0	0	0	0	0
0	0	0	0	0
0	0	0	0	0
0	0	0	0	0
0	0	0	0	0
0	0	0	0	0
0	0	0	0	0
0	0	0	0	0
0	0	0	0	0
0	0	0	0	0
0	0	0	0	0
0	0	0	0	0
0	0	0	0	0
0	0	0	0	0
0	0	0	0	0
0	0	0	0	0
0	0	0	0	0

0	0	0	0	0
0	0	0	0	0
0	0	0	0	0
0	0	0	0	0
0	0	0	0	0
0	0	0	0	0
0	0	0	0	0
0	0	0	0	0
0	0	0	0	0
0	0	0	0	0
0	0	0	0	0
0	0	0	0	0
0	0	0	0	0
0	0	0	0	0
0	0	0	0	0
0	0	0	0	0
0	0	0	0	0
0	0	0	0	0
0	0	0	0	0
0	0	0	0	0
0	0	0	0	0
0	0	0	0	0
0	0	0	0	0
0	0	0	0	0
0	0	0	0	0
0	0	0	0	0
0	0	0	0	0
0	0	0	0	0
0	0	0	0	0
0	0	0	0	0
0	0	0	0	0
0	0	0	0	0
0	0	0	0	0
0	0	0	0	0
0	0	0	0	0
0	0	0	0	0
0	0	0	0	0
0	0	0	0	0
0	0	0	0	0
0	0	0	0	0
0	0	0	0	0
0	0	0	0	0
0	0	0	0	0
0	0	0	0	0

0	0	0	0	0
0	0	0	0	0
0	0	0	0	0
0	0	0	0	0
0	0	0	0	0
0	0	0	0	0
0	0	0	0	0
0	0	0	0	0
0	0	0	0	0
0	0	0	0	0
0	0	0	0	0
0	0	0	0	0
0	0	0	0	0
0	0	0	0	0
0	0	0	0	0
0	0	0	0	0
0	0	0	0	0
0	0	0	0	0
0	0	0	0	0
0	0	0	0	0
0	0	0	0	0
0	0	0	0	0
0	0	0	0	0
0	0	0	0	0
0	0	0	0	0
0	0	0	0	0
0	0	0	0	0
0	0	0	0	0
0	0	0	0	0
0	0	0	0	0
0	0	0	0	0
0	0	0	0	0
0	0	0	0	0
0	0	0	0	0
0	0	0	0	0
0	0	0	0	0
0	0	0	0	0
0	0	0	0	0
0	0	0	0	0
0	0	0	0	0
0	0	0	0	0
0	0	0	0	0
0	0	0	0	0
0	0	0	0	0

0	0	0	0	0
0	0	0	0	0
0	0	0	0	0
0	0	0	0	0
0	0	0	0	0
0	0	0	0	0
0	0	0	0	0
0	0	0	0	0
0	0	0	0	0
0	0	0	0	0
0	0	0	0	0
0	0	0	0	0
0	0	0	0	0
0	0	0	0	0
0	0	0	0	0
0	0	0	0	0
0	0	0	0	0
0	0	0	0	0
0	0	0	0	0
0	0	0	0	0
0	0	0	0	0
0	0	0	0	0
0	0	0	0	0
0	0	0	0	0
0	0	0	0	0
0	0	0	0	0
0	0	0	0	0
0	0	0	0	0
0	0	0	0	0
0	0	0	0	0
0	0	0	0	0
0	0	0	0	0
0	0	0	0	0
0	0	0	0	0
0	0	0	0	0
0	0	0	0	0
0	0	0	0	0
0	0	0	0	0
0	0	0	0	0
0	0	0	0	0
0	0	0	0	0
0	0	0	0	0
0	0	0	0	0
0	0	0	0	0

0	0	0	0	0
0	0	0	0	0
0	0	0	0	0
0	0	0	0	0
0	0	0	0	0
0	0	0	0	0
0	0	0	0	0
0	0	0	0	0
0	0	0	0	0
0	0	0	0	0
0	0	0	0	0
0	0	0	0	0
0	0	0	0	0
0	0	0	0	0
0	0	0	0	0
0	0	0	0	0
0	0	0	0	0
0	0	0	0	0
0	0	0	0	0
0	0	0	0	0
0	0	0	0	0
0	0	0	0	0
0	0	0	0	0
0	0	0	0	0
0	0	0	0	0
0	0	0	0	0
0	0	0	0	0
0	0	0	0	0
0	0	0	0	0
0	0	0	0	0
0	0	0	0	0
0	0	0	0	0
0	0	0	0	0
0	0	0	0	0
0	0	0	0	0
0	0	0	0	0
0	0	0	0	0
0	0	0	0	0
0	0	0	0	0
0	0	0	0	0
0	0	0	0	0
0	0	0	0	0
0	0	0	0	0
0	0	0	0	0

0	0	0	0	0
0	0	0	0	0
0	0	0	0	0
0	0	0	0	0
0	0	0	0	0
0	0	0	0	0
0	0	0	0	0
0	0	0	0	0
0	0	0	0	0
0	0	0	0	0
0	0	0	0	0
0	0	0	0	0
0	0	0	0	0
0	0	0	0	0
0	0	0	0	0
0	0	0	0	0
0	0	0	0	0
0	0	0	0	0
0	0	0	0	0
0	0	0	0	0
0	0	0	0	0
0	0	0	0	0
0	0	0	0	0
0	0	0	0	0
0	0	0	0	0
0	0	0	0	0
0	0	0	0	0
0	0	0	0	0
0	0	0	0	0
0	0	0	0	0
0	0	0	0	0
0	0	0	0	0
0	0	0	0	0
0	0	0	0	0
0	0	0	0	0
0	0	0	0	0
0	0	0	0	0
0	0	0	0	0
0	0	0	0	0
0	0	0	0	0
0	0	0	0	0
0	0	0	0	0
0	0	0	0	0
0	0	0	0	0

0	0	0	0	0
0	0	0	0	0
0	0	0	0	0
0	0	0	0	0
0	0	0	0	0
0	0	0	0	0
0	0	0	0	0
0	0	0	0	0
0	0	0	0	0
0	0	0	0	0
0	0	0	0	0
0	0	0	0	0
0	0	0	0	0
0	0	0	0	0
0	0	0	0	0
0	0	0	0	0
0	0	0	0	0
0	0	0	0	0
0	0	0	0	0
0	0	0	0	0
0	0	0	0	0
0	0	0	0	0
0	0	0	0	0
0	0	0	0	0
0	0	0	0	0
0	0	0	0	0
0	0	0	0	0
0	0	0	0	0
0	0	0	0	0
0	0	0	0	0
0	0	0	0	0
0	0	0	0	0
0	0	0	0	0
0	0	0	0	0
0	0	0	0	0
0	0	0	0	0
0	0	0	0	0
0	0	0	0	0
0	0	0	0	0
0	0	0	0	0
0	0	0	0	0
0	0	0	0	0
0	0	0	0	0
0	0	0	0	0

0	0	0	0	0
0	0	0	0	0
0	0	0	0	0
0	0	0	0	0
0	0	0	0	0
0	0	0	0	0
0	0	0	0	0
0	0	0	0	0
0	0	0	0	0
0	0	0	0	0
0	0	0	0	0
0	0	0	0	0
0	0	0	0	0
0	0	0	0	0
0	0	0	0	0
0	0	0	0	0
0	0	0	0	0
0	0	0	0	0
0	0	0	0	0
0	0	0	0	0
0	0	0	0	0
0	0	0	0	0
0	0	0	0	0
0	0	0	0	0
0	0	0	0	0
0	0	0	0	0
0	0	0	0	0
0	0	0	0	0
0	0	0	0	0
0	0	0	0	0
0	0	0	0	0
0	0	0	0	0
0	0	0	0	0
0	0	0	0	0
0	0	0	0	0
0	0	0	0	0
0	0	0	0	0
0	0	0	0	0
0	0	0	0	0
0	0	0	0	0
0	0	0	0	0
0	0	0	0	0
0	0	0	0	0
0	0	0	0	0

0	0	0	0	0
0	0	0	0	0
0	0	0	0	0
0	0	0	0	0
0	0	0	0	0
0	0	0	0	0
0	0	0	0	0
0	0	0	0	0
0	0	0	0	0
0	0	0	0	0
0	0	0	0	0
0	0	0	0	0
0	0	0	0	0
0	0	0	0	0
0	0	0	0	0
0	0	0	0	0
0	0	0	0	0
0	0	0	0	0
0	0	0	0	0
0	0	0	0	0
0	0	0	0	0
0	0	0	0	0
0	0	0	0	0
0	0	0	0	0
0	0	0	0	0
0	0	0	0	0
0	0	0	0	0
0	0	0	0	0
0	0	0	0	0
0	0	0	0	0
0	0	0	0	0
0	0	0	0	0
0	0	0	0	0
0	0	0	0	0
0	0	0	0	0
0	0	0	0	0
0	0	0	0	0
0	0	0	0	0
0	0	0	0	0
0	0	0	0	0
0	0	0	0	0
0	0	0	0	0
0	0	0	0	0
0	0	0	0	0

0	0	0	0	0
0	0	0	0	0
0	0	0	0	0
0	0	0	0	0
0	0	0	0	0
0	0	0	0	0
0	0	0	0	0
0	0	0	0	0
0	0	0	0	0
0	0	0	0	0
0	0	0	0	0
0	0	0	0	0
0	0	0	0	0
0	0	0	0	0
0	0	0	0	0
0	0	0	0	0
0	0	0	0	0
0	0	0	0	0
0	0	0	0	0
0	0	0	0	0
0	0	0	0	0
0	0	0	0	0
0	0	0	0	0
0	0	0	0	0
0	0	0	0	0
0	0	0	0	0
0	0	0	0	0
0	0	0	0	0
0	0	0	0	0
0	0	0	0	0
0	0	0	0	0
0	0	0	0	0
0	0	0	0	0
0	0	0	0	0
0	0	0	0	0
0	0	0	0	0
0	0	0	0	0
0	0	0	0	0
0	0	0	0	0
0	0	0	0	0
0	0	0	0	0
0	0	0	0	0
0	0	0	0	0
0	0	0	0	0

0	0	0	0	0
0	0	0	0	0
0	0	0	0	0
0	0	0	0	0
0	0	0	0	0
0	0	0	0	0
0	0	0	0	0
0	0	0	0	0
0	0	0	0	0
0	0	0	0	0
0	0	0	0	0
0	0	0	0	0
0	0	0	0	0
0	0	0	0	0
0	0	0	0	0
0	0	0	0	0
0	0	0	0	0
0	0	0	0	0
0	0	0	0	0
0	0	0	0	0
0	0	0	0	0
0	0	0	0	0
0	0	0	0	0
0	0	0	0	0
0	0	0	0	0
0	0	0	0	0
0	0	0	0	0
0	0	0	0	0
0	0	0	0	0
0	0	0	0	0
0	0	0	0	0
0	0	0	0	0
0	0	0	0	0
0	0	0	0	0
0	0	0	0	0
0	0	0	0	0
